# Role of Lamin A/C as Candidate Biomarker of Aggressiveness and Tumorigenicity in Glioblastoma Multiforme

**DOI:** 10.3390/biomedicines9101343

**Published:** 2021-09-28

**Authors:** Giuliana Gatti, Laura Vilardo, Carla Musa, Chiara Di Pietro, Fabrizio Bonaventura, Ferdinando Scavizzi, Alessio Torcinaro, Barbara Bucci, Raffaele Saporito, Ivan Arisi, Francesca De Santa, Marcello Raspa, Loredana Guglielmi, Igea D’Agnano

**Affiliations:** 1 Department of Biotechnology and Translational Medicine, University of Milan, 20129 Milan, Italy; giuliana.gatti@unimi.it; 2 Institute for Biomedical Technologies (ITB), CNR, 20054 Segrate, Italy; laura.vilardo@itb.cnr.it (L.V.); carla.musa85@gmail.com (C.M.); 3 Institute of Biochemistry and Cell Biology (IBBC), CNR, 00015 Monterotondo, Italy; chiara.dipietro@cnr.it (C.D.P.); fabrizio.bonaventura@emma.cnr.it (F.B.); ferdinando.scavizzi@cnr.it (F.S.); alessio.torcinaro@libero.it (A.T.); francesca.desanta@cnr.it (F.D.S.); marcello.raspa@cnr.it (M.R.); 4 UOC Clinical Pathology, San Pietro Hospital FBF, 00189 Rome, Italy; bucci.barbara@fbfrm.it (B.B.); saporito.raffaele@fbfrm.it (R.S.); 5 Bioinformatics Facility, European Brain Research Institute (EBRI) “Rita Levi Montalcini”, 00161 Rome, Italy; i.arisi@ebri.it

**Keywords:** Lamin A/C, glioblastoma, Rictor

## Abstract

Nuclear lamina components have long been regarded as scaffolding proteins, forming a dense fibrillar structure necessary for the maintenance of the nucleus shape in all the animal kingdom. More recently, mutations, aberrant localisation and deregulation of these proteins have been linked to several diseases, including cancer. Using publicly available data we found that the increased expression levels of the nuclear protein Lamin A/C correlate with a reduced overall survival in The Cancer Genome Atlas Research Network (TCGA) patients affected by glioblastoma multiforme (GBM). We show that the expression of the *LMNA* gene is linked to the enrichment of cancer-related pathways, particularly pathways related to cell adhesion and cell migration. Mimicking the modulation of *LMNA* in a GBM preclinical cancer model, we confirmed both in vitro and in vivo that the increased expression of *LMNA* is associated with an increased aggressiveness and tumorigenicity. In addition, delving into the possible mechanism behind *LMNA*-induced GBM aggressiveness and tumorigenicity, we found that the mTORC2 component, Rictor, plays a central role in mediating these effects.

## 1. Introduction

Glioblastoma multiforme (GBM) is the most common and one of the most aggressive primary brain tumours with dismal prognosis both in children and adults [1].

GBM was the first cancer type to be extensively screened by The Cancer Genome Atlas Research Network (TCGA). Its heterogeneity, mainly evident at the level of histological appearance of the tumour cells, has been systematically investigated also at the molecular level, leading to the identification of several glioblastoma subclasses [2,3,4]. Defined biological subgroups of GBM differ for age distribution, tumour location, patient survival, genetic and epigenetics characteristics [4]. However, despite the increasing efforts in defining distinctive patient profiles, patient-specific therapies have not yet been discovered and developed [5,6]. Currently, only a few biomarkers are routinely used in clinical practice and the standard of sick care for patients remains surgery followed by radiation therapy or combined radiation and chemotherapy. Therefore, advances in both scientific and clinical fronts are needed. In this scenario, multi-dimensional profiling studies [2,3,4,7,8,9,10] provide a valid and useful platform to investigate the role of new molecular markers as diagnostic or prognostic factors.

Here we present clear evidence that the nuclear protein Lamin A/C, constituting the nuclear envelope, plays a crucial role in GBM pathogenesis. It is now evident that nuclear lamina components are not only structural proteins ensuring a well-defined nuclear architecture, but they play extensive roles in diverse cellular mechanisms [11]. While mutations in nuclear proteins are responsible of the so-called laminopathies [12], misregulation in their expression is generally linked to cancer development and progression [13]. Whether overexpression or downregulation of a nuclear component correlate with a poorer prognosis is strictly dependent on the tumour type is considered. As an example, Lamin A/C overexpression has been associated with growth, invasion and migration of prostate cancer [14]. Nevertheless, loss of Lamin A/C expression in stage II and III colon cancer is associated with disease recurrence [15]. The complexity of the structure and function of the nuclear Lamins is such that many of the interesting properties of these proteins remain to be determined [16].

## 2. Materials and Methods

### 2.1. Analysis of Transcriptomic Data

Transcriptomic data, and the corresponding clinical metadata including survival time, were retrieved from the freely available GDC Data Portal (https://portal.gdc.cancer.gov/, accessed on 30 March 2017): the RNA-Seq data correspond to the TCGA-GBM project (TCGA Glioblastoma Multiforme) and the microarray expression data are related to a transcriptomic study on Glioblatoma subtypes [8]. As to the TCGA-GBM dataset, only one primary tumor sample was considered for each patient, resulting in a (51,305 genes × 152 subjects) data matrix, where expression values are Log2-transformed FPKM units as Log2(FPKM + 0.0001); recurrent tumour and normal tissue samples were excluded from the analysis. Genes with less than four non-zero values were filtered out. The microarray dataset from [8] is a (11,863 genes × 202 subjects) normalized data matrix, including different tumour subtypes: Classical (n = 54), Neural (n = 33), Proneural (n = 57), Mesenchymal (n = 58). Correlation analysis, survival analysis, and Kaplan-Meier curve plots (survival time vs survival probability) were obtained using R-Bioconductor [17,18,19] and the “survival” package [20]. To associate the survival time to the expression level of selected genes, the subjects were divided in two subpopulations based on a specific quartile of expression values and two survival Kaplan-Meier curves were plotted for each of the two subpopulations: if a statistically significant difference was found between the curves, the gene was considered associated to the survival probability. Kaplan-Meier curves were compared using the Log-rank test. Gene Set Enrichment Analysis (https://www.gsea-msigdb.org/gsea/index.jsp, accessed on 11 September 2017) tools [21,22].

### 2.2. Cell Cultures and Treatments

T98G human GBM cell line (KCLB Cat# 21690) was cultured in Dulbecco’s modified Eagle’s Medium (DMEM) supplemented with 10% FBS, 2mM L-glutamine and 1% penicillin/streptomycin. Before using, the identity of the T98G cell clones was confirmed and certified by analyzing the genetic characteristics of the cell line by PCR-single-locus-technology. The Short Tandem Repeat (STR) analysis of the microsatellite motifs was performed using the Power Plex 16HSM system by Promega. The PCR products were run in the ABI 3130XL capillary electrophoresis and the electropherograms analysed using the GeneMapper IDx software (GeneMapper; https://www.thermofisher.com/order/catalog/product/4475073, accessed on 5 August 2018). The 16 loci analysed were: D5S818, D7S820, D8S1179, D13S317, D16S539, D18S51, CSF1PO, Penta D, TH01, vWA, D21S11, Amelogenin, Penta E, vWA, TPOX, FGA, plus a mouse marker to detect any cross-contamination with mouse DNA. The PCR amplification and CE steps were performed by the external company Genetica in the US. The Cancer Research UK Cambridge institute, University of Cambridge, performed the analysis. Knock-down of *LMNA* gene in T98G cells was obtained by transducing pLenti6/V5-GW/EmGFP-miR-*LMNA* (*LMNA*-KD) or pLenti6/V5-GW/EmGFP-miR#Neg (mock, control cells) lentiviral vectors as described in [23]. Overexpression of *LMNA* gene in T98G cells was obtained by transducing EX-Z3407-Lv105 (*LMNA^+^)* or EX-EGFP-Lv105 (mock, control cells) lentiviral vectors using the same procedures as in [23]. Silencing of Rictor protein in *LMNA*^+^ T98G cells was achieved by transfecting siRNA Rictor (Cell Signaling Technology, Beverly, MA, USA) and Scrambled siRNA control (SCR; SMARTpool, Horizon Discovery [formerly Dharmacon]; Lafayette, CO, USA). Lipofectamine 3000 (Life Technologies, Carlsbad, CA, USA) was used as transfection reagent according to manufacturer’s instructions. The final concentration of siRNA Rictor and SCR siRNA control was set at 100 nM. After 24 h from cell seeding, cells were transfected with both siRNAs and samples were harvested after 48 h to perform western blot analysis and CFA.

### 2.3. In Vivo Experiments

Animals (Mus musculus NOD.Cg-Prkdc^scid^ Il2rg^tm1Wjl^/SzJ) used in this study and bred at the National Research Council–Institute of Biochemistry and Cell Biology (CNR-IBBC), Infrafrontier/ESFRI–European Mouse Mutant Archive (EMMA), specific pathogen-free (SPF) barrier unit (Monterotondo Scalo, Rome, Italy), were housed in individually ventilated caging systems (Tecniplast, Gazzada, Italy) at a temperature (T) of 21 ± 2 °C and relative humidity (RH) of 55 ± 15% with 50–70 air changes per hour (ACH) and under controlled (12:12 h) light–dark cycle (7 a.m.−7 p.m.). Mice had ad libitum access to water and a standard rodent diet (Emma 23, Mucedola, Settimo Milanese, Italy). Each experimental group included six mice. Cells in the exponential growth phase were harvested from the culture, washed with medium and resuspended in Matrigel (2.5 mg/mL; BD Biosciences), and 106 cells implanted subcutaneously in the flank of the mice. When a tumor mass was evident, the tumor sizes were measured by a caliper, and the tumor volume was calculated using the following formula: (a × b^2^)/2, where a and b are the long and short diameters of the tumor, respectively. Three different experiments were performed.

### 2.4. Ethics Statement

Animal work was performed in accordance with a protocol approved by the Italian Ministry of Health (Authorization n.67/2016-PR 21 January 2016). Experimental procedures were also agreed upon, reviewed, and approved by local animal welfare oversight bodies and were performed with the approval and direct supervision of the CNR-IBBC/Infrafrontier—Animal Welfare and Ethical Review Body (AWERB), in accordance with general guidelines regarding animal experimentation, approved by the Italian Ministry of Health, in compliance with the Legislative Decree 26/2014, transposing the 2010/63/EU Directive on protection of animals used in research. This work was also conducted based on recommendations from both ARRIVE and PREPARE guidelines.

### 2.5. Western Blot Analysis

Total proteins were isolated from cells as previously described [23], and 30 µg were subjected to SDS–polyacrylamide gel electrophoresis. The blots were incubated with the following primary anti-human antibodies: anti-Lamin A/C monoclonal (Millipore Temecula, CA, USA; Cat# MAB3211); anti-phospho-mTOR polyclonal (Ser2448) (Cell Signaling Technology Cat# 2971); anti-hTOR monoclonal (Millipore Cat# OP97); anti-phospho-Rictor monoclonal (Thr 1135) (Cell Signaling Technology Cat# 3806); anti-Rictor monoclonal (Cell Signaling Technology Cat# 2114); anti-phospho-Akt monoclonal (Ser473) (Cell Signaling Technology Cat# 4060); anti-pan-Akt monoclonal (Cell Signaling Technology Cat# 4691) and anti-GAPDH monoclonal (Millipore Cat# MAB374). The membranes were then incubated with secondary goat antibodies, anti-mouse or anti-rabbit IgG HRP conjugated (Biorad Laboratories, Segrate, Italy) and were analyzed using the Chemidoc XRS+ Image System (Biorad). Densitometry of each band was performed by ImageJ software (Version 1.47v; NIH, Bethesda, MD, USA).

### 2.6. Colony Forming Assay

One thousand cells in each experimental condition were seeded at clonal density on a 35-mm Ø dish in complete medium (five replicates). Ten days after seeding, the cells were fixed for 1 h with a solution of 2% Methylene Blue in ethanol. The dishes were then accurately washed with double distilled H_2_O and the colonies were counted. Cell survival following treatments is expressed as the percentage of colonies formed out of cells seeded compared to untreated controls (% cell survival). A total of three independent experiments were performed.

### 2.7. Confocal Microscopy

*LMNA*^+^, KD and Control cells were seeded on cover-glass supports in complete medium and allowed to grow up to 70% confluence. To study Lamin A/C expression, the cells were fixed in absolute methanol for 5 min at −20 °C. Non-specific binding was blocked with 5% non-fat dry milk in PBS. Lamin A/C was detected using an anti-Lamin A/C monoclonal antibody (Jol2; Chemicon). Alexa Fluor 594 goat anti-mouse was used as a secondary antibody. For F-actin localization the cells were fixed in 2% paraformaldehyde in PBS containing 2% sucrose for 30 min, were permeabilized in PBS containing 0.2% Tween-20 and were blocked with 5% non-fat dry milk in PBS. Cells were then incubated for 30 min with 5 μg/mL TRITC-phalloidin (Sigma) in PBS. In both cases the nuclei were stained with 1 µg/mL 4′,6-diamidino-2-phenylindole (DAPI) for 5 min in PBS. Finally, the cells were washed in PBS and briefly rinsed in double distilled H_2_O and glass coverslips were mounted in ProLong Gold anti-Fade Reagent (Molecular Probes). Images were acquired using a confocal laser scanning microscope (Olympus FV1200 Laser Scanning Microscope). The brightness and contrast of the acquired images were adjusted, and the figures were generated using Adobe Photoshop CS3 (Version 10.0; San Jose, CA, USA).

### 2.8. Total RNA Preparation

Total RNA was isolated from each cell line using a Total RNA purification plus kit and following manufacturer’s instructions (Norgen Biotek, Thorold, ON, Canada). The RNA concentration and purity was determined by absorbance at 260 nm and A260/280 ratio, respectively, using a NanoDrop UV-VIS spectrophotometer.

### 2.9. Real Time RT-PCR

Expression levels of *LMNA* in the four cell clones (T98G mock, T98G eGFP, T98G *LMNA*^+^, T98G KD) were analyzed using real-time qPCR. RNA was reverse transcribed with a High-Capacity cDNA Reverse Transcription Kit (Applied Biosystem LifeTech #4368814 or #4374966) according to the manufacturer’s instructions. Each reverse transcriptase reaction contains 125 ng of total RNA in 10 µL of water plus 10 µL of 2X RT master mix. Real Time PCR reactions was performed using Applied Biosystems 7500 thermal cycler and the amplifications were done using the SYBR Green PCR Master Mix (Applied Biosystems). 1.5 ng cDNA were amplified as follows: 95 °C for 10 min, 95 °C for 15 s, 60 °C for 60 s. Steps 2 and 3 were repeated for 40 cycles. The experiments were carried out in quadruplicate for each data point. The relative quantification in gene expression was determined using the 2-ΔΔCt method [24]. The GAPDH was used as an internal control to normalize all data and the line T98G mock was chosen as the calibrator. *LMNA* primers: Sense 5′-AGCAAAGTGCGTGAGGAGTT-3′ Antisense 5′-AGGTCACCCTCCTTCTTGGT-3′; GADPH primers: Sense 5′-AGCCACATCGCTCAGACA-3′ Antisense 5′-GCCCAATACGACCAAATCC-3′. TaqMan assay ID and probe sequences: GAPDH (Hs99999905) GGCGCCTGGTCACCAGGGCTGCTT; *COL12A1* (Hs00189184) AGCCTACAGCAGACCTACACCCAAA; *FN1* (Hs01549976) CTGCACAGACCACACTGTTTTGGTT; *ITGA1* (Hs00235006) GGAAAATGGGTGCTTATTGGTTCTC; *ITGA2* (Hs00158127) CTCAGTCAAGGCATTTTAAATTGTT; *ITGA5* (Hs01547673) GGAAGTGTTTGGGGAGCAGAACCAT; *ITGA6* (Hs01041011) ATGCAGGCACTCAGGTTCGAGTGAC; *ITGA8* (Hs00233321) AAAGTTGTGGCCTGTGCTCCTTTAT; *ITGAL* (Hs00158218) GATCGTGCTGAGCTCCCGGCCCGTG; *ITGAM* (Hs00355885) GGCTAAGAGAAGGACAGATCCAGAG; *ITGB3* (Hs01001478) CAAGATTGGAGACACGGTGAGCTTC; *ITGB4* (Hs00174009) GCAACCGGGACTACATCCCCGTGGA; *ITGB5* (Hs00174435) GCAGCACCAAGAGAGATTGCGTCGA; *LAMB1* (Hs01055971) ACTTCGATTGAGTCTGAAACAGCAG; *MMP11* (Hs00968295) GTTCTTCCAAGGTGCTCAGTACTGG; *MMP12* (Hs00899662) ATATCACCTACAGAATCAATAATTA; *MMP14* (Hs01037009) TCATGGGCAGCGATGAAGTCTTCAC; *MMP2* (Hs01548727) ACCAGATCACATACAGGATCATTGG; *PECAM1* (Hs00169777) GAAAGCTGTCCCTGATGCCGTGGAA; *THBS3* (Hs00200157) ATCCACAGTGCAGTGACCAATGCAC; *CLEC3B* (Hs00162844) TGCAGACGGTCTGCCTGAAGGGGAC; *VCAM1* (Hs01003372) TCAATGTTGCCCCCAGAGATACAAC.

### 2.10. Wound Healing Assay

Cells from the three different clones were seeded in Culture-Insert 2 Well (ibidi) in p35 Petri dishes, cultured until 80–90% cell density was achieved and then the insert removed. Cell cultures were washed with PBS, the complete medium was added, and the wound size recorded at 0, 18, 24 and 48 h using an inverted light microscope (Motic AE31 equipped with Cool LED PE-100; Richmond, BC, Canada) and a MOTIC Wi-Fi camera. The wound width was then measured by using ImageJ (Version 1.47v).

### 2.11. Statistical Analysis

Student’s *t* test (unpaired, two-tailed) was used for statistical comparison of different data groups. If there were more than two groups, we used the one-way ANOVA test.

## 3. Results

### 3.1. Expression of LMNA Gene Correlates with a Reduced Overall Survival and Tumorigenic Pathways in Glioblastoma Multiforme Patients

We previously reported that low expression levels of the protein Lamin A/C correlate with a less differentiated phenotype in neuroblastoma cell models [23,25], identifying a subset of neuroblastoma cells with tumour initiating properties [26].

**Figure 1:**
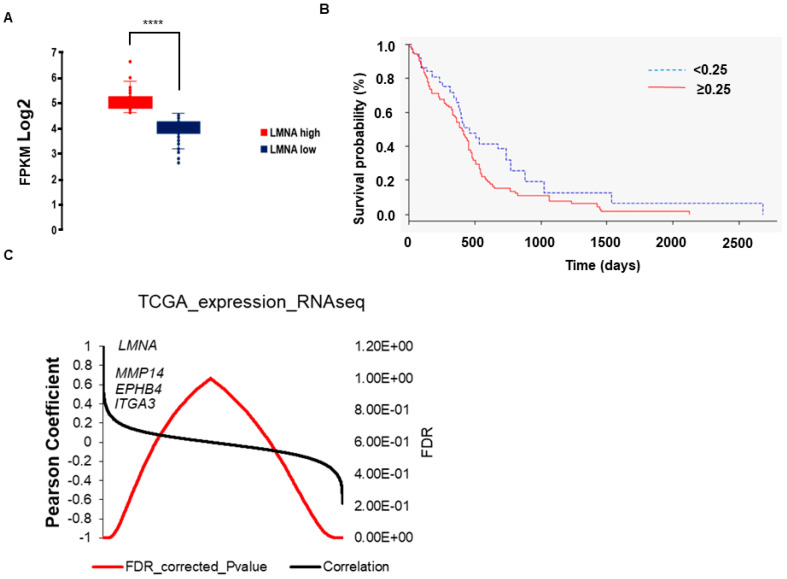
Analysis of *LMNA* gene expression in GBM patients from The Cancer Genome Atlas (TCGA) RNAseq data. (**A**) Distribution of *LMNA* gene expression in 152 GBM patients from TCGA RNAseq data. FPKM (Fragments per kilo base per million mapped reads). Statistical significance: *LMNA* high vs *LMNA* low **** *p* < 0.0001. (**B**) Kaplan-Meier overall survival curves comparing *LMNA* high (≥0.25) and low (<0.25) GBM patients from the TCGA database (Z = 2.1; *p* = 3.5 × 10^−2^). (**C**) Correlation analysis between *LMNA* gene and all other reported in the TCGA RNAseq data. Correlation data are reported in Pearson coefficient and error data in FDR.

With the aim to investigate the role of Lamin A/C in other nervous system tumours, we set out to analyse the expression pattern of the nuclear gene *LMNA* in patients with GBM. RNA-seq data were retrieved from the TCGA database and used to compare the expression of *LMNA* across 152 cases of primary GBM tumours. We found a binary pattern of *LMNA* expression (Figure 1A), with 45% of the patients showing relatively higher expression of *LMNA* gene (*p* < 10^−32^). When comparing this binary pattern with the survival data available from the TCGA, we found that *LMNA* overexpression correlated with a poorer overall survival (*p* = 3.5 × 10^−2^; Figure 1B). We then analysed the correlation between the expression of *LMNA* gene and all other genes reported in the TCGA. Among the genes positively correlated with the expression of *LMNA* we found cell-to-cell and cell-ECM interaction regulators such as metalloproteinases (e.g., *MMP14*), integrins (e.g., *ITGA3*), and ephrin receptors (e.g., *EPHB4*) (Figure 1C and Supplementary Material Table S1).

Gene set enrichment analysis (GSEA) was carried out to explore core molecular pathways directly related to the expression of *LMNA* in patients with GBM. KEGG pathways such as ‘Glioma’, ‘pathways in cancer’, ‘ECM-receptor interaction’, ‘focal adhesion’, ‘cell adhesion molecules’ and ‘regulation of actin cytoskeleton’ resulted enriched, providing further evidence that the expression of Lamin A/C is linked to cancer-related mechanisms in GBM (Supplementary Material Figure S1 and Table S2). Similar categories were found using the Reactome database (Supplementary Material Table S3).

**Figure 2:**
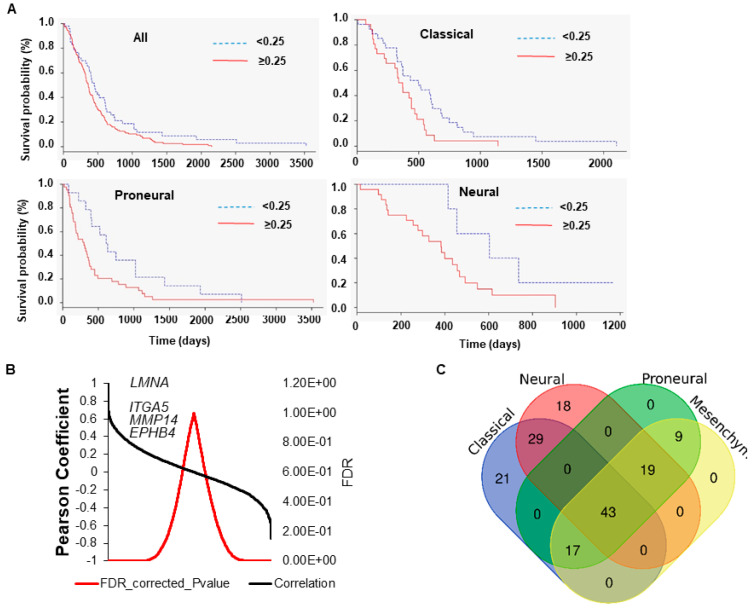
Analysis of *LMNA* gene expression in GBM patients from [8] microarray data. (**A**) Kaplan-Meier overall survival curves comparing *LMNA* high (≥0.25) and low (<0.25) from the TCGA database in GBM all (Z = 2.0; Log-Rank *p*-value = 4.1 × 10^−2^), classical (Z = 2.0; Log-Rank *p*-value = 4.4 × 10^−2^), proneural (Z = 2.3; Log-Rank *p*-value = 2.0 × 10^−2^) and neural (Z = 2.0; Log-Rank *p*-value = 4.0 × 10^−2^) group of patients. (**B**) Correlation analysis between *LMNA* gene and all other reported in the [8] microarray data. Correlation data are reported in Pearson coefficient and error data in FDR. (**C**) Venn diagram showing the enriched KEGG pathways in the different GBM subtypes of the [8] microarray database.

Different profiles of overall survival and tumorigenic pathways may be linked to the expression of the *LMNA* gene in different GBM subtypes. We questioned if our findings could be related to one or more subtypes of GBM. To this aim, we used a second independent dataset which stratified patients by GBM subtypes [8]. First, we verified that the expression of *LMNA* correlates, in general, with a poorer prognosis. We observed that *LMNA* gene expression correlates with a poorer overall survival, pooling the cancer subgroups in Classical, Neural and Proneural (Figure 2A) but not in Mesenchymal GBM subtypes.

Consistent with our previous observations, we found that genes showing a positive correlation with the expression of *LMNA* belonged to cell-to-cell and cell-ECM interactions regulators (Figure 2B and Supplementary Material Table S4). GSEA analysis confirmed the correlation between the expression of the *LMNA* gene and the regulation of pathways involved in cancer and ECM-cytoskeletal-nucleoskeletal interactions also in this second dataset (Supplementary Material Figure S2; see Figure S1 for comparison). We then cross-compared the GSEA analysis of the different GBM subtypes. Figure 2C shows the results of this analysis in a Venn diagram. The classical and the neural subtypes were characterised by 21 and 18 specific KEGG pathways, respectively. The classical subtype presented pathways related to cytotoxicity and immunogenicity, while the neural subtype was specifically enriched with pathways related to cellular metabolism (Supplementary Material Table S5). The proneural and mesenchymal subtypes did not show any specific pathways (Figure 2C). Interestingly, the KEGG pathway ‘Glioma’ was not enriched in this second dataset (Supplementary Material Figure S2). We therefore investigated whether this was true for all GBM subtypes and found that the expression of *LMNA* positively correlates with genes that are involved in ‘Glioma’ pathways specifically in the classical subtype (Supplementary Material Table S6).

### 3.2. Modulation of LMNA Gene Expression Levels in a GBM Cell Model

**Figure 3:**
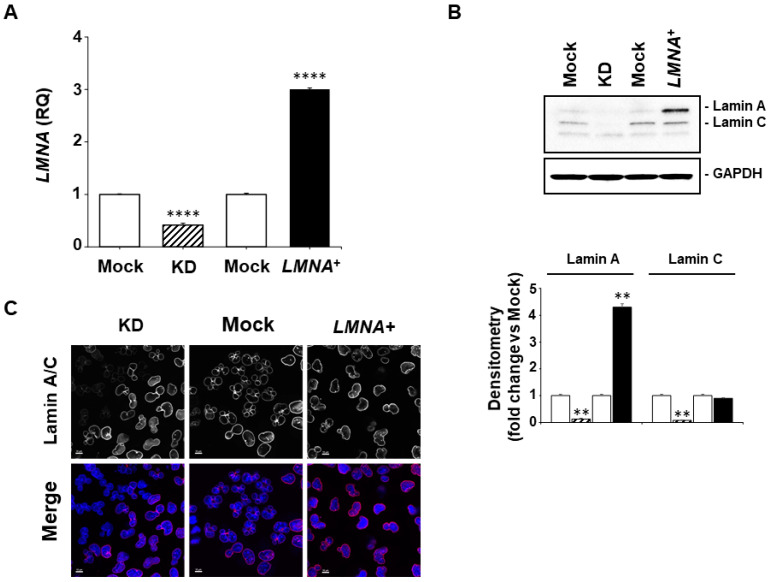
Modulation of *LMNA* gene expression in GBM T98G cells. (**A**) Relative quantification (RQ) of *LMNA* gene in *LMNA*-KD (dashed) and *LMNA*^+^ overexpressed (black) T98G cells as analysed by qRT-PCR. The data are reported as the level of mRNA relative to the respective transfected vector control cells and are the means + SD (n = 3). Statistical significance: *LMNA^+^* and KD vs mock **** *p* < 0.0001. (**B**) Top, representative western blot of the Lamin A/C protein in *LMNA*-KD (KD) and *LMNA*^+^ T98G cells; bottom, blots densitometry as analyzed by ImageJ software. Values are averages + s.d. (n = 3), relative to mock cells (white) of three independent experiments with similar results. *LMNA*-KD (dashed), *LMNA+* (black). GAPDH was used as loading control.). Statistical significance: *LMNA*^+^ and KD vs mock ** *p* < 0.01. (**C**) Representative confocal images of mock, *LMNA*-KD (KD) and *LMNA^+^* cells. Red, Lamin A/C immunostaining; blue, DAPI. Scale bar: 10 microns.

To investigate the correlation between the expression of *LMNA* and the aggressiveness and tumorigenicity observed in our in-silico analysis, we chose the T98G GBM cell line as our cellular in vitro model. This cell line is known to be weakly tumorigenic in nude mice [27] and to express moderate, therefore modulable, levels of Lamin A/C, which are comparable to the level of the protein expression in the normal astrocytes (data not shown). These constituted the optimal baseline characteristics to modulate the expression of the *LMNA* gene in this cellular model and generate multiple cell-line clones that would simulate the conditions of low- and high-expressing Lamin A/C tumors observed in vivo. We silenced the *LMNA* gene by infecting the T98G GBM cell line with a lentiviral vector that allows the simultaneous expression of the EmGFP reporter gene and of an artificial miRNA targeting the *LMNA* mRNA [23]. The *LMNA*-knock down cells (KD) showed a reduction of the *LMNA* transcript of more than 50% and of the protein levels of approximately 8-fold for the Lamin A isoform and 14-fold for the Lamin C, compared with mock cells infected with the control lentiviral vector carrying a non-targeting artificial miRNA (Figure 3A,B).

We overexpressed the *LMNA* gene by infecting the T98G cells with the lentiviral vector pEZ-Lv105 (#EX-Z3407, Genecopoeia) carrying the human transcript variant 1 of the *LMNA* gene. The *LMNA*^+^ cells showed about a threefold increase of the *LMNA* transcript and about a fivefold induction of the Lamin A/C protein levels compared with the mock cells infected with the control lentiviral vector (#EX-EGFP-Lv105, Genecopoeia) (Figure 3A,B). The increase observed at the protein levels were much more evident for the Lamin A compared with the Lamin C isoform (Figure 3B). Since the two control-vector-infected cell clones showed comparable levels of expression of *LMNA* gene and Lamin A/C protein we used only one mock control clone in the subsequent experiments. The modulation of the Lamin A/C expression was also visualised in the KD, *LMNA*^+^ and control cells by confocal microscopy with its peculiar perinuclear localization (Figure 3C).

### 3.3. Upregulation of LMNA Gene Increase the Aggressiveness of T98G

**Figure 4:**
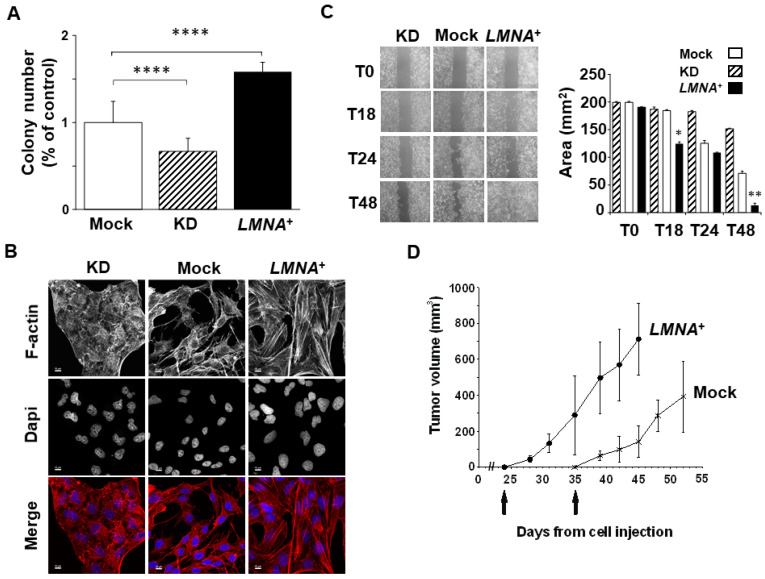
Overexpression of *LMNA* gene increases GBM aggressiveness and tumorigenesis. (**A**) Graphic quantification of colonies as analyzed by a colony-forming assay, showing that *LMNA* overexpression increased T98G cell colonies. The data are presented as the mean ± SEM of three independent experiments. Statistical significance: *LMNA^+^* vs mock cells **** *p* < 0.0001; KD vs mock cells **** *p* < 0.0001. (**B**) Representative confocal images of mock *LMNA^+^* and *LMNA*-KD (KD) cells. Red, F-actin; blue, DAPI. Scale bar: 10 microns. It is worthwhile to note that F-actin fibers are more organized and increased in number in *LMNA^+^* cell clone. (**C**) Cell migration as analyzed by a wound healing assay. *LMNA* expression has effect on migratory capacity as *LMNA* overexpression clearly increases wound healing at 48 h compared to the control cells. Left panel, representative images were captured with a phase-contrast microscope (20X) at 18, 24 and 48 h after wound. Scale bar (50 µm) is the same for all the images and is shown in the right bottom image. Right panel, values of wound area (mm2) ± SEM (n = 3). mock cells (white), KD cells (dashed), *LMNA*^+^ cells (black). Statistical significance: *LMNA^+^* vs Mock cells * *p* < 0.05, ** *p* < 0.01. (**D**) mock (*), *LMNA^+^* (•) and *LMNA*-KD cells were injected subcutaneously into the flanks of immunosuppressed mice at 10^6^ cells/mouse in 200 μL of Matrigel. When a tumor mass was evident, the tumor sizes were measured, and the tumor volumes were calculated using the following formula: (a × b^2^)/2, where a and b are the long and short diameters of the tumor, respectively. Injection of KD cells did not give rise to any tumour in the mouse up to 54 days post-injection, when also mice injected with mock cells were sacrificed. The mean ± SD tumor volumes are reported (n = 6).

In order to test whether the modulation of Lamin A/C could affect the aggressiveness of the T98G cells, we first performed a colony-forming assay in vitro. The analysis of the plating efficiency confirmed that an increased expression of Lamin A/C is associated with an aggressive phenotype, corresponding to an ability to form colonies in vitro around 50% higher than the control cells. A reduced expression of Lamin A/C resulted in a moderate decrease of the plating efficiency percentage corresponding to less than 25% decrease of the ability to form colonies in vitro compared to control cells (Figure 4A,B).

Tumour aggressiveness is known to be associated with a higher tumour invasion capacity, which typically involves rearrangements and reorganisation of the cytoskeleton. We investigated the intracellular organisation of F-actin filaments using phalloidin staining in our cell models. *LMNA*^+^ clones showed more and thicker cytoplasmic F-actin-containing fibers (Figure 4B). This observation was consistent with a higher migratory capacity of these cells, as evidenced by wound-healing assay, when compared with control cells (Figure 4C). Conversely, KD clones demonstrated a more disorganized structure of F-actin filaments and a reduced ability to migrate up to 48 h (Figure 4B,C). Our in vitro results were further corroborated by in vivo data that highlighted a significant increased tumorigenicity of *LMNA*^+^ cells in nude mice. In Figure 4D, we show the tumour appearance at 24 and 36 days after injecting the same number of *LMNA*^+^ and mock cells, respectively. Injection of KD cells did not give rise to any tumour in the mouse up to 54 days post-injection, when mice injected with mock cells were sacrificed (Figure 4D). The in vivo growth rate of the *LMNA*^+^ tumours was also significantly increased compared with mock tumours (about a threefold increase at the maximum differences; Figure 4D).

**Figure 5:**
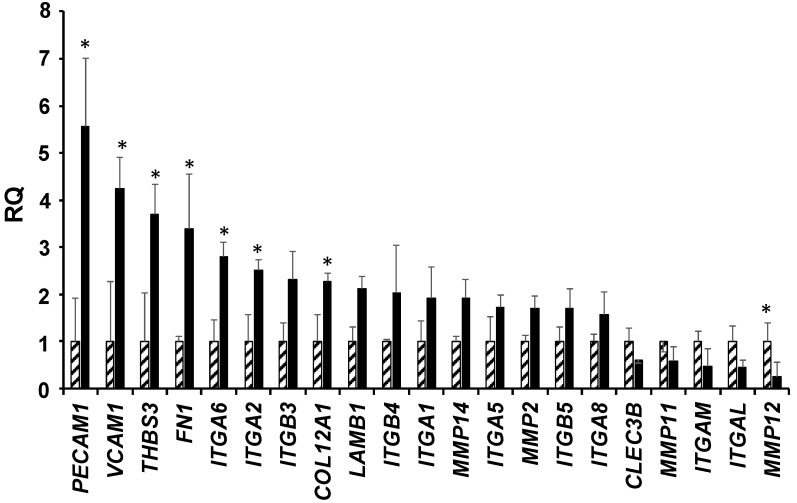
LMNA overexpression increases cell adhesion gene expression. Relative quantification (RQ) of the indicated genes in *LMNA^+^* (black) and *LMNA*-KD (dashed) T98G cells as analysed by qRT-PCR. The data are reported as the level of *LMNA^+^* clone mRNA relative to the *LMNA*-KD clone and are the means + SD (n = 3). Statistical significance: * *p* ≤ 0.05.

Our in silico, in vitro and in vivo analyses opened a possible scenario where the increased aggressiveness and tumorigenicity elicited by the overexpression of the *LMNA* gene is linked to altered adhesion/invasion cellular mechanisms in GBM. We confirmed by real time PCR analysis the expression of selected genes specifically involved in adhesion/invasion cellular mechanisms in our models. The genes that were differentially expressed between the *LMNA^+^* and KD cells are shown in Figure 5. Those overexpressed in the *LMNA^+^* cells are related to a more invasive phenotype and encode for integrins, metalloproteinases and other adhesion receptors. This result reflects what we previously observed in the GBM patient datasets used for our in-silico analysis, i.e., genes that highly correlated with the expression of the *LMNA* gene in patients with GBM are also highly correlated with the overexpression of *LMNA* gene in our GBM cell models (see Figure 5 and Supplementary Material Tables S1 and S4). By contrast, genes downregulated in *LMNA^+^* cells are mainly related to less malignant tumor characteristics (Figure 5).

**Figure 6:**
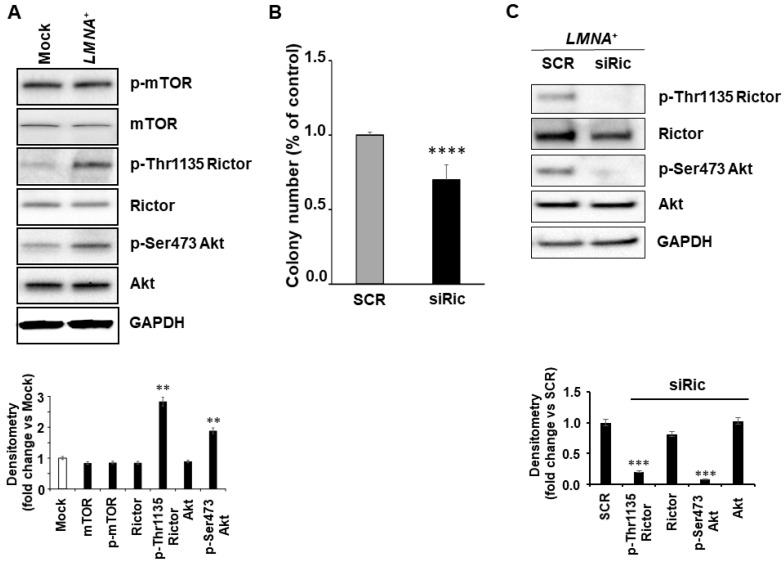
Rictor inhibition is associated with reduction of colony formation. (**A**) Top, representative western blot of the indicated proteins in CTRL and *LMNA^+^* T98G cells; bottom, blots densitometry as analyzed by ImageJ software. Values are averages + s.d. (n = 3), relative to mock cells of three independent experiments with similar results. GAPDH was used as loading control. Statistical significance: ** *p* < 0.01. (**B**) *LMNA^+^* clone was transfected with scrambled siRNA as control (SCR, 100 nM) or siRNA Rictor (siRic, 100 nM) and after 48 h from transfection the cells were analysed. Colony number as % of control are reported in siRic-treated (black) versus SCR-treated *LMNA+* cells (gray). Statistical significance: siRictor-treated vs SCR-treated *LMNA*^+^ cells, **** *p* < 0.0001. (**C**) Top panel, representative western blot of the indicated protein expression in *LMNA*^+^ cells transfected with scrambled (SCR) and siRNA Rictor (siRic); bottom panel, blots densitometry as analyzed by ImageJ software. Values are averages + s.d. (n = 3), relative to SCR-treated cells of three independent experiments with similar results. GAPDH was used as loading control. Statistical significance: *** *p* < 0.001.

Since it has been demonstrated that protein kinase B (AKT)/mTOR signaling can regulate adhesion pathways, we investigated the expression levels of proteins related to mTOR pathways in *LMNA^+^* cells. While the phosphorylation status of mTOR did not change, the phosphorylation of Rictor (Thr1135) and Akt (Ser473) proteins increased by about 3- and 2-folds, respectively, compared with control cells, indicating the activation of this signaling pathway (mTORC2; Figure 6A). The total protein level of Rictor and Akt remained unchanged. The other pathway of mTOR associated with Raptor (mTORC1) was not affected (data not shown). Conversely, KD cells showed a decreased phosphorylation of the same proteins (data not shown). To verify whether these changes could convey a biological effect we performed colony forming assay in *LMNA^+^* cells after inhibiting the whole Rictor protein by using siRNA interfering. The inhibition of Rictor produced a significant (*p* < 0.0001) reduction of colony formation in *LMNA^+^* cells, indicating that the activation of Rictor is necessary for the increased aggressiveness of the T98G GBM cells (Figure 6B). The inhibition of Rictor protein in *LMNA*+ cell clone was checked by WB (Figure 6C). Rictor protein levels and phosphorylation were reduced by about 20 and 80%, respectively. As well as Ser473, phosphorylation of Akt was reduced by more than 90%.

## 4. Discussion

The aberrant expression of Lamin A/C has been reported in a variety of cancer types with ambivalent roles in tumour progression [13]. Its decreased expression in breast, prostate, colon, ovarian, gastric, and endometrial cancer and osteosarcoma correlated with a reduction in overall patient survival and an increase in metastasis and tumour relapse [15,28,29,30]. In agreement with these works, in previous papers we also found that *LMNA* expression was related to a less malignant phenotype in human neuroblastoma [23,26]. By contrast, some studies revealed a link between increased *LMNA* expression and tumor progression in colorectal, prostate, ovarian and hepatocellular cancers [14,31,32,33]. This ambiguity in the *LMNA* gene role as a cancer biomarker is probably due to the numerous diverse functions that Lamins perform in the cell and their variable expression between cancer subtypes [13]. For these reasons, we decided to investigate whether *LMNA* could play a role in GBM progression.

GBM, the most frequent primary malignant brain tumour, is a complex cancer disease displaying an extremely heterogeneous phenotype and is lacking diagnostic/prognostic biomarkers [8,34,35]. Although some markers, such as IDH1 gene variants and MGMT promoter methylation, have been proposed to identify patients with better prognosis, they are applicable only to a restricted number of cases [36,37]. Molecular stratification of patients by expression [8] and methylome profiling [4] provided extensive evidence that different subgroups of GBM are characterised by the expression of distinct biomarkers and show different clinical outcomes. In the present study, we found that the overexpression of the *LMNA* gene could discriminate GBM patients with a poorer prognosis, specifically in tumours belonging to Classical, Neural and Proneural but not in Mesenchymal GBM subtypes. This suggests that Lamin A/C could play a crucial role in GBM.

In order to dissect the relevant role of Lamin A/C in GBM we developed a preclinical experimental model using a human glioblastoma cell line (T98G). We specifically chose the T98G cell line since these cells, among the tested cell lines, show levels of Lamin A/C expression similar to the normal astrocyte (data not shown). Modulating *LMNA* expression in these cells, we demonstrated that Lamin A/C overexpression induces a more malignant phenotype. Consistent with the poor prognosis shown by the patients with high levels of *LMNA* expression is the high tumorigenicity displayed in vivo by the T98G *LMNA*^+^ cells injected in nude mice. In fact, the *LMNA*^+^ cells formed tumours earlier and grew more rapidly compared with control cells. These data are further strengthened by the fact that *LMNA* knockdown cells lost their ability to form tumours in mice.

In addition, *LMNA*^+^ cells showed an increased clonogenic ability in vitro associated with a greater migratory capacity, compared with the KD cells. Lamin A/C, as a component of the nuclear lamina, belongs to the type V intermediate filament (IF) family of proteins that maintain the organisation of the cytoplasmic and nuclear architecture across cell types. Downregulation of Lamin A/C in our cellular model disrupted this organisation, as evidenced by the altered rearrangements of the actin filaments inside the cells. Conversely, by overexpressing Lamin A/C in our cellular model, the actin filaments appeared to form fibers, which besides giving structural support to the cells may favour cellular movement and migration. These findings are in keeping with the structural role of Lamin A/C in regulating ECM-cytoskeletal-nucleoskeletal interactions. These data are consistent with the increased expression in *LMNA*^+^ cells of genes encoding for known adhesion molecules that are involved in cell migration and invasion. The platelet endothelial cell adhesion molecule, PECAM1; the vascular cellular adhesion molecule-1, VCAM1; the gene for human thrombospondin 3, an extracellular matrix glycoprotein which mediates interactions between cells and the extracellular matrix, THBS3; fibronectin 1, FN1; laminin subunit beta 1, LAMB1, and collagen type XII α1 chain, COL12A1, are all stimulators of tumour progression linked to increased metastasis, motility and invasion in different cancer types [38,39,40,41,42,43]. These were found increased more than twofold in the *LMNA*^+^ cells (see Figure 5). We also observed an increased expression of some metalloproteinases such as MMP14 and MMP2, which are known mediators of cancer invasion [44] and a remarkable decrease of two metalloproteinases, MMP11 and MMP12, which are known to have a protective role against tumors [45,46]. Finally, the expression of the integrins also agreed with their significance in tumours. In *LMNA*^+^ cells, we found an increase in most of those integrins which are known to mediate migration, proliferation and survival in tumour cells, such as ITGA1, 2, 5, 6 and 8; as well as ITGB3, 4 and 5 [47]. Conversely, ITGAL and ITGAM were strongly decreased in *LMNA*^+^ cells and this is in accordance with the anti-tumour effects reported in the literature for these two integrins [48,49]. The results obtained in our cell models were largely in agreement with the analyses conducted in silico using RNA-seq and microarray expression data patients with GBM.

Overall, our findings pointed out that the increased malignancy and poor prognosis of GBM may be linked to altered ECM-cytoskeletal-nucleoskeletal interactions, specifically in the presence of high expression of the Lamin A/C protein. There is a delicate balance between the ECM and the organisation of the cytoskeletal and nucleoskeletal structures, often involving stimuli that are sent from the outside to the inside of the cell and vice versa. These are known to mediate the impact on several cellular events, including mitosis, cell polarisation and cell migration. In our paper, we focused on the mTORC2 complex that is involved in the control of cell growth via cytoskeleton remodelling, through modification of actin fiber and other phospho-activated proteins [50]. mTORC2 directly activates Akt by phosphorylating its hydrophobic motif (Ser473), a site required for its maximal activation [51]. mTORC2 has also been associated with tumorigenesis and progression of many different tumour types [52]. Rictor, an essential subunit of the mTORC2 complex, is overexpressed in numerous cancer types, including GBM, and is associated with poor patient survival. Wang et al., demonstrated that Rictor knockdown in renal cancer cells downregulates cell adhesion molecules such as ITGA5 and ITGA1 [47]. Our data agree with these authors, as we found that the overexpression of *LMNA* increased the levels of the same genes in T98G cells and the phosphorylation of Rictor, suggesting a positive regulatory role of mTORC2 in the control of cell migration. Rictor has also been demonstrated to promote cell growth in glioma cells [53]. Consistent with these data is the inhibition of clonogenic ability observed in the T98G *LMNA*^+^ cells after siRNA silencing of Rictor and not in *LMNA* KD clone.

Rictor and Akt are present not only in the cytoplasm but also in the nucleus. Studies with different approaches to immunoprecipitated cytoplasmic or nuclear mTOR and Rictor showed that mTORC2 component assembly occurs in both cell compartments [54]. We may speculate that the compartmentalisation of Rictor could influence GBM malignancy. In line with this, some researchers have shown that in patients with GBM there is a prevalence of Rictor in the nucleus [55]. Alternatively, Rictor itself may interact with some membrane proteins such as integrins and tetraspanins [56], or cytoskeletal proteins such as filamin A [57].

## 5. Conclusions

In conclusion, the data herein reported demonstrate that Lamin A/C expression play a relevant role in GBM and correlate with poor prognosis in all GBM subtypes except for Mesenchymal. The presence of Lamin A/C appears to be necessary for GBM tumour progression and may be linked to the altered regulation of specific adhesion/invasion cellular pathways. Therefore, Lamin A/C may represent a good candidate biomarker in this tumour type, bringing new possibilities to GBM targeted therapies and/or patients’ stratification.

## Data Availability

Transcriptomic data, and the corresponding clinical metadata including survival time, were retrieved from the freely available GDC Data Portal (https://portal.gdc.cancer.gov/, accessed on 30 March 2017): the RNA-Seq data correspond to the TCGA-GBM project (TCGA Glioblastoma Multiforme) and the microarray expression data are related to a transcriptomic study on Glioblatoma subtypes [8].

## Supplementary Materials

The following are available online at https://www.mdpi.com/article/10.3390/biomedicines9101343/s1, Table S1: List of genes correlated with *LMNA* gene expression in GBM patients from TCGA RNASeq data; Table S2: Gene set enrichment analysis of the core molecular KEGG pathways related to the expression of *LMNA* in GBM patients from TCGA RNASeq data; Table S3: Gene set enrichment analysis of the core molecular Reactome pathways related to the expression of *LMNA* in GBM patients from TCGA RNASeq data; Table S4: List of genes correlated with *LMNA* gene expression in GBM patients from microarray dataset; Table S5: Venn diagram breakdown of the core molecular KEGG pathways in relation to the GBM subtypes from microarray dataset; Table S6: Gene set enrichment analysis of the core molecular KEGG pathways in the GBM subtype patients from microarray dataset; Figure S1: Enrichment plots from Gene Set Enrichment Analysis (GSEA) of genes resulting from the analysis shown in Figure 1C; Figure S2: Enrichment plots from Gene Set Enrichment Analysis (GSEA) of genes resulting from the analysis shown in Figure 2B.
