# Supplementary material for: Role of Lamin A/C as Candidate Biomarker of Aggressiveness and Tumorigenicity in Glioblastoma Multiforme
Source: Biomedicines. 2021 Sep 28;9(10):1343. doi: 10.3390/biomedicines9101343 (PMC8533312; doi:10.3390/biomedicines9101343)
Supplement: Supplementary file 1 [file biomedicines-09-01343-s001.zip › biomedicines-1353075-suppl/biomedicines-1353075-suppl(figures).pdf]

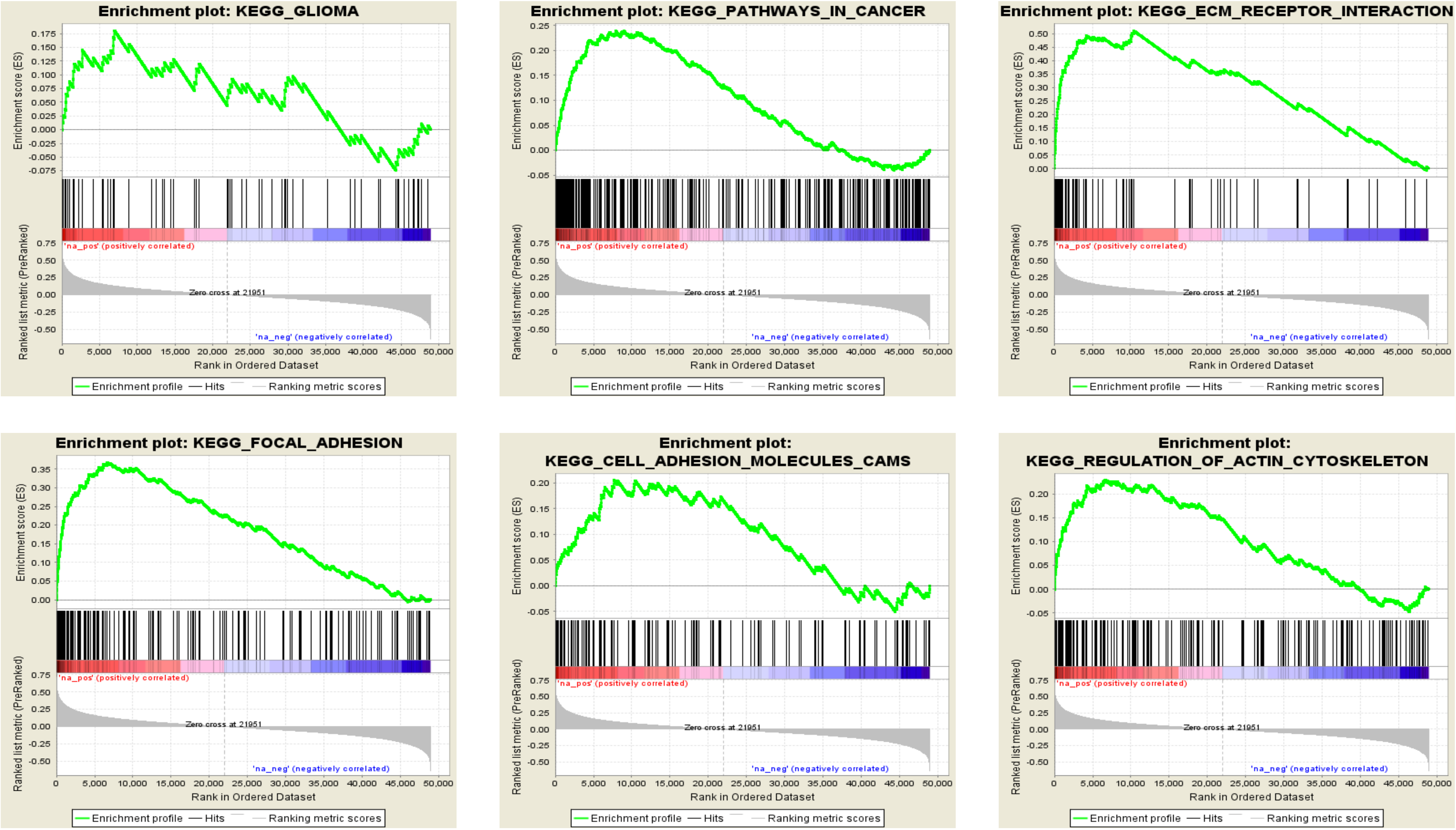

Supplementary Figure S1. Enrichment plots from Gene Set Enrichment Analysis (GSEA) of genes that resulted to be positively correlated with the expression of the LMNA gene based on the results shown in Figure 1C. The KEGG database was chosen to display the results of the enriched pathways.

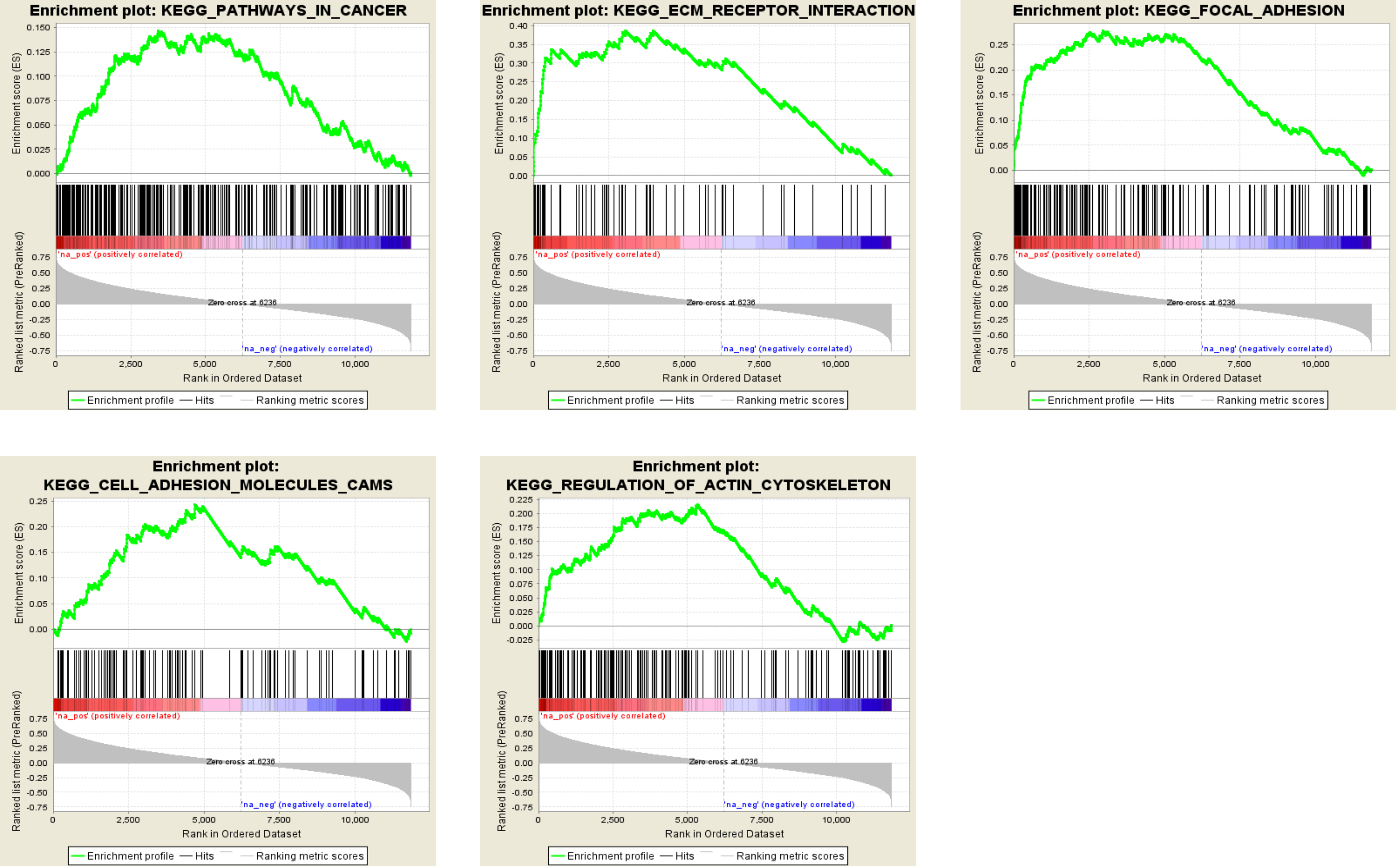

Supplementary Figure S2. Enrichment plots from Gene Set Enrichment Analysis (GSEA) of genes that resulted to be positively correlated with the expression of the LMNA gene based on the results shown in Figure 2B. The KEGG database was chosen to display the results of the enriched pathways.
