# Supplementary material for: Role of Lamin A/C as Candidate Biomarker of Aggressiveness and Tumorigenicity in Glioblastoma Multiforme
Source: Biomedicines. 2021 Sep 28;9(10):1343. doi: 10.3390/biomedicines9101343 (PMC8533312; doi:10.3390/biomedicines9101343)
Supplement: Supplementary file 1 [file biomedicines-09-01343-s001.zip › biomedicines-1353075-suppl/biomedicines-1353075-suppl(tables).zip!/supplementary tables/Supplementary Table 1.pdf]

Table S1. List of genes correlated with LMNA gene expression in GBM patients from TCGA RNASeq data.

var1	var2	corr	corr_CI5%	corr_CI95%	pvalue	FDR_corrected_Pval	Bonferroni_corrected_Pval
ENSG00000198804.2_MT-CO1	ENSG00000160789.18_LMNA	-0.07543	-0.231841961	0.084793727	3.56E-01	6.39E-01	1.00E+00
ENSG00000198712.1_MT-CO2	ENSG00000160789.18_LMNA	-0.08738	-0.243200158	0.072831177	2.84E-01	5.70E-01	1.00E+00
ENSG00000198938.2_MT-CO3	ENSG00000160789.18_LMNA	-0.10589	-0.26069709	0.054223593	1.94E-01	4.63E-01	1.00E+00
ENSG00000198886.2_MT-ND4	ENSG00000160789.18_LMNA	-0.04667	-0.204348304	0.113377256	5.68E-01	7.98E-01	1.00E+00
ENSG00000210082.2_MT-RNR2	ENSG00000160789.18_LMNA	-0.2448	-0.388849341	-0.08907397	2.37E-03	2.23E-02	1.00E+00
ENSG00000198899.2_MT-ATP6	ENSG00000160789.18_LMNA	-0.0679	-0.224675206	0.092295136	4.06E-01	6.81E-01	1.00E+00
ENSG00000087086.12_FTL	ENSG00000160789.18_LMNA	-0.01079	-0.169695688	0.14866961	8.95E-01	9.59E-01	1.00E+00
ENSG00000198727.2_MT-CYB	ENSG00000160789.18_LMNA	-0.0821	-0.238192233	0.078116798	3.15E-01	6.01E-01	1.00E+00
ENSG00000198840.2_MT-ND3	ENSG00000160789.18_LMNA	-0.20335	-0.351184778	-0.04562985	1.20E-02	7.47E-02	1.00E+00
ENSG00000198888.2_MT-ND1	ENSG00000160789.18_LMNA	-0.0862	-0.242075001	0.074020274	2.91E-01	5.76E-01	1.00E+00
ENSG00000131095.10_GFAP	ENSG00000160789.18_LMNA	-0.13738	-0.290230427	0.022311441	9.15E-02	2.95E-01	1.00E+00
ENSG00000198763.3_MT-ND2	ENSG00000160789.18_LMNA	-0.09697	-0.252278475	0.063203981	2.35E-01	5.12E-01	1.00E+00
ENSG00000075624.12_ACTB	ENSG00000160789.18_LMNA	0.37417	0.228580944	0.503381237	2.05E-06	8.15E-05	1.05E-01
ENSG00000034510.5_TMSB10	ENSG00000160789.18_LMNA	0.1625	0.003391312	0.313592103	4.55E-02	1.89E-01	1.00E+00
ENSG00000198695.2_MT-ND6	ENSG00000160789.18_LMNA	-0.04973	-0.20728873	0.110345181	5.43E-01	7.82E-01	1.00E+00
ENSG00000212907.2_MT-ND4L	ENSG00000160789.18_LMNA	-0.06853	-0.225274021	0.091669734	4.02E-01	6.78E-01	1.00E+00
ENSG00000228253.1_MT-ATP8	ENSG00000160789.18_LMNA	-0.13935	-0.292072688	0.020299462	8.69E-02	2.86E-01	1.00E+00
ENSG00000118785.12_SPP1	ENSG00000160789.18_LMNA	-0.00769	-0.166683346	0.151699723	9.25E-01	9.71E-01	1.00E+00
ENSG00000205542.9_TMSB4X	ENSG00000160789.18_LMNA	0.03292	-0.126949375	0.191115775	6.87E-01	8.65E-01	1.00E+00
ENSG00000211459.2_MT-RNR1	ENSG00000160789.18_LMNA	-0.30052	-0.438734046	-0.14842443	1.69E-04	2.86E-03	1.00E+00
ENSG00000120885.18_CLU	ENSG00000160789.18_LMNA	0.07476	-0.085456838	0.231209814	3.60E-01	6.42E-01	1.00E+00
ENSG00000111640.13_GAPDH	ENSG00000160789.18_LMNA	0.29525	0.142758697	0.434047951	2.22E-04	3.54E-03	1.00E+00
ENSG00000198786.2_MT-ND5	ENSG00000160789.18_LMNA	-0.02553	-0.1839824	0.134216741	7.55E-01	8.98E-01	1.00E+00
ENSG00000133048.11_CHI3L1	ENSG00000160789.18_LMNA	0.48448	0.352459879	0.597585359	2.55E-10	4.98E-08	1.31E-05
ENSG00000184009.8_ACTG1	ENSG00000160789.18_LMNA	0.32541	0.175292742	0.46074373	4.30E-05	9.49E-04	1.00E+00
ENSG00000156508.16_EEF1A1	ENSG00000160789.18_LMNA	-0.17853	-0.328393441	-0.01989079	2.78E-02	1.36E-01	1.00E+00
ENSG00000167552.12_TUBA1A	ENSG00000160789.18_LMNA	-0.34893	-0.481393725	-0.20089384	1.05E-05	3.04E-04	5.41E-01
ENSG00000113140.9_SPARC	ENSG00000160789.18_LMNA	0.16619	0.007181661	0.317005734	4.07E-02	1.76E-01	1.00E+00
ENSG00000142534.5_RPS11	ENSG00000160789.18_LMNA	-0.17678	-0.326779306	-0.01808327	2.94E-02	1.41E-01	1.00E+00
ENSG00000130203.8_APOE	ENSG00000160789.18_LMNA	-0.10662	-0.261380906	0.053491902	1.91E-01	4.59E-01	1.00E+00
ENSG00000204287.12_HLA-DRA	ENSG00000160789.18_LMNA	-0.04471	-0.202468612	0.11531244	5.84E-01	8.07E-01	1.00E+00

ENSG00000166710.16_B2M	ENSG00000160789.18_LMNA	0.06027	-0.099884464	0.217389389	4.61E-01	7.24E-01	1.00E+00
ENSG00000106153.12_CHCHD2	ENSG00000160789.18_LMNA	-0.04143	-0.199314576	0.118554178	6.12E-01	8.24E-01	1.00E+00
ENSG00000019582.13_CD74	ENSG00000160789.18_LMNA	0.06402	-0.096156052	0.220973119	4.33E-01	7.03E-01	1.00E+00
ENSG00000197746.12_PSAP	ENSG00000160789.18_LMNA	-0.04443	-0.202198497	0.115590331	5.87E-01	8.08E-01	1.00E+00
ENSG00000112306.7_RPS12	ENSG00000160789.18_LMNA	-0.17524	-0.325366627	-0.01650299	3.08E-02	1.46E-01	1.00E+00
ENSG00000231500.5_RPS18	ENSG00000160789.18_LMNA	-0.12002	-0.27398863	0.039940635	1.41E-01	3.83E-01	1.00E+00
ENSG00000026025.12_VIM	ENSG00000160789.18_LMNA	0.64748	0.544394412	0.731298451	1.99E-19	1.13E-15	1.02E-14
ENSG00000142541.15_RPL13A	ENSG00000160789.18_LMNA	-0.1275	-0.280993436	0.032361362	1.18E-01	3.43E-01	1.00E+00
ENSG00000248527.1_MTATP6P1	ENSG00000160789.18_LMNA	-0.1109	-0.265414696	0.04916881	1.74E-01	4.34E-01	1.00E+00
ENSG00000197956.8_S100A6	ENSG00000160789.18_LMNA	0.37524	0.229768343	0.50431631	1.90E-06	7.70E-05	9.77E-02
ENSG00000152583.11_SPARCL1	ENSG00000160789.18_LMNA	-0.28162	-0.421909294	-0.12817065	4.40E-04	6.11E-03	1.00E+00
ENSG00000177954.10_RPS27	ENSG00000160789.18_LMNA	-0.08695	-0.242786342	0.073268613	2.87E-01	5.72E-01	1.00E+00
ENSG00000105894.10_PTN	ENSG00000160789.18_LMNA	0.2032	0.04547209	0.351046182	1.20E-02	7.50E-02	1.00E+00
ENSG00000135916.14_ITM2C	ENSG00000160789.18_LMNA	-0.20808	-0.355504802	-0.05055477	1.01E-02	6.61E-02	1.00E+00
ENSG00000125148.6_MT2A	ENSG00000160789.18_LMNA	0.3297	0.179940029	0.464515358	3.36E-05	7.84E-04	1.00E+00
ENSG00000210196.2_MT-TP	ENSG00000160789.18_LMNA	-0.28146	-0.421760193	-0.12799225	4.44E-04	6.15E-03	1.00E+00
ENSG00000170315.12_UBB	ENSG00000160789.18_LMNA	-0.09348	-0.248974614	0.066714403	2.52E-01	5.33E-01	1.00E+00
ENSG00000162734.11_PEA15	ENSG00000160789.18_LMNA	-0.34513	-0.478067492	-0.19674405	1.33E-05	3.69E-04	6.84E-01
ENSG00000160307.8_S100B	ENSG00000160789.18_LMNA	-0.27833	-0.418967585	-0.12465439	5.17E-04	6.92E-03	1.00E+00
ENSG00000167658.14_EEF2	ENSG00000160789.18_LMNA	0.1105	-0.049570549	0.265040305	1.75E-01	4.36E-01	1.00E+00
ENSG00000101439.7_CST3	ENSG00000160789.18_LMNA	0.07293	-0.087287712	0.229463045	3.72E-01	6.52E-01	1.00E+00
ENSG00000161016.14_RPL8	ENSG00000160789.18_LMNA	0.00189	-0.157354496	0.161045795	9.82E-01	9.93E-01	1.00E+00
ENSG00000206503.10_HLA-A	ENSG00000160789.18_LMNA	0.26811	0.113770354	0.409822674	8.39E-04	1.01E-02	1.00E+00
ENSG00000109846.6_CRYAB	ENSG00000160789.18_LMNA	-0.4645	-0.580751083	-0.32967383	1.66E-09	2.37E-07	8.52E-05
ENSG00000163453.10_IGFBP7	ENSG00000160789.18_LMNA	0.19638	0.038383104	0.344804368	1.53E-02	8.92E-02	1.00E+00
ENSG00000074800.12_ENO1	ENSG00000160789.18_LMNA	0.31354	0.162443363	0.450261539	8.38E-05	1.63E-03	1.00E+00
ENSG00000234745.8_HLA-B	ENSG00000160789.18_LMNA	0.2449	0.089171936	0.38893316	2.36E-03	2.22E-02	1.00E+00
ENSG00000148303.15_RPL7A	ENSG00000160789.18_LMNA	-0.14563	-0.297927051	0.013888928	7.34E-02	2.58E-01	1.00E+00
ENSG00000100097.10_LGALS1	ENSG00000160789.18_LMNA	0.43522	0.29656801	0.555903874	2.10E-08	2.01E-06	1.08E-03
ENSG00000126709.13_IFI6	ENSG00000160789.18_LMNA	-0.06273	-0.219735177	0.097444978	4.43E-01	7.10E-01	1.00E+00
ENSG00000089157.14_RPLP0	ENSG00000160789.18_LMNA	0.01009	-0.149352247	0.169017573	9.02E-01	9.62E-01	1.00E+00
ENSG00000135404.10_CD63	ENSG00000160789.18_LMNA	0.36226	0.215491216	0.493030288	4.51E-06	1.53E-04	2.32E-01
ENSG00000087250.7_MT3	ENSG00000160789.18_LMNA	0.10095	-0.05920418	0.256033974	2.16E-01	4.90E-01	1.00E+00

ENSG00000137154.11_RPS6	ENSG00000160789.18_LMNA	-0.18171	-0.331324047	-0.02317768	2.51E-02	1.27E-01	1.00E+00
ENSG00000140988.14_RPS2	ENSG00000160789.18_LMNA	0.11884	-0.041142933	0.27287439	1.45E-01	3.90E-01	1.00E+00
ENSG00000132692.17_BCAN	ENSG00000160789.18_LMNA	-0.33341	-0.467778373	-0.18397089	2.70E-05	6.57E-04	1.00E+00
ENSG00000179218.12_CALR	ENSG00000160789.18_LMNA	0.41738	0.276550703	0.540651868	8.82E-08	6.47E-06	4.53E-03
ENSG00000274012.1_RN7SL2	ENSG00000160789.18_LMNA	-0.05763	-0.214857815	0.102512884	4.81E-01	7.38E-01	1.00E+00
ENSG00000240583.9_AQP1	ENSG00000160789.18_LMNA	-0.36383	-0.494397839	-0.21721491	4.07E-06	1.40E-04	2.09E-01
ENSG00000145425.8_RPS3A	ENSG00000160789.18_LMNA	-0.16074	-0.311956281	-0.00157812	4.79E-02	1.96E-01	1.00E+00
ENSG00000204525.13_HLA-C	ENSG00000160789.18_LMNA	0.17869	0.020064869	0.328548796	2.76E-02	1.36E-01	1.00E+00
ENSG00000167996.14_FTH1	ENSG00000160789.18_LMNA	0.04239	-0.117602354	0.200241332	6.04E-01	8.18E-01	1.00E+00
ENSG00000132432.12_SEC61G	ENSG00000160789.18_LMNA	0.17239	0.013557844	0.322730145	3.37E-02	1.55E-01	1.00E+00
ENSG00000176340.3_COX8A	ENSG00000160789.18_LMNA	-0.13832	-0.291108336	0.021352971	8.92E-02	2.91E-01	1.00E+00
ENSG00000065978.16_YBX1	ENSG00000160789.18_LMNA	0.02408	-0.135637588	0.182583821	7.68E-01	9.04E-01	1.00E+00
ENSG00000096384.18_HSP90AB	ENSG00000160789.18_LMNA	0.03016	-0.129659752	0.188459292	7.12E-01	8.78E-01	1.00E+00
ENSG00000102265.10_TIMP1	ENSG00000160789.18_LMNA	0.49482	0.364316976	0.606260693	9.18E-11	2.10E-08	4.71E-06
ENSG00000108298.8_RPL19	ENSG00000160789.18_LMNA	-0.23873	-0.383363984	-0.08267512	3.06E-03	2.71E-02	1.00E+00
ENSG00000171858.16_RPS21	ENSG00000160789.18_LMNA	-0.06658	-0.223413878	0.093611649	4.15E-01	6.88E-01	1.00E+00
ENSG00000108953.15_YWHAE	ENSG00000160789.18_LMNA	-0.32545	-0.460774229	-0.17533027	4.29E-05	9.48E-04	1.00E+00
ENSG00000168653.9_NDUFS5	ENSG00000160789.18_LMNA	-0.02081	-0.179412285	0.13885478	7.99E-01	9.19E-01	1.00E+00
ENSG00000117519.14_CNN3	ENSG00000160789.18_LMNA	0.1751	0.016359728	0.325238487	3.10E-02	1.46E-01	1.00E+00
ENSG00000173369.14_C1QB	ENSG00000160789.18_LMNA	0.00975	-0.149682985	0.168688917	9.05E-01	9.63E-01	1.00E+00
ENSG00000175130.6_MARCKSL1	ENSG00000160789.18_LMNA	-0.31564	-0.452118514	-0.1647126	7.46E-05	1.49E-03	1.00E+00
ENSG00000198755.9_RPL10A	ENSG00000160789.18_LMNA	-0.19011	-0.339049279	-0.03187412	1.90E-02	1.04E-01	1.00E+00
ENSG00000159189.10_C1QC	ENSG00000160789.18_LMNA	0.01743	-0.14216587	0.17614126	8.31E-01	9.34E-01	1.00E+00
ENSG00000142937.10_RPS8	ENSG00000160789.18_LMNA	-0.12453	-0.278214733	0.035372267	1.26E-01	3.59E-01	1.00E+00
ENSG00000166165.11_CKB	ENSG00000160789.18_LMNA	-0.18563	-0.33493215	-0.02723359	2.20E-02	1.16E-01	1.00E+00
ENSG00000136942.13_RPL35	ENSG00000160789.18_LMNA	-0.04673	-0.204409265	0.113314455	5.68E-01	7.98E-01	1.00E+00
ENSG00000198034.9_RPS4X	ENSG00000160789.18_LMNA	-0.04995	-0.207501256	0.110125801	5.41E-01	7.81E-01	1.00E+00
ENSG00000133112.15_TPT1	ENSG00000160789.18_LMNA	-0.11269	-0.267099407	0.047359775	1.67E-01	4.24E-01	1.00E+00
ENSG00000159335.14_PTMS	ENSG00000160789.18_LMNA	-0.05369	-0.211088641	0.106418054	5.11E-01	7.60E-01	1.00E+00
ENSG00000187653.11_TMSB4XP	ENSG00000160789.18_LMNA	0.05708	-0.103055752	0.214334417	4.85E-01	7.42E-01	1.00E+00
ENSG00000111669.13_TPI1	ENSG00000160789.18_LMNA	0.28068	0.127162839	0.421066787	4.61E-04	6.32E-03	1.00E+00
ENSG00000109971.12_HSPA8	ENSG00000160789.18_LMNA	-0.07149	-0.228097832	0.088717171	3.81E-01	6.60E-01	1.00E+00
ENSG00000233276.3_GPX1	ENSG00000160789.18_LMNA	-0.09398	-0.249448774	0.066211078	2.49E-01	5.30E-01	1.00E+00

ENSG00000229117.7_RPL41	ENSG00000160789.18_LMNA	-0.13045	-0.28375516	0.029363211	1.09E-01	3.29E-01	1.00E+00
ENSG00000131469.11_RPL27	ENSG00000160789.18_LMNA	-0.19545	-0.343951503	-0.03741687	1.58E-02	9.12E-02	1.00E+00
ENSG00000135744.7_AGT	ENSG00000160789.18_LMNA	-0.20796	-0.355393911	-0.05042817	1.01E-02	6.63E-02	1.00E+00
ENSG00000110955.7_ATP5B	ENSG00000160789.18_LMNA	-0.16885	-0.31946478	-0.00991761	3.76E-02	1.66E-01	1.00E+00
ENSG00000196126.9_HLA-DRB1	ENSG00000160789.18_LMNA	0.02751	-0.132265538	0.185900925	7.37E-01	8.89E-01	1.00E+00
ENSG00000196230.11_TUBB	ENSG00000160789.18_LMNA	0.10341	-0.056721735	0.258360019	2.05E-01	4.76E-01	1.00E+00
ENSG00000089220.4_PEBP1	ENSG00000160789.18_LMNA	-0.17841	-0.328286236	-0.01977068	2.79E-02	1.36E-01	1.00E+00
ENSG00000142089.14_IFITM3	ENSG00000160789.18_LMNA	0.3756	0.230156597	0.504621919	1.86E-06	7.59E-05	9.54E-02
ENSG00000137818.10_RPLP1	ENSG00000160789.18_LMNA	0.0436	-0.116404221	0.201407104	5.94E-01	8.12E-01	1.00E+00
ENSG00000122406.11_RPL5	ENSG00000160789.18_LMNA	-0.22362	-0.369660645	-0.06679735	5.62E-03	4.28E-02	1.00E+00
ENSG00000177600.7_RPLP2	ENSG00000160789.18_LMNA	0.03893	-0.121016062	0.196914887	6.34E-01	8.36E-01	1.00E+00
ENSG00000162244.9_RPL29	ENSG00000160789.18_LMNA	-0.1821	-0.331685832	-0.02358391	2.47E-02	1.25E-01	1.00E+00
ENSG00000108518.7_PFN1	ENSG00000160789.18_LMNA	0.3179	0.167155018	0.454114419	6.58E-05	1.34E-03	1.00E+00
ENSG00000202198.1_7SK	ENSG00000160789.18_LMNA	-0.14516	-0.29748881	0.014369692	7.44E-02	2.60E-01	1.00E+00
ENSG00000067560.9_RHOA	ENSG00000160789.18_LMNA	-0.1205	-0.274433432	0.039460426	1.39E-01	3.80E-01	1.00E+00
ENSG00000155368.15_DBI	ENSG00000160789.18_LMNA	-0.15883	-0.310184391	0.000383594	5.07E-02	2.03E-01	1.00E+00
ENSG00000172020.11_GAP43	ENSG00000160789.18_LMNA	0.1788	0.020172833	0.328645141	2.75E-02	1.35E-01	1.00E+00
ENSG00000244734.2_HBB	ENSG00000160789.18_LMNA	-0.13187	-0.285088443	0.027913767	1.05E-01	3.22E-01	1.00E+00
ENSG00000143878.9_RHOB	ENSG00000160789.18_LMNA	-0.18319	-0.332691135	-0.02471325	2.39E-02	1.23E-01	1.00E+00
ENSG00000166681.12_BEX3	ENSG00000160789.18_LMNA	-0.13704	-0.289917919	0.022652488	9.23E-02	2.97E-01	1.00E+00
ENSG00000109472.12_CPE	ENSG00000160789.18_LMNA	0.01401	-0.145512094	0.17282837	8.64E-01	9.47E-01	1.00E+00
ENSG00000147588.6_PMP2	ENSG00000160789.18_LMNA	-0.26463	-0.406699276	-0.11006918	9.86E-04	1.15E-02	1.00E+00
ENSG00000126432.12_PRDX5	ENSG00000160789.18_LMNA	-0.07691	-0.233250786	0.083314882	3.46E-01	6.31E-01	1.00E+00
ENSG00000118181.9_RPS25	ENSG00000160789.18_LMNA	-0.26422	-0.40633313	-0.10963584	1.00E-03	1.16E-02	1.00E+00
ENSG00000100316.14_RPL3	ENSG00000160789.18_LMNA	-0.14158	-0.294150248	0.018027469	8.19E-02	2.76E-01	1.00E+00
ENSG00000128524.4_ATP6V1F	ENSG00000160789.18_LMNA	-0.11846	-0.272524425	0.041520369	1.46E-01	3.91E-01	1.00E+00
ENSG00000163191.5_S100A11	ENSG00000160789.18_LMNA	0.35285	0.205178025	0.48481922	8.25E-06	2.49E-04	4.23E-01
ENSG00000173372.15_C1QA	ENSG00000160789.18_LMNA	0.0412	-0.118779483	0.199095121	6.14E-01	8.25E-01	1.00E+00
ENSG00000168028.12_RPSA	ENSG00000160789.18_LMNA	-0.22819	-0.373812874	-0.07159231	4.69E-03	3.74E-02	1.00E+00
ENSG00000142192.19_APP	ENSG00000160789.18_LMNA	0.29907	0.146857993	0.43744003	1.82E-04	3.03E-03	1.00E+00
ENSG00000164111.13_ANXA5	ENSG00000160789.18_LMNA	0.41219	0.27075281	0.536201549	1.32E-07	9.10E-06	6.76E-03
ENSG00000105193.7_RPS16	ENSG00000160789.18_LMNA	-0.13778	-0.290608431	0.02189882	9.05E-02	2.93E-01	1.00E+00
ENSG00000099795.5_NDUFB7	ENSG00000160789.18_LMNA	-0.1194	-0.27340191	0.040573837	1.43E-01	3.86E-01	1.00E+00

ENSG00000171885.12_AQP4	ENSG00000160789.18_LMNA	-0.22836	-0.373965875	-0.07176926	4.66E-03	3.72E-02	1.00E+00
ENSG00000166033.10_HTRA1	ENSG00000160789.18_LMNA	0.19584	0.037816078	0.344303932	1.56E-02	9.03E-02	1.00E+00
ENSG00000137285.9_TUBB2B	ENSG00000160789.18_LMNA	-0.03384	-0.192001491	0.126044633	6.79E-01	8.60E-01	1.00E+00
ENSG00000142676.11_RPL11	ENSG00000160789.18_LMNA	0.02211	-0.137576442	0.180673274	7.87E-01	9.13E-01	1.00E+00
ENSG00000147403.15_RPL10	ENSG00000160789.18_LMNA	-0.05843	-0.215627779	0.101713933	4.75E-01	7.34E-01	1.00E+00
ENSG00000204592.8_HLA-E	ENSG00000160789.18_LMNA	0.16014	0.000962592	0.311400547	4.88E-02	1.98E-01	1.00E+00
ENSG00000149925.15_ALDOA	ENSG00000160789.18_LMNA	0.32284	0.172504675	0.458476042	4.98E-05	1.07E-03	1.00E+00
ENSG00000117450.12_PRDX1	ENSG00000160789.18_LMNA	-0.01829	-0.17697661	0.141320965	8.23E-01	9.31E-01	1.00E+00
ENSG00000144713.11_RPL32	ENSG00000160789.18_LMNA	-0.22995	-0.375412101	-0.07344281	4.37E-03	3.54E-02	1.00E+00
ENSG00000129244.7_ATP1B2	ENSG00000160789.18_LMNA	0.16533	0.006290748	0.31620409	4.18E-02	1.79E-01	1.00E+00
ENSG00000149806.9_FAU	ENSG00000160789.18_LMNA	-0.12822	-0.281673078	0.03162406	1.15E-01	3.40E-01	1.00E+00
ENSG00000083845.7_RPS5	ENSG00000160789.18_LMNA	-0.10019	-0.255321156	0.059964148	2.19E-01	4.94E-01	1.00E+00
ENSG00000106211.8_HSPB1	ENSG00000160789.18_LMNA	0.49421	0.36361903	0.605751625	9.76E-11	2.20E-08	5.01E-06
ENSG00000080824.17_HSP90AA	ENSG00000160789.18_LMNA	0.12652	-0.033348085	0.280083388	1.20E-01	3.49E-01	1.00E+00
ENSG00000002586.16_CD99	ENSG00000160789.18_LMNA	0.23312	0.076772057	0.378284745	3.85E-03	3.22E-02	1.00E+00
ENSG00000158710.13_TAGLN2	ENSG00000160789.18_LMNA	0.58274	0.466850571	0.678952747	3.38E-15	5.83E-12	1.73E-10
ENSG00000105372.5_RPS19	ENSG00000160789.18_LMNA	-0.05992	-0.217053885	0.100233057	4.63E-01	7.26E-01	1.00E+00
ENSG00000169100.11_SLC25A6	ENSG00000160789.18_LMNA	0.07445	-0.085769883	0.230911294	3.62E-01	6.44E-01	1.00E+00
ENSG00000139644.11_TMBIM6	ENSG00000160789.18_LMNA	0.09586	-0.064319	0.251229862	2.40E-01	5.19E-01	1.00E+00
ENSG00000163682.14_RPL9	ENSG00000160789.18_LMNA	-0.22566	-0.371515794	-0.06893793	5.18E-03	4.04E-02	1.00E+00
ENSG00000092841.17_MYL6	ENSG00000160789.18_LMNA	0.00865	-0.150761335	0.167616866	9.16E-01	9.68E-01	1.00E+00
ENSG00000115268.8_RPS15	ENSG00000160789.18_LMNA	-0.04708	-0.204747574	0.112965888	5.65E-01	7.95E-01	1.00E+00
ENSG00000134590.12_FAM127A	ENSG00000160789.18_LMNA	-0.12168	-0.275542735	0.03826219	1.35E-01	3.74E-01	1.00E+00
ENSG00000228474.4_OST4	ENSG00000160789.18_LMNA	-0.09615	-0.251500133	0.064031689	2.39E-01	5.17E-01	1.00E+00
ENSG00000164733.19_CTSB	ENSG00000160789.18_LMNA	0.19415	0.036060105	0.342753068	1.65E-02	9.42E-02	1.00E+00
ENSG00000046653.13_GPM6B	ENSG00000160789.18_LMNA	-0.16743	-0.318150963	-0.00845527	3.92E-02	1.71E-01	1.00E+00
ENSG00000134308.12_YWHAQ	ENSG00000160789.18_LMNA	-0.31065	-0.447705167	-0.15932442	9.82E-05	1.85E-03	1.00E+00
ENSG00000111716.11_LDHB	ENSG00000160789.18_LMNA	-0.35364	-0.485503605	-0.20603525	7.85E-06	2.41E-04	4.03E-01
ENSG00000129562.9_DAD1	ENSG00000160789.18_LMNA	0.03688	-0.123041998	0.194937273	6.52E-01	8.46E-01	1.00E+00
ENSG00000115461.4_IGFBP5	ENSG00000160789.18_LMNA	0.13601	-0.023706785	0.288951424	9.48E-02	3.01E-01	1.00E+00
ENSG00000126267.7_COX6B1	ENSG00000160789.18_LMNA	-0.15222	-0.304047903	0.007159067	6.12E-02	2.29E-01	1.00E+00
ENSG00000150991.13_UBC	ENSG00000160789.18_LMNA	0.30309	0.151187376	0.441013545	1.47E-04	2.56E-03	1.00E+00
ENSG00000197958.11_RPL12	ENSG00000160789.18_LMNA	-0.03326	-0.191449106	0.126608945	6.84E-01	8.63E-01	1.00E+00

ENSG00000172757.11_CFL1	ENSG00000160789.18_LMNA	0.071	-0.089213192	0.227623814	3.85E-01	6.63E-01	1.00E+00
ENSG00000172270.17_BSG	ENSG00000160789.18_LMNA	0.21641	0.059251481	0.363101657	7.41E-03	5.25E-02	1.00E+00
ENSG00000196262.12_PPIA	ENSG00000160789.18_LMNA	0.07004	-0.090168971	0.226710006	3.91E-01	6.69E-01	1.00E+00
ENSG00000005022.5_SLC25A5	ENSG00000160789.18_LMNA	-0.06335	-0.220328758	0.096827083	4.38E-01	7.06E-01	1.00E+00
ENSG00000103363.13_TCEB2	ENSG00000160789.18_LMNA	-0.24847	-0.392154206	-0.09294118	2.02E-03	1.97E-02	1.00E+00
ENSG00000147604.12_RPL7	ENSG00000160789.18_LMNA	-0.13747	-0.290315569	0.022218511	9.12E-02	2.95E-01	1.00E+00
ENSG00000100979.13_PLTP	ENSG00000160789.18_LMNA	0.31441	0.163390489	0.451036898	7.98E-05	1.57E-03	1.00E+00
ENSG00000117984.11_CTSD	ENSG00000160789.18_LMNA	0.24622	0.090569759	0.39012854	2.23E-03	2.13E-02	1.00E+00
ENSG00000152661.7_GJA1	ENSG00000160789.18_LMNA	-0.05806	-0.21527501	0.102080034	4.77E-01	7.36E-01	1.00E+00
ENSG00000067225.16_PKM	ENSG00000160789.18_LMNA	0.44961	0.312797829	0.568143169	6.22E-09	7.20E-07	3.19E-04
ENSG00000112335.13_SNX3	ENSG00000160789.18_LMNA	-0.13738	-0.290236573	0.022304733	9.14E-02	2.95E-01	1.00E+00
ENSG00000117318.8_ID3	ENSG00000160789.18_LMNA	-0.23573	-0.380649556	-0.0795178	3.46E-03	2.98E-02	1.00E+00
ENSG00000169714.15_CNBP	ENSG00000160789.18_LMNA	-0.2814	-0.421705728	-0.12792709	4.45E-04	6.16E-03	1.00E+00
ENSG00000135446.15_CDK4	ENSG00000160789.18_LMNA	-0.27617	-0.417031363	-0.12234402	5.73E-04	7.49E-03	1.00E+00
ENSG00000187514.13_PTMA	ENSG00000160789.18_LMNA	0.03038	-0.129444646	0.188670292	7.10E-01	8.77E-01	1.00E+00
ENSG00000123416.14_TUBA1B	ENSG00000160789.18_LMNA	-0.04439	-0.202159929	0.115630005	5.87E-01	8.08E-01	1.00E+00
ENSG00000106278.10_PTPRZ1	ENSG00000160789.18_LMNA	-0.06422	-0.221160925	0.095960418	4.32E-01	7.01E-01	1.00E+00
ENSG00000173812.9_EIF1	ENSG00000160789.18_LMNA	-0.05592	-0.213219118	0.10421191	4.94E-01	7.49E-01	1.00E+00
ENSG00000175899.13_A2M	ENSG00000160789.18_LMNA	0.22991	0.073397743	0.375373171	4.38E-03	3.55E-02	1.00E+00
ENSG00000114391.11_RPL24	ENSG00000160789.18_LMNA	-0.20762	-0.355082047	-0.05007217	1.03E-02	6.68E-02	1.00E+00
ENSG00000008988.8_RPS20	ENSG00000160789.18_LMNA	-0.09887	-0.254066929	0.061300448	2.26E-01	5.02E-01	1.00E+00
ENSG00000079215.12_SLC1A3	ENSG00000160789.18_LMNA	0.07612	-0.084099968	0.232503037	3.51E-01	6.35E-01	1.00E+00
ENSG00000123560.12_PLP1	ENSG00000160789.18_LMNA	-0.52361	-0.630274363	-0.39755213	4.46E-12	1.82E-09	2.29E-07
ENSG00000143947.11_RPS27A	ENSG00000160789.18_LMNA	-0.20093	-0.348964527	-0.04310448	1.31E-02	7.94E-02	1.00E+00
ENSG00000133872.12_SARAF	ENSG00000160789.18_LMNA	-0.13669	-0.289591112	0.023009062	9.31E-02	2.98E-01	1.00E+00
ENSG00000122026.9_RPL21	ENSG00000160789.18_LMNA	-0.27814	-0.418798951	-0.12445304	5.21E-04	6.97E-03	1.00E+00
ENSG00000136238.16_RAC1	ENSG00000160789.18_LMNA	0.06609	-0.094103848	0.222942038	4.19E-01	6.91E-01	1.00E+00
ENSG00000167815.10_PRDX2	ENSG00000160789.18_LMNA	-0.28413	-0.424143944	-0.13084671	3.89E-04	5.54E-03	1.00E+00
ENSG00000110700.5_RPS13	ENSG00000160789.18_LMNA	-0.20736	-0.354850411	-0.0498078	1.04E-02	6.72E-02	1.00E+00
ENSG00000130066.15_SAT1	ENSG00000160789.18_LMNA	0.12105	-0.038903372	0.274949245	1.37E-01	3.78E-01	1.00E+00
ENSG00000254999.3_BRK1	ENSG00000160789.18_LMNA	-0.25052	-0.394010588	-0.0951174	1.85E-03	1.85E-02	1.00E+00
ENSG00000068697.6_LAPTM4A	ENSG00000160789.18_LMNA	0.18855	0.03026213	0.337620436	2.00E-02	1.08E-01	1.00E+00
ENSG00000174444.13_RPL4	ENSG00000160789.18_LMNA	-0.06603	-0.222887002	0.094161249	4.19E-01	6.91E-01	1.00E+00

ENSG00000044574.7_HSPA5	ENSG00000160789.18_LMNA	0.51934	0.392601134	0.626725084	7.12E-12	2.66E-09	3.65E-07
ENSG00000204628.10_RACK1	ENSG00000160789.18_LMNA	0.08195	-0.07826705	0.238049628	3.15E-01	6.02E-01	1.00E+00
ENSG00000135821.15_GLUL	ENSG00000160789.18_LMNA	0.12603	-0.0338477	0.279622382	1.22E-01	3.51E-01	1.00E+00
ENSG00000127022.13_CANX	ENSG00000160789.18_LMNA	0.20593	0.048313298	0.353540249	1.09E-02	6.98E-02	1.00E+00
ENSG00000164587.10_RPS14	ENSG00000160789.18_LMNA	-0.1826	-0.332141698	-0.02409592	2.43E-02	1.24E-01	1.00E+00
ENSG00000130255.11_RPL36	ENSG00000160789.18_LMNA	-0.0073	-0.16630898	0.152075879	9.29E-01	9.73E-01	1.00E+00
ENSG00000179010.13_MRFAP1	ENSG00000160789.18_LMNA	-0.13733	-0.290189671	0.022355923	9.16E-02	2.95E-01	1.00E+00
ENSG00000145284.10_SCD5	ENSG00000160789.18_LMNA	-0.31134	-0.448315381	-0.1600684	9.45E-05	1.81E-03	1.00E+00
ENSG00000104904.11_OAZ1	ENSG00000160789.18_LMNA	-0.06565	-0.222525451	0.094538281	4.22E-01	6.93E-01	1.00E+00
ENSG00000130208.8_APOC1	ENSG00000160789.18_LMNA	-0.17984	-0.329601901	-0.02124536	2.66E-02	1.32E-01	1.00E+00
ENSG00000107223.11_EDF1	ENSG00000160789.18_LMNA	0.13035	-0.029460068	0.283666021	1.09E-01	3.29E-01	1.00E+00
ENSG00000012223.11_LTF	ENSG00000160789.18_LMNA	0.19218	0.034020889	0.340949946	1.77E-02	9.88E-02	1.00E+00
ENSG00000188643.9_S100A16	ENSG00000160789.18_LMNA	0.40199	0.259388079	0.527435237	2.85E-07	1.69E-05	1.46E-02
ENSG00000143742.11_SRP9	ENSG00000160789.18_LMNA	-0.35417	-0.485966962	-0.20661588	7.59E-06	2.34E-04	3.89E-01
ENSG00000227097.5_RPS28P7	ENSG00000160789.18_LMNA	-0.13547	-0.288447318	0.024256411	9.61E-02	3.04E-01	1.00E+00
ENSG00000277443.1_MARCKS	ENSG00000160789.18_LMNA	-0.4093	-0.533723386	-0.26753258	1.64E-07	1.08E-05	8.43E-03
ENSG00000114942.12_EEF1B2	ENSG00000160789.18_LMNA	-0.19466	-0.343225305	-0.0365946	1.63E-02	9.30E-02	1.00E+00
ENSG00000132688.10_NES	ENSG00000160789.18_LMNA	0.46598	0.331364128	0.582007286	1.45E-09	2.13E-07	7.44E-05
ENSG00000183255.10_PTTG1IP	ENSG00000160789.18_LMNA	0.18765	0.029322088	0.336786543	2.06E-02	1.10E-01	1.00E+00
ENSG00000162511.7_LAPTM5	ENSG00000160789.18_LMNA	-0.00192	-0.161073643	0.157326614	9.81E-01	9.93E-01	1.00E+00
ENSG00000087460.22_GNAS	ENSG00000160789.18_LMNA	0.01126	-0.14820626	0.170155796	8.90E-01	9.57E-01	1.00E+00
ENSG00000198242.12_RPL23A	ENSG00000160789.18_LMNA	-0.11992	-0.273892146	0.04004478	1.41E-01	3.83E-01	1.00E+00
ENSG00000240342.3_RPS2P5	ENSG00000160789.18_LMNA	-0.03516	-0.193281668	0.12473602	6.67E-01	8.53E-01	1.00E+00
ENSG00000118680.11_MYL12B	ENSG00000160789.18_LMNA	0.00925	-0.150168084	0.168206745	9.10E-01	9.66E-01	1.00E+00
ENSG00000197747.7_S100A10	ENSG00000160789.18_LMNA	0.30492	0.153157305	0.442636507	1.34E-04	2.38E-03	1.00E+00
ENSG00000125534.8_PPDPF	ENSG00000160789.18_LMNA	0.25227	0.096966345	0.395585872	1.72E-03	1.74E-02	1.00E+00
ENSG00000149273.13_RPS3	ENSG00000160789.18_LMNA	-0.20604	-0.353637201	-0.04842384	1.09E-02	6.95E-02	1.00E+00
ENSG00000170889.12_RPS9	ENSG00000160789.18_LMNA	-0.09734	-0.252623386	0.062837055	2.33E-01	5.11E-01	1.00E+00
ENSG00000084207.14_GSTP1	ENSG00000160789.18_LMNA	0.202	0.044221036	0.349946605	1.26E-02	7.72E-02	1.00E+00
ENSG00000164929.15_BAALC	ENSG00000160789.18_LMNA	-0.32835	-0.463326527	-0.17847381	3.63E-05	8.34E-04	1.00E+00
ENSG00000182492.14_BGN	ENSG00000160789.18_LMNA	0.45747	0.321702506	0.574810747	3.12E-09	4.02E-07	1.60E-04
ENSG00000132507.16_EIF5A	ENSG00000160789.18_LMNA	0.02374	-0.135974312	0.182252186	7.72E-01	9.06E-01	1.00E+00
ENSG00000164434.10_FABP7	ENSG00000160789.18_LMNA	0.1508	-0.008603773	0.302736096	6.37E-02	2.35E-01	1.00E+00

ENSG00000146648.14_EGFR	ENSG00000160789.18_LMNA	0.06766	-0.092539539	0.224441128	4.08E-01	6.83E-01	1.00E+00
ENSG00000181449.3_SOX2	ENSG00000160789.18_LMNA	-0.12631	-0.279885039	0.033563064	1.21E-01	3.50E-01	1.00E+00
ENSG00000125730.15_C3	ENSG00000160789.18_LMNA	0.02494	-0.13479908	0.183409344	7.60E-01	9.00E-01	1.00E+00
ENSG00000122862.4_SRGN	ENSG00000160789.18_LMNA	-0.04124	-0.199128779	0.11874493	6.14E-01	8.25E-01	1.00E+00
ENSG00000124614.12_RPS10	ENSG00000160789.18_LMNA	-0.14006	-0.292734107	0.019576493	8.52E-02	2.83E-01	1.00E+00
ENSG00000084623.10_EIF3I	ENSG00000160789.18_LMNA	0.01618	-0.143393714	0.174926485	8.43E-01	9.39E-01	1.00E+00
ENSG00000102144.12_PGK1	ENSG00000160789.18_LMNA	0.19356	0.035454722	0.34221801	1.69E-02	9.56E-02	1.00E+00
ENSG00000063177.11_RPL18	ENSG00000160789.18_LMNA	-0.05469	-0.212046517	0.105426545	5.03E-01	7.55E-01	1.00E+00
ENSG00000146731.9_CCT6A	ENSG00000160789.18_LMNA	0.08516	-0.075054838	0.241095374	2.97E-01	5.83E-01	1.00E+00
ENSG00000075785.11_RAB7A	ENSG00000160789.18_LMNA	-0.0773	-0.233626586	0.082920169	3.44E-01	6.28E-01	1.00E+00
ENSG00000011600.10_TYROBP	ENSG00000160789.18_LMNA	-0.04422	-0.201994721	0.11579994	5.89E-01	8.09E-01	1.00E+00
ENSG00000172354.8_GNB2	ENSG00000160789.18_LMNA	0.39058	0.246721264	0.517596823	6.53E-07	3.25E-05	3.35E-02
ENSG00000166598.11_HSP90B1	ENSG00000160789.18_LMNA	0.39074	0.246899889	0.517736062	6.46E-07	3.22E-05	3.31E-02
ENSG00000109107.12_ALDOC	ENSG00000160789.18_LMNA	-0.52129	-0.628342005	-0.39485494	5.76E-12	2.25E-09	2.95E-07
ENSG00000154277.11_UCHL1	ENSG00000160789.18_LMNA	-0.40957	-0.533953212	-0.26783097	1.61E-07	1.06E-05	8.26E-03
ENSG00000143933.15_CALM2	ENSG00000160789.18_LMNA	-0.30168	-0.439756198	-0.14966281	1.59E-04	2.73E-03	1.00E+00
ENSG00000213719.7_CLIC1	ENSG00000160789.18_LMNA	0.41486	0.273737896	0.538494671	1.07E-07	7.65E-06	5.50E-03
ENSG00000174748.17_RPL15	ENSG00000160789.18_LMNA	-0.3727	-0.50210689	-0.22696403	2.26E-06	8.76E-05	1.16E-01
ENSG00000111775.2_COX6A1	ENSG00000160789.18_LMNA	-0.24736	-0.391156183	-0.09177238	2.12E-03	2.05E-02	1.00E+00
ENSG00000143761.12_ARF1	ENSG00000160789.18_LMNA	0.08166	-0.078566632	0.237765254	3.17E-01	6.04E-01	1.00E+00
ENSG00000167468.15_GPX4	ENSG00000160789.18_LMNA	-0.07401	-0.230497632	0.086203569	3.65E-01	6.46E-01	1.00E+00
ENSG00000188229.5_TUBB4B	ENSG00000160789.18_LMNA	0.31955	0.168940591	0.455571744	6.00E-05	1.24E-03	1.00E+00
ENSG00000110651.10_CD81	ENSG00000160789.18_LMNA	0.13534	-0.024381511	0.288332554	9.64E-02	3.05E-01	1.00E+00
ENSG00000140319.9_SRP14	ENSG00000160789.18_LMNA	-0.10395	-0.258862656	0.056184789	2.03E-01	4.73E-01	1.00E+00
ENSG00000255302.3_EID1	ENSG00000160789.18_LMNA	-0.03975	-0.197696046	0.120215086	6.27E-01	8.32E-01	1.00E+00
ENSG00000182117.5_NOP10	ENSG00000160789.18_LMNA	0.01065	-0.148805412	0.16956081	8.96E-01	9.60E-01	1.00E+00
ENSG00000169504.13_CLIC4	ENSG00000160789.18_LMNA	0.32936	0.179572006	0.464217056	3.42E-05	7.96E-04	1.00E+00
ENSG00000105640.11_RPL18A	ENSG00000160789.18_LMNA	-0.15074	-0.302679352	0.008666236	6.38E-02	2.35E-01	1.00E+00
ENSG00000145741.14_BTF3	ENSG00000160789.18_LMNA	-0.16941	-0.319977352	-0.01048848	3.69E-02	1.64E-01	1.00E+00
ENSG00000167526.12_RPL13	ENSG00000160789.18_LMNA	0.02847	-0.131321816	0.186827972	7.28E-01	8.85E-01	1.00E+00
ENSG00000118705.15_RPN2	ENSG00000160789.18_LMNA	0.36669	0.220356104	0.496886486	3.38E-06	1.21E-04	1.73E-01
ENSG00000101182.13_PSMA7	ENSG00000160789.18_LMNA	0.03631	-0.123605467	0.194386783	6.57E-01	8.48E-01	1.00E+00
ENSG00000125995.14_ROMO1	ENSG00000160789.18_LMNA	-0.06069	-0.217783731	0.099474637	4.58E-01	7.22E-01	1.00E+00

ENSG00000100427.14_MLC1	ENSG00000160789.18_LMNA	0.08277	-0.077448054	0.238826773	3.11E-01	5.97E-01	1.00E+00
ENSG00000132329.9_RAMP1	ENSG00000160789.18_LMNA	0.10015	-0.060004459	0.255283336	2.20E-01	4.95E-01	1.00E+00
ENSG00000169567.10_HINT1	ENSG00000160789.18_LMNA	-0.10924	-0.263850425	0.05084667	1.80E-01	4.44E-01	1.00E+00
ENSG00000182899.13_RPL35A	ENSG00000160789.18_LMNA	-0.18533	-0.334655283	-0.026922	2.23E-02	1.17E-01	1.00E+00
ENSG00000125743.9_SNRPD2	ENSG00000160789.18_LMNA	-0.01566	-0.17442424	0.143901075	8.48E-01	9.41E-01	1.00E+00
ENSG00000165119.17_HNRNPK	ENSG00000160789.18_LMNA	0.01605	-0.1435187	0.174802775	8.44E-01	9.39E-01	1.00E+00
ENSG00000113732.7_ATP6V0E1	ENSG00000160789.18_LMNA	-0.09489	-0.25031381	0.065292421	2.45E-01	5.25E-01	1.00E+00
ENSG00000160014.15_CALM3	ENSG00000160789.18_LMNA	-0.12828	-0.281727422	0.03156509	1.15E-01	3.39E-01	1.00E+00
ENSG00000165502.6_RPL36AL	ENSG00000160789.18_LMNA	0.02637	-0.133388042	0.184797517	7.47E-01	8.94E-01	1.00E+00
ENSG00000166925.7_TSC22D4	ENSG00000160789.18_LMNA	-0.20645	-0.354013406	-0.04885287	1.07E-02	6.88E-02	1.00E+00
ENSG00000169976.6_SF3B5	ENSG00000160789.18_LMNA	-0.14145	-0.294026026	0.018163406	8.22E-02	2.77E-01	1.00E+00
ENSG00000135486.16_HNRNPA1	ENSG00000160789.18_LMNA	-0.05135	-0.208839065	0.10874414	5.30E-01	7.73E-01	1.00E+00
ENSG00000132718.8_SYT11	ENSG00000160789.18_LMNA	-0.22873	-0.374304197	-0.07216061	4.59E-03	3.68E-02	1.00E+00
ENSG00000125835.16_SNRPB	ENSG00000160789.18_LMNA	0.07999	-0.080228014	0.236187224	3.27E-01	6.14E-01	1.00E+00
ENSG00000184221.11_OLIG1	ENSG00000160789.18_LMNA	-0.37258	-0.502006873	-0.2268372	2.28E-06	8.81E-05	1.17E-01
ENSG00000108679.11_LGALS3BI	ENSG00000160789.18_LMNA	0.30937	0.15794762	0.446575198	1.05E-04	1.96E-03	1.00E+00
ENSG00000070087.12_PFN2	ENSG00000160789.18_LMNA	-0.45851	-0.575689862	-0.32287993	2.85E-09	3.73E-07	1.46E-04
ENSG00000178719.15_GRINA	ENSG00000160789.18_LMNA	-0.00759	-0.166592854	0.151790655	9.26E-01	9.72E-01	1.00E+00
ENSG00000159377.9_PSMB4	ENSG00000160789.18_LMNA	0.04309	-0.116909033	0.200916037	5.98E-01	8.15E-01	1.00E+00
ENSG00000131094.3_C1QL1	ENSG00000160789.18_LMNA	-0.19809	-0.346370339	-0.04015872	1.44E-02	8.55E-02	1.00E+00
ENSG00000115457.8_IGFBP2	ENSG00000160789.18_LMNA	0.4278	0.288234933	0.549575706	3.85E-08	3.33E-06	1.98E-03
ENSG00000233927.4_RPS28	ENSG00000160789.18_LMNA	-0.05454	-0.211897118	0.105581231	5.05E-01	7.55E-01	1.00E+00
ENSG00000162512.14_SDC3	ENSG00000160789.18_LMNA	0.09072	-0.069480811	0.246365802	2.66E-01	5.50E-01	1.00E+00
ENSG00000142168.13_SOD1	ENSG00000160789.18_LMNA	-0.02885	-0.187194551	0.130948484	7.24E-01	8.83E-01	1.00E+00
ENSG00000232112.3_TMA7	ENSG00000160789.18_LMNA	-0.25776	-0.400524744	-0.10277658	1.35E-03	1.45E-02	1.00E+00
ENSG00000125356.6_NDUFA1	ENSG00000160789.18_LMNA	-0.15175	-0.303613488	0.007637636	6.20E-02	2.30E-01	1.00E+00
ENSG00000183291.14_SELENOF	ENSG00000160789.18_LMNA	-0.04402	-0.201810242	0.115989676	5.90E-01	8.10E-01	1.00E+00
ENSG00000221983.6_UBA52	ENSG00000160789.18_LMNA	-0.05558	-0.21289251	0.104550321	4.96E-01	7.51E-01	1.00E+00
ENSG00000133169.5_BEX1	ENSG00000160789.18_LMNA	-0.39965	-0.525419986	-0.25678594	3.38E-07	1.94E-05	1.74E-02
ENSG00000198830.9_HMGN2	ENSG00000160789.18_LMNA	-0.09666	-0.251980549	0.063520854	2.36E-01	5.14E-01	1.00E+00
ENSG00000168309.15_FAM107A	ENSG00000160789.18_LMNA	-0.30297	-0.440902502	-0.15105268	1.48E-04	2.57E-03	1.00E+00
ENSG00000135506.14_OS9	ENSG00000160789.18_LMNA	-0.11927	-0.273282071	0.040703139	1.43E-01	3.87E-01	1.00E+00
ENSG00000159840.14_ZYX	ENSG00000160789.18_LMNA	0.5167	0.389546225	0.624530219	9.46E-12	3.42E-09	4.85E-07

ENSG00000197728.8_RPS26	ENSG00000160789.18_LMNA	-0.1963	-0.344725764	-0.03829403	1.54E-02	8.93E-02	1.00E+00
ENSG00000150625.15_GPM6A	ENSG00000160789.18_LMNA	-0.38461	-0.512438117	-0.24011629	9.97E-07	4.59E-05	5.12E-02
ENSG00000035862.11_TIMP2	ENSG00000160789.18_LMNA	0.15668	-0.002580659	0.30819736	5.39E-02	2.11E-01	1.00E+00
ENSG00000168385.16_SEPT2	ENSG00000160789.18_LMNA	-0.26159	-0.403964274	-0.10683499	1.13E-03	1.27E-02	1.00E+00
ENSG00000105701.14_FKBP8	ENSG00000160789.18_LMNA	-0.00885	-0.167810766	0.150566352	9.14E-01	9.67E-01	1.00E+00
ENSG00000116288.11_PARK7	ENSG00000160789.18_LMNA	-0.12231	-0.276129387	0.037628141	1.33E-01	3.71E-01	1.00E+00
ENSG00000170540.13_ARL6IP1	ENSG00000160789.18_LMNA	-0.1785	-0.328368678	-0.01986305	2.78E-02	1.36E-01	1.00E+00
ENSG00000156482.9_RPL30	ENSG00000160789.18_LMNA	-0.20115	-0.349168262	-0.04333605	1.30E-02	7.90E-02	1.00E+00
ENSG00000070756.12_PABPC1	ENSG00000160789.18_LMNA	-0.0576	-0.214835498	0.102536035	4.81E-01	7.39E-01	1.00E+00
ENSG00000113657.11_DPYSL3	ENSG00000160789.18_LMNA	-0.0429	-0.200728525	0.11710175	6.00E-01	8.16E-01	1.00E+00
ENSG00000166794.4_PPIB	ENSG00000160789.18_LMNA	0.3522	0.204461822	0.484247164	8.60E-06	2.58E-04	4.41E-01
ENSG00000130303.11_BST2	ENSG00000160789.18_LMNA	0.16766	0.008693112	0.31836473	3.90E-02	1.70E-01	1.00E+00
ENSG00000105767.2_CADM4	ENSG00000160789.18_LMNA	-0.09992	-0.255061082	0.060241332	2.21E-01	4.96E-01	1.00E+00
ENSG00000186468.11_RPS23	ENSG00000160789.18_LMNA	-0.23494	-0.379931871	-0.07868403	3.57E-03	3.05E-02	1.00E+00
ENSG00000198668.9_CALM1	ENSG00000160789.18_LMNA	-0.04752	-0.20516836	0.112532234	5.61E-01	7.93E-01	1.00E+00
ENSG00000128272.13_ATF4	ENSG00000160789.18_LMNA	-0.15072	-0.302661752	0.008685609	6.38E-02	2.35E-01	1.00E+00
ENSG00000005486.15_RHBDD2	ENSG00000160789.18_LMNA	0.21711	0.0599853	0.363740829	7.21E-03	5.15E-02	1.00E+00
ENSG00000178952.7_TUFM	ENSG00000160789.18_LMNA	0.00118	-0.158051629	0.160349344	9.89E-01	9.96E-01	1.00E+00
ENSG00000126247.9_CAPNS1	ENSG00000160789.18_LMNA	0.19427	0.03618986	0.342867724	1.65E-02	9.39E-02	1.00E+00
ENSG00000189043.8_NDUFA4	ENSG00000160789.18_LMNA	-0.21449	-0.361353315	-0.05724592	7.96E-03	5.54E-02	1.00E+00
ENSG00000075618.16_FSCN1	ENSG00000160789.18_LMNA	0.08752	-0.07269647	0.243327568	2.84E-01	5.69E-01	1.00E+00
ENSG00000196683.9_TOMM7	ENSG00000160789.18_LMNA	0.02835	-0.131447632	0.186704412	7.29E-01	8.85E-01	1.00E+00
ENSG00000132475.7_H3F3B	ENSG00000160789.18_LMNA	-0.09385	-0.249322949	0.066344659	2.50E-01	5.31E-01	1.00E+00
ENSG00000111897.6_SERINC1	ENSG00000160789.18_LMNA	-0.15105	-0.302968006	0.008348462	6.32E-02	2.34E-01	1.00E+00
ENSG00000187608.7_ISG15	ENSG00000160789.18_LMNA	-0.05053	-0.208054896	0.109554162	5.36E-01	7.77E-01	1.00E+00
ENSG00000120738.7_EGR1	ENSG00000160789.18_LMNA	0.30914	0.157703771	0.446374971	1.07E-04	1.98E-03	1.00E+00
ENSG00000110321.14_EIF4G2	ENSG00000160789.18_LMNA	0.04497	-0.115054591	0.202719202	5.82E-01	8.06E-01	1.00E+00
ENSG00000143621.15_ILF2	ENSG00000160789.18_LMNA	-0.11129	-0.265784395	0.048772006	1.72E-01	4.31E-01	1.00E+00
ENSG00000117592.8_PRDX6	ENSG00000160789.18_LMNA	0.23011	0.073610257	0.375556718	4.34E-03	3.53E-02	1.00E+00
ENSG00000106624.7_AEBP1	ENSG00000160789.18_LMNA	0.39524	0.251888751	0.521619119	4.67E-07	2.49E-05	2.40E-02
ENSG00000137409.17_MTCH1	ENSG00000160789.18_LMNA	-0.25034	-0.393842705	-0.09492048	1.87E-03	1.86E-02	1.00E+00
ENSG00000122566.19_HNRNPA2	ENSG00000160789.18_LMNA	0.19381	0.035711959	0.342445389	1.67E-02	9.50E-02	1.00E+00
ENSG00000105854.11_PON2	ENSG00000160789.18_LMNA	-0.08561	-0.241523048	0.074603264	2.94E-01	5.80E-01	1.00E+00

ENSG00000189058.7_APOD	ENSG00000160789.18_LMNA	-0.10518	-0.260026658	0.054940635	1.97E-01	4.67E-01	1.00E+00
ENSG00000064886.12_CHI3L2	ENSG00000160789.18_LMNA	-0.009	-0.167956628	0.150419659	9.12E-01	9.67E-01	1.00E+00
ENSG00000134333.12_LDHA	ENSG00000160789.18_LMNA	0.33608	0.186878487	0.470127299	2.31E-05	5.79E-04	1.00E+00
ENSG00000203879.10_GDI1	ENSG00000160789.18_LMNA	-0.14509	-0.297418984	0.014446281	7.45E-02	2.60E-01	1.00E+00
ENSG00000277791.3_PSMB3	ENSG00000160789.18_LMNA	-0.14841	-0.300506932	0.011055796	6.80E-02	2.45E-01	1.00E+00
ENSG00000106682.13_EIF4H	ENSG00000160789.18_LMNA	0.38077	0.235868269	0.509109851	1.30E-06	5.72E-05	6.69E-02
ENSG00000182774.9_RPS17	ENSG00000160789.18_LMNA	-0.0365	-0.194572799	0.123415088	6.55E-01	8.47E-01	1.00E+00
ENSG00000170458.12_CD14	ENSG00000160789.18_LMNA	0.18159	0.023054432	0.331214268	2.52E-02	1.27E-01	1.00E+00
ENSG00000131238.15_PPT1	ENSG00000160789.18_LMNA	-0.14757	-0.299732791	0.011906455	6.96E-02	2.49E-01	1.00E+00
ENSG00000123349.12_PFDN5	ENSG00000160789.18_LMNA	-0.21123	-0.358377627	-0.05383809	9.00E-03	6.07E-02	1.00E+00
ENSG00000118816.8_CCNI	ENSG00000160789.18_LMNA	-0.21231	-0.359362878	-0.05496564	8.64E-03	5.89E-02	1.00E+00
ENSG00000181163.12_NPM1	ENSG00000160789.18_LMNA	-0.04253	-0.200378336	0.117461596	6.03E-01	8.18E-01	1.00E+00
ENSG00000204580.10_DDR1	ENSG00000160789.18_LMNA	0.0204	-0.139253498	0.179018764	8.03E-01	9.21E-01	1.00E+00
ENSG00000168710.16_AHCYL1	ENSG00000160789.18_LMNA	-0.16854	-0.319173777	-0.00959359	3.79E-02	1.67E-01	1.00E+00
ENSG00000089009.14_RPL6	ENSG00000160789.18_LMNA	-0.13391	-0.286990403	0.02584382	1.00E-01	3.12E-01	1.00E+00
ENSG00000142669.12_SH3BGRL	ENSG00000160789.18_LMNA	0.35278	0.205095036	0.484752946	8.29E-06	2.50E-04	4.25E-01
ENSG00000135940.5_COX5B	ENSG00000160789.18_LMNA	-0.22628	-0.372073462	-0.06958195	5.06E-03	3.96E-02	1.00E+00
ENSG00000088832.13_FKBP1A	ENSG00000160789.18_LMNA	0.27357	0.119579373	0.414710906	6.49E-04	8.27E-03	1.00E+00
ENSG00000161203.12_AP2M1	ENSG00000160789.18_LMNA	0.14372	-0.015841574	0.296146296	7.73E-02	2.66E-01	1.00E+00
ENSG00000143612.17_C1orf43	ENSG00000160789.18_LMNA	-0.14791	-0.30004107	0.011567759	6.90E-02	2.47E-01	1.00E+00
ENSG00000142156.13_COL6A1	ENSG00000160789.18_LMNA	0.57287	0.45517961	0.670883072	1.24E-14	1.63E-11	6.36E-10
ENSG00000164405.9_UQCRQ	ENSG00000160789.18_LMNA	-0.14728	-0.299457826	0.012208491	7.02E-02	2.50E-01	1.00E+00
ENSG00000197756.8_RPL37A	ENSG00000160789.18_LMNA	-0.17354	-0.323795492	-0.01474727	3.25E-02	1.51E-01	1.00E+00
ENSG00000132646.9_PCNA	ENSG00000160789.18_LMNA	0.04714	-0.112907022	0.2048047	5.64E-01	7.95E-01	1.00E+00
ENSG00000129538.12_RNASE1	ENSG00000160789.18_LMNA	0.02959	-0.130228088	0.187901667	7.17E-01	8.80E-01	1.00E+00
ENSG00000123562.15_MORF4L2	ENSG00000160789.18_LMNA	-0.20214	-0.350076195	-0.04436843	1.25E-02	7.70E-02	1.00E+00
ENSG00000172201.9_ID4	ENSG00000160789.18_LMNA	-0.19922	-0.347399409	-0.04132662	1.39E-02	8.29E-02	1.00E+00
ENSG00000198258.9_UBL5	ENSG00000160789.18_LMNA	-0.17743	-0.327378955	-0.01875453	2.88E-02	1.39E-01	1.00E+00
ENSG00000088986.9_DYNLL1	ENSG00000160789.18_LMNA	-0.20084	-0.348889057	-0.0430187	1.31E-02	7.96E-02	1.00E+00
ENSG00000225630.1_MTND2P28	ENSG00000160789.18_LMNA	-0.10453	-0.259411272	0.055598518	2.00E-01	4.70E-01	1.00E+00
ENSG00000075142.12_SRI	ENSG00000160789.18_LMNA	-0.06878	-0.225510803	0.09142237	4.00E-01	6.76E-01	1.00E+00
ENSG00000110958.14_PTGES3	ENSG00000160789.18_LMNA	-0.21486	-0.361693291	-0.05763573	7.85E-03	5.48E-02	1.00E+00
ENSG00000105088.7_OLFM2	ENSG00000160789.18_LMNA	-0.09622	-0.251563836	0.063963962	2.38E-01	5.17E-01	1.00E+00

ENSG00000126524.8_SBDS	ENSG00000160789.18_LMNA	0.06982	-0.090385942	0.226502485	3.93E-01	6.70E-01	1.00E+00
ENSG00000058262.8_SEC61A1	ENSG00000160789.18_LMNA	0.41502	0.273918519	0.538633299	1.06E-07	7.59E-06	5.44E-03
ENSG00000147065.15_MSN	ENSG00000160789.18_LMNA	0.51254	0.384734491	0.621065581	1.48E-11	4.67E-09	7.57E-07
ENSG00000010278.10_CD9	ENSG00000160789.18_LMNA	-0.05195	-0.209414293	0.108149678	5.25E-01	7.70E-01	1.00E+00
ENSG00000214253.7_FIS1	ENSG00000160789.18_LMNA	-0.13916	-0.291891084	0.020497909	8.73E-02	2.87E-01	1.00E+00
ENSG00000004059.9_ARF5	ENSG00000160789.18_LMNA	0.0935	-0.066694887	0.248993002	2.52E-01	5.33E-01	1.00E+00
ENSG00000170439.6_METTL7B	ENSG00000160789.18_LMNA	0.44013	0.302095912	0.560085263	1.40E-08	1.42E-06	7.17E-04
ENSG00000127184.9_COX7C	ENSG00000160789.18_LMNA	-0.22335	-0.369415984	-0.06651525	5.68E-03	4.31E-02	1.00E+00
ENSG00000115738.8_ID2	ENSG00000160789.18_LMNA	-0.28056	-0.420956714	-0.12703121	4.64E-04	6.35E-03	1.00E+00
ENSG00000131981.14_LGALS3	ENSG00000160789.18_LMNA	0.48866	0.357249293	0.601096493	1.69E-10	3.47E-08	8.68E-06
ENSG00000168374.9_ARF4	ENSG00000160789.18_LMNA	-0.07775	-0.234055732	0.082469303	3.41E-01	6.26E-01	1.00E+00
ENSG00000174021.9_GNG5	ENSG00000160789.18_LMNA	0.26612	0.111650903	0.408034932	9.20E-04	1.09E-02	1.00E+00
ENSG00000171863.11_RPS7	ENSG00000160789.18_LMNA	-0.19276	-0.341478589	-0.03461849	1.74E-02	9.74E-02	1.00E+00
ENSG00000101421.3_CHMP4B	ENSG00000160789.18_LMNA	0.28658	0.133468629	0.426329931	3.44E-04	5.00E-03	1.00E+00
ENSG00000174903.13_RAB1B	ENSG00000160789.18_LMNA	0.07179	-0.088424451	0.228377498	3.79E-01	6.58E-01	1.00E+00
ENSG00000123144.9_C19orf43	ENSG00000160789.18_LMNA	-0.03389	-0.19205627	0.125988659	6.78E-01	8.60E-01	1.00E+00
ENSG00000197696.8_NMB	ENSG00000160789.18_LMNA	-0.19963	-0.347779958	-0.04175871	1.37E-02	8.20E-02	1.00E+00
ENSG00000237550.5_RPL9P9	ENSG00000160789.18_LMNA	-0.06091	-0.217998626	0.099251259	4.56E-01	7.20E-01	1.00E+00
ENSG00000173660.10_UQCRH	ENSG00000160789.18_LMNA	-0.18291	-0.332431432	-0.02442143	2.41E-02	1.23E-01	1.00E+00
ENSG00000138326.17_RPS24	ENSG00000160789.18_LMNA	-0.20656	-0.354117777	-0.04897191	1.07E-02	6.86E-02	1.00E+00
ENSG00000135069.12_PSAT1	ENSG00000160789.18_LMNA	-0.39202	-0.518842503	-0.24831996	5.89E-07	3.00E-05	3.02E-02
ENSG00000105887.10_MTPN	ENSG00000160789.18_LMNA	0.12492	-0.034979554	0.278577458	1.25E-01	3.57E-01	1.00E+00
ENSG00000018625.13_ATP1A2	ENSG00000160789.18_LMNA	-0.29852	-0.436952356	-0.14626803	1.87E-04	3.10E-03	1.00E+00
ENSG00000170345.8_FOS	ENSG00000160789.18_LMNA	0.2193	0.062277714	0.365735728	6.64E-03	4.84E-02	1.00E+00
ENSG00000102316.15_MAGED2	ENSG00000160789.18_LMNA	-0.21953	-0.365938509	-0.06251092	6.58E-03	4.82E-02	1.00E+00
ENSG00000137312.13_FLOT1	ENSG00000160789.18_LMNA	0.25137	0.096012792	0.394773671	1.79E-03	1.79E-02	1.00E+00
ENSG00000198356.10_ASNA1	ENSG00000160789.18_LMNA	-0.1942	-0.34280443	-0.03611823	1.65E-02	9.41E-02	1.00E+00
ENSG00000095970.15_TREM2	ENSG00000160789.18_LMNA	-0.18423	-0.333648102	-0.02578902	2.31E-02	1.20E-01	1.00E+00
ENSG00000185624.13_P4HB	ENSG00000160789.18_LMNA	0.59699	0.483768843	0.690560294	4.77E-16	1.11E-12	2.45E-11
ENSG00000177700.5_POLR2L	ENSG00000160789.18_LMNA	0.14909	-0.010356166	0.301143321	6.68E-02	2.42E-01	1.00E+00
ENSG00000169020.8_ATP5I	ENSG00000160789.18_LMNA	-0.21285	-0.359860372	-0.05553528	8.47E-03	5.81E-02	1.00E+00
ENSG00000115380.17_EFEMP1	ENSG00000160789.18_LMNA	0.15746	-0.001787596	0.308914921	5.27E-02	2.08E-01	1.00E+00
ENSG00000111843.12_TMEM14C	ENSG00000160789.18_LMNA	-0.18834	-0.337427356	-0.03004442	2.01E-02	1.09E-01	1.00E+00

ENSG00000166595.10_FAM96B	ENSG00000160789.18_LMNA	-0.1116	-0.266074047	0.048461047	1.71E-01	4.30E-01	1.00E+00
ENSG00000166913.11_YWHAB	ENSG00000160789.18_LMNA	-0.22037	-0.36670683	-0.0633948	6.37E-03	4.70E-02	1.00E+00
ENSG00000185825.14_BCAP31	ENSG00000160789.18_LMNA	0.24973	0.094280236	0.393296748	1.92E-03	1.89E-02	1.00E+00
ENSG00000100632.9_ERH	ENSG00000160789.18_LMNA	-0.16234	-0.313442706	-0.00322563	4.57E-02	1.90E-01	1.00E+00
ENSG00000128245.13_YWHAH	ENSG00000160789.18_LMNA	-0.15804	-0.309451578	0.001194217	5.18E-02	2.06E-01	1.00E+00
ENSG00000171130.16_ATP6V0E	ENSG00000160789.18_LMNA	-0.09423	-0.249689275	0.065955722	2.48E-01	5.28E-01	1.00E+00
ENSG00000107317.10_PTGDS	ENSG00000160789.18_LMNA	-0.15736	-0.308824602	0.00188744	5.29E-02	2.08E-01	1.00E+00
ENSG00000184076.12_UQCR10	ENSG00000160789.18_LMNA	-0.13589	-0.288840783	0.023827433	9.51E-02	3.02E-01	1.00E+00
ENSG00000175334.6_BANF1	ENSG00000160789.18_LMNA	-0.16485	-0.315760211	-0.00579765	4.24E-02	1.80E-01	1.00E+00
ENSG00000092964.15_DPYSL2	ENSG00000160789.18_LMNA	-0.15193	-0.303780001	0.007454215	6.17E-02	2.30E-01	1.00E+00
ENSG00000072506.11_HSD17B1	ENSG00000160789.18_LMNA	0.03665	-0.123267307	0.194717177	6.54E-01	8.47E-01	1.00E+00
ENSG00000170027.6_YWHAG	ENSG00000160789.18_LMNA	0.10841	-0.051685421	0.263067836	1.84E-01	4.49E-01	1.00E+00
ENSG00000164032.10_H2AFZ	ENSG00000160789.18_LMNA	-0.0461	-0.20380094	0.113941028	5.73E-01	8.01E-01	1.00E+00
ENSG00000100823.10_APEX1	ENSG00000160789.18_LMNA	-0.08473	-0.240682806	0.07549034	2.99E-01	5.86E-01	1.00E+00
ENSG00000078369.16_GNB1	ENSG00000160789.18_LMNA	-0.03045	-0.188731428	0.129382314	7.10E-01	8.77E-01	1.00E+00
ENSG00000168899.4_VAMP5	ENSG00000160789.18_LMNA	0.19802	0.040079842	0.346300807	1.45E-02	8.57E-02	1.00E+00
ENSG00000109475.15_RPL34	ENSG00000160789.18_LMNA	-0.18251	-0.332065175	-0.02400996	2.44E-02	1.24E-01	1.00E+00
ENSG00000143543.13_JTB	ENSG00000160789.18_LMNA	-0.06594	-0.222797747	0.094254335	4.20E-01	6.91E-01	1.00E+00
ENSG00000053438.8_NNAT	ENSG00000160789.18_LMNA	-0.00883	-0.167790991	0.150586238	9.14E-01	9.67E-01	1.00E+00
ENSG00000184897.5_H1FX	ENSG00000160789.18_LMNA	-0.11849	-0.27254936	0.041493479	1.46E-01	3.91E-01	1.00E+00
ENSG00000136810.11_TXN	ENSG00000160789.18_LMNA	0.19314	0.035016455	0.34183053	1.71E-02	9.65E-02	1.00E+00
ENSG00000131236.15_CAP1	ENSG00000160789.18_LMNA	0.11031	-0.049763693	0.264860275	1.76E-01	4.37E-01	1.00E+00
ENSG00000182220.12_ATP6AP2	ENSG00000160789.18_LMNA	0.05665	-0.103485663	0.213919796	4.88E-01	7.44E-01	1.00E+00
ENSG00000084234.15_APLP2	ENSG00000160789.18_LMNA	0.3513	0.203484717	0.483466329	9.09E-06	2.70E-04	4.66E-01
ENSG00000104979.7_C19orf53	ENSG00000160789.18_LMNA	-0.05029	-0.207824941	0.109791619	5.38E-01	7.79E-01	1.00E+00
ENSG00000155659.13_VSIG4	ENSG00000160789.18_LMNA	0.00277	-0.156500119	0.161898904	9.73E-01	9.89E-01	1.00E+00
ENSG00000171867.15_PRNP	ENSG00000160789.18_LMNA	0.08178	-0.078442377	0.237883208	3.17E-01	6.03E-01	1.00E+00
ENSG00000071082.9_RPL31	ENSG00000160789.18_LMNA	-0.12154	-0.27541137	0.038404134	1.36E-01	3.75E-01	1.00E+00
ENSG00000163220.10_S100A9	ENSG00000160789.18_LMNA	0.09613	-0.064052161	0.251480877	2.39E-01	5.17E-01	1.00E+00
ENSG00000196419.11_XRCC6	ENSG00000160789.18_LMNA	-0.17193	-0.322312829	-0.01309216	3.42E-02	1.56E-01	1.00E+00
ENSG00000244038.8_DDOST	ENSG00000160789.18_LMNA	0.35049	0.202597447	0.482756898	9.57E-06	2.81E-04	4.91E-01
ENSG00000177606.6_JUN	ENSG00000160789.18_LMNA	0.42008	0.279581136	0.542972086	7.13E-08	5.49E-06	3.66E-03
ENSG00000070831.14_CDC42	ENSG00000160789.18_LMNA	-0.12489	-0.278547703	0.035011772	1.25E-01	3.58E-01	1.00E+00

ENSG00000101856.9_PGRMC1	ENSG00000160789.18_LMNA	-0.19087	-0.339746518	-0.03266131	1.85E-02	1.02E-01	1.00E+00
ENSG00000265972.4_TXNIP	ENSG00000160789.18_LMNA	-0.10203	-0.257054404	0.058115618	2.11E-01	4.84E-01	1.00E+00
ENSG00000187193.8_MT1X	ENSG00000160789.18_LMNA	-0.02561	-0.184061423	0.134136421	7.54E-01	8.98E-01	1.00E+00
ENSG00000115414.17_FN1	ENSG00000160789.18_LMNA	0.28289	0.129520162	0.423036652	4.14E-04	5.82E-03	1.00E+00
ENSG00000120129.5_DUSP1	ENSG00000160789.18_LMNA	0.0788	-0.081421827	0.235052273	3.35E-01	6.20E-01	1.00E+00
ENSG00000111328.5_CDK2AP1	ENSG00000160789.18_LMNA	-0.16608	-0.316902024	-0.00706637	4.09E-02	1.76E-01	1.00E+00
ENSG00000124733.3_MEA1	ENSG00000160789.18_LMNA	-0.02295	-0.181482878	0.136755141	7.79E-01	9.09E-01	1.00E+00
ENSG00000158869.9_FCER1G	ENSG00000160789.18_LMNA	0.03943	-0.120530681	0.197388307	6.30E-01	8.33E-01	1.00E+00
ENSG00000069535.13_MAOB	ENSG00000160789.18_LMNA	0.2651	0.110560547	0.407114338	9.65E-04	1.13E-02	1.00E+00
ENSG00000089597.15_GANAB	ENSG00000160789.18_LMNA	0.44028	0.302267572	0.560214899	1.38E-08	1.41E-06	7.07E-04
ENSG00000077549.16_CAPZB	ENSG00000160789.18_LMNA	0.01192	-0.147560786	0.170796524	8.84E-01	9.55E-01	1.00E+00
ENSG00000235174.1_RPL39P3	ENSG00000160789.18_LMNA	-0.09123	-0.246844067	0.068974018	2.64E-01	5.47E-01	1.00E+00
ENSG00000168542.11_COL3A1	ENSG00000160789.18_LMNA	0.36133	0.214467922	0.492217774	4.80E-06	1.61E-04	2.46E-01
ENSG00000119655.7_NPC2	ENSG00000160789.18_LMNA	0.0589	-0.101248669	0.216075982	4.71E-01	7.31E-01	1.00E+00
ENSG00000223865.9_HLA-DPB1	ENSG00000160789.18_LMNA	0.0232	-0.136500486	0.181733819	7.77E-01	9.08E-01	1.00E+00
ENSG00000136930.11_PSMB7	ENSG00000160789.18_LMNA	-0.03129	-0.189547226	0.128550321	7.02E-01	8.73E-01	1.00E+00
ENSG00000196205.8_EEF1A1P5	ENSG00000160789.18_LMNA	-0.18399	-0.33342058	-0.02553318	2.33E-02	1.20E-01	1.00E+00
ENSG00000215021.7_PHB2	ENSG00000160789.18_LMNA	-0.23491	-0.379906784	-0.07865489	3.58E-03	3.05E-02	1.00E+00
ENSG00000131171.11_SH3BGRL	ENSG00000160789.18_LMNA	-0.35549	-0.487122295	-0.20806446	6.98E-06	2.19E-04	3.58E-01
ENSG00000155366.15_RHOC	ENSG00000160789.18_LMNA	0.21335	0.056055606	0.360314645	8.31E-03	5.73E-02	1.00E+00
ENSG00000162430.15_SELENON	ENSG00000160789.18_LMNA	0.55072	0.42914188	0.652693728	1.97E-13	1.47E-10	1.01E-08
ENSG00000169564.6_PCBP1	ENSG00000160789.18_LMNA	0.27459	0.120662997	0.415620884	6.18E-04	7.97E-03	1.00E+00
ENSG00000136888.6_ATP6V1G1	ENSG00000160789.18_LMNA	-0.13237	-0.285555581	0.027405619	1.04E-01	3.19E-01	1.00E+00
ENSG00000162493.15_PDPN	ENSG00000160789.18_LMNA	0.35232	0.204597342	0.484355426	8.53E-06	2.56E-04	4.38E-01
ENSG00000189060.5_H1F0	ENSG00000160789.18_LMNA	-0.08199	-0.238079418	0.078235664	3.15E-01	6.02E-01	1.00E+00
ENSG00000146066.2_HIGD2A	ENSG00000160789.18_LMNA	-0.04754	-0.20519073	0.112509177	5.61E-01	7.93E-01	1.00E+00
ENSG00000108107.11_RPL28	ENSG00000160789.18_LMNA	0.11949	-0.040477724	0.273490983	1.43E-01	3.86E-01	1.00E+00
ENSG00000104267.8_CA2	ENSG00000160789.18_LMNA	-0.01067	-0.169580378	0.14878571	8.96E-01	9.59E-01	1.00E+00
ENSG00000133318.12_RTN3	ENSG00000160789.18_LMNA	-0.32406	-0.459552798	-0.17382795	4.65E-05	1.01E-03	1.00E+00
ENSG00000166441.11_RPL27A	ENSG00000160789.18_LMNA	-0.13855	-0.291320501	0.02112125	8.87E-02	2.90E-01	1.00E+00
ENSG00000144746.6_ARL6IP5	ENSG00000160789.18_LMNA	-0.14069	-0.293324717	0.018930645	8.38E-02	2.80E-01	1.00E+00
ENSG00000198502.5_HLA-DRB5	ENSG00000160789.18_LMNA	0.08741	-0.072807781	0.243222288	2.84E-01	5.70E-01	1.00E+00
ENSG00000030582.15_GRN	ENSG00000160789.18_LMNA	0.36864	0.222499659	0.498582135	2.97E-06	1.09E-04	1.52E-01

ENSG00000213639.8_PPP1CB	ENSG00000160789.18_LMNA	0.17733	0.018657374	0.32729218	2.88E-02	1.40E-01	1.00E+00
ENSG00000165795.19_NDRG2	ENSG00000160789.18_LMNA	-0.42007	-0.542955903	-0.27955998	7.14E-08	5.49E-06	3.67E-03
ENSG00000110492.14_MDK	ENSG00000160789.18_LMNA	0.4451	0.307705342	0.564314926	9.17E-09	9.94E-07	4.70E-04
ENSG00000104805.14_NUCB1	ENSG00000160789.18_LMNA	0.48253	0.350230498	0.59594778	3.07E-10	5.84E-08	1.58E-05
ENSG00000172809.11_RPL38	ENSG00000160789.18_LMNA	-0.17865	-0.328505964	-0.02001687	2.77E-02	1.36E-01	1.00E+00
ENSG00000105438.7_KDELR1	ENSG00000160789.18_LMNA	0.42725	0.287606806	0.549097485	4.03E-08	3.45E-06	2.07E-03
ENSG00000163902.10_RPN1	ENSG00000160789.18_LMNA	0.24407	0.08829955	0.388186607	2.44E-03	2.28E-02	1.00E+00
ENSG00000141522.10_ARHGDIA	ENSG00000160789.18_LMNA	0.41246	0.271054745	0.536433671	1.29E-07	8.93E-06	6.62E-03
ENSG00000168461.11_RAB31	ENSG00000160789.18_LMNA	0.0931	-0.067096108	0.248614926	2.54E-01	5.36E-01	1.00E+00
ENSG00000115758.11_ODC1	ENSG00000160789.18_LMNA	-0.21279	-0.359805733	-0.05547271	8.49E-03	5.82E-02	1.00E+00
ENSG00000196924.13_FLNA	ENSG00000160789.18_LMNA	0.58108	0.464881848	0.677595094	4.22E-15	6.91E-12	2.16E-10
ENSG00000125868.14_DSTN	ENSG00000160789.18_LMNA	-0.11543	-0.269675825	0.044589235	1.57E-01	4.09E-01	1.00E+00
ENSG00000101367.8_MAPRE1	ENSG00000160789.18_LMNA	-0.12084	-0.274758273	0.039109636	1.38E-01	3.79E-01	1.00E+00
ENSG00000125462.15_C1orf61	ENSG00000160789.18_LMNA	-0.25	-0.393535338	-0.09456	1.90E-03	1.88E-02	1.00E+00
ENSG00000146674.13_IGFBP3	ENSG00000160789.18_LMNA	0.28594	0.132788066	0.425762851	3.55E-04	5.13E-03	1.00E+00
ENSG00000197457.8_STMN3	ENSG00000160789.18_LMNA	0.07724	-0.082979248	0.233570344	3.44E-01	6.29E-01	1.00E+00
ENSG00000155660.9_PDIA4	ENSG00000160789.18_LMNA	0.56837	0.449874177	0.667197758	2.21E-14	2.70E-11	1.14E-09
ENSG00000170348.7_TMED10	ENSG00000160789.18_LMNA	0.28094	0.127441958	0.421300174	4.55E-04	6.26E-03	1.00E+00
ENSG00000161970.11_RPL26	ENSG00000160789.18_LMNA	-0.1416	-0.294164885	0.01801145	8.18E-02	2.76E-01	1.00E+00
ENSG00000205336.10_ADGRG1	ENSG00000160789.18_LMNA	0.00795	-0.15143879	0.166942982	9.23E-01	9.70E-01	1.00E+00
ENSG00000179222.16_MAGED1	ENSG00000160789.18_LMNA	-0.08049	-0.236661693	0.079728667	3.24E-01	6.11E-01	1.00E+00
ENSG00000147140.14_NONO	ENSG00000160789.18_LMNA	0.06617	-0.094021809	0.223020694	4.18E-01	6.90E-01	1.00E+00
ENSG00000114353.15_GNAI2	ENSG00000160789.18_LMNA	0.24785	0.092291973	0.391599943	2.08E-03	2.01E-02	1.00E+00
ENSG00000128016.5_ZFP36	ENSG00000160789.18_LMNA	0.22994	0.07342717	0.375398588	4.38E-03	3.55E-02	1.00E+00
ENSG00000147123.10_NDUFB11	ENSG00000160789.18_LMNA	-0.13128	-0.284534007	0.028516667	1.07E-01	3.25E-01	1.00E+00
ENSG00000124702.16_KLHDC3	ENSG00000160789.18_LMNA	-0.46797	-0.5836864	-0.33362599	1.21E-09	1.82E-07	6.20E-05
ENSG00000130726.10_TRIM28	ENSG00000160789.18_LMNA	0.16491	0.005861977	0.315818122	4.23E-02	1.80E-01	1.00E+00
ENSG00000135046.12_ANXA1	ENSG00000160789.18_LMNA	0.42681	0.287122257	0.54872846	4.18E-08	3.52E-06	2.14E-03
ENSG00000106366.8_SERPINE1	ENSG00000160789.18_LMNA	0.47888	0.346061505	0.592879977	4.36E-10	7.82E-08	2.24E-05
ENSG00000130522.5_JUND	ENSG00000160789.18_LMNA	0.15664	-0.002622608	0.308159394	5.40E-02	2.11E-01	1.00E+00
ENSG00000241468.6_ATP5J2	ENSG00000160789.18_LMNA	-0.04723	-0.204894283	0.112814706	5.63E-01	7.95E-01	1.00E+00
ENSG00000171223.5_JUNB	ENSG00000160789.18_LMNA	0.29667	0.144278816	0.435306781	2.06E-04	3.34E-03	1.00E+00
ENSG00000101160.12_CTSZ	ENSG00000160789.18_LMNA	0.30155	0.149523116	0.439640935	1.60E-04	2.74E-03	1.00E+00

ENSG00000102760.12_RGCC	ENSG00000160789.18_LMNA	-0.58092	-0.677464352	-0.46469237	4.31E-15	6.91E-12	2.21E-10
ENSG00000138085.15_ATRAID	ENSG00000160789.18_LMNA	0.03926	-0.12069078	0.19723217	6.31E-01	8.34E-01	1.00E+00
ENSG00000089693.9_MLF2	ENSG00000160789.18_LMNA	-0.19288	-0.341588587	-0.03474286	1.73E-02	9.72E-02	1.00E+00
ENSG00000112514.14_CUTA	ENSG00000160789.18_LMNA	-0.13074	-0.284027245	0.029067527	1.08E-01	3.28E-01	1.00E+00
ENSG00000175203.14_DCTN2	ENSG00000160789.18_LMNA	-0.38895	-0.516188687	-0.24491585	7.34E-07	3.58E-05	3.77E-02
ENSG00000054148.16_PHPT1	ENSG00000160789.18_LMNA	0.06771	-0.092487411	0.224491056	4.07E-01	6.82E-01	1.00E+00
ENSG00000140990.13_NDUFB10	ENSG00000160789.18_LMNA	-0.14287	-0.295351624	0.016712185	7.91E-02	2.70E-01	1.00E+00
ENSG00000109099.12_PMP22	ENSG00000160789.18_LMNA	0.36356	0.21690865	0.494154957	4.15E-06	1.43E-04	2.13E-01
ENSG00000119335.15_SET	ENSG00000160789.18_LMNA	0.05055	-0.109531439	0.208076899	5.36E-01	7.77E-01	1.00E+00
ENSG00000178913.7_TAF7	ENSG00000160789.18_LMNA	-0.16188	-0.313009292	-0.00274507	4.63E-02	1.91E-01	1.00E+00
ENSG00000184840.10_TMED9	ENSG00000160789.18_LMNA	0.48733	0.355728236	0.599982421	1.93E-10	3.89E-08	9.89E-06
ENSG00000173511.8_VEGFB	ENSG00000160789.18_LMNA	0.17214	0.013304422	0.322503059	3.40E-02	1.55E-01	1.00E+00
ENSG00000179091.4_CYC1	ENSG00000160789.18_LMNA	-0.11855	-0.272604533	0.04143398	1.46E-01	3.91E-01	1.00E+00
ENSG00000100292.15_HMOX1	ENSG00000160789.18_LMNA	0.11792	-0.042074033	0.272010907	1.48E-01	3.94E-01	1.00E+00
ENSG00000227063.5_RPL41P1	ENSG00000160789.18_LMNA	-0.13278	-0.285939953	0.026987381	1.03E-01	3.17E-01	1.00E+00
ENSG00000167004.11_PDIA3	ENSG00000160789.18_LMNA	0.5163	0.389078167	0.624193604	9.88E-12	3.47E-09	5.07E-07
ENSG00000108654.10_DDX5	ENSG00000160789.18_LMNA	-0.11284	-0.267240546	0.047208128	1.66E-01	4.23E-01	1.00E+00
ENSG00000115310.16_RTN4	ENSG00000160789.18_LMNA	-0.04682	-0.204497055	0.113224011	5.67E-01	7.97E-01	1.00E+00
ENSG00000236552.2_RPL13AP5	ENSG00000160789.18_LMNA	-0.07375	-0.230247664	0.08646558	3.67E-01	6.47E-01	1.00E+00
ENSG00000135624.14_CCT7	ENSG00000160789.18_LMNA	-0.1054	-0.260234933	0.054717915	1.96E-01	4.65E-01	1.00E+00
ENSG00000163399.14_ATP1A1	ENSG00000160789.18_LMNA	0.48094	0.348421021	0.594617135	3.58E-10	6.60E-08	1.84E-05
ENSG00000112308.11_C6orf62	ENSG00000160789.18_LMNA	0.09243	-0.067765428	0.247984003	2.57E-01	5.40E-01	1.00E+00
ENSG00000116729.12_WLS	ENSG00000160789.18_LMNA	0.10803	-0.052069777	0.262709078	1.85E-01	4.51E-01	1.00E+00
ENSG00000187957.7_DNER	ENSG00000160789.18_LMNA	-0.43055	-0.55191591	-0.29131196	3.09E-08	2.76E-06	1.58E-03
ENSG00000155876.5_RRAGA	ENSG00000160789.18_LMNA	0.03086	-0.128974471	0.189131387	7.06E-01	8.75E-01	1.00E+00
ENSG00000125691.11_RPL23	ENSG00000160789.18_LMNA	-0.23657	-0.381404904	-0.08039579	3.34E-03	2.90E-02	1.00E+00
ENSG00000135047.13_CTSL	ENSG00000160789.18_LMNA	0.17552	0.016786953	0.325620573	3.05E-02	1.45E-01	1.00E+00
ENSG00000146701.10_MDH2	ENSG00000160789.18_LMNA	0.1391	-0.020556999	0.291837005	8.74E-02	2.88E-01	1.00E+00
ENSG00000204387.11_C6orf48	ENSG00000160789.18_LMNA	-0.15984	-0.311127686	-0.00066046	4.92E-02	1.99E-01	1.00E+00
ENSG00000146729.8_GBAS	ENSG00000160789.18_LMNA	0.00799	-0.151401581	0.166980002	9.22E-01	9.70E-01	1.00E+00
ENSG00000169715.13_MT1E	ENSG00000160789.18_LMNA	0.16796	0.009000267	0.318640747	3.86E-02	1.69E-01	1.00E+00
ENSG00000099194.5_SCD	ENSG00000160789.18_LMNA	-0.04457	-0.202331589	0.115453413	5.86E-01	8.08E-01	1.00E+00
ENSG00000101474.10_APMAP	ENSG00000160789.18_LMNA	-0.07392	-0.230408754	0.086296734	3.65E-01	6.47E-01	1.00E+00

ENSG00000117362.11_APH1A	ENSG00000160789.18_LMNA	0.13975	-0.019897433	0.292440527	8.60E-02	2.85E-01	1.00E+00
ENSG00000170043.10_TRAPPC1	ENSG00000160789.18_LMNA	-0.02881	-0.187155294	0.130988469	7.25E-01	8.83E-01	1.00E+00
ENSG00000086061.14_DNAJA1	ENSG00000160789.18_LMNA	-0.04861	-0.20621249	0.111455652	5.52E-01	7.88E-01	1.00E+00
ENSG00000100906.9_NFKBIA	ENSG00000160789.18_LMNA	0.18317	0.024687287	0.332668034	2.39E-02	1.23E-01	1.00E+00
ENSG00000112096.15_SOD2	ENSG00000160789.18_LMNA	0.18255	0.024051633	0.332102272	2.44E-02	1.24E-01	1.00E+00
ENSG00000105404.9_RABAC1	ENSG00000160789.18_LMNA	0.01067	-0.148787051	0.169579046	8.96E-01	9.59E-01	1.00E+00
ENSG00000156298.11_TSPAN7	ENSG00000160789.18_LMNA	-0.32848	-0.463447518	-0.17862298	3.60E-05	8.29E-04	1.00E+00
ENSG00000112695.10_COX7A2	ENSG00000160789.18_LMNA	-0.25984	-0.402395398	-0.10498259	1.23E-03	1.35E-02	1.00E+00
ENSG00000171603.15_CLSTN1	ENSG00000160789.18_LMNA	0.13829	-0.021378985	0.291084515	8.93E-02	2.91E-01	1.00E+00
ENSG00000168036.15_CTNNB1	ENSG00000160789.18_LMNA	-0.09866	-0.253870264	0.06150988	2.27E-01	5.03E-01	1.00E+00
ENSG00000111348.7_ARHGDIB	ENSG00000160789.18_LMNA	-0.02808	-0.186443611	0.13171316	7.31E-01	8.87E-01	1.00E+00
ENSG00000136156.11_ITM2B	ENSG00000160789.18_LMNA	-0.19332	-0.341998277	-0.03520617	1.70E-02	9.61E-02	1.00E+00
ENSG00000132963.7_POMP	ENSG00000160789.18_LMNA	-0.16675	-0.317523101	-0.0077569	4.00E-02	1.74E-01	1.00E+00
ENSG00000143384.11_MCL1	ENSG00000160789.18_LMNA	0.41052	0.268890554	0.534768989	1.50E-07	1.00E-05	7.68E-03
ENSG00000137726.14_FXYD6	ENSG00000160789.18_LMNA	-0.42299	-0.545462965	-0.28284032	5.67E-08	4.52E-06	2.91E-03
ENSG00000023734.9_STRAP	ENSG00000160789.18_LMNA	-0.32572	-0.461016483	-0.17562839	4.22E-05	9.38E-04	1.00E+00
ENSG00000148248.12_SURF4	ENSG00000160789.18_LMNA	0.46012	0.324703929	0.577050582	2.46E-09	3.31E-07	1.26E-04
ENSG00000092820.16_EZR	ENSG00000160789.18_LMNA	-0.0482	-0.205819304	0.111861146	5.55E-01	7.89E-01	1.00E+00
ENSG00000117691.8_NENF	ENSG00000160789.18_LMNA	-0.02978	-0.188085971	0.130040267	7.16E-01	8.79E-01	1.00E+00
ENSG00000130287.12_NCAN	ENSG00000160789.18_LMNA	-0.24472	-0.388775424	-0.08898758	2.38E-03	2.23E-02	1.00E+00
ENSG00000203747.8_FCGR3A	ENSG00000160789.18_LMNA	0.06001	-0.100143539	0.21714005	4.63E-01	7.26E-01	1.00E+00
ENSG00000009307.14_CSDE1	ENSG00000160789.18_LMNA	-0.00702	-0.166033455	0.152352663	9.32E-01	9.73E-01	1.00E+00
ENSG00000104964.13_AES	ENSG00000160789.18_LMNA	-0.02509	-0.183561819	0.134644156	7.59E-01	8.99E-01	1.00E+00
ENSG00000188186.9_LAMTOR4	ENSG00000160789.18_LMNA	-0.1132	-0.267581078	0.04684218	1.65E-01	4.21E-01	1.00E+00
ENSG00000152137.5_HSPB8	ENSG00000160789.18_LMNA	-0.27415	-0.415225914	-0.12019257	6.31E-04	8.10E-03	1.00E+00
ENSG00000123131.11_PRDX4	ENSG00000160789.18_LMNA	0.2225	0.065621825	0.368640842	5.87E-03	4.43E-02	1.00E+00
ENSG00000140391.13_TSPAN3	ENSG00000160789.18_LMNA	-0.24643	-0.390318037	-0.09079146	2.21E-03	2.11E-02	1.00E+00
ENSG00000103260.7_METRN	ENSG00000160789.18_LMNA	-0.03609	-0.194172816	0.123824424	6.59E-01	8.49E-01	1.00E+00
ENSG00000142507.8_PSMB6	ENSG00000160789.18_LMNA	-0.10572	-0.26053621	0.054395687	1.95E-01	4.64E-01	1.00E+00
ENSG00000124562.8_SNRPC	ENSG00000160789.18_LMNA	-0.15236	-0.30418148	0.007011885	6.10E-02	2.28E-01	1.00E+00
ENSG00000008018.8_PSMB1	ENSG00000160789.18_LMNA	-0.06696	-0.223779878	0.09322975	4.12E-01	6.86E-01	1.00E+00
ENSG00000124767.6_GLO1	ENSG00000160789.18_LMNA	-0.14855	-0.300638208	0.010911499	6.78E-02	2.44E-01	1.00E+00
ENSG00000213593.8_TMX2	ENSG00000160789.18_LMNA	-0.17851	-0.328375544	-0.01987074	2.78E-02	1.36E-01	1.00E+00

ENSG00000260032.1_NORAD	ENSG00000160789.18_LMNA	0.17035	0.011458158	0.320847583	3.59E-02	1.61E-01	1.00E+00
ENSG00000171951.4_SCG2	ENSG00000160789.18_LMNA	0.32792	0.178013277	0.462952904	3.72E-05	8.49E-04	1.00E+00
ENSG00000142227.9_EMP3	ENSG00000160789.18_LMNA	0.58267	0.466769254	0.678896699	3.41E-15	5.83E-12	1.75E-10
ENSG00000128595.15_CALU	ENSG00000160789.18_LMNA	0.45895	0.32338086	0.576063699	2.74E-09	3.60E-07	1.40E-04
ENSG00000173113.5_TRMT112	ENSG00000160789.18_LMNA	0.02975	-0.130062011	0.188064636	7.16E-01	8.79E-01	1.00E+00
ENSG00000085063.13_CD59	ENSG00000160789.18_LMNA	0.0867	-0.07351996	0.242548516	2.88E-01	5.74E-01	1.00E+00
ENSG00000100380.11_ST13	ENSG00000160789.18_LMNA	-0.31111	-0.448114701	-0.15982369	9.57E-05	1.82E-03	1.00E+00
ENSG00000149557.11_FEZ1	ENSG00000160789.18_LMNA	-0.34564	-0.478507698	-0.19729267	1.29E-05	3.61E-04	6.63E-01
ENSG00000034713.6_GABARAPL	ENSG00000160789.18_LMNA	-0.37807	-0.506764485	-0.23288103	1.57E-06	6.66E-05	8.06E-02
ENSG00000041982.13_TNC	ENSG00000160789.18_LMNA	0.58865	0.473855497	0.683771691	1.52E-15	2.88E-12	7.78E-11
ENSG00000129103.16_SUMF2	ENSG00000160789.18_LMNA	0.0982	-0.061972936	0.253435342	2.29E-01	5.06E-01	1.00E+00
ENSG00000137267.5_TUBB2A	ENSG00000160789.18_LMNA	0.08162	-0.078604103	0.237729681	3.17E-01	6.04E-01	1.00E+00
ENSG00000185432.11_METTL7A	ENSG00000160789.18_LMNA	-0.16813	-0.318797492	-0.00917472	3.84E-02	1.69E-01	1.00E+00
ENSG00000173726.9_TOMM20	ENSG00000160789.18_LMNA	-0.33212	-0.466644014	-0.18256852	2.91E-05	6.96E-04	1.00E+00
ENSG00000102007.9_PLP2	ENSG00000160789.18_LMNA	0.56513	0.446059489	0.664541369	3.34E-14	3.72E-11	1.71E-09
ENSG00000226278.1_PSPHP1	ENSG00000160789.18_LMNA	-0.05638	-0.213665794	0.103748973	4.90E-01	7.46E-01	1.00E+00
ENSG00000105669.11_COPE	ENSG00000160789.18_LMNA	-0.10287	-0.257847995	0.057268521	2.07E-01	4.79E-01	1.00E+00
ENSG00000143321.17_HDGF	ENSG00000160789.18_LMNA	0.17957	0.020968693	0.329355157	2.69E-02	1.33E-01	1.00E+00
ENSG00000152518.6_ZFP36L2	ENSG00000160789.18_LMNA	0.417	0.276130163	0.540329568	9.08E-08	6.64E-06	4.66E-03
ENSG00000163468.13_CCT3	ENSG00000160789.18_LMNA	-0.11127	-0.265761277	0.048796822	1.72E-01	4.31E-01	1.00E+00
ENSG00000148672.8_GLUD1	ENSG00000160789.18_LMNA	-0.30292	-0.440864479	-0.15100656	1.49E-04	2.58E-03	1.00E+00
ENSG00000132341.10_RAN	ENSG00000160789.18_LMNA	-0.14948	-0.301508474	0.009954589	6.61E-02	2.40E-01	1.00E+00
ENSG00000008441.15_NFIX	ENSG00000160789.18_LMNA	-0.0956	-0.250979005	0.064585628	2.41E-01	5.21E-01	1.00E+00
ENSG00000156587.14_UBE2L6	ENSG00000160789.18_LMNA	-0.06388	-0.220835668	0.096299216	4.34E-01	7.03E-01	1.00E+00
ENSG00000165916.7_PSMC3	ENSG00000160789.18_LMNA	0.11921	-0.040761432	0.273228041	1.44E-01	3.87E-01	1.00E+00
ENSG00000125817.7_CENPB	ENSG00000160789.18_LMNA	0.43221	0.293180646	0.553335119	2.69E-08	2.49E-06	1.38E-03
ENSG00000213585.9_VDAC1	ENSG00000160789.18_LMNA	0.28734	0.134282345	0.42700766	3.32E-04	4.85E-03	1.00E+00
ENSG00000092199.16_HNRNPC	ENSG00000160789.18_LMNA	-0.09725	-0.252544538	0.062920943	2.33E-01	5.11E-01	1.00E+00
ENSG00000185236.10_RAB11B	ENSG00000160789.18_LMNA	-0.09729	-0.252577914	0.062885434	2.33E-01	5.11E-01	1.00E+00
ENSG00000149131.14_SERPING	ENSG00000160789.18_LMNA	0.26923	0.114959621	0.410824816	7.96E-04	9.69E-03	1.00E+00
ENSG00000241685.7_ARPC1A	ENSG00000160789.18_LMNA	0.23335	0.077005611	0.378486051	3.81E-03	3.20E-02	1.00E+00
ENSG00000111676.13_ATN1	ENSG00000160789.18_LMNA	0.07333	-0.086883381	0.229848978	3.69E-01	6.50E-01	1.00E+00
ENSG00000178980.13_SELENOW	ENSG00000160789.18_LMNA	-0.21988	-0.366259952	-0.06288065	6.49E-03	4.77E-02	1.00E+00

ENSG00000157150.4_TIMP4	ENSG00000160789.18_LMNA	-0.22442	-0.370391003	-0.06763975	5.44E-03	4.18E-02	1.00E+00
ENSG00000136938.8_ANP32B	ENSG00000160789.18_LMNA	0.07578	-0.08443963	0.232179413	3.53E-01	6.37E-01	1.00E+00
ENSG00000131143.7_COX4I1	ENSG00000160789.18_LMNA	-0.15126	-0.303162442	0.008134376	6.29E-02	2.33E-01	1.00E+00
ENSG00000175061.16_LRRC75A-	ENSG00000160789.18_LMNA	-0.30338	-0.441264848	-0.15149225	1.45E-04	2.53E-03	1.00E+00
ENSG00000108828.14_VAT1	ENSG00000160789.18_LMNA	0.15269	-0.006671536	0.30449032	6.04E-02	2.27E-01	1.00E+00
ENSG00000117525.12_F3	ENSG00000160789.18_LMNA	0.42845	0.288964213	0.550130724	3.66E-08	3.19E-06	1.88E-03
ENSG00000115484.13_CCT4	ENSG00000160789.18_LMNA	-0.18469	-0.334064275	-0.02625708	2.27E-02	1.18E-01	1.00E+00
ENSG00000132002.6_DNAJB1	ENSG00000160789.18_LMNA	0.2191	0.062067309	0.365552746	6.69E-03	4.87E-02	1.00E+00
ENSG00000168159.10_RNF187	ENSG00000160789.18_LMNA	0.02835	-0.131448413	0.186703645	7.29E-01	8.85E-01	1.00E+00
ENSG00000092010.13_PSME1	ENSG00000160789.18_LMNA	0.0619	-0.098270704	0.218941577	4.49E-01	7.15E-01	1.00E+00
ENSG00000119421.6_NDUFA8	ENSG00000160789.18_LMNA	-0.07818	-0.234463581	0.082040693	3.38E-01	6.24E-01	1.00E+00
ENSG00000099341.10_PSMD8	ENSG00000160789.18_LMNA	-0.01173	-0.170609098	0.147749629	8.86E-01	9.56E-01	1.00E+00
ENSG00000106565.16_TMEM176	ENSG00000160789.18_LMNA	0.00147	-0.157763041	0.160637688	9.86E-01	9.94E-01	1.00E+00
ENSG00000168002.10_POLR2G	ENSG00000160789.18_LMNA	-0.22243	-0.368574795	-0.06554572	5.88E-03	4.44E-02	1.00E+00
ENSG00000130724.7_CHMP2A	ENSG00000160789.18_LMNA	0.13619	-0.02352362	0.289119381	9.43E-02	3.01E-01	1.00E+00
ENSG00000105270.13_CLIP3	ENSG00000160789.18_LMNA	-0.24968	-0.393247013	-0.09422192	1.92E-03	1.89E-02	1.00E+00
ENSG00000124155.15_PIGT	ENSG00000160789.18_LMNA	0.45472	0.318585192	0.572480405	3.98E-09	4.93E-07	2.04E-04
ENSG00000164924.16_YWHAZ	ENSG00000160789.18_LMNA	0.06065	-0.0995102	0.217749516	4.58E-01	7.22E-01	1.00E+00
ENSG00000106367.12_AP1S1	ENSG00000160789.18_LMNA	0.02868	-0.131118297	0.187027821	7.26E-01	8.84E-01	1.00E+00
ENSG00000063660.7_GPC1	ENSG00000160789.18_LMNA	0.43207	0.293020789	0.553213771	2.73E-08	2.50E-06	1.40E-03
ENSG00000136240.8_KDELR2	ENSG00000160789.18_LMNA	0.34542	0.197057962	0.478319386	1.31E-05	3.64E-04	6.72E-01
ENSG00000166340.13_TPP1	ENSG00000160789.18_LMNA	0.36057	0.2136372	0.49155781	5.04E-06	1.67E-04	2.58E-01
ENSG00000113013.11_HSPA9	ENSG00000160789.18_LMNA	0.23072	0.074250274	0.376109352	4.24E-03	3.47E-02	1.00E+00
ENSG00000237973.1_MTCO1P12	ENSG00000160789.18_LMNA	-0.21697	-0.363613406	-0.05983898	7.25E-03	5.17E-02	1.00E+00
ENSG00000111229.14_ARPC3	ENSG00000160789.18_LMNA	-0.16142	-0.312587649	-0.0022777	4.70E-02	1.93E-01	1.00E+00
ENSG00000022267.15_FHL1	ENSG00000160789.18_LMNA	-0.20513	-0.35280968	-0.04748053	1.12E-02	7.12E-02	1.00E+00
ENSG00000144381.15_HSPD1	ENSG00000160789.18_LMNA	0.08556	-0.074659214	0.241470066	2.95E-01	5.80E-01	1.00E+00
ENSG00000167614.12_TTYH1	ENSG00000160789.18_LMNA	-0.17233	-0.32267779	-0.01349941	3.38E-02	1.55E-01	1.00E+00
ENSG00000178741.10_COX5A	ENSG00000160789.18_LMNA	-0.08182	-0.2379254	0.078397929	3.16E-01	6.03E-01	1.00E+00
ENSG00000105290.10_APLP1	ENSG00000160789.18_LMNA	-0.08288	-0.238924451	0.077345085	3.10E-01	5.96E-01	1.00E+00
ENSG00000177697.16_CD151	ENSG00000160789.18_LMNA	0.57519	0.457923464	0.672784865	9.17E-15	1.31E-11	4.70E-10
ENSG00000100804.17_PSMB5	ENSG00000160789.18_LMNA	0.01391	-0.145611402	0.17272994	8.65E-01	9.47E-01	1.00E+00
ENSG00000198918.7_RPL39	ENSG00000160789.18_LMNA	-0.13428	-0.287339294	0.025463824	9.91E-02	3.10E-01	1.00E+00

ENSG00000042753.10_AP2S1	ENSG00000160789.18_LMNA	0.12802	-0.031830395	0.281482911	1.16E-01	3.41E-01	1.00E+00
ENSG00000108551.4_RASD1	ENSG00000160789.18_LMNA	-0.02976	-0.18806745	0.130059142	7.16E-01	8.79E-01	1.00E+00
ENSG00000136295.13_TTYH3	ENSG00000160789.18_LMNA	0.41162	0.270122767	0.535717052	1.38E-07	9.42E-06	7.06E-03
ENSG00000168090.8_COPS6	ENSG00000160789.18_LMNA	0.15394	-0.005390702	0.30565199	5.83E-02	2.21E-01	1.00E+00
ENSG00000188486.3_H2AFX	ENSG00000160789.18_LMNA	-0.0391	-0.197075184	0.120851733	6.32E-01	8.35E-01	1.00E+00
ENSG00000142173.13_COL6A2	ENSG00000160789.18_LMNA	0.5312	0.406367954	0.636570276	1.92E-12	9.18E-10	9.83E-08
ENSG00000163346.15_PBXIP1	ENSG00000160789.18_LMNA	-0.00405	-0.163143339	0.15525298	9.61E-01	9.84E-01	1.00E+00
ENSG00000144485.9_HES6	ENSG00000160789.18_LMNA	-0.41895	-0.542004721	-0.27831702	7.80E-08	5.89E-06	4.00E-03
ENSG00000115053.14_NCL	ENSG00000160789.18_LMNA	0.25802	0.103055957	0.400761786	1.33E-03	1.44E-02	1.00E+00
ENSG00000143256.4_PFDN2	ENSG00000160789.18_LMNA	0.01802	-0.141587987	0.176712657	8.26E-01	9.32E-01	1.00E+00
ENSG00000101955.13_SRPX	ENSG00000160789.18_LMNA	0.31423	0.163195363	0.450877195	8.06E-05	1.58E-03	1.00E+00
ENSG00000198618.5_PPIAP22	ENSG00000160789.18_LMNA	0.10835	-0.051747465	0.263009929	1.84E-01	4.49E-01	1.00E+00
ENSG00000084733.9_RAB10	ENSG00000160789.18_LMNA	-0.06221	-0.219243281	0.097956838	4.46E-01	7.13E-01	1.00E+00
ENSG00000184602.5_SNN	ENSG00000160789.18_LMNA	-0.25377	-0.396930167	-0.0985458	1.61E-03	1.66E-02	1.00E+00
ENSG00000104341.15_LAPTM4B	ENSG00000160789.18_LMNA	-0.28925	-0.428711674	-0.13633002	3.01E-04	4.52E-03	1.00E+00
ENSG00000141753.6_IGFBP4	ENSG00000160789.18_LMNA	0.23871	0.082650688	0.383342996	3.06E-03	2.71E-02	1.00E+00
ENSG00000026508.15_CD44	ENSG00000160789.18_LMNA	0.43496	0.296274979	0.555681854	2.15E-08	2.05E-06	1.10E-03
ENSG00000163382.10_NAXE	ENSG00000160789.18_LMNA	-0.11985	-0.273822207	0.040120269	1.41E-01	3.84E-01	1.00E+00
ENSG00000175550.6_DRAP1	ENSG00000160789.18_LMNA	0.30631	0.154649353	0.443864497	1.24E-04	2.25E-03	1.00E+00
ENSG00000123989.12_CHPF	ENSG00000160789.18_LMNA	0.57742	0.460558515	0.674608576	6.84E-15	1.03E-11	3.51E-10
ENSG00000135052.15_GOLM1	ENSG00000160789.18_LMNA	-0.10247	-0.257473177	0.057668666	2.09E-01	4.82E-01	1.00E+00
ENSG00000101210.9_EEF1A2	ENSG00000160789.18_LMNA	-0.02031	-0.178936544	0.13933679	8.04E-01	9.21E-01	1.00E+00
ENSG00000050165.16_DKK3	ENSG00000160789.18_LMNA	0.00878	-0.150626416	0.167751038	9.14E-01	9.67E-01	1.00E+00
ENSG00000168906.11_MAT2A	ENSG00000160789.18_LMNA	-0.30335	-0.441244313	-0.15146734	1.45E-04	2.53E-03	1.00E+00
ENSG00000181061.12_HIGD1A	ENSG00000160789.18_LMNA	-0.27985	-0.420324395	-0.12627528	4.80E-04	6.53E-03	1.00E+00
ENSG00000130985.15_UBA1	ENSG00000160789.18_LMNA	0.2151	0.057877434	0.361904064	7.79E-03	5.44E-02	1.00E+00
ENSG00000175215.8_CTDSP2	ENSG00000160789.18_LMNA	-0.14239	-0.294906645	0.017199481	8.01E-02	2.72E-01	1.00E+00
ENSG00000169223.13_LMAN2	ENSG00000160789.18_LMNA	0.39536	0.252020181	0.521721266	4.63E-07	2.47E-05	2.38E-02
ENSG00000138760.7_SCARB2	ENSG00000160789.18_LMNA	-0.0251	-0.183563564	0.134642383	7.59E-01	8.99E-01	1.00E+00
ENSG00000004142.10_POLDIP2	ENSG00000160789.18_LMNA	-0.21144	-0.358572037	-0.05406052	8.92E-03	6.04E-02	1.00E+00
ENSG00000010256.9_UQCRC1	ENSG00000160789.18_LMNA	-0.04026	-0.198186476	0.119712003	6.22E-01	8.29E-01	1.00E+00
ENSG00000116459.9_ATP5F1	ENSG00000160789.18_LMNA	-0.32435	-0.459804669	-0.17413763	4.57E-05	1.00E-03	1.00E+00
ENSG00000125398.5_SOX9	ENSG00000160789.18_LMNA	0.30573	0.154021016	0.443347494	1.28E-04	2.31E-03	1.00E+00

ENSG00000138069.15_RAB1A	ENSG00000160789.18_LMNA	-0.14304	-0.295507622	0.016541317	7.88E-02	2.69E-01	1.00E+00
ENSG00000187498.13_COL4A1	ENSG00000160789.18_LMNA	0.36074	0.213814686	0.49169884	4.99E-06	1.65E-04	2.56E-01
ENSG00000100994.10_PYGB	ENSG00000160789.18_LMNA	0.25149	0.09614356	0.394885082	1.78E-03	1.79E-02	1.00E+00
ENSG00000021300.12_PLEKHB1	ENSG00000160789.18_LMNA	-0.35393	-0.485756604	-0.20635226	7.71E-06	2.37E-04	3.95E-01
ENSG00000131507.10_NDFIP1	ENSG00000160789.18_LMNA	-0.17437	-0.324561554	-0.0156031	3.17E-02	1.49E-01	1.00E+00
ENSG00000182004.11_SNRPE	ENSG00000160789.18_LMNA	-0.10874	-0.263376627	0.051354523	1.82E-01	4.47E-01	1.00E+00
ENSG00000164692.16_COL1A2	ENSG00000160789.18_LMNA	0.45955	0.324061573	0.576571538	2.59E-09	3.45E-07	1.33E-04
ENSG00000231389.6_HLA-DPA1	ENSG00000160789.18_LMNA	-0.0304	-0.188687931	0.129426662	7.10E-01	8.77E-01	1.00E+00
ENSG00000166226.11_CCT2	ENSG00000160789.18_LMNA	-0.10229	-0.257301634	0.057851768	2.10E-01	4.83E-01	1.00E+00
ENSG00000185222.7_TCEAL9	ENSG00000160789.18_LMNA	0.21331	0.056012185	0.360276741	8.32E-03	5.73E-02	1.00E+00
ENSG00000198937.8_CCDC167	ENSG00000160789.18_LMNA	-0.35053	-0.482788015	-0.20263635	9.54E-06	2.81E-04	4.90E-01
ENSG00000188536.11_HBA2	ENSG00000160789.18_LMNA	-0.03687	-0.194930129	0.123049311	6.52E-01	8.46E-01	1.00E+00
ENSG00000106803.8_SEC61B	ENSG00000160789.18_LMNA	0.07901	-0.081212219	0.235251608	3.33E-01	6.19E-01	1.00E+00
ENSG00000118418.13_HMGN3	ENSG00000160789.18_LMNA	-0.34356	-0.476686719	-0.19502436	1.47E-05	4.01E-04	7.53E-01
ENSG00000111639.6_MRPL51	ENSG00000160789.18_LMNA	-0.17967	-0.329449572	-0.02107455	2.68E-02	1.33E-01	1.00E+00
ENSG00000242372.5_EIF6	ENSG00000160789.18_LMNA	0.18699	0.028642764	0.336183627	2.11E-02	1.12E-01	1.00E+00
ENSG00000129824.14_RPS4Y1	ENSG00000160789.18_LMNA	-0.08246	-0.238531481	0.077759298	3.12E-01	5.99E-01	1.00E+00
ENSG00000084090.12_STARD7	ENSG00000160789.18_LMNA	-0.24247	-0.386738305	-0.08660844	2.62E-03	2.40E-02	1.00E+00
ENSG00000174684.6_B4GAT1	ENSG00000160789.18_LMNA	-0.15213	-0.303970111	0.007244776	6.13E-02	2.29E-01	1.00E+00
ENSG00000235655.3_H3F3AP4	ENSG00000160789.18_LMNA	-0.236	-0.380885986	-0.07979257	3.42E-03	2.95E-02	1.00E+00
ENSG00000117632.19_STMN1	ENSG00000160789.18_LMNA	-0.39801	-0.524011587	-0.25496969	3.82E-07	2.12E-05	1.96E-02
ENSG00000176903.4_PNMA1	ENSG00000160789.18_LMNA	0.0327	-0.127163967	0.19090562	6.89E-01	8.66E-01	1.00E+00
ENSG00000145293.13_ENOPH1	ENSG00000160789.18_LMNA	-0.19686	-0.345244379	-0.03888183	1.51E-02	8.81E-02	1.00E+00
ENSG00000135390.16_ATP5G2	ENSG00000160789.18_LMNA	-0.30806	-0.445417143	-0.15653776	1.13E-04	2.08E-03	1.00E+00
ENSG00000197451.9_HNRNPAB	ENSG00000160789.18_LMNA	0.14192	-0.01767954	0.294468141	8.11E-02	2.75E-01	1.00E+00
ENSG00000118523.5_CTGF	ENSG00000160789.18_LMNA	0.17795	0.019294605	0.327861237	2.83E-02	1.38E-01	1.00E+00
ENSG00000226608.3_FTLP3	ENSG00000160789.18_LMNA	-0.00302	-0.16214396	0.156254611	9.71E-01	9.89E-01	1.00E+00
ENSG00000111674.7_ENO2	ENSG00000160789.18_LMNA	0.12287	-0.037058269	0.276656459	1.32E-01	3.68E-01	1.00E+00
ENSG00000168003.15_SLC3A2	ENSG00000160789.18_LMNA	0.26564	0.111142779	0.407605996	9.41E-04	1.11E-02	1.00E+00
ENSG00000134153.8_EMC7	ENSG00000160789.18_LMNA	0.02878	-0.131021038	0.187123317	7.25E-01	8.83E-01	1.00E+00
ENSG00000100644.15_HIF1A	ENSG00000160789.18_LMNA	0.17879	0.020159336	0.328633097	2.75E-02	1.35E-01	1.00E+00
ENSG00000188612.10_SUMO2	ENSG00000160789.18_LMNA	-0.27218	-0.413464488	-0.11809625	6.93E-04	8.71E-03	1.00E+00
ENSG00000215030.5_RPL13P12	ENSG00000160789.18_LMNA	0.02311	-0.136590141	0.181645476	7.77E-01	9.09E-01	1.00E+00

ENSG00000159388.5_BTG2	ENSG00000160789.18_LMNA	-0.06262	-0.219630504	0.097553914	4.43E-01	7.11E-01	1.00E+00
ENSG00000115307.15_AUP1	ENSG00000160789.18_LMNA	0.01382	-0.145703407	0.172638744	8.66E-01	9.48E-01	1.00E+00
ENSG00000157227.11_MMP14	ENSG00000160789.18_LMNA	0.66328	0.563594746	0.743928187	1.27E-20	1.09E-16	6.54E-16
ENSG00000134686.15_PHC2	ENSG00000160789.18_LMNA	0.30014	0.148009939	0.438391755	1.72E-04	2.90E-03	1.00E+00
ENSG00000168894.8_RNF181	ENSG00000160789.18_LMNA	-0.05311	-0.210530156	0.106995858	5.16E-01	7.63E-01	1.00E+00
ENSG00000122705.15_CLTA	ENSG00000160789.18_LMNA	-0.08886	-0.244596277	0.07135447	2.76E-01	5.61E-01	1.00E+00
ENSG00000182054.8_IDH2	ENSG00000160789.18_LMNA	0.00728	-0.15209222	0.166292715	9.29E-01	9.73E-01	1.00E+00
ENSG00000069275.12_NUCKS1	ENSG00000160789.18_LMNA	0.11388	-0.046161507	0.268214269	1.62E-01	4.17E-01	1.00E+00
ENSG00000183283.14_DAZAP2	ENSG00000160789.18_LMNA	-0.29783	-0.436344211	-0.14553261	1.94E-04	3.19E-03	1.00E+00
ENSG00000117410.12_ATP6V0B	ENSG00000160789.18_LMNA	-0.07578	-0.232183908	0.084434912	3.53E-01	6.37E-01	1.00E+00
ENSG00000102409.9_BEX4	ENSG00000160789.18_LMNA	-0.36312	-0.493777844	-0.21643324	4.27E-06	1.46E-04	2.19E-01
ENSG00000102109.8_PCSK1N	ENSG00000160789.18_LMNA	-0.11565	-0.269880141	0.044369319	1.56E-01	4.07E-01	1.00E+00
ENSG00000091129.18_NRCAM	ENSG00000160789.18_LMNA	0.14671	-0.012792481	0.298926028	7.13E-02	2.53E-01	1.00E+00
ENSG00000204392.9_LSM2	ENSG00000160789.18_LMNA	-0.19002	-0.338966607	-0.03178081	1.90E-02	1.04E-01	1.00E+00
ENSG00000178531.5_CTXN1	ENSG00000160789.18_LMNA	-0.14096	-0.293568515	0.01866397	8.33E-02	2.79E-01	1.00E+00
ENSG00000163069.11_SGCB	ENSG00000160789.18_LMNA	0.04242	-0.117572416	0.200270473	6.04E-01	8.18E-01	1.00E+00
ENSG00000105518.12_TMEM205	ENSG00000160789.18_LMNA	0.16279	0.003682793	0.313854899	4.51E-02	1.88E-01	1.00E+00
ENSG00000086598.9_TMED2	ENSG00000160789.18_LMNA	-0.08371	-0.239719916	0.076506285	3.05E-01	5.92E-01	1.00E+00
ENSG00000154978.11_VOPP1	ENSG00000160789.18_LMNA	0.12028	-0.039684318	0.274226065	1.40E-01	3.82E-01	1.00E+00
ENSG00000005075.14_POLR2J	ENSG00000160789.18_LMNA	0.01943	-0.14020296	0.178081268	8.12E-01	9.26E-01	1.00E+00
ENSG00000134419.14_RPS15A	ENSG00000160789.18_LMNA	-0.16608	-0.316899987	-0.00706411	4.09E-02	1.76E-01	1.00E+00
ENSG00000150779.10_TIMM8B	ENSG00000160789.18_LMNA	-0.2472	-0.391010358	-0.09160167	2.14E-03	2.06E-02	1.00E+00
ENSG00000125901.5_MRPS26	ENSG00000160789.18_LMNA	-0.00593	-0.16497513	0.15341536	9.42E-01	9.77E-01	1.00E+00
ENSG00000233639.4_LINC01158	ENSG00000160789.18_LMNA	-0.08208	-0.238167208	0.078143166	3.15E-01	6.01E-01	1.00E+00
ENSG00000106636.6_YKT6	ENSG00000160789.18_LMNA	0.31679	0.165963027	0.453140701	7.00E-05	1.41E-03	1.00E+00
ENSG00000162545.5_CAMK2N1	ENSG00000160789.18_LMNA	0.10084	-0.059315105	0.255929953	2.16E-01	4.91E-01	1.00E+00
ENSG00000105223.17_PLD3	ENSG00000160789.18_LMNA	0.31804	0.167313421	0.454243764	6.53E-05	1.33E-03	1.00E+00
ENSG00000142657.19_PGD	ENSG00000160789.18_LMNA	0.04454	-0.115480371	0.202305386	5.86E-01	8.08E-01	1.00E+00
ENSG00000185787.13_MORF4L1	ENSG00000160789.18_LMNA	-0.20714	-0.354648813	-0.04957775	1.04E-02	6.76E-02	1.00E+00
ENSG00000105968.17_H2AFV	ENSG00000160789.18_LMNA	-0.14271	-0.295205685	0.016872019	7.94E-02	2.71E-01	1.00E+00
ENSG00000133321.9_RARRES3	ENSG00000160789.18_LMNA	0.00439	-0.15492332	0.163472115	9.57E-01	9.83E-01	1.00E+00
ENSG00000134287.8_ARF3	ENSG00000160789.18_LMNA	0.02373	-0.135985091	0.182241568	7.72E-01	9.06E-01	1.00E+00
ENSG00000140264.18_SERF2	ENSG00000160789.18_LMNA	0.07946	-0.08076015	0.235681432	3.30E-01	6.16E-01	1.00E+00

ENSG00000125971.15_DYNLRB1	ENSG00000160789.18_LMNA	-0.02799	-0.18635639	0.131801953	7.32E-01	8.87E-01	1.00E+00
ENSG00000165283.14_STOML2	ENSG00000160789.18_LMNA	-0.01217	-0.171038836	0.147316609	8.82E-01	9.54E-01	1.00E+00
ENSG00000165629.18_ATP5C1	ENSG00000160789.18_LMNA	-0.36815	-0.498157565	-0.22196269	3.07E-06	1.12E-04	1.57E-01
ENSG00000108821.12_COL1A1	ENSG00000160789.18_LMNA	0.40766	0.265707422	0.532316787	1.86E-07	1.20E-05	9.54E-03
ENSG00000172531.13_PPP1CA	ENSG00000160789.18_LMNA	0.02175	-0.137933056	0.180321605	7.90E-01	9.15E-01	1.00E+00
ENSG00000165678.18_GHITM	ENSG00000160789.18_LMNA	-0.32683	-0.461990344	-0.17682738	3.96E-05	8.94E-04	1.00E+00
ENSG00000137309.18_HMGA1	ENSG00000160789.18_LMNA	0.22234	0.065460327	0.36850068	5.90E-03	4.45E-02	1.00E+00
ENSG00000130175.8_PRKCSH	ENSG00000160789.18_LMNA	0.42851	0.289025288	0.550177195	3.64E-08	3.18E-06	1.87E-03
ENSG00000137575.10_SDCBP	ENSG00000160789.18_LMNA	0.14144	-0.018166675	0.294023039	8.22E-02	2.77E-01	1.00E+00
ENSG00000134871.16_COL4A2	ENSG00000160789.18_LMNA	0.40122	0.258534367	0.526774404	3.01E-07	1.77E-05	1.55E-02
ENSG00000106484.13_MEST	ENSG00000160789.18_LMNA	0.15931	0.000110125	0.310630539	4.99E-02	2.01E-01	1.00E+00
ENSG00000179085.7_DPM3	ENSG00000160789.18_LMNA	0.04636	-0.113680767	0.204053651	5.71E-01	7.99E-01	1.00E+00
ENSG00000136193.15_SCRN1	ENSG00000160789.18_LMNA	0.16332	0.004231953	0.314349886	4.44E-02	1.86E-01	1.00E+00
ENSG00000178982.8_EIF3K	ENSG00000160789.18_LMNA	-0.11379	-0.268131562	0.046250433	1.63E-01	4.18E-01	1.00E+00
ENSG00000105058.10_FAM32A	ENSG00000160789.18_LMNA	-0.24365	-0.38780837	-0.08785774	2.49E-03	2.31E-02	1.00E+00
ENSG00000116649.8_SRM	ENSG00000160789.18_LMNA	0.12188	-0.038056219	0.275733335	1.35E-01	3.73E-01	1.00E+00
ENSG00000106244.11_PDAP1	ENSG00000160789.18_LMNA	0.38348	0.238862546	0.511456676	1.08E-06	4.88E-05	5.54E-02
ENSG00000123975.4_CKS2	ENSG00000160789.18_LMNA	-0.06285	-0.219849065	0.097326444	4.42E-01	7.09E-01	1.00E+00
ENSG00000173786.15_CNP	ENSG00000160789.18_LMNA	-0.29341	-0.43241177	-0.14078495	2.44E-04	3.83E-03	1.00E+00
ENSG00000122545.16_SEPT7	ENSG00000160789.18_LMNA	-0.01705	-0.175773733	0.142537454	8.35E-01	9.36E-01	1.00E+00
ENSG00000240184.5_PCDHGC3	ENSG00000160789.18_LMNA	0.04501	-0.115019352	0.202753446	5.82E-01	8.05E-01	1.00E+00
ENSG00000156976.13_EIF4A2	ENSG00000160789.18_LMNA	-0.43142	-0.552660647	-0.29229232	2.87E-08	2.61E-06	1.47E-03
ENSG00000173915.11_USMG5	ENSG00000160789.18_LMNA	-0.26356	-0.40573492	-0.10892809	1.04E-03	1.19E-02	1.00E+00
ENSG00000122565.17_CBX3	ENSG00000160789.18_LMNA	-0.04504	-0.202789226	0.114982531	5.82E-01	8.05E-01	1.00E+00
ENSG00000159228.11_CBR1	ENSG00000160789.18_LMNA	0.26634	0.111886617	0.408233868	9.11E-04	1.08E-02	1.00E+00
ENSG00000171766.14_GATM	ENSG00000160789.18_LMNA	0.03741	-0.122525058	0.195442129	6.47E-01	8.43E-01	1.00E+00
ENSG00000130402.10_ACTN4	ENSG00000160789.18_LMNA	0.54171	0.418612835	0.645264474	5.73E-13	3.68E-10	2.94E-08
ENSG00000204308.7_RNF5	ENSG00000160789.18_LMNA	-0.32478	-0.460183342	-0.17460333	4.46E-05	9.79E-04	1.00E+00
ENSG00000135452.8_TSPAN31	ENSG00000160789.18_LMNA	-0.33923	-0.472892466	-0.19030769	1.91E-05	4.97E-04	9.79E-01
ENSG00000178498.14_DTX3	ENSG00000160789.18_LMNA	-0.37543	-0.504475418	-0.22997047	1.88E-06	7.64E-05	9.65E-02
ENSG00000173457.9_PPP1R14B	ENSG00000160789.18_LMNA	0.13085	-0.02895493	0.284130842	1.08E-01	3.27E-01	1.00E+00
ENSG00000173432.9_SAA1	ENSG00000160789.18_LMNA	0.02835	-0.131441211	0.186710719	7.29E-01	8.85E-01	1.00E+00
ENSG00000108561.7_C1QBP	ENSG00000160789.18_LMNA	-0.24996	-0.393499646	-0.09451815	1.90E-03	1.88E-02	1.00E+00

ENSG00000143774.15_GUK1	ENSG00000160789.18_LMNA	-0.03066	-0.188938639	0.129171032	7.08E-01	8.76E-01	1.00E+00
ENSG00000167460.13_TPM4	ENSG00000160789.18_LMNA	0.38171	0.236903168	0.509921434	1.22E-06	5.41E-05	6.27E-02
ENSG00000084754.9_HADHA	ENSG00000160789.18_LMNA	-0.07339	-0.229909256	0.086820221	3.69E-01	6.50E-01	1.00E+00
ENSG00000042493.14_CAPG	ENSG00000160789.18_LMNA	0.21287	0.055554875	0.359877481	8.46E-03	5.81E-02	1.00E+00
ENSG00000089063.13_TMEM230	ENSG00000160789.18_LMNA	-0.07222	-0.228786432	0.087996329	3.77E-01	6.56E-01	1.00E+00
ENSG00000137161.15_CNPY3	ENSG00000160789.18_LMNA	-0.15506	-0.30669021	0.004245119	5.65E-02	2.17E-01	1.00E+00
ENSG00000155115.6_GTF3C6	ENSG00000160789.18_LMNA	-0.20772	-0.355172924	-0.0501759	1.02E-02	6.66E-02	1.00E+00
ENSG00000170390.13_DCLK2	ENSG00000160789.18_LMNA	-0.26634	-0.408233836	-0.11188658	9.11E-04	1.08E-02	1.00E+00
ENSG00000138071.12_ACTR2	ENSG00000160789.18_LMNA	0.09496	-0.065221994	0.250380105	2.45E-01	5.24E-01	1.00E+00
ENSG00000074842.6_MYDGF	ENSG00000160789.18_LMNA	0.20815	0.0506321	0.355572528	1.01E-02	6.59E-02	1.00E+00
ENSG00000211445.10_GPX3	ENSG00000160789.18_LMNA	-0.09292	-0.248444863	0.067276549	2.55E-01	5.37E-01	1.00E+00
ENSG00000147400.8_CETN2	ENSG00000160789.18_LMNA	-0.05742	-0.214659236	0.102718872	4.82E-01	7.40E-01	1.00E+00
ENSG00000111341.8_MGP	ENSG00000160789.18_LMNA	0.05069	-0.109393404	0.208210555	5.35E-01	7.76E-01	1.00E+00
ENSG00000124762.12_CDKN1A	ENSG00000160789.18_LMNA	0.4059	0.263747349	0.530804569	2.12E-07	1.33E-05	1.09E-02
ENSG00000100348.8_TXN2	ENSG00000160789.18_LMNA	-0.19225	-0.341012581	-0.03409168	1.77E-02	9.87E-02	1.00E+00
ENSG00000108774.13_RAB5C	ENSG00000160789.18_LMNA	-0.09997	-0.255114768	0.060184119	2.20E-01	4.96E-01	1.00E+00
ENSG00000167191.10_GPRC5B	ENSG00000160789.18_LMNA	-0.17952	-0.329311121	-0.02091932	2.69E-02	1.33E-01	1.00E+00
ENSG00000242299.1_RP11-234A	ENSG00000160789.18_LMNA	-0.08822	-0.243989993	0.071995921	2.80E-01	5.65E-01	1.00E+00
ENSG00000243279.3_PRAF2	ENSG00000160789.18_LMNA	0.00153	-0.15771119	0.160689489	9.85E-01	9.94E-01	1.00E+00
ENSG00000005893.14_LAMP2	ENSG00000160789.18_LMNA	0.1778	0.019136317	0.327719905	2.84E-02	1.38E-01	1.00E+00
ENSG00000165280.14_VCP	ENSG00000160789.18_LMNA	0.14516	-0.014375369	0.297483634	7.44E-02	2.60E-01	1.00E+00
ENSG00000132434.8_LANCL2	ENSG00000160789.18_LMNA	-0.07148	-0.228080646	0.088735158	3.82E-01	6.60E-01	1.00E+00
ENSG00000185043.9_CIB1	ENSG00000160789.18_LMNA	0.19414	0.036058766	0.342751885	1.65E-02	9.42E-02	1.00E+00
ENSG00000184983.8_NDUFA6	ENSG00000160789.18_LMNA	-0.17319	-0.323468959	-0.01438261	3.29E-02	1.52E-01	1.00E+00
ENSG00000147684.6_NDUFB9	ENSG00000160789.18_LMNA	-0.32172	-0.457485489	-0.17128825	5.31E-05	1.13E-03	1.00E+00
ENSG00000175826.10_CTDNEP1	ENSG00000160789.18_LMNA	0.03641	-0.123509138	0.194480907	6.56E-01	8.48E-01	1.00E+00
ENSG00000111678.9_C12orf57	ENSG00000160789.18_LMNA	-0.11976	-0.27374008	0.040208907	1.42E-01	3.84E-01	1.00E+00
ENSG00000074071.12_MRPS34	ENSG00000160789.18_LMNA	-0.03029	-0.188578013	0.129538724	7.11E-01	8.77E-01	1.00E+00
ENSG00000141756.17_FKBP10	ENSG00000160789.18_LMNA	0.5051	0.376146547	0.614858883	3.22E-11	8.79E-09	1.65E-06
ENSG00000127884.4_ECHS1	ENSG00000160789.18_LMNA	-0.17676	-0.326762424	-0.01806438	2.94E-02	1.41E-01	1.00E+00
ENSG00000120509.9_PDZD11	ENSG00000160789.18_LMNA	-0.07819	-0.234474021	0.08202972	3.38E-01	6.24E-01	1.00E+00
ENSG00000203950.6_FAM127B	ENSG00000160789.18_LMNA	-0.05904	-0.216210424	0.101109081	4.70E-01	7.30E-01	1.00E+00
ENSG00000175166.15_PSMD2	ENSG00000160789.18_LMNA	0.13587	-0.023846761	0.288823057	9.51E-02	3.02E-01	1.00E+00

ENSG00000060762.17_MPC1	ENSG00000160789.18_LMNA	-0.16248	-0.313567913	-0.00336448	4.55E-02	1.89E-01	1.00E+00
ENSG00000159403.14_C1R	ENSG00000160789.18_LMNA	0.45208	0.315588153	0.5702361	5.02E-09	6.02E-07	2.58E-04
ENSG00000184900.14_SUMO3	ENSG00000160789.18_LMNA	0.02338	-0.136324198	0.181907511	7.75E-01	9.07E-01	1.00E+00
ENSG00000105677.10_TMEM147	ENSG00000160789.18_LMNA	0.08132	-0.078904353	0.237444611	3.19E-01	6.06E-01	1.00E+00
ENSG00000168077.12_SCARA3	ENSG00000160789.18_LMNA	0.25544	0.100322599	0.398440895	1.49E-03	1.56E-02	1.00E+00
ENSG00000131495.7_NDUFA2	ENSG00000160789.18_LMNA	-0.21436	-0.361233178	-0.0571082	8.00E-03	5.56E-02	1.00E+00
ENSG00000185896.10_LAMP1	ENSG00000160789.18_LMNA	0.21458	0.057338679	0.361434221	7.94E-03	5.53E-02	1.00E+00
ENSG00000092096.13_SLC22A17	ENSG00000160789.18_LMNA	-0.15713	-0.308611395	0.002123106	5.32E-02	2.09E-01	1.00E+00
ENSG00000149357.8_LAMTOR1	ENSG00000160789.18_LMNA	-0.19672	-0.34511284	-0.03873272	1.51E-02	8.84E-02	1.00E+00
ENSG00000078596.9_ITM2A	ENSG00000160789.18_LMNA	-0.40237	-0.527763949	-0.25981289	2.77E-07	1.65E-05	1.42E-02
ENSG00000079246.14_XRCC5	ENSG00000160789.18_LMNA	-0.05653	-0.213805252	0.10360441	4.89E-01	7.45E-01	1.00E+00
ENSG00000145824.11_CXCL14	ENSG00000160789.18_LMNA	0.28733	0.134275845	0.427002247	3.32E-04	4.85E-03	1.00E+00
ENSG00000104833.9_TUBB4A	ENSG00000160789.18_LMNA	-0.35323	-0.48514442	-0.2055853	8.06E-06	2.45E-04	4.13E-01
ENSG00000081154.10_PCNP	ENSG00000160789.18_LMNA	-0.16326	-0.314292984	-0.00416881	4.45E-02	1.86E-01	1.00E+00
ENSG00000143862.6_ARL8A	ENSG00000160789.18_LMNA	0.02285	-0.136849797	0.181389592	7.80E-01	9.10E-01	1.00E+00
ENSG00000244313.3_RP11-425L	ENSG00000160789.18_LMNA	-0.10168	-0.256724251	0.058467896	2.13E-01	4.86E-01	1.00E+00
ENSG00000213442.5_RPL18AP3	ENSG00000160789.18_LMNA	-0.13063	-0.283924046	0.029179683	1.09E-01	3.28E-01	1.00E+00
ENSG00000121653.10_MAPK8IP1	ENSG00000160789.18_LMNA	-0.10493	-0.259790278	0.055193371	1.98E-01	4.68E-01	1.00E+00
ENSG00000175197.9_DDIT3	ENSG00000160789.18_LMNA	-0.14565	-0.297942303	0.013872194	7.34E-02	2.58E-01	1.00E+00
ENSG00000198961.8_PJA2	ENSG00000160789.18_LMNA	0.17028	0.01138338	0.320780493	3.60E-02	1.61E-01	1.00E+00
ENSG00000102172.14_SMS	ENSG00000160789.18_LMNA	0.47695	0.343854588	0.591253086	5.23E-10	9.13E-08	2.69E-05
ENSG00000104695.11_PPP2CB	ENSG00000160789.18_LMNA	-0.09813	-0.25337334	0.062038939	2.29E-01	5.06E-01	1.00E+00
ENSG00000105568.16_PPP2R1A	ENSG00000160789.18_LMNA	0.03945	-0.120511809	0.197406712	6.29E-01	8.33E-01	1.00E+00
ENSG00000148834.11_GSTO1	ENSG00000160789.18_LMNA	-0.0867	-0.242557358	0.073510615	2.88E-01	5.74E-01	1.00E+00
ENSG00000100300.16_TSPO	ENSG00000160789.18_LMNA	0.16868	0.009743677	0.319308578	3.78E-02	1.67E-01	1.00E+00
ENSG00000148175.11_STOM	ENSG00000160789.18_LMNA	0.03874	-0.12120848	0.19672717	6.36E-01	8.37E-01	1.00E+00
ENSG00000099622.12_CIRBP	ENSG00000160789.18_LMNA	-0.07328	-0.229798104	0.086936687	3.70E-01	6.50E-01	1.00E+00
ENSG00000146007.9_ZMAT2	ENSG00000160789.18_LMNA	-0.18233	-0.331896488	-0.02382049	2.46E-02	1.25E-01	1.00E+00
ENSG00000134817.10_APLNR	ENSG00000160789.18_LMNA	-0.13193	-0.285137627	0.027860272	1.05E-01	3.22E-01	1.00E+00
ENSG00000179604.9_CDC42EP4	ENSG00000160789.18_LMNA	-0.04286	-0.200690583	0.117140742	6.00E-01	8.16E-01	1.00E+00
ENSG00000182718.15_ANXA2	ENSG00000160789.18_LMNA	0.56821	0.449682208	0.66706421	2.26E-14	2.70E-11	1.16E-09
ENSG00000115128.6_SF3B6	ENSG00000160789.18_LMNA	-0.09049	-0.246144381	0.069715384	2.68E-01	5.52E-01	1.00E+00
ENSG00000132589.14_FLOT2	ENSG00000160789.18_LMNA	0.26342	0.10877601	0.405606343	1.04E-03	1.19E-02	1.00E+00

ENSG00000177469.12_PTRF	ENSG00000160789.18_LMNA	0.7066	0.616777723	0.778251349	2.70E-24	6.92E-20	1.38E-19
ENSG00000049245.11_VAMP3	ENSG00000160789.18_LMNA	0.07346	-0.086756463	0.229970101	3.68E-01	6.49E-01	1.00E+00
ENSG00000031698.11_SARS	ENSG00000160789.18_LMNA	-0.06435	-0.221287266	0.095828797	4.31E-01	7.00E-01	1.00E+00
ENSG00000164096.12_C4orf3	ENSG00000160789.18_LMNA	-0.02677	-0.185181178	0.132997833	7.43E-01	8.92E-01	1.00E+00
ENSG00000124172.8_ATP5E	ENSG00000160789.18_LMNA	-0.03308	-0.191269416	0.126792471	6.86E-01	8.64E-01	1.00E+00
ENSG00000127540.10_UQCR11	ENSG00000160789.18_LMNA	-0.17819	-0.328087691	-0.01954826	2.81E-02	1.37E-01	1.00E+00
ENSG00000085662.12_AKR1B1	ENSG00000160789.18_LMNA	-0.27295	-0.41415139	-0.11891344	6.69E-04	8.46E-03	1.00E+00
ENSG00000266472.4_MRPS21	ENSG00000160789.18_LMNA	-0.1251	-0.27874447	0.034798701	1.25E-01	3.56E-01	1.00E+00
ENSG00000102804.13_TSC22D1	ENSG00000160789.18_LMNA	-0.47145	-0.58662284	-0.33758847	8.76E-10	1.41E-07	4.49E-05
ENSG00000071553.15_ATP6AP1	ENSG00000160789.18_LMNA	0.26302	0.108360697	0.405255158	1.06E-03	1.21E-02	1.00E+00
ENSG00000115541.9_HSPE1	ENSG00000160789.18_LMNA	-0.1483	-0.300411682	0.011160485	6.82E-02	2.45E-01	1.00E+00
ENSG00000197971.13_MBP	ENSG00000160789.18_LMNA	-0.32748	-0.462561724	-0.17753123	3.82E-05	8.68E-04	1.00E+00
ENSG00000088247.14_KHSRP	ENSG00000160789.18_LMNA	0.13533	-0.024392097	0.288322843	9.64E-02	3.05E-01	1.00E+00
ENSG00000110107.7_PRPF19	ENSG00000160789.18_LMNA	-0.04183	-0.199698742	0.118159692	6.09E-01	8.21E-01	1.00E+00
ENSG00000102317.16_RBM3	ENSG00000160789.18_LMNA	-0.03604	-0.19413116	0.123867047	6.59E-01	8.50E-01	1.00E+00
ENSG00000245910.7_SNHG6	ENSG00000160789.18_LMNA	-0.25074	-0.394209277	-0.0953505	1.83E-03	1.84E-02	1.00E+00
ENSG00000106615.8_RHEB	ENSG00000160789.18_LMNA	-0.07423	-0.230700642	0.085990747	3.63E-01	6.45E-01	1.00E+00
ENSG00000105835.10_NAMPT	ENSG00000160789.18_LMNA	0.29819	0.145916115	0.436661377	1.91E-04	3.15E-03	1.00E+00
ENSG00000220205.7_VAMP2	ENSG00000160789.18_LMNA	-0.25607	-0.399004105	-0.10098549	1.45E-03	1.53E-02	1.00E+00
ENSG00000104763.16_ASAH1	ENSG00000160789.18_LMNA	-0.33874	-0.472458939	-0.1897696	1.97E-05	5.10E-04	1.00E+00
ENSG00000159352.14_PSMD4	ENSG00000160789.18_LMNA	0.0857	-0.074519841	0.241602042	2.94E-01	5.80E-01	1.00E+00
ENSG00000134222.15_PSRC1	ENSG00000160789.18_LMNA	0.12037	-0.039589946	0.274313475	1.40E-01	3.81E-01	1.00E+00
ENSG00000129353.13_SLC44A2	ENSG00000160789.18_LMNA	0.16702	0.008036286	0.317774312	3.97E-02	1.73E-01	1.00E+00
ENSG00000013275.6_PSMC4	ENSG00000160789.18_LMNA	-0.0202	-0.178822381	0.139452436	8.05E-01	9.22E-01	1.00E+00
ENSG00000107874.9_CUEDC2	ENSG00000160789.18_LMNA	-0.42353	-0.545917074	-0.28343515	5.43E-08	4.38E-06	2.79E-03
ENSG00000185650.9_ZFP36L1	ENSG00000160789.18_LMNA	0.18006	0.021471607	0.329803645	2.64E-02	1.32E-01	1.00E+00
ENSG00000147533.15_GOLGA7	ENSG00000160789.18_LMNA	-0.28497	-0.424897577	-0.13175017	3.73E-04	5.35E-03	1.00E+00
ENSG00000198892.6_SHISA4	ENSG00000160789.18_LMNA	-0.17581	-0.325890624	-0.01708898	3.03E-02	1.44E-01	1.00E+00
ENSG00000063244.11_U2AF2	ENSG00000160789.18_LMNA	0.11723	-0.042767852	0.271367143	1.50E-01	3.98E-01	1.00E+00
ENSG00000154723.11_ATP5J	ENSG00000160789.18_LMNA	-0.15324	-0.305003901	0.006105397	5.94E-02	2.24E-01	1.00E+00
ENSG00000175756.12_AURKAIP1	ENSG00000160789.18_LMNA	0.09591	-0.064271703	0.251274358	2.40E-01	5.19E-01	1.00E+00
ENSG00000143222.10_UFC1	ENSG00000160789.18_LMNA	-0.27195	-0.413256573	-0.11784898	7.01E-04	8.79E-03	1.00E+00
ENSG00000225733.4_FGD5-AS1	ENSG00000160789.18_LMNA	-0.42396	-0.546290324	-0.28392422	5.25E-08	4.25E-06	2.69E-03

ENSG00000176101.10_SSNA1	ENSG00000160789.18_LMNA	0.07286	-0.08735472	0.229399077	3.72E-01	6.53E-01	1.00E+00
ENSG00000101265.14_RASSF2	ENSG00000160789.18_LMNA	-0.06717	-0.223979223	0.093021707	4.11E-01	6.85E-01	1.00E+00
ENSG00000141504.10_SAT2	ENSG00000160789.18_LMNA	-0.08782	-0.243613331	0.072394299	2.82E-01	5.67E-01	1.00E+00
ENSG00000105254.10_TBCB	ENSG00000160789.18_LMNA	-0.16306	-0.314109912	-0.00396569	4.47E-02	1.87E-01	1.00E+00
ENSG00000130725.6_UBE2M	ENSG00000160789.18_LMNA	0.13206	-0.027722601	0.285264198	1.05E-01	3.21E-01	1.00E+00
ENSG00000125991.17_ERGIC3	ENSG00000160789.18_LMNA	0.14265	-0.016938408	0.295145063	7.96E-02	2.71E-01	1.00E+00
ENSG00000152795.16_HNRNPDL	ENSG00000160789.18_LMNA	-0.13838	-0.29116284	0.021293446	8.91E-02	2.91E-01	1.00E+00
ENSG00000114646.8_CSPG5	ENSG00000160789.18_LMNA	-0.26056	-0.403038711	-0.10574191	1.19E-03	1.32E-02	1.00E+00
ENSG00000176978.12_DPP7	ENSG00000160789.18_LMNA	0.37358	0.227935712	0.502872849	2.13E-06	8.37E-05	1.09E-01
ENSG00000188846.12_RPL14	ENSG00000160789.18_LMNA	-0.18914	-0.338155428	-0.03086551	1.96E-02	1.06E-01	1.00E+00
ENSG00000142871.14_CYR61	ENSG00000160789.18_LMNA	0.34642	0.198149321	0.479194778	1.23E-05	3.47E-04	6.32E-01
ENSG00000115234.9_SNX17	ENSG00000160789.18_LMNA	-0.18367	-0.333129287	-0.0252057	2.35E-02	1.21E-01	1.00E+00
ENSG00000132471.10_WBP2	ENSG00000160789.18_LMNA	-0.02144	-0.180028231	0.138230492	7.93E-01	9.16E-01	1.00E+00
ENSG00000126698.9_DNAJC8	ENSG00000160789.18_LMNA	-0.20421	-0.351963848	-0.04651691	1.16E-02	7.29E-02	1.00E+00
ENSG00000027697.11_IFNGR1	ENSG00000160789.18_LMNA	-0.34792	-0.480504194	-0.19978308	1.12E-05	3.20E-04	5.76E-01
ENSG00000170142.10_UBE2E1	ENSG00000160789.18_LMNA	-0.40984	-0.534186771	-0.26813427	1.58E-07	1.04E-05	8.09E-03
ENSG00000176971.3_FIBIN	ENSG00000160789.18_LMNA	-0.05278	-0.21021264	0.107324262	5.18E-01	7.65E-01	1.00E+00
ENSG00000132965.8_ALOX5AP	ENSG00000160789.18_LMNA	0.04451	-0.115511228	0.202275392	5.86E-01	8.08E-01	1.00E+00
ENSG00000069849.9_ATP1B3	ENSG00000160789.18_LMNA	0.01154	-0.147934648	0.170425444	8.88E-01	9.56E-01	1.00E+00
ENSG00000131711.13_MAP1B	ENSG00000160789.18_LMNA	0.1588	-0.000409224	0.310161227	5.07E-02	2.03E-01	1.00E+00
ENSG00000198843.11_SELENOT	ENSG00000160789.18_LMNA	-0.26038	-0.402884255	-0.10555957	1.20E-03	1.33E-02	1.00E+00
ENSG00000101444.11_AHCY	ENSG00000160789.18_LMNA	0.07435	-0.085871459	0.230814418	3.63E-01	6.44E-01	1.00E+00
ENSG00000164054.14_SHISA5	ENSG00000160789.18_LMNA	0.37619	0.230808813	0.505135147	1.79E-06	7.33E-05	9.16E-02
ENSG00000152234.14_ATP5A1	ENSG00000160789.18_LMNA	-0.22482	-0.370754795	-0.06805951	5.36E-03	4.13E-02	1.00E+00
ENSG00000006125.15_AP2B1	ENSG00000160789.18_LMNA	-0.21898	-0.365437649	-0.06193498	6.72E-03	4.89E-02	1.00E+00
ENSG00000204472.11_AIF1	ENSG00000160789.18_LMNA	-0.10803	-0.262715495	0.052062903	1.85E-01	4.51E-01	1.00E+00
ENSG00000108523.14_RNF167	ENSG00000160789.18_LMNA	-0.00554	-0.164599331	0.153792535	9.46E-01	9.78E-01	1.00E+00
ENSG00000112473.15_SLC39A7	ENSG00000160789.18_LMNA	0.37237	0.22660726	0.501825543	2.31E-06	8.91E-05	1.19E-01
ENSG00000107281.8_NPDC1	ENSG00000160789.18_LMNA	0.06702	-0.093173654	0.223833631	4.12E-01	6.86E-01	1.00E+00
ENSG00000067167.6_TRAM1	ENSG00000160789.18_LMNA	0.15104	-0.008361373	0.30295628	6.32E-02	2.34E-01	1.00E+00
ENSG00000204264.7_PSMB8	ENSG00000160789.18_LMNA	0.12715	-0.032708785	0.280673075	1.19E-01	3.45E-01	1.00E+00
ENSG00000005513.9_SOX8	ENSG00000160789.18_LMNA	-0.42178	-0.544420135	-0.28147509	6.24E-08	4.93E-06	3.20E-03
ENSG00000007080.9_CCDC124	ENSG00000160789.18_LMNA	0.09043	-0.069779595	0.246083765	2.68E-01	5.52E-01	1.00E+00

ENSG00000205927.4_OLIG2	ENSG00000160789.18_LMNA	-0.35039	-0.482669878	-0.20248864	9.63E-06	2.83E-04	4.94E-01
ENSG00000105401.5_CDC37	ENSG00000160789.18_LMNA	0.29443	0.141875339	0.433315909	2.31E-04	3.67E-03	1.00E+00
ENSG00000183684.7_ALYREF	ENSG00000160789.18_LMNA	0.12508	-0.034815995	0.278728501	1.25E-01	3.56E-01	1.00E+00
ENSG00000116717.10_GADD45A	ENSG00000160789.18_LMNA	0.53154	0.406765186	0.636853242	1.84E-12	9.09E-10	9.45E-08
ENSG00000272398.4_CD24	ENSG00000160789.18_LMNA	-0.29821	-0.436676435	-0.14593432	1.90E-04	3.14E-03	1.00E+00
ENSG00000143870.11_PDIA6	ENSG00000160789.18_LMNA	0.37936	0.234311165	0.507887832	1.44E-06	6.21E-05	7.37E-02
ENSG00000104870.11_FCGRT	ENSG00000160789.18_LMNA	0.04051	-0.119460946	0.198431159	6.20E-01	8.28E-01	1.00E+00
ENSG00000265452.1_MIR3682	ENSG00000160789.18_LMNA	-0.24395	-0.388075227	-0.08816944	2.46E-03	2.29E-02	1.00E+00
ENSG00000179950.12_PUF60	ENSG00000160789.18_LMNA	-0.17513	-0.325261881	-0.01638588	3.09E-02	1.46E-01	1.00E+00
ENSG00000160213.5_CSTB	ENSG00000160789.18_LMNA	0.16798	0.009016324	0.318655175	3.86E-02	1.69E-01	1.00E+00
ENSG00000111481.8_COPZ1	ENSG00000160789.18_LMNA	-0.31965	-0.455665321	-0.16905531	5.96E-05	1.23E-03	1.00E+00
ENSG00000161547.13_SRSF2	ENSG00000160789.18_LMNA	-0.18442	-0.333820964	-0.02598341	2.29E-02	1.19E-01	1.00E+00
ENSG00000143553.9_SNAPIN	ENSG00000160789.18_LMNA	-0.2767	-0.417511587	-0.12291674	5.59E-04	7.35E-03	1.00E+00
ENSG00000141580.14_WDR45B	ENSG00000160789.18_LMNA	-0.11533	-0.269585226	0.044686743	1.57E-01	4.09E-01	1.00E+00
ENSG00000182952.4_HMGN4	ENSG00000160789.18_LMNA	-0.14758	-0.299741026	0.011897409	6.96E-02	2.49E-01	1.00E+00
ENSG00000103257.7_SLC7A5	ENSG00000160789.18_LMNA	-0.01139	-0.170278716	0.148082451	8.89E-01	9.57E-01	1.00E+00
ENSG00000132670.19_PTPRA	ENSG00000160789.18_LMNA	0.10308	-0.057057423	0.258045694	2.06E-01	4.78E-01	1.00E+00
ENSG00000178035.10_IMPDH2	ENSG00000160789.18_LMNA	0.01294	-0.146564888	0.171784571	8.74E-01	9.51E-01	1.00E+00
ENSG00000225972.1_MTND1P23	ENSG00000160789.18_LMNA	-0.14958	-0.301601001	0.009852816	6.59E-02	2.40E-01	1.00E+00
ENSG00000137207.10_YIPF3	ENSG00000160789.18_LMNA	0.04883	-0.111230832	0.206430441	5.50E-01	7.87E-01	1.00E+00
ENSG00000143570.16_SLC39A1	ENSG00000160789.18_LMNA	0.54384	0.421092254	0.647017809	4.47E-13	3.02E-10	2.29E-08
ENSG00000129625.11_REEP5	ENSG00000160789.18_LMNA	-0.10985	-0.264426186	0.050229307	1.78E-01	4.40E-01	1.00E+00
ENSG00000143753.11_DEGS1	ENSG00000160789.18_LMNA	0.09634	-0.0638374	0.251682871	2.38E-01	5.16E-01	1.00E+00
ENSG00000180992.6_MRPL14	ENSG00000160789.18_LMNA	-0.06252	-0.219537623	0.09765057	4.44E-01	7.11E-01	1.00E+00
ENSG00000177706.8_FAM20C	ENSG00000160789.18_LMNA	0.63423	0.528376253	0.720662918	1.76E-18	6.95E-15	9.04E-14
ENSG00000155959.9_VBP1	ENSG00000160789.18_LMNA	-0.33263	-0.467095321	-0.18312631	2.83E-05	6.80E-04	1.00E+00
ENSG00000104765.13_BNIP3L	ENSG00000160789.18_LMNA	-0.06996	-0.226639112	0.090243097	3.92E-01	6.70E-01	1.00E+00
ENSG00000101363.11_MANBAL	ENSG00000160789.18_LMNA	-0.1361	-0.289038576	0.023611744	9.45E-02	3.01E-01	1.00E+00
ENSG00000002549.11_LAP3	ENSG00000160789.18_LMNA	0.19531	0.037268253	0.343820275	1.59E-02	9.15E-02	1.00E+00
ENSG00000071575.10_TRIB2	ENSG00000160789.18_LMNA	0.1959	0.037879317	0.344359752	1.56E-02	9.01E-02	1.00E+00
ENSG00000123091.4_RNF11	ENSG00000160789.18_LMNA	-0.07358	-0.230080988	0.086640261	3.68E-01	6.48E-01	1.00E+00
ENSG00000101198.13_NKAIN4	ENSG00000160789.18_LMNA	-0.44437	-0.563694344	-0.3068812	9.76E-09	1.05E-06	5.00E-04
ENSG00000078549.13_ADCYAP1	ENSG00000160789.18_LMNA	-0.25008	-0.393605877	-0.09464272	1.89E-03	1.87E-02	1.00E+00

ENSG00000143149.11_ALDH9A1	ENSG00000160789.18_LMNA	-0.1833	-0.332791099	-0.02482559	2.38E-02	1.22E-01	1.00E+00
ENSG00000145592.12_RPL37	ENSG00000160789.18_LMNA	-0.15389	-0.305606166	0.005441246	5.84E-02	2.21E-01	1.00E+00
ENSG00000171604.10_CXXC5	ENSG00000160789.18_LMNA	0.09868	-0.061487744	0.253891051	2.26E-01	5.03E-01	1.00E+00
ENSG00000057608.15_GDI2	ENSG00000160789.18_LMNA	-0.29594	-0.43466448	-0.14350302	2.14E-04	3.44E-03	1.00E+00
ENSG00000082641.14_NFE2L1	ENSG00000160789.18_LMNA	0.27685	0.123076661	0.417645648	5.55E-04	7.31E-03	1.00E+00
ENSG00000134824.12_FADS2	ENSG00000160789.18_LMNA	-0.22431	-0.370283944	-0.06751624	5.47E-03	4.19E-02	1.00E+00
ENSG00000168913.6_ENHO	ENSG00000160789.18_LMNA	-0.40518	-0.530182216	-0.26294132	2.24E-07	1.39E-05	1.15E-02
ENSG00000090861.14_AARS	ENSG00000160789.18_LMNA	0.05131	-0.108782063	0.208802361	5.30E-01	7.73E-01	1.00E+00
ENSG00000156261.11_CCT8	ENSG00000160789.18_LMNA	0.1153	-0.044719351	0.269554927	1.57E-01	4.09E-01	1.00E+00
ENSG00000164104.10_HMGB2	ENSG00000160789.18_LMNA	0.03079	-0.129043107	0.189064085	7.06E-01	8.75E-01	1.00E+00
ENSG00000065518.7_NDUFB4	ENSG00000160789.18_LMNA	-0.17352	-0.32377639	-0.01472594	3.25E-02	1.51E-01	1.00E+00
ENSG00000102226.8_USP11	ENSG00000160789.18_LMNA	-0.09559	-0.2509675	0.064597855	2.41E-01	5.21E-01	1.00E+00
ENSG00000100138.12_SNU13	ENSG00000160789.18_LMNA	-0.2686	-0.4102564	-0.11428496	8.20E-04	9.91E-03	1.00E+00
ENSG00000121774.16_KHDRBS1	ENSG00000160789.18_LMNA	0.0917	-0.068501029	0.247290292	2.61E-01	5.44E-01	1.00E+00
ENSG00000189403.13_HMGB1	ENSG00000160789.18_LMNA	-0.1869	-0.336101902	-0.0285507	2.11E-02	1.12E-01	1.00E+00
ENSG00000106628.9_POLD2	ENSG00000160789.18_LMNA	0.17262	0.013798882	0.322946101	3.35E-02	1.54E-01	1.00E+00
ENSG00000126756.10_UXT	ENSG00000160789.18_LMNA	-0.17186	-0.322240821	-0.01301182	3.43E-02	1.56E-01	1.00E+00
ENSG00000105696.7_TMEM59L	ENSG00000160789.18_LMNA	-0.20571	-0.353339842	-0.04808481	1.10E-02	7.01E-02	1.00E+00
ENSG00000072778.18_ACADVL	ENSG00000160789.18_LMNA	0.18303	0.024548335	0.33254438	2.40E-02	1.23E-01	1.00E+00
ENSG00000122218.13_COPA	ENSG00000160789.18_LMNA	-0.02607	-0.184506296	0.133684166	7.50E-01	8.95E-01	1.00E+00
ENSG00000213553.4_RPLP0P6	ENSG00000160789.18_LMNA	0.02224	-0.137443847	0.18080401	7.86E-01	9.13E-01	1.00E+00
ENSG00000044115.19_CTNNA1	ENSG00000160789.18_LMNA	0.35181	0.204040744	0.483910721	8.81E-06	2.63E-04	4.52E-01
ENSG00000008282.6_SYPL1	ENSG00000160789.18_LMNA	0.18758	0.029253275	0.336725481	2.07E-02	1.10E-01	1.00E+00
ENSG00000143819.11_EPHX1	ENSG00000160789.18_LMNA	0.05845	-0.101697384	0.215643724	4.74E-01	7.34E-01	1.00E+00
ENSG00000105583.8_WDR83OS	ENSG00000160789.18_LMNA	-0.14432	-0.296705995	0.015228105	7.61E-02	2.64E-01	1.00E+00
ENSG00000162873.13_KLHDC8A	ENSG00000160789.18_LMNA	0.07646	-0.083758291	0.23282851	3.49E-01	6.33E-01	1.00E+00
ENSG00000143575.13_HAX1	ENSG00000160789.18_LMNA	-0.1754	-0.325508967	-0.01666215	3.07E-02	1.45E-01	1.00E+00
ENSG00000198856.11_OSTC	ENSG00000160789.18_LMNA	0.01673	-0.142850818	0.175463721	8.38E-01	9.37E-01	1.00E+00
ENSG00000169862.17_CTNND2	ENSG00000160789.18_LMNA	-0.22698	-0.372708485	-0.0703156	4.92E-03	3.87E-02	1.00E+00
ENSG00000172301.9_COPRS	ENSG00000160789.18_LMNA	-0.01513	-0.173908666	0.144421728	8.53E-01	9.42E-01	1.00E+00
ENSG00000168209.4_DDIT4	ENSG00000160789.18_LMNA	-0.03355	-0.191723676	0.126328473	6.82E-01	8.61E-01	1.00E+00
ENSG00000149932.15_TMEM219	ENSG00000160789.18_LMNA	-0.24248	-0.386748081	-0.08661985	2.61E-03	2.40E-02	1.00E+00
ENSG00000226958.1_	ENSG00000160789.18_LMNA	-0.01932	-0.177970272	0.140315333	8.13E-01	9.27E-01	1.00E+00

ENSG00000169813.15_HNRNPF	ENSG00000160789.18_LMNA	0.36472	0.218190801	0.495171486	3.84E-06	1.34E-04	1.97E-01
ENSG00000118640.9_VAMP8	ENSG00000160789.18_LMNA	-0.05203	-0.209495474	0.108065765	5.24E-01	7.70E-01	1.00E+00
ENSG00000006327.12_TNFRSF1:	ENSG00000160789.18_LMNA	0.599	0.486158322	0.692191164	3.59E-16	8.78E-13	1.84E-11
ENSG00000108830.9_RND2	ENSG00000160789.18_LMNA	-0.30011	-0.438365429	-0.14797806	1.72E-04	2.91E-03	1.00E+00
ENSG00000112977.14_DAP	ENSG00000160789.18_LMNA	0.14117	-0.01844361	0.29376994	8.28E-02	2.78E-01	1.00E+00
ENSG00000171388.11_APLN	ENSG00000160789.18_LMNA	-0.07222	-0.228792359	0.087990124	3.77E-01	6.56E-01	1.00E+00
ENSG00000116857.15_TMEM9	ENSG00000160789.18_LMNA	-0.14048	-0.293129417	0.019144239	8.43E-02	2.81E-01	1.00E+00
ENSG00000136158.9_SPRY2	ENSG00000160789.18_LMNA	0.4274	0.28778573	0.549233725	3.98E-08	3.42E-06	2.04E-03
ENSG00000130706.11_ADRM1	ENSG00000160789.18_LMNA	0.41052	0.268893896	0.534771561	1.50E-07	1.00E-05	7.68E-03
ENSG00000106665.14_CLIP2	ENSG00000160789.18_LMNA	0.20287	0.045125388	0.350741542	1.22E-02	7.56E-02	1.00E+00
ENSG00000143153.11_ATP1B1	ENSG00000160789.18_LMNA	-0.26409	-0.406213474	-0.10949425	1.01E-03	1.17E-02	1.00E+00
ENSG00000104852.13_SNRNP70	ENSG00000160789.18_LMNA	0.13645	-0.023258157	0.289362768	9.37E-02	2.99E-01	1.00E+00
ENSG00000126945.8_HNRNPH2	ENSG00000160789.18_LMNA	0.0812	-0.079020471	0.23733435	3.20E-01	6.06E-01	1.00E+00
ENSG00000134248.12_LAMTOR5	ENSG00000160789.18_LMNA	-0.16381	-0.314797868	-0.00472912	4.37E-02	1.84E-01	1.00E+00
ENSG00000101161.7_PRPF6	ENSG00000160789.18_LMNA	0.17939	0.020786791	0.329192907	2.70E-02	1.33E-01	1.00E+00
ENSG00000108691.8_CCL2	ENSG00000160789.18_LMNA	0.19122	0.033028379	0.340071528	1.83E-02	1.01E-01	1.00E+00
ENSG00000185262.8_UBALD2	ENSG00000160789.18_LMNA	0.19135	0.033159565	0.340187664	1.82E-02	1.01E-01	1.00E+00
ENSG00000183722.7_LHFP	ENSG00000160789.18_LMNA	0.17522	0.016483068	0.325348806	3.08E-02	1.46E-01	1.00E+00
ENSG00000105323.15_HNRNPUL	ENSG00000160789.18_LMNA	0.11048	-0.049587973	0.265024065	1.75E-01	4.36E-01	1.00E+00
ENSG00000149923.12_PPP4C	ENSG00000160789.18_LMNA	0.04208	-0.117905787	0.199945953	6.07E-01	8.20E-01	1.00E+00
ENSG00000117419.13_ERI3	ENSG00000160789.18_LMNA	-0.2375	-0.382247025	-0.08137519	3.22E-03	2.81E-02	1.00E+00
ENSG00000117748.8_RPA2	ENSG00000160789.18_LMNA	-0.13195	-0.285157368	0.027838801	1.05E-01	3.22E-01	1.00E+00
ENSG00000101608.11_MYL12A	ENSG00000160789.18_LMNA	0.23496	0.078708348	0.379952812	3.57E-03	3.04E-02	1.00E+00
ENSG00000012660.12_ELOVL5	ENSG00000160789.18_LMNA	0.10575	-0.054370445	0.260559808	1.95E-01	4.64E-01	1.00E+00
ENSG00000232573.1_RPL3P4	ENSG00000160789.18_LMNA	-0.1213	-0.275184263	0.038649499	1.37E-01	3.76E-01	1.00E+00
ENSG00000180964.15_TCEAL8	ENSG00000160789.18_LMNA	-0.21081	-0.357994557	-0.0533999	9.14E-03	6.14E-02	1.00E+00
ENSG00000171476.20_HOPX	ENSG00000160789.18_LMNA	0.05492	-0.10520196	0.212263399	5.02E-01	7.53E-01	1.00E+00
ENSG00000118971.6_CCND2	ENSG00000160789.18_LMNA	-0.08676	-0.242605491	0.073459749	2.88E-01	5.73E-01	1.00E+00
ENSG00000137168.7_PPIL1	ENSG00000160789.18_LMNA	-0.35453	-0.4862858	-0.20701552	7.42E-06	2.30E-04	3.81E-01
ENSG00000240972.1_MIF	ENSG00000160789.18_LMNA	0.03501	-0.124882531	0.193138394	6.68E-01	8.54E-01	1.00E+00
ENSG00000137547.7_MRPL15	ENSG00000160789.18_LMNA	-0.03554	-0.193646874	0.124362498	6.64E-01	8.52E-01	1.00E+00
ENSG00000145912.7_NHP2	ENSG00000160789.18_LMNA	-0.28881	-0.428321183	-0.13586055	3.08E-04	4.60E-03	1.00E+00
ENSG00000067182.6_TNFRSF1A	ENSG00000160789.18_LMNA	0.52063	0.394088572	0.627792423	6.19E-12	2.39E-09	3.17E-07

ENSG00000179262.8_RAD23A	ENSG00000160789.18_LMNA	0.11465	-0.045378314	0.268942493	1.60E-01	4.13E-01	1.00E+00
ENSG00000154146.11_NRGN	ENSG00000160789.18_LMNA	-0.05604	-0.213335563	0.104091239	4.93E-01	7.48E-01	1.00E+00
ENSG00000164683.15_HEY1	ENSG00000160789.18_LMNA	-0.19134	-0.340181492	-0.03315259	1.82E-02	1.01E-01	1.00E+00
ENSG00000167283.6_ATP5L	ENSG00000160789.18_LMNA	-0.29176	-0.430947374	-0.1390204	2.65E-04	4.08E-03	1.00E+00
ENSG00000114850.5_SSR3	ENSG00000160789.18_LMNA	0.07052	-0.089684352	0.227173414	3.88E-01	6.66E-01	1.00E+00
ENSG00000099804.7_CDC34	ENSG00000160789.18_LMNA	-0.04426	-0.20203428	0.115759251	5.88E-01	8.09E-01	1.00E+00
ENSG00000100201.17_DDX17	ENSG00000160789.18_LMNA	-0.17094	-0.321394495	-0.01206787	3.52E-02	1.59E-01	1.00E+00
ENSG00000131100.11_ATP6V1E1	ENSG00000160789.18_LMNA	-0.1738	-0.324032137	-0.0150116	3.22E-02	1.50E-01	1.00E+00
ENSG00000170144.17_HNRNPA3	ENSG00000160789.18_LMNA	0.04951	-0.110560872	0.207079747	5.45E-01	7.83E-01	1.00E+00
ENSG00000154096.12_THY1	ENSG00000160789.18_LMNA	-0.02194	-0.180509598	0.13774243	7.88E-01	9.14E-01	1.00E+00
ENSG00000182481.7_KPNA2	ENSG00000160789.18_LMNA	0.11083	-0.049240063	0.2653483	1.74E-01	4.34E-01	1.00E+00
ENSG00000179632.8_MAF1	ENSG00000160789.18_LMNA	-0.05169	-0.209165543	0.108406774	5.27E-01	7.72E-01	1.00E+00
ENSG00000010810.16_FYN	ENSG00000160789.18_LMNA	-0.1458	-0.298081047	0.013719956	7.31E-02	2.57E-01	1.00E+00
ENSG00000131174.4_COX7B	ENSG00000160789.18_LMNA	-0.22003	-0.366400319	-0.06304213	6.45E-03	4.75E-02	1.00E+00
ENSG00000177410.11_ZFAS1	ENSG00000160789.18_LMNA	-0.13513	-0.288127583	0.024604919	9.70E-02	3.06E-01	1.00E+00
ENSG00000162517.11_PEF1	ENSG00000160789.18_LMNA	0.01686	-0.142728185	0.175585051	8.37E-01	9.37E-01	1.00E+00
ENSG00000100991.10_TRPC4AP	ENSG00000160789.18_LMNA	-0.03597	-0.194061651	0.123938168	6.60E-01	8.50E-01	1.00E+00
ENSG00000131473.15_ACLY	ENSG00000160789.18_LMNA	0.13915	-0.020509443	0.291880529	8.73E-02	2.87E-01	1.00E+00
ENSG00000071127.15_WDR1	ENSG00000160789.18_LMNA	0.53668	0.41274912	0.64110839	1.03E-12	5.54E-10	5.27E-08
ENSG00000163344.5_PMVK	ENSG00000160789.18_LMNA	0.00955	-0.149880165	0.168492946	9.07E-01	9.64E-01	1.00E+00
ENSG00000105202.6_FBL	ENSG00000160789.18_LMNA	0.15415	-0.005173273	0.305849099	5.79E-02	2.20E-01	1.00E+00
ENSG00000104969.8_SGTA	ENSG00000160789.18_LMNA	-0.1464	-0.298637639	0.013109086	7.19E-02	2.54E-01	1.00E+00
ENSG00000172336.4_POP7	ENSG00000160789.18_LMNA	-0.00957	-0.168509986	0.149863021	9.07E-01	9.64E-01	1.00E+00
ENSG00000112531.15_QKI	ENSG00000160789.18_LMNA	-0.21406	-0.360958788	-0.05679369	8.10E-03	5.61E-02	1.00E+00
ENSG00000153187.15_HNRNPU	ENSG00000160789.18_LMNA	0.14941	-0.010031561	0.301438491	6.62E-02	2.40E-01	1.00E+00
ENSG00000124107.5_SLPI	ENSG00000160789.18_LMNA	0.03044	-0.129391837	0.188722088	7.10E-01	8.77E-01	1.00E+00
ENSG00000104853.14_CLPTM1	ENSG00000160789.18_LMNA	0.23358	0.077250244	0.378696874	3.78E-03	3.18E-02	1.00E+00
ENSG00000204227.4_RING1	ENSG00000160789.18_LMNA	0.03548	-0.124422044	0.19358866	6.64E-01	8.52E-01	1.00E+00
ENSG00000172115.7_CYCS	ENSG00000160789.18_LMNA	-0.08668	-0.242529188	0.073540385	2.88E-01	5.74E-01	1.00E+00
ENSG00000166508.16_MCM7	ENSG00000160789.18_LMNA	0.0834	-0.076817666	0.239424672	3.07E-01	5.93E-01	1.00E+00
ENSG00000099956.16_SMARCB1	ENSG00000160789.18_LMNA	-0.18989	-0.338846877	-0.03164568	1.91E-02	1.04E-01	1.00E+00
ENSG00000125304.8_TM9SF2	ENSG00000160789.18_LMNA	0.00437	-0.154933716	0.163461749	9.57E-01	9.83E-01	1.00E+00
ENSG00000146733.12_PSPH	ENSG00000160789.18_LMNA	0.00277	-0.156503079	0.16189595	9.73E-01	9.89E-01	1.00E+00

ENSG00000143545.7_RAB13	ENSG00000160789.18_LMNA	0.54947	0.427681674	0.651665974	2.29E-13	1.63E-10	1.17E-08
ENSG00000090273.12_NUDC	ENSG00000160789.18_LMNA	0.19852	0.040600453	0.346759648	1.42E-02	8.46E-02	1.00E+00
ENSG00000165264.9_NDUFB6	ENSG00000160789.18_LMNA	-0.18995	-0.338899724	-0.03170532	1.91E-02	1.04E-01	1.00E+00
ENSG00000123143.11_PKN1	ENSG00000160789.18_LMNA	0.267	0.112583516	0.408821864	8.84E-04	1.05E-02	1.00E+00
ENSG00000133110.13_POSTN	ENSG00000160789.18_LMNA	0.32248	0.172117305	0.458160678	5.08E-05	1.09E-03	1.00E+00
ENSG00000197858.9_GPAA1	ENSG00000160789.18_LMNA	0.37757	0.23233623	0.506336312	1.62E-06	6.82E-05	8.33E-02
ENSG00000136450.11_SRSF1	ENSG00000160789.18_LMNA	0.05253	-0.107571625	0.209973434	5.20E-01	7.66E-01	1.00E+00
ENSG00000196531.9_NACA	ENSG00000160789.18_LMNA	-0.22179	-0.367996414	-0.06487943	6.03E-03	4.52E-02	1.00E+00
ENSG00000204463.11_BAG6	ENSG00000160789.18_LMNA	0.11773	-0.042262374	0.271836181	1.49E-01	3.95E-01	1.00E+00
ENSG00000104112.7_SCG3	ENSG00000160789.18_LMNA	-0.40611	-0.53098298	-0.26397848	2.09E-07	1.32E-05	1.07E-02
ENSG00000158604.13_TMED4	ENSG00000160789.18_LMNA	0.20279	0.045039774	0.350666305	1.22E-02	7.58E-02	1.00E+00
ENSG00000143119.11_CD53	ENSG00000160789.18_LMNA	-0.0883	-0.244071562	0.071909636	2.79E-01	5.65E-01	1.00E+00
ENSG00000177156.9_TALDO1	ENSG00000160789.18_LMNA	-0.05954	-0.216687369	0.100613784	4.66E-01	7.28E-01	1.00E+00
ENSG00000157593.15_SLC35B2	ENSG00000160789.18_LMNA	0.05675	-0.103385373	0.214016529	4.87E-01	7.44E-01	1.00E+00
ENSG00000143314.11_MRPL24	ENSG00000160789.18_LMNA	-0.00935	-0.168295577	0.150078724	9.09E-01	9.65E-01	1.00E+00
ENSG00000146535.12_GNA12	ENSG00000160789.18_LMNA	0.25856	0.103626952	0.401246135	1.30E-03	1.41E-02	1.00E+00
ENSG00000241553.11_ARPC4	ENSG00000160789.18_LMNA	-0.07782	-0.234115878	0.082406103	3.41E-01	6.26E-01	1.00E+00
ENSG00000116030.15_SUMO1	ENSG00000160789.18_LMNA	-0.28746	-0.427114953	-0.1344112	3.30E-04	4.85E-03	1.00E+00
ENSG00000164687.9_FABP5	ENSG00000160789.18_LMNA	0.12451	-0.035394579	0.278194122	1.26E-01	3.59E-01	1.00E+00
ENSG00000161057.9_PSMC2	ENSG00000160789.18_LMNA	0.02989	-0.129931417	0.188192773	7.15E-01	8.79E-01	1.00E+00
ENSG00000183617.4_MRPL54	ENSG00000160789.18_LMNA	-0.20676	-0.35429727	-0.04917666	1.06E-02	6.83E-02	1.00E+00
ENSG00000132481.5_TRIM47	ENSG00000160789.18_LMNA	0.2805	0.126968398	0.420904181	4.65E-04	6.36E-03	1.00E+00
ENSG00000159200.16_RCAN1	ENSG00000160789.18_LMNA	0.29526	0.142765426	0.434053526	2.22E-04	3.54E-03	1.00E+00
ENSG00000125968.8_ID1	ENSG00000160789.18_LMNA	-0.00783	-0.16681847	0.151563929	9.24E-01	9.71E-01	1.00E+00
ENSG00000136160.13_EDNRB	ENSG00000160789.18_LMNA	-0.30114	-0.439280991	-0.14908696	1.63E-04	2.78E-03	1.00E+00
ENSG00000168066.19_SF1	ENSG00000160789.18_LMNA	0.04057	-0.119406166	0.198484544	6.20E-01	8.28E-01	1.00E+00
ENSG00000134809.7_TIMM10	ENSG00000160789.18_LMNA	-0.14509	-0.297423055	0.014441815	7.45E-02	2.60E-01	1.00E+00
ENSG00000197111.14_PCBP2	ENSG00000160789.18_LMNA	-0.15876	-0.310126501	0.000447646	5.07E-02	2.03E-01	1.00E+00
ENSG00000187840.4_EIF4EBP1	ENSG00000160789.18_LMNA	0.16538	0.006349052	0.316256565	4.17E-02	1.78E-01	1.00E+00
ENSG00000187837.3_HIST1H1C	ENSG00000160789.18_LMNA	-0.01907	-0.177728949	0.140559624	8.16E-01	9.28E-01	1.00E+00
ENSG00000165802.18_NSMF	ENSG00000160789.18_LMNA	-0.00127	-0.160438836	0.157962067	9.88E-01	9.95E-01	1.00E+00
ENSG00000148229.11_POLE3	ENSG00000160789.18_LMNA	-0.21262	-0.359648	-0.05529209	8.54E-03	5.85E-02	1.00E+00
ENSG00000213246.5_SUPT4H1	ENSG00000160789.18_LMNA	-0.3672	-0.497325575	-0.22091092	3.27E-06	1.18E-04	1.68E-01

ENSG00000125841.11_NRSN2	ENSG00000160789.18_LMNA	0.31965	0.16904977	0.455660802	5.97E-05	1.23E-03	1.00E+00
ENSG00000126254.10_RBM42	ENSG00000160789.18_LMNA	0.22544	0.068700219	0.371309898	5.23E-03	4.06E-02	1.00E+00
ENSG00000174080.9_CTSF	ENSG00000160789.18_LMNA	-0.0594	-0.216550559	0.100755875	4.67E-01	7.29E-01	1.00E+00
ENSG00000086065.12_CHMP5	ENSG00000160789.18_LMNA	-0.23547	-0.38041061	-0.07924016	3.50E-03	3.00E-02	1.00E+00
ENSG00000182307.11_C8orf33	ENSG00000160789.18_LMNA	-0.21511	-0.361914442	-0.05788934	7.78E-03	5.44E-02	1.00E+00
ENSG00000134531.8_EMP1	ENSG00000160789.18_LMNA	0.55958	0.439530557	0.659982044	6.68E-14	6.35E-11	3.43E-09
ENSG00000127837.8_AAMP	ENSG00000160789.18_LMNA	0.06426	-0.095925171	0.221194759	4.32E-01	7.01E-01	1.00E+00
ENSG00000116251.8_RPL22	ENSG00000160789.18_LMNA	-0.11961	-0.273598877	0.040361294	1.42E-01	3.85E-01	1.00E+00
ENSG00000166848.5_TERF2IP	ENSG00000160789.18_LMNA	-0.21807	-0.364615503	-0.06099003	6.96E-03	5.02E-02	1.00E+00
ENSG00000177383.4_MAGEF1	ENSG00000160789.18_LMNA	-0.09233	-0.247891044	0.067864021	2.58E-01	5.41E-01	1.00E+00
ENSG00000185201.15_IFITM2	ENSG00000160789.18_LMNA	0.27112	0.116965868	0.412513773	7.29E-04	9.04E-03	1.00E+00
ENSG00000119013.7_NDUFB3	ENSG00000160789.18_LMNA	-0.22402	-0.370025415	-0.06721802	5.53E-03	4.22E-02	1.00E+00
ENSG00000117394.18_SLC2A1	ENSG00000160789.18_LMNA	0.23476	0.078490506	0.379765245	3.60E-03	3.06E-02	1.00E+00
ENSG00000087077.10_TRIP6	ENSG00000160789.18_LMNA	0.47914	0.34635796	0.593098364	4.25E-10	7.65E-08	2.18E-05
ENSG00000080822.15_CLDND1	ENSG00000160789.18_LMNA	-0.34112	-0.474551288	-0.19236819	1.70E-05	4.52E-04	8.74E-01
ENSG00000130332.13_LSM7	ENSG00000160789.18_LMNA	-0.1298	-0.283150846	0.030019741	1.11E-01	3.32E-01	1.00E+00
ENSG00000177885.12_GRB2	ENSG00000160789.18_LMNA	-0.05139	-0.208879767	0.108702084	5.30E-01	7.73E-01	1.00E+00
ENSG00000128185.8_DGCR6L	ENSG00000160789.18_LMNA	-0.05127	-0.208768445	0.108817105	5.30E-01	7.73E-01	1.00E+00
ENSG00000182916.7_TCEAL7	ENSG00000160789.18_LMNA	-0.34733	-0.479991154	-0.19914278	1.16E-05	3.30E-04	5.97E-01
ENSG00000007520.3_TSR3	ENSG00000160789.18_LMNA	0.15482	-0.004492077	0.30646646	5.68E-02	2.18E-01	1.00E+00
ENSG00000144749.12_LRIG1	ENSG00000160789.18_LMNA	-0.09734	-0.252625142	0.062835186	2.33E-01	5.11E-01	1.00E+00
ENSG00000164258.10_NDUFS4	ENSG00000160789.18_LMNA	-0.26238	-0.404680626	-0.10768148	1.09E-03	1.24E-02	1.00E+00
ENSG00000178988.10_MRFAP1L	ENSG00000160789.18_LMNA	-0.35213	-0.484190166	-0.20439048	8.63E-06	2.59E-04	4.43E-01
ENSG00000083444.15_PLOD1	ENSG00000160789.18_LMNA	0.5116	0.383645347	0.62028007	1.63E-11	5.10E-09	8.37E-07
ENSG00000128739.19_SNRPN	ENSG00000160789.18_LMNA	-0.42883	-0.55045253	-0.2893872	3.55E-08	3.12E-06	1.82E-03
ENSG00000179271.2_GADD45GI	ENSG00000160789.18_LMNA	0.03252	-0.127336984	0.190736159	6.91E-01	8.67E-01	1.00E+00
ENSG00000121900.17_TMEM54	ENSG00000160789.18_LMNA	0.1244	-0.035507938	0.278089401	1.27E-01	3.60E-01	1.00E+00
ENSG00000114631.10_PODXL2	ENSG00000160789.18_LMNA	-0.32619	-0.461429286	-0.17613652	4.11E-05	9.20E-04	1.00E+00
ENSG00000188986.4_NELFB	ENSG00000160789.18_LMNA	0.26033	0.105504101	0.402837264	1.20E-03	1.33E-02	1.00E+00
ENSG00000122176.10_FMOD	ENSG00000160789.18_LMNA	0.16656	0.007554638	0.317341208	4.03E-02	1.74E-01	1.00E+00
ENSG00000118564.13_FBXL5	ENSG00000160789.18_LMNA	-0.33441	-0.468659042	-0.18506044	2.55E-05	6.26E-04	1.00E+00
ENSG00000139266.5_MARCH9	ENSG00000160789.18_LMNA	-0.28588	-0.425706539	-0.1327205	3.57E-04	5.15E-03	1.00E+00
ENSG00000221869.4_CEBPD	ENSG00000160789.18_LMNA	0.41156	0.270049048	0.535660352	1.38E-07	9.45E-06	7.10E-03

ENSG00000131116.10_ZNF428	ENSG00000160789.18_LMNA	-0.06298	-0.219972454	0.097198012	4.41E-01	7.09E-01	1.00E+00
ENSG00000105220.13_GPI	ENSG00000160789.18_LMNA	0.29186	0.139121569	0.431031376	2.64E-04	4.06E-03	1.00E+00
ENSG00000103187.7_COTL1	ENSG00000160789.18_LMNA	0.08114	-0.079083647	0.237274356	3.20E-01	6.07E-01	1.00E+00
ENSG00000218175.2_AC016739.2	ENSG00000160789.18_LMNA	0.03783	-0.122101353	0.195855804	6.44E-01	8.41E-01	1.00E+00
ENSG00000177105.9_RHOG	ENSG00000160789.18_LMNA	0.12767	-0.032180859	0.281159852	1.17E-01	3.42E-01	1.00E+00
ENSG00000004779.8_NDUFAB1	ENSG00000160789.18_LMNA	-0.26695	-0.408780729	-0.11253475	8.85E-04	1.05E-02	1.00E+00
ENSG00000187601.4_MAGEH1	ENSG00000160789.18_LMNA	-0.36498	-0.495397269	-0.21847571	3.78E-06	1.32E-04	1.94E-01
ENSG00000137710.13_RDX	ENSG00000160789.18_LMNA	-0.06804	-0.224802589	0.092162118	4.05E-01	6.81E-01	1.00E+00
ENSG00000086232.11_EIF2AK1	ENSG00000160789.18_LMNA	0.04056	-0.119407028	0.198483703	6.20E-01	8.28E-01	1.00E+00
ENSG00000170558.7_CDH2	ENSG00000160789.18_LMNA	0.34268	0.194065022	0.475915842	1.55E-05	4.20E-04	7.95E-01
ENSG00000159176.12_CSRP1	ENSG00000160789.18_LMNA	-0.02015	-0.178780763	0.139494592	8.05E-01	9.22E-01	1.00E+00
ENSG00000227081.5_RP11-543P	ENSG00000160789.18_LMNA	-0.08133	-0.237458246	0.078889994	3.19E-01	6.06E-01	1.00E+00
ENSG00000185885.14_IFITM1	ENSG00000160789.18_LMNA	0.13679	-0.022907911	0.289683826	9.29E-02	2.98E-01	1.00E+00
ENSG00000160932.9_LY6E	ENSG00000160789.18_LMNA	0.14185	-0.017751233	0.294402643	8.13E-02	2.75E-01	1.00E+00
ENSG00000122203.13_KIAA1191	ENSG00000160789.18_LMNA	-0.1719	-0.322285105	-0.01306123	3.42E-02	1.56E-01	1.00E+00
ENSG00000105355.7_PLIN3	ENSG00000160789.18_LMNA	0.39501	0.251630921	0.521418712	4.75E-07	2.52E-05	2.44E-02
ENSG00000184216.10_IRAK1	ENSG00000160789.18_LMNA	0.32797	0.178064468	0.462994438	3.71E-05	8.48E-04	1.00E+00
ENSG00000114956.18_DGUOK	ENSG00000160789.18_LMNA	-0.252	-0.395339116	-0.09667659	1.74E-03	1.76E-02	1.00E+00
ENSG00000234664.1_HMGN2P5	ENSG00000160789.18_LMNA	-0.08258	-0.23864399	0.077640718	3.12E-01	5.98E-01	1.00E+00
ENSG00000068903.18_SIRT2	ENSG00000160789.18_LMNA	-0.48954	-0.601832873	-0.35825539	1.55E-10	3.26E-08	7.96E-06
ENSG00000198053.10_SIRPA	ENSG00000160789.18_LMNA	0.07411	-0.086109903	0.230586983	3.64E-01	6.46E-01	1.00E+00
ENSG00000182578.12_CSF1R	ENSG00000160789.18_LMNA	0.02713	-0.132646968	0.185526074	7.40E-01	8.91E-01	1.00E+00
ENSG00000116752.5_BCAS2	ENSG00000160789.18_LMNA	-0.25205	-0.395385964	-0.09673159	1.73E-03	1.75E-02	1.00E+00
ENSG00000121766.13_ZCCHC17	ENSG00000160789.18_LMNA	-0.36096	-0.49189716	-0.2140643	4.91E-06	1.64E-04	2.52E-01
ENSG00000124422.10_USP22	ENSG00000160789.18_LMNA	-0.13026	-0.283580853	0.029552606	1.10E-01	3.30E-01	1.00E+00
ENSG00000099624.6_ATP5D	ENSG00000160789.18_LMNA	0.02749	-0.132291698	0.185875219	7.37E-01	8.89E-01	1.00E+00
ENSG00000146425.9_DYNLT1	ENSG00000160789.18_LMNA	-0.18509	-0.334434495	-0.02667357	2.24E-02	1.17E-01	1.00E+00
ENSG00000160710.14_ADAR	ENSG00000160789.18_LMNA	0.12887	-0.03096186	0.282283222	1.14E-01	3.37E-01	1.00E+00
ENSG00000122034.11_GTF3A	ENSG00000160789.18_LMNA	-0.22217	-0.368339052	-0.06527411	5.94E-03	4.47E-02	1.00E+00
ENSG00000113811.9_SELENOK	ENSG00000160789.18_LMNA	-0.30373	-0.441577751	-0.15187194	1.42E-04	2.50E-03	1.00E+00
ENSG00000197043.12_ANXA6	ENSG00000160789.18_LMNA	-0.25418	-0.397301672	-0.09898256	1.58E-03	1.63E-02	1.00E+00
ENSG00000149257.12_SERPINH1	ENSG00000160789.18_LMNA	0.55185	0.430470773	0.653628345	1.72E-13	1.34E-10	8.82E-09
ENSG00000166741.6_NNMT	ENSG00000160789.18_LMNA	0.1949	0.036840914	0.343442879	1.61E-02	9.24E-02	1.00E+00

ENSG00000162736.14_NCSTN	ENSG00000160789.18_LMNA	0.40146	0.258800021	0.526980073	2.96E-07	1.75E-05	1.52E-02
ENSG00000126457.19_PRMT1	ENSG00000160789.18_LMNA	-0.01543	-0.174200947	0.144126589	8.50E-01	9.41E-01	1.00E+00
ENSG00000175348.9_TMEM9B	ENSG00000160789.18_LMNA	-0.15217	-0.304005635	0.007205638	6.13E-02	2.29E-01	1.00E+00
ENSG00000115226.8_FNDC4	ENSG00000160789.18_LMNA	0.15837	-0.000849739	0.309763035	5.13E-02	2.05E-01	1.00E+00
ENSG00000159713.9_TPPP3	ENSG00000160789.18_LMNA	-0.02339	-0.181910008	0.136321663	7.75E-01	9.07E-01	1.00E+00
ENSG00000075415.11_SLC25A3	ENSG00000160789.18_LMNA	-0.13839	-0.291174122	0.021281125	8.91E-02	2.91E-01	1.00E+00
ENSG00000047849.20_MAP4	ENSG00000160789.18_LMNA	0.12679	-0.03308339	0.28032757	1.20E-01	3.47E-01	1.00E+00
ENSG00000130811.9_EIF3G	ENSG00000160789.18_LMNA	0.07653	-0.083693116	0.232890587	3.49E-01	6.33E-01	1.00E+00
ENSG00000123384.12_LRP1	ENSG00000160789.18_LMNA	0.40056	0.257804173	0.526208925	3.16E-07	1.83E-05	1.62E-02
ENSG00000166922.7_SCG5	ENSG00000160789.18_LMNA	-0.20045	-0.348526737	-0.04260698	1.33E-02	8.03E-02	1.00E+00
ENSG00000175445.13_LPL	ENSG00000160789.18_LMNA	0.09037	-0.069832409	0.246033906	2.68E-01	5.52E-01	1.00E+00
ENSG00000106003.11_LFNG	ENSG00000160789.18_LMNA	0.11458	-0.045449502	0.268876316	1.60E-01	4.13E-01	1.00E+00
ENSG00000150459.11_SAP18	ENSG00000160789.18_LMNA	-0.25322	-0.39643929	-0.09796888	1.65E-03	1.69E-02	1.00E+00
ENSG00000088826.16_SMOX	ENSG00000160789.18_LMNA	0.262	0.107270583	0.404332948	1.11E-03	1.26E-02	1.00E+00
ENSG00000163466.14_ARPC2	ENSG00000160789.18_LMNA	0.03484	-0.125057128	0.192967637	6.70E-01	8.55E-01	1.00E+00
ENSG00000100345.19_MYH9	ENSG00000160789.18_LMNA	0.5269	0.401371349	0.633005661	3.10E-12	1.38E-09	1.59E-07
ENSG00000126088.11_UROD	ENSG00000160789.18_LMNA	0.11035	-0.049727264	0.264894233	1.76E-01	4.37E-01	1.00E+00
ENSG00000108468.13_CBX1	ENSG00000160789.18_LMNA	-0.13339	-0.286509699	0.026367236	1.01E-01	3.14E-01	1.00E+00
ENSG00000174574.14_AKIRIN1	ENSG00000160789.18_LMNA	-0.01482	-0.173610343	0.144722909	8.56E-01	9.43E-01	1.00E+00
ENSG00000124785.7_NRN1	ENSG00000160789.18_LMNA	0.07781	-0.082413078	0.23410924	3.41E-01	6.26E-01	1.00E+00
ENSG00000130520.9_LSM4	ENSG00000160789.18_LMNA	-0.17152	-0.321934108	-0.01266966	3.46E-02	1.57E-01	1.00E+00
ENSG00000165672.6_PRDX3	ENSG00000160789.18_LMNA	-0.2516	-0.394976881	-0.09625132	1.77E-03	1.78E-02	1.00E+00
ENSG00000112697.14_TMEM30A	ENSG00000160789.18_LMNA	-0.12592	-0.279515002	0.03396405	1.22E-01	3.52E-01	1.00E+00
ENSG00000135926.11_TMBIM1	ENSG00000160789.18_LMNA	0.24118	0.085249587	0.385573475	2.76E-03	2.51E-02	1.00E+00
ENSG00000107742.11_SPOCK2	ENSG00000160789.18_LMNA	0.21648	0.059318919	0.363160409	7.39E-03	5.24E-02	1.00E+00
ENSG00000100353.16_EIF3D	ENSG00000160789.18_LMNA	-0.02492	-0.18339327	0.13481541	7.61E-01	9.00E-01	1.00E+00
ENSG00000133142.16_TCEAL4	ENSG00000160789.18_LMNA	-0.02278	-0.181322153	0.136918223	7.81E-01	9.10E-01	1.00E+00
ENSG00000244398.1_RP11-466H	ENSG00000160789.18_LMNA	-0.04539	-0.203124822	0.114637131	5.79E-01	8.04E-01	1.00E+00
ENSG00000148180.15_GSN	ENSG00000160789.18_LMNA	0.23449	0.078203901	0.379518432	3.64E-03	3.09E-02	1.00E+00
ENSG00000204469.11_PRRC2A	ENSG00000160789.18_LMNA	0.17419	0.015416848	0.324394874	3.19E-02	1.49E-01	1.00E+00
ENSG00000134440.10_NARS	ENSG00000160789.18_LMNA	0.05573	-0.104401783	0.213035876	4.95E-01	7.50E-01	1.00E+00
ENSG00000100243.19_CYB5R3	ENSG00000160789.18_LMNA	0.01507	-0.144479874	0.173851076	8.54E-01	9.43E-01	1.00E+00
ENSG00000101365.19_IDH3B	ENSG00000160789.18_LMNA	0.00978	-0.149649693	0.168722003	9.05E-01	9.63E-01	1.00E+00

ENSG00000072832.13_CRMP1	ENSG00000160789.18_LMNA	-0.2589	-0.401546885	-0.1039816	1.28E-03	1.39E-02	1.00E+00
ENSG00000167470.11_MIDN	ENSG00000160789.18_LMNA	0.13033	-0.029478844	0.283648741	1.10E-01	3.29E-01	1.00E+00
ENSG00000165475.12_CRYL1	ENSG00000160789.18_LMNA	-0.33259	-0.467063251	-0.18308667	2.83E-05	6.81E-04	1.00E+00
ENSG00000175582.18_RAB6A	ENSG00000160789.18_LMNA	-0.18233	-0.331898216	-0.02382243	2.46E-02	1.25E-01	1.00E+00
ENSG00000172428.9_COPS9	ENSG00000160789.18_LMNA	-0.24952	-0.39310249	-0.09405249	1.93E-03	1.90E-02	1.00E+00
ENSG00000249992.1_TMEM158	ENSG00000160789.18_LMNA	0.58925	0.474569941	0.684262157	1.40E-15	2.76E-12	7.17E-11
ENSG00000184007.16_PTP4A2	ENSG00000160789.18_LMNA	0.25563	0.100516904	0.398606006	1.48E-03	1.55E-02	1.00E+00
ENSG00000107175.9_CREB3	ENSG00000160789.18_LMNA	0.02105	-0.138612412	0.179651445	7.97E-01	9.18E-01	1.00E+00
ENSG00000180879.12_SSR4	ENSG00000160789.18_LMNA	-0.08915	-0.24487355	0.071061027	2.75E-01	5.59E-01	1.00E+00
ENSG00000142230.10_SAE1	ENSG00000160789.18_LMNA	0.15792	-0.001312206	0.309344883	5.20E-02	2.06E-01	1.00E+00
ENSG00000141985.8_SH3GL1	ENSG00000160789.18_LMNA	0.33779	0.188737044	0.471626652	2.08E-05	5.34E-04	1.00E+00
ENSG00000111667.12_USP5	ENSG00000160789.18_LMNA	-0.11668	-0.270853024	0.043321729	1.52E-01	4.01E-01	1.00E+00
ENSG00000181789.13_COPG1	ENSG00000160789.18_LMNA	0.32014	0.169583932	0.45609644	5.80E-05	1.21E-03	1.00E+00
ENSG00000225663.6_MCRIP1	ENSG00000160789.18_LMNA	-0.19784	-0.346138779	-0.03989604	1.46E-02	8.60E-02	1.00E+00
ENSG00000078808.15_SDF4	ENSG00000160789.18_LMNA	0.53179	0.407055899	0.637060292	1.79E-12	8.92E-10	9.19E-08
ENSG00000141367.10_CLTC	ENSG00000160789.18_LMNA	0.08777	-0.072441226	0.243568956	2.82E-01	5.68E-01	1.00E+00
ENSG00000145494.10_NDUFS6	ENSG00000160789.18_LMNA	0.2169	0.05976149	0.363545916	7.27E-03	5.18E-02	1.00E+00
ENSG00000184557.4_SOCS3	ENSG00000160789.18_LMNA	0.50123	0.371692022	0.61162781	4.80E-11	1.24E-08	2.46E-06
ENSG00000196338.11_NLGN3	ENSG00000160789.18_LMNA	-0.16243	-0.313525199	-0.00331711	4.56E-02	1.89E-01	1.00E+00
ENSG00000099860.7_GADD45B	ENSG00000160789.18_LMNA	0.33114	0.181506524	0.465784367	3.09E-05	7.31E-04	1.00E+00
ENSG00000120265.15_PCMT1	ENSG00000160789.18_LMNA	-0.30478	-0.442506947	-0.15299996	1.35E-04	2.39E-03	1.00E+00
ENSG00000159128.13_IFNGR2	ENSG00000160789.18_LMNA	0.32449	0.174290869	0.45992928	4.53E-05	9.93E-04	1.00E+00
ENSG00000104823.7_ECH1	ENSG00000160789.18_LMNA	-0.01905	-0.177711562	0.140577223	8.16E-01	9.28E-01	1.00E+00
ENSG00000275395.3_FCGBP	ENSG00000160789.18_LMNA	0.34129	0.192555589	0.47470206	1.69E-05	4.48E-04	8.65E-01
ENSG00000063046.16_EIF4B	ENSG00000160789.18_LMNA	-0.01064	-0.169555985	0.14881027	8.96E-01	9.60E-01	1.00E+00
ENSG00000187713.6_TMEM203	ENSG00000160789.18_LMNA	0.05221	-0.107887017	0.209668387	5.23E-01	7.68E-01	1.00E+00
ENSG00000124193.13_SRSF6	ENSG00000160789.18_LMNA	-0.13169	-0.284913908	0.028103583	1.06E-01	3.23E-01	1.00E+00
ENSG00000112081.15_SRSF3	ENSG00000160789.18_LMNA	-0.26439	-0.406480929	-0.10981075	9.97E-04	1.16E-02	1.00E+00
ENSG00000124126.12_PREX1	ENSG00000160789.18_LMNA	0.18139	0.022846071	0.331028654	2.53E-02	1.27E-01	1.00E+00
ENSG00000218426.5_RP11-475C	ENSG00000160789.18_LMNA	-0.11356	-0.267917262	0.046480823	1.64E-01	4.19E-01	1.00E+00
ENSG00000149591.15_TAGLN	ENSG00000160789.18_LMNA	0.32603	0.175961561	0.461287164	4.15E-05	9.25E-04	1.00E+00
ENSG00000100075.8_SLC25A1	ENSG00000160789.18_LMNA	0.12205	-0.037890033	0.275887101	1.34E-01	3.72E-01	1.00E+00
ENSG00000182087.11_TMEM259	ENSG00000160789.18_LMNA	0.16748	0.008504767	0.318195452	3.92E-02	1.71E-01	1.00E+00

ENSG00000132824.12_SERINC3	ENSG00000160789.18_LMNA	0.13592	-0.023791745	0.288873512	9.50E-02	3.02E-01	1.00E+00
ENSG00000088882.7_CPXM1	ENSG00000160789.18_LMNA	0.05735	-0.102790129	0.214590536	4.83E-01	7.40E-01	1.00E+00
ENSG00000115415.17_STAT1	ENSG00000160789.18_LMNA	0.04491	-0.115117863	0.202657715	5.83E-01	8.06E-01	1.00E+00
ENSG00000188257.9_PLA2G2A	ENSG00000160789.18_LMNA	0.0886	-0.071606245	0.244358335	2.78E-01	5.63E-01	1.00E+00
ENSG00000147224.9_PRPS1	ENSG00000160789.18_LMNA	-0.32949	-0.464332537	-0.17971447	3.40E-05	7.91E-04	1.00E+00
ENSG00000134108.11_ARL8B	ENSG00000160789.18_LMNA	-0.22369	-0.369721801	-0.06686787	5.60E-03	4.27E-02	1.00E+00
ENSG00000124145.6_SDC4	ENSG00000160789.18_LMNA	0.35441	0.206886712	0.486183044	7.47E-06	2.31E-04	3.83E-01
ENSG00000146072.6_TNFRSF21	ENSG00000160789.18_LMNA	0.00768	-0.151702921	0.166680163	9.25E-01	9.71E-01	1.00E+00
ENSG00000172037.12_LAMB2	ENSG00000160789.18_LMNA	0.50554	0.37665836	0.615229612	3.08E-11	8.49E-09	1.58E-06
ENSG00000275830.1_RP11-403A	ENSG00000160789.18_LMNA	0.05149	-0.108605362	0.208973373	5.29E-01	7.73E-01	1.00E+00
ENSG00000106609.15_TMEM248	ENSG00000160789.18_LMNA	0.29718	0.144836086	0.435767979	2.01E-04	3.27E-03	1.00E+00
ENSG00000116741.7_RGS2	ENSG00000160789.18_LMNA	-0.00242	-0.161557417	0.156842171	9.76E-01	9.91E-01	1.00E+00
ENSG00000079462.6_PAFAH1B3	ENSG00000160789.18_LMNA	-0.15543	-0.307029796	0.003870239	5.59E-02	2.16E-01	1.00E+00
ENSG00000119396.9_RAB14	ENSG00000160789.18_LMNA	-0.08061	-0.236772823	0.079611688	3.24E-01	6.10E-01	1.00E+00
ENSG00000116560.9_SFPQ	ENSG00000160789.18_LMNA	-0.02291	-0.181447756	0.13679078	7.79E-01	9.09E-01	1.00E+00
ENSG00000138029.12_HADHB	ENSG00000160789.18_LMNA	-0.20047	-0.348543749	-0.04262631	1.33E-02	8.03E-02	1.00E+00
ENSG00000165507.8_C10orf10	ENSG00000160789.18_LMNA	0.18848	0.030187637	0.337554372	2.00E-02	1.08E-01	1.00E+00
ENSG00000168216.9_LMBRD1	ENSG00000160789.18_LMNA	-0.15527	-0.306884463	0.004030687	5.61E-02	2.16E-01	1.00E+00
ENSG00000128564.6_VGF	ENSG00000160789.18_LMNA	0.03741	-0.122522564	0.195444564	6.47E-01	8.43E-01	1.00E+00
ENSG00000164754.11_RAD21	ENSG00000160789.18_LMNA	-0.28072	-0.421100235	-0.12720284	4.60E-04	6.32E-03	1.00E+00
ENSG00000198171.11_DDRGK1	ENSG00000160789.18_LMNA	0.42577	0.285955029	0.547839098	4.54E-08	3.77E-06	2.33E-03
ENSG00000131409.11_LRRC4B	ENSG00000160789.18_LMNA	-0.04335	-0.201161116	0.116657114	5.96E-01	8.14E-01	1.00E+00
ENSG00000143368.9_SF3B4	ENSG00000160789.18_LMNA	0.30422	0.152402777	0.4420151	1.39E-04	2.44E-03	1.00E+00
ENSG00000158526.7_TSR2	ENSG00000160789.18_LMNA	-0.05066	-0.208182999	0.109421864	5.35E-01	7.77E-01	1.00E+00
ENSG00000164919.9_COX6C	ENSG00000160789.18_LMNA	-0.20689	-0.354419955	-0.04931663	1.05E-02	6.81E-02	1.00E+00
ENSG00000120438.10_TCP1	ENSG00000160789.18_LMNA	-0.16528	-0.316166249	-0.00624871	4.19E-02	1.79E-01	1.00E+00
ENSG00000131508.14_UBE2D2	ENSG00000160789.18_LMNA	-0.28313	-0.423254117	-0.1297806	4.09E-04	5.76E-03	1.00E+00
ENSG00000082996.18_RNF13	ENSG00000160789.18_LMNA	-0.4353	-0.555967734	-0.2966523	2.09E-08	2.00E-06	1.07E-03
ENSG00000170075.8_GPR37L1	ENSG00000160789.18_LMNA	0.11295	-0.047095249	0.267345594	1.66E-01	4.23E-01	1.00E+00
ENSG00000113068.8_PFDN1	ENSG00000160789.18_LMNA	-0.27153	-0.412885431	-0.11740767	7.15E-04	8.91E-03	1.00E+00
ENSG00000186591.10_UBE2H	ENSG00000160789.18_LMNA	0.26897	0.114679281	0.41058865	8.06E-04	9.79E-03	1.00E+00
ENSG00000143977.12_SNRPG	ENSG00000160789.18_LMNA	-0.17974	-0.329509259	-0.02114148	2.67E-02	1.33E-01	1.00E+00
ENSG00000182809.9_CRIP2	ENSG00000160789.18_LMNA	0.19167	0.033491244	0.340481252	1.80E-02	1.00E-01	1.00E+00

ENSG00000169902.12_TPST1	ENSG00000160789.18_LMNA	0.18767	0.029347661	0.336809234	2.06E-02	1.10E-01	1.00E+00
ENSG00000108528.12_SLC25A11	ENSG00000160789.18_LMNA	-0.14585	-0.298124375	0.013672411	7.30E-02	2.57E-01	1.00E+00
ENSG00000022277.11_RTFDC1	ENSG00000160789.18_LMNA	-0.21372	-0.360653565	-0.05644391	8.20E-03	5.67E-02	1.00E+00
ENSG00000112759.15_SLC29A1	ENSG00000160789.18_LMNA	0.00409	-0.155209629	0.163186578	9.60E-01	9.84E-01	1.00E+00
ENSG00000173402.10_DAG1	ENSG00000160789.18_LMNA	0.23557	0.079345642	0.380501397	3.48E-03	2.99E-02	1.00E+00
ENSG00000113758.12_DBN1	ENSG00000160789.18_LMNA	-0.15554	-0.307139833	0.003748746	5.57E-02	2.15E-01	1.00E+00
ENSG00000162407.8_PLPP3	ENSG00000160789.18_LMNA	0.05616	-0.103970642	0.213451928	4.92E-01	7.47E-01	1.00E+00
ENSG00000089248.6_ERP29	ENSG00000160789.18_LMNA	0.26614	0.111669905	0.40805097	9.19E-04	1.09E-02	1.00E+00
ENSG00000135677.9_GNS	ENSG00000160789.18_LMNA	0.25764	0.102654698	0.400421317	1.35E-03	1.45E-02	1.00E+00
ENSG00000130821.14_SLC6A8	ENSG00000160789.18_LMNA	0.12031	-0.039647751	0.274259935	1.40E-01	3.81E-01	1.00E+00
ENSG00000135387.18_CAPRIN1	ENSG00000160789.18_LMNA	0.02685	-0.132919086	0.185258592	7.43E-01	8.92E-01	1.00E+00
ENSG00000137876.8_RSL24D1	ENSG00000160789.18_LMNA	-0.19065	-0.339543918	-0.03243253	1.86E-02	1.02E-01	1.00E+00
ENSG00000159346.11_ADIPOR1	ENSG00000160789.18_LMNA	0.2428	0.086962723	0.38704184	2.58E-03	2.38E-02	1.00E+00
ENSG00000112110.8_MRPL18	ENSG00000160789.18_LMNA	-0.14758	-0.299740055	0.011898476	6.96E-02	2.49E-01	1.00E+00
ENSG00000109919.8_MTCH2	ENSG00000160789.18_LMNA	-0.15837	-0.30975637	0.000857112	5.13E-02	2.05E-01	1.00E+00
ENSG00000198576.3_ARC	ENSG00000160789.18_LMNA	0.00516	-0.154162785	0.164230341	9.50E-01	9.80E-01	1.00E+00
ENSG00000140941.11_MAP1LC3	ENSG00000160789.18_LMNA	-0.23293	-0.378111131	-0.07657066	3.88E-03	3.24E-02	1.00E+00
ENSG00000111652.8_COPS7A	ENSG00000160789.18_LMNA	-0.09999	-0.255131572	0.060166209	2.20E-01	4.95E-01	1.00E+00
ENSG00000161921.13_CXCL16	ENSG00000160789.18_LMNA	0.13648	-0.023223008	0.289394991	9.36E-02	2.99E-01	1.00E+00
ENSG00000110711.8_AIP	ENSG00000160789.18_LMNA	-0.17268	-0.322997539	-0.0138563	3.34E-02	1.54E-01	1.00E+00
ENSG00000256235.1_SMIM3	ENSG00000160789.18_LMNA	0.26196	0.107235109	0.404302927	1.11E-03	1.26E-02	1.00E+00
ENSG00000075461.5_CACNG4	ENSG00000160789.18_LMNA	-0.22466	-0.3706024	-0.06788365	5.39E-03	4.15E-02	1.00E+00
ENSG00000197982.13_C1orf122	ENSG00000160789.18_LMNA	-0.0669	-0.223718843	0.093293443	4.13E-01	6.86E-01	1.00E+00
ENSG00000184640.16_SEPT9	ENSG00000160789.18_LMNA	0.33985	0.190985611	0.473438453	1.84E-05	4.81E-04	9.43E-01
ENSG00000183978.7_COA3	ENSG00000160789.18_LMNA	-0.38324	-0.511246169	-0.23859375	1.10E-06	4.95E-05	5.63E-02
ENSG00000147274.13_RBMX	ENSG00000160789.18_LMNA	-0.23051	-0.375913679	-0.07402363	4.28E-03	3.49E-02	1.00E+00
ENSG00000167863.10_ATP5H	ENSG00000160789.18_LMNA	-0.14347	-0.29591043	0.016100028	7.78E-02	2.67E-01	1.00E+00
ENSG00000135829.15_DHX9	ENSG00000160789.18_LMNA	0.0298	-0.130018981	0.188106857	7.16E-01	8.79E-01	1.00E+00
ENSG00000124102.4_PI3	ENSG00000160789.18_LMNA	0.16348	0.004398329	0.314499816	4.42E-02	1.85E-01	1.00E+00
ENSG00000170296.8_GABARAP	ENSG00000160789.18_LMNA	-0.30153	-0.439624387	-0.14950306	1.60E-04	2.74E-03	1.00E+00
ENSG00000128989.9_ARPP19	ENSG00000160789.18_LMNA	-0.05595	-0.213254278	0.104175475	4.94E-01	7.49E-01	1.00E+00
ENSG00000183726.9_TMEM50A	ENSG00000160789.18_LMNA	0.00155	-0.157690109	0.16071055	9.85E-01	9.94E-01	1.00E+00
ENSG00000099783.10_HNRNPM	ENSG00000160789.18_LMNA	0.17427	0.015494881	0.324464711	3.18E-02	1.49E-01	1.00E+00

ENSG00000164106.6_SCRG1	ENSG00000160789.18_LMNA	-0.42636	-0.548342331	-0.28661539	4.33E-08	3.61E-06	2.22E-03
ENSG00000174917.7_C19orf70	ENSG00000160789.18_LMNA	-0.0421	-0.199959039	0.117892345	6.07E-01	8.20E-01	1.00E+00
ENSG00000167792.10_NDUFV1	ENSG00000160789.18_LMNA	0.10015	-0.060008566	0.255279482	2.20E-01	4.95E-01	1.00E+00
ENSG00000204356.10_NELFE	ENSG00000160789.18_LMNA	-0.13451	-0.287553505	0.025230469	9.85E-02	3.09E-01	1.00E+00
ENSG00000170525.17_PFKFB3	ENSG00000160789.18_LMNA	0.2161	0.05893123	0.362822622	7.50E-03	5.29E-02	1.00E+00
ENSG00000113312.9_TTC1	ENSG00000160789.18_LMNA	-0.23891	-0.383523758	-0.08286116	3.03E-03	2.70E-02	1.00E+00
ENSG00000114115.8_RBP1	ENSG00000160789.18_LMNA	0.2006	0.042766833	0.348667417	1.32E-02	8.00E-02	1.00E+00
ENSG00000160781.14_PAQR6	ENSG00000160789.18_LMNA	-0.11365	-0.267998349	0.046393652	1.63E-01	4.19E-01	1.00E+00
ENSG00000110880.9_CORO1C	ENSG00000160789.18_LMNA	-0.25224	-0.395553375	-0.09692818	1.72E-03	1.74E-02	1.00E+00
ENSG00000113558.17_SKP1	ENSG00000160789.18_LMNA	-0.33625	-0.47027738	-0.18706443	2.28E-05	5.75E-04	1.00E+00
ENSG00000100216.5_TOMM22	ENSG00000160789.18_LMNA	-0.28804	-0.427635007	-0.13503592	3.20E-04	4.73E-03	1.00E+00
ENSG00000171159.4_C9orf16	ENSG00000160789.18_LMNA	-0.07858	-0.234839948	0.081645063	3.36E-01	6.22E-01	1.00E+00
ENSG00000114867.18_EIF4G1	ENSG00000160789.18_LMNA	0.34347	0.194927363	0.476608795	1.48E-05	4.03E-04	7.58E-01
ENSG00000131165.13_CHMP1A	ENSG00000160789.18_LMNA	0.20432	0.046640181	0.352072079	1.16E-02	7.27E-02	1.00E+00
ENSG00000163479.12_SSR2	ENSG00000160789.18_LMNA	0.04675	-0.113294051	0.204429071	5.67E-01	7.97E-01	1.00E+00
ENSG00000135334.8_AKIRIN2	ENSG00000160789.18_LMNA	-0.13081	-0.28409369	0.028995311	1.08E-01	3.27E-01	1.00E+00
ENSG00000138448.10_ITGAV	ENSG00000160789.18_LMNA	0.08881	-0.071400716	0.244552575	2.77E-01	5.61E-01	1.00E+00
ENSG00000119383.18_PTPA	ENSG00000160789.18_LMNA	0.34239	0.193755439	0.475666984	1.58E-05	4.23E-04	8.09E-01
ENSG00000078668.12_VDAC3	ENSG00000160789.18_LMNA	-0.30075	-0.438936026	-0.14866907	1.67E-04	2.83E-03	1.00E+00
ENSG00000234741.6_GAS5	ENSG00000160789.18_LMNA	-0.1404	-0.293046968	0.019234403	8.45E-02	2.82E-01	1.00E+00
ENSG00000197894.9_ADH5	ENSG00000160789.18_LMNA	-0.17221	-0.322567774	-0.01337664	3.39E-02	1.55E-01	1.00E+00
ENSG00000173905.7_GOLIM4	ENSG00000160789.18_LMNA	0.05289	-0.107217558	0.210315814	5.18E-01	7.64E-01	1.00E+00
ENSG00000115241.10_PPM1G	ENSG00000160789.18_LMNA	0.2783	0.124619302	0.418938201	5.17E-04	6.93E-03	1.00E+00
ENSG00000066322.11_ELOVL1	ENSG00000160789.18_LMNA	0.16321	0.004120262	0.314249227	4.45E-02	1.86E-01	1.00E+00
ENSG00000151552.10_QDPR	ENSG00000160789.18_LMNA	-0.23373	-0.378833982	-0.07740936	3.75E-03	3.16E-02	1.00E+00
ENSG00000135424.14_ITGA7	ENSG00000160789.18_LMNA	0.51432	0.386785737	0.622543689	1.22E-11	4.04E-09	6.27E-07
ENSG00000168734.12_PKIG	ENSG00000160789.18_LMNA	0.0045	-0.154812554	0.163582568	9.56E-01	9.82E-01	1.00E+00
ENSG00000198816.5_ZNF358	ENSG00000160789.18_LMNA	0.13357	-0.026192641	0.286670064	1.01E-01	3.13E-01	1.00E+00
ENSG00000162191.12_UBXN1	ENSG00000160789.18_LMNA	-0.20941	-0.356718476	-0.05194106	9.62E-03	6.38E-02	1.00E+00
ENSG00000188042.7_ARL4C	ENSG00000160789.18_LMNA	0.46507	0.330321794	0.58123278	1.58E-09	2.29E-07	8.09E-05
ENSG00000106245.8_BUD31	ENSG00000160789.18_LMNA	-0.05371	-0.211104135	0.106402021	5.11E-01	7.60E-01	1.00E+00
ENSG00000189159.14_HN1	ENSG00000160789.18_LMNA	-0.33383	-0.468148437	-0.18442864	2.63E-05	6.45E-04	1.00E+00
ENSG00000119318.11_RAD23B	ENSG00000160789.18_LMNA	0.1055	-0.054622751	0.260323916	1.96E-01	4.65E-01	1.00E+00

ENSG00000204257.13_HLA-DMA	ENSG00000160789.18_LMNA	0.02053	-0.139126683	0.179143937	8.02E-01	9.20E-01	1.00E+00
ENSG00000266412.4_NCOA4	ENSG00000160789.18_LMNA	-0.39523	-0.521612174	-0.25187982	4.67E-07	2.49E-05	2.40E-02
ENSG00000105698.14_USF2	ENSG00000160789.18_LMNA	-0.11463	-0.268927729	0.045394195	1.60E-01	4.13E-01	1.00E+00
ENSG00000095139.12_ARCN1	ENSG00000160789.18_LMNA	0.12469	-0.035205149	0.2783691	1.26E-01	3.59E-01	1.00E+00
ENSG00000124214.18_STAU1	ENSG00000160789.18_LMNA	0.24771	0.092145099	0.391474518	2.09E-03	2.02E-02	1.00E+00
ENSG00000142864.13_SERBP1	ENSG00000160789.18_LMNA	0.29796	0.145668774	0.436456828	1.93E-04	3.17E-03	1.00E+00
ENSG00000143799.11_PARP1	ENSG00000160789.18_LMNA	0.0347	-0.125188484	0.192839158	6.71E-01	8.56E-01	1.00E+00
ENSG00000178449.7_COX14	ENSG00000160789.18_LMNA	-0.2355	-0.380438851	-0.07927297	3.49E-03	3.00E-02	1.00E+00
ENSG00000106538.8_RARRES2	ENSG00000160789.18_LMNA	0.31443	0.163413192	0.451055478	7.98E-05	1.57E-03	1.00E+00
ENSG00000125651.12_GTF2F1	ENSG00000160789.18_LMNA	0.24048	0.084513428	0.38494203	2.84E-03	2.57E-02	1.00E+00
ENSG00000230202.1_RP11-632C	ENSG00000160789.18_LMNA	-0.07808	-0.234371457	0.082137517	3.39E-01	6.24E-01	1.00E+00
ENSG00000135245.9_HILPDA	ENSG00000160789.18_LMNA	0.14523	-0.014304548	0.2975482	7.42E-02	2.60E-01	1.00E+00
ENSG00000078804.11_TP53INP2	ENSG00000160789.18_LMNA	-0.01872	-0.177394003	0.140898624	8.19E-01	9.30E-01	1.00E+00
ENSG00000175416.11_CLTB	ENSG00000160789.18_LMNA	0.13378	-0.025975963	0.286869059	1.00E-01	3.12E-01	1.00E+00
ENSG00000109180.13_OCIAD1	ENSG00000160789.18_LMNA	-0.31416	-0.450814632	-0.16311893	8.10E-05	1.59E-03	1.00E+00
ENSG00000160948.12_VPS28	ENSG00000160789.18_LMNA	-0.18256	-0.332108454	-0.02405858	2.44E-02	1.24E-01	1.00E+00
ENSG00000103647.11_CORO2B	ENSG00000160789.18_LMNA	0.1721	0.013262742	0.322465707	3.40E-02	1.56E-01	1.00E+00
ENSG00000099800.6_TIMM13	ENSG00000160789.18_LMNA	-0.06325	-0.220233206	0.096926566	4.39E-01	7.07E-01	1.00E+00
ENSG00000130770.16_ATPIF1	ENSG00000160789.18_LMNA	-0.30254	-0.440526685	-0.15059688	1.52E-04	2.62E-03	1.00E+00
ENSG00000113575.8_PPP2CA	ENSG00000160789.18_LMNA	-0.21433	-0.361204825	-0.0570757	8.01E-03	5.57E-02	1.00E+00
ENSG00000181817.5_LSM10	ENSG00000160789.18_LMNA	0.01039	-0.149058451	0.16930946	8.99E-01	9.61E-01	1.00E+00
ENSG00000152558.13_TMEM123	ENSG00000160789.18_LMNA	-0.11848	-0.272536983	0.041506826	1.46E-01	3.91E-01	1.00E+00
ENSG00000196154.10_S100A4	ENSG00000160789.18_LMNA	0.26872	0.114414136	0.410365249	8.16E-04	9.86E-03	1.00E+00
ENSG00000130741.9_EIF2S3	ENSG00000160789.18_LMNA	0.16838	0.009433674	0.319030129	3.81E-02	1.68E-01	1.00E+00
ENSG00000116774.10_OLFML3	ENSG00000160789.18_LMNA	0.04089	-0.119090753	0.198791881	6.17E-01	8.26E-01	1.00E+00
ENSG00000125977.6_EIF2S2	ENSG00000160789.18_LMNA	0.07731	-0.082915026	0.233631482	3.44E-01	6.28E-01	1.00E+00
ENSG00000108424.8_KPNB1	ENSG00000160789.18_LMNA	0.18984	0.031596298	0.338803124	1.92E-02	1.05E-01	1.00E+00
ENSG00000158773.13_USF1	ENSG00000160789.18_LMNA	-0.25806	-0.400791853	-0.1030914	1.33E-03	1.43E-02	1.00E+00
ENSG00000142634.11_EFHD2	ENSG00000160789.18_LMNA	0.13526	-0.024468775	0.288252495	9.66E-02	3.05E-01	1.00E+00
ENSG00000121966.6_CXCR4	ENSG00000160789.18_LMNA	0.11437	-0.045665483	0.26867552	1.61E-01	4.15E-01	1.00E+00
ENSG00000152082.12_MZT2B	ENSG00000160789.18_LMNA	0.21622	0.059052714	0.362928477	7.46E-03	5.28E-02	1.00E+00
ENSG00000171530.12_TBCA	ENSG00000160789.18_LMNA	-0.07991	-0.236104282	0.080315288	3.28E-01	6.14E-01	1.00E+00
ENSG00000156639.10_ZFAND3	ENSG00000160789.18_LMNA	0.15036	-0.009056152	0.302325088	6.45E-02	2.36E-01	1.00E+00

ENSG00000214078.10_CPNE1	ENSG00000160789.18_LMNA	0.03734	-0.122584931	0.195383664	6.48E-01	8.43E-01	1.00E+00
ENSG00000108588.12_CCDC47	ENSG00000160789.18_LMNA	0.20481	0.047150539	0.352520081	1.14E-02	7.18E-02	1.00E+00
ENSG00000182326.13_C1S	ENSG00000160789.18_LMNA	0.32229	0.171913742	0.457994925	5.14E-05	1.10E-03	1.00E+00
ENSG00000158470.5_B4GALT5	ENSG00000160789.18_LMNA	0.36454	0.217990357	0.495012618	3.89E-06	1.35E-04	2.00E-01
ENSG00000167775.9_CD320	ENSG00000160789.18_LMNA	0.18861	0.030316546	0.337668693	2.00E-02	1.08E-01	1.00E+00
ENSG00000143653.9_SCCPDH	ENSG00000160789.18_LMNA	-0.10137	-0.256433555	0.058778009	2.14E-01	4.88E-01	1.00E+00
ENSG00000145919.9_BOD1	ENSG00000160789.18_LMNA	-0.19992	-0.348040354	-0.04205445	1.35E-02	8.14E-02	1.00E+00
ENSG00000149547.13_EI24	ENSG00000160789.18_LMNA	-0.0542	-0.211574424	0.105915293	5.07E-01	7.57E-01	1.00E+00
ENSG00000171148.12_TADA3	ENSG00000160789.18_LMNA	0.21582	0.058638711	0.362567702	7.58E-03	5.33E-02	1.00E+00
ENSG00000113578.16_FGF1	ENSG00000160789.18_LMNA	0.02084	-0.138825439	0.17944124	7.99E-01	9.19E-01	1.00E+00
ENSG00000002330.12_BAD	ENSG00000160789.18_LMNA	-0.03508	-0.193201869	0.124817623	6.68E-01	8.54E-01	1.00E+00
ENSG00000179051.12_RCC2	ENSG00000160789.18_LMNA	0.07718	-0.083038512	0.233513923	3.45E-01	6.29E-01	1.00E+00
ENSG00000113384.12_GOLPH3	ENSG00000160789.18_LMNA	0.00891	-0.15050266	0.167874099	9.13E-01	9.67E-01	1.00E+00
ENSG00000176871.7_WSB2	ENSG00000160789.18_LMNA	-0.2457	-0.389656401	-0.09001752	2.28E-03	2.16E-02	1.00E+00
ENSG00000116586.10_LAMTOR2	ENSG00000160789.18_LMNA	0.1813	0.02275709	0.330949379	2.54E-02	1.28E-01	1.00E+00
ENSG00000171314.8_PGAM1	ENSG00000160789.18_LMNA	0.01632	-0.143256986	0.175061805	8.42E-01	9.38E-01	1.00E+00
ENSG00000087245.11_MMP2	ENSG00000160789.18_LMNA	0.1466	-0.012898067	0.298829859	7.15E-02	2.53E-01	1.00E+00
ENSG00000114784.3_EIF1B	ENSG00000160789.18_LMNA	-0.38673	-0.514264811	-0.24245223	8.59E-07	4.03E-05	4.41E-02
ENSG00000124570.16_SERPINB6	ENSG00000160789.18_LMNA	0.30292	0.15100036	0.44085937	1.49E-04	2.58E-03	1.00E+00
ENSG00000177575.11_CD163	ENSG00000160789.18_LMNA	0.25586	0.100766164	0.398817785	1.46E-03	1.54E-02	1.00E+00
ENSG00000197780.8_TAF13	ENSG00000160789.18_LMNA	0.01623	-0.143336478	0.174983133	8.43E-01	9.39E-01	1.00E+00
ENSG00000104419.13_NDRG1	ENSG00000160789.18_LMNA	0.22133	0.06439643	0.367576997	6.14E-03	4.58E-02	1.00E+00
ENSG00000214022.10_REPIN1	ENSG00000160789.18_LMNA	0.07897	-0.081252162	0.235213625	3.34E-01	6.19E-01	1.00E+00
ENSG00000129596.4_CDO1	ENSG00000160789.18_LMNA	-0.24402	-0.388137911	-0.08824266	2.45E-03	2.29E-02	1.00E+00
ENSG00000168288.11_MMADHC	ENSG00000160789.18_LMNA	-0.21331	-0.360277374	-0.05601291	8.32E-03	5.73E-02	1.00E+00
ENSG00000091483.6_FH	ENSG00000160789.18_LMNA	0.06747	-0.092729487	0.22425918	4.09E-01	6.83E-01	1.00E+00
ENSG00000116521.9_SCAMP3	ENSG00000160789.18_LMNA	0.27285	0.118809534	0.414064071	6.72E-04	8.50E-03	1.00E+00
ENSG00000179431.6_FJX1	ENSG00000160789.18_LMNA	0.24644	0.090805613	0.390330136	2.21E-03	2.11E-02	1.00E+00
ENSG00000126351.11_THRA	ENSG00000160789.18_LMNA	-0.4068	-0.531575557	-0.2647464	1.99E-07	1.26E-05	1.02E-02
ENSG00000096092.5_TMEM14A	ENSG00000160789.18_LMNA	-0.23775	-0.382477112	-0.08164288	3.18E-03	2.79E-02	1.00E+00
ENSG00000119801.11_YPEL5	ENSG00000160789.18_LMNA	-0.42322	-0.545659595	-0.28309786	5.56E-08	4.46E-06	2.85E-03
ENSG00000101400.5_SNTA1	ENSG00000160789.18_LMNA	0.10666	-0.053445621	0.261424148	1.91E-01	4.59E-01	1.00E+00
ENSG00000168439.15_STIP1	ENSG00000160789.18_LMNA	0.18057	0.022002351	0.3302768	2.60E-02	1.30E-01	1.00E+00

ENSG00000204568.10_MRPS18B	ENSG00000160789.18_LMNA	-0.15373	-0.305453722	0.005609381	5.86E-02	2.22E-01	1.00E+00
ENSG00000105258.7_POLR2I	ENSG00000160789.18_LMNA	-0.10157	-0.256622277	0.058576688	2.13E-01	4.87E-01	1.00E+00
ENSG00000117758.12_STX12	ENSG00000160789.18_LMNA	-0.34342	-0.476561635	-0.19486866	1.48E-05	4.03E-04	7.60E-01
ENSG00000100997.17_ABHD12	ENSG00000160789.18_LMNA	0.11803	-0.041958705	0.272117887	1.48E-01	3.94E-01	1.00E+00
ENSG00000136802.10_LRRC8A	ENSG00000160789.18_LMNA	0.08036	-0.079857467	0.236539325	3.25E-01	6.12E-01	1.00E+00
ENSG00000105520.9_PLPPR2	ENSG00000160789.18_LMNA	0.0798	-0.080422795	0.236002106	3.28E-01	6.15E-01	1.00E+00
ENSG00000105953.13_OGDH	ENSG00000160789.18_LMNA	0.28704	0.133963272	0.426741949	3.37E-04	4.90E-03	1.00E+00
ENSG00000163430.8_FSTL1	ENSG00000160789.18_LMNA	0.33235	0.182827207	0.466853336	2.87E-05	6.88E-04	1.00E+00
ENSG00000167085.10_PHB	ENSG00000160789.18_LMNA	-0.09215	-0.247717828	0.068047717	2.59E-01	5.42E-01	1.00E+00
ENSG00000135441.6_BLOC1S1	ENSG00000160789.18_LMNA	-0.18134	-0.330985887	-0.02279807	2.54E-02	1.28E-01	1.00E+00
ENSG00000170515.12_PA2G4	ENSG00000160789.18_LMNA	-0.03664	-0.194706661	0.123278071	6.54E-01	8.47E-01	1.00E+00
ENSG00000231925.10_TAPBP	ENSG00000160789.18_LMNA	0.37524	0.229767696	0.5043158	1.90E-06	7.70E-05	9.77E-02
ENSG00000126878.11_AIF1L	ENSG00000160789.18_LMNA	-0.19568	-0.344156608	-0.03764919	1.57E-02	9.06E-02	1.00E+00
ENSG00000103202.11_NME4	ENSG00000160789.18_LMNA	0.01599	-0.143579962	0.174742134	8.45E-01	9.40E-01	1.00E+00
ENSG00000179115.9_FARSA	ENSG00000160789.18_LMNA	0.04282	-0.117175579	0.200656683	6.00E-01	8.16E-01	1.00E+00
ENSG00000079785.13_DDX1	ENSG00000160789.18_LMNA	0.01336	-0.146151509	0.172194504	8.70E-01	9.50E-01	1.00E+00
ENSG00000183011.12_NAA38	ENSG00000160789.18_LMNA	-0.09619	-0.251540399	0.06398888	2.38E-01	5.17E-01	1.00E+00
ENSG00000172992.10_DCAKD	ENSG00000160789.18_LMNA	-0.12259	-0.276399881	0.037335708	1.32E-01	3.69E-01	1.00E+00
ENSG00000128283.6_CDC42EP1	ENSG00000160789.18_LMNA	0.1983	0.040368789	0.346555489	1.43E-02	8.51E-02	1.00E+00
ENSG00000120708.15_TGFBI	ENSG00000160789.18_LMNA	0.30645	0.154802275	0.443990294	1.23E-04	2.24E-03	1.00E+00
ENSG00000213722.7_DDAH2	ENSG00000160789.18_LMNA	0.11016	-0.049913917	0.264720236	1.77E-01	4.38E-01	1.00E+00
ENSG00000178252.16_WDR6	ENSG00000160789.18_LMNA	-0.02717	-0.185572804	0.132599423	7.40E-01	8.91E-01	1.00E+00
ENSG00000112715.19_VEGFA	ENSG00000160789.18_LMNA	0.28729	0.134225505	0.426960329	3.32E-04	4.86E-03	1.00E+00
ENSG00000164091.10_WDR82	ENSG00000160789.18_LMNA	-0.09844	-0.253664718	0.061728739	2.28E-01	5.04E-01	1.00E+00
ENSG00000111276.9_CDKN1B	ENSG00000160789.18_LMNA	-0.35573	-0.487329481	-0.20832436	6.88E-06	2.17E-04	3.53E-01
ENSG00000057757.8_PITHD1	ENSG00000160789.18_LMNA	-0.17731	-0.327276907	-0.01864027	2.89E-02	1.40E-01	1.00E+00
ENSG00000099817.10_POLR2E	ENSG00000160789.18_LMNA	0.025	-0.13473733	0.183470119	7.60E-01	9.00E-01	1.00E+00
ENSG00000115649.14_CNPPD1	ENSG00000160789.18_LMNA	0.14249	-0.017101934	0.294995731	7.99E-02	2.72E-01	1.00E+00
ENSG00000169045.16_HNRNPH1	ENSG00000160789.18_LMNA	-0.02525	-0.18371165	0.134491904	7.57E-01	8.99E-01	1.00E+00
ENSG00000122642.9_FKBP9	ENSG00000160789.18_LMNA	0.56108	0.441295541	0.661216182	5.55E-14	5.49E-11	2.85E-09
ENSG00000122694.14_GLIPR2	ENSG00000160789.18_LMNA	0.03153	-0.128314306	0.189778567	7.00E-01	8.72E-01	1.00E+00
ENSG00000125652.6_ALKBH7	ENSG00000160789.18_LMNA	-0.06417	-0.221110376	0.096013076	4.32E-01	7.02E-01	1.00E+00
ENSG00000089280.17_FUS	ENSG00000160789.18_LMNA	0.1102	-0.049878018	0.264753703	1.77E-01	4.38E-01	1.00E+00

ENSG00000106991.12_ENG	ENSG00000160789.18_LMNA	0.30415	0.152326497	0.441952262	1.39E-04	2.45E-03	1.00E+00
ENSG00000063322.12_MED29	ENSG00000160789.18_LMNA	-0.03834	-0.196346379	0.121598733	6.39E-01	8.39E-01	1.00E+00
ENSG00000167978.15_SRRM2	ENSG00000160789.18_LMNA	0.08863	-0.071584539	0.24437885	2.78E-01	5.63E-01	1.00E+00
ENSG00000125656.7_CLPP	ENSG00000160789.18_LMNA	0.12058	-0.039373919	0.274513547	1.39E-01	3.80E-01	1.00E+00
ENSG00000104408.8_EIF3E	ENSG00000160789.18_LMNA	-0.19735	-0.345693207	-0.0393907	1.48E-02	8.71E-02	1.00E+00
ENSG00000093010.10_COMT	ENSG00000160789.18_LMNA	0.07639	-0.083832195	0.232758118	3.50E-01	6.33E-01	1.00E+00
ENSG00000169738.6_DCXR	ENSG00000160789.18_LMNA	-0.18006	-0.329808558	-0.02147712	2.64E-02	1.32E-01	1.00E+00
ENSG00000170275.13_CRTAP	ENSG00000160789.18_LMNA	0.19463	0.036561453	0.343196026	1.63E-02	9.31E-02	1.00E+00
ENSG00000198898.11_CAPZA2	ENSG00000160789.18_LMNA	-0.09517	-0.250578243	0.065011489	2.43E-01	5.23E-01	1.00E+00
ENSG00000176788.8_BASP1	ENSG00000160789.18_LMNA	-0.35784	-0.489175875	-0.21064231	6.01E-06	1.93E-04	3.08E-01
ENSG00000211450.8_SELENOH	ENSG00000160789.18_LMNA	-0.10894	-0.2635685	0.051148879	1.82E-01	4.46E-01	1.00E+00
ENSG00000143013.11_LMO4	ENSG00000160789.18_LMNA	0.04173	-0.11825488	0.199606053	6.10E-01	8.22E-01	1.00E+00
ENSG00000171552.11_BCL2L1	ENSG00000160789.18_LMNA	0.17718	0.018497324	0.327149218	2.90E-02	1.40E-01	1.00E+00
ENSG00000159461.13_AMFR	ENSG00000160789.18_LMNA	0.12298	-0.036945846	0.276760417	1.31E-01	3.67E-01	1.00E+00
ENSG00000161011.18_SQSTM1	ENSG00000160789.18_LMNA	0.32062	0.170100939	0.456517958	5.65E-05	1.18E-03	1.00E+00
ENSG00000077585.12_GPR137B	ENSG00000160789.18_LMNA	-0.15797	-0.309390521	0.001261738	5.19E-02	2.06E-01	1.00E+00
ENSG00000184113.9_CLDN5	ENSG00000160789.18_LMNA	-0.23495	-0.379935624	-0.07868838	3.57E-03	3.05E-02	1.00E+00
ENSG00000068912.12_ERLEC1	ENSG00000160789.18_LMNA	-0.10182	-0.256853084	0.058330438	2.12E-01	4.86E-01	1.00E+00
ENSG00000106105.12_GARS	ENSG00000160789.18_LMNA	0.28219	0.128769498	0.422409671	4.28E-04	5.98E-03	1.00E+00
ENSG00000174851.13_YIF1A	ENSG00000160789.18_LMNA	-0.00766	-0.16666037	0.151722811	9.25E-01	9.71E-01	1.00E+00
ENSG00000104915.13_STX10	ENSG00000160789.18_LMNA	0.05483	-0.105290751	0.212177657	5.02E-01	7.54E-01	1.00E+00
ENSG00000143549.18_TPM3	ENSG00000160789.18_LMNA	0.35332	0.205693571	0.485230857	8.01E-06	2.44E-04	4.11E-01
ENSG00000100227.16_POLDIP3	ENSG00000160789.18_LMNA	-0.13812	-0.290919823	0.021558831	8.97E-02	2.92E-01	1.00E+00
ENSG00000197694.12_SPTAN1	ENSG00000160789.18_LMNA	0.03759	-0.12234219	0.195620682	6.46E-01	8.43E-01	1.00E+00
ENSG00000148053.14_NTRK2	ENSG00000160789.18_LMNA	-0.3434	-0.476548029	-0.19485173	1.48E-05	4.04E-04	7.61E-01
ENSG00000182195.7_LDOC1	ENSG00000160789.18_LMNA	-0.14434	-0.296726708	0.015205398	7.60E-02	2.64E-01	1.00E+00
ENSG00000105612.7_DNASE2	ENSG00000160789.18_LMNA	0.16648	0.007472578	0.317267406	4.04E-02	1.74E-01	1.00E+00
ENSG00000013306.14_SLC25A39	ENSG00000160789.18_LMNA	-0.00042	-0.159607395	0.158793954	9.96E-01	9.98E-01	1.00E+00
ENSG00000149600.10_COMMD7	ENSG00000160789.18_LMNA	-0.25054	-0.394028973	-0.09513897	1.85E-03	1.85E-02	1.00E+00
ENSG00000102119.9_EMD	ENSG00000160789.18_LMNA	0.23847	0.0823938	0.383122334	3.09E-03	2.73E-02	1.00E+00
ENSG00000205531.11_NAP1L4	ENSG00000160789.18_LMNA	0.19011	0.031878716	0.339053352	1.90E-02	1.04E-01	1.00E+00
ENSG00000144028.13_SNRNP200	ENSG00000160789.18_LMNA	0.10591	-0.054207933	0.260711729	1.94E-01	4.63E-01	1.00E+00
ENSG00000257698.1_RP11-620J1	ENSG00000160789.18_LMNA	-0.4108	-0.53501074	-0.26920468	1.47E-07	9.86E-06	7.52E-03

ENSG00000108946.13_PRKAR1A	ENSG00000160789.18_LMNA	0.03808	-0.121863978	0.19608751	6.41E-01	8.40E-01	1.00E+00
ENSG00000123080.9_CDKN2C	ENSG00000160789.18_LMNA	0.04443	-0.115592364	0.202196521	5.87E-01	8.08E-01	1.00E+00
ENSG00000117448.12_AKR1A1	ENSG00000160789.18_LMNA	-0.06303	-0.220025892	0.097142386	4.40E-01	7.08E-01	1.00E+00
ENSG00000259976.1_MIR568	ENSG00000160789.18_LMNA	0.0308	-0.129030969	0.189075987	7.06E-01	8.75E-01	1.00E+00
ENSG00000116489.11_CAPZA1	ENSG00000160789.18_LMNA	0.11338	-0.046663545	0.267747278	1.64E-01	4.20E-01	1.00E+00
ENSG00000170035.14_UBE2E3	ENSG00000160789.18_LMNA	-0.35206	-0.484128529	-0.20431333	8.67E-06	2.60E-04	4.45E-01
ENSG00000149136.6_SSRP1	ENSG00000160789.18_LMNA	0.12369	-0.036219886	0.277431534	1.29E-01	3.64E-01	1.00E+00
ENSG00000197448.12_GSTK1	ENSG00000160789.18_LMNA	0.23221	0.075812695	0.377457553	3.99E-03	3.31E-02	1.00E+00
ENSG00000134291.10_TMEM106	ENSG00000160789.18_LMNA	0.00834	-0.151059681	0.167320129	9.19E-01	9.69E-01	1.00E+00
ENSG00000172380.5_GNG12	ENSG00000160789.18_LMNA	0.17737	0.018694105	0.327324988	2.88E-02	1.39E-01	1.00E+00
ENSG00000214194.7_LINC00998	ENSG00000160789.18_LMNA	-0.14185	-0.294402528	0.017751358	8.13E-02	2.75E-01	1.00E+00
ENSG00000117118.8_SDHB	ENSG00000160789.18_LMNA	-0.1368	-0.28968837	0.022902954	9.29E-02	2.98E-01	1.00E+00
ENSG00000116871.14_MAP7D1	ENSG00000160789.18_LMNA	0.21727	0.060153267	0.363887091	7.17E-03	5.12E-02	1.00E+00
ENSG00000204310.9_AGPAT1	ENSG00000160789.18_LMNA	0.12168	-0.038264165	0.275540907	1.35E-01	3.74E-01	1.00E+00
ENSG00000138413.12_IDH1	ENSG00000160789.18_LMNA	0.15405	-0.005280727	0.305751691	5.81E-02	2.21E-01	1.00E+00
ENSG00000055070.15_SZRD1	ENSG00000160789.18_LMNA	0.18603	0.02764448	0.335297169	2.18E-02	1.15E-01	1.00E+00
ENSG00000131669.8_NINJ1	ENSG00000160789.18_LMNA	-0.1138	-0.268138348	0.046243137	1.63E-01	4.18E-01	1.00E+00
ENSG00000049540.15_ELN	ENSG00000160789.18_LMNA	0.26055	0.10573346	0.403031552	1.19E-03	1.32E-02	1.00E+00
ENSG00000204388.6_HSPA1B	ENSG00000160789.18_LMNA	0.22077	0.063814738	0.367071714	6.27E-03	4.65E-02	1.00E+00
ENSG00000108344.13_PSMD3	ENSG00000160789.18_LMNA	-0.1206	-0.274533698	0.03935216	1.39E-01	3.80E-01	1.00E+00
ENSG00000133265.9_HSPBP1	ENSG00000160789.18_LMNA	0.12756	-0.032297215	0.281052579	1.17E-01	3.43E-01	1.00E+00
ENSG00000138801.7_PAPSS1	ENSG00000160789.18_LMNA	-0.21256	-0.359588697	-0.05522418	8.56E-03	5.85E-02	1.00E+00
ENSG00000175063.15_UBE2C	ENSG00000160789.18_LMNA	0.06687	-0.093323665	0.223689881	4.13E-01	6.86E-01	1.00E+00
ENSG00000102898.10_NUTF2	ENSG00000160789.18_LMNA	-0.23351	-0.378633398	-0.07717658	3.79E-03	3.18E-02	1.00E+00
ENSG00000127990.14_SGCE	ENSG00000160789.18_LMNA	0.01533	-0.144218199	0.17411023	8.51E-01	9.42E-01	1.00E+00
ENSG00000053371.11_AKR7A2	ENSG00000160789.18_LMNA	0.08559	-0.074624921	0.24150254	2.94E-01	5.80E-01	1.00E+00
ENSG00000144567.9_FAM134A	ENSG00000160789.18_LMNA	0.15744	-0.001808539	0.308895977	5.27E-02	2.08E-01	1.00E+00
ENSG00000158864.11_NDUFS2	ENSG00000160789.18_LMNA	0.07601	-0.084208793	0.232399358	3.52E-01	6.36E-01	1.00E+00
ENSG00000149294.15_NCAM1	ENSG00000160789.18_LMNA	-0.39264	-0.519371447	-0.24899925	5.64E-07	2.89E-05	2.89E-02
ENSG00000106537.7_TSPAN13	ENSG00000160789.18_LMNA	-0.16018	-0.311442299	-0.00100883	4.87E-02	1.98E-01	1.00E+00
ENSG00000147955.15_SIGMAR1	ENSG00000160789.18_LMNA	0.02101	-0.138654782	0.179609638	7.97E-01	9.18E-01	1.00E+00
ENSG00000159199.12_ATP5G1	ENSG00000160789.18_LMNA	-0.28978	-0.42918015	-0.13689341	2.93E-04	4.42E-03	1.00E+00
ENSG00000175573.6_C11orf68	ENSG00000160789.18_LMNA	0.04958	-0.110495079	0.207143497	5.44E-01	7.82E-01	1.00E+00

ENSG00000134243.10_SORT1	ENSG00000160789.18_LMNA	0.2003	0.042456375	0.348394175	1.34E-02	8.06E-02	1.00E+00
ENSG00000158195.9_WASF2	ENSG00000160789.18_LMNA	0.2524	0.097096705	0.395696872	1.71E-03	1.73E-02	1.00E+00
ENSG00000116209.10_TMEM59	ENSG00000160789.18_LMNA	-0.18947	-0.338460668	-0.03120987	1.94E-02	1.06E-01	1.00E+00
ENSG00000116786.10_PLEKHM2	ENSG00000160789.18_LMNA	0.24851	0.092983973	0.392190734	2.02E-03	1.97E-02	1.00E+00
ENSG00000103145.9_HCFC1R1	ENSG00000160789.18_LMNA	-0.06435	-0.221286346	0.095829755	4.31E-01	7.00E-01	1.00E+00
ENSG00000143162.7_CREG1	ENSG00000160789.18_LMNA	-0.12033	-0.274273899	0.039632675	1.40E-01	3.81E-01	1.00E+00
ENSG00000100664.9_EIF5	ENSG00000160789.18_LMNA	-0.02292	-0.181457582	0.136780809	7.79E-01	9.09E-01	1.00E+00
ENSG00000105722.8_ERF	ENSG00000160789.18_LMNA	0.25549	0.100369124	0.398480431	1.49E-03	1.56E-02	1.00E+00
ENSG00000144043.10_TEX261	ENSG00000160789.18_LMNA	0.26508	0.110544457	0.407100749	9.65E-04	1.13E-02	1.00E+00
ENSG00000132563.14_REEP2	ENSG00000160789.18_LMNA	-0.09572	-0.251094509	0.064462868	2.41E-01	5.20E-01	1.00E+00
ENSG00000065427.13_KARS	ENSG00000160789.18_LMNA	0.10463	-0.055492456	0.2595105	2.00E-01	4.70E-01	1.00E+00
ENSG00000011304.15_PTBP1	ENSG00000160789.18_LMNA	0.35043	0.202530916	0.482703687	9.60E-06	2.82E-04	4.93E-01
ENSG00000110917.6_MLEC	ENSG00000160789.18_LMNA	0.41947	0.278890271	0.542443486	7.49E-08	5.70E-06	3.84E-03
ENSG00000006712.13_PAF1	ENSG00000160789.18_LMNA	0.09054	-0.069662096	0.246194684	2.67E-01	5.51E-01	1.00E+00
ENSG00000100220.10_RTCB	ENSG00000160789.18_LMNA	-0.23103	-0.37638727	-0.07457223	4.19E-03	3.44E-02	1.00E+00
ENSG00000088930.7_XRN2	ENSG00000160789.18_LMNA	0.08457	-0.075652323	0.240529324	3.00E-01	5.87E-01	1.00E+00
ENSG00000132639.11_SNAP25	ENSG00000160789.18_LMNA	-0.21244	-0.359480971	-0.05510084	8.60E-03	5.87E-02	1.00E+00
ENSG00000132823.9_OSER1	ENSG00000160789.18_LMNA	-0.12908	-0.282474566	0.030754135	1.13E-01	3.36E-01	1.00E+00
ENSG00000239672.6_NME1	ENSG00000160789.18_LMNA	-0.17094	-0.321392971	-0.01206617	3.52E-02	1.59E-01	1.00E+00
ENSG00000104824.15_HNRNPL	ENSG00000160789.18_LMNA	-0.15992	-0.311200243	-0.00074079	4.91E-02	1.99E-01	1.00E+00
ENSG00000145022.4_TCTA	ENSG00000160789.18_LMNA	-0.13945	-0.292163936	0.020199742	8.66E-02	2.86E-01	1.00E+00
ENSG00000089289.14_IGBP1	ENSG00000160789.18_LMNA	-0.17008	-0.320599271	-0.01118141	3.62E-02	1.62E-01	1.00E+00
ENSG00000100412.14_ACO2	ENSG00000160789.18_LMNA	-0.21667	-0.363334223	-0.05951845	7.34E-03	5.21E-02	1.00E+00
ENSG00000087191.11_PSMC5	ENSG00000160789.18_LMNA	0.16088	0.001728185	0.312091737	4.77E-02	1.95E-01	1.00E+00
ENSG00000134853.10_PDGFRA	ENSG00000160789.18_LMNA	-0.13665	-0.289553302	0.023050311	9.32E-02	2.99E-01	1.00E+00
ENSG00000121005.7_CRISPLD1	ENSG00000160789.18_LMNA	-0.14812	-0.300243252	0.011345589	6.86E-02	2.46E-01	1.00E+00
ENSG00000111642.13_CHD4	ENSG00000160789.18_LMNA	0.17009	0.011191762	0.32060856	3.62E-02	1.62E-01	1.00E+00
ENSG00000155096.12_AZIN1	ENSG00000160789.18_LMNA	-0.42983	-0.551306143	-0.29050969	3.27E-08	2.88E-06	1.68E-03
ENSG00000162729.12_IGSF8	ENSG00000160789.18_LMNA	0.01051	-0.148935638	0.169431458	8.98E-01	9.60E-01	1.00E+00
ENSG00000169230.8_PRELID1	ENSG00000160789.18_LMNA	0.10262	-0.057520951	0.257611554	2.08E-01	4.81E-01	1.00E+00
ENSG00000166482.10_MFAP4	ENSG00000160789.18_LMNA	0.13839	-0.021276263	0.291178574	8.91E-02	2.91E-01	1.00E+00
ENSG00000090104.10_RGS1	ENSG00000160789.18_LMNA	-0.01281	-0.171663027	0.146687432	8.76E-01	9.52E-01	1.00E+00
ENSG00000078018.18_MAP2	ENSG00000160789.18_LMNA	-0.36401	-0.49454817	-0.2174045	4.03E-06	1.39E-04	2.07E-01

ENSG00000130638.14_ATXN10	ENSG00000160789.18_LMNA	-0.37539	-0.504447623	-0.22993516	1.88E-06	7.66E-05	9.67E-02
ENSG00000113583.7_C5orf15	ENSG00000160789.18_LMNA	-0.09163	-0.247228153	0.068566903	2.62E-01	5.45E-01	1.00E+00
ENSG00000197622.11_CDC42SE	ENSG00000160789.18_LMNA	-0.01424	-0.173045273	0.14529323	8.62E-01	9.46E-01	1.00E+00
ENSG00000205544.3_TMEM256	ENSG00000160789.18_LMNA	-0.18087	-0.330555486	-0.02231504	2.57E-02	1.29E-01	1.00E+00
ENSG00000107372.11_ZFAND5	ENSG00000160789.18_LMNA	-0.01256	-0.171413125	0.146939362	8.78E-01	9.53E-01	1.00E+00
ENSG00000197903.7_HIST1H2Bk	ENSG00000160789.18_LMNA	0.08286	-0.077365954	0.238904655	3.10E-01	5.97E-01	1.00E+00
ENSG00000171222.9_SCAND1	ENSG00000160789.18_LMNA	0.0742	-0.086018785	0.230673898	3.64E-01	6.45E-01	1.00E+00
ENSG00000138279.14_ANXA7	ENSG00000160789.18_LMNA	-0.19061	-0.339512245	-0.03239677	1.87E-02	1.03E-01	1.00E+00
ENSG00000159069.12_FBXW5	ENSG00000160789.18_LMNA	0.43097	0.291786475	0.552276432	2.98E-08	2.68E-06	1.53E-03
ENSG00000092621.10_PHGDH	ENSG00000160789.18_LMNA	-0.22006	-0.366420652	-0.06306553	6.45E-03	4.75E-02	1.00E+00
ENSG00000116863.10_ADPRHL2	ENSG00000160789.18_LMNA	0.17573	0.017005137	0.325815664	3.03E-02	1.44E-01	1.00E+00
ENSG00000157514.15_TSC22D3	ENSG00000160789.18_LMNA	-0.14392	-0.29632778	0.015642681	7.69E-02	2.66E-01	1.00E+00
ENSG00000162368.12_CMPK1	ENSG00000160789.18_LMNA	0.23076	0.074293356	0.376146545	4.23E-03	3.46E-02	1.00E+00
ENSG00000138107.10_ACTR1A	ENSG00000160789.18_LMNA	-0.32132	-0.457133424	-0.17085612	5.43E-05	1.15E-03	1.00E+00
ENSG00000141552.16_ANAPC11	ENSG00000160789.18_LMNA	-0.11196	-0.266410027	0.048100278	1.70E-01	4.28E-01	1.00E+00
ENSG00000232533.1_AC093673.5	ENSG00000160789.18_LMNA	0.43046	0.291216788	0.551843588	3.11E-08	2.77E-06	1.59E-03
ENSG00000148290.8_SURF1	ENSG00000160789.18_LMNA	-0.11121	-0.265710731	0.048851079	1.73E-01	4.32E-01	1.00E+00
ENSG00000101843.17_PSMD10	ENSG00000160789.18_LMNA	-0.3643	-0.494801071	-0.21772348	3.95E-06	1.37E-04	2.03E-01
ENSG00000061794.11_MRPS35	ENSG00000160789.18_LMNA	-0.26645	-0.408332266	-0.11200322	9.06E-04	1.07E-02	1.00E+00
ENSG00000265354.3_TIMM23	ENSG00000160789.18_LMNA	-0.22915	-0.374682618	-0.07259845	4.52E-03	3.63E-02	1.00E+00
ENSG00000155380.10_SLC16A1	ENSG00000160789.18_LMNA	0.12869	-0.031147058	0.282112609	1.14E-01	3.37E-01	1.00E+00
ENSG00000014641.16_MDH1	ENSG00000160789.18_LMNA	-0.30128	-0.439405713	-0.14923808	1.62E-04	2.77E-03	1.00E+00
ENSG00000183207.11_RUVBL2	ENSG00000160789.18_LMNA	0.13988	-0.019761234	0.292565122	8.57E-02	2.84E-01	1.00E+00
ENSG00000132591.10_ERAL1	ENSG00000160789.18_LMNA	-0.26932	-0.4108995	-0.11504828	7.93E-04	9.67E-03	1.00E+00
ENSG00000166780.9_C16orf45	ENSG00000160789.18_LMNA	-0.14706	-0.299256631	0.012429457	7.06E-02	2.51E-01	1.00E+00
ENSG00000115866.9_DARS	ENSG00000160789.18_LMNA	-0.08536	-0.2412817	0.074858116	2.96E-01	5.82E-01	1.00E+00
ENSG00000091542.8_ALKBH5	ENSG00000160789.18_LMNA	0.37719	0.231912445	0.506003153	1.67E-06	6.95E-05	8.56E-02
ENSG00000129055.11_ANAPC13	ENSG00000160789.18_LMNA	-0.37788	-0.506601199	-0.23267325	1.59E-06	6.70E-05	8.16E-02
ENSG00000135535.13_CD164	ENSG00000160789.18_LMNA	0.17388	0.015093883	0.324105798	3.22E-02	1.50E-01	1.00E+00
ENSG00000115677.15_HDLBP	ENSG00000160789.18_LMNA	0.37276	0.227034656	0.502162575	2.25E-06	8.75E-05	1.16E-01
ENSG00000174780.14_SRP72	ENSG00000160789.18_LMNA	-0.15548	-0.30707866	0.003816289	5.58E-02	2.16E-01	1.00E+00
ENSG00000215301.8_DDX3X	ENSG00000160789.18_LMNA	0.19585	0.037831454	0.344317504	1.56E-02	9.02E-02	1.00E+00
ENSG00000114686.7_MRPL3	ENSG00000160789.18_LMNA	-0.10935	-0.26395334	0.050736337	1.80E-01	4.43E-01	1.00E+00

ENSG00000124766.5_SOX4	ENSG00000160789.18_LMNA	-0.21864	-0.365128355	-0.06157942	6.81E-03	4.93E-02	1.00E+00
ENSG00000139289.12_PHLDA1	ENSG00000160789.18_LMNA	0.03807	-0.121872017	0.196079664	6.41E-01	8.40E-01	1.00E+00
ENSG00000120727.11_PAIP2	ENSG00000160789.18_LMNA	-0.40989	-0.534229433	-0.26818967	1.57E-07	1.04E-05	8.06E-03
ENSG00000178695.5_KCTD12	ENSG00000160789.18_LMNA	0.10349	-0.056641898	0.258434766	2.05E-01	4.76E-01	1.00E+00
ENSG00000073921.16_PICALM	ENSG00000160789.18_LMNA	0.02924	-0.130570204	0.187565899	7.21E-01	8.81E-01	1.00E+00
ENSG00000213190.3_MLLT11	ENSG00000160789.18_LMNA	-0.297	-0.43560342	-0.14463723	2.03E-04	3.30E-03	1.00E+00
ENSG00000004776.10_HSPB6	ENSG00000160789.18_LMNA	0.37157	0.22572346	0.501128337	2.44E-06	9.29E-05	1.25E-01
ENSG00000174238.13_PITPNA	ENSG00000160789.18_LMNA	-0.04527	-0.203008587	0.114756771	5.80E-01	8.04E-01	1.00E+00
ENSG00000183172.8_SMDT1	ENSG00000160789.18_LMNA	-0.43968	-0.55970157	-0.30158794	1.45E-08	1.47E-06	7.44E-04
ENSG00000180354.14_MTURN	ENSG00000160789.18_LMNA	-0.40252	-0.527895833	-0.25998336	2.74E-07	1.64E-05	1.40E-02
ENSG00000184752.11_NDUFA12	ENSG00000160789.18_LMNA	-0.26184	-0.404188554	-0.10709996	1.12E-03	1.26E-02	1.00E+00
ENSG00000137076.17_TLN1	ENSG00000160789.18_LMNA	0.39839	0.255383469	0.524332577	3.71E-07	2.08E-05	1.90E-02
ENSG00000168101.13_NUDT16L1	ENSG00000160789.18_LMNA	-0.27041	-0.411881005	-0.11621395	7.54E-04	9.28E-03	1.00E+00
ENSG00000147669.9_POLR2K	ENSG00000160789.18_LMNA	-0.27156	-0.412909828	-0.11743668	7.14E-04	8.90E-03	1.00E+00
ENSG00000125818.16_PSMF1	ENSG00000160789.18_LMNA	0.07399	-0.086224741	0.230477435	3.65E-01	6.46E-01	1.00E+00
ENSG00000122042.9_UBL3	ENSG00000160789.18_LMNA	-0.20334	-0.351171984	-0.04561528	1.20E-02	7.47E-02	1.00E+00
ENSG00000213741.7_RPS29	ENSG00000160789.18_LMNA	-0.17973	-0.329506805	-0.02113873	2.67E-02	1.33E-01	1.00E+00
ENSG00000182103.4_FAM181B	ENSG00000160789.18_LMNA	-0.28231	-0.42251842	-0.12889968	4.26E-04	5.95E-03	1.00E+00
ENSG00000102241.10_HTATSF1	ENSG00000160789.18_LMNA	0.0183	-0.141313713	0.176983778	8.23E-01	9.31E-01	1.00E+00
ENSG00000135363.10_LMO2	ENSG00000160789.18_LMNA	0.24902	0.093530464	0.392657124	1.98E-03	1.93E-02	1.00E+00
ENSG00000010404.16_IDS	ENSG00000160789.18_LMNA	0.15793	-0.001301308	0.309354738	5.20E-02	2.06E-01	1.00E+00
ENSG00000126261.11_UBA2	ENSG00000160789.18_LMNA	-0.1574	-0.308863078	0.001844907	5.28E-02	2.08E-01	1.00E+00
ENSG00000161642.16_ZNF385A	ENSG00000160789.18_LMNA	-0.09604	-0.251395299	0.064143139	2.39E-01	5.18E-01	1.00E+00
ENSG00000180573.9_HIST1H2AC	ENSG00000160789.18_LMNA	-0.1544	-0.306080111	0.004918408	5.75E-02	2.20E-01	1.00E+00
ENSG00000127483.16_HP1BP3	ENSG00000160789.18_LMNA	-0.07901	-0.235253441	0.081210292	3.33E-01	6.19E-01	1.00E+00
ENSG00000133134.10_BEX2	ENSG00000160789.18_LMNA	-0.50104	-0.611468175	-0.37147223	4.89E-11	1.25E-08	2.51E-06
ENSG00000196976.6_LAGE3	ENSG00000160789.18_LMNA	-0.00263	-0.161765823	0.15663343	9.74E-01	9.90E-01	1.00E+00
ENSG00000143420.16_ENSA	ENSG00000160789.18_LMNA	-0.05031	-0.207845869	0.10977001	5.38E-01	7.78E-01	1.00E+00
ENSG00000119632.3_IFI27L2	ENSG00000160789.18_LMNA	0.0035	-0.155790568	0.162607041	9.66E-01	9.87E-01	1.00E+00
ENSG00000185475.7_TMEM179B	ENSG00000160789.18_LMNA	0.00065	-0.158562295	0.159838976	9.94E-01	9.97E-01	1.00E+00
ENSG00000119333.10_WDR34	ENSG00000160789.18_LMNA	0.34019	0.191351954	0.47373341	1.80E-05	4.73E-04	9.24E-01
ENSG00000168394.10_TAP1	ENSG00000160789.18_LMNA	0.26267	0.107985006	0.4049374	1.08E-03	1.23E-02	1.00E+00
ENSG00000102103.14_PQBP1	ENSG00000160789.18_LMNA	-0.21817	-0.364706416	-0.06109449	6.93E-03	5.01E-02	1.00E+00

ENSG00000140740.9_UQCRC2	ENSG00000160789.18_LMNA	-0.39705	-0.523178729	-0.25389655	4.09E-07	2.24E-05	2.10E-02
ENSG00000115073.7_ACTR1B	ENSG00000160789.18_LMNA	0.01854	-0.141081941	0.177212848	8.21E-01	9.30E-01	1.00E+00
ENSG00000169689.13_CENPX	ENSG00000160789.18_LMNA	-0.12569	-0.279304791	0.034191797	1.23E-01	3.53E-01	1.00E+00
ENSG00000076604.13_TRAF4	ENSG00000160789.18_LMNA	-0.16285	-0.313914446	-0.00374885	4.50E-02	1.88E-01	1.00E+00
ENSG00000168615.10_ADAM9	ENSG00000160789.18_LMNA	0.45474	0.318601515	0.572492618	3.98E-09	4.93E-07	2.04E-04
ENSG00000104960.14_PTOV1	ENSG00000160789.18_LMNA	-0.13491	-0.287928121	0.024822293	9.75E-02	3.07E-01	1.00E+00
ENSG00000011028.12_MRC2	ENSG00000160789.18_LMNA	0.62361	0.515594879	0.712111084	9.40E-18	3.01E-14	4.82E-13
ENSG00000105605.6_CACNG7	ENSG00000160789.18_LMNA	-0.23667	-0.381499884	-0.08050622	3.33E-03	2.89E-02	1.00E+00
ENSG00000143772.8_ITPKB	ENSG00000160789.18_LMNA	0.18662	0.028260839	0.335844549	2.13E-02	1.13E-01	1.00E+00
ENSG00000128050.7_PAICS	ENSG00000160789.18_LMNA	0.12061	-0.039344371	0.274540911	1.39E-01	3.80E-01	1.00E+00
ENSG00000123297.15_TSFM	ENSG00000160789.18_LMNA	-0.31972	-0.455723678	-0.16912686	5.94E-05	1.23E-03	1.00E+00
ENSG00000100983.8_GSS	ENSG00000160789.18_LMNA	0.26539	0.110877333	0.407381865	9.52E-04	1.12E-02	1.00E+00
ENSG00000124299.12_PEPD	ENSG00000160789.18_LMNA	0.10112	-0.059032162	0.256195273	2.15E-01	4.89E-01	1.00E+00
ENSG00000204256.11_BRD2	ENSG00000160789.18_LMNA	0.11293	-0.047114565	0.267327618	1.66E-01	4.23E-01	1.00E+00
ENSG00000207770.1_	ENSG00000160789.18_LMNA	0.04633	-0.113706046	0.204029107	5.71E-01	7.99E-01	1.00E+00
ENSG00000165478.6_HEPACAM	ENSG00000160789.18_LMNA	-0.10198	-0.257003505	0.058169933	2.11E-01	4.85E-01	1.00E+00
ENSG00000090932.9_DLL3	ENSG00000160789.18_LMNA	-0.375	-0.504108094	-0.22950387	1.94E-06	7.78E-05	9.93E-02
ENSG00000189077.9_TMEM120A	ENSG00000160789.18_LMNA	0.14091	-0.018712082	0.293524532	8.34E-02	2.79E-01	1.00E+00
ENSG00000134970.13_TMED7	ENSG00000160789.18_LMNA	-0.04036	-0.19828762	0.119608229	6.22E-01	8.29E-01	1.00E+00
ENSG00000152102.16_FAM168B	ENSG00000160789.18_LMNA	0.02939	-0.130422215	0.187711151	7.19E-01	8.81E-01	1.00E+00
ENSG00000170906.14_NDUFA3	ENSG00000160789.18_LMNA	-0.11007	-0.264632536	0.050007988	1.77E-01	4.39E-01	1.00E+00
ENSG00000116016.12_EPAS1	ENSG00000160789.18_LMNA	0.19348	0.035368765	0.342142022	1.69E-02	9.57E-02	1.00E+00
ENSG00000110436.10_SLC1A2	ENSG00000160789.18_LMNA	-0.06272	-0.219728753	0.097451665	4.43E-01	7.10E-01	1.00E+00
ENSG00000100129.16_EIF3L	ENSG00000160789.18_LMNA	-0.29044	-0.429767722	-0.1376003	2.84E-04	4.30E-03	1.00E+00
ENSG00000087087.17_SRRT	ENSG00000160789.18_LMNA	0.13662	-0.023086202	0.289520402	9.33E-02	2.99E-01	1.00E+00
ENSG00000100650.14_SRSF5	ENSG00000160789.18_LMNA	-0.11171	-0.266177696	0.04834976	1.71E-01	4.30E-01	1.00E+00
ENSG00000103024.6_NME3	ENSG00000160789.18_LMNA	0.23596	0.079757197	0.380855552	3.43E-03	2.95E-02	1.00E+00
ENSG00000165006.12_UBAP1	ENSG00000160789.18_LMNA	-0.17789	-0.327806213	-0.01923298	2.83E-02	1.38E-01	1.00E+00
ENSG00000179195.14_ZNF664	ENSG00000160789.18_LMNA	-0.13723	-0.290096085	0.02245806	9.18E-02	2.96E-01	1.00E+00
ENSG00000139684.12_ESD	ENSG00000160789.18_LMNA	-0.25678	-0.399645522	-0.10174075	1.41E-03	1.50E-02	1.00E+00
ENSG00000100410.6_PHF5A	ENSG00000160789.18_LMNA	-0.18164	-0.331261167	-0.02310708	2.51E-02	1.27E-01	1.00E+00
ENSG00000079739.14_PGM1	ENSG00000160789.18_LMNA	-0.14115	-0.293749984	0.018465443	8.28E-02	2.78E-01	1.00E+00
ENSG00000186577.10_C6orf1	ENSG00000160789.18_LMNA	0.0994	-0.060758907	0.254575337	2.23E-01	4.99E-01	1.00E+00

ENSG00000205581.9_HMGN1	ENSG00000160789.18_LMNA	0.00117	-0.158061352	0.160339628	9.89E-01	9.96E-01	1.00E+00
ENSG00000172500.11_FIBP	ENSG00000160789.18_LMNA	0.09542	-0.064767805	0.250807578	2.42E-01	5.21E-01	1.00E+00
ENSG00000079999.12_KEAP1	ENSG00000160789.18_LMNA	0.15059	-0.008826866	0.30253342	6.41E-02	2.36E-01	1.00E+00
ENSG00000090266.11_NDUFB2	ENSG00000160789.18_LMNA	-0.06065	-0.217752796	0.099506791	4.58E-01	7.22E-01	1.00E+00
ENSG00000095059.14_DHPS	ENSG00000160789.18_LMNA	-0.15952	-0.3108302	-0.00033112	4.96E-02	2.00E-01	1.00E+00
ENSG00000111540.14_RAB5B	ENSG00000160789.18_LMNA	-0.30086	-0.439030138	-0.14878307	1.66E-04	2.82E-03	1.00E+00
ENSG00000146966.11_DENND2A	ENSG00000160789.18_LMNA	0.25623	0.101154738	0.399147868	1.44E-03	1.52E-02	1.00E+00
ENSG00000151500.13_THYN1	ENSG00000160789.18_LMNA	-0.36408	-0.494610615	-0.21748325	4.01E-06	1.39E-04	2.06E-01
ENSG00000197157.9_SND1	ENSG00000160789.18_LMNA	0.33222	0.182678306	0.466732856	2.90E-05	6.93E-04	1.00E+00
ENSG00000269893.5_SNHG8	ENSG00000160789.18_LMNA	-0.16301	-0.31405863	-0.0039088	4.48E-02	1.87E-01	1.00E+00
ENSG00000108829.9_LRRC59	ENSG00000160789.18_LMNA	0.22301	0.066157738	0.369105853	5.75E-03	4.36E-02	1.00E+00
ENSG00000110108.8_TMEM109	ENSG00000160789.18_LMNA	0.29312	0.140478844	0.432157842	2.47E-04	3.86E-03	1.00E+00
ENSG00000143418.18_CERS2	ENSG00000160789.18_LMNA	0.30591	0.154224336	0.443514809	1.27E-04	2.29E-03	1.00E+00
ENSG00000148362.9_C9orf142	ENSG00000160789.18_LMNA	0.03335	-0.126521071	0.191535137	6.83E-01	8.62E-01	1.00E+00
ENSG00000116221.14_MRPL37	ENSG00000160789.18_LMNA	0.23809	0.081997701	0.382782025	3.14E-03	2.76E-02	1.00E+00
ENSG00000170876.7_TMEM43	ENSG00000160789.18_LMNA	0.38243	0.237697977	0.510544405	1.16E-06	5.19E-05	5.96E-02
ENSG00000167615.15_LENG8	ENSG00000160789.18_LMNA	0.08495	-0.075264325	0.240896934	2.98E-01	5.84E-01	1.00E+00
ENSG00000169217.7_CD2BP2	ENSG00000160789.18_LMNA	0.03061	-0.12921868	0.188891911	7.08E-01	8.76E-01	1.00E+00
ENSG00000176087.13_SLC35A4	ENSG00000160789.18_LMNA	0.23248	0.076090968	0.377697539	3.95E-03	3.29E-02	1.00E+00
ENSG00000119408.15_NEK6	ENSG00000160789.18_LMNA	0.50755	0.378966403	0.616900121	2.50E-11	7.16E-09	1.28E-06
ENSG00000091527.14_CDV3	ENSG00000160789.18_LMNA	0.16648	0.007472552	0.317267383	4.04E-02	1.74E-01	1.00E+00
ENSG00000126067.10_PSMB2	ENSG00000160789.18_LMNA	0.0138	-0.145719538	0.172622755	8.66E-01	9.48E-01	1.00E+00
ENSG00000247596.7_TWF2	ENSG00000160789.18_LMNA	0.34554	0.197187211	0.478423087	1.30E-05	3.62E-04	6.67E-01
ENSG00000152291.12_TGOLN2	ENSG00000160789.18_LMNA	0.213	0.055688218	0.359993909	8.42E-03	5.79E-02	1.00E+00
ENSG00000198931.9_APRT	ENSG00000160789.18_LMNA	0.22143	0.064500676	0.367667531	6.12E-03	4.56E-02	1.00E+00
ENSG00000233762.3_AC007969.5	ENSG00000160789.18_LMNA	-0.09875	-0.253953634	0.061421101	2.26E-01	5.02E-01	1.00E+00
ENSG00000237949.1_LINC00844	ENSG00000160789.18_LMNA	-0.57392	-0.671745398	-0.45642324	1.08E-14	1.50E-11	5.55E-10
ENSG00000099797.10_TECR	ENSG00000160789.18_LMNA	0.12208	-0.037856535	0.275918093	1.34E-01	3.72E-01	1.00E+00
ENSG00000106605.9_BLVRA	ENSG00000160789.18_LMNA	0.0734	-0.08681979	0.229909667	3.69E-01	6.50E-01	1.00E+00
ENSG00000141349.7_G6PC3	ENSG00000160789.18_LMNA	0.20001	0.042152431	0.348126617	1.35E-02	8.13E-02	1.00E+00
ENSG00000082014.15_SMARCD3	ENSG00000160789.18_LMNA	-0.16931	-0.319884336	-0.01038487	3.71E-02	1.65E-01	1.00E+00
ENSG00000116350.14_SRSF4	ENSG00000160789.18_LMNA	0.26708	0.112674086	0.408898263	8.80E-04	1.05E-02	1.00E+00
ENSG00000122786.18_CALD1	ENSG00000160789.18_LMNA	0.38429	0.239756214	0.512156319	1.02E-06	4.67E-05	5.23E-02

ENSG00000182154.7_MRPL41	ENSG00000160789.18_LMNA	0.04806	-0.111999212	0.205685405	5.57E-01	7.90E-01	1.00E+00
ENSG00000174695.8_TMEM167A	ENSG00000160789.18_LMNA	-0.44849	-0.567197051	-0.3115379	6.85E-09	7.83E-07	3.51E-04
ENSG00000173486.11_FKBP2	ENSG00000160789.18_LMNA	0.23325	0.076907315	0.378401331	3.83E-03	3.21E-02	1.00E+00
ENSG00000101150.16_TPD52L2	ENSG00000160789.18_LMNA	0.30375	0.151897455	0.441598775	1.42E-04	2.49E-03	1.00E+00
ENSG00000240694.7_PNMA2	ENSG00000160789.18_LMNA	-0.04761	-0.205253653	0.112444319	5.60E-01	7.93E-01	1.00E+00
ENSG00000064601.15_CTSA	ENSG00000160789.18_LMNA	0.49349	0.362790729	0.605147222	1.05E-10	2.33E-08	5.38E-06
ENSG00000125648.13_SLC25A23	ENSG00000160789.18_LMNA	-0.18675	-0.335958371	-0.02838903	2.12E-02	1.13E-01	1.00E+00
ENSG00000105373.17_GLTSCR2	ENSG00000160789.18_LMNA	0.13373	-0.026020786	0.286827895	1.00E-01	3.12E-01	1.00E+00
ENSG00000140931.18_CMTM3	ENSG00000160789.18_LMNA	0.45027	0.313548199	0.568706322	5.87E-09	6.91E-07	3.01E-04
ENSG00000077097.12_TOP2B	ENSG00000160789.18_LMNA	-0.19657	-0.344971432	-0.03857244	1.52E-02	8.88E-02	1.00E+00
ENSG00000134769.20_DTNA	ENSG00000160789.18_LMNA	-0.10562	-0.260441626	0.054496856	1.95E-01	4.64E-01	1.00E+00
ENSG00000100600.13_LGMN	ENSG00000160789.18_LMNA	0.01281	-0.146687288	0.171663171	8.76E-01	9.52E-01	1.00E+00
ENSG00000136830.10_FAM129B	ENSG00000160789.18_LMNA	0.48848	0.357049019	0.600949859	1.72E-10	3.52E-08	8.83E-06
ENSG00000070413.17_DGCR2	ENSG00000160789.18_LMNA	-0.04325	-0.201068286	0.116752539	5.97E-01	8.14E-01	1.00E+00
ENSG00000125166.11_GOT2	ENSG00000160789.18_LMNA	-0.05853	-0.215718322	0.101619955	4.74E-01	7.33E-01	1.00E+00
ENSG00000141030.11_COPS3	ENSG00000160789.18_LMNA	-0.24951	-0.393094795	-0.09404347	1.94E-03	1.90E-02	1.00E+00
ENSG00000120742.9_SERP1	ENSG00000160789.18_LMNA	-0.37639	-0.505307882	-0.23102838	1.76E-06	7.26E-05	9.04E-02
ENSG00000235363.1_SNRPGP10	ENSG00000160789.18_LMNA	-0.11823	-0.2723081	0.041753629	1.47E-01	3.93E-01	1.00E+00
ENSG00000149658.16_YTHDF1	ENSG00000160789.18_LMNA	0.18593	0.027546597	0.335210221	2.18E-02	1.15E-01	1.00E+00
ENSG00000145050.14_MANF	ENSG00000160789.18_LMNA	0.28544	0.132245667	0.42531073	3.65E-04	5.24E-03	1.00E+00
ENSG00000115875.17_SRSF7	ENSG00000160789.18_LMNA	-0.16456	-0.315496634	-0.00550492	4.28E-02	1.81E-01	1.00E+00
ENSG00000159164.8_SV2A	ENSG00000160789.18_LMNA	0.02794	-0.131845389	0.186313721	7.33E-01	8.87E-01	1.00E+00
ENSG00000149541.8_B3GAT3	ENSG00000160789.18_LMNA	0.25738	0.102375024	0.400183964	1.37E-03	1.47E-02	1.00E+00
ENSG00000099785.9_MARCH2	ENSG00000160789.18_LMNA	-0.12401	-0.277731629	0.035895161	1.28E-01	3.62E-01	1.00E+00
ENSG00000116688.15_MFN2	ENSG00000160789.18_LMNA	0.1073	-0.05280393	0.262023576	1.88E-01	4.55E-01	1.00E+00
ENSG00000259330.1_INAFM2	ENSG00000160789.18_LMNA	0.2369	0.080740487	0.381701345	3.30E-03	2.87E-02	1.00E+00
ENSG00000260260.1_SNHG19	ENSG00000160789.18_LMNA	-0.09693	-0.252235683	0.063249499	2.35E-01	5.13E-01	1.00E+00
ENSG00000225648.4_SBDSP1	ENSG00000160789.18_LMNA	0.05267	-0.10743263	0.21010785	5.19E-01	7.65E-01	1.00E+00
ENSG00000172053.13_QARS	ENSG00000160789.18_LMNA	0.09554	-0.064648493	0.250919851	2.42E-01	5.21E-01	1.00E+00
ENSG00000179958.7_DCTPP1	ENSG00000160789.18_LMNA	-0.15559	-0.307183498	0.003700533	5.56E-02	2.15E-01	1.00E+00
ENSG00000126458.3_RRAS	ENSG00000160789.18_LMNA	0.26311	0.108453404	0.405333557	1.06E-03	1.21E-02	1.00E+00
ENSG00000139579.11_NABP2	ENSG00000160789.18_LMNA	-0.22533	-0.371213737	-0.06858921	5.25E-03	4.07E-02	1.00E+00
ENSG00000119917.12_IFIT3	ENSG00000160789.18_LMNA	-0.16172	-0.312866151	-0.00258639	4.65E-02	1.92E-01	1.00E+00

ENSG00000049541.9_RFC2	ENSG00000160789.18_LMNA	0.2204	0.063422267	0.366730696	6.36E-03	4.70E-02	1.00E+00
ENSG00000204370.7_SDHD	ENSG00000160789.18_LMNA	-0.18295	-0.332470621	-0.02446546	2.41E-02	1.23E-01	1.00E+00
ENSG00000071859.13_FAM50A	ENSG00000160789.18_LMNA	0.36211	0.215317608	0.492892474	4.56E-06	1.55E-04	2.34E-01
ENSG00000172379.17_ARNT2	ENSG00000160789.18_LMNA	-0.01616	-0.17491084	0.143409521	8.43E-01	9.39E-01	1.00E+00
ENSG00000113387.10_SUB1	ENSG00000160789.18_LMNA	-0.35243	-0.484453017	-0.20471951	8.47E-06	2.55E-04	4.35E-01
ENSG00000101460.11_MAP1LC3.	ENSG00000160789.18_LMNA	0.16504	0.005997927	0.315940512	4.22E-02	1.80E-01	1.00E+00
ENSG00000068078.16_FGFR3	ENSG00000160789.18_LMNA	0.08772	-0.072497141	0.24351608	2.83E-01	5.68E-01	1.00E+00
ENSG00000123159.14_GIPC1	ENSG00000160789.18_LMNA	-0.0579	-0.215122173	0.102238619	4.79E-01	7.37E-01	1.00E+00
ENSG00000171617.12_ENC1	ENSG00000160789.18_LMNA	-0.04663	-0.204313078	0.113413544	5.68E-01	7.98E-01	1.00E+00
ENSG00000054965.9_FAM168A	ENSG00000160789.18_LMNA	0.03	-0.129821047	0.188301059	7.14E-01	8.78E-01	1.00E+00
ENSG00000189067.11_LITAF	ENSG00000160789.18_LMNA	0.21741	0.060298029	0.364013135	7.13E-03	5.11E-02	1.00E+00
ENSG00000124207.15_CSE1L	ENSG00000160789.18_LMNA	-0.0357	-0.193800458	0.124205391	6.62E-01	8.51E-01	1.00E+00
ENSG00000169429.9_CXCL8	ENSG00000160789.18_LMNA	0.09256	-0.067640157	0.248102108	2.57E-01	5.39E-01	1.00E+00
ENSG00000179295.14_PTPN11	ENSG00000160789.18_LMNA	0.00357	-0.155719818	0.162677633	9.65E-01	9.86E-01	1.00E+00
ENSG00000117862.10_TXNDC12	ENSG00000160789.18_LMNA	0.13211	-0.027676029	0.285307013	1.05E-01	3.21E-01	1.00E+00
ENSG00000204619.6_PPP1R11	ENSG00000160789.18_LMNA	-0.07489	-0.231333611	0.085326999	3.59E-01	6.42E-01	1.00E+00
ENSG00000177807.6_KCNJ10	ENSG00000160789.18_LMNA	-0.29768	-0.436210291	-0.14537071	1.96E-04	3.21E-03	1.00E+00
ENSG00000105974.10_CAV1	ENSG00000160789.18_LMNA	0.34594	0.197621855	0.478771761	1.27E-05	3.55E-04	6.51E-01
ENSG00000136522.12_MRPL47	ENSG00000160789.18_LMNA	-0.2126	-0.359630902	-0.05527251	8.55E-03	5.85E-02	1.00E+00
ENSG00000198732.9_SMOC1	ENSG00000160789.18_LMNA	-0.40348	-0.528719127	-0.2610479	2.55E-07	1.55E-05	1.31E-02
ENSG00000107796.11_ACTA2	ENSG00000160789.18_LMNA	0.2886	0.135630251	0.428129583	3.11E-04	4.64E-03	1.00E+00
ENSG00000100591.6_AHSA1	ENSG00000160789.18_LMNA	0.01286	-0.146637962	0.171712095	8.75E-01	9.51E-01	1.00E+00
ENSG00000141959.15_PFKL	ENSG00000160789.18_LMNA	0.44983	0.313043017	0.568327209	6.11E-09	7.14E-07	3.13E-04
ENSG00000196576.13_PLXNB2	ENSG00000160789.18_LMNA	0.47949	0.346760244	0.593394653	4.11E-10	7.48E-08	2.11E-05
ENSG00000155980.10_KIF5A	ENSG00000160789.18_LMNA	-0.39354	-0.520148838	-0.24999808	5.28E-07	2.74E-05	2.71E-02
ENSG00000162706.11_CADM3	ENSG00000160789.18_LMNA	-0.24643	-0.390321511	-0.09079552	2.21E-03	2.11E-02	1.00E+00
ENSG00000162975.4_KCNF1	ENSG00000160789.18_LMNA	0.36824	0.222055373	0.498230859	3.05E-06	1.11E-04	1.56E-01
ENSG00000136859.8_ANGPTL2	ENSG00000160789.18_LMNA	0.10136	-0.058788778	0.256423459	2.14E-01	4.88E-01	1.00E+00
ENSG00000183044.10_ABAT	ENSG00000160789.18_LMNA	-0.39882	-0.524703438	-0.25586166	3.60E-07	2.03E-05	1.85E-02
ENSG00000077380.14_DYNC1I2	ENSG00000160789.18_LMNA	-0.31256	-0.4493998	-0.16139134	8.84E-05	1.71E-03	1.00E+00
ENSG00000140262.16_TCF12	ENSG00000160789.18_LMNA	-0.21353	-0.360475031	-0.05623935	8.26E-03	5.70E-02	1.00E+00
ENSG00000168056.13_LTBP3	ENSG00000160789.18_LMNA	0.42841	0.288914228	0.55009269	3.67E-08	3.19E-06	1.88E-03
ENSG00000101224.16_CDC25B	ENSG00000160789.18_LMNA	0.36337	0.216709992	0.493997387	4.20E-06	1.44E-04	2.15E-01

ENSG00000232388.4_LINC00493	ENSG00000160789.18_LMNA	-0.11742	-0.271540438	0.042581112	1.50E-01	3.97E-01	1.00E+00
ENSG00000162496.7_DHRS3	ENSG00000160789.18_LMNA	-0.07489	-0.231332763	0.085327888	3.59E-01	6.42E-01	1.00E+00
ENSG00000187416.10_LHFPL3	ENSG00000160789.18_LMNA	-0.28118	-0.421510877	-0.12769399	4.50E-04	6.21E-03	1.00E+00
ENSG00000006118.13_TMEM132	ENSG00000160789.18_LMNA	0.07812	-0.082097817	0.23440923	3.39E-01	6.24E-01	1.00E+00
ENSG00000213619.8_NDUFS3	ENSG00000160789.18_LMNA	-0.13364	-0.286738409	0.026118226	1.01E-01	3.13E-01	1.00E+00
ENSG00000185627.16_PSMD13	ENSG00000160789.18_LMNA	-0.05976	-0.216900622	0.100392274	4.65E-01	7.27E-01	1.00E+00
ENSG00000145247.10_OCIAD2	ENSG00000160789.18_LMNA	0.24264	0.086787007	0.386891301	2.60E-03	2.39E-02	1.00E+00
ENSG00000164615.4_CAMLG	ENSG00000160789.18_LMNA	-0.34001	-0.473573552	-0.1911534	1.82E-05	4.78E-04	9.35E-01
ENSG00000168610.13_STAT3	ENSG00000160789.18_LMNA	0.42792	0.288364795	0.549674554	3.82E-08	3.30E-06	1.96E-03
ENSG00000150093.17_ITGB1	ENSG00000160789.18_LMNA	0.3185	0.167806563	0.454646367	6.36E-05	1.30E-03	1.00E+00
ENSG00000163950.11_SLBP	ENSG00000160789.18_LMNA	0.14999	-0.009439742	0.301976489	6.51E-02	2.38E-01	1.00E+00
ENSG00000133639.4_BTG1	ENSG00000160789.18_LMNA	-0.12676	-0.28030519	0.033107652	1.20E-01	3.48E-01	1.00E+00
ENSG00000185721.10_DRG1	ENSG00000160789.18_LMNA	-0.18927	-0.33827485	-0.03100023	1.95E-02	1.06E-01	1.00E+00
ENSG00000242265.4_PEG10	ENSG00000160789.18_LMNA	0.01582	-0.143741631	0.174582094	8.47E-01	9.40E-01	1.00E+00
ENSG00000022840.14_RNF10	ENSG00000160789.18_LMNA	0.19993	0.042064556	0.348049252	1.35E-02	8.14E-02	1.00E+00
ENSG00000139433.8_GLTP	ENSG00000160789.18_LMNA	-0.36194	-0.49275	-0.21513815	4.61E-06	1.56E-04	2.37E-01
ENSG00000134107.4_BHLHE40	ENSG00000160789.18_LMNA	0.55957	0.439526231	0.659979018	6.69E-14	6.35E-11	3.43E-09
ENSG00000185651.13_UBE2L3	ENSG00000160789.18_LMNA	-0.04212	-0.199975946	0.117874978	6.06E-01	8.20E-01	1.00E+00
ENSG00000067829.17_IDH3G	ENSG00000160789.18_LMNA	0.0078	-0.151590277	0.166792253	9.24E-01	9.71E-01	1.00E+00
ENSG00000197324.7_LRP10	ENSG00000160789.18_LMNA	0.41268	0.271298218	0.53662082	1.27E-07	8.81E-06	6.51E-03
ENSG00000126934.12_MAP2K2	ENSG00000160789.18_LMNA	0.13497	-0.024763052	0.287982483	9.73E-02	3.07E-01	1.00E+00
ENSG00000141424.11_SLC39A6	ENSG00000160789.18_LMNA	-0.14105	-0.293659803	0.018564104	8.30E-02	2.78E-01	1.00E+00
ENSG00000154721.13_JAM2	ENSG00000160789.18_LMNA	0.01057	-0.148886056	0.169480708	8.97E-01	9.60E-01	1.00E+00
ENSG00000112667.11_DNPH1	ENSG00000160789.18_LMNA	0.17408	0.015304725	0.324294523	3.20E-02	1.49E-01	1.00E+00
ENSG00000169740.12_ZNF32	ENSG00000160789.18_LMNA	-0.3968	-0.522961333	-0.25361654	4.17E-07	2.28E-05	2.14E-02
ENSG00000188157.12_AGRN	ENSG00000160789.18_LMNA	0.23104	0.074583546	0.376397037	4.19E-03	3.44E-02	1.00E+00
ENSG00000100280.15_AP1B1	ENSG00000160789.18_LMNA	0.0963	-0.063879788	0.251643005	2.38E-01	5.16E-01	1.00E+00
ENSG00000154518.8_ATP5G3	ENSG00000160789.18_LMNA	-0.15677	-0.308278909	0.002490549	5.38E-02	2.11E-01	1.00E+00
ENSG00000241837.5_ATP5O	ENSG00000160789.18_LMNA	-0.20299	-0.350855349	-0.0452549	1.21E-02	7.54E-02	1.00E+00
ENSG00000138629.14_UBL7	ENSG00000160789.18_LMNA	-0.006	-0.165046419	0.1533438	9.41E-01	9.77E-01	1.00E+00
ENSG00000132661.3_NXT1	ENSG00000160789.18_LMNA	0.08516	-0.075062821	0.241087813	2.97E-01	5.83E-01	1.00E+00
ENSG00000090097.19_PCBP4	ENSG00000160789.18_LMNA	-0.2323	-0.377539997	-0.07590829	3.98E-03	3.30E-02	1.00E+00
ENSG00000242485.4_MRPL20	ENSG00000160789.18_LMNA	-0.08799	-0.24377367	0.072224727	2.81E-01	5.67E-01	1.00E+00

ENSG00000024422.10_EHD2	ENSG00000160789.18_LMNA	0.64859	0.545745953	0.73219169	1.65E-19	1.07E-15	8.45E-15
ENSG00000147164.10_SNX12	ENSG00000160789.18_LMNA	-0.03131	-0.189567899	0.128529232	7.02E-01	8.72E-01	1.00E+00
ENSG00000104435.12_STMN2	ENSG00000160789.18_LMNA	-0.31749	-0.453756482	-0.16671675	6.73E-05	1.37E-03	1.00E+00
ENSG00000168994.12_PXDC1	ENSG00000160789.18_LMNA	0.33205	0.182501172	0.466589518	2.92E-05	6.98E-04	1.00E+00
ENSG00000276171.1_	ENSG00000160789.18_LMNA	-0.25945	-0.402047837	-0.1045725	1.25E-03	1.37E-02	1.00E+00
ENSG00000135617.3_PRADC1	ENSG00000160789.18_LMNA	-0.20519	-0.352860734	-0.04753871	1.12E-02	7.11E-02	1.00E+00
ENSG00000132613.13_MTSS1L	ENSG00000160789.18_LMNA	-0.0263	-0.184728013	0.133458721	7.48E-01	8.95E-01	1.00E+00
ENSG00000150753.10_CCT5	ENSG00000160789.18_LMNA	-0.09709	-0.25238856	0.063086879	2.34E-01	5.12E-01	1.00E+00
ENSG00000162521.17_RBBP4	ENSG00000160789.18_LMNA	-0.19968	-0.347825192	-0.04181008	1.36E-02	8.19E-02	1.00E+00
ENSG00000146540.13_C7orf50	ENSG00000160789.18_LMNA	0.10228	-0.057865341	0.257288916	2.10E-01	4.83E-01	1.00E+00
ENSG00000162889.9_MAPKAPK2	ENSG00000160789.18_LMNA	0.40483	0.262554847	0.529883708	2.30E-07	1.42E-05	1.18E-02
ENSG00000072110.12_ACTN1	ENSG00000160789.18_LMNA	0.55198	0.430622127	0.65373475	1.69E-13	1.34E-10	8.69E-09
ENSG00000086504.14_MRPL28	ENSG00000160789.18_LMNA	-0.08196	-0.238055596	0.078260763	3.15E-01	6.02E-01	1.00E+00
ENSG00000125877.11_ITPA	ENSG00000160789.18_LMNA	0.15357	-0.005767683	0.305310178	5.89E-02	2.23E-01	1.00E+00
ENSG00000168701.17_TMEM208	ENSG00000160789.18_LMNA	0.07958	-0.080639302	0.235796312	3.30E-01	6.16E-01	1.00E+00
ENSG00000101361.13_NOP56	ENSG00000160789.18_LMNA	0.01308	-0.14642742	0.171920905	8.73E-01	9.51E-01	1.00E+00
ENSG00000104388.13_RAB2A	ENSG00000160789.18_LMNA	-0.41804	-0.541217631	-0.27728916	8.38E-08	6.19E-06	4.30E-03
ENSG00000087365.13_SF3B2	ENSG00000160789.18_LMNA	0.17368	0.014887348	0.323920904	3.24E-02	1.51E-01	1.00E+00
ENSG00000115306.14_SPTBN1	ENSG00000160789.18_LMNA	0.05277	-0.107336724	0.21020059	5.19E-01	7.65E-01	1.00E+00
ENSG00000182199.9_SHMT2	ENSG00000160789.18_LMNA	0.06955	-0.090656872	0.226243315	3.95E-01	6.72E-01	1.00E+00
ENSG00000141698.15_NT5C3B	ENSG00000160789.18_LMNA	-0.23966	-0.384198849	-0.08364742	2.94E-03	2.64E-02	1.00E+00
ENSG00000126062.3_TMEM115	ENSG00000160789.18_LMNA	0.35013	0.202199337	0.482438464	9.79E-06	2.86E-04	5.02E-01
ENSG00000002834.16_LASP1	ENSG00000160789.18_LMNA	0.2949	0.142388694	0.433741376	2.26E-04	3.60E-03	1.00E+00
ENSG00000205364.3_MT1M	ENSG00000160789.18_LMNA	0.1597	0.000510172	0.310991941	4.94E-02	2.00E-01	1.00E+00
ENSG00000185633.9_NDUFA4L2	ENSG00000160789.18_LMNA	0.14713	-0.012362655	0.299317458	7.05E-02	2.51E-01	1.00E+00
ENSG00000167797.6_CDK2AP2	ENSG00000160789.18_LMNA	0.23253	0.076146969	0.377745829	3.94E-03	3.28E-02	1.00E+00
ENSG00000136026.12_CKAP4	ENSG00000160789.18_LMNA	0.38471	0.240218404	0.512518021	9.91E-07	4.57E-05	5.08E-02
ENSG00000109332.18_UBE2D3	ENSG00000160789.18_LMNA	-0.25668	-0.399554662	-0.10163374	1.41E-03	1.50E-02	1.00E+00
ENSG00000129351.16_ILF3	ENSG00000160789.18_LMNA	0.0757	-0.084516298	0.232106355	3.54E-01	6.37E-01	1.00E+00
ENSG00000144476.5_ACKR3	ENSG00000160789.18_LMNA	-0.20908	-0.356416223	-0.05159571	9.74E-03	6.44E-02	1.00E+00
ENSG00000068323.15_TFE3	ENSG00000160789.18_LMNA	0.36748	0.221221824	0.497571565	3.21E-06	1.16E-04	1.64E-01
ENSG00000107798.16_LIPA	ENSG00000160789.18_LMNA	-0.30804	-0.445398477	-0.15651504	1.13E-04	2.08E-03	1.00E+00
ENSG00000113845.8_TIMMDC1	ENSG00000160789.18_LMNA	-0.28207	-0.422306244	-0.1286457	4.30E-04	6.00E-03	1.00E+00

ENSG00000150551.10_LYPD1	ENSG00000160789.18_LMNA	0.1307	-0.029109959	0.283988202	1.09E-01	3.28E-01	1.00E+00
ENSG00000181104.6_F2R	ENSG00000160789.18_LMNA	0.1521	-0.007274865	0.3039428	6.14E-02	2.29E-01	1.00E+00
ENSG00000148908.13_RGS10	ENSG00000160789.18_LMNA	-0.23358	-0.378698515	-0.07725215	3.78E-03	3.18E-02	1.00E+00
ENSG00000171298.11_GAA	ENSG00000160789.18_LMNA	0.31177	0.160537266	0.448699809	9.23E-05	1.77E-03	1.00E+00
ENSG00000144566.9_RAB5A	ENSG00000160789.18_LMNA	-0.04799	-0.205622787	0.112063775	5.57E-01	7.91E-01	1.00E+00
ENSG00000105329.8_TGFB1	ENSG00000160789.18_LMNA	0.35419	0.206642958	0.485988569	7.58E-06	2.34E-04	3.89E-01
ENSG00000130429.11_ARPC1B	ENSG00000160789.18_LMNA	0.32601	0.175940617	0.46127015	4.15E-05	9.26E-04	1.00E+00
ENSG00000167671.10_UBXN6	ENSG00000160789.18_LMNA	0.03381	-0.126073678	0.191973064	6.79E-01	8.60E-01	1.00E+00
ENSG00000184117.10_NIPSNAP1	ENSG00000160789.18_LMNA	-0.15836	-0.309754288	0.000859415	5.13E-02	2.05E-01	1.00E+00
ENSG00000108179.12_PPIF	ENSG00000160789.18_LMNA	-0.31461	-0.45121079	-0.16360298	7.90E-05	1.56E-03	1.00E+00
ENSG00000051523.9_CYBA	ENSG00000160789.18_LMNA	0.00813	-0.151270871	0.167110043	9.21E-01	9.70E-01	1.00E+00
ENSG00000167772.10_ANGPTL4	ENSG00000160789.18_LMNA	0.25704	0.102015478	0.399878767	1.39E-03	1.48E-02	1.00E+00
ENSG00000136235.14_GPNMB	ENSG00000160789.18_LMNA	0.10014	-0.060013575	0.255274782	2.20E-01	4.95E-01	1.00E+00
ENSG00000006451.6_RALA	ENSG00000160789.18_LMNA	0.01269	-0.146809621	0.171541827	8.77E-01	9.52E-01	1.00E+00
ENSG00000144647.5_POMGNT2	ENSG00000160789.18_LMNA	-0.34151	-0.474894778	-0.19279516	1.66E-05	4.43E-04	8.53E-01
ENSG00000115524.14_SF3B1	ENSG00000160789.18_LMNA	-0.09919	-0.254371637	0.060975904	2.24E-01	5.00E-01	1.00E+00
ENSG00000173418.10_NAA20	ENSG00000160789.18_LMNA	-0.06005	-0.217179181	0.100102882	4.62E-01	7.25E-01	1.00E+00
ENSG00000185883.9_ATP6V0C	ENSG00000160789.18_LMNA	-0.02638	-0.184802755	0.133382715	7.47E-01	8.94E-01	1.00E+00
ENSG00000183648.8_NDUFB1	ENSG00000160789.18_LMNA	-0.18155	-0.331180271	-0.02301627	2.52E-02	1.27E-01	1.00E+00
ENSG00000165389.6_SPTSSA	ENSG00000160789.18_LMNA	0.05952	-0.100634681	0.216667249	4.66E-01	7.28E-01	1.00E+00
ENSG00000169021.5_UQCRFS1	ENSG00000160789.18_LMNA	-0.20802	-0.355446389	-0.05048808	1.01E-02	6.62E-02	1.00E+00
ENSG00000131467.9_PSME3	ENSG00000160789.18_LMNA	-0.07134	-0.227948822	0.088873115	3.82E-01	6.61E-01	1.00E+00
ENSG00000106333.11_PCOLCE	ENSG00000160789.18_LMNA	0.46185	0.326673409	0.578518262	2.11E-09	2.88E-07	1.08E-04
ENSG00000243147.6_MRPL33	ENSG00000160789.18_LMNA	-0.25702	-0.399857405	-0.10199032	1.39E-03	1.48E-02	1.00E+00
ENSG00000167193.7_CRK	ENSG00000160789.18_LMNA	0.14254	-0.017051849	0.295041471	7.98E-02	2.72E-01	1.00E+00
ENSG00000173674.9_EIF1AX	ENSG00000160789.18_LMNA	-0.10966	-0.264248085	0.050420303	1.79E-01	4.41E-01	1.00E+00
ENSG00000105671.10_DDX49	ENSG00000160789.18_LMNA	-0.06792	-0.224691391	0.092278236	4.06E-01	6.81E-01	1.00E+00
ENSG00000100911.12_PSME2	ENSG00000160789.18_LMNA	0.14643	-0.013079225	0.298664841	7.18E-02	2.54E-01	1.00E+00
ENSG00000084070.10_SMAP2	ENSG00000160789.18_LMNA	-0.25199	-0.395333336	-0.0966698	1.74E-03	1.76E-02	1.00E+00
ENSG00000166986.11_MARS	ENSG00000160789.18_LMNA	-0.26995	-0.411470368	-0.11572616	7.70E-04	9.42E-03	1.00E+00
ENSG00000144224.15_UBXN4	ENSG00000160789.18_LMNA	-0.00804	-0.16702821	0.151353127	9.22E-01	9.70E-01	1.00E+00
ENSG00000165637.12_VDAC2	ENSG00000160789.18_LMNA	-0.18033	-0.330059172	-0.02175821	2.62E-02	1.31E-01	1.00E+00
ENSG00000228232.1_GAPDHP1	ENSG00000160789.18_LMNA	0.28106	0.12756526	0.421403261	4.52E-04	6.24E-03	1.00E+00

ENSG00000264364.2_DYNLL2	ENSG00000160789.18_LMNA	-0.06936	-0.226068988	0.090839083	3.96E-01	6.73E-01	1.00E+00
ENSG00000101335.8_MYL9	ENSG00000160789.18_LMNA	0.38762	0.243443396	0.515039157	8.07E-07	3.83E-05	4.14E-02
ENSG00000178057.13_NDUFAF3	ENSG00000160789.18_LMNA	-0.03401	-0.192167273	0.12587523	6.77E-01	8.59E-01	1.00E+00
ENSG00000087274.15_ADD1	ENSG00000160789.18_LMNA	-0.10973	-0.264314689	0.050348879	1.78E-01	4.40E-01	1.00E+00
ENSG00000102158.18_MAGT1	ENSG00000160789.18_LMNA	0.03503	-0.124867356	0.193153234	6.68E-01	8.54E-01	1.00E+00
ENSG00000116133.10_DHCR24	ENSG00000160789.18_LMNA	0.18296	0.02447021	0.33247485	2.41E-02	1.23E-01	1.00E+00
ENSG00000146278.10_PNRC1	ENSG00000160789.18_LMNA	-0.13747	-0.29031305	0.02222126	9.13E-02	2.95E-01	1.00E+00
ENSG00000182175.12_RGMA	ENSG00000160789.18_LMNA	0.0077	-0.151683327	0.166699661	9.25E-01	9.71E-01	1.00E+00
ENSG00000112118.16_MCM3	ENSG00000160789.18_LMNA	0.25504	0.099893506	0.398076206	1.52E-03	1.59E-02	1.00E+00
ENSG00000052802.11_MSMO1	ENSG00000160789.18_LMNA	0.0799	-0.080325092	0.236094965	3.28E-01	6.14E-01	1.00E+00
ENSG00000169992.8_NLGN2	ENSG00000160789.18_LMNA	-0.08299	-0.23903171	0.077232009	3.09E-01	5.96E-01	1.00E+00
ENSG00000155189.10_AGPAT5	ENSG00000160789.18_LMNA	-0.019	-0.177659853	0.140629562	8.16E-01	9.28E-01	1.00E+00
ENSG00000133243.7_BTBD2	ENSG00000160789.18_LMNA	0.03229	-0.127563426	0.190514342	6.93E-01	8.68E-01	1.00E+00
ENSG00000087088.18_BAX	ENSG00000160789.18_LMNA	0.29996	0.147813906	0.438229841	1.74E-04	2.93E-03	1.00E+00
ENSG00000109079.8_TNFAIP1	ENSG00000160789.18_LMNA	-0.04731	-0.204962598	0.112744304	5.63E-01	7.94E-01	1.00E+00
ENSG00000141858.10_SAMD1	ENSG00000160789.18_LMNA	0.01507	-0.144481858	0.173849111	8.54E-01	9.43E-01	1.00E+00
ENSG00000123136.13_DDX39A	ENSG00000160789.18_LMNA	0.08501	-0.075205592	0.240952572	2.98E-01	5.84E-01	1.00E+00
ENSG00000103855.16_CD276	ENSG00000160789.18_LMNA	0.62675	0.51936658	0.714640754	5.77E-18	1.97E-14	2.96E-13
ENSG00000166311.8_SMPD1	ENSG00000160789.18_LMNA	0.31924	0.168609276	0.455301452	6.10E-05	1.26E-03	1.00E+00
ENSG00000055130.14_CUL1	ENSG00000160789.18_LMNA	0.29333	0.140696762	0.432338618	2.45E-04	3.84E-03	1.00E+00
ENSG00000196937.9_FAM3C	ENSG00000160789.18_LMNA	0.34182	0.193126738	0.475161465	1.63E-05	4.36E-04	8.38E-01
ENSG00000167553.13_TUBA1C	ENSG00000160789.18_LMNA	0.4069	0.264860965	0.531663945	1.97E-07	1.25E-05	1.01E-02
ENSG00000100258.16_LMF2	ENSG00000160789.18_LMNA	0.45938	0.323859373	0.576420709	2.63E-09	3.49E-07	1.35E-04
ENSG00000124795.13_DEK	ENSG00000160789.18_LMNA	0.14501	-0.014528238	0.297344258	7.47E-02	2.60E-01	1.00E+00
ENSG00000174231.15_PRPF8	ENSG00000160789.18_LMNA	0.14638	-0.013128864	0.298619621	7.19E-02	2.54E-01	1.00E+00
ENSG00000225921.5_NOL7	ENSG00000160789.18_LMNA	-0.0524	-0.209854284	0.107694823	5.21E-01	7.67E-01	1.00E+00
ENSG00000090382.5_LYZ	ENSG00000160789.18_LMNA	-0.00343	-0.162545441	0.155852304	9.67E-01	9.87E-01	1.00E+00
ENSG00000138668.17_HNRNPD	ENSG00000160789.18_LMNA	0.11641	-0.043594285	0.270599966	1.53E-01	4.03E-01	1.00E+00
ENSG00000257103.7_LSM14A	ENSG00000160789.18_LMNA	0.04162	-0.118361951	0.199501786	6.11E-01	8.22E-01	1.00E+00
ENSG00000198900.5_TOP1	ENSG00000160789.18_LMNA	0.19315	0.035025139	0.34183821	1.71E-02	9.65E-02	1.00E+00
ENSG00000136271.9_DDX56	ENSG00000160789.18_LMNA	0.19897	0.041065592	0.347169475	1.40E-02	8.35E-02	1.00E+00
ENSG00000171659.12_GPR34	ENSG00000160789.18_LMNA	-0.26845	-0.410120946	-0.11412423	8.26E-04	9.97E-03	1.00E+00
ENSG00000124491.14_F13A1	ENSG00000160789.18_LMNA	0.17477	0.016019458	0.324934098	3.13E-02	1.47E-01	1.00E+00

ENSG00000137100.14_DCTN3	ENSG00000160789.18_LMNA	-0.24918	-0.392799451	-0.09369727	1.96E-03	1.92E-02	1.00E+00
ENSG00000171202.5_TMEM126A	ENSG00000160789.18_LMNA	-0.2472	-0.391012874	-0.09160462	2.14E-03	2.06E-02	1.00E+00
ENSG00000171135.12_JAGN1	ENSG00000160789.18_LMNA	-0.17415	-0.324361371	-0.01537941	3.19E-02	1.49E-01	1.00E+00
ENSG00000010270.12_STARD3N	ENSG00000160789.18_LMNA	-0.09633	-0.251668119	0.063853085	2.38E-01	5.16E-01	1.00E+00
ENSG00000265241.5_RBM8A	ENSG00000160789.18_LMNA	-0.05804	-0.215250524	0.102105441	4.78E-01	7.36E-01	1.00E+00
ENSG00000276293.3_PIP4K2B	ENSG00000160789.18_LMNA	-0.18199	-0.331581506	-0.02346676	2.48E-02	1.26E-01	1.00E+00
ENSG00000105576.14_TNPO2	ENSG00000160789.18_LMNA	0.01422	-0.14530536	0.173033253	8.62E-01	9.46E-01	1.00E+00
ENSG00000130731.14_METTL26	ENSG00000160789.18_LMNA	0.05372	-0.106393155	0.211112703	5.11E-01	7.60E-01	1.00E+00
ENSG00000140105.16_WARS	ENSG00000160789.18_LMNA	0.22762	0.070996155	0.37329729	4.80E-03	3.80E-02	1.00E+00
ENSG00000103502.12_CDIPT	ENSG00000160789.18_LMNA	0.29257	0.139884005	0.43166427	2.55E-04	3.96E-03	1.00E+00
ENSG00000101220.16_C20orf27	ENSG00000160789.18_LMNA	0.15823	-0.000990835	0.309635472	5.15E-02	2.05E-01	1.00E+00
ENSG00000116962.13_NID1	ENSG00000160789.18_LMNA	0.28379	0.130485756	0.423842738	3.96E-04	5.62E-03	1.00E+00
ENSG00000181924.6_COA4	ENSG00000160789.18_LMNA	-0.19531	-0.343819449	-0.03726732	1.59E-02	9.15E-02	1.00E+00
ENSG00000168268.9_NT5DC2	ENSG00000160789.18_LMNA	0.05296	-0.107146126	0.210384878	5.17E-01	7.64E-01	1.00E+00
ENSG00000226950.5_DANCR	ENSG00000160789.18_LMNA	-0.38611	-0.51373102	-0.2417693	8.98E-07	4.19E-05	4.61E-02
ENSG00000134825.12_TMEM258	ENSG00000160789.18_LMNA	-0.17956	-0.329346934	-0.02095947	2.69E-02	1.33E-01	1.00E+00
ENSG00000132635.15_PCED1A	ENSG00000160789.18_LMNA	0.02839	-0.13140878	0.186742569	7.28E-01	8.85E-01	1.00E+00
ENSG00000090013.8_BLVRB	ENSG00000160789.18_LMNA	0.31939	0.168773011	0.455435036	6.05E-05	1.25E-03	1.00E+00
ENSG00000173852.12_DPY19L1	ENSG00000160789.18_LMNA	0.326	0.175927208	0.461259256	4.16E-05	9.26E-04	1.00E+00
ENSG00000213676.9_ATF6B	ENSG00000160789.18_LMNA	0.12892	-0.030911618	0.282329505	1.13E-01	3.36E-01	1.00E+00
ENSG00000196562.13_SULF2	ENSG00000160789.18_LMNA	-0.0134	-0.172233255	0.146112425	8.70E-01	9.49E-01	1.00E+00
ENSG00000125445.9_MRPS7	ENSG00000160789.18_LMNA	-0.29974	-0.438040024	-0.14758412	1.76E-04	2.95E-03	1.00E+00
ENSG00000171206.12_TRIM8	ENSG00000160789.18_LMNA	0.08194	-0.078280741	0.238036634	3.16E-01	6.02E-01	1.00E+00
ENSG00000102882.10_MAPK3	ENSG00000160789.18_LMNA	0.02679	-0.132976892	0.185201766	7.43E-01	8.92E-01	1.00E+00
ENSG00000124479.8_NDP	ENSG00000160789.18_LMNA	-0.03328	-0.191461482	0.126596305	6.84E-01	8.63E-01	1.00E+00
ENSG00000197977.3_ELOVL2	ENSG00000160789.18_LMNA	0.32661	0.176594372	0.461801143	4.01E-05	9.05E-04	1.00E+00
ENSG00000111142.12_METAP2	ENSG00000160789.18_LMNA	-0.12306	-0.276836956	0.036863069	1.31E-01	3.67E-01	1.00E+00
ENSG00000176907.4_C8orf4	ENSG00000160789.18_LMNA	0.13546	-0.024265639	0.288438853	9.61E-02	3.04E-01	1.00E+00
ENSG00000149792.7_MRPL49	ENSG00000160789.18_LMNA	-0.14576	-0.298043319	0.013761355	7.32E-02	2.57E-01	1.00E+00
ENSG00000184831.12_APOO	ENSG00000160789.18_LMNA	-0.06753	-0.224321613	0.092664311	4.08E-01	6.83E-01	1.00E+00
ENSG00000196465.9_MYL6B	ENSG00000160789.18_LMNA	-0.22956	-0.375052817	-0.0730269	4.44E-03	3.58E-02	1.00E+00
ENSG00000146834.12_MEPCE	ENSG00000160789.18_LMNA	0.28133	0.127852087	0.421643035	4.46E-04	6.17E-03	1.00E+00
ENSG00000145730.19_PAM	ENSG00000160789.18_LMNA	0.27029	0.116083309	0.411771042	7.58E-04	9.31E-03	1.00E+00

ENSG00000125826.18_RBCK1	ENSG00000160789.18_LMNA	0.25151	0.096158161	0.394897521	1.77E-03	1.79E-02	1.00E+00
ENSG00000230989.5_HSBP1	ENSG00000160789.18_LMNA	-0.39032	-0.517373238	-0.24643447	6.66E-07	3.30E-05	3.42E-02
ENSG00000182934.10_SRPRA	ENSG00000160789.18_LMNA	0.39185	0.248122882	0.518689006	5.97E-07	3.03E-05	3.06E-02
ENSG00000131446.14_MGAT1	ENSG00000160789.18_LMNA	0.26124	0.106465269	0.403651284	1.15E-03	1.29E-02	1.00E+00
ENSG00000130005.10_GAMT	ENSG00000160789.18_LMNA	0.06312	-0.097059467	0.220105547	4.40E-01	7.08E-01	1.00E+00
ENSG00000109390.10_NDUFC1	ENSG00000160789.18_LMNA	0.00324	-0.156045983	0.162352173	9.68E-01	9.88E-01	1.00E+00
ENSG00000100505.12_TRIM9	ENSG00000160789.18_LMNA	0.1106	-0.049469301	0.26513467	1.75E-01	4.36E-01	1.00E+00
ENSG00000184524.5_CEND1	ENSG00000160789.18_LMNA	-0.15062	-0.30256149	0.00879597	6.40E-02	2.35E-01	1.00E+00
ENSG00000064726.8_BTBD1	ENSG00000160789.18_LMNA	0.02824	-0.131551925	0.186601982	7.30E-01	8.86E-01	1.00E+00
ENSG00000225840.2_AC010970.[illegible]	ENSG00000160789.18_LMNA	-0.10139	-0.256452818	0.058757461	2.14E-01	4.88E-01	1.00E+00
ENSG00000058668.13_ATP2B4	ENSG00000160789.18_LMNA	0.06462	-0.095567037	0.221538494	4.29E-01	6.99E-01	1.00E+00
ENSG00000117751.16_PPP1R8	ENSG00000160789.18_LMNA	-0.20331	-0.351143287	-0.04558262	1.20E-02	7.48E-02	1.00E+00
ENSG00000135679.20_MDM2	ENSG00000160789.18_LMNA	0.05237	-0.10772867	0.209821547	5.22E-01	7.67E-01	1.00E+00
ENSG00000168936.9_TMEM129	ENSG00000160789.18_LMNA	0.13504	-0.024693256	0.288046529	9.72E-02	3.06E-01	1.00E+00
ENSG00000101166.14_PRELID3[illegible]	ENSG00000160789.18_LMNA	0.08669	-0.073530242	0.242538785	2.88E-01	5.74E-01	1.00E+00
ENSG00000147677.9_EIF3H	ENSG00000160789.18_LMNA	-0.17084	-0.321305963	-0.01196915	3.53E-02	1.60E-01	1.00E+00
ENSG00000144834.11_TAGLN3	ENSG00000160789.18_LMNA	-0.40496	-0.529992516	-0.26269571	2.28E-07	1.41E-05	1.17E-02
ENSG00000129083.11_COPB1	ENSG00000160789.18_LMNA	0.12277	-0.037159942	0.276562437	1.32E-01	3.68E-01	1.00E+00
ENSG00000092330.14_TINF2	ENSG00000160789.18_LMNA	-0.00911	-0.168068643	0.150306996	9.11E-01	9.66E-01	1.00E+00
ENSG00000211460.10_TSN	ENSG00000160789.18_LMNA	-0.34768	-0.480299835	-0.199528	1.14E-05	3.24E-04	5.85E-01
ENSG00000124226.9_RNF114	ENSG00000160789.18_LMNA	0.0252	-0.134541522	0.183662823	7.58E-01	8.99E-01	1.00E+00
ENSG00000166734.17_CASC4	ENSG00000160789.18_LMNA	0.05863	-0.101515746	0.215818715	4.73E-01	7.33E-01	1.00E+00
ENSG00000146457.13_WTAP	ENSG00000160789.18_LMNA	-0.05263	-0.210066126	0.107475777	5.20E-01	7.66E-01	1.00E+00
ENSG00000139737.20_SLAIN1	ENSG00000160789.18_LMNA	-0.37039	-0.50010546	-0.22442765	2.64E-06	9.88E-05	1.35E-01
ENSG00000213995.10_NAXD	ENSG00000160789.18_LMNA	-0.16653	-0.317316289	-0.00752693	4.03E-02	1.74E-01	1.00E+00
ENSG00000134352.18_IL6ST	ENSG00000160789.18_LMNA	0.08955	-0.070655266	0.245256867	2.73E-01	5.57E-01	1.00E+00
ENSG00000153904.17_DDAH1	ENSG00000160789.18_LMNA	0.00772	-0.151667443	0.166715468	9.25E-01	9.71E-01	1.00E+00
ENSG00000050405.12_LIMA1	ENSG00000160789.18_LMNA	-0.05411	-0.21148904	0.106003672	5.08E-01	7.58E-01	1.00E+00
ENSG00000243317.6_C7orf73	ENSG00000160789.18_LMNA	-0.05837	-0.215567173	0.101776835	4.75E-01	7.34E-01	1.00E+00
ENSG00000100167.18_SEPT3	ENSG00000160789.18_LMNA	-0.41312	-0.53700541	-0.27179866	1.23E-07	8.56E-06	6.29E-03
ENSG00000062485.17_CS	ENSG00000160789.18_LMNA	-0.09734	-0.252624684	0.062835674	2.33E-01	5.11E-01	1.00E+00
ENSG00000099942.11_CRKL	ENSG00000160789.18_LMNA	-0.11321	-0.267588765	0.046833919	1.65E-01	4.21E-01	1.00E+00
ENSG00000101152.9_DNAJC5	ENSG00000160789.18_LMNA	0.21644	0.059285581	0.363131365	7.40E-03	5.24E-02	1.00E+00

ENSG00000157827.18_FMNL2	ENSG00000160789.18_LMNA	-0.17689	-0.326883923	-0.01820036	2.93E-02	1.41E-01	1.00E+00
ENSG00000074696.11_HACD3	ENSG00000160789.18_LMNA	-0.2363	-0.381162796	-0.08011432	3.38E-03	2.92E-02	1.00E+00
ENSG00000107341.4_UBE2R2	ENSG00000160789.18_LMNA	-0.09177	-0.247361134	0.068425925	2.61E-01	5.44E-01	1.00E+00
ENSG00000111786.7_SRSF9	ENSG00000160789.18_LMNA	0.06297	-0.097209741	0.219961186	4.41E-01	7.09E-01	1.00E+00
ENSG00000204272.9_LINC01420	ENSG00000160789.18_LMNA	-0.16277	-0.313837577	-0.00366358	4.51E-02	1.88E-01	1.00E+00
ENSG00000175220.10_ARHGAP1	ENSG00000160789.18_LMNA	0.12852	-0.031317538	0.281955535	1.15E-01	3.38E-01	1.00E+00
ENSG00000117115.11_PADI2	ENSG00000160789.18_LMNA	-0.16833	-0.318984168	-0.00938251	3.82E-02	1.68E-01	1.00E+00
ENSG00000115762.15_PLEKHB2	ENSG00000160789.18_LMNA	-0.27797	-0.418644694	-0.12426888	5.26E-04	7.01E-03	1.00E+00
ENSG00000147416.9_ATP6V1B2	ENSG00000160789.18_LMNA	-0.18359	-0.333055836	-0.02512314	2.36E-02	1.21E-01	1.00E+00
ENSG00000037241.6_RPL26L1	ENSG00000160789.18_LMNA	-0.15565	-0.30723975	0.003638419	5.55E-02	2.15E-01	1.00E+00
ENSG00000113141.14_IK	ENSG00000160789.18_LMNA	0.07113	-0.089080432	0.2277507	3.84E-01	6.62E-01	1.00E+00
ENSG00000164896.18_FASTK	ENSG00000160789.18_LMNA	0.35568	0.208271734	0.487287529	6.90E-06	2.18E-04	3.54E-01
ENSG00000182628.11_SKA2	ENSG00000160789.18_LMNA	-0.31461	-0.451209068	-0.16360087	7.90E-05	1.56E-03	1.00E+00
ENSG00000162909.16_CAPN2	ENSG00000160789.18_LMNA	0.48401	0.351932102	0.597197868	2.66E-10	5.17E-08	1.37E-05
ENSG00000160789.18_LMNA	ENSG00000160789.18_LMNA	1	1	1	0.00E+00	0.00E+00	0.00E+00
ENSG00000130717.11_UCK1	ENSG00000160789.18_LMNA	-0.00224	-0.161383604	0.157016242	9.78E-01	9.92E-01	1.00E+00
ENSG00000112245.8_PTP4A1	ENSG00000160789.18_LMNA	-0.07057	-0.227219181	0.089636481	3.88E-01	6.66E-01	1.00E+00
ENSG00000144021.2_CIAO1	ENSG00000160789.18_LMNA	-0.15383	-0.305551258	0.005501808	5.85E-02	2.22E-01	1.00E+00
ENSG00000054118.12_THRAP3	ENSG00000160789.18_LMNA	0.2643	0.109720138	0.406404365	1.00E-03	1.16E-02	1.00E+00
ENSG00000037897.15_METTL1	ENSG00000160789.18_LMNA	-0.21466	-0.36150975	-0.05742527	7.91E-03	5.52E-02	1.00E+00
ENSG00000170606.12_HSPA4	ENSG00000160789.18_LMNA	0.12992	-0.029902768	0.283258534	1.11E-01	3.32E-01	1.00E+00
ENSG00000104897.8_SF3A2	ENSG00000160789.18_LMNA	0.10683	-0.053273599	0.261584864	1.90E-01	4.58E-01	1.00E+00
ENSG00000109062.8_SLC9A3R1	ENSG00000160789.18_LMNA	-0.14653	-0.298760118	0.012974632	7.16E-02	2.53E-01	1.00E+00
ENSG00000128590.4_DNAJB9	ENSG00000160789.18_LMNA	-0.1341	-0.287173973	0.025643895	9.95E-02	3.11E-01	1.00E+00
ENSG00000056097.14_ZFR	ENSG00000160789.18_LMNA	-0.31818	-0.454364962	-0.16746186	6.48E-05	1.32E-03	1.00E+00
ENSG00000189266.10_PNRC2	ENSG00000160789.18_LMNA	-0.22598	-0.371807189	-0.06927441	5.12E-03	4.00E-02	1.00E+00
ENSG00000163485.14_ADORA1	ENSG00000160789.18_LMNA	0.37519	0.229714824	0.504274178	1.91E-06	7.71E-05	9.80E-02
ENSG00000116670.13_MAD2L2	ENSG00000160789.18_LMNA	-0.05162	-0.209103324	0.108471073	5.28E-01	7.72E-01	1.00E+00
ENSG00000129675.14_ARHGEF6	ENSG00000160789.18_LMNA	0.06997	-0.090238833	0.22664319	3.92E-01	6.70E-01	1.00E+00
ENSG00000090581.8_GNPTG	ENSG00000160789.18_LMNA	0.10652	-0.05359109	0.261288227	1.92E-01	4.59E-01	1.00E+00
ENSG00000204389.9_HSPA1A	ENSG00000160789.18_LMNA	0.24377	0.087986102	0.387918275	2.47E-03	2.30E-02	1.00E+00
ENSG00000174437.15_ATP2A2	ENSG00000160789.18_LMNA	0.18578	0.027386026	0.335067578	2.19E-02	1.15E-01	1.00E+00
ENSG00000102178.11_UBL4A	ENSG00000160789.18_LMNA	0.26481	0.110258062	0.406858842	9.78E-04	1.14E-02	1.00E+00

ENSG00000132305.19_IMMT	ENSG00000160789.18_LMNA	0.07198	-0.088230897	0.228562391	3.78E-01	6.57E-01	1.00E+00
ENSG00000102181.18_CD99L2	ENSG00000160789.18_LMNA	0.01374	-0.145776372	0.172566417	8.67E-01	9.48E-01	1.00E+00
ENSG00000130312.5_MRPL34	ENSG00000160789.18_LMNA	-0.10813	-0.262806194	0.05196574	1.85E-01	4.50E-01	1.00E+00
ENSG00000198612.9_COPS8	ENSG00000160789.18_LMNA	-0.10107	-0.256146562	0.059084112	2.15E-01	4.89E-01	1.00E+00
ENSG00000120705.11_ETF1	ENSG00000160789.18_LMNA	0.2961	0.143670407	0.434803087	2.12E-04	3.41E-03	1.00E+00
ENSG00000170899.9_GSTA4	ENSG00000160789.18_LMNA	-0.53235	-0.637524266	-0.40770752	1.68E-12	8.46E-10	8.63E-08
ENSG00000055211.11_GINM1	ENSG00000160789.18_LMNA	0.08356	-0.076660242	0.239573945	3.06E-01	5.93E-01	1.00E+00
ENSG00000097033.13_SH3GLB1	ENSG00000160789.18_LMNA	0.21903	0.061988293	0.365484022	6.71E-03	4.88E-02	1.00E+00
ENSG00000100387.8_RBX1	ENSG00000160789.18_LMNA	-0.2428	-0.387040004	-0.08696058	2.58E-03	2.38E-02	1.00E+00
ENSG00000135924.14_DNAJB2	ENSG00000160789.18_LMNA	0.10005	-0.06010686	0.255187259	2.20E-01	4.95E-01	1.00E+00
ENSG00000174243.8_DDX23	ENSG00000160789.18_LMNA	0.08227	-0.077950355	0.238350188	3.14E-01	6.00E-01	1.00E+00
ENSG00000100439.9_ABHD4	ENSG00000160789.18_LMNA	0.15816	-0.00107064	0.309563317	5.16E-02	2.06E-01	1.00E+00
ENSG00000189171.12_S100A13	ENSG00000160789.18_LMNA	0.32979	0.18003846	0.464595131	3.34E-05	7.81E-04	1.00E+00
ENSG00000143158.9_MPC2	ENSG00000160789.18_LMNA	-0.2191	-0.365553172	-0.0620678	6.69E-03	4.87E-02	1.00E+00
ENSG00000136485.13_DCAF7	ENSG00000160789.18_LMNA	-0.11868	-0.272727346	0.04130153	1.45E-01	3.90E-01	1.00E+00
ENSG00000141741.10_MIEN1	ENSG00000160789.18_LMNA	-0.3352	-0.469355159	-0.18592215	2.43E-05	6.04E-04	1.00E+00
ENSG00000175193.11_PARL	ENSG00000160789.18_LMNA	-0.2445	-0.388576591	-0.08875521	2.40E-03	2.25E-02	1.00E+00
ENSG00000172086.7_KRCC1	ENSG00000160789.18_LMNA	-0.22223	-0.368400363	-0.06534475	5.93E-03	4.46E-02	1.00E+00
ENSG00000000419.11_DPM1	ENSG00000160789.18_LMNA	-0.01279	-0.17164249	0.146708138	8.76E-01	9.52E-01	1.00E+00
ENSG00000196743.7_GM2A	ENSG00000160789.18_LMNA	0.04081	-0.119160863	0.198723572	6.18E-01	8.27E-01	1.00E+00
ENSG00000118508.4_RAB32	ENSG00000160789.18_LMNA	0.1137	-0.046336145	0.26805184	1.63E-01	4.18E-01	1.00E+00
ENSG00000105185.10_PDCD5	ENSG00000160789.18_LMNA	0.01545	-0.144103653	0.174223658	8.50E-01	9.41E-01	1.00E+00
ENSG00000189241.6_TSPYL1	ENSG00000160789.18_LMNA	-0.32037	-0.456297275	-0.16983024	5.73E-05	1.19E-03	1.00E+00
ENSG00000130382.8_MLLT1	ENSG00000160789.18_LMNA	0.2124	0.055061971	0.359447021	8.61E-03	5.88E-02	1.00E+00
ENSG00000134996.11_OSTF1	ENSG00000160789.18_LMNA	0.06144	-0.098723418	0.218506298	4.52E-01	7.17E-01	1.00E+00
ENSG00000213923.9_CSNK1E	ENSG00000160789.18_LMNA	-0.1432	-0.29566403	0.016369982	7.84E-02	2.69E-01	1.00E+00
ENSG00000154640.13_BTG3	ENSG00000160789.18_LMNA	0.14719	-0.012300641	0.299373924	7.04E-02	2.50E-01	1.00E+00
ENSG00000170855.3_TRIAP1	ENSG00000160789.18_LMNA	-0.11037	-0.264912987	0.049707144	1.76E-01	4.37E-01	1.00E+00
ENSG00000033050.6_ABCF2	ENSG00000160789.18_LMNA	0.25509	0.099947074	0.398121739	1.52E-03	1.58E-02	1.00E+00
ENSG00000120306.8_CYSTM1	ENSG00000160789.18_LMNA	0.03908	-0.120871162	0.197056232	6.33E-01	8.35E-01	1.00E+00
ENSG00000184014.6_DENND5A	ENSG00000160789.18_LMNA	-0.07722	-0.233550029	0.083000587	3.44E-01	6.29E-01	1.00E+00
ENSG00000101310.13_SEC23B	ENSG00000160789.18_LMNA	0.11541	-0.044611089	0.26965552	1.57E-01	4.09E-01	1.00E+00
ENSG00000157778.7_PSMG3	ENSG00000160789.18_LMNA	0.16911	0.010187901	0.319707493	3.73E-02	1.65E-01	1.00E+00

ENSG00000146677.7_AC004453.8	ENSG00000160789.18_LMNA	-0.13712	-0.289993714	0.022569778	9.21E-02	2.96E-01	1.00E+00
ENSG00000074201.7_CLNS1A	ENSG00000160789.18_LMNA	-0.31159	-0.448537697	-0.16033953	9.32E-05	1.79E-03	1.00E+00
ENSG00000125870.9_SNRPB2	ENSG00000160789.18_LMNA	-0.10583	-0.260641371	0.054283198	1.94E-01	4.63E-01	1.00E+00
ENSG00000117411.15_B4GALT2	ENSG00000160789.18_LMNA	0.15072	-0.008689933	0.302657825	6.38E-02	2.35E-01	1.00E+00
ENSG00000145860.10_RNF145	ENSG00000160789.18_LMNA	0.13451	-0.025231272	0.287552768	9.85E-02	3.09E-01	1.00E+00
ENSG00000263266.2_RPS7P1	ENSG00000160789.18_LMNA	-0.18463	-0.334012599	-0.02619895	2.28E-02	1.18E-01	1.00E+00
ENSG00000160255.15_ITGB2	ENSG00000160789.18_LMNA	0.12185	-0.038088695	0.275703284	1.35E-01	3.73E-01	1.00E+00
ENSG00000157933.9_SKI	ENSG00000160789.18_LMNA	0.35335	0.205717979	0.485250343	8.00E-06	2.44E-04	4.10E-01
ENSG00000186298.10_PPP1CC	ENSG00000160789.18_LMNA	-0.2765	-0.417333078	-0.12270383	5.64E-04	7.40E-03	1.00E+00
ENSG00000130309.9_COLGALT1	ENSG00000160789.18_LMNA	0.50129	0.371756153	0.611674384	4.77E-11	1.24E-08	2.45E-06
ENSG00000229119.3_CTB-63M22	ENSG00000160789.18_LMNA	0.01041	-0.149035562	0.169332198	8.99E-01	9.61E-01	1.00E+00
ENSG00000129255.13_MPDU1	ENSG00000160789.18_LMNA	0.00617	-0.153180555	0.165209032	9.40E-01	9.76E-01	1.00E+00
ENSG00000118363.10_SPCS2	ENSG00000160789.18_LMNA	-0.19013	-0.339071654	-0.03189938	1.90E-02	1.04E-01	1.00E+00
ENSG00000114200.8_BCHE	ENSG00000160789.18_LMNA	-0.36514	-0.495539098	-0.21865471	3.74E-06	1.31E-04	1.92E-01
ENSG00000198561.11_CTNND1	ENSG00000160789.18_LMNA	0.16411	0.005046094	0.315083405	4.33E-02	1.83E-01	1.00E+00
ENSG00000138080.12_EMILIN1	ENSG00000160789.18_LMNA	0.47762	0.344620952	0.591818261	4.91E-10	8.72E-08	2.52E-05
ENSG00000136045.10_PWP1	ENSG00000160789.18_LMNA	-0.23007	-0.375513498	-0.07356021	4.35E-03	3.54E-02	1.00E+00
ENSG00000117298.13_ECE1	ENSG00000160789.18_LMNA	0.56403	0.444763494	0.663637633	3.83E-14	4.10E-11	1.97E-09
ENSG00000172775.15_FAM192A	ENSG00000160789.18_LMNA	-0.22833	-0.373934872	-0.0717334	4.67E-03	3.72E-02	1.00E+00
ENSG00000198744.5_MTCO3P12	ENSG00000160789.18_LMNA	-0.14612	-0.298375967	0.013396305	7.25E-02	2.55E-01	1.00E+00
ENSG00000086289.10_EPDR1	ENSG00000160789.18_LMNA	0.07889	-0.081335074	0.235134777	3.34E-01	6.20E-01	1.00E+00
ENSG00000103034.13_NDRG4	ENSG00000160789.18_LMNA	-0.22236	-0.368518867	-0.06548128	5.90E-03	4.45E-02	1.00E+00
ENSG00000100028.10_SNRPD3	ENSG00000160789.18_LMNA	0.0057	-0.153636808	0.164754501	9.44E-01	9.78E-01	1.00E+00
ENSG00000132383.10_RPA1	ENSG00000160789.18_LMNA	0.07679	-0.083429368	0.233141768	3.47E-01	6.31E-01	1.00E+00
ENSG00000198176.11_TFDP1	ENSG00000160789.18_LMNA	0.03103	-0.128807294	0.189295302	7.04E-01	8.74E-01	1.00E+00
ENSG00000181222.13_POLR2A	ENSG00000160789.18_LMNA	0.17679	0.018098959	0.326793324	2.93E-02	1.41E-01	1.00E+00
ENSG00000080493.12_SLC4A4	ENSG00000160789.18_LMNA	0.01883	-0.140793663	0.177497717	8.18E-01	9.29E-01	1.00E+00
ENSG00000168264.9_IRF2BP2	ENSG00000160789.18_LMNA	0.0446	-0.115423048	0.202361105	5.85E-01	8.07E-01	1.00E+00
ENSG00000138381.8_ASNSD1	ENSG00000160789.18_LMNA	-0.361	-0.491924946	-0.21409928	4.90E-06	1.64E-04	2.51E-01
ENSG00000163930.8_BAP1	ENSG00000160789.18_LMNA	0.09154	-0.06865871	0.247141549	2.62E-01	5.45E-01	1.00E+00
ENSG00000103769.8_RAB11A	ENSG00000160789.18_LMNA	-0.15456	-0.306221681	0.0047622	5.73E-02	2.19E-01	1.00E+00
ENSG00000010017.12_RANBP9	ENSG00000160789.18_LMNA	0.27356	0.119568025	0.414701373	6.49E-04	8.27E-03	1.00E+00
ENSG00000077312.7_SNRPA	ENSG00000160789.18_LMNA	0.00912	-0.15029585	0.168079725	9.11E-01	9.66E-01	1.00E+00

ENSG00000139168.6_ZCRB1	ENSG00000160789.18_LMNA	-0.2835	-0.423585992	-0.13017814	4.01E-04	5.67E-03	1.00E+00
ENSG00000135919.11_SERPINE2	ENSG00000160789.18_LMNA	-0.02502	-0.183487066	0.134720111	7.60E-01	9.00E-01	1.00E+00
ENSG00000102401.18_ARMCX3	ENSG00000160789.18_LMNA	0.07683	-0.083390829	0.233178467	3.47E-01	6.31E-01	1.00E+00
ENSG00000130222.9_GADD45G	ENSG00000160789.18_LMNA	-0.37414	-0.50335555	-0.22854834	2.05E-06	8.16E-05	1.05E-01
ENSG00000176485.9_PLA2G16	ENSG00000160789.18_LMNA	0.1502	-0.009224292	0.302172296	6.48E-02	2.37E-01	1.00E+00
ENSG00000106066.12_CPVL	ENSG00000160789.18_LMNA	0.0744	-0.085813212	0.230869971	3.62E-01	6.44E-01	1.00E+00
ENSG00000175183.8_CSRP2	ENSG00000160789.18_LMNA	0.10998	-0.050099958	0.26454679	1.77E-01	4.39E-01	1.00E+00
ENSG00000167862.8_MRPL58	ENSG00000160789.18_LMNA	-0.20662	-0.354168408	-0.04902967	1.07E-02	6.85E-02	1.00E+00
ENSG00000129151.7_BBOX1	ENSG00000160789.18_LMNA	-0.07624	-0.232615492	0.083981923	3.51E-01	6.34E-01	1.00E+00
ENSG00000143183.15_TMCO1	ENSG00000160789.18_LMNA	0.00364	-0.155651199	0.162746094	9.65E-01	9.86E-01	1.00E+00
ENSG00000243449.5_C4orf48	ENSG00000160789.18_LMNA	-0.0974	-0.252683745	0.062772834	2.33E-01	5.10E-01	1.00E+00
ENSG00000164776.8_PHKG1	ENSG00000160789.18_LMNA	-0.01412	-0.172934777	0.145404729	8.63E-01	9.46E-01	1.00E+00
ENSG00000068079.6_IFI35	ENSG00000160789.18_LMNA	0.12429	-0.035616517	0.277989089	1.27E-01	3.61E-01	1.00E+00
ENSG00000100603.12_SNW1	ENSG00000160789.18_LMNA	0.21031	0.052884679	0.357544009	9.30E-03	6.22E-02	1.00E+00
ENSG00000174775.15_HRAS	ENSG00000160789.18_LMNA	0.27983	0.126256438	0.420308627	4.80E-04	6.53E-03	1.00E+00
ENSG00000161202.16_DVL3	ENSG00000160789.18_LMNA	0.06782	-0.092373582	0.224600078	4.06E-01	6.82E-01	1.00E+00
ENSG00000103035.9_PSMD7	ENSG00000160789.18_LMNA	0.06775	-0.092452015	0.224524958	4.07E-01	6.82E-01	1.00E+00
ENSG00000066336.10_SPI1	ENSG00000160789.18_LMNA	0.07968	-0.080540164	0.235890548	3.29E-01	6.15E-01	1.00E+00
ENSG00000204103.3_MAFB	ENSG00000160789.18_LMNA	0.06038	-0.099778536	0.217491324	4.60E-01	7.23E-01	1.00E+00
ENSG00000179918.16_SEPHS2	ENSG00000160789.18_LMNA	0.22916	0.07261163	0.374694005	4.51E-03	3.63E-02	1.00E+00
ENSG00000160691.17_SHC1	ENSG00000160789.18_LMNA	0.57614	0.459038661	0.673557008	8.10E-15	1.19E-11	4.16E-10
ENSG00000106397.10_PLOD3	ENSG00000160789.18_LMNA	0.69113	0.597695217	0.766045902	6.56E-23	9.95E-19	3.36E-18
ENSG00000108819.10_PPP1R9B	ENSG00000160789.18_LMNA	0.23761	0.081493094	0.382348372	3.20E-03	2.80E-02	1.00E+00
ENSG00000114503.9_NCBP2	ENSG00000160789.18_LMNA	-0.06489	-0.221801472	0.095292988	4.27E-01	6.98E-01	1.00E+00
ENSG00000164105.3_SAP30	ENSG00000160789.18_LMNA	0.03005	-0.129772479	0.188348706	7.13E-01	8.78E-01	1.00E+00
ENSG00000131779.9_PEX11B	ENSG00000160789.18_LMNA	-0.2036	-0.351405862	-0.04588153	1.19E-02	7.42E-02	1.00E+00
ENSG00000004455.15_AK2	ENSG00000160789.18_LMNA	0.16849	0.009549036	0.319133755	3.80E-02	1.67E-01	1.00E+00
ENSG00000111653.18_ING4	ENSG00000160789.18_LMNA	-0.50908	-0.618178363	-0.38073447	2.13E-11	6.33E-09	1.09E-06
ENSG00000151366.11_NDUFC2	ENSG00000160789.18_LMNA	-0.40713	-0.531858821	-0.26511359	1.94E-07	1.23E-05	9.94E-03
ENSG00000071054.14_MAP4K4	ENSG00000160789.18_LMNA	0.00043	-0.158785429	0.159615918	9.96E-01	9.98E-01	1.00E+00
ENSG00000196704.10_AMZ2	ENSG00000160789.18_LMNA	-0.1499	-0.301898252	0.009525819	6.53E-02	2.38E-01	1.00E+00
ENSG00000007168.11_PAFAH1B	ENSG00000160789.18_LMNA	-0.04444	-0.202209242	0.115579278	5.87E-01	8.08E-01	1.00E+00
ENSG00000205155.6_PSENEN	ENSG00000160789.18_LMNA	-0.06208	-0.219110515	0.098094964	4.47E-01	7.14E-01	1.00E+00

ENSG00000224114.1_RP11-343H	ENSG00000160789.18_LMNA	0.07848	-0.08173895	0.234750641	3.37E-01	6.22E-01	1.00E+00
ENSG00000138073.12_PREB	ENSG00000160789.18_LMNA	-0.07688	-0.23323008	0.083336628	3.46E-01	6.31E-01	1.00E+00
ENSG00000141510.14_TP53	ENSG00000160789.18_LMNA	0.24502	0.089304047	0.38904618	2.35E-03	2.21E-02	1.00E+00
ENSG00000240771.5_ARHGEF25	ENSG00000160789.18_LMNA	-0.34244	-0.475704714	-0.19380237	1.57E-05	4.23E-04	8.07E-01
ENSG00000165943.4_MOAP1	ENSG00000160789.18_LMNA	-0.30116	-0.439297331	-0.14910676	1.63E-04	2.78E-03	1.00E+00
ENSG00000149480.5_MTA2	ENSG00000160789.18_LMNA	0.07842	-0.08180045	0.234692138	3.37E-01	6.22E-01	1.00E+00
ENSG00000106236.3_NPTX2	ENSG00000160789.18_LMNA	0.10821	-0.051882991	0.262883435	1.85E-01	4.50E-01	1.00E+00
ENSG00000144579.6_CTDSP1	ENSG00000160789.18_LMNA	0.37466	0.229130075	0.503813756	1.98E-06	7.92E-05	1.02E-01
ENSG00000168393.11_DTYMK	ENSG00000160789.18_LMNA	-0.04173	-0.19960205	0.118258991	6.10E-01	8.22E-01	1.00E+00
ENSG00000162236.10_STX5	ENSG00000160789.18_LMNA	-0.13953	-0.292237155	0.020119721	8.65E-02	2.86E-01	1.00E+00
ENSG00000106263.16_EIF3B	ENSG00000160789.18_LMNA	0.35345	0.20583428	0.485343184	7.94E-06	2.43E-04	4.07E-01
ENSG00000164985.13_PSIP1	ENSG00000160789.18_LMNA	-0.02396	-0.182468782	0.135754401	7.69E-01	9.05E-01	1.00E+00
ENSG00000166313.17_APBB1	ENSG00000160789.18_LMNA	-0.26151	-0.403893416	-0.10675128	1.14E-03	1.28E-02	1.00E+00
ENSG00000129187.13_DCTD	ENSG00000160789.18_LMNA	0.35924	0.212173716	0.490394373	5.49E-06	1.79E-04	2.82E-01
ENSG00000132912.11_DCTN4	ENSG00000160789.18_LMNA	-0.13583	-0.288785389	0.023887833	9.52E-02	3.02E-01	1.00E+00
ENSG00000122884.11_P4HA1	ENSG00000160789.18_LMNA	0.22979	0.073274647	0.375266842	4.40E-03	3.56E-02	1.00E+00
ENSG00000108773.9_KAT2A	ENSG00000160789.18_LMNA	-0.0069	-0.165919947	0.152466675	9.33E-01	9.74E-01	1.00E+00
ENSG00000102978.11_POLR2C	ENSG00000160789.18_LMNA	-0.03419	-0.192339151	0.125699579	6.76E-01	8.58E-01	1.00E+00
ENSG00000187735.11_TCEA1	ENSG00000160789.18_LMNA	-0.02465	-0.183127695	0.13508521	7.63E-01	9.01E-01	1.00E+00
ENSG00000169413.2_RNASE6	ENSG00000160789.18_LMNA	-0.05236	-0.209815112	0.107735324	5.22E-01	7.67E-01	1.00E+00
ENSG00000143393.15_PI4KB	ENSG00000160789.18_LMNA	0.26096	0.106168238	0.403399777	1.17E-03	1.30E-02	1.00E+00
ENSG00000135211.5_TMEM60	ENSG00000160789.18_LMNA	0.07467	-0.085549251	0.231121696	3.61E-01	6.43E-01	1.00E+00
ENSG00000164897.11_TMUB1	ENSG00000160789.18_LMNA	0.42734	0.287707945	0.549174498	4.00E-08	3.43E-06	2.05E-03
ENSG00000090432.6_MUL1	ENSG00000160789.18_LMNA	0.38308	0.238422138	0.511111752	1.11E-06	5.00E-05	5.69E-02
ENSG00000187778.12_MCRS1	ENSG00000160789.18_LMNA	-0.30948	-0.446671218	-0.15806457	1.05E-04	1.96E-03	1.00E+00
ENSG00000106635.6_BCL7B	ENSG00000160789.18_LMNA	0.21224	0.054895939	0.359301994	8.66E-03	5.90E-02	1.00E+00
ENSG00000180398.10_MCFD2	ENSG00000160789.18_LMNA	0.20882	0.051329368	0.356183074	9.83E-03	6.48E-02	1.00E+00
ENSG00000264350.1_SNRPGP2	ENSG00000160789.18_LMNA	-0.16428	-0.315237387	-0.00521705	4.31E-02	1.82E-01	1.00E+00
ENSG00000165457.12_FOLR2	ENSG00000160789.18_LMNA	-0.05218	-0.209643179	0.107913076	5.23E-01	7.69E-01	1.00E+00
ENSG00000164879.6_CA3	ENSG00000160789.18_LMNA	-0.04838	-0.20599805	0.111676818	5.54E-01	7.88E-01	1.00E+00
ENSG00000136628.16_EPRS	ENSG00000160789.18_LMNA	0.08904	-0.071165733	0.24477462	2.75E-01	5.60E-01	1.00E+00
ENSG00000125734.12_GPR108	ENSG00000160789.18_LMNA	0.17525	0.016512477	0.325375109	3.08E-02	1.46E-01	1.00E+00
ENSG00000131475.5_VPS25	ENSG00000160789.18_LMNA	-0.15789	-0.309314973	0.001345281	5.20E-02	2.07E-01	1.00E+00

ENSG00000175567.7_UCP2	ENSG00000160789.18_LMNA	-0.08017	-0.236358637	0.080047632	3.26E-01	6.13E-01	1.00E+00
ENSG00000233913.7_RPL10P9	ENSG00000160789.18_LMNA	-0.01711	-0.175829142	0.14248144	8.34E-01	9.36E-01	1.00E+00
ENSG00000100442.9_FKBP3	ENSG00000160789.18_LMNA	-0.07827	-0.234547202	0.081952801	3.38E-01	6.23E-01	1.00E+00
ENSG00000196365.10_LONP1	ENSG00000160789.18_LMNA	0.356	0.208618736	0.487564106	6.76E-06	2.14E-04	3.47E-01
ENSG00000108349.13_CASC3	ENSG00000160789.18_LMNA	-0.33465	-0.468868153	-0.18531925	2.51E-05	6.20E-04	1.00E+00
ENSG00000115446.10_UNC50	ENSG00000160789.18_LMNA	-0.25104	-0.394474922	-0.09566219	1.81E-03	1.82E-02	1.00E+00
ENSG00000227615.1_RP11-864N	ENSG00000160789.18_LMNA	-0.16189	-0.313025267	-0.00276278	4.63E-02	1.91E-01	1.00E+00
ENSG00000182132.11_KCNIP1	ENSG00000160789.18_LMNA	0.00713	-0.152241286	0.166144332	9.31E-01	9.73E-01	1.00E+00
ENSG00000183513.7_COA5	ENSG00000160789.18_LMNA	-0.45673	-0.574184237	-0.32086388	3.33E-09	4.23E-07	1.71E-04
ENSG00000155304.5_HSPA13	ENSG00000160789.18_LMNA	-0.0237	-0.182215111	0.136011951	7.72E-01	9.06E-01	1.00E+00
ENSG00000169727.11_GPS1	ENSG00000160789.18_LMNA	-0.20563	-0.353266728	-0.04800147	1.10E-02	7.03E-02	1.00E+00
ENSG00000085117.10_CD82	ENSG00000160789.18_LMNA	0.18354	0.025068655	0.333007364	2.36E-02	1.21E-01	1.00E+00
ENSG00000095321.15_CRAT	ENSG00000160789.18_LMNA	0.34973	0.201768098	0.482093446	1.00E-05	2.92E-04	5.15E-01
ENSG00000143436.9_MRPL9	ENSG00000160789.18_LMNA	-0.02069	-0.179296801	0.1389718	8.00E-01	9.20E-01	1.00E+00
ENSG00000148337.18_CIZ1	ENSG00000160789.18_LMNA	0.23852	0.082455108	0.383174999	3.08E-03	2.73E-02	1.00E+00
ENSG00000213465.6_ARL2	ENSG00000160789.18_LMNA	-0.21824	-0.36476877	-0.06116615	6.91E-03	5.00E-02	1.00E+00
ENSG00000162735.17_PEX19	ENSG00000160789.18_LMNA	-0.3927	-0.519425904	-0.2490692	5.61E-07	2.88E-05	2.88E-02
ENSG00000175745.10_NR2F1	ENSG00000160789.18_LMNA	-0.01572	-0.174487477	0.143837203	8.48E-01	9.40E-01	1.00E+00
ENSG00000101079.19_NDRG3	ENSG00000160789.18_LMNA	-0.46695	-0.582823429	-0.33246316	1.33E-09	1.96E-07	6.81E-05
ENSG00000132406.10_TMEM128	ENSG00000160789.18_LMNA	-0.32728	-0.462387369	-0.17731642	3.86E-05	8.75E-04	1.00E+00
ENSG00000063180.7_CA11	ENSG00000160789.18_LMNA	-0.09168	-0.247270056	0.068522482	2.61E-01	5.45E-01	1.00E+00
ENSG00000230629.2_RPS23P8	ENSG00000160789.18_LMNA	-0.16569	-0.316538648	-0.0066625	4.14E-02	1.77E-01	1.00E+00
ENSG00000153395.8_LPCAT1	ENSG00000160789.18_LMNA	0.24982	0.094373481	0.393376274	1.91E-03	1.89E-02	1.00E+00
ENSG00000150316.10_CWC15	ENSG00000160789.18_LMNA	-0.34363	-0.476752728	-0.19510653	1.46E-05	4.00E-04	7.50E-01
ENSG00000224631.4_RPS27AP1	ENSG00000160789.18_LMNA	-0.15198	-0.303833701	0.007395058	6.16E-02	2.29E-01	1.00E+00
ENSG00000198858.8_R3HDM4	ENSG00000160789.18_LMNA	0.20925	0.05177585	0.356573889	9.67E-03	6.41E-02	1.00E+00
ENSG00000104889.4_RNASEH2A	ENSG00000160789.18_LMNA	-0.00632	-0.165358269	0.153030724	9.38E-01	9.76E-01	1.00E+00
ENSG00000122756.13_CNTFR	ENSG00000160789.18_LMNA	-0.27593	-0.416821723	-0.12209406	5.80E-04	7.57E-03	1.00E+00
ENSG00000162434.10_JAK1	ENSG00000160789.18_LMNA	-0.05263	-0.210068628	0.107473189	5.20E-01	7.66E-01	1.00E+00
ENSG00000137965.9_IFI44	ENSG00000160789.18_LMNA	-0.08224	-0.238320344	0.077981804	3.14E-01	6.00E-01	1.00E+00
ENSG00000166562.7_SEC11C	ENSG00000160789.18_LMNA	-0.299	-0.437384434	-0.14679072	1.83E-04	3.04E-03	1.00E+00
ENSG00000106400.10_ZNHIT1	ENSG00000160789.18_LMNA	-0.07997	-0.236162151	0.080254397	3.27E-01	6.14E-01	1.00E+00
ENSG00000101457.11_DNTTIP1	ENSG00000160789.18_LMNA	0.16405	0.004977536	0.31502165	4.34E-02	1.83E-01	1.00E+00

ENSG00000163754.16_GYG1	ENSG00000160789.18_LMNA	-0.39159	-0.51846927	-0.2478408	6.08E-07	3.06E-05	3.12E-02
ENSG00000101347.8_SAMHD1	ENSG00000160789.18_LMNA	-0.0317	-0.189942077	0.128147471	6.98E-01	8.71E-01	1.00E+00
ENSG00000146112.10_PPP1R18	ENSG00000160789.18_LMNA	0.5239	0.397890687	0.630516714	4.32E-12	1.77E-09	2.22E-07
ENSG00000184887.12_BTBD6	ENSG00000160789.18_LMNA	0.27312	0.119096993	0.414305636	6.63E-04	8.40E-03	1.00E+00
ENSG00000109113.16_RAB34	ENSG00000160789.18_LMNA	0.4602	0.324800589	0.577122652	2.45E-09	3.29E-07	1.25E-04
ENSG00000176890.14_TYMS	ENSG00000160789.18_LMNA	0.11519	-0.044832968	0.269449351	1.58E-01	4.10E-01	1.00E+00
ENSG00000089486.15_CDIP1	ENSG00000160789.18_LMNA	-0.30387	-0.441702917	-0.15202385	1.41E-04	2.48E-03	1.00E+00
ENSG00000113552.14_GNPDA1	ENSG00000160789.18_LMNA	0.02267	-0.137021691	0.181220172	7.82E-01	9.10E-01	1.00E+00
ENSG00000143546.8_S100A8	ENSG00000160789.18_LMNA	0.08985	-0.0703568	0.24553876	2.71E-01	5.55E-01	1.00E+00
ENSG00000144867.10_SRPRB	ENSG00000160789.18_LMNA	-0.08895	-0.244687666	0.071257757	2.76E-01	5.60E-01	1.00E+00
ENSG00000124098.9_FAM210B	ENSG00000160789.18_LMNA	0.18415	0.025700196	0.333569115	2.31E-02	1.20E-01	1.00E+00
ENSG00000056050.6_HPF1	ENSG00000160789.18_LMNA	-0.20615	-0.353740598	-0.04854175	1.08E-02	6.94E-02	1.00E+00
ENSG00000171051.7_FPR1	ENSG00000160789.18_LMNA	-0.01979	-0.178427758	0.139852115	8.09E-01	9.24E-01	1.00E+00
ENSG00000072042.11_RDH11	ENSG00000160789.18_LMNA	-0.09883	-0.254037977	0.061331281	2.26E-01	5.02E-01	1.00E+00
ENSG00000165424.6_ZCCHC24	ENSG00000160789.18_LMNA	0.01369	-0.145826887	0.172516341	8.67E-01	9.48E-01	1.00E+00
ENSG00000148926.8_ADM	ENSG00000160789.18_LMNA	0.34519	0.19680973	0.478120198	1.33E-05	3.68E-04	6.82E-01
ENSG00000104131.11_EIF3J	ENSG00000160789.18_LMNA	-0.01483	-0.173625112	0.144708	8.56E-01	9.43E-01	1.00E+00
ENSG00000184905.7_TCEAL2	ENSG00000160789.18_LMNA	-0.29457	-0.433444316	-0.14203026	2.30E-04	3.65E-03	1.00E+00
ENSG00000173141.4_MRPL57	ENSG00000160789.18_LMNA	-0.13153	-0.284769684	0.028260417	1.06E-01	3.24E-01	1.00E+00
ENSG00000161671.15_EMC10	ENSG00000160789.18_LMNA	0.03818	-0.121757183	0.196191743	6.40E-01	8.39E-01	1.00E+00
ENSG00000090487.9_SPG21	ENSG00000160789.18_LMNA	-0.04264	-0.20048	0.117357137	6.02E-01	8.17E-01	1.00E+00
ENSG00000105409.14_ATP1A3	ENSG00000160789.18_LMNA	-0.38178	-0.509983179	-0.23698193	1.22E-06	5.38E-05	6.23E-02
ENSG00000076706.13_MCAM	ENSG00000160789.18_LMNA	0.18754	0.029207271	0.336684658	2.07E-02	1.10E-01	1.00E+00
ENSG00000205339.8_IPO7	ENSG00000160789.18_LMNA	0.03807	-0.121867829	0.196083751	6.41E-01	8.40E-01	1.00E+00
ENSG00000172239.12_PAIP1	ENSG00000160789.18_LMNA	-0.15746	-0.30891502	0.001787487	5.27E-02	2.08E-01	1.00E+00
ENSG00000141480.16_ARRB2	ENSG00000160789.18_LMNA	-0.0847	-0.240655356	0.075519312	3.00E-01	5.86E-01	1.00E+00
ENSG00000218283.2_MORF4L1P	ENSG00000160789.18_LMNA	-0.14751	-0.299671436	0.011973856	6.98E-02	2.49E-01	1.00E+00
ENSG00000163527.8_STT3B	ENSG00000160789.18_LMNA	0.16459	0.005536299	0.315524888	4.27E-02	1.81E-01	1.00E+00
ENSG00000239789.4_MRPS17	ENSG00000160789.18_LMNA	0.02473	-0.135002422	0.183209192	7.62E-01	9.01E-01	1.00E+00
ENSG00000151465.12_CDC123	ENSG00000160789.18_LMNA	-0.36075	-0.491713734	-0.21383343	4.98E-06	1.65E-04	2.55E-01
ENSG00000198794.10_SCAMP5	ENSG00000160789.18_LMNA	-0.32299	-0.458611825	-0.17267149	4.94E-05	1.07E-03	1.00E+00
ENSG00000079459.11_FDFT1	ENSG00000160789.18_LMNA	-0.23797	-0.382673725	-0.08187167	3.16E-03	2.77E-02	1.00E+00
ENSG00000111237.17_VPS29	ENSG00000160789.18_LMNA	-0.23906	-0.383657499	-0.08301689	3.02E-03	2.69E-02	1.00E+00

ENSG00000006468.12_ETV1	ENSG00000160789.18_LMNA	-0.15976	-0.311053464	-0.00057828	4.93E-02	1.99E-01	1.00E+00
ENSG00000138796.14_HADH	ENSG00000160789.18_LMNA	-0.26625	-0.408147435	-0.1117842	9.15E-04	1.08E-02	1.00E+00
ENSG00000115216.12_NRBP1	ENSG00000160789.18_LMNA	0.32082	0.170317415	0.456694413	5.58E-05	1.17E-03	1.00E+00
ENSG00000114902.12_SPCS1	ENSG00000160789.18_LMNA	-0.19498	-0.343519527	-0.0369277	1.61E-02	9.22E-02	1.00E+00
ENSG00000165458.12_INPPL1	ENSG00000160789.18_LMNA	0.21342	0.056128691	0.36037844	8.29E-03	5.72E-02	1.00E+00
ENSG00000198860.10_TSEN15	ENSG00000160789.18_LMNA	-0.23358	-0.378702158	-0.07725638	3.78E-03	3.18E-02	1.00E+00
ENSG00000105700.9_KXD1	ENSG00000160789.18_LMNA	0.0627	-0.09746956	0.219711558	4.43E-01	7.10E-01	1.00E+00
ENSG00000071967.10_CYBRD1	ENSG00000160789.18_LMNA	0.20924	0.051768511	0.356567466	9.68E-03	6.41E-02	1.00E+00
ENSG00000155438.10_NIFK	ENSG00000160789.18_LMNA	-0.02746	-0.185852723	0.132314592	7.37E-01	8.89E-01	1.00E+00
ENSG00000102054.16_RBBP7	ENSG00000160789.18_LMNA	0.05021	-0.109869836	0.207749187	5.39E-01	7.79E-01	1.00E+00
ENSG00000105426.13_PTPRS	ENSG00000160789.18_LMNA	-0.12296	-0.27674028	0.036967624	1.31E-01	3.67E-01	1.00E+00
ENSG00000088448.13_ANKRD10	ENSG00000160789.18_LMNA	-0.06019	-0.217311659	0.099965234	4.61E-01	7.25E-01	1.00E+00
ENSG00000109736.13_MFSD10	ENSG00000160789.18_LMNA	0.4251	0.285197844	0.547261849	4.79E-08	3.93E-06	2.46E-03
ENSG00000164402.12_SEPT8	ENSG00000160789.18_LMNA	-0.13673	-0.289622252	0.022975089	9.30E-02	2.98E-01	1.00E+00
ENSG00000112559.12_MDFI	ENSG00000160789.18_LMNA	-0.12643	-0.279998352	0.033440254	1.21E-01	3.49E-01	1.00E+00
ENSG00000278970.1_HEIH	ENSG00000160789.18_LMNA	-0.0402	-0.198128216	0.119771774	6.23E-01	8.30E-01	1.00E+00
ENSG00000116044.14_NFE2L2	ENSG00000160789.18_LMNA	0.34872	0.200664171	0.481209842	1.07E-05	3.07E-04	5.48E-01
ENSG00000034677.10_RNF19A	ENSG00000160789.18_LMNA	-0.09474	-0.250166638	0.065448753	2.46E-01	5.25E-01	1.00E+00
ENSG00000131462.6_TUBG1	ENSG00000160789.18_LMNA	0.18005	0.021467646	0.329800114	2.64E-02	1.32E-01	1.00E+00
ENSG00000151239.12_TWF1	ENSG00000160789.18_LMNA	-0.12385	-0.277577057	0.036062428	1.28E-01	3.63E-01	1.00E+00
ENSG00000163661.3_PTX3	ENSG00000160789.18_LMNA	0.37355	0.227899751	0.502844509	2.14E-06	8.38E-05	1.10E-01
ENSG00000053372.4_MRTO4	ENSG00000160789.18_LMNA	-0.04187	-0.199737563	0.118119822	6.09E-01	8.21E-01	1.00E+00
ENSG00000105393.14_BABAM1	ENSG00000160789.18_LMNA	-0.21594	-0.36267108	-0.05875733	7.54E-03	5.32E-02	1.00E+00
ENSG00000100612.12_DHRS7	ENSG00000160789.18_LMNA	-0.25569	-0.398665131	-0.10058649	1.48E-03	1.55E-02	1.00E+00
ENSG00000176533.11_GNG7	ENSG00000160789.18_LMNA	-0.00495	-0.16402532	0.154368468	9.52E-01	9.81E-01	1.00E+00
ENSG00000163565.17_IFI16	ENSG00000160789.18_LMNA	0.26057	0.105751583	0.403046903	1.19E-03	1.32E-02	1.00E+00
ENSG00000106299.7_WASL	ENSG00000160789.18_LMNA	0.2309	0.074435814	0.37626952	4.21E-03	3.45E-02	1.00E+00
ENSG00000114354.11_TFG	ENSG00000160789.18_LMNA	0.22088	0.063930372	0.367172173	6.25E-03	4.63E-02	1.00E+00
ENSG00000156642.15_NPTN	ENSG00000160789.18_LMNA	0.1042	-0.05593262	0.259098654	2.01E-01	4.72E-01	1.00E+00
ENSG00000164305.16_CASP3	ENSG00000160789.18_LMNA	0.188	0.029688738	0.337111849	2.04E-02	1.09E-01	1.00E+00
ENSG00000172638.11_EFEMP2	ENSG00000160789.18_LMNA	0.64419	0.540411536	0.728662415	3.45E-19	1.77E-15	1.77E-14
ENSG00000128567.15_PODXL	ENSG00000160789.18_LMNA	0.30616	0.154490081	0.443733465	1.25E-04	2.26E-03	1.00E+00
ENSG00000260807.5_RP11-161M	ENSG00000160789.18_LMNA	-0.43758	-0.557916309	-0.29922633	1.73E-08	1.70E-06	8.87E-04

ENSG00000105618.12_PRPF31	ENSG00000160789.18_LMNA	0.26282	0.108148279	0.405075505	1.07E-03	1.22E-02	1.00E+00
ENSG00000132470.12_ITGB4	ENSG00000160789.18_LMNA	0.25589	0.100793143	0.398840706	1.46E-03	1.54E-02	1.00E+00
ENSG00000244187.6_TMEM141	ENSG00000160789.18_LMNA	-0.00871	-0.167673685	0.150704201	9.15E-01	9.68E-01	1.00E+00
ENSG00000163931.14_TKT	ENSG00000160789.18_LMNA	0.16695	0.00796385	0.317709185	3.98E-02	1.73E-01	1.00E+00
ENSG00000160752.13_FDPS	ENSG00000160789.18_LMNA	0.0592	-0.100952078	0.216361626	4.69E-01	7.30E-01	1.00E+00
ENSG00000169919.15_GUSB	ENSG00000160789.18_LMNA	0.54401	0.421293105	0.647159738	4.38E-13	3.00E-10	2.25E-08
ENSG00000105559.10_PLEKHA4	ENSG00000160789.18_LMNA	0.49512	0.364668782	0.606517218	8.91E-11	2.06E-08	4.57E-06
ENSG00000149428.17_HYOU1	ENSG00000160789.18_LMNA	0.26285	0.10817315	0.40509654	1.07E-03	1.22E-02	1.00E+00
ENSG00000177733.6_HNRNPA0	ENSG00000160789.18_LMNA	-0.0674	-0.224198389	0.092792945	4.09E-01	6.83E-01	1.00E+00
ENSG00000169604.18_ANTXR1	ENSG00000160789.18_LMNA	-0.12665	-0.280200284	0.033221374	1.20E-01	3.48E-01	1.00E+00
ENSG00000135472.7_FAIM2	ENSG00000160789.18_LMNA	-0.36725	-0.497373349	-0.2209713	3.25E-06	1.17E-04	1.67E-01
ENSG00000183431.10_SF3A3	ENSG00000160789.18_LMNA	0.11141	-0.048658427	0.265890198	1.72E-01	4.31E-01	1.00E+00
ENSG00000163041.8_H3F3A	ENSG00000160789.18_LMNA	-0.30293	-0.440871291	-0.15101482	1.49E-04	2.58E-03	1.00E+00
ENSG00000125970.10_RALY	ENSG00000160789.18_LMNA	0.17529	0.016548822	0.325407615	3.08E-02	1.46E-01	1.00E+00
ENSG00000106723.15_SPIN1	ENSG00000160789.18_LMNA	-0.08337	-0.239392401	0.076851697	3.07E-01	5.94E-01	1.00E+00
ENSG00000068308.12_OTUD5	ENSG00000160789.18_LMNA	0.19173	0.033555883	0.34053846	1.80E-02	9.99E-02	1.00E+00
ENSG00000171421.11_MRPL36	ENSG00000160789.18_LMNA	0.15329	-0.006063234	0.305042144	5.94E-02	2.24E-01	1.00E+00
ENSG00000112799.7_LY86	ENSG00000160789.18_LMNA	-0.16014	-0.311399518	-0.00096145	4.88E-02	1.98E-01	1.00E+00
ENSG00000104679.9_R3HCC1	ENSG00000160789.18_LMNA	-0.12213	-0.27596254	0.037808493	1.34E-01	3.72E-01	1.00E+00
ENSG00000163131.9_CTSS	ENSG00000160789.18_LMNA	-0.07345	-0.22995883	0.086768273	3.69E-01	6.49E-01	1.00E+00
ENSG00000068394.10_GPKOW	ENSG00000160789.18_LMNA	0.08117	-0.07905395	0.237302558	3.20E-01	6.07E-01	1.00E+00
ENSG00000136247.13_ZDHHC4	ENSG00000160789.18_LMNA	0.08061	-0.079609384	0.236775012	3.24E-01	6.10E-01	1.00E+00
ENSG00000178605.11_GTPBP6	ENSG00000160789.18_LMNA	0.08914	-0.071067618	0.244867323	2.75E-01	5.59E-01	1.00E+00
ENSG00000062716.9_VMP1	ENSG00000160789.18_LMNA	0.15355	-0.005791101	0.305288942	5.89E-02	2.23E-01	1.00E+00
ENSG00000025772.7_TOMM34	ENSG00000160789.18_LMNA	0.10681	-0.053298053	0.261562019	1.90E-01	4.58E-01	1.00E+00
ENSG00000130313.5_PGLS	ENSG00000160789.18_LMNA	0.2507	0.095301717	0.3941677	1.84E-03	1.84E-02	1.00E+00
ENSG00000157020.16_SEC13	ENSG00000160789.18_LMNA	-0.07252	-0.229078801	0.087690171	3.75E-01	6.55E-01	1.00E+00
ENSG00000256043.2_CTSO	ENSG00000160789.18_LMNA	-0.00148	-0.160640438	0.157760288	9.86E-01	9.94E-01	1.00E+00
ENSG00000067057.15_PFKP	ENSG00000160789.18_LMNA	0.23255	0.076168763	0.377764622	3.94E-03	3.28E-02	1.00E+00
ENSG00000076248.9_UNG	ENSG00000160789.18_LMNA	-0.04739	-0.205039176	0.112665382	5.62E-01	7.94E-01	1.00E+00
ENSG00000166974.11_MAPRE2	ENSG00000160789.18_LMNA	-0.19668	-0.34507639	-0.03869141	1.52E-02	8.85E-02	1.00E+00
ENSG00000112640.13_PPP2R5D	ENSG00000160789.18_LMNA	-0.15378	-0.305503003	0.00555503	5.85E-02	2.22E-01	1.00E+00
ENSG00000115468.10_EFHD1	ENSG00000160789.18_LMNA	-0.30372	-0.441567528	-0.15185953	1.42E-04	2.50E-03	1.00E+00

ENSG00000177889.8_UBE2N	ENSG00000160789.18_LMNA	-0.35876	-0.489976175	-0.21164797	5.66E-06	1.84E-04	2.91E-01
ENSG00000135862.5_LAMC1	ENSG00000160789.18_LMNA	0.54234	0.419346806	0.645783754	5.33E-13	3.46E-10	2.73E-08
ENSG00000123095.5_BHLHE41	ENSG00000160789.18_LMNA	0.07186	-0.088353433	0.228445341	3.79E-01	6.58E-01	1.00E+00
ENSG00000206418.3_RAB12	ENSG00000160789.18_LMNA	0.02879	-0.131015227	0.187129023	7.25E-01	8.83E-01	1.00E+00
ENSG00000149218.4_ENDOD1	ENSG00000160789.18_LMNA	0.27214	0.11805063	0.413426133	6.95E-04	8.72E-03	1.00E+00
ENSG00000203875.9_SNHG5	ENSG00000160789.18_LMNA	-0.07891	-0.235156959	0.081311749	3.34E-01	6.20E-01	1.00E+00
ENSG00000000003.13_TSPAN6	ENSG00000160789.18_LMNA	-0.02632	-0.184743481	0.133442992	7.48E-01	8.95E-01	1.00E+00
ENSG00000198663.15_C6orf89	ENSG00000160789.18_LMNA	-0.06745	-0.224238717	0.092750848	4.09E-01	6.83E-01	1.00E+00
ENSG00000130511.14_SSBP4	ENSG00000160789.18_LMNA	0.44201	0.304221671	0.561689727	1.19E-08	1.25E-06	6.11E-04
ENSG00000182287.12_AP1S2	ENSG00000160789.18_LMNA	0.13522	-0.024511584	0.288213219	9.67E-02	3.05E-01	1.00E+00
ENSG00000185437.12_SH3BGR	ENSG00000160789.18_LMNA	-0.17631	-0.326350077	-0.01760296	2.98E-02	1.43E-01	1.00E+00
ENSG00000065548.16_ZC3H15	ENSG00000160789.18_LMNA	-0.18298	-0.332493219	-0.02449085	2.40E-02	1.23E-01	1.00E+00
ENSG00000164062.11_APEH	ENSG00000160789.18_LMNA	0.08283	-0.077386684	0.238884991	3.10E-01	5.97E-01	1.00E+00
ENSG00000270647.4_TAF15	ENSG00000160789.18_LMNA	0.18415	0.025698808	0.333567881	2.31E-02	1.20E-01	1.00E+00
ENSG00000197102.9_DYNC1H1	ENSG00000160789.18_LMNA	0.14066	-0.018965288	0.293293043	8.39E-02	2.80E-01	1.00E+00
ENSG00000132541.9_RIDA	ENSG00000160789.18_LMNA	-0.24583	-0.389774163	-0.09015524	2.27E-03	2.15E-02	1.00E+00
ENSG00000121691.4_CAT	ENSG00000160789.18_LMNA	-0.19235	-0.341109248	-0.03420095	1.76E-02	9.84E-02	1.00E+00
ENSG00000170291.13_ELP5	ENSG00000160789.18_LMNA	-0.1457	-0.297986487	0.013823714	7.33E-02	2.57E-01	1.00E+00
ENSG00000173692.11_PSMD1	ENSG00000160789.18_LMNA	0.11241	-0.047644984	0.266833927	1.68E-01	4.26E-01	1.00E+00
ENSG00000131724.9_IL13RA1	ENSG00000160789.18_LMNA	0.26149	0.106733723	0.403878555	1.14E-03	1.28E-02	1.00E+00
ENSG00000023330.13_ALAS1	ENSG00000160789.18_LMNA	-0.01829	-0.176971971	0.141325658	8.23E-01	9.31E-01	1.00E+00
ENSG00000181524.6_RPL24P4	ENSG00000160789.18_LMNA	-0.15809	-0.309499992	0.001140675	5.17E-02	2.06E-01	1.00E+00
ENSG00000166086.11_JAM3	ENSG00000160789.18_LMNA	0.04976	-0.110319347	0.207313758	5.43E-01	7.82E-01	1.00E+00
ENSG00000002933.6_TMEM176A	ENSG00000160789.18_LMNA	-0.02921	-0.187542932	0.130593603	7.21E-01	8.82E-01	1.00E+00
ENSG00000136842.12_TMOD1	ENSG00000160789.18_LMNA	-0.23261	-0.377815023	-0.07622721	3.93E-03	3.28E-02	1.00E+00
ENSG00000149260.13_CAPN5	ENSG00000160789.18_LMNA	0.16945	0.010529835	0.320014476	3.69E-02	1.64E-01	1.00E+00
ENSG00000181191.11_PJA1	ENSG00000160789.18_LMNA	-0.1035	-0.258437778	0.05663868	2.04E-01	4.76E-01	1.00E+00
ENSG00000116574.5_RHOU	ENSG00000160789.18_LMNA	-0.26792	-0.40964636	-0.11356121	8.47E-04	1.02E-02	1.00E+00
ENSG00000134884.12_ARGLU1	ENSG00000160789.18_LMNA	-0.19414	-0.342744602	-0.03605052	1.65E-02	9.42E-02	1.00E+00
ENSG00000067113.15_PLPP1	ENSG00000160789.18_LMNA	-0.33551	-0.469628213	-0.18626028	2.38E-05	5.96E-04	1.00E+00
ENSG00000085998.12_POMGNT1	ENSG00000160789.18_LMNA	0.15183	-0.007556709	0.303686958	6.19E-02	2.30E-01	1.00E+00
ENSG00000108639.6_SYNGR2	ENSG00000160789.18_LMNA	-0.08226	-0.23834372	0.07795717	3.14E-01	6.00E-01	1.00E+00
ENSG00000166908.16_PIP4K2C	ENSG00000160789.18_LMNA	-0.18435	-0.333751662	-0.02590547	2.30E-02	1.19E-01	1.00E+00

ENSG00000101052.11_IFT52	ENSG00000160789.18_LMNA	-0.21048	-0.3576906	-0.05305229	9.25E-03	6.19E-02	1.00E+00
ENSG00000126461.13_SCAF1	ENSG00000160789.18_LMNA	0.27945	0.12584406	0.419963557	4.89E-04	6.62E-03	1.00E+00
ENSG00000139352.3_ASCL1	ENSG00000160789.18_LMNA	-0.32645	-0.461654954	-0.17641436	4.05E-05	9.12E-04	1.00E+00
ENSG00000088356.5_PDRG1	ENSG00000160789.18_LMNA	-0.12697	-0.280499147	0.032897373	1.19E-01	3.46E-01	1.00E+00
ENSG00000148700.12_ADD3	ENSG00000160789.18_LMNA	-0.46417	-0.580475424	-0.32930313	1.71E-09	2.41E-07	8.77E-05
ENSG00000198914.2_POU3F3	ENSG00000160789.18_LMNA	0.05638	-0.103747123	0.213667579	4.90E-01	7.46E-01	1.00E+00
ENSG00000130159.12_ECSIT	ENSG00000160789.18_LMNA	-0.21546	-0.362233754	-0.05825559	7.68E-03	5.39E-02	1.00E+00
ENSG00000143569.17_UBAP2L	ENSG00000160789.18_LMNA	0.18592	0.027531671	0.335196962	2.18E-02	1.15E-01	1.00E+00
ENSG00000114383.8_TUSC2	ENSG00000160789.18_LMNA	-0.37472	-0.503864827	-0.22919493	1.97E-06	7.90E-05	1.01E-01
ENSG00000134294.12_SLC38A2	ENSG00000160789.18_LMNA	-0.04225	-0.200100044	0.117747502	6.05E-01	8.19E-01	1.00E+00
ENSG00000165175.14_MID1IP1	ENSG00000160789.18_LMNA	-0.00032	-0.159514749	0.158886623	9.97E-01	9.99E-01	1.00E+00
ENSG00000134057.13_CCNB1	ENSG00000160789.18_LMNA	0.04571	-0.114326238	0.203426827	5.76E-01	8.02E-01	1.00E+00
ENSG00000143401.13_ANP32E	ENSG00000160789.18_LMNA	0.28068	0.127164392	0.421068086	4.61E-04	6.32E-03	1.00E+00
ENSG00000149564.10_ESAM	ENSG00000160789.18_LMNA	-0.09137	-0.246981723	0.068828121	2.63E-01	5.46E-01	1.00E+00
ENSG00000167118.9_URM1	ENSG00000160789.18_LMNA	0.00999	-0.149452258	0.168918199	9.03E-01	9.62E-01	1.00E+00
ENSG00000100982.10_PCIF1	ENSG00000160789.18_LMNA	-0.03054	-0.188820733	0.129291258	7.09E-01	8.76E-01	1.00E+00
ENSG00000116685.14_KIAA2013	ENSG00000160789.18_LMNA	0.38762	0.243443535	0.515039265	8.07E-07	3.83E-05	4.14E-02
ENSG00000101337.14_TM9SF4	ENSG00000160789.18_LMNA	0.3646	0.218057199	0.495065598	3.88E-06	1.35E-04	1.99E-01
ENSG00000108106.12_UBE2S	ENSG00000160789.18_LMNA	0.10039	-0.059770868	0.255502476	2.19E-01	4.93E-01	1.00E+00
ENSG00000004487.14_KDM1A	ENSG00000160789.18_LMNA	-0.19578	-0.344253467	-0.03775891	1.56E-02	9.03E-02	1.00E+00
ENSG00000090971.4_NAT14	ENSG00000160789.18_LMNA	0.24298	0.087150985	0.387203111	2.56E-03	2.36E-02	1.00E+00
ENSG00000115107.18_STEAP3	ENSG00000160789.18_LMNA	0.46222	0.327086772	0.578826098	2.04E-09	2.81E-07	1.05E-04
ENSG00000226976.3_COX6A1P2	ENSG00000160789.18_LMNA	-0.16996	-0.320486133	-0.01105533	3.63E-02	1.62E-01	1.00E+00
ENSG00000159658.9_EFCAB14	ENSG00000160789.18_LMNA	0.18533	0.026925892	0.334658739	2.23E-02	1.17E-01	1.00E+00
ENSG00000119689.13_DLST	ENSG00000160789.18_LMNA	0.15479	-0.00452537	0.306436293	5.69E-02	2.18E-01	1.00E+00
ENSG00000054116.10_TRAPPC3	ENSG00000160789.18_LMNA	-0.07785	-0.234150039	0.082370206	3.40E-01	6.26E-01	1.00E+00
ENSG00000103710.9_RASL12	ENSG00000160789.18_LMNA	-0.22578	-0.37161988	-0.06905812	5.16E-03	4.02E-02	1.00E+00
ENSG00000131386.16_GALNT15	ENSG00000160789.18_LMNA	-0.18912	-0.338138912	-0.03084688	1.96E-02	1.06E-01	1.00E+00
ENSG00000186395.6_KRT10	ENSG00000160789.18_LMNA	-0.15008	-0.302063969	0.009343489	6.50E-02	2.37E-01	1.00E+00
ENSG00000165443.10_PHYHIPL	ENSG00000160789.18_LMNA	-0.59228	-0.686724733	-0.47816127	9.21E-16	1.89E-12	4.73E-11
ENSG00000188706.11_ZDHHC9	ENSG00000160789.18_LMNA	0.10413	-0.056001224	0.259034453	2.02E-01	4.72E-01	1.00E+00
ENSG00000164970.13_FAM219A	ENSG00000160789.18_LMNA	-0.08381	-0.239807523	0.076413878	3.05E-01	5.91E-01	1.00E+00
ENSG00000099995.17_SF3A1	ENSG00000160789.18_LMNA	0.09194	-0.068258109	0.247519414	2.60E-01	5.43E-01	1.00E+00

ENSG00000103495.12_MAZ	ENSG00000160789.18_LMNA	0.07433	-0.085886458	0.230800113	3.63E-01	6.44E-01	1.00E+00
ENSG00000128463.11_EMC4	ENSG00000160789.18_LMNA	-0.13152	-0.284757023	0.028274184	1.06E-01	3.24E-01	1.00E+00
ENSG00000158615.8_PPP1R15B	ENSG00000160789.18_LMNA	-0.11237	-0.266794776	0.04768704	1.68E-01	4.26E-01	1.00E+00
ENSG00000110799.12_VWF	ENSG00000160789.18_LMNA	0.22036	0.06338439	0.366697781	6.37E-03	4.71E-02	1.00E+00
ENSG00000100985.7_MMP9	ENSG00000160789.18_LMNA	0.20903	0.051541193	0.356368503	9.76E-03	6.45E-02	1.00E+00
ENSG00000065000.14_AP3D1	ENSG00000160789.18_LMNA	0.26913	0.114853319	0.410735269	8.00E-04	9.73E-03	1.00E+00
ENSG00000088298.11_EDEM2	ENSG00000160789.18_LMNA	0.18973	0.031475041	0.338695674	1.92E-02	1.05E-01	1.00E+00
ENSG00000077152.8_UBE2T	ENSG00000160789.18_LMNA	-0.11937	-0.273372383	0.040605697	1.43E-01	3.86E-01	1.00E+00
ENSG00000197006.12_METTL9	ENSG00000160789.18_LMNA	-0.34433	-0.47736072	-0.19586358	1.40E-05	3.86E-04	7.19E-01
ENSG00000125355.14_TMEM255	ENSG00000160789.18_LMNA	-0.02286	-0.181396463	0.136842825	7.80E-01	9.10E-01	1.00E+00
ENSG00000151929.8_BAG3	ENSG00000160789.18_LMNA	0.22664	0.069966857	0.372406659	4.99E-03	3.92E-02	1.00E+00
ENSG00000213281.4_NRAS	ENSG00000160789.18_LMNA	-0.0305	-0.188786809	0.129325848	7.09E-01	8.76E-01	1.00E+00
ENSG00000128335.12_APOL2	ENSG00000160789.18_LMNA	0.11693	-0.043076922	0.27108028	1.51E-01	4.00E-01	1.00E+00
ENSG00000197582.5_GPX1P1	ENSG00000160789.18_LMNA	-0.02229	-0.180850978	0.137396208	7.85E-01	9.12E-01	1.00E+00
ENSG00000143294.13_PRCC	ENSG00000160789.18_LMNA	0.08234	-0.077880953	0.238416046	3.13E-01	6.00E-01	1.00E+00
ENSG00000163541.10_SUCLG1	ENSG00000160789.18_LMNA	-0.38708	-0.514568447	-0.24284082	8.38E-07	3.94E-05	4.30E-02
ENSG00000182872.14_RBM10	ENSG00000160789.18_LMNA	0.18851	0.030218244	0.337581516	2.00E-02	1.08E-01	1.00E+00
ENSG00000177674.14_AGTRAP	ENSG00000160789.18_LMNA	0.26154	0.106783553	0.403920737	1.14E-03	1.27E-02	1.00E+00
ENSG00000130300.7_PLVAP	ENSG00000160789.18_LMNA	0.21252	0.055189432	0.359558347	8.57E-03	5.86E-02	1.00E+00
ENSG00000183258.10_DDX41	ENSG00000160789.18_LMNA	0.27673	0.122942911	0.417533525	5.58E-04	7.34E-03	1.00E+00
ENSG00000137509.9_PRCP	ENSG00000160789.18_LMNA	-0.19939	-0.347559845	-0.04150877	1.38E-02	8.25E-02	1.00E+00
ENSG00000270170.1_NCBP2-AS2	ENSG00000160789.18_LMNA	-0.11333	-0.267700424	0.046713907	1.64E-01	4.20E-01	1.00E+00
ENSG00000162910.17_MRPL55	ENSG00000160789.18_LMNA	0.03636	-0.123555585	0.194435524	6.57E-01	8.48E-01	1.00E+00
ENSG00000168329.12_CX3CR1	ENSG00000160789.18_LMNA	-0.18165	-0.331266672	-0.02311326	2.51E-02	1.27E-01	1.00E+00
ENSG00000125520.12_SLC2A4R	ENSG00000160789.18_LMNA	0.53766	0.4138872	0.641916086	9.17E-13	5.29E-10	4.71E-08
ENSG00000105695.13_MAG	ENSG00000160789.18_LMNA	-0.30068	-0.438870222	-0.14858936	1.67E-04	2.84E-03	1.00E+00
ENSG00000112078.12_KCTD20	ENSG00000160789.18_LMNA	-0.17869	-0.328543084	-0.02005847	2.76E-02	1.36E-01	1.00E+00
ENSG00000048544.5_MRPS10	ENSG00000160789.18_LMNA	-0.22488	-0.370807839	-0.06812072	5.35E-03	4.12E-02	1.00E+00
ENSG00000170759.10_KIF5B	ENSG00000160789.18_LMNA	-0.03141	-0.189661428	0.128433817	7.01E-01	8.72E-01	1.00E+00
ENSG00000162840.4_MT2P1	ENSG00000160789.18_LMNA	0.32782	0.177907065	0.462866722	3.74E-05	8.51E-04	1.00E+00
ENSG00000004478.7_FKBP4	ENSG00000160789.18_LMNA	-0.09524	-0.250640604	0.06494523	2.43E-01	5.23E-01	1.00E+00
ENSG00000070761.6_CFAP20	ENSG00000160789.18_LMNA	-0.3072	-0.444650253	-0.15560476	1.18E-04	2.17E-03	1.00E+00
ENSG00000119280.15_C1orf198	ENSG00000160789.18_LMNA	-0.15231	-0.304139617	0.007058013	6.10E-02	2.28E-01	1.00E+00

ENSG00000196507.9_TCEAL3	ENSG00000160789.18_LMNA	-0.06286	-0.219857267	0.097317907	4.42E-01	7.09E-01	1.00E+00
ENSG00000064115.9_TM7SF3	ENSG00000160789.18_LMNA	-0.09932	-0.254495933	0.060843498	2.23E-01	4.99E-01	1.00E+00
ENSG00000198467.12_TPM2	ENSG00000160789.18_LMNA	0.41819	0.277456405	0.54134573	8.28E-08	6.13E-06	4.25E-03
ENSG00000278845.3_MRPL45	ENSG00000160789.18_LMNA	-0.38047	-0.508852024	-0.23553963	1.33E-06	5.82E-05	6.83E-02
ENSG00000134247.9_PTGFRN	ENSG00000160789.18_LMNA	0.39658	0.253373884	0.52277291	4.24E-07	2.31E-05	2.17E-02
ENSG00000142875.18_PRKACB	ENSG00000160789.18_LMNA	-0.46175	-0.578432988	-0.32655892	2.13E-09	2.90E-07	1.09E-04
ENSG00000136003.14_ISCU	ENSG00000160789.18_LMNA	-0.30206	-0.440093485	-0.15007164	1.56E-04	2.68E-03	1.00E+00
ENSG00000160888.6_IER2	ENSG00000160789.18_LMNA	0.2157	0.058506704	0.362452648	7.61E-03	5.35E-02	1.00E+00
ENSG00000204128.5_C2orf72	ENSG00000160789.18_LMNA	-0.06364	-0.22060765	0.096536683	4.36E-01	7.04E-01	1.00E+00
ENSG00000012963.12_UBR7	ENSG00000160789.18_LMNA	0.23416	0.077856821	0.379219484	3.69E-03	3.12E-02	1.00E+00
ENSG00000116120.9_FARSB	ENSG00000160789.18_LMNA	-0.03974	-0.197689075	0.120222236	6.27E-01	8.32E-01	1.00E+00
ENSG00000255690.2_TRIL	ENSG00000160789.18_LMNA	0.03872	-0.121230787	0.196705406	6.36E-01	8.37E-01	1.00E+00
ENSG00000106049.7_HIBADH	ENSG00000160789.18_LMNA	0.00815	-0.151250305	0.167130503	9.21E-01	9.70E-01	1.00E+00
ENSG00000170891.9_CYTL1	ENSG00000160789.18_LMNA	-0.17217	-0.322533321	-0.01333819	3.39E-02	1.55E-01	1.00E+00
ENSG00000138434.15_SSFA2	ENSG00000160789.18_LMNA	0.22276	0.065900718	0.368882856	5.81E-03	4.39E-02	1.00E+00
ENSG00000167770.10_OTUB1	ENSG00000160789.18_LMNA	-0.31964	-0.455655224	-0.16904293	5.97E-05	1.23E-03	1.00E+00
ENSG00000234648.1_AL162151.3	ENSG00000160789.18_LMNA	-0.06567	-0.222543088	0.094519891	4.22E-01	6.93E-01	1.00E+00
ENSG00000164292.11_RHOBTB3	ENSG00000160789.18_LMNA	-0.08712	-0.242952078	0.073093432	2.86E-01	5.71E-01	1.00E+00
ENSG00000101144.11_BMP7	ENSG00000160789.18_LMNA	-0.15297	-0.304749404	0.006385964	5.99E-02	2.25E-01	1.00E+00
ENSG00000064490.12_RFXANK	ENSG00000160789.18_LMNA	0.14198	-0.017620098	0.294522445	8.10E-02	2.74E-01	1.00E+00
ENSG00000198492.13_YTHDF2	ENSG00000160789.18_LMNA	0.04471	-0.115313187	0.202467886	5.84E-01	8.07E-01	1.00E+00
ENSG00000164978.16_NUDT2	ENSG00000160789.18_LMNA	-0.2166	-0.363274597	-0.05945	7.36E-03	5.22E-02	1.00E+00
ENSG00000102225.14_CDK16	ENSG00000160789.18_LMNA	0.32364	0.173370745	0.459180863	4.76E-05	1.03E-03	1.00E+00
ENSG00000245526.7_LINC00461	ENSG00000160789.18_LMNA	-0.16504	-0.315942797	-0.00600047	4.22E-02	1.80E-01	1.00E+00
ENSG00000133606.9_MKRN1	ENSG00000160789.18_LMNA	0.09862	-0.06155156	0.253831121	2.27E-01	5.03E-01	1.00E+00
ENSG00000160271.13_RALGDS	ENSG00000160789.18_LMNA	0.07953	-0.080696182	0.235742242	3.30E-01	6.16E-01	1.00E+00
ENSG00000001036.12_FUCA2	ENSG00000160789.18_LMNA	0.36097	0.214068127	0.491900198	4.91E-06	1.64E-04	2.52E-01
ENSG00000154589.5_LY96	ENSG00000160789.18_LMNA	0.07727	-0.082946726	0.233601304	3.44E-01	6.29E-01	1.00E+00
ENSG00000135439.10_AGAP2	ENSG00000160789.18_LMNA	-0.28918	-0.428645558	-0.13625052	3.02E-04	4.53E-03	1.00E+00
ENSG00000136636.11_KCTD3	ENSG00000160789.18_LMNA	0.03716	-0.122771096	0.195201863	6.49E-01	8.44E-01	1.00E+00
ENSG00000187325.4_TAF9B	ENSG00000160789.18_LMNA	-0.23342	-0.378551258	-0.07708127	3.80E-03	3.19E-02	1.00E+00
ENSG00000097007.16_ABL1	ENSG00000160789.18_LMNA	0.22032	0.06334207	0.366661003	6.38E-03	4.71E-02	1.00E+00
ENSG00000104671.6_DCTN6	ENSG00000160789.18_LMNA	-0.35476	-0.48648823	-0.2072693	7.31E-06	2.27E-04	3.75E-01

ENSG00000106330.10_MOSPD3	ENSG00000160789.18_LMNA	0.08549	-0.074730234	0.24140281	2.95E-01	5.81E-01	1.00E+00
ENSG00000243678.10_NME2	ENSG00000160789.18_LMNA	0.03781	-0.122129012	0.195828803	6.44E-01	8.42E-01	1.00E+00
ENSG00000160131.12_VMA21	ENSG00000160789.18_LMNA	-0.13672	-0.289620399	0.02297711	9.30E-02	2.98E-01	1.00E+00
ENSG00000171960.9_PPIH	ENSG00000160789.18_LMNA	-0.13921	-0.291939041	0.020445507	8.72E-02	2.87E-01	1.00E+00
ENSG00000185608.7_MRPL40	ENSG00000160789.18_LMNA	-0.12896	-0.282368117	0.030869701	1.13E-01	3.36E-01	1.00E+00
ENSG00000167244.16_IGF2	ENSG00000160789.18_LMNA	-0.10636	-0.261140287	0.053749406	1.92E-01	4.60E-01	1.00E+00
ENSG00000234797.5_RPS3AP6	ENSG00000160789.18_LMNA	-0.11437	-0.268677423	0.045663437	1.61E-01	4.15E-01	1.00E+00
ENSG00000138449.9_SLC40A1	ENSG00000160789.18_LMNA	-0.25681	-0.399672416	-0.10177242	1.40E-03	1.49E-02	1.00E+00
ENSG00000198952.8_SMG5	ENSG00000160789.18_LMNA	0.27131	0.117166026	0.412682164	7.22E-04	8.98E-03	1.00E+00
ENSG00000141905.16_NFIC	ENSG00000160789.18_LMNA	0.4251	0.28519778	0.5472618	4.79E-08	3.93E-06	2.46E-03
ENSG00000213625.7_LEPROT	ENSG00000160789.18_LMNA	0.0446	-0.115420149	0.202363923	5.85E-01	8.07E-01	1.00E+00
ENSG00000121753.11_ADGRB2	ENSG00000160789.18_LMNA	0.1855	0.027097523	0.33481125	2.21E-02	1.16E-01	1.00E+00
ENSG00000272579.1_RP11-101E	ENSG00000160789.18_LMNA	-0.09569	-0.251065635	0.064493556	2.41E-01	5.20E-01	1.00E+00
ENSG00000125249.6_RAP2A	ENSG00000160789.18_LMNA	-0.52108	-0.62817259	-0.39461867	5.89E-12	2.29E-09	3.02E-07
ENSG00000116906.10_GNPAT	ENSG00000160789.18_LMNA	-0.20119	-0.349202118	-0.04337453	1.29E-02	7.89E-02	1.00E+00
ENSG00000084073.7_ZMPSTE24	ENSG00000160789.18_LMNA	0.02718	-0.132596344	0.18557583	7.40E-01	8.91E-01	1.00E+00
ENSG00000130294.13_KIF1A	ENSG00000160789.18_LMNA	-0.20604	-0.353637429	-0.0484241	1.09E-02	6.95E-02	1.00E+00
ENSG00000175602.3_CCDC85B	ENSG00000160789.18_LMNA	0.30064	0.148545234	0.438833788	1.68E-04	2.84E-03	1.00E+00
ENSG00000148110.14_MFSD14B	ENSG00000160789.18_LMNA	0.2899	0.137018411	0.429284068	2.92E-04	4.40E-03	1.00E+00
ENSG00000051108.13_HERPUD1	ENSG00000160789.18_LMNA	0.03993	-0.120028943	0.197877526	6.25E-01	8.31E-01	1.00E+00
ENSG00000016864.15_GLT8D1	ENSG00000160789.18_LMNA	0.08491	-0.075306491	0.240856987	2.98E-01	5.84E-01	1.00E+00
ENSG00000008952.15_SEC62	ENSG00000160789.18_LMNA	-0.19919	-0.347378438	-0.04130281	1.39E-02	8.30E-02	1.00E+00
ENSG00000171490.11_RSL1D1	ENSG00000160789.18_LMNA	-0.0627	-0.219705221	0.097476155	4.43E-01	7.10E-01	1.00E+00
ENSG00000203880.10_PCMTD2	ENSG00000160789.18_LMNA	-0.15321	-0.304969719	0.006143084	5.95E-02	2.24E-01	1.00E+00
ENSG00000140632.15_GLYR1	ENSG00000160789.18_LMNA	-0.16677	-0.317543038	-0.00777907	4.00E-02	1.73E-01	1.00E+00
ENSG00000166181.11_API5	ENSG00000160789.18_LMNA	0.06093	-0.099236356	0.218012962	4.56E-01	7.20E-01	1.00E+00
ENSG00000257704.3_INAFM1	ENSG00000160789.18_LMNA	0.30957	0.158168397	0.446756456	1.04E-04	1.95E-03	1.00E+00
ENSG00000234851.4_RPL23AP42	ENSG00000160789.18_LMNA	-0.05851	-0.215698287	0.101640751	4.74E-01	7.34E-01	1.00E+00
ENSG00000067840.11_PDZD4	ENSG00000160789.18_LMNA	-0.12203	-0.27587	0.037908516	1.34E-01	3.72E-01	1.00E+00
ENSG00000006007.10_GDE1	ENSG00000160789.18_LMNA	-0.35315	-0.485076985	-0.20550084	8.10E-06	2.46E-04	4.15E-01
ENSG00000167778.7_SPRYD3	ENSG00000160789.18_LMNA	-0.01166	-0.170539925	0.147819318	8.87E-01	9.56E-01	1.00E+00
ENSG00000197461.12_PDGFA	ENSG00000160789.18_LMNA	0.28391	0.130610571	0.4239469	3.93E-04	5.59E-03	1.00E+00
ENSG00000145349.15_CAMK2D	ENSG00000160789.18_LMNA	0.47813	0.345203924	0.592248027	4.68E-10	8.34E-08	2.40E-05

ENSG00000169439.10_SDC2	ENSG00000160789.18_LMNA	0.14681	-0.012682787	0.299025934	7.11E-02	2.52E-01	1.00E+00
ENSG00000123595.6_RAB9A	ENSG00000160789.18_LMNA	-0.06478	-0.221697251	0.095401602	4.28E-01	6.98E-01	1.00E+00
ENSG00000204209.9_DAXX	ENSG00000160789.18_LMNA	0.126	-0.033879169	0.279593339	1.22E-01	3.51E-01	1.00E+00
ENSG00000065150.17_IPO5	ENSG00000160789.18_LMNA	-0.17159	-0.3219978	-0.01274071	3.45E-02	1.57E-01	1.00E+00
ENSG00000156411.8_C14orf2	ENSG00000160789.18_LMNA	-0.17622	-0.326266945	-0.01750995	2.99E-02	1.43E-01	1.00E+00
ENSG00000177963.11_RIC8A	ENSG00000160789.18_LMNA	0.3453	0.196921756	0.478210095	1.32E-05	3.67E-04	6.77E-01
ENSG00000064995.15_TAF11	ENSG00000160789.18_LMNA	-0.34066	-0.474143053	-0.19186087	1.75E-05	4.63E-04	8.99E-01
ENSG00000229344.1_MTCO2P12	ENSG00000160789.18_LMNA	-0.11797	-0.272062645	0.042018258	1.48E-01	3.94E-01	1.00E+00
ENSG00000082153.16_BZW1	ENSG00000160789.18_LMNA	0.00046	-0.158754682	0.159646657	9.96E-01	9.98E-01	1.00E+00
ENSG00000167986.12_DDB1	ENSG00000160789.18_LMNA	-0.08472	-0.240673827	0.075499817	2.99E-01	5.86E-01	1.00E+00
ENSG00000134575.8_ACP2	ENSG00000160789.18_LMNA	0.39819	0.255161338	0.524160266	3.77E-07	2.10E-05	1.93E-02
ENSG00000114125.12_RNF7	ENSG00000160789.18_LMNA	-0.10638	-0.261158368	0.053730057	1.92E-01	4.60E-01	1.00E+00
ENSG00000104325.5_DECR1	ENSG00000160789.18_LMNA	-0.10823	-0.262902263	0.051862819	1.84E-01	4.50E-01	1.00E+00
ENSG00000114023.14_FAM162A	ENSG00000160789.18_LMNA	-0.07636	-0.232729874	0.083861846	3.50E-01	6.33E-01	1.00E+00
ENSG00000107130.8_NCS1	ENSG00000160789.18_LMNA	0.02782	-0.131966717	0.186194528	7.34E-01	8.88E-01	1.00E+00
ENSG00000143811.15_PYCR2	ENSG00000160789.18_LMNA	0.08261	-0.07761233	0.238670924	3.12E-01	5.98E-01	1.00E+00
ENSG00000071051.12_NCK2	ENSG00000160789.18_LMNA	0.14952	-0.009918388	0.301541386	6.60E-02	2.40E-01	1.00E+00
ENSG00000255737.2_AGAP2-AS1	ENSG00000160789.18_LMNA	0.05916	-0.100993192	0.216322033	4.69E-01	7.30E-01	1.00E+00
ENSG00000163634.10_THOC7	ENSG00000160789.18_LMNA	-0.20895	-0.356295626	-0.05145794	9.78E-03	6.46E-02	1.00E+00
ENSG00000176171.10_BNIP3	ENSG00000160789.18_LMNA	0.02485	-0.134879772	0.183329921	7.61E-01	9.00E-01	1.00E+00
ENSG00000188243.11_COMMD6	ENSG00000160789.18_LMNA	-0.19791	-0.346202576	-0.03996841	1.45E-02	8.59E-02	1.00E+00
ENSG00000113369.7_ARRDC3	ENSG00000160789.18_LMNA	0.06415	-0.096027857	0.221096187	4.32E-01	7.02E-01	1.00E+00
ENSG00000183864.4_TOB2	ENSG00000160789.18_LMNA	-0.20868	-0.356055724	-0.0511839	9.88E-03	6.50E-02	1.00E+00
ENSG00000009954.9_BAZ1B	ENSG00000160789.18_LMNA	0.32796	0.178055703	0.462987327	3.71E-05	8.48E-04	1.00E+00
ENSG00000131477.9_RAMP2	ENSG00000160789.18_LMNA	-0.24529	-0.389289797	-0.08958885	2.32E-03	2.19E-02	1.00E+00
ENSG00000174871.9_CNIH2	ENSG00000160789.18_LMNA	-0.10324	-0.258194101	0.056898939	2.06E-01	4.77E-01	1.00E+00
ENSG00000137804.11_NUSAP1	ENSG00000160789.18_LMNA	0.0669	-0.093298179	0.223714304	4.13E-01	6.86E-01	1.00E+00
ENSG00000265681.5_RPL17	ENSG00000160789.18_LMNA	-0.15963	-0.31093032	-0.00044196	4.95E-02	2.00E-01	1.00E+00
ENSG00000170290.3_SLN	ENSG00000160789.18_LMNA	-0.08288	-0.238925768	0.077343697	3.10E-01	5.96E-01	1.00E+00
ENSG00000108465.13_CDK5RAP	ENSG00000160789.18_LMNA	0.07751	-0.082706547	0.233829932	3.43E-01	6.27E-01	1.00E+00
ENSG00000086589.10_RBM22	ENSG00000160789.18_LMNA	-0.19275	-0.341473738	-0.034613	1.74E-02	9.74E-02	1.00E+00
ENSG00000106554.10_CHCHD3	ENSG00000160789.18_LMNA	0.20977	0.052313624	0.357044472	9.49E-03	6.32E-02	1.00E+00
ENSG00000091592.14_NLRP1	ENSG00000160789.18_LMNA	-0.16929	-0.319869826	-0.01036871	3.71E-02	1.65E-01	1.00E+00

ENSG00000119471.13_HSDL2	ENSG00000160789.18_LMNA	0.02307	-0.136628509	0.181607668	7.78E-01	9.09E-01	1.00E+00
ENSG00000233476.3_EEF1A1P6	ENSG00000160789.18_LMNA	-0.12413	-0.277844745	0.035772743	1.28E-01	3.62E-01	1.00E+00
ENSG00000187109.12_NAP1L1	ENSG00000160789.18_LMNA	-0.28583	-0.425662161	-0.13266726	3.57E-04	5.16E-03	1.00E+00
ENSG00000100567.11_PSMA3	ENSG00000160789.18_LMNA	0.02816	-0.13162896	0.186526317	7.31E-01	8.86E-01	1.00E+00
ENSG00000103266.9_STUB1	ENSG00000160789.18_LMNA	0.01653	-0.143046416	0.175270184	8.40E-01	9.38E-01	1.00E+00
ENSG00000117133.9_RPF1	ENSG00000160789.18_LMNA	-0.08163	-0.237740326	0.07859289	3.17E-01	6.04E-01	1.00E+00
ENSG00000182149.19_IST1	ENSG00000160789.18_LMNA	-0.13789	-0.290706521	0.021791729	9.03E-02	2.93E-01	1.00E+00
ENSG00000197093.9_GAL3ST4	ENSG00000160789.18_LMNA	0.17005	0.011154729	0.320575329	3.62E-02	1.62E-01	1.00E+00
ENSG00000105723.10_GSK3A	ENSG00000160789.18_LMNA	0.09049	-0.069712269	0.246147322	2.68E-01	5.52E-01	1.00E+00
ENSG00000160877.5_NACC1	ENSG00000160789.18_LMNA	0.26838	0.114052376	0.410060385	8.29E-04	9.99E-03	1.00E+00
ENSG00000160803.7_UBQLN4	ENSG00000160789.18_LMNA	-0.19978	-0.347914028	-0.04191097	1.36E-02	8.17E-02	1.00E+00
ENSG00000120896.12_SORBS3	ENSG00000160789.18_LMNA	0.28074	0.127225072	0.421118827	4.59E-04	6.32E-03	1.00E+00
ENSG00000155506.15_LARP1	ENSG00000160789.18_LMNA	0.2451	0.089385281	0.389115672	2.34E-03	2.21E-02	1.00E+00
ENSG00000160310.15_PRMT2	ENSG00000160789.18_LMNA	0.22334	0.066505478	0.369407506	5.68E-03	4.31E-02	1.00E+00
ENSG00000188191.13_PRKAR1B	ENSG00000160789.18_LMNA	-0.09652	-0.251852444	0.063657086	2.37E-01	5.15E-01	1.00E+00
ENSG00000175928.5_LRRN1	ENSG00000160789.18_LMNA	-0.2954	-0.434185623	-0.14292488	2.20E-04	3.52E-03	1.00E+00
ENSG00000155463.11_OXA1L	ENSG00000160789.18_LMNA	0.25447	0.099290549	0.397563589	1.56E-03	1.62E-02	1.00E+00
ENSG00000077721.14_UBE2A	ENSG00000160789.18_LMNA	-0.12607	-0.279661108	0.033805736	1.22E-01	3.51E-01	1.00E+00
ENSG00000165168.7_CYBB	ENSG00000160789.18_LMNA	0.02323	-0.136470931	0.18176294	7.76E-01	9.08E-01	1.00E+00
ENSG00000196235.12_SUPT5H	ENSG00000160789.18_LMNA	0.0733	-0.086914577	0.229819206	3.69E-01	6.50E-01	1.00E+00
ENSG00000244405.6_ETV5	ENSG00000160789.18_LMNA	0.27422	0.120274323	0.41529456	6.29E-04	8.08E-03	1.00E+00
ENSG00000001629.8_ANKIB1	ENSG00000160789.18_LMNA	-0.06625	-0.223096723	0.093942506	4.17E-01	6.90E-01	1.00E+00
ENSG00000154719.12_MRPL39	ENSG00000160789.18_LMNA	0.04733	-0.112722557	0.204983699	5.63E-01	7.94E-01	1.00E+00
ENSG00000130560.7_UBAC1	ENSG00000160789.18_LMNA	0.00416	-0.155140967	0.16325506	9.59E-01	9.84E-01	1.00E+00
ENSG00000124181.13_PLCG1	ENSG00000160789.18_LMNA	0.22384	0.067027365	0.369860106	5.57E-03	4.25E-02	1.00E+00
ENSG00000117228.9_GBP1	ENSG00000160789.18_LMNA	0.27764	0.123915407	0.418348567	5.34E-04	7.10E-03	1.00E+00
ENSG00000136807.10_CDK9	ENSG00000160789.18_LMNA	0.13764	-0.022041085	0.290478112	9.08E-02	2.94E-01	1.00E+00
ENSG00000204574.11_ABCF1	ENSG00000160789.18_LMNA	0.12762	-0.032239003	0.281106248	1.17E-01	3.43E-01	1.00E+00
ENSG00000085719.10_CPNE3	ENSG00000160789.18_LMNA	-0.02338	-0.181904227	0.136327531	7.75E-01	9.07E-01	1.00E+00
ENSG00000139726.9_DENR	ENSG00000160789.18_LMNA	0.02333	-0.136381292	0.181851259	7.75E-01	9.08E-01	1.00E+00
ENSG00000168488.17_ATXN2L	ENSG00000160789.18_LMNA	0.18811	0.029800038	0.337210584	2.03E-02	1.09E-01	1.00E+00
ENSG00000158164.6_TMSB15A	ENSG00000160789.18_LMNA	-0.30691	-0.444400003	-0.15530042	1.20E-04	2.19E-03	1.00E+00
ENSG00000139318.7_DUSP6	ENSG00000160789.18_LMNA	0.32304	0.172724099	0.458654647	4.92E-05	1.06E-03	1.00E+00

ENSG00000129559.11_NEDD8	ENSG00000160789.18_LMNA	-0.09166	-0.247256422	0.068536935	2.61E-01	5.45E-01	1.00E+00
ENSG00000113328.17_CCNG1	ENSG00000160789.18_LMNA	-0.04957	-0.207136201	0.110502609	5.44E-01	7.82E-01	1.00E+00
ENSG00000164548.9_TRA2A	ENSG00000160789.18_LMNA	-0.13893	-0.291678049	0.02073067	8.78E-02	2.88E-01	1.00E+00
ENSG00000110717.9_NDUFS8	ENSG00000160789.18_LMNA	0.08529	-0.074932737	0.241211026	2.96E-01	5.82E-01	1.00E+00
ENSG00000168802.11_CHTF8	ENSG00000160789.18_LMNA	0.02346	-0.136251335	0.181979295	7.74E-01	9.07E-01	1.00E+00
ENSG00000143641.9_GALNT2	ENSG00000160789.18_LMNA	0.49207	0.361157757	0.60395484	1.21E-10	2.62E-08	6.20E-06
ENSG00000205352.9_PRR13	ENSG00000160789.18_LMNA	-0.06935	-0.226057415	0.090851179	3.96E-01	6.73E-01	1.00E+00
ENSG00000171860.4_C3AR1	ENSG00000160789.18_LMNA	-0.02244	-0.180997151	0.137247939	7.84E-01	9.11E-01	1.00E+00
ENSG00000132676.14_DAP3	ENSG00000160789.18_LMNA	0.13247	-0.027305538	0.285647567	1.04E-01	3.19E-01	1.00E+00
ENSG00000156515.20_HK1	ENSG00000160789.18_LMNA	0.36605	0.219651881	0.496328953	3.52E-06	1.25E-04	1.81E-01
ENSG00000085733.14_CTTN	ENSG00000160789.18_LMNA	0.22377	0.066959738	0.369801465	5.58E-03	4.26E-02	1.00E+00
ENSG00000020129.14_NCDN	ENSG00000160789.18_LMNA	-0.00697	-0.165986709	0.152399618	9.32E-01	9.73E-01	1.00E+00
ENSG00000116478.10_HDAC1	ENSG00000160789.18_LMNA	0.26279	0.108111617	0.405044495	1.07E-03	1.22E-02	1.00E+00
ENSG00000170989.8_S1PR1	ENSG00000160789.18_LMNA	-0.27036	-0.411836944	-0.1161616	7.55E-04	9.29E-03	1.00E+00
ENSG00000163938.15_GNL3	ENSG00000160789.18_LMNA	-0.06597	-0.222826317	0.094224539	4.19E-01	6.91E-01	1.00E+00
ENSG00000049449.7_RCN1	ENSG00000160789.18_LMNA	0.27252	0.118462468	0.413772362	6.82E-04	8.60E-03	1.00E+00
ENSG00000124541.6_RRP36	ENSG00000160789.18_LMNA	-0.06761	-0.224397337	0.092585257	4.08E-01	6.83E-01	1.00E+00
ENSG00000120333.4_MRPS14	ENSG00000160789.18_LMNA	-0.33774	-0.471588039	-0.18868916	2.09E-05	5.35E-04	1.00E+00
ENSG00000131747.13_TOP2A	ENSG00000160789.18_LMNA	-0.02514	-0.18360309	0.13460222	7.59E-01	8.99E-01	1.00E+00
ENSG00000086300.14_SNX10	ENSG00000160789.18_LMNA	0.02672	-0.133046525	0.185133309	7.44E-01	8.93E-01	1.00E+00
ENSG00000100836.9_PABPN1	ENSG00000160789.18_LMNA	0.01734	-0.142251322	0.176056749	8.32E-01	9.35E-01	1.00E+00
ENSG00000142188.15_TMEM50B	ENSG00000160789.18_LMNA	-0.04224	-0.200097743	0.117749866	6.05E-01	8.19E-01	1.00E+00
ENSG00000106052.12_TAX1BP1	ENSG00000160789.18_LMNA	0.08382	-0.076403706	0.239817166	3.05E-01	5.91E-01	1.00E+00
ENSG00000147027.3_TMEM47	ENSG00000160789.18_LMNA	-0.03911	-0.197082014	0.12084473	6.32E-01	8.35E-01	1.00E+00
ENSG00000087074.7_PPP1R15A	ENSG00000160789.18_LMNA	0.43147	0.292349992	0.552704449	2.86E-08	2.61E-06	1.47E-03
ENSG00000011009.9_LYPLA2	ENSG00000160789.18_LMNA	0.32859	0.178736712	0.463539764	3.58E-05	8.25E-04	1.00E+00
ENSG00000186010.17_NDUFA13	ENSG00000160789.18_LMNA	-0.16197	-0.313094639	-0.00283969	4.62E-02	1.91E-01	1.00E+00
ENSG00000182944.16_EWSR1	ENSG00000160789.18_LMNA	-0.0084	-0.167377607	0.151001896	9.18E-01	9.69E-01	1.00E+00
ENSG00000168286.2_THAP11	ENSG00000160789.18_LMNA	-0.11806	-0.272140992	0.041933796	1.47E-01	3.94E-01	1.00E+00
ENSG00000264281.3_CTD-2031P	ENSG00000160789.18_LMNA	-0.19715	-0.345508244	-0.03918097	1.49E-02	8.75E-02	1.00E+00
ENSG00000105619.12_TFPT	ENSG00000160789.18_LMNA	0.15371	-0.005630771	0.305434327	5.87E-02	2.22E-01	1.00E+00
ENSG00000127337.5_YEATS4	ENSG00000160789.18_LMNA	-0.25412	-0.397246077	-0.09891719	1.58E-03	1.63E-02	1.00E+00
ENSG00000157191.18_NECAP2	ENSG00000160789.18_LMNA	-0.10116	-0.256233664	0.058991216	2.15E-01	4.89E-01	1.00E+00

ENSG00000114767.6_RRP9	ENSG00000160789.18_LMNA	0.07314	-0.087071364	0.229669562	3.71E-01	6.51E-01	1.00E+00
ENSG00000212802.4_RPL15P3	ENSG00000160789.18_LMNA	-0.22754	-0.373221062	-0.07090803	4.81E-03	3.81E-02	1.00E+00
ENSG00000136448.10_NMT1	ENSG00000160789.18_LMNA	-0.01845	-0.177134305	0.141161414	8.21E-01	9.30E-01	1.00E+00
ENSG00000066117.13_SMARCD1	ENSG00000160789.18_LMNA	-0.08788	-0.243675995	0.072328029	2.82E-01	5.67E-01	1.00E+00
ENSG00000084652.14_TXLNA	ENSG00000160789.18_LMNA	0.40831	0.266423415	0.53286876	1.77E-07	1.15E-05	9.09E-03
ENSG00000162704.14_ARPC5	ENSG00000160789.18_LMNA	-0.03391	-0.192070284	0.12597434	6.78E-01	8.60E-01	1.00E+00
ENSG00000135929.7_CYP27A1	ENSG00000160789.18_LMNA	0.29475	0.142219457	0.433601126	2.28E-04	3.62E-03	1.00E+00
ENSG00000198721.11_ECI2	ENSG00000160789.18_LMNA	0.02216	-0.137523688	0.18072529	7.86E-01	9.13E-01	1.00E+00
ENSG00000023191.15_RNH1	ENSG00000160789.18_LMNA	0.41367	0.272408741	0.537474108	1.18E-07	8.26E-06	6.03E-03
ENSG00000149532.14_CPSF7	ENSG00000160789.18_LMNA	-0.11493	-0.269207967	0.045092705	1.59E-01	4.12E-01	1.00E+00
ENSG00000142186.15_SCYL1	ENSG00000160789.18_LMNA	0.28057	0.127040994	0.420964894	4.63E-04	6.35E-03	1.00E+00
ENSG00000005194.13_CIAPIN1	ENSG00000160789.18_LMNA	-0.23942	-0.383982994	-0.08339598	2.97E-03	2.66E-02	1.00E+00
ENSG00000124380.9_SNRNP27	ENSG00000160789.18_LMNA	-0.29924	-0.437596587	-0.14704743	1.80E-04	3.01E-03	1.00E+00
ENSG00000109220.9_CHIC2	ENSG00000160789.18_LMNA	-0.17154	-0.321948909	-0.01268617	3.46E-02	1.57E-01	1.00E+00
ENSG00000169245.5_CXCL10	ENSG00000160789.18_LMNA	0.11666	-0.043350155	0.270826633	1.52E-01	4.02E-01	1.00E+00
ENSG00000165949.11_IFI27	ENSG00000160789.18_LMNA	-0.10514	-0.259990343	0.054979465	1.97E-01	4.67E-01	1.00E+00
ENSG00000100528.10_CNIH1	ENSG00000160789.18_LMNA	-0.14123	-0.293827249	0.018380908	8.26E-02	2.78E-01	1.00E+00
ENSG00000072062.12_PRKACA	ENSG00000160789.18_LMNA	0.32788	0.177964538	0.462913357	3.73E-05	8.50E-04	1.00E+00
ENSG00000141543.8_EIF4A3	ENSG00000160789.18_LMNA	0.16389	0.004814533	0.314874813	4.36E-02	1.84E-01	1.00E+00
ENSG00000132561.12_MATN2	ENSG00000160789.18_LMNA	-0.00119	-0.160355494	0.158045474	9.88E-01	9.96E-01	1.00E+00
ENSG00000010610.8_CD4	ENSG00000160789.18_LMNA	0.03306	-0.126806486	0.191255693	6.86E-01	8.64E-01	1.00E+00
ENSG00000188010.11_MORN2	ENSG00000160789.18_LMNA	-0.20175	-0.349720294	-0.04396367	1.27E-02	7.77E-02	1.00E+00
ENSG00000124333.13_VAMP7	ENSG00000160789.18_LMNA	-0.06441	-0.221345341	0.095768291	4.30E-01	7.00E-01	1.00E+00
ENSG00000175221.13_MED16	ENSG00000160789.18_LMNA	0.15889	-0.000322964	0.310239187	5.06E-02	2.03E-01	1.00E+00
ENSG00000130558.17_OLFM1	ENSG00000160789.18_LMNA	-0.1397	-0.292395687	0.019946446	8.61E-02	2.85E-01	1.00E+00
ENSG00000262655.3_SPON1	ENSG00000160789.18_LMNA	-0.24453	-0.388603789	-0.08878699	2.40E-03	2.25E-02	1.00E+00
ENSG00000164611.11_PTTG1	ENSG00000160789.18_LMNA	0.04103	-0.118946536	0.198932386	6.16E-01	8.26E-01	1.00E+00
ENSG00000135018.12_UBQLN1	ENSG00000160789.18_LMNA	0.07055	-0.089653618	0.227202798	3.88E-01	6.66E-01	1.00E+00
ENSG00000136908.16_DPM2	ENSG00000160789.18_LMNA	0.11135	-0.048711537	0.265840725	1.72E-01	4.31E-01	1.00E+00
ENSG00000174307.6_PHLDA3	ENSG00000160789.18_LMNA	0.39067	0.246818803	0.517672857	6.49E-07	3.23E-05	3.33E-02
ENSG00000129084.16_PSMA1	ENSG00000160789.18_LMNA	-0.0545	-0.211860983	0.105618643	5.05E-01	7.56E-01	1.00E+00
ENSG00000170881.4_RNF139	ENSG00000160789.18_LMNA	-0.27568	-0.416597766	-0.12182708	5.87E-04	7.65E-03	1.00E+00
ENSG00000273749.3_CYFIP1	ENSG00000160789.18_LMNA	0.08571	-0.074505913	0.241615231	2.94E-01	5.80E-01	1.00E+00

ENSG00000180900.15_SCRIB	ENSG00000160789.18_LMNA	0.20548	0.047848247	0.353132315	1.11E-02	7.05E-02	1.00E+00
ENSG00000119048.6_UBE2B	ENSG00000160789.18_LMNA	-0.23837	-0.383032826	-0.08228961	3.10E-03	2.74E-02	1.00E+00
ENSG00000139970.15_RTN1	ENSG00000160789.18_LMNA	-0.2744	-0.415452606	-0.12046256	6.24E-04	8.03E-03	1.00E+00
ENSG00000068745.13_IP6K2	ENSG00000160789.18_LMNA	-0.22142	-0.367663657	-0.06449622	6.12E-03	4.56E-02	1.00E+00
ENSG00000101266.15_CSNK2A1	ENSG00000160789.18_LMNA	-0.00332	-0.162435512	0.15596247	9.68E-01	9.87E-01	1.00E+00
ENSG00000221823.9_PPP3R1	ENSG00000160789.18_LMNA	-0.35875	-0.489965737	-0.21163485	5.67E-06	1.84E-04	2.91E-01
ENSG00000179344.15_HLA-DQB1	ENSG00000160789.18_LMNA	0.12097	-0.038981272	0.274877124	1.38E-01	3.78E-01	1.00E+00
ENSG00000133597.8_ADCK2	ENSG00000160789.18_LMNA	0.24662	0.090990842	0.39048844	2.19E-03	2.10E-02	1.00E+00
ENSG00000161981.9_SNRNP25	ENSG00000160789.18_LMNA	-0.21735	-0.363960998	-0.06023815	7.15E-03	5.12E-02	1.00E+00
ENSG00000237441.8_RGL2	ENSG00000160789.18_LMNA	0.05019	-0.109891609	0.207728098	5.39E-01	7.79E-01	1.00E+00
ENSG00000166228.7_PCBD1	ENSG00000160789.18_LMNA	-0.01395	-0.172762474	0.145578579	8.65E-01	9.47E-01	1.00E+00
ENSG00000048828.15_FAM120A	ENSG00000160789.18_LMNA	0.34938	0.201384817	0.481786725	1.03E-05	2.97E-04	5.26E-01
ENSG00000158109.13_TPRG1L	ENSG00000160789.18_LMNA	-0.23017	-0.37560424	-0.07366528	4.34E-03	3.52E-02	1.00E+00
ENSG00000173546.7_CSPG4	ENSG00000160789.18_LMNA	0.26629	0.111829796	0.408185915	9.13E-04	1.08E-02	1.00E+00
ENSG00000096080.10_MRPS18A	ENSG00000160789.18_LMNA	0.02614	-0.13361819	0.184571184	7.49E-01	8.95E-01	1.00E+00
ENSG00000224877.3_NDUFAF8	ENSG00000160789.18_LMNA	-0.09304	-0.248560861	0.067153475	2.54E-01	5.36E-01	1.00E+00
ENSG00000158716.7_DUSP23	ENSG00000160789.18_LMNA	0.15601	-0.003272102	0.307571455	5.49E-02	2.14E-01	1.00E+00
ENSG00000127948.12_POR	ENSG00000160789.18_LMNA	0.34246	0.193830394	0.475727241	1.57E-05	4.22E-04	8.05E-01
ENSG00000221978.10_CCNL2	ENSG00000160789.18_LMNA	-0.02607	-0.184506344	0.133684117	7.50E-01	8.95E-01	1.00E+00
ENSG00000146005.3_PSD2	ENSG00000160789.18_LMNA	-0.35567	-0.487276665	-0.20825811	6.90E-06	2.18E-04	3.54E-01
ENSG00000018408.13_WWTR1	ENSG00000160789.18_LMNA	0.50356	0.374369296	0.613570721	3.78E-11	1.01E-08	1.94E-06
ENSG00000119487.15_MAPKAP1	ENSG00000160789.18_LMNA	0.08292	-0.07730174	0.238965568	3.10E-01	5.96E-01	1.00E+00
ENSG00000184489.10_PTP4A3	ENSG00000160789.18_LMNA	0.27753	0.123797782	0.418250011	5.37E-04	7.13E-03	1.00E+00
ENSG00000065154.10_OAT	ENSG00000160789.18_LMNA	-0.27814	-0.418799299	-0.12445346	5.21E-04	6.97E-03	1.00E+00
ENSG00000169032.8_MAP2K1	ENSG00000160789.18_LMNA	0.12777	-0.032085209	0.28124803	1.17E-01	3.42E-01	1.00E+00
ENSG00000179526.15_SHARPIN	ENSG00000160789.18_LMNA	0.22045	0.063472258	0.366774138	6.35E-03	4.70E-02	1.00E+00
ENSG00000241258.5_CRCP	ENSG00000160789.18_LMNA	0.11606	-0.043952648	0.270267174	1.54E-01	4.05E-01	1.00E+00
ENSG00000132386.9_SERPINF1	ENSG00000160789.18_LMNA	0.13007	-0.02974914	0.283399954	1.10E-01	3.31E-01	1.00E+00
ENSG00000104687.11_GSR	ENSG00000160789.18_LMNA	0.12227	-0.037666605	0.276093805	1.33E-01	3.71E-01	1.00E+00
ENSG00000182013.16_PNMAL1	ENSG00000160789.18_LMNA	-0.0782	-0.234484598	0.082018603	3.38E-01	6.24E-01	1.00E+00
ENSG00000100030.13_MAPK1	ENSG00000160789.18_LMNA	-0.00015	-0.159346319	0.159055078	9.99E-01	9.99E-01	1.00E+00
ENSG00000162076.11_FLYWCH2	ENSG00000160789.18_LMNA	0.06542	-0.09476506	0.222307943	4.23E-01	6.94E-01	1.00E+00
ENSG00000103449.10_SALL1	ENSG00000160789.18_LMNA	0.12347	-0.036451512	0.27721744	1.30E-01	3.65E-01	1.00E+00

ENSG00000173039.17_RELA	ENSG00000160789.18_LMNA	0.26273	0.108044736	0.404987924	1.08E-03	1.23E-02	1.00E+00
ENSG00000067704.9_IARS2	ENSG00000160789.18_LMNA	0.24045	0.084481216	0.384914394	2.85E-03	2.57E-02	1.00E+00
ENSG00000049860.12_HEXB	ENSG00000160789.18_LMNA	0.39036	0.246476293	0.517405843	6.64E-07	3.29E-05	3.41E-02
ENSG00000140612.12_SEC11A	ENSG00000160789.18_LMNA	0.10512	-0.055003509	0.259967857	1.97E-01	4.67E-01	1.00E+00
ENSG00000182180.12_MRPS16	ENSG00000160789.18_LMNA	-0.20355	-0.351360579	-0.04582998	1.19E-02	7.43E-02	1.00E+00
ENSG00000178381.10_ZFAND2A	ENSG00000160789.18_LMNA	-0.03789	-0.195907971	0.122047913	6.43E-01	8.41E-01	1.00E+00
ENSG00000151729.9_SLC25A4	ENSG00000160789.18_LMNA	-0.2784	-0.419027983	-0.12472651	5.15E-04	6.90E-03	1.00E+00
ENSG00000120063.8_GNA13	ENSG00000160789.18_LMNA	-0.07509	-0.231518608	0.085132954	3.58E-01	6.41E-01	1.00E+00
ENSG00000136720.6_HS6ST1	ENSG00000160789.18_LMNA	0.3863	0.241977116	0.513893475	8.86E-07	4.14E-05	4.54E-02
ENSG00000103051.17_COG4	ENSG00000160789.18_LMNA	-0.00888	-0.167839044	0.150537914	9.14E-01	9.67E-01	1.00E+00
ENSG00000091513.13_TF	ENSG00000160789.18_LMNA	-0.37804	-0.506739746	-0.23284954	1.57E-06	6.66E-05	8.07E-02
ENSG00000250317.7_SMIM20	ENSG00000160789.18_LMNA	-0.26996	-0.411472234	-0.11572838	7.70E-04	9.42E-03	1.00E+00
ENSG00000184371.12_CSF1	ENSG00000160789.18_LMNA	0.33796	0.188924077	0.471777446	2.06E-05	5.29E-04	1.00E+00
ENSG00000163875.14_MEAF6	ENSG00000160789.18_LMNA	-0.15228	-0.304106333	0.007094687	6.11E-02	2.28E-01	1.00E+00
ENSG00000204262.10_COL5A2	ENSG00000160789.18_LMNA	0.44652	0.309310035	0.565522435	8.12E-09	9.07E-07	4.16E-04
ENSG00000109046.13_WSB1	ENSG00000160789.18_LMNA	-0.15972	-0.311014525	-0.00053517	4.93E-02	2.00E-01	1.00E+00
ENSG00000242808.6_SOX2-OT	ENSG00000160789.18_LMNA	-0.41452	-0.538203782	-0.27335895	1.10E-07	7.83E-06	5.65E-03
ENSG00000154917.9_RAB6B	ENSG00000160789.18_LMNA	-0.39949	-0.525281476	-0.25660724	3.42E-07	1.96E-05	1.76E-02
ENSG00000179241.11_LDLRAD3	ENSG00000160789.18_LMNA	0.05163	-0.108466882	0.20910738	5.28E-01	7.72E-01	1.00E+00
ENSG00000164713.8_BRI3	ENSG00000160789.18_LMNA	0.17478	0.016025477	0.324939483	3.13E-02	1.47E-01	1.00E+00
ENSG00000179403.11_VWA1	ENSG00000160789.18_LMNA	0.35325	0.205617836	0.485170394	8.04E-06	2.45E-04	4.13E-01
ENSG00000128656.12_CHN1	ENSG00000160789.18_LMNA	-0.10458	-0.259463508	0.055542685	2.00E-01	4.70E-01	1.00E+00
ENSG00000170775.2_GPR37	ENSG00000160789.18_LMNA	-0.03735	-0.19538891	0.122579559	6.48E-01	8.43E-01	1.00E+00
ENSG00000112033.12_PPARD	ENSG00000160789.18_LMNA	0.31539	0.164450517	0.451904169	7.56E-05	1.50E-03	1.00E+00
ENSG00000251562.6_MALAT1	ENSG00000160789.18_LMNA	0.04582	-0.114213804	0.203536031	5.75E-01	8.02E-01	1.00E+00
ENSG00000109572.12_CLCN3	ENSG00000160789.18_LMNA	-0.12893	-0.282334126	0.030906601	1.13E-01	3.36E-01	1.00E+00
ENSG00000214717.8_ZBED1	ENSG00000160789.18_LMNA	0.01782	-0.141782286	0.176520563	8.27E-01	9.32E-01	1.00E+00
ENSG00000262814.5_MRPL12	ENSG00000160789.18_LMNA	0.00371	-0.155580634	0.162816494	9.64E-01	9.86E-01	1.00E+00
ENSG00000078618.18_NRDC	ENSG00000160789.18_LMNA	0.29959	0.147424984	0.437908553	1.77E-04	2.97E-03	1.00E+00
ENSG00000139637.12_C12orf10	ENSG00000160789.18_LMNA	-0.09358	-0.24907449	0.066608398	2.51E-01	5.32E-01	1.00E+00
ENSG00000172216.5_CEBPB	ENSG00000160789.18_LMNA	0.39195	0.248237281	0.51877811	5.92E-07	3.01E-05	3.04E-02
ENSG00000171204.11_TMEM126	ENSG00000160789.18_LMNA	-0.30025	-0.438490825	-0.1481299	1.71E-04	2.89E-03	1.00E+00
ENSG00000183386.8_FHL3	ENSG00000160789.18_LMNA	0.36257	0.215824354	0.493294701	4.43E-06	1.51E-04	2.27E-01

ENSG00000239697.9_TNFSF12	ENSG00000160789.18_LMNA	0.14697	-0.012524907	0.299169713	7.08E-02	2.51E-01	1.00E+00
ENSG00000227500.8_SCAMP4	ENSG00000160789.18_LMNA	0.22367	0.066852126	0.369708147	5.60E-03	4.27E-02	1.00E+00
ENSG00000100242.14_SUN2	ENSG00000160789.18_LMNA	0.05562	-0.104508769	0.212932616	4.96E-01	7.50E-01	1.00E+00
ENSG00000125821.10_DTD1	ENSG00000160789.18_LMNA	-0.24646	-0.39034583	-0.09082397	2.21E-03	2.11E-02	1.00E+00
ENSG00000130202.8_NECTIN2	ENSG00000160789.18_LMNA	0.50107	0.371504209	0.611491404	4.88E-11	1.25E-08	2.50E-06
ENSG00000115365.10_LANCL1	ENSG00000160789.18_LMNA	-0.52007	-0.627327126	-0.39343999	6.58E-12	2.50E-09	3.37E-07
ENSG00000131408.12_NR1H2	ENSG00000160789.18_LMNA	0.28963	0.136732896	0.429046693	2.96E-04	4.44E-03	1.00E+00
ENSG00000101294.15_HM13	ENSG00000160789.18_LMNA	0.25321	0.097955308	0.396427741	1.65E-03	1.69E-02	1.00E+00
ENSG00000197965.10_MPZL1	ENSG00000160789.18_LMNA	-0.25043	-0.393925588	-0.0950177	1.86E-03	1.85E-02	1.00E+00
ENSG00000065268.9_WDR18	ENSG00000160789.18_LMNA	-0.01989	-0.178527737	0.139750865	8.08E-01	9.23E-01	1.00E+00
ENSG00000176095.10_IP6K1	ENSG00000160789.18_LMNA	-0.035	-0.193124128	0.124897119	6.69E-01	8.54E-01	1.00E+00
ENSG00000146409.9_SLC18B1	ENSG00000160789.18_LMNA	-0.14799	-0.300118875	0.011482266	6.88E-02	2.47E-01	1.00E+00
ENSG00000182636.5_NDN	ENSG00000160789.18_LMNA	-0.2542	-0.397318236	-0.09900203	1.58E-03	1.63E-02	1.00E+00
ENSG00000160679.11_CHTOP	ENSG00000160789.18_LMNA	-0.13795	-0.290763499	0.02172952	9.01E-02	2.92E-01	1.00E+00
ENSG00000143320.7_CRABP2	ENSG00000160789.18_LMNA	0.38735	0.243148236	0.514808611	8.22E-07	3.88E-05	4.22E-02
ENSG00000119669.4_IRF2BPL	ENSG00000160789.18_LMNA	0.42545	0.285596098	0.547565494	4.66E-08	3.85E-06	2.39E-03
ENSG00000033100.13_CHPF2	ENSG00000160789.18_LMNA	0.53812	0.414425124	0.642297676	8.70E-13	5.13E-10	4.46E-08
ENSG00000179314.12_WSCD1	ENSG00000160789.18_LMNA	-0.01956	-0.178207036	0.14007562	8.11E-01	9.26E-01	1.00E+00
ENSG00000110696.8_C11orf58	ENSG00000160789.18_LMNA	-0.27708	-0.417844521	-0.12331392	5.49E-04	7.26E-03	1.00E+00
ENSG00000096746.16_HNRNPH3	ENSG00000160789.18_LMNA	0.02779	-0.131991918	0.186169769	7.34E-01	8.88E-01	1.00E+00
ENSG00000139112.9_GABARAPL	ENSG00000160789.18_LMNA	-0.23913	-0.383724406	-0.08309481	3.01E-03	2.68E-02	1.00E+00
ENSG00000166797.9_FAM96A	ENSG00000160789.18_LMNA	-0.09133	-0.246937501	0.068874991	2.63E-01	5.46E-01	1.00E+00
ENSG00000145901.13_TNIP1	ENSG00000160789.18_LMNA	0.24865	0.093134765	0.392319439	2.01E-03	1.96E-02	1.00E+00
ENSG00000154945.6_ANKRD40	ENSG00000160789.18_LMNA	-0.16595	-0.316780565	-0.00693137	4.10E-02	1.76E-01	1.00E+00
ENSG00000143727.14_ACP1	ENSG00000160789.18_LMNA	-0.19112	-0.339978779	-0.03292362	1.83E-02	1.01E-01	1.00E+00
ENSG00000172465.12_TCEAL1	ENSG00000160789.18_LMNA	-0.39499	-0.521401973	-0.25160939	4.76E-07	2.52E-05	2.44E-02
ENSG00000167657.10_DAPK3	ENSG00000160789.18_LMNA	0.39035	0.246462568	0.517395142	6.64E-07	3.29E-05	3.41E-02
ENSG00000110092.3_CCND1	ENSG00000160789.18_LMNA	-0.05776	-0.214981264	0.102384815	4.80E-01	7.38E-01	1.00E+00
ENSG00000133313.13_CNDP2	ENSG00000160789.18_LMNA	-0.00945	-0.168395388	0.149978314	9.08E-01	9.65E-01	1.00E+00
ENSG00000184588.16_PDE4B	ENSG00000160789.18_LMNA	-0.03167	-0.189914465	0.128175646	6.99E-01	8.71E-01	1.00E+00
ENSG00000198911.10_SREBF2	ENSG00000160789.18_LMNA	0.01024	-0.149207387	0.169161498	9.00E-01	9.61E-01	1.00E+00
ENSG00000179094.12_PER1	ENSG00000160789.18_LMNA	0.02643	-0.133328539	0.184856027	7.47E-01	8.94E-01	1.00E+00
ENSG00000114573.8_ATP6V1A	ENSG00000160789.18_LMNA	-0.32405	-0.459547555	-0.1738215	4.65E-05	1.01E-03	1.00E+00

ENSG00000113621.13_TXNDC15	ENSG00000160789.18_LMNA	0.03298	-0.126887016	0.19117684	6.87E-01	8.64E-01	1.00E+00
ENSG00000128791.10_TWSG1	ENSG00000160789.18_LMNA	0.19445	0.036370893	0.343027676	1.64E-02	9.35E-02	1.00E+00
ENSG00000055208.16_TAB2	ENSG00000160789.18_LMNA	-0.05649	-0.213769067	0.10364192	4.89E-01	7.46E-01	1.00E+00
ENSG00000180304.13_OAZ2	ENSG00000160789.18_LMNA	-0.17389	-0.324115597	-0.01510483	3.22E-02	1.50E-01	1.00E+00
ENSG00000127947.14_PTPN12	ENSG00000160789.18_LMNA	0.4939	0.363262057	0.60549118	1.01E-10	2.25E-08	5.17E-06
ENSG00000180370.9_PAK2	ENSG00000160789.18_LMNA	0.04488	-0.115144148	0.202632171	5.83E-01	8.06E-01	1.00E+00
ENSG00000110876.9_SELPLG	ENSG00000160789.18_LMNA	-0.0579	-0.215116214	0.102244803	4.79E-01	7.37E-01	1.00E+00
ENSG00000166963.11_MAP1A	ENSG00000160789.18_LMNA	0.01464	-0.144903081	0.173431852	8.58E-01	9.44E-01	1.00E+00
ENSG00000107404.16_DVL1	ENSG00000160789.18_LMNA	0.19728	0.039314069	0.345625626	1.48E-02	8.73E-02	1.00E+00
ENSG00000151348.12_EXT2	ENSG00000160789.18_LMNA	0.32554	0.175430821	0.460855941	4.27E-05	9.45E-04	1.00E+00
ENSG00000136521.11_NDUFB5	ENSG00000160789.18_LMNA	-0.29409	-0.433020276	-0.14151873	2.35E-04	3.72E-03	1.00E+00
ENSG00000136261.13_BZW2	ENSG00000160789.18_LMNA	-0.07508	-0.231509355	0.08514266	3.58E-01	6.41E-01	1.00E+00
ENSG00000143155.11_TIPRL	ENSG00000160789.18_LMNA	-0.23815	-0.382836734	-0.08206137	3.13E-03	2.76E-02	1.00E+00
ENSG00000167106.10_FAM102A	ENSG00000160789.18_LMNA	0.25061	0.095205675	0.394085833	1.85E-03	1.84E-02	1.00E+00
ENSG00000144659.9_SLC25A38	ENSG00000160789.18_LMNA	-0.2192	-0.36564427	-0.06217255	6.66E-03	4.86E-02	1.00E+00
ENSG00000170581.12_STAT2	ENSG00000160789.18_LMNA	0.10736	-0.052741453	0.262081925	1.88E-01	4.55E-01	1.00E+00
ENSG00000185339.7_TCN2	ENSG00000160789.18_LMNA	0.14553	-0.013993863	0.297831409	7.36E-02	2.58E-01	1.00E+00
ENSG00000187189.10_TSPYL4	ENSG00000160789.18_LMNA	-0.34419	-0.477241686	-0.19571534	1.41E-05	3.89E-04	7.25E-01
ENSG00000042445.12_RETSAT	ENSG00000160789.18_LMNA	0.04582	-0.114210459	0.203539281	5.75E-01	8.02E-01	1.00E+00
ENSG00000237190.3_CDKN2AIPI	ENSG00000160789.18_LMNA	-0.20763	-0.355094829	-0.05008676	1.03E-02	6.68E-02	1.00E+00
ENSG00000165030.3_NFIL3	ENSG00000160789.18_LMNA	0.06071	-0.099449488	0.217807927	4.57E-01	7.22E-01	1.00E+00
ENSG00000131051.19_RBM39	ENSG00000160789.18_LMNA	0.05669	-0.103447311	0.213956788	4.88E-01	7.44E-01	1.00E+00
ENSG00000112511.16_PHF1	ENSG00000160789.18_LMNA	0.09104	-0.069159855	0.246668709	2.65E-01	5.48E-01	1.00E+00
ENSG00000157916.17_RER1	ENSG00000160789.18_LMNA	0.18095	0.022398999	0.330630309	2.57E-02	1.29E-01	1.00E+00
ENSG00000144118.12_RALB	ENSG00000160789.18_LMNA	0.19989	0.042028429	0.348017445	1.35E-02	8.14E-02	1.00E+00
ENSG00000011465.15_DCN	ENSG00000160789.18_LMNA	0.14623	-0.013275369	0.298486152	7.22E-02	2.55E-01	1.00E+00
ENSG00000017483.13_SLC38A5	ENSG00000160789.18_LMNA	-0.22199	-0.368179064	-0.06508981	5.98E-03	4.50E-02	1.00E+00
ENSG00000198189.9_HSD17B11	ENSG00000160789.18_LMNA	-0.17429	-0.324490857	-0.0155241	3.17E-02	1.49E-01	1.00E+00
ENSG00000100462.14_PRMT5	ENSG00000160789.18_LMNA	0.11246	-0.047596692	0.266878882	1.68E-01	4.26E-01	1.00E+00
ENSG00000177225.15_PDDC1	ENSG00000160789.18_LMNA	-0.0518	-0.209270845	0.108297944	5.26E-01	7.71E-01	1.00E+00
ENSG00000100842.11_EFS	ENSG00000160789.18_LMNA	-0.05876	-0.215943165	0.101386556	4.72E-01	7.32E-01	1.00E+00
ENSG00000177731.14_FLII	ENSG00000160789.18_LMNA	0.46299	0.327958533	0.579475073	1.90E-09	2.65E-07	9.77E-05
ENSG00000162385.9_MAGOH	ENSG00000160789.18_LMNA	0.17019	0.011296424	0.320702472	3.61E-02	1.62E-01	1.00E+00

ENSG00000100401.18_RANGAP1	ENSG00000160789.18_LMNA	-0.02519	-0.183649495	0.134555065	7.58E-01	8.99E-01	1.00E+00
ENSG00000131435.11_PDLIM4	ENSG00000160789.18_LMNA	0.37816	0.232986179	0.506847112	1.56E-06	6.63E-05	8.00E-02
ENSG00000140497.15_SCAMP2	ENSG00000160789.18_LMNA	0.12063	-0.039325503	0.274558383	1.39E-01	3.80E-01	1.00E+00
ENSG00000164904.14_ALDH7A1	ENSG00000160789.18_LMNA	0.08355	-0.076669016	0.239565626	3.06E-01	5.93E-01	1.00E+00
ENSG00000113916.16_BCL6	ENSG00000160789.18_LMNA	0.06604	-0.094150101	0.222897691	4.19E-01	6.91E-01	1.00E+00
ENSG00000174938.13_SEZ6L2	ENSG00000160789.18_LMNA	0.04139	-0.118592447	0.199277303	6.13E-01	8.24E-01	1.00E+00
ENSG00000176014.11_TUBB6	ENSG00000160789.18_LMNA	0.37708	0.231797338	0.505912648	1.68E-06	6.98E-05	8.62E-02
ENSG00000168883.18_USP39	ENSG00000160789.18_LMNA	-0.18172	-0.331333298	-0.02318806	2.51E-02	1.27E-01	1.00E+00
ENSG00000162576.15_MXRA8	ENSG00000160789.18_LMNA	0.40525	0.26301852	0.530241835	2.23E-07	1.39E-05	1.14E-02
ENSG00000196517.10_SLC6A9	ENSG00000160789.18_LMNA	0.10326	-0.05687601	0.25821557	2.06E-01	4.77E-01	1.00E+00
ENSG00000121851.11_POLR3GL	ENSG00000160789.18_LMNA	0.0157	-0.143856411	0.17446846	8.48E-01	9.41E-01	1.00E+00
ENSG00000179820.14_MYADM	ENSG00000160789.18_LMNA	0.55022	0.428561721	0.652285486	2.09E-13	1.53E-10	1.07E-08
ENSG00000120686.10_UFM1	ENSG00000160789.18_LMNA	-0.05307	-0.210490841	0.107036525	5.16E-01	7.63E-01	1.00E+00
ENSG00000165527.6_ARF6	ENSG00000160789.18_LMNA	0.51122	0.383212885	0.619968039	1.70E-11	5.21E-09	8.71E-07
ENSG00000160714.8_UBE2Q1	ENSG00000160789.18_LMNA	0.15186	-0.007526645	0.30371425	6.18E-02	2.30E-01	1.00E+00
ENSG00000122515.13_ZMIZ2	ENSG00000160789.18_LMNA	0.05382	-0.106289279	0.211213082	5.10E-01	7.59E-01	1.00E+00
ENSG00000151502.9_VPS26B	ENSG00000160789.18_LMNA	-0.14126	-0.293854706	0.018350866	8.26E-02	2.78E-01	1.00E+00
ENSG00000172057.8_ORMDL3	ENSG00000160789.18_LMNA	0.00147	-0.157765191	0.16063554	9.86E-01	9.94E-01	1.00E+00
ENSG00000128591.14_FLNC	ENSG00000160789.18_LMNA	0.21768	0.06057906	0.364257797	7.06E-03	5.07E-02	1.00E+00
ENSG00000114650.17_SCAP	ENSG00000160789.18_LMNA	0.10758	-0.052521562	0.262287269	1.87E-01	4.53E-01	1.00E+00
ENSG00000159111.11_MRPL10	ENSG00000160789.18_LMNA	-0.17302	-0.323316706	-0.01421261	3.30E-02	1.52E-01	1.00E+00
ENSG00000132819.15_RBM38	ENSG00000160789.18_LMNA	0.14478	-0.014761805	0.297131281	7.51E-02	2.62E-01	1.00E+00
ENSG00000102024.16_PLS3	ENSG00000160789.18_LMNA	0.4607	0.325367562	0.577545311	2.34E-09	3.17E-07	1.20E-04
ENSG00000143248.11_RGS5	ENSG00000160789.18_LMNA	-0.20983	-0.357105336	-0.05238319	9.47E-03	6.30E-02	1.00E+00
ENSG00000025770.17_NCAPH2	ENSG00000160789.18_LMNA	-0.03123	-0.189486749	0.128612015	7.03E-01	8.73E-01	1.00E+00
ENSG00000275400.1_RP4-756H1	ENSG00000160789.18_LMNA	0.1552	-0.004101928	0.306819929	5.62E-02	2.17E-01	1.00E+00
ENSG00000125827.7_TMX4	ENSG00000160789.18_LMNA	-0.16199	-0.313114132	-0.0028613	4.62E-02	1.91E-01	1.00E+00
ENSG00000141429.12_GALNT1	ENSG00000160789.18_LMNA	-0.15179	-0.303650686	0.007596663	6.19E-02	2.30E-01	1.00E+00
ENSG00000169957.9_ZNF768	ENSG00000160789.18_LMNA	0.32217	0.171784208	0.457889441	5.17E-05	1.11E-03	1.00E+00
ENSG00000100605.15_ITPK1	ENSG00000160789.18_LMNA	-0.02512	-0.183588206	0.134617344	7.59E-01	8.99E-01	1.00E+00
ENSG00000148411.6_NACC2	ENSG00000160789.18_LMNA	0.36452	0.217972573	0.494998522	3.89E-06	1.35E-04	2.00E-01
ENSG00000130600.14_H19	ENSG00000160789.18_LMNA	0.21945	0.062430589	0.365868664	6.60E-03	4.82E-02	1.00E+00
ENSG00000161714.10_PLCD3	ENSG00000160789.18_LMNA	-0.0002	-0.159400476	0.159000914	9.98E-01	9.99E-01	1.00E+00

ENSG00000104812.13_GYS1	ENSG00000160789.18_LMNA	0.38933	0.245338821	0.516518715	7.14E-07	3.51E-05	3.66E-02
ENSG00000138757.13_G3BP2	ENSG00000160789.18_LMNA	-0.29624	-0.434932374	-0.14382655	2.11E-04	3.40E-03	1.00E+00
ENSG00000131899.9_LLGL1	ENSG00000160789.18_LMNA	-0.20951	-0.356805384	-0.05204038	9.59E-03	6.36E-02	1.00E+00
ENSG00000242574.7_HLA-DMB	ENSG00000160789.18_LMNA	-0.13726	-0.290123636	0.022427992	9.17E-02	2.95E-01	1.00E+00
ENSG00000125844.14_RRBP1	ENSG00000160789.18_LMNA	0.53699	0.413104874	0.641360923	9.91E-13	5.53E-10	5.08E-08
ENSG00000168958.18_MFF	ENSG00000160789.18_LMNA	-0.50642	-0.615958669	-0.37766529	2.81E-11	7.88E-09	1.44E-06
ENSG00000078061.11_ARAF	ENSG00000160789.18_LMNA	0.38884	0.244788814	0.51608955	7.40E-07	3.60E-05	3.80E-02
ENSG00000107833.9_NPM3	ENSG00000160789.18_LMNA	-0.03789	-0.19590885	0.122047012	6.43E-01	8.41E-01	1.00E+00
ENSG00000240376.1_RP11-36C2	ENSG00000160789.18_LMNA	-0.09761	-0.252878562	0.062565537	2.32E-01	5.09E-01	1.00E+00
ENSG00000172331.10_BPGM	ENSG00000160789.18_LMNA	-0.27356	-0.414697437	-0.11956334	6.49E-04	8.27E-03	1.00E+00
ENSG00000135900.3_MRPL44	ENSG00000160789.18_LMNA	-0.29499	-0.433816144	-0.14247892	2.25E-04	3.58E-03	1.00E+00
ENSG00000108262.14_GIT1	ENSG00000160789.18_LMNA	0.00324	-0.156039599	0.162358544	9.68E-01	9.88E-01	1.00E+00
ENSG00000129515.17_SNX6	ENSG00000160789.18_LMNA	-0.10248	-0.257482573	0.057658638	2.09E-01	4.82E-01	1.00E+00
ENSG00000148291.8_SURF2	ENSG00000160789.18_LMNA	-0.00295	-0.162077639	0.156321059	9.71E-01	9.89E-01	1.00E+00
ENSG00000173918.13_C1QTNF1	ENSG00000160789.18_LMNA	0.28774	0.134709204	0.427363051	3.25E-04	4.79E-03	1.00E+00
ENSG00000152556.14_PFKM	ENSG00000160789.18_LMNA	-0.14793	-0.300062813	0.011543869	6.89E-02	2.47E-01	1.00E+00
ENSG00000129518.7_EAPP	ENSG00000160789.18_LMNA	-0.29338	-0.432387915	-0.14075619	2.44E-04	3.83E-03	1.00E+00
ENSG00000177971.8_IMP3	ENSG00000160789.18_LMNA	-0.08996	-0.245642854	0.070246573	2.70E-01	5.55E-01	1.00E+00
ENSG00000253683.1_CTB-79E8.3	ENSG00000160789.18_LMNA	-0.1769	-0.326896847	-0.01821483	2.92E-02	1.41E-01	1.00E+00
ENSG00000164543.5_STK17A	ENSG00000160789.18_LMNA	0.25526	0.10013169	0.398278652	1.50E-03	1.57E-02	1.00E+00
ENSG00000117408.9_IPO13	ENSG00000160789.18_LMNA	0.04303	-0.116976625	0.200850272	5.99E-01	8.15E-01	1.00E+00
ENSG00000130204.11_TOMM40	ENSG00000160789.18_LMNA	0.32384	0.173592289	0.459361102	4.70E-05	1.02E-03	1.00E+00
ENSG00000135241.15_PNPLA8	ENSG00000160789.18_LMNA	-0.2627	-0.404961506	-0.1080135	1.08E-03	1.23E-02	1.00E+00
ENSG00000187446.10_CHP1	ENSG00000160789.18_LMNA	0.1881	0.029794613	0.337205771	2.03E-02	1.09E-01	1.00E+00
ENSG00000184432.8_COPB2	ENSG00000160789.18_LMNA	0.20573	0.048107837	0.353360037	1.10E-02	7.01E-02	1.00E+00
ENSG00000064961.17_HMG20B	ENSG00000160789.18_LMNA	0.23596	0.079750849	0.38085009	3.43E-03	2.95E-02	1.00E+00
ENSG00000167325.13_RRM1	ENSG00000160789.18_LMNA	0.15549	-0.003808164	0.307086019	5.58E-02	2.16E-01	1.00E+00
ENSG00000090238.10_YPEL3	ENSG00000160789.18_LMNA	-0.17615	-0.326198485	-0.01743336	3.00E-02	1.43E-01	1.00E+00
ENSG00000114520.9_SNX4	ENSG00000160789.18_LMNA	-0.34835	-0.480880519	-0.20025291	1.09E-05	3.13E-04	5.61E-01
ENSG00000038427.14_VCAN	ENSG00000160789.18_LMNA	-0.16234	-0.313437314	-0.00321965	4.57E-02	1.90E-01	1.00E+00
ENSG00000116161.16_CACYBP	ENSG00000160789.18_LMNA	-0.31532	-0.451842961	-0.16437568	7.59E-05	1.51E-03	1.00E+00
ENSG00000115806.11_GORASP2	ENSG00000160789.18_LMNA	-0.18372	-0.33317947	-0.02526212	2.35E-02	1.21E-01	1.00E+00
ENSG00000139329.4_LUM	ENSG00000160789.18_LMNA	0.25821	0.103250013	0.400926413	1.32E-03	1.43E-02	1.00E+00

ENSG00000111361.11_EIF2B1	ENSG00000160789.18_LMNA	-0.20564	-0.353274949	-0.04801084	1.10E-02	7.03E-02	1.00E+00
ENSG00000167645.15_YIF1B	ENSG00000160789.18_LMNA	0.0019	-0.157348317	0.161051966	9.81E-01	9.93E-01	1.00E+00
ENSG00000104635.12_SLC39A14	ENSG00000160789.18_LMNA	0.30917	0.157727885	0.446394772	1.06E-04	1.98E-03	1.00E+00
ENSG00000116898.10_MRPS15	ENSG00000160789.18_LMNA	-0.0636	-0.220564132	0.096582	4.36E-01	7.04E-01	1.00E+00
ENSG00000141560.13_FN3KRP	ENSG00000160789.18_LMNA	-0.06468	-0.221600375	0.095502556	4.29E-01	6.99E-01	1.00E+00
ENSG00000114978.16_MOB1A	ENSG00000160789.18_LMNA	0.0624	-0.097772819	0.219420142	4.45E-01	7.12E-01	1.00E+00
ENSG00000117399.12_CDC20	ENSG00000160789.18_LMNA	0.15637	-0.002900717	0.307907671	5.44E-02	2.12E-01	1.00E+00
ENSG00000088325.14_TPX2	ENSG00000160789.18_LMNA	0.14766	-0.011818174	0.29981315	6.95E-02	2.48E-01	1.00E+00
ENSG00000104872.9_PIH1D1	ENSG00000160789.18_LMNA	-0.09021	-0.245877185	0.069998405	2.69E-01	5.53E-01	1.00E+00
ENSG00000129654.7_FOXJ1	ENSG00000160789.18_LMNA	0.13452	-0.025220954	0.287562239	9.85E-02	3.09E-01	1.00E+00
ENSG00000146576.11_C7orf26	ENSG00000160789.18_LMNA	0.17819	0.019547791	0.328087274	2.81E-02	1.37E-01	1.00E+00
ENSG00000175216.13_CKAP5	ENSG00000160789.18_LMNA	0.07639	-0.083828866	0.232761288	3.50E-01	6.33E-01	1.00E+00
ENSG00000138385.14_SSB	ENSG00000160789.18_LMNA	-0.01048	-0.169395132	0.148972208	8.98E-01	9.60E-01	1.00E+00
ENSG00000091140.11_DLD	ENSG00000160789.18_LMNA	0.15704	-0.002219235	0.308524419	5.33E-02	2.10E-01	1.00E+00
ENSG00000136451.7_VEZF1	ENSG00000160789.18_LMNA	-0.28661	-0.426358301	-0.13350268	3.44E-04	4.99E-03	1.00E+00
ENSG00000125505.15_MBOAT7	ENSG00000160789.18_LMNA	0.44104	0.303124797	0.560862079	1.29E-08	1.34E-06	6.64E-04
ENSG00000125944.17_HNRNPR	ENSG00000160789.18_LMNA	0.02831	-0.131486936	0.186665811	7.29E-01	8.85E-01	1.00E+00
ENSG00000015676.16_NUDCD3	ENSG00000160789.18_LMNA	0.10918	-0.050905951	0.263795127	1.81E-01	4.44E-01	1.00E+00
ENSG00000123983.12_ACSL3	ENSG00000160789.18_LMNA	0.25007	0.094642159	0.393605398	1.89E-03	1.87E-02	1.00E+00
ENSG00000113296.13_THBS4	ENSG00000160789.18_LMNA	0.00906	-0.150354261	0.168021651	9.12E-01	9.66E-01	1.00E+00
ENSG00000159348.11_CYB5R1	ENSG00000160789.18_LMNA	0.15972	0.000528319	0.311008332	4.94E-02	2.00E-01	1.00E+00
ENSG00000058799.12_YIPF1	ENSG00000160789.18_LMNA	0.10975	-0.050332458	0.264330002	1.78E-01	4.40E-01	1.00E+00
ENSG00000159202.16_UBE2Z	ENSG00000160789.18_LMNA	0.2865	0.13337988	0.426255994	3.46E-04	5.02E-03	1.00E+00
ENSG00000198736.10_MSRB1	ENSG00000160789.18_LMNA	-0.04524	-0.202982082	0.11478405	5.80E-01	8.04E-01	1.00E+00
ENSG00000118292.7_C1orf54	ENSG00000160789.18_LMNA	0.01636	-0.143217655	0.175100729	8.41E-01	9.38E-01	1.00E+00
ENSG00000072274.11_TFRC	ENSG00000160789.18_LMNA	0.18163	0.023095507	0.331250856	2.51E-02	1.27E-01	1.00E+00
ENSG00000128602.8_SMO	ENSG00000160789.18_LMNA	0.24743	0.091844771	0.391218016	2.12E-03	2.04E-02	1.00E+00
ENSG00000213654.8_GPSM3	ENSG00000160789.18_LMNA	-0.0676	-0.224387661	0.092595359	4.08E-01	6.83E-01	1.00E+00
ENSG00000125912.9_NCLN	ENSG00000160789.18_LMNA	0.40104	0.258332088	0.526617778	3.05E-07	1.79E-05	1.57E-02
ENSG00000089006.15_SNX5	ENSG00000160789.18_LMNA	0.02531	-0.134436133	0.18376653	7.57E-01	8.99E-01	1.00E+00
ENSG00000166123.12_GPT2	ENSG00000160789.18_LMNA	-0.12481	-0.278475614	0.035089827	1.26E-01	3.58E-01	1.00E+00
ENSG00000196305.16_IARS	ENSG00000160789.18_LMNA	0.16192	0.002793074	0.313052589	4.63E-02	1.91E-01	1.00E+00
ENSG00000101057.14_MYBL2	ENSG00000160789.18_LMNA	0.00977	-0.149662565	0.168709211	9.05E-01	9.63E-01	1.00E+00

ENSG00000140545.13_MFGE8	ENSG00000160789.18_LMNA	0.35064	0.202757536	0.482884927	9.48E-06	2.79E-04	4.86E-01
ENSG00000125037.11_EMC3	ENSG00000160789.18_LMNA	-0.11097	-0.26548419	0.049094228	1.73E-01	4.33E-01	1.00E+00
ENSG00000131067.15_GGT7	ENSG00000160789.18_LMNA	-0.06191	-0.218951289	0.098260601	4.49E-01	7.15E-01	1.00E+00
ENSG00000136718.8_IMP4	ENSG00000160789.18_LMNA	-0.12287	-0.276662267	0.037051989	1.32E-01	3.68E-01	1.00E+00
ENSG00000074319.11_TSG101	ENSG00000160789.18_LMNA	-0.27083	-0.412259913	-0.11666416	7.39E-04	9.13E-03	1.00E+00
ENSG00000147649.8_MTDH	ENSG00000160789.18_LMNA	0.17924	0.02062295	0.32904675	2.71E-02	1.34E-01	1.00E+00
ENSG00000166197.15_NOLC1	ENSG00000160789.18_LMNA	0.02848	-0.131315105	0.186834563	7.28E-01	8.85E-01	1.00E+00
ENSG00000089685.13_BIRC5	ENSG00000160789.18_LMNA	-0.04973	-0.207292806	0.110340973	5.43E-01	7.82E-01	1.00E+00
ENSG00000101134.10_DOK5	ENSG00000160789.18_LMNA	0.02758	-0.132201567	0.185963784	7.36E-01	8.89E-01	1.00E+00
ENSG00000140259.6_MFAP1	ENSG00000160789.18_LMNA	0.2052	0.047557657	0.352877356	1.12E-02	7.11E-02	1.00E+00
ENSG00000198792.11_TMEM184	ENSG00000160789.18_LMNA	0.12137	-0.038574025	0.275254125	1.36E-01	3.76E-01	1.00E+00
ENSG00000164050.11_PLXNB1	ENSG00000160789.18_LMNA	-0.23777	-0.382493844	-0.08166235	3.18E-03	2.79E-02	1.00E+00
ENSG00000137491.13_SLCO2B1	ENSG00000160789.18_LMNA	-0.06135	-0.218416646	0.098816645	4.53E-01	7.18E-01	1.00E+00
ENSG00000160075.11_SSU72	ENSG00000160789.18_LMNA	-0.01503	-0.173814909	0.144516389	8.54E-01	9.43E-01	1.00E+00
ENSG00000167965.16_MLST8	ENSG00000160789.18_LMNA	-0.12897	-0.282376406	0.030860702	1.13E-01	3.36E-01	1.00E+00
ENSG00000168389.16_MFSD2A	ENSG00000160789.18_LMNA	-0.15354	-0.305281679	0.00579911	5.89E-02	2.23E-01	1.00E+00
ENSG00000185803.7_SLC52A2	ENSG00000160789.18_LMNA	0.40476	0.262471988	0.529819701	2.31E-07	1.43E-05	1.19E-02
ENSG00000006625.16_GGCT	ENSG00000160789.18_LMNA	0.28221	0.128800064	0.422435206	4.27E-04	5.97E-03	1.00E+00
ENSG00000069345.10_DNAJA2	ENSG00000160789.18_LMNA	-0.15944	-0.310751363	-0.00024386	4.98E-02	2.01E-01	1.00E+00
ENSG00000135316.16_SYNCRIP	ENSG00000160789.18_LMNA	0.17149	0.012635835	0.32190378	3.46E-02	1.57E-01	1.00E+00
ENSG00000167740.8_CYB5D2	ENSG00000160789.18_LMNA	-0.07932	-0.235544987	0.080903671	3.31E-01	6.17E-01	1.00E+00
ENSG00000255112.2_CHMP1B	ENSG00000160789.18_LMNA	-0.27049	-0.41194942	-0.11629523	7.51E-04	9.25E-03	1.00E+00
ENSG00000122140.9_MRPS2	ENSG00000160789.18_LMNA	0.19596	0.037947699	0.344420111	1.55E-02	9.00E-02	1.00E+00
ENSG00000119414.10_PPP6C	ENSG00000160789.18_LMNA	-0.19716	-0.345517105	-0.03919102	1.49E-02	8.75E-02	1.00E+00
ENSG00000169180.10_XPO6	ENSG00000160789.18_LMNA	0.14226	-0.017331621	0.294785956	8.04E-02	2.73E-01	1.00E+00
ENSG00000101193.7_GID8	ENSG00000160789.18_LMNA	0.05726	-0.102877991	0.214505823	4.83E-01	7.41E-01	1.00E+00
ENSG00000174282.10_ZBTB4	ENSG00000160789.18_LMNA	0.15203	-0.007344035	0.303880016	6.15E-02	2.29E-01	1.00E+00
ENSG00000106028.9_SSBP1	ENSG00000160789.18_LMNA	0.04435	-0.115666547	0.202124405	5.87E-01	8.08E-01	1.00E+00
ENSG00000157851.15_DPYSL5	ENSG00000160789.18_LMNA	-0.02379	-0.182296186	0.135929641	7.71E-01	9.05E-01	1.00E+00
ENSG00000197249.11_SERPINA1	ENSG00000160789.18_LMNA	0.17013	0.011232866	0.320645443	3.61E-02	1.62E-01	1.00E+00
ENSG00000187678.8_SPRY4	ENSG00000160789.18_LMNA	0.38661	0.242327568	0.51416739	8.66E-07	4.06E-05	4.44E-02
ENSG00000236876.3_TMSB4XP1	ENSG00000160789.18_LMNA	0.04974	-0.110331321	0.207302158	5.43E-01	7.82E-01	1.00E+00
ENSG00000159423.15_ALDH4A1	ENSG00000160789.18_LMNA	-0.02586	-0.184304358	0.133889471	7.52E-01	8.97E-01	1.00E+00

ENSG00000213523.8_SRA1	ENSG00000160789.18_LMNA	-0.05924	-0.21639873	0.100913548	4.68E-01	7.29E-01	1.00E+00
ENSG00000185189.14_NRBP2	ENSG00000160789.18_LMNA	0.0245	-0.135231159	0.182984012	7.65E-01	9.02E-01	1.00E+00
ENSG00000188636.3_LDOC1L	ENSG00000160789.18_LMNA	-0.12775	-0.281226387	0.032108686	1.17E-01	3.42E-01	1.00E+00
ENSG00000155508.12_CNOT8	ENSG00000160789.18_LMNA	-0.40735	-0.53205104	-0.26536281	1.90E-07	1.22E-05	9.77E-03
ENSG00000034053.13_APBA2	ENSG00000160789.18_LMNA	-0.07738	-0.233698051	0.082845097	3.43E-01	6.28E-01	1.00E+00
ENSG00000141232.4_TOB1	ENSG00000160789.18_LMNA	0.16878	0.009839542	0.319394675	3.77E-02	1.66E-01	1.00E+00
ENSG00000136717.13_BIN1	ENSG00000160789.18_LMNA	-0.28177	-0.422035784	-0.12832202	4.37E-04	6.08E-03	1.00E+00
ENSG00000221890.2_NPTXR	ENSG00000160789.18_LMNA	0.10373	-0.05639838	0.258662734	2.03E-01	4.74E-01	1.00E+00
ENSG00000186501.13_TMEM222	ENSG00000160789.18_LMNA	0.04329	-0.116716576	0.201103272	5.96E-01	8.14E-01	1.00E+00
ENSG00000164442.9_CITED2	ENSG00000160789.18_LMNA	0.03053	-0.129301018	0.188811161	7.09E-01	8.76E-01	1.00E+00
ENSG00000073111.12_MCM2	ENSG00000160789.18_LMNA	0.13451	-0.025228483	0.287555328	9.85E-02	3.09E-01	1.00E+00
ENSG00000091317.7_CMTM6	ENSG00000160789.18_LMNA	-0.05687	-0.214136049	0.10326145	4.86E-01	7.43E-01	1.00E+00
ENSG00000253719.3_ATXN7L3B	ENSG00000160789.18_LMNA	-0.38426	-0.512134369	-0.23972817	1.02E-06	4.68E-05	5.24E-02
ENSG00000108312.13_UBTF	ENSG00000160789.18_LMNA	0.13055	-0.029256366	0.283853483	1.09E-01	3.28E-01	1.00E+00
ENSG00000003056.6_M6PR	ENSG00000160789.18_LMNA	-0.01539	-0.1741596	0.144168343	8.51E-01	9.41E-01	1.00E+00
ENSG00000084463.6_WBP11	ENSG00000160789.18_LMNA	-0.01159	-0.170475852	0.147883868	8.87E-01	9.56E-01	1.00E+00
ENSG00000117395.9_EBNA1BP2	ENSG00000160789.18_LMNA	0.05998	-0.100181719	0.217103301	4.63E-01	7.26E-01	1.00E+00
ENSG00000124225.14_PMEPA1	ENSG00000160789.18_LMNA	0.43907	0.300896839	0.559179381	1.53E-08	1.54E-06	7.83E-04
ENSG00000089737.14_DDX24	ENSG00000160789.18_LMNA	0.08651	-0.073706425	0.242372056	2.89E-01	5.75E-01	1.00E+00
ENSG00000135956.7_TMEM127	ENSG00000160789.18_LMNA	0.29398	0.141397038	0.432919378	2.37E-04	3.74E-03	1.00E+00
ENSG00000213699.7_SLC35F6	ENSG00000160789.18_LMNA	0.40255	0.260011802	0.527917837	2.73E-07	1.64E-05	1.40E-02
ENSG00000127445.12_PIN1	ENSG00000160789.18_LMNA	-0.14833	-0.300439831	0.011129547	6.82E-02	2.45E-01	1.00E+00
ENSG00000071564.13_TCF3	ENSG00000160789.18_LMNA	0.16411	0.005045522	0.31508289	4.33E-02	1.83E-01	1.00E+00
ENSG00000144744.15_UBA3	ENSG00000160789.18_LMNA	-0.40613	-0.530996458	-0.26399594	2.09E-07	1.32E-05	1.07E-02
ENSG00000099901.15_RANBP1	ENSG00000160789.18_LMNA	-0.07479	-0.231237028	0.085428297	3.60E-01	6.42E-01	1.00E+00
ENSG00000106683.13_LIMK1	ENSG00000160789.18_LMNA	0.47316	0.339539388	0.588066186	7.47E-10	1.23E-07	3.83E-05
ENSG00000133706.16_LARS	ENSG00000160789.18_LMNA	-0.10432	-0.259215875	0.055807352	2.01E-01	4.71E-01	1.00E+00
ENSG00000018236.13_CNTN1	ENSG00000160789.18_LMNA	-0.52741	-0.633428523	-0.40196336	2.93E-12	1.33E-09	1.50E-07
ENSG00000160445.9_ZER1	ENSG00000160789.18_LMNA	-0.03726	-0.195298076	0.122672576	6.49E-01	8.44E-01	1.00E+00
ENSG00000162738.5_VANGL2	ENSG00000160789.18_LMNA	-0.097	-0.252304934	0.063175836	2.35E-01	5.12E-01	1.00E+00
ENSG00000167005.12_NUDT21	ENSG00000160789.18_LMNA	0.04378	-0.116230543	0.201576016	5.92E-01	8.11E-01	1.00E+00
ENSG00000115266.10_APC2	ENSG00000160789.18_LMNA	-0.16209	-0.313204667	-0.00296168	4.60E-02	1.91E-01	1.00E+00
ENSG00000014216.14_CAPN1	ENSG00000160789.18_LMNA	0.30788	0.156341326	0.445255717	1.14E-04	2.10E-03	1.00E+00

ENSG00000267100.1_ILF3-AS1	ENSG00000160789.18_LMNA	-0.28834	-0.427902354	-0.13535717	3.15E-04	4.68E-03	1.00E+00
ENSG00000120948.14_TARDBP	ENSG00000160789.18_LMNA	0.10506	-0.055060474	0.259914579	1.98E-01	4.67E-01	1.00E+00
ENSG00000064419.12_TNPO3	ENSG00000160789.18_LMNA	0.24801	0.092464175	0.391746982	2.06E-03	2.00E-02	1.00E+00
ENSG00000149657.18_LSM14B	ENSG00000160789.18_LMNA	0.04819	-0.11186737	0.205813268	5.55E-01	7.89E-01	1.00E+00
ENSG00000167969.11_ECI1	ENSG00000160789.18_LMNA	0.01555	-0.144008801	0.174317579	8.49E-01	9.41E-01	1.00E+00
ENSG00000143387.11_CTSK	ENSG00000160789.18_LMNA	0.09125	-0.068954661	0.246862331	2.64E-01	5.47E-01	1.00E+00
ENSG00000100941.7_PNN	ENSG00000160789.18_LMNA	0.12559	-0.034292577	0.279211761	1.23E-01	3.54E-01	1.00E+00
ENSG00000130755.11_GMFG	ENSG00000160789.18_LMNA	-0.07089	-0.227524425	0.089317174	3.85E-01	6.64E-01	1.00E+00
ENSG00000176490.4_DIRAS1	ENSG00000160789.18_LMNA	0.06456	-0.095621002	0.221486703	4.29E-01	6.99E-01	1.00E+00
ENSG00000116754.12_SRSF11	ENSG00000160789.18_LMNA	-0.00887	-0.167834023	0.150542963	9.14E-01	9.67E-01	1.00E+00
ENSG00000138663.7_COPS4	ENSG00000160789.18_LMNA	-0.31693	-0.453259005	-0.16610781	6.95E-05	1.40E-03	1.00E+00
ENSG00000221821.3_C6orf226	ENSG00000160789.18_LMNA	-0.17942	-0.329216917	-0.02081371	2.70E-02	1.33E-01	1.00E+00
ENSG00000171703.15_TCEA2	ENSG00000160789.18_LMNA	-0.01416	-0.17296997	0.145369218	8.63E-01	9.46E-01	1.00E+00
ENSG00000101126.14_ADNP	ENSG00000160789.18_LMNA	-0.05375	-0.211147965	0.106356666	5.11E-01	7.59E-01	1.00E+00
ENSG00000040633.11_PHF23	ENSG00000160789.18_LMNA	0.04142	-0.118561057	0.199307876	6.12E-01	8.24E-01	1.00E+00
ENSG00000131016.15_AKAP12	ENSG00000160789.18_LMNA	0.39098	0.247162965	0.517941105	6.35E-07	3.18E-05	3.26E-02
ENSG00000124160.10_NCOA5	ENSG00000160789.18_LMNA	-0.08358	-0.239592985	0.076640161	3.06E-01	5.93E-01	1.00E+00
ENSG00000175970.8_UNC119B	ENSG00000160789.18_LMNA	0.18706	0.028714477	0.336247285	2.10E-02	1.12E-01	1.00E+00
ENSG00000091640.6_SPAG7	ENSG00000160789.18_LMNA	-0.00753	-0.166530967	0.151852841	9.27E-01	9.72E-01	1.00E+00
ENSG00000037042.8_TUBG2	ENSG00000160789.18_LMNA	0.03415	-0.125729715	0.192309664	6.76E-01	8.58E-01	1.00E+00
ENSG00000103507.12_BCKDK	ENSG00000160789.18_LMNA	0.34251	0.193882106	0.475768811	1.57E-05	4.22E-04	8.03E-01
ENSG00000167635.10_ZNF146	ENSG00000160789.18_LMNA	0.0093	-0.150123122	0.168251442	9.09E-01	9.65E-01	1.00E+00
ENSG00000107263.17_RAPGEF1	ENSG00000160789.18_LMNA	0.11962	-0.040345646	0.273613377	1.42E-01	3.85E-01	1.00E+00
ENSG00000272888.4_LINC01578	ENSG00000160789.18_LMNA	-0.08637	-0.242236968	0.073849158	2.90E-01	5.75E-01	1.00E+00
ENSG00000204569.8_PPP1R10	ENSG00000160789.18_LMNA	0.06962	-0.090586183	0.22631094	3.94E-01	6.72E-01	1.00E+00
ENSG00000147853.15_AK3	ENSG00000160789.18_LMNA	-0.18476	-0.334128043	-0.02632881	2.27E-02	1.18E-01	1.00E+00
ENSG00000185818.7_NAT8L	ENSG00000160789.18_LMNA	-0.02958	-0.187897039	0.130232804	7.18E-01	8.80E-01	1.00E+00
ENSG00000173114.11_LRRN3	ENSG00000160789.18_LMNA	0.10268	-0.057462184	0.257666602	2.08E-01	4.81E-01	1.00E+00
ENSG00000163001.10_CFAP36	ENSG00000160789.18_LMNA	-0.13765	-0.290483061	0.022035683	9.08E-02	2.94E-01	1.00E+00
ENSG00000159140.16_SON	ENSG00000160789.18_LMNA	0.11415	-0.045880046	0.268476017	1.61E-01	4.16E-01	1.00E+00
ENSG00000159363.16_ATP13A2	ENSG00000160789.18_LMNA	0.13613	-0.023584523	0.289063536	9.45E-02	3.01E-01	1.00E+00
ENSG00000174807.3_CD248	ENSG00000160789.18_LMNA	0.41994	0.279423074	0.542851167	7.21E-08	5.53E-06	3.70E-03
ENSG00000210140.1_MT-TC	ENSG00000160789.18_LMNA	-0.19354	-0.342194787	-0.03542845	1.69E-02	9.56E-02	1.00E+00

ENSG00000131043.10_AAR2	ENSG00000160789.18_LMNA	0.10161	-0.058538793	0.256657798	2.13E-01	4.87E-01	1.00E+00
ENSG00000169313.9_P2RY12	ENSG00000160789.18_LMNA	-0.42876	-0.550388441	-0.28930295	3.57E-08	3.13E-06	1.83E-03
ENSG00000240065.6_PSMB9	ENSG00000160789.18_LMNA	0.13394	-0.025809627	0.287021801	9.99E-02	3.11E-01	1.00E+00
ENSG00000114315.3_HES1	ENSG00000160789.18_LMNA	0.2348	0.078530573	0.379799745	3.59E-03	3.06E-02	1.00E+00
ENSG00000107551.19_RASSF4	ENSG00000160789.18_LMNA	-0.25046	-0.393951671	-0.09504829	1.86E-03	1.85E-02	1.00E+00
ENSG00000134202.9_GSTM3	ENSG00000160789.18_LMNA	-0.29484	-0.433686359	-0.1423223	2.27E-04	3.60E-03	1.00E+00
ENSG00000067082.13_KLF6	ENSG00000160789.18_LMNA	0.35201	0.204253289	0.484080556	8.70E-06	2.60E-04	4.46E-01
ENSG00000129128.11_SPCS3	ENSG00000160789.18_LMNA	-0.1942	-0.342799257	-0.03611238	1.65E-02	9.41E-02	1.00E+00
ENSG00000196821.8_C6orf106	ENSG00000160789.18_LMNA	0.28634	0.133207347	0.426112244	3.49E-04	5.05E-03	1.00E+00
ENSG00000133027.16_PEMT	ENSG00000160789.18_LMNA	-0.1865	-0.33573072	-0.02813265	2.14E-02	1.13E-01	1.00E+00
ENSG00000134986.12_NREP	ENSG00000160789.18_LMNA	-0.31289	-0.449693601	-0.16174994	8.68E-05	1.68E-03	1.00E+00
ENSG00000137216.17_TMEM63B	ENSG00000160789.18_LMNA	0.15778	-0.001460496	0.309210777	5.22E-02	2.07E-01	1.00E+00
ENSG00000072803.16_FBXW11	ENSG00000160789.18_LMNA	-0.15848	-0.309867437	0.000734253	5.12E-02	2.04E-01	1.00E+00
ENSG00000120992.16_LYPLA1	ENSG00000160789.18_LMNA	-0.09034	-0.245998512	0.069869899	2.68E-01	5.52E-01	1.00E+00
ENSG00000149823.6_VPS51	ENSG00000160789.18_LMNA	-0.10033	-0.255451779	0.059824911	2.19E-01	4.94E-01	1.00E+00
ENSG00000139239.7_RPL14P1	ENSG00000160789.18_LMNA	-0.10401	-0.258921927	0.056121461	2.02E-01	4.73E-01	1.00E+00
ENSG00000274523.3_RCC1L	ENSG00000160789.18_LMNA	0.40312	0.260650874	0.528412135	2.62E-07	1.58E-05	1.34E-02
ENSG00000073712.12_FERMT2	ENSG00000160789.18_LMNA	0.21018	0.052748429	0.357424838	9.35E-03	6.24E-02	1.00E+00
ENSG00000188554.12_NBR1	ENSG00000160789.18_LMNA	-0.06357	-0.220541091	0.096605993	4.37E-01	7.05E-01	1.00E+00
ENSG00000198121.12_LPAR1	ENSG00000160789.18_LMNA	-0.02221	-0.180768202	0.137480166	7.86E-01	9.13E-01	1.00E+00
ENSG00000103152.10_MPG	ENSG00000160789.18_LMNA	0.00835	-0.151054856	0.167324928	9.19E-01	9.69E-01	1.00E+00
ENSG00000278730.1_RP11-147L	ENSG00000160789.18_LMNA	0.06625	-0.09393829	0.223100765	4.17E-01	6.90E-01	1.00E+00
ENSG00000138363.13_ATIC	ENSG00000160789.18_LMNA	0.10419	-0.055933894	0.259097461	2.01E-01	4.72E-01	1.00E+00
ENSG00000140848.15_CPNE2	ENSG00000160789.18_LMNA	0.17595	0.017230969	0.326017567	3.01E-02	1.44E-01	1.00E+00
ENSG00000130177.13_CDC16	ENSG00000160789.18_LMNA	-0.06114	-0.218218964	0.099022189	4.54E-01	7.19E-01	1.00E+00
ENSG00000161013.15_MGAT4B	ENSG00000160789.18_LMNA	0.55595	0.43527594	0.657002169	1.04E-13	9.08E-11	5.36E-09
ENSG00000184575.10_XPOT	ENSG00000160789.18_LMNA	-0.08028	-0.236454632	0.079946605	3.26E-01	6.12E-01	1.00E+00
ENSG00000141002.17_TCF25	ENSG00000160789.18_LMNA	0.1148	-0.045228223	0.269082008	1.59E-01	4.12E-01	1.00E+00
ENSG00000135720.11_DYNC1LI2	ENSG00000160789.18_LMNA	-0.02107	-0.179671074	0.138592518	7.97E-01	9.18E-01	1.00E+00
ENSG00000131653.11_TRAF7	ENSG00000160789.18_LMNA	0.25448	0.09929968	0.397571353	1.56E-03	1.62E-02	1.00E+00
ENSG00000126947.10_ARMCX1	ENSG00000160789.18_LMNA	-0.24933	-0.392934305	-0.09385534	1.95E-03	1.92E-02	1.00E+00
ENSG00000204231.9_RXRB	ENSG00000160789.18_LMNA	-0.32876	-0.463694571	-0.1789276	3.54E-05	8.19E-04	1.00E+00
ENSG00000138758.10_SEPT11	ENSG00000160789.18_LMNA	0.31699	0.166179983	0.45331798	6.92E-05	1.40E-03	1.00E+00

ENSG00000115091.10_ACTR3	ENSG00000160789.18_LMNA	0.17044	0.0115521	0.320931863	3.58E-02	1.61E-01	1.00E+00
ENSG00000111269.2_CREBL2	ENSG00000160789.18_LMNA	-0.03304	-0.191231332	0.126831366	6.86E-01	8.64E-01	1.00E+00
ENSG00000054793.12_ATP9A	ENSG00000160789.18_LMNA	-0.18797	-0.337081059	-0.02965403	2.04E-02	1.09E-01	1.00E+00
ENSG00000148308.16_GTF3C5	ENSG00000160789.18_LMNA	-0.03812	-0.196128401	0.121822082	6.41E-01	8.40E-01	1.00E+00
ENSG00000123496.6_IL13RA2	ENSG00000160789.18_LMNA	-0.13686	-0.289746719	0.022839291	9.27E-02	2.98E-01	1.00E+00
ENSG00000204843.11_DCTN1	ENSG00000160789.18_LMNA	0.01428	-0.145252841	0.173085298	8.61E-01	9.46E-01	1.00E+00
ENSG00000179163.11_FUCA1	ENSG00000160789.18_LMNA	0.06354	-0.096639406	0.220509004	4.37E-01	7.05E-01	1.00E+00
ENSG00000086666.17_ZFAND6	ENSG00000160789.18_LMNA	-0.07235	-0.228916364	0.087860276	3.76E-01	6.56E-01	1.00E+00
ENSG00000204435.12_CSNK2B	ENSG00000160789.18_LMNA	0.00026	-0.158948326	0.159453058	9.97E-01	9.99E-01	1.00E+00
ENSG00000137343.16_ATAT1	ENSG00000160789.18_LMNA	-0.37805	-0.506755466	-0.23286955	1.57E-06	6.66E-05	8.06E-02
ENSG00000068001.12_HYAL2	ENSG00000160789.18_LMNA	0.11553	-0.044486639	0.269771147	1.56E-01	4.08E-01	1.00E+00
ENSG00000182165.16_TP53TG1	ENSG00000160789.18_LMNA	-0.07491	-0.231353025	0.085306637	3.59E-01	6.42E-01	1.00E+00
ENSG00000135932.9_CAB39	ENSG00000160789.18_LMNA	0.02584	-0.133914551	0.184279688	7.52E-01	8.97E-01	1.00E+00
ENSG00000128626.10_MRPS12	ENSG00000160789.18_LMNA	0.04908	-0.110992454	0.206661503	5.48E-01	7.85E-01	1.00E+00
ENSG00000132155.10_RAF1	ENSG00000160789.18_LMNA	-0.21539	-0.36217514	-0.05818835	7.70E-03	5.40E-02	1.00E+00
ENSG00000115350.10_POLE4	ENSG00000160789.18_LMNA	-0.2031	-0.350952079	-0.04536499	1.21E-02	7.52E-02	1.00E+00
ENSG00000116157.5_GPX7	ENSG00000160789.18_LMNA	0.02841	-0.131380235	0.186770602	7.28E-01	8.85E-01	1.00E+00
ENSG00000119729.9_RHOQ	ENSG00000160789.18_LMNA	-0.1235	-0.27725381	0.036412166	1.30E-01	3.65E-01	1.00E+00
ENSG00000218227.3_RP11-889L	ENSG00000160789.18_LMNA	-0.14767	-0.299823281	0.011807045	6.94E-02	2.48E-01	1.00E+00
ENSG00000168092.12_PAFAH1B	ENSG00000160789.18_LMNA	-0.15506	-0.306688156	0.004247386	5.65E-02	2.17E-01	1.00E+00
ENSG00000100554.10_ATP6V1D	ENSG00000160789.18_LMNA	-0.04578	-0.203495585	0.114255447	5.75E-01	8.02E-01	1.00E+00
ENSG00000234518.2_PTGES3P1	ENSG00000160789.18_LMNA	0.02915	-0.130661059	0.187476717	7.22E-01	8.82E-01	1.00E+00
ENSG00000232559.3_GS1-124K5	ENSG00000160789.18_LMNA	0.16635	0.007340573	0.317148677	4.05E-02	1.75E-01	1.00E+00
ENSG00000133393.11_FOPNL	ENSG00000160789.18_LMNA	-0.20332	-0.351158491	-0.04559993	1.20E-02	7.48E-02	1.00E+00
ENSG00000154856.11_APCDD1	ENSG00000160789.18_LMNA	-0.51023	-0.619136428	-0.38206081	1.89E-11	5.72E-09	9.67E-07
ENSG00000148400.9_NOTCH1	ENSG00000160789.18_LMNA	0.11966	-0.040304402	0.273651595	1.42E-01	3.85E-01	1.00E+00
ENSG00000142949.15_PTPRF	ENSG00000160789.18_LMNA	0.22652	0.069840948	0.372297674	5.01E-03	3.93E-02	1.00E+00
ENSG00000219507.4_FTH1P8	ENSG00000160789.18_LMNA	0.04282	-0.117182005	0.20065043	6.00E-01	8.16E-01	1.00E+00
ENSG00000148484.16_RSU1	ENSG00000160789.18_LMNA	0.06563	-0.094562017	0.222502687	4.22E-01	6.93E-01	1.00E+00
ENSG00000198833.6_UBE2J1	ENSG00000160789.18_LMNA	-0.06526	-0.222155195	0.094924297	4.24E-01	6.95E-01	1.00E+00
ENSG00000102038.14_SMARCA1	ENSG00000160789.18_LMNA	0.05512	-0.105004741	0.212453827	5.00E-01	7.53E-01	1.00E+00
ENSG00000141540.9_TTYH2	ENSG00000160789.18_LMNA	-0.33616	-0.470197588	-0.18696557	2.29E-05	5.77E-04	1.00E+00
ENSG00000172893.14_DHCR7	ENSG00000160789.18_LMNA	0.28632	0.13319272	0.426100057	3.49E-04	5.05E-03	1.00E+00

ENSG00000103342.11_GSPT1	ENSG00000160789.18_LMNA	0.04979	-0.110283514	0.207348473	5.42E-01	7.81E-01	1.00E+00
ENSG00000070214.14_SLC44A1	ENSG00000160789.18_LMNA	-0.39325	-0.519904598	-0.24968421	5.39E-07	2.79E-05	2.77E-02
ENSG00000104368.16_PLAT	ENSG00000160789.18_LMNA	0.39113	0.24733059	0.518071736	6.28E-07	3.15E-05	3.22E-02
ENSG00000169136.7_ATF5	ENSG00000160789.18_LMNA	0.15259	-0.006775749	0.304395761	6.06E-02	2.27E-01	1.00E+00
ENSG00000159842.13_ABR	ENSG00000160789.18_LMNA	0.05343	-0.106675701	0.210839634	5.13E-01	7.61E-01	1.00E+00
ENSG00000173545.4_ZNF622	ENSG00000160789.18_LMNA	-0.03786	-0.195882404	0.122074104	6.43E-01	8.41E-01	1.00E+00
ENSG00000011260.12_UTP18	ENSG00000160789.18_LMNA	-0.19375	-0.342393356	-0.03565309	1.68E-02	9.51E-02	1.00E+00
ENSG00000105248.14_CCDC94	ENSG00000160789.18_LMNA	-0.09632	-0.251661626	0.063859989	2.38E-01	5.16E-01	1.00E+00
ENSG00000241839.8_PLEKHO2	ENSG00000160789.18_LMNA	0.20703	0.049454372	0.354540686	1.05E-02	6.78E-02	1.00E+00
ENSG00000105402.6_NAPA	ENSG00000160789.18_LMNA	0.05103	-0.109061487	0.208531895	5.32E-01	7.75E-01	1.00E+00
ENSG00000169710.6_FASN	ENSG00000160789.18_LMNA	0.14899	-0.01045845	0.3010503	6.70E-02	2.42E-01	1.00E+00
ENSG00000105755.6_ETHE1	ENSG00000160789.18_LMNA	0.16772	0.008750255	0.318416084	3.89E-02	1.70E-01	1.00E+00
ENSG00000184678.9_HIST2H2BE	ENSG00000160789.18_LMNA	-0.10873	-0.263376316	0.051354856	1.82E-01	4.47E-01	1.00E+00
ENSG00000196776.13_CD47	ENSG00000160789.18_LMNA	0.02572	-0.134032334	0.184163822	7.53E-01	8.97E-01	1.00E+00
ENSG00000259781.1_HMGB1P6	ENSG00000160789.18_LMNA	-0.0657	-0.222575434	0.094486164	4.21E-01	6.93E-01	1.00E+00
ENSG00000102390.9_PBDC1	ENSG00000160789.18_LMNA	0.29831	0.146046358	0.436769076	1.89E-04	3.13E-03	1.00E+00
ENSG00000162073.12_PAQR4	ENSG00000160789.18_LMNA	-0.18749	-0.336638502	-0.02915526	2.07E-02	1.11E-01	1.00E+00
ENSG00000128228.4_SDF2L1	ENSG00000160789.18_LMNA	0.36341	0.216752324	0.494030965	4.19E-06	1.44E-04	2.15E-01
ENSG00000170382.10_LRRN2	ENSG00000160789.18_LMNA	-0.10377	-0.258693165	0.056365869	2.03E-01	4.74E-01	1.00E+00
ENSG00000197045.11_GMFB	ENSG00000160789.18_LMNA	-0.27766	-0.418363899	-0.12393371	5.34E-04	7.10E-03	1.00E+00
ENSG00000253352.7_TUG1	ENSG00000160789.18_LMNA	0.06908	-0.091122555	0.225797741	3.98E-01	6.75E-01	1.00E+00
ENSG00000072210.17_ALDH3A2	ENSG00000160789.18_LMNA	-0.07453	-0.230986038	0.085691508	3.62E-01	6.43E-01	1.00E+00
ENSG00000149182.13_ARFGAP2	ENSG00000160789.18_LMNA	-0.04246	-0.200310361	0.117531435	6.03E-01	8.18E-01	1.00E+00
ENSG00000105855.8_ITGB8	ENSG00000160789.18_LMNA	0.21397	0.05670033	0.360877323	8.12E-03	5.63E-02	1.00E+00
ENSG00000164182.9_NDUFAF2	ENSG00000160789.18_LMNA	-0.12622	-0.279795551	0.033660046	1.21E-01	3.50E-01	1.00E+00
ENSG00000155093.16_PTPRN2	ENSG00000160789.18_LMNA	-0.18247	-0.332022812	-0.02396238	2.45E-02	1.25E-01	1.00E+00
ENSG00000156875.12_MFSD14A	ENSG00000160789.18_LMNA	0.02686	-0.132909891	0.185267632	7.43E-01	8.92E-01	1.00E+00
ENSG00000131401.10_NAPSB	ENSG00000160789.18_LMNA	0.02625	-0.1335118	0.184675814	7.48E-01	8.95E-01	1.00E+00
ENSG00000120697.7_ALG5	ENSG00000160789.18_LMNA	-0.17273	-0.3230441	-0.01390827	3.33E-02	1.53E-01	1.00E+00
ENSG00000250479.7_CHCHD10	ENSG00000160789.18_LMNA	0.1217	-0.038240058	0.275563217	1.35E-01	3.74E-01	1.00E+00
ENSG00000167674.13_CTB-50L1	ENSG00000160789.18_LMNA	0.18656	0.028201696	0.335792034	2.14E-02	1.13E-01	1.00E+00
ENSG00000063015.18_SEZ6	ENSG00000160789.18_LMNA	-0.05682	-0.214088594	0.103310654	4.87E-01	7.43E-01	1.00E+00
ENSG00000169085.10_C8orf46	ENSG00000160789.18_LMNA	-0.43113	-0.552415167	-0.29196911	2.94E-08	2.66E-06	1.51E-03

ENSG00000139921.11_TMX1	ENSG00000160789.18_LMNA	0.22266	0.065792377	0.368788846	5.83E-03	4.41E-02	1.00E+00
ENSG00000136997.13_MYC	ENSG00000160789.18_LMNA	0.09209	-0.068113988	0.247655333	2.59E-01	5.42E-01	1.00E+00
ENSG00000047315.13_POLR2B	ENSG00000160789.18_LMNA	-0.10804	-0.262722859	0.052055013	1.85E-01	4.51E-01	1.00E+00
ENSG00000169189.15_NSMCE1	ENSG00000160789.18_LMNA	-0.08649	-0.242356192	0.073723187	2.89E-01	5.75E-01	1.00E+00
ENSG00000112576.11_CCND3	ENSG00000160789.18_LMNA	-0.02048	-0.179099302	0.139171905	8.02E-01	9.21E-01	1.00E+00
ENSG00000127418.13_FGFRL1	ENSG00000160789.18_LMNA	0.57965	0.463198401	0.676433018	5.10E-15	7.92E-12	2.61E-10
ENSG00000140450.8_ARRDC4	ENSG00000160789.18_LMNA	0.05417	-0.105942809	0.211547841	5.07E-01	7.57E-01	1.00E+00
ENSG00000196498.12_NCOR2	ENSG00000160789.18_LMNA	0.20551	0.047871214	0.353152464	1.11E-02	7.05E-02	1.00E+00
ENSG00000137038.7_TMEM261	ENSG00000160789.18_LMNA	-0.09725	-0.252538424	0.062927447	2.33E-01	5.11E-01	1.00E+00
ENSG00000185115.4_NSMCE3	ENSG00000160789.18_LMNA	0.09101	-0.069191341	0.246638996	2.65E-01	5.48E-01	1.00E+00
ENSG00000166902.4_MRPL16	ENSG00000160789.18_LMNA	-0.1034	-0.258347634	0.056734964	2.05E-01	4.76E-01	1.00E+00
ENSG00000178464.6_RPL10P16	ENSG00000160789.18_LMNA	-0.00772	-0.166718888	0.151664006	9.25E-01	9.71E-01	1.00E+00
ENSG00000163320.9_CGGBP1	ENSG00000160789.18_LMNA	-0.30009	-0.438345145	-0.14795351	1.73E-04	2.91E-03	1.00E+00
ENSG00000141551.13_CSNK1D	ENSG00000160789.18_LMNA	0.26818	0.11384489	0.409885502	8.36E-04	1.01E-02	1.00E+00
ENSG00000163348.3_PYGO2	ENSG00000160789.18_LMNA	0.09349	-0.066707818	0.248980819	2.52E-01	5.33E-01	1.00E+00
ENSG00000147471.10_PROSC	ENSG00000160789.18_LMNA	-0.00864	-0.167612938	0.150765285	9.16E-01	9.68E-01	1.00E+00
ENSG00000180447.6_GAS1	ENSG00000160789.18_LMNA	0.1148	-0.045222693	0.269087148	1.59E-01	4.12E-01	1.00E+00
ENSG00000158186.11_MRAS	ENSG00000160789.18_LMNA	-0.06924	-0.225954051	0.090959207	3.97E-01	6.73E-01	1.00E+00
ENSG00000167977.7_KCTD5	ENSG00000160789.18_LMNA	-0.01278	-0.171632161	0.146718551	8.76E-01	9.52E-01	1.00E+00
ENSG00000078319.8_PMS2P1	ENSG00000160789.18_LMNA	-0.01127	-0.170164207	0.148197789	8.90E-01	9.57E-01	1.00E+00
ENSG00000159692.14_CTBP1	ENSG00000160789.18_LMNA	0.13104	-0.028760141	0.284310046	1.08E-01	3.26E-01	1.00E+00
ENSG00000112972.13_HMGCS1	ENSG00000160789.18_LMNA	-0.05875	-0.215933457	0.101396634	4.72E-01	7.32E-01	1.00E+00
ENSG00000181929.10_PRKAG1	ENSG00000160789.18_LMNA	-0.2405	-0.384961922	-0.08453661	2.84E-03	2.56E-02	1.00E+00
ENSG00000174744.12_BRMS1	ENSG00000160789.18_LMNA	0.28186	0.128419625	0.422117348	4.35E-04	6.05E-03	1.00E+00
ENSG00000184486.8_POU3F2	ENSG00000160789.18_LMNA	0.16392	0.004847006	0.314904066	4.36E-02	1.84E-01	1.00E+00
ENSG00000182272.10_B4GALNT	ENSG00000160789.18_LMNA	0.0842	-0.07601819	0.240182599	3.02E-01	5.89E-01	1.00E+00
ENSG00000144230.15_GPR17	ENSG00000160789.18_LMNA	-0.42683	-0.548740864	-0.28713854	4.17E-08	3.52E-06	2.14E-03
ENSG00000110077.13_MS4A6A	ENSG00000160789.18_LMNA	-0.06189	-0.218933249	0.098279367	4.49E-01	7.15E-01	1.00E+00
ENSG00000166886.11_NAB2	ENSG00000160789.18_LMNA	0.55779	0.437438678	0.658517788	8.33E-14	7.77E-11	4.27E-09
ENSG00000090863.10_GLG1	ENSG00000160789.18_LMNA	0.48085	0.348308183	0.594534113	3.61E-10	6.62E-08	1.85E-05
ENSG00000213977.6_TAX1BP3	ENSG00000160789.18_LMNA	0.03179	-0.128054129	0.19003355	6.97E-01	8.70E-01	1.00E+00
ENSG00000090621.12_PABPC4	ENSG00000160789.18_LMNA	0.24956	0.094098552	0.393141781	1.93E-03	1.90E-02	1.00E+00
ENSG00000183624.12_HMCES	ENSG00000160789.18_LMNA	0.2688	0.114500785	0.41043826	8.13E-04	9.84E-03	1.00E+00

ENSG00000172340.13_SUCLG2	ENSG00000160789.18_LMNA	0.15582	-0.003469734	0.307392507	5.52E-02	2.14E-01	1.00E+00
ENSG00000134910.11_STT3A	ENSG00000160789.18_LMNA	0.33872	0.189747533	0.472441157	1.97E-05	5.10E-04	1.00E+00
ENSG00000065320.7_NTN1	ENSG00000160789.18_LMNA	0.09154	-0.068664868	0.247135739	2.62E-01	5.45E-01	1.00E+00
ENSG00000211895.4_IGHA1	ENSG00000160789.18_LMNA	0.01242	-0.14707122	0.171282311	8.79E-01	9.53E-01	1.00E+00
ENSG00000100029.16_PES1	ENSG00000160789.18_LMNA	0.08055	-0.07966844	0.23671891	3.24E-01	6.11E-01	1.00E+00
ENSG00000256393.1_RPL41P5	ENSG00000160789.18_LMNA	-0.14236	-0.294881117	0.017227431	8.02E-02	2.73E-01	1.00E+00
ENSG00000121933.16_TMIGD3	ENSG00000160789.18_LMNA	-0.15936	-0.310675617	-0.00016002	4.99E-02	2.01E-01	1.00E+00
ENSG00000100266.16_PACSIN2	ENSG00000160789.18_LMNA	0.25945	0.104569424	0.402045227	1.25E-03	1.37E-02	1.00E+00
ENSG00000100504.15_PYGL	ENSG00000160789.18_LMNA	0.53598	0.411928348	0.640525572	1.11E-12	5.95E-10	5.71E-08
ENSG00000156804.6_FBXO32	ENSG00000160789.18_LMNA	0.26076	0.105953745	0.403218129	1.18E-03	1.31E-02	1.00E+00
ENSG00000082146.11_STRADB	ENSG00000160789.18_LMNA	-0.12003	-0.273991317	0.039937734	1.41E-01	3.83E-01	1.00E+00
ENSG00000116473.13_RAP1A	ENSG00000160789.18_LMNA	-0.09906	-0.254253489	0.061101752	2.25E-01	5.01E-01	1.00E+00
ENSG00000129116.16_PALLD	ENSG00000160789.18_LMNA	0.29845	0.146192419	0.436889844	1.88E-04	3.11E-03	1.00E+00
ENSG00000100938.16_GMPR2	ENSG00000160789.18_LMNA	-0.08158	-0.237696782	0.078638757	3.18E-01	6.04E-01	1.00E+00
ENSG00000111726.11_CMAS	ENSG00000160789.18_LMNA	-0.06904	-0.225757653	0.091164445	3.98E-01	6.75E-01	1.00E+00
ENSG00000214087.7_ARL16	ENSG00000160789.18_LMNA	-0.30621	-0.443774656	-0.15454015	1.25E-04	2.26E-03	1.00E+00
ENSG00000146223.13_RPL7L1	ENSG00000160789.18_LMNA	-0.20466	-0.352378033	-0.0469887	1.14E-02	7.21E-02	1.00E+00
ENSG00000115275.10_MOGS	ENSG00000160789.18_LMNA	0.37081	0.22489014	0.500470627	2.57E-06	9.64E-05	1.32E-01
ENSG00000017797.10_RALBP1	ENSG00000160789.18_LMNA	0.23277	0.076398668	0.377962855	3.90E-03	3.26E-02	1.00E+00
ENSG00000180817.10_PPA1	ENSG00000160789.18_LMNA	-0.42625	-0.548247271	-0.28649063	4.37E-08	3.64E-06	2.24E-03
ENSG00000198522.12_GPN1	ENSG00000160789.18_LMNA	-0.12586	-0.27946309	0.034020295	1.22E-01	3.52E-01	1.00E+00
ENSG00000178209.13_PLEC	ENSG00000160789.18_LMNA	0.29228	0.139577418	0.431409808	2.58E-04	4.00E-03	1.00E+00
ENSG00000099864.16_PALM	ENSG00000160789.18_LMNA	-0.20314	-0.350992272	-0.04541073	1.21E-02	7.51E-02	1.00E+00
ENSG00000099256.17_PRTFDC1	ENSG00000160789.18_LMNA	-0.43761	-0.557939143	-0.29925652	1.72E-08	1.70E-06	8.85E-04
ENSG00000132780.15_NASP	ENSG00000160789.18_LMNA	0.16665	0.007649765	0.317426758	4.02E-02	1.74E-01	1.00E+00
ENSG00000157103.9_SLC6A1	ENSG00000160789.18_LMNA	-0.24794	-0.391676068	-0.09238112	2.07E-03	2.01E-02	1.00E+00
ENSG00000075945.11_KIFAP3	ENSG00000160789.18_LMNA	-0.12707	-0.280593289	0.032795298	1.19E-01	3.46E-01	1.00E+00
ENSG00000008294.19_SPAG9	ENSG00000160789.18_LMNA	-0.15768	-0.309120602	0.001560201	5.24E-02	2.07E-01	1.00E+00
ENSG00000160211.14_G6PD	ENSG00000160789.18_LMNA	0.42686	0.28717565	0.548769129	4.16E-08	3.52E-06	2.13E-03
ENSG00000166997.6_CNPY4	ENSG00000160789.18_LMNA	0.36449	0.217930503	0.494965175	3.90E-06	1.36E-04	2.00E-01
ENSG00000084764.9_MAPRE3	ENSG00000160789.18_LMNA	-0.20436	-0.352102912	-0.0466753	1.16E-02	7.27E-02	1.00E+00
ENSG00000105819.12_PMPCB	ENSG00000160789.18_LMNA	-0.00723	-0.166240317	0.15214486	9.30E-01	9.73E-01	1.00E+00
ENSG00000090054.12_SPTLC1	ENSG00000160789.18_LMNA	0.05657	-0.103559889	0.213848198	4.89E-01	7.45E-01	1.00E+00

ENSG00000101158.11_NELFCD	ENSG00000160789.18_LMNA	0.01484	-0.144707339	0.173625767	8.56E-01	9.43E-01	1.00E+00
ENSG00000203778.6_FAM229B	ENSG00000160789.18_LMNA	-0.38076	-0.509098372	-0.23585364	1.30E-06	5.72E-05	6.69E-02
ENSG00000070610.13_GBA2	ENSG00000160789.18_LMNA	0.08022	-0.079998242	0.236405567	3.26E-01	6.12E-01	1.00E+00
ENSG00000171302.15_CANT1	ENSG00000160789.18_LMNA	0.30602	0.154342864	0.443612338	1.26E-04	2.28E-03	1.00E+00
ENSG00000113368.10_LMNB1	ENSG00000160789.18_LMNA	0.07283	-0.087388004	0.229367302	3.73E-01	6.53E-01	1.00E+00
ENSG00000170852.9_KBTBD2	ENSG00000160789.18_LMNA	0.29467	0.142133544	0.433529924	2.29E-04	3.63E-03	1.00E+00
ENSG00000159720.10_ATP6V0D1	ENSG00000160789.18_LMNA	-0.08466	-0.240620617	0.075555976	3.00E-01	5.86E-01	1.00E+00
ENSG00000277394.1_	ENSG00000160789.18_LMNA	-0.24365	-0.387810802	-0.08786058	2.49E-03	2.31E-02	1.00E+00
ENSG00000167972.12_ABCA3	ENSG00000160789.18_LMNA	-0.01657	-0.175309797	0.143006384	8.39E-01	9.38E-01	1.00E+00
ENSG00000156052.9_GNAQ	ENSG00000160789.18_LMNA	-0.00016	-0.159354131	0.159047265	9.98E-01	9.99E-01	1.00E+00
ENSG00000250510.6_GPR162	ENSG00000160789.18_LMNA	-0.35787	-0.489195421	-0.21066686	6.00E-06	1.93E-04	3.08E-01
ENSG00000223551.1_TMSB4XP4	ENSG00000160789.18_LMNA	-0.0555	-0.21282089	0.10462452	4.97E-01	7.51E-01	1.00E+00
ENSG00000177628.14_GBA	ENSG00000160789.18_LMNA	0.32985	0.180110837	0.464653786	3.33E-05	7.79E-04	1.00E+00
ENSG00000175390.11_EIF3F	ENSG00000160789.18_LMNA	-0.01732	-0.176032393	0.142275948	8.32E-01	9.35E-01	1.00E+00
ENSG00000121769.6_FABP3	ENSG00000160789.18_LMNA	-0.15417	-0.305859203	0.005162127	5.79E-02	2.20E-01	1.00E+00
ENSG00000068971.12_PPP2R5B	ENSG00000160789.18_LMNA	-0.05448	-0.211841092	0.105639236	5.05E-01	7.56E-01	1.00E+00
ENSG00000101346.10_POFUT1	ENSG00000160789.18_LMNA	0.46198	0.32681185	0.578621369	2.09E-09	2.86E-07	1.07E-04
ENSG00000103194.14_USP10	ENSG00000160789.18_LMNA	0.03144	-0.128400058	0.189694517	7.01E-01	8.72E-01	1.00E+00
ENSG00000116679.14_IVNS1ABP	ENSG00000160789.18_LMNA	0.01684	-0.142744717	0.175568695	8.37E-01	9.37E-01	1.00E+00
ENSG00000158941.15_CCAR2	ENSG00000160789.18_LMNA	0.13465	-0.025083824	0.287688104	9.81E-02	3.08E-01	1.00E+00
ENSG00000182534.12_MXRA7	ENSG00000160789.18_LMNA	0.14809	-0.011376457	0.300215164	6.86E-02	2.46E-01	1.00E+00
ENSG00000164967.8_RPP25L	ENSG00000160789.18_LMNA	-0.13712	-0.289988766	0.022575178	9.21E-02	2.96E-01	1.00E+00
ENSG00000133398.3_MED10	ENSG00000160789.18_LMNA	0.15384	-0.00549659	0.305555989	5.85E-02	2.22E-01	1.00E+00
ENSG00000176619.9_LMNB2	ENSG00000160789.18_LMNA	0.22432	0.067530236	0.370296078	5.47E-03	4.19E-02	1.00E+00
ENSG00000133983.13_COX16	ENSG00000160789.18_LMNA	-0.11602	-0.270232431	0.043990056	1.55E-01	4.05E-01	1.00E+00
ENSG00000160094.13_ZNF362	ENSG00000160789.18_LMNA	0.00832	-0.151077295	0.167302608	9.19E-01	9.69E-01	1.00E+00
ENSG00000177830.16_CHID1	ENSG00000160789.18_LMNA	0.29861	0.146371428	0.43703784	1.86E-04	3.09E-03	1.00E+00
ENSG00000158042.8_MRPL17	ENSG00000160789.18_LMNA	0.20097	0.043151028	0.349005485	1.30E-02	7.93E-02	1.00E+00
ENSG00000138430.14_OLA1	ENSG00000160789.18_LMNA	-0.27886	-0.419435665	-0.1252134	5.04E-04	6.78E-03	1.00E+00
ENSG00000165821.10_SALL2	ENSG00000160789.18_LMNA	-0.05151	-0.208998205	0.108579701	5.29E-01	7.72E-01	1.00E+00
ENSG00000140374.14_ETFA	ENSG00000160789.18_LMNA	-0.20614	-0.35373314	-0.04853324	1.08E-02	6.94E-02	1.00E+00
ENSG00000160058.17_BSDC1	ENSG00000160789.18_LMNA	-0.07909	-0.235326127	0.081133853	3.33E-01	6.18E-01	1.00E+00
ENSG00000177666.14_PNPLA2	ENSG00000160789.18_LMNA	0.3769	0.231598576	0.505756352	1.70E-06	7.05E-05	8.72E-02

ENSG00000161999.10_JMJD8	ENSG00000160789.18_LMNA	0.24747	0.091893142	0.391259331	2.11E-03	2.04E-02	1.00E+00
ENSG00000160917.13_CPSF4	ENSG00000160789.18_LMNA	0.17465	0.015891367	0.324819497	3.14E-02	1.48E-01	1.00E+00
ENSG00000140350.14_ANP32A	ENSG00000160789.18_LMNA	-0.00154	-0.160703984	0.157696682	9.85E-01	9.94E-01	1.00E+00
ENSG00000213339.7_QTRT1	ENSG00000160789.18_LMNA	0.09395	-0.066236532	0.249424799	2.50E-01	5.30E-01	1.00E+00
ENSG00000133275.14_CSNK1G2	ENSG00000160789.18_LMNA	0.33171	0.182124035	0.466284287	2.98E-05	7.11E-04	1.00E+00
ENSG00000167397.13_VKORC1	ENSG00000160789.18_LMNA	0.22985	0.07333537	0.375319295	4.39E-03	3.55E-02	1.00E+00
ENSG00000163956.9_LRPAP1	ENSG00000160789.18_LMNA	0.33807	0.18904175	0.471872311	2.05E-05	5.27E-04	1.00E+00
ENSG00000205707.9_ETFRF1	ENSG00000160789.18_LMNA	-0.36738	-0.497488166	-0.22111641	3.23E-06	1.17E-04	1.65E-01
ENSG00000236609.3_ZNF853	ENSG00000160789.18_LMNA	0.03987	-0.120094993	0.197813134	6.26E-01	8.31E-01	1.00E+00
ENSG00000170266.14_GLB1	ENSG00000160789.18_LMNA	0.29254	0.139847912	0.431634315	2.55E-04	3.96E-03	1.00E+00
ENSG00000179134.13_SAMD4B	ENSG00000160789.18_LMNA	0.2594	0.104518369	0.402001952	1.25E-03	1.37E-02	1.00E+00
ENSG00000160813.5_PPP1R35	ENSG00000160789.18_LMNA	0.21981	0.062805068	0.366194247	6.51E-03	4.78E-02	1.00E+00
ENSG00000262902.1_MTCO1P40	ENSG00000160789.18_LMNA	-0.15912	-0.310452082	8.74E-05	5.02E-02	2.02E-01	1.00E+00
ENSG00000161677.10_JOSD2	ENSG00000160789.18_LMNA	0.24477	0.089035943	0.388816808	2.37E-03	2.23E-02	1.00E+00
ENSG00000150540.12_HNMT	ENSG00000160789.18_LMNA	-0.34522	-0.478144163	-0.19683959	1.33E-05	3.68E-04	6.80E-01
ENSG00000196961.11_AP2A1	ENSG00000160789.18_LMNA	0.26055	0.10573843	0.403035762	1.19E-03	1.32E-02	1.00E+00
ENSG00000078902.14_TOLLIP	ENSG00000160789.18_LMNA	0.13597	-0.023747484	0.288914101	9.49E-02	3.02E-01	1.00E+00
ENSG00000122861.14_PLAU	ENSG00000160789.18_LMNA	0.51774	0.39074575	0.625392486	8.46E-12	3.12E-09	4.34E-07
ENSG00000204394.11_VARS	ENSG00000160789.18_LMNA	0.12517	-0.034717774	0.278819198	1.24E-01	3.56E-01	1.00E+00
ENSG00000158417.9_EIF5B	ENSG00000160789.18_LMNA	0.11277	-0.047278007	0.267175511	1.67E-01	4.24E-01	1.00E+00
ENSG00000172922.7_RNASEH2C	ENSG00000160789.18_LMNA	-0.14815	-0.300267691	0.011318732	6.85E-02	2.46E-01	1.00E+00
ENSG00000089818.15_NECAP1	ENSG00000160789.18_LMNA	-0.41518	-0.53876576	-0.27409112	1.05E-07	7.52E-06	5.37E-03
ENSG00000213760.9_ATP6V1G2	ENSG00000160789.18_LMNA	-0.43704	-0.55745461	-0.2986161	1.81E-08	1.77E-06	9.28E-04
ENSG00000172725.12_CORO1B	ENSG00000160789.18_LMNA	0.25275	0.097473134	0.396017346	1.68E-03	1.71E-02	1.00E+00
ENSG00000077147.13_TM9SF3	ENSG00000160789.18_LMNA	0.04515	-0.114879547	0.202889295	5.81E-01	8.05E-01	1.00E+00
ENSG00000109270.11_LAMTOR3	ENSG00000160789.18_LMNA	-0.25363	-0.396809247	-0.09840367	1.62E-03	1.66E-02	1.00E+00
ENSG00000103811.14_CTSH	ENSG00000160789.18_LMNA	-0.12654	-0.280100932	0.033329069	1.20E-01	3.49E-01	1.00E+00
ENSG00000127952.15_STYXL1	ENSG00000160789.18_LMNA	0.15595	-0.00333723	0.307512486	5.50E-02	2.14E-01	1.00E+00
ENSG00000214389.2_RPS3AP26	ENSG00000160789.18_LMNA	-0.14595	-0.298225431	0.013561514	7.28E-02	2.56E-01	1.00E+00
ENSG00000117153.14_KLHL12	ENSG00000160789.18_LMNA	0.10795	-0.052143013	0.262640709	1.86E-01	4.51E-01	1.00E+00
ENSG00000120805.12_ARL1	ENSG00000160789.18_LMNA	-0.09824	-0.253472219	0.06193368	2.29E-01	5.05E-01	1.00E+00
ENSG00000069509.5_FUNDC1	ENSG00000160789.18_LMNA	-0.16916	-0.319751741	-0.01023718	3.72E-02	1.65E-01	1.00E+00
ENSG00000107581.11_EIF3A	ENSG00000160789.18_LMNA	0.20478	0.047111898	0.352486166	1.14E-02	7.18E-02	1.00E+00

ENSG00000155324.8_GRAMD3	ENSG00000160789.18_LMNA	-0.27988	-0.420353209	-0.12630972	4.79E-04	6.52E-03	1.00E+00
ENSG00000144655.13_CSRNP1	ENSG00000160789.18_LMNA	-0.01501	-0.173790782	0.144540748	8.54E-01	9.43E-01	1.00E+00
ENSG00000178585.13_CTNNBIP1	ENSG00000160789.18_LMNA	-0.17958	-0.329363708	-0.02097828	2.68E-02	1.33E-01	1.00E+00
ENSG00000104331.7_IMPAD1	ENSG00000160789.18_LMNA	0.00677	-0.15259981	0.165787391	9.34E-01	9.74E-01	1.00E+00
ENSG00000055917.14_PUM2	ENSG00000160789.18_LMNA	-0.07887	-0.235115712	0.081355122	3.34E-01	6.20E-01	1.00E+00
ENSG00000131871.13_SELENOS	ENSG00000160789.18_LMNA	0.22447	0.067685025	0.370430247	5.43E-03	4.17E-02	1.00E+00
ENSG00000170892.9_TSEN34	ENSG00000160789.18_LMNA	0.28744	0.13438569	0.427093711	3.30E-04	4.85E-03	1.00E+00
ENSG00000099331.12_MYO9B	ENSG00000160789.18_LMNA	0.2899	0.137021874	0.429286947	2.92E-04	4.40E-03	1.00E+00
ENSG00000104738.15_MCM4	ENSG00000160789.18_LMNA	0.204	0.046299771	0.351773183	1.17E-02	7.34E-02	1.00E+00
ENSG00000100379.16_KCTD17	ENSG00000160789.18_LMNA	0.11448	-0.04555304	0.268780061	1.60E-01	4.14E-01	1.00E+00
ENSG00000158467.15_AHCYL2	ENSG00000160789.18_LMNA	-0.1249	-0.278557224	0.035001463	1.25E-01	3.58E-01	1.00E+00
ENSG00000119900.7_OGFRL1	ENSG00000160789.18_LMNA	-0.20104	-0.349069892	-0.04322423	1.30E-02	7.92E-02	1.00E+00
ENSG00000122033.13_MTIF3	ENSG00000160789.18_LMNA	-0.20683	-0.354363571	-0.0492523	1.06E-02	6.81E-02	1.00E+00
ENSG00000180353.9_HCLS1	ENSG00000160789.18_LMNA	0.07173	-0.08848193	0.228322586	3.80E-01	6.59E-01	1.00E+00
ENSG00000204386.9_NEU1	ENSG00000160789.18_LMNA	0.13443	-0.025310916	0.287479661	9.87E-02	3.09E-01	1.00E+00
ENSG00000170915.8_PAQR8	ENSG00000160789.18_LMNA	-0.33322	-0.467612744	-0.18376606	2.73E-05	6.63E-04	1.00E+00
ENSG00000153130.16_SCOC	ENSG00000160789.18_LMNA	-0.29548	-0.434256292	-0.14301019	2.19E-04	3.51E-03	1.00E+00
ENSG00000104976.10_SNAPC2	ENSG00000160789.18_LMNA	0.27654	0.122743298	0.417366171	5.63E-04	7.39E-03	1.00E+00
ENSG00000134375.9_TIMM17A	ENSG00000160789.18_LMNA	0.00424	-0.15506398	0.163331841	9.59E-01	9.83E-01	1.00E+00
ENSG00000189343.7_RPS2P46	ENSG00000160789.18_LMNA	0.09891	-0.061251344	0.254113036	2.25E-01	5.01E-01	1.00E+00
ENSG00000198431.14_TXNRD1	ENSG00000160789.18_LMNA	0.02322	-0.136489775	0.181744372	7.76E-01	9.08E-01	1.00E+00
ENSG00000135070.12_ISCA1	ENSG00000160789.18_LMNA	-0.28083	-0.421203182	-0.12732595	4.57E-04	6.29E-03	1.00E+00
ENSG00000215305.8_VPS16	ENSG00000160789.18_LMNA	0.23101	0.074554385	0.376371868	4.19E-03	3.44E-02	1.00E+00
ENSG00000162595.4_DIRAS3	ENSG00000160789.18_LMNA	0.41874	0.278072492	0.541817511	7.93E-08	5.95E-06	4.07E-03
ENSG00000169908.9_TM4SF1	ENSG00000160789.18_LMNA	-0.1723	-0.322648454	-0.01346667	3.38E-02	1.55E-01	1.00E+00
ENSG00000130764.8_LRRC47	ENSG00000160789.18_LMNA	-0.01556	-0.174325077	0.144001229	8.49E-01	9.41E-01	1.00E+00
ENSG00000188021.8_UBQLN2	ENSG00000160789.18_LMNA	-0.10841	-0.263073818	0.051679011	1.84E-01	4.49E-01	1.00E+00
ENSG00000140564.9_FURIN	ENSG00000160789.18_LMNA	0.56873	0.450296521	0.667491521	2.11E-14	2.70E-11	1.08E-09
ENSG00000279483.1_AC090498.	ENSG00000160789.18_LMNA	-0.23318	-0.378338469	-0.07683438	3.84E-03	3.21E-02	1.00E+00
ENSG00000157601.12_MX1	ENSG00000160789.18_LMNA	0.08728	-0.072930626	0.243106089	2.85E-01	5.70E-01	1.00E+00
ENSG00000126768.11_TIMM17B	ENSG00000160789.18_LMNA	-0.10847	-0.263129782	0.051619046	1.83E-01	4.48E-01	1.00E+00
ENSG00000115694.13_STK25	ENSG00000160789.18_LMNA	0.02946	-0.130356672	0.187775477	7.19E-01	8.80E-01	1.00E+00
ENSG00000144369.11_FAM171B	ENSG00000160789.18_LMNA	-0.43364	-0.554557191	-0.29479135	2.40E-08	2.25E-06	1.23E-03

ENSG00000100575.12_TIMM9	ENSG00000160789.18_LMNA	-0.03583	-0.193924896	0.124078086	6.61E-01	8.50E-01	1.00E+00
ENSG00000136754.15_ABI1	ENSG00000160789.18_LMNA	-0.37344	-0.502748209	-0.22777756	2.15E-06	8.43E-05	1.10E-01
ENSG00000114988.10_LMAN2L	ENSG00000160789.18_LMNA	-0.15069	-0.302626283	0.008724652	6.39E-02	2.35E-01	1.00E+00
ENSG00000158457.5_TSPAN33	ENSG00000160789.18_LMNA	-0.06891	-0.225631129	0.09129665	3.99E-01	6.76E-01	1.00E+00
ENSG00000101558.12_VAPA	ENSG00000160789.18_LMNA	-0.18436	-0.333766805	-0.0259225	2.30E-02	1.19E-01	1.00E+00
ENSG00000182544.8_MFSD5	ENSG00000160789.18_LMNA	0.16606	0.007048238	0.316885708	4.09E-02	1.76E-01	1.00E+00
ENSG00000090372.13_STRN4	ENSG00000160789.18_LMNA	0.14378	-0.015785999	0.296197009	7.72E-02	2.66E-01	1.00E+00
ENSG00000167654.16_ATCAY	ENSG00000160789.18_LMNA	-0.38053	-0.508903611	-0.23560538	1.32E-06	5.80E-05	6.80E-02
ENSG00000123505.13_AMD1	ENSG00000160789.18_LMNA	-0.08698	-0.24281447	0.073238884	2.87E-01	5.72E-01	1.00E+00
ENSG00000107438.7_PDLIM1	ENSG00000160789.18_LMNA	0.25479	0.099625584	0.397848449	1.54E-03	1.60E-02	1.00E+00
ENSG00000205250.7_E2F4	ENSG00000160789.18_LMNA	0.15795	-0.001280861	0.309373228	5.20E-02	2.06E-01	1.00E+00
ENSG00000122644.11_ARL4A	ENSG00000160789.18_LMNA	0.14346	-0.016108802	0.295902423	7.79E-02	2.67E-01	1.00E+00
ENSG00000168496.3_FEN1	ENSG00000160789.18_LMNA	0.20564	0.048014649	0.353278293	1.10E-02	7.02E-02	1.00E+00
ENSG00000088682.12_COQ9	ENSG00000160789.18_LMNA	0.05327	-0.106836587	0.210684122	5.15E-01	7.62E-01	1.00E+00
ENSG00000168118.10_RAB4A	ENSG00000160789.18_LMNA	-0.32621	-0.461449631	-0.17616157	4.11E-05	9.20E-04	1.00E+00
ENSG00000122679.7_RAMP3	ENSG00000160789.18_LMNA	0.03359	-0.126281148	0.19177	6.81E-01	8.61E-01	1.00E+00
ENSG00000168575.8_SLC20A2	ENSG00000160789.18_LMNA	0.14962	-0.009814822	0.301635542	6.58E-02	2.40E-01	1.00E+00
ENSG00000167272.9_POP5	ENSG00000160789.18_LMNA	-0.25506	-0.398096218	-0.09991705	1.52E-03	1.58E-02	1.00E+00
ENSG00000242071.3_RPL7AP6	ENSG00000160789.18_LMNA	-0.12009	-0.274047171	0.039877443	1.41E-01	3.82E-01	1.00E+00
ENSG00000148730.6_EIF4EBP2	ENSG00000160789.18_LMNA	-0.17305	-0.323341093	-0.01423984	3.30E-02	1.52E-01	1.00E+00
ENSG00000175040.5_CHST2	ENSG00000160789.18_LMNA	0.41776	0.276983779	0.540983694	8.56E-08	6.32E-06	4.39E-03
ENSG00000153048.9_CARHSP1	ENSG00000160789.18_LMNA	0.06464	-0.095546934	0.221557786	4.29E-01	6.99E-01	1.00E+00
ENSG00000177030.15_DEAF1	ENSG00000160789.18_LMNA	-0.06194	-0.218983556	0.098227037	4.48E-01	7.15E-01	1.00E+00
ENSG00000007237.17_GAS7	ENSG00000160789.18_LMNA	0.04463	-0.115391789	0.202391488	5.85E-01	8.07E-01	1.00E+00
ENSG00000171033.11_PKIA	ENSG00000160789.18_LMNA	-0.39402	-0.520565401	-0.25053354	5.10E-07	2.66E-05	2.62E-02
ENSG00000228300.12_C19orf24	ENSG00000160789.18_LMNA	0.23865	0.082586167	0.383287576	3.07E-03	2.72E-02	1.00E+00
ENSG00000170604.4_IRF2BP1	ENSG00000160789.18_LMNA	0.26491	0.110364986	0.406949162	9.73E-04	1.14E-02	1.00E+00
ENSG00000143106.11_PSMA5	ENSG00000160789.18_LMNA	-0.10156	-0.256613304	0.058586261	2.13E-01	4.87E-01	1.00E+00
ENSG00000132485.11_ZRANB2	ENSG00000160789.18_LMNA	-0.27342	-0.414578714	-0.11942201	6.54E-04	8.32E-03	1.00E+00
ENSG00000062194.14_GPBP1	ENSG00000160789.18_LMNA	-0.25684	-0.399699815	-0.1018047	1.40E-03	1.49E-02	1.00E+00
ENSG00000168291.11_PDHB	ENSG00000160789.18_LMNA	-0.29216	-0.431297709	-0.13944237	2.60E-04	4.02E-03	1.00E+00
ENSG00000188338.13_SLC38A3	ENSG00000160789.18_LMNA	-0.09962	-0.254782972	0.060537687	2.22E-01	4.97E-01	1.00E+00
ENSG00000123933.13_MXD4	ENSG00000160789.18_LMNA	-0.39344	-0.520062975	-0.24988773	5.32E-07	2.76E-05	2.73E-02

ENSG00000115204.13_MPV17	ENSG00000160789.18_LMNA	-0.15876	-0.310118086	0.000456957	5.08E-02	2.03E-01	1.00E+00
ENSG00000101464.9_PIGU	ENSG00000160789.18_LMNA	0.07206	-0.088151156	0.228638558	3.78E-01	6.57E-01	1.00E+00
ENSG00000247627.2_MTND4P12	ENSG00000160789.18_LMNA	-0.08076	-0.236916435	0.079460504	3.23E-01	6.09E-01	1.00E+00
ENSG00000261236.4_BOP1	ENSG00000160789.18_LMNA	0.03055	-0.129279041	0.188832715	7.09E-01	8.76E-01	1.00E+00
ENSG00000143333.6_RGS16	ENSG00000160789.18_LMNA	0.09771	-0.062460312	0.252977441	2.31E-01	5.09E-01	1.00E+00
ENSG00000124783.11_SSR1	ENSG00000160789.18_LMNA	0.20991	0.052462007	0.357174289	9.44E-03	6.29E-02	1.00E+00
ENSG00000103415.10_HMOX2	ENSG00000160789.18_LMNA	-0.18166	-0.331280138	-0.02312838	2.51E-02	1.27E-01	1.00E+00
ENSG00000149100.11_EIF3M	ENSG00000160789.18_LMNA	-0.02837	-0.186726724	0.131424914	7.29E-01	8.85E-01	1.00E+00
ENSG00000178896.6_EXOSC4	ENSG00000160789.18_LMNA	0.00304	-0.15623345	0.16216508	9.70E-01	9.88E-01	1.00E+00
ENSG00000076003.4_MCM6	ENSG00000160789.18_LMNA	0.17626	0.017552249	0.326304756	2.98E-02	1.43E-01	1.00E+00
ENSG00000114993.14_RTKN	ENSG00000160789.18_LMNA	-0.32512	-0.460486973	-0.17497683	4.37E-05	9.63E-04	1.00E+00
ENSG00000163879.10_DNALI1	ENSG00000160789.18_LMNA	0.10167	-0.05847379	0.256718727	2.13E-01	4.86E-01	1.00E+00
ENSG00000107738.18_C10orf54	ENSG00000160789.18_LMNA	-0.15633	-0.307869569	0.002942809	5.44E-02	2.12E-01	1.00E+00
ENSG00000204655.10_MOG	ENSG00000160789.18_LMNA	-0.38239	-0.510512129	-0.23765679	1.16E-06	5.19E-05	5.98E-02
ENSG00000179456.10_ZBTB18	ENSG00000160789.18_LMNA	-0.29984	-0.438130165	-0.14769324	1.75E-04	2.94E-03	1.00E+00
ENSG00000175283.7_DOLK	ENSG00000160789.18_LMNA	0.26263	0.107937969	0.404897612	1.08E-03	1.23E-02	1.00E+00
ENSG00000178397.11_FAM220A	ENSG00000160789.18_LMNA	-0.04645	-0.204136795	0.113595129	5.70E-01	7.99E-01	1.00E+00
ENSG00000175467.13_SART1	ENSG00000160789.18_LMNA	0.22212	0.065230577	0.36830126	5.95E-03	4.48E-02	1.00E+00
ENSG00000092201.8_SUPT16H	ENSG00000160789.18_LMNA	0.22154	0.064616167	0.367767823	6.09E-03	4.55E-02	1.00E+00
ENSG00000122687.16_MRM2	ENSG00000160789.18_LMNA	0.24373	0.087942791	0.387881194	2.48E-03	2.31E-02	1.00E+00
ENSG00000163659.11_TIPARP	ENSG00000160789.18_LMNA	0.0606	-0.099557099	0.217704394	4.58E-01	7.22E-01	1.00E+00
ENSG00000160408.13_ST6GALN	ENSG00000160789.18_LMNA	0.21401	0.056743326	0.360914841	8.11E-03	5.62E-02	1.00E+00
ENSG00000140307.9_GTF2A2	ENSG00000160789.18_LMNA	-0.16128	-0.312460814	-0.00213714	4.71E-02	1.94E-01	1.00E+00
ENSG00000162961.12_DPY30	ENSG00000160789.18_LMNA	-0.28551	-0.425378073	-0.13232645	3.63E-04	5.22E-03	1.00E+00
ENSG00000133935.6_C14orf1	ENSG00000160789.18_LMNA	0.15973	0.000541222	0.311019987	4.93E-02	2.00E-01	1.00E+00
ENSG00000176165.9_FOXG1	ENSG00000160789.18_LMNA	0.02446	-0.135262828	0.182952833	7.65E-01	9.02E-01	1.00E+00
ENSG00000120053.10_GOT1	ENSG00000160789.18_LMNA	-0.26321	-0.40542285	-0.108559	1.05E-03	1.20E-02	1.00E+00
ENSG00000157259.6_GATAD1	ENSG00000160789.18_LMNA	0.16036	0.001187809	0.311603911	4.84E-02	1.97E-01	1.00E+00
ENSG00000135318.10_NT5E	ENSG00000160789.18_LMNA	0.23903	0.082987702	0.383632432	3.02E-03	2.69E-02	1.00E+00
ENSG00000196182.9_STK40	ENSG00000160789.18_LMNA	0.47377	0.340226552	0.588574194	7.06E-10	1.18E-07	3.62E-05
ENSG00000188725.6_SMIM15	ENSG00000160789.18_LMNA	0.17126	0.012398485	0.321690975	3.49E-02	1.58E-01	1.00E+00
ENSG00000164889.11_SLC4A2	ENSG00000160789.18_LMNA	0.42115	0.280770475	0.543881593	6.56E-08	5.16E-06	3.37E-03
ENSG00000187051.7_RPS19BP1	ENSG00000160789.18_LMNA	-0.02972	-0.188026898	0.13010047	7.16E-01	8.79E-01	1.00E+00

ENSG00000088256.7_GNA11	ENSG00000160789.18_LMNA	0.2441	0.088327674	0.388210681	2.44E-03	2.28E-02	1.00E+00
ENSG00000131966.12_ACTR10	ENSG00000160789.18_LMNA	-0.13768	-0.290512385	0.022003672	9.07E-02	2.94E-01	1.00E+00
ENSG00000175324.8_LSM1	ENSG00000160789.18_LMNA	-0.17674	-0.326749225	-0.01804961	2.94E-02	1.41E-01	1.00E+00
ENSG00000111783.11_RFX4	ENSG00000160789.18_LMNA	-0.20551	-0.353159789	-0.04787956	1.11E-02	7.05E-02	1.00E+00
ENSG00000169783.11_LINGO1	ENSG00000160789.18_LMNA	-0.1975	-0.345823559	-0.03953852	1.47E-02	8.67E-02	1.00E+00
ENSG00000144895.10_EIF2A	ENSG00000160789.18_LMNA	-0.27259	-0.413827716	-0.11852832	6.80E-04	8.58E-03	1.00E+00
ENSG00000212864.3_RNF208	ENSG00000160789.18_LMNA	-0.0476	-0.205246366	0.11245183	5.60E-01	7.93E-01	1.00E+00
ENSG00000239521.6_GATS	ENSG00000160789.18_LMNA	-0.38759	-0.515014844	-0.24341227	8.08E-07	3.83E-05	4.15E-02
ENSG00000205937.10_RNPS1	ENSG00000160789.18_LMNA	0.00779	-0.15160228	0.166780309	9.24E-01	9.71E-01	1.00E+00
ENSG00000108861.7_DUSP3	ENSG00000160789.18_LMNA	0.3499	0.201949175	0.48223833	9.93E-06	2.90E-04	5.09E-01
ENSG00000129925.9_TMEM8A	ENSG00000160789.18_LMNA	0.4089	0.267089724	0.533382226	1.69E-07	1.10E-05	8.69E-03
ENSG00000176973.7_FAM89B	ENSG00000160789.18_LMNA	0.02466	-0.135071282	0.183141406	7.63E-01	9.01E-01	1.00E+00
ENSG00000153147.5_SMARCA5	ENSG00000160789.18_LMNA	0.01604	-0.143524714	0.174796821	8.44E-01	9.39E-01	1.00E+00
ENSG00000164332.7_UBLCP1	ENSG00000160789.18_LMNA	-0.2659	-0.407836004	-0.11141523	9.30E-04	1.10E-02	1.00E+00
ENSG00000211584.12_SLC48A1	ENSG00000160789.18_LMNA	-0.33365	-0.467991618	-0.18423465	2.66E-05	6.51E-04	1.00E+00
ENSG00000101199.11_ARFGAP1	ENSG00000160789.18_LMNA	0.16065	0.001488744	0.3118756	4.80E-02	1.96E-01	1.00E+00
ENSG00000127616.16_SMARCA4	ENSG00000160789.18_LMNA	0.07376	-0.08645845	0.230254466	3.66E-01	6.47E-01	1.00E+00
ENSG00000207652.1_MIR621	ENSG00000160789.18_LMNA	-0.0475	-0.205153148	0.112547914	5.61E-01	7.93E-01	1.00E+00
ENSG00000070669.15_ASNS	ENSG00000160789.18_LMNA	-0.08726	-0.243081878	0.072956221	2.85E-01	5.71E-01	1.00E+00
ENSG00000119138.4_KLF9	ENSG00000160789.18_LMNA	0.12367	-0.036247903	0.277405639	1.29E-01	3.64E-01	1.00E+00
ENSG00000164530.12_PI16	ENSG00000160789.18_LMNA	-0.44038	-0.560294844	-0.30237344	1.37E-08	1.40E-06	7.02E-04
ENSG00000023697.11_DERA	ENSG00000160789.18_LMNA	-0.25871	-0.401380667	-0.10378559	1.29E-03	1.40E-02	1.00E+00
ENSG00000184500.13_PROS1	ENSG00000160789.18_LMNA	0.47487	0.341483601	0.589502999	6.37E-10	1.08E-07	3.27E-05
ENSG00000102753.8_KPNA3	ENSG00000160789.18_LMNA	-0.40427	-0.52940146	-0.26193067	2.40E-07	1.48E-05	1.23E-02
ENSG00000135148.10_TRAFD1	ENSG00000160789.18_LMNA	-0.05602	-0.213315839	0.10411168	4.93E-01	7.48E-01	1.00E+00
ENSG00000082781.10_ITGB5	ENSG00000160789.18_LMNA	0.43359	0.294737053	0.554516011	2.40E-08	2.26E-06	1.23E-03
ENSG00000145675.13_PIK3R1	ENSG00000160789.18_LMNA	-0.30618	-0.443748728	-0.15450863	1.25E-04	2.26E-03	1.00E+00
ENSG00000132879.12_FBXO44	ENSG00000160789.18_LMNA	-0.26507	-0.407093962	-0.11053642	9.66E-04	1.13E-02	1.00E+00
ENSG00000070614.13_NDST1	ENSG00000160789.18_LMNA	0.07834	-0.081882945	0.234613659	3.37E-01	6.23E-01	1.00E+00
ENSG00000151176.6_PLBD2	ENSG00000160789.18_LMNA	0.33401	0.184627839	0.468309443	2.61E-05	6.39E-04	1.00E+00
ENSG00000255198.4_SNHG9	ENSG00000160789.18_LMNA	-0.21411	-0.361001639	-0.05684281	8.08E-03	5.60E-02	1.00E+00
ENSG00000108406.8_DHX40	ENSG00000160789.18_LMNA	-0.24446	-0.388543268	-0.08871627	2.40E-03	2.25E-02	1.00E+00
ENSG00000149743.12_TRPT1	ENSG00000160789.18_LMNA	0.03301	-0.126855755	0.19120745	6.86E-01	8.64E-01	1.00E+00

ENSG00000103653.15_CSK	ENSG00000160789.18_LMNA	0.18766	0.029331503	0.336794897	2.06E-02	1.10E-01	1.00E+00
ENSG00000128311.12_TST	ENSG00000160789.18_LMNA	-0.13772	-0.290550847	0.021961685	9.06E-02	2.93E-01	1.00E+00
ENSG00000198369.8_SPRED2	ENSG00000160789.18_LMNA	0.45732	0.321525309	0.574678394	3.16E-09	4.04E-07	1.62E-04
ENSG00000169242.10_EFNA1	ENSG00000160789.18_LMNA	0.15009	-0.009329493	0.30207669	6.49E-02	2.37E-01	1.00E+00
ENSG00000127946.15_HIP1	ENSG00000160789.18_LMNA	0.20208	0.044307607	0.350022721	1.25E-02	7.71E-02	1.00E+00
ENSG00000167747.12_C19orf48	ENSG00000160789.18_LMNA	0.12642	-0.033454445	0.279985259	1.21E-01	3.49E-01	1.00E+00
ENSG00000006831.9_ADIPOR2	ENSG00000160789.18_LMNA	-0.27166	-0.412995152	-0.11753812	7.11E-04	8.88E-03	1.00E+00
ENSG00000102879.14_CORO1A	ENSG00000160789.18_LMNA	0.04243	-0.11756763	0.200275132	6.04E-01	8.18E-01	1.00E+00
ENSG00000165689.15_SDCCAG3	ENSG00000160789.18_LMNA	0.09482	-0.06537198	0.250238914	2.45E-01	5.25E-01	1.00E+00
ENSG00000108947.4_EFNB3	ENSG00000160789.18_LMNA	-0.09663	-0.251955438	0.063547559	2.36E-01	5.15E-01	1.00E+00
ENSG00000116237.14_ICMT	ENSG00000160789.18_LMNA	0.42718	0.287531505	0.549040144	4.05E-08	3.46E-06	2.08E-03
ENSG00000156471.11_PTDSS1	ENSG00000160789.18_LMNA	-0.0889	-0.244641856	0.071306237	2.76E-01	5.61E-01	1.00E+00
ENSG00000163444.10_TMEM183	ENSG00000160789.18_LMNA	-0.18858	-0.337639433	-0.03028355	2.00E-02	1.08E-01	1.00E+00
ENSG00000185950.8_IRS2	ENSG00000160789.18_LMNA	0.3716	0.225753834	0.501152304	2.44E-06	9.28E-05	1.25E-01
ENSG00000113721.12_PDGFRB	ENSG00000160789.18_LMNA	0.35373	0.206139786	0.485587037	7.80E-06	2.40E-04	4.00E-01
ENSG00000186318.15_BACE1	ENSG00000160789.18_LMNA	0.10912	-0.050968214	0.263737045	1.81E-01	4.44E-01	1.00E+00
ENSG00000250722.4_SELENOP	ENSG00000160789.18_LMNA	-0.15895	-0.310296911	0.000259091	5.05E-02	2.02E-01	1.00E+00
ENSG00000164022.15_AIMP1	ENSG00000160789.18_LMNA	-0.02324	-0.181766853	0.136466959	7.76E-01	9.08E-01	1.00E+00
ENSG00000128951.12_DUT	ENSG00000160789.18_LMNA	-0.07502	-0.231456759	0.08519783	3.58E-01	6.41E-01	1.00E+00
ENSG00000136044.10_APPL2	ENSG00000160789.18_LMNA	-0.3406	-0.474097902	-0.19180477	1.76E-05	4.64E-04	9.02E-01
ENSG00000136819.13_C9orf78	ENSG00000160789.18_LMNA	-0.06673	-0.22355245	0.093467069	4.14E-01	6.87E-01	1.00E+00
ENSG00000108784.8_NAGLU	ENSG00000160789.18_LMNA	0.29931	0.147119788	0.437656379	1.80E-04	3.00E-03	1.00E+00
ENSG00000152092.14_ASTN1	ENSG00000160789.18_LMNA	-0.05382	-0.211208126	0.106294409	5.10E-01	7.59E-01	1.00E+00
ENSG00000160563.12_MED27	ENSG00000160789.18_LMNA	-0.05757	-0.214805763	0.10256688	4.81E-01	7.39E-01	1.00E+00
ENSG00000094916.12_CBX5	ENSG00000160789.18_LMNA	-0.11937	-0.273377577	0.040600092	1.43E-01	3.86E-01	1.00E+00
ENSG00000137106.16_GRHPR	ENSG00000160789.18_LMNA	-0.20428	-0.352028941	-0.04659105	1.16E-02	7.28E-02	1.00E+00
ENSG00000111711.8_GOLT1B	ENSG00000160789.18_LMNA	-0.17641	-0.326446056	-0.01771035	2.97E-02	1.42E-01	1.00E+00
ENSG00000130165.9_ELOF1	ENSG00000160789.18_LMNA	0.02024	-0.139408578	0.178865678	8.05E-01	9.22E-01	1.00E+00
ENSG00000138942.14_RNF185	ENSG00000160789.18_LMNA	-0.21189	-0.358982773	-0.05453054	8.78E-03	5.96E-02	1.00E+00
ENSG00000186469.7_GNG2	ENSG00000160789.18_LMNA	-0.30677	-0.444274142	-0.15514738	1.21E-04	2.20E-03	1.00E+00
ENSG00000140526.15_ABHD2	ENSG00000160789.18_LMNA	0.33837	0.189371689	0.472138264	2.01E-05	5.18E-04	1.00E+00
ENSG00000124006.13_OBSL1	ENSG00000160789.18_LMNA	0.3441	0.195612781	0.477159328	1.42E-05	3.91E-04	7.29E-01
ENSG00000148516.20_ZEB1	ENSG00000160789.18_LMNA	-0.12211	-0.275950911	0.037821063	1.34E-01	3.72E-01	1.00E+00

ENSG00000164880.14_INTS1	ENSG00000160789.18_LMNA	0.28714	0.134065292	0.426826913	3.35E-04	4.89E-03	1.00E+00
ENSG00000197226.11_TBC1D9B	ENSG00000160789.18_LMNA	0.21702	0.059886109	0.363654448	7.24E-03	5.16E-02	1.00E+00
ENSG00000114166.7_KAT2B	ENSG00000160789.18_LMNA	-0.2031	-0.350952787	-0.04536579	1.21E-02	7.52E-02	1.00E+00
ENSG00000168275.13_COA6	ENSG00000160789.18_LMNA	-0.07792	-0.234211437	0.082305686	3.40E-01	6.25E-01	1.00E+00
ENSG00000105737.8_GRIK5	ENSG00000160789.18_LMNA	0.04513	-0.114895998	0.20287331	5.81E-01	8.05E-01	1.00E+00
ENSG00000107331.15_ABCA2	ENSG00000160789.18_LMNA	-0.01766	-0.176368744	0.141935831	8.29E-01	9.33E-01	1.00E+00
ENSG00000176946.10_THAP4	ENSG00000160789.18_LMNA	0.30867	0.15718865	0.445951903	1.09E-04	2.03E-03	1.00E+00
ENSG00000182551.12_ADI1	ENSG00000160789.18_LMNA	-0.1978	-0.346101788	-0.03985408	1.46E-02	8.61E-02	1.00E+00
ENSG00000174173.6_TRMT10C	ENSG00000160789.18_LMNA	-0.21707	-0.363705659	-0.05994491	7.23E-03	5.15E-02	1.00E+00
ENSG00000132153.13_DHX30	ENSG00000160789.18_LMNA	0.05139	-0.108697554	0.208884151	5.29E-01	7.73E-01	1.00E+00
ENSG00000163882.8_POLR2H	ENSG00000160789.18_LMNA	0.0193	-0.140328329	0.177957435	8.13E-01	9.27E-01	1.00E+00
ENSG00000141503.14_MINK1	ENSG00000160789.18_LMNA	-0.04484	-0.202590939	0.115186575	5.83E-01	8.06E-01	1.00E+00
ENSG00000179761.10_PIPOX	ENSG00000160789.18_LMNA	0.32883	0.179000508	0.463753697	3.53E-05	8.17E-04	1.00E+00
ENSG00000123472.11_ATPAF1	ENSG00000160789.18_LMNA	-0.10928	-0.263885779	0.050808769	1.80E-01	4.43E-01	1.00E+00
ENSG00000119777.17_TMEM214	ENSG00000160789.18_LMNA	0.49477	0.364263967	0.606222036	9.23E-11	2.10E-08	4.73E-06
ENSG00000140474.11_ULK3	ENSG00000160789.18_LMNA	0.07751	-0.082714232	0.233822618	3.43E-01	6.27E-01	1.00E+00
ENSG00000071655.16_MBD3	ENSG00000160789.18_LMNA	0.17281	0.013991311	0.323118484	3.33E-02	1.53E-01	1.00E+00
ENSG00000130881.12_LRP3	ENSG00000160789.18_LMNA	-0.00405	-0.163148303	0.155248004	9.60E-01	9.84E-01	1.00E+00
ENSG00000108826.14_MRPL27	ENSG00000160789.18_LMNA	-0.21978	-0.366170154	-0.06277735	6.52E-03	4.78E-02	1.00E+00
ENSG00000162627.15_SNX7	ENSG00000160789.18_LMNA	0.07042	-0.089786779	0.227075481	3.89E-01	6.67E-01	1.00E+00
ENSG00000158796.15_DEDD	ENSG00000160789.18_LMNA	-0.01108	-0.169983937	0.148379346	8.92E-01	9.58E-01	1.00E+00
ENSG00000170860.3_LSM3	ENSG00000160789.18_LMNA	-0.40087	-0.526469449	-0.25814055	3.09E-07	1.80E-05	1.59E-02
ENSG00000174547.12_MRPL11	ENSG00000160789.18_LMNA	-0.07738	-0.233700814	0.082842193	3.43E-01	6.28E-01	1.00E+00
ENSG00000169764.13_UGP2	ENSG00000160789.18_LMNA	0.31569	0.164775683	0.452170097	7.44E-05	1.48E-03	1.00E+00
ENSG00000173456.4_RNF26	ENSG00000160789.18_LMNA	0.15267	-0.006689505	0.304474015	6.04E-02	2.27E-01	1.00E+00
ENSG00000125753.12_VASP	ENSG00000160789.18_LMNA	0.46438	0.329544044	0.580654577	1.68E-09	2.37E-07	8.61E-05
ENSG00000163481.6_RNF25	ENSG00000160789.18_LMNA	0.04346	-0.116549704	0.201265598	5.95E-01	8.13E-01	1.00E+00
ENSG00000125931.9_CITED1	ENSG00000160789.18_LMNA	0.22419	0.067397179	0.370180735	5.49E-03	4.20E-02	1.00E+00
ENSG00000172590.17_MRPL52	ENSG00000160789.18_LMNA	-0.03332	-0.191509213	0.12654755	6.84E-01	8.63E-01	1.00E+00
ENSG00000130733.9_YIPF2	ENSG00000160789.18_LMNA	0.00389	-0.155409624	0.162987091	9.62E-01	9.85E-01	1.00E+00
ENSG00000115944.13_COX7A2L	ENSG00000160789.18_LMNA	-0.27799	-0.41865966	-0.12428675	5.25E-04	7.01E-03	1.00E+00
ENSG00000196396.8_PTPN1	ENSG00000160789.18_LMNA	0.31334	0.16223545	0.450091275	8.47E-05	1.65E-03	1.00E+00
ENSG00000136518.15_ACTL6A	ENSG00000160789.18_LMNA	-0.20305	-0.350911695	-0.04531903	1.21E-02	7.53E-02	1.00E+00

ENSG00000146122.15_DAAM2	ENSG00000160789.18_LMNA	-0.45078	-0.569141114	-0.31412775	5.62E-09	6.64E-07	2.88E-04
ENSG00000172081.12_MOB3A	ENSG00000160789.18_LMNA	0.35184	0.20406822	0.483932678	8.79E-06	2.62E-04	4.51E-01
ENSG00000141639.10_MAPK4	ENSG00000160789.18_LMNA	0.10729	-0.052810655	0.262017294	1.88E-01	4.55E-01	1.00E+00
ENSG00000138674.15_SEC31A	ENSG00000160789.18_LMNA	0.27282	0.118773047	0.414033407	6.73E-04	8.51E-03	1.00E+00
ENSG00000013561.16_RNF14	ENSG00000160789.18_LMNA	-0.16395	-0.314935051	-0.0048814	4.36E-02	1.84E-01	1.00E+00
ENSG00000067955.12_CBFB	ENSG00000160789.18_LMNA	0.22734	0.070696369	0.373037949	4.85E-03	3.83E-02	1.00E+00
ENSG00000121064.11_SCPEP1	ENSG00000160789.18_LMNA	0.0741	-0.086111822	0.230585153	3.64E-01	6.46E-01	1.00E+00
ENSG00000087076.7_HSD17B14	ENSG00000160789.18_LMNA	-0.0086	-0.167568735	0.150809731	9.16E-01	9.68E-01	1.00E+00
ENSG00000204681.9_GABBR1	ENSG00000160789.18_LMNA	-0.28915	-0.428619907	-0.13621968	3.03E-04	4.53E-03	1.00E+00
ENSG00000155287.9_SLC25A28	ENSG00000160789.18_LMNA	-0.08235	-0.238423739	0.077872845	3.13E-01	6.00E-01	1.00E+00
ENSG00000204237.4_OXLD1	ENSG00000160789.18_LMNA	0.04727	-0.112782677	0.204925363	5.63E-01	7.94E-01	1.00E+00
ENSG00000152620.11_NADK2	ENSG00000160789.18_LMNA	-0.09959	-0.254749364	0.060573496	2.22E-01	4.98E-01	1.00E+00
ENSG00000136143.12_SUCLA2	ENSG00000160789.18_LMNA	-0.36633	-0.496566623	-0.21995205	3.46E-06	1.23E-04	1.77E-01
ENSG00000078140.12_UBE2K	ENSG00000160789.18_LMNA	-0.16739	-0.31811024	-0.00840996	3.93E-02	1.71E-01	1.00E+00
ENSG00000227057.6_WDR46	ENSG00000160789.18_LMNA	0.16013	0.000949825	0.311389018	4.88E-02	1.98E-01	1.00E+00
ENSG00000196376.9_SLC35F1	ENSG00000160789.18_LMNA	-0.16216	-0.313271935	-0.00303627	4.59E-02	1.90E-01	1.00E+00
ENSG00000196072.10_BLOC1S2	ENSG00000160789.18_LMNA	-0.27376	-0.414879225	-0.11977976	6.43E-04	8.23E-03	1.00E+00
ENSG00000106305.8_AIMP2	ENSG00000160789.18_LMNA	-0.00673	-0.165752189	0.152635163	9.34E-01	9.74E-01	1.00E+00
ENSG00000167641.9_PPP1R14A	ENSG00000160789.18_LMNA	-0.32706	-0.462198516	-0.17708378	3.91E-05	8.85E-04	1.00E+00
ENSG00000142166.11_IFNAR1	ENSG00000160789.18_LMNA	0.36	0.213006934	0.491056884	5.23E-06	1.72E-04	2.68E-01
ENSG00000125457.12_MIF4GD	ENSG00000160789.18_LMNA	-0.27105	-0.412453653	-0.11689441	7.31E-04	9.06E-03	1.00E+00
ENSG00000130748.6_TMEM160	ENSG00000160789.18_LMNA	-0.06192	-0.218967	0.098244259	4.49E-01	7.15E-01	1.00E+00
ENSG00000165886.4_UBTD1	ENSG00000160789.18_LMNA	0.49344	0.36273031	0.605103124	1.05E-10	2.33E-08	5.41E-06
ENSG00000007255.9_TRAPPC6A	ENSG00000160789.18_LMNA	-0.11522	-0.269477079	0.044803129	1.58E-01	4.10E-01	1.00E+00
ENSG00000176393.9_RNPEP	ENSG00000160789.18_LMNA	0.10682	-0.053283008	0.261576074	1.90E-01	4.58E-01	1.00E+00
ENSG00000134851.11_TMEM165	ENSG00000160789.18_LMNA	0.02992	-0.129902665	0.188220983	7.14E-01	8.79E-01	1.00E+00
ENSG00000205403.11_CFI	ENSG00000160789.18_LMNA	0.40066	0.257908324	0.526289596	3.14E-07	1.82E-05	1.61E-02
ENSG00000188313.11_PLSCR1	ENSG00000160789.18_LMNA	0.18747	0.029140709	0.336625589	2.07E-02	1.11E-01	1.00E+00
ENSG00000118855.17_MFSD1	ENSG00000160789.18_LMNA	0.14663	-0.01287014	0.298855296	7.14E-02	2.53E-01	1.00E+00
ENSG00000165801.8_ARHGEF40	ENSG00000160789.18_LMNA	0.28809	0.135079513	0.427671287	3.19E-04	4.72E-03	1.00E+00
ENSG00000091136.12_LAMB1	ENSG00000160789.18_LMNA	0.46974	0.335639475	0.585179334	1.03E-09	1.61E-07	5.27E-05
ENSG00000007202.13_KIAA0100	ENSG00000160789.18_LMNA	0.10958	-0.050501937	0.264171955	1.79E-01	4.41E-01	1.00E+00
ENSG00000173581.6_CCDC106	ENSG00000160789.18_LMNA	0.19562	0.037589085	0.344103547	1.57E-02	9.07E-02	1.00E+00

ENSG00000122378.12_FAM213A	ENSG00000160789.18_LMNA	-0.06933	-0.226039203	0.090870213	3.96E-01	6.73E-01	1.00E+00
ENSG00000101384.10_JAG1	ENSG00000160789.18_LMNA	0.29157	0.138810003	0.430772665	2.68E-04	4.11E-03	1.00E+00
ENSG00000008838.16_MED24	ENSG00000160789.18_LMNA	-0.07902	-0.235257159	0.081206382	3.33E-01	6.19E-01	1.00E+00
ENSG00000156467.8_UQCRB	ENSG00000160789.18_LMNA	-0.24077	-0.385202327	-0.08481685	2.81E-03	2.54E-02	1.00E+00
ENSG00000166272.15_WBP1L	ENSG00000160789.18_LMNA	-0.22175	-0.367961212	-0.06483888	6.04E-03	4.52E-02	1.00E+00
ENSG00000018189.11_RUFY3	ENSG00000160789.18_LMNA	-0.38738	-0.514834723	-0.24318166	8.20E-07	3.88E-05	4.21E-02
ENSG00000171720.8_HDAC3	ENSG00000160789.18_LMNA	0.0366	-0.123314568	0.194671006	6.54E-01	8.47E-01	1.00E+00
ENSG00000106511.5_MEOX2	ENSG00000160789.18_LMNA	0.40435	0.262018026	0.529468964	2.39E-07	1.47E-05	1.22E-02
ENSG00000273841.3_TAF9	ENSG00000160789.18_LMNA	-0.1556	-0.307188711	0.003694776	5.56E-02	2.15E-01	1.00E+00
ENSG00000136827.11_TOR1A	ENSG00000160789.18_LMNA	0.18336	0.024889183	0.332847686	2.37E-02	1.22E-01	1.00E+00
ENSG00000177728.13_TMEM94	ENSG00000160789.18_LMNA	0.00945	-0.149972599	0.168401069	9.08E-01	9.65E-01	1.00E+00
ENSG00000110871.13_COQ5	ENSG00000160789.18_LMNA	-0.3666	-0.496807062	-0.22025577	3.40E-06	1.21E-04	1.74E-01
ENSG00000168591.14_TMUB2	ENSG00000160789.18_LMNA	-0.21191	-0.358995148	-0.05454471	8.77E-03	5.96E-02	1.00E+00
ENSG00000197586.11_ENTPD6	ENSG00000160789.18_LMNA	0.10103	-0.059122647	0.256110429	2.16E-01	4.90E-01	1.00E+00
ENSG00000187479.5_C11orf96	ENSG00000160789.18_LMNA	0.14274	-0.016848055	0.295227566	7.94E-02	2.71E-01	1.00E+00
ENSG00000137692.10_DCUN1D5	ENSG00000160789.18_LMNA	0.00056	-0.158657946	0.159743361	9.95E-01	9.98E-01	1.00E+00
ENSG00000147872.8_PLIN2	ENSG00000160789.18_LMNA	0.15702	-0.002235135	0.308510032	5.34E-02	2.10E-01	1.00E+00
ENSG00000102359.5_SRPX2	ENSG00000160789.18_LMNA	0.50727	0.378647971	0.616669775	2.57E-11	7.29E-09	1.32E-06
ENSG00000137947.10_GTF2B	ENSG00000160789.18_LMNA	-0.28059	-0.420985746	-0.12706593	4.63E-04	6.34E-03	1.00E+00
ENSG00000244509.3_APOBEC3C	ENSG00000160789.18_LMNA	0.19728	0.03931493	0.345626385	1.48E-02	8.73E-02	1.00E+00
ENSG00000082898.15_XPO1	ENSG00000160789.18_LMNA	-0.05405	-0.211429089	0.106065725	5.08E-01	7.58E-01	1.00E+00
ENSG00000125144.12_MT1G	ENSG00000160789.18_LMNA	0.06552	-0.094669575	0.222399528	4.23E-01	6.94E-01	1.00E+00
ENSG00000198728.9_LDB1	ENSG00000160789.18_LMNA	-0.23416	-0.379225821	-0.07786418	3.69E-03	3.12E-02	1.00E+00
ENSG00000119865.7_CNRIP1	ENSG00000160789.18_LMNA	-0.4987	-0.609505868	-0.3687726	6.21E-11	1.54E-08	3.19E-06
ENSG00000205302.5_SNX2	ENSG00000160789.18_LMNA	-0.1478	-0.299944292	0.011674094	6.92E-02	2.48E-01	1.00E+00
ENSG00000130826.14_DKC1	ENSG00000160789.18_LMNA	0.06342	-0.096759881	0.220393302	4.38E-01	7.06E-01	1.00E+00
ENSG00000166295.7_ANAPC16	ENSG00000160789.18_LMNA	-0.26189	-0.404234092	-0.10715377	1.12E-03	1.26E-02	1.00E+00
ENSG00000134882.14_UBAC2	ENSG00000160789.18_LMNA	-0.18243	-0.331987651	-0.02392288	2.45E-02	1.25E-01	1.00E+00
ENSG00000117697.13_NSL1	ENSG00000160789.18_LMNA	-0.40428	-0.529411228	-0.26194331	2.40E-07	1.48E-05	1.23E-02
ENSG00000164885.11_CDK5	ENSG00000160789.18_LMNA	-0.02976	-0.188070326	0.130056212	7.16E-01	8.79E-01	1.00E+00
ENSG00000184867.12_ARMCX2	ENSG00000160789.18_LMNA	0.26474	0.110185845	0.406797836	9.81E-04	1.14E-02	1.00E+00
ENSG00000086475.13_SEPHS1	ENSG00000160789.18_LMNA	-0.28852	-0.428062487	-0.13554961	3.12E-04	4.65E-03	1.00E+00
ENSG00000131584.17_ACAP3	ENSG00000160789.18_LMNA	-0.09775	-0.253014452	0.062420925	2.31E-01	5.09E-01	1.00E+00

ENSG00000177868.10_SVBP	ENSG00000160789.18_LMNA	-0.22468	-0.370622173	-0.06790647	5.39E-03	4.15E-02	1.00E+00
ENSG00000128928.7_IVD	ENSG00000160789.18_LMNA	0.05885	-0.10129527	0.216031095	4.71E-01	7.31E-01	1.00E+00
ENSG00000015592.15_STMN4	ENSG00000160789.18_LMNA	-0.415	-0.538611837	-0.27389055	1.06E-07	7.59E-06	5.45E-03
ENSG00000139200.12_PIANP	ENSG00000160789.18_LMNA	-0.28706	-0.426753366	-0.13397698	3.36E-04	4.90E-03	1.00E+00
ENSG00000028137.15_TNFRSF1	ENSG00000160789.18_LMNA	0.18701	0.028662967	0.336201561	2.11E-02	1.12E-01	1.00E+00
ENSG00000170017.11_ALCAM	ENSG00000160789.18_LMNA	-0.50188	-0.612170296	-0.37243915	4.49E-11	1.17E-08	2.30E-06
ENSG00000271601.3_LIX1L	ENSG00000160789.18_LMNA	0.12983	-0.02999214	0.283176256	1.11E-01	3.32E-01	1.00E+00
ENSG00000149489.7_ROM1	ENSG00000160789.18_LMNA	0.01349	-0.146026716	0.172318234	8.69E-01	9.49E-01	1.00E+00
ENSG00000074181.7_NOTCH3	ENSG00000160789.18_LMNA	0.46256	0.32747354	0.579114063	1.98E-09	2.74E-07	1.02E-04
ENSG00000166136.14_NDUFB8	ENSG00000160789.18_LMNA	-0.27157	-0.41291951	-0.11744819	7.13E-04	8.90E-03	1.00E+00
ENSG00000151135.8_TMEM263	ENSG00000160789.18_LMNA	-0.08368	-0.239689128	0.076538759	3.05E-01	5.92E-01	1.00E+00
ENSG00000224550.1_RP11-270C	ENSG00000160789.18_LMNA	-0.17616	-0.326215053	-0.01745189	2.99E-02	1.43E-01	1.00E+00
ENSG00000148334.13_PTGES2	ENSG00000160789.18_LMNA	-0.02857	-0.186918037	0.131230101	7.27E-01	8.84E-01	1.00E+00
ENSG00000010327.9_STAB1	ENSG00000160789.18_LMNA	0.26581	0.111323973	0.407758967	9.33E-04	1.10E-02	1.00E+00
ENSG00000263465.3_SRSF8	ENSG00000160789.18_LMNA	-0.33689	-0.470838533	-0.18775986	2.20E-05	5.57E-04	1.00E+00
ENSG00000197568.12_HHLA3	ENSG00000160789.18_LMNA	0.01847	-0.141150386	0.177145205	8.21E-01	9.30E-01	1.00E+00
ENSG00000139687.12_RB1	ENSG00000160789.18_LMNA	-0.25257	-0.395854634	-0.097282	1.69E-03	1.72E-02	1.00E+00
ENSG00000124222.20_STX16	ENSG00000160789.18_LMNA	0.14772	-0.011758181	0.299867757	6.93E-02	2.48E-01	1.00E+00
ENSG00000173264.12_GPR137	ENSG00000160789.18_LMNA	0.22563	0.068906543	0.371488608	5.19E-03	4.04E-02	1.00E+00
ENSG00000158623.13_COPG2	ENSG00000160789.18_LMNA	-0.08332	-0.239347126	0.07689944	3.07E-01	5.94E-01	1.00E+00
ENSG00000173391.7_OLR1	ENSG00000160789.18_LMNA	-0.15605	-0.307609511	0.00323007	5.49E-02	2.14E-01	1.00E+00
ENSG00000116691.9_MIIP	ENSG00000160789.18_LMNA	-0.20123	-0.349240144	-0.04341776	1.29E-02	7.88E-02	1.00E+00
ENSG00000125945.13_ZNF436	ENSG00000160789.18_LMNA	0.49909	0.36922441	0.609834485	5.97E-11	1.50E-08	3.06E-06
ENSG00000138867.15_GUCD1	ENSG00000160789.18_LMNA	0.17984	0.021249966	0.329606008	2.66E-02	1.32E-01	1.00E+00
ENSG00000198408.12_MGEA5	ENSG00000160789.18_LMNA	-0.15968	-0.310970817	-0.00048679	4.94E-02	2.00E-01	1.00E+00
ENSG00000004399.11_PLXND1	ENSG00000160789.18_LMNA	0.40691	0.264871098	0.531671762	1.97E-07	1.25E-05	1.01E-02
ENSG00000230897.1_RPS18P12	ENSG00000160789.18_LMNA	-0.0782	-0.234478295	0.082025228	3.38E-01	6.24E-01	1.00E+00
ENSG00000153774.7_CFDP1	ENSG00000160789.18_LMNA	0.00598	-0.153368185	0.165022127	9.42E-01	9.77E-01	1.00E+00
ENSG00000116171.15_SCP2	ENSG00000160789.18_LMNA	-0.03775	-0.195770056	0.122189189	6.44E-01	8.42E-01	1.00E+00
ENSG00000100095.17_SEZ6L	ENSG00000160789.18_LMNA	-0.30177	-0.439835699	-0.14975916	1.58E-04	2.72E-03	1.00E+00
ENSG00000013364.17_MVP	ENSG00000160789.18_LMNA	0.51141	0.383423549	0.620120047	1.66E-11	5.14E-09	8.54E-07
ENSG00000101972.17_STAG2	ENSG00000160789.18_LMNA	0.00931	-0.150116177	0.168258346	9.09E-01	9.65E-01	1.00E+00
ENSG00000186660.14_ZFP91	ENSG00000160789.18_LMNA	-0.08015	-0.236331402	0.080076293	3.26E-01	6.13E-01	1.00E+00

ENSG00000136146.13_MED4	ENSG00000160789.18_LMNA	-0.18529	-0.334616923	-0.02687884	2.23E-02	1.17E-01	1.00E+00
ENSG00000242247.9_ARFGAP3	ENSG00000160789.18_LMNA	-0.0141	-0.172914311	0.14542538	8.63E-01	9.46E-01	1.00E+00
ENSG00000115963.12_RND3	ENSG00000160789.18_LMNA	-0.00599	-0.165029231	0.153361053	9.42E-01	9.77E-01	1.00E+00
ENSG00000185122.9_HSF1	ENSG00000160789.18_LMNA	0.19566	0.037628726	0.344138543	1.57E-02	9.07E-02	1.00E+00
ENSG00000166582.8_CENPV	ENSG00000160789.18_LMNA	-0.15972	-0.311014753	-0.00053543	4.93E-02	2.00E-01	1.00E+00
ENSG00000105364.12_MRPL4	ENSG00000160789.18_LMNA	0.09411	-0.066081339	0.249570971	2.49E-01	5.29E-01	1.00E+00
ENSG00000134121.8_CHL1	ENSG00000160789.18_LMNA	0.15554	-0.00375857	0.307130936	5.57E-02	2.15E-01	1.00E+00
ENSG00000109501.12_WFS1	ENSG00000160789.18_LMNA	0.03398	-0.125902913	0.192140183	6.78E-01	8.59E-01	1.00E+00
ENSG00000088833.16_NSFL1C	ENSG00000160789.18_LMNA	0.06735	-0.092843495	0.224149962	4.10E-01	6.84E-01	1.00E+00
ENSG00000129250.10_KIF1C	ENSG00000160789.18_LMNA	0.11094	-0.049130587	0.265450312	1.74E-01	4.33E-01	1.00E+00
ENSG00000135097.5_MSI1	ENSG00000160789.18_LMNA	0.06552	-0.094667126	0.222401877	4.23E-01	6.94E-01	1.00E+00
ENSG00000012061.14_ERCC1	ENSG00000160789.18_LMNA	0.02832	-0.131469922	0.186682521	7.29E-01	8.85E-01	1.00E+00
ENSG00000163159.10_VPS72	ENSG00000160789.18_LMNA	-0.195	-0.343534883	-0.03694508	1.61E-02	9.22E-02	1.00E+00
ENSG00000119977.19_TCTN3	ENSG00000160789.18_LMNA	0.20209	0.044319561	0.350033231	1.25E-02	7.70E-02	1.00E+00
ENSG00000185641.6_CTD-2287C	ENSG00000160789.18_LMNA	-0.19676	-0.345147318	-0.0387718	1.51E-02	8.84E-02	1.00E+00
ENSG00000059573.7_ALDH18A1	ENSG00000160789.18_LMNA	0.0216	-0.138078946	0.180177715	7.92E-01	9.16E-01	1.00E+00
ENSG00000114853.12_ZBTB47	ENSG00000160789.18_LMNA	-0.08413	-0.24011679	0.076087623	3.03E-01	5.89E-01	1.00E+00
ENSG00000079931.13_MOXD1	ENSG00000160789.18_LMNA	0.21497	0.057743161	0.36178698	7.82E-03	5.46E-02	1.00E+00
ENSG00000134001.11_EIF2S1	ENSG00000160789.18_LMNA	0.16684	0.007844852	0.317602188	3.99E-02	1.73E-01	1.00E+00
ENSG00000179933.5_C14orf119	ENSG00000160789.18_LMNA	-0.01317	-0.172014645	0.146332893	8.72E-01	9.50E-01	1.00E+00
ENSG00000167114.11_SLC27A4	ENSG00000160789.18_LMNA	0.06537	-0.094819671	0.222255559	4.24E-01	6.95E-01	1.00E+00
ENSG00000122557.8_HERPUD2	ENSG00000160789.18_LMNA	0.13041	-0.029396499	0.283724525	1.09E-01	3.29E-01	1.00E+00
ENSG00000144136.9_SLC20A1	ENSG00000160789.18_LMNA	0.48886	0.357477231	0.60126336	1.66E-10	3.43E-08	8.51E-06
ENSG00000053501.11_USE1	ENSG00000160789.18_LMNA	-0.10066	-0.255759923	0.059496402	2.17E-01	4.92E-01	1.00E+00
ENSG00000137198.8_GMPR	ENSG00000160789.18_LMNA	0.00817	-0.151223138	0.167157529	9.20E-01	9.70E-01	1.00E+00
ENSG00000198951.10_NAGA	ENSG00000160789.18_LMNA	0.15387	-0.00546628	0.30558347	5.84E-02	2.22E-01	1.00E+00
ENSG00000128654.12_MTX2	ENSG00000160789.18_LMNA	-0.18091	-0.330587334	-0.02235077	2.57E-02	1.29E-01	1.00E+00
ENSG00000075975.14_MKRN2	ENSG00000160789.18_LMNA	-0.3068	-0.444301145	-0.15518021	1.21E-04	2.20E-03	1.00E+00
ENSG00000095906.15_NUBP2	ENSG00000160789.18_LMNA	-0.17333	-0.323604353	-0.01453381	3.27E-02	1.51E-01	1.00E+00
ENSG00000067064.9_IDI1	ENSG00000160789.18_LMNA	-0.32817	-0.463171041	-0.17828214	3.67E-05	8.41E-04	1.00E+00
ENSG00000139620.11_KANSL2	ENSG00000160789.18_LMNA	-0.2495	-0.393086158	-0.09403334	1.94E-03	1.91E-02	1.00E+00
ENSG00000079819.15_EPB41L2	ENSG00000160789.18_LMNA	-0.29148	-0.430691152	-0.13871185	2.69E-04	4.13E-03	1.00E+00
ENSG00000099875.13_MKNK2	ENSG00000160789.18_LMNA	0.42357	0.283481549	0.545952487	5.41E-08	4.37E-06	2.78E-03

ENSG00000162607.11_USP1	ENSG00000160789.18_LMNA	0.10594	-0.054170859	0.260746384	1.94E-01	4.63E-01	1.00E+00
ENSG00000132463.12_GRSF1	ENSG00000160789.18_LMNA	-0.07918	-0.235415583	0.081039773	3.32E-01	6.18E-01	1.00E+00
ENSG00000117640.16_MTFR1L	ENSG00000160789.18_LMNA	-0.17737	-0.327330523	-0.0187003	2.88E-02	1.39E-01	1.00E+00
ENSG00000188290.9_HES4	ENSG00000160789.18_LMNA	-0.15859	-0.309969308	0.000621558	5.10E-02	2.04E-01	1.00E+00
ENSG00000105429.11_MEGF8	ENSG00000160789.18_LMNA	0.28102	0.127521419	0.421366608	4.53E-04	6.25E-03	1.00E+00
ENSG00000145781.7_COMMD10	ENSG00000160789.18_LMNA	-0.20172	-0.349689914	-0.04392912	1.27E-02	7.78E-02	1.00E+00
ENSG00000147100.8_SLC16A2	ENSG00000160789.18_LMNA	0.1011	-0.05905075	0.256177843	2.15E-01	4.89E-01	1.00E+00
ENSG00000132581.8_SDF2	ENSG00000160789.18_LMNA	-0.17032	-0.320825799	-0.01143388	3.59E-02	1.61E-01	1.00E+00
ENSG00000062598.16_ELMO2	ENSG00000160789.18_LMNA	-0.05052	-0.208047849	0.10956144	5.37E-01	7.77E-01	1.00E+00
ENSG00000261371.4_PECAM1	ENSG00000160789.18_LMNA	0.04891	-0.11115286	0.206506025	5.50E-01	7.86E-01	1.00E+00
ENSG00000134717.16_BTF3L4	ENSG00000160789.18_LMNA	-0.40823	-0.532799242	-0.26633322	1.78E-07	1.15E-05	9.15E-03
ENSG00000115685.13_PPP1R7	ENSG00000160789.18_LMNA	0.06773	-0.092468694	0.224508984	4.07E-01	6.82E-01	1.00E+00
ENSG00000137200.11_CMTR1	ENSG00000160789.18_LMNA	-0.06062	-0.217715458	0.099545599	4.58E-01	7.22E-01	1.00E+00
ENSG00000260549.1_MT1L	ENSG00000160789.18_LMNA	0.25172	0.096377082	0.395084015	1.76E-03	1.77E-02	1.00E+00
ENSG00000198355.4_PIM3	ENSG00000160789.18_LMNA	0.27806	0.124365222	0.418725394	5.23E-04	6.99E-03	1.00E+00
ENSG00000180901.9_KCTD2	ENSG00000160789.18_LMNA	0.01465	-0.144884957	0.173449808	8.58E-01	9.44E-01	1.00E+00
ENSG00000169599.11_NFU1	ENSG00000160789.18_LMNA	-0.26073	-0.403197563	-0.10592946	1.18E-03	1.31E-02	1.00E+00
ENSG00000147862.13_NFIB	ENSG00000160789.18_LMNA	-0.16414	-0.315110089	-0.00507572	4.33E-02	1.83E-01	1.00E+00
ENSG00000104442.8_ARMC1	ENSG00000160789.18_LMNA	-0.46439	-0.58066583	-0.32955918	1.68E-09	2.37E-07	8.60E-05
ENSG00000140367.10_UBE2Q2	ENSG00000160789.18_LMNA	-0.13762	-0.290459679	0.022061208	9.09E-02	2.94E-01	1.00E+00
ENSG00000132970.11_WASF3	ENSG00000160789.18_LMNA	-0.25809	-0.40082431	-0.10312965	1.33E-03	1.43E-02	1.00E+00
ENSG00000136149.6_RPL13AP25	ENSG00000160789.18_LMNA	-0.20572	-0.353344953	-0.04809064	1.10E-02	7.01E-02	1.00E+00
ENSG00000164949.6_GEM	ENSG00000160789.18_LMNA	0.31087	0.159571244	0.447907641	9.69E-05	1.84E-03	1.00E+00
ENSG00000186340.13_THBS2	ENSG00000160789.18_LMNA	0.27912	0.125500472	0.419675984	4.97E-04	6.71E-03	1.00E+00
ENSG00000156171.13_DRAM2	ENSG00000160789.18_LMNA	-0.23399	-0.379070622	-0.07768402	3.71E-03	3.14E-02	1.00E+00
ENSG00000198876.11_DCAF12	ENSG00000160789.18_LMNA	-0.13997	-0.292646271	0.019672522	8.55E-02	2.84E-01	1.00E+00
ENSG00000234975.6_FTH1P2	ENSG00000160789.18_LMNA	0.01696	-0.142625071	0.175687061	8.36E-01	9.36E-01	1.00E+00
ENSG00000128581.14_IFT22	ENSG00000160789.18_LMNA	0.1436	-0.015966644	0.296032161	7.76E-02	2.67E-01	1.00E+00
ENSG00000049656.12_CLPTM1L	ENSG00000160789.18_LMNA	0.32789	0.177976876	0.462923368	3.73E-05	8.50E-04	1.00E+00
ENSG00000166987.13_MBD6	ENSG00000160789.18_LMNA	-0.20124	-0.349249192	-0.04342804	1.29E-02	7.88E-02	1.00E+00
ENSG00000106009.14_BRAT1	ENSG00000160789.18_LMNA	0.33902	0.190083511	0.472711866	1.93E-05	5.02E-04	9.92E-01
ENSG00000135250.15_SRPK2	ENSG00000160789.18_LMNA	-0.27363	-0.414762791	-0.11964114	6.47E-04	8.26E-03	1.00E+00
ENSG00000196363.8_WDR5	ENSG00000160789.18_LMNA	0.24117	0.085238374	0.385563859	2.76E-03	2.51E-02	1.00E+00

ENSG00000013374.14_NUB1	ENSG00000160789.18_LMNA	0.17879	0.020161453	0.328634987	2.75E-02	1.35E-01	1.00E+00
ENSG00000223501.7_VPS52	ENSG00000160789.18_LMNA	-0.13944	-0.2921556	0.020208853	8.67E-02	2.86E-01	1.00E+00
ENSG00000114738.9_MAPKAPK3	ENSG00000160789.18_LMNA	-0.02615	-0.184583635	0.13360553	7.49E-01	8.95E-01	1.00E+00
ENSG00000171792.9_RHNO1	ENSG00000160789.18_LMNA	-0.06179	-0.218834512	0.098382069	4.50E-01	7.15E-01	1.00E+00
ENSG00000225792.1_AC004540.4	ENSG00000160789.18_LMNA	-0.31512	-0.4516613	-0.1641536	7.68E-05	1.52E-03	1.00E+00
ENSG00000185745.9_IFIT1	ENSG00000160789.18_LMNA	-0.23598	-0.380875903	-0.07978085	3.42E-03	2.95E-02	1.00E+00
ENSG00000108848.14_LUC7L3	ENSG00000160789.18_LMNA	-0.10081	-0.255905471	0.05934121	2.17E-01	4.91E-01	1.00E+00
ENSG00000206341.7_HLA-H	ENSG00000160789.18_LMNA	0.17052	0.011632032	0.32100357	3.57E-02	1.61E-01	1.00E+00
ENSG00000123427.14_METTL21B	ENSG00000160789.18_LMNA	-0.1648	-0.315720209	-0.00575322	4.25E-02	1.80E-01	1.00E+00
ENSG00000132612.15_VPS4A	ENSG00000160789.18_LMNA	0.00154	-0.157700943	0.160699726	9.85E-01	9.94E-01	1.00E+00
ENSG00000117280.11_RAB29	ENSG00000160789.18_LMNA	0.06638	-0.093811678	0.223222141	4.16E-01	6.89E-01	1.00E+00
ENSG00000131778.16_CHD1L	ENSG00000160789.18_LMNA	0.06207	-0.098103769	0.219102051	4.47E-01	7.14E-01	1.00E+00
ENSG00000164898.11_FMC1	ENSG00000160789.18_LMNA	-0.18993	-0.338887062	-0.03169103	1.91E-02	1.04E-01	1.00E+00
ENSG00000133574.8_GIMAP4	ENSG00000160789.18_LMNA	-0.02988	-0.188185968	0.129938352	7.15E-01	8.79E-01	1.00E+00
ENSG00000205138.3_SDHAF1	ENSG00000160789.18_LMNA	-0.15652	-0.308046272	0.002747595	5.41E-02	2.12E-01	1.00E+00
ENSG00000060237.15_WNK1	ENSG00000160789.18_LMNA	0.04115	-0.118825477	0.199050318	6.15E-01	8.25E-01	1.00E+00
ENSG00000103978.14_TMEM87A	ENSG00000160789.18_LMNA	0.00927	-0.150151308	0.168223422	9.10E-01	9.66E-01	1.00E+00
ENSG00000133835.13_HSD17B4	ENSG00000160789.18_LMNA	-0.36894	-0.498843607	-0.22283044	2.91E-06	1.07E-04	1.49E-01
ENSG00000091986.14_CCDC80	ENSG00000160789.18_LMNA	0.01662	-0.142960388	0.175355309	8.39E-01	9.37E-01	1.00E+00
ENSG00000183010.15_PYCR1	ENSG00000160789.18_LMNA	-0.03001	-0.188309405	0.129812539	7.14E-01	8.78E-01	1.00E+00
ENSG00000108883.11_EFTUD2	ENSG00000160789.18_LMNA	0.26376	0.109141629	0.405915436	1.03E-03	1.18E-02	1.00E+00
ENSG00000102974.13_CTCF	ENSG00000160789.18_LMNA	-0.00571	-0.164760012	0.153631277	9.44E-01	9.78E-01	1.00E+00
ENSG00000137563.10_GGH	ENSG00000160789.18_LMNA	-0.06978	-0.226460363	0.090429979	3.93E-01	6.71E-01	1.00E+00
ENSG00000109536.10_FRG1	ENSG00000160789.18_LMNA	-0.23865	-0.383287597	-0.08258619	3.07E-03	2.72E-02	1.00E+00
ENSG00000080845.16_DLGAP4	ENSG00000160789.18_LMNA	0.27338	0.119379046	0.414542616	6.55E-04	8.33E-03	1.00E+00
ENSG00000196290.13_NIF3L1	ENSG00000160789.18_LMNA	-0.35854	-0.489780439	-0.21140195	5.75E-06	1.86E-04	2.95E-01
ENSG00000149084.10_HSD17B12	ENSG00000160789.18_LMNA	-0.14954	-0.301565697	0.009891648	6.59E-02	2.40E-01	1.00E+00
ENSG00000111737.10_RAB35	ENSG00000160789.18_LMNA	0.19659	0.038599759	0.344995534	1.52E-02	8.87E-02	1.00E+00
ENSG00000159784.16_FAM131B	ENSG00000160789.18_LMNA	-0.00427	-0.163356614	0.15503914	9.58E-01	9.83E-01	1.00E+00
ENSG00000090565.14_RAB11FIP	ENSG00000160789.18_LMNA	0.14772	-0.011755566	0.299870137	6.93E-02	2.48E-01	1.00E+00
ENSG00000135077.7_HAVCR2	ENSG00000160789.18_LMNA	-0.11277	-0.267175009	0.047278546	1.67E-01	4.24E-01	1.00E+00
ENSG00000076242.13_MLH1	ENSG00000160789.18_LMNA	-0.24302	-0.387241508	-0.08719581	2.55E-03	2.36E-02	1.00E+00
ENSG00000100003.16_SEC14L2	ENSG00000160789.18_LMNA	0.29248	0.139782992	0.431580436	2.56E-04	3.97E-03	1.00E+00

ENSG00000121039.8_RDH10	ENSG00000160789.18_LMNA	0.24844	0.092916018	0.392132729	2.03E-03	1.97E-02	1.00E+00
ENSG00000121579.11_NAA50	ENSG00000160789.18_LMNA	0.0263	-0.133455536	0.184731146	7.48E-01	8.95E-01	1.00E+00
ENSG00000107719.8_PALD1	ENSG00000160789.18_LMNA	-0.04753	-0.205174401	0.112526008	5.61E-01	7.93E-01	1.00E+00
ENSG00000189091.11_SF3B3	ENSG00000160789.18_LMNA	0.12982	-0.030002413	0.283166799	1.11E-01	3.32E-01	1.00E+00
ENSG00000126860.10_EVI2A	ENSG00000160789.18_LMNA	-0.37363	-0.502919803	-0.22799529	2.12E-06	8.35E-05	1.09E-01
ENSG00000172586.7_CHCHD1	ENSG00000160789.18_LMNA	-0.2302	-0.375634616	-0.07370046	4.33E-03	3.52E-02	1.00E+00
ENSG00000110330.7_BIRC2	ENSG00000160789.18_LMNA	-0.10049	-0.255605151	0.059661411	2.18E-01	4.93E-01	1.00E+00
ENSG00000139832.4_RAB20	ENSG00000160789.18_LMNA	0.18901	0.030730499	0.338035738	1.97E-02	1.07E-01	1.00E+00
ENSG00000141101.11_NOB1	ENSG00000160789.18_LMNA	0.19646	0.038466962	0.344878363	1.53E-02	8.90E-02	1.00E+00
ENSG00000163513.16_TGFBR2	ENSG00000160789.18_LMNA	0.14502	-0.014518599	0.297353047	7.47E-02	2.60E-01	1.00E+00
ENSG00000129009.11_ISLR	ENSG00000160789.18_LMNA	0.14983	-0.009595681	0.30183475	6.54E-02	2.39E-01	1.00E+00
ENSG00000127220.5_ABHD8	ENSG00000160789.18_LMNA	0.16253	0.003417265	0.313615504	4.54E-02	1.89E-01	1.00E+00
ENSG00000124587.12_PEX6	ENSG00000160789.18_LMNA	0.14862	-0.010838931	0.300704224	6.77E-02	2.44E-01	1.00E+00
ENSG00000143226.12_FCGR2A	ENSG00000160789.18_LMNA	0.13172	-0.028067569	0.284947024	1.06E-01	3.23E-01	1.00E+00
ENSG00000100221.9_JOSD1	ENSG00000160789.18_LMNA	0.10015	-0.060004591	0.255283212	2.20E-01	4.95E-01	1.00E+00
ENSG00000124659.6_TBCC	ENSG00000160789.18_LMNA	-0.24564	-0.389603449	-0.0899556	2.29E-03	2.17E-02	1.00E+00
ENSG00000116212.13_LRRC42	ENSG00000160789.18_LMNA	0.13202	-0.027760044	0.285229775	1.05E-01	3.21E-01	1.00E+00
ENSG00000111666.9_CHPT1	ENSG00000160789.18_LMNA	0.13661	-0.02309599	0.28951143	9.33E-02	2.99E-01	1.00E+00
ENSG00000139910.18_NOVA1	ENSG00000160789.18_LMNA	-0.18794	-0.337052009	-0.02962129	2.04E-02	1.10E-01	1.00E+00
ENSG00000169490.15_TM2D2	ENSG00000160789.18_LMNA	0.03337	-0.126504769	0.191551096	6.83E-01	8.62E-01	1.00E+00
ENSG00000168175.13_MAPK1IP1	ENSG00000160789.18_LMNA	0.31117	0.159887759	0.448167243	9.54E-05	1.82E-03	1.00E+00
ENSG00000119878.5_CRIPT	ENSG00000160789.18_LMNA	-0.49821	-0.60909853	-0.36821272	6.53E-11	1.59E-08	3.35E-06
ENSG00000234287.1_RP11-761N	ENSG00000160789.18_LMNA	-0.03524	-0.193351922	0.124664173	6.66E-01	8.53E-01	1.00E+00
ENSG00000137824.14_RMDN3	ENSG00000160789.18_LMNA	0.2487	0.093187927	0.392364812	2.00E-03	1.96E-02	1.00E+00
ENSG00000115526.9_CHST10	ENSG00000160789.18_LMNA	-0.03199	-0.190223426	0.127860358	6.96E-01	8.69E-01	1.00E+00
ENSG00000171700.12_RGS19	ENSG00000160789.18_LMNA	0.16887	0.009933379	0.319478945	3.76E-02	1.66E-01	1.00E+00
ENSG00000126767.16_ELK1	ENSG00000160789.18_LMNA	0.18749	0.029154782	0.336638078	2.07E-02	1.11E-01	1.00E+00
ENSG00000215717.5_TMEM167B	ENSG00000160789.18_LMNA	-0.10263	-0.257619198	0.057512791	2.08E-01	4.81E-01	1.00E+00
ENSG00000148358.18_GPR107	ENSG00000160789.18_LMNA	0.18901	0.030732641	0.338037636	1.97E-02	1.07E-01	1.00E+00
ENSG00000128789.19_PSMG2	ENSG00000160789.18_LMNA	-0.14406	-0.296463917	0.015493469	7.66E-02	2.65E-01	1.00E+00
ENSG00000104774.11_MAN2B1	ENSG00000160789.18_LMNA	0.36911	0.223009113	0.498984826	2.88E-06	1.06E-04	1.48E-01
ENSG00000177352.9_CCDC71	ENSG00000160789.18_LMNA	-0.10375	-0.25867614	0.056384058	2.03E-01	4.74E-01	1.00E+00
ENSG00000101084.15_C20orf24	ENSG00000160789.18_LMNA	0.12257	-0.037356054	0.276381063	1.32E-01	3.69E-01	1.00E+00

ENSG00000106290.13_TAF6	ENSG00000160789.18_LMNA	0.2595	0.104623637	0.402091179	1.24E-03	1.37E-02	1.00E+00
ENSG00000116584.16_ARHGEF2	ENSG00000160789.18_LMNA	0.11136	-0.048708563	0.265843495	1.72E-01	4.31E-01	1.00E+00
ENSG00000235501.4_RP4-639F2	ENSG00000160789.18_LMNA	0.05532	-0.104804085	0.21264755	4.98E-01	7.52E-01	1.00E+00
ENSG00000168078.8_PBK	ENSG00000160789.18_LMNA	0.02423	-0.135491724	0.182727459	7.67E-01	9.03E-01	1.00E+00
ENSG00000068438.13_FTSJ1	ENSG00000160789.18_LMNA	0.1764	0.017698904	0.326435831	2.97E-02	1.42E-01	1.00E+00
ENSG00000010322.14_NISCH	ENSG00000160789.18_LMNA	0.01639	-0.143182351	0.175135667	8.41E-01	9.38E-01	1.00E+00
ENSG00000185340.14_GAS2L1	ENSG00000160789.18_LMNA	0.11536	-0.044663563	0.269606764	1.57E-01	4.09E-01	1.00E+00
ENSG00000213024.9_NUP62	ENSG00000160789.18_LMNA	0.24798	0.092423474	0.39171223	2.07E-03	2.00E-02	1.00E+00
ENSG00000134684.9_YARS	ENSG00000160789.18_LMNA	0.08938	-0.070831844	0.245090068	2.74E-01	5.58E-01	1.00E+00
ENSG00000107863.15_ARHGAP2	ENSG00000160789.18_LMNA	-0.05772	-0.21494884	0.102418454	4.80E-01	7.38E-01	1.00E+00
ENSG00000106524.7_ANKMY2	ENSG00000160789.18_LMNA	-0.11916	-0.273179731	0.040813552	1.44E-01	3.88E-01	1.00E+00
ENSG00000197345.11_MRPL21	ENSG00000160789.18_LMNA	-0.22158	-0.367806913	-0.06466118	6.08E-03	4.54E-02	1.00E+00
ENSG00000198719.8_DLL1	ENSG00000160789.18_LMNA	-0.24732	-0.391123524	-0.09173415	2.13E-03	2.05E-02	1.00E+00
ENSG00000138768.13_USO1	ENSG00000160789.18_LMNA	0.18312	0.024642087	0.332627812	2.39E-02	1.23E-01	1.00E+00
ENSG00000272106.1_RP11-345P	ENSG00000160789.18_LMNA	-0.18017	-0.329912081	-0.02159323	2.63E-02	1.31E-01	1.00E+00
ENSG00000074695.5_LMAN1	ENSG00000160789.18_LMNA	0.38693	0.242675869	0.514439569	8.47E-07	3.98E-05	4.35E-02
ENSG00000051128.17_HOMER3	ENSG00000160789.18_LMNA	0.27358	0.119588273	0.414718382	6.49E-04	8.27E-03	1.00E+00
ENSG00000083937.7_CHMP2B	ENSG00000160789.18_LMNA	-0.15446	-0.306133791	0.004859179	5.74E-02	2.19E-01	1.00E+00
ENSG00000263146.2_RP11-849I1	ENSG00000160789.18_LMNA	-0.36467	-0.495123556	-0.21813033	3.86E-06	1.35E-04	1.98E-01
ENSG00000109606.11_DHX15	ENSG00000160789.18_LMNA	-0.06982	-0.226507798	0.090380388	3.93E-01	6.70E-01	1.00E+00
ENSG00000188976.9_NOC2L	ENSG00000160789.18_LMNA	0.22922	0.072676271	0.374749864	4.50E-03	3.63E-02	1.00E+00
ENSG00000185989.10_RASA3	ENSG00000160789.18_LMNA	0.24869	0.093180658	0.392358608	2.01E-03	1.96E-02	1.00E+00
ENSG00000211592.5_IGKC	ENSG00000160789.18_LMNA	0.00518	-0.15415071	0.164242376	9.50E-01	9.80E-01	1.00E+00
ENSG00000134644.14_PUM1	ENSG00000160789.18_LMNA	0.08342	-0.076796524	0.23944472	3.07E-01	5.93E-01	1.00E+00
ENSG00000140575.11_IQGAP1	ENSG00000160789.18_LMNA	0.54906	0.427201023	0.651327491	2.41E-13	1.67E-10	1.23E-08
ENSG00000118096.6_IFT46	ENSG00000160789.18_LMNA	-0.28645	-0.426217933	-0.1333342	3.47E-04	5.02E-03	1.00E+00
ENSG00000226415.1_TPI1P1	ENSG00000160789.18_LMNA	0.22681	0.070146487	0.37256213	4.95E-03	3.90E-02	1.00E+00
ENSG00000166068.11_SPRED1	ENSG00000160789.18_LMNA	0.33876	0.189798993	0.472482623	1.96E-05	5.09E-04	1.00E+00
ENSG00000174705.10_SH3PXD2	ENSG00000160789.18_LMNA	0.40567	0.263485796	0.53060265	2.16E-07	1.35E-05	1.11E-02
ENSG00000076201.13_PTPN23	ENSG00000160789.18_LMNA	0.06339	-0.096785787	0.220368421	4.38E-01	7.06E-01	1.00E+00
ENSG00000107164.15_FUBP3	ENSG00000160789.18_LMNA	0.13181	-0.027975978	0.285031242	1.06E-01	3.22E-01	1.00E+00
ENSG00000167930.14_FAM234A	ENSG00000160789.18_LMNA	0.34503	0.196627451	0.477973914	1.34E-05	3.71E-04	6.89E-01
ENSG00000182093.13_WRB	ENSG00000160789.18_LMNA	-0.20056	-0.348629131	-0.04272333	1.32E-02	8.01E-02	1.00E+00

ENSG00000162998.4_FRZB	ENSG00000160789.18_LMNA	-0.09966	-0.254819621	0.060498637	2.22E-01	4.97E-01	1.00E+00
ENSG00000138814.15_PPP3CA	ENSG00000160789.18_LMNA	-0.17209	-0.322452381	-0.01324787	3.40E-02	1.56E-01	1.00E+00
ENSG00000177879.13_AP3S1	ENSG00000160789.18_LMNA	-0.06304	-0.220032944	0.097135045	4.40E-01	7.08E-01	1.00E+00
ENSG00000073969.17_NSF	ENSG00000160789.18_LMNA	-0.25377	-0.396935272	-0.0985518	1.61E-03	1.66E-02	1.00E+00
ENSG00000213626.10_LBH	ENSG00000160789.18_LMNA	0.14258	-0.017010516	0.295079216	7.97E-02	2.72E-01	1.00E+00
ENSG00000173020.9_GRK2	ENSG00000160789.18_LMNA	0.26841	0.114085023	0.410087901	8.27E-04	9.98E-03	1.00E+00
ENSG00000142546.12_NOSIP	ENSG00000160789.18_LMNA	-0.09561	-0.250994867	0.064568769	2.41E-01	5.20E-01	1.00E+00
ENSG00000105197.9_TIMM50	ENSG00000160789.18_LMNA	-0.22334	-0.369405457	-0.06650312	5.68E-03	4.31E-02	1.00E+00
ENSG00000176108.8_CHMP6	ENSG00000160789.18_LMNA	-0.17166	-0.322059622	-0.01280967	3.45E-02	1.57E-01	1.00E+00
ENSG00000111845.4_PAK1IP1	ENSG00000160789.18_LMNA	-0.15636	-0.307895945	0.002913672	5.44E-02	2.12E-01	1.00E+00
ENSG00000065600.11_TMEM206	ENSG00000160789.18_LMNA	-0.45733	-0.574692929	-0.32154477	3.16E-09	4.04E-07	1.62E-04
ENSG00000157954.13_WIPI2	ENSG00000160789.18_LMNA	0.07907	-0.081155237	0.235305793	3.33E-01	6.19E-01	1.00E+00
ENSG00000105135.14_ILVBL	ENSG00000160789.18_LMNA	-0.00084	-0.160020524	0.15838066	9.92E-01	9.97E-01	1.00E+00
ENSG00000112290.11_WASF1	ENSG00000160789.18_LMNA	-0.38516	-0.512913396	-0.24072376	9.59E-07	4.44E-05	4.92E-02
ENSG00000118515.10_SGK1	ENSG00000160789.18_LMNA	-0.31032	-0.447419528	-0.15897628	9.99E-05	1.88E-03	1.00E+00
ENSG00000184281.13_TSSC4	ENSG00000160789.18_LMNA	0.24973	0.094273596	0.393291085	1.92E-03	1.89E-02	1.00E+00
ENSG00000197019.4_SERTAD1	ENSG00000160789.18_LMNA	0.31058	0.159257679	0.447650411	9.85E-05	1.86E-03	1.00E+00
ENSG00000113719.14_ERGIC1	ENSG00000160789.18_LMNA	0.2827	0.129320257	0.422869712	4.17E-04	5.86E-03	1.00E+00
ENSG00000125952.17_MAX	ENSG00000160789.18_LMNA	0.12778	-0.032074157	0.281258218	1.17E-01	3.42E-01	1.00E+00
ENSG00000119403.12_PHF19	ENSG00000160789.18_LMNA	0.36611	0.219711537	0.496376191	3.51E-06	1.25E-04	1.80E-01
ENSG00000198218.9_QRICH1	ENSG00000160789.18_LMNA	-0.08245	-0.23852071	0.07777065	3.13E-01	5.99E-01	1.00E+00
ENSG00000110104.10_CCDC86	ENSG00000160789.18_LMNA	0.1976	0.039642181	0.345914961	1.47E-02	8.65E-02	1.00E+00
ENSG00000181218.5_HIST3H2A	ENSG00000160789.18_LMNA	-0.33235	-0.466851628	-0.1828251	2.87E-05	6.88E-04	1.00E+00
ENSG00000107020.8_PLGRKT	ENSG00000160789.18_LMNA	-0.00229	-0.161429907	0.156969873	9.78E-01	9.92E-01	1.00E+00
ENSG00000169385.2_RNASE2	ENSG00000160789.18_LMNA	0.18224	0.023729121	0.331815136	2.46E-02	1.25E-01	1.00E+00
ENSG00000139613.10_SMARCC2	ENSG00000160789.18_LMNA	-0.07121	-0.227825648	0.089002009	3.83E-01	6.62E-01	1.00E+00
ENSG00000174109.4_C16orf91	ENSG00000160789.18_LMNA	-0.09446	-0.249905204	0.065726422	2.47E-01	5.27E-01	1.00E+00
ENSG00000167601.10_AXL	ENSG00000160789.18_LMNA	0.19457	0.036503367	0.343144712	1.63E-02	9.32E-02	1.00E+00
ENSG00000182512.4_GLRX5	ENSG00000160789.18_LMNA	-0.09344	-0.248937268	0.066754039	2.52E-01	5.33E-01	1.00E+00
ENSG00000226564.1_FTH1P20	ENSG00000160789.18_LMNA	-0.02733	-0.18571822	0.132451462	7.38E-01	8.90E-01	1.00E+00
ENSG00000175224.15_ATG13	ENSG00000160789.18_LMNA	-0.12797	-0.28143968	0.031877298	1.16E-01	3.41E-01	1.00E+00
ENSG00000167695.13_FAM57A	ENSG00000160789.18_LMNA	0.2871	0.134028461	0.42679624	3.35E-04	4.90E-03	1.00E+00
ENSG00000145354.8_CISD2	ENSG00000160789.18_LMNA	-0.1657	-0.316546844	-0.00667161	4.13E-02	1.77E-01	1.00E+00

ENSG00000147383.9_NSDHL	ENSG00000160789.18_LMNA	0.03769	-0.122247055	0.195713563	6.45E-01	8.42E-01	1.00E+00
ENSG00000166770.9_ZNF667-AS	ENSG00000160789.18_LMNA	-0.18294	-0.332458901	-0.02445229	2.41E-02	1.23E-01	1.00E+00
ENSG00000152952.10_PLOD2	ENSG00000160789.18_LMNA	0.34469	0.196262421	0.47768092	1.37E-05	3.78E-04	7.03E-01
ENSG00000178537.8_SLC25A20	ENSG00000160789.18_LMNA	0.02764	-0.132147006	0.186017394	7.35E-01	8.89E-01	1.00E+00
ENSG00000128563.12_PRKRIP1	ENSG00000160789.18_LMNA	0.1837	0.02523544	0.33315574	2.35E-02	1.21E-01	1.00E+00
ENSG00000109654.13_TRIM2	ENSG00000160789.18_LMNA	-0.33995	-0.473524679	-0.1910927	1.83E-05	4.79E-04	9.38E-01
ENSG00000139197.9_PEX5	ENSG00000160789.18_LMNA	-0.28808	-0.427663313	-0.13506993	3.20E-04	4.72E-03	1.00E+00
ENSG00000127838.12_PNKD	ENSG00000160789.18_LMNA	-0.20942	-0.356726097	-0.05194977	9.62E-03	6.38E-02	1.00E+00
ENSG00000239306.4_RBM14	ENSG00000160789.18_LMNA	0.09041	-0.069796183	0.246068105	2.68E-01	5.52E-01	1.00E+00
ENSG00000136161.11_RCBTB2	ENSG00000160789.18_LMNA	-0.45523	-0.572912946	-0.31916339	3.81E-09	4.77E-07	1.95E-04
ENSG00000185565.10_LSAMP	ENSG00000160789.18_LMNA	-0.40623	-0.53108653	-0.26411265	2.07E-07	1.31E-05	1.06E-02
ENSG00000153879.7_CEBPG	ENSG00000160789.18_LMNA	0.02104	-0.138623358	0.179640644	7.97E-01	9.18E-01	1.00E+00
ENSG00000204065.2_TCEAL5	ENSG00000160789.18_LMNA	-0.23595	-0.380846729	-0.07974694	3.43E-03	2.95E-02	1.00E+00
ENSG00000139641.11_ESYT1	ENSG00000160789.18_LMNA	0.37547	0.230018827	0.504513483	1.87E-06	7.63E-05	9.62E-02
ENSG00000159479.15_MED8	ENSG00000160789.18_LMNA	0.15742	-0.001827941	0.308878425	5.28E-02	2.08E-01	1.00E+00
ENSG00000100813.13_ACIN1	ENSG00000160789.18_LMNA	0.20535	0.047713947	0.353014488	1.11E-02	7.08E-02	1.00E+00
ENSG00000038274.15_MAT2B	ENSG00000160789.18_LMNA	-0.20702	-0.354535752	-0.04944874	1.05E-02	6.78E-02	1.00E+00
ENSG00000142686.7_C1orf216	ENSG00000160789.18_LMNA	-0.13679	-0.289678564	0.022913652	9.29E-02	2.98E-01	1.00E+00
ENSG00000127920.5_GNG11	ENSG00000160789.18_LMNA	0.10587	-0.054240054	0.260681703	1.94E-01	4.63E-01	1.00E+00
ENSG00000131828.12_PDHA1	ENSG00000160789.18_LMNA	-0.03253	-0.190740894	0.127332149	6.91E-01	8.67E-01	1.00E+00
ENSG00000213058.3_RP4-765C7	ENSG00000160789.18_LMNA	0.08739	-0.072821399	0.243209406	2.84E-01	5.70E-01	1.00E+00
ENSG00000089327.13_FXYD5	ENSG00000160789.18_LMNA	0.17279	0.01397861	0.323107106	3.33E-02	1.53E-01	1.00E+00
ENSG00000104936.16_DMPK	ENSG00000160789.18_LMNA	0.20051	0.042669775	0.348582	1.33E-02	8.02E-02	1.00E+00
ENSG00000149115.12_TNKS1BP	ENSG00000160789.18_LMNA	0.51271	0.384930395	0.62120682	1.45E-11	4.62E-09	7.44E-07
ENSG00000106025.7_TSPAN12	ENSG00000160789.18_LMNA	-0.18215	-0.331731647	-0.02363536	2.47E-02	1.25E-01	1.00E+00
ENSG00000021355.11_SERPINB1	ENSG00000160789.18_LMNA	0.05201	-0.108085914	0.209475982	5.25E-01	7.70E-01	1.00E+00
ENSG00000180228.11_PRKRA	ENSG00000160789.18_LMNA	-0.34988	-0.482224335	-0.20193168	9.94E-06	2.90E-04	5.10E-01
ENSG00000169221.12_TBC1D10I	ENSG00000160789.18_LMNA	0.15004	-0.00938185	0.302029105	6.50E-02	2.38E-01	1.00E+00
ENSG00000132669.11_RIN2	ENSG00000160789.18_LMNA	-0.09487	-0.250285828	0.065322145	2.45E-01	5.25E-01	1.00E+00
ENSG00000130227.15_XPO7	ENSG00000160789.18_LMNA	-0.00832	-0.167296637	0.151083299	9.19E-01	9.69E-01	1.00E+00
ENSG00000143398.18_PIP5K1A	ENSG00000160789.18_LMNA	0.18071	0.022144325	0.330403344	2.59E-02	1.30E-01	1.00E+00
ENSG00000106268.14_NUDT1	ENSG00000160789.18_LMNA	0.01276	-0.14673514	0.171615707	8.76E-01	9.52E-01	1.00E+00
ENSG00000165704.13_HPRT1	ENSG00000160789.18_LMNA	0.00979	-0.149643625	0.168728033	9.05E-01	9.63E-01	1.00E+00

ENSG00000154813.8_DPH3	ENSG00000160789.18_LMNA	-0.31876	-0.454873642	-0.16808501	6.27E-05	1.29E-03	1.00E+00
ENSG00000186480.11_INSIG1	ENSG00000160789.18_LMNA	0.22629	0.069600488	0.372089513	5.06E-03	3.96E-02	1.00E+00
ENSG00000185909.13_KLHDC8B	ENSG00000160789.18_LMNA	-0.07093	-0.227558108	0.089281935	3.85E-01	6.64E-01	1.00E+00
ENSG00000255248.5_MIR100HG	ENSG00000160789.18_LMNA	-0.17153	-0.321934929	-0.01267058	3.46E-02	1.57E-01	1.00E+00
ENSG00000066027.10_PPP2R5A	ENSG00000160789.18_LMNA	-0.11156	-0.266031721	0.048506491	1.71E-01	4.30E-01	1.00E+00
ENSG00000166619.11_BLCAP	ENSG00000160789.18_LMNA	-0.24953	-0.393118424	-0.09407117	1.93E-03	1.90E-02	1.00E+00
ENSG00000041357.14_PSMA4	ENSG00000160789.18_LMNA	-0.17358	-0.323835675	-0.01479215	3.25E-02	1.51E-01	1.00E+00
ENSG00000154174.7_TOMM70	ENSG00000160789.18_LMNA	-0.1795	-0.329293718	-0.02089981	2.69E-02	1.33E-01	1.00E+00
ENSG00000066044.12_ELAVL1	ENSG00000160789.18_LMNA	0.13396	-0.025787762	0.287041878	9.99E-02	3.11E-01	1.00E+00
ENSG00000147162.12_OGT	ENSG00000160789.18_LMNA	-0.13987	-0.292559112	0.019767804	8.57E-02	2.84E-01	1.00E+00
ENSG00000162384.12_C1orf123	ENSG00000160789.18_LMNA	-0.15553	-0.307125218	0.003764884	5.57E-02	2.15E-01	1.00E+00
ENSG00000154582.15_TCEB1	ENSG00000160789.18_LMNA	-0.14896	-0.301023925	0.010487451	6.70E-02	2.42E-01	1.00E+00
ENSG00000017260.18_ATP2C1	ENSG00000160789.18_LMNA	-0.0074	-0.166407911	0.151976484	9.28E-01	9.72E-01	1.00E+00
ENSG00000164040.15_PGRMC2	ENSG00000160789.18_LMNA	0.14618	-0.013331187	0.298435297	7.23E-02	2.55E-01	1.00E+00
ENSG00000160360.10_GPSM1	ENSG00000160789.18_LMNA	-0.07485	-0.231296224	0.085366212	3.59E-01	6.42E-01	1.00E+00
ENSG00000155827.10_RNF20	ENSG00000160789.18_LMNA	0.06829	-0.09190606	0.225047769	4.03E-01	6.79E-01	1.00E+00
ENSG00000183160.8_TMEM119	ENSG00000160789.18_LMNA	-0.115	-0.269274539	0.045021075	1.58E-01	4.11E-01	1.00E+00
ENSG00000186283.12_TOR3A	ENSG00000160789.18_LMNA	0.14893	-0.01052192	0.300992575	6.71E-02	2.43E-01	1.00E+00
ENSG00000076924.10_XAB2	ENSG00000160789.18_LMNA	0.10839	-0.051700474	0.263053787	1.84E-01	4.49E-01	1.00E+00
ENSG00000151640.11_DPYSL4	ENSG00000160789.18_LMNA	-0.20577	-0.353392792	-0.04814518	1.10E-02	7.00E-02	1.00E+00
ENSG00000196449.3_YRDC	ENSG00000160789.18_LMNA	-0.05774	-0.214960783	0.102406063	4.80E-01	7.38E-01	1.00E+00
ENSG00000145817.15_YIPF5	ENSG00000160789.18_LMNA	-0.04981	-0.207362626	0.110268905	5.42E-01	7.81E-01	1.00E+00
ENSG00000104522.14_TSTA3	ENSG00000160789.18_LMNA	0.12202	-0.037918893	0.275860398	1.34E-01	3.72E-01	1.00E+00
ENSG00000204371.10_EHMT2	ENSG00000160789.18_LMNA	0.08278	-0.077440704	0.238833745	3.11E-01	5.97E-01	1.00E+00
ENSG00000011485.13_PPP5C	ENSG00000160789.18_LMNA	0.0507	-0.109378739	0.208224755	5.35E-01	7.76E-01	1.00E+00
ENSG00000176894.8_PXMP2	ENSG00000160789.18_LMNA	-0.15883	-0.310187903	0.000379709	5.06E-02	2.03E-01	1.00E+00
ENSG00000155760.2_FZD7	ENSG00000160789.18_LMNA	0.5541	0.433103362	0.655477848	1.31E-13	1.08E-10	6.72E-09
ENSG00000095637.19_SORBS1	ENSG00000160789.18_LMNA	0.07029	-0.089917747	0.226950252	3.90E-01	6.68E-01	1.00E+00
ENSG00000229391.6_HLA-DRB6	ENSG00000160789.18_LMNA	-0.07647	-0.232838093	0.08374823	3.49E-01	6.33E-01	1.00E+00
ENSG00000142208.14_AKT1	ENSG00000160789.18_LMNA	0.38203	0.237258834	0.51020024	1.19E-06	5.30E-05	6.13E-02
ENSG00000104825.15_NFKBIB	ENSG00000160789.18_LMNA	0.18812	0.029814714	0.337223602	2.03E-02	1.09E-01	1.00E+00
ENSG00000006210.6_CX3CL1	ENSG00000160789.18_LMNA	0.16291	0.003803119	0.31396337	4.49E-02	1.88E-01	1.00E+00
ENSG00000149212.9_SESN3	ENSG00000160789.18_LMNA	-0.20785	-0.355294332	-0.05031449	1.02E-02	6.64E-02	1.00E+00

ENSG00000142039.3_CCDC97	ENSG00000160789.18_LMNA	0.12401	-0.035903221	0.277724181	1.28E-01	3.62E-01	1.00E+00
ENSG00000132535.17_DLG4	ENSG00000160789.18_LMNA	-0.024	-0.182505676	0.135716939	7.69E-01	9.04E-01	1.00E+00
ENSG00000255717.5_SNHG1	ENSG00000160789.18_LMNA	-0.28652	-0.426274147	-0.13340167	3.45E-04	5.01E-03	1.00E+00
ENSG00000113643.7_RARS	ENSG00000160789.18_LMNA	0.28592	0.132760907	0.425740215	3.56E-04	5.14E-03	1.00E+00
ENSG00000068120.13_COASY	ENSG00000160789.18_LMNA	0.26472	0.110165812	0.406780913	9.81E-04	1.14E-02	1.00E+00
ENSG00000114923.15_SLC4A3	ENSG00000160789.18_LMNA	0.4424	0.304659527	0.562019969	1.15E-08	1.22E-06	5.92E-04
ENSG00000198818.8_SFT2D1	ENSG00000160789.18_LMNA	-0.11245	-0.266874263	0.047601654	1.68E-01	4.26E-01	1.00E+00
ENSG00000162231.12_NXF1	ENSG00000160789.18_LMNA	-0.02228	-0.180837137	0.137410247	7.85E-01	9.12E-01	1.00E+00
ENSG00000169251.11_NMD3	ENSG00000160789.18_LMNA	-0.03054	-0.188823448	0.129288489	7.09E-01	8.76E-01	1.00E+00
ENSG00000132274.14_TRIM22	ENSG00000160789.18_LMNA	0.0871	-0.073112037	0.242934477	2.86E-01	5.71E-01	1.00E+00
ENSG00000138175.8_ARL3	ENSG00000160789.18_LMNA	-0.43664	-0.557114785	-0.29816708	1.87E-08	1.82E-06	9.59E-04
ENSG00000015171.17_ZMYND11	ENSG00000160789.18_LMNA	-0.247	-0.390833689	-0.09139488	2.16E-03	2.07E-02	1.00E+00
ENSG00000223802.6_CERS1	ENSG00000160789.18_LMNA	-0.0635	-0.22047102	0.096678958	4.37E-01	7.05E-01	1.00E+00
ENSG00000236104.2_ZBTB22	ENSG00000160789.18_LMNA	-0.00314	-0.162260075	0.156138268	9.69E-01	9.88E-01	1.00E+00
ENSG00000144040.11_SFXN5	ENSG00000160789.18_LMNA	0.19866	0.040752862	0.346893946	1.41E-02	8.42E-02	1.00E+00
ENSG00000243646.7_IL10RB	ENSG00000160789.18_LMNA	0.30189	0.1498985	0.439950654	1.57E-04	2.70E-03	1.00E+00
ENSG00000188938.14_FAM120A(	ENSG00000160789.18_LMNA	0.05123	-0.108859233	0.20872767	5.31E-01	7.74E-01	1.00E+00
ENSG00000071894.13_CPSF1	ENSG00000160789.18_LMNA	0.20868	0.051181554	0.356053666	9.88E-03	6.50E-02	1.00E+00
ENSG00000169750.7_RAC3	ENSG00000160789.18_LMNA	-0.14209	-0.294627063	0.017505574	8.08E-02	2.74E-01	1.00E+00
ENSG00000162783.10_IER5	ENSG00000160789.18_LMNA	0.47242	0.338694341	0.587441189	8.00E-10	1.30E-07	4.11E-05
ENSG00000186868.14_MAPT	ENSG00000160789.18_LMNA	-0.30137	-0.439487811	-0.14933756	1.61E-04	2.76E-03	1.00E+00
ENSG00000103549.20_RNF40	ENSG00000160789.18_LMNA	0.19671	0.038722661	0.345103965	1.51E-02	8.84E-02	1.00E+00
ENSG00000116514.15_RNF19B	ENSG00000160789.18_LMNA	0.31401	0.162955007	0.450680448	8.16E-05	1.59E-03	1.00E+00
ENSG00000164751.13_PEX2	ENSG00000160789.18_LMNA	-0.2683	-0.409990644	-0.11396963	8.32E-04	1.00E-02	1.00E+00
ENSG00000148218.14_ALAD	ENSG00000160789.18_LMNA	0.00531	-0.154016103	0.164376534	9.48E-01	9.79E-01	1.00E+00
ENSG00000088280.17_ASAP3	ENSG00000160789.18_LMNA	0.24223	0.086356176	0.386522131	2.64E-03	2.42E-02	1.00E+00
ENSG00000144827.7_ABHD10	ENSG00000160789.18_LMNA	-0.5391	-0.64310847	-0.41556862	7.77E-13	4.74E-10	3.98E-08
ENSG00000134056.10_MRPS36	ENSG00000160789.18_LMNA	-0.22762	-0.37329661	-0.07099537	4.80E-03	3.80E-02	1.00E+00
ENSG00000006282.18_SPATA20	ENSG00000160789.18_LMNA	0.31333	0.162216721	0.450075936	8.48E-05	1.65E-03	1.00E+00
ENSG00000136877.13_FPGS	ENSG00000160789.18_LMNA	0.25016	0.094727941	0.393678544	1.88E-03	1.87E-02	1.00E+00
ENSG00000140983.12_RHOT2	ENSG00000160789.18_LMNA	0.12143	-0.038511253	0.275312226	1.36E-01	3.76E-01	1.00E+00
ENSG00000171310.9_CHST11	ENSG00000160789.18_LMNA	0.08982	-0.070389375	0.245507997	2.71E-01	5.55E-01	1.00E+00
ENSG00000074410.12_CA12	ENSG00000160789.18_LMNA	0.39654	0.253326861	0.522736394	4.25E-07	2.31E-05	2.18E-02

ENSG00000175854.10_SWI5	ENSG00000160789.18_LMNA	0.1716	0.012746148	0.322002675	3.45E-02	1.57E-01	1.00E+00
ENSG00000102003.9_SYP	ENSG00000160789.18_LMNA	-0.31024	-0.447345259	-0.15888577	1.00E-04	1.89E-03	1.00E+00
ENSG00000089057.13_SLC23A2	ENSG00000160789.18_LMNA	-0.062	-0.219036736	0.098171716	4.48E-01	7.14E-01	1.00E+00
ENSG00000137404.13_NRM	ENSG00000160789.18_LMNA	0.35358	0.205976576	0.485456769	7.88E-06	2.42E-04	4.04E-01
ENSG00000113712.15_CSNK1A1	ENSG00000160789.18_LMNA	0.14035	-0.019281288	0.293004093	8.46E-02	2.82E-01	1.00E+00
ENSG00000136270.12_TBRG4	ENSG00000160789.18_LMNA	0.26255	0.107862137	0.404833463	1.08E-03	1.23E-02	1.00E+00
ENSG00000138459.7_SLC35A5	ENSG00000160789.18_LMNA	-0.36805	-0.498071005	-0.22185323	3.09E-06	1.12E-04	1.58E-01
ENSG00000101350.7_KIF3B	ENSG00000160789.18_LMNA	0.1313	-0.028499519	0.28454978	1.07E-01	3.25E-01	1.00E+00
ENSG00000105397.12_TYK2	ENSG00000160789.18_LMNA	0.22065	0.063689391	0.366962809	6.30E-03	4.66E-02	1.00E+00
ENSG00000113407.12_TARS	ENSG00000160789.18_LMNA	0.08946	-0.07075296	0.245164586	2.73E-01	5.58E-01	1.00E+00
ENSG00000171566.10_PLRG1	ENSG00000160789.18_LMNA	-0.07129	-0.227906374	0.088917535	3.83E-01	6.61E-01	1.00E+00
ENSG00000137414.5_FAM8A1	ENSG00000160789.18_LMNA	-0.20083	-0.348878543	-0.04300676	1.31E-02	7.96E-02	1.00E+00
ENSG00000065911.10_MTHFD2	ENSG00000160789.18_LMNA	-0.35545	-0.487087218	-0.20802046	7.00E-06	2.20E-04	3.59E-01
ENSG00000134748.12_PRPF38A	ENSG00000160789.18_LMNA	-0.0934	-0.248904186	0.06678915	2.52E-01	5.33E-01	1.00E+00
ENSG00000139343.9_SNRPF	ENSG00000160789.18_LMNA	-0.13566	-0.288628173	0.024059246	9.56E-02	3.03E-01	1.00E+00
ENSG00000123992.17_DNPEP	ENSG00000160789.18_LMNA	0.3771	0.231820916	0.505931187	1.68E-06	6.97E-05	8.60E-02
ENSG00000104946.9_TBC1D17	ENSG00000160789.18_LMNA	0.02311	-0.136595243	0.181640448	7.77E-01	9.09E-01	1.00E+00
ENSG00000184162.13_NR2C2AP	ENSG00000160789.18_LMNA	0.02786	-0.131929956	0.186230643	7.33E-01	8.88E-01	1.00E+00
ENSG00000134668.11_SPOCD1	ENSG00000160789.18_LMNA	0.37916	0.234091941	0.507715696	1.46E-06	6.28E-05	7.47E-02
ENSG00000152240.11_HAUS1	ENSG00000160789.18_LMNA	-0.1059	-0.260703815	0.054216398	1.94E-01	4.63E-01	1.00E+00
ENSG00000101940.16_WDR13	ENSG00000160789.18_LMNA	0.1413	-0.0183126	0.29388968	8.25E-02	2.77E-01	1.00E+00
ENSG00000075790.9_BCAP29	ENSG00000160789.18_LMNA	0.12979	-0.030026885	0.283144269	1.11E-01	3.32E-01	1.00E+00
ENSG00000172466.14_ZNF24	ENSG00000160789.18_LMNA	-0.19706	-0.345421563	-0.0390827	1.50E-02	8.77E-02	1.00E+00
ENSG00000134569.8_LRP4	ENSG00000160789.18_LMNA	-0.13858	-0.291352723	0.021086055	8.86E-02	2.90E-01	1.00E+00
ENSG00000196843.14_ARID5A	ENSG00000160789.18_LMNA	0.31391	0.162846515	0.45059163	8.21E-05	1.60E-03	1.00E+00
ENSG00000198231.11_DDX42	ENSG00000160789.18_LMNA	0.17053	0.011643871	0.321014191	3.57E-02	1.61E-01	1.00E+00
ENSG00000198700.8_IPO9	ENSG00000160789.18_LMNA	0.1249	-0.034994986	0.278563206	1.25E-01	3.58E-01	1.00E+00
ENSG00000083720.11_OXCT1	ENSG00000160789.18_LMNA	-0.14547	-0.297775681	0.014055003	7.37E-02	2.58E-01	1.00E+00
ENSG00000135108.13_FBXO21	ENSG00000160789.18_LMNA	-0.22999	-0.375448022	-0.0734844	4.37E-03	3.54E-02	1.00E+00
ENSG00000182827.8_ACBD3	ENSG00000160789.18_LMNA	0.21848	0.0614125	0.364983131	6.85E-03	4.96E-02	1.00E+00
ENSG00000008324.9_SS18L2	ENSG00000160789.18_LMNA	-0.38003	-0.508464497	-0.23504578	1.37E-06	5.97E-05	7.04E-02
ENSG00000130723.16_PRRC2B	ENSG00000160789.18_LMNA	0.12213	-0.037803702	0.275966973	1.34E-01	3.71E-01	1.00E+00
ENSG00000136167.12_LCP1	ENSG00000160789.18_LMNA	-0.00967	-0.168610786	0.149761602	9.06E-01	9.64E-01	1.00E+00

ENSG00000110079.15_MS4A4A	ENSG00000160789.18_LMNA	0.04598	-0.114052417	0.203692769	5.74E-01	8.01E-01	1.00E+00
ENSG00000069329.14_VPS35	ENSG00000160789.18_LMNA	-0.09027	-0.245931744	0.06994062	2.69E-01	5.53E-01	1.00E+00
ENSG00000077348.7_EXOSC5	ENSG00000160789.18_LMNA	-0.16405	-0.315019808	-0.00497549	4.34E-02	1.83E-01	1.00E+00
ENSG00000105325.12_FZR1	ENSG00000160789.18_LMNA	0.17418	0.015407724	0.324386708	3.19E-02	1.49E-01	1.00E+00
ENSG00000218739.8_CEBPZOS	ENSG00000160789.18_LMNA	-0.23035	-0.375767169	-0.07385396	4.30E-03	3.50E-02	1.00E+00
ENSG00000163156.10_SCNM1	ENSG00000160789.18_LMNA	-0.11892	-0.272955396	0.041055556	1.44E-01	3.89E-01	1.00E+00
ENSG00000119950.19_MXI1	ENSG00000160789.18_LMNA	-0.42525	-0.547388831	-0.28536438	4.73E-08	3.91E-06	2.43E-03
ENSG00000136279.17_DBNL	ENSG00000160789.18_LMNA	0.20606	0.048449731	0.353659904	1.09E-02	6.95E-02	1.00E+00
ENSG00000169093.13_ASMTL	ENSG00000160789.18_LMNA	0.24811	0.09256824	0.391835834	2.06E-03	1.99E-02	1.00E+00
ENSG00000159792.8_PSKH1	ENSG00000160789.18_LMNA	0.12244	-0.037490099	0.276257079	1.33E-01	3.70E-01	1.00E+00
ENSG00000154832.13_CXXC1	ENSG00000160789.18_LMNA	0.00202	-0.157232424	0.161167715	9.80E-01	9.93E-01	1.00E+00
ENSG00000054523.15_KIF1B	ENSG00000160789.18_LMNA	-0.10107	-0.256146464	0.059084216	2.15E-01	4.89E-01	1.00E+00
ENSG00000116001.14_TIA1	ENSG00000160789.18_LMNA	-0.31359	-0.450311322	-0.16250416	8.35E-05	1.63E-03	1.00E+00
ENSG00000166887.14_VPS39	ENSG00000160789.18_LMNA	-0.02931	-0.187634918	0.130499887	7.20E-01	8.81E-01	1.00E+00
ENSG00000127585.10_FBXL16	ENSG00000160789.18_LMNA	-0.25208	-0.395416031	-0.0967669	1.73E-03	1.75E-02	1.00E+00
ENSG00000131378.12_RFTN1	ENSG00000160789.18_LMNA	0.20934	0.051868287	0.356654788	9.64E-03	6.39E-02	1.00E+00
ENSG00000182985.15_CADM1	ENSG00000160789.18_LMNA	0.00909	-0.150327605	0.168048153	9.11E-01	9.66E-01	1.00E+00
ENSG00000127125.8_PPCS	ENSG00000160789.18_LMNA	0.09949	-0.060675331	0.254653784	2.23E-01	4.98E-01	1.00E+00
ENSG00000032444.14_PNPLA6	ENSG00000160789.18_LMNA	0.24206	0.086176699	0.386368312	2.66E-03	2.43E-02	1.00E+00
ENSG00000129657.13_SEC14L1	ENSG00000160789.18_LMNA	0.18571	0.027320744	0.335009581	2.20E-02	1.15E-01	1.00E+00
ENSG00000176022.4_B3GALT6	ENSG00000160789.18_LMNA	0.39849	0.255499305	0.524422424	3.68E-07	2.07E-05	1.89E-02
ENSG00000163873.8_GRIK3	ENSG00000160789.18_LMNA	-0.10833	-0.262994239	0.051764276	1.84E-01	4.49E-01	1.00E+00
ENSG00000161281.9_COX7A1	ENSG00000160789.18_LMNA	-0.03221	-0.190438223	0.127641125	6.94E-01	8.68E-01	1.00E+00
ENSG00000247077.5_PGAM5	ENSG00000160789.18_LMNA	-0.13635	-0.289269914	0.023359438	9.39E-02	3.00E-01	1.00E+00
ENSG00000186111.7_PIP5K1C	ENSG00000160789.18_LMNA	0.18893	0.03065522	0.337968996	1.97E-02	1.07E-01	1.00E+00
ENSG00000055044.9_NOP58	ENSG00000160789.18_LMNA	0.08464	-0.075578374	0.240599394	3.00E-01	5.86E-01	1.00E+00
ENSG00000083312.16_TNPO1	ENSG00000160789.18_LMNA	0.09997	-0.060184027	0.255114854	2.20E-01	4.96E-01	1.00E+00
ENSG00000172936.11_MYD88	ENSG00000160789.18_LMNA	0.29402	0.141444147	0.432958438	2.36E-04	3.73E-03	1.00E+00
ENSG00000100647.7_SUSD6	ENSG00000160789.18_LMNA	0.20674	0.049157472	0.354280446	1.06E-02	6.83E-02	1.00E+00
ENSG00000184702.16_SEPT5	ENSG00000160789.18_LMNA	0.13331	-0.026457099	0.286427152	1.02E-01	3.15E-01	1.00E+00
ENSG00000143126.7_CELSR2	ENSG00000160789.18_LMNA	0.13893	-0.020724682	0.29168353	8.78E-02	2.88E-01	1.00E+00
ENSG00000156973.12_PDE6D	ENSG00000160789.18_LMNA	-0.38863	-0.515909719	-0.2445584	7.51E-07	3.64E-05	3.85E-02
ENSG00000168137.14_SETD5	ENSG00000160789.18_LMNA	-0.07493	-0.231374069	0.085284565	3.59E-01	6.42E-01	1.00E+00

ENSG00000185090.13_MANEAL	ENSG00000160789.18_LMNA	-0.14161	-0.294181315	0.017993469	8.18E-02	2.76E-01	1.00E+00
ENSG00000170270.4_GON7	ENSG00000160789.18_LMNA	0.01143	-0.148037213	0.170323627	8.89E-01	9.57E-01	1.00E+00
ENSG00000158435.6_CNOT11	ENSG00000160789.18_LMNA	-0.05835	-0.215552628	0.101791931	4.75E-01	7.34E-01	1.00E+00
ENSG00000219438.7_FAM19A5	ENSG00000160789.18_LMNA	-0.40658	-0.531387696	-0.26450291	2.02E-07	1.28E-05	1.04E-02
ENSG00000196476.10_C20orf96	ENSG00000160789.18_LMNA	-0.00517	-0.164239191	0.154153906	9.50E-01	9.80E-01	1.00E+00
ENSG00000146282.16_RARS2	ENSG00000160789.18_LMNA	-0.10412	-0.259024669	0.056011679	2.02E-01	4.72E-01	1.00E+00
ENSG00000205629.10_LCMT1	ENSG00000160789.18_LMNA	-0.2555	-0.398489568	-0.10037988	1.49E-03	1.56E-02	1.00E+00
ENSG00000072071.15_ADGRL1	ENSG00000160789.18_LMNA	-0.00738	-0.166387452	0.151997039	9.28E-01	9.72E-01	1.00E+00
ENSG00000107021.14_TBC1D13	ENSG00000160789.18_LMNA	0.13935	-0.020302937	0.292069508	8.69E-02	2.86E-01	1.00E+00
ENSG00000141759.13_TXNL4A	ENSG00000160789.18_LMNA	-0.12227	-0.276093423	0.037667018	1.33E-01	3.71E-01	1.00E+00
ENSG00000112651.10_MRPL2	ENSG00000160789.18_LMNA	0.04215	-0.117842137	0.200007918	6.06E-01	8.20E-01	1.00E+00
ENSG00000060688.11_SNRNP40	ENSG00000160789.18_LMNA	-0.05298	-0.210404763	0.10712556	5.17E-01	7.64E-01	1.00E+00
ENSG00000196781.12_TLE1	ENSG00000160789.18_LMNA	0.24912	0.093637404	0.392748371	1.97E-03	1.93E-02	1.00E+00
ENSG00000152154.9_TMEM178A	ENSG00000160789.18_LMNA	-0.23582	-0.380725375	-0.07960591	3.45E-03	2.97E-02	1.00E+00
ENSG00000262919.6_FAM58A	ENSG00000160789.18_LMNA	-0.13465	-0.287680712	0.025091878	9.82E-02	3.08E-01	1.00E+00
ENSG00000164056.9_SPRY1	ENSG00000160789.18_LMNA	0.41408	0.272869965	0.537828337	1.14E-07	8.05E-06	5.84E-03
ENSG00000166889.13_PATL1	ENSG00000160789.18_LMNA	0.24022	0.084236793	0.384704672	2.87E-03	2.59E-02	1.00E+00
ENSG00000197744.5_PTMAP2	ENSG00000160789.18_LMNA	0.07455	-0.085671663	0.231004963	3.61E-01	6.43E-01	1.00E+00
ENSG00000102393.8_GLA	ENSG00000160789.18_LMNA	-0.03822	-0.196226871	0.12172119	6.40E-01	8.39E-01	1.00E+00
ENSG00000089351.13_GRAMD1A	ENSG00000160789.18_LMNA	0.06546	-0.094730763	0.22234084	4.23E-01	6.94E-01	1.00E+00
ENSG00000077942.16_FBLN1	ENSG00000160789.18_LMNA	0.28551	0.132320451	0.425373075	3.63E-04	5.22E-03	1.00E+00
ENSG00000104660.16_LEPROTL	ENSG00000160789.18_LMNA	-0.34391	-0.476997571	-0.19541136	1.44E-05	3.95E-04	7.37E-01
ENSG00000235552.4_RPL6P27	ENSG00000160789.18_LMNA	-0.12549	-0.279112759	0.03439982	1.23E-01	3.54E-01	1.00E+00
ENSG00000145721.10_LIX1	ENSG00000160789.18_LMNA	-0.37563	-0.504649092	-0.23019112	1.86E-06	7.58E-05	9.52E-02
ENSG00000143537.12_ADAM15	ENSG00000160789.18_LMNA	0.43243	0.293435049	0.553528213	2.64E-08	2.45E-06	1.36E-03
ENSG00000182871.13_COL18A1	ENSG00000160789.18_LMNA	0.45982	0.324367762	0.576799903	2.53E-09	3.38E-07	1.30E-04
ENSG00000164346.8_NSA2	ENSG00000160789.18_LMNA	-0.10374	-0.258668137	0.056392608	2.03E-01	4.74E-01	1.00E+00
ENSG00000116132.10_PRRX1	ENSG00000160789.18_LMNA	0.29651	0.144110016	0.435167052	2.08E-04	3.36E-03	1.00E+00
ENSG00000011275.17_RNF216	ENSG00000160789.18_LMNA	0.26025	0.105417709	0.402764075	1.20E-03	1.33E-02	1.00E+00
ENSG00000204498.9_NFKBIL1	ENSG00000160789.18_LMNA	0.12319	-0.036731185	0.276958893	1.31E-01	3.66E-01	1.00E+00
ENSG00000081721.10_DUSP12	ENSG00000160789.18_LMNA	-0.02395	-0.182455484	0.135767904	7.70E-01	9.05E-01	1.00E+00
ENSG00000167130.16_DOLPP1	ENSG00000160789.18_LMNA	-0.08299	-0.239033601	0.077230016	3.09E-01	5.96E-01	1.00E+00
ENSG00000106483.10_SFRP4	ENSG00000160789.18_LMNA	0.20134	0.043538576	0.349346422	1.29E-02	7.86E-02	1.00E+00

ENSG00000130024.13_PHF10	ENSG00000160789.18_LMNA	-0.04772	-0.205357719	0.112337045	5.59E-01	7.92E-01	1.00E+00
ENSG00000224051.5_CPTP	ENSG00000160789.18_LMNA	0.28124	0.127762998	0.421568565	4.48E-04	6.19E-03	1.00E+00
ENSG00000131459.11_GFPT2	ENSG00000160789.18_LMNA	0.18674	0.028384476	0.335954324	2.12E-02	1.13E-01	1.00E+00
ENSG00000167315.16_ACAA2	ENSG00000160789.18_LMNA	0.02413	-0.135591781	0.18262893	7.68E-01	9.04E-01	1.00E+00
ENSG00000186310.9_NAP1L3	ENSG00000160789.18_LMNA	-0.39888	-0.524757442	-0.2559313	3.58E-07	2.03E-05	1.84E-02
ENSG00000005243.8_COPZ2	ENSG00000160789.18_LMNA	0.1305	-0.029308001	0.283805967	1.09E-01	3.29E-01	1.00E+00
ENSG00000125458.5_NT5C	ENSG00000160789.18_LMNA	0.05181	-0.108283531	0.209284791	5.26E-01	7.71E-01	1.00E+00
ENSG00000162188.5_GNG3	ENSG00000160789.18_LMNA	-0.17857	-0.328434374	-0.01993666	2.77E-02	1.36E-01	1.00E+00
ENSG00000135457.8_TFCP2	ENSG00000160789.18_LMNA	-0.11714	-0.271281433	0.042860203	1.51E-01	3.99E-01	1.00E+00
ENSG00000163166.12_IWS1	ENSG00000160789.18_LMNA	-0.0084	-0.167375349	0.151004167	9.18E-01	9.69E-01	1.00E+00
ENSG00000157870.13_FAM213B	ENSG00000160789.18_LMNA	0.2099	0.052448918	0.357162838	9.45E-03	6.29E-02	1.00E+00
ENSG00000006715.14_VPS41	ENSG00000160789.18_LMNA	-0.17082	-0.321283223	-0.0119438	3.54E-02	1.60E-01	1.00E+00
ENSG00000187091.12_PLCD1	ENSG00000160789.18_LMNA	-0.03737	-0.195409113	0.122558869	6.48E-01	8.43E-01	1.00E+00
ENSG00000160741.15_CRTC2	ENSG00000160789.18_LMNA	0.21775	0.060652298	0.36432155	7.04E-03	5.07E-02	1.00E+00
ENSG00000115902.9_SLC1A4	ENSG00000160789.18_LMNA	-0.22629	-0.372082672	-0.06959259	5.06E-03	3.96E-02	1.00E+00
ENSG00000013810.17_TACC3	ENSG00000160789.18_LMNA	0.15663	-0.00263572	0.308147527	5.40E-02	2.11E-01	1.00E+00
ENSG00000250182.3_EEF1A1P13	ENSG00000160789.18_LMNA	-0.1567	-0.308216219	0.00255982	5.39E-02	2.11E-01	1.00E+00
ENSG00000177951.16_BET1L	ENSG00000160789.18_LMNA	0.46305	0.328028322	0.579527013	1.89E-09	2.64E-07	9.71E-05
ENSG00000188895.10_MSL1	ENSG00000160789.18_LMNA	-0.08914	-0.244868848	0.071066004	2.75E-01	5.59E-01	1.00E+00
ENSG00000100418.7_DESI1	ENSG00000160789.18_LMNA	-0.2696	-0.411156014	-0.11535285	7.83E-04	9.56E-03	1.00E+00
ENSG00000128607.12_KLHDC10	ENSG00000160789.18_LMNA	0.20835	0.050836801	0.355751796	1.00E-02	6.56E-02	1.00E+00
ENSG00000064393.14_HIPK2	ENSG00000160789.18_LMNA	-0.1599	-0.311180518	-0.00071895	4.91E-02	1.99E-01	1.00E+00
ENSG00000029725.15_RABEP1	ENSG00000160789.18_LMNA	-0.21871	-0.365194804	-0.0616558	6.79E-03	4.92E-02	1.00E+00
ENSG00000241343.8_RPL36A	ENSG00000160789.18_LMNA	-0.08633	-0.242197212	0.073891161	2.90E-01	5.76E-01	1.00E+00
ENSG00000108671.8_PSMD11	ENSG00000160789.18_LMNA	0.03668	-0.123242466	0.194741446	6.54E-01	8.47E-01	1.00E+00
ENSG00000223891.4_OSER1-AS	ENSG00000160789.18_LMNA	-0.38099	-0.509298888	-0.23610927	1.28E-06	5.64E-05	6.59E-02
ENSG00000162745.9_OLFML2B	ENSG00000160789.18_LMNA	0.21136	0.053979482	0.358501212	8.95E-03	6.05E-02	1.00E+00
ENSG00000135632.10_SMYD5	ENSG00000160789.18_LMNA	-0.10331	-0.258262566	0.056825818	2.05E-01	4.77E-01	1.00E+00
ENSG00000136854.16_STXBP1	ENSG00000160789.18_LMNA	-0.16905	-0.319647476	-0.01012106	3.73E-02	1.65E-01	1.00E+00
ENSG00000112312.8_GMNN	ENSG00000160789.18_LMNA	-0.18225	-0.331826224	-0.02374157	2.46E-02	1.25E-01	1.00E+00
ENSG00000100422.12_CERK	ENSG00000160789.18_LMNA	-0.15478	-0.306426996	0.004535629	5.69E-02	2.18E-01	1.00E+00
ENSG00000075426.10_FOSL2	ENSG00000160789.18_LMNA	0.50731	0.378695268	0.616703991	2.56E-11	7.29E-09	1.31E-06
ENSG00000104957.12_CCDC130	ENSG00000160789.18_LMNA	0.09195	-0.068252146	0.247525038	2.60E-01	5.43E-01	1.00E+00

ENSG00000025708.11_TYMP	ENSG00000160789.18_LMNA	0.21418	0.056919215	0.361068304	8.06E-03	5.59E-02	1.00E+00
ENSG00000091409.13_ITGA6	ENSG00000160789.18_LMNA	0.00375	-0.15554609	0.162850955	9.63E-01	9.85E-01	1.00E+00
ENSG00000135766.8_EGLN1	ENSG00000160789.18_LMNA	-0.07363	-0.230129676	0.086589236	3.67E-01	6.48E-01	1.00E+00
ENSG00000128699.12_ORMDL1	ENSG00000160789.18_LMNA	-0.23524	-0.380198565	-0.07899381	3.53E-03	3.02E-02	1.00E+00
ENSG00000071246.9_VASH1	ENSG00000160789.18_LMNA	0.05466	-0.105462466	0.212011825	5.04E-01	7.55E-01	1.00E+00
ENSG00000116455.12_WDR77	ENSG00000160789.18_LMNA	-0.17771	-0.32763936	-0.01904612	2.85E-02	1.38E-01	1.00E+00
ENSG00000111911.6_HINT3	ENSG00000160789.18_LMNA	-0.36711	-0.497250816	-0.22081645	3.28E-06	1.18E-04	1.69E-01
ENSG00000198417.6_MT1F	ENSG00000160789.18_LMNA	0.11763	-0.042369084	0.271737176	1.49E-01	3.96E-01	1.00E+00
ENSG00000102531.15_FNDC3A	ENSG00000160789.18_LMNA	0.04838	-0.111684233	0.20599086	5.54E-01	7.88E-01	1.00E+00
ENSG00000121390.16_PSPC1	ENSG00000160789.18_LMNA	-0.23352	-0.378645363	-0.07719047	3.79E-03	3.18E-02	1.00E+00
ENSG00000133895.13_MEN1	ENSG00000160789.18_LMNA	0.18662	0.028261871	0.335845465	2.13E-02	1.13E-01	1.00E+00
ENSG00000078967.11_UBE2D4	ENSG00000160789.18_LMNA	-0.22106	-0.36732917	-0.0641111	6.20E-03	4.61E-02	1.00E+00
ENSG00000169446.5_MMGT1	ENSG00000160789.18_LMNA	-0.17546	-0.325566305	-0.01672627	3.06E-02	1.45E-01	1.00E+00
ENSG00000123689.5_G0S2	ENSG00000160789.18_LMNA	0.1905	0.032277793	0.339406868	1.87E-02	1.03E-01	1.00E+00
ENSG00000108175.15_ZMIZ1	ENSG00000160789.18_LMNA	-0.00035	-0.159544332	0.158857034	9.97E-01	9.99E-01	1.00E+00
ENSG00000029993.13_HMGB3	ENSG00000160789.18_LMNA	-0.04187	-0.199736503	0.118120911	6.09E-01	8.21E-01	1.00E+00
ENSG00000167699.12_GLOD4	ENSG00000160789.18_LMNA	-0.15529	-0.306902329	0.004010964	5.61E-02	2.16E-01	1.00E+00
ENSG00000164172.17_MOCS2	ENSG00000160789.18_LMNA	-0.28263	-0.422810188	-0.12924899	4.19E-04	5.87E-03	1.00E+00
ENSG00000143344.14_RGL1	ENSG00000160789.18_LMNA	0.0932	-0.066998008	0.248707376	2.53E-01	5.35E-01	1.00E+00
ENSG00000124688.12_MAD2L1B	ENSG00000160789.18_LMNA	-0.14808	-0.300200647	0.011392409	6.87E-02	2.46E-01	1.00E+00
ENSG00000146085.7_MUT	ENSG00000160789.18_LMNA	-0.21508	-0.361892562	-0.05786424	7.79E-03	5.45E-02	1.00E+00
ENSG00000170091.9_HMP19	ENSG00000160789.18_LMNA	-0.40897	-0.533440172	-0.26716493	1.68E-07	1.10E-05	8.64E-03
ENSG00000141699.9_FAM134C	ENSG00000160789.18_LMNA	-0.00139	-0.160554096	0.157846709	9.86E-01	9.95E-01	1.00E+00
ENSG00000113194.11_FAF2	ENSG00000160789.18_LMNA	0.21756	0.060449699	0.364145182	7.09E-03	5.09E-02	1.00E+00
ENSG00000153406.12_NMRAL1	ENSG00000160789.18_LMNA	0.232	0.075594421	0.377269282	4.03E-03	3.33E-02	1.00E+00
ENSG00000156136.8_DCK	ENSG00000160789.18_LMNA	-0.28918	-0.428644793	-0.1362496	3.02E-04	4.53E-03	1.00E+00
ENSG00000125285.5_SOX21	ENSG00000160789.18_LMNA	-0.1281	-0.281556638	0.031750403	1.16E-01	3.40E-01	1.00E+00
ENSG00000168310.9_IRF2	ENSG00000160789.18_LMNA	0.25921	0.104315774	0.401830211	1.26E-03	1.38E-02	1.00E+00
ENSG00000105676.12_ARMC6	ENSG00000160789.18_LMNA	-0.11954	-0.273534166	0.040431125	1.42E-01	3.85E-01	1.00E+00
ENSG00000204590.11_GNL1	ENSG00000160789.18_LMNA	0.18016	0.021574842	0.329895691	2.64E-02	1.31E-01	1.00E+00
ENSG00000206053.11_HN1L	ENSG00000160789.18_LMNA	0.13692	-0.022779055	0.289801926	9.26E-02	2.97E-01	1.00E+00
ENSG00000129245.10_FXR2	ENSG00000160789.18_LMNA	0.01585	-0.143717058	0.174606421	8.46E-01	9.40E-01	1.00E+00
ENSG00000105245.8_NUMBL	ENSG00000160789.18_LMNA	0.44782	0.310770183	0.566620223	7.26E-09	8.26E-07	3.73E-04

ENSG00000117528.10_ABCD3	ENSG00000160789.18_LMNA	-0.08562	-0.241528571	0.074597432	2.94E-01	5.80E-01	1.00E+00
ENSG00000090989.16_EXOC1	ENSG00000160789.18_LMNA	-0.10966	-0.264244681	0.050423953	1.79E-01	4.41E-01	1.00E+00
ENSG00000110048.10_OSBP	ENSG00000160789.18_LMNA	0.1156	-0.044413747	0.269838867	1.56E-01	4.08E-01	1.00E+00
ENSG00000121067.16_SPOP	ENSG00000160789.18_LMNA	-0.39422	-0.520743369	-0.25076235	5.03E-07	2.63E-05	2.58E-02
ENSG00000154328.14_NEIL2	ENSG00000160789.18_LMNA	-0.0582	-0.215404427	0.101945736	4.76E-01	7.35E-01	1.00E+00
ENSG00000156858.10_PRR14	ENSG00000160789.18_LMNA	0.12216	-0.037778506	0.275990283	1.34E-01	3.71E-01	1.00E+00
ENSG00000178445.8_GLDC	ENSG00000160789.18_LMNA	0.09508	-0.065102912	0.250492194	2.44E-01	5.23E-01	1.00E+00
ENSG00000137806.7_NDUFAF1	ENSG00000160789.18_LMNA	-0.00557	-0.16462306	0.153768722	9.46E-01	9.78E-01	1.00E+00
ENSG00000086189.8_DIMT1	ENSG00000160789.18_LMNA	-0.5343	-0.639132091	-0.40996743	1.35E-12	6.92E-10	6.92E-08
ENSG00000090661.10_CERS4	ENSG00000160789.18_LMNA	-0.20783	-0.355277375	-0.05029513	1.02E-02	6.64E-02	1.00E+00
ENSG00000112851.13_ERBIN	ENSG00000160789.18_LMNA	0.30917	0.157733808	0.446399636	1.06E-04	1.98E-03	1.00E+00
ENSG00000094914.11_AAAS	ENSG00000160789.18_LMNA	-0.09591	-0.251277955	0.064267879	2.40E-01	5.19E-01	1.00E+00
ENSG00000116285.11_ERRFI1	ENSG00000160789.18_LMNA	0.39649	0.253281698	0.522701321	4.26E-07	2.32E-05	2.19E-02
ENSG00000120802.12_TMPO	ENSG00000160789.18_LMNA	-0.10337	-0.258323027	0.056761245	2.05E-01	4.76E-01	1.00E+00
ENSG00000103591.11_AAGAB	ENSG00000160789.18_LMNA	0.06157	-0.098598024	0.218626875	4.51E-01	7.17E-01	1.00E+00
ENSG00000171992.11_SYNPO	ENSG00000160789.18_LMNA	0.33434	0.184986505	0.4685993	2.56E-05	6.28E-04	1.00E+00
ENSG00000111802.12_TDP2	ENSG00000160789.18_LMNA	-0.26703	-0.408851148	-0.11261823	8.82E-04	1.05E-02	1.00E+00
ENSG00000013441.14_CLK1	ENSG00000160789.18_LMNA	-0.27125	-0.412628858	-0.11710266	7.24E-04	9.00E-03	1.00E+00
ENSG00000187555.13_USP7	ENSG00000160789.18_LMNA	0.02931	-0.130498423	0.187636354	7.20E-01	8.81E-01	1.00E+00
ENSG00000104907.11_TRMT1	ENSG00000160789.18_LMNA	0.06838	-0.091823056	0.225127239	4.03E-01	6.78E-01	1.00E+00
ENSG00000186834.3_HEXIM1	ENSG00000160789.18_LMNA	0.25791	0.10293351	0.400657899	1.34E-03	1.44E-02	1.00E+00
ENSG00000173409.12_ARV1	ENSG00000160789.18_LMNA	-0.35799	-0.489301076	-0.2107996	5.95E-06	1.92E-04	3.05E-01
ENSG00000086544.2_ITPKC	ENSG00000160789.18_LMNA	0.38923	0.245226424	0.516431024	7.20E-07	3.53E-05	3.69E-02
ENSG00000137033.10_IL33	ENSG00000160789.18_LMNA	-0.21964	-0.366040059	-0.06262771	6.55E-03	4.80E-02	1.00E+00
ENSG00000136875.11_PRPF4	ENSG00000160789.18_LMNA	0.15881	-0.000404487	0.310165509	5.07E-02	2.03E-01	1.00E+00
ENSG00000148154.8_UGCG	ENSG00000160789.18_LMNA	0.11745	-0.042548445	0.271570751	1.50E-01	3.97E-01	1.00E+00
ENSG00000172046.17_USP19	ENSG00000160789.18_LMNA	0.05412	-0.105997608	0.2114949	5.08E-01	7.58E-01	1.00E+00
ENSG00000143514.15_TP53BP2	ENSG00000160789.18_LMNA	0.04815	-0.111909812	0.205772108	5.56E-01	7.90E-01	1.00E+00
ENSG00000127472.9_PLA2G5	ENSG00000160789.18_LMNA	0.35111	0.203273783	0.483297707	9.20E-06	2.73E-04	4.72E-01
ENSG00000079432.6_CIC	ENSG00000160789.18_LMNA	0.26424	0.10965265	0.406347337	1.00E-03	1.16E-02	1.00E+00
ENSG00000105063.17_PPP6R1	ENSG00000160789.18_LMNA	0.38232	0.237581023	0.510452754	1.17E-06	5.21E-05	6.00E-02
ENSG00000055950.15_MRPL43	ENSG00000160789.18_LMNA	-0.30839	-0.445706497	-0.15688992	1.11E-04	2.05E-03	1.00E+00
ENSG00000066455.11_GOLGA5	ENSG00000160789.18_LMNA	0.13555	-0.024176803	0.288520343	9.59E-02	3.04E-01	1.00E+00

ENSG00000122958.13_VPS26A	ENSG00000160789.18_LMNA	-0.25991	-0.402453889	-0.10505162	1.22E-03	1.35E-02	1.00E+00
ENSG00000158828.5_PINK1	ENSG00000160789.18_LMNA	-0.07367	-0.230171287	0.086545627	3.67E-01	6.48E-01	1.00E+00
ENSG00000237039.1_AC018738.:	ENSG00000160789.18_LMNA	0.05494	-0.105184091	0.212280653	5.01E-01	7.53E-01	1.00E+00
ENSG00000125266.6_EFNB2	ENSG00000160789.18_LMNA	0.33363	0.18421861	0.467978649	2.66E-05	6.51E-04	1.00E+00
ENSG00000160469.15_BRSK1	ENSG00000160789.18_LMNA	-0.12875	-0.282166219	0.031088867	1.14E-01	3.37E-01	1.00E+00
ENSG00000185127.6_C6orf120	ENSG00000160789.18_LMNA	-0.00867	-0.167640351	0.15073772	9.16E-01	9.68E-01	1.00E+00
ENSG00000144580.12_CNOT9	ENSG00000160789.18_LMNA	-0.10813	-0.262808173	0.05196362	1.85E-01	4.50E-01	1.00E+00
ENSG00000101412.12_E2F1	ENSG00000160789.18_LMNA	0.13008	-0.02974139	0.283407088	1.10E-01	3.31E-01	1.00E+00
ENSG00000198752.8_CDC42BPB	ENSG00000160789.18_LMNA	0.14133	-0.018281113	0.293918456	8.24E-02	2.77E-01	1.00E+00
ENSG00000065054.12_SLC9A3R:	ENSG00000160789.18_LMNA	0.15236	-0.007006114	0.304186716	6.09E-02	2.28E-01	1.00E+00
ENSG00000170619.9_COMMD5	ENSG00000160789.18_LMNA	-0.11622	-0.270414021	0.043794527	1.54E-01	4.04E-01	1.00E+00
ENSG00000116005.10_PCYOX1	ENSG00000160789.18_LMNA	0.11466	-0.045366313	0.268953649	1.60E-01	4.13E-01	1.00E+00
ENSG00000184787.17_UBE2G2	ENSG00000160789.18_LMNA	0.08788	-0.072333832	0.243670507	2.82E-01	5.67E-01	1.00E+00
ENSG00000081791.7_KIAA0141	ENSG00000160789.18_LMNA	-0.21994	-0.366316192	-0.06294535	6.48E-03	4.76E-02	1.00E+00
ENSG00000171824.12_EXOSC10	ENSG00000160789.18_LMNA	0.02799	-0.131795478	0.18636275	7.32E-01	8.87E-01	1.00E+00
ENSG00000100239.14_PPP6R2	ENSG00000160789.18_LMNA	-0.0149	-0.173686483	0.144646045	8.55E-01	9.43E-01	1.00E+00
ENSG00000099246.15_RAB18	ENSG00000160789.18_LMNA	-0.38328	-0.511286117	-0.23864476	1.09E-06	4.94E-05	5.61E-02
ENSG00000005007.11_UPF1	ENSG00000160789.18_LMNA	0.21291	0.05559277	0.35991057	8.45E-03	5.80E-02	1.00E+00
ENSG00000110881.10_ASIC1	ENSG00000160789.18_LMNA	-0.30333	-0.441227115	-0.15144647	1.45E-04	2.53E-03	1.00E+00
ENSG00000157216.14_SSBP3	ENSG00000160789.18_LMNA	-0.22132	-0.367569704	-0.06438803	6.14E-03	4.58E-02	1.00E+00
ENSG00000165512.4_ZNF22	ENSG00000160789.18_LMNA	-0.34291	-0.476118566	-0.19431726	1.53E-05	4.15E-04	7.84E-01
ENSG00000091732.14_ZC3HC1	ENSG00000160789.18_LMNA	0.12976	-0.030060568	0.283113257	1.11E-01	3.32E-01	1.00E+00
ENSG00000155090.13_KLF10	ENSG00000160789.18_LMNA	0.36131	0.214440082	0.492195662	4.80E-06	1.61E-04	2.46E-01
ENSG00000137210.12_TMEM14B	ENSG00000160789.18_LMNA	-0.22494	-0.370860969	-0.06818204	5.33E-03	4.12E-02	1.00E+00
ENSG00000165410.13_CFL2	ENSG00000160789.18_LMNA	-0.20591	-0.353524137	-0.04829493	1.09E-02	6.98E-02	1.00E+00
ENSG00000130958.10_SLC35D2	ENSG00000160789.18_LMNA	0.18277	0.024278567	0.33230428	2.42E-02	1.24E-01	1.00E+00
ENSG00000106348.15_IMPDH1	ENSG00000160789.18_LMNA	0.33142	0.181807889	0.466028366	3.04E-05	7.21E-04	1.00E+00
ENSG00000105176.16_URI1	ENSG00000160789.18_LMNA	-0.2048	-0.352509542	-0.04713853	1.14E-02	7.18E-02	1.00E+00
ENSG00000160087.19_UBE2J2	ENSG00000160789.18_LMNA	0.07524	-0.084983198	0.231661364	3.57E-01	6.40E-01	1.00E+00
ENSG00000126391.12_FRMD8	ENSG00000160789.18_LMNA	0.46739	0.332960982	0.583192948	1.28E-09	1.90E-07	6.54E-05
ENSG00000100883.10_SRP54	ENSG00000160789.18_LMNA	0.15922	2.38E-05	0.310552573	5.01E-02	2.01E-01	1.00E+00
ENSG00000198925.9_ATG9A	ENSG00000160789.18_LMNA	0.22576	0.06903745	0.371601983	5.17E-03	4.03E-02	1.00E+00
ENSG00000180891.11_CUEDC1	ENSG00000160789.18_LMNA	-0.03171	-0.189951028	0.128138337	6.98E-01	8.71E-01	1.00E+00

ENSG00000178927.15_C17orf62	ENSG00000160789.18_LMNA	0.17891	0.020288067	0.328747967	2.74E-02	1.35E-01	1.00E+00
ENSG00000163884.3_KLF15	ENSG00000160789.18_LMNA	-0.35868	-0.489909785	-0.21156452	5.69E-06	1.85E-04	2.92E-01
ENSG00000234127.7_TRIM26	ENSG00000160789.18_LMNA	0.11022	-0.049856874	0.264773414	1.76E-01	4.38E-01	1.00E+00
ENSG00000110076.17_NRXN2	ENSG00000160789.18_LMNA	-0.26212	-0.404441017	-0.10739829	1.11E-03	1.25E-02	1.00E+00
ENSG00000118518.14_RNF146	ENSG00000160789.18_LMNA	-0.22369	-0.369722721	-0.06686893	5.60E-03	4.27E-02	1.00E+00
ENSG00000138698.13_RAP1GDS	ENSG00000160789.18_LMNA	-0.38704	-0.514537996	-0.24280184	8.40E-07	3.95E-05	4.31E-02
ENSG00000108960.6_MMD	ENSG00000160789.18_LMNA	-0.38449	-0.512334564	-0.23998396	1.01E-06	4.62E-05	5.16E-02
ENSG00000198563.12_DDX39B	ENSG00000160789.18_LMNA	-0.03099	-0.189260539	0.128842751	7.05E-01	8.74E-01	1.00E+00
ENSG00000117054.12_ACADM	ENSG00000160789.18_LMNA	-0.07486	-0.231304903	0.085357109	3.59E-01	6.42E-01	1.00E+00
ENSG00000132128.15_LRRC41	ENSG00000160789.18_LMNA	0.32623	0.176175542	0.461460984	4.10E-05	9.20E-04	1.00E+00
ENSG00000160862.11_AZGP1	ENSG00000160789.18_LMNA	-0.00603	-0.165074888	0.153315222	9.41E-01	9.77E-01	1.00E+00
ENSG00000169255.12_B3GALNT	ENSG00000160789.18_LMNA	-0.10314	-0.258102409	0.056996858	2.06E-01	4.78E-01	1.00E+00
ENSG00000119392.13_GLE1	ENSG00000160789.18_LMNA	0.11046	-0.049609763	0.265003755	1.75E-01	4.36E-01	1.00E+00
ENSG00000198890.7_PRMT6	ENSG00000160789.18_LMNA	-0.02853	-0.18687881	0.131270047	7.27E-01	8.85E-01	1.00E+00
ENSG00000114744.7_COMMD2	ENSG00000160789.18_LMNA	-0.4405	-0.560400436	-0.30251328	1.35E-08	1.39E-06	6.95E-04
ENSG00000127419.15_TMEM175	ENSG00000160789.18_LMNA	-0.01075	-0.169658155	0.148707401	8.95E-01	9.59E-01	1.00E+00
ENSG00000136783.9_NIPSNAP3A	ENSG00000160789.18_LMNA	-0.10823	-0.262900944	0.051864232	1.84E-01	4.50E-01	1.00E+00
ENSG00000171155.7_C1GALT1C	ENSG00000160789.18_LMNA	0.06475	-0.09543478	0.221665414	4.28E-01	6.99E-01	1.00E+00
ENSG00000177732.7_SOX12	ENSG00000160789.18_LMNA	0.08782	-0.072392572	0.243614964	2.82E-01	5.67E-01	1.00E+00
ENSG00000010292.11_NCAPD2	ENSG00000160789.18_LMNA	#######	-0.1592761	0.159125302	9.99E-01	1.00E+00	1.00E+00
ENSG00000023318.7_ERP44	ENSG00000160789.18_LMNA	0.25847	0.10352539	0.401159997	1.30E-03	1.42E-02	1.00E+00
ENSG00000141380.12_SS18	ENSG00000160789.18_LMNA	0.17285	0.01404052	0.323162563	3.32E-02	1.53E-01	1.00E+00
ENSG00000176105.12_YES1	ENSG00000160789.18_LMNA	0.09146	-0.068744273	0.247060829	2.62E-01	5.46E-01	1.00E+00
ENSG00000146386.7_ABRACL	ENSG00000160789.18_LMNA	-0.11337	-0.267738555	0.046672922	1.64E-01	4.20E-01	1.00E+00
ENSG00000219928.2_RP11-40C6	ENSG00000160789.18_LMNA	-0.16976	-0.320303296	-0.0108516	3.65E-02	1.63E-01	1.00E+00
ENSG00000137193.12_PIM1	ENSG00000160789.18_LMNA	0.3057	0.153998915	0.443329305	1.28E-04	2.31E-03	1.00E+00
ENSG00000067141.15_NEO1	ENSG00000160789.18_LMNA	0.11809	-0.041895923	0.272176121	1.47E-01	3.93E-01	1.00E+00
ENSG00000115317.10_HTRA2	ENSG00000160789.18_LMNA	-0.06862	-0.22536062	0.091579269	4.01E-01	6.77E-01	1.00E+00
ENSG00000092758.14_COL9A3	ENSG00000160789.18_LMNA	0.13102	-0.028782531	0.284289448	1.08E-01	3.26E-01	1.00E+00
ENSG00000115233.10_PSMD14	ENSG00000160789.18_LMNA	-0.14283	-0.29531463	0.016752703	7.92E-02	2.70E-01	1.00E+00
ENSG00000240409.1_MTATP8P1	ENSG00000160789.18_LMNA	-0.15545	-0.307051277	0.003846522	5.58E-02	2.16E-01	1.00E+00
ENSG00000106591.3_MRPL32	ENSG00000160789.18_LMNA	-0.02371	-0.182219085	0.136007917	7.72E-01	9.06E-01	1.00E+00
ENSG00000145780.7_FEM1C	ENSG00000160789.18_LMNA	0.39705	0.253896636	0.523178799	4.09E-07	2.24E-05	2.10E-02

ENSG00000133083.13_DCLK1	ENSG00000160789.18_LMNA	-0.10651	-0.261276527	0.053603611	1.92E-01	4.59E-01	1.00E+00
ENSG00000108100.16_CCNY	ENSG00000160789.18_LMNA	-0.27755	-0.418273327	-0.12382561	5.36E-04	7.13E-03	1.00E+00
ENSG00000157869.13_RAB28	ENSG00000160789.18_LMNA	-0.1738	-0.324039173	-0.01501946	3.22E-02	1.50E-01	1.00E+00
ENSG00000023902.12_PLEKHO1	ENSG00000160789.18_LMNA	-0.08504	-0.240979253	0.075177427	2.98E-01	5.83E-01	1.00E+00
ENSG00000160209.17_PDXK	ENSG00000160789.18_LMNA	0.24022	0.08424218	0.384709295	2.87E-03	2.59E-02	1.00E+00
ENSG00000106638.14_TBL2	ENSG00000160789.18_LMNA	0.29327	0.140631874	0.432284792	2.46E-04	3.84E-03	1.00E+00
ENSG00000136933.15_RABEPK	ENSG00000160789.18_LMNA	-0.13205	-0.285256864	0.027730578	1.05E-01	3.21E-01	1.00E+00
ENSG00000071462.10_WBSCR22	ENSG00000160789.18_LMNA	0.12269	-0.037236927	0.276491239	1.32E-01	3.69E-01	1.00E+00
ENSG00000109790.15_KLHL5	ENSG00000160789.18_LMNA	-0.29889	-0.437282544	-0.14666745	1.84E-04	3.05E-03	1.00E+00
ENSG00000231064.4_MIR92B	ENSG00000160789.18_LMNA	-0.02616	-0.184590724	0.133598323	7.49E-01	8.95E-01	1.00E+00
ENSG00000158985.12_CDC42SE	ENSG00000160789.18_LMNA	-0.312	-0.448899754	-0.16078118	9.12E-05	1.75E-03	1.00E+00
ENSG00000168246.5_UBTD2	ENSG00000160789.18_LMNA	-0.04447	-0.202235055	0.115552724	5.86E-01	8.08E-01	1.00E+00
ENSG00000230715.3_RP11-274B	ENSG00000160789.18_LMNA	0.1294	-0.030422631	0.282779876	1.12E-01	3.34E-01	1.00E+00
ENSG00000147155.9_EBP	ENSG00000160789.18_LMNA	0.09225	-0.067945561	0.247814158	2.58E-01	5.41E-01	1.00E+00
ENSG00000173757.8_STAT5B	ENSG00000160789.18_LMNA	-0.00591	-0.164953552	0.15343702	9.42E-01	9.77E-01	1.00E+00
ENSG00000163584.16_RPL22L1	ENSG00000160789.18_LMNA	-0.05621	-0.213498262	0.10392262	4.92E-01	7.47E-01	1.00E+00
ENSG00000103429.9_BFAR	ENSG00000160789.18_LMNA	0.05521	-0.10491616	0.21253935	4.99E-01	7.52E-01	1.00E+00
ENSG00000146830.9_GIGYF1	ENSG00000160789.18_LMNA	0.14221	-0.017385096	0.294737112	8.05E-02	2.73E-01	1.00E+00
ENSG00000137970.7_RPL7P9	ENSG00000160789.18_LMNA	-0.11049	-0.265032238	0.049579204	1.75E-01	4.36E-01	1.00E+00
ENSG00000258289.6_CHURC1	ENSG00000160789.18_LMNA	-0.13849	-0.291271444	0.021174832	8.88E-02	2.90E-01	1.00E+00
ENSG00000100241.19_SBF1	ENSG00000160789.18_LMNA	0.04933	-0.110741234	0.206904973	5.46E-01	7.84E-01	1.00E+00
ENSG00000008130.14_NADK	ENSG00000160789.18_LMNA	0.25611	0.101032627	0.399044146	1.45E-03	1.53E-02	1.00E+00
ENSG00000125124.10_BBS2	ENSG00000160789.18_LMNA	0.02521	-0.134526572	0.183677534	7.58E-01	8.99E-01	1.00E+00
ENSG00000163832.14_ELP6	ENSG00000160789.18_LMNA	-0.28158	-0.42187071	-0.12812449	4.41E-04	6.12E-03	1.00E+00
ENSG00000167123.17_CERCAM	ENSG00000160789.18_LMNA	0.14402	-0.015534394	0.296426578	7.67E-02	2.65E-01	1.00E+00
ENSG00000109084.12_TMEM97	ENSG00000160789.18_LMNA	-0.19222	-0.340985598	-0.03406118	1.77E-02	9.87E-02	1.00E+00
ENSG00000064607.15_SUGP2	ENSG00000160789.18_LMNA	-0.12665	-0.280205552	0.033215664	1.20E-01	3.48E-01	1.00E+00
ENSG00000123179.12_EBPL	ENSG00000160789.18_LMNA	-0.20826	-0.355664339	-0.05073693	1.00E-02	6.58E-02	1.00E+00
ENSG00000006744.17_ELAC2	ENSG00000160789.18_LMNA	-0.03	-0.188301841	0.129820249	7.14E-01	8.78E-01	1.00E+00
ENSG00000181904.8_C5orf24	ENSG00000160789.18_LMNA	-0.2294	-0.374905299	-0.07285616	4.47E-03	3.61E-02	1.00E+00
ENSG00000157837.14_SPPL3	ENSG00000160789.18_LMNA	-0.2973	-0.435868906	-0.14495806	2.00E-04	3.26E-03	1.00E+00
ENSG00000005059.14_MCUB	ENSG00000160789.18_LMNA	0.37179	0.225967951	0.501321245	2.40E-06	9.18E-05	1.23E-01
ENSG00000276023.3_DUSP14	ENSG00000160789.18_LMNA	-0.05548	-0.212802484	0.104643587	4.97E-01	7.51E-01	1.00E+00

ENSG00000227051.5_C14orf132	ENSG00000160789.18_LMNA	-0.11053	-0.265067148	0.049541748	1.75E-01	4.36E-01	1.00E+00
ENSG00000147419.15_CCDC25	ENSG00000160789.18_LMNA	-0.01571	-0.174473062	0.143851763	8.48E-01	9.41E-01	1.00E+00
ENSG00000096060.13_FKBP5	ENSG00000160789.18_LMNA	0.12302	-0.03690143	0.276801486	1.31E-01	3.67E-01	1.00E+00
ENSG00000100100.11_PIK3IP1	ENSG00000160789.18_LMNA	-0.39824	-0.5242029	-0.2552163	3.75E-07	2.10E-05	1.93E-02
ENSG00000169733.10_RFNG	ENSG00000160789.18_LMNA	0.11949	-0.04048543	0.273483841	1.43E-01	3.86E-01	1.00E+00
ENSG00000142694.6_EVA1B	ENSG00000160789.18_LMNA	0.44862	0.311677457	0.567301884	6.78E-09	7.76E-07	3.48E-04
ENSG00000177119.14_ANO6	ENSG00000160789.18_LMNA	0.22192	0.065019235	0.368117793	6.00E-03	4.50E-02	1.00E+00
ENSG00000162298.15_SYVN1	ENSG00000160789.18_LMNA	0.29888	0.146662137	0.437278151	1.84E-04	3.05E-03	1.00E+00
ENSG00000089159.14_PXN	ENSG00000160789.18_LMNA	0.45809	0.322402568	0.575333513	2.95E-09	3.83E-07	1.52E-04
ENSG00000069998.11_CECR5	ENSG00000160789.18_LMNA	-0.16181	-0.312948506	-0.00267769	4.64E-02	1.92E-01	1.00E+00
ENSG00000167900.10_TK1	ENSG00000160789.18_LMNA	0.12708	-0.032783678	0.280604006	1.19E-01	3.46E-01	1.00E+00
ENSG00000138095.17_LRPPRC	ENSG00000160789.18_LMNA	-0.10679	-0.261548063	0.053312991	1.90E-01	4.58E-01	1.00E+00
ENSG00000104324.14_CPQ	ENSG00000160789.18_LMNA	0.2255	0.068768697	0.371369214	5.22E-03	4.05E-02	1.00E+00
ENSG00000163374.18_YY1AP1	ENSG00000160789.18_LMNA	0.18844	0.030144578	0.337516184	2.01E-02	1.08E-01	1.00E+00
ENSG00000099203.5_TMED1	ENSG00000160789.18_LMNA	0.15965	0.000465952	0.310951996	4.94E-02	2.00E-01	1.00E+00
ENSG00000119203.12_CPSF3	ENSG00000160789.18_LMNA	-0.14123	-0.293827305	0.018380846	8.26E-02	2.78E-01	1.00E+00
ENSG00000228343.1_RP11-1148	ENSG00000160789.18_LMNA	-0.03609	-0.194173383	0.123823843	6.59E-01	8.49E-01	1.00E+00
ENSG00000167088.9_SNRPD1	ENSG00000160789.18_LMNA	-0.20034	-0.348430959	-0.04249817	1.33E-02	8.06E-02	1.00E+00
ENSG00000131558.13_EXOC4	ENSG00000160789.18_LMNA	0.07474	-0.085475057	0.231192442	3.60E-01	6.42E-01	1.00E+00
ENSG00000106799.11_TGFBR1	ENSG00000160789.18_LMNA	-0.11113	-0.265626967	0.048940988	1.73E-01	4.32E-01	1.00E+00
ENSG00000100211.9_CBY1	ENSG00000160789.18_LMNA	-0.09111	-0.246734901	0.06908971	2.64E-01	5.48E-01	1.00E+00
ENSG00000127863.14_TNFRSF1	ENSG00000160789.18_LMNA	0.15702	-0.002232859	0.308512092	5.34E-02	2.10E-01	1.00E+00
ENSG00000168397.15_ATG4B	ENSG00000160789.18_LMNA	-0.00674	-0.165759421	0.1526279	9.34E-01	9.74E-01	1.00E+00
ENSG00000198276.12_UCKL1	ENSG00000160789.18_LMNA	0.13317	-0.026591995	0.286303231	1.02E-01	3.15E-01	1.00E+00
ENSG00000238227.6_C9orf69	ENSG00000160789.18_LMNA	0.39311	0.249529415	0.519784132	5.45E-07	2.81E-05	2.80E-02
ENSG00000182253.13_SYNM	ENSG00000160789.18_LMNA	0.15695	-0.002304688	0.308447097	5.35E-02	2.10E-01	1.00E+00
ENSG00000196141.11_SPATS2L	ENSG00000160789.18_LMNA	0.50795	0.379429466	0.617235018	2.40E-11	6.98E-09	1.23E-06
ENSG00000105649.8_RAB3A	ENSG00000160789.18_LMNA	-0.29978	-0.438077512	-0.1476295	1.75E-04	2.94E-03	1.00E+00
ENSG00000085832.15_EPS15	ENSG00000160789.18_LMNA	-0.05703	-0.214284872	0.103107131	4.85E-01	7.42E-01	1.00E+00
ENSG00000079332.13_SAR1A	ENSG00000160789.18_LMNA	-0.23115	-0.376496782	-0.07469911	4.17E-03	3.43E-02	1.00E+00
ENSG00000131748.14_STARD3	ENSG00000160789.18_LMNA	0.01355	-0.145964691	0.172379727	8.68E-01	9.49E-01	1.00E+00
ENSG00000136205.15_TNS3	ENSG00000160789.18_LMNA	-0.13825	-0.291049313	0.021417428	8.94E-02	2.91E-01	1.00E+00
ENSG00000167491.16_GATAD2A	ENSG00000160789.18_LMNA	0.22967	0.073139236	0.375149868	4.42E-03	3.57E-02	1.00E+00

ENSG00000115816.12_CEBPZ	ENSG00000160789.18_LMNA	-0.0188	-0.17746864	0.14082309	8.18E-01	9.29E-01	1.00E+00
ENSG00000075239.12_ACAT1	ENSG00000160789.18_LMNA	-0.35634	-0.48786574	-0.20899726	6.61E-06	2.10E-04	3.39E-01
ENSG00000025039.13_RRAGD	ENSG00000160789.18_LMNA	-0.17803	-0.327935502	-0.01937779	2.82E-02	1.37E-01	1.00E+00
ENSG00000114790.11_ARHGEF2	ENSG00000160789.18_LMNA	0.08498	-0.075235721	0.240924031	2.98E-01	5.84E-01	1.00E+00
ENSG00000136274.8_NACAD	ENSG00000160789.18_LMNA	0.01023	-0.149214269	0.169154661	9.00E-01	9.61E-01	1.00E+00
ENSG00000006652.12_IFRD1	ENSG00000160789.18_LMNA	0.07758	-0.082637517	0.233895636	3.42E-01	6.27E-01	1.00E+00
ENSG00000165169.9_DYNLT3	ENSG00000160789.18_LMNA	0.33885	0.189895521	0.472560402	1.95E-05	5.07E-04	1.00E+00
ENSG00000198805.10_PNP	ENSG00000160789.18_LMNA	0.16939	0.010472964	0.319963423	3.70E-02	1.64E-01	1.00E+00
ENSG00000243927.4_MRPS6	ENSG00000160789.18_LMNA	0.07812	-0.082104511	0.234402861	3.39E-01	6.24E-01	1.00E+00
ENSG00000117713.16_ARID1A	ENSG00000160789.18_LMNA	0.06613	-0.094060154	0.222983931	4.18E-01	6.90E-01	1.00E+00
ENSG00000162772.15_ATF3	ENSG00000160789.18_LMNA	0.23234	0.075947001	0.377573385	3.97E-03	3.30E-02	1.00E+00
ENSG00000138078.14_PREPL	ENSG00000160789.18_LMNA	-0.36369	-0.494274496	-0.21705938	4.11E-06	1.42E-04	2.11E-01
ENSG00000130226.15_DPP6	ENSG00000160789.18_LMNA	-0.115	-0.2692711	0.045024775	1.58E-01	4.11E-01	1.00E+00
ENSG00000115946.6_PNO1	ENSG00000160789.18_LMNA	-0.15046	-0.302418875	0.008952936	6.43E-02	2.36E-01	1.00E+00
ENSG00000107745.15_MICU1	ENSG00000160789.18_LMNA	-0.40104	-0.526618843	-0.25833346	3.05E-07	1.79E-05	1.57E-02
ENSG00000163870.13_TPRA1	ENSG00000160789.18_LMNA	0.26149	0.106733994	0.403878785	1.14E-03	1.28E-02	1.00E+00
ENSG00000214753.2_HNRNPUL2	ENSG00000160789.18_LMNA	0.10221	-0.057937933	0.257220901	2.10E-01	4.83E-01	1.00E+00
ENSG00000177432.6_NAP1L5	ENSG00000160789.18_LMNA	-0.27901	-0.419573557	-0.12537811	5.00E-04	6.74E-03	1.00E+00
ENSG00000087258.12_GNAO1	ENSG00000160789.18_LMNA	-0.26039	-0.402890298	-0.10556671	1.20E-03	1.33E-02	1.00E+00
ENSG00000028528.13_SNX1	ENSG00000160789.18_LMNA	-0.06479	-0.221702383	0.095396254	4.28E-01	6.98E-01	1.00E+00
ENSG00000125875.12_TBC1D20	ENSG00000160789.18_LMNA	0.17677	0.018076711	0.326773445	2.94E-02	1.41E-01	1.00E+00
ENSG00000162441.10_LZIC	ENSG00000160789.18_LMNA	-0.28966	-0.429076562	-0.13676882	2.95E-04	4.44E-03	1.00E+00
ENSG00000182670.12_TTC3	ENSG00000160789.18_LMNA	-0.14789	-0.300030744	0.011579106	6.90E-02	2.47E-01	1.00E+00
ENSG00000104067.15_TJP1	ENSG00000160789.18_LMNA	0.03973	-0.12022699	0.19768444	6.27E-01	8.32E-01	1.00E+00
ENSG00000277258.3_PCGF2	ENSG00000160789.18_LMNA	-0.04817	-0.205792027	0.111889274	5.56E-01	7.90E-01	1.00E+00
ENSG00000085365.16_SCAMP1	ENSG00000160789.18_LMNA	-0.32385	-0.4593641	-0.17359597	4.70E-05	1.02E-03	1.00E+00
ENSG00000162599.14_NFIA	ENSG00000160789.18_LMNA	-0.20866	-0.356032513	-0.05115739	9.89E-03	6.51E-02	1.00E+00
ENSG00000253982.1_CTD-2336C	ENSG00000160789.18_LMNA	-0.12483	-0.278498065	0.035065519	1.25E-01	3.58E-01	1.00E+00
ENSG00000126012.10_KDM5C	ENSG00000160789.18_LMNA	0.25909	0.104187481	0.401721445	1.27E-03	1.39E-02	1.00E+00
ENSG00000100299.16_ARSA	ENSG00000160789.18_LMNA	0.15251	-0.006860356	0.304318988	6.07E-02	2.27E-01	1.00E+00
ENSG00000149090.10_PAMR1	ENSG00000160789.18_LMNA	0.17393	0.015149038	0.324155169	3.21E-02	1.50E-01	1.00E+00
ENSG00000038382.16_TRIO	ENSG00000160789.18_LMNA	0.21922	0.062190231	0.36565965	6.66E-03	4.85E-02	1.00E+00
ENSG00000132254.11_ARFIP2	ENSG00000160789.18_LMNA	-0.02144	-0.180022402	0.138236402	7.93E-01	9.16E-01	1.00E+00

ENSG00000162433.13_AK4	ENSG00000160789.18_LMNA	0.24258	0.086732407	0.38684452	2.60E-03	2.39E-02	1.00E+00
ENSG00000140157.13_NIPA2	ENSG00000160789.18_LMNA	0.24782	0.092261025	0.391573515	2.08E-03	2.02E-02	1.00E+00
ENSG00000137133.9_HINT2	ENSG00000160789.18_LMNA	0.06041	-0.099746339	0.217522307	4.60E-01	7.23E-01	1.00E+00
ENSG00000141425.16_RPRD1A	ENSG00000160789.18_LMNA	-0.51693	-0.624721196	-0.38981183	9.23E-12	3.36E-09	4.74E-07
ENSG00000196368.4_NUDT11	ENSG00000160789.18_LMNA	-0.49903	-0.609787109	-0.36915927	6.00E-11	1.50E-08	3.08E-06
ENSG00000156603.13_MED19	ENSG00000160789.18_LMNA	-0.27166	-0.412999366	-0.11754313	7.10E-04	8.88E-03	1.00E+00
ENSG00000088766.10_CRLS1	ENSG00000160789.18_LMNA	-0.20101	-0.349043169	-0.04319386	1.30E-02	7.93E-02	1.00E+00
ENSG00000281383.1_CH507-513	ENSG00000160789.18_LMNA	-0.06622	-0.223068286	0.093972168	4.18E-01	6.90E-01	1.00E+00
ENSG00000004777.17_ARHGAP3	ENSG00000160789.18_LMNA	-0.14892	-0.300984413	0.010530894	6.71E-02	2.43E-01	1.00E+00
ENSG00000127054.17_CPSF3L	ENSG00000160789.18_LMNA	0.24386	0.088075543	0.387994847	2.47E-03	2.30E-02	1.00E+00
ENSG00000170265.10_ZNF282	ENSG00000160789.18_LMNA	0.31152	0.16026724	0.448478424	9.36E-05	1.79E-03	1.00E+00
ENSG00000130311.9_DDA1	ENSG00000160789.18_LMNA	-0.19242	-0.341171215	-0.03427099	1.75E-02	9.82E-02	1.00E+00
ENSG00000184990.11_SIVA1	ENSG00000160789.18_LMNA	0.10891	-0.051175912	0.263543278	1.82E-01	4.46E-01	1.00E+00
ENSG00000152377.11_SPOCK1	ENSG00000160789.18_LMNA	-0.13385	-0.286935533	0.025903574	1.00E-01	3.12E-01	1.00E+00
ENSG00000153823.17_PID1	ENSG00000160789.18_LMNA	-0.30316	-0.441074373	-0.15126117	1.47E-04	2.55E-03	1.00E+00
ENSG00000116096.5_SPR	ENSG00000160789.18_LMNA	0.03421	-0.125671284	0.192366837	6.76E-01	8.58E-01	1.00E+00
ENSG00000153786.11_ZDHHC7	ENSG00000160789.18_LMNA	0.28069	0.127175084	0.421077027	4.61E-04	6.32E-03	1.00E+00
ENSG00000112658.7_SRF	ENSG00000160789.18_LMNA	0.40043	0.257648896	0.526088644	3.20E-07	1.85E-05	1.64E-02
ENSG00000015475.17_BID	ENSG00000160789.18_LMNA	-0.37391	-0.503160249	-0.22830044	2.08E-06	8.22E-05	1.07E-01
ENSG00000160695.13_VPS11	ENSG00000160789.18_LMNA	-0.03268	-0.190885352	0.127184661	6.89E-01	8.66E-01	1.00E+00
ENSG00000136816.14_TOR1B	ENSG00000160789.18_LMNA	0.27309	0.119060627	0.414275078	6.64E-04	8.41E-03	1.00E+00
ENSG00000160285.13_LSS	ENSG00000160789.18_LMNA	0.29323	0.140590872	0.432250778	2.46E-04	3.85E-03	1.00E+00
ENSG00000106355.8_LSM5	ENSG00000160789.18_LMNA	-0.03796	-0.195980828	0.121973274	6.42E-01	8.41E-01	1.00E+00
ENSG00000275052.3_PPP4R3B	ENSG00000160789.18_LMNA	-0.1905	-0.339409965	-0.03228129	1.87E-02	1.03E-01	1.00E+00
ENSG00000167779.6_IGFBP6	ENSG00000160789.18_LMNA	0.28983	0.136952405	0.429229195	2.92E-04	4.42E-03	1.00E+00
ENSG00000130489.11_SCO2	ENSG00000160789.18_LMNA	0.07931	-0.080911516	0.235537528	3.31E-01	6.17E-01	1.00E+00
ENSG00000186432.7_KPNA4	ENSG00000160789.18_LMNA	-0.00342	-0.162535048	0.15586272	9.67E-01	9.87E-01	1.00E+00
ENSG00000134086.7_VHL	ENSG00000160789.18_LMNA	-0.22851	-0.37409885	-0.07192307	4.63E-03	3.70E-02	1.00E+00
ENSG00000072134.14_EPN2	ENSG00000160789.18_LMNA	-0.03508	-0.193205907	0.124813495	6.68E-01	8.54E-01	1.00E+00
ENSG00000008394.11_MGST1	ENSG00000160789.18_LMNA	-0.00475	-0.163825077	0.15456933	9.54E-01	9.81E-01	1.00E+00
ENSG00000100336.16_APOL4	ENSG00000160789.18_LMNA	-0.10509	-0.259945385	0.055027536	1.98E-01	4.67E-01	1.00E+00
ENSG00000161800.11_RACGAP1	ENSG00000160789.18_LMNA	-0.04887	-0.206467959	0.111192129	5.50E-01	7.86E-01	1.00E+00
ENSG00000229833.8_PET100	ENSG00000160789.18_LMNA	-0.31998	-0.455950124	-0.16940451	5.86E-05	1.22E-03	1.00E+00

ENSG00000164039.13_BDH2	ENSG00000160789.18_LMNA	-0.14163	-0.294199903	0.017973127	8.18E-02	2.76E-01	1.00E+00
ENSG00000010361.12_FUZ	ENSG00000160789.18_LMNA	0.00139	-0.157849331	0.160551477	9.86E-01	9.95E-01	1.00E+00
ENSG00000108788.10_MLX	ENSG00000160789.18_LMNA	-0.16482	-0.315733007	-0.00576744	4.24E-02	1.80E-01	1.00E+00
ENSG00000029559.6_IBSP	ENSG00000160789.18_LMNA	0.21344	0.056145557	0.360393161	8.29E-03	5.72E-02	1.00E+00
ENSG00000168280.15_KIF5C	ENSG00000160789.18_LMNA	-0.29311	-0.432144687	-0.14046299	2.48E-04	3.87E-03	1.00E+00
ENSG00000066032.17_CTNNA2	ENSG00000160789.18_LMNA	-0.15921	-0.310543055	-1.33E-05	5.01E-02	2.02E-01	1.00E+00
ENSG00000164163.9_ABCE1	ENSG00000160789.18_LMNA	0.05798	-0.102163535	0.215194538	4.78E-01	7.36E-01	1.00E+00
ENSG00000033627.13_ATP6V0A1	ENSG00000160789.18_LMNA	-0.06689	-0.223709671	0.093303014	4.13E-01	6.86E-01	1.00E+00
ENSG00000145029.10_NICN1	ENSG00000160789.18_LMNA	-0.20019	-0.348291096	-0.04233927	1.34E-02	8.08E-02	1.00E+00
ENSG00000137815.13_RTF1	ENSG00000160789.18_LMNA	0.04852	-0.111539222	0.206131465	5.53E-01	7.88E-01	1.00E+00
ENSG00000008056.11_SYN1	ENSG00000160789.18_LMNA	-0.22565	-0.371502054	-0.06892207	5.19E-03	4.04E-02	1.00E+00
ENSG00000084731.12_KIF3C	ENSG00000160789.18_LMNA	-0.00139	-0.160557092	0.157843711	9.86E-01	9.95E-01	1.00E+00
ENSG00000197724.9_PHF2	ENSG00000160789.18_LMNA	0.0408	-0.119176211	0.198708617	6.18E-01	8.27E-01	1.00E+00
ENSG00000111275.11_ALDH2	ENSG00000160789.18_LMNA	-0.39166	-0.518532356	-0.24792178	6.05E-07	3.05E-05	3.10E-02
ENSG00000129946.9_SHC2	ENSG00000160789.18_LMNA	-0.01248	-0.171337371	0.147015721	8.79E-01	9.53E-01	1.00E+00
ENSG00000183020.12_AP2A2	ENSG00000160789.18_LMNA	0.18722	0.028883383	0.336397211	2.09E-02	1.11E-01	1.00E+00
ENSG00000058673.14_ZC3H11A	ENSG00000160789.18_LMNA	0.07038	-0.089824692	0.227039231	3.89E-01	6.67E-01	1.00E+00
ENSG00000132640.13_BTBD3	ENSG00000160789.18_LMNA	-0.17573	-0.325811115	-0.01700005	3.04E-02	1.44E-01	1.00E+00
ENSG00000168884.13_TNIP2	ENSG00000160789.18_LMNA	0.25208	0.096757567	0.395408083	1.73E-03	1.75E-02	1.00E+00
ENSG00000104886.8_PLEKHJ1	ENSG00000160789.18_LMNA	-0.03205	-0.190283746	0.127798795	6.95E-01	8.69E-01	1.00E+00
ENSG00000103275.17_UBE2I	ENSG00000160789.18_LMNA	-0.03491	-0.193034628	0.124988633	6.69E-01	8.55E-01	1.00E+00
ENSG00000080503.18_SMARCA2	ENSG00000160789.18_LMNA	0.0932	-0.06699386	0.248711285	2.53E-01	5.35E-01	1.00E+00
ENSG00000112578.8_BYSL	ENSG00000160789.18_LMNA	-0.03952	-0.197477751	0.120438961	6.29E-01	8.33E-01	1.00E+00
ENSG00000154079.5_SDHAF4	ENSG00000160789.18_LMNA	-0.15275	-0.304541264	0.006615387	6.03E-02	2.26E-01	1.00E+00
ENSG00000107159.11_CA9	ENSG00000160789.18_LMNA	0.3459	0.197577018	0.478735797	1.27E-05	3.55E-04	6.53E-01
ENSG00000120733.12_KDM3B	ENSG00000160789.18_LMNA	-0.13407	-0.287142529	0.025678142	9.96E-02	3.11E-01	1.00E+00
ENSG00000181038.12_METTL23	ENSG00000160789.18_LMNA	-0.03423	-0.19237759	0.125660294	6.76E-01	8.58E-01	1.00E+00
ENSG00000145555.13_MYO10	ENSG00000160789.18_LMNA	-0.14477	-0.297124129	0.014769649	7.52E-02	2.62E-01	1.00E+00
ENSG00000147889.15_CDKN2A	ENSG00000160789.18_LMNA	-0.05332	-0.210729807	0.106789325	5.14E-01	7.62E-01	1.00E+00
ENSG00000134013.14_LOXL2	ENSG00000160789.18_LMNA	0.37836	0.233211592	0.507024221	1.54E-06	6.55E-05	7.89E-02
ENSG00000014824.12_SLC30A9	ENSG00000160789.18_LMNA	-0.3664	-0.496633885	-0.22003701	3.44E-06	1.23E-04	1.77E-01
ENSG00000065717.13_TLE2	ENSG00000160789.18_LMNA	0.01211	-0.147378594	0.170977328	8.82E-01	9.54E-01	1.00E+00
ENSG00000155097.10_ATP6V1C1	ENSG00000160789.18_LMNA	-0.06075	-0.217848348	0.099407473	4.57E-01	7.21E-01	1.00E+00

ENSG00000171848.12_RRM2	ENSG00000160789.18_LMNA	0.1466	-0.012902748	0.298825595	7.15E-02	2.53E-01	1.00E+00
ENSG00000131844.14_MCCC2	ENSG00000160789.18_LMNA	-0.0217	-0.180275861	0.137979437	7.91E-01	9.15E-01	1.00E+00
ENSG00000086758.14_HUWE1	ENSG00000160789.18_LMNA	0.11991	-0.040053568	0.273884005	1.41E-01	3.83E-01	1.00E+00
ENSG00000164081.11_TEX264	ENSG00000160789.18_LMNA	0.17233	0.013498873	0.322677305	3.38E-02	1.55E-01	1.00E+00
ENSG00000105251.9_SHD	ENSG00000160789.18_LMNA	-0.42655	-0.548503474	-0.2868269	4.26E-08	3.58E-06	2.19E-03
ENSG00000232888.4_RPS11P5	ENSG00000160789.18_LMNA	-0.14651	-0.298739902	0.012996825	7.17E-02	2.53E-01	1.00E+00
ENSG00000148300.10_REXO4	ENSG00000160789.18_LMNA	0.19655	0.038551532	0.344952983	1.52E-02	8.88E-02	1.00E+00
ENSG00000118432.12_CNR1	ENSG00000160789.18_LMNA	0.06958	-0.090625188	0.226273626	3.94E-01	6.72E-01	1.00E+00
ENSG00000074317.9_SNCB	ENSG00000160789.18_LMNA	-0.16017	-0.311425436	-0.00099015	4.87E-02	1.98E-01	1.00E+00
ENSG00000228058.1_RP11-552D	ENSG00000160789.18_LMNA	-0.3191	-0.455180384	-0.1684609	6.15E-05	1.27E-03	1.00E+00
ENSG00000124535.14_WRNIP1	ENSG00000160789.18_LMNA	0.17411	0.015338199	0.324324483	3.19E-02	1.49E-01	1.00E+00
ENSG00000129195.14_FAM64A	ENSG00000160789.18_LMNA	-0.06763	-0.224419001	0.09256264	4.08E-01	6.83E-01	1.00E+00
ENSG00000126456.14_IRF3	ENSG00000160789.18_LMNA	0.37903	0.23395071	0.507604789	1.47E-06	6.31E-05	7.54E-02
ENSG00000163795.12_ZNF513	ENSG00000160789.18_LMNA	0.04734	-0.112708096	0.204997731	5.62E-01	7.94E-01	1.00E+00
ENSG00000154380.15_ENAH	ENSG00000160789.18_LMNA	-0.10316	-0.258121973	0.056975966	2.06E-01	4.78E-01	1.00E+00
ENSG00000116957.11_TBCE	ENSG00000160789.18_LMNA	-0.09404	-0.249506816	0.066149455	2.49E-01	5.30E-01	1.00E+00
ENSG00000117500.11_TMED5	ENSG00000160789.18_LMNA	-0.11039	-0.264934488	0.049684077	1.76E-01	4.37E-01	1.00E+00
ENSG00000172534.12_HCFC1	ENSG00000160789.18_LMNA	0.13616	-0.023549274	0.289095857	9.44E-02	3.01E-01	1.00E+00
ENSG00000159210.8_SNF8	ENSG00000160789.18_LMNA	0.04908	-0.110983699	0.206669988	5.48E-01	7.85E-01	1.00E+00
ENSG00000099385.10_BCL7C	ENSG00000160789.18_LMNA	0.19139	0.03320103	0.340224371	1.82E-02	1.01E-01	1.00E+00
ENSG00000013297.9_CLDN11	ENSG00000160789.18_LMNA	-0.25201	-0.395351046	-0.09669059	1.74E-03	1.76E-02	1.00E+00
ENSG00000105655.17_ISYNA1	ENSG00000160789.18_LMNA	0.12375	-0.036167965	0.277479521	1.29E-01	3.64E-01	1.00E+00
ENSG00000142784.14_WDTC1	ENSG00000160789.18_LMNA	0.04967	-0.110403956	0.207231786	5.43E-01	7.82E-01	1.00E+00
ENSG00000204220.8_PFDN6	ENSG00000160789.18_LMNA	-0.20982	-0.357093627	-0.05236981	9.47E-03	6.31E-02	1.00E+00
ENSG00000111875.7_ASF1A	ENSG00000160789.18_LMNA	-0.31744	-0.453715971	-0.16666715	6.75E-05	1.37E-03	1.00E+00
ENSG00000109111.13_SUPT6H	ENSG00000160789.18_LMNA	0.17284	0.014023602	0.323147409	3.32E-02	1.53E-01	1.00E+00
ENSG00000117614.8_SYF2	ENSG00000160789.18_LMNA	0.10561	-0.054508445	0.26043079	1.95E-01	4.64E-01	1.00E+00
ENSG00000250742.1_RP11-834C	ENSG00000160789.18_LMNA	-0.33669	-0.470665399	-0.18754527	2.22E-05	5.63E-04	1.00E+00
ENSG00000049089.12_COL9A2	ENSG00000160789.18_LMNA	-0.07987	-0.236066611	0.080354925	3.28E-01	6.14E-01	1.00E+00
ENSG00000161920.8_MED11	ENSG00000160789.18_LMNA	-0.15267	-0.304470802	0.006693047	6.04E-02	2.27E-01	1.00E+00
ENSG00000110367.10_DDX6	ENSG00000160789.18_LMNA	-0.07993	-0.236127253	0.080291117	3.28E-01	6.14E-01	1.00E+00
ENSG00000198648.9_STK39	ENSG00000160789.18_LMNA	-0.07336	-0.229879544	0.086851354	3.69E-01	6.50E-01	1.00E+00
ENSG00000232187.1_FTH1P7	ENSG00000160789.18_LMNA	0.01411	-0.145414042	0.172925547	8.63E-01	9.46E-01	1.00E+00

ENSG00000167881.13_SRP68	ENSG00000160789.18_LMNA	0.19271	0.034572956	0.341438319	1.74E-02	9.75E-02	1.00E+00
ENSG00000174886.11_NDUFA11	ENSG00000160789.18_LMNA	-0.17039	-0.320884077	-0.01149883	3.58E-02	1.61E-01	1.00E+00
ENSG00000097021.18_ACOT7	ENSG00000160789.18_LMNA	-0.01105	-0.169954887	0.148408602	8.92E-01	9.58E-01	1.00E+00
ENSG00000090006.16_LTBP4	ENSG00000160789.18_LMNA	0.14505	-0.014485556	0.297383175	7.46E-02	2.60E-01	1.00E+00
ENSG00000226221.1_RPL26P19	ENSG00000160789.18_LMNA	-0.09204	-0.247610747	0.068161266	2.59E-01	5.43E-01	1.00E+00
ENSG00000166452.10_AKIP1	ENSG00000160789.18_LMNA	0.06775	-0.092444738	0.224531928	4.07E-01	6.82E-01	1.00E+00
ENSG00000126107.13_HECTD3	ENSG00000160789.18_LMNA	0.41092	0.26933427	0.535110461	1.45E-07	9.80E-06	7.45E-03
ENSG00000047249.15_ATP6V1H	ENSG00000160789.18_LMNA	-0.21338	-0.360337981	-0.05608234	8.30E-03	5.73E-02	1.00E+00
ENSG00000235084.3_CHCHD2P6	ENSG00000160789.18_LMNA	-0.03154	-0.189792265	0.12830033	7.00E-01	8.71E-01	1.00E+00
ENSG00000071889.15_FAM3A	ENSG00000160789.18_LMNA	0.20808	0.050555189	0.355505166	1.01E-02	6.61E-02	1.00E+00
ENSG00000162613.15_FUBP1	ENSG00000160789.18_LMNA	-0.05512	-0.212452811	0.105005793	5.00E-01	7.53E-01	1.00E+00
ENSG00000100811.9_YY1	ENSG00000160789.18_LMNA	0.13799	-0.021683721	0.290805445	9.00E-02	2.92E-01	1.00E+00
ENSG00000126785.11_RHOJ	ENSG00000160789.18_LMNA	0.40283	0.260319178	0.528155603	2.67E-07	1.61E-05	1.37E-02
ENSG00000113648.15_H2AFY	ENSG00000160789.18_LMNA	-0.16588	-0.316715705	-0.00685928	4.11E-02	1.77E-01	1.00E+00
ENSG00000073578.15_SDHA	ENSG00000160789.18_LMNA	0.19928	0.04139367	0.34745847	1.38E-02	8.28E-02	1.00E+00
ENSG00000158850.13_B4GALT3	ENSG00000160789.18_LMNA	0.06328	-0.096899872	0.220258846	4.39E-01	7.07E-01	1.00E+00
ENSG00000120451.9_SNX19	ENSG00000160789.18_LMNA	-0.0758	-0.232200491	0.084417509	3.53E-01	6.37E-01	1.00E+00
ENSG00000103512.13_NOMO1	ENSG00000160789.18_LMNA	-0.00964	-0.168586001	0.149786539	9.06E-01	9.64E-01	1.00E+00
ENSG00000136950.12_ARPC5L	ENSG00000160789.18_LMNA	0.18823	0.029929195	0.337325151	2.02E-02	1.09E-01	1.00E+00
ENSG00000121073.12_SLC35B1	ENSG00000160789.18_LMNA	-0.0146	-0.173401749	0.144933466	8.58E-01	9.44E-01	1.00E+00
ENSG00000136732.13_GYPC	ENSG00000160789.18_LMNA	-0.09395	-0.249415989	0.066245885	2.50E-01	5.30E-01	1.00E+00
ENSG00000100350.13_FOXRED2	ENSG00000160789.18_LMNA	-0.01759	-0.176301567	0.142003766	8.30E-01	9.34E-01	1.00E+00
ENSG00000118242.14_MREG	ENSG00000160789.18_LMNA	0.16778	0.008815339	0.318474573	3.88E-02	1.70E-01	1.00E+00
ENSG00000141576.13_RNF157	ENSG00000160789.18_LMNA	0.02259	-0.137105269	0.18113779	7.82E-01	9.11E-01	1.00E+00
ENSG00000123146.18_ADGRE5	ENSG00000160789.18_LMNA	0.52714	0.401646882	0.633202485	3.02E-12	1.36E-09	1.55E-07
ENSG00000136868.10_SLC31A1	ENSG00000160789.18_LMNA	0.02648	-0.133285871	0.184897983	7.46E-01	8.94E-01	1.00E+00
ENSG00000087152.14_ATXN7L3	ENSG00000160789.18_LMNA	0.00766	-0.151727152	0.16665605	9.25E-01	9.71E-01	1.00E+00
ENSG00000213015.7_ZNF580	ENSG00000160789.18_LMNA	0.01178	-0.147700947	0.170657417	8.86E-01	9.56E-01	1.00E+00
ENSG00000100116.15_GCAT	ENSG00000160789.18_LMNA	-0.13774	-0.290569183	0.021941667	9.06E-02	2.93E-01	1.00E+00
ENSG00000197170.8_PSMD12	ENSG00000160789.18_LMNA	0.08673	-0.07348557	0.242581058	2.88E-01	5.74E-01	1.00E+00
ENSG00000107897.17_ACBD5	ENSG00000160789.18_LMNA	-0.22442	-0.370386659	-0.06763474	5.44E-03	4.18E-02	1.00E+00
ENSG00000132017.9_DCAF15	ENSG00000160789.18_LMNA	0.0995	-0.060662619	0.254665716	2.23E-01	4.98E-01	1.00E+00
ENSG00000153936.15_HS2ST1	ENSG00000160789.18_LMNA	0.28706	0.133985302	0.426760297	3.36E-04	4.90E-03	1.00E+00

ENSG00000120694.18_HSPH1	ENSG00000160789.18_LMNA	0.04823	-0.11182595	0.205853436	5.55E-01	7.89E-01	1.00E+00
ENSG00000196735.10_HLA-DQA:	ENSG00000160789.18_LMNA	0.0256	-0.134147003	0.184051012	7.54E-01	8.98E-01	1.00E+00
ENSG00000184924.5_PTRHD1	ENSG00000160789.18_LMNA	-0.22916	-0.374690434	-0.0726075	4.51E-03	3.63E-02	1.00E+00
ENSG00000137944.15_KYAT3	ENSG00000160789.18_LMNA	1.68E-05	-0.15918436	0.159217044	1.00E+00	1.00E+00	1.00E+00
ENSG00000182446.12_NPLOC4	ENSG00000160789.18_LMNA	0.27373	0.119747434	0.414852074	6.44E-04	8.23E-03	1.00E+00
ENSG00000160678.10_S100A1	ENSG00000160789.18_LMNA	-0.29314	-0.432173124	-0.14049726	2.47E-04	3.86E-03	1.00E+00
ENSG00000196361.8_ELAVL3	ENSG00000160789.18_LMNA	-0.29771	-0.436237748	-0.1454039	1.95E-04	3.20E-03	1.00E+00
ENSG00000140688.15_C16orf58	ENSG00000160789.18_LMNA	0.05073	-0.109354505	0.208248218	5.35E-01	7.76E-01	1.00E+00
ENSG00000129968.14_ABHD17A	ENSG00000160789.18_LMNA	0.03862	-0.121321986	0.196616426	6.37E-01	8.37E-01	1.00E+00
ENSG00000133026.11_MYH10	ENSG00000160789.18_LMNA	0.07562	-0.084602552	0.232024159	3.55E-01	6.38E-01	1.00E+00
ENSG00000101577.8_LPIN2	ENSG00000160789.18_LMNA	0.09908	-0.061084429	0.254269753	2.25E-01	5.01E-01	1.00E+00
ENSG00000149485.15_FADS1	ENSG00000160789.18_LMNA	-0.12394	-0.277659098	0.035973651	1.28E-01	3.62E-01	1.00E+00
ENSG00000168243.9_GNG4	ENSG00000160789.18_LMNA	-0.36196	-0.492769465	-0.21516267	4.60E-06	1.56E-04	2.36E-01
ENSG00000166337.8_TAF10	ENSG00000160789.18_LMNA	0.1976	0.039650357	0.34592217	1.47E-02	8.65E-02	1.00E+00
ENSG00000160633.11_SAFB	ENSG00000160789.18_LMNA	0.18799	0.029675942	0.337100497	2.04E-02	1.09E-01	1.00E+00
ENSG00000029364.10_SLC39A9	ENSG00000160789.18_LMNA	-0.04582	-0.203539071	0.114210674	5.75E-01	8.02E-01	1.00E+00
ENSG00000005884.16_ITGA3	ENSG00000160789.18_LMNA	0.57373	0.456202686	0.671592508	1.11E-14	1.50E-11	5.69E-10
ENSG00000136527.16_TRA2B	ENSG00000160789.18_LMNA	-0.09689	-0.252197543	0.063290066	2.35E-01	5.13E-01	1.00E+00
ENSG00000059804.14_SLC2A3	ENSG00000160789.18_LMNA	0.34052	0.19170849	0.474020411	1.77E-05	4.66E-04	9.06E-01
ENSG00000212747.4_FAM127C	ENSG00000160789.18_LMNA	-0.0516	-0.209080904	0.108494243	5.28E-01	7.72E-01	1.00E+00
ENSG00000158290.15_CUL4B	ENSG00000160789.18_LMNA	0.0662	-0.093994468	0.223046907	4.18E-01	6.90E-01	1.00E+00
ENSG00000139722.5_VPS37B	ENSG00000160789.18_LMNA	0.08396	-0.076256803	0.239956428	3.04E-01	5.90E-01	1.00E+00
ENSG00000161638.9_ITGA5	ENSG00000160789.18_LMNA	0.49581	0.365450543	0.607087071	8.32E-11	1.95E-08	4.27E-06
ENSG00000148468.15_FAM171A:	ENSG00000160789.18_LMNA	-0.30855	-0.445847269	-0.15706127	1.10E-04	2.03E-03	1.00E+00
ENSG00000105341.17_ATP5SL	ENSG00000160789.18_LMNA	-0.0077	-0.166697674	0.151685324	9.25E-01	9.71E-01	1.00E+00
ENSG00000239900.10_ADSL	ENSG00000160789.18_LMNA	-0.21992	-0.366295121	-0.06292111	6.48E-03	4.77E-02	1.00E+00
ENSG00000196715.5_VKORC1L1	ENSG00000160789.18_LMNA	0.15183	-0.007550571	0.30369253	6.19E-02	2.30E-01	1.00E+00
ENSG00000235106.7_LINC00094	ENSG00000160789.18_LMNA	-0.03473	-0.192865693	0.125161355	6.71E-01	8.56E-01	1.00E+00
ENSG00000114446.4_IFT57	ENSG00000160789.18_LMNA	0.15605	-0.00322609	0.307613115	5.49E-02	2.14E-01	1.00E+00
ENSG00000116991.9_SIPA1L2	ENSG00000160789.18_LMNA	0.20227	0.044497772	0.350189907	1.25E-02	7.68E-02	1.00E+00
ENSG00000249915.6_PDCD6	ENSG00000160789.18_LMNA	-0.2254	-0.371277234	-0.06866251	5.24E-03	4.06E-02	1.00E+00
ENSG00000180957.16_PITPNB	ENSG00000160789.18_LMNA	-0.09509	-0.250499917	0.065094707	2.44E-01	5.23E-01	1.00E+00
ENSG00000122122.9_SASH3	ENSG00000160789.18_LMNA	-0.01466	-0.17345172	0.144883027	8.58E-01	9.44E-01	1.00E+00

ENSG00000074416.12_MGLL	ENSG00000160789.18_LMNA	-0.018	-0.1766936	0.141607265	8.26E-01	9.32E-01	1.00E+00
ENSG00000143622.9_RIT1	ENSG00000160789.18_LMNA	0.11643	-0.043580517	0.270612751	1.53E-01	4.03E-01	1.00E+00
ENSG00000132467.3_UTP3	ENSG00000160789.18_LMNA	0.33893	0.189985432	0.472632845	1.94E-05	5.05E-04	9.97E-01
ENSG00000185728.15_YTHDF3	ENSG00000160789.18_LMNA	-0.0428	-0.200630983	0.117201989	6.01E-01	8.16E-01	1.00E+00
ENSG00000103018.15_CYB5B	ENSG00000160789.18_LMNA	0.17678	0.018088843	0.326784284	2.94E-02	1.41E-01	1.00E+00
ENSG00000132313.13_MRPL35	ENSG00000160789.18_LMNA	-0.14138	-0.293962463	0.01823296	8.23E-02	2.77E-01	1.00E+00
ENSG00000119711.11_ALDH6A1	ENSG00000160789.18_LMNA	-0.08077	-0.2369251	0.079451381	3.23E-01	6.09E-01	1.00E+00
ENSG00000058091.15_CDK14	ENSG00000160789.18_LMNA	0.09035	-0.069859699	0.246008142	2.68E-01	5.52E-01	1.00E+00
ENSG00000110315.5_RNF141	ENSG00000160789.18_LMNA	-0.28004	-0.42049668	-0.12648121	4.75E-04	6.48E-03	1.00E+00
ENSG00000111530.11_CAND1	ENSG00000160789.18_LMNA	-0.00174	-0.160892905	0.157507563	9.83E-01	9.94E-01	1.00E+00
ENSG00000130707.16_ASS1	ENSG00000160789.18_LMNA	0.06791	-0.092290853	0.224679308	4.06E-01	6.81E-01	1.00E+00
ENSG00000132792.17_CTNNBL1	ENSG00000160789.18_LMNA	0.15872	-0.000488677	0.310089416	5.08E-02	2.03E-01	1.00E+00
ENSG00000171067.9_C11orf24	ENSG00000160789.18_LMNA	0.28072	0.127206952	0.421103676	4.60E-04	6.32E-03	1.00E+00
ENSG00000092969.10_TGFB2	ENSG00000160789.18_LMNA	0.12601	-0.033867998	0.279603649	1.22E-01	3.51E-01	1.00E+00
ENSG00000141582.13_CBX4	ENSG00000160789.18_LMNA	0.07366	-0.086556755	0.230160669	3.67E-01	6.48E-01	1.00E+00
ENSG00000173621.8_LRFN4	ENSG00000160789.18_LMNA	0.13857	-0.0210977	0.291342062	8.87E-02	2.90E-01	1.00E+00
ENSG00000197405.6_C5AR1	ENSG00000160789.18_LMNA	0.24713	0.091525613	0.390945379	2.15E-03	2.06E-02	1.00E+00
ENSG00000168283.12_BMI1	ENSG00000160789.18_LMNA	-0.52967	-0.635298331	-0.40458346	2.28E-12	1.07E-09	1.17E-07
ENSG00000168785.6_TSPAN5	ENSG00000160789.18_LMNA	0.02297	-0.136730644	0.181507019	7.79E-01	9.09E-01	1.00E+00
ENSG00000089335.19_ZNF302	ENSG00000160789.18_LMNA	-0.24631	-0.390206724	-0.09066122	2.22E-03	2.12E-02	1.00E+00
ENSG00000165487.12_MICU2	ENSG00000160789.18_LMNA	-0.19356	-0.342218911	-0.03545574	1.69E-02	9.56E-02	1.00E+00
ENSG00000183696.12_UPP1	ENSG00000160789.18_LMNA	0.401	0.258287921	0.526583577	3.06E-07	1.79E-05	1.57E-02
ENSG00000101557.13_USP14	ENSG00000160789.18_LMNA	-0.08003	-0.236221824	0.080191604	3.27E-01	6.14E-01	1.00E+00
ENSG00000141696.11_P3H4	ENSG00000160789.18_LMNA	0.40166	0.259021235	0.527151314	2.92E-07	1.73E-05	1.50E-02
ENSG00000162692.9_VCAM1	ENSG00000160789.18_LMNA	0.02243	-0.137264542	0.180980783	7.84E-01	9.12E-01	1.00E+00
ENSG00000133104.11_SPG20	ENSG00000160789.18_LMNA	-0.13325	-0.286371224	0.026517982	1.02E-01	3.15E-01	1.00E+00
ENSG00000010165.18_METTL13	ENSG00000160789.18_LMNA	0.13393	-0.025819254	0.287012961	1.00E-01	3.11E-01	1.00E+00
ENSG00000101782.13_RIOK3	ENSG00000160789.18_LMNA	-0.00854	-0.167511985	0.150866791	9.17E-01	9.68E-01	1.00E+00
ENSG00000189046.9_ALKBH2	ENSG00000160789.18_LMNA	-0.23847	-0.38312307	-0.08239466	3.09E-03	2.73E-02	1.00E+00
ENSG00000167515.9_TRAPPC2L	ENSG00000160789.18_LMNA	-0.15012	-0.302098628	0.009305353	6.49E-02	2.37E-01	1.00E+00
ENSG00000164125.14_FAM198B	ENSG00000160789.18_LMNA	-0.03395	-0.192113042	0.125930648	6.78E-01	8.59E-01	1.00E+00
ENSG00000131504.14_DIAPH1	ENSG00000160789.18_LMNA	0.33977	0.190895573	0.47336595	1.85E-05	4.83E-04	9.48E-01
ENSG00000100403.11_ZC3H7B	ENSG00000160789.18_LMNA	0.04721	-0.112836714	0.204872927	5.64E-01	7.95E-01	1.00E+00

ENSG00000087111.19_PIGS	ENSG00000160789.18_LMNA	0.0685	-0.091699742	0.225245293	4.02E-01	6.78E-01	1.00E+00
ENSG00000117143.12_UAP1	ENSG00000160789.18_LMNA	0.24367	0.087874349	0.387822594	2.49E-03	2.31E-02	1.00E+00
ENSG00000060491.15_OGFR	ENSG00000160789.18_LMNA	0.37206	0.226261389	0.501552737	2.36E-06	9.05E-05	1.21E-01
ENSG00000137996.11_RTCA	ENSG00000160789.18_LMNA	0.12872	-0.031116512	0.282140751	1.14E-01	3.37E-01	1.00E+00
ENSG00000081913.12_PHLPP1	ENSG00000160789.18_LMNA	-0.25021	-0.39372747	-0.09478532	1.88E-03	1.87E-02	1.00E+00
ENSG00000187244.9_BCAM	ENSG00000160789.18_LMNA	0.40226	0.259689086	0.52766816	2.79E-07	1.66E-05	1.43E-02
ENSG00000198954.5_KIF1BP	ENSG00000160789.18_LMNA	-0.17369	-0.323936448	-0.01490471	3.24E-02	1.51E-01	1.00E+00
ENSG00000146833.14_TRIM4	ENSG00000160789.18_LMNA	0.17669	0.017991635	0.326697426	2.94E-02	1.41E-01	1.00E+00
ENSG00000114316.11_USP4	ENSG00000160789.18_LMNA	0.14754	-0.011940947	0.299701393	6.97E-02	2.49E-01	1.00E+00
ENSG00000198797.6_BRINP2	ENSG00000160789.18_LMNA	-0.0762	-0.232580937	0.084018197	3.51E-01	6.34E-01	1.00E+00
ENSG00000111962.7_UST	ENSG00000160789.18_LMNA	-0.49827	-0.609146599	-0.36827878	6.49E-11	1.59E-08	3.33E-06
ENSG00000213445.7_SIPA1	ENSG00000160789.18_LMNA	0.32448	0.174279792	0.459920273	4.54E-05	9.93E-04	1.00E+00
ENSG00000163703.16_CRELD1	ENSG00000160789.18_LMNA	0.0042	-0.155106009	0.163289925	9.59E-01	9.84E-01	1.00E+00
ENSG00000121644.17_DESI2	ENSG00000160789.18_LMNA	0.0661	-0.094091338	0.222954033	4.18E-01	6.90E-01	1.00E+00
ENSG00000136631.11_VPS45	ENSG00000160789.18_LMNA	-0.25377	-0.396933046	-0.09854918	1.61E-03	1.66E-02	1.00E+00
ENSG00000253729.6_PRKDC	ENSG00000160789.18_LMNA	0.16877	0.009829139	0.319385331	3.77E-02	1.66E-01	1.00E+00
ENSG00000173473.9_SMARCC1	ENSG00000160789.18_LMNA	-0.00419	-0.163285129	0.155110818	9.59E-01	9.84E-01	1.00E+00
ENSG00000196510.11_ANAPC7	ENSG00000160789.18_LMNA	-0.33105	-0.465703507	-0.18140667	3.10E-05	7.33E-04	1.00E+00
ENSG00000173992.7_CCS	ENSG00000160789.18_LMNA	0.20973	0.05227199	0.357008045	9.51E-03	6.32E-02	1.00E+00
ENSG00000105137.11_SYDE1	ENSG00000160789.18_LMNA	0.4724	0.33867055	0.587423589	8.02E-10	1.30E-07	4.11E-05
ENSG00000245060.5_LINC00847	ENSG00000160789.18_LMNA	-0.20138	-0.349383992	-0.04358129	1.29E-02	7.85E-02	1.00E+00
ENSG00000104980.6_TIMM44	ENSG00000160789.18_LMNA	0.01507	-0.144480803	0.173850155	8.54E-01	9.43E-01	1.00E+00
ENSG00000177479.18_ARIH2	ENSG00000160789.18_LMNA	-0.06693	-0.223745609	0.093265512	4.13E-01	6.86E-01	1.00E+00
ENSG00000142512.13_SIGLEC10	ENSG00000160789.18_LMNA	-0.00747	-0.166473637	0.151910446	9.27E-01	9.72E-01	1.00E+00
ENSG00000214160.8_ALG3	ENSG00000160789.18_LMNA	0.24663	0.091002094	0.390498056	2.19E-03	2.10E-02	1.00E+00
ENSG00000099821.12_POLRMT	ENSG00000160789.18_LMNA	0.25072	0.095327496	0.394189673	1.84E-03	1.84E-02	1.00E+00
ENSG00000197256.9_KANK2	ENSG00000160789.18_LMNA	0.56549	0.446482986	0.664836547	3.19E-14	3.64E-11	1.64E-09
ENSG00000213672.6_NCKIPSD	ENSG00000160789.18_LMNA	0.06155	-0.098618675	0.218607018	4.51E-01	7.17E-01	1.00E+00
ENSG00000124772.10_CPNE5	ENSG00000160789.18_LMNA	-0.06278	-0.219787789	0.097390222	4.42E-01	7.10E-01	1.00E+00
ENSG00000112079.8_STK38	ENSG00000160789.18_LMNA	0.17015	0.011251453	0.32066212	3.61E-02	1.62E-01	1.00E+00
ENSG00000132388.11_UBE2G1	ENSG00000160789.18_LMNA	-0.16142	-0.312589058	-0.00227927	4.69E-02	1.93E-01	1.00E+00
ENSG00000024862.15_CCDC28A	ENSG00000160789.18_LMNA	-0.14637	-0.298608924	0.013140607	7.20E-02	2.54E-01	1.00E+00
ENSG00000074219.12_TEAD2	ENSG00000160789.18_LMNA	0.18289	0.024402768	0.332414826	2.41E-02	1.23E-01	1.00E+00

ENSG00000131634.12_TMEM204	ENSG00000160789.18_LMNA	-0.06764	-0.224423305	0.092558146	4.08E-01	6.83E-01	1.00E+00
ENSG00000073050.10_XRCC1	ENSG00000160789.18_LMNA	0.18938	0.031117012	0.338378367	1.95E-02	1.06E-01	1.00E+00
ENSG00000162645.11_GBP2	ENSG00000160789.18_LMNA	0.23798	0.08187928	0.382680267	3.15E-03	2.77E-02	1.00E+00
ENSG00000181790.9_ADGRB1	ENSG00000160789.18_LMNA	-0.17256	-0.322893385	-0.01374004	3.35E-02	1.54E-01	1.00E+00
ENSG00000169105.7_CHST14	ENSG00000160789.18_LMNA	0.45412	0.317906866	0.57197278	4.20E-09	5.15E-07	2.15E-04
ENSG00000132109.9_TRIM21	ENSG00000160789.18_LMNA	0.19207	0.03390112	0.340843974	1.78E-02	9.92E-02	1.00E+00
ENSG00000261455.1_LINC01003	ENSG00000160789.18_LMNA	-0.06625	-0.223096429	0.093942812	4.17E-01	6.90E-01	1.00E+00
ENSG00000122692.8_SMU1	ENSG00000160789.18_LMNA	-0.04346	-0.201266996	0.116548267	5.95E-01	8.13E-01	1.00E+00
ENSG00000266017.1_MIR4477B	ENSG00000160789.18_LMNA	-0.18164	-0.331265088	-0.02311149	2.51E-02	1.27E-01	1.00E+00
ENSG00000136710.8_CCDC115	ENSG00000160789.18_LMNA	-0.37856	-0.507193938	-0.23342763	1.52E-06	6.48E-05	7.79E-02
ENSG00000107140.14_TESK1	ENSG00000160789.18_LMNA	0.22561	0.068881599	0.371467003	5.20E-03	4.04E-02	1.00E+00
ENSG00000170248.12_PDCD6IP	ENSG00000160789.18_LMNA	0.13944	-0.020212494	0.292152268	8.67E-02	2.86E-01	1.00E+00
ENSG00000145495.13_MARCH6	ENSG00000160789.18_LMNA	-0.15179	-0.303651817	0.007595417	6.19E-02	2.30E-01	1.00E+00
ENSG00000157557.10_ETS2	ENSG00000160789.18_LMNA	-0.19858	-0.346820049	-0.040669	1.42E-02	8.45E-02	1.00E+00
ENSG00000100568.9_VTI1B	ENSG00000160789.18_LMNA	-0.07987	-0.23606716	0.080354347	3.28E-01	6.14E-01	1.00E+00
ENSG00000153485.5_TMEM251	ENSG00000160789.18_LMNA	-0.06596	-0.222825578	0.09422531	4.19E-01	6.91E-01	1.00E+00
ENSG00000126602.9_TRAP1	ENSG00000160789.18_LMNA	-0.05613	-0.213425489	0.103998043	4.92E-01	7.48E-01	1.00E+00
ENSG00000215845.9_TSTD1	ENSG00000160789.18_LMNA	0.16504	0.006001932	0.315944117	4.22E-02	1.80E-01	1.00E+00
ENSG00000136444.8_RSAD1	ENSG00000160789.18_LMNA	-0.05871	-0.215894822	0.101436742	4.72E-01	7.32E-01	1.00E+00
ENSG00000260804.3_PKI55	ENSG00000160789.18_LMNA	-0.18577	-0.33505864	-0.02737596	2.19E-02	1.15E-01	1.00E+00
ENSG00000130254.10_SAFB2	ENSG00000160789.18_LMNA	0.13193	-0.027859721	0.285138134	1.05E-01	3.22E-01	1.00E+00
ENSG00000198715.10_GLMP	ENSG00000160789.18_LMNA	0.41537	0.27431158	0.538934923	1.03E-07	7.43E-06	5.29E-03
ENSG00000159063.11_ALG8	ENSG00000160789.18_LMNA	0.01718	-0.142412751	0.175897085	8.34E-01	9.35E-01	1.00E+00
ENSG00000178096.8_BOLA1	ENSG00000160789.18_LMNA	-0.15965	-0.310948317	-0.00046188	4.95E-02	2.00E-01	1.00E+00
ENSG00000125810.9_CD93	ENSG00000160789.18_LMNA	0.28935	0.136432458	0.428796867	3.00E-04	4.50E-03	1.00E+00
ENSG00000111206.11_FOXM1	ENSG00000160789.18_LMNA	0.13106	-0.028736788	0.284331528	1.08E-01	3.26E-01	1.00E+00
ENSG00000039523.16_FAM65A	ENSG00000160789.18_LMNA	0.23311	0.076758469	0.378273033	3.85E-03	3.22E-02	1.00E+00
ENSG00000087470.16_DNM1L	ENSG00000160789.18_LMNA	-0.27869	-0.419290108	-0.12503954	5.08E-04	6.82E-03	1.00E+00
ENSG00000089053.11_ANAPC5	ENSG00000160789.18_LMNA	-0.35106	-0.483252742	-0.20321754	9.23E-06	2.73E-04	4.74E-01
ENSG00000115539.12_PDCL3	ENSG00000160789.18_LMNA	0.05105	-0.10904002	0.208552676	5.32E-01	7.75E-01	1.00E+00
ENSG00000147586.8_MRPS28	ENSG00000160789.18_LMNA	-0.36934	-0.499187527	-0.22326561	2.83E-06	1.05E-04	1.45E-01
ENSG00000072310.15_SREBF1	ENSG00000160789.18_LMNA	0.21149	0.054114999	0.358619653	8.91E-03	6.03E-02	1.00E+00
ENSG00000137478.13_FCHSD2	ENSG00000160789.18_LMNA	-0.41324	-0.53710618	-0.27192981	1.22E-07	8.51E-06	6.24E-03

ENSG00000090776.5_EFNB1	ENSG00000160789.18_LMNA	0.29323	0.14058859	0.432248886	2.46E-04	3.85E-03	1.00E+00
ENSG00000198756.9_COLGALT2	ENSG00000160789.18_LMNA	0.08611	-0.074110679	0.241989421	2.92E-01	5.77E-01	1.00E+00
ENSG00000143198.11_MGST3	ENSG00000160789.18_LMNA	-0.23465	-0.379667133	-0.07837657	3.62E-03	3.07E-02	1.00E+00
ENSG00000117906.12_RCN2	ENSG00000160789.18_LMNA	-0.11951	-0.273511626	0.040455448	1.42E-01	3.85E-01	1.00E+00
ENSG00000002919.13_SNX11	ENSG00000160789.18_LMNA	-0.28977	-0.429169055	-0.13688007	2.93E-04	4.42E-03	1.00E+00
ENSG00000132394.9_EEFSEC	ENSG00000160789.18_LMNA	0.03863	-0.121319163	0.196619181	6.37E-01	8.37E-01	1.00E+00
ENSG00000006015.16_C19orf60	ENSG00000160789.18_LMNA	0.0166	-0.142979306	0.175336591	8.39E-01	9.38E-01	1.00E+00
ENSG00000155957.15_TMBIM4	ENSG00000160789.18_LMNA	-0.19531	-0.343824269	-0.03727278	1.59E-02	9.15E-02	1.00E+00
ENSG00000170310.13_STX8	ENSG00000160789.18_LMNA	-0.31951	-0.455539677	-0.16890128	6.01E-05	1.24E-03	1.00E+00
ENSG00000181788.3_SIAH2	ENSG00000160789.18_LMNA	0.16492	0.005873352	0.315828363	4.23E-02	1.80E-01	1.00E+00
ENSG00000119760.14_SUPT7L	ENSG00000160789.18_LMNA	-0.13819	-0.290992117	0.021479887	8.95E-02	2.91E-01	1.00E+00
ENSG00000089127.11_OAS1	ENSG00000160789.18_LMNA	-0.01507	-0.173853756	0.144477168	8.54E-01	9.43E-01	1.00E+00
ENSG00000117868.14_ESYT2	ENSG00000160789.18_LMNA	0.29498	0.142474479	0.433812461	2.25E-04	3.58E-03	1.00E+00
ENSG00000108439.8_PNPO	ENSG00000160789.18_LMNA	-0.15159	-0.303470479	0.00779515	6.23E-02	2.31E-01	1.00E+00
ENSG00000161204.10_ABCF3	ENSG00000160789.18_LMNA	0.12924	-0.030594165	0.282621904	1.13E-01	3.35E-01	1.00E+00
ENSG00000160049.10_DFFA	ENSG00000160789.18_LMNA	-0.12252	-0.2763309	0.037410289	1.33E-01	3.69E-01	1.00E+00
ENSG00000196923.12_PDLIM7	ENSG00000160789.18_LMNA	0.49042	0.359271881	0.602576436	1.42E-10	3.02E-08	7.30E-06
ENSG00000139514.11_SLC7A1	ENSG00000160789.18_LMNA	0.0217	-0.137979521	0.180275779	7.91E-01	9.15E-01	1.00E+00
ENSG00000122550.16_KLHL7	ENSG00000160789.18_LMNA	-0.31566	-0.452139432	-0.16473818	7.45E-05	1.49E-03	1.00E+00
ENSG00000204351.10_SKIV2L	ENSG00000160789.18_LMNA	0.22521	0.068465718	0.371106754	5.28E-03	4.08E-02	1.00E+00
ENSG00000177239.13_MAN1B1	ENSG00000160789.18_LMNA	0.50518	0.376235117	0.614923046	3.20E-11	8.77E-09	1.64E-06
ENSG00000183779.6_ZNF703	ENSG00000160789.18_LMNA	0.14736	-0.012121498	0.299537027	7.00E-02	2.50E-01	1.00E+00
ENSG00000168961.15_LGALS9	ENSG00000160789.18_LMNA	-0.04141	-0.199301231	0.118567879	6.12E-01	8.24E-01	1.00E+00
ENSG00000087302.7_C14orf166	ENSG00000160789.18_LMNA	-0.17235	-0.322699772	-0.01352395	3.37E-02	1.55E-01	1.00E+00
ENSG00000186940.6_CHCHD2P9	ENSG00000160789.18_LMNA	-0.00398	-0.163076722	0.155319768	9.61E-01	9.84E-01	1.00E+00
ENSG00000060138.11_YBX3	ENSG00000160789.18_LMNA	0.44198	0.304177414	0.561656343	1.20E-08	1.25E-06	6.13E-04
ENSG00000162813.16_BPNT1	ENSG00000160789.18_LMNA	-0.33176	-0.466329246	-0.18217958	2.98E-05	7.09E-04	1.00E+00
ENSG00000108798.7_ABI3	ENSG00000160789.18_LMNA	-0.03546	-0.19356996	0.124441171	6.64E-01	8.52E-01	1.00E+00
ENSG00000146707.13_POMZP3	ENSG00000160789.18_LMNA	-0.05619	-0.213485058	0.103936306	4.92E-01	7.47E-01	1.00E+00
ENSG00000164961.14_KIAA0196	ENSG00000160789.18_LMNA	-0.0293	-0.187625562	0.130509419	7.20E-01	8.81E-01	1.00E+00
ENSG00000091947.8_TMEM101	ENSG00000160789.18_LMNA	-0.1037	-0.258627745	0.056435758	2.04E-01	4.75E-01	1.00E+00
ENSG00000029363.14_BCLAF1	ENSG00000160789.18_LMNA	-0.01568	-0.174441634	0.143883506	8.48E-01	9.41E-01	1.00E+00
ENSG00000114450.8_GNB4	ENSG00000160789.18_LMNA	-0.21172	-0.358824025	-0.05434886	8.83E-03	5.99E-02	1.00E+00

ENSG00000119705.8_SLIRP	ENSG00000160789.18_LMNA	-0.08982	-0.245512683	0.070384413	2.71E-01	5.55E-01	1.00E+00
ENSG00000108479.10_GALK1	ENSG00000160789.18_LMNA	0.27675	0.122965984	0.417552867	5.58E-04	7.34E-03	1.00E+00
ENSG00000235750.8_KIAA0040	ENSG00000160789.18_LMNA	0.32274	0.172393595	0.458385617	5.01E-05	1.08E-03	1.00E+00
ENSG00000165688.10_PMPCA	ENSG00000160789.18_LMNA	0.15216	-0.007219493	0.303993059	6.13E-02	2.29E-01	1.00E+00
ENSG00000089050.13_RBBP9	ENSG00000160789.18_LMNA	-0.1811	-0.33076703	-0.02255243	2.56E-02	1.28E-01	1.00E+00
ENSG00000159399.8_HK2	ENSG00000160789.18_LMNA	0.12693	-0.032936866	0.280462721	1.19E-01	3.47E-01	1.00E+00
ENSG00000145907.13_G3BP1	ENSG00000160789.18_LMNA	0.11404	-0.045995286	0.268368854	1.62E-01	4.17E-01	1.00E+00
ENSG00000077254.13_USP33	ENSG00000160789.18_LMNA	-0.25642	-0.399320024	-0.10135744	1.43E-03	1.51E-02	1.00E+00
ENSG00000136463.7_TACO1	ENSG00000160789.18_LMNA	-0.13767	-0.290506195	0.02201043	9.08E-02	2.94E-01	1.00E+00
ENSG00000101290.12_CDS2	ENSG00000160789.18_LMNA	-0.131	-0.284272123	0.028801363	1.08E-01	3.26E-01	1.00E+00
ENSG00000137288.8_UQCC2	ENSG00000160789.18_LMNA	-0.24085	-0.385280261	-0.08490771	2.80E-03	2.53E-02	1.00E+00
ENSG00000167700.7_MFSD3	ENSG00000160789.18_LMNA	0.05535	-0.104771611	0.212678899	4.98E-01	7.51E-01	1.00E+00
ENSG00000035687.9_ADSS	ENSG00000160789.18_LMNA	0.07125	-0.088959488	0.227866283	3.83E-01	6.62E-01	1.00E+00
ENSG00000133065.9_SLC41A1	ENSG00000160789.18_LMNA	0.08836	-0.071852913	0.244125182	2.79E-01	5.64E-01	1.00E+00
ENSG00000172432.17_GTPBP2	ENSG00000160789.18_LMNA	-0.00101	-0.160180695	0.158220395	9.90E-01	9.96E-01	1.00E+00
ENSG00000204304.10_PBX2	ENSG00000160789.18_LMNA	0.10755	-0.052546218	0.262264246	1.87E-01	4.54E-01	1.00E+00
ENSG00000110057.6_UNC93B1	ENSG00000160789.18_LMNA	0.29803	0.145747353	0.436521816	1.92E-04	3.17E-03	1.00E+00
ENSG00000176444.17_CLK2	ENSG00000160789.18_LMNA	0.17373	0.014943868	0.323971504	3.23E-02	1.50E-01	1.00E+00
ENSG00000136536.13_MARCH7	ENSG00000160789.18_LMNA	-0.0917	-0.247291809	0.068499421	2.61E-01	5.44E-01	1.00E+00
ENSG00000152904.10_GGPS1	ENSG00000160789.18_LMNA	-0.15436	-0.306036682	0.004966324	5.76E-02	2.20E-01	1.00E+00
ENSG00000267321.1_RP11-1094	ENSG00000160789.18_LMNA	-0.30981	-0.446967888	-0.15842596	1.03E-04	1.93E-03	1.00E+00
ENSG00000113269.12_RNF130	ENSG00000160789.18_LMNA	-0.2229	-0.369009259	-0.0660464	5.78E-03	4.37E-02	1.00E+00
ENSG00000132024.16_CC2D1A	ENSG00000160789.18_LMNA	0.25014	0.094711767	0.393664753	1.88E-03	1.87E-02	1.00E+00
ENSG00000169018.5_FEM1B	ENSG00000160789.18_LMNA	-0.05052	-0.208052038	0.109557113	5.36E-01	7.77E-01	1.00E+00
ENSG00000033327.11_GAB2	ENSG00000160789.18_LMNA	0.02494	-0.134793615	0.183414722	7.60E-01	9.00E-01	1.00E+00
ENSG00000124067.15_SLC12A4	ENSG00000160789.18_LMNA	0.38954	0.245566986	0.516696711	7.04E-07	3.47E-05	3.61E-02
ENSG00000164932.11_CTHRC1	ENSG00000160789.18_LMNA	0.16547	0.00644039	0.316338768	4.16E-02	1.78E-01	1.00E+00
ENSG00000137331.11_IER3	ENSG00000160789.18_LMNA	0.16217	0.003052407	0.31328649	4.59E-02	1.90E-01	1.00E+00
ENSG00000035141.6_FAM136A	ENSG00000160789.18_LMNA	0.20932	0.051849408	0.356638266	9.65E-03	6.39E-02	1.00E+00
ENSG00000159593.13_NAE1	ENSG00000160789.18_LMNA	-0.2114	-0.358530083	-0.05401251	8.94E-03	6.05E-02	1.00E+00
ENSG00000171161.11_ZNF672	ENSG00000160789.18_LMNA	0.25734	0.102336336	0.400151127	1.37E-03	1.47E-02	1.00E+00
ENSG00000129235.9_TXNDC17	ENSG00000160789.18_LMNA	0.01534	-0.144216473	0.17411194	8.51E-01	9.42E-01	1.00E+00
ENSG00000214941.6_ZSWIM7	ENSG00000160789.18_LMNA	-0.25968	-0.402250938	-0.10481213	1.23E-03	1.36E-02	1.00E+00

ENSG00000168256.16_NKIRAS2	ENSG00000160789.18_LMNA	-0.05848	-0.215673152	0.101666839	4.74E-01	7.34E-01	1.00E+00
ENSG00000113048.15_MRPS27	ENSG00000160789.18_LMNA	-0.32552	-0.460835463	-0.17540562	4.27E-05	9.46E-04	1.00E+00
ENSG00000041880.13_PARP3	ENSG00000160789.18_LMNA	0.14961	-0.009827398	0.301624109	6.58E-02	2.40E-01	1.00E+00
ENSG00000079616.11_KIF22	ENSG00000160789.18_LMNA	-0.04571	-0.203431964	0.114320949	5.76E-01	8.02E-01	1.00E+00
ENSG00000184436.10_THAP7	ENSG00000160789.18_LMNA	-0.06601	-0.222868694	0.094180343	4.19E-01	6.91E-01	1.00E+00
ENSG00000134313.13_KIDINS220	ENSG00000160789.18_LMNA	-0.05334	-0.210750095	0.106768336	5.14E-01	7.62E-01	1.00E+00
ENSG00000105726.15_ATP13A1	ENSG00000160789.18_LMNA	0.28563	0.132454807	0.425485077	3.61E-04	5.20E-03	1.00E+00
ENSG00000187773.8_FAM69C	ENSG00000160789.18_LMNA	-0.17251	-0.322842654	-0.01368342	3.36E-02	1.54E-01	1.00E+00
ENSG00000056736.8_IL17RB	ENSG00000160789.18_LMNA	0.05006	-0.110017146	0.207606505	5.40E-01	7.80E-01	1.00E+00
ENSG00000100814.16_CCNB1IP1	ENSG00000160789.18_LMNA	-0.16312	-0.314160684	-0.00402202	4.47E-02	1.87E-01	1.00E+00
ENSG00000092421.15_SEMA6A	ENSG00000160789.18_LMNA	-0.05462	-0.211979104	0.105496345	5.04E-01	7.55E-01	1.00E+00
ENSG00000136108.13_CKAP2	ENSG00000160789.18_LMNA	-0.24441	-0.388490965	-0.08865515	2.41E-03	2.26E-02	1.00E+00
ENSG00000204228.3_HSD17B8	ENSG00000160789.18_LMNA	0.13465	-0.02509338	0.287679333	9.82E-02	3.08E-01	1.00E+00
ENSG00000149196.14_HIKESHI	ENSG00000160789.18_LMNA	-0.35206	-0.484124523	-0.20430832	8.67E-06	2.60E-04	4.45E-01
ENSG00000142453.10_CARM1	ENSG00000160789.18_LMNA	0.19547	0.037430999	0.343963975	1.58E-02	9.11E-02	1.00E+00
ENSG00000269958.1_RP11-73M1	ENSG00000160789.18_LMNA	0.26496	0.110422085	0.406997392	9.71E-04	1.13E-02	1.00E+00
ENSG00000204252.11_HLA-DOA	ENSG00000160789.18_LMNA	-0.06843	-0.225176725	0.091771366	4.02E-01	6.78E-01	1.00E+00
ENSG00000204673.9_AKT1S1	ENSG00000160789.18_LMNA	0.27961	0.126019829	0.420110648	4.85E-04	6.58E-03	1.00E+00
ENSG00000160216.17_AGPAT3	ENSG00000160789.18_LMNA	0.19032	0.032095661	0.33924554	1.88E-02	1.03E-01	1.00E+00
ENSG00000162819.10_BROX	ENSG00000160789.18_LMNA	-0.13347	-0.286580807	0.026289819	1.01E-01	3.14E-01	1.00E+00
ENSG00000139624.11_CERS5	ENSG00000160789.18_LMNA	-0.06324	-0.220224271	0.096935868	4.39E-01	7.07E-01	1.00E+00
ENSG00000100726.13_TELO2	ENSG00000160789.18_LMNA	0.1741	0.015326458	0.324313974	3.19E-02	1.49E-01	1.00E+00
ENSG00000206527.8_HACD2	ENSG00000160789.18_LMNA	0.0275	-0.132282173	0.185884579	7.37E-01	8.89E-01	1.00E+00
ENSG00000175115.10_PACS1	ENSG00000160789.18_LMNA	0.27253	0.118465495	0.413774906	6.82E-04	8.60E-03	1.00E+00
ENSG00000148123.13_PLPPR1	ENSG00000160789.18_LMNA	-0.50912	-0.618209122	-0.38077704	2.12E-11	6.33E-09	1.09E-06
ENSG00000132286.10_TIMM10B	ENSG00000160789.18_LMNA	-0.12928	-0.282666394	0.030545857	1.12E-01	3.35E-01	1.00E+00
ENSG00000170473.15_PYM1	ENSG00000160789.18_LMNA	-0.19032	-0.339243025	-0.03209282	1.88E-02	1.03E-01	1.00E+00
ENSG00000107819.12_SFXN3	ENSG00000160789.18_LMNA	0.28742	0.134369533	0.427080258	3.30E-04	4.85E-03	1.00E+00
ENSG00000112186.10_CAP2	ENSG00000160789.18_LMNA	-0.06362	-0.22058454	0.096560749	4.36E-01	7.04E-01	1.00E+00
ENSG00000160688.17_FLAD1	ENSG00000160789.18_LMNA	-0.15365	-0.305379487	0.00569125	5.88E-02	2.22E-01	1.00E+00
ENSG00000215861.5_WI2-1896O	ENSG00000160789.18_LMNA	-0.24432	-0.388409289	-0.08855972	2.42E-03	2.26E-02	1.00E+00
ENSG00000244462.6_RBM12	ENSG00000160789.18_LMNA	-0.05535	-0.212672324	0.104778423	4.98E-01	7.52E-01	1.00E+00
ENSG00000005448.15_WDR54	ENSG00000160789.18_LMNA	0.10086	-0.059291076	0.255952487	2.16E-01	4.91E-01	1.00E+00

ENSG00000166454.8_ATMIN	ENSG00000160789.18_LMNA	-0.04006	-0.197999538	0.119903784	6.24E-01	8.31E-01	1.00E+00
ENSG00000267541.1_MTCO2P2	ENSG00000160789.18_LMNA	-0.12982	-0.283172305	0.029996432	1.11E-01	3.32E-01	1.00E+00
ENSG00000084676.14_NCOA1	ENSG00000160789.18_LMNA	-0.03669	-0.194749929	0.123233781	6.54E-01	8.47E-01	1.00E+00
ENSG00000123610.4_TNFAIP6	ENSG00000160789.18_LMNA	0.24167	0.08577322	0.386022454	2.70E-03	2.47E-02	1.00E+00
ENSG00000110011.12_DNAJC4	ENSG00000160789.18_LMNA	0.18627	0.027896473	0.335520985	2.16E-02	1.14E-01	1.00E+00
ENSG00000117643.13_MAN1C1	ENSG00000160789.18_LMNA	0.39012	0.246210991	0.517198983	6.75E-07	3.34E-05	3.46E-02
ENSG00000152492.12_CCDC50	ENSG00000160789.18_LMNA	-0.1453	-0.297614488	0.014231835	7.41E-02	2.59E-01	1.00E+00
ENSG00000141971.11_MVB12A	ENSG00000160789.18_LMNA	0.06471	-0.095478413	0.221623543	4.28E-01	6.99E-01	1.00E+00
ENSG00000116815.14_CD58	ENSG00000160789.18_LMNA	0.14227	-0.01731814	0.294798269	8.04E-02	2.73E-01	1.00E+00
ENSG00000130787.12_HIP1R	ENSG00000160789.18_LMNA	-0.19172	-0.34052367	-0.03353917	1.80E-02	9.99E-02	1.00E+00
ENSG00000005206.15_SPPL2B	ENSG00000160789.18_LMNA	0.11236	-0.047689803	0.266792203	1.68E-01	4.26E-01	1.00E+00
ENSG00000069248.11_NUP133	ENSG00000160789.18_LMNA	-0.14784	-0.299979619	0.011635279	6.91E-02	2.48E-01	1.00E+00
ENSG00000111145.6_ELK3	ENSG00000160789.18_LMNA	0.36304	0.216338485	0.493702669	4.29E-06	1.47E-04	2.20E-01
ENSG00000139405.14_RITA1	ENSG00000160789.18_LMNA	0.03132	-0.128524304	0.18957273	7.02E-01	8.72E-01	1.00E+00
ENSG00000175606.9_TMEM70	ENSG00000160789.18_LMNA	0.05455	-0.105562985	0.211914741	5.04E-01	7.55E-01	1.00E+00
ENSG00000166927.11_MS4A7	ENSG00000160789.18_LMNA	-0.08963	-0.245326058	0.070582012	2.72E-01	5.57E-01	1.00E+00
ENSG00000075151.18_EIF4G3	ENSG00000160789.18_LMNA	0.07377	-0.086443581	0.230268653	3.66E-01	6.47E-01	1.00E+00
ENSG00000120656.10_TAF12	ENSG00000160789.18_LMNA	0.13609	-0.023621401	0.28902972	9.46E-02	3.01E-01	1.00E+00
ENSG00000136758.17_YME1L1	ENSG00000160789.18_LMNA	0.00129	-0.157938322	0.160462562	9.87E-01	9.95E-01	1.00E+00
ENSG00000073849.13_ST6GAL1	ENSG00000160789.18_LMNA	-0.27346	-0.414607703	-0.11945652	6.53E-04	8.31E-03	1.00E+00
ENSG00000147457.12_CHMP7	ENSG00000160789.18_LMNA	0.09811	-0.062056093	0.253357225	2.29E-01	5.06E-01	1.00E+00
ENSG00000149499.10_EML3	ENSG00000160789.18_LMNA	0.28017	0.126613558	0.42060739	4.72E-04	6.45E-03	1.00E+00
ENSG00000145623.11_OSMR	ENSG00000160789.18_LMNA	0.49624	0.365945547	0.607447769	7.96E-11	1.87E-08	4.09E-06
ENSG00000090060.16_PAPOLA	ENSG00000160789.18_LMNA	0.10469	-0.055438296	0.259561167	1.99E-01	4.70E-01	1.00E+00
ENSG00000160783.18_PMF1	ENSG00000160789.18_LMNA	-0.0907	-0.246339254	0.069508938	2.66E-01	5.50E-01	1.00E+00
ENSG00000185298.11_CCDC137	ENSG00000160789.18_LMNA	0.35954	0.212504332	0.490657292	5.39E-06	1.76E-04	2.76E-01
ENSG00000135372.7_NAT10	ENSG00000160789.18_LMNA	0.21094	0.053537958	0.358115258	9.09E-03	6.12E-02	1.00E+00
ENSG00000196655.8_TRAPPC4	ENSG00000160789.18_LMNA	-0.19733	-0.34566882	-0.03936305	1.48E-02	8.72E-02	1.00E+00
ENSG00000205220.10_PSMB10	ENSG00000160789.18_LMNA	0.05596	-0.104172542	0.213257108	4.94E-01	7.49E-01	1.00E+00
ENSG00000064787.11_BCAS1	ENSG00000160789.18_LMNA	-0.35558	-0.48719989	-0.20816179	6.94E-06	2.18E-04	3.56E-01
ENSG00000150867.12_PIP4K2A	ENSG00000160789.18_LMNA	-0.19481	-0.343363741	-0.03675132	1.62E-02	9.26E-02	1.00E+00
ENSG00000162923.13_WDR26	ENSG00000160789.18_LMNA	0.0954	-0.064782635	0.250793622	2.42E-01	5.22E-01	1.00E+00
ENSG00000108395.12_TRIM37	ENSG00000160789.18_LMNA	-0.13941	-0.292130445	0.020236344	8.67E-02	2.86E-01	1.00E+00

ENSG00000131148.7_EMC8	ENSG00000160789.18_LMNA	-0.07996	-0.236152527	0.080264523	3.27E-01	6.14E-01	1.00E+00
ENSG00000184203.6_PPP1R2	ENSG00000160789.18_LMNA	-0.13846	-0.291241912	0.021207087	8.89E-02	2.90E-01	1.00E+00
ENSG00000146859.6_TMEM140	ENSG00000160789.18_LMNA	0.00175	-0.157494758	0.160905695	9.83E-01	9.94E-01	1.00E+00
ENSG00000108797.10_CNTNAP1	ENSG00000160789.18_LMNA	0.18293	0.024442509	0.332450196	2.41E-02	1.23E-01	1.00E+00
ENSG00000178764.7_ZHX2	ENSG00000160789.18_LMNA	-0.20701	-0.354528829	-0.04944084	1.05E-02	6.78E-02	1.00E+00
ENSG00000159131.15_GART	ENSG00000160789.18_LMNA	0.02698	-0.132793839	0.185381712	7.41E-01	8.91E-01	1.00E+00
ENSG00000111605.15_CPSF6	ENSG00000160789.18_LMNA	-0.02513	-0.183599742	0.134605622	7.59E-01	8.99E-01	1.00E+00
ENSG00000088367.19_EPB41L1	ENSG00000160789.18_LMNA	-0.1564	-0.307930117	0.002875921	5.43E-02	2.12E-01	1.00E+00
ENSG00000188674.9_C2orf80	ENSG00000160789.18_LMNA	-0.19836	-0.346615746	-0.04043716	1.43E-02	8.49E-02	1.00E+00
ENSG00000061337.14_LZTS1	ENSG00000160789.18_LMNA	0.51051	0.382391216	0.619374979	1.83E-11	5.59E-09	9.39E-07
ENSG00000113594.8_LIFR	ENSG00000160789.18_LMNA	-0.22235	-0.368505685	-0.06546609	5.90E-03	4.45E-02	1.00E+00
ENSG00000149313.9_AASDHPPT	ENSG00000160789.18_LMNA	-0.41392	-0.537685988	-0.27268461	1.15E-07	8.13E-06	5.92E-03
ENSG00000133884.8_DPF2	ENSG00000160789.18_LMNA	-0.05166	-0.209140803	0.108432341	5.27E-01	7.72E-01	1.00E+00
ENSG00000138614.13_VWA9	ENSG00000160789.18_LMNA	0.07944	-0.080782468	0.235660215	3.31E-01	6.17E-01	1.00E+00
ENSG00000126803.9_HSPA2	ENSG00000160789.18_LMNA	0.00973	-0.14970138	0.168670636	9.05E-01	9.64E-01	1.00E+00
ENSG00000155561.13_NUP205	ENSG00000160789.18_LMNA	0.17931	0.020700292	0.329115746	2.71E-02	1.34E-01	1.00E+00
ENSG00000109445.9_ZNF330	ENSG00000160789.18_LMNA	0.21938	0.062362134	0.365809139	6.62E-03	4.83E-02	1.00E+00
ENSG00000064545.13_TMEM161	ENSG00000160789.18_LMNA	0.23158	0.075154391	0.376889663	4.10E-03	3.37E-02	1.00E+00
ENSG00000104142.9_VPS18	ENSG00000160789.18_LMNA	0.39417	0.250704336	0.520698246	5.05E-07	2.64E-05	2.59E-02
ENSG00000100897.16_DCAF11	ENSG00000160789.18_LMNA	0.03084	-0.128993817	0.189112418	7.06E-01	8.75E-01	1.00E+00
ENSG00000109956.11_B3GAT1	ENSG00000160789.18_LMNA	-0.20097	-0.349001805	-0.04314685	1.30E-02	7.93E-02	1.00E+00
ENSG00000115520.7_COQ10B	ENSG00000160789.18_LMNA	0.03016	-0.129667205	0.188451982	7.12E-01	8.78E-01	1.00E+00
ENSG00000099204.17_ABLIM1	ENSG00000160789.18_LMNA	-0.10259	-0.257579091	0.057555606	2.09E-01	4.81E-01	1.00E+00
ENSG00000156469.7_MTERF3	ENSG00000160789.18_LMNA	-0.11385	-0.268185359	0.046192592	1.63E-01	4.18E-01	1.00E+00
ENSG00000167842.14_MIS12	ENSG00000160789.18_LMNA	-0.20419	-0.351948497	-0.04649943	1.16E-02	7.30E-02	1.00E+00
ENSG00000171621.12_SPSB1	ENSG00000160789.18_LMNA	0.26781	0.113444002	0.409547546	8.51E-04	1.02E-02	1.00E+00
ENSG00000168214.19_RBPJ	ENSG00000160789.18_LMNA	-0.36675	-0.496936677	-0.22041951	3.36E-06	1.21E-04	1.73E-01
ENSG00000153310.17_FAM49B	ENSG00000160789.18_LMNA	-0.3546	-0.486347366	-0.2070927	7.39E-06	2.29E-04	3.79E-01
ENSG00000176845.11_METRNL	ENSG00000160789.18_LMNA	0.55216	0.430830874	0.653881488	1.66E-13	1.34E-10	8.50E-09
ENSG00000149781.11_FERMT3	ENSG00000160789.18_LMNA	0.15451	-0.004807266	0.30618084	5.73E-02	2.19E-01	1.00E+00
ENSG00000104613.10_INTS10	ENSG00000160789.18_LMNA	-0.16575	-0.316599785	-0.00673045	4.13E-02	1.77E-01	1.00E+00
ENSG00000133612.17_AGAP3	ENSG00000160789.18_LMNA	0.01584	-0.143719198	0.174604302	8.46E-01	9.40E-01	1.00E+00
ENSG00000130714.14_POMT1	ENSG00000160789.18_LMNA	-0.01537	-0.174144083	0.144184013	8.51E-01	9.41E-01	1.00E+00

ENSG00000114745.12_GORASP1	ENSG00000160789.18_LMNA	-0.27016	-0.411652323	-0.11594228	7.63E-04	9.35E-03	1.00E+00
ENSG00000141456.13_PELP1	ENSG00000160789.18_LMNA	0.09938	-0.060778177	0.254557249	2.23E-01	4.99E-01	1.00E+00
ENSG00000183798.4_EMILIN3	ENSG00000160789.18_LMNA	-0.02912	-0.187449652	0.130688631	7.22E-01	8.82E-01	1.00E+00
ENSG00000143363.14_PRUNE1	ENSG00000160789.18_LMNA	-0.18465	-0.334034488	-0.02622357	2.28E-02	1.18E-01	1.00E+00
ENSG00000171055.13_FEZ2	ENSG00000160789.18_LMNA	-0.07163	-0.228223702	0.088585433	3.81E-01	6.59E-01	1.00E+00
ENSG00000149809.13_TM7SF2	ENSG00000160789.18_LMNA	-0.10578	-0.260595405	0.054332368	1.95E-01	4.63E-01	1.00E+00
ENSG00000120725.11_SIL1	ENSG00000160789.18_LMNA	0.39225	0.248575602	0.51904159	5.80E-07	2.96E-05	2.97E-02
ENSG00000164252.11_AGGF1	ENSG00000160789.18_LMNA	0.1117	-0.048355962	0.266171919	1.71E-01	4.30E-01	1.00E+00
ENSG00000172458.4_IL17D	ENSG00000160789.18_LMNA	-0.46475	-0.580969784	-0.32996799	1.62E-09	2.33E-07	8.32E-05
ENSG00000148925.9_BTBD10	ENSG00000160789.18_LMNA	-0.11928	-0.2732896	0.040695016	1.43E-01	3.87E-01	1.00E+00
ENSG00000011295.14_TTC19	ENSG00000160789.18_LMNA	-0.26108	-0.403513586	-0.10630264	1.16E-03	1.29E-02	1.00E+00
ENSG00000031823.13_RANBP3	ENSG00000160789.18_LMNA	0.04777	-0.112289493	0.205403847	5.59E-01	7.92E-01	1.00E+00
ENSG00000041802.9_LSG1	ENSG00000160789.18_LMNA	0.15876	-0.000455439	0.310119458	5.08E-02	2.03E-01	1.00E+00
ENSG00000161091.11_MFSD12	ENSG00000160789.18_LMNA	0.38499	0.24053225	0.512763576	9.71E-07	4.48E-05	4.98E-02
ENSG00000120860.9_CCDC53	ENSG00000160789.18_LMNA	-0.17682	-0.326823158	-0.01813235	2.93E-02	1.41E-01	1.00E+00
ENSG00000131652.12_THOC6	ENSG00000160789.18_LMNA	0.00995	-0.149489994	0.168880702	9.03E-01	9.63E-01	1.00E+00
ENSG00000116791.12_CRYZ	ENSG00000160789.18_LMNA	0.14549	-0.014040299	0.297789083	7.37E-02	2.58E-01	1.00E+00
ENSG00000114209.13_PDCD10	ENSG00000160789.18_LMNA	-0.21098	-0.358148552	-0.05357604	9.08E-03	6.11E-02	1.00E+00
ENSG00000275700.3_AATF	ENSG00000160789.18_LMNA	0.1107	-0.049375259	0.265222312	1.75E-01	4.35E-01	1.00E+00
ENSG00000178053.16_MLF1	ENSG00000160789.18_LMNA	-0.14879	-0.30086304	0.010664337	6.73E-02	2.43E-01	1.00E+00
ENSG00000130956.12_HABP4	ENSG00000160789.18_LMNA	0.04458	-0.115444474	0.202340278	5.86E-01	8.08E-01	1.00E+00
ENSG00000184967.5_NOC4L	ENSG00000160789.18_LMNA	0.12453	-0.035367058	0.278219544	1.26E-01	3.59E-01	1.00E+00
ENSG00000138433.14_CIR1	ENSG00000160789.18_LMNA	-0.20443	-0.352172313	-0.04675435	1.15E-02	7.25E-02	1.00E+00
ENSG00000106077.17_ABHD11	ENSG00000160789.18_LMNA	0.25884	0.103922252	0.40149656	1.28E-03	1.40E-02	1.00E+00
ENSG00000012232.7_EXTL3	ENSG00000160789.18_LMNA	0.26145	0.106693633	0.403844618	1.14E-03	1.28E-02	1.00E+00
ENSG00000105011.7_ASF1B	ENSG00000160789.18_LMNA	0.17426	0.01548696	0.324457622	3.18E-02	1.49E-01	1.00E+00
ENSG00000173153.12_ESRRA	ENSG00000160789.18_LMNA	0.23474	0.078469083	0.379746797	3.60E-03	3.06E-02	1.00E+00
ENSG00000153827.12_TRIP12	ENSG00000160789.18_LMNA	0.20323	0.045505346	0.351075399	1.20E-02	7.49E-02	1.00E+00
ENSG00000090520.9_DNAJB11	ENSG00000160789.18_LMNA	0.24418	0.088415822	0.388286131	2.43E-03	2.27E-02	1.00E+00
ENSG00000134594.4_RAB33A	ENSG00000160789.18_LMNA	-0.47325	-0.588141551	-0.33964132	7.41E-10	1.23E-07	3.80E-05
ENSG00000251442.4_LINC01094	ENSG00000160789.18_LMNA	-0.28268	-0.42285319	-0.12930047	4.18E-04	5.86E-03	1.00E+00
ENSG00000172977.11_KAT5	ENSG00000160789.18_LMNA	-0.13823	-0.291026914	0.021441889	8.94E-02	2.91E-01	1.00E+00
ENSG00000188765.7_TMSB4XP2	ENSG00000160789.18_LMNA	0.00741	-0.151966593	0.166417756	9.28E-01	9.72E-01	1.00E+00

ENSG00000099977.12_DDT	ENSG00000160789.18_LMNA	0.0705	-0.08970689	0.227151865	3.88E-01	6.66E-01	1.00E+00
ENSG00000064666.13_CNN2	ENSG00000160789.18_LMNA	0.36001	0.213021591	0.491068535	5.22E-06	1.72E-04	2.68E-01
ENSG00000038945.13_MSR1	ENSG00000160789.18_LMNA	0.00533	-0.154003426	0.164389169	9.48E-01	9.79E-01	1.00E+00
ENSG00000196636.7_SDHAF3	ENSG00000160789.18_LMNA	-0.16508	-0.315972537	-0.0060335	4.21E-02	1.80E-01	1.00E+00
ENSG00000135454.12_B4GALNT	ENSG00000160789.18_LMNA	-0.28097	-0.421325474	-0.12747222	4.54E-04	6.26E-03	1.00E+00
ENSG00000146676.6_PURB	ENSG00000160789.18_LMNA	0.15336	-0.005990735	0.305107897	5.93E-02	2.24E-01	1.00E+00
ENSG00000142583.16_SLC2A5	ENSG00000160789.18_LMNA	-0.02301	-0.181545315	0.136691783	7.78E-01	9.09E-01	1.00E+00
ENSG00000107625.11_DDX50	ENSG00000160789.18_LMNA	-0.05725	-0.214496455	0.102887706	4.84E-01	7.41E-01	1.00E+00
ENSG00000004534.13_RBM6	ENSG00000160789.18_LMNA	-0.00892	-0.16788531	0.150491385	9.13E-01	9.67E-01	1.00E+00
ENSG00000248275.1_TRIM52-AS	ENSG00000160789.18_LMNA	-0.25047	-0.393964371	-0.09506319	1.86E-03	1.85E-02	1.00E+00
ENSG00000137959.14_IFI44L	ENSG00000160789.18_LMNA	-0.13065	-0.283941926	0.029160252	1.09E-01	3.28E-01	1.00E+00
ENSG00000205929.8_C21orf62	ENSG00000160789.18_LMNA	0.25427	0.099082546	0.397386707	1.57E-03	1.63E-02	1.00E+00
ENSG00000166292.10_TMEM100	ENSG00000160789.18_LMNA	-0.45166	-0.569887563	-0.31512317	5.20E-09	6.18E-07	2.67E-04
ENSG00000112902.10_SEMA5A	ENSG00000160789.18_LMNA	0.02237	-0.137320027	0.180926084	7.84E-01	9.12E-01	1.00E+00
ENSG00000113391.15_FAM172A	ENSG00000160789.18_LMNA	-0.03022	-0.188511722	0.129606305	7.12E-01	8.77E-01	1.00E+00
ENSG00000114480.11_GBE1	ENSG00000160789.18_LMNA	0.26208	0.107359873	0.404408507	1.11E-03	1.25E-02	1.00E+00
ENSG00000148450.11_MSRB2	ENSG00000160789.18_LMNA	-0.25785	-0.400606601	-0.10287305	1.34E-03	1.44E-02	1.00E+00
ENSG00000185414.18_MRPL30	ENSG00000160789.18_LMNA	-0.25475	-0.39781572	-0.09958709	1.54E-03	1.60E-02	1.00E+00
ENSG00000111331.11_OAS3	ENSG00000160789.18_LMNA	0.18158	0.023041114	0.331202405	2.52E-02	1.27E-01	1.00E+00
ENSG00000130479.9_MAP1S	ENSG00000160789.18_LMNA	0.19207	0.033908441	0.340850452	1.78E-02	9.91E-02	1.00E+00
ENSG00000116337.14_AMPD2	ENSG00000160789.18_LMNA	0.23232	0.075930961	0.377559552	3.97E-03	3.30E-02	1.00E+00
ENSG00000101463.5_SYNDIG1	ENSG00000160789.18_LMNA	-0.30488	-0.442593851	-0.1531055	1.34E-04	2.38E-03	1.00E+00
ENSG00000130055.12_GDPD2	ENSG00000160789.18_LMNA	0.17852	0.019883185	0.328386649	2.78E-02	1.36E-01	1.00E+00
ENSG00000158019.19_BRE	ENSG00000160789.18_LMNA	-0.10521	-0.260050905	0.054914708	1.97E-01	4.67E-01	1.00E+00
ENSG00000133874.1_RNF122	ENSG00000160789.18_LMNA	-0.23192	-0.377190137	-0.07550267	4.04E-03	3.34E-02	1.00E+00
ENSG00000012822.14_CALCOC	ENSG00000160789.18_LMNA	-0.30705	-0.444518435	-0.15544445	1.19E-04	2.18E-03	1.00E+00
ENSG00000170502.11_NUDT9	ENSG00000160789.18_LMNA	0.15528	-0.004018106	0.306895859	5.61E-02	2.16E-01	1.00E+00
ENSG00000108055.9_SMC3	ENSG00000160789.18_LMNA	0.04808	-0.111972904	0.20571092	5.56E-01	7.90E-01	1.00E+00
ENSG00000106689.9_LHX2	ENSG00000160789.18_LMNA	0.14321	-0.016363108	0.295670304	7.84E-02	2.69E-01	1.00E+00
ENSG00000123240.15_OPTN	ENSG00000160789.18_LMNA	-0.08711	-0.24294327	0.073102743	2.86E-01	5.71E-01	1.00E+00
ENSG00000103510.18_KAT8	ENSG00000160789.18_LMNA	-0.13527	-0.288260996	0.024459509	9.66E-02	3.05E-01	1.00E+00
ENSG00000157911.8_PEX10	ENSG00000160789.18_LMNA	0.03075	-0.129078983	0.189028905	7.07E-01	8.75E-01	1.00E+00
ENSG00000072135.11_PTPN18	ENSG00000160789.18_LMNA	0.17342	0.014619436	0.32368103	3.26E-02	1.51E-01	1.00E+00

ENSG00000164237.7_CMBL	ENSG00000160789.18_LMNA	-0.19929	-0.347466207	-0.04140246	1.38E-02	8.28E-02	1.00E+00
ENSG00000138777.18_PPA2	ENSG00000160789.18_LMNA	-0.12031	-0.274257496	0.039650385	1.40E-01	3.81E-01	1.00E+00
ENSG00000119326.13_CTNNAL1	ENSG00000160789.18_LMNA	0.18064	0.022075225	0.330341755	2.59E-02	1.30E-01	1.00E+00
ENSG00000181019.11_NQO1	ENSG00000160789.18_LMNA	0.19664	0.038644423	0.34503494	1.52E-02	8.86E-02	1.00E+00
ENSG00000138495.5_COX17	ENSG00000160789.18_LMNA	-0.14286	-0.295342598	0.016722071	7.91E-02	2.70E-01	1.00E+00
ENSG00000090263.14_MRPS33	ENSG00000160789.18_LMNA	-0.20561	-0.353250273	-0.04798271	1.10E-02	7.03E-02	1.00E+00
ENSG00000204482.9_LST1	ENSG00000160789.18_LMNA	-0.17417	-0.3243734	-0.01539285	3.19E-02	1.49E-01	1.00E+00
ENSG00000156453.12_PCDH1	ENSG00000160789.18_LMNA	0.20246	0.044698492	0.35036635	1.24E-02	7.64E-02	1.00E+00
ENSG00000080573.6_COL5A3	ENSG00000160789.18_LMNA	0.34142	0.192697732	0.474816407	1.67E-05	4.45E-04	8.58E-01
ENSG00000160410.13_SHKBP1	ENSG00000160789.18_LMNA	0.13279	-0.026978712	0.285947918	1.03E-01	3.17E-01	1.00E+00
ENSG00000189369.8_GSPT2	ENSG00000160789.18_LMNA	-0.13929	-0.29201421	0.020363366	8.70E-02	2.87E-01	1.00E+00
ENSG00000056998.17_GYG2	ENSG00000160789.18_LMNA	0.07008	-0.090125345	0.226751729	3.91E-01	6.69E-01	1.00E+00
ENSG00000112983.16_BRD8	ENSG00000160789.18_LMNA	-0.29463	-0.433501483	-0.14209923	2.29E-04	3.64E-03	1.00E+00
ENSG00000159596.6_TMEM69	ENSG00000160789.18_LMNA	-0.02626	-0.184687566	0.133499851	7.48E-01	8.95E-01	1.00E+00
ENSG00000165775.16_FUNDC2	ENSG00000160789.18_LMNA	-0.38762	-0.515042248	-0.24344735	8.06E-07	3.83E-05	4.14E-02
ENSG00000116260.15_QSOX1	ENSG00000160789.18_LMNA	0.39684	0.253666503	0.523000129	4.16E-07	2.27E-05	2.13E-02
ENSG00000174720.14_LARP7	ENSG00000160789.18_LMNA	-0.05048	-0.208006799	0.10960383	5.37E-01	7.78E-01	1.00E+00
ENSG00000171425.8_ZNF581	ENSG00000160789.18_LMNA	0.1158	-0.044217872	0.270020827	1.55E-01	4.07E-01	1.00E+00
ENSG00000119523.9_ALG2	ENSG00000160789.18_LMNA	0.32577	0.175680844	0.4610591	4.21E-05	9.36E-04	1.00E+00
ENSG00000050130.16_JKAMP	ENSG00000160789.18_LMNA	-0.11857	-0.272623895	0.041413099	1.46E-01	3.91E-01	1.00E+00
ENSG00000109787.11_KLF3	ENSG00000160789.18_LMNA	0.17069	0.011806459	0.321160036	3.55E-02	1.60E-01	1.00E+00
ENSG00000132768.12_DPH2	ENSG00000160789.18_LMNA	0.09188	-0.068316994	0.247463877	2.60E-01	5.43E-01	1.00E+00
ENSG00000256073.3_URB1-AS1	ENSG00000160789.18_LMNA	-0.10673	-0.261488248	0.053377014	1.91E-01	4.58E-01	1.00E+00
ENSG00000077782.18_FGFR1	ENSG00000160789.18_LMNA	0.55567	0.434948352	0.656772443	1.08E-13	9.24E-11	5.55E-09
ENSG00000232472.1_EEF1B2P3	ENSG00000160789.18_LMNA	-0.15725	-0.308721923	0.00200094	5.30E-02	2.09E-01	1.00E+00
ENSG00000085721.11_RRN3	ENSG00000160789.18_LMNA	-0.02974	-0.188054395	0.130072447	7.16E-01	8.79E-01	1.00E+00
ENSG00000108592.15_FTSJ3	ENSG00000160789.18_LMNA	0.29063	0.137807496	0.429939899	2.81E-04	4.27E-03	1.00E+00
ENSG00000122779.15_TRIM24	ENSG00000160789.18_LMNA	0.2056	0.047964268	0.353234097	1.11E-02	7.03E-02	1.00E+00
ENSG00000189306.9_RRP7A	ENSG00000160789.18_LMNA	-0.05822	-0.215424576	0.101924826	4.76E-01	7.35E-01	1.00E+00
ENSG00000123064.11_DDX54	ENSG00000160789.18_LMNA	0.28847	0.13549453	0.428016657	3.13E-04	4.65E-03	1.00E+00
ENSG00000166896.6_ATP23	ENSG00000160789.18_LMNA	-0.30967	-0.446843719	-0.1582747	1.03E-04	1.94E-03	1.00E+00
ENSG00000109775.9_UFSP2	ENSG00000160789.18_LMNA	-0.03226	-0.190484251	0.127594142	6.93E-01	8.68E-01	1.00E+00
ENSG00000125352.5_RNF113A	ENSG00000160789.18_LMNA	-0.06472	-0.221637152	0.095464231	4.28E-01	6.99E-01	1.00E+00

ENSG00000060982.13_BCAT1	ENSG00000160789.18_LMNA	0.25628	0.101213819	0.399198048	1.44E-03	1.52E-02	1.00E+00
ENSG00000197713.13_RPE	ENSG00000160789.18_LMNA	0.02976	-0.130059498	0.188067101	7.16E-01	8.79E-01	1.00E+00
ENSG00000118200.13_CAMSAP2	ENSG00000160789.18_LMNA	-0.2254	-0.371280431	-0.0686662	5.24E-03	4.06E-02	1.00E+00
ENSG00000185880.11_TRIM69	ENSG00000160789.18_LMNA	-0.14085	-0.293465732	0.018776403	8.35E-02	2.79E-01	1.00E+00
ENSG00000140694.15_PARN	ENSG00000160789.18_LMNA	0.22155	0.064625703	0.367776104	6.09E-03	4.55E-02	1.00E+00
ENSG00000164088.16_PPM1M	ENSG00000160789.18_LMNA	0.11029	-0.049788668	0.264836995	1.76E-01	4.37E-01	1.00E+00
ENSG00000144231.9_POLR2D	ENSG00000160789.18_LMNA	-0.23698	-0.381775807	-0.08082708	3.29E-03	2.86E-02	1.00E+00
ENSG00000074657.12_ZNF532	ENSG00000160789.18_LMNA	0.13182	-0.027962991	0.285043184	1.05E-01	3.22E-01	1.00E+00
ENSG00000164902.12_PHAX	ENSG00000160789.18_LMNA	0.05954	-0.100618348	0.216682974	4.66E-01	7.28E-01	1.00E+00
ENSG00000135002.10_RFK	ENSG00000160789.18_LMNA	-0.00442	-0.163502786	0.154892563	9.57E-01	9.83E-01	1.00E+00
ENSG00000210144.1_MT-TY	ENSG00000160789.18_LMNA	-0.22889	-0.374448538	-0.0723276	4.56E-03	3.66E-02	1.00E+00
ENSG00000100359.19_SGSM3	ENSG00000160789.18_LMNA	-0.07012	-0.226791782	0.090083463	3.91E-01	6.69E-01	1.00E+00
ENSG00000197872.10_FAM49A	ENSG00000160789.18_LMNA	-0.23843	-0.383086676	-0.08235229	3.10E-03	2.74E-02	1.00E+00
ENSG00000165915.12_SLC39A13	ENSG00000160789.18_LMNA	0.38248	0.23775829	0.510591666	1.16E-06	5.18E-05	5.94E-02
ENSG00000135643.4_KCNMB4	ENSG00000160789.18_LMNA	-0.11194	-0.266391865	0.048119783	1.70E-01	4.28E-01	1.00E+00
ENSG00000137497.16_NUMA1	ENSG00000160789.18_LMNA	0.13418	-0.025563509	0.287247776	9.93E-02	3.10E-01	1.00E+00
ENSG00000161526.13_SAP30BP	ENSG00000160789.18_LMNA	-0.16042	-0.311665326	-0.00125583	4.83E-02	1.97E-01	1.00E+00
ENSG00000102471.12_NDFIP2	ENSG00000160789.18_LMNA	-0.3332	-0.467595604	-0.18374486	2.73E-05	6.64E-04	1.00E+00
ENSG00000135972.7_MRPS9	ENSG00000160789.18_LMNA	-0.13628	-0.289206701	0.023428384	9.41E-02	3.00E-01	1.00E+00
ENSG00000114698.13_PLSCR4	ENSG00000160789.18_LMNA	-0.07948	-0.235694457	0.080746448	3.30E-01	6.16E-01	1.00E+00
ENSG00000170632.12_ARMC10	ENSG00000160789.18_LMNA	0.05011	-0.109964694	0.207657311	5.40E-01	7.80E-01	1.00E+00
ENSG00000064300.7_NGFR	ENSG00000160789.18_LMNA	0.03169	-0.1281592	0.189930583	6.98E-01	8.71E-01	1.00E+00
ENSG00000135678.10_CPM	ENSG00000160789.18_LMNA	-0.20745	-0.354927509	-0.04989579	1.03E-02	6.71E-02	1.00E+00
ENSG00000116698.19_SMG7	ENSG00000160789.18_LMNA	0.19912	0.041224378	0.347309353	1.39E-02	8.31E-02	1.00E+00
ENSG00000099991.15_CABIN1	ENSG00000160789.18_LMNA	0.11875	-0.04122804	0.272795485	1.45E-01	3.90E-01	1.00E+00
ENSG00000114544.14_SLC41A3	ENSG00000160789.18_LMNA	-0.12023	-0.274181257	0.039732693	1.40E-01	3.82E-01	1.00E+00
ENSG00000085231.12_AK6	ENSG00000160789.18_LMNA	0.05158	-0.10851502	0.209060797	5.28E-01	7.72E-01	1.00E+00
ENSG00000122490.17_PQLC1	ENSG00000160789.18_LMNA	0.17453	0.015769262	0.324710243	3.15E-02	1.48E-01	1.00E+00
ENSG00000070423.16_RNF126	ENSG00000160789.18_LMNA	0.11082	-0.049254533	0.265334816	1.74E-01	4.34E-01	1.00E+00
ENSG00000079805.15_DNM2	ENSG00000160789.18_LMNA	0.15084	-0.008566792	0.30276969	6.36E-02	2.35E-01	1.00E+00
ENSG00000104529.16_EEF1D	ENSG00000160789.18_LMNA	-0.00761	-0.166607745	0.151775692	9.26E-01	9.72E-01	1.00E+00
ENSG00000078246.14_TULP3	ENSG00000160789.18_LMNA	0.24842	0.09289451	0.39211437	2.03E-03	1.97E-02	1.00E+00
ENSG00000167851.12_CD300A	ENSG00000160789.18_LMNA	-0.04027	-0.198197461	0.119700733	6.22E-01	8.29E-01	1.00E+00

ENSG00000213398.6_LCAT	ENSG00000160789.18_LMNA	-0.10224	-0.257257713	0.057898644	2.10E-01	4.83E-01	1.00E+00
ENSG00000146433.8_TMEM181	ENSG00000160789.18_LMNA	0.06843	-0.091769519	0.225178493	4.02E-01	6.78E-01	1.00E+00
ENSG00000197329.10_PELI1	ENSG00000160789.18_LMNA	-0.42158	-0.54425062	-0.28125327	6.34E-08	5.00E-06	3.25E-03
ENSG00000063978.14_RNF4	ENSG00000160789.18_LMNA	0.21949	0.06247666	0.365908723	6.59E-03	4.82E-02	1.00E+00
ENSG00000204348.8_DXO	ENSG00000160789.18_LMNA	-0.08216	-0.238248072	0.07805796	3.14E-01	6.01E-01	1.00E+00
ENSG00000227766.1_HCG4P5	ENSG00000160789.18_LMNA	0.22349	0.066657908	0.369539714	5.65E-03	4.30E-02	1.00E+00
ENSG00000184205.13_TSPYL2	ENSG00000160789.18_LMNA	-0.16658	-0.3173671	-0.00758343	4.02E-02	1.74E-01	1.00E+00
ENSG00000140092.13_FBLN5	ENSG00000160789.18_LMNA	0.16399	0.004919529	0.314969398	4.35E-02	1.84E-01	1.00E+00
ENSG00000104549.10_SQLE	ENSG00000160789.18_LMNA	0.00121	-0.158024032	0.16037692	9.88E-01	9.96E-01	1.00E+00
ENSG00000144034.13_TPRKB	ENSG00000160789.18_LMNA	-0.3086	-0.445898062	-0.15712311	1.10E-04	2.03E-03	1.00E+00
ENSG00000184743.11_ATL3	ENSG00000160789.18_LMNA	0.35399	0.20642028	0.485810886	7.68E-06	2.37E-04	3.94E-01
ENSG00000119139.15_TJP2	ENSG00000160789.18_LMNA	-0.20507	-0.352752187	-0.04741501	1.13E-02	7.13E-02	1.00E+00
ENSG00000143499.12_SMYD2	ENSG00000160789.18_LMNA	0.12761	-0.032247132	0.281098754	1.17E-01	3.43E-01	1.00E+00
ENSG00000279118.1_RP11-517I3	ENSG00000160789.18_LMNA	-0.21752	-0.364108251	-0.06040728	7.10E-03	5.10E-02	1.00E+00
ENSG00000179041.3_RRS1	ENSG00000160789.18_LMNA	0.27429	0.120344067	0.415353122	6.27E-04	8.06E-03	1.00E+00
ENSG00000110047.16_EHD1	ENSG00000160789.18_LMNA	0.10605	-0.054067176	0.260843299	1.93E-01	4.62E-01	1.00E+00
ENSG00000172005.9_MAL	ENSG00000160789.18_LMNA	-0.26503	-0.407060004	-0.11049622	9.68E-04	1.13E-02	1.00E+00
ENSG00000182902.12_SLC25A18	ENSG00000160789.18_LMNA	-0.10348	-0.258420341	0.056657305	2.05E-01	4.76E-01	1.00E+00
ENSG00000160325.13_CACFD1	ENSG00000160789.18_LMNA	0.01865	-0.14096444	0.177328966	8.20E-01	9.30E-01	1.00E+00
ENSG00000187068.2_C3orf70	ENSG00000160789.18_LMNA	-0.23361	-0.378723838	-0.07728154	3.77E-03	3.18E-02	1.00E+00
ENSG00000114302.14_PRKAR2A	ENSG00000160789.18_LMNA	-0.13419	-0.287250213	0.025560855	9.93E-02	3.10E-01	1.00E+00
ENSG00000164828.16_SUN1	ENSG00000160789.18_LMNA	0.22014	0.063148993	0.3664932	6.43E-03	4.74E-02	1.00E+00
ENSG00000025796.12_SEC63	ENSG00000160789.18_LMNA	0.04128	-0.118705068	0.199167607	6.14E-01	8.24E-01	1.00E+00
ENSG00000142552.6_RCN3	ENSG00000160789.18_LMNA	0.38735	0.243140483	0.514802555	8.22E-07	3.88E-05	4.22E-02
ENSG00000066583.10_ISOC1	ENSG00000160789.18_LMNA	-0.11122	-0.265713376	0.04884824	1.73E-01	4.32E-01	1.00E+00
ENSG00000163110.13_PDLIM5	ENSG00000160789.18_LMNA	0.16192	0.002787757	0.313047793	4.63E-02	1.91E-01	1.00E+00
ENSG00000136144.10_RCBTB1	ENSG00000160789.18_LMNA	-0.48234	-0.595793179	-0.35002017	3.13E-10	5.92E-08	1.60E-05
ENSG00000143207.18_RFWD2	ENSG00000160789.18_LMNA	-0.20954	-0.356833388	-0.05207238	9.57E-03	6.36E-02	1.00E+00
ENSG00000149179.12_C11orf49	ENSG00000160789.18_LMNA	-0.08396	-0.239953281	0.076260122	3.04E-01	5.90E-01	1.00E+00
ENSG00000234782.3_TPT1P9	ENSG00000160789.18_LMNA	-0.08256	-0.238628249	0.077657309	3.12E-01	5.98E-01	1.00E+00
ENSG00000163132.6_MSX1	ENSG00000160789.18_LMNA	-0.08784	-0.243633742	0.072372713	2.82E-01	5.67E-01	1.00E+00
ENSG00000157637.11_SLC38A10	ENSG00000160789.18_LMNA	0.36585	0.219434621	0.496156902	3.57E-06	1.27E-04	1.83E-01
ENSG00000168938.5_PPIC	ENSG00000160789.18_LMNA	0.34829	0.200184965	0.480826099	1.10E-05	3.14E-04	5.63E-01

ENSG00000160007.16_ARHGAP3	ENSG00000160789.18_LMNA	0.09568	-0.064501358	0.251058294	2.41E-01	5.20E-01	1.00E+00
ENSG00000183520.10_UTP11	ENSG00000160789.18_LMNA	0.03248	-0.127377893	0.190696087	6.91E-01	8.67E-01	1.00E+00
ENSG00000026297.14_RNASET2	ENSG00000160789.18_LMNA	-0.01839	-0.177076067	0.141220339	8.22E-01	9.30E-01	1.00E+00
ENSG00000133731.8_IMPA1	ENSG00000160789.18_LMNA	-0.31029	-0.447387991	-0.15893785	1.00E-04	1.88E-03	1.00E+00
ENSG00000095319.13_NUP188	ENSG00000160789.18_LMNA	0.2643	0.109715216	0.406400206	1.00E-03	1.16E-02	1.00E+00
ENSG00000234171.2_RNASEH1-/	ENSG00000160789.18_LMNA	-0.17689	-0.326881837	-0.01819803	2.93E-02	1.41E-01	1.00E+00
ENSG00000108604.14_SMARCD2	ENSG00000160789.18_LMNA	0.12618	-0.033702544	0.279756335	1.21E-01	3.51E-01	1.00E+00
ENSG00000218510.5_LINC00339	ENSG00000160789.18_LMNA	-0.02781	-0.186189809	0.131971519	7.34E-01	8.88E-01	1.00E+00
ENSG00000104164.9_BLOC1S6	ENSG00000160789.18_LMNA	-0.22167	-0.367890828	-0.06475782	6.06E-03	4.53E-02	1.00E+00
ENSG00000119328.10_FAM206A	ENSG00000160789.18_LMNA	0.00484	-0.154475933	0.16391819	9.53E-01	9.81E-01	1.00E+00
ENSG00000181852.16_RNF41	ENSG00000160789.18_LMNA	-0.35493	-0.486632272	-0.20744991	7.23E-06	2.26E-04	3.71E-01
ENSG00000144848.9_ATG3	ENSG00000160789.18_LMNA	-0.10509	-0.259942331	0.055030801	1.98E-01	4.67E-01	1.00E+00
ENSG00000144909.7_OSBPL11	ENSG00000160789.18_LMNA	-0.27696	-0.417738677	-0.12318764	5.52E-04	7.29E-03	1.00E+00
ENSG00000106399.10_RPA3	ENSG00000160789.18_LMNA	-0.00699	-0.166009952	0.152376271	9.32E-01	9.73E-01	1.00E+00
ENSG00000088881.19_EBF4	ENSG00000160789.18_LMNA	0.01901	-0.140613067	0.17767615	8.16E-01	9.28E-01	1.00E+00
ENSG00000165029.14_ABCA1	ENSG00000160789.18_LMNA	0.21181	0.054448199	0.358910824	8.80E-03	5.97E-02	1.00E+00
ENSG00000122068.11_FYTTD1	ENSG00000160789.18_LMNA	-0.2029	-0.35077207	-0.04516013	1.22E-02	7.56E-02	1.00E+00
ENSG00000167182.12_SP2	ENSG00000160789.18_LMNA	0.04621	-0.113828278	0.203910424	5.72E-01	8.00E-01	1.00E+00
ENSG00000166170.9_BAG5	ENSG00000160789.18_LMNA	0.21051	0.053092597	0.357725844	9.24E-03	6.19E-02	1.00E+00
ENSG00000147010.16_SH3KBP1	ENSG00000160789.18_LMNA	0.10362	-0.056514411	0.258554116	2.04E-01	4.75E-01	1.00E+00
ENSG00000166128.11_RAB8B	ENSG00000160789.18_LMNA	-0.03959	-0.197549042	0.120365852	6.28E-01	8.33E-01	1.00E+00
ENSG00000088888.16_MAVS	ENSG00000160789.18_LMNA	0.21575	0.058559822	0.362498945	7.60E-03	5.35E-02	1.00E+00
ENSG00000171492.13_LRRC8D	ENSG00000160789.18_LMNA	-0.35146	-0.4836053	-0.20365858	9.00E-06	2.67E-04	4.62E-01
ENSG00000144711.12_IQSEC1	ENSG00000160789.18_LMNA	0.19952	0.041637465	0.347673184	1.37E-02	8.23E-02	1.00E+00
ENSG00000077420.14_APBB1IP	ENSG00000160789.18_LMNA	-0.09876	-0.253970535	0.061403103	2.26E-01	5.02E-01	1.00E+00
ENSG00000140995.15_DEF8	ENSG00000160789.18_LMNA	0.05426	-0.105855765	0.211631932	5.07E-01	7.57E-01	1.00E+00
ENSG00000176396.9_EID2	ENSG00000160789.18_LMNA	0.06786	-0.09233689	0.224635219	4.06E-01	6.82E-01	1.00E+00
ENSG00000156599.9_ZDHHC5	ENSG00000160789.18_LMNA	0.35469	0.207187442	0.486422936	7.34E-06	2.28E-04	3.77E-01
ENSG00000186141.7_POLR3C	ENSG00000160789.18_LMNA	-0.07664	-0.232995525	0.083582933	3.48E-01	6.32E-01	1.00E+00
ENSG00000143390.16_RFX5	ENSG00000160789.18_LMNA	-0.14103	-0.293633847	0.018592499	8.31E-02	2.79E-01	1.00E+00
ENSG00000115525.15_ST3GAL5	ENSG00000160789.18_LMNA	-0.17563	-0.3257196	-0.0168977	3.04E-02	1.45E-01	1.00E+00
ENSG00000102858.11_MGRN1	ENSG00000160789.18_LMNA	0.11258	-0.047473816	0.266993261	1.67E-01	4.25E-01	1.00E+00
ENSG00000102030.14_NAA10	ENSG00000160789.18_LMNA	0.0164	-0.143169731	0.175148156	8.41E-01	9.38E-01	1.00E+00

ENSG00000117543.18_DPH5	ENSG00000160789.18_LMNA	-0.31413	-0.450790428	-0.16308936	8.11E-05	1.59E-03	1.00E+00
ENSG00000145362.15_ANK2	ENSG00000160789.18_LMNA	0.00786	-0.151530663	0.166851571	9.23E-01	9.71E-01	1.00E+00
ENSG00000102580.13_DNAJC3	ENSG00000160789.18_LMNA	0.28799	0.134977531	0.427586406	3.21E-04	4.74E-03	1.00E+00
ENSG00000105419.16_MEIS3	ENSG00000160789.18_LMNA	-0.07592	-0.23231724	0.084294981	3.53E-01	6.36E-01	1.00E+00
ENSG00000088812.16_ATRN	ENSG00000160789.18_LMNA	0.1388	-0.020860026	0.291559643	8.81E-02	2.89E-01	1.00E+00
ENSG00000141750.6_STAC2	ENSG00000160789.18_LMNA	0.05518	-0.104939665	0.212516657	5.00E-01	7.52E-01	1.00E+00
ENSG00000136541.13_ERMN	ENSG00000160789.18_LMNA	-0.36903	-0.498920882	-0.22292821	2.89E-06	1.07E-04	1.48E-01
ENSG00000167588.11_GPD1	ENSG00000160789.18_LMNA	-0.46867	-0.584277223	-0.33442255	1.13E-09	1.73E-07	5.81E-05
ENSG00000123374.9_CDK2	ENSG00000160789.18_LMNA	0.17805	0.019397157	0.327952797	2.82E-02	1.37E-01	1.00E+00
ENSG00000181915.4_ADO	ENSG00000160789.18_LMNA	-0.08402	-0.240007542	0.07620288	3.03E-01	5.90E-01	1.00E+00
ENSG00000153317.13_ASAP1	ENSG00000160789.18_LMNA	0.18405	0.025604446	0.333483961	2.32E-02	1.20E-01	1.00E+00
ENSG00000254986.6_DPP3	ENSG00000160789.18_LMNA	0.21286	0.055539655	0.359864191	8.47E-03	5.81E-02	1.00E+00
ENSG00000109911.16_ELP4	ENSG00000160789.18_LMNA	-0.14229	-0.294810104	0.017305183	8.03E-02	2.73E-01	1.00E+00
ENSG00000156860.14_FBRS	ENSG00000160789.18_LMNA	0.28806	0.135049837	0.427646588	3.20E-04	4.73E-03	1.00E+00
ENSG00000137693.12_YAP1	ENSG00000160789.18_LMNA	0.32792	0.178008652	0.462949151	3.72E-05	8.49E-04	1.00E+00
ENSG00000114395.9_CYB561D2	ENSG00000160789.18_LMNA	0.03449	-0.125402208	0.192630091	6.73E-01	8.57E-01	1.00E+00
ENSG00000146463.10_ZMYM4	ENSG00000160789.18_LMNA	-0.03719	-0.195236706	0.122735418	6.49E-01	8.44E-01	1.00E+00
ENSG00000232119.6_MCTS1	ENSG00000160789.18_LMNA	-0.03893	-0.196914282	0.121016682	6.34E-01	8.36E-01	1.00E+00
ENSG00000154059.8_IMPACT	ENSG00000160789.18_LMNA	-0.07421	-0.230690015	0.086001888	3.64E-01	6.45E-01	1.00E+00
ENSG00000164638.9_SLC29A4	ENSG00000160789.18_LMNA	-0.01375	-0.172569462	0.1457733	8.67E-01	9.48E-01	1.00E+00
ENSG00000167113.9_COQ4	ENSG00000160789.18_LMNA	0.04631	-0.113732213	0.204003701	5.71E-01	8.00E-01	1.00E+00
ENSG00000113282.12_CLINT1	ENSG00000160789.18_LMNA	0.10335	-0.056787295	0.258298637	2.05E-01	4.77E-01	1.00E+00
ENSG00000167220.10_HDHD2	ENSG00000160789.18_LMNA	-0.26883	-0.410464892	-0.11453239	8.11E-04	9.83E-03	1.00E+00
ENSG00000183087.13_GAS6	ENSG00000160789.18_LMNA	0.27331	0.11929808	0.414474593	6.57E-04	8.35E-03	1.00E+00
ENSG00000100324.12_TAB1	ENSG00000160789.18_LMNA	-0.08254	-0.238606418	0.077680319	3.12E-01	5.98E-01	1.00E+00
ENSG00000100225.16_FBXO7	ENSG00000160789.18_LMNA	-0.1261	-0.279682365	0.033782702	1.22E-01	3.51E-01	1.00E+00
ENSG00000160993.3_ALKBH4	ENSG00000160789.18_LMNA	0.13875	-0.020915234	0.291509105	8.82E-02	2.89E-01	1.00E+00
ENSG00000168476.10_REEP4	ENSG00000160789.18_LMNA	0.34652	0.198253035	0.479277939	1.22E-05	3.45E-04	6.28E-01
ENSG00000128606.11_LRRC17	ENSG00000160789.18_LMNA	0.07574	-0.084476739	0.232144051	3.54E-01	6.37E-01	1.00E+00
ENSG00000090339.7_ICAM1	ENSG00000160789.18_LMNA	0.44661	0.309405774	0.565594443	8.06E-09	9.05E-07	4.13E-04
ENSG00000224870.5_RP4-758J1[illegible]	ENSG00000160789.18_LMNA	0.08414	-0.076080428	0.240123609	3.03E-01	5.89E-01	1.00E+00
ENSG00000169084.11_DHRSX	ENSG00000160789.18_LMNA	0.11626	-0.043745853	0.270459222	1.54E-01	4.04E-01	1.00E+00
ENSG00000182179.9_UBA7	ENSG00000160789.18_LMNA	0.02297	-0.136727411	0.181510205	7.79E-01	9.09E-01	1.00E+00

ENSG00000104490.16_NCALD	ENSG00000160789.18_LMNA	-0.36383	-0.49439615	-0.21721278	4.08E-06	1.40E-04	2.09E-01
ENSG00000143815.13_LBR	ENSG00000160789.18_LMNA	-0.09753	-0.252804716	0.062644117	2.32E-01	5.10E-01	1.00E+00
ENSG00000131771.12_PPP1R1B	ENSG00000160789.18_LMNA	-0.36344	-0.494058905	-0.21678755	4.18E-06	1.44E-04	2.14E-01
ENSG00000176046.8_NUPR1	ENSG00000160789.18_LMNA	-0.05547	-0.212789061	0.104657493	4.97E-01	7.51E-01	1.00E+00
ENSG00000138382.12_METTL5	ENSG00000160789.18_LMNA	-0.27483	-0.41583513	-0.12091823	6.11E-04	7.91E-03	1.00E+00
ENSG00000143157.10_POGK	ENSG00000160789.18_LMNA	0.01741	-0.142181038	0.176126259	8.31E-01	9.34E-01	1.00E+00
ENSG00000198894.6_CIPC	ENSG00000160789.18_LMNA	-0.18403	-0.333458805	-0.02557616	2.32E-02	1.20E-01	1.00E+00
ENSG00000164609.8_SLU7	ENSG00000160789.18_LMNA	-0.13639	-0.289305501	0.023320622	9.38E-02	3.00E-01	1.00E+00
ENSG00000182095.13_TNRC18	ENSG00000160789.18_LMNA	0.26627	0.111805224	0.408165178	9.14E-04	1.08E-02	1.00E+00
ENSG00000165156.13_ZHX1	ENSG00000160789.18_LMNA	-0.02844	-0.186791992	0.131358454	7.28E-01	8.85E-01	1.00E+00
ENSG00000075399.11_VPS9D1	ENSG00000160789.18_LMNA	0.21244	0.055107964	0.359487193	8.60E-03	5.87E-02	1.00E+00
ENSG00000185085.2_INTS5	ENSG00000160789.18_LMNA	0.1134	-0.046643779	0.267765667	1.64E-01	4.20E-01	1.00E+00
ENSG00000120963.10_ZNF706	ENSG00000160789.18_LMNA	-0.19177	-0.340569673	-0.03359115	1.79E-02	9.98E-02	1.00E+00
ENSG00000137801.10_THBS1	ENSG00000160789.18_LMNA	0.33601	0.186797179	0.470061667	2.32E-05	5.81E-04	1.00E+00
ENSG00000076043.8_REXO2	ENSG00000160789.18_LMNA	0.24422	0.088456735	0.388321148	2.43E-03	2.27E-02	1.00E+00
ENSG00000125675.16_GRIA3	ENSG00000160789.18_LMNA	-0.20662	-0.354169595	-0.04903102	1.07E-02	6.85E-02	1.00E+00
ENSG00000186665.8_C17orf58	ENSG00000160789.18_LMNA	-0.08695	-0.242792027	0.073262605	2.87E-01	5.72E-01	1.00E+00
ENSG00000164168.6_TMEM184C	ENSG00000160789.18_LMNA	0.00171	-0.157530832	0.160869662	9.83E-01	9.94E-01	1.00E+00
ENSG00000100342.19_APOL1	ENSG00000160789.18_LMNA	0.18219	0.023679864	0.331771277	2.47E-02	1.25E-01	1.00E+00
ENSG00000111906.16_HDDC2	ENSG00000160789.18_LMNA	-0.33127	-0.465898351	-0.1816473	3.06E-05	7.26E-04	1.00E+00
ENSG00000063241.6_ISOC2	ENSG00000160789.18_LMNA	0.04262	-0.117374747	0.200462861	6.02E-01	8.17E-01	1.00E+00
ENSG00000259956.1_RBM15B	ENSG00000160789.18_LMNA	0.18876	0.030475369	0.33780953	1.99E-02	1.07E-01	1.00E+00
ENSG00000125869.8_LAMP5	ENSG00000160789.18_LMNA	-0.16198	-0.313108079	-0.00285459	4.62E-02	1.91E-01	1.00E+00
ENSG00000136436.13_CALCOCO	ENSG00000160789.18_LMNA	0.00396	-0.155342603	0.163053944	9.61E-01	9.85E-01	1.00E+00
ENSG00000066135.11_KDM4A	ENSG00000160789.18_LMNA	0.10258	-0.057558756	0.25757614	2.09E-01	4.81E-01	1.00E+00
ENSG00000138386.15_NAB1	ENSG00000160789.18_LMNA	-0.01426	-0.173069336	0.145268948	8.62E-01	9.46E-01	1.00E+00
ENSG00000229638.1_RPL4P4	ENSG00000160789.18_LMNA	-0.01606	-0.174809299	0.143512108	8.44E-01	9.39E-01	1.00E+00
ENSG00000242616.3_GNG10	ENSG00000160789.18_LMNA	-0.12545	-0.279078734	0.034436675	1.24E-01	3.54E-01	1.00E+00
ENSG00000231991.4_ANXA2P2	ENSG00000160789.18_LMNA	0.51835	0.391450329	0.6258987	7.92E-12	2.95E-09	4.07E-07
ENSG00000007392.15_LUC7L	ENSG00000160789.18_LMNA	-0.1615	-0.312657494	-0.00235511	4.68E-02	1.93E-01	1.00E+00
ENSG00000187239.15_FNBP1	ENSG00000160789.18_LMNA	0.14735	-0.012130956	0.299528416	7.00E-02	2.50E-01	1.00E+00
ENSG00000186222.4_BLOC1S4	ENSG00000160789.18_LMNA	0.13789	-0.021790407	0.290707733	9.03E-02	2.93E-01	1.00E+00
ENSG00000146842.15_TMEM209	ENSG00000160789.18_LMNA	0.24733	0.091739197	0.391127836	2.13E-03	2.05E-02	1.00E+00

ENSG00000069956.10_MAPK6	ENSG00000160789.18_LMNA	0.09913	-0.061038932	0.254312466	2.24E-01	5.00E-01	1.00E+00
ENSG00000182979.16_MTA1	ENSG00000160789.18_LMNA	0.14101	-0.018611244	0.293616712	8.31E-02	2.79E-01	1.00E+00
ENSG00000005020.11_SKAP2	ENSG00000160789.18_LMNA	-0.09743	-0.252715254	0.062739308	2.32E-01	5.10E-01	1.00E+00
ENSG00000137094.13_DNAJB5	ENSG00000160789.18_LMNA	-0.01904	-0.177698882	0.140590058	8.16E-01	9.28E-01	1.00E+00
ENSG00000011243.16_AKAP8L	ENSG00000160789.18_LMNA	0.04661	-0.113427957	0.204299087	5.68E-01	7.98E-01	1.00E+00
ENSG00000113716.11_HMGXB3	ENSG00000160789.18_LMNA	0.41851	0.277820718	0.54162473	8.07E-08	6.02E-06	4.14E-03
ENSG00000137504.12_CREBZF	ENSG00000160789.18_LMNA	-0.05717	-0.214417638	0.102969448	4.84E-01	7.41E-01	1.00E+00
ENSG00000169019.10_COMMD8	ENSG00000160789.18_LMNA	-0.18532	-0.334647115	-0.02691281	2.23E-02	1.17E-01	1.00E+00
ENSG00000162944.9_RFTN2	ENSG00000160789.18_LMNA	-0.49062	-0.602742373	-0.3594988	1.39E-10	2.98E-08	7.16E-06
ENSG00000115561.13_CHMP3	ENSG00000160789.18_LMNA	-0.01174	-0.170625373	0.147733232	8.86E-01	9.56E-01	1.00E+00
ENSG00000134049.4_IER3IP1	ENSG00000160789.18_LMNA	-0.0675	-0.224288313	0.092699074	4.09E-01	6.83E-01	1.00E+00
ENSG00000103254.8_FAM173A	ENSG00000160789.18_LMNA	0.07341	-0.086806992	0.229921881	3.69E-01	6.50E-01	1.00E+00
ENSG00000179021.8_C3orf38	ENSG00000160789.18_LMNA	-0.24714	-0.390958493	-0.09154096	2.14E-03	2.06E-02	1.00E+00
ENSG00000119922.8_IFIT2	ENSG00000160789.18_LMNA	-0.12085	-0.274762175	0.039105422	1.38E-01	3.79E-01	1.00E+00
ENSG00000137656.10_BUD13	ENSG00000160789.18_LMNA	-0.09071	-0.246352993	0.069494382	2.66E-01	5.50E-01	1.00E+00
ENSG00000092203.12_TOX4	ENSG00000160789.18_LMNA	0.07816	-0.082057367	0.234447717	3.38E-01	6.24E-01	1.00E+00
ENSG00000186918.12_ZNF395	ENSG00000160789.18_LMNA	0.28392	0.130619762	0.42395457	3.93E-04	5.59E-03	1.00E+00
ENSG00000087269.14_NOP14	ENSG00000160789.18_LMNA	0.24214	0.086260807	0.386440398	2.65E-03	2.43E-02	1.00E+00
ENSG00000163840.8_DTX3L	ENSG00000160789.18_LMNA	0.27519	0.121307828	0.416162103	6.01E-04	7.79E-03	1.00E+00
ENSG00000163683.10_SMIM14	ENSG00000160789.18_LMNA	-0.28038	-0.420799935	-0.12684375	4.68E-04	6.39E-03	1.00E+00
ENSG00000102032.11_RENBP	ENSG00000160789.18_LMNA	0.23525	0.07901306	0.380215135	3.53E-03	3.02E-02	1.00E+00
ENSG00000197785.12_ATAD3A	ENSG00000160789.18_LMNA	0.30426	0.152446253	0.442050912	1.38E-04	2.44E-03	1.00E+00
ENSG00000006042.10_TMEM98	ENSG00000160789.18_LMNA	-0.16919	-0.319779838	-0.01026848	3.72E-02	1.65E-01	1.00E+00
ENSG00000100325.13_ASCC2	ENSG00000160789.18_LMNA	0.22114	0.064204344	0.367410163	6.18E-03	4.60E-02	1.00E+00
ENSG00000205246.7_	ENSG00000160789.18_LMNA	-0.15705	-0.308536288	0.002206117	5.33E-02	2.10E-01	1.00E+00
ENSG00000279528.1_RP11-1148	ENSG00000160789.18_LMNA	-0.06636	-0.223200284	0.093834479	4.17E-01	6.89E-01	1.00E+00
ENSG00000132361.15_CLUH	ENSG00000160789.18_LMNA	0.23253	0.076149425	0.377747947	3.94E-03	3.28E-02	1.00E+00
ENSG00000137054.14_POLR1E	ENSG00000160789.18_LMNA	0.13305	-0.026720855	0.286184845	1.02E-01	3.16E-01	1.00E+00
ENSG00000010295.18_IFFO1	ENSG00000160789.18_LMNA	0.20646	0.048867002	0.354025796	1.07E-02	6.88E-02	1.00E+00
ENSG00000103496.13_STX4	ENSG00000160789.18_LMNA	0.37686	0.231552069	0.505719779	1.71E-06	7.06E-05	8.75E-02
ENSG00000160570.12_DEDD2	ENSG00000160789.18_LMNA	0.25235	0.09705117	0.3956581	1.71E-03	1.74E-02	1.00E+00
ENSG00000164649.18_CDCA7L	ENSG00000160789.18_LMNA	0.15639	-0.00288734	0.307919781	5.44E-02	2.12E-01	1.00E+00
ENSG00000115207.12_GTF3C2	ENSG00000160789.18_LMNA	-0.0531	-0.21051677	0.107009704	5.16E-01	7.63E-01	1.00E+00

ENSG00000115966.15_ATF2	ENSG00000160789.18_LMNA	-0.12126	-0.275153621	0.038682603	1.37E-01	3.76E-01	1.00E+00
ENSG00000165724.5_ZMYND19	ENSG00000160789.18_LMNA	0.25342	0.098183634	0.396622036	1.63E-03	1.68E-02	1.00E+00
ENSG00000130414.10_NDUFA10	ENSG00000160789.18_LMNA	-0.06221	-0.219240577	0.097959652	4.46E-01	7.13E-01	1.00E+00
ENSG00000141279.14_NPEPPS	ENSG00000160789.18_LMNA	-0.24708	-0.390908588	-0.09148255	2.15E-03	2.07E-02	1.00E+00
ENSG00000150337.12_FCGR1A	ENSG00000160789.18_LMNA	-0.01845	-0.17713029	0.141165477	8.22E-01	9.30E-01	1.00E+00
ENSG00000148341.16_SH3GLB2	ENSG00000160789.18_LMNA	-0.16466	-0.315591632	-0.00561042	4.26E-02	1.81E-01	1.00E+00
ENSG00000173744.16_AGFG1	ENSG00000160789.18_LMNA	-0.24871	-0.392372243	-0.09319663	2.00E-03	1.95E-02	1.00E+00
ENSG00000142599.16_RERE	ENSG00000160789.18_LMNA	0.07827	-0.08194829	0.234551493	3.38E-01	6.23E-01	1.00E+00
ENSG00000134250.16_NOTCH2	ENSG00000160789.18_LMNA	0.23321	0.076866442	0.378366101	3.83E-03	3.21E-02	1.00E+00
ENSG00000175662.16_TOM1L2	ENSG00000160789.18_LMNA	-0.02426	-0.182753124	0.13546566	7.67E-01	9.03E-01	1.00E+00
ENSG00000174607.9_UGT8	ENSG00000160789.18_LMNA	-0.43162	-0.552831949	-0.29251789	2.83E-08	2.58E-06	1.45E-03
ENSG00000154153.12_FAM134B	ENSG00000160789.18_LMNA	-0.01899	-0.177658239	0.140631196	8.16E-01	9.28E-01	1.00E+00
ENSG00000132716.17_DCAF8	ENSG00000160789.18_LMNA	-0.16332	-0.3143479	-0.00422975	4.44E-02	1.86E-01	1.00E+00
ENSG00000173163.9_COMMD1	ENSG00000160789.18_LMNA	-0.24581	-0.389755009	-0.09013284	2.27E-03	2.16E-02	1.00E+00
ENSG00000007376.6_RPUSD1	ENSG00000160789.18_LMNA	0.10799	-0.052102904	0.262678153	1.85E-01	4.51E-01	1.00E+00
ENSG00000164466.11_SFXN1	ENSG00000160789.18_LMNA	-0.03644	-0.19451371	0.123475566	6.56E-01	8.48E-01	1.00E+00
ENSG00000141258.11_SGSM2	ENSG00000160789.18_LMNA	0.10382	-0.056312924	0.258742724	2.03E-01	4.74E-01	1.00E+00
ENSG00000137275.12_RIPK1	ENSG00000160789.18_LMNA	0.40401	0.261636457	0.529174089	2.45E-07	1.50E-05	1.26E-02
ENSG00000214046.7_SMIM7	ENSG00000160789.18_LMNA	-0.30317	-0.441082395	-0.1512709	1.47E-04	2.55E-03	1.00E+00
ENSG00000182768.8_NGRN	ENSG00000160789.18_LMNA	-0.1799	-0.329654726	-0.0213046	2.66E-02	1.32E-01	1.00E+00
ENSG00000165092.11_ALDH1A1	ENSG00000160789.18_LMNA	-0.11338	-0.267750086	0.046660527	1.64E-01	4.20E-01	1.00E+00
ENSG00000161533.10_ACOX1	ENSG00000160789.18_LMNA	-0.06107	-0.218147405	0.099096587	4.55E-01	7.20E-01	1.00E+00
ENSG00000213694.3_S1PR3	ENSG00000160789.18_LMNA	-0.00699	-0.166009511	0.152376715	9.32E-01	9.73E-01	1.00E+00
ENSG00000249353.2_NPM1P27	ENSG00000160789.18_LMNA	0.03567	-0.124237908	0.193768672	6.63E-01	8.51E-01	1.00E+00
ENSG00000166171.11_DPCD	ENSG00000160789.18_LMNA	-0.05462	-0.211980424	0.105494978	5.04E-01	7.55E-01	1.00E+00
ENSG00000181588.16_MEX3D	ENSG00000160789.18_LMNA	0.19874	0.040832591	0.346964196	1.41E-02	8.40E-02	1.00E+00
ENSG00000116147.15_TNR	ENSG00000160789.18_LMNA	-0.421	-0.543760154	-0.28061163	6.63E-08	5.20E-06	3.40E-03
ENSG00000196642.14_RABL6	ENSG00000160789.18_LMNA	0.20841	0.050894299	0.355802147	9.98E-03	6.55E-02	1.00E+00
ENSG00000172667.9_ZMAT3	ENSG00000160789.18_LMNA	0.0644	-0.095778474	0.221335567	4.31E-01	7.00E-01	1.00E+00
ENSG00000038002.7_AGA	ENSG00000160789.18_LMNA	0.07858	-0.081640026	0.234844739	3.36E-01	6.22E-01	1.00E+00
ENSG00000163349.20_HIPK1	ENSG00000160789.18_LMNA	0.12759	-0.032270528	0.281077184	1.17E-01	3.43E-01	1.00E+00
ENSG00000130816.13_DNMT1	ENSG00000160789.18_LMNA	0.21343	0.056139662	0.360388016	8.29E-03	5.72E-02	1.00E+00
ENSG00000205903.5_ZNF316	ENSG00000160789.18_LMNA	0.26314	0.10848012	0.40535615	1.06E-03	1.21E-02	1.00E+00

ENSG00000011007.11_TCEB3	ENSG00000160789.18_LMNA	0.16778	0.008814128	0.318473484	3.88E-02	1.70E-01	1.00E+00
ENSG00000136986.8_DERL1	ENSG00000160789.18_LMNA	0.00412	-0.155179984	0.163216145	9.60E-01	9.84E-01	1.00E+00
ENSG00000175792.10_RUVBL1	ENSG00000160789.18_LMNA	0.0207	-0.138962642	0.179305839	8.00E-01	9.20E-01	1.00E+00
ENSG00000135823.12_STX6	ENSG00000160789.18_LMNA	0.08225	-0.077974534	0.238327243	3.14E-01	6.00E-01	1.00E+00
ENSG00000148660.19_CAMK2G	ENSG00000160789.18_LMNA	-0.40296	-0.528271558	-0.2604691	2.65E-07	1.60E-05	1.36E-02
ENSG00000106976.17_DNM1	ENSG00000160789.18_LMNA	0.08734	-0.07287543	0.2431583	2.85E-01	5.70E-01	1.00E+00
ENSG00000003756.15_RBM5	ENSG00000160789.18_LMNA	-0.08912	-0.244850377	0.071085554	2.75E-01	5.59E-01	1.00E+00
ENSG00000171497.4_PPID	ENSG00000160789.18_LMNA	-0.1783	-0.328182539	-0.01965451	2.80E-02	1.37E-01	1.00E+00
ENSG00000133030.18_MPRIP	ENSG00000160789.18_LMNA	0.10835	-0.051740671	0.263016271	1.84E-01	4.49E-01	1.00E+00
ENSG00000243943.8_ZNF512	ENSG00000160789.18_LMNA	-0.25041	-0.393908417	-0.09499756	1.86E-03	1.85E-02	1.00E+00
ENSG00000137942.15_FNBP1L	ENSG00000160789.18_LMNA	-0.00471	-0.163790319	0.154604193	9.54E-01	9.82E-01	1.00E+00
ENSG00000141644.16_MBD1	ENSG00000160789.18_LMNA	0.10507	-0.055047424	0.259926784	1.98E-01	4.67E-01	1.00E+00
ENSG00000174306.20_ZHX3	ENSG00000160789.18_LMNA	0.07997	-0.080250244	0.236166097	3.27E-01	6.14E-01	1.00E+00
ENSG00000114019.13_AMOTL2	ENSG00000160789.18_LMNA	-0.333	-0.467424315	-0.18353306	2.77E-05	6.69E-04	1.00E+00
ENSG00000010244.15_ZNF207	ENSG00000160789.18_LMNA	-0.27535	-0.416301066	-0.12147343	5.96E-04	7.75E-03	1.00E+00
ENSG00000114779.18_ABHD14B	ENSG00000160789.18_LMNA	0.12382	-0.036094107	0.27754778	1.29E-01	3.63E-01	1.00E+00
ENSG00000148572.13_NRBF2	ENSG00000160789.18_LMNA	0.14226	-0.017332186	0.29478544	8.04E-02	2.73E-01	1.00E+00
ENSG00000143258.14_USP21	ENSG00000160789.18_LMNA	-0.05252	-0.209969609	0.10757558	5.20E-01	7.66E-01	1.00E+00
ENSG00000119185.11_ITGB1BP1	ENSG00000160789.18_LMNA	-0.04477	-0.202523222	0.115256252	5.84E-01	8.07E-01	1.00E+00
ENSG00000110925.5_CSRNP2	ENSG00000160789.18_LMNA	-0.12836	-0.281806068	0.031479747	1.15E-01	3.39E-01	1.00E+00
ENSG00000085433.14_WDR47	ENSG00000160789.18_LMNA	-0.1749	-0.325053578	-0.01615301	3.11E-02	1.47E-01	1.00E+00
ENSG00000067836.11_ROGDI	ENSG00000160789.18_LMNA	-0.29407	-0.433000435	-0.1414948	2.36E-04	3.72E-03	1.00E+00
ENSG00000151468.10_CCDC3	ENSG00000160789.18_LMNA	0.05267	-0.107428627	0.210111721	5.19E-01	7.65E-01	1.00E+00
ENSG00000061938.15_TNK2	ENSG00000160789.18_LMNA	-0.11962	-0.273607841	0.040351621	1.42E-01	3.85E-01	1.00E+00
ENSG00000185862.6_EVI2B	ENSG00000160789.18_LMNA	-0.13011	-0.283440438	0.02970516	1.10E-01	3.31E-01	1.00E+00
ENSG00000103168.15_TAF1C	ENSG00000160789.18_LMNA	-0.02185	-0.180426142	0.137827058	7.89E-01	9.14E-01	1.00E+00
ENSG00000164117.11_FBXO8	ENSG00000160789.18_LMNA	-0.17928	-0.329090415	-0.0206719	2.71E-02	1.34E-01	1.00E+00
ENSG00000186635.13_ARAP1	ENSG00000160789.18_LMNA	0.20785	0.0503114	0.355291627	1.02E-02	6.64E-02	1.00E+00
ENSG00000139410.13_SDSL	ENSG00000160789.18_LMNA	0.10091	-0.059237812	0.256002436	2.16E-01	4.90E-01	1.00E+00
ENSG00000131759.16_RARA	ENSG00000160789.18_LMNA	0.26378	0.109160061	0.405931017	1.03E-03	1.18E-02	1.00E+00
ENSG00000103423.12_DNAJA3	ENSG00000160789.18_LMNA	-0.02837	-0.186730773	0.131420791	7.29E-01	8.85E-01	1.00E+00
ENSG00000185215.7_TNFAIP2	ENSG00000160789.18_LMNA	0.30779	0.1562429	0.445174825	1.15E-04	2.11E-03	1.00E+00
ENSG00000185504.15_FAAP100	ENSG00000160789.18_LMNA	0.14764	-0.011836964	0.299796047	6.95E-02	2.48E-01	1.00E+00

ENSG00000140553.15_UNC45A	ENSG00000160789.18_LMNA	0.51481	0.387357802	0.622955614	1.16E-11	3.89E-09	5.95E-07
ENSG00000171729.12_TMEM51	ENSG00000160789.18_LMNA	0.16211	0.002988669	0.313229006	4.60E-02	1.90E-01	1.00E+00
ENSG00000177556.10_ATOX1	ENSG00000160789.18_LMNA	-0.09584	-0.251208498	0.064341709	2.40E-01	5.19E-01	1.00E+00
ENSG00000137364.4_TPMT	ENSG00000160789.18_LMNA	-0.07109	-0.227717038	0.089115653	3.84E-01	6.63E-01	1.00E+00
ENSG00000101000.4_PROCR	ENSG00000160789.18_LMNA	0.08646	-0.073752285	0.242328654	2.90E-01	5.75E-01	1.00E+00
ENSG00000088899.13_LZTS3	ENSG00000160789.18_LMNA	-0.18765	-0.336792908	-0.02932926	2.06E-02	1.10E-01	1.00E+00
ENSG00000011201.9_ANOS1	ENSG00000160789.18_LMNA	-0.27961	-0.420113739	-0.12602352	4.85E-04	6.58E-03	1.00E+00
ENSG00000186522.13_SEPT10	ENSG00000160789.18_LMNA	-0.04959	-0.207150575	0.110487774	5.44E-01	7.82E-01	1.00E+00
ENSG00000113083.11_LOX	ENSG00000160789.18_LMNA	0.34901	0.200976635	0.481460002	1.05E-05	3.03E-04	5.38E-01
ENSG00000004864.12_SLC25A13	ENSG00000160789.18_LMNA	-0.0787	-0.234956193	0.081522848	3.35E-01	6.21E-01	1.00E+00
ENSG00000111832.11_RWDD1	ENSG00000160789.18_LMNA	-0.29379	-0.432751807	-0.14119495	2.39E-04	3.76E-03	1.00E+00
ENSG00000196405.11_EVL	ENSG00000160789.18_LMNA	-0.17965	-0.329425244	-0.02104727	2.68E-02	1.33E-01	1.00E+00
ENSG00000093144.17_ECHDC1	ENSG00000160789.18_LMNA	-0.13294	-0.286089806	0.026824296	1.03E-01	3.17E-01	1.00E+00
ENSG00000138834.11_MAPK8IP3	ENSG00000160789.18_LMNA	-0.09698	-0.252281754	0.063200493	2.35E-01	5.12E-01	1.00E+00
ENSG00000048140.16_TSPAN17	ENSG00000160789.18_LMNA	0.22371	0.06688701	0.369738398	5.60E-03	4.27E-02	1.00E+00
ENSG00000217555.11_CKLF	ENSG00000160789.18_LMNA	-0.00197	-0.161122788	0.157277408	9.81E-01	9.93E-01	1.00E+00
ENSG00000125871.12_MGME1	ENSG00000160789.18_LMNA	0.09694	-0.063240279	0.25224435	2.35E-01	5.13E-01	1.00E+00
ENSG00000167323.8_STIM1	ENSG00000160789.18_LMNA	0.26121	0.106437499	0.403627772	1.15E-03	1.29E-02	1.00E+00
ENSG00000177981.9_ASB8	ENSG00000160789.18_LMNA	-0.3259	-0.461170201	-0.17581759	4.18E-05	9.30E-04	1.00E+00
ENSG00000148396.17_SEC16A	ENSG00000160789.18_LMNA	0.26603	0.11155617	0.407954972	9.24E-04	1.09E-02	1.00E+00
ENSG00000155511.16_GRIA1	ENSG00000160789.18_LMNA	-0.0448	-0.202557539	0.115220942	5.84E-01	8.06E-01	1.00E+00
ENSG00000085872.13_CHERP	ENSG00000160789.18_LMNA	0.1561	-0.003181582	0.30765341	5.48E-02	2.13E-01	1.00E+00
ENSG00000170846.14_AC093323	ENSG00000160789.18_LMNA	-0.2383	-0.382972511	-0.0822194	3.11E-03	2.75E-02	1.00E+00
ENSG00000177565.14_TBL1XR1	ENSG00000160789.18_LMNA	-0.20902	-0.356361293	-0.05153296	9.76E-03	6.45E-02	1.00E+00
ENSG00000168679.16_SLC16A4	ENSG00000160789.18_LMNA	0.03741	-0.122520341	0.195446734	6.47E-01	8.43E-01	1.00E+00
ENSG00000124942.12_AHNAK	ENSG00000160789.18_LMNA	0.31273	0.161575189	0.449550433	8.76E-05	1.70E-03	1.00E+00
ENSG00000165782.9_TMEM55B	ENSG00000160789.18_LMNA	-0.00862	-0.167589538	0.150788813	9.16E-01	9.68E-01	1.00E+00
ENSG00000048392.10_RRM2B	ENSG00000160789.18_LMNA	0.02582	-0.133935354	0.184259224	7.52E-01	8.97E-01	1.00E+00
ENSG00000011451.16_WIZ	ENSG00000160789.18_LMNA	0.11536	-0.044657222	0.269612656	1.57E-01	4.09E-01	1.00E+00
ENSG00000105519.11_CAPS	ENSG00000160789.18_LMNA	0.04344	-0.116566189	0.201249563	5.95E-01	8.13E-01	1.00E+00
ENSG00000228638.1_FCF1P2	ENSG00000160789.18_LMNA	-0.133	-0.286146515	0.026762575	1.02E-01	3.16E-01	1.00E+00
ENSG00000181610.11_MRPS23	ENSG00000160789.18_LMNA	-0.28068	-0.421068784	-0.12716523	4.61E-04	6.32E-03	1.00E+00
ENSG00000078747.11_ITCH	ENSG00000160789.18_LMNA	0.07932	-0.080905379	0.235543363	3.31E-01	6.17E-01	1.00E+00

ENSG00000115129.12_TP53I3	ENSG00000160789.18_LMNA	0.12165	-0.038295259	0.275512132	1.35E-01	3.74E-01	1.00E+00
ENSG00000123575.8_FAM199X	ENSG00000160789.18_LMNA	-0.09214	-0.247707933	0.06805821	2.59E-01	5.42E-01	1.00E+00
ENSG00000100413.15_POLR3H	ENSG00000160789.18_LMNA	-0.25367	-0.396846107	-0.09844699	1.61E-03	1.66E-02	1.00E+00
ENSG00000204899.5_MZT1	ENSG00000160789.18_LMNA	-0.24347	-0.387649726	-0.08767246	2.51E-03	2.33E-02	1.00E+00
ENSG00000120833.12_SOCS2	ENSG00000160789.18_LMNA	0.12584	-0.034047769	0.279437733	1.22E-01	3.52E-01	1.00E+00
ENSG00000025800.12_KPNA6	ENSG00000160789.18_LMNA	0.17905	0.020430849	0.328875365	2.73E-02	1.35E-01	1.00E+00
ENSG00000151726.12_ACSL1	ENSG00000160789.18_LMNA	-0.07158	-0.228181304	0.088629808	3.81E-01	6.60E-01	1.00E+00
ENSG00000186470.12_BTN3A2	ENSG00000160789.18_LMNA	0.10886	-0.051228259	0.263494438	1.82E-01	4.46E-01	1.00E+00
ENSG00000188760.9_TMEM198	ENSG00000160789.18_LMNA	0.0354	-0.124498107	0.193514295	6.65E-01	8.52E-01	1.00E+00
ENSG00000228989.1_	ENSG00000160789.18_LMNA	0.1252	-0.034689331	0.278845461	1.24E-01	3.56E-01	1.00E+00
ENSG00000077235.16_GTF3C1	ENSG00000160789.18_LMNA	0.18552	0.027120258	0.334831451	2.21E-02	1.16E-01	1.00E+00
ENSG00000234608.6_MAPKAPK5	ENSG00000160789.18_LMNA	-0.37681	-0.505672337	-0.23149174	1.71E-06	7.08E-05	8.78E-02
ENSG00000147130.13_ZMYM3	ENSG00000160789.18_LMNA	0.15036	-0.009059246	0.302322277	6.45E-02	2.36E-01	1.00E+00
ENSG00000101336.11_HCK	ENSG00000160789.18_LMNA	0.04636	-0.113675151	0.204059103	5.71E-01	7.99E-01	1.00E+00
ENSG00000266469.1_CTB-131K1	ENSG00000160789.18_LMNA	0.03656	-0.123359743	0.194626871	6.55E-01	8.47E-01	1.00E+00
ENSG00000169855.18_ROBO1	ENSG00000160789.18_LMNA	0.05023	-0.109846396	0.207771889	5.39E-01	7.79E-01	1.00E+00
ENSG00000074964.15_ARHGEF1	ENSG00000160789.18_LMNA	0.00437	-0.15493937	0.16345611	9.57E-01	9.83E-01	1.00E+00
ENSG00000153814.10_JAZF1	ENSG00000160789.18_LMNA	-0.16032	-0.311567793	-0.00114781	4.85E-02	1.97E-01	1.00E+00
ENSG00000169756.15_LIMS1	ENSG00000160789.18_LMNA	0.26822	0.113883556	0.409918094	8.35E-04	1.00E-02	1.00E+00
ENSG00000154845.14_PPP4R1	ENSG00000160789.18_LMNA	-0.09456	-0.24999339	0.065632765	2.47E-01	5.27E-01	1.00E+00
ENSG00000103160.10_HSDL1	ENSG00000160789.18_LMNA	-0.30899	-0.446240012	-0.15753943	1.07E-04	2.00E-03	1.00E+00
ENSG00000145685.12_LHFPL2	ENSG00000160789.18_LMNA	0.09741	-0.06275931	0.252696456	2.33E-01	5.10E-01	1.00E+00
ENSG00000136848.15_DAB2IP	ENSG00000160789.18_LMNA	-0.0121	-0.170969795	0.147386185	8.82E-01	9.54E-01	1.00E+00
ENSG00000173207.11_CKS1B	ENSG00000160789.18_LMNA	-0.06683	-0.223646928	0.093368486	4.13E-01	6.87E-01	1.00E+00
ENSG00000182247.8_UBE2E2	ENSG00000160789.18_LMNA	-0.45834	-0.575546789	-0.32268826	2.89E-09	3.77E-07	1.48E-04
ENSG00000214517.7_PPME1	ENSG00000160789.18_LMNA	0.07831	-0.081915371	0.234582812	3.38E-01	6.23E-01	1.00E+00
ENSG00000215114.6_UBXN2B	ENSG00000160789.18_LMNA	-0.22885	-0.374407667	-0.07228032	4.57E-03	3.66E-02	1.00E+00
ENSG00000061676.13_NCKAP1	ENSG00000160789.18_LMNA	-0.11521	-0.269466831	0.044814157	1.58E-01	4.10E-01	1.00E+00
ENSG00000101911.11_PRPS2	ENSG00000160789.18_LMNA	0.42866	0.289195959	0.550307045	3.60E-08	3.14E-06	1.85E-03
ENSG00000169169.13_CPT1C	ENSG00000160789.18_LMNA	-0.06546	-0.222342909	0.094728605	4.23E-01	6.94E-01	1.00E+00
ENSG00000116661.9_FBXO2	ENSG00000160789.18_LMNA	-0.28236	-0.422562274	-0.12895217	4.24E-04	5.94E-03	1.00E+00
ENSG00000100364.17_KIAA0930	ENSG00000160789.18_LMNA	-0.12458	-0.278260688	0.035322518	1.26E-01	3.59E-01	1.00E+00
ENSG00000231767.3_RP11-92K2	ENSG00000160789.18_LMNA	-0.17653	-0.326556858	-0.01783433	2.96E-02	1.42E-01	1.00E+00

ENSG00000177646.16_ACAD9	ENSG00000160789.18_LMNA	0.19195	0.033783954	0.340740297	1.78E-02	9.94E-02	1.00E+00
ENSG00000257727.4_CNPY2	ENSG00000160789.18_LMNA	-0.14932	-0.301354799	0.010123605	6.64E-02	2.41E-01	1.00E+00
ENSG00000180834.6_MAP6D1	ENSG00000160789.18_LMNA	-0.30654	-0.44407032	-0.15489956	1.23E-04	2.23E-03	1.00E+00
ENSG00000198917.10_SPOUT1	ENSG00000160789.18_LMNA	0.11667	-0.043338004	0.270837914	1.52E-01	4.02E-01	1.00E+00
ENSG00000198815.7_FOXJ3	ENSG00000160789.18_LMNA	0.2677	0.113327479	0.409449299	8.55E-04	1.03E-02	1.00E+00
ENSG00000134954.13_ETS1	ENSG00000160789.18_LMNA	0.01225	-0.147235698	0.17111912	8.81E-01	9.54E-01	1.00E+00
ENSG00000124357.11_NAGK	ENSG00000160789.18_LMNA	-0.13907	-0.291810935	0.020585483	8.75E-02	2.88E-01	1.00E+00
ENSG00000122643.17_NT5C3A	ENSG00000160789.18_LMNA	0.05085	-0.109238055	0.208360962	5.34E-01	7.76E-01	1.00E+00
ENSG00000105281.11_SLC1A5	ENSG00000160789.18_LMNA	0.29069	0.137871144	0.429992784	2.80E-04	4.26E-03	1.00E+00
ENSG00000228502.1_EEF1A1P1	ENSG00000160789.18_LMNA	-0.16125	-0.312431388	-0.00210453	4.72E-02	1.94E-01	1.00E+00
ENSG00000089154.9_GCN1	ENSG00000160789.18_LMNA	0.2107	0.0532835	0.357892779	9.17E-03	6.16E-02	1.00E+00
ENSG00000168140.4_VASN	ENSG00000160789.18_LMNA	0.54914	0.427299372	0.651396758	2.38E-13	1.67E-10	1.22E-08
ENSG00000137500.8_CCDC90B	ENSG00000160789.18_LMNA	-0.30873	-0.446011745	-0.1572615	1.09E-04	2.02E-03	1.00E+00
ENSG00000117226.10_GBP3	ENSG00000160789.18_LMNA	0.12209	-0.037842857	0.275930748	1.34E-01	3.72E-01	1.00E+00
ENSG00000140067.6_FAM181A	ENSG00000160789.18_LMNA	0.1012	-0.058947157	0.256274973	2.15E-01	4.89E-01	1.00E+00
ENSG00000235173.5_HGH1	ENSG00000160789.18_LMNA	0.26586	0.111371153	0.407798795	9.31E-04	1.10E-02	1.00E+00
ENSG00000120889.11_TNFRSF1	ENSG00000160789.18_LMNA	0.38865	0.244581941	0.515928093	7.50E-07	3.64E-05	3.85E-02
ENSG00000112149.8_CD83	ENSG00000160789.18_LMNA	0.0017	-0.157544507	0.160856001	9.83E-01	9.94E-01	1.00E+00
ENSG00000154229.10_PRKCA	ENSG00000160789.18_LMNA	0.06109	-0.099077936	0.218165345	4.55E-01	7.19E-01	1.00E+00
ENSG00000100714.14_MTHFD1	ENSG00000160789.18_LMNA	0.35395	0.206373778	0.485773777	7.70E-06	2.37E-04	3.95E-01
ENSG00000197798.7_FAM118B	ENSG00000160789.18_LMNA	-0.41127	-0.535416151	-0.26973159	1.41E-07	9.59E-06	7.25E-03
ENSG00000249936.3_RAC1P2	ENSG00000160789.18_LMNA	0.07686	-0.083357873	0.233209849	3.47E-01	6.31E-01	1.00E+00
ENSG00000130766.4_SESN2	ENSG00000160789.18_LMNA	0.26343	0.108794583	0.405622046	1.04E-03	1.19E-02	1.00E+00
ENSG00000018610.12_CXorf56	ENSG00000160789.18_LMNA	-0.0925	-0.248050468	0.067694932	2.57E-01	5.39E-01	1.00E+00
ENSG00000178188.13_SH2B1	ENSG00000160789.18_LMNA	0.02396	-0.135757599	0.182465633	7.70E-01	9.05E-01	1.00E+00
ENSG00000167112.9_TRUB2	ENSG00000160789.18_LMNA	-0.22748	-0.373165377	-0.07084366	4.83E-03	3.81E-02	1.00E+00
ENSG00000158669.10_GPAT4	ENSG00000160789.18_LMNA	0.30548	0.153752678	0.443126643	1.30E-04	2.33E-03	1.00E+00
ENSG00000174132.8_FAM174A	ENSG00000160789.18_LMNA	0.2361	0.079907233	0.380984641	3.41E-03	2.94E-02	1.00E+00
ENSG00000176956.11_LY6H	ENSG00000160789.18_LMNA	-0.20278	-0.350657822	-0.04503012	1.22E-02	7.58E-02	1.00E+00
ENSG00000134779.13_TPGS2	ENSG00000160789.18_LMNA	-0.06444	-0.221368202	0.095744472	4.30E-01	7.00E-01	1.00E+00
ENSG00000111412.5_C12orf49	ENSG00000160789.18_LMNA	0.11739	-0.042608454	0.271515067	1.50E-01	3.98E-01	1.00E+00
ENSG00000214706.9_IFRD2	ENSG00000160789.18_LMNA	0.26777	0.113402728	0.409512746	8.53E-04	1.02E-02	1.00E+00
ENSG00000171453.16_POLR1C	ENSG00000160789.18_LMNA	-0.16291	-0.313972143	-0.00381285	4.49E-02	1.88E-01	1.00E+00

ENSG00000183741.10_CBX6	ENSG00000160789.18_LMNA	0.24301	0.087186996	0.387233957	2.56E-03	2.36E-02	1.00E+00
ENSG00000136379.10_ABHD17C	ENSG00000160789.18_LMNA	0.23679	0.080633412	0.381609267	3.31E-03	2.88E-02	1.00E+00
ENSG00000107731.11_UNC5B	ENSG00000160789.18_LMNA	0.21084	0.053431774	0.358022423	9.13E-03	6.13E-02	1.00E+00
ENSG00000143337.17_TOR1AIP1	ENSG00000160789.18_LMNA	0.01164	-0.147833868	0.170525483	8.87E-01	9.56E-01	1.00E+00
ENSG00000127526.12_SLC35E1	ENSG00000160789.18_LMNA	0.25893	0.104016917	0.401576829	1.28E-03	1.39E-02	1.00E+00
ENSG00000149187.16_CELF1	ENSG00000160789.18_LMNA	0.2045	0.046825025	0.352234355	1.15E-02	7.24E-02	1.00E+00
ENSG00000100908.12_EMC9	ENSG00000160789.18_LMNA	-0.00214	-0.161288668	0.157111311	9.79E-01	9.92E-01	1.00E+00
ENSG00000126705.12_AHDC1	ENSG00000160789.18_LMNA	0.17781	0.019148758	0.327731014	2.84E-02	1.38E-01	1.00E+00
ENSG00000136897.7_MRPL50	ENSG00000160789.18_LMNA	-0.20269	-0.350580617	-0.04494227	1.23E-02	7.59E-02	1.00E+00
ENSG00000060971.16_ACAA1	ENSG00000160789.18_LMNA	-0.19427	-0.342864063	-0.03618572	1.65E-02	9.39E-02	1.00E+00
ENSG00000170638.8_TRABD	ENSG00000160789.18_LMNA	0.19115	0.03295073	0.340002782	1.83E-02	1.01E-01	1.00E+00
ENSG00000169139.10_UBE2V2	ENSG00000160789.18_LMNA	-0.2385	-0.383154359	-0.08243108	3.09E-03	2.73E-02	1.00E+00
ENSG00000004961.13_HCCS	ENSG00000160789.18_LMNA	0.14052	-0.019108169	0.2931624	8.42E-02	2.81E-01	1.00E+00
ENSG00000196998.14_WDR45	ENSG00000160789.18_LMNA	0.04198	-0.118007929	0.199846509	6.08E-01	8.21E-01	1.00E+00
ENSG00000170873.17_MTSS1	ENSG00000160789.18_LMNA	-0.39632	-0.522546795	-0.25308273	4.32E-07	2.34E-05	2.22E-02
ENSG00000140829.10_DHX38	ENSG00000160789.18_LMNA	0.22461	0.067839802	0.370564395	5.40E-03	4.15E-02	1.00E+00
ENSG00000081087.13_OSTM1	ENSG00000160789.18_LMNA	0.00941	-0.150012972	0.168360937	9.08E-01	9.65E-01	1.00E+00
ENSG00000131873.5_CHSY1	ENSG00000160789.18_LMNA	0.50772	0.379171727	0.617048627	2.45E-11	7.11E-09	1.26E-06
ENSG00000225973.3_PIGBOS1	ENSG00000160789.18_LMNA	-0.03098	-0.189245287	0.128858306	7.05E-01	8.74E-01	1.00E+00
ENSG00000248333.6_CDK11B	ENSG00000160789.18_LMNA	0.16879	0.009858291	0.319411512	3.76E-02	1.66E-01	1.00E+00
ENSG00000126368.5_NR1D1	ENSG00000160789.18_LMNA	0.21618	0.059014289	0.362894997	7.47E-03	5.28E-02	1.00E+00
ENSG00000204347.3_BTBD17	ENSG00000160789.18_LMNA	-0.3722	-0.501673755	-0.22641481	2.34E-06	8.98E-05	1.20E-01
ENSG00000175701.9_LINC00116	ENSG00000160789.18_LMNA	0.01881	-0.140817104	0.177474555	8.18E-01	9.29E-01	1.00E+00
ENSG00000140905.8_GCSH	ENSG00000160789.18_LMNA	-0.21382	-0.360737866	-0.05654051	8.17E-03	5.65E-02	1.00E+00
ENSG00000166224.15_SGPL1	ENSG00000160789.18_LMNA	-0.02475	-0.183224873	0.134986492	7.62E-01	9.01E-01	1.00E+00
ENSG00000167925.14_GHDC	ENSG00000160789.18_LMNA	0.31124	0.159961324	0.448227573	9.51E-05	1.81E-03	1.00E+00
ENSG00000185000.8_DGAT1	ENSG00000160789.18_LMNA	-0.02823	-0.186590552	0.131563562	7.30E-01	8.86E-01	1.00E+00
ENSG00000204070.8_SYS1	ENSG00000160789.18_LMNA	-0.02926	-0.187582612	0.130553177	7.21E-01	8.81E-01	1.00E+00
ENSG00000259291.2_RP11-617F	ENSG00000160789.18_LMNA	-0.09798	-0.253228486	0.062193126	2.30E-01	5.07E-01	1.00E+00
ENSG00000167771.5_RCOR2	ENSG00000160789.18_LMNA	-0.25309	-0.396319027	-0.09782757	1.66E-03	1.69E-02	1.00E+00
ENSG00000102981.8_PARD6A	ENSG00000160789.18_LMNA	-0.20321	-0.351054938	-0.04548206	1.20E-02	7.50E-02	1.00E+00
ENSG00000064989.11_CALCRL	ENSG00000160789.18_LMNA	-0.3345	-0.468742949	-0.18516428	2.53E-05	6.24E-04	1.00E+00
ENSG00000185359.11_HGS	ENSG00000160789.18_LMNA	0.19698	0.0390031	0.345351354	1.50E-02	8.79E-02	1.00E+00

ENSG00000162980.15_ARL5A	ENSG00000160789.18_LMNA	-0.2715	-0.412852074	-0.11736801	7.16E-04	8.92E-03	1.00E+00
ENSG00000171533.10_MAP6	ENSG00000160789.18_LMNA	-0.21306	-0.360050008	-0.05575247	8.40E-03	5.78E-02	1.00E+00
ENSG00000067445.19_TRO	ENSG00000160789.18_LMNA	-0.25653	-0.39942109	-0.10147645	1.42E-03	1.51E-02	1.00E+00
ENSG00000163539.14_CLASP2	ENSG00000160789.18_LMNA	-0.6127	-0.703302154	-0.50252007	4.91E-17	1.48E-13	2.52E-12
ENSG00000245532.5_NEAT1	ENSG00000160789.18_LMNA	0.24932	0.093849386	0.392929229	1.95E-03	1.92E-02	1.00E+00
ENSG00000198551.8_ZNF627	ENSG00000160789.18_LMNA	-0.32071	-0.456597789	-0.17019887	5.62E-05	1.18E-03	1.00E+00
ENSG00000003249.12_DBNDD1	ENSG00000160789.18_LMNA	0.28207	0.128648841	0.422308868	4.30E-04	6.00E-03	1.00E+00
ENSG00000005469.10_CROT	ENSG00000160789.18_LMNA	0.14242	-0.017171908	0.294931826	8.01E-02	2.72E-01	1.00E+00
ENSG00000147475.13_ERLIN2	ENSG00000160789.18_LMNA	0.11673	-0.043279188	0.270892518	1.52E-01	4.01E-01	1.00E+00
ENSG00000237264.4_FTH1P11	ENSG00000160789.18_LMNA	0.02351	-0.136199349	0.18203051	7.74E-01	9.07E-01	1.00E+00
ENSG00000129071.8_MBD4	ENSG00000160789.18_LMNA	0.03335	-0.126526922	0.191529408	6.83E-01	8.62E-01	1.00E+00
ENSG00000159322.16_ADPGK	ENSG00000160789.18_LMNA	0.213	0.055682893	0.35998926	8.42E-03	5.79E-02	1.00E+00
ENSG00000253948.1_RP11-410L	ENSG00000160789.18_LMNA	-0.20256	-0.350456959	-0.04480158	1.23E-02	7.62E-02	1.00E+00
ENSG00000229453.2_SPINK8	ENSG00000160789.18_LMNA	-0.2081	-0.355524152	-0.05057687	1.01E-02	6.60E-02	1.00E+00
ENSG00000103249.16_CLCN7	ENSG00000160789.18_LMNA	0.17674	0.018042378	0.326742767	2.94E-02	1.41E-01	1.00E+00
ENSG00000164574.14_GALNT10	ENSG00000160789.18_LMNA	0.27325	0.119236501	0.414422855	6.59E-04	8.36E-03	1.00E+00
ENSG00000101997.11_CCDC22	ENSG00000160789.18_LMNA	0.13172	-0.02807085	0.284944007	1.06E-01	3.23E-01	1.00E+00
ENSG00000106780.8_MEGF9	ENSG00000160789.18_LMNA	-0.09802	-0.253268507	0.062150528	2.30E-01	5.07E-01	1.00E+00
ENSG00000136243.15_NUPL2	ENSG00000160789.18_LMNA	-0.21378	-0.360705077	-0.05650294	8.18E-03	5.66E-02	1.00E+00
ENSG00000140395.7_WDR61	ENSG00000160789.18_LMNA	-0.17259	-0.322918526	-0.0137681	3.35E-02	1.54E-01	1.00E+00
ENSG00000176248.8_ANAPC2	ENSG00000160789.18_LMNA	0.19602	0.038006129	0.344471683	1.55E-02	8.99E-02	1.00E+00
ENSG00000073060.14_SCARB1	ENSG00000160789.18_LMNA	-0.17534	-0.325458205	-0.01660539	3.07E-02	1.46E-01	1.00E+00
ENSG00000134597.12_RBMX2	ENSG00000160789.18_LMNA	-0.02482	-0.183295787	0.13491445	7.61E-01	9.01E-01	1.00E+00
ENSG00000179364.12_PACS2	ENSG00000160789.18_LMNA	-0.03912	-0.19709102	0.120835498	6.32E-01	8.35E-01	1.00E+00
ENSG00000108578.13_BLMH	ENSG00000160789.18_LMNA	-0.15862	-0.309988779	0.000600017	5.10E-02	2.04E-01	1.00E+00
ENSG00000170004.15_CHD3	ENSG00000160789.18_LMNA	-0.01662	-0.175352374	0.142963355	8.39E-01	9.37E-01	1.00E+00
ENSG00000138496.15_PARP9	ENSG00000160789.18_LMNA	0.15873	-0.000482869	0.310094665	5.08E-02	2.03E-01	1.00E+00
ENSG00000176783.13_RUFY1	ENSG00000160789.18_LMNA	0.40552	0.263316398	0.530471858	2.19E-07	1.36E-05	1.12E-02
ENSG00000111231.7_GPN3	ENSG00000160789.18_LMNA	-0.33845	-0.472205568	-0.1894552	2.00E-05	5.17E-04	1.00E+00
ENSG00000122547.9_EEPD1	ENSG00000160789.18_LMNA	-0.25655	-0.399437722	-0.10149603	1.42E-03	1.51E-02	1.00E+00
ENSG00000162642.12_C1orf52	ENSG00000160789.18_LMNA	-0.25838	-0.401079342	-0.1034303	1.31E-03	1.42E-02	1.00E+00
ENSG00000215039.5_CD27-AS1	ENSG00000160789.18_LMNA	-0.21713	-0.363757779	-0.06000477	7.21E-03	5.15E-02	1.00E+00
ENSG00000123815.10_COQ8B	ENSG00000160789.18_LMNA	0.09762	-0.06255459	0.252888849	2.32E-01	5.09E-01	1.00E+00

ENSG00000109184.13_DCUN1D4	ENSG00000160789.18_LMNA	-0.1122	-0.266636832	0.047856692	1.69E-01	4.27E-01	1.00E+00
ENSG00000143374.13_TARS2	ENSG00000160789.18_LMNA	0.08921	-0.070994572	0.244936336	2.74E-01	5.59E-01	1.00E+00
ENSG00000118985.13_ELL2	ENSG00000160789.18_LMNA	0.01908	-0.140549407	0.177739042	8.16E-01	9.28E-01	1.00E+00
ENSG00000156504.15_FAM122B	ENSG00000160789.18_LMNA	0.04596	-0.114075231	0.203670613	5.74E-01	8.01E-01	1.00E+00
ENSG00000171475.12_WIPF2	ENSG00000160789.18_LMNA	0.0146	-0.144937431	0.173397821	8.58E-01	9.44E-01	1.00E+00
ENSG00000113070.7_HBEGF	ENSG00000160789.18_LMNA	0.32694	0.17694559	0.462086325	3.94E-05	8.90E-04	1.00E+00
ENSG00000151491.11_EPS8	ENSG00000160789.18_LMNA	-0.01081	-0.169722914	0.148642196	8.95E-01	9.59E-01	1.00E+00
ENSG00000141076.16_UTP4	ENSG00000160789.18_LMNA	-0.00747	-0.16647517	0.151908906	9.27E-01	9.72E-01	1.00E+00
ENSG00000139182.12_CLSTN3	ENSG00000160789.18_LMNA	0.03846	-0.121480095	0.19646215	6.38E-01	8.38E-01	1.00E+00
ENSG00000162302.11_RPS6KA4	ENSG00000160789.18_LMNA	0.28601	0.132856039	0.425819499	3.54E-04	5.12E-03	1.00E+00
ENSG00000130517.12_PGPEP1	ENSG00000160789.18_LMNA	0.34062	0.191823633	0.474113085	1.76E-05	4.64E-04	9.01E-01
ENSG00000197976.9_AKAP17A	ENSG00000160789.18_LMNA	0.26845	0.114125972	0.410122412	8.26E-04	9.97E-03	1.00E+00
ENSG00000125820.5_NKX2-2	ENSG00000160789.18_LMNA	-0.23807	-0.382763123	-0.0819757	3.14E-03	2.77E-02	1.00E+00
ENSG00000134453.14_RBM17	ENSG00000160789.18_LMNA	-0.20478	-0.352485305	-0.04711092	1.14E-02	7.18E-02	1.00E+00
ENSG00000105221.15_AKT2	ENSG00000160789.18_LMNA	0.15101	-0.008390585	0.302929747	6.33E-02	2.34E-01	1.00E+00
ENSG00000136840.17_ST6GALN	ENSG00000160789.18_LMNA	0.3003	0.148181702	0.438533607	1.71E-04	2.89E-03	1.00E+00
ENSG00000143376.11_SNX27	ENSG00000160789.18_LMNA	0.01691	-0.14267376	0.175638894	8.36E-01	9.37E-01	1.00E+00
ENSG00000198585.10_NUDT16	ENSG00000160789.18_LMNA	0.31488	0.163900254	0.451454034	7.78E-05	1.54E-03	1.00E+00
ENSG00000008256.14_CYTH3	ENSG00000160789.18_LMNA	0.12963	-0.030191316	0.282992876	1.11E-01	3.33E-01	1.00E+00
ENSG00000123728.8_RAP2C	ENSG00000160789.18_LMNA	0.16534	0.006309534	0.316220997	4.18E-02	1.79E-01	1.00E+00
ENSG00000130935.8_NOL11	ENSG00000160789.18_LMNA	-0.16015	-0.311410992	-0.00097416	4.87E-02	1.98E-01	1.00E+00
ENSG00000129472.11_RAB2B	ENSG00000160789.18_LMNA	-0.13815	-0.290953211	0.021522372	8.96E-02	2.92E-01	1.00E+00
ENSG00000070770.7_CSNK2A2	ENSG00000160789.18_LMNA	0.15456	-0.004754378	0.306228769	5.73E-02	2.19E-01	1.00E+00
ENSG00000280206.1_CTB-193M1	ENSG00000160789.18_LMNA	0.20794	0.050404623	0.355373286	1.02E-02	6.63E-02	1.00E+00
ENSG00000152944.7_MED21	ENSG00000160789.18_LMNA	-0.35439	-0.48616293	-0.2068615	7.48E-06	2.32E-04	3.84E-01
ENSG00000165322.16_ARHGAP1	ENSG00000160789.18_LMNA	-0.36184	-0.492657867	-0.21502211	4.64E-06	1.57E-04	2.38E-01
ENSG00000204713.9_TRIM27	ENSG00000160789.18_LMNA	-0.05945	-0.216602708	0.100701715	4.67E-01	7.28E-01	1.00E+00
ENSG00000183580.9_FBXL7	ENSG00000160789.18_LMNA	0.00995	-0.149483419	0.168887235	9.03E-01	9.63E-01	1.00E+00
ENSG00000197766.6_CFD	ENSG00000160789.18_LMNA	0.06944	-0.090763125	0.226141662	3.95E-01	6.73E-01	1.00E+00
ENSG00000152977.8_ZIC1	ENSG00000160789.18_LMNA	0.06493	-0.095256816	0.221836179	4.27E-01	6.97E-01	1.00E+00
ENSG00000150768.14_DLAT	ENSG00000160789.18_LMNA	0.07236	-0.087849186	0.228926955	3.76E-01	6.56E-01	1.00E+00
ENSG00000128609.13_NDUFA5	ENSG00000160789.18_LMNA	-0.20058	-0.34864514	-0.04274152	1.32E-02	8.01E-02	1.00E+00
ENSG00000136937.11_NCBP1	ENSG00000160789.18_LMNA	-0.03125	-0.189511078	0.128587197	7.02E-01	8.73E-01	1.00E+00

ENSG00000110536.12_PTPMT1	ENSG00000160789.18_LMNA	-0.04358	-0.201383411	0.116428581	5.94E-01	8.13E-01	1.00E+00
ENSG00000205763.12_RP9P	ENSG00000160789.18_LMNA	0.16354	0.004458919	0.314554414	4.41E-02	1.85E-01	1.00E+00
ENSG00000107862.4_GBF1	ENSG00000160789.18_LMNA	0.08621	-0.074009084	0.242085593	2.91E-01	5.76E-01	1.00E+00
ENSG00000178028.12_DMAP1	ENSG00000160789.18_LMNA	0.06386	-0.096323533	0.22081232	4.34E-01	7.03E-01	1.00E+00
ENSG00000030110.11_BAK1	ENSG00000160789.18_LMNA	0.14874	-0.010716006	0.300816042	6.74E-02	2.43E-01	1.00E+00
ENSG00000075234.15_TTC38	ENSG00000160789.18_LMNA	0.13428	-0.025463205	0.287339862	9.91E-02	3.10E-01	1.00E+00
ENSG00000169288.16_MRPL1	ENSG00000160789.18_LMNA	-0.11108	-0.265586171	0.048984775	1.73E-01	4.33E-01	1.00E+00
ENSG00000071537.12_SEL1L	ENSG00000160789.18_LMNA	0.14977	-0.009656953	0.301779054	6.55E-02	2.39E-01	1.00E+00
ENSG00000133226.15_SRRM1	ENSG00000160789.18_LMNA	0.10346	-0.056672141	0.258406451	2.05E-01	4.76E-01	1.00E+00
ENSG00000185033.13_SEMA4B	ENSG00000160789.18_LMNA	0.12235	-0.037579771	0.276174131	1.33E-01	3.70E-01	1.00E+00
ENSG00000103274.9_NUBP1	ENSG00000160789.18_LMNA	-0.05078	-0.208295253	0.109305925	5.34E-01	7.76E-01	1.00E+00
ENSG00000112992.15_NNT	ENSG00000160789.18_LMNA	-0.13786	-0.290678858	0.021821931	9.03E-02	2.93E-01	1.00E+00
ENSG00000102606.16_ARHGEF7	ENSG00000160789.18_LMNA	-0.35981	-0.490887607	-0.212794	5.29E-06	1.74E-04	2.72E-01
ENSG00000144908.12_ALDH1L1	ENSG00000160789.18_LMNA	-0.03808	-0.19609001	0.121861416	6.41E-01	8.40E-01	1.00E+00
ENSG00000256148.1_RP11-809N	ENSG00000160789.18_LMNA	-0.11309	-0.267470835	0.046960661	1.65E-01	4.22E-01	1.00E+00
ENSG00000178202.11_KDELC2	ENSG00000160789.18_LMNA	0.56132	0.441575692	0.661411965	5.38E-14	5.49E-11	2.76E-09
ENSG00000158793.12_NIT1	ENSG00000160789.18_LMNA	-0.13823	-0.291027233	0.02144154	8.94E-02	2.91E-01	1.00E+00
ENSG00000134371.9_CDC73	ENSG00000160789.18_LMNA	0.07553	-0.084684621	0.231945946	3.55E-01	6.39E-01	1.00E+00
ENSG00000181467.3_RAP2B	ENSG00000160789.18_LMNA	0.2278	0.071180212	0.373456492	4.76E-03	3.78E-02	1.00E+00
ENSG00000083896.11_YTHDC1	ENSG00000160789.18_LMNA	-0.01595	-0.174709078	0.143613356	8.45E-01	9.40E-01	1.00E+00
ENSG00000103485.16_QPRT	ENSG00000160789.18_LMNA	0.03481	-0.1250848	0.192940573	6.70E-01	8.55E-01	1.00E+00
ENSG00000138750.13_NUP54	ENSG00000160789.18_LMNA	0.06857	-0.091627435	0.225314513	4.01E-01	6.78E-01	1.00E+00
ENSG00000205981.5_DNAJC19	ENSG00000160789.18_LMNA	-0.3473	-0.479960014	-0.19910392	1.17E-05	3.30E-04	5.99E-01
ENSG00000100311.15_PDGFB	ENSG00000160789.18_LMNA	0.02446	-0.135268805	0.182946949	7.65E-01	9.02E-01	1.00E+00
ENSG00000007541.13_PIGQ	ENSG00000160789.18_LMNA	0.13161	-0.028179846	0.284843778	1.06E-01	3.23E-01	1.00E+00
ENSG00000104320.12_NBN	ENSG00000160789.18_LMNA	-0.22533	-0.371212351	-0.06858761	5.25E-03	4.07E-02	1.00E+00
ENSG00000270039.1_RP11-571M	ENSG00000160789.18_LMNA	-0.35048	-0.482747893	-0.20258619	9.57E-06	2.81E-04	4.91E-01
ENSG00000169692.11_AGPAT2	ENSG00000160789.18_LMNA	0.49247	0.361622187	0.604294074	1.16E-10	2.52E-08	5.96E-06
ENSG00000090674.14_MCOLN1	ENSG00000160789.18_LMNA	0.16014	0.000968431	0.31140582	4.87E-02	1.98E-01	1.00E+00
ENSG00000057663.11_ATG5	ENSG00000160789.18_LMNA	-0.16929	-0.319872621	-0.01037182	3.71E-02	1.65E-01	1.00E+00
ENSG00000132376.18_INPP5K	ENSG00000160789.18_LMNA	-0.16412	-0.315085224	-0.00504811	4.33E-02	1.83E-01	1.00E+00
ENSG00000100297.14_MCM5	ENSG00000160789.18_LMNA	0.15456	-0.004756205	0.306227114	5.73E-02	2.19E-01	1.00E+00
ENSG00000139793.17_MBNL2	ENSG00000160789.18_LMNA	-0.21063	-0.357831706	-0.05321366	9.20E-03	6.17E-02	1.00E+00

ENSG00000110442.10_COMMD9	ENSG00000160789.18_LMNA	-0.22198	-0.368171182	-0.06508073	5.99E-03	4.50E-02	1.00E+00
ENSG00000083750.11_RRAGB	ENSG00000160789.18_LMNA	-0.37262	-0.502039234	-0.22687823	2.27E-06	8.80E-05	1.17E-01
ENSG00000129219.12_PLD2	ENSG00000160789.18_LMNA	0.13669	-0.023006713	0.289593265	9.31E-02	2.98E-01	1.00E+00
ENSG00000174446.11_SNAPC5	ENSG00000160789.18_LMNA	-0.11365	-0.268003108	0.046388535	1.63E-01	4.19E-01	1.00E+00
ENSG00000172009.13_THOP1	ENSG00000160789.18_LMNA	0.05829	-0.101857312	0.215489631	4.76E-01	7.34E-01	1.00E+00
ENSG00000102100.13_SLC35A2	ENSG00000160789.18_LMNA	0.39176	0.248033233	0.518619175	6.00E-07	3.04E-05	3.08E-02
ENSG00000088205.11_DDX18	ENSG00000160789.18_LMNA	-0.15369	-0.305415461	0.005651577	5.87E-02	2.22E-01	1.00E+00
ENSG00000165476.11_REEP3	ENSG00000160789.18_LMNA	0.01791	-0.141695344	0.176606522	8.27E-01	9.32E-01	1.00E+00
ENSG00000180198.14_RCC1	ENSG00000160789.18_LMNA	0.1368	-0.022898748	0.289692224	9.28E-02	2.98E-01	1.00E+00
ENSG00000108840.14_HDAC5	ENSG00000160789.18_LMNA	-0.01682	-0.17554911	0.142764513	8.37E-01	9.37E-01	1.00E+00
ENSG00000103353.14_UBFD1	ENSG00000160789.18_LMNA	0.01421	-0.145320572	0.173018179	8.62E-01	9.46E-01	1.00E+00
ENSG00000112294.11_ALDH5A1	ENSG00000160789.18_LMNA	-0.18686	-0.3360657	-0.02850993	2.12E-02	1.12E-01	1.00E+00
ENSG00000100105.16_PATZ1	ENSG00000160789.18_LMNA	-0.16502	-0.315917419	-0.00597227	4.22E-02	1.80E-01	1.00E+00
ENSG00000129691.14_ASH2L	ENSG00000160789.18_LMNA	-0.05122	-0.208715863	0.108871432	5.31E-01	7.74E-01	1.00E+00
ENSG00000178467.16_P4HTM	ENSG00000160789.18_LMNA	0.09834	-0.061830129	0.253569487	2.28E-01	5.05E-01	1.00E+00
ENSG00000143771.10_CNIH4	ENSG00000160789.18_LMNA	0.06988	-0.090329247	0.226556714	3.92E-01	6.70E-01	1.00E+00
ENSG00000114054.12_PCCB	ENSG00000160789.18_LMNA	-0.23845	-0.383111878	-0.08238163	3.09E-03	2.73E-02	1.00E+00
ENSG00000143303.10_RRNAD1	ENSG00000160789.18_LMNA	-0.00047	-0.159662349	0.158738985	9.95E-01	9.98E-01	1.00E+00
ENSG00000157224.14_CLDN12	ENSG00000160789.18_LMNA	0.09745	-0.062718875	0.252734458	2.32E-01	5.10E-01	1.00E+00
ENSG00000124641.13_MED20	ENSG00000160789.18_LMNA	-0.24833	-0.392028123	-0.09279348	2.04E-03	1.98E-02	1.00E+00
ENSG00000156709.12_AIFM1	ENSG00000160789.18_LMNA	0.1102	-0.049873248	0.264758149	1.77E-01	4.38E-01	1.00E+00
ENSG00000122952.15_ZWINT	ENSG00000160789.18_LMNA	0.0288	-0.131005836	0.187138243	7.25E-01	8.83E-01	1.00E+00
ENSG00000198901.12_PRC1	ENSG00000160789.18_LMNA	0.19314	0.035012701	0.341827211	1.71E-02	9.65E-02	1.00E+00
ENSG00000163947.10_ARHGEF3	ENSG00000160789.18_LMNA	0.09131	-0.068894133	0.246919441	2.63E-01	5.47E-01	1.00E+00
ENSG00000177917.9_ARL6IP6	ENSG00000160789.18_LMNA	0.09148	-0.068720881	0.247082897	2.62E-01	5.46E-01	1.00E+00
ENSG00000118777.9_ABCG2	ENSG00000160789.18_LMNA	-0.34883	-0.481301678	-0.20077887	1.06E-05	3.06E-04	5.44E-01
ENSG00000159579.12_RSPRY1	ENSG00000160789.18_LMNA	0.17229	0.013454538	0.322637579	3.38E-02	1.55E-01	1.00E+00
ENSG00000093072.14_CECR1	ENSG00000160789.18_LMNA	0.04351	-0.116500797	0.201313169	5.95E-01	8.13E-01	1.00E+00
ENSG00000275832.3_ARHGAP23	ENSG00000160789.18_LMNA	0.09267	-0.067530365	0.248205612	2.56E-01	5.38E-01	1.00E+00
ENSG00000177370.4_TIMM22	ENSG00000160789.18_LMNA	0.07694	-0.083279652	0.233284332	3.46E-01	6.30E-01	1.00E+00
ENSG00000247315.3_ZCCHC3	ENSG00000160789.18_LMNA	0.12192	-0.038021837	0.275765149	1.35E-01	3.73E-01	1.00E+00
ENSG00000178429.9_RPS3AP5	ENSG00000160789.18_LMNA	-0.11559	-0.269826333	0.044427238	1.56E-01	4.08E-01	1.00E+00
ENSG00000121022.12_COPS5	ENSG00000160789.18_LMNA	-0.35094	-0.483144267	-0.20308186	9.30E-06	2.75E-04	4.77E-01

ENSG00000099899.13_TRMT2A	ENSG00000160789.18_LMNA	0.06571	-0.094476448	0.222584751	4.21E-01	6.93E-01	1.00E+00
ENSG00000092020.9_PPP2R3C	ENSG00000160789.18_LMNA	-0.23111	-0.376460938	-0.07465758	4.17E-03	3.43E-02	1.00E+00
ENSG00000165271.15_NOL6	ENSG00000160789.18_LMNA	0.12566	-0.034220415	0.279278374	1.23E-01	3.53E-01	1.00E+00
ENSG00000125863.16_MKKS	ENSG00000160789.18_LMNA	-0.23639	-0.3812439	-0.0802086	3.37E-03	2.92E-02	1.00E+00
ENSG00000185834.10_RPL12P4	ENSG00000160789.18_LMNA	-0.00053	-0.159716724	0.158684593	9.95E-01	9.98E-01	1.00E+00
ENSG00000100209.8_HSCB	ENSG00000160789.18_LMNA	-0.21187	-0.358960269	-0.05450479	8.78E-03	5.97E-02	1.00E+00
ENSG00000232368.1_FTLP2	ENSG00000160789.18_LMNA	-0.00755	-0.166551851	0.151831856	9.26E-01	9.72E-01	1.00E+00
ENSG00000148204.10_CRB2	ENSG00000160789.18_LMNA	0.23362	0.077289999	0.378731131	3.77E-03	3.17E-02	1.00E+00
ENSG00000276045.1_ORAI1	ENSG00000160789.18_LMNA	0.3088	0.157329229	0.446067373	1.09E-04	2.02E-03	1.00E+00
ENSG00000146416.15_AIG1	ENSG00000160789.18_LMNA	-0.15988	-0.311160579	-0.00069688	4.91E-02	1.99E-01	1.00E+00
ENSG00000163605.13_PPP4R2	ENSG00000160789.18_LMNA	0.00134	-0.157889931	0.160510911	9.87E-01	9.95E-01	1.00E+00
ENSG00000136715.16_SAP130	ENSG00000160789.18_LMNA	0.07633	-0.083887769	0.232705181	3.50E-01	6.34E-01	1.00E+00
ENSG00000040487.11_PQLC2	ENSG00000160789.18_LMNA	0.50904	0.380693161	0.618148509	2.14E-11	6.33E-09	1.10E-06
ENSG00000185722.15_ANKFY1	ENSG00000160789.18_LMNA	-0.00454	-0.163624284	0.154770718	9.56E-01	9.82E-01	1.00E+00
ENSG00000094880.9_CDC23	ENSG00000160789.18_LMNA	-0.1627	-0.313769496	-0.00358806	4.52E-02	1.88E-01	1.00E+00
ENSG00000100263.12_RHBDD3	ENSG00000160789.18_LMNA	0.24104	0.08510707	0.385451252	2.78E-03	2.52E-02	1.00E+00
ENSG00000013583.7_HEBP1	ENSG00000160789.18_LMNA	0.18172	0.023188174	0.331333396	2.51E-02	1.27E-01	1.00E+00
ENSG00000234160.1_RP11-613M	ENSG00000160789.18_LMNA	0.14553	-0.013994741	0.297830609	7.36E-02	2.58E-01	1.00E+00
ENSG00000141127.13_PRPSAP2	ENSG00000160789.18_LMNA	-0.55143	-0.653277831	-0.42997228	1.81E-13	1.37E-10	9.29E-09
ENSG00000063245.13_EPN1	ENSG00000160789.18_LMNA	0.2919	0.139163546	0.431066228	2.63E-04	4.06E-03	1.00E+00
ENSG00000165732.11_DDX21	ENSG00000160789.18_LMNA	0.06472	-0.095462092	0.221639205	4.28E-01	6.99E-01	1.00E+00
ENSG00000048740.16_CELF2	ENSG00000160789.18_LMNA	-0.27056	-0.412010865	-0.11636823	7.48E-04	9.23E-03	1.00E+00
ENSG00000132478.8_UNK	ENSG00000160789.18_LMNA	-0.18037	-0.330093328	-0.02179653	2.62E-02	1.31E-01	1.00E+00
ENSG00000142327.10_RNPEPL1	ENSG00000160789.18_LMNA	0.42433	0.284338095	0.546606107	5.09E-08	4.14E-06	2.61E-03
ENSG00000106070.16_GRB10	ENSG00000160789.18_LMNA	0.40176	0.259138749	0.527242271	2.89E-07	1.71E-05	1.48E-02
ENSG00000146109.4_ABT1	ENSG00000160789.18_LMNA	-0.09671	-0.25203392	0.063464093	2.36E-01	5.14E-01	1.00E+00
ENSG00000142655.11_PEX14	ENSG00000160789.18_LMNA	0.11148	-0.048579315	0.26596389	1.72E-01	4.31E-01	1.00E+00
ENSG00000166526.15_ZNF3	ENSG00000160789.18_LMNA	-0.11148	-0.265958186	0.048585439	1.72E-01	4.31E-01	1.00E+00
ENSG00000239779.5_WBP1	ENSG00000160789.18_LMNA	-0.20899	-0.356334642	-0.05150251	9.77E-03	6.45E-02	1.00E+00
ENSG00000169299.12_PGM2	ENSG00000160789.18_LMNA	0.21484	0.057609967	0.361670828	7.86E-03	5.48E-02	1.00E+00
ENSG00000111077.16_TNS2	ENSG00000160789.18_LMNA	-0.02891	-0.187250373	0.130891627	7.24E-01	8.83E-01	1.00E+00
ENSG00000142687.16_KIAA0319	ENSG00000160789.18_LMNA	0.18829	0.029989888	0.337378985	2.02E-02	1.09E-01	1.00E+00
ENSG00000078070.10_MCCC1	ENSG00000160789.18_LMNA	-0.22324	-0.369317084	-0.06640123	5.70E-03	4.33E-02	1.00E+00

ENSG00000131097.5_HIGD1B	ENSG00000160789.18_LMNA	-0.2533	-0.396515093	-0.09805796	1.64E-03	1.68E-02	1.00E+00
ENSG00000168758.9_SEMA4C	ENSG00000160789.18_LMNA	0.02735	-0.132430704	0.185738619	7.38E-01	8.90E-01	1.00E+00
ENSG00000225573.4_RPL35P5	ENSG00000160789.18_LMNA	-0.03201	-0.190243357	0.127840017	6.95E-01	8.69E-01	1.00E+00
ENSG00000096401.7_CDC5L	ENSG00000160789.18_LMNA	-0.11778	-0.271881686	0.042213326	1.48E-01	3.95E-01	1.00E+00
ENSG00000130340.13_SNX9	ENSG00000160789.18_LMNA	0.41886	0.278213441	0.541925423	7.85E-08	5.92E-06	4.03E-03
ENSG00000269131.1_	ENSG00000160789.18_LMNA	0.05702	-0.103114915	0.214277365	4.85E-01	7.42E-01	1.00E+00
ENSG00000157456.6_CCNB2	ENSG00000160789.18_LMNA	0.00832	-0.151084864	0.167295079	9.19E-01	9.69E-01	1.00E+00
ENSG00000054179.10_ENTPD2	ENSG00000160789.18_LMNA	0.03165	-0.128195802	0.189894711	6.99E-01	8.71E-01	1.00E+00
ENSG00000109929.8_SC5D	ENSG00000160789.18_LMNA	-0.37394	-0.503184853	-0.22833167	2.08E-06	8.22E-05	1.07E-01
ENSG00000171443.6_ZNF524	ENSG00000160789.18_LMNA	0.21552	0.058322726	0.362292282	7.66E-03	5.38E-02	1.00E+00
ENSG00000185386.13_MAPK11	ENSG00000160789.18_LMNA	0.13717	-0.022520506	0.290038865	9.20E-02	2.96E-01	1.00E+00
ENSG00000164144.13_ARFIP1	ENSG00000160789.18_LMNA	-0.03384	-0.192007329	0.126038667	6.79E-01	8.60E-01	1.00E+00
ENSG00000009335.16_UBE3C	ENSG00000160789.18_LMNA	0.31841	0.167709057	0.454566771	6.39E-05	1.31E-03	1.00E+00
ENSG00000223612.3_RP11-289I1	ENSG00000160789.18_LMNA	0.11809	-0.041897896	0.272174291	1.47E-01	3.93E-01	1.00E+00
ENSG00000070785.15_EIF2B3	ENSG00000160789.18_LMNA	-0.04005	-0.197986881	0.119916767	6.24E-01	8.31E-01	1.00E+00
ENSG00000187122.15_SLIT1	ENSG00000160789.18_LMNA	-0.31724	-0.453535575	-0.16644632	6.83E-05	1.38E-03	1.00E+00
ENSG00000214026.9_MRPL23	ENSG00000160789.18_LMNA	0.05959	-0.100560634	0.21673854	4.66E-01	7.28E-01	1.00E+00
ENSG00000204922.4_UQCC3	ENSG00000160789.18_LMNA	-0.00086	-0.160035155	0.158366022	9.92E-01	9.97E-01	1.00E+00
ENSG00000133704.8_IPO8	ENSG00000160789.18_LMNA	-0.07649	-0.232856921	0.083728462	3.49E-01	6.33E-01	1.00E+00
ENSG00000127511.8_SIN3B	ENSG00000160789.18_LMNA	0.1255	-0.034383282	0.279128026	1.23E-01	3.54E-01	1.00E+00
ENSG00000160293.15_VAV2	ENSG00000160789.18_LMNA	0.4575	0.321733516	0.574833908	3.11E-09	4.02E-07	1.60E-04
ENSG00000176749.7_CDK5R1	ENSG00000160789.18_LMNA	-0.32088	-0.456744684	-0.17037909	5.57E-05	1.17E-03	1.00E+00
ENSG00000142549.9_IGLON5	ENSG00000160789.18_LMNA	-0.21598	-0.362705719	-0.05879708	7.53E-03	5.31E-02	1.00E+00
ENSG00000131013.3_PPIL4	ENSG00000160789.18_LMNA	-0.0089	-0.167865028	0.150511783	9.13E-01	9.67E-01	1.00E+00
ENSG00000105255.9_FSD1	ENSG00000160789.18_LMNA	-0.25299	-0.396228811	-0.09772157	1.66E-03	1.70E-02	1.00E+00
ENSG00000119541.8_VPS4B	ENSG00000160789.18_LMNA	-0.03212	-0.190344366	0.127736924	6.94E-01	8.69E-01	1.00E+00
ENSG00000108387.13_SEPT4	ENSG00000160789.18_LMNA	-0.18961	-0.338587691	-0.03135319	1.93E-02	1.05E-01	1.00E+00
ENSG00000198832.9_SELENOM	ENSG00000160789.18_LMNA	0.07865	-0.08157208	0.234909367	3.35E-01	6.21E-01	1.00E+00
ENSG00000126861.4_OMG	ENSG00000160789.18_LMNA	-0.52604	-0.632286688	-0.40036522	3.41E-12	1.47E-09	1.75E-07
ENSG00000169499.13_PLEKHA2	ENSG00000160789.18_LMNA	0.10471	-0.055411421	0.259586309	1.99E-01	4.70E-01	1.00E+00
ENSG00000178878.11_APOLD1	ENSG00000160789.18_LMNA	-0.07269	-0.229235799	0.087525743	3.74E-01	6.53E-01	1.00E+00
ENSG00000204560.8_DHX16	ENSG00000160789.18_LMNA	0.23873	0.082672717	0.383361916	3.06E-03	2.71E-02	1.00E+00
ENSG00000099282.8_TSPAN15	ENSG00000160789.18_LMNA	-0.36173	-0.492563048	-0.21490269	4.67E-06	1.58E-04	2.40E-01

ENSG00000177542.9_SLC25A22	ENSG00000160789.18_LMNA	0.13532	-0.02440742	0.288308785	9.65E-02	3.05E-01	1.00E+00
ENSG00000145191.10_EIF2B5	ENSG00000160789.18_LMNA	-0.17897	-0.328804658	-0.0203516	2.74E-02	1.35E-01	1.00E+00
ENSG00000113161.14_HMGCR	ENSG00000160789.18_LMNA	-0.1617	-0.312842966	-0.00256069	4.66E-02	1.92E-01	1.00E+00
ENSG00000133433.9_GSTT2B	ENSG00000160789.18_LMNA	-0.08095	-0.237091897	0.079275771	3.22E-01	6.08E-01	1.00E+00
ENSG00000243679.1_RP11-274B	ENSG00000160789.18_LMNA	0.16446	0.005398081	0.315400422	4.29E-02	1.82E-01	1.00E+00
ENSG00000166477.11_LEO1	ENSG00000160789.18_LMNA	0.26516	0.110631582	0.407174332	9.62E-04	1.13E-02	1.00E+00
ENSG00000118946.10_PCDH17	ENSG00000160789.18_LMNA	-0.1624	-0.313496995	-0.00328584	4.56E-02	1.89E-01	1.00E+00
ENSG00000040531.13_CTNS	ENSG00000160789.18_LMNA	0.31203	0.160815378	0.448927784	9.10E-05	1.75E-03	1.00E+00
ENSG00000183576.11_SETD3	ENSG00000160789.18_LMNA	0.04771	-0.112343785	0.205351181	5.59E-01	7.92E-01	1.00E+00
ENSG00000144847.11_IGSF11	ENSG00000160789.18_LMNA	-0.35479	-0.486507194	-0.20729308	7.30E-06	2.27E-04	3.75E-01
ENSG00000125629.13_INSIG2	ENSG00000160789.18_LMNA	0.04321	-0.116794671	0.201027299	5.97E-01	8.14E-01	1.00E+00
ENSG00000159592.9_GPBP1L1	ENSG00000160789.18_LMNA	0.09147	-0.068731461	0.247072916	2.62E-01	5.46E-01	1.00E+00
ENSG00000183527.10_PSMG1	ENSG00000160789.18_LMNA	0.05793	-0.102214937	0.215144999	4.78E-01	7.37E-01	1.00E+00
ENSG00000167755.12_KLK6	ENSG00000160789.18_LMNA	-0.26343	-0.405618587	-0.10879049	1.04E-03	1.19E-02	1.00E+00
ENSG00000115170.12_ACVR1	ENSG00000160789.18_LMNA	-0.03306	-0.191255104	0.126807088	6.86E-01	8.64E-01	1.00E+00
ENSG00000272391.4_POM121C	ENSG00000160789.18_LMNA	0.32577	0.175681152	0.46105935	4.21E-05	9.36E-04	1.00E+00
ENSG00000129636.11_ITFG1	ENSG00000160789.18_LMNA	0.04206	-0.117930624	0.199921772	6.07E-01	8.20E-01	1.00E+00
ENSG00000233270.1_SNRPEP4	ENSG00000160789.18_LMNA	-0.06437	-0.22130229	0.095813144	4.31E-01	7.00E-01	1.00E+00
ENSG00000082701.13_GSK3B	ENSG00000160789.18_LMNA	-0.13196	-0.285167	0.027828324	1.05E-01	3.22E-01	1.00E+00
ENSG00000112592.11_TBP	ENSG00000160789.18_LMNA	-0.10662	-0.261384996	0.053487525	1.91E-01	4.59E-01	1.00E+00
ENSG00000115839.16_RAB3GAP	ENSG00000160789.18_LMNA	0.04746	-0.112591669	0.205110696	5.61E-01	7.93E-01	1.00E+00
ENSG00000116266.9_STXBP3	ENSG00000160789.18_LMNA	-0.01496	-0.173742458	0.144589536	8.55E-01	9.43E-01	1.00E+00
ENSG00000168005.7_C11orf84	ENSG00000160789.18_LMNA	0.13807	-0.02160794	0.290874849	8.98E-02	2.92E-01	1.00E+00
ENSG00000159231.5_CBR3	ENSG00000160789.18_LMNA	0.20503	0.04737264	0.352715001	1.13E-02	7.14E-02	1.00E+00
ENSG00000143324.12_XPR1	ENSG00000160789.18_LMNA	0.13636	-0.023348816	0.289279652	9.39E-02	3.00E-01	1.00E+00
ENSG00000153113.22_CAST	ENSG00000160789.18_LMNA	0.39819	0.255163397	0.524161864	3.77E-07	2.10E-05	1.93E-02
ENSG00000214975.4_PPIAP29	ENSG00000160789.18_LMNA	0.02574	-0.134007975	0.184187786	7.53E-01	8.97E-01	1.00E+00
ENSG00000007516.12_BAIAP3	ENSG00000160789.18_LMNA	-0.05738	-0.214618518	0.102761106	4.83E-01	7.40E-01	1.00E+00
ENSG00000106351.11_AGFG2	ENSG00000160789.18_LMNA	0.05516	-0.104957524	0.212499414	5.00E-01	7.53E-01	1.00E+00
ENSG00000149930.16_TAOK2	ENSG00000160789.18_LMNA	0.17982	0.021225146	0.329583874	2.66E-02	1.32E-01	1.00E+00
ENSG00000254682.1_RP11-660L	ENSG00000160789.18_LMNA	-0.15859	-0.309967025	0.000624083	5.10E-02	2.04E-01	1.00E+00
ENSG00000105472.11_CLEC11A	ENSG00000160789.18_LMNA	0.26784	0.113483095	0.409580506	8.50E-04	1.02E-02	1.00E+00
ENSG00000172831.10_CES2	ENSG00000160789.18_LMNA	0.26818	0.113841039	0.409882256	8.36E-04	1.01E-02	1.00E+00

ENSG00000072501.16_SMC1A	ENSG00000160789.18_LMNA	0.14699	-0.012498886	0.299193408	7.07E-02	2.51E-01	1.00E+00
ENSG00000168824.13_NSG1	ENSG00000160789.18_LMNA	-0.18615	-0.33541503	-0.02777717	2.17E-02	1.14E-01	1.00E+00
ENSG00000132382.13_MYBBP1A	ENSG00000160789.18_LMNA	0.20981	0.052357012	0.357082433	9.48E-03	6.31E-02	1.00E+00
ENSG00000115325.12_DOK1	ENSG00000160789.18_LMNA	0.39467	0.251260575	0.521130795	4.87E-07	2.56E-05	2.50E-02
ENSG00000155254.11_MARVELD	ENSG00000160789.18_LMNA	0.1675	0.008526921	0.318215364	3.91E-02	1.71E-01	1.00E+00
ENSG00000005483.18_KMT2E	ENSG00000160789.18_LMNA	0.11682	-0.043188669	0.270976549	1.52E-01	4.01E-01	1.00E+00
ENSG00000084636.16_COL16A1	ENSG00000160789.18_LMNA	0.30694	0.155333224	0.444426976	1.20E-04	2.19E-03	1.00E+00
ENSG00000068796.15_KIF2A	ENSG00000160789.18_LMNA	-0.23538	-0.380326418	-0.07914234	3.51E-03	3.01E-02	1.00E+00
ENSG00000256338.2_RPL41P2	ENSG00000160789.18_LMNA	-0.14676	-0.298973677	0.012740164	7.12E-02	2.52E-01	1.00E+00
ENSG00000108961.12_RANGRF	ENSG00000160789.18_LMNA	-0.09911	-0.254296016	0.061056454	2.24E-01	5.00E-01	1.00E+00
ENSG00000148339.11_SLC25A25	ENSG00000160789.18_LMNA	0.37546	0.230007657	0.50450469	1.88E-06	7.63E-05	9.63E-02
ENSG00000150776.16_C11orf57	ENSG00000160789.18_LMNA	-0.25614	-0.399071257	-0.10106454	1.45E-03	1.53E-02	1.00E+00
ENSG00000240344.7_PPIL3	ENSG00000160789.18_LMNA	-0.14379	-0.296207392	0.015774619	7.72E-02	2.66E-01	1.00E+00
ENSG00000091972.17_CD200	ENSG00000160789.18_LMNA	-0.49412	-0.605678781	-0.36351918	9.84E-11	2.21E-08	5.05E-06
ENSG00000237172.3_B3GNT9	ENSG00000160789.18_LMNA	0.52497	0.399130322	0.631403699	3.84E-12	1.62E-09	1.97E-07
ENSG00000149639.13_SOGA1	ENSG00000160789.18_LMNA	0.01809	-0.1415144	0.176785403	8.25E-01	9.31E-01	1.00E+00
ENSG00000184178.14_SCFD2	ENSG00000160789.18_LMNA	0.00565	-0.153691215	0.164700291	9.45E-01	9.78E-01	1.00E+00
ENSG00000103148.14_NPRL3	ENSG00000160789.18_LMNA	0.0314	-0.128440763	0.189654619	7.01E-01	8.72E-01	1.00E+00
ENSG00000140943.15_MBTPS1	ENSG00000160789.18_LMNA	0.16623	0.007219495	0.317039768	4.07E-02	1.75E-01	1.00E+00
ENSG00000172889.14_EGFL7	ENSG00000160789.18_LMNA	-0.02956	-0.187875733	0.130254515	7.18E-01	8.80E-01	1.00E+00
ENSG00000184232.7_OAF	ENSG00000160789.18_LMNA	0.40134	0.258671396	0.526880496	2.99E-07	1.76E-05	1.53E-02
ENSG00000173404.4_INSM1	ENSG00000160789.18_LMNA	-0.24729	-0.391096086	-0.09170203	2.13E-03	2.05E-02	1.00E+00
ENSG00000107902.12_LHPP	ENSG00000160789.18_LMNA	-0.30998	-0.447112577	-0.15860225	1.02E-04	1.91E-03	1.00E+00
ENSG00000135407.9_AVIL	ENSG00000160789.18_LMNA	-0.25897	-0.401610735	-0.10405691	1.27E-03	1.39E-02	1.00E+00
ENSG00000100271.15_TTLL1	ENSG00000160789.18_LMNA	-0.13135	-0.28460057	0.028444297	1.07E-01	3.25E-01	1.00E+00
ENSG00000134186.10_PRPF38B	ENSG00000160789.18_LMNA	0.00369	-0.155600957	0.162796219	9.64E-01	9.86E-01	1.00E+00
ENSG00000160606.9_TLCD1	ENSG00000160789.18_LMNA	0.33837	0.189373456	0.472139688	2.01E-05	5.18E-04	1.00E+00
ENSG00000115514.10_TXNDC9	ENSG00000160789.18_LMNA	-0.21921	-0.365653123	-0.06218273	6.66E-03	4.85E-02	1.00E+00
ENSG00000124486.11_USP9X	ENSG00000160789.18_LMNA	0.23202	0.075614647	0.377286729	4.02E-03	3.33E-02	1.00E+00
ENSG00000183908.5_LRRC55	ENSG00000160789.18_LMNA	-0.28662	-0.426362021	-0.13350715	3.44E-04	4.99E-03	1.00E+00
ENSG00000140939.13_NOL3	ENSG00000160789.18_LMNA	0.25264	0.097351912	0.395914151	1.69E-03	1.72E-02	1.00E+00
ENSG00000169398.18_PTK2	ENSG00000160789.18_LMNA	-0.17422	-0.324421163	-0.01544622	3.18E-02	1.49E-01	1.00E+00
ENSG00000162413.15_KLHL21	ENSG00000160789.18_LMNA	0.34091	0.192134163	0.474362986	1.73E-05	4.57E-04	8.85E-01

ENSG00000117360.11_PRPF3	ENSG00000160789.18_LMNA	0.08523	-0.074985179	0.241161355	2.96E-01	5.82E-01	1.00E+00
ENSG00000125503.11_PPP1R12(	ENSG00000160789.18_LMNA	0.26421	0.109619229	0.406319094	1.00E-03	1.16E-02	1.00E+00
ENSG00000075413.16_MARK3	ENSG00000160789.18_LMNA	0.18398	0.025524464	0.333412825	2.33E-02	1.20E-01	1.00E+00
ENSG00000100796.16_PPP4R3A	ENSG00000160789.18_LMNA	0.19473	0.036667758	0.343289933	1.62E-02	9.28E-02	1.00E+00
ENSG00000278133.1_RP11-196G	ENSG00000160789.18_LMNA	-0.07632	-0.232693385	0.083900153	3.50E-01	6.34E-01	1.00E+00
ENSG00000110721.10_CHKA	ENSG00000160789.18_LMNA	-0.05037	-0.207899988	0.109714127	5.38E-01	7.78E-01	1.00E+00
ENSG00000257093.5_KIAA1147	ENSG00000160789.18_LMNA	-0.15754	-0.308995422	0.0016986	5.26E-02	2.08E-01	1.00E+00
ENSG00000129355.6_CDKN2D	ENSG00000160789.18_LMNA	-0.1158	-0.270019961	0.044218805	1.55E-01	4.07E-01	1.00E+00
ENSG00000140854.11_KATNB1	ENSG00000160789.18_LMNA	-0.04924	-0.206820005	0.110828911	5.47E-01	7.84E-01	1.00E+00
ENSG00000226084.5_RP4-706A1	ENSG00000160789.18_LMNA	-0.09423	-0.24968319	0.065962184	2.48E-01	5.28E-01	1.00E+00
ENSG00000170445.11_HARS	ENSG00000160789.18_LMNA	0.11881	-0.041166497	0.272852544	1.45E-01	3.90E-01	1.00E+00
ENSG00000119812.17_FAM98A	ENSG00000160789.18_LMNA	-0.00032	-0.15951439	0.158886982	9.97E-01	9.99E-01	1.00E+00
ENSG00000137075.16_RNF38	ENSG00000160789.18_LMNA	-0.0173	-0.176019417	0.142289068	8.32E-01	9.35E-01	1.00E+00
ENSG00000178685.12_PARP10	ENSG00000160789.18_LMNA	0.26996	0.115731952	0.411475242	7.70E-04	9.42E-03	1.00E+00
ENSG00000142444.6_TIMM29	ENSG00000160789.18_LMNA	0.01687	-0.142710191	0.175602852	8.37E-01	9.37E-01	1.00E+00
ENSG00000110719.8_TCIRG1	ENSG00000160789.18_LMNA	0.38718	0.242958474	0.51466037	8.32E-07	3.92E-05	4.27E-02
ENSG00000211829.5_TRDC	ENSG00000160789.18_LMNA	0.12136	-0.038581489	0.275247216	1.36E-01	3.76E-01	1.00E+00
ENSG00000140365.14_COMMD4	ENSG00000160789.18_LMNA	0.24529	0.089586666	0.389287931	2.32E-03	2.19E-02	1.00E+00
ENSG00000100284.19_TOM1	ENSG00000160789.18_LMNA	0.12851	-0.03132725	0.281946587	1.15E-01	3.38E-01	1.00E+00
ENSG00000127554.12_GFER	ENSG00000160789.18_LMNA	0.00893	-0.150486344	0.167890322	9.13E-01	9.67E-01	1.00E+00
ENSG00000074582.11_BCS1L	ENSG00000160789.18_LMNA	-0.12645	-0.280018158	0.033418788	1.21E-01	3.49E-01	1.00E+00
ENSG00000135521.8_LTV1	ENSG00000160789.18_LMNA	0.05617	-0.10396451	0.213457844	4.92E-01	7.47E-01	1.00E+00
ENSG00000106261.15_ZKSCAN1	ENSG00000160789.18_LMNA	0.06786	-0.09234128	0.224631014	4.06E-01	6.82E-01	1.00E+00
ENSG00000100519.10_PSMC6	ENSG00000160789.18_LMNA	-0.14971	-0.301723146	0.009718457	6.56E-02	2.39E-01	1.00E+00
ENSG00000196950.12_SLC39A1(	ENSG00000160789.18_LMNA	-0.23333	-0.37846935	-0.07698623	3.82E-03	3.20E-02	1.00E+00
ENSG00000257267.2_ZNF271P	ENSG00000160789.18_LMNA	-0.24906	-0.392688368	-0.09356708	1.97E-03	1.93E-02	1.00E+00
ENSG00000129473.8_BCL2L2	ENSG00000160789.18_LMNA	0.17731	0.018632503	0.327269966	2.89E-02	1.40E-01	1.00E+00
ENSG00000182796.11_TMEM198	ENSG00000160789.18_LMNA	-0.16448	-0.315418414	-0.00541806	4.29E-02	1.82E-01	1.00E+00
ENSG00000168672.3_FAM84B	ENSG00000160789.18_LMNA	-0.18979	-0.338750894	-0.03153735	1.92E-02	1.05E-01	1.00E+00
ENSG00000183688.4_RFLNB	ENSG00000160789.18_LMNA	0.22134	0.064406331	0.367585596	6.14E-03	4.57E-02	1.00E+00
ENSG00000126264.8_HCST	ENSG00000160789.18_LMNA	0.0568	-0.103332595	0.214067434	4.87E-01	7.44E-01	1.00E+00
ENSG00000121057.11_AKAP1	ENSG00000160789.18_LMNA	-0.14476	-0.297114869	0.014779803	7.52E-02	2.62E-01	1.00E+00
ENSG00000148331.10_ASB6	ENSG00000160789.18_LMNA	0.33283	0.183345532	0.467272645	2.79E-05	6.75E-04	1.00E+00

ENSG00000106462.9_EZH2	ENSG00000160789.18_LMNA	0.06451	-0.09567212	0.221437644	4.30E-01	7.00E-01	1.00E+00
ENSG00000184613.9_NELL2	ENSG00000160789.18_LMNA	-0.10353	-0.258468889	0.056605449	2.04E-01	4.76E-01	1.00E+00
ENSG00000152382.5_TADA1	ENSG00000160789.18_LMNA	-0.25463	-0.397708057	-0.09946046	1.55E-03	1.61E-02	1.00E+00
ENSG00000055163.17_CYFIP2	ENSG00000160789.18_LMNA	-0.29117	-0.430420724	-0.13838625	2.73E-04	4.18E-03	1.00E+00
ENSG00000085377.12_PREP	ENSG00000160789.18_LMNA	-0.06122	-0.218288954	0.098949419	4.54E-01	7.19E-01	1.00E+00
ENSG00000183605.15_SFXN4	ENSG00000160789.18_LMNA	-0.22455	-0.370502216	-0.06776806	5.42E-03	4.16E-02	1.00E+00
ENSG00000116604.16_MEF2D	ENSG00000160789.18_LMNA	0.22144	0.064515173	0.36768012	6.11E-03	4.56E-02	1.00E+00
ENSG00000110911.13_SLC11A2	ENSG00000160789.18_LMNA	-0.45419	-0.572030921	-0.31798455	4.17E-09	5.13E-07	2.14E-04
ENSG00000111358.12_GTF2H3	ENSG00000160789.18_LMNA	8.11E-05	-0.159121614	0.159279788	9.99E-01	1.00E+00	1.00E+00
ENSG00000117266.14_CDK18	ENSG00000160789.18_LMNA	-0.18639	-0.33563304	-0.02802265	2.15E-02	1.14E-01	1.00E+00
ENSG00000134184.11_GSTM1	ENSG00000160789.18_LMNA	0.09162	-0.06858119	0.247214676	2.62E-01	5.45E-01	1.00E+00
ENSG00000095303.13_PTGS1	ENSG00000160789.18_LMNA	0.15219	-0.007188317	0.304021356	6.12E-02	2.29E-01	1.00E+00
ENSG00000055332.15_EIF2AK2	ENSG00000160789.18_LMNA	0.01714	-0.142449735	0.175860503	8.34E-01	9.35E-01	1.00E+00
ENSG00000146826.13_C7orf43	ENSG00000160789.18_LMNA	0.14912	-0.010321932	0.301174453	6.67E-02	2.42E-01	1.00E+00
ENSG00000125107.15_CNOT1	ENSG00000160789.18_LMNA	0.15999	0.000806627	0.311259699	4.90E-02	1.99E-01	1.00E+00
ENSG00000142252.9_GEMIN7	ENSG00000160789.18_LMNA	-0.08316	-0.239192667	0.077062307	3.08E-01	5.95E-01	1.00E+00
ENSG00000156110.12_ADK	ENSG00000160789.18_LMNA	-0.35455	-0.486300438	-0.20703387	7.41E-06	2.30E-04	3.80E-01
ENSG00000127463.12_EMC1	ENSG00000160789.18_LMNA	0.23415	0.077847823	0.379211733	3.69E-03	3.12E-02	1.00E+00
ENSG00000166855.8_CLPX	ENSG00000160789.18_LMNA	0.02187	-0.137814577	0.18043845	7.89E-01	9.14E-01	1.00E+00
ENSG00000196639.6_HRH1	ENSG00000160789.18_LMNA	0.5024	0.373032035	0.612600626	4.26E-11	1.13E-08	2.18E-06
ENSG00000168743.11_NPNT	ENSG00000160789.18_LMNA	0.34626	0.197972896	0.479053303	1.24E-05	3.49E-04	6.38E-01
ENSG00000106772.16_PRUNE2	ENSG00000160789.18_LMNA	0.09622	-0.063957143	0.25157025	2.38E-01	5.17E-01	1.00E+00
ENSG00000131773.12_KHDRBS3	ENSG00000160789.18_LMNA	-0.2625	-0.404781484	-0.10780069	1.09E-03	1.23E-02	1.00E+00
ENSG00000037474.13_NSUN2	ENSG00000160789.18_LMNA	0.19422	0.036133566	0.342817982	1.65E-02	9.40E-02	1.00E+00
ENSG00000185507.18_IRF7	ENSG00000160789.18_LMNA	0.16176	0.002628183	0.312903849	4.65E-02	1.92E-01	1.00E+00
ENSG00000102271.12_KLHL4	ENSG00000160789.18_LMNA	0.16266	0.003548928	0.313734214	4.53E-02	1.88E-01	1.00E+00
ENSG00000135336.13_ORC3	ENSG00000160789.18_LMNA	-0.17083	-0.321293138	-0.01195485	3.54E-02	1.60E-01	1.00E+00
ENSG00000008405.10_CRY1	ENSG00000160789.18_LMNA	0.04252	-0.117477222	0.200363127	6.03E-01	8.18E-01	1.00E+00
ENSG00000134014.15_ELP3	ENSG00000160789.18_LMNA	-0.0613	-0.218372074	0.098862991	4.53E-01	7.18E-01	1.00E+00
ENSG00000161036.9_LRWD1	ENSG00000160789.18_LMNA	0.34793	0.199791235	0.480510724	1.12E-05	3.20E-04	5.76E-01
ENSG00000196700.6_ZNF512B	ENSG00000160789.18_LMNA	0.2102	0.052768622	0.357442501	9.34E-03	6.24E-02	1.00E+00
ENSG00000162694.12_EXTL2	ENSG00000160789.18_LMNA	-0.23039	-0.375804792	-0.07389753	4.30E-03	3.50E-02	1.00E+00
ENSG00000236618.2_PITPNA-AS	ENSG00000160789.18_LMNA	-0.08902	-0.244753763	0.071187806	2.75E-01	5.60E-01	1.00E+00

ENSG00000077463.13_SIRT6	ENSG00000160789.18_LMNA	0.06957	-0.090636794	0.226262524	3.94E-01	6.72E-01	1.00E+00
ENSG00000196628.12_TCF4	ENSG00000160789.18_LMNA	-0.20178	-0.349742057	-0.04398841	1.27E-02	7.77E-02	1.00E+00
ENSG00000155893.10_PXYLP1	ENSG00000160789.18_LMNA	0.17716	0.018482757	0.327136206	2.90E-02	1.40E-01	1.00E+00
ENSG00000137642.11_SORL1	ENSG00000160789.18_LMNA	-0.06388	-0.220839031	0.096295713	4.34E-01	7.03E-01	1.00E+00
ENSG00000115993.10_TRAK2	ENSG00000160789.18_LMNA	-0.07517	-0.231602452	0.085045	3.57E-01	6.40E-01	1.00E+00
ENSG00000196453.7_ZNF777	ENSG00000160789.18_LMNA	0.27876	0.125111889	0.41935068	5.06E-04	6.81E-03	1.00E+00
ENSG00000167721.9_TSR1	ENSG00000160789.18_LMNA	0.22664	0.069958691	0.372399591	4.99E-03	3.92E-02	1.00E+00
ENSG00000154217.13_PITPNC1	ENSG00000160789.18_LMNA	-0.08365	-0.239654479	0.076575304	3.06E-01	5.92E-01	1.00E+00
ENSG00000158106.11_RHPN1	ENSG00000160789.18_LMNA	-0.06012	-0.21724143	0.100038206	4.62E-01	7.25E-01	1.00E+00
ENSG00000136002.15_ARHGEF4	ENSG00000160789.18_LMNA	-0.03339	-0.191575502	0.126479838	6.83E-01	8.62E-01	1.00E+00
ENSG00000199753.1_SNORD104	ENSG00000160789.18_LMNA	-0.06661	-0.223438678	0.093585775	4.15E-01	6.88E-01	1.00E+00
ENSG00000132004.11_FBXW9	ENSG00000160789.18_LMNA	0.08673	-0.073484347	0.242582215	2.88E-01	5.74E-01	1.00E+00
ENSG00000198677.9_TTC37	ENSG00000160789.18_LMNA	0.02904	-0.130765069	0.187374617	7.22E-01	8.83E-01	1.00E+00
ENSG00000100417.10_PMM1	ENSG00000160789.18_LMNA	-0.07369	-0.23018918	0.086526875	3.67E-01	6.48E-01	1.00E+00
ENSG00000119699.6_TGFB3	ENSG00000160789.18_LMNA	0.3473	0.199111426	0.47996603	1.17E-05	3.30E-04	5.99E-01
ENSG00000124228.13_DDX27	ENSG00000160789.18_LMNA	0.25517	0.100031152	0.398193202	1.51E-03	1.58E-02	1.00E+00
ENSG00000167619.10_TMEM145	ENSG00000160789.18_LMNA	-0.23964	-0.384187345	-0.08363402	2.94E-03	2.64E-02	1.00E+00
ENSG00000102897.8_LYRM1	ENSG00000160789.18_LMNA	-0.29801	-0.436505248	-0.14572732	1.92E-04	3.17E-03	1.00E+00
ENSG00000232442.1_CTD-3184A	ENSG00000160789.18_LMNA	-0.06317	-0.220157999	0.097004862	4.39E-01	7.07E-01	1.00E+00
ENSG00000161980.5_POLR3K	ENSG00000160789.18_LMNA	-0.24419	-0.388293458	-0.08842438	2.43E-03	2.27E-02	1.00E+00
ENSG00000127252.5_HRASLS	ENSG00000160789.18_LMNA	-0.38708	-0.514568771	-0.24284123	8.38E-07	3.94E-05	4.30E-02
ENSG00000165716.8_FAM69B	ENSG00000160789.18_LMNA	-0.15685	-0.308348778	0.002413342	5.36E-02	2.10E-01	1.00E+00
ENSG00000099822.2_HCN2	ENSG00000160789.18_LMNA	-0.14477	-0.297127239	0.014766238	7.51E-02	2.62E-01	1.00E+00
ENSG00000137872.14_SEMA6D	ENSG00000160789.18_LMNA	0.07359	-0.086626022	0.230094575	3.68E-01	6.48E-01	1.00E+00
ENSG00000172348.13_RCAN2	ENSG00000160789.18_LMNA	-0.07313	-0.229658323	0.087083139	3.71E-01	6.51E-01	1.00E+00
ENSG00000141994.14_DUS3L	ENSG00000160789.18_LMNA	0.01012	-0.149316499	0.169053092	9.01E-01	9.62E-01	1.00E+00
ENSG00000087263.15_OGFOD1	ENSG00000160789.18_LMNA	0.16456	0.005507746	0.315499176	4.28E-02	1.81E-01	1.00E+00
ENSG00000120437.8_ACAT2	ENSG00000160789.18_LMNA	-0.11761	-0.271721216	0.042386286	1.49E-01	3.96E-01	1.00E+00
ENSG00000115211.14_EIF2B4	ENSG00000160789.18_LMNA	-0.03337	-0.191553936	0.126501867	6.83E-01	8.62E-01	1.00E+00
ENSG00000259943.1_RP1-39G22	ENSG00000160789.18_LMNA	-0.29047	-0.42979466	-0.13763272	2.83E-04	4.30E-03	1.00E+00
ENSG00000012171.16_SEMA3B	ENSG00000160789.18_LMNA	0.0688	-0.091402062	0.225530239	4.00E-01	6.76E-01	1.00E+00
ENSG00000135698.8_MPHOSPH	ENSG00000160789.18_LMNA	-0.24813	-0.391854925	-0.0925906	2.05E-03	1.99E-02	1.00E+00
ENSG00000035928.13_RFC1	ENSG00000160789.18_LMNA	0.09446	-0.065734045	0.249898026	2.47E-01	5.27E-01	1.00E+00

ENSG00000109610.5_SOD3	ENSG00000160789.18_LMNA	0.23806	0.08196355	0.382752679	3.14E-03	2.77E-02	1.00E+00
ENSG00000134077.14_THUMPD3	ENSG00000160789.18_LMNA	-0.11723	-0.271369791	0.042764999	1.50E-01	3.98E-01	1.00E+00
ENSG00000198689.8_SLC9A6	ENSG00000160789.18_LMNA	-0.32527	-0.460620598	-0.17514124	4.33E-05	9.56E-04	1.00E+00
ENSG00000167081.15_PBX3	ENSG00000160789.18_LMNA	-0.02781	-0.186189004	0.131972339	7.34E-01	8.88E-01	1.00E+00
ENSG00000260708.1_CTA-29F11	ENSG00000160789.18_LMNA	-0.16279	-0.313853561	-0.00368131	4.51E-02	1.88E-01	1.00E+00
ENSG00000108309.11_RUNDC3A	ENSG00000160789.18_LMNA	-0.49151	-0.603485815	-0.36051583	1.28E-10	2.75E-08	6.55E-06
ENSG00000076928.16_ARHGEF1	ENSG00000160789.18_LMNA	0.272	0.117905396	0.413304016	6.99E-04	8.77E-03	1.00E+00
ENSG00000245848.2_CEBPA	ENSG00000160789.18_LMNA	-0.09639	-0.251725297	0.063792289	2.37E-01	5.16E-01	1.00E+00
ENSG00000143110.10_C1orf162	ENSG00000160789.18_LMNA	0.0293	-0.130506243	0.187628679	7.20E-01	8.81E-01	1.00E+00
ENSG00000166200.13_COPS2	ENSG00000160789.18_LMNA	0.05413	-0.105982845	0.211509162	5.08E-01	7.58E-01	1.00E+00
ENSG00000185800.10_DMWD	ENSG00000160789.18_LMNA	0.19326	0.035145281	0.341944439	1.71E-02	9.63E-02	1.00E+00
ENSG00000164300.14_SERINC5	ENSG00000160789.18_LMNA	-0.10733	-0.262054841	0.052770453	1.88E-01	4.55E-01	1.00E+00
ENSG00000106460.17_TMEM106	ENSG00000160789.18_LMNA	-0.24148	-0.385850658	-0.08557284	2.73E-03	2.48E-02	1.00E+00
ENSG00000172172.6_MRPL13	ENSG00000160789.18_LMNA	-0.19517	-0.343692112	-0.03712312	1.60E-02	9.18E-02	1.00E+00
ENSG00000117154.10_IGSF21	ENSG00000160789.18_LMNA	-0.26924	-0.410834	-0.11497052	7.96E-04	9.69E-03	1.00E+00
ENSG00000134758.12_RNF138	ENSG00000160789.18_LMNA	-0.15285	-0.304637721	0.00650907	6.01E-02	2.26E-01	1.00E+00
ENSG00000237541.3_HLA-DQA2	ENSG00000160789.18_LMNA	0.00231	-0.156946664	0.161453082	9.77E-01	9.91E-01	1.00E+00
ENSG00000072954.5_TMEM38A	ENSG00000160789.18_LMNA	-0.2704	-0.411872725	-0.11620411	7.54E-04	9.28E-03	1.00E+00
ENSG00000099940.10_SNAP29	ENSG00000160789.18_LMNA	-0.01165	-0.170533842	0.147825447	8.87E-01	9.56E-01	1.00E+00
ENSG00000134982.15_APC	ENSG00000160789.18_LMNA	-0.18213	-0.331716093	-0.02361789	2.47E-02	1.25E-01	1.00E+00
ENSG00000188342.10_GTF2F2	ENSG00000160789.18_LMNA	-0.1431	-0.295569069	0.016474008	7.86E-02	2.69E-01	1.00E+00
ENSG00000176731.10_C8orf59	ENSG00000160789.18_LMNA	-0.14935	-0.301384208	0.010091262	6.63E-02	2.41E-01	1.00E+00
ENSG00000166971.15_AKTIP	ENSG00000160789.18_LMNA	-0.37236	-0.501810074	-0.22658765	2.31E-06	8.91E-05	1.19E-01
ENSG00000163812.12_ZDHHC3	ENSG00000160789.18_LMNA	-0.28734	-0.427007055	-0.13428162	3.32E-04	4.85E-03	1.00E+00
ENSG00000130147.14_SH3BP4	ENSG00000160789.18_LMNA	0.05343	-0.106676078	0.21083927	5.13E-01	7.61E-01	1.00E+00
ENSG00000122126.14_OCRL	ENSG00000160789.18_LMNA	-0.00174	-0.160894231	0.157506236	9.83E-01	9.94E-01	1.00E+00
ENSG00000131941.6_RHPN2	ENSG00000160789.18_LMNA	0.20338	0.045654016	0.351206009	1.20E-02	7.47E-02	1.00E+00
ENSG00000178149.15_DALRD3	ENSG00000160789.18_LMNA	0.09391	-0.066284366	0.249379743	2.50E-01	5.30E-01	1.00E+00
ENSG00000101146.11_RAE1	ENSG00000160789.18_LMNA	-0.04029	-0.198221029	0.119676553	6.22E-01	8.29E-01	1.00E+00
ENSG00000120318.14_ARAP3	ENSG00000160789.18_LMNA	0.32311	0.172794664	0.458712079	4.91E-05	1.06E-03	1.00E+00
ENSG00000075043.16_KCNQ2	ENSG00000160789.18_LMNA	-0.18045	-0.330163496	-0.02187524	2.61E-02	1.30E-01	1.00E+00
ENSG00000115956.9_PLEK	ENSG00000160789.18_LMNA	-0.03127	-0.189531595	0.128566266	7.02E-01	8.73E-01	1.00E+00
ENSG00000248487.7_ABHD14A	ENSG00000160789.18_LMNA	-0.21091	-0.358087868	-0.05350663	9.10E-03	6.12E-02	1.00E+00

ENSG00000105607.11_GCDH	ENSG00000160789.18_LMNA	-0.31128	-0.448270101	-0.16001318	9.48E-05	1.81E-03	1.00E+00
ENSG00000197343.9_ZNF655	ENSG00000160789.18_LMNA	-0.0023	-0.161443054	0.156956706	9.78E-01	9.92E-01	1.00E+00
ENSG00000176623.10_RMDN1	ENSG00000160789.18_LMNA	0.05982	-0.100338323	0.216952557	4.64E-01	7.26E-01	1.00E+00
ENSG00000179967.11_PPP1R14I	ENSG00000160789.18_LMNA	0.12652	-0.033354911	0.28007709	1.20E-01	3.49E-01	1.00E+00
ENSG00000131269.14_ABCB7	ENSG00000160789.18_LMNA	0.11206	-0.048001914	0.266501619	1.69E-01	4.28E-01	1.00E+00
ENSG00000151923.16_TIAL1	ENSG00000160789.18_LMNA	-0.34409	-0.477156383	-0.19560911	1.42E-05	3.91E-04	7.29E-01
ENSG00000143353.10_LYPLAL1	ENSG00000160789.18_LMNA	-0.24277	-0.387009249	-0.08692468	2.58E-03	2.38E-02	1.00E+00
ENSG00000143889.14_HNRNPLL	ENSG00000160789.18_LMNA	-0.2714	-0.412768812	-0.11726903	7.19E-04	8.95E-03	1.00E+00
ENSG00000132604.9_TERF2	ENSG00000160789.18_LMNA	-0.03062	-0.18889609	0.12921442	7.08E-01	8.76E-01	1.00E+00
ENSG00000031691.6_CENPQ	ENSG00000160789.18_LMNA	-0.22672	-0.372475116	-0.07004595	4.97E-03	3.91E-02	1.00E+00
ENSG00000100918.11_REC8	ENSG00000160789.18_LMNA	-0.13381	-0.286901709	0.025940408	1.00E-01	3.12E-01	1.00E+00
ENSG00000075240.15_GRAMD4	ENSG00000160789.18_LMNA	0.08985	-0.07035561	0.245539884	2.71E-01	5.55E-01	1.00E+00
ENSG00000197021.7_CXorf40B	ENSG00000160789.18_LMNA	0.0304	-0.129430983	0.188683693	7.10E-01	8.77E-01	1.00E+00
ENSG00000226360.5_RPL10AP6	ENSG00000160789.18_LMNA	-0.16441	-0.315356594	-0.00534941	4.30E-02	1.82E-01	1.00E+00
ENSG00000135144.6_DTX1	ENSG00000160789.18_LMNA	-0.10516	-0.260012077	0.054956226	1.97E-01	4.67E-01	1.00E+00
ENSG00000221968.7_FADS3	ENSG00000160789.18_LMNA	0.16932	0.010395218	0.319893627	3.70E-02	1.64E-01	1.00E+00
ENSG00000049323.14_LTBP1	ENSG00000160789.18_LMNA	0.39234	0.248667513	0.519113161	5.76E-07	2.94E-05	2.96E-02
ENSG00000154511.10_FAM69A	ENSG00000160789.18_LMNA	-0.38873	-0.516000517	-0.24467473	7.45E-07	3.62E-05	3.82E-02
ENSG00000106266.7_SNX8	ENSG00000160789.18_LMNA	0.47306	0.339418714	0.587976954	7.54E-10	1.24E-07	3.87E-05
ENSG00000184194.5_GPR173	ENSG00000160789.18_LMNA	-0.17611	-0.326167326	-0.0173985	3.00E-02	1.43E-01	1.00E+00
ENSG00000213178.3_RP11-641D	ENSG00000160789.18_LMNA	-0.0071	-0.166116547	0.152269196	9.31E-01	9.73E-01	1.00E+00
ENSG00000277632.1_CCL3	ENSG00000160789.18_LMNA	-0.16019	-0.311452497	-0.00102012	4.87E-02	1.98E-01	1.00E+00
ENSG00000163702.17_IL17RC	ENSG00000160789.18_LMNA	0.37013	0.224133974	0.499873536	2.69E-06	1.00E-04	1.38E-01
ENSG00000196199.12_MPHOSPH	ENSG00000160789.18_LMNA	-0.07988	-0.23608205	0.08033868	3.28E-01	6.14E-01	1.00E+00
ENSG00000226479.3_TMEM185B	ENSG00000160789.18_LMNA	0.23413	0.077827146	0.379193921	3.69E-03	3.13E-02	1.00E+00
ENSG00000072364.11_AFF4	ENSG00000160789.18_LMNA	0.06717	-0.093022474	0.223978488	4.11E-01	6.85E-01	1.00E+00
ENSG00000197136.4_PCNX3	ENSG00000160789.18_LMNA	0.2555	0.100381517	0.398490963	1.49E-03	1.56E-02	1.00E+00
ENSG00000107262.15_BAG1	ENSG00000160789.18_LMNA	-0.13605	-0.288995796	0.023658397	9.47E-02	3.01E-01	1.00E+00
ENSG00000036054.11_TBC1D23	ENSG00000160789.18_LMNA	-0.05911	-0.216277374	0.101039565	4.69E-01	7.30E-01	1.00E+00
ENSG00000188529.13_SRSF10	ENSG00000160789.18_LMNA	0.03749	-0.122439149	0.195526013	6.47E-01	8.43E-01	1.00E+00
ENSG00000107758.14_PPP3CB	ENSG00000160789.18_LMNA	-0.40779	-0.532421882	-0.26584372	1.84E-07	1.19E-05	9.46E-03
ENSG00000141867.16_BRD4	ENSG00000160789.18_LMNA	0.2482	0.092665085	0.391918516	2.05E-03	1.99E-02	1.00E+00
ENSG00000236439.4_RP11-175B	ENSG00000160789.18_LMNA	-0.09386	-0.249331816	0.066335245	2.50E-01	5.31E-01	1.00E+00

ENSG00000080815.17_PSEN1	ENSG00000160789.18_LMNA	0.0068	-0.152568264	0.165818801	9.34E-01	9.74E-01	1.00E+00
ENSG00000163161.11_ERCC3	ENSG00000160789.18_LMNA	-0.09533	-0.25072815	0.064852207	2.43E-01	5.22E-01	1.00E+00
ENSG00000176428.5_VPS37D	ENSG00000160789.18_LMNA	-0.19272	-0.341440193	-0.03457507	1.74E-02	9.75E-02	1.00E+00
ENSG00000104412.6_EMC2	ENSG00000160789.18_LMNA	0.0804	-0.07982208	0.236572946	3.25E-01	6.12E-01	1.00E+00
ENSG00000004975.10_DVL2	ENSG00000160789.18_LMNA	0.13033	-0.029481794	0.283646026	1.10E-01	3.29E-01	1.00E+00
ENSG00000130508.9_PXDN	ENSG00000160789.18_LMNA	0.31837	0.16767155	0.454536153	6.41E-05	1.31E-03	1.00E+00
ENSG00000154319.13_FAM167A	ENSG00000160789.18_LMNA	-0.18389	-0.333330479	-0.02543188	2.33E-02	1.21E-01	1.00E+00
ENSG00000146083.10_RNF44	ENSG00000160789.18_LMNA	-0.0484	-0.206011641	0.111662802	5.54E-01	7.88E-01	1.00E+00
ENSG00000103066.11_PLA2G15	ENSG00000160789.18_LMNA	0.20534	0.047695555	0.352998351	1.12E-02	7.08E-02	1.00E+00
ENSG00000169062.13_UPF3A	ENSG00000160789.18_LMNA	-0.07321	-0.229731829	0.087006126	3.70E-01	6.50E-01	1.00E+00
ENSG00000015285.9_WAS	ENSG00000160789.18_LMNA	0.08196	-0.078264685	0.238051872	3.15E-01	6.02E-01	1.00E+00
ENSG00000156928.4_MALSU1	ENSG00000160789.18_LMNA	0.16723	0.008250231	0.317966653	3.95E-02	1.72E-01	1.00E+00
ENSG00000167543.14_TP53I13	ENSG00000160789.18_LMNA	0.51299	0.385255543	0.621441205	1.41E-11	4.54E-09	7.22E-07
ENSG00000120709.9_FAM53C	ENSG00000160789.18_LMNA	-0.06295	-0.219942032	0.097229678	4.41E-01	7.09E-01	1.00E+00
ENSG00000072182.11_ASIC4	ENSG00000160789.18_LMNA	-0.24601	-0.389935615	-0.09034408	2.25E-03	2.14E-02	1.00E+00
ENSG00000066557.5_LRRC40	ENSG00000160789.18_LMNA	-0.31544	-0.451943147	-0.16449817	7.54E-05	1.50E-03	1.00E+00
ENSG00000170634.11_ACYP2	ENSG00000160789.18_LMNA	-0.42991	-0.551373972	-0.29059891	3.25E-08	2.87E-06	1.67E-03
ENSG00000101811.12_CSTF2	ENSG00000160789.18_LMNA	0.22271	0.065838629	0.368828981	5.82E-03	4.40E-02	1.00E+00
ENSG00000155099.6_TMEM55A	ENSG00000160789.18_LMNA	0.02397	-0.135753019	0.182470143	7.69E-01	9.05E-01	1.00E+00
ENSG00000168569.7_TMEM223	ENSG00000160789.18_LMNA	-0.06398	-0.220927101	0.096203984	4.34E-01	7.03E-01	1.00E+00
ENSG00000149636.14_DSN1	ENSG00000160789.18_LMNA	0.10091	-0.059244552	0.255996115	2.16E-01	4.90E-01	1.00E+00
ENSG00000129347.18_KRI1	ENSG00000160789.18_LMNA	0.14919	-0.010252216	0.301237852	6.66E-02	2.41E-01	1.00E+00
ENSG00000108733.8_PEX12	ENSG00000160789.18_LMNA	-0.17281	-0.323119388	-0.01399232	3.33E-02	1.53E-01	1.00E+00
ENSG00000109133.11_TMEM33	ENSG00000160789.18_LMNA	-0.1689	-0.319506433	-0.00996399	3.75E-02	1.66E-01	1.00E+00
ENSG00000205084.9_TMEM231	ENSG00000160789.18_LMNA	-0.05699	-0.214243585	0.103149943	4.86E-01	7.42E-01	1.00E+00
ENSG00000144560.12_VGLL4	ENSG00000160789.18_LMNA	0.22867	0.07209242	0.374245253	4.60E-03	3.68E-02	1.00E+00
ENSG00000163918.9_RFC4	ENSG00000160789.18_LMNA	-0.11197	-0.266420633	0.048088888	1.70E-01	4.28E-01	1.00E+00
ENSG00000102699.5_PARP4	ENSG00000160789.18_LMNA	0.21196	0.054601989	0.359045195	8.75E-03	5.95E-02	1.00E+00
ENSG00000071794.14_HLTF	ENSG00000160789.18_LMNA	-0.19875	-0.346968765	-0.04083778	1.41E-02	8.40E-02	1.00E+00
ENSG00000254858.8_MPV17L2	ENSG00000160789.18_LMNA	-0.06981	-0.22649103	0.090397918	3.93E-01	6.70E-01	1.00E+00
ENSG00000198730.6_CTR9	ENSG00000160789.18_LMNA	0.18763	0.029300796	0.33676765	2.06E-02	1.10E-01	1.00E+00
ENSG00000166166.11_TRMT61A	ENSG00000160789.18_LMNA	0.30129	0.149251796	0.439417034	1.62E-04	2.77E-03	1.00E+00
ENSG00000148296.6_SURF6	ENSG00000160789.18_LMNA	0.15924	3.90E-05	0.310566257	5.00E-02	2.01E-01	1.00E+00

ENSG00000196814.13_MVB12B	ENSG00000160789.18_LMNA	-0.28136	-0.421672137	-0.1278869	4.46E-04	6.17E-03	1.00E+00
ENSG00000163923.8_RPL39L	ENSG00000160789.18_LMNA	0.07748	-0.082746248	0.233792144	3.43E-01	6.27E-01	1.00E+00
ENSG00000236801.1_RPL24P8	ENSG00000160789.18_LMNA	-0.15042	-0.302379309	0.008996481	6.44E-02	2.36E-01	1.00E+00
ENSG00000205060.9_SLC35B4	ENSG00000160789.18_LMNA	-0.01175	-0.170636082	0.147722443	8.86E-01	9.56E-01	1.00E+00
ENSG00000174915.10_PTDSS2	ENSG00000160789.18_LMNA	0.11389	-0.046146128	0.268228572	1.62E-01	4.17E-01	1.00E+00
ENSG00000102977.12_ACD	ENSG00000160789.18_LMNA	0.09927	-0.060888934	0.254453281	2.24E-01	4.99E-01	1.00E+00
ENSG00000112855.13_HARS2	ENSG00000160789.18_LMNA	0.23392	0.077611724	0.379008339	3.73E-03	3.14E-02	1.00E+00
ENSG00000149091.14_DGKZ	ENSG00000160789.18_LMNA	0.25946	0.104581688	0.402055623	1.25E-03	1.37E-02	1.00E+00
ENSG00000158882.11_TOMM40L	ENSG00000160789.18_LMNA	-0.09275	-0.248288522	0.067442412	2.56E-01	5.38E-01	1.00E+00
ENSG00000101255.9_TRIB3	ENSG00000160789.18_LMNA	0.03371	-0.126163726	0.191884931	6.80E-01	8.60E-01	1.00E+00
ENSG00000119446.12_RBM18	ENSG00000160789.18_LMNA	-0.20296	-0.350828353	-0.04522418	1.21E-02	7.55E-02	1.00E+00
ENSG00000130772.12_MED18	ENSG00000160789.18_LMNA	-0.09834	-0.25356739	0.061832361	2.28E-01	5.05E-01	1.00E+00
ENSG00000079308.15_TNS1	ENSG00000160789.18_LMNA	0.21376	0.056484859	0.360689297	8.19E-03	5.66E-02	1.00E+00
ENSG00000120699.11_EXOSC8	ENSG00000160789.18_LMNA	-0.23714	-0.38192036	-0.0809952	3.27E-03	2.85E-02	1.00E+00
ENSG00000153944.9_MSI2	ENSG00000160789.18_LMNA	0.03473	-0.125167275	0.192859903	6.71E-01	8.56E-01	1.00E+00
ENSG00000158092.5_NCK1	ENSG00000160789.18_LMNA	-0.11568	-0.269906028	0.044341453	1.56E-01	4.07E-01	1.00E+00
ENSG00000116273.5_PHF13	ENSG00000160789.18_LMNA	0.34254	0.193911169	0.475792173	1.56E-05	4.21E-04	8.02E-01
ENSG00000115540.13_MOB4	ENSG00000160789.18_LMNA	-0.38784	-0.515229487	-0.24368711	7.94E-07	3.78E-05	4.07E-02
ENSG00000109519.11_GRPEL1	ENSG00000160789.18_LMNA	0.0088	-0.150608548	0.167768806	9.14E-01	9.67E-01	1.00E+00
ENSG00000076864.18_RAP1GAP	ENSG00000160789.18_LMNA	-0.13127	-0.284521041	0.028530763	1.07E-01	3.25E-01	1.00E+00
ENSG00000122873.10_CISD1	ENSG00000160789.18_LMNA	-0.36082	-0.491768641	-0.21390254	4.96E-06	1.65E-04	2.54E-01
ENSG00000138593.7_SECISBP2L	ENSG00000160789.18_LMNA	-0.08159	-0.237700463	0.07863488	3.18E-01	6.04E-01	1.00E+00
ENSG00000011426.9_ANLN	ENSG00000160789.18_LMNA	-0.07627	-0.232643491	0.083952531	3.50E-01	6.34E-01	1.00E+00
ENSG00000166847.8_DCTN5	ENSG00000160789.18_LMNA	-0.27369	-0.414820863	-0.11971028	6.45E-04	8.24E-03	1.00E+00
ENSG00000139842.13_CUL4A	ENSG00000160789.18_LMNA	0.11486	-0.045166966	0.269138945	1.59E-01	4.12E-01	1.00E+00
ENSG00000117899.9_MESDC2	ENSG00000160789.18_LMNA	0.30764	0.156080622	0.445041445	1.16E-04	2.12E-03	1.00E+00
ENSG00000276043.3_UHRF1	ENSG00000160789.18_LMNA	0.16745	0.008472073	0.318166065	3.92E-02	1.71E-01	1.00E+00
ENSG00000160256.11_FAM207A	ENSG00000160789.18_LMNA	0.33694	0.187812206	0.470880762	2.19E-05	5.56E-04	1.00E+00
ENSG00000148384.11_INPP5E	ENSG00000160789.18_LMNA	0.23841	0.082335449	0.383072206	3.10E-03	2.74E-02	1.00E+00
ENSG00000145386.8_CCNA2	ENSG00000160789.18_LMNA	0.02207	-0.137610198	0.18063999	7.87E-01	9.13E-01	1.00E+00
ENSG00000237310.1_GS1-124K5	ENSG00000160789.18_LMNA	0.03668	-0.123243232	0.194740697	6.54E-01	8.47E-01	1.00E+00
ENSG00000023228.12_NDUFS1	ENSG00000160789.18_LMNA	-0.23926	-0.383837639	-0.08322668	2.99E-03	2.67E-02	1.00E+00
ENSG00000112763.14_BTN2A1	ENSG00000160789.18_LMNA	0.1217	-0.038242242	0.275561195	1.35E-01	3.74E-01	1.00E+00

ENSG00000076716.8_GPC4	ENSG00000160789.18_LMNA	0.36727	0.220985393	0.497384501	3.25E-06	1.17E-04	1.67E-01
ENSG00000161956.11_SENP3	ENSG00000160789.18_LMNA	-0.12638	-0.279951775	0.033490736	1.21E-01	3.49E-01	1.00E+00
ENSG00000137310.10_TCF19	ENSG00000160789.18_LMNA	0.34873	0.200667823	0.481212766	1.07E-05	3.07E-04	5.48E-01
ENSG00000109065.10_NAT9	ENSG00000160789.18_LMNA	-0.12399	-0.277704945	0.035924038	1.28E-01	3.62E-01	1.00E+00
ENSG00000101236.15_RNF24	ENSG00000160789.18_LMNA	0.33719	0.188086469	0.471101999	2.16E-05	5.50E-04	1.00E+00
ENSG00000221914.7_PPP2R2A	ENSG00000160789.18_LMNA	-0.27197	-0.413273174	-0.11786872	7.00E-04	8.78E-03	1.00E+00
ENSG00000049239.11_H6PD	ENSG00000160789.18_LMNA	0.45594	0.319966325	0.573513375	3.57E-09	4.51E-07	1.83E-04
ENSG00000143409.14_FAM63A	ENSG00000160789.18_LMNA	-0.24641	-0.390295848	-0.0907655	2.21E-03	2.11E-02	1.00E+00
ENSG00000241127.6_YAE1D1	ENSG00000160789.18_LMNA	-0.2651	-0.407118093	-0.11056499	9.65E-04	1.13E-02	1.00E+00
ENSG00000178904.17_DPY19L3	ENSG00000160789.18_LMNA	0.13402	-0.025731924	0.287093148	9.97E-02	3.11E-01	1.00E+00
ENSG00000111252.9_SH2B3	ENSG00000160789.18_LMNA	0.35492	0.207440327	0.486624631	7.24E-06	2.26E-04	3.71E-01
ENSG00000136813.13_KIAA0368	ENSG00000160789.18_LMNA	0.29702	0.144655848	0.43561883	2.03E-04	3.29E-03	1.00E+00
ENSG00000138650.8_PCDH10	ENSG00000160789.18_LMNA	-0.03191	-0.190144209	0.127941203	6.96E-01	8.70E-01	1.00E+00
ENSG00000196504.14_PRPF40A	ENSG00000160789.18_LMNA	0.04306	-0.116941184	0.200884755	5.98E-01	8.15E-01	1.00E+00
ENSG00000198837.8_DENND4B	ENSG00000160789.18_LMNA	0.137	-0.02270027	0.28987413	9.24E-02	2.97E-01	1.00E+00
ENSG00000150456.9_EEF1AKMT	ENSG00000160789.18_LMNA	-0.09373	-0.249212833	0.066461552	2.51E-01	5.31E-01	1.00E+00
ENSG00000107854.5_TNKS2	ENSG00000160789.18_LMNA	-0.11007	-0.264637574	0.050002584	1.77E-01	4.39E-01	1.00E+00
ENSG00000101888.10_NXT2	ENSG00000160789.18_LMNA	-0.23154	-0.376846305	-0.07510414	4.10E-03	3.38E-02	1.00E+00
ENSG00000095002.11_MSH2	ENSG00000160789.18_LMNA	0.01495	-0.144592803	0.173739221	8.55E-01	9.43E-01	1.00E+00
ENSG00000138802.10_SEC24B	ENSG00000160789.18_LMNA	0.19235	0.034199285	0.341107778	1.76E-02	9.84E-02	1.00E+00
ENSG00000093183.12_SEC22C	ENSG00000160789.18_LMNA	-0.138	-0.290808633	0.021680241	9.00E-02	2.92E-01	1.00E+00
ENSG00000143614.7_GATAD2B	ENSG00000160789.18_LMNA	0.0084	-0.151005566	0.167373956	9.18E-01	9.69E-01	1.00E+00
ENSG00000115286.18_NDUFS7	ENSG00000160789.18_LMNA	-0.11255	-0.266967923	0.047501036	1.67E-01	4.25E-01	1.00E+00
ENSG00000162670.9_BRINP3	ENSG00000160789.18_LMNA	-0.17182	-0.322206112	-0.0129731	3.43E-02	1.56E-01	1.00E+00
ENSG00000109861.14_CTSC	ENSG00000160789.18_LMNA	0.17246	0.01363725	0.322801292	3.36E-02	1.54E-01	1.00E+00
ENSG00000143842.13_SOX13	ENSG00000160789.18_LMNA	0.03151	-0.128333882	0.18975938	7.00E-01	8.72E-01	1.00E+00
ENSG00000177098.7_SCN4B	ENSG00000160789.18_LMNA	-0.00178	-0.160939172	0.157461244	9.83E-01	9.94E-01	1.00E+00
ENSG00000119938.8_PPP1R3C	ENSG00000160789.18_LMNA	-0.0077	-0.166700957	0.151682024	9.25E-01	9.71E-01	1.00E+00
ENSG00000197265.7_GTF2E2	ENSG00000160789.18_LMNA	0.16649	0.007487073	0.317280443	4.04E-02	1.74E-01	1.00E+00
ENSG00000101132.8_PFDN4	ENSG00000160789.18_LMNA	-0.05751	-0.214743343	0.10263163	4.82E-01	7.39E-01	1.00E+00
ENSG00000111450.12_STX2	ENSG00000160789.18_LMNA	0.11535	-0.044674301	0.269596787	1.57E-01	4.09E-01	1.00E+00
ENSG00000137124.6_ALDH1B1	ENSG00000160789.18_LMNA	0.06814	-0.092056653	0.224903578	4.04E-01	6.80E-01	1.00E+00
ENSG00000177485.6_ZBTB33	ENSG00000160789.18_LMNA	-0.12128	-0.275172187	0.038662545	1.37E-01	3.76E-01	1.00E+00

ENSG00000132294.12_EFR3A	ENSG00000160789.18_LMNA	-0.01699	-0.175716577	0.142595233	8.35E-01	9.36E-01	1.00E+00
ENSG00000073169.12_SELENOC	ENSG00000160789.18_LMNA	0.02924	-0.130564674	0.187571327	7.21E-01	8.81E-01	1.00E+00
ENSG00000237649.6_KIFC1	ENSG00000160789.18_LMNA	0.0453	-0.114726143	0.203038344	5.79E-01	8.04E-01	1.00E+00
ENSG00000115661.12_STK16	ENSG00000160789.18_LMNA	-0.07058	-0.227230436	0.089624709	3.88E-01	6.66E-01	1.00E+00
ENSG00000225177.4_RP11-390P	ENSG00000160789.18_LMNA	0.18414	0.025689711	0.333559791	2.32E-02	1.20E-01	1.00E+00
ENSG00000011454.15_RABGAP1	ENSG00000160789.18_LMNA	-0.08281	-0.238859575	0.077413476	3.10E-01	5.97E-01	1.00E+00
ENSG00000184182.17_UBE2F	ENSG00000160789.18_LMNA	-0.14159	-0.294160503	0.018016246	8.19E-02	2.76E-01	1.00E+00
ENSG00000168564.5_CDKN2AIP	ENSG00000160789.18_LMNA	-0.22124	-0.367492956	-0.06429967	6.16E-03	4.59E-02	1.00E+00
ENSG00000147119.3_CHST7	ENSG00000160789.18_LMNA	0.45142	0.314848396	0.569681554	5.31E-09	6.30E-07	2.73E-04
ENSG00000050393.10_MCUR1	ENSG00000160789.18_LMNA	0.0698	-0.090408995	0.226480435	3.93E-01	6.71E-01	1.00E+00
ENSG00000158373.8_HIST1H2BI	ENSG00000160789.18_LMNA	0.08597	-0.074247871	0.241859545	2.92E-01	5.78E-01	1.00E+00
ENSG00000272841.1_RP3-428L1	ENSG00000160789.18_LMNA	0.01126	-0.148210183	0.170151902	8.91E-01	9.57E-01	1.00E+00
ENSG00000120253.12_NUP43	ENSG00000160789.18_LMNA	-0.10359	-0.258529343	0.056540874	2.04E-01	4.75E-01	1.00E+00
ENSG00000130813.16_C19orf66	ENSG00000160789.18_LMNA	0.33461	0.185277408	0.468834349	2.52E-05	6.21E-04	1.00E+00
ENSG00000102218.5_RP2	ENSG00000160789.18_LMNA	-0.16548	-0.316345032	-0.00644735	4.16E-02	1.78E-01	1.00E+00
ENSG00000132680.9_KIAA0907	ENSG00000160789.18_LMNA	-0.10921	-0.263825969	0.050872888	1.80E-01	4.44E-01	1.00E+00
ENSG00000035403.15_VCL	ENSG00000160789.18_LMNA	0.43116	0.292003446	0.552441247	2.94E-08	2.66E-06	1.51E-03
ENSG00000198960.9_ARMCX6	ENSG00000160789.18_LMNA	-0.18826	-0.337349199	-0.02995631	2.02E-02	1.09E-01	1.00E+00
ENSG00000100060.16_MFNG	ENSG00000160789.18_LMNA	-0.2424	-0.386674501	-0.08653398	2.62E-03	2.40E-02	1.00E+00
ENSG00000153574.8_RPIA	ENSG00000160789.18_LMNA	0.14249	-0.017102112	0.294995569	7.99E-02	2.72E-01	1.00E+00
ENSG00000177683.12_THAP5	ENSG00000160789.18_LMNA	-0.17025	-0.320757136	-0.01135735	3.60E-02	1.62E-01	1.00E+00
ENSG00000177613.8_CSTF2T	ENSG00000160789.18_LMNA	-0.10107	-0.256153794	0.059076399	2.15E-01	4.89E-01	1.00E+00
ENSG00000160991.14_ORAI2	ENSG00000160789.18_LMNA	0.44073	0.302776825	0.560599408	1.33E-08	1.37E-06	6.81E-04
ENSG00000078304.18_PPP2R5C	ENSG00000160789.18_LMNA	-0.13393	-0.287008053	0.0258246	1.00E-01	3.11E-01	1.00E+00
ENSG00000141219.14_C17orf80	ENSG00000160789.18_LMNA	-0.37114	-0.500749819	-0.22524383	2.51E-06	9.48E-05	1.29E-01
ENSG00000138032.19_PPM1B	ENSG00000160789.18_LMNA	-0.18028	-0.330008571	-0.02170145	2.62E-02	1.31E-01	1.00E+00
ENSG00000255135.3_RP11-111M	ENSG00000160789.18_LMNA	-0.13812	-0.290925928	0.021552164	8.97E-02	2.92E-01	1.00E+00
ENSG00000169895.5_SYAP1	ENSG00000160789.18_LMNA	0.32986	0.180115987	0.464657959	3.33E-05	7.79E-04	1.00E+00
ENSG00000121680.14_PEX16	ENSG00000160789.18_LMNA	0.00813	-0.151270907	0.167110008	9.21E-01	9.70E-01	1.00E+00
ENSG00000164823.8_OSGIN2	ENSG00000160789.18_LMNA	-0.18198	-0.331573812	-0.02345811	2.48E-02	1.26E-01	1.00E+00
ENSG00000153885.13_KCTD15	ENSG00000160789.18_LMNA	-0.08783	-0.243620969	0.072386221	2.82E-01	5.67E-01	1.00E+00
ENSG00000180776.14_ZDHHC20	ENSG00000160789.18_LMNA	-0.0863	-0.242175648	0.073913943	2.90E-01	5.76E-01	1.00E+00
ENSG00000183155.4_RABIF	ENSG00000160789.18_LMNA	-0.01828	-0.176969229	0.141328433	8.23E-01	9.31E-01	1.00E+00

ENSG00000113658.15_SMAD5	ENSG00000160789.18_LMNA	-0.0604	-0.217504566	0.099764775	4.60E-01	7.23E-01	1.00E+00
ENSG00000105443.12_CYTH2	ENSG00000160789.18_LMNA	0.21182	0.054456145	0.358917767	8.80E-03	5.97E-02	1.00E+00
ENSG00000139178.9_C1RL	ENSG00000160789.18_LMNA	0.46996	0.335893256	0.585367383	1.01E-09	1.58E-07	5.16E-05
ENSG00000268205.1_CTC-444N2	ENSG00000160789.18_LMNA	0.19836	0.040435491	0.346614275	1.43E-02	8.49E-02	1.00E+00
ENSG00000086015.19_MAST2	ENSG00000160789.18_LMNA	0.3426	0.19398042	0.475847839	1.56E-05	4.20E-04	7.99E-01
ENSG00000101189.6_MRGBP	ENSG00000160789.18_LMNA	0.41316	0.271836284	0.537034319	1.22E-07	8.55E-06	6.28E-03
ENSG00000092094.9_OSGEP	ENSG00000160789.18_LMNA	0.00734	-0.152033625	0.166351038	9.28E-01	9.73E-01	1.00E+00
ENSG00000111725.9_PRKAB1	ENSG00000160789.18_LMNA	-0.16416	-0.315123574	-0.00509069	4.33E-02	1.83E-01	1.00E+00
ENSG00000158552.11_ZFAND2B	ENSG00000160789.18_LMNA	-0.05801	-0.215222205	0.102134826	4.78E-01	7.36E-01	1.00E+00
ENSG00000176887.6_SOX11	ENSG00000160789.18_LMNA	-0.19087	-0.339749077	-0.0326642	1.85E-02	1.02E-01	1.00E+00
ENSG00000080189.13_SLC35C2	ENSG00000160789.18_LMNA	0.16148	0.002341314	0.312645043	4.69E-02	1.93E-01	1.00E+00
ENSG00000196588.13_MKL1	ENSG00000160789.18_LMNA	0.07794	-0.082281578	0.234234378	3.40E-01	6.25E-01	1.00E+00
ENSG00000149929.14_HIRIP3	ENSG00000160789.18_LMNA	-0.01738	-0.176097133	0.142210489	8.32E-01	9.34E-01	1.00E+00
ENSG00000180329.12_CCDC43	ENSG00000160789.18_LMNA	-0.03675	-0.194810437	0.123171842	6.53E-01	8.46E-01	1.00E+00
ENSG00000050820.15_BCAR1	ENSG00000160789.18_LMNA	0.16269	0.003582989	0.313764922	4.52E-02	1.88E-01	1.00E+00
ENSG00000166091.18_CMTM5	ENSG00000160789.18_LMNA	-0.38353	-0.511500656	-0.23891871	1.08E-06	4.87E-05	5.52E-02
ENSG00000165948.9_IFI27L1	ENSG00000160789.18_LMNA	0.01142	-0.148047424	0.17031349	8.89E-01	9.57E-01	1.00E+00
ENSG00000102901.11_CENPT	ENSG00000160789.18_LMNA	-0.12949	-0.282862797	0.030332583	1.12E-01	3.34E-01	1.00E+00
ENSG00000085760.13_MTIF2	ENSG00000160789.18_LMNA	-0.07623	-0.232606454	0.083991411	3.51E-01	6.34E-01	1.00E+00
ENSG00000121905.8_HPCA	ENSG00000160789.18_LMNA	-0.20025	-0.348348568	-0.04240456	1.34E-02	8.07E-02	1.00E+00
ENSG00000118257.15_NRP2	ENSG00000160789.18_LMNA	0.37303	0.227334227	0.502398759	2.21E-06	8.62E-05	1.13E-01
ENSG00000134690.9_CDCA8	ENSG00000160789.18_LMNA	0.06354	-0.096640242	0.220508201	4.37E-01	7.05E-01	1.00E+00
ENSG00000125386.13_FAM193A	ENSG00000160789.18_LMNA	0.05257	-0.107532595	0.21001118	5.20E-01	7.66E-01	1.00E+00
ENSG00000068028.16_RASSF1	ENSG00000160789.18_LMNA	0.20069	0.042861281	0.348750533	1.32E-02	7.99E-02	1.00E+00
ENSG00000177303.8_CASKIN2	ENSG00000160789.18_LMNA	0.015	-0.144541634	0.173789904	8.54E-01	9.43E-01	1.00E+00
ENSG00000146938.13_NLGN4X	ENSG00000160789.18_LMNA	0.22435	0.067563212	0.370324662	5.46E-03	4.18E-02	1.00E+00
ENSG00000136940.12_PDCL	ENSG00000160789.18_LMNA	-0.22098	-0.367260299	-0.06403182	6.22E-03	4.62E-02	1.00E+00
ENSG00000163154.5_TNFAIP8L2	ENSG00000160789.18_LMNA	-0.08813	-0.243911773	0.072078659	2.80E-01	5.66E-01	1.00E+00
ENSG00000167565.11_SERTAD3	ENSG00000160789.18_LMNA	0.23173	0.075304433	0.377019118	4.07E-03	3.36E-02	1.00E+00
ENSG00000167110.15_GOLGA2	ENSG00000160789.18_LMNA	0.28808	0.13507668	0.42766893	3.19E-04	4.72E-03	1.00E+00
ENSG00000128487.15_SPECC1	ENSG00000160789.18_LMNA	0.04144	-0.11854616	0.199322385	6.12E-01	8.24E-01	1.00E+00
ENSG00000178573.6_MAF	ENSG00000160789.18_LMNA	0.19504	0.036984213	0.343569442	1.60E-02	9.21E-02	1.00E+00
ENSG00000119559.14_C19orf25	ENSG00000160789.18_LMNA	-0.23426	-0.379314924	-0.07796762	3.67E-03	3.11E-02	1.00E+00

ENSG00000111641.9_NOP2	ENSG00000160789.18_LMNA	0.2181	0.061019425	0.364641088	6.95E-03	5.02E-02	1.00E+00
ENSG00000176624.10_MEX3C	ENSG00000160789.18_LMNA	0.23194	0.075523177	0.377207827	4.04E-03	3.34E-02	1.00E+00
ENSG00000204642.12_HLA-F	ENSG00000160789.18_LMNA	0.14263	-0.016955269	0.295129667	7.96E-02	2.71E-01	1.00E+00
ENSG00000066136.18_NFYC	ENSG00000160789.18_LMNA	0.04421	-0.115805607	0.201989212	5.89E-01	8.09E-01	1.00E+00
ENSG00000081177.17_EXD2	ENSG00000160789.18_LMNA	0.0119	-0.14757609	0.170781336	8.84E-01	9.55E-01	1.00E+00
ENSG00000110075.13_PPP6R3	ENSG00000160789.18_LMNA	0.0324	-0.127456688	0.190618904	6.92E-01	8.67E-01	1.00E+00
ENSG00000182473.19_EXOC7	ENSG00000160789.18_LMNA	0.01873	-0.140894188	0.177398386	8.19E-01	9.30E-01	1.00E+00
ENSG00000170903.9_MSANTD4	ENSG00000160789.18_LMNA	-0.20051	-0.348586971	-0.04267542	1.33E-02	8.02E-02	1.00E+00
ENSG00000107295.9_SH3GL2	ENSG00000160789.18_LMNA	-0.40369	-0.528897179	-0.26127821	2.51E-07	1.53E-05	1.29E-02
ENSG00000103197.15_TSC2	ENSG00000160789.18_LMNA	0.01954	-0.140092106	0.178190754	8.11E-01	9.26E-01	1.00E+00
ENSG00000185052.10_SLC24A3	ENSG00000160789.18_LMNA	-0.19477	-0.343325629	-0.03670817	1.62E-02	9.27E-02	1.00E+00
ENSG00000138600.8_SPPL2A	ENSG00000160789.18_LMNA	0.21929	0.062257957	0.365718547	6.64E-03	4.85E-02	1.00E+00
ENSG00000125450.9_NUP85	ENSG00000160789.18_LMNA	0.03899	-0.120956757	0.196972739	6.33E-01	8.36E-01	1.00E+00
ENSG00000168522.11_FNTA	ENSG00000160789.18_LMNA	-0.37237	-0.501820631	-0.22660103	2.31E-06	8.91E-05	1.19E-01
ENSG00000139679.14_LPAR6	ENSG00000160789.18_LMNA	-0.01359	-0.17242247	0.145921578	8.68E-01	9.48E-01	1.00E+00
ENSG00000010626.13_LRRC23	ENSG00000160789.18_LMNA	-0.09258	-0.248122517	0.067618508	2.57E-01	5.39E-01	1.00E+00
ENSG00000184384.12_MAML2	ENSG00000160789.18_LMNA	-0.07749	-0.233808565	0.082728995	3.43E-01	6.27E-01	1.00E+00
ENSG00000099968.16_BCL2L13	ENSG00000160789.18_LMNA	0.03311	-0.126759094	0.191302098	6.86E-01	8.64E-01	1.00E+00
ENSG00000139645.9_ANKRD52	ENSG00000160789.18_LMNA	0.32815	0.178257322	0.463150904	3.67E-05	8.42E-04	1.00E+00
ENSG00000141295.12_SCRN2	ENSG00000160789.18_LMNA	0.04291	-0.117087495	0.200742395	6.00E-01	8.16E-01	1.00E+00
ENSG00000273344.1_PAXIP1-AS	ENSG00000160789.18_LMNA	0.00686	-0.152506935	0.165879863	9.33E-01	9.74E-01	1.00E+00
ENSG00000112062.9_MAPK14	ENSG00000160789.18_LMNA	-0.0236	-0.18211939	0.136109125	7.73E-01	9.06E-01	1.00E+00
ENSG00000226243.1_RPL37AP1	ENSG00000160789.18_LMNA	-0.12659	-0.280141784	0.033284787	1.20E-01	3.48E-01	1.00E+00
ENSG00000101343.13_CRNKL1	ENSG00000160789.18_LMNA	0.05778	-0.102360826	0.215004386	4.80E-01	7.38E-01	1.00E+00
ENSG00000064655.17_EYA2	ENSG00000160789.18_LMNA	0.28956	0.136661287	0.428987151	2.97E-04	4.46E-03	1.00E+00
ENSG00000124789.10_NUP153	ENSG00000160789.18_LMNA	0.21699	0.059852378	0.363625072	7.25E-03	5.17E-02	1.00E+00
ENSG00000070961.13_ATP2B1	ENSG00000160789.18_LMNA	0.09998	-0.060179358	0.255119235	2.20E-01	4.96E-01	1.00E+00
ENSG00000111144.8_LTA4H	ENSG00000160789.18_LMNA	0.03447	-0.125422716	0.192610029	6.73E-01	8.57E-01	1.00E+00
ENSG00000160194.16_NDUFV3	ENSG00000160789.18_LMNA	-0.01084	-0.169750932	0.148613984	8.95E-01	9.59E-01	1.00E+00
ENSG00000124201.13_ZNFX1	ENSG00000160789.18_LMNA	0.25395	0.098739507	0.397094946	1.59E-03	1.65E-02	1.00E+00
ENSG00000026950.15_BTN3A1	ENSG00000160789.18_LMNA	-0.11297	-0.267361545	0.047078107	1.66E-01	4.23E-01	1.00E+00
ENSG00000146963.16_LUC7L2	ENSG00000160789.18_LMNA	0.10353	-0.056604043	0.258470206	2.04E-01	4.76E-01	1.00E+00
ENSG00000165233.16_CARD19	ENSG00000160789.18_LMNA	0.29074	0.137918687	0.430032287	2.79E-04	4.25E-03	1.00E+00

ENSG00000117523.14_PRRC2C	ENSG00000160789.18_LMNA	0.12959	-0.030233443	0.282954087	1.12E-01	3.33E-01	1.00E+00
ENSG00000100564.7_PIGH	ENSG00000160789.18_LMNA	-0.22318	-0.369262607	-0.06633843	5.71E-03	4.33E-02	1.00E+00
ENSG00000004897.10_CDC27	ENSG00000160789.18_LMNA	-0.06128	-0.218350701	0.098885215	4.53E-01	7.18E-01	1.00E+00
ENSG00000197555.8_SIPA1L1	ENSG00000160789.18_LMNA	0.43853	0.300298477	0.558727096	1.60E-08	1.59E-06	8.19E-04
ENSG00000164176.11_EDIL3	ENSG00000160789.18_LMNA	-0.216	-0.362726526	-0.05882095	7.53E-03	5.31E-02	1.00E+00
ENSG00000182541.16_LIMK2	ENSG00000160789.18_LMNA	-0.05731	-0.214555116	0.102826866	4.83E-01	7.40E-01	1.00E+00
ENSG00000154553.12_PDLIM3	ENSG00000160789.18_LMNA	0.08939	-0.070817761	0.245103372	2.73E-01	5.58E-01	1.00E+00
ENSG00000169926.8_KLF13	ENSG00000160789.18_LMNA	0.03789	-0.122047252	0.195908616	6.43E-01	8.41E-01	1.00E+00
ENSG00000055118.13_KCNH2	ENSG00000160789.18_LMNA	0.0046	-0.154713014	0.16368182	9.55E-01	9.82E-01	1.00E+00
ENSG00000254087.6_LYN	ENSG00000160789.18_LMNA	-0.04965	-0.207209269	0.110427196	5.44E-01	7.82E-01	1.00E+00
ENSG00000074855.9_ANO8	ENSG00000160789.18_LMNA	0.00165	-0.157591291	0.160809267	9.84E-01	9.94E-01	1.00E+00
ENSG00000214535.3_RPS15AP1	ENSG00000160789.18_LMNA	-0.14535	-0.297664784	0.014176661	7.40E-02	2.59E-01	1.00E+00
ENSG00000186063.11_AIDA	ENSG00000160789.18_LMNA	0.17072	0.011844094	0.321193794	3.55E-02	1.60E-01	1.00E+00
ENSG00000130734.8_ATG4D	ENSG00000160789.18_LMNA	0.01165	-0.147829215	0.170530101	8.87E-01	9.56E-01	1.00E+00
ENSG00000130775.14_THEMIS2	ENSG00000160789.18_LMNA	0.06118	-0.098981557	0.218258044	4.54E-01	7.19E-01	1.00E+00
ENSG00000091164.11_TXNL1	ENSG00000160789.18_LMNA	-0.22139	-0.367636651	-0.06446512	6.12E-03	4.57E-02	1.00E+00
ENSG00000109534.15_GAR1	ENSG00000160789.18_LMNA	0.25419	0.098999875	0.397316399	1.58E-03	1.63E-02	1.00E+00
ENSG00000170312.14_CDK1	ENSG00000160789.18_LMNA	-0.00646	-0.165495022	0.152893412	9.37E-01	9.75E-01	1.00E+00
ENSG00000277977.1_CTD-3018C	ENSG00000160789.18_LMNA	0.03129	-0.128554262	0.189543363	7.02E-01	8.73E-01	1.00E+00
ENSG00000147044.19_CASK	ENSG00000160789.18_LMNA	-0.04766	-0.205307427	0.112388888	5.60E-01	7.92E-01	1.00E+00
ENSG00000106785.13_TRIM14	ENSG00000160789.18_LMNA	0.21792	0.060824988	0.364471864	7.00E-03	5.04E-02	1.00E+00
ENSG00000090470.13_PDCD7	ENSG00000160789.18_LMNA	0.19045	0.032225328	0.339360397	1.88E-02	1.03E-01	1.00E+00
ENSG00000187079.13_TEAD1	ENSG00000160789.18_LMNA	0.21036	0.052931837	0.357585253	9.29E-03	6.22E-02	1.00E+00
ENSG00000200087.1_SNORA73B	ENSG00000160789.18_LMNA	-0.13545	-0.288433507	0.024271466	9.61E-02	3.04E-01	1.00E+00
ENSG00000105928.12_DFNA5	ENSG00000160789.18_LMNA	0.01472	-0.144823437	0.173510756	8.57E-01	9.44E-01	1.00E+00
ENSG00000136425.11_CIB2	ENSG00000160789.18_LMNA	0.02511	-0.134629179	0.183576559	7.59E-01	8.99E-01	1.00E+00
ENSG00000197771.11_MCMBP	ENSG00000160789.18_LMNA	0.08595	-0.074261969	0.241846198	2.92E-01	5.78E-01	1.00E+00
ENSG00000075856.10_SART3	ENSG00000160789.18_LMNA	0.05082	-0.10926449	0.20833537	5.34E-01	7.76E-01	1.00E+00
ENSG00000188641.11_DPYD	ENSG00000160789.18_LMNA	0.38924	0.245241122	0.516442492	7.19E-07	3.53E-05	3.69E-02
ENSG00000115318.10_LOXL3	ENSG00000160789.18_LMNA	0.49911	0.369245769	0.609850017	5.96E-11	1.50E-08	3.06E-06
ENSG00000140463.12_BBS4	ENSG00000160789.18_LMNA	-0.07308	-0.229613193	0.08713042	3.71E-01	6.51E-01	1.00E+00
ENSG00000125733.16_TRIP10	ENSG00000160789.18_LMNA	0.59378	0.479950747	0.68794999	7.47E-16	1.60E-12	3.83E-11
ENSG00000135930.12_EIF4E2	ENSG00000160789.18_LMNA	-0.06797	-0.224741209	0.092226214	4.05E-01	6.81E-01	1.00E+00

ENSG00000277586.1_NEFL	ENSG00000160789.18_LMNA	-0.10966	-0.26424639	0.05042212	1.79E-01	4.41E-01	1.00E+00
ENSG00000074054.16_CLASP1	ENSG00000160789.18_LMNA	0.11172	-0.048337464	0.266189147	1.71E-01	4.29E-01	1.00E+00
ENSG00000226210.3_WASH7P	ENSG00000160789.18_LMNA	0.08242	-0.077806537	0.238486659	3.13E-01	5.99E-01	1.00E+00
ENSG00000160294.9_MCM3AP	ENSG00000160789.18_LMNA	0.30301	0.151102792	0.440943816	1.48E-04	2.57E-03	1.00E+00
ENSG00000166747.11_AP1G1	ENSG00000160789.18_LMNA	0.01218	-0.1473069	0.17104847	8.82E-01	9.54E-01	1.00E+00
ENSG00000105810.8_CDK6	ENSG00000160789.18_LMNA	0.13028	-0.029538195	0.283594117	1.10E-01	3.30E-01	1.00E+00
ENSG00000101203.15_COL20A1	ENSG00000160789.18_LMNA	-0.18935	-0.338349793	-0.03108477	1.95E-02	1.06E-01	1.00E+00
ENSG00000169231.12_THBS3	ENSG00000160789.18_LMNA	0.42095	0.280545953	0.543709945	6.67E-08	5.22E-06	3.42E-03
ENSG00000169188.4_APEX2	ENSG00000160789.18_LMNA	0.34199	0.193310351	0.475309122	1.62E-05	4.33E-04	8.29E-01
ENSG00000120688.8_WBP4	ENSG00000160789.18_LMNA	-0.29784	-0.436346881	-0.14553584	1.94E-04	3.19E-03	1.00E+00
ENSG00000099998.16_GGT5	ENSG00000160789.18_LMNA	0.24757	0.091994955	0.39134629	2.10E-03	2.03E-02	1.00E+00
ENSG00000106771.11_TMEM245	ENSG00000160789.18_LMNA	-0.06373	-0.22068891	0.09645206	4.35E-01	7.04E-01	1.00E+00
ENSG00000197180.2_CH17-340M	ENSG00000160789.18_LMNA	-0.17242	-0.322758098	-0.01358904	3.37E-02	1.55E-01	1.00E+00
ENSG00000161217.10_PCYT1A	ENSG00000160789.18_LMNA	0.29816	0.14588053	0.436631951	1.91E-04	3.15E-03	1.00E+00
ENSG00000095787.20_WAC	ENSG00000160789.18_LMNA	-0.2105	-0.35771389	-0.05307893	9.24E-03	6.19E-02	1.00E+00
ENSG00000160953.13_MUM1	ENSG00000160789.18_LMNA	-0.13513	-0.288133634	0.024598325	9.69E-02	3.06E-01	1.00E+00
ENSG00000137817.15_PARP6	ENSG00000160789.18_LMNA	0.01546	-0.144098812	0.174228453	8.50E-01	9.41E-01	1.00E+00
ENSG00000131089.12_ARHGEF9	ENSG00000160789.18_LMNA	-0.33467	-0.468891823	-0.18534855	2.51E-05	6.19E-04	1.00E+00
ENSG00000196123.11_KIAA0895	ENSG00000160789.18_LMNA	-0.1736	-0.323850994	-0.01480926	3.24E-02	1.51E-01	1.00E+00
ENSG00000175895.3_PLEKHF2	ENSG00000160789.18_LMNA	0.0089	-0.15051296	0.167863857	9.13E-01	9.67E-01	1.00E+00
ENSG00000001631.13_KRIT1	ENSG00000160789.18_LMNA	0.0442	-0.115814768	0.201980305	5.89E-01	8.09E-01	1.00E+00
ENSG00000099949.17_LZTR1	ENSG00000160789.18_LMNA	0.12021	-0.039752512	0.274162899	1.40E-01	3.82E-01	1.00E+00
ENSG00000139546.9_TARBP2	ENSG00000160789.18_LMNA	-0.1699	-0.32043626	-0.01099976	3.64E-02	1.62E-01	1.00E+00
ENSG00000196372.11_ASB13	ENSG00000160789.18_LMNA	-0.44085	-0.560701181	-0.30291164	1.31E-08	1.36E-06	6.74E-04
ENSG00000156256.13_USP16	ENSG00000160789.18_LMNA	0.03075	-0.129080331	0.189027584	7.07E-01	8.75E-01	1.00E+00
ENSG00000120690.12_ELF1	ENSG00000160789.18_LMNA	-0.03126	-0.189515855	0.128582323	7.02E-01	8.73E-01	1.00E+00
ENSG00000108010.10_GLRX3	ENSG00000160789.18_LMNA	-0.18445	-0.333845327	-0.02601081	2.29E-02	1.19E-01	1.00E+00
ENSG00000160113.5_NR2F6	ENSG00000160789.18_LMNA	0.42087	0.280463948	0.543647246	6.70E-08	5.23E-06	3.44E-03
ENSG00000152049.6_KCNE4	ENSG00000160789.18_LMNA	0.22438	0.067593574	0.37035098	5.45E-03	4.18E-02	1.00E+00
ENSG00000269293.2_ZSCAN16-A	ENSG00000160789.18_LMNA	-0.20177	-0.349737255	-0.04398295	1.27E-02	7.77E-02	1.00E+00
ENSG00000152601.16_MBNL1	ENSG00000160789.18_LMNA	-0.02683	-0.185236379	0.132941683	7.43E-01	8.92E-01	1.00E+00
ENSG00000159685.9_CHCHD6	ENSG00000160789.18_LMNA	-0.2548	-0.39786246	-0.09964207	1.54E-03	1.60E-02	1.00E+00
ENSG00000185104.18_FAF1	ENSG00000160789.18_LMNA	0.08409	-0.076134065	0.24007277	3.03E-01	5.90E-01	1.00E+00

ENSG00000235587.2_GAPDHP65	ENSG00000160789.18_LMNA	0.2452	0.089489021	0.389204411	2.33E-03	2.20E-02	1.00E+00
ENSG00000173598.12_NUDT4	ENSG00000160789.18_LMNA	-0.26105	-0.403485747	-0.10626976	1.16E-03	1.29E-02	1.00E+00
ENSG00000241973.9_PI4KA	ENSG00000160789.18_LMNA	0.02986	-0.129962473	0.188162302	7.15E-01	8.79E-01	1.00E+00
ENSG00000117616.16_RSRP1	ENSG00000160789.18_LMNA	-0.03045	-0.188739962	0.129373613	7.10E-01	8.77E-01	1.00E+00
ENSG00000235297.3_FAUP1	ENSG00000160789.18_LMNA	-0.08871	-0.244454587	0.071504402	2.77E-01	5.62E-01	1.00E+00
ENSG00000104888.8_SLC17A7	ENSG00000160789.18_LMNA	-0.16957	-0.320127795	-0.01065607	3.68E-02	1.64E-01	1.00E+00
ENSG00000094975.12_SUCO	ENSG00000160789.18_LMNA	0.03177	-0.128076826	0.190011308	6.98E-01	8.70E-01	1.00E+00
ENSG00000120533.11_ENY2	ENSG00000160789.18_LMNA	-0.10464	-0.259513587	0.055489157	2.00E-01	4.70E-01	1.00E+00
ENSG00000205765.7_C5orf51	ENSG00000160789.18_LMNA	0.04342	-0.116590761	0.201225661	5.95E-01	8.13E-01	1.00E+00
ENSG00000160785.12_SLC25A44	ENSG00000160789.18_LMNA	0.0653	-0.094882616	0.222195179	4.24E-01	6.95E-01	1.00E+00
ENSG00000141569.9_TRIM65	ENSG00000160789.18_LMNA	0.29638	0.14396866	0.435050029	2.09E-04	3.38E-03	1.00E+00
ENSG00000163811.10_WDR43	ENSG00000160789.18_LMNA	0.02305	-0.136652089	0.181584432	7.78E-01	9.09E-01	1.00E+00
ENSG00000034152.17_MAP2K3	ENSG00000160789.18_LMNA	0.6766	0.579863294	0.754529553	1.10E-21	1.13E-17	5.65E-17
ENSG00000173947.12_PIFO	ENSG00000160789.18_LMNA	-0.00731	-0.166316978	0.152067844	9.29E-01	9.73E-01	1.00E+00
ENSG00000135063.16_FAM189A2	ENSG00000160789.18_LMNA	-0.12669	-0.280239857	0.033178476	1.20E-01	3.48E-01	1.00E+00
ENSG00000170385.9_SLC30A1	ENSG00000160789.18_LMNA	0.11239	-0.047660275	0.266819693	1.68E-01	4.26E-01	1.00E+00
ENSG00000001497.15_LAS1L	ENSG00000160789.18_LMNA	0.07681	-0.083408923	0.233161237	3.47E-01	6.31E-01	1.00E+00
ENSG00000157500.9_APPL1	ENSG00000160789.18_LMNA	-0.11187	-0.266323378	0.048193328	1.70E-01	4.29E-01	1.00E+00
ENSG00000166275.14_BORCS7	ENSG00000160789.18_LMNA	-0.33623	-0.470260738	-0.18704381	2.28E-05	5.75E-04	1.00E+00
ENSG00000180185.10_FAHD1	ENSG00000160789.18_LMNA	0.00683	-0.15253639	0.165850536	9.33E-01	9.74E-01	1.00E+00
ENSG00000160753.14_RUSC1	ENSG00000160789.18_LMNA	0.32607	0.1760082	0.461325051	4.14E-05	9.24E-04	1.00E+00
ENSG00000166225.7_FRS2	ENSG00000160789.18_LMNA	-0.17948	-0.329269502	-0.02087266	2.69E-02	1.33E-01	1.00E+00
ENSG00000114388.11_NPRL2	ENSG00000160789.18_LMNA	-0.15452	-0.306186195	0.004801357	5.73E-02	2.19E-01	1.00E+00
ENSG00000174276.6_ZNHIT2	ENSG00000160789.18_LMNA	0.20943	0.051962892	0.356737579	9.61E-03	6.38E-02	1.00E+00
ENSG00000185009.11_AP3M1	ENSG00000160789.18_LMNA	-0.2701	-0.411599232	-0.11587922	7.65E-04	9.37E-03	1.00E+00
ENSG00000162972.9_MAIP1	ENSG00000160789.18_LMNA	-0.10574	-0.260552891	0.054377845	1.95E-01	4.64E-01	1.00E+00
ENSG00000057252.11_SOAT1	ENSG00000160789.18_LMNA	0.24208	0.086205164	0.386392708	2.66E-03	2.43E-02	1.00E+00
ENSG00000107537.12_PHYH	ENSG00000160789.18_LMNA	0.03615	-0.123764014	0.194231851	6.58E-01	8.49E-01	1.00E+00
ENSG00000151725.10_CENPU	ENSG00000160789.18_LMNA	-0.02682	-0.185230735	0.132947423	7.43E-01	8.92E-01	1.00E+00
ENSG00000198753.10_PLXNB3	ENSG00000160789.18_LMNA	-0.06796	-0.224729035	0.092238927	4.05E-01	6.81E-01	1.00E+00
ENSG00000138035.13_PNPT1	ENSG00000160789.18_LMNA	-0.19944	-0.347601977	-0.04155661	1.38E-02	8.24E-02	1.00E+00
ENSG00000128266.8_GNAZ	ENSG00000160789.18_LMNA	-0.19087	-0.339751347	-0.03266676	1.85E-02	1.02E-01	1.00E+00
ENSG00000105856.12_HBP1	ENSG00000160789.18_LMNA	-0.10197	-0.256996759	0.058177132	2.11E-01	4.85E-01	1.00E+00

ENSG00000108509.19_CAMTA2	ENSG00000160789.18_LMNA	0.11571	-0.044308439	0.269936696	1.56E-01	4.07E-01	1.00E+00
ENSG00000179941.6_BBS10	ENSG00000160789.18_LMNA	-0.4638	-0.580158937	-0.32887762	1.77E-09	2.48E-07	9.08E-05
ENSG00000124356.14_STAMBP	ENSG00000160789.18_LMNA	-0.41165	-0.535737815	-0.27014976	1.37E-07	9.41E-06	7.05E-03
ENSG00000165898.12_ISCA2	ENSG00000160789.18_LMNA	-0.08521	-0.241143368	0.075004169	2.97E-01	5.82E-01	1.00E+00
ENSG00000145740.17_SLC30A5	ENSG00000160789.18_LMNA	0.28937	0.136453087	0.428814022	2.99E-04	4.50E-03	1.00E+00
ENSG00000121060.13_TRIM25	ENSG00000160789.18_LMNA	0.21248	0.055144814	0.359519378	8.59E-03	5.87E-02	1.00E+00
ENSG00000172732.10_MUS81	ENSG00000160789.18_LMNA	0.06277	-0.097404027	0.219774524	4.42E-01	7.10E-01	1.00E+00
ENSG00000047410.12_TPR	ENSG00000160789.18_LMNA	0.25229	0.09698631	0.395602873	1.71E-03	1.74E-02	1.00E+00
ENSG00000004799.7_PDK4	ENSG00000160789.18_LMNA	-0.29586	-0.434587586	-0.14341017	2.15E-04	3.45E-03	1.00E+00
ENSG00000083123.13_BCKDHB	ENSG00000160789.18_LMNA	-0.22644	-0.372225129	-0.06975714	5.03E-03	3.94E-02	1.00E+00
ENSG00000168487.16_BMP1	ENSG00000160789.18_LMNA	0.46368	0.328747305	0.580061993	1.79E-09	2.50E-07	9.17E-05
ENSG00000247556.5_OIP5-AS1	ENSG00000160789.18_LMNA	-0.12196	-0.275805989	0.037977698	1.34E-01	3.73E-01	1.00E+00
ENSG00000130305.15_NSUN5	ENSG00000160789.18_LMNA	0.34177	0.193077284	0.475121693	1.64E-05	4.37E-04	8.40E-01
ENSG00000144597.12_EAF1	ENSG00000160789.18_LMNA	0.05393	-0.10618354	0.211315255	5.09E-01	7.59E-01	1.00E+00
ENSG00000149269.8_PAK1	ENSG00000160789.18_LMNA	-0.10714	-0.261873629	0.052964473	1.89E-01	4.56E-01	1.00E+00
ENSG00000115355.14_CCDC88A	ENSG00000160789.18_LMNA	-0.11828	-0.272347847	0.041710772	1.47E-01	3.92E-01	1.00E+00
ENSG00000104518.9_GSDMD	ENSG00000160789.18_LMNA	0.31619	0.165308003	0.452605331	7.24E-05	1.45E-03	1.00E+00
ENSG00000240024.4_LINC00888	ENSG00000160789.18_LMNA	-0.51352	-0.621880981	-0.38586578	1.33E-11	4.32E-09	6.82E-07
ENSG00000100246.11_DNAL4	ENSG00000160789.18_LMNA	-0.12139	-0.275270699	0.038556119	1.36E-01	3.76E-01	1.00E+00
ENSG00000204439.3_C6orf47	ENSG00000160789.18_LMNA	0.30173	0.149719392	0.439802886	1.58E-04	2.72E-03	1.00E+00
ENSG00000151694.11_ADAM17	ENSG00000160789.18_LMNA	0.14121	-0.018407092	0.293803317	8.27E-02	2.78E-01	1.00E+00
ENSG00000148153.12_INIP	ENSG00000160789.18_LMNA	-0.28882	-0.428328097	-0.13586886	3.08E-04	4.60E-03	1.00E+00
ENSG00000171456.15_ASXL1	ENSG00000160789.18_LMNA	0.13085	-0.028951284	0.284134197	1.08E-01	3.27E-01	1.00E+00
ENSG00000171861.9_MRM3	ENSG00000160789.18_LMNA	0.046	-0.114035128	0.203709559	5.74E-01	8.01E-01	1.00E+00
ENSG00000131876.15_SNRPA1	ENSG00000160789.18_LMNA	0.03783	-0.122101956	0.195855215	6.44E-01	8.41E-01	1.00E+00
ENSG00000162976.11_PQLC3	ENSG00000160789.18_LMNA	0.29117	0.138379576	0.430415177	2.73E-04	4.18E-03	1.00E+00
ENSG00000130193.7_THEM6	ENSG00000160789.18_LMNA	0.05985	-0.100307332	0.216982389	4.64E-01	7.26E-01	1.00E+00
ENSG00000136280.14_CCM2	ENSG00000160789.18_LMNA	0.46112	0.325833878	0.577892833	2.25E-09	3.07E-07	1.16E-04
ENSG00000010803.15_SCMH1	ENSG00000160789.18_LMNA	0.40015	0.257340746	0.525849913	3.26E-07	1.88E-05	1.67E-02
ENSG00000132603.12_NIP7	ENSG00000160789.18_LMNA	0.08836	-0.071848729	0.244129136	2.79E-01	5.64E-01	1.00E+00
ENSG00000163406.9_SLC15A2	ENSG00000160789.18_LMNA	-0.28134	-0.421652577	-0.1278635	4.46E-04	6.17E-03	1.00E+00
ENSG00000228109.1_MELTF-AS1	ENSG00000160789.18_LMNA	0.1137	-0.046336874	0.268051161	1.63E-01	4.18E-01	1.00E+00
ENSG00000099904.14_ZDHHC8	ENSG00000160789.18_LMNA	0.15881	-0.000397853	0.310171504	5.07E-02	2.03E-01	1.00E+00

ENSG00000188483.7_IER5L	ENSG00000160789.18_LMNA	0.44774	0.310681602	0.56655365	7.31E-09	8.30E-07	3.75E-04
ENSG00000165609.11_NUDT5	ENSG00000160789.18_LMNA	-0.19312	-0.341807363	-0.03499025	1.71E-02	9.66E-02	1.00E+00
ENSG00000235043.3_TECRP1	ENSG00000160789.18_LMNA	0.15365	-0.005688043	0.305382395	5.88E-02	2.22E-01	1.00E+00
ENSG00000224531.5_SMIM13	ENSG00000160789.18_LMNA	-0.07324	-0.22976623	0.086970083	3.70E-01	6.50E-01	1.00E+00
ENSG00000109066.12_TMEM104	ENSG00000160789.18_LMNA	0.29003	0.137159279	0.42940117	2.90E-04	4.38E-03	1.00E+00
ENSG00000242802.5_AP5Z1	ENSG00000160789.18_LMNA	0.3164	0.165533656	0.452789787	7.15E-05	1.44E-03	1.00E+00
ENSG00000140937.12_CDH11	ENSG00000160789.18_LMNA	0.30778	0.156232082	0.445165934	1.15E-04	2.11E-03	1.00E+00
ENSG00000087053.17_MTMR2	ENSG00000160789.18_LMNA	-0.14999	-0.301984104	0.009431363	6.51E-02	2.38E-01	1.00E+00
ENSG00000124074.10_ENKD1	ENSG00000160789.18_LMNA	0.26498	0.110439606	0.40701219	9.70E-04	1.13E-02	1.00E+00
ENSG00000129911.7_KLF16	ENSG00000160789.18_LMNA	0.51606	0.388806811	0.623998413	1.01E-11	3.50E-09	5.20E-07
ENSG00000280237.1_MIR4697H(	ENSG00000160789.18_LMNA	-0.15819	-0.309597698	0.001032615	5.16E-02	2.05E-01	1.00E+00
ENSG00000086730.15_LAT2	ENSG00000160789.18_LMNA	-0.07598	-0.232369851	0.084239763	3.52E-01	6.36E-01	1.00E+00
ENSG00000152133.13_GPATCH1	ENSG00000160789.18_LMNA	-0.24031	-0.38478631	-0.08433194	2.86E-03	2.58E-02	1.00E+00
ENSG00000108846.14_ABCC3	ENSG00000160789.18_LMNA	0.4021	0.259507146	0.527527378	2.82E-07	1.68E-05	1.45E-02
ENSG00000180155.17_LYNX1	ENSG00000160789.18_LMNA	0.23326	0.076918055	0.378410588	3.83E-03	3.21E-02	1.00E+00
ENSG00000136870.9_ZNF189	ENSG00000160789.18_LMNA	-0.16888	-0.319491476	-0.00994733	3.75E-02	1.66E-01	1.00E+00
ENSG00000204178.8_TMEM57	ENSG00000160789.18_LMNA	-0.16651	-0.317300486	-0.00750936	4.03E-02	1.74E-01	1.00E+00
ENSG00000138785.13_INTS12	ENSG00000160789.18_LMNA	-0.1357	-0.288666148	0.024017844	9.55E-02	3.03E-01	1.00E+00
ENSG00000224861.1_YBX1P1	ENSG00000160789.18_LMNA	0.07207	-0.088141535	0.228647747	3.78E-01	6.57E-01	1.00E+00
ENSG00000169410.8_PTPN9	ENSG00000160789.18_LMNA	0.53532	0.411162665	0.639981635	1.20E-12	6.28E-10	6.15E-08
ENSG00000163531.14_NFASC	ENSG00000160789.18_LMNA	-0.1446	-0.296961743	0.01494771	7.55E-02	2.62E-01	1.00E+00
ENSG00000213598.4_RP11-112J3	ENSG00000160789.18_LMNA	-0.08781	-0.243602705	0.072405536	2.82E-01	5.67E-01	1.00E+00
ENSG00000168556.6_ING2	ENSG00000160789.18_LMNA	0.02529	-0.134449432	0.183753444	7.57E-01	8.99E-01	1.00E+00
ENSG00000186866.15_POFUT2	ENSG00000160789.18_LMNA	0.43531	0.296665963	0.555978081	2.09E-08	2.00E-06	1.07E-03
ENSG00000164089.7_ETNPPL	ENSG00000160789.18_LMNA	-0.45833	-0.575533108	-0.32266993	2.89E-09	3.77E-07	1.48E-04
ENSG00000101773.15_RBBP8	ENSG00000160789.18_LMNA	0.08927	-0.070939395	0.244988465	2.74E-01	5.59E-01	1.00E+00
ENSG00000136770.9_DNAJC1	ENSG00000160789.18_LMNA	0.0229	-0.136802493	0.181436212	7.79E-01	9.09E-01	1.00E+00
ENSG00000152642.9_GPD1L	ENSG00000160789.18_LMNA	-0.30342	-0.441307343	-0.15154381	1.45E-04	2.53E-03	1.00E+00
ENSG00000114742.12_WDR48	ENSG00000160789.18_LMNA	-0.4163	-0.539732349	-0.27535117	9.59E-08	6.99E-06	4.92E-03
ENSG00000100304.12_TTLL12	ENSG00000160789.18_LMNA	0.10667	-0.053442929	0.261426663	1.91E-01	4.59E-01	1.00E+00
ENSG00000198060.8_MARCH5	ENSG00000160789.18_LMNA	-0.42758	-0.54938477	-0.28798412	3.92E-08	3.38E-06	2.01E-03
ENSG00000188322.4_SBK1	ENSG00000160789.18_LMNA	-0.26421	-0.406321157	-0.10962167	1.00E-03	1.16E-02	1.00E+00
ENSG00000134330.17_IAH1	ENSG00000160789.18_LMNA	0.04619	-0.113850658	0.203888693	5.72E-01	8.00E-01	1.00E+00

ENSG00000066084.11_DIP2B	ENSG00000160789.18_LMNA	-0.19558	-0.344067965	-0.03754878	1.57E-02	9.08E-02	1.00E+00
ENSG00000130349.8_C6orf203	ENSG00000160789.18_LMNA	-0.09612	-0.251472665	0.064060891	2.39E-01	5.18E-01	1.00E+00
ENSG00000080603.15_SRCAP	ENSG00000160789.18_LMNA	0.16978	0.010872345	0.320321911	3.65E-02	1.63E-01	1.00E+00
ENSG00000154473.16_BUB3	ENSG00000160789.18_LMNA	-0.25597	-0.398917441	-0.10088347	1.46E-03	1.54E-02	1.00E+00
ENSG00000136560.12_TANK	ENSG00000160789.18_LMNA	0.1551	-0.004206026	0.306725626	5.64E-02	2.17E-01	1.00E+00
ENSG00000105127.7_AKAP8	ENSG00000160789.18_LMNA	0.18352	0.025049894	0.332990674	2.36E-02	1.22E-01	1.00E+00
ENSG00000117335.17_CD46	ENSG00000160789.18_LMNA	0.08181	-0.078409031	0.237914861	3.16E-01	6.03E-01	1.00E+00
ENSG00000126858.15_RHOT1	ENSG00000160789.18_LMNA	-0.41961	-0.542568937	-0.27905421	7.40E-08	5.65E-06	3.80E-03
ENSG00000139116.16_KIF21A	ENSG00000160789.18_LMNA	-0.25974	-0.402309209	-0.10488089	1.23E-03	1.35E-02	1.00E+00
ENSG00000175931.11_UBE2O	ENSG00000160789.18_LMNA	-0.04769	-0.205332171	0.112363382	5.60E-01	7.92E-01	1.00E+00
ENSG00000106853.15_PTGR1	ENSG00000160789.18_LMNA	0.28419	0.130907934	0.424195029	3.88E-04	5.53E-03	1.00E+00
ENSG00000132466.16_ANKRD17	ENSG00000160789.18_LMNA	0.13615	-0.023560336	0.289085715	9.44E-02	3.01E-01	1.00E+00
ENSG00000164830.16_OXR1	ENSG00000160789.18_LMNA	-0.28573	-0.425574197	-0.13256172	3.59E-04	5.17E-03	1.00E+00
ENSG00000173599.12_PC	ENSG00000160789.18_LMNA	-0.14203	-0.294572395	0.01756542	8.09E-02	2.74E-01	1.00E+00
ENSG00000138623.8_SEMA7A	ENSG00000160789.18_LMNA	0.14981	-0.009624722	0.301808352	6.55E-02	2.39E-01	1.00E+00
ENSG00000163823.3_CCR1	ENSG00000160789.18_LMNA	0.03875	-0.121202238	0.19673326	6.36E-01	8.37E-01	1.00E+00
ENSG00000170633.15_RNF34	ENSG00000160789.18_LMNA	-0.10729	-0.262013788	0.05281441	1.88E-01	4.55E-01	1.00E+00
ENSG00000182704.7_TSKU	ENSG00000160789.18_LMNA	0.39908	0.256152668	0.524929085	3.53E-07	2.00E-05	1.81E-02
ENSG00000110237.3_ARHGEF17	ENSG00000160789.18_LMNA	0.1681	0.00914082	0.318767034	3.84E-02	1.69E-01	1.00E+00
ENSG00000196586.12_MYO6	ENSG00000160789.18_LMNA	-0.0795	-0.235719008	0.080720622	3.30E-01	6.16E-01	1.00E+00
ENSG00000163536.11_SERPINI1	ENSG00000160789.18_LMNA	-0.24136	-0.385739473	-0.08544317	2.74E-03	2.49E-02	1.00E+00
ENSG00000132300.17_PTCD3	ENSG00000160789.18_LMNA	-0.31372	-0.450427065	-0.16264551	8.29E-05	1.62E-03	1.00E+00
ENSG00000092445.10_TYRO3	ENSG00000160789.18_LMNA	0.25273	0.097453259	0.396000427	1.68E-03	1.72E-02	1.00E+00
ENSG00000143442.20_POGZ	ENSG00000160789.18_LMNA	-0.00115	-0.160316901	0.158084096	9.89E-01	9.96E-01	1.00E+00
ENSG00000117682.15_DHDDS	ENSG00000160789.18_LMNA	0.33289	0.1834065	0.467321958	2.78E-05	6.73E-04	1.00E+00
ENSG00000070061.13_IKBKAP	ENSG00000160789.18_LMNA	0.00028	-0.158926016	0.159475363	9.97E-01	9.99E-01	1.00E+00
ENSG00000103490.13_PYCARD	ENSG00000160789.18_LMNA	0.03952	-0.120440801	0.197475957	6.29E-01	8.33E-01	1.00E+00
ENSG00000227036.5_LINC00511	ENSG00000160789.18_LMNA	-0.25384	-0.397000723	-0.09862874	1.60E-03	1.65E-02	1.00E+00
ENSG00000066697.13_MSANTD3	ENSG00000160789.18_LMNA	0.06503	-0.095154203	0.221934633	4.26E-01	6.97E-01	1.00E+00
ENSG00000104973.13_MED25	ENSG00000160789.18_LMNA	0.12144	-0.038505873	0.275317206	1.36E-01	3.76E-01	1.00E+00
ENSG00000131626.14_PPFIA1	ENSG00000160789.18_LMNA	0.11897	-0.041012279	0.272995516	1.44E-01	3.89E-01	1.00E+00
ENSG00000104894.10_CD37	ENSG00000160789.18_LMNA	-0.04565	-0.203369033	0.114385738	5.77E-01	8.03E-01	1.00E+00
ENSG00000107362.12_ABHD17B	ENSG00000160789.18_LMNA	-0.34732	-0.479978752	-0.1991273	1.17E-05	3.30E-04	5.98E-01

ENSG00000105379.8_ETFB	ENSG00000160789.18_LMNA	0.18048	0.021909279	0.330193838	2.61E-02	1.30E-01	1.00E+00
ENSG00000166164.14_BRD7	ENSG00000160789.18_LMNA	-0.01581	-0.17456657	0.143757312	8.47E-01	9.40E-01	1.00E+00
ENSG00000054983.15_GALC	ENSG00000160789.18_LMNA	0.16683	0.007838711	0.317596666	3.99E-02	1.73E-01	1.00E+00
ENSG00000047932.12_GOPC	ENSG00000160789.18_LMNA	-0.13605	-0.288987237	0.023667731	9.47E-02	3.01E-01	1.00E+00
ENSG00000124164.14_VAPB	ENSG00000160789.18_LMNA	0.06235	-0.097818616	0.219376128	4.45E-01	7.12E-01	1.00E+00
ENSG00000113851.12_CRBN	ENSG00000160789.18_LMNA	-0.49257	-0.604379372	-0.36173898	1.15E-10	2.51E-08	5.90E-06
ENSG00000178307.8_TMEM11	ENSG00000160789.18_LMNA	0.0255	-0.134249546	0.183950124	7.55E-01	8.98E-01	1.00E+00
ENSG00000111602.10_TIMELESS	ENSG00000160789.18_LMNA	0.07883	-0.081387851	0.235084586	3.34E-01	6.20E-01	1.00E+00
ENSG00000197063.9_MAFG	ENSG00000160789.18_LMNA	0.27465	0.120722922	0.415671188	6.17E-04	7.96E-03	1.00E+00
ENSG00000112685.12_EXOC2	ENSG00000160789.18_LMNA	-0.03919	-0.19715776	0.120767073	6.32E-01	8.35E-01	1.00E+00
ENSG00000131381.11_RBSN	ENSG00000160789.18_LMNA	-0.3144	-0.451023487	-0.1633741	7.99E-05	1.57E-03	1.00E+00
ENSG00000075089.8_ACTR6	ENSG00000160789.18_LMNA	-0.25447	-0.397569052	-0.09929697	1.56E-03	1.62E-02	1.00E+00
ENSG00000118217.5_ATF6	ENSG00000160789.18_LMNA	0.12579	-0.034090983	0.279397846	1.23E-01	3.53E-01	1.00E+00
ENSG00000176076.7_KCNE5	ENSG00000160789.18_LMNA	-0.14057	-0.293205423	0.019061118	8.41E-02	2.81E-01	1.00E+00
ENSG00000167720.11_SRR	ENSG00000160789.18_LMNA	-0.1572	-0.308676795	0.002050821	5.31E-02	2.09E-01	1.00E+00
ENSG00000269028.3_MTRNR2L1	ENSG00000160789.18_LMNA	-0.2158	-0.362542645	-0.05860996	7.58E-03	5.34E-02	1.00E+00
ENSG00000107949.15_BCCIP	ENSG00000160789.18_LMNA	-0.22767	-0.373343186	-0.07104921	4.79E-03	3.80E-02	1.00E+00
ENSG00000163964.12_PIGX	ENSG00000160789.18_LMNA	0.06766	-0.092540673	0.224440042	4.08E-01	6.83E-01	1.00E+00
ENSG00000135838.12_NPL	ENSG00000160789.18_LMNA	-0.10102	-0.256099849	0.05913393	2.16E-01	4.90E-01	1.00E+00
ENSG00000164818.14_DNAAF5	ENSG00000160789.18_LMNA	0.38587	0.241499622	0.513520175	9.13E-07	4.26E-05	4.69E-02
ENSG00000118162.12_KPTN	ENSG00000160789.18_LMNA	0.0512	-0.108887233	0.208700568	5.31E-01	7.74E-01	1.00E+00
ENSG00000214391.3_TUBAP2	ENSG00000160789.18_LMNA	-0.12394	-0.27766097	0.035971625	1.28E-01	3.62E-01	1.00E+00
ENSG00000240731.1_RP5-890O3	ENSG00000160789.18_LMNA	0.07311	-0.08710174	0.229640568	3.71E-01	6.51E-01	1.00E+00
ENSG00000175161.12_CADM2	ENSG00000160789.18_LMNA	-0.50579	-0.615437444	-0.37694535	3.00E-11	8.31E-09	1.54E-06
ENSG00000110031.11_LPXN	ENSG00000160789.18_LMNA	-0.00723	-0.166241684	0.152143487	9.30E-01	9.73E-01	1.00E+00
ENSG00000112769.17_LAMA4	ENSG00000160789.18_LMNA	0.39668	0.253491428	0.522864188	4.21E-07	2.29E-05	2.16E-02
ENSG00000126821.7_SGPP1	ENSG00000160789.18_LMNA	-0.01277	-0.171621471	0.146729328	8.76E-01	9.52E-01	1.00E+00
ENSG00000198959.10_TGM2	ENSG00000160789.18_LMNA	0.26449	0.109920881	0.406573985	9.92E-04	1.15E-02	1.00E+00
ENSG00000275023.3_MLLT6	ENSG00000160789.18_LMNA	-0.0446	-0.20236051	0.115423659	5.85E-01	8.07E-01	1.00E+00
ENSG00000134046.10_MBD2	ENSG00000160789.18_LMNA	0.39086	0.247030691	0.517838013	6.41E-07	3.20E-05	3.29E-02
ENSG00000164610.7_RP9	ENSG00000160789.18_LMNA	0.1253	-0.034588106	0.278938925	1.24E-01	3.55E-01	1.00E+00
ENSG00000170464.8_DNAJC18	ENSG00000160789.18_LMNA	-0.52193	-0.628876534	-0.39560064	5.37E-12	2.13E-09	2.75E-07
ENSG00000274712.1_RP11-147L	ENSG00000160789.18_LMNA	-0.19699	-0.345359749	-0.03901262	1.50E-02	8.79E-02	1.00E+00

ENSG00000086062.11_B4GALT1	ENSG00000160789.18_LMNA	0.33498	0.185686539	0.469164858	2.46E-05	6.11E-04	1.00E+00
ENSG00000100321.13_SYNGR1	ENSG00000160789.18_LMNA	-0.21865	-0.365136593	-0.06158889	6.81E-03	4.93E-02	1.00E+00
ENSG00000173465.6_SSSCA1	ENSG00000160789.18_LMNA	-0.01603	-0.174788187	0.143533437	8.45E-01	9.39E-01	1.00E+00
ENSG00000121413.11_ZSCAN18	ENSG00000160789.18_LMNA	-0.01607	-0.174822825	0.143498443	8.44E-01	9.39E-01	1.00E+00
ENSG00000204253.4_HNRNPCP2	ENSG00000160789.18_LMNA	-0.04748	-0.205132081	0.112569628	5.61E-01	7.93E-01	1.00E+00
ENSG00000185591.8_SP1	ENSG00000160789.18_LMNA	0.24924	0.093755092	0.392848782	1.96E-03	1.92E-02	1.00E+00
ENSG00000128708.11_HAT1	ENSG00000160789.18_LMNA	0.03938	-0.120572338	0.197347683	6.30E-01	8.34E-01	1.00E+00
ENSG00000237214.3_RP11-12M9	ENSG00000160789.18_LMNA	-0.04826	-0.205882321	0.111796164	5.55E-01	7.89E-01	1.00E+00
ENSG00000136699.18_SMPD4	ENSG00000160789.18_LMNA	0.17113	0.012262004	0.321568594	3.50E-02	1.59E-01	1.00E+00
ENSG00000172845.12_SP3	ENSG00000160789.18_LMNA	0.09531	-0.064871741	0.250709767	2.43E-01	5.22E-01	1.00E+00
ENSG00000112584.12_FAM120B	ENSG00000160789.18_LMNA	-0.01852	-0.177198956	0.141095998	8.21E-01	9.30E-01	1.00E+00
ENSG00000276269.1_RP11-403A	ENSG00000160789.18_LMNA	0.06006	-0.100099937	0.217182016	4.62E-01	7.25E-01	1.00E+00
ENSG00000155229.19_MMS19	ENSG00000160789.18_LMNA	0.21939	0.062365299	0.365811891	6.61E-03	4.83E-02	1.00E+00
ENSG00000132702.11_HAPLN2	ENSG00000160789.18_LMNA	-0.21544	-0.362218979	-0.05823864	7.69E-03	5.39E-02	1.00E+00
ENSG00000005812.9_FBXL3	ENSG00000160789.18_LMNA	-0.24931	-0.392919533	-0.09383802	1.95E-03	1.92E-02	1.00E+00
ENSG00000182810.6_DDX28	ENSG00000160789.18_LMNA	-0.08018	-0.236368086	0.080037687	3.26E-01	6.13E-01	1.00E+00
ENSG00000100335.11_MIEF1	ENSG00000160789.18_LMNA	-0.07427	-0.230744631	0.085944629	3.63E-01	6.45E-01	1.00E+00
ENSG00000164024.10_METAP1	ENSG00000160789.18_LMNA	-0.1118	-0.266256943	0.048264667	1.70E-01	4.29E-01	1.00E+00
ENSG00000143797.10_MBOAT2	ENSG00000160789.18_LMNA	-0.12843	-0.281864221	0.031416639	1.15E-01	3.39E-01	1.00E+00
ENSG00000144191.10_CNGA3	ENSG00000160789.18_LMNA	0.0438	-0.116212177	0.201593877	5.92E-01	8.11E-01	1.00E+00
ENSG00000198853.10_RUSC2	ENSG00000160789.18_LMNA	-0.02166	-0.180238088	0.138017736	7.91E-01	9.15E-01	1.00E+00
ENSG00000164758.6_MED30	ENSG00000160789.18_LMNA	-0.17188	-0.322259785	-0.01303298	3.42E-02	1.56E-01	1.00E+00
ENSG00000158856.16_DMTN	ENSG00000160789.18_LMNA	-0.05461	-0.211968656	0.105507162	5.04E-01	7.55E-01	1.00E+00
ENSG00000108256.7_NUFIP2	ENSG00000160789.18_LMNA	0.24928	0.09380264	0.392889349	1.95E-03	1.92E-02	1.00E+00
ENSG00000136811.15_ODF2	ENSG00000160789.18_LMNA	0.26732	0.112921114	0.409106619	8.71E-04	1.04E-02	1.00E+00
ENSG00000160208.12_RRP1B	ENSG00000160789.18_LMNA	0.17333	0.014533463	0.323604046	3.27E-02	1.51E-01	1.00E+00
ENSG00000110090.11_CPT1A	ENSG00000160789.18_LMNA	0.02018	-0.139468405	0.178806616	8.05E-01	9.22E-01	1.00E+00
ENSG00000064042.16_LIMCH1	ENSG00000160789.18_LMNA	-0.24649	-0.39037141	-0.0908539	2.20E-03	2.11E-02	1.00E+00
ENSG00000172939.7_OXSR1	ENSG00000160789.18_LMNA	-0.01507	-0.173855302	0.144475607	8.54E-01	9.43E-01	1.00E+00
ENSG00000068400.12_GRIPAP1	ENSG00000160789.18_LMNA	0.14933	-0.010106119	0.301370699	6.63E-02	2.41E-01	1.00E+00
ENSG00000233929.1_MT1XP1	ENSG00000160789.18_LMNA	0.11838	-0.041607258	0.272443848	1.46E-01	3.92E-01	1.00E+00
ENSG00000151414.13_NEK7	ENSG00000160789.18_LMNA	-0.3208	-0.45668153	-0.17030161	5.59E-05	1.17E-03	1.00E+00
ENSG00000186815.11_TPCN1	ENSG00000160789.18_LMNA	0.11843	-0.041552634	0.272494504	1.46E-01	3.92E-01	1.00E+00

ENSG00000155329.10_ZCCHC10	ENSG00000160789.18_LMNA	-0.23768	-0.382411259	-0.08156626	3.19E-03	2.80E-02	1.00E+00
ENSG00000114554.10_PLXNA1	ENSG00000160789.18_LMNA	0.4597	0.324226521	0.576694566	2.56E-09	3.41E-07	1.31E-04
ENSG00000214548.13_MEG3	ENSG00000160789.18_LMNA	0.022	-0.137679354	0.180571798	7.88E-01	9.14E-01	1.00E+00
ENSG00000134802.16_SLC43A3	ENSG00000160789.18_LMNA	0.44593	0.30863972	0.565018166	8.54E-09	9.44E-07	4.38E-04
ENSG00000119929.11_CUTC	ENSG00000160789.18_LMNA	-0.3657	-0.496025405	-0.21926859	3.60E-06	1.28E-04	1.85E-01
ENSG00000153208.15_MERTK	ENSG00000160789.18_LMNA	-0.10658	-0.261344946	0.053530388	1.91E-01	4.59E-01	1.00E+00
ENSG00000163686.12_ABHD6	ENSG00000160789.18_LMNA	-0.43461	-0.555382659	-0.29588017	2.21E-08	2.11E-06	1.13E-03
ENSG00000249264.1_EEF1A1P9	ENSG00000160789.18_LMNA	-0.16273	-0.31380469	-0.0036271	4.52E-02	1.88E-01	1.00E+00
ENSG00000023041.10_ZDHHC6	ENSG00000160789.18_LMNA	-0.01272	-0.171572196	0.146779005	8.76E-01	9.52E-01	1.00E+00
ENSG00000070047.10_PHRF1	ENSG00000160789.18_LMNA	0.35339	0.20576153	0.485285109	7.98E-06	2.44E-04	4.09E-01
ENSG00000181704.10_YIPF6	ENSG00000160789.18_LMNA	-0.25984	-0.402395885	-0.10498317	1.23E-03	1.35E-02	1.00E+00
ENSG00000153066.11_TXNDC11	ENSG00000160789.18_LMNA	0.14812	-0.011344453	0.300244286	6.86E-02	2.46E-01	1.00E+00
ENSG00000047457.12_CP	ENSG00000160789.18_LMNA	0.10766	-0.052440568	0.262362898	1.87E-01	4.53E-01	1.00E+00
ENSG00000187147.16_RNF220	ENSG00000160789.18_LMNA	-0.04056	-0.198476798	0.119414114	6.20E-01	8.28E-01	1.00E+00
ENSG00000128872.8_TMOD2	ENSG00000160789.18_LMNA	-0.20831	-0.355715404	-0.05079524	1.00E-02	6.57E-02	1.00E+00
ENSG00000088543.13_C3orf18	ENSG00000160789.18_LMNA	-0.09887	-0.254072813	0.061294182	2.26E-01	5.02E-01	1.00E+00
ENSG00000187676.7_B3GLCT	ENSG00000160789.18_LMNA	-0.10673	-0.261483026	0.053382603	1.91E-01	4.58E-01	1.00E+00
ENSG00000189339.10_SLC35E2E	ENSG00000160789.18_LMNA	-0.08791	-0.243695696	0.072307194	2.82E-01	5.67E-01	1.00E+00
ENSG00000141562.16_NARF	ENSG00000160789.18_LMNA	-0.12871	-0.282127709	0.031130667	1.14E-01	3.37E-01	1.00E+00
ENSG00000165102.13_HGSNAT	ENSG00000160789.18_LMNA	0.33808	0.189053905	0.47188211	2.05E-05	5.27E-04	1.00E+00
ENSG00000151689.11_INPP1	ENSG00000160789.18_LMNA	-0.53883	-0.64287915	-0.41524513	8.02E-13	4.78E-10	4.11E-08
ENSG00000167733.12_HSD11B1l	ENSG00000160789.18_LMNA	-0.279	-0.41956337	-0.12536594	5.00E-04	6.74E-03	1.00E+00
ENSG00000094631.17_HDAC6	ENSG00000160789.18_LMNA	0.08425	-0.075968373	0.240229815	3.02E-01	5.89E-01	1.00E+00
ENSG00000065057.6_NTHL1	ENSG00000160789.18_LMNA	-0.16489	-0.315799097	-0.00584084	4.24E-02	1.80E-01	1.00E+00
ENSG00000007171.15_NOS2	ENSG00000160789.18_LMNA	0.11486	-0.045171203	0.269135007	1.59E-01	4.12E-01	1.00E+00
ENSG00000069399.11_BCL3	ENSG00000160789.18_LMNA	0.62718	0.519883657	0.714987159	5.39E-18	1.97E-14	2.77E-13
ENSG00000025156.11_HSF2	ENSG00000160789.18_LMNA	-0.35681	-0.488274789	-0.2095107	6.42E-06	2.04E-04	3.29E-01
ENSG00000112739.15_PRPF4B	ENSG00000160789.18_LMNA	0.01296	-0.146539077	0.17181017	8.74E-01	9.51E-01	1.00E+00
ENSG00000131374.13_TBC1D5	ENSG00000160789.18_LMNA	-0.10047	-0.255585018	0.059682875	2.18E-01	4.93E-01	1.00E+00
ENSG00000109083.12_IFT20	ENSG00000160789.18_LMNA	-0.37281	-0.50220658	-0.22709047	2.24E-06	8.73E-05	1.15E-01
ENSG00000141447.15_OSBPL1A	ENSG00000160789.18_LMNA	-0.20729	-0.354783602	-0.04973156	1.04E-02	6.73E-02	1.00E+00
ENSG00000031081.9_ARHGAP31	ENSG00000160789.18_LMNA	-0.19444	-0.34301835	-0.03636034	1.64E-02	9.35E-02	1.00E+00
ENSG00000124571.16_XPO5	ENSG00000160789.18_LMNA	0.04473	-0.115291153	0.202489301	5.84E-01	8.07E-01	1.00E+00

ENSG00000203993.4_ARRDC1-A	ENSG00000160789.18_LMNA	-0.06524	-0.222136922	0.094943346	4.25E-01	6.95E-01	1.00E+00
ENSG00000068305.16_MEF2A	ENSG00000160789.18_LMNA	0.06674	-0.093451743	0.223567138	4.14E-01	6.87E-01	1.00E+00
ENSG00000233016.5_SNHG7	ENSG00000160789.18_LMNA	-0.00581	-0.164853573	0.153537373	9.43E-01	9.77E-01	1.00E+00
ENSG00000170242.16_USP47	ENSG00000160789.18_LMNA	0.04636	-0.113674974	0.204059275	5.71E-01	7.99E-01	1.00E+00
ENSG00000147526.18_TACC1	ENSG00000160789.18_LMNA	0.28447	0.131213679	0.424450106	3.82E-04	5.46E-03	1.00E+00
ENSG00000159079.17_C21orf59	ENSG00000160789.18_LMNA	0.33011	0.180384112	0.464875226	3.28E-05	7.68E-04	1.00E+00
ENSG00000146828.16_SLC12A9	ENSG00000160789.18_LMNA	0.337	0.187882526	0.47093749	2.18E-05	5.54E-04	1.00E+00
ENSG00000169592.13_INO80E	ENSG00000160789.18_LMNA	0.05798	-0.10215879	0.215199111	4.78E-01	7.36E-01	1.00E+00
ENSG00000080546.12_SESN1	ENSG00000160789.18_LMNA	-0.41616	-0.539614174	-0.27519707	9.69E-08	7.03E-06	4.97E-03
ENSG00000137714.2_FDX1	ENSG00000160789.18_LMNA	-0.02472	-0.183200973	0.135010771	7.62E-01	9.01E-01	1.00E+00
ENSG00000111445.12_RFC5	ENSG00000160789.18_LMNA	-0.15703	-0.308515276	0.00222934	5.34E-02	2.10E-01	1.00E+00
ENSG00000139192.10_TAPBPL	ENSG00000160789.18_LMNA	0.13578	-0.023939252	0.288738231	9.53E-02	3.02E-01	1.00E+00
ENSG00000162373.11_BEND5	ENSG00000160789.18_LMNA	-0.20226	-0.350183147	-0.04449008	1.25E-02	7.68E-02	1.00E+00
ENSG00000108582.10_CPD	ENSG00000160789.18_LMNA	0.20893	0.051443863	0.356283304	9.79E-03	6.46E-02	1.00E+00
ENSG00000143457.9_GOLPH3L	ENSG00000160789.18_LMNA	-0.05644	-0.21372082	0.103691935	4.90E-01	7.46E-01	1.00E+00
ENSG00000231584.7_FAHD2CP	ENSG00000160789.18_LMNA	-0.14358	-0.296015948	0.01598441	7.76E-02	2.67E-01	1.00E+00
ENSG00000071242.10_RPS6KA2	ENSG00000160789.18_LMNA	0.20296	0.045217491	0.350822477	1.22E-02	7.55E-02	1.00E+00
ENSG00000197601.11_FAR1	ENSG00000160789.18_LMNA	-0.13094	-0.284218964	0.028859147	1.08E-01	3.27E-01	1.00E+00
ENSG00000114416.16_FXR1	ENSG00000160789.18_LMNA	-0.00962	-0.168565291	0.149807376	9.06E-01	9.64E-01	1.00E+00
ENSG00000164209.15_SLC25A46	ENSG00000160789.18_LMNA	-0.33441	-0.468663329	-0.18506574	2.54E-05	6.26E-04	1.00E+00
ENSG00000105993.13_DNAJB6	ENSG00000160789.18_LMNA	0.08497	-0.075246498	0.240913821	2.98E-01	5.84E-01	1.00E+00
ENSG00000130635.14_COL5A1	ENSG00000160789.18_LMNA	0.48209	0.349727629	0.595578119	3.21E-10	6.05E-08	1.64E-05
ENSG00000121481.9_RNF2	ENSG00000160789.18_LMNA	-0.21476	-0.361594729	-0.05752271	7.89E-03	5.50E-02	1.00E+00
ENSG00000160214.11_RRP1	ENSG00000160789.18_LMNA	0.32933	0.179539454	0.464190667	3.43E-05	7.97E-04	1.00E+00
ENSG00000198791.10_CNOT7	ENSG00000160789.18_LMNA	-0.37518	-0.504263763	-0.2297016	1.91E-06	7.71E-05	9.81E-02
ENSG00000126005.14_MMP24-AS	ENSG00000160789.18_LMNA	0.05928	-0.100875694	0.216435183	4.68E-01	7.29E-01	1.00E+00
ENSG00000126603.7_GLIS2	ENSG00000160789.18_LMNA	0.10153	-0.058622183	0.256579632	2.13E-01	4.87E-01	1.00E+00
ENSG00000125755.17_SYMPK	ENSG00000160789.18_LMNA	0.19502	0.036971501	0.343558215	1.61E-02	9.21E-02	1.00E+00
ENSG00000009844.14_VTA1	ENSG00000160789.18_LMNA	-0.30174	-0.439810924	-0.14972913	1.58E-04	2.72E-03	1.00E+00
ENSG00000126903.14_SLC10A3	ENSG00000160789.18_LMNA	0.50193	0.372498657	0.612213493	4.46E-11	1.17E-08	2.29E-06
ENSG00000101407.11_TTI1	ENSG00000160789.18_LMNA	0.1162	-0.043806437	0.270402962	1.54E-01	4.04E-01	1.00E+00
ENSG00000103479.13_RBL2	ENSG00000160789.18_LMNA	-0.07854	-0.234807936	0.081678717	3.36E-01	6.22E-01	1.00E+00
ENSG00000152127.7_MGAT5	ENSG00000160789.18_LMNA	0.00083	-0.158388874	0.160012315	9.92E-01	9.97E-01	1.00E+00

ENSG00000114982.16_KANSL3	ENSG00000160789.18_LMNA	-0.00854	-0.16750891	0.150869883	9.17E-01	9.68E-01	1.00E+00
ENSG00000177108.5_ZDHHC22	ENSG00000160789.18_LMNA	-0.38911	-0.516328856	-0.24509548	7.26E-07	3.55E-05	3.72E-02
ENSG00000175137.10_SH3BP5L	ENSG00000160789.18_LMNA	0.09546	-0.064720187	0.250852388	2.42E-01	5.21E-01	1.00E+00
ENSG00000239887.4_C1orf226	ENSG00000160789.18_LMNA	-0.04933	-0.206901647	0.110744667	5.46E-01	7.84E-01	1.00E+00
ENSG00000198783.5_ZNF830	ENSG00000160789.18_LMNA	0.13314	-0.026621269	0.286276337	1.02E-01	3.15E-01	1.00E+00
ENSG00000135164.17_DMTF1	ENSG00000160789.18_LMNA	-0.0164	-0.175140166	0.143177805	8.41E-01	9.38E-01	1.00E+00
ENSG00000171368.11_TPPP	ENSG00000160789.18_LMNA	-0.18234	-0.331905747	-0.02383089	2.46E-02	1.25E-01	1.00E+00
ENSG00000186818.11_LILRB4	ENSG00000160789.18_LMNA	-0.08634	-0.242209462	0.073878219	2.90E-01	5.76E-01	1.00E+00
ENSG00000102870.5_ZNF629	ENSG00000160789.18_LMNA	0.21729	0.060166168	0.363898325	7.17E-03	5.12E-02	1.00E+00
ENSG00000134697.11_GNL2	ENSG00000160789.18_LMNA	0.29978	0.147629619	0.438077611	1.75E-04	2.94E-03	1.00E+00
ENSG00000176853.14_FAM91A1	ENSG00000160789.18_LMNA	0.20337	0.045644743	0.351197863	1.20E-02	7.47E-02	1.00E+00
ENSG00000186625.12_KATNA1	ENSG00000160789.18_LMNA	-0.00197	-0.161119665	0.157280535	9.81E-01	9.93E-01	1.00E+00
ENSG00000169241.16_SLC50A1	ENSG00000160789.18_LMNA	0.20145	0.043646176	0.349441066	1.28E-02	7.84E-02	1.00E+00
ENSG00000163563.7_MNDA	ENSG00000160789.18_LMNA	-0.02737	-0.185764732	0.132404133	7.38E-01	8.90E-01	1.00E+00
ENSG00000101019.20_UQCC1	ENSG00000160789.18_LMNA	-0.15494	-0.306581815	0.004364761	5.66E-02	2.18E-01	1.00E+00
ENSG00000132164.8_SLC6A11	ENSG00000160789.18_LMNA	-0.08076	-0.236912294	0.079464864	3.23E-01	6.09E-01	1.00E+00
ENSG00000166479.8_TMX3	ENSG00000160789.18_LMNA	0.27631	0.122496205	0.417158985	5.69E-04	7.45E-03	1.00E+00
ENSG00000142459.7_EVI5L	ENSG00000160789.18_LMNA	-0.12401	-0.277731158	0.03589567	1.28E-01	3.62E-01	1.00E+00
ENSG00000088038.16_CNOT3	ENSG00000160789.18_LMNA	0.27145	0.117323566	0.412814687	7.17E-04	8.94E-03	1.00E+00
ENSG00000197062.10_ZSCAN26	ENSG00000160789.18_LMNA	-0.37253	-0.501963967	-0.22678278	2.29E-06	8.83E-05	1.17E-01
ENSG00000175182.12_FAM131A	ENSG00000160789.18_LMNA	-0.02643	-0.184854792	0.133329795	7.47E-01	8.94E-01	1.00E+00
ENSG00000175906.4_ARL4D	ENSG00000160789.18_LMNA	-0.0068	-0.165819962	0.152567098	9.34E-01	9.74E-01	1.00E+00
ENSG00000277972.1_CISD3	ENSG00000160789.18_LMNA	0.06069	-0.099474282	0.217784073	4.58E-01	7.22E-01	1.00E+00
ENSG00000100320.21_RBFOX2	ENSG00000160789.18_LMNA	-0.09968	-0.254836408	0.06048075	2.22E-01	4.97E-01	1.00E+00
ENSG00000228929.1_RPS13P2	ENSG00000160789.18_LMNA	-0.16164	-0.312796058	-0.0025087	4.66E-02	1.92E-01	1.00E+00
ENSG00000173110.7_HSPA6	ENSG00000160789.18_LMNA	0.31302	0.161891916	0.449809901	8.62E-05	1.67E-03	1.00E+00
ENSG00000142945.11_KIF2C	ENSG00000160789.18_LMNA	0.11376	-0.046275356	0.268108381	1.63E-01	4.18E-01	1.00E+00
ENSG00000138074.13_SLC5A6	ENSG00000160789.18_LMNA	0.22368	0.066858273	0.369713478	5.60E-03	4.27E-02	1.00E+00
ENSG00000163807.5_KIAA1143	ENSG00000160789.18_LMNA	-0.14217	-0.294697836	0.017428096	8.06E-02	2.73E-01	1.00E+00
ENSG00000148335.13_NTMT1	ENSG00000160789.18_LMNA	0.08697	-0.073249722	0.242804216	2.87E-01	5.72E-01	1.00E+00
ENSG00000197442.9_MAP3K5	ENSG00000160789.18_LMNA	0.01922	-0.140409695	0.177877061	8.14E-01	9.27E-01	1.00E+00
ENSG00000164323.11_CFAP97	ENSG00000160789.18_LMNA	0.01525	-0.144301153	0.17402808	8.52E-01	9.42E-01	1.00E+00
ENSG00000063854.11_HAGH	ENSG00000160789.18_LMNA	-0.34202	-0.475340583	-0.19334948	1.61E-05	4.32E-04	8.27E-01

ENSG00000100226.14_GTPBP1	ENSG00000160789.18_LMNA	-0.03578	-0.193878751	0.124125296	6.62E-01	8.51E-01	1.00E+00
ENSG00000116809.10_ZBTB17	ENSG00000160789.18_LMNA	0.13714	-0.022556345	0.290006023	9.20E-02	2.96E-01	1.00E+00
ENSG00000085449.13_WDFY1	ENSG00000160789.18_LMNA	0.12773	-0.032128652	0.281207981	1.17E-01	3.42E-01	1.00E+00
ENSG00000228716.5_DHFR	ENSG00000160789.18_LMNA	-0.06843	-0.225177791	0.091770253	4.02E-01	6.78E-01	1.00E+00
ENSG00000281649.1_EBLN3P	ENSG00000160789.18_LMNA	-0.12207	-0.27590613	0.037869466	1.34E-01	3.72E-01	1.00E+00
ENSG00000109458.7_GAB1	ENSG00000160789.18_LMNA	-0.08912	-0.244842752	0.071093624	2.75E-01	5.59E-01	1.00E+00
ENSG00000196547.13_MAN2A2	ENSG00000160789.18_LMNA	0.0675	-0.092692015	0.224295075	4.09E-01	6.83E-01	1.00E+00
ENSG00000100365.13_NCF4	ENSG00000160789.18_LMNA	0.02809	-0.131695272	0.186461183	7.31E-01	8.87E-01	1.00E+00
ENSG00000162222.12_TTC9C	ENSG00000160789.18_LMNA	-0.16482	-0.315733927	-0.00576846	4.24E-02	1.80E-01	1.00E+00
ENSG00000171724.2_VAT1L	ENSG00000160789.18_LMNA	-0.24979	-0.393347373	-0.09433959	1.91E-03	1.89E-02	1.00E+00
ENSG00000112367.9_FIG4	ENSG00000160789.18_LMNA	-0.33496	-0.469144212	-0.18566098	2.46E-05	6.12E-04	1.00E+00
ENSG00000171103.9_TRMT61B	ENSG00000160789.18_LMNA	-0.36679	-0.496973628	-0.2204662	3.35E-06	1.20E-04	1.72E-01
ENSG00000198795.9_ZNF521	ENSG00000160789.18_LMNA	0.14141	-0.018202711	0.293990107	8.23E-02	2.77E-01	1.00E+00
ENSG00000124786.9_SLC35B3	ENSG00000160789.18_LMNA	0.19398	0.035884607	0.342597977	1.66E-02	9.46E-02	1.00E+00
ENSG00000108389.8_MTMR4	ENSG00000160789.18_LMNA	-0.00906	-0.16801833	0.150357601	9.12E-01	9.66E-01	1.00E+00
ENSG00000046647.12_GEMIN8	ENSG00000160789.18_LMNA	-0.00937	-0.168318144	0.150056023	9.09E-01	9.65E-01	1.00E+00
ENSG00000099910.15_KLHL22	ENSG00000160789.18_LMNA	-0.00121	-0.160382607	0.15801834	9.88E-01	9.96E-01	1.00E+00
ENSG00000115159.14_GPD2	ENSG00000160789.18_LMNA	0.06027	-0.099886608	0.217387326	4.61E-01	7.24E-01	1.00E+00
ENSG00000107816.16_LZTS2	ENSG00000160789.18_LMNA	0.20384	0.046135423	0.351628856	1.18E-02	7.37E-02	1.00E+00
ENSG00000154429.9_CCSAP	ENSG00000160789.18_LMNA	-0.23448	-0.379512521	-0.07819704	3.64E-03	3.09E-02	1.00E+00
ENSG00000126777.16_KTN1	ENSG00000160789.18_LMNA	0.25083	0.095443263	0.394288345	1.83E-03	1.83E-02	1.00E+00
ENSG00000134278.13_SPIRE1	ENSG00000160789.18_LMNA	-0.01816	-0.176848014	0.141451062	8.24E-01	9.31E-01	1.00E+00
ENSG00000122971.7_ACADS	ENSG00000160789.18_LMNA	0.17016	0.011266389	0.320675522	3.61E-02	1.62E-01	1.00E+00
ENSG00000176407.16_KCMF1	ENSG00000160789.18_LMNA	0.03145	-0.128396144	0.189698354	7.01E-01	8.72E-01	1.00E+00
ENSG00000116954.7_RRAGC	ENSG00000160789.18_LMNA	0.09611	-0.064067064	0.251466859	2.39E-01	5.18E-01	1.00E+00
ENSG00000161395.11_PGAP3	ENSG00000160789.18_LMNA	0.11887	-0.041112418	0.272902681	1.45E-01	3.89E-01	1.00E+00
ENSG00000211896.5_IGHG1	ENSG00000160789.18_LMNA	0.07294	-0.087270118	0.229479841	3.72E-01	6.52E-01	1.00E+00
ENSG00000168890.12_TMEM150	ENSG00000160789.18_LMNA	0.13275	-0.027025355	0.285905058	1.03E-01	3.18E-01	1.00E+00
ENSG00000176209.10_SMIM19	ENSG00000160789.18_LMNA	-0.08886	-0.244598809	0.07135179	2.76E-01	5.61E-01	1.00E+00
ENSG00000128513.13_POT1	ENSG00000160789.18_LMNA	-0.01768	-0.176386591	0.141917781	8.29E-01	9.33E-01	1.00E+00
ENSG00000100347.13_SAMM50	ENSG00000160789.18_LMNA	-0.20151	-0.34949962	-0.04371275	1.28E-02	7.83E-02	1.00E+00
ENSG00000122863.5_CHST3	ENSG00000160789.18_LMNA	0.24526	0.089555591	0.389261351	2.32E-03	2.19E-02	1.00E+00
ENSG00000198055.9_GRK6	ENSG00000160789.18_LMNA	0.11607	-0.043940736	0.270278237	1.54E-01	4.05E-01	1.00E+00

ENSG00000164253.11_WDR41	ENSG00000160789.18_LMNA	0.11677	-0.043231999	0.270936325	1.52E-01	4.01E-01	1.00E+00
ENSG00000130713.14_EXOSC2	ENSG00000160789.18_LMNA	-0.09021	-0.245879571	0.069995878	2.69E-01	5.53E-01	1.00E+00
ENSG00000184164.13_CRELD2	ENSG00000160789.18_LMNA	0.46446	0.329636777	0.58072353	1.67E-09	2.37E-07	8.54E-05
ENSG00000147535.15_PLPP5	ENSG00000160789.18_LMNA	0.06006	-0.100098598	0.217183305	4.62E-01	7.25E-01	1.00E+00
ENSG00000163032.10_VSNL1	ENSG00000160789.18_LMNA	-0.04135	-0.199241861	0.118628835	6.13E-01	8.24E-01	1.00E+00
ENSG00000186132.13_C2orf76	ENSG00000160789.18_LMNA	-0.34839	-0.480916981	-0.20029844	1.09E-05	3.13E-04	5.60E-01
ENSG00000071994.9_PDCD2	ENSG00000160789.18_LMNA	-0.10969	-0.264279317	0.050386811	1.79E-01	4.41E-01	1.00E+00
ENSG00000014919.11_COX15	ENSG00000160789.18_LMNA	-0.35053	-0.482792268	-0.20264167	9.54E-06	2.81E-04	4.90E-01
ENSG00000065882.14_TBC1D1	ENSG00000160789.18_LMNA	0.3	0.147861157	0.438268869	1.73E-04	2.92E-03	1.00E+00
ENSG00000011422.10_PLAUR	ENSG00000160789.18_LMNA	0.3917	0.247960709	0.518562681	6.03E-07	3.05E-05	3.09E-02
ENSG00000018280.15_SLC11A1	ENSG00000160789.18_LMNA	0.12178	-0.038162067	0.275635389	1.35E-01	3.74E-01	1.00E+00
ENSG00000037757.12_MRI1	ENSG00000160789.18_LMNA	0.05009	-0.109986276	0.207636406	5.40E-01	7.80E-01	1.00E+00
ENSG00000143751.9_SDE2	ENSG00000160789.18_LMNA	0.04182	-0.118168037	0.199690616	6.09E-01	8.21E-01	1.00E+00
ENSG00000182518.12_FAM104B	ENSG00000160789.18_LMNA	-0.39823	-0.524193309	-0.25520393	3.76E-07	2.10E-05	1.93E-02
ENSG00000117676.12_RPS6KA1	ENSG00000160789.18_LMNA	-0.00463	-0.163714287	0.154680452	9.55E-01	9.82E-01	1.00E+00
ENSG00000139505.11_MTMR6	ENSG00000160789.18_LMNA	-0.31848	-0.454633859	-0.16779124	6.37E-05	1.30E-03	1.00E+00
ENSG00000145982.10_FARS2	ENSG00000160789.18_LMNA	0.06558	-0.094607112	0.222459437	4.22E-01	6.93E-01	1.00E+00
ENSG00000151332.17_MBIP	ENSG00000160789.18_LMNA	-0.14348	-0.295923391	0.016085828	7.78E-02	2.67E-01	1.00E+00
ENSG00000162618.11_ADGRL4	ENSG00000160789.18_LMNA	-0.10405	-0.258956947	0.056084042	2.02E-01	4.73E-01	1.00E+00
ENSG00000109814.10_UGDH	ENSG00000160789.18_LMNA	0.06223	-0.097940946	0.219258556	4.46E-01	7.13E-01	1.00E+00
ENSG00000135503.11_ACVR1B	ENSG00000160789.18_LMNA	-0.17199	-0.322366371	-0.0131519	3.41E-02	1.56E-01	1.00E+00
ENSG00000221838.8_AP4M1	ENSG00000160789.18_LMNA	0.22456	0.067778849	0.370511567	5.41E-03	4.16E-02	1.00E+00
ENSG00000154767.13_XPC	ENSG00000160789.18_LMNA	0.19453	0.036455116	0.343102085	1.63E-02	9.33E-02	1.00E+00
ENSG00000143252.13_SDHC	ENSG00000160789.18_LMNA	-0.32041	-0.456330289	-0.16987074	5.72E-05	1.19E-03	1.00E+00
ENSG00000143319.15_ISG20L2	ENSG00000160789.18_LMNA	0.22543	0.068689808	0.37130088	5.23E-03	4.06E-02	1.00E+00
ENSG00000008382.14_MPND	ENSG00000160789.18_LMNA	-0.06997	-0.226646116	0.090235774	3.92E-01	6.70E-01	1.00E+00
ENSG00000091879.12_ANGPT2	ENSG00000160789.18_LMNA	0.18653	0.028166499	0.335760781	2.14E-02	1.13E-01	1.00E+00
ENSG00000167962.11_ZNF598	ENSG00000160789.18_LMNA	0.2938	0.14119962	0.432755677	2.39E-04	3.76E-03	1.00E+00
ENSG00000095739.10_BAMBI	ENSG00000160789.18_LMNA	-0.11088	-0.265400173	0.049184396	1.74E-01	4.34E-01	1.00E+00
ENSG00000102302.7_FGD1	ENSG00000160789.18_LMNA	0.12107	-0.038877831	0.274972891	1.37E-01	3.77E-01	1.00E+00
ENSG00000100865.13_CINP	ENSG00000160789.18_LMNA	-0.053	-0.210425088	0.107104537	5.17E-01	7.63E-01	1.00E+00
ENSG00000102910.12_LONP2	ENSG00000160789.18_LMNA	0.05832	-0.101830321	0.215515638	4.75E-01	7.34E-01	1.00E+00
ENSG00000180581.7_SRP9P1	ENSG00000160789.18_LMNA	-0.18044	-0.330160175	-0.02187151	2.61E-02	1.30E-01	1.00E+00

ENSG00000130513.6_GDF15	ENSG00000160789.18_LMNA	0.55432	0.433361357	0.655658958	1.28E-13	1.07E-10	6.54E-09
ENSG00000151322.17_NPAS3	ENSG00000160789.18_LMNA	0.09917	-0.060994235	0.254354428	2.24E-01	5.00E-01	1.00E+00
ENSG00000157184.5_CPT2	ENSG00000160789.18_LMNA	0.10169	-0.058460913	0.256730796	2.13E-01	4.86E-01	1.00E+00
ENSG00000110080.17_ST3GAL4	ENSG00000160789.18_LMNA	0.19143	0.033243989	0.340262399	1.82E-02	1.01E-01	1.00E+00
ENSG00000152332.14_UHMK1	ENSG00000160789.18_LMNA	0.12706	-0.032801298	0.280587756	1.19E-01	3.46E-01	1.00E+00
ENSG00000131788.14_PIAS3	ENSG00000160789.18_LMNA	0.23675	0.080589174	0.381571223	3.32E-03	2.88E-02	1.00E+00
ENSG00000122584.11_NXPH1	ENSG00000160789.18_LMNA	-0.35554	-0.487161794	-0.20811401	6.96E-06	2.19E-04	3.57E-01
ENSG00000143845.13_ETNK2	ENSG00000160789.18_LMNA	0.02739	-0.132385492	0.18578305	7.38E-01	8.90E-01	1.00E+00
ENSG00000204564.10_C6orf136	ENSG00000160789.18_LMNA	-0.19853	-0.346774027	-0.04061677	1.42E-02	8.46E-02	1.00E+00
ENSG00000115364.12_MRPL19	ENSG00000160789.18_LMNA	-0.03648	-0.194555252	0.123433047	6.55E-01	8.47E-01	1.00E+00
ENSG00000078674.16_PCM1	ENSG00000160789.18_LMNA	0.12883	-0.031007012	0.282241628	1.14E-01	3.37E-01	1.00E+00
ENSG00000161904.10_LEMD2	ENSG00000160789.18_LMNA	0.30654	0.154896934	0.444068157	1.23E-04	2.23E-03	1.00E+00
ENSG00000118689.13_FOXO3	ENSG00000160789.18_LMNA	0.03074	-0.129094578	0.189013613	7.07E-01	8.75E-01	1.00E+00
ENSG00000164631.17_ZNF12	ENSG00000160789.18_LMNA	0.22043	0.063455092	0.366759221	6.36E-03	4.70E-02	1.00E+00
ENSG00000115271.9_GCA	ENSG00000160789.18_LMNA	-0.08319	-0.239226803	0.077026314	3.08E-01	5.95E-01	1.00E+00
ENSG00000111335.11_OAS2	ENSG00000160789.18_LMNA	0.04075	-0.119225081	0.198661	6.18E-01	8.27E-01	1.00E+00
ENSG00000116663.9_FBXO6	ENSG00000160789.18_LMNA	0.09755	-0.062623188	0.252824384	2.32E-01	5.10E-01	1.00E+00
ENSG00000132122.10_SPATA6	ENSG00000160789.18_LMNA	0.13826	-0.021417022	0.291049685	8.94E-02	2.91E-01	1.00E+00
ENSG00000165792.16_METTL17	ENSG00000160789.18_LMNA	-0.06359	-0.220560122	0.096586176	4.36E-01	7.04E-01	1.00E+00
ENSG00000173334.3_TRIB1	ENSG00000160789.18_LMNA	0.33701	0.187890932	0.47094427	2.18E-05	5.54E-04	1.00E+00
ENSG00000167363.12_FN3K	ENSG00000160789.18_LMNA	-0.10906	-0.263686237	0.051022677	1.81E-01	4.45E-01	1.00E+00
ENSG00000198841.3_KTI12	ENSG00000160789.18_LMNA	0.23064	0.074158921	0.376030485	4.25E-03	3.47E-02	1.00E+00
ENSG00000127922.8_SHFM1	ENSG00000160789.18_LMNA	0.07748	-0.082739466	0.233798599	3.43E-01	6.27E-01	1.00E+00
ENSG00000230330.1_HMGN2P3	ENSG00000160789.18_LMNA	-0.06241	-0.219434447	0.097757933	4.45E-01	7.12E-01	1.00E+00
ENSG00000174996.10_KLC2	ENSG00000160789.18_LMNA	0.02678	-0.132987904	0.18519094	7.43E-01	8.92E-01	1.00E+00
ENSG00000184207.8_PGP	ENSG00000160789.18_LMNA	-0.0978	-0.253061817	0.062370517	2.31E-01	5.08E-01	1.00E+00
ENSG00000140682.17_TGFB1I1	ENSG00000160789.18_LMNA	0.53484	0.410598428	0.639580657	1.27E-12	6.57E-10	6.51E-08
ENSG00000064932.14_SBNO2	ENSG00000160789.18_LMNA	0.54076	0.417504798	0.644480146	6.40E-13	4.01E-10	3.29E-08
ENSG00000184220.9_CMSS1	ENSG00000160789.18_LMNA	-0.29596	-0.434676843	-0.14351795	2.14E-04	3.43E-03	1.00E+00
ENSG00000139163.14_ETNK1	ENSG00000160789.18_LMNA	-0.43858	-0.558766649	-0.3003508	1.59E-08	1.59E-06	8.16E-04
ENSG00000186998.14_EMID1	ENSG00000160789.18_LMNA	0.37119	0.225305227	0.50079828	2.50E-06	9.47E-05	1.28E-01
ENSG00000155256.16_ZFYVE27	ENSG00000160789.18_LMNA	-0.15497	-0.30660859	0.004335209	5.66E-02	2.17E-01	1.00E+00
ENSG00000141873.9_SLC39A3	ENSG00000160789.18_LMNA	0.1154	-0.044623848	0.269643665	1.57E-01	4.09E-01	1.00E+00

ENSG00000256673.1_RP11-599J1	ENSG00000160789.18_LMNA	-0.23739	-0.382147482	-0.08125939	3.23E-03	2.82E-02	1.00E+00
ENSG00000110900.13_TSPAN11	ENSG00000160789.18_LMNA	0.00621	-0.153137538	0.16525188	9.39E-01	9.76E-01	1.00E+00
ENSG00000176435.6_CLEC14A	ENSG00000160789.18_LMNA	0.05968	-0.10047521	0.216820781	4.65E-01	7.27E-01	1.00E+00
ENSG00000143061.16_IGSF3	ENSG00000160789.18_LMNA	0.04729	-0.11275504	0.204952181	5.63E-01	7.94E-01	1.00E+00
ENSG00000125703.13_ATG4C	ENSG00000160789.18_LMNA	-0.36712	-0.497255606	-0.2208225	3.28E-06	1.18E-04	1.68E-01
ENSG00000111581.8_NUP107	ENSG00000160789.18_LMNA	-0.02701	-0.185415458	0.132759507	7.41E-01	8.91E-01	1.00E+00
ENSG00000198625.11_MDM4	ENSG00000160789.18_LMNA	-0.1811	-0.330762136	-0.02254694	2.56E-02	1.28E-01	1.00E+00
ENSG00000197380.9_DACT3	ENSG00000160789.18_LMNA	-0.32615	-0.461394777	-0.17609403	4.12E-05	9.21E-04	1.00E+00
ENSG00000181291.5_TMEM132E	ENSG00000160789.18_LMNA	-0.00758	-0.166576631	0.151806957	9.26E-01	9.72E-01	1.00E+00
ENSG00000116667.11_C1orf21	ENSG00000160789.18_LMNA	0.0612	-0.098961282	0.218277544	4.54E-01	7.19E-01	1.00E+00
ENSG00000121361.3_KCNJ8	ENSG00000160789.18_LMNA	-0.01871	-0.177377774	0.140915048	8.19E-01	9.30E-01	1.00E+00
ENSG00000119718.9_EIF2B2	ENSG00000160789.18_LMNA	0.09754	-0.062629638	0.252818323	2.32E-01	5.10E-01	1.00E+00
ENSG00000163636.9_PSMD6	ENSG00000160789.18_LMNA	-0.2012	-0.349218423	-0.04339307	1.29E-02	7.89E-02	1.00E+00
ENSG00000186193.8_SAPCD2	ENSG00000160789.18_LMNA	-0.12477	-0.278435745	0.035132994	1.26E-01	3.58E-01	1.00E+00
ENSG00000166471.9_TMEM41B	ENSG00000160789.18_LMNA	0.01696	-0.142624525	0.1756876	8.36E-01	9.36E-01	1.00E+00
ENSG00000163608.13_NEPRO	ENSG00000160789.18_LMNA	-0.28722	-0.426903819	-0.13415764	3.33E-04	4.87E-03	1.00E+00
ENSG00000186350.9_RXRA	ENSG00000160789.18_LMNA	0.2008	0.042976504	0.348851924	1.31E-02	7.96E-02	1.00E+00
ENSG00000256537.3_SMIM10L1	ENSG00000160789.18_LMNA	-0.20428	-0.352029888	-0.04659212	1.16E-02	7.28E-02	1.00E+00
ENSG00000166938.11_DIS3L	ENSG00000160789.18_LMNA	0.02513	-0.134607588	0.183597807	7.59E-01	8.99E-01	1.00E+00
ENSG00000152700.12_SAR1B	ENSG00000160789.18_LMNA	-0.08237	-0.238443434	0.07785209	3.13E-01	6.00E-01	1.00E+00
ENSG00000197712.10_FAM114A1	ENSG00000160789.18_LMNA	0.36604	0.219638956	0.496318717	3.53E-06	1.25E-04	1.81E-01
ENSG00000100372.13_SLC25A17	ENSG00000160789.18_LMNA	-0.2577	-0.400469031	-0.10271093	1.35E-03	1.45E-02	1.00E+00
ENSG00000104859.13_CLASRP	ENSG00000160789.18_LMNA	0.07867	-0.081554382	0.2349262	3.35E-01	6.21E-01	1.00E+00
ENSG00000052723.10_SIKE1	ENSG00000160789.18_LMNA	-0.0628	-0.219806458	0.097370791	4.42E-01	7.10E-01	1.00E+00
ENSG00000213753.9_CENPBD1P	ENSG00000160789.18_LMNA	0.15764	-0.00160409	0.309080907	5.24E-02	2.07E-01	1.00E+00
ENSG00000109089.7_CDR2L	ENSG00000160789.18_LMNA	0.20004	0.04218428	0.348154656	1.35E-02	8.12E-02	1.00E+00
ENSG00000254726.2_MEX3A	ENSG00000160789.18_LMNA	-0.15855	-0.309929155	0.000665978	5.11E-02	2.04E-01	1.00E+00
ENSG00000143554.12_SLC27A3	ENSG00000160789.18_LMNA	0.55648	0.435897184	0.657437712	9.79E-14	8.66E-11	5.02E-09
ENSG00000039123.14_SKIV2L2	ENSG00000160789.18_LMNA	-0.10371	-0.258642495	0.056420001	2.04E-01	4.74E-01	1.00E+00
ENSG00000173226.15_IQCB1	ENSG00000160789.18_LMNA	-0.0931	-0.248618546	0.067092268	2.54E-01	5.36E-01	1.00E+00
ENSG00000186184.14_POLR1D	ENSG00000160789.18_LMNA	0.07025	-0.089952154	0.226917351	3.90E-01	6.68E-01	1.00E+00
ENSG00000136878.11_USP20	ENSG00000160789.18_LMNA	0.02427	-0.135453807	0.182764795	7.67E-01	9.03E-01	1.00E+00
ENSG00000127824.12_TUBA4A	ENSG00000160789.18_LMNA	0.1762	0.017492219	0.3262511	2.99E-02	1.43E-01	1.00E+00

ENSG00000103111.13_MON1B	ENSG00000160789.18_LMNA	0.10557	-0.054542773	0.260398695	1.95E-01	4.64E-01	1.00E+00
ENSG00000169718.16_DUS1L	ENSG00000160789.18_LMNA	0.18223	0.023715646	0.331803138	2.46E-02	1.25E-01	1.00E+00
ENSG00000155363.17_MOV10	ENSG00000160789.18_LMNA	0.32905	0.17923382	0.46394288	3.49E-05	8.09E-04	1.00E+00
ENSG00000137074.17_APTX	ENSG00000160789.18_LMNA	-0.11497	-0.269246736	0.04505099	1.58E-01	4.11E-01	1.00E+00
ENSG00000144591.16_GMPPA	ENSG00000160789.18_LMNA	0.49485	0.364358615	0.606291057	9.15E-11	2.10E-08	4.69E-06
ENSG00000158062.19_UBXN11	ENSG00000160789.18_LMNA	0.21499	0.057762739	0.361804053	7.82E-03	5.46E-02	1.00E+00
ENSG00000141526.13_SLC16A3	ENSG00000160789.18_LMNA	0.36416	0.217569143	0.494678711	3.99E-06	1.38E-04	2.05E-01
ENSG00000174177.11_CTU2	ENSG00000160789.18_LMNA	-0.03323	-0.191415961	0.126642799	6.84E-01	8.63E-01	1.00E+00
ENSG00000198824.5_CHAMP1	ENSG00000160789.18_LMNA	-0.01587	-0.174630252	0.143692984	8.46E-01	9.40E-01	1.00E+00
ENSG00000110514.17_MADD	ENSG00000160789.18_LMNA	0.05027	-0.109813616	0.207803637	5.39E-01	7.79E-01	1.00E+00
ENSG00000119537.14_KDSR	ENSG00000160789.18_LMNA	0.07974	-0.080482981	0.235944901	3.29E-01	6.15E-01	1.00E+00
ENSG00000119616.10_FCF1	ENSG00000160789.18_LMNA	0.10587	-0.054246832	0.260675367	1.94E-01	4.63E-01	1.00E+00
ENSG00000198722.11_UNC13B	ENSG00000160789.18_LMNA	0.1378	-0.021884776	0.290621295	9.05E-02	2.93E-01	1.00E+00
ENSG00000169508.6_GPR183	ENSG00000160789.18_LMNA	0.02688	-0.132890876	0.185286324	7.42E-01	8.92E-01	1.00E+00
ENSG00000136021.16_SCYL2	ENSG00000160789.18_LMNA	0.00709	-0.152279538	0.166106253	9.31E-01	9.73E-01	1.00E+00
ENSG00000234147.1_RP3-460G2	ENSG00000160789.18_LMNA	0.01299	-0.146508838	0.17184016	8.74E-01	9.51E-01	1.00E+00
ENSG00000144524.16_COPS7B	ENSG00000160789.18_LMNA	0.05374	-0.106371102	0.211134014	5.11E-01	7.60E-01	1.00E+00
ENSG00000073008.13_PVR	ENSG00000160789.18_LMNA	0.53888	0.415312691	0.642927046	7.96E-13	4.78E-10	4.09E-08
ENSG00000130830.13_MPP1	ENSG00000160789.18_LMNA	-0.07072	-0.227362138	0.089486946	3.87E-01	6.65E-01	1.00E+00
ENSG00000130803.13_ZNF317	ENSG00000160789.18_LMNA	0.09257	-0.067630772	0.248110956	2.57E-01	5.39E-01	1.00E+00
ENSG00000106992.16_AK1	ENSG00000160789.18_LMNA	-0.1108	-0.265321809	0.049268491	1.74E-01	4.34E-01	1.00E+00
ENSG00000163428.3_LRRC58	ENSG00000160789.18_LMNA	-0.01645	-0.175188428	0.143129035	8.41E-01	9.38E-01	1.00E+00
ENSG00000198836.7_OPA1	ENSG00000160789.18_LMNA	-0.02865	-0.186993037	0.131153722	7.26E-01	8.84E-01	1.00E+00
ENSG00000237457.5_LINC01351	ENSG00000160789.18_LMNA	-0.28544	-0.425316427	-0.1322525	3.64E-04	5.24E-03	1.00E+00
ENSG00000257923.8_CUX1	ENSG00000160789.18_LMNA	0.17331	0.014509736	0.323582798	3.27E-02	1.51E-01	1.00E+00
ENSG00000196116.7_TDRD7	ENSG00000160789.18_LMNA	-0.14051	-0.29315376	0.019117618	8.42E-02	2.81E-01	1.00E+00
ENSG00000213866.3_YBX1P10	ENSG00000160789.18_LMNA	0.0384	-0.121548044	0.196395845	6.39E-01	8.38E-01	1.00E+00
ENSG00000253341.1_RP11-730G	ENSG00000160789.18_LMNA	-0.10866	-0.263308834	0.051427175	1.83E-01	4.47E-01	1.00E+00
ENSG00000129484.12_PARP2	ENSG00000160789.18_LMNA	-0.04948	-0.207047568	0.110594083	5.45E-01	7.83E-01	1.00E+00
ENSG00000138166.5_DUSP5	ENSG00000160789.18_LMNA	0.49903	0.369152427	0.609782134	6.01E-11	1.50E-08	3.08E-06
ENSG00000100852.11_ARHGAP5	ENSG00000160789.18_LMNA	-0.02816	-0.186528293	0.131626949	7.31E-01	8.86E-01	1.00E+00
ENSG00000176410.7_DNAJC30	ENSG00000160789.18_LMNA	0.17308	0.014272254	0.323370122	3.30E-02	1.52E-01	1.00E+00
ENSG00000180104.14_EXOC3	ENSG00000160789.18_LMNA	0.07024	-0.089965409	0.226904676	3.90E-01	6.68E-01	1.00E+00

ENSG00000127995.15_CASD1	ENSG00000160789.18_LMNA	-0.18643	-0.335671503	-0.02806596	2.15E-02	1.13E-01	1.00E+00
ENSG00000106546.11_AHR	ENSG00000160789.18_LMNA	0.12146	-0.038488947	0.275332872	1.36E-01	3.75E-01	1.00E+00
ENSG00000221829.8_FANCG	ENSG00000160789.18_LMNA	-0.00688	-0.165901679	0.152485024	9.33E-01	9.74E-01	1.00E+00
ENSG00000168924.13_LETM1	ENSG00000160789.18_LMNA	0.25285	0.097581503	0.396109594	1.67E-03	1.71E-02	1.00E+00
ENSG00000228205.1_RP11-778D	ENSG00000160789.18_LMNA	-0.16643	-0.317221389	-0.00742141	4.04E-02	1.75E-01	1.00E+00
ENSG00000232346.1_SC22CB-1E	ENSG00000160789.18_LMNA	0.00875	-0.15066207	0.167715582	9.15E-01	9.68E-01	1.00E+00
ENSG00000131943.16_C19orf12	ENSG00000160789.18_LMNA	-0.07675	-0.233105864	0.083467071	3.47E-01	6.31E-01	1.00E+00
ENSG00000116641.14_DOCK7	ENSG00000160789.18_LMNA	0.20079	0.042961016	0.348838296	1.31E-02	7.97E-02	1.00E+00
ENSG00000135249.6_RINT1	ENSG00000160789.18_LMNA	0.05772	-0.102422363	0.214945071	4.80E-01	7.38E-01	1.00E+00
ENSG00000207554.1_MIR647	ENSG00000160789.18_LMNA	0.03841	-0.121531387	0.196412099	6.38E-01	8.38E-01	1.00E+00
ENSG00000214199.3_EEF1A1P12	ENSG00000160789.18_LMNA	-0.1551	-0.306726771	0.004204762	5.64E-02	2.17E-01	1.00E+00
ENSG00000007047.13_MARK4	ENSG00000160789.18_LMNA	0.13586	-0.023859334	0.288811526	9.51E-02	3.02E-01	1.00E+00
ENSG00000169122.10_FAM110B	ENSG00000160789.18_LMNA	-0.30812	-0.445468135	-0.15659981	1.13E-04	2.08E-03	1.00E+00
ENSG00000110619.15_CARS	ENSG00000160789.18_LMNA	0.32123	0.170758922	0.457054227	5.46E-05	1.15E-03	1.00E+00
ENSG00000129194.6_SOX15	ENSG00000160789.18_LMNA	0.036	-0.123910447	0.194088745	6.60E-01	8.50E-01	1.00E+00
ENSG00000173221.12_GLRX	ENSG00000160789.18_LMNA	-0.02038	-0.178998502	0.139274025	8.03E-01	9.21E-01	1.00E+00
ENSG00000138138.12_ATAD1	ENSG00000160789.18_LMNA	0.00841	-0.150992832	0.167386623	9.18E-01	9.69E-01	1.00E+00
ENSG00000095261.12_PSMD5	ENSG00000160789.18_LMNA	0.08189	-0.078331257	0.237988685	3.16E-01	6.03E-01	1.00E+00
ENSG00000167680.14_SEMA6B	ENSG00000160789.18_LMNA	-0.07585	-0.232247656	0.084368011	3.53E-01	6.37E-01	1.00E+00
ENSG00000116199.10_FAM20B	ENSG00000160789.18_LMNA	0.002	-0.157252837	0.161147328	9.81E-01	9.93E-01	1.00E+00
ENSG00000174943.8_KCTD13	ENSG00000160789.18_LMNA	-0.10676	-0.261511334	0.053352304	1.91E-01	4.58E-01	1.00E+00
ENSG00000157833.11_GAREM2	ENSG00000160789.18_LMNA	-0.09666	-0.251982413	0.063518872	2.36E-01	5.14E-01	1.00E+00
ENSG00000198720.11_ANKRD13	ENSG00000160789.18_LMNA	-0.26726	-0.409059619	-0.11286539	8.73E-04	1.04E-02	1.00E+00
ENSG00000125351.9_UPF3B	ENSG00000160789.18_LMNA	-0.00623	-0.16526342	0.153125952	9.39E-01	9.76E-01	1.00E+00
ENSG00000179151.10_EDC3	ENSG00000160789.18_LMNA	0.01127	-0.148197191	0.170164801	8.90E-01	9.57E-01	1.00E+00
ENSG00000186716.18_BCR	ENSG00000160789.18_LMNA	-0.04186	-0.199729619	0.118127981	6.09E-01	8.21E-01	1.00E+00
ENSG00000101040.18_ZMYND8	ENSG00000160789.18_LMNA	0.09955	-0.060615092	0.254710323	2.22E-01	4.98E-01	1.00E+00
ENSG00000130640.12_TUBGCP2	ENSG00000160789.18_LMNA	0.15618	-0.003099972	0.307727295	5.47E-02	2.13E-01	1.00E+00
ENSG00000176102.10_CSTF3	ENSG00000160789.18_LMNA	-0.19778	-0.346086129	-0.03983632	1.46E-02	8.62E-02	1.00E+00
ENSG00000163482.10_STK36	ENSG00000160789.18_LMNA	0.17386	0.015078865	0.324092354	3.22E-02	1.50E-01	1.00E+00
ENSG00000099330.7_OCEL1	ENSG00000160789.18_LMNA	0.15326	-0.006085558	0.305021896	5.94E-02	2.24E-01	1.00E+00
ENSG00000150907.6_FOXO1	ENSG00000160789.18_LMNA	-0.08737	-0.243187051	0.072845033	2.84E-01	5.70E-01	1.00E+00
ENSG00000105879.10_CBLL1	ENSG00000160789.18_LMNA	0.07876	-0.081463948	0.235012213	3.35E-01	6.21E-01	1.00E+00

ENSG00000137814.8_HAUS2	ENSG00000160789.18_LMNA	0.1353	-0.024424295	0.288293303	9.65E-02	3.05E-01	1.00E+00
ENSG00000198746.11_GPATCH3	ENSG00000160789.18_LMNA	0.0076	-0.151786437	0.166597052	9.26E-01	9.72E-01	1.00E+00
ENSG00000248008.2_NRAV	ENSG00000160789.18_LMNA	0.03937	-0.120588511	0.197331911	6.30E-01	8.34E-01	1.00E+00
ENSG00000100124.11_ANKRD54	ENSG00000160789.18_LMNA	-0.09323	-0.248742216	0.066961037	2.53E-01	5.35E-01	1.00E+00
ENSG00000214485.6_RPL7P1	ENSG00000160789.18_LMNA	-0.05072	-0.208242822	0.109360079	5.35E-01	7.76E-01	1.00E+00
ENSG00000162337.10_LRP5	ENSG00000160789.18_LMNA	0.1018	-0.058343801	0.256840561	2.12E-01	4.86E-01	1.00E+00
ENSG00000211893.3_IGHG2	ENSG00000160789.18_LMNA	0.04079	-0.11918185	0.198703123	6.18E-01	8.27E-01	1.00E+00
ENSG00000169964.5_TMEM42	ENSG00000160789.18_LMNA	-0.16326	-0.314289483	-0.00416493	4.45E-02	1.86E-01	1.00E+00
ENSG00000115884.9_SDC1	ENSG00000160789.18_LMNA	0.49709	0.366924746	0.608160994	7.31E-11	1.76E-08	3.75E-06
ENSG00000164197.10_RNF180	ENSG00000160789.18_LMNA	0.05833	-0.101815339	0.215530074	4.75E-01	7.34E-01	1.00E+00
ENSG00000175264.6_CHST1	ENSG00000160789.18_LMNA	0.01218	-0.147309935	0.171045458	8.82E-01	9.54E-01	1.00E+00
ENSG00000163104.16_SMARCAD	ENSG00000160789.18_LMNA	-0.27793	-0.418609819	-0.12422725	5.27E-04	7.02E-03	1.00E+00
ENSG00000099917.16_MED15	ENSG00000160789.18_LMNA	0.35744	0.210202102	0.488825413	6.16E-06	1.97E-04	3.16E-01
ENSG00000115504.13_EHBP1	ENSG00000160789.18_LMNA	0.23021	0.073706399	0.375639747	4.33E-03	3.52E-02	1.00E+00
ENSG00000102931.6_ARL2BP	ENSG00000160789.18_LMNA	-0.05177	-0.209247773	0.108321789	5.26E-01	7.71E-01	1.00E+00
ENSG00000160446.17_ZDHHC12	ENSG00000160789.18_LMNA	0.39302	0.249424332	0.519702346	5.49E-07	2.82E-05	2.81E-02
ENSG00000141452.8_C18orf8	ENSG00000160789.18_LMNA	0.06871	-0.091496231	0.225440105	4.00E-01	6.77E-01	1.00E+00
ENSG00000113580.13_NR3C1	ENSG00000160789.18_LMNA	-0.02413	-0.182625591	0.135595173	7.68E-01	9.04E-01	1.00E+00
ENSG00000172315.5_TP53RK	ENSG00000160789.18_LMNA	0.00023	-0.158977875	0.159423513	9.98E-01	9.99E-01	1.00E+00
ENSG00000118046.13_STK11	ENSG00000160789.18_LMNA	0.32129	0.170826672	0.457109432	5.44E-05	1.15E-03	1.00E+00
ENSG00000115392.10_FANCL	ENSG00000160789.18_LMNA	-0.33765	-0.471505751	-0.1885871	2.10E-05	5.38E-04	1.00E+00
ENSG00000087266.14_SH3BP2	ENSG00000160789.18_LMNA	0.3023	0.150334486	0.440310287	1.54E-04	2.65E-03	1.00E+00
ENSG00000137955.14_RABGGTB	ENSG00000160789.18_LMNA	-0.16012	-0.31138169	-0.00094171	4.88E-02	1.98E-01	1.00E+00
ENSG00000166199.11_ALKBH3	ENSG00000160789.18_LMNA	0.0103	-0.149149582	0.169218927	9.00E-01	9.61E-01	1.00E+00
ENSG00000102996.4_MMP15	ENSG00000160789.18_LMNA	-0.02899	-0.187326052	0.130814539	7.23E-01	8.83E-01	1.00E+00
ENSG00000126091.18_ST3GAL3	ENSG00000160789.18_LMNA	0.00615	-0.153202591	0.165187082	9.40E-01	9.76E-01	1.00E+00
ENSG00000154122.11_ANKH	ENSG00000160789.18_LMNA	0.1041	-0.056029057	0.259008405	2.02E-01	4.73E-01	1.00E+00
ENSG00000135596.16_MICAL1	ENSG00000160789.18_LMNA	-0.15362	-0.305351087	0.00572257	5.88E-02	2.23E-01	1.00E+00
ENSG00000140718.17_FTO	ENSG00000160789.18_LMNA	-0.13822	-0.291015683	0.021454153	8.95E-02	2.91E-01	1.00E+00
ENSG00000117305.13_HMGCL	ENSG00000160789.18_LMNA	0.2364	0.080218989	0.381252832	3.37E-03	2.92E-02	1.00E+00
ENSG00000109917.9_ZPR1	ENSG00000160789.18_LMNA	0.1581	-0.001131405	0.309508374	5.17E-02	2.06E-01	1.00E+00
ENSG00000123130.15_ACOT9	ENSG00000160789.18_LMNA	0.25038	0.094963848	0.39387968	1.86E-03	1.85E-02	1.00E+00
ENSG00000130304.15_SLC27A1	ENSG00000160789.18_LMNA	0.38814	0.244022806	0.515491613	7.77E-07	3.74E-05	3.99E-02

ENSG00000187954.11_CYHR1	ENSG00000160789.18_LMNA	0.08717	-0.073039594	0.243003009	2.86E-01	5.71E-01	1.00E+00
ENSG00000123395.13_ATG101	ENSG00000160789.18_LMNA	0.07463	-0.085585143	0.23108747	3.61E-01	6.43E-01	1.00E+00
ENSG00000133789.13_SWAP70	ENSG00000160789.18_LMNA	0.47087	0.336930846	0.586135952	9.24E-10	1.47E-07	4.74E-05
ENSG00000163888.3_CAMK2N2	ENSG00000160789.18_LMNA	-0.08541	-0.241324641	0.074812775	2.95E-01	5.81E-01	1.00E+00
ENSG00000174652.16_ZNF266	ENSG00000160789.18_LMNA	-0.14224	-0.294764677	0.017354918	8.05E-02	2.73E-01	1.00E+00
ENSG00000163629.11_PTPN13	ENSG00000160789.18_LMNA	0.13317	-0.026590553	0.286304556	1.02E-01	3.15E-01	1.00E+00
ENSG00000082684.13_SEMA5B	ENSG00000160789.18_LMNA	-0.11253	-0.266952808	0.047517275	1.67E-01	4.25E-01	1.00E+00
ENSG00000099250.16_NRP1	ENSG00000160789.18_LMNA	0.44976	0.312968144	0.568271011	6.14E-09	7.14E-07	3.15E-04
ENSG00000166484.18_MAPK7	ENSG00000160789.18_LMNA	0.13532	-0.024403601	0.288312288	9.65E-02	3.05E-01	1.00E+00
ENSG00000120899.16_PTK2B	ENSG00000160789.18_LMNA	0.23596	0.079751134	0.380850336	3.43E-03	2.95E-02	1.00E+00
ENSG00000123353.8_ORMDL2	ENSG00000160789.18_LMNA	-0.08614	-0.242019516	0.074078888	2.91E-01	5.77E-01	1.00E+00
ENSG00000152284.4_TCF7L1	ENSG00000160789.18_LMNA	-0.18697	-0.336164382	-0.02862108	2.11E-02	1.12E-01	1.00E+00
ENSG00000153046.16_CDYL	ENSG00000160789.18_LMNA	-0.07213	-0.228700758	0.088086033	3.77E-01	6.57E-01	1.00E+00
ENSG00000131368.6_MRPS25	ENSG00000160789.18_LMNA	-0.10961	-0.264204724	0.0504668	1.79E-01	4.41E-01	1.00E+00
ENSG00000115935.15_WIPF1	ENSG00000160789.18_LMNA	0.20242	0.044659613	0.350332174	1.24E-02	7.65E-02	1.00E+00
ENSG00000106608.15_URGCP	ENSG00000160789.18_LMNA	0.25817	0.10321396	0.400895829	1.32E-03	1.43E-02	1.00E+00
ENSG00000103365.14_GGA2	ENSG00000160789.18_LMNA	0.02166	-0.138016239	0.180239564	7.91E-01	9.15E-01	1.00E+00
ENSG00000112146.15_FBXO9	ENSG00000160789.18_LMNA	-0.25318	-0.396405449	-0.09792911	1.65E-03	1.69E-02	1.00E+00
ENSG00000166822.11_TMEM170	ENSG00000160789.18_LMNA	-0.02808	-0.186444973	0.131711775	7.31E-01	8.87E-01	1.00E+00
ENSG00000242732.4_RGAG4	ENSG00000160789.18_LMNA	0.3044	0.15259695	0.442175041	1.37E-04	2.43E-03	1.00E+00
ENSG00000146232.13_NFKBIE	ENSG00000160789.18_LMNA	0.28186	0.128420523	0.422118099	4.35E-04	6.05E-03	1.00E+00
ENSG00000163257.9_DCAF16	ENSG00000160789.18_LMNA	-0.25816	-0.400881624	-0.10319721	1.32E-03	1.43E-02	1.00E+00
ENSG00000161542.15_PRPSAP1	ENSG00000160789.18_LMNA	-0.36489	-0.495319233	-0.21837723	3.80E-06	1.33E-04	1.95E-01
ENSG00000184584.11_TMEM173	ENSG00000160789.18_LMNA	0.11981	-0.040152576	0.273792273	1.41E-01	3.84E-01	1.00E+00
ENSG00000178999.11_AURKB	ENSG00000160789.18_LMNA	-0.02173	-0.180310436	0.137944381	7.90E-01	9.15E-01	1.00E+00
ENSG00000034693.13_PEX3	ENSG00000160789.18_LMNA	-0.22983	-0.375295277	-0.07330756	4.39E-03	3.55E-02	1.00E+00
ENSG00000135317.11_SNX14	ENSG00000160789.18_LMNA	-0.20639	-0.353956972	-0.04878851	1.07E-02	6.89E-02	1.00E+00
ENSG00000122386.9_ZNF205	ENSG00000160789.18_LMNA	0.17676	0.018066557	0.326764372	2.94E-02	1.41E-01	1.00E+00
ENSG00000148985.18_PGAP2	ENSG00000160789.18_LMNA	0.10937	-0.050715561	0.263972718	1.80E-01	4.43E-01	1.00E+00
ENSG00000188566.10_NDOR1	ENSG00000160789.18_LMNA	0.24856	0.093037711	0.392236602	2.02E-03	1.96E-02	1.00E+00
ENSG00000134058.9_CDK7	ENSG00000160789.18_LMNA	0.0379	-0.122040727	0.195914985	6.43E-01	8.41E-01	1.00E+00
ENSG00000150938.8_CRIM1	ENSG00000160789.18_LMNA	0.3283	0.178421827	0.463284356	3.64E-05	8.35E-04	1.00E+00
ENSG00000103994.15_ZNF106	ENSG00000160789.18_LMNA	0.11585	-0.044163366	0.270071457	1.55E-01	4.06E-01	1.00E+00

ENSG00000091039.15_OSBPL8	ENSG00000160789.18_LMNA	-0.26264	-0.404911522	-0.10795441	1.08E-03	1.23E-02	1.00E+00
ENSG00000277531.1_LLNLF-187I	ENSG00000160789.18_LMNA	-0.16683	-0.317591088	-0.00783251	4.00E-02	1.73E-01	1.00E+00
ENSG00000143801.15_PSEN2	ENSG00000160789.18_LMNA	0.21117	0.053778457	0.358325501	9.01E-03	6.08E-02	1.00E+00
ENSG00000187049.8_TMEM216	ENSG00000160789.18_LMNA	-0.1282	-0.281654709	0.031643992	1.15E-01	3.40E-01	1.00E+00
ENSG00000148219.15_ASTN2	ENSG00000160789.18_LMNA	-0.18459	-0.333977609	-0.02615959	2.28E-02	1.19E-01	1.00E+00
ENSG00000184634.14_MED12	ENSG00000160789.18_LMNA	0.17658	0.017884058	0.326601297	2.95E-02	1.42E-01	1.00E+00
ENSG00000108039.16_XPNPEP1	ENSG00000160789.18_LMNA	0.03999	-0.119975189	0.19792993	6.25E-01	8.31E-01	1.00E+00
ENSG00000240857.1_RDH14	ENSG00000160789.18_LMNA	0.10579	-0.054324968	0.260602323	1.95E-01	4.63E-01	1.00E+00
ENSG00000158863.20_FAM160B2	ENSG00000160789.18_LMNA	0.16536	0.006321443	0.316231716	4.18E-02	1.79E-01	1.00E+00
ENSG00000175274.17_TP53I11	ENSG00000160789.18_LMNA	0.05448	-0.10563595	0.211844265	5.05E-01	7.56E-01	1.00E+00
ENSG00000084693.14_AGBL5	ENSG00000160789.18_LMNA	0.0205	-0.139150662	0.17912027	8.02E-01	9.21E-01	1.00E+00
ENSG00000118762.6_PKD2	ENSG00000160789.18_LMNA	0.43841	0.300157315	0.558620373	1.61E-08	1.61E-06	8.28E-04
ENSG00000198642.6_KLHL9	ENSG00000160789.18_LMNA	-0.19283	-0.341544053	-0.0346925	1.73E-02	9.73E-02	1.00E+00
ENSG00000064102.13_ASUN	ENSG00000160789.18_LMNA	-0.11384	-0.268178563	0.046199898	1.63E-01	4.18E-01	1.00E+00
ENSG00000134480.12_CCNH	ENSG00000160789.18_LMNA	-0.27987	-0.420345137	-0.12630008	4.79E-04	6.52E-03	1.00E+00
ENSG00000159899.13_NPR2	ENSG00000160789.18_LMNA	0.27347	0.119470976	0.414619847	6.52E-04	8.30E-03	1.00E+00
ENSG00000078237.5_TIGAR	ENSG00000160789.18_LMNA	-0.08854	-0.244300585	0.071667346	2.78E-01	5.63E-01	1.00E+00
ENSG00000084774.12_CAD	ENSG00000160789.18_LMNA	0.30359	0.151726684	0.441458052	1.43E-04	2.51E-03	1.00E+00
ENSG00000067334.12_DNTTIP2	ENSG00000160789.18_LMNA	0.08612	-0.074096668	0.242002685	2.91E-01	5.77E-01	1.00E+00
ENSG00000076554.14_TPD52	ENSG00000160789.18_LMNA	-0.21143	-0.358562776	-0.05404992	8.93E-03	6.04E-02	1.00E+00
ENSG00000256269.5_HMBS	ENSG00000160789.18_LMNA	-0.23046	-0.375872501	-0.07397594	4.28E-03	3.49E-02	1.00E+00
ENSG00000137776.15_SLTM	ENSG00000160789.18_LMNA	0.27142	0.117287989	0.412784761	7.19E-04	8.95E-03	1.00E+00
ENSG00000223496.2_EXOSC6	ENSG00000160789.18_LMNA	0.00711	-0.152267796	0.166117942	9.31E-01	9.73E-01	1.00E+00
ENSG00000173706.11_HEG1	ENSG00000160789.18_LMNA	0.24848	0.092953642	0.392164845	2.02E-03	1.97E-02	1.00E+00
ENSG00000110435.10_PDHX	ENSG00000160789.18_LMNA	-0.09703	-0.252329399	0.063149812	2.34E-01	5.12E-01	1.00E+00
ENSG00000105204.12_DYRK1B	ENSG00000160789.18_LMNA	-0.11037	-0.264912992	0.049707139	1.76E-01	4.37E-01	1.00E+00
ENSG00000158480.9_SPATA2	ENSG00000160789.18_LMNA	-0.07231	-0.228876688	0.087901824	3.76E-01	6.56E-01	1.00E+00
ENSG00000164815.9_ORC5	ENSG00000160789.18_LMNA	-0.03987	-0.197814062	0.12009404	6.26E-01	8.31E-01	1.00E+00
ENSG00000012779.9_ALOX5	ENSG00000160789.18_LMNA	0.10699	-0.053111192	0.261736581	1.90E-01	4.57E-01	1.00E+00
ENSG00000186106.10_ANKRD46	ENSG00000160789.18_LMNA	-0.63737	-0.723185588	-0.5321633	1.06E-18	4.86E-15	5.44E-14
ENSG00000211455.6_STK38L	ENSG00000160789.18_LMNA	-0.13852	-0.291295572	0.021148479	8.88E-02	2.90E-01	1.00E+00
ENSG00000164307.11_ERAP1	ENSG00000160789.18_LMNA	0.21625	0.059079692	0.362951984	7.46E-03	5.27E-02	1.00E+00
ENSG00000104381.11_GDAP1	ENSG00000160789.18_LMNA	-0.35332	-0.485223893	-0.20568485	8.01E-06	2.44E-04	4.11E-01

ENSG00000176386.7_CDC26	ENSG00000160789.18_LMNA	0.02215	-0.137536214	0.180712939	7.87E-01	9.13E-01	1.00E+00
ENSG00000127951.5_FGL2	ENSG00000160789.18_LMNA	-0.07728	-0.233609882	0.082937715	3.44E-01	6.29E-01	1.00E+00
ENSG00000175376.7_EIF1AD	ENSG00000160789.18_LMNA	0.30532	0.15358	0.442984505	1.31E-04	2.34E-03	1.00E+00
ENSG00000111266.7_DUSP16	ENSG00000160789.18_LMNA	-0.04641	-0.2041059	0.113626952	5.70E-01	7.99E-01	1.00E+00
ENSG00000023287.11_RB1CC1	ENSG00000160789.18_LMNA	-0.1269	-0.280433183	0.032968891	1.19E-01	3.47E-01	1.00E+00
ENSG00000147642.15_SYBU	ENSG00000160789.18_LMNA	-0.25834	-0.401045104	-0.10338993	1.31E-03	1.42E-02	1.00E+00
ENSG00000143416.19_SELENBP	ENSG00000160789.18_LMNA	-0.03454	-0.192676943	0.125354315	6.73E-01	8.56E-01	1.00E+00
ENSG00000139990.16_DCAF5	ENSG00000160789.18_LMNA	0.14233	-0.017257726	0.294853448	8.02E-02	2.73E-01	1.00E+00
ENSG00000128973.10_CLN6	ENSG00000160789.18_LMNA	0.41932	0.278731354	0.542321865	7.57E-08	5.75E-06	3.89E-03
ENSG00000203724.9_C1orf53	ENSG00000160789.18_LMNA	-0.16624	-0.317051027	-0.00723201	4.07E-02	1.75E-01	1.00E+00
ENSG00000100888.11_CHD8	ENSG00000160789.18_LMNA	0.12871	-0.03112668	0.282131383	1.14E-01	3.37E-01	1.00E+00
ENSG00000226137.5_BAIAP2-AS	ENSG00000160789.18_LMNA	0.17119	0.012325368	0.321625413	3.50E-02	1.59E-01	1.00E+00
ENSG00000110218.7_PANX1	ENSG00000160789.18_LMNA	0.0085	-0.150900988	0.167477974	9.17E-01	9.69E-01	1.00E+00
ENSG00000119431.8_HDHD3	ENSG00000160789.18_LMNA	0.26032	0.105496467	0.402830796	1.20E-03	1.33E-02	1.00E+00
ENSG00000125447.15_GGA3	ENSG00000160789.18_LMNA	-0.02732	-0.185714607	0.132455137	7.38E-01	8.90E-01	1.00E+00
ENSG00000237976.1_RP11-126K	ENSG00000160789.18_LMNA	-0.40981	-0.534163548	-0.26810411	1.58E-07	1.04E-05	8.11E-03
ENSG00000155849.14_ELMO1	ENSG00000160789.18_LMNA	-0.37075	-0.500417375	-0.22482269	2.58E-06	9.67E-05	1.32E-01
ENSG00000160050.13_CCDC28B	ENSG00000160789.18_LMNA	-0.24609	-0.390009137	-0.09043008	2.24E-03	2.14E-02	1.00E+00
ENSG00000144320.12_LNPK	ENSG00000160789.18_LMNA	-0.00201	-0.161162088	0.157238058	9.80E-01	9.93E-01	1.00E+00
ENSG00000011105.10_TSPAN9	ENSG00000160789.18_LMNA	0.37111	0.225213898	0.500726196	2.52E-06	9.49E-05	1.29E-01
ENSG00000133943.19_C14orf159	ENSG00000160789.18_LMNA	0.12388	-0.036029205	0.27760776	1.28E-01	3.63E-01	1.00E+00
ENSG00000180628.13_PCGF5	ENSG00000160789.18_LMNA	-0.06219	-0.219220088	0.097980968	4.47E-01	7.13E-01	1.00E+00
ENSG00000163946.12_FAM208A	ENSG00000160789.18_LMNA	0.02088	-0.138780032	0.179486047	7.98E-01	9.19E-01	1.00E+00
ENSG00000169612.3_FAM103A1	ENSG00000160789.18_LMNA	0.02001	-0.139630746	0.178646339	8.07E-01	9.23E-01	1.00E+00
ENSG00000101473.15_ACOT8	ENSG00000160789.18_LMNA	-0.10173	-0.25676784	0.058421391	2.12E-01	4.86E-01	1.00E+00
ENSG00000011376.8_LARS2	ENSG00000160789.18_LMNA	0.12697	-0.032892402	0.280503732	1.19E-01	3.46E-01	1.00E+00
ENSG00000113734.16_BNIP1	ENSG00000160789.18_LMNA	-0.23501	-0.379991333	-0.07875309	3.56E-03	3.04E-02	1.00E+00
ENSG00000121058.4_COIL	ENSG00000160789.18_LMNA	-0.38448	-0.512324083	-0.23997057	1.01E-06	4.62E-05	5.16E-02
ENSG00000106610.13_STAG3L4	ENSG00000160789.18_LMNA	-0.07682	-0.233169153	0.083400611	3.47E-01	6.31E-01	1.00E+00
ENSG00000143373.16_ZNF687	ENSG00000160789.18_LMNA	0.16689	0.007899278	0.317651126	3.99E-02	1.73E-01	1.00E+00
ENSG00000060339.12_CCAR1	ENSG00000160789.18_LMNA	-0.08336	-0.239387811	0.076856537	3.07E-01	5.94E-01	1.00E+00
ENSG00000144233.8_AMMECR1I	ENSG00000160789.18_LMNA	-0.01717	-0.175890272	0.142419639	8.34E-01	9.35E-01	1.00E+00
ENSG00000166341.7_DCHS1	ENSG00000160789.18_LMNA	0.21437	0.057123123	0.361246194	8.00E-03	5.56E-02	1.00E+00

ENSG00000245556.2_SCAMP1-A	ENSG00000160789.18_LMNA	-0.27394	-0.415044278	-0.11997628	6.38E-04	8.17E-03	1.00E+00
ENSG00000125814.16_NAPB	ENSG00000160789.18_LMNA	-0.24909	-0.392714876	-0.09359815	1.97E-03	1.93E-02	1.00E+00
ENSG00000213025.2_COX20P1	ENSG00000160789.18_LMNA	-0.15324	-0.305001448	0.006108103	5.95E-02	2.24E-01	1.00E+00
ENSG00000126522.15_ASL	ENSG00000160789.18_LMNA	0.35243	0.204719119	0.484452703	8.47E-06	2.55E-04	4.35E-01
ENSG00000229604.2_MTATP8P2	ENSG00000160789.18_LMNA	-0.12832	-0.281764277	0.031525098	1.15E-01	3.39E-01	1.00E+00
ENSG00000153561.11_RMND5A	ENSG00000160789.18_LMNA	-0.32744	-0.462532731	-0.17749551	3.83E-05	8.68E-04	1.00E+00
ENSG00000072518.19_MARK2	ENSG00000160789.18_LMNA	0.11391	-0.04613207	0.268241647	1.62E-01	4.17E-01	1.00E+00
ENSG00000178234.11_GALNT11	ENSG00000160789.18_LMNA	0.36858	0.222435949	0.498531769	2.98E-06	1.09E-04	1.53E-01
ENSG00000136928.4_GABBR2	ENSG00000160789.18_LMNA	-0.17376	-0.323999825	-0.0149755	3.23E-02	1.50E-01	1.00E+00
ENSG00000198680.4_TUSC1	ENSG00000160789.18_LMNA	-0.08017	-0.236355626	0.0800508	3.26E-01	6.13E-01	1.00E+00
ENSG00000109466.12_KLHL2	ENSG00000160789.18_LMNA	-0.03671	-0.194770817	0.1232124	6.53E-01	8.47E-01	1.00E+00
ENSG00000139278.8_GLIPR1	ENSG00000160789.18_LMNA	0.16581	0.006785509	0.316649331	4.12E-02	1.77E-01	1.00E+00
ENSG00000170365.8_SMAD1	ENSG00000160789.18_LMNA	0.27135	0.117215907	0.412724126	7.21E-04	8.97E-03	1.00E+00
ENSG00000172269.15_DPAGT1	ENSG00000160789.18_LMNA	0.2337	0.077378039	0.378806995	3.76E-03	3.17E-02	1.00E+00
ENSG00000089041.15_P2RX7	ENSG00000160789.18_LMNA	-0.51643	-0.624303805	-0.38923139	9.74E-12	3.47E-09	5.00E-07
ENSG00000165118.13_C9orf64	ENSG00000160789.18_LMNA	0.37862	0.23349008	0.507242998	1.51E-06	6.47E-05	7.76E-02
ENSG00000173272.12_MZT2A	ENSG00000160789.18_LMNA	0.24571	0.090026447	0.389664034	2.28E-03	2.16E-02	1.00E+00
ENSG00000182580.2_EPHB3	ENSG00000160789.18_LMNA	0.04275	-0.117252522	0.200581808	6.01E-01	8.17E-01	1.00E+00
ENSG00000087995.14_METTL2A	ENSG00000160789.18_LMNA	-0.28691	-0.426626644	-0.13382483	3.39E-04	4.93E-03	1.00E+00
ENSG00000137040.9_RANBP6	ENSG00000160789.18_LMNA	-0.07203	-0.228610302	0.088180738	3.78E-01	6.57E-01	1.00E+00
ENSG00000008735.13_MAPK8IP2	ENSG00000160789.18_LMNA	-0.26682	-0.408658138	-0.11238944	8.91E-04	1.06E-02	1.00E+00
ENSG00000159884.10_CCDC107	ENSG00000160789.18_LMNA	0.22437	0.067583979	0.370342663	5.45E-03	4.18E-02	1.00E+00
ENSG00000196083.8_IL1RAP	ENSG00000160789.18_LMNA	0.26459	0.110021652	0.406659124	9.88E-04	1.15E-02	1.00E+00
ENSG00000115761.14_NOL10	ENSG00000160789.18_LMNA	0.19578	0.037755052	0.344250062	1.56E-02	9.03E-02	1.00E+00
ENSG00000168887.9_C2orf68	ENSG00000160789.18_LMNA	-0.12618	-0.279757974	0.033700768	1.21E-01	3.51E-01	1.00E+00
ENSG00000197122.10_SRC	ENSG00000160789.18_LMNA	-0.09348	-0.24897929	0.06670944	2.52E-01	5.33E-01	1.00E+00
ENSG00000171307.17_ZDHHC16	ENSG00000160789.18_LMNA	0.11077	-0.049296468	0.265295738	1.74E-01	4.35E-01	1.00E+00
ENSG00000117600.11_PLPPR4	ENSG00000160789.18_LMNA	-0.06361	-0.220576206	0.096569427	4.36E-01	7.04E-01	1.00E+00
ENSG00000166260.9_COX11	ENSG00000160789.18_LMNA	-0.39281	-0.519520396	-0.24919058	5.57E-07	2.86E-05	2.86E-02
ENSG00000163528.11_CHCHD4	ENSG00000160789.18_LMNA	-0.05669	-0.213963607	0.103440242	4.88E-01	7.44E-01	1.00E+00
ENSG00000175206.9_NPPA	ENSG00000160789.18_LMNA	-0.42321	-0.545647143	-0.28308155	5.57E-08	4.46E-06	2.86E-03
ENSG00000137462.6_TLR2	ENSG00000160789.18_LMNA	7.25E-05	-0.159130031	0.159271371	9.99E-01	1.00E+00	1.00E+00
ENSG00000227376.1_FTH1P16	ENSG00000160789.18_LMNA	-0.01549	-0.174261786	0.144065148	8.50E-01	9.41E-01	1.00E+00

ENSG00000101138.10_CSTF1	ENSG00000160789.18_LMNA	0.1233	-0.036623747	0.277058221	1.30E-01	3.66E-01	1.00E+00
ENSG00000165055.14_METTL2B	ENSG00000160789.18_LMNA	0.01156	-0.147917671	0.170442297	8.88E-01	9.56E-01	1.00E+00
ENSG00000131791.7_PRKAB2	ENSG00000160789.18_LMNA	-0.28526	-0.425155658	-0.13205967	3.68E-04	5.27E-03	1.00E+00
ENSG00000264148.1_RP11-848P	ENSG00000160789.18_LMNA	-0.25514	-0.398172203	-0.10000645	1.51E-03	1.58E-02	1.00E+00
ENSG00000142409.5_ZNF787	ENSG00000160789.18_LMNA	0.39536	0.252027275	0.521726779	4.63E-07	2.47E-05	2.37E-02
ENSG00000151498.10_ACAD8	ENSG00000160789.18_LMNA	-0.33361	-0.467957299	-0.1841922	2.67E-05	6.52E-04	1.00E+00
ENSG00000123545.5_NDUFAF4	ENSG00000160789.18_LMNA	-0.30441	-0.442185409	-0.15260954	1.37E-04	2.43E-03	1.00E+00
ENSG00000187391.16_MAGI2	ENSG00000160789.18_LMNA	-0.22211	-0.368291612	-0.06521946	5.96E-03	4.48E-02	1.00E+00
ENSG00000185049.11_NELFA	ENSG00000160789.18_LMNA	0.13858	-0.02108945	0.291349615	8.86E-02	2.90E-01	1.00E+00
ENSG00000153015.14_CWC27	ENSG00000160789.18_LMNA	-0.12805	-0.281507551	0.031803661	1.16E-01	3.40E-01	1.00E+00
ENSG00000148399.10_DPH7	ENSG00000160789.18_LMNA	-0.06445	-0.221381125	0.095731009	4.30E-01	7.00E-01	1.00E+00
ENSG00000103544.13_C16orf62	ENSG00000160789.18_LMNA	-0.01795	-0.176648416	0.141652969	8.26E-01	9.32E-01	1.00E+00
ENSG00000143369.13_ECM1	ENSG00000160789.18_LMNA	0.37096	0.225049316	0.500596283	2.54E-06	9.58E-05	1.30E-01
ENSG00000119638.11_NEK9	ENSG00000160789.18_LMNA	0.30688	0.155267593	0.444373005	1.20E-04	2.20E-03	1.00E+00
ENSG00000161847.12_RAVER1	ENSG00000160789.18_LMNA	0.19832	0.040394277	0.346577953	1.43E-02	8.50E-02	1.00E+00
ENSG00000105705.14_SUGP1	ENSG00000160789.18_LMNA	-0.02195	-0.180520831	0.137731039	7.88E-01	9.14E-01	1.00E+00
ENSG00000129757.11_CDKN1C	ENSG00000160789.18_LMNA	-0.23729	-0.382059225	-0.08115672	3.24E-03	2.83E-02	1.00E+00
ENSG00000171163.14_ZNF692	ENSG00000160789.18_LMNA	-0.03067	-0.188950866	0.129158564	7.08E-01	8.76E-01	1.00E+00
ENSG00000132323.7_ILKAP	ENSG00000160789.18_LMNA	-0.27694	-0.417719274	-0.12316449	5.53E-04	7.29E-03	1.00E+00
ENSG00000160818.15_GPATCH4	ENSG00000160789.18_LMNA	0.25069	0.095290477	0.394158119	1.84E-03	1.84E-02	1.00E+00
ENSG00000162869.14_PPP1R21	ENSG00000160789.18_LMNA	-0.40123	-0.526778536	-0.2585397	3.01E-07	1.77E-05	1.55E-02
ENSG00000203485.11_INF2	ENSG00000160789.18_LMNA	0.29214	0.139424085	0.431282527	2.60E-04	4.02E-03	1.00E+00
ENSG00000106415.11_GLCCI1	ENSG00000160789.18_LMNA	-0.37871	-0.507321099	-0.23358951	1.50E-06	6.43E-05	7.71E-02
ENSG00000110583.11_NAA40	ENSG00000160789.18_LMNA	-0.18042	-0.330135514	-0.02184385	2.61E-02	1.31E-01	1.00E+00
ENSG00000168259.13_DNAJC7	ENSG00000160789.18_LMNA	-0.02296	-0.181497013	0.136740798	7.79E-01	9.09E-01	1.00E+00
ENSG00000144306.12_SCRN3	ENSG00000160789.18_LMNA	-0.29824	-0.436706878	-0.14597114	1.90E-04	3.14E-03	1.00E+00
ENSG00000129158.9_SERGEF	ENSG00000160789.18_LMNA	0.01643	-0.143141688	0.175175908	8.41E-01	9.38E-01	1.00E+00
ENSG00000106560.9_GIMAP2	ENSG00000160789.18_LMNA	0.02905	-0.130757043	0.187382496	7.22E-01	8.82E-01	1.00E+00
ENSG00000228499.1_TMSB10P1	ENSG00000160789.18_LMNA	0.11358	-0.046464995	0.267931985	1.64E-01	4.19E-01	1.00E+00
ENSG00000105366.14_SIGLEC8	ENSG00000160789.18_LMNA	-0.30124	-0.439370461	-0.14919537	1.62E-04	2.77E-03	1.00E+00
ENSG00000047579.18_DTNBP1	ENSG00000160789.18_LMNA	0.00708	-0.152289088	0.166096746	9.31E-01	9.73E-01	1.00E+00
ENSG00000127452.7_FBXL12	ENSG00000160789.18_LMNA	0.14539	-0.014142145	0.297696248	7.39E-02	2.59E-01	1.00E+00
ENSG00000204438.9_GPANK1	ENSG00000160789.18_LMNA	-0.08244	-0.238512947	0.077778831	3.13E-01	5.99E-01	1.00E+00

ENSG00000066926.9_FECH	ENSG00000160789.18_LMNA	-0.11535	-0.269601699	0.044669015	1.57E-01	4.09E-01	1.00E+00
ENSG00000072958.7_AP1M1	ENSG00000160789.18_LMNA	0.15341	-0.005936227	0.305157332	5.92E-02	2.24E-01	1.00E+00
ENSG00000110422.10_HIPK3	ENSG00000160789.18_LMNA	0.13404	-0.025713234	0.287110309	9.97E-02	3.11E-01	1.00E+00
ENSG00000167291.14_TBC1D16	ENSG00000160789.18_LMNA	0.18755	0.029221364	0.336697164	2.07E-02	1.10E-01	1.00E+00
ENSG00000176595.3_KBTBD11	ENSG00000160789.18_LMNA	-0.15231	-0.304132219	0.007066165	6.10E-02	2.28E-01	1.00E+00
ENSG00000085871.7_MGST2	ENSG00000160789.18_LMNA	-0.03515	-0.193269428	0.124748537	6.67E-01	8.53E-01	1.00E+00
ENSG00000008710.16_PKD1	ENSG00000160789.18_LMNA	0.22488	0.068116844	0.370804479	5.35E-03	4.12E-02	1.00E+00
ENSG00000113504.18_SLC12A7	ENSG00000160789.18_LMNA	0.47532	0.34199727	0.589882349	6.10E-10	1.05E-07	3.13E-05
ENSG00000138231.11_DBR1	ENSG00000160789.18_LMNA	-0.15476	-0.3064086	0.004555931	5.69E-02	2.18E-01	1.00E+00
ENSG00000162458.11_FBLIM1	ENSG00000160789.18_LMNA	0.40406	0.261697071	0.529220935	2.44E-07	1.49E-05	1.25E-02
ENSG00000184060.9_ADAP2	ENSG00000160789.18_LMNA	0.01568	-0.143883925	0.17444122	8.48E-01	9.41E-01	1.00E+00
ENSG00000136936.9_XPA	ENSG00000160789.18_LMNA	-0.15279	-0.304584082	0.006568193	6.02E-02	2.26E-01	1.00E+00
ENSG00000100711.12_ZFYVE21	ENSG00000160789.18_LMNA	0.05217	-0.107923263	0.209633325	5.23E-01	7.69E-01	1.00E+00
ENSG00000014123.9_UFL1	ENSG00000160789.18_LMNA	-0.08523	-0.241157419	0.074989334	2.96E-01	5.82E-01	1.00E+00
ENSG00000140521.10_POLG	ENSG00000160789.18_LMNA	0.35757	0.210348274	0.488941795	6.11E-06	1.96E-04	3.14E-01
ENSG00000006530.14_AGK	ENSG00000160789.18_LMNA	0.17309	0.014286976	0.323383307	3.30E-02	1.52E-01	1.00E+00
ENSG00000141428.15_C18orf21	ENSG00000160789.18_LMNA	-0.08459	-0.240552911	0.075627431	3.00E-01	5.87E-01	1.00E+00
ENSG00000101966.11_XIAP	ENSG00000160789.18_LMNA	0.09737	-0.062808231	0.252650477	2.33E-01	5.11E-01	1.00E+00
ENSG00000102893.14_PHKB	ENSG00000160789.18_LMNA	-0.16506	-0.315953616	-0.00601248	4.21E-02	1.80E-01	1.00E+00
ENSG00000113812.12_ACTR8	ENSG00000160789.18_LMNA	-0.06693	-0.223750455	0.093260454	4.13E-01	6.86E-01	1.00E+00
ENSG00000138398.14_PPIG	ENSG00000160789.18_LMNA	0.0409	-0.119074221	0.198807988	6.17E-01	8.26E-01	1.00E+00
ENSG00000101938.13_CHRDL1	ENSG00000160789.18_LMNA	-0.37803	-0.506732524	-0.23284035	1.57E-06	6.66E-05	8.08E-02
ENSG00000156475.17_PPP2R2B	ENSG00000160789.18_LMNA	-0.32064	-0.456538392	-0.17012601	5.64E-05	1.18E-03	1.00E+00
ENSG00000115368.8_WDR75	ENSG00000160789.18_LMNA	-0.00084	-0.160017892	0.158383293	9.92E-01	9.97E-01	1.00E+00
ENSG00000101413.10_RPRD1B	ENSG00000160789.18_LMNA	0.15982	0.000632036	0.311102014	4.92E-02	1.99E-01	1.00E+00
ENSG00000186575.16_NF2	ENSG00000160789.18_LMNA	0.16372	0.00464261	0.314719923	4.39E-02	1.85E-01	1.00E+00
ENSG00000185324.20_CDK10	ENSG00000160789.18_LMNA	0.11615	-0.043865339	0.27034826	1.54E-01	4.05E-01	1.00E+00
ENSG00000206337.9_HCP5	ENSG00000160789.18_LMNA	0.14796	-0.011516488	0.300087731	6.89E-02	2.47E-01	1.00E+00
ENSG00000197562.8_RAB40C	ENSG00000160789.18_LMNA	0.13193	-0.027858592	0.285139172	1.05E-01	3.22E-01	1.00E+00
ENSG00000197119.11_SLC25A29	ENSG00000160789.18_LMNA	0.06269	-0.097484428	0.219697272	4.43E-01	7.10E-01	1.00E+00
ENSG00000129480.11_DTD2	ENSG00000160789.18_LMNA	-0.32861	-0.463559732	-0.17876133	3.58E-05	8.24E-04	1.00E+00
ENSG00000102524.10_TNFSF13B	ENSG00000160789.18_LMNA	-0.09499	-0.25040589	0.065194601	2.44E-01	5.24E-01	1.00E+00
ENSG00000165661.14_QSOX2	ENSG00000160789.18_LMNA	0.11171	-0.048352809	0.266174857	1.71E-01	4.30E-01	1.00E+00

ENSG00000125772.11_GPCPD1	ENSG00000160789.18_LMNA	-0.14349	-0.29592994	0.016078652	7.78E-02	2.67E-01	1.00E+00
ENSG00000072849.9_DERL2	ENSG00000160789.18_LMNA	-0.01069	-0.16960506	0.14876086	8.96E-01	9.59E-01	1.00E+00
ENSG00000166685.10_COG1	ENSG00000160789.18_LMNA	0.08775	-0.072465712	0.2435458	2.82E-01	5.68E-01	1.00E+00
ENSG00000073584.17_SMARCE1	ENSG00000160789.18_LMNA	-0.37479	-0.503920678	-0.22926585	1.96E-06	7.87E-05	1.01E-01
ENSG00000198075.8_SULT1C4	ENSG00000160789.18_LMNA	-0.30289	-0.440831375	-0.1509664	1.49E-04	2.58E-03	1.00E+00
ENSG00000058804.11_NDC1	ENSG00000160789.18_LMNA	-0.03386	-0.192026107	0.12601948	6.79E-01	8.60E-01	1.00E+00
ENSG00000011021.20_CLCN6	ENSG00000160789.18_LMNA	-0.04096	-0.198867876	0.119012751	6.16E-01	8.26E-01	1.00E+00
ENSG00000145358.5_DDIT4L	ENSG00000160789.18_LMNA	0.22643	0.06974493	0.372214557	5.03E-03	3.94E-02	1.00E+00
ENSG00000030066.12_NUP160	ENSG00000160789.18_LMNA	0.00331	-0.155968503	0.162429492	9.68E-01	9.87E-01	1.00E+00
ENSG00000207547.1_MIR25	ENSG00000160789.18_LMNA	-0.02991	-0.188211987	0.129911833	7.15E-01	8.79E-01	1.00E+00
ENSG00000136504.10_KAT7	ENSG00000160789.18_LMNA	-0.05629	-0.213576499	0.10384153	4.91E-01	7.47E-01	1.00E+00
ENSG00000213859.4_KCTD11	ENSG00000160789.18_LMNA	0.20634	0.048744184	0.353918111	1.08E-02	6.90E-02	1.00E+00
ENSG00000121775.16_TMEM39B	ENSG00000160789.18_LMNA	0.25559	0.100474161	0.398569687	1.48E-03	1.56E-02	1.00E+00
ENSG00000164808.15_SPIDR	ENSG00000160789.18_LMNA	0.1881	0.029792622	0.337204005	2.03E-02	1.09E-01	1.00E+00
ENSG00000109920.11_FNBP4	ENSG00000160789.18_LMNA	0.03328	-0.126588308	0.191469311	6.84E-01	8.63E-01	1.00E+00
ENSG00000068615.15_REEP1	ENSG00000160789.18_LMNA	-0.40198	-0.527431433	-0.25938316	2.85E-07	1.69E-05	1.46E-02
ENSG00000027847.12_B4GALT7	ENSG00000160789.18_LMNA	0.24858	0.093063073	0.39225825	2.01E-03	1.96E-02	1.00E+00
ENSG00000140416.18_TPM1	ENSG00000160789.18_LMNA	0.08203	-0.078189949	0.238122806	3.15E-01	6.01E-01	1.00E+00
ENSG00000170677.5_SOCS6	ENSG00000160789.18_LMNA	-0.01742	-0.176136292	0.142170894	8.31E-01	9.34E-01	1.00E+00
ENSG00000114030.11_KPNA1	ENSG00000160789.18_LMNA	-0.14421	-0.296605656	0.015338101	7.63E-02	2.64E-01	1.00E+00
ENSG00000181804.13_SLC9A9	ENSG00000160789.18_LMNA	-0.23344	-0.378567979	-0.07710067	3.80E-03	3.19E-02	1.00E+00
ENSG00000153446.14_C16orf89	ENSG00000160789.18_LMNA	-0.19806	-0.346337055	-0.04012096	1.44E-02	8.56E-02	1.00E+00
ENSG00000156671.11_SAMD8	ENSG00000160789.18_LMNA	-0.09696	-0.25227009	0.0632129	2.35E-01	5.13E-01	1.00E+00
ENSG00000013016.13_EHD3	ENSG00000160789.18_LMNA	-0.23875	-0.383380832	-0.08269474	3.05E-03	2.71E-02	1.00E+00
ENSG00000168061.12_SAC3D1	ENSG00000160789.18_LMNA	0.1466	-0.012903688	0.298824739	7.15E-02	2.53E-01	1.00E+00
ENSG00000144283.20_PKP4	ENSG00000160789.18_LMNA	-0.4141	-0.537840446	-0.27288573	1.14E-07	8.05E-06	5.84E-03
ENSG00000112655.14_PTK7	ENSG00000160789.18_LMNA	0.44527	0.307898229	0.564460129	9.03E-09	9.90E-07	4.63E-04
ENSG00000258056.2_RP11-644F	ENSG00000160789.18_LMNA	-0.22652	-0.372294146	-0.06983687	5.01E-03	3.93E-02	1.00E+00
ENSG00000111961.15_SASH1	ENSG00000160789.18_LMNA	-0.0572	-0.214449081	0.102936839	4.84E-01	7.41E-01	1.00E+00
ENSG00000254122.2_PCDHGB7	ENSG00000160789.18_LMNA	0.04218	-0.117810351	0.200038862	6.06E-01	8.20E-01	1.00E+00
ENSG00000126653.14_NSRP1	ENSG00000160789.18_LMNA	0.21669	0.059538589	0.363351768	7.33E-03	5.21E-02	1.00E+00
ENSG00000135549.13_PKIB	ENSG00000160789.18_LMNA	0.17679	0.018103467	0.326797351	2.93E-02	1.41E-01	1.00E+00
ENSG00000107521.17_HPS1	ENSG00000160789.18_LMNA	0.18098	0.022426371	0.330654701	2.57E-02	1.29E-01	1.00E+00

ENSG00000198435.3_NRARP	ENSG00000160789.18_LMNA	0.09327	-0.066921152	0.248779801	2.53E-01	5.34E-01	1.00E+00
ENSG00000137522.16_RNF121	ENSG00000160789.18_LMNA	0.06263	-0.097540029	0.219643846	4.43E-01	7.11E-01	1.00E+00
ENSG00000061918.11_GUCY1B3	ENSG00000160789.18_LMNA	-0.11985	-0.27382897	0.040112969	1.41E-01	3.84E-01	1.00E+00
ENSG00000164535.13_DAGLB	ENSG00000160789.18_LMNA	0.04584	-0.114191198	0.203557988	5.75E-01	8.02E-01	1.00E+00
ENSG00000234009.1_RPL5P34	ENSG00000160789.18_LMNA	-0.14549	-0.297795261	0.014033522	7.37E-02	2.58E-01	1.00E+00
ENSG00000083857.12_FAT1	ENSG00000160789.18_LMNA	0.2081	0.050576422	0.355523763	1.01E-02	6.60E-02	1.00E+00
ENSG00000163904.11_SENP2	ENSG00000160789.18_LMNA	-0.30791	-0.445281214	-0.15637235	1.14E-04	2.10E-03	1.00E+00
ENSG00000197702.10_PARVA	ENSG00000160789.18_LMNA	0.25299	0.097730497	0.396236412	1.66E-03	1.70E-02	1.00E+00
ENSG00000236939.2_BAALC-AS2	ENSG00000160789.18_LMNA	-0.13269	-0.2858519	0.027083202	1.03E-01	3.18E-01	1.00E+00
ENSG00000108559.10_NUP88	ENSG00000160789.18_LMNA	-0.19114	-0.339995477	-0.03294248	1.83E-02	1.01E-01	1.00E+00
ENSG00000183828.13_NUDT14	ENSG00000160789.18_LMNA	0.00819	-0.151209069	0.167171526	9.20E-01	9.70E-01	1.00E+00
ENSG00000113360.15_DROSHA	ENSG00000160789.18_LMNA	0.07589	-0.084328339	0.232285457	3.53E-01	6.36E-01	1.00E+00
ENSG00000154928.15_EPHB1	ENSG00000160789.18_LMNA	-0.51632	-0.624210991	-0.38910234	9.86E-12	3.47E-09	5.06E-07
ENSG00000014164.6_ZC3H3	ENSG00000160789.18_LMNA	0.17943	0.020828676	0.329230268	2.70E-02	1.33E-01	1.00E+00
ENSG00000117533.13_VAMP4	ENSG00000160789.18_LMNA	-0.05736	-0.214605006	0.102775121	4.83E-01	7.40E-01	1.00E+00
ENSG00000137752.21_CASP1	ENSG00000160789.18_LMNA	0.16356	0.00447625	0.31457003	4.41E-02	1.85E-01	1.00E+00
ENSG00000143093.13_STRIP1	ENSG00000160789.18_LMNA	-0.05491	-0.212251911	0.105213856	5.02E-01	7.53E-01	1.00E+00
ENSG00000211456.9_SACM1L	ENSG00000160789.18_LMNA	-0.20874	-0.356106584	-0.051242	9.86E-03	6.49E-02	1.00E+00
ENSG00000165698.14_SPACA9	ENSG00000160789.18_LMNA	-0.07573	-0.232132336	0.084489033	3.54E-01	6.37E-01	1.00E+00
ENSG00000234964.4_FABP5P7	ENSG00000160789.18_LMNA	0.12188	-0.038057023	0.275732591	1.35E-01	3.73E-01	1.00E+00
ENSG00000184574.8_LPAR5	ENSG00000160789.18_LMNA	-0.11889	-0.272923133	0.041090358	1.45E-01	3.89E-01	1.00E+00
ENSG00000128340.13_RAC2	ENSG00000160789.18_LMNA	0.15149	-0.007905892	0.303369925	6.25E-02	2.32E-01	1.00E+00
ENSG00000146670.8_CDCA5	ENSG00000160789.18_LMNA	0.03973	-0.120235238	0.197676398	6.27E-01	8.32E-01	1.00E+00
ENSG00000124532.13_MRS2	ENSG00000160789.18_LMNA	-0.09687	-0.252179334	0.063309434	2.35E-01	5.13E-01	1.00E+00
ENSG00000105939.11_ZC3HAV1	ENSG00000160789.18_LMNA	0.30337	0.151484408	0.441258382	1.45E-04	2.53E-03	1.00E+00
ENSG00000092036.15_HAUS4	ENSG00000160789.18_LMNA	-0.00253	-0.16166301	0.156736411	9.75E-01	9.91E-01	1.00E+00
ENSG00000167703.13_SLC43A2	ENSG00000160789.18_LMNA	0.03994	-0.120025852	0.197880539	6.25E-01	8.31E-01	1.00E+00
ENSG00000214174.7_AMZ2P1	ENSG00000160789.18_LMNA	-0.49088	-0.60296241	-0.35979975	1.36E-10	2.92E-08	6.97E-06
ENSG00000121871.3_SLITRK3	ENSG00000160789.18_LMNA	-0.01662	-0.175352787	0.142962937	8.39E-01	9.37E-01	1.00E+00
ENSG00000140280.12_LYSMD2	ENSG00000160789.18_LMNA	-0.24471	-0.38876598	-0.08897654	2.38E-03	2.23E-02	1.00E+00
ENSG00000117791.14_MARC2	ENSG00000160789.18_LMNA	0.22753	0.070898745	0.373213027	4.82E-03	3.81E-02	1.00E+00
ENSG00000110931.17_CAMKK2	ENSG00000160789.18_LMNA	-0.14	-0.292679827	0.019635836	8.54E-02	2.83E-01	1.00E+00
ENSG00000104472.8_CHRAC1	ENSG00000160789.18_LMNA	0.03656	-0.123354287	0.194632202	6.55E-01	8.47E-01	1.00E+00

ENSG00000145423.4_SFRP2	ENSG00000160789.18_LMNA	-0.02205	-0.180617984	0.137632515	7.87E-01	9.13E-01	1.00E+00
ENSG00000164329.12_PAPD4	ENSG00000160789.18_LMNA	-0.01032	-0.169238848	0.14912953	9.00E-01	9.61E-01	1.00E+00
ENSG00000059758.6_CDK17	ENSG00000160789.18_LMNA	-0.2362	-0.381072445	-0.08000929	3.39E-03	2.93E-02	1.00E+00
ENSG00000115756.11_HPCAL1	ENSG00000160789.18_LMNA	0.0928	-0.067399095	0.248329354	2.55E-01	5.38E-01	1.00E+00
ENSG00000153989.7_NUS1	ENSG00000160789.18_LMNA	-0.00137	-0.160536432	0.157864389	9.87E-01	9.95E-01	1.00E+00
ENSG00000143164.14_DCAF6	ENSG00000160789.18_LMNA	0.14039	-0.019235918	0.293045582	8.45E-02	2.82E-01	1.00E+00
ENSG00000110713.14_NUP98	ENSG00000160789.18_LMNA	0.2703	0.116092522	0.411778797	7.58E-04	9.31E-03	1.00E+00
ENSG00000130827.6_PLXNA3	ENSG00000160789.18_LMNA	0.2202	0.063216546	0.366551912	6.41E-03	4.73E-02	1.00E+00
ENSG00000151806.12_GUF1	ENSG00000160789.18_LMNA	-0.18018	-0.329915275	-0.02159681	2.63E-02	1.31E-01	1.00E+00
ENSG00000167136.6_ENDOG	ENSG00000160789.18_LMNA	0.10111	-0.059039526	0.256188368	2.15E-01	4.89E-01	1.00E+00
ENSG00000084092.6_NOA1	ENSG00000160789.18_LMNA	-0.05757	-0.214799528	0.102573348	4.81E-01	7.39E-01	1.00E+00
ENSG00000253764.1_RP11-439C	ENSG00000160789.18_LMNA	-0.11525	-0.269507172	0.044770744	1.57E-01	4.10E-01	1.00E+00
ENSG00000126950.7_TMEM35A	ENSG00000160789.18_LMNA	-0.44298	-0.562510445	-0.30531003	1.10E-08	1.17E-06	5.63E-04
ENSG00000236675.1_MTX1P1	ENSG00000160789.18_LMNA	0.39443	0.250984657	0.52091625	4.96E-07	2.60E-05	2.54E-02
ENSG00000132664.10_POLR3F	ENSG00000160789.18_LMNA	-0.13084	-0.284123776	0.028962611	1.08E-01	3.27E-01	1.00E+00
ENSG00000164414.15_SLC35A1	ENSG00000160789.18_LMNA	-0.28739	-0.427051774	-0.13433532	3.31E-04	4.85E-03	1.00E+00
ENSG00000135999.10_EPC2	ENSG00000160789.18_LMNA	-0.09374	-0.249226096	0.066447473	2.51E-01	5.31E-01	1.00E+00
ENSG00000166716.8_ZNF592	ENSG00000160789.18_LMNA	0.18133	0.022791795	0.3309803	2.54E-02	1.28E-01	1.00E+00
ENSG00000135341.16_MAP3K7	ENSG00000160789.18_LMNA	-0.08163	-0.237738398	0.078594921	3.17E-01	6.04E-01	1.00E+00
ENSG00000146067.14_FAM193B	ENSG00000160789.18_LMNA	0.03956	-0.120398708	0.197517003	6.28E-01	8.33E-01	1.00E+00
ENSG00000145782.11_ATG12	ENSG00000160789.18_LMNA	-0.3266	-0.461793849	-0.17658539	4.02E-05	9.05E-04	1.00E+00
ENSG00000119242.7_CCDC92	ENSG00000160789.18_LMNA	-0.03549	-0.193600306	0.124410132	6.64E-01	8.52E-01	1.00E+00
ENSG00000148356.12_LRSAM1	ENSG00000160789.18_LMNA	0.07976	-0.080461891	0.235964946	3.29E-01	6.15E-01	1.00E+00
ENSG00000173914.10_RBM4B	ENSG00000160789.18_LMNA	-0.33721	-0.471116275	-0.18810417	2.16E-05	5.50E-04	1.00E+00
ENSG00000133315.9_MACROD1	ENSG00000160789.18_LMNA	-0.0896	-0.245302856	0.070606577	2.72E-01	5.57E-01	1.00E+00
ENSG00000165819.10_METTL3	ENSG00000160789.18_LMNA	-0.01468	-0.173474167	0.14486037	8.58E-01	9.44E-01	1.00E+00
ENSG00000127666.9_TICAM1	ENSG00000160789.18_LMNA	0.41835	0.277640651	0.541486838	8.17E-08	6.07E-06	4.19E-03
ENSG00000143740.13_SNAP47	ENSG00000160789.18_LMNA	-0.05536	-0.21269096	0.104759118	4.98E-01	7.51E-01	1.00E+00
ENSG00000111790.12_FGFR1OP	ENSG00000160789.18_LMNA	-0.2546	-0.397685462	-0.09943388	1.55E-03	1.61E-02	1.00E+00
ENSG00000122359.16_ANXA11	ENSG00000160789.18_LMNA	0.18531	0.026898031	0.33463398	2.23E-02	1.17E-01	1.00E+00
ENSG00000142751.13_GPN2	ENSG00000160789.18_LMNA	0.18504	0.026628127	0.334394106	2.25E-02	1.17E-01	1.00E+00
ENSG00000164011.16_ZNF691	ENSG00000160789.18_LMNA	-0.03439	-0.192539842	0.125494458	6.74E-01	8.57E-01	1.00E+00
ENSG00000167985.5_SDHAF2	ENSG00000160789.18_LMNA	-0.22337	-0.369429872	-0.06653126	5.67E-03	4.31E-02	1.00E+00

ENSG00000237765.5_FAM200B	ENSG00000160789.18_LMNA	-0.49474	-0.606194285	-0.36422591	9.26E-11	2.10E-08	4.75E-06
ENSG00000103043.13_VAC14	ENSG00000160789.18_LMNA	0.1799	0.021311779	0.329661129	2.66E-02	1.32E-01	1.00E+00
ENSG00000121892.13_PDS5A	ENSG00000160789.18_LMNA	0.0598	-0.100356893	0.216934682	4.64E-01	7.27E-01	1.00E+00
ENSG00000181026.14_AEN	ENSG00000160789.18_LMNA	0.27681	0.123029985	0.41760652	5.56E-04	7.32E-03	1.00E+00
ENSG00000065243.17_PKN2	ENSG00000160789.18_LMNA	0.01337	-0.146138519	0.172207383	8.70E-01	9.50E-01	1.00E+00
ENSG00000067715.12_SYT1	ENSG00000160789.18_LMNA	-0.2031	-0.350948623	-0.04536105	1.21E-02	7.52E-02	1.00E+00
ENSG00000120251.17_GRIA2	ENSG00000160789.18_LMNA	-0.39117	-0.518107161	-0.24737605	6.26E-07	3.14E-05	3.21E-02
ENSG00000118849.8_RARRES1	ENSG00000160789.18_LMNA	0.19773	0.039779369	0.346035918	1.46E-02	8.62E-02	1.00E+00
ENSG00000140379.7_BCL2A1	ENSG00000160789.18_LMNA	0.01375	-0.145773645	0.17256912	8.67E-01	9.48E-01	1.00E+00
ENSG00000153044.8_CENPH	ENSG00000160789.18_LMNA	-0.06997	-0.226645334	0.090236591	3.92E-01	6.70E-01	1.00E+00
ENSG00000166548.14_TK2	ENSG00000160789.18_LMNA	0.09778	-0.062389102	0.253044354	2.31E-01	5.08E-01	1.00E+00
ENSG00000254531.1_FLJ20021	ENSG00000160789.18_LMNA	-0.15937	-0.310692226	-0.0001784	4.99E-02	2.01E-01	1.00E+00
ENSG00000143624.12_INTS3	ENSG00000160789.18_LMNA	0.2061	0.048488273	0.353693704	1.09E-02	6.94E-02	1.00E+00
ENSG00000100393.9_EP300	ENSG00000160789.18_LMNA	0.07059	-0.089620598	0.227234367	3.88E-01	6.66E-01	1.00E+00
ENSG00000204138.11_PHACTR4	ENSG00000160789.18_LMNA	-0.1224	-0.276216678	0.037533776	1.33E-01	3.70E-01	1.00E+00
ENSG00000136051.12_KIAA1033	ENSG00000160789.18_LMNA	-0.06005	-0.21717723	0.100104909	4.62E-01	7.25E-01	1.00E+00
ENSG00000102738.7_MRPS31	ENSG00000160789.18_LMNA	-0.26092	-0.403368583	-0.1061314	1.17E-03	1.30E-02	1.00E+00
ENSG00000021574.10_SPAST	ENSG00000160789.18_LMNA	-0.53126	-0.636620097	-0.40643789	1.90E-12	9.18E-10	9.76E-08
ENSG00000126581.11_BECN1	ENSG00000160789.18_LMNA	0.14236	-0.017229493	0.294879234	8.02E-02	2.73E-01	1.00E+00
ENSG00000151617.14_EDNRA	ENSG00000160789.18_LMNA	0.10404	-0.056094262	0.258947382	2.02E-01	4.73E-01	1.00E+00
ENSG00000203760.7_CENPW	ENSG00000160789.18_LMNA	-0.04022	-0.1981554	0.119743886	6.23E-01	8.30E-01	1.00E+00
ENSG00000013375.14_PGM3	ENSG00000160789.18_LMNA	-0.07269	-0.229238307	0.087523116	3.73E-01	6.53E-01	1.00E+00
ENSG00000214357.7_NEURL1B	ENSG00000160789.18_LMNA	0.02204	-0.137641899	0.180608731	7.88E-01	9.13E-01	1.00E+00
ENSG00000128594.6_LRRC4	ENSG00000160789.18_LMNA	-0.34876	-0.481241188	-0.20070332	1.07E-05	3.07E-04	5.47E-01
ENSG00000213563.5_C8orf82	ENSG00000160789.18_LMNA	0.06256	-0.097617585	0.21956932	4.44E-01	7.11E-01	1.00E+00
ENSG00000182667.13_NTM	ENSG00000160789.18_LMNA	-0.28737	-0.427037334	-0.13431798	3.31E-04	4.85E-03	1.00E+00
ENSG00000128482.14_RNF112	ENSG00000160789.18_LMNA	-0.24266	-0.386909371	-0.0868081	2.59E-03	2.39E-02	1.00E+00
ENSG00000136738.13_STAM	ENSG00000160789.18_LMNA	-0.3695	-0.499327917	-0.22344328	2.80E-06	1.04E-04	1.44E-01
ENSG00000197496.5_SLC2A10	ENSG00000160789.18_LMNA	0.41501	0.273901639	0.538620344	1.06E-07	7.59E-06	5.44E-03
ENSG00000137103.15_TMEM8B	ENSG00000160789.18_LMNA	-0.08708	-0.242910363	0.073137526	2.86E-01	5.72E-01	1.00E+00
ENSG00000150593.14_PDCD4	ENSG00000160789.18_LMNA	-0.30708	-0.444543577	-0.15547502	1.19E-04	2.18E-03	1.00E+00
ENSG00000152990.12_ADGRA3	ENSG00000160789.18_LMNA	0.00486	-0.154456345	0.163937717	9.53E-01	9.81E-01	1.00E+00
ENSG00000132694.17_ARHGEF1	ENSG00000160789.18_LMNA	0.07457	-0.085643507	0.231031813	3.61E-01	6.43E-01	1.00E+00

ENSG00000088970.14_KIZ	ENSG00000160789.18_LMNA	-0.25843	-0.401126636	-0.10348606	1.31E-03	1.42E-02	1.00E+00
ENSG00000131263.11_RLIM	ENSG00000160789.18_LMNA	0.1005	-0.059657394	0.255608919	2.18E-01	4.93E-01	1.00E+00
ENSG00000219545.8_UMAD1	ENSG00000160789.18_LMNA	-0.08574	-0.241642572	0.074477037	2.94E-01	5.79E-01	1.00E+00
ENSG00000150782.10_IL18	ENSG00000160789.18_LMNA	-0.14758	-0.299734202	0.011904905	6.96E-02	2.49E-01	1.00E+00
ENSG00000147180.15_ZNF711	ENSG00000160789.18_LMNA	-0.36526	-0.495638615	-0.21878031	3.71E-06	1.31E-04	1.90E-01
ENSG00000159055.3_MIS18A	ENSG00000160789.18_LMNA	0.07911	-0.081108282	0.235350442	3.33E-01	6.18E-01	1.00E+00
ENSG00000173846.11_PLK3	ENSG00000160789.18_LMNA	0.46705	0.33257626	0.58290739	1.32E-09	1.95E-07	6.75E-05
ENSG00000087884.13_AAMDC	ENSG00000160789.18_LMNA	-0.03605	-0.194134487	0.123863643	6.59E-01	8.50E-01	1.00E+00
ENSG00000130749.8_ZC3H4	ENSG00000160789.18_LMNA	0.17832	0.019681872	0.328206963	2.79E-02	1.36E-01	1.00E+00
ENSG00000176953.9_NFATC2IP	ENSG00000160789.18_LMNA	0.11795	-0.042041004	0.272041546	1.48E-01	3.94E-01	1.00E+00
ENSG00000179242.14_CDH4	ENSG00000160789.18_LMNA	0.26999	0.115765191	0.411503228	7.69E-04	9.42E-03	1.00E+00
ENSG00000111011.16_RSRC2	ENSG00000160789.18_LMNA	0.02384	-0.135876083	0.182348938	7.71E-01	9.05E-01	1.00E+00
ENSG00000013288.7_MAN2B2	ENSG00000160789.18_LMNA	0.32563	0.175528087	0.460934979	4.25E-05	9.41E-04	1.00E+00
ENSG00000166979.11_EVA1C	ENSG00000160789.18_LMNA	0.32338	0.173094937	0.458956446	4.83E-05	1.05E-03	1.00E+00
ENSG00000133119.11_RFC3	ENSG00000160789.18_LMNA	-0.14566	-0.297947227	0.013866791	7.34E-02	2.58E-01	1.00E+00
ENSG00000100399.14_CHADL	ENSG00000160789.18_LMNA	-0.37089	-0.500535232	-0.22497198	2.55E-06	9.61E-05	1.31E-01
ENSG00000130988.11_RGN	ENSG00000160789.18_LMNA	-0.06425	-0.22118633	0.095933952	4.32E-01	7.01E-01	1.00E+00
ENSG00000135473.13_PAN2	ENSG00000160789.18_LMNA	-0.13363	-0.286728452	0.026129067	1.01E-01	3.13E-01	1.00E+00
ENSG00000168490.12_PHYHIP	ENSG00000160789.18_LMNA	-0.15461	-0.306271747	0.004706954	5.72E-02	2.19E-01	1.00E+00
ENSG00000242861.1_RP11-285F	ENSG00000160789.18_LMNA	0.01796	-0.141650556	0.176650802	8.26E-01	9.32E-01	1.00E+00
ENSG00000241878.8_PISD	ENSG00000160789.18_LMNA	-0.11038	-0.264926406	0.049692748	1.76E-01	4.37E-01	1.00E+00
ENSG00000114948.11_ADAM23	ENSG00000160789.18_LMNA	-0.16588	-0.316712171	-0.00685535	4.11E-02	1.77E-01	1.00E+00
ENSG00000106823.11_ECM2	ENSG00000160789.18_LMNA	0.10772	-0.052377379	0.262421898	1.87E-01	4.53E-01	1.00E+00
ENSG00000185088.11_RPS27L	ENSG00000160789.18_LMNA	0.03816	-0.121781428	0.19616808	6.41E-01	8.40E-01	1.00E+00
ENSG00000155252.13_PI4K2A	ENSG00000160789.18_LMNA	0.16089	0.001737112	0.312099794	4.77E-02	1.95E-01	1.00E+00
ENSG00000130592.12_LSP1	ENSG00000160789.18_LMNA	0.22893	0.072365957	0.374481689	4.56E-03	3.66E-02	1.00E+00
ENSG00000050426.14_LETMD1	ENSG00000160789.18_LMNA	-0.15748	-0.30893871	0.001761297	5.27E-02	2.08E-01	1.00E+00
ENSG00000166352.14_C11orf74	ENSG00000160789.18_LMNA	-0.25741	-0.400211407	-0.10240736	1.37E-03	1.47E-02	1.00E+00
ENSG00000236060.2_HSPB1P1	ENSG00000160789.18_LMNA	0.42672	0.287019614	0.548650276	4.21E-08	3.54E-06	2.16E-03
ENSG00000070010.17_UFD1L	ENSG00000160789.18_LMNA	-0.15918	-0.310508171	2.53E-05	5.01E-02	2.02E-01	1.00E+00
ENSG00000270885.1_RASL10B	ENSG00000160789.18_LMNA	-0.29983	-0.438118058	-0.14767858	1.75E-04	2.94E-03	1.00E+00
ENSG00000141458.11_NPC1	ENSG00000160789.18_LMNA	0.2074	0.049842797	0.354881078	1.04E-02	6.72E-02	1.00E+00
ENSG00000001167.13_NFYA	ENSG00000160789.18_LMNA	0.00178	-0.157467592	0.160932831	9.83E-01	9.94E-01	1.00E+00

ENSG00000197912.12_SPG7	ENSG00000160789.18_LMNA	0.11239	-0.047665841	0.266814511	1.68E-01	4.26E-01	1.00E+00
ENSG00000162851.7_TFB2M	ENSG00000160789.18_LMNA	-0.11105	-0.26555884	0.04901411	1.73E-01	4.33E-01	1.00E+00
ENSG00000100526.18_CDKN3	ENSG00000160789.18_LMNA	0.14999	-0.009438177	0.301977911	6.51E-02	2.38E-01	1.00E+00
ENSG00000129460.14_NGDN	ENSG00000160789.18_LMNA	-0.02514	-0.183608845	0.134596372	7.58E-01	8.99E-01	1.00E+00
ENSG00000148943.10_LIN7C	ENSG00000160789.18_LMNA	-0.32349	-0.459053958	-0.17321477	4.80E-05	1.04E-03	1.00E+00
ENSG00000134321.10_RSAD2	ENSG00000160789.18_LMNA	-0.03112	-0.189378472	0.128722462	7.04E-01	8.73E-01	1.00E+00
ENSG00000164877.17_MICALL2	ENSG00000160789.18_LMNA	0.49543	0.365016988	0.606771069	8.64E-11	2.01E-08	4.43E-06
ENSG00000087448.8_KLHL42	ENSG00000160789.18_LMNA	-0.01452	-0.17331703	0.145018974	8.59E-01	9.45E-01	1.00E+00
ENSG00000133878.7_DUSP26	ENSG00000160789.18_LMNA	-0.4772	-0.591468004	-0.34414597	5.11E-10	8.95E-08	2.62E-05
ENSG00000132424.13_PNISR	ENSG00000160789.18_LMNA	-0.13744	-0.290293141	0.022242992	9.13E-02	2.95E-01	1.00E+00
ENSG00000213123.9_TCTEX1D2	ENSG00000160789.18_LMNA	-0.0003	-0.159490761	0.158910615	9.97E-01	9.99E-01	1.00E+00
ENSG00000070808.14_CAMK2A	ENSG00000160789.18_LMNA	-0.03869	-0.196678497	0.121258369	6.36E-01	8.37E-01	1.00E+00
ENSG00000136869.13_TLR4	ENSG00000160789.18_LMNA	-0.19007	-0.339008793	-0.03182842	1.90E-02	1.04E-01	1.00E+00
ENSG00000160799.10_CCDC12	ENSG00000160789.18_LMNA	-0.06833	-0.225084901	0.091867277	4.03E-01	6.79E-01	1.00E+00
ENSG00000197355.9_UAP1L1	ENSG00000160789.18_LMNA	0.30672	0.155096506	0.444232303	1.21E-04	2.21E-03	1.00E+00
ENSG00000036672.14_USP2	ENSG00000160789.18_LMNA	-0.30331	-0.44120485	-0.15141946	1.46E-04	2.53E-03	1.00E+00
ENSG00000103742.10_IGDCC4	ENSG00000160789.18_LMNA	0.31142	0.16015454	0.448386015	9.41E-05	1.80E-03	1.00E+00
ENSG00000152778.8_IFIT5	ENSG00000160789.18_LMNA	-0.28869	-0.428208252	-0.13572481	3.10E-04	4.62E-03	1.00E+00
ENSG00000157600.10_TMEM164	ENSG00000160789.18_LMNA	0.29646	0.144062328	0.435127575	2.08E-04	3.37E-03	1.00E+00
ENSG00000178996.11_SNX18	ENSG00000160789.18_LMNA	-0.02747	-0.185862087	0.132305062	7.37E-01	8.89E-01	1.00E+00
ENSG00000177854.7_TMEM187	ENSG00000160789.18_LMNA	0.04263	-0.117363641	0.200473669	6.02E-01	8.17E-01	1.00E+00
ENSG00000184515.9_BEX5	ENSG00000160789.18_LMNA	-0.25364	-0.396819241	-0.09841541	1.62E-03	1.66E-02	1.00E+00
ENSG00000164938.12_TP53INP1	ENSG00000160789.18_LMNA	-0.30237	-0.44037288	-0.15041038	1.53E-04	2.64E-03	1.00E+00
ENSG00000087095.11_NLK	ENSG00000160789.18_LMNA	-0.07895	-0.235195917	0.081270784	3.34E-01	6.19E-01	1.00E+00
ENSG00000198933.8_TBKBP1	ENSG00000160789.18_LMNA	-0.14775	-0.299900375	0.011722345	6.93E-02	2.48E-01	1.00E+00
ENSG00000220848.4_RPS18P9	ENSG00000160789.18_LMNA	-0.10658	-0.261343194	0.053532264	1.91E-01	4.59E-01	1.00E+00
ENSG00000168765.14_GSTM4	ENSG00000160789.18_LMNA	0.11026	-0.049812477	0.264814801	1.76E-01	4.38E-01	1.00E+00
ENSG00000048162.18_NOP16	ENSG00000160789.18_LMNA	0.18916	0.030888045	0.338175406	1.96E-02	1.06E-01	1.00E+00
ENSG00000170260.7_ZNF212	ENSG00000160789.18_LMNA	0.14347	-0.016100489	0.29591001	7.78E-02	2.67E-01	1.00E+00
ENSG00000087502.16_ERGIC2	ENSG00000160789.18_LMNA	-0.09834	-0.253569438	0.061830181	2.28E-01	5.05E-01	1.00E+00
ENSG00000119899.12_SLC17A5	ENSG00000160789.18_LMNA	-0.07784	-0.234138143	0.082382707	3.40E-01	6.26E-01	1.00E+00
ENSG00000196943.11_NOP9	ENSG00000160789.18_LMNA	0.20956	0.052096542	0.356854532	9.57E-03	6.35E-02	1.00E+00
ENSG00000167994.10_RAB3IL1	ENSG00000160789.18_LMNA	0.1485	-0.0109601	0.300593994	6.79E-02	2.44E-01	1.00E+00

ENSG00000204217.11_BMPR2	ENSG00000160789.18_LMNA	-0.02102	-0.179617524	0.13864679	7.97E-01	9.18E-01	1.00E+00
ENSG00000105849.5_TWISTNB	ENSG00000160789.18_LMNA	0.16124	0.002087944	0.312416419	4.72E-02	1.94E-01	1.00E+00
ENSG00000082212.10_ME2	ENSG00000160789.18_LMNA	-0.25733	-0.400138935	-0.10232197	1.37E-03	1.47E-02	1.00E+00
ENSG00000159086.13_PAXBP1	ENSG00000160789.18_LMNA	0.00725	-0.152124745	0.16626034	9.29E-01	9.73E-01	1.00E+00
ENSG00000101654.16_RNMT	ENSG00000160789.18_LMNA	0.03033	-0.129493489	0.188622384	7.11E-01	8.77E-01	1.00E+00
ENSG00000277203.1_F8A1	ENSG00000160789.18_LMNA	0.11176	-0.04830182	0.266222343	1.70E-01	4.29E-01	1.00E+00
ENSG00000103264.14_FBXO31	ENSG00000160789.18_LMNA	0.0372	-0.122731608	0.195240427	6.49E-01	8.44E-01	1.00E+00
ENSG00000033170.15_FUT8	ENSG00000160789.18_LMNA	0.04391	-0.116100412	0.201702563	5.91E-01	8.11E-01	1.00E+00
ENSG00000173120.13_KDM2A	ENSG00000160789.18_LMNA	0.25864	0.103713157	0.401319244	1.29E-03	1.41E-02	1.00E+00
ENSG00000157181.13_C1orf27	ENSG00000160789.18_LMNA	0.05131	-0.108779045	0.208805282	5.30E-01	7.73E-01	1.00E+00
ENSG00000259623.1_RP11-156E	ENSG00000160789.18_LMNA	-0.26522	-0.40722954	-0.11069695	9.59E-04	1.12E-02	1.00E+00
ENSG00000132182.10_NUP210	ENSG00000160789.18_LMNA	0.25856	0.103625991	0.40124532	1.30E-03	1.41E-02	1.00E+00
ENSG00000132356.10_PRKAA1	ENSG00000160789.18_LMNA	-0.00175	-0.160906925	0.157493528	9.83E-01	9.94E-01	1.00E+00
ENSG00000197150.11_ABCB8	ENSG00000160789.18_LMNA	0.29138	0.138610237	0.430606762	2.70E-04	4.14E-03	1.00E+00
ENSG00000174939.9_ASPHD1	ENSG00000160789.18_LMNA	0.05497	-0.105150484	0.212313104	5.01E-01	7.53E-01	1.00E+00
ENSG00000092051.15_JPH4	ENSG00000160789.18_LMNA	-0.37452	-0.503688503	-0.22897103	2.00E-06	7.98E-05	1.03E-01
ENSG00000162755.12_KLHDC9	ENSG00000160789.18_LMNA	0.04539	-0.114640006	0.203122029	5.79E-01	8.04E-01	1.00E+00
ENSG00000170871.10_KIAA0232	ENSG00000160789.18_LMNA	-0.13891	-0.29166299	0.020747123	8.79E-02	2.88E-01	1.00E+00
ENSG00000139370.9_SLC15A4	ENSG00000160789.18_LMNA	-0.00797	-0.166960126	0.151421559	9.22E-01	9.70E-01	1.00E+00
ENSG00000127241.15_MASP1	ENSG00000160789.18_LMNA	-0.35995	-0.491013833	-0.21295278	5.24E-06	1.72E-04	2.69E-01
ENSG00000161277.10_THAP8	ENSG00000160789.18_LMNA	-0.07378	-0.230271338	0.086440767	3.66E-01	6.47E-01	1.00E+00
ENSG00000076984.16_MAP2K7	ENSG00000160789.18_LMNA	0.094	-0.066195636	0.249463319	2.49E-01	5.30E-01	1.00E+00
ENSG00000141385.8_AFG3L2	ENSG00000160789.18_LMNA	0.21141	0.054031924	0.358547047	8.93E-03	6.04E-02	1.00E+00
ENSG00000135974.8_C2orf49	ENSG00000160789.18_LMNA	-0.37886	-0.507450095	-0.23375374	1.49E-06	6.38E-05	7.63E-02
ENSG00000175806.13_MSRA	ENSG00000160789.18_LMNA	-0.028	-0.186369855	0.131788246	7.32E-01	8.87E-01	1.00E+00
ENSG00000156959.8_LHFPL4	ENSG00000160789.18_LMNA	-0.09876	-0.253966132	0.061407792	2.26E-01	5.02E-01	1.00E+00
ENSG00000249855.1_EEF1A1P1!	ENSG00000160789.18_LMNA	-0.13188	-0.285093233	0.027908557	1.05E-01	3.22E-01	1.00E+00
ENSG00000150787.6_PTS	ENSG00000160789.18_LMNA	-0.18374	-0.333198521	-0.02528353	2.35E-02	1.21E-01	1.00E+00
ENSG00000129933.19_MAU2	ENSG00000160789.18_LMNA	0.03825	-0.121695963	0.196251491	6.40E-01	8.39E-01	1.00E+00
ENSG00000145431.9_PDGFC	ENSG00000160789.18_LMNA	0.07141	-0.088795715	0.228022783	3.82E-01	6.61E-01	1.00E+00
ENSG00000102547.17_CAB39L	ENSG00000160789.18_LMNA	-0.36817	-0.498169821	-0.22197819	3.06E-06	1.12E-04	1.57E-01
ENSG00000105447.11_GRWD1	ENSG00000160789.18_LMNA	0.31852	0.167831229	0.454666501	6.35E-05	1.30E-03	1.00E+00
ENSG00000154310.15_TNIK	ENSG00000160789.18_LMNA	-0.08585	-0.24174349	0.074370452	2.93E-01	5.79E-01	1.00E+00

ENSG00000110851.10_PRDM4	ENSG00000160789.18_LMNA	0.17093	0.012057886	0.321385544	3.52E-02	1.59E-01	1.00E+00
ENSG00000164576.10_SAP30L	ENSG00000160789.18_LMNA	-0.02304	-0.18157714	0.136659489	7.78E-01	9.09E-01	1.00E+00
ENSG00000140830.7_TXNL4B	ENSG00000160789.18_LMNA	-0.11629	-0.270484543	0.043718586	1.54E-01	4.04E-01	1.00E+00
ENSG00000185238.11_PRMT3	ENSG00000160789.18_LMNA	-0.17804	-0.327940932	-0.01938387	2.82E-02	1.37E-01	1.00E+00
ENSG00000133250.12_ZNF414	ENSG00000160789.18_LMNA	0.04878	-0.111286961	0.206376031	5.51E-01	7.87E-01	1.00E+00
ENSG00000181991.14_MRPS11	ENSG00000160789.18_LMNA	-0.15046	-0.302414729	0.0089575	6.43E-02	2.36E-01	1.00E+00
ENSG00000111707.10_SUDS3	ENSG00000160789.18_LMNA	-0.01301	-0.171850381	0.146498533	8.74E-01	9.51E-01	1.00E+00
ENSG00000162174.11_ASRGL1	ENSG00000160789.18_LMNA	-0.28632	-0.426100089	-0.13319276	3.49E-04	5.05E-03	1.00E+00
ENSG00000242960.1_FTH1P23	ENSG00000160789.18_LMNA	0.02037	-0.139280232	0.178992375	8.03E-01	9.21E-01	1.00E+00
ENSG00000261221.3_ZNF865	ENSG00000160789.18_LMNA	0.33785	0.188803081	0.471679896	2.07E-05	5.33E-04	1.00E+00
ENSG00000173273.14_TNKS	ENSG00000160789.18_LMNA	-0.0124	-0.171260272	0.147093434	8.80E-01	9.53E-01	1.00E+00
ENSG00000089048.13_ESF1	ENSG00000160789.18_LMNA	0.25901	0.104100321	0.401647547	1.27E-03	1.39E-02	1.00E+00
ENSG00000066654.11_THUMPD1	ENSG00000160789.18_LMNA	-0.16532	-0.316197272	-0.00628317	4.18E-02	1.79E-01	1.00E+00
ENSG00000183955.11_KMT5A	ENSG00000160789.18_LMNA	0.26851	0.114188712	0.410175287	8.24E-04	9.95E-03	1.00E+00
ENSG00000198586.12_TLK1	ENSG00000160789.18_LMNA	0.19003	0.031795273	0.338979425	1.90E-02	1.04E-01	1.00E+00
ENSG00000167632.13_TRAPPC9	ENSG00000160789.18_LMNA	-0.01142	-0.170310652	0.148050283	8.89E-01	9.57E-01	1.00E+00
ENSG00000095380.10_NANS	ENSG00000160789.18_LMNA	0.23836	0.082283881	0.383027904	3.10E-03	2.74E-02	1.00E+00
ENSG00000169006.6_NTSR2	ENSG00000160789.18_LMNA	-0.25925	-0.401865621	-0.10435754	1.26E-03	1.38E-02	1.00E+00
ENSG00000145016.12_RUBCN	ENSG00000160789.18_LMNA	0.07064	-0.089570543	0.22728222	3.87E-01	6.66E-01	1.00E+00
ENSG00000167461.10_RAB8A	ENSG00000160789.18_LMNA	0.16445	0.005392028	0.31539497	4.29E-02	1.82E-01	1.00E+00
ENSG00000105287.11_PRKD2	ENSG00000160789.18_LMNA	0.44862	0.311678691	0.567302811	6.78E-09	7.76E-07	3.48E-04
ENSG00000116701.13_NCF2	ENSG00000160789.18_LMNA	0.04946	-0.110610089	0.207032057	5.45E-01	7.83E-01	1.00E+00
ENSG00000107960.9_STN1	ENSG00000160789.18_LMNA	0.07591	-0.084313155	0.232299925	3.53E-01	6.36E-01	1.00E+00
ENSG00000090316.14_MAEA	ENSG00000160789.18_LMNA	0.04161	-0.118370807	0.199493162	6.11E-01	8.22E-01	1.00E+00
ENSG00000139531.11_SUOX	ENSG00000160789.18_LMNA	-0.23228	-0.377519727	-0.07588478	3.98E-03	3.30E-02	1.00E+00
ENSG00000163932.12_PRKCD	ENSG00000160789.18_LMNA	0.06449	-0.095688814	0.221421622	4.30E-01	7.00E-01	1.00E+00
ENSG00000171160.16_MORN4	ENSG00000160789.18_LMNA	-0.35597	-0.487538649	-0.20858679	6.77E-06	2.14E-04	3.47E-01
ENSG00000162981.13_FAM84A	ENSG00000160789.18_LMNA	0.3471	0.198891432	0.479789715	1.18E-05	3.34E-04	6.06E-01
ENSG00000261061.1_RP11-303E	ENSG00000160789.18_LMNA	0.12689	-0.032972398	0.280429948	1.19E-01	3.47E-01	1.00E+00
ENSG00000100083.17_GGA1	ENSG00000160789.18_LMNA	-0.04655	-0.204238959	0.113489895	5.69E-01	7.98E-01	1.00E+00
ENSG00000151778.9_SERP2	ENSG00000160789.18_LMNA	-0.47384	-0.588640368	-0.34031608	7.01E-10	1.17E-07	3.60E-05
ENSG00000108061.10_SHOC2	ENSG00000160789.18_LMNA	-0.14335	-0.295800329	0.01622066	7.81E-02	2.68E-01	1.00E+00
ENSG00000114120.10_SLC25A36	ENSG00000160789.18_LMNA	-0.29096	-0.430235022	-0.13816271	2.76E-04	4.21E-03	1.00E+00

ENSG00000143756.10_FBXO28	ENSG00000160789.18_LMNA	-0.05743	-0.214666958	0.102710863	4.82E-01	7.40E-01	1.00E+00
ENSG00000039650.8_PNKP	ENSG00000160789.18_LMNA	0.27317	0.119151631	0.414351545	6.61E-04	8.39E-03	1.00E+00
ENSG00000253716.4_MINCR	ENSG00000160789.18_LMNA	-0.19855	-0.346789353	-0.04063416	1.42E-02	8.45E-02	1.00E+00
ENSG00000166851.13_PLK1	ENSG00000160789.18_LMNA	0.1148	-0.04522662	0.269083499	1.59E-01	4.12E-01	1.00E+00
ENSG00000111371.14_SLC38A1	ENSG00000160789.18_LMNA	-0.22992	-0.37538037	-0.07340608	4.38E-03	3.55E-02	1.00E+00
ENSG00000101003.9_GINS1	ENSG00000160789.18_LMNA	0.01505	-0.144500722	0.173830427	8.54E-01	9.43E-01	1.00E+00
ENSG00000146757.12_ZNF92	ENSG00000160789.18_LMNA	-0.07157	-0.228168888	0.088642804	3.81E-01	6.60E-01	1.00E+00
ENSG00000135090.12_TAOK3	ENSG00000160789.18_LMNA	-0.05795	-0.215165769	0.102193386	4.78E-01	7.36E-01	1.00E+00
ENSG00000156795.5_WDYHV1	ENSG00000160789.18_LMNA	-0.18309	-0.332595108	-0.02460534	2.40E-02	1.23E-01	1.00E+00
ENSG00000117020.15_AKT3	ENSG00000160789.18_LMNA	-0.21273	-0.359742955	-0.05540082	8.51E-03	5.83E-02	1.00E+00
ENSG00000150760.11_DOCK1	ENSG00000160789.18_LMNA	0.1945	0.036429276	0.343079256	1.63E-02	9.33E-02	1.00E+00
ENSG00000112304.9_ACOT13	ENSG00000160789.18_LMNA	-0.08745	-0.243261146	0.072766697	2.84E-01	5.70E-01	1.00E+00
ENSG00000204852.14_TCTN1	ENSG00000160789.18_LMNA	0.11715	-0.042847327	0.271293384	1.51E-01	3.99E-01	1.00E+00
ENSG00000164331.8_ANKRA2	ENSG00000160789.18_LMNA	-0.26384	-0.405987585	-0.10922698	1.02E-03	1.18E-02	1.00E+00
ENSG00000177409.10_SAMD9L	ENSG00000160789.18_LMNA	0.17074	0.011861899	0.321209764	3.55E-02	1.60E-01	1.00E+00
ENSG00000147592.7_LACTB2	ENSG00000160789.18_LMNA	-0.17023	-0.320740175	-0.01133844	3.60E-02	1.62E-01	1.00E+00
ENSG00000167508.9_MVD	ENSG00000160789.18_LMNA	0.14306	-0.016519015	0.295527982	7.87E-02	2.69E-01	1.00E+00
ENSG00000196411.8_EPHB4	ENSG00000160789.18_LMNA	0.60108	0.488643726	0.693885276	2.67E-16	6.85E-13	1.37E-11
ENSG00000111801.14_BTN3A3	ENSG00000160789.18_LMNA	0.06207	-0.098102645	0.219103132	4.47E-01	7.14E-01	1.00E+00
ENSG00000156304.13_SCAF4	ENSG00000160789.18_LMNA	0.3001	0.147968513	0.43835754	1.72E-04	2.91E-03	1.00E+00
ENSG00000169562.9_GJB1	ENSG00000160789.18_LMNA	-0.30308	-0.441004696	-0.15117664	1.47E-04	2.56E-03	1.00E+00
ENSG00000169860.6_P2RY1	ENSG00000160789.18_LMNA	0.15119	-0.00820524	0.303098085	6.30E-02	2.33E-01	1.00E+00
ENSG00000272821.1_CTA-384D8	ENSG00000160789.18_LMNA	0.01934	-0.140290038	0.177995258	8.13E-01	9.26E-01	1.00E+00
ENSG00000269044.2_CTC-429P9	ENSG00000160789.18_LMNA	-0.17846	-0.328330982	-0.01982082	2.78E-02	1.36E-01	1.00E+00
ENSG00000177519.3_RPRM	ENSG00000160789.18_LMNA	-0.47526	-0.589835912	-0.34193438	6.13E-10	1.05E-07	3.15E-05
ENSG00000160551.8_TAOK1	ENSG00000160789.18_LMNA	-0.04075	-0.198657313	0.119228865	6.18E-01	8.27E-01	1.00E+00
ENSG00000085514.14_PILRA	ENSG00000160789.18_LMNA	-0.03574	-0.193841063	0.124163852	6.62E-01	8.51E-01	1.00E+00
ENSG00000166173.10_LARP6	ENSG00000160789.18_LMNA	-0.06111	-0.218185046	0.099057454	4.55E-01	7.19E-01	1.00E+00
ENSG00000175832.11_ETV4	ENSG00000160789.18_LMNA	0.47426	0.340785748	0.588987452	6.74E-10	1.13E-07	3.46E-05
ENSG00000160284.13_SPATC1L	ENSG00000160789.18_LMNA	0.18532	0.026912486	0.334646825	2.23E-02	1.17E-01	1.00E+00
ENSG00000131069.18_ACSS2	ENSG00000160789.18_LMNA	0.25106	0.095688376	0.394497239	1.81E-03	1.81E-02	1.00E+00
ENSG00000223803.1_RPS20P14	ENSG00000160789.18_LMNA	-0.07127	-0.227888011	0.088936751	3.83E-01	6.61E-01	1.00E+00
ENSG00000107104.17_KANK1	ENSG00000160789.18_LMNA	0.049	-0.111070104	0.20658624	5.49E-01	7.86E-01	1.00E+00

ENSG00000177875.4_CCDC184	ENSG00000160789.18_LMNA	0.05309	-0.107017974	0.210508775	5.16E-01	7.63E-01	1.00E+00
ENSG00000117114.18_ADGRL2	ENSG00000160789.18_LMNA	0.10528	-0.054837835	0.260122794	1.97E-01	4.66E-01	1.00E+00
ENSG00000123739.9_PLA2G12A	ENSG00000160789.18_LMNA	-0.07576	-0.232164991	0.084454765	3.54E-01	6.37E-01	1.00E+00
ENSG00000170271.9_FAXDC2	ENSG00000160789.18_LMNA	-0.05554	-0.212858837	0.104585207	4.97E-01	7.51E-01	1.00E+00
ENSG00000127528.5_KLF2	ENSG00000160789.18_LMNA	-0.10997	-0.264540402	0.05010681	1.77E-01	4.39E-01	1.00E+00
ENSG00000163781.11_TOPBP1	ENSG00000160789.18_LMNA	-0.00655	-0.165582208	0.152805865	9.36E-01	9.75E-01	1.00E+00
ENSG00000136478.6_TEX2	ENSG00000160789.18_LMNA	0.08584	-0.074376756	0.241737522	2.93E-01	5.79E-01	1.00E+00
ENSG00000122299.10_ZC3H7A	ENSG00000160789.18_LMNA	-0.04566	-0.203378042	0.114376463	5.76E-01	8.03E-01	1.00E+00
ENSG00000151445.14_VIPAS39	ENSG00000160789.18_LMNA	-0.13514	-0.288144926	0.024586017	9.69E-02	3.06E-01	1.00E+00
ENSG00000100395.13_L3MBTL2	ENSG00000160789.18_LMNA	-0.04096	-0.198865252	0.119015445	6.16E-01	8.26E-01	1.00E+00
ENSG00000108651.8_UTP6	ENSG00000160789.18_LMNA	-0.22253	-0.368670008	-0.06565543	5.86E-03	4.43E-02	1.00E+00
ENSG00000142002.15_DPP9	ENSG00000160789.18_LMNA	0.43206	0.293009328	0.553205071	2.73E-08	2.50E-06	1.40E-03
ENSG00000134265.11_NAPG	ENSG00000160789.18_LMNA	-0.2048	-0.352508149	-0.04713694	1.14E-02	7.18E-02	1.00E+00
ENSG00000161888.10_SPC24	ENSG00000160789.18_LMNA	-0.01348	-0.172309275	0.146035752	8.69E-01	9.49E-01	1.00E+00
ENSG00000104517.11_UBR5	ENSG00000160789.18_LMNA	0.1063	-0.053811602	0.261082163	1.92E-01	4.61E-01	1.00E+00
ENSG00000196367.11_TRRAP	ENSG00000160789.18_LMNA	0.29771	0.14540039	0.436234842	1.95E-04	3.20E-03	1.00E+00
ENSG00000112237.11_CCNC	ENSG00000160789.18_LMNA	-0.31296	-0.449754941	-0.16182482	8.65E-05	1.68E-03	1.00E+00
ENSG00000179144.4_GIMAP7	ENSG00000160789.18_LMNA	-0.03769	-0.195711665	0.122248999	6.45E-01	8.42E-01	1.00E+00
ENSG00000155926.12_SLA	ENSG00000160789.18_LMNA	-0.02793	-0.186305925	0.131853325	7.33E-01	8.87E-01	1.00E+00
ENSG00000171681.11_ATF7IP	ENSG00000160789.18_LMNA	-0.17827	-0.328157933	-0.01962694	2.80E-02	1.37E-01	1.00E+00
ENSG00000117222.12_RBBP5	ENSG00000160789.18_LMNA	-0.12134	-0.275221064	0.038609743	1.36E-01	3.76E-01	1.00E+00
ENSG00000109103.10_UNC119	ENSG00000160789.18_LMNA	-0.35993	-0.490997406	-0.21293211	5.25E-06	1.72E-04	2.69E-01
ENSG00000197070.12_ARRDC1	ENSG00000160789.18_LMNA	0.22011	0.063121936	0.366469683	6.43E-03	4.74E-02	1.00E+00
ENSG00000140400.13_MAN2C1	ENSG00000160789.18_LMNA	0.11542	-0.04459701	0.269668601	1.57E-01	4.09E-01	1.00E+00
ENSG00000145604.14_SKP2	ENSG00000160789.18_LMNA	0.05496	-0.105161227	0.212302731	5.01E-01	7.53E-01	1.00E+00
ENSG00000122585.6_NPY	ENSG00000160789.18_LMNA	-0.28249	-0.422684157	-0.12909809	4.22E-04	5.91E-03	1.00E+00
ENSG00000144635.7_DYNC1LI1	ENSG00000160789.18_LMNA	-0.21146	-0.358590595	-0.05408175	8.92E-03	6.04E-02	1.00E+00
ENSG00000043514.14_TRIT1	ENSG00000160789.18_LMNA	-0.25744	-0.400238402	-0.10243916	1.37E-03	1.46E-02	1.00E+00
ENSG00000156162.15_DPY19L4	ENSG00000160789.18_LMNA	-0.11853	-0.272591279	0.041448273	1.46E-01	3.91E-01	1.00E+00
ENSG00000118707.8_TGIF2	ENSG00000160789.18_LMNA	0.01778	-0.141822571	0.176480732	8.28E-01	9.33E-01	1.00E+00
ENSG00000105617.3_LENG1	ENSG00000160789.18_LMNA	0.08162	-0.078604072	0.23772971	3.17E-01	6.04E-01	1.00E+00
ENSG00000135845.8_PIGC	ENSG00000160789.18_LMNA	-0.19729	-0.345636049	-0.03932589	1.48E-02	8.73E-02	1.00E+00
ENSG00000144354.12_CDCA7	ENSG00000160789.18_LMNA	-0.01811	-0.176799188	0.141500456	8.25E-01	9.31E-01	1.00E+00

ENSG00000090924.13_PLEKHG2	ENSG00000160789.18_LMNA	0.29429	0.141731107	0.433196346	2.33E-04	3.69E-03	1.00E+00
ENSG00000099889.12_ARVCF	ENSG00000160789.18_LMNA	-0.15659	-0.308112852	0.002674032	5.40E-02	2.11E-01	1.00E+00
ENSG00000226396.1_RP5-1056L:	ENSG00000160789.18_LMNA	-0.03049	-0.18877207	0.129340876	7.09E-01	8.76E-01	1.00E+00
ENSG00000171793.12_CTPS1	ENSG00000160789.18_LMNA	0.06243	-0.097741183	0.219450545	4.45E-01	7.12E-01	1.00E+00
ENSG00000215006.4_CHCHD2P2	ENSG00000160789.18_LMNA	-0.0347	-0.192830844	0.125196983	6.71E-01	8.56E-01	1.00E+00
ENSG00000168301.11_KCTD6	ENSG00000160789.18_LMNA	-0.32041	-0.456331886	-0.1698727	5.72E-05	1.19E-03	1.00E+00
ENSG00000110002.14_VWA5A	ENSG00000160789.18_LMNA	0.22826	0.071665309	0.373875997	4.68E-03	3.73E-02	1.00E+00
ENSG00000185963.12_BICD2	ENSG00000160789.18_LMNA	0.24005	0.084066482	0.384558522	2.89E-03	2.60E-02	1.00E+00
ENSG00000122877.12_EGR2	ENSG00000160789.18_LMNA	0.35982	0.212804736	0.490896141	5.29E-06	1.74E-04	2.71E-01
ENSG00000204856.10_FAM216A	ENSG00000160789.18_LMNA	-0.36866	-0.498598847	-0.2225208	2.96E-06	1.09E-04	1.52E-01
ENSG00000116918.12_TSNAX	ENSG00000160789.18_LMNA	-0.26953	-0.411086807	-0.11527067	7.85E-04	9.59E-03	1.00E+00
ENSG00000265415.1_CTD-2510F	ENSG00000160789.18_LMNA	0.07609	-0.084125186	0.232479012	3.51E-01	6.35E-01	1.00E+00
ENSG00000165861.12_ZFYVE1	ENSG00000160789.18_LMNA	0.33202	0.18246836	0.466562965	2.93E-05	6.99E-04	1.00E+00
ENSG00000136960.11_ENPP2	ENSG00000160789.18_LMNA	-0.30445	-0.442219404	-0.15265081	1.37E-04	2.42E-03	1.00E+00
ENSG00000067248.8_DHX29	ENSG00000160789.18_LMNA	0.16261	0.003501969	0.313691875	4.53E-02	1.89E-01	1.00E+00
ENSG00000132842.12_AP3B1	ENSG00000160789.18_LMNA	0.17755	0.018881559	0.327492407	2.86E-02	1.39E-01	1.00E+00
ENSG00000065559.13_MAP2K4	ENSG00000160789.18_LMNA	-0.40005	-0.525762955	-0.25722852	3.29E-07	1.89E-05	1.69E-02
ENSG00000133561.14_GIMAP6	ENSG00000160789.18_LMNA	-0.01305	-0.171897508	0.146451013	8.73E-01	9.51E-01	1.00E+00
ENSG00000238142.1_RP11-108M	ENSG00000160789.18_LMNA	0.04996	-0.110118518	0.207508311	5.41E-01	7.81E-01	1.00E+00
ENSG00000166002.5_SMCO4	ENSG00000160789.18_LMNA	-0.17182	-0.322203447	-0.01297013	3.43E-02	1.56E-01	1.00E+00
ENSG00000011405.12_PIK3C2A	ENSG00000160789.18_LMNA	0.017	-0.142586095	0.175725617	8.35E-01	9.36E-01	1.00E+00
ENSG00000214655.9_ZSWIM8	ENSG00000160789.18_LMNA	0.06206	-0.098111512	0.219094608	4.48E-01	7.14E-01	1.00E+00
ENSG00000104312.7_RIPK2	ENSG00000160789.18_LMNA	-0.05349	-0.210896055	0.106617326	5.13E-01	7.61E-01	1.00E+00
ENSG00000116747.11_TROVE2	ENSG00000160789.18_LMNA	-0.01674	-0.175473825	0.142840606	8.38E-01	9.37E-01	1.00E+00
ENSG00000166326.6_TRIM44	ENSG00000160789.18_LMNA	0.12974	-0.0300799	0.283095459	1.11E-01	3.32E-01	1.00E+00
ENSG00000184144.8_CNTN2	ENSG00000160789.18_LMNA	-0.25589	-0.398846687	-0.10080018	1.46E-03	1.54E-02	1.00E+00
ENSG00000168818.8_STX18	ENSG00000160789.18_LMNA	-0.23645	-0.381294147	-0.08026702	3.36E-03	2.91E-02	1.00E+00
ENSG00000068366.18_ACSL4	ENSG00000160789.18_LMNA	-0.02057	-0.179184479	0.139085607	8.01E-01	9.20E-01	1.00E+00
ENSG00000007384.14_RHBDF1	ENSG00000160789.18_LMNA	0.32347	0.173182965	0.459028076	4.81E-05	1.04E-03	1.00E+00
ENSG00000163510.12_CWC22	ENSG00000160789.18_LMNA	0.06798	-0.092220245	0.224746926	4.05E-01	6.81E-01	1.00E+00
ENSG00000151692.13_RNF144A	ENSG00000160789.18_LMNA	-0.19345	-0.34211056	-0.03533318	1.69E-02	9.58E-02	1.00E+00
ENSG00000138443.14_ABI2	ENSG00000160789.18_LMNA	-0.05257	-0.210012295	0.107531442	5.20E-01	7.66E-01	1.00E+00
ENSG00000121864.8_ZNF639	ENSG00000160789.18_LMNA	-0.30153	-0.439631151	-0.14951126	1.60E-04	2.74E-03	1.00E+00

ENSG00000181585.3_TMIE	ENSG00000160789.18_LMNA	-0.08168	-0.237791217	0.078539283	3.17E-01	6.04E-01	1.00E+00
ENSG00000198874.11_TYW1	ENSG00000160789.18_LMNA	0.31712	0.166320486	0.453432775	6.87E-05	1.39E-03	1.00E+00
ENSG00000119673.13_ACOT2	ENSG00000160789.18_LMNA	0.19928	0.04139189	0.347456901	1.38E-02	8.28E-02	1.00E+00
ENSG00000155906.15_RMND1	ENSG00000160789.18_LMNA	-0.13635	-0.289269113	0.023360312	9.39E-02	3.00E-01	1.00E+00
ENSG00000135631.14_RAB11FIP	ENSG00000160789.18_LMNA	0.31739	0.166609595	0.453668957	6.77E-05	1.37E-03	1.00E+00
ENSG00000105229.5_PIAS4	ENSG00000160789.18_LMNA	0.11555	-0.044468703	0.26978781	1.56E-01	4.08E-01	1.00E+00
ENSG00000175110.10_MRPS22	ENSG00000160789.18_LMNA	-0.29632	-0.435000492	-0.14390882	2.10E-04	3.39E-03	1.00E+00
ENSG00000144840.7_RABL3	ENSG00000160789.18_LMNA	-0.0209	-0.179499086	0.138766818	7.98E-01	9.19E-01	1.00E+00
ENSG00000196313.10_POM121	ENSG00000160789.18_LMNA	0.24004	0.084050176	0.384544528	2.90E-03	2.60E-02	1.00E+00
ENSG00000163655.14_GMPS	ENSG00000160789.18_LMNA	0.27523	0.121344595	0.416192956	6.00E-04	7.79E-03	1.00E+00
ENSG00000177169.8_ULK1	ENSG00000160789.18_LMNA	0.13385	-0.025905987	0.286933317	1.00E-01	3.12E-01	1.00E+00
ENSG00000141736.12_ERBB2	ENSG00000160789.18_LMNA	0.37929	0.234234383	0.507827545	1.44E-06	6.23E-05	7.41E-02
ENSG00000068724.14_TTC7A	ENSG00000160789.18_LMNA	0.17335	0.014550507	0.323619308	3.27E-02	1.51E-01	1.00E+00
ENSG00000155465.17_SLC7A7	ENSG00000160789.18_LMNA	-0.01468	-0.173477834	0.144856668	8.58E-01	9.44E-01	1.00E+00
ENSG00000102934.8_PLLP	ENSG00000160789.18_LMNA	-0.23951	-0.384062843	-0.08348899	2.96E-03	2.65E-02	1.00E+00
ENSG00000176476.7_SGF29	ENSG00000160789.18_LMNA	-0.18371	-0.333171028	-0.02525263	2.35E-02	1.21E-01	1.00E+00
ENSG00000120837.6_NFYB	ENSG00000160789.18_LMNA	-0.18236	-0.331924434	-0.02385188	2.45E-02	1.25E-01	1.00E+00
ENSG00000166881.8_NEMP1	ENSG00000160789.18_LMNA	0.10333	-0.056809363	0.258277974	2.05E-01	4.77E-01	1.00E+00
ENSG00000148297.14_MED22	ENSG00000160789.18_LMNA	0.22589	0.069174827	0.371720952	5.14E-03	4.01E-02	1.00E+00
ENSG00000102125.14_TAZ	ENSG00000160789.18_LMNA	0.00103	-0.158193838	0.160207235	9.90E-01	9.96E-01	1.00E+00
ENSG00000011566.13_MAP4K3	ENSG00000160789.18_LMNA	-0.20352	-0.351334359	-0.04580013	1.19E-02	7.44E-02	1.00E+00
ENSG00000158717.9_RNF166	ENSG00000160789.18_LMNA	0.09775	-0.062417088	0.253018057	2.31E-01	5.09E-01	1.00E+00
ENSG00000103005.10_USB1	ENSG00000160789.18_LMNA	0.30429	0.152471414	0.442071639	1.38E-04	2.44E-03	1.00E+00
ENSG00000198363.14_ASPH	ENSG00000160789.18_LMNA	0.10958	-0.050506661	0.264167549	1.79E-01	4.41E-01	1.00E+00
ENSG00000122729.17_ACO1	ENSG00000160789.18_LMNA	0.03078	-0.129055964	0.189051478	7.07E-01	8.75E-01	1.00E+00
ENSG00000105875.12_WDR91	ENSG00000160789.18_LMNA	0.12698	-0.032889009	0.280506861	1.19E-01	3.46E-01	1.00E+00
ENSG00000100294.11_MCAT	ENSG00000160789.18_LMNA	-0.01088	-0.169782944	0.14858175	8.94E-01	9.59E-01	1.00E+00
ENSG00000138794.8_CASP6	ENSG00000160789.18_LMNA	0.27899	0.125360945	0.419559188	5.00E-04	6.74E-03	1.00E+00
ENSG00000119688.19_ABCD4	ENSG00000160789.18_LMNA	0.13394	-0.025811739	0.287019862	9.99E-02	3.11E-01	1.00E+00
ENSG00000124383.7_MPHOSPH	ENSG00000160789.18_LMNA	0.08856	-0.071651939	0.244315147	2.78E-01	5.63E-01	1.00E+00
ENSG00000173193.12_PARP14	ENSG00000160789.18_LMNA	0.21202	0.054669363	0.359104058	8.73E-03	5.94E-02	1.00E+00
ENSG00000114013.14_CD86	ENSG00000160789.18_LMNA	-0.08249	-0.238561398	0.077727768	3.12E-01	5.99E-01	1.00E+00
ENSG00000157240.3_FZD1	ENSG00000160789.18_LMNA	0.48816	0.356678669	0.60067866	1.78E-10	3.62E-08	9.12E-06

ENSG00000032389.11_TSSC1	ENSG00000160789.18_LMNA	-0.35118	-0.483358524	-0.20334986	9.16E-06	2.72E-04	4.70E-01
ENSG00000089876.10_DHX32	ENSG00000160789.18_LMNA	0.1058	-0.054313692	0.260612864	1.95E-01	4.63E-01	1.00E+00
ENSG00000155100.9_OTUD6B	ENSG00000160789.18_LMNA	-0.13622	-0.289148912	0.023491412	9.43E-02	3.01E-01	1.00E+00
ENSG00000116353.14_MECR	ENSG00000160789.18_LMNA	#######	-0.159252152	0.159149251	9.99E-01	1.00E+00	1.00E+00
ENSG00000217094.2_PPIAP31	ENSG00000160789.18_LMNA	0.09331	-0.066881522	0.248817145	2.53E-01	5.34E-01	1.00E+00
ENSG00000138613.12_APH1B	ENSG00000160789.18_LMNA	-0.2639	-0.406042875	-0.1092924	1.02E-03	1.18E-02	1.00E+00
ENSG00000182606.13_TRAK1	ENSG00000160789.18_LMNA	-0.15519	-0.306811489	0.004111245	5.62E-02	2.17E-01	1.00E+00
ENSG00000150433.8_TMEM218	ENSG00000160789.18_LMNA	-0.34153	-0.474910178	-0.19281431	1.66E-05	4.43E-04	8.52E-01
ENSG00000188783.5_PRELP	ENSG00000160789.18_LMNA	-0.0975	-0.252774792	0.062675958	2.32E-01	5.10E-01	1.00E+00
ENSG00000173436.12_MINOS1	ENSG00000160789.18_LMNA	-0.07048	-0.227127747	0.089732116	3.88E-01	6.66E-01	1.00E+00
ENSG00000156831.6_NSMCE2	ENSG00000160789.18_LMNA	-0.13778	-0.29060665	0.021900764	9.05E-02	2.93E-01	1.00E+00
ENSG00000154997.8_SEPT14	ENSG00000160789.18_LMNA	0.02223	-0.137453387	0.180794604	7.86E-01	9.13E-01	1.00E+00
ENSG00000128159.10_TUBGCP6	ENSG00000160789.18_LMNA	-0.01598	-0.174733746	0.143588436	8.45E-01	9.40E-01	1.00E+00
ENSG00000233429.8_HOTAIRM1	ENSG00000160789.18_LMNA	-0.0158	-0.174564892	0.143759007	8.47E-01	9.40E-01	1.00E+00
ENSG00000072163.17_LIMS2	ENSG00000160789.18_LMNA	-0.37125	-0.500845918	-0.22536558	2.49E-06	9.45E-05	1.28E-01
ENSG00000165474.5_GJB2	ENSG00000160789.18_LMNA	0.17133	0.012473815	0.321758518	3.48E-02	1.58E-01	1.00E+00
ENSG00000129521.12_EGLN3	ENSG00000160789.18_LMNA	-0.07487	-0.231309305	0.085352492	3.59E-01	6.42E-01	1.00E+00
ENSG00000163697.15_APBB2	ENSG00000160789.18_LMNA	-0.28633	-0.426104727	-0.13319832	3.49E-04	5.05E-03	1.00E+00
ENSG00000167766.17_ZNF83	ENSG00000160789.18_LMNA	-0.05585	-0.213152109	0.104281346	4.94E-01	7.49E-01	1.00E+00
ENSG00000100949.13_RABGGTA	ENSG00000160789.18_LMNA	0.18429	0.025849758	0.333702118	2.30E-02	1.19E-01	1.00E+00
ENSG00000108599.13_AKAP10	ENSG00000160789.18_LMNA	-0.18801	-0.337120416	-0.0296984	2.04E-02	1.09E-01	1.00E+00
ENSG00000139880.18_CDH24	ENSG00000160789.18_LMNA	0.14129	-0.018327138	0.293876393	8.25E-02	2.77E-01	1.00E+00
ENSG00000257218.4_GATC	ENSG00000160789.18_LMNA	-0.34019	-0.473730387	-0.1913482	1.80E-05	4.73E-04	9.25E-01
ENSG00000198301.10_SDAD1	ENSG00000160789.18_LMNA	0.12283	-0.037098282	0.276619458	1.32E-01	3.68E-01	1.00E+00
ENSG00000243422.1_RPL23AP49	ENSG00000160789.18_LMNA	-0.19706	-0.345421681	-0.03908283	1.50E-02	8.77E-02	1.00E+00
ENSG00000184304.13_PRKD1	ENSG00000160789.18_LMNA	0.00755	-0.151832383	0.166551328	9.26E-01	9.72E-01	1.00E+00
ENSG00000265808.3_SEC22B	ENSG00000160789.18_LMNA	0.03755	-0.122378341	0.195585385	6.46E-01	8.43E-01	1.00E+00
ENSG00000272990.1_RP11-305K	ENSG00000160789.18_LMNA	-0.14448	-0.296849759	0.015070492	7.58E-02	2.63E-01	1.00E+00
ENSG00000062725.8_APPBP2	ENSG00000160789.18_LMNA	-0.31137	-0.448346491	-0.16010634	9.44E-05	1.80E-03	1.00E+00
ENSG00000178567.7_EPM2AIP1	ENSG00000160789.18_LMNA	-0.28016	-0.420597226	-0.12660141	4.73E-04	6.45E-03	1.00E+00
ENSG00000126249.6_PDCD2L	ENSG00000160789.18_LMNA	-0.20836	-0.355759614	-0.05084573	1.00E-02	6.56E-02	1.00E+00
ENSG00000157734.12_SNX22	ENSG00000160789.18_LMNA	-0.34431	-0.477349174	-0.1958492	1.40E-05	3.86E-04	7.19E-01
ENSG00000197879.13_MYO1C	ENSG00000160789.18_LMNA	0.52579	0.40008145	0.63208384	3.51E-12	1.50E-09	1.80E-07

ENSG00000188820.11_FAM26F	ENSG00000160789.18_LMNA	-0.03784	-0.195856734	0.1221004	6.44E-01	8.41E-01	1.00E+00
ENSG00000048540.13_LMO3	ENSG00000160789.18_LMNA	-0.14506	-0.297394283	0.014473372	7.46E-02	2.60E-01	1.00E+00
ENSG00000123415.13_SMUG1	ENSG00000160789.18_LMNA	-0.30474	-0.442477239	-0.15296389	1.35E-04	2.39E-03	1.00E+00
ENSG00000222041.9_CYTOR	ENSG00000160789.18_LMNA	0.34956	0.201573392	0.481937642	1.01E-05	2.94E-04	5.20E-01
ENSG00000181830.8_SLC35C1	ENSG00000160789.18_LMNA	0.4809	0.3483696	0.594579302	3.59E-10	6.61E-08	1.84E-05
ENSG00000142892.13_PIGK	ENSG00000160789.18_LMNA	-0.00464	-0.163719681	0.154675042	9.55E-01	9.82E-01	1.00E+00
ENSG00000171877.18_FRMD5	ENSG00000160789.18_LMNA	-0.12143	-0.2753065	0.03851744	1.36E-01	3.76E-01	1.00E+00
ENSG00000101098.11_RIMS4	ENSG00000160789.18_LMNA	-0.19484	-0.343387665	-0.0367784	1.62E-02	9.26E-02	1.00E+00
ENSG00000178602.6_OTOS	ENSG00000160789.18_LMNA	-0.10465	-0.259526156	0.05547572	1.99E-01	4.70E-01	1.00E+00
ENSG00000065883.13_CDK13	ENSG00000160789.18_LMNA	0.26331	0.108665067	0.405512539	1.05E-03	1.20E-02	1.00E+00
ENSG00000272034.1_SNORD14A	ENSG00000160789.18_LMNA	-0.00251	-0.161645193	0.156754257	9.76E-01	9.91E-01	1.00E+00
ENSG00000254004.5_ZNF260	ENSG00000160789.18_LMNA	-0.22865	-0.374231417	-0.07207641	4.61E-03	3.68E-02	1.00E+00
ENSG00000239559.2_RPL37P2	ENSG00000160789.18_LMNA	-0.12026	-0.274214919	0.039696351	1.40E-01	3.82E-01	1.00E+00
ENSG00000124209.3_RAB22A	ENSG00000160789.18_LMNA	0.21794	0.060845713	0.364489902	6.99E-03	5.04E-02	1.00E+00
ENSG00000008517.15_IL32	ENSG00000160789.18_LMNA	0.08533	-0.074891504	0.241250078	2.96E-01	5.82E-01	1.00E+00
ENSG00000131188.10_PRR7	ENSG00000160789.18_LMNA	0.30622	0.154555532	0.443787312	1.25E-04	2.26E-03	1.00E+00
ENSG00000179776.16_CDH5	ENSG00000160789.18_LMNA	0.18439	0.025949368	0.333790692	2.30E-02	1.19E-01	1.00E+00
ENSG00000063587.13_ZNF275	ENSG00000160789.18_LMNA	0.00675	-0.152614422	0.165772841	9.34E-01	9.74E-01	1.00E+00
ENSG00000173230.14_GOLGB1	ENSG00000160789.18_LMNA	0.08692	-0.073297248	0.24275925	2.87E-01	5.72E-01	1.00E+00
ENSG00000160959.6_LRRC14	ENSG00000160789.18_LMNA	0.09757	-0.062599997	0.252846178	2.32E-01	5.09E-01	1.00E+00
ENSG00000143702.14_CEP170	ENSG00000160789.18_LMNA	-0.16328	-0.314306315	-0.00418361	4.44E-02	1.86E-01	1.00E+00
ENSG00000167613.14_LAIR1	ENSG00000160789.18_LMNA	0.08962	-0.070586004	0.245322288	2.72E-01	5.57E-01	1.00E+00
ENSG00000272523.1_LINC01023	ENSG00000160789.18_LMNA	-0.0077	-0.166691605	0.151691423	9.25E-01	9.71E-01	1.00E+00
ENSG00000101181.16_MTG2	ENSG00000160789.18_LMNA	0.17501	0.016266081	0.325154721	3.10E-02	1.47E-01	1.00E+00
ENSG00000141556.19_TBCD	ENSG00000160789.18_LMNA	0.09629	-0.063888011	0.251635272	2.38E-01	5.17E-01	1.00E+00
ENSG00000231234.2_SKP1P1	ENSG00000160789.18_LMNA	-0.23562	-0.3805454	-0.07939677	3.48E-03	2.99E-02	1.00E+00
ENSG00000134759.12_ELP2	ENSG00000160789.18_LMNA	-0.28161	-0.421900077	-0.12815963	4.40E-04	6.11E-03	1.00E+00
ENSG00000011198.6_ABHD5	ENSG00000160789.18_LMNA	-0.10612	-0.260916187	0.053989193	1.93E-01	4.62E-01	1.00E+00
ENSG00000139998.13_RAB15	ENSG00000160789.18_LMNA	0.2611	0.10631924	0.403527641	1.16E-03	1.29E-02	1.00E+00
ENSG00000090020.9_SLC9A1	ENSG00000160789.18_LMNA	0.5262	0.400554521	0.632421995	3.35E-12	1.46E-09	1.72E-07
ENSG00000162378.11_ZYG11B	ENSG00000160789.18_LMNA	-0.21977	-0.366158118	-0.06276351	6.52E-03	4.79E-02	1.00E+00
ENSG00000105983.18_LMBR1	ENSG00000160789.18_LMNA	0.0284	-0.131391306	0.18675973	7.28E-01	8.85E-01	1.00E+00
ENSG00000083099.9_LYRM2	ENSG00000160789.18_LMNA	-0.28742	-0.427080209	-0.13436947	3.30E-04	4.85E-03	1.00E+00

ENSG00000182362.12_YBEY	ENSG00000160789.18_LMNA	-0.09201	-0.247583263	0.068190409	2.60E-01	5.43E-01	1.00E+00
ENSG00000140299.10_BNIP2	ENSG00000160789.18_LMNA	0.23653	0.080352522	0.381367691	3.35E-03	2.90E-02	1.00E+00
ENSG00000115840.12_SLC25A12	ENSG00000160789.18_LMNA	-0.18646	-0.335692182	-0.02808925	2.14E-02	1.13E-01	1.00E+00
ENSG00000198646.12_NCOA6	ENSG00000160789.18_LMNA	0.154	-0.0053265	0.305710195	5.82E-02	2.21E-01	1.00E+00
ENSG00000063601.15_MTMR1	ENSG00000160789.18_LMNA	0.04444	-0.115576333	0.202212104	5.87E-01	8.08E-01	1.00E+00
ENSG00000145216.14_FIP1L1	ENSG00000160789.18_LMNA	-0.01975	-0.178388433	0.139891939	8.09E-01	9.24E-01	1.00E+00
ENSG00000112167.8_SAYSD1	ENSG00000160789.18_LMNA	-0.20687	-0.354401219	-0.04929525	1.06E-02	6.81E-02	1.00E+00
ENSG00000137411.15_VARS2	ENSG00000160789.18_LMNA	-0.01528	-0.174055539	0.144273426	8.52E-01	9.42E-01	1.00E+00
ENSG00000173801.15_JUP	ENSG00000160789.18_LMNA	0.18937	0.031103505	0.338366395	1.95E-02	1.06E-01	1.00E+00
ENSG00000162616.8_DNAJB4	ENSG00000160789.18_LMNA	-0.08148	-0.237599845	0.078740861	3.18E-01	6.05E-01	1.00E+00
ENSG00000109854.12_HTATIP2	ENSG00000160789.18_LMNA	0.02981	-0.130005785	0.188119805	7.15E-01	8.79E-01	1.00E+00
ENSG00000204576.10_PRR3	ENSG00000160789.18_LMNA	-0.18106	-0.330728506	-0.0225092	2.56E-02	1.28E-01	1.00E+00
ENSG00000035681.6_NSMAF	ENSG00000160789.18_LMNA	0.08168	-0.078546095	0.23778475	3.17E-01	6.04E-01	1.00E+00
ENSG00000131373.13_HACL1	ENSG00000160789.18_LMNA	-0.27967	-0.420159639	-0.12607838	4.84E-04	6.58E-03	1.00E+00
ENSG00000101104.11_PABPC1L	ENSG00000160789.18_LMNA	0.16066	0.00149446	0.311880761	4.80E-02	1.96E-01	1.00E+00
ENSG00000166402.7_TUB	ENSG00000160789.18_LMNA	-0.369	-0.498896753	-0.22289768	2.90E-06	1.07E-04	1.49E-01
ENSG00000101542.8_CDH20	ENSG00000160789.18_LMNA	-0.45442	-0.572224273	-0.3182429	4.09E-09	5.04E-07	2.10E-04
ENSG00000105662.14_CRTC1	ENSG00000160789.18_LMNA	-0.08324	-0.239273209	0.076977383	3.08E-01	5.95E-01	1.00E+00
ENSG00000130021.12_PUDP	ENSG00000160789.18_LMNA	0.11395	-0.046083629	0.268286697	1.62E-01	4.17E-01	1.00E+00
ENSG00000011478.10_QPCTL	ENSG00000160789.18_LMNA	0.23881	0.08275671	0.383434057	3.05E-03	2.71E-02	1.00E+00
ENSG00000146376.9_ARHGAP18	ENSG00000160789.18_LMNA	0.09737	-0.062803973	0.252654479	2.33E-01	5.11E-01	1.00E+00
ENSG00000107829.12_FBXW4	ENSG00000160789.18_LMNA	0.01814	-0.14146721	0.176832052	8.24E-01	9.31E-01	1.00E+00
ENSG00000233369.6_GTF2IP4	ENSG00000160789.18_LMNA	-0.17708	-0.327061921	-0.0183996	2.91E-02	1.40E-01	1.00E+00
ENSG00000145715.13_RASA1	ENSG00000160789.18_LMNA	0.11828	-0.041703905	0.272354217	1.47E-01	3.92E-01	1.00E+00
ENSG00000136159.3_NUDT15	ENSG00000160789.18_LMNA	-0.15755	-0.308999998	0.001693541	5.26E-02	2.08E-01	1.00E+00
ENSG00000171612.6_SLC25A33	ENSG00000160789.18_LMNA	-0.25056	-0.39404234	-0.09515465	1.85E-03	1.85E-02	1.00E+00
ENSG00000120910.13_PPP3CC	ENSG00000160789.18_LMNA	0.00592	-0.153426814	0.16496372	9.42E-01	9.77E-01	1.00E+00
ENSG00000127955.14_GNAI1	ENSG00000160789.18_LMNA	-0.35306	-0.485003771	-0.20540914	8.14E-06	2.47E-04	4.18E-01
ENSG00000164051.12_CCDC51	ENSG00000160789.18_LMNA	-0.0261	-0.184536029	0.133653935	7.50E-01	8.95E-01	1.00E+00
ENSG00000220842.6_RPL21P16	ENSG00000160789.18_LMNA	-0.27867	-0.419273126	-0.12501926	5.08E-04	6.82E-03	1.00E+00
ENSG00000147813.14_NAPRT	ENSG00000160789.18_LMNA	0.21018	0.052747594	0.357424108	9.35E-03	6.24E-02	1.00E+00
ENSG00000160685.12_ZBTB7B	ENSG00000160789.18_LMNA	0.42843	0.288940677	0.550112815	3.66E-08	3.19E-06	1.88E-03
ENSG00000065809.12_FAM107B	ENSG00000160789.18_LMNA	-0.49014	-0.60233933	-0.35894768	1.46E-10	3.09E-08	7.50E-06

ENSG00000171109.17_MFN1	ENSG00000160789.18_LMNA	-0.21725	-0.36386415	-0.06012692	7.18E-03	5.13E-02	1.00E+00
ENSG00000089234.14_BRAP	ENSG00000160789.18_LMNA	-0.06467	-0.221587098	0.095516391	4.29E-01	6.99E-01	1.00E+00
ENSG00000070404.8_FSTL3	ENSG00000160789.18_LMNA	0.29157	0.138817061	0.430778526	2.68E-04	4.11E-03	1.00E+00
ENSG00000235605.1_RP5-827C2	ENSG00000160789.18_LMNA	0.03157	-0.128272865	0.189819183	6.99E-01	8.71E-01	1.00E+00
ENSG00000092108.19_SCFD1	ENSG00000160789.18_LMNA	0.02136	-0.138310709	0.1799491	7.94E-01	9.17E-01	1.00E+00
ENSG00000158966.12_CACHD1	ENSG00000160789.18_LMNA	-0.08042	-0.23659076	0.07980333	3.25E-01	6.11E-01	1.00E+00
ENSG00000105697.6_HAMP	ENSG00000160789.18_LMNA	0.1004	-0.059759227	0.255513397	2.18E-01	4.93E-01	1.00E+00
ENSG00000155016.16_CYP2U1	ENSG00000160789.18_LMNA	-0.20112	-0.349145929	-0.04331066	1.30E-02	7.90E-02	1.00E+00
ENSG00000158301.17_GPRASP2	ENSG00000160789.18_LMNA	-0.26117	-0.40359411	-0.10639774	1.15E-03	1.29E-02	1.00E+00
ENSG00000137492.6_THAP12	ENSG00000160789.18_LMNA	-0.36524	-0.495621741	-0.21875901	3.72E-06	1.31E-04	1.91E-01
ENSG00000108799.11_EZH1	ENSG00000160789.18_LMNA	-0.05878	-0.215958224	0.101370923	4.72E-01	7.32E-01	1.00E+00
ENSG00000165140.8_FBP1	ENSG00000160789.18_LMNA	0.02946	-0.13035636	0.187775783	7.19E-01	8.80E-01	1.00E+00
ENSG00000103335.18_PIEZO1	ENSG00000160789.18_LMNA	0.52172	0.395360684	0.628704554	5.49E-12	2.17E-09	2.82E-07
ENSG00000215251.3_FASTKD5	ENSG00000160789.18_LMNA	0.14604	-0.013474155	0.298305033	7.26E-02	2.56E-01	1.00E+00
ENSG00000184939.14_ZFP90	ENSG00000160789.18_LMNA	-0.11193	-0.266382932	0.048129376	1.70E-01	4.28E-01	1.00E+00
ENSG00000230777.1_RPS29P5	ENSG00000160789.18_LMNA	-0.14427	-0.296658245	0.015280451	7.62E-02	2.64E-01	1.00E+00
ENSG00000115825.8_PRKD3	ENSG00000160789.18_LMNA	-0.13558	-0.288554275	0.024139811	9.58E-02	3.04E-01	1.00E+00
ENSG00000104524.12_PYCRL	ENSG00000160789.18_LMNA	0.01628	-0.143288399	0.175030716	8.42E-01	9.39E-01	1.00E+00
ENSG00000163359.14_COL6A3	ENSG00000160789.18_LMNA	0.32094	0.170450517	0.456802896	5.55E-05	1.17E-03	1.00E+00
ENSG00000188493.13_C19orf54	ENSG00000160789.18_LMNA	0.16659	0.007586663	0.31737001	4.02E-02	1.74E-01	1.00E+00
ENSG00000167670.14_CHAF1A	ENSG00000160789.18_LMNA	0.21638	0.059220816	0.36307494	7.42E-03	5.25E-02	1.00E+00
ENSG00000261526.2_CTB-31O20	ENSG00000160789.18_LMNA	-0.16117	-0.312356022	-0.00202102	4.73E-02	1.94E-01	1.00E+00
ENSG00000099284.12_H2AFY2	ENSG00000160789.18_LMNA	-0.23577	-0.3806791	-0.07955213	3.45E-03	2.97E-02	1.00E+00
ENSG00000197852.9_FAM212B	ENSG00000160789.18_LMNA	-0.14058	-0.293218923	0.019046352	8.41E-02	2.81E-01	1.00E+00
ENSG00000221909.2_FAM200A	ENSG00000160789.18_LMNA	-0.13056	-0.283864183	0.029244738	1.09E-01	3.28E-01	1.00E+00
ENSG00000109689.13_STIM2	ENSG00000160789.18_LMNA	0.16677	0.007776737	0.317540939	4.00E-02	1.73E-01	1.00E+00
ENSG00000181481.12_RNF135	ENSG00000160789.18_LMNA	0.28432	0.131052134	0.424315338	3.85E-04	5.50E-03	1.00E+00
ENSG00000165804.14_ZNF219	ENSG00000160789.18_LMNA	0.16851	0.009561233	0.319144711	3.80E-02	1.67E-01	1.00E+00
ENSG00000204381.10_LAYN	ENSG00000160789.18_LMNA	-0.01338	-0.172211335	0.146134533	8.70E-01	9.50E-01	1.00E+00
ENSG00000181555.18_SETD2	ENSG00000160789.18_LMNA	-0.0356	-0.193701074	0.124307057	6.63E-01	8.51E-01	1.00E+00
ENSG00000139291.12_TMEM19	ENSG00000160789.18_LMNA	-0.31943	-0.455469762	-0.16881558	6.04E-05	1.25E-03	1.00E+00
ENSG00000144029.10_MRPS5	ENSG00000160789.18_LMNA	0.08457	-0.075651224	0.240530365	3.00E-01	5.87E-01	1.00E+00
ENSG00000125449.5_ARMC7	ENSG00000160789.18_LMNA	-0.00612	-0.16516288	0.153226889	9.40E-01	9.76E-01	1.00E+00

ENSG00000253910.2_PCDHGB2	ENSG00000160789.18_LMNA	0.00771	-0.151676655	0.1667063	9.25E-01	9.71E-01	1.00E+00
ENSG00000154305.15_MIA3	ENSG00000160789.18_LMNA	0.0485	-0.111561801	0.206109573	5.53E-01	7.88E-01	1.00E+00
ENSG00000160124.8_CCDC58	ENSG00000160789.18_LMNA	-0.11895	-0.272977192	0.041032045	1.44E-01	3.89E-01	1.00E+00
ENSG00000166407.12_LMO1	ENSG00000160789.18_LMNA	-0.17343	-0.32369405	-0.01463398	3.26E-02	1.51E-01	1.00E+00
ENSG00000168538.14_TRAPPC1	ENSG00000160789.18_LMNA	0.00406	-0.155237399	0.16315888	9.60E-01	9.84E-01	1.00E+00
ENSG00000168040.4_FADD	ENSG00000160789.18_LMNA	0.30681	0.155188099	0.444307631	1.21E-04	2.20E-03	1.00E+00
ENSG00000167987.9_VPS37C	ENSG00000160789.18_LMNA	0.21204	0.054687139	0.359119588	8.73E-03	5.94E-02	1.00E+00
ENSG00000197498.11_RPF2	ENSG00000160789.18_LMNA	0.07683	-0.083392772	0.233176617	3.47E-01	6.31E-01	1.00E+00
ENSG00000106144.18_CASP2	ENSG00000160789.18_LMNA	0.24958	0.094121808	0.393161619	1.93E-03	1.90E-02	1.00E+00
ENSG00000157014.9_TATDN2	ENSG00000160789.18_LMNA	0.08209	-0.078136074	0.238173938	3.15E-01	6.01E-01	1.00E+00
ENSG00000215256.3_DHRS4-AS	ENSG00000160789.18_LMNA	-0.07698	-0.233323865	0.083238133	3.46E-01	6.30E-01	1.00E+00
ENSG00000092531.8_SNAP23	ENSG00000160789.18_LMNA	-0.11674	-0.270902971	0.043267928	1.52E-01	4.01E-01	1.00E+00
ENSG00000005882.10_PDK2	ENSG00000160789.18_LMNA	-0.11194	-0.266390911	0.048120807	1.70E-01	4.28E-01	1.00E+00
ENSG00000204261.7_PSMB8-AS	ENSG00000160789.18_LMNA	0.07146	-0.088748153	0.228068229	3.82E-01	6.60E-01	1.00E+00
ENSG00000023516.8_AKAP11	ENSG00000160789.18_LMNA	-0.24573	-0.389683779	-0.09004954	2.28E-03	2.16E-02	1.00E+00
ENSG00000162129.11_CLPB	ENSG00000160789.18_LMNA	0.14496	-0.01457735	0.297299478	7.48E-02	2.61E-01	1.00E+00
ENSG00000044090.7_CUL7	ENSG00000160789.18_LMNA	0.4683	0.334004882	0.583967466	1.17E-09	1.77E-07	6.01E-05
ENSG00000136824.17_SMC2	ENSG00000160789.18_LMNA	0.02503	-0.134707301	0.183499674	7.60E-01	9.00E-01	1.00E+00
ENSG00000186566.10_GPATCH8	ENSG00000160789.18_LMNA	0.00865	-0.150755305	0.167622863	9.16E-01	9.68E-01	1.00E+00
ENSG00000004866.17_ST7	ENSG00000160789.18_LMNA	-0.09082	-0.246459035	0.06938203	2.66E-01	5.50E-01	1.00E+00
ENSG00000157540.18_DYRK1A	ENSG00000160789.18_LMNA	0.06141	-0.098757206	0.218473806	4.52E-01	7.18E-01	1.00E+00
ENSG00000278611.1_CTC-543D1	ENSG00000160789.18_LMNA	-0.27035	-0.41182493	-0.11614733	7.56E-04	9.30E-03	1.00E+00
ENSG00000105771.12_SMG9	ENSG00000160789.18_LMNA	0.12844	-0.031401878	0.281877822	1.15E-01	3.38E-01	1.00E+00
ENSG00000119707.12_RBM25	ENSG00000160789.18_LMNA	0.08397	-0.076253764	0.239959308	3.04E-01	5.90E-01	1.00E+00
ENSG00000168916.14_ZNF608	ENSG00000160789.18_LMNA	0.09427	-0.065918984	0.249723873	2.48E-01	5.28E-01	1.00E+00
ENSG00000136856.16_SLC2A8	ENSG00000160789.18_LMNA	0.03633	-0.123590207	0.194401694	6.57E-01	8.48E-01	1.00E+00
ENSG00000197930.11_ERO1A	ENSG00000160789.18_LMNA	0.35502	0.207545839	0.486708775	7.19E-06	2.25E-04	3.69E-01
ENSG00000163798.12_SLC4A1AI	ENSG00000160789.18_LMNA	0.14245	-0.017142728	0.294958476	8.00E-02	2.72E-01	1.00E+00
ENSG00000153443.11_UBALD1	ENSG00000160789.18_LMNA	-0.04598	-0.203686601	0.114058769	5.74E-01	8.01E-01	1.00E+00
ENSG00000182118.5_FAM89A	ENSG00000160789.18_LMNA	-0.39309	-0.519765921	-0.24950602	5.46E-07	2.81E-05	2.80E-02
ENSG00000251580.1_RP11-539L	ENSG00000160789.18_LMNA	-0.15347	-0.305211756	0.005876216	5.91E-02	2.23E-01	1.00E+00
ENSG00000087842.9_PIR	ENSG00000160789.18_LMNA	0.09769	-0.062484649	0.252954572	2.31E-01	5.09E-01	1.00E+00
ENSG00000162377.5_COA7	ENSG00000160789.18_LMNA	0.14453	-0.015014987	0.296900384	7.56E-02	2.63E-01	1.00E+00

ENSG00000249119.1_MTND6P4	ENSG00000160789.18_LMNA	-0.12457	-0.278249224	0.035334928	1.26E-01	3.59E-01	1.00E+00
ENSG00000251022.5_THAP9-AS1	ENSG00000160789.18_LMNA	0.00506	-0.15426109	0.164132356	9.51E-01	9.80E-01	1.00E+00
ENSG00000259969.1_RP11-999E	ENSG00000160789.18_LMNA	-0.28136	-0.421671381	-0.127886	4.46E-04	6.17E-03	1.00E+00
ENSG00000243554.1_AC004967.7	ENSG00000160789.18_LMNA	0.02305	-0.136655423	0.181581147	7.78E-01	9.09E-01	1.00E+00
ENSG00000163719.17_MTMR14	ENSG00000160789.18_LMNA	-0.00435	-0.163441955	0.154953565	9.58E-01	9.83E-01	1.00E+00
ENSG00000087157.17_PGS1	ENSG00000160789.18_LMNA	-0.0432	-0.201018629	0.116803582	5.97E-01	8.14E-01	1.00E+00
ENSG00000168763.14_CNNM3	ENSG00000160789.18_LMNA	0.04003	-0.119938745	0.197965457	6.24E-01	8.31E-01	1.00E+00
ENSG00000128342.4_LIF	ENSG00000160789.18_LMNA	0.56157	0.441874224	0.661620559	5.22E-14	5.46E-11	2.68E-09
ENSG00000164087.6_POC1A	ENSG00000160789.18_LMNA	0.01376	-0.145757621	0.172585004	8.66E-01	9.48E-01	1.00E+00
ENSG00000181192.10_DHTKD1	ENSG00000160789.18_LMNA	-0.20974	-0.357017869	-0.05228322	9.50E-03	6.32E-02	1.00E+00
ENSG00000142082.13_SIRT3	ENSG00000160789.18_LMNA	0.15707	-0.002184476	0.308555869	5.33E-02	2.09E-01	1.00E+00
ENSG00000179029.13_TMEM107	ENSG00000160789.18_LMNA	0.16587	0.006848039	0.316705592	4.11E-02	1.77E-01	1.00E+00
ENSG00000148719.13_DNAJB12	ENSG00000160789.18_LMNA	-0.02509	-0.183556719	0.134649338	7.59E-01	8.99E-01	1.00E+00
ENSG00000227008.2_RP3-417G1	ENSG00000160789.18_LMNA	-0.12411	-0.277820844	0.03579861	1.28E-01	3.62E-01	1.00E+00
ENSG00000051009.9_FAM160A2	ENSG00000160789.18_LMNA	0.23859	0.082527783	0.383237427	3.07E-03	2.72E-02	1.00E+00
ENSG00000153071.13_DAB2	ENSG00000160789.18_LMNA	0.07587	-0.084353981	0.232261025	3.53E-01	6.37E-01	1.00E+00
ENSG00000144048.9_DUSP11	ENSG00000160789.18_LMNA	-0.22694	-0.372677553	-0.07027986	4.93E-03	3.88E-02	1.00E+00
ENSG00000100461.16_RBM23	ENSG00000160789.18_LMNA	0.01865	-0.140966152	0.177327274	8.20E-01	9.30E-01	1.00E+00
ENSG00000061273.16_HDAC7	ENSG00000160789.18_LMNA	0.43435	0.29559292	0.555164937	2.26E-08	2.14E-06	1.16E-03
ENSG00000254615.2_RP11-395G	ENSG00000160789.18_LMNA	0.22106	0.064118512	0.367335608	6.20E-03	4.61E-02	1.00E+00
ENSG00000132405.17_TBC1D14	ENSG00000160789.18_LMNA	-0.1892	-0.338213017	-0.03093047	1.96E-02	1.06E-01	1.00E+00
ENSG00000184408.8_KCND2	ENSG00000160789.18_LMNA	-0.31423	-0.450877082	-0.16319523	8.06E-05	1.58E-03	1.00E+00
ENSG00000236819.1_LINC01563	ENSG00000160789.18_LMNA	-0.3071	-0.444569117	-0.15550608	1.19E-04	2.18E-03	1.00E+00
ENSG00000168993.13_CPLX1	ENSG00000160789.18_LMNA	-0.27527	-0.416227191	-0.12138539	5.99E-04	7.77E-03	1.00E+00
ENSG00000165417.10_GTF2A1	ENSG00000160789.18_LMNA	-0.04983	-0.207381151	0.110249783	5.42E-01	7.81E-01	1.00E+00
ENSG00000203705.9_TATDN3	ENSG00000160789.18_LMNA	-0.36925	-0.49911212	-0.22317019	2.85E-06	1.06E-04	1.46E-01
ENSG00000163155.10_LYSMD1	ENSG00000160789.18_LMNA	-0.23627	-0.381136148	-0.08008334	3.38E-03	2.93E-02	1.00E+00
ENSG00000096070.18_BRPF3	ENSG00000160789.18_LMNA	0.01562	-0.143938946	0.174386744	8.49E-01	9.41E-01	1.00E+00
ENSG00000115652.13_UXS1	ENSG00000160789.18_LMNA	-0.17332	-0.323594232	-0.0145225	3.27E-02	1.51E-01	1.00E+00
ENSG00000179562.2_GCC1	ENSG00000160789.18_LMNA	0.3909	0.247071156	0.517869552	6.39E-07	3.19E-05	3.28E-02
ENSG00000147654.13_EBAG9	ENSG00000160789.18_LMNA	-0.23903	-0.383636378	-0.0829923	3.02E-03	2.69E-02	1.00E+00
ENSG00000161791.12_FMNL3	ENSG00000160789.18_LMNA	0.10697	-0.053139689	0.261709961	1.90E-01	4.57E-01	1.00E+00
ENSG00000112679.13_DUSP22	ENSG00000160789.18_LMNA	-0.01778	-0.176479962	0.14182335	8.28E-01	9.33E-01	1.00E+00

ENSG00000078124.10_ACER3	ENSG00000160789.18_LMNA	-0.1025	-0.257498661	0.057641464	2.09E-01	4.82E-01	1.00E+00
ENSG00000113838.11_TBCCD1	ENSG00000160789.18_LMNA	-0.21239	-0.359439586	-0.05505346	8.61E-03	5.88E-02	1.00E+00
ENSG00000129667.11_RHBDF2	ENSG00000160789.18_LMNA	0.14688	-0.01261711	0.299085747	7.10E-02	2.52E-01	1.00E+00
ENSG00000177453.6_NIM1K	ENSG00000160789.18_LMNA	-0.20458	-0.352306877	-0.04690764	1.15E-02	7.22E-02	1.00E+00
ENSG00000155307.16_SAMSN1	ENSG00000160789.18_LMNA	-0.08796	-0.243745598	0.072254417	2.81E-01	5.67E-01	1.00E+00
ENSG00000206172.7_HBA1	ENSG00000160789.18_LMNA	-0.0926	-0.248137892	0.0676022	2.57E-01	5.39E-01	1.00E+00
ENSG00000135966.11_TGFBRAP	ENSG00000160789.18_LMNA	0.07769	-0.08252977	0.233998185	3.41E-01	6.26E-01	1.00E+00
ENSG00000115289.11_PCGF1	ENSG00000160789.18_LMNA	0.1702	0.011301813	0.320707308	3.61E-02	1.62E-01	1.00E+00
ENSG00000163714.16_U2SURP	ENSG00000160789.18_LMNA	-0.10184	-0.256874725	0.058307348	2.12E-01	4.86E-01	1.00E+00
ENSG00000185813.9_PCYT2	ENSG00000160789.18_LMNA	0.14967	-0.009760165	0.30168523	6.57E-02	2.39E-01	1.00E+00
ENSG00000151292.16_CSNK1G3	ENSG00000160789.18_LMNA	-0.17159	-0.321998375	-0.01274135	3.45E-02	1.57E-01	1.00E+00
ENSG00000163162.7_RNF149	ENSG00000160789.18_LMNA	0.07542	-0.084800502	0.231835503	3.56E-01	6.39E-01	1.00E+00
ENSG00000172731.12_LRRC20	ENSG00000160789.18_LMNA	-0.17231	-0.322663823	-0.01348383	3.38E-02	1.55E-01	1.00E+00
ENSG00000103671.8_TRIP4	ENSG00000160789.18_LMNA	0.36627	0.219896186	0.496522395	3.47E-06	1.24E-04	1.78E-01
ENSG00000155961.4_RAB39B	ENSG00000160789.18_LMNA	-0.23593	-0.380827124	-0.07972416	3.43E-03	2.96E-02	1.00E+00
ENSG00000154727.9_GABPA	ENSG00000160789.18_LMNA	0.01332	-0.146194501	0.172151875	8.71E-01	9.50E-01	1.00E+00
ENSG00000166532.14_RIMKLB	ENSG00000160789.18_LMNA	-0.24628	-0.390178332	-0.09062801	2.22E-03	2.12E-02	1.00E+00
ENSG00000081307.11_UBA5	ENSG00000160789.18_LMNA	-0.15297	-0.304746329	0.006389353	5.99E-02	2.25E-01	1.00E+00
ENSG00000176986.13_SEC24C	ENSG00000160789.18_LMNA	0.00628	-0.153075585	0.165313587	9.39E-01	9.76E-01	1.00E+00
ENSG00000165025.13_SYK	ENSG00000160789.18_LMNA	0.00089	-0.158330649	0.160070508	9.91E-01	9.97E-01	1.00E+00
ENSG00000106012.16_IQCE	ENSG00000160789.18_LMNA	0.18361	0.025145444	0.333075679	2.36E-02	1.21E-01	1.00E+00
ENSG00000179889.17_PDXDC1	ENSG00000160789.18_LMNA	0.14777	-0.011704302	0.299916797	6.92E-02	2.48E-01	1.00E+00
ENSG00000089639.9_GMIP	ENSG00000160789.18_LMNA	-0.02658	-0.185002579	0.133179492	7.45E-01	8.93E-01	1.00E+00
ENSG00000114062.16_UBE3A	ENSG00000160789.18_LMNA	0.1144	-0.045632741	0.268705962	1.61E-01	4.14E-01	1.00E+00
ENSG00000233223.2_AC113189.5	ENSG00000160789.18_LMNA	-0.2156	-0.362361269	-0.05840187	7.64E-03	5.37E-02	1.00E+00
ENSG00000164603.10_BMT2	ENSG00000160789.18_LMNA	-0.32804	-0.463054722	-0.17813877	3.70E-05	8.46E-04	1.00E+00
ENSG00000148444.14_COMMD3	ENSG00000160789.18_LMNA	-0.471	-0.586243162	-0.33707563	9.14E-10	1.46E-07	4.69E-05
ENSG00000104884.13_ERCC2	ENSG00000160789.18_LMNA	0.16223	0.003105314	0.313334204	4.58E-02	1.90E-01	1.00E+00
ENSG00000254545.1_RP11-84A1	ENSG00000160789.18_LMNA	0.3931	0.249512201	0.519770735	5.45E-07	2.81E-05	2.80E-02
ENSG00000130702.12_LAMA5	ENSG00000160789.18_LMNA	0.48323	0.351029939	0.59653524	2.87E-10	5.52E-08	1.47E-05
ENSG00000179912.18_R3HDM2	ENSG00000160789.18_LMNA	-0.1302	-0.283528099	0.029609922	1.10E-01	3.30E-01	1.00E+00
ENSG00000135040.14_NAA35	ENSG00000160789.18_LMNA	0.06199	-0.098176716	0.21903193	4.48E-01	7.14E-01	1.00E+00
ENSG00000143437.19_ARNT	ENSG00000160789.18_LMNA	0.26032	0.105494624	0.402829235	1.20E-03	1.33E-02	1.00E+00

ENSG00000140320.10_BAHD1	ENSG00000160789.18_LMNA	0.23422	0.077921569	0.379275258	3.68E-03	3.12E-02	1.00E+00
ENSG00000264918.1_	ENSG00000160789.18_LMNA	0.02087	-0.13878829	0.179477898	7.99E-01	9.19E-01	1.00E+00
ENSG00000109738.9_GLRB	ENSG00000160789.18_LMNA	-0.31764	-0.453884308	-0.16687325	6.68E-05	1.36E-03	1.00E+00
ENSG00000215908.6_CROCCP2	ENSG00000160789.18_LMNA	-0.11302	-0.267409007	0.047027105	1.66E-01	4.22E-01	1.00E+00
ENSG00000171823.6_FBXL14	ENSG00000160789.18_LMNA	0.06571	-0.094480535	0.222580832	4.21E-01	6.93E-01	1.00E+00
ENSG00000260025.1_RP11-490M	ENSG00000160789.18_LMNA	-0.17564	-0.325733399	-0.01691313	3.04E-02	1.45E-01	1.00E+00
ENSG00000182700.4_IGIP	ENSG00000160789.18_LMNA	-0.04759	-0.205235794	0.112462727	5.60E-01	7.93E-01	1.00E+00
ENSG00000124784.7_RIOK1	ENSG00000160789.18_LMNA	-0.07547	-0.231888862	0.084744517	3.55E-01	6.39E-01	1.00E+00
ENSG00000244716.3_RP11-20O2	ENSG00000160789.18_LMNA	-0.07932	-0.235548677	0.08089979	3.31E-01	6.17E-01	1.00E+00
ENSG00000158156.7_XKR8	ENSG00000160789.18_LMNA	0.27691	0.123140805	0.417699417	5.53E-04	7.30E-03	1.00E+00
ENSG00000166483.9_WEE1	ENSG00000160789.18_LMNA	0.35563	0.208218833	0.48724536	6.92E-06	2.18E-04	3.55E-01
ENSG00000054282.14_SDCCAG8	ENSG00000160789.18_LMNA	-0.18761	-0.336752976	-0.02928426	2.06E-02	1.10E-01	1.00E+00
ENSG00000130669.16_PAK4	ENSG00000160789.18_LMNA	0.23524	0.079000108	0.380203986	3.53E-03	3.02E-02	1.00E+00
ENSG00000153560.10_UBP1	ENSG00000160789.18_LMNA	-0.06289	-0.219892879	0.09728084	4.41E-01	7.09E-01	1.00E+00
ENSG00000230733.2_AC092171.4	ENSG00000160789.18_LMNA	0.19114	0.032943195	0.339996111	1.83E-02	1.01E-01	1.00E+00
ENSG00000174738.11_NR1D2	ENSG00000160789.18_LMNA	-0.25245	-0.395746813	-0.09715536	1.70E-03	1.73E-02	1.00E+00
ENSG00000068137.13_PLEKHH3	ENSG00000160789.18_LMNA	0.23869	0.082627179	0.383322804	3.06E-03	2.72E-02	1.00E+00
ENSG00000125611.14_CHCHD5	ENSG00000160789.18_LMNA	-0.13252	-0.285692289	0.027256879	1.04E-01	3.19E-01	1.00E+00
ENSG00000129932.6_DOHH	ENSG00000160789.18_LMNA	0.21447	0.057221314	0.361331848	7.97E-03	5.55E-02	1.00E+00
ENSG00000177426.19_TGIF1	ENSG00000160789.18_LMNA	0.3488	0.200751677	0.481279905	1.06E-05	3.06E-04	5.45E-01
ENSG00000112333.10_NR2E1	ENSG00000160789.18_LMNA	0.28232	0.128915339	0.422531505	4.25E-04	5.95E-03	1.00E+00
ENSG00000125812.14_GZF1	ENSG00000160789.18_LMNA	0.03871	-0.12123311	0.19670314	6.36E-01	8.37E-01	1.00E+00
ENSG00000160999.10_SH2B2	ENSG00000160789.18_LMNA	0.14728	-0.012210297	0.299456181	7.02E-02	2.50E-01	1.00E+00
ENSG00000230199.1_RP11-331N	ENSG00000160789.18_LMNA	-0.11993	-0.2738988	0.040037598	1.41E-01	3.83E-01	1.00E+00
ENSG00000090615.11_GOLGA3	ENSG00000160789.18_LMNA	0.26971	0.115469767	0.411254475	7.79E-04	9.52E-03	1.00E+00
ENSG00000127774.6_EMC6	ENSG00000160789.18_LMNA	0.082	-0.078224013	0.238090476	3.15E-01	6.02E-01	1.00E+00
ENSG00000274956.2_UG0898H09	ENSG00000160789.18_LMNA	-0.34384	-0.476934249	-0.19533252	1.44E-05	3.96E-04	7.41E-01
ENSG00000168918.12_INPP5D	ENSG00000160789.18_LMNA	-0.03902	-0.196995068	0.120933866	6.33E-01	8.35E-01	1.00E+00
ENSG00000273014.1_RP11-225B	ENSG00000160789.18_LMNA	0.17284	0.014022722	0.32314662	3.32E-02	1.53E-01	1.00E+00
ENSG00000230076.1_RPL10P6	ENSG00000160789.18_LMNA	-0.07637	-0.23274074	0.083850439	3.50E-01	6.33E-01	1.00E+00
ENSG00000110060.7_PUS3	ENSG00000160789.18_LMNA	-0.31107	-0.448076592	-0.15977723	9.59E-05	1.82E-03	1.00E+00
ENSG00000124194.14_GDAP1L1	ENSG00000160789.18_LMNA	-0.43534	-0.556008866	-0.2967066	2.08E-08	2.00E-06	1.07E-03
ENSG00000168612.4_ZSWIM1	ENSG00000160789.18_LMNA	-0.08848	-0.244237641	0.07173394	2.78E-01	5.64E-01	1.00E+00

ENSG00000131153.7_GINS2	ENSG00000160789.18_LMNA	-0.00103	-0.160202645	0.158198431	9.90E-01	9.96E-01	1.00E+00
ENSG00000119650.11_IFT43	ENSG00000160789.18_LMNA	0.15014	-0.009283177	0.302118783	6.49E-02	2.37E-01	1.00E+00
ENSG00000257365.6_FNTB	ENSG00000160789.18_LMNA	0.02766	-0.132122883	0.186041096	7.35E-01	8.89E-01	1.00E+00
ENSG00000185619.16_PCGF3	ENSG00000160789.18_LMNA	0.00624	-0.153115884	0.165273449	9.39E-01	9.76E-01	1.00E+00
ENSG00000128309.15_MPST	ENSG00000160789.18_LMNA	-0.18269	-0.332231174	-0.02419644	2.43E-02	1.24E-01	1.00E+00
ENSG00000102081.12_FMR1	ENSG00000160789.18_LMNA	-0.07229	-0.228855111	0.087924417	3.76E-01	6.56E-01	1.00E+00
ENSG00000180425.9_C11orf71	ENSG00000160789.18_LMNA	-0.35155	-0.48367934	-0.20375122	8.95E-06	2.66E-04	4.59E-01
ENSG00000198742.8_SMURF1	ENSG00000160789.18_LMNA	0.23223	0.075833556	0.377475545	3.99E-03	3.31E-02	1.00E+00
ENSG00000058404.18_CAMK2B	ENSG00000160789.18_LMNA	0.22374	0.066923305	0.369769872	5.59E-03	4.27E-02	1.00E+00
ENSG00000154639.17_CXADR	ENSG00000160789.18_LMNA	-0.05603	-0.213323467	0.104103774	4.93E-01	7.48E-01	1.00E+00
ENSG00000145920.13_CPLX2	ENSG00000160789.18_LMNA	-0.24814	-0.391859865	-0.09259639	2.05E-03	1.99E-02	1.00E+00
ENSG00000169258.6_GPRIN1	ENSG00000160789.18_LMNA	0.00783	-0.151562908	0.166819486	9.24E-01	9.71E-01	1.00E+00
ENSG00000115548.15_KDM3A	ENSG00000160789.18_LMNA	-0.05133	-0.208827448	0.108756143	5.30E-01	7.73E-01	1.00E+00
ENSG00000134574.10_DDB2	ENSG00000160789.18_LMNA	0.37292	0.227213829	0.502303841	2.23E-06	8.68E-05	1.14E-01
ENSG00000237928.4_NFIA-AS2	ENSG00000160789.18_LMNA	-0.28901	-0.428494724	-0.13606918	3.05E-04	4.56E-03	1.00E+00
ENSG00000134042.11_MRO	ENSG00000160789.18_LMNA	-0.27195	-0.413257636	-0.11785024	7.01E-04	8.79E-03	1.00E+00
ENSG00000164306.9_PRIMPOL	ENSG00000160789.18_LMNA	-0.07028	-0.226943927	0.089924362	3.90E-01	6.68E-01	1.00E+00
ENSG00000105556.9_MIER2	ENSG00000160789.18_LMNA	0.26273	0.108049547	0.404991994	1.08E-03	1.23E-02	1.00E+00
ENSG00000169925.15_BRD3	ENSG00000160789.18_LMNA	-0.03721	-0.195251012	0.122720769	6.49E-01	8.44E-01	1.00E+00
ENSG00000130559.17_CAMSAP1	ENSG00000160789.18_LMNA	0.1025	-0.057645139	0.257495218	2.09E-01	4.82E-01	1.00E+00
ENSG00000145632.13_PLK2	ENSG00000160789.18_LMNA	0.04505	-0.114976738	0.202794856	5.82E-01	8.05E-01	1.00E+00
ENSG00000175287.17_PHYHD1	ENSG00000160789.18_LMNA	-0.07427	-0.230743919	0.085945375	3.63E-01	6.45E-01	1.00E+00
ENSG00000094841.12_UPRT	ENSG00000160789.18_LMNA	0.17834	0.019694595	0.32821832	2.79E-02	1.36E-01	1.00E+00
ENSG00000180867.10_PDIA3P1	ENSG00000160789.18_LMNA	0.40718	0.265167739	0.531900586	1.93E-07	1.23E-05	9.90E-03
ENSG00000121417.12_ZNF211	ENSG00000160789.18_LMNA	0.11813	-0.041860987	0.272208526	1.47E-01	3.93E-01	1.00E+00
ENSG00000065526.9_SPEN	ENSG00000160789.18_LMNA	0.06344	-0.096735296	0.220416914	4.37E-01	7.06E-01	1.00E+00
ENSG00000185515.13_BRCC3	ENSG00000160789.18_LMNA	0.1219	-0.038039425	0.275748874	1.35E-01	3.73E-01	1.00E+00
ENSG00000081870.10_HSPB11	ENSG00000160789.18_LMNA	0.04786	-0.112195465	0.205495055	5.58E-01	7.91E-01	1.00E+00
ENSG00000163909.7_HEYL	ENSG00000160789.18_LMNA	0.09381	-0.066385986	0.249284019	2.50E-01	5.31E-01	1.00E+00
ENSG00000183036.9_PCP4	ENSG00000160789.18_LMNA	-0.37471	-0.503853333	-0.22918033	1.97E-06	7.90E-05	1.01E-01
ENSG00000213079.8_SCAF8	ENSG00000160789.18_LMNA	0.03004	-0.129777618	0.188343665	7.13E-01	8.78E-01	1.00E+00
ENSG00000067596.9_DHX8	ENSG00000160789.18_LMNA	0.19852	0.040604771	0.346763453	1.42E-02	8.46E-02	1.00E+00
ENSG00000154734.13_ADAMTS1	ENSG00000160789.18_LMNA	0.47428	0.340814336	0.589008576	6.73E-10	1.13E-07	3.45E-05

ENSG00000154133.13_ROBO4	ENSG00000160789.18_LMNA	-0.0047	-0.163779946	0.154614597	9.54E-01	9.82E-01	1.00E+00
ENSG00000182379.9_NXPH4	ENSG00000160789.18_LMNA	0.1469	-0.012593181	0.299107538	7.09E-02	2.52E-01	1.00E+00
ENSG00000234961.1_RP11-124N	ENSG00000160789.18_LMNA	0.55741	0.43698459	0.658199717	8.74E-14	8.00E-11	4.48E-09
ENSG00000117461.13_PIK3R3	ENSG00000160789.18_LMNA	-0.32427	-0.45973813	-0.17405582	4.59E-05	1.00E-03	1.00E+00
ENSG00000116675.14_DNAJC6	ENSG00000160789.18_LMNA	-0.32386	-0.459379191	-0.17361453	4.70E-05	1.02E-03	1.00E+00
ENSG00000121957.11_GPSM2	ENSG00000160789.18_LMNA	-0.27771	-0.418415356	-0.12399512	5.32E-04	7.08E-03	1.00E+00
ENSG00000204366.3_ZBTB12	ENSG00000160789.18_LMNA	-0.02446	-0.18295188	0.135263796	7.65E-01	9.02E-01	1.00E+00
ENSG00000272686.1_RP11-390E	ENSG00000160789.18_LMNA	-0.25571	-0.398684634	-0.10060944	1.47E-03	1.55E-02	1.00E+00
ENSG00000092148.11_HECTD1	ENSG00000160789.18_LMNA	0.13686	-0.022833621	0.289751916	9.27E-02	2.98E-01	1.00E+00
ENSG00000149474.12_KAT14	ENSG00000160789.18_LMNA	0.04938	-0.110690121	0.206954505	5.46E-01	7.84E-01	1.00E+00
ENSG00000171451.13_DSEL	ENSG00000160789.18_LMNA	-0.22865	-0.374232607	-0.07207779	4.61E-03	3.68E-02	1.00E+00
ENSG00000134072.9_CAMK1	ENSG00000160789.18_LMNA	0.24444	0.088692106	0.38852259	2.41E-03	2.25E-02	1.00E+00
ENSG00000138031.13_ADCY3	ENSG00000160789.18_LMNA	0.31081	0.159500262	0.447849415	9.73E-05	1.84E-03	1.00E+00
ENSG00000110697.11_PITPNM1	ENSG00000160789.18_LMNA	0.38251	0.237784827	0.51061246	1.16E-06	5.18E-05	5.93E-02
ENSG00000116128.8_BCL9	ENSG00000160789.18_LMNA	0.22225	0.065357068	0.368411056	5.92E-03	4.46E-02	1.00E+00
ENSG00000153822.12_KCNJ16	ENSG00000160789.18_LMNA	-0.18372	-0.333174471	-0.0252565	2.35E-02	1.21E-01	1.00E+00
ENSG00000149782.10_PLCB3	ENSG00000160789.18_LMNA	0.43592	0.297350095	0.556496253	1.99E-08	1.92E-06	1.02E-03
ENSG00000036257.11_CUL3	ENSG00000160789.18_LMNA	-0.15755	-0.308999558	0.001694028	5.26E-02	2.08E-01	1.00E+00
ENSG00000174365.18_SNHG11	ENSG00000160789.18_LMNA	0.17852	0.019879344	0.328383221	2.78E-02	1.36E-01	1.00E+00
ENSG00000119574.11_ZBTB45	ENSG00000160789.18_LMNA	0.25821	0.103255544	0.400931106	1.32E-03	1.43E-02	1.00E+00
ENSG00000167702.10_KIFC2	ENSG00000160789.18_LMNA	0.11608	-0.043932127	0.270286233	1.54E-01	4.05E-01	1.00E+00
ENSG00000166189.7_HPS6	ENSG00000160789.18_LMNA	0.22485	0.068085368	0.370777203	5.35E-03	4.13E-02	1.00E+00
ENSG00000149761.7_NUDT22	ENSG00000160789.18_LMNA	0.35225	0.204521076	0.484294501	8.57E-06	2.57E-04	4.40E-01
ENSG00000149577.14_SIDT2	ENSG00000160789.18_LMNA	0.20427	0.046586965	0.352025358	1.16E-02	7.28E-02	1.00E+00
ENSG00000198793.11_MTOR	ENSG00000160789.18_LMNA	0.07319	-0.087028337	0.22971063	3.70E-01	6.51E-01	1.00E+00
ENSG00000038358.13_EDC4	ENSG00000160789.18_LMNA	0.12509	-0.034808429	0.278735487	1.25E-01	3.56E-01	1.00E+00
ENSG00000138303.16_ASCC1	ENSG00000160789.18_LMNA	-0.50872	-0.617880762	-0.38032268	2.21E-11	6.48E-09	1.13E-06
ENSG00000143379.11_SETDB1	ENSG00000160789.18_LMNA	-0.05704	-0.214291858	0.103099886	4.85E-01	7.42E-01	1.00E+00
ENSG00000110660.13_SLC35F2	ENSG00000160789.18_LMNA	0.23214	0.075738039	0.377393162	4.00E-03	3.32E-02	1.00E+00
ENSG00000196591.10_HDAC2	ENSG00000160789.18_LMNA	-0.2167	-0.363364033	-0.05955267	7.33E-03	5.21E-02	1.00E+00
ENSG00000137337.13_MDC1	ENSG00000160789.18_LMNA	0.08217	-0.078052045	0.238253685	3.14E-01	6.01E-01	1.00E+00
ENSG00000157510.12_AFAP1L1	ENSG00000160789.18_LMNA	0.13044	-0.029370323	0.283748614	1.09E-01	3.29E-01	1.00E+00
ENSG00000168917.8_SLC35G2	ENSG00000160789.18_LMNA	0.13441	-0.025328781	0.287463261	9.87E-02	3.09E-01	1.00E+00

ENSG00000100485.10_SOS2	ENSG00000160789.18_LMNA	-0.0939	-0.249372469	0.066292088	2.50E-01	5.30E-01	1.00E+00
ENSG00000169403.10_PTAFR	ENSG00000160789.18_LMNA	-0.07234	-0.228906479	0.087870627	3.76E-01	6.56E-01	1.00E+00
ENSG00000180098.8_TRNAU1AF	ENSG00000160789.18_LMNA	-0.11419	-0.268512432	0.045840885	1.61E-01	4.16E-01	1.00E+00
ENSG00000272779.1_LL22NC03-	ENSG00000160789.18_LMNA	-0.00846	-0.167438025	0.150941153	9.18E-01	9.69E-01	1.00E+00
ENSG00000124104.17_SNX21	ENSG00000160789.18_LMNA	0.36462	0.21807748	0.495081673	3.87E-06	1.35E-04	1.99E-01
ENSG00000276855.1_CTD-3157E	ENSG00000160789.18_LMNA	0.07057	-0.089633315	0.227222209	3.88E-01	6.66E-01	1.00E+00
ENSG00000173805.14_HAP1	ENSG00000160789.18_LMNA	0.24743	0.091851158	0.391223471	2.12E-03	2.04E-02	1.00E+00
ENSG00000267317.2_CTB-25B13	ENSG00000160789.18_LMNA	0.44974	0.312940645	0.568250371	6.15E-09	7.14E-07	3.16E-04
ENSG00000100934.13_SEC23A	ENSG00000160789.18_LMNA	0.23948	0.083459904	0.384037874	2.96E-03	2.65E-02	1.00E+00
ENSG00000071626.15_DAZAP1	ENSG00000160789.18_LMNA	0.1828	0.024305513	0.332328264	2.42E-02	1.24E-01	1.00E+00
ENSG00000217027.1_TPT1P4	ENSG00000160789.18_LMNA	-0.0651	-0.221998214	0.095087931	4.26E-01	6.96E-01	1.00E+00
ENSG00000117505.11_DR1	ENSG00000160789.18_LMNA	-0.10278	-0.257759408	0.057363104	2.08E-01	4.80E-01	1.00E+00
ENSG00000164109.12_MAD2L1	ENSG00000160789.18_LMNA	-0.18027	-0.330001005	-0.02169297	2.63E-02	1.31E-01	1.00E+00
ENSG00000164347.16_GFM2	ENSG00000160789.18_LMNA	-0.37335	-0.502673318	-0.22768254	2.16E-06	8.46E-05	1.11E-01
ENSG00000152942.17_RAD17	ENSG00000160789.18_LMNA	-0.23471	-0.379719298	-0.07843715	3.61E-03	3.07E-02	1.00E+00
ENSG00000154359.11_LONRF1	ENSG00000160789.18_LMNA	0.03146	-0.128380869	0.189713326	7.00E-01	8.72E-01	1.00E+00
ENSG00000181666.16_HKR1	ENSG00000160789.18_LMNA	-0.01926	-0.177911843	0.140374484	8.14E-01	9.27E-01	1.00E+00
ENSG00000178802.16_MPI	ENSG00000160789.18_LMNA	0.07876	-0.081459478	0.235016465	3.35E-01	6.21E-01	1.00E+00
ENSG00000126464.12_PRR12	ENSG00000160789.18_LMNA	0.21651	0.059351712	0.363188977	7.38E-03	5.23E-02	1.00E+00
ENSG00000105486.12_LIG1	ENSG00000160789.18_LMNA	0.11357	-0.046466308	0.267930764	1.64E-01	4.19E-01	1.00E+00
ENSG00000166912.15_MTMR10	ENSG00000160789.18_LMNA	-0.08975	-0.24544644	0.070454554	2.71E-01	5.56E-01	1.00E+00
ENSG00000168872.14_DDX19A	ENSG00000160789.18_LMNA	0.21091	0.053506101	0.358087407	9.10E-03	6.12E-02	1.00E+00
ENSG00000151353.13_TMEM18	ENSG00000160789.18_LMNA	-0.17896	-0.32879669	-0.02034267	2.74E-02	1.35E-01	1.00E+00
ENSG00000019549.7_SNAI2	ENSG00000160789.18_LMNA	0.20227	0.044502371	0.35019395	1.25E-02	7.68E-02	1.00E+00
ENSG00000138379.4_MSTN	ENSG00000160789.18_LMNA	-0.50001	-0.610607012	-0.37028699	5.43E-11	1.39E-08	2.79E-06
ENSG00000014914.18_MTMR11	ENSG00000160789.18_LMNA	0.37635	0.230990921	0.505278414	1.77E-06	7.27E-05	9.06E-02
ENSG00000169554.15_ZEB2	ENSG00000160789.18_LMNA	-0.22322	-0.369295835	-0.06637674	5.70E-03	4.33E-02	1.00E+00
ENSG00000168827.13_GFM1	ENSG00000160789.18_LMNA	0.02712	-0.13265079	0.185522317	7.40E-01	8.91E-01	1.00E+00
ENSG00000164494.10_PDSS2	ENSG00000160789.18_LMNA	0.03444	-0.125445064	0.192588166	6.74E-01	8.57E-01	1.00E+00
ENSG00000116711.9_PLA2G4A	ENSG00000160789.18_LMNA	-0.30512	-0.442814122	-0.15337303	1.32E-04	2.36E-03	1.00E+00
ENSG00000117859.17_OSBPL9	ENSG00000160789.18_LMNA	-0.01816	-0.176848808	0.141450259	8.24E-01	9.31E-01	1.00E+00
ENSG00000225791.5_TRAM2-AS	ENSG00000160789.18_LMNA	-0.19944	-0.347605164	-0.04156023	1.38E-02	8.24E-02	1.00E+00
ENSG00000163660.10_CCNL1	ENSG00000160789.18_LMNA	-0.01557	-0.174337415	0.143988768	8.49E-01	9.41E-01	1.00E+00

ENSG00000120254.14_MTHFD1L	ENSG00000160789.18_LMNA	0.10639	-0.053722398	0.261165526	1.92E-01	4.60E-01	1.00E+00
ENSG00000075336.10_TIMM21	ENSG00000160789.18_LMNA	-0.1815	-0.331129443	-0.02295921	2.52E-02	1.27E-01	1.00E+00
ENSG00000118579.10_MED28	ENSG00000160789.18_LMNA	-0.3644	-0.494894496	-0.21784134	3.93E-06	1.36E-04	2.01E-01
ENSG00000100162.13_CENPM	ENSG00000160789.18_LMNA	-0.01121	-0.170105789	0.148256627	8.91E-01	9.57E-01	1.00E+00
ENSG00000165304.6_MELK	ENSG00000160789.18_LMNA	0.16559	0.006559819	0.316446247	4.15E-02	1.78E-01	1.00E+00
ENSG00000205356.8_TECPR1	ENSG00000160789.18_LMNA	0.17369	0.014898307	0.323930715	3.24E-02	1.51E-01	1.00E+00
ENSG00000224578.4_HNRNPA1F	ENSG00000160789.18_LMNA	0.05804	-0.102107597	0.215248447	4.78E-01	7.36E-01	1.00E+00
ENSG00000166801.14_FAM111A	ENSG00000160789.18_LMNA	0.15917	-3.59E-05	0.310498622	5.02E-02	2.02E-01	1.00E+00
ENSG00000102805.12_CLN5	ENSG00000160789.18_LMNA	-0.15268	-0.30447598	0.00668734	6.04E-02	2.27E-01	1.00E+00
ENSG00000156253.5_RWDD2B	ENSG00000160789.18_LMNA	-0.08347	-0.239486531	0.076752431	3.07E-01	5.93E-01	1.00E+00
ENSG00000154930.13_ACSS1	ENSG00000160789.18_LMNA	-0.0842	-0.240185235	0.076015409	3.02E-01	5.89E-01	1.00E+00
ENSG00000110344.8_UBE4A	ENSG00000160789.18_LMNA	-0.02422	-0.182713967	0.135505425	7.67E-01	9.03E-01	1.00E+00
ENSG00000121749.14_TBC1D15	ENSG00000160789.18_LMNA	-0.23118	-0.376520421	-0.0747265	4.16E-03	3.42E-02	1.00E+00
ENSG00000117385.14_P3H1	ENSG00000160789.18_LMNA	0.54264	0.419699741	0.64603338	5.14E-13	3.38E-10	2.64E-08
ENSG00000138463.8_DIRC2	ENSG00000160789.18_LMNA	-0.01384	-0.17266044	0.14568152	8.66E-01	9.47E-01	1.00E+00
ENSG00000171469.9_ZNF561	ENSG00000160789.18_LMNA	-0.01817	-0.17685737	0.141441598	8.24E-01	9.31E-01	1.00E+00
ENSG00000167447.11_SMG8	ENSG00000160789.18_LMNA	-0.10138	-0.256440085	0.058771044	2.14E-01	4.88E-01	1.00E+00
ENSG00000079313.10_REXO1	ENSG00000160789.18_LMNA	0.10034	-0.059816327	0.255459832	2.19E-01	4.94E-01	1.00E+00
ENSG00000130348.10_QRSL1	ENSG00000160789.18_LMNA	-0.19231	-0.34106996	-0.03415654	1.76E-02	9.85E-02	1.00E+00
ENSG00000126243.7_LRFN3	ENSG00000160789.18_LMNA	0.19611	0.038093702	0.344548974	1.55E-02	8.98E-02	1.00E+00
ENSG00000185551.11_NR2F2	ENSG00000160789.18_LMNA	0.20117	0.043354246	0.349184271	1.30E-02	7.89E-02	1.00E+00
ENSG00000204099.10_NEU4	ENSG00000160789.18_LMNA	-0.37556	-0.504593304	-0.23012024	1.86E-06	7.60E-05	9.56E-02
ENSG00000214110.3_LDHAP4	ENSG00000160789.18_LMNA	0.30461	0.15282474	0.44236265	1.36E-04	2.40E-03	1.00E+00
ENSG00000139438.5_FAM222A	ENSG00000160789.18_LMNA	-0.38039	-0.508776168	-0.23544295	1.34E-06	5.85E-05	6.87E-02
ENSG00000107959.14_PITRM1	ENSG00000160789.18_LMNA	-0.06369	-0.220654549	0.096487844	4.36E-01	7.04E-01	1.00E+00
ENSG00000149582.14_TMEM25	ENSG00000160789.18_LMNA	-0.30492	-0.442633916	-0.15315416	1.34E-04	2.38E-03	1.00E+00
ENSG00000159147.16_DONSON	ENSG00000160789.18_LMNA	0.32572	0.175626072	0.461014596	4.22E-05	9.38E-04	1.00E+00
ENSG00000110042.6_DTX4	ENSG00000160789.18_LMNA	-0.28765	-0.427283021	-0.13461307	3.26E-04	4.81E-03	1.00E+00
ENSG00000004700.14_RECQL	ENSG00000160789.18_LMNA	0.19429	0.036212322	0.342887571	1.65E-02	9.39E-02	1.00E+00
ENSG00000103966.8_EHD4	ENSG00000160789.18_LMNA	0.4186	0.277918063	0.54169927	8.02E-08	5.99E-06	4.11E-03
ENSG00000123737.11_EXOSC9	ENSG00000160789.18_LMNA	0.07198	-0.088235923	0.22855759	3.78E-01	6.57E-01	1.00E+00
ENSG00000150347.13_ARID5B	ENSG00000160789.18_LMNA	0.37533	0.229860909	0.504389178	1.89E-06	7.68E-05	9.71E-02
ENSG00000183723.11_CMTM4	ENSG00000160789.18_LMNA	-0.11891	-0.272942747	0.0410692	1.45E-01	3.89E-01	1.00E+00

ENSG00000070718.10_AP3M2	ENSG00000160789.18_LMNA	-0.29158	-0.43078615	-0.13882624	2.68E-04	4.11E-03	1.00E+00
ENSG00000141027.19_NCOR1	ENSG00000160789.18_LMNA	0.04599	-0.114044746	0.203700219	5.74E-01	8.01E-01	1.00E+00
ENSG00000140948.10_ZCCHC14	ENSG00000160789.18_LMNA	-0.01384	-0.172661215	0.145680737	8.66E-01	9.47E-01	1.00E+00
ENSG00000111859.15_NEDD9	ENSG00000160789.18_LMNA	0.42225	0.282002386	0.544823004	6.01E-08	4.77E-06	3.09E-03
ENSG00000155897.8_ADCY8	ENSG00000160789.18_LMNA	-0.13086	-0.284145825	0.028938646	1.08E-01	3.27E-01	1.00E+00
ENSG00000139636.14_LMBR1L	ENSG00000160789.18_LMNA	-0.26298	-0.405217381	-0.10831603	1.06E-03	1.21E-02	1.00E+00
ENSG00000105643.8_ARRDC2	ENSG00000160789.18_LMNA	0.01557	-0.143984095	0.174342042	8.49E-01	9.41E-01	1.00E+00
ENSG00000169871.11_TRIM56	ENSG00000160789.18_LMNA	0.45828	0.322617253	0.575493786	2.90E-09	3.77E-07	1.49E-04
ENSG00000114346.12_ECT2	ENSG00000160789.18_LMNA	-0.05047	-0.207995516	0.109615482	5.37E-01	7.78E-01	1.00E+00
ENSG00000174891.11_RSRC1	ENSG00000160789.18_LMNA	-0.19248	-0.341220192	-0.03432636	1.75E-02	9.81E-02	1.00E+00
ENSG00000138119.15_MYOF	ENSG00000160789.18_LMNA	0.54056	0.417273444	0.644316321	6.55E-13	4.05E-10	3.36E-08
ENSG00000155393.11_HEATR3	ENSG00000160789.18_LMNA	0.06229	-0.097881664	0.219315533	4.46E-01	7.12E-01	1.00E+00
ENSG00000124615.16_MOCS1	ENSG00000160789.18_LMNA	-0.01677	-0.175497827	0.142816347	8.38E-01	9.37E-01	1.00E+00
ENSG00000028203.16_VEZT	ENSG00000160789.18_LMNA	-0.26426	-0.406367138	-0.10967608	1.00E-03	1.16E-02	1.00E+00
ENSG00000137513.8_NARS2	ENSG00000160789.18_LMNA	-0.38766	-0.515070571	-0.24348362	8.05E-07	3.83E-05	4.13E-02
ENSG00000166444.16_ST5	ENSG00000160789.18_LMNA	0.27283	0.118786243	0.414044497	6.72E-04	8.50E-03	1.00E+00
ENSG00000187866.7_FAM122A	ENSG00000160789.18_LMNA	0.06077	-0.09939266	0.217862599	4.57E-01	7.21E-01	1.00E+00
ENSG00000213064.8_SFT2D2	ENSG00000160789.18_LMNA	0.20507	0.047416636	0.35275361	1.13E-02	7.13E-02	1.00E+00
ENSG00000165572.7_KBTBD6	ENSG00000160789.18_LMNA	-0.36793	-0.497961329	-0.22171456	3.11E-06	1.13E-04	1.60E-01
ENSG00000095794.18_CREM	ENSG00000160789.18_LMNA	-0.04138	-0.199270837	0.118599086	6.13E-01	8.24E-01	1.00E+00
ENSG00000275342.3_SGK223	ENSG00000160789.18_LMNA	0.38579	0.241412626	0.513452151	9.18E-07	4.27E-05	4.71E-02
ENSG00000178691.9_SUZ12	ENSG00000160789.18_LMNA	0.11898	-0.040997207	0.273009488	1.44E-01	3.89E-01	1.00E+00
ENSG00000164933.10_SLC25A32	ENSG00000160789.18_LMNA	0.0825	-0.07772434	0.238564651	3.12E-01	5.99E-01	1.00E+00
ENSG00000165097.12_KDM1B	ENSG00000160789.18_LMNA	-0.02377	-0.182284666	0.135941337	7.71E-01	9.05E-01	1.00E+00
ENSG00000182173.11_TSEN54	ENSG00000160789.18_LMNA	-0.14775	-0.299897295	0.011725729	6.93E-02	2.48E-01	1.00E+00
ENSG00000138821.11_SLC39A8	ENSG00000160789.18_LMNA	0.09934	-0.060817935	0.254519929	2.23E-01	4.99E-01	1.00E+00
ENSG00000133193.11_FAM104A	ENSG00000160789.18_LMNA	-0.09361	-0.249101554	0.066579672	2.51E-01	5.32E-01	1.00E+00
ENSG00000275620.1_FLJ16779	ENSG00000160789.18_LMNA	-0.37711	-0.505931939	-0.23182187	1.68E-06	6.97E-05	8.60E-02
ENSG00000110446.8_SLC15A3	ENSG00000160789.18_LMNA	0.12222	-0.037715574	0.276048503	1.34E-01	3.71E-01	1.00E+00
ENSG00000198912.9_C1orf174	ENSG00000160789.18_LMNA	-0.10466	-0.259535282	0.055465966	1.99E-01	4.70E-01	1.00E+00
ENSG00000102312.19_PORCN	ENSG00000160789.18_LMNA	0.15734	-0.001904707	0.308808982	5.29E-02	2.09E-01	1.00E+00
ENSG00000173267.12_SNCG	ENSG00000160789.18_LMNA	-0.25794	-0.4006881	-0.10296911	1.34E-03	1.44E-02	1.00E+00
ENSG00000152253.7_SPC25	ENSG00000160789.18_LMNA	-0.08611	-0.24199382	0.074106033	2.91E-01	5.77E-01	1.00E+00

ENSG00000256463.7_SALL3	ENSG00000160789.18_LMNA	-0.26759	-0.409349789	-0.11320947	8.60E-04	1.03E-02	1.00E+00
ENSG00000151779.11_NBAS	ENSG00000160789.18_LMNA	0.17904	0.020424801	0.328869969	2.73E-02	1.35E-01	1.00E+00
ENSG00000152578.11_GRIA4	ENSG00000160789.18_LMNA	-0.44602	-0.565092833	-0.30873896	8.48E-09	9.39E-07	4.35E-04
ENSG00000065491.8_TBC1D22B	ENSG00000160789.18_LMNA	0.05773	-0.102408948	0.214958002	4.80E-01	7.38E-01	1.00E+00
ENSG00000127993.13_RBM48	ENSG00000160789.18_LMNA	-0.09871	-0.253917579	0.061459496	2.26E-01	5.03E-01	1.00E+00
ENSG00000151116.15_UEVLD	ENSG00000160789.18_LMNA	-0.05459	-0.211946989	0.105529597	5.04E-01	7.55E-01	1.00E+00
ENSG00000075914.11_EXOSC7	ENSG00000160789.18_LMNA	-0.24314	-0.387344883	-0.0873165	2.54E-03	2.35E-02	1.00E+00
ENSG00000125633.9_CCDC93	ENSG00000160789.18_LMNA	0.07714	-0.083078651	0.23347571	3.45E-01	6.29E-01	1.00E+00
ENSG00000008283.14_CYB561	ENSG00000160789.18_LMNA	0.37242	0.226655414	0.501863519	2.31E-06	8.89E-05	1.18E-01
ENSG00000076108.10_BAZ2A	ENSG00000160789.18_LMNA	0.11513	-0.044892511	0.269394018	1.58E-01	4.11E-01	1.00E+00
ENSG00000137221.13_TJAP1	ENSG00000160789.18_LMNA	-0.05132	-0.208816537	0.108767416	5.30E-01	7.73E-01	1.00E+00
ENSG00000189221.8_MAOA	ENSG00000160789.18_LMNA	-0.15344	-0.30518559	0.005905068	5.91E-02	2.23E-01	1.00E+00
ENSG00000025293.14_PHF20	ENSG00000160789.18_LMNA	0.01131	-0.148156249	0.170205449	8.90E-01	9.57E-01	1.00E+00
ENSG00000183578.5_TNFAIP8L3	ENSG00000160789.18_LMNA	-0.08418	-0.240165934	0.076035773	3.02E-01	5.89E-01	1.00E+00
ENSG00000188070.9_C11orf95	ENSG00000160789.18_LMNA	-0.15038	-0.302338396	0.009041507	6.44E-02	2.36E-01	1.00E+00
ENSG00000271122.1_RP11-379H	ENSG00000160789.18_LMNA	0.12094	-0.039014164	0.27484667	1.38E-01	3.78E-01	1.00E+00
ENSG00000110074.9_FOXRED1	ENSG00000160789.18_LMNA	-0.17511	-0.325244866	-0.01636686	3.09E-02	1.46E-01	1.00E+00
ENSG00000171224.8_C10orf35	ENSG00000160789.18_LMNA	-0.00385	-0.162952509	0.155444291	9.62E-01	9.85E-01	1.00E+00
ENSG00000213326.4_RPS7P11	ENSG00000160789.18_LMNA	-0.1563	-0.307843366	0.002971756	5.45E-02	2.13E-01	1.00E+00
ENSG00000186162.9_CIDECP	ENSG00000160789.18_LMNA	-0.13986	-0.292549055	0.019778798	8.57E-02	2.84E-01	1.00E+00
ENSG00000059122.15_FLYWCH1	ENSG00000160789.18_LMNA	0.05477	-0.105344405	0.212125843	5.03E-01	7.54E-01	1.00E+00
ENSG00000066468.19_FGFR2	ENSG00000160789.18_LMNA	-0.33268	-0.46713854	-0.18317974	2.82E-05	6.80E-04	1.00E+00
ENSG00000123096.10_SSPN	ENSG00000160789.18_LMNA	-0.01455	-0.173348641	0.14498707	8.59E-01	9.44E-01	1.00E+00
ENSG00000160746.11_ANO10	ENSG00000160789.18_LMNA	-0.06045	-0.217554917	0.099712449	4.59E-01	7.23E-01	1.00E+00
ENSG00000065029.13_ZNF76	ENSG00000160789.18_LMNA	-0.12456	-0.278242009	0.035342739	1.26E-01	3.59E-01	1.00E+00
ENSG00000122783.15_C7orf49	ENSG00000160789.18_LMNA	0.19373	0.035625256	0.342368754	1.68E-02	9.52E-02	1.00E+00
ENSG00000126226.20_PCID2	ENSG00000160789.18_LMNA	-0.13747	-0.290316372	0.022217635	9.12E-02	2.95E-01	1.00E+00
ENSG00000135547.7_HEY2	ENSG00000160789.18_LMNA	-0.1502	-0.302172369	0.009224212	6.48E-02	2.37E-01	1.00E+00
ENSG00000133773.10_CCDC59	ENSG00000160789.18_LMNA	-0.12133	-0.275214976	0.038616319	1.36E-01	3.76E-01	1.00E+00
ENSG00000101190.11_TCFL5	ENSG00000160789.18_LMNA	0.08066	-0.079559057	0.23682282	3.23E-01	6.10E-01	1.00E+00
ENSG00000184517.10_ZFP1	ENSG00000160789.18_LMNA	-0.22532	-0.371209124	-0.06858389	5.25E-03	4.07E-02	1.00E+00
ENSG00000174579.3_MSL2	ENSG00000160789.18_LMNA	-0.04462	-0.202383349	0.115400163	5.85E-01	8.07E-01	1.00E+00
ENSG00000115145.8_STAM2	ENSG00000160789.18_LMNA	-0.16941	-0.319982364	-0.01049406	3.69E-02	1.64E-01	1.00E+00

ENSG00000167371.15_PRRT2	ENSG00000160789.18_LMNA	-0.06738	-0.224180525	0.092811592	4.09E-01	6.84E-01	1.00E+00
ENSG00000168495.11_POLR3D	ENSG00000160789.18_LMNA	0.15262	-0.006748964	0.304420065	6.05E-02	2.27E-01	1.00E+00
ENSG00000165526.7_RPUSD4	ENSG00000160789.18_LMNA	-0.27926	-0.419798166	-0.12564644	4.94E-04	6.67E-03	1.00E+00
ENSG00000230979.3_AC079250.1	ENSG00000160789.18_LMNA	-0.1761	-0.32615598	-0.0173858	3.00E-02	1.43E-01	1.00E+00
ENSG00000239569.3_KMT2E-AS1	ENSG00000160789.18_LMNA	-0.04581	-0.20352352	0.114226685	5.75E-01	8.02E-01	1.00E+00
ENSG00000225472.1_RP11-120J1	ENSG00000160789.18_LMNA	-0.37517	-0.504250245	-0.22968442	1.91E-06	7.71E-05	9.82E-02
ENSG00000023572.7_GLRX2	ENSG00000160789.18_LMNA	0.17906	0.020441814	0.328885148	2.73E-02	1.35E-01	1.00E+00
ENSG00000111679.15_PTPN6	ENSG00000160789.18_LMNA	0.06041	-0.099750712	0.217518098	4.60E-01	7.23E-01	1.00E+00
ENSG00000119402.15_FBXW2	ENSG00000160789.18_LMNA	0.28009	0.126531554	0.420538792	4.74E-04	6.47E-03	1.00E+00
ENSG00000135842.15_FAM129A	ENSG00000160789.18_LMNA	0.37963	0.234611234	0.508123413	1.41E-06	6.12E-05	7.23E-02
ENSG00000187513.8_GJA4	ENSG00000160789.18_LMNA	0.11085	-0.04921853	0.265368366	1.74E-01	4.34E-01	1.00E+00
ENSG00000102362.14_SYTL4	ENSG00000160789.18_LMNA	0.14425	-0.015299359	0.296640997	7.62E-02	2.64E-01	1.00E+00
ENSG00000174028.6_FAM3C2	ENSG00000160789.18_LMNA	0.36627	0.219886484	0.496514713	3.47E-06	1.24E-04	1.78E-01
ENSG00000005339.11_CREBBP	ENSG00000160789.18_LMNA	0.10584	-0.054277121	0.260647052	1.94E-01	4.63E-01	1.00E+00
ENSG00000131375.8_CAPN7	ENSG00000160789.18_LMNA	-0.23631	-0.381171317	-0.08012423	3.38E-03	2.92E-02	1.00E+00
ENSG00000182853.10_VMO1	ENSG00000160789.18_LMNA	0.08565	-0.074563955	0.241560271	2.94E-01	5.80E-01	1.00E+00
ENSG00000213614.8_HEXA	ENSG00000160789.18_LMNA	0.25727	0.102259026	0.400085507	1.38E-03	1.47E-02	1.00E+00
ENSG00000169826.7_CSGALNAC	ENSG00000160789.18_LMNA	0.14187	-0.017727865	0.294423992	8.12E-02	2.75E-01	1.00E+00
ENSG00000141627.12_DYM	ENSG00000160789.18_LMNA	-0.08567	-0.241576125	0.074547212	2.94E-01	5.80E-01	1.00E+00
ENSG00000130758.6_MAP3K10	ENSG00000160789.18_LMNA	-0.04421	-0.201991141	0.115803623	5.89E-01	8.09E-01	1.00E+00
ENSG00000145916.17_RMND5B	ENSG00000160789.18_LMNA	-0.08979	-0.245485561	0.070413132	2.71E-01	5.56E-01	1.00E+00
ENSG00000253368.3_TRNP1	ENSG00000160789.18_LMNA	-0.02281	-0.181353725	0.136886189	7.80E-01	9.10E-01	1.00E+00
ENSG00000113742.11_CPEB4	ENSG00000160789.18_LMNA	-0.16075	-0.311967046	-0.00159005	4.79E-02	1.96E-01	1.00E+00
ENSG00000127603.22_MACF1	ENSG00000160789.18_LMNA	0.35312	0.205470828	0.485053023	8.11E-06	2.46E-04	4.16E-01
ENSG00000261829.1_RP11-223I1	ENSG00000160789.18_LMNA	-0.02881	-0.187149945	0.130993917	7.25E-01	8.83E-01	1.00E+00
ENSG00000138172.9_CALHM2	ENSG00000160789.18_LMNA	0.33797	0.188939824	0.471790141	2.06E-05	5.29E-04	1.00E+00
ENSG00000174456.12_C12orf76	ENSG00000160789.18_LMNA	-0.47325	-0.5881389	-0.33963773	7.41E-10	1.23E-07	3.80E-05
ENSG00000143786.6_CNIH3	ENSG00000160789.18_LMNA	0.50246	0.373103513	0.612652497	4.23E-11	1.12E-08	2.17E-06
ENSG00000070540.11_WIPI1	ENSG00000160789.18_LMNA	0.34705	0.198829599	0.479740154	1.19E-05	3.35E-04	6.08E-01
ENSG00000134030.12_CTIF	ENSG00000160789.18_LMNA	-0.21804	-0.364589655	-0.06096033	6.96E-03	5.02E-02	1.00E+00
ENSG00000146476.9_ARMT1	ENSG00000160789.18_LMNA	0.09549	-0.064698655	0.25087265	2.42E-01	5.21E-01	1.00E+00
ENSG00000106459.13_NRF1	ENSG00000160789.18_LMNA	0.08703	-0.073186657	0.242863882	2.86E-01	5.72E-01	1.00E+00
ENSG00000205363.5_C15orf59	ENSG00000160789.18_LMNA	-0.03614	-0.194225105	0.123770918	6.58E-01	8.49E-01	1.00E+00

ENSG00000174233.10_ADCY6	ENSG00000160789.18_LMNA	0.20491	0.047246406	0.352604219	1.13E-02	7.16E-02	1.00E+00
ENSG00000005238.18_FAM214B	ENSG00000160789.18_LMNA	0.28805	0.135045874	0.42764329	3.20E-04	4.73E-03	1.00E+00
ENSG00000263001.4_GTF2I	ENSG00000160789.18_LMNA	0.04296	-0.11704221	0.200786459	5.99E-01	8.16E-01	1.00E+00
ENSG00000162604.11_TM2D1	ENSG00000160789.18_LMNA	-0.1854	-0.334717242	-0.02699173	2.22E-02	1.16E-01	1.00E+00
ENSG00000162227.6_TAF6L	ENSG00000160789.18_LMNA	-0.07797	-0.2342647	0.082249713	3.40E-01	6.25E-01	1.00E+00
ENSG00000086827.7_ZW10	ENSG00000160789.18_LMNA	0.08833	-0.071879564	0.244099989	2.79E-01	5.65E-01	1.00E+00
ENSG00000189114.6_BLOC1S3	ENSG00000160789.18_LMNA	0.23584	0.079625894	0.380742571	3.44E-03	2.97E-02	1.00E+00
ENSG00000196652.10_ZKSCAN5	ENSG00000160789.18_LMNA	0.13739	-0.022295744	0.290244809	9.14E-02	2.95E-01	1.00E+00
ENSG00000136935.12_GOLGA1	ENSG00000160789.18_LMNA	0.1181	-0.041885981	0.272185343	1.47E-01	3.93E-01	1.00E+00
ENSG00000186594.11_MIR22HG	ENSG00000160789.18_LMNA	0.20056	0.042720081	0.348626273	1.32E-02	8.01E-02	1.00E+00
ENSG00000182158.13_CREB3L2	ENSG00000160789.18_LMNA	0.2308	0.074328191	0.376176616	4.23E-03	3.46E-02	1.00E+00
ENSG00000170779.10_CDCA4	ENSG00000160789.18_LMNA	0.20822	0.050698736	0.355630887	1.00E-02	6.58E-02	1.00E+00
ENSG00000162551.12_ALPL	ENSG00000160789.18_LMNA	-0.04412	-0.201899547	0.115897829	5.89E-01	8.09E-01	1.00E+00
ENSG00000117593.9_DARS2	ENSG00000160789.18_LMNA	0.12808	-0.031764132	0.281543984	1.16E-01	3.40E-01	1.00E+00
ENSG00000155816.18_FMN2	ENSG00000160789.18_LMNA	-0.00064	-0.159823144	0.158578133	9.94E-01	9.98E-01	1.00E+00
ENSG00000198300.11_PEG3	ENSG00000160789.18_LMNA	-0.25871	-0.401380953	-0.10378592	1.29E-03	1.40E-02	1.00E+00
ENSG00000278540.3_ACACA	ENSG00000160789.18_LMNA	-0.06221	-0.219237108	0.09796326	4.46E-01	7.13E-01	1.00E+00
ENSG00000162733.15_DDR2	ENSG00000160789.18_LMNA	0.0222	-0.137481843	0.180766549	7.86E-01	9.13E-01	1.00E+00
ENSG00000143603.17_KCNN3	ENSG00000160789.18_LMNA	-0.3205	-0.456414972	-0.16997461	5.69E-05	1.19E-03	1.00E+00
ENSG00000184785.5_SMIM10	ENSG00000160789.18_LMNA	0.26534	0.110816264	0.407330296	9.54E-04	1.12E-02	1.00E+00
ENSG00000167513.7_CDT1	ENSG00000160789.18_LMNA	0.14456	-0.014988769	0.296924295	7.56E-02	2.63E-01	1.00E+00
ENSG00000097046.11_CDC7	ENSG00000160789.18_LMNA	-0.07205	-0.228624995	0.088165355	3.78E-01	6.57E-01	1.00E+00
ENSG00000133739.14_LRRCC1	ENSG00000160789.18_LMNA	0.1626	0.003488217	0.313679477	4.53E-02	1.89E-01	1.00E+00
ENSG00000157538.12_DSCR3	ENSG00000160789.18_LMNA	0.09207	-0.068129814	0.247640408	2.59E-01	5.42E-01	1.00E+00
ENSG00000173065.12_FAM222B	ENSG00000160789.18_LMNA	0.02647	-0.133288297	0.184895597	7.46E-01	8.94E-01	1.00E+00
ENSG00000118961.13_LDAH	ENSG00000160789.18_LMNA	-0.30675	-0.44425809	-0.15512786	1.21E-04	2.21E-03	1.00E+00
ENSG00000165023.5_DIRAS2	ENSG00000160789.18_LMNA	-0.18197	-0.331567673	-0.02345122	2.48E-02	1.26E-01	1.00E+00
ENSG00000181045.13_SLC26A11	ENSG00000160789.18_LMNA	0.05223	-0.107866499	0.209688233	5.23E-01	7.68E-01	1.00E+00
ENSG00000110768.10_GTF2H1	ENSG00000160789.18_LMNA	-0.10242	-0.257420947	0.057724418	2.09E-01	4.82E-01	1.00E+00
ENSG00000099381.15_SETD1A	ENSG00000160789.18_LMNA	0.20049	0.042654524	0.348568577	1.33E-02	8.02E-02	1.00E+00
ENSG00000155755.17_TMEM237	ENSG00000160789.18_LMNA	-0.13497	-0.287982019	0.024763557	9.73E-02	3.07E-01	1.00E+00
ENSG00000019144.15_PHLDB1	ENSG00000160789.18_LMNA	0.1012	-0.058949282	0.256272981	2.15E-01	4.89E-01	1.00E+00
ENSG00000166266.12_CUL5	ENSG00000160789.18_LMNA	-0.18633	-0.335576967	-0.02795951	2.15E-02	1.14E-01	1.00E+00

ENSG00000108771.11_DHX58	ENSG00000160789.18_LMNA	0.18798	0.029663252	0.337089239	2.04E-02	1.09E-01	1.00E+00
ENSG00000129197.13_RPAIN	ENSG00000160789.18_LMNA	-0.42038	-0.543228235	-0.27991601	6.97E-08	5.40E-06	3.57E-03
ENSG00000120594.15_PLXDC2	ENSG00000160789.18_LMNA	-0.01501	-0.173798478	0.144532978	8.54E-01	9.43E-01	1.00E+00
ENSG00000101544.7_ADNP2	ENSG00000160789.18_LMNA	-0.01897	-0.177629482	0.140660302	8.17E-01	9.28E-01	1.00E+00
ENSG00000158792.14_SPATA2L	ENSG00000160789.18_LMNA	0.41624	0.275286282	0.539682588	9.63E-08	7.00E-06	4.94E-03
ENSG00000185614.4_FAM212A	ENSG00000160789.18_LMNA	-0.22155	-0.367782698	-0.0646333	6.09E-03	4.55E-02	1.00E+00
ENSG00000176720.4_BOK	ENSG00000160789.18_LMNA	-0.30488	-0.442596165	-0.15310831	1.34E-04	2.38E-03	1.00E+00
ENSG00000122482.19_ZNF644	ENSG00000160789.18_LMNA	-0.04157	-0.199448239	0.118416935	6.11E-01	8.23E-01	1.00E+00
ENSG00000145248.6_SLC10A4	ENSG00000160789.18_LMNA	0.13469	-0.025046866	0.287722024	9.80E-02	3.08E-01	1.00E+00
ENSG00000166130.13_IKBIP	ENSG00000160789.18_LMNA	0.33625	0.18705829	0.470272423	2.28E-05	5.75E-04	1.00E+00
ENSG00000168297.14_PXK	ENSG00000160789.18_LMNA	-0.03466	-0.192796187	0.125232414	6.72E-01	8.56E-01	1.00E+00
ENSG00000104365.12_IKBKB	ENSG00000160789.18_LMNA	0.11808	-0.041910875	0.272162253	1.47E-01	3.93E-01	1.00E+00
ENSG00000217716.3_RPS10P3	ENSG00000160789.18_LMNA	-0.11543	-0.269671327	0.044594077	1.57E-01	4.09E-01	1.00E+00
ENSG00000100319.12_ZMAT5	ENSG00000160789.18_LMNA	-0.16918	-0.319768908	-0.0102563	3.72E-02	1.65E-01	1.00E+00
ENSG00000100596.5_SPTLC2	ENSG00000160789.18_LMNA	-0.06917	-0.225882612	0.091033864	3.97E-01	6.74E-01	1.00E+00
ENSG00000225200.2_AB019441.2	ENSG00000160789.18_LMNA	-0.09875	-0.253959365	0.061414998	2.26E-01	5.02E-01	1.00E+00
ENSG00000143079.13_CTTNBP2	ENSG00000160789.18_LMNA	0.17413	0.015352134	0.324336955	3.19E-02	1.49E-01	1.00E+00
ENSG00000198373.11_WWP2	ENSG00000160789.18_LMNA	-0.1487	-0.30077675	0.010759202	6.75E-02	2.44E-01	1.00E+00
ENSG00000124444.14_ZNF576	ENSG00000160789.18_LMNA	0.09197	-0.068232677	0.247543399	2.60E-01	5.43E-01	1.00E+00
ENSG00000182973.17_CNOT10	ENSG00000160789.18_LMNA	-0.04565	-0.203376374	0.11437818	5.77E-01	8.03E-01	1.00E+00
ENSG00000169857.6_AVEN	ENSG00000160789.18_LMNA	0.17293	0.014114668	0.323228979	3.31E-02	1.53E-01	1.00E+00
ENSG00000183853.16_KIRREL	ENSG00000160789.18_LMNA	0.41402	0.272796127	0.537771634	1.14E-07	8.08E-06	5.87E-03
ENSG00000119787.12_ATL2	ENSG00000160789.18_LMNA	-0.19301	-0.341714403	-0.03488513	1.72E-02	9.69E-02	1.00E+00
ENSG00000116922.13_C1orf109	ENSG00000160789.18_LMNA	-0.06398	-0.220930417	0.096200529	4.34E-01	7.03E-01	1.00E+00
ENSG00000108590.9_MED31	ENSG00000160789.18_LMNA	-0.1905	-0.339404144	-0.03227472	1.87E-02	1.03E-01	1.00E+00
ENSG00000256053.6_APOPT1	ENSG00000160789.18_LMNA	0.10794	-0.05215784	0.262626867	1.86E-01	4.52E-01	1.00E+00
ENSG00000109016.16_DHRS7B	ENSG00000160789.18_LMNA	-0.19829	-0.346552236	-0.0403651	1.43E-02	8.51E-02	1.00E+00
ENSG00000126746.16_ZNF384	ENSG00000160789.18_LMNA	0.00274	-0.156526932	0.161872138	9.73E-01	9.90E-01	1.00E+00
ENSG00000232956.7_SNHG15	ENSG00000160789.18_LMNA	-0.06873	-0.225460605	0.091474814	4.00E-01	6.77E-01	1.00E+00
ENSG00000183751.13_TBL3	ENSG00000160789.18_LMNA	0.1794	0.020797595	0.329202544	2.70E-02	1.33E-01	1.00E+00
ENSG00000136731.11_UGGT1	ENSG00000160789.18_LMNA	0.25568	0.100574767	0.39865517	1.48E-03	1.55E-02	1.00E+00
ENSG00000158122.10_AAED1	ENSG00000160789.18_LMNA	0.3426	0.193983045	0.475849949	1.56E-05	4.20E-04	7.99E-01
ENSG00000090889.11_KIF4A	ENSG00000160789.18_LMNA	0.14068	-0.018940053	0.293316116	8.39E-02	2.80E-01	1.00E+00

ENSG00000175634.13_RPS6KB2	ENSG00000160789.18_LMNA	0.10176	-0.058390649	0.256796653	2.12E-01	4.86E-01	1.00E+00
ENSG00000108852.13_MPP2	ENSG00000160789.18_LMNA	-0.23786	-0.382576704	-0.08175877	3.17E-03	2.78E-02	1.00E+00
ENSG00000007923.14_DNAJC11	ENSG00000160789.18_LMNA	0.08164	-0.078584616	0.237748181	3.17E-01	6.04E-01	1.00E+00
ENSG00000119682.15_AREL1	ENSG00000160789.18_LMNA	0.17528	0.016538924	0.325398762	3.08E-02	1.46E-01	1.00E+00
ENSG00000113108.16_APBB3	ENSG00000160789.18_LMNA	-0.11076	-0.265285904	0.049307021	1.74E-01	4.35E-01	1.00E+00
ENSG00000121940.14_CLCC1	ENSG00000160789.18_LMNA	0.05908	-0.10106761	0.216250365	4.70E-01	7.30E-01	1.00E+00
ENSG00000168795.4_ZBTB5	ENSG00000160789.18_LMNA	-0.20467	-0.352385445	-0.04699715	1.14E-02	7.21E-02	1.00E+00
ENSG00000173171.13_MTX1	ENSG00000160789.18_LMNA	0.09014	-0.070069965	0.245809619	2.69E-01	5.54E-01	1.00E+00
ENSG00000186908.13_ZDHHC17	ENSG00000160789.18_LMNA	-0.30367	-0.441529544	-0.15181344	1.43E-04	2.50E-03	1.00E+00
ENSG00000273611.3_ZNHIT3	ENSG00000160789.18_LMNA	-0.3657	-0.496024068	-0.2192669	3.60E-06	1.28E-04	1.85E-01
ENSG00000005100.11_DHX33	ENSG00000160789.18_LMNA	0.03251	-0.127352603	0.19072086	6.91E-01	8.67E-01	1.00E+00
ENSG00000127870.15_RNF6	ENSG00000160789.18_LMNA	0.10993	-0.050152691	0.264497623	1.78E-01	4.39E-01	1.00E+00
ENSG00000164008.12_C1orf50	ENSG00000160789.18_LMNA	-0.11492	-0.269194642	0.045107041	1.59E-01	4.12E-01	1.00E+00
ENSG00000141646.12_SMAD4	ENSG00000160789.18_LMNA	-0.13561	-0.288584124	0.02410727	9.57E-02	3.03E-01	1.00E+00
ENSG00000166783.18_KIAA0430	ENSG00000160789.18_LMNA	-0.09841	-0.253641039	0.06175395	2.28E-01	5.04E-01	1.00E+00
ENSG00000112941.11_PAPD7	ENSG00000160789.18_LMNA	0.13087	-0.028932579	0.284151406	1.08E-01	3.27E-01	1.00E+00
ENSG00000180035.9_ZNF48	ENSG00000160789.18_LMNA	-0.00411	-0.1631994	0.155196773	9.60E-01	9.84E-01	1.00E+00
ENSG00000104723.19_TUSC3	ENSG00000160789.18_LMNA	-0.11272	-0.267131617	0.047325168	1.67E-01	4.24E-01	1.00E+00
ENSG00000269190.4_FBXO17	ENSG00000160789.18_LMNA	0.30616	0.154494409	0.443737025	1.25E-04	2.26E-03	1.00E+00
ENSG00000138135.6_CH25H	ENSG00000160789.18_LMNA	0.07742	-0.082803878	0.233737286	3.43E-01	6.28E-01	1.00E+00
ENSG00000116983.11_HPCAL4	ENSG00000160789.18_LMNA	-0.39801	-0.52400617	-0.25496271	3.82E-07	2.12E-05	1.96E-02
ENSG00000126016.12_AMOT	ENSG00000160789.18_LMNA	-0.23395	-0.379032876	-0.07764021	3.72E-03	3.14E-02	1.00E+00
ENSG00000128512.18_DOCK4	ENSG00000160789.18_LMNA	-0.10964	-0.264232594	0.050436914	1.79E-01	4.41E-01	1.00E+00
ENSG00000204160.10_ZDHHC18	ENSG00000160789.18_LMNA	0.49764	0.367553418	0.608618699	6.92E-11	1.67E-08	3.55E-06
ENSG00000075711.19_DLG1	ENSG00000160789.18_LMNA	0.03259	-0.127267678	0.190804043	6.90E-01	8.67E-01	1.00E+00
ENSG00000183665.5_TRMT12	ENSG00000160789.18_LMNA	-0.16022	-0.311477764	-0.0010481	4.86E-02	1.98E-01	1.00E+00
ENSG00000111596.10_CNOT2	ENSG00000160789.18_LMNA	0.01482	-0.144721048	0.173612186	8.56E-01	9.43E-01	1.00E+00
ENSG00000100196.9_KDELR3	ENSG00000160789.18_LMNA	0.48097	0.348455444	0.594642462	3.57E-10	6.60E-08	1.83E-05
ENSG00000164975.14_SNAPC3	ENSG00000160789.18_LMNA	-0.03906	-0.197037178	0.120890697	6.33E-01	8.35E-01	1.00E+00
ENSG00000265519.1_CTD-3157E	ENSG00000160789.18_LMNA	0.08019	-0.080034409	0.236371201	3.26E-01	6.13E-01	1.00E+00
ENSG00000196526.9_AFAP1	ENSG00000160789.18_LMNA	0.16996	0.011061246	0.32049144	3.63E-02	1.62E-01	1.00E+00
ENSG00000108091.10_CCDC6	ENSG00000160789.18_LMNA	0.02295	-0.136749967	0.181487977	7.79E-01	9.09E-01	1.00E+00
ENSG00000140749.8_IGSF6	ENSG00000160789.18_LMNA	-0.24267	-0.386926493	-0.08682808	2.59E-03	2.39E-02	1.00E+00

ENSG00000091490.9_SEL1L3	ENSG00000160789.18_LMNA	0.31492	0.163935152	0.451482586	7.77E-05	1.54E-03	1.00E+00
ENSG00000157985.16_AGAP1	ENSG00000160789.18_LMNA	0.0669	-0.093290138	0.22372201	4.13E-01	6.86E-01	1.00E+00
ENSG00000136100.11_VPS36	ENSG00000160789.18_LMNA	-0.20906	-0.356398095	-0.051575	9.74E-03	6.44E-02	1.00E+00
ENSG00000163788.12_SNRK	ENSG00000160789.18_LMNA	-0.20174	-0.34971203	-0.04395427	1.27E-02	7.77E-02	1.00E+00
ENSG00000075568.15_TMEM131	ENSG00000160789.18_LMNA	0.31238	0.161197474	0.449240939	8.93E-05	1.72E-03	1.00E+00
ENSG00000133678.12_TMEM254	ENSG00000160789.18_LMNA	-0.07501	-0.231443477	0.085211762	3.58E-01	6.41E-01	1.00E+00
ENSG00000175768.11_TOMM5	ENSG00000160789.18_LMNA	-0.08476	-0.240713973	0.075457445	2.99E-01	5.86E-01	1.00E+00
ENSG00000170802.14_FOXN2	ENSG00000160789.18_LMNA	-0.44976	-0.568273674	-0.31297169	6.14E-09	7.14E-07	3.15E-04
ENSG00000101391.19_CDK5RAP	ENSG00000160789.18_LMNA	-0.05756	-0.214791614	0.102581558	4.81E-01	7.39E-01	1.00E+00
ENSG00000150403.16_TMCO3	ENSG00000160789.18_LMNA	0.17265	0.013826962	0.322971257	3.34E-02	1.54E-01	1.00E+00
ENSG00000064652.9_SNX24	ENSG00000160789.18_LMNA	-0.0883	-0.244067634	0.07191379	2.79E-01	5.65E-01	1.00E+00
ENSG00000114439.17_BBX	ENSG00000160789.18_LMNA	-0.03918	-0.197146369	0.120778752	6.32E-01	8.35E-01	1.00E+00
ENSG00000062822.11_POLD1	ENSG00000160789.18_LMNA	0.25054	0.095130208	0.394021502	1.85E-03	1.85E-02	1.00E+00
ENSG00000271869.1_RP11-51J9.	ENSG00000160789.18_LMNA	-0.28401	-0.424037163	-0.13071874	3.91E-04	5.57E-03	1.00E+00
ENSG00000150756.12_FAM173B	ENSG00000160789.18_LMNA	-0.31139	-0.448360141	-0.16012299	9.43E-05	1.80E-03	1.00E+00
ENSG00000169249.11_ZRSR2	ENSG00000160789.18_LMNA	0.24952	0.094055619	0.39310516	1.93E-03	1.90E-02	1.00E+00
ENSG00000176261.14_ZBTB8OS	ENSG00000160789.18_LMNA	-0.05049	-0.208019385	0.109590834	5.37E-01	7.78E-01	1.00E+00
ENSG00000164167.8_LSM6	ENSG00000160789.18_LMNA	0.03448	-0.125404221	0.192628122	6.73E-01	8.57E-01	1.00E+00
ENSG00000182957.14_SPATA13	ENSG00000160789.18_LMNA	0.19597	0.037953356	0.344425104	1.55E-02	9.00E-02	1.00E+00
ENSG00000106004.4_HOXA5	ENSG00000160789.18_LMNA	-0.11974	-0.273720559	0.040229975	1.42E-01	3.84E-01	1.00E+00
ENSG00000163785.11_RYK	ENSG00000160789.18_LMNA	-0.10649	-0.261257485	0.053623989	1.92E-01	4.59E-01	1.00E+00
ENSG00000214063.9_TSPAN4	ENSG00000160789.18_LMNA	0.50449	0.375440931	0.614347601	3.43E-11	9.32E-09	1.76E-06
ENSG00000087586.16_AURKA	ENSG00000160789.18_LMNA	0.17471	0.015952059	0.324873798	3.13E-02	1.48E-01	1.00E+00
ENSG00000100109.15_TFIP11	ENSG00000160789.18_LMNA	0.14792	-0.011553175	0.300054343	6.90E-02	2.47E-01	1.00E+00
ENSG00000120256.8_LRP11	ENSG00000160789.18_LMNA	0.17441	0.015642291	0.324596627	3.16E-02	1.48E-01	1.00E+00
ENSG00000121671.10_CRY2	ENSG00000160789.18_LMNA	-0.14113	-0.293729583	0.018487763	8.29E-02	2.78E-01	1.00E+00
ENSG00000109320.10_NFKB1	ENSG00000160789.18_LMNA	0.46234	0.327227253	0.5789307	2.02E-09	2.79E-07	1.04E-04
ENSG00000086102.17_NFX1	ENSG00000160789.18_LMNA	0.05731	-0.10282906	0.214553001	4.83E-01	7.40E-01	1.00E+00
ENSG00000080608.9_PUM3	ENSG00000160789.18_LMNA	0.36272	0.215986518	0.493423393	4.38E-06	1.49E-04	2.25E-01
ENSG00000076321.9_KLHL20	ENSG00000160789.18_LMNA	-0.04663	-0.204315771	0.11341077	5.68E-01	7.98E-01	1.00E+00
ENSG00000198887.8_SMC5	ENSG00000160789.18_LMNA	0.31122	0.159948154	0.448216772	9.51E-05	1.81E-03	1.00E+00
ENSG00000152684.10_PELO	ENSG00000160789.18_LMNA	0.29815	0.145872211	0.436625071	1.91E-04	3.15E-03	1.00E+00
ENSG00000171208.8_NETO2	ENSG00000160789.18_LMNA	-0.02942	-0.187737213	0.130395661	7.19E-01	8.81E-01	1.00E+00

ENSG00000169972.10_PUSL1	ENSG00000160789.18_LMNA	0.15558	-0.003707979	0.307176755	5.56E-02	2.15E-01	1.00E+00
ENSG00000248593.3_DSTNP2	ENSG00000160789.18_LMNA	0.11067	-0.049403644	0.265195859	1.75E-01	4.35E-01	1.00E+00
ENSG00000166888.9_STAT6	ENSG00000160789.18_LMNA	0.14154	-0.01807104	0.294110433	8.20E-02	2.76E-01	1.00E+00
ENSG00000063176.14_SPHK2	ENSG00000160789.18_LMNA	0.04531	-0.114721673	0.203042687	5.79E-01	8.04E-01	1.00E+00
ENSG00000168434.11_COG7	ENSG00000160789.18_LMNA	0.13859	-0.021077712	0.291360362	8.86E-02	2.90E-01	1.00E+00
ENSG00000174953.12_DHX36	ENSG00000160789.18_LMNA	-0.11462	-0.268915987	0.045406827	1.60E-01	4.13E-01	1.00E+00
ENSG00000235912.1_RP1-159A1	ENSG00000160789.18_LMNA	-0.12514	-0.27878597	0.034753759	1.25E-01	3.56E-01	1.00E+00
ENSG00000279088.1_RP11-574K	ENSG00000160789.18_LMNA	-0.15979	-0.311073383	-0.00060034	4.93E-02	1.99E-01	1.00E+00
ENSG00000196227.9_FAM217B	ENSG00000160789.18_LMNA	-0.41424	-0.537965766	-0.27304894	1.13E-07	7.97E-06	5.77E-03
ENSG00000140577.14_CRTC3	ENSG00000160789.18_LMNA	0.20814	0.050613282	0.355556046	1.01E-02	6.60E-02	1.00E+00
ENSG00000164284.13_GRPEL2	ENSG00000160789.18_LMNA	0.0761	-0.084120598	0.232483383	3.51E-01	6.35E-01	1.00E+00
ENSG00000082213.16_C5orf22	ENSG00000160789.18_LMNA	-0.05402	-0.211400417	0.106095401	5.09E-01	7.58E-01	1.00E+00
ENSG00000219747.1_RP1-292B1	ENSG00000160789.18_LMNA	-0.09933	-0.254502526	0.060836475	2.23E-01	4.99E-01	1.00E+00
ENSG00000182400.13_TRAPPC6	ENSG00000160789.18_LMNA	-0.15705	-0.308540493	0.00220147	5.33E-02	2.10E-01	1.00E+00
ENSG00000170037.12_CNTROB	ENSG00000160789.18_LMNA	0.1659	0.006883435	0.31673744	4.11E-02	1.77E-01	1.00E+00
ENSG00000134900.10_TPP2	ENSG00000160789.18_LMNA	-0.16509	-0.315982382	-0.00604444	4.21E-02	1.80E-01	1.00E+00
ENSG00000157077.13_ZFYVE9	ENSG00000160789.18_LMNA	-0.04108	-0.198983676	0.118893887	6.15E-01	8.26E-01	1.00E+00
ENSG00000116745.6_RPE65	ENSG00000160789.18_LMNA	-0.10215	-0.257167586	0.057994832	2.10E-01	4.84E-01	1.00E+00
ENSG00000175489.9_LRRC25	ENSG00000160789.18_LMNA	0.09048	-0.069721146	0.246138942	2.68E-01	5.52E-01	1.00E+00
ENSG00000160584.14_SIK3	ENSG00000160789.18_LMNA	-0.03974	-0.197694126	0.120217056	6.27E-01	8.32E-01	1.00E+00
ENSG00000158545.14_ZC3H18	ENSG00000160789.18_LMNA	0.2123	0.054952151	0.359351096	8.65E-03	5.90E-02	1.00E+00
ENSG00000164976.8_KIAA1161	ENSG00000160789.18_LMNA	-0.11572	-0.269948914	0.044295287	1.56E-01	4.07E-01	1.00E+00
ENSG00000123810.6_B9D2	ENSG00000160789.18_LMNA	0.07817	-0.082050825	0.234453941	3.38E-01	6.24E-01	1.00E+00
ENSG00000115084.11_SLC35F5	ENSG00000160789.18_LMNA	0.10864	-0.051453054	0.263284686	1.83E-01	4.47E-01	1.00E+00
ENSG00000248092.6_NNT-AS1	ENSG00000160789.18_LMNA	-0.28736	-0.427023754	-0.13430167	3.31E-04	4.85E-03	1.00E+00
ENSG00000196422.9_PPP1R26	ENSG00000160789.18_LMNA	0.19536	0.0373242	0.343869676	1.59E-02	9.14E-02	1.00E+00
ENSG00000169371.12_SNUPN	ENSG00000160789.18_LMNA	0.08529	-0.074930969	0.2412127	2.96E-01	5.82E-01	1.00E+00
ENSG00000247903.1_RP11-421F	ENSG00000160789.18_LMNA	-0.00244	-0.161577623	0.156821934	9.76E-01	9.91E-01	1.00E+00
ENSG00000143891.15_GALM	ENSG00000160789.18_LMNA	0.22978	0.073260023	0.37525421	4.40E-03	3.56E-02	1.00E+00
ENSG00000152219.4_ARL14EP	ENSG00000160789.18_LMNA	-0.41104	-0.535213136	-0.26946771	1.44E-07	9.74E-06	7.39E-03
ENSG00000117308.13_GALE	ENSG00000160789.18_LMNA	0.14831	-0.011156129	0.300415645	6.82E-02	2.45E-01	1.00E+00
ENSG00000121068.12_TBX2	ENSG00000160789.18_LMNA	0.39492	0.251534351	0.521343641	4.78E-07	2.53E-05	2.45E-02
ENSG00000011332.18_DPF1	ENSG00000160789.18_LMNA	-0.24228	-0.386569467	-0.08641141	2.64E-03	2.41E-02	1.00E+00

ENSG00000160767.19_FAM189B	ENSG00000160789.18_LMNA	0.25689	0.101852091	0.399740055	1.40E-03	1.49E-02	1.00E+00
ENSG00000186687.14_LYRM7	ENSG00000160789.18_LMNA	-0.51362	-0.621966609	-0.38598462	1.32E-11	4.32E-09	6.75E-07
ENSG00000237991.3_RPL35P1	ENSG00000160789.18_LMNA	-0.05903	-0.216197056	0.101122962	4.70E-01	7.30E-01	1.00E+00
ENSG00000137767.12_SQRDL	ENSG00000160789.18_LMNA	0.07644	-0.083778138	0.232809607	3.49E-01	6.33E-01	1.00E+00
ENSG00000105171.8_POP4	ENSG00000160789.18_LMNA	-0.10533	-0.260167581	0.054789941	1.97E-01	4.66E-01	1.00E+00
ENSG00000184857.7_TMEM186	ENSG00000160789.18_LMNA	-0.25433	-0.397436608	-0.09914122	1.57E-03	1.62E-02	1.00E+00
ENSG00000081189.12_MEF2C	ENSG00000160789.18_LMNA	-0.26226	-0.404573266	-0.10755459	1.10E-03	1.25E-02	1.00E+00
ENSG00000104228.11_TRIM35	ENSG00000160789.18_LMNA	0.16368	0.004602043	0.314683373	4.39E-02	1.85E-01	1.00E+00
ENSG00000165660.7_FAM175B	ENSG00000160789.18_LMNA	-0.21725	-0.363868914	-0.06013239	7.18E-03	5.13E-02	1.00E+00
ENSG00000118454.11_ANKRD13	ENSG00000160789.18_LMNA	0.15235	-0.007017865	0.304176052	6.10E-02	2.28E-01	1.00E+00
ENSG00000214578.5_HMGN2P15	ENSG00000160789.18_LMNA	-0.31873	-0.454847494	-0.16805298	6.28E-05	1.29E-03	1.00E+00
ENSG00000130449.5_ZSWIM6	ENSG00000160789.18_LMNA	-0.19547	-0.343969167	-0.03743688	1.58E-02	9.11E-02	1.00E+00
ENSG00000163083.5_INHBB	ENSG00000160789.18_LMNA	0.25646	0.101398261	0.399354694	1.43E-03	1.51E-02	1.00E+00
ENSG00000153201.14_RANBP2	ENSG00000160789.18_LMNA	0.10042	-0.059733063	0.255537939	2.18E-01	4.93E-01	1.00E+00
ENSG00000047230.13_CTPS2	ENSG00000160789.18_LMNA	-0.05709	-0.214341784	0.103048113	4.85E-01	7.42E-01	1.00E+00
ENSG00000198825.10_INPP5F	ENSG00000160789.18_LMNA	0.02041	-0.139242592	0.179029529	8.03E-01	9.21E-01	1.00E+00
ENSG00000142733.13_MAP3K6	ENSG00000160789.18_LMNA	0.55202	0.430667939	0.653766954	1.68E-13	1.34E-10	8.64E-09
ENSG00000099364.15_FBXL19	ENSG00000160789.18_LMNA	0.19529	0.03724511	0.34379984	1.59E-02	9.15E-02	1.00E+00
ENSG00000137055.13_PLAA	ENSG00000160789.18_LMNA	0.00353	-0.155760133	0.162637409	9.66E-01	9.87E-01	1.00E+00
ENSG00000143502.13_SUSD4	ENSG00000160789.18_LMNA	-0.17988	-0.32964501	-0.0212937	2.66E-02	1.32E-01	1.00E+00
ENSG00000197530.11_MIB2	ENSG00000160789.18_LMNA	0.22324	0.066396412	0.369312901	5.70E-03	4.33E-02	1.00E+00
ENSG00000164010.12_ERMAP	ENSG00000160789.18_LMNA	0.08411	-0.076108162	0.240097323	3.03E-01	5.89E-01	1.00E+00
ENSG00000137574.9_TGS1	ENSG00000160789.18_LMNA	0.03438	-0.125508155	0.192526442	6.74E-01	8.57E-01	1.00E+00
ENSG00000087206.15_UIMC1	ENSG00000160789.18_LMNA	0.0092	-0.150217399	0.168157719	9.10E-01	9.66E-01	1.00E+00
ENSG00000047621.10_C12orf4	ENSG00000160789.18_LMNA	-0.31983	-0.455825833	-0.1692521	5.90E-05	1.23E-03	1.00E+00
ENSG00000158321.14_AUTS2	ENSG00000160789.18_LMNA	0.09812	-0.062047796	0.253365019	2.29E-01	5.06E-01	1.00E+00
ENSG00000104967.6_NOVA2	ENSG00000160789.18_LMNA	-0.05647	-0.213749894	0.103661796	4.90E-01	7.46E-01	1.00E+00
ENSG00000106443.13_PHF14	ENSG00000160789.18_LMNA	0.006	-0.153345989	0.165044238	9.42E-01	9.77E-01	1.00E+00
ENSG00000138642.13_HERC6	ENSG00000160789.18_LMNA	-0.04373	-0.201531915	0.11627589	5.93E-01	8.12E-01	1.00E+00
ENSG00000150471.14_ADGRL3	ENSG00000160789.18_LMNA	-0.14601	-0.298277003	0.013504918	7.27E-02	2.56E-01	1.00E+00
ENSG00000163125.14_RPRD2	ENSG00000160789.18_LMNA	0.07014	-0.090066928	0.226807595	3.91E-01	6.69E-01	1.00E+00
ENSG00000198863.6_RUNDC1	ENSG00000160789.18_LMNA	0.02092	-0.138744725	0.179520888	7.98E-01	9.19E-01	1.00E+00
ENSG00000108094.13_CUL2	ENSG00000160789.18_LMNA	-0.31056	-0.447627027	-0.15922918	9.86E-05	1.86E-03	1.00E+00

ENSG00000213366.11_GSTM2	ENSG00000160789.18_LMNA	-0.2242	-0.370187986	-0.06740554	5.49E-03	4.20E-02	1.00E+00
ENSG00000011638.9_TMEM159	ENSG00000160789.18_LMNA	0.1259	-0.033977488	0.279502599	1.22E-01	3.52E-01	1.00E+00
ENSG00000151287.15_TEX30	ENSG00000160789.18_LMNA	-0.2401	-0.384598523	-0.08411309	2.89E-03	2.60E-02	1.00E+00
ENSG00000120029.11_C10orf76	ENSG00000160789.18_LMNA	-0.20998	-0.357240475	-0.05253766	9.42E-03	6.28E-02	1.00E+00
ENSG00000076513.15_ANKRD13	ENSG00000160789.18_LMNA	-0.28758	-0.42722209	-0.13453988	3.28E-04	4.82E-03	1.00E+00
ENSG00000089195.13_TRMT6	ENSG00000160789.18_LMNA	-0.01521	-0.173984828	0.144344827	8.53E-01	9.42E-01	1.00E+00
ENSG00000229809.7_ZNF688	ENSG00000160789.18_LMNA	-0.10156	-0.256608786	0.058591081	2.13E-01	4.87E-01	1.00E+00
ENSG00000133422.11_MORC2	ENSG00000160789.18_LMNA	0.16993	0.011022608	0.320456766	3.64E-02	1.62E-01	1.00E+00
ENSG00000213420.6_GPC2	ENSG00000160789.18_LMNA	-0.21747	-0.364066409	-0.06035922	7.12E-03	5.10E-02	1.00E+00
ENSG00000112242.13_E2F3	ENSG00000160789.18_LMNA	-0.00265	-0.161785541	0.156613679	9.74E-01	9.90E-01	1.00E+00
ENSG00000100532.10_CGRRF1	ENSG00000160789.18_LMNA	-0.2487	-0.392362673	-0.09318542	2.00E-03	1.96E-02	1.00E+00
ENSG00000178715.8_RP11-169K	ENSG00000160789.18_LMNA	-0.14683	-0.299038877	0.012668575	7.11E-02	2.52E-01	1.00E+00
ENSG00000272333.4_KMT2B	ENSG00000160789.18_LMNA	0.15452	-0.004798399	0.306188876	5.73E-02	2.19E-01	1.00E+00
ENSG00000170145.4_SIK2	ENSG00000160789.18_LMNA	0.11243	-0.047620959	0.266856291	1.68E-01	4.26E-01	1.00E+00
ENSG00000169129.13_AFAP1L2	ENSG00000160789.18_LMNA	-0.28358	-0.423652999	-0.13025842	4.00E-04	5.66E-03	1.00E+00
ENSG00000013523.8_ANGEL1	ENSG00000160789.18_LMNA	-0.1771	-0.327082009	-0.01842209	2.91E-02	1.40E-01	1.00E+00
ENSG00000148057.14_IDNK	ENSG00000160789.18_LMNA	-0.09871	-0.253924478	0.061452149	2.26E-01	5.03E-01	1.00E+00
ENSG00000171100.13_MTM1	ENSG00000160789.18_LMNA	0.1115	-0.048559441	0.265982402	1.71E-01	4.31E-01	1.00E+00
ENSG00000018510.11_AGPS	ENSG00000160789.18_LMNA	-0.03256	-0.190770098	0.127302334	6.90E-01	8.67E-01	1.00E+00
ENSG00000122390.16_NAA60	ENSG00000160789.18_LMNA	0.04467	-0.115353449	0.202428753	5.85E-01	8.07E-01	1.00E+00
ENSG00000120159.10_CAAP1	ENSG00000160789.18_LMNA	-0.00031	-0.159502054	0.15889932	9.97E-01	9.99E-01	1.00E+00
ENSG00000078401.6_EDN1	ENSG00000160789.18_LMNA	-0.08037	-0.236541808	0.079854854	3.25E-01	6.12E-01	1.00E+00
ENSG00000205423.10_CNEP1R1	ENSG00000160789.18_LMNA	-0.11623	-0.270428084	0.043779384	1.54E-01	4.04E-01	1.00E+00
ENSG00000198315.9_ZKSCAN8	ENSG00000160789.18_LMNA	-0.03569	-0.1937854	0.124220795	6.62E-01	8.51E-01	1.00E+00
ENSG00000120942.12_UBIAD1	ENSG00000160789.18_LMNA	-0.04078	-0.198692626	0.119192624	6.18E-01	8.27E-01	1.00E+00
ENSG00000213144.2_RP11-64B1	ENSG00000160789.18_LMNA	-0.07719	-0.233521127	0.083030946	3.45E-01	6.29E-01	1.00E+00
ENSG00000119321.7_FKBP15	ENSG00000160789.18_LMNA	0.20225	0.044486111	0.350179655	1.25E-02	7.68E-02	1.00E+00
ENSG00000084072.15_PPIE	ENSG00000160789.18_LMNA	-0.0003	-0.159497933	0.158903442	9.97E-01	9.99E-01	1.00E+00
ENSG00000175356.11_SCUBE2	ENSG00000160789.18_LMNA	-0.22506	-0.370966387	-0.0683037	5.31E-03	4.10E-02	1.00E+00
ENSG00000113300.10_CNOT6	ENSG00000160789.18_LMNA	-0.12985	-0.283192118	0.029974911	1.11E-01	3.32E-01	1.00E+00
ENSG00000065802.10_ASB1	ENSG00000160789.18_LMNA	0.08596	-0.074261637	0.241846512	2.92E-01	5.78E-01	1.00E+00
ENSG00000240225.9_ZNF542P	ENSG00000160789.18_LMNA	-0.18832	-0.337403167	-0.03001715	2.02E-02	1.09E-01	1.00E+00
ENSG00000230124.5_ACBD6	ENSG00000160789.18_LMNA	0.02148	-0.13819006	0.180068115	7.93E-01	9.16E-01	1.00E+00

ENSG00000263753.5_LINC00667	ENSG00000160789.18_LMNA	-0.16203	-0.313156045	-0.00290777	4.61E-02	1.91E-01	1.00E+00
ENSG00000255529.6_POLR2M	ENSG00000160789.18_LMNA	-0.1006	-0.255707805	0.059551969	2.17E-01	4.92E-01	1.00E+00
ENSG00000139131.11_YARS2	ENSG00000160789.18_LMNA	0.112	-0.048061178	0.266446436	1.70E-01	4.28E-01	1.00E+00
ENSG00000134452.18_FBXO18	ENSG00000160789.18_LMNA	0.00735	-0.152026481	0.166358148	9.28E-01	9.72E-01	1.00E+00
ENSG00000135093.11_USP30	ENSG00000160789.18_LMNA	-0.38834	-0.515662647	-0.24424188	7.66E-07	3.70E-05	3.93E-02
ENSG00000143486.14_EIF2D	ENSG00000160789.18_LMNA	0.08361	-0.076609238	0.239622305	3.06E-01	5.92E-01	1.00E+00
ENSG00000150687.10_PRSS23	ENSG00000160789.18_LMNA	0.31545	0.164514848	0.451956784	7.54E-05	1.50E-03	1.00E+00
ENSG00000169016.15_E2F6	ENSG00000160789.18_LMNA	-0.04571	-0.203427475	0.114325571	5.76E-01	8.02E-01	1.00E+00
ENSG00000116406.17_EDEM3	ENSG00000160789.18_LMNA	0.32847	0.17861314	0.463439539	3.60E-05	8.29E-04	1.00E+00
ENSG00000008853.15_RHOBTB2	ENSG00000160789.18_LMNA	0.08207	-0.078154366	0.238156578	3.15E-01	6.01E-01	1.00E+00
ENSG00000196914.7_ARHGEF12	ENSG00000160789.18_LMNA	0.11716	-0.042841378	0.271298905	1.51E-01	3.99E-01	1.00E+00
ENSG00000228623.3_ZNF883	ENSG00000160789.18_LMNA	-0.21087	-0.358054588	-0.05346856	9.11E-03	6.13E-02	1.00E+00
ENSG00000163050.15_COQ8A	ENSG00000160789.18_LMNA	0.12555	-0.034337085	0.279170674	1.23E-01	3.54E-01	1.00E+00
ENSG00000068354.14_TBC1D25	ENSG00000160789.18_LMNA	0.21585	0.058667736	0.362592998	7.57E-03	5.33E-02	1.00E+00
ENSG00000143493.11_INTS7	ENSG00000160789.18_LMNA	-0.21359	-0.360527541	-0.05629952	8.24E-03	5.69E-02	1.00E+00
ENSG00000163517.13_HDAC11	ENSG00000160789.18_LMNA	-0.22499	-0.370905735	-0.0682337	5.32E-03	4.11E-02	1.00E+00
ENSG00000108669.15_CYTH1	ENSG00000160789.18_LMNA	-0.18755	-0.336697833	-0.02922212	2.07E-02	1.10E-01	1.00E+00
ENSG00000144677.13_CTDSPL	ENSG00000160789.18_LMNA	-0.12725	-0.280764819	0.032609299	1.18E-01	3.45E-01	1.00E+00
ENSG00000079257.6_LXN	ENSG00000160789.18_LMNA	-0.04803	-0.20565814	0.112027325	5.57E-01	7.90E-01	1.00E+00
ENSG00000123200.15_ZC3H13	ENSG00000160789.18_LMNA	-0.00878	-0.167743844	0.15063365	9.15E-01	9.67E-01	1.00E+00
ENSG00000197008.8_ZNF138	ENSG00000160789.18_LMNA	-0.21025	-0.357480428	-0.05281198	9.33E-03	6.23E-02	1.00E+00
ENSG00000163872.14_YEATS2	ENSG00000160789.18_LMNA	-0.01628	-0.175030826	0.143288288	8.42E-01	9.39E-01	1.00E+00
ENSG00000167186.9_COQ7	ENSG00000160789.18_LMNA	-0.28352	-0.423597835	-0.13019233	4.01E-04	5.67E-03	1.00E+00
ENSG00000178974.8_FBXO34	ENSG00000160789.18_LMNA	-0.01542	-0.174196507	0.144131073	8.50E-01	9.41E-01	1.00E+00
ENSG00000137496.16_IL18BP	ENSG00000160789.18_LMNA	0.02036	-0.139291693	0.178981061	8.03E-01	9.21E-01	1.00E+00
ENSG00000101752.10_MIB1	ENSG00000160789.18_LMNA	-0.14676	-0.29897428	0.012739503	7.12E-02	2.52E-01	1.00E+00
ENSG00000232815.1_DUX4L50	ENSG00000160789.18_LMNA	-0.14059	-0.293232131	0.019031907	8.41E-02	2.81E-01	1.00E+00
ENSG00000182749.5_PAQR7	ENSG00000160789.18_LMNA	0.19183	0.033656202	0.340627243	1.79E-02	9.97E-02	1.00E+00
ENSG00000213160.8_KLHL23	ENSG00000160789.18_LMNA	-0.52741	-0.633425729	-0.40195945	2.93E-12	1.33E-09	1.50E-07
ENSG00000173011.11_TADA2B	ENSG00000160789.18_LMNA	0.04722	-0.11282833	0.204881063	5.63E-01	7.95E-01	1.00E+00
ENSG00000196756.10_SNHG17	ENSG00000160789.18_LMNA	0.1416	-0.018003242	0.294172385	8.18E-02	2.76E-01	1.00E+00
ENSG00000137070.16_IL11RA	ENSG00000160789.18_LMNA	-0.24321	-0.387410833	-0.0873935	2.53E-03	2.35E-02	1.00E+00
ENSG00000169442.7_CD52	ENSG00000160789.18_LMNA	-0.0682	-0.224955658	0.092002263	4.04E-01	6.80E-01	1.00E+00

ENSG00000196767.6_POU3F4	ENSG00000160789.18_LMNA	-0.18263	-0.332168693	-0.02412625	2.43E-02	1.24E-01	1.00E+00
ENSG00000006756.14_ARSD	ENSG00000160789.18_LMNA	0.47644	0.343281712	0.590830445	5.49E-10	9.51E-08	2.82E-05
ENSG00000123609.9_NMI	ENSG00000160789.18_LMNA	0.16392	0.004840787	0.314898464	4.36E-02	1.84E-01	1.00E+00
ENSG00000265298.1_RP13-104F	ENSG00000160789.18_LMNA	-0.02954	-0.187857738	0.130272851	7.18E-01	8.80E-01	1.00E+00
ENSG00000270001.1_RP11-218C	ENSG00000160789.18_LMNA	-0.06639	-0.223227115	0.093806489	4.16E-01	6.89E-01	1.00E+00
ENSG00000198133.7_TMEM229B	ENSG00000160789.18_LMNA	-0.18499	-0.334346769	-0.02657487	2.25E-02	1.17E-01	1.00E+00
ENSG00000165434.7_PGM2L1	ENSG00000160789.18_LMNA	0.27014	0.115925929	0.411638555	7.63E-04	9.36E-03	1.00E+00
ENSG00000116062.13_MSH6	ENSG00000160789.18_LMNA	0.11728	-0.042713801	0.271417305	1.50E-01	3.98E-01	1.00E+00
ENSG00000126804.12_ZBTB1	ENSG00000160789.18_LMNA	0.20903	0.051545794	0.35637253	9.75E-03	6.45E-02	1.00E+00
ENSG00000133703.10_KRAS	ENSG00000160789.18_LMNA	-0.28105	-0.421393482	-0.12755356	4.53E-04	6.24E-03	1.00E+00
ENSG00000163743.12_RCHY1	ENSG00000160789.18_LMNA	-0.3958	-0.522101578	-0.25250961	4.49E-07	2.41E-05	2.30E-02
ENSG00000154654.13_NCAM2	ENSG00000160789.18_LMNA	-0.21727	-0.363886051	-0.06015207	7.17E-03	5.12E-02	1.00E+00
ENSG00000048991.15_R3HDM1	ENSG00000160789.18_LMNA	-0.15076	-0.302697169	0.008646624	6.37E-02	2.35E-01	1.00E+00
ENSG00000166348.16_USP54	ENSG00000160789.18_LMNA	-0.32649	-0.461691251	-0.17645905	4.04E-05	9.10E-04	1.00E+00
ENSG00000213315.5_CTD-3035D	ENSG00000160789.18_LMNA	-0.08451	-0.240478475	0.075705984	3.01E-01	5.87E-01	1.00E+00
ENSG00000133316.14_WDR74	ENSG00000160789.18_LMNA	0.02114	-0.138527226	0.179735493	7.96E-01	9.18E-01	1.00E+00
ENSG00000232677.5_LINC00665	ENSG00000160789.18_LMNA	-0.22632	-0.372116035	-0.06963112	5.05E-03	3.95E-02	1.00E+00
ENSG00000114796.14_KLHL24	ENSG00000160789.18_LMNA	-0.37794	-0.506659513	-0.23274745	1.58E-06	6.69E-05	8.12E-02
ENSG00000273559.3_CWC25	ENSG00000160789.18_LMNA	0.07912	-0.081106956	0.235351702	3.33E-01	6.18E-01	1.00E+00
ENSG00000026103.18_FAS	ENSG00000160789.18_LMNA	0.24034	0.084369153	0.384818244	2.86E-03	2.58E-02	1.00E+00
ENSG00000185630.17_PBX1	ENSG00000160789.18_LMNA	-0.2485	-0.392182499	-0.09297432	2.02E-03	1.97E-02	1.00E+00
ENSG00000243056.1_EIF4EBP3	ENSG00000160789.18_LMNA	0.0702	-0.090002243	0.226869453	3.90E-01	6.68E-01	1.00E+00
ENSG00000269834.4_ZNF528-AS	ENSG00000160789.18_LMNA	-0.06777	-0.224548528	0.092427407	4.07E-01	6.82E-01	1.00E+00
ENSG00000123094.14_RASSF8	ENSG00000160789.18_LMNA	0.38255	0.237835162	0.510651901	1.15E-06	5.16E-05	5.91E-02
ENSG00000147799.10_ARHGAP3	ENSG00000160789.18_LMNA	0.03324	-0.126627314	0.191431123	6.84E-01	8.63E-01	1.00E+00
ENSG00000107290.12_SETX	ENSG00000160789.18_LMNA	0.18254	0.024033134	0.332085804	2.44E-02	1.24E-01	1.00E+00
ENSG00000205309.12_NT5M	ENSG00000160789.18_LMNA	0.03616	-0.123751985	0.194243608	6.58E-01	8.49E-01	1.00E+00
ENSG00000113595.13_TRIM23	ENSG00000160789.18_LMNA	-0.32416	-0.45964051	-0.17393579	4.62E-05	1.01E-03	1.00E+00
ENSG00000243302.3_RP11-274B	ENSG00000160789.18_LMNA	0.1346	-0.02514053	0.287636058	9.83E-02	3.08E-01	1.00E+00
ENSG00000121440.13_PDZRN3	ENSG00000160789.18_LMNA	-0.17492	-0.325067341	-0.0161684	3.11E-02	1.47E-01	1.00E+00
ENSG00000128944.12_KNSTRN	ENSG00000160789.18_LMNA	-0.02625	-0.18468163	0.133505887	7.48E-01	8.95E-01	1.00E+00
ENSG00000171262.10_FAM98B	ENSG00000160789.18_LMNA	0.10985	-0.050231721	0.264423935	1.78E-01	4.40E-01	1.00E+00
ENSG00000105829.10_BET1	ENSG00000160789.18_LMNA	0.06528	-0.094906199	0.222172557	4.24E-01	6.95E-01	1.00E+00

ENSG00000106588.9_PSMA2	ENSG00000160789.18_LMNA	-0.16269	-0.313763141	-0.00358101	4.52E-02	1.88E-01	1.00E+00
ENSG00000161513.10_FDXR	ENSG00000160789.18_LMNA	0.17972	0.021122656	0.329492473	2.67E-02	1.33E-01	1.00E+00
ENSG00000101986.11_ABCD1	ENSG00000160789.18_LMNA	0.31572	0.164797832	0.452188209	7.43E-05	1.48E-03	1.00E+00
ENSG00000166949.14_SMAD3	ENSG00000160789.18_LMNA	0.19281	0.034673633	0.341527363	1.73E-02	9.73E-02	1.00E+00
ENSG00000110013.11_SIAE	ENSG00000160789.18_LMNA	0.24969	0.09423653	0.393259471	1.92E-03	1.89E-02	1.00E+00
ENSG00000226525.5_RPS7P10	ENSG00000160789.18_LMNA	-0.16138	-0.312548891	-0.00223475	4.70E-02	1.93E-01	1.00E+00
ENSG00000198420.8_TCAF1	ENSG00000160789.18_LMNA	0.09362	-0.066570053	0.249110616	2.51E-01	5.32E-01	1.00E+00
ENSG00000145743.14_FBXL17	ENSG00000160789.18_LMNA	-0.26259	-0.404862071	-0.10789596	1.08E-03	1.23E-02	1.00E+00
ENSG00000152683.13_SLC30A6	ENSG00000160789.18_LMNA	-0.07859	-0.23484973	0.081634779	3.36E-01	6.22E-01	1.00E+00
ENSG00000231770.4_TMEM44-A:	ENSG00000160789.18_LMNA	-0.06035	-0.217464512	0.099806399	4.60E-01	7.24E-01	1.00E+00
ENSG00000183018.7_SPNS2	ENSG00000160789.18_LMNA	-0.15262	-0.304427184	0.006741119	6.05E-02	2.27E-01	1.00E+00
ENSG00000138660.10_AP1AR	ENSG00000160789.18_LMNA	-0.25472	-0.397785474	-0.09955151	1.54E-03	1.60E-02	1.00E+00
ENSG00000175213.2_ZNF408	ENSG00000160789.18_LMNA	0.23837	0.082295993	0.38303831	3.10E-03	2.74E-02	1.00E+00
ENSG00000224543.4_SNRPGP15	ENSG00000160789.18_LMNA	-0.17512	-0.325252506	-0.0163754	3.09E-02	1.46E-01	1.00E+00
ENSG00000172346.13_CSDC2	ENSG00000160789.18_LMNA	-0.48128	-0.59490197	-0.3488082	3.46E-10	6.44E-08	1.78E-05
ENSG00000275302.1_CCL4	ENSG00000160789.18_LMNA	-0.15593	-0.307500622	0.003350334	5.51E-02	2.14E-01	1.00E+00
ENSG00000138435.13_CHRNA1	ENSG00000160789.18_LMNA	0.12269	-0.037235773	0.276492307	1.32E-01	3.69E-01	1.00E+00
ENSG00000198040.9_ZNF84	ENSG00000160789.18_LMNA	-0.25866	-0.401333848	-0.10373038	1.29E-03	1.41E-02	1.00E+00
ENSG00000183735.8_TBK1	ENSG00000160789.18_LMNA	0.05636	-0.103771412	0.213644146	4.90E-01	7.46E-01	1.00E+00
ENSG00000107566.12_ERLIN1	ENSG00000160789.18_LMNA	0.13095	-0.028856338	0.284221548	1.08E-01	3.27E-01	1.00E+00
ENSG00000182022.16_CHST15	ENSG00000160789.18_LMNA	-0.03718	-0.195221121	0.122751377	6.49E-01	8.44E-01	1.00E+00
ENSG00000125347.12_IRF1	ENSG00000160789.18_LMNA	0.32008	0.169520569	0.456044772	5.82E-05	1.21E-03	1.00E+00
ENSG00000064999.13_ANKS1A	ENSG00000160789.18_LMNA	-0.08111	-0.237246052	0.079113452	3.21E-01	6.07E-01	1.00E+00
ENSG00000205213.12_LGR4	ENSG00000160789.18_LMNA	0.01367	-0.145849929	0.172493499	8.67E-01	9.48E-01	1.00E+00
ENSG00000172888.10_ZNF621	ENSG00000160789.18_LMNA	-0.08965	-0.245347527	0.070559282	2.72E-01	5.57E-01	1.00E+00
ENSG00000235238.1_SUMO2P1	ENSG00000160789.18_LMNA	-0.19886	-0.3470755	-0.04095892	1.40E-02	8.38E-02	1.00E+00
ENSG00000167074.13_TEF	ENSG00000160789.18_LMNA	-0.25256	-0.395848937	-0.09727531	1.69E-03	1.72E-02	1.00E+00
ENSG00000129422.12_MTUS1	ENSG00000160789.18_LMNA	-0.09473	-0.250157409	0.065458556	2.46E-01	5.25E-01	1.00E+00
ENSG00000145687.14_SSBP2	ENSG00000160789.18_LMNA	0.08401	-0.076210596	0.240000228	3.03E-01	5.90E-01	1.00E+00
ENSG00000103528.15_SYT17	ENSG00000160789.18_LMNA	0.00938	-0.150043435	0.168330656	9.09E-01	9.65E-01	1.00E+00
ENSG00000196455.6_PIK3R4	ENSG00000160789.18_LMNA	0.13038	-0.029436111	0.283688069	1.09E-01	3.29E-01	1.00E+00
ENSG00000160957.11_RECQL4	ENSG00000160789.18_LMNA	0.17995	0.021362214	0.329706103	2.65E-02	1.32E-01	1.00E+00
ENSG00000188807.11_TMEM201	ENSG00000160789.18_LMNA	0.12187	-0.038069048	0.275721463	1.35E-01	3.73E-01	1.00E+00

ENSG00000107185.9_RGP1	ENSG00000160789.18_LMNA	0.21128	0.053888152	0.358421386	8.98E-03	6.07E-02	1.00E+00
ENSG00000151876.11_FBXO4	ENSG00000160789.18_LMNA	0.09646	-0.063716666	0.251796415	2.37E-01	5.16E-01	1.00E+00
ENSG00000112320.10_SOBP	ENSG00000160789.18_LMNA	-0.17439	-0.324575485	-0.01561867	3.17E-02	1.49E-01	1.00E+00
ENSG00000105246.5_EBI3	ENSG00000160789.18_LMNA	-0.08894	-0.244679214	0.071266702	2.76E-01	5.61E-01	1.00E+00
ENSG00000213762.9_ZNF134	ENSG00000160789.18_LMNA	0.09627	-0.063914163	0.251610675	2.38E-01	5.17E-01	1.00E+00
ENSG00000259775.1_RP11-45P1	ENSG00000160789.18_LMNA	0.09157	-0.068633818	0.24716503	2.62E-01	5.45E-01	1.00E+00
ENSG00000198324.13_FAM109A	ENSG00000160789.18_LMNA	0.18672	0.028367244	0.335939025	2.13E-02	1.13E-01	1.00E+00
ENSG00000180537.11_RNF182	ENSG00000160789.18_LMNA	-0.28098	-0.421333019	-0.12748124	4.54E-04	6.26E-03	1.00E+00
ENSG00000253276.2_CCDC71L	ENSG00000160789.18_LMNA	0.08125	-0.078973323	0.237379121	3.20E-01	6.06E-01	1.00E+00
ENSG00000274561.1_RP11-147L	ENSG00000160789.18_LMNA	0.09466	-0.065527959	0.250092068	2.46E-01	5.26E-01	1.00E+00
ENSG00000203805.9_PLPP4	ENSG00000160789.18_LMNA	-0.11737	-0.271495213	0.042629849	1.50E-01	3.98E-01	1.00E+00
ENSG00000197223.10_C1D	ENSG00000160789.18_LMNA	-0.3064	-0.443949578	-0.15475278	1.23E-04	2.24E-03	1.00E+00
ENSG00000184708.16_EIF4ENIF1	ENSG00000160789.18_LMNA	-0.0749	-0.231344352	0.085315733	3.59E-01	6.42E-01	1.00E+00
ENSG00000264247.1_LINC00909	ENSG00000160789.18_LMNA	-0.27729	-0.418036609	-0.12354312	5.43E-04	7.20E-03	1.00E+00
ENSG00000116106.10_EPHA4	ENSG00000160789.18_LMNA	-0.09632	-0.251665511	0.063855858	2.38E-01	5.16E-01	1.00E+00
ENSG00000184986.9_TMEM121	ENSG00000160789.18_LMNA	0.04729	-0.112757277	0.20495001	5.63E-01	7.94E-01	1.00E+00
ENSG00000213763.4_ACTBP2	ENSG00000160789.18_LMNA	0.32262	0.17226983	0.458284859	5.04E-05	1.08E-03	1.00E+00
ENSG00000072415.7_MPP5	ENSG00000160789.18_LMNA	0.00705	-0.15232646	0.166059541	9.31E-01	9.73E-01	1.00E+00
ENSG00000130939.17_UBE4B	ENSG00000160789.18_LMNA	0.01666	-0.142919181	0.175396082	8.39E-01	9.37E-01	1.00E+00
ENSG00000197296.5_FITM2	ENSG00000160789.18_LMNA	-0.10828	-0.262952099	0.051809426	1.84E-01	4.50E-01	1.00E+00
ENSG00000135272.8_MDFIC	ENSG00000160789.18_LMNA	0.29789	0.14559074	0.436392289	1.94E-04	3.18E-03	1.00E+00
ENSG00000100023.16_PPIL2	ENSG00000160789.18_LMNA	0.07373	-0.086485482	0.230228674	3.67E-01	6.48E-01	1.00E+00
ENSG00000013619.12_MAMLD1	ENSG00000160789.18_LMNA	-0.00786	-0.166852845	0.151529382	9.23E-01	9.71E-01	1.00E+00
ENSG00000090686.14_USP48	ENSG00000160789.18_LMNA	0.21433	0.057079945	0.361208527	8.01E-03	5.57E-02	1.00E+00
ENSG00000054356.12_PTPRN	ENSG00000160789.18_LMNA	-0.00316	-0.162276999	0.15612131	9.69E-01	9.88E-01	1.00E+00
ENSG00000185985.8_SLITRK2	ENSG00000160789.18_LMNA	-0.26929	-0.41087232	-0.11501602	7.94E-04	9.68E-03	1.00E+00
ENSG00000220472.1_RP11-69L1	ENSG00000160789.18_LMNA	-0.14661	-0.298836226	0.012891076	7.15E-02	2.53E-01	1.00E+00
ENSG00000080802.17_CNOT4	ENSG00000160789.18_LMNA	0.14434	-0.015205183	0.296726904	7.60E-02	2.64E-01	1.00E+00
ENSG00000164654.14_MIOS	ENSG00000160789.18_LMNA	-0.03455	-0.192691762	0.125339166	6.73E-01	8.56E-01	1.00E+00
ENSG00000175592.7_FOSL1	ENSG00000160789.18_LMNA	0.64854	0.545682391	0.732149697	1.66E-19	1.07E-15	8.52E-15
ENSG00000204315.3_FKBPL	ENSG00000160789.18_LMNA	0.0115	-0.147969692	0.170390657	8.88E-01	9.57E-01	1.00E+00
ENSG00000163170.10_BOLA3	ENSG00000160789.18_LMNA	0.02333	-0.136377568	0.181854929	7.75E-01	9.08E-01	1.00E+00
ENSG00000159788.17_RGS12	ENSG00000160789.18_LMNA	0.07727	-0.082947275	0.233600782	3.44E-01	6.29E-01	1.00E+00

ENSG00000107201.8_DDX58	ENSG00000160789.18_LMNA	-0.05473	-0.212085011	0.105386686	5.03E-01	7.54E-01	1.00E+00
ENSG00000207383.1_Y_RNA	ENSG00000160789.18_LMNA	-0.0999	-0.25504105	0.060262681	2.21E-01	4.96E-01	1.00E+00
ENSG00000227671.4_RP11-488L	ENSG00000160789.18_LMNA	-0.16046	-0.311695106	-0.00128882	4.83E-02	1.97E-01	1.00E+00
ENSG00000181523.11_SGSH	ENSG00000160789.18_LMNA	0.3993	0.256399347	0.525120327	3.47E-07	1.98E-05	1.78E-02
ENSG00000270607.1_RP11-359E	ENSG00000160789.18_LMNA	0.34718	0.198981473	0.479861882	1.18E-05	3.32E-04	6.03E-01
ENSG00000197081.11_IGF2R	ENSG00000160789.18_LMNA	0.26651	0.112061903	0.408381785	9.04E-04	1.07E-02	1.00E+00
ENSG00000070367.14_EXOC5	ENSG00000160789.18_LMNA	-0.006	-0.165045206	0.153345017	9.41E-01	9.77E-01	1.00E+00
ENSG00000127527.12_EPS15L1	ENSG00000160789.18_LMNA	0.11002	-0.050058517	0.264585428	1.77E-01	4.39E-01	1.00E+00
ENSG00000205704.6_LINC00634	ENSG00000160789.18_LMNA	-0.51496	-0.623079594	-0.38753002	1.14E-11	3.85E-09	5.85E-07
ENSG00000255284.1_AP006621.!	ENSG00000160789.18_LMNA	0.08879	-0.071417254	0.244536946	2.77E-01	5.61E-01	1.00E+00
ENSG00000168778.10_TCTN2	ENSG00000160789.18_LMNA	0.32916	0.1793568	0.464042589	3.46E-05	8.04E-04	1.00E+00
ENSG00000095485.15_CWF19L1	ENSG00000160789.18_LMNA	-0.35328	-0.485190423	-0.20564292	8.03E-06	2.44E-04	4.12E-01
ENSG00000166398.11_KIAA0355	ENSG00000160789.18_LMNA	0.02914	-0.130670239	0.187467706	7.22E-01	8.82E-01	1.00E+00
ENSG00000178921.12_PFAS	ENSG00000160789.18_LMNA	0.08107	-0.079148506	0.237212763	3.21E-01	6.07E-01	1.00E+00
ENSG00000139350.10_NEDD1	ENSG00000160789.18_LMNA	0.07016	-0.090046831	0.226826814	3.90E-01	6.69E-01	1.00E+00
ENSG00000167920.7_TMEM99	ENSG00000160789.18_LMNA	-0.32643	-0.461637906	-0.17639337	4.06E-05	9.12E-04	1.00E+00
ENSG00000145220.12_LYAR	ENSG00000160789.18_LMNA	0.14483	-0.014709108	0.297179335	7.50E-02	2.61E-01	1.00E+00
ENSG00000167578.15_RAB4B	ENSG00000160789.18_LMNA	-0.1956	-0.344085552	-0.0375687	1.57E-02	9.08E-02	1.00E+00
ENSG00000197885.9_NKIRAS1	ENSG00000160789.18_LMNA	-0.5428	-0.646161305	-0.41988064	5.05E-13	3.36E-10	2.59E-08
ENSG00000146918.18_NCAPG2	ENSG00000160789.18_LMNA	0.21109	0.053697519	0.358254749	9.04E-03	6.10E-02	1.00E+00
ENSG00000178951.7_ZBTB7A	ENSG00000160789.18_LMNA	0.36864	0.222499816	0.49858226	2.97E-06	1.09E-04	1.52E-01
ENSG00000151690.13_MFSD6	ENSG00000160789.18_LMNA	-0.21177	-0.358871558	-0.05440326	8.82E-03	5.98E-02	1.00E+00
ENSG00000113460.11_BRIX1	ENSG00000160789.18_LMNA	-0.0057	-0.164750805	0.153640518	9.44E-01	9.78E-01	1.00E+00
ENSG00000258199.1_RP11-977G	ENSG00000160789.18_LMNA	-0.20741	-0.354892249	-0.04985555	1.03E-02	6.72E-02	1.00E+00
ENSG00000110046.11_ATG2A	ENSG00000160789.18_LMNA	0.16041	0.001239864	0.31165091	4.84E-02	1.97E-01	1.00E+00
ENSG00000132773.10_TOE1	ENSG00000160789.18_LMNA	0.14357	-0.015993521	0.296007633	7.76E-02	2.67E-01	1.00E+00
ENSG00000059377.14_TBXAS1	ENSG00000160789.18_LMNA	-0.0054	-0.164457304	0.153935058	9.47E-01	9.79E-01	1.00E+00
ENSG00000161618.8_ALDH16A1	ENSG00000160789.18_LMNA	0.29549	0.143021539	0.434265696	2.19E-04	3.51E-03	1.00E+00
ENSG00000271447.4_MMP28	ENSG00000160789.18_LMNA	-0.14943	-0.301459061	0.010008937	6.61E-02	2.40E-01	1.00E+00
ENSG00000108510.8_MED13	ENSG00000160789.18_LMNA	0.00882	-0.150591205	0.167786052	9.14E-01	9.67E-01	1.00E+00
ENSG00000136603.12_SKIL	ENSG00000160789.18_LMNA	-0.19317	-0.341855612	-0.03504482	1.71E-02	9.65E-02	1.00E+00
ENSG00000160972.8_PPP1R16A	ENSG00000160789.18_LMNA	0.32133	0.17086908	0.457143987	5.43E-05	1.15E-03	1.00E+00
ENSG00000107672.13_NSMCE4A	ENSG00000160789.18_LMNA	-0.41289	-0.536808559	-0.27154249	1.25E-07	8.68E-06	6.40E-03

ENSG00000141568.18_FOXK2	ENSG00000160789.18_LMNA	0.19611	0.038095706	0.344550742	1.55E-02	8.98E-02	1.00E+00
ENSG00000104856.12_RELB	ENSG00000160789.18_LMNA	0.48318	0.350975975	0.596495593	2.89E-10	5.52E-08	1.48E-05
ENSG00000182208.11_MOB2	ENSG00000160789.18_LMNA	0.22109	0.064149978	0.36736294	6.20E-03	4.61E-02	1.00E+00
ENSG00000106078.16_COBL	ENSG00000160789.18_LMNA	0.11003	-0.050045746	0.264597334	1.77E-01	4.39E-01	1.00E+00
ENSG00000160539.4_PLPP7	ENSG00000160789.18_LMNA	-0.37942	-0.507941362	-0.23437934	1.43E-06	6.20E-05	7.34E-02
ENSG00000136048.12_DRAM1	ENSG00000160789.18_LMNA	0.34434	0.195873441	0.477368635	1.40E-05	3.86E-04	7.18E-01
ENSG00000174839.11_DENND6A	ENSG00000160789.18_LMNA	-0.09546	-0.250851055	0.064721603	2.42E-01	5.21E-01	1.00E+00
ENSG00000050748.16_MAPK9	ENSG00000160789.18_LMNA	-0.31851	-0.454659989	-0.16782325	6.36E-05	1.30E-03	1.00E+00
ENSG00000155111.13_CDK19	ENSG00000160789.18_LMNA	-0.15117	-0.303076763	0.008228717	6.30E-02	2.33E-01	1.00E+00
ENSG00000122257.17_RBBP6	ENSG00000160789.18_LMNA	0.04785	-0.112202518	0.205488214	5.58E-01	7.91E-01	1.00E+00
ENSG00000110400.9_NECTIN1	ENSG00000160789.18_LMNA	-0.11993	-0.273902582	0.040033516	1.41E-01	3.83E-01	1.00E+00
ENSG00000001084.9_GCLC	ENSG00000160789.18_LMNA	-0.4683	-0.583964816	-0.33400131	1.17E-09	1.77E-07	6.02E-05
ENSG00000134780.8_DAGLA	ENSG00000160789.18_LMNA	0.00429	-0.155015865	0.163379825	9.58E-01	9.83E-01	1.00E+00
ENSG00000266032.1_	ENSG00000160789.18_LMNA	-0.21532	-0.362109704	-0.05811329	7.72E-03	5.41E-02	1.00E+00
ENSG00000128191.12_DGCR8	ENSG00000160789.18_LMNA	0.11291	-0.047138722	0.267305137	1.66E-01	4.23E-01	1.00E+00
ENSG00000087085.12_ACHE	ENSG00000160789.18_LMNA	0.14664	-0.012864491	0.298860441	7.14E-02	2.53E-01	1.00E+00
ENSG00000184271.14_POU6F1	ENSG00000160789.18_LMNA	-0.11976	-0.273741868	0.040206978	1.42E-01	3.84E-01	1.00E+00
ENSG00000075292.17_ZNF638	ENSG00000160789.18_LMNA	0.03018	-0.129644282	0.188474469	7.12E-01	8.78E-01	1.00E+00
ENSG00000233901.4_LINC01503	ENSG00000160789.18_LMNA	0.29412	0.141549537	0.433045818	2.35E-04	3.72E-03	1.00E+00
ENSG00000143224.16_PPOX	ENSG00000160789.18_LMNA	-0.18751	-0.336662494	-0.0291823	2.07E-02	1.11E-01	1.00E+00
ENSG00000198223.13_CSF2RA	ENSG00000160789.18_LMNA	-0.24604	-0.389968182	-0.09038218	2.25E-03	2.14E-02	1.00E+00
ENSG00000173041.10_ZNF680	ENSG00000160789.18_LMNA	-0.24568	-0.389636535	-0.08999429	2.28E-03	2.16E-02	1.00E+00
ENSG00000137507.10_LRRC32	ENSG00000160789.18_LMNA	0.27463	0.120708095	0.415658742	6.17E-04	7.96E-03	1.00E+00
ENSG00000111670.13_GNPTAB	ENSG00000160789.18_LMNA	-0.21658	-0.363256321	-0.05942902	7.36E-03	5.22E-02	1.00E+00
ENSG00000183248.10_PRR36	ENSG00000160789.18_LMNA	-0.23114	-0.376482992	-0.07468313	4.17E-03	3.43E-02	1.00E+00
ENSG00000165282.12_PIGO	ENSG00000160789.18_LMNA	0.29008	0.137216589	0.429448808	2.89E-04	4.37E-03	1.00E+00
ENSG00000233670.6_PIRT	ENSG00000160789.18_LMNA	0.00833	-0.151070836	0.167309033	9.19E-01	9.69E-01	1.00E+00
ENSG00000133794.16_ARNTL	ENSG00000160789.18_LMNA	0.19549	0.037458836	0.343988553	1.58E-02	9.11E-02	1.00E+00
ENSG00000147408.13_CSGALNA	ENSG00000160789.18_LMNA	-0.08091	-0.237060821	0.07930849	3.22E-01	6.08E-01	1.00E+00
ENSG00000159714.9_ZDHHC1	ENSG00000160789.18_LMNA	0.40613	0.263994833	0.5309956	2.09E-07	1.32E-05	1.07E-02
ENSG00000166133.16_RPUSD2	ENSG00000160789.18_LMNA	0.09827	-0.061897702	0.253506014	2.28E-01	5.05E-01	1.00E+00
ENSG00000278311.3_GGNBP2	ENSG00000160789.18_LMNA	0.06465	-0.095536976	0.221567343	4.29E-01	6.99E-01	1.00E+00
ENSG00000157881.12_PANK4	ENSG00000160789.18_LMNA	-0.01509	-0.173872205	0.144458541	8.54E-01	9.43E-01	1.00E+00

ENSG00000242259.7_C22orf39	ENSG00000160789.18_LMNA	-0.3347	-0.46891613	-0.18537863	2.50E-05	6.18E-04	1.00E+00
ENSG00000099365.8_STX1B	ENSG00000160789.18_LMNA	-0.23269	-0.377890271	-0.07631448	3.92E-03	3.27E-02	1.00E+00
ENSG00000040731.9_CDH10	ENSG00000160789.18_LMNA	-0.20804	-0.355466032	-0.05051051	1.01E-02	6.61E-02	1.00E+00
ENSG00000070882.11_OSBPL3	ENSG00000160789.18_LMNA	0.37791	0.232708929	0.506629243	1.59E-06	6.69E-05	8.14E-02
ENSG00000132846.5_ZBED3	ENSG00000160789.18_LMNA	-0.13283	-0.285984008	0.026939438	1.03E-01	3.17E-01	1.00E+00
ENSG00000136643.10_RPS6KC1	ENSG00000160789.18_LMNA	-0.1875	-0.336654894	-0.02917373	2.07E-02	1.11E-01	1.00E+00
ENSG00000227157.1_AC068535.:	ENSG00000160789.18_LMNA	-0.1682	-0.318859067	-0.00924326	3.83E-02	1.68E-01	1.00E+00
ENSG00000065665.19_SEC61A2	ENSG00000160789.18_LMNA	-0.43042	-0.551810163	-0.2911728	3.12E-08	2.77E-06	1.60E-03
ENSG00000172765.15_TMCC1	ENSG00000160789.18_LMNA	-0.16564	-0.316498755	-0.00661817	4.14E-02	1.77E-01	1.00E+00
ENSG00000112773.14_FAM46A	ENSG00000160789.18_LMNA	0.35525	0.207800579	0.486911907	7.09E-06	2.22E-04	3.64E-01
ENSG00000171853.14_TRAPPC1	ENSG00000160789.18_LMNA	-0.05508	-0.212416428	0.105043476	5.00E-01	7.53E-01	1.00E+00
ENSG00000265763.3_ZNF488	ENSG00000160789.18_LMNA	-0.51154	-0.620232938	-0.38358002	1.64E-11	5.10E-09	8.42E-07
ENSG00000101928.11_MOSPD1	ENSG00000160789.18_LMNA	-0.17561	-0.325706338	-0.01688287	3.05E-02	1.45E-01	1.00E+00
ENSG00000159921.13_GNE	ENSG00000160789.18_LMNA	-0.09703	-0.252328698	0.063150558	2.34E-01	5.12E-01	1.00E+00
ENSG00000109685.16_WHSC1	ENSG00000160789.18_LMNA	0.05956	-0.100593036	0.216707345	4.66E-01	7.28E-01	1.00E+00
ENSG00000165516.9_KLHDC2	ENSG00000160789.18_LMNA	-0.27928	-0.419811345	-0.12566219	4.93E-04	6.67E-03	1.00E+00
ENSG00000143952.18_VPS54	ENSG00000160789.18_LMNA	-0.05881	-0.215985541	0.101342564	4.72E-01	7.32E-01	1.00E+00
ENSG00000096093.13_EFHC1	ENSG00000160789.18_LMNA	0.10839	-0.051701459	0.263052868	1.84E-01	4.49E-01	1.00E+00
ENSG00000109171.13_SLAIN2	ENSG00000160789.18_LMNA	0.36976	0.22372903	0.499553672	2.75E-06	1.02E-04	1.41E-01
ENSG00000158747.12_NBL1	ENSG00000160789.18_LMNA	0.14271	-0.016872489	0.295205256	7.94E-02	2.71E-01	1.00E+00
ENSG00000089820.14_ARHGAP4	ENSG00000160789.18_LMNA	0.17953	0.020931147	0.329321669	2.69E-02	1.33E-01	1.00E+00
ENSG00000174059.15_CD34	ENSG00000160789.18_LMNA	0.17404	0.015265534	0.324259445	3.20E-02	1.50E-01	1.00E+00
ENSG00000228589.3_SPCS2P4	ENSG00000160789.18_LMNA	-0.07471	-0.231156921	0.085512309	3.60E-01	6.43E-01	1.00E+00
ENSG00000090857.12_PDPR	ENSG00000160789.18_LMNA	0.12572	-0.034161054	0.279333168	1.23E-01	3.53E-01	1.00E+00
ENSG00000136490.7_LIMD2	ENSG00000160789.18_LMNA	-0.22645	-0.372233678	-0.06976702	5.03E-03	3.94E-02	1.00E+00
ENSG00000125484.10_GTF3C4	ENSG00000160789.18_LMNA	0.04855	-0.111515978	0.206154002	5.53E-01	7.88E-01	1.00E+00
ENSG00000261115.4_TMEM178B	ENSG00000160789.18_LMNA	-0.04965	-0.207208285	0.110428212	5.44E-01	7.82E-01	1.00E+00
ENSG00000082516.8_GEMIN5	ENSG00000160789.18_LMNA	0.0712	-0.089008022	0.227819901	3.83E-01	6.62E-01	1.00E+00
ENSG00000233954.6_UQCRHL	ENSG00000160789.18_LMNA	-0.19777	-0.34607652	-0.03982542	1.46E-02	8.62E-02	1.00E+00
ENSG00000153140.7_CETN3	ENSG00000160789.18_LMNA	-0.23353	-0.378657027	-0.077204	3.78E-03	3.18E-02	1.00E+00
ENSG00000178966.14_RMI1	ENSG00000160789.18_LMNA	-0.13945	-0.292164207	0.020199446	8.66E-02	2.86E-01	1.00E+00
ENSG00000203644.3_RP11-332M	ENSG00000160789.18_LMNA	0.05303	-0.107074906	0.210453735	5.16E-01	7.63E-01	1.00E+00
ENSG00000067048.15_DDX3Y	ENSG00000160789.18_LMNA	-0.06535	-0.222234509	0.094841616	4.24E-01	6.95E-01	1.00E+00

ENSG00000204267.12_TAP2	ENSG00000160789.18_LMNA	0.15652	-0.002744304	0.30804925	5.41E-02	2.12E-01	1.00E+00
ENSG00000189319.12_FAM53B	ENSG00000160789.18_LMNA	-0.05018	-0.207720875	0.109899067	5.39E-01	7.79E-01	1.00E+00
ENSG00000165506.13_DNAAF2	ENSG00000160789.18_LMNA	-0.19953	-0.347683991	-0.04164974	1.37E-02	8.23E-02	1.00E+00
ENSG00000093000.17_NUP50	ENSG00000160789.18_LMNA	0.02975	-0.13007137	0.188055452	7.16E-01	8.79E-01	1.00E+00
ENSG00000018699.10_TTC27	ENSG00000160789.18_LMNA	-0.00458	-0.163657451	0.154737454	9.55E-01	9.82E-01	1.00E+00
ENSG00000145391.12_SETD7	ENSG00000160789.18_LMNA	0.01803	-0.141581851	0.176718724	8.26E-01	9.32E-01	1.00E+00
ENSG00000163815.5_CLEC3B	ENSG00000160789.18_LMNA	-0.08106	-0.237195747	0.079166423	3.21E-01	6.07E-01	1.00E+00
ENSG00000124920.12_MYRF	ENSG00000160789.18_LMNA	-0.25248	-0.395776171	-0.09718984	1.70E-03	1.73E-02	1.00E+00
ENSG00000164294.12_GPX8	ENSG00000160789.18_LMNA	0.43626	0.297736081	0.556788516	1.93E-08	1.87E-06	9.90E-04
ENSG00000213088.8_ACKR1	ENSG00000160789.18_LMNA	-0.1306	-0.283893368	0.029213022	1.09E-01	3.28E-01	1.00E+00
ENSG00000145439.10_CBR4	ENSG00000160789.18_LMNA	-0.45627	-0.573789013	-0.32033505	3.47E-09	4.39E-07	1.78E-04
ENSG00000272047.1_GTF2H5	ENSG00000160789.18_LMNA	-0.25335	-0.396557501	-0.09810779	1.64E-03	1.68E-02	1.00E+00
ENSG00000148950.8_IMMP1L	ENSG00000160789.18_LMNA	-0.30485	-0.442573476	-0.15308076	1.34E-04	2.38E-03	1.00E+00
ENSG00000163516.12_ANKZF1	ENSG00000160789.18_LMNA	-0.01814	-0.176831539	0.141467729	8.24E-01	9.31E-01	1.00E+00
ENSG00000170185.8_USP38	ENSG00000160789.18_LMNA	0.1166	-0.04341085	0.270770283	1.53E-01	4.02E-01	1.00E+00
ENSG00000069122.17_ADGRF5	ENSG00000160789.18_LMNA	0.00662	-0.152738596	0.165649195	9.35E-01	9.75E-01	1.00E+00
ENSG00000113212.6_PCDHB7	ENSG00000160789.18_LMNA	-0.01821	-0.176896082	0.141402434	8.24E-01	9.31E-01	1.00E+00
ENSG00000279117.1_CTD-2562J	ENSG00000160789.18_LMNA	-0.25381	-0.396971604	-0.09859451	1.60E-03	1.65E-02	1.00E+00
ENSG00000245694.7_CRNDE	ENSG00000160789.18_LMNA	-0.08816	-0.243934333	0.072054796	2.80E-01	5.66E-01	1.00E+00
ENSG00000163866.8_SMIM12	ENSG00000160789.18_LMNA	-0.02652	-0.184939432	0.133243716	7.46E-01	8.94E-01	1.00E+00
ENSG00000141469.15_SLC14A1	ENSG00000160789.18_LMNA	-0.38156	-0.509791717	-0.23673772	1.23E-06	5.46E-05	6.33E-02
ENSG00000138685.11_FGF2	ENSG00000160789.18_LMNA	0.07466	-0.08556101	0.231110482	3.61E-01	6.43E-01	1.00E+00
ENSG00000104219.11_ZDHHC2	ENSG00000160789.18_LMNA	-0.34917	-0.481601965	-0.20115398	1.04E-05	3.00E-04	5.33E-01
ENSG00000104885.16_DOT1L	ENSG00000160789.18_LMNA	0.15221	-0.007165856	0.304041741	6.12E-02	2.29E-01	1.00E+00
ENSG00000172663.7_TMEM134	ENSG00000160789.18_LMNA	0.12967	-0.030157644	0.283023879	1.11E-01	3.33E-01	1.00E+00
ENSG00000110063.7_DCPS	ENSG00000160789.18_LMNA	0.32871	0.178868677	0.463646789	3.56E-05	8.21E-04	1.00E+00
ENSG00000182552.13_RWDD4	ENSG00000160789.18_LMNA	-0.17363	-0.323881951	-0.01484384	3.24E-02	1.51E-01	1.00E+00
ENSG00000141026.5_MED9	ENSG00000160789.18_LMNA	0.12293	-0.036991242	0.27671844	1.31E-01	3.67E-01	1.00E+00
ENSG00000171735.17_CAMTA1	ENSG00000160789.18_LMNA	-0.31139	-0.44836278	-0.1601262	9.43E-05	1.80E-03	1.00E+00
ENSG00000101246.18_ARFRP1	ENSG00000160789.18_LMNA	0.36203	0.215239689	0.492830616	4.58E-06	1.55E-04	2.35E-01
ENSG00000133657.13_ATP13A3	ENSG00000160789.18_LMNA	0.35203	0.204280124	0.484101996	8.69E-06	2.60E-04	4.46E-01
ENSG00000176182.5_MYPOP	ENSG00000160789.18_LMNA	0.28968	0.136784734	0.429089794	2.95E-04	4.44E-03	1.00E+00
ENSG00000015532.8_XYLT2	ENSG00000160789.18_LMNA	0.49696	0.366775382	0.608052226	7.41E-11	1.77E-08	3.80E-06

ENSG00000242498.6_ARPIN	ENSG00000160789.18_LMNA	0.26987	0.115639263	0.411397199	7.73E-04	9.46E-03	1.00E+00
ENSG00000031003.9_FAM13B	ENSG00000160789.18_LMNA	-0.1879	-0.337015802	-0.02958048	2.04E-02	1.10E-01	1.00E+00
ENSG00000161813.19_LARP4	ENSG00000160789.18_LMNA	0.02263	-0.137066886	0.181175624	7.82E-01	9.11E-01	1.00E+00
ENSG00000166037.9_CEP57	ENSG00000160789.18_LMNA	-0.2306	-0.376000591	-0.0741243	4.26E-03	3.48E-02	1.00E+00
ENSG00000276234.3_TADA2A	ENSG00000160789.18_LMNA	-0.28275	-0.422911521	-0.12937032	4.16E-04	5.85E-03	1.00E+00
ENSG00000227372.9_TP73-AS1	ENSG00000160789.18_LMNA	0.29903	0.146820574	0.437409104	1.82E-04	3.04E-03	1.00E+00
ENSG00000151376.15_ME3	ENSG00000160789.18_LMNA	-0.27939	-0.419911962	-0.12578241	4.91E-04	6.64E-03	1.00E+00
ENSG00000183196.7_CHST6	ENSG00000160789.18_LMNA	0.17006	0.011164823	0.320584386	3.62E-02	1.62E-01	1.00E+00
ENSG00000082074.14_FYB	ENSG00000160789.18_LMNA	-0.06772	-0.224499102	0.092479012	4.07E-01	6.82E-01	1.00E+00
ENSG00000143507.16_DUSP10	ENSG00000160789.18_LMNA	0.0138	-0.145718931	0.172623356	8.66E-01	9.48E-01	1.00E+00
ENSG00000157326.17_DHRS4	ENSG00000160789.18_LMNA	0.30934	0.157915563	0.446548877	1.05E-04	1.97E-03	1.00E+00
ENSG00000132967.9_HMGB1P5	ENSG00000160789.18_LMNA	-0.08033	-0.23650484	0.079893762	3.25E-01	6.12E-01	1.00E+00
ENSG00000107789.14_MINPP1	ENSG00000160789.18_LMNA	0.03883	-0.12111596	0.196817433	6.35E-01	8.36E-01	1.00E+00
ENSG00000166025.16_AMOTL1	ENSG00000160789.18_LMNA	0.14389	-0.015670633	0.296302276	7.70E-02	2.66E-01	1.00E+00
ENSG00000068878.13_PSME4	ENSG00000160789.18_LMNA	0.12459	-0.035315545	0.278267129	1.26E-01	3.59E-01	1.00E+00
ENSG00000213965.3_NUDT19	ENSG00000160789.18_LMNA	0.49537	0.364953684	0.606724922	8.69E-11	2.02E-08	4.46E-06
ENSG00000132821.10_VSTM2L	ENSG00000160789.18_LMNA	-0.18287	-0.33238958	-0.0243744	2.41E-02	1.23E-01	1.00E+00
ENSG00000188690.11_UROS	ENSG00000160789.18_LMNA	-0.29331	-0.432324939	-0.14068027	2.45E-04	3.84E-03	1.00E+00
ENSG00000163933.8_RFT1	ENSG00000160789.18_LMNA	0.2063	0.048700806	0.353880075	1.08E-02	6.91E-02	1.00E+00
ENSG00000181396.11_OGFOD3	ENSG00000160789.18_LMNA	0.26251	0.107812072	0.40479111	1.09E-03	1.23E-02	1.00E+00
ENSG00000137845.13_ADAM10	ENSG00000160789.18_LMNA	0.24939	0.093916215	0.392986241	1.95E-03	1.91E-02	1.00E+00
ENSG00000172594.11_SMPDL3A	ENSG00000160789.18_LMNA	0.01455	-0.144988158	0.173347563	8.59E-01	9.44E-01	1.00E+00
ENSG00000110066.13_KMT5B	ENSG00000160789.18_LMNA	-0.16942	-0.31999347	-0.01050644	3.69E-02	1.64E-01	1.00E+00
ENSG00000142867.11_BCL10	ENSG00000160789.18_LMNA	0.31109	0.15980569	0.448099935	9.58E-05	1.82E-03	1.00E+00
ENSG00000274828.1_RP11-567M	ENSG00000160789.18_LMNA	-0.2972	-0.435783197	-0.14485448	2.01E-04	3.27E-03	1.00E+00
ENSG00000126775.8_ATG14	ENSG00000160789.18_LMNA	-0.05551	-0.212825128	0.104620128	4.97E-01	7.51E-01	1.00E+00
ENSG00000115828.14_QPCT	ENSG00000160789.18_LMNA	-0.07764	-0.233946681	0.082583886	3.42E-01	6.27E-01	1.00E+00
ENSG00000077522.11_ACTN2	ENSG00000160789.18_LMNA	-0.03136	-0.189615875	0.128480289	7.01E-01	8.72E-01	1.00E+00
ENSG00000139625.11_MAP3K12	ENSG00000160789.18_LMNA	0.08755	-0.072664943	0.243357386	2.83E-01	5.69E-01	1.00E+00
ENSG00000166682.9_TMPRSS5	ENSG00000160789.18_LMNA	-0.30333	-0.441221424	-0.15143957	1.45E-04	2.53E-03	1.00E+00
ENSG00000112561.16_TFEB	ENSG00000160789.18_LMNA	0.02725	-0.132528809	0.185642205	7.39E-01	8.90E-01	1.00E+00
ENSG00000246705.4_H2AFJ	ENSG00000160789.18_LMNA	0.1172	-0.042803712	0.271333863	1.50E-01	3.99E-01	1.00E+00
ENSG00000126883.15_NUP214	ENSG00000160789.18_LMNA	0.13278	-0.026988646	0.285938791	1.03E-01	3.17E-01	1.00E+00

ENSG00000122882.9_ECD	ENSG00000160789.18_LMNA	-0.09053	-0.246186467	0.069670801	2.67E-01	5.51E-01	1.00E+00
ENSG00000178104.18_PDE4DIP	ENSG00000160789.18_LMNA	-0.17015	-0.320660153	-0.01124926	3.61E-02	1.62E-01	1.00E+00
ENSG00000175866.14_BAIAP2	ENSG00000160789.18_LMNA	0.36083	0.213922037	0.491784134	4.95E-06	1.65E-04	2.54E-01
ENSG00000144730.15_IL17RD	ENSG00000160789.18_LMNA	0.02891	-0.130890097	0.187251875	7.24E-01	8.83E-01	1.00E+00
ENSG00000056972.17_TRAF3IP2	ENSG00000160789.18_LMNA	0.15674	-0.002527365	0.308245591	5.38E-02	2.11E-01	1.00E+00
ENSG00000180182.9_MED14	ENSG00000160789.18_LMNA	0.26188	0.107143038	0.404225009	1.12E-03	1.26E-02	1.00E+00
ENSG00000154001.12_PPP2R5E	ENSG00000160789.18_LMNA	0.05574	-0.104387144	0.213050005	4.95E-01	7.50E-01	1.00E+00
ENSG00000007866.17_TEAD3	ENSG00000160789.18_LMNA	0.5166	0.389422585	0.624441309	9.57E-12	3.43E-09	4.91E-07
ENSG00000241370.4_RPP21	ENSG00000160789.18_LMNA	-0.05221	-0.209668743	0.107886648	5.23E-01	7.68E-01	1.00E+00
ENSG00000153037.11_SRP19	ENSG00000160789.18_LMNA	-0.13943	-0.29214583	0.02021953	8.67E-02	2.86E-01	1.00E+00
ENSG00000156735.9_BAG4	ENSG00000160789.18_LMNA	-0.16983	-0.320369797	-0.0109257	3.65E-02	1.63E-01	1.00E+00
ENSG00000122986.12_HVCN1	ENSG00000160789.18_LMNA	0.07951	-0.080716873	0.235722573	3.30E-01	6.16E-01	1.00E+00
ENSG00000001561.6_ENPP4	ENSG00000160789.18_LMNA	-0.25234	-0.395646072	-0.09703704	1.71E-03	1.74E-02	1.00E+00
ENSG00000180448.9_ARHGAP45	ENSG00000160789.18_LMNA	0.20378	0.046078501	0.351578865	1.18E-02	7.38E-02	1.00E+00
ENSG00000132423.9_COQ3	ENSG00000160789.18_LMNA	-0.38377	-0.511704999	-0.23917968	1.06E-06	4.81E-05	5.43E-02
ENSG00000227077.3_AC107983.4	ENSG00000160789.18_LMNA	-0.02074	-0.179343831	0.138924145	8.00E-01	9.20E-01	1.00E+00
ENSG00000005302.16_MSL3	ENSG00000160789.18_LMNA	-0.179	-0.328832408	-0.0203827	2.73E-02	1.35E-01	1.00E+00
ENSG00000120685.18_PROSER1	ENSG00000160789.18_LMNA	-0.1307	-0.283994584	0.029103023	1.08E-01	3.28E-01	1.00E+00
ENSG00000112874.8_NUDT12	ENSG00000160789.18_LMNA	-0.19869	-0.346915794	-0.04077766	1.41E-02	8.42E-02	1.00E+00
ENSG00000100139.12_MICALL1	ENSG00000160789.18_LMNA	0.11806	-0.041928974	0.272145464	1.47E-01	3.94E-01	1.00E+00
ENSG00000164934.12_DCAF13	ENSG00000160789.18_LMNA	-0.13972	-0.292417693	0.019922392	8.60E-02	2.85E-01	1.00E+00
ENSG00000267519.3_MIR24-2	ENSG00000160789.18_LMNA	0.2792	0.12558156	0.419743858	4.95E-04	6.68E-03	1.00E+00
ENSG00000116539.9_ASH1L	ENSG00000160789.18_LMNA	0.11328	-0.046762675	0.267655052	1.65E-01	4.21E-01	1.00E+00
ENSG00000105298.12_CACTIN	ENSG00000160789.18_LMNA	0.23102	0.074560143	0.376376838	4.19E-03	3.44E-02	1.00E+00
ENSG00000135469.12_COQ10A	ENSG00000160789.18_LMNA	-0.25117	-0.394595512	-0.0958037	1.80E-03	1.81E-02	1.00E+00
ENSG00000165449.10_SLC16A9	ENSG00000160789.18_LMNA	-0.45252	-0.570612397	-0.3160903	4.83E-09	5.83E-07	2.48E-04
ENSG00000119820.9_YIPF4	ENSG00000160789.18_LMNA	-0.44218	-0.561829688	-0.30440723	1.18E-08	1.24E-06	6.03E-04
ENSG00000147883.10_CDKN2B	ENSG00000160789.18_LMNA	0.00023	-0.158976203	0.159425184	9.98E-01	9.99E-01	1.00E+00
ENSG00000244165.1_P2RY11	ENSG00000160789.18_LMNA	0.25962	0.104746586	0.402195387	1.24E-03	1.36E-02	1.00E+00
ENSG00000139567.11_ACVRL1	ENSG00000160789.18_LMNA	0.08513	-0.075086459	0.241065423	2.97E-01	5.83E-01	1.00E+00
ENSG00000132952.10_USPL1	ENSG00000160789.18_LMNA	-0.1195	-0.273492922	0.040475632	1.43E-01	3.86E-01	1.00E+00
ENSG00000143793.11_C1orf35	ENSG00000160789.18_LMNA	0.08869	-0.071517182	0.244442509	2.77E-01	5.62E-01	1.00E+00
ENSG00000135414.8_GDF11	ENSG00000160789.18_LMNA	-0.17048	-0.320969066	-0.01159357	3.57E-02	1.61E-01	1.00E+00

ENSG00000059378.11_PARP12	ENSG00000160789.18_LMNA	0.15499	-0.004315897	0.306626087	5.66E-02	2.17E-01	1.00E+00
ENSG00000075340.21_ADD2	ENSG00000160789.18_LMNA	-0.24557	-0.389545323	-0.08988762	2.29E-03	2.17E-02	1.00E+00
ENSG00000215712.9_TMEM242	ENSG00000160789.18_LMNA	-0.1966	-0.344998264	-0.03860285	1.52E-02	8.87E-02	1.00E+00
ENSG00000087008.14_ACOX3	ENSG00000160789.18_LMNA	0.17705	0.018368182	0.327033853	2.91E-02	1.40E-01	1.00E+00
ENSG00000115267.5_IFIH1	ENSG00000160789.18_LMNA	0.06363	-0.096552517	0.220592445	4.36E-01	7.04E-01	1.00E+00
ENSG00000113593.10_PPWD1	ENSG00000160789.18_LMNA	0.06262	-0.097557908	0.219626666	4.43E-01	7.11E-01	1.00E+00
ENSG00000101849.14_TBL1X	ENSG00000160789.18_LMNA	0.28976	0.136875691	0.429165417	2.94E-04	4.42E-03	1.00E+00
ENSG00000121152.8_NCAPH	ENSG00000160789.18_LMNA	0.00641	-0.152943351	0.165445288	9.37E-01	9.75E-01	1.00E+00
ENSG00000080819.5_CPOX	ENSG00000160789.18_LMNA	0.07354	-0.086672471	0.230050252	3.68E-01	6.49E-01	1.00E+00
ENSG00000113231.12_PDE8B	ENSG00000160789.18_LMNA	-0.147	-0.299195904	0.012496145	7.07E-02	2.51E-01	1.00E+00
ENSG00000142235.7_LMTK3	ENSG00000160789.18_LMNA	-0.04379	-0.201585696	0.116220589	5.92E-01	8.11E-01	1.00E+00
ENSG00000173327.6_MAP3K11	ENSG00000160789.18_LMNA	0.28138	0.127911717	0.421692878	4.45E-04	6.16E-03	1.00E+00
ENSG00000184056.13_VPS33B	ENSG00000160789.18_LMNA	-0.01311	-0.171954847	0.146393194	8.73E-01	9.51E-01	1.00E+00
ENSG00000115904.11_SOS1	ENSG00000160789.18_LMNA	-0.0506	-0.208127236	0.109479454	5.36E-01	7.77E-01	1.00E+00
ENSG00000162402.11_USP24	ENSG00000160789.18_LMNA	0.01296	-0.146538551	0.171810692	8.74E-01	9.51E-01	1.00E+00
ENSG00000168314.16_MOBP	ENSG00000160789.18_LMNA	-0.38104	-0.509345901	-0.23616921	1.28E-06	5.63E-05	6.56E-02
ENSG00000164916.10_FOXK1	ENSG00000160789.18_LMNA	0.31724	0.166448042	0.453536985	6.83E-05	1.38E-03	1.00E+00
ENSG00000166140.16_ZFYVE19	ENSG00000160789.18_LMNA	0.13793	-0.021746962	0.290747524	9.02E-02	2.92E-01	1.00E+00
ENSG00000006704.9_GTF2IRD1	ENSG00000160789.18_LMNA	0.13161	-0.028181009	0.284842709	1.06E-01	3.23E-01	1.00E+00
ENSG00000102871.14_TRADD	ENSG00000160789.18_LMNA	0.43807	0.299780152	0.558335185	1.66E-08	1.64E-06	8.51E-04
ENSG00000005249.11_PRKAR2B	ENSG00000160789.18_LMNA	-0.06223	-0.219260073	0.097939368	4.46E-01	7.13E-01	1.00E+00
ENSG00000154447.13_SH3RF1	ENSG00000160789.18_LMNA	0.08463	-0.075591116	0.240587321	3.00E-01	5.86E-01	1.00E+00
ENSG00000236287.6_ZBED5	ENSG00000160789.18_LMNA	-0.28284	-0.422992745	-0.12946758	4.15E-04	5.83E-03	1.00E+00
ENSG00000075303.11_SLC25A40	ENSG00000160789.18_LMNA	-0.01341	-0.172247158	0.146098404	8.70E-01	9.49E-01	1.00E+00
ENSG00000233328.3_PFN1P1	ENSG00000160789.18_LMNA	0.20499	0.04733493	0.352681908	1.13E-02	7.14E-02	1.00E+00
ENSG00000146802.11_TMEM168	ENSG00000160789.18_LMNA	-0.00549	-0.164548407	0.153843638	9.46E-01	9.79E-01	1.00E+00
ENSG00000239305.5_RNF103	ENSG00000160789.18_LMNA	-0.221	-0.367277938	-0.06405212	6.22E-03	4.62E-02	1.00E+00
ENSG00000011143.15_MKS1	ENSG00000160789.18_LMNA	-0.11861	-0.272658117	0.041376194	1.46E-01	3.91E-01	1.00E+00
ENSG00000151914.16_DST	ENSG00000160789.18_LMNA	-0.00744	-0.166442655	0.151941575	9.28E-01	9.72E-01	1.00E+00
ENSG00000198093.9_ZNF649	ENSG00000160789.18_LMNA	-0.15428	-0.305965939	0.005044374	5.77E-02	2.20E-01	1.00E+00
ENSG00000101417.10_PXMP4	ENSG00000160789.18_LMNA	0.08046	-0.079759298	0.236632593	3.24E-01	6.11E-01	1.00E+00
ENSG00000163249.8_CCNYL1	ENSG00000160789.18_LMNA	0.15454	-0.004780616	0.306204991	5.73E-02	2.19E-01	1.00E+00
ENSG00000148082.8_SHC3	ENSG00000160789.18_LMNA	-0.12148	-0.275355753	0.038464226	1.36E-01	3.75E-01	1.00E+00

ENSG00000141564.12_RPTOR	ENSG00000160789.18_LMNA	0.28232	0.128913367	0.422529857	4.25E-04	5.95E-03	1.00E+00
ENSG00000197535.13_MYO5A	ENSG00000160789.18_LMNA	-0.08148	-0.237601917	0.078738678	3.18E-01	6.05E-01	1.00E+00
ENSG00000145945.5_FAM50B	ENSG00000160789.18_LMNA	0.11004	-0.050042009	0.264600818	1.77E-01	4.39E-01	1.00E+00
ENSG00000244005.11_NFS1	ENSG00000160789.18_LMNA	0.05917	-0.100979789	0.216334941	4.69E-01	7.30E-01	1.00E+00
ENSG00000128641.16_MYO1B	ENSG00000160789.18_LMNA	0.22334	0.066503313	0.369405628	5.68E-03	4.31E-02	1.00E+00
ENSG00000254635.4_WAC-AS1	ENSG00000160789.18_LMNA	-0.05731	-0.214556705	0.102825218	4.83E-01	7.40E-01	1.00E+00
ENSG00000157045.7_NTAN1	ENSG00000160789.18_LMNA	0.38376	0.23917046	0.511697779	1.06E-06	4.81E-05	5.43E-02
ENSG00000146592.15_CREB5	ENSG00000160789.18_LMNA	0.03779	-0.122145888	0.195812328	6.44E-01	8.42E-01	1.00E+00
ENSG00000142798.15_HSPG2	ENSG00000160789.18_LMNA	0.37693	0.231625054	0.505777173	1.70E-06	7.04E-05	8.71E-02
ENSG00000196850.5_PPTC7	ENSG00000160789.18_LMNA	0.05015	-0.109925033	0.207695726	5.39E-01	7.79E-01	1.00E+00
ENSG00000129038.14_LOXL1	ENSG00000160789.18_LMNA	0.50443	0.375373369	0.614298636	3.45E-11	9.32E-09	1.77E-06
ENSG00000049130.12_KITLG	ENSG00000160789.18_LMNA	0.08559	-0.074632181	0.241495665	2.94E-01	5.80E-01	1.00E+00
ENSG00000198162.11_MAN1A2	ENSG00000160789.18_LMNA	-0.06418	-0.221123828	0.095999062	4.32E-01	7.01E-01	1.00E+00
ENSG00000200714.1_Y_RNA	ENSG00000160789.18_LMNA	-0.10983	-0.264404953	0.050252078	1.78E-01	4.40E-01	1.00E+00
ENSG00000152443.11_ZNF776	ENSG00000160789.18_LMNA	-0.0899	-0.245585794	0.070306996	2.71E-01	5.55E-01	1.00E+00
ENSG00000168386.17_FILIP1L	ENSG00000160789.18_LMNA	0.1231	-0.036824215	0.276872881	1.31E-01	3.67E-01	1.00E+00
ENSG00000164597.12_COG5	ENSG00000160789.18_LMNA	0.06207	-0.098104317	0.219101524	4.47E-01	7.14E-01	1.00E+00
ENSG00000187474.4_FPR3	ENSG00000160789.18_LMNA	0.01508	-0.144469899	0.173860956	8.54E-01	9.43E-01	1.00E+00
ENSG00000168273.6_SMIM4	ENSG00000160789.18_LMNA	0.11174	-0.048321077	0.266204409	1.71E-01	4.29E-01	1.00E+00
ENSG00000143318.11_CASQ1	ENSG00000160789.18_LMNA	-0.32541	-0.46074319	-0.17529208	4.30E-05	9.49E-04	1.00E+00
ENSG00000184481.15_FOXO4	ENSG00000160789.18_LMNA	-0.20505	-0.352732086	-0.04739211	1.13E-02	7.13E-02	1.00E+00
ENSG00000110328.5_GALNT18	ENSG00000160789.18_LMNA	0.13592	-0.023794459	0.288871023	9.50E-02	3.02E-01	1.00E+00
ENSG00000107771.14_CCSER2	ENSG00000160789.18_LMNA	-0.30116	-0.439296149	-0.14910533	1.63E-04	2.78E-03	1.00E+00
ENSG00000142700.10_DMRTA2	ENSG00000160789.18_LMNA	0.40361	0.261192097	0.528830607	2.52E-07	1.54E-05	1.29E-02
ENSG00000110906.11_KCTD10	ENSG00000160789.18_LMNA	0.08797	-0.072246907	0.243752699	2.81E-01	5.67E-01	1.00E+00
ENSG00000188015.8_S100A3	ENSG00000160789.18_LMNA	0.09723	-0.062946511	0.252520505	2.33E-01	5.11E-01	1.00E+00
ENSG00000172932.13_ANKRD13	ENSG00000160789.18_LMNA	0.25908	0.104177132	0.401712671	1.27E-03	1.39E-02	1.00E+00
ENSG00000183655.12_KLHL25	ENSG00000160789.18_LMNA	-0.10146	-0.256516119	0.058689936	2.14E-01	4.88E-01	1.00E+00
ENSG00000107186.15_MPDZ	ENSG00000160789.18_LMNA	-0.01221	-0.171074966	0.147280197	8.81E-01	9.54E-01	1.00E+00
ENSG00000115486.10_GGCX	ENSG00000160789.18_LMNA	0.22559	0.068864173	0.37145191	5.20E-03	4.04E-02	1.00E+00
ENSG00000167967.14_E4F1	ENSG00000160789.18_LMNA	0.11605	-0.04396522	0.270255498	1.55E-01	4.05E-01	1.00E+00
ENSG00000077279.15_DCX	ENSG00000160789.18_LMNA	-0.33014	-0.464905483	-0.18042145	3.27E-05	7.67E-04	1.00E+00
ENSG00000125686.10_MED1	ENSG00000160789.18_LMNA	0.00619	-0.153165022	0.165224505	9.40E-01	9.76E-01	1.00E+00

ENSG00000170471.13_RALGAPB	ENSG00000160789.18_LMNA	0.12963	-0.030191261	0.282992926	1.11E-01	3.33E-01	1.00E+00
ENSG00000204397.6_CARD16	ENSG00000160789.18_LMNA	0.19593	0.037914537	0.34439084	1.56E-02	9.01E-02	1.00E+00
ENSG00000247271.5_ZBED5-AS1	ENSG00000160789.18_LMNA	-0.23037	-0.375788968	-0.0738792	4.30E-03	3.50E-02	1.00E+00
ENSG00000075188.7_NUP37	ENSG00000160789.18_LMNA	0.0475	-0.11254979	0.205151328	5.61E-01	7.93E-01	1.00E+00
ENSG00000262484.1_CCER2	ENSG00000160789.18_LMNA	-0.24883	-0.39248422	-0.09332784	1.99E-03	1.95E-02	1.00E+00
ENSG00000165752.15_STK32C	ENSG00000160789.18_LMNA	-0.25009	-0.393623192	-0.09466303	1.89E-03	1.87E-02	1.00E+00
ENSG00000140678.15_ITGAX	ENSG00000160789.18_LMNA	-0.09185	-0.247436935	0.06834556	2.60E-01	5.43E-01	1.00E+00
ENSG00000109339.17_MAPK10	ENSG00000160789.18_LMNA	-0.38833	-0.51564929	-0.24422477	7.67E-07	3.70E-05	3.94E-02
ENSG00000280800.1_CH507-513	ENSG00000160789.18_LMNA	-0.04148	-0.199364383	0.118503038	6.12E-01	8.23E-01	1.00E+00
ENSG00000205643.9_CDPF1	ENSG00000160789.18_LMNA	-0.0502	-0.207743267	0.109875948	5.39E-01	7.79E-01	1.00E+00
ENSG00000137171.13_KLC4	ENSG00000160789.18_LMNA	-0.17118	-0.321618171	-0.01231729	3.50E-02	1.59E-01	1.00E+00
ENSG00000106089.10_STX1A	ENSG00000160789.18_LMNA	0.00232	-0.156942313	0.161457426	9.77E-01	9.91E-01	1.00E+00
ENSG00000128268.11_MGAT3	ENSG00000160789.18_LMNA	-0.10643	-0.261201954	0.053683415	1.92E-01	4.60E-01	1.00E+00
ENSG00000177042.13_TMEM80	ENSG00000160789.18_LMNA	0.05164	-0.10845112	0.209122632	5.27E-01	7.72E-01	1.00E+00
ENSG00000221988.11_PPT2	ENSG00000160789.18_LMNA	-0.09402	-0.249490226	0.066167068	2.49E-01	5.30E-01	1.00E+00
ENSG00000169375.14_SIN3A	ENSG00000160789.18_LMNA	0.23061	0.07413261	0.376007769	4.26E-03	3.48E-02	1.00E+00
ENSG00000213516.8_RBMXL1	ENSG00000160789.18_LMNA	-0.15785	-0.309275097	0.001389376	5.21E-02	2.07E-01	1.00E+00
ENSG00000161179.12_YDJC	ENSG00000160789.18_LMNA	0.36928	0.223197383	0.499133612	2.84E-06	1.05E-04	1.46E-01
ENSG00000151093.6_OXSM	ENSG00000160789.18_LMNA	-0.29916	-0.437520781	-0.1469557	1.81E-04	3.02E-03	1.00E+00
ENSG00000119596.16_YLPM1	ENSG00000160789.18_LMNA	0.14082	-0.018802384	0.29344198	8.36E-02	2.80E-01	1.00E+00
ENSG00000173950.14_XXYLT1	ENSG00000160789.18_LMNA	0.13267	-0.027106446	0.285830539	1.03E-01	3.18E-01	1.00E+00
ENSG00000180211.5_RP1-278E1	ENSG00000160789.18_LMNA	-0.14902	-0.301079471	0.010426375	6.69E-02	2.42E-01	1.00E+00
ENSG00000173848.17_NET1	ENSG00000160789.18_LMNA	-0.39046	-0.517489127	-0.24658312	6.59E-07	3.27E-05	3.38E-02
ENSG00000167685.13_ZNF444	ENSG00000160789.18_LMNA	0.15481	-0.004502224	0.306457265	5.69E-02	2.18E-01	1.00E+00
ENSG00000145833.14_DDX46	ENSG00000160789.18_LMNA	-0.04514	-0.202885754	0.114883191	5.81E-01	8.05E-01	1.00E+00
ENSG00000229097.1_CALM2P2	ENSG00000160789.18_LMNA	-0.16942	-0.31999095	-0.01050363	3.69E-02	1.64E-01	1.00E+00
ENSG00000105552.13_BCAT2	ENSG00000160789.18_LMNA	0.2871	0.134030211	0.426797698	3.35E-04	4.90E-03	1.00E+00
ENSG00000139746.14_RBM26	ENSG00000160789.18_LMNA	-0.0706	-0.227249356	0.089604919	3.87E-01	6.66E-01	1.00E+00
ENSG00000140743.6_CDR2	ENSG00000160789.18_LMNA	0.37908	0.233998356	0.507642206	1.46E-06	6.30E-05	7.52E-02
ENSG00000137764.18_MAP2K5	ENSG00000160789.18_LMNA	-0.05454	-0.21190133	0.10557687	5.05E-01	7.55E-01	1.00E+00
ENSG00000187164.16_SHTN1	ENSG00000160789.18_LMNA	-0.33526	-0.469406306	-0.18598549	2.42E-05	6.03E-04	1.00E+00
ENSG00000134369.14_NAV1	ENSG00000160789.18_LMNA	-0.0984	-0.253627611	0.061768247	2.28E-01	5.04E-01	1.00E+00
ENSG00000235437.6_LINC01278	ENSG00000160789.18_LMNA	-0.01373	-0.172556567	0.145786308	8.67E-01	9.48E-01	1.00E+00

ENSG00000157303.9_SUSD3	ENSG00000160789.18_LMNA	-0.1483	-0.30040642	0.011166268	6.83E-02	2.45E-01	1.00E+00
ENSG00000169814.11_BTD	ENSG00000160789.18_LMNA	-0.44653	-0.565528001	-0.30931744	8.11E-09	9.07E-07	4.16E-04
ENSG00000169762.15_TAPT1	ENSG00000160789.18_LMNA	-0.41883	-0.54190069	-0.27818114	7.87E-08	5.92E-06	4.04E-03
ENSG00000090905.16_TNRC6A	ENSG00000160789.18_LMNA	0.10964	-0.050440322	0.264229416	1.79E-01	4.41E-01	1.00E+00
ENSG00000145908.11_ZNF300	ENSG00000160789.18_LMNA	-0.17777	-0.327693774	-0.01910705	2.84E-02	1.38E-01	1.00E+00
ENSG00000164151.10_ICE1	ENSG00000160789.18_LMNA	0.16318	0.004085197	0.314217624	4.46E-02	1.87E-01	1.00E+00
ENSG00000187257.13_RSBN1L	ENSG00000160789.18_LMNA	-0.24283	-0.38706723	-0.08699236	2.58E-03	2.38E-02	1.00E+00
ENSG00000123836.13_PFKFB2	ENSG00000160789.18_LMNA	-0.29124	-0.430479668	-0.13845722	2.72E-04	4.17E-03	1.00E+00
ENSG00000187630.13_DHRS4L2	ENSG00000160789.18_LMNA	0.24544	0.089750117	0.389427727	2.31E-03	2.18E-02	1.00E+00
ENSG00000158270.11_COLEC12	ENSG00000160789.18_LMNA	0.21215	0.054796441	0.359215076	8.69E-03	5.92E-02	1.00E+00
ENSG00000130023.14_ERMARD	ENSG00000160789.18_LMNA	-0.00022	-0.159410379	0.158991011	9.98E-01	9.99E-01	1.00E+00
ENSG00000125247.14_TMTC4	ENSG00000160789.18_LMNA	-0.17365	-0.323892709	-0.01485586	3.24E-02	1.51E-01	1.00E+00
ENSG00000096063.13_SRPK1	ENSG00000160789.18_LMNA	-0.15354	-0.305275587	0.005805828	5.90E-02	2.23E-01	1.00E+00
ENSG00000165494.9_PCF11	ENSG00000160789.18_LMNA	0.02822	-0.131573308	0.186580979	7.30E-01	8.86E-01	1.00E+00
ENSG00000143924.17_EML4	ENSG00000160789.18_LMNA	0.04639	-0.113651209	0.204082349	5.70E-01	7.99E-01	1.00E+00
ENSG00000147454.12_SLC25A37	ENSG00000160789.18_LMNA	0.17751	0.018845455	0.327460163	2.87E-02	1.39E-01	1.00E+00
ENSG00000180389.6_ATP5EP2	ENSG00000160789.18_LMNA	-0.03458	-0.192717103	0.125313262	6.72E-01	8.56E-01	1.00E+00
ENSG00000139631.17_CSAD	ENSG00000160789.18_LMNA	-0.08291	-0.238960972	0.077306585	3.10E-01	5.96E-01	1.00E+00
ENSG00000156802.11_ATAD2	ENSG00000160789.18_LMNA	0.08712	-0.073094856	0.24295073	2.86E-01	5.71E-01	1.00E+00
ENSG00000120675.5_DNAJC15	ENSG00000160789.18_LMNA	-0.24655	-0.390422928	-0.09091418	2.20E-03	2.10E-02	1.00E+00
ENSG00000117139.15_KDM5B	ENSG00000160789.18_LMNA	-0.04412	-0.201898423	0.115898985	5.89E-01	8.09E-01	1.00E+00
ENSG00000149177.11_PTPRJ	ENSG00000160789.18_LMNA	0.04372	-0.11629497	0.201513359	5.93E-01	8.12E-01	1.00E+00
ENSG00000127328.20_RAB3IP	ENSG00000160789.18_LMNA	-0.20075	-0.34880532	-0.04292354	1.31E-02	7.97E-02	1.00E+00
ENSG00000272667.1_RP11-395A	ENSG00000160789.18_LMNA	-0.13825	-0.291042365	0.021425015	8.94E-02	2.91E-01	1.00E+00
ENSG00000086712.11_TXLNG	ENSG00000160789.18_LMNA	0.09209	-0.068113363	0.247655923	2.59E-01	5.42E-01	1.00E+00
ENSG00000101945.15_SUV39H1	ENSG00000160789.18_LMNA	0.2268	0.070128402	0.372546477	4.96E-03	3.90E-02	1.00E+00
ENSG00000134874.16_DZIP1	ENSG00000160789.18_LMNA	-0.06467	-0.221586984	0.095516509	4.29E-01	6.99E-01	1.00E+00
ENSG00000181472.4_ZBTB2	ENSG00000160789.18_LMNA	0.2151	0.057880785	0.361906986	7.78E-03	5.44E-02	1.00E+00
ENSG00000023892.10_DEF6	ENSG00000160789.18_LMNA	0.08958	-0.070631478	0.245279336	2.72E-01	5.57E-01	1.00E+00
ENSG00000154222.13_CC2D1B	ENSG00000160789.18_LMNA	0.27254	0.118483556	0.413790088	6.81E-04	8.59E-03	1.00E+00
ENSG00000138646.7_HERC5	ENSG00000160789.18_LMNA	0.1031	-0.057034499	0.258067161	2.06E-01	4.78E-01	1.00E+00
ENSG00000214455.4_RCN1P2	ENSG00000160789.18_LMNA	0.10066	-0.059493788	0.255762374	2.17E-01	4.92E-01	1.00E+00
ENSG00000112406.4_HECA	ENSG00000160789.18_LMNA	-0.051	-0.208512315	0.109081713	5.33E-01	7.75E-01	1.00E+00

ENSG00000271895.2_RP4-635E1	ENSG00000160789.18_LMNA	-0.16118	-0.312369069	-0.00203547	4.73E-02	1.94E-01	1.00E+00
ENSG00000125967.15_NECAB3	ENSG00000160789.18_LMNA	0.26027	0.105437353	0.402780717	1.20E-03	1.33E-02	1.00E+00
ENSG00000105880.4_DLX5	ENSG00000160789.18_LMNA	-0.30149	-0.439595096	-0.14946757	1.60E-04	2.74E-03	1.00E+00
ENSG00000198380.11_GFPT1	ENSG00000160789.18_LMNA	-0.05069	-0.208215254	0.109388551	5.35E-01	7.76E-01	1.00E+00
ENSG00000081320.9_STK17B	ENSG00000160789.18_LMNA	-0.16003	-0.311300103	-0.00085137	4.89E-02	1.98E-01	1.00E+00
ENSG00000132906.16_CASP9	ENSG00000160789.18_LMNA	-0.27664	-0.417459215	-0.12285427	5.60E-04	7.36E-03	1.00E+00
ENSG00000139083.9_ETV6	ENSG00000160789.18_LMNA	0.37605	0.230663709	0.505020981	1.80E-06	7.39E-05	9.24E-02
ENSG00000085644.12_ZNF213	ENSG00000160789.18_LMNA	0.03305	-0.126820733	0.191241744	6.86E-01	8.64E-01	1.00E+00
ENSG00000118873.14_RAB3GAP	ENSG00000160789.18_LMNA	0.22882	0.072254776	0.374385592	4.57E-03	3.66E-02	1.00E+00
ENSG00000170653.17_ATF7	ENSG00000160789.18_LMNA	0.14945	-0.009988871	0.301477305	6.61E-02	2.40E-01	1.00E+00
ENSG00000114268.10_PFKFB4	ENSG00000160789.18_LMNA	0.27051	0.116319649	0.411969976	7.50E-04	9.25E-03	1.00E+00
ENSG00000269837.1_IPO5P1	ENSG00000160789.18_LMNA	-0.51244	-0.620983225	-0.38462027	1.49E-11	4.69E-09	7.65E-07
ENSG00000100744.13_GSKIP	ENSG00000160789.18_LMNA	-0.1767	-0.326710611	-0.01800639	2.94E-02	1.41E-01	1.00E+00
ENSG00000183077.14_AFMID	ENSG00000160789.18_LMNA	-0.33662	-0.470603035	-0.18746798	2.23E-05	5.64E-04	1.00E+00
ENSG00000109881.15_CCDC34	ENSG00000160789.18_LMNA	-0.04832	-0.205937785	0.111738967	5.54E-01	7.89E-01	1.00E+00
ENSG00000162490.6_DRAXIN	ENSG00000160789.18_LMNA	0.0985	-0.061669329	0.253720517	2.27E-01	5.04E-01	1.00E+00
ENSG00000244486.6_SCARF2	ENSG00000160789.18_LMNA	0.31893	0.168273118	0.455027154	6.21E-05	1.28E-03	1.00E+00
ENSG00000109118.12_PHF12	ENSG00000160789.18_LMNA	0.09576	-0.064421526	0.251133406	2.41E-01	5.20E-01	1.00E+00
ENSG00000118655.4_DCLRE1B	ENSG00000160789.18_LMNA	0.18537	0.026964856	0.334693363	2.22E-02	1.16E-01	1.00E+00
ENSG00000175581.12_MRPL48	ENSG00000160789.18_LMNA	-0.42012	-0.543005371	-0.27962465	7.11E-08	5.48E-06	3.65E-03
ENSG00000099290.14_FAM21A	ENSG00000160789.18_LMNA	0.07682	-0.083401711	0.233168105	3.47E-01	6.31E-01	1.00E+00
ENSG00000171385.8_KCND3	ENSG00000160789.18_LMNA	-0.04237	-0.200224232	0.117619922	6.04E-01	8.18E-01	1.00E+00
ENSG00000116035.2_VAX2	ENSG00000160789.18_LMNA	-0.33234	-0.466839647	-0.18281029	2.88E-05	6.88E-04	1.00E+00
ENSG00000106688.10_SLC1A1	ENSG00000160789.18_LMNA	-0.27591	-0.41679927	-0.12206729	5.81E-04	7.57E-03	1.00E+00
ENSG00000163463.10_KRTCAP2	ENSG00000160789.18_LMNA	0.10462	-0.055502842	0.259500783	2.00E-01	4.70E-01	1.00E+00
ENSG00000243335.7_KCTD7	ENSG00000160789.18_LMNA	-0.06085	-0.217940767	0.099311405	4.56E-01	7.21E-01	1.00E+00
ENSG00000164244.19_PRRC1	ENSG00000160789.18_LMNA	0.20946	0.051987869	0.356759436	9.60E-03	6.37E-02	1.00E+00
ENSG00000196535.13_MYO18A	ENSG00000160789.18_LMNA	0.04245	-0.117540376	0.200301659	6.04E-01	8.18E-01	1.00E+00
ENSG00000106603.16_COA1	ENSG00000160789.18_LMNA	-0.18118	-0.330837424	-0.02263143	2.55E-02	1.28E-01	1.00E+00
ENSG00000260428.2_SCX	ENSG00000160789.18_LMNA	0.0379	-0.122032518	0.195922999	6.43E-01	8.41E-01	1.00E+00
ENSG00000000971.14_CFH	ENSG00000160789.18_LMNA	0.14992	-0.009506054	0.301916218	6.53E-02	2.38E-01	1.00E+00
ENSG00000275494.1_RP11-266L	ENSG00000160789.18_LMNA	-0.18521	-0.334542255	-0.02679482	2.24E-02	1.17E-01	1.00E+00
ENSG00000119630.12_PGF	ENSG00000160789.18_LMNA	-0.25961	-0.402192461	-0.10474313	1.24E-03	1.36E-02	1.00E+00

ENSG00000052841.13_TTC17	ENSG00000160789.18_LMNA	0.12857	-0.031275267	0.281994484	1.14E-01	3.38E-01	1.00E+00
ENSG00000033011.10_ALG1	ENSG00000160789.18_LMNA	0.26251	0.107818721	0.404796735	1.09E-03	1.23E-02	1.00E+00
ENSG00000144649.7_FAM198A	ENSG00000160789.18_LMNA	-0.16869	-0.319311894	-0.00974737	3.78E-02	1.67E-01	1.00E+00
ENSG00000164077.12_MON1A	ENSG00000160789.18_LMNA	-0.2094	-0.356705611	-0.05192636	9.62E-03	6.38E-02	1.00E+00
ENSG00000170322.13_NFRKB	ENSG00000160789.18_LMNA	-0.00029	-0.15948598	0.158915398	9.97E-01	9.99E-01	1.00E+00
ENSG00000223768.1_LINC00205	ENSG00000160789.18_LMNA	0.18434	0.025903435	0.333749848	2.30E-02	1.19E-01	1.00E+00
ENSG00000112701.16_SENP6	ENSG00000160789.18_LMNA	0.00593	-0.1534115	0.164978976	9.42E-01	9.77E-01	1.00E+00
ENSG00000140332.14_TLE3	ENSG00000160789.18_LMNA	-0.08733	-0.243151512	0.072882606	2.85E-01	5.70E-01	1.00E+00
ENSG00000180818.4_HOXC10	ENSG00000160789.18_LMNA	-0.03258	-0.190792154	0.127279816	6.90E-01	8.67E-01	1.00E+00
ENSG00000166012.13_TAF1D	ENSG00000160789.18_LMNA	-0.25334	-0.396548655	-0.0980974	1.64E-03	1.68E-02	1.00E+00
ENSG00000100228.11_RAB36	ENSG00000160789.18_LMNA	0.37887	0.233771164	0.50746378	1.49E-06	6.38E-05	7.62E-02
ENSG00000043462.10_LCP2	ENSG00000160789.18_LMNA	0.05719	-0.102951343	0.214435096	4.84E-01	7.41E-01	1.00E+00
ENSG00000171444.16_MCC	ENSG00000160789.18_LMNA	0.2669	0.112474238	0.408729679	8.88E-04	1.06E-02	1.00E+00
ENSG00000196569.10_LAMA2	ENSG00000160789.18_LMNA	0.42086	0.280451199	0.543637498	6.71E-08	5.23E-06	3.44E-03
ENSG00000188917.13_TRMT2B	ENSG00000160789.18_LMNA	0.19945	0.041572589	0.34761605	1.38E-02	8.24E-02	1.00E+00
ENSG00000127980.14_PEX1	ENSG00000160789.18_LMNA	-0.06467	-0.221587658	0.095515807	4.29E-01	6.99E-01	1.00E+00
ENSG00000170340.10_B3GNT2	ENSG00000160789.18_LMNA	0.07991	-0.080308919	0.236110335	3.28E-01	6.14E-01	1.00E+00
ENSG00000106993.10_CDC37L1	ENSG00000160789.18_LMNA	-0.15336	-0.30511591	0.0059819	5.92E-02	2.24E-01	1.00E+00
ENSG00000229043.2_AC091729.!	ENSG00000160789.18_LMNA	0.03228	-0.127572435	0.190505516	6.93E-01	8.68E-01	1.00E+00
ENSG00000280789.1_PAGR1	ENSG00000160789.18_LMNA	0.17289	0.014075358	0.323193768	3.32E-02	1.53E-01	1.00E+00
ENSG00000249115.7_HAUS5	ENSG00000160789.18_LMNA	0.05901	-0.101145324	0.216175518	4.70E-01	7.31E-01	1.00E+00
ENSG00000106479.9_ZNF862	ENSG00000160789.18_LMNA	0.13979	-0.019852284	0.29248183	8.59E-02	2.84E-01	1.00E+00
ENSG00000120278.13_PLEKHG1	ENSG00000160789.18_LMNA	0.40739	0.265405298	0.532083806	1.90E-07	1.22E-05	9.74E-03
ENSG00000196204.10_RNF216P1	ENSG00000160789.18_LMNA	0.08828	-0.07192844	0.244053786	2.79E-01	5.65E-01	1.00E+00
ENSG00000110497.13_AMBRA1	ENSG00000160789.18_LMNA	0.10374	-0.056390328	0.25867027	2.03E-01	4.74E-01	1.00E+00
ENSG00000149231.10_CCDC82	ENSG00000160789.18_LMNA	-0.27808	-0.418743825	-0.12438723	5.23E-04	6.99E-03	1.00E+00
ENSG00000197579.6_TOPORS	ENSG00000160789.18_LMNA	-0.00706	-0.166078475	0.152307441	9.31E-01	9.73E-01	1.00E+00
ENSG00000130244.11_FAM98C	ENSG00000160789.18_LMNA	0.03204	-0.127808784	0.190273959	6.95E-01	8.69E-01	1.00E+00
ENSG00000076382.15_SPAG5	ENSG00000160789.18_LMNA	0.0573	-0.102836597	0.214545734	4.83E-01	7.41E-01	1.00E+00
ENSG00000158201.8_ABHD3	ENSG00000160789.18_LMNA	0.13356	-0.02620002	0.286663286	1.01E-01	3.13E-01	1.00E+00
ENSG00000163558.11_PRKCI	ENSG00000160789.18_LMNA	0.09031	-0.06989755	0.245972406	2.69E-01	5.53E-01	1.00E+00
ENSG00000057019.14_DCBLD2	ENSG00000160789.18_LMNA	0.44944	0.312607504	0.568000292	6.31E-09	7.29E-07	3.24E-04
ENSG00000140525.16_FANCI	ENSG00000160789.18_LMNA	0.07185	-0.088366286	0.228433063	3.79E-01	6.58E-01	1.00E+00

ENSG00000184402.13_SS18L1	ENSG00000160789.18_LMNA	-0.15446	-0.306133604	0.004859386	5.74E-02	2.19E-01	1.00E+00
ENSG00000159110.18_IFNAR2	ENSG00000160789.18_LMNA	0.00902	-0.150399569	0.167976603	9.12E-01	9.67E-01	1.00E+00
ENSG00000132256.17_TRIM5	ENSG00000160789.18_LMNA	0.2511	0.095726284	0.394529543	1.81E-03	1.81E-02	1.00E+00
ENSG00000223705.8_NSUN5P1	ENSG00000160789.18_LMNA	-0.05436	-0.211728671	0.105755621	5.06E-01	7.57E-01	1.00E+00
ENSG00000102900.11_NUP93	ENSG00000160789.18_LMNA	0.03626	-0.123653777	0.194339577	6.57E-01	8.49E-01	1.00E+00
ENSG00000169660.14_HEXDC	ENSG00000160789.18_LMNA	0.02942	-0.13039152	0.187741276	7.19E-01	8.81E-01	1.00E+00
ENSG00000112996.8_MRPS30	ENSG00000160789.18_LMNA	0.08338	-0.076841087	0.239402462	3.07E-01	5.94E-01	1.00E+00
ENSG00000240849.9_TMEM189	ENSG00000160789.18_LMNA	0.23888	0.08283368	0.383500162	3.04E-03	2.70E-02	1.00E+00
ENSG00000179902.11_C1orf194	ENSG00000160789.18_LMNA	-0.12562	-0.279240413	0.034261539	1.23E-01	3.53E-01	1.00E+00
ENSG00000134318.12_ROCK2	ENSG00000160789.18_LMNA	0.21309	0.055778118	0.3600724	8.39E-03	5.77E-02	1.00E+00
ENSG00000118246.12_FASTKD2	ENSG00000160789.18_LMNA	-0.01171	-0.170590585	0.147768281	8.86E-01	9.56E-01	1.00E+00
ENSG00000166803.9_KIAA0101	ENSG00000160789.18_LMNA	0.04988	-0.110194122	0.207435072	5.42E-01	7.81E-01	1.00E+00
ENSG00000104691.13_UBXN8	ENSG00000160789.18_LMNA	-0.14202	-0.294556444	0.01758288	8.09E-02	2.74E-01	1.00E+00
ENSG00000101191.15_DIDO1	ENSG00000160789.18_LMNA	0.21589	0.0587087	0.362628699	7.56E-03	5.32E-02	1.00E+00
ENSG00000155975.8_VPS37A	ENSG00000160789.18_LMNA	0.00749	-0.151894823	0.166489186	9.27E-01	9.72E-01	1.00E+00
ENSG00000012983.10_MAP4K5	ENSG00000160789.18_LMNA	-0.01772	-0.176424883	0.141879055	8.28E-01	9.33E-01	1.00E+00
ENSG00000102921.6_N4BP1	ENSG00000160789.18_LMNA	0.16471	0.005657576	0.315634091	4.26E-02	1.81E-01	1.00E+00
ENSG00000112984.10_KIF20A	ENSG00000160789.18_LMNA	0.15244	-0.00692646	0.304259002	6.08E-02	2.28E-01	1.00E+00
ENSG00000185986.11_SDHAP3	ENSG00000160789.18_LMNA	-0.09949	-0.254656524	0.060672412	2.23E-01	4.98E-01	1.00E+00
ENSG00000178950.15_GAK	ENSG00000160789.18_LMNA	0.23248	0.076091325	0.377697846	3.95E-03	3.29E-02	1.00E+00
ENSG00000131437.14_KIF3A	ENSG00000160789.18_LMNA	-0.39716	-0.523275393	-0.25402106	4.06E-07	2.23E-05	2.08E-02
ENSG00000065970.7_FOXJ2	ENSG00000160789.18_LMNA	0.12057	-0.039384659	0.274503601	1.39E-01	3.80E-01	1.00E+00
ENSG00000224470.6_ATXN1L	ENSG00000160789.18_LMNA	0.15872	-0.000495131	0.310083583	5.08E-02	2.03E-01	1.00E+00
ENSG00000181751.8_C5orf30	ENSG00000160789.18_LMNA	-0.34376	-0.476863385	-0.19524429	1.45E-05	3.98E-04	7.44E-01
ENSG00000123268.7_ATF1	ENSG00000160789.18_LMNA	-0.03543	-0.193534981	0.124476949	6.65E-01	8.52E-01	1.00E+00
ENSG00000173209.21_AHSA2	ENSG00000160789.18_LMNA	0.06824	-0.091958779	0.224997293	4.04E-01	6.79E-01	1.00E+00
ENSG00000148737.14_TCF7L2	ENSG00000160789.18_LMNA	-0.12918	-0.282568707	0.030651925	1.13E-01	3.35E-01	1.00E+00
ENSG00000239467.4_AC007405.(	ENSG00000160789.18_LMNA	-0.01817	-0.176858084	0.141440876	8.24E-01	9.31E-01	1.00E+00
ENSG00000122678.13_POLM	ENSG00000160789.18_LMNA	0.23838	0.082297861	0.383039915	3.10E-03	2.74E-02	1.00E+00
ENSG00000171246.5_NPTX1	ENSG00000160789.18_LMNA	-0.02564	-0.184088808	0.134108585	7.54E-01	8.98E-01	1.00E+00
ENSG00000213839.4_TMX2P1	ENSG00000160789.18_LMNA	0.02065	-0.139009164	0.179259925	8.01E-01	9.20E-01	1.00E+00
ENSG00000137573.12_SULF1	ENSG00000160789.18_LMNA	-0.16959	-0.320147678	-0.01067823	3.67E-02	1.64E-01	1.00E+00
ENSG00000166833.18_NAV2	ENSG00000160789.18_LMNA	0.25817	0.103207985	0.40089076	1.32E-03	1.43E-02	1.00E+00

ENSG00000267390.1_RP11-635N	ENSG00000160789.18_LMNA	-0.22578	-0.371623298	-0.06906206	5.16E-03	4.02E-02	1.00E+00
ENSG00000168481.7_LGI3	ENSG00000160789.18_LMNA	-0.3749	-0.504014364	-0.22938483	1.95E-06	7.83E-05	1.00E-01
ENSG00000230896.1_RP11-767N	ENSG00000160789.18_LMNA	-0.06242	-0.219437679	0.097754571	4.45E-01	7.12E-01	1.00E+00
ENSG00000154134.13_ROBO3	ENSG00000160789.18_LMNA	0.02112	-0.138543806	0.179719135	7.96E-01	9.18E-01	1.00E+00
ENSG00000104299.13_INTS9	ENSG00000160789.18_LMNA	-0.06915	-0.225859682	0.091057827	3.97E-01	6.74E-01	1.00E+00
ENSG00000122824.9_NUDT10	ENSG00000160789.18_LMNA	-0.45185	-0.570048146	-0.31533739	5.12E-09	6.11E-07	2.63E-04
ENSG00000170955.9_PRKCDBP	ENSG00000160789.18_LMNA	0.36029	0.213328565	0.491312534	5.13E-06	1.69E-04	2.63E-01
ENSG00000006194.9_ZNF263	ENSG00000160789.18_LMNA	0.13034	-0.029472337	0.28365473	1.09E-01	3.29E-01	1.00E+00
ENSG00000101298.12_SNPH	ENSG00000160789.18_LMNA	-0.00549	-0.164551035	0.153841001	9.46E-01	9.79E-01	1.00E+00
ENSG00000132964.10_CDK8	ENSG00000160789.18_LMNA	-0.35074	-0.482970967	-0.20286513	9.42E-06	2.78E-04	4.83E-01
ENSG00000215041.8_NEURL4	ENSG00000160789.18_LMNA	0.02488	-0.134859726	0.183349652	7.61E-01	9.00E-01	1.00E+00
ENSG00000138375.11_SMARCAL	ENSG00000160789.18_LMNA	0.28042	0.126884536	0.420834044	4.67E-04	6.38E-03	1.00E+00
ENSG00000105971.13_CAV2	ENSG00000160789.18_LMNA	0.16521	0.006167583	0.31609323	4.20E-02	1.79E-01	1.00E+00
ENSG00000218891.4_ZNF579	ENSG00000160789.18_LMNA	0.26432	0.109735772	0.406417576	1.00E-03	1.16E-02	1.00E+00
ENSG00000175395.14_ZNF25	ENSG00000160789.18_LMNA	-0.39335	-0.519989547	-0.24979337	5.36E-07	2.77E-05	2.75E-02
ENSG00000115183.12_TANC1	ENSG00000160789.18_LMNA	0.04595	-0.114083174	0.2036629	5.74E-01	8.01E-01	1.00E+00
ENSG00000108433.14_GOSR2	ENSG00000160789.18_LMNA	-0.16164	-0.31278728	-0.00249897	4.67E-02	1.92E-01	1.00E+00
ENSG00000157570.10_TSPAN18	ENSG00000160789.18_LMNA	-0.07549	-0.231900019	0.08473281	3.55E-01	6.39E-01	1.00E+00
ENSG00000204837.3_FGF7P3	ENSG00000160789.18_LMNA	-0.16694	-0.317698472	-0.00795194	3.98E-02	1.73E-01	1.00E+00
ENSG00000137273.3_FOXF2	ENSG00000160789.18_LMNA	0.05431	-0.105801352	0.211684495	5.06E-01	7.57E-01	1.00E+00
ENSG00000243749.1_TMEM35B	ENSG00000160789.18_LMNA	0.19155	0.033361709	0.3403666	1.81E-02	1.00E-01	1.00E+00
ENSG00000120137.6_PANK3	ENSG00000160789.18_LMNA	-0.12576	-0.2793656	0.034125918	1.23E-01	3.53E-01	1.00E+00
ENSG00000027001.8_MIPEP	ENSG00000160789.18_LMNA	0.1178	-0.042188794	0.271904444	1.48E-01	3.95E-01	1.00E+00
ENSG00000167566.15_NCKAP5L	ENSG00000160789.18_LMNA	0.06727	-0.092928826	0.224068211	4.10E-01	6.84E-01	1.00E+00
ENSG00000155629.13_PIK3AP1	ENSG00000160789.18_LMNA	-0.0336	-0.191774079	0.12627698	6.81E-01	8.61E-01	1.00E+00
ENSG00000247828.6_TMEM161B	ENSG00000160789.18_LMNA	-0.24277	-0.387015443	-0.08693191	2.58E-03	2.38E-02	1.00E+00
ENSG00000041988.14_THAP3	ENSG00000160789.18_LMNA	-0.12869	-0.282109227	0.031150728	1.14E-01	3.37E-01	1.00E+00
ENSG00000198056.12_PRIM1	ENSG00000160789.18_LMNA	-0.13973	-0.292422373	0.019917277	8.60E-02	2.85E-01	1.00E+00
ENSG00000153815.15_CMIP	ENSG00000160789.18_LMNA	0.09942	-0.060745296	0.254588113	2.23E-01	4.99E-01	1.00E+00
ENSG00000103356.14_EARS2	ENSG00000160789.18_LMNA	0.09142	-0.068779077	0.247027994	2.63E-01	5.46E-01	1.00E+00
ENSG00000119231.9_SENP5	ENSG00000160789.18_LMNA	-0.05591	-0.213208643	0.104222765	4.94E-01	7.49E-01	1.00E+00
ENSG00000108963.16_DPH1	ENSG00000160789.18_LMNA	-0.00802	-0.167009043	0.151372393	9.22E-01	9.70E-01	1.00E+00
ENSG00000119401.10_TRIM32	ENSG00000160789.18_LMNA	-0.01134	-0.170228894	0.148132635	8.90E-01	9.57E-01	1.00E+00

ENSG00000118507.14_AKAP7	ENSG00000160789.18_LMNA	-0.22478	-0.370715634	-0.06801432	5.37E-03	4.13E-02	1.00E+00
ENSG00000148798.8_INA	ENSG00000160789.18_LMNA	-0.36403	-0.494565249	-0.21742604	4.02E-06	1.39E-04	2.06E-01
ENSG00000115419.11_GLS	ENSG00000160789.18_LMNA	-0.10233	-0.257342363	0.057808297	2.10E-01	4.82E-01	1.00E+00
ENSG00000168528.10_SERINC2	ENSG00000160789.18_LMNA	0.01596	-0.143603351	0.174718982	8.45E-01	9.40E-01	1.00E+00
ENSG00000114107.7_CEP70	ENSG00000160789.18_LMNA	-0.37394	-0.50318286	-0.22832914	2.08E-06	8.22E-05	1.07E-01
ENSG00000125354.21_SEPT6	ENSG00000160789.18_LMNA	0.00947	-0.149958698	0.168414887	9.08E-01	9.65E-01	1.00E+00
ENSG00000240509.1_RPL34P18	ENSG00000160789.18_LMNA	-0.10876	-0.263402002	0.051327328	1.82E-01	4.47E-01	1.00E+00
ENSG00000009830.10_POMT2	ENSG00000160789.18_LMNA	0.18607	0.027686054	0.335334097	2.17E-02	1.14E-01	1.00E+00
ENSG00000198846.5_TOX	ENSG00000160789.18_LMNA	-0.15067	-0.302610036	0.008742536	6.39E-02	2.35E-01	1.00E+00
ENSG00000237854.3_LINC00674	ENSG00000160789.18_LMNA	-0.05766	-0.214886496	0.102483131	4.80E-01	7.38E-01	1.00E+00
ENSG00000119862.11_LGALSL	ENSG00000160789.18_LMNA	-0.07613	-0.232511379	0.084091212	3.51E-01	6.35E-01	1.00E+00
ENSG00000266028.6_SRGAP2	ENSG00000160789.18_LMNA	0.15307	-0.006282115	0.304843609	5.97E-02	2.25E-01	1.00E+00
ENSG00000150712.9_MTMR12	ENSG00000160789.18_LMNA	-0.13039	-0.283702457	0.029420478	1.09E-01	3.29E-01	1.00E+00
ENSG00000070495.13_JMJD6	ENSG00000160789.18_LMNA	0.25584	0.100737702	0.398793605	1.47E-03	1.54E-02	1.00E+00
ENSG00000224032.5_EPB41L4A-	ENSG00000160789.18_LMNA	-0.24425	-0.388345952	-0.08848571	2.43E-03	2.27E-02	1.00E+00
ENSG00000108641.13_B9D1	ENSG00000160789.18_LMNA	-0.09591	-0.25127694	0.064268958	2.40E-01	5.19E-01	1.00E+00
ENSG00000141574.6_SECTM1	ENSG00000160789.18_LMNA	0.37638	0.231022852	0.505303532	1.76E-06	7.26E-05	9.04E-02
ENSG00000125834.11_STK35	ENSG00000160789.18_LMNA	0.28884	0.135890834	0.428346373	3.07E-04	4.60E-03	1.00E+00
ENSG00000151208.15_DLG5	ENSG00000160789.18_LMNA	0.21016	0.052725051	0.35740439	9.36E-03	6.24E-02	1.00E+00
ENSG00000071243.14_ING3	ENSG00000160789.18_LMNA	-0.32948	-0.464328708	-0.17970975	3.40E-05	7.91E-04	1.00E+00
ENSG00000177189.11_RPS6KA3	ENSG00000160789.18_LMNA	0.34743	0.199245173	0.480073211	1.16E-05	3.29E-04	5.94E-01
ENSG00000152117.16_AC093838	ENSG00000160789.18_LMNA	-0.04484	-0.20259017	0.115187366	5.83E-01	8.06E-01	1.00E+00
ENSG00000096654.14_ZNF184	ENSG00000160789.18_LMNA	-0.14133	-0.293918182	0.018281413	8.24E-02	2.77E-01	1.00E+00
ENSG00000181754.6_AMIGO1	ENSG00000160789.18_LMNA	-0.06234	-0.21936097	0.097834388	4.45E-01	7.12E-01	1.00E+00
ENSG00000152484.12_USP12	ENSG00000160789.18_LMNA	0.04804	-0.112021021	0.205664253	5.57E-01	7.90E-01	1.00E+00
ENSG00000128294.14_TPST2	ENSG00000160789.18_LMNA	0.2441	0.088331828	0.388214236	2.44E-03	2.28E-02	1.00E+00
ENSG00000196663.14_TECPR2	ENSG00000160789.18_LMNA	0.10024	-0.059917711	0.255364721	2.19E-01	4.94E-01	1.00E+00
ENSG00000272886.4_DCP1A	ENSG00000160789.18_LMNA	-0.12045	-0.274392037	0.039505123	1.39E-01	3.81E-01	1.00E+00
ENSG00000124302.11_CHST8	ENSG00000160789.18_LMNA	-0.07727	-0.233599691	0.082948421	3.44E-01	6.29E-01	1.00E+00
ENSG00000226328.5_NUP50-AS1	ENSG00000160789.18_LMNA	-0.27461	-0.415638348	-0.1206838	6.18E-04	7.97E-03	1.00E+00
ENSG00000170584.9_NUDCD2	ENSG00000160789.18_LMNA	-0.20539	-0.353044885	-0.04774859	1.11E-02	7.07E-02	1.00E+00
ENSG00000253797.2_UTP14C	ENSG00000160789.18_LMNA	-0.26589	-0.407831834	-0.11141029	9.30E-04	1.10E-02	1.00E+00
ENSG00000177363.4_LRRN4CL	ENSG00000160789.18_LMNA	0.16385	0.004772877	0.314837285	4.37E-02	1.84E-01	1.00E+00

ENSG00000188002.9_RP11-43F1	ENSG00000160789.18_LMNA	-0.12493	-0.278593749	0.034961914	1.25E-01	3.57E-01	1.00E+00
ENSG00000077713.17_SLC25A43	ENSG00000160789.18_LMNA	0.41007	0.268389555	0.534383329	1.55E-07	1.03E-05	7.95E-03
ENSG00000168411.12_RFWD3	ENSG00000160789.18_LMNA	0.18548	0.027081711	0.3347972	2.21E-02	1.16E-01	1.00E+00
ENSG00000139146.12_FAM60A	ENSG00000160789.18_LMNA	-0.29639	-0.435060048	-0.14398076	2.09E-04	3.38E-03	1.00E+00
ENSG00000122965.9_RBM19	ENSG00000160789.18_LMNA	0.16311	0.004012862	0.314152428	4.47E-02	1.87E-01	1.00E+00
ENSG00000137449.14_CPEB2	ENSG00000160789.18_LMNA	0.09134	-0.068865919	0.246946061	2.63E-01	5.46E-01	1.00E+00
ENSG00000177302.13_TOP3A	ENSG00000160789.18_LMNA	0.15267	-0.006693242	0.304470625	6.04E-02	2.27E-01	1.00E+00
ENSG00000103932.10_RPAP1	ENSG00000160789.18_LMNA	0.18684	0.028490447	0.336048407	2.12E-02	1.12E-01	1.00E+00
ENSG00000110324.8_IL10RA	ENSG00000160789.18_LMNA	0.04766	-0.112389131	0.205307191	5.60E-01	7.92E-01	1.00E+00
ENSG00000157152.15_SYN2	ENSG00000160789.18_LMNA	-0.22898	-0.374528615	-0.07242025	4.55E-03	3.65E-02	1.00E+00
ENSG00000185163.8_DDX51	ENSG00000160789.18_LMNA	0.07874	-0.081477797	0.234999041	3.35E-01	6.21E-01	1.00E+00
ENSG00000160179.17_ABCG1	ENSG00000160789.18_LMNA	-0.29275	-0.431821856	-0.1400739	2.52E-04	3.93E-03	1.00E+00
ENSG00000280120.1_RP11-546D	ENSG00000160789.18_LMNA	-0.29283	-0.431892849	-0.14015946	2.51E-04	3.91E-03	1.00E+00
ENSG00000008311.13_AASS	ENSG00000160789.18_LMNA	0.13125	-0.028545516	0.284507472	1.07E-01	3.25E-01	1.00E+00
ENSG00000125630.14_POLR1B	ENSG00000160789.18_LMNA	-0.04294	-0.200765004	0.11706426	5.99E-01	8.16E-01	1.00E+00
ENSG00000135365.14_PHF21A	ENSG00000160789.18_LMNA	0.0639	-0.096284427	0.220849868	4.34E-01	7.03E-01	1.00E+00
ENSG00000118298.9_CA14	ENSG00000160789.18_LMNA	-0.30637	-0.443922522	-0.15471989	1.24E-04	2.24E-03	1.00E+00
ENSG00000182378.11_PLCXD1	ENSG00000160789.18_LMNA	0.12213	-0.037801694	0.275968831	1.34E-01	3.71E-01	1.00E+00
ENSG00000276550.3_HERC2P2	ENSG00000160789.18_LMNA	-0.15693	-0.308421216	0.00233329	5.35E-02	2.10E-01	1.00E+00
ENSG00000124275.13_MTRR	ENSG00000160789.18_LMNA	-0.05272	-0.210159549	0.107379167	5.19E-01	7.65E-01	1.00E+00
ENSG00000127249.13_ATP13A4	ENSG00000160789.18_LMNA	-0.00791	-0.166903314	0.151478659	9.23E-01	9.70E-01	1.00E+00
ENSG00000124831.17_LRRFIP1	ENSG00000160789.18_LMNA	0.38751	0.243315673	0.514939399	8.13E-07	3.85E-05	4.17E-02
ENSG00000163364.8_LINC01116	ENSG00000160789.18_LMNA	-0.24604	-0.389962126	-0.09037509	2.25E-03	2.14E-02	1.00E+00
ENSG00000101546.11_RBFA	ENSG00000160789.18_LMNA	-0.05715	-0.214400115	0.10298762	4.84E-01	7.41E-01	1.00E+00
ENSG00000136114.14_THSD1	ENSG00000160789.18_LMNA	0.16177	0.002635051	0.312910044	4.65E-02	1.92E-01	1.00E+00
ENSG00000078295.14_ADCY2	ENSG00000160789.18_LMNA	-0.35347	-0.485354213	-0.2058481	7.94E-06	2.43E-04	4.07E-01
ENSG00000173653.6_RCE1	ENSG00000160789.18_LMNA	0.22833	0.071734045	0.373935428	4.67E-03	3.72E-02	1.00E+00
ENSG00000100084.13_HIRA	ENSG00000160789.18_LMNA	0.08748	-0.072735405	0.243290743	2.84E-01	5.69E-01	1.00E+00
ENSG00000059915.15_PSD	ENSG00000160789.18_LMNA	-0.26691	-0.408741702	-0.11248849	8.87E-04	1.06E-02	1.00E+00
ENSG00000100604.11_CHGA	ENSG00000160789.18_LMNA	-0.23545	-0.380387984	-0.07921387	3.50E-03	3.00E-02	1.00E+00
ENSG00000167785.7_ZNF558	ENSG00000160789.18_LMNA	-0.02526	-0.183724103	0.134479249	7.57E-01	8.99E-01	1.00E+00
ENSG00000125482.11_TTF1	ENSG00000160789.18_LMNA	-0.04011	-0.198043463	0.119858723	6.24E-01	8.30E-01	1.00E+00
ENSG00000166188.2_ZNF319	ENSG00000160789.18_LMNA	0.14919	-0.010255575	0.301234797	6.66E-02	2.41E-01	1.00E+00

ENSG00000171806.10_METTL18	ENSG00000160789.18_LMNA	-0.08072	-0.236881145	0.079497656	3.23E-01	6.09E-01	1.00E+00
ENSG00000104133.13_SPG11	ENSG00000160789.18_LMNA	0.15006	-0.009361988	0.302047157	6.50E-02	2.38E-01	1.00E+00
ENSG00000156983.14_BRPF1	ENSG00000160789.18_LMNA	0.16815	0.00919816	0.318818551	3.84E-02	1.69E-01	1.00E+00
ENSG00000108854.14_SMURF2	ENSG00000160789.18_LMNA	0.0762	-0.084024261	0.23257516	3.51E-01	6.34E-01	1.00E+00
ENSG00000117475.12_BLZF1	ENSG00000160789.18_LMNA	-0.00081	-0.159990497	0.158410703	9.92E-01	9.97E-01	1.00E+00
ENSG00000157873.16_TNFRSF14	ENSG00000160789.18_LMNA	0.25977	0.104907505	0.402331765	1.23E-03	1.35E-02	1.00E+00
ENSG00000188404.7_SELL	ENSG00000160789.18_LMNA	-0.14598	-0.298253499	0.013530711	7.27E-02	2.56E-01	1.00E+00
ENSG00000175505.10_CLCF1	ENSG00000160789.18_LMNA	0.52628	0.400652351	0.632491913	3.32E-12	1.46E-09	1.70E-07
ENSG00000025434.17_NR1H3	ENSG00000160789.18_LMNA	0.0788	-0.081417259	0.235056618	3.35E-01	6.20E-01	1.00E+00
ENSG00000229152.2_ANKRD10-I	ENSG00000160789.18_LMNA	-0.01663	-0.175368578	0.142946978	8.39E-01	9.37E-01	1.00E+00
ENSG00000132510.9_KDM6B	ENSG00000160789.18_LMNA	0.03423	-0.125650929	0.192386753	6.75E-01	8.58E-01	1.00E+00
ENSG00000140859.14_KIFC3	ENSG00000160789.18_LMNA	0.3209	0.170401846	0.456763229	5.56E-05	1.17E-03	1.00E+00
ENSG00000100014.18_SPECC1L	ENSG00000160789.18_LMNA	0.17993	0.021339221	0.329685599	2.65E-02	1.32E-01	1.00E+00
ENSG00000165566.12_AMER2	ENSG00000160789.18_LMNA	-0.46478	-0.580992686	-0.3299988	1.62E-09	2.33E-07	8.30E-05
ENSG00000188599.16_NPIPP1	ENSG00000160789.18_LMNA	0.00905	-0.15036253	0.16801343	9.12E-01	9.66E-01	1.00E+00
ENSG00000173917.10_HOXB2	ENSG00000160789.18_LMNA	0.02629	-0.133467333	0.184719544	7.48E-01	8.95E-01	1.00E+00
ENSG00000068383.17_INPP5A	ENSG00000160789.18_LMNA	-0.22271	-0.368836726	-0.06584755	5.82E-03	4.40E-02	1.00E+00
ENSG00000133818.11_RRAS2	ENSG00000160789.18_LMNA	-0.17861	-0.328467499	-0.01997377	2.77E-02	1.36E-01	1.00E+00
ENSG00000134323.10_MYCN	ENSG00000160789.18_LMNA	-0.24586	-0.38980086	-0.09018647	2.26E-03	2.15E-02	1.00E+00
ENSG00000062582.12_MRPS24	ENSG00000160789.18_LMNA	0.12856	-0.031277203	0.281992701	1.14E-01	3.38E-01	1.00E+00
ENSG00000066248.13_NGEF	ENSG00000160789.18_LMNA	-0.02095	-0.179549408	0.138715822	7.98E-01	9.19E-01	1.00E+00
ENSG00000127415.11_IDUA	ENSG00000160789.18_LMNA	0.26937	0.115103921	0.410946363	7.91E-04	9.65E-03	1.00E+00
ENSG00000172164.12_SNTB1	ENSG00000160789.18_LMNA	0.06266	-0.097514803	0.219668086	4.43E-01	7.10E-01	1.00E+00
ENSG00000160191.16_PDE9A	ENSG00000160789.18_LMNA	-0.14712	-0.299313074	0.01236747	7.05E-02	2.51E-01	1.00E+00
ENSG00000175938.6_ORAI3	ENSG00000160789.18_LMNA	0.14905	-0.010402176	0.301101479	6.69E-02	2.42E-01	1.00E+00
ENSG00000170954.10_ZNF415	ENSG00000160789.18_LMNA	-0.13486	-0.287882679	0.024871811	9.76E-02	3.07E-01	1.00E+00
ENSG00000110756.16_HPS5	ENSG00000160789.18_LMNA	-0.1063	-0.261081238	0.053812592	1.92E-01	4.61E-01	1.00E+00
ENSG00000172830.11_SSH3	ENSG00000160789.18_LMNA	0.35637	0.20903365	0.487894739	6.60E-06	2.09E-04	3.39E-01
ENSG00000171723.14_GPHN	ENSG00000160789.18_LMNA	-0.13998	-0.292662941	0.019654296	8.54E-02	2.84E-01	1.00E+00
ENSG00000197818.10_SLC9A8	ENSG00000160789.18_LMNA	0.14199	-0.01760835	0.294533177	8.10E-02	2.74E-01	1.00E+00
ENSG00000113649.10_TCERG1	ENSG00000160789.18_LMNA	-0.07539	-0.231804096	0.084833454	3.56E-01	6.39E-01	1.00E+00
ENSG00000156531.15_PHF6	ENSG00000160789.18_LMNA	-0.17648	-0.326510664	-0.01778264	2.96E-02	1.42E-01	1.00E+00
ENSG00000104626.13_ERI1	ENSG00000160789.18_LMNA	0.51935	0.392614193	0.626734459	7.11E-12	2.66E-09	3.65E-07

ENSG00000171574.16_ZNF584	ENSG00000160789.18_LMNA	0.13762	-0.022069145	0.290452407	9.09E-02	2.94E-01	1.00E+00
ENSG00000168303.6_MPLKIP	ENSG00000160789.18_LMNA	0.02769	-0.132096319	0.186067195	7.35E-01	8.89E-01	1.00E+00
ENSG00000166821.7_PEX11A	ENSG00000160789.18_LMNA	-0.05484	-0.212188436	0.105279588	5.02E-01	7.54E-01	1.00E+00
ENSG00000272288.4_RP11-140K	ENSG00000160789.18_LMNA	-0.21511	-0.36192031	-0.05789607	7.78E-03	5.44E-02	1.00E+00
ENSG00000188647.11_PTAR1	ENSG00000160789.18_LMNA	0.12774	-0.032108725	0.281226352	1.17E-01	3.42E-01	1.00E+00
ENSG00000119522.14_DENND1A	ENSG00000160789.18_LMNA	0.27245	0.118389076	0.413710668	6.84E-04	8.62E-03	1.00E+00
ENSG00000149679.10_CABLES2	ENSG00000160789.18_LMNA	0.17418	0.015401797	0.324381404	3.19E-02	1.49E-01	1.00E+00
ENSG00000253570.1_RNF5P1	ENSG00000160789.18_LMNA	-0.27841	-0.419038203	-0.12473871	5.15E-04	6.90E-03	1.00E+00
ENSG00000142207.6_URB1	ENSG00000160789.18_LMNA	0.15557	-0.003727948	0.307158669	5.56E-02	2.15E-01	1.00E+00
ENSG00000197386.9_HTT	ENSG00000160789.18_LMNA	0.20888	0.05138323	0.356230226	9.81E-03	6.47E-02	1.00E+00
ENSG00000189184.10_PCDH18	ENSG00000160789.18_LMNA	0.25893	0.104015588	0.401575702	1.28E-03	1.39E-02	1.00E+00
ENSG00000123154.10_WDR83	ENSG00000160789.18_LMNA	-0.03836	-0.196366115	0.121578509	6.39E-01	8.39E-01	1.00E+00
ENSG00000173442.10_EHBP1L1	ENSG00000160789.18_LMNA	0.4161	0.27512002	0.539555083	9.75E-08	7.06E-06	5.00E-03
ENSG00000125779.20_PANK2	ENSG00000160789.18_LMNA	0.29351	0.140896414	0.432504219	2.43E-04	3.81E-03	1.00E+00
ENSG00000070501.10_POLB	ENSG00000160789.18_LMNA	-0.40364	-0.528858937	-0.26122874	2.52E-07	1.53E-05	1.29E-02
ENSG00000124608.4_AARS2	ENSG00000160789.18_LMNA	0.20675	0.049166286	0.354288173	1.06E-02	6.83E-02	1.00E+00
ENSG00000114251.12_WNT5A	ENSG00000160789.18_LMNA	-0.23541	-0.380354801	-0.07917532	3.51E-03	3.01E-02	1.00E+00
ENSG00000089060.10_SLC8B1	ENSG00000160789.18_LMNA	0.25143	0.09607291	0.394824891	1.78E-03	1.79E-02	1.00E+00
ENSG00000113638.11_TTC33	ENSG00000160789.18_LMNA	-0.39247	-0.519229399	-0.2488168	5.71E-07	2.92E-05	2.93E-02
ENSG00000156697.11_UTP14A	ENSG00000160789.18_LMNA	0.29553	0.143063968	0.434300842	2.19E-04	3.50E-03	1.00E+00
ENSG00000171862.8_PTEN	ENSG00000160789.18_LMNA	-0.028	-0.18637317	0.131784871	7.32E-01	8.87E-01	1.00E+00
ENSG00000237350.1_CDC42P6	ENSG00000160789.18_LMNA	-0.00295	-0.162076563	0.156322136	9.71E-01	9.89E-01	1.00E+00
ENSG00000143179.11_UCK2	ENSG00000160789.18_LMNA	0.22186	0.064956249	0.36806311	6.01E-03	4.51E-02	1.00E+00
ENSG00000170175.9_CHRNB1	ENSG00000160789.18_LMNA	0.0205	-0.139153837	0.179117136	8.02E-01	9.21E-01	1.00E+00
ENSG00000128285.4_MCHR1	ENSG00000160789.18_LMNA	0.17075	0.011867798	0.321215055	3.54E-02	1.60E-01	1.00E+00
ENSG00000262089.1_RP11-589P	ENSG00000160789.18_LMNA	-0.04254	-0.20038654	0.117453166	6.03E-01	8.18E-01	1.00E+00
ENSG00000117122.12_MFAP2	ENSG00000160789.18_LMNA	0.1669	0.007909646	0.317660448	3.99E-02	1.73E-01	1.00E+00
ENSG00000254612.2_RP11-676M	ENSG00000160789.18_LMNA	-0.02024	-0.178869067	0.139405145	8.04E-01	9.22E-01	1.00E+00
ENSG00000185291.9_IL3RA	ENSG00000160789.18_LMNA	0.06847	-0.091727475	0.225218745	4.02E-01	6.78E-01	1.00E+00
ENSG00000100425.17_BRD1	ENSG00000160789.18_LMNA	0.06223	-0.097938978	0.219260447	4.46E-01	7.13E-01	1.00E+00
ENSG00000175575.11_PAAF1	ENSG00000160789.18_LMNA	-0.30483	-0.442550636	-0.15305302	1.34E-04	2.39E-03	1.00E+00
ENSG00000133055.7_MYBPH	ENSG00000160789.18_LMNA	0.41971	0.279163482	0.542652553	7.35E-08	5.62E-06	3.77E-03
ENSG00000167378.7_IRGQ	ENSG00000160789.18_LMNA	0.10598	-0.054135564	0.260779376	1.94E-01	4.62E-01	1.00E+00

ENSG00000173575.17_CHD2	ENSG00000160789.18_LMNA	0.15982	0.000634758	0.311104473	4.92E-02	1.99E-01	1.00E+00
ENSG00000243477.4_NAT6	ENSG00000160789.18_LMNA	-0.13607	-0.289014253	0.023638269	9.46E-02	3.01E-01	1.00E+00
ENSG00000020577.12_SAMD4A	ENSG00000160789.18_LMNA	0.27235	0.118277923	0.413617227	6.88E-04	8.65E-03	1.00E+00
ENSG00000068097.13_HEATR6	ENSG00000160789.18_LMNA	0.01479	-0.144753477	0.173580062	8.57E-01	9.43E-01	1.00E+00
ENSG00000170961.6_HAS2	ENSG00000160789.18_LMNA	0.15626	-0.003013642	0.307805448	5.45E-02	2.13E-01	1.00E+00
ENSG00000143036.15_SLC44A3	ENSG00000160789.18_LMNA	-0.04865	-0.206249282	0.111417703	5.52E-01	7.87E-01	1.00E+00
ENSG00000123009.4_NME2P1	ENSG00000160789.18_LMNA	-0.05143	-0.208916219	0.108664419	5.29E-01	7.73E-01	1.00E+00
ENSG00000077238.12_IL4R	ENSG00000160789.18_LMNA	0.21755	0.060446729	0.364142597	7.09E-03	5.09E-02	1.00E+00
ENSG00000138081.18_FBXO11	ENSG00000160789.18_LMNA	-0.0687	-0.225431174	0.091505561	4.00E-01	6.77E-01	1.00E+00
ENSG00000230798.4_FOXD3-AS1	ENSG00000160789.18_LMNA	-0.06695	-0.223764461	0.093245839	4.12E-01	6.86E-01	1.00E+00
ENSG00000271605.4_MILR1	ENSG00000160789.18_LMNA	-0.07372	-0.230219638	0.086494953	3.67E-01	6.48E-01	1.00E+00
ENSG00000204859.10_ZBTB48	ENSG00000160789.18_LMNA	0.09172	-0.068484469	0.247305912	2.61E-01	5.44E-01	1.00E+00
ENSG00000006757.10_PNPLA4	ENSG00000160789.18_LMNA	0.11953	-0.040435454	0.273530155	1.42E-01	3.85E-01	1.00E+00
ENSG00000179292.4_TMEM151A	ENSG00000160789.18_LMNA	-0.30641	-0.443954401	-0.15475864	1.23E-04	2.24E-03	1.00E+00
ENSG00000108587.13_GOSR1	ENSG00000160789.18_LMNA	0.03218	-0.1276776	0.190402488	6.94E-01	8.68E-01	1.00E+00
ENSG00000167840.12_ZNF232	ENSG00000160789.18_LMNA	-0.12688	-0.280419304	0.032983938	1.19E-01	3.47E-01	1.00E+00
ENSG00000137700.15_SLC37A4	ENSG00000160789.18_LMNA	0.08599	-0.074229051	0.241877362	2.92E-01	5.78E-01	1.00E+00
ENSG00000085978.20_ATG16L1	ENSG00000160789.18_LMNA	0.22665	0.069978459	0.372416701	4.99E-03	3.91E-02	1.00E+00
ENSG00000172071.10_EIF2AK3	ENSG00000160789.18_LMNA	0.13247	-0.027303645	0.285649307	1.04E-01	3.19E-01	1.00E+00
ENSG00000265491.4_RNF115	ENSG00000160789.18_LMNA	-0.18565	-0.334954527	-0.02725878	2.20E-02	1.16E-01	1.00E+00
ENSG00000161265.13_U2AF1L4	ENSG00000160789.18_LMNA	-0.0719	-0.22848419	0.088312764	3.79E-01	6.58E-01	1.00E+00
ENSG00000154803.11_FLCN	ENSG00000160789.18_LMNA	0.01357	-0.145943933	0.172400307	8.68E-01	9.48E-01	1.00E+00
ENSG00000166529.13_ZSCAN21	ENSG00000160789.18_LMNA	0.0216	-0.138077562	0.18017908	7.92E-01	9.16E-01	1.00E+00
ENSG00000139180.9_NDUFA9	ENSG00000160789.18_LMNA	-0.24237	-0.386655206	-0.08651147	2.63E-03	2.41E-02	1.00E+00
ENSG00000140464.18_PML	ENSG00000160789.18_LMNA	0.46681	0.332302819	0.582704392	1.34E-09	1.98E-07	6.90E-05
ENSG00000154781.14_CCDC174	ENSG00000160789.18_LMNA	-0.00453	-0.163611847	0.154783191	9.56E-01	9.82E-01	1.00E+00
ENSG00000118482.10_PHF3	ENSG00000160789.18_LMNA	0.13907	-0.020584298	0.29181202	8.75E-02	2.88E-01	1.00E+00
ENSG00000119953.11_SMNDC1	ENSG00000160789.18_LMNA	-0.13813	-0.290929837	0.021547896	8.97E-02	2.92E-01	1.00E+00
ENSG00000109189.11_USP46	ENSG00000160789.18_LMNA	-0.30658	-0.44410337	-0.15493974	1.22E-04	2.22E-03	1.00E+00
ENSG00000164124.9_TMEM144	ENSG00000160789.18_LMNA	-0.39812	-0.524102121	-0.25508639	3.79E-07	2.11E-05	1.94E-02
ENSG00000198042.9_MAK16	ENSG00000160789.18_LMNA	0.02625	-0.133507508	0.184680036	7.48E-01	8.95E-01	1.00E+00
ENSG00000117625.12_RCOR3	ENSG00000160789.18_LMNA	-0.17341	-0.323673294	-0.0146108	3.26E-02	1.51E-01	1.00E+00
ENSG00000121858.9_TNFSF10	ENSG00000160789.18_LMNA	0.01514	-0.144407982	0.17392228	8.53E-01	9.42E-01	1.00E+00

ENSG00000005175.8_RPAP3	ENSG00000160789.18_LMNA	0.0015	-0.157738583	0.160662123	9.85E-01	9.94E-01	1.00E+00
ENSG00000023909.8_GCLM	ENSG00000160789.18_LMNA	0.27742	0.123686397	0.418156678	5.40E-04	7.15E-03	1.00E+00
ENSG00000180914.9_OXTR	ENSG00000160789.18_LMNA	0.26182	0.107087371	0.404177895	1.12E-03	1.26E-02	1.00E+00
ENSG00000151327.11_FAM177A1	ENSG00000160789.18_LMNA	-0.06151	-0.218572599	0.098654469	4.52E-01	7.17E-01	1.00E+00
ENSG00000148481.12_FAM188A	ENSG00000160789.18_LMNA	-0.47828	-0.592378192	-0.34538053	4.61E-10	8.24E-08	2.37E-05
ENSG00000272760.1_RP11-5C23	ENSG00000160789.18_LMNA	0.19578	0.037759452	0.344253946	1.56E-02	9.03E-02	1.00E+00
ENSG00000068654.14_POLR1A	ENSG00000160789.18_LMNA	0.11498	-0.045043551	0.26925365	1.58E-01	4.11E-01	1.00E+00
ENSG00000114670.12_NEK11	ENSG00000160789.18_LMNA	0.07962	-0.080597821	0.235835743	3.29E-01	6.15E-01	1.00E+00
ENSG00000128059.7_PPAT	ENSG00000160789.18_LMNA	0.02767	-0.132110373	0.186053388	7.35E-01	8.89E-01	1.00E+00
ENSG00000126790.10_L3HYPDH	ENSG00000160789.18_LMNA	0.09481	-0.065375812	0.250235307	2.45E-01	5.25E-01	1.00E+00
ENSG00000156103.14_MMP16	ENSG00000160789.18_LMNA	-0.20241	-0.350318681	-0.04464426	1.24E-02	7.65E-02	1.00E+00
ENSG00000103343.11_ZNF174	ENSG00000160789.18_LMNA	-0.23671	-0.381534402	-0.08054636	3.32E-03	2.89E-02	1.00E+00
ENSG00000163755.7_HPS3	ENSG00000160789.18_LMNA	-0.06413	-0.221071849	0.09605321	4.33E-01	7.02E-01	1.00E+00
ENSG00000131931.7_THAP1	ENSG00000160789.18_LMNA	-0.1519	-0.30375715	0.007479389	6.17E-02	2.30E-01	1.00E+00
ENSG00000176049.14_JAKMIP2	ENSG00000160789.18_LMNA	-0.06093	-0.218012122	0.099237229	4.56E-01	7.20E-01	1.00E+00
ENSG00000124370.9_MCEE	ENSG00000160789.18_LMNA	-0.27872	-0.419313975	-0.12506805	5.07E-04	6.82E-03	1.00E+00
ENSG00000102096.9_PIM2	ENSG00000160789.18_LMNA	0.19607	0.038056258	0.344515927	1.55E-02	8.98E-02	1.00E+00
ENSG00000089199.8_CHGB	ENSG00000160789.18_LMNA	-0.20069	-0.348748366	-0.04285882	1.32E-02	7.99E-02	1.00E+00
ENSG00000143850.11_PLEKHA6	ENSG00000160789.18_LMNA	-0.17525	-0.325372359	-0.0165094	3.08E-02	1.46E-01	1.00E+00
ENSG00000147548.15_WHSC1L1	ENSG00000160789.18_LMNA	0.01592	-0.143643061	0.174679673	8.46E-01	9.40E-01	1.00E+00
ENSG00000164308.15_ERAP2	ENSG00000160789.18_LMNA	0.16902	0.010091413	0.319620855	3.74E-02	1.65E-01	1.00E+00
ENSG00000179409.9_GEMIN4	ENSG00000160789.18_LMNA	0.08405	-0.076165979	0.24004252	3.03E-01	5.90E-01	1.00E+00
ENSG00000103126.13_AXIN1	ENSG00000160789.18_LMNA	0.29509	0.142589572	0.433907827	2.24E-04	3.57E-03	1.00E+00
ENSG00000164283.11_ESM1	ENSG00000160789.18_LMNA	0.23502	0.078765222	0.380001778	3.56E-03	3.04E-02	1.00E+00
ENSG00000276291.3_FRG1HP	ENSG00000160789.18_LMNA	-0.2332	-0.378355203	-0.0768538	3.84E-03	3.21E-02	1.00E+00
ENSG00000065135.8_GNAI3	ENSG00000160789.18_LMNA	0.10564	-0.054480359	0.260457049	1.95E-01	4.64E-01	1.00E+00
ENSG00000149503.11_INCENP	ENSG00000160789.18_LMNA	0.0849	-0.075320447	0.240843766	2.98E-01	5.84E-01	1.00E+00
ENSG00000214114.7_MYCBP	ENSG00000160789.18_LMNA	0.16339	0.004297401	0.314408867	4.43E-02	1.86E-01	1.00E+00
ENSG00000139218.16_SCAF11	ENSG00000160789.18_LMNA	0.04558	-0.114455155	0.203301603	5.77E-01	8.03E-01	1.00E+00
ENSG00000268087.1_CTC-429P9	ENSG00000160789.18_LMNA	-0.15603	-0.307587503	0.003254378	5.49E-02	2.14E-01	1.00E+00
ENSG00000143748.16_NVL	ENSG00000160789.18_LMNA	-0.04495	-0.202700334	0.115074007	5.82E-01	8.06E-01	1.00E+00
ENSG00000133111.3_RFXAP	ENSG00000160789.18_LMNA	-0.30462	-0.44236862	-0.15283199	1.36E-04	2.40E-03	1.00E+00
ENSG00000187522.12_HSPA14	ENSG00000160789.18_LMNA	-0.33486	-0.469058534	-0.18555491	2.48E-05	6.14E-04	1.00E+00

ENSG00000115947.12_ORC4	ENSG00000160789.18_LMNA	-0.53713	-0.641475258	-0.41326596	9.75E-13	5.53E-10	5.00E-08
ENSG00000125538.10_IL1B	ENSG00000160789.18_LMNA	0.04922	-0.110847545	0.206801947	5.47E-01	7.84E-01	1.00E+00
ENSG00000180694.12_TMEM64	ENSG00000160789.18_LMNA	0.18942	0.031157637	0.338414375	1.94E-02	1.06E-01	1.00E+00
ENSG00000117724.11_CENPF	ENSG00000160789.18_LMNA	0.15076	-0.008644897	0.302698738	6.37E-02	2.35E-01	1.00E+00
ENSG00000114098.16_ARMC8	ENSG00000160789.18_LMNA	-0.22869	-0.374268486	-0.0721193	4.60E-03	3.68E-02	1.00E+00
ENSG00000174013.7_FBXO45	ENSG00000160789.18_LMNA	-0.10326	-0.258215177	0.05687643	2.06E-01	4.77E-01	1.00E+00
ENSG00000166788.8_SAAL1	ENSG00000160789.18_LMNA	-0.03484	-0.192969317	0.125055411	6.70E-01	8.55E-01	1.00E+00
ENSG00000128039.9_SRD5A3	ENSG00000160789.18_LMNA	0.09474	-0.065452568	0.250163046	2.46E-01	5.25E-01	1.00E+00
ENSG00000158006.12_PAFAH2	ENSG00000160789.18_LMNA	0.03439	-0.125502119	0.192532347	6.74E-01	8.57E-01	1.00E+00
ENSG00000122376.10_FAM35A	ENSG00000160789.18_LMNA	-0.14417	-0.296567926	0.01537946	7.64E-02	2.64E-01	1.00E+00
ENSG00000089169.13_RPH3A	ENSG00000160789.18_LMNA	-0.14821	-0.300328359	0.01125206	6.84E-02	2.46E-01	1.00E+00
ENSG00000228106.4_RP11-452F	ENSG00000160789.18_LMNA	-0.26787	-0.4096015	-0.113508	8.49E-04	1.02E-02	1.00E+00
ENSG00000101844.16_ATG4A	ENSG00000160789.18_LMNA	-0.10781	-0.26250364	0.052289831	1.86E-01	4.52E-01	1.00E+00
ENSG00000088451.9_TGDS	ENSG00000160789.18_LMNA	-0.27536	-0.41631432	-0.12148923	5.96E-04	7.75E-03	1.00E+00
ENSG00000120693.12_SMAD9	ENSG00000160789.18_LMNA	-0.43171	-0.552909814	-0.29262043	2.81E-08	2.57E-06	1.44E-03
ENSG00000100055.19_CYTH4	ENSG00000160789.18_LMNA	0.1053	-0.054819978	0.260139493	1.97E-01	4.66E-01	1.00E+00
ENSG00000144199.10_FAHD2B	ENSG00000160789.18_LMNA	-0.0236	-0.182115363	0.136113213	7.73E-01	9.06E-01	1.00E+00
ENSG00000174106.2_LEMD3	ENSG00000160789.18_LMNA	-0.06322	-0.220204307	0.096956652	4.39E-01	7.07E-01	1.00E+00
ENSG00000162585.15_FAAP20	ENSG00000160789.18_LMNA	0.17113	0.012259973	0.321566772	3.50E-02	1.59E-01	1.00E+00
ENSG00000171984.13_C20orf196	ENSG00000160789.18_LMNA	-0.38899	-0.516226427	-0.24496421	7.32E-07	3.58E-05	3.75E-02
ENSG00000072864.11_NDE1	ENSG00000160789.18_LMNA	0.10324	-0.05689566	0.258197171	2.06E-01	4.77E-01	1.00E+00
ENSG00000158406.3_HIST1H4H	ENSG00000160789.18_LMNA	-0.04793	-0.205561746	0.112126709	5.58E-01	7.91E-01	1.00E+00
ENSG00000187699.9_C2orf88	ENSG00000160789.18_LMNA	-0.42611	-0.548126069	-0.28633157	4.42E-08	3.67E-06	2.27E-03
ENSG00000180190.10_TDRP	ENSG00000160789.18_LMNA	-0.17503	-0.325168424	-0.0162814	3.10E-02	1.47E-01	1.00E+00
ENSG00000111752.9_PHC1	ENSG00000160789.18_LMNA	-0.13994	-0.292625016	0.019695758	8.55E-02	2.84E-01	1.00E+00
ENSG00000162522.9_KIAA1522	ENSG00000160789.18_LMNA	0.35526	0.207807758	0.486917631	7.09E-06	2.22E-04	3.64E-01
ENSG00000047644.16_WWC3	ENSG00000160789.18_LMNA	0.30478	0.153000145	0.442507098	1.35E-04	2.39E-03	1.00E+00
ENSG00000135540.10_NHSL1	ENSG00000160789.18_LMNA	0.11191	-0.048153484	0.266360482	1.70E-01	4.29E-01	1.00E+00
ENSG00000132781.16_MUTYH	ENSG00000160789.18_LMNA	0.18888	0.030595788	0.337916302	1.98E-02	1.07E-01	1.00E+00
ENSG00000101216.9_GMEB2	ENSG00000160789.18_LMNA	0.30268	0.150746891	0.440650384	1.51E-04	2.60E-03	1.00E+00
ENSG00000137441.7_FGFBP2	ENSG00000160789.18_LMNA	-0.25658	-0.399459965	-0.10152222	1.42E-03	1.51E-02	1.00E+00
ENSG00000112309.9_B3GAT2	ENSG00000160789.18_LMNA	-0.39456	-0.521034786	-0.2511371	4.91E-07	2.58E-05	2.52E-02
ENSG00000136861.16_CDK5RAP	ENSG00000160789.18_LMNA	0.18783	0.029511215	0.336954352	2.05E-02	1.10E-01	1.00E+00

ENSG00000152214.11_RIT2	ENSG00000160789.18_LMNA	-0.31596	-0.452400383	-0.16505732	7.33E-05	1.47E-03	1.00E+00
ENSG00000164951.14_PDP1	ENSG00000160789.18_LMNA	0.00133	-0.157904132	0.160496723	9.87E-01	9.95E-01	1.00E+00
ENSG00000065457.9_ADAT1	ENSG00000160789.18_LMNA	-0.02666	-0.185072991	0.133107877	7.44E-01	8.93E-01	1.00E+00
ENSG00000111731.11_C2CD5	ENSG00000160789.18_LMNA	-0.20974	-0.35701607	-0.05228116	9.50E-03	6.32E-02	1.00E+00
ENSG00000144674.15_GOLGA4	ENSG00000160789.18_LMNA	0.13887	-0.020786346	0.291627087	8.80E-02	2.89E-01	1.00E+00
ENSG00000138696.9_BMPR1B	ENSG00000160789.18_LMNA	-0.06712	-0.223924151	0.093079184	4.11E-01	6.85E-01	1.00E+00
ENSG00000108219.13_TSPAN14	ENSG00000160789.18_LMNA	-0.08682	-0.242666096	0.0733957	2.88E-01	5.73E-01	1.00E+00
ENSG00000147905.16_ZCCHC7	ENSG00000160789.18_LMNA	-0.14284	-0.295322102	0.01674452	7.92E-02	2.70E-01	1.00E+00
ENSG00000106868.15_SUSD1	ENSG00000160789.18_LMNA	-0.11256	-0.266977754	0.047490475	1.67E-01	4.25E-01	1.00E+00
ENSG00000083168.8_KAT6A	ENSG00000160789.18_LMNA	0.05546	-0.104666828	0.21278005	4.97E-01	7.51E-01	1.00E+00
ENSG00000123352.16_SPATS2	ENSG00000160789.18_LMNA	-0.13822	-0.291020374	0.021449031	8.95E-02	2.91E-01	1.00E+00
ENSG00000092208.15_GEMIN2	ENSG00000160789.18_LMNA	-0.19219	-0.340956713	-0.03402854	1.77E-02	9.88E-02	1.00E+00
ENSG00000227640.2_SOX21-AS1	ENSG00000160789.18_LMNA	-0.2427	-0.386950415	-0.08685601	2.59E-03	2.38E-02	1.00E+00
ENSG00000092470.10_WDR76	ENSG00000160789.18_LMNA	0.16214	0.003017823	0.3132553	4.60E-02	1.90E-01	1.00E+00
ENSG00000196792.10_STRN3	ENSG00000160789.18_LMNA	0.02813	-0.131658188	0.186497609	7.31E-01	8.86E-01	1.00E+00
ENSG00000166946.12_CCNDBP1	ENSG00000160789.18_LMNA	-0.15589	-0.307455753	0.003399886	5.51E-02	2.14E-01	1.00E+00
ENSG00000162702.7_ZNF281	ENSG00000160789.18_LMNA	-0.17444	-0.324629315	-0.01567882	3.16E-02	1.48E-01	1.00E+00
ENSG00000114904.11_NEK4	ENSG00000160789.18_LMNA	0.05653	-0.103600963	0.213808577	4.89E-01	7.45E-01	1.00E+00
ENSG00000040341.16_STAU2	ENSG00000160789.18_LMNA	-0.41848	-0.541600907	-0.27778961	8.09E-08	6.02E-06	4.15E-03
ENSG00000180530.8_NRIP1	ENSG00000160789.18_LMNA	0.17317	0.014362126	0.32345061	3.29E-02	1.52E-01	1.00E+00
ENSG00000144724.17_PTPRG	ENSG00000160789.18_LMNA	-0.12016	-0.27411715	0.039801899	1.40E-01	3.82E-01	1.00E+00
ENSG00000144959.8_NCEH1	ENSG00000160789.18_LMNA	0.19721	0.039238483	0.345558965	1.49E-02	8.74E-02	1.00E+00
ENSG00000130347.11_RTN4IP1	ENSG00000160789.18_LMNA	-0.0513	-0.208794967	0.108789702	5.30E-01	7.73E-01	1.00E+00
ENSG00000167716.17_WDR81	ENSG00000160789.18_LMNA	0.29643	0.144024009	0.435095852	2.09E-04	3.37E-03	1.00E+00
ENSG00000001461.15_NIPAL3	ENSG00000160789.18_LMNA	0.12288	-0.037043168	0.276670424	1.32E-01	3.68E-01	1.00E+00
ENSG00000179833.4_SERTAD2	ENSG00000160789.18_LMNA	0.1741	0.015321155	0.324309228	3.19E-02	1.49E-01	1.00E+00
ENSG00000081041.8_CXCL2	ENSG00000160789.18_LMNA	0.12367	-0.036247857	0.277405682	1.29E-01	3.64E-01	1.00E+00
ENSG00000072401.13_UBE2D1	ENSG00000160789.18_LMNA	-0.24218	-0.386480585	-0.0863077	2.65E-03	2.42E-02	1.00E+00
ENSG00000188573.7_FBLL1	ENSG00000160789.18_LMNA	-0.34561	-0.478488176	-0.19726834	1.29E-05	3.61E-04	6.64E-01
ENSG00000242125.3_SNHG3	ENSG00000160789.18_LMNA	-0.10281	-0.257795916	0.057324126	2.08E-01	4.80E-01	1.00E+00
ENSG00000157107.12_FCHO2	ENSG00000160789.18_LMNA	-0.05384	-0.211231318	0.106270408	5.10E-01	7.59E-01	1.00E+00
ENSG00000136147.15_PHF11	ENSG00000160789.18_LMNA	0.06991	-0.090293102	0.226591285	3.92E-01	6.70E-01	1.00E+00
ENSG00000143515.15_ATP8B2	ENSG00000160789.18_LMNA	0.34264	0.194019811	0.475879502	1.55E-05	4.20E-04	7.97E-01

ENSG00000162654.8_GBP4	ENSG00000160789.18_LMNA	0.0857	-0.074519091	0.241602752	2.94E-01	5.80E-01	1.00E+00
ENSG00000162714.11_ZNF496	ENSG00000160789.18_LMNA	0.14066	-0.018963978	0.293294241	8.39E-02	2.80E-01	1.00E+00
ENSG00000161682.13_FAM171A2	ENSG00000160789.18_LMNA	-0.14003	-0.292705529	0.019607737	8.53E-02	2.83E-01	1.00E+00
ENSG00000280670.1_CCDC163	ENSG00000160789.18_LMNA	-0.27423	-0.415300272	-0.12028112	6.29E-04	8.08E-03	1.00E+00
ENSG00000088854.12_C20orf194	ENSG00000160789.18_LMNA	-0.06182	-0.218870254	0.098344893	4.49E-01	7.15E-01	1.00E+00
ENSG00000113522.12_RAD50	ENSG00000160789.18_LMNA	0.10856	-0.051534348	0.263208824	1.83E-01	4.48E-01	1.00E+00
ENSG00000269242.1_CTD-2192J	ENSG00000160789.18_LMNA	-0.13171	-0.284936922	0.028078555	1.06E-01	3.23E-01	1.00E+00
ENSG00000160908.14_ZNF394	ENSG00000160789.18_LMNA	0.28431	0.131038162	0.424303681	3.86E-04	5.50E-03	1.00E+00
ENSG00000101311.14_FERMT1	ENSG00000160789.18_LMNA	-0.42319	-0.545633359	-0.28306349	5.58E-08	4.46E-06	2.86E-03
ENSG00000163602.9_RYBP	ENSG00000160789.18_LMNA	-0.02949	-0.18780489	0.130326702	7.18E-01	8.80E-01	1.00E+00
ENSG00000158292.6_GPR153	ENSG00000160789.18_LMNA	-0.00614	-0.165177906	0.153211804	9.40E-01	9.76E-01	1.00E+00
ENSG00000127580.14_WDR24	ENSG00000160789.18_LMNA	0.19068	0.032464122	0.339571895	1.86E-02	1.02E-01	1.00E+00
ENSG00000125676.18_THOC2	ENSG00000160789.18_LMNA	0.16588	0.006858081	0.316714628	4.11E-02	1.77E-01	1.00E+00
ENSG00000100522.7_GNPNAT1	ENSG00000160789.18_LMNA	0.23472	0.078450835	0.379731084	3.61E-03	3.07E-02	1.00E+00
ENSG00000136999.4_NOV	ENSG00000160789.18_LMNA	0.1274	-0.032457301	0.280904977	1.18E-01	3.44E-01	1.00E+00
ENSG00000108064.9_TFAM	ENSG00000160789.18_LMNA	-0.30408	-0.441886415	-0.15224657	1.40E-04	2.46E-03	1.00E+00
ENSG00000231663.1_RP5-827C2	ENSG00000160789.18_LMNA	-0.16316	-0.314198176	-0.00406362	4.46E-02	1.87E-01	1.00E+00
ENSG00000138764.12_CCNG2	ENSG00000160789.18_LMNA	-0.32134	-0.457154036	-0.17088141	5.42E-05	1.15E-03	1.00E+00
ENSG00000250571.5_GLI4	ENSG00000160789.18_LMNA	-0.01257	-0.171427985	0.146924382	8.78E-01	9.53E-01	1.00E+00
ENSG00000151012.12_SLC7A11	ENSG00000160789.18_LMNA	0.10855	-0.051546125	0.263197834	1.83E-01	4.48E-01	1.00E+00
ENSG00000068489.11_PRR11	ENSG00000160789.18_LMNA	0.05746	-0.102675841	0.214700722	4.82E-01	7.40E-01	1.00E+00
ENSG00000133624.12_ZNF767P	ENSG00000160789.18_LMNA	-0.02944	-0.187763318	0.130369062	7.19E-01	8.81E-01	1.00E+00
ENSG00000120324.6_PCDHB10	ENSG00000160789.18_LMNA	-0.01457	-0.173366765	0.144968777	8.59E-01	9.44E-01	1.00E+00
ENSG00000279672.1_CMB9-55F2	ENSG00000160789.18_LMNA	0.12897	-0.030865366	0.28237211	1.13E-01	3.36E-01	1.00E+00
ENSG00000173875.12_ZNF791	ENSG00000160789.18_LMNA	-0.12296	-0.276743448	0.036964197	1.31E-01	3.67E-01	1.00E+00
ENSG00000198932.11_GPRASP1	ENSG00000160789.18_LMNA	0.10418	-0.055945079	0.259086994	2.01E-01	4.72E-01	1.00E+00
ENSG00000156273.14_BACH1	ENSG00000160789.18_LMNA	0.26825	0.113914533	0.409944204	8.34E-04	1.00E-02	1.00E+00
ENSG00000066379.13_ZNRD1	ENSG00000160789.18_LMNA	-0.13187	-0.285086022	0.027916399	1.05E-01	3.22E-01	1.00E+00
ENSG00000152147.9_GEMIN6	ENSG00000160789.18_LMNA	-0.20845	-0.355839267	-0.05093669	9.96E-03	6.54E-02	1.00E+00
ENSG00000164403.13_SHROOM1	ENSG00000160789.18_LMNA	0.2972	0.144856748	0.435785076	2.01E-04	3.27E-03	1.00E+00
ENSG00000187535.12_IFT140	ENSG00000160789.18_LMNA	0.09106	-0.069142978	0.246684635	2.65E-01	5.48E-01	1.00E+00
ENSG00000062650.16_WAPL	ENSG00000160789.18_LMNA	0.02775	-0.132030928	0.186131442	7.34E-01	8.88E-01	1.00E+00
ENSG00000204536.12_CCHCR1	ENSG00000160789.18_LMNA	0.31436	0.163330084	0.450987461	8.01E-05	1.57E-03	1.00E+00

ENSG00000118260.13_CREB1	ENSG00000160789.18_LMNA	-0.11647	-0.270654894	0.043535129	1.53E-01	4.03E-01	1.00E+00
ENSG00000198160.13_MIER1	ENSG00000160789.18_LMNA	-0.07785	-0.23414602	0.08237443	3.40E-01	6.26E-01	1.00E+00
ENSG00000163026.10_WDCP	ENSG00000160789.18_LMNA	-0.13685	-0.289738902	0.02284782	9.27E-02	2.98E-01	1.00E+00
ENSG00000180008.8_SOCS4	ENSG00000160789.18_LMNA	-0.01834	-0.177022232	0.141274808	8.23E-01	9.31E-01	1.00E+00
ENSG00000126003.6_PLAGL2	ENSG00000160789.18_LMNA	0.23001	0.073503211	0.375464266	4.36E-03	3.54E-02	1.00E+00
ENSG00000164944.10_KIAA1429	ENSG00000160789.18_LMNA	0.04245	-0.117549604	0.200292677	6.04E-01	8.18E-01	1.00E+00
ENSG00000157613.9_CREB3L1	ENSG00000160789.18_LMNA	0.26398	0.109378681	0.406115802	1.02E-03	1.17E-02	1.00E+00
ENSG00000006634.6_DBF4	ENSG00000160789.18_LMNA	0.00165	-0.157587281	0.160813273	9.84E-01	9.94E-01	1.00E+00
ENSG00000093009.8_CDC45	ENSG00000160789.18_LMNA	0.02475	-0.13498174	0.18322955	7.62E-01	9.01E-01	1.00E+00
ENSG00000115109.12_EPB41L5	ENSG00000160789.18_LMNA	-0.15923	-0.310558131	-3.00E-05	5.01E-02	2.01E-01	1.00E+00
ENSG00000214293.7_APTR	ENSG00000160789.18_LMNA	-0.06021	-0.217326138	0.099950188	4.61E-01	7.25E-01	1.00E+00
ENSG00000170836.10_PPM1D	ENSG00000160789.18_LMNA	-0.20633	-0.353906247	-0.04873065	1.08E-02	6.91E-02	1.00E+00
ENSG00000221923.7_ZNF880	ENSG00000160789.18_LMNA	0.23566	0.079438659	0.380581449	3.47E-03	2.98E-02	1.00E+00
ENSG00000196730.11_DAPK1	ENSG00000160789.18_LMNA	-0.2371	-0.381889514	-0.08095933	3.27E-03	2.85E-02	1.00E+00
ENSG00000127481.13_UBR4	ENSG00000160789.18_LMNA	0.14142	-0.018185774	0.294005585	8.22E-02	2.77E-01	1.00E+00
ENSG00000055483.18_USP36	ENSG00000160789.18_LMNA	0.27933	0.125715274	0.419855774	4.92E-04	6.66E-03	1.00E+00
ENSG00000144589.19_STK11IP	ENSG00000160789.18_LMNA	0.12265	-0.037282534	0.27644906	1.32E-01	3.69E-01	1.00E+00
ENSG00000106080.9_FKBP14	ENSG00000160789.18_LMNA	0.19717	0.039201787	0.345526601	1.49E-02	8.75E-02	1.00E+00
ENSG00000197429.9_IPP	ENSG00000160789.18_LMNA	0.01529	-0.144259558	0.174069273	8.52E-01	9.42E-01	1.00E+00
ENSG00000163939.17_PBRM1	ENSG00000160789.18_LMNA	-0.16189	-0.313022372	-0.00275957	4.63E-02	1.91E-01	1.00E+00
ENSG00000182325.9_FBXL6	ENSG00000160789.18_LMNA	0.16893	0.009997796	0.319536791	3.75E-02	1.66E-01	1.00E+00
ENSG00000121931.14_LRIF1	ENSG00000160789.18_LMNA	-0.1019	-0.256934054	0.058244042	2.12E-01	4.85E-01	1.00E+00
ENSG00000143228.11_NUF2	ENSG00000160789.18_LMNA	-0.03898	-0.196961053	0.120968737	6.33E-01	8.36E-01	1.00E+00
ENSG00000174606.11_ANGEL2	ENSG00000160789.18_LMNA	-0.23253	-0.377749367	-0.07615107	3.94E-03	3.28E-02	1.00E+00
ENSG00000197860.8_SGTB	ENSG00000160789.18_LMNA	-0.12782	-0.281301265	0.03202746	1.17E-01	3.42E-01	1.00E+00
ENSG00000148840.9_PPRC1	ENSG00000160789.18_LMNA	0.22186	0.064955612	0.368062557	6.01E-03	4.51E-02	1.00E+00
ENSG00000123178.13_SPRYD7	ENSG00000160789.18_LMNA	-0.23959	-0.384138788	-0.08357746	2.95E-03	2.64E-02	1.00E+00
ENSG00000112029.8_FBXO5	ENSG00000160789.18_LMNA	-0.0136	-0.172431344	0.145912626	8.68E-01	9.48E-01	1.00E+00
ENSG00000271503.4_CCL5	ENSG00000160789.18_LMNA	0.13457	-0.025169648	0.287609331	9.83E-02	3.08E-01	1.00E+00
ENSG00000101447.12_FAM83D	ENSG00000160789.18_LMNA	0.06752	-0.092674057	0.224312277	4.08E-01	6.83E-01	1.00E+00
ENSG00000131725.12_WDR44	ENSG00000160789.18_LMNA	0.23301	0.076654931	0.378183782	3.87E-03	3.23E-02	1.00E+00
ENSG00000138795.8_LEF1	ENSG00000160789.18_LMNA	0.37837	0.233219926	0.507030768	1.54E-06	6.55E-05	7.89E-02
ENSG00000152128.13_TMEM163	ENSG00000160789.18_LMNA	-0.22244	-0.36859158	-0.06556506	5.88E-03	4.44E-02	1.00E+00

ENSG00000175229.6_GAL3ST3	ENSG00000160789.18_LMNA	-0.19449	-0.343066594	-0.03641494	1.64E-02	9.34E-02	1.00E+00
ENSG00000037637.9_FBXO42	ENSG00000160789.18_LMNA	0.04334	-0.116662197	0.201156172	5.96E-01	8.14E-01	1.00E+00
ENSG00000173085.12_COQ2	ENSG00000160789.18_LMNA	0.08706	-0.073152294	0.242896392	2.86E-01	5.72E-01	1.00E+00
ENSG00000169683.6_LRRC45	ENSG00000160789.18_LMNA	0.2973	0.144955727	0.435866974	2.00E-04	3.26E-03	1.00E+00
ENSG00000102309.11_PIN4	ENSG00000160789.18_LMNA	-0.06477	-0.221687932	0.095411314	4.28E-01	6.98E-01	1.00E+00
ENSG00000177051.5_FBXO46	ENSG00000160789.18_LMNA	0.24758	0.092010719	0.391359753	2.10E-03	2.03E-02	1.00E+00
ENSG00000133619.16_KRBA1	ENSG00000160789.18_LMNA	0.27799	0.124294859	0.418666455	5.25E-04	7.01E-03	1.00E+00
ENSG00000074603.17_DPP8	ENSG00000160789.18_LMNA	-0.0307	-0.188973654	0.129135326	7.07E-01	8.76E-01	1.00E+00
ENSG00000071073.11_MGAT4A	ENSG00000160789.18_LMNA	-0.19304	-0.341739551	-0.03491357	1.72E-02	9.68E-02	1.00E+00
ENSG00000197989.12_SNHG12	ENSG00000160789.18_LMNA	-0.05311	-0.210528653	0.106997413	5.16E-01	7.63E-01	1.00E+00
ENSG00000257270.1_RP11-521B	ENSG00000160789.18_LMNA	0.03512	-0.124782353	0.19323636	6.68E-01	8.54E-01	1.00E+00
ENSG00000135912.9_TTLL4	ENSG00000160789.18_LMNA	0.23005	0.073539681	0.375495764	4.36E-03	3.54E-02	1.00E+00
ENSG00000165806.18_CASP7	ENSG00000160789.18_LMNA	0.4187	0.278035429	0.541789134	7.95E-08	5.96E-06	4.08E-03
ENSG00000135119.13_RNFT2	ENSG00000160789.18_LMNA	-0.24823	-0.391939568	-0.09268974	2.05E-03	1.99E-02	1.00E+00
ENSG00000152894.13_PTPRK	ENSG00000160789.18_LMNA	-0.06719	-0.223996034	0.093004161	4.11E-01	6.85E-01	1.00E+00
ENSG00000181220.14_ZNF746	ENSG00000160789.18_LMNA	0.23327	0.076920919	0.378413056	3.83E-03	3.21E-02	1.00E+00
ENSG00000182919.13_C11orf54	ENSG00000160789.18_LMNA	-0.42648	-0.548443983	-0.28674881	4.29E-08	3.58E-06	2.20E-03
ENSG00000269246.1_CTC-246B1	ENSG00000160789.18_LMNA	-0.14319	-0.295654239	0.016380708	7.84E-02	2.69E-01	1.00E+00
ENSG00000070081.14_NUCB2	ENSG00000160789.18_LMNA	0.24733	0.091741895	0.39113014	2.13E-03	2.05E-02	1.00E+00
ENSG00000095209.10_TMEM38B	ENSG00000160789.18_LMNA	-0.09947	-0.254637252	0.060692944	2.23E-01	4.98E-01	1.00E+00
ENSG00000114126.16_TFDP2	ENSG00000160789.18_LMNA	-0.19726	-0.345607627	-0.03929366	1.49E-02	8.73E-02	1.00E+00
ENSG00000261295.1_RP11-524D	ENSG00000160789.18_LMNA	0.56105	0.441264596	0.661194555	5.56E-14	5.49E-11	2.86E-09
ENSG00000244045.9_TMEM199	ENSG00000160789.18_LMNA	-0.35581	-0.487397807	-0.20841008	6.84E-06	2.16E-04	3.51E-01
ENSG00000171150.7_SOCS5	ENSG00000160789.18_LMNA	0.02399	-0.135728817	0.182493978	7.69E-01	9.04E-01	1.00E+00
ENSG00000174989.11_FBXW8	ENSG00000160789.18_LMNA	-0.00421	-0.163297574	0.155098339	9.59E-01	9.84E-01	1.00E+00
ENSG00000054611.12_TBC1D22/	ENSG00000160789.18_LMNA	0.06344	-0.0967417	0.220410763	4.38E-01	7.06E-01	1.00E+00
ENSG00000067177.13_PHKA1	ENSG00000160789.18_LMNA	0.04665	-0.113395933	0.204330174	5.68E-01	7.98E-01	1.00E+00
ENSG00000175482.7_POLD4	ENSG00000160789.18_LMNA	0.22971	0.073188824	0.375192706	4.41E-03	3.57E-02	1.00E+00
ENSG00000267454.4_ZNF582-AS	ENSG00000160789.18_LMNA	-0.23709	-0.38187319	-0.08094034	3.27E-03	2.85E-02	1.00E+00
ENSG00000175093.4_SPSB4	ENSG00000160789.18_LMNA	-0.12504	-0.27869103	0.034856572	1.25E-01	3.57E-01	1.00E+00
ENSG00000151023.15_ENKUR	ENSG00000160789.18_LMNA	-0.17571	-0.325793407	-0.01698024	3.04E-02	1.44E-01	1.00E+00
ENSG00000104866.9_PPP1R37	ENSG00000160789.18_LMNA	0.33703	0.187913476	0.470962457	2.18E-05	5.54E-04	1.00E+00
ENSG00000114999.7_TTL	ENSG00000160789.18_LMNA	0.07995	-0.080272313	0.236145124	3.28E-01	6.14E-01	1.00E+00

ENSG00000092068.17_SLC7A8	ENSG00000160789.18_LMNA	0.01502	-0.144521802	0.173809547	8.54E-01	9.43E-01	1.00E+00
ENSG00000002822.14_MAD1L1	ENSG00000160789.18_LMNA	0.22871	0.072139811	0.374286219	4.59E-03	3.68E-02	1.00E+00
ENSG00000196670.12_ZFP62	ENSG00000160789.18_LMNA	-0.01986	-0.178494347	0.139784681	8.08E-01	9.24E-01	1.00E+00
ENSG00000100281.12_HMGXB4	ENSG00000160789.18_LMNA	-0.10591	-0.260717604	0.054201647	1.94E-01	4.63E-01	1.00E+00
ENSG00000103245.12_NARFL	ENSG00000160789.18_LMNA	-0.09253	-0.248080204	0.067663391	2.57E-01	5.39E-01	1.00E+00
ENSG00000185246.16_PRPF39	ENSG00000160789.18_LMNA	-0.05606	-0.213359148	0.104066797	4.93E-01	7.48E-01	1.00E+00
ENSG00000103319.10_EEF2K	ENSG00000160789.18_LMNA	0.33165	0.18206685	0.466237999	2.99E-05	7.12E-04	1.00E+00
ENSG00000099326.7_MZF1	ENSG00000160789.18_LMNA	0.04504	-0.114984341	0.202787467	5.82E-01	8.05E-01	1.00E+00
ENSG00000084628.8_NKAIN1	ENSG00000160789.18_LMNA	-0.40863	-0.533144195	-0.26678081	1.73E-07	1.12E-05	8.87E-03
ENSG00000156990.13_RPUSD3	ENSG00000160789.18_LMNA	-0.16419	-0.315152853	-0.0051232	4.32E-02	1.83E-01	1.00E+00
ENSG00000170364.11_SETMAR	ENSG00000160789.18_LMNA	-0.45503	-0.572739092	-0.31893097	3.87E-09	4.85E-07	1.99E-04
ENSG00000060642.9_PIGV	ENSG00000160789.18_LMNA	0.04565	-0.114378213	0.203376342	5.77E-01	8.03E-01	1.00E+00
ENSG00000155545.18_MIER3	ENSG00000160789.18_LMNA	-0.11711	-0.271257395	0.042886103	1.51E-01	3.99E-01	1.00E+00
ENSG00000118503.13_TNFAIP3	ENSG00000160789.18_LMNA	0.28156	0.128100359	0.421850546	4.41E-04	6.12E-03	1.00E+00
ENSG00000203883.6_SOX18	ENSG00000160789.18_LMNA	-0.01636	-0.175100377	0.143218011	8.41E-01	9.38E-01	1.00E+00
ENSG00000260822.1_GS1-358P8	ENSG00000160789.18_LMNA	0.27399	0.120020708	0.41508159	6.36E-04	8.15E-03	1.00E+00
ENSG00000136273.10_HUS1	ENSG00000160789.18_LMNA	0.09656	-0.063613913	0.251893043	2.37E-01	5.15E-01	1.00E+00
ENSG00000198771.9_RCSD1	ENSG00000160789.18_LMNA	-0.14275	-0.295237612	0.016837054	7.94E-02	2.71E-01	1.00E+00
ENSG00000174721.9_FGFBP3	ENSG00000160789.18_LMNA	-0.00227	-0.161416619	0.15698318	9.78E-01	9.92E-01	1.00E+00
ENSG00000170542.5_SERPINB9	ENSG00000160789.18_LMNA	0.03898	-0.120974971	0.196954972	6.34E-01	8.36E-01	1.00E+00
ENSG00000118900.13_UBN1	ENSG00000160789.18_LMNA	0.20098	0.043157528	0.349011204	1.30E-02	7.93E-02	1.00E+00
ENSG00000277075.1_HIST1H2AE	ENSG00000160789.18_LMNA	-0.04914	-0.20672698	0.110924897	5.48E-01	7.85E-01	1.00E+00
ENSG00000111671.8_SPSB2	ENSG00000160789.18_LMNA	0.10422	-0.055907888	0.259121798	2.01E-01	4.72E-01	1.00E+00
ENSG00000198682.11_PAPSS2	ENSG00000160789.18_LMNA	0.03178	-0.128067001	0.190020937	6.98E-01	8.70E-01	1.00E+00
ENSG00000184979.9_USP18	ENSG00000160789.18_LMNA	0.03544	-0.12445903	0.1935525	6.65E-01	8.52E-01	1.00E+00
ENSG00000197283.11_SYNGAP1	ENSG00000160789.18_LMNA	0.02124	-0.138432422	0.179829027	7.95E-01	9.18E-01	1.00E+00
ENSG00000177595.16_PIDD1	ENSG00000160789.18_LMNA	0.15709	-0.002168671	0.308570169	5.33E-02	2.09E-01	1.00E+00
ENSG00000101109.10_STK4	ENSG00000160789.18_LMNA	0.04305	-0.116949204	0.200876952	5.98E-01	8.15E-01	1.00E+00
ENSG00000181264.7_TMEM136	ENSG00000160789.18_LMNA	-0.41002	-0.53434275	-0.26833685	1.56E-07	1.03E-05	7.98E-03
ENSG00000235354.1_RPS29P16	ENSG00000160789.18_LMNA	-0.13464	-0.28767469	0.025098439	9.82E-02	3.08E-01	1.00E+00
ENSG00000163848.17_ZNF148	ENSG00000160789.18_LMNA	0.08109	-0.079134043	0.237226498	3.21E-01	6.07E-01	1.00E+00
ENSG00000110987.7_BCL7A	ENSG00000160789.18_LMNA	-0.34866	-0.481151598	-0.20059143	1.07E-05	3.08E-04	5.50E-01
ENSG00000046651.13_OFD1	ENSG00000160789.18_LMNA	0.06618	-0.094008003	0.223033931	4.18E-01	6.90E-01	1.00E+00

ENSG00000179981.9_TSHZ1	ENSG00000160789.18_LMNA	-0.17593	-0.325998804	-0.01720998	3.02E-02	1.44E-01	1.00E+00
ENSG00000165655.15_ZNF503	ENSG00000160789.18_LMNA	0.27469	0.120770235	0.415710905	6.15E-04	7.95E-03	1.00E+00
ENSG00000198081.9_ZBTB14	ENSG00000160789.18_LMNA	-0.15476	-0.306412421	0.004551715	5.69E-02	2.18E-01	1.00E+00
ENSG00000171812.9_COL8A2	ENSG00000160789.18_LMNA	0.27652	0.122726506	0.417352093	5.64E-04	7.40E-03	1.00E+00
ENSG00000145335.14_SNCA	ENSG00000160789.18_LMNA	-0.21547	-0.362243722	-0.05826702	7.68E-03	5.39E-02	1.00E+00
ENSG00000213463.4_SYNJ2BP	ENSG00000160789.18_LMNA	0.00702	-0.152353241	0.166032879	9.32E-01	9.73E-01	1.00E+00
ENSG00000105321.11_CCDC9	ENSG00000160789.18_LMNA	0.5205	0.393946279	0.627690356	6.27E-12	2.40E-09	3.22E-07
ENSG00000166924.7_NYAP1	ENSG00000160789.18_LMNA	-0.20257	-0.350468406	-0.0448146	1.23E-02	7.62E-02	1.00E+00
ENSG00000173548.8_SNX33	ENSG00000160789.18_LMNA	0.4891	0.357758216	0.601469033	1.62E-10	3.36E-08	8.31E-06
ENSG00000262877.4_RP11-1055	ENSG00000160789.18_LMNA	-0.13787	-0.29068805	0.021811896	9.03E-02	2.93E-01	1.00E+00
ENSG00000278768.1_BACE1-AS	ENSG00000160789.18_LMNA	0.00793	-0.15146488	0.166917023	9.23E-01	9.70E-01	1.00E+00
ENSG00000099308.9_MAST3	ENSG00000160789.18_LMNA	-0.15811	-0.309520285	0.001118231	5.17E-02	2.06E-01	1.00E+00
ENSG00000110395.4_CBL	ENSG00000160789.18_LMNA	0.14681	-0.012682244	0.299026428	7.11E-02	2.52E-01	1.00E+00
ENSG00000144455.12_SUMF1	ENSG00000160789.18_LMNA	0.22316	0.066317154	0.369244149	5.72E-03	4.34E-02	1.00E+00
ENSG00000255389.1_C6orf3	ENSG00000160789.18_LMNA	-0.33541	-0.469541485	-0.18615288	2.40E-05	5.99E-04	1.00E+00
ENSG00000136381.11_IREB2	ENSG00000160789.18_LMNA	0.01669	-0.142892135	0.175422843	8.38E-01	9.37E-01	1.00E+00
ENSG00000121741.15_ZMYM2	ENSG00000160789.18_LMNA	-0.27006	-0.411566428	-0.11584026	7.66E-04	9.39E-03	1.00E+00
ENSG00000169228.12_RAB24	ENSG00000160789.18_LMNA	-0.0029	-0.162027482	0.156371309	9.72E-01	9.89E-01	1.00E+00
ENSG00000058272.14_PPP1R12A	ENSG00000160789.18_LMNA	-0.01735	-0.176061676	0.142246341	8.32E-01	9.35E-01	1.00E+00
ENSG00000100614.16_PPM1A	ENSG00000160789.18_LMNA	-0.16807	-0.318737974	-0.00910848	3.85E-02	1.69E-01	1.00E+00
ENSG00000119314.14_PTBP3	ENSG00000160789.18_LMNA	0.06901	-0.09119608	0.225727379	3.98E-01	6.75E-01	1.00E+00
ENSG00000232640.1_RP1-266L2	ENSG00000160789.18_LMNA	-0.28276	-0.422918865	-0.12937911	4.16E-04	5.85E-03	1.00E+00
ENSG00000101442.9_ACTR5	ENSG00000160789.18_LMNA	-0.07867	-0.234929948	0.081550442	3.35E-01	6.21E-01	1.00E+00
ENSG00000165832.5_TRUB1	ENSG00000160789.18_LMNA	-0.40552	-0.530474353	-0.26331963	2.19E-07	1.36E-05	1.12E-02
ENSG00000139155.7_SLCO1C1	ENSG00000160789.18_LMNA	-0.32105	-0.456899148	-0.17056862	5.51E-05	1.16E-03	1.00E+00
ENSG00000161654.8_LSM12	ENSG00000160789.18_LMNA	0.11312	-0.046922916	0.267505956	1.65E-01	4.22E-01	1.00E+00
ENSG00000153902.12_LGI4	ENSG00000160789.18_LMNA	-0.05897	-0.216142653	0.101179448	4.70E-01	7.31E-01	1.00E+00
ENSG00000126453.8_BCL2L12	ENSG00000160789.18_LMNA	0.41033	0.268682676	0.534608982	1.52E-07	1.01E-05	7.79E-03
ENSG00000011523.12_CEP68	ENSG00000160789.18_LMNA	-0.20045	-0.348532031	-0.042613	1.33E-02	8.03E-02	1.00E+00
ENSG00000113615.11_SEC24A	ENSG00000160789.18_LMNA	0.20223	0.044465456	0.350161497	1.25E-02	7.68E-02	1.00E+00
ENSG00000100151.14_PICK1	ENSG00000160789.18_LMNA	0.09273	-0.067469195	0.248263276	2.56E-01	5.38E-01	1.00E+00
ENSG00000135723.12_FHOD1	ENSG00000160789.18_LMNA	0.4012	0.258513676	0.526758384	3.02E-07	1.78E-05	1.55E-02
ENSG00000181458.9_TMEM45A	ENSG00000160789.18_LMNA	0.1071	-0.053005939	0.261834898	1.89E-01	4.56E-01	1.00E+00

ENSG00000118965.13_WDR35	ENSG00000160789.18_LMNA	0.02715	-0.132627145	0.185545557	7.40E-01	8.91E-01	1.00E+00
ENSG00000104863.10_LIN7B	ENSG00000160789.18_LMNA	-0.1755	-0.325605779	-0.01677041	3.06E-02	1.45E-01	1.00E+00
ENSG00000146281.5_PM20D2	ENSG00000160789.18_LMNA	-0.19227	-0.341033687	-0.03411554	1.76E-02	9.86E-02	1.00E+00
ENSG00000136891.12_TEX10	ENSG00000160789.18_LMNA	-0.16795	-0.31863168	-0.00899018	3.86E-02	1.69E-01	1.00E+00
ENSG00000135392.14_DNAJC14	ENSG00000160789.18_LMNA	0.08124	-0.078984087	0.2373689	3.20E-01	6.06E-01	1.00E+00
ENSG00000083814.11_ZNF671	ENSG00000160789.18_LMNA	-0.08899	-0.244723962	0.071219346	2.76E-01	5.60E-01	1.00E+00
ENSG00000116885.17_OSCP1	ENSG00000160789.18_LMNA	0.07406	-0.086154954	0.230544009	3.65E-01	6.46E-01	1.00E+00
ENSG00000223361.5_FTH1P10	ENSG00000160789.18_LMNA	-0.02806	-0.186430117	0.131726898	7.31E-01	8.87E-01	1.00E+00
ENSG00000117174.9_ZNHIT6	ENSG00000160789.18_LMNA	0.07234	-0.087874505	0.228902776	3.76E-01	6.56E-01	1.00E+00
ENSG00000272143.1_FGF14-AS2	ENSG00000160789.18_LMNA	-0.26545	-0.407428507	-0.11093257	9.49E-04	1.12E-02	1.00E+00
ENSG00000253696.2_KBTBD11-(	ENSG00000160789.18_LMNA	-0.19594	-0.344395811	-0.03792017	1.56E-02	9.01E-02	1.00E+00
ENSG00000171105.12_INSR	ENSG00000160789.18_LMNA	0.16506	0.006016039	0.315956816	4.21E-02	1.80E-01	1.00E+00
ENSG00000164741.13_DLC1	ENSG00000160789.18_LMNA	0.45217	0.315695927	0.570316872	4.98E-09	5.98E-07	2.55E-04
ENSG00000254685.5_FPGT	ENSG00000160789.18_LMNA	-0.02745	-0.185834805	0.132332826	7.37E-01	8.89E-01	1.00E+00
ENSG00000249751.3_ECSCR	ENSG00000160789.18_LMNA	-0.10508	-0.25992776	0.055046381	1.98E-01	4.67E-01	1.00E+00
ENSG00000174227.14_PIGG	ENSG00000160789.18_LMNA	0.16611	0.007099086	0.316931452	4.08E-02	1.76E-01	1.00E+00
ENSG00000164850.13_GPER1	ENSG00000160789.18_LMNA	0.03543	-0.124474937	0.193536948	6.65E-01	8.52E-01	1.00E+00
ENSG00000123338.11_NCKAP1L	ENSG00000160789.18_LMNA	0.00659	-0.152768778	0.165619139	9.36E-01	9.75E-01	1.00E+00
ENSG00000175084.10_DES	ENSG00000160789.18_LMNA	0.3002	0.14808063	0.438450137	1.72E-04	2.89E-03	1.00E+00
ENSG00000103042.7_SLC38A7	ENSG00000160789.18_LMNA	0.27133	0.117195956	0.412707342	7.21E-04	8.97E-03	1.00E+00
ENSG00000269386.4_RAB11B-AS	ENSG00000160789.18_LMNA	-0.24849	-0.392175691	-0.09296635	2.02E-03	1.97E-02	1.00E+00
ENSG00000197323.9_TRIM33	ENSG00000160789.18_LMNA	-0.12712	-0.280638546	0.032746225	1.19E-01	3.46E-01	1.00E+00
ENSG00000263740.2_RN7SL4P	ENSG00000160789.18_LMNA	0.00508	-0.154242804	0.164150583	9.50E-01	9.80E-01	1.00E+00
ENSG00000105732.11_ZNF574	ENSG00000160789.18_LMNA	0.22822	0.071623311	0.373839682	4.69E-03	3.73E-02	1.00E+00
ENSG00000147121.14_KRBOX4	ENSG00000160789.18_LMNA	-0.116	-0.270206836	0.044017614	1.55E-01	4.06E-01	1.00E+00
ENSG00000115042.8_FAHD2A	ENSG00000160789.18_LMNA	-0.19144	-0.340269189	-0.03325166	1.81E-02	1.01E-01	1.00E+00
ENSG00000120162.9_MOB3B	ENSG00000160789.18_LMNA	-0.43665	-0.557119398	-0.29817317	1.87E-08	1.82E-06	9.59E-04
ENSG00000151131.8_C12orf45	ENSG00000160789.18_LMNA	0.03698	-0.122948279	0.195028813	6.51E-01	8.45E-01	1.00E+00
ENSG00000184083.10_FAM120C	ENSG00000160789.18_LMNA	0.19375	0.035646996	0.34238797	1.68E-02	9.51E-02	1.00E+00
ENSG00000172366.18_MCRIP2	ENSG00000160789.18_LMNA	-0.02001	-0.178639805	0.139637364	8.07E-01	9.23E-01	1.00E+00
ENSG00000135045.6_C9orf40	ENSG00000160789.18_LMNA	0.12994	-0.029877386	0.283281899	1.11E-01	3.32E-01	1.00E+00
ENSG00000228798.1_AP000473.!	ENSG00000160789.18_LMNA	-0.08398	-0.239972629	0.076239711	3.04E-01	5.90E-01	1.00E+00
ENSG00000161048.10_NAPEPLD	ENSG00000160789.18_LMNA	-0.03134	-0.189591388	0.12850527	7.02E-01	8.72E-01	1.00E+00

ENSG00000142627.12_EPHA2	ENSG00000160789.18_LMNA	0.51287	0.385111507	0.621337381	1.43E-11	4.57E-09	7.32E-07
ENSG00000242612.5_DECR2	ENSG00000160789.18_LMNA	-0.02946	-0.187783948	0.130348041	7.19E-01	8.80E-01	1.00E+00
ENSG00000186185.12_KIF18B	ENSG00000160789.18_LMNA	0.0184	-0.141211886	0.177084423	8.22E-01	9.30E-01	1.00E+00
ENSG00000138246.14_DNAJC13	ENSG00000160789.18_LMNA	0.17106	0.012196157	0.321509545	3.51E-02	1.59E-01	1.00E+00
ENSG00000169515.5_CCDC8	ENSG00000160789.18_LMNA	0.35701	0.209726681	0.488446816	6.34E-06	2.02E-04	3.25E-01
ENSG00000164134.11_NAA15	ENSG00000160789.18_LMNA	0.21664	0.059486414	0.36330632	7.35E-03	5.22E-02	1.00E+00
ENSG00000264229.1_RNU4ATAC	ENSG00000160789.18_LMNA	-0.18164	-0.33126005	-0.02310583	2.51E-02	1.27E-01	1.00E+00
ENSG00000148343.17_MIGA2	ENSG00000160789.18_LMNA	0.12189	-0.038053656	0.275735707	1.35E-01	3.73E-01	1.00E+00
ENSG00000157349.14_DDX19B	ENSG00000160789.18_LMNA	-0.03334	-0.191523672	0.126532781	6.83E-01	8.63E-01	1.00E+00
ENSG00000133641.16_C12orf29	ENSG00000160789.18_LMNA	-0.30523	-0.442909104	-0.15348841	1.31E-04	2.35E-03	1.00E+00
ENSG00000167522.13_ANKRD11	ENSG00000160789.18_LMNA	0.23329	0.076951981	0.378439828	3.82E-03	3.20E-02	1.00E+00
ENSG00000122512.13_PMS2	ENSG00000160789.18_LMNA	0.127	-0.032867754	0.280526464	1.19E-01	3.46E-01	1.00E+00
ENSG00000006062.12_MAP3K14	ENSG00000160789.18_LMNA	0.21253	0.055193175	0.359561616	8.57E-03	5.86E-02	1.00E+00
ENSG00000107937.17_GTPBP4	ENSG00000160789.18_LMNA	-0.02016	-0.178787283	0.139487988	8.05E-01	9.22E-01	1.00E+00
ENSG00000237758.1_BANF1P3	ENSG00000160789.18_LMNA	-0.10299	-0.257963884	0.057144782	2.07E-01	4.79E-01	1.00E+00
ENSG00000253738.1_OTUD6B-AS	ENSG00000160789.18_LMNA	-0.26196	-0.404302706	-0.10723485	1.11E-03	1.26E-02	1.00E+00
ENSG00000243829.1_CTB-33G10	ENSG00000160789.18_LMNA	-0.15278	-0.304574488	0.006578768	6.02E-02	2.26E-01	1.00E+00
ENSG00000112787.11_FBRSL1	ENSG00000160789.18_LMNA	-0.01856	-0.17724181	0.141052635	8.20E-01	9.30E-01	1.00E+00
ENSG00000007944.13_MYLIP	ENSG00000160789.18_LMNA	-0.29219	-0.431327997	-0.13947886	2.60E-04	4.01E-03	1.00E+00
ENSG00000185610.6_DBX2	ENSG00000160789.18_LMNA	0.17968	0.021080392	0.32945478	2.68E-02	1.33E-01	1.00E+00
ENSG00000140382.13_HMG20A	ENSG00000160789.18_LMNA	-0.14789	-0.300022717	0.011587925	6.90E-02	2.47E-01	1.00E+00
ENSG00000114026.20_OGG1	ENSG00000160789.18_LMNA	-0.14239	-0.294902352	0.017204181	8.01E-02	2.72E-01	1.00E+00
ENSG00000080986.11_NDC80	ENSG00000160789.18_LMNA	0.07331	-0.086900778	0.229832375	3.69E-01	6.50E-01	1.00E+00
ENSG00000280798.1_LINC00294	ENSG00000160789.18_LMNA	-0.29368	-0.432653963	-0.14107697	2.40E-04	3.78E-03	1.00E+00
ENSG00000198001.12_IRAK4	ENSG00000160789.18_LMNA	-0.08793	-0.24372192	0.072279459	2.81E-01	5.67E-01	1.00E+00
ENSG00000247774.5_PCED1B-AS	ENSG00000160789.18_LMNA	0.01263	-0.146863872	0.171488012	8.77E-01	9.52E-01	1.00E+00
ENSG00000103091.13_WDR59	ENSG00000160789.18_LMNA	-0.11212	-0.266563237	0.047935736	1.69E-01	4.28E-01	1.00E+00
ENSG00000235957.1_COX7CP1	ENSG00000160789.18_LMNA	-0.06913	-0.225847851	0.091070191	3.97E-01	6.74E-01	1.00E+00
ENSG00000271870.1_RP11-97C1	ENSG00000160789.18_LMNA	-0.20683	-0.354363695	-0.04925244	1.06E-02	6.81E-02	1.00E+00
ENSG00000214530.6_STARD10	ENSG00000160789.18_LMNA	-0.34134	-0.474744457	-0.19260829	1.68E-05	4.47E-04	8.62E-01
ENSG00000188428.15_BLOC1S5	ENSG00000160789.18_LMNA	-0.00183	-0.160985769	0.157414593	9.82E-01	9.93E-01	1.00E+00
ENSG00000124787.12_RPP40	ENSG00000160789.18_LMNA	-0.10864	-0.263284348	0.051453415	1.83E-01	4.47E-01	1.00E+00
ENSG00000183309.10_ZNF623	ENSG00000160789.18_LMNA	-0.0053	-0.164360858	0.154031833	9.48E-01	9.79E-01	1.00E+00

ENSG00000067601.7_PMS2P4	ENSG00000160789.18_LMNA	-0.19764	-0.345959765	-0.039693	1.47E-02	8.64E-02	1.00E+00
ENSG00000135597.17_REPS1	ENSG00000160789.18_LMNA	-0.01716	-0.175877943	0.142432104	8.34E-01	9.35E-01	1.00E+00
ENSG00000039560.12_RAI14	ENSG00000160789.18_LMNA	0.18205	0.023534856	0.331642151	2.48E-02	1.26E-01	1.00E+00
ENSG00000104177.16_MYEF2	ENSG00000160789.18_LMNA	-0.17905	-0.328872156	-0.02042725	2.73E-02	1.35E-01	1.00E+00
ENSG00000185798.7_WDR53	ENSG00000160789.18_LMNA	-0.01057	-0.169482787	0.148883963	8.97E-01	9.60E-01	1.00E+00
ENSG00000125740.12_FOSB	ENSG00000160789.18_LMNA	0.06415	-0.096029749	0.221094371	4.32E-01	7.02E-01	1.00E+00
ENSG00000139428.10_MMAB	ENSG00000160789.18_LMNA	-0.14606	-0.298321858	0.01345569	7.26E-02	2.56E-01	1.00E+00
ENSG00000197217.11_ENTPD4	ENSG00000160789.18_LMNA	0.07981	-0.080408691	0.23601551	3.28E-01	6.14E-01	1.00E+00
ENSG00000213280.2_RP11-212P	ENSG00000160789.18_LMNA	-0.08074	-0.236896683	0.079481299	3.23E-01	6.09E-01	1.00E+00
ENSG00000073417.13_PDE8A	ENSG00000160789.18_LMNA	0.0596	-0.100557826	0.216741244	4.66E-01	7.28E-01	1.00E+00
ENSG00000148090.10_AUH	ENSG00000160789.18_LMNA	-0.23813	-0.382816847	-0.08203823	3.13E-03	2.76E-02	1.00E+00
ENSG00000143643.11_TTC13	ENSG00000160789.18_LMNA	-0.13467	-0.287706629	0.02506364	9.81E-02	3.08E-01	1.00E+00
ENSG00000171940.12_ZNF217	ENSG00000160789.18_LMNA	0.45732	0.321531695	0.574683164	3.16E-09	4.04E-07	1.62E-04
ENSG00000231503.4_PTMAP4	ENSG00000160789.18_LMNA	0.04667	-0.113377443	0.204348123	5.68E-01	7.98E-01	1.00E+00
ENSG00000172159.14_FRMD3	ENSG00000160789.18_LMNA	0.13537	-0.024353643	0.28835812	9.63E-02	3.05E-01	1.00E+00
ENSG00000140750.15_ARHGAP1	ENSG00000160789.18_LMNA	0.17444	0.01567254	0.324623695	3.16E-02	1.48E-01	1.00E+00
ENSG00000104415.12_WISP1	ENSG00000160789.18_LMNA	0.07227	-0.087938243	0.228841906	3.76E-01	6.56E-01	1.00E+00
ENSG00000188997.6_KCTD21	ENSG00000160789.18_LMNA	0.04935	-0.110720425	0.206925139	5.46E-01	7.84E-01	1.00E+00
ENSG00000112039.3_FANCE	ENSG00000160789.18_LMNA	-0.01115	-0.170044761	0.14831809	8.92E-01	9.57E-01	1.00E+00
ENSG00000147324.9_MFHAS1	ENSG00000160789.18_LMNA	0.32424	0.174024304	0.459712501	4.60E-05	1.00E-03	1.00E+00
ENSG00000138018.16_SELENOI	ENSG00000160789.18_LMNA	0.01801	-0.141592872	0.176707829	8.26E-01	9.32E-01	1.00E+00
ENSG00000171097.12_KYAT1	ENSG00000160789.18_LMNA	-0.14421	-0.296600908	0.015343305	7.63E-02	2.64E-01	1.00E+00
ENSG00000114473.12_IQCG	ENSG00000160789.18_LMNA	0.15435	-0.004972872	0.306030747	5.76E-02	2.20E-01	1.00E+00
ENSG00000137842.5_TMEM62	ENSG00000160789.18_LMNA	-0.04249	-0.200339114	0.117501894	6.03E-01	8.18E-01	1.00E+00
ENSG00000149531.13_FRG1BP	ENSG00000160789.18_LMNA	-0.06752	-0.224306956	0.092679612	4.09E-01	6.83E-01	1.00E+00
ENSG00000172409.5_CLP1	ENSG00000160789.18_LMNA	0.03564	-0.124270359	0.193736949	6.63E-01	8.51E-01	1.00E+00
ENSG00000136514.2_RTP4	ENSG00000160789.18_LMNA	-0.02725	-0.185643962	0.132527021	7.39E-01	8.90E-01	1.00E+00
ENSG00000169896.15_ITGAM	ENSG00000160789.18_LMNA	0.16121	0.00206138	0.312392448	4.72E-02	1.94E-01	1.00E+00
ENSG00000023608.4_SNAPC1	ENSG00000160789.18_LMNA	-0.02504	-0.183513925	0.134692821	7.59E-01	9.00E-01	1.00E+00
ENSG00000111885.6_MAN1A1	ENSG00000160789.18_LMNA	0.10444	-0.055688338	0.259327234	2.00E-01	4.71E-01	1.00E+00
ENSG00000131149.16_GSE1	ENSG00000160789.18_LMNA	-0.0964	-0.251736048	0.063780857	2.37E-01	5.16E-01	1.00E+00
ENSG00000181631.6_P2RY13	ENSG00000160789.18_LMNA	-0.21824	-0.364771416	-0.06116919	6.91E-03	5.00E-02	1.00E+00
ENSG00000198517.8_MAFK	ENSG00000160789.18_LMNA	0.49839	0.368424803	0.609252845	6.41E-11	1.58E-08	3.29E-06

ENSG00000172965.13_MIR4435-2	ENSG00000160789.18_LMNA	0.31912	0.168473579	0.455190732	6.15E-05	1.27E-03	1.00E+00
ENSG00000128585.16_MKLN1	ENSG00000160789.18_LMNA	0.04694	-0.113104948	0.204612616	5.66E-01	7.96E-01	1.00E+00
ENSG00000137501.15_SYTL2	ENSG00000160789.18_LMNA	0.11878	-0.041197526	0.272823776	1.45E-01	3.90E-01	1.00E+00
ENSG00000121897.12_LIAS	ENSG00000160789.18_LMNA	-0.38447	-0.512317036	-0.23996157	1.01E-06	4.62E-05	5.17E-02
ENSG00000149418.9_ST14	ENSG00000160789.18_LMNA	0.06666	-0.09353208	0.223490142	4.15E-01	6.88E-01	1.00E+00
ENSG00000133961.18_NUMB	ENSG00000160789.18_LMNA	0.2545	0.099323353	0.397591482	1.56E-03	1.61E-02	1.00E+00
ENSG00000186130.4_ZBTB6	ENSG00000160789.18_LMNA	-0.19254	-0.341281259	-0.03439539	1.75E-02	9.79E-02	1.00E+00
ENSG00000157350.11_ST3GAL2	ENSG00000160789.18_LMNA	0.39977	0.256923159	0.525526332	3.35E-07	1.92E-05	1.72E-02
ENSG00000159409.13_CELF3	ENSG00000160789.18_LMNA	-0.34221	-0.475507643	-0.19355725	1.59E-05	4.27E-04	8.18E-01
ENSG00000128052.8_KDR	ENSG00000160789.18_LMNA	0.13366	-0.026096504	0.286758359	1.01E-01	3.13E-01	1.00E+00
ENSG00000100916.12_BRMS1L	ENSG00000160789.18_LMNA	-0.2249	-0.370824152	-0.06813955	5.34E-03	4.12E-02	1.00E+00
ENSG00000116704.7_SLC35D1	ENSG00000160789.18_LMNA	0.44784	0.310800998	0.56664338	7.25E-09	8.26E-07	3.72E-04
ENSG00000057704.9_TMCC3	ENSG00000160789.18_LMNA	-0.37486	-0.503983258	-0.22934532	1.95E-06	7.84E-05	1.00E-01
ENSG00000111247.13_RAD51AP	ENSG00000160789.18_LMNA	-0.22661	-0.372379372	-0.06993533	4.99E-03	3.92E-02	1.00E+00
ENSG00000060718.17_COL11A1	ENSG00000160789.18_LMNA	-0.15563	-0.307215087	0.003665652	5.56E-02	2.15E-01	1.00E+00
ENSG00000144278.13_GALNT13	ENSG00000160789.18_LMNA	-0.36218	-0.492961172	-0.21540415	4.54E-06	1.54E-04	2.33E-01
ENSG00000177427.11_MIEF2	ENSG00000160789.18_LMNA	-0.04286	-0.200693396	0.117137851	6.00E-01	8.16E-01	1.00E+00
ENSG00000260027.4_HOXB7	ENSG00000160789.18_LMNA	0.13551	-0.024209669	0.288490195	9.60E-02	3.04E-01	1.00E+00
ENSG00000221963.5_APOL6	ENSG00000160789.18_LMNA	0.14339	-0.016181161	0.295836381	7.80E-02	2.68E-01	1.00E+00
ENSG00000178074.5_C2orf69	ENSG00000160789.18_LMNA	-0.09133	-0.246936073	0.068876505	2.63E-01	5.46E-01	1.00E+00
ENSG00000238103.4_RPL9P7	ENSG00000160789.18_LMNA	-0.17569	-0.32577677	-0.01696164	3.04E-02	1.45E-01	1.00E+00
ENSG00000123104.10_ITPR2	ENSG00000160789.18_LMNA	0.15545	-0.003847897	0.307050031	5.58E-02	2.16E-01	1.00E+00
ENSG00000160226.14_C21orf2	ENSG00000160789.18_LMNA	0.17013	0.011236373	0.32064859	3.61E-02	1.62E-01	1.00E+00
ENSG00000150656.13_CNDP1	ENSG00000160789.18_LMNA	-0.22555	-0.371416068	-0.06882279	5.21E-03	4.05E-02	1.00E+00
ENSG00000116525.12_TRIM62	ENSG00000160789.18_LMNA	-0.1201	-0.274061863	0.039861583	1.41E-01	3.82E-01	1.00E+00
ENSG00000124802.10_EEF1E1	ENSG00000160789.18_LMNA	-0.23402	-0.379099879	-0.07771798	3.71E-03	3.13E-02	1.00E+00
ENSG00000165912.14_PACSIN3	ENSG00000160789.18_LMNA	0.38823	0.244113825	0.515562675	7.73E-07	3.72E-05	3.96E-02
ENSG00000145996.10_CDKAL1	ENSG00000160789.18_LMNA	-0.10206	-0.257079847	0.058088466	2.11E-01	4.84E-01	1.00E+00
ENSG00000077044.8_DGKD	ENSG00000160789.18_LMNA	0.11866	-0.041323846	0.272706654	1.45E-01	3.90E-01	1.00E+00
ENSG00000120327.6_PCDHB14	ENSG00000160789.18_LMNA	0.09163	-0.068571302	0.247224003	2.62E-01	5.45E-01	1.00E+00
ENSG00000117000.8_RLF	ENSG00000160789.18_LMNA	0.05305	-0.107051849	0.210476026	5.16E-01	7.63E-01	1.00E+00
ENSG00000116141.14_MARK1	ENSG00000160789.18_LMNA	-0.11794	-0.272028996	0.042054533	1.48E-01	3.94E-01	1.00E+00
ENSG00000130703.14_OSBPL2	ENSG00000160789.18_LMNA	-0.08969	-0.245390463	0.070513823	2.72E-01	5.56E-01	1.00E+00

ENSG00000137338.5_PGBD1	ENSG00000160789.18_LMNA	-0.17504	-0.325174911	-0.01628865	3.10E-02	1.47E-01	1.00E+00
ENSG00000242419.4_PCDHGC4	ENSG00000160789.18_LMNA	-0.04834	-0.205958666	0.111717434	5.54E-01	7.89E-01	1.00E+00
ENSG00000173960.11_UBXN2A	ENSG00000160789.18_LMNA	-0.02477	-0.183243634	0.134967433	7.62E-01	9.01E-01	1.00E+00
ENSG00000084710.12_EFR3B	ENSG00000160789.18_LMNA	0.02542	-0.134326957	0.183873958	7.56E-01	8.98E-01	1.00E+00
ENSG00000183401.10_CCDC159	ENSG00000160789.18_LMNA	-0.19328	-0.341955185	-0.03515743	1.70E-02	9.63E-02	1.00E+00
ENSG00000079974.16_RABL2B	ENSG00000160789.18_LMNA	-0.03714	-0.19518863	0.122784646	6.50E-01	8.44E-01	1.00E+00
ENSG00000162426.13_SLC45A1	ENSG00000160789.18_LMNA	0.00091	-0.158309456	0.160091688	9.91E-01	9.97E-01	1.00E+00
ENSG00000153767.8_GTF2E1	ENSG00000160789.18_LMNA	-0.2432	-0.38740301	-0.08738437	2.54E-03	2.35E-02	1.00E+00
ENSG00000171189.15_GRIK1	ENSG00000160789.18_LMNA	0.14452	-0.015023901	0.296892254	7.57E-02	2.63E-01	1.00E+00
ENSG00000181409.10_AATK	ENSG00000160789.18_LMNA	-0.28068	-0.421064507	-0.12716011	4.61E-04	6.32E-03	1.00E+00
ENSG00000186153.15_WWOX	ENSG00000160789.18_LMNA	-0.03878	-0.196768505	0.121166112	6.35E-01	8.37E-01	1.00E+00
ENSG00000128596.15_CCDC136	ENSG00000160789.18_LMNA	-0.24821	-0.391926959	-0.09267498	2.05E-03	1.99E-02	1.00E+00
ENSG00000225507.1_AC069282.(	ENSG00000160789.18_LMNA	0.11066	-0.049407518	0.265192249	1.75E-01	4.35E-01	1.00E+00
ENSG00000153339.12_TRAPPC8	ENSG00000160789.18_LMNA	-0.36504	-0.49544453	-0.21853535	3.77E-06	1.32E-04	1.93E-01
ENSG00000172508.9_CARNS1	ENSG00000160789.18_LMNA	-0.2713	-0.412674226	-0.11715659	7.23E-04	8.98E-03	1.00E+00
ENSG00000105821.13_DNAJC2	ENSG00000160789.18_LMNA	0.30047	0.148367819	0.438687298	1.69E-04	2.86E-03	1.00E+00
ENSG00000162928.8_PEX13	ENSG00000160789.18_LMNA	-0.06262	-0.219628053	0.097556465	4.43E-01	7.11E-01	1.00E+00
ENSG00000005801.15_ZNF195	ENSG00000160789.18_LMNA	-0.32136	-0.457174433	-0.17090645	5.42E-05	1.15E-03	1.00E+00
ENSG00000163644.13_PPM1K	ENSG00000160789.18_LMNA	-0.44403	-0.563402432	-0.30649367	1.00E-08	1.08E-06	5.15E-04
ENSG00000174521.7_TTC9B	ENSG00000160789.18_LMNA	-0.28745	-0.427108096	-0.13440297	3.30E-04	4.85E-03	1.00E+00
ENSG00000111321.9_LTBR	ENSG00000160789.18_LMNA	0.17363	0.014838188	0.323876892	3.24E-02	1.51E-01	1.00E+00
ENSG00000167173.17_C15orf39	ENSG00000160789.18_LMNA	0.25925	0.104356826	0.401865012	1.26E-03	1.38E-02	1.00E+00
ENSG00000135185.10_TMEM243	ENSG00000160789.18_LMNA	0.09502	-0.065163231	0.250435418	2.44E-01	5.24E-01	1.00E+00
ENSG00000186517.12_ARHGAP3	ENSG00000160789.18_LMNA	0.13585	-0.023863048	0.28880812	9.51E-02	3.02E-01	1.00E+00
ENSG00000106392.9_C1GALT1	ENSG00000160789.18_LMNA	-0.11703	-0.271177237	0.042972466	1.51E-01	4.00E-01	1.00E+00
ENSG00000014138.7_POLA2	ENSG00000160789.18_LMNA	0.05777	-0.102367492	0.214997961	4.80E-01	7.38E-01	1.00E+00
ENSG00000152953.11_STK32B	ENSG00000160789.18_LMNA	0.16418	0.005110648	0.31514155	4.33E-02	1.83E-01	1.00E+00
ENSG00000138036.17_DYNC2LI1	ENSG00000160789.18_LMNA	-0.11279	-0.267190697	0.04726169	1.67E-01	4.24E-01	1.00E+00
ENSG00000274605.1_RP11-12G1	ENSG00000160789.18_LMNA	0.02109	-0.13857422	0.179689127	7.96E-01	9.18E-01	1.00E+00
ENSG00000221886.3_ZBED8	ENSG00000160789.18_LMNA	-0.20928	-0.35659828	-0.05180372	9.67E-03	6.40E-02	1.00E+00
ENSG00000150990.6_DHX37	ENSG00000160789.18_LMNA	0.2935	0.140877568	0.432488589	2.43E-04	3.81E-03	1.00E+00
ENSG00000120925.12_RNF170	ENSG00000160789.18_LMNA	-0.26691	-0.408744208	-0.11249146	8.87E-04	1.06E-02	1.00E+00
ENSG00000205758.10_CRYZL1	ENSG00000160789.18_LMNA	-0.33446	-0.468702464	-0.18511418	2.54E-05	6.25E-04	1.00E+00

ENSG00000197961.10_ZNF121	ENSG00000160789.18_LMNA	-0.0354	-0.193508346	0.124504192	6.65E-01	8.52E-01	1.00E+00
ENSG00000180340.6_FZD2	ENSG00000160789.18_LMNA	0.15055	-0.008859126	0.30250411	6.41E-02	2.36E-01	1.00E+00
ENSG00000135451.11_TROAP	ENSG00000160789.18_LMNA	0.00166	-0.157579122	0.160821423	9.84E-01	9.94E-01	1.00E+00
ENSG00000145545.10_SRD5A1	ENSG00000160789.18_LMNA	0.22338	0.066550417	0.369446484	5.67E-03	4.31E-02	1.00E+00
ENSG00000078114.17_NEBL	ENSG00000160789.18_LMNA	-0.16744	-0.318163389	-0.0084691	3.92E-02	1.71E-01	1.00E+00
ENSG00000244026.5_FAM86DP	ENSG00000160789.18_LMNA	0.19783	0.039886837	0.346130665	1.46E-02	8.61E-02	1.00E+00
ENSG00000188060.6_RAB42	ENSG00000160789.18_LMNA	0.10844	-0.051656163	0.263095141	1.84E-01	4.49E-01	1.00E+00
ENSG00000119041.9_GTF3C3	ENSG00000160789.18_LMNA	-0.03848	-0.196479885	0.121461921	6.38E-01	8.38E-01	1.00E+00
ENSG00000197050.9_ZNF420	ENSG00000160789.18_LMNA	-0.35023	-0.482524109	-0.2023064	9.73E-06	2.85E-04	4.99E-01
ENSG00000120832.8_MTERF2	ENSG00000160789.18_LMNA	-0.23396	-0.379045791	-0.0776552	3.72E-03	3.14E-02	1.00E+00
ENSG00000104290.9_FZD3	ENSG00000160789.18_LMNA	-0.01301	-0.171850291	0.146498623	8.74E-01	9.51E-01	1.00E+00
ENSG00000198948.10_MFAP3L	ENSG00000160789.18_LMNA	0.04047	-0.119499003	0.19839407	6.21E-01	8.28E-01	1.00E+00
ENSG00000165501.15_LRR1	ENSG00000160789.18_LMNA	-0.02194	-0.180507681	0.137744374	7.88E-01	9.14E-01	1.00E+00
ENSG00000127804.11_METTL16	ENSG00000160789.18_LMNA	0.00812	-0.151280394	0.16710057	9.21E-01	9.70E-01	1.00E+00
ENSG00000161955.15_TNFSF13	ENSG00000160789.18_LMNA	0.09102	-0.069182351	0.24664748	2.65E-01	5.48E-01	1.00E+00
ENSG00000259865.1_RP11-488L	ENSG00000160789.18_LMNA	-0.17682	-0.326824256	-0.01813358	2.93E-02	1.41E-01	1.00E+00
ENSG00000182511.10_FES	ENSG00000160789.18_LMNA	0.41575	0.274734481	0.539259369	1.00E-07	7.23E-06	5.14E-03
ENSG00000223969.4_AC002456.:	ENSG00000160789.18_LMNA	0.0569	-0.103235006	0.214161552	4.86E-01	7.43E-01	1.00E+00
ENSG00000114405.9_C3orf14	ENSG00000160789.18_LMNA	-0.16244	-0.313529699	-0.0033221	4.56E-02	1.89E-01	1.00E+00
ENSG00000077150.16_NFKB2	ENSG00000160789.18_LMNA	0.43315	0.294236847	0.554136604	2.49E-08	2.33E-06	1.28E-03
ENSG00000115998.6_C2orf42	ENSG00000160789.18_LMNA	-0.1899	-0.338855348	-0.03165524	1.91E-02	1.04E-01	1.00E+00
ENSG00000105053.9_VRK3	ENSG00000160789.18_LMNA	-0.08697	-0.242808548	0.073245143	2.87E-01	5.72E-01	1.00E+00
ENSG00000123485.10_HJURP	ENSG00000160789.18_LMNA	0.16694	0.007949541	0.317696319	3.98E-02	1.73E-01	1.00E+00
ENSG00000112796.8_ENPP5	ENSG00000160789.18_LMNA	-0.17631	-0.326349451	-0.01760226	2.98E-02	1.43E-01	1.00E+00
ENSG00000249307.4_LINC01088	ENSG00000160789.18_LMNA	-0.33265	-0.467117213	-0.18315338	2.82E-05	6.80E-04	1.00E+00
ENSG00000205659.9_LIN52	ENSG00000160789.18_LMNA	-0.10724	-0.261970349	0.05286092	1.88E-01	4.55E-01	1.00E+00
ENSG00000166579.14_NDEL1	ENSG00000160789.18_LMNA	0.16923	0.010302701	0.319810566	3.71E-02	1.65E-01	1.00E+00
ENSG00000198513.10_ATL1	ENSG00000160789.18_LMNA	-0.42084	-0.543623298	-0.28043263	6.72E-08	5.23E-06	3.45E-03
ENSG00000156853.10_ZNF689	ENSG00000160789.18_LMNA	-0.08638	-0.242245978	0.073839638	2.90E-01	5.75E-01	1.00E+00
ENSG00000169131.9_ZNF354A	ENSG00000160789.18_LMNA	-0.06892	-0.225640913	0.091286428	3.99E-01	6.76E-01	1.00E+00
ENSG00000107984.8_DKK1	ENSG00000160789.18_LMNA	0.20509	0.047442868	0.352776629	1.13E-02	7.13E-02	1.00E+00
ENSG00000130150.10_MOSPD2	ENSG00000160789.18_LMNA	0.02789	-0.131897628	0.186262401	7.33E-01	8.88E-01	1.00E+00
ENSG00000156931.14_VPS8	ENSG00000160789.18_LMNA	-0.26051	-0.403000575	-0.10569689	1.19E-03	1.32E-02	1.00E+00

ENSG00000099625.11_CBARP	ENSG00000160789.18_LMNA	0.20647	0.048876243	0.354033899	1.07E-02	6.88E-02	1.00E+00
ENSG00000134901.11_KDELC1	ENSG00000160789.18_LMNA	0.01273	-0.146763827	0.171587252	8.76E-01	9.52E-01	1.00E+00
ENSG00000162437.13_RAVER2	ENSG00000160789.18_LMNA	-0.11658	-0.270757303	0.043424831	1.53E-01	4.02E-01	1.00E+00
ENSG00000185112.5_FAM43A	ENSG00000160789.18_LMNA	0.23954	0.083519991	0.384089457	2.96E-03	2.65E-02	1.00E+00
ENSG00000176454.12_LPCAT4	ENSG00000160789.18_LMNA	0.1453	-0.014227981	0.297618001	7.41E-02	2.59E-01	1.00E+00
ENSG00000100749.6_VRK1	ENSG00000160789.18_LMNA	-0.03449	-0.192634205	0.125398003	6.73E-01	8.57E-01	1.00E+00
ENSG00000140598.12_EFL1	ENSG00000160789.18_LMNA	0.27996	0.126393023	0.4204229	4.77E-04	6.50E-03	1.00E+00
ENSG00000180011.6_ZADH2	ENSG00000160789.18_LMNA	-0.34616	-0.478969309	-0.19786816	1.25E-05	3.51E-04	6.42E-01
ENSG00000142396.9_ERVK3-1	ENSG00000160789.18_LMNA	-0.1678	-0.318494029	-0.00883699	3.88E-02	1.70E-01	1.00E+00
ENSG00000077232.15_DNAJC10	ENSG00000160789.18_LMNA	0.17613	0.017417193	0.326184038	3.00E-02	1.43E-01	1.00E+00
ENSG00000093167.16_LRRFIP2	ENSG00000160789.18_LMNA	0.01577	-0.143789844	0.174534363	8.47E-01	9.40E-01	1.00E+00
ENSG00000138002.13_IFT172	ENSG00000160789.18_LMNA	0.09318	-0.067019488	0.248687134	2.54E-01	5.35E-01	1.00E+00
ENSG00000156502.12_SUPV3L1	ENSG00000160789.18_LMNA	-0.16071	-0.311930751	-0.00154984	4.79E-02	1.96E-01	1.00E+00
ENSG00000010282.13_HHATL	ENSG00000160789.18_LMNA	-0.32124	-0.457067291	-0.17077495	5.45E-05	1.15E-03	1.00E+00
ENSG00000112357.11_PEX7	ENSG00000160789.18_LMNA	-0.21464	-0.361491197	-0.057404	7.92E-03	5.52E-02	1.00E+00
ENSG00000107249.20_GLIS3	ENSG00000160789.18_LMNA	0.2074	0.049842054	0.354880427	1.04E-02	6.72E-02	1.00E+00
ENSG00000091127.12_PUS7	ENSG00000160789.18_LMNA	0.15099	-0.00841456	0.302907971	6.33E-02	2.34E-01	1.00E+00
ENSG00000053254.14_FOXN3	ENSG00000160789.18_LMNA	-0.03671	-0.194776208	0.123206881	6.53E-01	8.47E-01	1.00E+00
ENSG00000102230.12_PCYT1B	ENSG00000160789.18_LMNA	-0.13972	-0.292414646	0.019925723	8.60E-02	2.85E-01	1.00E+00
ENSG00000126216.11_TUBGCP3	ENSG00000160789.18_LMNA	-0.03488	-0.193005921	0.125017986	6.70E-01	8.55E-01	1.00E+00
ENSG00000204120.13_GIGYF2	ENSG00000160789.18_LMNA	0.09032	-0.069889194	0.245980296	2.68E-01	5.53E-01	1.00E+00
ENSG00000174977.8_AC026271.5	ENSG00000160789.18_LMNA	-0.05841	-0.215603528	0.101739104	4.75E-01	7.34E-01	1.00E+00
ENSG00000106404.12_CLDN15	ENSG00000160789.18_LMNA	0.06096	-0.099201553	0.21804644	4.56E-01	7.20E-01	1.00E+00
ENSG00000113456.17_RAD1	ENSG00000160789.18_LMNA	-0.23778	-0.382500946	-0.08167062	3.18E-03	2.79E-02	1.00E+00
ENSG00000141084.9_RANBP10	ENSG00000160789.18_LMNA	0.16633	0.007323873	0.317133656	4.06E-02	1.75E-01	1.00E+00
ENSG00000129315.8_CCNT1	ENSG00000160789.18_LMNA	-0.04652	-0.204211648	0.113518027	5.69E-01	7.99E-01	1.00E+00
ENSG00000110675.11_ELMOD1	ENSG00000160789.18_LMNA	0.16467	0.005615309	0.315596032	4.26E-02	1.81E-01	1.00E+00
ENSG00000122507.19_BBS9	ENSG00000160789.18_LMNA	0.07461	-0.085611609	0.231062232	3.61E-01	6.43E-01	1.00E+00
ENSG00000123124.12_WWP1	ENSG00000160789.18_LMNA	0.11355	-0.046489818	0.267908894	1.64E-01	4.19E-01	1.00E+00
ENSG00000072786.11_STK10	ENSG00000160789.18_LMNA	0.22627	0.069571041	0.372064019	5.06E-03	3.96E-02	1.00E+00
ENSG00000132003.8_ZSWIM4	ENSG00000160789.18_LMNA	0.15509	-0.004219141	0.306713745	5.64E-02	2.17E-01	1.00E+00
ENSG00000224389.7_C4B	ENSG00000160789.18_LMNA	0.11107	-0.048999568	0.265572388	1.73E-01	4.33E-01	1.00E+00
ENSG00000135074.14_ADAM19	ENSG00000160789.18_LMNA	0.39199	0.248283753	0.518814305	5.91E-07	3.00E-05	3.03E-02

ENSG00000134815.17_DHX34	ENSG00000160789.18_LMNA	0.17094	0.01206274	0.321389897	3.52E-02	1.59E-01	1.00E+00
ENSG00000172661.16_FAM21C	ENSG00000160789.18_LMNA	0.02374	-0.135973971	0.182252522	7.72E-01	9.06E-01	1.00E+00
ENSG00000107562.15_CXCL12	ENSG00000160789.18_LMNA	-0.01003	-0.168964996	0.149405162	9.02E-01	9.62E-01	1.00E+00
ENSG00000153707.14_PTPRD	ENSG00000160789.18_LMNA	-0.22734	-0.373041069	-0.07069998	4.85E-03	3.83E-02	1.00E+00
ENSG00000066056.12_TIE1	ENSG00000160789.18_LMNA	-0.07514	-0.231572472	0.08507645	3.58E-01	6.41E-01	1.00E+00
ENSG00000124279.10_FASTKD3	ENSG00000160789.18_LMNA	-0.34619	-0.478995136	-0.19790036	1.25E-05	3.50E-04	6.41E-01
ENSG00000055147.16_FAM114A2	ENSG00000160789.18_LMNA	-0.11842	-0.272485036	0.041562843	1.46E-01	3.92E-01	1.00E+00
ENSG00000066651.16_TRMT11	ENSG00000160789.18_LMNA	-0.09908	-0.254274631	0.061079233	2.25E-01	5.01E-01	1.00E+00
ENSG00000135049.14_AGTPBP1	ENSG00000160789.18_LMNA	-0.22986	-0.375325944	-0.07334307	4.39E-03	3.55E-02	1.00E+00
ENSG00000197149.5_RP11-452G	ENSG00000160789.18_LMNA	-0.03583	-0.193927124	0.124075807	6.61E-01	8.50E-01	1.00E+00
ENSG00000013392.7_RWDD2A	ENSG00000160789.18_LMNA	-0.43256	-0.553638664	-0.29358058	2.62E-08	2.43E-06	1.34E-03
ENSG00000102078.14_SLC25A14	ENSG00000160789.18_LMNA	-0.209	-0.356345649	-0.05151509	9.76E-03	6.45E-02	1.00E+00
ENSG00000077514.7_POLD3	ENSG00000160789.18_LMNA	0.04851	-0.111552527	0.206118565	5.53E-01	7.88E-01	1.00E+00
ENSG00000055609.16_KMT2C	ENSG00000160789.18_LMNA	0.16411	0.005036764	0.315075002	4.34E-02	1.83E-01	1.00E+00
ENSG00000005700.13_IBTK	ENSG00000160789.18_LMNA	-0.00619	-0.165228301	0.15316121	9.40E-01	9.76E-01	1.00E+00
ENSG00000189050.13_RNFT1	ENSG00000160789.18_LMNA	-0.17645	-0.326481241	-0.01774972	2.97E-02	1.42E-01	1.00E+00
ENSG00000141314.11_RHBDL3	ENSG00000160789.18_LMNA	-0.28436	-0.424350487	-0.13109427	3.85E-04	5.49E-03	1.00E+00
ENSG00000170791.16_CHCHD7	ENSG00000160789.18_LMNA	-0.00911	-0.168062313	0.150313363	9.11E-01	9.66E-01	1.00E+00
ENSG00000188321.12_ZNF559	ENSG00000160789.18_LMNA	-0.2098	-0.357069786	-0.05234256	9.48E-03	6.31E-02	1.00E+00
ENSG00000156345.16_CDK20	ENSG00000160789.18_LMNA	0.07454	-0.085677753	0.230999155	3.61E-01	6.43E-01	1.00E+00
ENSG00000171865.8_RNASEH1	ENSG00000160789.18_LMNA	0.07798	-0.082236885	0.234276906	3.40E-01	6.25E-01	1.00E+00
ENSG00000172469.13_MANEA	ENSG00000160789.18_LMNA	-0.15818	-0.309583588	0.00104822	5.16E-02	2.06E-01	1.00E+00
ENSG00000065183.14_WDR3	ENSG00000160789.18_LMNA	0.03135	-0.128493716	0.189602713	7.01E-01	8.72E-01	1.00E+00
ENSG00000198919.11_DZIP3	ENSG00000160789.18_LMNA	-0.31022	-0.447326184	-0.15886253	1.00E-04	1.89E-03	1.00E+00
ENSG00000036549.11_ZZZ3	ENSG00000160789.18_LMNA	-0.07696	-0.233305946	0.083256952	3.46E-01	6.30E-01	1.00E+00
ENSG00000221946.6_FXYD7	ENSG00000160789.18_LMNA	-0.29919	-0.437553433	-0.14699521	1.81E-04	3.02E-03	1.00E+00
ENSG00000142347.15_MYO1F	ENSG00000160789.18_LMNA	0.12018	-0.039785543	0.274132301	1.40E-01	3.82E-01	1.00E+00
ENSG00000088035.14_ALG6	ENSG00000160789.18_LMNA	-0.13392	-0.287004073	0.025828933	1.00E-01	3.11E-01	1.00E+00
ENSG00000141013.13_GAS8	ENSG00000160789.18_LMNA	0.32187	0.171452572	0.45761934	5.26E-05	1.12E-03	1.00E+00
ENSG00000106829.17_TLE4	ENSG00000160789.18_LMNA	0.03581	-0.124096883	0.193906523	6.61E-01	8.50E-01	1.00E+00
ENSG00000085982.12_USP40	ENSG00000160789.18_LMNA	0.17422	0.015448664	0.324423349	3.18E-02	1.49E-01	1.00E+00
ENSG00000235065.1_RPL24P2	ENSG00000160789.18_LMNA	-0.15153	-0.303413483	0.007857922	6.24E-02	2.31E-01	1.00E+00
ENSG00000164086.9_DUSP7	ENSG00000160789.18_LMNA	-0.02173	-0.180307936	0.137946916	7.90E-01	9.15E-01	1.00E+00

ENSG00000137673.7_MMP7	ENSG00000160789.18_LMNA	0.1373	-0.022394247	0.290154557	9.17E-02	2.95E-01	1.00E+00
ENSG00000143443.9_C1orf56	ENSG00000160789.18_LMNA	0.05039	-0.109695136	0.20791838	5.38E-01	7.78E-01	1.00E+00
ENSG00000095015.5_MAP3K1	ENSG00000160789.18_LMNA	-0.05115	-0.208650632	0.108938824	5.31E-01	7.74E-01	1.00E+00
ENSG00000127191.16_TRAF2	ENSG00000160789.18_LMNA	0.34047	0.191659921	0.473981319	1.77E-05	4.67E-04	9.09E-01
ENSG00000223804.4_CH17-472G	ENSG00000160789.18_LMNA	-0.17713	-0.327109396	-0.01845275	2.90E-02	1.40E-01	1.00E+00
ENSG00000060558.3_GNA15	ENSG00000160789.18_LMNA	0.21795	0.060859254	0.364501688	6.99E-03	5.04E-02	1.00E+00
ENSG00000163913.10_IFT122	ENSG00000160789.18_LMNA	0.25982	0.104957562	0.402374186	1.23E-03	1.35E-02	1.00E+00
ENSG00000232480.1_TGFB2-AS1	ENSG00000160789.18_LMNA	0.03677	-0.123151224	0.194830578	6.53E-01	8.46E-01	1.00E+00
ENSG00000108443.12_RPS6KB1	ENSG00000160789.18_LMNA	0.01379	-0.145731076	0.172611318	8.66E-01	9.48E-01	1.00E+00
ENSG00000061936.8_SFSWAP	ENSG00000160789.18_LMNA	0.06401	-0.096167892	0.220961752	4.33E-01	7.03E-01	1.00E+00
ENSG00000135905.17_DOCK10	ENSG00000160789.18_LMNA	-0.12361	-0.277353568	0.03630424	1.29E-01	3.64E-01	1.00E+00
ENSG00000175426.9_PCSK1	ENSG00000160789.18_LMNA	0.20038	0.042533014	0.348461632	1.33E-02	8.05E-02	1.00E+00
ENSG00000141378.14_PTRH2	ENSG00000160789.18_LMNA	-0.12296	-0.276744837	0.036962695	1.31E-01	3.67E-01	1.00E+00
ENSG00000204219.8_TCEA3	ENSG00000160789.18_LMNA	0.39732	0.254193378	0.523409149	4.02E-07	2.21E-05	2.06E-02
ENSG00000126814.6_TRMT5	ENSG00000160789.18_LMNA	-0.24322	-0.387422685	-0.08740734	2.53E-03	2.35E-02	1.00E+00
ENSG00000196151.9_WDSUB1	ENSG00000160789.18_LMNA	-0.28858	-0.428108884	-0.13560537	3.12E-04	4.64E-03	1.00E+00
ENSG00000116750.12_UCHL5	ENSG00000160789.18_LMNA	-0.09424	-0.24969591	0.065948678	2.48E-01	5.28E-01	1.00E+00
ENSG00000085788.12_DDHD2	ENSG00000160789.18_LMNA	-0.25835	-0.401057373	-0.1034044	1.31E-03	1.42E-02	1.00E+00
ENSG00000137770.12_CTDSPL2	ENSG00000160789.18_LMNA	-0.05514	-0.212479201	0.10497846	5.00E-01	7.53E-01	1.00E+00
ENSG00000126870.14_WDR60	ENSG00000160789.18_LMNA	0.16891	0.009976489	0.319517658	3.75E-02	1.66E-01	1.00E+00
ENSG00000177463.14_NR2C2	ENSG00000160789.18_LMNA	-0.08767	-0.243472131	0.072543614	2.83E-01	5.68E-01	1.00E+00
ENSG00000160097.14_FNDC5	ENSG00000160789.18_LMNA	-0.39489	-0.521313747	-0.2514959	4.79E-07	2.54E-05	2.46E-02
ENSG00000100601.8_ALKBH1	ENSG00000160789.18_LMNA	0.00399	-0.155314021	0.163082454	9.61E-01	9.84E-01	1.00E+00
ENSG00000249242.6_TMEM150C	ENSG00000160789.18_LMNA	-0.04469	-0.202452777	0.115328732	5.85E-01	8.07E-01	1.00E+00
ENSG00000259038.1_CTD-2325P	ENSG00000160789.18_LMNA	-0.03014	-0.188440596	0.129678811	7.12E-01	8.78E-01	1.00E+00
ENSG00000235217.6_TSPY26P	ENSG00000160789.18_LMNA	-0.02663	-0.185046136	0.133135192	7.45E-01	8.93E-01	1.00E+00
ENSG00000179387.8_ELMOD2	ENSG00000160789.18_LMNA	0.11796	-0.042029387	0.272052322	1.48E-01	3.94E-01	1.00E+00
ENSG00000118197.12_DDX59	ENSG00000160789.18_LMNA	-0.08446	-0.240429285	0.075757893	3.01E-01	5.87E-01	1.00E+00
ENSG00000143195.11_ILDR2	ENSG00000160789.18_LMNA	0.09716	-0.063017712	0.252453578	2.34E-01	5.11E-01	1.00E+00
ENSG00000243517.1_RP11-627K	ENSG00000160789.18_LMNA	-0.11036	-0.264906896	0.049713679	1.76E-01	4.37E-01	1.00E+00
ENSG00000166503.7_RP11-382A	ENSG00000160789.18_LMNA	-0.29243	-0.431543011	-0.1397379	2.56E-04	3.97E-03	1.00E+00
ENSG00000060749.13_QSER1	ENSG00000160789.18_LMNA	0.04655	-0.113490821	0.20423806	5.69E-01	7.98E-01	1.00E+00
ENSG00000228409.4_CCT6P1	ENSG00000160789.18_LMNA	-0.09349	-0.248989016	0.066699118	2.52E-01	5.33E-01	1.00E+00

ENSG00000100889.10_PCK2	ENSG00000160789.18_LMNA	0.15666	-0.002601588	0.308178418	5.39E-02	2.11E-01	1.00E+00
ENSG00000120370.11_GORAB	ENSG00000160789.18_LMNA	0.10612	-0.05399561	0.26091019	1.93E-01	4.62E-01	1.00E+00
ENSG00000110200.7_ANAPC15	ENSG00000160789.18_LMNA	-0.18752	-0.336673633	-0.02919485	2.07E-02	1.11E-01	1.00E+00
ENSG00000173281.4_PPP1R3B	ENSG00000160789.18_LMNA	0.40877	0.266939795	0.533266706	1.71E-07	1.11E-05	8.78E-03
ENSG00000130396.19_AFDN	ENSG00000160789.18_LMNA	-0.04282	-0.200648862	0.117183616	6.00E-01	8.16E-01	1.00E+00
ENSG00000009780.14_FAM76A	ENSG00000160789.18_LMNA	-0.02309	-0.181618329	0.13661769	7.78E-01	9.09E-01	1.00E+00
ENSG00000186417.12_GLDN	ENSG00000160789.18_LMNA	-0.13734	-0.2901961	0.022348907	9.16E-02	2.95E-01	1.00E+00
ENSG00000232931.4_LINC00342	ENSG00000160789.18_LMNA	0.01492	-0.144621115	0.173711177	8.55E-01	9.43E-01	1.00E+00
ENSG00000244274.6_DBNDD2	ENSG00000160789.18_LMNA	-0.24022	-0.384708562	-0.08424133	2.87E-03	2.59E-02	1.00E+00
ENSG00000064313.10_TAF2	ENSG00000160789.18_LMNA	0.07205	-0.088166023	0.228624357	3.78E-01	6.57E-01	1.00E+00
ENSG00000125744.10_RTN2	ENSG00000160789.18_LMNA	-0.18944	-0.338434085	-0.03117988	1.94E-02	1.06E-01	1.00E+00
ENSG00000177663.12_IL17RA	ENSG00000160789.18_LMNA	0.21735	0.060237368	0.363960319	7.15E-03	5.12E-02	1.00E+00
ENSG00000151490.12_PTPRO	ENSG00000160789.18_LMNA	-0.35015	-0.482453197	-0.20221775	9.78E-06	2.86E-04	5.02E-01
ENSG00000026652.12_AGPAT4	ENSG00000160789.18_LMNA	-0.23717	-0.381948325	-0.08102773	3.26E-03	2.84E-02	1.00E+00
ENSG00000124459.10_ZNF45	ENSG00000160789.18_LMNA	0.07552	-0.08469642	0.231934701	3.55E-01	6.39E-01	1.00E+00
ENSG00000276529.1_AP001505.1	ENSG00000160789.18_LMNA	0.17029	0.01139727	0.320792954	3.60E-02	1.61E-01	1.00E+00
ENSG00000079482.12_OPHN1	ENSG00000160789.18_LMNA	-0.29794	-0.43643948	-0.1456478	1.93E-04	3.18E-03	1.00E+00
ENSG00000130779.18_CLIP1	ENSG00000160789.18_LMNA	0.2865	0.133386444	0.426261463	3.46E-04	5.02E-03	1.00E+00
ENSG00000177311.9_ZBTB38	ENSG00000160789.18_LMNA	0.24525	0.089542542	0.38925019	2.32E-03	2.19E-02	1.00E+00
ENSG00000234456.6_MAGI2-AS3	ENSG00000160789.18_LMNA	0.03945	-0.120507736	0.197410683	6.29E-01	8.33E-01	1.00E+00
ENSG00000082258.11_CCNT2	ENSG00000160789.18_LMNA	-0.27416	-0.415233976	-0.12020217	6.31E-04	8.10E-03	1.00E+00
ENSG00000079277.18_MKNK1	ENSG00000160789.18_LMNA	0.03194	-0.127914277	0.190170594	6.96E-01	8.70E-01	1.00E+00
ENSG00000138185.15_ENTPD1	ENSG00000160789.18_LMNA	-0.16218	-0.313294453	-0.00306124	4.59E-02	1.90E-01	1.00E+00
ENSG00000187094.10_CCK	ENSG00000160789.18_LMNA	-0.3186	-0.454734317	-0.16791431	6.33E-05	1.30E-03	1.00E+00
ENSG00000163002.11_NUP35	ENSG00000160789.18_LMNA	-0.38494	-0.512720387	-0.24047705	9.74E-07	4.50E-05	5.00E-02
ENSG00000205413.6_SAMD9	ENSG00000160789.18_LMNA	0.17015	0.011253539	0.320663992	3.61E-02	1.62E-01	1.00E+00
ENSG00000172602.8_RND1	ENSG00000160789.18_LMNA	-0.32884	-0.463765491	-0.17901505	3.53E-05	8.17E-04	1.00E+00
ENSG00000169991.9_IFFO2	ENSG00000160789.18_LMNA	0.31878	0.168111268	0.454895069	6.26E-05	1.29E-03	1.00E+00
ENSG00000166167.16_BTRC	ENSG00000160789.18_LMNA	-0.26533	-0.407325236	-0.11081027	9.54E-04	1.12E-02	1.00E+00
ENSG00000181404.15_WASH1	ENSG00000160789.18_LMNA	0.07647	-0.08375392	0.232832674	3.49E-01	6.33E-01	1.00E+00
ENSG00000166428.11_PLD4	ENSG00000160789.18_LMNA	-0.29608	-0.434785591	-0.14364928	2.13E-04	3.42E-03	1.00E+00
ENSG00000056586.14_RC3H2	ENSG00000160789.18_LMNA	-0.07525	-0.231677127	0.084966661	3.57E-01	6.40E-01	1.00E+00
ENSG00000247134.5_RP11-11N9	ENSG00000160789.18_LMNA	-0.15904	-0.310380171	0.000166958	5.03E-02	2.02E-01	1.00E+00

ENSG00000175309.13_PHYKPL	ENSG00000160789.18_LMNA	0.10364	-0.05648989	0.258577071	2.04E-01	4.75E-01	1.00E+00
ENSG00000257621.6_PSMA3-AS	ENSG00000160789.18_LMNA	-0.03641	-0.194484469	0.123505493	6.56E-01	8.48E-01	1.00E+00
ENSG00000181027.9_FKRP	ENSG00000160789.18_LMNA	0.29336	0.140733681	0.432369241	2.44E-04	3.83E-03	1.00E+00
ENSG00000260018.1_RP11-505K	ENSG00000160789.18_LMNA	0.00935	-0.150072862	0.168301405	9.09E-01	9.65E-01	1.00E+00
ENSG00000125843.9_AP5S1	ENSG00000160789.18_LMNA	0.11259	-0.047463929	0.267002463	1.67E-01	4.25E-01	1.00E+00
ENSG00000073536.16_NLE1	ENSG00000160789.18_LMNA	-0.02157	-0.180149962	0.138107083	7.92E-01	9.16E-01	1.00E+00
ENSG00000121289.16_CEP89	ENSG00000160789.18_LMNA	0.18136	0.022821223	0.331006517	2.53E-02	1.28E-01	1.00E+00
ENSG00000171634.15_BPTF	ENSG00000160789.18_LMNA	-0.02629	-0.184721474	0.13346537	7.48E-01	8.95E-01	1.00E+00
ENSG00000183943.5_PRKX	ENSG00000160789.18_LMNA	-0.2877	-0.427329359	-0.13466873	3.26E-04	4.80E-03	1.00E+00
ENSG00000101868.9_POLA1	ENSG00000160789.18_LMNA	0.13877	-0.020887805	0.291534214	8.82E-02	2.89E-01	1.00E+00
ENSG00000170222.11_ADPRM	ENSG00000160789.18_LMNA	-0.34242	-0.475688337	-0.193782	1.57E-05	4.23E-04	8.08E-01
ENSG00000214182.5_PTMAP5	ENSG00000160789.18_LMNA	0.07342	-0.086798513	0.229929972	3.69E-01	6.50E-01	1.00E+00
ENSG00000254415.3_SIGLEC14	ENSG00000160789.18_LMNA	0.0076	-0.151780821	0.166602641	9.26E-01	9.72E-01	1.00E+00
ENSG00000143776.17_CDC42BP	ENSG00000160789.18_LMNA	0.20447	0.046788903	0.352202645	1.15E-02	7.24E-02	1.00E+00
ENSG00000164076.15_CAMKV	ENSG00000160789.18_LMNA	-0.40528	-0.530264055	-0.26304729	2.23E-07	1.39E-05	1.14E-02
ENSG00000157450.14_RNF111	ENSG00000160789.18_LMNA	0.10493	-0.055197373	0.259786535	1.98E-01	4.68E-01	1.00E+00
ENSG00000119986.6_AVPI1	ENSG00000160789.18_LMNA	-0.2026	-0.35049836	-0.04484868	1.23E-02	7.61E-02	1.00E+00
ENSG00000127663.13_KDM4B	ENSG00000160789.18_LMNA	0.00687	-0.152492942	0.165893795	9.33E-01	9.74E-01	1.00E+00
ENSG00000169302.13_STK32A	ENSG00000160789.18_LMNA	-0.07986	-0.236060726	0.080361117	3.28E-01	6.14E-01	1.00E+00
ENSG00000138092.9_CENPO	ENSG00000160789.18_LMNA	0.03024	-0.129586964	0.188530694	7.12E-01	8.77E-01	1.00E+00
ENSG00000185305.9_ARL15	ENSG00000160789.18_LMNA	-0.44487	-0.564114935	-0.30743971	9.35E-09	1.01E-06	4.80E-04
ENSG00000103326.9_CAPN15	ENSG00000160789.18_LMNA	0.26248	0.107787557	0.404770371	1.09E-03	1.23E-02	1.00E+00
ENSG00000144747.13_TMF1	ENSG00000160789.18_LMNA	0.16601	0.006991269	0.316834456	4.10E-02	1.76E-01	1.00E+00
ENSG00000070476.13_ZXDC	ENSG00000160789.18_LMNA	0.0955	-0.064686184	0.250884385	2.42E-01	5.21E-01	1.00E+00
ENSG00000073464.10_CLCN4	ENSG00000160789.18_LMNA	-0.22257	-0.368707261	-0.06569836	5.85E-03	4.42E-02	1.00E+00
ENSG00000124120.9_TTPAL	ENSG00000160789.18_LMNA	0.17846	0.019825556	0.328335213	2.78E-02	1.36E-01	1.00E+00
ENSG00000188242.4_PP7080	ENSG00000160789.18_LMNA	0.20282	0.045076236	0.350698348	1.22E-02	7.57E-02	1.00E+00
ENSG00000140022.8_STON2	ENSG00000160789.18_LMNA	-0.05833	-0.215531512	0.101813847	4.75E-01	7.34E-01	1.00E+00
ENSG00000121316.9_PLBD1	ENSG00000160789.18_LMNA	0.10367	-0.056457661	0.258607241	2.04E-01	4.75E-01	1.00E+00
ENSG00000065833.8_ME1	ENSG00000160789.18_LMNA	-0.06373	-0.220691421	0.096449444	4.35E-01	7.04E-01	1.00E+00
ENSG00000225138.6_CTD-2228K	ENSG00000160789.18_LMNA	0.11464	-0.045388065	0.268933429	1.60E-01	4.13E-01	1.00E+00
ENSG00000126561.15_STAT5A	ENSG00000160789.18_LMNA	0.22534	0.068600899	0.371223863	5.25E-03	4.07E-02	1.00E+00
ENSG00000174165.6_ZDHHC24	ENSG00000160789.18_LMNA	0.26575	0.11125329	0.407699295	9.36E-04	1.10E-02	1.00E+00

ENSG00000144857.13_BOC	ENSG00000160789.18_LMNA	0.27838	0.124710817	0.419014843	5.15E-04	6.91E-03	1.00E+00
ENSG00000144868.12_TMEM108	ENSG00000160789.18_LMNA	-0.30354	-0.441413335	-0.15167242	1.44E-04	2.51E-03	1.00E+00
ENSG00000148803.10_FUOM	ENSG00000160789.18_LMNA	-0.01302	-0.171866279	0.146482502	8.73E-01	9.51E-01	1.00E+00
ENSG00000163638.12_ADAMTS9	ENSG00000160789.18_LMNA	0.31907	0.168426065	0.455151963	6.16E-05	1.27E-03	1.00E+00
ENSG00000244242.1_IFITM10	ENSG00000160789.18_LMNA	0.00896	-0.150452514	0.16792396	9.13E-01	9.67E-01	1.00E+00
ENSG00000110888.16_CAPRIN2	ENSG00000160789.18_LMNA	0.12216	-0.037779285	0.275989562	1.34E-01	3.71E-01	1.00E+00
ENSG00000179922.5_ZNF784	ENSG00000160789.18_LMNA	0.23378	0.077462449	0.378879726	3.75E-03	3.16E-02	1.00E+00
ENSG00000129450.7_SIGLEC9	ENSG00000160789.18_LMNA	0.11562	-0.044400545	0.269851131	1.56E-01	4.08E-01	1.00E+00
ENSG00000215018.8_COL28A1	ENSG00000160789.18_LMNA	-0.13838	-0.291162227	0.021294115	8.91E-02	2.91E-01	1.00E+00
ENSG00000260766.1_RP11-226L	ENSG00000160789.18_LMNA	-0.14236	-0.294880889	0.017227682	8.02E-02	2.73E-01	1.00E+00
ENSG00000172716.15_SLFN11	ENSG00000160789.18_LMNA	0.04658	-0.113461186	0.204266829	5.69E-01	7.98E-01	1.00E+00
ENSG00000132334.15_PTPRE	ENSG00000160789.18_LMNA	0.25118	0.095808562	0.394599655	1.80E-03	1.81E-02	1.00E+00
ENSG00000156299.11_TIAM1	ENSG00000160789.18_LMNA	0.05122	-0.108873093	0.208714254	5.31E-01	7.74E-01	1.00E+00
ENSG00000150281.6_CTF1	ENSG00000160789.18_LMNA	0.29618	0.143760715	0.434877863	2.11E-04	3.41E-03	1.00E+00
ENSG00000128829.10_EIF2AK4	ENSG00000160789.18_LMNA	0.22191	0.065009904	0.368109693	6.00E-03	4.50E-02	1.00E+00
ENSG00000272086.1_CTD-2186M	ENSG00000160789.18_LMNA	-0.22971	-0.375187826	-0.07318318	4.42E-03	3.57E-02	1.00E+00
ENSG00000123213.21_NLN	ENSG00000160789.18_LMNA	0.24356	0.087765728	0.38772959	2.50E-03	2.32E-02	1.00E+00
ENSG00000184277.11_TM2D3	ENSG00000160789.18_LMNA	-0.22598	-0.37180771	-0.06927502	5.12E-03	4.00E-02	1.00E+00
ENSG00000111261.12_MANSC1	ENSG00000160789.18_LMNA	-0.15296	-0.304744006	0.006391914	5.99E-02	2.25E-01	1.00E+00
ENSG00000212978.6_AC016747.3	ENSG00000160789.18_LMNA	-0.0797	-0.23590484	0.080525127	3.29E-01	6.15E-01	1.00E+00
ENSG00000138160.5_KIF11	ENSG00000160789.18_LMNA	0.02959	-0.130223721	0.187905952	7.17E-01	8.80E-01	1.00E+00
ENSG00000004838.12_ZMYND10	ENSG00000160789.18_LMNA	0.17475	0.015995457	0.324912625	3.13E-02	1.47E-01	1.00E+00
ENSG00000204520.11_MICA	ENSG00000160789.18_LMNA	0.36494	0.218434939	0.49536496	3.79E-06	1.33E-04	1.94E-01
ENSG00000043355.9_ZIC2	ENSG00000160789.18_LMNA	0.11667	-0.043334615	0.270841061	1.52E-01	4.02E-01	1.00E+00
ENSG00000180787.5_ZFP3	ENSG00000160789.18_LMNA	-0.09923	-0.254407999	0.060937171	2.24E-01	5.00E-01	1.00E+00
ENSG00000148841.14_ITPRIP	ENSG00000160789.18_LMNA	0.47408	0.34057947	0.588835023	6.86E-10	1.15E-07	3.52E-05
ENSG00000196653.10_ZNF502	ENSG00000160789.18_LMNA	-0.25707	-0.399908121	-0.10205006	1.39E-03	1.48E-02	1.00E+00
ENSG00000136250.10_AOAH	ENSG00000160789.18_LMNA	-0.1198	-0.273781453	0.040164254	1.42E-01	3.84E-01	1.00E+00
ENSG00000100445.15_SDR39U1	ENSG00000160789.18_LMNA	-0.12689	-0.280425336	0.032977398	1.19E-01	3.47E-01	1.00E+00
ENSG00000184863.9_RBM33	ENSG00000160789.18_LMNA	0.11554	-0.044478255	0.269778936	1.56E-01	4.08E-01	1.00E+00
ENSG00000164038.13_SLC9B2	ENSG00000160789.18_LMNA	-0.29036	-0.429694195	-0.13751183	2.85E-04	4.32E-03	1.00E+00
ENSG00000187688.13_TRPV2	ENSG00000160789.18_LMNA	0.16943	0.010510348	0.319996983	3.69E-02	1.64E-01	1.00E+00
ENSG00000064692.17_SNCAIP	ENSG00000160789.18_LMNA	-0.32871	-0.463645067	-0.17886655	3.56E-05	8.21E-04	1.00E+00

ENSG00000229132.2_EIF4A1P10	ENSG00000160789.18_LMNA	0.04947	-0.110600383	0.207041463	5.45E-01	7.83E-01	1.00E+00
ENSG00000165699.12_TSC1	ENSG00000160789.18_LMNA	-0.03917	-0.197144098	0.12078108	6.32E-01	8.35E-01	1.00E+00
ENSG00000165684.3_SNAPC4	ENSG00000160789.18_LMNA	0.15505	-0.004255164	0.30668111	5.65E-02	2.17E-01	1.00E+00
ENSG00000234545.6_FAM133B	ENSG00000160789.18_LMNA	-0.10927	-0.263880124	0.050814831	1.80E-01	4.43E-01	1.00E+00
ENSG00000159256.11_MORC3	ENSG00000160789.18_LMNA	0.01097	-0.148489357	0.169874696	8.93E-01	9.58E-01	1.00E+00
ENSG00000170734.10_POLH	ENSG00000160789.18_LMNA	0.08934	-0.070871355	0.245052743	2.74E-01	5.58E-01	1.00E+00
ENSG00000255571.5_MIR9-3HG	ENSG00000160789.18_LMNA	-0.30491	-0.442625627	-0.15314409	1.34E-04	2.38E-03	1.00E+00
ENSG00000110811.18_P3H3	ENSG00000160789.18_LMNA	0.41215	0.2707152	0.536172632	1.32E-07	9.11E-06	6.78E-03
ENSG00000154975.12_CA10	ENSG00000160789.18_LMNA	-0.41707	-0.540387225	-0.27620539	9.04E-08	6.61E-06	4.64E-03
ENSG00000153982.9_GDPD1	ENSG00000160789.18_LMNA	-0.4816	-0.595169461	-0.34917188	3.36E-10	6.29E-08	1.72E-05
ENSG00000136068.13_FLNB	ENSG00000160789.18_LMNA	0.09609	-0.064093292	0.251442188	2.39E-01	5.18E-01	1.00E+00
ENSG00000141577.12_CEP131	ENSG00000160789.18_LMNA	0.17498	0.016234381	0.325126365	3.11E-02	1.47E-01	1.00E+00
ENSG00000183049.11_CAMK1D	ENSG00000160789.18_LMNA	-0.1655	-0.316363641	-0.00646803	4.16E-02	1.78E-01	1.00E+00
ENSG00000255823.2_MTRNR2L8	ENSG00000160789.18_LMNA	-0.25865	-0.401325973	-0.10372109	1.29E-03	1.41E-02	1.00E+00
ENSG00000149476.13_TKFC	ENSG00000160789.18_LMNA	0.11298	-0.047068486	0.267370498	1.66E-01	4.23E-01	1.00E+00
ENSG00000172493.19_AFF1	ENSG00000160789.18_LMNA	0.09163	-0.068571066	0.247224227	2.62E-01	5.45E-01	1.00E+00
ENSG00000082805.18_ERC1	ENSG00000160789.18_LMNA	-0.00748	-0.166483542	0.151900494	9.27E-01	9.72E-01	1.00E+00
ENSG00000113810.14_SMC4	ENSG00000160789.18_LMNA	0.2043	0.046617885	0.352052504	1.16E-02	7.28E-02	1.00E+00
ENSG00000080371.5_RAB21	ENSG00000160789.18_LMNA	0.09986	-0.060299848	0.255006174	2.21E-01	4.96E-01	1.00E+00
ENSG00000170893.3_TRH	ENSG00000160789.18_LMNA	0.04183	-0.118156867	0.199701492	6.09E-01	8.21E-01	1.00E+00
ENSG00000161835.9_GRASP	ENSG00000160789.18_LMNA	-0.08603	-0.241917005	0.074187176	2.92E-01	5.78E-01	1.00E+00
ENSG00000244567.1_	ENSG00000160789.18_LMNA	-0.08276	-0.238817073	0.077458279	3.11E-01	5.97E-01	1.00E+00
ENSG00000100056.10_DGCR14	ENSG00000160789.18_LMNA	-0.01821	-0.176898224	0.141400267	8.24E-01	9.31E-01	1.00E+00
ENSG00000113761.10_ZNF346	ENSG00000160789.18_LMNA	-0.22606	-0.37187312	-0.06935056	5.10E-03	3.99E-02	1.00E+00
ENSG00000198909.6_MAP3K3	ENSG00000160789.18_LMNA	0.05076	-0.109327984	0.208273896	5.35E-01	7.76E-01	1.00E+00
ENSG00000151881.13_TMEM267	ENSG00000160789.18_LMNA	-0.12507	-0.278721684	0.034823377	1.25E-01	3.56E-01	1.00E+00
ENSG00000113555.5_PCDH12	ENSG00000160789.18_LMNA	0.12338	-0.036536777	0.277138621	1.30E-01	3.65E-01	1.00E+00
ENSG00000266402.3_SNHG25	ENSG00000160789.18_LMNA	-0.15497	-0.306608361	0.004335462	5.66E-02	2.17E-01	1.00E+00
ENSG00000073792.14_IGF2BP2	ENSG00000160789.18_LMNA	0.21905	0.062009459	0.365502432	6.70E-03	4.88E-02	1.00E+00
ENSG00000219023.1_RP3-340B1	ENSG00000160789.18_LMNA	-0.14752	-0.299682842	0.011961326	6.97E-02	2.49E-01	1.00E+00
ENSG00000220583.1_RPL35P2	ENSG00000160789.18_LMNA	0.0803	-0.079921492	0.236478493	3.25E-01	6.12E-01	1.00E+00
ENSG00000043093.12_DCUN1D1	ENSG00000160789.18_LMNA	-0.44162	-0.561356695	-0.30378022	1.23E-08	1.28E-06	6.32E-04
ENSG00000261685.2_	ENSG00000160789.18_LMNA	-0.29704	-0.435640251	-0.14468173	2.02E-04	3.29E-03	1.00E+00

ENSG00000196091.11_MYBPC1	ENSG00000160789.18_LMNA	-0.26946	-0.411032263	-0.11520591	7.88E-04	9.61E-03	1.00E+00
ENSG00000115282.18_TTC31	ENSG00000160789.18_LMNA	0.08128	-0.078940201	0.237410572	3.19E-01	6.06E-01	1.00E+00
ENSG00000114491.12_UMPS	ENSG00000160789.18_LMNA	0.02688	-0.132890083	0.185287103	7.42E-01	8.92E-01	1.00E+00
ENSG00000247081.6_BAALC-AS1	ENSG00000160789.18_LMNA	-0.1745	-0.324678179	-0.01573343	3.15E-02	1.48E-01	1.00E+00
ENSG00000170647.3_TMEM133	ENSG00000160789.18_LMNA	-0.06241	-0.219433114	0.097759321	4.45E-01	7.12E-01	1.00E+00
ENSG00000113163.14_COL4A3B	ENSG00000160789.18_LMNA	0.03954	-0.120422967	0.197493347	6.29E-01	8.33E-01	1.00E+00
ENSG00000172985.10_SH3RF3	ENSG00000160789.18_LMNA	-0.08159	-0.23770781	0.07862714	3.18E-01	6.04E-01	1.00E+00
ENSG00000145041.14_DCAF1	ENSG00000160789.18_LMNA	0.04334	-0.116661139	0.2011572	5.96E-01	8.14E-01	1.00E+00
ENSG00000277161.1_PIGW	ENSG00000160789.18_LMNA	-0.04287	-0.200697944	0.117133177	6.00E-01	8.16E-01	1.00E+00
ENSG00000183114.7_FAM43B	ENSG00000160789.18_LMNA	0.29316	0.140517373	0.432189806	2.47E-04	3.86E-03	1.00E+00
ENSG00000178458.5_H3F3AP6	ENSG00000160789.18_LMNA	-0.14622	-0.298471018	0.01329198	7.23E-02	2.55E-01	1.00E+00
ENSG00000176018.11_LYSMD3	ENSG00000160789.18_LMNA	0.01901	-0.140620475	0.177668831	8.16E-01	9.28E-01	1.00E+00
ENSG00000130590.12_SAMD10	ENSG00000160789.18_LMNA	-0.00237	-0.161508372	0.156891291	9.77E-01	9.91E-01	1.00E+00
ENSG00000119737.5_GPR75	ENSG00000160789.18_LMNA	-0.15659	-0.308114208	0.002672535	5.40E-02	2.11E-01	1.00E+00
ENSG00000188747.7_NOXA1	ENSG00000160789.18_LMNA	0.13092	-0.028887285	0.284193077	1.08E-01	3.27E-01	1.00E+00
ENSG00000177096.7_FAM109B	ENSG00000160789.18_LMNA	0.21715	0.060019991	0.363771038	7.21E-03	5.14E-02	1.00E+00
ENSG00000272325.1_NUDT3	ENSG00000160789.18_LMNA	-0.22759	-0.373270207	-0.07096485	4.80E-03	3.80E-02	1.00E+00
ENSG00000163877.9_SNIP1	ENSG00000160789.18_LMNA	0.08962	-0.070584124	0.245324064	2.72E-01	5.57E-01	1.00E+00
ENSG00000120526.9_NUDCD1	ENSG00000160789.18_LMNA	0.01689	-0.14269224	0.175620612	8.36E-01	9.37E-01	1.00E+00
ENSG00000132205.9_EMILIN2	ENSG00000160789.18_LMNA	0.25572	0.100613397	0.398687993	1.47E-03	1.55E-02	1.00E+00
ENSG00000175265.16_GOLGA8A	ENSG00000160789.18_LMNA	-0.07586	-0.232253581	0.084361792	3.53E-01	6.37E-01	1.00E+00
ENSG00000140548.8_ZNF710	ENSG00000160789.18_LMNA	0.16672	0.007720202	0.3174901	4.01E-02	1.74E-01	1.00E+00
ENSG00000196812.4_ZSCAN16	ENSG00000160789.18_LMNA	-0.34742	-0.480067415	-0.19923794	1.16E-05	3.29E-04	5.94E-01
ENSG00000205593.10_DENND6B	ENSG00000160789.18_LMNA	0.01456	-0.144973614	0.173361972	8.59E-01	9.44E-01	1.00E+00
ENSG00000256806.3_C17orf100	ENSG00000160789.18_LMNA	-0.34274	-0.475967488	-0.19412928	1.54E-05	4.19E-04	7.92E-01
ENSG00000133800.7_LYVE1	ENSG00000160789.18_LMNA	0.10266	-0.057481954	0.257648083	2.08E-01	4.81E-01	1.00E+00
ENSG00000163818.15_LZTFL1	ENSG00000160789.18_LMNA	-0.17864	-0.328496557	-0.02000633	2.77E-02	1.36E-01	1.00E+00
ENSG00000256164.1_CCND2-AS	ENSG00000160789.18_LMNA	-0.29047	-0.429795742	-0.13763402	2.83E-04	4.30E-03	1.00E+00
ENSG00000153207.13_AHCTF1	ENSG00000160789.18_LMNA	0.18857	0.030275146	0.337631979	2.00E-02	1.08E-01	1.00E+00
ENSG00000151612.14_ZNF827	ENSG00000160789.18_LMNA	0.0057	-0.153642653	0.164748677	9.44E-01	9.78E-01	1.00E+00
ENSG00000151240.14_DIP2C	ENSG00000160789.18_LMNA	-0.05566	-0.212975379	0.104464464	4.96E-01	7.50E-01	1.00E+00
ENSG00000175727.12_MLXIP	ENSG00000160789.18_LMNA	0.15258	-0.006785536	0.30438688	6.06E-02	2.27E-01	1.00E+00
ENSG00000197121.13_PGAP1	ENSG00000160789.18_LMNA	-0.06671	-0.223532301	0.093488092	4.14E-01	6.87E-01	1.00E+00

ENSG00000135801.8_TAF5L	ENSG00000160789.18_LMNA	-0.11825	-0.272323743	0.041736763	1.47E-01	3.93E-01	1.00E+00
ENSG00000166073.7_GPR176	ENSG00000160789.18_LMNA	0.12819	-0.03165232	0.281647033	1.16E-01	3.40E-01	1.00E+00
ENSG00000128908.14_INO80	ENSG00000160789.18_LMNA	0.31213	0.160921083	0.449014425	9.05E-05	1.75E-03	1.00E+00
ENSG00000139496.14_NUP58	ENSG00000160789.18_LMNA	-0.05901	-0.21618129	0.101139332	4.70E-01	7.31E-01	1.00E+00
ENSG00000146006.7_LRRTM2	ENSG00000160789.18_LMNA	-0.21504	-0.361855727	-0.057822	7.80E-03	5.45E-02	1.00E+00
ENSG00000135709.11_KIAA0513	ENSG00000160789.18_LMNA	-0.07546	-0.23187476	0.084759313	3.56E-01	6.39E-01	1.00E+00
ENSG00000108591.8_DRG2	ENSG00000160789.18_LMNA	0.23317	0.076817562	0.378323969	3.84E-03	3.22E-02	1.00E+00
ENSG00000127314.16_RAP1B	ENSG00000160789.18_LMNA	0.02039	-0.139264636	0.179007769	8.03E-01	9.21E-01	1.00E+00
ENSG00000100034.12_PPM1F	ENSG00000160789.18_LMNA	0.21753	0.060420869	0.364120083	7.10E-03	5.10E-02	1.00E+00
ENSG00000176058.10_TPRN	ENSG00000160789.18_LMNA	0.00791	-0.151482342	0.16689965	9.23E-01	9.70E-01	1.00E+00
ENSG00000112981.4_NME5	ENSG00000160789.18_LMNA	-0.18376	-0.333216043	-0.02530323	2.34E-02	1.21E-01	1.00E+00
ENSG00000086200.15_IPO11	ENSG00000160789.18_LMNA	-0.24205	-0.386359631	-0.08616657	2.66E-03	2.43E-02	1.00E+00
ENSG00000259431.4_THTPA	ENSG00000160789.18_LMNA	-0.16834	-0.318989797	-0.00938878	3.82E-02	1.68E-01	1.00E+00
ENSG00000164164.14_OTUD4	ENSG00000160789.18_LMNA	0.22545	0.068720264	0.371327261	5.23E-03	4.06E-02	1.00E+00
ENSG00000123684.11_LPGAT1	ENSG00000160789.18_LMNA	0.00713	-0.152238794	0.166146812	9.30E-01	9.73E-01	1.00E+00
ENSG00000092871.15_RFFL	ENSG00000160789.18_LMNA	0.21031	0.052875307	0.357535812	9.31E-03	6.22E-02	1.00E+00
ENSG00000230084.4_RP4-613B2	ENSG00000160789.18_LMNA	-0.36565	-0.495978293	-0.21920911	3.62E-06	1.28E-04	1.86E-01
ENSG00000099377.12_HSD3B7	ENSG00000160789.18_LMNA	0.48531	0.35341734	0.598288029	2.35E-10	4.63E-08	1.20E-05
ENSG00000113597.16_TRAPPC1	ENSG00000160789.18_LMNA	-0.38842	-0.515726152	-0.24432323	7.62E-07	3.69E-05	3.91E-02
ENSG00000171311.11_EXOSC1	ENSG00000160789.18_LMNA	-0.17078	-0.321243132	-0.0118991	3.54E-02	1.60E-01	1.00E+00
ENSG00000104728.14_ARHGEF1	ENSG00000160789.18_LMNA	0.15837	-0.000856497	0.309756926	5.13E-02	2.05E-01	1.00E+00
ENSG00000136152.13_COG3	ENSG00000160789.18_LMNA	-0.194	-0.342622259	-0.03591208	1.66E-02	9.45E-02	1.00E+00
ENSG00000125845.6_BMP2	ENSG00000160789.18_LMNA	-0.30352	-0.441388455	-0.15164223	1.44E-04	2.52E-03	1.00E+00
ENSG00000112144.14_ICK	ENSG00000160789.18_LMNA	-0.24684	-0.390684664	-0.09122047	2.17E-03	2.08E-02	1.00E+00
ENSG00000174007.7_CEP19	ENSG00000160789.18_LMNA	0.09062	-0.069581407	0.24627085	2.67E-01	5.51E-01	1.00E+00
ENSG00000137841.10_PLCB2	ENSG00000160789.18_LMNA	0.0161	-0.143472222	0.174848779	8.44E-01	9.39E-01	1.00E+00
ENSG00000082068.7_WDR70	ENSG00000160789.18_LMNA	-0.22989	-0.375351136	-0.07337223	4.38E-03	3.55E-02	1.00E+00
ENSG00000117155.15_SSX2IP	ENSG00000160789.18_LMNA	0.31832	0.167618492	0.454492839	6.42E-05	1.31E-03	1.00E+00
ENSG00000181826.8_RELL1	ENSG00000160789.18_LMNA	0.07281	-0.08740662	0.229349529	3.73E-01	6.53E-01	1.00E+00
ENSG00000198146.4_ZNF770	ENSG00000160789.18_LMNA	0.02531	-0.134433977	0.183768652	7.57E-01	8.99E-01	1.00E+00
ENSG00000134716.8_CYP2J2	ENSG00000160789.18_LMNA	-0.07174	-0.228333658	0.088470341	3.80E-01	6.59E-01	1.00E+00
ENSG00000119906.10_SLF2	ENSG00000160789.18_LMNA	-0.01305	-0.171889117	0.146459474	8.73E-01	9.51E-01	1.00E+00
ENSG00000163762.5_TM4SF18	ENSG00000160789.18_LMNA	-0.15436	-0.306037572	0.004965342	5.76E-02	2.20E-01	1.00E+00

ENSG00000082515.15_MRPL22	ENSG00000160789.18_LMNA	-0.14048	-0.293121734	0.019152642	8.43E-02	2.81E-01	1.00E+00
ENSG00000144036.13_EXOC6B	ENSG00000160789.18_LMNA	-0.11469	-0.268982185	0.045335614	1.59E-01	4.13E-01	1.00E+00
ENSG00000175785.11_PRIMA1	ENSG00000160789.18_LMNA	-0.15016	-0.302133495	0.009266988	6.48E-02	2.37E-01	1.00E+00
ENSG00000134873.8_CLDN10	ENSG00000160789.18_LMNA	0.02466	-0.135067908	0.183144728	7.63E-01	9.01E-01	1.00E+00
ENSG00000143971.7_ETAA1	ENSG00000160789.18_LMNA	0.07689	-0.083330674	0.233235749	3.46E-01	6.31E-01	1.00E+00
ENSG00000117479.11_SLC19A2	ENSG00000160789.18_LMNA	-0.18481	-0.33418275	-0.02639035	2.26E-02	1.18E-01	1.00E+00
ENSG00000137266.13_SLC22A23	ENSG00000160789.18_LMNA	0.25133	0.095970394	0.394737547	1.79E-03	1.80E-02	1.00E+00
ENSG00000134109.9_EDEM1	ENSG00000160789.18_LMNA	0.29888	0.146660357	0.43727668	1.84E-04	3.05E-03	1.00E+00
ENSG00000058729.9_RIOK2	ENSG00000160789.18_LMNA	0.0064	-0.152953947	0.165434736	9.38E-01	9.75E-01	1.00E+00
ENSG00000177150.11_FAM210A	ENSG00000160789.18_LMNA	0.03784	-0.122098131	0.195858949	6.43E-01	8.41E-01	1.00E+00
ENSG00000163138.17_PACRGL	ENSG00000160789.18_LMNA	-0.26736	-0.409148801	-0.11297113	8.69E-04	1.04E-02	1.00E+00
ENSG00000151748.13_SAV1	ENSG00000160789.18_LMNA	0.17714	0.018460147	0.327116008	2.90E-02	1.40E-01	1.00E+00
ENSG00000105656.11_ELL	ENSG00000160789.18_LMNA	0.30886	0.157400084	0.446125569	1.08E-04	2.01E-03	1.00E+00
ENSG00000116497.16_S100PBP	ENSG00000160789.18_LMNA	0.05505	-0.105070507	0.212390328	5.01E-01	7.53E-01	1.00E+00
ENSG00000149262.15_INTS4	ENSG00000160789.18_LMNA	0.10167	-0.058473922	0.256718603	2.13E-01	4.86E-01	1.00E+00
ENSG00000151917.16_BEND6	ENSG00000160789.18_LMNA	-0.21043	-0.357646315	-0.05300166	9.27E-03	6.20E-02	1.00E+00
ENSG00000048342.14_CC2D2A	ENSG00000160789.18_LMNA	-0.22635	-0.372143766	-0.06966316	5.05E-03	3.95E-02	1.00E+00
ENSG00000146063.17_TRIM41	ENSG00000160789.18_LMNA	0.12836	-0.031479072	0.28180669	1.15E-01	3.39E-01	1.00E+00
ENSG00000068650.17_ATP11A	ENSG00000160789.18_LMNA	0.16481	0.005765087	0.315730891	4.24E-02	1.80E-01	1.00E+00
ENSG00000114626.16_ABTB1	ENSG00000160789.18_LMNA	-0.02298	-0.181517636	0.136719871	7.79E-01	9.09E-01	1.00E+00
ENSG00000118960.11_HS1BP3	ENSG00000160789.18_LMNA	0.09364	-0.066550705	0.249128844	2.51E-01	5.32E-01	1.00E+00
ENSG00000132600.15_PRMT7	ENSG00000160789.18_LMNA	-0.06299	-0.219984875	0.097185083	4.41E-01	7.08E-01	1.00E+00
ENSG00000183597.14_TANGO2	ENSG00000160789.18_LMNA	0.00375	-0.155543876	0.162853165	9.63E-01	9.85E-01	1.00E+00
ENSG00000152520.12_PAN3	ENSG00000160789.18_LMNA	-0.13933	-0.292048348	0.020326061	8.69E-02	2.86E-01	1.00E+00
ENSG00000273619.1_RP5-908M1	ENSG00000160789.18_LMNA	0.01622	-0.143346306	0.174973406	8.43E-01	9.39E-01	1.00E+00
ENSG00000057935.12_MTA3	ENSG00000160789.18_LMNA	-0.19722	-0.345574831	-0.03925647	1.49E-02	8.73E-02	1.00E+00
ENSG00000133069.13_TMCC2	ENSG00000160789.18_LMNA	-0.30126	-0.439391125	-0.1492204	1.62E-04	2.77E-03	1.00E+00
ENSG00000273576.1_RP11-390P	ENSG00000160789.18_LMNA	-0.09331	-0.248812465	0.066886489	2.53E-01	5.34E-01	1.00E+00
ENSG00000107872.11_FBXL15	ENSG00000160789.18_LMNA	-0.07701	-0.233349882	0.083210808	3.46E-01	6.30E-01	1.00E+00
ENSG00000148498.14_PARD3	ENSG00000160789.18_LMNA	0.08919	-0.071018735	0.244913507	2.75E-01	5.59E-01	1.00E+00
ENSG00000065308.4_TRAM2	ENSG00000160789.18_LMNA	0.53581	0.411725269	0.640381328	1.14E-12	6.00E-10	5.82E-08
ENSG00000179152.17_TCAIM	ENSG00000160789.18_LMNA	-0.42734	-0.549180963	-0.28771643	4.00E-08	3.43E-06	2.05E-03
ENSG00000204136.9_GGTA1P	ENSG00000160789.18_LMNA	-0.30253	-0.440513071	-0.15058037	1.52E-04	2.62E-03	1.00E+00

ENSG00000197563.8_PIGN	ENSG00000160789.18_LMNA	-0.18729	-0.336462125	-0.02895652	2.09E-02	1.11E-01	1.00E+00
ENSG00000270504.1_RP11-420L	ENSG00000160789.18_LMNA	0.42872	0.289264174	0.550358942	3.58E-08	3.13E-06	1.84E-03
ENSG00000170832.11_USP32	ENSG00000160789.18_LMNA	0.1079	-0.052192941	0.262594098	1.86E-01	4.52E-01	1.00E+00
ENSG00000076685.17_NT5C2	ENSG00000160789.18_LMNA	-0.30662	-0.444136627	-0.15498018	1.22E-04	2.22E-03	1.00E+00
ENSG00000087338.4_GMCL1	ENSG00000160789.18_LMNA	-0.17545	-0.325556197	-0.01671496	3.06E-02	1.45E-01	1.00E+00
ENSG00000258227.5_CLEC5A	ENSG00000160789.18_LMNA	0.3384	0.189404694	0.472164865	2.01E-05	5.18E-04	1.00E+00
ENSG00000069424.13_KCNAB2	ENSG00000160789.18_LMNA	0.01601	-0.143553345	0.174768481	8.45E-01	9.39E-01	1.00E+00
ENSG00000235162.7_C12orf75	ENSG00000160789.18_LMNA	-0.0758	-0.232196037	0.084422184	3.53E-01	6.37E-01	1.00E+00
ENSG00000204054.10_LINC0096	ENSG00000160789.18_LMNA	0.13585	-0.023869652	0.288802063	9.52E-02	3.02E-01	1.00E+00
ENSG00000113645.12_WWC1	ENSG00000160789.18_LMNA	-0.09553	-0.250910696	0.064658223	2.42E-01	5.21E-01	1.00E+00
ENSG00000168792.4_ABHD15	ENSG00000160789.18_LMNA	0.36039	0.213432126	0.49139484	5.10E-06	1.69E-04	2.62E-01
ENSG00000250222.1_CTC-338M1	ENSG00000160789.18_LMNA	-0.10418	-0.259087563	0.055944472	2.01E-01	4.72E-01	1.00E+00
ENSG00000183161.4_FANCF	ENSG00000160789.18_LMNA	0.08093	-0.079287796	0.237080476	3.22E-01	6.08E-01	1.00E+00
ENSG00000169967.15_MAP3K2	ENSG00000160789.18_LMNA	0.04347	-0.116538427	0.201276567	5.95E-01	8.13E-01	1.00E+00
ENSG00000165219.20_GAPVD1	ENSG00000160789.18_LMNA	0.0247	-0.135030079	0.183181967	7.63E-01	9.01E-01	1.00E+00
ENSG00000101596.13_SMCHD1	ENSG00000160789.18_LMNA	0.11473	-0.045295428	0.26901954	1.59E-01	4.13E-01	1.00E+00
ENSG00000157379.12_DHRS1	ENSG00000160789.18_LMNA	-0.00383	-0.162927449	0.155469412	9.63E-01	9.85E-01	1.00E+00
ENSG00000139372.13_TDG	ENSG00000160789.18_LMNA	-0.25725	-0.400061793	-0.10223109	1.38E-03	1.47E-02	1.00E+00
ENSG00000256771.2_ZNF253	ENSG00000160789.18_LMNA	-0.40088	-0.526480787	-0.25815519	3.09E-07	1.80E-05	1.59E-02
ENSG00000112530.10_PACRG	ENSG00000160789.18_LMNA	-0.23381	-0.37891097	-0.07749871	3.74E-03	3.16E-02	1.00E+00
ENSG00000229320.3_KRT8P12	ENSG00000160789.18_LMNA	0.44011	0.302068904	0.560064866	1.40E-08	1.42E-06	7.18E-04
ENSG00000196378.10_ZNF34	ENSG00000160789.18_LMNA	-0.21976	-0.366148752	-0.06275274	6.52E-03	4.79E-02	1.00E+00
ENSG00000111647.11_UHRF1BP	ENSG00000160789.18_LMNA	-0.16016	-0.311420291	-0.00098446	4.87E-02	1.98E-01	1.00E+00
ENSG00000198839.8_ZNF277	ENSG00000160789.18_LMNA	-0.15409	-0.305784719	0.005244293	5.80E-02	2.21E-01	1.00E+00
ENSG00000166401.12_SERPINB8	ENSG00000160789.18_LMNA	0.2631	0.108446697	0.405327886	1.06E-03	1.21E-02	1.00E+00
ENSG00000118894.13_EEF2KMT	ENSG00000160789.18_LMNA	-0.00532	-0.164380216	0.154012409	9.48E-01	9.79E-01	1.00E+00
ENSG00000198498.8_TMA16	ENSG00000160789.18_LMNA	0.02001	-0.139637597	0.178639575	8.07E-01	9.23E-01	1.00E+00
ENSG00000205791.3_LOH12CR2	ENSG00000160789.18_LMNA	-0.32395	-0.459456149	-0.17370913	4.67E-05	1.02E-03	1.00E+00
ENSG00000104231.9_ZFAND1	ENSG00000160789.18_LMNA	-0.22029	-0.366629678	-0.06330602	6.39E-03	4.71E-02	1.00E+00
ENSG00000144120.11_TMEM177	ENSG00000160789.18_LMNA	-0.01436	-0.173164609	0.145172803	8.61E-01	9.45E-01	1.00E+00
ENSG00000169981.9_ZNF35	ENSG00000160789.18_LMNA	-0.25701	-0.399853771	-0.10198603	1.39E-03	1.48E-02	1.00E+00
ENSG00000184208.10_C22orf46	ENSG00000160789.18_LMNA	0.1832	0.024719909	0.332697064	2.39E-02	1.23E-01	1.00E+00
ENSG00000221944.4_TIGD1	ENSG00000160789.18_LMNA	-0.16903	-0.319629024	-0.01010051	3.74E-02	1.65E-01	1.00E+00

ENSG00000167280.15_ENGASE	ENSG00000160789.18_LMNA	0.08862	-0.071586223	0.244377258	2.78E-01	5.63E-01	1.00E+00
ENSG00000028310.16_BRD9	ENSG00000160789.18_LMNA	0.03903	-0.120926263	0.197002485	6.33E-01	8.35E-01	1.00E+00
ENSG00000198134.3_RP11-267J:	ENSG00000160789.18_LMNA	0.0891	-0.071107095	0.244830024	2.75E-01	5.59E-01	1.00E+00
ENSG00000139190.15_VAMP1	ENSG00000160789.18_LMNA	-0.17397	-0.324195166	-0.01519372	3.21E-02	1.50E-01	1.00E+00
ENSG00000000938.11_FGR	ENSG00000160789.18_LMNA	0.00728	-0.152095462	0.166289487	9.29E-01	9.73E-01	1.00E+00
ENSG00000264107.1_RP11-848P	ENSG00000160789.18_LMNA	-0.15495	-0.306587821	0.004358132	5.66E-02	2.18E-01	1.00E+00
ENSG00000006747.13_SCIN	ENSG00000160789.18_LMNA	-0.08619	-0.242069507	0.074026078	2.91E-01	5.77E-01	1.00E+00
ENSG00000142556.17_ZNF614	ENSG00000160789.18_LMNA	-0.22956	-0.375054618	-0.07302898	4.44E-03	3.58E-02	1.00E+00
ENSG00000084444.12_FAM234B	ENSG00000160789.18_LMNA	-0.05949	-0.216634676	0.100668512	4.67E-01	7.28E-01	1.00E+00
ENSG00000256060.2_TRAPPC2B	ENSG00000160789.18_LMNA	0.0531	-0.107008683	0.210517757	5.16E-01	7.63E-01	1.00E+00
ENSG00000161021.10_MAML1	ENSG00000160789.18_LMNA	0.21656	0.059409887	0.363239656	7.37E-03	5.23E-02	1.00E+00
ENSG00000119844.13_AFTPH	ENSG00000160789.18_LMNA	-0.12253	-0.276343041	0.037397162	1.33E-01	3.69E-01	1.00E+00
ENSG00000233426.3_EIF3FP3	ENSG00000160789.18_LMNA	0.02189	-0.137788535	0.180464132	7.89E-01	9.14E-01	1.00E+00
ENSG00000157823.15_AP3S2	ENSG00000160789.18_LMNA	-0.04511	-0.202853985	0.114915886	5.81E-01	8.05E-01	1.00E+00
ENSG00000160345.11_C9orf116	ENSG00000160789.18_LMNA	0.16304	0.003936877	0.314083939	4.48E-02	1.87E-01	1.00E+00
ENSG00000135048.12_TMEM2	ENSG00000160789.18_LMNA	0.37028	0.224299175	0.500004006	2.66E-06	9.93E-05	1.37E-01
ENSG00000101452.13_DHX35	ENSG00000160789.18_LMNA	0.01956	-0.140080169	0.178202543	8.11E-01	9.26E-01	1.00E+00
ENSG00000225439.2_BOLA3-AS:	ENSG00000160789.18_LMNA	-0.13108	-0.284344187	0.028723027	1.07E-01	3.26E-01	1.00E+00
ENSG00000089902.9_RCOR1	ENSG00000160789.18_LMNA	0.27167	0.117557127	0.413011136	7.10E-04	8.88E-03	1.00E+00
ENSG00000109686.15_SH3D19	ENSG00000160789.18_LMNA	-0.08811	-0.243890056	0.072101629	2.80E-01	5.66E-01	1.00E+00
ENSG00000145337.4_PYURF	ENSG00000160789.18_LMNA	-0.1279	-0.281374102	0.031948443	1.16E-01	3.41E-01	1.00E+00
ENSG00000214401.4_KANSL1-AS	ENSG00000160789.18_LMNA	-0.06411	-0.221052296	0.096073577	4.33E-01	7.02E-01	1.00E+00
ENSG00000101017.12_CD40	ENSG00000160789.18_LMNA	0.17121	0.012343819	0.321641958	3.49E-02	1.58E-01	1.00E+00
ENSG00000115255.9_REEP6	ENSG00000160789.18_LMNA	0.09333	-0.066867389	0.248830464	2.53E-01	5.34E-01	1.00E+00
ENSG00000172943.17_PHF8	ENSG00000160789.18_LMNA	0.09118	-0.06902295	0.246797896	2.64E-01	5.47E-01	1.00E+00
ENSG00000138399.16_FASTKD1	ENSG00000160789.18_LMNA	-0.23867	-0.383307586	-0.08260946	3.06E-03	2.72E-02	1.00E+00
ENSG00000144810.14_COL8A1	ENSG00000160789.18_LMNA	0.42707	0.287415583	0.548951865	4.09E-08	3.48E-06	2.10E-03
ENSG00000007062.10_PROM1	ENSG00000160789.18_LMNA	-0.18413	-0.333556382	-0.02568588	2.32E-02	1.20E-01	1.00E+00
ENSG00000174669.10_SLC29A2	ENSG00000160789.18_LMNA	-0.05193	-0.209404092	0.108160222	5.25E-01	7.70E-01	1.00E+00
ENSG00000100902.9_PSMA6	ENSG00000160789.18_LMNA	-0.10868	-0.263324237	0.051410669	1.83E-01	4.47E-01	1.00E+00
ENSG00000159082.16_SYNJ1	ENSG00000160789.18_LMNA	-0.0547	-0.212052056	0.105420809	5.03E-01	7.55E-01	1.00E+00
ENSG00000260563.3_RP13-516M	ENSG00000160789.18_LMNA	0.10602	-0.054092794	0.260819354	1.94E-01	4.62E-01	1.00E+00
ENSG00000185022.10_MAFF	ENSG00000160789.18_LMNA	0.39792	0.254865733	0.523930928	3.84E-07	2.13E-05	1.97E-02

ENSG00000105711.9_SCN1B	ENSG00000160789.18_LMNA	-0.00508	-0.164148787	0.154244605	9.50E-01	9.80E-01	1.00E+00
ENSG00000111880.14_RNGTT	ENSG00000160789.18_LMNA	-0.19621	-0.344646021	-0.03820367	1.54E-02	8.95E-02	1.00E+00
ENSG00000185585.18_OLFML2A	ENSG00000160789.18_LMNA	0.39001	0.246087321	0.517102545	6.81E-07	3.36E-05	3.49E-02
ENSG00000176142.11_TMEM39A	ENSG00000160789.18_LMNA	-0.01138	-0.170276305	0.14808488	8.89E-01	9.57E-01	1.00E+00
ENSG00000142102.14_PGGHG	ENSG00000160789.18_LMNA	0.24984	0.094391986	0.393392056	1.91E-03	1.89E-02	1.00E+00
ENSG00000251322.6_SHANK3	ENSG00000160789.18_LMNA	-0.0699	-0.226583251	0.090301502	3.92E-01	6.70E-01	1.00E+00
ENSG00000196975.13_ANXA4	ENSG00000160789.18_LMNA	0.0658	-0.094387728	0.222669833	4.21E-01	6.92E-01	1.00E+00
ENSG00000239922.1_RP11-71N1	ENSG00000160789.18_LMNA	0.1492	-0.010247424	0.301242209	6.66E-02	2.41E-01	1.00E+00
ENSG00000081237.17_PTPRC	ENSG00000160789.18_LMNA	-0.03245	-0.190669397	0.127405142	6.91E-01	8.67E-01	1.00E+00
ENSG00000134262.11_AP4B1	ENSG00000160789.18_LMNA	-0.18552	-0.334831294	-0.02712008	2.21E-02	1.16E-01	1.00E+00
ENSG00000157483.7_MYO1E	ENSG00000160789.18_LMNA	0.40413	0.261766968	0.529274955	2.43E-07	1.49E-05	1.25E-02
ENSG00000107371.11_EXOSC3	ENSG00000160789.18_LMNA	0.10344	-0.056692236	0.258387638	2.05E-01	4.76E-01	1.00E+00
ENSG00000008277.13_ADAM22	ENSG00000160789.18_LMNA	-0.37613	-0.505082396	-0.23074177	1.79E-06	7.36E-05	9.20E-02
ENSG00000274211.3_SOCS7	ENSG00000160789.18_LMNA	-0.06225	-0.219276557	0.097922217	4.46E-01	7.13E-01	1.00E+00
ENSG00000165671.17_NSD1	ENSG00000160789.18_LMNA	0.14427	-0.015277579	0.296660865	7.62E-02	2.64E-01	1.00E+00
ENSG00000117152.12_RGS4	ENSG00000160789.18_LMNA	0.06474	-0.095449015	0.221651754	4.28E-01	6.99E-01	1.00E+00
ENSG00000100599.14_RIN3	ENSG00000160789.18_LMNA	0.10115	-0.059004866	0.256220866	2.15E-01	4.89E-01	1.00E+00
ENSG00000260081.1_RP11-66N1	ENSG00000160789.18_LMNA	-0.23225	-0.377492467	-0.07585318	3.99E-03	3.31E-02	1.00E+00
ENSG00000175455.13_CCDC14	ENSG00000160789.18_LMNA	-0.08332	-0.239344755	0.07690194	3.07E-01	5.94E-01	1.00E+00
ENSG00000066855.14_MTFR1	ENSG00000160789.18_LMNA	-0.14698	-0.299179886	0.012513735	7.08E-02	2.51E-01	1.00E+00
ENSG00000230037.1_UBBP1	ENSG00000160789.18_LMNA	0.00828	-0.151121442	0.167258694	9.19E-01	9.69E-01	1.00E+00
ENSG00000139508.13_SLC46A3	ENSG00000160789.18_LMNA	-0.03355	-0.191728526	0.126323518	6.82E-01	8.61E-01	1.00E+00
ENSG00000157693.13_TMEM268	ENSG00000160789.18_LMNA	-0.11374	-0.268083224	0.046302403	1.63E-01	4.18E-01	1.00E+00
ENSG00000131115.14_ZNF227	ENSG00000160789.18_LMNA	0.01667	-0.142912934	0.175402263	8.39E-01	9.37E-01	1.00E+00
ENSG00000151276.22_MAGI1	ENSG00000160789.18_LMNA	-0.17479	-0.324950905	-0.01603824	3.13E-02	1.47E-01	1.00E+00
ENSG00000166333.12_ILK	ENSG00000160789.18_LMNA	0.18622	0.027842425	0.335472984	2.16E-02	1.14E-01	1.00E+00
ENSG00000140968.9_IRF8	ENSG00000160789.18_LMNA	-0.13438	-0.287429731	0.025365308	9.88E-02	3.09E-01	1.00E+00
ENSG00000081377.15_CDC14B	ENSG00000160789.18_LMNA	-0.06826	-0.225012954	0.091942423	4.03E-01	6.79E-01	1.00E+00
ENSG00000186166.7_CCDC84	ENSG00000160789.18_LMNA	-0.07687	-0.233218475	0.083348815	3.47E-01	6.31E-01	1.00E+00
ENSG00000174136.10_RGMB	ENSG00000160789.18_LMNA	-0.08982	-0.24551331	0.070383749	2.71E-01	5.55E-01	1.00E+00
ENSG00000239246.3_RP11-464D	ENSG00000160789.18_LMNA	-0.04536	-0.203098845	0.114663869	5.79E-01	8.04E-01	1.00E+00
ENSG00000087301.7_TXNDC16	ENSG00000160789.18_LMNA	-0.24535	-0.389345337	-0.08965378	2.31E-03	2.19E-02	1.00E+00
ENSG00000263563.4_UBBP4	ENSG00000160789.18_LMNA	-0.1466	-0.298824157	0.012904327	7.15E-02	2.53E-01	1.00E+00

ENSG00000077684.14_JADE1	ENSG00000160789.18_LMNA	0.05085	-0.109235582	0.208363357	5.34E-01	7.76E-01	1.00E+00
ENSG00000185681.11_MORN5	ENSG00000160789.18_LMNA	-0.17064	-0.32111491	-0.01175615	3.56E-02	1.60E-01	1.00E+00
ENSG00000270419.1_CAHM	ENSG00000160789.18_LMNA	-0.28582	-0.425653868	-0.13265731	3.58E-04	5.16E-03	1.00E+00
ENSG00000171450.5_CDK5R2	ENSG00000160789.18_LMNA	-0.14884	-0.300907834	0.01061509	6.72E-02	2.43E-01	1.00E+00
ENSG00000234465.9_PINLYP	ENSG00000160789.18_LMNA	0.18893	0.030647028	0.337961733	1.98E-02	1.07E-01	1.00E+00
ENSG00000121578.11_B4GALT4	ENSG00000160789.18_LMNA	0.1305	-0.029307951	0.283806013	1.09E-01	3.29E-01	1.00E+00
ENSG00000196104.9_SPOCK3	ENSG00000160789.18_LMNA	-0.38239	-0.510511264	-0.23765569	1.16E-06	5.19E-05	5.98E-02
ENSG00000116852.13_KIF21B	ENSG00000160789.18_LMNA	-0.3883	-0.515626241	-0.24419525	7.69E-07	3.71E-05	3.94E-02
ENSG00000114770.15_ABCC5	ENSG00000160789.18_LMNA	-0.28261	-0.422789521	-0.12922424	4.19E-04	5.88E-03	1.00E+00
ENSG00000110852.4_CLEC2B	ENSG00000160789.18_LMNA	0.23275	0.076383689	0.37794994	3.91E-03	3.26E-02	1.00E+00
ENSG00000104899.5_AMH	ENSG00000160789.18_LMNA	-0.09632	-0.251658772	0.063863024	2.38E-01	5.16E-01	1.00E+00
ENSG00000198246.7_SLC29A3	ENSG00000160789.18_LMNA	-0.23824	-0.382913894	-0.08215118	3.12E-03	2.75E-02	1.00E+00
ENSG00000185338.4_SOCS1	ENSG00000160789.18_LMNA	0.306	0.154321828	0.44359503	1.26E-04	2.28E-03	1.00E+00
ENSG00000124151.17_NCOA3	ENSG00000160789.18_LMNA	0.07517	-0.085044851	0.231602595	3.57E-01	6.40E-01	1.00E+00
ENSG00000141499.15_WRAP53	ENSG00000160789.18_LMNA	0.00415	-0.155150358	0.163245694	9.60E-01	9.84E-01	1.00E+00
ENSG00000134283.16_PPHLN1	ENSG00000160789.18_LMNA	-0.32134	-0.457151201	-0.17087793	5.42E-05	1.15E-03	1.00E+00
ENSG00000123358.18_NR4A1	ENSG00000160789.18_LMNA	-0.0754	-0.231813283	0.084823815	3.56E-01	6.39E-01	1.00E+00
ENSG00000131845.13_ZNF304	ENSG00000160789.18_LMNA	0.02999	-0.129825739	0.188296455	7.14E-01	8.78E-01	1.00E+00
ENSG00000234912.8_SNHG20	ENSG00000160789.18_LMNA	-0.17842	-0.328291252	-0.0197763	2.79E-02	1.36E-01	1.00E+00
ENSG00000134152.9_KATNBL1	ENSG00000160789.18_LMNA	-0.2179	-0.364461154	-0.06081268	7.00E-03	5.04E-02	1.00E+00
ENSG00000162396.5_PARS2	ENSG00000160789.18_LMNA	0.06661	-0.093587133	0.223437376	4.15E-01	6.88E-01	1.00E+00
ENSG00000146872.16_TLK2	ENSG00000160789.18_LMNA	0.00642	-0.152941652	0.16544698	9.37E-01	9.75E-01	1.00E+00
ENSG00000204301.6_NOTCH4	ENSG00000160789.18_LMNA	0.06269	-0.097483702	0.21969797	4.43E-01	7.10E-01	1.00E+00
ENSG00000173540.11_GMPPB	ENSG00000160789.18_LMNA	0.24215	0.086280028	0.386456871	2.65E-03	2.42E-02	1.00E+00
ENSG00000160404.16_TOR2A	ENSG00000160789.18_LMNA	-0.03213	-0.190352495	0.127728627	6.94E-01	8.69E-01	1.00E+00
ENSG00000115163.13_CENPA	ENSG00000160789.18_LMNA	0.061	-0.099163507	0.218083037	4.55E-01	7.20E-01	1.00E+00
ENSG00000153179.10_RASSF3	ENSG00000160789.18_LMNA	0.12822	-0.031621818	0.281675144	1.15E-01	3.40E-01	1.00E+00
ENSG00000119782.12_FKBP1B	ENSG00000160789.18_LMNA	-0.10749	-0.262203507	0.052611261	1.87E-01	4.54E-01	1.00E+00
ENSG00000157796.16_WDR19	ENSG00000160789.18_LMNA	0.08307	-0.077152193	0.239107415	3.09E-01	5.95E-01	1.00E+00
ENSG00000079950.12_STX7	ENSG00000160789.18_LMNA	-0.35349	-0.485379237	-0.20587944	7.92E-06	2.43E-04	4.06E-01
ENSG00000213757.3_CTC-451P1	ENSG00000160789.18_LMNA	-0.16652	-0.317309968	-0.0075199	4.03E-02	1.74E-01	1.00E+00
ENSG00000266916.4_ZNF793-AS	ENSG00000160789.18_LMNA	-0.41209	-0.536117331	-0.27064328	1.33E-07	9.15E-06	6.81E-03
ENSG00000184545.9_DUSP8	ENSG00000160789.18_LMNA	-0.14527	-0.297585888	0.014263207	7.42E-02	2.59E-01	1.00E+00

ENSG00000109436.7_TBC1D9	ENSG00000160789.18_LMNA	-0.10886	-0.26349376	0.051228986	1.82E-01	4.46E-01	1.00E+00
ENSG00000196757.6_ZNF700	ENSG00000160789.18_LMNA	0.00574	-0.153601834	0.164789348	9.44E-01	9.78E-01	1.00E+00
ENSG00000105976.13_MET	ENSG00000160789.18_LMNA	0.26382	0.109210699	0.40597382	1.02E-03	1.18E-02	1.00E+00
ENSG00000225470.5_JPX	ENSG00000160789.18_LMNA	0.01022	-0.149219245	0.169149717	9.01E-01	9.61E-01	1.00E+00
ENSG00000217130.1_RP3-375P9	ENSG00000160789.18_LMNA	-0.08338	-0.239405521	0.076837861	3.07E-01	5.94E-01	1.00E+00
ENSG00000171503.10_ETFDH	ENSG00000160789.18_LMNA	-0.04632	-0.204018424	0.113717049	5.71E-01	7.99E-01	1.00E+00
ENSG00000267080.4_ASB16-AS1	ENSG00000160789.18_LMNA	-0.0523	-0.209757504	0.107794884	5.22E-01	7.68E-01	1.00E+00
ENSG00000139154.13_AEBP2	ENSG00000160789.18_LMNA	0.21498	0.057759582	0.361801299	7.82E-03	5.46E-02	1.00E+00
ENSG00000134897.12_BIVM	ENSG00000160789.18_LMNA	-0.26059	-0.403064696	-0.10577259	1.19E-03	1.32E-02	1.00E+00
ENSG00000156011.15_PSD3	ENSG00000160789.18_LMNA	8.88E-05	-0.159114155	0.159287246	9.99E-01	1.00E+00	1.00E+00
ENSG00000153214.8_TMEM87B	ENSG00000160789.18_LMNA	0.17877	0.020139715	0.328615588	2.76E-02	1.35E-01	1.00E+00
ENSG00000150961.13_SEC24D	ENSG00000160789.18_LMNA	0.34273	0.194116221	0.475956994	1.55E-05	4.19E-04	7.93E-01
ENSG00000260257.2_RP5-1085F	ENSG00000160789.18_LMNA	0.00211	-0.157139322	0.161260695	9.79E-01	9.92E-01	1.00E+00
ENSG00000130164.10_LDLR	ENSG00000160789.18_LMNA	0.51461	0.387124157	0.62278739	1.18E-11	3.95E-09	6.08E-07
ENSG00000172795.14_DCP2	ENSG00000160789.18_LMNA	-0.14438	-0.296761102	0.015167692	7.60E-02	2.64E-01	1.00E+00
ENSG00000163214.19_DHX57	ENSG00000160789.18_LMNA	-0.07376	-0.230257145	0.086455642	3.66E-01	6.47E-01	1.00E+00
ENSG00000263470.1_	ENSG00000160789.18_LMNA	-0.15277	-0.304567738	0.006586208	6.02E-02	2.26E-01	1.00E+00
ENSG00000198483.11_ANKRD35	ENSG00000160789.18_LMNA	0.10733	-0.05277323	0.262052248	1.88E-01	4.55E-01	1.00E+00
ENSG00000135776.4_ABCB10	ENSG00000160789.18_LMNA	0.14538	-0.014150296	0.297688818	7.39E-02	2.59E-01	1.00E+00
ENSG00000234327.6_AC012146.7	ENSG00000160789.18_LMNA	-0.1585	-0.30988426	0.000715642	5.11E-02	2.04E-01	1.00E+00
ENSG00000152404.14_CWF19L2	ENSG00000160789.18_LMNA	-0.08744	-0.243255612	0.072772548	2.84E-01	5.70E-01	1.00E+00
ENSG00000154957.12_ZNF18	ENSG00000160789.18_LMNA	0.1832	0.024715178	0.332692854	2.39E-02	1.23E-01	1.00E+00
ENSG00000100764.12_PSMC1	ENSG00000160789.18_LMNA	0.06097	-0.099192931	0.218054734	4.56E-01	7.20E-01	1.00E+00
ENSG00000101901.9_ALG13	ENSG00000160789.18_LMNA	-0.33438	-0.46863514	-0.18503086	2.55E-05	6.27E-04	1.00E+00
ENSG00000250786.1_SNHG18	ENSG00000160789.18_LMNA	0.09211	-0.068093304	0.247674839	2.59E-01	5.42E-01	1.00E+00
ENSG00000261474.1_RP11-452L	ENSG00000160789.18_LMNA	0.05345	-0.106660692	0.210854142	5.13E-01	7.61E-01	1.00E+00
ENSG00000197299.9_BLM	ENSG00000160789.18_LMNA	0.23513	0.078884421	0.380104397	3.55E-03	3.03E-02	1.00E+00
ENSG00000151092.15_NGLY1	ENSG00000160789.18_LMNA	-0.1204	-0.274346327	0.039554476	1.40E-01	3.81E-01	1.00E+00
ENSG00000243199.1_RP11-408P	ENSG00000160789.18_LMNA	-0.09624	-0.251581447	0.063945238	2.38E-01	5.17E-01	1.00E+00
ENSG00000219200.9_RNASEK	ENSG00000160789.18_LMNA	0.02179	-0.13788575	0.18036826	7.90E-01	9.15E-01	1.00E+00
ENSG00000169679.13_BUB1	ENSG00000160789.18_LMNA	0.07858	-0.081642478	0.234842407	3.36E-01	6.22E-01	1.00E+00
ENSG00000188811.11_NHLRC3	ENSG00000160789.18_LMNA	-0.07549	-0.231901403	0.084731358	3.55E-01	6.39E-01	1.00E+00
ENSG00000082196.19_C1QTNF3	ENSG00000160789.18_LMNA	-0.2544	-0.397500623	-0.0992165	1.56E-03	1.62E-02	1.00E+00

ENSG00000116874.10_WARS2	ENSG00000160789.18_LMNA	-0.05981	-0.216941447	0.100349865	4.64E-01	7.27E-01	1.00E+00
ENSG00000085274.14_MYNN	ENSG00000160789.18_LMNA	-0.21183	-0.358923876	-0.05446314	8.80E-03	5.97E-02	1.00E+00
ENSG00000033178.11_UBA6	ENSG00000160789.18_LMNA	-0.02514	-0.183601847	0.134603482	7.59E-01	8.99E-01	1.00E+00
ENSG00000204334.7_ERICH2	ENSG00000160789.18_LMNA	0.15421	-0.005111323	0.305905254	5.78E-02	2.20E-01	1.00E+00
ENSG00000187135.7_VSTM2B	ENSG00000160789.18_LMNA	-0.44549	-0.56464063	-0.30813804	8.87E-09	9.77E-07	4.55E-04
ENSG00000186462.8_NAP1L2	ENSG00000160789.18_LMNA	-0.29771	-0.436233881	-0.14539923	1.95E-04	3.20E-03	1.00E+00
ENSG00000077080.8_ACTL6B	ENSG00000160789.18_LMNA	-0.35837	-0.489633241	-0.21121697	5.81E-06	1.88E-04	2.98E-01
ENSG00000153029.13_MR1	ENSG00000160789.18_LMNA	0.11142	-0.048641299	0.265906153	1.72E-01	4.31E-01	1.00E+00
ENSG00000251493.3_FOXD1	ENSG00000160789.18_LMNA	-0.1531	-0.304871571	0.006251288	5.97E-02	2.25E-01	1.00E+00
ENSG00000186814.11_ZSCAN30	ENSG00000160789.18_LMNA	-0.07875	-0.23500709	0.081469334	3.35E-01	6.21E-01	1.00E+00
ENSG00000187742.13_SECISBP2	ENSG00000160789.18_LMNA	-0.07554	-0.23195261	0.084677628	3.55E-01	6.38E-01	1.00E+00
ENSG00000279673.1_RP11-185E	ENSG00000160789.18_LMNA	-0.30692	-0.444403668	-0.15530488	1.20E-04	2.19E-03	1.00E+00
ENSG00000145882.9_PCYOX1L	ENSG00000160789.18_LMNA	0.13588	-0.023838685	0.288830464	9.51E-02	3.02E-01	1.00E+00
ENSG00000102189.15_EEA1	ENSG00000160789.18_LMNA	0.22533	0.068594115	0.371217986	5.25E-03	4.07E-02	1.00E+00
ENSG00000167912.5_RP11-25K1	ENSG00000160789.18_LMNA	-0.23872	-0.383349583	-0.08265836	3.06E-03	2.71E-02	1.00E+00
ENSG00000024048.9_UBR2	ENSG00000160789.18_LMNA	-0.05086	-0.208373248	0.109225365	5.34E-01	7.76E-01	1.00E+00
ENSG00000152464.13_RPP38	ENSG00000160789.18_LMNA	-0.17356	-0.323809509	-0.01476293	3.25E-02	1.51E-01	1.00E+00
ENSG00000197905.7_TEAD4	ENSG00000160789.18_LMNA	0.15392	-0.005409462	0.305634982	5.83E-02	2.21E-01	1.00E+00
ENSG00000184154.11_LRTOMT	ENSG00000160789.18_LMNA	-0.20294	-0.350803318	-0.04519569	1.22E-02	7.55E-02	1.00E+00
ENSG00000137166.13_FOXP4	ENSG00000160789.18_LMNA	0.16314	0.004042266	0.31417893	4.46E-02	1.87E-01	1.00E+00
ENSG00000083817.8_ZNF416	ENSG00000160789.18_LMNA	0.03419	-0.125689947	0.192348576	6.76E-01	8.58E-01	1.00E+00
ENSG00000168497.4_SDPR	ENSG00000160789.18_LMNA	-0.23421	-0.379266552	-0.07791146	3.68E-03	3.12E-02	1.00E+00
ENSG00000197892.11_KIF13B	ENSG00000160789.18_LMNA	0.33108	0.18144658	0.465735827	3.10E-05	7.32E-04	1.00E+00
ENSG00000187231.12_SESTD1	ENSG00000160789.18_LMNA	-0.30085	-0.439026874	-0.14877911	1.66E-04	2.82E-03	1.00E+00
ENSG00000276180.1_HIST1H4I	ENSG00000160789.18_LMNA	0.0189	-0.140721591	0.177568928	8.17E-01	9.29E-01	1.00E+00
ENSG00000100416.11_TRMU	ENSG00000160789.18_LMNA	-0.1074	-0.2621228	0.052697685	1.88E-01	4.54E-01	1.00E+00
ENSG00000147894.13_C9orf72	ENSG00000160789.18_LMNA	-0.27325	-0.414421892	-0.11923536	6.59E-04	8.36E-03	1.00E+00
ENSG00000179388.8_EGR3	ENSG00000160789.18_LMNA	0.10659	-0.053523545	0.26135134	1.91E-01	4.59E-01	1.00E+00
ENSG00000144451.17_SPAG16	ENSG00000160789.18_LMNA	-0.14228	-0.294803793	0.017312092	8.04E-02	2.73E-01	1.00E+00
ENSG00000071539.12_TRIP13	ENSG00000160789.18_LMNA	0.07188	-0.088335265	0.228462696	3.79E-01	6.58E-01	1.00E+00
ENSG00000021762.18_OSBPL5	ENSG00000160789.18_LMNA	0.42523	0.2853419	0.547371691	4.74E-08	3.91E-06	2.43E-03
ENSG00000280088.1_RP11-135F	ENSG00000160789.18_LMNA	-0.1829	-0.332425863	-0.02441517	2.41E-02	1.23E-01	1.00E+00
ENSG00000067369.12_TP53BP1	ENSG00000160789.18_LMNA	0.17364	0.014849324	0.323886862	3.24E-02	1.51E-01	1.00E+00

ENSG00000188677.13_PARVB	ENSG00000160789.18_LMNA	0.2067	0.049118346	0.354246148	1.06E-02	6.84E-02	1.00E+00
ENSG00000167333.11_TRIM68	ENSG00000160789.18_LMNA	-0.09227	-0.247828842	0.067929988	2.58E-01	5.41E-01	1.00E+00
ENSG00000161381.12_PLXDC1	ENSG00000160789.18_LMNA	0.13885	-0.020814606	0.291601219	8.80E-02	2.89E-01	1.00E+00
ENSG00000233830.2_EIF4HP1	ENSG00000160789.18_LMNA	0.25028	0.094861156	0.393792127	1.87E-03	1.86E-02	1.00E+00
ENSG00000197024.7_ZNF398	ENSG00000160789.18_LMNA	0.23165	0.075222651	0.376948559	4.08E-03	3.37E-02	1.00E+00
ENSG00000154144.11_TBRG1	ENSG00000160789.18_LMNA	-0.11485	-0.269129544	0.045177081	1.59E-01	4.12E-01	1.00E+00
ENSG00000172262.10_ZNF131	ENSG00000160789.18_LMNA	-0.15787	-0.309301302	0.001360398	5.21E-02	2.07E-01	1.00E+00
ENSG00000140691.15_ARMC5	ENSG00000160789.18_LMNA	0.15243	-0.006934347	0.304251845	6.08E-02	2.28E-01	1.00E+00
ENSG00000145569.5_FAM105A	ENSG00000160789.18_LMNA	-0.29416	-0.433079184	-0.14158978	2.35E-04	3.71E-03	1.00E+00
ENSG00000083457.10_ITGAE	ENSG00000160789.18_LMNA	-0.30775	-0.445141208	-0.156202	1.15E-04	2.11E-03	1.00E+00
ENSG00000147601.12_TERF1	ENSG00000160789.18_LMNA	-0.23959	-0.384139457	-0.08357824	2.95E-03	2.64E-02	1.00E+00
ENSG00000159239.10_C2orf81	ENSG00000160789.18_LMNA	0.17907	0.020455796	0.328897623	2.73E-02	1.34E-01	1.00E+00
ENSG00000178127.11_NDUFV2	ENSG00000160789.18_LMNA	-0.13182	-0.285039268	0.027967249	1.05E-01	3.22E-01	1.00E+00
ENSG00000016391.9_CHDH	ENSG00000160789.18_LMNA	0.1537	-0.005638494	0.305427325	5.87E-02	2.22E-01	1.00E+00
ENSG00000204934.9_ATP6V0E2-	ENSG00000160789.18_LMNA	0.04002	-0.119945647	0.197958728	6.24E-01	8.31E-01	1.00E+00
ENSG00000166478.8_ZNF143	ENSG00000160789.18_LMNA	-0.11815	-0.272232561	0.041835074	1.47E-01	3.93E-01	1.00E+00
ENSG00000149256.13_TENM4	ENSG00000160789.18_LMNA	0.14213	-0.017469625	0.294659901	8.07E-02	2.74E-01	1.00E+00
ENSG00000163625.14_WDFY3	ENSG00000160789.18_LMNA	-0.11973	-0.273716794	0.040234039	1.42E-01	3.84E-01	1.00E+00
ENSG00000139651.10_ZNF740	ENSG00000160789.18_LMNA	-0.08553	-0.241440041	0.07469092	2.95E-01	5.81E-01	1.00E+00
ENSG00000203797.8_DDO	ENSG00000160789.18_LMNA	0.02915	-0.130658585	0.187479146	7.21E-01	8.82E-01	1.00E+00
ENSG00000078053.15_AMPH	ENSG00000160789.18_LMNA	-0.37079	-0.500451662	-0.22486612	2.57E-06	9.65E-05	1.32E-01
ENSG00000170854.16_MINA	ENSG00000160789.18_LMNA	0.00895	-0.150465178	0.167911368	9.13E-01	9.67E-01	1.00E+00
ENSG00000148688.12_RPP30	ENSG00000160789.18_LMNA	-0.25142	-0.394820143	-0.09606734	1.78E-03	1.79E-02	1.00E+00
ENSG00000171227.6_TMEM37	ENSG00000160789.18_LMNA	0.12534	-0.034545964	0.278977834	1.24E-01	3.55E-01	1.00E+00
ENSG00000163728.9_TTC14	ENSG00000160789.18_LMNA	-0.23529	-0.380246115	-0.07904905	3.52E-03	3.02E-02	1.00E+00
ENSG00000259407.1_RP11-158M	ENSG00000160789.18_LMNA	-0.32249	-0.458170533	-0.17212941	5.08E-05	1.09E-03	1.00E+00
ENSG00000073803.12_MAP3K13	ENSG00000160789.18_LMNA	-0.34066	-0.474149318	-0.19186865	1.75E-05	4.63E-04	8.98E-01
ENSG00000011132.10_APBA3	ENSG00000160789.18_LMNA	0.13213	-0.027652293	0.285328833	1.05E-01	3.21E-01	1.00E+00
ENSG00000253250.2_C8orf88	ENSG00000160789.18_LMNA	-0.12227	-0.276094188	0.037666191	1.33E-01	3.71E-01	1.00E+00
ENSG00000164073.8_MFSD8	ENSG00000160789.18_LMNA	-0.01114	-0.170038389	0.148324508	8.92E-01	9.57E-01	1.00E+00
ENSG00000100360.13_IFT27	ENSG00000160789.18_LMNA	-0.22553	-0.371396992	-0.06880077	5.21E-03	4.05E-02	1.00E+00
ENSG00000165406.14_MARCH8	ENSG00000160789.18_LMNA	-0.21006	-0.357308516	-0.05261545	9.39E-03	6.26E-02	1.00E+00
ENSG00000119640.7_ACYP1	ENSG00000160789.18_LMNA	-0.00811	-0.167092654	0.15128835	9.21E-01	9.70E-01	1.00E+00

ENSG00000138061.10_CYP1B1	ENSG00000160789.18_LMNA	0.22747	0.070837685	0.373160205	4.83E-03	3.82E-02	1.00E+00
ENSG00000144357.15_UBR3	ENSG00000160789.18_LMNA	-0.00914	-0.168100493	0.15027496	9.11E-01	9.66E-01	1.00E+00
ENSG00000168395.13_ING5	ENSG00000160789.18_LMNA	0.01399	-0.145536618	0.172804063	8.64E-01	9.47E-01	1.00E+00
ENSG00000131323.13_TRAF3	ENSG00000160789.18_LMNA	0.29746	0.14512699	0.43600867	1.98E-04	3.24E-03	1.00E+00
ENSG00000163328.12_GPR155	ENSG00000160789.18_LMNA	-0.30682	-0.444318186	-0.15520093	1.21E-04	2.20E-03	1.00E+00
ENSG00000119535.16_CSF3R	ENSG00000160789.18_LMNA	-0.01677	-0.175502904	0.142811216	8.38E-01	9.37E-01	1.00E+00
ENSG00000120875.7_DUSP4	ENSG00000160789.18_LMNA	0.41938	0.278794405	0.54237012	7.54E-08	5.73E-06	3.87E-03
ENSG00000164418.18_GRIK2	ENSG00000160789.18_LMNA	-0.27701	-0.417788013	-0.1232465	5.50E-04	7.28E-03	1.00E+00
ENSG00000206560.9_ANKRD28	ENSG00000160789.18_LMNA	-0.14721	-0.299396327	0.012276035	7.03E-02	2.50E-01	1.00E+00
ENSG00000165934.11_CPSF2	ENSG00000160789.18_LMNA	0.10784	-0.052256873	0.26253441	1.86E-01	4.52E-01	1.00E+00
ENSG00000111785.17_RIC8B	ENSG00000160789.18_LMNA	-0.25648	-0.399377368	-0.10142496	1.42E-03	1.51E-02	1.00E+00
ENSG00000113569.14_NUP155	ENSG00000160789.18_LMNA	0.04313	-0.116876747	0.200947449	5.98E-01	8.15E-01	1.00E+00
ENSG00000221990.4_EXOC3-AS	ENSG00000160789.18_LMNA	0.01643	-0.143143197	0.175174414	8.41E-01	9.38E-01	1.00E+00
ENSG00000112339.13_HBS1L	ENSG00000160789.18_LMNA	-0.15468	-0.30634115	0.004630366	5.71E-02	2.18E-01	1.00E+00
ENSG00000071189.20_SNX13	ENSG00000160789.18_LMNA	-0.01981	-0.178444996	0.139834658	8.09E-01	9.24E-01	1.00E+00
ENSG00000075218.17_GTSE1	ENSG00000160789.18_LMNA	0.06458	-0.095606444	0.221500675	4.29E-01	6.99E-01	1.00E+00
ENSG00000106617.12_PRKAG2	ENSG00000160789.18_LMNA	-0.33702	-0.470949241	-0.18789709	2.18E-05	5.54E-04	1.00E+00
ENSG00000196456.7_ZNF775	ENSG00000160789.18_LMNA	-0.0738	-0.230296914	0.086413961	3.66E-01	6.47E-01	1.00E+00
ENSG00000149483.10_TMEM138	ENSG00000160789.18_LMNA	0.01212	-0.147367226	0.170988608	8.82E-01	9.54E-01	1.00E+00
ENSG00000008083.12_JARID2	ENSG00000160789.18_LMNA	-0.05959	-0.216731397	0.100568054	4.66E-01	7.28E-01	1.00E+00
ENSG00000181894.13_ZNF329	ENSG00000160789.18_LMNA	-0.09539	-0.250778663	0.064798531	2.42E-01	5.22E-01	1.00E+00
ENSG00000082397.14_EPB41L3	ENSG00000160789.18_LMNA	-0.13646	-0.289372961	0.023247039	9.37E-02	2.99E-01	1.00E+00
ENSG00000196954.11_CASP4	ENSG00000160789.18_LMNA	0.19645	0.038453085	0.344866119	1.53E-02	8.90E-02	1.00E+00
ENSG00000133059.15_DSTYK	ENSG00000160789.18_LMNA	0.01784	-0.141763341	0.176539295	8.27E-01	9.32E-01	1.00E+00
ENSG00000269888.1_RP11-3P17	ENSG00000160789.18_LMNA	-0.16235	-0.313450834	-0.00323464	4.57E-02	1.90E-01	1.00E+00
ENSG00000198169.7_ZNF251	ENSG00000160789.18_LMNA	-0.149	-0.301056719	0.010451393	6.69E-02	2.42E-01	1.00E+00
ENSG00000125846.14_ZNF133	ENSG00000160789.18_LMNA	0.00126	-0.157971812	0.160429099	9.88E-01	9.95E-01	1.00E+00
ENSG00000114859.13_CLCN2	ENSG00000160789.18_LMNA	0.17762	0.018950494	0.327553969	2.86E-02	1.39E-01	1.00E+00
ENSG00000165275.8_TRMT10B	ENSG00000160789.18_LMNA	-0.12039	-0.274334666	0.039567067	1.40E-01	3.81E-01	1.00E+00
ENSG00000176390.11_CRLF3	ENSG00000160789.18_LMNA	-0.15407	-0.305773344	0.005256842	5.81E-02	2.21E-01	1.00E+00
ENSG00000081692.11_JMJD4	ENSG00000160789.18_LMNA	-0.03997	-0.197913365	0.11999218	6.25E-01	8.31E-01	1.00E+00
ENSG00000075131.8_TIPIN	ENSG00000160789.18_LMNA	0.24517	0.089464481	0.38918342	2.33E-03	2.20E-02	1.00E+00
ENSG00000135587.7_SMPD2	ENSG00000160789.18_LMNA	-0.0861	-0.24198148	0.074119068	2.92E-01	5.77E-01	1.00E+00

ENSG00000258441.1_LINC00641	ENSG00000160789.18_LMNA	-0.30238	-0.440378066	-0.15041667	1.53E-04	2.64E-03	1.00E+00
ENSG00000114021.10_NIT2	ENSG00000160789.18_LMNA	-0.24938	-0.392977432	-0.09390589	1.95E-03	1.91E-02	1.00E+00
ENSG00000214612.3_RPS19P1	ENSG00000160789.18_LMNA	-0.02726	-0.185652134	0.132518706	7.39E-01	8.90E-01	1.00E+00
ENSG00000123965.13_PMS2P5	ENSG00000160789.18_LMNA	0.02058	-0.139076005	0.179193956	8.01E-01	9.20E-01	1.00E+00
ENSG00000148773.11_MKI67	ENSG00000160789.18_LMNA	0.1482	-0.011269252	0.300312716	6.84E-02	2.46E-01	1.00E+00
ENSG00000189376.10_C8orf76	ENSG00000160789.18_LMNA	-0.08852	-0.244276189	0.071693157	2.78E-01	5.63E-01	1.00E+00
ENSG00000151746.12_BICD1	ENSG00000160789.18_LMNA	0.34654	0.198273313	0.479294198	1.22E-05	3.45E-04	6.28E-01
ENSG00000079150.16_FKBP7	ENSG00000160789.18_LMNA	-0.00503	-0.164100703	0.154292845	9.51E-01	9.80E-01	1.00E+00
ENSG00000095574.10_IKZF5	ENSG00000160789.18_LMNA	-0.50875	-0.617900371	-0.38034981	2.20E-11	6.48E-09	1.13E-06
ENSG00000235508.3_RPS2P7	ENSG00000160789.18_LMNA	-0.00459	-0.163667689	0.154727187	9.55E-01	9.82E-01	1.00E+00
ENSG00000166278.13_C2	ENSG00000160789.18_LMNA	0.01124	-0.148226749	0.170135454	8.91E-01	9.57E-01	1.00E+00
ENSG00000178726.6_THBD	ENSG00000160789.18_LMNA	0.26576	0.111269472	0.407712956	9.36E-04	1.10E-02	1.00E+00
ENSG00000155868.7_MED7	ENSG00000160789.18_LMNA	-0.17847	-0.328341373	-0.01983246	2.78E-02	1.36E-01	1.00E+00
ENSG00000187556.7_NANOS3	ENSG00000160789.18_LMNA	-0.21645	-0.363136357	-0.05929131	7.40E-03	5.24E-02	1.00E+00
ENSG00000246695.6_RASSF8-AS	ENSG00000160789.18_LMNA	-0.0493	-0.206879714	0.1107673	5.46E-01	7.84E-01	1.00E+00
ENSG00000136040.7_PLXNC1	ENSG00000160789.18_LMNA	-0.21847	-0.364978084	-0.0614067	6.85E-03	4.96E-02	1.00E+00
ENSG00000134326.10_CMPK2	ENSG00000160789.18_LMNA	-0.06561	-0.222490164	0.094575075	4.22E-01	6.93E-01	1.00E+00
ENSG00000163710.6_PCOLCE2	ENSG00000160789.18_LMNA	-0.00399	-0.163086675	0.15530979	9.61E-01	9.84E-01	1.00E+00
ENSG00000109756.7_RAPGEF2	ENSG00000160789.18_LMNA	-0.24755	-0.391330463	-0.09197642	2.11E-03	2.03E-02	1.00E+00
ENSG00000103222.17_ABCC1	ENSG00000160789.18_LMNA	0.16775	0.008780307	0.31844309	3.89E-02	1.70E-01	1.00E+00
ENSG00000213885.3_RPL13AP7	ENSG00000160789.18_LMNA	-0.1495	-0.301520656	0.009941189	6.60E-02	2.40E-01	1.00E+00
ENSG00000143476.16_DTL	ENSG00000160789.18_LMNA	0.06896	-0.091243788	0.225681721	3.99E-01	6.75E-01	1.00E+00
ENSG00000185753.11_CXorf38	ENSG00000160789.18_LMNA	0.22492	0.06815951	0.370841449	5.34E-03	4.12E-02	1.00E+00
ENSG00000177548.11_RABEP2	ENSG00000160789.18_LMNA	0.27898	0.125351685	0.419551436	5.00E-04	6.74E-03	1.00E+00
ENSG00000128694.10_OSGEPL1	ENSG00000160789.18_LMNA	-0.46564	-0.581714002	-0.33096935	1.50E-09	2.19E-07	7.68E-05
ENSG00000198000.10_NOL8	ENSG00000160789.18_LMNA	0.16592	0.006906425	0.316758123	4.11E-02	1.76E-01	1.00E+00
ENSG00000114331.11_ACAP2	ENSG00000160789.18_LMNA	-0.09265	-0.248189658	0.067547289	2.56E-01	5.39E-01	1.00E+00
ENSG00000263422.1_	ENSG00000160789.18_LMNA	-0.01914	-0.177796528	0.140491218	8.15E-01	9.28E-01	1.00E+00
ENSG00000198464.12_ZNF480	ENSG00000160789.18_LMNA	-0.08274	-0.238793538	0.077483087	3.11E-01	5.97E-01	1.00E+00
ENSG00000108666.8_C17orf75	ENSG00000160789.18_LMNA	-0.38067	-0.50902312	-0.23575771	1.31E-06	5.75E-05	6.73E-02
ENSG00000115020.15_PIKFYVE	ENSG00000160789.18_LMNA	0.03516	-0.124740622	0.193277167	6.67E-01	8.53E-01	1.00E+00
ENSG00000225377.4_NRSN2-AS	ENSG00000160789.18_LMNA	0.04248	-0.117513805	0.20032752	6.03E-01	8.18E-01	1.00E+00
ENSG00000109576.12_AADAT	ENSG00000160789.18_LMNA	-0.15409	-0.30578483	0.00524417	5.80E-02	2.21E-01	1.00E+00

ENSG00000181035.12_SLC25A42	ENSG00000160789.18_LMNA	-0.13858	-0.291357312	0.021081042	8.86E-02	2.90E-01	1.00E+00
ENSG00000170776.18_AKAP13	ENSG00000160789.18_LMNA	0.30023	0.148106387	0.438471409	1.71E-04	2.89E-03	1.00E+00
ENSG00000122970.14_IFT81	ENSG00000160789.18_LMNA	0.01753	-0.142067833	0.176238212	8.30E-01	9.34E-01	1.00E+00
ENSG00000197147.11_LRRC8B	ENSG00000160789.18_LMNA	-0.31164	-0.448585177	-0.16039744	9.30E-05	1.78E-03	1.00E+00
ENSG00000258297.1_RP11-658F	ENSG00000160789.18_LMNA	-0.28851	-0.428050079	-0.1355347	3.13E-04	4.65E-03	1.00E+00
ENSG00000169840.4_GSX1	ENSG00000160789.18_LMNA	-0.20518	-0.352853902	-0.04753093	1.12E-02	7.11E-02	1.00E+00
ENSG00000066777.7_ARFGEF1	ENSG00000160789.18_LMNA	-0.03405	-0.192208862	0.12583273	6.77E-01	8.59E-01	1.00E+00
ENSG00000160188.8_RSPH1	ENSG00000160789.18_LMNA	-0.03244	-0.190655513	0.127419315	6.92E-01	8.67E-01	1.00E+00
ENSG00000124198.8_ARFGEF2	ENSG00000160789.18_LMNA	0.0604	-0.099760105	0.217509061	4.60E-01	7.23E-01	1.00E+00
ENSG00000048028.10_USP28	ENSG00000160789.18_LMNA	-0.0572	-0.214450875	0.102934978	4.84E-01	7.41E-01	1.00E+00
ENSG00000173821.18_RNF213	ENSG00000160789.18_LMNA	0.21563	0.058434116	0.362389378	7.63E-03	5.36E-02	1.00E+00
ENSG00000162542.12_TMCO4	ENSG00000160789.18_LMNA	0.37513	0.229641676	0.50421659	1.92E-06	7.73E-05	9.85E-02
ENSG00000099219.12_ERMP1	ENSG00000160789.18_LMNA	-0.15199	-0.303837418	0.007390963	6.16E-02	2.29E-01	1.00E+00
ENSG00000104332.10_SFRP1	ENSG00000160789.18_LMNA	-0.22076	-0.367062443	-0.06380407	6.27E-03	4.65E-02	1.00E+00
ENSG00000237187.7_NR2F1-AS1	ENSG00000160789.18_LMNA	-0.32783	-0.462875292	-0.17791763	3.74E-05	8.51E-04	1.00E+00
ENSG00000081019.12_RSBN1	ENSG00000160789.18_LMNA	-0.30914	-0.446368114	-0.15769542	1.07E-04	1.98E-03	1.00E+00
ENSG00000070778.11_PTPN21	ENSG00000160789.18_LMNA	0.15529	-0.004012548	0.306900894	5.61E-02	2.16E-01	1.00E+00
ENSG00000147679.10_UTP23	ENSG00000160789.18_LMNA	-0.29346	-0.432456088	-0.14083838	2.43E-04	3.82E-03	1.00E+00
ENSG00000200534.1_SNORA33	ENSG00000160789.18_LMNA	-0.05123	-0.208725448	0.108861528	5.31E-01	7.74E-01	1.00E+00
ENSG00000099814.14_CEP170B	ENSG00000160789.18_LMNA	0.23401	0.077704165	0.379087978	3.71E-03	3.14E-02	1.00E+00
ENSG00000169359.12_SLC33A1	ENSG00000160789.18_LMNA	0.10244	-0.057701827	0.257442111	2.09E-01	4.82E-01	1.00E+00
ENSG00000141524.14_TMC6	ENSG00000160789.18_LMNA	-0.21733	-0.363934276	-0.06020746	7.16E-03	5.12E-02	1.00E+00
ENSG00000121542.10_SEC22A	ENSG00000160789.18_LMNA	-0.23097	-0.376335814	-0.07451262	4.20E-03	3.44E-02	1.00E+00
ENSG00000181513.13_ACBD4	ENSG00000160789.18_LMNA	0.06282	-0.097359288	0.219817509	4.42E-01	7.09E-01	1.00E+00
ENSG00000234498.3_RPL13AP20	ENSG00000160789.18_LMNA	-0.09959	-0.254751185	0.060571555	2.22E-01	4.98E-01	1.00E+00
ENSG00000153250.16_RBMS1	ENSG00000160789.18_LMNA	0.3894	0.245408957	0.516573432	7.11E-07	3.50E-05	3.65E-02
ENSG00000216775.2_RP1-152L7	ENSG00000160789.18_LMNA	-0.15544	-0.307038987	0.003860091	5.59E-02	2.16E-01	1.00E+00
ENSG00000166451.12_CENPN	ENSG00000160789.18_LMNA	-0.02037	-0.178989474	0.13928317	8.03E-01	9.21E-01	1.00E+00
ENSG00000019169.10_MARCO	ENSG00000160789.18_LMNA	0.24614	0.090488131	0.390058761	2.24E-03	2.13E-02	1.00E+00
ENSG00000157087.15_ATP2B2	ENSG00000160789.18_LMNA	-0.28315	-0.423270506	-0.12980023	4.08E-04	5.75E-03	1.00E+00
ENSG00000235859.5_AC006978.(	ENSG00000160789.18_LMNA	0.07089	-0.0893185	0.227523157	3.85E-01	6.64E-01	1.00E+00
ENSG00000112305.13_SMAP1	ENSG00000160789.18_LMNA	-0.14777	-0.299914826	0.011706468	6.93E-02	2.48E-01	1.00E+00
ENSG00000135945.8_REV1	ENSG00000160789.18_LMNA	-0.10963	-0.264216416	0.050454262	1.79E-01	4.41E-01	1.00E+00

ENSG00000183298.5_RPSAP19	ENSG00000160789.18_LMNA	-0.18504	-0.334391738	-0.02662546	2.25E-02	1.17E-01	1.00E+00
ENSG00000164190.15_NIPBL	ENSG00000160789.18_LMNA	0.04922	-0.110851913	0.206797713	5.47E-01	7.85E-01	1.00E+00
ENSG00000175029.15_CTBP2	ENSG00000160789.18_LMNA	-0.26121	-0.40362658	-0.10643609	1.15E-03	1.29E-02	1.00E+00
ENSG00000128604.17_IRF5	ENSG00000160789.18_LMNA	-0.02545	-0.183906587	0.134293795	7.56E-01	8.98E-01	1.00E+00
ENSG00000109805.8_NCAPG	ENSG00000160789.18_LMNA	0.06301	-0.097164499	0.220004648	4.41E-01	7.08E-01	1.00E+00
ENSG00000142303.12_ADAMTS1	ENSG00000160789.18_LMNA	0.00838	-0.151021122	0.167358483	9.18E-01	9.69E-01	1.00E+00
ENSG00000213362.3_FTH1P12	ENSG00000160789.18_LMNA	0.02454	-0.135189233	0.183025288	7.64E-01	9.02E-01	1.00E+00
ENSG00000151665.11_PIGF	ENSG00000160789.18_LMNA	-0.34096	-0.474406691	-0.19218848	1.72E-05	4.56E-04	8.83E-01
ENSG00000169181.11_GSG1L	ENSG00000160789.18_LMNA	-0.30297	-0.440908113	-0.15105948	1.48E-04	2.57E-03	1.00E+00
ENSG00000100697.13_DICER1	ENSG00000160789.18_LMNA	0.14102	-0.018594598	0.293631929	8.31E-02	2.79E-01	1.00E+00
ENSG00000147874.9_HAUS6	ENSG00000160789.18_LMNA	0.00042	-0.158795228	0.159606122	9.96E-01	9.98E-01	1.00E+00
ENSG00000156265.14_MAP3K7C	ENSG00000160789.18_LMNA	0.17102	0.012151421	0.321469428	3.51E-02	1.59E-01	1.00E+00
ENSG00000170962.11_PDGFD	ENSG00000160789.18_LMNA	0.14028	-0.019353308	0.29293823	8.48E-02	2.82E-01	1.00E+00
ENSG00000158769.16_F11R	ENSG00000160789.18_LMNA	0.30787	0.15633016	0.445246541	1.14E-04	2.10E-03	1.00E+00
ENSG00000171988.16_JMJD1C	ENSG00000160789.18_LMNA	-0.14732	-0.299492	0.012170955	7.01E-02	2.50E-01	1.00E+00
ENSG00000122691.11_TWIST1	ENSG00000160789.18_LMNA	0.04196	-0.118032139	0.199822936	6.08E-01	8.21E-01	1.00E+00
ENSG00000177464.4_GPR4	ENSG00000160789.18_LMNA	0.20423	0.046542551	0.351986362	1.16E-02	7.29E-02	1.00E+00
ENSG00000122574.9_WIPF3	ENSG00000160789.18_LMNA	-0.00391	-0.163008769	0.155387892	9.62E-01	9.85E-01	1.00E+00
ENSG00000167555.12_ZNF528	ENSG00000160789.18_LMNA	-0.02987	-0.188179697	0.129944744	7.15E-01	8.79E-01	1.00E+00
ENSG00000168300.12_PCMTD1	ENSG00000160789.18_LMNA	-0.24401	-0.388131215	-0.08823484	2.45E-03	2.29E-02	1.00E+00
ENSG00000087495.15_PHACTR3	ENSG00000160789.18_LMNA	-0.52648	-0.63265111	-0.40087512	3.25E-12	1.44E-09	1.67E-07
ENSG00000117650.11_NEK2	ENSG00000160789.18_LMNA	0.03789	-0.122045561	0.195910267	6.43E-01	8.41E-01	1.00E+00
ENSG00000072952.17_MRVI1	ENSG00000160789.18_LMNA	-0.27315	-0.414329422	-0.1191253	6.62E-04	8.39E-03	1.00E+00
ENSG00000179523.4_EIF3J-AS1	ENSG00000160789.18_LMNA	-0.17593	-0.326002181	-0.01721376	3.02E-02	1.44E-01	1.00E+00
ENSG00000104343.18_UBE2W	ENSG00000160789.18_LMNA	-0.24136	-0.385740976	-0.08544492	2.74E-03	2.49E-02	1.00E+00
ENSG00000127399.13_LRRC61	ENSG00000160789.18_LMNA	0.06116	-0.099005944	0.218234589	4.54E-01	7.19E-01	1.00E+00
ENSG00000173926.5_MARCH3	ENSG00000160789.18_LMNA	-0.32427	-0.459740821	-0.17405912	4.59E-05	1.00E-03	1.00E+00
ENSG00000213853.8_EMP2	ENSG00000160789.18_LMNA	0.32443	0.174226001	0.459876531	4.55E-05	9.96E-04	1.00E+00
ENSG00000229644.6_NAMPTP1	ENSG00000160789.18_LMNA	0.29541	0.142929959	0.434189833	2.20E-04	3.52E-03	1.00E+00
ENSG00000243964.1_RPL23AP6!	ENSG00000160789.18_LMNA	-0.05053	-0.20806156	0.10954728	5.36E-01	7.77E-01	1.00E+00
ENSG00000280649.1_AC245100.:	ENSG00000160789.18_LMNA	0.10041	-0.059741022	0.255530474	2.18E-01	4.93E-01	1.00E+00
ENSG00000083290.18_ULK2	ENSG00000160789.18_LMNA	-0.14641	-0.298652008	0.013093313	7.19E-02	2.54E-01	1.00E+00
ENSG00000152760.8_TCTEX1D1	ENSG00000160789.18_LMNA	0.08448	-0.07574124	0.240445066	3.01E-01	5.87E-01	1.00E+00

ENSG00000100104.11_SRRD	ENSG00000160789.18_LMNA	-0.13062	-0.283916118	0.029188299	1.09E-01	3.28E-01	1.00E+00
ENSG00000230916.2_MTCO1P53	ENSG00000160789.18_LMNA	-0.09231	-0.247868408	0.067888027	2.58E-01	5.41E-01	1.00E+00
ENSG00000123685.7_BATF3	ENSG00000160789.18_LMNA	0.253	0.097733626	0.396239075	1.66E-03	1.70E-02	1.00E+00
ENSG00000049769.11_PPP1R3F	ENSG00000160789.18_LMNA	0.01872	-0.140900878	0.177391775	8.19E-01	9.30E-01	1.00E+00
ENSG00000111727.10_HCFC2	ENSG00000160789.18_LMNA	-0.2893	-0.428754831	-0.13638191	3.00E-04	4.51E-03	1.00E+00
ENSG00000124596.15_OARD1	ENSG00000160789.18_LMNA	-0.26655	-0.408416381	-0.1121029	9.02E-04	1.07E-02	1.00E+00
ENSG00000133997.10_MED6	ENSG00000160789.18_LMNA	0.0837	-0.076522601	0.239704448	3.05E-01	5.92E-01	1.00E+00
ENSG00000240682.8_ISY1	ENSG00000160789.18_LMNA	0.28755	0.13450979	0.427197036	3.28E-04	4.83E-03	1.00E+00
ENSG00000245937.6_LINC01184	ENSG00000160789.18_LMNA	-0.13893	-0.291678912	0.020729728	8.78E-02	2.88E-01	1.00E+00
ENSG00000266282.1_UBL5P2	ENSG00000160789.18_LMNA	-0.07354	-0.230044279	0.08667873	3.68E-01	6.49E-01	1.00E+00
ENSG00000213551.4_DNAJC9	ENSG00000160789.18_LMNA	-0.01953	-0.178175693	0.140107355	8.11E-01	9.26E-01	1.00E+00
ENSG00000150401.13_DCUN1D2	ENSG00000160789.18_LMNA	-0.33777	-0.471609718	-0.18871604	2.08E-05	5.34E-04	1.00E+00
ENSG00000173542.7_MOB1B	ENSG00000160789.18_LMNA	0.13731	-0.022379502	0.290168067	9.16E-02	2.95E-01	1.00E+00
ENSG00000179104.7_TMTC2	ENSG00000160789.18_LMNA	0.0365	-0.12342181	0.194566232	6.55E-01	8.47E-01	1.00E+00
ENSG00000167257.9_RNF214	ENSG00000160789.18_LMNA	0.00316	-0.156119128	0.162279177	9.69E-01	9.88E-01	1.00E+00
ENSG00000051382.7_PIK3CB	ENSG00000160789.18_LMNA	0.05908	-0.101072674	0.216245488	4.70E-01	7.30E-01	1.00E+00
ENSG00000184319.14_RPL23AP8	ENSG00000160789.18_LMNA	-0.07855	-0.234812918	0.08167348	3.36E-01	6.22E-01	1.00E+00
ENSG00000129028.7_THAP10	ENSG00000160789.18_LMNA	-0.13052	-0.283820784	0.029291899	1.09E-01	3.29E-01	1.00E+00
ENSG00000269640.4_	ENSG00000160789.18_LMNA	0.01934	-0.140288513	0.177996764	8.13E-01	9.26E-01	1.00E+00
ENSG00000146950.11_SHROOM2	ENSG00000160789.18_LMNA	0.24055	0.084592846	0.385010165	2.83E-03	2.56E-02	1.00E+00
ENSG00000217128.10_FNIP1	ENSG00000160789.18_LMNA	-0.02153	-0.180109076	0.138148533	7.92E-01	9.16E-01	1.00E+00
ENSG00000174840.8_PDE12	ENSG00000160789.18_LMNA	-0.03828	-0.196284496	0.121662144	6.40E-01	8.39E-01	1.00E+00
ENSG00000244731.6_C4A	ENSG00000160789.18_LMNA	0.14235	-0.01724383	0.29486614	8.02E-02	2.73E-01	1.00E+00
ENSG00000126787.11_DLGAP5	ENSG00000160789.18_LMNA	0.12904	-0.030792788	0.282438964	1.13E-01	3.36E-01	1.00E+00
ENSG00000178026.11_LRRC75B	ENSG00000160789.18_LMNA	-0.02186	-0.180434666	0.137818415	7.89E-01	9.14E-01	1.00E+00
ENSG00000138594.11_TMOD3	ENSG00000160789.18_LMNA	0.43046	0.291221754	0.551847362	3.11E-08	2.77E-06	1.59E-03
ENSG00000155008.12_APOOL	ENSG00000160789.18_LMNA	-0.16119	-0.312372656	-0.00203945	4.73E-02	1.94E-01	1.00E+00
ENSG00000169760.16_NLGN1	ENSG00000160789.18_LMNA	-0.18593	-0.33520842	-0.02754457	2.18E-02	1.15E-01	1.00E+00
ENSG00000172613.6_RAD9A	ENSG00000160789.18_LMNA	0.0783	-0.081923698	0.234574889	3.38E-01	6.23E-01	1.00E+00
ENSG00000183715.12_OPCML	ENSG00000160789.18_LMNA	-0.44034	-0.560262026	-0.30232998	1.37E-08	1.40E-06	7.04E-04
ENSG00000168062.8_BATF2	ENSG00000160789.18_LMNA	0.16533	0.006294792	0.316207729	4.18E-02	1.79E-01	1.00E+00
ENSG00000151148.12_UBE3B	ENSG00000160789.18_LMNA	0.10637	-0.053745327	0.261144098	1.92E-01	4.60E-01	1.00E+00
ENSG00000196981.3_WDR5B	ENSG00000160789.18_LMNA	-0.22169	-0.367906892	-0.06477632	6.05E-03	4.53E-02	1.00E+00

ENSG00000133056.12_PIK3C2B	ENSG00000160789.18_LMNA	-0.24248	-0.386754246	-0.08662705	2.61E-03	2.40E-02	1.00E+00
ENSG00000167380.15_ZNF226	ENSG00000160789.18_LMNA	-0.09003	-0.245706459	0.070179216	2.70E-01	5.54E-01	1.00E+00
ENSG00000004468.11_CD38	ENSG00000160789.18_LMNA	-0.37118	-0.500792258	-0.2252976	2.50E-06	9.47E-05	1.28E-01
ENSG00000104221.11_BRF2	ENSG00000160789.18_LMNA	-0.121	-0.274904644	0.038951548	1.38E-01	3.78E-01	1.00E+00
ENSG00000244701.1_RP5-894A1	ENSG00000160789.18_LMNA	-0.10159	-0.256641489	0.058556193	2.13E-01	4.87E-01	1.00E+00
ENSG00000160326.12_SLC2A6	ENSG00000160789.18_LMNA	0.0212	-0.138471866	0.179790112	7.95E-01	9.18E-01	1.00E+00
ENSG00000148843.12_PDCD11	ENSG00000160789.18_LMNA	0.07258	-0.087632634	0.22913374	3.74E-01	6.54E-01	1.00E+00
ENSG00000165312.6_OTUD1	ENSG00000160789.18_LMNA	-0.20944	-0.356749982	-0.05197706	9.61E-03	6.37E-02	1.00E+00
ENSG00000169379.14_ARL13B	ENSG00000160789.18_LMNA	0.27337	0.119360595	0.414527115	6.55E-04	8.33E-03	1.00E+00
ENSG00000003096.12_KLHL13	ENSG00000160789.18_LMNA	-0.18246	-0.332019785	-0.02395898	2.45E-02	1.25E-01	1.00E+00
ENSG00000172974.11_AC007318	ENSG00000160789.18_LMNA	-0.08821	-0.243980135	0.072006348	2.80E-01	5.65E-01	1.00E+00
ENSG00000110429.12_FBXO3	ENSG00000160789.18_LMNA	-0.25186	-0.395215239	-0.09653114	1.75E-03	1.77E-02	1.00E+00
ENSG00000139826.5_ABHD13	ENSG00000160789.18_LMNA	-0.21974	-0.366130932	-0.06273224	6.53E-03	4.79E-02	1.00E+00
ENSG00000135094.9_SDS	ENSG00000160789.18_LMNA	-0.07805	-0.234342326	0.082168132	3.39E-01	6.24E-01	1.00E+00
ENSG00000176974.16_SHMT1	ENSG00000160789.18_LMNA	0.1224	-0.037533643	0.276216801	1.33E-01	3.70E-01	1.00E+00
ENSG00000145794.15_MEGF10	ENSG00000160789.18_LMNA	-0.09165	-0.247243554	0.068550576	2.61E-01	5.45E-01	1.00E+00
ENSG00000166448.13_TMEM130	ENSG00000160789.18_LMNA	-0.16823	-0.318888385	-0.00927589	3.83E-02	1.68E-01	1.00E+00
ENSG00000117036.10_ETV3	ENSG00000160789.18_LMNA	0.19343	0.035316	0.342095375	1.70E-02	9.58E-02	1.00E+00
ENSG00000197921.5_HES5	ENSG00000160789.18_LMNA	-0.34856	-0.481069994	-0.20048952	1.08E-05	3.10E-04	5.53E-01
ENSG00000164930.10_FZD6	ENSG00000160789.18_LMNA	0.08472	-0.075500334	0.240673337	2.99E-01	5.86E-01	1.00E+00
ENSG00000225190.7_PLEKHM1	ENSG00000160789.18_LMNA	0.18956	0.031308426	0.338548018	1.93E-02	1.05E-01	1.00E+00
ENSG00000115808.10_STRN	ENSG00000160789.18_LMNA	-0.24745	-0.391241667	-0.09187246	2.12E-03	2.04E-02	1.00E+00
ENSG00000118263.13_KLF7	ENSG00000160789.18_LMNA	0.14556	-0.013962163	0.297860303	7.36E-02	2.58E-01	1.00E+00
ENSG00000258947.5_TUBB3	ENSG00000160789.18_LMNA	0.11872	-0.041255936	0.272769621	1.45E-01	3.90E-01	1.00E+00
ENSG00000165480.14_SKA3	ENSG00000160789.18_LMNA	-0.00337	-0.162484928	0.155912949	9.67E-01	9.87E-01	1.00E+00
ENSG00000279453.1_RP3-425C1	ENSG00000160789.18_LMNA	-0.0783	-0.2345746	0.081924002	3.38E-01	6.23E-01	1.00E+00
ENSG00000103740.8_ACSBG1	ENSG00000160789.18_LMNA	0.04134	-0.118638032	0.199232903	6.13E-01	8.24E-01	1.00E+00
ENSG00000078699.20_CBFA2T2	ENSG00000160789.18_LMNA	0.01594	-0.143625307	0.174697248	8.45E-01	9.40E-01	1.00E+00
ENSG00000171649.10_ZIK1	ENSG00000160789.18_LMNA	-0.04804	-0.205668518	0.112016624	5.57E-01	7.90E-01	1.00E+00
ENSG00000157800.16_SLC37A3	ENSG00000160789.18_LMNA	0.10439	-0.055741098	0.259277868	2.01E-01	4.71E-01	1.00E+00
ENSG00000184903.8_IMMP2L	ENSG00000160789.18_LMNA	-0.06796	-0.224733754	0.092234	4.05E-01	6.81E-01	1.00E+00
ENSG00000163040.13_CCDC74A	ENSG00000160789.18_LMNA	0.2418	0.085905821	0.386136127	2.69E-03	2.45E-02	1.00E+00
ENSG00000204344.13_STK19	ENSG00000160789.18_LMNA	0.09303	-0.067161732	0.248553079	2.54E-01	5.36E-01	1.00E+00

ENSG00000180902.15_D2HGDH	ENSG00000160789.18_LMNA	0.14599	-0.013527768	0.298256181	7.27E-02	2.56E-01	1.00E+00
ENSG00000145214.12_DGKQ	ENSG00000160789.18_LMNA	0.19069	0.032470146	0.33957723	1.86E-02	1.02E-01	1.00E+00
ENSG00000236603.2_RANP1	ENSG00000160789.18_LMNA	-0.03718	-0.195227036	0.122745321	6.49E-01	8.44E-01	1.00E+00
ENSG00000164187.6_LMBRD2	ENSG00000160789.18_LMNA	-0.17362	-0.32386808	-0.01482835	3.24E-02	1.51E-01	1.00E+00
ENSG00000135298.12_ADGRB3	ENSG00000160789.18_LMNA	-0.2504	-0.393902114	-0.09499016	1.86E-03	1.85E-02	1.00E+00
ENSG00000119772.15_DNMT3A	ENSG00000160789.18_LMNA	0.21032	0.052893151	0.357551419	9.30E-03	6.22E-02	1.00E+00
ENSG00000198855.5_FICD	ENSG00000160789.18_LMNA	0.19514	0.037093309	0.343665789	1.60E-02	9.19E-02	1.00E+00
ENSG00000158714.9_SLAMF8	ENSG00000160789.18_LMNA	0.12511	-0.034782006	0.278759887	1.25E-01	3.56E-01	1.00E+00
ENSG00000135778.10_NTPCR	ENSG00000160789.18_LMNA	-0.40713	-0.531860907	-0.2651163	1.94E-07	1.23E-05	9.93E-03
ENSG00000143412.8_ANXA9	ENSG00000160789.18_LMNA	-0.22813	-0.373758643	-0.07152959	4.70E-03	3.74E-02	1.00E+00
ENSG00000130299.15_GTPBP3	ENSG00000160789.18_LMNA	0.08692	-0.073292996	0.242763273	2.87E-01	5.72E-01	1.00E+00
ENSG00000185955.4_C7orf61	ENSG00000160789.18_LMNA	-0.26034	-0.40284512	-0.10551337	1.20E-03	1.33E-02	1.00E+00
ENSG00000116213.14_WRAP73	ENSG00000160789.18_LMNA	0.02531	-0.13443163	0.183770961	7.57E-01	8.99E-01	1.00E+00
ENSG00000011347.8_SYT7	ENSG00000160789.18_LMNA	0.02965	-0.130161966	0.187966554	7.17E-01	8.80E-01	1.00E+00
ENSG00000214113.9_LYRM4	ENSG00000160789.18_LMNA	-0.29612	-0.434822196	-0.14369349	2.12E-04	3.41E-03	1.00E+00
ENSG00000067900.6_ROCK1	ENSG00000160789.18_LMNA	0.15236	-0.007009406	0.304183729	6.09E-02	2.28E-01	1.00E+00
ENSG00000138442.8_WDR12	ENSG00000160789.18_LMNA	-0.21027	-0.35750669	-0.05284201	9.32E-03	6.23E-02	1.00E+00
ENSG00000005889.14_ZFX	ENSG00000160789.18_LMNA	0.16142	0.002278895	0.312588723	4.69E-02	1.93E-01	1.00E+00
ENSG00000167487.10_KLHL26	ENSG00000160789.18_LMNA	0.20308	0.045342987	0.350932749	1.21E-02	7.52E-02	1.00E+00
ENSG00000133216.15_EPHB2	ENSG00000160789.18_LMNA	-0.03697	-0.195022155	0.122955096	6.51E-01	8.45E-01	1.00E+00
ENSG00000120008.14_WDR11	ENSG00000160789.18_LMNA	-0.19254	-0.34127942	-0.03439331	1.75E-02	9.79E-02	1.00E+00
ENSG00000123444.12_KBTBD4	ENSG00000160789.18_LMNA	-0.09987	-0.255019915	0.060285204	2.21E-01	4.96E-01	1.00E+00
ENSG00000188026.10_RILPL1	ENSG00000160789.18_LMNA	0.30087	0.148792504	0.439037929	1.66E-04	2.82E-03	1.00E+00
ENSG00000188603.15_CLN3	ENSG00000160789.18_LMNA	0.24036	0.08438646	0.384833094	2.86E-03	2.58E-02	1.00E+00
ENSG00000112249.12_ASCC3	ENSG00000160789.18_LMNA	0.21787	0.060774763	0.364428148	7.01E-03	5.05E-02	1.00E+00
ENSG00000196388.7_INCA1	ENSG00000160789.18_LMNA	-0.06259	-0.219600763	0.097584864	4.44E-01	7.11E-01	1.00E+00
ENSG00000124177.13_CHD6	ENSG00000160789.18_LMNA	0.01737	-0.142219193	0.176088525	8.32E-01	9.35E-01	1.00E+00
ENSG00000153487.12_ING1	ENSG00000160789.18_LMNA	-0.02924	-0.187569584	0.13056645	7.21E-01	8.81E-01	1.00E+00
ENSG00000227502.2_LINC01268	ENSG00000160789.18_LMNA	-0.2714	-0.412769429	-0.11726976	7.19E-04	8.95E-03	1.00E+00
ENSG00000165675.15_ENOX2	ENSG00000160789.18_LMNA	-0.02999	-0.188287231	0.12983514	7.14E-01	8.78E-01	1.00E+00
ENSG00000180357.8_ZNF609	ENSG00000160789.18_LMNA	-0.03648	-0.194553966	0.123434363	6.55E-01	8.47E-01	1.00E+00
ENSG00000257151.1_PWAR6	ENSG00000160789.18_LMNA	-0.32216	-0.45787931	-0.17177177	5.18E-05	1.11E-03	1.00E+00
ENSG00000151503.11_NCAPD3	ENSG00000160789.18_LMNA	-0.00276	-0.161891324	0.156507713	9.73E-01	9.90E-01	1.00E+00

ENSG00000178922.15_HYI	ENSG00000160789.18_LMNA	0.17919	0.020575267	0.329004211	2.72E-02	1.34E-01	1.00E+00
ENSG00000001617.10_SEMA3F	ENSG00000160789.18_LMNA	0.30144	0.149412811	0.439549913	1.61E-04	2.75E-03	1.00E+00
ENSG00000076641.4_PAG1	ENSG00000160789.18_LMNA	-0.17894	-0.328770264	-0.02031305	2.74E-02	1.35E-01	1.00E+00
ENSG00000140455.15_USP3	ENSG00000160789.18_LMNA	-0.27413	-0.415213454	-0.12017773	6.32E-04	8.10E-03	1.00E+00
ENSG00000138028.13_CGREF1	ENSG00000160789.18_LMNA	-0.18266	-0.332195865	-0.02415677	2.43E-02	1.24E-01	1.00E+00
ENSG00000205090.7_TMEM240	ENSG00000160789.18_LMNA	-0.04195	-0.199815215	0.11804007	6.08E-01	8.21E-01	1.00E+00
ENSG00000188916.7_FAM196A	ENSG00000160789.18_LMNA	0.20453	0.04685639	0.352261888	1.15E-02	7.23E-02	1.00E+00
ENSG00000204071.8_TCEAL6	ENSG00000160789.18_LMNA	-0.24458	-0.388647436	-0.088838	2.39E-03	2.24E-02	1.00E+00
ENSG00000243364.6_EFNA4	ENSG00000160789.18_LMNA	0.27158	0.117454364	0.412924705	7.13E-04	8.90E-03	1.00E+00
ENSG00000164983.7_TMEM65	ENSG00000160789.18_LMNA	0.10193	-0.058211571	0.256964484	2.11E-01	4.85E-01	1.00E+00
ENSG00000174672.14_BRSK2	ENSG00000160789.18_LMNA	-0.28545	-0.425324426	-0.13226209	3.64E-04	5.23E-03	1.00E+00
ENSG00000159167.10_STC1	ENSG00000160789.18_LMNA	0.27232	0.118242119	0.413587127	6.89E-04	8.66E-03	1.00E+00
ENSG00000109323.7_MANBA	ENSG00000160789.18_LMNA	0.2875	0.134457875	0.427153812	3.29E-04	4.84E-03	1.00E+00
ENSG00000164188.7_RANBP3L	ENSG00000160789.18_LMNA	-0.31235	-0.449214907	-0.16116571	8.94E-05	1.73E-03	1.00E+00
ENSG00000151117.7_TMEM86A	ENSG00000160789.18_LMNA	-0.12738	-0.280888696	0.032474958	1.18E-01	3.44E-01	1.00E+00
ENSG00000196968.9_FUT11	ENSG00000160789.18_LMNA	0.20328	0.045553718	0.351117896	1.20E-02	7.49E-02	1.00E+00
ENSG00000120071.11_KANSL1	ENSG00000160789.18_LMNA	-0.08222	-0.238299125	0.078004164	3.14E-01	6.00E-01	1.00E+00
ENSG00000118007.11_STAG1	ENSG00000160789.18_LMNA	0.10105	-0.059105721	0.256126299	2.15E-01	4.90E-01	1.00E+00
ENSG00000125459.13_MSTO1	ENSG00000160789.18_LMNA	0.31688	0.166061815	0.453221425	6.96E-05	1.40E-03	1.00E+00
ENSG00000047056.13_WDR37	ENSG00000160789.18_LMNA	-0.06699	-0.223806835	0.093201619	4.12E-01	6.86E-01	1.00E+00
ENSG00000196922.9_ZNF252P	ENSG00000160789.18_LMNA	-0.30345	-0.441333708	-0.1515758	1.44E-04	2.52E-03	1.00E+00
ENSG00000145868.15_FBXO38	ENSG00000160789.18_LMNA	-0.03838	-0.196379345	0.121564951	6.39E-01	8.39E-01	1.00E+00
ENSG00000092931.10_MFSD11	ENSG00000160789.18_LMNA	-0.20783	-0.355278739	-0.05029669	1.02E-02	6.64E-02	1.00E+00
ENSG00000239911.2_PRKAG2-A	ENSG00000160789.18_LMNA	-0.26271	-0.404969589	-0.10802306	1.08E-03	1.23E-02	1.00E+00
ENSG00000149451.16_ADAM33	ENSG00000160789.18_LMNA	0.14546	-0.014064385	0.297767129	7.38E-02	2.58E-01	1.00E+00
ENSG00000133067.16_LGR6	ENSG00000160789.18_LMNA	0.15264	-0.006726227	0.304440696	6.05E-02	2.27E-01	1.00E+00
ENSG00000163281.10_GNPDA2	ENSG00000160789.18_LMNA	-0.25333	-0.396537669	-0.09808449	1.64E-03	1.68E-02	1.00E+00
ENSG00000158560.13_DYNC1I1	ENSG00000160789.18_LMNA	-0.39773	-0.523768272	-0.25465611	3.90E-07	2.16E-05	2.00E-02
ENSG00000181649.5_PHLDA2	ENSG00000160789.18_LMNA	0.46953	0.335401487	0.585002961	1.05E-09	1.63E-07	5.37E-05
ENSG00000091428.16_RAPGEF4	ENSG00000160789.18_LMNA	-0.39738	-0.523465706	-0.25426624	4.00E-07	2.20E-05	2.05E-02
ENSG00000164068.14_RNF123	ENSG00000160789.18_LMNA	0.0359	-0.124006981	0.193994396	6.61E-01	8.50E-01	1.00E+00
ENSG00000224152.1_AC009506.1	ENSG00000160789.18_LMNA	-0.23534	-0.380291022	-0.07910122	3.52E-03	3.01E-02	1.00E+00
ENSG00000164761.7_TNFRSF11I	ENSG00000160789.18_LMNA	0.3289	0.179077448	0.463816087	3.52E-05	8.15E-04	1.00E+00

ENSG00000010219.12_DYRK4	ENSG00000160789.18_LMNA	-0.30407	-0.441883588	-0.15224314	1.40E-04	2.46E-03	1.00E+00
ENSG00000047578.11_KIAA0556	ENSG00000160789.18_LMNA	-0.03753	-0.195561938	0.122402355	6.46E-01	8.43E-01	1.00E+00
ENSG00000198015.11_MRPL42	ENSG00000160789.18_LMNA	-0.31066	-0.447718597	-0.15934079	9.81E-05	1.85E-03	1.00E+00
ENSG00000198729.4_PPP1R14C	ENSG00000160789.18_LMNA	0.01402	-0.145504942	0.172835458	8.64E-01	9.47E-01	1.00E+00
ENSG00000111364.14_DDX55	ENSG00000160789.18_LMNA	-0.0508	-0.208317737	0.109282703	5.34E-01	7.76E-01	1.00E+00
ENSG00000162852.12_CNST	ENSG00000160789.18_LMNA	-0.2159	-0.362639389	-0.05872097	7.55E-03	5.32E-02	1.00E+00
ENSG00000182372.6_CLN8	ENSG00000160789.18_LMNA	-0.19099	-0.339859337	-0.03278872	1.84E-02	1.02E-01	1.00E+00
ENSG00000183891.5_TTC32	ENSG00000160789.18_LMNA	-0.07031	-0.226968579	0.089898581	3.89E-01	6.68E-01	1.00E+00
ENSG00000132436.10_FIGNL1	ENSG00000160789.18_LMNA	-0.01651	-0.175253781	0.143062993	8.40E-01	9.38E-01	1.00E+00
ENSG00000101445.8_PPP1R16B	ENSG00000160789.18_LMNA	-0.34944	-0.481836128	-0.20144654	1.02E-05	2.96E-04	5.24E-01
ENSG00000008869.10_HEATR5B	ENSG00000160789.18_LMNA	-0.08683	-0.242674539	0.073386777	2.87E-01	5.73E-01	1.00E+00
ENSG00000197841.13_ZNF181	ENSG00000160789.18_LMNA	-0.20415	-0.35191121	-0.04645696	1.16E-02	7.31E-02	1.00E+00
ENSG00000146094.12_DOK3	ENSG00000160789.18_LMNA	0.20236	0.044592609	0.350273276	1.24E-02	7.66E-02	1.00E+00
ENSG00000065485.16_PDIA5	ENSG00000160789.18_LMNA	0.40391	0.261528532	0.529090673	2.47E-07	1.51E-05	1.27E-02
ENSG00000205269.5_TMEM170B	ENSG00000160789.18_LMNA	-0.39436	-0.520859755	-0.25091201	4.98E-07	2.61E-05	2.55E-02
ENSG00000275074.1_NUDT18	ENSG00000160789.18_LMNA	-0.07515	-0.231579148	0.085069446	3.57E-01	6.41E-01	1.00E+00
ENSG00000165495.14_PKNOX2	ENSG00000160789.18_LMNA	-0.32268	-0.458334305	-0.17233057	5.03E-05	1.08E-03	1.00E+00
ENSG00000100099.19_HPS4	ENSG00000160789.18_LMNA	-0.09504	-0.250448678	0.065149144	2.44E-01	5.24E-01	1.00E+00
ENSG00000103642.10_LACTB	ENSG00000160789.18_LMNA	0.12843	-0.03141416	0.281866506	1.15E-01	3.39E-01	1.00E+00
ENSG00000013563.12_DNASE1L	ENSG00000160789.18_LMNA	0.22751	0.070878311	0.37319535	4.82E-03	3.81E-02	1.00E+00
ENSG00000226781.1_TBCAP1	ENSG00000160789.18_LMNA	-0.02071	-0.179321037	0.138947242	8.00E-01	9.20E-01	1.00E+00
ENSG00000204842.13_ATXN2	ENSG00000160789.18_LMNA	0.11039	-0.049685531	0.264933132	1.76E-01	4.37E-01	1.00E+00
ENSG00000167705.10_RILP	ENSG00000160789.18_LMNA	0.14172	-0.017888511	0.294277218	8.16E-02	2.75E-01	1.00E+00
ENSG00000106733.19_NMRK1	ENSG00000160789.18_LMNA	-0.00713	-0.16614507	0.152240545	9.30E-01	9.73E-01	1.00E+00
ENSG00000160703.14_NLRX1	ENSG00000160789.18_LMNA	0.21206	0.054708346	0.359138115	8.72E-03	5.94E-02	1.00E+00
ENSG00000134905.15_CARS2	ENSG00000160789.18_LMNA	0.05065	-0.109431434	0.208173733	5.35E-01	7.77E-01	1.00E+00
ENSG00000164647.7_STEAP1	ENSG00000160789.18_LMNA	0.20895	0.051462444	0.35629957	9.78E-03	6.46E-02	1.00E+00
ENSG00000092098.15_RNF31	ENSG00000160789.18_LMNA	0.22819	0.071590097	0.373810963	4.69E-03	3.74E-02	1.00E+00
ENSG00000160193.10_WDR4	ENSG00000160789.18_LMNA	0.42028	0.279796688	0.543136968	7.03E-08	5.43E-06	3.60E-03
ENSG00000242173.7_ARHGDIG	ENSG00000160789.18_LMNA	-0.23075	-0.37613744	-0.07428281	4.23E-03	3.46E-02	1.00E+00
ENSG00000140263.12_SORD	ENSG00000160789.18_LMNA	-0.06087	-0.217958692	0.099292771	4.56E-01	7.21E-01	1.00E+00
ENSG00000158161.14_EYA3	ENSG00000160789.18_LMNA	0.16207	0.002946452	0.313190931	4.61E-02	1.91E-01	1.00E+00
ENSG00000124313.11_IQSEC2	ENSG00000160789.18_LMNA	-0.02686	-0.185263753	0.132913836	7.43E-01	8.92E-01	1.00E+00

ENSG00000197859.8_ADAMTSL2	ENSG00000160789.18_LMNA	-0.07164	-0.228235464	0.088573122	3.80E-01	6.59E-01	1.00E+00
ENSG00000153006.13_SREK1IP1	ENSG00000160789.18_LMNA	-0.31427	-0.450906699	-0.16323141	8.05E-05	1.58E-03	1.00E+00
ENSG00000158411.9_MITD1	ENSG00000160789.18_LMNA	-0.0932	-0.2487089	0.066996391	2.53E-01	5.35E-01	1.00E+00
ENSG00000114857.16_NKTR	ENSG00000160789.18_LMNA	-0.10543	-0.260264916	0.054685849	1.96E-01	4.65E-01	1.00E+00
ENSG00000163389.9_POGLUT1	ENSG00000160789.18_LMNA	-0.01061	-0.169526553	0.148839901	8.97E-01	9.60E-01	1.00E+00
ENSG00000122591.10_FAM126A	ENSG00000160789.18_LMNA	0.15893	-0.000282335	0.310275905	5.05E-02	2.03E-01	1.00E+00
ENSG00000140006.10_WDR89	ENSG00000160789.18_LMNA	0.0331	-0.126764334	0.191296967	6.86E-01	8.64E-01	1.00E+00
ENSG00000125775.13_SDCBP2	ENSG00000160789.18_LMNA	0.01217	-0.147311787	0.171043621	8.82E-01	9.54E-01	1.00E+00
ENSG00000176170.12_SPHK1	ENSG00000160789.18_LMNA	0.20799	0.050462804	0.355424248	1.01E-02	6.62E-02	1.00E+00
ENSG00000247095.2_MIR210HG	ENSG00000160789.18_LMNA	0.36227	0.215502738	0.493039434	4.51E-06	1.53E-04	2.31E-01
ENSG00000196387.8_ZNF140	ENSG00000160789.18_LMNA	-0.20152	-0.349507139	-0.0437213	1.28E-02	7.83E-02	1.00E+00
ENSG00000170909.12_OSCAR	ENSG00000160789.18_LMNA	0.12469	-0.035206219	0.278368112	1.26E-01	3.59E-01	1.00E+00
ENSG00000166411.12_IDH3A	ENSG00000160789.18_LMNA	0.05258	-0.107522384	0.210021054	5.20E-01	7.66E-01	1.00E+00
ENSG00000143315.6_PIGM	ENSG00000160789.18_LMNA	-0.17476	-0.324921004	-0.01600482	3.13E-02	1.47E-01	1.00E+00
ENSG00000173898.10_SPTBN2	ENSG00000160789.18_LMNA	-0.18087	-0.330551827	-0.02231093	2.58E-02	1.29E-01	1.00E+00
ENSG00000128534.6_LSM8	ENSG00000160789.18_LMNA	0.15414	-0.005192427	0.305831736	5.80E-02	2.21E-01	1.00E+00
ENSG00000137713.14_PPP2R1B	ENSG00000160789.18_LMNA	-0.14783	-0.299975107	0.011640237	6.91E-02	2.48E-01	1.00E+00
ENSG00000047365.10_ARAP2	ENSG00000160789.18_LMNA	0.03917	-0.120782249	0.197142958	6.32E-01	8.35E-01	1.00E+00
ENSG00000198910.11_L1CAM	ENSG00000160789.18_LMNA	-0.16693	-0.317689687	-0.00794216	3.98E-02	1.73E-01	1.00E+00
ENSG00000101825.7_MXRA5	ENSG00000160789.18_LMNA	0.39568	0.252375825	0.521997633	4.52E-07	2.43E-05	2.32E-02
ENSG00000278615.3_C11orf98	ENSG00000160789.18_LMNA	-0.34732	-0.479979586	-0.19912834	1.17E-05	3.30E-04	5.98E-01
ENSG00000256968.1_SNRPEP2	ENSG00000160789.18_LMNA	-0.05566	-0.2129766	0.104463199	4.96E-01	7.50E-01	1.00E+00
ENSG00000050555.16_LAMC3	ENSG00000160789.18_LMNA	0.17946	0.020859399	0.329257673	2.69E-02	1.33E-01	1.00E+00
ENSG00000187730.7_GABRD	ENSG00000160789.18_LMNA	-0.06033	-0.217446994	0.099824604	4.60E-01	7.24E-01	1.00E+00
ENSG00000165244.6_ZNF367	ENSG00000160789.18_LMNA	0.17758	0.018914424	0.327521757	2.86E-02	1.39E-01	1.00E+00
ENSG00000109381.18_ELF2	ENSG00000160789.18_LMNA	0.04661	-0.113431069	0.204296066	5.69E-01	7.98E-01	1.00E+00
ENSG00000149798.4_CDC42EP2	ENSG00000160789.18_LMNA	-0.00371	-0.162818139	0.155578985	9.64E-01	9.86E-01	1.00E+00
ENSG00000077063.9_CTTNBP2	ENSG00000160789.18_LMNA	-0.40338	-0.528633179	-0.26093674	2.57E-07	1.56E-05	1.32E-02
ENSG00000136982.5_DSCC1	ENSG00000160789.18_LMNA	-0.06845	-0.225194118	0.091753198	4.02E-01	6.78E-01	1.00E+00
ENSG00000163512.12_AZI2	ENSG00000160789.18_LMNA	-0.23478	-0.37978864	-0.07851768	3.60E-03	3.06E-02	1.00E+00
ENSG00000105778.16_AVL9	ENSG00000160789.18_LMNA	0.27789	0.124178546	0.41856902	5.28E-04	7.03E-03	1.00E+00
ENSG00000155729.11_KCTD18	ENSG00000160789.18_LMNA	-0.08595	-0.241838864	0.074269715	2.92E-01	5.78E-01	1.00E+00
ENSG00000272674.3_PCDHB16	ENSG00000160789.18_LMNA	0.02867	-0.131130442	0.187015896	7.26E-01	8.84E-01	1.00E+00

ENSG00000180233.10_ZNRF2	ENSG00000160789.18_LMNA	-0.07408	-0.230560114	0.086138071	3.64E-01	6.46E-01	1.00E+00
ENSG00000198157.9_HMGN5	ENSG00000160789.18_LMNA	-0.18838	-0.337458053	-0.03007903	2.01E-02	1.08E-01	1.00E+00
ENSG00000144741.16_SLC25A26	ENSG00000160789.18_LMNA	-0.28158	-0.421869565	-0.12812312	4.41E-04	6.12E-03	1.00E+00
ENSG00000197548.11_ATG7	ENSG00000160789.18_LMNA	-0.01822	-0.176910426	0.141387924	8.24E-01	9.31E-01	1.00E+00
ENSG00000116731.21_PRDM2	ENSG00000160789.18_LMNA	-0.1113	-0.265787925	0.048768216	1.72E-01	4.31E-01	1.00E+00
ENSG00000130921.6_C12orf65	ENSG00000160789.18_LMNA	-0.17911	-0.328932409	-0.02049479	2.73E-02	1.34E-01	1.00E+00
ENSG00000048405.8_ZNF800	ENSG00000160789.18_LMNA	0.33437	0.185014475	0.468621902	2.55E-05	6.27E-04	1.00E+00
ENSG00000133131.13_MORC4	ENSG00000160789.18_LMNA	0.23274	0.076371582	0.377939502	3.91E-03	3.26E-02	1.00E+00
ENSG00000136297.13_MMD2	ENSG00000160789.18_LMNA	-0.41947	-0.542444784	-0.27889197	7.49E-08	5.70E-06	3.84E-03
ENSG00000265531.2_FCGR1CP	ENSG00000160789.18_LMNA	-0.05697	-0.214226999	0.103167143	4.86E-01	7.42E-01	1.00E+00
ENSG00000133740.9_E2F5	ENSG00000160789.18_LMNA	-0.08419	-0.240171178	0.076030241	3.02E-01	5.89E-01	1.00E+00
ENSG00000120915.12_EPHX2	ENSG00000160789.18_LMNA	-0.00088	-0.160061305	0.158339857	9.91E-01	9.97E-01	1.00E+00
ENSG00000148848.13_ADAM12	ENSG00000160789.18_LMNA	0.52234	0.396079133	0.629219414	5.13E-12	2.06E-09	2.63E-07
ENSG00000113209.8_PCDHB5	ENSG00000160789.18_LMNA	0.00958	-0.149852999	0.168519946	9.07E-01	9.64E-01	1.00E+00
ENSG00000113272.12_THG1L	ENSG00000160789.18_LMNA	-0.02414	-0.182640841	0.135579686	7.68E-01	9.04E-01	1.00E+00
ENSG00000132522.14_GPS2	ENSG00000160789.18_LMNA	-0.03221	-0.190435141	0.12764427	6.94E-01	8.68E-01	1.00E+00
ENSG00000145703.14_IQGAP2	ENSG00000160789.18_LMNA	0.25322	0.097973927	0.396443586	1.65E-03	1.69E-02	1.00E+00
ENSG00000123066.6_MED13L	ENSG00000160789.18_LMNA	-0.06858	-0.225318789	0.091622968	4.01E-01	6.78E-01	1.00E+00
ENSG00000188153.11_COL4A5	ENSG00000160789.18_LMNA	-0.02618	-0.184608989	0.133579751	7.49E-01	8.95E-01	1.00E+00
ENSG00000175354.17_PTPN2	ENSG00000160789.18_LMNA	0.07111	-0.089100073	0.227731928	3.84E-01	6.63E-01	1.00E+00
ENSG00000075420.11_FNDC3B	ENSG00000160789.18_LMNA	0.46909	0.33490072	0.584631767	1.09E-09	1.68E-07	5.59E-05
ENSG00000169955.7_ZNF747	ENSG00000160789.18_LMNA	0.07699	-0.08322622	0.233335208	3.46E-01	6.30E-01	1.00E+00
ENSG00000230910.2_RP3-525N1	ENSG00000160789.18_LMNA	-0.27453	-0.415571403	-0.12060406	6.20E-04	7.99E-03	1.00E+00
ENSG00000085511.18_MAP3K4	ENSG00000160789.18_LMNA	-0.01457	-0.173373128	0.144962354	8.59E-01	9.44E-01	1.00E+00
ENSG00000149089.11_APIP	ENSG00000160789.18_LMNA	-0.15559	-0.307184611	0.003699304	5.56E-02	2.15E-01	1.00E+00
ENSG00000182963.8_GJC1	ENSG00000160789.18_LMNA	0.16837	0.009419351	0.319017262	3.81E-02	1.68E-01	1.00E+00
ENSG00000163864.13_NMNAT3	ENSG00000160789.18_LMNA	0.07979	-0.080437279	0.235988339	3.29E-01	6.15E-01	1.00E+00
ENSG00000249774.1_CTD-2206G	ENSG00000160789.18_LMNA	0.02669	-0.133079884	0.185100513	7.44E-01	8.93E-01	1.00E+00
ENSG00000006576.15_PHTF2	ENSG00000160789.18_LMNA	0.00453	-0.15478332	0.163611718	9.56E-01	9.82E-01	1.00E+00
ENSG00000269352.1_PTOV1-AS2	ENSG00000160789.18_LMNA	0.04362	-0.11638484	0.201425954	5.94E-01	8.12E-01	1.00E+00
ENSG00000085415.14_SEH1L	ENSG00000160789.18_LMNA	-0.0424	-0.200253598	0.117589752	6.04E-01	8.18E-01	1.00E+00
ENSG00000174842.15_GLMN	ENSG00000160789.18_LMNA	-0.12358	-0.277324783	0.036335383	1.29E-01	3.64E-01	1.00E+00
ENSG00000176915.13_ANKLE2	ENSG00000160789.18_LMNA	0.26363	0.109001301	0.405796811	1.03E-03	1.19E-02	1.00E+00

ENSG00000105708.8_ZNF14	ENSG00000160789.18_LMNA	-0.17656	-0.326581147	-0.01786151	2.96E-02	1.42E-01	1.00E+00
ENSG00000139718.9_SETD1B	ENSG00000160789.18_LMNA	0.12877	-0.03106972	0.282183859	1.14E-01	3.37E-01	1.00E+00
ENSG00000179886.5_TIGD5	ENSG00000160789.18_LMNA	0.20347	0.045749043	0.351289487	1.19E-02	7.45E-02	1.00E+00
ENSG00000133103.15_COG6	ENSG00000160789.18_LMNA	-0.225	-0.370918567	-0.06824851	5.32E-03	4.11E-02	1.00E+00
ENSG00000253676.1_TAGLN2P1	ENSG00000160789.18_LMNA	0.51603	0.388763222	0.623967055	1.02E-11	3.50E-09	5.22E-07
ENSG00000134698.10_38200	ENSG00000160789.18_LMNA	-0.17411	-0.324320417	-0.01533366	3.19E-02	1.49E-01	1.00E+00
ENSG00000256087.5_ZNF432	ENSG00000160789.18_LMNA	-0.03843	-0.196430462	0.121512569	6.38E-01	8.38E-01	1.00E+00
ENSG00000064763.9_FAR2	ENSG00000160789.18_LMNA	0.05899	-0.101158209	0.216163108	4.70E-01	7.31E-01	1.00E+00
ENSG00000158234.11_FAIM	ENSG00000160789.18_LMNA	-0.12071	-0.274633711	0.039244158	1.39E-01	3.79E-01	1.00E+00
ENSG00000136709.10_WDR33	ENSG00000160789.18_LMNA	0.07357	-0.086640716	0.230080554	3.68E-01	6.48E-01	1.00E+00
ENSG00000117016.8_RIMS3	ENSG00000160789.18_LMNA	-0.25721	-0.400029952	-0.10219358	1.38E-03	1.48E-02	1.00E+00
ENSG00000095370.18_SH2D3C	ENSG00000160789.18_LMNA	-0.06243	-0.219446986	0.097744886	4.45E-01	7.12E-01	1.00E+00
ENSG00000100580.7_TMED8	ENSG00000160789.18_LMNA	0.0198	-0.139839476	0.178440239	8.09E-01	9.24E-01	1.00E+00
ENSG00000058600.14_POLR3E	ENSG00000160789.18_LMNA	0.04418	-0.115832472	0.201963093	5.89E-01	8.09E-01	1.00E+00
ENSG00000179743.3_FLJ37453	ENSG00000160789.18_LMNA	-0.29418	-0.433100613	-0.14161563	2.34E-04	3.71E-03	1.00E+00
ENSG00000135775.12_COG2	ENSG00000160789.18_LMNA	-0.37776	-0.506500395	-0.23254498	1.60E-06	6.74E-05	8.23E-02
ENSG00000137936.15_BCAR3	ENSG00000160789.18_LMNA	0.19712	0.039150045	0.345480965	1.49E-02	8.76E-02	1.00E+00
ENSG00000198692.8_EIF1AY	ENSG00000160789.18_LMNA	-0.08766	-0.243458966	0.072557534	2.83E-01	5.68E-01	1.00E+00
ENSG00000175087.8_PDIK1L	ENSG00000160789.18_LMNA	-0.11594	-0.270157261	0.044070989	1.55E-01	4.06E-01	1.00E+00
ENSG00000270696.1_RP11-342K	ENSG00000160789.18_LMNA	0.04174	-0.11824486	0.19961581	6.10E-01	8.22E-01	1.00E+00
ENSG00000154645.12_CHODL	ENSG00000160789.18_LMNA	0.06683	-0.09336625	0.223649071	4.13E-01	6.87E-01	1.00E+00
ENSG00000163106.9_HPGDS	ENSG00000160789.18_LMNA	-0.17984	-0.329607017	-0.0212511	2.66E-02	1.32E-01	1.00E+00
ENSG00000115568.14_ZNF142	ENSG00000160789.18_LMNA	0.09089	-0.06931329	0.24652391	2.65E-01	5.49E-01	1.00E+00
ENSG00000143434.14_SEMA6C	ENSG00000160789.18_LMNA	-0.04291	-0.20073912	0.117090861	6.00E-01	8.16E-01	1.00E+00
ENSG00000106346.10_USP42	ENSG00000160789.18_LMNA	0.12666	-0.033212078	0.28020886	1.20E-01	3.48E-01	1.00E+00
ENSG00000267565.1_CTC-559E9	ENSG00000160789.18_LMNA	-0.43961	-0.559646342	-0.30151483	1.46E-08	1.48E-06	7.48E-04
ENSG00000157895.10_C12orf43	ENSG00000160789.18_LMNA	-0.3105	-0.447572155	-0.1591623	9.90E-05	1.87E-03	1.00E+00
ENSG00000096717.10_SIRT1	ENSG00000160789.18_LMNA	-0.22607	-0.371886191	-0.06936565	5.10E-03	3.99E-02	1.00E+00
ENSG00000131732.10_ZCCHC9	ENSG00000160789.18_LMNA	0.18091	0.022357188	0.33059305	2.57E-02	1.29E-01	1.00E+00
ENSG00000205129.7_C4orf47	ENSG00000160789.18_LMNA	0.20664	0.049057804	0.354193074	1.06E-02	6.85E-02	1.00E+00
ENSG00000143768.10_LEFTY2	ENSG00000160789.18_LMNA	0.09091	-0.069295533	0.246540669	2.65E-01	5.49E-01	1.00E+00
ENSG00000140044.11_JDP2	ENSG00000160789.18_LMNA	0.00667	-0.152697084	0.165690531	9.35E-01	9.74E-01	1.00E+00
ENSG00000188343.11_FAM92A	ENSG00000160789.18_LMNA	-0.1209	-0.274808023	0.039055906	1.38E-01	3.78E-01	1.00E+00

ENSG00000167693.15_NXN	ENSG00000160789.18_LMNA	-0.20503	-0.35272064	-0.04737907	1.13E-02	7.14E-02	1.00E+00
ENSG00000137177.17_KIF13A	ENSG00000160789.18_LMNA	-0.04676	-0.204437114	0.113285764	5.67E-01	7.97E-01	1.00E+00
ENSG00000205133.10_TRIQK	ENSG00000160789.18_LMNA	-0.25285	-0.396109187	-0.09758103	1.67E-03	1.71E-02	1.00E+00
ENSG00000267534.2_S1PR2	ENSG00000160789.18_LMNA	0.24769	0.092122751	0.391455433	2.09E-03	2.02E-02	1.00E+00
ENSG00000039319.15_ZFYVE16	ENSG00000160789.18_LMNA	-0.36656	-0.496769178	-0.22020791	3.41E-06	1.22E-04	1.75E-01
ENSG00000230521.1_HCG4P7	ENSG00000160789.18_LMNA	0.24333	0.087524673	0.387523168	2.52E-03	2.34E-02	1.00E+00
ENSG00000163472.17_TMEM79	ENSG00000160789.18_LMNA	0.2224	0.065524159	0.36855608	5.89E-03	4.44E-02	1.00E+00
ENSG00000230844.2_ZNF674-AS	ENSG00000160789.18_LMNA	-0.13486	-0.28787847	0.024876397	9.76E-02	3.07E-01	1.00E+00
ENSG00000148948.6_LRRC4C	ENSG00000160789.18_LMNA	-0.09704	-0.252345808	0.063132357	2.34E-01	5.12E-01	1.00E+00
ENSG00000198478.7_SH3BGRL2	ENSG00000160789.18_LMNA	-0.41984	-0.542762765	-0.27930753	7.27E-08	5.57E-06	3.73E-03
ENSG00000198131.12_ZNF544	ENSG00000160789.18_LMNA	-0.09326	-0.248763563	0.066938385	2.53E-01	5.35E-01	1.00E+00
ENSG00000133195.10_SLC39A11	ENSG00000160789.18_LMNA	-0.16342	-0.314444482	-0.00433692	4.42E-02	1.86E-01	1.00E+00
ENSG00000169436.15_COL22A1	ENSG00000160789.18_LMNA	0.30242	0.150465163	0.440418061	1.53E-04	2.64E-03	1.00E+00
ENSG00000164162.11_ANAPC10	ENSG00000160789.18_LMNA	-0.04365	-0.201452605	0.116357438	5.93E-01	8.12E-01	1.00E+00
ENSG00000113273.14_ARSB	ENSG00000160789.18_LMNA	0.21086	0.05345708	0.358044548	9.12E-03	6.13E-02	1.00E+00
ENSG00000121297.6_TSHZ3	ENSG00000160789.18_LMNA	0.22711	0.070458391	0.372832043	4.90E-03	3.86E-02	1.00E+00
ENSG00000179943.7_FIZ1	ENSG00000160789.18_LMNA	0.09341	-0.06677993	0.248912873	2.52E-01	5.33E-01	1.00E+00
ENSG00000095066.10_HOOK2	ENSG00000160789.18_LMNA	0.13877	-0.020889937	0.291532263	8.82E-02	2.89E-01	1.00E+00
ENSG00000275964.1_RP11-61K9	ENSG00000160789.18_LMNA	-0.01378	-0.172601004	0.14574148	8.66E-01	9.48E-01	1.00E+00
ENSG00000132005.7_RFX1	ENSG00000160789.18_LMNA	0.28963	0.136733779	0.429047427	2.96E-04	4.44E-03	1.00E+00
ENSG00000128045.6_RASL11B	ENSG00000160789.18_LMNA	-0.38404	-0.511941338	-0.23948156	1.04E-06	4.74E-05	5.33E-02
ENSG00000204822.6_MRPL53	ENSG00000160789.18_LMNA	-0.11076	-0.265282767	0.049310387	1.74E-01	4.35E-01	1.00E+00
ENSG00000064703.10_DDX20	ENSG00000160789.18_LMNA	-0.00705	-0.166059968	0.152326031	9.31E-01	9.73E-01	1.00E+00
ENSG00000159674.10_SPON2	ENSG00000160789.18_LMNA	0.36686	0.220539194	0.497031401	3.34E-06	1.20E-04	1.71E-01
ENSG00000138030.11_KHK	ENSG00000160789.18_LMNA	-0.00483	-0.163902511	0.15449166	9.53E-01	9.81E-01	1.00E+00
ENSG00000172687.12_ZNF738	ENSG00000160789.18_LMNA	-0.38667	-0.514216564	-0.24239049	8.63E-07	4.04E-05	4.43E-02
ENSG00000213380.12_COG8	ENSG00000160789.18_LMNA	0.08508	-0.075135889	0.2410186	2.97E-01	5.83E-01	1.00E+00
ENSG00000065361.13_ERBB3	ENSG00000160789.18_LMNA	-0.36312	-0.493773237	-0.21642743	4.27E-06	1.46E-04	2.19E-01
ENSG00000165349.10_SLC7A3	ENSG00000160789.18_LMNA	-0.12122	-0.27511147	0.038728137	1.37E-01	3.77E-01	1.00E+00
ENSG00000146587.16_RBAK	ENSG00000160789.18_LMNA	0.26661	0.11216964	0.408472694	9.00E-04	1.07E-02	1.00E+00
ENSG00000124731.11_TREM1	ENSG00000160789.18_LMNA	0.13201	-0.027778602	0.285212714	1.05E-01	3.21E-01	1.00E+00
ENSG00000228532.4_RP1-241P1	ENSG00000160789.18_LMNA	-0.0487	-0.206305109	0.111360118	5.51E-01	7.87E-01	1.00E+00
ENSG00000184677.15_ZBTB40	ENSG00000160789.18_LMNA	0.0047	-0.154619951	0.163774608	9.54E-01	9.82E-01	1.00E+00

ENSG00000196872.9_KIAA1211L	ENSG00000160789.18_LMNA	0.28019	0.126640778	0.420630159	4.72E-04	6.44E-03	1.00E+00
ENSG00000171953.14_ATPAF2	ENSG00000160789.18_LMNA	-0.16351	-0.314522099	-0.00442306	4.41E-02	1.85E-01	1.00E+00
ENSG00000009413.14_REV3L	ENSG00000160789.18_LMNA	-0.13193	-0.285139196	0.027858565	1.05E-01	3.22E-01	1.00E+00
ENSG00000163597.13_SNHG16	ENSG00000160789.18_LMNA	-0.1104	-0.264947746	0.049669854	1.76E-01	4.37E-01	1.00E+00
ENSG00000162300.10_ZFPL1	ENSG00000160789.18_LMNA	-0.04248	-0.200328099	0.117513211	6.03E-01	8.18E-01	1.00E+00
ENSG00000083520.13_DIS3	ENSG00000160789.18_LMNA	-0.05376	-0.211152794	0.106351669	5.11E-01	7.59E-01	1.00E+00
ENSG00000181744.7_C3orf58	ENSG00000160789.18_LMNA	0.19301	0.034883267	0.341712757	1.72E-02	9.69E-02	1.00E+00
ENSG00000143156.12_NME7	ENSG00000160789.18_LMNA	-0.05132	-0.208816209	0.108767755	5.30E-01	7.73E-01	1.00E+00
ENSG00000113532.11_ST8SIA4	ENSG00000160789.18_LMNA	0.13162	-0.028168905	0.28485384	1.06E-01	3.23E-01	1.00E+00
ENSG00000100441.8_KHNYN	ENSG00000160789.18_LMNA	0.45842	0.322781136	0.57561612	2.87E-09	3.75E-07	1.47E-04
ENSG00000257017.7_HP	ENSG00000160789.18_LMNA	0.06368	-0.096495792	0.220646916	4.36E-01	7.04E-01	1.00E+00
ENSG00000106692.12_FKTN	ENSG00000160789.18_LMNA	0.11135	-0.048718447	0.265834288	1.72E-01	4.31E-01	1.00E+00
ENSG00000076067.10_RBMS2	ENSG00000160789.18_LMNA	0.10156	-0.058585942	0.256613603	2.13E-01	4.87E-01	1.00E+00
ENSG00000015413.8_DPEP1	ENSG00000160789.18_LMNA	-0.07484	-0.231287501	0.085375361	3.59E-01	6.42E-01	1.00E+00
ENSG00000165355.7_FBXO33	ENSG00000160789.18_LMNA	-0.19078	-0.339661897	-0.03256575	1.86E-02	1.02E-01	1.00E+00
ENSG00000198130.13_HIBCH	ENSG00000160789.18_LMNA	-0.34931	-0.481723515	-0.20130584	1.03E-05	2.98E-04	5.28E-01
ENSG00000126106.12_TMEM53	ENSG00000160789.18_LMNA	0.32832	0.178450352	0.463307495	3.64E-05	8.35E-04	1.00E+00
ENSG00000174348.12_PODN	ENSG00000160789.18_LMNA	-0.118	-0.272090534	0.041988193	1.48E-01	3.94E-01	1.00E+00
ENSG00000267673.5_FDX2	ENSG00000160789.18_LMNA	-0.09825	-0.253481874	0.061923401	2.29E-01	5.05E-01	1.00E+00
ENSG00000130818.9_ZNF426	ENSG00000160789.18_LMNA	-0.04015	-0.198080765	0.119820455	6.23E-01	8.30E-01	1.00E+00
ENSG00000223313.1_RNU6-516F	ENSG00000160789.18_LMNA	-0.09189	-0.247469696	0.068310825	2.60E-01	5.43E-01	1.00E+00
ENSG00000167107.11_ACSF2	ENSG00000160789.18_LMNA	0.13797	-0.021703417	0.290787406	9.01E-02	2.92E-01	1.00E+00
ENSG00000169057.18_MECP2	ENSG00000160789.18_LMNA	0.1055	-0.054615229	0.260330949	1.96E-01	4.65E-01	1.00E+00
ENSG00000273437.1_RP11-434H	ENSG00000160789.18_LMNA	-0.08366	-0.239667812	0.076561242	3.06E-01	5.92E-01	1.00E+00
ENSG00000156970.11_BUB1B	ENSG00000160789.18_LMNA	0.10913	-0.05095967	0.263745016	1.81E-01	4.44E-01	1.00E+00
ENSG00000121486.10_TRMT1L	ENSG00000160789.18_LMNA	-0.18946	-0.33845003	-0.03119786	1.94E-02	1.06E-01	1.00E+00
ENSG00000182771.16_GRID1	ENSG00000160789.18_LMNA	-0.15713	-0.30860877	0.002126008	5.32E-02	2.09E-01	1.00E+00
ENSG00000254027.1_RP11-363E	ENSG00000160789.18_LMNA	0.14258	-0.017002559	0.295086482	7.97E-02	2.71E-01	1.00E+00
ENSG00000112130.15_RNF8	ENSG00000160789.18_LMNA	-0.06297	-0.2199641	0.097206708	4.41E-01	7.09E-01	1.00E+00
ENSG00000142961.13_MOB3C	ENSG00000160789.18_LMNA	0.22167	0.064757645	0.367890673	6.06E-03	4.53E-02	1.00E+00
ENSG00000162408.10_NOL9	ENSG00000160789.18_LMNA	0.1077	-0.052399042	0.262401671	1.87E-01	4.53E-01	1.00E+00
ENSG00000182108.8_DEXI	ENSG00000160789.18_LMNA	-0.10458	-0.259464003	0.055542156	2.00E-01	4.70E-01	1.00E+00
ENSG00000152818.17_UTRN	ENSG00000160789.18_LMNA	0.23059	0.074112844	0.375990704	4.26E-03	3.48E-02	1.00E+00

ENSG00000253954.3_HMGN1P38	ENSG00000160789.18_LMNA	0.1665	0.007500392	0.317292422	4.03E-02	1.74E-01	1.00E+00
ENSG00000101493.9_ZNF516	ENSG00000160789.18_LMNA	-0.08681	-0.242659461	0.073402712	2.88E-01	5.73E-01	1.00E+00
ENSG00000113739.9_STC2	ENSG00000160789.18_LMNA	-0.02841	-0.186765358	0.131385574	7.28E-01	8.85E-01	1.00E+00
ENSG00000156873.14_PHKG2	ENSG00000160789.18_LMNA	0.00598	-0.153369044	0.165021271	9.42E-01	9.77E-01	1.00E+00
ENSG00000141012.11_GALNS	ENSG00000160789.18_LMNA	0.46891	0.334697551	0.584481138	1.11E-09	1.70E-07	5.69E-05
ENSG00000028839.8_TBPL1	ENSG00000160789.18_LMNA	-0.46948	-0.584961063	-0.33534496	1.05E-09	1.63E-07	5.39E-05
ENSG00000068784.11_SRBD1	ENSG00000160789.18_LMNA	-0.07099	-0.227620334	0.089216832	3.85E-01	6.63E-01	1.00E+00
ENSG00000101222.11_SPEF1	ENSG00000160789.18_LMNA	0.06372	-0.096463289	0.220678127	4.35E-01	7.04E-01	1.00E+00
ENSG00000059769.18_DNAJC25	ENSG00000160789.18_LMNA	0.12932	-0.030506135	0.282702977	1.12E-01	3.34E-01	1.00E+00
ENSG00000103852.11_TTC23	ENSG00000160789.18_LMNA	0.29764	0.145330524	0.436177049	1.96E-04	3.21E-03	1.00E+00
ENSG00000174442.10_ZWILCH	ENSG00000160789.18_LMNA	0.03124	-0.128598953	0.189499553	7.02E-01	8.73E-01	1.00E+00
ENSG00000049618.20_ARID1B	ENSG00000160789.18_LMNA	-0.02625	-0.184674419	0.133513219	7.48E-01	8.95E-01	1.00E+00
ENSG00000065613.12_SLK	ENSG00000160789.18_LMNA	0.23795	0.081854139	0.382658662	3.16E-03	2.78E-02	1.00E+00
ENSG00000162695.10_SLC30A7	ENSG00000160789.18_LMNA	0.32943	0.179655038	0.464284363	3.41E-05	7.93E-04	1.00E+00
ENSG00000230204.1_FTH1P5	ENSG00000160789.18_LMNA	0.01086	-0.148601107	0.169763721	8.94E-01	9.59E-01	1.00E+00
ENSG00000274180.1_NATD1	ENSG00000160789.18_LMNA	0.15191	-0.00747151	0.303764301	6.17E-02	2.30E-01	1.00E+00
ENSG00000104147.7_OIP5	ENSG00000160789.18_LMNA	0.00272	-0.15655308	0.161846036	9.74E-01	9.90E-01	1.00E+00
ENSG00000176222.8_ZNF404	ENSG00000160789.18_LMNA	-0.14318	-0.295645662	0.016390104	7.84E-02	2.69E-01	1.00E+00
ENSG00000233325.3_MIPEPP3	ENSG00000160789.18_LMNA	-0.14663	-0.298854348	0.012871181	7.14E-02	2.53E-01	1.00E+00
ENSG00000227946.1_AC007383.3	ENSG00000160789.18_LMNA	0.04519	-0.114835476	0.202932116	5.80E-01	8.04E-01	1.00E+00
ENSG00000247626.4_MARS2	ENSG00000160789.18_LMNA	-0.10658	-0.261343313	0.053532136	1.91E-01	4.59E-01	1.00E+00
ENSG00000174600.12_CMKLR1	ENSG00000160789.18_LMNA	0.06009	-0.100070006	0.217210823	4.62E-01	7.25E-01	1.00E+00
ENSG00000073614.10_KDM5A	ENSG00000160789.18_LMNA	0.12207	-0.037868686	0.275906851	1.34E-01	3.72E-01	1.00E+00
ENSG00000231414.1_AC016700.(	ENSG00000160789.18_LMNA	-0.05134	-0.208834566	0.108748788	5.30E-01	7.73E-01	1.00E+00
ENSG00000237493.3_RP11-603J	ENSG00000160789.18_LMNA	0.45455	0.318384245	0.572330047	4.04E-09	5.00E-07	2.07E-04
ENSG00000165733.7_BMS1	ENSG00000160789.18_LMNA	0.2265	0.069812858	0.372273359	5.02E-03	3.93E-02	1.00E+00
ENSG00000256745.1_RP11-680H	ENSG00000160789.18_LMNA	-0.12375	-0.277485492	0.036161504	1.29E-01	3.64E-01	1.00E+00
ENSG00000269867.1_CTD-2583A	ENSG00000160789.18_LMNA	0.00371	-0.155581705	0.162815425	9.64E-01	9.86E-01	1.00E+00
ENSG00000040933.14_INPP4A	ENSG00000160789.18_LMNA	-0.01937	-0.17802422	0.140260717	8.13E-01	9.26E-01	1.00E+00
ENSG00000141098.11_GFOD2	ENSG00000160789.18_LMNA	-0.06178	-0.218830723	0.098386011	4.50E-01	7.15E-01	1.00E+00
ENSG00000117480.14_FAAH	ENSG00000160789.18_LMNA	-0.35699	-0.488430309	-0.20970595	6.34E-06	2.02E-04	3.25E-01
ENSG00000105613.8_MAST1	ENSG00000160789.18_LMNA	-0.28716	-0.426844403	-0.13408629	3.35E-04	4.89E-03	1.00E+00
ENSG00000185019.15_UBOX5	ENSG00000160789.18_LMNA	0.10298	-0.057162126	0.257947641	2.07E-01	4.79E-01	1.00E+00

ENSG00000224318.4_CHL1-AS2	ENSG00000160789.18_LMNA	0.06941	-0.090793062	0.22611302	3.95E-01	6.73E-01	1.00E+00
ENSG00000003436.13_TFPI	ENSG00000160789.18_LMNA	-0.10398	-0.258892011	0.056153425	2.02E-01	4.73E-01	1.00E+00
ENSG00000105738.9_SIPA1L3	ENSG00000160789.18_LMNA	0.15787	-0.001360339	0.309301355	5.21E-02	2.07E-01	1.00E+00
ENSG00000230487.6_PSMG3-AS	ENSG00000160789.18_LMNA	0.13752	-0.022170568	0.290359492	9.11E-02	2.94E-01	1.00E+00
ENSG00000197629.5_MPEG1	ENSG00000160789.18_LMNA	-0.02594	-0.184380278	0.133812288	7.51E-01	8.96E-01	1.00E+00
ENSG00000205208.4_C4orf46	ENSG00000160789.18_LMNA	-0.13387	-0.286957785	0.025879342	1.00E-01	3.12E-01	1.00E+00
ENSG00000264538.5_SUZ12P1	ENSG00000160789.18_LMNA	-0.35668	-0.488156935	-0.20936275	6.47E-06	2.06E-04	3.32E-01
ENSG00000004766.14_VPS50	ENSG00000160789.18_LMNA	-0.13838	-0.291165489	0.021290554	8.91E-02	2.91E-01	1.00E+00
ENSG00000276085.1_CCL3L3	ENSG00000160789.18_LMNA	-0.10405	-0.258958118	0.05608279	2.02E-01	4.73E-01	1.00E+00
ENSG00000178761.13_FAM219B	ENSG00000160789.18_LMNA	-0.14545	-0.297754871	0.014077833	7.38E-02	2.58E-01	1.00E+00
ENSG00000020181.16_ADGRA2	ENSG00000160789.18_LMNA	0.28055	0.127024007	0.420950687	4.64E-04	6.35E-03	1.00E+00
ENSG00000137185.10_ZSCAN9	ENSG00000160789.18_LMNA	-0.14996	-0.301952086	0.00946659	6.52E-02	2.38E-01	1.00E+00
ENSG00000074590.12_NUAK1	ENSG00000160789.18_LMNA	-0.07356	-0.230069678	0.086652113	3.68E-01	6.48E-01	1.00E+00
ENSG00000159208.14_CIART	ENSG00000160789.18_LMNA	-0.08145	-0.237566387	0.0787761	3.19E-01	6.05E-01	1.00E+00
ENSG00000138111.13_MFSD13A	ENSG00000160789.18_LMNA	-0.24369	-0.387843297	-0.08789853	2.48E-03	2.31E-02	1.00E+00
ENSG00000170522.8_ELOVL6	ENSG00000160789.18_LMNA	0.36117	0.214290913	0.492077176	4.85E-06	1.62E-04	2.49E-01
ENSG00000107815.6_TWNK	ENSG00000160789.18_LMNA	-0.14392	-0.296331858	0.015638212	7.69E-02	2.66E-01	1.00E+00
ENSG00000173145.10_NOC3L	ENSG00000160789.18_LMNA	-0.09134	-0.246947605	0.068864283	2.63E-01	5.46E-01	1.00E+00
ENSG00000104883.6_PEX11G	ENSG00000160789.18_LMNA	0.04022	-0.119743039	0.198156225	6.23E-01	8.30E-01	1.00E+00
ENSG00000110195.10_FOLR1	ENSG00000160789.18_LMNA	0.07548	-0.084740423	0.231892763	3.55E-01	6.39E-01	1.00E+00
ENSG00000276600.3_RAB7B	ENSG00000160789.18_LMNA	0.31622	0.165347717	0.452637796	7.22E-05	1.45E-03	1.00E+00
ENSG00000163017.12_ACTG2	ENSG00000160789.18_LMNA	0.26556	0.111054965	0.407531853	9.44E-04	1.11E-02	1.00E+00
ENSG00000166347.17_CYB5A	ENSG00000160789.18_LMNA	-0.27021	-0.411696081	-0.11599426	7.61E-04	9.34E-03	1.00E+00
ENSG00000166265.10_CYYR1	ENSG00000160789.18_LMNA	-0.12462	-0.278295057	0.03528531	1.26E-01	3.59E-01	1.00E+00
ENSG00000131470.13_PSMC3IP	ENSG00000160789.18_LMNA	0.14138	-0.018228987	0.293966095	8.23E-02	2.77E-01	1.00E+00
ENSG00000148826.8_NKX6-2	ENSG00000160789.18_LMNA	-0.27573	-0.416641504	-0.12187921	5.85E-04	7.63E-03	1.00E+00
ENSG00000188785.10_ZNF548	ENSG00000160789.18_LMNA	0.06963	-0.090577434	0.22631931	3.94E-01	6.72E-01	1.00E+00
ENSG00000080709.13_KCNN2	ENSG00000160789.18_LMNA	-0.35363	-0.485500765	-0.2060317	7.85E-06	2.41E-04	4.03E-01
ENSG00000229413.2_RP11-274B	ENSG00000160789.18_LMNA	0.14343	-0.016144006	0.295870292	7.79E-02	2.68E-01	1.00E+00
ENSG00000184922.12_FMNL1	ENSG00000160789.18_LMNA	0.19603	0.038013905	0.344478546	1.55E-02	8.99E-02	1.00E+00
ENSG00000214135.7_AC024560.:	ENSG00000160789.18_LMNA	-0.03573	-0.193832662	0.124172446	6.62E-01	8.51E-01	1.00E+00
ENSG00000170113.14_NIPA1	ENSG00000160789.18_LMNA	0.00916	-0.150256375	0.16811897	9.11E-01	9.66E-01	1.00E+00
ENSG00000164221.11_CCDC112	ENSG00000160789.18_LMNA	0.02396	-0.13576246	0.182460845	7.70E-01	9.05E-01	1.00E+00

ENSG00000198740.7_ZNF652	ENSG00000160789.18_LMNA	-0.18217	-0.331749373	-0.02365526	2.47E-02	1.25E-01	1.00E+00
ENSG00000188549.11_C15orf52	ENSG00000160789.18_LMNA	0.20916	0.051677476	0.35648779	9.71E-03	6.43E-02	1.00E+00
ENSG00000198690.8_FAN1	ENSG00000160789.18_LMNA	-0.0736	-0.230099975	0.086620363	3.68E-01	6.48E-01	1.00E+00
ENSG00000107669.16_ATE1	ENSG00000160789.18_LMNA	-0.31606	-0.452496377	-0.16517473	7.29E-05	1.46E-03	1.00E+00
ENSG00000175048.15_ZDHHC14	ENSG00000160789.18_LMNA	0.02572	-0.134029235	0.184166871	7.53E-01	8.97E-01	1.00E+00
ENSG00000120314.17_WDR55	ENSG00000160789.18_LMNA	0.31004	0.158666548	0.447165351	1.01E-04	1.90E-03	1.00E+00
ENSG00000111012.8_CYP27B1	ENSG00000160789.18_LMNA	-0.20686	-0.354387148	-0.0492792	1.06E-02	6.81E-02	1.00E+00
ENSG00000125966.9_MMP24	ENSG00000160789.18_LMNA	-0.17378	-0.324012269	-0.0149894	3.23E-02	1.50E-01	1.00E+00
ENSG00000006695.9_COX10	ENSG00000160789.18_LMNA	-0.0837	-0.239705294	0.076521708	3.05E-01	5.92E-01	1.00E+00
ENSG00000215190.7_LINC00680	ENSG00000160789.18_LMNA	-0.28818	-0.427755059	-0.13518017	3.18E-04	4.71E-03	1.00E+00
ENSG00000170949.16_ZNF160	ENSG00000160789.18_LMNA	0.18242	0.023908796	0.331975109	2.45E-02	1.25E-01	1.00E+00
ENSG00000020426.9_MNAT1	ENSG00000160789.18_LMNA	-0.06466	-0.221582985	0.095520677	4.29E-01	6.99E-01	1.00E+00
ENSG00000108384.13_RAD51C	ENSG00000160789.18_LMNA	-0.14752	-0.299678225	0.011966398	6.97E-02	2.49E-01	1.00E+00
ENSG00000160055.18_TMEM234	ENSG00000160789.18_LMNA	0.13109	-0.028705588	0.284360229	1.07E-01	3.26E-01	1.00E+00
ENSG00000103351.11_CLUAP1	ENSG00000160789.18_LMNA	0.05281	-0.107290568	0.21024522	5.18E-01	7.65E-01	1.00E+00
ENSG00000171587.13_DSCAM	ENSG00000160789.18_LMNA	-0.27266	-0.413890783	-0.11860335	6.78E-04	8.56E-03	1.00E+00
ENSG00000134324.10_LPIN1	ENSG00000160789.18_LMNA	-0.06666	-0.223490168	0.093532052	4.15E-01	6.88E-01	1.00E+00
ENSG00000116198.11_CEP104	ENSG00000160789.18_LMNA	0.16931	0.010389008	0.319888051	3.70E-02	1.65E-01	1.00E+00
ENSG00000087253.10_LPCAT2	ENSG00000160789.18_LMNA	-0.14837	-0.300471193	0.011095078	6.81E-02	2.45E-01	1.00E+00
ENSG00000102445.17_RUBCNL	ENSG00000160789.18_LMNA	0.2023	0.044532833	0.350220728	1.24E-02	7.67E-02	1.00E+00
ENSG00000147257.12_GPC3	ENSG00000160789.18_LMNA	-0.14038	-0.293029574	0.019253424	8.45E-02	2.82E-01	1.00E+00
ENSG00000204954.8_C12orf73	ENSG00000160789.18_LMNA	-0.29384	-0.432796563	-0.14124892	2.39E-04	3.76E-03	1.00E+00
ENSG00000167861.14_HID1	ENSG00000160789.18_LMNA	-0.28377	-0.423819415	-0.13045781	3.96E-04	5.62E-03	1.00E+00
ENSG00000212664.5_RP11-592N	ENSG00000160789.18_LMNA	-0.11218	-0.266614904	0.047880244	1.69E-01	4.27E-01	1.00E+00
ENSG00000125746.13_EML2	ENSG00000160789.18_LMNA	0.28279	0.129410826	0.422945348	4.16E-04	5.84E-03	1.00E+00
ENSG00000162066.13_AMDHD2	ENSG00000160789.18_LMNA	0.23058	0.074102786	0.37598202	4.26E-03	3.48E-02	1.00E+00
ENSG00000119661.13_DNAL1	ENSG00000160789.18_LMNA	0.12289	-0.037036417	0.276676666	1.31E-01	3.68E-01	1.00E+00
ENSG00000177034.13_MTX3	ENSG00000160789.18_LMNA	0.17708	0.018401528	0.327063643	2.91E-02	1.40E-01	1.00E+00
ENSG00000102575.9_ACP5	ENSG00000160789.18_LMNA	-0.01779	-0.176486255	0.141816986	8.28E-01	9.33E-01	1.00E+00
ENSG00000188766.11_SPRED3	ENSG00000160789.18_LMNA	0.47932	0.346565864	0.593251497	4.18E-10	7.56E-08	2.14E-05
ENSG00000012174.10_MBTPS2	ENSG00000160789.18_LMNA	0.17384	0.015059269	0.324074812	3.22E-02	1.50E-01	1.00E+00
ENSG00000244754.7_N4BP2L2	ENSG00000160789.18_LMNA	-0.19793	-0.346218074	-0.03998599	1.45E-02	8.59E-02	1.00E+00
ENSG00000206562.10_METTL6	ENSG00000160789.18_LMNA	-0.37739	-0.50618231	-0.23214032	1.64E-06	6.89E-05	8.44E-02

ENSG00000275066.3_SYNRG	ENSG00000160789.18_LMNA	-0.14523	-0.297548983	0.014303689	7.42E-02	2.60E-01	1.00E+00
ENSG00000130540.12_SULT4A1	ENSG00000160789.18_LMNA	-0.11009	-0.264654425	0.049984509	1.77E-01	4.38E-01	1.00E+00
ENSG00000133466.12_C1QTNF6	ENSG00000160789.18_LMNA	0.40916	0.267380499	0.53360624	1.66E-07	1.09E-05	8.52E-03
ENSG00000174791.9_RIN1	ENSG00000160789.18_LMNA	0.69029	0.596661588	0.765381293	7.76E-23	9.95E-19	3.98E-18
ENSG00000157353.15_FUK	ENSG00000160789.18_LMNA	0.10578	-0.054334444	0.260593465	1.95E-01	4.63E-01	1.00E+00
ENSG00000108557.16_RAI1	ENSG00000160789.18_LMNA	0.25063	0.095224954	0.394102267	1.84E-03	1.84E-02	1.00E+00
ENSG00000225217.1_HSPA7	ENSG00000160789.18_LMNA	0.3364	0.187230679	0.470411548	2.26E-05	5.71E-04	1.00E+00
ENSG00000103089.7_FA2H	ENSG00000160789.18_LMNA	-0.3947	-0.521153537	-0.25128983	4.86E-07	2.56E-05	2.49E-02
ENSG00000122435.8_TRMT13	ENSG00000160789.18_LMNA	-0.18999	-0.338939046	-0.0317497	1.91E-02	1.04E-01	1.00E+00
ENSG00000102710.18_SUPT20H	ENSG00000160789.18_LMNA	-0.10878	-0.263423153	0.05130466	1.82E-01	4.46E-01	1.00E+00
ENSG00000274265.3_CH17-189H	ENSG00000160789.18_LMNA	-0.06574	-0.22261386	0.094446095	4.21E-01	6.93E-01	1.00E+00
ENSG00000070018.7_LRP6	ENSG00000160789.18_LMNA	-0.25905	-0.401687694	-0.10414767	1.27E-03	1.39E-02	1.00E+00
ENSG00000166557.11_TMED3	ENSG00000160789.18_LMNA	0.16187	0.002740879	0.313005508	4.63E-02	1.91E-01	1.00E+00
ENSG00000131389.15_SLC6A6	ENSG00000160789.18_LMNA	0.18412	0.025669197	0.333541547	2.32E-02	1.20E-01	1.00E+00
ENSG00000064933.15_PMS1	ENSG00000160789.18_LMNA	-0.18191	-0.331512307	-0.02338905	2.49E-02	1.26E-01	1.00E+00
ENSG00000104756.14_KCTD9	ENSG00000160789.18_LMNA	0.28738	0.134322261	0.427040897	3.31E-04	4.85E-03	1.00E+00
ENSG00000151461.18_UPF2	ENSG00000160789.18_LMNA	-0.02477	-0.183249142	0.134961837	7.62E-01	9.01E-01	1.00E+00
ENSG00000006016.9_CRLF1	ENSG00000160789.18_LMNA	-0.03071	-0.188989256	0.129119416	7.07E-01	8.76E-01	1.00E+00
ENSG00000255224.1_CTD-3065J	ENSG00000160789.18_LMNA	-0.09771	-0.252973692	0.062464303	2.31E-01	5.09E-01	1.00E+00
ENSG00000113318.9_MSH3	ENSG00000160789.18_LMNA	0.18247	0.023966287	0.332026293	2.44E-02	1.25E-01	1.00E+00
ENSG00000174405.12_LIG4	ENSG00000160789.18_LMNA	-0.25708	-0.399917262	-0.10206082	1.39E-03	1.48E-02	1.00E+00
ENSG00000182986.11_ZNF320	ENSG00000160789.18_LMNA	-0.09078	-0.246417364	0.069426181	2.66E-01	5.50E-01	1.00E+00
ENSG00000053900.9_ANAPC4	ENSG00000160789.18_LMNA	-0.09386	-0.249338146	0.066328525	2.50E-01	5.31E-01	1.00E+00
ENSG00000246022.2_ALDH1L1-A	ENSG00000160789.18_LMNA	-0.02236	-0.180919146	0.137327064	7.84E-01	9.12E-01	1.00E+00
ENSG00000204946.8_ZNF783	ENSG00000160789.18_LMNA	0.14201	-0.017588017	0.294551752	8.09E-02	2.74E-01	1.00E+00
ENSG00000110934.9_BIN2	ENSG00000160789.18_LMNA	-0.06815	-0.224911106	0.092048792	4.04E-01	6.80E-01	1.00E+00
ENSG00000261701.5_HPR	ENSG00000160789.18_LMNA	-0.29256	-0.431659842	-0.13987867	2.55E-04	3.96E-03	1.00E+00
ENSG00000037749.10_MFAP3	ENSG00000160789.18_LMNA	-0.02637	-0.184799174	0.133386356	7.47E-01	8.94E-01	1.00E+00
ENSG00000186260.15_MKL2	ENSG00000160789.18_LMNA	0.15897	-0.000235508	0.310318223	5.04E-02	2.02E-01	1.00E+00
ENSG00000140104.12_C14orf79	ENSG00000160789.18_LMNA	0.22162	0.064706595	0.367846346	6.07E-03	4.54E-02	1.00E+00
ENSG00000214274.8_ANG	ENSG00000160789.18_LMNA	0.34255	0.193921158	0.475800202	1.56E-05	4.21E-04	8.01E-01
ENSG00000166432.13_ZMAT1	ENSG00000160789.18_LMNA	-0.04693	-0.204599843	0.113118108	5.66E-01	7.96E-01	1.00E+00
ENSG00000168116.12_KIAA1586	ENSG00000160789.18_LMNA	-0.30052	-0.438726951	-0.14841584	1.69E-04	2.86E-03	1.00E+00

ENSG00000198142.4_SOWAHC	ENSG00000160789.18_LMNA	0.10555	-0.054562862	0.260379913	1.96E-01	4.65E-01	1.00E+00
ENSG00000184508.9_HDDC3	ENSG00000160789.18_LMNA	0.1288	-0.03103735	0.282213679	1.14E-01	3.37E-01	1.00E+00
ENSG00000138468.14_SENP7	ENSG00000160789.18_LMNA	-0.29045	-0.429776839	-0.13761127	2.84E-04	4.30E-03	1.00E+00
ENSG00000187398.10_LUZP2	ENSG00000160789.18_LMNA	-0.33236	-0.466860748	-0.18283637	2.87E-05	6.88E-04	1.00E+00
ENSG00000166289.5_PLEKHF1	ENSG00000160789.18_LMNA	0.36056	0.213620887	0.491544847	5.04E-06	1.67E-04	2.59E-01
ENSG00000072756.15_TRNT1	ENSG00000160789.18_LMNA	-0.29682	-0.435445497	-0.14444641	2.05E-04	3.32E-03	1.00E+00
ENSG00000164251.4_F2RL1	ENSG00000160789.18_LMNA	0.1979	0.039955552	0.346191243	1.45E-02	8.59E-02	1.00E+00
ENSG00000139977.12_NAA30	ENSG00000160789.18_LMNA	0.0622	-0.097973857	0.219226923	4.47E-01	7.13E-01	1.00E+00
ENSG00000114279.12_FGF12	ENSG00000160789.18_LMNA	-0.37337	-0.502687422	-0.22770043	2.16E-06	8.46E-05	1.11E-01
ENSG00000128731.14_HERC2	ENSG00000160789.18_LMNA	0.14534	-0.014192751	0.297650116	7.40E-02	2.59E-01	1.00E+00
ENSG00000127989.12_MTERF1	ENSG00000160789.18_LMNA	-0.12428	-0.277980865	0.035625418	1.27E-01	3.61E-01	1.00E+00
ENSG00000129493.13_HEATR5A	ENSG00000160789.18_LMNA	-0.02334	-0.181869675	0.136362601	7.75E-01	9.08E-01	1.00E+00
ENSG00000138376.9_BARD1	ENSG00000160789.18_LMNA	0.11732	-0.042676453	0.271451964	1.50E-01	3.98E-01	1.00E+00
ENSG00000169193.10_CCDC126	ENSG00000160789.18_LMNA	-0.23203	-0.377294738	-0.07562393	4.02E-03	3.33E-02	1.00E+00
ENSG00000126749.13_EMG1	ENSG00000160789.18_LMNA	-0.18122	-0.33087612	-0.02267486	2.55E-02	1.28E-01	1.00E+00
ENSG00000223547.8_ZNF844	ENSG00000160789.18_LMNA	-0.1586	-0.309977265	0.000612754	5.10E-02	2.04E-01	1.00E+00
ENSG00000137825.9_ITPKA	ENSG00000160789.18_LMNA	0.28662	0.133515902	0.426369313	3.44E-04	4.99E-03	1.00E+00
ENSG00000065989.14_PDE4A	ENSG00000160789.18_LMNA	0.02784	-0.131944577	0.186216278	7.33E-01	8.88E-01	1.00E+00
ENSG00000128709.11_HOXD9	ENSG00000160789.18_LMNA	-0.10176	-0.256800549	0.058386492	2.12E-01	4.86E-01	1.00E+00
ENSG00000152766.5_ANKRD22	ENSG00000160789.18_LMNA	-0.19325	-0.341927492	-0.03512611	1.71E-02	9.63E-02	1.00E+00
ENSG00000267855.4_NDUFA7	ENSG00000160789.18_LMNA	-0.17454	-0.324715938	-0.01577563	3.15E-02	1.48E-01	1.00E+00
ENSG00000157426.12_AASDH	ENSG00000160789.18_LMNA	-0.20387	-0.351657317	-0.04616783	1.18E-02	7.37E-02	1.00E+00
ENSG00000163521.14_GLB1L	ENSG00000160789.18_LMNA	0.25317	0.097919255	0.396397059	1.65E-03	1.69E-02	1.00E+00
ENSG00000106477.17_CEP41	ENSG00000160789.18_LMNA	0.10576	-0.054353624	0.260575534	1.95E-01	4.63E-01	1.00E+00
ENSG00000147133.14_TAF1	ENSG00000160789.18_LMNA	0.20552	0.047886513	0.353165885	1.11E-02	7.05E-02	1.00E+00
ENSG00000182197.9_EXT1	ENSG00000160789.18_LMNA	0.27107	0.116910889	0.412467516	7.31E-04	9.06E-03	1.00E+00
ENSG00000173210.18_ABLIM3	ENSG00000160789.18_LMNA	-0.15906	-0.310401838	0.000142981	5.03E-02	2.02E-01	1.00E+00
ENSG00000198252.10_STYX	ENSG00000160789.18_LMNA	0.24153	0.085622237	0.385893011	2.72E-03	2.48E-02	1.00E+00
ENSG00000171467.14_ZNF318	ENSG00000160789.18_LMNA	0.03295	-0.126912824	0.191151567	6.87E-01	8.64E-01	1.00E+00
ENSG00000196353.10_CPNE4	ENSG00000160789.18_LMNA	0.21349	0.056197821	0.360438781	8.27E-03	5.71E-02	1.00E+00
ENSG00000141968.6_VAV1	ENSG00000160789.18_LMNA	0.06959	-0.090616289	0.22628214	3.94E-01	6.72E-01	1.00E+00
ENSG00000149016.14_TUT1	ENSG00000160789.18_LMNA	0.0884	-0.071809282	0.244166425	2.79E-01	5.64E-01	1.00E+00
ENSG00000126970.14_ZC4H2	ENSG00000160789.18_LMNA	-0.28917	-0.428636725	-0.1362399	3.02E-04	4.53E-03	1.00E+00

ENSG00000177200.14_CHD9	ENSG00000160789.18_LMNA	-0.02606	-0.184494069	0.133696598	7.50E-01	8.96E-01	1.00E+00
ENSG00000134987.10_WDR36	ENSG00000160789.18_LMNA	0.16683	0.007839007	0.317596932	3.99E-02	1.73E-01	1.00E+00
ENSG00000186312.9_CA5BP1	ENSG00000160789.18_LMNA	0.14469	-0.014856835	0.29704462	7.53E-02	2.62E-01	1.00E+00
ENSG00000198868.3_MTND4LP3	ENSG00000160789.18_LMNA	-0.15281	-0.304599396	0.006551314	6.02E-02	2.26E-01	1.00E+00
ENSG00000151247.11_EIF4E	ENSG00000160789.18_LMNA	-0.19452	-0.343092961	-0.03644479	1.63E-02	9.33E-02	1.00E+00
ENSG00000166169.15_POLL	ENSG00000160789.18_LMNA	-0.10645	-0.261227892	0.053655658	1.92E-01	4.60E-01	1.00E+00
ENSG00000197183.11_NOL4L	ENSG00000160789.18_LMNA	-0.1201	-0.274063929	0.039859352	1.41E-01	3.82E-01	1.00E+00
ENSG00000116761.10_CTH	ENSG00000160789.18_LMNA	0.08001	-0.080208436	0.236205829	3.27E-01	6.14E-01	1.00E+00
ENSG00000156966.6_B3GNT7	ENSG00000160789.18_LMNA	0.2021	0.044325015	0.350038026	1.25E-02	7.70E-02	1.00E+00
ENSG00000119147.8_C2orf40	ENSG00000160789.18_LMNA	-0.15946	-0.310772061	-0.00026677	4.97E-02	2.01E-01	1.00E+00
ENSG00000147687.15_TATDN1	ENSG00000160789.18_LMNA	-0.29758	-0.436118683	-0.14525997	1.97E-04	3.22E-03	1.00E+00
ENSG00000167202.10_TBC1D2B	ENSG00000160789.18_LMNA	0.23577	0.079551928	0.380678923	3.45E-03	2.97E-02	1.00E+00
ENSG00000164972.11_C9orf24	ENSG00000160789.18_LMNA	-0.02816	-0.186526591	0.131628681	7.31E-01	8.86E-01	1.00E+00
ENSG00000115425.12_PECR	ENSG00000160789.18_LMNA	-0.00846	-0.167430854	0.150948362	9.18E-01	9.69E-01	1.00E+00
ENSG00000168876.7_ANKRD49	ENSG00000160789.18_LMNA	-0.37429	-0.503486659	-0.22871477	2.03E-06	8.09E-05	1.04E-01
ENSG00000244055.1_AC007566.1	ENSG00000160789.18_LMNA	-0.04849	-0.206099952	0.111571723	5.53E-01	7.88E-01	1.00E+00
ENSG00000182575.7_NXPH3	ENSG00000160789.18_LMNA	-0.09552	-0.25090922	0.064659792	2.42E-01	5.21E-01	1.00E+00
ENSG00000181274.6_FRAT2	ENSG00000160789.18_LMNA	-0.16449	-0.315431596	-0.0054327	4.29E-02	1.82E-01	1.00E+00
ENSG00000015153.13_YAF2	ENSG00000160789.18_LMNA	-0.52807	-0.633974285	-0.40272773	2.72E-12	1.27E-09	1.40E-07
ENSG00000204977.8_TRIM13	ENSG00000160789.18_LMNA	-0.4265	-0.548458743	-0.28676819	4.28E-08	3.58E-06	2.20E-03
ENSG00000279348.1_RP11-566E	ENSG00000160789.18_LMNA	-0.25257	-0.395850384	-0.09727701	1.69E-03	1.72E-02	1.00E+00
ENSG00000137720.6_C11orf1	ENSG00000160789.18_LMNA	-0.27665	-0.417464604	-0.1228607	5.60E-04	7.36E-03	1.00E+00
ENSG00000136404.14_TM6SF1	ENSG00000160789.18_LMNA	-0.20585	-0.353467129	-0.04822993	1.10E-02	6.99E-02	1.00E+00
ENSG00000176994.10_SMCR8	ENSG00000160789.18_LMNA	0.08374	-0.076475992	0.239748636	3.05E-01	5.92E-01	1.00E+00
ENSG00000128849.10_CGNL1	ENSG00000160789.18_LMNA	0.2785	0.124828855	0.41911369	5.12E-04	6.88E-03	1.00E+00
ENSG00000167528.11_ZNF641	ENSG00000160789.18_LMNA	-0.2119	-0.358987234	-0.05453565	8.77E-03	5.96E-02	1.00E+00
ENSG00000103227.17_LMF1	ENSG00000160789.18_LMNA	0.1027	-0.057442673	0.257684878	2.08E-01	4.80E-01	1.00E+00
ENSG00000197557.6_TTC30A	ENSG00000160789.18_LMNA	-0.03066	-0.188940637	0.129168995	7.08E-01	8.76E-01	1.00E+00
ENSG00000115942.7_ORC2	ENSG00000160789.18_LMNA	-0.1091	-0.263718373	0.05098823	1.81E-01	4.44E-01	1.00E+00
ENSG00000197362.12_ZNF786	ENSG00000160789.18_LMNA	0.26755	0.113169005	0.409315671	8.61E-04	1.03E-02	1.00E+00
ENSG00000143033.16_MTF2	ENSG00000160789.18_LMNA	-0.11923	-0.27324174	0.040746652	1.43E-01	3.87E-01	1.00E+00
ENSG00000196187.10_TMEM63A	ENSG00000160789.18_LMNA	-0.10431	-0.25920865	0.055815072	2.01E-01	4.71E-01	1.00E+00
ENSG00000003393.13_ALS2	ENSG00000160789.18_LMNA	0.17173	0.012884965	0.322127113	3.44E-02	1.57E-01	1.00E+00

ENSG00000076356.6_PLXNA2	ENSG00000160789.18_LMNA	0.16646	0.007457646	0.317253977	4.04E-02	1.74E-01	1.00E+00
ENSG00000198604.9_BAZ1A	ENSG00000160789.18_LMNA	0.18717	0.028823649	0.336344191	2.09E-02	1.11E-01	1.00E+00
ENSG00000198026.7_ZNF335	ENSG00000160789.18_LMNA	0.21592	0.058738483	0.362654655	7.55E-03	5.32E-02	1.00E+00
ENSG00000218582.2_GAPDHP63	ENSG00000160789.18_LMNA	0.22918	0.072629021	0.374709034	4.51E-03	3.63E-02	1.00E+00
ENSG00000251050.1_RP11-168A	ENSG00000160789.18_LMNA	-0.11847	-0.2725336	0.041510474	1.46E-01	3.91E-01	1.00E+00
ENSG00000067221.12_STOML1	ENSG00000160789.18_LMNA	0.0909	-0.069305949	0.246530838	2.65E-01	5.49E-01	1.00E+00
ENSG00000281490.1_CICP14	ENSG00000160789.18_LMNA	0.10411	-0.056022427	0.25901461	2.02E-01	4.72E-01	1.00E+00
ENSG00000112893.8_MAN2A1	ENSG00000160789.18_LMNA	0.25444	0.09926154	0.397538921	1.56E-03	1.62E-02	1.00E+00
ENSG00000167642.11_SPINT2	ENSG00000160789.18_LMNA	-0.10575	-0.260563038	0.05436699	1.95E-01	4.64E-01	1.00E+00
ENSG00000139324.10_TMTC3	ENSG00000160789.18_LMNA	0.18021	0.021627846	0.329942948	2.63E-02	1.31E-01	1.00E+00
ENSG00000226742.3_HSBP1L1	ENSG00000160789.18_LMNA	-0.13841	-0.291194916	0.021258414	8.90E-02	2.90E-01	1.00E+00
ENSG00000278053.3_DDX52	ENSG00000160789.18_LMNA	-0.22481	-0.370740502	-0.06804301	5.36E-03	4.13E-02	1.00E+00
ENSG00000138604.8_GLCE	ENSG00000160789.18_LMNA	0.00631	-0.153040478	0.165348553	9.38E-01	9.76E-01	1.00E+00
ENSG00000174343.5_CHRNA9	ENSG00000160789.18_LMNA	0.42575	0.285926288	0.547817192	4.55E-08	3.77E-06	2.33E-03
ENSG00000140443.12_IGF1R	ENSG00000160789.18_LMNA	0.17889	0.020265218	0.32872758	2.74E-02	1.35E-01	1.00E+00
ENSG00000141391.12_PRELID3A	ENSG00000160789.18_LMNA	0.02697	-0.132800408	0.185375254	7.42E-01	8.92E-01	1.00E+00
ENSG00000163808.15_KIF15	ENSG00000160789.18_LMNA	-0.03016	-0.188454902	0.129664228	7.12E-01	8.78E-01	1.00E+00
ENSG00000156968.8_MPV17L	ENSG00000160789.18_LMNA	-0.02545	-0.183905929	0.134294463	7.56E-01	8.98E-01	1.00E+00
ENSG00000150764.12_DIXDC1	ENSG00000160789.18_LMNA	-0.2316	-0.376901117	-0.07516767	4.09E-03	3.37E-02	1.00E+00
ENSG00000143942.4_CHAC2	ENSG00000160789.18_LMNA	-0.27754	-0.4182599	-0.12380958	5.37E-04	7.13E-03	1.00E+00
ENSG00000140396.11_NCOA2	ENSG00000160789.18_LMNA	-0.2363	-0.381160259	-0.08011137	3.38E-03	2.92E-02	1.00E+00
ENSG00000225770.1_RPS29P3	ENSG00000160789.18_LMNA	-0.12858	-0.28200384	0.031265113	1.14E-01	3.38E-01	1.00E+00
ENSG00000240356.5_RPL23AP7	ENSG00000160789.18_LMNA	0.06539	-0.094802047	0.222272465	4.24E-01	6.95E-01	1.00E+00
ENSG00000089472.15_HEPH	ENSG00000160789.18_LMNA	0.09187	-0.068333422	0.247448383	2.60E-01	5.43E-01	1.00E+00
ENSG00000269374.1_CTB-50E14	ENSG00000160789.18_LMNA	0.07221	-0.088004049	0.22877906	3.77E-01	6.56E-01	1.00E+00
ENSG00000162817.6_C1orf115	ENSG00000160789.18_LMNA	-0.05095	-0.208456199	0.109139682	5.33E-01	7.75E-01	1.00E+00
ENSG00000064651.12_SLC12A2	ENSG00000160789.18_LMNA	-0.00325	-0.162367796	0.156030328	9.68E-01	9.88E-01	1.00E+00
ENSG00000074755.13_ZZEF1	ENSG00000160789.18_LMNA	0.14268	-0.016901864	0.295178433	7.95E-02	2.71E-01	1.00E+00
ENSG00000153253.14_SCN3A	ENSG00000160789.18_LMNA	-0.48675	-0.599491466	-0.35505834	2.04E-10	4.09E-08	1.05E-05
ENSG00000182240.14_BACE2	ENSG00000160789.18_LMNA	0.26875	0.114442624	0.410389253	8.15E-04	9.85E-03	1.00E+00
ENSG00000128203.6_ASPHD2	ENSG00000160789.18_LMNA	0.17559	0.016863279	0.325688823	3.05E-02	1.45E-01	1.00E+00
ENSG00000169905.11_TOR1AIP2	ENSG00000160789.18_LMNA	0.2457	0.090022298	0.389660486	2.28E-03	2.16E-02	1.00E+00
ENSG00000109586.10_GALNT7	ENSG00000160789.18_LMNA	0.19394	0.035848842	0.342566369	1.67E-02	9.46E-02	1.00E+00

ENSG00000152193.7_RNF219	ENSG00000160789.18_LMNA	-0.26356	-0.405738084	-0.10893183	1.04E-03	1.19E-02	1.00E+00
ENSG00000177283.5_FZD8	ENSG00000160789.18_LMNA	0.25239	0.097093017	0.395693732	1.71E-03	1.73E-02	1.00E+00
ENSG00000088992.16_TESC	ENSG00000160789.18_LMNA	-0.05486	-0.212207288	0.105260066	5.02E-01	7.54E-01	1.00E+00
ENSG00000196741.5_LINC01560	ENSG00000160789.18_LMNA	-0.29755	-0.43609295	-0.14522886	1.97E-04	3.22E-03	1.00E+00
ENSG00000141854.6_MISP3	ENSG00000160789.18_LMNA	0.0368	-0.123124056	0.194857117	6.53E-01	8.46E-01	1.00E+00
ENSG00000145246.12_ATP10D	ENSG00000160789.18_LMNA	0.18536	0.026954316	0.334683997	2.22E-02	1.16E-01	1.00E+00
ENSG00000273270.1_RP11-212P	ENSG00000160789.18_LMNA	0.0561	-0.104029022	0.213395598	4.92E-01	7.48E-01	1.00E+00
ENSG00000279413.1_AC112497.:	ENSG00000160789.18_LMNA	0.23235	0.07596262	0.377586855	3.97E-03	3.30E-02	1.00E+00
ENSG00000083635.7_NUFIP1	ENSG00000160789.18_LMNA	-0.2034	-0.351227874	-0.0456789	1.20E-02	7.46E-02	1.00E+00
ENSG00000051620.9_HEBP2	ENSG00000160789.18_LMNA	0.12238	-0.037553389	0.276198536	1.33E-01	3.70E-01	1.00E+00
ENSG00000130844.15_ZNF331	ENSG00000160789.18_LMNA	-0.04066	-0.19857114	0.119317301	6.19E-01	8.27E-01	1.00E+00
ENSG00000139719.8_VPS33A	ENSG00000160789.18_LMNA	-0.28881	-0.428321153	-0.13586052	3.08E-04	4.60E-03	1.00E+00
ENSG00000196990.7_FAM163B	ENSG00000160789.18_LMNA	-0.24944	-0.393032283	-0.09397019	1.94E-03	1.91E-02	1.00E+00
ENSG00000179277.9_MEIS3P1	ENSG00000160789.18_LMNA	0.08477	-0.075452692	0.240718476	2.99E-01	5.86E-01	1.00E+00
ENSG00000124507.9_PACSIN1	ENSG00000160789.18_LMNA	-0.22501	-0.370927642	-0.06825899	5.32E-03	4.11E-02	1.00E+00
ENSG00000139697.10_SBNO1	ENSG00000160789.18_LMNA	0.01688	-0.142703678	0.175609295	8.36E-01	9.37E-01	1.00E+00
ENSG00000153975.8_ZUFSP	ENSG00000160789.18_LMNA	-0.19664	-0.345035104	-0.03864461	1.52E-02	8.86E-02	1.00E+00
ENSG00000134070.4_IRAK2	ENSG00000160789.18_LMNA	0.48755	0.355976589	0.600164387	1.89E-10	3.83E-08	9.68E-06
ENSG00000172243.16_CLEC7A	ENSG00000160789.18_LMNA	-0.04179	-0.199660742	0.118198717	6.09E-01	8.22E-01	1.00E+00
ENSG00000136932.12_TRMO	ENSG00000160789.18_LMNA	-0.22101	-0.367290268	-0.06406632	6.21E-03	4.61E-02	1.00E+00
ENSG00000205730.6_ITPRIPL2	ENSG00000160789.18_LMNA	0.43687	0.298424259	0.557309434	1.83E-08	1.80E-06	9.41E-04
ENSG00000102781.12_KATNAL1	ENSG00000160789.18_LMNA	0.15042	-0.008996657	0.302379149	6.44E-02	2.36E-01	1.00E+00
ENSG00000064199.5_SPA17	ENSG00000160789.18_LMNA	-0.0066	-0.165621923	0.152765983	9.36E-01	9.75E-01	1.00E+00
ENSG00000259417.2_LINC01314	ENSG00000160789.18_LMNA	-0.11015	-0.264710092	0.049924799	1.77E-01	4.38E-01	1.00E+00
ENSG00000151835.12_SACS	ENSG00000160789.18_LMNA	0.05747	-0.102666729	0.214709506	4.82E-01	7.40E-01	1.00E+00
ENSG00000160392.12_C19orf47	ENSG00000160789.18_LMNA	0.04733	-0.11272091	0.204985297	5.63E-01	7.94E-01	1.00E+00
ENSG00000163961.4_RNF168	ENSG00000160789.18_LMNA	0.17772	0.019059924	0.32765169	2.85E-02	1.38E-01	1.00E+00
ENSG00000163960.10_UBXN7	ENSG00000160789.18_LMNA	0.08015	-0.080070698	0.236336719	3.26E-01	6.13E-01	1.00E+00
ENSG00000141446.9_ESCO1	ENSG00000160789.18_LMNA	-0.02268	-0.181223866	0.137017943	7.82E-01	9.10E-01	1.00E+00
ENSG00000162623.14_TYW3	ENSG00000160789.18_LMNA	0.13673	-0.022969996	0.28962692	9.30E-02	2.98E-01	1.00E+00
ENSG00000245067.5_IGFBP7-AS	ENSG00000160789.18_LMNA	0.05433	-0.105789598	0.21169585	5.06E-01	7.57E-01	1.00E+00
ENSG00000067208.13_EVI5	ENSG00000160789.18_LMNA	-0.05797	-0.215185102	0.102173326	4.78E-01	7.36E-01	1.00E+00
ENSG00000250321.1_RP11-478C	ENSG00000160789.18_LMNA	-0.0883	-0.244068113	0.071913284	2.79E-01	5.65E-01	1.00E+00

ENSG00000213453.3_FTH1P3	ENSG00000160789.18_LMNA	-0.02097	-0.179574719	0.13869017	7.98E-01	9.19E-01	1.00E+00
ENSG00000180801.12_ARSJ	ENSG00000160789.18_LMNA	0.60754	0.496347896	0.699122227	1.05E-16	3.00E-13	5.39E-12
ENSG00000133401.14_PDZD2	ENSG00000160789.18_LMNA	0.03267	-0.127192852	0.19087733	6.89E-01	8.66E-01	1.00E+00
ENSG00000107651.11_SEC23IP	ENSG00000160789.18_LMNA	0.1565	-0.002764851	0.308030652	5.42E-02	2.12E-01	1.00E+00
ENSG00000116977.17_LGALS8	ENSG00000160789.18_LMNA	0.31116	0.159882524	0.448162949	9.54E-05	1.82E-03	1.00E+00
ENSG00000268006.1_PTOV1-AS1	ENSG00000160789.18_LMNA	0.21712	0.059993002	0.363747535	7.21E-03	5.15E-02	1.00E+00
ENSG00000198743.5_SLC5A3	ENSG00000160789.18_LMNA	0.27233	0.118259024	0.413601339	6.88E-04	8.66E-03	1.00E+00
ENSG00000140265.11_ZSCAN29	ENSG00000160789.18_LMNA	0.02865	-0.131146944	0.186999692	7.26E-01	8.84E-01	1.00E+00
ENSG00000212694.7_LINC01089	ENSG00000160789.18_LMNA	-0.31561	-0.452094039	-0.16468268	7.47E-05	1.49E-03	1.00E+00
ENSG00000175198.13_PCCA	ENSG00000160789.18_LMNA	-0.32887	-0.463788639	-0.1790436	3.52E-05	8.16E-04	1.00E+00
ENSG00000102755.9_FLT1	ENSG00000160789.18_LMNA	0.15512	-0.00418098	0.306748316	5.64E-02	2.17E-01	1.00E+00
ENSG00000168779.18_SHOX2	ENSG00000160789.18_LMNA	0.13209	-0.027697715	0.285287076	1.05E-01	3.21E-01	1.00E+00
ENSG00000240204.2_SMKR1	ENSG00000160789.18_LMNA	0.07359	-0.086628591	0.230092123	3.68E-01	6.48E-01	1.00E+00
ENSG00000147854.15_UHRF2	ENSG00000160789.18_LMNA	0.22633	0.069634653	0.372119091	5.05E-03	3.95E-02	1.00E+00
ENSG00000233184.5_RP11-421L1	ENSG00000160789.18_LMNA	-0.31314	-0.449907664	-0.16201127	8.57E-05	1.66E-03	1.00E+00
ENSG00000166900.13_STX3	ENSG00000160789.18_LMNA	0.09213	-0.068064685	0.247701827	2.59E-01	5.42E-01	1.00E+00
ENSG00000182903.14_ZNF721	ENSG00000160789.18_LMNA	0.06049	-0.099667388	0.217598276	4.59E-01	7.23E-01	1.00E+00
ENSG00000125454.10_SLC25A19	ENSG00000160789.18_LMNA	0.09622	-0.0639567	0.251570667	2.38E-01	5.17E-01	1.00E+00
ENSG00000185917.12_SETD4	ENSG00000160789.18_LMNA	-0.03981	-0.197754879	0.120154744	6.26E-01	8.32E-01	1.00E+00
ENSG00000103707.8_MTFMT	ENSG00000160789.18_LMNA	0.06086	-0.099301025	0.217950752	4.56E-01	7.21E-01	1.00E+00
ENSG00000128011.4_LRFN1	ENSG00000160789.18_LMNA	-0.05695	-0.214208011	0.103186833	4.86E-01	7.43E-01	1.00E+00
ENSG00000115459.16_ELMOD3	ENSG00000160789.18_LMNA	-0.14147	-0.294050553	0.018136567	8.21E-02	2.77E-01	1.00E+00
ENSG00000083799.16_CYLD	ENSG00000160789.18_LMNA	0.12631	-0.033567013	0.279881395	1.21E-01	3.50E-01	1.00E+00
ENSG00000092847.9_37104	ENSG00000160789.18_LMNA	0.06142	-0.098743113	0.218487359	4.52E-01	7.17E-01	1.00E+00
ENSG00000137274.11_BPHL	ENSG00000160789.18_LMNA	-0.26609	-0.408009775	-0.1116211	9.21E-04	1.09E-02	1.00E+00
ENSG00000213609.3_RPL7AP50	ENSG00000160789.18_LMNA	-0.17147	-0.321880893	-0.01261031	3.47E-02	1.57E-01	1.00E+00
ENSG00000138587.5_MNS1	ENSG00000160789.18_LMNA	0.12725	-0.032612132	0.280762206	1.18E-01	3.45E-01	1.00E+00
ENSG00000166816.12_LDHD	ENSG00000160789.18_LMNA	-0.14107	-0.29367896	0.018543146	8.30E-02	2.78E-01	1.00E+00
ENSG00000186364.10_NUDT17	ENSG00000160789.18_LMNA	-0.07414	-0.230614747	0.086080797	3.64E-01	6.46E-01	1.00E+00
ENSG00000213930.10_GALT	ENSG00000160789.18_LMNA	-0.228	-0.373640698	-0.0713932	4.73E-03	3.76E-02	1.00E+00
ENSG00000087903.11_RFX2	ENSG00000160789.18_LMNA	0.30896	0.157510084	0.446215911	1.08E-04	2.00E-03	1.00E+00
ENSG00000175643.8_RMI2	ENSG00000160789.18_LMNA	0.16112	0.001967283	0.31230753	4.74E-02	1.94E-01	1.00E+00
ENSG00000105499.12_PLA2G4C	ENSG00000160789.18_LMNA	-0.21925	-0.365682564	-0.06221658	6.65E-03	4.85E-02	1.00E+00

ENSG00000277998.1_RP11-452N	ENSG00000160789.18_LMNA	-0.09549	-0.250879715	0.064691147	2.42E-01	5.21E-01	1.00E+00
ENSG00000183495.12_EP400	ENSG00000160789.18_LMNA	0.13391	-0.025839116	0.286994723	1.00E-01	3.12E-01	1.00E+00
ENSG00000034533.10_ASTE1	ENSG00000160789.18_LMNA	0.04823	-0.111824916	0.205854438	5.55E-01	7.89E-01	1.00E+00
ENSG00000120049.17_KCNIP2	ENSG00000160789.18_LMNA	-0.44236	-0.561981415	-0.3046084	1.16E-08	1.22E-06	5.94E-04
ENSG00000048707.12_VPS13D	ENSG00000160789.18_LMNA	-0.1246	-0.278277247	0.035304591	1.26E-01	3.59E-01	1.00E+00
ENSG00000112210.10_RAB23	ENSG00000160789.18_LMNA	-0.00749	-0.16649688	0.151887092	9.27E-01	9.72E-01	1.00E+00
ENSG00000140406.3_MESDC1	ENSG00000160789.18_LMNA	0.21359	0.056298915	0.360527016	8.24E-03	5.69E-02	1.00E+00
ENSG00000104427.10_ZC2HC1A	ENSG00000160789.18_LMNA	-0.31433	-0.45095968	-0.16329614	8.02E-05	1.58E-03	1.00E+00
ENSG00000159267.13_HLCS	ENSG00000160789.18_LMNA	0.35941	0.212357207	0.490540298	5.43E-06	1.78E-04	2.79E-01
ENSG00000184209.14_SNRNP35	ENSG00000160789.18_LMNA	-0.1316	-0.284836751	0.028187488	1.06E-01	3.23E-01	1.00E+00
ENSG00000163794.6_UCN	ENSG00000160789.18_LMNA	-0.07046	-0.227112491	0.089748071	3.88E-01	6.67E-01	1.00E+00
ENSG00000177839.6_PCDHB9	ENSG00000160789.18_LMNA	0.04513	-0.114900105	0.202869319	5.81E-01	8.05E-01	1.00E+00
ENSG00000206535.6_LNP1	ENSG00000160789.18_LMNA	-0.29928	-0.437626579	-0.14708373	1.80E-04	3.01E-03	1.00E+00
ENSG00000142856.15_ITGB3BP	ENSG00000160789.18_LMNA	-0.18957	-0.338552023	-0.03131294	1.93E-02	1.05E-01	1.00E+00
ENSG00000132275.9_RRP8	ENSG00000160789.18_LMNA	0.21115	0.053755627	0.358305544	9.02E-03	6.09E-02	1.00E+00
ENSG00000253661.1_ZFHX4-AS1	ENSG00000160789.18_LMNA	-0.0759	-0.232293495	0.084319902	3.53E-01	6.36E-01	1.00E+00
ENSG00000103154.8_NECAB2	ENSG00000160789.18_LMNA	-0.26472	-0.406779276	-0.11016387	9.82E-04	1.14E-02	1.00E+00
ENSG00000103876.10_FAH	ENSG00000160789.18_LMNA	0.17067	0.011788309	0.321143756	3.55E-02	1.60E-01	1.00E+00
ENSG00000221866.8_PLXNA4	ENSG00000160789.18_LMNA	-0.00734	-0.166343571	0.152041126	9.29E-01	9.73E-01	1.00E+00
ENSG00000186001.11_LRCH3	ENSG00000160789.18_LMNA	-0.09045	-0.246109617	0.06975221	2.68E-01	5.52E-01	1.00E+00
ENSG00000164941.12_INTS8	ENSG00000160789.18_LMNA	-0.00884	-0.16780549	0.150571658	9.14E-01	9.67E-01	1.00E+00
ENSG00000171608.14_PIK3CD	ENSG00000160789.18_LMNA	0.1548	-0.00451138	0.306448968	5.69E-02	2.18E-01	1.00E+00
ENSG00000067066.15_SP100	ENSG00000160789.18_LMNA	0.10939	-0.050696888	0.263990134	1.80E-01	4.43E-01	1.00E+00
ENSG00000173269.12_MMRN2	ENSG00000160789.18_LMNA	0.12432	-0.035580362	0.278022492	1.27E-01	3.61E-01	1.00E+00
ENSG00000204305.12_AGER	ENSG00000160789.18_LMNA	0.00315	-0.156130734	0.162267594	9.69E-01	9.88E-01	1.00E+00
ENSG00000135763.8_URB2	ENSG00000160789.18_LMNA	0.098	-0.06216702	0.253253014	2.30E-01	5.07E-01	1.00E+00
ENSG00000167536.12_DHRS13	ENSG00000160789.18_LMNA	0.16469	0.005637823	0.315616305	4.26E-02	1.81E-01	1.00E+00
ENSG00000054690.12_PLEKHH1	ENSG00000160789.18_LMNA	-0.34664	-0.4793854	-0.19838706	1.22E-05	3.43E-04	6.24E-01
ENSG00000072195.13_SPEG	ENSG00000160789.18_LMNA	0.2457	0.090019581	0.389658163	2.28E-03	2.16E-02	1.00E+00
ENSG00000274070.1_GATSL2	ENSG00000160789.18_LMNA	-0.07857	-0.234837432	0.081647709	3.36E-01	6.22E-01	1.00E+00
ENSG00000134909.17_ARHGAP3	ENSG00000160789.18_LMNA	-0.04143	-0.199319196	0.118549435	6.12E-01	8.24E-01	1.00E+00
ENSG00000266490.1_CTD-2349P	ENSG00000160789.18_LMNA	-0.1956	-0.344082953	-0.03756576	1.57E-02	9.08E-02	1.00E+00
ENSG00000153914.14_SREK1	ENSG00000160789.18_LMNA	0.10982	-0.050261268	0.264396384	1.78E-01	4.40E-01	1.00E+00

ENSG00000112282.16_MED23	ENSG00000160789.18_LMNA	-0.08286	-0.238911149	0.077359109	3.10E-01	5.97E-01	1.00E+00
ENSG00000100207.17_TCF20	ENSG00000160789.18_LMNA	0.00638	-0.152980961	0.165407831	9.38E-01	9.75E-01	1.00E+00
ENSG00000204104.10_TRAF3IP1	ENSG00000160789.18_LMNA	0.24095	0.085013973	0.385371406	2.79E-03	2.53E-02	1.00E+00
ENSG00000213347.9_MXD3	ENSG00000160789.18_LMNA	-0.04956	-0.207124322	0.110514869	5.44E-01	7.82E-01	1.00E+00
ENSG00000197037.9_ZSCAN25	ENSG00000160789.18_LMNA	0.2344	0.078112753	0.37943993	3.65E-03	3.10E-02	1.00E+00
ENSG00000176401.5_EID2B	ENSG00000160789.18_LMNA	-0.17043	-0.320926443	-0.01154606	3.58E-02	1.61E-01	1.00E+00
ENSG00000081803.14_CADPS2	ENSG00000160789.18_LMNA	0.25486	0.099701101	0.397912648	1.53E-03	1.60E-02	1.00E+00
ENSG00000100426.6_ZBED4	ENSG00000160789.18_LMNA	-0.01103	-0.169935348	0.148428279	8.93E-01	9.58E-01	1.00E+00
ENSG00000084093.14_REST	ENSG00000160789.18_LMNA	0.3261	0.176042657	0.461353042	4.13E-05	9.23E-04	1.00E+00
ENSG00000139323.12_POC1B	ENSG00000160789.18_LMNA	-0.14568	-0.297971381	0.01384029	7.33E-02	2.57E-01	1.00E+00
ENSG00000232838.3_PET117	ENSG00000160789.18_LMNA	-0.00561	-0.164662803	0.153728837	9.45E-01	9.78E-01	1.00E+00
ENSG00000108176.13_DNAJC12	ENSG00000160789.18_LMNA	-0.47059	-0.585898792	-0.33661061	9.49E-10	1.50E-07	4.87E-05
ENSG00000153832.10_FBXO36	ENSG00000160789.18_LMNA	-0.08916	-0.244885852	0.071048007	2.75E-01	5.59E-01	1.00E+00
ENSG00000137218.9_FRS3	ENSG00000160789.18_LMNA	-0.16137	-0.312543362	-0.00222862	4.70E-02	1.93E-01	1.00E+00
ENSG00000115750.15_TAF1B	ENSG00000160789.18_LMNA	-0.1199	-0.273868786	0.040069994	1.41E-01	3.84E-01	1.00E+00
ENSG00000163319.9_MRPS18C	ENSG00000160789.18_LMNA	-0.18194	-0.331533493	-0.02341284	2.49E-02	1.26E-01	1.00E+00
ENSG00000176054.6_RPL23P2	ENSG00000160789.18_LMNA	0.00159	-0.157648145	0.160752471	9.84E-01	9.94E-01	1.00E+00
ENSG00000241741.1_RPL7AP30	ENSG00000160789.18_LMNA	-0.10443	-0.259317678	0.055698551	2.00E-01	4.71E-01	1.00E+00
ENSG00000198168.7_SVIP	ENSG00000160789.18_LMNA	-0.04897	-0.206559825	0.111097356	5.49E-01	7.86E-01	1.00E+00
ENSG00000085224.19_ATRX	ENSG00000160789.18_LMNA	0.08682	-0.073398412	0.24266353	2.88E-01	5.73E-01	1.00E+00
ENSG00000174460.3_ZCCHC12	ENSG00000160789.18_LMNA	-0.22235	-0.368508094	-0.06546887	5.90E-03	4.45E-02	1.00E+00
ENSG00000261799.1_RP11-283I3	ENSG00000160789.18_LMNA	-0.09189	-0.247472305	0.068308058	2.60E-01	5.43E-01	1.00E+00
ENSG00000164715.5_LMTK2	ENSG00000160789.18_LMNA	0.11654	-0.043471213	0.270714239	1.53E-01	4.02E-01	1.00E+00
ENSG00000167874.6_TMEM88	ENSG00000160789.18_LMNA	-0.22708	-0.372807109	-0.07042958	4.90E-03	3.86E-02	1.00E+00
ENSG00000235823.1_OLMALINC	ENSG00000160789.18_LMNA	-0.12587	-0.279465902	0.034017249	1.22E-01	3.52E-01	1.00E+00
ENSG00000116580.17_GON4L	ENSG00000160789.18_LMNA	0.2104	0.052971605	0.357620033	9.28E-03	6.21E-02	1.00E+00
ENSG00000137601.14_NEK1	ENSG00000160789.18_LMNA	0.03119	-0.128653449	0.18944613	7.03E-01	8.73E-01	1.00E+00
ENSG00000105186.13_ANKRD27	ENSG00000160789.18_LMNA	0.04423	-0.115782694	0.202011489	5.88E-01	8.09E-01	1.00E+00
ENSG00000197620.9_CXorf40A	ENSG00000160789.18_LMNA	0.31107	0.159783879	0.448082047	9.59E-05	1.82E-03	1.00E+00
ENSG00000184992.10_BRI3BP	ENSG00000160789.18_LMNA	-0.00751	-0.166513666	0.151870225	9.27E-01	9.72E-01	1.00E+00
ENSG00000205808.5_PLPP6	ENSG00000160789.18_LMNA	-0.04758	-0.20522499	0.112473864	5.61E-01	7.93E-01	1.00E+00
ENSG00000064547.12_LPAR2	ENSG00000160789.18_LMNA	-0.09284	-0.24836588	0.067360345	2.55E-01	5.37E-01	1.00E+00
ENSG00000211899.6_IGHM	ENSG00000160789.18_LMNA	0.05486	-0.105260871	0.212206511	5.02E-01	7.54E-01	1.00E+00

ENSG00000214765.7_SEPT7P2	ENSG00000160789.18_LMNA	-0.05383	-0.211219216	0.106282932	5.10E-01	7.59E-01	1.00E+00
ENSG00000151304.5_SRFBP1	ENSG00000160789.18_LMNA	-0.09083	-0.246469155	0.069371307	2.66E-01	5.50E-01	1.00E+00
ENSG00000144791.8_LIMD1	ENSG00000160789.18_LMNA	0.01467	-0.14486569	0.173468897	8.58E-01	9.44E-01	1.00E+00
ENSG00000137145.19_DENND4C	ENSG00000160789.18_LMNA	0.03737	-0.122556335	0.195411587	6.48E-01	8.43E-01	1.00E+00
ENSG00000173614.12_NMNAT1	ENSG00000160789.18_LMNA	0.05273	-0.107371326	0.210167131	5.19E-01	7.65E-01	1.00E+00
ENSG00000139364.9_TMEM132B	ENSG00000160789.18_LMNA	-0.3404	-0.47392141	-0.19158549	1.78E-05	4.68E-04	9.13E-01
ENSG00000267510.1_CTC-325H2	ENSG00000160789.18_LMNA	-0.03662	-0.194688787	0.123296367	6.54E-01	8.47E-01	1.00E+00
ENSG00000237181.1_AC147651.4	ENSG00000160789.18_LMNA	0.22852	0.071935657	0.374109736	4.63E-03	3.70E-02	1.00E+00
ENSG00000048649.12_RSF1	ENSG00000160789.18_LMNA	-0.02022	-0.17884602	0.139428491	8.05E-01	9.22E-01	1.00E+00
ENSG00000171316.10_CHD7	ENSG00000160789.18_LMNA	-0.21288	-0.359885954	-0.05556458	8.46E-03	5.81E-02	1.00E+00
ENSG00000180917.15_CMTR2	ENSG00000160789.18_LMNA	-0.09045	-0.246104288	0.069757855	2.68E-01	5.52E-01	1.00E+00
ENSG00000119285.9_HEATR1	ENSG00000160789.18_LMNA	0.14603	-0.013483542	0.29829648	7.26E-02	2.56E-01	1.00E+00
ENSG00000171606.16_ZNF274	ENSG00000160789.18_LMNA	0.09256	-0.067632689	0.248109149	2.57E-01	5.39E-01	1.00E+00
ENSG00000254416.4_RP11-347E	ENSG00000160789.18_LMNA	-0.10496	-0.259819104	0.055162552	1.98E-01	4.68E-01	1.00E+00
ENSG00000165533.17_TTC8	ENSG00000160789.18_LMNA	-0.08834	-0.244104026	0.071875293	2.79E-01	5.65E-01	1.00E+00
ENSG00000155850.7_SLC26A2	ENSG00000160789.18_LMNA	0.24598	0.090316705	0.38991221	2.25E-03	2.14E-02	1.00E+00
ENSG00000127586.15_CHTF18	ENSG00000160789.18_LMNA	0.08141	-0.078816091	0.237528416	3.19E-01	6.05E-01	1.00E+00
ENSG00000053918.14_KCNQ1	ENSG00000160789.18_LMNA	0.07815	-0.082068607	0.234437023	3.39E-01	6.24E-01	1.00E+00
ENSG00000254901.6_BORCS8	ENSG00000160789.18_LMNA	-0.19974	-0.347876388	-0.04186822	1.36E-02	8.18E-02	1.00E+00
ENSG00000135525.17_MAP7	ENSG00000160789.18_LMNA	-0.13419	-0.28725142	0.025559541	9.93E-02	3.10E-01	1.00E+00
ENSG00000183072.9_NKX2-5	ENSG00000160789.18_LMNA	0.21509	0.05786835	0.361896143	7.79E-03	5.45E-02	1.00E+00
ENSG00000196839.11_ADA	ENSG00000160789.18_LMNA	0.07821	-0.082010938	0.23449189	3.38E-01	6.24E-01	1.00E+00
ENSG00000145476.14_CYP4V2	ENSG00000160789.18_LMNA	0.2369	0.080743161	0.381703645	3.30E-03	2.87E-02	1.00E+00
ENSG00000263874.1_LINC00672	ENSG00000160789.18_LMNA	-0.35753	-0.488898537	-0.21029394	6.13E-06	1.96E-04	3.15E-01
ENSG00000021776.9_AQR	ENSG00000160789.18_LMNA	0.13297	-0.026795639	0.286116136	1.02E-01	3.16E-01	1.00E+00
ENSG00000166825.12_ANPEP	ENSG00000160789.18_LMNA	0.36447	0.217914431	0.494952436	3.91E-06	1.36E-04	2.01E-01
ENSG00000171928.12_TVP23B	ENSG00000160789.18_LMNA	0.09817	-0.061994929	0.253414683	2.29E-01	5.06E-01	1.00E+00
ENSG00000081386.11_ZNF510	ENSG00000160789.18_LMNA	-0.13003	-0.283361556	0.029790855	1.10E-01	3.31E-01	1.00E+00
ENSG00000074266.16_EED	ENSG00000160789.18_LMNA	-0.14622	-0.298471254	0.013291721	7.23E-02	2.55E-01	1.00E+00
ENSG00000169372.11_CRADD	ENSG00000160789.18_LMNA	-0.2726	-0.413837747	-0.11854025	6.80E-04	8.58E-03	1.00E+00
ENSG00000169116.10_PARM1	ENSG00000160789.18_LMNA	0.05294	-0.107159704	0.21037175	5.17E-01	7.64E-01	1.00E+00
ENSG00000198521.10_ZNF43	ENSG00000160789.18_LMNA	-0.18454	-0.333927894	-0.02610368	2.29E-02	1.19E-01	1.00E+00
ENSG00000175538.9_KCNE3	ENSG00000160789.18_LMNA	-0.00985	-0.168781527	0.149589796	9.04E-01	9.63E-01	1.00E+00

ENSG00000111058.6_ACSS3	ENSG00000160789.18_LMNA	0.36759	0.221339973	0.497665033	3.18E-06	1.15E-04	1.63E-01
ENSG00000174099.9_MSRB3	ENSG00000160789.18_LMNA	0.13362	-0.026132237	0.286725541	1.01E-01	3.13E-01	1.00E+00
ENSG00000128923.9_FAM63B	ENSG00000160789.18_LMNA	0.25687	0.101833669	0.399724414	1.40E-03	1.49E-02	1.00E+00
ENSG00000053770.10_AP5M1	ENSG00000160789.18_LMNA	-0.12917	-0.282558815	0.030662665	1.13E-01	3.35E-01	1.00E+00
ENSG00000105866.12_SP4	ENSG00000160789.18_LMNA	-0.22175	-0.367956346	-0.06483328	6.04E-03	4.52E-02	1.00E+00
ENSG00000182831.10_C16orf72	ENSG00000160789.18_LMNA	0.29628	0.143865934	0.434964981	2.10E-04	3.39E-03	1.00E+00
ENSG00000186897.4_C1QL4	ENSG00000160789.18_LMNA	0.06595	-0.094243601	0.222808039	4.20E-01	6.91E-01	1.00E+00
ENSG00000006025.10_OSBPL7	ENSG00000160789.18_LMNA	-0.04013	-0.198064383	0.119837262	6.24E-01	8.30E-01	1.00E+00
ENSG00000134255.12_CEPT1	ENSG00000160789.18_LMNA	-0.192	-0.340782678	-0.03383185	1.78E-02	9.93E-02	1.00E+00
ENSG00000003509.14_NDUFAF7	ENSG00000160789.18_LMNA	-0.12912	-0.282515253	0.030709961	1.13E-01	3.35E-01	1.00E+00
ENSG00000240087.3_RPSAP12	ENSG00000160789.18_LMNA	-0.19419	-0.342796644	-0.03610942	1.65E-02	9.41E-02	1.00E+00
ENSG00000272645.2_GTF2IP20	ENSG00000160789.18_LMNA	-0.02831	-0.1866732	0.131479413	7.29E-01	8.85E-01	1.00E+00
ENSG00000124788.16_ATXN1	ENSG00000160789.18_LMNA	0.32055	0.170028892	0.456459225	5.67E-05	1.19E-03	1.00E+00
ENSG00000226067.5_LINC00623	ENSG00000160789.18_LMNA	0.08035	-0.07987615	0.236521574	3.25E-01	6.12E-01	1.00E+00
ENSG00000133028.9_SCO1	ENSG00000160789.18_LMNA	-0.00343	-0.16253817	0.155859592	9.67E-01	9.87E-01	1.00E+00
ENSG00000228594.3_FNDC10	ENSG00000160789.18_LMNA	-0.05492	-0.212260286	0.105205183	5.02E-01	7.53E-01	1.00E+00
ENSG00000117151.11_CTBS	ENSG00000160789.18_LMNA	0.11864	-0.041341521	0.272690266	1.45E-01	3.91E-01	1.00E+00
ENSG00000050344.8_NFE2L3	ENSG00000160789.18_LMNA	0.15898	-0.000228146	0.310324876	5.04E-02	2.02E-01	1.00E+00
ENSG00000135362.12_PRR5L	ENSG00000160789.18_LMNA	-0.03699	-0.19504089	0.122935915	6.51E-01	8.45E-01	1.00E+00
ENSG00000185453.11_C19orf68	ENSG00000160789.18_LMNA	0.28165	0.128202749	0.421936115	4.39E-04	6.10E-03	1.00E+00
ENSG00000270223.1_RP13-36G1	ENSG00000160789.18_LMNA	-0.12102	-0.274923658	0.03893101	1.37E-01	3.78E-01	1.00E+00
ENSG00000135686.11_KLHL36	ENSG00000160789.18_LMNA	0.2364	0.080219149	0.381252971	3.37E-03	2.92E-02	1.00E+00
ENSG00000155313.14_USP25	ENSG00000160789.18_LMNA	0.12397	-0.035936151	0.277693752	1.28E-01	3.62E-01	1.00E+00
ENSG00000059728.9_MXD1	ENSG00000160789.18_LMNA	-0.01231	-0.171179705	0.147174637	8.80E-01	9.54E-01	1.00E+00
ENSG00000101871.13_MID1	ENSG00000160789.18_LMNA	0.25157	0.096224602	0.394954124	1.77E-03	1.78E-02	1.00E+00
ENSG00000162688.14_AGL	ENSG00000160789.18_LMNA	-0.1009	-0.25598964	0.059251458	2.16E-01	4.90E-01	1.00E+00
ENSG00000223658.6_C1GALT1C	ENSG00000160789.18_LMNA	-0.15755	-0.308997372	0.001696445	5.26E-02	2.08E-01	1.00E+00
ENSG00000120458.8_MSANTD2	ENSG00000160789.18_LMNA	-0.07803	-0.234320611	0.082190954	3.39E-01	6.25E-01	1.00E+00
ENSG00000259045.1_MTCO1P2	ENSG00000160789.18_LMNA	-0.1245	-0.278190969	0.035397992	1.26E-01	3.59E-01	1.00E+00
ENSG00000112562.17_SMOC2	ENSG00000160789.18_LMNA	0.03021	-0.12961114	0.188506979	7.12E-01	8.77E-01	1.00E+00
ENSG00000176381.5_PRR18	ENSG00000160789.18_LMNA	-0.24903	-0.392666985	-0.09354202	1.98E-03	1.93E-02	1.00E+00
ENSG00000137628.15_DDX60	ENSG00000160789.18_LMNA	0.13066	-0.029151404	0.283950067	1.09E-01	3.28E-01	1.00E+00
ENSG00000236859.5_NIFK-AS1	ENSG00000160789.18_LMNA	-0.4453	-0.564481563	-0.3079267	9.01E-09	9.90E-07	4.62E-04

ENSG00000072422.15_RHOBTB1	ENSG00000160789.18_LMNA	0.07524	-0.084975897	0.231668324	3.57E-01	6.40E-01	1.00E+00
ENSG00000242992.3_FTH1P4	ENSG00000160789.18_LMNA	0.00305	-0.156228136	0.162170384	9.70E-01	9.88E-01	1.00E+00
ENSG00000267152.1_CTD-2528L	ENSG00000160789.18_LMNA	-0.19006	-0.339004227	-0.03182327	1.90E-02	1.04E-01	1.00E+00
ENSG00000225492.5_GBP1P1	ENSG00000160789.18_LMNA	0.08448	-0.075735862	0.240450163	3.01E-01	5.87E-01	1.00E+00
ENSG00000196132.10_MYT1	ENSG00000160789.18_LMNA	-0.37186	-0.501376697	-0.22603824	2.39E-06	9.16E-05	1.23E-01
ENSG00000174928.14_C3orf33	ENSG00000160789.18_LMNA	-0.25697	-0.399810003	-0.10193448	1.39E-03	1.49E-02	1.00E+00
ENSG00000232177.1_MTND4P24	ENSG00000160789.18_LMNA	-0.06197	-0.219009052	0.098200514	4.48E-01	7.14E-01	1.00E+00
ENSG00000131023.11_LATS1	ENSG00000160789.18_LMNA	-0.1069	-0.261645239	0.053208972	1.90E-01	4.57E-01	1.00E+00
ENSG00000182685.6_BRICD5	ENSG00000160789.18_LMNA	-0.09863	-0.253845879	0.061535846	2.27E-01	5.03E-01	1.00E+00
ENSG00000149599.14_DUSP15	ENSG00000160789.18_LMNA	-0.046	-0.203705001	0.114039822	5.74E-01	8.01E-01	1.00E+00
ENSG00000217930.6_PAM16	ENSG00000160789.18_LMNA	-0.16164	-0.312793377	-0.00250573	4.66E-02	1.92E-01	1.00E+00
ENSG00000076053.9_RBM7	ENSG00000160789.18_LMNA	0.02821	-0.13157839	0.186575988	7.30E-01	8.86E-01	1.00E+00
ENSG00000090530.8_P3H2	ENSG00000160789.18_LMNA	0.33102	0.181374707	0.465677627	3.11E-05	7.33E-04	1.00E+00
ENSG00000091436.15_AC013461	ENSG00000160789.18_LMNA	0.18736	0.029028659	0.336526149	2.08E-02	1.11E-01	1.00E+00
ENSG00000273271.1_AP000254.8	ENSG00000160789.18_LMNA	-0.02368	-0.182193508	0.136033882	7.72E-01	9.06E-01	1.00E+00
ENSG00000173727.10_CMB9-22P	ENSG00000160789.18_LMNA	-0.01587	-0.174630314	0.143692922	8.46E-01	9.40E-01	1.00E+00
ENSG00000196659.9_TTC30B	ENSG00000160789.18_LMNA	-0.06163	-0.218687952	0.098534503	4.51E-01	7.16E-01	1.00E+00
ENSG00000231327.1_AC016700.5	ENSG00000160789.18_LMNA	-0.30571	-0.443337146	-0.15400844	1.28E-04	2.31E-03	1.00E+00
ENSG00000276966.1_HIST1H4E	ENSG00000160789.18_LMNA	-0.07919	-0.235427028	0.081027736	3.32E-01	6.18E-01	1.00E+00
ENSG00000122966.12_CIT	ENSG00000160789.18_LMNA	-0.16388	-0.314867653	-0.00480658	4.36E-02	1.84E-01	1.00E+00
ENSG00000268713.1_CTC-444N2	ENSG00000160789.18_LMNA	0.24885	0.093346679	0.392500294	1.99E-03	1.95E-02	1.00E+00
ENSG00000139233.5_LLPH	ENSG00000160789.18_LMNA	-0.12455	-0.278237113	0.035348039	1.26E-01	3.59E-01	1.00E+00
ENSG00000070814.16_TCOF1	ENSG00000160789.18_LMNA	0.21013	0.052696322	0.357379261	9.37E-03	6.25E-02	1.00E+00
ENSG00000159214.11_CCDC24	ENSG00000160789.18_LMNA	0.08767	-0.072545437	0.243470406	2.83E-01	5.68E-01	1.00E+00
ENSG00000145020.13_AMT	ENSG00000160789.18_LMNA	0.0374	-0.122530075	0.19543723	6.47E-01	8.43E-01	1.00E+00
ENSG00000178665.13_ZNF713	ENSG00000160789.18_LMNA	-0.0254	-0.183861047	0.134340078	7.56E-01	8.98E-01	1.00E+00
ENSG00000137831.13_UACA	ENSG00000160789.18_LMNA	0.33015	0.18043596	0.464917236	3.27E-05	7.67E-04	1.00E+00
ENSG00000183397.5_C19orf71	ENSG00000160789.18_LMNA	0.16333	0.004241024	0.314358061	4.44E-02	1.86E-01	1.00E+00
ENSG00000128714.5_HOXD13	ENSG00000160789.18_LMNA	-0.12862	-0.282044752	0.031220709	1.14E-01	3.38E-01	1.00E+00
ENSG00000100503.22_NIN	ENSG00000160789.18_LMNA	0.01657	-0.143011762	0.175304476	8.39E-01	9.38E-01	1.00E+00
ENSG00000029639.9_TFB1M	ENSG00000160789.18_LMNA	-0.02049	-0.179102591	0.139168573	8.02E-01	9.21E-01	1.00E+00
ENSG00000111196.8_MAGOHB	ENSG00000160789.18_LMNA	-0.08474	-0.240692458	0.075480153	2.99E-01	5.86E-01	1.00E+00
ENSG00000197363.8_ZNF517	ENSG00000160789.18_LMNA	0.00992	-0.149512553	0.168858285	9.03E-01	9.63E-01	1.00E+00

ENSG00000187626.8_ZKSCAN4	ENSG00000160789.18_LMNA	-0.11897	-0.273002313	0.041004947	1.44E-01	3.89E-01	1.00E+00
ENSG00000165060.10_FXN	ENSG00000160789.18_LMNA	-0.18731	-0.336478579	-0.02897506	2.08E-02	1.11E-01	1.00E+00
ENSG00000185418.14_TARSL2	ENSG00000160789.18_LMNA	-0.07491	-0.231351446	0.085308292	3.59E-01	6.42E-01	1.00E+00
ENSG00000163431.12_LMOD1	ENSG00000160789.18_LMNA	-0.03175	-0.18999409	0.128094396	6.98E-01	8.71E-01	1.00E+00
ENSG00000122741.14_DCAF10	ENSG00000160789.18_LMNA	-0.0151	-0.17388587	0.144444745	8.53E-01	9.43E-01	1.00E+00
ENSG00000173276.12_ZBTB21	ENSG00000160789.18_LMNA	0.31044	0.159099015	0.447520234	9.93E-05	1.87E-03	1.00E+00
ENSG00000183150.6_GPR19	ENSG00000160789.18_LMNA	-0.32619	-0.461426394	-0.17613296	4.11E-05	9.20E-04	1.00E+00
ENSG00000167535.6_CACNB3	ENSG00000160789.18_LMNA	0.04574	-0.114292172	0.203459915	5.76E-01	8.02E-01	1.00E+00
ENSG00000155792.8_DEPTOR	ENSG00000160789.18_LMNA	-0.29294	-0.431995946	-0.14028371	2.50E-04	3.89E-03	1.00E+00
ENSG00000277283.1_RP1-267D1	ENSG00000160789.18_LMNA	-0.14852	-0.300613117	0.01093908	6.78E-02	2.44E-01	1.00E+00
ENSG00000257303.1_RP11-977G	ENSG00000160789.18_LMNA	-0.21839	-0.364900638	-0.06131769	6.87E-03	4.97E-02	1.00E+00
ENSG00000125149.10_C16orf70	ENSG00000160789.18_LMNA	-0.07994	-0.2361365	0.080281388	3.28E-01	6.14E-01	1.00E+00
ENSG00000184898.6_RBM43	ENSG00000160789.18_LMNA	-0.06344	-0.220416632	0.096735589	4.37E-01	7.06E-01	1.00E+00
ENSG00000102743.13_SLC25A15	ENSG00000160789.18_LMNA	-0.07806	-0.23434566	0.082164629	3.39E-01	6.24E-01	1.00E+00
ENSG00000121310.15_ECHDC2	ENSG00000160789.18_LMNA	0.25421	0.099020597	0.397334023	1.58E-03	1.63E-02	1.00E+00
ENSG00000150977.10_RILPL2	ENSG00000160789.18_LMNA	0.21486	0.057628848	0.361687294	7.86E-03	5.48E-02	1.00E+00
ENSG00000163637.10_PRICKLE2	ENSG00000160789.18_LMNA	0.17013	0.011237378	0.320649491	3.61E-02	1.62E-01	1.00E+00
ENSG00000143590.12_EFNA3	ENSG00000160789.18_LMNA	-0.22887	-0.374432201	-0.0723087	4.57E-03	3.66E-02	1.00E+00
ENSG00000171914.13_TLN2	ENSG00000160789.18_LMNA	0.1761	0.017388923	0.326158768	3.00E-02	1.43E-01	1.00E+00
ENSG00000138686.8_BBS7	ENSG00000160789.18_LMNA	-0.05965	-0.216789495	0.100507707	4.65E-01	7.27E-01	1.00E+00
ENSG00000241852.8_C8orf58	ENSG00000160789.18_LMNA	0.38845	0.244355547	0.515751379	7.61E-07	3.69E-05	3.90E-02
ENSG00000232818.2_RPS2P32	ENSG00000160789.18_LMNA	0.16566	0.006636727	0.316515454	4.14E-02	1.77E-01	1.00E+00
ENSG00000251495.1_PPIAP11	ENSG00000160789.18_LMNA	0.06127	-0.09889988	0.218336598	4.53E-01	7.18E-01	1.00E+00
ENSG00000176473.12_WDR25	ENSG00000160789.18_LMNA	-0.07856	-0.234824325	0.081661487	3.36E-01	6.22E-01	1.00E+00
ENSG00000144468.15_RHBDD1	ENSG00000160789.18_LMNA	0.00809	-0.151307149	0.167073952	9.21E-01	9.70E-01	1.00E+00
ENSG00000249780.1_RP11-352E	ENSG00000160789.18_LMNA	-0.03405	-0.192205539	0.125836126	6.77E-01	8.59E-01	1.00E+00
ENSG00000118496.4_FBXO30	ENSG00000160789.18_LMNA	-0.15122	-0.30312687	0.008173545	6.29E-02	2.33E-01	1.00E+00
ENSG00000130158.12_DOCK6	ENSG00000160789.18_LMNA	0.20729	0.049726526	0.354779194	1.04E-02	6.73E-02	1.00E+00
ENSG00000152242.9_C18orf25	ENSG00000160789.18_LMNA	0.15999	0.000808907	0.311261758	4.90E-02	1.99E-01	1.00E+00
ENSG00000185774.13_KCNIP4	ENSG00000160789.18_LMNA	-0.42668	-0.548611183	-0.28696829	4.22E-08	3.54E-06	2.17E-03
ENSG00000059588.8_TARBP1	ENSG00000160789.18_LMNA	-0.09296	-0.248481002	0.067238207	2.55E-01	5.36E-01	1.00E+00
ENSG00000119042.15_SATB2	ENSG00000160789.18_LMNA	0.0784	-0.081825407	0.234668396	3.37E-01	6.22E-01	1.00E+00
ENSG00000005436.12_GCFC2	ENSG00000160789.18_LMNA	-0.0794	-0.235625261	0.080819235	3.31E-01	6.17E-01	1.00E+00

ENSG00000130695.12_CEP85	ENSG00000160789.18_LMNA	-0.06056	-0.217664334	0.099598734	4.59E-01	7.22E-01	1.00E+00
ENSG00000054267.19_ARID4B	ENSG00000160789.18_LMNA	-0.06969	-0.226383396	0.090510441	3.94E-01	6.71E-01	1.00E+00
ENSG00000133106.13_EPSTI1	ENSG00000160789.18_LMNA	-0.10173	-0.256768315	0.058420884	2.12E-01	4.86E-01	1.00E+00
ENSG00000166575.15_TMEM135	ENSG00000160789.18_LMNA	-0.20643	-0.353993511	-0.04883018	1.07E-02	6.89E-02	1.00E+00
ENSG00000110841.12_PPFIBP1	ENSG00000160789.18_LMNA	0.0614	-0.098769659	0.218461831	4.52E-01	7.18E-01	1.00E+00
ENSG00000186020.11_ZNF529	ENSG00000160789.18_LMNA	-0.34585	-0.478691116	-0.19752132	1.28E-05	3.56E-04	6.55E-01
ENSG00000166704.10_ZNF606	ENSG00000160789.18_LMNA	-0.04433	-0.202102723	0.11568885	5.88E-01	8.08E-01	1.00E+00
ENSG00000064225.11_ST3GAL6	ENSG00000160789.18_LMNA	-0.43947	-0.559525738	-0.3013552	1.48E-08	1.49E-06	7.57E-04
ENSG00000107882.10_SUFU	ENSG00000160789.18_LMNA	-0.10123	-0.256297776	0.058922836	2.15E-01	4.89E-01	1.00E+00
ENSG00000052749.12_RRP12	ENSG00000160789.18_LMNA	0.2998	0.147651386	0.438095592	1.75E-04	2.94E-03	1.00E+00
ENSG00000095397.12_WHRN	ENSG00000160789.18_LMNA	0.22557	0.068837294	0.371428629	5.20E-03	4.05E-02	1.00E+00
ENSG00000272849.1_RP11-347I1	ENSG00000160789.18_LMNA	-0.15806	-0.309476129	0.001167066	5.18E-02	2.06E-01	1.00E+00
ENSG00000275835.3_TUBGCP5	ENSG00000160789.18_LMNA	0.20138	0.043572767	0.349376497	1.29E-02	7.85E-02	1.00E+00
ENSG00000040275.15_SPDL1	ENSG00000160789.18_LMNA	-0.04526	-0.202998711	0.114766936	5.80E-01	8.04E-01	1.00E+00
ENSG00000141542.9_RAB40B	ENSG00000160789.18_LMNA	-0.29939	-0.43772982	-0.14720866	1.79E-04	2.99E-03	1.00E+00
ENSG00000279496.1_	ENSG00000160789.18_LMNA	0.09134	-0.068863852	0.246948011	2.63E-01	5.46E-01	1.00E+00
ENSG00000149554.11_CHEK1	ENSG00000160789.18_LMNA	0.09101	-0.069197515	0.24663317	2.65E-01	5.49E-01	1.00E+00
ENSG00000104643.8_MTMR9	ENSG00000160789.18_LMNA	-0.25129	-0.394704668	-0.09593181	1.79E-03	1.80E-02	1.00E+00
ENSG00000145332.12_KLHL8	ENSG00000160789.18_LMNA	-0.1217	-0.275565318	0.038237788	1.35E-01	3.74E-01	1.00E+00
ENSG00000181773.6_GPR3	ENSG00000160789.18_LMNA	0.60321	0.491171964	0.695606256	1.97E-16	5.32E-13	1.01E-11
ENSG00000181090.16_EHMT1	ENSG00000160789.18_LMNA	0.22482	0.068055036	0.37075092	5.36E-03	4.13E-02	1.00E+00
ENSG00000188185.10_LINC0026	ENSG00000160789.18_LMNA	-0.03693	-0.19498538	0.122992747	6.51E-01	8.46E-01	1.00E+00
ENSG00000258655.2_ARHGAP5-	ENSG00000160789.18_LMNA	-0.32615	-0.461396615	-0.1760963	4.12E-05	9.21E-04	1.00E+00
ENSG00000123908.10_37469	ENSG00000160789.18_LMNA	0.15078	-0.008629276	0.302712928	6.37E-02	2.35E-01	1.00E+00
ENSG00000211448.10_DIO2	ENSG00000160789.18_LMNA	-0.03989	-0.197838271	0.120069209	6.26E-01	8.31E-01	1.00E+00
ENSG00000163449.9_TMEM169	ENSG00000160789.18_LMNA	-0.31437	-0.450995369	-0.16333975	8.01E-05	1.57E-03	1.00E+00
ENSG00000215386.9_MIR99AHG	ENSG00000160789.18_LMNA	-0.24505	-0.389072957	-0.08933535	2.34E-03	2.21E-02	1.00E+00
ENSG00000147421.16_HMBOX1	ENSG00000160789.18_LMNA	-0.03941	-0.197375391	0.120543926	6.30E-01	8.33E-01	1.00E+00
ENSG00000135299.15_ANKRD6	ENSG00000160789.18_LMNA	-0.40268	-0.528033287	-0.26016104	2.70E-07	1.63E-05	1.39E-02
ENSG00000197208.5_SLC22A4	ENSG00000160789.18_LMNA	0.13829	-0.021377788	0.291085612	8.93E-02	2.91E-01	1.00E+00
ENSG00000161010.13_MRNIP	ENSG00000160789.18_LMNA	-0.04399	-0.201775765	0.116025133	5.91E-01	8.10E-01	1.00E+00
ENSG00000163219.10_ARHGAP2	ENSG00000160789.18_LMNA	-0.00348	-0.162586953	0.155810701	9.66E-01	9.87E-01	1.00E+00
ENSG00000198406.7_BZW1P2	ENSG00000160789.18_LMNA	0.05044	-0.109645651	0.207966301	5.37E-01	7.78E-01	1.00E+00

ENSG00000198924.6_DCLRE1A	ENSG00000160789.18_LMNA	-0.29084	-0.430121784	-0.1380264	2.78E-04	4.24E-03	1.00E+00
ENSG00000140403.11_DNAJA4	ENSG00000160789.18_LMNA	-0.0327	-0.190906166	0.127163409	6.89E-01	8.66E-01	1.00E+00
ENSG00000168772.10_CXXC4	ENSG00000160789.18_LMNA	-0.42053	-0.543355651	-0.28008262	6.89E-08	5.34E-06	3.53E-03
ENSG00000214870.7_AC004540.!	ENSG00000160789.18_LMNA	-0.25795	-0.400692032	-0.10297374	1.33E-03	1.44E-02	1.00E+00
ENSG00000197013.8_ZNF429	ENSG00000160789.18_LMNA	-0.56476	-0.664239146	-0.44562599	3.50E-14	3.82E-11	1.79E-09
ENSG00000267523.1_CTD-2537I!	ENSG00000160789.18_LMNA	0.07202	-0.088190649	0.228600835	3.78E-01	6.57E-01	1.00E+00
ENSG00000261478.1_RP11-429B	ENSG00000160789.18_LMNA	-0.35469	-0.486420503	-0.20718439	7.35E-06	2.28E-04	3.77E-01
ENSG00000152359.13_POC5	ENSG00000160789.18_LMNA	-0.25717	-0.399991361	-0.10214811	1.38E-03	1.48E-02	1.00E+00
ENSG00000107779.10_BMPR1A	ENSG00000160789.18_LMNA	-0.11183	-0.266289985	0.048229187	1.70E-01	4.29E-01	1.00E+00
ENSG00000205683.10_DPF3	ENSG00000160789.18_LMNA	0.31998	0.169412059	0.455956284	5.85E-05	1.22E-03	1.00E+00
ENSG00000160199.13_PKNOX1	ENSG00000160789.18_LMNA	0.04541	-0.114619514	0.203141938	5.79E-01	8.04E-01	1.00E+00
ENSG00000153094.20_BCL2L11	ENSG00000160789.18_LMNA	0.05951	-0.100644572	0.216657726	4.66E-01	7.28E-01	1.00E+00
ENSG00000271971.1_CTD-2006H	ENSG00000160789.18_LMNA	0.01695	-0.142639198	0.175673085	8.36E-01	9.36E-01	1.00E+00
ENSG00000214784.4_AC010468.:	ENSG00000160789.18_LMNA	-0.13296	-0.28610434	0.026808476	1.02E-01	3.17E-01	1.00E+00
ENSG00000225968.6_ELFN1	ENSG00000160789.18_LMNA	0.08358	-0.076643718	0.239589613	3.06E-01	5.93E-01	1.00E+00
ENSG00000272335.1_RP11-53O1	ENSG00000160789.18_LMNA	0.1398	-0.019842791	0.292490515	8.58E-02	2.84E-01	1.00E+00
ENSG00000267280.4_TBX2-AS1	ENSG00000160789.18_LMNA	0.13765	-0.022037671	0.290481239	9.08E-02	2.94E-01	1.00E+00
ENSG00000169744.11_LDB2	ENSG00000160789.18_LMNA	-0.15322	-0.304984152	0.006127172	5.95E-02	2.24E-01	1.00E+00
ENSG00000112659.12_CUL9	ENSG00000160789.18_LMNA	0.0713	-0.088914422	0.227909349	3.83E-01	6.61E-01	1.00E+00
ENSG00000143578.14_CREB3L4	ENSG00000160789.18_LMNA	0.05418	-0.105930355	0.211559873	5.07E-01	7.57E-01	1.00E+00
ENSG00000122778.8_KIAA1549	ENSG00000160789.18_LMNA	-0.21231	-0.359361061	-0.05496356	8.64E-03	5.89E-02	1.00E+00
ENSG00000263272.1_CTC-524C5	ENSG00000160789.18_LMNA	-0.14877	-0.300841064	0.010688497	6.74E-02	2.43E-01	1.00E+00
ENSG00000235863.3_B3GALT4	ENSG00000160789.18_LMNA	0.1061	-0.054008238	0.260898387	1.93E-01	4.62E-01	1.00E+00
ENSG00000171448.8_ZBTB26	ENSG00000160789.18_LMNA	-0.25707	-0.39990639	-0.10204802	1.39E-03	1.48E-02	1.00E+00
ENSG00000105699.15_LSR	ENSG00000160789.18_LMNA	-0.26086	-0.403310547	-0.10606287	1.17E-03	1.30E-02	1.00E+00
ENSG00000100307.11_CBX7	ENSG00000160789.18_LMNA	-0.08106	-0.237200964	0.07916093	3.21E-01	6.07E-01	1.00E+00
ENSG00000140326.11_CDAN1	ENSG00000160789.18_LMNA	0.15321	-0.00614424	0.304968671	5.95E-02	2.24E-01	1.00E+00
ENSG00000177337.6_DLGAP1-AS	ENSG00000160789.18_LMNA	0.06446	-0.095721978	0.221389793	4.30E-01	7.00E-01	1.00E+00
ENSG00000132950.17_ZMYM5	ENSG00000160789.18_LMNA	0.00237	-0.156890303	0.161509358	9.77E-01	9.91E-01	1.00E+00
ENSG00000186812.11_ZNF397	ENSG00000160789.18_LMNA	-0.14157	-0.294138159	0.018040698	8.19E-02	2.76E-01	1.00E+00
ENSG00000079691.16_CARMIL1	ENSG00000160789.18_LMNA	0.3396	0.190708638	0.473215409	1.87E-05	4.87E-04	9.58E-01
ENSG00000133627.16_ACTR3B	ENSG00000160789.18_LMNA	0.06643	-0.09376341	0.22326841	4.16E-01	6.89E-01	1.00E+00
ENSG00000214106.6_PAXIP1-AS	ENSG00000160789.18_LMNA	0.23103	0.07456692	0.376382687	4.19E-03	3.44E-02	1.00E+00

ENSG00000277358.1_RP11-321C	ENSG00000160789.18_LMNA	0.1479	-0.011570862	0.300038246	6.90E-02	2.47E-01	1.00E+00
ENSG00000155542.10_SETD9	ENSG00000160789.18_LMNA	-0.10626	-0.261042125	0.053854444	1.93E-01	4.61E-01	1.00E+00
ENSG00000167393.15_PPP2R3B	ENSG00000160789.18_LMNA	-0.08902	-0.244753153	0.071188452	2.75E-01	5.60E-01	1.00E+00
ENSG00000156172.5_C8orf37	ENSG00000160789.18_LMNA	-0.16429	-0.315244329	-0.00522476	4.31E-02	1.82E-01	1.00E+00
ENSG00000038210.12_PI4K2B	ENSG00000160789.18_LMNA	0.47739	0.344364116	0.591628878	5.02E-10	8.88E-08	2.57E-05
ENSG00000160447.6_PKN3	ENSG00000160789.18_LMNA	0.24146	0.085547593	0.385829012	2.73E-03	2.48E-02	1.00E+00
ENSG00000158859.9_ADAMTS4	ENSG00000160789.18_LMNA	0.21265	0.055320623	0.359672922	8.53E-03	5.84E-02	1.00E+00
ENSG00000179119.13_SPTY2D1	ENSG00000160789.18_LMNA	0.26521	0.110680808	0.407215906	9.60E-04	1.12E-02	1.00E+00
ENSG00000115760.12_BIRC6	ENSG00000160789.18_LMNA	-0.01059	-0.169505405	0.148861193	8.97E-01	9.60E-01	1.00E+00
ENSG00000263335.1_AF001548.5	ENSG00000160789.18_LMNA	-0.0021	-0.161243574	0.157156466	9.80E-01	9.92E-01	1.00E+00
ENSG00000152954.10_NRSN1	ENSG00000160789.18_LMNA	-0.29565	-0.434402374	-0.14318655	2.17E-04	3.48E-03	1.00E+00
ENSG00000164171.9_ITGA2	ENSG00000160789.18_LMNA	0.32983	0.180082682	0.464630969	3.33E-05	7.79E-04	1.00E+00
ENSG00000254093.7_PINX1	ENSG00000160789.18_LMNA	-0.13848	-0.291255796	0.021191923	8.89E-02	2.90E-01	1.00E+00
ENSG00000116793.14_PHTF1	ENSG00000160789.18_LMNA	0.27319	0.1191702	0.414367147	6.61E-04	8.38E-03	1.00E+00
ENSG00000148459.14_PDSS1	ENSG00000160789.18_LMNA	-0.33167	-0.466250786	-0.18208265	2.99E-05	7.12E-04	1.00E+00
ENSG00000122085.15_MTERF4	ENSG00000160789.18_LMNA	-0.25002	-0.39355631	-0.09458459	1.89E-03	1.88E-02	1.00E+00
ENSG00000183111.10_ARHGEF3	ENSG00000160789.18_LMNA	-0.01395	-0.172762956	0.145578093	8.65E-01	9.47E-01	1.00E+00
ENSG00000144182.15_LIPT1	ENSG00000160789.18_LMNA	-0.27341	-0.414563951	-0.11940444	6.54E-04	8.32E-03	1.00E+00
ENSG00000163626.15_COX18	ENSG00000160789.18_LMNA	-0.24245	-0.386723495	-0.08659116	2.62E-03	2.40E-02	1.00E+00
ENSG00000125485.16_DDX31	ENSG00000160789.18_LMNA	0.13375	-0.026003501	0.286843769	1.00E-01	3.12E-01	1.00E+00
ENSG00000160299.15_PCNT	ENSG00000160789.18_LMNA	0.15938	0.000184878	0.310698078	4.98E-02	2.01E-01	1.00E+00
ENSG00000100968.12_NFATC4	ENSG00000160789.18_LMNA	0.39478	0.251383448	0.521226327	4.83E-07	2.55E-05	2.48E-02
ENSG00000173933.18_RBM4	ENSG00000160789.18_LMNA	-0.31071	-0.447759681	-0.15939087	9.78E-05	1.85E-03	1.00E+00
ENSG00000247092.5_SNHG10	ENSG00000160789.18_LMNA	-0.10484	-0.259703094	0.055286577	1.99E-01	4.69E-01	1.00E+00
ENSG00000261934.2_PCDHGA9	ENSG00000160789.18_LMNA	0.21313	0.055822266	0.360110943	8.38E-03	5.77E-02	1.00E+00
ENSG00000105948.12_TTC26	ENSG00000160789.18_LMNA	0.29179	0.139052696	0.430974191	2.65E-04	4.07E-03	1.00E+00
ENSG00000183475.11_ASB7	ENSG00000160789.18_LMNA	0.12633	-0.033549811	0.279897268	1.21E-01	3.50E-01	1.00E+00
ENSG00000198496.9_NBR2	ENSG00000160789.18_LMNA	-0.12	-0.273968046	0.039962854	1.41E-01	3.83E-01	1.00E+00
ENSG00000100092.19_SH3BP1	ENSG00000160789.18_LMNA	0.02432	-0.135407253	0.182810634	7.66E-01	9.03E-01	1.00E+00
ENSG00000114648.10_KLHL18	ENSG00000160789.18_LMNA	0.0779	-0.082321428	0.234196457	3.40E-01	6.25E-01	1.00E+00
ENSG00000105483.15_CARD8	ENSG00000160789.18_LMNA	0.1061	-0.054013863	0.260893129	1.93E-01	4.62E-01	1.00E+00
ENSG00000123106.9_CCDC91	ENSG00000160789.18_LMNA	-0.18111	-0.330775101	-0.02256149	2.56E-02	1.28E-01	1.00E+00
ENSG00000129696.11_TTI2	ENSG00000160789.18_LMNA	-0.1999	-0.348022204	-0.04203383	1.35E-02	8.14E-02	1.00E+00

ENSG00000161798.6_AQP5	ENSG00000160789.18_LMNA	0.0726	-0.087611516	0.229153904	3.74E-01	6.54E-01	1.00E+00
ENSG00000169641.12_LUZP1	ENSG00000160789.18_LMNA	0.29729	0.144945182	0.435858249	2.00E-04	3.26E-03	1.00E+00
ENSG00000215217.5_C5orf49	ENSG00000160789.18_LMNA	-0.06473	-0.221648578	0.095452325	4.28E-01	6.99E-01	1.00E+00
ENSG00000224858.5_RPL29P11	ENSG00000160789.18_LMNA	-0.14515	-0.297480298	0.014379029	7.44E-02	2.60E-01	1.00E+00
ENSG00000091009.7_RBM27	ENSG00000160789.18_LMNA	0.03571	-0.124194812	0.193810799	6.62E-01	8.51E-01	1.00E+00
ENSG00000175782.9_SLC35E3	ENSG00000160789.18_LMNA	-0.06744	-0.224237616	0.092751997	4.09E-01	6.83E-01	1.00E+00
ENSG00000171763.16_SPATA5L1	ENSG00000160789.18_LMNA	0.20211	0.044331018	0.350043304	1.25E-02	7.70E-02	1.00E+00
ENSG00000175741.5_RWDD4P2	ENSG00000160789.18_LMNA	-0.11219	-0.26663305	0.047860754	1.69E-01	4.27E-01	1.00E+00
ENSG00000138286.13_FAM149B1	ENSG00000160789.18_LMNA	-0.30872	-0.445997092	-0.15724366	1.09E-04	2.02E-03	1.00E+00
ENSG00000149403.10_GRIK4	ENSG00000160789.18_LMNA	-0.20284	-0.350712349	-0.04509217	1.22E-02	7.57E-02	1.00E+00
ENSG00000258890.5_CEP95	ENSG00000160789.18_LMNA	-0.15424	-0.305926944	0.005087394	5.78E-02	2.20E-01	1.00E+00
ENSG00000163013.10_FBXO41	ENSG00000160789.18_LMNA	-0.02054	-0.179158414	0.139112016	8.02E-01	9.20E-01	1.00E+00
ENSG00000162572.18_SCNN1D	ENSG00000160789.18_LMNA	0.01737	-0.142224579	0.176083198	8.32E-01	9.35E-01	1.00E+00
ENSG00000205871.5_RPS3AP47	ENSG00000160789.18_LMNA	-0.15873	-0.31009361	0.000484037	5.08E-02	2.03E-01	1.00E+00
ENSG00000225649.4_AC064875.1	ENSG00000160789.18_LMNA	0.22907	0.072511929	0.374607844	4.53E-03	3.64E-02	1.00E+00
ENSG00000163064.6_EN1	ENSG00000160789.18_LMNA	-0.10622	-0.261010381	0.05388841	1.93E-01	4.61E-01	1.00E+00
ENSG00000084112.13_SSH1	ENSG00000160789.18_LMNA	0.17774	0.019074889	0.327665053	2.85E-02	1.38E-01	1.00E+00
ENSG00000147099.18_HDAC8	ENSG00000160789.18_LMNA	0.07922	-0.08099842	0.235454902	3.32E-01	6.18E-01	1.00E+00
ENSG00000171241.7_SHCBP1	ENSG00000160789.18_LMNA	0.31482	0.16382967	0.451396284	7.81E-05	1.55E-03	1.00E+00
ENSG00000153922.9_CHD1	ENSG00000160789.18_LMNA	0.25773	0.102744812	0.400497786	1.35E-03	1.45E-02	1.00E+00
ENSG00000254910.1_RP11-326C	ENSG00000160789.18_LMNA	0.23081	0.074337825	0.376184934	4.23E-03	3.46E-02	1.00E+00
ENSG00000187531.12_SIRT7	ENSG00000160789.18_LMNA	0.16415	0.005086124	0.315119461	4.33E-02	1.83E-01	1.00E+00
ENSG00000216866.5_RPS2P55	ENSG00000160789.18_LMNA	0.13482	-0.024920322	0.287838161	9.77E-02	3.07E-01	1.00E+00
ENSG00000103723.11_AP3B2	ENSG00000160789.18_LMNA	-0.18738	-0.336538389	-0.02904245	2.08E-02	1.11E-01	1.00E+00
ENSG00000259456.3_ADNP-AS1	ENSG00000160789.18_LMNA	-0.17928	-0.329091383	-0.02067298	2.71E-02	1.34E-01	1.00E+00
ENSG00000104447.10_TRPS1	ENSG00000160789.18_LMNA	0.22555	0.068815456	0.371409715	5.21E-03	4.05E-02	1.00E+00
ENSG00000215218.3_UBE2QL1	ENSG00000160789.18_LMNA	-0.39837	-0.524322325	-0.25537025	3.72E-07	2.08E-05	1.91E-02
ENSG00000189337.14_KAZN	ENSG00000160789.18_LMNA	-0.04041	-0.198332251	0.119562436	6.21E-01	8.29E-01	1.00E+00
ENSG00000079156.15_OSBPL6	ENSG00000160789.18_LMNA	0.24122	0.08529833	0.385615275	2.76E-03	2.50E-02	1.00E+00
ENSG00000142920.15_AZIN2	ENSG00000160789.18_LMNA	-0.06488	-0.221790209	0.095304726	4.27E-01	6.98E-01	1.00E+00
ENSG00000116984.11_MTR	ENSG00000160789.18_LMNA	0.20959	0.052127896	0.356881967	9.56E-03	6.35E-02	1.00E+00
ENSG00000198382.7_UVRAG	ENSG00000160789.18_LMNA	-0.2746	-0.415630735	-0.12067473	6.18E-04	7.97E-03	1.00E+00
ENSG00000184227.6_ACOT1	ENSG00000160789.18_LMNA	0.12529	-0.034601138	0.278926892	1.24E-01	3.55E-01	1.00E+00

ENSG00000118939.16_UCHL3	ENSG00000160789.18_LMNA	-0.17521	-0.325338674	-0.01647174	3.08E-02	1.46E-01	1.00E+00
ENSG00000164684.12_ZNF704	ENSG00000160789.18_LMNA	-0.20663	-0.354182317	-0.04904553	1.06E-02	6.85E-02	1.00E+00
ENSG00000156384.13_SFR1	ENSG00000160789.18_LMNA	-0.31661	-0.452981763	-0.16576854	7.07E-05	1.42E-03	1.00E+00
ENSG00000130589.15_HELZ2	ENSG00000160789.18_LMNA	0.36435	0.2177827	0.494848014	3.94E-06	1.37E-04	2.02E-01
ENSG00000264112.1_RP11-159D	ENSG00000160789.18_LMNA	0.02623	-0.133529097	0.184658805	7.48E-01	8.95E-01	1.00E+00
ENSG00000183337.15_BCOR	ENSG00000160789.18_LMNA	-0.05216	-0.209616366	0.107940795	5.23E-01	7.69E-01	1.00E+00
ENSG00000010818.7_HIVEP2	ENSG00000160789.18_LMNA	0.11775	-0.04223815	0.271858655	1.49E-01	3.95E-01	1.00E+00
ENSG00000175691.8_ZNF77	ENSG00000160789.18_LMNA	-0.20147	-0.349460974	-0.04366881	1.28E-02	7.83E-02	1.00E+00
ENSG00000079134.10_THOC1	ENSG00000160789.18_LMNA	-0.04954	-0.207110011	0.110529639	5.44E-01	7.83E-01	1.00E+00
ENSG00000172007.5_RAB33B	ENSG00000160789.18_LMNA	-0.00678	-0.165802306	0.15258483	9.34E-01	9.74E-01	1.00E+00
ENSG00000138744.13_NAAA	ENSG00000160789.18_LMNA	0.1262	-0.033677699	0.279779262	1.21E-01	3.51E-01	1.00E+00
ENSG00000157617.15_C2CD2	ENSG00000160789.18_LMNA	0.20755	0.050005608	0.355023733	1.03E-02	6.69E-02	1.00E+00
ENSG00000112031.14_MTRF1L	ENSG00000160789.18_LMNA	-0.0757	-0.232102853	0.084519973	3.54E-01	6.38E-01	1.00E+00
ENSG00000085563.13_ABCB1	ENSG00000160789.18_LMNA	-0.11759	-0.271707442	0.042401131	1.49E-01	3.96E-01	1.00E+00
ENSG00000139597.15_N4BP2L1	ENSG00000160789.18_LMNA	-0.3274	-0.462496694	-0.17745111	3.83E-05	8.69E-04	1.00E+00
ENSG00000076344.14_RGS11	ENSG00000160789.18_LMNA	-0.0284	-0.186755517	0.131395595	7.28E-01	8.85E-01	1.00E+00
ENSG00000213199.6_ASIC3	ENSG00000160789.18_LMNA	-0.09205	-0.247621897	0.068149443	2.59E-01	5.42E-01	1.00E+00
ENSG00000197162.9_ZNF785	ENSG00000160789.18_LMNA	-0.15513	-0.306753751	0.004174981	5.63E-02	2.17E-01	1.00E+00
ENSG00000135124.13_P2RX4	ENSG00000160789.18_LMNA	0.05289	-0.107216785	0.210316561	5.18E-01	7.64E-01	1.00E+00
ENSG00000186416.11_NKRF	ENSG00000160789.18_LMNA	-0.17143	-0.321847908	-0.01257352	3.47E-02	1.58E-01	1.00E+00
ENSG00000078725.11_BRINP1	ENSG00000160789.18_LMNA	-0.23274	-0.377932805	-0.07636381	3.91E-03	3.26E-02	1.00E+00
ENSG00000164048.12_ZNF589	ENSG00000160789.18_LMNA	-0.12129	-0.275178243	0.038656003	1.37E-01	3.76E-01	1.00E+00
ENSG00000231721.5_LINC-PINT	ENSG00000160789.18_LMNA	-0.17348	-0.323741906	-0.01468742	3.26E-02	1.51E-01	1.00E+00
ENSG00000213862.4_CTD-2270N	ENSG00000160789.18_LMNA	-0.04607	-0.203776433	0.113966265	5.73E-01	8.01E-01	1.00E+00
ENSG00000185252.16_ZNF74	ENSG00000160789.18_LMNA	-0.13631	-0.289233914	0.023398703	9.40E-02	3.00E-01	1.00E+00
ENSG00000228288.5_PCAT6	ENSG00000160789.18_LMNA	-0.05212	-0.209582825	0.107975468	5.24E-01	7.69E-01	1.00E+00
ENSG00000205189.10_ZBTB10	ENSG00000160789.18_LMNA	-0.02429	-0.182779927	0.135438439	7.66E-01	9.03E-01	1.00E+00
ENSG00000147050.13_KDM6A	ENSG00000160789.18_LMNA	0.10119	-0.058960992	0.256262002	2.15E-01	4.89E-01	1.00E+00
ENSG00000070366.12_SMG6	ENSG00000160789.18_LMNA	0.16741	0.00843136	0.318129471	3.93E-02	1.71E-01	1.00E+00
ENSG00000174151.13_CYB561D1	ENSG00000160789.18_LMNA	0.20397	0.046268636	0.351745842	1.17E-02	7.34E-02	1.00E+00
ENSG00000240764.3_PCDHGC5	ENSG00000160789.18_LMNA	0.20363	0.045921603	0.351441061	1.19E-02	7.41E-02	1.00E+00
ENSG00000111729.11_CLEC4A	ENSG00000160789.18_LMNA	-0.15118	-0.303089633	0.008214547	6.30E-02	2.33E-01	1.00E+00
ENSG00000113361.11_CDH6	ENSG00000160789.18_LMNA	0.19195	0.033782429	0.340738947	1.78E-02	9.94E-02	1.00E+00

ENSG00000275367.1_RP11-266K	ENSG00000160789.18_LMNA	-0.0894	-0.245113102	0.070807462	2.73E-01	5.58E-01	1.00E+00
ENSG00000170500.11_LONRF2	ENSG00000160789.18_LMNA	0.14301	-0.016566307	0.295484808	7.88E-02	2.69E-01	1.00E+00
ENSG00000025423.10_HSD17B6	ENSG00000160789.18_LMNA	0.13849	-0.021179693	0.291266993	8.88E-02	2.90E-01	1.00E+00
ENSG00000175414.6_ARL10	ENSG00000160789.18_LMNA	-0.04739	-0.205040146	0.112664382	5.62E-01	7.94E-01	1.00E+00
ENSG00000144712.10_CAND2	ENSG00000160789.18_LMNA	-0.02029	-0.178908777	0.139364919	8.04E-01	9.22E-01	1.00E+00
ENSG00000186174.11_BCL9L	ENSG00000160789.18_LMNA	0.14213	-0.017467266	0.294662056	8.07E-02	2.74E-01	1.00E+00
ENSG00000250318.1_CTA-963H5	ENSG00000160789.18_LMNA	0.0827	-0.077520782	0.238757777	3.11E-01	5.98E-01	1.00E+00
ENSG00000168675.17_LDLRAD4	ENSG00000160789.18_LMNA	-0.38467	-0.512483522	-0.24017432	9.93E-07	4.57E-05	5.10E-02
ENSG00000013503.8_POLR3B	ENSG00000160789.18_LMNA	-0.32872	-0.463657213	-0.17888153	3.55E-05	8.21E-04	1.00E+00
ENSG00000135736.5_CCDC102A	ENSG00000160789.18_LMNA	0.48334	0.351154885	0.596627031	2.84E-10	5.48E-08	1.46E-05
ENSG00000078142.10_PIK3C3	ENSG00000160789.18_LMNA	-0.17015	-0.320666008	-0.01125579	3.61E-02	1.62E-01	1.00E+00
ENSG00000125898.11_FAM110A	ENSG00000160789.18_LMNA	-0.25966	-0.402229859	-0.10478726	1.24E-03	1.36E-02	1.00E+00
ENSG00000111271.13_ACAD10	ENSG00000160789.18_LMNA	0.11462	-0.045405489	0.268917231	1.60E-01	4.13E-01	1.00E+00
ENSG00000245205.3_EEF1A1P4	ENSG00000160789.18_LMNA	-0.15151	-0.303392184	0.007881379	6.24E-02	2.32E-01	1.00E+00
ENSG00000185499.15_MUC1	ENSG00000160789.18_LMNA	0.42695	0.287273239	0.548843457	4.13E-08	3.51E-06	2.12E-03
ENSG00000197177.14_ADGRA1	ENSG00000160789.18_LMNA	-0.17624	-0.32628707	-0.01753246	2.99E-02	1.43E-01	1.00E+00
ENSG00000182732.15_RGS6	ENSG00000160789.18_LMNA	0.29626	0.14384719	0.434949462	2.11E-04	3.39E-03	1.00E+00
ENSG00000198908.10_BHLHB9	ENSG00000160789.18_LMNA	-0.18831	-0.337394519	-0.0300074	2.02E-02	1.09E-01	1.00E+00
ENSG00000118402.5_ELOVL4	ENSG00000160789.18_LMNA	-0.24698	-0.390810647	-0.09136791	2.16E-03	2.07E-02	1.00E+00
ENSG00000163312.9_HELQ	ENSG00000160789.18_LMNA	-0.16863	-0.319260127	-0.00968973	3.78E-02	1.67E-01	1.00E+00
ENSG00000156170.11_NDUFAF6	ENSG00000160789.18_LMNA	-0.30276	-0.440719617	-0.15083086	1.50E-04	2.60E-03	1.00E+00
ENSG00000156374.13_PCGF6	ENSG00000160789.18_LMNA	-0.16604	-0.316861709	-0.00702156	4.09E-02	1.76E-01	1.00E+00
ENSG00000133256.11_PDE6B	ENSG00000160789.18_LMNA	0.01246	-0.147034067	0.171319171	8.79E-01	9.53E-01	1.00E+00
ENSG00000182568.15_SATB1	ENSG00000160789.18_LMNA	-0.33644	-0.470445398	-0.18727262	2.26E-05	5.70E-04	1.00E+00
ENSG00000038532.13_CLEC16A	ENSG00000160789.18_LMNA	0.00962	-0.149808102	0.16856457	9.06E-01	9.64E-01	1.00E+00
ENSG00000104998.3_IL27RA	ENSG00000160789.18_LMNA	0.33518	0.185904043	0.469340532	2.43E-05	6.05E-04	1.00E+00
ENSG00000060069.15_CTDP1	ENSG00000160789.18_LMNA	0.22513	0.068379903	0.371032408	5.29E-03	4.09E-02	1.00E+00
ENSG00000168067.10_MAP4K2	ENSG00000160789.18_LMNA	0.05952	-0.100630696	0.216671086	4.66E-01	7.28E-01	1.00E+00
ENSG00000133812.13_SBF2	ENSG00000160789.18_LMNA	0.05915	-0.100997344	0.216318035	4.69E-01	7.30E-01	1.00E+00
ENSG00000092607.12_TBX15	ENSG00000160789.18_LMNA	0.02426	-0.135464233	0.182754529	7.67E-01	9.03E-01	1.00E+00
ENSG00000146216.10_TTBK1	ENSG00000160789.18_LMNA	-0.07737	-0.233693773	0.08284959	3.43E-01	6.28E-01	1.00E+00
ENSG00000066422.4_ZBTB11	ENSG00000160789.18_LMNA	-0.16182	-0.312954737	-0.00268459	4.64E-02	1.92E-01	1.00E+00
ENSG00000169248.11_CXCL11	ENSG00000160789.18_LMNA	0.07644	-0.083775606	0.232812019	3.49E-01	6.33E-01	1.00E+00

ENSG00000267481.1_CTC-559E9	ENSG00000160789.18_LMNA	-0.26683	-0.408670587	-0.11240419	8.91E-04	1.06E-02	1.00E+00
ENSG00000204314.9_PRRT1	ENSG00000160789.18_LMNA	-0.22603	-0.371850764	-0.06932474	5.11E-03	3.99E-02	1.00E+00
ENSG00000115295.18_CLIP4	ENSG00000160789.18_LMNA	-0.13125	-0.284506793	0.028546254	1.07E-01	3.25E-01	1.00E+00
ENSG00000148344.10_PTGES	ENSG00000160789.18_LMNA	0.28867	0.135708238	0.428194467	3.10E-04	4.62E-03	1.00E+00
ENSG00000105514.6_RAB3D	ENSG00000160789.18_LMNA	0.45189	0.315375292	0.570076556	5.10E-09	6.10E-07	2.62E-04
ENSG00000075539.12_FRYL	ENSG00000160789.18_LMNA	0.05021	-0.109873699	0.207745445	5.39E-01	7.79E-01	1.00E+00
ENSG00000002587.8_HS3ST1	ENSG00000160789.18_LMNA	-0.195	-0.343531762	-0.03694155	1.61E-02	9.22E-02	1.00E+00
ENSG00000148482.10_SLC39A12	ENSG00000160789.18_LMNA	-0.17396	-0.324182708	-0.0151798	3.21E-02	1.50E-01	1.00E+00
ENSG00000229953.1_RP11-284F	ENSG00000160789.18_LMNA	-0.02866	-0.187005323	0.13114121	7.26E-01	8.84E-01	1.00E+00
ENSG00000159459.10_UBR1	ENSG00000160789.18_LMNA	0.11745	-0.0425452	0.271573762	1.50E-01	3.97E-01	1.00E+00
ENSG00000196136.15_SERPINA3	ENSG00000160789.18_LMNA	0.22486	0.068094374	0.370785008	5.35E-03	4.13E-02	1.00E+00
ENSG00000148143.11_ZNF462	ENSG00000160789.18_LMNA	-0.01729	-0.176010195	0.142298392	8.33E-01	9.35E-01	1.00E+00
ENSG00000168172.7_HOOK3	ENSG00000160789.18_LMNA	-0.0604	-0.217510507	0.099758601	4.60E-01	7.23E-01	1.00E+00
ENSG00000254837.2_AP001372.2	ENSG00000160789.18_LMNA	-0.32693	-0.462076357	-0.17693331	3.94E-05	8.90E-04	1.00E+00
ENSG00000107338.9_SHB	ENSG00000160789.18_LMNA	0.21938	0.062352001	0.365800327	6.62E-03	4.83E-02	1.00E+00
ENSG00000175470.17_PPP2R2D	ENSG00000160789.18_LMNA	-0.18408	-0.33350586	-0.02562907	2.32E-02	1.20E-01	1.00E+00
ENSG00000179915.19_NRXN1	ENSG00000160789.18_LMNA	-0.47001	-0.585406018	-0.3359454	1.00E-09	1.58E-07	5.14E-05
ENSG00000170419.9_VSTM2A	ENSG00000160789.18_LMNA	-0.24685	-0.390700308	-0.09123878	2.17E-03	2.08E-02	1.00E+00
ENSG00000159445.11_THEM4	ENSG00000160789.18_LMNA	0.03754	-0.122393847	0.195570246	6.46E-01	8.43E-01	1.00E+00
ENSG00000141642.7_ELAC1	ENSG00000160789.18_LMNA	-0.1752	-0.325323886	-0.01645521	3.09E-02	1.46E-01	1.00E+00
ENSG00000164303.9_ENPP6	ENSG00000160789.18_LMNA	-0.48466	-0.597740892	-0.35267177	2.50E-10	4.91E-08	1.28E-05
ENSG00000042429.9_MED17	ENSG00000160789.18_LMNA	-0.10305	-0.258019317	0.057085589	2.06E-01	4.78E-01	1.00E+00
ENSG00000141570.9_CBX8	ENSG00000160789.18_LMNA	-0.19761	-0.345925715	-0.03965438	1.47E-02	8.65E-02	1.00E+00
ENSG00000167100.13_SAMD14	ENSG00000160789.18_LMNA	-0.18761	-0.336753021	-0.02928431	2.06E-02	1.10E-01	1.00E+00
ENSG00000185008.16_ROBO2	ENSG00000160789.18_LMNA	-0.25409	-0.397218051	-0.09888424	1.58E-03	1.64E-02	1.00E+00
ENSG00000170430.9_MGMT	ENSG00000160789.18_LMNA	-0.05732	-0.214563934	0.10281772	4.83E-01	7.40E-01	1.00E+00
ENSG00000136104.17_RNASEH2	ENSG00000160789.18_LMNA	-0.36031	-0.491327152	-0.21334696	5.12E-06	1.69E-04	2.63E-01
ENSG00000186187.10_ZNRF1	ENSG00000160789.18_LMNA	0.0028	-0.15647076	0.161928212	9.73E-01	9.89E-01	1.00E+00
ENSG00000198546.13_ZNF511	ENSG00000160789.18_LMNA	-0.0386	-0.196590963	0.121348083	6.37E-01	8.38E-01	1.00E+00
ENSG00000111684.9_LPCAT3	ENSG00000160789.18_LMNA	0.01569	-0.143869629	0.174455373	8.48E-01	9.41E-01	1.00E+00
ENSG00000275632.1_RP5-967N2	ENSG00000160789.18_LMNA	-0.07365	-0.230149097	0.086568883	3.67E-01	6.48E-01	1.00E+00
ENSG00000267939.1_CTD-2325M	ENSG00000160789.18_LMNA	-0.08173	-0.237839638	0.078488275	3.17E-01	6.04E-01	1.00E+00
ENSG00000196352.12_CD55	ENSG00000160789.18_LMNA	-0.04597	-0.203683294	0.114062174	5.74E-01	8.01E-01	1.00E+00

ENSG00000103037.10_SETD6	ENSG00000160789.18_LMNA	0.04934	-0.110726756	0.206919004	5.46E-01	7.84E-01	1.00E+00
ENSG00000067992.11_PDK3	ENSG00000160789.18_LMNA	0.33587	0.186647948	0.469941202	2.33E-05	5.85E-04	1.00E+00
ENSG00000204599.13_TRIM39	ENSG00000160789.18_LMNA	0.20204	0.044260391	0.349981207	1.26E-02	7.71E-02	1.00E+00
ENSG00000213920.7_MDP1	ENSG00000160789.18_LMNA	-0.15891	-0.310265882	0.000293426	5.05E-02	2.03E-01	1.00E+00
ENSG00000183963.17_SMTN	ENSG00000160789.18_LMNA	0.16431	0.005248719	0.315265908	4.31E-02	1.82E-01	1.00E+00
ENSG00000204444.9_APOM	ENSG00000160789.18_LMNA	-0.17582	-0.325897107	-0.01709623	3.03E-02	1.44E-01	1.00E+00
ENSG00000176244.6_ACBD7	ENSG00000160789.18_LMNA	-0.30958	-0.446765003	-0.15817881	1.04E-04	1.95E-03	1.00E+00
ENSG00000165416.13_SUGT1	ENSG00000160789.18_LMNA	-0.24281	-0.387048407	-0.08697039	2.58E-03	2.38E-02	1.00E+00
ENSG00000083093.8_PALB2	ENSG00000160789.18_LMNA	0.02544	-0.134302111	0.183898404	7.56E-01	8.98E-01	1.00E+00
ENSG00000120798.15_NR2C1	ENSG00000160789.18_LMNA	-0.31527	-0.451791753	-0.16431308	7.62E-05	1.51E-03	1.00E+00
ENSG00000117481.9_NSUN4	ENSG00000160789.18_LMNA	0.01277	-0.14672447	0.17162629	8.76E-01	9.52E-01	1.00E+00
ENSG00000011258.14_MBTD1	ENSG00000160789.18_LMNA	-0.32069	-0.456578603	-0.17017534	5.63E-05	1.18E-03	1.00E+00
ENSG00000033800.12_PIAS1	ENSG00000160789.18_LMNA	-0.09689	-0.252204813	0.063282334	2.35E-01	5.13E-01	1.00E+00
ENSG00000183876.8_ARSI	ENSG00000160789.18_LMNA	0.43407	0.295273095	0.55492248	2.31E-08	2.18E-06	1.19E-03
ENSG00000165997.4_ARL5B	ENSG00000160789.18_LMNA	-0.20992	-0.357181133	-0.05246983	9.44E-03	6.29E-02	1.00E+00
ENSG00000131080.13_EDA2R	ENSG00000160789.18_LMNA	0.26459	0.110024073	0.406661169	9.87E-04	1.15E-02	1.00E+00
ENSG00000095564.12_BTAF1	ENSG00000160789.18_LMNA	-0.00803	-0.167016563	0.151364833	9.22E-01	9.70E-01	1.00E+00
ENSG00000213462.4_ERV3-1	ENSG00000160789.18_LMNA	-0.0001	-0.159298621	0.15910278	9.99E-01	1.00E+00	1.00E+00
ENSG00000033867.15_SLC4A7	ENSG00000160789.18_LMNA	0.28034	0.126802541	0.420765465	4.68E-04	6.41E-03	1.00E+00
ENSG00000006128.10_TAC1	ENSG00000160789.18_LMNA	-0.15785	-0.309278317	0.001385815	5.21E-02	2.07E-01	1.00E+00
ENSG00000274020.1_LINC01138	ENSG00000160789.18_LMNA	0.00638	-0.152974867	0.1654139	9.38E-01	9.75E-01	1.00E+00
ENSG00000094804.8_CDC6	ENSG00000160789.18_LMNA	0.1267	-0.033171535	0.28024626	1.20E-01	3.48E-01	1.00E+00
ENSG00000181938.12_GINS3	ENSG00000160789.18_LMNA	-0.23546	-0.380403683	-0.07923211	3.50E-03	3.00E-02	1.00E+00
ENSG00000174529.7_TMEM81	ENSG00000160789.18_LMNA	0.04005	-0.119917718	0.197985955	6.24E-01	8.31E-01	1.00E+00
ENSG00000151065.12_DCP1B	ENSG00000160789.18_LMNA	-0.01461	-0.173404525	0.144930664	8.58E-01	9.44E-01	1.00E+00
ENSG00000258754.6_LINC01579	ENSG00000160789.18_LMNA	-0.034	-0.192160799	0.125881846	6.78E-01	8.59E-01	1.00E+00
ENSG00000165209.17_STRBP	ENSG00000160789.18_LMNA	-0.10467	-0.259542209	0.055458562	1.99E-01	4.70E-01	1.00E+00
ENSG00000270067.1_CTC-487M2	ENSG00000160789.18_LMNA	-0.18277	-0.332304134	-0.0242784	2.42E-02	1.24E-01	1.00E+00
ENSG00000166813.13_KIF7	ENSG00000160789.18_LMNA	0.26131	0.106540285	0.403714796	1.15E-03	1.28E-02	1.00E+00
ENSG00000183346.6_C10orf107	ENSG00000160789.18_LMNA	0.04256	-0.117431542	0.200407586	6.03E-01	8.18E-01	1.00E+00
ENSG00000107554.14_DNMBP	ENSG00000160789.18_LMNA	0.35663	0.209314088	0.488118167	6.49E-06	2.06E-04	3.33E-01
ENSG00000183354.10_KIAA2026	ENSG00000160789.18_LMNA	0.0318	-0.128051352	0.190036272	6.97E-01	8.70E-01	1.00E+00
ENSG00000137502.8_RAB30	ENSG00000160789.18_LMNA	-0.28632	-0.426102579	-0.13319575	3.49E-04	5.05E-03	1.00E+00

ENSG00000144681.9_STAC	ENSG00000160789.18_LMNA	0.2198	0.062792566	0.366183379	6.51E-03	4.78E-02	1.00E+00
ENSG00000142330.18_CAPN10	ENSG00000160789.18_LMNA	0.14267	-0.016918738	0.295163025	7.95E-02	2.71E-01	1.00E+00
ENSG00000277782.1_RP11-775C	ENSG00000160789.18_LMNA	0.1658	0.006773186	0.316638243	4.12E-02	1.77E-01	1.00E+00
ENSG00000111203.10_ITFG2	ENSG00000160789.18_LMNA	-0.10351	-0.258451605	0.056623911	2.04E-01	4.76E-01	1.00E+00
ENSG00000127564.15_PKMYT1	ENSG00000160789.18_LMNA	-0.02869	-0.187038374	0.131107549	7.26E-01	8.84E-01	1.00E+00
ENSG00000108469.13_RECQL5	ENSG00000160789.18_LMNA	0.09328	-0.066909914	0.248790392	2.53E-01	5.34E-01	1.00E+00
ENSG00000249087.5_ZNF436-AS	ENSG00000160789.18_LMNA	0.29377	0.1411736	0.4327341	2.39E-04	3.77E-03	1.00E+00
ENSG00000187266.12_EPOR	ENSG00000160789.18_LMNA	0.24443	0.088679289	0.388511621	2.41E-03	2.25E-02	1.00E+00
ENSG00000259065.1_RP5-1021I2	ENSG00000160789.18_LMNA	-0.02257	-0.181122262	0.137121021	7.83E-01	9.11E-01	1.00E+00
ENSG00000185361.7_TNFAIP8L1	ENSG00000160789.18_LMNA	0.1706	0.011713725	0.321076853	3.56E-02	1.60E-01	1.00E+00
ENSG00000151893.13_CACUL1	ENSG00000160789.18_LMNA	-0.13353	-0.286634068	0.026231833	1.01E-01	3.13E-01	1.00E+00
ENSG00000158158.10_CNNM4	ENSG00000160789.18_LMNA	0.23033	0.073835328	0.375751084	4.31E-03	3.51E-02	1.00E+00
ENSG00000203685.8_STUM	ENSG00000160789.18_LMNA	-0.25687	-0.399721438	-0.10183016	1.40E-03	1.49E-02	1.00E+00
ENSG00000198862.12_LTN1	ENSG00000160789.18_LMNA	0.06	-0.100156947	0.217127144	4.63E-01	7.26E-01	1.00E+00
ENSG00000128683.12_GAD1	ENSG00000160789.18_LMNA	-0.19475	-0.343304693	-0.03668447	1.62E-02	9.28E-02	1.00E+00
ENSG00000133985.2_TTC9	ENSG00000160789.18_LMNA	0.00904	-0.150376081	0.167999957	9.12E-01	9.66E-01	1.00E+00
ENSG00000138592.12_USP8	ENSG00000160789.18_LMNA	0.16605	0.007035076	0.316873867	4.09E-02	1.76E-01	1.00E+00
ENSG00000138767.11_CNOT6L	ENSG00000160789.18_LMNA	-0.04717	-0.204829774	0.112881184	5.64E-01	7.95E-01	1.00E+00
ENSG00000281327.1_LINC01338	ENSG00000160789.18_LMNA	-0.45033	-0.568756271	-0.31361477	5.84E-09	6.89E-07	3.00E-04
ENSG00000010671.14_BTK	ENSG00000160789.18_LMNA	-0.13611	-0.289044053	0.023605771	9.45E-02	3.01E-01	1.00E+00
ENSG00000124508.15_BTN2A2	ENSG00000160789.18_LMNA	0.01084	-0.148613985	0.169750931	8.95E-01	9.59E-01	1.00E+00
ENSG00000168306.11_ACOX2	ENSG00000160789.18_LMNA	0.16615	0.007136588	0.316965188	4.08E-02	1.76E-01	1.00E+00
ENSG00000165138.15_ANKS6	ENSG00000160789.18_LMNA	0.16435	0.005289386	0.315302533	4.30E-02	1.82E-01	1.00E+00
ENSG00000137941.15_TTLL7	ENSG00000160789.18_LMNA	-0.17995	-0.329702911	-0.02135863	2.65E-02	1.32E-01	1.00E+00
ENSG00000187953.9_PMS2CL	ENSG00000160789.18_LMNA	0.09311	-0.067088591	0.248622011	2.54E-01	5.36E-01	1.00E+00
ENSG00000198826.9_ARHGAP11	ENSG00000160789.18_LMNA	0.2009	0.043076652	0.348940045	1.31E-02	7.94E-02	1.00E+00
ENSG00000099985.3_OSM	ENSG00000160789.18_LMNA	0.08732	-0.072896024	0.24313882	2.85E-01	5.70E-01	1.00E+00
ENSG00000152926.13_ZNF117	ENSG00000160789.18_LMNA	-0.02947	-0.187784977	0.130346993	7.19E-01	8.80E-01	1.00E+00
ENSG00000279812.1_CTD-2545G	ENSG00000160789.18_LMNA	0.06273	-0.097441413	0.219738602	4.43E-01	7.10E-01	1.00E+00
ENSG00000153551.12_CMTM7	ENSG00000160789.18_LMNA	-0.02796	-0.186330853	0.131827949	7.32E-01	8.87E-01	1.00E+00
ENSG00000179862.6_CITED4	ENSG00000160789.18_LMNA	0.06475	-0.095430942	0.221669097	4.28E-01	6.99E-01	1.00E+00
ENSG00000157036.11_EXOG	ENSG00000160789.18_LMNA	-0.27349	-0.414639813	-0.11949474	6.51E-04	8.30E-03	1.00E+00
ENSG00000170468.7_C14orf169	ENSG00000160789.18_LMNA	0.38403	0.239470208	0.511932447	1.04E-06	4.74E-05	5.33E-02

ENSG00000101665.7_SMAD7	ENSG00000160789.18_LMNA	-0.10479	-0.259662495	0.055329979	1.99E-01	4.69E-01	1.00E+00
ENSG00000002016.15_RAD52	ENSG00000160789.18_LMNA	-0.1339	-0.286980445	0.025854665	1.00E-01	3.12E-01	1.00E+00
ENSG00000145416.12_MARCH1	ENSG00000160789.18_LMNA	-0.33015	-0.464914475	-0.18043255	3.27E-05	7.67E-04	1.00E+00
ENSG00000184470.18_TXNRD2	ENSG00000160789.18_LMNA	0.06884	-0.091359296	0.225571172	3.99E-01	6.76E-01	1.00E+00
ENSG00000117620.11_SLC35A3	ENSG00000160789.18_LMNA	0.01872	-0.140898093	0.177394528	8.19E-01	9.30E-01	1.00E+00
ENSG00000174640.11_SLCO2A1	ENSG00000160789.18_LMNA	0.10187	-0.058271042	0.25690875	2.12E-01	4.85E-01	1.00E+00
ENSG00000162728.4_KCNJ9	ENSG00000160789.18_LMNA	-0.31814	-0.454332682	-0.16742232	6.49E-05	1.32E-03	1.00E+00
ENSG00000112699.9_GMDS	ENSG00000160789.18_LMNA	0.20422	0.046530216	0.351975532	1.16E-02	7.29E-02	1.00E+00
ENSG00000112936.17_C7	ENSG00000160789.18_LMNA	0.16937	0.010446479	0.319939646	3.70E-02	1.64E-01	1.00E+00
ENSG00000116138.11_DNAJC16	ENSG00000160789.18_LMNA	0.01531	-0.144245065	0.174083625	8.52E-01	9.42E-01	1.00E+00
ENSG00000128710.5_HOXD10	ENSG00000160789.18_LMNA	-0.07433	-0.230799936	0.085886644	3.63E-01	6.44E-01	1.00E+00
ENSG00000272711.1_RP11-259N	ENSG00000160789.18_LMNA	0.04281	-0.117186071	0.200646474	6.00E-01	8.16E-01	1.00E+00
ENSG00000187123.13_LYPD6	ENSG00000160789.18_LMNA	0.00889	-0.150522783	0.167854089	9.13E-01	9.67E-01	1.00E+00
ENSG00000105339.9_DENND3	ENSG00000160789.18_LMNA	0.0586	-0.101545993	0.215789576	4.73E-01	7.33E-01	1.00E+00
ENSG00000174516.13_PELI3	ENSG00000160789.18_LMNA	-0.02598	-0.184413165	0.133778854	7.51E-01	8.96E-01	1.00E+00
ENSG00000170921.13_TANC2	ENSG00000160789.18_LMNA	0.0344	-0.125486501	0.192547627	6.74E-01	8.57E-01	1.00E+00
ENSG00000152422.14_XRCC4	ENSG00000160789.18_LMNA	-0.25492	-0.397971036	-0.09976978	1.53E-03	1.59E-02	1.00E+00
ENSG00000066629.15_EML1	ENSG00000160789.18_LMNA	0.22083	0.063876864	0.367125688	6.26E-03	4.64E-02	1.00E+00
ENSG00000272831.1_RP11-792A	ENSG00000160789.18_LMNA	-0.05021	-0.207745798	0.109873335	5.39E-01	7.79E-01	1.00E+00
ENSG00000158825.5_CDA	ENSG00000160789.18_LMNA	-0.1132	-0.267576774	0.046846807	1.65E-01	4.21E-01	1.00E+00
ENSG00000103121.7_CMC2	ENSG00000160789.18_LMNA	-0.26057	-0.403053236	-0.10575906	1.19E-03	1.32E-02	1.00E+00
ENSG00000160305.16_DIP2A	ENSG00000160789.18_LMNA	0.11325	-0.046795775	0.267624255	1.65E-01	4.21E-01	1.00E+00
ENSG00000142185.15_TRPM2	ENSG00000160789.18_LMNA	0.09251	-0.067691989	0.248053242	2.57E-01	5.39E-01	1.00E+00
ENSG00000102572.13_STK24	ENSG00000160789.18_LMNA	0.12415	-0.035753628	0.277862407	1.28E-01	3.62E-01	1.00E+00
ENSG00000159259.7_CHAF1B	ENSG00000160789.18_LMNA	0.29387	0.141276965	0.432819815	2.38E-04	3.75E-03	1.00E+00
ENSG00000175104.13_TRAF6	ENSG00000160789.18_LMNA	0.08352	-0.076701673	0.239534661	3.06E-01	5.93E-01	1.00E+00
ENSG00000164366.3_CCDC127	ENSG00000160789.18_LMNA	0.03928	-0.120676383	0.197246212	6.31E-01	8.34E-01	1.00E+00
ENSG00000104450.11_SPAG1	ENSG00000160789.18_LMNA	0.034	-0.125878926	0.192163656	6.77E-01	8.59E-01	1.00E+00
ENSG00000243498.2_UBA52P5	ENSG00000160789.18_LMNA	0.09852	-0.061647856	0.253740684	2.27E-01	5.04E-01	1.00E+00
ENSG00000197928.9_ZNF677	ENSG00000160789.18_LMNA	-0.15768	-0.309119883	0.001560996	5.24E-02	2.07E-01	1.00E+00
ENSG00000278619.3_MRM1	ENSG00000160789.18_LMNA	0.21344	0.056149901	0.360396953	8.28E-03	5.72E-02	1.00E+00
ENSG00000065534.17_MYLK	ENSG00000160789.18_LMNA	0.03091	-0.128928008	0.189176946	7.05E-01	8.75E-01	1.00E+00
ENSG00000080839.10_RBL1	ENSG00000160789.18_LMNA	0.14589	-0.013631863	0.298161326	7.29E-02	2.56E-01	1.00E+00

ENSG00000186448.13_ZNF197	ENSG00000160789.18_LMNA	-0.06074	-0.217829927	0.099426621	4.57E-01	7.22E-01	1.00E+00
ENSG00000174125.6_TLR1	ENSG00000160789.18_LMNA	-0.01087	-0.169774039	0.148590717	8.94E-01	9.59E-01	1.00E+00
ENSG00000146909.6_NOM1	ENSG00000160789.18_LMNA	0.37083	0.224905289	0.500482586	2.56E-06	9.64E-05	1.32E-01
ENSG00000078403.15_MLLT10	ENSG00000160789.18_LMNA	-0.19327	-0.341951645	-0.03515343	1.70E-02	9.63E-02	1.00E+00
ENSG00000186642.14_PDE2A	ENSG00000160789.18_LMNA	-0.336	-0.470056695	-0.18679102	2.32E-05	5.81E-04	1.00E+00
ENSG00000272870.1_RP11-798M	ENSG00000160789.18_LMNA	-0.13855	-0.291320611	0.02112113	8.87E-02	2.90E-01	1.00E+00
ENSG00000167264.16_DUS2	ENSG00000160789.18_LMNA	0.0496	-0.110475803	0.207162174	5.44E-01	7.82E-01	1.00E+00
ENSG00000163735.6_CXCL5	ENSG00000160789.18_LMNA	0.22106	0.064115758	0.367333215	6.20E-03	4.61E-02	1.00E+00
ENSG00000134138.18_MEIS2	ENSG00000160789.18_LMNA	-0.12268	-0.27648458	0.037244128	1.32E-01	3.69E-01	1.00E+00
ENSG00000183856.9_IQGAP3	ENSG00000160789.18_LMNA	0.2711	0.116951623	0.412501788	7.29E-04	9.04E-03	1.00E+00
ENSG00000260231.1_JHDM1D-A	ENSG00000160789.18_LMNA	-0.14236	-0.294874057	0.017235162	8.02E-02	2.73E-01	1.00E+00
ENSG00000273492.4_AP000230.	ENSG00000160789.18_LMNA	-0.18364	-0.333104341	-0.02517766	2.35E-02	1.21E-01	1.00E+00
ENSG00000119227.6_PIGZ	ENSG00000160789.18_LMNA	-0.04639	-0.204084697	0.113648791	5.70E-01	7.99E-01	1.00E+00
ENSG00000077458.11_FAM76B	ENSG00000160789.18_LMNA	-0.22165	-0.367867121	-0.06473052	6.06E-03	4.53E-02	1.00E+00
ENSG00000279207.1_RP11-159D	ENSG00000160789.18_LMNA	0.02244	-0.137246472	0.180998597	7.84E-01	9.11E-01	1.00E+00
ENSG00000124440.14_HIF3A	ENSG00000160789.18_LMNA	-0.2207	-0.367002581	-0.06373517	6.29E-03	4.66E-02	1.00E+00
ENSG00000139173.8_TMEM117	ENSG00000160789.18_LMNA	-0.28819	-0.427765045	-0.13519217	3.18E-04	4.71E-03	1.00E+00
ENSG00000130119.14_GNL3L	ENSG00000160789.18_LMNA	0.11036	-0.049716856	0.264903934	1.76E-01	4.37E-01	1.00E+00
ENSG00000043143.19_JADE2	ENSG00000160789.18_LMNA	0.27031	0.116105218	0.411789484	7.57E-04	9.31E-03	1.00E+00
ENSG00000162664.15_ZNF326	ENSG00000160789.18_LMNA	-0.05644	-0.213718986	0.103693836	4.90E-01	7.46E-01	1.00E+00
ENSG00000080298.14_RFX3	ENSG00000160789.18_LMNA	-0.08906	-0.24479455	0.07114464	2.75E-01	5.60E-01	1.00E+00
ENSG00000269858.4_EGLN2	ENSG00000160789.18_LMNA	0.21783	0.060738253	0.364396369	7.02E-03	5.05E-02	1.00E+00
ENSG00000136925.13_TSTD2	ENSG00000160789.18_LMNA	-0.17101	-0.32145949	-0.01214034	3.52E-02	1.59E-01	1.00E+00
ENSG00000048471.12_SNX29	ENSG00000160789.18_LMNA	-0.02316	-0.181695395	0.13653948	7.77E-01	9.08E-01	1.00E+00
ENSG00000125170.9_DOK4	ENSG00000160789.18_LMNA	0.0339	-0.125976202	0.192068461	6.78E-01	8.60E-01	1.00E+00
ENSG00000136111.11_TBC1D4	ENSG00000160789.18_LMNA	-0.27193	-0.413241946	-0.11783158	7.01E-04	8.79E-03	1.00E+00
ENSG00000198598.5_MMP17	ENSG00000160789.18_LMNA	-0.1919	-0.340694237	-0.03373191	1.79E-02	9.95E-02	1.00E+00
ENSG00000156030.11_ELMSAN1	ENSG00000160789.18_LMNA	0.32476	0.174584192	0.46016778	4.46E-05	9.80E-04	1.00E+00
ENSG00000168944.14_CEP120	ENSG00000160789.18_LMNA	-0.05742	-0.214653956	0.102724349	4.82E-01	7.40E-01	1.00E+00
ENSG00000052795.11_FNIP2	ENSG00000160789.18_LMNA	-0.25555	-0.398536432	-0.10043503	1.49E-03	1.56E-02	1.00E+00
ENSG00000106571.11_GLI3	ENSG00000160789.18_LMNA	0.25721	0.102197511	0.400033292	1.38E-03	1.48E-02	1.00E+00
ENSG00000272655.1_POLR2J4	ENSG00000160789.18_LMNA	-0.01169	-0.170575223	0.147783758	8.86E-01	9.56E-01	1.00E+00
ENSG00000213846.4_AC098614.	ENSG00000160789.18_LMNA	0.23571	0.079489351	0.380625073	3.46E-03	2.98E-02	1.00E+00

ENSG00000196544.7_BORCS6	ENSG00000160789.18_LMNA	0.07886	-0.081362095	0.235109081	3.34E-01	6.20E-01	1.00E+00
ENSG00000103047.6_TANGO6	ENSG00000160789.18_LMNA	0.12881	-0.031022588	0.282227279	1.14E-01	3.37E-01	1.00E+00
ENSG00000054598.6_FOXC1	ENSG00000160789.18_LMNA	0.27825	0.124572648	0.418899128	5.18E-04	6.94E-03	1.00E+00
ENSG00000155962.11_CLIC2	ENSG00000160789.18_LMNA	-0.04209	-0.199949137	0.117902516	6.07E-01	8.20E-01	1.00E+00
ENSG00000163704.10_PRRT3	ENSG00000160789.18_LMNA	0.00508	-0.15424941	0.164143998	9.51E-01	9.80E-01	1.00E+00
ENSG00000187961.12_KLHL17	ENSG00000160789.18_LMNA	-0.09568	-0.251059875	0.064499678	2.41E-01	5.20E-01	1.00E+00
ENSG00000179859.7_AC025335.1	ENSG00000160789.18_LMNA	-0.07139	-0.22799523	0.088824549	3.82E-01	6.61E-01	1.00E+00
ENSG00000233966.1_UBE2SP1	ENSG00000160789.18_LMNA	0.07685	-0.083368663	0.233199574	3.47E-01	6.31E-01	1.00E+00
ENSG00000136237.17_RAPGEF5	ENSG00000160789.18_LMNA	-0.28797	-0.42756444	-0.13495114	3.21E-04	4.74E-03	1.00E+00
ENSG00000185129.5_PURA	ENSG00000160789.18_LMNA	-0.09511	-0.250520293	0.065073059	2.44E-01	5.23E-01	1.00E+00
ENSG00000141040.13_ZNF287	ENSG00000160789.18_LMNA	-0.09379	-0.249265069	0.066406102	2.50E-01	5.31E-01	1.00E+00
ENSG00000170801.8_HTRA3	ENSG00000160789.18_LMNA	0.32054	0.170020567	0.456452439	5.67E-05	1.19E-03	1.00E+00
ENSG00000215375.5_MYL5	ENSG00000160789.18_LMNA	0.24353	0.087730235	0.387699198	2.50E-03	2.32E-02	1.00E+00
ENSG00000177721.4_ANXA2R	ENSG00000160789.18_LMNA	0.16328	0.0041881	0.314310366	4.44E-02	1.86E-01	1.00E+00
ENSG00000197646.7_PDCD1LG2	ENSG00000160789.18_LMNA	0.14624	-0.013267597	0.298493233	7.22E-02	2.55E-01	1.00E+00
ENSG00000150457.8_LATS2	ENSG00000160789.18_LMNA	0.48305	0.350832966	0.596390521	2.92E-10	5.57E-08	1.50E-05
ENSG00000169951.8_ZNF764	ENSG00000160789.18_LMNA	-0.03905	-0.19702662	0.120901521	6.33E-01	8.35E-01	1.00E+00
ENSG00000118600.10_TMEM5	ENSG00000160789.18_LMNA	-0.1381	-0.290905672	0.021574283	8.98E-02	2.92E-01	1.00E+00
ENSG00000101882.8_NKAP	ENSG00000160789.18_LMNA	-0.02596	-0.184402588	0.133789607	7.51E-01	8.96E-01	1.00E+00
ENSG00000263050.1_RP11-667K	ENSG00000160789.18_LMNA	-0.21215	-0.359219939	-0.05480201	8.69E-03	5.92E-02	1.00E+00
ENSG00000162144.8_CYB561A3	ENSG00000160789.18_LMNA	0.14706	-0.012433321	0.299253112	7.06E-02	2.51E-01	1.00E+00
ENSG00000278834.1_RP11-458J1	ENSG00000160789.18_LMNA	-0.27193	-0.413243632	-0.11783359	7.01E-04	8.79E-03	1.00E+00
ENSG00000109265.11_KIAA1211	ENSG00000160789.18_LMNA	-0.05314	-0.210556846	0.10696825	5.16E-01	7.63E-01	1.00E+00
ENSG00000170425.3_ADORA2B	ENSG00000160789.18_LMNA	0.23976	0.083754584	0.384290832	2.93E-03	2.63E-02	1.00E+00
ENSG00000163297.15_ANTXR2	ENSG00000160789.18_LMNA	0.25906	0.10415848	0.401696857	1.27E-03	1.39E-02	1.00E+00
ENSG00000163618.16_CADPS	ENSG00000160789.18_LMNA	-0.1869	-0.336097038	-0.02854522	2.11E-02	1.12E-01	1.00E+00
ENSG00000188295.13_ZNF669	ENSG00000160789.18_LMNA	-0.15268	-0.304475574	0.006687788	6.04E-02	2.27E-01	1.00E+00
ENSG00000145388.13_METTL14	ENSG00000160789.18_LMNA	0.00102	-0.158204108	0.160196971	9.90E-01	9.96E-01	1.00E+00
ENSG00000182134.14_TDRKH	ENSG00000160789.18_LMNA	-0.17433	-0.324519729	-0.01555636	3.17E-02	1.49E-01	1.00E+00
ENSG00000198739.9_LRRTM3	ENSG00000160789.18_LMNA	-0.246	-0.389933435	-0.09034153	2.25E-03	2.14E-02	1.00E+00
ENSG00000102383.12_ZDHHC15	ENSG00000160789.18_LMNA	-0.05996	-0.217084715	0.100201028	4.63E-01	7.26E-01	1.00E+00
ENSG00000083642.17_PDS5B	ENSG00000160789.18_LMNA	-0.00713	-0.166144059	0.15224156	9.31E-01	9.73E-01	1.00E+00
ENSG00000154114.11_TBCEL	ENSG00000160789.18_LMNA	-0.16996	-0.3204903	-0.01105998	3.63E-02	1.62E-01	1.00E+00

ENSG00000127561.13_SYNGR3	ENSG00000160789.18_LMNA	-0.13136	-0.284604313	0.028440228	1.07E-01	3.25E-01	1.00E+00
ENSG00000144843.10_ADPRH	ENSG00000160789.18_LMNA	0.18021	0.021626117	0.329941407	2.63E-02	1.31E-01	1.00E+00
ENSG00000186907.6_RTN4RL2	ENSG00000160789.18_LMNA	-0.14707	-0.299265097	0.012420159	7.06E-02	2.51E-01	1.00E+00
ENSG00000179772.7_FOXS1	ENSG00000160789.18_LMNA	0.19759	0.039634694	0.345908359	1.47E-02	8.65E-02	1.00E+00
ENSG00000227199.1_ST7-AS1	ENSG00000160789.18_LMNA	-0.11681	-0.270972257	0.043193292	1.52E-01	4.01E-01	1.00E+00
ENSG00000156239.10_N6AMT1	ENSG00000160789.18_LMNA	-0.17884	-0.328686404	-0.02021907	2.75E-02	1.35E-01	1.00E+00
ENSG00000165113.11_GKAP1	ENSG00000160789.18_LMNA	-0.34872	-0.48120992	-0.20066427	1.07E-05	3.07E-04	5.48E-01
ENSG00000273702.1_RP11-758H	ENSG00000160789.18_LMNA	-0.37518	-0.504259532	-0.22969622	1.91E-06	7.71E-05	9.81E-02
ENSG00000186198.3_SLC51B	ENSG00000160789.18_LMNA	-0.33761	-0.471469581	-0.18854225	2.10E-05	5.39E-04	1.00E+00
ENSG00000171291.7_ZNF439	ENSG00000160789.18_LMNA	-0.2026	-0.350498503	-0.04484884	1.23E-02	7.61E-02	1.00E+00
ENSG00000124588.18_NQO2	ENSG00000160789.18_LMNA	0.15312	-0.006236472	0.304885011	5.97E-02	2.25E-01	1.00E+00
ENSG00000134955.10_SLC37A2	ENSG00000160789.18_LMNA	-0.02257	-0.181117793	0.137125555	7.83E-01	9.11E-01	1.00E+00
ENSG00000185482.6_STAC3	ENSG00000160789.18_LMNA	0.04823	-0.111833081	0.205846521	5.55E-01	7.89E-01	1.00E+00
ENSG00000196810.4_CTBP1-AS2	ENSG00000160789.18_LMNA	0.20818	0.050654812	0.355592419	1.01E-02	6.59E-02	1.00E+00
ENSG00000139211.6_AMIGO2	ENSG00000160789.18_LMNA	0.06704	-0.093156775	0.223849805	4.12E-01	6.86E-01	1.00E+00
ENSG00000198920.8_KIAA0753	ENSG00000160789.18_LMNA	0.12736	-0.032496355	0.280868967	1.18E-01	3.44E-01	1.00E+00
ENSG00000266338.5_NBPF15	ENSG00000160789.18_LMNA	0.18484	0.026419377	0.334208555	2.26E-02	1.18E-01	1.00E+00
ENSG00000232859.8_LYRM9	ENSG00000160789.18_LMNA	-0.28656	-0.426311772	-0.13344683	3.45E-04	5.00E-03	1.00E+00
ENSG00000166592.10_RRAD	ENSG00000160789.18_LMNA	0.18811	0.029802174	0.337212478	2.03E-02	1.09E-01	1.00E+00
ENSG00000240036.4_RP11-587D	ENSG00000160789.18_LMNA	-0.2421	-0.386407441	-0.08622235	2.66E-03	2.43E-02	1.00E+00
ENSG00000196646.10_ZNF136	ENSG00000160789.18_LMNA	-0.10106	-0.256135076	0.059096361	2.15E-01	4.90E-01	1.00E+00
ENSG00000171840.10_NINJ2	ENSG00000160789.18_LMNA	-0.30611	-0.443686728	-0.15443328	1.25E-04	2.27E-03	1.00E+00
ENSG00000215440.10_NPEPL1	ENSG00000160789.18_LMNA	0.31437	0.163342388	0.450997531	8.00E-05	1.57E-03	1.00E+00
ENSG00000235568.5_NFAM1	ENSG00000160789.18_LMNA	0.08449	-0.075724552	0.24046088	3.01E-01	5.87E-01	1.00E+00
ENSG00000100296.12_THOC5	ENSG00000160789.18_LMNA	-0.03898	-0.196962863	0.120966882	6.33E-01	8.36E-01	1.00E+00
ENSG00000164465.17_DCBLD1	ENSG00000160789.18_LMNA	-0.02702	-0.185426357	0.132748419	7.41E-01	8.91E-01	1.00E+00
ENSG00000185658.12_BRWD1	ENSG00000160789.18_LMNA	-0.2743	-0.415362161	-0.12035483	6.27E-04	8.06E-03	1.00E+00
ENSG00000137393.9_RNF144B	ENSG00000160789.18_LMNA	0.06695	-0.093249013	0.223761419	4.13E-01	6.86E-01	1.00E+00
ENSG00000137802.12_MAPKBP1	ENSG00000160789.18_LMNA	0.21253	0.055192488	0.359561016	8.57E-03	5.86E-02	1.00E+00
ENSG00000250462.7_LRRC37BP	ENSG00000160789.18_LMNA	-0.20709	-0.354598915	-0.04952081	1.05E-02	6.77E-02	1.00E+00
ENSG00000213341.9_CHUK	ENSG00000160789.18_LMNA	-0.1928	-0.341517673	-0.03466268	1.73E-02	9.73E-02	1.00E+00
ENSG00000272010.1_CTD-3025N	ENSG00000160789.18_LMNA	-0.14458	-0.296949682	0.014960935	7.55E-02	2.63E-01	1.00E+00
ENSG00000140199.10_SLC12A6	ENSG00000160789.18_LMNA	-0.01864	-0.177312246	0.140981359	8.20E-01	9.30E-01	1.00E+00

ENSG00000259877.2_RP11-46C2	ENSG00000160789.18_LMNA	-0.20785	-0.355295512	-0.05031583	1.02E-02	6.64E-02	1.00E+00
ENSG00000106948.15_AKNA	ENSG00000160789.18_LMNA	0.072	-0.088211195	0.22858121	3.78E-01	6.57E-01	1.00E+00
ENSG00000020256.18_ZFP64	ENSG00000160789.18_LMNA	0.12104	-0.038912659	0.274940647	1.37E-01	3.78E-01	1.00E+00
ENSG00000151553.13_FAM160B1	ENSG00000160789.18_LMNA	-0.20657	-0.354120357	-0.04897486	1.07E-02	6.86E-02	1.00E+00
ENSG00000215252.10_GOLGA8B	ENSG00000160789.18_LMNA	-0.06234	-0.219364402	0.097830817	4.45E-01	7.12E-01	1.00E+00
ENSG00000062096.13_ARSF	ENSG00000160789.18_LMNA	0.08402	-0.076201441	0.240008906	3.03E-01	5.90E-01	1.00E+00
ENSG00000263072.4_ZNF213-AS	ENSG00000160789.18_LMNA	-0.13582	-0.288776047	0.02389802	9.52E-02	3.02E-01	1.00E+00
ENSG00000182141.8_ZNF708	ENSG00000160789.18_LMNA	-0.45314	-0.57113465	-0.31678746	4.58E-09	5.56E-07	2.35E-04
ENSG00000196459.12_TRAPPC2	ENSG00000160789.18_LMNA	-0.19988	-0.348004921	-0.04201421	1.36E-02	8.15E-02	1.00E+00
ENSG00000101846.6_STS	ENSG00000160789.18_LMNA	0.26468	0.110115124	0.406738092	9.84E-04	1.14E-02	1.00E+00
ENSG00000087303.15_NID2	ENSG00000160789.18_LMNA	0.15767	-0.001566211	0.309115167	5.24E-02	2.07E-01	1.00E+00
ENSG00000187994.12_RINL	ENSG00000160789.18_LMNA	-0.00935	-0.168303764	0.150070489	9.09E-01	9.65E-01	1.00E+00
ENSG00000104064.15_GABPB1	ENSG00000160789.18_LMNA	-0.1332	-0.286329189	0.026563739	1.02E-01	3.15E-01	1.00E+00
ENSG00000243547.1_HNRNPKP4	ENSG00000160789.18_LMNA	0.04416	-0.115856634	0.2019396	5.89E-01	8.09E-01	1.00E+00
ENSG00000234906.7_APOC2	ENSG00000160789.18_LMNA	-0.20331	-0.351148245	-0.04558826	1.20E-02	7.48E-02	1.00E+00
ENSG00000125375.13_ATP5S	ENSG00000160789.18_LMNA	-0.27965	-0.420148721	-0.12606533	4.84E-04	6.58E-03	1.00E+00
ENSG00000236698.1_EIF1AXP1	ENSG00000160789.18_LMNA	0.08849	-0.071722227	0.244248712	2.78E-01	5.64E-01	1.00E+00
ENSG00000164045.10_CDC25A	ENSG00000160789.18_LMNA	-0.10942	-0.264025419	0.050659058	1.80E-01	4.42E-01	1.00E+00
ENSG00000183340.6_JRKL	ENSG00000160789.18_LMNA	0.17797	0.019319814	0.327883745	2.83E-02	1.38E-01	1.00E+00
ENSG00000103021.8_CCDC113	ENSG00000160789.18_LMNA	0.04537	-0.114662057	0.203100607	5.79E-01	8.04E-01	1.00E+00
ENSG00000054967.11_RELT	ENSG00000160789.18_LMNA	0.27497	0.12106771	0.415960591	6.07E-04	7.86E-03	1.00E+00
ENSG00000245680.8_ZNF585B	ENSG00000160789.18_LMNA	0.01126	-0.148209179	0.170152899	8.91E-01	9.57E-01	1.00E+00
ENSG00000151967.17_SCHIP1	ENSG00000160789.18_LMNA	-0.39309	-0.519766339	-0.24950655	5.46E-07	2.81E-05	2.80E-02
ENSG00000278784.1_RP11-468E	ENSG00000160789.18_LMNA	-0.03472	-0.192850615	0.125176771	6.71E-01	8.56E-01	1.00E+00
ENSG00000137073.19_UBAP2	ENSG00000160789.18_LMNA	0.00565	-0.153688119	0.164703376	9.45E-01	9.78E-01	1.00E+00
ENSG00000089094.15_KDM2B	ENSG00000160789.18_LMNA	-0.07771	-0.23401821	0.08250873	3.41E-01	6.26E-01	1.00E+00
ENSG00000157193.13_LRP8	ENSG00000160789.18_LMNA	-0.01387	-0.172687723	0.145653995	8.65E-01	9.47E-01	1.00E+00
ENSG00000164211.11_STARD4	ENSG00000160789.18_LMNA	-0.03271	-0.190919769	0.12714952	6.89E-01	8.66E-01	1.00E+00
ENSG00000126773.11_PCNX4	ENSG00000160789.18_LMNA	0.04224	-0.117750478	0.200097147	6.05E-01	8.19E-01	1.00E+00
ENSG00000067533.5_RRP15	ENSG00000160789.18_LMNA	0.03779	-0.122150406	0.195807918	6.44E-01	8.42E-01	1.00E+00
ENSG00000047346.11_FAM214A	ENSG00000160789.18_LMNA	-0.24781	-0.391561066	-0.09224645	2.08E-03	2.02E-02	1.00E+00
ENSG00000183098.9_GPC6	ENSG00000160789.18_LMNA	-0.15379	-0.305511577	0.005545573	5.85E-02	2.22E-01	1.00E+00
ENSG00000119004.13_CYP20A1	ENSG00000160789.18_LMNA	-0.10134	-0.256403099	0.058810495	2.14E-01	4.88E-01	1.00E+00

ENSG00000128617.2_OPN1SW	ENSG00000160789.18_LMNA	0.22495	0.068190107	0.370867962	5.33E-03	4.11E-02	1.00E+00
ENSG00000231167.3_YBX1P2	ENSG00000160789.18_LMNA	0.03167	-0.12818019	0.189910011	6.99E-01	8.71E-01	1.00E+00
ENSG00000144355.13_DLX1	ENSG00000160789.18_LMNA	-0.11813	-0.272213044	0.041856116	1.47E-01	3.93E-01	1.00E+00
ENSG00000177888.7_ZBTB41	ENSG00000160789.18_LMNA	-0.10182	-0.256860828	0.058322176	2.12E-01	4.86E-01	1.00E+00
ENSG00000105357.14_MYH14	ENSG00000160789.18_LMNA	0.03656	-0.12335926	0.194627343	6.55E-01	8.47E-01	1.00E+00
ENSG00000136918.6_WDR38	ENSG00000160789.18_LMNA	0.08422	-0.075996774	0.240202897	3.02E-01	5.89E-01	1.00E+00
ENSG00000188130.12_MAPK12	ENSG00000160789.18_LMNA	-0.02474	-0.183223224	0.134988167	7.62E-01	9.01E-01	1.00E+00
ENSG00000122035.6_RASL11A	ENSG00000160789.18_LMNA	-0.29432	-0.433221191	-0.14176108	2.33E-04	3.69E-03	1.00E+00
ENSG00000111696.10_NT5DC3	ENSG00000160789.18_LMNA	-0.13539	-0.288375888	0.024334276	9.63E-02	3.05E-01	1.00E+00
ENSG00000111912.17_NCOA7	ENSG00000160789.18_LMNA	-0.17047	-0.320962591	-0.01158635	3.57E-02	1.61E-01	1.00E+00
ENSG00000163093.10_BBS5	ENSG00000160789.18_LMNA	0.22029	0.063305763	0.36662945	6.39E-03	4.71E-02	1.00E+00
ENSG00000032219.17_ARID4A	ENSG00000160789.18_LMNA	-0.08124	-0.237374999	0.078977663	3.20E-01	6.06E-01	1.00E+00
ENSG00000198538.9_ZNF28	ENSG00000160789.18_LMNA	-0.09326	-0.248769734	0.066931836	2.53E-01	5.35E-01	1.00E+00
ENSG00000198556.12_ZNF789	ENSG00000160789.18_LMNA	-0.08104	-0.237180062	0.079182939	3.21E-01	6.07E-01	1.00E+00
ENSG00000172292.13_CERS6	ENSG00000160789.18_LMNA	-0.0725	-0.229054183	0.087715952	3.75E-01	6.55E-01	1.00E+00
ENSG00000107968.8_MAP3K8	ENSG00000160789.18_LMNA	0.04846	-0.111601658	0.206070928	5.53E-01	7.88E-01	1.00E+00
ENSG00000175879.8_HOXD8	ENSG00000160789.18_LMNA	-0.32615	-0.461392116	-0.17609076	4.12E-05	9.21E-04	1.00E+00
ENSG00000235098.7_ANKRD65	ENSG00000160789.18_LMNA	0.14292	-0.0166585	0.29540064	7.90E-02	2.70E-01	1.00E+00
ENSG00000168438.13_CDC40	ENSG00000160789.18_LMNA	-0.18944	-0.338437409	-0.03118363	1.94E-02	1.06E-01	1.00E+00
ENSG00000102034.15_ELF4	ENSG00000160789.18_LMNA	0.42478	0.284837826	0.546987298	4.92E-08	4.02E-06	2.52E-03
ENSG00000156381.7_ANKRD9	ENSG00000160789.18_LMNA	0.38791	0.243758082	0.515284912	7.91E-07	3.77E-05	4.06E-02
ENSG00000168874.11_ATOH8	ENSG00000160789.18_LMNA	-0.38527	-0.513006734	-0.24084309	9.52E-07	4.42E-05	4.88E-02
ENSG00000147251.14_DOCK11	ENSG00000160789.18_LMNA	-0.07171	-0.22830792	0.088497282	3.80E-01	6.59E-01	1.00E+00
ENSG00000219451.3_AC005251.3	ENSG00000160789.18_LMNA	-0.18593	-0.335207946	-0.02754404	2.18E-02	1.15E-01	1.00E+00
ENSG00000245573.6_BDNF-AS	ENSG00000160789.18_LMNA	-0.34935	-0.481760683	-0.20135228	1.03E-05	2.98E-04	5.27E-01
ENSG00000199024.1_MIR103A2	ENSG00000160789.18_LMNA	0.03011	-0.129712695	0.188407356	7.13E-01	8.78E-01	1.00E+00
ENSG00000120784.14_ZFP30	ENSG00000160789.18_LMNA	-0.20935	-0.356664268	-0.05187912	9.64E-03	6.39E-02	1.00E+00
ENSG00000137269.13_LRRC1	ENSG00000160789.18_LMNA	-0.35491	-0.486615853	-0.20742932	7.24E-06	2.26E-04	3.72E-01
ENSG00000138180.14_CEP55	ENSG00000160789.18_LMNA	0.28175	0.128308998	0.422024906	4.37E-04	6.08E-03	1.00E+00
ENSG00000250067.10_YJEFN3	ENSG00000160789.18_LMNA	-0.11336	-0.267733427	0.046678433	1.64E-01	4.20E-01	1.00E+00
ENSG00000123342.14_MMP19	ENSG00000160789.18_LMNA	0.22873	0.072152106	0.374296847	4.59E-03	3.68E-02	1.00E+00
ENSG00000185219.14_ZNF445	ENSG00000160789.18_LMNA	-0.06864	-0.225374629	0.091564634	4.01E-01	6.77E-01	1.00E+00
ENSG00000154309.8_DISP1	ENSG00000160789.18_LMNA	-0.03083	-0.189106021	0.129000341	7.06E-01	8.75E-01	1.00E+00

ENSG00000240463.1_RPS19P3	ENSG00000160789.18_LMNA	0.0474	-0.112654069	0.205050152	5.62E-01	7.94E-01	1.00E+00
ENSG00000159023.17_EPB41	ENSG00000160789.18_LMNA	-0.22881	-0.374376183	-0.07224389	4.58E-03	3.67E-02	1.00E+00
ENSG00000105374.8_NKG7	ENSG00000160789.18_LMNA	0.04296	-0.117040921	0.200787713	5.99E-01	8.16E-01	1.00E+00
ENSG00000174206.11_C12orf66	ENSG00000160789.18_LMNA	-0.10007	-0.255203249	0.060089817	2.20E-01	4.95E-01	1.00E+00
ENSG00000171466.8_ZNF562	ENSG00000160789.18_LMNA	-0.1872	-0.336373904	-0.02885712	2.09E-02	1.11E-01	1.00E+00
ENSG00000204556.4_CTD-2514C	ENSG00000160789.18_LMNA	-0.04769	-0.205327276	0.112368428	5.60E-01	7.92E-01	1.00E+00
ENSG00000108448.19_TRIM16L	ENSG00000160789.18_LMNA	-0.03218	-0.190401447	0.127678663	6.94E-01	8.68E-01	1.00E+00
ENSG00000095713.12_CRTAC1	ENSG00000160789.18_LMNA	-0.32249	-0.458171739	-0.17213089	5.08E-05	1.09E-03	1.00E+00
ENSG00000152503.8_TRIM36	ENSG00000160789.18_LMNA	-0.24558	-0.389552524	-0.08989605	2.29E-03	2.17E-02	1.00E+00
ENSG00000256525.5_POLG2	ENSG00000160789.18_LMNA	-0.16467	-0.315600011	-0.00561973	4.26E-02	1.81E-01	1.00E+00
ENSG00000252965.1_Y_RNA	ENSG00000160789.18_LMNA	-0.26345	-0.405637988	-0.10881344	1.04E-03	1.19E-02	1.00E+00
ENSG00000196323.10_ZBTB44	ENSG00000160789.18_LMNA	-0.30832	-0.445643458	-0.15681319	1.11E-04	2.06E-03	1.00E+00
ENSG00000259051.1_HNRNPUP1	ENSG00000160789.18_LMNA	0.18361	0.02514343	0.333073887	2.36E-02	1.21E-01	1.00E+00
ENSG00000072571.18_HMMR	ENSG00000160789.18_LMNA	0.02683	-0.1329348	0.185243145	7.43E-01	8.92E-01	1.00E+00
ENSG00000185904.10_LINC0083	ENSG00000160789.18_LMNA	-0.20896	-0.356303775	-0.05146725	9.78E-03	6.46E-02	1.00E+00
ENSG00000070950.8_RAD18	ENSG00000160789.18_LMNA	0.03526	-0.12464408	0.193371569	6.66E-01	8.53E-01	1.00E+00
ENSG00000120647.8_CCDC77	ENSG00000160789.18_LMNA	-0.09298	-0.248506783	0.067210853	2.55E-01	5.36E-01	1.00E+00
ENSG00000138606.18_SHF	ENSG00000160789.18_LMNA	-0.31779	-0.454019034	-0.16703821	6.62E-05	1.35E-03	1.00E+00
ENSG00000248508.5_SRP14-AS1	ENSG00000160789.18_LMNA	-0.1115	-0.26597778	0.048564402	1.71E-01	4.31E-01	1.00E+00
ENSG00000061656.8_SPAG4	ENSG00000160789.18_LMNA	0.32117	0.170699496	0.457005802	5.47E-05	1.16E-03	1.00E+00
ENSG00000196911.8_KPNA5	ENSG00000160789.18_LMNA	-0.41188	-0.535938031	-0.2704101	1.35E-07	9.26E-06	6.92E-03
ENSG00000204316.11_MRPL38	ENSG00000160789.18_LMNA	-0.13366	-0.286757646	0.026097279	1.01E-01	3.13E-01	1.00E+00
ENSG00000224272.2_AC114730.3	ENSG00000160789.18_LMNA	-0.33307	-0.467484574	-0.18360757	2.75E-05	6.67E-04	1.00E+00
ENSG00000123119.10_NECAB1	ENSG00000160789.18_LMNA	-0.37675	-0.505620722	-0.23142611	1.72E-06	7.09E-05	8.82E-02
ENSG00000100592.14_DAAM1	ENSG00000160789.18_LMNA	0.04587	-0.114160006	0.203588282	5.75E-01	8.02E-01	1.00E+00
ENSG00000163291.13_PAQR3	ENSG00000160789.18_LMNA	-0.15354	-0.305280502	0.005800408	5.89E-02	2.23E-01	1.00E+00
ENSG00000261188.1_CTA-445C9	ENSG00000160789.18_LMNA	-0.31855	-0.454691794	-0.16786222	6.34E-05	1.30E-03	1.00E+00
ENSG00000157064.9_NMNAT2	ENSG00000160789.18_LMNA	-0.2398	-0.384325036	-0.08379443	2.92E-03	2.62E-02	1.00E+00
ENSG00000161914.8_ZNF653	ENSG00000160789.18_LMNA	0.08545	-0.074763419	0.241371383	2.95E-01	5.81E-01	1.00E+00
ENSG00000188419.12_CHM	ENSG00000160789.18_LMNA	0.09569	-0.064495688	0.25106363	2.41E-01	5.20E-01	1.00E+00
ENSG00000108021.18_FAM208B	ENSG00000160789.18_LMNA	-0.10651	-0.261283483	0.053596167	1.92E-01	4.59E-01	1.00E+00
ENSG00000083535.14_PIBF1	ENSG00000160789.18_LMNA	-0.11543	-0.269673782	0.044591434	1.57E-01	4.09E-01	1.00E+00
ENSG00000270804.1_CTD-2583A	ENSG00000160789.18_LMNA	0.02522	-0.134517437	0.183686524	7.58E-01	8.99E-01	1.00E+00

ENSG00000112852.6_PCDHB2	ENSG00000160789.18_LMNA	-0.00728	-0.166292679	0.152092256	9.29E-01	9.73E-01	1.00E+00
ENSG00000196428.11_TSC22D2	ENSG00000160789.18_LMNA	-0.05987	-0.216998684	0.100290404	4.64E-01	7.26E-01	1.00E+00
ENSG00000173894.9_CBX2	ENSG00000160789.18_LMNA	-0.10777	-0.262472111	0.0523236	1.86E-01	4.52E-01	1.00E+00
ENSG00000243244.4_STON1	ENSG00000160789.18_LMNA	-0.1333	-0.286426452	0.026457861	1.02E-01	3.15E-01	1.00E+00
ENSG00000103489.10_XYLT1	ENSG00000160789.18_LMNA	0.04965	-0.110425982	0.207210445	5.44E-01	7.82E-01	1.00E+00
ENSG00000134376.13_CRB1	ENSG00000160789.18_LMNA	-0.40006	-0.525772247	-0.25724051	3.28E-07	1.89E-05	1.68E-02
ENSG00000058453.15_CROCC	ENSG00000160789.18_LMNA	0.14558	-0.013947934	0.297873272	7.35E-02	2.58E-01	1.00E+00
ENSG00000169696.14_ASPSCR1	ENSG00000160789.18_LMNA	-0.11075	-0.265272599	0.049321299	1.74E-01	4.35E-01	1.00E+00
ENSG00000089022.12_MAPKAPK	ENSG00000160789.18_LMNA	-0.13521	-0.288202757	0.024522987	9.68E-02	3.05E-01	1.00E+00
ENSG00000213928.7_IRF9	ENSG00000160789.18_LMNA	0.00295	-0.156326873	0.162071836	9.71E-01	9.89E-01	1.00E+00
ENSG00000181350.10_LRRC75A	ENSG00000160789.18_LMNA	0.07332	-0.086899443	0.229833649	3.69E-01	6.50E-01	1.00E+00
ENSG00000112624.11_GLTSCR1	ENSG00000160789.18_LMNA	0.02189	-0.137790446	0.180462248	7.89E-01	9.14E-01	1.00E+00
ENSG00000157404.14_KIT	ENSG00000160789.18_LMNA	-0.45287	-0.570913761	-0.31649256	4.68E-09	5.66E-07	2.40E-04
ENSG00000264575.1_LINC00526	ENSG00000160789.18_LMNA	-0.28469	-0.424646429	-0.13144904	3.78E-04	5.41E-03	1.00E+00
ENSG00000240950.1_RP11-372E	ENSG00000160789.18_LMNA	0.05107	-0.109013591	0.208578259	5.32E-01	7.75E-01	1.00E+00
ENSG00000056558.9_TRAF1	ENSG00000160789.18_LMNA	0.27748	0.123742104	0.418203358	5.38E-04	7.14E-03	1.00E+00
ENSG00000140153.16_WDR20	ENSG00000160789.18_LMNA	0.1008	-0.059352135	0.255895226	2.17E-01	4.91E-01	1.00E+00
ENSG00000118922.15_KLF12	ENSG00000160789.18_LMNA	-0.18831	-0.337398041	-0.03001137	2.02E-02	1.09E-01	1.00E+00
ENSG00000058866.13_DGKG	ENSG00000160789.18_LMNA	-0.06424	-0.22117538	0.095945359	4.32E-01	7.01E-01	1.00E+00
ENSG00000140543.12_DET1	ENSG00000160789.18_LMNA	-0.24342	-0.387601764	-0.08761645	2.51E-03	2.33E-02	1.00E+00
ENSG00000188488.12_SERPINA5	ENSG00000160789.18_LMNA	0.21556	0.058365722	0.362329762	7.65E-03	5.37E-02	1.00E+00
ENSG00000226532.1_RP5-1052M	ENSG00000160789.18_LMNA	-0.11152	-0.266000401	0.048540117	1.71E-01	4.31E-01	1.00E+00
ENSG00000198822.9_GRM3	ENSG00000160789.18_LMNA	-0.39869	-0.524589727	-0.25571502	3.63E-07	2.05E-05	1.86E-02
ENSG00000234882.1_EIF3EP1	ENSG00000160789.18_LMNA	-0.21156	-0.358682297	-0.05418668	8.88E-03	6.02E-02	1.00E+00
ENSG00000100376.10_FAM118A	ENSG00000160789.18_LMNA	0.01638	-0.143196412	0.175121751	8.41E-01	9.38E-01	1.00E+00
ENSG00000225783.5_MIAT	ENSG00000160789.18_LMNA	-0.02608	-0.184519436	0.133670806	7.50E-01	8.95E-01	1.00E+00
ENSG00000158805.10_ZNF276	ENSG00000160789.18_LMNA	-0.00742	-0.166421292	0.15196304	9.28E-01	9.72E-01	1.00E+00
ENSG00000121879.3_PIK3CA	ENSG00000160789.18_LMNA	0.02858	-0.131212952	0.186934876	7.27E-01	8.84E-01	1.00E+00
ENSG00000099840.12_IZUMO4	ENSG00000160789.18_LMNA	-0.13585	-0.288804311	0.023867201	9.52E-02	3.02E-01	1.00E+00
ENSG00000196712.15_NF1	ENSG00000160789.18_LMNA	-0.16658	-0.317367017	-0.00758334	4.03E-02	1.74E-01	1.00E+00
ENSG00000260314.2_MRC1	ENSG00000160789.18_LMNA	0.16835	0.009406274	0.319005516	3.81E-02	1.68E-01	1.00E+00
ENSG00000272172.1_RP13-582O	ENSG00000160789.18_LMNA	0.10906	-0.051030212	0.263679209	1.81E-01	4.45E-01	1.00E+00
ENSG00000162878.11_PKDCC	ENSG00000160789.18_LMNA	-0.17924	-0.329052772	-0.0206297	2.71E-02	1.34E-01	1.00E+00

ENSG00000068885.13_IFT80	ENSG00000160789.18_LMNA	-0.04023	-0.198161002	0.119738139	6.23E-01	8.30E-01	1.00E+00
ENSG00000106344.7_RBM28	ENSG00000160789.18_LMNA	0.19632	0.038312139	0.344741746	1.54E-02	8.93E-02	1.00E+00
ENSG00000241680.1_RPL31P49	ENSG00000160789.18_LMNA	-0.07202	-0.228602849	0.088188541	3.78E-01	6.57E-01	1.00E+00
ENSG00000171115.3_GIMAP8	ENSG00000160789.18_LMNA	0.09113	-0.069071461	0.246752121	2.64E-01	5.48E-01	1.00E+00
ENSG00000271976.1_RP11-884K	ENSG00000160789.18_LMNA	-0.14949	-0.301516227	0.009946062	6.60E-02	2.40E-01	1.00E+00
ENSG00000260442.4_ATP2A1-AS	ENSG00000160789.18_LMNA	0.00596	-0.153386038	0.165004342	9.42E-01	9.77E-01	1.00E+00
ENSG00000147576.14_ADHFE1	ENSG00000160789.18_LMNA	-0.25736	-0.400167726	-0.10235589	1.37E-03	1.47E-02	1.00E+00
ENSG00000113966.8_ARL6	ENSG00000160789.18_LMNA	-0.31665	-0.453015295	-0.16580957	7.05E-05	1.42E-03	1.00E+00
ENSG00000140992.17_PDPK1	ENSG00000160789.18_LMNA	0.06142	-0.098750285	0.218480461	4.52E-01	7.18E-01	1.00E+00
ENSG00000082482.12_KCNK2	ENSG00000160789.18_LMNA	-0.27658	-0.417403602	-0.12278794	5.62E-04	7.38E-03	1.00E+00
ENSG00000127325.17_BEST3	ENSG00000160789.18_LMNA	-0.26874	-0.410380965	-0.11443279	8.15E-04	9.86E-03	1.00E+00
ENSG00000183444.10_OR7E38P	ENSG00000160789.18_LMNA	-0.10118	-0.256255474	0.058967954	2.15E-01	4.89E-01	1.00E+00
ENSG00000032742.16_IFT88	ENSG00000160789.18_LMNA	-0.17765	-0.327584029	-0.01898415	2.86E-02	1.39E-01	1.00E+00
ENSG00000141034.8_GID4	ENSG00000160789.18_LMNA	0.07897	-0.081247503	0.235218055	3.33E-01	6.19E-01	1.00E+00
ENSG00000164169.11_PRMT9	ENSG00000160789.18_LMNA	-0.14318	-0.295638794	0.016397628	7.85E-02	2.69E-01	1.00E+00
ENSG00000130052.12_STARD8	ENSG00000160789.18_LMNA	-0.05057	-0.208094878	0.109512872	5.36E-01	7.77E-01	1.00E+00
ENSG00000180979.8_LRRC57	ENSG00000160789.18_LMNA	-0.12422	-0.277924051	0.035686911	1.27E-01	3.61E-01	1.00E+00
ENSG00000164219.8_PGGT1B	ENSG00000160789.18_LMNA	0.14147	-0.018135276	0.294051733	8.21E-02	2.77E-01	1.00E+00
ENSG00000203761.5_MSTO2P	ENSG00000160789.18_LMNA	0.05522	-0.104901468	0.212553535	4.99E-01	7.52E-01	1.00E+00
ENSG00000107957.15_SH3PXD2.	ENSG00000160789.18_LMNA	0.06896	-0.091239177	0.225686134	3.99E-01	6.75E-01	1.00E+00
ENSG00000263528.6_IKBKE	ENSG00000160789.18_LMNA	0.11985	-0.040114827	0.273827249	1.41E-01	3.84E-01	1.00E+00
ENSG00000144802.10_NFKBIZ	ENSG00000160789.18_LMNA	0.3755	0.230053746	0.504540967	1.87E-06	7.62E-05	9.60E-02
ENSG00000139926.14_FRMD6	ENSG00000160789.18_LMNA	0.09515	-0.065040135	0.250551282	2.44E-01	5.23E-01	1.00E+00
ENSG00000237749.4_RP3-423B2	ENSG00000160789.18_LMNA	-0.11381	-0.268151989	0.046228471	1.63E-01	4.18E-01	1.00E+00
ENSG00000044446.10_PHKA2	ENSG00000160789.18_LMNA	0.13654	-0.023164478	0.289448647	9.35E-02	2.99E-01	1.00E+00
ENSG00000168000.13_BSCL2	ENSG00000160789.18_LMNA	0.14539	-0.014137064	0.297700879	7.39E-02	2.59E-01	1.00E+00
ENSG00000127589.4_TUBBP1	ENSG00000160789.18_LMNA	0.08675	-0.073469391	0.242596367	2.88E-01	5.73E-01	1.00E+00
ENSG00000146143.16_PRIM2	ENSG00000160789.18_LMNA	-0.08385	-0.239853284	0.076365608	3.04E-01	5.91E-01	1.00E+00
ENSG00000164114.17_MAP9	ENSG00000160789.18_LMNA	-0.0735	-0.230005345	0.086719531	3.68E-01	6.49E-01	1.00E+00
ENSG00000107929.13_LARP4B	ENSG00000160789.18_LMNA	0.09472	-0.065466153	0.250150257	2.46E-01	5.25E-01	1.00E+00
ENSG00000106819.10_ASPN	ENSG00000160789.18_LMNA	0.20427	0.046585778	0.352024315	1.16E-02	7.28E-02	1.00E+00
ENSG00000072682.17_P4HA2	ENSG00000160789.18_LMNA	0.43072	0.291507547	0.552064522	3.04E-08	2.72E-06	1.56E-03
ENSG00000154839.8_SKA1	ENSG00000160789.18_LMNA	0.06389	-0.096285164	0.220849159	4.34E-01	7.03E-01	1.00E+00

ENSG00000164080.12_RAD54L2	ENSG00000160789.18_LMNA	0.11115	-0.048911527	0.265654415	1.73E-01	4.32E-01	1.00E+00
ENSG00000264522.4_OTUD7B	ENSG00000160789.18_LMNA	0.27877	0.125126415	0.419362842	5.06E-04	6.80E-03	1.00E+00
ENSG00000197769.5_MAP1LC3C	ENSG00000160789.18_LMNA	0.3346	0.18526646	0.468825504	2.52E-05	6.21E-04	1.00E+00
ENSG00000122696.11_SLC25A51	ENSG00000160789.18_LMNA	-0.03937	-0.197336306	0.120584004	6.30E-01	8.34E-01	1.00E+00
ENSG00000126953.5_TIMM8A	ENSG00000160789.18_LMNA	-0.06686	-0.223678715	0.093335317	4.13E-01	6.86E-01	1.00E+00
ENSG00000231768.1_LINC01354	ENSG00000160789.18_LMNA	-0.19354	-0.342199734	-0.03543405	1.69E-02	9.56E-02	1.00E+00
ENSG00000120800.4_UTP20	ENSG00000160789.18_LMNA	0.16991	0.011003077	0.320439239	3.64E-02	1.62E-01	1.00E+00
ENSG00000137413.14_TAF8	ENSG00000160789.18_LMNA	-0.11109	-0.265594759	0.048975558	1.73E-01	4.33E-01	1.00E+00
ENSG00000172878.12_METAP1D	ENSG00000160789.18_LMNA	-0.22418	-0.370167531	-0.06738195	5.50E-03	4.20E-02	1.00E+00
ENSG00000229994.1_RPL5P4	ENSG00000160789.18_LMNA	-0.13728	-0.290141825	0.022408142	9.17E-02	2.95E-01	1.00E+00
ENSG00000074356.15_NCBP3	ENSG00000160789.18_LMNA	0.02026	-0.139385998	0.178887968	8.04E-01	9.22E-01	1.00E+00
ENSG00000070444.13_MNT	ENSG00000160789.18_LMNA	0.23525	0.07901292	0.380215015	3.53E-03	3.02E-02	1.00E+00
ENSG00000083838.14_ZNF446	ENSG00000160789.18_LMNA	0.15468	-0.004633472	0.306338336	5.71E-02	2.18E-01	1.00E+00
ENSG00000177380.12_PPFIA3	ENSG00000160789.18_LMNA	-0.13606	-0.289002802	0.023650756	9.46E-02	3.01E-01	1.00E+00
ENSG00000189423.10_USP32P3	ENSG00000160789.18_LMNA	-0.14314	-0.29560101	0.016439018	7.85E-02	2.69E-01	1.00E+00
ENSG00000115274.13_INO80B	ENSG00000160789.18_LMNA	-0.06218	-0.21921165	0.097989747	4.47E-01	7.13E-01	1.00E+00
ENSG00000142046.13_TMEM91	ENSG00000160789.18_LMNA	-0.02078	-0.179382809	0.138884649	7.99E-01	9.19E-01	1.00E+00
ENSG00000165868.11_HSPA12A	ENSG00000160789.18_LMNA	-0.22558	-0.371443734	-0.06885473	5.20E-03	4.04E-02	1.00E+00
ENSG00000172113.7_NME6	ENSG00000160789.18_LMNA	-0.14098	-0.293590076	0.018640383	8.32E-02	2.79E-01	1.00E+00
ENSG00000226403.1_RP11-31F1	ENSG00000160789.18_LMNA	-0.27363	-0.414758843	-0.11963644	6.47E-04	8.26E-03	1.00E+00
ENSG00000178700.6_DHFR2	ENSG00000160789.18_LMNA	-0.05665	-0.213920966	0.103484449	4.88E-01	7.44E-01	1.00E+00
ENSG00000102221.12_JADE3	ENSG00000160789.18_LMNA	-0.35198	-0.484052532	-0.20421822	8.72E-06	2.60E-04	4.47E-01
ENSG00000173482.15_PTPRM	ENSG00000160789.18_LMNA	-0.21542	-0.362201993	-0.05821915	7.69E-03	5.40E-02	1.00E+00
ENSG00000144339.10_TMEFF2	ENSG00000160789.18_LMNA	-0.29978	-0.438075985	-0.14762765	1.75E-04	2.94E-03	1.00E+00
ENSG00000182600.8_C2orf82	ENSG00000160789.18_LMNA	-0.26588	-0.407821712	-0.1113983	9.30E-04	1.10E-02	1.00E+00
ENSG00000110171.17_TRIM3	ENSG00000160789.18_LMNA	0.05697	-0.10316367	0.214230348	4.86E-01	7.42E-01	1.00E+00
ENSG00000124217.4_MOCS3	ENSG00000160789.18_LMNA	0.1024	-0.057738839	0.257407437	2.09E-01	4.82E-01	1.00E+00
ENSG00000229891.1_LINC01315	ENSG00000160789.18_LMNA	-0.42249	-0.545032192	-0.28227624	5.90E-08	4.69E-06	3.03E-03
ENSG00000143633.11_C1orf131	ENSG00000160789.18_LMNA	-0.0715	-0.228099665	0.088715253	3.81E-01	6.60E-01	1.00E+00
ENSG00000239713.6_APOBEC3C	ENSG00000160789.18_LMNA	0.16761	0.008643419	0.318320069	3.90E-02	1.71E-01	1.00E+00
ENSG00000160051.10_IQCC	ENSG00000160789.18_LMNA	0.05884	-0.101306045	0.216020717	4.71E-01	7.31E-01	1.00E+00
ENSG00000003402.18_CFLAR	ENSG00000160789.18_LMNA	0.28238	0.128974874	0.422581236	4.24E-04	5.94E-03	1.00E+00
ENSG00000149926.12_FAM57B	ENSG00000160789.18_LMNA	-0.37258	-0.50200269	-0.22683189	2.28E-06	8.81E-05	1.17E-01

ENSG00000118058.19_KMT2A	ENSG00000160789.18_LMNA	-0.00847	-0.16744556	0.150933577	9.18E-01	9.69E-01	1.00E+00
ENSG00000106123.10_EPHB6	ENSG00000160789.18_LMNA	-0.06773	-0.224508505	0.092469194	4.07E-01	6.82E-01	1.00E+00
ENSG00000167850.3_CD300C	ENSG00000160789.18_LMNA	0.03325	-0.126617493	0.191440738	6.84E-01	8.63E-01	1.00E+00
ENSG00000167548.13_KMT2D	ENSG00000160789.18_LMNA	0.1587	-0.000512493	0.31006789	5.08E-02	2.03E-01	1.00E+00
ENSG00000247516.4_MIR4458HG	ENSG00000160789.18_LMNA	-0.04972	-0.207278472	0.110355769	5.43E-01	7.82E-01	1.00E+00
ENSG00000189057.9_FAM111B	ENSG00000160789.18_LMNA	0.16014	0.000968733	0.311406092	4.87E-02	1.98E-01	1.00E+00
ENSG00000166261.9_ZNF202	ENSG00000160789.18_LMNA	-0.10451	-0.259396362	0.055614454	2.00E-01	4.70E-01	1.00E+00
ENSG00000106018.12_VIPR2	ENSG00000160789.18_LMNA	-0.49835	-0.609213687	-0.36837098	6.44E-11	1.58E-08	3.30E-06
ENSG00000146250.6_PRSS35	ENSG00000160789.18_LMNA	-0.28921	-0.428671135	-0.13628127	3.02E-04	4.53E-03	1.00E+00
ENSG00000104983.7_CCDC61	ENSG00000160789.18_LMNA	0.28312	0.129769864	0.42324515	4.09E-04	5.76E-03	1.00E+00
ENSG00000105173.12_CCNE1	ENSG00000160789.18_LMNA	0.1746	0.015840205	0.324773721	3.14E-02	1.48E-01	1.00E+00
ENSG00000125741.4_OPA3	ENSG00000160789.18_LMNA	0.36255	0.215803921	0.493278484	4.43E-06	1.51E-04	2.27E-01
ENSG00000111224.12_PARP11	ENSG00000160789.18_LMNA	-0.28599	-0.425805318	-0.13283902	3.55E-04	5.12E-03	1.00E+00
ENSG00000108423.13_TUBD1	ENSG00000160789.18_LMNA	-0.37939	-0.507912863	-0.23434305	1.43E-06	6.21E-05	7.36E-02
ENSG00000134285.9_FKBP11	ENSG00000160789.18_LMNA	0.29416	0.141591077	0.433080258	2.35E-04	3.71E-03	1.00E+00
ENSG00000198814.11_GK	ENSG00000160789.18_LMNA	0.00653	-0.152829227	0.165558942	9.36E-01	9.75E-01	1.00E+00
ENSG00000133424.19_LARGE1	ENSG00000160789.18_LMNA	-0.17032	-0.320820494	-0.01142796	3.59E-02	1.61E-01	1.00E+00
ENSG00000214021.14_TTLL3	ENSG00000160789.18_LMNA	0.01387	-0.145648627	0.172693043	8.65E-01	9.47E-01	1.00E+00
ENSG00000266274.2_RN7SL138P	ENSG00000160789.18_LMNA	0.07107	-0.089143759	0.227690176	3.84E-01	6.63E-01	1.00E+00
ENSG00000121552.3_CSTA	ENSG00000160789.18_LMNA	0.17157	0.012718513	0.321977901	3.46E-02	1.57E-01	1.00E+00
ENSG00000115594.10_IL1R1	ENSG00000160789.18_LMNA	0.19997	0.042108475	0.348087919	1.35E-02	8.13E-02	1.00E+00
ENSG00000067191.14_CACNB1	ENSG00000160789.18_LMNA	-0.019	-0.17766001	0.140629404	8.16E-01	9.28E-01	1.00E+00
ENSG00000183763.7_TRAIP	ENSG00000160789.18_LMNA	-0.1522	-0.304030458	0.007178288	6.12E-02	2.29E-01	1.00E+00
ENSG00000170234.11_PWWP2A	ENSG00000160789.18_LMNA	0.0569	-0.103237478	0.214159168	4.86E-01	7.43E-01	1.00E+00
ENSG00000204084.11_INPP5B	ENSG00000160789.18_LMNA	0.12206	-0.037875383	0.275900654	1.34E-01	3.72E-01	1.00E+00
ENSG00000242814.2_RP11-436H	ENSG00000160789.18_LMNA	0.15392	-0.005411205	0.305633402	5.83E-02	2.21E-01	1.00E+00
ENSG00000090975.11_PITPNM2	ENSG00000160789.18_LMNA	0.17208	0.013240247	0.322445546	3.40E-02	1.56E-01	1.00E+00
ENSG00000134852.13_CLOCK	ENSG00000160789.18_LMNA	0.11447	-0.045559485	0.26877407	1.60E-01	4.14E-01	1.00E+00
ENSG00000138617.13_PARP16	ENSG00000160789.18_LMNA	0.12001	-0.039957574	0.273972937	1.41E-01	3.83E-01	1.00E+00
ENSG00000234268.1_AP000936.1	ENSG00000160789.18_LMNA	-0.01648	-0.175221092	0.143096027	8.40E-01	9.38E-01	1.00E+00
ENSG00000091651.7_ORC6	ENSG00000160789.18_LMNA	-0.09807	-0.253316385	0.062099565	2.29E-01	5.06E-01	1.00E+00
ENSG00000243680.1_RPL37P23	ENSG00000160789.18_LMNA	-0.04924	-0.206822378	0.110826463	5.47E-01	7.84E-01	1.00E+00
ENSG00000239268.2_RP11-384F	ENSG00000160789.18_LMNA	-0.02308	-0.181614374	0.136621704	7.78E-01	9.09E-01	1.00E+00

ENSG00000185559.12_DLK1	ENSG00000160789.18_LMNA	-0.03676	-0.194824129	0.123157826	6.53E-01	8.46E-01	1.00E+00
ENSG00000175175.5_PPM1E	ENSG00000160789.18_LMNA	-0.28936	-0.428809506	-0.13644766	3.00E-04	4.50E-03	1.00E+00
ENSG00000138835.21_RGS3	ENSG00000160789.18_LMNA	0.18964	0.031382543	0.338613703	1.93E-02	1.05E-01	1.00E+00
ENSG00000171791.11_BCL2	ENSG00000160789.18_LMNA	-0.20702	-0.354537378	-0.0494506	1.05E-02	6.78E-02	1.00E+00
ENSG00000082458.10_DLG3	ENSG00000160789.18_LMNA	0.06364	-0.09653636	0.22060796	4.36E-01	7.04E-01	1.00E+00
ENSG00000171169.8_NAIF1	ENSG00000160789.18_LMNA	0.12285	-0.037074995	0.276640992	1.32E-01	3.68E-01	1.00E+00
ENSG00000102796.9_DHRS12	ENSG00000160789.18_LMNA	-0.18634	-0.335588558	-0.02797256	2.15E-02	1.14E-01	1.00E+00
ENSG00000196139.10_AKR1C3	ENSG00000160789.18_LMNA	-0.30402	-0.441834167	-0.15218315	1.40E-04	2.46E-03	1.00E+00
ENSG00000197808.10_ZNF461	ENSG00000160789.18_LMNA	-0.25871	-0.401376254	-0.10378038	1.29E-03	1.40E-02	1.00E+00
ENSG00000152465.16_NMT2	ENSG00000160789.18_LMNA	-0.32268	-0.458334432	-0.17233072	5.03E-05	1.08E-03	1.00E+00
ENSG00000158711.12_ELK4	ENSG00000160789.18_LMNA	0.23418	0.077883324	0.379242314	3.69E-03	3.12E-02	1.00E+00
ENSG00000161940.9_BCL6B	ENSG00000160789.18_LMNA	-0.0155	-0.174269861	0.144056993	8.50E-01	9.41E-01	1.00E+00
ENSG00000203497.2_PDCD4-AS	ENSG00000160789.18_LMNA	-0.329	-0.463901896	-0.17918327	3.50E-05	8.11E-04	1.00E+00
ENSG00000159216.17_RUNX1	ENSG00000160789.18_LMNA	0.54141	0.418260504	0.645015126	5.94E-13	3.76E-10	3.05E-08
ENSG00000119912.14_IDE	ENSG00000160789.18_LMNA	0.12084	-0.039111253	0.274756777	1.38E-01	3.79E-01	1.00E+00
ENSG00000213139.6_CRYGS	ENSG00000160789.18_LMNA	-0.29889	-0.437282276	-0.14666713	1.84E-04	3.05E-03	1.00E+00
ENSG00000204789.4_ZNF204P	ENSG00000160789.18_LMNA	0.02841	-0.131382902	0.186767983	7.28E-01	8.85E-01	1.00E+00
ENSG00000231419.5_LINC00689	ENSG00000160789.18_LMNA	-0.48337	-0.596652476	-0.35118952	2.83E-10	5.48E-08	1.45E-05
ENSG00000135114.11_OASL	ENSG00000160789.18_LMNA	0.02444	-0.135286126	0.182929895	7.65E-01	9.02E-01	1.00E+00
ENSG00000050438.15_SLC4A8	ENSG00000160789.18_LMNA	-0.03186	-0.190100068	0.12798625	6.97E-01	8.70E-01	1.00E+00
ENSG00000065923.8_SLC9A7	ENSG00000160789.18_LMNA	0.20753	0.049976106	0.354997884	1.03E-02	6.70E-02	1.00E+00
ENSG00000172890.10_NADSYN1	ENSG00000160789.18_LMNA	0.17733	0.018652321	0.327287667	2.88E-02	1.40E-01	1.00E+00
ENSG00000160336.13_ZNF761	ENSG00000160789.18_LMNA	0.14795	-0.011523363	0.300081474	6.89E-02	2.47E-01	1.00E+00
ENSG00000224616.1_RTCA-AS1	ENSG00000160789.18_LMNA	-0.27608	-0.416952594	-0.1222501	5.76E-04	7.52E-03	1.00E+00
ENSG00000112742.8_TTK	ENSG00000160789.18_LMNA	-0.03298	-0.191174564	0.12688934	6.87E-01	8.64E-01	1.00E+00
ENSG00000049167.12_ERCC8	ENSG00000160789.18_LMNA	-0.24453	-0.38860488	-0.08878827	2.40E-03	2.25E-02	1.00E+00
ENSG00000137486.15_ARRB1	ENSG00000160789.18_LMNA	-0.34238	-0.475653303	-0.19373842	1.58E-05	4.23E-04	8.10E-01
ENSG00000008513.13_ST3GAL1	ENSG00000160789.18_LMNA	0.2125	0.055167911	0.359539551	8.58E-03	5.86E-02	1.00E+00
ENSG00000167395.10_ZNF646	ENSG00000160789.18_LMNA	0.1099	-0.050181849	0.264470437	1.78E-01	4.40E-01	1.00E+00
ENSG00000117598.10_PLPPR5	ENSG00000160789.18_LMNA	-0.13883	-0.291588938	0.020828022	8.80E-02	2.89E-01	1.00E+00
ENSG00000156232.7_WHAMM	ENSG00000160789.18_LMNA	0.34346	0.194915227	0.476599045	1.48E-05	4.03E-04	7.58E-01
ENSG00000089123.14_TASP1	ENSG00000160789.18_LMNA	0.34988	0.201922425	0.482216927	9.94E-06	2.90E-04	5.10E-01
ENSG00000170412.15_GPRC5C	ENSG00000160789.18_LMNA	-0.01238	-0.171247225	0.147106584	8.80E-01	9.53E-01	1.00E+00

ENSG00000269556.6_TMEM185A	ENSG00000160789.18_LMNA	-0.06915	-0.225866721	0.091050471	3.97E-01	6.74E-01	1.00E+00
ENSG00000248180.1_GAPDHP60	ENSG00000160789.18_LMNA	0.25211	0.096793811	0.395438949	1.73E-03	1.75E-02	1.00E+00
ENSG00000102543.13_CDADC1	ENSG00000160789.18_LMNA	-0.23947	-0.384032769	-0.08345396	2.96E-03	2.65E-02	1.00E+00
ENSG00000006534.14_ALDH3B1	ENSG00000160789.18_LMNA	0.32407	0.173839513	0.459562204	4.64E-05	1.01E-03	1.00E+00
ENSG00000258986.5_TMEM179	ENSG00000160789.18_LMNA	-0.08525	-0.241179919	0.074965579	2.96E-01	5.82E-01	1.00E+00
ENSG00000254893.4_AC113404.1	ENSG00000160789.18_LMNA	0.00387	-0.155423618	0.162973131	9.62E-01	9.85E-01	1.00E+00
ENSG00000236773.1_RP11-365C	ENSG00000160789.18_LMNA	0.00179	-0.157451635	0.160948771	9.82E-01	9.94E-01	1.00E+00
ENSG00000176463.12_SLCO3A1	ENSG00000160789.18_LMNA	-0.1207	-0.27462104	0.039257841	1.39E-01	3.79E-01	1.00E+00
ENSG00000133805.14_AMPD3	ENSG00000160789.18_LMNA	-0.03967	-0.197625069	0.120287881	6.27E-01	8.32E-01	1.00E+00
ENSG00000138780.13_GSTCD	ENSG00000160789.18_LMNA	-0.20479	-0.352501262	-0.0471291	1.14E-02	7.18E-02	1.00E+00
ENSG00000179627.9_ZBTB42	ENSG00000160789.18_LMNA	0.50755	0.378973576	0.61690531	2.50E-11	7.16E-09	1.28E-06
ENSG00000188315.6_C3orf62	ENSG00000160789.18_LMNA	-0.22335	-0.369419246	-0.06651901	5.67E-03	4.31E-02	1.00E+00
ENSG00000104972.13_LILRB1	ENSG00000160789.18_LMNA	0.03435	-0.125538521	0.192496733	6.74E-01	8.57E-01	1.00E+00
ENSG00000180953.10_ST20	ENSG00000160789.18_LMNA	0.11881	-0.041171739	0.272847684	1.45E-01	3.90E-01	1.00E+00
ENSG00000267002.1_RP11-242D	ENSG00000160789.18_LMNA	-0.2428	-0.387041292	-0.08696208	2.58E-03	2.38E-02	1.00E+00
ENSG00000088827.11_SIGLEC1	ENSG00000160789.18_LMNA	-0.00663	-0.165654377	0.152733392	9.35E-01	9.75E-01	1.00E+00
ENSG00000187764.10_SEMA4D	ENSG00000160789.18_LMNA	-0.28221	-0.422431813	-0.128796	4.28E-04	5.97E-03	1.00E+00
ENSG00000134444.12_KIAA1468	ENSG00000160789.18_LMNA	-0.20342	-0.351243503	-0.0456967	1.20E-02	7.46E-02	1.00E+00
ENSG00000198185.10_ZNF334	ENSG00000160789.18_LMNA	0.18726	0.028917868	0.336427819	2.09E-02	1.11E-01	1.00E+00
ENSG00000135837.14_CEP350	ENSG00000160789.18_LMNA	0.07275	-0.087463175	0.229295536	3.73E-01	6.53E-01	1.00E+00
ENSG00000166831.7_RBPMS2	ENSG00000160789.18_LMNA	0.31637	0.165507751	0.452768613	7.16E-05	1.44E-03	1.00E+00
ENSG00000129003.14_VPS13C	ENSG00000160789.18_LMNA	0.09083	-0.069369866	0.246470515	2.66E-01	5.50E-01	1.00E+00
ENSG00000131849.10_ZNF132	ENSG00000160789.18_LMNA	-0.00999	-0.168919988	0.149450458	9.03E-01	9.62E-01	1.00E+00
ENSG00000138964.15_PARVG	ENSG00000160789.18_LMNA	-0.00189	-0.161040957	0.15735934	9.82E-01	9.93E-01	1.00E+00
ENSG00000249014.2_HMGN2P4	ENSG00000160789.18_LMNA	-0.01231	-0.171177814	0.147176543	8.80E-01	9.54E-01	1.00E+00
ENSG00000214212.7_C19orf38	ENSG00000160789.18_LMNA	-0.18385	-0.333291033	-0.02538754	2.34E-02	1.21E-01	1.00E+00
ENSG00000088538.12_DOCK3	ENSG00000160789.18_LMNA	-0.27199	-0.413293165	-0.11789249	7.00E-04	8.78E-03	1.00E+00
ENSG00000135324.5_MRAP2	ENSG00000160789.18_LMNA	-0.03651	-0.194576576	0.123411222	6.55E-01	8.47E-01	1.00E+00
ENSG00000134215.14_VAV3	ENSG00000160789.18_LMNA	0.38372	0.239128217	0.511664705	1.06E-06	4.81E-05	5.45E-02
ENSG00000103381.10_CPPED1	ENSG00000160789.18_LMNA	0.03604	-0.123866537	0.194131658	6.59E-01	8.50E-01	1.00E+00
ENSG00000100302.6_RASD2	ENSG00000160789.18_LMNA	-0.02412	-0.18261469	0.135606242	7.68E-01	9.04E-01	1.00E+00
ENSG00000274104.1_CTD-2553L	ENSG00000160789.18_LMNA	-0.25599	-0.39893522	-0.1009044	1.46E-03	1.54E-02	1.00E+00
ENSG00000120279.6_MYCT1	ENSG00000160789.18_LMNA	-0.08219	-0.238273188	0.078031494	3.14E-01	6.00E-01	1.00E+00

ENSG00000186088.14_GSAP	ENSG00000160789.18_LMNA	0.25286	0.097587646	0.396114822	1.67E-03	1.71E-02	1.00E+00
ENSG00000066294.13_CD84	ENSG00000160789.18_LMNA	-0.11463	-0.268919828	0.045402695	1.60E-01	4.13E-01	1.00E+00
ENSG00000159885.12_ZNF222	ENSG00000160789.18_LMNA	-0.11669	-0.270862465	0.043311559	1.52E-01	4.01E-01	1.00E+00
ENSG00000204604.8_ZNF468	ENSG00000160789.18_LMNA	-0.02713	-0.185530332	0.132642636	7.40E-01	8.91E-01	1.00E+00
ENSG00000172273.11_HINFP	ENSG00000160789.18_LMNA	-0.32029	-0.456230684	-0.16974857	5.75E-05	1.20E-03	1.00E+00
ENSG00000177000.9_MTHFR	ENSG00000160789.18_LMNA	0.1253	-0.034595755	0.278931863	1.24E-01	3.55E-01	1.00E+00
ENSG00000161082.11_CELF5	ENSG00000160789.18_LMNA	-0.32272	-0.458367331	-0.17237113	5.02E-05	1.08E-03	1.00E+00
ENSG00000130477.13_UNC13A	ENSG00000160789.18_LMNA	-0.21261	-0.359635355	-0.05527761	8.55E-03	5.85E-02	1.00E+00
ENSG00000126882.11_FAM78A	ENSG00000160789.18_LMNA	-0.00198	-0.161125843	0.157274349	9.81E-01	9.93E-01	1.00E+00
ENSG00000241511.1_RPS15AP24	ENSG00000160789.18_LMNA	-0.13262	-0.285782911	0.027158273	1.03E-01	3.18E-01	1.00E+00
ENSG00000273314.1_RP5-1136G	ENSG00000160789.18_LMNA	0.26002	0.105172519	0.402556335	1.22E-03	1.34E-02	1.00E+00
ENSG00000125510.14_OPRL1	ENSG00000160789.18_LMNA	0.10683	-0.053275319	0.261583257	1.90E-01	4.58E-01	1.00E+00
ENSG00000228834.1_RP11-249L	ENSG00000160789.18_LMNA	-0.11357	-0.267925685	0.046471768	1.64E-01	4.19E-01	1.00E+00
ENSG00000162063.11_CCNF	ENSG00000160789.18_LMNA	0.07428	-0.085939988	0.230749057	3.63E-01	6.45E-01	1.00E+00
ENSG00000254198.1_RP11-598P	ENSG00000160789.18_LMNA	0.05468	-0.105441895	0.212031691	5.03E-01	7.55E-01	1.00E+00
ENSG00000167889.11_MGAT5B	ENSG00000160789.18_LMNA	-0.12249	-0.276307034	0.037436091	1.33E-01	3.70E-01	1.00E+00
ENSG00000226179.4_LINC00685	ENSG00000160789.18_LMNA	0.11163	-0.048431608	0.266101467	1.71E-01	4.30E-01	1.00E+00
ENSG00000122481.15_RWDD3	ENSG00000160789.18_LMNA	-0.13595	-0.288898556	0.023764435	9.49E-02	3.02E-01	1.00E+00
ENSG00000140030.5_GPR65	ENSG00000160789.18_LMNA	-0.03527	-0.193380136	0.124635319	6.66E-01	8.53E-01	1.00E+00
ENSG00000196664.4_TLR7	ENSG00000160789.18_LMNA	-0.10358	-0.258519006	0.056551915	2.04E-01	4.75E-01	1.00E+00
ENSG00000152455.14_SUV39H2	ENSG00000160789.18_LMNA	-0.27834	-0.418972355	-0.12466008	5.16E-04	6.92E-03	1.00E+00
ENSG00000169607.11_CKAP2L	ENSG00000160789.18_LMNA	0.03458	-0.125314681	0.192715714	6.72E-01	8.56E-01	1.00E+00
ENSG00000241081.1_RP11-488C	ENSG00000160789.18_LMNA	0.08013	-0.080094149	0.236314434	3.26E-01	6.13E-01	1.00E+00
ENSG00000186532.10_SMYD4	ENSG00000160789.18_LMNA	0.16695	0.007961631	0.31770719	3.98E-02	1.73E-01	1.00E+00
ENSG00000170456.13_DENND5B	ENSG00000160789.18_LMNA	-0.04493	-0.202683473	0.115091358	5.83E-01	8.06E-01	1.00E+00
ENSG00000132016.10_C19orf57	ENSG00000160789.18_LMNA	-0.03398	-0.192142227	0.125900824	6.78E-01	8.59E-01	1.00E+00
ENSG00000101327.7_PDYN	ENSG00000160789.18_LMNA	-0.17992	-0.329678769	-0.02133156	2.66E-02	1.32E-01	1.00E+00
ENSG00000196177.11_ACADSB	ENSG00000160789.18_LMNA	-0.37855	-0.50718774	-0.23341974	1.52E-06	6.48E-05	7.79E-02
ENSG00000116903.7_EXOC8	ENSG00000160789.18_LMNA	0.08638	-0.073838476	0.242247078	2.90E-01	5.75E-01	1.00E+00
ENSG00000108239.8_TBC1D12	ENSG00000160789.18_LMNA	-0.33153	-0.466132741	-0.18193682	3.02E-05	7.17E-04	1.00E+00
ENSG00000246985.6_SOCS2-AS1	ENSG00000160789.18_LMNA	0.10433	-0.055794309	0.259228079	2.01E-01	4.71E-01	1.00E+00
ENSG00000147124.11_ZNF41	ENSG00000160789.18_LMNA	-0.05631	-0.213598727	0.10381849	4.91E-01	7.47E-01	1.00E+00
ENSG00000079387.12_SENP1	ENSG00000160789.18_LMNA	0.0116	-0.147870666	0.170488956	8.87E-01	9.56E-01	1.00E+00

ENSG00000198353.7_HOXC4	ENSG00000160789.18_LMNA	-0.29381	-0.43277099	-0.14121809	2.39E-04	3.76E-03	1.00E+00
ENSG00000177576.9_C18orf32	ENSG00000160789.18_LMNA	-0.47127	-0.586471155	-0.33738357	8.91E-10	1.42E-07	4.57E-05
ENSG00000225194.2_LINC00092	ENSG00000160789.18_LMNA	0.0311	-0.128735994	0.189365205	7.04E-01	8.74E-01	1.00E+00
ENSG00000166676.13_TVP23A	ENSG00000160789.18_LMNA	-0.06653	-0.22336207	0.0936657	4.15E-01	6.88E-01	1.00E+00
ENSG00000157978.10_LDLRAP1	ENSG00000160789.18_LMNA	0.11462	-0.045409048	0.268913923	1.60E-01	4.13E-01	1.00E+00
ENSG00000160801.12_PTH1R	ENSG00000160789.18_LMNA	-0.03513	-0.193253015	0.124765321	6.67E-01	8.54E-01	1.00E+00
ENSG00000162600.10_OMA1	ENSG00000160789.18_LMNA	0.07983	-0.080395277	0.23602826	3.28E-01	6.14E-01	1.00E+00
ENSG00000104093.12_DMXL2	ENSG00000160789.18_LMNA	-0.11636	-0.270549778	0.043648335	1.53E-01	4.04E-01	1.00E+00
ENSG00000042088.12_TDP1	ENSG00000160789.18_LMNA	0.08714	-0.07307419	0.242970281	2.86E-01	5.71E-01	1.00E+00
ENSG00000227345.7_PARG	ENSG00000160789.18_LMNA	-0.11107	-0.265574125	0.048997704	1.73E-01	4.33E-01	1.00E+00
ENSG00000065609.13_SNAP91	ENSG00000160789.18_LMNA	-0.41892	-0.541974582	-0.27827765	7.82E-08	5.90E-06	4.01E-03
ENSG00000178538.8_CA8	ENSG00000160789.18_LMNA	-0.38112	-0.509415166	-0.23625752	1.27E-06	5.61E-05	6.52E-02
ENSG00000164031.15_DNAJB14	ENSG00000160789.18_LMNA	-0.11419	-0.268513123	0.045840142	1.61E-01	4.16E-01	1.00E+00
ENSG00000196656.7_AC004057.1	ENSG00000160789.18_LMNA	-0.1746	-0.324774781	-0.01584139	3.14E-02	1.48E-01	1.00E+00
ENSG00000156521.12_TYSND1	ENSG00000160789.18_LMNA	-0.01295	-0.171795685	0.146553682	8.74E-01	9.51E-01	1.00E+00
ENSG00000163607.13_GTPBP8	ENSG00000160789.18_LMNA	-0.30049	-0.438702268	-0.14838595	1.69E-04	2.86E-03	1.00E+00
ENSG00000100154.13_TTC28	ENSG00000160789.18_LMNA	0.00533	-0.154003837	0.164388759	9.48E-01	9.79E-01	1.00E+00
ENSG00000185716.10_C16orf52	ENSG00000160789.18_LMNA	-0.18648	-0.335711267	-0.02811074	2.14E-02	1.13E-01	1.00E+00
ENSG00000119681.10_LTBP2	ENSG00000160789.18_LMNA	0.32946	0.17968594	0.464309412	3.40E-05	7.92E-04	1.00E+00
ENSG00000174804.3_FZD4	ENSG00000160789.18_LMNA	0.01999	-0.139659046	0.178618398	8.07E-01	9.23E-01	1.00E+00
ENSG00000107643.14_MAPK8	ENSG00000160789.18_LMNA	-0.48867	-0.601105044	-0.35726097	1.69E-10	3.47E-08	8.67E-06
ENSG00000204149.8_AGAP6	ENSG00000160789.18_LMNA	-0.0398	-0.197743648	0.120166264	6.26E-01	8.32E-01	1.00E+00
ENSG00000232352.1_SEMA3B-A	ENSG00000160789.18_LMNA	-0.17678	-0.326782069	-0.01808636	2.94E-02	1.41E-01	1.00E+00
ENSG00000104081.12_BMF	ENSG00000160789.18_LMNA	-0.10232	-0.257328247	0.057823363	2.10E-01	4.83E-01	1.00E+00
ENSG00000225022.1_UBE2D3P1	ENSG00000160789.18_LMNA	-0.02533	-0.183788408	0.1344139	7.57E-01	8.99E-01	1.00E+00
ENSG00000072201.12_LNX1	ENSG00000160789.18_LMNA	-0.27255	-0.413793728	-0.11848789	6.81E-04	8.59E-03	1.00E+00
ENSG00000144115.15_THNSL2	ENSG00000160789.18_LMNA	0.16504	0.006001463	0.315943694	4.22E-02	1.80E-01	1.00E+00
ENSG00000168661.13_ZNF30	ENSG00000160789.18_LMNA	-0.15807	-0.309486226	0.001155898	5.18E-02	2.06E-01	1.00E+00
ENSG00000163739.4_CXCL1	ENSG00000160789.18_LMNA	0.14396	-0.015597408	0.296369086	7.68E-02	2.65E-01	1.00E+00
ENSG00000177989.12_ODF3B	ENSG00000160789.18_LMNA	0.08917	-0.071038354	0.244894972	2.75E-01	5.59E-01	1.00E+00
ENSG00000120158.10_RCL1	ENSG00000160789.18_LMNA	-0.20279	-0.350669492	-0.0450434	1.22E-02	7.58E-02	1.00E+00
ENSG00000272505.1_RP11-981G	ENSG00000160789.18_LMNA	-0.17775	-0.327674944	-0.01908597	2.85E-02	1.38E-01	1.00E+00
ENSG00000132622.9_HSPA12B	ENSG00000160789.18_LMNA	-0.03126	-0.189521488	0.128576577	7.02E-01	8.73E-01	1.00E+00

ENSG00000215105.4_TTC3P1	ENSG00000160789.18_LMNA	0.02347	-0.136241427	0.181989056	7.74E-01	9.07E-01	1.00E+00
ENSG00000083223.16_ZCCHC6	ENSG00000160789.18_LMNA	0.29337	0.140746466	0.432379846	2.44E-04	3.83E-03	1.00E+00
ENSG00000172819.15_RARG	ENSG00000160789.18_LMNA	0.31665	0.165803685	0.453010485	7.06E-05	1.42E-03	1.00E+00
ENSG00000234106.3_RP11-288E	ENSG00000160789.18_LMNA	-0.04908	-0.206670058	0.110983627	5.48E-01	7.85E-01	1.00E+00
ENSG00000163545.7_NUAK2	ENSG00000160789.18_LMNA	0.4335	0.294627521	0.55443294	2.42E-08	2.27E-06	1.24E-03
ENSG00000233170.4_RP11-863K	ENSG00000160789.18_LMNA	-0.10085	-0.255939289	0.059305149	2.16E-01	4.91E-01	1.00E+00
ENSG00000121089.4_NACA3P	ENSG00000160789.18_LMNA	-0.04846	-0.20606863	0.111604027	5.53E-01	7.88E-01	1.00E+00
ENSG00000165630.12_PRPF18	ENSG00000160789.18_LMNA	-0.10429	-0.259187298	0.055837891	2.01E-01	4.71E-01	1.00E+00
ENSG00000215012.7_C22orf29	ENSG00000160789.18_LMNA	0.10584	-0.054280118	0.26064425	1.94E-01	4.63E-01	1.00E+00
ENSG00000005810.16_MYCBP2	ENSG00000160789.18_LMNA	-0.19435	-0.342939983	-0.03627164	1.64E-02	9.37E-02	1.00E+00
ENSG00000140839.10_CLEC18B	ENSG00000160789.18_LMNA	0.20008	0.042225872	0.34819127	1.35E-02	8.11E-02	1.00E+00
ENSG00000187624.8_C17orf97	ENSG00000160789.18_LMNA	0.18226	0.023746319	0.331830448	2.46E-02	1.25E-01	1.00E+00
ENSG00000181827.13_RFX7	ENSG00000160789.18_LMNA	-0.00746	-0.166464731	0.151919394	9.27E-01	9.72E-01	1.00E+00
ENSG00000076650.5_GPATCH1	ENSG00000160789.18_LMNA	0.08063	-0.079590981	0.236792494	3.23E-01	6.10E-01	1.00E+00
ENSG00000157368.9_IL34	ENSG00000160789.18_LMNA	-0.03832	-0.196320023	0.12162574	6.39E-01	8.39E-01	1.00E+00
ENSG00000185634.10_SHC4	ENSG00000160789.18_LMNA	0.14035	-0.019282114	0.293003337	8.46E-02	2.82E-01	1.00E+00
ENSG00000146556.13_WASH2P	ENSG00000160789.18_LMNA	-0.0242	-0.182695848	0.135523826	7.67E-01	9.04E-01	1.00E+00
ENSG00000233621.1_LINC01137	ENSG00000160789.18_LMNA	0.16939	0.010475057	0.319965302	3.70E-02	1.64E-01	1.00E+00
ENSG00000141068.12_KSR1	ENSG00000160789.18_LMNA	-0.0604	-0.217504537	0.099764806	4.60E-01	7.23E-01	1.00E+00
ENSG00000177106.13_EPS8L2	ENSG00000160789.18_LMNA	0.27875	0.125101862	0.419342285	5.06E-04	6.81E-03	1.00E+00
ENSG00000198885.8_ITPRIPL1	ENSG00000160789.18_LMNA	0.27856	0.124893414	0.419167751	5.11E-04	6.86E-03	1.00E+00
ENSG00000268471.4_MIR4453	ENSG00000160789.18_LMNA	-0.10841	-0.26307334	0.051679524	1.84E-01	4.49E-01	1.00E+00
ENSG00000127957.15_PMS2P3	ENSG00000160789.18_LMNA	0.00347	-0.15581493	0.162582733	9.66E-01	9.87E-01	1.00E+00
ENSG00000145348.15_TBCK	ENSG00000160789.18_LMNA	-0.1999	-0.348024776	-0.04203676	1.35E-02	8.14E-02	1.00E+00
ENSG00000185875.11_THNSL1	ENSG00000160789.18_LMNA	-0.33991	-0.473492293	-0.19105248	1.83E-05	4.79E-04	9.40E-01
ENSG00000151458.10_ANKRD50	ENSG00000160789.18_LMNA	0.10749	-0.052615437	0.262199607	1.87E-01	4.54E-01	1.00E+00
ENSG00000236494.1_RP11-89N1	ENSG00000160789.18_LMNA	-0.42476	-0.546969139	-0.28481402	4.92E-08	4.02E-06	2.53E-03
ENSG00000120696.8_KBTBD7	ENSG00000160789.18_LMNA	-0.34976	-0.482116934	-0.20179745	1.00E-05	2.92E-04	5.14E-01
ENSG00000173137.10_ADCK5	ENSG00000160789.18_LMNA	0.13865	-0.021012023	0.291420499	8.85E-02	2.90E-01	1.00E+00
ENSG00000166106.3_ADAMTS15	ENSG00000160789.18_LMNA	0.00825	-0.151145444	0.167234818	9.20E-01	9.69E-01	1.00E+00
ENSG00000213096.8_ZNF254	ENSG00000160789.18_LMNA	-0.21147	-0.358594919	-0.0540867	8.92E-03	6.04E-02	1.00E+00
ENSG00000135205.13_CCDC146	ENSG00000160789.18_LMNA	-0.00308	-0.162205088	0.156193365	9.70E-01	9.88E-01	1.00E+00
ENSG00000232629.7_HLA-DQB2	ENSG00000160789.18_LMNA	-0.03364	-0.191817022	0.126233108	6.81E-01	8.61E-01	1.00E+00

ENSG00000152413.13_HOMER1	ENSG00000160789.18_LMNA	0.16771	0.008742262	0.3184089	3.89E-02	1.70E-01	1.00E+00
ENSG00000162086.13_ZNF75A	ENSG00000160789.18_LMNA	-0.10133	-0.256398985	0.058814884	2.14E-01	4.88E-01	1.00E+00
ENSG00000196754.9_S100A2	ENSG00000160789.18_LMNA	0.18202	0.023501107	0.331612098	2.48E-02	1.26E-01	1.00E+00
ENSG00000160712.11_IL6R	ENSG00000160789.18_LMNA	0.16625	0.007243153	0.317061049	4.07E-02	1.75E-01	1.00E+00
ENSG00000198964.12_SGMS1	ENSG00000160789.18_LMNA	-0.36281	-0.493507153	-0.21609207	4.36E-06	1.49E-04	2.23E-01
ENSG00000196247.10_ZNF107	ENSG00000160789.18_LMNA	-0.07446	-0.230927274	0.085753127	3.62E-01	6.44E-01	1.00E+00
ENSG00000132359.12_RAP1GAP	ENSG00000160789.18_LMNA	0.27442	0.120479485	0.415466821	6.23E-04	8.03E-03	1.00E+00
ENSG00000156026.13_MCU	ENSG00000160789.18_LMNA	-0.16269	-0.313760804	-0.00357842	4.52E-02	1.88E-01	1.00E+00
ENSG00000065060.15_UHRF1BP	ENSG00000160789.18_LMNA	-0.0272	-0.185597173	0.132574628	7.39E-01	8.91E-01	1.00E+00
ENSG00000175893.10_ZDHHC21	ENSG00000160789.18_LMNA	-0.16819	-0.318857383	-0.00924138	3.83E-02	1.68E-01	1.00E+00
ENSG00000170837.2_GPR27	ENSG00000160789.18_LMNA	-0.24348	-0.387652756	-0.087676	2.51E-03	2.33E-02	1.00E+00
ENSG00000147439.10_BIN3	ENSG00000160789.18_LMNA	0.26342	0.108777094	0.40560726	1.04E-03	1.19E-02	1.00E+00
ENSG00000137807.12_KIF23	ENSG00000160789.18_LMNA	0.23111	0.074657851	0.376461171	4.17E-03	3.43E-02	1.00E+00
ENSG00000254473.1_RP11-522I2	ENSG00000160789.18_LMNA	-0.16978	-0.320320703	-0.010871	3.65E-02	1.63E-01	1.00E+00
ENSG00000204624.7_DISP3	ENSG00000160789.18_LMNA	-0.30109	-0.439232871	-0.14902866	1.64E-04	2.79E-03	1.00E+00
ENSG00000162065.10_TBC1D24	ENSG00000160789.18_LMNA	-0.11269	-0.267096984	0.047362378	1.67E-01	4.24E-01	1.00E+00
ENSG00000051180.15_RAD51	ENSG00000160789.18_LMNA	0.02775	-0.132036465	0.186126002	7.34E-01	8.88E-01	1.00E+00
ENSG00000170088.12_TMEM192	ENSG00000160789.18_LMNA	-0.00378	-0.162882276	0.155514695	9.63E-01	9.85E-01	1.00E+00
ENSG00000059691.10_GATB	ENSG00000160789.18_LMNA	-0.23499	-0.379979478	-0.07873932	3.57E-03	3.04E-02	1.00E+00
ENSG00000165650.10_PDZD8	ENSG00000160789.18_LMNA	0.05459	-0.105531852	0.211944811	5.04E-01	7.55E-01	1.00E+00
ENSG00000152804.9_HHEX	ENSG00000160789.18_LMNA	-0.16324	-0.314274698	-0.00414852	4.45E-02	1.86E-01	1.00E+00
ENSG00000074660.14_SCARF1	ENSG00000160789.18_LMNA	0.12922	-0.030611748	0.28260571	1.13E-01	3.35E-01	1.00E+00
ENSG00000152454.3_ZNF256	ENSG00000160789.18_LMNA	0.08575	-0.074462813	0.24165604	2.94E-01	5.79E-01	1.00E+00
ENSG00000154822.14_PLCL2	ENSG00000160789.18_LMNA	-0.26009	-0.402617305	-0.10524448	1.21E-03	1.34E-02	1.00E+00
ENSG00000170469.9_SPATA24	ENSG00000160789.18_LMNA	-0.16251	-0.31359822	-0.0033981	4.55E-02	1.89E-01	1.00E+00
ENSG00000224046.1_AC005076.5	ENSG00000160789.18_LMNA	-0.21269	-0.359712876	-0.05536637	8.52E-03	5.84E-02	1.00E+00
ENSG00000173156.5_RHOD	ENSG00000160789.18_LMNA	0.21527	0.058059652	0.362062939	7.73E-03	5.42E-02	1.00E+00
ENSG00000121211.6_MND1	ENSG00000160789.18_LMNA	-0.06754	-0.224324216	0.092661593	4.08E-01	6.83E-01	1.00E+00
ENSG00000241399.5_CD302	ENSG00000160789.18_LMNA	-0.11274	-0.267149714	0.047305724	1.67E-01	4.24E-01	1.00E+00
ENSG00000178814.14_OPLAH	ENSG00000160789.18_LMNA	0.29246	0.139765205	0.431565673	2.56E-04	3.97E-03	1.00E+00
ENSG00000198455.4_ZXDB	ENSG00000160789.18_LMNA	-0.09522	-0.250625049	0.064961757	2.43E-01	5.23E-01	1.00E+00
ENSG00000154027.17_AK5	ENSG00000160789.18_LMNA	-0.19801	-0.346298003	-0.04007666	1.45E-02	8.57E-02	1.00E+00
ENSG00000123219.11_CENPK	ENSG00000160789.18_LMNA	0.03692	-0.123004805	0.194973601	6.52E-01	8.46E-01	1.00E+00

ENSG00000132031.11_MATN3	ENSG00000160789.18_LMNA	0.03843	-0.12151604	0.196427075	6.38E-01	8.38E-01	1.00E+00
ENSG00000270673.1_YTHDF3-AS	ENSG00000160789.18_LMNA	-0.08534	-0.241265131	0.07487561	2.96E-01	5.82E-01	1.00E+00
ENSG00000100731.14_PCNX1	ENSG00000160789.18_LMNA	0.27369	0.119709763	0.414820433	6.45E-04	8.24E-03	1.00E+00
ENSG00000213300.5_HNRNPA3P	ENSG00000160789.18_LMNA	0.12282	-0.037102214	0.276615821	1.32E-01	3.68E-01	1.00E+00
ENSG00000268001.1_CARD8-AS	ENSG00000160789.18_LMNA	-0.23748	-0.38222543	-0.08135006	3.22E-03	2.82E-02	1.00E+00
ENSG00000103540.15_CCP110	ENSG00000160789.18_LMNA	-0.11403	-0.26835505	0.046010129	1.62E-01	4.17E-01	1.00E+00
ENSG00000103569.8_AQP9	ENSG00000160789.18_LMNA	0.11171	-0.04834825	0.266179102	1.71E-01	4.30E-01	1.00E+00
ENSG00000270728.1_RP4-657E1	ENSG00000160789.18_LMNA	-0.00682	-0.165836805	0.152550182	9.34E-01	9.74E-01	1.00E+00
ENSG00000227986.1_TRIM60P18	ENSG00000160789.18_LMNA	-0.09548	-0.250868405	0.064703166	2.42E-01	5.21E-01	1.00E+00
ENSG00000126214.19_KLC1	ENSG00000160789.18_LMNA	0.15535	-0.003951371	0.306956309	5.60E-02	2.16E-01	1.00E+00
ENSG00000197619.12_ZNF615	ENSG00000160789.18_LMNA	0.05558	-0.104541339	0.212901179	4.96E-01	7.51E-01	1.00E+00
ENSG00000151743.9_AMN1	ENSG00000160789.18_LMNA	-0.58697	-0.68240613	-0.47186779	1.91E-15	3.49E-12	9.78E-11
ENSG00000134201.9_GSTM5	ENSG00000160789.18_LMNA	0.02438	-0.135341654	0.182875223	7.66E-01	9.03E-01	1.00E+00
ENSG00000266173.4_STRADA	ENSG00000160789.18_LMNA	-0.20148	-0.34947572	-0.04368558	1.28E-02	7.83E-02	1.00E+00
ENSG00000101574.13_METTL4	ENSG00000160789.18_LMNA	-0.14842	-0.300517739	0.011043918	6.80E-02	2.45E-01	1.00E+00
ENSG00000149633.10_KIAA1755	ENSG00000160789.18_LMNA	-0.34476	-0.477740827	-0.19633705	1.36E-05	3.77E-04	7.00E-01
ENSG00000255197.4_RP11-750H	ENSG00000160789.18_LMNA	-0.08259	-0.238651449	0.077632857	3.12E-01	5.98E-01	1.00E+00
ENSG00000235453.6_TOPORS-A	ENSG00000160789.18_LMNA	-0.15075	-0.302683292	0.008661899	6.38E-02	2.35E-01	1.00E+00
ENSG00000270953.1_RP11-2E11	ENSG00000160789.18_LMNA	0.07312	-0.08709174	0.229650113	3.71E-01	6.51E-01	1.00E+00
ENSG00000078687.15_TNRC6C	ENSG00000160789.18_LMNA	-0.08851	-0.244273221	0.071696297	2.78E-01	5.63E-01	1.00E+00
ENSG00000105072.7_C19orf44	ENSG00000160789.18_LMNA	0.17298	0.014171402	0.323279795	3.31E-02	1.53E-01	1.00E+00
ENSG00000248626.1_GAPDHP40	ENSG00000160789.18_LMNA	0.22884	0.072269858	0.374398628	4.57E-03	3.66E-02	1.00E+00
ENSG00000125257.12_ABCC4	ENSG00000160789.18_LMNA	-0.29017	-0.429530516	-0.13731489	2.88E-04	4.35E-03	1.00E+00
ENSG00000178971.12_CTC1	ENSG00000160789.18_LMNA	-0.00169	-0.160850641	0.157549873	9.83E-01	9.94E-01	1.00E+00
ENSG00000103269.12_RHBDL1	ENSG00000160789.18_LMNA	0.04861	-0.111456423	0.206211742	5.52E-01	7.88E-01	1.00E+00
ENSG00000058063.14_ATP11B	ENSG00000160789.18_LMNA	0.10485	-0.055271965	0.259716763	1.99E-01	4.69E-01	1.00E+00
ENSG00000131055.4_COX4I2	ENSG00000160789.18_LMNA	-0.07448	-0.230943482	0.085736132	3.62E-01	6.44E-01	1.00E+00
ENSG00000103550.12_KNOP1	ENSG00000160789.18_LMNA	-0.12767	-0.281158251	0.032182595	1.17E-01	3.42E-01	1.00E+00
ENSG00000163462.16_TRIM46	ENSG00000160789.18_LMNA	-0.03577	-0.193863894	0.124140495	6.62E-01	8.51E-01	1.00E+00
ENSG00000197142.9_ACSL5	ENSG00000160789.18_LMNA	-0.07452	-0.230984306	0.085693324	3.62E-01	6.43E-01	1.00E+00
ENSG00000179796.10_LRRC3B	ENSG00000160789.18_LMNA	-0.24292	-0.387149505	-0.0870884	2.57E-03	2.37E-02	1.00E+00
ENSG00000240652.1_RP11-832N	ENSG00000160789.18_LMNA	0.00741	-0.151973841	0.166410541	9.28E-01	9.72E-01	1.00E+00
ENSG00000223478.1_RP11-545E	ENSG00000160789.18_LMNA	0.15193	-0.00745199	0.303782022	6.17E-02	2.30E-01	1.00E+00

ENSG00000235449.2_RP11-480I1	ENSG00000160789.18_LMNA	-0.15414	-0.305831101	0.005193128	5.80E-02	2.21E-01	1.00E+00
ENSG00000165195.12_PIGA	ENSG00000160789.18_LMNA	0.06849	-0.091706892	0.225238449	4.02E-01	6.78E-01	1.00E+00
ENSG00000273142.1_RP11-458F	ENSG00000160789.18_LMNA	0.15305	-0.006299389	0.304827939	5.98E-02	2.25E-01	1.00E+00
ENSG00000163900.9_TMEM41A	ENSG00000160789.18_LMNA	0.00553	-0.153805218	0.164586693	9.46E-01	9.78E-01	1.00E+00
ENSG00000100483.12_VCPKMT	ENSG00000160789.18_LMNA	-0.03974	-0.19768952	0.120221779	6.27E-01	8.32E-01	1.00E+00
ENSG00000205978.5_NYNRIN	ENSG00000160789.18_LMNA	-0.08457	-0.240529616	0.075652014	3.00E-01	5.87E-01	1.00E+00
ENSG00000144228.7_SPOPL	ENSG00000160789.18_LMNA	-0.10887	-0.263499817	0.051222494	1.82E-01	4.46E-01	1.00E+00
ENSG00000095383.18_TBC1D2	ENSG00000160789.18_LMNA	0.14169	-0.017918647	0.294249683	8.16E-02	2.76E-01	1.00E+00
ENSG00000109670.12_FBXW7	ENSG00000160789.18_LMNA	-0.21832	-0.364841657	-0.06124991	6.89E-03	4.98E-02	1.00E+00
ENSG00000170191.4_NANP	ENSG00000160789.18_LMNA	0.22331	0.066470154	0.369376866	5.69E-03	4.32E-02	1.00E+00
ENSG00000159640.13_ACE	ENSG00000160789.18_LMNA	0.16218	0.003062927	0.313295978	4.59E-02	1.90E-01	1.00E+00
ENSG00000196196.2_HRCT1	ENSG00000160789.18_LMNA	0.0665	-0.093689888	0.223338885	4.16E-01	6.88E-01	1.00E+00
ENSG00000180638.16_SLC47A2	ENSG00000160789.18_LMNA	0.07033	-0.08987809	0.226988173	3.89E-01	6.67E-01	1.00E+00
ENSG00000275180.1_RP11-631N	ENSG00000160789.18_LMNA	-0.13353	-0.286638252	0.026227277	1.01E-01	3.13E-01	1.00E+00
ENSG00000035115.20_SH3YL1	ENSG00000160789.18_LMNA	-0.35533	-0.486987009	-0.20789477	7.05E-06	2.21E-04	3.62E-01
ENSG00000109794.12_FAM149A	ENSG00000160789.18_LMNA	-0.24971	-0.393275929	-0.09425583	1.92E-03	1.89E-02	1.00E+00
ENSG00000167904.13_TMEM68	ENSG00000160789.18_LMNA	-0.14909	-0.301138373	0.010361607	6.68E-02	2.42E-01	1.00E+00
ENSG00000145819.14_ARHGAP2	ENSG00000160789.18_LMNA	0.22477	0.06799773	0.37070126	5.37E-03	4.13E-02	1.00E+00
ENSG00000224597.8_SVIL-AS1	ENSG00000160789.18_LMNA	-0.09069	-0.246329981	0.069518762	2.67E-01	5.50E-01	1.00E+00
ENSG00000156050.7_FAM161B	ENSG00000160789.18_LMNA	-0.05586	-0.213162078	0.104271017	4.94E-01	7.49E-01	1.00E+00
ENSG00000185596.15_WASH3P	ENSG00000160789.18_LMNA	-0.03469	-0.192826822	0.125201095	6.71E-01	8.56E-01	1.00E+00
ENSG00000215193.11_PEX26	ENSG00000160789.18_LMNA	-0.10196	-0.256993356	0.058180763	2.11E-01	4.85E-01	1.00E+00
ENSG00000162374.15_ELAVL4	ENSG00000160789.18_LMNA	-0.35393	-0.485763099	-0.2063604	7.70E-06	2.37E-04	3.95E-01
ENSG00000161996.16_WDR90	ENSG00000160789.18_LMNA	0.15403	-0.005305715	0.305729038	5.81E-02	2.21E-01	1.00E+00
ENSG00000168724.13_DNAJC21	ENSG00000160789.18_LMNA	0.16705	0.008068105	0.31780292	3.97E-02	1.73E-01	1.00E+00
ENSG00000111554.13_MDM1	ENSG00000160789.18_LMNA	-0.20798	-0.355410366	-0.05044695	1.01E-02	6.62E-02	1.00E+00
ENSG00000101638.12_ST8SIA5	ENSG00000160789.18_LMNA	0.09052	-0.069685198	0.246172877	2.67E-01	5.51E-01	1.00E+00
ENSG00000113119.11_TMCO6	ENSG00000160789.18_LMNA	0.11253	-0.047518779	0.266951408	1.67E-01	4.25E-01	1.00E+00
ENSG00000233527.7_ZNF529-AS	ENSG00000160789.18_LMNA	-0.09256	-0.248108817	0.067633041	2.57E-01	5.39E-01	1.00E+00
ENSG00000111615.11_KRR1	ENSG00000160789.18_LMNA	-0.05715	-0.214396342	0.102991534	4.84E-01	7.41E-01	1.00E+00
ENSG00000255310.2_AF131215.2	ENSG00000160789.18_LMNA	-0.22769	-0.373358261	-0.07106664	4.79E-03	3.79E-02	1.00E+00
ENSG00000141179.12_PCTP	ENSG00000160789.18_LMNA	-0.28877	-0.428278828	-0.13580964	3.09E-04	4.60E-03	1.00E+00
ENSG00000148288.10_GBGT1	ENSG00000160789.18_LMNA	0.01998	-0.139668212	0.178609347	8.07E-01	9.23E-01	1.00E+00

ENSG00000165617.13_DACT1	ENSG00000160789.18_LMNA	0.17174	0.012894683	0.322135824	3.44E-02	1.57E-01	1.00E+00
ENSG00000134253.8_TRIM45	ENSG00000160789.18_LMNA	0.10041	-0.059750163	0.255521899	2.18E-01	4.93E-01	1.00E+00
ENSG00000144815.13_NXPE3	ENSG00000160789.18_LMNA	-0.14795	-0.300084197	0.011520371	6.89E-02	2.47E-01	1.00E+00
ENSG00000273893.1_RP1-101A2	ENSG00000160789.18_LMNA	-0.02585	-0.184287531	0.133906577	7.52E-01	8.97E-01	1.00E+00
ENSG00000205268.9_PDE7A	ENSG00000160789.18_LMNA	-0.12925	-0.282637445	0.03057729	1.13E-01	3.35E-01	1.00E+00
ENSG00000174718.10_KIAA1551	ENSG00000160789.18_LMNA	0.07385	-0.08636507	0.230343559	3.66E-01	6.47E-01	1.00E+00
ENSG00000115970.17_THADA	ENSG00000160789.18_LMNA	0.17565	0.016926752	0.325745578	3.04E-02	1.45E-01	1.00E+00
ENSG00000120539.13_MASTL	ENSG00000160789.18_LMNA	-0.08718	-0.243010744	0.073031418	2.86E-01	5.71E-01	1.00E+00
ENSG00000179832.16_MROH1	ENSG00000160789.18_LMNA	0.28358	0.130262784	0.42365664	4.00E-04	5.66E-03	1.00E+00
ENSG00000174567.7_GOLT1A	ENSG00000160789.18_LMNA	-0.04186	-0.19973442	0.11812305	6.09E-01	8.21E-01	1.00E+00
ENSG00000137434.10_C6orf52	ENSG00000160789.18_LMNA	-0.03629	-0.194372521	0.123620062	6.57E-01	8.48E-01	1.00E+00
ENSG00000004848.6_ARX	ENSG00000160789.18_LMNA	0.13832	-0.021355052	0.29110643	8.92E-02	2.91E-01	1.00E+00
ENSG00000135314.11_KHDC1	ENSG00000160789.18_LMNA	-0.07824	-0.234517118	0.081984421	3.38E-01	6.24E-01	1.00E+00
ENSG00000213939.4_RP11-314A	ENSG00000160789.18_LMNA	-0.1482	-0.300319297	0.011262018	6.84E-02	2.46E-01	1.00E+00
ENSG00000171798.16_KNDC1	ENSG00000160789.18_LMNA	0.10957	-0.050511407	0.264163124	1.79E-01	4.41E-01	1.00E+00
ENSG00000149289.9_ZC3H12C	ENSG00000160789.18_LMNA	-0.11364	-0.26799107	0.046401476	1.63E-01	4.19E-01	1.00E+00
ENSG00000184792.14_OSBP2	ENSG00000160789.18_LMNA	0.28663	0.133521025	0.42637358	3.44E-04	4.99E-03	1.00E+00
ENSG00000213390.9_ARHGAP19	ENSG00000160789.18_LMNA	0.19396	0.035863613	0.342579424	1.67E-02	9.46E-02	1.00E+00
ENSG00000077454.14_LRCH4	ENSG00000160789.18_LMNA	0.12838	-0.031463574	0.281820971	1.15E-01	3.39E-01	1.00E+00
ENSG00000164619.7_BMPER	ENSG00000160789.18_LMNA	-0.25064	-0.394117321	-0.09524261	1.84E-03	1.84E-02	1.00E+00
ENSG00000255455.2_RP11-890B	ENSG00000160789.18_LMNA	-0.04693	-0.204606257	0.113111499	5.66E-01	7.96E-01	1.00E+00
ENSG00000163820.13_FYCO1	ENSG00000160789.18_LMNA	0.27182	0.117708988	0.413138853	7.05E-04	8.83E-03	1.00E+00
ENSG00000270093.1_AP000473.8	ENSG00000160789.18_LMNA	-0.12326	-0.277024008	0.036660754	1.30E-01	3.66E-01	1.00E+00
ENSG00000184381.17_PLA2G6	ENSG00000160789.18_LMNA	-0.26426	-0.406362579	-0.10967069	1.00E-03	1.16E-02	1.00E+00
ENSG00000198182.11_ZNF607	ENSG00000160789.18_LMNA	-0.02929	-0.187619654	0.130515438	7.20E-01	8.81E-01	1.00E+00
ENSG00000165121.10_RP11-213	ENSG00000160789.18_LMNA	-0.14959	-0.301607886	0.009845243	6.59E-02	2.40E-01	1.00E+00
ENSG00000198265.10_HELZ	ENSG00000160789.18_LMNA	0.05209	-0.108001771	0.209557381	5.24E-01	7.69E-01	1.00E+00
ENSG00000162650.14_ATXN7L2	ENSG00000160789.18_LMNA	-0.04644	-0.204134417	0.113597578	5.70E-01	7.99E-01	1.00E+00
ENSG00000120658.11_ENOX1	ENSG00000160789.18_LMNA	-0.35885	-0.490054927	-0.21174696	5.63E-06	1.83E-04	2.89E-01
ENSG00000101247.16_NDUFAF5	ENSG00000160789.18_LMNA	-0.33998	-0.473549286	-0.19112326	1.82E-05	4.78E-04	9.36E-01
ENSG00000181004.8_BBS12	ENSG00000160789.18_LMNA	-0.02684	-0.185251234	0.132926571	7.43E-01	8.92E-01	1.00E+00
ENSG00000115464.13_USP34	ENSG00000160789.18_LMNA	0.02747	-0.13230739	0.1858598	7.37E-01	8.89E-01	1.00E+00
ENSG00000186446.10_ZNF501	ENSG00000160789.18_LMNA	-0.21995	-0.366326614	-0.06295734	6.47E-03	4.76E-02	1.00E+00

ENSG00000231889.6_TRAF3IP2-/	ENSG00000160789.18_LMNA	-0.42274	-0.54524886	-0.28255994	5.78E-08	4.61E-06	2.97E-03
ENSG00000270015.1_RP11-540B	ENSG00000160789.18_LMNA	0.1093	-0.05078882	0.263904387	1.80E-01	4.43E-01	1.00E+00
ENSG00000140853.14_NLRC5	ENSG00000160789.18_LMNA	0.24495	0.089224681	0.388978284	2.35E-03	2.22E-02	1.00E+00
ENSG00000180773.13_SLC36A4	ENSG00000160789.18_LMNA	0.03947	-0.120483642	0.197434179	6.29E-01	8.33E-01	1.00E+00
ENSG00000128578.8_STRIP2	ENSG00000160789.18_LMNA	-0.12549	-0.279109917	0.034402899	1.23E-01	3.54E-01	1.00E+00
ENSG00000261461.1_UBE2MP1	ENSG00000160789.18_LMNA	0.1551	-0.004203859	0.306727589	5.64E-02	2.17E-01	1.00E+00
ENSG00000132744.6_ACY3	ENSG00000160789.18_LMNA	-0.18078	-0.330472748	-0.0222222	2.58E-02	1.29E-01	1.00E+00
ENSG00000249673.5_NOP14-AS1	ENSG00000160789.18_LMNA	-0.04314	-0.200963093	0.116860667	5.98E-01	8.14E-01	1.00E+00
ENSG00000131187.8_F12	ENSG00000160789.18_LMNA	0.08433	-0.075886996	0.240306938	3.02E-01	5.88E-01	1.00E+00
ENSG00000147234.9_FRMPD3	ENSG00000160789.18_LMNA	-0.2617	-0.404061806	-0.10695021	1.13E-03	1.27E-02	1.00E+00
ENSG00000063169.9_GLTSCR1	ENSG00000160789.18_LMNA	0.18605	0.027667273	0.335317415	2.17E-02	1.15E-01	1.00E+00
ENSG00000108352.10_RAPGEFL	ENSG00000160789.18_LMNA	-0.10758	-0.262284326	0.052524714	1.87E-01	4.53E-01	1.00E+00
ENSG00000242294.5_STAG3L5P	ENSG00000160789.18_LMNA	0.05589	-0.104233516	0.213198268	4.94E-01	7.49E-01	1.00E+00
ENSG00000163053.9_SLC16A14	ENSG00000160789.18_LMNA	-0.40732	-0.532022977	-0.26532642	1.91E-07	1.22E-05	9.79E-03
ENSG00000132763.13_MMACHC	ENSG00000160789.18_LMNA	-0.04817	-0.205787899	0.11189353	5.56E-01	7.90E-01	1.00E+00
ENSG00000176912.3_TYMSOS	ENSG00000160789.18_LMNA	-0.00878	-0.167746822	0.150630656	9.15E-01	9.67E-01	1.00E+00
ENSG00000147113.15_CXorf36	ENSG00000160789.18_LMNA	-0.0056	-0.16465212	0.153739558	9.45E-01	9.78E-01	1.00E+00
ENSG00000220749.4_RPL21P28	ENSG00000160789.18_LMNA	-0.23787	-0.382584273	-0.08176757	3.17E-03	2.78E-02	1.00E+00
ENSG00000166135.12_HIF1AN	ENSG00000160789.18_LMNA	0.04979	-0.110280026	0.207351852	5.42E-01	7.81E-01	1.00E+00
ENSG00000047188.14_YTHDC2	ENSG00000160789.18_LMNA	-0.07718	-0.233512629	0.083039871	3.45E-01	6.29E-01	1.00E+00
ENSG00000206573.7_THUMPD3-	ENSG00000160789.18_LMNA	-0.26006	-0.40259507	-0.10521823	1.21E-03	1.34E-02	1.00E+00
ENSG00000105926.14_MPP6	ENSG00000160789.18_LMNA	-0.00794	-0.166930451	0.151451385	9.23E-01	9.70E-01	1.00E+00
ENSG00000101639.17_CEP192	ENSG00000160789.18_LMNA	-0.0122	-0.171067017	0.147288208	8.81E-01	9.54E-01	1.00E+00
ENSG00000204271.9_SPIN3	ENSG00000160789.18_LMNA	-0.4133	-0.537155336	-0.27199379	1.21E-07	8.48E-06	6.21E-03
ENSG00000130224.13_LRCH2	ENSG00000160789.18_LMNA	-0.3535	-0.485384281	-0.20588576	7.92E-06	2.43E-04	4.06E-01
ENSG00000224415.1_RP11-401L	ENSG00000160789.18_LMNA	-0.1883	-0.337388475	-0.03000059	2.02E-02	1.09E-01	1.00E+00
ENSG00000103657.12_HERC1	ENSG00000160789.18_LMNA	-0.01752	-0.176230721	0.142075408	8.30E-01	9.34E-01	1.00E+00
ENSG00000164291.15_ARSK	ENSG00000160789.18_LMNA	0.15935	0.000156324	0.31067228	4.99E-02	2.01E-01	1.00E+00
ENSG00000077092.17_RARB	ENSG00000160789.18_LMNA	-0.28275	-0.422912779	-0.12937183	4.16E-04	5.85E-03	1.00E+00
ENSG00000216624.2_GAPDHP72	ENSG00000160789.18_LMNA	0.25575	0.100648856	0.39871812	1.47E-03	1.55E-02	1.00E+00
ENSG00000187955.10_COL14A1	ENSG00000160789.18_LMNA	0.10697	-0.053132993	0.261716216	1.90E-01	4.57E-01	1.00E+00
ENSG00000170370.11_EMX2	ENSG00000160789.18_LMNA	-0.03749	-0.19552055	0.122444744	6.47E-01	8.43E-01	1.00E+00
ENSG00000254470.2_AP5B1	ENSG00000160789.18_LMNA	0.24086	0.084912353	0.385284246	2.80E-03	2.53E-02	1.00E+00

ENSG00000188352.11_FOCAD	ENSG00000160789.18_LMNA	-0.00791	-0.166900454	0.151481533	9.23E-01	9.70E-01	1.00E+00
ENSG00000173064.9_HECTD4	ENSG00000160789.18_LMNA	-0.08628	-0.242150751	0.073940247	2.91E-01	5.76E-01	1.00E+00
ENSG00000166965.11_RCCD1	ENSG00000160789.18_LMNA	0.10115	-0.059000622	0.256224845	2.15E-01	4.89E-01	1.00E+00
ENSG00000226752.6_PSMD5-AS	ENSG00000160789.18_LMNA	0.00534	-0.153988803	0.164403742	9.48E-01	9.79E-01	1.00E+00
ENSG00000147255.16_IGSF1	ENSG00000160789.18_LMNA	-0.12593	-0.279526684	0.033951392	1.22E-01	3.52E-01	1.00E+00
ENSG00000175886.10_RPL7AP6(	ENSG00000160789.18_LMNA	-0.12909	-0.28248207	0.030745988	1.13E-01	3.36E-01	1.00E+00
ENSG00000104903.4_LYL1	ENSG00000160789.18_LMNA	-0.04421	-0.201991303	0.115803456	5.89E-01	8.09E-01	1.00E+00
ENSG00000204130.11_RUFY2	ENSG00000160789.18_LMNA	-0.27996	-0.420421685	-0.12639157	4.77E-04	6.50E-03	1.00E+00
ENSG00000188859.6_FAM78B	ENSG00000160789.18_LMNA	0.12402	-0.035888521	0.277737765	1.28E-01	3.62E-01	1.00E+00
ENSG00000141298.16_SSH2	ENSG00000160789.18_LMNA	-0.26671	-0.408561326	-0.11227469	8.96E-04	1.06E-02	1.00E+00
ENSG00000266074.7_BAHCC1	ENSG00000160789.18_LMNA	-0.02063	-0.179238616	0.139030756	8.01E-01	9.20E-01	1.00E+00
ENSG00000243069.6_ARHGEF26	ENSG00000160789.18_LMNA	-0.41728	-0.540570193	-0.27644412	8.89E-08	6.51E-06	4.56E-03
ENSG00000096968.11_JAK2	ENSG00000160789.18_LMNA	-0.05624	-0.213529768	0.103889965	4.91E-01	7.47E-01	1.00E+00
ENSG00000188786.9_MTF1	ENSG00000160789.18_LMNA	0.23091	0.074442447	0.376275246	4.21E-03	3.45E-02	1.00E+00
ENSG00000172171.9_TEFM	ENSG00000160789.18_LMNA	-0.35826	-0.489542693	-0.21110318	5.85E-06	1.89E-04	3.00E-01
ENSG00000100276.9_RASL10A	ENSG00000160789.18_LMNA	-0.41089	-0.535088096	-0.26930521	1.46E-07	9.80E-06	7.47E-03
ENSG00000013573.15_DDX11	ENSG00000160789.18_LMNA	0.01831	-0.141303396	0.176993976	8.23E-01	9.31E-01	1.00E+00
ENSG00000241764.3_AC002467.7	ENSG00000160789.18_LMNA	-0.08676	-0.242606178	0.073459023	2.88E-01	5.73E-01	1.00E+00
ENSG00000186787.7_SPIN2B	ENSG00000160789.18_LMNA	-0.30405	-0.441864114	-0.1522195	1.40E-04	2.46E-03	1.00E+00
ENSG00000123570.3_RAB9B	ENSG00000160789.18_LMNA	-0.38799	-0.51535893	-0.24385287	7.86E-07	3.76E-05	4.03E-02
ENSG00000228459.3_LINC01546	ENSG00000160789.18_LMNA	-0.33483	-0.469028316	-0.1855175	2.48E-05	6.15E-04	1.00E+00
ENSG00000148158.15_SNX30	ENSG00000160789.18_LMNA	-0.10681	-0.261561222	0.053298906	1.90E-01	4.58E-01	1.00E+00
ENSG00000172766.17_NAA16	ENSG00000160789.18_LMNA	-0.25548	-0.398471055	-0.10035809	1.49E-03	1.56E-02	1.00E+00
ENSG00000234093.3_RPS15AP1	ENSG00000160789.18_LMNA	-0.06513	-0.222029048	0.095055793	4.25E-01	6.96E-01	1.00E+00
ENSG00000219626.7_FAM228B	ENSG00000160789.18_LMNA	-0.13868	-0.291448209	0.020981755	8.84E-02	2.89E-01	1.00E+00
ENSG00000188051.6_TMEM221	ENSG00000160789.18_LMNA	0.2834	0.130066863	0.4234931	4.03E-04	5.70E-03	1.00E+00
ENSG00000103199.12_ZNF500	ENSG00000160789.18_LMNA	-0.01896	-0.177624112	0.140665738	8.17E-01	9.28E-01	1.00E+00
ENSG00000108950.10_FAM20A	ENSG00000160789.18_LMNA	0.21704	0.059912954	0.363677826	7.23E-03	5.16E-02	1.00E+00
ENSG00000262049.1_RP13-1032	ENSG00000160789.18_LMNA	-0.07445	-0.230909153	0.085772128	3.62E-01	6.44E-01	1.00E+00
ENSG00000184047.14_DIABLO	ENSG00000160789.18_LMNA	-0.30972	-0.446887973	-0.15832861	1.03E-04	1.94E-03	1.00E+00
ENSG00000178105.8_DDX10	ENSG00000160789.18_LMNA	0.00167	-0.157570779	0.160829757	9.84E-01	9.94E-01	1.00E+00
ENSG00000103174.10_NAGPA	ENSG00000160789.18_LMNA	0.30359	0.151721272	0.441453592	1.43E-04	2.51E-03	1.00E+00
ENSG00000272455.1_RP4-758J18	ENSG00000160789.18_LMNA	0.0279	-0.131887315	0.186272534	7.33E-01	8.88E-01	1.00E+00

ENSG00000101751.9_POLI	ENSG00000160789.18_LMNA	-0.05157	-0.209050396	0.108525769	5.28E-01	7.72E-01	1.00E+00
ENSG00000248049.5_UBA6-AS1	ENSG00000160789.18_LMNA	-0.19195	-0.340737321	-0.03378059	1.78E-02	9.94E-02	1.00E+00
ENSG00000176597.10_B3GNT5	ENSG00000160789.18_LMNA	0.41154	0.270034391	0.535649079	1.38E-07	9.45E-06	7.10E-03
ENSG00000189129.12_PLAC9	ENSG00000160789.18_LMNA	0.03518	-0.124715165	0.193302061	6.67E-01	8.53E-01	1.00E+00
ENSG00000124574.13_ABCC10	ENSG00000160789.18_LMNA	0.12216	-0.037780387	0.275988543	1.34E-01	3.71E-01	1.00E+00
ENSG00000175664.8_TEX26	ENSG00000160789.18_LMNA	0.22923	0.072678133	0.374751473	4.50E-03	3.63E-02	1.00E+00
ENSG00000148606.11_POLR3A	ENSG00000160789.18_LMNA	0.0856	-0.074619952	0.241507245	2.94E-01	5.80E-01	1.00E+00
ENSG00000197948.9_FCHSD1	ENSG00000160789.18_LMNA	0.26415	0.109558175	0.406267499	1.01E-03	1.17E-02	1.00E+00
ENSG00000136052.8_SLC41A2	ENSG00000160789.18_LMNA	0.08702	-0.073198806	0.242852388	2.86E-01	5.72E-01	1.00E+00
ENSG00000151651.14_ADAM8	ENSG00000160789.18_LMNA	0.22658	0.069899696	0.372348527	5.00E-03	3.92E-02	1.00E+00
ENSG00000270189.1_RP11-258C	ENSG00000160789.18_LMNA	-0.13137	-0.284621095	0.028421982	1.07E-01	3.25E-01	1.00E+00
ENSG00000261652.2_C15orf65	ENSG00000160789.18_LMNA	-0.01603	-0.174787849	0.143533779	8.45E-01	9.39E-01	1.00E+00
ENSG00000103888.14_CEMIP	ENSG00000160789.18_LMNA	0.49641	0.366141902	0.60759082	7.83E-11	1.85E-08	4.02E-06
ENSG00000140511.10_HAPLN3	ENSG00000160789.18_LMNA	0.2245	0.067722795	0.370462985	5.43E-03	4.17E-02	1.00E+00
ENSG00000257815.4_LINC01481	ENSG00000160789.18_LMNA	-0.1614	-0.312566287	-0.00225403	4.70E-02	1.93E-01	1.00E+00
ENSG00000058056.7_USP13	ENSG00000160789.18_LMNA	-0.02941	-0.187729512	0.130403507	7.19E-01	8.81E-01	1.00E+00
ENSG00000147394.17_ZNF185	ENSG00000160789.18_LMNA	0.02487	-0.13486244	0.183346981	7.61E-01	9.00E-01	1.00E+00
ENSG00000275764.1_RP11-582E	ENSG00000160789.18_LMNA	-0.12596	-0.279557909	0.03391756	1.22E-01	3.52E-01	1.00E+00
ENSG00000110693.14_SOX6	ENSG00000160789.18_LMNA	-0.41745	-0.540714473	-0.2766324	8.77E-08	6.45E-06	4.50E-03
ENSG00000068976.12_PYGM	ENSG00000160789.18_LMNA	-0.1128	-0.267204347	0.047247024	1.66E-01	4.24E-01	1.00E+00
ENSG00000100122.5_CRYBB1	ENSG00000160789.18_LMNA	-0.15802	-0.309436356	0.001211051	5.19E-02	2.06E-01	1.00E+00
ENSG00000152409.8_JMY	ENSG00000160789.18_LMNA	-0.15457	-0.306235508	0.004746942	5.73E-02	2.19E-01	1.00E+00
ENSG00000142197.11_DOPEY2	ENSG00000160789.18_LMNA	0.11202	-0.048033343	0.266472354	1.69E-01	4.28E-01	1.00E+00
ENSG00000123643.11_SLC36A1	ENSG00000160789.18_LMNA	0.29207	0.139347033	0.431218562	2.61E-04	4.03E-03	1.00E+00
ENSG00000176834.12_VSIG10	ENSG00000160789.18_LMNA	0.15014	-0.009284871	0.302117243	6.49E-02	2.37E-01	1.00E+00
ENSG00000102935.10_ZNF423	ENSG00000160789.18_LMNA	-0.21164	-0.358751124	-0.05426544	8.86E-03	6.00E-02	1.00E+00
ENSG00000160883.9_HK3	ENSG00000160789.18_LMNA	0.23656	0.080387239	0.381397551	3.34E-03	2.90E-02	1.00E+00
ENSG00000012817.14_KDM5D	ENSG00000160789.18_LMNA	-0.06849	-0.22523841	0.091706932	4.02E-01	6.78E-01	1.00E+00
ENSG00000144935.13_TRPC1	ENSG00000160789.18_LMNA	-0.11028	-0.264826723	0.049799687	1.76E-01	4.38E-01	1.00E+00
ENSG00000268089.2_GABRQ	ENSG00000160789.18_LMNA	-0.24382	-0.387960494	-0.08803542	2.47E-03	2.30E-02	1.00E+00
ENSG00000234444.8_ZNF736	ENSG00000160789.18_LMNA	0.00552	-0.153817186	0.164574766	9.46E-01	9.78E-01	1.00E+00
ENSG00000185920.14_PTCH1	ENSG00000160789.18_LMNA	-0.13928	-0.292003138	0.020375466	8.70E-02	2.87E-01	1.00E+00
ENSG00000179178.9_TMEM125	ENSG00000160789.18_LMNA	-0.3716	-0.501152152	-0.22575364	2.44E-06	9.28E-05	1.25E-01

ENSG00000177192.12_PUS1	ENSG00000160789.18_LMNA	0.20824	0.050719046	0.355648674	1.00E-02	6.58E-02	1.00E+00
ENSG00000099999.13_RNF215	ENSG00000160789.18_LMNA	0.12985	-0.029973573	0.28319335	1.11E-01	3.32E-01	1.00E+00
ENSG00000143995.18_MEIS1	ENSG00000160789.18_LMNA	-0.0215	-0.180088571	0.138169322	7.93E-01	9.16E-01	1.00E+00
ENSG00000147127.7_RAB41	ENSG00000160789.18_LMNA	-0.23619	-0.381061537	-0.07999661	3.40E-03	2.93E-02	1.00E+00
ENSG00000146247.13_PHIP	ENSG00000160789.18_LMNA	-0.10032	-0.255441717	0.059835637	2.19E-01	4.94E-01	1.00E+00
ENSG00000196152.9_ZNF79	ENSG00000160789.18_LMNA	0.16813	0.00917543	0.31879813	3.84E-02	1.69E-01	1.00E+00
ENSG00000168646.11_AXIN2	ENSG00000160789.18_LMNA	-0.20618	-0.353768183	-0.0485732	1.08E-02	6.93E-02	1.00E+00
ENSG00000166405.13_RIC3	ENSG00000160789.18_LMNA	-0.29501	-0.433832064	-0.14249814	2.25E-04	3.58E-03	1.00E+00
ENSG00000143147.13_GPR161	ENSG00000160789.18_LMNA	0.07712	-0.08309907	0.233456269	3.45E-01	6.29E-01	1.00E+00
ENSG00000116985.9_BMP8B	ENSG00000160789.18_LMNA	-0.01463	-0.173424742	0.144910258	8.58E-01	9.44E-01	1.00E+00
ENSG00000213621.3_RPSAP54	ENSG00000160789.18_LMNA	-0.18711	-0.336294776	-0.02876798	2.10E-02	1.12E-01	1.00E+00
ENSG00000134207.13_SYT6	ENSG00000160789.18_LMNA	-0.35314	-0.485066411	-0.2054876	8.10E-06	2.46E-04	4.16E-01
ENSG00000151693.8_ASAP2	ENSG00000160789.18_LMNA	0.21706	0.059934752	0.36369681	7.23E-03	5.15E-02	1.00E+00
ENSG00000111052.6_LIN7A	ENSG00000160789.18_LMNA	0.04846	-0.111600497	0.206072054	5.53E-01	7.88E-01	1.00E+00
ENSG00000196437.9_ZNF569	ENSG00000160789.18_LMNA	-0.11156	-0.266038259	0.048499472	1.71E-01	4.30E-01	1.00E+00
ENSG00000140945.14_CDH13	ENSG00000160789.18_LMNA	-0.33079	-0.465478112	-0.18112834	3.15E-05	7.42E-04	1.00E+00
ENSG00000163520.12_FBLN2	ENSG00000160789.18_LMNA	0.1023	-0.057840564	0.257312131	2.10E-01	4.83E-01	1.00E+00
ENSG00000153291.14_SLC25A27	ENSG00000160789.18_LMNA	-0.2689	-0.410522191	-0.1146004	8.09E-04	9.81E-03	1.00E+00
ENSG00000020922.11_MRE11	ENSG00000160789.18_LMNA	-0.19828	-0.346538109	-0.04034907	1.43E-02	8.51E-02	1.00E+00
ENSG00000060656.18_PTPRU	ENSG00000160789.18_LMNA	0.33088	0.181222299	0.465554204	3.13E-05	7.39E-04	1.00E+00
ENSG00000184985.15_SORCS2	ENSG00000160789.18_LMNA	0.22429	0.067500691	0.370270467	5.47E-03	4.19E-02	1.00E+00
ENSG00000166233.11_ARIH1	ENSG00000160789.18_LMNA	0.21077	0.053356448	0.357956563	9.15E-03	6.15E-02	1.00E+00
ENSG00000242615.1_CTC-359D2	ENSG00000160789.18_LMNA	-0.11718	-0.27131917	0.042819543	1.51E-01	3.99E-01	1.00E+00
ENSG00000135622.11_SEMA4F	ENSG00000160789.18_LMNA	0.1334	-0.026356358	0.28651969	1.01E-01	3.14E-01	1.00E+00
ENSG00000160766.13_GBAP1	ENSG00000160789.18_LMNA	-0.01953	-0.178179664	0.140103335	8.11E-01	9.26E-01	1.00E+00
ENSG00000163378.12_EOGT	ENSG00000160789.18_LMNA	0.33313	0.18367382	0.467538153	2.74E-05	6.65E-04	1.00E+00
ENSG00000160072.18_ATAD3B	ENSG00000160789.18_LMNA	0.10449	-0.055635847	0.259376346	2.00E-01	4.71E-01	1.00E+00
ENSG00000246560.2_RP11-10L1	ENSG00000160789.18_LMNA	-0.18	-0.32974823	-0.02140946	2.65E-02	1.32E-01	1.00E+00
ENSG00000171943.10_SRGAP2C	ENSG00000160789.18_LMNA	0.16718	0.008199965	0.317921465	3.95E-02	1.72E-01	1.00E+00
ENSG00000110274.13_CEP164	ENSG00000160789.18_LMNA	0.23971	0.083702565	0.384246182	2.94E-03	2.63E-02	1.00E+00
ENSG00000168096.13_ANKS3	ENSG00000160789.18_LMNA	0.02847	-0.131323037	0.186826773	7.28E-01	8.85E-01	1.00E+00
ENSG00000197044.9_ZNF441	ENSG00000160789.18_LMNA	-0.16713	-0.317871654	-0.00814456	3.96E-02	1.72E-01	1.00E+00
ENSG00000101745.14_ANKRD12	ENSG00000160789.18_LMNA	-0.08735	-0.243165168	0.072868168	2.85E-01	5.70E-01	1.00E+00

ENSG00000272913.1_RP11-440D	ENSG00000160789.18_LMNA	-0.04066	-0.198572059	0.119316357	6.19E-01	8.27E-01	1.00E+00
ENSG00000044524.9_EPHA3	ENSG00000160789.18_LMNA	0.02328	-0.136423915	0.181809265	7.76E-01	9.08E-01	1.00E+00
ENSG00000232098.3_CTD-2619J	ENSG00000160789.18_LMNA	0.26357	0.108940519	0.405745427	1.03E-03	1.19E-02	1.00E+00
ENSG00000269918.1_AF131215.9	ENSG00000160789.18_LMNA	-0.24642	-0.39030586	-0.09077721	2.21E-03	2.11E-02	1.00E+00
ENSG00000157764.11_BRAF	ENSG00000160789.18_LMNA	0.10282	-0.057323823	0.2577962	2.07E-01	4.80E-01	1.00E+00
ENSG00000240489.1_SETP14	ENSG00000160789.18_LMNA	0.04488	-0.115147455	0.202628957	5.83E-01	8.06E-01	1.00E+00
ENSG00000173818.15_ENDOV	ENSG00000160789.18_LMNA	-0.06856	-0.225305782	0.091636556	4.01E-01	6.78E-01	1.00E+00
ENSG00000068831.17_RASGRP2	ENSG00000160789.18_LMNA	-0.02384	-0.18234617	0.135878894	7.71E-01	9.05E-01	1.00E+00
ENSG00000258645.2_HSPE1P2	ENSG00000160789.18_LMNA	-0.12771	-0.281191648	0.032146369	1.17E-01	3.42E-01	1.00E+00
ENSG00000241990.4_PRR34-AS1	ENSG00000160789.18_LMNA	-0.09554	-0.250924067	0.064644013	2.42E-01	5.21E-01	1.00E+00
ENSG00000074527.10_NTN4	ENSG00000160789.18_LMNA	-0.38747	-0.514905745	-0.24327259	8.16E-07	3.86E-05	4.18E-02
ENSG00000122674.10_CCZ1	ENSG00000160789.18_LMNA	0.01842	-0.141197633	0.177098509	8.22E-01	9.30E-01	1.00E+00
ENSG00000007372.19_PAX6	ENSG00000160789.18_LMNA	-0.07539	-0.23181123	0.084825969	3.56E-01	6.39E-01	1.00E+00
ENSG00000204947.7_ZNF425	ENSG00000160789.18_LMNA	0.12088	-0.039074676	0.274790643	1.38E-01	3.78E-01	1.00E+00
ENSG00000145014.16_TMEM44	ENSG00000160789.18_LMNA	0.3535	0.205885955	0.485384434	7.92E-06	2.43E-04	4.06E-01
ENSG00000242114.4_MTFP1	ENSG00000160789.18_LMNA	0.26444	0.109866876	0.406528354	9.94E-04	1.15E-02	1.00E+00
ENSG00000273080.1_RP11-301O	ENSG00000160789.18_LMNA	-0.12661	-0.28016363	0.033261107	1.20E-01	3.48E-01	1.00E+00
ENSG00000136874.9_STX17	ENSG00000160789.18_LMNA	0.04844	-0.111626251	0.206047082	5.53E-01	7.88E-01	1.00E+00
ENSG00000220563.1_PKMP3	ENSG00000160789.18_LMNA	-0.29208	-0.431226427	-0.13935651	2.61E-04	4.03E-03	1.00E+00
ENSG00000111665.10_CDCA3	ENSG00000160789.18_LMNA	-0.00723	-0.166241744	0.152143427	9.30E-01	9.73E-01	1.00E+00
ENSG00000185187.11_SIGIRR	ENSG00000160789.18_LMNA	0.06633	-0.093858524	0.223177233	4.17E-01	6.89E-01	1.00E+00
ENSG00000111404.5_RERGL	ENSG00000160789.18_LMNA	-0.31082	-0.447860469	-0.15951374	9.72E-05	1.84E-03	1.00E+00
ENSG00000171813.12_PWWP2B	ENSG00000160789.18_LMNA	0.04747	-0.112582482	0.20511961	5.61E-01	7.93E-01	1.00E+00
ENSG00000151702.15_FLI1	ENSG00000160789.18_LMNA	-0.03517	-0.193290077	0.124727421	6.67E-01	8.53E-01	1.00E+00
ENSG00000158887.14_MPZ	ENSG00000160789.18_LMNA	-0.21091	-0.358088579	-0.05350744	9.10E-03	6.12E-02	1.00E+00
ENSG00000268996.3_MAN1B1-AS	ENSG00000160789.18_LMNA	-0.11387	-0.268210908	0.046165121	1.62E-01	4.17E-01	1.00E+00
ENSG00000172575.10_RASGRP1	ENSG00000160789.18_LMNA	0.14737	-0.012112894	0.29954486	7.00E-02	2.50E-01	1.00E+00
ENSG00000173517.9_PEAK1	ENSG00000160789.18_LMNA	0.08607	-0.074146709	0.241955314	2.92E-01	5.77E-01	1.00E+00
ENSG00000168806.7_LCMT2	ENSG00000160789.18_LMNA	0.00682	-0.152550451	0.165836536	9.34E-01	9.74E-01	1.00E+00
ENSG00000100802.13_C14orf93	ENSG00000160789.18_LMNA	-0.06005	-0.2171745	0.100107746	4.62E-01	7.25E-01	1.00E+00
ENSG00000240137.4_ERICH6-AS	ENSG00000160789.18_LMNA	-0.07409	-0.230571508	0.086126127	3.64E-01	6.46E-01	1.00E+00
ENSG00000138621.10_PPCDC	ENSG00000160789.18_LMNA	0.05508	-0.105045273	0.212414693	5.00E-01	7.53E-01	1.00E+00
ENSG00000164746.12_C7orf57	ENSG00000160789.18_LMNA	0.11045	-0.049619805	0.264994395	1.76E-01	4.36E-01	1.00E+00

ENSG00000266865.5_RP11-848P	ENSG00000160789.18_LMNA	-0.05887	-0.216048847	0.101276841	4.71E-01	7.31E-01	1.00E+00
ENSG00000167258.12_CDK12	ENSG00000160789.18_LMNA	0.11486	-0.04516337	0.269142288	1.59E-01	4.12E-01	1.00E+00
ENSG00000140538.15_NTRK3	ENSG00000160789.18_LMNA	-0.05553	-0.212853717	0.10459051	4.97E-01	7.51E-01	1.00E+00
ENSG00000228452.1_RP5-994D1	ENSG00000160789.18_LMNA	-0.26405	-0.406172303	-0.10944553	1.01E-03	1.17E-02	1.00E+00
ENSG00000258675.1_RP11-299L	ENSG00000160789.18_LMNA	-0.21732	-0.363926576	-0.06019861	7.16E-03	5.12E-02	1.00E+00
ENSG00000102984.13_ZNF821	ENSG00000160789.18_LMNA	-0.32103	-0.456878437	-0.17054321	5.52E-05	1.16E-03	1.00E+00
ENSG00000162104.8_ADCY9	ENSG00000160789.18_LMNA	0.25286	0.097587938	0.396115071	1.67E-03	1.71E-02	1.00E+00
ENSG00000103253.16_HAGHL	ENSG00000160789.18_LMNA	0.04221	-0.117777659	0.200070688	6.06E-01	8.19E-01	1.00E+00
ENSG00000237506.3_RPSAP15	ENSG00000160789.18_LMNA	-0.18478	-0.334151459	-0.02635515	2.27E-02	1.18E-01	1.00E+00
ENSG00000246067.6_RAB30-AS1	ENSG00000160789.18_LMNA	-0.26004	-0.402578489	-0.10519866	1.21E-03	1.34E-02	1.00E+00
ENSG00000167523.12_SPATA33	ENSG00000160789.18_LMNA	0.11075	-0.049320728	0.265273131	1.74E-01	4.35E-01	1.00E+00
ENSG00000128917.6_DLL4	ENSG00000160789.18_LMNA	0.17979	0.021197045	0.329558814	2.67E-02	1.32E-01	1.00E+00
ENSG00000145681.9_HAPLN1	ENSG00000160789.18_LMNA	-0.09705	-0.252348355	0.063129647	2.34E-01	5.12E-01	1.00E+00
ENSG00000204611.5_ZNF616	ENSG00000160789.18_LMNA	0.04452	-0.115497491	0.202288744	5.86E-01	8.08E-01	1.00E+00
ENSG00000119686.8_FLVCR2	ENSG00000160789.18_LMNA	0.00673	-0.152634	0.165753347	9.34E-01	9.74E-01	1.00E+00
ENSG00000091073.18_DTX2	ENSG00000160789.18_LMNA	0.40612	0.263990241	0.530992056	2.09E-07	1.32E-05	1.07E-02
ENSG00000154642.9_C21orf91	ENSG00000160789.18_LMNA	-0.24087	-0.3852946	-0.08492443	2.80E-03	2.53E-02	1.00E+00
ENSG00000061987.13_MON2	ENSG00000160789.18_LMNA	-0.19331	-0.341989644	-0.03519641	1.70E-02	9.62E-02	1.00E+00
ENSG00000142528.14_ZNF473	ENSG00000160789.18_LMNA	0.09161	-0.068592264	0.24720423	2.62E-01	5.45E-01	1.00E+00
ENSG00000168807.15_SNTB2	ENSG00000160789.18_LMNA	0.45982	0.324360578	0.576794546	2.53E-09	3.38E-07	1.30E-04
ENSG00000248485.1_PCP4L1	ENSG00000160789.18_LMNA	-0.25623	-0.399152889	-0.10116065	1.44E-03	1.52E-02	1.00E+00
ENSG00000253978.1_CTB-178M2	ENSG00000160789.18_LMNA	-0.21668	-0.36334365	-0.05952927	7.33E-03	5.21E-02	1.00E+00
ENSG00000240616.1_RPS6P25	ENSG00000160789.18_LMNA	-0.23086	-0.376230046	-0.07439008	4.22E-03	3.45E-02	1.00E+00
ENSG00000211897.6_IGHG3	ENSG00000160789.18_LMNA	-0.01397	-0.172785232	0.145555618	8.64E-01	9.47E-01	1.00E+00
ENSG00000184260.5_HIST2H2AC	ENSG00000160789.18_LMNA	0.07456	-0.085655196	0.231020666	3.61E-01	6.43E-01	1.00E+00
ENSG00000188994.11_ZNF292	ENSG00000160789.18_LMNA	-0.12066	-0.274588674	0.039292793	1.39E-01	3.80E-01	1.00E+00
ENSG00000149308.15_NPAT	ENSG00000160789.18_LMNA	-0.0748	-0.231245493	0.085419419	3.60E-01	6.42E-01	1.00E+00
ENSG00000259802.1_CTD-2256P	ENSG00000160789.18_LMNA	-0.23265	-0.377851688	-0.07626974	3.92E-03	3.27E-02	1.00E+00
ENSG00000175073.7_VCPIP1	ENSG00000160789.18_LMNA	0.0538	-0.106311926	0.211191199	5.10E-01	7.59E-01	1.00E+00
ENSG00000257740.1_RP11-977G	ENSG00000160789.18_LMNA	-0.23006	-0.375506174	-0.07355173	4.35E-03	3.54E-02	1.00E+00
ENSG00000144736.12_SHQ1	ENSG00000160789.18_LMNA	0.05504	-0.105081217	0.212379987	5.01E-01	7.53E-01	1.00E+00
ENSG00000256940.1_RP11-783K	ENSG00000160789.18_LMNA	0.26175	0.107013707	0.404115549	1.12E-03	1.27E-02	1.00E+00
ENSG00000139675.11_HNRNPA1	ENSG00000160789.18_LMNA	-0.03019	-0.18848644	0.129632078	7.12E-01	8.78E-01	1.00E+00

ENSG00000100577.17_GSTZ1	ENSG00000160789.18_LMNA	-0.01671	-0.175444519	0.142870226	8.38E-01	9.37E-01	1.00E+00
ENSG00000213713.3_PIGCP1	ENSG00000160789.18_LMNA	-0.19621	-0.344648597	-0.03820659	1.54E-02	8.95E-02	1.00E+00
ENSG00000123329.16_ARHGAP9	ENSG00000160789.18_LMNA	0.09051	-0.0696999	0.246158998	2.67E-01	5.52E-01	1.00E+00
ENSG00000198934.4_MAGEE1	ENSG00000160789.18_LMNA	-0.16015	-0.311407903	-0.00097074	4.87E-02	1.98E-01	1.00E+00
ENSG00000146090.14_RASGEF1	ENSG00000160789.18_LMNA	-0.41627	-0.539707397	-0.27531863	9.61E-08	6.99E-06	4.93E-03
ENSG00000132881.10_RSG1	ENSG00000160789.18_LMNA	0.0745	-0.085716656	0.230962056	3.62E-01	6.44E-01	1.00E+00
ENSG00000184428.11_TOP1MT	ENSG00000160789.18_LMNA	0.19425	0.036172273	0.342852184	1.65E-02	9.39E-02	1.00E+00
ENSG00000109762.14_SNX25	ENSG00000160789.18_LMNA	0.23746	0.081329778	0.382207992	3.22E-03	2.82E-02	1.00E+00
ENSG00000100767.14_PAPLN	ENSG00000160789.18_LMNA	0.20595	0.048329682	0.353554619	1.09E-02	6.97E-02	1.00E+00
ENSG00000267119.1_RPL10P15	ENSG00000160789.18_LMNA	-0.01013	-0.169061651	0.149307885	9.01E-01	9.62E-01	1.00E+00
ENSG00000151883.15_PARP8	ENSG00000160789.18_LMNA	-0.22872	-0.37429604	-0.07215117	4.59E-03	3.68E-02	1.00E+00
ENSG00000134504.11_KCTD1	ENSG00000160789.18_LMNA	-0.2217	-0.367911054	-0.06478112	6.05E-03	4.53E-02	1.00E+00
ENSG00000278974.1_RP11-756P	ENSG00000160789.18_LMNA	-0.03836	-0.196362301	0.121582417	6.39E-01	8.39E-01	1.00E+00
ENSG00000049759.15_NEDD4L	ENSG00000160789.18_LMNA	-0.13843	-0.291217148	0.021234134	8.90E-02	2.90E-01	1.00E+00
ENSG00000197647.10_ZNF433	ENSG00000160789.18_LMNA	-0.1525	-0.304311489	0.006868621	6.07E-02	2.27E-01	1.00E+00
ENSG00000272267.2_RP11-375N	ENSG00000160789.18_LMNA	0.18686	0.028506233	0.336062422	2.12E-02	1.12E-01	1.00E+00
ENSG00000174004.5_NRROS	ENSG00000160789.18_LMNA	0.03438	-0.125507178	0.192527397	6.74E-01	8.57E-01	1.00E+00
ENSG00000132740.7_IGHMBP2	ENSG00000160789.18_LMNA	0.21871	0.061656499	0.365195411	6.79E-03	4.92E-02	1.00E+00
ENSG00000128242.11_GAL3ST1	ENSG00000160789.18_LMNA	-0.32541	-0.46074001	-0.17528816	4.30E-05	9.49E-04	1.00E+00
ENSG00000225131.2_PSME2P2	ENSG00000160789.18_LMNA	0.15362	-0.005719504	0.305353867	5.88E-02	2.23E-01	1.00E+00
ENSG00000267106.4_ZNF561-AS	ENSG00000160789.18_LMNA	-0.32289	-0.458522264	-0.17256146	4.97E-05	1.07E-03	1.00E+00
ENSG00000164338.8_UTP15	ENSG00000160789.18_LMNA	0.15068	-0.008732648	0.302619018	6.39E-02	2.35E-01	1.00E+00
ENSG00000117425.12_PTCH2	ENSG00000160789.18_LMNA	0.16094	0.001783229	0.312141419	4.76E-02	1.95E-01	1.00E+00
ENSG00000130413.14_STK33	ENSG00000160789.18_LMNA	-0.12288	-0.276664136	0.037049968	1.32E-01	3.68E-01	1.00E+00
ENSG00000201302.1_SNORA65	ENSG00000160789.18_LMNA	-0.00329	-0.162405037	0.155993009	9.68E-01	9.87E-01	1.00E+00
ENSG00000120093.10_HOXB3	ENSG00000160789.18_LMNA	0.23788	0.081774715	0.382590409	3.17E-03	2.78E-02	1.00E+00
ENSG00000249042.4_CTD-2015H	ENSG00000160789.18_LMNA	-0.09029	-0.245959037	0.069911711	2.69E-01	5.53E-01	1.00E+00
ENSG00000170293.7_CMTM8	ENSG00000160789.18_LMNA	0.05631	-0.103819989	0.213597281	4.91E-01	7.47E-01	1.00E+00
ENSG00000163684.10_RPP14	ENSG00000160789.18_LMNA	-0.19376	-0.342399907	-0.0356605	1.68E-02	9.51E-02	1.00E+00
ENSG00000153391.14_INO80C	ENSG00000160789.18_LMNA	-0.29279	-0.431859828	-0.14011966	2.52E-04	3.92E-03	1.00E+00
ENSG00000167414.4_GNG8	ENSG00000160789.18_LMNA	-0.20118	-0.349197583	-0.04336938	1.29E-02	7.89E-02	1.00E+00
ENSG00000279332.1_RP11-799B	ENSG00000160789.18_LMNA	0.04893	-0.111136853	0.20652154	5.49E-01	7.86E-01	1.00E+00
ENSG00000138050.13_THUMPD2	ENSG00000160789.18_LMNA	-0.10168	-0.256729632	0.058462156	2.13E-01	4.86E-01	1.00E+00

ENSG00000168010.9_ATG16L2	ENSG00000160789.18_LMNA	-0.02684	-0.185249076	0.132928767	7.43E-01	8.92E-01	1.00E+00
ENSG00000248923.1_MTND5P11	ENSG00000160789.18_LMNA	-0.07501	-0.231448558	0.085206432	3.58E-01	6.41E-01	1.00E+00
ENSG00000230207.1_RPL4P5	ENSG00000160789.18_LMNA	-0.06197	-0.21901321	0.09819619	4.48E-01	7.14E-01	1.00E+00
ENSG00000180611.6_MB21D2	ENSG00000160789.18_LMNA	-0.31193	-0.448841549	-0.16071017	9.15E-05	1.76E-03	1.00E+00
ENSG00000104881.13_PPP1R13I	ENSG00000160789.18_LMNA	0.34956	0.201580427	0.481943271	1.01E-05	2.94E-04	5.20E-01
ENSG00000185274.10_WBSCR17	ENSG00000160789.18_LMNA	-0.07608	-0.232463714	0.084141244	3.52E-01	6.35E-01	1.00E+00
ENSG00000178947.8_SMIM10L2A	ENSG00000160789.18_LMNA	-0.19737	-0.345708429	-0.03940796	1.48E-02	8.71E-02	1.00E+00
ENSG00000144134.17_RABL2A	ENSG00000160789.18_LMNA	-0.13447	-0.287518932	0.025268134	9.86E-02	3.09E-01	1.00E+00
ENSG00000162415.6_ZSWIM5	ENSG00000160789.18_LMNA	-0.20904	-0.356382618	-0.05155732	9.75E-03	6.44E-02	1.00E+00
ENSG00000167302.8_TEPSIN	ENSG00000160789.18_LMNA	0.02265	-0.137045164	0.181197035	7.82E-01	9.10E-01	1.00E+00
ENSG00000277734.3_TRAC	ENSG00000160789.18_LMNA	0.26713	0.112720042	0.408937027	8.78E-04	1.05E-02	1.00E+00
ENSG00000238072.1_RP11-305M	ENSG00000160789.18_LMNA	-0.02577	-0.184212099	0.133983259	7.53E-01	8.97E-01	1.00E+00
ENSG00000184226.13_PCDH9	ENSG00000160789.18_LMNA	0.15247	-0.006900262	0.304282776	6.08E-02	2.28E-01	1.00E+00
ENSG00000086619.12_ERO1B	ENSG00000160789.18_LMNA	-0.27271	-0.413941863	-0.11866413	6.76E-04	8.54E-03	1.00E+00
ENSG00000150636.14_CCDC102	ENSG00000160789.18_LMNA	0.16375	0.004672248	0.314746626	4.38E-02	1.84E-01	1.00E+00
ENSG00000197261.10_C6orf141	ENSG00000160789.18_LMNA	0.37141	0.225542651	0.500985658	2.47E-06	9.36E-05	1.27E-01
ENSG00000225127.2_LINC00237	ENSG00000160789.18_LMNA	-0.41828	-0.541424703	-0.27755952	8.22E-08	6.09E-06	4.22E-03
ENSG00000176125.4_UFSP1	ENSG00000160789.18_LMNA	0.26303	0.108369127	0.405262287	1.06E-03	1.21E-02	1.00E+00
ENSG00000272970.1_RP11-329B	ENSG00000160789.18_LMNA	-0.09046	-0.246120505	0.069740676	2.68E-01	5.52E-01	1.00E+00
ENSG00000038219.11_BOD1L1	ENSG00000160789.18_LMNA	0.06104	-0.099124469	0.218120587	4.55E-01	7.20E-01	1.00E+00
ENSG00000139946.8_PELI2	ENSG00000160789.18_LMNA	-0.12824	-0.281692957	0.031602489	1.15E-01	3.39E-01	1.00E+00
ENSG00000280128.1_XXbac-BPG	ENSG00000160789.18_LMNA	-0.10878	-0.263418767	0.051309361	1.82E-01	4.46E-01	1.00E+00
ENSG00000224646.2_AC007387.1	ENSG00000160789.18_LMNA	-0.06318	-0.220168299	0.09699414	4.39E-01	7.07E-01	1.00E+00
ENSG00000136098.15_NEK3	ENSG00000160789.18_LMNA	-0.32722	-0.462335901	-0.17725302	3.88E-05	8.77E-04	1.00E+00
ENSG00000115827.12_DCAF17	ENSG00000160789.18_LMNA	-0.11616	-0.270363985	0.043848406	1.54E-01	4.05E-01	1.00E+00
ENSG00000145979.16_TBC1D7	ENSG00000160789.18_LMNA	-0.23198	-0.377252485	-0.07557495	4.03E-03	3.33E-02	1.00E+00
ENSG00000188559.12_RALGAPA	ENSG00000160789.18_LMNA	0.09718	-0.062996201	0.252473798	2.34E-01	5.11E-01	1.00E+00
ENSG00000196263.6_ZNF471	ENSG00000160789.18_LMNA	0.08723	-0.072988439	0.243051401	2.85E-01	5.71E-01	1.00E+00
ENSG00000180543.4_TSPYL5	ENSG00000160789.18_LMNA	-0.1325	-0.285676293	0.027274282	1.04E-01	3.19E-01	1.00E+00
ENSG00000169155.8_ZBTB43	ENSG00000160789.18_LMNA	0.20486	0.047203422	0.352566495	1.13E-02	7.17E-02	1.00E+00
ENSG00000125430.7_HS3ST3B1	ENSG00000160789.18_LMNA	0.31477	0.163772088	0.451349169	7.83E-05	1.55E-03	1.00E+00
ENSG00000151025.9_GPR158	ENSG00000160789.18_LMNA	-0.2969	-0.435516329	-0.14453199	2.04E-04	3.31E-03	1.00E+00
ENSG00000110628.12_SLC22A18	ENSG00000160789.18_LMNA	0.51591	0.388630517	0.623871585	1.03E-11	3.52E-09	5.29E-07

ENSG00000141376.19_BCAS3	ENSG00000160789.18_LMNA	-0.29545	-0.434224887	-0.14297227	2.20E-04	3.51E-03	1.00E+00
ENSG00000174599.5_TRAM1L1	ENSG00000160789.18_LMNA	-0.34106	-0.474497924	-0.19230186	1.71E-05	4.53E-04	8.77E-01
ENSG00000281896.1_RP11-274B	ENSG00000160789.18_LMNA	0.10113	-0.059017307	0.2562092	2.15E-01	4.89E-01	1.00E+00
ENSG00000230409.3_TCEA1P2	ENSG00000160789.18_LMNA	0.03321	-0.126663993	0.191395211	6.85E-01	8.63E-01	1.00E+00
ENSG00000240230.4_COX19	ENSG00000160789.18_LMNA	0.19781	0.039861989	0.346108759	1.46E-02	8.61E-02	1.00E+00
ENSG00000181418.7_DDN	ENSG00000160789.18_LMNA	-0.05553	-0.212848359	0.104596062	4.97E-01	7.51E-01	1.00E+00
ENSG00000078487.16_ZCWPW1	ENSG00000160789.18_LMNA	-0.00952	-0.168467902	0.149905361	9.07E-01	9.64E-01	1.00E+00
ENSG00000214300.7_SPDYE3	ENSG00000160789.18_LMNA	0.21112	0.053727788	0.358281209	9.03E-03	6.09E-02	1.00E+00
ENSG00000147364.15_FBXO25	ENSG00000160789.18_LMNA	-0.20773	-0.355188257	-0.0501934	1.02E-02	6.66E-02	1.00E+00
ENSG00000165152.7_TMEM246	ENSG00000160789.18_LMNA	-0.36081	-0.491763882	-0.21389655	4.96E-06	1.65E-04	2.55E-01
ENSG00000246263.2_UBR5-AS1	ENSG00000160789.18_LMNA	-0.25432	-0.397430319	-0.09913383	1.57E-03	1.62E-02	1.00E+00
ENSG00000139354.9_GAS2L3	ENSG00000160789.18_LMNA	-0.07952	-0.235733596	0.080705277	3.30E-01	6.16E-01	1.00E+00
ENSG00000183199.6_HSP90AB3	ENSG00000160789.18_LMNA	0.01368	-0.145842966	0.172500402	8.67E-01	9.48E-01	1.00E+00
ENSG00000236017.6_ASMTL-AS1	ENSG00000160789.18_LMNA	0.09661	-0.063564177	0.251939812	2.36E-01	5.15E-01	1.00E+00
ENSG00000180644.6_PRF1	ENSG00000160789.18_LMNA	0.25326	0.098010297	0.396474537	1.64E-03	1.69E-02	1.00E+00
ENSG00000134744.12_ZCCHC11	ENSG00000160789.18_LMNA	0.00882	-0.150587408	0.167789828	9.14E-01	9.67E-01	1.00E+00
ENSG00000144152.11_FBLN7	ENSG00000160789.18_LMNA	0.59579	0.482341227	0.689584911	5.64E-16	1.26E-12	2.90E-11
ENSG00000119927.12_GPAM	ENSG00000160789.18_LMNA	-0.09717	-0.252461296	0.063009501	2.34E-01	5.11E-01	1.00E+00
ENSG00000203666.11_EFCAB2	ENSG00000160789.18_LMNA	-0.14731	-0.299489248	0.012173977	7.01E-02	2.50E-01	1.00E+00
ENSG00000137992.13_DBT	ENSG00000160789.18_LMNA	-0.3142	-0.450852464	-0.16316515	8.08E-05	1.58E-03	1.00E+00
ENSG00000162630.5_B3GALT2	ENSG00000160789.18_LMNA	-0.41841	-0.541535438	-0.27770411	8.14E-08	6.05E-06	4.18E-03
ENSG00000138688.14_KIAA1109	ENSG00000160789.18_LMNA	-0.00872	-0.167683622	0.150694209	9.15E-01	9.68E-01	1.00E+00
ENSG00000143322.18_ABL2	ENSG00000160789.18_LMNA	0.32184	0.171424387	0.457596382	5.27E-05	1.12E-03	1.00E+00
ENSG00000198835.3_GJC2	ENSG00000160789.18_LMNA	-0.21598	-0.362705707	-0.05879706	7.53E-03	5.31E-02	1.00E+00
ENSG00000075223.12_SEMA3C	ENSG00000160789.18_LMNA	-0.02583	-0.184270709	0.133923678	7.52E-01	8.97E-01	1.00E+00
ENSG00000130810.18_PPAN	ENSG00000160789.18_LMNA	0.28637	0.133240998	0.426140283	3.48E-04	5.04E-03	1.00E+00
ENSG00000120868.12_APAF1	ENSG00000160789.18_LMNA	-0.18107	-0.330738418	-0.02252032	2.56E-02	1.28E-01	1.00E+00
ENSG00000160961.10_ZNF333	ENSG00000160789.18_LMNA	-0.16593	-0.316764462	-0.00691347	4.10E-02	1.76E-01	1.00E+00
ENSG00000117707.14_PROX1	ENSG00000160789.18_LMNA	0.04449	-0.115524625	0.202262369	5.86E-01	8.08E-01	1.00E+00
ENSG00000278126.1_RP11-454E	ENSG00000160789.18_LMNA	-0.4148	-0.538443946	-0.27367181	1.08E-07	7.68E-06	5.53E-03
ENSG00000168453.13_HR	ENSG00000160789.18_LMNA	-0.14712	-0.299312146	0.01236849	7.05E-02	2.51E-01	1.00E+00
ENSG00000234017.1_RP11-214N	ENSG00000160789.18_LMNA	-0.13963	-0.292332091	0.020015959	8.62E-02	2.85E-01	1.00E+00
ENSG00000223382.3_RP5-1125N	ENSG00000160789.18_LMNA	-0.22384	-0.369864292	-0.06703219	5.57E-03	4.25E-02	1.00E+00

ENSG00000198113.2_TOR4A	ENSG00000160789.18_LMNA	0.36832	0.22214094	0.49829852	3.03E-06	1.11E-04	1.56E-01
ENSG00000121104.6_FAM117A	ENSG00000160789.18_LMNA	0.15491	-0.004399479	0.30655036	5.67E-02	2.18E-01	1.00E+00
ENSG00000186889.8_TMEM17	ENSG00000160789.18_LMNA	-0.21916	-0.365600357	-0.06212205	6.67E-03	4.86E-02	1.00E+00
ENSG00000276026.1_RP4-545L1	ENSG00000160789.18_LMNA	-0.08034	-0.236515213	0.079882844	3.25E-01	6.12E-01	1.00E+00
ENSG00000105793.14_GTPBP10	ENSG00000160789.18_LMNA	-0.02368	-0.182196821	0.136030519	7.72E-01	9.06E-01	1.00E+00
ENSG00000237668.1_RPS15AP3	ENSG00000160789.18_LMNA	-0.15675	-0.308260805	0.002510553	5.38E-02	2.11E-01	1.00E+00
ENSG00000273253.2_RP3-402G1	ENSG00000160789.18_LMNA	-0.10271	-0.257692884	0.057434126	2.08E-01	4.80E-01	1.00E+00
ENSG00000109255.10_NMU	ENSG00000160789.18_LMNA	-0.22897	-0.374515957	-0.07240561	4.55E-03	3.65E-02	1.00E+00
ENSG00000132196.12_HSD17B7	ENSG00000160789.18_LMNA	-0.09643	-0.25176474	0.063750348	2.37E-01	5.16E-01	1.00E+00
ENSG00000181284.2_TMEM102	ENSG00000160789.18_LMNA	0.30945	0.158037942	0.446649356	1.05E-04	1.96E-03	1.00E+00
ENSG00000278107.1_RP11-162A	ENSG00000160789.18_LMNA	0.1887	0.030413834	0.337754965	1.99E-02	1.08E-01	1.00E+00
ENSG00000085840.11_ORC1	ENSG00000160789.18_LMNA	0.03723	-0.122698207	0.195273046	6.49E-01	8.44E-01	1.00E+00
ENSG00000274471.1_RP11-228M	ENSG00000160789.18_LMNA	-0.08399	-0.239977212	0.076234876	3.04E-01	5.90E-01	1.00E+00
ENSG00000230291.5_RP11-244J	ENSG00000160789.18_LMNA	-0.12284	-0.276632352	0.037084338	1.32E-01	3.68E-01	1.00E+00
ENSG00000182923.15_CEP63	ENSG00000160789.18_LMNA	-0.17254	-0.322869905	-0.01371383	3.35E-02	1.54E-01	1.00E+00
ENSG00000223745.6_CCDC18-AS	ENSG00000160789.18_LMNA	-0.10019	-0.255317454	0.059968093	2.19E-01	4.94E-01	1.00E+00
ENSG00000182109.6_RP11-69E1	ENSG00000160789.18_LMNA	0.08675	-0.073465461	0.242600086	2.88E-01	5.73E-01	1.00E+00
ENSG00000085999.10_RAD54L	ENSG00000160789.18_LMNA	0.09472	-0.06547194	0.250144809	2.46E-01	5.25E-01	1.00E+00
ENSG00000103316.9_CRYM	ENSG00000160789.18_LMNA	-0.20477	-0.352478386	-0.04710303	1.14E-02	7.18E-02	1.00E+00
ENSG00000099849.13_RASSF7	ENSG00000160789.18_LMNA	0.28661	0.133500839	0.426356764	3.44E-04	4.99E-03	1.00E+00
ENSG00000170579.13_DLGAP1	ENSG00000160789.18_LMNA	-0.39158	-0.518464239	-0.24783434	6.08E-07	3.06E-05	3.12E-02
ENSG00000269176.2_RP11-727F	ENSG00000160789.18_LMNA	-0.05311	-0.210533006	0.10699291	5.16E-01	7.63E-01	1.00E+00
ENSG00000078269.12_SYNJ2	ENSG00000160789.18_LMNA	0.23794	0.08184269	0.382648824	3.16E-03	2.78E-02	1.00E+00
ENSG00000124216.3_SNAI1	ENSG00000160789.18_LMNA	0.30705	0.155448021	0.444521373	1.19E-04	2.18E-03	1.00E+00
ENSG00000265735.2_RN7SL5P	ENSG00000160789.18_LMNA	-0.00228	-0.161422707	0.156977083	9.78E-01	9.92E-01	1.00E+00
ENSG00000139974.14_SLC38A6	ENSG00000160789.18_LMNA	0.10643	-0.053679525	0.261205589	1.92E-01	4.60E-01	1.00E+00
ENSG00000164674.14_SYTL3	ENSG00000160789.18_LMNA	-0.0073	-0.166304233	0.152080648	9.29E-01	9.73E-01	1.00E+00
ENSG00000132825.6_PPP1R3D	ENSG00000160789.18_LMNA	0.2177	0.06060289	0.36427854	7.05E-03	5.07E-02	1.00E+00
ENSG00000270558.1_CTD-2124B	ENSG00000160789.18_LMNA	-0.06729	-0.224089889	0.0929062	4.10E-01	6.84E-01	1.00E+00
ENSG00000267049.1_AC002398.	ENSG00000160789.18_LMNA	0.24176	0.085864069	0.386100335	2.69E-03	2.46E-02	1.00E+00
ENSG00000220785.6_MTMR9LP	ENSG00000160789.18_LMNA	-0.14964	-0.301658076	0.009790035	6.58E-02	2.39E-01	1.00E+00
ENSG00000139437.16_TCHP	ENSG00000160789.18_LMNA	-0.011	-0.169902736	0.148461121	8.93E-01	9.58E-01	1.00E+00
ENSG00000167964.11_RAB26	ENSG00000160789.18_LMNA	-0.31659	-0.452963979	-0.16574678	7.08E-05	1.42E-03	1.00E+00

ENSG00000033030.12_ZCCHC8	ENSG00000160789.18_LMNA	0.11216	-0.047897969	0.266598401	1.69E-01	4.27E-01	1.00E+00
ENSG00000166501.11_PRKCB	ENSG00000160789.18_LMNA	-0.1665	-0.317293598	-0.0075017	4.03E-02	1.74E-01	1.00E+00
ENSG00000113763.9_UNC5A	ENSG00000160789.18_LMNA	-0.19126	-0.340102629	-0.03306351	1.83E-02	1.01E-01	1.00E+00
ENSG00000023171.13_GRAMD1E	ENSG00000160789.18_LMNA	-0.18864	-0.337702959	-0.03035519	1.99E-02	1.08E-01	1.00E+00
ENSG00000184730.9_APOBR	ENSG00000160789.18_LMNA	0.27105	0.116892557	0.412452092	7.31E-04	9.06E-03	1.00E+00
ENSG00000137463.4_MGARP	ENSG00000160789.18_LMNA	0.10984	-0.050236701	0.264419292	1.78E-01	4.40E-01	1.00E+00
ENSG00000139263.10_LRIG3	ENSG00000160789.18_LMNA	0.08257	-0.077651597	0.238633669	3.12E-01	5.98E-01	1.00E+00
ENSG00000104218.12_CSPP1	ENSG00000160789.18_LMNA	-0.00947	-0.168412962	0.149960634	9.08E-01	9.65E-01	1.00E+00
ENSG00000133247.12_KMT5C	ENSG00000160789.18_LMNA	0.09474	-0.065446113	0.250169124	2.46E-01	5.25E-01	1.00E+00
ENSG00000267023.4_LRRC37A1	ENSG00000160789.18_LMNA	0.00899	-0.150429056	0.167947285	9.13E-01	9.67E-01	1.00E+00
ENSG00000187534.6_PRR13P5	ENSG00000160789.18_LMNA	-0.02605	-0.184483164	0.133707685	7.50E-01	8.96E-01	1.00E+00
ENSG00000128284.18_APOL3	ENSG00000160789.18_LMNA	0.12051	-0.039451086	0.274442083	1.39E-01	3.80E-01	1.00E+00
ENSG00000107560.9_RAB11FIP2	ENSG00000160789.18_LMNA	-0.17703	-0.327017985	-0.01835042	2.91E-02	1.40E-01	1.00E+00
ENSG00000162444.11_RBP7	ENSG00000160789.18_LMNA	-0.23546	-0.380396938	-0.07922427	3.50E-03	3.00E-02	1.00E+00
ENSG00000185344.12_ATP6V0A2	ENSG00000160789.18_LMNA	-0.14148	-0.294059925	0.018126311	8.21E-02	2.77E-01	1.00E+00
ENSG00000090539.14_CHRD	ENSG00000160789.18_LMNA	0.03488	-0.125010888	0.193012863	6.70E-01	8.55E-01	1.00E+00
ENSG00000232434.2_C9orf172	ENSG00000160789.18_LMNA	0.19078	0.032568288	0.339664144	1.86E-02	1.02E-01	1.00E+00
ENSG00000166016.5_ABTB2	ENSG00000160789.18_LMNA	0.1947	0.036639779	0.343265217	1.62E-02	9.29E-02	1.00E+00
ENSG00000270028.1_RP11-380L	ENSG00000160789.18_LMNA	0.04272	-0.117278016	0.200556999	6.01E-01	8.17E-01	1.00E+00
ENSG00000113240.11_CLK4	ENSG00000160789.18_LMNA	-0.20088	-0.348919988	-0.04305386	1.31E-02	7.95E-02	1.00E+00
ENSG00000166147.12_FBN1	ENSG00000160789.18_LMNA	0.37371	0.22807339	0.502981344	2.11E-06	8.31E-05	1.08E-01
ENSG00000003147.16_ICA1	ENSG00000160789.18_LMNA	-0.43273	-0.553781026	-0.29376819	2.58E-08	2.40E-06	1.32E-03
ENSG00000166024.12_R3HCC1L	ENSG00000160789.18_LMNA	-0.08948	-0.245184787	0.070731574	2.73E-01	5.57E-01	1.00E+00
ENSG00000145194.16_ECE2	ENSG00000160789.18_LMNA	-0.09633	-0.251669582	0.06385153	2.38E-01	5.16E-01	1.00E+00
ENSG00000103460.15_TOX3	ENSG00000160789.18_LMNA	-0.21937	-0.365798516	-0.06234992	6.62E-03	4.83E-02	1.00E+00
ENSG00000130962.16_PRRG1	ENSG00000160789.18_LMNA	-0.14574	-0.298029441	0.013776583	7.32E-02	2.57E-01	1.00E+00
ENSG00000163029.14_SMC6	ENSG00000160789.18_LMNA	0.14733	-0.01215508	0.299506453	7.01E-02	2.50E-01	1.00E+00
ENSG00000113441.14_LNPEP	ENSG00000160789.18_LMNA	0.17328	0.014473738	0.323550563	3.28E-02	1.52E-01	1.00E+00
ENSG00000186792.14_HYAL3	ENSG00000160789.18_LMNA	0.05723	-0.102905248	0.214479541	4.84E-01	7.41E-01	1.00E+00
ENSG00000267896.1_	ENSG00000160789.18_LMNA	0.04944	-0.110635318	0.207007611	5.45E-01	7.83E-01	1.00E+00
ENSG00000005379.14_TSPOAP1	ENSG00000160789.18_LMNA	-0.23916	-0.383746442	-0.08312047	3.00E-03	2.68E-02	1.00E+00
ENSG00000167799.8_NUDT8	ENSG00000160789.18_LMNA	0.2433	0.087489005	0.387492622	2.52E-03	2.34E-02	1.00E+00
ENSG00000167196.12_FBXO22	ENSG00000160789.18_LMNA	0.17398	0.01519839	0.324199345	3.21E-02	1.50E-01	1.00E+00

ENSG00000011114.13_BTBD7	ENSG00000160789.18_LMNA	0.25002	0.094581399	0.393553587	1.89E-03	1.88E-02	1.00E+00
ENSG00000176714.9_CCDC121	ENSG00000160789.18_LMNA	-0.32631	-0.461535718	-0.17626755	4.08E-05	9.17E-04	1.00E+00
ENSG00000186017.13_ZNF566	ENSG00000160789.18_LMNA	-0.27681	-0.417609057	-0.12303301	5.56E-04	7.32E-03	1.00E+00
ENSG00000254719.1_RP11-351I2	ENSG00000160789.18_LMNA	-0.03225	-0.190477509	0.127601024	6.93E-01	8.68E-01	1.00E+00
ENSG00000185973.9_TMLHE	ENSG00000160789.18_LMNA	-0.25527	-0.398287848	-0.10014251	1.50E-03	1.57E-02	1.00E+00
ENSG00000251867.3_RP11-48B3	ENSG00000160789.18_LMNA	-0.21538	-0.362165277	-0.05817704	7.70E-03	5.40E-02	1.00E+00
ENSG00000204428.11_LY6G5C	ENSG00000160789.18_LMNA	-0.19309	-0.341786383	-0.03496653	1.72E-02	9.67E-02	1.00E+00
ENSG00000117091.8_CD48	ENSG00000160789.18_LMNA	-0.05707	-0.21432076	0.103069915	4.85E-01	7.42E-01	1.00E+00
ENSG00000224094.1_RPS24P8	ENSG00000160789.18_LMNA	-0.10504	-0.25989889	0.055077249	1.98E-01	4.67E-01	1.00E+00
ENSG00000183826.15_BTBD9	ENSG00000160789.18_LMNA	0.08667	-0.073547468	0.242522485	2.88E-01	5.74E-01	1.00E+00
ENSG00000100058.11_CRYBB2P	ENSG00000160789.18_LMNA	0.00492	-0.154398035	0.163995846	9.52E-01	9.81E-01	1.00E+00
ENSG00000171045.13_TSNARE1	ENSG00000160789.18_LMNA	-0.08052	-0.236684866	0.079704275	3.24E-01	6.11E-01	1.00E+00
ENSG00000171786.5_NHLH1	ENSG00000160789.18_LMNA	-0.25147	-0.394864156	-0.096119	1.78E-03	1.79E-02	1.00E+00
ENSG00000197927.11_C2orf27A	ENSG00000160789.18_LMNA	-0.42068	-0.543484632	-0.28025128	6.80E-08	5.29E-06	3.49E-03
ENSG00000177614.8_PGBD5	ENSG00000160789.18_LMNA	-0.17923	-0.329037577	-0.02061267	2.72E-02	1.34E-01	1.00E+00
ENSG00000124406.15_ATP8A1	ENSG00000160789.18_LMNA	-0.5098	-0.618778105	-0.38156464	1.97E-11	5.95E-09	1.01E-06
ENSG00000168014.15_C2CD3	ENSG00000160789.18_LMNA	0.09647	-0.063706052	0.251806397	2.37E-01	5.16E-01	1.00E+00
ENSG00000277072.3_STAG3L2	ENSG00000160789.18_LMNA	-0.06459	-0.221511455	0.095595212	4.29E-01	6.99E-01	1.00E+00
ENSG00000278535.3_DHRS11	ENSG00000160789.18_LMNA	0.11982	-0.040142423	0.27380168	1.41E-01	3.84E-01	1.00E+00
ENSG00000185250.14_PPIL6	ENSG00000160789.18_LMNA	-0.00366	-0.162769664	0.155627574	9.64E-01	9.86E-01	1.00E+00
ENSG00000063761.14_ADCK1	ENSG00000160789.18_LMNA	-0.03471	-0.192842192	0.125185382	6.71E-01	8.56E-01	1.00E+00
ENSG00000183530.12_PRR14L	ENSG00000160789.18_LMNA	0.01247	-0.147022783	0.171330365	8.79E-01	9.53E-01	1.00E+00
ENSG00000230424.1_RP1-43E13	ENSG00000160789.18_LMNA	-0.17097	-0.321418524	-0.01209466	3.52E-02	1.59E-01	1.00E+00
ENSG00000147509.12_RGS20	ENSG00000160789.18_LMNA	0.1038	-0.056328899	0.258727771	2.03E-01	4.74E-01	1.00E+00
ENSG00000268357.1_VN1R81P	ENSG00000160789.18_LMNA	-0.24471	-0.388763666	-0.08897383	2.38E-03	2.23E-02	1.00E+00
ENSG00000270547.4_LINC01235	ENSG00000160789.18_LMNA	-0.38258	-0.510672667	-0.23786166	1.15E-06	5.16E-05	5.90E-02
ENSG00000204634.11_TBC1D8	ENSG00000160789.18_LMNA	0.07696	-0.083256931	0.233305966	3.46E-01	6.30E-01	1.00E+00
ENSG00000269378.1_ITGB1P1	ENSG00000160789.18_LMNA	0.27799	0.124291904	0.418663979	5.25E-04	7.01E-03	1.00E+00
ENSG00000274565.1_CTD-3035K	ENSG00000160789.18_LMNA	0.43733	0.298944345	0.557702979	1.76E-08	1.73E-06	9.05E-04
ENSG00000129566.11_TEP1	ENSG00000160789.18_LMNA	0.23185	0.075430024	0.377127467	4.05E-03	3.35E-02	1.00E+00
ENSG00000047634.13_SCML1	ENSG00000160789.18_LMNA	0.14051	-0.019121645	0.293150078	8.42E-02	2.81E-01	1.00E+00
ENSG00000080345.16_RIF1	ENSG00000160789.18_LMNA	0.08406	-0.076160825	0.240047405	3.03E-01	5.90E-01	1.00E+00
ENSG00000230074.1_RP11-195F	ENSG00000160789.18_LMNA	0.02506	-0.134678077	0.183528436	7.59E-01	9.00E-01	1.00E+00

ENSG00000260095.1_RP11-715J:	ENSG00000160789.18_LMNA	0.07851	-0.08171162	0.234776638	3.36E-01	6.22E-01	1.00E+00
ENSG00000241061.3_RPL5P1	ENSG00000160789.18_LMNA	-0.18649	-0.335727101	-0.02812857	2.14E-02	1.13E-01	1.00E+00
ENSG00000231684.3_EIF1P3	ENSG00000160789.18_LMNA	0.14719	-0.012298983	0.299375434	7.04E-02	2.50E-01	1.00E+00
ENSG00000155636.13_RBM45	ENSG00000160789.18_LMNA	-0.15946	-0.310766828	-0.00026098	4.97E-02	2.01E-01	1.00E+00
ENSG00000133246.10_PRAM1	ENSG00000160789.18_LMNA	-0.02712	-0.185524107	0.132648969	7.40E-01	8.91E-01	1.00E+00
ENSG00000197056.8_ZMYM1	ENSG00000160789.18_LMNA	-0.05982	-0.216956342	0.100334391	4.64E-01	7.26E-01	1.00E+00
ENSG00000171004.16_HS6ST2	ENSG00000160789.18_LMNA	-0.12026	-0.274213701	0.039697667	1.40E-01	3.82E-01	1.00E+00
ENSG00000110801.12_PSMD9	ENSG00000160789.18_LMNA	0.04853	-0.111528844	0.206141528	5.53E-01	7.88E-01	1.00E+00
ENSG00000143479.14_DYRK3	ENSG00000160789.18_LMNA	0.38986	0.245923687	0.516974932	6.88E-07	3.40E-05	3.53E-02
ENSG00000165392.8_WRN	ENSG00000160789.18_LMNA	0.05136	-0.10873435	0.208848539	5.30E-01	7.73E-01	1.00E+00
ENSG00000198003.10_CCDC151	ENSG00000160789.18_LMNA	0.23631	0.080128127	0.381174673	3.38E-03	2.92E-02	1.00E+00
ENSG00000132801.6_ZSWIM3	ENSG00000160789.18_LMNA	-0.22024	-0.366586283	-0.06325609	6.40E-03	4.72E-02	1.00E+00
ENSG00000175105.5_ZNF654	ENSG00000160789.18_LMNA	0.14313	-0.016448496	0.295592358	7.86E-02	2.69E-01	1.00E+00
ENSG00000105327.14_BBC3	ENSG00000160789.18_LMNA	0.11348	-0.046566009	0.267838017	1.64E-01	4.20E-01	1.00E+00
ENSG00000134363.10_FST	ENSG00000160789.18_LMNA	0.06951	-0.090697852	0.226204111	3.95E-01	6.72E-01	1.00E+00
ENSG00000259891.1_CTA-204B4	ENSG00000160789.18_LMNA	-0.03598	-0.194072354	0.123927218	6.60E-01	8.50E-01	1.00E+00
ENSG00000167625.9_ZNF526	ENSG00000160789.18_LMNA	0.31935	0.168726539	0.455397123	6.07E-05	1.25E-03	1.00E+00
ENSG00000273456.1_RP11-686O	ENSG00000160789.18_LMNA	-0.27137	-0.412735302	-0.11722919	7.20E-04	8.96E-03	1.00E+00
ENSG00000165757.8_KIAA1462	ENSG00000160789.18_LMNA	-0.124	-0.277713789	0.035914466	1.28E-01	3.62E-01	1.00E+00
ENSG00000187902.10_SHISA7	ENSG00000160789.18_LMNA	-0.30838	-0.44570041	-0.15688251	1.11E-04	2.05E-03	1.00E+00
ENSG00000173083.13_HPSE	ENSG00000160789.18_LMNA	-0.03659	-0.194657386	0.123328509	6.54E-01	8.47E-01	1.00E+00
ENSG00000267904.1_CTC-429P9	ENSG00000160789.18_LMNA	-0.13385	-0.286939377	0.025899389	1.00E-01	3.12E-01	1.00E+00
ENSG00000224945.1_RP11-82L1	ENSG00000160789.18_LMNA	-0.02018	-0.178806939	0.139468077	8.05E-01	9.22E-01	1.00E+00
ENSG00000118369.11_USP35	ENSG00000160789.18_LMNA	0.03678	-0.123143971	0.194837663	6.53E-01	8.46E-01	1.00E+00
ENSG00000162636.14_FAM102B	ENSG00000160789.18_LMNA	-0.16213	-0.31324617	-0.0030077	4.60E-02	1.90E-01	1.00E+00
ENSG00000083812.10_ZNF324	ENSG00000160789.18_LMNA	0.20925	0.051769416	0.356568258	9.68E-03	6.41E-02	1.00E+00
ENSG00000121210.14_KIAA0922	ENSG00000160789.18_LMNA	0.01526	-0.144293529	0.174035631	8.52E-01	9.42E-01	1.00E+00
ENSG00000225616.2_RP4-604A2	ENSG00000160789.18_LMNA	-0.13789	-0.290706397	0.021791865	9.03E-02	2.93E-01	1.00E+00
ENSG00000147231.12_CXorf57	ENSG00000160789.18_LMNA	-0.17911	-0.328929886	-0.02049196	2.73E-02	1.34E-01	1.00E+00
ENSG00000092439.12_TRPM7	ENSG00000160789.18_LMNA	0.15913	-7.48E-05	0.310463425	5.02E-02	2.02E-01	1.00E+00
ENSG00000135636.12_DYSF	ENSG00000160789.18_LMNA	0.08756	-0.072652419	0.243369231	2.83E-01	5.69E-01	1.00E+00
ENSG00000204060.5_FOXO6	ENSG00000160789.18_LMNA	-0.1637	-0.314701751	-0.00462244	4.39E-02	1.85E-01	1.00E+00
ENSG00000251333.3_RTN3P1	ENSG00000160789.18_LMNA	-0.22419	-0.370182325	-0.06739901	5.49E-03	4.20E-02	1.00E+00

ENSG00000069974.14_RAB27A	ENSG00000160789.18_LMNA	0.41142	0.26990013	0.535545806	1.40E-07	9.51E-06	7.17E-03
ENSG00000073756.10_PTGS2	ENSG00000160789.18_LMNA	0.15909	-0.000111127	0.310430623	5.03E-02	2.02E-01	1.00E+00
ENSG00000072121.14_ZFYVE26	ENSG00000160789.18_LMNA	0.24223	0.086361074	0.386526327	2.64E-03	2.42E-02	1.00E+00
ENSG00000154889.15_MPPE1	ENSG00000160789.18_LMNA	-0.03593	-0.194017879	0.123982955	6.60E-01	8.50E-01	1.00E+00
ENSG00000100314.3_CABP7	ENSG00000160789.18_LMNA	-0.17933	-0.329135449	-0.02072238	2.71E-02	1.34E-01	1.00E+00
ENSG00000144554.9_FANCD2	ENSG00000160789.18_LMNA	-0.119	-0.273030997	0.040974005	1.44E-01	3.89E-01	1.00E+00
ENSG00000241547.1_ACTG1P20	ENSG00000160789.18_LMNA	0.1901	0.031867382	0.33904331	1.90E-02	1.04E-01	1.00E+00
ENSG00000099992.14_TBC1D10A	ENSG00000160789.18_LMNA	-0.05182	-0.209296672	0.108271251	5.26E-01	7.71E-01	1.00E+00
ENSG00000183621.14_ZNF438	ENSG00000160789.18_LMNA	-0.10538	-0.260214155	0.054740135	1.96E-01	4.66E-01	1.00E+00
ENSG00000104951.14_IL4I1	ENSG00000160789.18_LMNA	0.11917	-0.040804671	0.273187963	1.44E-01	3.88E-01	1.00E+00
ENSG00000007545.14_CRAMP1	ENSG00000160789.18_LMNA	0.05966	-0.100498606	0.216798257	4.65E-01	7.27E-01	1.00E+00
ENSG00000100815.11_TRIP11	ENSG00000160789.18_LMNA	0.22918	0.072633939	0.374713283	4.51E-03	3.63E-02	1.00E+00
ENSG00000010072.14_SPRTN	ENSG00000160789.18_LMNA	-0.24524	-0.389245592	-0.08953717	2.33E-03	2.19E-02	1.00E+00
ENSG00000051596.8_THOC3	ENSG00000160789.18_LMNA	0.01687	-0.142714133	0.175598952	8.37E-01	9.37E-01	1.00E+00
ENSG00000172375.11_C2CD2L	ENSG00000160789.18_LMNA	-0.07988	-0.236082528	0.080338177	3.28E-01	6.14E-01	1.00E+00
ENSG00000029153.13_ARNTL2	ENSG00000160789.18_LMNA	0.40977	0.268056309	0.534126741	1.59E-07	1.05E-05	8.13E-03
ENSG00000197128.10_ZNF772	ENSG00000160789.18_LMNA	-0.11907	-0.273093892	0.040906156	1.44E-01	3.88E-01	1.00E+00
ENSG00000196470.10_SIAH1	ENSG00000160789.18_LMNA	-0.19699	-0.345363917	-0.03901734	1.50E-02	8.79E-02	1.00E+00
ENSG00000244119.1_PDCL3P4	ENSG00000160789.18_LMNA	-0.04884	-0.206436951	0.111224117	5.50E-01	7.87E-01	1.00E+00
ENSG00000147481.12_SNTG1	ENSG00000160789.18_LMNA	-0.17958	-0.32936454	-0.02097921	2.68E-02	1.33E-01	1.00E+00
ENSG00000254860.4_TMEM9B-A	ENSG00000160789.18_LMNA	0.16823	0.009281975	0.318893851	3.83E-02	1.68E-01	1.00E+00
ENSG00000126217.19_MCF2L	ENSG00000160789.18_LMNA	-0.28328	-0.423387809	-0.12994074	4.06E-04	5.72E-03	1.00E+00
ENSG00000104213.11_PDGFRL	ENSG00000160789.18_LMNA	0.14765	-0.01182977	0.299802595	6.95E-02	2.48E-01	1.00E+00
ENSG00000204815.7_TTC25	ENSG00000160789.18_LMNA	0.13171	-0.028084493	0.284931462	1.06E-01	3.23E-01	1.00E+00
ENSG00000101898.5_MCTS2P	ENSG00000160789.18_LMNA	-0.13365	-0.2867474	0.026108436	1.01E-01	3.13E-01	1.00E+00
ENSG00000185686.16_PRAME	ENSG00000160789.18_LMNA	-0.03514	-0.193261593	0.124756549	6.67E-01	8.53E-01	1.00E+00
ENSG00000182968.4_SOX1	ENSG00000160789.18_LMNA	-0.30555	-0.443193239	-0.15383359	1.29E-04	2.32E-03	1.00E+00
ENSG00000154370.12_TRIM11	ENSG00000160789.18_LMNA	0.09409	-0.066102658	0.249550892	2.49E-01	5.29E-01	1.00E+00
ENSG00000238058.1_RP11-432J2	ENSG00000160789.18_LMNA	0.11022	-0.049855268	0.26477491	1.76E-01	4.38E-01	1.00E+00
ENSG00000160949.15_TONSL	ENSG00000160789.18_LMNA	0.17972	0.021128394	0.32949759	2.67E-02	1.33E-01	1.00E+00
ENSG00000168228.13_ZCCHC4	ENSG00000160789.18_LMNA	-0.09959	-0.254754704	0.060567806	2.22E-01	4.98E-01	1.00E+00
ENSG00000253293.4_HOXA10	ENSG00000160789.18_LMNA	-0.26461	-0.406679777	-0.1100461	9.87E-04	1.15E-02	1.00E+00
ENSG00000188803.13_SHISA6	ENSG00000160789.18_LMNA	0.00397	-0.155328595	0.163067917	9.61E-01	9.85E-01	1.00E+00

ENSG00000147852.14_VLDLR	ENSG00000160789.18_LMNA	0.16775	0.008783833	0.318446259	3.89E-02	1.70E-01	1.00E+00
ENSG00000133114.16_GPALPP1	ENSG00000160789.18_LMNA	-0.32738	-0.462476216	-0.17742588	3.84E-05	8.70E-04	1.00E+00
ENSG00000123607.13_TTC21B	ENSG00000160789.18_LMNA	-0.15254	-0.304348055	0.006828324	6.06E-02	2.27E-01	1.00E+00
ENSG00000261159.1_RP11-723C	ENSG00000160789.18_LMNA	-0.22552	-0.371389629	-0.06879227	5.21E-03	4.05E-02	1.00E+00
ENSG00000085491.14_SLC25A24	ENSG00000160789.18_LMNA	0.28247	0.129075074	0.422664933	4.22E-04	5.92E-03	1.00E+00
ENSG00000120334.14_CENPL	ENSG00000160789.18_LMNA	-0.06299	-0.219988134	0.09718169	4.41E-01	7.08E-01	1.00E+00
ENSG00000141682.11_PMAIP1	ENSG00000160789.18_LMNA	0.04626	-0.113778021	0.203959223	5.71E-01	8.00E-01	1.00E+00
ENSG00000163596.15_ICA1L	ENSG00000160789.18_LMNA	-0.21631	-0.363008276	-0.0591443	7.44E-03	5.26E-02	1.00E+00
ENSG00000265688.1_MAFG-AS1	ENSG00000160789.18_LMNA	0.23382	0.077505049	0.37891643	3.74E-03	3.16E-02	1.00E+00
ENSG00000177707.9_NECTIN3	ENSG00000160789.18_LMNA	0.02082	-0.138839175	0.179427684	7.99E-01	9.19E-01	1.00E+00
ENSG00000166839.15_ANKDD1A	ENSG00000160789.18_LMNA	0.15804	-0.00119204	0.309453546	5.18E-02	2.06E-01	1.00E+00
ENSG00000254941.1_RP11-677M	ENSG00000160789.18_LMNA	0.02491	-0.134825222	0.183383613	7.61E-01	9.00E-01	1.00E+00
ENSG00000165644.9_COMTD1	ENSG00000160789.18_LMNA	0.21996	0.06296861	0.366336412	6.47E-03	4.76E-02	1.00E+00
ENSG00000162341.14_TPCN2	ENSG00000160789.18_LMNA	-0.03188	-0.190119217	0.127966708	6.97E-01	8.70E-01	1.00E+00
ENSG00000174485.13_DENND4A	ENSG00000160789.18_LMNA	-0.05412	-0.211498054	0.105994343	5.08E-01	7.58E-01	1.00E+00
ENSG00000213949.7_ITGA1	ENSG00000160789.18_LMNA	0.18739	0.029051756	0.336546647	2.08E-02	1.11E-01	1.00E+00
ENSG00000225265.1_TAF1A-AS1	ENSG00000160789.18_LMNA	-0.22601	-0.371834877	-0.06930639	5.11E-03	3.99E-02	1.00E+00
ENSG00000147316.11_MCPH1	ENSG00000160789.18_LMNA	-0.178	-0.327911928	-0.01935138	2.82E-02	1.37E-01	1.00E+00
ENSG00000145365.10_TIFA	ENSG00000160789.18_LMNA	-0.00569	-0.164739961	0.153651401	9.45E-01	9.78E-01	1.00E+00
ENSG00000231084.2_CTA-253N1	ENSG00000160789.18_LMNA	-0.04862	-0.206223911	0.111443871	5.52E-01	7.88E-01	1.00E+00
ENSG00000010704.17_HFE	ENSG00000160789.18_LMNA	0.27367	0.119680833	0.414796133	6.46E-04	8.25E-03	1.00E+00
ENSG00000221926.10_TRIM16	ENSG00000160789.18_LMNA	-0.07909	-0.235326387	0.081133579	3.33E-01	6.18E-01	1.00E+00
ENSG00000189308.9_LIN54	ENSG00000160789.18_LMNA	-0.02593	-0.184368934	0.133823821	7.51E-01	8.96E-01	1.00E+00
ENSG00000165633.11_VSTM4	ENSG00000160789.18_LMNA	0.1102	-0.049871834	0.264759468	1.77E-01	4.38E-01	1.00E+00
ENSG00000234618.1_RPSAP9	ENSG00000160789.18_LMNA	-0.07564	-0.232043718	0.084582028	3.54E-01	6.38E-01	1.00E+00
ENSG00000151320.9_AKAP6	ENSG00000160789.18_LMNA	-0.19205	-0.34082667	-0.03388156	1.78E-02	9.92E-02	1.00E+00
ENSG00000138101.17_DTNB	ENSG00000160789.18_LMNA	-0.15627	-0.307815476	0.003002565	5.45E-02	2.13E-01	1.00E+00
ENSG00000111300.8_NAA25	ENSG00000160789.18_LMNA	0.08571	-0.0745056	0.241615527	2.94E-01	5.80E-01	1.00E+00
ENSG00000138193.13_PLCE1	ENSG00000160789.18_LMNA	0.10931	-0.050774785	0.263917478	1.80E-01	4.43E-01	1.00E+00
ENSG00000163235.14_TGFA	ENSG00000160789.18_LMNA	-0.23512	-0.380094985	-0.07887349	3.55E-03	3.03E-02	1.00E+00
ENSG00000218208.1_RPS27AP1	ENSG00000160789.18_LMNA	-0.1287	-0.2821173	0.031141965	1.14E-01	3.37E-01	1.00E+00
ENSG00000197020.9_ZNF100	ENSG00000160789.18_LMNA	-0.37875	-0.507361498	-0.23364094	1.50E-06	6.42E-05	7.68E-02
ENSG00000197774.11_EME2	ENSG00000160789.18_LMNA	0.02615	-0.133606604	0.184582579	7.49E-01	8.95E-01	1.00E+00

ENSG00000248161.4_RP11-499E	ENSG00000160789.18_LMNA	-0.04975	-0.20730593	0.110327427	5.43E-01	7.82E-01	1.00E+00
ENSG00000161267.10_BDH1	ENSG00000160789.18_LMNA	0.18482	0.026392464	0.334184631	2.26E-02	1.18E-01	1.00E+00
ENSG00000099994.10_SUSD2	ENSG00000160789.18_LMNA	0.34995	0.202007553	0.482285035	9.89E-06	2.89E-04	5.08E-01
ENSG00000186076.5_RP11-887P	ENSG00000160789.18_LMNA	0.05626	-0.103872359	0.213546755	4.91E-01	7.47E-01	1.00E+00
ENSG00000179715.11_PCED1B	ENSG00000160789.18_LMNA	0.09868	-0.061484467	0.253894129	2.26E-01	5.03E-01	1.00E+00
ENSG00000143630.8_HCN3	ENSG00000160789.18_LMNA	-0.18254	-0.33208639	-0.02403379	2.44E-02	1.24E-01	1.00E+00
ENSG00000108506.10_INTS2	ENSG00000160789.18_LMNA	-0.12439	-0.278083375	0.035514461	1.27E-01	3.60E-01	1.00E+00
ENSG00000157106.15_SMG1	ENSG00000160789.18_LMNA	0.06472	-0.095468383	0.221633168	4.28E-01	6.99E-01	1.00E+00
ENSG00000157890.16_MEGF11	ENSG00000160789.18_LMNA	-0.29992	-0.438201613	-0.14777973	1.74E-04	2.93E-03	1.00E+00
ENSG00000173531.14_MST1	ENSG00000160789.18_LMNA	0.03681	-0.123114841	0.194866119	6.53E-01	8.46E-01	1.00E+00
ENSG00000104722.12_NEFM	ENSG00000160789.18_LMNA	-0.09142	-0.247025089	0.068782156	2.63E-01	5.46E-01	1.00E+00
ENSG00000121964.13_GTDC1	ENSG00000160789.18_LMNA	-0.36128	-0.492173515	-0.2144122	4.81E-06	1.61E-04	2.47E-01
ENSG00000275160.1_RP11-187C	ENSG00000160789.18_LMNA	-0.29139	-0.430610984	-0.13861532	2.70E-04	4.14E-03	1.00E+00
ENSG00000105516.9_DBP	ENSG00000160789.18_LMNA	-0.08085	-0.236999334	0.079373227	3.22E-01	6.09E-01	1.00E+00
ENSG00000122254.6_HS3ST2	ENSG00000160789.18_LMNA	-0.06853	-0.225270627	0.091673278	4.02E-01	6.78E-01	1.00E+00
ENSG00000035499.11_DEPDC1B	ENSG00000160789.18_LMNA	-0.11644	-0.270624093	0.043568302	1.53E-01	4.03E-01	1.00E+00
ENSG00000104731.12_KLHDC4	ENSG00000160789.18_LMNA	0.10899	-0.051101565	0.263612641	1.81E-01	4.45E-01	1.00E+00
ENSG00000168803.13_ADAL	ENSG00000160789.18_LMNA	-0.00382	-0.162919498	0.155477383	9.63E-01	9.85E-01	1.00E+00
ENSG00000069482.6_GAL	ENSG00000160789.18_LMNA	0.08793	-0.072284015	0.243717613	2.81E-01	5.67E-01	1.00E+00
ENSG00000177932.6_ZNF354C	ENSG00000160789.18_LMNA	-0.07504	-0.231472126	0.085181711	3.58E-01	6.41E-01	1.00E+00
ENSG00000136141.13_LRCH1	ENSG00000160789.18_LMNA	0.04197	-0.118022161	0.199832651	6.08E-01	8.21E-01	1.00E+00
ENSG00000185730.6_ZNF696	ENSG00000160789.18_LMNA	0.12717	-0.032688936	0.280691379	1.18E-01	3.45E-01	1.00E+00
ENSG00000115657.11_ABCB6	ENSG00000160789.18_LMNA	0.00908	-0.150333692	0.168042101	9.12E-01	9.66E-01	1.00E+00
ENSG00000125848.9_FLRT3	ENSG00000160789.18_LMNA	-0.11198	-0.266428929	0.048079979	1.70E-01	4.28E-01	1.00E+00
ENSG00000166257.7_SCN3B	ENSG00000160789.18_LMNA	-0.33283	-0.467269651	-0.18334183	2.79E-05	6.75E-04	1.00E+00
ENSG00000197951.7_ZNF71	ENSG00000160789.18_LMNA	0.08221	-0.078016653	0.238287273	3.14E-01	6.00E-01	1.00E+00
ENSG00000120616.14_EPC1	ENSG00000160789.18_LMNA	-0.29713	-0.435717265	-0.1447748	2.01E-04	3.28E-03	1.00E+00
ENSG00000102763.14_VWA8	ENSG00000160789.18_LMNA	-0.10806	-0.262736793	0.052040087	1.85E-01	4.51E-01	1.00E+00
ENSG00000203965.11_EFCAB7	ENSG00000160789.18_LMNA	-0.39384	-0.520414042	-0.25033896	5.17E-07	2.69E-05	2.65E-02
ENSG00000132326.10_PER2	ENSG00000160789.18_LMNA	-0.02729	-0.185688382	0.132481823	7.39E-01	8.90E-01	1.00E+00
ENSG00000167554.13_ZNF610	ENSG00000160789.18_LMNA	-0.12037	-0.274317457	0.039585647	1.40E-01	3.81E-01	1.00E+00
ENSG00000224307.1_RP11-344B	ENSG00000160789.18_LMNA	-0.0837	-0.239702303	0.076524863	3.05E-01	5.92E-01	1.00E+00
ENSG00000273156.1_RP11-127B	ENSG00000160789.18_LMNA	-0.29954	-0.437861668	-0.14736824	1.78E-04	2.97E-03	1.00E+00

ENSG00000266094.5_RASSF5	ENSG00000160789.18_LMNA	0.18203	0.023514185	0.331623744	2.48E-02	1.26E-01	1.00E+00
ENSG00000067606.14_PRKCZ	ENSG00000160789.18_LMNA	-0.38515	-0.51290239	-0.24070969	9.60E-07	4.44E-05	4.93E-02
ENSG00000104129.8_DNAJC17	ENSG00000160789.18_LMNA	0.26768	0.11330768	0.409432605	8.56E-04	1.03E-02	1.00E+00
ENSG00000115421.11_PAPOLG	ENSG00000160789.18_LMNA	-0.33729	-0.471192654	-0.18819886	2.14E-05	5.48E-04	1.00E+00
ENSG00000165525.16_NEMF	ENSG00000160789.18_LMNA	0.04381	-0.116199811	0.201605902	5.92E-01	8.11E-01	1.00E+00
ENSG00000116883.8_RP11-268J1	ENSG00000160789.18_LMNA	0.23992	0.083927205	0.384438992	2.91E-03	2.61E-02	1.00E+00
ENSG00000134532.14_SOX5	ENSG00000160789.18_LMNA	-0.18586	-0.335145665	-0.02747393	2.19E-02	1.15E-01	1.00E+00
ENSG00000255857.4_PXN-AS1	ENSG00000160789.18_LMNA	-0.28114	-0.421475446	-0.12765161	4.51E-04	6.22E-03	1.00E+00
ENSG00000223960.5_AC009948.5	ENSG00000160789.18_LMNA	-0.30654	-0.444068905	-0.15489784	1.23E-04	2.23E-03	1.00E+00
ENSG00000165194.13_PCDH19	ENSG00000160789.18_LMNA	0.15749	-0.001750407	0.308948561	5.26E-02	2.08E-01	1.00E+00
ENSG00000081181.6_ARG2	ENSG00000160789.18_LMNA	-0.21565	-0.362406489	-0.05845375	7.63E-03	5.36E-02	1.00E+00
ENSG00000156535.12_CD109	ENSG00000160789.18_LMNA	0.33598	0.186770364	0.470040023	2.32E-05	5.81E-04	1.00E+00
ENSG00000272654.1_RP11-422P	ENSG00000160789.18_LMNA	-0.2366	-0.381434567	-0.08043028	3.34E-03	2.90E-02	1.00E+00
ENSG00000148120.13_C9orf3	ENSG00000160789.18_LMNA	-0.02528	-0.183738722	0.134464392	7.57E-01	8.99E-01	1.00E+00
ENSG00000147118.9_ZNF182	ENSG00000160789.18_LMNA	0.00487	-0.154451645	0.163942403	9.53E-01	9.81E-01	1.00E+00
ENSG00000186352.7_ANKRD37	ENSG00000160789.18_LMNA	0.09956	-0.060598688	0.25472572	2.22E-01	4.98E-01	1.00E+00
ENSG00000227775.3_RP1-283E3	ENSG00000160789.18_LMNA	-0.0287	-0.187042854	0.131102987	7.26E-01	8.84E-01	1.00E+00
ENSG00000223722.3_RP11-467L	ENSG00000160789.18_LMNA	0.34499	0.196581188	0.477936784	1.35E-05	3.72E-04	6.90E-01
ENSG00000196220.14_SRGAP3	ENSG00000160789.18_LMNA	-0.28968	-0.429091278	-0.13678652	2.95E-04	4.44E-03	1.00E+00
ENSG00000158050.4_DUSP2	ENSG00000160789.18_LMNA	0.07777	-0.082448365	0.234075659	3.41E-01	6.26E-01	1.00E+00
ENSG00000133858.14_ZFC3H1	ENSG00000160789.18_LMNA	-0.06564	-0.222517896	0.094546159	4.22E-01	6.93E-01	1.00E+00
ENSG00000241556.1_RP11-490G	ENSG00000160789.18_LMNA	-0.1906	-0.339494734	-0.032377	1.87E-02	1.03E-01	1.00E+00
ENSG00000213707.2_HMGB1P10	ENSG00000160789.18_LMNA	-0.16623	-0.317040952	-0.00722081	4.07E-02	1.75E-01	1.00E+00
ENSG00000132953.15_XPO4	ENSG00000160789.18_LMNA	-0.03677	-0.194833976	0.123147746	6.53E-01	8.46E-01	1.00E+00
ENSG00000258427.3_RBM8B	ENSG00000160789.18_LMNA	0.18733	0.028995362	0.336496597	2.08E-02	1.11E-01	1.00E+00
ENSG00000277462.1_ZNF670	ENSG00000160789.18_LMNA	-0.34472	-0.477701167	-0.19628764	1.37E-05	3.78E-04	7.02E-01
ENSG00000245970.2_KB-1208A1	ENSG00000160789.18_LMNA	-0.2129	-0.359898627	-0.05557909	8.45E-03	5.80E-02	1.00E+00
ENSG00000143970.15_ASXL2	ENSG00000160789.18_LMNA	0.06791	-0.092287881	0.224682154	4.06E-01	6.81E-01	1.00E+00
ENSG00000197016.10_ZNF470	ENSG00000160789.18_LMNA	0.16514	0.006096864	0.316029573	4.20E-02	1.79E-01	1.00E+00
ENSG00000119979.14_FAM45A	ENSG00000160789.18_LMNA	-0.24049	-0.384948692	-0.08452119	2.84E-03	2.57E-02	1.00E+00
ENSG00000141441.14_GAREM1	ENSG00000160789.18_LMNA	-0.02741	-0.185804144	0.132364027	7.37E-01	8.90E-01	1.00E+00
ENSG00000267254.4_ZNF790-AS	ENSG00000160789.18_LMNA	-0.17956	-0.329345169	-0.0209575	2.69E-02	1.33E-01	1.00E+00
ENSG00000168995.12_SIGLEC7	ENSG00000160789.18_LMNA	0.01151	-0.14796611	0.170394212	8.88E-01	9.57E-01	1.00E+00

ENSG00000112425.12_EPM2A	ENSG00000160789.18_LMNA	-0.24463	-0.388692509	-0.08889067	2.39E-03	2.24E-02	1.00E+00
ENSG00000196793.12_ZNF239	ENSG00000160789.18_LMNA	-0.1812	-0.330860585	-0.02265743	2.55E-02	1.28E-01	1.00E+00
ENSG00000162601.8_MYSM1	ENSG00000160789.18_LMNA	0.00799	-0.151400114	0.166981462	9.22E-01	9.70E-01	1.00E+00
ENSG00000261824.5_LINC00662	ENSG00000160789.18_LMNA	-0.31746	-0.453729048	-0.16668316	6.74E-05	1.37E-03	1.00E+00
ENSG00000136826.13_KLF4	ENSG00000160789.18_LMNA	0.0515	-0.108592577	0.208985745	5.29E-01	7.73E-01	1.00E+00
ENSG00000235194.7_PPP1R3E	ENSG00000160789.18_LMNA	-0.08609	-0.241972338	0.074128726	2.92E-01	5.77E-01	1.00E+00
ENSG00000201772.1_SNORA5C	ENSG00000160789.18_LMNA	0.06929	-0.090918328	0.225993166	3.96E-01	6.73E-01	1.00E+00
ENSG00000161544.8_CYGB	ENSG00000160789.18_LMNA	0.17002	0.011116655	0.320541163	3.63E-02	1.62E-01	1.00E+00
ENSG00000158246.7_FAM46B	ENSG00000160789.18_LMNA	0.3034	0.151520944	0.441288495	1.45E-04	2.53E-03	1.00E+00
ENSG00000213087.3_RP11-613F	ENSG00000160789.18_LMNA	-0.14079	-0.293410345	0.018836987	8.36E-02	2.80E-01	1.00E+00
ENSG00000272602.4_ZNF595	ENSG00000160789.18_LMNA	-0.04498	-0.202732705	0.115040696	5.82E-01	8.06E-01	1.00E+00
ENSG00000162062.13_C16orf59	ENSG00000160789.18_LMNA	0.0207	-0.138963	0.179305486	8.00E-01	9.20E-01	1.00E+00
ENSG00000135519.6_KCNH3	ENSG00000160789.18_LMNA	-0.01312	-0.171962288	0.146385691	8.73E-01	9.51E-01	1.00E+00
ENSG00000163681.13_SLMAP	ENSG00000160789.18_LMNA	0.13841	-0.021258794	0.291194568	8.90E-02	2.90E-01	1.00E+00
ENSG00000238278.3_ALG1L6P	ENSG00000160789.18_LMNA	0.19945	0.041571761	0.34761532	1.38E-02	8.24E-02	1.00E+00
ENSG00000234645.3_YWHAEP5	ENSG00000160789.18_LMNA	-0.19566	-0.344140214	-0.03763062	1.57E-02	9.07E-02	1.00E+00
ENSG00000112137.15_PHACTR1	ENSG00000160789.18_LMNA	-0.29958	-0.437899358	-0.14741385	1.77E-04	2.97E-03	1.00E+00
ENSG00000086205.15_FOLH1	ENSG00000160789.18_LMNA	-0.25763	-0.400406006	-0.10263666	1.35E-03	1.46E-02	1.00E+00
ENSG00000151233.9_GXYLT1	ENSG00000160789.18_LMNA	0.21407	0.056806387	0.360969863	8.09E-03	5.61E-02	1.00E+00
ENSG00000241494.1_RP11-796G	ENSG00000160789.18_LMNA	-0.08696	-0.242796645	0.073257724	2.87E-01	5.72E-01	1.00E+00
ENSG00000172175.11_MALT1	ENSG00000160789.18_LMNA	0.12883	-0.031011657	0.282237349	1.14E-01	3.37E-01	1.00E+00
ENSG00000102967.10_DHODH	ENSG00000160789.18_LMNA	0.1617	0.002565219	0.312847049	4.66E-02	1.92E-01	1.00E+00
ENSG00000176884.13_GRIN1	ENSG00000160789.18_LMNA	-0.17208	-0.322446182	-0.01324096	3.40E-02	1.56E-01	1.00E+00
ENSG00000187824.7_TMEM220	ENSG00000160789.18_LMNA	-0.01631	-0.175057687	0.143261147	8.42E-01	9.38E-01	1.00E+00
ENSG00000130699.15_TAF4	ENSG00000160789.18_LMNA	0.12478	-0.035115601	0.278451809	1.26E-01	3.58E-01	1.00E+00
ENSG00000141338.12_ABCA8	ENSG00000160789.18_LMNA	0.00715	-0.152228392	0.166157167	9.30E-01	9.73E-01	1.00E+00
ENSG00000198087.7_CD2AP	ENSG00000160789.18_LMNA	0.34903	0.200999918	0.481478641	1.05E-05	3.03E-04	5.38E-01
ENSG00000186666.5_BCDIN3D	ENSG00000160789.18_LMNA	-0.22042	-0.366750194	-0.0634447	6.36E-03	4.70E-02	1.00E+00
ENSG00000119684.14_MLH3	ENSG00000160789.18_LMNA	0.30506	0.15330833	0.442760853	1.33E-04	2.37E-03	1.00E+00
ENSG00000243701.4_DUBR	ENSG00000160789.18_LMNA	-0.10078	-0.255875549	0.059373115	2.17E-01	4.91E-01	1.00E+00
ENSG00000163535.16_SGO2	ENSG00000160789.18_LMNA	0.04682	-0.113222655	0.20449837	5.67E-01	7.97E-01	1.00E+00
ENSG00000227939.1_RPL3P2	ENSG00000160789.18_LMNA	-0.08807	-0.243850758	0.072143195	2.81E-01	5.66E-01	1.00E+00
ENSG00000116194.11_ANGPTL1	ENSG00000160789.18_LMNA	-0.05888	-0.216058718	0.101266592	4.71E-01	7.31E-01	1.00E+00

ENSG00000136319.10_TTC5	ENSG00000160789.18_LMNA	-0.22227	-0.368429772	-0.06537863	5.92E-03	4.46E-02	1.00E+00
ENSG00000003989.15_SLC7A2	ENSG00000160789.18_LMNA	-0.24527	-0.389269765	-0.08956543	2.32E-03	2.19E-02	1.00E+00
ENSG00000174498.12_IGDCC3	ENSG00000160789.18_LMNA	-0.11963	-0.273618386	0.04034024	1.42E-01	3.85E-01	1.00E+00
ENSG00000135447.15_PPP1R1A	ENSG00000160789.18_LMNA	-0.40896	-0.533434156	-0.26715713	1.69E-07	1.10E-05	8.65E-03
ENSG00000273820.1_USP27X	ENSG00000160789.18_LMNA	0.07148	-0.088735357	0.228080456	3.82E-01	6.60E-01	1.00E+00
ENSG00000258634.3_RP4-773N1	ENSG00000160789.18_LMNA	0.02558	-0.134171954	0.184026464	7.54E-01	8.98E-01	1.00E+00
ENSG00000197608.10_ZNF841	ENSG00000160789.18_LMNA	0.14587	-0.013650391	0.298144442	7.30E-02	2.56E-01	1.00E+00
ENSG00000109618.10_SEPSECS	ENSG00000160789.18_LMNA	-0.17382	-0.324054355	-0.01503642	3.22E-02	1.50E-01	1.00E+00
ENSG00000049246.13_PER3	ENSG00000160789.18_LMNA	-0.05228	-0.209736614	0.107816481	5.22E-01	7.68E-01	1.00E+00
ENSG00000095585.15_BLNK	ENSG00000160789.18_LMNA	-0.13996	-0.29264354	0.019675506	8.55E-02	2.84E-01	1.00E+00
ENSG00000112378.11_PERP	ENSG00000160789.18_LMNA	0.1766	0.01790563	0.326620574	2.95E-02	1.42E-01	1.00E+00
ENSG00000123411.13_IKZF4	ENSG00000160789.18_LMNA	-0.0917	-0.247293737	0.068497377	2.61E-01	5.44E-01	1.00E+00
ENSG00000081665.12_ZNF506	ENSG00000160789.18_LMNA	-0.33353	-0.467882408	-0.18409957	2.68E-05	6.54E-04	1.00E+00
ENSG00000108379.8_WNT3	ENSG00000160789.18_LMNA	-0.10991	-0.264486182	0.050164962	1.78E-01	4.39E-01	1.00E+00
ENSG00000135269.16_TES	ENSG00000160789.18_LMNA	0.13487	-0.024869352	0.287884936	9.76E-02	3.07E-01	1.00E+00
ENSG00000198624.11_CCDC69	ENSG00000160789.18_LMNA	-0.1209	-0.274813265	0.039050244	1.38E-01	3.78E-01	1.00E+00
ENSG00000170448.10_NFXL1	ENSG00000160789.18_LMNA	-0.06467	-0.221590343	0.095513009	4.29E-01	6.99E-01	1.00E+00
ENSG00000165895.16_ARHGAP4	ENSG00000160789.18_LMNA	-0.0207	-0.179307463	0.138960997	8.00E-01	9.20E-01	1.00E+00
ENSG00000250982.2_GAPDHP35	ENSG00000160789.18_LMNA	0.23635	0.080160949	0.381202907	3.37E-03	2.92E-02	1.00E+00
ENSG00000165714.9_BORCS5	ENSG00000160789.18_LMNA	-0.14785	-0.299993736	0.011619769	6.91E-02	2.48E-01	1.00E+00
ENSG00000168135.4_KCNJ4	ENSG00000160789.18_LMNA	-0.08561	-0.241516329	0.07461036	2.94E-01	5.80E-01	1.00E+00
ENSG00000247416.2_RP11-629G	ENSG00000160789.18_LMNA	-0.2115	-0.358628299	-0.05412489	8.90E-03	6.03E-02	1.00E+00
ENSG00000261786.1_RP4-555D2	ENSG00000160789.18_LMNA	-0.30442	-0.442189057	-0.15261397	1.37E-04	2.43E-03	1.00E+00
ENSG00000155903.10_RASA2	ENSG00000160789.18_LMNA	0.13018	-0.029638834	0.283501487	1.10E-01	3.30E-01	1.00E+00
ENSG00000125388.18_GRK4	ENSG00000160789.18_LMNA	-0.1283	-0.281745574	0.031545393	1.15E-01	3.39E-01	1.00E+00
ENSG00000167384.9_ZNF180	ENSG00000160789.18_LMNA	0.13121	-0.028592529	0.284464229	1.07E-01	3.25E-01	1.00E+00
ENSG00000203667.8_COX20	ENSG00000160789.18_LMNA	-0.21468	-0.361522595	-0.05744	7.91E-03	5.51E-02	1.00E+00
ENSG00000130529.14_TRPM4	ENSG00000160789.18_LMNA	0.38118	0.236319678	0.509463912	1.27E-06	5.59E-05	6.50E-02
ENSG00000083844.9_ZNF264	ENSG00000160789.18_LMNA	0.18834	0.030042054	0.337425255	2.01E-02	1.09E-01	1.00E+00
ENSG00000103248.16_MTHFSD	ENSG00000160789.18_LMNA	0.145	-0.014539224	0.297334242	7.47E-02	2.60E-01	1.00E+00
ENSG00000225889.6_AC074289.1	ENSG00000160789.18_LMNA	-0.35526	-0.486921039	-0.20781203	7.08E-06	2.22E-04	3.63E-01
ENSG00000250616.2_RP11-455F	ENSG00000160789.18_LMNA	-0.25516	-0.398182453	-0.10001851	1.51E-03	1.58E-02	1.00E+00
ENSG00000078043.14_PIAS2	ENSG00000160789.18_LMNA	-0.08418	-0.240159529	0.076042531	3.03E-01	5.89E-01	1.00E+00

ENSG00000241058.2_NSUN6	ENSG00000160789.18_LMNA	-0.31953	-0.455553817	-0.16891861	6.01E-05	1.24E-03	1.00E+00
ENSG00000268412.2_TRMT112P	ENSG00000160789.18_LMNA	0.13895	-0.020705173	0.291701387	8.78E-02	2.88E-01	1.00E+00
ENSG00000125089.15_SH3TC1	ENSG00000160789.18_LMNA	0.12185	-0.038086906	0.275704939	1.35E-01	3.73E-01	1.00E+00
ENSG00000130584.9_ZBTB46	ENSG00000160789.18_LMNA	0.22277	0.065907581	0.36888881	5.81E-03	4.39E-02	1.00E+00
ENSG00000256223.4_ZNF10	ENSG00000160789.18_LMNA	-0.31572	-0.452189241	-0.16479909	7.43E-05	1.48E-03	1.00E+00
ENSG00000120662.14_MTRF1	ENSG00000160789.18_LMNA	-0.27074	-0.412171152	-0.11655869	7.42E-04	9.17E-03	1.00E+00
ENSG00000126215.12_XRCC3	ENSG00000160789.18_LMNA	0.21365	0.056363225	0.360583145	8.22E-03	5.68E-02	1.00E+00
ENSG00000257605.2_RP11-680A	ENSG00000160789.18_LMNA	0.18564	0.027242832	0.334940359	2.20E-02	1.16E-01	1.00E+00
ENSG00000189180.14_ZNF33A	ENSG00000160789.18_LMNA	-0.18675	-0.335961043	-0.02839204	2.12E-02	1.13E-01	1.00E+00
ENSG00000129534.12_MIS18BP1	ENSG00000160789.18_LMNA	-0.03375	-0.191923522	0.126124298	6.80E-01	8.60E-01	1.00E+00
ENSG00000167617.2_CDC42EP5	ENSG00000160789.18_LMNA	0.27484	0.120931912	0.415846614	6.11E-04	7.91E-03	1.00E+00
ENSG00000171408.12_PDE7B	ENSG00000160789.18_LMNA	-0.01713	-0.175847631	0.142462748	8.34E-01	9.36E-01	1.00E+00
ENSG00000146574.14_CCZ1B	ENSG00000160789.18_LMNA	-0.08254	-0.238604158	0.077682701	3.12E-01	5.98E-01	1.00E+00
ENSG00000188171.13_ZNF626	ENSG00000160789.18_LMNA	-0.20801	-0.355444717	-0.05048617	1.01E-02	6.62E-02	1.00E+00
ENSG00000182858.12_ALG12	ENSG00000160789.18_LMNA	0.22916	0.072608465	0.37469127	4.51E-03	3.63E-02	1.00E+00
ENSG00000275557.1_RP11-353N	ENSG00000160789.18_LMNA	0.08625	-0.073961631	0.24213051	2.91E-01	5.76E-01	1.00E+00
ENSG00000271737.1_CTB-113I2(	ENSG00000160789.18_LMNA	0.01067	-0.148788023	0.169578081	8.96E-01	9.59E-01	1.00E+00
ENSG00000124635.8_HIST1H2BJ	ENSG00000160789.18_LMNA	-0.03518	-0.193299794	0.124717483	6.67E-01	8.53E-01	1.00E+00
ENSG00000278238.1_RP11-245D	ENSG00000160789.18_LMNA	-0.35821	-0.489493454	-0.21104131	5.87E-06	1.89E-04	3.01E-01
ENSG00000123600.17_METTL8	ENSG00000160789.18_LMNA	0.02466	-0.135071982	0.183140718	7.63E-01	9.01E-01	1.00E+00
ENSG00000111907.19_TPD52L1	ENSG00000160789.18_LMNA	-0.13596	-0.288907923	0.023754221	9.49E-02	3.02E-01	1.00E+00
ENSG00000169087.9_HSPBAP1	ENSG00000160789.18_LMNA	-0.30091	-0.43908026	-0.14884378	1.65E-04	2.81E-03	1.00E+00
ENSG00000110921.10_MVK	ENSG00000160789.18_LMNA	0.08109	-0.079127415	0.237232792	3.21E-01	6.07E-01	1.00E+00
ENSG00000161960.13_EIF4A1	ENSG00000160789.18_LMNA	0.06351	-0.09666529	0.220484146	4.37E-01	7.05E-01	1.00E+00
ENSG00000074935.12_TUBE1	ENSG00000160789.18_LMNA	-0.18531	-0.334636436	-0.02690079	2.23E-02	1.17E-01	1.00E+00
ENSG00000088727.11_KIF9	ENSG00000160789.18_LMNA	-0.05864	-0.215826092	0.101508089	4.73E-01	7.33E-01	1.00E+00
ENSG00000155265.9_GOLGA7B	ENSG00000160789.18_LMNA	-0.14974	-0.301746901	0.009692325	6.56E-02	2.39E-01	1.00E+00
ENSG00000126001.14_CEP250	ENSG00000160789.18_LMNA	0.25715	0.102132106	0.399977772	1.38E-03	1.48E-02	1.00E+00
ENSG00000162419.11_GMEB1	ENSG00000160789.18_LMNA	0.10248	-0.057662491	0.257478963	2.09E-01	4.82E-01	1.00E+00
ENSG00000185010.12_F8	ENSG00000160789.18_LMNA	-0.02628	-0.184710715	0.133476312	7.48E-01	8.95E-01	1.00E+00
ENSG00000179979.8_CRIPAK	ENSG00000160789.18_LMNA	0.19587	0.037846004	0.344330348	1.56E-02	9.02E-02	1.00E+00
ENSG00000172840.5_PDP2	ENSG00000160789.18_LMNA	-0.11687	-0.271031093	0.043129911	1.52E-01	4.00E-01	1.00E+00
ENSG00000147912.11_FBXO10	ENSG00000160789.18_LMNA	-0.10497	-0.2598246	0.055156676	1.98E-01	4.68E-01	1.00E+00

ENSG00000256683.5_ZNF350	ENSG00000160789.18_LMNA	-0.0055	-0.164558318	0.153833693	9.46E-01	9.79E-01	1.00E+00
ENSG00000004660.13_CAMKK1	ENSG00000160789.18_LMNA	-0.27698	-0.417756674	-0.12320911	5.51E-04	7.29E-03	1.00E+00
ENSG00000272425.1_RP11-363E	ENSG00000160789.18_LMNA	0.16481	0.005761474	0.315727638	4.25E-02	1.80E-01	1.00E+00
ENSG00000244879.4_GABPB1-AS	ENSG00000160789.18_LMNA	-0.07609	-0.232475829	0.084128527	3.51E-01	6.35E-01	1.00E+00
ENSG00000081026.17_MAGI3	ENSG00000160789.18_LMNA	-0.16056	-0.311788277	-0.00139202	4.82E-02	1.96E-01	1.00E+00
ENSG00000163590.12_PPM1L	ENSG00000160789.18_LMNA	-0.28028	-0.420706113	-0.12673158	4.70E-04	6.42E-03	1.00E+00
ENSG00000255320.1_RP11-755F	ENSG00000160789.18_LMNA	0.11723	-0.042764739	0.271370032	1.50E-01	3.98E-01	1.00E+00
ENSG00000168237.16_GLYCTK	ENSG00000160789.18_LMNA	-0.21636	-0.363054376	-0.05919721	7.42E-03	5.26E-02	1.00E+00
ENSG00000189079.14_ARID2	ENSG00000160789.18_LMNA	-0.11092	-0.265428914	0.049153551	1.74E-01	4.34E-01	1.00E+00
ENSG00000225697.9_SLC26A6	ENSG00000160789.18_LMNA	0.14226	-0.017336125	0.294781842	8.04E-02	2.73E-01	1.00E+00
ENSG00000139537.9_CCDC65	ENSG00000160789.18_LMNA	-0.0301	-0.188401285	0.129718883	7.13E-01	8.78E-01	1.00E+00
ENSG00000142544.6_CTU1	ENSG00000160789.18_LMNA	0.23197	0.075562855	0.377242054	4.03E-03	3.33E-02	1.00E+00
ENSG00000088387.16_DOCK9	ENSG00000160789.18_LMNA	-0.19931	-0.347481551	-0.04141988	1.38E-02	8.28E-02	1.00E+00
ENSG00000135750.13_KCNK1	ENSG00000160789.18_LMNA	-0.31316	-0.449930751	-0.16203946	8.55E-05	1.66E-03	1.00E+00
ENSG00000267809.1_NDUFV2P1	ENSG00000160789.18_LMNA	-0.09873	-0.253942373	0.061433093	2.26E-01	5.02E-01	1.00E+00
ENSG00000079435.8_LIPE	ENSG00000160789.18_LMNA	-0.28989	-0.429279348	-0.13701273	2.92E-04	4.40E-03	1.00E+00
ENSG00000235314.1_LINC00957	ENSG00000160789.18_LMNA	0.01133	-0.148140652	0.170220935	8.90E-01	9.57E-01	1.00E+00
ENSG00000235776.2_AC000089.3	ENSG00000160789.18_LMNA	-0.14362	-0.296051978	0.01594493	7.75E-02	2.67E-01	1.00E+00
ENSG00000050767.14_COL23A1	ENSG00000160789.18_LMNA	0.09527	-0.064916037	0.250668079	2.43E-01	5.22E-01	1.00E+00
ENSG00000162769.11_FLVCR1	ENSG00000160789.18_LMNA	-0.13889	-0.2916397	0.020772567	8.79E-02	2.88E-01	1.00E+00
ENSG00000213189.5_BTF3L4P2	ENSG00000160789.18_LMNA	-0.2004	-0.348485461	-0.04256009	1.33E-02	8.04E-02	1.00E+00
ENSG00000262246.4_CORO7	ENSG00000160789.18_LMNA	-0.12938	-0.282757809	0.030446594	1.12E-01	3.34E-01	1.00E+00
ENSG00000164035.8_EMCN	ENSG00000160789.18_LMNA	-0.23572	-0.380633206	-0.0794988	3.46E-03	2.98E-02	1.00E+00
ENSG00000157399.13_ARSE	ENSG00000160789.18_LMNA	0.24138	0.085468447	0.385761149	2.74E-03	2.49E-02	1.00E+00
ENSG00000124523.13_SIRT5	ENSG00000160789.18_LMNA	-0.05539	-0.212719625	0.104729424	4.98E-01	7.51E-01	1.00E+00
ENSG00000245849.5_RAD51-AS1	ENSG00000160789.18_LMNA	-0.12459	-0.278273616	0.035308522	1.26E-01	3.59E-01	1.00E+00
ENSG00000205726.12_ITSN1	ENSG00000160789.18_LMNA	-0.00147	-0.160632411	0.157768323	9.86E-01	9.94E-01	1.00E+00
ENSG00000132357.12_CARD6	ENSG00000160789.18_LMNA	0.01717	-0.14241883	0.175891072	8.34E-01	9.35E-01	1.00E+00
ENSG00000171680.19_PLEKHG5	ENSG00000160789.18_LMNA	0.13203	-0.027751429	0.285237696	1.05E-01	3.21E-01	1.00E+00
ENSG00000277494.1_GPIHBP1	ENSG00000160789.18_LMNA	-0.32364	-0.459186087	-0.17337717	4.76E-05	1.03E-03	1.00E+00
ENSG00000280123.1_RP11-267M	ENSG00000160789.18_LMNA	-0.12755	-0.281046258	0.032304072	1.17E-01	3.43E-01	1.00E+00
ENSG00000179965.10_ZNF771	ENSG00000160789.18_LMNA	0.0183	-0.141317159	0.176980372	8.23E-01	9.31E-01	1.00E+00
ENSG00000105538.7_RASIP1	ENSG00000160789.18_LMNA	0.00149	-0.157743433	0.160657278	9.85E-01	9.94E-01	1.00E+00

ENSG00000134461.14_ANKRD16	ENSG00000160789.18_LMNA	-0.20651	-0.354069775	-0.04891716	1.07E-02	6.87E-02	1.00E+00
ENSG00000135870.10_RC3H1	ENSG00000160789.18_LMNA	-0.00895	-0.167912618	0.150463921	9.13E-01	9.67E-01	1.00E+00
ENSG00000101443.16_WFDC2	ENSG00000160789.18_LMNA	0.15296	-0.006395214	0.304741012	5.99E-02	2.25E-01	1.00E+00
ENSG00000183307.3_CECR6	ENSG00000160789.18_LMNA	-0.26221	-0.404520322	-0.10749201	1.10E-03	1.25E-02	1.00E+00
ENSG00000165632.7_TAF3	ENSG00000160789.18_LMNA	-0.05931	-0.216465225	0.100844495	4.68E-01	7.29E-01	1.00E+00
ENSG00000165730.13_STOX1	ENSG00000160789.18_LMNA	-0.36052	-0.491507139	-0.21357344	5.06E-06	1.67E-04	2.59E-01
ENSG00000228962.1_HCG23	ENSG00000160789.18_LMNA	-0.16402	-0.314996585	-0.00494971	4.35E-02	1.83E-01	1.00E+00
ENSG00000062370.15_ZNF112	ENSG00000160789.18_LMNA	-0.10016	-0.255287239	0.060000298	2.20E-01	4.95E-01	1.00E+00
ENSG00000136244.10_IL6	ENSG00000160789.18_LMNA	0.14274	-0.016844838	0.295230504	7.94E-02	2.71E-01	1.00E+00
ENSG00000164542.11_KIAA0895	ENSG00000160789.18_LMNA	0.07479	-0.085431447	0.231234024	3.60E-01	6.42E-01	1.00E+00
ENSG00000149970.13_CNKSR2	ENSG00000160789.18_LMNA	-0.09269	-0.24822843	0.06750616	2.56E-01	5.38E-01	1.00E+00
ENSG00000188906.12_LRRK2	ENSG00000160789.18_LMNA	-0.30628	-0.443837902	-0.15461702	1.24E-04	2.25E-03	1.00E+00
ENSG00000264278.1_RP11-162A	ENSG00000160789.18_LMNA	0.24988	0.094434713	0.393428495	1.90E-03	1.88E-02	1.00E+00
ENSG00000130338.11_TULP4	ENSG00000160789.18_LMNA	-0.04716	-0.204826677	0.112884375	5.64E-01	7.95E-01	1.00E+00
ENSG00000130363.10_RSPH3	ENSG00000160789.18_LMNA	0.09455	-0.065633758	0.249992456	2.47E-01	5.27E-01	1.00E+00
ENSG00000105825.10_TFPI2	ENSG00000160789.18_LMNA	0.17611	0.017394212	0.326163496	3.00E-02	1.43E-01	1.00E+00
ENSG00000196214.9_ZNF766	ENSG00000160789.18_LMNA	0.14308	-0.016494414	0.295550441	7.87E-02	2.69E-01	1.00E+00
ENSG00000138439.11_FAM117B	ENSG00000160789.18_LMNA	-0.43897	-0.559102408	-0.30079499	1.54E-08	1.55E-06	7.89E-04
ENSG00000171827.9_ZNF570	ENSG00000160789.18_LMNA	-0.22609	-0.371905337	-0.06938776	5.10E-03	3.98E-02	1.00E+00
ENSG00000151470.11_C4orf33	ENSG00000160789.18_LMNA	-0.41089	-0.535090017	-0.2693077	1.46E-07	9.80E-06	7.47E-03
ENSG00000164118.11_CEP44	ENSG00000160789.18_LMNA	-0.25877	-0.401431043	-0.10384499	1.29E-03	1.40E-02	1.00E+00
ENSG00000105865.9_DUS4L	ENSG00000160789.18_LMNA	-0.08393	-0.239921331	0.076293826	3.04E-01	5.91E-01	1.00E+00
ENSG00000122008.14_POLK	ENSG00000160789.18_LMNA	-0.0679	-0.224675901	0.09229441	4.06E-01	6.81E-01	1.00E+00
ENSG00000236144.5_TMEM147-/	ENSG00000160789.18_LMNA	-0.13409	-0.287160332	0.025658753	9.96E-02	3.11E-01	1.00E+00
ENSG00000076944.13_STXBP2	ENSG00000160789.18_LMNA	0.04411	-0.115903695	0.201893844	5.89E-01	8.09E-01	1.00E+00
ENSG00000171295.11_ZNF440	ENSG00000160789.18_LMNA	-0.22131	-0.367556213	-0.0643725	6.14E-03	4.58E-02	1.00E+00
ENSG00000179598.5_PLD6	ENSG00000160789.18_LMNA	-0.28859	-0.428121994	-0.13562113	3.11E-04	4.64E-03	1.00E+00
ENSG00000214367.6_HAUS3	ENSG00000160789.18_LMNA	-0.10182	-0.256858526	0.058324632	2.12E-01	4.86E-01	1.00E+00
ENSG00000164953.14_TMEM67	ENSG00000160789.18_LMNA	0.03183	-0.128022966	0.190064089	6.97E-01	8.70E-01	1.00E+00
ENSG00000279457.2_FO538757.:	ENSG00000160789.18_LMNA	0.04043	-0.119540369	0.198353757	6.21E-01	8.28E-01	1.00E+00
ENSG00000133121.19_STARD13	ENSG00000160789.18_LMNA	0.16187	0.002735659	0.3130008	4.63E-02	1.91E-01	1.00E+00
ENSG00000172954.12_LCLAT1	ENSG00000160789.18_LMNA	-0.12395	-0.277669745	0.035962129	1.28E-01	3.62E-01	1.00E+00
ENSG00000169253.3_RP11-220D	ENSG00000160789.18_LMNA	0.01829	-0.141326514	0.176971125	8.23E-01	9.31E-01	1.00E+00

ENSG00000272168.4_CASC15	ENSG00000160789.18_LMNA	-0.40589	-0.530795237	-0.26373526	2.13E-07	1.33E-05	1.09E-02
ENSG00000138755.5_CXCL9	ENSG00000160789.18_LMNA	0.05379	-0.106321272	0.211182167	5.10E-01	7.59E-01	1.00E+00
ENSG00000119965.11_C10orf88	ENSG00000160789.18_LMNA	-0.32385	-0.459370666	-0.17360405	4.70E-05	1.02E-03	1.00E+00
ENSG00000132549.17_VPS13B	ENSG00000160789.18_LMNA	-0.00184	-0.160995285	0.157405066	9.82E-01	9.93E-01	1.00E+00
ENSG00000018869.15_ZNF582	ENSG00000160789.18_LMNA	-0.23874	-0.38336842	-0.08268029	3.06E-03	2.71E-02	1.00E+00
ENSG00000172318.5_B3GALT1	ENSG00000160789.18_LMNA	0.04563	-0.114406247	0.203349111	5.77E-01	8.03E-01	1.00E+00
ENSG00000130544.10_ZNF557	ENSG00000160789.18_LMNA	-0.16132	-0.312492684	-0.00217246	4.71E-02	1.93E-01	1.00E+00
ENSG00000216895.7_AC009403.:	ENSG00000160789.18_LMNA	0.09434	-0.065849303	0.249789492	2.48E-01	5.28E-01	1.00E+00
ENSG00000141497.12_ZMYND15	ENSG00000160789.18_LMNA	0.01271	-0.146791664	0.171559639	8.77E-01	9.52E-01	1.00E+00
ENSG00000096872.14_IFT74	ENSG00000160789.18_LMNA	0.06326	-0.096915496	0.220243839	4.39E-01	7.07E-01	1.00E+00
ENSG00000165246.11_NLGN4Y	ENSG00000160789.18_LMNA	-0.04194	-0.199806661	0.118048856	6.08E-01	8.21E-01	1.00E+00
ENSG00000258584.1_FAM181A-A	ENSG00000160789.18_LMNA	-0.03626	-0.19434247	0.123650816	6.57E-01	8.49E-01	1.00E+00
ENSG00000173715.14_C11orf80	ENSG00000160789.18_LMNA	0.18266	0.024162609	0.332201062	2.43E-02	1.24E-01	1.00E+00
ENSG00000117594.8_HSD11B1	ENSG00000160789.18_LMNA	0.00202	-0.157234106	0.161166035	9.80E-01	9.93E-01	1.00E+00
ENSG00000235082.2_SUMO1P3	ENSG00000160789.18_LMNA	-0.17061	-0.321090756	-0.01172922	3.56E-02	1.60E-01	1.00E+00
ENSG00000228214.5_	ENSG00000160789.18_LMNA	-0.55164	-0.65345502	-0.43022426	1.76E-13	1.35E-10	9.05E-09
ENSG00000153956.14_CACNA2D	ENSG00000160789.18_LMNA	-0.18568	-0.334975671	-0.02728258	2.20E-02	1.16E-01	1.00E+00
ENSG00000239213.4_NCK1-AS1	ENSG00000160789.18_LMNA	-0.29538	-0.434163184	-0.14289779	2.20E-04	3.52E-03	1.00E+00
ENSG00000183765.19_CHEK2	ENSG00000160789.18_LMNA	-0.1477	-0.299850914	0.011776686	6.94E-02	2.48E-01	1.00E+00
ENSG00000068024.15_HDAC4	ENSG00000160789.18_LMNA	-0.12144	-0.275318628	0.038504337	1.36E-01	3.76E-01	1.00E+00
ENSG00000105136.18_ZNF419	ENSG00000160789.18_LMNA	-0.0906	-0.246251828	0.06960156	2.67E-01	5.51E-01	1.00E+00
ENSG00000112276.12_BVES	ENSG00000160789.18_LMNA	-0.0995	-0.254671128	0.060656853	2.23E-01	4.98E-01	1.00E+00
ENSG00000154920.13_EME1	ENSG00000160789.18_LMNA	-0.00548	-0.164539045	0.153853033	9.47E-01	9.79E-01	1.00E+00
ENSG00000065615.12_CYB5R4	ENSG00000160789.18_LMNA	-0.31106	-0.448075069	-0.15977537	9.59E-05	1.82E-03	1.00E+00
ENSG00000234224.2_TMEM229A	ENSG00000160789.18_LMNA	-0.0309	-0.189174715	0.128930282	7.05E-01	8.75E-01	1.00E+00
ENSG00000156017.11_CARNMT1	ENSG00000160789.18_LMNA	0.17768	0.019015817	0.327612303	2.85E-02	1.38E-01	1.00E+00
ENSG00000184988.7_TMEM106A	ENSG00000160789.18_LMNA	0.06658	-0.093615891	0.223409813	4.15E-01	6.88E-01	1.00E+00
ENSG00000172062.15_SMN1	ENSG00000160789.18_LMNA	-0.00945	-0.168394718	0.149978988	9.08E-01	9.65E-01	1.00E+00
ENSG00000071282.10_LMCD1	ENSG00000160789.18_LMNA	0.08621	-0.074004715	0.242089729	2.91E-01	5.76E-01	1.00E+00
ENSG00000173638.17_SLC19A1	ENSG00000160789.18_LMNA	0.21637	0.059207677	0.363063494	7.42E-03	5.25E-02	1.00E+00
ENSG00000005844.16_ITGAL	ENSG00000160789.18_LMNA	-0.05475	-0.212103333	0.105367714	5.03E-01	7.54E-01	1.00E+00
ENSG00000179750.14_APOBEC3	ENSG00000160789.18_LMNA	0.30563	0.153918774	0.443263349	1.29E-04	2.31E-03	1.00E+00
ENSG00000270532.1_PEBP1P2	ENSG00000160789.18_LMNA	-0.06315	-0.220139334	0.097024293	4.40E-01	7.08E-01	1.00E+00

ENSG00000277363.3_SRCIN1	ENSG00000160789.18_LMNA	-0.30589	-0.443495227	-0.15420054	1.27E-04	2.29E-03	1.00E+00
ENSG00000233846.2_RP11-307L	ENSG00000160789.18_LMNA	-0.1574	-0.308859452	0.001848915	5.28E-02	2.08E-01	1.00E+00
ENSG00000227540.1_DNAJC9-AS	ENSG00000160789.18_LMNA	-0.24902	-0.392654901	-0.09352786	1.98E-03	1.93E-02	1.00E+00
ENSG00000198208.10_RPS6KL1	ENSG00000160789.18_LMNA	-0.35942	-0.490549812	-0.21236917	5.43E-06	1.77E-04	2.78E-01
ENSG00000266962.2_RP11-400F	ENSG00000160789.18_LMNA	-0.00094	-0.160112507	0.158288626	9.91E-01	9.97E-01	1.00E+00
ENSG00000263004.1_RP11-166P	ENSG00000160789.18_LMNA	-0.2739	-0.41500041	-0.11992405	6.39E-04	8.18E-03	1.00E+00
ENSG00000175352.9_NRIP3	ENSG00000160789.18_LMNA	-0.25803	-0.400765132	-0.1030599	1.33E-03	1.44E-02	1.00E+00
ENSG00000269910.1_RP11-73M1	ENSG00000160789.18_LMNA	0.24241	0.086549989	0.386688217	2.62E-03	2.40E-02	1.00E+00
ENSG00000273403.1_RP11-329B	ENSG00000160789.18_LMNA	-0.16191	-0.313045692	-0.00278543	4.63E-02	1.91E-01	1.00E+00
ENSG00000143847.14_PPFIA4	ENSG00000160789.18_LMNA	0.03939	-0.120563807	0.197356002	6.30E-01	8.34E-01	1.00E+00
ENSG00000137760.13_ALKBH8	ENSG00000160789.18_LMNA	0.09537	-0.064818496	0.250759874	2.43E-01	5.22E-01	1.00E+00
ENSG00000046889.17_PREX2	ENSG00000160789.18_LMNA	-0.10501	-0.259866123	0.055112283	1.98E-01	4.68E-01	1.00E+00
ENSG00000170571.10_EMB	ENSG00000160789.18_LMNA	0.0852	-0.075022439	0.241126063	2.97E-01	5.83E-01	1.00E+00
ENSG00000256229.6_ZNF486	ENSG00000160789.18_LMNA	-0.22064	-0.366955353	-0.06368081	6.30E-03	4.66E-02	1.00E+00
ENSG00000012048.18_BRCA1	ENSG00000160789.18_LMNA	0.02348	-0.136225548	0.182004699	7.74E-01	9.07E-01	1.00E+00
ENSG00000277744.1_CTC-435M1	ENSG00000160789.18_LMNA	-0.04106	-0.198963013	0.118915097	6.15E-01	8.26E-01	1.00E+00
ENSG00000153714.5_LURAP1L	ENSG00000160789.18_LMNA	-0.1098	-0.264377074	0.050281977	1.78E-01	4.40E-01	1.00E+00
ENSG00000151014.5_NOCT	ENSG00000160789.18_LMNA	0.37996	0.234970433	0.508405362	1.38E-06	5.99E-05	7.07E-02
ENSG00000169220.16_RGS14	ENSG00000160789.18_LMNA	0.19282	0.034683268	0.341535885	1.73E-02	9.73E-02	1.00E+00
ENSG00000198198.12_SZT2	ENSG00000160789.18_LMNA	0.18024	0.021662297	0.329973662	2.63E-02	1.31E-01	1.00E+00
ENSG00000100926.13_TM9SF1	ENSG00000160789.18_LMNA	0.5161	0.388850273	0.624029677	1.01E-11	3.50E-09	5.18E-07
ENSG00000219481.9_NBPF1	ENSG00000160789.18_LMNA	-0.06737	-0.224167039	0.09282567	4.10E-01	6.84E-01	1.00E+00
ENSG00000270091.1_RP11-78O7	ENSG00000160789.18_LMNA	-0.07642	-0.232787174	0.083801689	3.49E-01	6.33E-01	1.00E+00
ENSG00000186104.9_CYP2R1	ENSG00000160789.18_LMNA	-0.19457	-0.343138327	-0.03649614	1.63E-02	9.32E-02	1.00E+00
ENSG00000154124.4_OTULIN	ENSG00000160789.18_LMNA	0.16354	0.004453826	0.314549824	4.41E-02	1.85E-01	1.00E+00
ENSG00000228305.2_AC016734.2	ENSG00000160789.18_LMNA	0.12307	-0.036849107	0.276849865	1.31E-01	3.67E-01	1.00E+00
ENSG00000005156.10_LIG3	ENSG00000160789.18_LMNA	-0.08077	-0.236927704	0.07944864	3.23E-01	6.09E-01	1.00E+00
ENSG00000083544.12_TDRD3	ENSG00000160789.18_LMNA	-0.12581	-0.279411539	0.034076148	1.22E-01	3.52E-01	1.00E+00
ENSG00000137968.15_SLC44A5	ENSG00000160789.18_LMNA	-0.24131	-0.38569441	-0.08539061	2.75E-03	2.50E-02	1.00E+00
ENSG00000102595.17_UGGT2	ENSG00000160789.18_LMNA	0.09811	-0.062064508	0.253349319	2.29E-01	5.06E-01	1.00E+00
ENSG00000152217.15_SETBP1	ENSG00000160789.18_LMNA	0.12404	-0.035867474	0.277757213	1.28E-01	3.62E-01	1.00E+00
ENSG00000177738.3_CTD-2201E	ENSG00000160789.18_LMNA	0.05348	-0.106626464	0.210887223	5.13E-01	7.61E-01	1.00E+00
ENSG00000181444.11_ZNF467	ENSG00000160789.18_LMNA	0.08944	-0.070768347	0.245150051	2.73E-01	5.58E-01	1.00E+00

ENSG00000108306.10_FBXL20	ENSG00000160789.18_LMNA	-0.26162	-0.403991358	-0.10686698	1.13E-03	1.27E-02	1.00E+00
ENSG00000166707.9_ZCCHC18	ENSG00000160789.18_LMNA	-0.47216	-0.58721949	-0.33839468	8.20E-10	1.33E-07	4.21E-05
ENSG00000141252.18_VPS53	ENSG00000160789.18_LMNA	0.05577	-0.104354088	0.213081908	4.95E-01	7.50E-01	1.00E+00
ENSG00000151576.9_QTRT2	ENSG00000160789.18_LMNA	0.25869	0.103759748	0.401358757	1.29E-03	1.40E-02	1.00E+00
ENSG00000180596.7_HIST1H2B(	ENSG00000160789.18_LMNA	0.03544	-0.124467778	0.193543947	6.65E-01	8.52E-01	1.00E+00
ENSG00000132872.10_SYT4	ENSG00000160789.18_LMNA	-0.33345	-0.467818966	-0.1840211	2.69E-05	6.56E-04	1.00E+00
ENSG00000138641.14_HERC3	ENSG00000160789.18_LMNA	-0.03246	-0.190679012	0.127395326	6.91E-01	8.67E-01	1.00E+00
ENSG00000138823.11_MTTP	ENSG00000160789.18_LMNA	0.17722	0.018538495	0.327185995	2.90E-02	1.40E-01	1.00E+00
ENSG00000198466.11_ZNF587	ENSG00000160789.18_LMNA	0.00113	-0.158101133	0.160299876	9.89E-01	9.96E-01	1.00E+00
ENSG00000187605.14_TET3	ENSG00000160789.18_LMNA	-0.01962	-0.178261921	0.140020046	8.10E-01	9.25E-01	1.00E+00
ENSG00000069966.17_GNB5	ENSG00000160789.18_LMNA	0.1	-0.060153581	0.255143421	2.20E-01	4.95E-01	1.00E+00
ENSG00000271133.4_CTA-293F1	ENSG00000160789.18_LMNA	-0.12362	-0.277363781	0.036293191	1.29E-01	3.64E-01	1.00E+00
ENSG00000275111.3_ZNF2	ENSG00000160789.18_LMNA	-0.03663	-0.19469473	0.123290285	6.54E-01	8.47E-01	1.00E+00
ENSG00000174371.15_EXO1	ENSG00000160789.18_LMNA	0.03749	-0.122446158	0.19551917	6.47E-01	8.43E-01	1.00E+00
ENSG00000233461.4_RP11-295G	ENSG00000160789.18_LMNA	0.04759	-0.112458717	0.205239684	5.60E-01	7.93E-01	1.00E+00
ENSG00000107282.6_APBA1	ENSG00000160789.18_LMNA	-0.12637	-0.279942325	0.033500978	1.21E-01	3.50E-01	1.00E+00
ENSG00000251994.1_RNU2-27P	ENSG00000160789.18_LMNA	0.06209	-0.098084911	0.219120178	4.47E-01	7.14E-01	1.00E+00
ENSG00000182504.9_CEP97	ENSG00000160789.18_LMNA	-0.30561	-0.443243023	-0.15389408	1.29E-04	2.31E-03	1.00E+00
ENSG00000239523.4_MYLK-AS1	ENSG00000160789.18_LMNA	-0.29111	-0.430364559	-0.13831864	2.74E-04	4.19E-03	1.00E+00
ENSG00000152767.14_FARP1	ENSG00000160789.18_LMNA	0.01375	-0.145770039	0.172572695	8.66E-01	9.48E-01	1.00E+00
ENSG00000179399.12_GPC5	ENSG00000160789.18_LMNA	-0.06672	-0.223542801	0.093477136	4.14E-01	6.87E-01	1.00E+00
ENSG00000272556.1_GTF2IP13	ENSG00000160789.18_LMNA	0.10505	-0.055071926	0.259903868	1.98E-01	4.67E-01	1.00E+00
ENSG00000213433.5_RPLP1P6	ENSG00000160789.18_LMNA	0.04332	-0.116690313	0.201128821	5.96E-01	8.14E-01	1.00E+00
ENSG00000117569.17_PTBP2	ENSG00000160789.18_LMNA	-0.48184	-0.595370566	-0.34944535	3.28E-10	6.17E-08	1.68E-05
ENSG00000085185.14_BCORL1	ENSG00000160789.18_LMNA	0.1402	-0.019431376	0.292866833	8.49E-02	2.82E-01	1.00E+00
ENSG00000228393.3_LINC01004	ENSG00000160789.18_LMNA	0.04808	-0.111980422	0.205703629	5.56E-01	7.90E-01	1.00E+00
ENSG00000138792.8_ENPEP	ENSG00000160789.18_LMNA	0.24968	0.094220238	0.393245575	1.92E-03	1.89E-02	1.00E+00
ENSG00000268516.2_CTD-3138B	ENSG00000160789.18_LMNA	0.43452	0.295777836	0.555305101	2.23E-08	2.11E-06	1.14E-03
ENSG00000110427.13_KIAA1549	ENSG00000160789.18_LMNA	-0.0165	-0.175240069	0.14307685	8.40E-01	9.38E-01	1.00E+00
ENSG00000134899.16_ERCC5	ENSG00000160789.18_LMNA	-0.05326	-0.210675141	0.106845879	5.15E-01	7.62E-01	1.00E+00
ENSG00000139117.12_CPNE8	ENSG00000160789.18_LMNA	0.23752	0.081396833	0.382265631	3.21E-03	2.81E-02	1.00E+00
ENSG00000227203.3_SUB1P1	ENSG00000160789.18_LMNA	-0.21899	-0.36545218	-0.06195168	6.72E-03	4.88E-02	1.00E+00
ENSG00000175387.14_SMAD2	ENSG00000160789.18_LMNA	-0.02209	-0.180652366	0.137597647	7.87E-01	9.13E-01	1.00E+00

ENSG00000149346.13_SLX4IP	ENSG00000160789.18_LMNA	0.04979	-0.110289733	0.207342449	5.42E-01	7.81E-01	1.00E+00
ENSG00000167434.8_CA4	ENSG00000160789.18_LMNA	-0.33723	-0.471134863	-0.18812721	2.15E-05	5.49E-04	1.00E+00
ENSG00000256124.4_LINC01152	ENSG00000160789.18_LMNA	-0.10505	-0.259905218	0.055070483	1.98E-01	4.67E-01	1.00E+00
ENSG00000114541.13_FRMD4B	ENSG00000160789.18_LMNA	-0.17568	-0.325766725	-0.0169504	3.04E-02	1.45E-01	1.00E+00
ENSG00000170049.8_KCNAB3	ENSG00000160789.18_LMNA	0.09152	-0.068679469	0.247121965	2.62E-01	5.45E-01	1.00E+00
ENSG00000177946.5_CENPBD1	ENSG00000160789.18_LMNA	0.12795	-0.031896424	0.281422051	1.16E-01	3.41E-01	1.00E+00
ENSG00000144057.14_ST6GAL2	ENSG00000160789.18_LMNA	-0.29855	-0.436978768	-0.14629997	1.87E-04	3.10E-03	1.00E+00
ENSG00000267751.4_AC009005.:	ENSG00000160789.18_LMNA	0.24766	0.092090663	0.391428029	2.10E-03	2.03E-02	1.00E+00
ENSG00000258824.2_CTD-2555C	ENSG00000160789.18_LMNA	-0.13173	-0.284958619	0.028054959	1.06E-01	3.23E-01	1.00E+00
ENSG00000259917.1_HNRNPLP2	ENSG00000160789.18_LMNA	-0.01269	-0.171545326	0.146806093	8.77E-01	9.52E-01	1.00E+00
ENSG00000164691.15_TAGAP	ENSG00000160789.18_LMNA	-0.20016	-0.348266994	-0.04231189	1.34E-02	8.09E-02	1.00E+00
ENSG00000100722.17_ZC3H14	ENSG00000160789.18_LMNA	0.18335	0.024879947	0.332839468	2.38E-02	1.22E-01	1.00E+00
ENSG00000143669.12_LYST	ENSG00000160789.18_LMNA	0.17548	0.016750709	0.325588162	3.06E-02	1.45E-01	1.00E+00
ENSG00000123243.13_ITIH5	ENSG00000160789.18_LMNA	-0.02557	-0.184025161	0.134173278	7.54E-01	8.98E-01	1.00E+00
ENSG00000259494.1_MRPL46	ENSG00000160789.18_LMNA	-0.16223	-0.313341934	-0.00311389	4.58E-02	1.90E-01	1.00E+00
ENSG00000198879.10_SFMBT2	ENSG00000160789.18_LMNA	-0.10529	-0.260131181	0.054828866	1.97E-01	4.66E-01	1.00E+00
ENSG00000279375.1_CH17-140K	ENSG00000160789.18_LMNA	-0.0002	-0.159393112	0.15900828	9.98E-01	9.99E-01	1.00E+00
ENSG00000099617.3_EFNA2	ENSG00000160789.18_LMNA	-0.08958	-0.245284767	0.070625729	2.72E-01	5.57E-01	1.00E+00
ENSG00000151422.11_FER	ENSG00000160789.18_LMNA	0.006	-0.153345577	0.165044649	9.42E-01	9.77E-01	1.00E+00
ENSG00000226696.4_LENG8-AS1	ENSG00000160789.18_LMNA	0.00854	-0.15086401	0.167514751	9.17E-01	9.68E-01	1.00E+00
ENSG00000166823.5_MESP1	ENSG00000160789.18_LMNA	0.00169	-0.157551283	0.160849233	9.83E-01	9.94E-01	1.00E+00
ENSG00000102878.14_HSF4	ENSG00000160789.18_LMNA	0.01272	-0.146782145	0.171569082	8.76E-01	9.52E-01	1.00E+00
ENSG00000260747.1_RP11-421N	ENSG00000160789.18_LMNA	-0.19234	-0.341095928	-0.03418589	1.76E-02	9.85E-02	1.00E+00
ENSG00000127334.10_DYRK2	ENSG00000160789.18_LMNA	0.08077	-0.079449296	0.236927081	3.23E-01	6.09E-01	1.00E+00
ENSG00000215067.8_ALOX12-AS	ENSG00000160789.18_LMNA	-0.22755	-0.373229277	-0.07091753	4.81E-03	3.81E-02	1.00E+00
ENSG00000148187.16_MRRF	ENSG00000160789.18_LMNA	0.08679	-0.073430007	0.242633634	2.88E-01	5.73E-01	1.00E+00
ENSG00000115041.11_KCNIP3	ENSG00000160789.18_LMNA	-0.32783	-0.462876384	-0.17791897	3.74E-05	8.51E-04	1.00E+00
ENSG00000197635.8_DPP4	ENSG00000160789.18_LMNA	0.21018	0.052743847	0.357420831	9.35E-03	6.24E-02	1.00E+00
ENSG00000180423.4_HARBI1	ENSG00000160789.18_LMNA	-0.31472	-0.45130912	-0.16372314	7.85E-05	1.55E-03	1.00E+00
ENSG00000186074.17_CD300LF	ENSG00000160789.18_LMNA	-0.1052	-0.260049367	0.054916353	1.97E-01	4.67E-01	1.00E+00
ENSG00000158528.10_PPP1R9A	ENSG00000160789.18_LMNA	-0.17171	-0.322102519	-0.01285753	3.44E-02	1.57E-01	1.00E+00
ENSG00000229671.1_LINC01150	ENSG00000160789.18_LMNA	-0.12876	-0.282178125	0.031075944	1.14E-01	3.37E-01	1.00E+00
ENSG00000163738.17_MTHFD2L	ENSG00000160789.18_LMNA	-0.35272	-0.484699784	-0.20502847	8.32E-06	2.51E-04	4.27E-01

ENSG00000152061.20_RABGAP1	ENSG00000160789.18_LMNA	-0.15391	-0.305624553	0.005420966	5.83E-02	2.21E-01	1.00E+00
ENSG00000251661.3_RP11-326C	ENSG00000160789.18_LMNA	0.1362	-0.023511525	0.28913047	9.43E-02	3.01E-01	1.00E+00
ENSG00000204650.12_CRHR1-IT	ENSG00000160789.18_LMNA	-0.47518	-0.58976448	-0.34183765	6.18E-10	1.05E-07	3.17E-05
ENSG00000135541.19_AHI1	ENSG00000160789.18_LMNA	-0.0253	-0.183759263	0.134443518	7.57E-01	8.99E-01	1.00E+00
ENSG00000185158.11_LRRC37B	ENSG00000160789.18_LMNA	-0.19349	-0.342151588	-0.03537959	1.69E-02	9.57E-02	1.00E+00
ENSG00000273604.1_C17orf96	ENSG00000160789.18_LMNA	-0.09684	-0.252151529	0.063339008	2.35E-01	5.13E-01	1.00E+00
ENSG00000188732.9_FAM221A	ENSG00000160789.18_LMNA	-0.04032	-0.198244677	0.11965229	6.22E-01	8.29E-01	1.00E+00
ENSG00000150893.10_FREM2	ENSG00000160789.18_LMNA	0.11338	-0.046661046	0.267749604	1.64E-01	4.20E-01	1.00E+00
ENSG00000059145.17_UNKL	ENSG00000160789.18_LMNA	-0.04257	-0.200414466	0.117424473	6.03E-01	8.18E-01	1.00E+00
ENSG00000196867.6_ZFP28	ENSG00000160789.18_LMNA	0.06312	-0.097054975	0.220109861	4.40E-01	7.08E-01	1.00E+00
ENSG00000143367.14_TUFT1	ENSG00000160789.18_LMNA	0.17083	0.01195094	0.321289627	3.54E-02	1.60E-01	1.00E+00
ENSG00000182263.12_FIGN	ENSG00000160789.18_LMNA	-0.15051	-0.302461075	0.008906491	6.42E-02	2.36E-01	1.00E+00
ENSG00000165178.9_NCF1C	ENSG00000160789.18_LMNA	0.02749	-0.132293087	0.185873854	7.37E-01	8.89E-01	1.00E+00
ENSG00000100558.7_PLEK2	ENSG00000160789.18_LMNA	0.1343	-0.025448869	0.287353022	9.90E-02	3.10E-01	1.00E+00
ENSG00000154743.16_TSEN2	ENSG00000160789.18_LMNA	-0.21945	-0.365870809	-0.06243306	6.60E-03	4.82E-02	1.00E+00
ENSG00000144401.13_METTL21A	ENSG00000160789.18_LMNA	-0.13304	-0.286182774	0.02672311	1.02E-01	3.16E-01	1.00E+00
ENSG00000163935.12_SFMBT1	ENSG00000160789.18_LMNA	0.03794	-0.12199621	0.19595844	6.43E-01	8.41E-01	1.00E+00
ENSG00000185485.13_SDHAP1	ENSG00000160789.18_LMNA	0.17211	0.013277503	0.322478934	3.40E-02	1.56E-01	1.00E+00
ENSG00000164742.13_ADCY1	ENSG00000160789.18_LMNA	0.08245	-0.077770099	0.238521233	3.13E-01	5.99E-01	1.00E+00
ENSG00000108924.12_HLF	ENSG00000160789.18_LMNA	-0.3973	-0.523394348	-0.25417431	4.02E-07	2.21E-05	2.06E-02
ENSG00000224837.1_GCSHP5	ENSG00000160789.18_LMNA	-0.17738	-0.327336993	-0.01870755	2.88E-02	1.39E-01	1.00E+00
ENSG00000186231.15_KLHL32	ENSG00000160789.18_LMNA	-0.46461	-0.580846101	-0.32980163	1.64E-09	2.36E-07	8.43E-05
ENSG00000188452.12_CERKL	ENSG00000160789.18_LMNA	0.00442	-0.154884912	0.163510416	9.57E-01	9.83E-01	1.00E+00
ENSG00000127903.13_ZNF835	ENSG00000160789.18_LMNA	-0.0118	-0.170675597	0.14768263	8.85E-01	9.56E-01	1.00E+00
ENSG00000139971.14_C14orf37	ENSG00000160789.18_LMNA	-0.08297	-0.239016864	0.077247661	3.09E-01	5.96E-01	1.00E+00
ENSG00000165879.8_FRAT1	ENSG00000160789.18_LMNA	-0.28062	-0.421011036	-0.12709617	4.62E-04	6.33E-03	1.00E+00
ENSG00000100626.15_GALNT16	ENSG00000160789.18_LMNA	0.05624	-0.103886871	0.213532754	4.91E-01	7.47E-01	1.00E+00
ENSG00000167595.13_PROSER3	ENSG00000160789.18_LMNA	0.24331	0.087498065	0.387500381	2.52E-03	2.34E-02	1.00E+00
ENSG00000151849.13_CENPJ	ENSG00000160789.18_LMNA	-0.16893	-0.319540476	-0.0100019	3.75E-02	1.66E-01	1.00E+00
ENSG00000233593.5_RP4-665J2	ENSG00000160789.18_LMNA	0.12528	-0.034607809	0.278920733	1.24E-01	3.55E-01	1.00E+00
ENSG00000196503.2_ARL9	ENSG00000160789.18_LMNA	0.15986	0.000678914	0.311144355	4.91E-02	1.99E-01	1.00E+00
ENSG00000113211.5_PCDHB6	ENSG00000160789.18_LMNA	0.01727	-0.142326494	0.175982401	8.33E-01	9.35E-01	1.00E+00
ENSG00000144445.14_KANSL1L	ENSG00000160789.18_LMNA	-0.30179	-0.439854945	-0.14978249	1.58E-04	2.72E-03	1.00E+00

ENSG00000198963.9_RORB	ENSG00000160789.18_LMNA	0.17007	0.011166773	0.320586136	3.62E-02	1.62E-01	1.00E+00
ENSG00000237651.5_C2orf74	ENSG00000160789.18_LMNA	-0.13046	-0.283764772	0.029352766	1.09E-01	3.29E-01	1.00E+00
ENSG00000185947.13_ZNF267	ENSG00000160789.18_LMNA	0.17605	0.017332701	0.326108511	3.00E-02	1.43E-01	1.00E+00
ENSG00000267475.1_CTD-2538C	ENSG00000160789.18_LMNA	0.09788	-0.062295145	0.253132636	2.30E-01	5.08E-01	1.00E+00
ENSG00000253251.2_CTC-534A2	ENSG00000160789.18_LMNA	-0.33614	-0.470178479	-0.18694189	2.30E-05	5.77E-04	1.00E+00
ENSG00000100478.13_AP4S1	ENSG00000160789.18_LMNA	-0.19157	-0.340385534	-0.0333831	1.81E-02	1.00E-01	1.00E+00
ENSG00000228439.4_TSTD3	ENSG00000160789.18_LMNA	-0.14677	-0.298983433	0.012729453	7.12E-02	2.52E-01	1.00E+00
ENSG00000228887.3_EEF1DP1	ENSG00000160789.18_LMNA	-0.00365	-0.162753587	0.155643688	9.64E-01	9.86E-01	1.00E+00
ENSG00000117597.16_DIEXF	ENSG00000160789.18_LMNA	0.20584	0.048224121	0.353462033	1.10E-02	6.99E-02	1.00E+00
ENSG00000273015.2_RP11-352M	ENSG00000160789.18_LMNA	-0.3456	-0.47847532	-0.19725232	1.30E-05	3.61E-04	6.65E-01
ENSG00000223309.1_RNU6-722F	ENSG00000160789.18_LMNA	-0.00785	-0.166841447	0.151540838	9.24E-01	9.71E-01	1.00E+00
ENSG00000167858.11_TEKT1	ENSG00000160789.18_LMNA	0.10578	-0.054337696	0.260590424	1.95E-01	4.63E-01	1.00E+00
ENSG00000238832.1_snoU109	ENSG00000160789.18_LMNA	0.27541	0.121543381	0.416359755	5.94E-04	7.73E-03	1.00E+00
ENSG00000082269.15_FAM135A	ENSG00000160789.18_LMNA	-0.12919	-0.282580444	0.030639181	1.13E-01	3.35E-01	1.00E+00
ENSG00000272173.1_U47924.31	ENSG00000160789.18_LMNA	-0.05496	-0.212299943	0.105164114	5.01E-01	7.53E-01	1.00E+00
ENSG00000069011.14_PITX1	ENSG00000160789.18_LMNA	0.16344	0.004351435	0.31445756	4.42E-02	1.86E-01	1.00E+00
ENSG00000164296.6_TIGD6	ENSG00000160789.18_LMNA	0.19454	0.03646553	0.343111285	1.63E-02	9.33E-02	1.00E+00
ENSG00000241015.2_TPM3P9	ENSG00000160789.18_LMNA	0.02877	-0.13103242	0.187112142	7.25E-01	8.83E-01	1.00E+00
ENSG00000148019.11_CEP78	ENSG00000160789.18_LMNA	-0.06401	-0.220960426	0.096169273	4.33E-01	7.03E-01	1.00E+00
ENSG00000221883.2_ARIH2OS	ENSG00000160789.18_LMNA	-0.26889	-0.410517777	-0.11459516	8.09E-04	9.81E-03	1.00E+00
ENSG00000180346.3_TIGD2	ENSG00000160789.18_LMNA	-0.13448	-0.287526402	0.025259996	9.86E-02	3.09E-01	1.00E+00
ENSG00000214541.3_RPS4XP3	ENSG00000160789.18_LMNA	-0.02727	-0.185665852	0.132504747	7.39E-01	8.90E-01	1.00E+00
ENSG00000147789.14_ZNF7	ENSG00000160789.18_LMNA	-0.05701	-0.214268742	0.103123857	4.85E-01	7.42E-01	1.00E+00
ENSG00000164440.13_TXLNB	ENSG00000160789.18_LMNA	0.16463	0.005575889	0.315560538	4.27E-02	1.81E-01	1.00E+00
ENSG00000232320.6_AC009299.5	ENSG00000160789.18_LMNA	0.194	0.0359077	0.342618386	1.66E-02	9.45E-02	1.00E+00
ENSG00000154188.8_ANGPT1	ENSG00000160789.18_LMNA	0.20213	0.044359939	0.350068731	1.25E-02	7.70E-02	1.00E+00
ENSG00000262165.1_RP11-81A2	ENSG00000160789.18_LMNA	-0.06279	-0.219792183	0.097385648	4.42E-01	7.10E-01	1.00E+00
ENSG00000174428.15_GTF2IRD2	ENSG00000160789.18_LMNA	-0.15586	-0.307435165	0.003422623	5.52E-02	2.14E-01	1.00E+00
ENSG00000197863.7_ZNF790	ENSG00000160789.18_LMNA	0.19956	0.041684005	0.347714168	1.37E-02	8.22E-02	1.00E+00
ENSG00000275457.1_RP1-198K1	ENSG00000160789.18_LMNA	0.38815	0.244033756	0.515500162	7.77E-07	3.74E-05	3.99E-02
ENSG00000214309.4_MBLAC1	ENSG00000160789.18_LMNA	0.12488	-0.035019596	0.278540478	1.25E-01	3.58E-01	1.00E+00
ENSG00000129292.19_PHF20L1	ENSG00000160789.18_LMNA	-0.06742	-0.224214976	0.09277563	4.09E-01	6.83E-01	1.00E+00
ENSG00000166863.10_TAC3	ENSG00000160789.18_LMNA	-0.13442	-0.287471949	0.025319317	9.87E-02	3.09E-01	1.00E+00

ENSG00000074211.12_PPP2R2C	ENSG00000160789.18_LMNA	-0.32227	-0.457976898	-0.1718916	5.14E-05	1.10E-03	1.00E+00
ENSG00000163171.7_CDC42EP3	ENSG00000160789.18_LMNA	0.00207	-0.157182938	0.161217136	9.80E-01	9.93E-01	1.00E+00
ENSG00000042286.13_AIFM2	ENSG00000160789.18_LMNA	0.47239	0.338654088	0.587411411	8.03E-10	1.30E-07	4.12E-05
ENSG00000167995.14_BEST1	ENSG00000160789.18_LMNA	-0.35017	-0.482477723	-0.20224841	9.76E-06	2.86E-04	5.01E-01
ENSG00000102057.8_KCND1	ENSG00000160789.18_LMNA	-0.05471	-0.212059556	0.105413043	5.03E-01	7.54E-01	1.00E+00
ENSG00000106809.9_OGN	ENSG00000160789.18_LMNA	0.01805	-0.141557812	0.176742488	8.25E-01	9.32E-01	1.00E+00
ENSG00000197959.12_DNM3	ENSG00000160789.18_LMNA	-0.3186	-0.454734038	-0.16791397	6.33E-05	1.30E-03	1.00E+00
ENSG00000187650.3_VMAC	ENSG00000160789.18_LMNA	0.01288	-0.14662063	0.171729286	8.75E-01	9.51E-01	1.00E+00
ENSG00000171016.10_PYGO1	ENSG00000160789.18_LMNA	-0.0366	-0.19466349	0.123322262	6.54E-01	8.47E-01	1.00E+00
ENSG00000164070.10_HSPA4L	ENSG00000160789.18_LMNA	0.06061	-0.099551144	0.217710122	4.58E-01	7.22E-01	1.00E+00
ENSG00000277806.1_RP11-15A1	ENSG00000160789.18_LMNA	-0.08721	-0.243039581	0.073000934	2.85E-01	5.71E-01	1.00E+00
ENSG00000128915.10_ICE2	ENSG00000160789.18_LMNA	-0.1889	-0.337933315	-0.03061498	1.98E-02	1.07E-01	1.00E+00
ENSG00000185839.3_RP4-592A1	ENSG00000160789.18_LMNA	0.0945	-0.065691395	0.249938185	2.47E-01	5.27E-01	1.00E+00
ENSG00000158169.10_FANCC	ENSG00000160789.18_LMNA	0.08717	-0.073049088	0.242994028	2.86E-01	5.71E-01	1.00E+00
ENSG00000184731.5_FAM110C	ENSG00000160789.18_LMNA	0.0227	-0.136993349	0.181248107	7.81E-01	9.10E-01	1.00E+00
ENSG00000279059.1_RP11-257C	ENSG00000160789.18_LMNA	-0.25816	-0.400881468	-0.10319703	1.32E-03	1.43E-02	1.00E+00
ENSG00000164845.15_FAM86FP	ENSG00000160789.18_LMNA	0.2203	0.063317652	0.366639783	6.39E-03	4.71E-02	1.00E+00
ENSG00000141506.12_PIK3R5	ENSG00000160789.18_LMNA	0.02747	-0.132308999	0.185858219	7.37E-01	8.89E-01	1.00E+00
ENSG00000165669.12_FAM204A	ENSG00000160789.18_LMNA	-0.3935	-0.52011571	-0.2499555	5.30E-07	2.75E-05	2.72E-02
ENSG00000187546.12_AGMO	ENSG00000160789.18_LMNA	0.29461	0.142074105	0.43348066	2.29E-04	3.64E-03	1.00E+00
ENSG00000109680.9_TBC1D19	ENSG00000160789.18_LMNA	0.09933	-0.060834299	0.254504568	2.23E-01	4.99E-01	1.00E+00
ENSG00000253846.2_PCDHGA10	ENSG00000160789.18_LMNA	-0.05568	-0.212993546	0.104445642	4.96E-01	7.50E-01	1.00E+00
ENSG00000136213.9_CHST12	ENSG00000160789.18_LMNA	0.35506	0.207591711	0.486745356	7.17E-06	2.24E-04	3.68E-01
ENSG00000161551.11_ZNF577	ENSG00000160789.18_LMNA	-0.02559	-0.184044677	0.134153441	7.54E-01	8.98E-01	1.00E+00
ENSG00000231789.2_PIK3CD-AS	ENSG00000160789.18_LMNA	0.09669	-0.063485134	0.252014136	2.36E-01	5.14E-01	1.00E+00
ENSG00000163507.12_KIAA1524	ENSG00000160789.18_LMNA	-0.03209	-0.190320328	0.127761458	6.95E-01	8.69E-01	1.00E+00
ENSG00000163734.4_CXCL3	ENSG00000160789.18_LMNA	0.25114	0.095770623	0.394567326	1.80E-03	1.81E-02	1.00E+00
ENSG00000263002.6_ZNF234	ENSG00000160789.18_LMNA	0.02078	-0.138884975	0.179382487	7.99E-01	9.19E-01	1.00E+00
ENSG00000149328.13_GLB1L2	ENSG00000160789.18_LMNA	-0.03207	-0.190295964	0.127786325	6.95E-01	8.69E-01	1.00E+00
ENSG00000281332.1_LINC00997	ENSG00000160789.18_LMNA	0.27065	0.116472656	0.412098751	7.45E-04	9.20E-03	1.00E+00
ENSG00000197857.12_ZNF44	ENSG00000160789.18_LMNA	-0.05044	-0.207975054	0.109636613	5.37E-01	7.78E-01	1.00E+00
ENSG00000180667.9_YOD1	ENSG00000160789.18_LMNA	-0.02113	-0.179729088	0.138533718	7.96E-01	9.18E-01	1.00E+00
ENSG00000158258.14_CLSTN2	ENSG00000160789.18_LMNA	-0.31847	-0.454625011	-0.1677804	6.37E-05	1.30E-03	1.00E+00

ENSG00000276075.1_CTD-2012K	ENSG00000160789.18_LMNA	-0.02398	-0.182488835	0.135734039	7.69E-01	9.05E-01	1.00E+00
ENSG00000134508.11_CABLES1	ENSG00000160789.18_LMNA	-0.25793	-0.400682299	-0.10296227	1.34E-03	1.44E-02	1.00E+00
ENSG00000158402.17_CDC25C	ENSG00000160789.18_LMNA	-0.0494	-0.206976313	0.110667616	5.46E-01	7.83E-01	1.00E+00
ENSG00000186767.5_SPIN4	ENSG00000160789.18_LMNA	-0.04944	-0.207006857	0.110636095	5.45E-01	7.83E-01	1.00E+00
ENSG00000234616.7_JRK	ENSG00000160789.18_LMNA	0.01412	-0.145409423	0.172930125	8.63E-01	9.46E-01	1.00E+00
ENSG00000104497.12_SNX16	ENSG00000160789.18_LMNA	-0.06991	-0.226587736	0.090296812	3.92E-01	6.70E-01	1.00E+00
ENSG00000219891.2_ZSCAN12P	ENSG00000160789.18_LMNA	-0.00773	-0.166721965	0.151660913	9.25E-01	9.71E-01	1.00E+00
ENSG00000142731.9_PLK4	ENSG00000160789.18_LMNA	0.10239	-0.057749421	0.257397523	2.09E-01	4.82E-01	1.00E+00
ENSG00000230415.1_RP5-902P8	ENSG00000160789.18_LMNA	-0.04404	-0.201822771	0.115976791	5.90E-01	8.10E-01	1.00E+00
ENSG00000019995.6_ZRANB1	ENSG00000160789.18_LMNA	-0.10416	-0.25906733	0.055966093	2.02E-01	4.72E-01	1.00E+00
ENSG00000185404.15_SP140L	ENSG00000160789.18_LMNA	0.29312	0.140478554	0.432157601	2.47E-04	3.86E-03	1.00E+00
ENSG00000237729.2_AC002075.4	ENSG00000160789.18_LMNA	-0.0519	-0.20936855	0.10819696	5.25E-01	7.70E-01	1.00E+00
ENSG00000241464.3_RPL39P38	ENSG00000160789.18_LMNA	-0.07525	-0.231672042	0.084971996	3.57E-01	6.40E-01	1.00E+00
ENSG00000221716.1_SNORA11	ENSG00000160789.18_LMNA	-0.27678	-0.417582295	-0.12300109	5.57E-04	7.33E-03	1.00E+00
ENSG00000169598.14_DFFB	ENSG00000160789.18_LMNA	-0.15787	-0.309298367	0.001363643	5.21E-02	2.07E-01	1.00E+00
ENSG00000112234.7_FBXL4	ENSG00000160789.18_LMNA	-0.2119	-0.358992685	-0.05454189	8.77E-03	5.96E-02	1.00E+00
ENSG00000275441.1_RP11-666O	ENSG00000160789.18_LMNA	-0.11208	-0.266521739	0.047980305	1.69E-01	4.28E-01	1.00E+00
ENSG00000115641.17_FHL2	ENSG00000160789.18_LMNA	0.36518	0.218688791	0.495566104	3.73E-06	1.31E-04	1.91E-01
ENSG00000183793.12_NPIPA5	ENSG00000160789.18_LMNA	-0.12604	-0.279627899	0.033841721	1.22E-01	3.51E-01	1.00E+00
ENSG00000140398.12_NEIL1	ENSG00000160789.18_LMNA	0.00329	-0.155989519	0.16240852	9.68E-01	9.87E-01	1.00E+00
ENSG00000186960.9_LINC01551	ENSG00000160789.18_LMNA	0.11874	-0.041238039	0.272786214	1.45E-01	3.90E-01	1.00E+00
ENSG00000239998.4_LILRA2	ENSG00000160789.18_LMNA	-0.17814	-0.328041729	-0.01949677	2.81E-02	1.37E-01	1.00E+00
ENSG00000277476.1_RP11-147L	ENSG00000160789.18_LMNA	0.26133	0.106567764	0.403738059	1.15E-03	1.28E-02	1.00E+00
ENSG00000072840.11_EVC	ENSG00000160789.18_LMNA	0.44469	0.307238728	0.563963599	9.50E-09	1.03E-06	4.87E-04
ENSG00000147650.10_LRP12	ENSG00000160789.18_LMNA	0.02328	-0.136426487	0.18180673	7.76E-01	9.08E-01	1.00E+00
ENSG00000141933.8_TPGS1	ENSG00000160789.18_LMNA	0.11992	-0.040043969	0.273892897	1.41E-01	3.83E-01	1.00E+00
ENSG00000197483.9_ZNF628	ENSG00000160789.18_LMNA	0.26258	0.10789428	0.404860654	1.08E-03	1.23E-02	1.00E+00
ENSG00000042980.11_ADAM28	ENSG00000160789.18_LMNA	-0.11366	-0.268011056	0.046379991	1.63E-01	4.19E-01	1.00E+00
ENSG00000158423.15_RIBC1	ENSG00000160789.18_LMNA	0.00529	-0.154043398	0.164349332	9.48E-01	9.79E-01	1.00E+00
ENSG00000141401.10_IMPA2	ENSG00000160789.18_LMNA	0.29974	0.147579891	0.43803653	1.76E-04	2.95E-03	1.00E+00
ENSG00000196878.11_LAMB3	ENSG00000160789.18_LMNA	0.35895	0.211856369	0.490141958	5.59E-06	1.82E-04	2.87E-01
ENSG00000260136.4_CTD-2270L	ENSG00000160789.18_LMNA	-0.08099	-0.23713268	0.07923283	3.21E-01	6.08E-01	1.00E+00
ENSG00000259032.2_ENSAP2	ENSG00000160789.18_LMNA	0.12661	-0.03326523	0.280159827	1.20E-01	3.48E-01	1.00E+00

ENSG00000267796.6_LIN37	ENSG00000160789.18_LMNA	-0.15157	-0.30344879	0.007819037	6.23E-02	2.31E-01	1.00E+00
ENSG00000167971.15_CASKIN1	ENSG00000160789.18_LMNA	-0.2395	-0.384054143	-0.08347885	2.96E-03	2.65E-02	1.00E+00
ENSG00000169118.14_CSNK1G1	ENSG00000160789.18_LMNA	0.31145	0.160193006	0.448417556	9.39E-05	1.80E-03	1.00E+00
ENSG00000226332.2_RP11-157P	ENSG00000160789.18_LMNA	0.31974	0.169149642	0.455742263	5.93E-05	1.23E-03	1.00E+00
ENSG00000183549.9_ACSM5	ENSG00000160789.18_LMNA	-0.33852	-0.47227066	-0.18953596	1.99E-05	5.15E-04	1.00E+00
ENSG00000169397.3_RNASE3	ENSG00000160789.18_LMNA	0.06593	-0.094261181	0.222791182	4.20E-01	6.91E-01	1.00E+00
ENSG00000213399.3_AC022210.:	ENSG00000160789.18_LMNA	0.04651	-0.113534819	0.204195347	5.69E-01	7.99E-01	1.00E+00
ENSG00000172869.13_DMXL1	ENSG00000160789.18_LMNA	-0.07157	-0.228171005	0.088640588	3.81E-01	6.60E-01	1.00E+00
ENSG00000114127.9_XRN1	ENSG00000160789.18_LMNA	-0.14379	-0.296213699	0.015767707	7.72E-02	2.66E-01	1.00E+00
ENSG00000083828.14_ZNF586	ENSG00000160789.18_LMNA	-0.15694	-0.308437845	0.002314913	5.35E-02	2.10E-01	1.00E+00
ENSG00000006607.12_FARP2	ENSG00000160789.18_LMNA	0.02587	-0.133884878	0.184308876	7.52E-01	8.97E-01	1.00E+00
ENSG00000145088.7_EAF2	ENSG00000160789.18_LMNA	-0.14368	-0.296109182	0.015882245	7.74E-02	2.67E-01	1.00E+00
ENSG00000235919.4_ASH1L-AS1	ENSG00000160789.18_LMNA	-0.13816	-0.290958714	0.021516364	8.96E-02	2.92E-01	1.00E+00
ENSG00000138400.11_MDH1B	ENSG00000160789.18_LMNA	-0.06743	-0.224219229	0.092771191	4.09E-01	6.83E-01	1.00E+00
ENSG00000168566.11_SNRNP48	ENSG00000160789.18_LMNA	-0.03609	-0.194177355	0.123819779	6.59E-01	8.49E-01	1.00E+00
ENSG00000184445.10_KNTC1	ENSG00000160789.18_LMNA	0.14816	-0.011307277	0.300278115	6.85E-02	2.46E-01	1.00E+00
ENSG00000177943.12_MAMDC4	ENSG00000160789.18_LMNA	0.15435	-0.00497608	0.30602784	5.76E-02	2.20E-01	1.00E+00
ENSG00000158691.13_ZSCAN12	ENSG00000160789.18_LMNA	-0.101	-0.256082282	0.059152663	2.16E-01	4.90E-01	1.00E+00
ENSG00000112200.15_ZNF451	ENSG00000160789.18_LMNA	-0.20168	-0.349653438	-0.04388764	1.27E-02	7.79E-02	1.00E+00
ENSG00000166860.2_ZBTB39	ENSG00000160789.18_LMNA	-0.04549	-0.203219146	0.114540037	5.78E-01	8.03E-01	1.00E+00
ENSG00000107036.10_RIC1	ENSG00000160789.18_LMNA	0.08309	-0.077129807	0.239128648	3.09E-01	5.95E-01	1.00E+00
ENSG00000166920.9_C15orf48	ENSG00000160789.18_LMNA	0.07998	-0.080238186	0.236177556	3.27E-01	6.14E-01	1.00E+00
ENSG00000136371.8_MTHFS	ENSG00000160789.18_LMNA	0.21887	0.061818556	0.365336384	6.75E-03	4.90E-02	1.00E+00
ENSG00000100234.11_TIMP3	ENSG00000160789.18_LMNA	0.07165	-0.088563404	0.228244748	3.80E-01	6.59E-01	1.00E+00
ENSG00000005108.14_THSD7A	ENSG00000160789.18_LMNA	-0.37604	-0.505008085	-0.23064732	1.80E-06	7.39E-05	9.25E-02
ENSG00000115507.8_OTX1	ENSG00000160789.18_LMNA	0.18166	0.02312893	0.331280627	2.51E-02	1.27E-01	1.00E+00
ENSG00000246731.2_MGC16275	ENSG00000160789.18_LMNA	-0.17443	-0.324614431	-0.01566219	3.16E-02	1.48E-01	1.00E+00
ENSG00000180998.10_GPR137C	ENSG00000160789.18_LMNA	-0.25175	-0.395112977	-0.09641108	1.76E-03	1.77E-02	1.00E+00
ENSG00000139668.8_WDFY2	ENSG00000160789.18_LMNA	0.10695	-0.053151705	0.261698737	1.90E-01	4.57E-01	1.00E+00
ENSG00000146070.15_PLA2G7	ENSG00000160789.18_LMNA	-0.17796	-0.32787119	-0.01930575	2.83E-02	1.38E-01	1.00E+00
ENSG00000157625.14_TAB3	ENSG00000160789.18_LMNA	0.0175	-0.142091907	0.176214405	8.31E-01	9.34E-01	1.00E+00
ENSG00000225093.1_RPL3P7	ENSG00000160789.18_LMNA	-0.12542	-0.27905154	0.034466131	1.24E-01	3.55E-01	1.00E+00
ENSG00000252481.1_SCARNA13	ENSG00000160789.18_LMNA	-0.15444	-0.306110026	0.004885401	5.75E-02	2.19E-01	1.00E+00

ENSG00000122335.12_SERAC1	ENSG00000160789.18_LMNA	-0.19922	-0.347404597	-0.04133251	1.39E-02	8.29E-02	1.00E+00
ENSG00000214460.3_TPT1P6	ENSG00000160789.18_LMNA	-0.12606	-0.279651549	0.033816094	1.22E-01	3.51E-01	1.00E+00
ENSG00000181982.16_CCDC149	ENSG00000160789.18_LMNA	0.04947	-0.110597882	0.207043885	5.45E-01	7.83E-01	1.00E+00
ENSG00000119599.15_DCAF4	ENSG00000160789.18_LMNA	0.08878	-0.071428504	0.244526315	2.77E-01	5.62E-01	1.00E+00
ENSG00000180488.13_MIGA1	ENSG00000160789.18_LMNA	-0.07001	-0.22668565	0.090194438	3.91E-01	6.69E-01	1.00E+00
ENSG00000122707.10_RECK	ENSG00000160789.18_LMNA	0.1068	-0.053308233	0.261552508	1.90E-01	4.58E-01	1.00E+00
ENSG00000168813.15_ZNF507	ENSG00000160789.18_LMNA	0.01795	-0.141651848	0.176649523	8.26E-01	9.32E-01	1.00E+00
ENSG00000134595.7_SOX3	ENSG00000160789.18_LMNA	-0.10094	-0.256025562	0.05921315	2.16E-01	4.90E-01	1.00E+00
ENSG00000066185.11_ZMYND12	ENSG00000160789.18_LMNA	-0.03346	-0.191635586	0.126418462	6.82E-01	8.62E-01	1.00E+00
ENSG00000154548.8_SRSF12	ENSG00000160789.18_LMNA	-0.27808	-0.418739013	-0.12438148	5.23E-04	6.99E-03	1.00E+00
ENSG00000005189.18_AC004381	ENSG00000160789.18_LMNA	-0.25271	-0.395983361	-0.09743321	1.68E-03	1.72E-02	1.00E+00
ENSG00000234589.4_RP11-253E	ENSG00000160789.18_LMNA	-0.10428	-0.259176879	0.055849026	2.01E-01	4.72E-01	1.00E+00
ENSG00000168826.14_ZBTB49	ENSG00000160789.18_LMNA	-0.14858	-0.300669186	0.010877448	6.77E-02	2.44E-01	1.00E+00
ENSG00000281404.1_LINC01176	ENSG00000160789.18_LMNA	-0.07976	-0.235962937	0.080464005	3.29E-01	6.15E-01	1.00E+00
ENSG00000111817.15_DSE	ENSG00000160789.18_LMNA	0.16262	0.003509038	0.31369825	4.53E-02	1.89E-01	1.00E+00
ENSG00000225067.4_RPL23AP2	ENSG00000160789.18_LMNA	-0.0563	-0.213585355	0.103832351	4.91E-01	7.47E-01	1.00E+00
ENSG00000265458.1_RP13-20L1	ENSG00000160789.18_LMNA	0.0093	-0.150124477	0.168250096	9.09E-01	9.65E-01	1.00E+00
ENSG00000167895.13_TMC8	ENSG00000160789.18_LMNA	0.01627	-0.143298436	0.175020783	8.42E-01	9.39E-01	1.00E+00
ENSG00000172935.8_MRGPRF	ENSG00000160789.18_LMNA	0.18036	0.021789926	0.330087444	2.62E-02	1.31E-01	1.00E+00
ENSG00000239961.2_LILRA4	ENSG00000160789.18_LMNA	-0.32028	-0.456221481	-0.16973728	5.76E-05	1.20E-03	1.00E+00
ENSG00000188542.8_DUSP28	ENSG00000160789.18_LMNA	-0.46763	-0.583397734	-0.33323693	1.25E-09	1.87E-07	6.40E-05
ENSG00000138640.13_FAM13A	ENSG00000160789.18_LMNA	-0.24112	-0.385520718	-0.08518807	2.77E-03	2.51E-02	1.00E+00
ENSG00000137962.11_ARHGAP2	ENSG00000160789.18_LMNA	0.42088	0.280472851	0.543654053	6.70E-08	5.23E-06	3.44E-03
ENSG00000182983.13_ZNF662	ENSG00000160789.18_LMNA	-0.08469	-0.240643011	0.07553234	3.00E-01	5.86E-01	1.00E+00
ENSG00000278318.3_ZNF229	ENSG00000160789.18_LMNA	0.07442	-0.085795289	0.230887064	3.62E-01	6.44E-01	1.00E+00
ENSG00000126500.3_FLRT1	ENSG00000160789.18_LMNA	-0.29225	-0.431379003	-0.13954031	2.59E-04	4.01E-03	1.00E+00
ENSG00000129474.14_AJUBA	ENSG00000160789.18_LMNA	0.44343	0.305816286	0.562892029	1.06E-08	1.13E-06	5.42E-04
ENSG00000229944.3_EIF4EP2	ENSG00000160789.18_LMNA	-0.03136	-0.189611816	0.12848443	7.01E-01	8.72E-01	1.00E+00
ENSG00000197430.9_OPALIN	ENSG00000160789.18_LMNA	-0.37234	-0.501796659	-0.22657064	2.32E-06	8.91E-05	1.19E-01
ENSG00000137098.12_SPAG8	ENSG00000160789.18_LMNA	-0.10768	-0.262384999	0.052416898	1.87E-01	4.53E-01	1.00E+00
ENSG00000155621.13_C9orf85	ENSG00000160789.18_LMNA	-0.16463	-0.315559484	-0.00557472	4.27E-02	1.81E-01	1.00E+00
ENSG00000074047.19_GLI2	ENSG00000160789.18_LMNA	0.31391	0.16285293	0.450596882	8.21E-05	1.60E-03	1.00E+00
ENSG00000205579.3_DYNLL1P1	ENSG00000160789.18_LMNA	-0.19463	-0.343192511	-0.03655747	1.63E-02	9.31E-02	1.00E+00

ENSG00000136828.17_RALGPS1	ENSG00000160789.18_LMNA	-0.34788	-0.480473683	-0.199745	1.13E-05	3.21E-04	5.77E-01
ENSG00000152749.7_GPR180	ENSG00000160789.18_LMNA	-0.37121	-0.500811856	-0.22532243	2.50E-06	9.47E-05	1.28E-01
ENSG00000111728.9_ST8SIA1	ENSG00000160789.18_LMNA	-0.0776	-0.233914409	0.082617794	3.42E-01	6.27E-01	1.00E+00
ENSG00000236735.3_RPL31P63	ENSG00000160789.18_LMNA	-0.02336	-0.181885052	0.136346994	7.75E-01	9.07E-01	1.00E+00
ENSG00000088836.11_SLC4A11	ENSG00000160789.18_LMNA	0.2286	0.072017023	0.374180076	4.62E-03	3.69E-02	1.00E+00
ENSG00000163412.11_EIF4E3	ENSG00000160789.18_LMNA	-0.17422	-0.324426134	-0.01545178	3.18E-02	1.49E-01	1.00E+00
ENSG00000183690.12_EFHC2	ENSG00000160789.18_LMNA	0.16113	0.001978974	0.312318082	4.74E-02	1.94E-01	1.00E+00
ENSG00000134028.13_ADAMDE(	ENSG00000160789.18_LMNA	-0.14744	-0.299605582	0.012046195	6.99E-02	2.49E-01	1.00E+00
ENSG00000239763.2_AC009120.:	ENSG00000160789.18_LMNA	-0.05408	-0.2114594	0.106034352	5.08E-01	7.58E-01	1.00E+00
ENSG00000183323.11_CCDC125	ENSG00000160789.18_LMNA	0.02879	-0.131015431	0.187128822	7.25E-01	8.83E-01	1.00E+00
ENSG00000174628.15_IQCK	ENSG00000160789.18_LMNA	-0.02149	-0.180071654	0.138186472	7.93E-01	9.16E-01	1.00E+00
ENSG00000268751.1_SCGB1B2P	ENSG00000160789.18_LMNA	-0.03962	-0.197574323	0.120339924	6.28E-01	8.33E-01	1.00E+00
ENSG00000279382.1_RP11-449J:	ENSG00000160789.18_LMNA	-0.0179	-0.176602068	0.141699849	8.27E-01	9.32E-01	1.00E+00
ENSG00000230747.1_AC021188.4	ENSG00000160789.18_LMNA	0.01952	-0.140119691	0.17816351	8.11E-01	9.26E-01	1.00E+00
ENSG00000275765.3_RP11-54C4	ENSG00000160789.18_LMNA	-0.11753	-0.271651542	0.042461377	1.49E-01	3.97E-01	1.00E+00
ENSG00000198873.11_GRK5	ENSG00000160789.18_LMNA	0.28808	0.135073432	0.427666227	3.19E-04	4.72E-03	1.00E+00
ENSG00000149311.16_ATM	ENSG00000160789.18_LMNA	-0.13749	-0.290337895	0.022194142	9.12E-02	2.94E-01	1.00E+00
ENSG00000269427.1_CTC-429P9	ENSG00000160789.18_LMNA	-0.15007	-0.302057384	0.009350735	6.50E-02	2.38E-01	1.00E+00
ENSG00000276259.1_RP11-481J:	ENSG00000160789.18_LMNA	-0.1476	-0.299755029	0.011882025	6.96E-02	2.49E-01	1.00E+00
ENSG00000177103.12_DSCAML1	ENSG00000160789.18_LMNA	-0.39623	-0.522475337	-0.25299073	4.35E-07	2.35E-05	2.23E-02
ENSG00000213942.3_RP1-102E2	ENSG00000160789.18_LMNA	-0.09668	-0.252000589	0.063499542	2.36E-01	5.14E-01	1.00E+00
ENSG00000072609.16_CHFR	ENSG00000160789.18_LMNA	0.11293	-0.04711794	0.267324477	1.66E-01	4.23E-01	1.00E+00
ENSG00000227544.7_AC018647.:	ENSG00000160789.18_LMNA	-0.43306	-0.554063205	-0.2941401	2.51E-08	2.34E-06	1.29E-03
ENSG00000198580.7_RP11-611L	ENSG00000160789.18_LMNA	-0.00513	-0.164200439	0.154192786	9.50E-01	9.80E-01	1.00E+00
ENSG00000163331.9_DAPL1	ENSG00000160789.18_LMNA	-0.36857	-0.498517261	-0.2224176	2.98E-06	1.09E-04	1.53E-01
ENSG00000197124.10_ZNF682	ENSG00000160789.18_LMNA	-0.23119	-0.376529779	-0.07473735	4.16E-03	3.42E-02	1.00E+00
ENSG00000166750.8_SLFN5	ENSG00000160789.18_LMNA	0.13427	-0.02547233	0.287331485	9.91E-02	3.10E-01	1.00E+00
ENSG00000145649.7_GZMA	ENSG00000160789.18_LMNA	0.02309	-0.136608236	0.181627645	7.78E-01	9.09E-01	1.00E+00
ENSG00000185164.13_NOMO2	ENSG00000160789.18_LMNA	0.10533	-0.054789831	0.260167685	1.97E-01	4.66E-01	1.00E+00
ENSG00000008300.13_CELSR3	ENSG00000160789.18_LMNA	-0.19086	-0.339735376	-0.03264873	1.85E-02	1.02E-01	1.00E+00
ENSG00000173530.5_TNFRSF10I	ENSG00000160789.18_LMNA	0.24027	0.084296664	0.384756046	2.87E-03	2.58E-02	1.00E+00
ENSG00000144161.11_ZC3H8	ENSG00000160789.18_LMNA	-0.30941	-0.446608336	-0.15798798	1.05E-04	1.96E-03	1.00E+00
ENSG00000267128.1_RP11-449J:	ENSG00000160789.18_LMNA	-0.0622	-0.219231876	0.097968704	4.46E-01	7.13E-01	1.00E+00

ENSG00000159761.13_C16orf86	ENSG00000160789.18_LMNA	0.02829	-0.13150326	0.186649779	7.29E-01	8.86E-01	1.00E+00
ENSG00000125885.12_MCM8	ENSG00000160789.18_LMNA	0.0599	-0.100254657	0.217033094	4.64E-01	7.26E-01	1.00E+00
ENSG00000170089.14_RP11-423	ENSG00000160789.18_LMNA	0.00258	-0.156683265	0.16171607	9.75E-01	9.90E-01	1.00E+00
ENSG00000251615.3_RP11-774O	ENSG00000160789.18_LMNA	-0.13994	-0.292620642	0.01970054	8.55E-02	2.84E-01	1.00E+00
ENSG00000250988.6_SNHG21	ENSG00000160789.18_LMNA	-0.057	-0.214253299	0.103139871	4.86E-01	7.42E-01	1.00E+00
ENSG00000273117.1_AC144652.1	ENSG00000160789.18_LMNA	0.04006	-0.11990739	0.197996022	6.24E-01	8.31E-01	1.00E+00
ENSG00000134548.8_SPX	ENSG00000160789.18_LMNA	-0.35246	-0.484474284	-0.20474614	8.46E-06	2.55E-04	4.34E-01
ENSG00000106246.16_PTCD1	ENSG00000160789.18_LMNA	0.23924	0.083208505	0.383822033	2.99E-03	2.67E-02	1.00E+00
ENSG00000280216.1_RP3-333H2	ENSG00000160789.18_LMNA	-0.22776	-0.373423234	-0.07114176	4.77E-03	3.79E-02	1.00E+00
ENSG00000133136.4_GNG5P2	ENSG00000160789.18_LMNA	0.19921	0.041315179	0.347389335	1.39E-02	8.29E-02	1.00E+00
ENSG00000132530.15_XAF1	ENSG00000160789.18_LMNA	0.08984	-0.070366452	0.245529645	2.71E-01	5.55E-01	1.00E+00
ENSG00000104611.10_SH2D4A	ENSG00000160789.18_LMNA	0.31074	0.15942864	0.447790663	9.76E-05	1.85E-03	1.00E+00
ENSG00000164398.11_ACSL6	ENSG00000160789.18_LMNA	-0.47728	-0.591536528	-0.34423889	5.07E-10	8.91E-08	2.60E-05
ENSG00000229358.3_DPY19L1P1	ENSG00000160789.18_LMNA	0.1031	-0.057034338	0.258067311	2.06E-01	4.78E-01	1.00E+00
ENSG00000160190.12_SLC37A1	ENSG00000160789.18_LMNA	-0.12142	-0.275295433	0.038529397	1.36E-01	3.76E-01	1.00E+00
ENSG00000261534.1_RP11-244O	ENSG00000160789.18_LMNA	-0.06579	-0.222662654	0.094395214	4.21E-01	6.92E-01	1.00E+00
ENSG00000145936.7_KCNMB1	ENSG00000160789.18_LMNA	0.14802	-0.011448505	0.300149599	6.88E-02	2.47E-01	1.00E+00
ENSG00000152223.11_EPG5	ENSG00000160789.18_LMNA	0.03915	-0.120803567	0.197122165	6.32E-01	8.35E-01	1.00E+00
ENSG00000244363.3_RPL7P23	ENSG00000160789.18_LMNA	-0.1221	-0.275939931	0.037832931	1.34E-01	3.72E-01	1.00E+00
ENSG00000276846.1_CTD-3220F	ENSG00000160789.18_LMNA	-0.17949	-0.329282899	-0.02088768	2.69E-02	1.33E-01	1.00E+00
ENSG00000255142.1_AP006621.6	ENSG00000160789.18_LMNA	0.15838	-0.000837274	0.309774305	5.13E-02	2.05E-01	1.00E+00
ENSG00000241506.1_PSMC1P1	ENSG00000160789.18_LMNA	0.09353	-0.066661447	0.249024509	2.52E-01	5.33E-01	1.00E+00
ENSG00000234177.4_LINC01114	ENSG00000160789.18_LMNA	0.03829	-0.121657195	0.196289326	6.40E-01	8.39E-01	1.00E+00
ENSG00000222009.7_BTBD19	ENSG00000160789.18_LMNA	0.27646	0.122661403	0.417297506	5.65E-04	7.41E-03	1.00E+00
ENSG00000171843.14_MLLT3	ENSG00000160789.18_LMNA	-0.13946	-0.29217484	0.020187826	8.66E-02	2.86E-01	1.00E+00
ENSG00000255769.6_GOLGA2P1	ENSG00000160789.18_LMNA	-0.05002	-0.207568548	0.110056333	5.41E-01	7.80E-01	1.00E+00
ENSG00000156574.8_NODAL	ENSG00000160789.18_LMNA	-0.14255	-0.295056027	0.01703591	7.98E-02	2.72E-01	1.00E+00
ENSG00000167232.12_ZNF91	ENSG00000160789.18_LMNA	-0.21725	-0.363864978	-0.06012787	7.18E-03	5.13E-02	1.00E+00
ENSG00000134460.14_IL2RA	ENSG00000160789.18_LMNA	0.22241	0.065530945	0.36856197	5.89E-03	4.44E-02	1.00E+00
ENSG00000277511.1_CTD-2095E	ENSG00000160789.18_LMNA	-0.23362	-0.378738834	-0.07729894	3.77E-03	3.17E-02	1.00E+00
ENSG00000169083.14_AR	ENSG00000160789.18_LMNA	-0.10086	-0.25595427	0.059289174	2.16E-01	4.91E-01	1.00E+00
ENSG00000203896.8_LIME1	ENSG00000160789.18_LMNA	0.06387	-0.096313972	0.2208215	4.34E-01	7.03E-01	1.00E+00
ENSG00000231747.1_AC079922.2	ENSG00000160789.18_LMNA	-0.04162	-0.199503386	0.118360308	6.11E-01	8.22E-01	1.00E+00

ENSG00000151229.11_SLC2A13	ENSG00000160789.18_LMNA	-0.18869	-0.337741143	-0.03039825	1.99E-02	1.08E-01	1.00E+00
ENSG00000139436.19_GIT2	ENSG00000160789.18_LMNA	0.07727	-0.082954112	0.233594273	3.44E-01	6.29E-01	1.00E+00
ENSG00000163874.8_ZC3H12A	ENSG00000160789.18_LMNA	0.38792	0.243769583	0.515293892	7.90E-07	3.77E-05	4.05E-02
ENSG00000179335.17_CLK3	ENSG00000160789.18_LMNA	0.16609	0.007080165	0.31691443	4.08E-02	1.76E-01	1.00E+00
ENSG00000227854.1_RP11-307C	ENSG00000160789.18_LMNA	-0.11728	-0.271417672	0.042713405	1.50E-01	3.98E-01	1.00E+00
ENSG00000088876.10_ZNF343	ENSG00000160789.18_LMNA	0.33068	0.181005381	0.465378521	3.17E-05	7.46E-04	1.00E+00
ENSG00000001630.14_CYP51A1	ENSG00000160789.18_LMNA	0.17654	0.017843785	0.326565308	2.96E-02	1.42E-01	1.00E+00
ENSG00000270084.1_GAS5-AS1	ENSG00000160789.18_LMNA	-0.20027	-0.348361077	-0.04241877	1.34E-02	8.07E-02	1.00E+00
ENSG00000131015.4_ULBP2	ENSG00000160789.18_LMNA	0.26093	0.106133323	0.40337021	1.17E-03	1.30E-02	1.00E+00
ENSG00000131591.16_C1orf159	ENSG00000160789.18_LMNA	0.02573	-0.13401611	0.184179782	7.53E-01	8.97E-01	1.00E+00
ENSG00000271646.1_RP11-326I1	ENSG00000160789.18_LMNA	0.14625	-0.013256688	0.298503172	7.22E-02	2.55E-01	1.00E+00
ENSG00000223529.1_EEF1A1P8	ENSG00000160789.18_LMNA	-0.09098	-0.246612035	0.069219911	2.65E-01	5.49E-01	1.00E+00
ENSG00000153363.11_LINC0046	ENSG00000160789.18_LMNA	-0.20504	-0.352730652	-0.04739047	1.13E-02	7.13E-02	1.00E+00
ENSG00000267355.2_RPL9P29	ENSG00000160789.18_LMNA	-0.11392	-0.268256054	0.046116579	1.62E-01	4.17E-01	1.00E+00
ENSG00000135968.18_GCC2	ENSG00000160789.18_LMNA	-0.05128	-0.208779916	0.108805253	5.30E-01	7.73E-01	1.00E+00
ENSG00000178184.14_PARD6G	ENSG00000160789.18_LMNA	-0.10485	-0.259718832	0.055269753	1.99E-01	4.69E-01	1.00E+00
ENSG00000230163.1_RP5-997D1	ENSG00000160789.18_LMNA	0.28292	0.129554441	0.423065277	4.13E-04	5.81E-03	1.00E+00
ENSG00000260244.1_RP11-588K	ENSG00000160789.18_LMNA	-0.09324	-0.248750241	0.066952522	2.53E-01	5.35E-01	1.00E+00
ENSG00000138190.15_EXOC6	ENSG00000160789.18_LMNA	-0.28745	-0.427107106	-0.13440178	3.30E-04	4.85E-03	1.00E+00
ENSG00000242262.1_RP11-100N	ENSG00000160789.18_LMNA	-0.18723	-0.336407524	-0.028895	2.09E-02	1.11E-01	1.00E+00
ENSG00000158483.14_FAM86C1	ENSG00000160789.18_LMNA	0.26823	0.113888313	0.409922104	8.35E-04	1.00E-02	1.00E+00
ENSG00000178338.9_ZNF354B	ENSG00000160789.18_LMNA	0.01563	-0.143928427	0.174397159	8.48E-01	9.41E-01	1.00E+00
ENSG00000121989.13_ACVR2A	ENSG00000160789.18_LMNA	-0.37747	-0.506251206	-0.23222796	1.64E-06	6.85E-05	8.39E-02
ENSG00000154330.11_PGM5	ENSG00000160789.18_LMNA	-0.26395	-0.406087168	-0.1093448	1.02E-03	1.17E-02	1.00E+00
ENSG00000104361.8_NIPAL2	ENSG00000160789.18_LMNA	0.04	-0.119963406	0.197941416	6.25E-01	8.31E-01	1.00E+00
ENSG00000204519.9_ZNF551	ENSG00000160789.18_LMNA	-0.06546	-0.222347455	0.094723866	4.23E-01	6.94E-01	1.00E+00
ENSG00000196236.11_XPNPEP3	ENSG00000160789.18_LMNA	-0.09802	-0.253268786	0.062150232	2.30E-01	5.07E-01	1.00E+00
ENSG00000104313.16_EYA1	ENSG00000160789.18_LMNA	-0.30596	-0.443553548	-0.15427141	1.26E-04	2.28E-03	1.00E+00
ENSG00000204851.6_PNMAL2	ENSG00000160789.18_LMNA	-0.17566	-0.32574945	-0.01693108	3.04E-02	1.45E-01	1.00E+00
ENSG00000185615.14_PDIA2	ENSG00000160789.18_LMNA	-0.22191	-0.368108031	-0.06500799	6.00E-03	4.50E-02	1.00E+00
ENSG00000163617.9_CCDC191	ENSG00000160789.18_LMNA	0.14396	-0.015598288	0.296368284	7.68E-02	2.65E-01	1.00E+00
ENSG00000231113.2_RP3-475N1	ENSG00000160789.18_LMNA	-0.08154	-0.237660159	0.078677333	3.18E-01	6.05E-01	1.00E+00
ENSG00000166897.13_ELFN2	ENSG00000160789.18_LMNA	-0.29448	-0.433365761	-0.14193548	2.31E-04	3.66E-03	1.00E+00

ENSG00000160716.4_CHRNB2	ENSG00000160789.18_LMNA	-0.28485	-0.424784812	-0.13161495	3.75E-04	5.37E-03	1.00E+00
ENSG00000273058.2_RP11-385F	ENSG00000160789.18_LMNA	-0.04369	-0.201492039	0.116316892	5.93E-01	8.12E-01	1.00E+00
ENSG00000173641.16_HSPB7	ENSG00000160789.18_LMNA	-0.01966	-0.178304139	0.139977296	8.10E-01	9.25E-01	1.00E+00
ENSG00000215447.6_BX322557.1	ENSG00000160789.18_LMNA	0.15604	-0.003240786	0.307599809	5.49E-02	2.14E-01	1.00E+00
ENSG00000266964.4_FXYD1	ENSG00000160789.18_LMNA	0.01043	-0.149017956	0.169349687	8.99E-01	9.61E-01	1.00E+00
ENSG00000212719.9_C17orf51	ENSG00000160789.18_LMNA	-0.10056	-0.255666049	0.059596486	2.18E-01	4.93E-01	1.00E+00
ENSG00000170011.12_MYRIP	ENSG00000160789.18_LMNA	-0.22674	-0.372496776	-0.07007098	4.97E-03	3.90E-02	1.00E+00
ENSG00000250144.1_RP11-553P	ENSG00000160789.18_LMNA	-0.12905	-0.282444258	0.030787039	1.13E-01	3.36E-01	1.00E+00
ENSG00000255883.1_FUNDC2P1	ENSG00000160789.18_LMNA	-0.28397	-0.424006368	-0.13068183	3.92E-04	5.57E-03	1.00E+00
ENSG00000162148.9_PPP1R32	ENSG00000160789.18_LMNA	-0.12295	-0.276730069	0.036978666	1.31E-01	3.67E-01	1.00E+00
ENSG00000101746.14_NOL4	ENSG00000160789.18_LMNA	-0.43525	-0.555928952	-0.29660111	2.10E-08	2.01E-06	1.08E-03
ENSG00000135828.10_RNASEL	ENSG00000160789.18_LMNA	0.18778	0.029457134	0.336906369	2.05E-02	1.10E-01	1.00E+00
ENSG00000226824.5_RP4-756H1	ENSG00000160789.18_LMNA	-0.00157	-0.160727144	0.157673498	9.85E-01	9.94E-01	1.00E+00
ENSG00000269899.1_RP11-589N	ENSG00000160789.18_LMNA	0.06743	-0.092763547	0.224226551	4.09E-01	6.83E-01	1.00E+00
ENSG00000118412.11_CASP8AP	ENSG00000160789.18_LMNA	0.00663	-0.152728914	0.165658836	9.35E-01	9.75E-01	1.00E+00
ENSG00000215271.7_HOMEZ	ENSG00000160789.18_LMNA	0.12349	-0.036424295	0.277242598	1.30E-01	3.65E-01	1.00E+00
ENSG00000154080.11_CHST9	ENSG00000160789.18_LMNA	-0.32889	-0.46380485	-0.17906359	3.52E-05	8.15E-04	1.00E+00
ENSG00000085382.10_HACE1	ENSG00000160789.18_LMNA	-0.23641	-0.381260275	-0.08022764	3.36E-03	2.92E-02	1.00E+00
ENSG00000145012.11_LPP	ENSG00000160789.18_LMNA	0.41304	0.271701876	0.53693104	1.23E-07	8.60E-06	6.33E-03
ENSG00000268912.1_CTD-2619J	ENSG00000160789.18_LMNA	-0.03633	-0.194408972	0.123582758	6.57E-01	8.48E-01	1.00E+00
ENSG00000157110.14_RBPMS	ENSG00000160789.18_LMNA	0.25399	0.098779941	0.397129339	1.59E-03	1.64E-02	1.00E+00
ENSG00000175155.7_YPEL2	ENSG00000160789.18_LMNA	-0.31613	-0.452553961	-0.16524517	7.26E-05	1.46E-03	1.00E+00
ENSG00000170631.13_ZNF16	ENSG00000160789.18_LMNA	0.02715	-0.132624565	0.185548093	7.40E-01	8.91E-01	1.00E+00
ENSG00000165071.13_TMEM71	ENSG00000160789.18_LMNA	0.18418	0.025732985	0.333598275	2.31E-02	1.20E-01	1.00E+00
ENSG00000162775.13_RBM15	ENSG00000160789.18_LMNA	0.17577	0.017043295	0.325849781	3.03E-02	1.44E-01	1.00E+00
ENSG00000163635.16_ATXN7	ENSG00000160789.18_LMNA	0.18363	0.025168981	0.333096619	2.35E-02	1.21E-01	1.00E+00
ENSG00000152076.17_CCDC74B	ENSG00000160789.18_LMNA	0.20322	0.045491966	0.351063644	1.20E-02	7.50E-02	1.00E+00
ENSG00000056487.14_PHF21B	ENSG00000160789.18_LMNA	-0.21752	-0.364108832	-0.06040795	7.10E-03	5.10E-02	1.00E+00
ENSG00000108370.14_RGS9	ENSG00000160789.18_LMNA	-0.39647	-0.522677412	-0.25325091	4.27E-07	2.32E-05	2.19E-02
ENSG00000197114.10_ZGPAT	ENSG00000160789.18_LMNA	0.24601	0.090343895	0.389935455	2.25E-03	2.14E-02	1.00E+00
ENSG00000197763.11_TXNRD3	ENSG00000160789.18_LMNA	0.08011	-0.080107993	0.236301279	3.27E-01	6.13E-01	1.00E+00
ENSG00000105497.6_ZNF175	ENSG00000160789.18_LMNA	-0.08979	-0.24548145	0.070417484	2.71E-01	5.56E-01	1.00E+00
ENSG00000091157.12_WDR7	ENSG00000160789.18_LMNA	-0.15849	-0.309868149	0.000733465	5.12E-02	2.04E-01	1.00E+00

ENSG00000173208.3_ABCD2	ENSG00000160789.18_LMNA	-0.08979	-0.245478759	0.070420334	2.71E-01	5.56E-01	1.00E+00
ENSG00000173175.13_ADCY5	ENSG00000160789.18_LMNA	-0.30742	-0.444851945	-0.15585009	1.17E-04	2.14E-03	1.00E+00
ENSG00000143498.16_TAF1A	ENSG00000160789.18_LMNA	0.08654	-0.073680434	0.242396653	2.89E-01	5.74E-01	1.00E+00
ENSG00000179454.12_KLHL28	ENSG00000160789.18_LMNA	-0.21199	-0.359076845	-0.05463821	8.74E-03	5.95E-02	1.00E+00
ENSG00000173705.7_SUSD5	ENSG00000160789.18_LMNA	-0.36838	-0.498353496	-0.22221047	3.02E-06	1.10E-04	1.55E-01
ENSG00000189042.12_ZNF567	ENSG00000160789.18_LMNA	-0.10288	-0.257857503	0.05725837	2.07E-01	4.79E-01	1.00E+00
ENSG00000139572.3_GPR84	ENSG00000160789.18_LMNA	0.25335	0.098107658	0.396557387	1.64E-03	1.68E-02	1.00E+00
ENSG00000100479.11_POLE2	ENSG00000160789.18_LMNA	0.10947	-0.050610806	0.264070422	1.79E-01	4.42E-01	1.00E+00
ENSG00000178233.16_TMEM151	ENSG00000160789.18_LMNA	-0.30311	-0.441029498	-0.15120673	1.47E-04	2.56E-03	1.00E+00
ENSG00000204427.10_ABHD16A	ENSG00000160789.18_LMNA	0.0546	-0.105521745	0.211954572	5.04E-01	7.55E-01	1.00E+00
ENSG00000106086.17_PLEKHA8	ENSG00000160789.18_LMNA	0.01005	-0.149394064	0.168976023	9.02E-01	9.62E-01	1.00E+00
ENSG00000092929.10_UNC13D	ENSG00000160789.18_LMNA	0.14576	-0.013759009	0.298045457	7.32E-02	2.57E-01	1.00E+00
ENSG00000006638.10_TBXA2R	ENSG00000160789.18_LMNA	0.30608	0.154400073	0.44365941	1.26E-04	2.27E-03	1.00E+00
ENSG00000129990.13_SYT5	ENSG00000160789.18_LMNA	-0.14984	-0.30184	0.009589906	6.54E-02	2.39E-01	1.00E+00
ENSG00000260541.1_LA16c-429E	ENSG00000160789.18_LMNA	-0.16446	-0.315400231	-0.00539787	4.29E-02	1.82E-01	1.00E+00
ENSG00000196150.12_ZNF250	ENSG00000160789.18_LMNA	-0.16125	-0.312430106	-0.00210311	4.72E-02	1.94E-01	1.00E+00
ENSG00000119685.18_TTLL5	ENSG00000160789.18_LMNA	0.26473	0.110175206	0.406788849	9.81E-04	1.14E-02	1.00E+00
ENSG00000262879.4_RP11-156P	ENSG00000160789.18_LMNA	-0.24813	-0.391853598	-0.09258905	2.05E-03	1.99E-02	1.00E+00
ENSG00000180855.14_ZNF443	ENSG00000160789.18_LMNA	-0.13754	-0.290379042	0.022149229	9.11E-02	2.94E-01	1.00E+00
ENSG00000270959.1_LPP-AS2	ENSG00000160789.18_LMNA	0.11606	-0.043954369	0.270265576	1.54E-01	4.05E-01	1.00E+00
ENSG00000255153.1_TOLLIP-AS	ENSG00000160789.18_LMNA	-0.07154	-0.228143067	0.088669829	3.81E-01	6.60E-01	1.00E+00
ENSG00000173013.5_CCDC96	ENSG00000160789.18_LMNA	0.24628	0.090635457	0.390184698	2.22E-03	2.12E-02	1.00E+00
ENSG00000198270.11_TMEM116	ENSG00000160789.18_LMNA	0.00082	-0.158400689	0.160000506	9.92E-01	9.97E-01	1.00E+00
ENSG00000274602.3_PI4KAP1	ENSG00000160789.18_LMNA	-0.10444	-0.259329891	0.055685499	2.00E-01	4.71E-01	1.00E+00
ENSG00000280254.1_RP11-81A2	ENSG00000160789.18_LMNA	-0.01455	-0.173350085	0.144985612	8.59E-01	9.44E-01	1.00E+00
ENSG00000198718.11_FAM179B	ENSG00000160789.18_LMNA	0.09168	-0.068522963	0.247269602	2.61E-01	5.45E-01	1.00E+00
ENSG00000273226.1_RP11-513M	ENSG00000160789.18_LMNA	-0.22158	-0.367807648	-0.06466203	6.08E-03	4.54E-02	1.00E+00
ENSG00000120913.22_PDLIM2	ENSG00000160789.18_LMNA	0.20237	0.044607301	0.350286191	1.24E-02	7.66E-02	1.00E+00
ENSG00000197933.11_ZNF823	ENSG00000160789.18_LMNA	-0.25378	-0.396940032	-0.0985574	1.61E-03	1.66E-02	1.00E+00
ENSG00000244921.2_MTCYBP18	ENSG00000160789.18_LMNA	-0.04601	-0.20372148	0.114022853	5.74E-01	8.01E-01	1.00E+00
ENSG00000135749.17_PCNX2	ENSG00000160789.18_LMNA	0.21051	0.053090626	0.357724121	9.24E-03	6.19E-02	1.00E+00
ENSG00000135655.12_USP15	ENSG00000160789.18_LMNA	-0.15693	-0.3084256	0.002328445	5.35E-02	2.10E-01	1.00E+00
ENSG00000222011.7_FAM185A	ENSG00000160789.18_LMNA	-0.01367	-0.172499294	0.145844084	8.67E-01	9.48E-01	1.00E+00

ENSG00000183496.5_MEX3B	ENSG00000160789.18_LMNA	-0.23886	-0.383475738	-0.08280524	3.04E-03	2.70E-02	1.00E+00
ENSG00000128274.14_A4GALT	ENSG00000160789.18_LMNA	0.25629	0.101224312	0.399206961	1.44E-03	1.52E-02	1.00E+00
ENSG00000232858.1_RPL34P27	ENSG00000160789.18_LMNA	-0.1032	-0.258161028	0.056934259	2.06E-01	4.77E-01	1.00E+00
ENSG00000172733.11_PURG	ENSG00000160789.18_LMNA	-0.41687	-0.540217342	-0.27598375	9.18E-08	6.70E-06	4.71E-03
ENSG00000239719.1_RP4-800G7	ENSG00000160789.18_LMNA	-0.04621	-0.203908866	0.113829882	5.72E-01	8.00E-01	1.00E+00
ENSG00000037280.14_FLT4	ENSG00000160789.18_LMNA	0.03968	-0.120284365	0.197628497	6.27E-01	8.32E-01	1.00E+00
ENSG00000267372.2_AC005330.:	ENSG00000160789.18_LMNA	0.01601	-0.143554768	0.174767072	8.45E-01	9.39E-01	1.00E+00
ENSG00000268225.1_CTD-2331H	ENSG00000160789.18_LMNA	-0.13537	-0.288360402	0.024351155	9.63E-02	3.05E-01	1.00E+00
ENSG00000277978.1_RP11-403P	ENSG00000160789.18_LMNA	-0.07802	-0.234314839	0.08219702	3.39E-01	6.25E-01	1.00E+00
ENSG00000163814.6_CDCP1	ENSG00000160789.18_LMNA	0.3353	0.186033967	0.469445459	2.41E-05	6.02E-04	1.00E+00
ENSG00000178860.8_MSC	ENSG00000160789.18_LMNA	0.12676	-0.033104223	0.280308353	1.20E-01	3.48E-01	1.00E+00
ENSG00000122912.13_SLC25A16	ENSG00000160789.18_LMNA	-0.21338	-0.360343281	-0.05608841	8.30E-03	5.73E-02	1.00E+00
ENSG00000203952.8_CCDC160	ENSG00000160789.18_LMNA	-0.33131	-0.465938152	-0.18169646	3.05E-05	7.25E-04	1.00E+00
ENSG00000226499.1_RP5-882O7	ENSG00000160789.18_LMNA	-0.07393	-0.230417032	0.086288057	3.65E-01	6.47E-01	1.00E+00
ENSG00000185379.19_RAD51D	ENSG00000160789.18_LMNA	0.19202	0.033859059	0.340806756	1.78E-02	9.92E-02	1.00E+00
ENSG00000124097.7_HMGB1P1	ENSG00000160789.18_LMNA	-0.07775	-0.234049478	0.082475875	3.41E-01	6.26E-01	1.00E+00
ENSG00000083097.13_DOPEY1	ENSG00000160789.18_LMNA	-0.17839	-0.32826994	-0.01975243	2.79E-02	1.36E-01	1.00E+00
ENSG00000198799.10_LRIG2	ENSG00000160789.18_LMNA	-0.04434	-0.202111391	0.115679934	5.88E-01	8.08E-01	1.00E+00
ENSG00000170946.13_DNAJC24	ENSG00000160789.18_LMNA	-0.3158	-0.452263002	-0.1648893	7.39E-05	1.48E-03	1.00E+00
ENSG00000019505.6_SYT13	ENSG00000160789.18_LMNA	-0.2513	-0.394707662	-0.09593532	1.79E-03	1.80E-02	1.00E+00
ENSG00000179406.6_LINC00174	ENSG00000160789.18_LMNA	0.05536	-0.104763384	0.212686842	4.98E-01	7.51E-01	1.00E+00
ENSG00000126822.14_PLEKHG3	ENSG00000160789.18_LMNA	-0.06912	-0.225833028	0.09108568	3.97E-01	6.74E-01	1.00E+00
ENSG00000136099.12_PCDH8	ENSG00000160789.18_LMNA	0.06875	-0.091450053	0.225484306	4.00E-01	6.77E-01	1.00E+00
ENSG00000183628.11_DGCR6	ENSG00000160789.18_LMNA	-0.07887	-0.235118985	0.08135168	3.34E-01	6.20E-01	1.00E+00
ENSG00000205485.12_AC004980	ENSG00000160789.18_LMNA	0.16055	0.001386274	0.311783094	4.82E-02	1.96E-01	1.00E+00
ENSG00000066382.15_MPPED2	ENSG00000160789.18_LMNA	-0.4455	-0.564653322	-0.3081549	8.86E-09	9.77E-07	4.55E-04
ENSG00000235706.6_DICER1-AS	ENSG00000160789.18_LMNA	-0.17691	-0.326902887	-0.01822159	2.92E-02	1.41E-01	1.00E+00
ENSG00000272077.1_RP11-348P	ENSG00000160789.18_LMNA	-0.20726	-0.35475575	-0.04969977	1.04E-02	6.74E-02	1.00E+00
ENSG00000109743.9_BST1	ENSG00000160789.18_LMNA	0.02176	-0.137918372	0.180336088	7.90E-01	9.15E-01	1.00E+00
ENSG00000204516.8_MICB	ENSG00000160789.18_LMNA	0.04689	-0.113156286	0.204562789	5.66E-01	7.97E-01	1.00E+00
ENSG00000137494.12_ANKRD42	ENSG00000160789.18_LMNA	0.26154	0.10678741	0.403924002	1.14E-03	1.27E-02	1.00E+00
ENSG00000090612.19_ZNF268	ENSG00000160789.18_LMNA	0.02488	-0.134859542	0.183349833	7.61E-01	9.00E-01	1.00E+00
ENSG00000177084.15_POLE	ENSG00000160789.18_LMNA	0.14925	-0.010190534	0.301293941	6.65E-02	2.41E-01	1.00E+00

ENSG00000154127.8_UBASH3B	ENSG00000160789.18_LMNA	0.24068	0.084729381	0.385127295	2.82E-03	2.55E-02	1.00E+00
ENSG00000228118.3_MYL6P5	ENSG00000160789.18_LMNA	0.01176	-0.147720296	0.170638212	8.86E-01	9.56E-01	1.00E+00
ENSG00000224411.3_HSP90AA2	ENSG00000160789.18_LMNA	0.09696	-0.063216812	0.252266412	2.35E-01	5.13E-01	1.00E+00
ENSG00000235944.6_ZNF815P	ENSG00000160789.18_LMNA	-0.20029	-0.348380796	-0.04244118	1.34E-02	8.07E-02	1.00E+00
ENSG00000230478.1_RP1-52D1.:	ENSG00000160789.18_LMNA	0.17364	0.014852974	0.32389013	3.24E-02	1.51E-01	1.00E+00
ENSG00000136630.12_HLX	ENSG00000160789.18_LMNA	0.18183	0.023304598	0.331437091	2.50E-02	1.26E-01	1.00E+00
ENSG00000203791.12_METTL10	ENSG00000160789.18_LMNA	-0.26544	-0.407420563	-0.11092316	9.50E-04	1.12E-02	1.00E+00
ENSG00000127914.15_AKAP9	ENSG00000160789.18_LMNA	0.08627	-0.073945382	0.242145891	2.91E-01	5.76E-01	1.00E+00
ENSG00000235734.4_HMGN1P36	ENSG00000160789.18_LMNA	0.06895	-0.091256185	0.225669857	3.99E-01	6.75E-01	1.00E+00
ENSG00000224924.5_LINC00320	ENSG00000160789.18_LMNA	-0.40592	-0.530814311	-0.26375997	2.12E-07	1.33E-05	1.09E-02
ENSG00000101276.13_SLC52A3	ENSG00000160789.18_LMNA	-0.23621	-0.381079699	-0.08001773	3.39E-03	2.93E-02	1.00E+00
ENSG00000234118.1_RPL13AP6	ENSG00000160789.18_LMNA	-0.13864	-0.291411865	0.021021455	8.85E-02	2.90E-01	1.00E+00
ENSG00000230383.1_AC009245.:	ENSG00000160789.18_LMNA	-0.0864	-0.242264739	0.073819816	2.90E-01	5.75E-01	1.00E+00
ENSG00000183718.5_TRIM52	ENSG00000160789.18_LMNA	0.02583	-0.133924061	0.184270333	7.52E-01	8.97E-01	1.00E+00
ENSG00000129680.14_MAP7D3	ENSG00000160789.18_LMNA	0.28473	0.131490403	0.424680931	3.78E-04	5.40E-03	1.00E+00
ENSG00000215482.3_CALM2P3	ENSG00000160789.18_LMNA	-0.27667	-0.417483466	-0.1228832	5.60E-04	7.36E-03	1.00E+00
ENSG00000198947.13_DMD	ENSG00000160789.18_LMNA	-0.00688	-0.165897827	0.152488893	9.33E-01	9.74E-01	1.00E+00
ENSG00000108958.4_AC016292.:	ENSG00000160789.18_LMNA	-0.17276	-0.323071699	-0.01393908	3.33E-02	1.53E-01	1.00E+00
ENSG00000111834.11_RSPH4A	ENSG00000160789.18_LMNA	-0.08823	-0.244002197	0.071983012	2.80E-01	5.65E-01	1.00E+00
ENSG00000165695.8_AK8	ENSG00000160789.18_LMNA	-0.06946	-0.226157235	0.090746848	3.95E-01	6.72E-01	1.00E+00
ENSG00000170745.10_KCNS3	ENSG00000160789.18_LMNA	-0.23899	-0.383599938	-0.08294986	3.02E-03	2.69E-02	1.00E+00
ENSG00000153790.10_C7orf31	ENSG00000160789.18_LMNA	-0.03028	-0.188567223	0.129549725	7.11E-01	8.77E-01	1.00E+00
ENSG00000174197.15_MGA	ENSG00000160789.18_LMNA	0.04981	-0.110266339	0.207365112	5.42E-01	7.81E-01	1.00E+00
ENSG00000179909.14_ZNF154	ENSG00000160789.18_LMNA	-0.17047	-0.320960525	-0.01158405	3.58E-02	1.61E-01	1.00E+00
ENSG00000091656.14_ZFHX4	ENSG00000160789.18_LMNA	0.1327	-0.027070173	0.285863873	1.03E-01	3.18E-01	1.00E+00
ENSG00000105642.14_KCNN1	ENSG00000160789.18_LMNA	-0.26042	-0.402914132	-0.10559484	1.19E-03	1.33E-02	1.00E+00
ENSG00000198589.9_LRBA	ENSG00000160789.18_LMNA	0.18793	0.02961814	0.337049217	2.04E-02	1.10E-01	1.00E+00
ENSG00000228436.2_RP5-864K1	ENSG00000160789.18_LMNA	-0.02876	-0.187100627	0.131044148	7.25E-01	8.83E-01	1.00E+00
ENSG00000272777.1_RP11-571L	ENSG00000160789.18_LMNA	-0.36889	-0.498800808	-0.22277629	2.92E-06	1.07E-04	1.50E-01
ENSG00000107099.14_DOCK8	ENSG00000160789.18_LMNA	-0.02042	-0.179043276	0.139228665	8.03E-01	9.21E-01	1.00E+00
ENSG00000214222.2_TUBBP2	ENSG00000160789.18_LMNA	0.02519	-0.134553135	0.183651395	7.58E-01	8.99E-01	1.00E+00
ENSG00000115009.10_CCL20	ENSG00000160789.18_LMNA	0.10762	-0.052484275	0.262322087	1.87E-01	4.53E-01	1.00E+00
ENSG00000134516.14_DOCK2	ENSG00000160789.18_LMNA	0.01549	-0.144064133	0.174262792	8.50E-01	9.41E-01	1.00E+00

ENSG00000006459.9_KDM7A	ENSG00000160789.18_LMNA	-0.08668	-0.242537459	0.073531644	2.88E-01	5.74E-01	1.00E+00
ENSG00000231884.1_NDUFB1P1	ENSG00000160789.18_LMNA	-0.11144	-0.265922803	0.048623424	1.72E-01	4.31E-01	1.00E+00
ENSG00000160796.15_NBEAL2	ENSG00000160789.18_LMNA	0.24937	0.093893269	0.392966666	1.95E-03	1.91E-02	1.00E+00
ENSG00000176024.15_ZNF613	ENSG00000160789.18_LMNA	-0.07314	-0.229664468	0.087076701	3.71E-01	6.51E-01	1.00E+00
ENSG00000175305.15_CCNE2	ENSG00000160789.18_LMNA	-0.09988	-0.255025217	0.060279553	2.21E-01	4.96E-01	1.00E+00
ENSG00000168234.11_TTC39C	ENSG00000160789.18_LMNA	0.12489	-0.035007656	0.278551505	1.25E-01	3.58E-01	1.00E+00
ENSG00000111799.19_COL12A1	ENSG00000160789.18_LMNA	0.34841	0.200316008	0.480931048	1.09E-05	3.12E-04	5.59E-01
ENSG00000048052.20_HDAC9	ENSG00000160789.18_LMNA	-0.07927	-0.2354964	0.080954774	3.32E-01	6.17E-01	1.00E+00
ENSG00000213700.3_RPL17P50	ENSG00000160789.18_LMNA	0.05415	-0.105961891	0.211529407	5.08E-01	7.58E-01	1.00E+00
ENSG00000223749.6_MIR503HG	ENSG00000160789.18_LMNA	-0.03834	-0.196345638	0.121599492	6.39E-01	8.39E-01	1.00E+00
ENSG00000176593.6_CTD-2368P	ENSG00000160789.18_LMNA	0.01057	-0.148882976	0.169483767	8.97E-01	9.60E-01	1.00E+00
ENSG00000168904.13_LRRC28	ENSG00000160789.18_LMNA	-0.17085	-0.321312789	-0.01197676	3.53E-02	1.60E-01	1.00E+00
ENSG00000279329.1_CTD-2553L	ENSG00000160789.18_LMNA	-0.16421	-0.315169943	-0.00514217	4.32E-02	1.83E-01	1.00E+00
ENSG00000092850.10_TEKT2	ENSG00000160789.18_LMNA	0.04441	-0.115607019	0.202182274	5.87E-01	8.08E-01	1.00E+00
ENSG00000188873.4_RPL10AP2	ENSG00000160789.18_LMNA	-0.10269	-0.257682628	0.057445076	2.08E-01	4.80E-01	1.00E+00
ENSG00000163251.3_FZD5	ENSG00000160789.18_LMNA	0.46916	0.334976026	0.584687595	1.08E-09	1.67E-07	5.56E-05
ENSG00000101187.14_SLCO4A1	ENSG00000160789.18_LMNA	0.03326	-0.1266145	0.191443668	6.84E-01	8.63E-01	1.00E+00
ENSG00000161243.7_FBXO27	ENSG00000160789.18_LMNA	-0.02751	-0.185894711	0.132271862	7.37E-01	8.89E-01	1.00E+00
ENSG00000176148.14_TCP11L1	ENSG00000160789.18_LMNA	0.01664	-0.142942324	0.175373183	8.39E-01	9.37E-01	1.00E+00
ENSG00000162139.8_NEU3	ENSG00000160789.18_LMNA	-0.29267	-0.431752274	-0.13999005	2.53E-04	3.94E-03	1.00E+00
ENSG00000164463.11_CREBRF	ENSG00000160789.18_LMNA	-0.19719	-0.345545256	-0.03922294	1.49E-02	8.74E-02	1.00E+00
ENSG00000173480.9_ZNF417	ENSG00000160789.18_LMNA	-0.01128	-0.170178465	0.148183428	8.90E-01	9.57E-01	1.00E+00
ENSG00000130643.7_CALY	ENSG00000160789.18_LMNA	-0.21298	-0.359976741	-0.05566855	8.43E-03	5.79E-02	1.00E+00
ENSG00000142065.12_ZFP14	ENSG00000160789.18_LMNA	-0.40251	-0.527881715	-0.25996511	2.74E-07	1.64E-05	1.41E-02
ENSG00000177125.5_ZBTB34	ENSG00000160789.18_LMNA	-0.02142	-0.18000588	0.13825315	7.93E-01	9.16E-01	1.00E+00
ENSG00000160298.16_C21orf58	ENSG00000160789.18_LMNA	0.16104	0.001888193	0.312236153	4.75E-02	1.94E-01	1.00E+00
ENSG00000083807.8_SLC27A5	ENSG00000160789.18_LMNA	-0.09105	-0.246679596	0.069148318	2.65E-01	5.48E-01	1.00E+00
ENSG00000217241.1_CBX3P9	ENSG00000160789.18_LMNA	-0.0008	-0.159982882	0.158418322	9.92E-01	9.97E-01	1.00E+00
ENSG00000187609.14_EXD3	ENSG00000160789.18_LMNA	0.10417	-0.055960285	0.259072765	2.02E-01	4.72E-01	1.00E+00
ENSG00000131831.16_RAI2	ENSG00000160789.18_LMNA	-0.46844	-0.584085274	-0.33416372	1.16E-09	1.76E-07	5.94E-05
ENSG00000134775.14_FHOD3	ENSG00000160789.18_LMNA	-0.1101	-0.264658537	0.049980099	1.77E-01	4.38E-01	1.00E+00
ENSG00000112365.4_ZBTB24	ENSG00000160789.18_LMNA	-0.03089	-0.189161521	0.128943739	7.06E-01	8.75E-01	1.00E+00
ENSG00000117010.14_ZNF684	ENSG00000160789.18_LMNA	-0.23395	-0.379037535	-0.07764561	3.72E-03	3.14E-02	1.00E+00

ENSG00000170485.15_NPAS2	ENSG00000160789.18_LMNA	0.2224	0.065518673	0.36855132	5.89E-03	4.44E-02	1.00E+00
ENSG00000196865.4_NHLRC2	ENSG00000160789.18_LMNA	-0.07478	-0.231226457	0.085439384	3.60E-01	6.42E-01	1.00E+00
ENSG00000116990.10_MYCL	ENSG00000160789.18_LMNA	-0.37264	-0.5020573	-0.22690114	2.27E-06	8.79E-05	1.16E-01
ENSG00000259972.2_AC009120.(	ENSG00000160789.18_LMNA	-0.03757	-0.19559706	0.122366383	6.46E-01	8.43E-01	1.00E+00
ENSG00000144285.14_SCN1A	ENSG00000160789.18_LMNA	-0.21271	-0.359730749	-0.05538684	8.51E-03	5.83E-02	1.00E+00
ENSG00000185100.9_ADSSL1	ENSG00000160789.18_LMNA	0.33532	0.186053422	0.46946117	2.41E-05	6.01E-04	1.00E+00
ENSG00000177025.3_C19orf18	ENSG00000160789.18_LMNA	0.06483	-0.095351114	0.221745698	4.27E-01	6.98E-01	1.00E+00
ENSG00000188215.8_DCUN1D3	ENSG00000160789.18_LMNA	0.29151	0.138750575	0.430723313	2.69E-04	4.12E-03	1.00E+00
ENSG00000262223.5_RP11-1055I	ENSG00000160789.18_LMNA	-0.39907	-0.524919924	-0.25614085	3.53E-07	2.00E-05	1.81E-02
ENSG00000270441.1_RP11-694I1	ENSG00000160789.18_LMNA	-0.04224	-0.20009181	0.117755961	6.05E-01	8.19E-01	1.00E+00
ENSG00000135931.16_ARMC9	ENSG00000160789.18_LMNA	0.29001	0.137142085	0.429386877	2.90E-04	4.38E-03	1.00E+00
ENSG00000224189.5_HAGLR	ENSG00000160789.18_LMNA	-0.39737	-0.523456542	-0.25425444	4.00E-07	2.20E-05	2.05E-02
ENSG00000244245.1_RP11-120B	ENSG00000160789.18_LMNA	-0.13039	-0.283704503	0.029418254	1.09E-01	3.29E-01	1.00E+00
ENSG00000261716.1_RP11-196G	ENSG00000160789.18_LMNA	-0.1294	-0.282779871	0.030422636	1.12E-01	3.34E-01	1.00E+00
ENSG00000270820.4_RP11-355B	ENSG00000160789.18_LMNA	-0.27444	-0.415489372	-0.12050635	6.23E-04	8.02E-03	1.00E+00
ENSG00000164116.15_GUCY1A3	ENSG00000160789.18_LMNA	-0.12286	-0.276646077	0.037069497	1.32E-01	3.68E-01	1.00E+00
ENSG00000237676.1_RPL30P4	ENSG00000160789.18_LMNA	-0.13547	-0.288450455	0.024252991	9.61E-02	3.04E-01	1.00E+00
ENSG00000156395.11_SORCS3	ENSG00000160789.18_LMNA	-0.38803	-0.515395484	-0.24389969	7.83E-07	3.76E-05	4.02E-02
ENSG00000176715.14_ACSF3	ENSG00000160789.18_LMNA	0.28646	0.13333979	0.426222593	3.46E-04	5.02E-03	1.00E+00
ENSG00000145725.18_PPIP5K2	ENSG00000160789.18_LMNA	-0.20033	-0.34842014	-0.04248587	1.33E-02	8.06E-02	1.00E+00
ENSG00000066739.10_ATG2B	ENSG00000160789.18_LMNA	0.03613	-0.123785166	0.194211181	6.59E-01	8.49E-01	1.00E+00
ENSG00000263011.1_RP11-473M	ENSG00000160789.18_LMNA	-0.13662	-0.289523248	0.023083096	9.33E-02	2.99E-01	1.00E+00
ENSG00000211772.7_TRBC2	ENSG00000160789.18_LMNA	0.07441	-0.08580545	0.230877374	3.62E-01	6.44E-01	1.00E+00
ENSG00000232065.1_LINC01063	ENSG00000160789.18_LMNA	-0.24123	-0.385620973	-0.08530497	2.75E-03	2.50E-02	1.00E+00
ENSG00000214654.6_RP11-27I1.4	ENSG00000160789.18_LMNA	-0.10596	-0.260758292	0.05415812	1.94E-01	4.63E-01	1.00E+00
ENSG00000228175.3_GEMIN8P4	ENSG00000160789.18_LMNA	-0.33043	-0.465156832	-0.1807317	3.22E-05	7.56E-04	1.00E+00
ENSG00000272927.1_RP11-1191.	ENSG00000160789.18_LMNA	0.05307	-0.107034802	0.210492507	5.16E-01	7.63E-01	1.00E+00
ENSG00000231549.1_USMG5P1	ENSG00000160789.18_LMNA	-0.10435	-0.25924771	0.055773329	2.01E-01	4.71E-01	1.00E+00
ENSG00000231551.5_RP11-495P	ENSG00000160789.18_LMNA	-0.33111	-0.465763195	-0.18148038	3.09E-05	7.31E-04	1.00E+00
ENSG00000197935.6_ZNF311	ENSG00000160789.18_LMNA	0.05391	-0.10620077	0.211298607	5.09E-01	7.59E-01	1.00E+00
ENSG00000119509.11_INVS	ENSG00000160789.18_LMNA	0.19328	0.035164208	0.341961174	1.70E-02	9.62E-02	1.00E+00
ENSG00000152689.16_RASGRP3	ENSG00000160789.18_LMNA	-0.20252	-0.350423468	-0.04476347	1.23E-02	7.63E-02	1.00E+00
ENSG00000156650.11_KAT6B	ENSG00000160789.18_LMNA	-0.08754	-0.243349755	0.072673011	2.84E-01	5.69E-01	1.00E+00

ENSG00000150995.16_ITPR1	ENSG00000160789.18_LMNA	0.05497	-0.105155811	0.212307961	5.01E-01	7.53E-01	1.00E+00
ENSG00000105464.3_GRIN2D	ENSG00000160789.18_LMNA	0.09189	-0.068308412	0.247471971	2.60E-01	5.43E-01	1.00E+00
ENSG00000092140.13_G2E3	ENSG00000160789.18_LMNA	-0.12805	-0.281512127	0.031798696	1.16E-01	3.40E-01	1.00E+00
ENSG00000087494.14_PTHLH	ENSG00000160789.18_LMNA	0.24862	0.093100868	0.392290509	2.01E-03	1.96E-02	1.00E+00
ENSG00000161929.13_SCIMP	ENSG00000160789.18_LMNA	-0.0213	-0.179893326	0.138367246	7.94E-01	9.17E-01	1.00E+00
ENSG00000108231.10_LGI1	ENSG00000160789.18_LMNA	-0.09997	-0.255109743	0.060189474	2.20E-01	4.96E-01	1.00E+00
ENSG00000166387.10_PPFIBP2	ENSG00000160789.18_LMNA	-0.14454	-0.296911727	0.01500255	7.56E-02	2.63E-01	1.00E+00
ENSG00000198554.10_WDHD1	ENSG00000160789.18_LMNA	0.20576	0.048135362	0.353384181	1.10E-02	7.01E-02	1.00E+00
ENSG00000154118.11_JPH3	ENSG00000160789.18_LMNA	-0.36307	-0.493733763	-0.21637768	4.28E-06	1.46E-04	2.20E-01
ENSG00000125962.13_ARMCX5	ENSG00000160789.18_LMNA	-0.17422	-0.324421958	-0.01544711	3.18E-02	1.49E-01	1.00E+00
ENSG00000182901.14_RGS7	ENSG00000160789.18_LMNA	-0.28021	-0.420649493	-0.12666389	4.71E-04	6.44E-03	1.00E+00
ENSG00000233230.1_AC079807.:	ENSG00000160789.18_LMNA	-0.23823	-0.382905152	-0.082141	3.12E-03	2.75E-02	1.00E+00
ENSG00000186654.19_PRR5	ENSG00000160789.18_LMNA	0.09448	-0.065711682	0.249919083	2.47E-01	5.27E-01	1.00E+00
ENSG00000261840.2_RP11-146F	ENSG00000160789.18_LMNA	-0.12216	-0.275990803	0.037777944	1.34E-01	3.71E-01	1.00E+00
ENSG00000183186.6_C2CD4C	ENSG00000160789.18_LMNA	0.02432	-0.135404926	0.182812926	7.66E-01	9.03E-01	1.00E+00
ENSG00000099260.9_PALMD	ENSG00000160789.18_LMNA	-0.13725	-0.290113431	0.02243913	9.18E-02	2.95E-01	1.00E+00
ENSG00000179071.4_CCDC89	ENSG00000160789.18_LMNA	-0.02796	-0.186330255	0.131828557	7.32E-01	8.87E-01	1.00E+00
ENSG00000137821.10_LRRC49	ENSG00000160789.18_LMNA	-0.30492	-0.442633245	-0.15315334	1.34E-04	2.38E-03	1.00E+00
ENSG00000169914.5_OTUD3	ENSG00000160789.18_LMNA	0.20694	0.049369213	0.354466047	1.05E-02	6.80E-02	1.00E+00
ENSG00000023445.12_BIRC3	ENSG00000160789.18_LMNA	0.24871	0.093196376	0.392372023	2.00E-03	1.95E-02	1.00E+00
ENSG00000178235.7_SLITRK1	ENSG00000160789.18_LMNA	-0.41382	-0.537599408	-0.27257187	1.16E-07	8.18E-06	5.97E-03
ENSG00000160791.13_CCR5	ENSG00000160789.18_LMNA	0.14088	-0.018744811	0.293494613	8.34E-02	2.79E-01	1.00E+00
ENSG00000180769.7_WDFY3-AS	ENSG00000160789.18_LMNA	-0.30688	-0.444370782	-0.15526489	1.20E-04	2.20E-03	1.00E+00
ENSG00000273466.1_RP11-548H	ENSG00000160789.18_LMNA	0.14515	-0.014379696	0.29747969	7.44E-02	2.60E-01	1.00E+00
ENSG00000178078.10_STAP2	ENSG00000160789.18_LMNA	0.16173	0.002596689	0.312875438	4.65E-02	1.92E-01	1.00E+00
ENSG00000188375.4_H3F3C	ENSG00000160789.18_LMNA	0.03976	-0.120198858	0.197711869	6.27E-01	8.32E-01	1.00E+00
ENSG00000140807.5_NKD1	ENSG00000160789.18_LMNA	-0.39487	-0.521302457	-0.25148137	4.80E-07	2.54E-05	2.46E-02
ENSG00000108622.9_ICAM2	ENSG00000160789.18_LMNA	-0.23658	-0.381413461	-0.08040574	3.34E-03	2.90E-02	1.00E+00
ENSG00000152256.12_PDK1	ENSG00000160789.18_LMNA	0.06699	-0.093204663	0.223803917	4.12E-01	6.86E-01	1.00E+00
ENSG00000184661.12_CDCA2	ENSG00000160789.18_LMNA	0.17156	0.012702044	0.321963136	3.46E-02	1.57E-01	1.00E+00
ENSG00000239528.1_RPS14P8	ENSG00000160789.18_LMNA	0.07796	-0.082266534	0.234248694	3.40E-01	6.25E-01	1.00E+00
ENSG00000176809.9_LRRC37A3	ENSG00000160789.18_LMNA	0.12795	-0.031896531	0.281421952	1.16E-01	3.41E-01	1.00E+00
ENSG00000250896.1_RNPS1P1	ENSG00000160789.18_LMNA	0.03116	-0.128674808	0.18942519	7.03E-01	8.73E-01	1.00E+00

ENSG00000223797.4_ENTPD3-AS	ENSG00000160789.18_LMNA	-0.35844	-0.489693339	-0.21129249	5.78E-06	1.87E-04	2.97E-01
ENSG00000065357.18_DGKA	ENSG00000160789.18_LMNA	0.10666	-0.053443977	0.261425684	1.91E-01	4.59E-01	1.00E+00
ENSG00000111110.10_PPM1H	ENSG00000160789.18_LMNA	-0.31087	-0.447903476	-0.15956617	9.70E-05	1.84E-03	1.00E+00
ENSG00000105996.6_HOXA2	ENSG00000160789.18_LMNA	-0.13732	-0.290179528	0.022366994	9.16E-02	2.95E-01	1.00E+00
ENSG00000154814.12_OXNAD1	ENSG00000160789.18_LMNA	-0.06942	-0.226121257	0.090784453	3.95E-01	6.73E-01	1.00E+00
ENSG00000164659.13_KIAA1324	ENSG00000160789.18_LMNA	-0.46445	-0.580712071	-0.32962137	1.67E-09	2.37E-07	8.55E-05
ENSG00000089177.16_KIF16B	ENSG00000160789.18_LMNA	0.41527	0.27420197	0.538850818	1.04E-07	7.47E-06	5.33E-03
ENSG00000272894.4_RP5-1159O	ENSG00000160789.18_LMNA	-0.07483	-0.231277982	0.085385344	3.60E-01	6.42E-01	1.00E+00
ENSG00000186376.13_ZNF75D	ENSG00000160789.18_LMNA	0.00434	-0.154969308	0.163426255	9.58E-01	9.83E-01	1.00E+00
ENSG00000130035.5_GALNT8	ENSG00000160789.18_LMNA	-0.36669	-0.496880904	-0.22034905	3.38E-06	1.21E-04	1.73E-01
ENSG00000212916.4_MAP10	ENSG00000160789.18_LMNA	0.06578	-0.094406095	0.222652219	4.21E-01	6.92E-01	1.00E+00
ENSG00000234354.3_RPS26P47	ENSG00000160789.18_LMNA	-0.14338	-0.295825452	0.016193135	7.80E-02	2.68E-01	1.00E+00
ENSG00000228775.6_WEE2-AS1	ENSG00000160789.18_LMNA	-0.08345	-0.239466943	0.076773088	3.07E-01	5.93E-01	1.00E+00
ENSG00000164764.10_SBSPON	ENSG00000160789.18_LMNA	-0.07786	-0.234160686	0.082359018	3.40E-01	6.25E-01	1.00E+00
ENSG00000162992.3_NEUROD1	ENSG00000160789.18_LMNA	-0.34157	-0.474945868	-0.19285868	1.66E-05	4.42E-04	8.50E-01
ENSG00000178301.3_AQP11	ENSG00000160789.18_LMNA	-0.22299	-0.369087627	-0.06613673	5.76E-03	4.36E-02	1.00E+00
ENSG00000207357.1_RNU6-2	ENSG00000160789.18_LMNA	-0.13092	-0.284201487	0.028878144	1.08E-01	3.27E-01	1.00E+00
ENSG00000175279.20_CENPS	ENSG00000160789.18_LMNA	0.01952	-0.140119195	0.178163999	8.11E-01	9.26E-01	1.00E+00
ENSG00000261662.1_RP5-1042I8	ENSG00000160789.18_LMNA	0.09916	-0.061004673	0.254344629	2.24E-01	5.00E-01	1.00E+00
ENSG00000245317.2_CTC-241N9	ENSG00000160789.18_LMNA	-0.07103	-0.227654882	0.089180687	3.85E-01	6.63E-01	1.00E+00
ENSG00000075702.15_WDR62	ENSG00000160789.18_LMNA	0.06105	-0.099118975	0.218125872	4.55E-01	7.20E-01	1.00E+00
ENSG00000218502.3_H2AFZP3	ENSG00000160789.18_LMNA	-0.0046	-0.163685246	0.154709579	9.55E-01	9.82E-01	1.00E+00
ENSG00000198225.5_FKBP1C	ENSG00000160789.18_LMNA	0.17356	0.014768296	0.323814316	3.25E-02	1.51E-01	1.00E+00
ENSG00000172059.9_KLF11	ENSG00000160789.18_LMNA	0.00102	-0.158206075	0.160195005	9.90E-01	9.96E-01	1.00E+00
ENSG00000183807.7_FAM162B	ENSG00000160789.18_LMNA	-0.19259	-0.341325259	-0.03444513	1.74E-02	9.78E-02	1.00E+00
ENSG00000171757.14_LRRC34	ENSG00000160789.18_LMNA	-0.05097	-0.208482615	0.109112394	5.33E-01	7.75E-01	1.00E+00
ENSG00000181315.9_ZNF322	ENSG00000160789.18_LMNA	-0.18387	-0.333313707	-0.02541303	2.34E-02	1.21E-01	1.00E+00
ENSG00000227850.1_SEPT2P1	ENSG00000160789.18_LMNA	-0.0943	-0.249753152	0.065887893	2.48E-01	5.28E-01	1.00E+00
ENSG00000241255.1_RP1-89D4.1	ENSG00000160789.18_LMNA	-0.20253	-0.35043474	-0.0447763	1.23E-02	7.63E-02	1.00E+00
ENSG00000163694.13_RBM47	ENSG00000160789.18_LMNA	0.33505	0.185753063	0.469218591	2.45E-05	6.09E-04	1.00E+00
ENSG00000000457.12_SCYL3	ENSG00000160789.18_LMNA	-0.26626	-0.408157633	-0.11179628	9.14E-04	1.08E-02	1.00E+00
ENSG00000159314.10_ARHGAP2	ENSG00000160789.18_LMNA	0.09827	-0.061897077	0.253506601	2.28E-01	5.05E-01	1.00E+00
ENSG00000042062.10_FAM65C	ENSG00000160789.18_LMNA	0.34144	0.192715112	0.474830388	1.67E-05	4.45E-04	8.57E-01

ENSG00000036530.7_CYP46A1	ENSG00000160789.18_LMNA	-0.22407	-0.370071293	-0.06727094	5.52E-03	4.22E-02	1.00E+00
ENSG00000065413.15_ANKRD44	ENSG00000160789.18_LMNA	-0.39844	-0.52438227	-0.25544754	3.70E-07	2.08E-05	1.90E-02
ENSG00000117477.11_CCDC181	ENSG00000160789.18_LMNA	-0.15077	-0.302709091	0.0086335	6.37E-02	2.35E-01	1.00E+00
ENSG00000244617.2_ASPRV1	ENSG00000160789.18_LMNA	-0.24123	-0.385621613	-0.08530572	2.75E-03	2.50E-02	1.00E+00
ENSG00000110172.10_CHORDC1	ENSG00000160789.18_LMNA	0.02353	-0.136183586	0.182046038	7.74E-01	9.07E-01	1.00E+00
ENSG00000128965.10_CHAC1	ENSG00000160789.18_LMNA	0.20052	0.042679713	0.348590746	1.33E-02	8.02E-02	1.00E+00
ENSG00000101096.18_NFATC2	ENSG00000160789.18_LMNA	0.27927	0.125661064	0.419810402	4.93E-04	6.67E-03	1.00E+00
ENSG00000007968.6_E2F2	ENSG00000160789.18_LMNA	-0.05012	-0.207667033	0.109954657	5.40E-01	7.80E-01	1.00E+00
ENSG00000145414.7_NAF1	ENSG00000160789.18_LMNA	0.11094	-0.04913027	0.265450607	1.74E-01	4.33E-01	1.00E+00
ENSG00000152208.10_GRID2	ENSG00000160789.18_LMNA	-0.46475	-0.580962398	-0.32995806	1.62E-09	2.33E-07	8.33E-05
ENSG00000250303.3_RP11-356J5	ENSG00000160789.18_LMNA	-0.32246	-0.458138774	-0.1720904	5.09E-05	1.09E-03	1.00E+00
ENSG00000235961.5_PNMA6A	ENSG00000160789.18_LMNA	-0.1286	-0.282026629	0.031240379	1.14E-01	3.38E-01	1.00E+00
ENSG00000148832.13_PAOX	ENSG00000160789.18_LMNA	0.22964	0.073115343	0.375129227	4.43E-03	3.57E-02	1.00E+00
ENSG00000186205.11_MARC1	ENSG00000160789.18_LMNA	-0.13545	-0.288434549	0.02427033	9.61E-02	3.04E-01	1.00E+00
ENSG00000197106.6_SLC6A17	ENSG00000160789.18_LMNA	-0.11871	-0.272759254	0.041267116	1.45E-01	3.90E-01	1.00E+00
ENSG00000143919.13_CAMKMT	ENSG00000160789.18_LMNA	-0.15768	-0.309120078	0.001560781	5.24E-02	2.07E-01	1.00E+00
ENSG00000119397.15_CNTRL	ENSG00000160789.18_LMNA	0.14965	-0.009786275	0.301661494	6.57E-02	2.39E-01	1.00E+00
ENSG00000188523.7_CFAP77	ENSG00000160789.18_LMNA	0.12212	-0.037818016	0.27595373	1.34E-01	3.72E-01	1.00E+00
ENSG00000173451.5_THAP2	ENSG00000160789.18_LMNA	-0.29705	-0.435648951	-0.14469225	2.02E-04	3.29E-03	1.00E+00
ENSG00000124593.13_PRICKLE4	ENSG00000160789.18_LMNA	0.03327	-0.126599157	0.19145869	6.84E-01	8.63E-01	1.00E+00
ENSG00000112139.13_MDGA1	ENSG00000160789.18_LMNA	0.23485	0.078583994	0.379845743	3.59E-03	3.06E-02	1.00E+00
ENSG00000126778.8_SIX1	ENSG00000160789.18_LMNA	-0.1557	-0.30728397	0.00358959	5.54E-02	2.15E-01	1.00E+00
ENSG00000183801.6_OLFML1	ENSG00000160789.18_LMNA	-0.0592	-0.216365124	0.100948446	4.69E-01	7.30E-01	1.00E+00
ENSG00000271576.1_RP11-486G	ENSG00000160789.18_LMNA	-0.25657	-0.399458199	-0.10152014	1.42E-03	1.51E-02	1.00E+00
ENSG00000260114.2_CTD-2574D	ENSG00000160789.18_LMNA	-0.2073	-0.354791859	-0.04974098	1.04E-02	6.73E-02	1.00E+00
ENSG00000125434.9_SLC25A35	ENSG00000160789.18_LMNA	0.05056	-0.109520661	0.208087336	5.36E-01	7.77E-01	1.00E+00
ENSG00000179593.14_ALOX15B	ENSG00000160789.18_LMNA	-0.03266	-0.190872092	0.1271982	6.90E-01	8.66E-01	1.00E+00
ENSG00000268521.1_VN1R83P	ENSG00000160789.18_LMNA	-0.21571	-0.3624601	-0.05851525	7.61E-03	5.35E-02	1.00E+00
ENSG00000106327.11_TFR2	ENSG00000160789.18_LMNA	0.25025	0.094826019	0.393762169	1.87E-03	1.86E-02	1.00E+00
ENSG00000024526.15_DEPDC1	ENSG00000160789.18_LMNA	0.18299	0.024506552	0.332507195	2.40E-02	1.23E-01	1.00E+00
ENSG00000147168.11_IL2RG	ENSG00000160789.18_LMNA	0.04006	-0.119905001	0.197998352	6.24E-01	8.31E-01	1.00E+00
ENSG00000221916.3_C19orf73	ENSG00000160789.18_LMNA	-0.17227	-0.322623368	-0.01343868	3.38E-02	1.55E-01	1.00E+00
ENSG00000086848.13_ALG9	ENSG00000160789.18_LMNA	-0.24943	-0.393022425	-0.09395863	1.94E-03	1.91E-02	1.00E+00

ENSG00000248925.1_CTD-2083E	ENSG00000160789.18_LMNA	-0.23768	-0.38240709	-0.08156141	3.19E-03	2.80E-02	1.00E+00
ENSG00000126246.8_IGFLR1	ENSG00000160789.18_LMNA	0.03751	-0.122422739	0.195542036	6.46E-01	8.43E-01	1.00E+00
ENSG00000269940.1_RP11-73M1	ENSG00000160789.18_LMNA	0.24065	0.084697188	0.385099678	2.82E-03	2.55E-02	1.00E+00
ENSG00000069869.14_NEDD4	ENSG00000160789.18_LMNA	0.1201	-0.039863666	0.274059933	1.41E-01	3.82E-01	1.00E+00
ENSG00000178163.6_ZNF518B	ENSG00000160789.18_LMNA	-0.10342	-0.258363466	0.056718054	2.05E-01	4.76E-01	1.00E+00
ENSG00000171970.11_ZNF57	ENSG00000160789.18_LMNA	0.03668	-0.123236459	0.194747314	6.54E-01	8.47E-01	1.00E+00
ENSG00000240370.5_U47924.6	ENSG00000160789.18_LMNA	0.10045	-0.059707995	0.255561454	2.18E-01	4.93E-01	1.00E+00
ENSG00000115165.8_CYTIP	ENSG00000160789.18_LMNA	0.07814	-0.082081458	0.234424795	3.39E-01	6.24E-01	1.00E+00
ENSG00000274750.1_HIST1H3E	ENSG00000160789.18_LMNA	0.00465	-0.154662016	0.163732668	9.55E-01	9.82E-01	1.00E+00
ENSG00000272525.1_RP11-79P5	ENSG00000160789.18_LMNA	-0.20545	-0.353103457	-0.04781535	1.11E-02	7.06E-02	1.00E+00
ENSG00000203772.7_SPRN	ENSG00000160789.18_LMNA	0.02116	-0.13850775	0.179754709	7.96E-01	9.18E-01	1.00E+00
ENSG00000164342.11_TLR3	ENSG00000160789.18_LMNA	-0.07045	-0.227101241	0.089759837	3.88E-01	6.67E-01	1.00E+00
ENSG00000198399.13_ITSN2	ENSG00000160789.18_LMNA	0.15168	-0.007711062	0.303546825	6.21E-02	2.31E-01	1.00E+00
ENSG00000040608.12_RTN4R	ENSG00000160789.18_LMNA	-0.08602	-0.241908254	0.07419642	2.92E-01	5.78E-01	1.00E+00
ENSG00000184916.7_JAG2	ENSG00000160789.18_LMNA	0.1373	-0.022394705	0.290154137	9.17E-02	2.95E-01	1.00E+00
ENSG00000259366.1_CTD-2647L	ENSG00000160789.18_LMNA	0.01272	-0.14677328	0.171577875	8.76E-01	9.52E-01	1.00E+00
ENSG00000120217.12_CD274	ENSG00000160789.18_LMNA	0.40525	0.263018675	0.530241956	2.23E-07	1.39E-05	1.14E-02
ENSG00000178718.6_RPP25	ENSG00000160789.18_LMNA	0.14447	-0.015076139	0.296844608	7.58E-02	2.63E-01	1.00E+00
ENSG00000127084.16_FGD3	ENSG00000160789.18_LMNA	-0.23211	-0.377367917	-0.07570877	4.01E-03	3.32E-02	1.00E+00
ENSG00000197191.4_CYSRT1	ENSG00000160789.18_LMNA	0.12983	-0.029990046	0.283178184	1.11E-01	3.32E-01	1.00E+00
ENSG00000072736.17_NFATC3	ENSG00000160789.18_LMNA	0.08722	-0.072992182	0.24304786	2.85E-01	5.71E-01	1.00E+00
ENSG00000092978.9_GPATCH2	ENSG00000160789.18_LMNA	0.04122	-0.118763275	0.199110909	6.14E-01	8.25E-01	1.00E+00
ENSG00000182310.11_SPACA6	ENSG00000160789.18_LMNA	0.03856	-0.121383245	0.196556654	6.37E-01	8.38E-01	1.00E+00
ENSG00000243746.1_EEF1A1P1(	ENSG00000160789.18_LMNA	-0.11316	-0.267537473	0.046889045	1.65E-01	4.21E-01	1.00E+00
ENSG00000258926.1_RP11-47I22	ENSG00000160789.18_LMNA	-0.35616	-0.487705305	-0.20879591	6.69E-06	2.12E-04	3.43E-01
ENSG00000131351.13_HAUS8	ENSG00000160789.18_LMNA	0.06516	-0.095027478	0.222056212	4.25E-01	6.96E-01	1.00E+00
ENSG00000100077.13_GRK3	ENSG00000160789.18_LMNA	-0.31965	-0.455664916	-0.16905481	5.96E-05	1.23E-03	1.00E+00
ENSG00000188372.13_ZP3	ENSG00000160789.18_LMNA	0.12118	-0.038772245	0.275070639	1.37E-01	3.77E-01	1.00E+00
ENSG00000165185.13_KIAA1958	ENSG00000160789.18_LMNA	-0.05146	-0.208945217	0.108634455	5.29E-01	7.73E-01	1.00E+00
ENSG00000214290.6_COLCA2	ENSG00000160789.18_LMNA	-0.11717	-0.271307504	0.042832113	1.51E-01	3.99E-01	1.00E+00
ENSG00000153132.11_CLGN	ENSG00000160789.18_LMNA	-0.33061	-0.465323688	-0.18093768	3.18E-05	7.48E-04	1.00E+00
ENSG00000101974.13_ATP11C	ENSG00000160789.18_LMNA	0.21542	0.058220184	0.362202892	7.69E-03	5.40E-02	1.00E+00
ENSG00000204390.9_HSPA1L	ENSG00000160789.18_LMNA	0.05014	-0.109940396	0.207680845	5.40E-01	7.79E-01	1.00E+00

ENSG00000101004.13_NINL	ENSG00000160789.18_LMNA	0.08937	-0.070834955	0.245087129	2.74E-01	5.58E-01	1.00E+00
ENSG00000116668.11_SWT1	ENSG00000160789.18_LMNA	-0.24504	-0.389062711	-0.08932337	2.35E-03	2.21E-02	1.00E+00
ENSG00000120322.3_PCDHB8	ENSG00000160789.18_LMNA	0.16736	0.008385855	0.318088568	3.93E-02	1.71E-01	1.00E+00
ENSG00000100429.16_HDAC10	ENSG00000160789.18_LMNA	0.14142	-0.018194928	0.29399722	8.22E-02	2.77E-01	1.00E+00
ENSG00000172361.5_CFAP53	ENSG00000160789.18_LMNA	0.06167	-0.098493736	0.21872715	4.50E-01	7.16E-01	1.00E+00
ENSG00000273899.3_NOL12	ENSG00000160789.18_LMNA	-0.22017	-0.366524394	-0.06318488	6.42E-03	4.73E-02	1.00E+00
ENSG00000064012.20_CASP8	ENSG00000160789.18_LMNA	0.25897	0.104055279	0.401609357	1.28E-03	1.39E-02	1.00E+00
ENSG00000236058.3_RPL17P36	ENSG00000160789.18_LMNA	-0.10259	-0.257587624	0.057546497	2.08E-01	4.81E-01	1.00E+00
ENSG00000264577.1_AC010761.8	ENSG00000160789.18_LMNA	-0.04458	-0.202342491	0.115442197	5.86E-01	8.08E-01	1.00E+00
ENSG00000197937.11_ZNF347	ENSG00000160789.18_LMNA	0.21335	0.056054466	0.360313649	8.31E-03	5.73E-02	1.00E+00
ENSG00000270175.1_RP11-793H	ENSG00000160789.18_LMNA	-0.28868	-0.428204383	-0.13572016	3.10E-04	4.62E-03	1.00E+00
ENSG00000181638.16_ZFP41	ENSG00000160789.18_LMNA	0.11151	-0.048553588	0.265987853	1.71E-01	4.31E-01	1.00E+00
ENSG00000119636.14_BBOF1	ENSG00000160789.18_LMNA	0.26114	0.106364032	0.403565568	1.16E-03	1.29E-02	1.00E+00
ENSG00000142698.13_C1orf94	ENSG00000160789.18_LMNA	-0.01637	-0.175116021	0.143202203	8.41E-01	9.38E-01	1.00E+00
ENSG00000260918.1_RP11-731J8	ENSG00000160789.18_LMNA	-0.22217	-0.368339611	-0.06527476	5.94E-03	4.47E-02	1.00E+00
ENSG00000233057.1_EEF1A1P14	ENSG00000160789.18_LMNA	-0.11004	-0.264602264	0.050040458	1.77E-01	4.39E-01	1.00E+00
ENSG00000183814.14_LIN9	ENSG00000160789.18_LMNA	-0.13094	-0.28421879	0.028859337	1.08E-01	3.27E-01	1.00E+00
ENSG00000105988.6_NHP2P1	ENSG00000160789.18_LMNA	-0.16407	-0.315037285	-0.00499489	4.34E-02	1.83E-01	1.00E+00
ENSG00000235280.2_MCF2L-AS1	ENSG00000160789.18_LMNA	-0.0651	-0.221997825	0.095088337	4.26E-01	6.96E-01	1.00E+00
ENSG00000112159.10_MDN1	ENSG00000160789.18_LMNA	-0.04815	-0.205768694	0.111913333	5.56E-01	7.90E-01	1.00E+00
ENSG00000004139.12_SARM1	ENSG00000160789.18_LMNA	-0.08782	-0.243611017	0.072396746	2.82E-01	5.67E-01	1.00E+00
ENSG00000251369.7_ZNF550	ENSG00000160789.18_LMNA	-0.02863	-0.186975282	0.131171804	7.26E-01	8.84E-01	1.00E+00
ENSG00000152315.4_KCNK13	ENSG00000160789.18_LMNA	-0.13921	-0.291938137	0.020446494	8.72E-02	2.87E-01	1.00E+00
ENSG00000120156.19_TEK	ENSG00000160789.18_LMNA	-0.06182	-0.218867203	0.098348066	4.49E-01	7.15E-01	1.00E+00
ENSG00000254595.1_CTD-2010I1	ENSG00000160789.18_LMNA	-0.0323	-0.190523547	0.12755403	6.93E-01	8.68E-01	1.00E+00
ENSG00000157212.17_PAXIP1	ENSG00000160789.18_LMNA	0.30425	0.152432824	0.44203985	1.39E-04	2.44E-03	1.00E+00
ENSG00000213222.3_AC093724.2	ENSG00000160789.18_LMNA	-0.13545	-0.28842657	0.024279028	9.62E-02	3.04E-01	1.00E+00
ENSG00000169026.11_MFSD7	ENSG00000160789.18_LMNA	0.24155	0.085646988	0.385914232	2.72E-03	2.48E-02	1.00E+00
ENSG00000188735.11_TMEM120	ENSG00000160789.18_LMNA	0.05914	-0.101016454	0.216299631	4.69E-01	7.30E-01	1.00E+00
ENSG00000187815.8_ZFP69	ENSG00000160789.18_LMNA	-0.26662	-0.408485387	-0.11218468	8.99E-04	1.07E-02	1.00E+00
ENSG00000196312.10_MFSD14C	ENSG00000160789.18_LMNA	0.17698	0.018298805	0.326971874	2.92E-02	1.41E-01	1.00E+00
ENSG00000269694.1_AC005197.2	ENSG00000160789.18_LMNA	-0.12439	-0.278088422	0.035508998	1.27E-01	3.60E-01	1.00E+00
ENSG00000204685.6_STARD7-AS	ENSG00000160789.18_LMNA	0.14388	-0.015675324	0.296297995	7.70E-02	2.66E-01	1.00E+00

ENSG00000164074.13_ABHD18	ENSG00000160789.18_LMNA	-0.17623	-0.3262767	-0.01752086	2.99E-02	1.43E-01	1.00E+00
ENSG00000160219.10_GAB3	ENSG00000160789.18_LMNA	0.06557	-0.094621549	0.222445591	4.22E-01	6.94E-01	1.00E+00
ENSG00000145734.17_BDP1	ENSG00000160789.18_LMNA	0.08782	-0.072388542	0.243618774	2.82E-01	5.67E-01	1.00E+00
ENSG00000183048.10_SLC25A1(	ENSG00000160789.18_LMNA	-0.03247	-0.190685845	0.127388349	6.91E-01	8.67E-01	1.00E+00
ENSG00000113248.5_PCDHB15	ENSG00000160789.18_LMNA	-0.05279	-0.210227608	0.107308783	5.18E-01	7.65E-01	1.00E+00
ENSG00000069702.9_TGFBR3	ENSG00000160789.18_LMNA	-0.1476	-0.299754288	0.011882839	6.96E-02	2.49E-01	1.00E+00
ENSG00000105122.11_RASAL3	ENSG00000160789.18_LMNA	0.10606	-0.054058176	0.260851711	1.93E-01	4.62E-01	1.00E+00
ENSG00000081818.3_PCDHB4	ENSG00000160789.18_LMNA	-0.12347	-0.277218145	0.036450749	1.30E-01	3.65E-01	1.00E+00
ENSG00000077984.5_CST7	ENSG00000160789.18_LMNA	0.12841	-0.031430297	0.281851635	1.15E-01	3.39E-01	1.00E+00
ENSG00000184616.8_SPDYE12P	ENSG00000160789.18_LMNA	0.08582	-0.074392516	0.241722599	2.93E-01	5.79E-01	1.00E+00
ENSG00000273230.1_RP11-1246	ENSG00000160789.18_LMNA	0.34633	0.198054359	0.47911863	1.24E-05	3.48E-04	6.35E-01
ENSG00000141198.12_TOM1L1	ENSG00000160789.18_LMNA	0.25995	0.105094449	0.402490184	1.22E-03	1.34E-02	1.00E+00
ENSG00000158636.15_EMSY	ENSG00000160789.18_LMNA	-0.13807	-0.290877947	0.021604557	8.98E-02	2.92E-01	1.00E+00
ENSG00000009950.14_MLXIPL	ENSG00000160789.18_LMNA	-0.11446	-0.268759584	0.045575066	1.60E-01	4.14E-01	1.00E+00
ENSG00000198780.10_FAM169A	ENSG00000160789.18_LMNA	-0.35844	-0.489694131	-0.21129349	5.78E-06	1.87E-04	2.97E-01
ENSG00000175054.13_ATR	ENSG00000160789.18_LMNA	0.04846	-0.111606284	0.206066442	5.53E-01	7.88E-01	1.00E+00
ENSG00000247400.3_DNAJC3-AS	ENSG00000160789.18_LMNA	-0.32082	-0.456693522	-0.17031632	5.58E-05	1.17E-03	1.00E+00
ENSG00000272696.1_RP11-339B	ENSG00000160789.18_LMNA	-0.23224	-0.377485632	-0.07584525	3.99E-03	3.31E-02	1.00E+00
ENSG00000124374.8_PAIP2B	ENSG00000160789.18_LMNA	-0.44177	-0.561481169	-0.30394521	1.22E-08	1.27E-06	6.24E-04
ENSG00000273680.1_RP11-996F	ENSG00000160789.18_LMNA	-0.13276	-0.285917914	0.027011365	1.03E-01	3.18E-01	1.00E+00
ENSG00000253159.2_PCDHGA12	ENSG00000160789.18_LMNA	0.00154	-0.157699399	0.160701269	9.85E-01	9.94E-01	1.00E+00
ENSG00000112343.10_TRIM38	ENSG00000160789.18_LMNA	0.13226	-0.027518217	0.285452082	1.04E-01	3.20E-01	1.00E+00
ENSG00000125851.8_PCSK2	ENSG00000160789.18_LMNA	-0.37338	-0.502696041	-0.22771137	2.16E-06	8.46E-05	1.11E-01
ENSG00000280077.1_RP11-87H9	ENSG00000160789.18_LMNA	-0.17249	-0.322830109	-0.01366941	3.36E-02	1.54E-01	1.00E+00
ENSG00000176155.17_CCDC57	ENSG00000160789.18_LMNA	0.04593	-0.114100752	0.203645828	5.74E-01	8.01E-01	1.00E+00
ENSG00000260418.1_RP3-406A7	ENSG00000160789.18_LMNA	-0.08386	-0.239857972	0.076360663	3.04E-01	5.91E-01	1.00E+00
ENSG00000124191.16_TOX2	ENSG00000160789.18_LMNA	-0.02386	-0.18236894	0.135855775	7.70E-01	9.05E-01	1.00E+00
ENSG00000280153.1_RP11-876N	ENSG00000160789.18_LMNA	0.12113	-0.038816322	0.275029835	1.37E-01	3.77E-01	1.00E+00
ENSG00000213865.6_C8orf44	ENSG00000160789.18_LMNA	-0.25489	-0.397941625	-0.09973519	1.53E-03	1.59E-02	1.00E+00
ENSG00000167216.15_KATNAL2	ENSG00000160789.18_LMNA	0.21789	0.060796473	0.364447044	7.00E-03	5.05E-02	1.00E+00
ENSG00000144535.18_DIS3L2	ENSG00000160789.18_LMNA	0.05144	-0.108654703	0.208925622	5.29E-01	7.73E-01	1.00E+00
ENSG00000246089.3_RP11-115C	ENSG00000160789.18_LMNA	-0.221	-0.367279314	-0.06405371	6.22E-03	4.62E-02	1.00E+00
ENSG00000172339.9_ALG14	ENSG00000160789.18_LMNA	-0.13933	-0.292048684	0.020325694	8.69E-02	2.86E-01	1.00E+00

ENSG00000175595.13_ERCC4	ENSG00000160789.18_LMNA	-0.12814	-0.281599235	0.031704184	1.16E-01	3.40E-01	1.00E+00
ENSG00000137822.11_TUBGCP4	ENSG00000160789.18_LMNA	0.03657	-0.123349534	0.194636846	6.55E-01	8.47E-01	1.00E+00
ENSG00000105639.17_JAK3	ENSG00000160789.18_LMNA	0.18795	0.029634401	0.337063643	2.04E-02	1.09E-01	1.00E+00
ENSG00000100147.12_CCDC134	ENSG00000160789.18_LMNA	0.07797	-0.082253754	0.234260854	3.40E-01	6.25E-01	1.00E+00
ENSG00000149716.11_ORAOV1	ENSG00000160789.18_LMNA	-0.00939	-0.168336006	0.150038054	9.09E-01	9.65E-01	1.00E+00
ENSG00000186272.11_ZNF17	ENSG00000160789.18_LMNA	0.17384	0.015053423	0.324069579	3.22E-02	1.50E-01	1.00E+00
ENSG00000168505.6_GBX2	ENSG00000160789.18_LMNA	-0.24527	-0.389271931	-0.08956796	2.32E-03	2.19E-02	1.00E+00
ENSG00000231764.7_DLX6-AS1	ENSG00000160789.18_LMNA	-0.29493	-0.433760089	-0.14241128	2.26E-04	3.59E-03	1.00E+00
ENSG00000235423.7_RP11-282C	ENSG00000160789.18_LMNA	-0.23492	-0.379910767	-0.07865951	3.58E-03	3.05E-02	1.00E+00
ENSG00000248690.5_HAS2-AS1	ENSG00000160789.18_LMNA	-0.20843	-0.355828117	-0.05092396	9.97E-03	6.55E-02	1.00E+00
ENSG00000089916.16_GPATCH2	ENSG00000160789.18_LMNA	0.07534	-0.084881098	0.231758685	3.56E-01	6.40E-01	1.00E+00
ENSG00000273447.1_AC004067.5	ENSG00000160789.18_LMNA	0.28063	0.127105193	0.421018582	4.62E-04	6.33E-03	1.00E+00
ENSG00000164327.11_RICTOR	ENSG00000160789.18_LMNA	-0.46784	-0.583573815	-0.33347424	1.22E-09	1.84E-07	6.28E-05
ENSG00000278259.3_MYO19	ENSG00000160789.18_LMNA	0.21692	0.059782074	0.363563844	7.27E-03	5.17E-02	1.00E+00
ENSG00000107951.11_MTPAP	ENSG00000160789.18_LMNA	-0.25678	-0.399644188	-0.10173918	1.41E-03	1.50E-02	1.00E+00
ENSG00000107443.14_CCNJ	ENSG00000160789.18_LMNA	-0.11342	-0.267782837	0.046625323	1.64E-01	4.20E-01	1.00E+00
ENSG00000260916.4_CCPG1	ENSG00000160789.18_LMNA	0.26648	0.112035061	0.408359135	9.05E-04	1.07E-02	1.00E+00
ENSG00000100285.9_NEFH	ENSG00000160789.18_LMNA	-0.20702	-0.354531766	-0.04944419	1.05E-02	6.78E-02	1.00E+00
ENSG00000188827.9_SLX4	ENSG00000160789.18_LMNA	0.08537	-0.074849444	0.241289913	2.96E-01	5.82E-01	1.00E+00
ENSG00000067365.13_METTL22	ENSG00000160789.18_LMNA	-0.147	-0.299197967	0.012493879	7.07E-02	2.51E-01	1.00E+00
ENSG00000137474.18_MYO7A	ENSG00000160789.18_LMNA	0.04513	-0.114891471	0.202877708	5.81E-01	8.05E-01	1.00E+00
ENSG00000158715.5_SLC45A3	ENSG00000160789.18_LMNA	-0.21225	-0.359306466	-0.05490106	8.66E-03	5.90E-02	1.00E+00
ENSG00000236850.4_BMS1P20	ENSG00000160789.18_LMNA	-0.07509	-0.231526352	0.08512483	3.58E-01	6.41E-01	1.00E+00
ENSG00000172006.10_ZNF554	ENSG00000160789.18_LMNA	-0.0769	-0.23324249	0.083323594	3.46E-01	6.31E-01	1.00E+00
ENSG00000262001.1_DLGAP1-AS	ENSG00000160789.18_LMNA	0.24088	0.084939458	0.385307495	2.79E-03	2.53E-02	1.00E+00
ENSG00000269946.1_RP11-2B6.3	ENSG00000160789.18_LMNA	0.34515	0.196762605	0.478082381	1.33E-05	3.69E-04	6.83E-01
ENSG00000196678.12_ERI2	ENSG00000160789.18_LMNA	-0.18849	-0.337565021	-0.03019964	2.00E-02	1.08E-01	1.00E+00
ENSG00000152475.6_ZNF837	ENSG00000160789.18_LMNA	0.32051	0.16998587	0.456424153	5.68E-05	1.19E-03	1.00E+00
ENSG00000184254.15_ALDH1A3	ENSG00000160789.18_LMNA	0.11466	-0.045371268	0.268949042	1.60E-01	4.13E-01	1.00E+00
ENSG00000145428.13_RNF175	ENSG00000160789.18_LMNA	-0.29806	-0.436545119	-0.14577553	1.92E-04	3.16E-03	1.00E+00
ENSG00000169184.5_MN1	ENSG00000160789.18_LMNA	-0.11918	-0.27320135	0.040790228	1.44E-01	3.87E-01	1.00E+00
ENSG00000170264.11_FAM161A	ENSG00000160789.18_LMNA	-0.06753	-0.224316176	0.092669987	4.08E-01	6.83E-01	1.00E+00
ENSG00000272829.1_XXbac-B135	ENSG00000160789.18_LMNA	-0.09393	-0.249403576	0.066259063	2.50E-01	5.30E-01	1.00E+00

ENSG00000161896.9_IP6K3	ENSG00000160789.18_LMNA	-0.27987	-0.420342245	-0.12629662	4.79E-04	6.52E-03	1.00E+00
ENSG00000136267.12_DGKB	ENSG00000160789.18_LMNA	-0.56848	-0.667288868	-0.45000516	2.18E-14	2.70E-11	1.12E-09
ENSG00000150681.8_RGS18	ENSG00000160789.18_LMNA	-0.12392	-0.277639934	0.035994389	1.28E-01	3.63E-01	1.00E+00
ENSG00000138311.14_ZNF365	ENSG00000160789.18_LMNA	-0.1451	-0.297430298	0.014433871	7.45E-02	2.60E-01	1.00E+00
ENSG00000228801.5_RP11-110G	ENSG00000160789.18_LMNA	-0.10256	-0.257554329	0.05758204	2.09E-01	4.81E-01	1.00E+00
ENSG00000234743.1_EIF5AP4	ENSG00000160789.18_LMNA	-0.05552	-0.212838835	0.104605928	4.97E-01	7.51E-01	1.00E+00
ENSG00000169519.18_METTL15	ENSG00000160789.18_LMNA	0.07637	-0.083852603	0.232738678	3.50E-01	6.33E-01	1.00E+00
ENSG00000179044.14_EXOC3L1	ENSG00000160789.18_LMNA	0.13253	-0.027240932	0.285706944	1.04E-01	3.19E-01	1.00E+00
ENSG00000196605.6_ZNF846	ENSG00000160789.18_LMNA	-0.1629	-0.313954665	-0.00379346	4.49E-02	1.88E-01	1.00E+00
ENSG00000179101.5_RP11-349N	ENSG00000160789.18_LMNA	-0.1958	-0.344266525	-0.0377737	1.56E-02	9.03E-02	1.00E+00
ENSG00000196345.11_ZKSCAN7	ENSG00000160789.18_LMNA	-0.30861	-0.44590206	-0.15712797	1.10E-04	2.03E-03	1.00E+00
ENSG00000106633.14_GCK	ENSG00000160789.18_LMNA	0.16958	0.010663269	0.320134254	3.67E-02	1.64E-01	1.00E+00
ENSG00000255633.4_MTRNR2L9	ENSG00000160789.18_LMNA	-0.23548	-0.380421621	-0.07925295	3.50E-03	3.00E-02	1.00E+00
ENSG00000119723.14_COQ6	ENSG00000160789.18_LMNA	-0.10206	-0.257084397	0.058083611	2.11E-01	4.84E-01	1.00E+00
ENSG00000177054.12_ZDHHC13	ENSG00000160789.18_LMNA	-0.10541	-0.260246463	0.054705584	1.96E-01	4.65E-01	1.00E+00
ENSG00000266714.5_MYO15B	ENSG00000160789.18_LMNA	0.02441	-0.135312837	0.182903596	7.65E-01	9.03E-01	1.00E+00
ENSG00000168071.20_CCDC88B	ENSG00000160789.18_LMNA	0.10154	-0.058606934	0.256593926	2.13E-01	4.87E-01	1.00E+00
ENSG00000272405.1_RP11-284F	ENSG00000160789.18_LMNA	0.1989	0.040996873	0.347108936	1.40E-02	8.37E-02	1.00E+00
ENSG00000215612.6_HMX1	ENSG00000160789.18_LMNA	-0.35162	-0.483741091	-0.20382848	8.91E-06	2.65E-04	4.57E-01
ENSG00000278129.1_ZNF8	ENSG00000160789.18_LMNA	-0.09015	-0.245827387	0.070051147	2.69E-01	5.54E-01	1.00E+00
ENSG00000151090.16_THRB	ENSG00000160789.18_LMNA	-0.14765	-0.299799899	0.011832733	6.95E-02	2.48E-01	1.00E+00
ENSG00000168016.12_TRANK1	ENSG00000160789.18_LMNA	0.23733	0.081201613	0.382097816	3.24E-03	2.83E-02	1.00E+00
ENSG00000172530.18_BANP	ENSG00000160789.18_LMNA	0.22731	0.070662431	0.373008586	4.86E-03	3.83E-02	1.00E+00
ENSG00000228981.3_RP11-364L4	ENSG00000160789.18_LMNA	-0.02013	-0.178758225	0.139517421	8.06E-01	9.22E-01	1.00E+00
ENSG00000154274.13_C4orf19	ENSG00000160789.18_LMNA	-0.03138	-0.189629418	0.128466472	7.01E-01	8.72E-01	1.00E+00
ENSG00000138709.16_LARP1B	ENSG00000160789.18_LMNA	0.1441	-0.015452985	0.296500851	7.65E-02	2.65E-01	1.00E+00
ENSG00000267532.3_MIR497HG	ENSG00000160789.18_LMNA	-0.19437	-0.342953675	-0.03628714	1.64E-02	9.37E-02	1.00E+00
ENSG00000103196.10_CRISPLD2	ENSG00000160789.18_LMNA	0.33125	0.181622742	0.465878467	3.07E-05	7.27E-04	1.00E+00
ENSG00000164620.7_RELL2	ENSG00000160789.18_LMNA	-0.07637	-0.232742096	0.083849015	3.50E-01	6.33E-01	1.00E+00
ENSG00000173320.8_STOX2	ENSG00000160789.18_LMNA	-0.28234	-0.422547423	-0.12893439	4.25E-04	5.95E-03	1.00E+00
ENSG00000244687.10_UBE2V1	ENSG00000160789.18_LMNA	0.03166	-0.128184998	0.189905299	6.99E-01	8.71E-01	1.00E+00
ENSG00000145390.10_USP53	ENSG00000160789.18_LMNA	0.1257	-0.034181911	0.279313916	1.23E-01	3.53E-01	1.00E+00
ENSG00000259612.1_EEF1A1P22	ENSG00000160789.18_LMNA	-0.13369	-0.286790758	0.026061224	1.01E-01	3.12E-01	1.00E+00

ENSG00000188011.5_RTP5	ENSG00000160789.18_LMNA	-0.33327	-0.467657577	-0.1838215	2.72E-05	6.62E-04	1.00E+00
ENSG00000116095.9_PLEKHA3	ENSG00000160789.18_LMNA	-0.06241	-0.219432111	0.097760364	4.45E-01	7.12E-01	1.00E+00
ENSG00000184635.12_ZNF93	ENSG00000160789.18_LMNA	-0.14843	-0.300528013	0.011032625	6.80E-02	2.45E-01	1.00E+00
ENSG00000102904.13_TSNAXIP1	ENSG00000160789.18_LMNA	0.01328	-0.146234668	0.172112046	8.71E-01	9.50E-01	1.00E+00
ENSG00000185670.7_ZBTB3	ENSG00000160789.18_LMNA	-0.16779	-0.318482657	-0.00882434	3.88E-02	1.70E-01	1.00E+00
ENSG00000187867.7_PALM3	ENSG00000160789.18_LMNA	0.47735	0.344315948	0.591593357	5.04E-10	8.88E-08	2.58E-05
ENSG00000155034.17_FBXL18	ENSG00000160789.18_LMNA	0.1766	0.017904905	0.326619926	2.95E-02	1.42E-01	1.00E+00
ENSG00000168004.8_HRASLS5	ENSG00000160789.18_LMNA	0.08901	-0.071194981	0.244746984	2.75E-01	5.60E-01	1.00E+00
ENSG00000115556.12_PLCD4	ENSG00000160789.18_LMNA	-0.08312	-0.239153219	0.0771039	3.09E-01	5.95E-01	1.00E+00
ENSG00000156113.19_KCNMA1	ENSG00000160789.18_LMNA	-0.07072	-0.227359245	0.089489972	3.87E-01	6.65E-01	1.00E+00
ENSG00000250966.2_RP11-402J	ENSG00000160789.18_LMNA	-0.2652	-0.40720995	-0.11067376	9.60E-04	1.12E-02	1.00E+00
ENSG00000012211.11_PRICKLE3	ENSG00000160789.18_LMNA	0.53672	0.412785102	0.641133934	1.02E-12	5.54E-10	5.25E-08
ENSG00000121350.14_PYROXD1	ENSG00000160789.18_LMNA	-0.255	-0.398040588	-0.0998516	1.52E-03	1.59E-02	1.00E+00
ENSG00000171132.12_PRKCE	ENSG00000160789.18_LMNA	-0.33666	-0.470639123	-0.1875127	2.23E-05	5.63E-04	1.00E+00
ENSG00000187800.12_PEAR1	ENSG00000160789.18_LMNA	0.01309	-0.146415326	0.171932898	8.73E-01	9.51E-01	1.00E+00
ENSG00000177045.7_SIX5	ENSG00000160789.18_LMNA	0.53698	0.413093407	0.641352784	9.92E-13	5.53E-10	5.09E-08
ENSG00000232134.1_RPS15AP1	ENSG00000160789.18_LMNA	-0.15219	-0.304027209	0.007181867	6.12E-02	2.29E-01	1.00E+00
ENSG00000237036.4_ZEB1-AS1	ENSG00000160789.18_LMNA	-0.18136	-0.331001466	-0.02281555	2.53E-02	1.28E-01	1.00E+00
ENSG00000118276.10_B4GALT6	ENSG00000160789.18_LMNA	-0.40081	-0.526417574	-0.25807356	3.11E-07	1.81E-05	1.59E-02
ENSG00000213903.7_LTB4R	ENSG00000160789.18_LMNA	0.20555	0.047922311	0.35319729	1.11E-02	7.04E-02	1.00E+00
ENSG00000231609.4_AC009501.4	ENSG00000160789.18_LMNA	0.08095	-0.079268298	0.237098994	3.21E-01	6.08E-01	1.00E+00
ENSG00000257842.4_NOVA1-AS	ENSG00000160789.18_LMNA	-0.41443	-0.538122331	-0.27325285	1.11E-07	7.87E-06	5.69E-03
ENSG00000128805.13_ARHGAP2	ENSG00000160789.18_LMNA	-0.09011	-0.245780741	0.070100549	2.70E-01	5.54E-01	1.00E+00
ENSG00000269743.2_SLC25A53	ENSG00000160789.18_LMNA	-0.37731	-0.506107264	-0.23204486	1.65E-06	6.91E-05	8.49E-02
ENSG00000213799.9_ZNF845	ENSG00000160789.18_LMNA	0.09691	-0.063266469	0.252219728	2.35E-01	5.13E-01	1.00E+00
ENSG00000182484.13_WASH6P	ENSG00000160789.18_LMNA	0.1771	0.01842078	0.327080841	2.91E-02	1.40E-01	1.00E+00
ENSG00000105085.9_MED26	ENSG00000160789.18_LMNA	0.14157	-0.018037862	0.294140751	8.19E-02	2.76E-01	1.00E+00
ENSG00000162946.19_DISC1	ENSG00000160789.18_LMNA	-0.14027	-0.292931347	0.019360835	8.48E-02	2.82E-01	1.00E+00
ENSG00000260920.2_RP1-228H1	ENSG00000160789.18_LMNA	-0.02133	-0.17992313	0.138337035	7.94E-01	9.17E-01	1.00E+00
ENSG00000260219.2_RP11-347C	ENSG00000160789.18_LMNA	-0.01398	-0.172796959	0.145543786	8.64E-01	9.47E-01	1.00E+00
ENSG00000170276.5_HSPB2	ENSG00000160789.18_LMNA	-0.10073	-0.255824528	0.059427518	2.17E-01	4.92E-01	1.00E+00
ENSG00000157343.7_ARMC12	ENSG00000160789.18_LMNA	-0.0923	-0.247859769	0.067897189	2.58E-01	5.41E-01	1.00E+00
ENSG00000164778.4_EN2	ENSG00000160789.18_LMNA	0.00823	-0.151166069	0.167214302	9.20E-01	9.69E-01	1.00E+00

ENSG00000154678.15_PDE1C	ENSG00000160789.18_LMNA	0.10547	-0.054647152	0.2603011	1.96E-01	4.65E-01	1.00E+00
ENSG00000151623.13_NR3C2	ENSG00000160789.18_LMNA	-0.0296	-0.18791985	0.130209559	7.17E-01	8.80E-01	1.00E+00
ENSG00000088808.15_PPP1R13I	ENSG00000160789.18_LMNA	0.02931	-0.130501044	0.187633782	7.20E-01	8.81E-01	1.00E+00
ENSG00000213983.10_AP1G2	ENSG00000160789.18_LMNA	0.05892	-0.101226591	0.216097246	4.71E-01	7.31E-01	1.00E+00
ENSG00000279369.1_RP11-51F1	ENSG00000160789.18_LMNA	-0.12072	-0.274641546	0.039235697	1.38E-01	3.79E-01	1.00E+00
ENSG00000255517.5_CTD-3074C	ENSG00000160789.18_LMNA	-0.12305	-0.276823354	0.03687778	1.31E-01	3.67E-01	1.00E+00
ENSG00000198939.6_ZFP2	ENSG00000160789.18_LMNA	-0.19203	-0.340814346	-0.03386764	1.78E-02	9.92E-02	1.00E+00
ENSG00000232044.6_LINC01105	ENSG00000160789.18_LMNA	-0.45664	-0.574105253	-0.32075818	3.36E-09	4.26E-07	1.72E-04
ENSG00000204116.10_CHIC1	ENSG00000160789.18_LMNA	-0.0552	-0.212534551	0.104921131	4.99E-01	7.52E-01	1.00E+00
ENSG00000131944.8_FAAP24	ENSG00000160789.18_LMNA	0.04472	-0.115305374	0.202475479	5.84E-01	8.07E-01	1.00E+00
ENSG00000172748.11_ZNF596	ENSG00000160789.18_LMNA	-0.01889	-0.177555589	0.140735091	8.17E-01	9.29E-01	1.00E+00
ENSG00000123473.14_STIL	ENSG00000160789.18_LMNA	0.12209	-0.037850636	0.27592355	1.34E-01	3.72E-01	1.00E+00
ENSG00000260088.1_RP11-92G1	ENSG00000160789.18_LMNA	-0.02962	-0.187932604	0.130196562	7.17E-01	8.80E-01	1.00E+00
ENSG00000158104.10_HPD	ENSG00000160789.18_LMNA	0.14436	-0.01518941	0.296741292	7.60E-02	2.64E-01	1.00E+00
ENSG00000166845.12_C18orf54	ENSG00000160789.18_LMNA	-0.0947	-0.250126715	0.065491158	2.46E-01	5.26E-01	1.00E+00
ENSG00000231369.1_RP1-40G4F	ENSG00000160789.18_LMNA	-0.17497	-0.32511778	-0.01622478	3.11E-02	1.47E-01	1.00E+00
ENSG00000110455.12_ACCS	ENSG00000160789.18_LMNA	0.22809	0.071485768	0.373720747	4.71E-03	3.75E-02	1.00E+00
ENSG00000272153.1_RP1-286D6	ENSG00000160789.18_LMNA	-0.02706	-0.185463264	0.132710871	7.41E-01	8.91E-01	1.00E+00
ENSG00000245571.5_AP001258.4	ENSG00000160789.18_LMNA	-0.00539	-0.164445265	0.153947138	9.47E-01	9.79E-01	1.00E+00
ENSG00000141837.17_CACNA1A	ENSG00000160789.18_LMNA	-0.16836	-0.319008511	-0.00940961	3.81E-02	1.68E-01	1.00E+00
ENSG00000212123.3_PRR22	ENSG00000160789.18_LMNA	-0.11339	-0.267753732	0.046656608	1.64E-01	4.20E-01	1.00E+00
ENSG00000172456.15_FGGY	ENSG00000160789.18_LMNA	0.11785	-0.042145082	0.271944996	1.48E-01	3.95E-01	1.00E+00
ENSG00000268222.1_EEF1A1P7	ENSG00000160789.18_LMNA	-0.13799	-0.290806034	0.021683078	9.00E-02	2.92E-01	1.00E+00
ENSG00000125895.5_TMEM74B	ENSG00000160789.18_LMNA	-0.18218	-0.331761818	-0.02366924	2.47E-02	1.25E-01	1.00E+00
ENSG00000177855.8_CACYBPP2	ENSG00000160789.18_LMNA	-0.18487	-0.334234817	-0.02644892	2.26E-02	1.18E-01	1.00E+00
ENSG00000125319.13_C17orf53	ENSG00000160789.18_LMNA	0.13387	-0.025887024	0.286950731	1.00E-01	3.12E-01	1.00E+00
ENSG00000131196.16_NFATC1	ENSG00000160789.18_LMNA	0.12433	-0.035575876	0.278026636	1.27E-01	3.61E-01	1.00E+00
ENSG00000228519.3_RP11-298C	ENSG00000160789.18_LMNA	-0.04742	-0.205073305	0.112630207	5.62E-01	7.93E-01	1.00E+00
ENSG00000178075.18_GRAMD1C	ENSG00000160789.18_LMNA	-0.0328	-0.191003182	0.127064349	6.88E-01	8.66E-01	1.00E+00
ENSG00000135406.12_PRPH	ENSG00000160789.18_LMNA	0.09261	-0.067586854	0.248152359	2.56E-01	5.39E-01	1.00E+00
ENSG00000169946.12_ZFPM2	ENSG00000160789.18_LMNA	-0.35931	-0.490457903	-0.2122536	5.47E-06	1.78E-04	2.80E-01
ENSG00000161692.16_DBF4B	ENSG00000160789.18_LMNA	-0.10628	-0.261060068	0.053835245	1.93E-01	4.61E-01	1.00E+00
ENSG00000263731.1_RP11-498C	ENSG00000160789.18_LMNA	0.02916	-0.130645721	0.187491773	7.21E-01	8.82E-01	1.00E+00

ENSG00000164002.10_EXO5	ENSG00000160789.18_LMNA	-0.1371	-0.289969562	0.022596133	9.21E-02	2.96E-01	1.00E+00
ENSG00000253305.2_PCDHGB6	ENSG00000160789.18_LMNA	-0.06262	-0.219635744	0.09754846	4.43E-01	7.11E-01	1.00E+00
ENSG00000263956.4_NBPF11	ENSG00000160789.18_LMNA	0.00594	-0.153410225	0.164980246	9.42E-01	9.77E-01	1.00E+00
ENSG00000170085.16_SIMC1	ENSG00000160789.18_LMNA	0.16123	0.002077794	0.312407259	4.72E-02	1.94E-01	1.00E+00
ENSG00000106804.7_C5	ENSG00000160789.18_LMNA	0.22914	0.072592335	0.374677331	4.52E-03	3.63E-02	1.00E+00
ENSG00000276980.1_CTD-3128G	ENSG00000160789.18_LMNA	-0.03401	-0.192170677	0.125871752	6.77E-01	8.59E-01	1.00E+00
ENSG00000229932.3_YWHAZP3	ENSG00000160789.18_LMNA	0.0153	-0.14425612	0.174072677	8.52E-01	9.42E-01	1.00E+00
ENSG00000163827.11_LRRC2	ENSG00000160789.18_LMNA	0.07002	-0.090182319	0.22669724	3.91E-01	6.69E-01	1.00E+00
ENSG00000140043.10_PTGR2	ENSG00000160789.18_LMNA	-0.07802	-0.234314845	0.082197014	3.39E-01	6.25E-01	1.00E+00
ENSG00000256594.6_RP11-705C	ENSG00000160789.18_LMNA	-0.25807	-0.400805458	-0.10310743	1.33E-03	1.43E-02	1.00E+00
ENSG00000108785.7_HSD17B1P	ENSG00000160789.18_LMNA	-0.01553	-0.174303614	0.144022905	8.49E-01	9.41E-01	1.00E+00
ENSG00000110786.16_PTPN5	ENSG00000160789.18_LMNA	-0.18113	-0.330788463	-0.02257648	2.55E-02	1.28E-01	1.00E+00
ENSG00000010539.10_ZNF200	ENSG00000160789.18_LMNA	-0.00451	-0.163588392	0.154806714	9.56E-01	9.82E-01	1.00E+00
ENSG00000157856.9_DRC1	ENSG00000160789.18_LMNA	0.18846	0.030164453	0.337533812	2.01E-02	1.08E-01	1.00E+00
ENSG00000147144.11_CCDC120	ENSG00000160789.18_LMNA	-0.20849	-0.355877724	-0.05098061	9.95E-03	6.54E-02	1.00E+00
ENSG00000227473.1_TSSK5P	ENSG00000160789.18_LMNA	0.13362	-0.026137324	0.286720869	1.01E-01	3.13E-01	1.00E+00
ENSG00000236992.2_RPL12P12	ENSG00000160789.18_LMNA	-0.16188	-0.31301481	-0.00275119	4.63E-02	1.91E-01	1.00E+00
ENSG00000147256.9_ARHGAP36	ENSG00000160789.18_LMNA	-0.12289	-0.276673541	0.037039797	1.31E-01	3.68E-01	1.00E+00
ENSG00000257511.1_RP11-278C	ENSG00000160789.18_LMNA	-0.13797	-0.290785066	0.021705972	9.01E-02	2.92E-01	1.00E+00
ENSG00000142684.7_ZNF593	ENSG00000160789.18_LMNA	0.08885	-0.071361946	0.244589212	2.76E-01	5.61E-01	1.00E+00
ENSG00000248489.1_CTD-2007H	ENSG00000160789.18_LMNA	-0.10822	-0.262890195	0.051875749	1.84E-01	4.50E-01	1.00E+00
ENSG00000263142.4_LRRC37A17	ENSG00000160789.18_LMNA	0.06941	-0.09079542	0.226110764	3.95E-01	6.73E-01	1.00E+00
ENSG00000258016.1_HIGD1AP1	ENSG00000160789.18_LMNA	-0.24057	-0.385027816	-0.08461342	2.83E-03	2.56E-02	1.00E+00
ENSG00000185046.17_ANKS1B	ENSG00000160789.18_LMNA	-0.33404	-0.468333174	-0.1846572	2.60E-05	6.38E-04	1.00E+00
ENSG00000178401.13_DNAJC22	ENSG00000160789.18_LMNA	0.3362	0.187003604	0.470228285	2.29E-05	5.76E-04	1.00E+00
ENSG00000179583.16_CIITA	ENSG00000160789.18_LMNA	0.1941	0.036013001	0.342711443	1.66E-02	9.43E-02	1.00E+00
ENSG00000103241.6_FOXF1	ENSG00000160789.18_LMNA	-0.09185	-0.247429755	0.068353172	2.60E-01	5.44E-01	1.00E+00
ENSG00000185024.14_BRF1	ENSG00000160789.18_LMNA	0.13654	-0.023160006	0.289452747	9.35E-02	2.99E-01	1.00E+00
ENSG00000146242.7_TPBG	ENSG00000160789.18_LMNA	0.09284	-0.06735607	0.24836991	2.55E-01	5.37E-01	1.00E+00
ENSG00000151474.18_FRMD4A	ENSG00000160789.18_LMNA	-0.08396	-0.239957891	0.076255258	3.04E-01	5.90E-01	1.00E+00
ENSG00000183137.13_CEP57L1	ENSG00000160789.18_LMNA	-0.1111	-0.265602366	0.048967393	1.73E-01	4.33E-01	1.00E+00
ENSG00000156313.11_RPGR	ENSG00000160789.18_LMNA	0.23623	0.080038669	0.381097717	3.39E-03	2.93E-02	1.00E+00
ENSG00000184307.12_ZDHHC23	ENSG00000160789.18_LMNA	0.16355	0.004466567	0.314561305	4.41E-02	1.85E-01	1.00E+00

ENSG00000166377.18_ATP9B	ENSG00000160789.18_LMNA	-0.03276	-0.190962471	0.127105918	6.89E-01	8.66E-01	1.00E+00
ENSG00000243445.1_RP11-715J	ENSG00000160789.18_LMNA	-0.0959	-0.251262919	0.064283862	2.40E-01	5.19E-01	1.00E+00
ENSG00000165323.14_FAT3	ENSG00000160789.18_LMNA	-0.12108	-0.274981733	0.038868281	1.37E-01	3.77E-01	1.00E+00
ENSG00000204248.9_COL11A2	ENSG00000160789.18_LMNA	-0.16966	-0.320209661	-0.01074728	3.67E-02	1.63E-01	1.00E+00
ENSG00000266208.1_CTD-2267D	ENSG00000160789.18_LMNA	0.24678	0.091160446	0.390633376	2.18E-03	2.09E-02	1.00E+00
ENSG00000166596.13_CFAP52	ENSG00000160789.18_LMNA	-0.07561	-0.232020203	0.084606703	3.55E-01	6.38E-01	1.00E+00
ENSG00000142494.12_SLC47A1	ENSG00000160789.18_LMNA	0.00053	-0.158683662	0.159717655	9.95E-01	9.98E-01	1.00E+00
ENSG00000232453.4_RP4-794H1	ENSG00000160789.18_LMNA	0.00993	-0.149504072	0.168866713	9.03E-01	9.63E-01	1.00E+00
ENSG00000072694.17_FCGR2B	ENSG00000160789.18_LMNA	0.27522	0.121335851	0.416185618	6.00E-04	7.79E-03	1.00E+00
ENSG00000140386.11_SCAPER	ENSG00000160789.18_LMNA	-0.0986	-0.253819924	0.061563484	2.27E-01	5.03E-01	1.00E+00
ENSG00000261737.1_RP4-612B1	ENSG00000160789.18_LMNA	-0.06693	-0.223746193	0.093264902	4.13E-01	6.86E-01	1.00E+00
ENSG00000232176.1_RP11-146N	ENSG00000160789.18_LMNA	-0.15422	-0.305913798	0.005101898	5.78E-02	2.20E-01	1.00E+00
ENSG00000256211.1_RP11-1018	ENSG00000160789.18_LMNA	-0.18958	-0.338560996	-0.03132307	1.93E-02	1.05E-01	1.00E+00
ENSG00000124212.5_PTGIS	ENSG00000160789.18_LMNA	0.05945	-0.100701642	0.216602777	4.67E-01	7.28E-01	1.00E+00
ENSG00000142408.2_CACNG8	ENSG00000160789.18_LMNA	0.02087	-0.138792611	0.179473634	7.99E-01	9.19E-01	1.00E+00
ENSG00000137691.11_C11orf70	ENSG00000160789.18_LMNA	0.03218	-0.127677357	0.190402726	6.94E-01	8.68E-01	1.00E+00
ENSG00000125122.13_LRRC29	ENSG00000160789.18_LMNA	-0.00356	-0.162666927	0.155730548	9.65E-01	9.86E-01	1.00E+00
ENSG00000224831.3_RP11-651P	ENSG00000160789.18_LMNA	-0.04062	-0.198539811	0.119349451	6.19E-01	8.28E-01	1.00E+00
ENSG00000174032.15_SLC25A30	ENSG00000160789.18_LMNA	-0.072	-0.228576432	0.088216197	3.78E-01	6.57E-01	1.00E+00
ENSG00000171962.16_DRC3	ENSG00000160789.18_LMNA	-0.10111	-0.256185265	0.059042835	2.15E-01	4.89E-01	1.00E+00
ENSG00000052126.13_PLEKHA5	ENSG00000160789.18_LMNA	-0.1031	-0.258061415	0.057040635	2.06E-01	4.78E-01	1.00E+00
ENSG00000138771.13_SHROOM	ENSG00000160789.18_LMNA	0.43646	0.297962397	0.556959849	1.90E-08	1.84E-06	9.74E-04
ENSG00000230359.4_TPI1P2	ENSG00000160789.18_LMNA	0.0584	-0.101751422	0.21559166	4.75E-01	7.34E-01	1.00E+00
ENSG00000167711.12_SERPINF	ENSG00000160789.18_LMNA	-0.12713	-0.280650119	0.032733677	1.19E-01	3.46E-01	1.00E+00
ENSG00000075213.9_SEMA3A	ENSG00000160789.18_LMNA	0.20024	0.042392749	0.34833817	1.34E-02	8.07E-02	1.00E+00
ENSG00000234742.1_AC144530.	ENSG00000160789.18_LMNA	-0.08589	-0.241780078	0.074331808	2.93E-01	5.79E-01	1.00E+00
ENSG00000131042.12_LILRB2	ENSG00000160789.18_LMNA	0.19963	0.041760745	0.347781746	1.37E-02	8.20E-02	1.00E+00
ENSG00000156127.6_BATF	ENSG00000160789.18_LMNA	0.13767	-0.022009788	0.290506782	9.08E-02	2.94E-01	1.00E+00
ENSG00000245958.5_RP11-33B1	ENSG00000160789.18_LMNA	-0.28265	-0.422823973	-0.12926549	4.18E-04	5.87E-03	1.00E+00
ENSG00000268043.6_NBPF12	ENSG00000160789.18_LMNA	-0.02615	-0.18458316	0.133606013	7.49E-01	8.95E-01	1.00E+00
ENSG00000129295.7_LRRC6	ENSG00000160789.18_LMNA	0.0584	-0.101750889	0.215592173	4.75E-01	7.34E-01	1.00E+00
ENSG00000136866.12_ZFP37	ENSG00000160789.18_LMNA	-0.13805	-0.290858707	0.021625566	8.99E-02	2.92E-01	1.00E+00
ENSG00000149150.7_SLC43A1	ENSG00000160789.18_LMNA	-0.04282	-0.200653435	0.117178918	6.00E-01	8.16E-01	1.00E+00

ENSG00000215788.8_TNFRSF25	ENSG00000160789.18_LMNA	0.08182	-0.078400669	0.237922799	3.16E-01	6.03E-01	1.00E+00
ENSG00000233514.1_RP11-570P	ENSG00000160789.18_LMNA	-0.08782	-0.243615197	0.072392325	2.82E-01	5.67E-01	1.00E+00
ENSG00000238150.1_AC008753.3	ENSG00000160789.18_LMNA	0.10427	-0.055859692	0.259166898	2.01E-01	4.72E-01	1.00E+00
ENSG00000259985.1_RP11-549B	ENSG00000160789.18_LMNA	-0.38404	-0.511936689	-0.23947563	1.04E-06	4.74E-05	5.33E-02
ENSG00000164199.14_ADGRV1	ENSG00000160789.18_LMNA	-0.30156	-0.439655423	-0.14954067	1.60E-04	2.74E-03	1.00E+00
ENSG00000260290.2_CTD-2033A	ENSG00000160789.18_LMNA	0.0779	-0.082323743	0.234194254	3.40E-01	6.25E-01	1.00E+00
ENSG00000143190.20_POU2F1	ENSG00000160789.18_LMNA	-0.00702	-0.166033809	0.152352308	9.32E-01	9.73E-01	1.00E+00
ENSG00000158008.8_EXTL1	ENSG00000160789.18_LMNA	-0.34198	-0.475299491	-0.19329837	1.62E-05	4.33E-04	8.30E-01
ENSG00000182318.5_ZSCAN22	ENSG00000160789.18_LMNA	0.24676	0.091135694	0.390612225	2.18E-03	2.09E-02	1.00E+00
ENSG00000182621.15_PLCB1	ENSG00000160789.18_LMNA	-0.46503	-0.58119961	-0.33027717	1.58E-09	2.29E-07	8.12E-05
ENSG00000275888.1_RP13-516M	ENSG00000160789.18_LMNA	0.16968	0.010769728	0.320229809	3.66E-02	1.63E-01	1.00E+00
ENSG00000054654.14_SYNE2	ENSG00000160789.18_LMNA	0.14574	-0.013779745	0.298026559	7.32E-02	2.57E-01	1.00E+00
ENSG00000127124.12_HIVEP3	ENSG00000160789.18_LMNA	0.63694	0.531652633	0.722845714	1.14E-18	4.86E-15	5.83E-14
ENSG00000146858.7_ZC3HAV1L	ENSG00000160789.18_LMNA	0.12876	-0.031073771	0.282180126	1.14E-01	3.37E-01	1.00E+00
ENSG00000007341.17_ST7L	ENSG00000160789.18_LMNA	-0.06823	-0.22498536	0.091971242	4.04E-01	6.79E-01	1.00E+00
ENSG00000224078.11_SNHG14	ENSG00000160789.18_LMNA	-0.11973	-0.273714654	0.040236348	1.42E-01	3.84E-01	1.00E+00
ENSG00000104356.9_POP1	ENSG00000160789.18_LMNA	0.01823	-0.141376502	0.176921715	8.24E-01	9.31E-01	1.00E+00
ENSG00000278058.1_RP11-70D2	ENSG00000160789.18_LMNA	0.02838	-0.13141147	0.186739927	7.29E-01	8.85E-01	1.00E+00
ENSG00000279095.1_AC092066.1	ENSG00000160789.18_LMNA	0.34007	0.191217515	0.473625176	1.82E-05	4.76E-04	9.31E-01
ENSG00000198865.8_CCDC152	ENSG00000160789.18_LMNA	-0.06627	-0.223120878	0.09391731	4.17E-01	6.90E-01	1.00E+00
ENSG00000162520.13_SYNC	ENSG00000160789.18_LMNA	0.33198	0.18242237	0.466525746	2.94E-05	7.01E-04	1.00E+00
ENSG00000223959.7_AFG3L1P	ENSG00000160789.18_LMNA	0.09563	-0.064554024	0.251008741	2.41E-01	5.20E-01	1.00E+00
ENSG00000128000.14_ZNF780B	ENSG00000160789.18_LMNA	-0.24958	-0.393158895	-0.09411862	1.93E-03	1.90E-02	1.00E+00
ENSG00000197321.13_SVIL	ENSG00000160789.18_LMNA	0.38835	0.244245884	0.515665773	7.66E-07	3.70E-05	3.93E-02
ENSG00000163362.9_C1orf106	ENSG00000160789.18_LMNA	-0.20899	-0.356330682	-0.05149799	9.77E-03	6.45E-02	1.00E+00
ENSG00000183023.17_SLC8A1	ENSG00000160789.18_LMNA	-0.03504	-0.193159851	0.12486059	6.68E-01	8.54E-01	1.00E+00
ENSG00000148429.13_USP6NL	ENSG00000160789.18_LMNA	-0.15012	-0.302102839	0.00930072	6.49E-02	2.37E-01	1.00E+00
ENSG00000213261.3_EEF1B2P6	ENSG00000160789.18_LMNA	-0.13204	-0.285248719	0.027739438	1.05E-01	3.21E-01	1.00E+00
ENSG00000124613.7_ZNF391	ENSG00000160789.18_LMNA	-0.0693	-0.226003695	0.090907324	3.96E-01	6.73E-01	1.00E+00
ENSG00000184110.13_EIF3C	ENSG00000160789.18_LMNA	0.13498	-0.024756412	0.287988576	9.73E-02	3.07E-01	1.00E+00
ENSG00000006283.16_CACNA1G	ENSG00000160789.18_LMNA	0.05085	-0.109238314	0.208360711	5.34E-01	7.76E-01	1.00E+00
ENSG00000270681.1_RP11-372K	ENSG00000160789.18_LMNA	-0.27652	-0.417350969	-0.12272517	5.64E-04	7.40E-03	1.00E+00
ENSG00000176658.15_MYO1D	ENSG00000160789.18_LMNA	0.04803	-0.112022742	0.205662584	5.57E-01	7.90E-01	1.00E+00

ENSG00000113205.4_PCDHB3	ENSG00000160789.18_LMNA	0.03746	-0.122471653	0.195494275	6.47E-01	8.43E-01	1.00E+00
ENSG00000183647.9_ZNF530	ENSG00000160789.18_LMNA	-0.13812	-0.29092771	0.021550219	8.97E-02	2.92E-01	1.00E+00
ENSG00000240759.1_RP11-25I15	ENSG00000160789.18_LMNA	-0.03673	-0.194790937	0.123191804	6.53E-01	8.47E-01	1.00E+00
ENSG00000100578.13_KIAA0586	ENSG00000160789.18_LMNA	0.10244	-0.057701189	0.257442709	2.09E-01	4.82E-01	1.00E+00
ENSG00000188283.10_ZNF383	ENSG00000160789.18_LMNA	-0.22198	-0.368170949	-0.06508046	5.99E-03	4.50E-02	1.00E+00
ENSG00000258920.1_FOXN3-AS1	ENSG00000160789.18_LMNA	-0.01812	-0.176812099	0.141487394	8.25E-01	9.31E-01	1.00E+00
ENSG00000069943.8_PIGB	ENSG00000160789.18_LMNA	0.24714	0.091538847	0.390956685	2.14E-03	2.06E-02	1.00E+00
ENSG00000138316.9_ADAMTS14	ENSG00000160789.18_LMNA	0.35048	0.202577805	0.482741189	9.58E-06	2.81E-04	4.91E-01
ENSG00000213337.7_ANKRD39	ENSG00000160789.18_LMNA	-0.21971	-0.366105745	-0.06270327	6.53E-03	4.79E-02	1.00E+00
ENSG00000176531.9_PHLDB3	ENSG00000160789.18_LMNA	0.33693	0.187804857	0.470874833	2.19E-05	5.56E-04	1.00E+00
ENSG00000170537.11_TMC7	ENSG00000160789.18_LMNA	0.10089	-0.059264257	0.255977637	2.16E-01	4.91E-01	1.00E+00
ENSG00000204514.8_ZNF814	ENSG00000160789.18_LMNA	-0.05638	-0.21366512	0.103749672	4.90E-01	7.46E-01	1.00E+00
ENSG00000228408.4_RP1-111D6	ENSG00000160789.18_LMNA	-0.38241	-0.510533417	-0.23768396	1.16E-06	5.19E-05	5.97E-02
ENSG00000174514.11_MFSD4A	ENSG00000160789.18_LMNA	-0.26123	-0.403641632	-0.10645387	1.15E-03	1.29E-02	1.00E+00
ENSG00000187097.11_ENTPD5	ENSG00000160789.18_LMNA	0.23847	0.082402736	0.38313001	3.09E-03	2.73E-02	1.00E+00
ENSG00000168152.11_THAP9	ENSG00000160789.18_LMNA	-0.25086	-0.394310826	-0.09546964	1.83E-03	1.83E-02	1.00E+00
ENSG00000129951.17_PLPPR3	ENSG00000160789.18_LMNA	-0.29669	-0.435330458	-0.14430742	2.06E-04	3.34E-03	1.00E+00
ENSG00000180257.11_ZNF816	ENSG00000160789.18_LMNA	0.03849	-0.121450236	0.196491287	6.38E-01	8.38E-01	1.00E+00
ENSG00000280187.1_CTC-351M1	ENSG00000160789.18_LMNA	-0.06468	-0.221594317	0.095508868	4.29E-01	6.99E-01	1.00E+00
ENSG00000270231.3_NBPF8	ENSG00000160789.18_LMNA	0.10308	-0.057060586	0.258042731	2.06E-01	4.78E-01	1.00E+00
ENSG00000184809.11_B3GALT5-	ENSG00000160789.18_LMNA	-0.12424	-0.27794368	0.035665665	1.27E-01	3.61E-01	1.00E+00
ENSG00000240449.1_RP4-584D1	ENSG00000160789.18_LMNA	-0.14851	-0.300602471	0.010950782	6.79E-02	2.44E-01	1.00E+00
ENSG00000109674.3_NEIL3	ENSG00000160789.18_LMNA	0.04437	-0.115649679	0.202140803	5.87E-01	8.08E-01	1.00E+00
ENSG00000158079.13_PTPDC1	ENSG00000160789.18_LMNA	0.08517	-0.075044758	0.241104922	2.97E-01	5.83E-01	1.00E+00
ENSG00000129810.13_SGO1	ENSG00000160789.18_LMNA	-0.09823	-0.253462292	0.061944247	2.29E-01	5.06E-01	1.00E+00
ENSG00000135482.5_ZC3H10	ENSG00000160789.18_LMNA	-0.22558	-0.371442668	-0.0688535	5.20E-03	4.04E-02	1.00E+00
ENSG00000170381.11_SEMA3E	ENSG00000160789.18_LMNA	-0.09825	-0.25348747	0.061917443	2.28E-01	5.05E-01	1.00E+00
ENSG00000163867.15_ZMYM6	ENSG00000160789.18_LMNA	-0.18378	-0.33323327	-0.0253226	2.34E-02	1.21E-01	1.00E+00
ENSG00000198453.11_ZNF568	ENSG00000160789.18_LMNA	-0.05417	-0.211546644	0.105944048	5.07E-01	7.57E-01	1.00E+00
ENSG00000171522.5_PTGER4	ENSG00000160789.18_LMNA	0.11358	-0.046462989	0.267933852	1.64E-01	4.19E-01	1.00E+00
ENSG00000198429.8_ZNF69	ENSG00000160789.18_LMNA	-0.22851	-0.374105762	-0.07193106	4.63E-03	3.70E-02	1.00E+00
ENSG00000144362.10_PHOSPHO	ENSG00000160789.18_LMNA	-0.2986	-0.437025887	-0.14635697	1.87E-04	3.09E-03	1.00E+00
ENSG00000273391.1_RP11-634H	ENSG00000160789.18_LMNA	0.05992	-0.100235922	0.217051128	4.63E-01	7.26E-01	1.00E+00

ENSG00000134020.7_PEBP4	ENSG00000160789.18_LMNA	-0.32695	-0.462102244	-0.1769652	3.93E-05	8.89E-04	1.00E+00
ENSG00000146910.10_CNPY1	ENSG00000160789.18_LMNA	-0.06116	-0.218237911	0.099002489	4.54E-01	7.19E-01	1.00E+00
ENSG00000217648.1_RP1-95L4.4	ENSG00000160789.18_LMNA	-0.01618	-0.174925849	0.143394356	8.43E-01	9.39E-01	1.00E+00
ENSG00000176225.11_RTTN	ENSG00000160789.18_LMNA	-0.17256	-0.322893778	-0.01374048	3.35E-02	1.54E-01	1.00E+00
ENSG00000124243.16_BCAS4	ENSG00000160789.18_LMNA	-0.04966	-0.207218873	0.110417284	5.44E-01	7.82E-01	1.00E+00
ENSG00000104783.10_KCNN4	ENSG00000160789.18_LMNA	0.49443	0.363875223	0.605938511	9.54E-11	2.16E-08	4.90E-06
ENSG00000122733.12_PHF24	ENSG00000160789.18_LMNA	-0.15067	-0.302615493	0.008736529	6.39E-02	2.35E-01	1.00E+00
ENSG00000267419.1_CTC-559E9	ENSG00000160789.18_LMNA	-0.18478	-0.334150578	-0.02635416	2.27E-02	1.18E-01	1.00E+00
ENSG00000249637.1_CTC-329D1	ENSG00000160789.18_LMNA	-0.26877	-0.410411427	-0.11446894	8.14E-04	9.85E-03	1.00E+00
ENSG00000142910.14_TINAGL1	ENSG00000160789.18_LMNA	0.02452	-0.135209776	0.183005064	7.64E-01	9.02E-01	1.00E+00
ENSG00000100150.15_DEPDC5	ENSG00000160789.18_LMNA	-0.03599	-0.194082344	0.123916996	6.60E-01	8.50E-01	1.00E+00
ENSG00000082512.13_TRAF5	ENSG00000160789.18_LMNA	0.24673	0.091110298	0.390590524	2.18E-03	2.09E-02	1.00E+00
ENSG00000163577.6_EIF5A2	ENSG00000160789.18_LMNA	-0.15505	-0.306680673	0.004255646	5.65E-02	2.17E-01	1.00E+00
ENSG00000223508.5_RPL23AP53	ENSG00000160789.18_LMNA	0.14157	-0.018033375	0.29414485	8.19E-02	2.76E-01	1.00E+00
ENSG00000070269.12_TMEM260	ENSG00000160789.18_LMNA	0.16234	0.003222193	0.313439605	4.57E-02	1.90E-01	1.00E+00
ENSG00000173991.5_TCAP	ENSG00000160789.18_LMNA	-0.10182	-0.256854042	0.058329417	2.12E-01	4.86E-01	1.00E+00
ENSG00000120280.5_CXorf21	ENSG00000160789.18_LMNA	-0.09681	-0.252125148	0.063367067	2.35E-01	5.13E-01	1.00E+00
ENSG00000238165.1_AC007560.1	ENSG00000160789.18_LMNA	0.0162	-0.143368722	0.17495122	8.43E-01	9.39E-01	1.00E+00
ENSG00000114737.14_CISH	ENSG00000160789.18_LMNA	0.41113	0.269578233	0.535298171	1.43E-07	9.68E-06	7.33E-03
ENSG00000155592.14_ZKSCAN2	ENSG00000160789.18_LMNA	-0.06749	-0.224280092	0.092707656	4.09E-01	6.83E-01	1.00E+00
ENSG00000189164.13_ZNF527	ENSG00000160789.18_LMNA	-0.19849	-0.346730847	-0.04056777	1.42E-02	8.47E-02	1.00E+00
ENSG00000196724.11_ZNF418	ENSG00000160789.18_LMNA	-0.06086	-0.217953781	0.099297877	4.56E-01	7.21E-01	1.00E+00
ENSG00000130176.6_CNN1	ENSG00000160789.18_LMNA	0.33811	0.189089897	0.471911124	2.04E-05	5.26E-04	1.00E+00
ENSG00000115977.17_AAK1	ENSG00000160789.18_LMNA	0.03987	-0.12009031	0.197817699	6.26E-01	8.31E-01	1.00E+00
ENSG00000167083.5_GNGT2	ENSG00000160789.18_LMNA	-0.12513	-0.278775856	0.034764712	1.25E-01	3.56E-01	1.00E+00
ENSG00000272669.1_RP3-508I15	ENSG00000160789.18_LMNA	-0.00562	-0.164676477	0.153715114	9.45E-01	9.78E-01	1.00E+00
ENSG00000198286.8_CARD11	ENSG00000160789.18_LMNA	0.01666	-0.142921936	0.175393356	8.39E-01	9.37E-01	1.00E+00
ENSG00000272275.1_RP11-791G	ENSG00000160789.18_LMNA	0.10489	-0.055228634	0.259757294	1.98E-01	4.68E-01	1.00E+00
ENSG00000196358.9_NTNG2	ENSG00000160789.18_LMNA	-0.26381	-0.405964705	-0.10919992	1.02E-03	1.18E-02	1.00E+00
ENSG00000196436.8_NPIPB15	ENSG00000160789.18_LMNA	-0.08316	-0.23919634	0.077058435	3.08E-01	5.95E-01	1.00E+00
ENSG00000067646.10_ZFY	ENSG00000160789.18_LMNA	-0.08202	-0.238115182	0.078197983	3.15E-01	6.01E-01	1.00E+00
ENSG00000131398.12_KCNC3	ENSG00000160789.18_LMNA	0.02576	-0.133994864	0.184200683	7.53E-01	8.97E-01	1.00E+00
ENSG00000100354.19_TNRC6B	ENSG00000160789.18_LMNA	0.03108	-0.128755929	0.189345661	7.04E-01	8.74E-01	1.00E+00

ENSG00000272663.1_RP11-191L	ENSG00000160789.18_LMNA	-0.22165	-0.367868949	-0.06473263	6.06E-03	4.53E-02	1.00E+00
ENSG00000107821.13_KAZALD1	ENSG00000160789.18_LMNA	0.23128	0.074835251	0.376614274	4.15E-03	3.41E-02	1.00E+00
ENSG00000073150.12_PANX2	ENSG00000160789.18_LMNA	0.32639	0.176356553	0.461608005	4.06E-05	9.14E-04	1.00E+00
ENSG00000213080.3_RP11-312J	ENSG00000160789.18_LMNA	0.4126	0.271216283	0.536557843	1.28E-07	8.85E-06	6.55E-03
ENSG00000169418.9_NPR1	ENSG00000160789.18_LMNA	0.24912	0.093627683	0.392740077	1.97E-03	1.93E-02	1.00E+00
ENSG00000215414.4_PSMA6P1	ENSG00000160789.18_LMNA	-0.04638	-0.204072425	0.11366143	5.70E-01	7.99E-01	1.00E+00
ENSG00000214223.4_HNRNPA1F	ENSG00000160789.18_LMNA	-0.10097	-0.256053118	0.059183765	2.16E-01	4.90E-01	1.00E+00
ENSG00000227992.1_AC108463.:	ENSG00000160789.18_LMNA	0.39132	0.247542357	0.51823675	6.20E-07	3.12E-05	3.18E-02
ENSG00000214263.2_RPSAP53	ENSG00000160789.18_LMNA	-0.10942	-0.264025454	0.050659019	1.80E-01	4.42E-01	1.00E+00
ENSG00000136383.6_ALPK3	ENSG00000160789.18_LMNA	0.16881	0.00987391	0.319425538	3.76E-02	1.66E-01	1.00E+00
ENSG00000277969.1_CTB-58E17	ENSG00000160789.18_LMNA	-0.18597	-0.335244164	-0.02758481	2.18E-02	1.15E-01	1.00E+00
ENSG00000131979.17_GCH1	ENSG00000160789.18_LMNA	0.03016	-0.129666565	0.18845261	7.12E-01	8.78E-01	1.00E+00
ENSG00000227034.1_RP11-234N	ENSG00000160789.18_LMNA	-0.13674	-0.28963538	0.022960766	9.30E-02	2.98E-01	1.00E+00
ENSG00000087237.9_CETP	ENSG00000160789.18_LMNA	-0.15365	-0.305381376	0.005689167	5.88E-02	2.22E-01	1.00E+00
ENSG00000007174.16_DNAH9	ENSG00000160789.18_LMNA	0.21762	0.060515841	0.364202763	7.08E-03	5.08E-02	1.00E+00
ENSG00000153896.16_ZNF599	ENSG00000160789.18_LMNA	-0.13531	-0.288303383	0.024413308	9.65E-02	3.05E-01	1.00E+00
ENSG00000155886.10_SLC24A2	ENSG00000160789.18_LMNA	-0.19486	-0.343404055	-0.03679696	1.61E-02	9.25E-02	1.00E+00
ENSG00000150722.9_PPP1R1C	ENSG00000160789.18_LMNA	-0.24863	-0.392304308	-0.09311704	2.01E-03	1.96E-02	1.00E+00
ENSG00000269892.1_RP4-761J1	ENSG00000160789.18_LMNA	-0.05964	-0.216784448	0.10051295	4.65E-01	7.27E-01	1.00E+00
ENSG00000162882.13_HAAO	ENSG00000160789.18_LMNA	-0.02018	-0.178809295	0.139465691	8.05E-01	9.22E-01	1.00E+00
ENSG00000073910.18_FRY	ENSG00000160789.18_LMNA	-0.41201	-0.536048589	-0.27055388	1.34E-07	9.19E-06	6.86E-03
ENSG00000275131.1_CH17-472G	ENSG00000160789.18_LMNA	-0.23323	-0.378385382	-0.07688881	3.83E-03	3.21E-02	1.00E+00
ENSG00000181381.12_DDX60L	ENSG00000160789.18_LMNA	0.07463	-0.085590893	0.231081987	3.61E-01	6.43E-01	1.00E+00
ENSG00000102886.13_GDPD3	ENSG00000160789.18_LMNA	0.0035	-0.155782851	0.162614741	9.66E-01	9.87E-01	1.00E+00
ENSG00000185946.14_RNPC3	ENSG00000160789.18_LMNA	-0.20728	-0.354769135	-0.04971505	1.04E-02	6.73E-02	1.00E+00
ENSG00000143382.12_ADAMTSL	ENSG00000160789.18_LMNA	0.34243	0.193790296	0.475695006	1.57E-05	4.23E-04	8.07E-01
ENSG00000181896.10_ZNF101	ENSG00000160789.18_LMNA	-0.21666	-0.363326533	-0.05950962	7.34E-03	5.21E-02	1.00E+00
ENSG00000070759.15_TESK2	ENSG00000160789.18_LMNA	-0.26123	-0.403645176	-0.10645806	1.15E-03	1.29E-02	1.00E+00
ENSG00000180626.9_ZNF594	ENSG00000160789.18_LMNA	0.13398	-0.025774128	0.287054397	9.99E-02	3.11E-01	1.00E+00
ENSG00000198707.13_CEP290	ENSG00000160789.18_LMNA	0.10145	-0.058698012	0.256508549	2.14E-01	4.88E-01	1.00E+00
ENSG00000125378.14_BMP4	ENSG00000160789.18_LMNA	-0.18196	-0.331559922	-0.02344252	2.49E-02	1.26E-01	1.00E+00
ENSG00000073670.12_ADAM11	ENSG00000160789.18_LMNA	-0.17938	-0.329175665	-0.02076746	2.70E-02	1.34E-01	1.00E+00
ENSG00000077157.19_PPP1R12I	ENSG00000160789.18_LMNA	-0.11912	-0.273145312	0.040850685	1.44E-01	3.88E-01	1.00E+00

ENSG00000158517.12_NCF1	ENSG00000160789.18_LMNA	0.0448	-0.115225405	0.202553201	5.84E-01	8.06E-01	1.00E+00
ENSG00000172915.17_NBEA	ENSG00000160789.18_LMNA	-0.23003	-0.375476213	-0.07351704	4.36E-03	3.54E-02	1.00E+00
ENSG00000161911.10_TREML1	ENSG00000160789.18_LMNA	-0.02557	-0.184017695	0.134180867	7.55E-01	8.98E-01	1.00E+00
ENSG00000155970.10_MICU3	ENSG00000160789.18_LMNA	-0.39637	-0.522594777	-0.25314451	4.30E-07	2.33E-05	2.21E-02
ENSG00000051825.13_MPHOSPH	ENSG00000160789.18_LMNA	-0.13455	-0.287591116	0.025189493	9.84E-02	3.09E-01	1.00E+00
ENSG00000249072.1_RP11-777B	ENSG00000160789.18_LMNA	-0.2307	-0.376091244	-0.0742293	4.24E-03	3.47E-02	1.00E+00
ENSG00000198105.10_ZNF248	ENSG00000160789.18_LMNA	-0.41919	-0.54220609	-0.27858009	7.65E-08	5.80E-06	3.93E-03
ENSG00000261087.1_KB-1460A1	ENSG00000160789.18_LMNA	-0.108	-0.262685973	0.052094527	1.85E-01	4.51E-01	1.00E+00
ENSG00000174010.9_KLHL15	ENSG00000160789.18_LMNA	0.11221	-0.047844228	0.266648436	1.69E-01	4.27E-01	1.00E+00
ENSG00000272341.1_RP1-151F1	ENSG00000160789.18_LMNA	0.05977	-0.100385841	0.216906815	4.64E-01	7.27E-01	1.00E+00
ENSG00000213203.2_GIMAP1	ENSG00000160789.18_LMNA	0.15892	-0.00029282	0.310266429	5.05E-02	2.03E-01	1.00E+00
ENSG00000248367.1_CTB-129O4	ENSG00000160789.18_LMNA	-0.05955	-0.216695799	0.100605028	4.66E-01	7.28E-01	1.00E+00
ENSG00000241352.3_RP11-392P	ENSG00000160789.18_LMNA	-0.15748	-0.30893276	0.001767875	5.27E-02	2.08E-01	1.00E+00
ENSG00000028277.19_POU2F2	ENSG00000160789.18_LMNA	-0.03057	-0.188854938	0.129256381	7.08E-01	8.76E-01	1.00E+00
ENSG00000042317.15_SPATA7	ENSG00000160789.18_LMNA	-0.17947	-0.329264338	-0.02086687	2.69E-02	1.33E-01	1.00E+00
ENSG00000226686.6_LINC01535	ENSG00000160789.18_LMNA	-0.17915	-0.328965718	-0.02053212	2.72E-02	1.34E-01	1.00E+00
ENSG00000249249.1_AC010226.4	ENSG00000160789.18_LMNA	0.066	-0.094194782	0.222854849	4.19E-01	6.91E-01	1.00E+00
ENSG00000196972.7_SMIM10L2B	ENSG00000160789.18_LMNA	-0.12572	-0.279332781	0.034161473	1.23E-01	3.53E-01	1.00E+00
ENSG00000173275.11_ZNF449	ENSG00000160789.18_LMNA	0.03622	-0.123692977	0.19430127	6.58E-01	8.49E-01	1.00E+00
ENSG00000036448.8_MYOM2	ENSG00000160789.18_LMNA	-0.27827	-0.418910366	-0.12458607	5.18E-04	6.94E-03	1.00E+00
ENSG00000158458.18_NRG2	ENSG00000160789.18_LMNA	-0.1154	-0.269650859	0.044616106	1.57E-01	4.09E-01	1.00E+00
ENSG00000273107.1_RP11-165A	ENSG00000160789.18_LMNA	-0.16589	-0.316728719	-0.00687374	4.11E-02	1.77E-01	1.00E+00
ENSG00000105997.21_HOXA3	ENSG00000160789.18_LMNA	-0.01757	-0.176273899	0.142031745	8.30E-01	9.34E-01	1.00E+00
ENSG00000101489.17_CELF4	ENSG00000160789.18_LMNA	-0.2798	-0.420280621	-0.12622297	4.81E-04	6.54E-03	1.00E+00
ENSG00000115290.8_GRB14	ENSG00000160789.18_LMNA	-0.25381	-0.396973528	-0.09859677	1.60E-03	1.65E-02	1.00E+00
ENSG00000214013.8_GANC	ENSG00000160789.18_LMNA	-0.06474	-0.221654997	0.095445635	4.28E-01	6.99E-01	1.00E+00
ENSG00000233971.1_RPS20P10	ENSG00000160789.18_LMNA	-0.03811	-0.196117103	0.121833659	6.41E-01	8.40E-01	1.00E+00
ENSG00000161860.7_SYCE2	ENSG00000160789.18_LMNA	-0.1607	-0.31191694	-0.00153454	4.80E-02	1.96E-01	1.00E+00
ENSG00000254332.1_GS1-44D20	ENSG00000160789.18_LMNA	0.30047	0.148368593	0.438687938	1.69E-04	2.86E-03	1.00E+00
ENSG00000134490.12_TMEM241	ENSG00000160789.18_LMNA	-0.15906	-0.310400404	0.000144568	5.03E-02	2.02E-01	1.00E+00
ENSG00000181234.9_TMEM132C	ENSG00000160789.18_LMNA	-0.25617	-0.399094322	-0.1010917	1.44E-03	1.53E-02	1.00E+00
ENSG00000188580.12_NKAIN2	ENSG00000160789.18_LMNA	-0.40808	-0.53267481	-0.2661718	1.80E-07	1.17E-05	9.25E-03
ENSG00000130201.6_EXOC3L2	ENSG00000160789.18_LMNA	0.33673	0.187587168	0.470699206	2.22E-05	5.62E-04	1.00E+00

ENSG00000138639.16_ARHGAP2	ENSG00000160789.18_LMNA	-0.02157	-0.180155601	0.138101366	7.92E-01	9.16E-01	1.00E+00
ENSG00000177679.15_SRRM3	ENSG00000160789.18_LMNA	-0.227	-0.372729234	-0.07033958	4.92E-03	3.87E-02	1.00E+00
ENSG00000178752.14_ERFE	ENSG00000160789.18_LMNA	0.03909	-0.120864703	0.197062533	6.33E-01	8.35E-01	1.00E+00
ENSG00000196209.11_SIRPB2	ENSG00000160789.18_LMNA	-0.00703	-0.166049708	0.152336337	9.31E-01	9.73E-01	1.00E+00
ENSG00000250299.4_MRPS31P4	ENSG00000160789.18_LMNA	-0.31444	-0.45105885	-0.16341731	7.97E-05	1.57E-03	1.00E+00
ENSG00000242445.1_RPL7AP11	ENSG00000160789.18_LMNA	-0.14911	-0.301163255	0.010334246	6.67E-02	2.42E-01	1.00E+00
ENSG00000269793.4_ZIM2-AS1	ENSG00000160789.18_LMNA	-0.24317	-0.38737873	-0.08735602	2.54E-03	2.35E-02	1.00E+00
ENSG00000225706.1_PTPRD-AS	ENSG00000160789.18_LMNA	-0.27643	-0.417265396	-0.12262311	5.66E-04	7.42E-03	1.00E+00
ENSG00000162994.14_CLHC1	ENSG00000160789.18_LMNA	-0.25617	-0.39909329	-0.10109048	1.44E-03	1.53E-02	1.00E+00
ENSG00000167992.11_VWCE	ENSG00000160789.18_LMNA	-0.30005	-0.438316873	-0.14791928	1.73E-04	2.91E-03	1.00E+00
ENSG00000260238.4_PMF1-BGL	ENSG00000160789.18_LMNA	-0.19041	-0.339327048	-0.03218768	1.88E-02	1.03E-01	1.00E+00
ENSG00000165490.11_DDIAS	ENSG00000160789.18_LMNA	-0.11261	-0.267019965	0.047445127	1.67E-01	4.25E-01	1.00E+00
ENSG00000224020.1_MIR181A2H	ENSG00000160789.18_LMNA	-0.29295	-0.432000384	-0.14028906	2.50E-04	3.89E-03	1.00E+00
ENSG00000154764.5_WNT7A	ENSG00000160789.18_LMNA	-0.13742	-0.290272517	0.022265502	9.14E-02	2.95E-01	1.00E+00
ENSG00000162639.14_HENMT1	ENSG00000160789.18_LMNA	-0.22987	-0.375332078	-0.07335017	4.39E-03	3.55E-02	1.00E+00
ENSG00000166578.8_IQCD	ENSG00000160789.18_LMNA	-0.07036	-0.227015044	0.089849987	3.89E-01	6.67E-01	1.00E+00
ENSG00000164180.12_TMEM161	ENSG00000160789.18_LMNA	-0.09204	-0.247616644	0.068155012	2.59E-01	5.42E-01	1.00E+00
ENSG00000140600.15_SH3GL3	ENSG00000160789.18_LMNA	-0.42338	-0.54579271	-0.28327223	5.50E-08	4.42E-06	2.82E-03
ENSG00000152969.15_JAKMIP1	ENSG00000160789.18_LMNA	-0.30812	-0.445465211	-0.15659625	1.13E-04	2.08E-03	1.00E+00
ENSG00000162836.10_ACP6	ENSG00000160789.18_LMNA	0.32457	0.174377438	0.459999674	4.51E-05	9.89E-04	1.00E+00
ENSG00000101306.9_MYLK2	ENSG00000160789.18_LMNA	-0.26485	-0.406892184	-0.11029753	9.76E-04	1.14E-02	1.00E+00
ENSG00000221994.9_ZNF630	ENSG00000160789.18_LMNA	-0.05788	-0.215094144	0.102267702	4.79E-01	7.37E-01	1.00E+00
ENSG00000236682.1_AC068282.3	ENSG00000160789.18_LMNA	-0.14951	-0.301528946	0.009932072	6.60E-02	2.40E-01	1.00E+00
ENSG00000279252.1_	ENSG00000160789.18_LMNA	-0.07196	-0.228539169	0.088255209	3.78E-01	6.58E-01	1.00E+00
ENSG00000232630.1_PRPS1P2	ENSG00000160789.18_LMNA	-0.04053	-0.198446181	0.119445532	6.20E-01	8.28E-01	1.00E+00
ENSG00000171903.15_CYP4F11	ENSG00000160789.18_LMNA	0.03344	-0.126430914	0.191623396	6.83E-01	8.62E-01	1.00E+00
ENSG00000215093.3_EEF1A1P29	ENSG00000160789.18_LMNA	-0.14801	-0.300136021	0.011463425	6.88E-02	2.47E-01	1.00E+00
ENSG00000174796.11_THAP6	ENSG00000160789.18_LMNA	-0.11963	-0.273614876	0.040344028	1.42E-01	3.85E-01	1.00E+00
ENSG00000247796.2_CTD-2366F	ENSG00000160789.18_LMNA	-0.23447	-0.379507422	-0.07819112	3.64E-03	3.09E-02	1.00E+00
ENSG00000160460.14_SPTBN4	ENSG00000160789.18_LMNA	0.00585	-0.153493437	0.164897345	9.43E-01	9.77E-01	1.00E+00
ENSG00000232024.2_LSM12P1	ENSG00000160789.18_LMNA	0.03181	-0.128042755	0.190044697	6.97E-01	8.70E-01	1.00E+00
ENSG00000153234.12_NR4A2	ENSG00000160789.18_LMNA	-0.05152	-0.209002662	0.108575095	5.28E-01	7.72E-01	1.00E+00
ENSG00000183250.10_LINC0154	ENSG00000160789.18_LMNA	0.10846	-0.051634689	0.263115182	1.84E-01	4.48E-01	1.00E+00

ENSG00000009694.12_TENM1	ENSG00000160789.18_LMNA	-0.00892	-0.167884894	0.150491803	9.13E-01	9.67E-01	1.00E+00
ENSG00000104835.13_SARS2	ENSG00000160789.18_LMNA	-0.03034	-0.188630708	0.129485003	7.11E-01	8.77E-01	1.00E+00
ENSG00000141519.13_CCDC40	ENSG00000160789.18_LMNA	0.12532	-0.034569251	0.278956333	1.24E-01	3.55E-01	1.00E+00
ENSG00000112214.9_FHL5	ENSG00000160789.18_LMNA	-0.16704	-0.317790037	-0.00805378	3.97E-02	1.73E-01	1.00E+00
ENSG00000140950.14_TLDC1	ENSG00000160789.18_LMNA	0.2711	0.116944493	0.412495789	7.30E-04	9.05E-03	1.00E+00
ENSG00000229939.1_RP11-111F	ENSG00000160789.18_LMNA	-0.0137	-0.172524419	0.145818739	8.67E-01	9.48E-01	1.00E+00
ENSG00000148690.11_FRA10AC	ENSG00000160789.18_LMNA	-0.15118	-0.303085724	0.008218851	6.30E-02	2.33E-01	1.00E+00
ENSG00000281468.1_CTC-459F4	ENSG00000160789.18_LMNA	-0.20714	-0.354647333	-0.04957606	1.05E-02	6.76E-02	1.00E+00
ENSG00000213023.8_SYT3	ENSG00000160789.18_LMNA	-0.07751	-0.233829091	0.082707432	3.43E-01	6.27E-01	1.00E+00
ENSG00000189227.5_C15orf61	ENSG00000160789.18_LMNA	-0.2014	-0.349396853	-0.04359591	1.28E-02	7.85E-02	1.00E+00
ENSG00000161558.9_TMEM143	ENSG00000160789.18_LMNA	0.05978	-0.100377817	0.216914539	4.64E-01	7.27E-01	1.00E+00
ENSG00000226124.5_FTCDNL1	ENSG00000160789.18_LMNA	-0.25916	-0.401784282	-0.1042616	1.26E-03	1.38E-02	1.00E+00
ENSG00000248668.2_OXCT1-AS:	ENSG00000160789.18_LMNA	-0.27622	-0.417077779	-0.12239937	5.72E-04	7.48E-03	1.00E+00
ENSG00000028116.15_VRK2	ENSG00000160789.18_LMNA	-0.0721	-0.228676255	0.088111688	3.77E-01	6.57E-01	1.00E+00
ENSG00000150510.14_FAM124A	ENSG00000160789.18_LMNA	-0.31414	-0.45079915	-0.16310002	8.10E-05	1.59E-03	1.00E+00
ENSG00000057657.13_PRDM1	ENSG00000160789.18_LMNA	0.24821	0.092669942	0.391922662	2.05E-03	1.99E-02	1.00E+00
ENSG00000173588.13_CEP83	ENSG00000160789.18_LMNA	-0.15142	-0.303308655	0.007973368	6.26E-02	2.32E-01	1.00E+00
ENSG00000237436.1_RP11-312B	ENSG00000160789.18_LMNA	-0.14604	-0.298305599	0.013473534	7.26E-02	2.56E-01	1.00E+00
ENSG00000176343.5_RPL37AP8	ENSG00000160789.18_LMNA	-0.13042	-0.283727378	0.029393399	1.09E-01	3.29E-01	1.00E+00
ENSG00000198944.5_SOWAHA	ENSG00000160789.18_LMNA	-0.21281	-0.359821033	-0.05549023	8.48E-03	5.82E-02	1.00E+00
ENSG00000137871.18_ZNF280D	ENSG00000160789.18_LMNA	-0.19331	-0.341983818	-0.03518982	1.70E-02	9.62E-02	1.00E+00
ENSG00000136231.12_IGF2BP3	ENSG00000160789.18_LMNA	-0.00059	-0.159778856	0.158622438	9.94E-01	9.98E-01	1.00E+00
ENSG00000174799.9_CEP135	ENSG00000160789.18_LMNA	0.01936	-0.140277894	0.178007253	8.13E-01	9.26E-01	1.00E+00
ENSG00000230082.1_PRRT3-AS:	ENSG00000160789.18_LMNA	0.09254	-0.06765991	0.248083486	2.57E-01	5.39E-01	1.00E+00
ENSG00000272644.1_RP11-33O4	ENSG00000160789.18_LMNA	0.17812	0.019475893	0.328023089	2.81E-02	1.37E-01	1.00E+00
ENSG00000099953.8_MMP11	ENSG00000160789.18_LMNA	0.427	0.287329094	0.548885997	4.11E-08	3.50E-06	2.11E-03
ENSG00000105383.13_CD33	ENSG00000160789.18_LMNA	-0.00816	-0.167144044	0.151236693	9.21E-01	9.70E-01	1.00E+00
ENSG00000169239.11_CA5B	ENSG00000160789.18_LMNA	-0.09522	-0.25062524	0.064961555	2.43E-01	5.23E-01	1.00E+00
ENSG00000204991.9_SPIRE2	ENSG00000160789.18_LMNA	0.06626	-0.093926665	0.22311191	4.17E-01	6.90E-01	1.00E+00
ENSG00000101440.8_ASIP	ENSG00000160789.18_LMNA	0.05027	-0.109812395	0.207804819	5.39E-01	7.79E-01	1.00E+00
ENSG00000145241.9_CENPC	ENSG00000160789.18_LMNA	-0.14714	-0.299324985	0.012354389	7.05E-02	2.51E-01	1.00E+00
ENSG00000069020.17_MAST4	ENSG00000160789.18_LMNA	0.24379	0.088005803	0.387935142	2.47E-03	2.30E-02	1.00E+00
ENSG00000112379.8_ARFGEF3	ENSG00000160789.18_LMNA	-0.28627	-0.42604965	-0.13313222	3.50E-04	5.06E-03	1.00E+00

ENSG00000102452.14_NALCN	ENSG00000160789.18_LMNA	-0.49704	-0.608118862	-0.36686689	7.35E-11	1.76E-08	3.77E-06
ENSG00000197444.8_OGDHL	ENSG00000160789.18_LMNA	0.0535	-0.10660748	0.210905572	5.13E-01	7.61E-01	1.00E+00
ENSG00000260426.1_RP5-912I13	ENSG00000160789.18_LMNA	-0.02125	-0.179838755	0.138422562	7.95E-01	9.17E-01	1.00E+00
ENSG00000197375.11_SLC22A5	ENSG00000160789.18_LMNA	0.03165	-0.128197024	0.189893513	6.99E-01	8.71E-01	1.00E+00
ENSG00000054803.3_CBLN4	ENSG00000160789.18_LMNA	0.19792	0.039980241	0.346213007	1.45E-02	8.59E-02	1.00E+00
ENSG00000131242.14_RAB11FIP	ENSG00000160789.18_LMNA	-0.30178	-0.439845833	-0.14977145	1.58E-04	2.72E-03	1.00E+00
ENSG00000147202.16_DIAPH2	ENSG00000160789.18_LMNA	0.02013	-0.139517201	0.178758443	8.06E-01	9.22E-01	1.00E+00
ENSG00000198019.11_FCGR1B	ENSG00000160789.18_LMNA	-0.11216	-0.266596846	0.047899639	1.69E-01	4.27E-01	1.00E+00
ENSG00000236534.1_H3F3BP1	ENSG00000160789.18_LMNA	-0.28349	-0.423575656	-0.13016576	4.01E-04	5.68E-03	1.00E+00
ENSG00000135476.10_ESPL1	ENSG00000160789.18_LMNA	0.08078	-0.079441075	0.23693489	3.22E-01	6.09E-01	1.00E+00
ENSG00000238113.5_LINC01410	ENSG00000160789.18_LMNA	-0.1222	-0.276034133	0.037731108	1.34E-01	3.71E-01	1.00E+00
ENSG00000260664.2_AC004158.3	ENSG00000160789.18_LMNA	-0.11657	-0.270748827	0.04343396	1.53E-01	4.02E-01	1.00E+00
ENSG00000225071.1_GS1-184P1	ENSG00000160789.18_LMNA	-0.18695	-0.336143694	-0.02859778	2.11E-02	1.12E-01	1.00E+00
ENSG00000166689.13_PLEKHA7	ENSG00000160789.18_LMNA	0.2091	0.051620317	0.35643776	9.73E-03	6.43E-02	1.00E+00
ENSG00000100106.18_TRIOBP	ENSG00000160789.18_LMNA	0.30186	0.149863635	0.439921891	1.57E-04	2.71E-03	1.00E+00
ENSG00000228794.7_LINC01128	ENSG00000160789.18_LMNA	0.08658	-0.073632925	0.242441614	2.89E-01	5.74E-01	1.00E+00
ENSG00000183808.10_RBM12B	ENSG00000160789.18_LMNA	0.06416	-0.096021349	0.221102434	4.32E-01	7.02E-01	1.00E+00
ENSG00000276148.1_RP11-278C	ENSG00000160789.18_LMNA	-0.17635	-0.326388735	-0.01764621	2.98E-02	1.42E-01	1.00E+00
ENSG00000214193.8_SH3D21	ENSG00000160789.18_LMNA	0.35173	0.203944598	0.48383389	8.86E-06	2.64E-04	4.54E-01
ENSG00000121797.9_CCRL2	ENSG00000160789.18_LMNA	-0.18439	-0.333788136	-0.02594649	2.30E-02	1.19E-01	1.00E+00
ENSG00000113327.13_GABRG2	ENSG00000160789.18_LMNA	-0.24313	-0.38733608	-0.08730623	2.54E-03	2.35E-02	1.00E+00
ENSG00000196458.9_ZNF605	ENSG00000160789.18_LMNA	-0.07519	-0.231619863	0.085026735	3.57E-01	6.40E-01	1.00E+00
ENSG00000113790.9_EHHADH	ENSG00000160789.18_LMNA	-0.08352	-0.239538226	0.076697913	3.06E-01	5.93E-01	1.00E+00
ENSG00000237753.1_AC079922.3	ENSG00000160789.18_LMNA	0.15936	0.000162512	0.310677871	4.99E-02	2.01E-01	1.00E+00
ENSG00000163995.17_ABLIM2	ENSG00000160789.18_LMNA	-0.25248	-0.395770203	-0.09718283	1.70E-03	1.73E-02	1.00E+00
ENSG00000177842.10_ZNF620	ENSG00000160789.18_LMNA	-0.25745	-0.400245025	-0.10244697	1.36E-03	1.46E-02	1.00E+00
ENSG00000278828.1_HIST1H3H	ENSG00000160789.18_LMNA	0.01368	-0.145838415	0.172504913	8.67E-01	9.48E-01	1.00E+00
ENSG00000254064.1_CTD-2530N	ENSG00000160789.18_LMNA	0.07516	-0.085055263	0.231592669	3.57E-01	6.40E-01	1.00E+00
ENSG00000121716.17_PILRB	ENSG00000160789.18_LMNA	0.06738	-0.092818424	0.224173981	4.10E-01	6.84E-01	1.00E+00
ENSG00000270157.1_RP5-894A1	ENSG00000160789.18_LMNA	-0.07287	-0.22940856	0.087344787	3.72E-01	6.52E-01	1.00E+00
ENSG00000197312.10_DDI2	ENSG00000160789.18_LMNA	0.11967	-0.040301272	0.273654495	1.42E-01	3.85E-01	1.00E+00
ENSG00000162711.15_NLRP3	ENSG00000160789.18_LMNA	-0.04304	-0.20086489	0.116961602	5.99E-01	8.15E-01	1.00E+00
ENSG00000089163.4_SIRT4	ENSG00000160789.18_LMNA	-0.33924	-0.472903354	-0.19032121	1.91E-05	4.97E-04	9.79E-01

ENSG00000203668.1_CHML	ENSG00000160789.18_LMNA	-0.027	-0.185399327	0.132775917	7.41E-01	8.91E-01	1.00E+00
ENSG00000269896.2_RP4-740C4	ENSG00000160789.18_LMNA	0.20713	0.049559231	0.354632586	1.05E-02	6.76E-02	1.00E+00
ENSG00000109906.12_ZBTB16	ENSG00000160789.18_LMNA	-0.18123	-0.330882356	-0.02268186	2.55E-02	1.28E-01	1.00E+00
ENSG00000139714.11_MORN3	ENSG00000160789.18_LMNA	0.10536	-0.054763737	0.260192085	1.96E-01	4.66E-01	1.00E+00
ENSG00000125650.4_PSPN	ENSG00000160789.18_LMNA	0.18418	0.025738384	0.333603076	2.31E-02	1.20E-01	1.00E+00
ENSG00000204161.12_C10orf128	ENSG00000160789.18_LMNA	-0.25784	-0.400599569	-0.10286477	1.34E-03	1.44E-02	1.00E+00
ENSG00000271147.6_ARMCX5-G	ENSG00000160789.18_LMNA	-0.17646	-0.326490121	-0.01775965	2.97E-02	1.42E-01	1.00E+00
ENSG00000228506.1_RP11-98I9.4	ENSG00000160789.18_LMNA	-0.24908	-0.392704356	-0.09358582	1.97E-03	1.93E-02	1.00E+00
ENSG00000234636.2_MED14OS	ENSG00000160789.18_LMNA	-0.07121	-0.227829956	0.088997501	3.83E-01	6.62E-01	1.00E+00
ENSG00000185607.5_ACTBP7	ENSG00000160789.18_LMNA	0.37275	0.227021201	0.502151967	2.25E-06	8.75E-05	1.16E-01
ENSG00000168447.9_SCNN1B	ENSG00000160789.18_LMNA	0.2768	0.123022672	0.41760039	5.56E-04	7.32E-03	1.00E+00
ENSG00000204653.8_ASPDH	ENSG00000160789.18_LMNA	-0.18948	-0.338472813	-0.03122357	1.94E-02	1.06E-01	1.00E+00
ENSG00000279716.1_AC006128.2	ENSG00000160789.18_LMNA	-0.01679	-0.175517776	0.142796184	8.37E-01	9.37E-01	1.00E+00
ENSG00000132199.17_ENOSF1	ENSG00000160789.18_LMNA	-0.03276	-0.190966915	0.127101381	6.89E-01	8.66E-01	1.00E+00
ENSG00000154710.14_RABGEF1	ENSG00000160789.18_LMNA	0.07503	-0.085191501	0.231462793	3.58E-01	6.41E-01	1.00E+00
ENSG00000250312.5_ZNF718	ENSG00000160789.18_LMNA	0.02705	-0.132719938	0.185454352	7.41E-01	8.91E-01	1.00E+00
ENSG00000150455.12_TIRAP	ENSG00000160789.18_LMNA	0.20428	0.046593482	0.352031079	1.16E-02	7.28E-02	1.00E+00
ENSG00000171873.7_ADRA1D	ENSG00000160789.18_LMNA	0.54997	0.428266829	0.652077929	2.16E-13	1.56E-10	1.11E-08
ENSG00000134864.9_GGACT	ENSG00000160789.18_LMNA	0.1229	-0.037030191	0.276682424	1.31E-01	3.68E-01	1.00E+00
ENSG00000230216.1_HSPB1P2	ENSG00000160789.18_LMNA	0.43201	0.292962771	0.553169727	2.74E-08	2.51E-06	1.40E-03
ENSG00000218980.2_FTH1P15	ENSG00000160789.18_LMNA	0.04481	-0.115213943	0.202564341	5.84E-01	8.06E-01	1.00E+00
ENSG00000244968.5_LIFR-AS1	ENSG00000160789.18_LMNA	-0.42244	-0.544984512	-0.28221382	5.92E-08	4.70E-06	3.04E-03
ENSG00000124496.11_TRERF1	ENSG00000160789.18_LMNA	-0.13411	-0.287178082	0.02563942	9.95E-02	3.11E-01	1.00E+00
ENSG00000225828.1_FAM229A	ENSG00000160789.18_LMNA	0.1025	-0.057645957	0.257494452	2.09E-01	4.82E-01	1.00E+00
ENSG00000216819.1_TUBB2BP1	ENSG00000160789.18_LMNA	0.06121	-0.098958089	0.218280615	4.54E-01	7.19E-01	1.00E+00
ENSG00000182742.5_HOXB4	ENSG00000160789.18_LMNA	0.17512	0.016372789	0.32525017	3.09E-02	1.46E-01	1.00E+00
ENSG00000116544.10_DLGAP3	ENSG00000160789.18_LMNA	-0.2446	-0.388663675	-0.08885698	2.39E-03	2.24E-02	1.00E+00
ENSG00000261557.1_EEF1A1P38	ENSG00000160789.18_LMNA	-0.17959	-0.329372314	-0.02098793	2.68E-02	1.33E-01	1.00E+00
ENSG00000078900.13_TP73	ENSG00000160789.18_LMNA	0.02016	-0.139486802	0.178788453	8.05E-01	9.22E-01	1.00E+00
ENSG00000176909.10_MAMSTR	ENSG00000160789.18_LMNA	0.11924	-0.040730826	0.273256409	1.43E-01	3.87E-01	1.00E+00
ENSG00000233247.3_GS1-257G1	ENSG00000160789.18_LMNA	-0.24408	-0.388196453	-0.08831105	2.44E-03	2.28E-02	1.00E+00
ENSG00000173889.14_PHC3	ENSG00000160789.18_LMNA	0.13273	-0.027045751	0.285886315	1.03E-01	3.18E-01	1.00E+00
ENSG00000144026.10_ZNF514	ENSG00000160789.18_LMNA	0.06721	-0.09298633	0.224013118	4.11E-01	6.85E-01	1.00E+00

ENSG00000105750.13_ZNF85	ENSG00000160789.18_LMNA	-0.27375	-0.414873792	-0.11977329	6.43E-04	8.23E-03	1.00E+00
ENSG00000158296.12_SLC13A3	ENSG00000160789.18_LMNA	0.04025	-0.119717189	0.198181422	6.22E-01	8.29E-01	1.00E+00
ENSG00000109819.7_PPARGC1A	ENSG00000160789.18_LMNA	0.02141	-0.138262618	0.179996541	7.93E-01	9.16E-01	1.00E+00
ENSG00000164743.4_C8orf48	ENSG00000160789.18_LMNA	0.13975	-0.019891025	0.292446389	8.59E-02	2.85E-01	1.00E+00
ENSG00000149571.9_KIRREL3	ENSG00000160789.18_LMNA	-0.26361	-0.405778106	-0.10897917	1.03E-03	1.19E-02	1.00E+00
ENSG00000261678.2_SCRT1	ENSG00000160789.18_LMNA	-0.31505	-0.451601698	-0.16408074	7.71E-05	1.53E-03	1.00E+00
ENSG00000008516.15_MMP25	ENSG00000160789.18_LMNA	-0.02499	-0.183461628	0.134745958	7.60E-01	9.00E-01	1.00E+00
ENSG00000156140.7_ADAMTS3	ENSG00000160789.18_LMNA	0.32031	0.169765088	0.456244152	5.75E-05	1.20E-03	1.00E+00
ENSG00000229122.1_AGBL5-IT1	ENSG00000160789.18_LMNA	-0.04555	-0.203277324	0.114480149	5.77E-01	8.03E-01	1.00E+00
ENSG00000160172.9_FAM86C2P	ENSG00000160789.18_LMNA	0.17562	0.016896162	0.325718226	3.04E-02	1.45E-01	1.00E+00
ENSG00000008118.8_CAMK1G	ENSG00000160789.18_LMNA	-0.14555	-0.297844679	0.013979304	7.36E-02	2.58E-01	1.00E+00
ENSG00000197372.8_ZNF675	ENSG00000160789.18_LMNA	-0.40265	-0.52800352	-0.26012256	2.71E-07	1.63E-05	1.39E-02
ENSG00000128692.8_EIF2S2P4	ENSG00000160789.18_LMNA	0.00944	-0.149985084	0.168388659	9.08E-01	9.65E-01	1.00E+00
ENSG00000118620.11_ZNF430	ENSG00000160789.18_LMNA	-0.22085	-0.367140854	-0.06389432	6.25E-03	4.64E-02	1.00E+00
ENSG00000257702.3_LBX2-AS1	ENSG00000160789.18_LMNA	0.42682	0.287133183	0.548736783	4.17E-08	3.52E-06	2.14E-03
ENSG00000214413.6_BBIP1	ENSG00000160789.18_LMNA	-0.43094	-0.552256941	-0.29176082	2.99E-08	2.68E-06	1.53E-03
ENSG00000273373.1_RP5-1074L	ENSG00000160789.18_LMNA	-0.09572	-0.251093368	0.064464081	2.41E-01	5.20E-01	1.00E+00
ENSG00000113448.15_PDE4D	ENSG00000160789.18_LMNA	-0.11221	-0.266650888	0.047841595	1.69E-01	4.27E-01	1.00E+00
ENSG00000116830.10_TTF2	ENSG00000160789.18_LMNA	-0.09628	-0.251625617	0.063898276	2.38E-01	5.17E-01	1.00E+00
ENSG00000123700.4_KCNJ2	ENSG00000160789.18_LMNA	-0.16083	-0.312040693	-0.00167163	4.78E-02	1.95E-01	1.00E+00
ENSG00000187607.14_ZNF286A	ENSG00000160789.18_LMNA	-0.21826	-0.364782399	-0.06118181	6.91E-03	4.99E-02	1.00E+00
ENSG00000246451.2_RP11-894P	ENSG00000160789.18_LMNA	0.03346	-0.126412373	0.191641546	6.82E-01	8.62E-01	1.00E+00
ENSG00000225855.5_RUSC1-AS	ENSG00000160789.18_LMNA	0.10086	-0.059297529	0.255946435	2.16E-01	4.91E-01	1.00E+00
ENSG00000224126.2_UBE2SP2	ENSG00000160789.18_LMNA	0.06988	-0.09032332	0.226562383	3.92E-01	6.70E-01	1.00E+00
ENSG00000092054.12_MYH7	ENSG00000160789.18_LMNA	-0.04038	-0.198306592	0.119588764	6.21E-01	8.29E-01	1.00E+00
ENSG00000164318.16_EGFLAM	ENSG00000160789.18_LMNA	0.18805	0.029736452	0.337154177	2.03E-02	1.09E-01	1.00E+00
ENSG00000172244.7_C5orf34	ENSG00000160789.18_LMNA	-0.06052	-0.217624009	0.099640644	4.59E-01	7.23E-01	1.00E+00
ENSG00000240053.8_LY6G5B	ENSG00000160789.18_LMNA	-0.08974	-0.245437517	0.070464002	2.72E-01	5.56E-01	1.00E+00
ENSG00000228653.2_HNRNPCP7	ENSG00000160789.18_LMNA	0.00648	-0.152877776	0.165510594	9.37E-01	9.75E-01	1.00E+00
ENSG00000145723.15_GIN1	ENSG00000160789.18_LMNA	-0.36149	-0.49235789	-0.21464434	4.75E-06	1.60E-04	2.44E-01
ENSG00000223668.1_EEF1A1P24	ENSG00000160789.18_LMNA	-0.11362	-0.267971998	0.04642198	1.63E-01	4.19E-01	1.00E+00
ENSG00000214016.3_RPSAP61	ENSG00000160789.18_LMNA	-0.14272	-0.295210037	0.016867253	7.94E-02	2.71E-01	1.00E+00
ENSG00000140961.11_OSGIN1	ENSG00000160789.18_LMNA	0.27691	0.123137949	0.417697023	5.53E-04	7.30E-03	1.00E+00

ENSG00000197603.12_C5orf42	ENSG00000160789.18_LMNA	0.0504	-0.109678437	0.207934551	5.37E-01	7.78E-01	1.00E+00
ENSG00000108381.9_ASPA	ENSG00000160789.18_LMNA	-0.35288	-0.48484486	-0.20521013	8.23E-06	2.49E-04	4.22E-01
ENSG00000247679.2_RP11-1277.	ENSG00000160789.18_LMNA	0.08353	-0.076687355	0.239548237	3.06E-01	5.93E-01	1.00E+00
ENSG00000173376.12_NDNF	ENSG00000160789.18_LMNA	-0.1639	-0.314884321	-0.00482509	4.36E-02	1.84E-01	1.00E+00
ENSG00000169851.14_PCDH7	ENSG00000160789.18_LMNA	-0.29883	-0.437232753	-0.14660721	1.84E-04	3.06E-03	1.00E+00
ENSG00000248124.6_RRN3P1	ENSG00000160789.18_LMNA	-0.18449	-0.333884132	-0.02605446	2.29E-02	1.19E-01	1.00E+00
ENSG00000159263.14_SIM2	ENSG00000160789.18_LMNA	-0.25798	-0.400719743	-0.1030064	1.33E-03	1.44E-02	1.00E+00
ENSG00000196705.7_ZNF431	ENSG00000160789.18_LMNA	-0.36853	-0.498487692	-0.2223802	2.99E-06	1.09E-04	1.53E-01
ENSG00000272345.1_RP1-30M3.	ENSG00000160789.18_LMNA	-0.00526	-0.164326037	0.154066771	9.49E-01	9.79E-01	1.00E+00
ENSG00000174373.14_RALGAPA	ENSG00000160789.18_LMNA	-0.10291	-0.257887873	0.057225943	2.07E-01	4.79E-01	1.00E+00
ENSG00000253506.2_NACA2	ENSG00000160789.18_LMNA	-0.06908	-0.225798597	0.091121661	3.98E-01	6.75E-01	1.00E+00
ENSG00000253549.4_CA3-AS1	ENSG00000160789.18_LMNA	0.05227	-0.107824024	0.209729318	5.22E-01	7.68E-01	1.00E+00
ENSG00000114423.17_CBLB	ENSG00000160789.18_LMNA	-0.09766	-0.252932081	0.062508584	2.31E-01	5.09E-01	1.00E+00
ENSG00000261609.3_GAN	ENSG00000160789.18_LMNA	-0.01613	-0.174877256	0.143443452	8.44E-01	9.39E-01	1.00E+00
ENSG00000262692.1_CTD-3195I	ENSG00000160789.18_LMNA	-0.16784	-0.318531574	-0.00887877	3.87E-02	1.70E-01	1.00E+00
ENSG00000235109.6_ZSCAN31	ENSG00000160789.18_LMNA	-0.37798	-0.506692642	-0.2327896	1.58E-06	6.67E-05	8.10E-02
ENSG00000236753.4_MKLN1-AS	ENSG00000160789.18_LMNA	0.0099	-0.14953834	0.16883266	9.04E-01	9.63E-01	1.00E+00
ENSG00000225125.2_RANP4	ENSG00000160789.18_LMNA	-0.24435	-0.388440425	-0.0885961	2.41E-03	2.26E-02	1.00E+00
ENSG00000128891.14_C15orf57	ENSG00000160789.18_LMNA	-0.25151	-0.394903099	-0.09616471	1.77E-03	1.79E-02	1.00E+00
ENSG00000142530.9_FAM71E1	ENSG00000160789.18_LMNA	-0.11837	-0.272432946	0.041619013	1.46E-01	3.92E-01	1.00E+00
ENSG00000108515.16_ENO3	ENSG00000160789.18_LMNA	0.09479	-0.065394186	0.250218009	2.45E-01	5.25E-01	1.00E+00
ENSG00000131370.14_SH3BP5	ENSG00000160789.18_LMNA	0.21261	0.055285385	0.359642148	8.54E-03	5.85E-02	1.00E+00
ENSG00000112303.12_VNN2	ENSG00000160789.18_LMNA	-0.07383	-0.23032378	0.086385801	3.66E-01	6.47E-01	1.00E+00
ENSG00000202058.1_RN7SKP80	ENSG00000160789.18_LMNA	-0.04905	-0.206632678	0.111022193	5.48E-01	7.85E-01	1.00E+00
ENSG00000165548.9_TMEM63C	ENSG00000160789.18_LMNA	-0.29116	-0.43041027	-0.13837367	2.73E-04	4.18E-03	1.00E+00
ENSG00000121895.7_TMEM156	ENSG00000160789.18_LMNA	-0.30876	-0.446035395	-0.1572903	1.09E-04	2.02E-03	1.00E+00
ENSG00000173681.15_CXorf23	ENSG00000160789.18_LMNA	-0.00656	-0.16558421	0.152803855	9.36E-01	9.75E-01	1.00E+00
ENSG00000213780.9_GTF2H4	ENSG00000160789.18_LMNA	-0.0903	-0.245965182	0.069905202	2.69E-01	5.53E-01	1.00E+00
ENSG00000258738.1_RP11-73E1	ENSG00000160789.18_LMNA	0.20952	0.052050457	0.356814205	9.58E-03	6.36E-02	1.00E+00
ENSG00000165891.14_E2F7	ENSG00000160789.18_LMNA	0.31685	0.166025976	0.453192141	6.98E-05	1.41E-03	1.00E+00
ENSG00000075391.15_RASAL2	ENSG00000160789.18_LMNA	0.06548	-0.094711966	0.222358869	4.23E-01	6.94E-01	1.00E+00
ENSG00000147443.11_DOK2	ENSG00000160789.18_LMNA	0.17955	0.020947368	0.329336137	2.69E-02	1.33E-01	1.00E+00
ENSG00000197776.6_KLHDC1	ENSG00000160789.18_LMNA	-0.15196	-0.303810688	0.007420411	6.16E-02	2.30E-01	1.00E+00

ENSG00000273137.1_RP3-402G1	ENSG00000160789.18_LMNA	0.03749	-0.122438162	0.195526977	6.47E-01	8.43E-01	1.00E+00
ENSG00000278962.1_RP11-399B	ENSG00000160789.18_LMNA	0.50717	0.378533207	0.616586748	2.60E-11	7.32E-09	1.33E-06
ENSG00000198089.13_SFI1	ENSG00000160789.18_LMNA	0.09457	-0.065616094	0.250009087	2.46E-01	5.27E-01	1.00E+00
ENSG00000178425.12_NT5DC1	ENSG00000160789.18_LMNA	-0.19578	-0.344251915	-0.03775715	1.56E-02	9.03E-02	1.00E+00
ENSG00000076351.11_SLC46A1	ENSG00000160789.18_LMNA	-0.07235	-0.228913809	0.087862952	3.76E-01	6.56E-01	1.00E+00
ENSG00000268049.1_CTD-2619J	ENSG00000160789.18_LMNA	-0.08026	-0.236435806	0.079966418	3.26E-01	6.12E-01	1.00E+00
ENSG00000065328.15_MCM10	ENSG00000160789.18_LMNA	-0.06138	-0.218448581	0.098783437	4.53E-01	7.18E-01	1.00E+00
ENSG00000263781.3_RP11-321A	ENSG00000160789.18_LMNA	-0.03254	-0.19074831	0.127324579	6.91E-01	8.67E-01	1.00E+00
ENSG00000258429.1_PDF	ENSG00000160789.18_LMNA	0.27637	0.122565987	0.417217499	5.68E-04	7.44E-03	1.00E+00
ENSG00000178229.7_ZNF543	ENSG00000160789.18_LMNA	0.08161	-0.078607234	0.237726709	3.18E-01	6.04E-01	1.00E+00
ENSG00000267493.3_CIRBP-AS1	ENSG00000160789.18_LMNA	0.05585	-0.104276382	0.2131569	4.94E-01	7.49E-01	1.00E+00
ENSG00000268403.2_CTD-2371C	ENSG00000160789.18_LMNA	-0.13262	-0.28578546	0.027155499	1.03E-01	3.18E-01	1.00E+00
ENSG00000099954.17_CECR2	ENSG00000160789.18_LMNA	-0.19421	-0.342808529	-0.03612287	1.65E-02	9.41E-02	1.00E+00
ENSG00000116117.16_PARD3B	ENSG00000160789.18_LMNA	0.02121	-0.138456265	0.179805504	7.95E-01	9.18E-01	1.00E+00
ENSG00000146678.8_IGFBP1	ENSG00000160789.18_LMNA	-0.11586	-0.270082249	0.044151748	1.55E-01	4.06E-01	1.00E+00
ENSG00000169047.5_IRS1	ENSG00000160789.18_LMNA	0.21183	0.054464847	0.35892537	8.80E-03	5.97E-02	1.00E+00
ENSG00000008128.21_CDK11A	ENSG00000160789.18_LMNA	0.10324	-0.056896224	0.258196643	2.06E-01	4.77E-01	1.00E+00
ENSG00000231074.7_HCG18	ENSG00000160789.18_LMNA	-0.24531	-0.389303586	-0.08960497	2.32E-03	2.19E-02	1.00E+00
ENSG00000110848.7_CD69	ENSG00000160789.18_LMNA	-0.14022	-0.292879213	0.019417839	8.49E-02	2.82E-01	1.00E+00
ENSG00000115523.15_GNLY	ENSG00000160789.18_LMNA	-0.06416	-0.221107062	0.096016528	4.32E-01	7.02E-01	1.00E+00
ENSG00000152763.15_WDR78	ENSG00000160789.18_LMNA	0.1117	-0.048363629	0.266164779	1.71E-01	4.30E-01	1.00E+00
ENSG00000105963.12_ADAP1	ENSG00000160789.18_LMNA	-0.23784	-0.38255705	-0.0817359	3.17E-03	2.78E-02	1.00E+00
ENSG00000241889.1_RP11-435F	ENSG00000160789.18_LMNA	0.08428	-0.075934729	0.240261701	3.02E-01	5.89E-01	1.00E+00
ENSG00000265666.1_RARA-AS1	ENSG00000160789.18_LMNA	0.04084	-0.119132621	0.198751089	6.17E-01	8.27E-01	1.00E+00
ENSG00000120457.10_KCNJ5	ENSG00000160789.18_LMNA	-0.07159	-0.228190185	0.088620514	3.81E-01	6.60E-01	1.00E+00
ENSG00000152348.14_ATG10	ENSG00000160789.18_LMNA	-0.04057	-0.198493488	0.119396987	6.20E-01	8.28E-01	1.00E+00
ENSG00000250565.5_ATP6V1E2	ENSG00000160789.18_LMNA	-0.29253	-0.431630754	-0.13984362	2.55E-04	3.96E-03	1.00E+00
ENSG00000279253.1_RP4-614O4	ENSG00000160789.18_LMNA	0.14986	-0.009573805	0.301854635	6.54E-02	2.39E-01	1.00E+00
ENSG00000227694.1_RPL23AP7	ENSG00000160789.18_LMNA	-0.00171	-0.160868391	0.157532104	9.83E-01	9.94E-01	1.00E+00
ENSG00000162614.17_NEXN	ENSG00000160789.18_LMNA	0.19702	0.039043418	0.345386917	1.50E-02	8.78E-02	1.00E+00
ENSG00000105376.4_ICAM5	ENSG00000160789.18_LMNA	-0.0016	-0.160756149	0.157644464	9.84E-01	9.94E-01	1.00E+00
ENSG00000271228.1_CTC-336P1	ENSG00000160789.18_LMNA	-0.46027	-0.577176976	-0.32487345	2.43E-09	3.28E-07	1.25E-04
ENSG00000073711.9_PPP2R3A	ENSG00000160789.18_LMNA	-0.25134	-0.394743813	-0.09597775	1.79E-03	1.80E-02	1.00E+00

ENSG00000081760.15_AACS	ENSG00000160789.18_LMNA	0.02807	-0.131721509	0.186435411	7.31E-01	8.87E-01	1.00E+00
ENSG00000276698.1_RP11-468E	ENSG00000160789.18_LMNA	0.0149	-0.144644929	0.173687588	8.55E-01	9.43E-01	1.00E+00
ENSG00000171488.13_LRRC8C	ENSG00000160789.18_LMNA	-0.07166	-0.228254723	0.088552964	3.80E-01	6.59E-01	1.00E+00
ENSG00000146555.17_SDK1	ENSG00000160789.18_LMNA	0.37631	0.230944155	0.505241623	1.77E-06	7.29E-05	9.08E-02
ENSG00000258608.1_DNAJC19P	ENSG00000160789.18_LMNA	-0.2517	-0.395068363	-0.09635871	1.76E-03	1.78E-02	1.00E+00
ENSG00000225137.1_DYNC1I2P1	ENSG00000160789.18_LMNA	-0.12868	-0.282101076	0.031159575	1.14E-01	3.37E-01	1.00E+00
ENSG00000206190.10_ATP10A	ENSG00000160789.18_LMNA	-0.01204	-0.170908274	0.147448181	8.83E-01	9.55E-01	1.00E+00
ENSG00000186951.15_PPARA	ENSG00000160789.18_LMNA	-0.02384	-0.182346318	0.135878743	7.71E-01	9.05E-01	1.00E+00
ENSG00000266967.5_AARSD1	ENSG00000160789.18_LMNA	-0.03307	-0.191267391	0.126794539	6.86E-01	8.64E-01	1.00E+00
ENSG00000020633.17_RUNX3	ENSG00000160789.18_LMNA	0.19096	0.032750001	0.339825054	1.84E-02	1.02E-01	1.00E+00
ENSG00000139132.13_FGD4	ENSG00000160789.18_LMNA	-0.19212	-0.340897808	-0.03396196	1.77E-02	9.90E-02	1.00E+00
ENSG00000168769.11_TET2	ENSG00000160789.18_LMNA	-0.11953	-0.273521062	0.040445265	1.42E-01	3.85E-01	1.00E+00
ENSG00000271918.1_CTD-2287C	ENSG00000160789.18_LMNA	-0.09518	-0.25058686	0.065002333	2.43E-01	5.23E-01	1.00E+00
ENSG00000260160.1_CTC-471J1	ENSG00000160789.18_LMNA	0.08928	-0.07092768	0.244999532	2.74E-01	5.59E-01	1.00E+00
ENSG00000176826.14_FKBP9P1	ENSG00000160789.18_LMNA	0.3181	0.167378297	0.454296736	6.51E-05	1.32E-03	1.00E+00
ENSG00000162929.12_KIAA1841	ENSG00000160789.18_LMNA	-0.08748	-0.243292637	0.072733403	2.84E-01	5.69E-01	1.00E+00
ENSG00000175857.7_GAPT	ENSG00000160789.18_LMNA	0.07098	-0.089227666	0.22760998	3.85E-01	6.63E-01	1.00E+00
ENSG00000269713.6_NBPF9	ENSG00000160789.18_LMNA	0.10316	-0.056971771	0.258125901	2.06E-01	4.78E-01	1.00E+00
ENSG00000182873.5_RP11-181G	ENSG00000160789.18_LMNA	-0.07798	-0.234276664	0.082237139	3.40E-01	6.25E-01	1.00E+00
ENSG00000182359.13_KBTBD3	ENSG00000160789.18_LMNA	-0.37915	-0.507703024	-0.2340758	1.46E-06	6.28E-05	7.48E-02
ENSG00000180739.13_S1PR5	ENSG00000160789.18_LMNA	-0.17468	-0.324850212	-0.0159257	3.14E-02	1.48E-01	1.00E+00
ENSG00000198039.10_ZNF273	ENSG00000160789.18_LMNA	-0.23681	-0.381622194	-0.08064844	3.31E-03	2.88E-02	1.00E+00
ENSG00000232611.1_RP11-1114	ENSG00000160789.18_LMNA	-0.18135	-0.330997614	-0.02281123	2.54E-02	1.28E-01	1.00E+00
ENSG00000162849.14_KIF26B	ENSG00000160789.18_LMNA	0.08876	-0.071447242	0.244508606	2.77E-01	5.62E-01	1.00E+00
ENSG00000187486.5_KCNJ11	ENSG00000160789.18_LMNA	-0.3327	-0.467157449	-0.18320312	2.82E-05	6.79E-04	1.00E+00
ENSG00000188460.4_ACTBP11	ENSG00000160789.18_LMNA	0.30502	0.15325988	0.442720963	1.33E-04	2.37E-03	1.00E+00
ENSG00000172803.16_SNX32	ENSG00000160789.18_LMNA	-0.15267	-0.304467712	0.006696452	6.04E-02	2.27E-01	1.00E+00
ENSG00000144559.9_TAMM41	ENSG00000160789.18_LMNA	-0.11776	-0.271865391	0.04223089	1.48E-01	3.95E-01	1.00E+00
ENSG00000081014.9_AP4E1	ENSG00000160789.18_LMNA	0.22396	0.0671543	0.369970166	5.54E-03	4.23E-02	1.00E+00
ENSG00000231252.1_RP11-436K	ENSG00000160789.18_LMNA	-0.47162	-0.586762912	-0.33777771	8.63E-10	1.39E-07	4.43E-05
ENSG00000116824.4_CD2	ENSG00000160789.18_LMNA	0.04294	-0.117061641	0.200767552	5.99E-01	8.16E-01	1.00E+00
ENSG00000163082.9_SGPP2	ENSG00000160789.18_LMNA	-0.25251	-0.395803633	-0.0972221	1.70E-03	1.73E-02	1.00E+00
ENSG00000111087.8_GLI1	ENSG00000160789.18_LMNA	0.12103	-0.03892179	0.274932194	1.37E-01	3.78E-01	1.00E+00

ENSG00000185621.10_LMLN	ENSG00000160789.18_LMNA	0.37117	0.225276453	0.50077557	2.51E-06	9.47E-05	1.29E-01
ENSG00000121621.6_KIF18A	ENSG00000160789.18_LMNA	0.12197	-0.03796318	0.275819422	1.34E-01	3.72E-01	1.00E+00
ENSG00000235225.1_AC016712.:	ENSG00000160789.18_LMNA	-0.02862	-0.186965954	0.131181303	7.26E-01	8.84E-01	1.00E+00
ENSG00000112319.16_EYA4	ENSG00000160789.18_LMNA	0.10662	-0.053486176	0.261386256	1.91E-01	4.59E-01	1.00E+00
ENSG00000163624.5_CDS1	ENSG00000160789.18_LMNA	-0.0814	-0.237519707	0.078825264	3.19E-01	6.06E-01	1.00E+00
ENSG00000232415.1_CTB-51J22	ENSG00000160789.18_LMNA	0.27336	0.119349657	0.414517925	6.56E-04	8.33E-03	1.00E+00
ENSG00000136110.11_LECT1	ENSG00000160789.18_LMNA	-0.05006	-0.207607968	0.110015636	5.40E-01	7.80E-01	1.00E+00
ENSG00000213235.3_EEF1A1P1(	ENSG00000160789.18_LMNA	-0.14136	-0.293949121	0.018247559	8.24E-02	2.77E-01	1.00E+00
ENSG00000150773.9_PIH1D2	ENSG00000160789.18_LMNA	-0.06329	-0.220274385	0.096883694	4.39E-01	7.07E-01	1.00E+00
ENSG00000179331.2_RAB39A	ENSG00000160789.18_LMNA	0.07459	-0.085627352	0.231047219	3.61E-01	6.43E-01	1.00E+00
ENSG00000244041.6_LINC01011	ENSG00000160789.18_LMNA	-0.12981	-0.283159184	0.030010684	1.11E-01	3.32E-01	1.00E+00
ENSG00000231409.3_RP11-83J1(	ENSG00000160789.18_LMNA	-0.09585	-0.251216302	0.064333414	2.40E-01	5.19E-01	1.00E+00
ENSG00000179455.7_MKRN3	ENSG00000160789.18_LMNA	-0.34321	-0.476380408	-0.1946431	1.50E-05	4.08E-04	7.70E-01
ENSG00000175600.14_SUGCT	ENSG00000160789.18_LMNA	0.13907	-0.020585662	0.291810772	8.75E-02	2.88E-01	1.00E+00
ENSG00000143127.11_ITGA10	ENSG00000160789.18_LMNA	0.13658	-0.023126427	0.289483528	9.34E-02	2.99E-01	1.00E+00
ENSG00000187118.11_CMC1	ENSG00000160789.18_LMNA	-0.21466	-0.361506601	-0.05742166	7.91E-03	5.52E-02	1.00E+00
ENSG00000206432.4_TMEM200C	ENSG00000160789.18_LMNA	-0.18917	-0.338187061	-0.03090119	1.96E-02	1.06E-01	1.00E+00
ENSG00000140876.10_NUDT7	ENSG00000160789.18_LMNA	-0.19888	-0.347095502	-0.04098163	1.40E-02	8.37E-02	1.00E+00
ENSG00000237440.7_ZNF737	ENSG00000160789.18_LMNA	-0.31066	-0.447718299	-0.15934043	9.81E-05	1.85E-03	1.00E+00
ENSG00000134256.11_CD101	ENSG00000160789.18_LMNA	0.06492	-0.095269426	0.22182408	4.27E-01	6.98E-01	1.00E+00
ENSG00000133816.12_MICAL2	ENSG00000160789.18_LMNA	0.24659	0.09096035	0.390462382	2.19E-03	2.10E-02	1.00E+00
ENSG00000164604.11_GPR85	ENSG00000160789.18_LMNA	-0.51751	-0.625201992	-0.39048068	8.67E-12	3.18E-09	4.45E-07
ENSG00000123636.16_BAZ2B	ENSG00000160789.18_LMNA	-0.2314	-0.376725491	-0.07496413	4.13E-03	3.40E-02	1.00E+00
ENSG00000160963.12_COL26A1	ENSG00000160789.18_LMNA	-0.22575	-0.371598432	-0.06903335	5.17E-03	4.03E-02	1.00E+00
ENSG00000167604.12_NFKBID	ENSG00000160789.18_LMNA	0.05604	-0.104086131	0.213340492	4.93E-01	7.48E-01	1.00E+00
ENSG00000214881.4_TMEM14DF	ENSG00000160789.18_LMNA	-0.12885	-0.282260268	0.030986778	1.14E-01	3.37E-01	1.00E+00
ENSG00000099251.13_HSD17B7I	ENSG00000160789.18_LMNA	-0.28894	-0.428432698	-0.13599461	3.06E-04	4.58E-03	1.00E+00
ENSG00000187186.13_RP11-195I	ENSG00000160789.18_LMNA	0.12287	-0.037058326	0.276656407	1.32E-01	3.68E-01	1.00E+00
ENSG00000281912.1_LINC01144	ENSG00000160789.18_LMNA	0.03059	-0.129244373	0.188866714	7.08E-01	8.76E-01	1.00E+00
ENSG00000235954.5_TTC28-AS1	ENSG00000160789.18_LMNA	-0.28791	-0.427514149	-0.13489072	3.22E-04	4.75E-03	1.00E+00
ENSG00000253626.3_EIF5AL1	ENSG00000160789.18_LMNA	0.15979	0.000605966	0.311078467	4.92E-02	1.99E-01	1.00E+00
ENSG00000189283.8_FHIT	ENSG00000160789.18_LMNA	-0.42101	-0.543768998	-0.28062319	6.63E-08	5.20E-06	3.40E-03
ENSG00000107518.15_ATRNL1	ENSG00000160789.18_LMNA	-0.44256	-0.562157065	-0.30484133	1.14E-08	1.21E-06	5.84E-04

ENSG00000269973.1_RP11-95D1	ENSG00000160789.18_LMNA	-0.17031	-0.320812177	-0.0114187	3.59E-02	1.61E-01	1.00E+00
ENSG00000181135.14_ZNF707	ENSG00000160789.18_LMNA	0.13729	-0.022398794	0.290150391	9.17E-02	2.95E-01	1.00E+00
ENSG00000278041.1_RP5-984P4	ENSG00000160789.18_LMNA	-0.23916	-0.383749831	-0.08312442	3.00E-03	2.68E-02	1.00E+00
ENSG00000152402.9_GUCY1A2	ENSG00000160789.18_LMNA	-0.31195	-0.448861454	-0.16073446	9.14E-05	1.76E-03	1.00E+00
ENSG00000066279.15_ASPM	ENSG00000160789.18_LMNA	0.13103	-0.028772575	0.284298607	1.08E-01	3.26E-01	1.00E+00
ENSG00000130822.14_PNCK	ENSG00000160789.18_LMNA	-0.13301	-0.28615556	0.02675273	1.02E-01	3.16E-01	1.00E+00
ENSG00000164061.4_BSN	ENSG00000160789.18_LMNA	-0.22899	-0.37453945	-0.07243279	4.54E-03	3.65E-02	1.00E+00
ENSG00000224908.1_TIMM8BP2	ENSG00000160789.18_LMNA	-0.22421	-0.370199601	-0.06741894	5.49E-03	4.20E-02	1.00E+00
ENSG00000095951.15_HIVEP1	ENSG00000160789.18_LMNA	0.0993	-0.060863412	0.25447724	2.24E-01	4.99E-01	1.00E+00
ENSG00000075407.16_ZNF37A	ENSG00000160789.18_LMNA	0.03825	-0.12169019	0.196257125	6.40E-01	8.39E-01	1.00E+00
ENSG00000162777.15_DENND2D	ENSG00000160789.18_LMNA	0.05677	-0.103369138	0.214032189	4.87E-01	7.44E-01	1.00E+00
ENSG00000152433.13_ZNF547	ENSG00000160789.18_LMNA	-0.13651	-0.289420523	0.023195157	9.35E-02	2.99E-01	1.00E+00
ENSG00000237429.1_RP1-159A1	ENSG00000160789.18_LMNA	-0.15017	-0.302151429	0.009247254	6.48E-02	2.37E-01	1.00E+00
ENSG00000230673.3_PABPC1P3	ENSG00000160789.18_LMNA	0.13531	-0.024418836	0.288298311	9.65E-02	3.05E-01	1.00E+00
ENSG00000135297.14_MTO1	ENSG00000160789.18_LMNA	-0.03488	-0.193010796	0.125013001	6.70E-01	8.55E-01	1.00E+00
ENSG00000107938.16_EDRF1	ENSG00000160789.18_LMNA	-0.15017	-0.302147235	0.009251869	6.48E-02	2.37E-01	1.00E+00
ENSG00000140451.11_PIF1	ENSG00000160789.18_LMNA	-0.01135	-0.1702468	0.148114599	8.90E-01	9.57E-01	1.00E+00
ENSG00000263345.1_RP1-59D14	ENSG00000160789.18_LMNA	0.06782	-0.092380722	0.22459324	4.06E-01	6.82E-01	1.00E+00
ENSG00000127081.12_ZNF484	ENSG00000160789.18_LMNA	-0.1414	-0.293980375	0.01821336	8.23E-02	2.77E-01	1.00E+00
ENSG00000281538.1_RP4-669P1	ENSG00000160789.18_LMNA	-0.17179	-0.322180465	-0.01294448	3.43E-02	1.57E-01	1.00E+00
ENSG00000198331.9_HYLS1	ENSG00000160789.18_LMNA	-0.12357	-0.277318883	0.036341766	1.29E-01	3.64E-01	1.00E+00
ENSG00000111913.14_FAM65B	ENSG00000160789.18_LMNA	0.01975	-0.139892346	0.178388031	8.09E-01	9.24E-01	1.00E+00
ENSG00000204682.5_CASC10	ENSG00000160789.18_LMNA	0.06636	-0.093833228	0.223201483	4.17E-01	6.89E-01	1.00E+00
ENSG00000267680.4_ZNF224	ENSG00000160789.18_LMNA	0.00771	-0.15167342	0.166709519	9.25E-01	9.71E-01	1.00E+00
ENSG00000138756.16_BMP2K	ENSG00000160789.18_LMNA	-0.00964	-0.168584244	0.149788306	9.06E-01	9.64E-01	1.00E+00
ENSG00000138207.11_RBP4	ENSG00000160789.18_LMNA	-0.04695	-0.204622739	0.113094517	5.66E-01	7.96E-01	1.00E+00
ENSG00000236942.1_RP11-6B6.3	ENSG00000160789.18_LMNA	-0.19123	-0.340073797	-0.03303094	1.83E-02	1.01E-01	1.00E+00
ENSG00000280332.1_CTD-2013N	ENSG00000160789.18_LMNA	0.00211	-0.157139966	0.161260051	9.79E-01	9.92E-01	1.00E+00
ENSG00000196935.7_SRGAP1	ENSG00000160789.18_LMNA	-0.01292	-0.17176299	0.146586647	8.75E-01	9.51E-01	1.00E+00
ENSG00000185480.10_PARPBP	ENSG00000160789.18_LMNA	-0.15173	-0.303597588	0.00765515	6.20E-02	2.31E-01	1.00E+00
ENSG00000164181.12_ELOVL7	ENSG00000160789.18_LMNA	-0.29185	-0.431027639	-0.13911707	2.64E-04	4.06E-03	1.00E+00
ENSG00000143194.11_MAEL	ENSG00000160789.18_LMNA	0.12575	-0.034130865	0.279361034	1.23E-01	3.53E-01	1.00E+00
ENSG00000242193.8_RP11-568K	ENSG00000160789.18_LMNA	0.19647	0.038472284	0.344883059	1.53E-02	8.90E-02	1.00E+00

ENSG00000166523.6_CLEC4E	ENSG00000160789.18_LMNA	-0.15034	-0.302309837	0.009072937	6.45E-02	2.36E-01	1.00E+00
ENSG00000228672.3_PROB1	ENSG00000160789.18_LMNA	0.37412	0.228527241	0.503338931	2.06E-06	8.16E-05	1.05E-01
ENSG00000197258.5_EIF4BP6	ENSG00000160789.18_LMNA	0.05057	-0.109510543	0.208097134	5.36E-01	7.77E-01	1.00E+00
ENSG00000239881.1_RPS27P25	ENSG00000160789.18_LMNA	0.01283	-0.14666735	0.171682946	8.75E-01	9.52E-01	1.00E+00
ENSG00000216285.5_RP11-490H	ENSG00000160789.18_LMNA	0.05265	-0.107455309	0.210085919	5.19E-01	7.66E-01	1.00E+00
ENSG00000183831.6_ANKRD45	ENSG00000160789.18_LMNA	0.14711	-0.012383697	0.299298298	7.05E-02	2.51E-01	1.00E+00
ENSG00000149634.4_SPATA25	ENSG00000160789.18_LMNA	-0.04898	-0.20656569	0.111091305	5.49E-01	7.86E-01	1.00E+00
ENSG00000054277.11_OPN3	ENSG00000160789.18_LMNA	0.15469	-0.004625224	0.30634581	5.71E-02	2.18E-01	1.00E+00
ENSG00000157741.13_UBN2	ENSG00000160789.18_LMNA	0.01632	-0.143252529	0.175066217	8.42E-01	9.38E-01	1.00E+00
ENSG00000198046.10_ZNF667	ENSG00000160789.18_LMNA	-0.01851	-0.177191245	0.141103801	8.21E-01	9.30E-01	1.00E+00
ENSG00000152467.8_ZSCAN1	ENSG00000160789.18_LMNA	-0.2498	-0.393359308	-0.09435359	1.91E-03	1.89E-02	1.00E+00
ENSG00000148541.11_FAM13C	ENSG00000160789.18_LMNA	-0.52271	-0.629522616	-0.39650236	4.93E-12	1.99E-09	2.53E-07
ENSG00000165914.13_TTC7B	ENSG00000160789.18_LMNA	0.06419	-0.095986923	0.221135481	4.32E-01	7.01E-01	1.00E+00
ENSG00000177853.13_ZNF518A	ENSG00000160789.18_LMNA	-0.21385	-0.360770437	-0.05657784	8.16E-03	5.65E-02	1.00E+00
ENSG00000172197.10_MBOAT1	ENSG00000160789.18_LMNA	0.2285	0.071912497	0.374089714	4.63E-03	3.70E-02	1.00E+00
ENSG00000135315.10_CEP162	ENSG00000160789.18_LMNA	0.03872	-0.12123129	0.196704916	6.36E-01	8.37E-01	1.00E+00
ENSG00000132849.17_PATJ	ENSG00000160789.18_LMNA	-0.03126	-0.189519622	0.12857848	7.02E-01	8.73E-01	1.00E+00
ENSG00000261496.1_RP13-514E	ENSG00000160789.18_LMNA	-0.42526	-0.547397997	-0.2853764	4.73E-08	3.91E-06	2.43E-03
ENSG00000136378.13_ADAMTS7	ENSG00000160789.18_LMNA	0.06761	-0.092591503	0.224391354	4.08E-01	6.83E-01	1.00E+00
ENSG00000170323.7_FABP4	ENSG00000160789.18_LMNA	-0.07541	-0.231830537	0.084805712	3.56E-01	6.39E-01	1.00E+00
ENSG00000228477.1_RP3-342P2	ENSG00000160789.18_LMNA	0.03351	-0.126369496	0.191683518	6.82E-01	8.62E-01	1.00E+00
ENSG00000144792.8_ZNF660	ENSG00000160789.18_LMNA	-0.1961	-0.344540995	-0.03808466	1.55E-02	8.98E-02	1.00E+00
ENSG00000158352.14_SHROOM	ENSG00000160789.18_LMNA	-0.11488	-0.26915821	0.04514624	1.59E-01	4.12E-01	1.00E+00
ENSG00000183837.9_PNMA3	ENSG00000160789.18_LMNA	-0.14178	-0.294339448	0.017820401	8.14E-02	2.75E-01	1.00E+00
ENSG00000261353.1_CTA-14H9.	ENSG00000160789.18_LMNA	-0.02212	-0.18068153	0.137568069	7.87E-01	9.13E-01	1.00E+00
ENSG00000185697.15_MYBL1	ENSG00000160789.18_LMNA	-0.07237	-0.228935732	0.087839995	3.76E-01	6.56E-01	1.00E+00
ENSG00000156958.13_GALK2	ENSG00000160789.18_LMNA	0.22997	0.073458463	0.375425617	4.37E-03	3.54E-02	1.00E+00
ENSG00000183666.15_GUSBP1	ENSG00000160789.18_LMNA	-0.37149	-0.501059948	-0.22563679	2.45E-06	9.33E-05	1.26E-01
ENSG00000000460.15_C1orf112	ENSG00000160789.18_LMNA	-0.13631	-0.289229759	0.023403235	9.40E-02	3.00E-01	1.00E+00
ENSG00000237846.1_RP11-216M	ENSG00000160789.18_LMNA	-0.02478	-0.183260102	0.134950703	7.62E-01	9.01E-01	1.00E+00
ENSG00000172986.11_GXYLT2	ENSG00000160789.18_LMNA	0.27405	0.120090438	0.415140149	6.34E-04	8.13E-03	1.00E+00
ENSG00000145331.12_TRMT10A	ENSG00000160789.18_LMNA	-0.18046	-0.330174324	-0.02188739	2.61E-02	1.30E-01	1.00E+00
ENSG00000135637.12_CCDC142	ENSG00000160789.18_LMNA	0.03613	-0.123780358	0.19421588	6.59E-01	8.49E-01	1.00E+00

ENSG00000108474.15_PIGL	ENSG00000160789.18_LMNA	-0.05763	-0.214857306	0.102513412	4.81E-01	7.38E-01	1.00E+00
ENSG00000189320.7_FAM180A	ENSG00000160789.18_LMNA	-0.05006	-0.20760237	0.110021415	5.40E-01	7.80E-01	1.00E+00
ENSG00000170325.13_PRDM10	ENSG00000160789.18_LMNA	0.01121	-0.148254345	0.170108055	8.91E-01	9.57E-01	1.00E+00
ENSG00000267811.1_RP11-727F	ENSG00000160789.18_LMNA	-0.01545	-0.174222805	0.144104515	8.50E-01	9.41E-01	1.00E+00
ENSG00000224975.1_INE1	ENSG00000160789.18_LMNA	0.02761	-0.132171197	0.185993625	7.36E-01	8.89E-01	1.00E+00
ENSG00000204052.4_LRRC73	ENSG00000160789.18_LMNA	0.12831	-0.031530211	0.281759564	1.15E-01	3.39E-01	1.00E+00
ENSG00000251474.5_RPL32P3	ENSG00000160789.18_LMNA	-0.15324	-0.305000516	0.00610913	5.95E-02	2.24E-01	1.00E+00
ENSG00000234883.3_MIR155HG	ENSG00000160789.18_LMNA	0.36365	0.217015241	0.494239494	4.12E-06	1.42E-04	2.12E-01
ENSG00000187741.13_FANCA	ENSG00000160789.18_LMNA	0.17358	0.014783258	0.323827712	3.25E-02	1.51E-01	1.00E+00
ENSG00000134061.5_CD180	ENSG00000160789.18_LMNA	-0.06363	-0.220593275	0.096551653	4.36E-01	7.04E-01	1.00E+00
ENSG00000116191.16_RALGPS2	ENSG00000160789.18_LMNA	-0.31098	-0.448002654	-0.15968708	9.64E-05	1.83E-03	1.00E+00
ENSG00000186301.8_MST1P2	ENSG00000160789.18_LMNA	0.08431	-0.075910707	0.240284467	3.02E-01	5.88E-01	1.00E+00
ENSG00000239445.4_ST3GAL6-A	ENSG00000160789.18_LMNA	-0.29892	-0.437305505	-0.14669523	1.83E-04	3.05E-03	1.00E+00
ENSG00000146700.8_SSC4D	ENSG00000160789.18_LMNA	0.07458	-0.085638919	0.231036188	3.61E-01	6.43E-01	1.00E+00
ENSG00000271344.1_RP11-274B	ENSG00000160789.18_LMNA	-0.11642	-0.270606484	0.043587265	1.53E-01	4.03E-01	1.00E+00
ENSG00000175170.13_FAM182B	ENSG00000160789.18_LMNA	-0.12024	-0.274196623	0.039716104	1.40E-01	3.82E-01	1.00E+00
ENSG00000272031.2_ANKRD34A	ENSG00000160789.18_LMNA	-0.03024	-0.188534459	0.129583126	7.11E-01	8.77E-01	1.00E+00
ENSG00000242060.1_RPS3AP49	ENSG00000160789.18_LMNA	-0.10406	-0.258970988	0.056069039	2.02E-01	4.73E-01	1.00E+00
ENSG00000130475.13_FCHO1	ENSG00000160789.18_LMNA	-0.20855	-0.35593172	-0.05104228	9.93E-03	6.53E-02	1.00E+00
ENSG00000181007.8_ZFP82	ENSG00000160789.18_LMNA	-0.15976	-0.311051557	-0.00057617	4.93E-02	1.99E-01	1.00E+00
ENSG00000230551.4_CTB-89H12	ENSG00000160789.18_LMNA	0.07852	-0.081703489	0.234784373	3.36E-01	6.22E-01	1.00E+00
ENSG00000256210.3_AC005255.3	ENSG00000160789.18_LMNA	-0.09712	-0.252415123	0.063058621	2.34E-01	5.12E-01	1.00E+00
ENSG00000076258.8_FMO4	ENSG00000160789.18_LMNA	0.03225	-0.127610233	0.190468487	6.93E-01	8.68E-01	1.00E+00
ENSG00000177508.11_IRX3	ENSG00000160789.18_LMNA	0.25512	0.099984163	0.398153263	1.51E-03	1.58E-02	1.00E+00
ENSG00000172728.14_FUT10	ENSG00000160789.18_LMNA	-0.20505	-0.352737096	-0.04739782	1.13E-02	7.13E-02	1.00E+00
ENSG00000115232.12_ITGA4	ENSG00000160789.18_LMNA	0.21301	0.055694615	0.359999495	8.42E-03	5.79E-02	1.00E+00
ENSG00000136689.17_IL1RN	ENSG00000160789.18_LMNA	0.09254	-0.067659347	0.248084016	2.57E-01	5.39E-01	1.00E+00
ENSG00000149575.5_SCN2B	ENSG00000160789.18_LMNA	-0.18939	-0.338390856	-0.0311311	1.94E-02	1.06E-01	1.00E+00
ENSG00000269335.4_IKBKG	ENSG00000160789.18_LMNA	0.21324	0.055941637	0.360215155	8.35E-03	5.75E-02	1.00E+00
ENSG00000169609.12_C15orf40	ENSG00000160789.18_LMNA	-0.12569	-0.279298252	0.03419888	1.23E-01	3.53E-01	1.00E+00
ENSG00000149292.15_TTC12	ENSG00000160789.18_LMNA	0.28596	0.13280602	0.425777814	3.55E-04	5.13E-03	1.00E+00
ENSG00000196584.2_XRCC2	ENSG00000160789.18_LMNA	0.07608	-0.084138088	0.232466721	3.52E-01	6.35E-01	1.00E+00
ENSG00000256663.1_RP11-424C	ENSG00000160789.18_LMNA	0.14411	-0.015440978	0.296511804	7.65E-02	2.65E-01	1.00E+00

ENSG00000273027.1_LL21NC02-	ENSG00000160789.18_LMNA	0.15522	-0.004079465	0.306840277	5.62E-02	2.17E-01	1.00E+00
ENSG00000104205.11_SGK3	ENSG00000160789.18_LMNA	-0.40319	-0.52846543	-0.26071979	2.60E-07	1.58E-05	1.34E-02
ENSG00000183691.4_NOG	ENSG00000160789.18_LMNA	-0.41064	-0.534871904	-0.26902427	1.48E-07	9.95E-06	7.61E-03
ENSG00000228247.1_UBBP2	ENSG00000160789.18_LMNA	-0.04532	-0.203057547	0.114706378	5.79E-01	8.04E-01	1.00E+00
ENSG00000188501.10_LCTL	ENSG00000160789.18_LMNA	0.31431	0.163274737	0.450942162	8.03E-05	1.58E-03	1.00E+00
ENSG00000183486.11_MX2	ENSG00000160789.18_LMNA	-0.03942	-0.197377548	0.120541715	6.30E-01	8.33E-01	1.00E+00
ENSG00000242951.1_RP11-507E	ENSG00000160789.18_LMNA	-0.18265	-0.332188695	-0.02414872	2.43E-02	1.24E-01	1.00E+00
ENSG00000260329.1_RP11-412D	ENSG00000160789.18_LMNA	-0.30285	-0.440796334	-0.1509239	1.49E-04	2.58E-03	1.00E+00
ENSG00000197381.14_ADARB1	ENSG00000160789.18_LMNA	0.17328	0.014474358	0.323551117	3.28E-02	1.52E-01	1.00E+00
ENSG00000277200.1_RP11-74E2	ENSG00000160789.18_LMNA	-0.30928	-0.44649834	-0.15785401	1.06E-04	1.97E-03	1.00E+00
ENSG00000107105.13_ELAVL2	ENSG00000160789.18_LMNA	-0.34167	-0.475035179	-0.19296972	1.65E-05	4.40E-04	8.45E-01
ENSG00000206077.9_ZDHHC11B	ENSG00000160789.18_LMNA	-0.11777	-0.271870021	0.042225899	1.48E-01	3.95E-01	1.00E+00
ENSG00000270195.1_RP11-572C	ENSG00000160789.18_LMNA	-0.06749	-0.224283637	0.092703955	4.09E-01	6.83E-01	1.00E+00
ENSG00000188206.6_	ENSG00000160789.18_LMNA	-0.03284	-0.19103794	0.127028857	6.88E-01	8.65E-01	1.00E+00
ENSG00000205105.6_COX17P1	ENSG00000160789.18_LMNA	-0.06945	-0.226151984	0.090752336	3.95E-01	6.72E-01	1.00E+00
ENSG00000253540.4_FAM86HP	ENSG00000160789.18_LMNA	0.12647	-0.033408138	0.280027983	1.21E-01	3.49E-01	1.00E+00
ENSG00000091844.6_RGS17	ENSG00000160789.18_LMNA	0.16867	0.009730739	0.319296958	3.78E-02	1.67E-01	1.00E+00
ENSG00000155330.8_C16orf87	ENSG00000160789.18_LMNA	-0.23172	-0.377012969	-0.07529731	4.07E-03	3.36E-02	1.00E+00
ENSG00000136169.15_SETDB2	ENSG00000160789.18_LMNA	-0.22532	-0.371204365	-0.06857839	5.26E-03	4.07E-02	1.00E+00
ENSG00000277149.3_TYW1B	ENSG00000160789.18_LMNA	0.16281	0.003704187	0.313874185	4.51E-02	1.88E-01	1.00E+00
ENSG00000118898.14_PPL	ENSG00000160789.18_LMNA	0.37767	0.232447822	0.506424027	1.61E-06	6.78E-05	8.28E-02
ENSG00000260917.1_RP11-57H1	ENSG00000160789.18_LMNA	-0.07655	-0.232911012	0.083671671	3.49E-01	6.33E-01	1.00E+00
ENSG00000113356.9_POLR3G	ENSG00000160789.18_LMNA	-0.16588	-0.316712493	-0.00685571	4.11E-02	1.77E-01	1.00E+00
ENSG00000154479.11_CCDC173	ENSG00000160789.18_LMNA	-0.10013	-0.25525784	0.060031633	2.20E-01	4.95E-01	1.00E+00
ENSG00000259746.1_HSPE1P3	ENSG00000160789.18_LMNA	-0.12155	-0.275416703	0.038398372	1.36E-01	3.75E-01	1.00E+00
ENSG00000173218.13_VANGL1	ENSG00000160789.18_LMNA	-0.07326	-0.229782126	0.086953428	3.70E-01	6.50E-01	1.00E+00
ENSG00000163945.14_UVSSA	ENSG00000160789.18_LMNA	0.12539	-0.034498691	0.279021479	1.24E-01	3.55E-01	1.00E+00
ENSG00000184005.10_ST6GALN	ENSG00000160789.18_LMNA	-0.51354	-0.621897224	-0.38588832	1.33E-11	4.32E-09	6.81E-07
ENSG00000254139.1_CTD-2339F	ENSG00000160789.18_LMNA	-0.31979	-0.455789648	-0.16920774	5.92E-05	1.23E-03	1.00E+00
ENSG00000163666.7_HESX1	ENSG00000160789.18_LMNA	-0.07756	-0.233873168	0.082661123	3.42E-01	6.27E-01	1.00E+00
ENSG00000249471.6_ZNF324B	ENSG00000160789.18_LMNA	0.28328	0.129937797	0.423385354	4.06E-04	5.72E-03	1.00E+00
ENSG00000197417.7_SHPK	ENSG00000160789.18_LMNA	0.16445	0.005388815	0.315392077	4.29E-02	1.82E-01	1.00E+00
ENSG00000276649.1_RP5-858L1	ENSG00000160789.18_LMNA	-0.14159	-0.294157354	0.018019692	8.19E-02	2.76E-01	1.00E+00

ENSG00000211451.10_GNRHR2	ENSG00000160789.18_LMNA	-0.3034	-0.441281898	-0.15151294	1.45E-04	2.53E-03	1.00E+00
ENSG00000186710.10_CFAP73	ENSG00000160789.18_LMNA	0.09643	-0.063749538	0.251765502	2.37E-01	5.16E-01	1.00E+00
ENSG00000260077.1_RP11-254F	ENSG00000160789.18_LMNA	-0.11277	-0.267169484	0.047284483	1.67E-01	4.24E-01	1.00E+00
ENSG00000242477.1_CTD-2161E	ENSG00000160789.18_LMNA	-0.1408	-0.293425669	0.018820226	8.36E-02	2.80E-01	1.00E+00
ENSG00000148824.17_MTG1	ENSG00000160789.18_LMNA	0.0175	-0.14209709	0.176209279	8.31E-01	9.34E-01	1.00E+00
ENSG00000152784.14_PRDM8	ENSG00000160789.18_LMNA	-0.16464	-0.315571797	-0.00558839	4.27E-02	1.81E-01	1.00E+00
ENSG00000271551.1_RP11-230C	ENSG00000160789.18_LMNA	-0.31336	-0.450101864	-0.16224838	8.46E-05	1.65E-03	1.00E+00
ENSG00000274272.1_RP11-44M6	ENSG00000160789.18_LMNA	0.15726	-0.001989975	0.308731843	5.30E-02	2.09E-01	1.00E+00
ENSG00000142959.4_BEST4	ENSG00000160789.18_LMNA	0.07632	-0.083899256	0.232694239	3.50E-01	6.34E-01	1.00E+00
ENSG00000152104.10_PTPN14	ENSG00000160789.18_LMNA	0.43639	0.297878993	0.55689671	1.91E-08	1.85E-06	9.80E-04
ENSG00000238000.1_RP11-274E	ENSG00000160789.18_LMNA	0.0965	-0.06368257	0.25182848	2.37E-01	5.15E-01	1.00E+00
ENSG00000248280.1_	ENSG00000160789.18_LMNA	-0.19662	-0.345016736	-0.03862379	1.52E-02	8.87E-02	1.00E+00
ENSG00000204291.9_COL15A1	ENSG00000160789.18_LMNA	0.29274	0.14006651	0.431815723	2.52E-04	3.93E-03	1.00E+00
ENSG00000135953.9_MFSD9	ENSG00000160789.18_LMNA	0.01329	-0.146219009	0.172127573	8.71E-01	9.50E-01	1.00E+00
ENSG00000168781.20_PPIP5K1	ENSG00000160789.18_LMNA	-0.04911	-0.206691604	0.110961397	5.48E-01	7.85E-01	1.00E+00
ENSG00000253200.1_RP11-582J1	ENSG00000160789.18_LMNA	0.16263	0.003523323	0.313711129	4.53E-02	1.88E-01	1.00E+00
ENSG00000121577.12_POPDC2	ENSG00000160789.18_LMNA	0.15435	-0.004969124	0.306034145	5.76E-02	2.20E-01	1.00E+00
ENSG00000178694.8_NSUN3	ENSG00000160789.18_LMNA	-0.30089	-0.439056825	-0.14881539	1.65E-04	2.81E-03	1.00E+00
ENSG00000197182.11_MIRLET7E	ENSG00000160789.18_LMNA	0.04191	-0.118077227	0.199779037	6.08E-01	8.21E-01	1.00E+00
ENSG00000260230.2_FRRS1L	ENSG00000160789.18_LMNA	-0.37572	-0.504726802	-0.23028986	1.84E-06	7.54E-05	9.46E-02
ENSG00000197782.13_ZNF780A	ENSG00000160789.18_LMNA	-0.00502	-0.164089058	0.154304527	9.51E-01	9.80E-01	1.00E+00
ENSG00000102385.11_DRP2	ENSG00000160789.18_LMNA	-0.34782	-0.480417119	-0.19967439	1.13E-05	3.22E-04	5.80E-01
ENSG00000164220.6_F2RL2	ENSG00000160789.18_LMNA	0.14515	-0.014384743	0.297475088	7.44E-02	2.60E-01	1.00E+00
ENSG00000135604.9_STX11	ENSG00000160789.18_LMNA	0.12922	-0.030614639	0.282603047	1.13E-01	3.35E-01	1.00E+00
ENSG00000102802.8_MEDAG	ENSG00000160789.18_LMNA	0.31317	0.162047928	0.44993769	8.55E-05	1.66E-03	1.00E+00
ENSG00000116205.9_TCEANC2	ENSG00000160789.18_LMNA	-0.04197	-0.199831311	0.118023538	6.08E-01	8.21E-01	1.00E+00
ENSG00000181908.5_AP003774.4	ENSG00000160789.18_LMNA	-0.13148	-0.2847167	0.02831803	1.06E-01	3.24E-01	1.00E+00
ENSG00000186230.6_ZNF749	ENSG00000160789.18_LMNA	-0.01982	-0.178457368	0.139822129	8.09E-01	9.24E-01	1.00E+00
ENSG00000278948.1_RP5-1039K	ENSG00000160789.18_LMNA	0.03431	-0.125576487	0.192459588	6.75E-01	8.58E-01	1.00E+00
ENSG00000165240.16_ATP7A	ENSG00000160789.18_LMNA	0.01487	-0.144676425	0.17365639	8.56E-01	9.43E-01	1.00E+00
ENSG00000100678.17_SLC8A3	ENSG00000160789.18_LMNA	-0.3283	-0.463290812	-0.17842979	3.64E-05	8.35E-04	1.00E+00
ENSG00000184828.8_ZBTB7C	ENSG00000160789.18_LMNA	-0.02008	-0.178711349	0.139564901	8.06E-01	9.23E-01	1.00E+00
ENSG00000220008.3_LINGO3	ENSG00000160789.18_LMNA	-0.00319	-0.162309829	0.156088414	9.69E-01	9.88E-01	1.00E+00

ENSG00000160218.11_TRAPPC1	ENSG00000160789.18_LMNA	0.14056	-0.019064178	0.293202625	8.41E-02	2.81E-01	1.00E+00
ENSG00000154175.15_ABI3BP	ENSG00000160789.18_LMNA	-0.17346	-0.323721001	-0.01466408	3.26E-02	1.51E-01	1.00E+00
ENSG00000066923.16_STAG3	ENSG00000160789.18_LMNA	-0.19362	-0.342272923	-0.03551684	1.68E-02	9.54E-02	1.00E+00
ENSG00000273033.2_RP11-67L2	ENSG00000160789.18_LMNA	0.06812	-0.092077724	0.224883402	4.04E-01	6.80E-01	1.00E+00
ENSG00000069712.10_KIAA1107	ENSG00000160789.18_LMNA	-0.2881	-0.4276845	-0.13509539	3.19E-04	4.72E-03	1.00E+00
ENSG00000226648.1_PLCG1-AS1	ENSG00000160789.18_LMNA	0.05667	-0.10346038	0.213944183	4.88E-01	7.44E-01	1.00E+00
ENSG00000139517.7_LNX2	ENSG00000160789.18_LMNA	0.12967	-0.030153227	0.283027946	1.11E-01	3.33E-01	1.00E+00
ENSG00000272899.2_RP11-309L	ENSG00000160789.18_LMNA	0.02092	-0.138738138	0.179527388	7.98E-01	9.19E-01	1.00E+00
ENSG00000148634.14_HERC4	ENSG00000160789.18_LMNA	-0.06355	-0.220518554	0.096629461	4.37E-01	7.05E-01	1.00E+00
ENSG00000128394.15_APOBEC3	ENSG00000160789.18_LMNA	0.241	0.085066107	0.385416121	2.78E-03	2.52E-02	1.00E+00
ENSG00000176055.9_MBLAC2	ENSG00000160789.18_LMNA	-0.45728	-0.574650974	-0.3214886	3.17E-09	4.04E-07	1.63E-04
ENSG00000168517.9_HEXIM2	ENSG00000160789.18_LMNA	-0.06882	-0.225547534	0.091383993	4.00E-01	6.76E-01	1.00E+00
ENSG00000168477.16_TNXB	ENSG00000160789.18_LMNA	0.26102	0.10623694	0.403457953	1.16E-03	1.30E-02	1.00E+00
ENSG00000128833.11_MYO5C	ENSG00000160789.18_LMNA	0.16082	0.001658591	0.312028919	4.78E-02	1.95E-01	1.00E+00
ENSG00000254505.7_CHMP4A	ENSG00000160789.18_LMNA	0.11811	-0.041878874	0.272191935	1.47E-01	3.93E-01	1.00E+00
ENSG00000089847.11_ANKRD24	ENSG00000160789.18_LMNA	0.19745	0.039487077	0.345778195	1.48E-02	8.68E-02	1.00E+00
ENSG00000101670.10_LIPG	ENSG00000160789.18_LMNA	0.03418	-0.125707422	0.192331478	6.76E-01	8.58E-01	1.00E+00
ENSG00000138722.8_MMRN1	ENSG00000160789.18_LMNA	-0.05513	-0.212467035	0.104991061	5.00E-01	7.53E-01	1.00E+00
ENSG00000258810.1_RP11-219E	ENSG00000160789.18_LMNA	-0.05418	-0.211560106	0.105930114	5.07E-01	7.57E-01	1.00E+00
ENSG00000121406.7_ZNF549	ENSG00000160789.18_LMNA	0.01621	-0.143356175	0.174963638	8.43E-01	9.39E-01	1.00E+00
ENSG00000240914.1_RPL15P2	ENSG00000160789.18_LMNA	-0.18039	-0.330112633	-0.02181818	2.62E-02	1.31E-01	1.00E+00
ENSG00000116039.10_ATP6V1B1	ENSG00000160789.18_LMNA	-0.17999	-0.329740613	-0.02140092	2.65E-02	1.32E-01	1.00E+00
ENSG00000187554.10_TLR5	ENSG00000160789.18_LMNA	-0.0739	-0.2303934	0.086312828	3.66E-01	6.47E-01	1.00E+00
ENSG00000120519.13_SLC10A7	ENSG00000160789.18_LMNA	-0.00319	-0.16230346	0.156094796	9.69E-01	9.88E-01	1.00E+00
ENSG00000135338.12_LCA5	ENSG00000160789.18_LMNA	-0.14919	-0.301235862	0.010254404	6.66E-02	2.41E-01	1.00E+00
ENSG00000266563.1_EIF1P5	ENSG00000160789.18_LMNA	-0.00059	-0.159773115	0.158628181	9.94E-01	9.98E-01	1.00E+00
ENSG00000172183.13_ISG20	ENSG00000160789.18_LMNA	0.43027	0.291005818	0.551683259	3.16E-08	2.80E-06	1.62E-03
ENSG00000261971.5_MMP25-AS	ENSG00000160789.18_LMNA	0.01768	-0.141924435	0.176380012	8.29E-01	9.33E-01	1.00E+00
ENSG00000019991.14_HGF	ENSG00000160789.18_LMNA	0.10511	-0.055009594	0.259962165	1.97E-01	4.67E-01	1.00E+00
ENSG00000151136.13_BTBD11	ENSG00000160789.18_LMNA	0.09604	-0.064140662	0.25139763	2.39E-01	5.18E-01	1.00E+00
ENSG00000197077.11_KIAA1671	ENSG00000160789.18_LMNA	0.14877	-0.010685795	0.300843522	6.74E-02	2.43E-01	1.00E+00
ENSG00000269481.1_CTD-2521M	ENSG00000160789.18_LMNA	-0.20516	-0.352836597	-0.04751121	1.12E-02	7.11E-02	1.00E+00
ENSG00000258733.4_CTD-2341M	ENSG00000160789.18_LMNA	0.02071	-0.138946689	0.179321583	8.00E-01	9.20E-01	1.00E+00

ENSG00000255150.1_EID3	ENSG00000160789.18_LMNA	0.18166	0.023129341	0.331280992	2.51E-02	1.27E-01	1.00E+00
ENSG00000240898.1_RP11-48B1	ENSG00000160789.18_LMNA	-0.18303	-0.332536802	-0.02453982	2.40E-02	1.23E-01	1.00E+00
ENSG00000229180.5_GS1-124K5	ENSG00000160789.18_LMNA	0.09209	-0.068112238	0.247656983	2.59E-01	5.42E-01	1.00E+00
ENSG00000117242.7_PINK1-AS	ENSG00000160789.18_LMNA	0.15826	-0.000969229	0.309655007	5.15E-02	2.05E-01	1.00E+00
ENSG00000145861.7_C1QTNF2	ENSG00000160789.18_LMNA	0.06202	-0.098146119	0.219061342	4.48E-01	7.14E-01	1.00E+00
ENSG00000182218.8_HHIPL1	ENSG00000160789.18_LMNA	0.11204	-0.048020815	0.266484019	1.69E-01	4.28E-01	1.00E+00
ENSG00000187098.13_MITF	ENSG00000160789.18_LMNA	-0.04094	-0.198848836	0.119032294	6.16E-01	8.26E-01	1.00E+00
ENSG00000257086.1_RP11-783K	ENSG00000160789.18_LMNA	0.08133	-0.078889934	0.237458303	3.19E-01	6.06E-01	1.00E+00
ENSG00000165929.11_TC2N	ENSG00000160789.18_LMNA	0.11921	-0.040765759	0.273224031	1.44E-01	3.87E-01	1.00E+00
ENSG00000270006.2_RP11-178L	ENSG00000160789.18_LMNA	-0.07549	-0.231899381	0.08473348	3.55E-01	6.39E-01	1.00E+00
ENSG00000167701.12_GPT	ENSG00000160789.18_LMNA	-0.05931	-0.216465834	0.100843863	4.68E-01	7.29E-01	1.00E+00
ENSG00000272660.1_RP11-145M	ENSG00000160789.18_LMNA	-0.18441	-0.333811292	-0.02597254	2.29E-02	1.19E-01	1.00E+00
ENSG00000233654.1_AC093388.3	ENSG00000160789.18_LMNA	0.28816	0.135156908	0.4277357	3.18E-04	4.72E-03	1.00E+00
ENSG00000160221.15_C21orf33	ENSG00000160789.18_LMNA	-0.00558	-0.164632545	0.153759203	9.46E-01	9.78E-01	1.00E+00
ENSG00000249210.1_GAPDHP38	ENSG00000160789.18_LMNA	0.21381	0.056535244	0.360733266	8.17E-03	5.65E-02	1.00E+00
ENSG00000100884.8_CPNE6	ENSG00000160789.18_LMNA	-0.26019	-0.402709236	-0.10535298	1.21E-03	1.33E-02	1.00E+00
ENSG00000047648.20_ARHGAP6	ENSG00000160789.18_LMNA	0.01717	-0.14242081	0.175889113	8.34E-01	9.35E-01	1.00E+00
ENSG00000234335.1_RPS4XP11	ENSG00000160789.18_LMNA	0.03298	-0.126891795	0.19117216	6.87E-01	8.64E-01	1.00E+00
ENSG00000278192.1_RP5-1056H	ENSG00000160789.18_LMNA	-0.50261	-0.612777834	-0.37327624	4.17E-11	1.11E-08	2.14E-06
ENSG00000188512.6_RP1-273G1	ENSG00000160789.18_LMNA	-0.09795	-0.253202888	0.062220373	2.30E-01	5.07E-01	1.00E+00
ENSG00000250486.3_FAM218A	ENSG00000160789.18_LMNA	-0.14066	-0.29329405	0.018964187	8.39E-02	2.80E-01	1.00E+00
ENSG00000260793.2_RP5-882C2	ENSG00000160789.18_LMNA	-0.06299	-0.219985036	0.097184915	4.41E-01	7.08E-01	1.00E+00
ENSG00000053702.13_NRIP2	ENSG00000160789.18_LMNA	-0.15702	-0.308510522	0.002234594	5.34E-02	2.10E-01	1.00E+00
ENSG00000239282.6_GATSL3	ENSG00000160789.18_LMNA	-0.13529	-0.288277777	0.024441219	9.66E-02	3.05E-01	1.00E+00
ENSG00000254343.2_RP11-760H	ENSG00000160789.18_LMNA	-0.34128	-0.474690426	-0.19254113	1.69E-05	4.48E-04	8.65E-01
ENSG00000167550.9_RHEBL1	ENSG00000160789.18_LMNA	0.01928	-0.140347013	0.177938979	8.14E-01	9.27E-01	1.00E+00
ENSG00000151532.12_VTI1A	ENSG00000160789.18_LMNA	-0.14202	-0.294559826	0.017579179	8.09E-02	2.74E-01	1.00E+00
ENSG00000088179.7_PTPN4	ENSG00000160789.18_LMNA	-0.39129	-0.518210343	-0.24750847	6.21E-07	3.12E-05	3.19E-02
ENSG00000115137.10_DNAJC27	ENSG00000160789.18_LMNA	-0.20616	-0.353748057	-0.04855025	1.08E-02	6.94E-02	1.00E+00
ENSG00000213169.3_RP11-393N	ENSG00000160789.18_LMNA	0.03413	-0.125752344	0.192287522	6.76E-01	8.59E-01	1.00E+00
ENSG00000224940.7_PRRT4	ENSG00000160789.18_LMNA	-0.10793	-0.262616868	0.05216855	1.86E-01	4.52E-01	1.00E+00
ENSG00000157927.15_RADIL	ENSG00000160789.18_LMNA	0.0961	-0.064081219	0.251453544	2.39E-01	5.18E-01	1.00E+00
ENSG00000266053.2_NDUFV2-AS	ENSG00000160789.18_LMNA	-0.06328	-0.220264769	0.096893705	4.39E-01	7.07E-01	1.00E+00

ENSG00000111879.17_FAM184A	ENSG00000160789.18_LMNA	0.0561	-0.104032272	0.213392462	4.92E-01	7.48E-01	1.00E+00
ENSG00000148600.13_CDHR1	ENSG00000160789.18_LMNA	-0.36954	-0.499363845	-0.22348876	2.80E-06	1.04E-04	1.43E-01
ENSG00000095539.14_SEMA4G	ENSG00000160789.18_LMNA	-0.107	-0.261743939	0.053103315	1.89E-01	4.57E-01	1.00E+00
ENSG00000151413.15_NUBPL	ENSG00000160789.18_LMNA	-0.22221	-0.368379709	-0.06532095	5.93E-03	4.47E-02	1.00E+00
ENSG00000162894.10_FCMR	ENSG00000160789.18_LMNA	-0.06052	-0.217620832	0.099643946	4.59E-01	7.23E-01	1.00E+00
ENSG00000118308.13_LRMP	ENSG00000160789.18_LMNA	-0.26185	-0.404205196	-0.10711963	1.12E-03	1.26E-02	1.00E+00
ENSG00000228501.2_RPL15P18	ENSG00000160789.18_LMNA	-0.22529	-0.371181755	-0.06855229	5.26E-03	4.07E-02	1.00E+00
ENSG00000137727.11_ARHGAP2	ENSG00000160789.18_LMNA	0.2119	0.054543938	0.358994475	8.77E-03	5.96E-02	1.00E+00
ENSG00000278231.1_RP5-906C1	ENSG00000160789.18_LMNA	-0.06337	-0.220343795	0.096811427	4.38E-01	7.06E-01	1.00E+00
ENSG00000204622.9_HLA-J	ENSG00000160789.18_LMNA	0.21942	0.062396895	0.365839366	6.61E-03	4.83E-02	1.00E+00
ENSG00000135502.15_SLC26A1(	ENSG00000160789.18_LMNA	-0.19626	-0.344687232	-0.03825036	1.54E-02	8.94E-02	1.00E+00
ENSG00000260317.1_RP11-48B3	ENSG00000160789.18_LMNA	0.10693	-0.053175054	0.261676925	1.90E-01	4.57E-01	1.00E+00
ENSG00000185347.16_C14orf80	ENSG00000160789.18_LMNA	0.09136	-0.068837769	0.24697262	2.63E-01	5.46E-01	1.00E+00
ENSG00000245648.1_RP11-277P	ENSG00000160789.18_LMNA	-0.40955	-0.533936148	-0.26780882	1.61E-07	1.06E-05	8.27E-03
ENSG00000185808.12_PIGP	ENSG00000160789.18_LMNA	0.03145	-0.128398428	0.189696116	7.01E-01	8.72E-01	1.00E+00
ENSG00000234284.5_ZNF879	ENSG00000160789.18_LMNA	-0.21028	-0.357510472	-0.05284633	9.32E-03	6.23E-02	1.00E+00
ENSG00000229692.3_SOS1-IT1	ENSG00000160789.18_LMNA	-0.03171	-0.189947271	0.128142171	6.98E-01	8.71E-01	1.00E+00
ENSG00000226853.2_AC010894.3	ENSG00000160789.18_LMNA	-0.06426	-0.221195068	0.095924849	4.32E-01	7.01E-01	1.00E+00
ENSG00000274964.1_RP11-817I4	ENSG00000160789.18_LMNA	0.39518	0.251824798	0.521569412	4.69E-07	2.49E-05	2.41E-02
ENSG00000270659.1_RP11-105N	ENSG00000160789.18_LMNA	-0.28844	-0.427988831	-0.13546109	3.14E-04	4.66E-03	1.00E+00
ENSG00000271780.1_RP11-1017(	ENSG00000160789.18_LMNA	-0.04522	-0.202957377	0.114809478	5.80E-01	8.04E-01	1.00E+00
ENSG00000269814.1_CTC-273B1	ENSG00000160789.18_LMNA	-0.23677	-0.381587069	-0.0806076	3.32E-03	2.88E-02	1.00E+00
ENSG00000251669.4_FAM86EP	ENSG00000160789.18_LMNA	0.35733	0.210081478	0.488729366	6.21E-06	1.98E-04	3.18E-01
ENSG00000121904.16_CSMD2	ENSG00000160789.18_LMNA	-0.02249	-0.181040846	0.137203614	7.83E-01	9.11E-01	1.00E+00
ENSG00000100031.17_GGT1	ENSG00000160789.18_LMNA	0.10692	-0.053181301	0.261671089	1.90E-01	4.57E-01	1.00E+00
ENSG00000273802.1_HIST1H2BC	ENSG00000160789.18_LMNA	-0.08187	-0.23797166	0.078349193	3.16E-01	6.03E-01	1.00E+00
ENSG00000260912.1_RP11-363E	ENSG00000160789.18_LMNA	0.02132	-0.138352603	0.179907772	7.94E-01	9.17E-01	1.00E+00
ENSG00000181450.16_ZNF678	ENSG00000160789.18_LMNA	-0.30665	-0.444167283	-0.15501745	1.22E-04	2.22E-03	1.00E+00
ENSG00000175267.13_VWA3A	ENSG00000160789.18_LMNA	-0.09554	-0.250922665	0.064645503	2.42E-01	5.21E-01	1.00E+00
ENSG00000258477.1_PPIAP6	ENSG00000160789.18_LMNA	0.06176	-0.09840366	0.218813755	4.50E-01	7.15E-01	1.00E+00
ENSG00000080854.13_IGSF9B	ENSG00000160789.18_LMNA	-0.13135	-0.284594856	0.02845051	1.07E-01	3.25E-01	1.00E+00
ENSG00000198945.6_L3MBTL3	ENSG00000160789.18_LMNA	0.13979	-0.019852538	0.292481598	8.59E-02	2.84E-01	1.00E+00
ENSG00000183773.14_AIFM3	ENSG00000160789.18_LMNA	-0.14005	-0.292726869	0.019584406	8.53E-02	2.83E-01	1.00E+00

ENSG00000267281.2_RP11-793H	ENSG00000160789.18_LMNA	-0.05809	-0.215300687	0.102053389	4.77E-01	7.36E-01	1.00E+00
ENSG00000228847.1_ATP5G2P4	ENSG00000160789.18_LMNA	-0.17502	-0.325157664	-0.01626937	3.10E-02	1.47E-01	1.00E+00
ENSG00000163630.9_SYNPR	ENSG00000160789.18_LMNA	-0.27603	-0.416909403	-0.1221986	5.77E-04	7.54E-03	1.00E+00
ENSG00000271936.1_RP11-443B	ENSG00000160789.18_LMNA	0.10538	-0.05473842	0.260215759	1.96E-01	4.66E-01	1.00E+00
ENSG00000141337.11_ARSG	ENSG00000160789.18_LMNA	0.29652	0.144120865	0.435176033	2.08E-04	3.36E-03	1.00E+00
ENSG00000158445.7_KCNB1	ENSG00000160789.18_LMNA	-0.41475	-0.538398126	-0.27361211	1.08E-07	7.70E-06	5.55E-03
ENSG00000132000.10_PODNL1	ENSG00000160789.18_LMNA	0.46718	0.332725794	0.583018388	1.30E-09	1.93E-07	6.67E-05
ENSG00000247240.6_UBL7-AS1	ENSG00000160789.18_LMNA	-0.21521	-0.362009496	-0.05799835	7.75E-03	5.43E-02	1.00E+00
ENSG00000183615.5_FAM167B	ENSG00000160789.18_LMNA	0.33933	0.190412973	0.472977272	1.90E-05	4.95E-04	9.74E-01
ENSG00000162631.17_NTNG1	ENSG00000160789.18_LMNA	-0.01838	-0.177059731	0.141236868	8.22E-01	9.31E-01	1.00E+00
ENSG00000229931.1_RP1-151F1	ENSG00000160789.18_LMNA	0.11869	-0.041294131	0.272734207	1.45E-01	3.90E-01	1.00E+00
ENSG00000235597.1_LINC01102	ENSG00000160789.18_LMNA	-0.27702	-0.417793939	-0.12325357	5.50E-04	7.28E-03	1.00E+00
ENSG00000279400.1_CTD-2353F	ENSG00000160789.18_LMNA	0.12111	-0.038841814	0.275006236	1.37E-01	3.77E-01	1.00E+00
ENSG00000276334.1_AL133243.1	ENSG00000160789.18_LMNA	-0.19685	-0.345228898	-0.03886428	1.51E-02	8.82E-02	1.00E+00
ENSG00000205078.5_SYCE1L	ENSG00000160789.18_LMNA	0.18148	0.02294098	0.331113204	2.52E-02	1.27E-01	1.00E+00
ENSG00000198440.8_ZNF583	ENSG00000160789.18_LMNA	0.03438	-0.125509248	0.192525373	6.74E-01	8.57E-01	1.00E+00
ENSG00000269001.1_CTD-2245F	ENSG00000160789.18_LMNA	-0.10044	-0.255556147	0.059713653	2.18E-01	4.93E-01	1.00E+00
ENSG00000184786.5_TCTE3	ENSG00000160789.18_LMNA	-0.14741	-0.299584158	0.012069728	6.99E-02	2.49E-01	1.00E+00
ENSG00000269936.3_	ENSG00000160789.18_LMNA	0.09299	-0.067209366	0.248508185	2.55E-01	5.36E-01	1.00E+00
ENSG00000027075.12_PRKCH	ENSG00000160789.18_LMNA	-0.01606	-0.174817253	0.143504073	8.44E-01	9.39E-01	1.00E+00
ENSG00000144218.17_AFF3	ENSG00000160789.18_LMNA	-0.30559	-0.443224497	-0.15387157	1.29E-04	2.32E-03	1.00E+00
ENSG00000146192.13_FGD2	ENSG00000160789.18_LMNA	-0.11036	-0.264904628	0.049716112	1.76E-01	4.37E-01	1.00E+00
ENSG00000237517.7_DGCR5	ENSG00000160789.18_LMNA	-0.22167	-0.367883953	-0.0647499	6.06E-03	4.53E-02	1.00E+00
ENSG00000261643.1_RP11-529E	ENSG00000160789.18_LMNA	-0.02652	-0.184938545	0.133244618	7.46E-01	8.94E-01	1.00E+00
ENSG00000148835.10_TAF5	ENSG00000160789.18_LMNA	-0.16773	-0.318429554	-0.00876524	3.89E-02	1.70E-01	1.00E+00
ENSG00000270587.1_RP11-51F1	ENSG00000160789.18_LMNA	-0.1268	-0.280346162	0.033063234	1.20E-01	3.47E-01	1.00E+00
ENSG00000185811.15_IKZF1	ENSG00000160789.18_LMNA	-0.00404	-0.163135962	0.155260376	9.61E-01	9.84E-01	1.00E+00
ENSG00000242100.2_RPL9P32	ENSG00000160789.18_LMNA	-0.194	-0.342620973	-0.03591063	1.66E-02	9.45E-02	1.00E+00
ENSG00000104047.13_DTWD1	ENSG00000160789.18_LMNA	-0.11361	-0.26796023	0.046434632	1.63E-01	4.19E-01	1.00E+00
ENSG00000166793.9_YPEL4	ENSG00000160789.18_LMNA	-0.29879	-0.437191221	-0.14655697	1.85E-04	3.06E-03	1.00E+00
ENSG00000131018.21_SYNE1	ENSG00000160789.18_LMNA	-0.04905	-0.206633805	0.111021031	5.48E-01	7.85E-01	1.00E+00
ENSG00000204406.10_MBD5	ENSG00000160789.18_LMNA	-0.13403	-0.287102212	0.025722052	9.97E-02	3.11E-01	1.00E+00
ENSG00000224383.6_PRR29	ENSG00000160789.18_LMNA	0.13906	-0.020597403	0.291800026	8.75E-02	2.88E-01	1.00E+00

ENSG00000188707.5_ZBED6CL	ENSG00000160789.18_LMNA	0.08784	-0.072368553	0.243637676	2.82E-01	5.67E-01	1.00E+00
ENSG00000010438.15_PRSS3	ENSG00000160789.18_LMNA	-0.32416	-0.45963894	-0.17393386	4.62E-05	1.01E-03	1.00E+00
ENSG00000117877.9_CD3EAP	ENSG00000160789.18_LMNA	0.21254	0.055205127	0.359572055	8.57E-03	5.86E-02	1.00E+00
ENSG00000129317.13_PUS7L	ENSG00000160789.18_LMNA	-0.20853	-0.355916116	-0.05102445	9.93E-03	6.53E-02	1.00E+00
ENSG00000249859.6_PVT1	ENSG00000160789.18_LMNA	0.20626	0.04865317	0.353838305	1.08E-02	6.92E-02	1.00E+00
ENSG00000102174.8_PHEX	ENSG00000160789.18_LMNA	-0.06687	-0.223687116	0.09332655	4.13E-01	6.86E-01	1.00E+00
ENSG00000124140.11_SLC12A5	ENSG00000160789.18_LMNA	-0.17756	-0.32750477	-0.0188954	2.86E-02	1.39E-01	1.00E+00
ENSG00000227082.1_CH17-437K	ENSG00000160789.18_LMNA	-0.04637	-0.204065215	0.113668857	5.71E-01	7.99E-01	1.00E+00
ENSG00000248866.1_USP46-AS1	ENSG00000160789.18_LMNA	-0.07909	-0.23532658	0.081133377	3.33E-01	6.18E-01	1.00E+00
ENSG00000223825.5_DAZAP2P1	ENSG00000160789.18_LMNA	-0.22551	-0.371379568	-0.06878065	5.22E-03	4.05E-02	1.00E+00
ENSG00000114735.8_HEMK1	ENSG00000160789.18_LMNA	-0.07805	-0.234342754	0.082167683	3.39E-01	6.24E-01	1.00E+00
ENSG00000117602.10_RCAN3	ENSG00000160789.18_LMNA	0.28327	0.12993165	0.423380222	4.06E-04	5.72E-03	1.00E+00
ENSG00000182150.14_ERCC6L2	ENSG00000160789.18_LMNA	-0.10427	-0.25916495	0.055861773	2.01E-01	4.72E-01	1.00E+00
ENSG00000163611.10_SPICE1	ENSG00000160789.18_LMNA	0.06219	-0.097982018	0.219219079	4.47E-01	7.13E-01	1.00E+00
ENSG00000178201.4_VN1R1	ENSG00000160789.18_LMNA	-0.08122	-0.237352581	0.079001272	3.20E-01	6.06E-01	1.00E+00
ENSG00000100027.13_YPEL1	ENSG00000160789.18_LMNA	-0.32955	-0.464386719	-0.17978131	3.39E-05	7.90E-04	1.00E+00
ENSG00000203706.7_SERTAD4-/	ENSG00000160789.18_LMNA	-0.00013	-0.159328645	0.159072753	9.99E-01	9.99E-01	1.00E+00
ENSG00000108932.10_SLC16A6	ENSG00000160789.18_LMNA	0.15407	-0.005264013	0.305766842	5.81E-02	2.21E-01	1.00E+00
ENSG00000204152.9_TIMM23B	ENSG00000160789.18_LMNA	-0.19353	-0.342190078	-0.03542313	1.69E-02	9.56E-02	1.00E+00
ENSG00000189298.12_ZKSCAN3	ENSG00000160789.18_LMNA	-0.11699	-0.27113628	0.043016592	1.51E-01	4.00E-01	1.00E+00
ENSG00000080823.20_MOK	ENSG00000160789.18_LMNA	0.10687	-0.053233512	0.261622314	1.90E-01	4.57E-01	1.00E+00
ENSG00000160352.14_ZNF714	ENSG00000160789.18_LMNA	-0.30872	-0.446003866	-0.15725191	1.09E-04	2.02E-03	1.00E+00
ENSG00000198482.9_ZNF808	ENSG00000160789.18_LMNA	0.04031	-0.119663063	0.198234176	6.22E-01	8.29E-01	1.00E+00
ENSG00000102908.19_NFAT5	ENSG00000160789.18_LMNA	0.12452	-0.035384731	0.278203219	1.26E-01	3.59E-01	1.00E+00
ENSG00000278291.1_RP11-172H	ENSG00000160789.18_LMNA	-0.3533	-0.48520918	-0.20566642	8.02E-06	2.44E-04	4.11E-01
ENSG00000243403.1_RP11-330L	ENSG00000160789.18_LMNA	-0.14945	-0.301475187	0.0099912	6.61E-02	2.40E-01	1.00E+00
ENSG00000136010.12_ALDH1L2	ENSG00000160789.18_LMNA	-0.0748	-0.231243706	0.085421293	3.60E-01	6.42E-01	1.00E+00
ENSG00000132429.8_POPDC3	ENSG00000160789.18_LMNA	-0.0999	-0.255040111	0.060263681	2.21E-01	4.96E-01	1.00E+00
ENSG00000205476.7_CCDC85C	ENSG00000160789.18_LMNA	0.00148	-0.157753634	0.160647086	9.86E-01	9.94E-01	1.00E+00
ENSG00000114861.17_FOXP1	ENSG00000160789.18_LMNA	0.05675	-0.103384599	0.214017276	4.87E-01	7.44E-01	1.00E+00
ENSG00000206149.9_HERC2P9	ENSG00000160789.18_LMNA	-0.09491	-0.25033185	0.065273256	2.45E-01	5.25E-01	1.00E+00
ENSG00000172031.6_EPHX4	ENSG00000160789.18_LMNA	-0.12377	-0.27750082	0.03614492	1.29E-01	3.63E-01	1.00E+00
ENSG00000164651.15_SP8	ENSG00000160789.18_LMNA	-0.10586	-0.260670506	0.054252031	1.94E-01	4.63E-01	1.00E+00

ENSG00000146426.16_TIAM2	ENSG00000160789.18_LMNA	0.04496	-0.115067098	0.202707049	5.82E-01	8.06E-01	1.00E+00
ENSG00000154240.15_CEP112	ENSG00000160789.18_LMNA	0.39911	0.256188107	0.524956561	3.52E-07	2.00E-05	1.81E-02
ENSG00000100033.15_PRODH	ENSG00000160789.18_LMNA	-0.13669	-0.289587408	0.023013102	9.31E-02	2.98E-01	1.00E+00
ENSG00000010030.12_ETV7	ENSG00000160789.18_LMNA	0.26225	0.107534196	0.404556014	1.10E-03	1.25E-02	1.00E+00
ENSG00000104154.6_SLC30A4	ENSG00000160789.18_LMNA	0.14234	-0.017252044	0.294858638	8.02E-02	2.73E-01	1.00E+00
ENSG00000112297.13_AIM1	ENSG00000160789.18_LMNA	0.37331	0.227638529	0.502638631	2.17E-06	8.48E-05	1.11E-01
ENSG00000106100.9_NOD1	ENSG00000160789.18_LMNA	0.36728	0.221004061	0.497399273	3.25E-06	1.17E-04	1.67E-01
ENSG00000225285.1_RP4-758J18	ENSG00000160789.18_LMNA	0.30082	0.148744742	0.4389985	1.66E-04	2.82E-03	1.00E+00
ENSG00000164663.12_USP49	ENSG00000160789.18_LMNA	-0.32549	-0.460814027	-0.17537924	4.28E-05	9.47E-04	1.00E+00
ENSG00000261612.1_SUB1P3	ENSG00000160789.18_LMNA	-0.22034	-0.36667883	-0.06336258	6.38E-03	4.71E-02	1.00E+00
ENSG00000175764.13_TTLL11	ENSG00000160789.18_LMNA	-0.12866	-0.28208427	0.031177817	1.14E-01	3.37E-01	1.00E+00
ENSG00000273084.1_RP11-1275	ENSG00000160789.18_LMNA	-0.01311	-0.171950196	0.146397884	8.73E-01	9.51E-01	1.00E+00
ENSG00000268970.1_CTD-3099C	ENSG00000160789.18_LMNA	-0.13727	-0.290133465	0.022417265	9.17E-02	2.95E-01	1.00E+00
ENSG00000255622.3_PCDHB17P	ENSG00000160789.18_LMNA	0.11199	-0.048070856	0.266437424	1.70E-01	4.28E-01	1.00E+00
ENSG00000205517.11_RGL3	ENSG00000160789.18_LMNA	0.21853	0.061465082	0.36502888	6.84E-03	4.95E-02	1.00E+00
ENSG00000204172.10_AGAP9	ENSG00000160789.18_LMNA	-0.12854	-0.281973559	0.031297977	1.15E-01	3.38E-01	1.00E+00
ENSG00000121274.11_PAPD5	ENSG00000160789.18_LMNA	0.06502	-0.095166356	0.221922973	4.26E-01	6.97E-01	1.00E+00
ENSG00000099974.7_DDTL	ENSG00000160789.18_LMNA	0.03067	-0.129163324	0.188946198	7.08E-01	8.76E-01	1.00E+00
ENSG00000229204.3_PTGES3P3	ENSG00000160789.18_LMNA	-0.18929	-0.338296537	-0.03102469	1.95E-02	1.06E-01	1.00E+00
ENSG00000184156.14_KCNQ3	ENSG00000160789.18_LMNA	-0.04595	-0.203658613	0.114087588	5.74E-01	8.01E-01	1.00E+00
ENSG00000152910.17_CNTNAP4	ENSG00000160789.18_LMNA	-0.29969	-0.437996881	-0.1475319	1.76E-04	2.96E-03	1.00E+00
ENSG00000251247.8_ZNF345	ENSG00000160789.18_LMNA	-0.1593	-0.310619017	-9.74E-05	5.00E-02	2.01E-01	1.00E+00
ENSG00000267169.1_CTB-55O6.	ENSG00000160789.18_LMNA	-0.06791	-0.224680458	0.092289651	4.06E-01	6.81E-01	1.00E+00
ENSG00000147536.10_GINS4	ENSG00000160789.18_LMNA	0.18958	0.03132515	0.338562841	1.93E-02	1.05E-01	1.00E+00
ENSG00000106701.10_FSD1L	ENSG00000160789.18_LMNA	-0.19002	-0.338968359	-0.03178278	1.90E-02	1.04E-01	1.00E+00
ENSG00000248098.9_BCKDHA	ENSG00000160789.18_LMNA	0.00279	-0.156475662	0.161923319	9.73E-01	9.89E-01	1.00E+00
ENSG00000241935.7_HOGA1	ENSG00000160789.18_LMNA	-0.19892	-0.347129772	-0.04102052	1.40E-02	8.36E-02	1.00E+00
ENSG00000230091.5_TMEM254-A	ENSG00000160789.18_LMNA	-0.36141	-0.492289565	-0.21455831	4.77E-06	1.60E-04	2.45E-01
ENSG00000233597.3_RP11-343B	ENSG00000160789.18_LMNA	-0.24995	-0.393492089	-0.09450928	1.90E-03	1.88E-02	1.00E+00
ENSG00000278882.1_	ENSG00000160789.18_LMNA	0.17518	0.016442221	0.325312272	3.09E-02	1.46E-01	1.00E+00
ENSG00000170214.3_ADRA1B	ENSG00000160789.18_LMNA	0.00683	-0.152537385	0.165849546	9.33E-01	9.74E-01	1.00E+00
ENSG00000100036.12_SLC35E4	ENSG00000160789.18_LMNA	0.48911	0.357769812	0.60147752	1.62E-10	3.36E-08	8.30E-06
ENSG00000160323.17_ADAMTS1	ENSG00000160789.18_LMNA	0.17015	0.011249461	0.320660333	3.61E-02	1.62E-01	1.00E+00

ENSG00000171084.14_FAM86JP	ENSG00000160789.18_LMNA	-0.00215	-0.16129498	0.15710499	9.79E-01	9.92E-01	1.00E+00
ENSG00000156876.9_SASS6	ENSG00000160789.18_LMNA	-0.08566	-0.241565445	0.074558491	2.94E-01	5.80E-01	1.00E+00
ENSG00000273061.1_CDC37L1-A	ENSG00000160789.18_LMNA	-0.13466	-0.287690592	0.025081113	9.81E-02	3.08E-01	1.00E+00
ENSG00000170100.12_ZNF778	ENSG00000160789.18_LMNA	-0.202	-0.34995059	-0.04422557	1.26E-02	7.72E-02	1.00E+00
ENSG00000144290.15_SLC4A10	ENSG00000160789.18_LMNA	-0.1985	-0.34674652	-0.04058556	1.42E-02	8.46E-02	1.00E+00
ENSG00000239969.4_RP11-163E	ENSG00000160789.18_LMNA	0.03876	-0.121184422	0.196750642	6.35E-01	8.37E-01	1.00E+00
ENSG00000162885.11_B3GALNT	ENSG00000160789.18_LMNA	0.13363	-0.02612756	0.286729836	1.01E-01	3.13E-01	1.00E+00
ENSG00000234072.1_AC074117.:	ENSG00000160789.18_LMNA	0.00121	-0.158016686	0.16038426	9.88E-01	9.96E-01	1.00E+00
ENSG00000115257.14_PCSK4	ENSG00000160789.18_LMNA	0.16733	0.008351497	0.318057683	3.94E-02	1.72E-01	1.00E+00
ENSG00000163975.10_MELTF	ENSG00000160789.18_LMNA	0.33568	0.186444114	0.469776641	2.36E-05	5.90E-04	1.00E+00
ENSG00000128346.9_C22orf23	ENSG00000160789.18_LMNA	-0.02769	-0.186072872	0.132090542	7.35E-01	8.89E-01	1.00E+00
ENSG00000274213.1_RP11-670E	ENSG00000160789.18_LMNA	0.05357	-0.106540863	0.210969956	5.12E-01	7.61E-01	1.00E+00
ENSG00000259250.1_RP11-50C1	ENSG00000160789.18_LMNA	0.25174	0.096406983	0.395109485	1.76E-03	1.77E-02	1.00E+00
ENSG00000223401.1_RP11-211G	ENSG00000160789.18_LMNA	0.04105	-0.118930397	0.198948108	6.16E-01	8.26E-01	1.00E+00
ENSG00000086991.11_NOX4	ENSG00000160789.18_LMNA	0.17258	0.013756487	0.32290812	3.35E-02	1.54E-01	1.00E+00
ENSG00000151364.14_KCTD14	ENSG00000160789.18_LMNA	0.17529	0.016554752	0.325412918	3.08E-02	1.46E-01	1.00E+00
ENSG00000234500.1_GS1-124K5	ENSG00000160789.18_LMNA	-0.0138	-0.172625465	0.145716804	8.66E-01	9.48E-01	1.00E+00
ENSG00000185345.17_PARK2	ENSG00000160789.18_LMNA	-0.15468	-0.306337477	0.00463442	5.71E-02	2.18E-01	1.00E+00
ENSG00000150627.14_WDR17	ENSG00000160789.18_LMNA	-0.40437	-0.529487759	-0.26204235	2.38E-07	1.47E-05	1.22E-02
ENSG00000168589.13_DYNLRB2	ENSG00000160789.18_LMNA	-0.07841	-0.234680446	0.081812741	3.37E-01	6.22E-01	1.00E+00
ENSG00000280852.1_CTD-2319I1	ENSG00000160789.18_LMNA	0.21748	0.060370967	0.364076638	7.11E-03	5.10E-02	1.00E+00
ENSG00000171357.5_LURAP1	ENSG00000160789.18_LMNA	-0.07551	-0.231919116	0.084712773	3.55E-01	6.39E-01	1.00E+00
ENSG00000279392.1_RP11-665C	ENSG00000160789.18_LMNA	-0.09753	-0.252802684	0.062646279	2.32E-01	5.10E-01	1.00E+00
ENSG00000114270.14_COL7A1	ENSG00000160789.18_LMNA	0.25803	0.103067236	0.400771355	1.33E-03	1.44E-02	1.00E+00
ENSG00000269194.1_AC006942.4	ENSG00000160789.18_LMNA	0.14189	-0.017711	0.2944394	8.12E-02	2.75E-01	1.00E+00
ENSG00000178662.14_CSRNP3	ENSG00000160789.18_LMNA	-0.3889	-0.516148497	-0.24486435	7.36E-07	3.59E-05	3.78E-02
ENSG00000254427.1_RP11-430H	ENSG00000160789.18_LMNA	-0.09734	-0.252626897	0.062833319	2.33E-01	5.11E-01	1.00E+00
ENSG00000144031.10_ANKRD53	ENSG00000160789.18_LMNA	0.08959	-0.070619864	0.245290306	2.72E-01	5.57E-01	1.00E+00
ENSG00000241269.1_AC093620.5	ENSG00000160789.18_LMNA	-0.10799	-0.262672348	0.052109122	1.85E-01	4.51E-01	1.00E+00
ENSG00000274818.1_RP1-292L2(	ENSG00000160789.18_LMNA	0.07388	-0.086334687	0.230372546	3.66E-01	6.47E-01	1.00E+00
ENSG00000236810.4_TCEB3-AS1	ENSG00000160789.18_LMNA	-0.10976	-0.264345812	0.050315503	1.78E-01	4.40E-01	1.00E+00
ENSG00000136122.14_BORA	ENSG00000160789.18_LMNA	-0.04301	-0.20083686	0.11699041	5.99E-01	8.15E-01	1.00E+00
ENSG00000188177.12_ZC3H6	ENSG00000160789.18_LMNA	-0.13965	-0.292353161	0.019992929	8.62E-02	2.85E-01	1.00E+00

ENSG00000089775.10_ZBTB25	ENSG00000160789.18_LMNA	-0.01115	-0.170046088	0.148316754	8.92E-01	9.57E-01	1.00E+00
ENSG00000196284.12_SUPT3H	ENSG00000160789.18_LMNA	-0.23001	-0.375463166	-0.07350194	4.36E-03	3.54E-02	1.00E+00
ENSG00000143171.11_RXRG	ENSG00000160789.18_LMNA	-0.52405	-0.630636852	-0.39805854	4.25E-12	1.77E-09	2.18E-07
ENSG00000165511.6_C10orf25	ENSG00000160789.18_LMNA	-0.32695	-0.462100782	-0.1769634	3.94E-05	8.89E-04	1.00E+00
ENSG00000196502.10_SULT1A1	ENSG00000160789.18_LMNA	-0.16977	-0.32031435	-0.01086392	3.65E-02	1.63E-01	1.00E+00
ENSG00000136531.12_SCN2A	ENSG00000160789.18_LMNA	-0.31391	-0.450592585	-0.16284768	8.21E-05	1.60E-03	1.00E+00
ENSG00000170919.14_TPT1-AS1	ENSG00000160789.18_LMNA	-0.22773	-0.373394619	-0.07110868	4.78E-03	3.79E-02	1.00E+00
ENSG00000118160.12_SLC8A2	ENSG00000160789.18_LMNA	-0.22558	-0.371441721	-0.06885241	5.20E-03	4.04E-02	1.00E+00
ENSG00000261485.1_PAN3-AS1	ENSG00000160789.18_LMNA	-0.23787	-0.382578736	-0.08176113	3.17E-03	2.78E-02	1.00E+00
ENSG00000188368.8_PRR19	ENSG00000160789.18_LMNA	0.00655	-0.152811636	0.16557646	9.36E-01	9.75E-01	1.00E+00
ENSG00000141505.10_ASGR1	ENSG00000160789.18_LMNA	0.01388	-0.145641647	0.172699962	8.65E-01	9.47E-01	1.00E+00
ENSG00000186648.13_CARMIL3	ENSG00000160789.18_LMNA	-0.24033	-0.38480871	-0.08435804	2.86E-03	2.58E-02	1.00E+00
ENSG00000066933.14_MYO9A	ENSG00000160789.18_LMNA	0.05451	-0.105602902	0.211876187	5.05E-01	7.56E-01	1.00E+00
ENSG00000268836.1_LA16c-OS1	ENSG00000160789.18_LMNA	-0.11321	-0.267583235	0.046839862	1.65E-01	4.21E-01	1.00E+00
ENSG00000133302.11_SLF1	ENSG00000160789.18_LMNA	0.07206	-0.088153319	0.228636491	3.78E-01	6.57E-01	1.00E+00
ENSG00000164099.3_PRSS12	ENSG00000160789.18_LMNA	-0.2681	-0.409807674	-0.11375256	8.40E-04	1.01E-02	1.00E+00
ENSG00000259495.2_RP11-210M	ENSG00000160789.18_LMNA	-0.05757	-0.214801519	0.102571283	4.81E-01	7.39E-01	1.00E+00
ENSG00000227398.3_KIF9-AS1	ENSG00000160789.18_LMNA	-0.03357	-0.191744664	0.126307031	6.81E-01	8.61E-01	1.00E+00
ENSG00000225912.1_RP13-258C	ENSG00000160789.18_LMNA	-0.08858	-0.244338821	0.071626891	2.78E-01	5.63E-01	1.00E+00
ENSG00000196739.13_COL27A1	ENSG00000160789.18_LMNA	0.33554	0.186292569	0.469654282	2.38E-05	5.95E-04	1.00E+00
ENSG00000228665.2_RP11-20O2	ENSG00000160789.18_LMNA	-0.07999	-0.236183551	0.080231879	3.27E-01	6.14E-01	1.00E+00
ENSG00000021826.13_CPS1	ENSG00000160789.18_LMNA	-0.23404	-0.379112165	-0.07773224	3.71E-03	3.13E-02	1.00E+00
ENSG00000224993.3_RPL29P12	ENSG00000160789.18_LMNA	-0.13806	-0.290869003	0.021614324	8.98E-02	2.92E-01	1.00E+00
ENSG00000273723.1_RP11-93H2	ENSG00000160789.18_LMNA	-0.27858	-0.419190643	-0.12492075	5.10E-04	6.85E-03	1.00E+00
ENSG00000166046.9_TCP11L2	ENSG00000160789.18_LMNA	-0.22606	-0.37187913	-0.0693575	5.10E-03	3.99E-02	1.00E+00
ENSG00000196632.9_WNK3	ENSG00000160789.18_LMNA	-0.20788	-0.355324504	-0.05034893	1.02E-02	6.64E-02	1.00E+00
ENSG00000178093.13_TSSK6	ENSG00000160789.18_LMNA	0.02566	-0.134088883	0.184108191	7.54E-01	8.98E-01	1.00E+00
ENSG00000267319.1_CTD-2528L	ENSG00000160789.18_LMNA	-0.20188	-0.349839069	-0.04409874	1.26E-02	7.75E-02	1.00E+00
ENSG00000170153.9_RNF150	ENSG00000160789.18_LMNA	-0.29142	-0.43064452	-0.1386557	2.70E-04	4.14E-03	1.00E+00
ENSG00000272338.2_RP11-722E	ENSG00000160789.18_LMNA	-0.21528	-0.362072889	-0.05807106	7.73E-03	5.42E-02	1.00E+00
ENSG00000217442.3_SYCE3	ENSG00000160789.18_LMNA	0.15811	-0.001122104	0.309516784	5.17E-02	2.06E-01	1.00E+00
ENSG00000178935.5_ZNF552	ENSG00000160789.18_LMNA	-0.08022	-0.236402173	0.080001814	3.26E-01	6.12E-01	1.00E+00
ENSG00000261342.1_AC006538.:	ENSG00000160789.18_LMNA	0.12408	-0.035830028	0.277791815	1.28E-01	3.62E-01	1.00E+00

ENSG00000167011.7_NAT16	ENSG00000160789.18_LMNA	-0.17856	-0.328423646	-0.01992464	2.77E-02	1.36E-01	1.00E+00
ENSG00000152527.12_PLEKHH2	ENSG00000160789.18_LMNA	0.17652	0.01782425	0.32654785	2.96E-02	1.42E-01	1.00E+00
ENSG00000278864.1_RP11-45M2	ENSG00000160789.18_LMNA	-0.01262	-0.171473331	0.14687867	8.77E-01	9.52E-01	1.00E+00
ENSG00000219607.3_PPP1R3G	ENSG00000160789.18_LMNA	0.26898	0.114693232	0.410600404	8.06E-04	9.79E-03	1.00E+00
ENSG00000122484.8_RPAP2	ENSG00000160789.18_LMNA	-0.16258	-0.313663899	-0.00347094	4.54E-02	1.89E-01	1.00E+00
ENSG00000185519.8_FAM131C	ENSG00000160789.18_LMNA	-0.39528	-0.52164966	-0.25192805	4.66E-07	2.48E-05	2.39E-02
ENSG00000262251.1_RP11-199F	ENSG00000160789.18_LMNA	-0.0821	-0.23818877	0.078120446	3.15E-01	6.01E-01	1.00E+00
ENSG00000170629.13_DPY19L2F	ENSG00000160789.18_LMNA	0.12387	-0.036036814	0.277600728	1.28E-01	3.63E-01	1.00E+00
ENSG00000172995.15_ARPP21	ENSG00000160789.18_LMNA	-0.37784	-0.506566435	-0.23262901	1.60E-06	6.71E-05	8.18E-02
ENSG00000177599.11_ZNF491	ENSG00000160789.18_LMNA	-0.33858	-0.472321759	-0.18959937	1.99E-05	5.14E-04	1.00E+00
ENSG00000262333.1_HNRNPA1F	ENSG00000160789.18_LMNA	0.00221	-0.157044771	0.161355116	9.78E-01	9.92E-01	1.00E+00
ENSG00000104059.4_FAM189A1	ENSG00000160789.18_LMNA	-0.03558	-0.193685029	0.124323469	6.63E-01	8.51E-01	1.00E+00
ENSG00000156219.15_ART3	ENSG00000160789.18_LMNA	0.00895	-0.150465206	0.16791134	9.13E-01	9.67E-01	1.00E+00
ENSG00000100523.13_DDHD1	ENSG00000160789.18_LMNA	-0.07525	-0.231677435	0.084966338	3.57E-01	6.40E-01	1.00E+00
ENSG00000198400.10_NTRK1	ENSG00000160789.18_LMNA	0.11531	-0.044715694	0.269558326	1.57E-01	4.09E-01	1.00E+00
ENSG00000225892.3_	ENSG00000160789.18_LMNA	0.17741	0.018739326	0.327365378	2.88E-02	1.39E-01	1.00E+00
ENSG00000064687.11_ABCA7	ENSG00000160789.18_LMNA	0.20205	0.044272015	0.349991428	1.26E-02	7.71E-02	1.00E+00
ENSG00000272994.1_RP11-332H	ENSG00000160789.18_LMNA	-0.15956	-0.310860468	-0.00036463	4.96E-02	2.00E-01	1.00E+00
ENSG00000254054.2_RP11-156K	ENSG00000160789.18_LMNA	-0.21434	-0.361214372	-0.05708665	8.01E-03	5.57E-02	1.00E+00
ENSG00000106069.19_CHN2	ENSG00000160789.18_LMNA	-0.41569	-0.53920298	-0.27466097	1.01E-07	7.26E-06	5.16E-03
ENSG00000170417.13_TMEM182	ENSG00000160789.18_LMNA	-0.34204	-0.475352347	-0.19336411	1.61E-05	4.32E-04	8.27E-01
ENSG00000213950.2_RPS10P2	ENSG00000160789.18_LMNA	-0.08351	-0.23952953	0.076707084	3.06E-01	5.93E-01	1.00E+00
ENSG00000185220.10_PGBD2	ENSG00000160789.18_LMNA	0.08716	-0.073051187	0.242992042	2.86E-01	5.71E-01	1.00E+00
ENSG00000226945.1_RP11-564A	ENSG00000160789.18_LMNA	-0.02274	-0.181281416	0.136959555	7.81E-01	9.10E-01	1.00E+00
ENSG00000139767.7_SRRM4	ENSG00000160789.18_LMNA	-0.36512	-0.495520106	-0.21863074	3.74E-06	1.31E-04	1.92E-01
ENSG00000073905.8_VDAC1P1	ENSG00000160789.18_LMNA	0.17289	0.014076052	0.32319439	3.32E-02	1.53E-01	1.00E+00
ENSG00000177181.13_RIMKLA	ENSG00000160789.18_LMNA	-0.25146	-0.394854727	-0.09610793	1.78E-03	1.79E-02	1.00E+00
ENSG00000075826.15_SEC31B	ENSG00000160789.18_LMNA	-0.22128	-0.367532971	-0.06434574	6.15E-03	4.58E-02	1.00E+00
ENSG00000228328.2_RP11-516A	ENSG00000160789.18_LMNA	0.15664	-0.002626166	0.308156174	5.40E-02	2.11E-01	1.00E+00
ENSG00000228692.2_RP5-826L7	ENSG00000160789.18_LMNA	-0.15658	-0.3081009	0.002687239	5.41E-02	2.11E-01	1.00E+00
ENSG00000162366.6_PDZK1IP1	ENSG00000160789.18_LMNA	0.03188	-0.12797331	0.190112748	6.97E-01	8.70E-01	1.00E+00
ENSG00000266904.4_CTC-559E9	ENSG00000160789.18_LMNA	-0.10772	-0.262423984	0.052375145	1.87E-01	4.53E-01	1.00E+00
ENSG00000275183.1_LENG9	ENSG00000160789.18_LMNA	0.48149	0.349039698	0.595072247	3.40E-10	6.34E-08	1.74E-05

ENSG00000177873.11_ZNF619	ENSG00000160789.18_LMNA	-0.0242	-0.182697014	0.135522642	7.67E-01	9.04E-01	1.00E+00
ENSG00000163072.13_NOSTRIN	ENSG00000160789.18_LMNA	-0.32499	-0.460370517	-0.17483357	4.41E-05	9.68E-04	1.00E+00
ENSG00000148935.9_GAS2	ENSG00000160789.18_LMNA	-0.04372	-0.201515945	0.116292311	5.93E-01	8.12E-01	1.00E+00
ENSG00000116127.16_ALMS1	ENSG00000160789.18_LMNA	0.12753	-0.032324717	0.281027223	1.17E-01	3.43E-01	1.00E+00
ENSG00000141622.12_RNF165	ENSG00000160789.18_LMNA	-0.29189	-0.431058938	-0.13915477	2.64E-04	4.06E-03	1.00E+00
ENSG00000142449.11_FBN3	ENSG00000160789.18_LMNA	-0.15774	-0.30917334	0.00150189	5.23E-02	2.07E-01	1.00E+00
ENSG00000270021.1_CTC-203F4	ENSG00000160789.18_LMNA	-0.27953	-0.420036558	-0.12593129	4.87E-04	6.60E-03	1.00E+00
ENSG00000183888.4_C1orf64	ENSG00000160789.18_LMNA	-0.28198	-0.422227951	-0.12855199	4.32E-04	6.02E-03	1.00E+00
ENSG00000131730.14_CKMT2	ENSG00000160789.18_LMNA	-0.14149	-0.294071326	0.018113835	8.21E-02	2.76E-01	1.00E+00
ENSG00000218336.6_TENM3	ENSG00000160789.18_LMNA	-0.02781	-0.186187475	0.131973895	7.34E-01	8.88E-01	1.00E+00
ENSG00000129226.12_CD68	ENSG00000160789.18_LMNA	0.1357	-0.024023726	0.288660753	9.55E-02	3.03E-01	1.00E+00
ENSG00000182196.12_ARL6IP4	ENSG00000160789.18_LMNA	0.03856	-0.121388759	0.196551274	6.37E-01	8.38E-01	1.00E+00
ENSG00000142794.17_NBPF3	ENSG00000160789.18_LMNA	0.00374	-0.155556951	0.162840121	9.64E-01	9.85E-01	1.00E+00
ENSG00000022567.8_SLC45A4	ENSG00000160789.18_LMNA	0.05559	-0.104535271	0.212907036	4.96E-01	7.51E-01	1.00E+00
ENSG00000135437.8_RDH5	ENSG00000160789.18_LMNA	0.23879	0.082733473	0.383414099	3.05E-03	2.71E-02	1.00E+00
ENSG00000007129.16_CEACAM2	ENSG00000160789.18_LMNA	-0.04067	-0.198589389	0.119298573	6.19E-01	8.27E-01	1.00E+00
ENSG00000151151.5_IPMK	ENSG00000160789.18_LMNA	-0.20624	-0.353819339	-0.04863154	1.08E-02	6.92E-02	1.00E+00
ENSG00000264920.1_RP11-6N17	ENSG00000160789.18_LMNA	-0.19598	-0.344430348	-0.0379593	1.55E-02	9.00E-02	1.00E+00
ENSG00000131697.16_NPHP4	ENSG00000160789.18_LMNA	0.06547	-0.09472276	0.222348516	4.23E-01	6.94E-01	1.00E+00
ENSG00000230555.2_RP11-517P	ENSG00000160789.18_LMNA	0.03511	-0.124783631	0.19323511	6.68E-01	8.54E-01	1.00E+00
ENSG00000254023.1_PKMP4	ENSG00000160789.18_LMNA	0.3365	0.187338854	0.470498842	2.25E-05	5.68E-04	1.00E+00
ENSG00000136682.13_CBWD2	ENSG00000160789.18_LMNA	0.05276	-0.107347029	0.210190625	5.19E-01	7.65E-01	1.00E+00
ENSG00000142149.7_HUNK	ENSG00000160789.18_LMNA	-0.05076	-0.208281581	0.109320046	5.35E-01	7.76E-01	1.00E+00
ENSG00000120820.11_GLT8D2	ENSG00000160789.18_LMNA	0.17189	0.013050338	0.322275343	3.42E-02	1.56E-01	1.00E+00
ENSG00000175066.14_GK5	ENSG00000160789.18_LMNA	-0.08511	-0.24104389	0.075109191	2.97E-01	5.83E-01	1.00E+00
ENSG00000139174.9_PRICKLE1	ENSG00000160789.18_LMNA	-0.14161	-0.294174261	0.018001189	8.18E-02	2.76E-01	1.00E+00
ENSG00000104375.14_STK3	ENSG00000160789.18_LMNA	0.10678	-0.053322636	0.261539052	1.90E-01	4.58E-01	1.00E+00
ENSG00000244073.1_RPS4XP6	ENSG00000160789.18_LMNA	-0.02047	-0.179085348	0.139186042	8.02E-01	9.21E-01	1.00E+00
ENSG00000259146.3_RP1-261D1	ENSG00000160789.18_LMNA	0.23762	0.081503329	0.382357169	3.20E-03	2.80E-02	1.00E+00
ENSG00000279227.1_AC009303.:	ENSG00000160789.18_LMNA	-0.24601	-0.389943235	-0.09035299	2.25E-03	2.14E-02	1.00E+00
ENSG00000236869.1_RP11-944L	ENSG00000160789.18_LMNA	-0.30521	-0.442886948	-0.15346149	1.32E-04	2.35E-03	1.00E+00
ENSG00000178977.3_LINC00324	ENSG00000160789.18_LMNA	-0.1197	-0.273683535	0.040269932	1.42E-01	3.85E-01	1.00E+00
ENSG00000263990.1_CTC-542B2	ENSG00000160789.18_LMNA	-0.10538	-0.260215876	0.054738294	1.96E-01	4.66E-01	1.00E+00

ENSG00000247572.6_CKMT2-AS	ENSG00000160789.18_LMNA	0.05127	-0.108819578	0.208766051	5.30E-01	7.73E-01	1.00E+00
ENSG00000163006.10_CCDC138	ENSG00000160789.18_LMNA	-0.34095	-0.474402398	-0.19218314	1.72E-05	4.56E-04	8.83E-01
ENSG00000224849.2_	ENSG00000160789.18_LMNA	-0.20286	-0.350732589	-0.0451152	1.22E-02	7.56E-02	1.00E+00
ENSG00000143458.10_GABPB2	ENSG00000160789.18_LMNA	0.09423	-0.065957846	0.249687276	2.48E-01	5.28E-01	1.00E+00
ENSG00000196557.9_CACNA1H	ENSG00000160789.18_LMNA	0.08006	-0.080164274	0.236247796	3.27E-01	6.13E-01	1.00E+00
ENSG00000196693.13_ZNF33B	ENSG00000160789.18_LMNA	-0.20111	-0.34912891	-0.04329132	1.30E-02	7.90E-02	1.00E+00
ENSG00000254966.1_RP11-1081	ENSG00000160789.18_LMNA	-0.20674	-0.354278641	-0.04915541	1.06E-02	6.83E-02	1.00E+00
ENSG00000267221.1_CTD-2132N	ENSG00000160789.18_LMNA	-0.15456	-0.306226143	0.004757277	5.73E-02	2.19E-01	1.00E+00
ENSG00000167394.11_ZNF668	ENSG00000160789.18_LMNA	0.1567	-0.002560201	0.308215875	5.39E-02	2.11E-01	1.00E+00
ENSG00000205181.5_LINC00654	ENSG00000160789.18_LMNA	-0.07178	-0.22836647	0.088435995	3.80E-01	6.58E-01	1.00E+00
ENSG00000151718.14_WWC2	ENSG00000160789.18_LMNA	0.10667	-0.053437803	0.261431453	1.91E-01	4.59E-01	1.00E+00
ENSG00000235565.1_	ENSG00000160789.18_LMNA	0.03823	-0.121710676	0.196237133	6.40E-01	8.39E-01	1.00E+00
ENSG00000087299.10_L2HGDH	ENSG00000160789.18_LMNA	-0.04882	-0.206417863	0.111243808	5.50E-01	7.87E-01	1.00E+00
ENSG00000138131.3_LOXL4	ENSG00000160789.18_LMNA	0.3924	0.248737737	0.519167841	5.74E-07	2.93E-05	2.94E-02
ENSG00000156711.15_MAPK13	ENSG00000160789.18_LMNA	0.17316	0.014354643	0.323443909	3.29E-02	1.52E-01	1.00E+00
ENSG00000258943.1_RP11-696D	ENSG00000160789.18_LMNA	0.20783	0.050295832	0.355277989	1.02E-02	6.64E-02	1.00E+00
ENSG00000132570.13_PCBD2	ENSG00000160789.18_LMNA	0.03554	-0.124366144	0.19364331	6.64E-01	8.52E-01	1.00E+00
ENSG00000111664.9_GNB3	ENSG00000160789.18_LMNA	-0.05939	-0.216543916	0.100762773	4.67E-01	7.29E-01	1.00E+00
ENSG00000099139.12_PCSK5	ENSG00000160789.18_LMNA	0.46989	0.335812772	0.585307748	1.01E-09	1.59E-07	5.19E-05
ENSG00000069667.14_RORA	ENSG00000160789.18_LMNA	0.17609	0.017374333	0.326145726	3.00E-02	1.43E-01	1.00E+00
ENSG00000112877.7_CEP72	ENSG00000160789.18_LMNA	-0.0071	-0.16611177	0.152273995	9.31E-01	9.73E-01	1.00E+00
ENSG00000137101.11_CD72	ENSG00000160789.18_LMNA	0.11834	-0.041645366	0.272408507	1.46E-01	3.92E-01	1.00E+00
ENSG00000177791.11_MYOZ1	ENSG00000160789.18_LMNA	-0.39251	-0.519265596	-0.24886329	5.69E-07	2.91E-05	2.92E-02
ENSG00000148814.16_LRRC27	ENSG00000160789.18_LMNA	-0.16874	-0.319357892	-0.00979859	3.77E-02	1.66E-01	1.00E+00
ENSG00000198838.10_RYR3	ENSG00000160789.18_LMNA	0.20261	0.044853544	0.350502634	1.23E-02	7.61E-02	1.00E+00
ENSG00000170545.15_SMAGP	ENSG00000160789.18_LMNA	0.30969	0.158293317	0.446859006	1.03E-04	1.94E-03	1.00E+00
ENSG00000179058.6_C9orf50	ENSG00000160789.18_LMNA	0.12231	-0.037621492	0.276135538	1.33E-01	3.71E-01	1.00E+00
ENSG00000235387.1_LINC00961	ENSG00000160789.18_LMNA	-0.07972	-0.235922724	0.080506313	3.29E-01	6.15E-01	1.00E+00
ENSG00000279789.1_CTD-2574D	ENSG00000160789.18_LMNA	-0.12086	-0.274777136	0.039089264	1.38E-01	3.79E-01	1.00E+00
ENSG00000198844.9_ARHGEF15	ENSG00000160789.18_LMNA	0.05654	-0.103592409	0.213816828	4.89E-01	7.45E-01	1.00E+00
ENSG00000106952.6_TNFSF8	ENSG00000160789.18_LMNA	-0.0698	-0.226479696	0.090409768	3.93E-01	6.71E-01	1.00E+00
ENSG00000187796.12_CARD9	ENSG00000160789.18_LMNA	0.23243	0.076040184	0.377653745	3.96E-03	3.29E-02	1.00E+00
ENSG00000185420.17_SMYD3	ENSG00000160789.18_LMNA	-0.20017	-0.348268237	-0.0423133	1.34E-02	8.09E-02	1.00E+00

ENSG00000269343.5_ZNF587B	ENSG00000160789.18_LMNA	0.15451	-0.004807563	0.30618057	5.73E-02	2.19E-01	1.00E+00
ENSG00000196440.10_ARMCX4	ENSG00000160789.18_LMNA	-0.12674	-0.280282729	0.033132001	1.20E-01	3.48E-01	1.00E+00
ENSG00000261269.1_RP11-389C	ENSG00000160789.18_LMNA	-0.12334	-0.277098863	0.036579784	1.30E-01	3.66E-01	1.00E+00
ENSG00000186994.10_KANK3	ENSG00000160789.18_LMNA	-0.11679	-0.270956581	0.043210179	1.52E-01	4.01E-01	1.00E+00
ENSG00000275202.1_RP11-156K	ENSG00000160789.18_LMNA	0.16392	0.004841528	0.314899132	4.36E-02	1.84E-01	1.00E+00
ENSG00000133460.18_SLC2A11	ENSG00000160789.18_LMNA	-0.12115	-0.275043146	0.038801943	1.37E-01	3.77E-01	1.00E+00
ENSG00000231025.1_	ENSG00000160789.18_LMNA	0.02342	-0.136292409	0.181938829	7.75E-01	9.07E-01	1.00E+00
ENSG00000165983.13_PTER	ENSG00000160789.18_LMNA	-0.09809	-0.253337616	0.062076966	2.29E-01	5.06E-01	1.00E+00
ENSG00000186827.9_TNFRSF4	ENSG00000160789.18_LMNA	0.06595	-0.094242766	0.22280884	4.20E-01	6.91E-01	1.00E+00
ENSG00000184599.12_FAM19A3	ENSG00000160789.18_LMNA	0.1691	0.010176903	0.319697617	3.73E-02	1.65E-01	1.00E+00
ENSG00000196542.7_SPTSSB	ENSG00000160789.18_LMNA	0.23516	0.078918181	0.380133461	3.54E-03	3.03E-02	1.00E+00
ENSG00000107954.9_NEURL1	ENSG00000160789.18_LMNA	-0.2474	-0.391191104	-0.09181327	2.12E-03	2.04E-02	1.00E+00
ENSG00000178177.13_LCORL	ENSG00000160789.18_LMNA	-0.29075	-0.430046616	-0.13793593	2.79E-04	4.25E-03	1.00E+00
ENSG00000136867.9_SLC31A2	ENSG00000160789.18_LMNA	-0.16525	-0.31613766	-0.00621694	4.19E-02	1.79E-01	1.00E+00
ENSG00000260118.1_RP6-201G1	ENSG00000160789.18_LMNA	-0.47558	-0.590103345	-0.34229658	5.95E-10	1.02E-07	3.05E-05
ENSG00000049883.13_PTCD2	ENSG00000160789.18_LMNA	-0.04586	-0.203570812	0.114177993	5.75E-01	8.02E-01	1.00E+00
ENSG00000226751.2_AF127936.5	ENSG00000160789.18_LMNA	-0.31399	-0.450664526	-0.16293556	8.17E-05	1.60E-03	1.00E+00
ENSG00000243970.1_PPIEL	ENSG00000160789.18_LMNA	0.03417	-0.125719287	0.192319868	6.76E-01	8.58E-01	1.00E+00
ENSG00000234936.1_AC010883.5	ENSG00000160789.18_LMNA	-0.0329	-0.191100058	0.126965425	6.87E-01	8.65E-01	1.00E+00
ENSG00000162004.15_CCDC78	ENSG00000160789.18_LMNA	0.02392	-0.135800124	0.18242375	7.70E-01	9.05E-01	1.00E+00
ENSG00000123360.10_PDE1B	ENSG00000160789.18_LMNA	-0.19858	-0.346818912	-0.04066771	1.42E-02	8.45E-02	1.00E+00
ENSG00000273355.1_RP11-672L	ENSG00000160789.18_LMNA	-0.11663	-0.270806535	0.043371803	1.52E-01	4.02E-01	1.00E+00
ENSG00000205744.8_DENND1C	ENSG00000160789.18_LMNA	0.05896	-0.101194724	0.21612794	4.71E-01	7.31E-01	1.00E+00
ENSG00000250251.5_PKD1P6	ENSG00000160789.18_LMNA	0.13176	-0.028026298	0.284984973	1.06E-01	3.23E-01	1.00E+00
ENSG00000179855.5_GIPC3	ENSG00000160789.18_LMNA	0.11699	-0.043015615	0.271137187	1.51E-01	4.00E-01	1.00E+00
ENSG00000171540.7_OTP	ENSG00000160789.18_LMNA	-0.14205	-0.29458767	0.017548698	8.09E-02	2.74E-01	1.00E+00
ENSG00000234771.3_SLC25A25-	ENSG00000160789.18_LMNA	-0.04574	-0.203457401	0.114294761	5.76E-01	8.02E-01	1.00E+00
ENSG00000242660.1_RP11-1094	ENSG00000160789.18_LMNA	-0.10442	-0.259314057	0.055702422	2.00E-01	4.71E-01	1.00E+00
ENSG00000153558.12_FBXL2	ENSG00000160789.18_LMNA	-0.35811	-0.489410812	-0.21093747	5.90E-06	1.90E-04	3.03E-01
ENSG00000006611.14_USH1C	ENSG00000160789.18_LMNA	-0.53131	-0.636662071	-0.40649681	1.89E-12	9.18E-10	9.70E-08
ENSG00000254221.2_PCDHGB1	ENSG00000160789.18_LMNA	-0.0353	-0.193413486	0.124601211	6.66E-01	8.53E-01	1.00E+00
ENSG00000108700.4_CCL8	ENSG00000160789.18_LMNA	0.09877	-0.061397571	0.25397573	2.26E-01	5.02E-01	1.00E+00
ENSG00000122477.11_LRRC39	ENSG00000160789.18_LMNA	-0.26714	-0.40894687	-0.11273171	8.78E-04	1.05E-02	1.00E+00

ENSG00000136059.13_VILL	ENSG00000160789.18_LMNA	-0.13563	-0.28859812	0.024092011	9.57E-02	3.03E-01	1.00E+00
ENSG00000104814.11_MAP4K1	ENSG00000160789.18_LMNA	0.11843	-0.041549262	0.272497631	1.46E-01	3.92E-01	1.00E+00
ENSG00000181656.6_GPR88	ENSG00000160789.18_LMNA	-0.29115	-0.430399108	-0.13836023	2.74E-04	4.18E-03	1.00E+00
ENSG00000262587.2_RP11-266L	ENSG00000160789.18_LMNA	-0.14346	-0.295898942	0.016112616	7.79E-02	2.67E-01	1.00E+00
ENSG00000149573.7_MPZL2	ENSG00000160789.18_LMNA	0.33257	0.183059723	0.46704145	2.84E-05	6.82E-04	1.00E+00
ENSG00000259020.3_RP11-529H	ENSG00000160789.18_LMNA	0.02676	-0.133009273	0.185169932	7.43E-01	8.92E-01	1.00E+00
ENSG00000278600.1_RP11-81A1	ENSG00000160789.18_LMNA	0.00033	-0.158874984	0.159526385	9.97E-01	9.99E-01	1.00E+00
ENSG00000114374.11_USP9Y	ENSG00000160789.18_LMNA	-0.08867	-0.24441754	0.071543601	2.77E-01	5.62E-01	1.00E+00
ENSG00000276182.1_RP11-872J	ENSG00000160789.18_LMNA	-0.38337	-0.511356747	-0.23873494	1.09E-06	4.92E-05	5.58E-02
ENSG00000272462.2_U91328.19	ENSG00000160789.18_LMNA	-0.16852	-0.319158102	-0.00957614	3.80E-02	1.67E-01	1.00E+00
ENSG00000269609.4_RPARP-AS	ENSG00000160789.18_LMNA	-0.32744	-0.46252705	-0.17748851	3.83E-05	8.68E-04	1.00E+00
ENSG00000140471.15_LINS1	ENSG00000160789.18_LMNA	0.03534	-0.124558242	0.193455499	6.66E-01	8.53E-01	1.00E+00
ENSG00000225938.1_RP4-575N6	ENSG00000160789.18_LMNA	-0.2406	-0.385052164	-0.0846418	2.83E-03	2.56E-02	1.00E+00
ENSG00000271581.1_XXbac-BPG	ENSG00000160789.18_LMNA	0.29607	0.143634721	0.434773537	2.13E-04	3.42E-03	1.00E+00
ENSG00000272368.2_RP4-605O3	ENSG00000160789.18_LMNA	-0.35298	-0.484927943	-0.20531418	8.18E-06	2.47E-04	4.20E-01
ENSG00000100288.18_CHKB	ENSG00000160789.18_LMNA	-0.1187	-0.272743498	0.04128411	1.45E-01	3.90E-01	1.00E+00
ENSG00000273183.1_RP11-5C23	ENSG00000160789.18_LMNA	0.29224	0.139528837	0.431369482	2.59E-04	4.01E-03	1.00E+00
ENSG00000196267.11_ZNF836	ENSG00000160789.18_LMNA	-0.02652	-0.184940075	0.133243062	7.46E-01	8.94E-01	1.00E+00
ENSG00000260083.1_MIR762HG	ENSG00000160789.18_LMNA	-0.20268	-0.350570768	-0.04493107	1.23E-02	7.60E-02	1.00E+00
ENSG00000224660.1_SH3BP5-AS	ENSG00000160789.18_LMNA	-0.0468	-0.204474041	0.113247721	5.67E-01	7.97E-01	1.00E+00
ENSG00000258789.1_RP11-507K	ENSG00000160789.18_LMNA	-0.34646	-0.47923236	-0.19819619	1.23E-05	3.46E-04	6.30E-01
ENSG00000146243.12_IRAK1BP1	ENSG00000160789.18_LMNA	-0.32864	-0.463583387	-0.1787905	3.57E-05	8.23E-04	1.00E+00
ENSG00000141664.8_ZCCHC2	ENSG00000160789.18_LMNA	0.07402	-0.08619281	0.230507896	3.65E-01	6.46E-01	1.00E+00
ENSG00000230734.1_RPL10P3	ENSG00000160789.18_LMNA	-0.02834	-0.186696698	0.131455487	7.29E-01	8.85E-01	1.00E+00
ENSG00000272842.1_RP11-513M	ENSG00000160789.18_LMNA	-0.32197	-0.457709708	-0.17156352	5.23E-05	1.12E-03	1.00E+00
ENSG00000177301.12_KCNA2	ENSG00000160789.18_LMNA	-0.24677	-0.390623369	-0.09114874	2.18E-03	2.09E-02	1.00E+00
ENSG00000168702.15_LRP1B	ENSG00000160789.18_LMNA	-0.04397	-0.201755976	0.116045484	5.91E-01	8.10E-01	1.00E+00
ENSG00000112280.14_COL9A1	ENSG00000160789.18_LMNA	-0.15188	-0.303736133	0.007502539	6.18E-02	2.30E-01	1.00E+00
ENSG00000272447.1_RP11-182L	ENSG00000160789.18_LMNA	-0.19192	-0.34071422	-0.03375449	1.78E-02	9.94E-02	1.00E+00
ENSG00000275055.1_CTC-471J1	ENSG00000160789.18_LMNA	-0.17736	-0.327319991	-0.01868851	2.88E-02	1.39E-01	1.00E+00
ENSG00000267194.1_RP1-193H1	ENSG00000160789.18_LMNA	-0.40866	-0.533174096	-0.26681961	1.72E-07	1.12E-05	8.85E-03
ENSG00000165995.17_CACNB2	ENSG00000160789.18_LMNA	-0.09898	-0.254176366	0.061183895	2.25E-01	5.01E-01	1.00E+00
ENSG00000232093.1_RP11-307C	ENSG00000160789.18_LMNA	0.20535	0.047712959	0.353013621	1.11E-02	7.08E-02	1.00E+00

ENSG00000213309.3_RPL9P18	ENSG00000160789.18_LMNA	-0.18845	-0.337524328	-0.03015376	2.01E-02	1.08E-01	1.00E+00
ENSG00000260966.1_RP11-690D	ENSG00000160789.18_LMNA	-0.35303	-0.484971199	-0.20536835	8.16E-06	2.47E-04	4.19E-01
ENSG00000228360.1_RP11-365F	ENSG00000160789.18_LMNA	-0.02401	-0.182517594	0.135704838	7.69E-01	9.04E-01	1.00E+00
ENSG00000257337.5_RP11-983P	ENSG00000160789.18_LMNA	-0.19516	-0.34368103	-0.03711057	1.60E-02	9.18E-02	1.00E+00
ENSG00000242282.5_AC108488.4	ENSG00000160789.18_LMNA	-0.07021	-0.226879139	0.089992114	3.90E-01	6.68E-01	1.00E+00
ENSG00000130751.8_NPAS1	ENSG00000160789.18_LMNA	0.17209	0.013254611	0.32245842	3.40E-02	1.56E-01	1.00E+00
ENSG00000206567.8_AC022007.5	ENSG00000160789.18_LMNA	0.04359	-0.11641911	0.201392623	5.94E-01	8.13E-01	1.00E+00
ENSG00000147804.8_SLC39A4	ENSG00000160789.18_LMNA	0.14855	-0.010908459	0.300640974	6.78E-02	2.44E-01	1.00E+00
ENSG00000240758.2_RP11-155G	ENSG00000160789.18_LMNA	0.18596	0.027570575	0.335231521	2.18E-02	1.15E-01	1.00E+00
ENSG00000115112.7_TFCP2L1	ENSG00000160789.18_LMNA	0.23884	0.082786468	0.383459615	3.04E-03	2.70E-02	1.00E+00
ENSG00000241644.2_INMT	ENSG00000160789.18_LMNA	-0.01807	-0.176762133	0.14153794	8.25E-01	9.31E-01	1.00E+00
ENSG00000184465.14_WDR27	ENSG00000160789.18_LMNA	-0.0537	-0.211096765	0.106409648	5.11E-01	7.60E-01	1.00E+00
ENSG00000272986.1_RP11-46J23	ENSG00000160789.18_LMNA	-0.10932	-0.263929347	0.050762059	1.80E-01	4.43E-01	1.00E+00
ENSG00000125246.14_CLYBL	ENSG00000160789.18_LMNA	-0.04806	-0.205684686	0.111999954	5.57E-01	7.90E-01	1.00E+00
ENSG00000212829.8_RPS26P3	ENSG00000160789.18_LMNA	-0.14686	-0.299070921	0.012633389	7.10E-02	2.52E-01	1.00E+00
ENSG00000270010.1_	ENSG00000160789.18_LMNA	-0.23328	-0.378428494	-0.07693883	3.82E-03	3.21E-02	1.00E+00
ENSG00000236540.6_AC006547.1	ENSG00000160789.18_LMNA	-0.01968	-0.178323484	0.139957708	8.10E-01	9.25E-01	1.00E+00
ENSG00000100368.12_CSF2RB	ENSG00000160789.18_LMNA	0.08975	-0.070458254	0.245442946	2.72E-01	5.56E-01	1.00E+00
ENSG00000164867.9_NOS3	ENSG00000160789.18_LMNA	0.17836	0.019714072	0.328235705	2.79E-02	1.36E-01	1.00E+00
ENSG00000271452.1_RP11-342K	ENSG00000160789.18_LMNA	-0.04529	-0.203028385	0.114736393	5.80E-01	8.04E-01	1.00E+00
ENSG00000138162.16_TACC2	ENSG00000160789.18_LMNA	-0.11174	-0.266207993	0.048317228	1.71E-01	4.29E-01	1.00E+00
ENSG00000236814.1_RP11-446E	ENSG00000160789.18_LMNA	-0.17671	-0.326716478	-0.01801296	2.94E-02	1.41E-01	1.00E+00
ENSG00000153012.10_LGI2	ENSG00000160789.18_LMNA	-0.07286	-0.229395068	0.08735892	3.72E-01	6.53E-01	1.00E+00
ENSG00000119943.9_PYROXD2	ENSG00000160789.18_LMNA	0.16616	0.007143417	0.316971332	4.08E-02	1.76E-01	1.00E+00
ENSG00000172461.9_FUT9	ENSG00000160789.18_LMNA	-0.39107	-0.518019419	-0.24726346	6.31E-07	3.16E-05	3.24E-02
ENSG00000156049.6_GNA14	ENSG00000160789.18_LMNA	0.15173	-0.007656486	0.303596374	6.20E-02	2.31E-01	1.00E+00
ENSG00000154783.9_FGD5	ENSG00000160789.18_LMNA	0.14355	-0.016020472	0.295983038	7.77E-02	2.67E-01	1.00E+00
ENSG00000180376.15_CCDC66	ENSG00000160789.18_LMNA	-0.22772	-0.373388498	-0.0711016	4.78E-03	3.79E-02	1.00E+00
ENSG00000237424.1_FOXD2-AS1	ENSG00000160789.18_LMNA	0.28154	0.128083662	0.421836591	4.42E-04	6.12E-03	1.00E+00
ENSG00000196381.9_ZNF781	ENSG00000160789.18_LMNA	-0.19985	-0.34797601	-0.04198137	1.36E-02	8.16E-02	1.00E+00
ENSG00000263400.5_TMEM220-A	ENSG00000160789.18_LMNA	0.09761	-0.062560893	0.252882926	2.32E-01	5.09E-01	1.00E+00
ENSG00000261663.1_RP11-304L	ENSG00000160789.18_LMNA	-0.08272	-0.238772528	0.077505234	3.11E-01	5.97E-01	1.00E+00
ENSG00000022355.13_GABRA1	ENSG00000160789.18_LMNA	-0.21771	-0.364287641	-0.06061334	7.05E-03	5.07E-02	1.00E+00

ENSG00000092853.12_CLSPN	ENSG00000160789.18_LMNA	0.19104	0.032843017	0.339907415	1.84E-02	1.02E-01	1.00E+00
ENSG00000203288.3_RP11-98D1	ENSG00000160789.18_LMNA	-0.4452	-0.564400785	-0.30781939	9.09E-09	9.92E-07	4.66E-04
ENSG00000078177.12_N4BP2	ENSG00000160789.18_LMNA	0.00355	-0.155740556	0.162656941	9.65E-01	9.86E-01	1.00E+00
ENSG00000151657.10_KIN	ENSG00000160789.18_LMNA	-0.2629	-0.405141252	-0.10822601	1.07E-03	1.22E-02	1.00E+00
ENSG00000085276.16_MECOM	ENSG00000160789.18_LMNA	-0.12969	-0.283043008	0.030136869	1.11E-01	3.33E-01	1.00E+00
ENSG00000169682.16_SPNS1	ENSG00000160789.18_LMNA	0.24771	0.092147206	0.391476318	2.09E-03	2.02E-02	1.00E+00
ENSG00000154620.5_TMSB4Y	ENSG00000160789.18_LMNA	-0.15578	-0.307357462	0.003508434	5.53E-02	2.15E-01	1.00E+00
ENSG00000213293.4_CTD-2666L	ENSG00000160789.18_LMNA	0.09326	-0.066937142	0.248764733	2.53E-01	5.35E-01	1.00E+00
ENSG00000224043.6_CCNT2-AS1	ENSG00000160789.18_LMNA	-0.31987	-0.455857141	-0.16929049	5.89E-05	1.22E-03	1.00E+00
ENSG00000179588.7_ZFPM1	ENSG00000160789.18_LMNA	0.307	0.155398689	0.444480809	1.20E-04	2.19E-03	1.00E+00
ENSG00000188856.6_RPSAP47	ENSG00000160789.18_LMNA	-0.14025	-0.292912616	0.019381316	8.48E-02	2.82E-01	1.00E+00
ENSG00000248079.2_DPH6-AS1	ENSG00000160789.18_LMNA	-0.05992	-0.217051247	0.100235798	4.63E-01	7.26E-01	1.00E+00
ENSG00000171777.14_RASGRP4	ENSG00000160789.18_LMNA	-0.00973	-0.168673292	0.149698708	9.05E-01	9.64E-01	1.00E+00
ENSG00000076826.8_CAMSAP3	ENSG00000160789.18_LMNA	-0.28891	-0.428405766	-0.13596223	3.06E-04	4.58E-03	1.00E+00
ENSG00000176422.12_SPRYD4	ENSG00000160789.18_LMNA	-0.30694	-0.444428079	-0.15533456	1.20E-04	2.19E-03	1.00E+00
ENSG00000241360.1_PDXP	ENSG00000160789.18_LMNA	-0.20118	-0.349200537	-0.04337274	1.29E-02	7.89E-02	1.00E+00
ENSG00000157470.10_FAM81A	ENSG00000160789.18_LMNA	0.01799	-0.14161794	0.176683046	8.26E-01	9.32E-01	1.00E+00
ENSG00000112539.13_C6orf118	ENSG00000160789.18_LMNA	0.08331	-0.076910565	0.239336576	3.08E-01	5.94E-01	1.00E+00
ENSG00000131044.15_TTLL9	ENSG00000160789.18_LMNA	-0.15364	-0.30536963	0.005702121	5.88E-02	2.23E-01	1.00E+00
ENSG00000229133.1_RPS7P4	ENSG00000160789.18_LMNA	-0.13369	-0.286787138	0.026065167	1.01E-01	3.12E-01	1.00E+00
ENSG00000258102.2_MAP1LC3B	ENSG00000160789.18_LMNA	-0.19498	-0.343518225	-0.03692622	1.61E-02	9.22E-02	1.00E+00
ENSG00000269696.1_AC007228.1	ENSG00000160789.18_LMNA	-0.01405	-0.172863044	0.145477108	8.64E-01	9.47E-01	1.00E+00
ENSG00000160318.5_CLDND2	ENSG00000160789.18_LMNA	-0.0657	-0.222571593	0.094490169	4.21E-01	6.93E-01	1.00E+00
ENSG00000232385.2_RPS3AP25	ENSG00000160789.18_LMNA	-0.08031	-0.236483308	0.079916424	3.25E-01	6.12E-01	1.00E+00
ENSG00000135083.13_CCNJL	ENSG00000160789.18_LMNA	0.01208	-0.147401276	0.17095482	8.83E-01	9.55E-01	1.00E+00
ENSG00000186300.10_ZNF555	ENSG00000160789.18_LMNA	-0.21133	-0.358469143	-0.05394279	8.96E-03	6.06E-02	1.00E+00
ENSG00000128881.15_TTBK2	ENSG00000160789.18_LMNA	-0.07174	-0.228335851	0.088468045	3.80E-01	6.59E-01	1.00E+00
ENSG00000224081.6_LINC01057	ENSG00000160789.18_LMNA	0.10333	-0.056809917	0.258277455	2.05E-01	4.77E-01	1.00E+00
ENSG00000226786.2_	ENSG00000160789.18_LMNA	-0.12217	-0.275998318	0.037769821	1.34E-01	3.71E-01	1.00E+00
ENSG00000224281.4_SLC25A5-A	ENSG00000160789.18_LMNA	-0.2888	-0.428312336	-0.13584992	3.08E-04	4.60E-03	1.00E+00
ENSG00000198520.9_C1orf228	ENSG00000160789.18_LMNA	0.04179	-0.118198166	0.199661279	6.09E-01	8.22E-01	1.00E+00
ENSG00000230177.1_RP5-1112D	ENSG00000160789.18_LMNA	0.01891	-0.140710337	0.177580047	8.17E-01	9.29E-01	1.00E+00
ENSG00000224546.2_EIF4BP3	ENSG00000160789.18_LMNA	0.08527	-0.074949903	0.241194767	2.96E-01	5.82E-01	1.00E+00

ENSG00000165171.9_WBSCR27	ENSG00000160789.18_LMNA	0.26758	0.113205549	0.409346486	8.60E-04	1.03E-02	1.00E+00
ENSG00000135218.16_CD36	ENSG00000160789.18_LMNA	-0.00184	-0.160990258	0.157410099	9.82E-01	9.93E-01	1.00E+00
ENSG00000231908.1_IDH1-AS1	ENSG00000160789.18_LMNA	-0.12113	-0.27502712	0.038819254	1.37E-01	3.77E-01	1.00E+00
ENSG00000240231.1_RPS27P29	ENSG00000160789.18_LMNA	-0.09658	-0.251910138	0.063595734	2.37E-01	5.15E-01	1.00E+00
ENSG00000131002.10_TXLNGY	ENSG00000160789.18_LMNA	-0.13475	-0.287781219	0.024982367	9.79E-02	3.08E-01	1.00E+00
ENSG00000171365.14_CLCN5	ENSG00000160789.18_LMNA	0.00861	-0.150795641	0.167582748	9.16E-01	9.68E-01	1.00E+00
ENSG00000087116.12_ADAMTS2	ENSG00000160789.18_LMNA	0.29958	0.147406188	0.437893024	1.77E-04	2.97E-03	1.00E+00
ENSG00000164430.14_MB21D1	ENSG00000160789.18_LMNA	0.23423	0.077935882	0.379287587	3.68E-03	3.12E-02	1.00E+00
ENSG00000130856.14_ZNF236	ENSG00000160789.18_LMNA	0.14556	-0.013959891	0.297862374	7.36E-02	2.58E-01	1.00E+00
ENSG00000166342.17_NETO1	ENSG00000160789.18_LMNA	-0.35375	-0.485601602	-0.20615804	7.80E-06	2.40E-04	4.00E-01
ENSG00000230982.1_DSTNP1	ENSG00000160789.18_LMNA	0.15742	-0.001826021	0.308880162	5.28E-02	2.08E-01	1.00E+00
ENSG00000179954.13_SSC5D	ENSG00000160789.18_LMNA	0.35667	0.209356104	0.488151638	6.47E-06	2.06E-04	3.32E-01
ENSG00000176293.18_ZNF135	ENSG00000160789.18_LMNA	-0.05668	-0.213954789	0.103449384	4.88E-01	7.44E-01	1.00E+00
ENSG00000213918.9_DNASE1	ENSG00000160789.18_LMNA	0.0088	-0.150610519	0.167766846	9.14E-01	9.67E-01	1.00E+00
ENSG00000125726.9_CD70	ENSG00000160789.18_LMNA	0.30539	0.153663259	0.443053041	1.30E-04	2.33E-03	1.00E+00
ENSG00000271855.1_RP11-214N	ENSG00000160789.18_LMNA	-0.3275	-0.462586464	-0.17756171	3.81E-05	8.67E-04	1.00E+00
ENSG00000237515.7_SHISA9	ENSG00000160789.18_LMNA	-0.12103	-0.274931213	0.038922849	1.37E-01	3.78E-01	1.00E+00
ENSG00000239617.1_RP11-302B	ENSG00000160789.18_LMNA	-0.10065	-0.255751456	0.059505428	2.17E-01	4.92E-01	1.00E+00
ENSG00000183060.14_LYSMD4	ENSG00000160789.18_LMNA	0.09485	-0.065338391	0.250270534	2.45E-01	5.25E-01	1.00E+00
ENSG00000157554.17_ERG	ENSG00000160789.18_LMNA	0.02435	-0.135377965	0.182839471	7.66E-01	9.03E-01	1.00E+00
ENSG00000167664.7_TMIGD2	ENSG00000160789.18_LMNA	-0.33992	-0.473494487	-0.1910552	1.83E-05	4.79E-04	9.40E-01
ENSG00000267309.1_CTD-2630F	ENSG00000160789.18_LMNA	-0.0043	-0.163388516	0.155007151	9.58E-01	9.83E-01	1.00E+00
ENSG00000007350.15_TKTL1	ENSG00000160789.18_LMNA	-0.38228	-0.510418818	-0.23753772	1.17E-06	5.22E-05	6.02E-02
ENSG00000272079.2_LA16c-380H	ENSG00000160789.18_LMNA	-0.11708	-0.271227184	0.042918653	1.51E-01	3.99E-01	1.00E+00
ENSG00000229950.1_TFAP2A-AS	ENSG00000160789.18_LMNA	-0.18429	-0.333702299	-0.02584996	2.30E-02	1.19E-01	1.00E+00
ENSG00000231365.4_RP11-418J1	ENSG00000160789.18_LMNA	-0.15955	-0.310852362	-0.00035566	4.96E-02	2.00E-01	1.00E+00
ENSG00000236565.3_HNRNPA3P	ENSG00000160789.18_LMNA	0.20687	0.049291345	0.354397795	1.06E-02	6.81E-02	1.00E+00
ENSG00000196295.10_AC005154	ENSG00000160789.18_LMNA	-0.03351	-0.19168534	0.126367636	6.82E-01	8.62E-01	1.00E+00
ENSG00000243802.2_RP11-390K	ENSG00000160789.18_LMNA	-0.00373	-0.16283331	0.155563777	9.64E-01	9.86E-01	1.00E+00
ENSG00000108187.14_PBLD	ENSG00000160789.18_LMNA	-0.10015	-0.255284109	0.060003635	2.20E-01	4.95E-01	1.00E+00
ENSG00000185015.6_CA13	ENSG00000160789.18_LMNA	0.13061	-0.029197252	0.283907879	1.09E-01	3.28E-01	1.00E+00
ENSG00000267980.1_AC007292.(	ENSG00000160789.18_LMNA	-0.14828	-0.300389298	0.011185086	6.83E-02	2.45E-01	1.00E+00
ENSG00000184451.5_CCR10	ENSG00000160789.18_LMNA	0.18188	0.02335957	0.33148605	2.49E-02	1.26E-01	1.00E+00

ENSG00000104714.12_ERICH1	ENSG00000160789.18_LMNA	0.23836	0.082281366	0.383025744	3.10E-03	2.74E-02	1.00E+00
ENSG00000234420.6_ZNF37BP	ENSG00000160789.18_LMNA	0.02611	-0.133643155	0.184546632	7.49E-01	8.95E-01	1.00E+00
ENSG00000076555.14_ACACB	ENSG00000160789.18_LMNA	0.13862	-0.021040679	0.291394266	8.85E-02	2.90E-01	1.00E+00
ENSG00000235475.1_LINC01372	ENSG00000160789.18_LMNA	-0.01969	-0.178328244	0.139952888	8.10E-01	9.25E-01	1.00E+00
ENSG00000235001.3_EIF4A1P2	ENSG00000160789.18_LMNA	0.08074	-0.079482566	0.236895479	3.23E-01	6.09E-01	1.00E+00
ENSG00000162804.12_SNED1	ENSG00000160789.18_LMNA	0.26224	0.10753159	0.404553808	1.10E-03	1.25E-02	1.00E+00
ENSG00000143067.4_ZNF697	ENSG00000160789.18_LMNA	-0.01221	-0.171080971	0.147274145	8.81E-01	9.54E-01	1.00E+00
ENSG00000270225.1_MTCO2P22	ENSG00000160789.18_LMNA	-0.1324	-0.285579811	0.027379256	1.04E-01	3.19E-01	1.00E+00
ENSG00000136546.12_SCN7A	ENSG00000160789.18_LMNA	0.06752	-0.092672066	0.224314184	4.08E-01	6.83E-01	1.00E+00
ENSG00000239374.1_RP11-407P	ENSG00000160789.18_LMNA	-0.22572	-0.371567049	-0.06899711	5.17E-03	4.03E-02	1.00E+00
ENSG00000006071.10_ABCC8	ENSG00000160789.18_LMNA	-0.24506	-0.389081231	-0.08934502	2.34E-03	2.21E-02	1.00E+00
ENSG00000236972.2_FABP5P1	ENSG00000160789.18_LMNA	0.09062	-0.069583745	0.246268643	2.67E-01	5.51E-01	1.00E+00
ENSG00000196189.11_SEMA4A	ENSG00000160789.18_LMNA	-0.11314	-0.267525071	0.046902373	1.65E-01	4.22E-01	1.00E+00
ENSG00000236901.4_MIR600HG	ENSG00000160789.18_LMNA	-0.06866	-0.225397859	0.091540365	4.01E-01	6.77E-01	1.00E+00
ENSG00000155666.10_KDM8	ENSG00000160789.18_LMNA	-0.19825	-0.346512635	-0.04032017	1.44E-02	8.51E-02	1.00E+00
ENSG00000273026.1_RP11-422P	ENSG00000160789.18_LMNA	0.09626	-0.063921419	0.25160385	2.38E-01	5.17E-01	1.00E+00
ENSG00000163803.11_PLB1	ENSG00000160789.18_LMNA	0.18643	0.028060079	0.335666279	2.15E-02	1.13E-01	1.00E+00
ENSG00000137812.18_KNL1	ENSG00000160789.18_LMNA	0.01898	-0.140641409	0.177648149	8.16E-01	9.28E-01	1.00E+00
ENSG00000258682.1_CTD-2002H	ENSG00000160789.18_LMNA	-0.00614	-0.165178529	0.153211178	9.40E-01	9.76E-01	1.00E+00
ENSG00000159733.12_ZFYVE28	ENSG00000160789.18_LMNA	0.16507	0.006025412	0.315965253	4.21E-02	1.80E-01	1.00E+00
ENSG00000013588.5_GPRC5A	ENSG00000160789.18_LMNA	0.45247	0.316036237	0.570571885	4.85E-09	5.84E-07	2.49E-04
ENSG00000269947.1_RP11-849F	ENSG00000160789.18_LMNA	0.05206	-0.108038831	0.20952153	5.24E-01	7.69E-01	1.00E+00
ENSG00000177058.10_SLC38A9	ENSG00000160789.18_LMNA	-0.32292	-0.458548137	-0.17259324	4.96E-05	1.07E-03	1.00E+00
ENSG00000273148.1_RP5-1068E	ENSG00000160789.18_LMNA	-0.14602	-0.298283579	0.013497701	7.27E-02	2.56E-01	1.00E+00
ENSG00000162367.10_TAL1	ENSG00000160789.18_LMNA	-0.28023	-0.420667179	-0.12668504	4.71E-04	6.43E-03	1.00E+00
ENSG00000246273.5_SBF2-AS1	ENSG00000160789.18_LMNA	0.14948	-0.009957883	0.301505479	6.61E-02	2.40E-01	1.00E+00
ENSG00000244151.1_RP11-148K	ENSG00000160789.18_LMNA	0.23985	0.083851537	0.384374049	2.92E-03	2.62E-02	1.00E+00
ENSG00000079335.16_CDC14A	ENSG00000160789.18_LMNA	-0.22289	-0.36899773	-0.06603311	5.78E-03	4.37E-02	1.00E+00
ENSG00000150967.16_ABCB9	ENSG00000160789.18_LMNA	0.13481	-0.024926601	0.287832398	9.77E-02	3.07E-01	1.00E+00
ENSG00000237004.3_ZNRF2P1	ENSG00000160789.18_LMNA	0.22279	0.065931797	0.368909823	5.80E-03	4.39E-02	1.00E+00
ENSG00000122417.14_ODF2L	ENSG00000160789.18_LMNA	-0.03713	-0.195173651	0.122799983	6.50E-01	8.44E-01	1.00E+00
ENSG00000026559.12_KCNG1	ENSG00000160789.18_LMNA	0.08547	-0.074749555	0.241384514	2.95E-01	5.81E-01	1.00E+00
ENSG00000165338.15_HECTD2	ENSG00000160789.18_LMNA	0.04976	-0.110314215	0.20731873	5.43E-01	7.82E-01	1.00E+00

ENSG00000182841.11_RRP7BP	ENSG00000160789.18_LMNA	-0.14106	-0.293666416	0.018556869	8.30E-02	2.78E-01	1.00E+00
ENSG00000167065.12_DUSP18	ENSG00000160789.18_LMNA	0.09075	-0.06944963	0.246395232	2.66E-01	5.50E-01	1.00E+00
ENSG00000153107.10_ANAPC1	ENSG00000160789.18_LMNA	0.07111	-0.089097938	0.227733969	3.84E-01	6.63E-01	1.00E+00
ENSG00000102466.14_FGF14	ENSG00000160789.18_LMNA	-0.19081	-0.339687887	-0.0325951	1.85E-02	1.02E-01	1.00E+00
ENSG00000233111.1_RAB1C	ENSG00000160789.18_LMNA	0.04279	-0.117205398	0.200627667	6.01E-01	8.16E-01	1.00E+00
ENSG00000267575.5_CTC-459F4	ENSG00000160789.18_LMNA	-0.15199	-0.303842279	0.007385608	6.16E-02	2.29E-01	1.00E+00
ENSG00000196268.10_ZNF493	ENSG00000160789.18_LMNA	-0.35375	-0.485600719	-0.20615693	7.80E-06	2.40E-04	4.00E-01
ENSG00000140836.13_ZFHX3	ENSG00000160789.18_LMNA	0.18033	0.021752093	0.330053717	2.62E-02	1.31E-01	1.00E+00
ENSG00000260572.1_RP11-16N1	ENSG00000160789.18_LMNA	-0.4468	-0.5657607	-0.30962684	7.92E-09	8.92E-07	4.07E-04
ENSG00000165966.13_PDZRN4	ENSG00000160789.18_LMNA	-0.40602	-0.530905432	-0.26387801	2.11E-07	1.32E-05	1.08E-02
ENSG00000079337.14_RAPGEF3	ENSG00000160789.18_LMNA	-0.30549	-0.44313751	-0.15376588	1.30E-04	2.32E-03	1.00E+00
ENSG00000204920.9_ZNF155	ENSG00000160789.18_LMNA	0.11193	-0.04812782	0.26638438	1.70E-01	4.28E-01	1.00E+00
ENSG00000226711.5_FAM66C	ENSG00000160789.18_LMNA	-0.36095	-0.491887632	-0.21405231	4.92E-06	1.64E-04	2.52E-01
ENSG00000236312.3_RPL34P34	ENSG00000160789.18_LMNA	-0.1341	-0.287166437	0.025652103	9.95E-02	3.11E-01	1.00E+00
ENSG00000233337.1_UBE2FP3	ENSG00000160789.18_LMNA	-0.28701	-0.426713671	-0.13392932	3.37E-04	4.91E-03	1.00E+00
ENSG00000249348.1_UGDH-AS1	ENSG00000160789.18_LMNA	-0.14503	-0.297366035	0.014504355	7.46E-02	2.60E-01	1.00E+00
ENSG00000182890.4_GLUD2	ENSG00000160789.18_LMNA	-0.11202	-0.266464814	0.048041441	1.69E-01	4.28E-01	1.00E+00
ENSG00000258741.3_RP11-386M	ENSG00000160789.18_LMNA	0.12888	-0.030956662	0.282288011	1.14E-01	3.37E-01	1.00E+00
ENSG00000256694.1_RP11-598F	ENSG00000160789.18_LMNA	0.05548	-0.104647668	0.212798545	4.97E-01	7.51E-01	1.00E+00
ENSG00000272953.1_RP11-1275	ENSG00000160789.18_LMNA	-0.18381	-0.33326241	-0.02535536	2.34E-02	1.21E-01	1.00E+00
ENSG00000231233.1_CFAP58-AS	ENSG00000160789.18_LMNA	-0.20412	-0.351889123	-0.0464318	1.17E-02	7.31E-02	1.00E+00
ENSG00000178150.7_ZNF114	ENSG00000160789.18_LMNA	0.0847	-0.075522161	0.240652656	3.00E-01	5.86E-01	1.00E+00
ENSG00000257907.2_EEF1A1P1	ENSG00000160789.18_LMNA	-0.10275	-0.257736587	0.057387468	2.08E-01	4.80E-01	1.00E+00
ENSG00000155858.5_LSM11	ENSG00000160789.18_LMNA	-0.06504	-0.221946573	0.095141758	4.26E-01	6.97E-01	1.00E+00
ENSG00000111788.10_RP11-22B	ENSG00000160789.18_LMNA	-0.09894	-0.25413555	0.061227366	2.25E-01	5.01E-01	1.00E+00
ENSG00000169570.8_DTWD2	ENSG00000160789.18_LMNA	-0.2596	-0.402183289	-0.10473231	1.24E-03	1.36E-02	1.00E+00
ENSG00000227347.1_HNRNPKP2	ENSG00000160789.18_LMNA	0.03705	-0.122873999	0.195101363	6.50E-01	8.45E-01	1.00E+00
ENSG00000183092.14_BEGAIN	ENSG00000160789.18_LMNA	0.07664	-0.083579472	0.232998821	3.48E-01	6.32E-01	1.00E+00
ENSG00000188064.8_WNT7B	ENSG00000160789.18_LMNA	-0.04453	-0.202298577	0.115487375	5.86E-01	8.08E-01	1.00E+00
ENSG00000166250.10_CLMP	ENSG00000160789.18_LMNA	0.1399	-0.019743731	0.292581133	8.56E-02	2.84E-01	1.00E+00
ENSG00000278376.1_RP11-158I9	ENSG00000160789.18_LMNA	-0.18476	-0.334130647	-0.02633174	2.27E-02	1.18E-01	1.00E+00
ENSG00000166192.13_SENP8	ENSG00000160789.18_LMNA	-0.31798	-0.454184985	-0.16724143	6.55E-05	1.33E-03	1.00E+00
ENSG00000103064.12_SLC7A6	ENSG00000160789.18_LMNA	-0.06429	-0.221224358	0.095894335	4.31E-01	7.01E-01	1.00E+00

ENSG00000261094.2_RP11-355C	ENSG00000160789.18_LMNA	-0.17499	-0.325130571	-0.01623908	3.11E-02	1.47E-01	1.00E+00
ENSG00000266910.1_CTC-448F2	ENSG00000160789.18_LMNA	-0.0703	-0.226964781	0.089902553	3.89E-01	6.68E-01	1.00E+00
ENSG00000103494.11_RPGRIP1I	ENSG00000160789.18_LMNA	-0.05893	-0.21610663	0.101216849	4.71E-01	7.31E-01	1.00E+00
ENSG00000120549.14_KIAA1217	ENSG00000160789.18_LMNA	0.30149	0.149466676	0.439594362	1.60E-04	2.74E-03	1.00E+00
ENSG00000150672.15_DLG2	ENSG00000160789.18_LMNA	-0.39122	-0.518150432	-0.24743158	6.24E-07	3.14E-05	3.20E-02
ENSG00000187240.12_DYNC2H1	ENSG00000160789.18_LMNA	-0.00138	-0.160546872	0.157853939	9.87E-01	9.95E-01	1.00E+00
ENSG00000237950.1_RP11-7O11	ENSG00000160789.18_LMNA	0.05329	-0.106820629	0.210699548	5.14E-01	7.62E-01	1.00E+00
ENSG00000219951.4_RP11-378G	ENSG00000160789.18_LMNA	0.20748	0.049933244	0.354960329	1.03E-02	6.70E-02	1.00E+00
ENSG00000229659.1_RPL26P6	ENSG00000160789.18_LMNA	-0.18201	-0.331604731	-0.02349284	2.48E-02	1.26E-01	1.00E+00
ENSG00000167286.8_CD3D	ENSG00000160789.18_LMNA	0.03792	-0.122019111	0.195936086	6.43E-01	8.41E-01	1.00E+00
ENSG00000276445.1_LLNLR-268	ENSG00000160789.18_LMNA	-0.05899	-0.216159303	0.101162161	4.70E-01	7.31E-01	1.00E+00
ENSG00000243753.4_HLA-L	ENSG00000160789.18_LMNA	0.20482	0.047154373	0.352523446	1.14E-02	7.18E-02	1.00E+00
ENSG00000198108.3_CHSY3	ENSG00000160789.18_LMNA	0.19051	0.032286671	0.339414731	1.87E-02	1.03E-01	1.00E+00
ENSG00000185442.11_FAM174B	ENSG00000160789.18_LMNA	-0.00234	-0.161482281	0.156917421	9.77E-01	9.91E-01	1.00E+00
ENSG00000234899.8_SOX9-AS1	ENSG00000160789.18_LMNA	-0.0863	-0.242177106	0.073912403	2.90E-01	5.76E-01	1.00E+00
ENSG00000040199.17_PHLPP2	ENSG00000160789.18_LMNA	0.06742	-0.092775608	0.224214997	4.09E-01	6.83E-01	1.00E+00
ENSG00000274536.3_MIR223	ENSG00000160789.18_LMNA	-0.09333	-0.248831182	0.066866627	2.53E-01	5.34E-01	1.00E+00
ENSG00000241431.1_RPL37P6	ENSG00000160789.18_LMNA	-0.02857	-0.186917631	0.131230515	7.27E-01	8.84E-01	1.00E+00
ENSG00000270127.2_RP11-526I2	ENSG00000160789.18_LMNA	-0.0656	-0.222476737	0.094589075	4.22E-01	6.93E-01	1.00E+00
ENSG00000255642.1_PABPC1P4	ENSG00000160789.18_LMNA	-0.02539	-0.183845438	0.134355941	7.56E-01	8.98E-01	1.00E+00
ENSG00000115363.12_EVA1A	ENSG00000160789.18_LMNA	0.03037	-0.129459933	0.188655298	7.10E-01	8.77E-01	1.00E+00
ENSG00000103061.10_SLC7A6OS	ENSG00000160789.18_LMNA	-0.15192	-0.30377772	0.007456728	6.17E-02	2.30E-01	1.00E+00
ENSG00000164330.15_EBF1	ENSG00000160789.18_LMNA	-0.00393	-0.16302635	0.155370267	9.62E-01	9.85E-01	1.00E+00
ENSG00000271270.4_TMCC1-AS	ENSG00000160789.18_LMNA	-0.30586	-0.443471016	-0.15417112	1.27E-04	2.29E-03	1.00E+00
ENSG00000280071.2_CH507-9B2	ENSG00000160789.18_LMNA	0.11502	-0.045001829	0.269292426	1.58E-01	4.11E-01	1.00E+00
ENSG00000165731.16_RET	ENSG00000160789.18_LMNA	-0.05105	-0.208560009	0.109032444	5.32E-01	7.75E-01	1.00E+00
ENSG00000236199.1_RP11-264I1	ENSG00000160789.18_LMNA	0.00583	-0.153517349	0.164873522	9.43E-01	9.77E-01	1.00E+00
ENSG00000140993.9_TIGD7	ENSG00000160789.18_LMNA	-0.07317	-0.229691813	0.087048052	3.70E-01	6.51E-01	1.00E+00
ENSG00000261740.4_RP11-345J4	ENSG00000160789.18_LMNA	-0.16737	-0.318090496	-0.008388	3.93E-02	1.71E-01	1.00E+00
ENSG00000143851.14_PTPN7	ENSG00000160789.18_LMNA	0.24584	0.090170078	0.389786846	2.27E-03	2.15E-02	1.00E+00
ENSG00000161681.14_SHANK1	ENSG00000160789.18_LMNA	-0.17178	-0.322168993	-0.01293169	3.43E-02	1.57E-01	1.00E+00
ENSG00000101331.14_CCM2L	ENSG00000160789.18_LMNA	-0.1634	-0.314418669	-0.00430828	4.43E-02	1.86E-01	1.00E+00
ENSG00000126070.17_37834	ENSG00000160789.18_LMNA	-0.16111	-0.312297488	-0.00195615	4.74E-02	1.94E-01	1.00E+00

ENSG00000154655.13_L3MBTL4	ENSG00000160789.18_LMNA	-0.13795	-0.290762021	0.021731134	9.01E-02	2.92E-01	1.00E+00
ENSG00000186675.6_MAGEE2	ENSG00000160789.18_LMNA	-0.19127	-0.340113394	-0.03307567	1.83E-02	1.01E-01	1.00E+00
ENSG00000167600.12_CYP2S1	ENSG00000160789.18_LMNA	0.21852	0.061458245	0.365022932	6.84E-03	4.95E-02	1.00E+00
ENSG00000112208.11_BAG2	ENSG00000160789.18_LMNA	-0.16539	-0.316265383	-0.00635885	4.17E-02	1.78E-01	1.00E+00
ENSG00000138380.16_CARF	ENSG00000160789.18_LMNA	-0.26555	-0.407518041	-0.11103861	9.45E-04	1.11E-02	1.00E+00
ENSG00000196357.10_ZNF565	ENSG00000160789.18_LMNA	-0.2464	-0.390294794	-0.09076426	2.21E-03	2.11E-02	1.00E+00
ENSG00000115896.14_PLCL1	ENSG00000160789.18_LMNA	-0.44333	-0.562805738	-0.30570179	1.07E-08	1.14E-06	5.47E-04
ENSG00000174327.6_SLC16A13	ENSG00000160789.18_LMNA	0.40036	0.257574957	0.526031366	3.21E-07	1.86E-05	1.65E-02
ENSG00000022976.14_ZNF839	ENSG00000160789.18_LMNA	0.03669	-0.123235311	0.194748435	6.54E-01	8.47E-01	1.00E+00
ENSG00000101935.8_AMMECR1	ENSG00000160789.18_LMNA	0.17862	0.019992537	0.328484244	2.77E-02	1.36E-01	1.00E+00
ENSG00000090447.10_TFAP4	ENSG00000160789.18_LMNA	-0.0938	-0.249274899	0.066395667	2.50E-01	5.31E-01	1.00E+00
ENSG00000096433.9_ITPR3	ENSG00000160789.18_LMNA	0.5368	0.412880059	0.641201343	1.01E-12	5.54E-10	5.20E-08
ENSG00000280374.1_RP11-337N	ENSG00000160789.18_LMNA	-0.26088	-0.40333386	-0.1060904	1.17E-03	1.30E-02	1.00E+00
ENSG00000240776.1_RP11-435F	ENSG00000160789.18_LMNA	-0.11472	-0.269006252	0.045309723	1.59E-01	4.13E-01	1.00E+00
ENSG00000224892.5_RPS4XP16	ENSG00000160789.18_LMNA	-0.04088	-0.198791458	0.119091188	6.17E-01	8.26E-01	1.00E+00
ENSG00000105784.14_RUNDC3E	ENSG00000160789.18_LMNA	-0.39624	-0.522484969	-0.25300313	4.34E-07	2.35E-05	2.23E-02
ENSG00000234546.2_RP3-510D1	ENSG00000160789.18_LMNA	0.29168	0.138935744	0.43087708	2.66E-04	4.09E-03	1.00E+00
ENSG00000203943.7_SAMD13	ENSG00000160789.18_LMNA	-0.25222	-0.395537345	-0.09690936	1.72E-03	1.74E-02	1.00E+00
ENSG00000257043.1_RP11-137N	ENSG00000160789.18_LMNA	-0.2077	-0.355153218	-0.0501534	1.02E-02	6.67E-02	1.00E+00
ENSG00000141294.8_LRRC46	ENSG00000160789.18_LMNA	0.07117	-0.089042978	0.227786494	3.84E-01	6.62E-01	1.00E+00
ENSG00000144214.8_LYG1	ENSG00000160789.18_LMNA	-0.18274	-0.332273417	-0.02424389	2.42E-02	1.24E-01	1.00E+00
ENSG00000255561.5_FDXACB1	ENSG00000160789.18_LMNA	-0.31271	-0.449533823	-0.16155492	8.77E-05	1.70E-03	1.00E+00
ENSG00000133687.14_TMTC1	ENSG00000160789.18_LMNA	0.11301	-0.047036751	0.267400031	1.66E-01	4.22E-01	1.00E+00
ENSG00000225975.5_LINC01534	ENSG00000160789.18_LMNA	-0.17515	-0.325279455	-0.01640553	3.09E-02	1.46E-01	1.00E+00
ENSG00000178568.12_ERBB4	ENSG00000160789.18_LMNA	-0.26497	-0.407003632	-0.11042947	9.70E-04	1.13E-02	1.00E+00
ENSG00000256294.6_ZNF225	ENSG00000160789.18_LMNA	0.00525	-0.154076937	0.164315905	9.49E-01	9.79E-01	1.00E+00
ENSG00000162951.9_LRRTM1	ENSG00000160789.18_LMNA	-0.39418	-0.520706539	-0.250715	5.04E-07	2.64E-05	2.59E-02
ENSG00000168502.16_MTCL1	ENSG00000160789.18_LMNA	0.2566	0.101550184	0.399483708	1.42E-03	1.50E-02	1.00E+00
ENSG00000272512.1_RP11-54O7	ENSG00000160789.18_LMNA	-0.12597	-0.279567639	0.033907017	1.22E-01	3.52E-01	1.00E+00
ENSG00000157021.9_FAM92A1P	ENSG00000160789.18_LMNA	-0.02987	-0.188176609	0.129947892	7.15E-01	8.79E-01	1.00E+00
ENSG00000089091.15_DZANK1	ENSG00000160789.18_LMNA	0.02664	-0.13312607	0.185055104	7.45E-01	8.93E-01	1.00E+00
ENSG00000235459.5_RPS26P31	ENSG00000160789.18_LMNA	-0.13009	-0.283425599	0.029721281	1.10E-01	3.31E-01	1.00E+00
ENSG00000198018.6_ENTPD7	ENSG00000160789.18_LMNA	0.22778	0.07116389	0.373442375	4.77E-03	3.78E-02	1.00E+00

ENSG00000160602.12_NEK8	ENSG00000160789.18_LMNA	0.23979	0.083789984	0.384321217	2.93E-03	2.62E-02	1.00E+00
ENSG00000105146.11_AURKC	ENSG00000160789.18_LMNA	-0.06009	-0.217215732	0.100064906	4.62E-01	7.25E-01	1.00E+00
ENSG00000228158.2_TLE1P1	ENSG00000160789.18_LMNA	0.10917	-0.050914168	0.263787462	1.81E-01	4.44E-01	1.00E+00
ENSG00000176387.6_HSD11B2	ENSG00000160789.18_LMNA	-0.08148	-0.23759548	0.078745458	3.18E-01	6.05E-01	1.00E+00
ENSG00000259918.1_NDUFA5P1	ENSG00000160789.18_LMNA	-0.05206	-0.209526375	0.108033823	5.24E-01	7.69E-01	1.00E+00
ENSG00000119508.16_NR4A3	ENSG00000160789.18_LMNA	0.0788	-0.081419888	0.235054116	3.35E-01	6.20E-01	1.00E+00
ENSG00000101353.13_MROH8	ENSG00000160789.18_LMNA	-0.13244	-0.285617125	0.02733866	1.04E-01	3.19E-01	1.00E+00
ENSG00000215769.7_RP13-104F	ENSG00000160789.18_LMNA	-0.07243	-0.228991744	0.08778134	3.75E-01	6.55E-01	1.00E+00
ENSG00000101624.9_CEP76	ENSG00000160789.18_LMNA	-0.27425	-0.415319205	-0.12030367	6.28E-04	8.07E-03	1.00E+00
ENSG00000170379.18_TCAF2	ENSG00000160789.18_LMNA	0.35763	0.210412309	0.488992777	6.09E-06	1.95E-04	3.12E-01
ENSG00000231999.5_FLJ27354	ENSG00000160789.18_LMNA	-0.5278	-0.633750535	-0.40241432	2.81E-12	1.30E-09	1.44E-07
ENSG00000134146.10_DPH6	ENSG00000160789.18_LMNA	-0.19636	-0.34478648	-0.03836283	1.53E-02	8.92E-02	1.00E+00
ENSG00000214121.4_PRDX1P1	ENSG00000160789.18_LMNA	-0.07704	-0.233377951	0.083181328	3.45E-01	6.30E-01	1.00E+00
ENSG00000204950.3_LRRC10B	ENSG00000160789.18_LMNA	-0.16193	-0.313059863	-0.00280114	4.63E-02	1.91E-01	1.00E+00
ENSG00000167261.12_DPEP2	ENSG00000160789.18_LMNA	-0.11665	-0.270816925	0.043360612	1.52E-01	4.02E-01	1.00E+00
ENSG00000220201.6_ZGLP1	ENSG00000160789.18_LMNA	-0.01161	-0.170498056	0.147861499	8.87E-01	9.56E-01	1.00E+00
ENSG00000198930.11_CSAG1	ENSG00000160789.18_LMNA	-0.17378	-0.324018491	-0.01499635	3.23E-02	1.50E-01	1.00E+00
ENSG00000225259.4_ST13P6	ENSG00000160789.18_LMNA	-0.06548	-0.222367064	0.094703422	4.23E-01	6.94E-01	1.00E+00
ENSG00000272807.1_RP11-260M	ENSG00000160789.18_LMNA	-0.13803	-0.290838542	0.021647584	8.99E-02	2.92E-01	1.00E+00
ENSG00000084453.15_SLCO1A2	ENSG00000160789.18_LMNA	-0.52494	-0.631377622	-0.39909386	3.85E-12	1.62E-09	1.98E-07
ENSG00000134463.13_ECHDC3	ENSG00000160789.18_LMNA	-0.10569	-0.260506382	0.054427593	1.95E-01	4.64E-01	1.00E+00
ENSG00000154102.9_C16orf74	ENSG00000160789.18_LMNA	-0.01143	-0.170317834	0.148043048	8.89E-01	9.57E-01	1.00E+00
ENSG00000158473.6_CD1D	ENSG00000160789.18_LMNA	0.07771	-0.082508795	0.234018148	3.41E-01	6.26E-01	1.00E+00
ENSG00000244560.5_RP4-800G7	ENSG00000160789.18_LMNA	0.07497	-0.0852467	0.231410168	3.59E-01	6.41E-01	1.00E+00
ENSG00000162723.8_SLAMF9	ENSG00000160789.18_LMNA	0.06013	-0.100025723	0.217253443	4.62E-01	7.25E-01	1.00E+00
ENSG00000231305.3_RP11-723C	ENSG00000160789.18_LMNA	-0.1524	-0.304222206	0.006967008	6.09E-02	2.28E-01	1.00E+00
ENSG00000217165.1_ANKRD18E	ENSG00000160789.18_LMNA	-0.09302	-0.248541075	0.067174469	2.54E-01	5.36E-01	1.00E+00
ENSG00000260778.3_MIR940	ENSG00000160789.18_LMNA	0.01498	-0.144562534	0.173769203	8.55E-01	9.43E-01	1.00E+00
ENSG00000148225.14_WDR31	ENSG00000160789.18_LMNA	0.11605	-0.043962835	0.270257713	1.55E-01	4.05E-01	1.00E+00
ENSG00000236480.1_PKMP1	ENSG00000160789.18_LMNA	0.37143	0.225564564	0.501002951	2.46E-06	9.36E-05	1.26E-01
ENSG00000255145.2_STX17-AS1	ENSG00000160789.18_LMNA	-0.19399	-0.342612427	-0.03590096	1.66E-02	9.45E-02	1.00E+00
ENSG00000171657.5_GPR82	ENSG00000160789.18_LMNA	-0.08466	-0.240622227	0.075554276	3.00E-01	5.86E-01	1.00E+00
ENSG00000143429.8_AC027612.(	ENSG00000160789.18_LMNA	-0.00844	-0.167418951	0.15096033	9.18E-01	9.69E-01	1.00E+00

ENSG00000213376.4_GAPDHP71	ENSG00000160789.18_LMNA	0.21108	0.053678669	0.358238271	9.05E-03	6.10E-02	1.00E+00
ENSG00000160145.14_KALRN	ENSG00000160789.18_LMNA	0.08971	-0.070494214	0.245408983	2.72E-01	5.56E-01	1.00E+00
ENSG00000241429.1_EEF1A1P25	ENSG00000160789.18_LMNA	-0.15015	-0.302125025	0.009276307	6.48E-02	2.37E-01	1.00E+00
ENSG00000077264.13_PAK3	ENSG00000160789.18_LMNA	-0.32337	-0.45894259	-0.17307791	4.83E-05	1.05E-03	1.00E+00
ENSG00000234036.4_TXNP6	ENSG00000160789.18_LMNA	0.13803	-0.021647265	0.290838834	8.99E-02	2.92E-01	1.00E+00
ENSG00000154556.16_SORBS2	ENSG00000160789.18_LMNA	-0.19065	-0.339541218	-0.03242948	1.86E-02	1.02E-01	1.00E+00
ENSG00000223460.1_GAPDHP69	ENSG00000160789.18_LMNA	0.27694	0.123171666	0.417725285	5.52E-04	7.29E-03	1.00E+00
ENSG00000219755.1_RP1-199J3.	ENSG00000160789.18_LMNA	-0.01195	-0.170826532	0.147530549	8.84E-01	9.55E-01	1.00E+00
ENSG00000184068.2_RP5-821D1	ENSG00000160789.18_LMNA	-0.00675	-0.165768389	0.152618894	9.34E-01	9.74E-01	1.00E+00
ENSG00000228335.1_AC073063.1	ENSG00000160789.18_LMNA	-0.17353	-0.323783689	-0.01473409	3.25E-02	1.51E-01	1.00E+00
ENSG00000267102.1_RP11-686D	ENSG00000160789.18_LMNA	0.1487	-0.010759818	0.30077619	6.75E-02	2.44E-01	1.00E+00
ENSG00000166439.5_RNF169	ENSG00000160789.18_LMNA	0.14188	-0.017720348	0.294430859	8.12E-02	2.75E-01	1.00E+00
ENSG00000230438.6_SERPINB9P	ENSG00000160789.18_LMNA	0.02044	-0.139217188	0.179054605	8.03E-01	9.21E-01	1.00E+00
ENSG00000104880.16_ARHGEF1	ENSG00000160789.18_LMNA	0.16208	0.002959302	0.31320252	4.60E-02	1.91E-01	1.00E+00
ENSG00000213020.7_ZNF611	ENSG00000160789.18_LMNA	-0.10714	-0.261877611	0.05296021	1.89E-01	4.56E-01	1.00E+00
ENSG00000075275.15_CELSR1	ENSG00000160789.18_LMNA	0.35404	0.206474852	0.485854433	7.65E-06	2.36E-04	3.93E-01
ENSG00000176723.8_ZNF843	ENSG00000160789.18_LMNA	-0.18767	-0.336810077	-0.02934861	2.06E-02	1.10E-01	1.00E+00
ENSG00000234585.5_CCT6P3	ENSG00000160789.18_LMNA	0.01717	-0.142421675	0.175888258	8.34E-01	9.35E-01	1.00E+00
ENSG00000135697.8_BCO1	ENSG00000160789.18_LMNA	-0.16101	-0.312207967	-0.00185696	4.75E-02	1.94E-01	1.00E+00
ENSG00000255794.5_RMST	ENSG00000160789.18_LMNA	-0.21022	-0.357456051	-0.05278411	9.34E-03	6.24E-02	1.00E+00
ENSG00000164749.10_HNF4G	ENSG00000160789.18_LMNA	0.06282	-0.097357381	0.219819342	4.42E-01	7.09E-01	1.00E+00
ENSG00000127329.13_PTPRB	ENSG00000160789.18_LMNA	-0.15835	-0.309740834	0.000874297	5.14E-02	2.05E-01	1.00E+00
ENSG00000152439.11_ZNF773	ENSG00000160789.18_LMNA	0.06828	-0.091923981	0.225030611	4.03E-01	6.79E-01	1.00E+00
ENSG00000104974.9_LILRA1	ENSG00000160789.18_LMNA	-0.08979	-0.245484556	0.070414196	2.71E-01	5.56E-01	1.00E+00
ENSG00000189223.12_PAX8-AS1	ENSG00000160789.18_LMNA	0.02519	-0.13455439	0.183650159	7.58E-01	8.99E-01	1.00E+00
ENSG00000171174.12_RBKS	ENSG00000160789.18_LMNA	0.02851	-0.131287995	0.186861185	7.27E-01	8.85E-01	1.00E+00
ENSG00000272087.1_RP11-379F	ENSG00000160789.18_LMNA	0.00589	-0.153456258	0.164934386	9.43E-01	9.77E-01	1.00E+00
ENSG00000176204.12_LRRTM4	ENSG00000160789.18_LMNA	-0.33037	-0.465106565	-0.18066965	3.23E-05	7.58E-04	1.00E+00
ENSG00000175874.8_CREG2	ENSG00000160789.18_LMNA	-0.09722	-0.252514676	0.062952713	2.33E-01	5.11E-01	1.00E+00
ENSG00000228878.6_SEPT7-AS1	ENSG00000160789.18_LMNA	-0.03881	-0.196792159	0.121141866	6.35E-01	8.36E-01	1.00E+00
ENSG00000204186.6_ZDBF2	ENSG00000160789.18_LMNA	-0.24737	-0.391169349	-0.0917878	2.12E-03	2.05E-02	1.00E+00
ENSG00000166436.14_TRIM66	ENSG00000160789.18_LMNA	0.0976	-0.062571612	0.252872852	2.32E-01	5.09E-01	1.00E+00
ENSG00000173928.2_SWSAP1	ENSG00000160789.18_LMNA	-0.04364	-0.201438653	0.116371783	5.93E-01	8.12E-01	1.00E+00

ENSG00000188681.10_TEKT4P2	ENSG00000160789.18_LMNA	0.01618	-0.143386977	0.174933152	8.43E-01	9.39E-01	1.00E+00
ENSG00000118473.20_SGIP1	ENSG00000160789.18_LMNA	-0.32279	-0.458434268	-0.17245336	4.99E-05	1.08E-03	1.00E+00
ENSG00000172123.11_SLFN12	ENSG00000160789.18_LMNA	0.00538	-0.153947942	0.164444463	9.48E-01	9.79E-01	1.00E+00
ENSG00000167524.13_SGK494	ENSG00000160789.18_LMNA	-0.13488	-0.287899122	0.024853893	9.76E-02	3.07E-01	1.00E+00
ENSG00000229806.1_RPS15P5	ENSG00000160789.18_LMNA	-0.0126	-0.171455362	0.146896785	8.78E-01	9.52E-01	1.00E+00
ENSG00000260625.2_RP11-452L	ENSG00000160789.18_LMNA	-0.28187	-0.422129715	-0.12843443	4.35E-04	6.05E-03	1.00E+00
ENSG00000162591.14_MEGF6	ENSG00000160789.18_LMNA	-0.18032	-0.330042597	-0.02173962	2.62E-02	1.31E-01	1.00E+00
ENSG00000256802.2_RP11-680F	ENSG00000160789.18_LMNA	0.27162	0.117500671	0.412963653	7.12E-04	8.89E-03	1.00E+00
ENSG00000145491.10_ROPN1L	ENSG00000160789.18_LMNA	0.01049	-0.148959977	0.169407281	8.98E-01	9.60E-01	1.00E+00
ENSG00000135111.13_TBX3	ENSG00000160789.18_LMNA	-0.22864	-0.37421777	-0.07206063	4.61E-03	3.68E-02	1.00E+00
ENSG00000242088.1_AC090602.:	ENSG00000160789.18_LMNA	-0.10547	-0.260295527	0.054653113	1.96E-01	4.65E-01	1.00E+00
ENSG00000271430.1_RP3-368A4	ENSG00000160789.18_LMNA	0.03871	-0.121234691	0.196701598	6.36E-01	8.37E-01	1.00E+00
ENSG00000138778.10_CENPE	ENSG00000160789.18_LMNA	0.16489	0.005839344	0.315797746	4.24E-02	1.80E-01	1.00E+00
ENSG00000270194.1_RP11-259K	ENSG00000160789.18_LMNA	0.14678	-0.012716853	0.298994909	7.12E-02	2.52E-01	1.00E+00
ENSG00000270906.1_MTND4P35	ENSG00000160789.18_LMNA	-0.04886	-0.206457164	0.111203265	5.50E-01	7.86E-01	1.00E+00
ENSG00000223791.1_UBE2E1-AS	ENSG00000160789.18_LMNA	-0.34274	-0.475972401	-0.19413539	1.54E-05	4.19E-04	7.92E-01
ENSG00000163071.9_SPATA18	ENSG00000160789.18_LMNA	0.14056	-0.019070939	0.293196442	8.41E-02	2.81E-01	1.00E+00
ENSG00000188039.12_NWD1	ENSG00000160789.18_LMNA	0.0103	-0.149149532	0.169218977	9.00E-01	9.61E-01	1.00E+00
ENSG00000262686.1_GLIS2-AS1	ENSG00000160789.18_LMNA	-0.12324	-0.277010444	0.036675426	1.30E-01	3.66E-01	1.00E+00
ENSG00000153162.8_BMP6	ENSG00000160789.18_LMNA	-0.25489	-0.397941315	-0.09973482	1.53E-03	1.59E-02	1.00E+00
ENSG00000229847.7_EMX2OS	ENSG00000160789.18_LMNA	-0.04761	-0.205258981	0.112438826	5.60E-01	7.93E-01	1.00E+00
ENSG00000175536.6_LIPT2	ENSG00000160789.18_LMNA	-0.08997	-0.245652156	0.070236722	2.70E-01	5.55E-01	1.00E+00
ENSG00000227232.5_WASH7P	ENSG00000160789.18_LMNA	0.068	-0.092203209	0.22476324	4.05E-01	6.81E-01	1.00E+00
ENSG00000151611.12_MMAA	ENSG00000160789.18_LMNA	0.18504	0.026627601	0.334393638	2.25E-02	1.17E-01	1.00E+00
ENSG00000260296.1_RP11-395I6	ENSG00000160789.18_LMNA	-0.19169	-0.340501321	-0.03351392	1.80E-02	1.00E-01	1.00E+00
ENSG00000186369.8_LINC00643	ENSG00000160789.18_LMNA	-0.48646	-0.599252395	-0.35473222	2.10E-10	4.18E-08	1.08E-05
ENSG00000224397.4_LINC01272	ENSG00000160789.18_LMNA	0.11512	-0.044904296	0.269383066	1.58E-01	4.11E-01	1.00E+00
ENSG00000188613.6_NANOS1	ENSG00000160789.18_LMNA	-0.24602	-0.389945824	-0.09035602	2.25E-03	2.14E-02	1.00E+00
ENSG00000183506.15_PI4KAP2	ENSG00000160789.18_LMNA	-0.01025	-0.169179265	0.149189504	9.00E-01	9.61E-01	1.00E+00
ENSG00000125637.14_PSD4	ENSG00000160789.18_LMNA	0.11001	-0.050064139	0.264580185	1.77E-01	4.39E-01	1.00E+00
ENSG00000229539.1_RP11-119B	ENSG00000160789.18_LMNA	-0.11802	-0.272110023	0.041967182	1.48E-01	3.94E-01	1.00E+00
ENSG00000167981.5_ZNF597	ENSG00000160789.18_LMNA	0.02296	-0.136744641	0.181493225	7.79E-01	9.09E-01	1.00E+00
ENSG00000262903.1_RP11-235E	ENSG00000160789.18_LMNA	0.10909	-0.05099447	0.263712552	1.81E-01	4.44E-01	1.00E+00

ENSG00000236088.8_COX10-AS1	ENSG00000160789.18_LMNA	-0.21071	-0.357904883	-0.05329734	9.17E-03	6.16E-02	1.00E+00
ENSG00000226964.1_RHEBP2	ENSG00000160789.18_LMNA	-0.2294	-0.374911055	-0.07286282	4.47E-03	3.60E-02	1.00E+00
ENSG00000132321.15_IQCA1	ENSG00000160789.18_LMNA	-0.15767	-0.309114916	0.001566488	5.24E-02	2.07E-01	1.00E+00
ENSG00000118193.10_KIF14	ENSG00000160789.18_LMNA	0.1214	-0.03855024	0.275276141	1.36E-01	3.76E-01	1.00E+00
ENSG00000172572.6_PDE3A	ENSG00000160789.18_LMNA	0.06235	-0.097818515	0.219376225	4.45E-01	7.12E-01	1.00E+00
ENSG00000255366.1_RP11-1134	ENSG00000160789.18_LMNA	-0.25824	-0.400955999	-0.10328489	1.32E-03	1.43E-02	1.00E+00
ENSG00000174776.9_WDR49	ENSG00000160789.18_LMNA	-0.12805	-0.28150773	0.031803467	1.16E-01	3.40E-01	1.00E+00
ENSG00000223822.2_EEF1A1P1	ENSG00000160789.18_LMNA	-0.07194	-0.228523467	0.088271646	3.78E-01	6.58E-01	1.00E+00
ENSG00000197083.10_ZNF300P1	ENSG00000160789.18_LMNA	-0.21418	-0.361065639	-0.05691616	8.06E-03	5.59E-02	1.00E+00
ENSG00000180616.7_SSTR2	ENSG00000160789.18_LMNA	-0.48011	-0.593918704	-0.34747199	3.87E-10	7.07E-08	1.99E-05
ENSG00000168546.9_GFRA2	ENSG00000160789.18_LMNA	-0.21443	-0.361296215	-0.05718046	7.98E-03	5.55E-02	1.00E+00
ENSG00000236120.5_RP11-733C	ENSG00000160789.18_LMNA	-0.15119	-0.303092648	0.008211227	6.30E-02	2.33E-01	1.00E+00
ENSG00000267871.4_CTC-444N2	ENSG00000160789.18_LMNA	-0.09738	-0.252666232	0.062791468	2.33E-01	5.11E-01	1.00E+00
ENSG00000280294.1_RP11-177H	ENSG00000160789.18_LMNA	-0.09632	-0.251657492	0.063864385	2.38E-01	5.16E-01	1.00E+00
ENSG00000277147.3_LINC00869	ENSG00000160789.18_LMNA	-0.19911	-0.347298411	-0.04121196	1.39E-02	8.32E-02	1.00E+00
ENSG00000230337.1_RP4-635E1	ENSG00000160789.18_LMNA	-0.08779	-0.243583786	0.072425543	2.82E-01	5.67E-01	1.00E+00
ENSG00000157657.13_ZNF618	ENSG00000160789.18_LMNA	0.11475	-0.045278583	0.269035198	1.59E-01	4.12E-01	1.00E+00
ENSG00000261556.7_SMG1P7	ENSG00000160789.18_LMNA	-0.05132	-0.208811011	0.108773126	5.30E-01	7.73E-01	1.00E+00
ENSG00000267249.1_RP11-973H	ENSG00000160789.18_LMNA	-0.29024	-0.429591354	-0.13738809	2.87E-04	4.34E-03	1.00E+00
ENSG00000135899.15_SP110	ENSG00000160789.18_LMNA	0.07511	-0.08511318	0.231537457	3.58E-01	6.41E-01	1.00E+00
ENSG00000103056.10_SMPD3	ENSG00000160789.18_LMNA	-0.37523	-0.504307745	-0.22975746	1.91E-06	7.70E-05	9.78E-02
ENSG00000142611.15_PRDM16	ENSG00000160789.18_LMNA	0.20805	0.05052336	0.355477288	1.01E-02	6.61E-02	1.00E+00
ENSG00000139800.8_ZIC5	ENSG00000160789.18_LMNA	0.15532	-0.003983732	0.306926996	5.60E-02	2.16E-01	1.00E+00
ENSG00000166206.12_GABRB3	ENSG00000160789.18_LMNA	-0.36908	-0.498960548	-0.22297839	2.88E-06	1.07E-04	1.48E-01
ENSG00000111181.11_SLC6A12	ENSG00000160789.18_LMNA	-0.28345	-0.423534834	-0.13011686	4.02E-04	5.69E-03	1.00E+00
ENSG00000280079.1_CTC-260E6	ENSG00000160789.18_LMNA	-0.15144	-0.303324953	0.00795542	6.25E-02	2.32E-01	1.00E+00
ENSG00000271914.1_RP4-789D1	ENSG00000160789.18_LMNA	-0.21655	-0.363231357	-0.05940036	7.37E-03	5.23E-02	1.00E+00
ENSG00000102384.12_CENPI	ENSG00000160789.18_LMNA	0.13554	-0.024184923	0.288512895	9.59E-02	3.04E-01	1.00E+00
ENSG00000166145.13_SPINT1	ENSG00000160789.18_LMNA	0.14957	-0.009865556	0.301589419	6.59E-02	2.40E-01	1.00E+00
ENSG00000163633.9_C4orf36	ENSG00000160789.18_LMNA	-0.12022	-0.274170749	0.039744037	1.40E-01	3.82E-01	1.00E+00
ENSG00000145287.9_PLAC8	ENSG00000160789.18_LMNA	-0.11183	-0.266293551	0.048225357	1.70E-01	4.29E-01	1.00E+00
ENSG00000117245.11_KIF17	ENSG00000160789.18_LMNA	-0.07186	-0.228448807	0.088349804	3.79E-01	6.58E-01	1.00E+00
ENSG00000235545.1_RP11-230B	ENSG00000160789.18_LMNA	-0.09271	-0.248247076	0.067486379	2.56E-01	5.38E-01	1.00E+00

ENSG00000232810.3_TNF	ENSG00000160789.18_LMNA	-0.14337	-0.295817036	0.016202355	7.81E-02	2.68E-01	1.00E+00
ENSG00000149654.8_CDH22	ENSG00000160789.18_LMNA	-0.28367	-0.423737537	-0.13035971	3.98E-04	5.64E-03	1.00E+00
ENSG00000236397.3_DDX11L2	ENSG00000160789.18_LMNA	-0.07735	-0.233674708	0.082869618	3.44E-01	6.28E-01	1.00E+00
ENSG00000272677.1_RP11-127B	ENSG00000160789.18_LMNA	-0.1561	-0.307654627	0.003180238	5.48E-02	2.13E-01	1.00E+00
ENSG00000270147.1_RP11-646I6	ENSG00000160789.18_LMNA	-0.12895	-0.282351203	0.030888063	1.13E-01	3.36E-01	1.00E+00
ENSG00000165238.15_WNK2	ENSG00000160789.18_LMNA	-0.34605	-0.478868918	-0.19774299	1.26E-05	3.53E-04	6.47E-01
ENSG00000258777.1_HIF1A-AS1	ENSG00000160789.18_LMNA	0.00627	-0.153083265	0.165305938	9.39E-01	9.76E-01	1.00E+00
ENSG00000149927.16_DOC2A	ENSG00000160789.18_LMNA	-0.17954	-0.329328373	-0.02093866	2.69E-02	1.33E-01	1.00E+00
ENSG00000144645.12_OSBPL10	ENSG00000160789.18_LMNA	0.52393	0.397925239	0.630541444	4.31E-12	1.77E-09	2.21E-07
ENSG00000257074.3_RPL29P33	ENSG00000160789.18_LMNA	-0.13211	-0.285311853	0.027670764	1.05E-01	3.21E-01	1.00E+00
ENSG00000234494.6_SP2-AS1	ENSG00000160789.18_LMNA	-0.33418	-0.468458363	-0.1848121	2.58E-05	6.33E-04	1.00E+00
ENSG00000231416.1_RP11-422P	ENSG00000160789.18_LMNA	-0.14585	-0.298131779	0.013664287	7.30E-02	2.56E-01	1.00E+00
ENSG00000152078.8_TMEM56	ENSG00000160789.18_LMNA	-0.08839	-0.244156656	0.071819617	2.79E-01	5.64E-01	1.00E+00
ENSG00000165288.10_BRWD3	ENSG00000160789.18_LMNA	-0.04621	-0.203907199	0.1138316	5.72E-01	8.00E-01	1.00E+00
ENSG00000198915.10_RASGEF1	ENSG00000160789.18_LMNA	0.11268	-0.0473726	0.267087471	1.67E-01	4.24E-01	1.00E+00
ENSG00000226833.4_AC097724.:	ENSG00000160789.18_LMNA	-0.25053	-0.394015013	-0.0951226	1.85E-03	1.85E-02	1.00E+00
ENSG00000227852.1_RPS29P17	ENSG00000160789.18_LMNA	-0.10309	-0.258052126	0.057050554	2.06E-01	4.78E-01	1.00E+00
ENSG00000264016.2_CTC-297N7	ENSG00000160789.18_LMNA	0.08182	-0.078400327	0.237923124	3.16E-01	6.03E-01	1.00E+00
ENSG00000129159.6_KCNC1	ENSG00000160789.18_LMNA	-0.23721	-0.381988201	-0.08107411	3.26E-03	2.84E-02	1.00E+00
ENSG00000111846.14_GCNT2	ENSG00000160789.18_LMNA	-0.19833	-0.346591198	-0.04040931	1.43E-02	8.50E-02	1.00E+00
ENSG00000160062.13_ZBTB8A	ENSG00000160789.18_LMNA	-0.19087	-0.339742517	-0.03265679	1.85E-02	1.02E-01	1.00E+00
ENSG00000223886.3_RP11-251G	ENSG00000160789.18_LMNA	-0.03824	-0.196246597	0.121700978	6.40E-01	8.39E-01	1.00E+00
ENSG00000010318.18_PHF7	ENSG00000160789.18_LMNA	0.0053	-0.154027958	0.16436472	9.48E-01	9.79E-01	1.00E+00
ENSG00000273259.2_SERPINA3	ENSG00000160789.18_LMNA	0.171	0.01212587	0.321446513	3.52E-02	1.59E-01	1.00E+00
ENSG00000213867.4_RP11-829H	ENSG00000160789.18_LMNA	-0.17828	-0.328170806	-0.01964137	2.80E-02	1.37E-01	1.00E+00
ENSG00000101680.12_LAMA1	ENSG00000160789.18_LMNA	0.04196	-0.118030341	0.199824687	6.08E-01	8.21E-01	1.00E+00
ENSG00000263013.1_RP11-876N	ENSG00000160789.18_LMNA	0.08276	-0.077457211	0.238818086	3.11E-01	5.97E-01	1.00E+00
ENSG00000179630.9_LACC1	ENSG00000160789.18_LMNA	-0.03248	-0.190697216	0.127376741	6.91E-01	8.67E-01	1.00E+00
ENSG00000187634.9_SAMD11	ENSG00000160789.18_LMNA	-0.15575	-0.307326845	0.003542244	5.54E-02	2.15E-01	1.00E+00
ENSG00000126583.9_PRKCG	ENSG00000160789.18_LMNA	-0.20698	-0.35450234	-0.04941062	1.05E-02	6.79E-02	1.00E+00
ENSG00000117262.17_GPR89A	ENSG00000160789.18_LMNA	0.11478	-0.04524435	0.269067018	1.59E-01	4.12E-01	1.00E+00
ENSG00000226085.3_UQCRFS1F	ENSG00000160789.18_LMNA	-0.06493	-0.221837759	0.09525517	4.27E-01	6.97E-01	1.00E+00
ENSG00000270055.1_CTD-3092A	ENSG00000160789.18_LMNA	0.26552	0.111008816	0.407492887	9.46E-04	1.11E-02	1.00E+00

ENSG00000116690.10_PRG4	ENSG00000160789.18_LMNA	0.23109	0.074631558	0.376438477	4.18E-03	3.43E-02	1.00E+00
ENSG00000140368.11_PSTPIP1	ENSG00000160789.18_LMNA	0.06927	-0.090932231	0.225979862	3.96E-01	6.73E-01	1.00E+00
ENSG00000233006.5_AC034220.:	ENSG00000160789.18_LMNA	-0.374	-0.503237887	-0.22839898	2.07E-06	8.20E-05	1.06E-01
ENSG00000244682.6_FCGR2C	ENSG00000160789.18_LMNA	0.33065	0.180977473	0.465355916	3.18E-05	7.47E-04	1.00E+00
ENSG00000178882.12_RFLNA	ENSG00000160789.18_LMNA	-0.19349	-0.342150191	-0.03537801	1.69E-02	9.57E-02	1.00E+00
ENSG00000260259.1_RP11-368I7	ENSG00000160789.18_LMNA	0.18908	0.03080403	0.338100926	1.97E-02	1.07E-01	1.00E+00
ENSG00000223482.6_NUTM2A-A	ENSG00000160789.18_LMNA	-0.2124	-0.35944966	-0.05506499	8.61E-03	5.88E-02	1.00E+00
ENSG00000251602.5_RP11-521B	ENSG00000160789.18_LMNA	0.13749	-0.022195716	0.290336454	9.12E-02	2.94E-01	1.00E+00
ENSG00000122870.10_BICC1	ENSG00000160789.18_LMNA	0.30868	0.157204228	0.445964699	1.09E-04	2.02E-03	1.00E+00
ENSG00000226855.1_RPSAP17	ENSG00000160789.18_LMNA	-0.175	-0.325138538	-0.01624799	3.11E-02	1.47E-01	1.00E+00
ENSG00000272323.1_CTD-2517C	ENSG00000160789.18_LMNA	-0.15256	-0.30437113	0.006802894	6.06E-02	2.27E-01	1.00E+00
ENSG00000223416.3_RPS26P15	ENSG00000160789.18_LMNA	-0.17263	-0.32295421	-0.01380793	3.34E-02	1.54E-01	1.00E+00
ENSG00000239470.3_RP11-16F1	ENSG00000160789.18_LMNA	-0.23027	-0.375696163	-0.07377173	4.32E-03	3.51E-02	1.00E+00
ENSG00000242588.5_RP11-274B	ENSG00000160789.18_LMNA	0.08708	-0.073137894	0.242910015	2.86E-01	5.72E-01	1.00E+00
ENSG00000238228.1_OR7E7P	ENSG00000160789.18_LMNA	-0.11133	-0.265823375	0.048730162	1.72E-01	4.31E-01	1.00E+00
ENSG00000248890.1_HHIP-AS1	ENSG00000160789.18_LMNA	-0.10933	-0.263940269	0.050750351	1.80E-01	4.43E-01	1.00E+00
ENSG00000276231.3_PIK3R6	ENSG00000160789.18_LMNA	0.02562	-0.134124184	0.184073461	7.54E-01	8.98E-01	1.00E+00
ENSG00000137203.9_TFAP2A	ENSG00000160789.18_LMNA	-0.19777	-0.346072633	-0.03982101	1.46E-02	8.62E-02	1.00E+00
ENSG00000176472.9_ZNF575	ENSG00000160789.18_LMNA	-0.02067	-0.179280232	0.138988589	8.00E-01	9.20E-01	1.00E+00
ENSG00000158865.11_SLC5A11	ENSG00000160789.18_LMNA	-0.26884	-0.410474974	-0.11454436	8.11E-04	9.83E-03	1.00E+00
ENSG00000279461.1_AC005480.:	ENSG00000160789.18_LMNA	-0.02831	-0.186666701	0.13148603	7.29E-01	8.85E-01	1.00E+00
ENSG00000196172.9_ZNF681	ENSG00000160789.18_LMNA	-0.13607	-0.289014476	0.023638026	9.46E-02	3.01E-01	1.00E+00
ENSG00000256667.5_KLRA1P	ENSG00000160789.18_LMNA	-0.18986	-0.338820841	-0.03161629	1.91E-02	1.05E-01	1.00E+00
ENSG00000267422.1_CTD-2554C	ENSG00000160789.18_LMNA	-0.05153	-0.209016558	0.108560736	5.28E-01	7.72E-01	1.00E+00
ENSG00000184838.13_PRR16	ENSG00000160789.18_LMNA	0.19599	0.037976502	0.344445533	1.55E-02	9.00E-02	1.00E+00
ENSG00000214826.5_DDX12P	ENSG00000160789.18_LMNA	-0.07759	-0.233903413	0.082629347	3.42E-01	6.27E-01	1.00E+00
ENSG00000123552.16_USP45	ENSG00000160789.18_LMNA	-0.27634	-0.417191076	-0.12253448	5.68E-04	7.45E-03	1.00E+00
ENSG00000276547.1_PCDHGB5	ENSG00000160789.18_LMNA	0.03337	-0.126500525	0.191555251	6.83E-01	8.62E-01	1.00E+00
ENSG00000259768.5_RP5-991G2	ENSG00000160789.18_LMNA	0.19273	0.0345875	0.341451183	1.74E-02	9.75E-02	1.00E+00
ENSG00000244219.5_GS1-259H1	ENSG00000160789.18_LMNA	-0.08466	-0.24061559	0.075561281	3.00E-01	5.86E-01	1.00E+00
ENSG00000198205.6_ZXDA	ENSG00000160789.18_LMNA	0.11118	-0.048888306	0.265676049	1.73E-01	4.32E-01	1.00E+00
ENSG00000176732.6_PFN4	ENSG00000160789.18_LMNA	0.11179	-0.048264831	0.26625679	1.70E-01	4.29E-01	1.00E+00
ENSG00000230939.1_RP11-314C	ENSG00000160789.18_LMNA	-0.2338	-0.378902225	-0.07748856	3.74E-03	3.16E-02	1.00E+00

ENSG00000174469.16_CNTNAP2	ENSG00000160789.18_LMNA	-0.36795	-0.497977046	-0.22173443	3.11E-06	1.13E-04	1.59E-01
ENSG00000172426.14_RSPH9	ENSG00000160789.18_LMNA	-0.04272	-0.200553418	0.117281696	6.01E-01	8.17E-01	1.00E+00
ENSG00000196418.11_ZNF124	ENSG00000160789.18_LMNA	-0.36718	-0.49730857	-0.22088943	3.27E-06	1.18E-04	1.68E-01
ENSG00000213772.3_EIF1P7	ENSG00000160789.18_LMNA	-0.01816	-0.176852369	0.141446657	8.24E-01	9.31E-01	1.00E+00
ENSG00000197291.7_RAMP2-AS	ENSG00000160789.18_LMNA	-0.33105	-0.46570655	-0.18141042	3.10E-05	7.33E-04	1.00E+00
ENSG00000152213.3_ARL11	ENSG00000160789.18_LMNA	-0.0483	-0.205915507	0.111761941	5.55E-01	7.89E-01	1.00E+00
ENSG00000213236.3_YWHAZP2	ENSG00000160789.18_LMNA	0.074	-0.086213044	0.230488594	3.65E-01	6.46E-01	1.00E+00
ENSG00000117069.13_ST6GALN	ENSG00000160789.18_LMNA	0.00057	-0.15864798	0.159753324	9.94E-01	9.98E-01	1.00E+00
ENSG00000215221.2_UBA52P6	ENSG00000160789.18_LMNA	0.14725	-0.012234426	0.299434212	7.02E-02	2.50E-01	1.00E+00
ENSG00000228701.1_TNKS2-AS	ENSG00000160789.18_LMNA	-0.10651	-0.261283518	0.05359613	1.92E-01	4.59E-01	1.00E+00
ENSG00000115844.9_DLX2	ENSG00000160789.18_LMNA	-0.12476	-0.27842618	0.03514335	1.26E-01	3.58E-01	1.00E+00
ENSG00000109771.14_LRP2BP	ENSG00000160789.18_LMNA	-0.17242	-0.322756705	-0.01358749	3.37E-02	1.55E-01	1.00E+00
ENSG00000117586.9_TNFSF4	ENSG00000160789.18_LMNA	0.31338	0.162270867	0.45012028	8.45E-05	1.64E-03	1.00E+00
ENSG00000134470.18_IL15RA	ENSG00000160789.18_LMNA	0.21066	0.053245838	0.357859847	9.19E-03	6.16E-02	1.00E+00
ENSG00000261338.2_RP11-378A	ENSG00000160789.18_LMNA	-0.21332	-0.360287937	-0.05602501	8.32E-03	5.73E-02	1.00E+00
ENSG00000127423.9_AUNIP	ENSG00000160789.18_LMNA	-0.06407	-0.221017085	0.096110255	4.33E-01	7.02E-01	1.00E+00
ENSG00000180479.12_ZNF571	ENSG00000160789.18_LMNA	-0.20463	-0.352347695	-0.04695414	1.14E-02	7.21E-02	1.00E+00
ENSG00000090376.7_IRAK3	ENSG00000160789.18_LMNA	-0.11547	-0.269713664	0.04454851	1.57E-01	4.09E-01	1.00E+00
ENSG00000275854.1_RP11-278C	ENSG00000160789.18_LMNA	-0.12501	-0.278669021	0.034880406	1.25E-01	3.57E-01	1.00E+00
ENSG00000267082.1_CTC-510F1	ENSG00000160789.18_LMNA	0.0243	-0.135427125	0.182791067	7.66E-01	9.03E-01	1.00E+00
ENSG00000249485.1_RBBP4P1	ENSG00000160789.18_LMNA	-0.09167	-0.247265556	0.068527252	2.61E-01	5.45E-01	1.00E+00
ENSG00000275476.1_RP11-996F	ENSG00000160789.18_LMNA	-0.24372	-0.387872369	-0.08793248	2.48E-03	2.31E-02	1.00E+00
ENSG00000253988.1_RP11-489C	ENSG00000160789.18_LMNA	-0.3546	-0.486341253	-0.20708504	7.39E-06	2.29E-04	3.79E-01
ENSG00000225159.1_NPM1P39	ENSG00000160789.18_LMNA	0.02658	-0.133181987	0.185000127	7.45E-01	8.93E-01	1.00E+00
ENSG00000043591.5_ADRB1	ENSG00000160789.18_LMNA	-0.04099	-0.19888902	0.118991048	6.16E-01	8.26E-01	1.00E+00
ENSG00000215915.8_ATAD3C	ENSG00000160789.18_LMNA	-0.07488	-0.231320516	0.085340733	3.59E-01	6.42E-01	1.00E+00
ENSG00000250568.1_RP11-333E	ENSG00000160789.18_LMNA	-0.10165	-0.25669404	0.058500128	2.13E-01	4.86E-01	1.00E+00
ENSG00000146267.11_FAXC	ENSG00000160789.18_LMNA	-0.3156	-0.452081888	-0.16466782	7.48E-05	1.49E-03	1.00E+00
ENSG00000085465.12_OVGP1	ENSG00000160789.18_LMNA	-0.20846	-0.355847158	-0.0509457	9.96E-03	6.54E-02	1.00E+00
ENSG00000167525.12_PROCA1	ENSG00000160789.18_LMNA	0.19201	0.033839808	0.34078972	1.78E-02	9.93E-02	1.00E+00
ENSG00000213888.3_LINC01521	ENSG00000160789.18_LMNA	-0.08259	-0.238656012	0.077628047	3.12E-01	5.98E-01	1.00E+00
ENSG00000197302.9_ZNF720	ENSG00000160789.18_LMNA	-0.11094	-0.2654529	0.04912781	1.74E-01	4.33E-01	1.00E+00
ENSG00000276805.1_RP11-291L	ENSG00000160789.18_LMNA	-0.03925	-0.197221975	0.120701234	6.31E-01	8.34E-01	1.00E+00

ENSG00000149596.6_JPH2	ENSG00000160789.18_LMNA	-0.09632	-0.25165898	0.063862802	2.38E-01	5.16E-01	1.00E+00
ENSG00000196505.9_GDAP2	ENSG00000160789.18_LMNA	0.16899	0.010063369	0.319595674	3.74E-02	1.66E-01	1.00E+00
ENSG00000138670.15_RASGEF1	ENSG00000160789.18_LMNA	-0.43454	-0.555326663	-0.29580628	2.22E-08	2.11E-06	1.14E-03
ENSG00000160613.11_PCSK7	ENSG00000160789.18_LMNA	0.26072	0.105913943	0.403184419	1.18E-03	1.31E-02	1.00E+00
ENSG00000254373.1_RP11-34P1	ENSG00000160789.18_LMNA	-0.03833	-0.196334141	0.121611273	6.39E-01	8.39E-01	1.00E+00
ENSG00000260293.2_RP11-715J	ENSG00000160789.18_LMNA	-0.02878	-0.187124521	0.131019811	7.25E-01	8.83E-01	1.00E+00
ENSG00000258311.4_RP11-644F	ENSG00000160789.18_LMNA	-0.01341	-0.172244201	0.146101385	8.70E-01	9.49E-01	1.00E+00
ENSG00000262468.4_LINC01569	ENSG00000160789.18_LMNA	0.12984	-0.029983716	0.283184012	1.11E-01	3.32E-01	1.00E+00
ENSG00000163376.10_KBTBD8	ENSG00000160789.18_LMNA	-0.33548	-0.469601621	-0.18622735	2.39E-05	5.96E-04	1.00E+00
ENSG00000272070.1_AC005618.(	ENSG00000160789.18_LMNA	-0.15403	-0.305738065	0.005295758	5.81E-02	2.21E-01	1.00E+00
ENSG00000124713.5_GNMT	ENSG00000160789.18_LMNA	-0.22244	-0.368585404	-0.06555795	5.88E-03	4.44E-02	1.00E+00
ENSG00000159882.11_ZNF230	ENSG00000160789.18_LMNA	-0.0159	-0.174654956	0.143668029	8.46E-01	9.40E-01	1.00E+00
ENSG00000069431.9_ABCC9	ENSG00000160789.18_LMNA	-0.08033	-0.236507924	0.079890516	3.25E-01	6.12E-01	1.00E+00
ENSG00000255468.5_RP11-867G	ENSG00000160789.18_LMNA	-0.01857	-0.177242506	0.14105193	8.20E-01	9.30E-01	1.00E+00
ENSG00000056277.14_ZNF280C	ENSG00000160789.18_LMNA	-0.00929	-0.168239122	0.150135516	9.10E-01	9.65E-01	1.00E+00
ENSG00000139187.8_KLRG1	ENSG00000160789.18_LMNA	-0.33405	-0.46834024	-0.18466594	2.60E-05	6.38E-04	1.00E+00
ENSG00000073737.15_DHRS9	ENSG00000160789.18_LMNA	-0.40737	-0.532065784	-0.26538193	1.90E-07	1.22E-05	9.76E-03
ENSG00000168398.6_BDKRB2	ENSG00000160789.18_LMNA	0.4473	0.310190786	0.566184721	7.59E-09	8.60E-07	3.89E-04
ENSG00000243989.6_ACY1	ENSG00000160789.18_LMNA	-0.08343	-0.239451134	0.07678976	3.07E-01	5.93E-01	1.00E+00
ENSG00000179611.3_DGKZP1	ENSG00000160789.18_LMNA	0.21885	0.061806107	0.365325555	6.75E-03	4.90E-02	1.00E+00
ENSG00000220793.5_RPL21P11(	ENSG00000160789.18_LMNA	-0.2348	-0.379807335	-0.07853939	3.59E-03	3.06E-02	1.00E+00
ENSG00000253958.1_CLDN23	ENSG00000160789.18_LMNA	0.38007	0.235099194	0.508506417	1.37E-06	5.96E-05	7.02E-02
ENSG00000242858.1_CTC-484M2	ENSG00000160789.18_LMNA	-0.07546	-0.231875186	0.084758866	3.56E-01	6.39E-01	1.00E+00
ENSG00000221955.9_SLC12A8	ENSG00000160789.18_LMNA	-0.06628	-0.223129817	0.093907986	4.17E-01	6.89E-01	1.00E+00
ENSG00000230953.2_RP4-631H1	ENSG00000160789.18_LMNA	0.06233	-0.09784442	0.219351329	4.46E-01	7.12E-01	1.00E+00
ENSG00000103404.13_USP31	ENSG00000160789.18_LMNA	0.13087	-0.028931355	0.284152533	1.08E-01	3.27E-01	1.00E+00
ENSG00000162543.5_UBXN10	ENSG00000160789.18_LMNA	0.09885	-0.061312217	0.254055878	2.26E-01	5.02E-01	1.00E+00
ENSG00000212994.5_RPS26P6	ENSG00000160789.18_LMNA	-0.14959	-0.301605057	0.009848355	6.59E-02	2.40E-01	1.00E+00
ENSG00000064309.13_CDON	ENSG00000160789.18_LMNA	-0.09152	-0.247124364	0.068676926	2.62E-01	5.45E-01	1.00E+00
ENSG00000233287.1_AC009362.:	ENSG00000160789.18_LMNA	-0.12304	-0.276822175	0.036879055	1.31E-01	3.67E-01	1.00E+00
ENSG00000155275.17_TRMT44	ENSG00000160789.18_LMNA	0.13873	-0.020932146	0.291493623	8.83E-02	2.89E-01	1.00E+00
ENSG00000139835.12_GRTP1	ENSG00000160789.18_LMNA	-0.00671	-0.165733381	0.152654052	9.35E-01	9.74E-01	1.00E+00
ENSG00000185838.12_GNB1L	ENSG00000160789.18_LMNA	0.09161	-0.068589629	0.247206716	2.62E-01	5.45E-01	1.00E+00

ENSG00000251141.4_RP11-53O1	ENSG00000160789.18_LMNA	-0.18074	-0.330436204	-0.02218119	2.59E-02	1.29E-01	1.00E+00
ENSG00000142632.15_ARHGEF1	ENSG00000160789.18_LMNA	0.0416	-0.11838128	0.199482963	6.11E-01	8.22E-01	1.00E+00
ENSG00000267778.1_AC004221.2	ENSG00000160789.18_LMNA	-0.14347	-0.295907319	0.016103438	7.79E-02	2.67E-01	1.00E+00
ENSG00000184497.11_TMEM255	ENSG00000160789.18_LMNA	0.19943	0.041550992	0.347597029	1.38E-02	8.24E-02	1.00E+00
ENSG00000182648.10_LINC0100	ENSG00000160789.18_LMNA	-0.20526	-0.352923938	-0.04761075	1.12E-02	7.10E-02	1.00E+00
ENSG00000177335.9_C8orf31	ENSG00000160789.18_LMNA	-0.06925	-0.225957541	0.090955559	3.97E-01	6.73E-01	1.00E+00
ENSG00000127507.16_ADGRE2	ENSG00000160789.18_LMNA	0.19346	0.035348809	0.34212438	1.69E-02	9.58E-02	1.00E+00
ENSG00000171631.13_P2RY6	ENSG00000160789.18_LMNA	0.21623	0.05906123	0.362935897	7.46E-03	5.27E-02	1.00E+00
ENSG00000106034.16_CPED1	ENSG00000160789.18_LMNA	-0.02416	-0.182657568	0.1355627	7.68E-01	9.04E-01	1.00E+00
ENSG00000229852.2_RP11-398K	ENSG00000160789.18_LMNA	-0.23822	-0.382903502	-0.08213908	3.12E-03	2.75E-02	1.00E+00
ENSG00000228451.3_SDAD1P1	ENSG00000160789.18_LMNA	0.02878	-0.131017656	0.187126638	7.25E-01	8.83E-01	1.00E+00
ENSG00000244251.1_RP11-64K1	ENSG00000160789.18_LMNA	-0.1001	-0.255235592	0.060055346	2.20E-01	4.95E-01	1.00E+00
ENSG00000272145.1_NFYC-AS1	ENSG00000160789.18_LMNA	-0.16038	-0.311625609	-0.00121184	4.84E-02	1.97E-01	1.00E+00
ENSG00000196967.9_ZNF585A	ENSG00000160789.18_LMNA	-0.08135	-0.237475938	0.078871361	3.19E-01	6.06E-01	1.00E+00
ENSG00000271380.1_RP11-307C	ENSG00000160789.18_LMNA	0.03103	-0.128802826	0.189299682	7.04E-01	8.74E-01	1.00E+00
ENSG00000258548.4_LINC00645	ENSG00000160789.18_LMNA	-0.33757	-0.471432918	-0.18849678	2.11E-05	5.40E-04	1.00E+00
ENSG00000041353.8_RAB27B	ENSG00000160789.18_LMNA	-0.37676	-0.505636381	-0.23144602	1.72E-06	7.09E-05	8.81E-02
ENSG00000247763.2_RP11-447H	ENSG00000160789.18_LMNA	-0.23636	-0.381211962	-0.08017148	3.37E-03	2.92E-02	1.00E+00
ENSG00000217026.3_RPL10P1	ENSG00000160789.18_LMNA	-0.04244	-0.200291806	0.117550498	6.04E-01	8.18E-01	1.00E+00
ENSG00000214593.3_RP11-65N1	ENSG00000160789.18_LMNA	-0.15634	-0.307878358	0.002933101	5.44E-02	2.12E-01	1.00E+00
ENSG00000271714.1_CTD-2377C	ENSG00000160789.18_LMNA	-0.3843	-0.512170036	-0.23977374	1.02E-06	4.67E-05	5.23E-02
ENSG00000132793.10_LPIN3	ENSG00000160789.18_LMNA	0.111	-0.04907087	0.265505955	1.73E-01	4.33E-01	1.00E+00
ENSG00000101438.3_SLC32A1	ENSG00000160789.18_LMNA	-0.17873	-0.328577403	-0.02009692	2.76E-02	1.35E-01	1.00E+00
ENSG00000215241.3_RP11-266K	ENSG00000160789.18_LMNA	-0.0867	-0.242549608	0.073518806	2.88E-01	5.74E-01	1.00E+00
ENSG00000234684.5_SDCBP2-AS	ENSG00000160789.18_LMNA	-0.16839	-0.319036252	-0.00944049	3.81E-02	1.68E-01	1.00E+00
ENSG00000151892.13_GFRA1	ENSG00000160789.18_LMNA	-0.30911	-0.446342959	-0.15766479	1.07E-04	1.99E-03	1.00E+00
ENSG00000255062.1_RP11-712L	ENSG00000160789.18_LMNA	0.17113	0.012260768	0.321567486	3.50E-02	1.59E-01	1.00E+00
ENSG00000238197.4_PAXBP1-AS	ENSG00000160789.18_LMNA	-0.33347	-0.467830861	-0.18403581	2.69E-05	6.56E-04	1.00E+00
ENSG00000164690.6_SHH	ENSG00000160789.18_LMNA	0.3226	0.172247117	0.458266368	5.05E-05	1.08E-03	1.00E+00
ENSG00000219133.2_RP11-203F	ENSG00000160789.18_LMNA	-0.18331	-0.332798773	-0.02483421	2.38E-02	1.22E-01	1.00E+00
ENSG00000171320.13_ESCO2	ENSG00000160789.18_LMNA	-0.08128	-0.237404414	0.078946686	3.20E-01	6.06E-01	1.00E+00
ENSG00000119771.13_KLHL29	ENSG00000160789.18_LMNA	0.27236	0.118293286	0.413630142	6.87E-04	8.65E-03	1.00E+00
ENSG00000225383.5_SFTA1P	ENSG00000160789.18_LMNA	-0.13086	-0.284138793	0.028946288	1.08E-01	3.27E-01	1.00E+00

ENSG00000116017.9_ARID3A	ENSG00000160789.18_LMNA	0.20313	0.04539347	0.350977105	1.21E-02	7.52E-02	1.00E+00
ENSG00000088320.3_REM1	ENSG00000160789.18_LMNA	-0.27966	-0.420154362	-0.12607207	4.84E-04	6.58E-03	1.00E+00
ENSG00000204652.6_RPS26P8	ENSG00000160789.18_LMNA	-0.1504	-0.302361725	0.009015833	6.44E-02	2.36E-01	1.00E+00
ENSG00000225338.1_RPL23AP18	ENSG00000160789.18_LMNA	-0.00494	-0.164007835	0.154386008	9.52E-01	9.81E-01	1.00E+00
ENSG00000261373.1_VPS9D1-AS	ENSG00000160789.18_LMNA	0.20464	0.046973001	0.35236425	1.14E-02	7.21E-02	1.00E+00
ENSG00000260400.1_RP11-119F	ENSG00000160789.18_LMNA	-0.10123	-0.256304507	0.058915657	2.15E-01	4.89E-01	1.00E+00
ENSG00000100330.14_MTMR3	ENSG00000160789.18_LMNA	-0.02027	-0.1788915	0.13938242	8.04E-01	9.22E-01	1.00E+00
ENSG00000099810.17_MTAP	ENSG00000160789.18_LMNA	-0.00239	-0.16153179	0.156867837	9.77E-01	9.91E-01	1.00E+00
ENSG00000267858.4_MZF1-AS1	ENSG00000160789.18_LMNA	0.12136	-0.038585925	0.27524311	1.36E-01	3.76E-01	1.00E+00
ENSG00000238041.3_RP11-59K5	ENSG00000160789.18_LMNA	-0.03138	-0.189634499	0.128461289	7.01E-01	8.72E-01	1.00E+00
ENSG00000186026.6_ZNF284	ENSG00000160789.18_LMNA	-0.03894	-0.196917592	0.12101329	6.34E-01	8.36E-01	1.00E+00
ENSG00000258900.1_HNRNPCP1	ENSG00000160789.18_LMNA	0.20121	0.043403218	0.349227352	1.29E-02	7.89E-02	1.00E+00
ENSG00000278922.1_AC002310.1	ENSG00000160789.18_LMNA	0.18082	0.022260949	0.330507283	2.58E-02	1.29E-01	1.00E+00
ENSG00000165359.13_INTS6L	ENSG00000160789.18_LMNA	-0.14452	-0.296889554	0.015026861	7.57E-02	2.63E-01	1.00E+00
ENSG00000175548.7_ALG10B	ENSG00000160789.18_LMNA	-0.19155	-0.340374573	-0.03337072	1.81E-02	1.00E-01	1.00E+00
ENSG00000272308.1_RP11-231G	ENSG00000160789.18_LMNA	0.03314	-0.126732782	0.19132786	6.85E-01	8.64E-01	1.00E+00
ENSG00000167208.13_SNX20	ENSG00000160789.18_LMNA	0.1503	-0.009117806	0.302269064	6.46E-02	2.37E-01	1.00E+00
ENSG00000213638.5_ADAT3	ENSG00000160789.18_LMNA	0.04623	-0.113807067	0.20393102	5.72E-01	8.00E-01	1.00E+00
ENSG00000158806.12_NPM2	ENSG00000160789.18_LMNA	-0.37226	-0.501728536	-0.22648426	2.33E-06	8.94E-05	1.20E-01
ENSG00000278492.1_RP11-15A1	ENSG00000160789.18_LMNA	-0.15018	-0.302154566	0.009243802	6.48E-02	2.37E-01	1.00E+00
ENSG00000204323.5_SMIM5	ENSG00000160789.18_LMNA	-0.09976	-0.254915452	0.060396524	2.21E-01	4.97E-01	1.00E+00
ENSG00000244932.2_RP11-529F	ENSG00000160789.18_LMNA	-0.00677	-0.165790711	0.152596475	9.34E-01	9.74E-01	1.00E+00
ENSG00000131061.12_ZNF341	ENSG00000160789.18_LMNA	0.12866	-0.031179838	0.282082408	1.14E-01	3.37E-01	1.00E+00
ENSG00000271614.1_LINC00936	ENSG00000160789.18_LMNA	-0.12851	-0.281938808	0.031335692	1.15E-01	3.38E-01	1.00E+00
ENSG00000180822.10_PSMG4	ENSG00000160789.18_LMNA	0.08235	-0.077873956	0.238422685	3.13E-01	6.00E-01	1.00E+00
ENSG00000278921.2_EPB41L4A-	ENSG00000160789.18_LMNA	-0.16746	-0.318175769	-0.00848287	3.92E-02	1.71E-01	1.00E+00
ENSG00000179348.10_GATA2	ENSG00000160789.18_LMNA	-0.17964	-0.329423339	-0.02104514	2.68E-02	1.33E-01	1.00E+00
ENSG00000111860.12_CEP85L	ENSG00000160789.18_LMNA	0.07767	-0.082555334	0.233973855	3.42E-01	6.26E-01	1.00E+00
ENSG00000103044.9_HAS3	ENSG00000160789.18_LMNA	0.39592	0.252644189	0.522206138	4.45E-07	2.39E-05	2.28E-02
ENSG00000106133.16_NSUN5P2	ENSG00000160789.18_LMNA	-0.11788	-0.271978622	0.042108835	1.48E-01	3.94E-01	1.00E+00
ENSG00000185684.11_EP400NL	ENSG00000160789.18_LMNA	-0.00053	-0.159718494	0.158682822	9.95E-01	9.98E-01	1.00E+00
ENSG00000243431.1_RPL5P30	ENSG00000160789.18_LMNA	-0.22417	-0.370162961	-0.06737668	5.50E-03	4.20E-02	1.00E+00
ENSG00000264885.1_RP11-815I9	ENSG00000160789.18_LMNA	-0.07137	-0.227979834	0.088840661	3.82E-01	6.61E-01	1.00E+00

ENSG00000273145.1_CITF22-92A	ENSG00000160789.18_LMNA	0.11638	-0.043632619	0.270564372	1.53E-01	4.03E-01	1.00E+00
ENSG00000266970.1_RP11-806H	ENSG00000160789.18_LMNA	0.28879	0.135832312	0.428297689	3.08E-04	4.60E-03	1.00E+00
ENSG00000204618.7_RNF39	ENSG00000160789.18_LMNA	-0.07975	-0.235951095	0.080476464	3.29E-01	6.15E-01	1.00E+00
ENSG00000197536.9_C5orf56	ENSG00000160789.18_LMNA	-0.15203	-0.303876241	0.007348194	6.15E-02	2.29E-01	1.00E+00
ENSG00000178409.12_BEND3	ENSG00000160789.18_LMNA	0.07656	-0.083660793	0.232921372	3.49E-01	6.33E-01	1.00E+00
ENSG00000271851.1_RP11-565F	ENSG00000160789.18_LMNA	-0.23065	-0.376045471	-0.07417628	4.25E-03	3.47E-02	1.00E+00
ENSG00000230580.1_AC021016.1	ENSG00000160789.18_LMNA	-0.18257	-0.332120035	-0.02407159	2.44E-02	1.24E-01	1.00E+00
ENSG00000003400.13_CASP10	ENSG00000160789.18_LMNA	-0.07528	-0.23170716	0.084935154	3.57E-01	6.40E-01	1.00E+00
ENSG00000173535.12_TNFRSF10	ENSG00000160789.18_LMNA	0.1409	-0.018720739	0.293516619	8.34E-02	2.79E-01	1.00E+00
ENSG00000115252.17_PDE1A	ENSG00000160789.18_LMNA	-0.35756	-0.488931139	-0.21033489	6.12E-06	1.96E-04	3.14E-01
ENSG00000118307.17_CASC1	ENSG00000160789.18_LMNA	-0.08963	-0.245330793	0.070577	2.72E-01	5.57E-01	1.00E+00
ENSG00000066735.13_KIF26A	ENSG00000160789.18_LMNA	-0.18809	-0.337192828	-0.02978002	2.03E-02	1.09E-01	1.00E+00
ENSG00000234825.3_XRCC6P2	ENSG00000160789.18_LMNA	-0.1164	-0.270590912	0.043604036	1.53E-01	4.03E-01	1.00E+00
ENSG00000080561.12_MID2	ENSG00000160789.18_LMNA	-0.05402	-0.211402855	0.106092877	5.09E-01	7.58E-01	1.00E+00
ENSG00000247746.4_USP51	ENSG00000160789.18_LMNA	-0.20001	-0.348122753	-0.04214804	1.35E-02	8.13E-02	1.00E+00
ENSG00000212443.1_SNORA53	ENSG00000160789.18_LMNA	-0.10401	-0.258922514	0.056120833	2.02E-01	4.73E-01	1.00E+00
ENSG00000115239.20_ASB3	ENSG00000160789.18_LMNA	-0.41255	-0.536509796	-0.27115378	1.28E-07	8.88E-06	6.58E-03
ENSG00000187912.10_CLEC17A	ENSG00000160789.18_LMNA	-0.138	-0.290809321	0.02167949	9.00E-02	2.92E-01	1.00E+00
ENSG00000196275.12_GTF2IRD2	ENSG00000160789.18_LMNA	-0.13459	-0.287623661	0.025154036	9.83E-02	3.08E-01	1.00E+00
ENSG00000230581.2_ACTG1P14	ENSG00000160789.18_LMNA	0.31121	0.159930919	0.448202638	9.52E-05	1.81E-03	1.00E+00
ENSG00000162746.13_FCRLB	ENSG00000160789.18_LMNA	0.05604	-0.104090088	0.213336674	4.93E-01	7.48E-01	1.00E+00
ENSG00000227603.1_RP11-165J3	ENSG00000160789.18_LMNA	-0.03395	-0.192115916	0.125927711	6.78E-01	8.59E-01	1.00E+00
ENSG00000137460.7_FHDC1	ENSG00000160789.18_LMNA	-0.04349	-0.2012978	0.116516598	5.95E-01	8.13E-01	1.00E+00
ENSG00000268034.1_AC005795.1	ENSG00000160789.18_LMNA	0.19373	0.035626487	0.342369842	1.68E-02	9.52E-02	1.00E+00
ENSG00000278175.2_GLIDR	ENSG00000160789.18_LMNA	-0.37997	-0.508412606	-0.23497966	1.38E-06	5.99E-05	7.07E-02
ENSG00000067798.12_NAV3	ENSG00000160789.18_LMNA	-0.325	-0.460377318	-0.17484194	4.40E-05	9.68E-04	1.00E+00
ENSG00000174586.9_ZNF497	ENSG00000160789.18_LMNA	0.16807	0.009113851	0.318742803	3.85E-02	1.69E-01	1.00E+00
ENSG00000228395.1_RP11-216B	ENSG00000160789.18_LMNA	-0.36821	-0.498204737	-0.22202234	3.05E-06	1.11E-04	1.57E-01
ENSG00000265542.4_RP11-60A2	ENSG00000160789.18_LMNA	-0.18759	-0.336733826	-0.02926268	2.07E-02	1.10E-01	1.00E+00
ENSG00000158315.9_RHBDL2	ENSG00000160789.18_LMNA	0.05533	-0.104794115	0.212657175	4.98E-01	7.52E-01	1.00E+00
ENSG00000119969.13_HELLS	ENSG00000160789.18_LMNA	0.17079	0.011912927	0.321255533	3.54E-02	1.60E-01	1.00E+00
ENSG00000186638.14_KIF24	ENSG00000160789.18_LMNA	0.04528	-0.114748505	0.203016618	5.80E-01	8.04E-01	1.00E+00
ENSG00000133101.8_CCNA1	ENSG00000160789.18_LMNA	-0.03487	-0.192995176	0.125028972	6.70E-01	8.55E-01	1.00E+00

ENSG00000123453.15_SARDH	ENSG00000160789.18_LMNA	0.3403	0.19147408	0.473831725	1.79E-05	4.71E-04	9.18E-01
ENSG00000171714.10_ANO5	ENSG00000160789.18_LMNA	-0.22155	-0.367776789	-0.06462649	6.09E-03	4.55E-02	1.00E+00
ENSG00000242667.1_RP11-745A	ENSG00000160789.18_LMNA	-0.10552	-0.260351043	0.054593738	1.96E-01	4.65E-01	1.00E+00
ENSG00000234176.1_HSPA8P1	ENSG00000160789.18_LMNA	-0.04718	-0.20484161	0.112868987	5.64E-01	7.95E-01	1.00E+00
ENSG00000234925.2_ATP5HP4	ENSG00000160789.18_LMNA	-0.06736	-0.224159408	0.092833635	4.10E-01	6.84E-01	1.00E+00
ENSG00000066827.14_ZFAT	ENSG00000160789.18_LMNA	0.17193	0.013089302	0.322310265	3.42E-02	1.56E-01	1.00E+00
ENSG00000196782.11_MAML3	ENSG00000160789.18_LMNA	0.10311	-0.057031132	0.258070314	2.06E-01	4.78E-01	1.00E+00
ENSG00000141934.8_PLPP2	ENSG00000160789.18_LMNA	-0.13152	-0.284763015	0.028267668	1.06E-01	3.24E-01	1.00E+00
ENSG00000246100.3_LINC00900	ENSG00000160789.18_LMNA	0.15982	0.000634588	0.311104319	4.92E-02	1.99E-01	1.00E+00
ENSG00000168282.5_MGAT2	ENSG00000160789.18_LMNA	0.26328	0.108633166	0.405485566	1.05E-03	1.20E-02	1.00E+00
ENSG00000239269.1_RPSAP4	ENSG00000160789.18_LMNA	-0.16352	-0.314535275	-0.00443768	4.41E-02	1.85E-01	1.00E+00
ENSG00000073605.17_GSDMB	ENSG00000160789.18_LMNA	-0.07624	-0.232614073	0.083983413	3.51E-01	6.34E-01	1.00E+00
ENSG00000256618.2_MTRNR2L1	ENSG00000160789.18_LMNA	-0.13042	-0.283730756	0.029389728	1.09E-01	3.29E-01	1.00E+00
ENSG00000276564.1_CTA-276F8	ENSG00000160789.18_LMNA	-0.18088	-0.330559509	-0.02231955	2.57E-02	1.29E-01	1.00E+00
ENSG00000224953.1_SRIP3	ENSG00000160789.18_LMNA	0.04396	-0.116053377	0.201748301	5.91E-01	8.10E-01	1.00E+00
ENSG00000131127.12_ZNF141	ENSG00000160789.18_LMNA	-0.06521	-0.222107	0.094974537	4.25E-01	6.96E-01	1.00E+00
ENSG00000146410.10_MTFR2	ENSG00000160789.18_LMNA	-0.05537	-0.212695025	0.104754906	4.98E-01	7.51E-01	1.00E+00
ENSG00000091106.17_NLRC4	ENSG00000160789.18_LMNA	-0.12806	-0.281521123	0.031788936	1.16E-01	3.40E-01	1.00E+00
ENSG00000173825.6_TIGD3	ENSG00000160789.18_LMNA	-0.16922	-0.319806535	-0.01029821	3.71E-02	1.65E-01	1.00E+00
ENSG00000176771.14_NCKAP5	ENSG00000160789.18_LMNA	-0.39251	-0.519260722	-0.24885703	5.69E-07	2.91E-05	2.92E-02
ENSG00000204282.4_TNRC6C-A	ENSG00000160789.18_LMNA	-0.30026	-0.438498651	-0.14813937	1.71E-04	2.89E-03	1.00E+00
ENSG00000174123.9_TLR10	ENSG00000160789.18_LMNA	-0.2599	-0.402452979	-0.10505054	1.22E-03	1.35E-02	1.00E+00
ENSG00000152932.7_RAB3C	ENSG00000160789.18_LMNA	-0.38898	-0.516212729	-0.24494666	7.33E-07	3.58E-05	3.76E-02
ENSG00000273568.1_RP11-417L	ENSG00000160789.18_LMNA	0.09542	-0.064764856	0.250810353	2.42E-01	5.21E-01	1.00E+00
ENSG00000248415.1_GAPDHP61	ENSG00000160789.18_LMNA	0.22713	0.070477174	0.372848296	4.89E-03	3.86E-02	1.00E+00
ENSG00000109944.9_C11orf63	ENSG00000160789.18_LMNA	0.25874	0.10381559	0.401406112	1.29E-03	1.40E-02	1.00E+00
ENSG00000267260.1_CTD-2162K	ENSG00000160789.18_LMNA	-0.11771	-0.271814543	0.042285697	1.49E-01	3.95E-01	1.00E+00
ENSG00000272933.1_RP11-47A8	ENSG00000160789.18_LMNA	-0.01615	-0.174903573	0.143416863	8.43E-01	9.39E-01	1.00E+00
ENSG00000140323.5_DISP2	ENSG00000160789.18_LMNA	-0.18719	-0.336371108	-0.02885397	2.09E-02	1.11E-01	1.00E+00
ENSG00000145990.9_GFOD1	ENSG00000160789.18_LMNA	-0.10166	-0.256705072	0.058488358	2.13E-01	4.86E-01	1.00E+00
ENSG00000138738.9_PRDM5	ENSG00000160789.18_LMNA	0.01291	-0.146595119	0.171754588	8.75E-01	9.51E-01	1.00E+00
ENSG00000180929.5_GPR62	ENSG00000160789.18_LMNA	-0.29639	-0.435061101	-0.14398203	2.09E-04	3.38E-03	1.00E+00
ENSG00000205559.3_CHKB-AS1	ENSG00000160789.18_LMNA	-0.09104	-0.246666816	0.06916186	2.65E-01	5.48E-01	1.00E+00

ENSG00000213290.4_PGK1P2	ENSG00000160789.18_LMNA	0.15284	-0.006522226	0.304625786	6.01E-02	2.26E-01	1.00E+00
ENSG00000259762.1_RP11-158M	ENSG00000160789.18_LMNA	-0.08463	-0.240584947	0.075593621	3.00E-01	5.86E-01	1.00E+00
ENSG00000228407.2_RP4-800M2	ENSG00000160789.18_LMNA	-0.07435	-0.230822974	0.085862489	3.63E-01	6.44E-01	1.00E+00
ENSG00000230508.1_RPL19P21	ENSG00000160789.18_LMNA	-0.15995	-0.311229502	-0.00077319	4.90E-02	1.99E-01	1.00E+00
ENSG00000165685.7_TMEM52B	ENSG00000160789.18_LMNA	-0.10553	-0.260352623	0.054592048	1.96E-01	4.65E-01	1.00E+00
ENSG00000133063.14_CHIT1	ENSG00000160789.18_LMNA	-0.06665	-0.223479044	0.093543659	4.15E-01	6.88E-01	1.00E+00
ENSG00000244349.1_HCG16	ENSG00000160789.18_LMNA	0.10874	-0.051353316	0.263377753	1.82E-01	4.47E-01	1.00E+00
ENSG00000188672.15_RHCE	ENSG00000160789.18_LMNA	0.02746	-0.132321472	0.185845961	7.37E-01	8.89E-01	1.00E+00
ENSG00000196183.5_RPS2P4	ENSG00000160789.18_LMNA	0.06184	-0.098324978	0.2188894	4.49E-01	7.15E-01	1.00E+00
ENSG00000272468.1_RP1-86C11	ENSG00000160789.18_LMNA	0.03619	-0.123723009	0.194271923	6.58E-01	8.49E-01	1.00E+00
ENSG00000162241.11_SLC25A45	ENSG00000160789.18_LMNA	0.10783	-0.052267378	0.262524603	1.86E-01	4.52E-01	1.00E+00
ENSG00000237883.1_DGUOK-AS	ENSG00000160789.18_LMNA	0.1846	0.026165644	0.333982987	2.28E-02	1.19E-01	1.00E+00
ENSG00000138829.9_FBN2	ENSG00000160789.18_LMNA	0.03173	-0.128121292	0.189967733	6.98E-01	8.71E-01	1.00E+00
ENSG00000187210.11_GCNT1	ENSG00000160789.18_LMNA	0.30865	0.157171852	0.445938105	1.09E-04	2.03E-03	1.00E+00
ENSG00000224057.1_EGFR-AS1	ENSG00000160789.18_LMNA	0.03457	-0.125321702	0.192708847	6.72E-01	8.56E-01	1.00E+00
ENSG00000146776.13_ATXN7L1	ENSG00000160789.18_LMNA	-0.01122	-0.170112506	0.148249862	8.91E-01	9.57E-01	1.00E+00
ENSG00000134533.5_RERG	ENSG00000160789.18_LMNA	-0.28943	-0.428871658	-0.1365224	2.98E-04	4.49E-03	1.00E+00
ENSG00000267508.4_ZNF285	ENSG00000160789.18_LMNA	-0.15381	-0.305529865	0.005525403	5.85E-02	2.22E-01	1.00E+00
ENSG00000124782.18_RREB1	ENSG00000160789.18_LMNA	0.36468	0.218139647	0.495130944	3.86E-06	1.35E-04	1.98E-01
ENSG00000279865.1_RP11-69M1	ENSG00000160789.18_LMNA	-0.2467	-0.390562298	-0.09107727	2.18E-03	2.09E-02	1.00E+00
ENSG00000203865.8_ATP1A1-AS	ENSG00000160789.18_LMNA	-0.35186	-0.483949582	-0.20408938	8.78E-06	2.62E-04	4.51E-01
ENSG00000118004.16_COLEC11	ENSG00000160789.18_LMNA	-0.16529	-0.316169909	-0.00625277	4.18E-02	1.79E-01	1.00E+00
ENSG00000226948.1_RPS4XP2	ENSG00000160789.18_LMNA	-0.05775	-0.214972623	0.10239378	4.80E-01	7.38E-01	1.00E+00
ENSG00000151790.7_TDO2	ENSG00000160789.18_LMNA	0.19181	0.033640043	0.340612943	1.79E-02	9.97E-02	1.00E+00
ENSG00000162687.15_KCNT2	ENSG00000160789.18_LMNA	-0.37433	-0.50352375	-0.22876186	2.03E-06	8.08E-05	1.04E-01
ENSG00000158163.13_DZIP1L	ENSG00000160789.18_LMNA	0.26651	0.112064272	0.408383785	9.04E-04	1.07E-02	1.00E+00
ENSG00000155744.8_FAM126B	ENSG00000160789.18_LMNA	-0.30585	-0.443454488	-0.15415103	1.27E-04	2.29E-03	1.00E+00
ENSG00000280149.1_RP4-751H1	ENSG00000160789.18_LMNA	0.19692	0.038940838	0.345296433	1.50E-02	8.80E-02	1.00E+00
ENSG00000260565.5_CTD-2270P	ENSG00000160789.18_LMNA	-0.11195	-0.266404353	0.048106372	1.70E-01	4.28E-01	1.00E+00
ENSG00000262766.1_RP11-196G	ENSG00000160789.18_LMNA	0.04431	-0.115707578	0.202084516	5.88E-01	8.08E-01	1.00E+00
ENSG00000272293.1_RP11-91J19	ENSG00000160789.18_LMNA	0.06399	-0.096189095	0.220941395	4.33E-01	7.03E-01	1.00E+00
ENSG00000163393.11_SLC22A15	ENSG00000160789.18_LMNA	-0.1148	-0.269084668	0.045225361	1.59E-01	4.12E-01	1.00E+00
ENSG00000187801.13_ZFP69B	ENSG00000160789.18_LMNA	-0.06248	-0.219495728	0.097694167	4.44E-01	7.12E-01	1.00E+00

ENSG00000213684.4_LDHBP2	ENSG00000160789.18_LMNA	-0.2245	-0.370456372	-0.06771517	5.43E-03	4.17E-02	1.00E+00
ENSG00000261428.2_RP11-16P6	ENSG00000160789.18_LMNA	-0.22744	-0.373132703	-0.07080589	4.83E-03	3.82E-02	1.00E+00
ENSG00000044459.13_CNTLN	ENSG00000160789.18_LMNA	0.1399	-0.019740114	0.292584441	8.56E-02	2.84E-01	1.00E+00
ENSG00000183833.15_MAATS1	ENSG00000160789.18_LMNA	0.19728	0.039308986	0.345621144	1.48E-02	8.73E-02	1.00E+00
ENSG00000233509.2_ZNF197-AS	ENSG00000160789.18_LMNA	-0.43146	-0.552694991	-0.29233754	2.86E-08	2.61E-06	1.47E-03
ENSG00000186280.6_KDM4D	ENSG00000160789.18_LMNA	-0.16571	-0.31655623	-0.00668204	4.13E-02	1.77E-01	1.00E+00
ENSG00000196371.3_FUT4	ENSG00000160789.18_LMNA	0.07021	-0.089999163	0.226872398	3.90E-01	6.68E-01	1.00E+00
ENSG00000262576.2_PCDHGA4	ENSG00000160789.18_LMNA	0.1424	-0.017190378	0.294914958	8.01E-02	2.72E-01	1.00E+00
ENSG00000179168.13_GGN	ENSG00000160789.18_LMNA	0.49273	0.361914134	0.604507275	1.13E-10	2.48E-08	5.81E-06
ENSG00000270060.1_RP11-390K	ENSG00000160789.18_LMNA	-0.02661	-0.185028446	0.133153183	7.45E-01	8.93E-01	1.00E+00
ENSG00000162757.4_C1orf74	ENSG00000160789.18_LMNA	0.01227	-0.147214264	0.171140387	8.81E-01	9.54E-01	1.00E+00
ENSG00000254585.2_MAGEL2	ENSG00000160789.18_LMNA	-0.20376	-0.351554588	-0.04605086	1.18E-02	7.39E-02	1.00E+00
ENSG00000196417.11_ZNF765	ENSG00000160789.18_LMNA	-0.02293	-0.181471961	0.136766218	7.79E-01	9.09E-01	1.00E+00
ENSG00000230191.1_RP4-725G1	ENSG00000160789.18_LMNA	0.25011	0.094683663	0.393640789	1.89E-03	1.87E-02	1.00E+00
ENSG00000272636.3_DOC2B	ENSG00000160789.18_LMNA	-0.24578	-0.389730068	-0.09010367	2.27E-03	2.16E-02	1.00E+00
ENSG00000145832.11_SLC25A48	ENSG00000160789.18_LMNA	-0.47639	-0.590782086	-0.34321618	5.52E-10	9.53E-08	2.83E-05
ENSG00000219470.1_RP3-337H4	ENSG00000160789.18_LMNA	0.0434	-0.116606385	0.201210463	5.95E-01	8.13E-01	1.00E+00
ENSG00000158055.14_GRHL3	ENSG00000160789.18_LMNA	-0.22444	-0.370403245	-0.06765387	5.44E-03	4.17E-02	1.00E+00
ENSG00000278383.1_RP4-694B1	ENSG00000160789.18_LMNA	0.10859	-0.051497623	0.263243095	1.83E-01	4.48E-01	1.00E+00
ENSG00000226284.1_ARPC3P1	ENSG00000160789.18_LMNA	-0.12375	-0.277483486	0.036163675	1.29E-01	3.64E-01	1.00E+00
ENSG00000242571.1_RPL21P11	ENSG00000160789.18_LMNA	-0.26127	-0.40368195	-0.10650149	1.15E-03	1.29E-02	1.00E+00
ENSG00000279536.1_RP11-445N	ENSG00000160789.18_LMNA	0.19403	0.035938684	0.342645768	1.66E-02	9.45E-02	1.00E+00
ENSG00000140025.14_EFCAB11	ENSG00000160789.18_LMNA	-0.20889	-0.356245017	-0.05140013	9.80E-03	6.47E-02	1.00E+00
ENSG00000119125.15_GDA	ENSG00000160789.18_LMNA	-0.087	-0.242837716	0.073214314	2.87E-01	5.72E-01	1.00E+00
ENSG00000090061.16_CCNK	ENSG00000160789.18_LMNA	0.05491	-0.105212082	0.212253625	5.02E-01	7.53E-01	1.00E+00
ENSG00000231043.3_AC007238.1	ENSG00000160789.18_LMNA	0.0485	-0.111562634	0.206108765	5.53E-01	7.88E-01	1.00E+00
ENSG00000185567.6_AHNAK2	ENSG00000160789.18_LMNA	0.53784	0.414097986	0.642065626	8.98E-13	5.24E-10	4.61E-08
ENSG00000261351.2_CTD-3185P	ENSG00000160789.18_LMNA	-0.19379	-0.342425942	-0.03568996	1.67E-02	9.50E-02	1.00E+00
ENSG00000212695.5_RP11-134K	ENSG00000160789.18_LMNA	-0.12717	-0.280688112	0.03269248	1.18E-01	3.45E-01	1.00E+00
ENSG00000149050.8_ZNF214	ENSG00000160789.18_LMNA	0.0392	-0.120750395	0.197174026	6.32E-01	8.35E-01	1.00E+00
ENSG00000173227.12_SYT12	ENSG00000160789.18_LMNA	0.3773	0.23203763	0.506101575	1.65E-06	6.91E-05	8.49E-02
ENSG00000272922.1_RP11-329B	ENSG00000160789.18_LMNA	-0.20609	-0.353682501	-0.0484755	1.09E-02	6.95E-02	1.00E+00
ENSG00000168405.13_CMAHP	ENSG00000160789.18_LMNA	0.16944	0.010521346	0.320006855	3.69E-02	1.64E-01	1.00E+00

ENSG00000111057.9_KRT18	ENSG00000160789.18_LMNA	0.38134	0.236492726	0.509599617	1.25E-06	5.54E-05	6.43E-02
ENSG00000116819.6_TFAP2E	ENSG00000160789.18_LMNA	-0.08896	-0.244692127	0.071253037	2.76E-01	5.60E-01	1.00E+00
ENSG00000261326.2_LINC01355	ENSG00000160789.18_LMNA	-0.03453	-0.192673947	0.125357378	6.73E-01	8.56E-01	1.00E+00
ENSG00000010319.5_SEMA3G	ENSG00000160789.18_LMNA	-0.0622	-0.219231515	0.09796908	4.46E-01	7.13E-01	1.00E+00
ENSG00000126259.18_KIRREL2	ENSG00000160789.18_LMNA	0.24093	0.084990051	0.385350888	2.79E-03	2.53E-02	1.00E+00
ENSG00000187792.4_ZNF70	ENSG00000160789.18_LMNA	0.0558	-0.10432826	0.213106834	4.95E-01	7.50E-01	1.00E+00
ENSG00000197566.8_ZNF624	ENSG00000160789.18_LMNA	-0.14097	-0.293583209	0.018647895	8.32E-02	2.79E-01	1.00E+00
ENSG00000109832.11_DDX25	ENSG00000160789.18_LMNA	-0.36867	-0.498603323	-0.22252646	2.96E-06	1.09E-04	1.52E-01
ENSG00000167306.17_MYO5B	ENSG00000160789.18_LMNA	0.15543	-0.003868817	0.307031084	5.59E-02	2.16E-01	1.00E+00
ENSG00000175497.15_DPP10	ENSG00000160789.18_LMNA	-0.55657	-0.657515107	-0.4360076	9.68E-14	8.66E-11	4.97E-09
ENSG00000272812.1_RP5-855D2	ENSG00000160789.18_LMNA	-0.07627	-0.232649564	0.083946156	3.50E-01	6.34E-01	1.00E+00
ENSG00000236264.4_RPL26P30	ENSG00000160789.18_LMNA	-0.05626	-0.213543507	0.103875726	4.91E-01	7.47E-01	1.00E+00
ENSG00000100890.14_KIAA0391	ENSG00000160789.18_LMNA	-0.10652	-0.261288277	0.053591036	1.92E-01	4.59E-01	1.00E+00
ENSG00000159871.13_LYPD5	ENSG00000160789.18_LMNA	-0.24906	-0.39268741	-0.09356596	1.97E-03	1.93E-02	1.00E+00
ENSG00000239486.1_RP11-163E	ENSG00000160789.18_LMNA	0.02553	-0.134219991	0.183979203	7.55E-01	8.98E-01	1.00E+00
ENSG00000179818.12_PCBP1-AS	ENSG00000160789.18_LMNA	-0.38034	-0.508733572	-0.23538866	1.34E-06	5.86E-05	6.89E-02
ENSG00000186047.9_DLEU7	ENSG00000160789.18_LMNA	-0.28581	-0.425639099	-0.13263959	3.58E-04	5.16E-03	1.00E+00
ENSG00000188878.15_FBF1	ENSG00000160789.18_LMNA	0.23106	0.074607153	0.376417413	4.18E-03	3.43E-02	1.00E+00
ENSG00000157214.12_STEAP2	ENSG00000160789.18_LMNA	0.0518	-0.108294664	0.209274018	5.26E-01	7.71E-01	1.00E+00
ENSG00000157782.8_CABP1	ENSG00000160789.18_LMNA	-0.1937	-0.342343128	-0.03559626	1.68E-02	9.52E-02	1.00E+00
ENSG00000115353.9_TACR1	ENSG00000160789.18_LMNA	-0.15608	-0.307633667	0.003203389	5.48E-02	2.13E-01	1.00E+00
ENSG00000276728.1_AC142472.(	ENSG00000160789.18_LMNA	-0.15659	-0.308113584	0.002673224	5.40E-02	2.11E-01	1.00E+00
ENSG00000138735.14_PDE5A	ENSG00000160789.18_LMNA	0.04993	-0.110145776	0.207481906	5.41E-01	7.81E-01	1.00E+00
ENSG00000267348.2_CTB-179K2	ENSG00000160789.18_LMNA	-0.06684	-0.223665459	0.09334915	4.13E-01	6.86E-01	1.00E+00
ENSG00000111850.9_SMIM8	ENSG00000160789.18_LMNA	-0.41077	-0.534988436	-0.2691757	1.47E-07	9.86E-06	7.54E-03
ENSG00000174326.10_SLC16A11	ENSG00000160789.18_LMNA	0.28198	0.128546884	0.422223682	4.32E-04	6.02E-03	1.00E+00
ENSG00000235245.1_RP11-122K	ENSG00000160789.18_LMNA	0.12959	-0.030235678	0.282952029	1.12E-01	3.33E-01	1.00E+00
ENSG00000239917.3_RPS10P16	ENSG00000160789.18_LMNA	-0.02974	-0.188048369	0.130078589	7.16E-01	8.79E-01	1.00E+00
ENSG00000270872.2_SRGAP2D	ENSG00000160789.18_LMNA	-0.09647	-0.251805926	0.063706553	2.37E-01	5.16E-01	1.00E+00
ENSG00000188921.13_HACD4	ENSG00000160789.18_LMNA	-0.011	-0.169898627	0.148465258	8.93E-01	9.58E-01	1.00E+00
ENSG00000214783.8_POLR2J4	ENSG00000160789.18_LMNA	0.03641	-0.12350926	0.194480788	6.56E-01	8.48E-01	1.00E+00
ENSG00000213397.9_HAUS7	ENSG00000160789.18_LMNA	0.04144	-0.118538013	0.19933032	6.12E-01	8.24E-01	1.00E+00
ENSG00000249565.2_SERBP1P5	ENSG00000160789.18_LMNA	0.26243	0.107733839	0.404724925	1.09E-03	1.24E-02	1.00E+00

ENSG00000178502.5_KLHL11	ENSG00000160789.18_LMNA	-0.19287	-0.341583448	-0.03473705	1.73E-02	9.72E-02	1.00E+00
ENSG00000174837.13_ADGRE1	ENSG00000160789.18_LMNA	0.09492	-0.065271154	0.250333829	2.45E-01	5.25E-01	1.00E+00
ENSG00000151466.10_SCLT1	ENSG00000160789.18_LMNA	-0.10198	-0.257012347	0.058160497	2.11E-01	4.85E-01	1.00E+00
ENSG00000224261.2_RPSAP18	ENSG00000160789.18_LMNA	-0.15337	-0.305118513	0.00597903	5.92E-02	2.24E-01	1.00E+00
ENSG00000129646.12_QRICH2	ENSG00000160789.18_LMNA	-0.11065	-0.265177583	0.049423255	1.75E-01	4.35E-01	1.00E+00
ENSG00000127720.6_METTL25	ENSG00000160789.18_LMNA	-0.01182	-0.170704315	0.147653695	8.85E-01	9.55E-01	1.00E+00
ENSG00000136895.17_GARNL3	ENSG00000160789.18_LMNA	-0.24436	-0.388447885	-0.08860481	2.41E-03	2.26E-02	1.00E+00
ENSG00000275580.1_RP11-295H	ENSG00000160789.18_LMNA	-0.11001	-0.264573547	0.050071259	1.77E-01	4.39E-01	1.00E+00
ENSG00000135917.12_SLC19A3	ENSG00000160789.18_LMNA	-0.11074	-0.265263416	0.049331153	1.74E-01	4.35E-01	1.00E+00
ENSG00000237149.5_ZNF503-AS	ENSG00000160789.18_LMNA	0.08827	-0.071942065	0.244040906	2.80E-01	5.65E-01	1.00E+00
ENSG00000183878.14_UTY	ENSG00000160789.18_LMNA	-0.07172	-0.228315726	0.088489111	3.80E-01	6.59E-01	1.00E+00
ENSG00000078098.12_FAP	ENSG00000160789.18_LMNA	0.28804	0.13502616	0.427626881	3.20E-04	4.73E-03	1.00E+00
ENSG00000178607.14_ERN1	ENSG00000160789.18_LMNA	0.2597	0.104837509	0.402272446	1.23E-03	1.36E-02	1.00E+00
ENSG00000134917.9_ADAMTS8	ENSG00000160789.18_LMNA	-0.24595	-0.389885218	-0.09028513	2.26E-03	2.14E-02	1.00E+00
ENSG00000115590.12_IL1R2	ENSG00000160789.18_LMNA	0.04358	-0.116429024	0.20138298	5.94E-01	8.13E-01	1.00E+00
ENSG00000091622.14_PITPNM3	ENSG00000160789.18_LMNA	0.10779	-0.052312238	0.262482719	1.86E-01	4.52E-01	1.00E+00
ENSG00000069493.13_CLEC2D	ENSG00000160789.18_LMNA	-0.1186	-0.272654595	0.041379992	1.46E-01	3.91E-01	1.00E+00
ENSG00000271335.4_RP11-324I2	ENSG00000160789.18_LMNA	-0.05376	-0.211150068	0.106354489	5.11E-01	7.59E-01	1.00E+00
ENSG00000184368.14_MAP7D2	ENSG00000160789.18_LMNA	-0.12035	-0.274297369	0.039607336	1.40E-01	3.81E-01	1.00E+00
ENSG00000185737.11_NRG3	ENSG00000160789.18_LMNA	-0.2108	-0.357991355	-0.05339624	9.14E-03	6.14E-02	1.00E+00
ENSG00000196118.10_CCDC189	ENSG00000160789.18_LMNA	0.14738	-0.012102954	0.299553909	7.00E-02	2.50E-01	1.00E+00
ENSG00000279312.1_RP3-331H2	ENSG00000160789.18_LMNA	-0.12799	-0.281460285	0.031854942	1.16E-01	3.41E-01	1.00E+00
ENSG00000260643.1_RP11-303E	ENSG00000160789.18_LMNA	-0.10749	-0.262203561	0.052611204	1.87E-01	4.54E-01	1.00E+00
ENSG00000234390.4_USP27X-AS	ENSG00000160789.18_LMNA	0.09191	-0.068294789	0.24748482	2.60E-01	5.43E-01	1.00E+00
ENSG00000204620.3_RP11-1148	ENSG00000160789.18_LMNA	0.08796	-0.072254631	0.243745396	2.81E-01	5.67E-01	1.00E+00
ENSG00000129437.8_KLK14	ENSG00000160789.18_LMNA	0.11711	-0.042893681	0.271250361	1.51E-01	3.99E-01	1.00E+00
ENSG00000267185.1_PTP4A2P1	ENSG00000160789.18_LMNA	0.27499	0.121089649	0.415979004	6.07E-04	7.86E-03	1.00E+00
ENSG00000186806.5_VSIG10L	ENSG00000160789.18_LMNA	0.14108	-0.018534053	0.293687271	8.30E-02	2.78E-01	1.00E+00
ENSG00000073331.16_ALPK1	ENSG00000160789.18_LMNA	0.1231	-0.036818817	0.276877872	1.31E-01	3.67E-01	1.00E+00
ENSG00000145375.7_SPATA5	ENSG00000160789.18_LMNA	0.08514	-0.075073701	0.241077507	2.97E-01	5.83E-01	1.00E+00
ENSG00000162461.7_SLC25A34	ENSG00000160789.18_LMNA	-0.00973	-0.168666656	0.149705385	9.05E-01	9.64E-01	1.00E+00
ENSG00000243014.1_PTMAP8	ENSG00000160789.18_LMNA	0.08096	-0.079259191	0.237107643	3.21E-01	6.08E-01	1.00E+00
ENSG00000074621.12_SLC24A1	ENSG00000160789.18_LMNA	0.25734	0.102329596	0.400145407	1.37E-03	1.47E-02	1.00E+00

ENSG00000240395.1_RPL5P23	ENSG00000160789.18_LMNA	-0.15098	-0.302901436	0.008421754	6.34E-02	2.34E-01	1.00E+00
ENSG00000272142.1_RP11-428J1	ENSG00000160789.18_LMNA	0.12235	-0.037584098	0.276170129	1.33E-01	3.70E-01	1.00E+00
ENSG00000196787.3_HIST1H2AC	ENSG00000160789.18_LMNA	-0.00377	-0.162869451	0.155527551	9.63E-01	9.85E-01	1.00E+00
ENSG00000171094.14_ALK	ENSG00000160789.18_LMNA	0.21052	0.053097123	0.357729802	9.23E-03	6.19E-02	1.00E+00
ENSG00000240970.1_RPL23AP64	ENSG00000160789.18_LMNA	-0.06988	-0.226557626	0.090328293	3.92E-01	6.70E-01	1.00E+00
ENSG00000214189.7_ZNF788	ENSG00000160789.18_LMNA	0.13556	-0.024161956	0.288533962	9.59E-02	3.04E-01	1.00E+00
ENSG00000162971.9_TYW5	ENSG00000160789.18_LMNA	-0.21112	-0.358277411	-0.05372344	9.03E-03	6.09E-02	1.00E+00
ENSG00000176371.12_ZSCAN2	ENSG00000160789.18_LMNA	-0.2088	-0.356159437	-0.05130237	9.84E-03	6.48E-02	1.00E+00
ENSG00000214575.8_CPEB1	ENSG00000160789.18_LMNA	-0.17826	-0.328149977	-0.01961803	2.80E-02	1.37E-01	1.00E+00
ENSG00000134297.6_PLEKHA8P	ENSG00000160789.18_LMNA	0.20849	0.050978737	0.355876085	9.95E-03	6.54E-02	1.00E+00
ENSG00000162747.8_FCGR3B	ENSG00000160789.18_LMNA	-0.05645	-0.213729934	0.103682486	4.90E-01	7.46E-01	1.00E+00
ENSG00000215397.3_SCRT2	ENSG00000160789.18_LMNA	-0.37799	-0.506700209	-0.23279923	1.58E-06	6.67E-05	8.10E-02
ENSG00000187116.12_LILRA5	ENSG00000160789.18_LMNA	0.07016	-0.09005109	0.226822741	3.90E-01	6.69E-01	1.00E+00
ENSG00000185669.5_SNAI3	ENSG00000160789.18_LMNA	-0.07438	-0.230850907	0.085833201	3.62E-01	6.44E-01	1.00E+00
ENSG00000147655.9_RSPO2	ENSG00000160789.18_LMNA	-0.0083	-0.167275286	0.151104763	9.19E-01	9.69E-01	1.00E+00
ENSG00000250588.5_	ENSG00000160789.18_LMNA	-0.21261	-0.359634358	-0.05527647	8.55E-03	5.85E-02	1.00E+00
ENSG00000259673.4_IQCH-AS1	ENSG00000160789.18_LMNA	-0.24689	-0.390733575	-0.09127771	2.17E-03	2.08E-02	1.00E+00
ENSG00000277290.1_RP11-326C	ENSG00000160789.18_LMNA	0.12441	-0.035489766	0.278106189	1.27E-01	3.60E-01	1.00E+00
ENSG00000260490.2_MYL12BP1	ENSG00000160789.18_LMNA	0.01186	-0.147615737	0.170741988	8.85E-01	9.55E-01	1.00E+00
ENSG00000231259.4_AC125232.1	ENSG00000160789.18_LMNA	-0.25402	-0.397155082	-0.09881021	1.59E-03	1.64E-02	1.00E+00
ENSG00000231120.3_BTF3P10	ENSG00000160789.18_LMNA	-0.15074	-0.302681419	0.008663961	6.38E-02	2.35E-01	1.00E+00
ENSG00000183426.14_NPIPA1	ENSG00000160789.18_LMNA	0.05706	-0.103075703	0.214315178	4.85E-01	7.42E-01	1.00E+00
ENSG00000214491.7_SEC14L6	ENSG00000160789.18_LMNA	-0.01444	-0.173244422	0.145092255	8.60E-01	9.45E-01	1.00E+00
ENSG00000124257.6_NEURL2	ENSG00000160789.18_LMNA	0.0534	-0.106712521	0.210804045	5.14E-01	7.62E-01	1.00E+00
ENSG00000144061.11_NPHP1	ENSG00000160789.18_LMNA	0.08837	-0.071839414	0.244137942	2.79E-01	5.64E-01	1.00E+00
ENSG00000156510.12_HKDC1	ENSG00000160789.18_LMNA	0.19327	0.035147301	0.341946225	1.71E-02	9.63E-02	1.00E+00
ENSG00000135835.9_KIAA1614	ENSG00000160789.18_LMNA	0.27774	0.124019898	0.418436112	5.32E-04	7.08E-03	1.00E+00
ENSG00000243725.5_TTC4	ENSG00000160789.18_LMNA	0.04805	-0.112003842	0.205680915	5.57E-01	7.90E-01	1.00E+00
ENSG00000099957.15_P2RX6	ENSG00000160789.18_LMNA	-0.02644	-0.18486068	0.133323808	7.46E-01	8.94E-01	1.00E+00
ENSG00000214756.6_METTL12	ENSG00000160789.18_LMNA	-0.23642	-0.381269748	-0.08023865	3.36E-03	2.92E-02	1.00E+00
ENSG00000267370.1_CTD-2623N	ENSG00000160789.18_LMNA	-0.20684	-0.354369683	-0.04925927	1.06E-02	6.81E-02	1.00E+00
ENSG00000121281.11_ADCY7	ENSG00000160789.18_LMNA	0.02734	-0.132438438	0.185731018	7.38E-01	8.90E-01	1.00E+00
ENSG00000134198.8_TSPAN2	ENSG00000160789.18_LMNA	-0.10123	-0.256300447	0.058919987	2.15E-01	4.89E-01	1.00E+00

ENSG00000140650.10_PMM2	ENSG00000160789.18_LMNA	0.2291	0.072547407	0.374638505	4.52E-03	3.64E-02	1.00E+00
ENSG00000198523.5_PLN	ENSG00000160789.18_LMNA	0.00313	-0.156145757	0.162252601	9.69E-01	9.88E-01	1.00E+00
ENSG00000110844.12_PRPF40B	ENSG00000160789.18_LMNA	0.00831	-0.151087983	0.167291977	9.19E-01	9.69E-01	1.00E+00
ENSG00000248932.4_RP11-319G	ENSG00000160789.18_LMNA	-0.12911	-0.282501083	0.030725346	1.13E-01	3.35E-01	1.00E+00
ENSG00000099337.4_KCNK6	ENSG00000160789.18_LMNA	0.21285	0.05552939	0.359855228	8.47E-03	5.81E-02	1.00E+00
ENSG00000231160.8_KLF3-AS1	ENSG00000160789.18_LMNA	-0.10266	-0.257648913	0.057481069	2.08E-01	4.81E-01	1.00E+00
ENSG00000104953.17_TLE6	ENSG00000160789.18_LMNA	0.06528	-0.094902753	0.222175863	4.24E-01	6.95E-01	1.00E+00
ENSG00000228265.4_RALY-AS1	ENSG00000160789.18_LMNA	0.0249	-0.134835901	0.183373103	7.61E-01	9.00E-01	1.00E+00
ENSG00000254270.1_ERHP1	ENSG00000160789.18_LMNA	-0.07651	-0.232871472	0.083713186	3.49E-01	6.33E-01	1.00E+00
ENSG00000273265.1_RP11-353K	ENSG00000160789.18_LMNA	-0.26188	-0.404228381	-0.10714702	1.12E-03	1.26E-02	1.00E+00
ENSG00000268858.2_RP4-591C2	ENSG00000160789.18_LMNA	0.1676	0.008629169	0.318307262	3.90E-02	1.71E-01	1.00E+00
ENSG00000196208.12_GREB1	ENSG00000160789.18_LMNA	0.06811	-0.092092337	0.22486941	4.04E-01	6.80E-01	1.00E+00
ENSG00000213860.4_RPL21P75	ENSG00000160789.18_LMNA	-0.23231	-0.377543871	-0.07591278	3.98E-03	3.30E-02	1.00E+00
ENSG00000176542.8_USF3	ENSG00000160789.18_LMNA	0.03441	-0.125482246	0.192551789	6.74E-01	8.57E-01	1.00E+00
ENSG00000130948.8_HSD17B3	ENSG00000160789.18_LMNA	0.00977	-0.149665423	0.168706371	9.05E-01	9.63E-01	1.00E+00
ENSG00000166446.13_CDYL2	ENSG00000160789.18_LMNA	-0.15815	-0.309558688	0.001075759	5.17E-02	2.06E-01	1.00E+00
ENSG00000255100.1_RP11-21L2	ENSG00000160789.18_LMNA	0.06806	-0.092135759	0.22482783	4.05E-01	6.80E-01	1.00E+00
ENSG00000164309.13_CMYA5	ENSG00000160789.18_LMNA	0.30442	0.152611837	0.442187303	1.37E-04	2.43E-03	1.00E+00
ENSG00000197969.10_VPS13A	ENSG00000160789.18_LMNA	0.04293	-0.117071111	0.200758337	5.99E-01	8.16E-01	1.00E+00
ENSG00000213976.4_CTD-2561J	ENSG00000160789.18_LMNA	-0.12483	-0.278498214	0.035065357	1.25E-01	3.58E-01	1.00E+00
ENSG00000172349.15_IL16	ENSG00000160789.18_LMNA	-0.04132	-0.199211565	0.118659939	6.13E-01	8.24E-01	1.00E+00
ENSG00000154265.14_ABCA5	ENSG00000160789.18_LMNA	0.00706	-0.152312247	0.166073691	9.31E-01	9.73E-01	1.00E+00
ENSG00000119778.13_ATAD2B	ENSG00000160789.18_LMNA	-0.0245	-0.182983103	0.135232082	7.65E-01	9.02E-01	1.00E+00
ENSG00000269439.4_CTD-3131K	ENSG00000160789.18_LMNA	-0.01102	-0.169924096	0.148439611	8.93E-01	9.58E-01	1.00E+00
ENSG00000156500.13_FAM122C	ENSG00000160789.18_LMNA	-0.17603	-0.326094514	-0.01731704	3.01E-02	1.43E-01	1.00E+00
ENSG00000159917.13_ZNF235	ENSG00000160789.18_LMNA	-0.13976	-0.292453695	0.019883039	8.59E-02	2.84E-01	1.00E+00
ENSG00000041515.14_MYO16	ENSG00000160789.18_LMNA	-0.16265	-0.313729751	-0.00354398	4.53E-02	1.88E-01	1.00E+00
ENSG00000230311.2_TOMM20P4	ENSG00000160789.18_LMNA	-0.0567	-0.213969413	0.103434222	4.88E-01	7.44E-01	1.00E+00
ENSG00000280161.1_CTC-205M6	ENSG00000160789.18_LMNA	-0.21453	-0.361389593	-0.05728751	7.95E-03	5.54E-02	1.00E+00
ENSG00000272795.1_RP11-602N	ENSG00000160789.18_LMNA	-0.27032	-0.411801528	-0.11611953	7.57E-04	9.31E-03	1.00E+00
ENSG00000272752.4_STAG3L5P-	ENSG00000160789.18_LMNA	0.03741	-0.122519013	0.195448031	6.47E-01	8.43E-01	1.00E+00
ENSG00000136286.13_MYO1G	ENSG00000160789.18_LMNA	0.28979	0.136907484	0.429191849	2.93E-04	4.42E-03	1.00E+00
ENSG00000237748.1_UQCRBP1	ENSG00000160789.18_LMNA	-0.06392	-0.220868789	0.096264719	4.34E-01	7.03E-01	1.00E+00

ENSG00000231006.1_RPL7P32	ENSG00000160789.18_LMNA	-0.12406	-0.277778003	0.035844975	1.28E-01	3.62E-01	1.00E+00
ENSG00000157766.14_ACAN	ENSG00000160789.18_LMNA	-0.00028	-0.159470726	0.158930654	9.97E-01	9.99E-01	1.00E+00
ENSG00000179299.15_NSUN7	ENSG00000160789.18_LMNA	0.20519	0.047540604	0.352862393	1.12E-02	7.11E-02	1.00E+00
ENSG00000260805.2_RP11-61J1!	ENSG00000160789.18_LMNA	-0.05118	-0.208678096	0.10891045	5.31E-01	7.74E-01	1.00E+00
ENSG00000239415.1_AP001469.!	ENSG00000160789.18_LMNA	-0.15952	-0.31082782	-0.00032849	4.96E-02	2.00E-01	1.00E+00
ENSG00000178295.13_36892	ENSG00000160789.18_LMNA	-0.03193	-0.19016545	0.127919526	6.96E-01	8.70E-01	1.00E+00
ENSG00000152229.17_PSTPIP2	ENSG00000160789.18_LMNA	0.01484	-0.144702004	0.173631051	8.56E-01	9.43E-01	1.00E+00
ENSG00000182870.11_GALNT9	ENSG00000160789.18_LMNA	-0.14138	-0.293960822	0.018234756	8.23E-02	2.77E-01	1.00E+00
ENSG00000281091.1_RP13-349C	ENSG00000160789.18_LMNA	0.04321	-0.116796055	0.201025952	5.97E-01	8.14E-01	1.00E+00
ENSG00000241057.1_AC004985.:	ENSG00000160789.18_LMNA	-0.01467	-0.173462873	0.14487177	8.58E-01	9.44E-01	1.00E+00
ENSG00000101278.6_RPS10P5	ENSG00000160789.18_LMNA	-0.17135	-0.321776487	-0.01249386	3.48E-02	1.58E-01	1.00E+00
ENSG00000272501.1_XXbac-BPG	ENSG00000160789.18_LMNA	-0.18271	-0.332242305	-0.02420894	2.43E-02	1.24E-01	1.00E+00
ENSG00000151655.16_ITIH2	ENSG00000160789.18_LMNA	-0.04092	-0.198827813	0.119053873	6.17E-01	8.26E-01	1.00E+00
ENSG00000168404.11_MLKL	ENSG00000160789.18_LMNA	0.14493	-0.014606134	0.297273233	7.48E-02	2.61E-01	1.00E+00
ENSG00000241155.1_ARHGAP31	ENSG00000160789.18_LMNA	-0.19828	-0.346538307	-0.04034929	1.43E-02	8.51E-02	1.00E+00
ENSG00000183067.5_IGSF5	ENSG00000160789.18_LMNA	-0.35102	-0.483216294	-0.20317195	9.26E-06	2.74E-04	4.75E-01
ENSG00000185760.14_KCNQ5	ENSG00000160789.18_LMNA	-0.24937	-0.392968968	-0.09389597	1.95E-03	1.91E-02	1.00E+00
ENSG00000166573.5_GALR1	ENSG00000160789.18_LMNA	-0.14348	-0.295922992	0.016086265	7.78E-02	2.67E-01	1.00E+00
ENSG00000256234.1_RP11-283G	ENSG00000160789.18_LMNA	0.10561	-0.054510696	0.260428686	1.95E-01	4.64E-01	1.00E+00
ENSG00000270714.1_MINOS1P2	ENSG00000160789.18_LMNA	0.06543	-0.094757933	0.222314778	4.23E-01	6.94E-01	1.00E+00
ENSG00000254528.6_RP11-728F	ENSG00000160789.18_LMNA	-0.35956	-0.490677108	-0.21252925	5.38E-06	1.76E-04	2.76E-01
ENSG00000225032.4_RP11-228B	ENSG00000160789.18_LMNA	0.0959	-0.06428315	0.251263589	2.40E-01	5.19E-01	1.00E+00
ENSG00000167562.10_ZNF701	ENSG00000160789.18_LMNA	-0.07762	-0.233933088	0.082598168	3.42E-01	6.27E-01	1.00E+00
ENSG00000265451.1_RP11-204L	ENSG00000160789.18_LMNA	0.0123	-0.147192692	0.171161791	8.80E-01	9.54E-01	1.00E+00
ENSG00000226015.2_CCT8P1	ENSG00000160789.18_LMNA	-0.12559	-0.279211246	0.034293134	1.23E-01	3.54E-01	1.00E+00
ENSG00000198346.9_ZNF813	ENSG00000160789.18_LMNA	-0.05845	-0.215645614	0.101695422	4.74E-01	7.34E-01	1.00E+00
ENSG00000102098.16_SCML2	ENSG00000160789.18_LMNA	-0.23225	-0.377496783	-0.07585818	3.99E-03	3.31E-02	1.00E+00
ENSG00000198393.6_ZNF26	ENSG00000160789.18_LMNA	-0.05955	-0.216693297	0.100607627	4.66E-01	7.28E-01	1.00E+00
ENSG00000197943.8_PLCG2	ENSG00000160789.18_LMNA	-0.00455	-0.16363223	0.154762749	9.56E-01	9.82E-01	1.00E+00
ENSG00000130653.14_PNPLA7	ENSG00000160789.18_LMNA	0.20522	0.04757146	0.352889467	1.12E-02	7.11E-02	1.00E+00
ENSG00000246859.2_STARD4-A!	ENSG00000160789.18_LMNA	-0.12035	-0.274295951	0.039608866	1.40E-01	3.81E-01	1.00E+00
ENSG00000145506.12_NKD2	ENSG00000160789.18_LMNA	-0.03434	-0.192492035	0.125543323	6.74E-01	8.57E-01	1.00E+00
ENSG00000114933.14_INO80D	ENSG00000160789.18_LMNA	-0.0037	-0.162801987	0.155595175	9.64E-01	9.86E-01	1.00E+00

ENSG00000238045.8_AC009133.1	ENSG00000160789.18_LMNA	0.23682	0.080657021	0.38162957	3.31E-03	2.88E-02	1.00E+00
ENSG00000169282.16_KCNAB1	ENSG00000160789.18_LMNA	-0.47282	-0.587777217	-0.33914863	7.71E-10	1.26E-07	3.96E-05
ENSG00000003137.7_CYP26B1	ENSG00000160789.18_LMNA	-0.16388	-0.314867823	-0.00480677	4.36E-02	1.84E-01	1.00E+00
ENSG00000115687.12_PASK	ENSG00000160789.18_LMNA	0.13205	-0.02773766	0.285250354	1.05E-01	3.21E-01	1.00E+00
ENSG00000241404.5_EGFL8	ENSG00000160789.18_LMNA	-0.03025	-0.188541196	0.129576258	7.11E-01	8.77E-01	1.00E+00
ENSG00000239830.1_RPS4XP22	ENSG00000160789.18_LMNA	-0.10385	-0.258774831	0.056278622	2.03E-01	4.74E-01	1.00E+00
ENSG00000081059.18_TCF7	ENSG00000160789.18_LMNA	0.05006	-0.110019567	0.207604161	5.40E-01	7.80E-01	1.00E+00
ENSG00000240038.5_AMY2B	ENSG00000160789.18_LMNA	-0.1352	-0.288193053	0.024533563	9.68E-02	3.05E-01	1.00E+00
ENSG00000172403.9_SYNPO2	ENSG00000160789.18_LMNA	-0.12371	-0.277446878	0.036203285	1.29E-01	3.64E-01	1.00E+00
ENSG00000177602.5_GSG2	ENSG00000160789.18_LMNA	0.04045	-0.119520446	0.198373174	6.21E-01	8.28E-01	1.00E+00
ENSG00000234409.6_CCDC188	ENSG00000160789.18_LMNA	0.11424	-0.045793406	0.26855658	1.61E-01	4.16E-01	1.00E+00
ENSG00000117013.13_KCNQ4	ENSG00000160789.18_LMNA	-0.03593	-0.194024511	0.123976169	6.60E-01	8.50E-01	1.00E+00
ENSG00000105967.14_TFEC	ENSG00000160789.18_LMNA	-0.11371	-0.268058734	0.046328732	1.63E-01	4.18E-01	1.00E+00
ENSG00000214402.6_LCNL1	ENSG00000160789.18_LMNA	-0.12265	-0.276450859	0.037280589	1.32E-01	3.69E-01	1.00E+00
ENSG00000260404.2_RP11-384K	ENSG00000160789.18_LMNA	0.15424	-0.005088088	0.305926315	5.78E-02	2.20E-01	1.00E+00
ENSG00000171428.12_NAT1	ENSG00000160789.18_LMNA	0.01267	-0.14682759	0.171524002	8.77E-01	9.52E-01	1.00E+00
ENSG00000224138.1_AC000123.4	ENSG00000160789.18_LMNA	0.1041	-0.056031857	0.259005785	2.02E-01	4.73E-01	1.00E+00
ENSG00000239264.7_TXNDC5	ENSG00000160789.18_LMNA	0.32845	0.178587543	0.463418776	3.61E-05	8.30E-04	1.00E+00
ENSG00000257660.4_RP11-579D	ENSG00000160789.18_LMNA	-0.21108	-0.358238746	-0.05367921	9.05E-03	6.10E-02	1.00E+00
ENSG00000162999.11_DUSP19	ENSG00000160789.18_LMNA	-0.37719	-0.506009088	-0.23191999	1.67E-06	6.95E-05	8.55E-02
ENSG00000184343.9_SRPK3	ENSG00000160789.18_LMNA	-0.16438	-0.315328679	-0.00531842	4.30E-02	1.82E-01	1.00E+00
ENSG00000174740.7_PABPC5	ENSG00000160789.18_LMNA	-0.33263	-0.467093855	-0.1831245	2.83E-05	6.80E-04	1.00E+00
ENSG00000105227.13_PRX	ENSG00000160789.18_LMNA	0.29075	0.13792985	0.430041562	2.79E-04	4.25E-03	1.00E+00
ENSG00000082438.14_COBLL1	ENSG00000160789.18_LMNA	-0.06223	-0.219253756	0.09794594	4.46E-01	7.13E-01	1.00E+00
ENSG00000185436.10_IFNLR1	ENSG00000160789.18_LMNA	-0.04557	-0.203299912	0.114456896	5.77E-01	8.03E-01	1.00E+00
ENSG00000279026.1_RP3-414A1	ENSG00000160789.18_LMNA	0.05938	-0.100769729	0.216537219	4.67E-01	7.29E-01	1.00E+00
ENSG00000280832.1_ST3GAL4-A	ENSG00000160789.18_LMNA	-0.07127	-0.227887977	0.088936786	3.83E-01	6.61E-01	1.00E+00
ENSG00000102890.13_ELMO3	ENSG00000160789.18_LMNA	-0.05239	-0.209844933	0.107704491	5.21E-01	7.67E-01	1.00E+00
ENSG00000173890.15_GPR160	ENSG00000160789.18_LMNA	-0.10434	-0.259235003	0.055786909	2.01E-01	4.71E-01	1.00E+00
ENSG00000232727.2_YWHAEP1	ENSG00000160789.18_LMNA	-0.15809	-0.309498859	0.001141927	5.18E-02	2.06E-01	1.00E+00
ENSG00000224856.2_RP4-796I17	ENSG00000160789.18_LMNA	-0.00028	-0.159477985	0.158923394	9.97E-01	9.99E-01	1.00E+00
ENSG00000075884.11_ARHGAP1	ENSG00000160789.18_LMNA	-0.04245	-0.200293261	0.117549004	6.04E-01	8.18E-01	1.00E+00
ENSG00000109758.7_HGFAC	ENSG00000160789.18_LMNA	-0.07441	-0.230878597	0.085804167	3.62E-01	6.44E-01	1.00E+00

ENSG00000200693.2_U3	ENSG00000160789.18_LMNA	-0.22355	-0.369599167	-0.06672646	5.63E-03	4.29E-02	1.00E+00
ENSG00000129173.11_E2F8	ENSG00000160789.18_LMNA	0.22175	0.064839792	0.367962	6.04E-03	4.52E-02	1.00E+00
ENSG00000183783.6_KCTD8	ENSG00000160789.18_LMNA	-0.30743	-0.444855312	-0.15585419	1.17E-04	2.14E-03	1.00E+00
ENSG00000257167.2_TMPO-AS1	ENSG00000160789.18_LMNA	0.05647	-0.103663459	0.21374829	4.90E-01	7.46E-01	1.00E+00
ENSG00000101916.11_TLR8	ENSG00000160789.18_LMNA	-0.07131	-0.227920269	0.088902994	3.83E-01	6.61E-01	1.00E+00
ENSG00000139734.16_DIAPH3	ENSG00000160789.18_LMNA	-0.01828	-0.176966719	0.141330972	8.23E-01	9.31E-01	1.00E+00
ENSG00000147145.11_LPAR4	ENSG00000160789.18_LMNA	-0.44644	-0.565451532	-0.30921577	8.18E-09	9.12E-07	4.19E-04
ENSG00000233205.1_AC108479.:	ENSG00000160789.18_LMNA	-0.10214	-0.257155956	0.058007244	2.11E-01	4.84E-01	1.00E+00
ENSG00000260000.2_RP3-467N1	ENSG00000160789.18_LMNA	-0.19761	-0.345927537	-0.03965644	1.47E-02	8.65E-02	1.00E+00
ENSG00000130720.11_FIBCD1	ENSG00000160789.18_LMNA	0.14221	-0.017383708	0.294738381	8.05E-02	2.73E-01	1.00E+00
ENSG00000180481.9_GLIPR1L2	ENSG00000160789.18_LMNA	-0.15173	-0.303593167	0.00766002	6.20E-02	2.31E-01	1.00E+00
ENSG00000054392.11_HHAT	ENSG00000160789.18_LMNA	0.10699	-0.053112109	0.261735725	1.90E-01	4.57E-01	1.00E+00
ENSG00000235436.9_DPY19L2P4	ENSG00000160789.18_LMNA	-0.2536	-0.396779645	-0.09836887	1.62E-03	1.66E-02	1.00E+00
ENSG00000275120.1_RP11-182J:	ENSG00000160789.18_LMNA	-0.35315	-0.485077454	-0.20550143	8.10E-06	2.46E-04	4.15E-01
ENSG00000065675.13_PRKCQ	ENSG00000160789.18_LMNA	-0.14257	-0.295069242	0.017021438	7.98E-02	2.72E-01	1.00E+00
ENSG00000230565.1_ZNF32-AS2	ENSG00000160789.18_LMNA	-0.12039	-0.274333541	0.039568282	1.40E-01	3.81E-01	1.00E+00
ENSG00000148655.13_C10orf11	ENSG00000160789.18_LMNA	-0.09092	-0.246553788	0.069281632	2.65E-01	5.49E-01	1.00E+00
ENSG00000130054.4_FAM155B	ENSG00000160789.18_LMNA	-0.41901	-0.542054306	-0.2783818	7.76E-08	5.87E-06	3.98E-03
ENSG00000254035.1_RP11-281C	ENSG00000160789.18_LMNA	0.01568	-0.14387531	0.174449749	8.48E-01	9.41E-01	1.00E+00
ENSG00000250305.7_KIAA1456	ENSG00000160789.18_LMNA	0.16568	0.006653633	0.316530667	4.14E-02	1.77E-01	1.00E+00
ENSG00000256103.2_ATP5J2P5	ENSG00000160789.18_LMNA	-0.04972	-0.207281354	0.110352793	5.43E-01	7.82E-01	1.00E+00
ENSG00000111490.11_TBC1D30	ENSG00000160789.18_LMNA	-0.19078	-0.339668085	-0.03257274	1.86E-02	1.02E-01	1.00E+00
ENSG00000230626.3_RP11-286H	ENSG00000160789.18_LMNA	0.06932	-0.090882355	0.226027586	3.96E-01	6.73E-01	1.00E+00
ENSG00000196876.12_SCN8A	ENSG00000160789.18_LMNA	-0.07516	-0.231591922	0.085056046	3.57E-01	6.40E-01	1.00E+00
ENSG00000273486.1_RP11-731C	ENSG00000160789.18_LMNA	-0.18211	-0.331691514	-0.02359029	2.47E-02	1.25E-01	1.00E+00
ENSG00000243156.6_MICAL3	ENSG00000160789.18_LMNA	-0.0848	-0.240746044	0.075423594	2.99E-01	5.85E-01	1.00E+00
ENSG00000250959.2_GLUD1P3	ENSG00000160789.18_LMNA	-0.25705	-0.399884601	-0.10202235	1.39E-03	1.48E-02	1.00E+00
ENSG00000279259.1_RP11-334C	ENSG00000160789.18_LMNA	-0.05643	-0.213710678	0.103702447	4.90E-01	7.46E-01	1.00E+00
ENSG00000260837.1_RP11-434B	ENSG00000160789.18_LMNA	-0.30558	-0.443217718	-0.15386333	1.29E-04	2.32E-03	1.00E+00
ENSG00000241014.1_RP11-244H	ENSG00000160789.18_LMNA	0.20225	0.044483286	0.350177172	1.25E-02	7.68E-02	1.00E+00
ENSG00000206417.7_H1FX-AS1	ENSG00000160789.18_LMNA	-0.03012	-0.188417448	0.129702408	7.13E-01	8.78E-01	1.00E+00
ENSG00000214796.7_RP11-480I1	ENSG00000160789.18_LMNA	-0.18958	-0.338566314	-0.03132907	1.93E-02	1.05E-01	1.00E+00
ENSG00000227001.3_NBPF2P	ENSG00000160789.18_LMNA	-0.10588	-0.260683181	0.054238473	1.94E-01	4.63E-01	1.00E+00

ENSG00000229605.5_RPL21P93	ENSG00000160789.18_LMNA	-0.14239	-0.294909401	0.017196463	8.01E-02	2.72E-01	1.00E+00
ENSG00000182487.11_NCF1B	ENSG00000160789.18_LMNA	0.00219	-0.157066189	0.161333728	9.79E-01	9.92E-01	1.00E+00
ENSG00000272430.1_RP11-38L1!	ENSG00000160789.18_LMNA	-0.20588	-0.353495696	-0.0482625	1.09E-02	6.98E-02	1.00E+00
ENSG00000212789.4_ST13P5	ENSG00000160789.18_LMNA	-0.07986	-0.236056561	0.0803655	3.28E-01	6.14E-01	1.00E+00
ENSG00000244331.1_CTB-47B8.:	ENSG00000160789.18_LMNA	0.0386	-0.121347577	0.196591456	6.37E-01	8.38E-01	1.00E+00
ENSG00000231312.5_AC007246.:	ENSG00000160789.18_LMNA	-0.10696	-0.261708829	0.053140901	1.90E-01	4.57E-01	1.00E+00
ENSG00000173040.11_EVC2	ENSG00000160789.18_LMNA	0.43885	0.300652937	0.558995041	1.56E-08	1.56E-06	7.98E-04
ENSG00000166394.13_CYB5R2	ENSG00000160789.18_LMNA	0.08319	-0.077028329	0.239224892	3.08E-01	5.95E-01	1.00E+00
ENSG00000272148.1_RP11-195B	ENSG00000160789.18_LMNA	-0.10404	-0.258952932	0.056088332	2.02E-01	4.73E-01	1.00E+00
ENSG00000272459.1_RP11-1277.	ENSG00000160789.18_LMNA	0.11064	-0.049427042	0.265174054	1.75E-01	4.35E-01	1.00E+00
ENSG00000153531.11_ADPRHL1	ENSG00000160789.18_LMNA	-0.20054	-0.34861468	-0.04270691	1.32E-02	8.01E-02	1.00E+00
ENSG00000261879.4_RP11-333E	ENSG00000160789.18_LMNA	-0.0805	-0.236667302	0.079722764	3.24E-01	6.11E-01	1.00E+00
ENSG00000168026.15_TTC21A	ENSG00000160789.18_LMNA	-0.05816	-0.215367816	0.101983729	4.77E-01	7.35E-01	1.00E+00
ENSG00000220875.1_HIST1H3PS	ENSG00000160789.18_LMNA	-0.24582	-0.389764585	-0.09014404	2.27E-03	2.15E-02	1.00E+00
ENSG00000150048.9_CLEC1A	ENSG00000160789.18_LMNA	-0.07208	-0.228660365	0.088128324	3.77E-01	6.57E-01	1.00E+00
ENSG00000138182.13_KIF20B	ENSG00000160789.18_LMNA	0.10497	-0.055156035	0.2598252	1.98E-01	4.68E-01	1.00E+00
ENSG00000138134.10_STAMBPL	ENSG00000160789.18_LMNA	-0.24565	-0.389617005	-0.08997145	2.28E-03	2.16E-02	1.00E+00
ENSG00000180938.5_ZNF572	ENSG00000160789.18_LMNA	-0.23936	-0.383926789	-0.08333051	2.98E-03	2.66E-02	1.00E+00
ENSG00000120645.10_IQSEC3	ENSG00000160789.18_LMNA	-0.16162	-0.312776477	-0.00248699	4.67E-02	1.92E-01	1.00E+00
ENSG00000273329.1_RP11-448A	ENSG00000160789.18_LMNA	0.01685	-0.142734014	0.175579284	8.37E-01	9.37E-01	1.00E+00
ENSG00000263155.4_MYZAP	ENSG00000160789.18_LMNA	-0.12084	-0.27475214	0.03911626	1.38E-01	3.79E-01	1.00E+00
ENSG00000177406.4_RP11-218M	ENSG00000160789.18_LMNA	0.04437	-0.115647178	0.202143235	5.87E-01	8.08E-01	1.00E+00
ENSG00000118495.17_PLAGL1	ENSG00000160789.18_LMNA	0.10237	-0.057777234	0.257371465	2.10E-01	4.82E-01	1.00E+00
ENSG00000224738.1_AC099850.:	ENSG00000160789.18_LMNA	-0.31466	-0.451255715	-0.16365788	7.88E-05	1.56E-03	1.00E+00
ENSG00000139899.9_CBLN3	ENSG00000160789.18_LMNA	0.45006	0.313305993	0.568524573	5.98E-09	7.02E-07	3.07E-04
ENSG00000276007.1_RP11-358L:	ENSG00000160789.18_LMNA	-0.19792	-0.34620935	-0.03997609	1.45E-02	8.59E-02	1.00E+00
ENSG00000186073.10_C15orf41	ENSG00000160789.18_LMNA	-0.11533	-0.269583564	0.044688532	1.57E-01	4.09E-01	1.00E+00
ENSG00000177990.10_DPY19L2	ENSG00000160789.18_LMNA	-0.11516	-0.269421046	0.044863426	1.58E-01	4.10E-01	1.00E+00
ENSG00000249572.1_CTD-2203K	ENSG00000160789.18_LMNA	-0.11668	-0.270853015	0.043321739	1.52E-01	4.01E-01	1.00E+00
ENSG00000152457.16_DCLRE1C	ENSG00000160789.18_LMNA	-0.27366	-0.414786859	-0.11966979	6.46E-04	8.25E-03	1.00E+00
ENSG00000213430.6_HSPD1P1	ENSG00000160789.18_LMNA	0.07171	-0.088499395	0.228305901	3.80E-01	6.59E-01	1.00E+00
ENSG00000276644.3_DACH1	ENSG00000160789.18_LMNA	-0.31104	-0.448056682	-0.15975295	9.61E-05	1.82E-03	1.00E+00
ENSG00000213970.3_RP11-264F	ENSG00000160789.18_LMNA	0.02548	-0.134260654	0.183939195	7.55E-01	8.98E-01	1.00E+00

ENSG00000089505.16_CMTM1	ENSG00000160789.18_LMNA	0.05146	-0.108633589	0.208946056	5.29E-01	7.73E-01	1.00E+00
ENSG00000204442.3_FAM155A	ENSG00000160789.18_LMNA	-0.27174	-0.413066501	-0.11762296	7.08E-04	8.86E-03	1.00E+00
ENSG00000164066.11_INTU	ENSG00000160789.18_LMNA	0.24044	0.084468406	0.384903403	2.85E-03	2.57E-02	1.00E+00
ENSG00000160808.8_MYL3	ENSG00000160789.18_LMNA	-0.13816	-0.290959538	0.021515464	8.96E-02	2.92E-01	1.00E+00
ENSG00000259863.1_SH3RF3-AS	ENSG00000160789.18_LMNA	-0.26409	-0.406215674	-0.10949685	1.01E-03	1.17E-02	1.00E+00
ENSG00000226823.1_SUGT1P1	ENSG00000160789.18_LMNA	-0.28814	-0.427724127	-0.135143	3.18E-04	4.72E-03	1.00E+00
ENSG00000170684.7_ZNF296	ENSG00000160789.18_LMNA	0.23879	0.082732426	0.3834132	3.05E-03	2.71E-02	1.00E+00
ENSG00000230042.1_AK3P3	ENSG00000160789.18_LMNA	-0.19701	-0.345381753	-0.03903756	1.50E-02	8.78E-02	1.00E+00
ENSG00000134627.10_PIWIL4	ENSG00000160789.18_LMNA	-0.01103	-0.169930952	0.148432706	8.93E-01	9.58E-01	1.00E+00
ENSG00000107077.16_KDM4C	ENSG00000160789.18_LMNA	0.1236	-0.036318921	0.277339999	1.29E-01	3.64E-01	1.00E+00
ENSG00000105889.13_STEAP1B	ENSG00000160789.18_LMNA	-0.22788	-0.373525021	-0.07125945	4.75E-03	3.78E-02	1.00E+00
ENSG00000273796.1_LL21NC02-	ENSG00000160789.18_LMNA	0.40356	0.26113563	0.528786954	2.53E-07	1.54E-05	1.30E-02
ENSG00000177374.11_HIC1	ENSG00000160789.18_LMNA	0.42685	0.287158201	0.548755838	4.16E-08	3.52E-06	2.14E-03
ENSG00000112182.13_BACH2	ENSG00000160789.18_LMNA	-0.06377	-0.220730487	0.09640876	4.35E-01	7.04E-01	1.00E+00
ENSG00000175611.10_LINC0047	ENSG00000160789.18_LMNA	-0.38659	-0.514149932	-0.24230523	8.67E-07	4.06E-05	4.45E-02
ENSG00000228084.1_RP5-884G6	ENSG00000160789.18_LMNA	-0.18409	-0.333520357	-0.02564537	2.32E-02	1.20E-01	1.00E+00
ENSG00000237451.3_CDK2AP2P	ENSG00000160789.18_LMNA	-0.20728	-0.3547695	-0.04971546	1.04E-02	6.73E-02	1.00E+00
ENSG00000224713.4_AC025165.8	ENSG00000160789.18_LMNA	-0.27038	-0.411856497	-0.11618483	7.54E-04	9.29E-03	1.00E+00
ENSG00000271032.1_CTD-2527I2	ENSG00000160789.18_LMNA	-0.00873	-0.16769341	0.150684366	9.15E-01	9.68E-01	1.00E+00
ENSG00000182389.17_CACNB4	ENSG00000160789.18_LMNA	-0.43838	-0.558596798	-0.30012613	1.62E-08	1.61E-06	8.30E-04
ENSG00000105048.15_TNNT1	ENSG00000160789.18_LMNA	0.232	0.075595272	0.377270017	4.03E-03	3.33E-02	1.00E+00
ENSG00000272604.1_RP11-251G	ENSG00000160789.18_LMNA	0.01507	-0.144481544	0.173849422	8.54E-01	9.43E-01	1.00E+00
ENSG00000254012.1_RPL10P8	ENSG00000160789.18_LMNA	-0.07389	-0.230383582	0.08632312	3.66E-01	6.47E-01	1.00E+00
ENSG00000232803.1_SLCO4A1-A	ENSG00000160789.18_LMNA	-0.04002	-0.197961466	0.119942838	6.24E-01	8.31E-01	1.00E+00
ENSG00000080200.8_CRYBG3	ENSG00000160789.18_LMNA	0.00952	-0.149906463	0.168466807	9.07E-01	9.64E-01	1.00E+00
ENSG00000169635.8_HIC2	ENSG00000160789.18_LMNA	0.05391	-0.106202684	0.211296758	5.09E-01	7.59E-01	1.00E+00
ENSG00000182405.5_PGBD4	ENSG00000160789.18_LMNA	-0.1894	-0.338399712	-0.03114109	1.94E-02	1.06E-01	1.00E+00
ENSG00000164946.18_FREM1	ENSG00000160789.18_LMNA	-0.0166	-0.175341438	0.142974407	8.39E-01	9.37E-01	1.00E+00
ENSG00000171847.9_FAM90A1	ENSG00000160789.18_LMNA	-0.12959	-0.282957433	0.030229808	1.12E-01	3.33E-01	1.00E+00
ENSG00000054938.14_CHRDL2	ENSG00000160789.18_LMNA	0.21661	0.059462233	0.363285256	7.35E-03	5.22E-02	1.00E+00
ENSG00000253833.1_RP11-367E	ENSG00000160789.18_LMNA	-0.06824	-0.225000639	0.091955284	4.03E-01	6.79E-01	1.00E+00
ENSG00000261795.1_RP11-90P1	ENSG00000160789.18_LMNA	-0.16582	-0.316660646	-0.00679809	4.12E-02	1.77E-01	1.00E+00
ENSG00000184454.6_NCMAP	ENSG00000160789.18_LMNA	0.08453	-0.075694147	0.240489692	3.01E-01	5.87E-01	1.00E+00

ENSG00000231848.1_AC012354.8	ENSG00000160789.18_LMNA	0.10109	-0.05906058	0.256168627	2.15E-01	4.89E-01	1.00E+00
ENSG00000183971.5_NPW	ENSG00000160789.18_LMNA	0.05692	-0.103215848	0.214180028	4.86E-01	7.43E-01	1.00E+00
ENSG00000046604.11_DSG2	ENSG00000160789.18_LMNA	0.26827	0.113940364	0.409965977	8.33E-04	1.00E-02	1.00E+00
ENSG00000100065.13_CARD10	ENSG00000160789.18_LMNA	0.17969	0.02109599	0.329468691	2.67E-02	1.33E-01	1.00E+00
ENSG00000228218.1_ATF4P3	ENSG00000160789.18_LMNA	-0.13857	-0.291346291	0.021093081	8.86E-02	2.90E-01	1.00E+00
ENSG00000179899.8_PHC1P1	ENSG00000160789.18_LMNA	0.00795	-0.151443503	0.166938293	9.23E-01	9.70E-01	1.00E+00
ENSG00000173611.16_SCAI	ENSG00000160789.18_LMNA	-0.20731	-0.354799944	-0.04975021	1.04E-02	6.73E-02	1.00E+00
ENSG00000131019.9_ULBP3	ENSG00000160789.18_LMNA	0.32545	0.175330567	0.460774469	4.29E-05	9.48E-04	1.00E+00
ENSG00000138346.13_DNA2	ENSG00000160789.18_LMNA	-0.17249	-0.322827231	-0.0136662	3.36E-02	1.54E-01	1.00E+00
ENSG00000279108.1_CTC-490E2	ENSG00000160789.18_LMNA	0.03802	-0.121917862	0.196034916	6.42E-01	8.40E-01	1.00E+00
ENSG00000239857.5_GET4	ENSG00000160789.18_LMNA	0.12801	-0.031836013	0.281477733	1.16E-01	3.41E-01	1.00E+00
ENSG00000238085.1_RP11-435F	ENSG00000160789.18_LMNA	-0.03799	-0.196002629	0.12195094	6.42E-01	8.41E-01	1.00E+00
ENSG00000092529.21_CAPN3	ENSG00000160789.18_LMNA	-0.35558	-0.487203836	-0.20816674	6.94E-06	2.18E-04	3.56E-01
ENSG00000228889.5_UBAC2-AS1	ENSG00000160789.18_LMNA	-0.42371	-0.546073586	-0.28364021	5.35E-08	4.33E-06	2.75E-03
ENSG00000107242.16_PIP5K1B	ENSG00000160789.18_LMNA	-0.37399	-0.503232727	-0.22839243	2.07E-06	8.20E-05	1.06E-01
ENSG00000230269.5_RP1-40E16	ENSG00000160789.18_LMNA	0.20278	0.04503341	0.350660712	1.22E-02	7.58E-02	1.00E+00
ENSG00000267277.1_CTD-2342J	ENSG00000160789.18_LMNA	-0.19319	-0.341879008	-0.03507128	1.71E-02	9.64E-02	1.00E+00
ENSG00000237248.4_LINC00987	ENSG00000160789.18_LMNA	-0.27533	-0.416284932	-0.12145421	5.97E-04	7.76E-03	1.00E+00
ENSG00000114757.17_PEX5L	ENSG00000160789.18_LMNA	-0.33448	-0.468718222	-0.18513368	2.54E-05	6.25E-04	1.00E+00
ENSG00000132170.18_PPARG	ENSG00000160789.18_LMNA	-0.06614	-0.222995173	0.094048429	4.18E-01	6.90E-01	1.00E+00
ENSG00000240399.1_RP1-228P1	ENSG00000160789.18_LMNA	-0.1664	-0.317193994	-0.00739096	4.05E-02	1.75E-01	1.00E+00
ENSG00000272764.1_RP11-318E	ENSG00000160789.18_LMNA	-0.0791	-0.235339067	0.081120244	3.33E-01	6.18E-01	1.00E+00
ENSG00000268182.4_SMIM17	ENSG00000160789.18_LMNA	-0.37274	-0.502144052	-0.22701116	2.26E-06	8.75E-05	1.16E-01
ENSG00000177511.5_ST8SIA3	ENSG00000160789.18_LMNA	-0.31693	-0.453259122	-0.16610795	6.95E-05	1.40E-03	1.00E+00
ENSG00000155158.19_TTC39B	ENSG00000160789.18_LMNA	0.30468	0.152894917	0.442420443	1.35E-04	2.40E-03	1.00E+00
ENSG00000118322.11_ATP10B	ENSG00000160789.18_LMNA	-0.21987	-0.366247378	-0.06286619	6.49E-03	4.77E-02	1.00E+00
ENSG00000099958.13_DERL3	ENSG00000160789.18_LMNA	0.33419	0.184821297	0.468465794	2.58E-05	6.33E-04	1.00E+00
ENSG00000262227.1_RP5-1050D	ENSG00000160789.18_LMNA	-0.02305	-0.181583563	0.136652971	7.78E-01	9.09E-01	1.00E+00
ENSG00000260233.3_SSSCA1-AS	ENSG00000160789.18_LMNA	-0.16148	-0.312641068	-0.00233691	4.69E-02	1.93E-01	1.00E+00
ENSG00000089682.15_RBM41	ENSG00000160789.18_LMNA	-0.05333	-0.210744896	0.106773716	5.14E-01	7.62E-01	1.00E+00
ENSG00000268201.1_CTD-3138B	ENSG00000160789.18_LMNA	-0.10296	-0.25793491	0.057175718	2.07E-01	4.79E-01	1.00E+00
ENSG00000273352.1_RP11-61L1	ENSG00000160789.18_LMNA	0.07072	-0.089485769	0.227363264	3.87E-01	6.65E-01	1.00E+00
ENSG00000179361.16_ARID3B	ENSG00000160789.18_LMNA	0.09716	-0.063017033	0.252454217	2.34E-01	5.11E-01	1.00E+00

ENSG00000214192.3_UBE2V1P2	ENSG00000160789.18_LMNA	0.19543	0.037394387	0.343931649	1.58E-02	9.12E-02	1.00E+00
ENSG00000213478.2_CFL1P2	ENSG00000160789.18_LMNA	0.0499	-0.110180219	0.207448541	5.42E-01	7.81E-01	1.00E+00
ENSG00000164506.13_STXBP5	ENSG00000160789.18_LMNA	-0.20289	-0.350758647	-0.04514485	1.22E-02	7.56E-02	1.00E+00
ENSG00000204241.6_RP11-713P	ENSG00000160789.18_LMNA	-0.44062	-0.560499363	-0.30264431	1.34E-08	1.38E-06	6.88E-04
ENSG00000165810.15_BTNL9	ENSG00000160789.18_LMNA	-0.2242	-0.370191616	-0.06740973	5.49E-03	4.20E-02	1.00E+00
ENSG00000197415.10_VEPH1	ENSG00000160789.18_LMNA	-0.37696	-0.505810167	-0.23166701	1.69E-06	7.03E-05	8.69E-02
ENSG00000169507.8_SLC38A11	ENSG00000160789.18_LMNA	-0.087	-0.242837023	0.073215046	2.87E-01	5.72E-01	1.00E+00
ENSG00000158987.18_RAPGEF6	ENSG00000160789.18_LMNA	-0.36038	-0.491388303	-0.2134239	5.10E-06	1.69E-04	2.62E-01
ENSG00000206561.11_COLQ	ENSG00000160789.18_LMNA	-0.29396	-0.432898013	-0.14137127	2.37E-04	3.74E-03	1.00E+00
ENSG00000247982.5_LINC00926	ENSG00000160789.18_LMNA	0.00816	-0.151238096	0.167142649	9.21E-01	9.70E-01	1.00E+00
ENSG00000146856.13_AGBL3	ENSG00000160789.18_LMNA	-0.17147	-0.32188643	-0.01261648	3.47E-02	1.57E-01	1.00E+00
ENSG00000260267.1_RP11-452L	ENSG00000160789.18_LMNA	0.16568	0.006654845	0.316531758	4.14E-02	1.77E-01	1.00E+00
ENSG00000180096.10_SEPT1	ENSG00000160789.18_LMNA	0.24377	0.087981128	0.387914017	2.48E-03	2.30E-02	1.00E+00
ENSG00000153930.9_ANKFN1	ENSG00000160789.18_LMNA	-0.09744	-0.252718781	0.062735556	2.32E-01	5.10E-01	1.00E+00
ENSG00000144668.10_ITGA9	ENSG00000160789.18_LMNA	-0.06703	-0.223843736	0.093163109	4.12E-01	6.86E-01	1.00E+00
ENSG00000180881.18_CAPS2	ENSG00000160789.18_LMNA	-0.24395	-0.388079878	-0.08817487	2.46E-03	2.29E-02	1.00E+00
ENSG00000235115.3_CHCHD2P8	ENSG00000160789.18_LMNA	-0.04612	-0.203827222	0.113913963	5.73E-01	8.00E-01	1.00E+00
ENSG00000241923.2_RPL14P3	ENSG00000160789.18_LMNA	-0.15443	-0.306103684	0.004892399	5.75E-02	2.19E-01	1.00E+00
ENSG00000177425.9_PAWR	ENSG00000160789.18_LMNA	0.00918	-0.150235542	0.168139682	9.11E-01	9.66E-01	1.00E+00
ENSG00000233225.3_AC004987.9	ENSG00000160789.18_LMNA	-0.11804	-0.272127898	0.041947912	1.48E-01	3.94E-01	1.00E+00
ENSG00000253854.1_RP11-10N2	ENSG00000160789.18_LMNA	0.01097	-0.148491304	0.169872763	8.93E-01	9.58E-01	1.00E+00
ENSG00000177692.10_DNAJC28	ENSG00000160789.18_LMNA	-0.19009	-0.339031789	-0.03185438	1.90E-02	1.04E-01	1.00E+00
ENSG00000225400.1_RAB28P5	ENSG00000160789.18_LMNA	-0.19462	-0.343190201	-0.03655486	1.63E-02	9.31E-02	1.00E+00
ENSG00000266405.3_CBX3P2	ENSG00000160789.18_LMNA	-0.02423	-0.18272753	0.135491652	7.67E-01	9.03E-01	1.00E+00
ENSG00000213026.4_CFL1P4	ENSG00000160789.18_LMNA	0.04663	-0.113417111	0.204309616	5.68E-01	7.98E-01	1.00E+00
ENSG00000204839.7_MROH6	ENSG00000160789.18_LMNA	0.35199	0.204238663	0.48406887	8.71E-06	2.60E-04	4.47E-01
ENSG00000274349.3_ZNF658	ENSG00000160789.18_LMNA	-0.03161	-0.189858323	0.12823293	6.99E-01	8.71E-01	1.00E+00
ENSG00000183208.11_GDPGP1	ENSG00000160789.18_LMNA	0.02529	-0.134455831	0.183747147	7.57E-01	8.99E-01	1.00E+00
ENSG00000146350.12_TBC1D32	ENSG00000160789.18_LMNA	-0.24548	-0.3894606	-0.08978856	2.30E-03	2.18E-02	1.00E+00
ENSG00000231050.1_RP1-140A9	ENSG00000160789.18_LMNA	-0.11167	-0.2661423	0.048387765	1.71E-01	4.30E-01	1.00E+00
ENSG00000196218.10_RYR1	ENSG00000160789.18_LMNA	-0.21432	-0.361196819	-0.05706652	8.02E-03	5.57E-02	1.00E+00
ENSG00000255435.5_RP11-770J1	ENSG00000160789.18_LMNA	-0.23576	-0.380674354	-0.07954662	3.46E-03	2.97E-02	1.00E+00
ENSG00000213066.10_FGFR1OP	ENSG00000160789.18_LMNA	-0.05911	-0.216271716	0.10104544	4.69E-01	7.30E-01	1.00E+00

ENSG00000180089.5_TMEM86B	ENSG00000160789.18_LMNA	0.26398	0.10937469	0.406112429	1.02E-03	1.17E-02	1.00E+00
ENSG00000178965.12_ERICH3	ENSG00000160789.18_LMNA	0.10516	-0.054961922	0.26000675	1.97E-01	4.67E-01	1.00E+00
ENSG00000185513.13_L3MBTL1	ENSG00000160789.18_LMNA	-0.12675	-0.280293219	0.03312063	1.20E-01	3.48E-01	1.00E+00
ENSG00000102287.15_GABRE	ENSG00000160789.18_LMNA	-0.07461	-0.231068077	0.085605479	3.61E-01	6.43E-01	1.00E+00
ENSG00000235272.1_FAM103A2I	ENSG00000160789.18_LMNA	0.08233	-0.077889634	0.238407808	3.13E-01	6.00E-01	1.00E+00
ENSG00000280047.1_CTC-463A1	ENSG00000160789.18_LMNA	-0.09245	-0.24799859	0.067749957	2.57E-01	5.40E-01	1.00E+00
ENSG00000188227.11_ZNF793	ENSG00000160789.18_LMNA	-0.12044	-0.274381963	0.039516	1.39E-01	3.81E-01	1.00E+00
ENSG00000196110.6_ZNF699	ENSG00000160789.18_LMNA	-0.03789	-0.195906261	0.122049665	6.43E-01	8.41E-01	1.00E+00
ENSG00000224376.1_AC017104.(	ENSG00000160789.18_LMNA	-0.01136	-0.170256022	0.148105311	8.89E-01	9.57E-01	1.00E+00
ENSG00000256894.1_RP11-283G	ENSG00000160789.18_LMNA	0.19321	0.035086498	0.341892464	1.71E-02	9.64E-02	1.00E+00
ENSG00000197245.4_FAM110D	ENSG00000160789.18_LMNA	0.02567	-0.134077253	0.184119633	7.54E-01	8.98E-01	1.00E+00
ENSG00000161647.17_MPP3	ENSG00000160789.18_LMNA	0.12069	-0.039263704	0.274615611	1.39E-01	3.79E-01	1.00E+00
ENSG00000180884.9_ZNF792	ENSG00000160789.18_LMNA	-0.03248	-0.19069364	0.127380392	6.91E-01	8.67E-01	1.00E+00
ENSG00000235652.6_RP11-545I5	ENSG00000160789.18_LMNA	-0.2339	-0.378986779	-0.0775867	3.73E-03	3.15E-02	1.00E+00
ENSG00000253320.4_AZIN1-AS1	ENSG00000160789.18_LMNA	-0.20025	-0.348347054	-0.04240284	1.34E-02	8.07E-02	1.00E+00
ENSG00000230630.2_DNM3OS	ENSG00000160789.18_LMNA	0.07136	-0.088849364	0.227971518	3.82E-01	6.61E-01	1.00E+00
ENSG00000113971.17_NPHP3	ENSG00000160789.18_LMNA	0.03495	-0.12495049	0.193071933	6.69E-01	8.54E-01	1.00E+00
ENSG00000237943.5_PRKCQ-AS	ENSG00000160789.18_LMNA	-0.2649	-0.406935829	-0.1103492	9.74E-04	1.14E-02	1.00E+00
ENSG00000269416.4_LINC01224	ENSG00000160789.18_LMNA	-0.13123	-0.284490294	0.028564191	1.07E-01	3.25E-01	1.00E+00
ENSG00000198851.8_CD3E	ENSG00000160789.18_LMNA	0.08694	-0.073277481	0.242777952	2.87E-01	5.72E-01	1.00E+00
ENSG00000184349.11_EFNA5	ENSG00000160789.18_LMNA	0.07093	-0.089276613	0.227563195	3.85E-01	6.64E-01	1.00E+00
ENSG00000129595.11_EPB41L4A	ENSG00000160789.18_LMNA	-0.09976	-0.254912868	0.060399276	2.21E-01	4.97E-01	1.00E+00
ENSG00000279265.1_AC000123.3	ENSG00000160789.18_LMNA	0.13927	-0.020379043	0.291999865	8.70E-02	2.87E-01	1.00E+00
ENSG00000162409.9_PRKAA2	ENSG00000160789.18_LMNA	-0.0666	-0.22342752	0.093597416	4.15E-01	6.88E-01	1.00E+00
ENSG00000255121.2_RP11-110I1	ENSG00000160789.18_LMNA	0.09987	-0.060288131	0.255017168	2.21E-01	4.96E-01	1.00E+00
ENSG00000185869.12_ZNF829	ENSG00000160789.18_LMNA	-0.06152	-0.218576593	0.098650316	4.52E-01	7.17E-01	1.00E+00
ENSG00000242970.2_AC068522.4	ENSG00000160789.18_LMNA	-0.24309	-0.387305197	-0.08727017	2.55E-03	2.35E-02	1.00E+00
ENSG00000236762.1_RPL19P16	ENSG00000160789.18_LMNA	-0.13176	-0.284982731	0.028028736	1.06E-01	3.23E-01	1.00E+00
ENSG00000152669.8_CCNO	ENSG00000160789.18_LMNA	0.17172	0.012874585	0.322117808	3.44E-02	1.57E-01	1.00E+00
ENSG00000218537.1_MIF-AS1	ENSG00000160789.18_LMNA	-0.00554	-0.164600165	0.153791698	9.46E-01	9.78E-01	1.00E+00
ENSG00000162959.12_MEMO1	ENSG00000160789.18_LMNA	-0.32083	-0.456704064	-0.17032926	5.58E-05	1.17E-03	1.00E+00
ENSG00000259953.1_RP11-4O1.2	ENSG00000160789.18_LMNA	-0.16276	-0.31382636	-0.00365114	4.51E-02	1.88E-01	1.00E+00
ENSG00000130684.12_ZNF337	ENSG00000160789.18_LMNA	0.16047	0.00130114	0.311706234	4.83E-02	1.97E-01	1.00E+00

ENSG00000240392.1_RPL9P3	ENSG00000160789.18_LMNA	-0.13434	-0.287395021	0.025403119	9.89E-02	3.10E-01	1.00E+00
ENSG00000170396.7_ZNF804A	ENSG00000160789.18_LMNA	-0.42393	-0.546266783	-0.28389337	5.26E-08	4.26E-06	2.70E-03
ENSG00000182327.7_GLTPD2	ENSG00000160789.18_LMNA	0.01934	-0.140295329	0.177990032	8.13E-01	9.26E-01	1.00E+00
ENSG00000151575.13_TEX9	ENSG00000160789.18_LMNA	0.04342	-0.116585134	0.201231134	5.95E-01	8.13E-01	1.00E+00
ENSG00000196466.9_ZNF799	ENSG00000160789.18_LMNA	-0.24928	-0.392889329	-0.09380262	1.95E-03	1.92E-02	1.00E+00
ENSG00000112419.13_PHACTR2	ENSG00000160789.18_LMNA	0.18524	0.026826447	0.334570364	2.23E-02	1.17E-01	1.00E+00
ENSG00000039068.17_CDH1	ENSG00000160789.18_LMNA	-0.13897	-0.291718784	0.020686166	8.77E-02	2.88E-01	1.00E+00
ENSG00000272871.1_RP11-408A	ENSG00000160789.18_LMNA	-0.05945	-0.216602401	0.100702032	4.67E-01	7.28E-01	1.00E+00
ENSG00000269952.1_RP11-324I2	ENSG00000160789.18_LMNA	-0.03025	-0.188539676	0.129577808	7.11E-01	8.77E-01	1.00E+00
ENSG00000111877.16_MCM9	ENSG00000160789.18_LMNA	-0.08131	-0.237439972	0.078909239	3.19E-01	6.06E-01	1.00E+00
ENSG00000161328.10_LRRC56	ENSG00000160789.18_LMNA	0.28539	0.132195081	0.425268557	3.65E-04	5.24E-03	1.00E+00
ENSG00000123892.10_RAB38	ENSG00000160789.18_LMNA	0.2474	0.091811809	0.391189861	2.12E-03	2.04E-02	1.00E+00
ENSG00000187566.4_NHLRC1	ENSG00000160789.18_LMNA	-0.16474	-0.315665783	-0.00569277	4.25E-02	1.81E-01	1.00E+00
ENSG00000181195.9_PENK	ENSG00000160789.18_LMNA	0.02284	-0.136855649	0.181383824	7.80E-01	9.10E-01	1.00E+00
ENSG00000223345.3_HIST2H2BA	ENSG00000160789.18_LMNA	0.00051	-0.15870653	0.159694794	9.95E-01	9.98E-01	1.00E+00
ENSG00000187323.10_DCC	ENSG00000160789.18_LMNA	-0.37146	-0.501035309	-0.22560557	2.46E-06	9.34E-05	1.26E-01
ENSG00000186496.9_ZNF396	ENSG00000160789.18_LMNA	-0.1592	-0.310531923	-9.81E-07	5.01E-02	2.02E-01	1.00E+00
ENSG00000072858.9_SIDT1	ENSG00000160789.18_LMNA	-0.35088	-0.483091081	-0.20301534	9.34E-06	2.76E-04	4.79E-01
ENSG00000164406.7_LEAP2	ENSG00000160789.18_LMNA	-0.1197	-0.27368251	0.040271039	1.42E-01	3.85E-01	1.00E+00
ENSG00000161509.12_GRIN2C	ENSG00000160789.18_LMNA	-0.29836	-0.436815191	-0.14610213	1.89E-04	3.12E-03	1.00E+00
ENSG00000138356.12_AOX1	ENSG00000160789.18_LMNA	-0.03018	-0.18847928	0.129639377	7.12E-01	8.78E-01	1.00E+00
ENSG00000198468.6_FLVCR1-AS	ENSG00000160789.18_LMNA	-0.15763	-0.30907467	0.001610985	5.24E-02	2.07E-01	1.00E+00
ENSG00000257576.1_HSPD1P4	ENSG00000160789.18_LMNA	0.07373	-0.086487649	0.230226607	3.67E-01	6.48E-01	1.00E+00
ENSG00000229729.5_	ENSG00000160789.18_LMNA	0.0363	-0.123612426	0.194379983	6.57E-01	8.48E-01	1.00E+00
ENSG00000180385.7_EMC3-AS1	ENSG00000160789.18_LMNA	0.05532	-0.104799637	0.212651844	4.98E-01	7.52E-01	1.00E+00
ENSG00000135378.3_PRRG4	ENSG00000160789.18_LMNA	-0.10559	-0.260409074	0.054531672	1.95E-01	4.64E-01	1.00E+00
ENSG00000156787.15_TBC1D31	ENSG00000160789.18_LMNA	-0.12569	-0.279304323	0.034192304	1.23E-01	3.53E-01	1.00E+00
ENSG00000272240.1_RP5-855D2	ENSG00000160789.18_LMNA	-0.06283	-0.219829734	0.097346564	4.42E-01	7.09E-01	1.00E+00
ENSG00000165626.15_BEND7	ENSG00000160789.18_LMNA	-0.25689	-0.399742585	-0.10185507	1.40E-03	1.49E-02	1.00E+00
ENSG00000237886.1_NALT1	ENSG00000160789.18_LMNA	0.02241	-0.137280054	0.180965491	7.84E-01	9.12E-01	1.00E+00
ENSG00000135116.8_HRK	ENSG00000160789.18_LMNA	-0.22429	-0.370273669	-0.06750438	5.47E-03	4.19E-02	1.00E+00
ENSG00000225234.1_TRAPPC12	ENSG00000160789.18_LMNA	0.00256	-0.156699845	0.161699518	9.75E-01	9.90E-01	1.00E+00
ENSG00000256092.2_RP13-942N	ENSG00000160789.18_LMNA	-0.03147	-0.189721783	0.128372241	7.00E-01	8.72E-01	1.00E+00

ENSG00000224167.1_RP3-522D1	ENSG00000160789.18_LMNA	-0.36556	-0.49589675	-0.21910616	3.64E-06	1.29E-04	1.87E-01
ENSG00000280255.1_RP5-1007F	ENSG00000160789.18_LMNA	-0.39828	-0.524242668	-0.25526756	3.74E-07	2.09E-05	1.92E-02
ENSG00000215237.6_RP11-54D1	ENSG00000160789.18_LMNA	-0.04194	-0.199802328	0.118053305	6.08E-01	8.21E-01	1.00E+00
ENSG00000242086.7_LINC00969	ENSG00000160789.18_LMNA	0.02369	-0.136025952	0.18220132	7.72E-01	9.06E-01	1.00E+00
ENSG00000213047.10_DENND1E	ENSG00000160789.18_LMNA	-0.23885	-0.383465035	-0.08279278	3.04E-03	2.70E-02	1.00E+00
ENSG00000176208.7_ATAD5	ENSG00000160789.18_LMNA	-0.105	-0.259858186	0.055120768	1.98E-01	4.68E-01	1.00E+00
ENSG00000169891.16_REPS2	ENSG00000160789.18_LMNA	-0.35426	-0.486050649	-0.20672076	7.55E-06	2.33E-04	3.87E-01
ENSG00000272768.1_RP4-673M1	ENSG00000160789.18_LMNA	0.2736	0.119604012	0.414731603	6.48E-04	8.27E-03	1.00E+00
ENSG00000243181.2_RP11-734J.	ENSG00000160789.18_LMNA	-0.24235	-0.386636142	-0.08648922	2.63E-03	2.41E-02	1.00E+00
ENSG00000279861.1_RP11-43N1	ENSG00000160789.18_LMNA	0.02592	-0.133830992	0.184361881	7.51E-01	8.96E-01	1.00E+00
ENSG00000237977.1_EIF4HP2	ENSG00000160789.18_LMNA	0.0006	-0.158612974	0.159788317	9.94E-01	9.98E-01	1.00E+00
ENSG00000254489.1_RP5-1024C	ENSG00000160789.18_LMNA	-0.18964	-0.33861519	-0.03138422	1.93E-02	1.05E-01	1.00E+00
ENSG00000214872.7_SMTNL1	ENSG00000160789.18_LMNA	-0.06844	-0.225187139	0.091760489	4.02E-01	6.78E-01	1.00E+00
ENSG00000258315.4_C17orf49	ENSG00000160789.18_LMNA	-0.03557	-0.193677024	0.124331658	6.63E-01	8.52E-01	1.00E+00
ENSG00000196369.9_SRGAP2B	ENSG00000160789.18_LMNA	0.03373	-0.126150053	0.191898314	6.80E-01	8.60E-01	1.00E+00
ENSG00000168811.5_IL12A	ENSG00000160789.18_LMNA	0.00671	-0.1526573	0.165730146	9.35E-01	9.74E-01	1.00E+00
ENSG00000100450.11_GZMH	ENSG00000160789.18_LMNA	0.09706	-0.063115139	0.252361994	2.34E-01	5.12E-01	1.00E+00
ENSG00000130812.9_ANGPTL6	ENSG00000160789.18_LMNA	-0.14914	-0.301191101	0.010303626	6.67E-02	2.42E-01	1.00E+00
ENSG00000236197.2_AC002429.!	ENSG00000160789.18_LMNA	-0.36242	-0.493164424	-0.21566021	4.47E-06	1.52E-04	2.29E-01
ENSG00000188985.6_DHFRP1	ENSG00000160789.18_LMNA	0.02071	-0.138952792	0.17931556	8.00E-01	9.20E-01	1.00E+00
ENSG00000171817.15_ZNF540	ENSG00000160789.18_LMNA	-0.43225	-0.55337088	-0.29322776	2.69E-08	2.48E-06	1.38E-03
ENSG00000270230.1_MTND6P22	ENSG00000160789.18_LMNA	-0.09177	-0.247353167	0.068434371	2.61E-01	5.44E-01	1.00E+00
ENSG00000268543.1_CTD-2619J	ENSG00000160789.18_LMNA	0.06559	-0.09459373	0.222472272	4.22E-01	6.93E-01	1.00E+00
ENSG00000136492.7_BRIP1	ENSG00000160789.18_LMNA	0.0308	-0.129032036	0.189074942	7.06E-01	8.75E-01	1.00E+00
ENSG00000237730.1_RP11-459A	ENSG00000160789.18_LMNA	-0.10605	-0.260848178	0.054061957	1.93E-01	4.62E-01	1.00E+00
ENSG00000249930.1_AC007016.:	ENSG00000160789.18_LMNA	0.27663	0.122834919	0.417442988	5.61E-04	7.37E-03	1.00E+00
ENSG00000213857.3_RP11-12M9	ENSG00000160789.18_LMNA	0.27164	0.117520033	0.412979938	7.11E-04	8.88E-03	1.00E+00
ENSG00000260372.5_AQP4-AS1	ENSG00000160789.18_LMNA	-0.28835	-0.427904624	-0.13535989	3.15E-04	4.68E-03	1.00E+00
ENSG00000214176.8_PLEKHM1P	ENSG00000160789.18_LMNA	0.10166	-0.058485886	0.256707389	2.13E-01	4.86E-01	1.00E+00
ENSG00000155367.14_PPM1J	ENSG00000160789.18_LMNA	0.31723	0.166438343	0.453529061	6.83E-05	1.38E-03	1.00E+00
ENSG00000269399.2_CTD-3222D	ENSG00000160789.18_LMNA	-0.13652	-0.289430561	0.023184207	9.35E-02	2.99E-01	1.00E+00
ENSG00000259420.4_RP11-307C	ENSG00000160789.18_LMNA	-0.3374	-0.471282878	-0.18831073	2.13E-05	5.45E-04	1.00E+00
ENSG00000118690.11_ARMC2	ENSG00000160789.18_LMNA	-0.02987	-0.188171854	0.129952738	7.15E-01	8.79E-01	1.00E+00

ENSG00000127080.8_IPPK	ENSG00000160789.18_LMNA	0.21901	0.061967719	0.365466128	6.71E-03	4.88E-02	1.00E+00
ENSG00000214203.4_RPS4XP1	ENSG00000160789.18_LMNA	0.01899	-0.140638484	0.177651039	8.16E-01	9.28E-01	1.00E+00
ENSG00000148604.12_RGR	ENSG00000160789.18_LMNA	-0.36104	-0.491965258	-0.21415002	4.89E-06	1.63E-04	2.51E-01
ENSG00000129465.14_RIPK3	ENSG00000160789.18_LMNA	-0.01631	-0.175055656	0.143263199	8.42E-01	9.38E-01	1.00E+00
ENSG00000204311.10_DFNB59	ENSG00000160789.18_LMNA	-0.14609	-0.298354487	0.01341988	7.25E-02	2.55E-01	1.00E+00
ENSG00000259070.4_LINC00639	ENSG00000160789.18_LMNA	0.15574	-0.003544608	0.307324705	5.54E-02	2.15E-01	1.00E+00
ENSG00000271743.1_CTD-2541M	ENSG00000160789.18_LMNA	-0.1977	-0.346009465	-0.03974937	1.46E-02	8.63E-02	1.00E+00
ENSG00000242110.6_AMACR	ENSG00000160789.18_LMNA	-0.05591	-0.21321191	0.104219379	4.94E-01	7.49E-01	1.00E+00
ENSG00000244485.1_RPL18P13	ENSG00000160789.18_LMNA	-0.01636	-0.175102435	0.143215931	8.41E-01	9.38E-01	1.00E+00
ENSG00000228071.4_RPL7P47	ENSG00000160789.18_LMNA	-0.06869	-0.225423216	0.091513875	4.00E-01	6.77E-01	1.00E+00
ENSG00000162482.4_AKR7A3	ENSG00000160789.18_LMNA	0.02943	-0.130377522	0.187755015	7.19E-01	8.81E-01	1.00E+00
ENSG00000187808.4_SOWAHD	ENSG00000160789.18_LMNA	-0.12291	-0.276695028	0.03701656	1.31E-01	3.68E-01	1.00E+00
ENSG00000240747.6_KRBOX1	ENSG00000160789.18_LMNA	-0.12719	-0.280705854	0.032673241	1.18E-01	3.45E-01	1.00E+00
ENSG00000260686.1_CTB-36H16	ENSG00000160789.18_LMNA	-0.46098	-0.577779519	-0.32568182	2.28E-09	3.10E-07	1.17E-04
ENSG00000149295.12_DRD2	ENSG00000160789.18_LMNA	-0.13318	-0.286313387	0.02658094	1.02E-01	3.15E-01	1.00E+00
ENSG00000232310.5_RP11-557H	ENSG00000160789.18_LMNA	-0.34595	-0.478777819	-0.19762941	1.27E-05	3.55E-04	6.51E-01
ENSG00000121236.18_TRIM6	ENSG00000160789.18_LMNA	0.11443	-0.045605834	0.268730978	1.60E-01	4.14E-01	1.00E+00
ENSG00000019485.11_PRDM11	ENSG00000160789.18_LMNA	-0.19183	-0.34062752	-0.03365651	1.79E-02	9.97E-02	1.00E+00
ENSG00000137868.17_STRA6	ENSG00000160789.18_LMNA	0.26882	0.114524196	0.410457985	8.12E-04	9.83E-03	1.00E+00
ENSG00000268061.4_NAPA-AS1	ENSG00000160789.18_LMNA	-0.25022	-0.393733004	-0.09479181	1.88E-03	1.86E-02	1.00E+00
ENSG00000214855.8_APOC1P1	ENSG00000160789.18_LMNA	-0.2879	-0.427502439	-0.13487665	3.22E-04	4.76E-03	1.00E+00
ENSG00000158555.13_GDPD5	ENSG00000160789.18_LMNA	0.0178	-0.141799173	0.176503867	8.28E-01	9.33E-01	1.00E+00
ENSG00000169989.2_TIGD4	ENSG00000160789.18_LMNA	-0.0847	-0.240657932	0.075516593	2.99E-01	5.86E-01	1.00E+00
ENSG00000264456.1_RP11-848P	ENSG00000160789.18_LMNA	0.05528	-0.10484771	0.212605434	4.99E-01	7.52E-01	1.00E+00
ENSG00000230825.1_AC005532.5	ENSG00000160789.18_LMNA	-0.12599	-0.27958025	0.033893351	1.22E-01	3.52E-01	1.00E+00
ENSG00000161653.9_NAGS	ENSG00000160789.18_LMNA	0.08677	-0.073445501	0.242618973	2.88E-01	5.73E-01	1.00E+00
ENSG00000104369.4_JPH1	ENSG00000160789.18_LMNA	0.26431	0.109728463	0.4064114	1.00E-03	1.16E-02	1.00E+00
ENSG00000188305.5_C19orf35	ENSG00000160789.18_LMNA	0.05112	-0.108969424	0.208621012	5.32E-01	7.74E-01	1.00E+00
ENSG00000256690.1_RP11-727F	ENSG00000160789.18_LMNA	0.08347	-0.076747346	0.239491353	3.07E-01	5.93E-01	1.00E+00
ENSG00000258559.2_AC005519.4	ENSG00000160789.18_LMNA	-0.04537	-0.203104052	0.11465851	5.79E-01	8.04E-01	1.00E+00
ENSG00000269921.1_RP11-646I6	ENSG00000160789.18_LMNA	-0.14874	-0.300818545	0.010713255	6.74E-02	2.43E-01	1.00E+00
ENSG00000259137.1_RP11-305B	ENSG00000160789.18_LMNA	-0.13521	-0.288207929	0.02451735	9.67E-02	3.05E-01	1.00E+00
ENSG00000165959.10_CLMN	ENSG00000160789.18_LMNA	0.16751	0.008533859	0.3182216	3.91E-02	1.71E-01	1.00E+00

ENSG00000124813.19_RUNX2	ENSG00000160789.18_LMNA	0.16834	0.009386713	0.318987943	3.82E-02	1.68E-01	1.00E+00
ENSG00000121207.10_LRAT	ENSG00000160789.18_LMNA	0.21125	0.053864663	0.358400855	8.99E-03	6.07E-02	1.00E+00
ENSG00000173406.14_DAB1	ENSG00000160789.18_LMNA	-0.21735	-0.363957885	-0.06023457	7.15E-03	5.12E-02	1.00E+00
ENSG00000224424.7_PRKAR2A-/	ENSG00000160789.18_LMNA	-0.12049	-0.274422287	0.039472461	1.39E-01	3.81E-01	1.00E+00
ENSG00000254944.1_ATP5F1P5	ENSG00000160789.18_LMNA	0.07824	-0.08198452	0.234517024	3.38E-01	6.24E-01	1.00E+00
ENSG00000076770.13_MBNL3	ENSG00000160789.18_LMNA	-0.07276	-0.229307985	0.087450135	3.73E-01	6.53E-01	1.00E+00
ENSG00000116254.16_CHD5	ENSG00000160789.18_LMNA	-0.08846	-0.244219478	0.071753155	2.78E-01	5.64E-01	1.00E+00
ENSG00000165028.10_NIPSNAP3	ENSG00000160789.18_LMNA	-0.2088	-0.356160923	-0.05130407	9.84E-03	6.48E-02	1.00E+00
ENSG00000230721.1_RP4-612B1	ENSG00000160789.18_LMNA	-0.10512	-0.259973752	0.054997205	1.97E-01	4.67E-01	1.00E+00
ENSG00000250132.5_RP11-359B	ENSG00000160789.18_LMNA	-0.31891	-0.455007886	-0.16824951	6.22E-05	1.28E-03	1.00E+00
ENSG00000185483.10_ROR1	ENSG00000160789.18_LMNA	0.23377	0.077450816	0.378869703	3.75E-03	3.16E-02	1.00E+00
ENSG00000153563.14_CD8A	ENSG00000160789.18_LMNA	0.04508	-0.114943156	0.202827487	5.81E-01	8.05E-01	1.00E+00
ENSG00000188868.12_ZNF563	ENSG00000160789.18_LMNA	-0.03646	-0.194526908	0.123462058	6.56E-01	8.48E-01	1.00E+00
ENSG00000225313.4_RP11-415J8	ENSG00000160789.18_LMNA	0.19316	0.035040594	0.341851875	1.71E-02	9.65E-02	1.00E+00
ENSG00000249489.1_GAPDHP70	ENSG00000160789.18_LMNA	0.23151	0.075079329	0.376824896	4.11E-03	3.38E-02	1.00E+00
ENSG00000227337.1_RPL23AP43	ENSG00000160789.18_LMNA	-0.06712	-0.223932138	0.093070848	4.11E-01	6.85E-01	1.00E+00
ENSG00000226314.6_ZNF192P1	ENSG00000160789.18_LMNA	-0.29102	-0.430288427	-0.13822699	2.75E-04	4.20E-03	1.00E+00
ENSG00000071205.10_ARHGAP1	ENSG00000160789.18_LMNA	0.08736	-0.072851982	0.243180479	2.85E-01	5.70E-01	1.00E+00
ENSG00000254614.2_AP003068.2	ENSG00000160789.18_LMNA	0.13225	-0.027532869	0.285438614	1.04E-01	3.20E-01	1.00E+00
ENSG00000259705.1_RP11-227D	ENSG00000160789.18_LMNA	0.05972	-0.100438356	0.21685626	4.65E-01	7.27E-01	1.00E+00
ENSG00000187860.9_CCDC157	ENSG00000160789.18_LMNA	-0.13204	-0.285240401	0.027748485	1.05E-01	3.21E-01	1.00E+00
ENSG00000233406.5_RP11-296A	ENSG00000160789.18_LMNA	0.07156	-0.088648697	0.228163257	3.81E-01	6.60E-01	1.00E+00
ENSG00000090554.11_FLT3LG	ENSG00000160789.18_LMNA	0.32059	0.17006394	0.456487796	5.66E-05	1.19E-03	1.00E+00
ENSG00000149922.9_TBX6	ENSG00000160789.18_LMNA	0.16729	0.008310405	0.318020745	3.94E-02	1.72E-01	1.00E+00
ENSG00000239223.3_RPL34P31	ENSG00000160789.18_LMNA	-0.1	-0.255141838	0.060155269	2.20E-01	4.95E-01	1.00E+00
ENSG00000267547.1_RP11-686D	ENSG00000160789.18_LMNA	0.07511	-0.085107165	0.231543192	3.58E-01	6.41E-01	1.00E+00
ENSG00000241170.2_RP11-147I3	ENSG00000160789.18_LMNA	-0.16358	-0.314586932	-0.00449501	4.40E-02	1.85E-01	1.00E+00
ENSG00000226754.1_RP5-1024G	ENSG00000160789.18_LMNA	-0.04556	-0.203288812	0.114468322	5.77E-01	8.03E-01	1.00E+00
ENSG00000267034.1_RP11-384C	ENSG00000160789.18_LMNA	-0.38126	-0.509533022	-0.2364078	1.26E-06	5.56E-05	6.46E-02
ENSG00000234031.1_RPS3AP44	ENSG00000160789.18_LMNA	-0.1312	-0.284461624	0.02859536	1.07E-01	3.25E-01	1.00E+00
ENSG00000144406.17_UNC80	ENSG00000160789.18_LMNA	-0.31034	-0.44743482	-0.15899492	9.98E-05	1.88E-03	1.00E+00
ENSG00000153976.2_HS3ST3A1	ENSG00000160789.18_LMNA	0.22923	0.072685312	0.374757676	4.50E-03	3.63E-02	1.00E+00
ENSG00000240291.1_RP11-499P	ENSG00000160789.18_LMNA	-0.32573	-0.461023841	-0.17563745	4.22E-05	9.38E-04	1.00E+00

ENSG00000196407.10_THEM5	ENSG00000160789.18_LMNA	0.13535	-0.024375986	0.288337623	9.64E-02	3.05E-01	1.00E+00
ENSG00000268941.2_RP5-907D1	ENSG00000160789.18_LMNA	0.5308	0.40589993	0.636236801	2.00E-12	9.52E-10	1.03E-07
ENSG00000136634.5_IL10	ENSG00000160789.18_LMNA	0.01684	-0.142744558	0.175568853	8.37E-01	9.37E-01	1.00E+00
ENSG00000160439.14_RDH13	ENSG00000160789.18_LMNA	0.21647	0.05931285	0.363155121	7.39E-03	5.24E-02	1.00E+00
ENSG00000184719.10_RNLS	ENSG00000160789.18_LMNA	-0.01617	-0.174916902	0.143403395	8.43E-01	9.39E-01	1.00E+00
ENSG00000178343.4_SHISA3	ENSG00000160789.18_LMNA	-0.30097	-0.439129728	-0.14890371	1.65E-04	2.81E-03	1.00E+00
ENSG00000265511.1_RP11-524F	ENSG00000160789.18_LMNA	-0.07129	-0.227900297	0.088923895	3.83E-01	6.61E-01	1.00E+00
ENSG00000001460.16_STPG1	ENSG00000160789.18_LMNA	0.25533	0.100200682	0.398337286	1.50E-03	1.57E-02	1.00E+00
ENSG00000230316.5_FEZF1-AS1	ENSG00000160789.18_LMNA	0.08609	-0.074131333	0.24196987	2.92E-01	5.77E-01	1.00E+00
ENSG00000274031.1_RP11-413H	ENSG00000160789.18_LMNA	-0.14224	-0.29476401	0.017355648	8.05E-02	2.73E-01	1.00E+00
ENSG00000189362.10_NEMP2	ENSG00000160789.18_LMNA	0.30922	0.157788033	0.446444163	1.06E-04	1.98E-03	1.00E+00
ENSG00000243660.8_ZNF487	ENSG00000160789.18_LMNA	-0.02427	-0.182768857	0.135449681	7.67E-01	9.03E-01	1.00E+00
ENSG00000187260.14_WDR86	ENSG00000160789.18_LMNA	-0.11617	-0.270368085	0.043843992	1.54E-01	4.05E-01	1.00E+00
ENSG00000240371.1_RPS4XP13	ENSG00000160789.18_LMNA	-0.0256	-0.184048322	0.134149737	7.54E-01	8.98E-01	1.00E+00
ENSG00000223403.3_MEG9	ENSG00000160789.18_LMNA	0.11415	-0.045882286	0.268473933	1.61E-01	4.16E-01	1.00E+00
ENSG00000272909.1_CTD-2555C	ENSG00000160789.18_LMNA	0.02943	-0.130378848	0.187753714	7.19E-01	8.81E-01	1.00E+00
ENSG00000260274.1_RP11-817C	ENSG00000160789.18_LMNA	0.0408	-0.119177596	0.198707268	6.18E-01	8.27E-01	1.00E+00
ENSG00000128815.16_WDFY4	ENSG00000160789.18_LMNA	0.02343	-0.136281176	0.181949896	7.75E-01	9.07E-01	1.00E+00
ENSG00000259088.1_CTD-2017C	ENSG00000160789.18_LMNA	-0.08018	-0.236364673	0.080041279	3.26E-01	6.13E-01	1.00E+00
ENSG00000103184.10_SEC14L5	ENSG00000160789.18_LMNA	-0.29535	-0.434134788	-0.14286351	2.21E-04	3.52E-03	1.00E+00
ENSG00000273136.4_NBPF26	ENSG00000160789.18_LMNA	0.20772	0.05017867	0.355175353	1.02E-02	6.66E-02	1.00E+00
ENSG00000229728.1_RP11-314N	ENSG00000160789.18_LMNA	-0.05775	-0.214974731	0.102391593	4.80E-01	7.38E-01	1.00E+00
ENSG00000237669.1_HCG4P3	ENSG00000160789.18_LMNA	0.03578	-0.124128084	0.193876025	6.62E-01	8.51E-01	1.00E+00
ENSG00000168685.13_IL7R	ENSG00000160789.18_LMNA	0.15347	-0.005869518	0.30521783	5.91E-02	2.23E-01	1.00E+00
ENSG00000131650.12_KREMEN2	ENSG00000160789.18_LMNA	-0.11023	-0.264782085	0.049847572	1.76E-01	4.38E-01	1.00E+00
ENSG00000256628.3_ZBTB11-AS	ENSG00000160789.18_LMNA	0.049	-0.111064859	0.206591324	5.49E-01	7.86E-01	1.00E+00
ENSG00000205085.7_FAM71F2	ENSG00000160789.18_LMNA	-0.01361	-0.172436044	0.145907885	8.68E-01	9.48E-01	1.00E+00
ENSG00000241043.1_GVQW1	ENSG00000160789.18_LMNA	-0.11932	-0.273332726	0.040648486	1.43E-01	3.87E-01	1.00E+00
ENSG00000230658.1_KLHL7-AS1	ENSG00000160789.18_LMNA	-0.22785	-0.373501559	-0.07123232	4.76E-03	3.78E-02	1.00E+00
ENSG00000225880.5_LINC00115	ENSG00000160789.18_LMNA	-0.01624	-0.17498593	0.143333652	8.43E-01	9.39E-01	1.00E+00
ENSG00000102786.13_INTS6	ENSG00000160789.18_LMNA	-0.05998	-0.217106893	0.100177987	4.63E-01	7.26E-01	1.00E+00
ENSG00000267107.5_PCAT19	ENSG00000160789.18_LMNA	-0.13947	-0.292184585	0.020177175	8.66E-02	2.86E-01	1.00E+00
ENSG00000260236.1_RP11-708J1	ENSG00000160789.18_LMNA	0.02246	-0.137234818	0.181010085	7.84E-01	9.11E-01	1.00E+00

ENSG00000260448.4_LCMT1-AS1	ENSG00000160789.18_LMNA	-0.34526	-0.478177997	-0.19688176	1.32E-05	3.67E-04	6.79E-01
ENSG00000233859.2_ADH5P4	ENSG00000160789.18_LMNA	-0.01148	-0.170370356	0.147990141	8.88E-01	9.57E-01	1.00E+00
ENSG00000196597.10_ZNF782	ENSG00000160789.18_LMNA	0.1264	-0.033476323	0.279965073	1.21E-01	3.49E-01	1.00E+00
ENSG00000272370.1_RP11-307L	ENSG00000160789.18_LMNA	-0.2976	-0.436134246	-0.14527878	1.97E-04	3.22E-03	1.00E+00
ENSG00000150630.3_VEGFC	ENSG00000160789.18_LMNA	0.2935	0.140885886	0.432495488	2.43E-04	3.81E-03	1.00E+00
ENSG00000187187.12_ZNF546	ENSG00000160789.18_LMNA	-0.0986	-0.253817637	0.061565919	2.27E-01	5.03E-01	1.00E+00
ENSG00000182168.13_UNC5C	ENSG00000160789.18_LMNA	0.15228	-0.00709333	0.304107565	6.11E-02	2.28E-01	1.00E+00
ENSG00000254806.4_SYS1-DBNI	ENSG00000160789.18_LMNA	0.03666	-0.123255766	0.194728452	6.54E-01	8.47E-01	1.00E+00
ENSG00000227354.5_RBM26-AS1	ENSG00000160789.18_LMNA	-0.22094	-0.367226993	-0.06399348	6.23E-03	4.62E-02	1.00E+00
ENSG00000253641.4_LINCR-0001	ENSG00000160789.18_LMNA	-0.12428	-0.27798423	0.035621777	1.27E-01	3.61E-01	1.00E+00
ENSG00000217624.2_RP11-204C	ENSG00000160789.18_LMNA	-0.02993	-0.188230294	0.129893174	7.14E-01	8.79E-01	1.00E+00
ENSG00000279722.1_RP11-44F1	ENSG00000160789.18_LMNA	-0.32983	-0.464630181	-0.18008171	3.33E-05	7.79E-04	1.00E+00
ENSG00000274026.1_FAM27E3	ENSG00000160789.18_LMNA	-0.07419	-0.23066695	0.08602607	3.64E-01	6.45E-01	1.00E+00
ENSG00000152207.6_CYSLTR2	ENSG00000160789.18_LMNA	-0.12654	-0.280102562	0.033327302	1.20E-01	3.49E-01	1.00E+00
ENSG00000227214.2_HCG15	ENSG00000160789.18_LMNA	0.08676	-0.073453175	0.242611712	2.88E-01	5.73E-01	1.00E+00
ENSG00000128298.15_BAIAP2L2	ENSG00000160789.18_LMNA	-0.06678	-0.223607869	0.093409242	4.14E-01	6.87E-01	1.00E+00
ENSG00000251400.1_ALDH7A1P	ENSG00000160789.18_LMNA	0.10165	-0.058492625	0.256701073	2.13E-01	4.86E-01	1.00E+00
ENSG00000165813.15_CCDC186	ENSG00000160789.18_LMNA	-0.13248	-0.285658274	0.027293888	1.04E-01	3.19E-01	1.00E+00
ENSG00000162927.12_PUS10	ENSG00000160789.18_LMNA	-0.17407	-0.324283539	-0.01529245	3.20E-02	1.49E-01	1.00E+00
ENSG00000176933.5_TOB2P1	ENSG00000160789.18_LMNA	0.13203	-0.027752671	0.285236553	1.05E-01	3.21E-01	1.00E+00
ENSG00000234062.6_RP11-308D	ENSG00000160789.18_LMNA	-0.04586	-0.203577378	0.114171233	5.75E-01	8.02E-01	1.00E+00
ENSG00000154493.16_C10orf90	ENSG00000160789.18_LMNA	-0.01023	-0.169157431	0.149211481	9.00E-01	9.61E-01	1.00E+00
ENSG00000138944.7_KIAA1644	ENSG00000160789.18_LMNA	-0.20887	-0.356224363	-0.05137653	9.81E-03	6.47E-02	1.00E+00
ENSG00000112599.8_GUCA1B	ENSG00000160789.18_LMNA	-0.02993	-0.188229737	0.129893742	7.14E-01	8.79E-01	1.00E+00
ENSG00000254461.1_RP11-755F	ENSG00000160789.18_LMNA	0.00728	-0.152093668	0.166291273	9.29E-01	9.73E-01	1.00E+00
ENSG00000277534.1_RP11-9E17	ENSG00000160789.18_LMNA	-0.059	-0.216166939	0.101154232	4.70E-01	7.31E-01	1.00E+00
ENSG00000279522.1_CTC-487M2	ENSG00000160789.18_LMNA	-0.27558	-0.416503445	-0.12171464	5.90E-04	7.68E-03	1.00E+00
ENSG00000258153.1_HSPE1P4	ENSG00000160789.18_LMNA	-0.12604	-0.279629633	0.033839843	1.22E-01	3.51E-01	1.00E+00
ENSG00000123870.10_ZNF137P	ENSG00000160789.18_LMNA	-0.1417	-0.294263512	0.017903512	8.16E-02	2.75E-01	1.00E+00
ENSG00000236562.4_RP11-396M	ENSG00000160789.18_LMNA	0.24685	0.091233419	0.39069573	2.17E-03	2.08E-02	1.00E+00
ENSG00000203326.8_ZNF525	ENSG00000160789.18_LMNA	0.14778	-0.01169541	0.299924891	6.92E-02	2.48E-01	1.00E+00
ENSG00000272910.1_RP11-15L13	ENSG00000160789.18_LMNA	-0.32227	-0.457978313	-0.17189334	5.14E-05	1.10E-03	1.00E+00
ENSG00000276900.1_RP11-467L	ENSG00000160789.18_LMNA	-0.18077	-0.330458297	-0.02220598	2.58E-02	1.29E-01	1.00E+00

ENSG00000123999.4_INHA	ENSG00000160789.18_LMNA	0.24583	0.090160365	0.389778541	2.27E-03	2.15E-02	1.00E+00
ENSG00000165072.9_MAMDC2	ENSG00000160789.18_LMNA	0.17652	0.017818451	0.326542668	2.96E-02	1.42E-01	1.00E+00
ENSG00000262050.1_RP11-74E2	ENSG00000160789.18_LMNA	0.12592	-0.03395713	0.279521388	1.22E-01	3.52E-01	1.00E+00
ENSG00000140534.12_TICRR	ENSG00000160789.18_LMNA	0.06664	-0.093551276	0.223471744	4.15E-01	6.88E-01	1.00E+00
ENSG00000203362.2_RP3-337H4	ENSG00000160789.18_LMNA	-0.20822	-0.35563439	-0.05070274	1.00E-02	6.58E-02	1.00E+00
ENSG00000110318.12_CEP126	ENSG00000160789.18_LMNA	-0.18273	-0.332266786	-0.02423645	2.42E-02	1.24E-01	1.00E+00
ENSG00000269982.1_RP11-1020	ENSG00000160789.18_LMNA	-0.16286	-0.313920153	-0.00375518	4.50E-02	1.88E-01	1.00E+00
ENSG00000163322.12_FAM175A	ENSG00000160789.18_LMNA	-0.18328	-0.332769467	-0.02480128	2.38E-02	1.22E-01	1.00E+00
ENSG00000178187.6_ZNF454	ENSG00000160789.18_LMNA	-0.03966	-0.197611903	0.120301384	6.28E-01	8.32E-01	1.00E+00
ENSG00000257354.2_RP11-631N	ENSG00000160789.18_LMNA	-0.08396	-0.239952311	0.076261146	3.04E-01	5.90E-01	1.00E+00
ENSG00000276791.1_CTD-2270P	ENSG00000160789.18_LMNA	-0.18509	-0.334439888	-0.02667964	2.24E-02	1.17E-01	1.00E+00
ENSG00000250091.2_DNAH10OS	ENSG00000160789.18_LMNA	0.05162	-0.108475022	0.209099503	5.28E-01	7.72E-01	1.00E+00
ENSG00000172543.6_CTSW	ENSG00000160789.18_LMNA	0.08877	-0.071437633	0.244517687	2.77E-01	5.62E-01	1.00E+00
ENSG00000281205.1_LINC00950	ENSG00000160789.18_LMNA	-0.16148	-0.312640194	-0.00233594	4.69E-02	1.93E-01	1.00E+00
ENSG00000227525.4_RPL7P6	ENSG00000160789.18_LMNA	-0.04857	-0.206178587	0.111490621	5.52E-01	7.88E-01	1.00E+00
ENSG00000183484.10_GPR132	ENSG00000160789.18_LMNA	0.17921	0.020598314	0.329024772	2.72E-02	1.34E-01	1.00E+00
ENSG00000131848.8_ZSCAN5A	ENSG00000160789.18_LMNA	-0.06715	-0.223955375	0.093046596	4.11E-01	6.85E-01	1.00E+00
ENSG00000215326.3_GPX1P2	ENSG00000160789.18_LMNA	-0.06843	-0.225179917	0.091768032	4.02E-01	6.78E-01	1.00E+00
ENSG00000162881.6_OXER1	ENSG00000160789.18_LMNA	0.10812	-0.051975813	0.262796792	1.85E-01	4.51E-01	1.00E+00
ENSG00000231940.1_RPS7P3	ENSG00000160789.18_LMNA	-0.13185	-0.285064415	0.027939899	1.05E-01	3.22E-01	1.00E+00
ENSG00000197358.9_BNIP3P1	ENSG00000160789.18_LMNA	0.06414	-0.096044536	0.221080175	4.32E-01	7.02E-01	1.00E+00
ENSG00000224195.1_RP11-574K	ENSG00000160789.18_LMNA	-0.29643	-0.435095989	-0.14402417	2.09E-04	3.37E-03	1.00E+00
ENSG00000239382.9_ALKBH6	ENSG00000160789.18_LMNA	-0.01395	-0.172763806	0.145577235	8.65E-01	9.47E-01	1.00E+00
ENSG00000214050.6_FBXO16	ENSG00000160789.18_LMNA	-0.28822	-0.427791662	-0.13522415	3.17E-04	4.71E-03	1.00E+00
ENSG00000163347.5_CLDN1	ENSG00000160789.18_LMNA	0.24993	0.094490738	0.393476273	1.90E-03	1.88E-02	1.00E+00
ENSG00000213996.11_TM6SF2	ENSG00000160789.18_LMNA	0.15734	-0.001913395	0.308801122	5.29E-02	2.09E-01	1.00E+00
ENSG00000204410.13_MSH5	ENSG00000160789.18_LMNA	0.01567	-0.143894545	0.174430705	8.48E-01	9.41E-01	1.00E+00
ENSG00000243716.9_NPIPB5	ENSG00000160789.18_LMNA	0.0574	-0.102743946	0.214635063	4.82E-01	7.40E-01	1.00E+00
ENSG00000259207.6_ITGB3	ENSG00000160789.18_LMNA	0.38179	0.236998936	0.509996512	1.21E-06	5.38E-05	6.23E-02
ENSG00000234513.1_AC073072.1	ENSG00000160789.18_LMNA	-0.1527	-0.304502806	0.006657774	6.04E-02	2.27E-01	1.00E+00
ENSG00000149548.13_CCDC15	ENSG00000160789.18_LMNA	0.049	-0.11106899	0.20658732	5.49E-01	7.86E-01	1.00E+00
ENSG00000227279.1_AC015933.2	ENSG00000160789.18_LMNA	0.13549	-0.024234087	0.288467796	9.60E-02	3.04E-01	1.00E+00
ENSG00000168952.14_STXBP6	ENSG00000160789.18_LMNA	-0.2318	-0.377082618	-0.07537804	4.06E-03	3.35E-02	1.00E+00

ENSG00000224914.2_LINC00863	ENSG00000160789.18_LMNA	-0.25439	-0.397490111	-0.09920414	1.56E-03	1.62E-02	1.00E+00
ENSG00000236021.1_RP11-195C	ENSG00000160789.18_LMNA	-0.2323	-0.377540805	-0.07590922	3.98E-03	3.30E-02	1.00E+00
ENSG00000230772.1_VN1R108P	ENSG00000160789.18_LMNA	-0.29671	-0.43534914	-0.14432999	2.06E-04	3.34E-03	1.00E+00
ENSG00000268895.4_A1BG-AS1	ENSG00000160789.18_LMNA	-0.01329	-0.172124038	0.146222575	8.71E-01	9.50E-01	1.00E+00
ENSG00000269038.1_AP001462.(	ENSG00000160789.18_LMNA	-0.06977	-0.226457181	0.090433306	3.93E-01	6.71E-01	1.00E+00
ENSG00000234785.1_EEF1GP5	ENSG00000160789.18_LMNA	-0.05668	-0.213950214	0.103454128	4.88E-01	7.44E-01	1.00E+00
ENSG00000188993.3_LRRC66	ENSG00000160789.18_LMNA	0.03216	-0.127696219	0.190384246	6.94E-01	8.69E-01	1.00E+00
ENSG00000228727.7_SAPCD1	ENSG00000160789.18_LMNA	-0.04493	-0.202676207	0.115098834	5.83E-01	8.06E-01	1.00E+00
ENSG00000144481.15_TRPM8	ENSG00000160789.18_LMNA	0.34422	0.195746644	0.477266822	1.41E-05	3.88E-04	7.24E-01
ENSG00000128262.7_POM121L9I	ENSG00000160789.18_LMNA	0.20906	0.051571167	0.356394739	9.74E-03	6.44E-02	1.00E+00
ENSG00000258839.2_MC1R	ENSG00000160789.18_LMNA	0.31794	0.167198855	0.454150216	6.57E-05	1.34E-03	1.00E+00
ENSG00000196668.3_LINC00173	ENSG00000160789.18_LMNA	-0.29679	-0.435414332	-0.14440875	2.05E-04	3.33E-03	1.00E+00
ENSG00000272081.1_CTD-2376I4	ENSG00000160789.18_LMNA	0.15315	-0.006201595	0.304916647	5.96E-02	2.25E-01	1.00E+00
ENSG00000108984.12_MAP2K6	ENSG00000160789.18_LMNA	-0.46229	-0.578890428	-0.32717317	2.03E-09	2.79E-07	1.04E-04
ENSG00000167637.15_ZNF283	ENSG00000160789.18_LMNA	0.01114	-0.148319512	0.170043349	8.92E-01	9.57E-01	1.00E+00
ENSG00000006837.10_CDKL3	ENSG00000160789.18_LMNA	-0.20974	-0.357019789	-0.05228541	9.50E-03	6.32E-02	1.00E+00
ENSG00000278356.1_RP11-372B	ENSG00000160789.18_LMNA	-0.22854	-0.374127317	-0.07195599	4.63E-03	3.70E-02	1.00E+00
ENSG00000116771.5_AGMAT	ENSG00000160789.18_LMNA	-0.12542	-0.279050906	0.034466817	1.24E-01	3.55E-01	1.00E+00
ENSG00000204778.4_CBWD4P	ENSG00000160789.18_LMNA	-0.03038	-0.188665297	0.129449738	7.10E-01	8.77E-01	1.00E+00
ENSG00000225591.2_RP11-360D	ENSG00000160789.18_LMNA	-0.02167	-0.180252951	0.138002666	7.91E-01	9.15E-01	1.00E+00
ENSG00000132141.12_CCT6B	ENSG00000160789.18_LMNA	-0.10014	-0.255269788	0.060018899	2.20E-01	4.95E-01	1.00E+00
ENSG00000231607.7_DLEU2	ENSG00000160789.18_LMNA	-0.2065	-0.354057578	-0.04890325	1.07E-02	6.87E-02	1.00E+00
ENSG00000172260.12_NEGR1	ENSG00000160789.18_LMNA	-0.1393	-0.292025929	0.02035056	8.70E-02	2.87E-01	1.00E+00
ENSG00000173868.10_PHOSPH(	ENSG00000160789.18_LMNA	-0.01165	-0.170529594	0.147829727	8.87E-01	9.56E-01	1.00E+00
ENSG00000267395.4_AC074212.(	ENSG00000160789.18_LMNA	0.24341	0.087601972	0.387589365	2.51E-03	2.33E-02	1.00E+00
ENSG00000255262.3_TCEB2P2	ENSG00000160789.18_LMNA	-0.16342	-0.314442918	-0.00433519	4.42E-02	1.86E-01	1.00E+00
ENSG00000165409.14_TSHR	ENSG00000160789.18_LMNA	-0.4692	-0.584724736	-0.33502613	1.08E-09	1.67E-07	5.54E-05
ENSG00000270248.1_CTC-471J1	ENSG00000160789.18_LMNA	-0.21136	-0.358493376	-0.05397052	8.95E-03	6.05E-02	1.00E+00
ENSG00000213574.2_LDHAP5	ENSG00000160789.18_LMNA	0.22467	0.067894351	0.37061167	5.39E-03	4.15E-02	1.00E+00
ENSG00000231007.5_CDC20P1	ENSG00000160789.18_LMNA	0.15609	-0.003190637	0.307645213	5.48E-02	2.13E-01	1.00E+00
ENSG00000171303.6_KCNK3	ENSG00000160789.18_LMNA	-0.1539	-0.305608957	0.005438168	5.84E-02	2.21E-01	1.00E+00
ENSG00000157680.14_DGKI	ENSG00000160789.18_LMNA	-0.26169	-0.404057879	-0.10694557	1.13E-03	1.27E-02	1.00E+00
ENSG00000213790.2_OLA1P1	ENSG00000160789.18_LMNA	-0.13782	-0.290639621	0.021864769	9.04E-02	2.93E-01	1.00E+00

ENSG00000272158.1_RP11-840I1	ENSG00000160789.18_LMNA	-0.0576	-0.214826245	0.102545633	4.81E-01	7.39E-01	1.00E+00
ENSG00000175711.7_B3GNTL1	ENSG00000160789.18_LMNA	0.12468	-0.035216612	0.278358512	1.26E-01	3.59E-01	1.00E+00
ENSG00000145934.14_TENM2	ENSG00000160789.18_LMNA	-0.07084	-0.22747683	0.089366966	3.86E-01	6.64E-01	1.00E+00
ENSG00000101333.15_PLCB4	ENSG00000160789.18_LMNA	-0.36933	-0.499181937	-0.22325854	2.83E-06	1.05E-04	1.45E-01
ENSG00000215790.5_SLC35E2	ENSG00000160789.18_LMNA	-0.15602	-0.30758138	0.003261141	5.49E-02	2.14E-01	1.00E+00
ENSG00000249592.4_RP11-440L	ENSG00000160789.18_LMNA	0.14994	-0.009486206	0.301934257	6.52E-02	2.38E-01	1.00E+00
ENSG00000008226.18_DLEC1	ENSG00000160789.18_LMNA	-0.23409	-0.379162661	-0.07779086	3.70E-03	3.13E-02	1.00E+00
ENSG00000156398.11_SFXN2	ENSG00000160789.18_LMNA	-0.22328	-0.369353604	-0.06644334	5.69E-03	4.32E-02	1.00E+00
ENSG00000237882.1_PPIAP13	ENSG00000160789.18_LMNA	0.00939	-0.150033151	0.168340879	9.09E-01	9.65E-01	1.00E+00
ENSG00000230513.1_THAP7-AS1	ENSG00000160789.18_LMNA	-0.10189	-0.25692038	0.058258633	2.12E-01	4.85E-01	1.00E+00
ENSG00000269397.1_CTB-92J24	ENSG00000160789.18_LMNA	-0.20893	-0.356283407	-0.05144398	9.79E-03	6.46E-02	1.00E+00
ENSG00000279267.1_RP11-395L	ENSG00000160789.18_LMNA	0.021	-0.13865969	0.179604795	7.97E-01	9.19E-01	1.00E+00
ENSG00000165046.11_LETM2	ENSG00000160789.18_LMNA	0.23939	0.08336882	0.383959676	2.97E-03	2.66E-02	1.00E+00
ENSG00000198929.11_NOS1AP	ENSG00000160789.18_LMNA	-0.14441	-0.296787478	0.015138775	7.59E-02	2.63E-01	1.00E+00
ENSG00000228938.3_SNRPD2P1	ENSG00000160789.18_LMNA	-0.04267	-0.200505246	0.117331195	6.02E-01	8.17E-01	1.00E+00
ENSG00000185518.10_SV2B	ENSG00000160789.18_LMNA	-0.12381	-0.277536468	0.036106347	1.29E-01	3.63E-01	1.00E+00
ENSG00000183960.7_KCNH8	ENSG00000160789.18_LMNA	-0.45488	-0.572615851	-0.31876623	3.92E-09	4.89E-07	2.01E-04
ENSG00000217239.3_RP1-182O1	ENSG00000160789.18_LMNA	0.0246	-0.13512851	0.183085069	7.64E-01	9.02E-01	1.00E+00
ENSG00000203780.9_FANK1	ENSG00000160789.18_LMNA	0.17912	0.02050817	0.328944351	2.72E-02	1.34E-01	1.00E+00
ENSG00000274897.2_PANO1	ENSG00000160789.18_LMNA	0.05929	-0.100858028	0.216452194	4.68E-01	7.29E-01	1.00E+00
ENSG00000103942.11_HOMER2	ENSG00000160789.18_LMNA	0.41194	0.270477695	0.535990009	1.34E-07	9.23E-06	6.89E-03
ENSG00000281183.1_NPTN-IT1	ENSG00000160789.18_LMNA	0.02317	-0.136529625	0.181705106	7.77E-01	9.08E-01	1.00E+00
ENSG00000226986.4_RP11-543B	ENSG00000160789.18_LMNA	0.06527	-0.094917476	0.222161739	4.24E-01	6.95E-01	1.00E+00
ENSG00000136153.18_LMO7	ENSG00000160789.18_LMNA	0.04937	-0.110702322	0.206942681	5.46E-01	7.84E-01	1.00E+00
ENSG00000164379.5_FOXQ1	ENSG00000160789.18_LMNA	-0.1506	-0.302547986	0.008810834	6.40E-02	2.36E-01	1.00E+00
ENSG00000086159.11_AQP6	ENSG00000160789.18_LMNA	-0.14611	-0.298374536	0.013397875	7.25E-02	2.55E-01	1.00E+00
ENSG00000234040.3_RPL10P12	ENSG00000160789.18_LMNA	-0.0478	-0.205436106	0.112256238	5.59E-01	7.92E-01	1.00E+00
ENSG00000271020.1_RP11-10C2	ENSG00000160789.18_LMNA	0.25664	0.101593027	0.399520089	1.41E-03	1.50E-02	1.00E+00
ENSG00000260285.1_RP11-600F	ENSG00000160789.18_LMNA	0.0969	-0.063281194	0.252205884	2.35E-01	5.13E-01	1.00E+00
ENSG00000189007.14_ADAT2	ENSG00000160789.18_LMNA	-0.05304	-0.210463354	0.107064957	5.16E-01	7.63E-01	1.00E+00
ENSG00000215421.8_ZNF407	ENSG00000160789.18_LMNA	0.19193	0.033765768	0.340724204	1.78E-02	9.94E-02	1.00E+00
ENSG00000268362.4_CTD-2017D	ENSG00000160789.18_LMNA	-0.19164	-0.34045227	-0.0334585	1.80E-02	1.00E-01	1.00E+00
ENSG00000273987.1_RP11-585P	ENSG00000160789.18_LMNA	-0.28125	-0.4215706	-0.12776543	4.48E-04	6.19E-03	1.00E+00

ENSG00000232587.1_EEF1A1P3	ENSG00000160789.18_LMNA	-0.10856	-0.263214094	0.0515287	1.83E-01	4.48E-01	1.00E+00
ENSG00000279519.1_RP11-288C	ENSG00000160789.18_LMNA	-0.07375	-0.230248794	0.086464395	3.67E-01	6.47E-01	1.00E+00
ENSG00000257027.1_RP11-705C	ENSG00000160789.18_LMNA	-0.15331	-0.305061176	0.00604225	5.93E-02	2.24E-01	1.00E+00
ENSG00000243504.1_RPS23P1	ENSG00000160789.18_LMNA	-0.11584	-0.270058962	0.044176818	1.55E-01	4.07E-01	1.00E+00
ENSG00000174963.16_ZIC4	ENSG00000160789.18_LMNA	0.01094	-0.148523442	0.169840849	8.94E-01	9.58E-01	1.00E+00
ENSG00000121690.8_DEPDC7	ENSG00000160789.18_LMNA	-0.36143	-0.492304045	-0.21457654	4.77E-06	1.60E-04	2.44E-01
ENSG00000137809.15_ITGA11	ENSG00000160789.18_LMNA	0.28204	0.128608962	0.422275549	4.31E-04	6.01E-03	1.00E+00
ENSG00000206228.4_HNRNPA1F	ENSG00000160789.18_LMNA	-0.01911	-0.177769047	0.140519035	8.15E-01	9.28E-01	1.00E+00
ENSG00000242687.2_AC004893.1	ENSG00000160789.18_LMNA	0.15919	-1.45E-05	0.310517971	5.01E-02	2.02E-01	1.00E+00
ENSG00000105851.9_PIK3CG	ENSG00000160789.18_LMNA	-0.01689	-0.175619385	0.14269348	8.36E-01	9.37E-01	1.00E+00
ENSG00000267470.4_ZNF571-AS	ENSG00000160789.18_LMNA	-0.26464	-0.40670996	-0.11008182	9.85E-04	1.15E-02	1.00E+00
ENSG00000150594.6_ADRA2A	ENSG00000160789.18_LMNA	-0.07879	-0.235045762	0.081428672	3.35E-01	6.20E-01	1.00E+00
ENSG00000250474.1_WBP1LP2	ENSG00000160789.18_LMNA	0.24991	0.09446709	0.393456107	1.90E-03	1.88E-02	1.00E+00
ENSG00000205609.11_EIF3CL	ENSG00000160789.18_LMNA	0.16611	0.007100701	0.316932905	4.08E-02	1.76E-01	1.00E+00
ENSG00000131797.11_CLUHP3	ENSG00000160789.18_LMNA	-0.17454	-0.324718742	-0.01577876	3.15E-02	1.48E-01	1.00E+00
ENSG00000139220.15_PPFIA2	ENSG00000160789.18_LMNA	-0.13834	-0.291131965	0.021327165	8.92E-02	2.91E-01	1.00E+00
ENSG00000128536.14_CDHR3	ENSG00000160789.18_LMNA	-0.10337	-0.258317798	0.056766829	2.05E-01	4.76E-01	1.00E+00
ENSG00000197520.9_FAM177B	ENSG00000160789.18_LMNA	-0.02329	-0.18181277	0.136420357	7.76E-01	9.08E-01	1.00E+00
ENSG00000233155.1_HMGA1P8	ENSG00000160789.18_LMNA	0.25815	0.103192175	0.400877348	1.32E-03	1.43E-02	1.00E+00
ENSG00000248208.1_RP11-153M	ENSG00000160789.18_LMNA	0.03649	-0.1234257	0.194562431	6.55E-01	8.47E-01	1.00E+00
ENSG00000279520.1_RP11-20I23	ENSG00000160789.18_LMNA	0.07154	-0.088670859	0.228142083	3.81E-01	6.60E-01	1.00E+00
ENSG00000007402.10_CACNA2D	ENSG00000160789.18_LMNA	-0.20374	-0.351538302	-0.04603232	1.18E-02	7.39E-02	1.00E+00
ENSG00000049192.13_ADAMTS6	ENSG00000160789.18_LMNA	-0.09516	-0.250561743	0.06502902	2.44E-01	5.23E-01	1.00E+00
ENSG00000213881.3_NPM1P6	ENSG00000160789.18_LMNA	0.07372	-0.086496848	0.23021783	3.67E-01	6.48E-01	1.00E+00
ENSG00000160401.13_CFAP157	ENSG00000160789.18_LMNA	0.08722	-0.07299513	0.243045071	2.85E-01	5.71E-01	1.00E+00
ENSG00000254703.2_SENCR	ENSG00000160789.18_LMNA	-0.15757	-0.309021383	0.001669899	5.25E-02	2.08E-01	1.00E+00
ENSG00000277447.1_RP11-193E	ENSG00000160789.18_LMNA	0.09108	-0.069119658	0.246706641	2.64E-01	5.48E-01	1.00E+00
ENSG00000234917.2_RP5-994D1	ENSG00000160789.18_LMNA	-0.44462	-0.5639059	-0.30716211	9.55E-09	1.03E-06	4.90E-04
ENSG00000225808.1_DNAJC19P	ENSG00000160789.18_LMNA	0.03264	-0.127218133	0.190852569	6.90E-01	8.66E-01	1.00E+00
ENSG00000137880.5_GCHFR	ENSG00000160789.18_LMNA	0.12374	-0.036177868	0.277470368	1.29E-01	3.64E-01	1.00E+00
ENSG00000239556.2_AC004951.5	ENSG00000160789.18_LMNA	0.10684	-0.053270553	0.26158771	1.90E-01	4.58E-01	1.00E+00
ENSG00000138411.9_HECW2	ENSG00000160789.18_LMNA	-0.13149	-0.284726872	0.028306969	1.06E-01	3.24E-01	1.00E+00
ENSG00000172794.18_RAB37	ENSG00000160789.18_LMNA	-0.04538	-0.203110633	0.114651736	5.79E-01	8.04E-01	1.00E+00

ENSG00000205464.10_ATP6AP1I	ENSG00000160789.18_LMNA	0.13553	-0.024195579	0.28850312	9.60E-02	3.04E-01	1.00E+00
ENSG00000151650.7_VENTX	ENSG00000160789.18_LMNA	0.2807	0.127187171	0.421087134	4.60E-04	6.32E-03	1.00E+00
ENSG00000107679.13_PLEKHA1	ENSG00000160789.18_LMNA	-0.31642	-0.452813856	-0.1655631	7.14E-05	1.44E-03	1.00E+00
ENSG00000273350.1_RP4-539M6	ENSG00000160789.18_LMNA	0.09451	-0.065674012	0.249954553	2.47E-01	5.27E-01	1.00E+00
ENSG00000272269.1_RP11-500C	ENSG00000160789.18_LMNA	0.3047	0.152916914	0.442438558	1.35E-04	2.40E-03	1.00E+00
ENSG00000169252.5_ADRB2	ENSG00000160789.18_LMNA	-0.21811	-0.364645996	-0.06102506	6.95E-03	5.02E-02	1.00E+00
ENSG00000206530.7_CFAP44	ENSG00000160789.18_LMNA	-0.09761	-0.25288418	0.062559558	2.32E-01	5.09E-01	1.00E+00
ENSG00000272491.1_RP5-1024N	ENSG00000160789.18_LMNA	0.20218	0.044410913	0.350113546	1.25E-02	7.69E-02	1.00E+00
ENSG00000111186.11_WNT5B	ENSG00000160789.18_LMNA	-0.04173	-0.199604069	0.118256917	6.10E-01	8.22E-01	1.00E+00
ENSG00000281398.1_SNHG4	ENSG00000160789.18_LMNA	-0.04971	-0.20727317	0.110361242	5.43E-01	7.82E-01	1.00E+00
ENSG00000013293.5_SLC7A14	ENSG00000160789.18_LMNA	-0.3151	-0.451641625	-0.16412955	7.69E-05	1.53E-03	1.00E+00
ENSG00000260947.1_RP11-384P	ENSG00000160789.18_LMNA	-0.34291	-0.476114831	-0.19431261	1.53E-05	4.15E-04	7.84E-01
ENSG00000114739.12_ACVR2B	ENSG00000160789.18_LMNA	-0.17038	-0.320877616	-0.01149163	3.59E-02	1.61E-01	1.00E+00
ENSG00000248278.1_SUMO2P17	ENSG00000160789.18_LMNA	-0.08024	-0.236421987	0.079980962	3.26E-01	6.12E-01	1.00E+00
ENSG00000177685.15_CRACR2E	ENSG00000160789.18_LMNA	0.3553	0.207861379	0.486960384	7.06E-06	2.22E-04	3.62E-01
ENSG00000105427.8_CNFN	ENSG00000160789.18_LMNA	0.04046	-0.119515285	0.198378203	6.21E-01	8.28E-01	1.00E+00
ENSG00000143178.11_TBX19	ENSG00000160789.18_LMNA	0.2088	0.051308264	0.356164599	9.84E-03	6.48E-02	1.00E+00
ENSG00000272277.1_RP1-40E16	ENSG00000160789.18_LMNA	-0.04602	-0.203730227	0.114013846	5.73E-01	8.01E-01	1.00E+00
ENSG00000243649.7_CFB	ENSG00000160789.18_LMNA	0.23035	0.073858329	0.375770945	4.30E-03	3.50E-02	1.00E+00
ENSG00000115339.12_GALNT3	ENSG00000160789.18_LMNA	-0.04347	-0.20127914	0.116535782	5.95E-01	8.13E-01	1.00E+00
ENSG00000224689.6_ZNF812P	ENSG00000160789.18_LMNA	-0.2154	-0.362183782	-0.05819826	7.70E-03	5.40E-02	1.00E+00
ENSG00000272905.1_RP11-265E	ENSG00000160789.18_LMNA	-0.11233	-0.266755967	0.047728727	1.68E-01	4.26E-01	1.00E+00
ENSG00000270210.1_RP11-373D	ENSG00000160789.18_LMNA	0.39485	0.251455423	0.521282283	4.81E-07	2.54E-05	2.47E-02
ENSG00000253552.6_HOXA-AS2	ENSG00000160789.18_LMNA	-0.08997	-0.245654018	0.07023475	2.70E-01	5.55E-01	1.00E+00
ENSG00000111254.6_AKAP3	ENSG00000160789.18_LMNA	-0.21871	-0.365196887	-0.0616582	6.79E-03	4.92E-02	1.00E+00
ENSG00000111644.6_ACRBP	ENSG00000160789.18_LMNA	-0.02093	-0.179528318	0.138737195	7.98E-01	9.19E-01	1.00E+00
ENSG00000238082.1_AC009948.1	ENSG00000160789.18_LMNA	0.09476	-0.065426202	0.250187868	2.46E-01	5.25E-01	1.00E+00
ENSG00000235560.3_AC002310.1	ENSG00000160789.18_LMNA	-0.32259	-0.45825781	-0.17223661	5.05E-05	1.08E-03	1.00E+00
ENSG00000236764.4_COX7A2P2	ENSG00000160789.18_LMNA	-0.11965	-0.273633786	0.040323621	1.42E-01	3.85E-01	1.00E+00
ENSG00000108001.12_EBF3	ENSG00000160789.18_LMNA	-0.27499	-0.415981592	-0.12109273	6.07E-04	7.86E-03	1.00E+00
ENSG00000196497.14_IPO4	ENSG00000160789.18_LMNA	0.396	0.252733358	0.52227541	4.42E-07	2.38E-05	2.27E-02
ENSG00000182463.14_TSHZ2	ENSG00000160789.18_LMNA	0.15478	-0.004531956	0.306430325	5.69E-02	2.18E-01	1.00E+00
ENSG00000135722.7_FBXL8	ENSG00000160789.18_LMNA	0.2873	0.134241407	0.426973571	3.32E-04	4.86E-03	1.00E+00

ENSG00000171462.13_DLK2	ENSG00000160789.18_LMNA	-0.12363	-0.27737446	0.036281637	1.29E-01	3.64E-01	1.00E+00
ENSG00000088305.17_DNMT3B	ENSG00000160789.18_LMNA	-0.01841	-0.17709639	0.141199778	8.22E-01	9.30E-01	1.00E+00
ENSG00000272750.1_RP11-378J1	ENSG00000160789.18_LMNA	0.09463	-0.065560852	0.250061098	2.46E-01	5.26E-01	1.00E+00
ENSG00000198848.11_CES1	ENSG00000160789.18_LMNA	0.18208	0.023561424	0.33166581	2.48E-02	1.26E-01	1.00E+00
ENSG00000229091.2_HSPA8P8	ENSG00000160789.18_LMNA	-0.06652	-0.223352378	0.093675811	4.16E-01	6.88E-01	1.00E+00
ENSG00000264589.1_MAPT-AS1	ENSG00000160789.18_LMNA	-0.44696	-0.565896113	-0.30980692	7.82E-09	8.81E-07	4.01E-04
ENSG00000186567.11_CEACAM1	ENSG00000160789.18_LMNA	0.00203	-0.157222028	0.161178098	9.80E-01	9.93E-01	1.00E+00
ENSG00000087589.15_CASS4	ENSG00000160789.18_LMNA	-0.07632	-0.232694324	0.083899167	3.50E-01	6.34E-01	1.00E+00
ENSG00000248785.1_HIGD1AP14	ENSG00000160789.18_LMNA	-0.25365	-0.396826239	-0.09842364	1.62E-03	1.66E-02	1.00E+00
ENSG00000070371.14_CLTCL1	ENSG00000160789.18_LMNA	0.074	-0.08621791	0.230483952	3.65E-01	6.46E-01	1.00E+00
ENSG00000074370.16_ATP2A3	ENSG00000160789.18_LMNA	0.17176	0.012908022	0.322147781	3.44E-02	1.57E-01	1.00E+00
ENSG00000214089.3_RPL9P21	ENSG00000160789.18_LMNA	-0.1077	-0.262397603	0.052403399	1.87E-01	4.53E-01	1.00E+00
ENSG00000226054.2_MEMO1P1	ENSG00000160789.18_LMNA	-0.12018	-0.274133103	0.039784678	1.40E-01	3.82E-01	1.00E+00
ENSG00000267251.2_AC139100.3	ENSG00000160789.18_LMNA	0.05352	-0.106588224	0.210924182	5.13E-01	7.61E-01	1.00E+00
ENSG00000139055.5_ERP27	ENSG00000160789.18_LMNA	-0.22711	-0.372832981	-0.07045947	4.90E-03	3.86E-02	1.00E+00
ENSG00000213432.2_RPL17P34	ENSG00000160789.18_LMNA	-0.07756	-0.23387158	0.082662792	3.42E-01	6.27E-01	1.00E+00
ENSG00000114841.16_DNAH1	ENSG00000160789.18_LMNA	-0.03775	-0.195774138	0.122185008	6.44E-01	8.42E-01	1.00E+00
ENSG00000246922.7_UBAP1L	ENSG00000160789.18_LMNA	-0.00081	-0.1599881	0.158413101	9.92E-01	9.97E-01	1.00E+00
ENSG00000227124.7_ZNF717	ENSG00000160789.18_LMNA	-0.14372	-0.296144593	0.01584344	7.73E-02	2.66E-01	1.00E+00
ENSG00000069696.6_DRD4	ENSG00000160789.18_LMNA	0.19026	0.032024895	0.339182852	1.89E-02	1.04E-01	1.00E+00
ENSG00000259448.2_RP11-16E1	ENSG00000160789.18_LMNA	-0.09897	-0.254164432	0.061196605	2.25E-01	5.01E-01	1.00E+00
ENSG00000241007.1_SEPT7P6	ENSG00000160789.18_LMNA	0.05133	-0.108763536	0.208820293	5.30E-01	7.73E-01	1.00E+00
ENSG00000213740.2_SERBP1P1	ENSG00000160789.18_LMNA	0.25821	0.103251501	0.400927676	1.32E-03	1.43E-02	1.00E+00
ENSG00000197471.10_SPN	ENSG00000160789.18_LMNA	-0.02444	-0.182928961	0.135287074	7.65E-01	9.02E-01	1.00E+00
ENSG00000272732.1_RP5-1159O	ENSG00000160789.18_LMNA	-0.14159	-0.294158231	0.018018732	8.19E-02	2.76E-01	1.00E+00
ENSG00000273343.1_XXbac-B44	ENSG00000160789.18_LMNA	-0.17052	-0.321007793	-0.01163674	3.57E-02	1.61E-01	1.00E+00
ENSG00000096996.14_IL12RB1	ENSG00000160789.18_LMNA	-0.03637	-0.194441484	0.123549485	6.56E-01	8.48E-01	1.00E+00
ENSG00000273478.1_RP11-465N	ENSG00000160789.18_LMNA	0.01861	-0.141005186	0.177288701	8.20E-01	9.30E-01	1.00E+00
ENSG00000227973.1_PIN4P1	ENSG00000160789.18_LMNA	-0.01895	-0.17761295	0.140677035	8.17E-01	9.28E-01	1.00E+00
ENSG00000229807.8_XIST	ENSG00000160789.18_LMNA	0.01845	-0.141162334	0.177133396	8.21E-01	9.30E-01	1.00E+00
ENSG00000188092.13_GPR89B	ENSG00000160789.18_LMNA	-0.09727	-0.252563484	0.062900786	2.33E-01	5.11E-01	1.00E+00
ENSG00000257179.1_HMGN2P6	ENSG00000160789.18_LMNA	-0.24128	-0.385662185	-0.08535303	2.75E-03	2.50E-02	1.00E+00
ENSG00000188659.8_SAXO2	ENSG00000160789.18_LMNA	-0.00714	-0.166155842	0.152229723	9.30E-01	9.73E-01	1.00E+00

ENSG00000151834.14_GABRA2	ENSG00000160789.18_LMNA	-0.21749	-0.364085908	-0.06038161	7.11E-03	5.10E-02	1.00E+00
ENSG00000144366.14_GULP1	ENSG00000160789.18_LMNA	-0.3271	-0.462234662	-0.1771283	3.90E-05	8.83E-04	1.00E+00
ENSG00000214552.4_COPS8P2	ENSG00000160789.18_LMNA	0.03029	-0.1295307	0.188585885	7.11E-01	8.77E-01	1.00E+00
ENSG00000275582.1_RP4-681N2	ENSG00000160789.18_LMNA	-0.00688	-0.16589537	0.152491361	9.33E-01	9.74E-01	1.00E+00
ENSG00000183935.5_HTR7P1	ENSG00000160789.18_LMNA	0.14231	-0.017287172	0.294826554	8.03E-02	2.73E-01	1.00E+00
ENSG00000278982.1_RP5-1031D	ENSG00000160789.18_LMNA	-0.0982	-0.253440734	0.061967197	2.29E-01	5.06E-01	1.00E+00
ENSG00000231969.1_AC144449.1	ENSG00000160789.18_LMNA	-0.31541	-0.451914383	-0.16446301	7.56E-05	1.50E-03	1.00E+00
ENSG00000172167.6_MTBP	ENSG00000160789.18_LMNA	-0.01927	-0.177924823	0.140361343	8.14E-01	9.27E-01	1.00E+00
ENSG00000175544.12_CABP4	ENSG00000160789.18_LMNA	0.12968	-0.030141813	0.283038455	1.11E-01	3.33E-01	1.00E+00
ENSG00000171611.8_PTCRA	ENSG00000160789.18_LMNA	-0.20318	-0.3510237	-0.0454465	1.21E-02	7.51E-02	1.00E+00
ENSG00000144504.14_ANKMY1	ENSG00000160789.18_LMNA	0.16268	0.003571119	0.313754221	4.52E-02	1.88E-01	1.00E+00
ENSG00000226624.1_CTA-351J1	ENSG00000160789.18_LMNA	-0.07521	-0.231639356	0.085006286	3.57E-01	6.40E-01	1.00E+00
ENSG00000139908.13_TSSK4	ENSG00000160789.18_LMNA	0.07454	-0.08567974	0.23099726	3.61E-01	6.43E-01	1.00E+00
ENSG00000229980.4_TOB1-AS1	ENSG00000160789.18_LMNA	-0.37507	-0.504168059	-0.22958003	1.93E-06	7.75E-05	9.88E-02
ENSG00000119866.19_BCL11A	ENSG00000160789.18_LMNA	-0.35648	-0.487988737	-0.20915163	6.55E-06	2.08E-04	3.36E-01
ENSG00000268568.1_AC007228.9	ENSG00000160789.18_LMNA	-0.18617	-0.335432693	-0.02779706	2.16E-02	1.14E-01	1.00E+00
ENSG00000119720.16_NRDE2	ENSG00000160789.18_LMNA	0.0995	-0.060657542	0.254670481	2.23E-01	4.98E-01	1.00E+00
ENSG00000227039.5_ITGB2-AS1	ENSG00000160789.18_LMNA	-0.02116	-0.17975283	0.138509655	7.96E-01	9.18E-01	1.00E+00
ENSG00000135903.17_PAX3	ENSG00000160789.18_LMNA	-0.10942	-0.264024111	0.05066046	1.80E-01	4.42E-01	1.00E+00
ENSG00000143867.6_OSR1	ENSG00000160789.18_LMNA	-0.12469	-0.278365872	0.035208645	1.26E-01	3.59E-01	1.00E+00
ENSG00000232362.1_ATP5LP2	ENSG00000160789.18_LMNA	-0.10474	-0.259612001	0.055383957	1.99E-01	4.69E-01	1.00E+00
ENSG00000155085.14_AK9	ENSG00000160789.18_LMNA	-0.44511	-0.564321374	-0.30771391	9.16E-09	9.94E-07	4.70E-04
ENSG00000139292.11_LGR5	ENSG00000160789.18_LMNA	-0.31176	-0.448690439	-0.16052584	9.24E-05	1.77E-03	1.00E+00
ENSG00000279660.1_RP11-849N	ENSG00000160789.18_LMNA	0.28287	0.129503144	0.423022441	4.14E-04	5.82E-03	1.00E+00
ENSG00000129535.11_NRL	ENSG00000160789.18_LMNA	-0.1455	-0.297803221	0.014024788	7.37E-02	2.58E-01	1.00E+00
ENSG00000272273.1_XXbac-BPG	ENSG00000160789.18_LMNA	-0.04402	-0.201803595	0.115996512	5.90E-01	8.10E-01	1.00E+00
ENSG00000268262.1_CTC-246B1	ENSG00000160789.18_LMNA	-0.08682	-0.24266251	0.07339949	2.88E-01	5.73E-01	1.00E+00
ENSG00000228274.3_RP3-508I15	ENSG00000160789.18_LMNA	-0.19004	-0.338988343	-0.03180534	1.90E-02	1.04E-01	1.00E+00
ENSG00000179476.6_C14orf28	ENSG00000160789.18_LMNA	-0.1484	-0.300498252	0.011065336	6.81E-02	2.45E-01	1.00E+00
ENSG00000182383.8_RPL27AP5	ENSG00000160789.18_LMNA	-0.09652	-0.251847545	0.063662296	2.37E-01	5.15E-01	1.00E+00
ENSG00000266920.1_ACTBP9	ENSG00000160789.18_LMNA	0.27744	0.123705661	0.41817282	5.39E-04	7.15E-03	1.00E+00
ENSG00000280809.1_LINC00836	ENSG00000160789.18_LMNA	-0.49898	-0.609744719	-0.36910098	6.03E-11	1.50E-08	3.10E-06
ENSG00000231878.1_SNRPFP1	ENSG00000160789.18_LMNA	-0.01455	-0.173347636	0.144988084	8.59E-01	9.44E-01	1.00E+00

ENSG00000272056.1_RP11-503P	ENSG00000160789.18_LMNA	-0.17803	-0.327939014	-0.01938172	2.82E-02	1.37E-01	1.00E+00
ENSG00000261490.1_RP11-448G	ENSG00000160789.18_LMNA	-0.06491	-0.221818778	0.095274951	4.27E-01	6.98E-01	1.00E+00
ENSG00000227855.3_DPY19L2P3	ENSG00000160789.18_LMNA	-0.10643	-0.261201305	0.05368411	1.92E-01	4.60E-01	1.00E+00
ENSG00000225171.2_DUTP6	ENSG00000160789.18_LMNA	0.06991	-0.090296087	0.22658843	3.92E-01	6.70E-01	1.00E+00
ENSG00000120729.8_MYOT	ENSG00000160789.18_LMNA	-0.48433	-0.597463757	-0.35229424	2.58E-10	5.03E-08	1.32E-05
ENSG00000243811.6_APOBEC3D	ENSG00000160789.18_LMNA	0.26396	0.109359404	0.406099509	1.02E-03	1.17E-02	1.00E+00
ENSG00000179988.12_PSTK	ENSG00000160789.18_LMNA	-0.44615	-0.565201418	-0.30888328	8.38E-09	9.31E-07	4.30E-04
ENSG00000263214.1_	ENSG00000160789.18_LMNA	-0.04594	-0.203648783	0.11409771	5.74E-01	8.01E-01	1.00E+00
ENSG00000261220.2_RP11-629O	ENSG00000160789.18_LMNA	0.03935	-0.12060617	0.197314688	6.30E-01	8.34E-01	1.00E+00
ENSG00000166535.18_A2ML1	ENSG00000160789.18_LMNA	-0.21422	-0.361102352	-0.05695824	8.05E-03	5.58E-02	1.00E+00
ENSG00000275993.2_CH507-42P	ENSG00000160789.18_LMNA	0.10519	-0.054933581	0.260033255	1.97E-01	4.67E-01	1.00E+00
ENSG00000100218.10_RSPH14	ENSG00000160789.18_LMNA	0.07631	-0.083907391	0.23268649	3.50E-01	6.34E-01	1.00E+00
ENSG00000230457.3_PA2G4P4	ENSG00000160789.18_LMNA	0.01918	-0.140451962	0.177835308	8.15E-01	9.27E-01	1.00E+00
ENSG00000165259.12_HDX	ENSG00000160789.18_LMNA	-0.25048	-0.393974244	-0.09507477	1.86E-03	1.85E-02	1.00E+00
ENSG00000271755.1_RP1-153G1	ENSG00000160789.18_LMNA	-0.26479	-0.406844354	-0.11024091	9.78E-04	1.14E-02	1.00E+00
ENSG00000227218.6_RP11-203J	ENSG00000160789.18_LMNA	0.17178	0.012935976	0.322172838	3.43E-02	1.57E-01	1.00E+00
ENSG00000173811.9_CCDC13-AS	ENSG00000160789.18_LMNA	-0.31849	-0.454635479	-0.16779323	6.37E-05	1.30E-03	1.00E+00
ENSG00000100583.4_SAMD15	ENSG00000160789.18_LMNA	0.20539	0.047754636	0.353050187	1.11E-02	7.07E-02	1.00E+00
ENSG00000271984.1_RP3-337O1	ENSG00000160789.18_LMNA	0.22178	0.064867323	0.367985904	6.03E-03	4.52E-02	1.00E+00
ENSG00000236533.1_AC009413.	ENSG00000160789.18_LMNA	-0.1573	-0.308768886	0.001949029	5.29E-02	2.09E-01	1.00E+00
ENSG00000172382.8_PRSS27	ENSG00000160789.18_LMNA	-0.04195	-0.199812978	0.118042367	6.08E-01	8.21E-01	1.00E+00
ENSG00000260121.1_RP5-1142A	ENSG00000160789.18_LMNA	0.24136	0.085437773	0.385734848	2.74E-03	2.49E-02	1.00E+00
ENSG00000162194.11_LBHD1	ENSG00000160789.18_LMNA	-0.05957	-0.216715844	0.100584208	4.66E-01	7.28E-01	1.00E+00
ENSG00000243071.1_RP11-613M	ENSG00000160789.18_LMNA	-0.21465	-0.361500313	-0.05741445	7.92E-03	5.52E-02	1.00E+00
ENSG00000146147.13_MLIP	ENSG00000160789.18_LMNA	-0.41762	-0.540863929	-0.27682746	8.65E-08	6.38E-06	4.44E-03
ENSG00000260526.1_RP11-73K9	ENSG00000160789.18_LMNA	-0.25208	-0.3954113	-0.09676134	1.73E-03	1.75E-02	1.00E+00
ENSG00000118804.8_STBD1	ENSG00000160789.18_LMNA	0.3411	0.192346038	0.474533469	1.71E-05	4.52E-04	8.75E-01
[truncated: 904,414 more chars]
