# Supplementary material for: Role of Lamin A/C as Candidate Biomarker of Aggressiveness and Tumorigenicity in Glioblastoma Multiforme
Source: Biomedicines. 2021 Sep 28;9(10):1343. doi: 10.3390/biomedicines9101343 (PMC8533312; doi:10.3390/biomedicines9101343)
Supplement: Supplementary file 1 [file biomedicines-09-01343-s001.zip › biomedicines-1353075-suppl/biomedicines-1353075-suppl(tables).zip!/supplementary tables/Supplementary Table 2.pdf]

Table S2. Gene set enrichment analysis of the core molecular KEGG pathways related to the expression of LMNA in GBM patients from TCGA RNASeq data.

NAME	GS<br>follow link to MSigDB	SIZE	ES	NES	NOM p-val	FDR q-val	FWER p-val	RANK AT MAX	LEADING EDGE
KEGG_GLYCOSAMINOGLYCAN_BIOSYNTHESIS_KERATAN_SULFATE	KEGG_GLYCOSAMINOGLYCAN_BIOSYNTHESIS_KERATAN_SULFATE	14	0.5707749	0.5707749	0.001012146	0.005897383	0.0045	21024	tags=100%, list=43%, signal=175%
KEGG_GLYCOSAMINOGLYCAN_DEGRADATION	KEGG_GLYCOSAMINOGLYCAN_DEGRADATION	21	0.5617917	0.5617917	0	0.004521676	0.007	5146	tags=67%, list=11%, signal=74%
KEGG_GLYCOSAMINOGLYCAN_BIOSYNTHESIS_CHONDROITIN_SULFATE	KEGG_GLYCOSAMINOGLYCAN_BIOSYNTHESIS_CHONDROITIN_SULFATE	22	0.5529131	0.5529131	0	0.003887325	0.009	6323	tags=68%, list=13%, signal=78%
KEGG_OTHER_GLYCAN_DEGRADATION	KEGG_OTHER_GLYCAN_DEGRADATION	16	0.54579383	0.54579383	0	0.003555518	0.011	13067	tags=81%, list=27%, signal=111%
KEGG_ECM_RECEPTOR_INTERACTION	KEGG_ECM_RECEPTOR_INTERACTION	83	0.51111627	0.51111627	0	0.00674333	0.026	10411	tags=72%, list=21%,
KEGG_GALACTOSE_METABOLISM	KEGG_GALACTOSE_METABOLISM	26	0.51001406	0.51001406	0	0.005733673	0.0265	7055	tags=65%, list=14%, signal=76%
KEGG_VALINE_LEUCINE_AND_ISOLEUCINE_BIOSYNTHESIS	KEGG_VALINE_LEUCINE_AND_ISOLEUCINE_BIOSYNTHESIS	11	0.49006304	0.49006304	0.010111224	0.007629179	0.0405	7168	tags=64%, list=15%, signal=75%
KEGG_GLYCOSPHINGOLIPID_BIOSYNTHESIS_GANGLIO_SERIES	KEGG_GLYCOSPHINGOLIPID_BIOSYNTHESIS_GANGLIO_SERIES	15	0.46108958	0.46108958	9.92E-04	0.011893394	0.0705	10072	tags=67%, list=21%, signal=84%
KEGG_AMINO_SUGAR_AND_NUCLEOTIDE_SUGAR_METABOLISM	KEGG_AMINO_SUGAR_AND_NUCLEOTIDE_SUGAR_METABOLISM	44	0.41718328	0.41718328	0	0.024909593	0.153	7411	tags=57%, list=15%, signal=67%
KEGG_ONE_CARBON_POOL_BY_FOLATE	KEGG_ONE_CARBON_POOL_BY_FOLATE	17	0.40005362	0.40005362	0.00483559	0.031165738	0.211	17861	tags=76%, list=36%, signal=120%
KEGG_NOTCH_SIGNALING_PATHWAY	KEGG_NOTCH_SIGNALING_PATHWAY	47	0.3713157	0.3713157	0	0.048369367	0.3345	8922	tags=55%, list=18%, signal=68%
KEGG_GLYCOSAMINOGLYCAN_BIOSYNTHESIS_HEPARAN_SULFATE	KEGG_GLYCOSAMINOGLYCAN_BIOSYNTHESIS_HEPARAN_SULFATE	26	0.37113473	0.37113473	0.001034126	0.04460336	0.3365	2552	tags=42%, list=5%, signal=45%
KEGG_O_GLYCAN_BIOSYNTHESIS	KEGG_O_GLYCAN_BIOSYNTHESIS	27	0.37069806	0.37069806	0	0.04154259	0.34	3620	tags=44%, list=7%, signal=48%
KEGG_FOCAL_ADHESION	KEGG_FOCAL_ADHESION	197	0.36790058	0.36790058	0	0.040333424	0.353	6664	tags=50%, list=14%, signal=58%
KEGG_PHENYLALANINE_METABOLISM	KEGG_PHENYLALANINE_METABOLISM	18	0.36671865	0.36671865	0.01003009	0.038857285	0.3625	9252	tags=56%, list=19%, signal=68%
KEGG_MISMATCH_REPAIR	KEGG_MISMATCH_REPAIR	23	0.36115423	0.36115423	0.003024194	0.040877532	0.3945	16386	tags=70%, list=33%, signal=105%
KEGG_PRION_DISEASES	KEGG_PRION_DISEASES	35	0.35352546	0.35352546	0	0.044632327	0.4425	20474	tags=77%, list=42%, signal=132%
KEGG_LYSOSOME	KEGG_LYSOSOME	119	0.35129255	0.35129255	0	0.044455867	0.4605	7528	tags=50%, list=15%, signal=59%
KEGG_N_GLYCAN_BIOSYNTHESIS	KEGG_N_GLYCAN_BIOSYNTHESIS	46	0.34807894	0.34807894	0	0.04503697	0.4825	3196	tags=41%, list=7%, signal=44%
KEGG_RENIN_ANGIOTENSIN_SYSTEM	KEGG_RENIN_ANGIOTENSIN_SYSTEM	17	0.34315288	0.34315288	0.024390243	0.047652006	0.52	9125	tags=53%, list=19%, signal=65%
KEGG_HOMOLOGOUS_RECOMBINATION	KEGG_HOMOLOGOUS_RECOMBINATION	27	0.34149027	0.34149027	0.002979146	0.04736493	0.532	17744	tags=70%, list=36%, signal=110%
KEGG_SMALL_CELL_LUNG_CANCER	KEGG_SMALL_CELL_LUNG_CANCER	84	0.34056348	0.34056348	0	0.0458273	0.5365	10749	tags=56%, list=22%, signal=72%
KEGG_AMINOACYL_TRNA_BIOSYNTHESIS	KEGG_AMINOACYL_TRNA_BIOSYNTHESIS	41	0.33934763	0.33934763	0	0.04497418	0.548	15642	tags=66%, list=32%, signal=97%
KEGG_BLADDER_CANCER	KEGG_BLADDER_CANCER	40	0.33908534	0.33908534	0	0.043325417	0.5495	10340	tags=55%, list=21%, signal=70%
KEGG_CIRCADIAN_RHYTHM_MAMMAL	KEGG_CIRCADIAN_RHYTHM_MAMMAL	13	0.3261531	0.3261531	0.10958904	0.054071195	0.646	17932	tags=69%, list=37%, signal=109%
KEGG_DNA_REPLICATION	KEGG_DNA_REPLICATION	36	0.32357487	0.32357487	0	0.054292593	0.664	15450	tags=64%, list=32%, signal=93%

KEGG_GLYCOSPHINGOLIPID_BIOSYNTHESIS_GLOBO_SERIES	KEGG_GLYCOSPHINGOLIPID_BIOSYNTHESIS_GLOBO_SERIES	14	0.31870648	0.31870648	0.07411168	0.05694878	0.694	5383	tags=43%, list=11%, signal=48%
KEGG_PATHOGENIC_ESCHERICHIA_COLI_INFECTION	KEGG_PATHOGENIC_ESCHERICHIA_COLI_INFECTION	56	0.31789142	0.31789142	0	0.05612397	0.7025	14180	tags=61%, list=29%, signal=85%
KEGG_NOD_LIKE_RECEPTOR_SIGNALING_PATHWAY	KEGG_NOD_LIKE_RECEPTOR_SIGNALING_PATHWAY	60	0.31417376	0.31417376	0	0.0590761	0.7295	10749	tags=53%, list=22%, signal=68%
KEGG_APOPTOSIS	KEGG_APOPTOSIS	86	0.3058642	0.3058642	0	0.06799298	0.7965	6115	tags=43%, list=12%, signal=49%
KEGG_HEMATOPOIETIC_CELL_LINEAGE	KEGG_HEMATOPOIETIC_CELL_LINEAGE	84	0.30288526	0.30288526	0	0.06964665	0.8155	17837	tags=67%, list=36%, signal=105%
KEGG_PENTOSE_PHOSPHATE_PATHWAY	KEGG_PENTOSE_PHOSPHATE_PATHWAY	26	0.30067965	0.30067965	0.016260162	0.07024792	0.8275	6000	tags=42%, list=12%, signal=48%
KEGG_CYTOKINE_CYTOKINE_RECEPTOR_INTERACTION	KEGG_CYTOKINE_CYTOKINE_RECEPTOR_INTERACTION	253	0.29452127	0.29452127	0	0.076147236	0.858	12554	tags=55%, list=26%, signal=74%
KEGG_FRUCTOSE_AND_MANNOSE_METABOLISM	KEGG_FRUCTOSE_AND_MANNOSE_METABOLISM	34	0.2937678	0.2937678	0.001998002	0.07501489	0.862	8667	tags=47%, list=18%, signal=57%
KEGG_GLYCOLYSIS_GLUCONEOGENESIS	KEGG_GLYCOLYSIS_GLUCONEOGENESIS	60	0.29372936	0.29372936	0	0.07292745	0.862	13381	tags=57%, list=27%, signal=78%
KEGG_STEROID_BIOSYNTHESIS	KEGG_STEROID_BIOSYNTHESIS	16	0.29142195	0.29142195	0.108778626	0.07410208	0.8705	13277	tags=56%, list=27%, signal=77%
KEGG_ARRHYTHMOGENIC_RIGHT_VENTRICULAR_CARDIOMYOPATHY_ARVC	KEGG_ARRHYTHMOGENIC_RIGHT_VENTRICULAR_CARDIOMYOPATHY_ARVC	73	0.2906476	0.2906476	0	0.07346807	0.875	3899	tags=37%, list=8%, signal=40%
KEGG_RIG_I_LIKE_RECEPTOR_SIGNALING_PATHWAY	KEGG_RIG_I_LIKE_RECEPTOR_SIGNALING PATHWAY	67	0.2890226	0.2890226	0	0.07339862	0.882	7791	tags=45%, list=16%, signal=53%
KEGG_ALLOGRAFT_REJECTION	KEGG_ALLOGRAFT_REJECTION	35	0.28694054	0.28694054	0.005785921	0.07447637	0.8935	22333	tags=74%, list=46%, signal=136%
KEGG_CHRONIC_MYELOID_LEUKEMIA	KEGG_CHRONIC_MYELOID_LEUKEMIA	73	0.2816025	0.2816025	0	0.080159724	0.9145	7695	tags=44%, list=16%, signal=52%
KEGG_GRAFT_VERSUS_HOST_DISEASE	KEGG_GRAFT_VERSUS_HOST_DISEASE	37	0.27381495	0.27381495	0.003838772	0.09004085	0.942	22333	tags=73%, list=46%, signal=134%
KEGG_PANTOTHENATE_AND_COA_BIOSYNTHESIS	KEGG_PANTOTHENATE_AND_COA_BIOSYNTHESIS	16	0.27190673	0.27190673	0.14565004	0.091334164	0.9505	1991	tags=31%, list=4%, signal=33%
KEGG_COMPLEMENT_AND_COAGULATION_CASCADES	KEGG_COMPLEMENT_AND_COAGULATION_CASCADES	69	0.27130255	0.27130255	0	0.09022862	0.9525	20796	tags=70%, list=42%, signal=121%
KEGG_ADHERENS_JUNCTION	KEGG_ADHERENS_JUNCTION	68	0.26347038	0.26347038	0	0.10228589	0.97	5838	tags=38%, list=12%, signal=43%
KEGG_ANTIGEN_PROCESSING_AND_PRESENTATION	KEGG_ANTIGEN_PROCESSING_AND_PRESENTATION	78	0.2629452	0.2629452	0	0.10102689	0.9715	21671	tags=71%, list=44%, signal=126%
KEGG_LEUKOCYTE_TRANSENDOTHELIAL_MIGRATION	KEGG_LEUKOCYTE_TRANSENDOTHELIAL_MIGRATION	114	0.26122954	0.26122954	0	0.1019268	0.9745	7425	tags=41%, list=15%, signal=48%
KEGG_PRIMARY_IMMUNODEFICIENCY	KEGG_PRIMARY_IMMUNODEFICIENCY	35	0.2586961	0.2586961	0.017924529	0.10439286	0.981	22316	tags=71%, list=46%, signal=131%
KEGG_NICOTINATE_AND_NICOTINAMIDE_METABOLISM	KEGG_NICOTINATE_AND_NICOTINAMIDE_METABOLISM	22	0.25616628	0.25616628	0.1002045	0.10682454	0.983	18621	tags=64%, list=38%, signal=103%
KEGG_LEISHMANIA_INFECTION	KEGG_LEISHMANIA_INFECTION	70	0.25167194	0.25167194	0	0.11405265	0.989	21969	tags=70%, list=45%, signal=127%
KEGG_CYTOSOLIC_DNA_SENSING_PATHW	KEGG_CYTOSOLIC_DNA_SENSING_PATH	52	0.24965757	0.24965757	0.003024194	0.115912095	0.99	12270	tags=50%, list=25%,
KEGG_B_CELL_RECEPTOR_SIGNALING_PATHWAY	KEGG_B_CELL_RECEPTOR_SIGNALING_PATHWAY	74	0.24860764	0.24860764	0	0.11626541	0.992	7695	tags=41%, list=16%, signal=48%
KEGG_DILATED_CARDIOMYOPATHY	KEGG_DILATED_CARDIOMYOPATHY	89	0.24664228	0.24664228	0	0.118488595	0.9925	3899	tags=33%, list=8%, signal=35%
KEGG_JAK_STAT_SIGNALING_PATHWAY	KEGG_JAK_STAT_SIGNALING_PATHWAY	147	0.24655522	0.24655522	0	0.11657327	0.9925	13611	tags=52%, list=28%,
KEGG_THYROID_CANCER	KEGG_THYROID_CANCER	29	0.24586862	0.24586862	0.03767821	0.11586282	0.993	9917	tags=45%, list=20%, signal=56%

KEGG_ENDOCYTOSIS	KEGG_ENDOCYTOSIS	176	0.24256493	0.24256493	0	0.121194586	0.995	12647	tags=50%, list=26%, signal=67%	
KEGG_GLUTATHIONE_METABOLISM	KEGG_GLUTATHIONE_METABOLISM	48	0.24181949	0.24181949	0.009081735	0.120522715	0.9955	8572	tags=42%, list=18%, signal=50%	
KEGG_PATHWAYS_IN_CANCER	KEGG_PATHWAYS_IN_CANCER	323	0.23958392	0.23958392	0	0.1235381	0.998	8962	tags=42%, list=18%, signal=51%	
KEGG_ACUTE_MYELOID_LEUKEMIA	KEGG_ACUTE_MYELOID_LEUKEMIA	57	0.23699278	0.23699278	9.97E-04	0.1275729	0.9985	9025	tags=42%, list=18%, signal=52%	
KEGG_PANCREATIC_CANCER	KEGG_PANCREATIC_CANCER	69	0.23446126	0.23446126	0	0.13158838	0.999	7695	tags=39%, list=16%, signal=46%	
KEGG_NATURAL_KILLER_CELL_MEDIATED_CYTOTOXICITY	KEGG_NATURAL_KILLER_CELL_MEDIATED_CYTOTOXICITY	129	0.23270607	0.23270607	0	0.13328154	0.999	14066	tags=52%, list=29%, signal=73%	
KEGG_TOLL_LIKE_RECEPTOR_SIGNALING_PATHWAY	KEGG_TOLL_LIKE_RECEPTOR_SIGNALING_PATHWAY	99	0.23225111	0.23225111	0	0.13216415	0.999	15358	tags=55%, list=31%, signal=79%	
KEGG_P53_SIGNALING_PATHWAY	KEGG_P53_SIGNALING_PATHWAY	66	0.23008187	0.23008187	0.001980198	0.13508774	0.9995	4328	tags=32%, list=9%, signal=35%	
KEGG_REGULATION_OF_ACTIN_CYTOSKELETON	KEGG_REGULATION_OF_ACTIN_CYTOSKELETON	211	0.22980155	0.22980155	0	0.13364965	0.9995	6664	tags=36%, list=14%, signal=42%	
KEGG_HYPERTROPHIC_CARDIOMYOPATHY	KEGG_HYPERTROPHIC_CARDIOMYOPATH	82	0.22560713	0.22560713	9.82E-04	0.14203712	0.9995	3899	tags=30%, list=8%, [illegible]	
KEGG_ABC_TRANSPORTERS	KEGG_ABC_TRANSPORTERS	44	0.22011603	0.22011603	0.02123357	0.15496477	1	8149	tags=39%, list=17%, signal=46%	
KEGG_T_CELL_RECEPTOR_SIGNALING_PATHWAY	KEGG_T_CELL_RECEPTOR_SIGNALING_PATHWAY	106	0.22003943	0.22003943	0	0.15284643	1	17888	tags=58%, list=37%, signal=92%	
KEGG_ADIPOCYTOKINE_SIGNALING_PATHWAY	KEGG_ADIPOCYTOKINE_SIGNALING_PATHWAY	67	0.21723624	0.21723624	0.00295858	0.15878147	1	3993	tags=30%, list=8%, signal=32%	
KEGG_GLYCOSPHINGOLIPID_BIOSYNTHESIS_LACTO_AND_NEOLACTO_SERIES	KEGG_GLYCOSPHINGOLIPID_BIOSYNTHESIS_LACTO_AND_NEOLACTO_SERIES	25	0.21279526	0.21279526	0.20101523	0.16945462	1	13088	tags=48%, list=27%, signal=65%	
KEGG_CHEMOKINE_SIGNALING_PATHWAY	KEGG_CHEMOKINE_SIGNALING_PATHWAY	184	0.21250992	0.21250992	0	0.16776825	1	9325	tags=40%, list=19%, signal=49%	
KEGG_AUTOIMMUNE_THYROID_DISEASE	KEGG_AUTOIMMUNE_THYROID_DISEASE	48	0.21075156	0.21075156	0.02098636	0.1709527	1	22333	tags=67%, list=46%, signal=122%	
KEGG_VIBRIO_CHOLERAE_INFECTION	KEGG_VIBRIO_CHOLERAE_INFECTION	53	0.20939955	0.20939955	0.019354839	0.17299344	1	2691	tags=26%, list=5%, signal=28%	
KEGG_CITRATE_CYCLE_TCA_CYCLE	KEGG_CITRATE_CYCLE_TCA_CYCLE	30	0.20651206	0.20651206	0.15915915	0.17967749	1	6215	tags=33%, list=13%, signal=38%	
KEGG_CELL_ADHESION_MOLECULES_CAMS	KEGG_CELL_ADHESION_MOLECULES_CAMS	127	0.20562582	0.20562582	9.93E-04	0.18025388	1	7692	tags=36%, list=16%, signal=43%	
KEGG_GLYCOSYLPHOSPHATIDYLINOSITOL_GPI_ANCHOR_BIOSYNTHESIS	KEGG_GLYCOSYLPHOSPHATIDYLINOSITOL_GPI_ANCHOR_BIOSYNTHESIS	24	0.2020043	0.2020043	0.22344689	0.18981017	1	12555	tags=46%, list=26%, signal=62%	
KEGG_EPITHELIAL_CELL_SIGNALING_IN_HELICOBACTER_PYLORI_INFECTION	KEGG_EPITHELIAL_CELL_SIGNALING_IN_HELICOBACTER_PYLORI_INFECTION	67	0.20131092	0.20131092	0.012695313	0.18962152	1	7695	tags=36%, list=16%, signal=42%	
KEGG_TYPE_I_DIABETES_MELLITUS	KEGG_TYPE_I_DIABETES_MELLITUS	41	0.20127633	0.20127633	0.057059962	0.18722332	1	22397	tags=66%, list=46%, signal=121%	
KEGG_VIRAL_MYOCARDITIS	KEGG_VIRAL_MYOCARDITIS	68	0.20037559	0.20037559	0.008196721	0.18821001	1	7483	tags=35%, list=15%, signal=42%	
KEGG_RENAL_CELL_CARCINOMA	KEGG_RENAL_CELL_CARCINOMA	68	0.20009227	0.20009227	0.006776379	0.18676144	1	8937	tags=38%, list=18%, signal=47%	
KEGG_PROSTATE_CANCER	KEGG_PROSTATE_CANCER	89	0.19984718	0.19984718	0.001012146	0.18515569	1	8937	tags=38%, list=18%, signal=47%	
KEGG_VEGF_SIGNALING_PATHWAY	KEGG_VEGF_SIGNALING_PATHWAY	75	0.19935638	0.19935638	0.007920792	0.18430574	1	7227	tags=35%, list=15%, signal=41%	
KEGG_DORSO_VENTRAL_AXIS_FORMATION	KEGG_DORSO_VENTRAL_AXIS_FORMATION	24	0.19932505	0.19932505	0.24616957	0.18214957	1	31048	tags=83%, list=63%, signal=228%	

KEGG_BASAL_CELL_CARCINOMA	KEGG_BASAL_CELL_CARCINOMA	55	0.1959443	0.1959443	0.031281535	0.19130553	1	5550	tags=31%, list=11%, signal=35%
KEGG_STARCH_AND_SUCROSE_METABOLISM	KEGG_STARCH_AND_SUCROSE_METABOLISM	47	0.19530034	0.19530034	0.054421768	0.19100228	1	2947	tags=26%, list=6%, signal=27%
KEGG_NEUROTROPHIN_SIGNALING_PATHWAY	KEGG_NEUROTROPHIN_SIGNALING_PATHWAY	125	0.1912711	0.1912711	0	0.2028899	1	7893	tags=35%, list=16%, signal=42%
KEGG_INTESTINAL_IMMUNE_NETWORK_FOR_IGA_PRODUCTION	KEGG_INTESTINAL_IMMUNE_NETWORK_FOR_IGA_PRODUCTION	46	0.19004308	0.19004308	0.07468477	0.20506701	1	34345	tags=89%, list=70%, signal=298%
KEGG_INSULIN_SIGNALING_PATHWAY	KEGG_INSULIN_SIGNALING_PATHWAY	137	0.18641376	0.18641376	0	0.21613605	1	7695	tags=34%, list=16%, signal=41%
KEGG_ALPHA_LINOLENIC_ACID_METABOLISM	KEGG_ALPHA_LINOLENIC_ACID_METABOLISM	19	0.18434495	0.18434495	0.46474677	0.22140309	1	14170	tags=47%, list=29%, signal=67%
KEGG_ERBB_SIGNALING_PATHWAY	KEGG_ERBB_SIGNALING_PATHWAY	86	0.18300483	0.18300483	0.005081301	0.2238749	1	6996	tags=33%, list=14%, signal=38%
KEGG_GLIOMA	KEGG_GLIOMA	65	0.18041843	0.18041843	0.029693486	0.2316829	1	6996	tags=32%, list=14%, signal=38%
KEGG_LYSINE_DEGRADATION	KEGG_LYSINE_DEGRADATION	41	0.17700425	0.17700425	0.14980158	0.24317455	1	8059	tags=34%, list=16%, signal=41%
KEGG_INOSITOL_PHOSPHATE_METABOLISM	KEGG_INOSITOL_PHOSPHATE_METABOLISM	54	0.17616956	0.17616956	0.051102206	0.24407972	1	4077	tags=26%, list=8%, signal=28%
KEGG_BIOSYNTHESIS_OF_UNSATURATED_FATTY_ACIDS	KEGG_BIOSYNTHESIS_OF_UNSATURATED_FATTY_ACIDS	20	0.17284197	0.17284197	0.56646216	0.25562945	1	8677	tags=35%, list=18%, signal=43%
KEGG_BASE_EXCISION_REPAIR	KEGG_BASE_EXCISION_REPAIR	35	0.17198038	0.17198038	0.21978022	0.2567461	1	8372	tags=34%, list=17%, signal=41%
KEGG_FC_GAMMA_R_MEDIATED_PHAGOCYTOSIS	KEGG_FC_GAMMA_R_MEDIATED_PHAGOCYTOSIS	93	0.16925143	0.16925143	0.010880317	0.26566455	1	6996	tags=31%, list=14%, signal=36%
KEGG_HEDGEHOG_SIGNALING_PATHWAY	KEGG_HEDGEHOG_SIGNALING_PATHWAY	56	0.1684864	0.1684864	0.060817547	0.26635736	1	9246	tags=36%, list=19%, signal=44%
KEGG_COLORECTAL_CANCER	KEGG_COLORECTAL_CANCER	62	0.16455351	0.16455351	0.051896207	0.28242758	1	7747	tags=32%, list=16%, signal=38%
KEGG_PYRIMIDINE_METABOLISM	KEGG_PYRIMIDINE_METABOLISM	96	0.16265254	0.16265254	0.007121058	0.28870386	1	8372	tags=33%, list=17%, signal=40%
KEGG_AXON_GUIDANCE	KEGG_AXON_GUIDANCE	127	0.1618852	0.1618852	0.003003003	0.2894131	1	8286	tags=33%, list=17%, signal=40%
KEGG_DRUG_METABOLISM_OTHER_ENZYMES	KEGG_DRUG_METABOLISM_OTHER_ENZYMES	46	0.16064088	0.16064088	0.16716717	0.2923025	1	7043	tags=30%, list=14%, signal=36%
KEGG_TGF_BETA_SIGNALING_PATHWAY	KEGG_TGF_BETA_SIGNALING_PATHWAY	85	0.15992348	0.15992348	0.022749752	0.2928523	1	12344	tags=41%, list=25%, signal=55%
KEGG_MAPK_SIGNALING_PATHWAY	KEGG_MAPK_SIGNALING_PATHWAY	265	0.1490233	0.1490233	0	0.34802884	1	7893	tags=31%, list=16%, signal=37%
KEGG_GLYCEROPHOSPHOLIPID_METABOLISM	KEGG_GLYCEROPHOSPHOLIPID_METABOLISM	71	0.14864607	0.14864607	0.07186858	0.3468184	1	8593	tags=32%, list=18%, signal=39%
KEGG_NUCLEOTIDE_EXCISION_REPAIR	KEGG_NUCLEOTIDE_EXCISION_REPAIR	44	0.14676353	0.14676353	0.2682927	0.35451967	1	15076	tags=45%, list=31%, signal=66%
KEGG_SYSTEMIC_LUPUS_ERYTHEMATOSUS	KEGG_SYSTEMIC_LUPUS_ERYTHEMATOSUS	132	0.1465513	0.1465513	0.007744434	0.35240236	1	31050	tags=78%, list=63%, signal=213%
KEGG_ENDOMETRIAL_CANCER	KEGG_ENDOMETRIAL_CANCER	52	0.1457183	0.1457183	0.22073922	0.35389972	1	6996	tags=29%, list=14%, signal=34%
KEGG_PHOSPHATIDYLINOSITOL_SIGNALING_SYSTEM	KEGG_PHOSPHATIDYLINOSITOL_SIGNALING_SYSTEM	76	0.14150934	0.14150934	0.079380445	0.3752617	1	5322	tags=25%, list=11%, signal=28%
KEGG_PROTEASOME	KEGG_PROTEASOME	46	0.13877021	0.13877021	0.32604373	0.38816062	1	17693	tags=50%, list=36%, signal=78%
KEGG_GNRH_SIGNALING_PATHWAY	KEGG_GNRH_SIGNALING_PATHWAY	101	0.13815673	0.13815673	0.0347567	0.3885226	1	15549	tags=46%, list=32%, signal=67%

KEGG_TYROSINE_METABOLISM	KEGG_TYROSINE_METABOLISM	42	0.13142422	0.13142422	0.42627883	0.42909488	1	9891	tags=33%, list=20%, signal=42%
KEGG_ASTHMA	KEGG_ASTHMA	28	0.13051477	0.13051477	0.6719841	0.43090236	1	21592	tags=57%, list=44%, signal=102%
KEGG_VASCULAR_SMOOTH_MUSCLE_CONTRACTION	KEGG_VASCULAR_SMOOTH_MUSCLE_CONTRACTION	115	0.12828177	0.12828177	0.039354187	0.4418992	1	14170	tags=42%, list=29%, signal=59%
KEGG_MELANOGENESIS	KEGG_MELANOGENESIS	101	0.1270473	0.1270473	0.07947686	0.44643047	1	3487	tags=20%, list=7%, signal=21%
KEGG_PPAR_SIGNALING_PATHWAY	KEGG_PPAR_SIGNALING_PATHWAY	69	0.127001	0.127001	0.20170757	0.44274196	1	5853	tags=25%, list=12%, signal=28%
KEGG_MELANOMA	KEGG_MELANOMA	71	0.124888726	0.12488873	0.20406091	0.4528833	1	6996	tags=27%, list=14%, signal=31%
KEGG_MTOR_SIGNALING_PATHWAY	KEGG_MTOR_SIGNALING_PATHWAY	51	0.12258611	0.12258611	0.36948177	0.46501824	1	5524	tags=24%, list=11%, signal=26%
KEGG_CELL_CYCLE	KEGG_CELL_CYCLE	124	0.121814124	0.12181412	0.046421662	0.46621928	1	8660	tags=30%, list=18%, signal=36%
KEGG_GAP_JUNCTION	KEGG_GAP_JUNCTION	90	0.1214902	0.1214902	0.12410256	0.4645354	1	2222	tags=17%, list=5%, signal=17%
KEGG_SPLICEOSOME	KEGG_SPLICEOSOME	123	0.11543792	0.11543792	0.079637095	0.50406617	1	8692	tags=29%, list=18%, signal=35%
KEGG_FC_EPSILON_RI_SIGNALING_PATHWAY	KEGG_FC_EPSILON_RI_SIGNALING_PATHWAY	79	0.1031607	0.1031607	0.34890655	0.59530956	1	14170	tags=39%, list=29%, signal=55%
KEGG_PEROXISOME	KEGG_PEROXISOME	78	0.096561775	0.09656178	0.47244897	0.64505506	1	6578	tags=23%, list=13%, signal=27%
KEGG_LINOLEIC_ACID_METABOLISM	KEGG_LINOLEIC_ACID_METABOLISM	29	0.089952804	0.0899528	0.9468085	0.6940002	1	14170	tags=38%, list=29%, signal=53%
KEGG_ARACHIDONIC_ACID_METABOLISM	KEGG_ARACHIDONIC_ACID_METABOLISM	58	0.07075287	0.07075287	0.9047619	0.8404326	1	35373	tags=79%, list=72%, signal=285%
