# Supplementary material for: Role of Lamin A/C as Candidate Biomarker of Aggressiveness and Tumorigenicity in Glioblastoma Multiforme
Source: Biomedicines. 2021 Sep 28;9(10):1343. doi: 10.3390/biomedicines9101343 (PMC8533312; doi:10.3390/biomedicines9101343)
Supplement: Supplementary file 1 [file biomedicines-09-01343-s001.zip › biomedicines-1353075-suppl/biomedicines-1353075-suppl(tables).zip!/supplementary tables/Supplementary Table 3.pdf]

Table S3. Gene set enrichment analysis of the core molecular Reactome pathways related to the expression of LMNA in GBM patients from TCGA RNASeq data.

NAME	GS<br>follow link to MSigDB	SIZE	ES	NES	NOM p-val	FDR q-val	FWER p-val	RANK AT MAX	LEADING EDGE
REACTOME_RUNX3 REGULATES RUNX1-MEDIATED	REACTOME_RUNX3 REGULATES RUNX1-MEDIATED TRANSCRIPTION	3	0.9241453	0.9241453	0	0.051445175	0.038	3716	tags=100%, list=8%, signal=108%
REACTOME_LOCALIZATION OF THE PINCH-ILK-PARVIN COMPLEX TO FOCAL ADHESIONS	REACTOME_LOCALIZATION OF THE PINCH-ILK-PARVIN COMPLEX TO FOCAL ADHESIONS	4	0.9207737	0.9207737	0	0.02892474	0.043	3882	tags=100%, list=8%, signal=109%
REACTOME_LACTOSE	REACTOME_LACTOSE SYNTHESIS	3	0.8843185	0.8843185	9.88E-04	0.0647311	0.1425	5666	tags=100%, list=12%, signal=113%
REACTOME_NEUROPHILIN INTERACTIONS WITH VEGF AND VEGFR	REACTOME_NEUROPHILIN INTERACTIONS WITH VEGF AND VEGFR	4	0.86562777	0.86562777	9.77E-04	0.07358983	0.2075	6582	tags=100%, list=13%, signal=116%
REACTOME_INTERCONVERSION OF POLYAMINES	REACTOME_INTERCONVERSION OF POLYAMINES	3	0.84733063	0.84733063	0.0071795	0.08763774	0.2865	7477	tags=100%, list=15%, signal=118%
REACTOME_LYSOSOMAL OLIGOSACCHARIDE CATABOLISM	REACTOME_LYSOSOMAL OLIGOSACCHARIDE CATABOLISM	4	0.8390147	0.8390147	0.0029851	0.08392301	0.3245	7885	tags=100%, list=16%, signal=119%
REACTOME_MET ACTIVATES	REACTOME_MET ACTIVATES STAT3	3	0.8215555	0.8215555	0.0077369	0.1000432	0.4235	8739	tags=100%, list=18%, signal=122%
REACTOME_PTK6 ACTIVATES	REACTOME_PTK6 ACTIVATES STAT3	4	0.80690753	0.80690753	9.93E-04	0.11036967	0.507	9457	tags=100%, list=19%, signal=124%
REACTOME_PTK6 DOWN-REGULATION	REACTOME_PTK6 DOWN-REGULATION	3	0.8068911	0.8068911	0.0101317	0.09810638	0.5075	9457	tags=100%, list=19%, signal=124%
REACTOME_CASP8 ACTIVITY IS INHIBITED	REACTOME_CASP8 ACTIVITY IS INHIBITED	11	0.7955558	0.7955558	0	0.106128596	0.5765	5567	tags=91%, list=11%, signal=103%
REACTOME_DIMERIZATION OF PROCASPASE-8	REACTOME_DIMERIZATION OF PROCASPASE-8	11	0.7955558	0.7955558	0	0.09648054	0.5765	5567	tags=91%, list=11%, signal=103%
REACTOME_REGULATION BY C-	REACTOME_REGULATION BY C-FLIP	11	0.7955558	0.7955558	0	0.0884405	0.5765	5567	tags=91%, list=11%, signal=103%
REACTOME_CROSSLINKING OF COLLAGEN FIBRILS	REACTOME_CROSSLINKING OF COLLAGEN FIBRILS	10	0.77763253	0.77763253	0	0.10739531	0.679	1102	tags=80%, list=2%, signal=82%
REACTOME_RUNX3 REGULATES CDKN1A TRANSCRIPTION	REACTOME_RUNX3 REGULATES CDKN1A TRANSCRIPTION	7	0.7736836	0.7736836	0	0.10527642	0.697	4091	tags=86%, list=8%, signal=94%
REACTOME_LAMININ	REACTOME_LAMININ INTERACTIONS	23	0.7646043	0.7646043	0	0.11314367	0.7425	5156	tags=87%, list=11%, signal=97%
REACTOME_ANTAGONISM OF ACTIVIN BY FOLLISTATIN	REACTOME_ANTAGONISM OF ACTIVIN BY FOLLISTATIN	4	0.74794227	0.74794227	0.0059821	0.13218437	0.818	12344	tags=100%, list=25%, signal=134%
REACTOME_INVADOPODIA FORMATION	REACTOME_INVADOPODIA FORMATION	4	0.7467372	0.7467372	0.0069307	0.12705012	0.8245	12403	tags=100%, list=25%, signal=134%
REACTOME_PLCG1 EVENTS IN ERBB2 SIGNALING	REACTOME_PLCG1 EVENTS IN ERBB2 SIGNALING	4	0.7436531	0.7436531	0.0060484	0.124367744	0.836	12554	tags=100%, list=26%, signal=134%
REACTOME_REGULATION OF CYTOSKELETAL REMODELING AND CELL SPREADING BY IPP COMPLEX COMPONENTS	REACTOME_REGULATION OF CYTOSKELETAL REMODELING AND CELL SPREADING BY IPP COMPLEX COMPONENTS	8	0.73836225	0.73836225	0.001006	0.12615298	0.85	12816	tags=100%, list=26%, signal=135%
REACTOME_CELL-EXTRACELLULAR MATRIX	REACTOME_CELL-EXTRACELLULAR MATRIX INTERACTIONS	16	0.73343813	0.73343813	0	0.12777638	0.8645	3882	tags=81%, list=8%, signal=88%
REACTOME_DEFECTIVE LFNG CAUSES SCDO3	REACTOME_DEFECTIVE LFNG CAUSES SCDO3	4	0.73131675	0.73131675	0.0130916	0.12502472	0.873	13158	tags=100%, list=27%, signal=137%
REACTOME_FORMATION OF THE ACTIVE COFACTOR, UDP-GLUCURONATE	REACTOME_FORMATION OF THE ACTIVE COFACTOR, UDP-GLUCURONATE	3	0.7302398	0.7302398	0.0346275	0.120628394	0.8755	13210	tags=100%, list=27%, signal=137%
REACTOME_IKBKG DEFICIENCY CAUSES ANHIDROTIC ECTODERMAL DYSPLASIA WITH IMMUNODEFICIENCY (EDA-ID) (VIA TLR)	REACTOME_IKBKG DEFICIENCY CAUSES ANHIDROTIC ECTODERMAL DYSPLASIA WITH IMMUNODEFICIENCY (EDA-ID) (VIA TLR)	8	0.7179438	0.7179438	0	0.13657485	0.915	7695	tags=88%, list=16%, signal=104%

REACTOME_ANTIGEN PRESENTATION: FOLDING, ASSEMBLY AND PEPTIDE LOADING OF CLASS I MHC	REACTOME_ANTIGEN PRESENTATION: FOLDING, ASSEMBLY AND PEPTIDE LOADING OF CLASS I MHC	25	0.7178913	0.7178913	0	0.13088423	0.915	5996	tags=84%, list=12%, signal=96%
REACTOME_STAT6-MEDIATED INDUCTION OF CHEMOKINES	REACTOME_STAT6-MEDIATED INDUCTION OF CHEMOKINES	3	0.715943	0.715943	0.0528893	0.12870412	0.9195	13910	tags=100%, list=28%, signal=140%
REACTOME_THE ACTIVATION OF ARYLSULFATASES	REACTOME_THE ACTIVATION OF ARYLSULFATASES	13	0.7044138	0.7044138	0	0.1420604	0.949	10715	tags=92%, list=22%, signal=118%
REACTOME_CONSTITUTIVE SIGNALING BY NOTCH1 T(7;9)(NOTCH1:M1580_K2555) TRANSLOCATION MUTANT	REACTOME_CONSTITUTIVE SIGNALING BY NOTCH1 T(7;9)(NOTCH1:M1580_K2555) TRANSLOCATION MUTANT	7	0.70260227	0.70260227	0.0019841	0.13984948	0.953	7571	tags=86%, list=15%, signal=101%
REACTOME_SIGNALING BY NOTCH1 T(7;9)(NOTCH1:M1580_K2555)	REACTOME_SIGNALING BY NOTCH1 T(7;9)(NOTCH1:M1580_K2555) TRANSLOCATION MUTANT	7	0.70260227	0.70260227	0	0.13485484	0.953	7571	tags=86%, list=15%, signal=101%
REACTOME_IRS ACTIVATION	REACTOME_IRS ACTIVATION	5	0.70194036	0.70194036	0.0048077	0.13139956	0.9545	4804	tags=80%, list=10%, signal=89%
REACTOME_IKBA VARIANT LEADS TO EDA-ID	REACTOME_IKBA VARIANT LEADS TO EDA-ID	7	0.7000695	0.7000695	0	0.12995693	0.957	7695	tags=86%, list=16%, signal=102%
REACTOME_OXYGEN-DEPENDENT ASPARAGINE HYDROXYLATION OF HYPOXIA-INDUCIBLE FACTOR ALPHA	REACTOME_OXYGEN-DEPENDENT ASPARAGINE HYDROXYLATION OF HYPOXIA-INDUCIBLE FACTOR ALPHA	3	0.6993587	0.6993587	0.0549451	0.12683098	0.9585	14722	tags=100%, list=30%, signal=143%
REACTOME_AMINE OXIDASE REACTIONS	REACTOME_AMINE OXIDASE REACTIONS	4	0.69797903	0.69797903	0.0107422	0.12487799	0.9635	2549	tags=75%, list=5%, signal=79%
REACTOME_HYALURONAN BIOSYNTHESIS AND EXPORT	REACTOME_HYALURONAN BIOSYNTHESIS AND EXPORT	5	0.69321895	0.69321895	0.0029383	0.12848996	0.9695	5231	tags=80%, list=11%, signal=90%
REACTOME_MET ACTIVATES PTK2 SIGNALING	REACTOME_MET ACTIVATES PTK2 SIGNALING	18	0.6924201	0.6924201	0	0.12614985	0.97	4191	tags=78%, list=9%, signal=85%
REACTOME_NOSTRIN MEDIATED ENOS TRAFFICKING	REACTOME_NOSTRIN MEDIATED ENOS TRAFFICKING	5	0.68703026	0.68703026	0.0047893	0.1307439	0.977	5534	tags=80%, list=11%, signal=90%
REACTOME_FASL/ CD95L	REACTOME_FASL/ CD95L SIGNALING	5	0.68635625	0.68635625	0.0121827	0.12802182	0.977	5567	tags=80%, list=11%, signal=90%
REACTOME_RUNX1 REGULATES TRANSCRIPTION OF GENES INVOLVED IN DIFFERENTIATION OF MYELOID CELLS	REACTOME_RUNX1 REGULATES TRANSCRIPTION OF GENES INVOLVED IN DIFFERENTIATION OF MYELOID CELLS	7	0.6803587	0.6803587	0	0.1328533	0.983	8660	tags=86%, list=18%, signal=104%
REACTOME_ADENOSINE P1 RECEPTORS	REACTOME_ADENOSINE P1 RECEPTORS	3	0.6795066	0.6795066	0.049904	0.1306613	0.983	15694	tags=100%, list=32%, signal=147%
REACTOME_TRAIL SIGNALING	REACTOME_TRAIL SIGNALING	7	0.67476207	0.67476207	0.0019802	0.13421275	0.986	1939	tags=71%, list=4%, signal=74%
REACTOME_REGULATION OF NECROPTOTIC CELL DEATH	REACTOME_REGULATION OF NECROPTOTIC CELL DEATH	14	0.67219263	0.67219263	0	0.13481751	0.987	5567	tags=79%, list=11%, signal=89%
REACTOME_CLEC7A/INFLAMMASOME PATHWAY	REACTOME_CLEC7A/INFLAMMASOME PATHWAY	6	0.6712964	0.6712964	0.0059055	0.13298735	0.9875	16098	tags=100%, list=33%, signal=149%
REACTOME_ACTIVATION OF	REACTOME_ACTIVATION OF AKT2	4	0.65484774	0.65484774	0.0451354	0.15667795	0.9935	16902	tags=100%, list=35%, signal=153%
REACTOME_BINDING AND ENTRY OF HIV VIRION	REACTOME_BINDING AND ENTRY OF HIV VIRION	4	0.6531525	0.6531525	0.0403226	0.15615384	0.994	16985	tags=100%, list=35%, signal=153%
REACTOME_METAL SEQUESTRATION BY	REACTOME_METAL SEQUESTRATION BY ANTIMICROBIAL PROTEINS	6	0.6523418	0.6523418	0.0020492	0.15409464	0.994	17026	tags=100%, list=35%, signal=153%
REACTOME_LIGAND-DEPENDENT CASPASE ACTIVATION	REACTOME_LIGAND-DEPENDENT CASPASE ACTIVATION	17	0.6512181	0.6512181	0	0.15266374	0.996	5567	tags=76%, list=11%, signal=86%
REACTOME_CHL1	REACTOME_CHL1 INTERACTIONS	9	0.6474771	0.6474771	0	0.15614267	0.9965	6385	tags=78%, list=13%, signal=89%
REACTOME_HDL ASSEMBLY	REACTOME_HDL ASSEMBLY	8	0.64580655	0.64580655	9.90E-04	0.15592112	0.9965	5106	tags=75%, list=10%, signal=84%
REACTOME_RUNX1 AND FOXP3 CONTROL THE DEVELOPMENT OF REGULATORY T LYMPHOCYTES (TREGS)	REACTOME_RUNX1 AND FOXP3 CONTROL THE DEVELOPMENT OF REGULATORY T LYMPHOCYTES (TREGS)	10	0.64033294	0.64033294	0	0.16375242	0.997	12720	tags=90%, list=26%, signal=122%

REACTOME_TRAF3 DEFICIENCY - HSE	REACTOME_TRAF3 DEFICIENCY - HSE	3	0.63974786	0.63974786	0.0913605	0.16140738	0.997	1319	tags=67%, list=3%, signal=69%
REACTOME_SODIUM-COUPLED PHOSPHATE COTRANSPORTERS	REACTOME_SODIUM-COUPLED PHOSPHATE COTRANSPORTERS	5	0.63713235	0.63713235	0.0238342	0.1635468	0.9975	7977	tags=80%, list=16%, signal=96%
REACTOME_PYRIMIDINE BIOSYNTHESIS	REACTOME_PYRIMIDINE BIOSYNTHESIS	3	0.6349414	0.6349414	0.0907217	0.16457468	0.9985	17876	tags=100%, list=37%, signal=157%
REACTOME_RIPK1-MEDIATED REGULATED NECROSIS	REACTOME_RIPK1-MEDIATED REGULATED NECROSIS	16	0.63081473	0.63081473	0	0.16914149	0.999	5845	tags=75%, list=12%, signal=85%
REACTOME_REGULATED	REACTOME_REGULATED NECROSIS	16	0.63081473	0.63081473	0	0.16595013	0.999	5845	tags=75%, list=12%, signal=85%
REACTOME_LOSS OF FUNCTION OF TGFBR2 IN CANCER	REACTOME_LOSS OF FUNCTION OF TGFBR2 IN CANCER	4	0.6308031	0.6308031	0.0448654	0.16290064	0.999	5838	tags=75%, list=12%, signal=85%
REACTOME_TGFBR1 LBD MUTANTS IN CANCER	REACTOME_TGFBR1 LBD MUTANTS IN CANCER	4	0.6308031	0.6308031	0.0469208	0.1599388	0.999	5838	tags=75%, list=12%, signal=85%
REACTOME_TGFBR2 KINASE DOMAIN MUTANTS IN CANCER	REACTOME_TGFBR2 KINASE DOMAIN MUTANTS IN CANCER	4	0.6308031	0.6308031	0.0398366	0.15708275	0.999	5838	tags=75%, list=12%, signal=85%
REACTOME_ALTERNATIVE COMPLEMENT ACTIVATION	REACTOME_ALTERNATIVE COMPLEMENT ACTIVATION	4	0.6290926	0.6290926	0.0443996	0.15764348	0.999	18163	tags=100%, list=37%, signal=159%
REACTOME_ANCHORING FIBRIL FORMATION	REACTOME_ANCHORING FIBRIL FORMATION	7	0.62876344	0.62876344	0.004065	0.15565756	0.999	4191	tags=71%, list=9%, signal=78%
REACTOME_TYPE II NA+/PI COTRANSPORTERS	REACTOME_TYPE II NA+/PI COTRANSPORTERS	3	0.6280381	0.6280381	0.123108	0.15419634	1	18214	tags=100%, list=37%, signal=159%
REACTOME_ENDOSOMAL/VACUOLAR PATHWAY	REACTOME_ENDOSOMAL/VACUOLAR PATHWAY	12	0.62767863	0.62767863	0	0.15198812	1	5996	tags=75%, list=12%, signal=85%
REACTOME_SYNTHESIS OF PI	REACTOME_SYNTHESIS OF PI	5	0.6264502	0.6264502	0.0172589	0.15180609	1	8500	tags=80%, list=17%, signal=97%
REACTOME_TNFR1-INDUCED PROAPOPTOTIC SIGNALING	REACTOME_TNFR1-INDUCED PROAPOPTOTIC SIGNALING	13	0.624538	0.624538	0	0.15284513	1	7092	tags=77%, list=14%, signal=90%
REACTOME_MET INTERACTS WITH TNS PROTEINS	REACTOME_MET INTERACTS WITH TNS PROTEINS	5	0.6215686	0.6215686	0.0184426	0.15609622	1	8739	tags=80%, list=18%, signal=97%
REACTOME_ARL13B-MEDIATED CILIARY TRAFFICKING OF INPP5E	REACTOME_ARL13B-MEDIATED CILIARY TRAFFICKING OF INPP5E	3	0.6188541	0.6188541	0.1215686	0.15885086	1	2342	tags=67%, list=5%, signal=70%
REACTOME_NR1D1 (REV-ERBA) REPRESSES GENE EXPRESSION	REACTOME_NR1D1 (REV-ERBA) REPRESSES GENE EXPRESSION	8	0.6187675	0.6187675	0	0.15658432	1	18671	tags=100%, list=38%, signal=162%
REACTOME_HHAT G278V ABROGATES PALMITOYLATION OF HH-NP	REACTOME_HHAT G278V ABROGATES PALMITOYLATION OF HH-NP	3	0.61873156	0.61873156	0.1261445	0.15427974	1	2348	tags=67%, list=5%, signal=70%
REACTOME_PTK6 EXPRESSION	REACTOME_PTK6 EXPRESSION	5	0.6069036	0.6069036	0.031	0.1740238	1	9457	tags=80%, list=19%, signal=99%
REACTOME_DEFECTIVE MISMATCH REPAIR ASSOCIATED	REACTOME_DEFECTIVE MISMATCH REPAIR ASSOCIATED WITH MSH2	3	0.5979331	0.5979331	0.1539225	0.1910249	1	19688	tags=100%, list=40%, signal=167%
REACTOME_SIGNALING BY	REACTOME_SIGNALING BY NOTCH3	11	0.59792686	0.59792686	0	0.18826577	1	6339	tags=73%, list=13%, signal=84%
REACTOME_RUNX1 REGULATES TRANSCRIPTION OF GENES INVOLVED IN WNT SIGNALING	REACTOME_RUNX1 REGULATES TRANSCRIPTION OF GENES INVOLVED IN WNT SIGNALING	6	0.5890507	0.5890507	0.0204082	0.20564455	1	3803	tags=67%, list=8%, signal=72%
REACTOME_CASPASE-MEDIATED CLEAVAGE OF CYTOSKELETAL PROTEINS	REACTOME_CASPASE-MEDIATED CLEAVAGE OF CYTOSKELETAL PROTEINS	12	0.58889824	0.58889824	0	0.2031457	1	3814	tags=67%, list=8%, signal=72%
REACTOME_TNF RECEPTOR SUPERFAMILY (TNFSF) MEMBERS MEDIATING NON-CANONICAL NF-KB PATHWAY	REACTOME_TNF RECEPTOR SUPERFAMILY (TNFSF) MEMBERS MEDIATING NON-CANONICAL NF-KB PATHWAY	17	0.58883977	0.58883977	0	0.200477	1	5740	tags=71%, list=12%, signal=80%
REACTOME_ACTIVATION OF C3 AND C5	REACTOME_ACTIVATION OF C3 AND C5	6	0.5861231	0.5861231	0.0172239	0.20382991	1	20268	tags=100%, list=41%, signal=171%
REACTOME_MITOCHONDRIAL ABC TRANSPORTERS	REACTOME_MITOCHONDRIAL ABC TRANSPORTERS	4	0.58360225	0.58360225	0.0777454	0.2070922	1	8149	tags=75%, list=17%, signal=90%
REACTOME_TYPE I HEMIDESMOSOME ASSEMBLY	REACTOME_TYPE I HEMIDESMOSOME ASSEMBLY	9	0.5811613	0.5811613	0.0019084	0.21058372	1	4191	tags=67%, list=9%, signal=73%

REACTOME_DISSOLUTION OF FIBRIN CLOT	REACTOME_DISSOLUTION OF FIBRIN CLOT	13	0.5776538	0.5776538	0	0.21647505	1	1854	tags=62%, list=4%, signal=64%
REACTOME_TRAF3-DEPENDENT IRF ACTIVATION PATHWAY	REACTOME_TRAF3-DEPENDENT IRF ACTIVATION PATHWAY	14	0.5732059	0.5732059	0	0.22429603	1	13910	tags=86%, list=28%, signal=120%
REACTOME_HDL CLEARANCE	REACTOME_HDL CLEARANCE	5	0.57148695	0.57148695	0.0432161	0.2257464	1	11191	tags=80%, list=23%, signal=104%
REACTOME_SYNTHESIS OF GDP-MANNOSE	REACTOME_SYNTHESIS OF GDP-MANNOSE	5	0.5700163	0.5700163	0.0406674	0.22646251	1	11263	tags=80%, list=23%, signal=104%
REACTOME_PRE-NOTCH PROCESSING IN THE ENDOPLASMIC RETICULUM	REACTOME_PRE-NOTCH PROCESSING IN THE ENDOPLASMIC RETICULUM	6	0.5646596	0.5646596	0.0179641	0.23712938	1	13158	tags=83%, list=27%, signal=114%
REACTOME_GLI PROTEINS BIND PROMOTERS OF HH RESPONSIVE GENES TO PROMOTE TRANSCRIPTION	REACTOME_GLI PROTEINS BIND PROMOTERS OF HH RESPONSIVE GENES TO PROMOTE TRANSCRIPTION	7	0.56160384	0.56160384	0.0114504	0.242047	1	7479	tags=71%, list=15%, signal=84%
REACTOME_NEF MEDIATED DOWNREGULATION OF MHC CLASS I COMPLEX CELL SURFACE EXPRESSION	REACTOME_NEF MEDIATED DOWNREGULATION OF MHC CLASS I COMPLEX CELL SURFACE EXPRESSION	10	0.55749154	0.55749154	0.005	0.24987137	1	21672	tags=100%, list=44%, signal=179%
REACTOME_METABOLISM OF VITAMIN K	REACTOME_METABOLISM OF VITAMIN K	3	0.5545389	0.5545389	0.2009756	0.2545208	1	5491	tags=67%, list=11%, signal=75%
REACTOME_GALACTOSE CATABOLISM	REACTOME_GALACTOSE CATABOLISM	6	0.55103934	0.55103934	0.0288557	0.26059273	1	5664	tags=67%, list=12%, signal=75%
REACTOME_LRR FLII-INTERACTING PROTEIN 1 (LRRFIP1) ACTIVATES TYPE I IFN	REACTOME_LRR FLII-INTERACTING PROTEIN 1 (LRRFIP1) ACTIVATES TYPE I IFN PRODUCTION	5	0.55085784	0.55085784	0.0669331	0.25799718	1	12201	tags=80%, list=25%, signal=107%
REACTOME_PLATELET ADHESION TO EXPOSED	REACTOME_PLATELET ADHESION TO EXPOSED COLLAGEN	12	0.55040044	0.55040044	0	0.25621033	1	9779	tags=75%, list=20%, signal=94%
REACTOME_HS-GAG	REACTOME_HS-GAG DEGRADATION	21	0.5494715	0.5494715	0	0.25570154	1	5749	tags=67%, list=12%, signal=76%
REACTOME_CHOLESTEROL BIOSYNTHESIS VIA	REACTOME_CHOLESTEROL BIOSYNTHESIS VIA DESMOSTEROL	4	0.5477574	0.5477574	0.1232742	0.25694463	1	9904	tags=75%, list=20%, signal=94%
REACTOME_CHOLESTEROL BIOSYNTHESIS VIA	REACTOME_CHOLESTEROL BIOSYNTHESIS VIA LATHOSTEROL	4	0.5477574	0.5477574	0.1436059	0.25405762	1	9904	tags=75%, list=20%, signal=94%
REACTOME_INSULIN-LIKE GROWTH FACTOR-2 MRNA BINDING PROTEINS	REACTOME_INSULIN-LIKE GROWTH FACTOR-2 MRNA BINDING PROTEINS (IGF2BPS/IMPS/VICKZS) BIND RNA	8	0.5475366	0.5475366	0.0091185	0.25185776	1	9917	tags=75%, list=20%, signal=94%
REACTOME_VEGF BINDS TO VEGFR LEADING TO RECEPTOR DIMERIZATION	REACTOME_VEGF BINDS TO VEGFR LEADING TO RECEPTOR DIMERIZATION	8	0.5467936	0.5467936	0.014	0.2509417	1	16074	tags=88%, list=33%, signal=130%
REACTOME_VEGF LIGAND-RECEPTOR INTERACTIONS	REACTOME_VEGF LIGAND-RECEPTOR INTERACTIONS	8	0.5467936	0.5467936	0.007913	0.24821408	1	16074	tags=88%, list=33%, signal=130%
REACTOME_SEMA3A-PLEXIN REPULSION SIGNALING BY INHIBITING INTEGRIN ADHESION	REACTOME_SEMA3A-PLEXIN REPULSION SIGNALING BY INHIBITING INTEGRIN ADHESION	14	0.5464138	0.5464138	0	0.2464687	1	4729	tags=64%, list=10%, signal=71%
REACTOME_CROSS-PRESENTATION OF PARTICULATE EXOGENOUS	REACTOME_CROSS-PRESENTATION OF PARTICULATE EXOGENOUS ANTIGENS (PHAGOSOMES)	8	0.54639786	0.54639786	0.0151057	0.24391584	1	22214	tags=100%, list=45%, signal=183%
REACTOME_SYNTHESIS OF UDP-N-ACETYL-GLUCOSAMINE	REACTOME_SYNTHESIS OF UDP-N-ACETYL-GLUCOSAMINE	8	0.54611444	0.54611444	0.0039254	0.2419243	1	3866	tags=63%, list=8%, signal=68%
REACTOME_NEGATIVE REGULATION OF TCF-DEPENDENT SIGNALING BY DVL-	REACTOME_NEGATIVE REGULATION OF TCF-DEPENDENT SIGNALING BY DVL-INTERACTING PROTEINS	5	0.54405636	0.54405636	0.0705522	0.24472003	1	12534	tags=80%, list=26%, signal=108%
REACTOME_FIBRONECTIN MATRIX FORMATION	REACTOME_FIBRONECTIN MATRIX FORMATION	6	0.5421918	0.5421918	0.0223124	0.24713701	1	14258	tags=83%, list=29%, signal=118%
REACTOME_PLC-MEDIATED HYDROLYSIS OF PIP2	REACTOME_PLC-MEDIATED HYDROLYSIS OF PIP2	4	0.5387758	0.5387758	0.1233831	0.25350112	1	22585	tags=100%, list=46%, signal=186%

REACTOME_FORMATION OF ANNULAR GAP JUNCTIONS	REACTOME_FORMATION OF ANNULAR GAP JUNCTIONS	9	0.53672457	0.53672457	0.0050505	0.25611737	1	11807	tags=78%, list=24%, signal=102%	
REACTOME_METALLOTHIONEINS BIND METALS	REACTOME_METALLOTHIONEINS BIND METALS	11	0.5354062	0.5354062	0.001001	0.2569884	1	13851	tags=82%, list=28%, signal=114%	
REACTOME_DEFECTIVE AVP CAUSES NEUROHYPOPHYSEAL DIABETES INSIPIDUS (NDI)	REACTOME_DEFECTIVE AVP CAUSES NEUROHYPOPHYSEAL DIABETES INSIPIDUS (NDI)	4	0.53348076	0.53348076	0.1166501	0.25966275	1	10603	tags=75%, list=22%, signal=96%	
REACTOME_CONDENSATION OF PROMETAPHASE	REACTOME_CONDENSATION OF PROMETAPHASE CHROMOSOMES	11	0.5330515	0.5330515	0.002994	0.25841528	1	22869	tags=100%, list=47%, signal=188%	
REACTOME_INTERLEUKIN-15 SIGNALING	REACTOME_INTERLEUKIN-15 SIGNALING	6	0.5321868	0.5321868	0.0308642	0.25833377	1	6587	tags=67%, list=13%, signal=77%	
REACTOME_COLLAGEN	REACTOME_COLLAGEN FORMATION	90	0.53166467	0.53166467	0	0.25711486	1	4481	tags=62%, list=9%, signal=68%	
REACTOME_TP53 REGULATES TRANSCRIPTION OF DEATH RECEPTORS AND LIGANDS	REACTOME_TP53 REGULATES TRANSCRIPTION OF DEATH RECEPTORS AND LIGANDS	12	0.5314809	0.5314809	0.0020182	0.25517616	1	18866	tags=92%, list=39%, signal=149%	
REACTOME_COLLAGEN BIOSYNTHESIS AND MODIFYING ENZYMES	REACTOME_COLLAGEN BIOSYNTHESIS AND MODIFYING ENZYMES	67	0.5308604	0.5308604	0	0.25447845	1	2543	tags=58%, list=5%, signal=61%	
REACTOME_SCAVENGING BY CLASS F RECEPTORS	REACTOME_SCAVENGING BY CLASS F RECEPTORS	6	0.5283026	0.5283026	0.0575968	0.25876683	1	14938	tags=83%, list=31%, signal=120%	
REACTOME_SYNDECAN INTERACTIONS	REACTOME_SYNDECAN INTERACTIONS	20	0.5274083	0.5274083	0	0.2586908	1	13357	tags=80%, list=27%, signal=110%	
REACTOME_FGFR1C AND KLOTHO LIGAND BINDING AND	REACTOME_FGFR1C AND KLOTHO LIGAND BINDING AND ACTIVATION	3	0.52694607	0.52694607	0.2763819	0.25742516	1	6842	tags=67%, list=14%, signal=77%	
REACTOME_ELASTIC FIBRE FORMATION	REACTOME_ELASTIC FIBRE FORMATION	41	0.5242985	0.5242985	0	0.26189452	1	8982	tags=71%, list=18%, signal=87%	
REACTOME_HYALURONAN METABOLISM	REACTOME_HYALURONAN METABOLISM	17	0.5231783	0.5231783	0	0.26245642	1	8954	tags=71%, list=18%, signal=86%	
REACTOME_TERMINAL PATHWAY OF COMPLEMENT	REACTOME_TERMINAL PATHWAY OF COMPLEMENT	8	0.5227113	0.5227113	0.0132248	0.26135218	1	17253	tags=88%, list=35%, signal=135%	
REACTOME_NF-KB ACTIVATION THROUGH FADD/RIP-1 PATHWAY MEDIATED BY CASPASE-8 AND -[illegible]	REACTOME_NF-KB ACTIVATION THROUGH FADD/RIP-1 PATHWAY MEDIATED BY CASPASE-8 AND -10	12	0.52174366	0.52174366	0	0.26158696	1	3021	tags=58%, list=6%, signal=62%	
REACTOME_COLLAGEN DEGRADATION	REACTOME_COLLAGEN DEGRADATION	40	0.52101433	0.52101433	0	0.2612079	1	10008	tags=73%, list=20%, signal=91%	
REACTOME_SYNTHESIS OF LIPOXINS (LX)	REACTOME_SYNTHESIS OF LIPOXINS (LX)	6	0.5184985	0.5184985	0.0504032	0.26547706	1	15418	tags=83%, list=31%, signal=122%	
REACTOME_TRIF-MEDIATED PROGRAMMED CELL DEATH	REACTOME_TRIF-MEDIATED PROGRAMMED CELL DEATH	10	0.517414	0.517414	0.0042328	0.26614916	1	4048	tags=60%, list=8%, signal=65%	
REACTOME_ASSEMBLY OF COLLAGEN FIBRILS AND OTHER MULTIMERIC STRUCTURES	REACTOME_ASSEMBLY OF COLLAGEN FIBRILS AND OTHER MULTIMERIC STRUCTURES	58	0.51633745	0.51633745	0	0.26692992	1	6827	tags=66%, list=14%, signal=76%	
REACTOME_SYNTHESIS OF DOLICHYL-PHOSPHATE [illegible]	REACTOME_SYNTHESIS OF DOLICHYL-PHOSPHATE MANNOSE	3	0.5123566	0.5123566	0.3123753	0.27516922	1	23878	tags=100%, list=49%, signal=195%	
REACTOME_A THIRD PROTEOLYTIC CLEAVAGE	REACTOME_A THIRD PROTEOLYTIC CLEAVAGE RELEASES NICD	9	0.5121197	0.5121197	0.0102145	0.27350482	1	7571	tags=67%, list=15%, signal=79%	
REACTOME_NON-INTEGRIN MEMBRANE-ECM INTERACTIONS	REACTOME_NON-INTEGRIN MEMBRANE-ECM INTERACTIONS	42	0.51006347	0.51006347	0	0.27676645	1	4191	tags=60%, list=9%, signal=65%	
REACTOME_PHOSPHORYLATION OF EMI1	REACTOME_PHOSPHORYLATION OF EMI1	6	0.50979394	0.50979394	0.0673171	0.2752341	1	24005	tags=100%, list=49%, signal=196%	
REACTOME_HISTAMINE	REACTOME_HISTAMINE RECEPTORS	4	0.50797063	0.50797063	0.1841564	0.27786925	1	11852	tags=75%, list=24%, signal=99%	
REACTOME_SIGNALING BY PLASMA MEMBRANE FGFR1	REACTOME_SIGNALING BY PLASMA MEMBRANE FGFR1 FUSIONS	3	0.50768626	0.50768626	0.3306773	0.27645156	1	7785	tags=67%, list=16%, signal=79%	

REACTOME_SIGNALING BY	REACTOME_SIGNALING BY NOTCH4	11	0.5069973	0.5069973	0.0040984	0.2760106	1	6339	tags=64%, list=13%, signal=73%
REACTOME_SIGNALLING TO	REACTOME_SIGNALLING TO STAT3	3	0.50548047	0.50548047	0.3212928	0.27801064	1	7893	tags=67%, list=16%, signal=79%
REACTOME_NEF MEDIATED CD8 DOWN-REGULATION	REACTOME_NEF MEDIATED CD8 DOWN-REGULATION	7	0.5049022	0.5049022	0.0385376	0.2773282	1	10255	tags=71%, list=21%, signal=90%
REACTOME_OPTINEURIN AND MYOSIN PHOSPHATASE NEGATIVELY REGULATE PLK1	REACTOME_OPTINEURIN AND MYOSIN PHOSPHATASE NEGATIVELY REGULATE PLK1	3	0.5048269	0.5048269	0.32999	0.27537686	1	7925	tags=67%, list=16%, signal=80%
REACTOME_ACTIVATION OF PUMA AND TRANSLOCATION TO MITOCHONDRIA	REACTOME_ACTIVATION OF PUMA AND TRANSLOCATION TO MITOCHONDRIA	9	0.50366545	0.50366545	0.0129225	0.27637482	1	18866	tags=89%, list=39%, signal=145%
REACTOME_SLC TRANSPORTER DISORDERS	REACTOME_SLC TRANSPORTER DISORDERS	7	0.49779406	0.49779406	0.0372984	0.29004017	1	10603	tags=71%, list=22%, signal=91%
REACTOME_NGF PROCESSING	REACTOME_NGF PROCESSING	4	0.49562916	0.49562916	0.1957619	0.2937462	1	215	tags=50%, list=0%, signal=50%
REACTOME_MOLECULES ASSOCIATED WITH ELASTIC	REACTOME_MOLECULES ASSOCIATED WITH ELASTIC FIBRES	30	0.4947107	0.4947107	0	0.29394174	1	11698	tags=73%, list=24%, signal=96%
REACTOME_GDP-FUCOSE BIOSYNTHESIS	REACTOME_GDP-FUCOSE BIOSYNTHESIS	6	0.48970228	0.48970228	0.0629302	0.3061298	1	8667	tags=67%, list=18%, signal=81%
REACTOME_RECEPTOR-LIGAND BINDING INITIATES THE SECOND PROTEOLYTIC CLEAVAGE OF NOTCH RECEPTOR	REACTOME_RECEPTOR-LIGAND BINDING INITIATES THE SECOND PROTEOLYTIC CLEAVAGE OF NOTCH RECEPTOR	14	0.48835573	0.48835573	0.0029098	0.3076914	1	7571	tags=64%, list=15%, signal=76%
REACTOME_KERATAN SULFATE DEGRADATION	REACTOME_KERATAN SULFATE DEGRADATION	12	0.48701647	0.48701647	0.0096339	0.30922753	1	4721	tags=58%, list=10%, signal=65%
REACTOME_PROGRESSIVE TRIMMING OF ALPHA-1,2-LINKED MANNOSE RESIDUES FROM MAN9/8/7GLCNAC2 TO PRODUCE MAN5GLCNAC2	REACTOME_PROGRESSIVE TRIMMING OF ALPHA-1,2-LINKED MANNOSE RESIDUES FROM MAN9/8/7GLCNAC2 TO PRODUCE MAN5GLCNAC2	3	0.48683333	0.48683333	0.361658	0.3074705	1	8806	tags=67%, list=18%, signal=81%
REACTOME_HYALURONAN UPTAKE AND DEGRADATION	REACTOME_HYALURONAN UPTAKE AND DEGRADATION	12	0.48389953	0.48389953	0.0039254	0.31397265	1	8954	tags=67%, list=18%, signal=82%
REACTOME_TRANSPORT AND SYNTHESIS OF PAPS	REACTOME_TRANSPORT AND SYNTHESIS OF PAPS	6	0.48328537	0.48328537	0.0782953	0.31351948	1	17142	tags=83%, list=35%, signal=128%
REACTOME_WNT5A-DEPENDENT INTERNALIZATION OF FZD2, FZD5 AND ROR2	REACTOME_WNT5A-DEPENDENT INTERNALIZATION OF FZD2, FZD5 AND ROR2	13	0.48140723	0.48140723	0.005	0.3170939	1	10332	tags=69%, list=21%, signal=88%
REACTOME_RUNX3 REGULATES YAP1-MEDIATED TRANSCRIPTION	REACTOME_RUNX3 REGULATES YAP1-MEDIATED TRANSCRIPTION	22	0.48125094	0.48125094	0	0.31534016	1	5379	tags=59%, list=11%, signal=66%
REACTOME_REGULATED PROTEOLYSIS OF P75NTR	REACTOME_REGULATED PROTEOLYSIS OF P75NTR	10	0.48055357	0.48055357	0.0119522	0.315104	1	10749	tags=70%, list=22%, signal=90%
REACTOME_CASPASE ACTIVATION VIA EXTRINSIC APOPTOTIC SIGNALLING	REACTOME_CASPASE ACTIVATION VIA EXTRINSIC APOPTOTIC SIGNALLING PATHWAY	27	0.47914293	0.47914293	0	0.31696254	1	5567	tags=59%, list=11%, signal=67%
REACTOME_REGULATION OF GENE EXPRESSION BY HYPOXIA-INDUCIBLE FACTOR	REACTOME_REGULATION OF GENE EXPRESSION BY HYPOXIA-INDUCIBLE FACTOR	11	0.47818175	0.47818175	0.0089374	0.31760788	1	12201	tags=73%, list=25%, signal=97%
REACTOME_MET PROMOTES CELL MOTILITY	REACTOME_MET PROMOTES CELL MOTILITY	29	0.47696003	0.47696003	0	0.31914815	1	8739	tags=66%, list=18%, signal=80%
REACTOME_REGULATION OF TNFR1 SIGNALING	REACTOME_REGULATION OF TNFR1 SIGNALING	33	0.4743138	0.4743138	0	0.32475746	1	10917	tags=70%, list=22%, signal=90%
REACTOME_TRANSPORT OF NUCLEOTIDE SUGARS	REACTOME_TRANSPORT OF NUCLEOTIDE SUGARS	9	0.47382912	0.47382912	0.0232558	0.32396805	1	4005	tags=56%, list=8%, signal=60%

REACTOME_PTK6 REGULATES RTKS AND THEIR EFFECTORS AKT1 AND DOK1	REACTOME_PTK6 REGULATES RTKS AND THEIR EFFECTORS AKT1 AND DOK1	9	0.47359535	0.47359535	0.0328358	0.32250014	1	9457	tags=67%, list=19%, signal=83%
REACTOME_INTERLEUKIN-1 PROCESSING	REACTOME_INTERLEUKIN-1 PROCESSING	7	0.47199643	0.47199643	0.0482283	0.32530847	1	4871	tags=57%, list=10%, signal=63%
REACTOME_CHONDROITIN SULFATE/DERMATAN SULFATE METABOLISM	REACTOME_CHONDROITIN SULFATE/DERMATAN SULFATE METABOLISM	50	0.47132778	0.47132778	0	0.32508138	1	6323	tags=60%, list=13%, signal=69%
REACTOME_CHONDROITIN SULFATE BIOSYNTHESIS	REACTOME_CHONDROITIN SULFATE BIOSYNTHESIS	20	0.47103894	0.47103894	0	0.32370687	1	6323	tags=60%, list=13%, signal=69%
REACTOME_SYNTHESIS OF DOLICHYL-PHOSPHATE	REACTOME_SYNTHESIS OF DOLICHYL-PHOSPHATE	6	0.46895024	0.46895024	0.0929773	0.3279084	1	9683	tags=67%, list=20%, signal=83%
REACTOME_RHO GTPASES ACTIVATE IQGAPS	REACTOME_RHO GTPASES ACTIVATE IQGAPS	11	0.46652332	0.46652332	0.008008	0.33355713	1	3869	tags=55%, list=8%, signal=59%
REACTOME_DEFECTIVE EXT1 CAUSES EXOSTOSES 1, TRPS2 AND CHDS	REACTOME_DEFECTIVE EXT1 CAUSES EXOSTOSES 1, TRPS2 AND CHDS	12	0.46601674	0.46601674	0.0098328	0.33288562	1	5749	tags=58%, list=12%, signal=66%
REACTOME_DEFECTIVE EXT2 CAUSES EXOSTOSES 2	REACTOME_DEFECTIVE EXT2 CAUSES EXOSTOSES 2	12	0.46601674	0.46601674	0.0051546	0.3307099	1	5749	tags=58%, list=12%, signal=66%
REACTOME_UNWINDING OF DNA	REACTOME_UNWINDING OF DNA	12	0.46535626	0.46535626	0.0110442	0.33066857	1	22103	tags=92%, list=45%, signal=167%
REACTOME_G2/M DNA REPLICATION CHECKPOINT	REACTOME_G2/M DNA REPLICATION CHECKPOINT	5	0.46343955	0.46343955	0.1661507	0.3346996	1	26274	tags=100%, list=54%, signal=216%
REACTOME_NEF-MEDIATES DOWN MODULATION OF CELL SURFACE RECEPTORS BY RECRUITING THEM TO CLATHRIN	REACTOME_NEF-MEDIATES DOWN MODULATION OF CELL SURFACE RECEPTORS BY RECRUITING THEM TO CLATHRIN ADAPTERS	21	0.46233794	0.46233794	0	0.33592865	1	21672	tags=90%, list=44%, signal=162%
REACTOME_ATTACHMENT OF GPI ANCHOR TO UPAR	REACTOME_ATTACHMENT OF GPI ANCHOR TO UPAR	7	0.460027	0.460027	0.0694864	0.34090436	1	12452	tags=71%, list=25%, signal=96%
REACTOME_MITOTIC TELOPHASE/CYTOKINESIS	REACTOME_MITOTIC TELOPHASE/CYTOKINESIS	14	0.45912245	0.45912245	0.0020429	0.34138077	1	22992	tags=93%, list=47%, signal=175%
REACTOME_GAP JUNCTION DEGRADATION	REACTOME_GAP JUNCTION DEGRADATION	10	0.45894188	0.45894188	0.0149402	0.33977878	1	11807	tags=70%, list=24%, signal=92%
REACTOME_NUCLEOBASE BIOSYNTHESIS	REACTOME_NUCLEOBASE BIOSYNTHESIS	15	0.45829082	0.45829082	0.004065	0.33982414	1	20002	tags=87%, list=41%, signal=146%
REACTOME_PTK6 PROMOTES HIF1A STABILIZATION	REACTOME_PTK6 PROMOTES HIF1A STABILIZATION	7	0.4579436	0.4579436	0.0733198	0.3388057	1	12554	tags=71%, list=26%, signal=96%
REACTOME_UPTAKE AND FUNCTION OF DIPHTHERIA TOXIN	REACTOME_UPTAKE AND FUNCTION OF DIPHTHERIA TOXIN	6	0.45740655	0.45740655	0.1115538	0.33833677	1	18409	tags=83%, list=38%, signal=134%
REACTOME_TRANSCRIPTIONAL ACTIVATION OF CELL CYCLE INHIBITOR P21	REACTOME_TRANSCRIPTIONAL ACTIVATION OF CELL CYCLE INHIBITOR P21	4	0.4549233	0.4549233	0.2941177	0.34431326	1	2208	tags=50%, list=5%, signal=52%
REACTOME_TRANSCRIPTIONAL ACTIVATION OF P53 RESPONSIVE GENES	REACTOME_TRANSCRIPTIONAL ACTIVATION OF P53 RESPONSIVE GENES	4	0.4549233	0.4549233	0.2621167	0.34221378	1	2208	tags=50%, list=5%, signal=52%
REACTOME_CS/DS	REACTOME_CS/DS DEGRADATION	14	0.4535556	0.4535556	0.0019474	0.34450197	1	5777	tags=57%, list=12%, signal=65%
REACTOME_LOSS OF FUNCTION OF TGFBR1 IN CANCER	REACTOME_LOSS OF FUNCTION OF TGFBR1 IN CANCER	7	0.45224482	0.45224482	0.0721443	0.34651455	1	5838	tags=57%, list=12%, signal=65%
REACTOME_TGFBR1 KD MUTANTS IN CANCER	REACTOME_TGFBR1 KD MUTANTS IN CANCER	7	0.45224482	0.45224482	0.0869565	0.3444396	1	5838	tags=57%, list=12%, signal=65%
REACTOME_PROTON-COUPLED MONOCARBOXYLATE TRANSPORT	REACTOME_PROTON-COUPLED MONOCARBOXYLATE TRANSPORT	6	0.45046538	0.45046538	0.1300403	0.34823865	1	10588	tags=67%, list=22%, signal=85%

REACTOME_VASOPRESSIN-LIKE RECEPTORS	REACTOME_VASOPRESSIN-LIKE RECEPTORS	6	0.45015898	0.45015898	0.1193416	0.34712338	1	10603	tags=67%, list=22%, signal=85%
REACTOME_TRAF6 MEDIATED IRF7 ACTIVATION	REACTOME_TRAF6 MEDIATED IRF7 ACTIVATION	30	0.4495079	0.4495079	0	0.34702203	1	13910	tags=73%, list=28%, signal=102%
REACTOME_ACTIVATION OF SMO	REACTOME_ACTIVATION OF SMO	18	0.44942603	0.44942603	0	0.34521106	1	7924	tags=61%, list=16%, signal=73%
REACTOME_PRE-NOTCH PROCESSING IN GOLGI	REACTOME_PRE-NOTCH PROCESSING IN GOLGI	18	0.4488744	0.4488744	0	0.34497017	1	7951	tags=61%, list=16%, signal=73%
REACTOME_ACTIVATION OF NOXA AND TRANSLOCATION TO MITOCHONDRIA	REACTOME_ACTIVATION OF NOXA AND TRANSLOCATION TO MITOCHONDRIA	5	0.44775328	0.44775328	0.2102102	0.3467182	1	17249	tags=80%, list=35%, signal=123%
REACTOME_NEUROFASCIN INTERACTIONS	REACTOME_NEUROFASCIN INTERACTIONS	7	0.44728136	0.44728136	0.0891473	0.34624007	1	6081	tags=57%, list=12%, signal=65%
REACTOME_RUNX3 REGULATES BCL2L11 (BIM) TRANSCRIPTION	REACTOME_RUNX3 REGULATES BCL2L11 (BIM) TRANSCRIPTION	5	0.4468546	0.4468546	0.1981707	0.34574997	1	17293	tags=80%, list=35%, signal=124%
REACTOME_RUNX1 REGULATES ESTROGEN RECEPTOR MEDIATED TRANSCRIPTION	REACTOME_RUNX1 REGULATES ESTROGEN RECEPTOR MEDIATED TRANSCRIPTION	6	0.44685352	0.44685352	0.1363184	0.3437855	1	2604	tags=50%, list=5%, signal=53%
REACTOME_PYRIMIDINE	REACTOME_PYRIMIDINE SALVAGE	11	0.4444692	0.4444692	0.0221519	0.34964767	1	9400	tags=64%, list=19%, signal=79%
REACTOME_G2 PHASE	REACTOME_G2 PHASE	5	0.443893	0.443893	0.1941177	0.34945968	1	27231	tags=100%, list=56%, signal=225%
REACTOME_PHOSPHORYLATION OF PROTEINS INVOLVED IN THE G2/M TRANSITION BY CYCLIN A:CDC2 COMPLEXES	REACTOME_PHOSPHORYLATION OF PROTEINS INVOLVED IN THE G2/M TRANSITION BY CYCLIN A:CDC2 COMPLEXES	3	0.44387487	0.44387487	0.4708786	0.3475607	1	27231	tags=100%, list=56%, signal=225%
REACTOME_LINOLEIC ACID (LA) METABOLISM	REACTOME_LINOLEIC ACID (LA) METABOLISM	8	0.44359592	0.44359592	0.0629515	0.34655014	1	8885	tags=63%, list=18%, signal=76%
REACTOME_NEF MEDIATED CD4 DOWN-REGULATION	REACTOME_NEF MEDIATED CD4 DOWN-REGULATION	9	0.44322112	0.44322112	0.0303644	0.34584716	1	10944	tags=67%, list=22%, signal=86%
REACTOME_ALKBH3 MEDIATED REVERSAL OF ALKYLATION	REACTOME_ALKBH3 MEDIATED REVERSAL OF ALKYLATION DAMAGE	4	0.44191295	0.44191295	0.2981557	0.34800676	1	2845	tags=50%, list=6%, signal=53%
REACTOME_DISEASES OF MISMATCH REPAIR (MMR)	REACTOME_DISEASES OF MISMATCH REPAIR (MMR)	5	0.44180965	0.44180965	0.2126607	0.3464265	1	7747	tags=60%, list=16%, signal=71%
REACTOME_COLLAGEN CHAIN TRIMERIZATION	REACTOME_COLLAGEN CHAIN TRIMERIZATION	44	0.43988362	0.43988362	0	0.35062072	1	9639	tags=64%, list=20%, signal=79%
REACTOME_DEFECTIVE AMN CAUSES HEREDITARY MEGALOBLASTIC ANEMIA 1	REACTOME_DEFECTIVE AMN CAUSES HEREDITARY MEGALOBLASTIC ANEMIA 1	3	0.4381221	0.4381221	0.4757185	0.3544044	1	11191	tags=67%, list=23%, signal=86%
REACTOME_DEFECTIVE CUBN CAUSES HEREDITARY MEGALOBLASTIC ANEMIA 1	REACTOME_DEFECTIVE CUBN CAUSES HEREDITARY MEGALOBLASTIC ANEMIA 1	3	0.4381221	0.4381221	0.4856294	0.352499	1	11191	tags=67%, list=23%, signal=86%
REACTOME_UPTAKE AND FUNCTION OF ANTHRAX TOXINS	REACTOME_UPTAKE AND FUNCTION OF ANTHRAX TOXINS	11	0.43723795	0.43723795	0.024558	0.35340998	1	9754	tags=64%, list=20%, signal=79%
REACTOME_FGFR1B LIGAND BINDING AND ACTIVATION	REACTOME_FGFR1B LIGAND BINDING AND ACTIVATION	6	0.4371447	0.4371447	0.1444333	0.35182458	1	19401	tags=83%, list=40%, signal=138%
REACTOME_VLDLR INTERNALISATION AND DEGRADATION	REACTOME_VLDLR INTERNALISATION AND DEGRADATION	12	0.43687487	0.43687487	0.0158259	0.35081342	1	11256	tags=67%, list=23%, signal=87%
REACTOME_PD-1 SIGNALING	REACTOME_PD-1 SIGNALING	24	0.4342244	0.4342244	0	0.357482	1	21592	tags=88%, list=44%, signal=156%
REACTOME_BETA-OXIDATION OF PRISTANOYL-COA	REACTOME_BETA-OXIDATION OF PRISTANOYL-COA	9	0.4329761	0.4329761	0.0482546	0.35953304	1	6005	tags=56%, list=12%, signal=63%
REACTOME_TP53 REGULATES TRANSCRIPTION OF GENES INVOLVED IN G1 CELL CYCLE ARREST	REACTOME_TP53 REGULATES TRANSCRIPTION OF GENES INVOLVED IN G1 CELL CYCLE ARREST	14	0.4323916	0.4323916	0.00611	0.35932982	1	6813	tags=57%, list=14%, signal=66%

REACTOME_RESPONSE TO METAL IONS	REACTOME_RESPONSE TO METAL IONS	14	0.4315132	0.4315132	0.0128079	0.36021245	1	13851	tags=71%, list=28%, signal=100%
REACTOME_TNFR1-INDUCED NFKAPPAB SIGNALING PATHWAY	REACTOME_TNFR1-INDUCED NFKAPPAB SIGNALING PATHWAY	26	0.4310995	0.4310995	0	0.35964105	1	10917	tags=65%, list=22%, signal=84%
REACTOME_COHESIN LOADING ONTO CHROMATIN	REACTOME_COHESIN LOADING ONTO CHROMATIN	10	0.4305076	0.4305076	0.048583	0.3596388	1	22992	tags=90%, list=47%, signal=170%
REACTOME_ENOS ACTIVATION	REACTOME_ENOS ACTIVATION	10	0.4302727	0.4302727	0.0389474	0.3584809	1	8314	tags=60%, list=17%, signal=72%
REACTOME_INHIBITION OF TSC COMPLEX FORMATION BY PKB	REACTOME_INHIBITION OF TSC COMPLEX FORMATION BY PKB	3	0.43006825	0.43006825	0.5300493	0.35732195	1	27907	tags=100%, list=57%, signal=233%
REACTOME_SMOOTH MUSCLE CONTRACTION	REACTOME_SMOOTH MUSCLE CONTRACTION	35	0.4289393	0.4289393	0	0.35920075	1	6991	tags=57%, list=14%, signal=67%
REACTOME_PURINE RIBONUCLEOSIDE MONOPHOSPHATE	REACTOME_PURINE RIBONUCLEOSIDE MONOPHOSPHATE BIOSYNTHESIS	12	0.4249212	0.4249212	0.0106486	0.37033302	1	20002	tags=83%, list=41%, signal=141%
REACTOME_SPERM MOTILITY AND TAXES	REACTOME_SPERM MOTILITY AND TAXES	9	0.42456266	0.42456266	0.0485537	0.36954698	1	17298	tags=78%, list=35%, signal=120%
REACTOME_RUNX3 REGULATES IMMUNE RESPONSE AND CELL MIGRATION	REACTOME_RUNX3 REGULATES IMMUNE RESPONSE AND CELL MIGRATION	6	0.42414063	0.42414063	0.170297	0.36913863	1	3716	tags=50%, list=8%, signal=54%
REACTOME_SCAVENGING BY CLASS A RECEPTORS	REACTOME_SCAVENGING BY CLASS A RECEPTORS	19	0.42367208	0.42367208	9.70E-04	0.36885548	1	5033	tags=53%, list=10%, signal=59%
REACTOME_EXTRACELLULAR MATRIX ORGANIZATION	REACTOME_EXTRACELLULAR MATRIX ORGANIZATION	295	0.42242044	0.42242044	0	0.3711149	1	6827	tags=56%, list=14%, signal=65%
REACTOME_PLASMA LIPOPROTEIN ASSEMBLY	REACTOME_PLASMA LIPOPROTEIN ASSEMBLY	19	0.42218062	0.42218062	0	0.3701236	1	5106	tags=53%, list=10%, signal=59%
REACTOME_A TETRASACCHARIDE LINKER SEQUENCE IS REQUIRED FOR	REACTOME_A TETRASACCHARIDE LINKER SEQUENCE IS REQUIRED FOR GAG SYNTHESIS	26	0.42068228	0.42068228	0	0.37321317	1	5777	tags=54%, list=12%, signal=61%
REACTOME_RUNX1 REGULATES TRANSCRIPTION OF GENES INVOLVED IN DIFFERENTIATION OF KERATINOCYTES	REACTOME_RUNX1 REGULATES TRANSCRIPTION OF GENES INVOLVED IN DIFFERENTIATION OF KERATINOCYTES	8	0.4206574	0.4206574	0.0843965	0.37149796	1	10008	tags=63%, list=20%, signal=79%
REACTOME_N-GLYCAN TRIMMING AND ELONGATION IN	REACTOME_N-GLYCAN TRIMMING AND ELONGATION IN THE CIS-GOLGI	5	0.42017975	0.42017975	0.2487258	0.37118647	1	8806	tags=60%, list=18%, signal=73%
REACTOME_PROCESSIVE SYNTHESIS ON THE C-STRAND OF THE TELOMERE	REACTOME_PROCESSIVE SYNTHESIS ON THE C-STRAND OF THE TELOMERE	11	0.41945317	0.41945317	0.0156709	0.37179115	1	15076	tags=73%, list=31%, signal=105%
REACTOME_CREB3 FACTORS ACTIVATE GENES	REACTOME_CREB3 FACTORS ACTIVATE GENES	9	0.41704342	0.41704342	0.0732422	0.3778764	1	6785	tags=56%, list=14%, signal=64%
REACTOME_LYSOSOMAL GLYCOGEN CATABOLISM	REACTOME_LYSOSOMAL GLYCOGEN CATABOLISM	3	0.41602334	0.41602334	0.5429418	0.37955466	1	12273	tags=67%, list=25%, signal=89%
REACTOME_MISSPLICED LRP5 MUTANTS HAVE ENHANCED BETA-CATENIN-DEPENDENT [illegible]	REACTOME_MISSPLICED LRP5 MUTANTS HAVE ENHANCED BETA-CATENIN-DEPENDENT SIGNALING	5	0.4157884	0.4157884	0.2731855	0.37856635	1	9021	tags=60%, list=18%, signal=74%
REACTOME_PTK6 REGULATES RHO GTPASES, RAS GTPASE AND MAP KINASES	REACTOME_PTK6 REGULATES RHO GTPASES, RAS GTPASE AND MAP KINASES	18	0.41498062	0.41498062	0.0019704	0.37955284	1	9610	tags=61%, list=20%, signal=76%
REACTOME_GLYCINE	REACTOME_GLYCINE DEGRADATION	4	0.41497827	0.41497827	0.3792757	0.37778014	1	16405	tags=75%, list=34%, signal=113%
REACTOME_THE ROLE OF NEF IN HIV-1 REPLICATION AND DISEASE PATHOGENESIS	REACTOME_THE ROLE OF NEF IN HIV-1 REPLICATION AND DISEASE PATHOGENESIS	28	0.4147578	0.4147578	0	0.37670675	1	21672	tags=86%, list=44%, signal=154%

REACTOME_TRANSLOCATION OF ZAP-70 TO IMMUNOLOGICAL SYNAPSE	REACTOME_TRANSLOCATION OF ZAP-70 TO IMMUNOLOGICAL SYNAPSE	20	0.41368884	0.41368884	9.64E-04	0.37880638	1	28716	tags=100%, list=59%, signal=242%
REACTOME_PHOSPHORYLATION OF PROTEINS INVOLVED IN G1/S TRANSITION BY ACTIVE CYCLIN E:CDK2 COMPLEXES	REACTOME_PHOSPHORYLATION OF PROTEINS INVOLVED IN G1/S TRANSITION BY ACTIVE CYCLIN E:CDK2 COMPLEXES	4	0.41162354	0.41162354	0.4040896	0.384208	1	4328	tags=50%, list=9%, signal=55%
REACTOME_CHREBP ACTIVATES METABOLIC GENE EXPRESSION	REACTOME_CHREBP ACTIVATES METABOLIC GENE EXPRESSION	8	0.41138396	0.41138396	0.1014199	0.3833146	1	10462	tags=63%, list=21%, signal=79%
REACTOME_ZINC INFLUX INTO CELLS BY THE SLC39 GENE	REACTOME_ZINC INFLUX INTO CELLS BY THE SLC39 GENE FAMILY	10	0.41064242	0.41064242	0.0460784	0.38403845	1	9275	tags=60%, list=19%, signal=74%
REACTOME_DEFECTIVE B3GALT6 CAUSES EDSP2 AND [illegible]	REACTOME_DEFECTIVE B3GALT6 CAUSES EDSP2 AND SEMDJL1	19	0.40847164	0.40847164	0	0.3895812	1	5777	tags=53%, list=12%, signal=60%
REACTOME_DEFECTIVE B3GAT3 CAUSES JDSSDHD	REACTOME_DEFECTIVE B3GAT3 CAUSES JDSSDHD	19	0.40847164	0.40847164	0.0031847	0.38781038	1	5777	tags=53%, list=12%, signal=60%
REACTOME_DEFECTIVE B4GALT7 CAUSES EDS, [illegible]	REACTOME_DEFECTIVE B4GALT7 CAUSES EDS, PROGEROID TYPE	19	0.40847164	0.40847164	0.0041494	0.38605556	1	5777	tags=53%, list=12%, signal=60%
REACTOME_RNF MUTANTS SHOW ENHANCED WNT SIGNALING AND PROLIFERATION	REACTOME_RNF MUTANTS SHOW ENHANCED WNT SIGNALING AND PROLIFERATION	8	0.40787068	0.40787068	0.1033797	0.38637233	1	10634	tags=63%, list=22%, signal=80%
REACTOME_KERATAN SULFATE/KERATIN METABOLISM	REACTOME_KERATAN SULFATE/KERATIN METABOLISM	33	0.40720147	0.40720147	0	0.38688856	1	6782	tags=55%, list=14%, signal=63%
REACTOME_SMAC BINDS TO [illegible]	REACTOME_SMAC BINDS TO IAPS	5	0.40700573	0.40700573	0.2781726	0.3858096	1	9451	tags=60%, list=19%, signal=74%
REACTOME_SMAC-MEDIATED APOPTOTIC RESPONSE	REACTOME_SMAC-MEDIATED APOPTOTIC RESPONSE	5	0.40700573	0.40700573	0.2918707	0.3840949	1	9451	tags=60%, list=19%, signal=74%
REACTOME_SMAC-MEDIATED DISSOCIATION OF IAP:CASPASE COMPLEXES	REACTOME_SMAC-MEDIATED DISSOCIATION OF IAP:CASPASE COMPLEXES	5	0.40700573	0.40700573	0.2821256	0.38239536	1	9451	tags=60%, list=19%, signal=74%
REACTOME_ECM	REACTOME_ECM PROTEOGLYCANS	55	0.40599614	0.40599614	0	0.384048	1	10411	tags=62%, list=21%, signal=78%
REACTOME_LDL CLEARANCE	REACTOME_LDL CLEARANCE	19	0.40582213	0.40582213	0	0.38292122	1	13638	tags=68%, list=28%, signal=95%
REACTOME_P75NTR SIGNALS VIA NF-KB	REACTOME_P75NTR SIGNALS VIA NF-KB	16	0.40558797	0.40558797	0.002994	0.38202682	1	10749	tags=63%, list=22%, signal=80%
REACTOME_SCAVENGING BY CLASS H RECEPTORS	REACTOME_SCAVENGING BY CLASS H RECEPTORS	4	0.4031474	0.4031474	0.4414683	0.38845712	1	4743	tags=50%, list=10%, signal=55%
REACTOME_TNFS BIND THEIR PHYSIOLOGICAL RECEPTORS	REACTOME_TNFS BIND THEIR PHYSIOLOGICAL RECEPTORS	27	0.40289223	0.40289223	0	0.387648	1	3858	tags=48%, list=8%, signal=52%
REACTOME_DEGRADATION OF THE EXTRACELLULAR MATRIX	REACTOME_DEGRADATION OF THE EXTRACELLULAR MATRIX	111	0.4023533	0.4023533	0	0.38771582	1	6810	tags=54%, list=14%, signal=63%
REACTOME_SIGNAL	REACTOME_SIGNAL ATTENUATION	10	0.40195078	0.40195078	0.074331	0.38739923	1	4804	tags=50%, list=10%, signal=55%
REACTOME_HUR (ELAVL1) BINDS AND STABILIZES MRNA	REACTOME_HUR (ELAVL1) BINDS AND STABILIZES MRNA	10	0.40131754	0.40131754	0.0653218	0.3879464	1	14628	tags=70%, list=30%, signal=100%
REACTOME_HEPARAN SULFATE/HEPARIN (HS-GAG) METABOLISM	REACTOME_HEPARAN SULFATE/HEPARIN (HS-GAG) METABOLISM	54	0.40095806	0.40095806	0	0.38745353	1	5777	tags=52%, list=12%, signal=59%
REACTOME_TP53 REGULATES TRANSCRIPTION OF SEVERAL ADDITIONAL CELL DEATH GENES WHOSE SPECIFIC ROLES IN P53-DEPENDENT APOPTOSIS REMAIN UNCERTAIN	REACTOME_TP53 REGULATES TRANSCRIPTION OF SEVERAL ADDITIONAL CELL DEATH GENES WHOSE SPECIFIC ROLES IN P53-DEPENDENT APOPTOSIS REMAIN UNCERTAIN	14	0.40051278	0.40051278	0.0166503	0.38729984	1	18866	tags=79%, list=39%, signal=128%

REACTOME_ATF6 (ATF6-ALPHA) ACTIVATES CHAPERONES	REACTOME_ATF6 (ATF6-ALPHA) ACTIVATES CHAPERONES	11	0.39995062	0.39995062	0.039666	0.38758382	1	7128	tags=55%, list=15%, signal=64%
REACTOME_MISMATCH REPAIR	REACTOME_MISMATCH REPAIR	15	0.39878792	0.39878792	0.0107527	0.3899788	1	16386	tags=73%, list=33%, signal=110%
REACTOME_INTEGRIN CELL SURFACE INTERACTIONS	REACTOME_INTEGRIN CELL SURFACE INTERACTIONS	66	0.39807874	0.39807874	0	0.39079052	1	10951	tags=62%, list=22%, signal=80%
REACTOME_TELOMERE EXTENSION BY TELOMERASE	REACTOME_TELOMERE EXTENSION BY TELOMERASE	6	0.39756057	0.39756057	0.2270434	0.39086103	1	21339	tags=83%, list=44%, signal=148%
REACTOME_TFAP2 (AP-2) FAMILY REGULATES TRANSCRIPTION OF CELL CYCLE FACTORS	REACTOME_TFAP2 (AP-2) FAMILY REGULATES TRANSCRIPTION OF CELL CYCLE FACTORS	5	0.39748776	0.39748776	0.3415113	0.3894936	1	9917	tags=60%, list=20%, signal=75%
REACTOME_CHYLOMICRON ASSEMBLY	REACTOME_CHYLOMICRON ASSEMBLY	10	0.397273	0.397273	0.0525803	0.38858214	1	5033	tags=50%, list=10%, signal=56%
REACTOME_DEATH RECEPTOR SIGNALLING	REACTOME_DEATH RECEPTOR SIGNALLING	50	0.3970009	0.3970009	0	0.3878593	1	10938	tags=62%, list=22%, signal=80%
REACTOME_ACYL CHAIN REMODELING OF DAG AND TAG	REACTOME_ACYL CHAIN REMODELING OF DAG AND TAG	8	0.3965342	0.3965342	0.1080808	0.3879151	1	11189	tags=63%, list=23%, signal=81%
REACTOME_TFAP2 (AP-2) FAMILY REGULATES TRANSCRIPTION OF OTHER TRANSCRIPTION	REACTOME_TFAP2 (AP-2) FAMILY REGULATES TRANSCRIPTION OF OTHER TRANSCRIPTION FACTORS	4	0.3961061	0.3961061	0.4540059	0.38775325	1	17329	tags=75%, list=35%, signal=116%
REACTOME_NF-KB IS ACTIVATED AND SIGNALS SURVIVAL	REACTOME_NF-KB IS ACTIVATED AND SIGNALS SURVIVAL	13	0.3959452	0.3959452	0.016129	0.38674057	1	10749	tags=62%, list=22%, signal=79%
REACTOME_VEGFR2 MEDIATED VASCULAR PERMEABILITY	REACTOME_VEGFR2 MEDIATED VASCULAR PERMEABILITY	27	0.3957532	0.3957532	9.79E-04	0.38583618	1	16902	tags=74%, list=35%, signal=113%
REACTOME_SEMA3A PAK DEPENDENT AXON REPULSION	REACTOME_SEMA3A PAK DEPENDENT AXON REPULSION	16	0.39501828	0.39501828	0.0099206	0.38672847	1	17387	tags=75%, list=36%, signal=116%
REACTOME_NOTCH2 ACTIVATION AND TRANSMISSION OF SIGNAL TO THE NUCLEUS	REACTOME_NOTCH2 ACTIVATION AND TRANSMISSION OF SIGNAL TO THE NUCLEUS	21	0.3944985	0.3944985	0.002006	0.38689154	1	6339	tags=52%, list=13%, signal=60%
REACTOME_RUNX3 REGULATES NOTCH SIGNALING	REACTOME_RUNX3 REGULATES NOTCH SIGNALING	14	0.39377135	0.39377135	0.0153218	0.38792565	1	12201	tags=64%, list=25%, signal=86%
REACTOME_POST-TRANSLATIONAL PROTEIN	REACTOME_POST-TRANSLATIONAL PROTEIN PHOSPHORYLATION	107	0.3937361	0.3937361	0	0.38652247	1	6846	tags=53%, list=14%, signal=62%
REACTOME_GP1B-IX-V ACTIVATION SIGNALLING	REACTOME_GP1B-IX-V ACTIVATION SIGNALLING	9	0.39278808	0.39278808	0.1060456	0.38822928	1	13413	tags=67%, list=27%, signal=92%
REACTOME_GLUTATHIONE SYNTHESIS AND RECYCLING	REACTOME_GLUTATHIONE SYNTHESIS AND RECYCLING	13	0.39238912	0.39238912	0.027027	0.38798055	1	3390	tags=46%, list=7%, signal=50%
REACTOME_REMOVAL OF THE FLAP INTERMEDIATE FROM THE	REACTOME_REMOVAL OF THE FLAP INTERMEDIATE FROM THE C-STRAND	10	0.3921663	0.3921663	0.0770732	0.3872302	1	15076	tags=70%, list=31%, signal=101%
REACTOME_PROSTANOID LIGAND RECEPTORS	REACTOME_PROSTANOID LIGAND RECEPTORS	9	0.39155114	0.39155114	0.0922053	0.3878809	1	8033	tags=56%, list=16%, signal=66%
REACTOME_ACTIVATION OF NIMA KINASES NEK9, NEK6, NEK7	REACTOME_ACTIVATION OF NIMA KINASES NEK9, NEK6, NEK7	7	0.39013037	0.39013037	0.2099609	0.39119905	1	22869	tags=86%, list=47%, signal=161%
REACTOME_REGULATION OF IFNA SIGNALING	REACTOME_REGULATION OF IFNA SIGNALING	23	0.38897777	0.38897777	0.0020471	0.3936926	1	21411	tags=83%, list=44%, signal=147%
REACTOME_CALNEXIN/CALRETIC ULIN CYCLE	REACTOME_CALNEXIN/CALRETICULIN CYCLE	26	0.38888758	0.38888758	0	0.39250958	1	7333	tags=54%, list=15%, signal=63%
REACTOME_PLASMA LIPOPROTEIN CLEARANCE	REACTOME_PLASMA LIPOPROTEIN CLEARANCE	33	0.38838252	0.38838252	0	0.39264196	1	13638	tags=67%, list=28%, signal=92%
REACTOME_MISMATCH REPAIR (MMR) DIRECTED BY MSH2:MSH3 (MUTSBETA)	REACTOME_MISMATCH REPAIR (MMR) DIRECTED BY MSH2:MSH3 (MUTSBETA)	14	0.388225	0.388225	0.0210843	0.39165986	1	22965	tags=86%, list=47%, signal=161%

REACTOME_MISMATCH REPAIR (MMR) DIRECTED BY MSH2:MSH6 (MUTSALPHA)	REACTOME_MISMATCH REPAIR (MMR) DIRECTED BY MSH2:MSH6 (MUTSALPHA)	14	0.388225	0.388225	0.0221774	0.39015925	1	22965	tags=86%, list=47%, signal=161%
REACTOME_GLYCOSAMINOGLYCAN METABOLISM	REACTOME_GLYCOSAMINOGLYCAN METABOLISM	122	0.3882072	0.3882072	0	0.38870472	1	6323	tags=52%, list=13%, signal=59%
REACTOME_FATTY ACIDS BOUND TO GPR40 (FFAR1) REGULATE INSULIN SECRETION	REACTOME_FATTY ACIDS BOUND TO GPR40 (FFAR1) REGULATE INSULIN SECRETION	8	0.38777906	0.38777906	0.1324245	0.3886077	1	5497	tags=50%, list=11%, signal=56%
REACTOME_FREE FATTY ACIDS REGULATE INSULIN SECRETION	REACTOME_FREE FATTY ACIDS REGULATE INSULIN SECRETION	8	0.38777906	0.38777906	0.1135647	0.3871357	1	5497	tags=50%, list=11%, signal=56%
REACTOME_AKT-MEDIATED INACTIVATION OF FOXO1A	REACTOME_AKT-MEDIATED INACTIVATION OF FOXO1A	4	0.38719594	0.38719594	0.4888889	0.3875344	1	5524	tags=50%, list=11%, signal=56%
REACTOME_ROLE OF ABL IN ROBO-SLIT SIGNALING	REACTOME_ROLE OF ABL IN ROBO-SLIT SIGNALING	9	0.3861177	0.3861177	0.097371	0.3895573	1	8299	tags=56%, list=17%, signal=67%
REACTOME_NEGATIVE FEEDBACK REGULATION OF	REACTOME_NEGATIVE FEEDBACK REGULATION OF MAPK PATHWAY	6	0.38469267	0.38469267	0.2530864	0.3928872	1	21969	tags=83%, list=45%, signal=151%
REACTOME_TNF SIGNALING	REACTOME_TNF SIGNALING	43	0.38158178	0.38158178	0	0.40220746	1	10938	tags=60%, list=22%, signal=78%
REACTOME_SIGNALING BY TGF-BETA RECEPTOR COMPLEX IN CANCER	REACTOME_SIGNALING BY TGF-BETA RECEPTOR COMPLEX IN CANCER	8	0.38081378	0.38081378	0.1438776	0.40345994	1	5838	tags=50%, list=12%, signal=57%
REACTOME_SMAD2/3 PHOSPHORYLATION MOTIF MUTANTS IN CANCER	REACTOME_SMAD2/3 PHOSPHORYLATION MOTIF MUTANTS IN CANCER	6	0.38079822	0.38079822	0.2864173	0.40201548	1	5838	tags=50%, list=12%, signal=57%
REACTOME_FORMATION OF SENESCENCE-ASSOCIATED HETEROCHROMATIN FOCI (SAHF)	REACTOME_FORMATION OF SENESCENCE-ASSOCIATED HETEROCHROMATIN FOCI (SAHF)	17	0.38059238	0.38059238	0.0121581	0.40121248	1	13053	tags=65%, list=27%, signal=88%
REACTOME_HEME DEGRADATION	REACTOME_HEME DEGRADATION	5	0.3805147	0.3805147	0.3501027	0.39999497	1	20541	tags=80%, list=42%, signal=138%
REACTOME_TRAF6 MEDIATED NF-KB ACTIVATION	REACTOME_TRAF6 MEDIATED NF-KB ACTIVATION	26	0.38017187	0.38017187	0	0.3997319	1	3993	tags=46%, list=8%, signal=50%
REACTOME_FORMYL PEPTIDE RECEPTORS BIND FORMYL PEPTIDES AND MANY OTHER LIGANDS	REACTOME_FORMYL PEPTIDE RECEPTORS BIND FORMYL PEPTIDES AND MANY OTHER LIGANDS	9	0.37908417	0.37908417	0.1151151	0.40210247	1	24965	tags=89%, list=51%, signal=181%
REACTOME_STING MEDIATED INDUCTION OF HOST IMMUNE RESPONSES	REACTOME_STING MEDIATED INDUCTION OF HOST IMMUNE RESPONSES	15	0.37897176	0.37897176	0.0219436	0.40102565	1	7563	tags=53%, list=15%, signal=63%
REACTOME_CONSTITUTIVE SIGNALING BY NOTCH1 HD DOMAIN MUTANTS	REACTOME_CONSTITUTIVE SIGNALING BY NOTCH1 HD DOMAIN MUTANTS	15	0.37880832	0.37880832	0.0194473	0.40010488	1	7571	tags=53%, list=15%, signal=63%
REACTOME_SIGNALING BY NOTCH1 HD DOMAIN MUTANTS IN	REACTOME_SIGNALING BY NOTCH1 HD DOMAIN MUTANTS IN CANCER	15	0.37880832	0.37880832	0.018664	0.39866045	1	7571	tags=53%, list=15%, signal=63%
REACTOME_MAPK3 (ERK1) ACTIVATION	REACTOME_MAPK3 (ERK1) ACTIVATION	10	0.37853128	0.37853128	0.083089	0.39820364	1	30433	tags=100%, list=62%, signal=264%
REACTOME_MAPK1 (ERK2) ACTIVATION	REACTOME_MAPK1 (ERK2) ACTIVATION	9	0.3785236	0.3785236	0.1295716	0.39680886	1	30433	tags=100%, list=62%, signal=264%
REACTOME_ELECTRIC TRANSMISSION ACROSS GAP JUNCTIONS	REACTOME_ELECTRIC TRANSMISSION ACROSS GAP JUNCTIONS	5	0.3782067	0.3782067	0.3721841	0.39651802	1	20654	tags=80%, list=42%, signal=138%
REACTOME_TRANSMISSION ACROSS ELECTRICAL SYNAPSES	REACTOME_TRANSMISSION ACROSS ELECTRICAL SYNAPSES	5	0.3782067	0.3782067	0.3892216	0.39510694	1	20654	tags=80%, list=42%, signal=138%
REACTOME_INTERLEUKIN-6 FAMILY SIGNALING	REACTOME_INTERLEUKIN-6 FAMILY SIGNALING	24	0.3782003	0.3782003	0.0020121	0.3937195	1	5972	tags=50%, list=12%, signal=57%

REACTOME_REGULATION OF INSULIN-LIKE GROWTH FACTOR (IGF) TRANSPORT AND UPTAKE BY INSULIN-LIKE GROWTH FACTOR BINDING PROTEINS	REACTOME_REGULATION OF INSULIN-LIKE GROWTH FACTOR (IGF) TRANSPORT AND UPTAKE BY INSULIN-LIKE GROWTH FACTOR BINDING PROTEINS (IGFBPS)	124	0.3772498	0.3772498	0	0.39553076	1	6846	tags=52%, list=14%, signal=60%
REACTOME_DISEASES ASSOCIATED WITH THE TLR	REACTOME_DISEASES ASSOCIATED WITH THE TLR SIGNALING CASCADE	24	0.37582582	0.37582582	0	0.39899176	1	4048	tags=46%, list=8%, signal=50%
REACTOME_DISEASES OF IMMUNE SYSTEM	REACTOME_DISEASES OF IMMUNE SYSTEM	24	0.37582582	0.37582582	0	0.39759177	1	4048	tags=46%, list=8%, signal=50%
REACTOME_YAP1- AND WWTR1 (TAZ)-STIMULATED GENE EXPRESSION	REACTOME_YAP1- AND WWTR1 (TAZ)-STIMULATED GENE EXPRESSION	29	0.37506482	0.37506482	9.96E-04	0.39873588	1	8660	tags=55%, list=18%, signal=67%
REACTOME_METABOLISM OF INGESTED H2SEO4 AND H2SEO3 INTO H2SE	REACTOME_METABOLISM OF INGESTED H2SEO4 AND H2SEO3 INTO H2SE	4	0.37404773	0.37404773	0.523301	0.40102962	1	18409	tags=75%, list=38%, signal=120%
REACTOME_LYSINE CATABOLISM	REACTOME_LYSINE CATABOLISM	12	0.37363052	0.37363052	0.0659341	0.40108514	1	14352	tags=67%, list=29%, signal=94%
REACTOME_NICOTINAMIDE SALVAGING	REACTOME_NICOTINAMIDE SALVAGING	19	0.37230936	0.37230936	0.0071356	0.4044372	1	7547	tags=53%, list=15%, signal=62%
REACTOME_MULTIFUNCTIONAL ANION EXCHANGERS	REACTOME_MULTIFUNCTIONAL ANION EXCHANGERS	9	0.37217775	0.37217775	0.131579	0.40350336	1	14422	tags=67%, list=29%, signal=94%
REACTOME_RSK ACTIVATION	REACTOME_RSK ACTIVATION	6	0.37180433	0.37180433	0.320398	0.40332735	1	22600	tags=83%, list=46%, signal=155%
REACTOME_SODIUM/PROTON EXCHANGERS	REACTOME_SODIUM/PROTON EXCHANGERS	9	0.3715536	0.3715536	0.1146045	0.40282798	1	9012	tags=56%, list=18%, signal=68%
REACTOME_IRE1ALPHA ACTIVATES CHAPERONES	REACTOME_IRE1ALPHA ACTIVATES CHAPERONES	55	0.37070313	0.37070313	0	0.4044703	1	5015	tags=47%, list=10%, signal=53%
REACTOME_ACETYLCHOLINE REGULATES INSULIN SECRETION	REACTOME_ACETYLCHOLINE REGULATES INSULIN SECRETION	10	0.3706976	0.3706976	0.0861723	0.40311414	1	6334	tags=50%, list=13%, signal=57%
REACTOME_REVERSAL OF ALKYLATION DAMAGE BY DNA DIOXYGENASES	REACTOME_REVERSAL OF ALKYLATION DAMAGE BY DNA DIOXYGENASES	7	0.37050128	0.37050128	0.2440538	0.4024193	1	2845	tags=43%, list=6%, signal=45%
REACTOME_PHOSPHORYLATION OF CD3 AND TCR ZETA CHAINS	REACTOME_PHOSPHORYLATION OF CD3 AND TCR ZETA CHAINS	23	0.37021554	0.37021554	0.00409	0.4020297	1	28716	tags=96%, list=59%, signal=231%
REACTOME_SEMA4D IN SEMAPHORIN SIGNALING	REACTOME_SEMA4D IN SEMAPHORIN SIGNALING	27	0.3694024	0.3694024	0.0019305	0.4034806	1	12751	tags=63%, list=26%, signal=85%
REACTOME_THE AIM2 INFLAMMASOME	REACTOME_THE AIM2 INFLAMMASOME	3	0.36932728	0.36932728	0.6880642	0.4023813	1	30881	tags=100%, list=63%, signal=271%
REACTOME_METABOLISM OF ANGIOTENSINOGEN TO ANGIOTENSINS	REACTOME_METABOLISM OF ANGIOTENSINOGEN TO ANGIOTENSINS	16	0.3688699	0.3688699	0.0194872	0.40270793	1	9486	tags=56%, list=19%, signal=70%
REACTOME_MITOCHONDRIAL TRNA AMINOACYLATION	REACTOME_MITOCHONDRIAL TRNA AMINOACYLATION	21	0.368244	0.368244	0.0077519	0.4036165	1	14619	tags=67%, list=30%, signal=95%
REACTOME_GLYCOSPHINGOLIPID METABOLISM	REACTOME_GLYCOSPHINGOLIPID METABOLISM	46	0.36726105	0.36726105	0	0.40577823	1	5451	tags=48%, list=11%, signal=54%
REACTOME_MET ACTIVATES RAS SIGNALING	REACTOME_MET ACTIVATES RAS SIGNALING	11	0.36704218	0.36704218	0.0506586	0.40523508	1	8739	tags=55%, list=18%, signal=66%
REACTOME_NRCAM	REACTOME_NRCAM INTERACTIONS	7	0.36639574	0.36639574	0.2237903	0.4062267	1	17036	tags=71%, list=35%, signal=110%
REACTOME_PHOSPHATE BOND HYDROLYSIS BY NTPDASE PROTEINS	REACTOME_PHOSPHATE BOND HYDROLYSIS BY NTPDASE PROTEINS	8	0.36621422	0.36621422	0.1819106	0.4055142	1	18794	tags=75%, list=38%, signal=122%
REACTOME_N-GLYCAN TRIMMING IN THE ER AND CALNEXIN/CALRETICULIN CYCLE	REACTOME_N-GLYCAN TRIMMING IN THE ER AND CALNEXIN/CALRETICULIN CYCLE	35	0.36212957	0.36212957	9.93E-04	0.41893065	1	10260	tags=57%, list=21%, signal=72%
REACTOME_CREB PHOSPHORYLATION	REACTOME_CREB PHOSPHORYLATION	7	0.36100334	0.36100334	0.2535787	0.42165908	1	3310	tags=43%, list=7%, signal=46%

REACTOME_DEX/H-BOX HELICASES ACTIVATE TYPE I IFN AND INFLAMMATORY CYTOKINES PRODUCTION	REACTOME_DEX/H-BOX HELICASES ACTIVATE TYPE I IFN AND INFLAMMATORY CYTOKINES PRODUCTION	13	0.3603986	0.3603986	0.0510204	0.42254743	1	4956	tags=46%, list=10%, signal=51%
REACTOME_RETROGRADE NEUROTROPHIN SIGNALLING	REACTOME_RETROGRADE NEUROTROPHIN SIGNALLING	14	0.3597884	0.3597884	0.0362538	0.42346293	1	10367	tags=57%, list=21%, signal=72%
REACTOME_DISEASES ASSOCIATED WITH VISUAL	REACTOME_DISEASES ASSOCIATED WITH VISUAL TRANSDUCTION	7	0.35943055	0.35943055	0.2632114	0.42341834	1	3387	tags=43%, list=7%, signal=46%
REACTOME_RETINOID CYCLE DISEASE EVENTS	REACTOME_RETINOID CYCLE DISEASE EVENTS	7	0.35943055	0.35943055	0.2306911	0.42205247	1	3387	tags=43%, list=7%, signal=46%
REACTOME_PROCESSIVE SYNTHESIS ON THE LAGGING STRAND	REACTOME_PROCESSIVE SYNTHESIS ON THE LAGGING STRAND	15	0.35886282	0.35886282	0.0319767	0.42271984	1	15076	tags=67%, list=31%, signal=96%
REACTOME_SIGNAL TRANSDUCTION BY L1	REACTOME_SIGNAL TRANSDUCTION BY L1	21	0.35877058	0.35877058	0.0050302	0.42170224	1	12751	tags=62%, list=26%, signal=84%
REACTOME_EICOSANOID LIGAND-BINDING RECEPTORS	REACTOME_EICOSANOID LIGAND-BINDING RECEPTORS	15	0.35672456	0.35672456	0.0363815	0.42784035	1	8652	tags=53%, list=18%, signal=65%
REACTOME_NICD TRAFFICS TO NUCLEUS	REACTOME_NICD TRAFFICS TO NUCLEUS	13	0.35632396	0.35632396	0.0546341	0.42792612	1	8922	tags=54%, list=18%, signal=66%
REACTOME_NOTCH-HLH TRANSCRIPTION PATHWAY	REACTOME_NOTCH-HLH TRANSCRIPTION PATHWAY	13	0.35632396	0.35632396	0.0608108	0.4265676	1	8922	tags=54%, list=18%, signal=66%
REACTOME_INTERACTION WITH THE ZONA PELLUCIDA	REACTOME_INTERACTION WITH THE ZONA PELLUCIDA	10	0.3550097	0.3550097	0.14	0.42982212	1	26688	tags=90%, list=55%, signal=198%
REACTOME_RIP-MEDIATED NFKB ACTIVATION VIA ZBP1	REACTOME_RIP-MEDIATED NFKB ACTIVATION VIA ZBP1	23	0.35338014	0.35338014	0.0050607	0.43449453	1	3993	tags=43%, list=8%, signal=47%
REACTOME_TERMINATION OF O-GLYCAN BIOSYNTHESIS	REACTOME_TERMINATION OF O-GLYCAN BIOSYNTHESIS	25	0.35332653	0.35332653	0.0020387	0.4333078	1	8169	tags=52%, list=17%, signal=62%
REACTOME_NOD1/2 SIGNALING PATHWAY	REACTOME_NOD1/2 SIGNALING PATHWAY	31	0.35192892	0.35192892	0	0.43715876	1	11209	tags=58%, list=23%, signal=75%
REACTOME_RUNX3 REGULATES P14-ARF	REACTOME_RUNX3 REGULATES P14-ARF	10	0.35087326	0.35087326	0.1150713	0.4396838	1	12201	tags=60%, list=25%, signal=80%
REACTOME_XBP1(S) ACTIVATES CHAPERONE GENES	REACTOME_XBP1(S) ACTIVATES CHAPERONE GENES	53	0.35076934	0.35076934	0	0.43874648	1	5015	tags=45%, list=10%, signal=50%
REACTOME_PLATELET DEGRANULATION	REACTOME_PLATELET DEGRANULATION	128	0.35021618	0.35021618	0	0.43938982	1	12351	tags=60%, list=25%, signal=80%
REACTOME_NEF AND SIGNAL TRANSDUCTION	REACTOME_NEF AND SIGNAL TRANSDUCTION	8	0.34938312	0.34938312	0.2492401	0.44109482	1	19618	tags=75%, list=40%, signal=125%
REACTOME_APOBEC3G MEDIATED RESISTANCE TO HIV-1	REACTOME_APOBEC3G MEDIATED RESISTANCE TO HIV-1 INFECTION	5	0.34932598	0.34932598	0.4582485	0.43997332	1	12275	tags=60%, list=25%, signal=80%
REACTOME_RUNX1 REGULATES EXPRESSION OF COMPONENTS OF TIGHT JUNCTIONS	REACTOME_RUNX1 REGULATES EXPRESSION OF COMPONENTS OF TIGHT JUNCTIONS	5	0.34683415	0.34683415	0.4556098	0.4479447	1	2604	tags=40%, list=5%, signal=42%
REACTOME_RUNX1 REGULATES TRANSCRIPTION OF GENES INVOLVED IN INTERLEUKIN SIGNALING	REACTOME_RUNX1 REGULATES TRANSCRIPTION OF GENES INVOLVED IN INTERLEUKIN SIGNALING	5	0.34683415	0.34683415	0.5	0.44657063	1	2604	tags=40%, list=5%, signal=42%
REACTOME_PHYSIOLOGICAL FACTORS	REACTOME_PHYSIOLOGICAL FACTORS	12	0.34611773	0.34611773	0.0765358	0.44795004	1	7538	tags=50%, list=15%, signal=59%
REACTOME_ACTIVATION OF MATRIX METALLOPROTEINASES	REACTOME_ACTIVATION OF MATRIX METALLOPROTEINASES	33	0.3459823	0.3459823	0.0010225	0.44709906	1	6810	tags=48%, list=14%, signal=56%
REACTOME_OVARIAN TUMOR DOMAIN PROTEASES	REACTOME_OVARIAN TUMOR DOMAIN PROTEASES	38	0.34594074	0.34594074	0	0.4458941	1	3690	tags=42%, list=8%, signal=46%

REACTOME_ACTIVATION OF IRF3/IRF7 MEDIATED BY TBK1/IKK EPSILON	REACTOME_ACTIVATION OF IRF3/IRF7 MEDIATED BY TBK1/IKK EPSILON	18	0.3457311	0.3457311	0.0243902	0.44534117	1	7559	tags=50%, list=15%, signal=59%
REACTOME_TP53 REGULATES TRANSCRIPTION OF CASPASE ACTIVATORS AND CASPASES	REACTOME_TP53 REGULATES TRANSCRIPTION OF CASPASE ACTIVATORS AND CASPASES	12	0.3454232	0.3454232	0.0833333	0.44519347	1	7572	tags=50%, list=15%, signal=59%
REACTOME_PENTOSE PHOSPHATE PATHWAY (HEXOSE MONOPHOSPHATE SHUNT)	REACTOME_PENTOSE PHOSPHATE PATHWAY (HEXOSE MONOPHOSPHATE SHUNT)	15	0.3442152	0.3442152	0.0486815	0.44852263	1	6000	tags=47%, list=12%, signal=53%
REACTOME_DISEASES ASSOCIATED WITH GLYCOSAMINOGLYCAN	REACTOME_DISEASES ASSOCIATED WITH GLYCOSAMINOGLYCAN METABOLISM	26	0.34371832	0.34371832	0.0010163	0.44906783	1	5777	tags=46%, list=12%, signal=52%
REACTOME_DEFECTIVE C1GALT1C1 CAUSES TN POLYAGGLUTINATION	REACTOME_DEFECTIVE C1GALT1C1 CAUSES TN POLYAGGLUTINATION SYNDROME (TNPS)	17	0.34331393	0.34331393	0.0240964	0.44924432	1	17758	tags=71%, list=36%, signal=111%
REACTOME_DEFECTIVE GALNT12 CAUSES COLORECTAL CANCER 1 (CRCS1)	REACTOME_DEFECTIVE GALNT12 CAUSES COLORECTAL CANCER 1 (CRCS1)	17	0.34331393	0.34331393	0.0300971	0.44790328	1	17758	tags=71%, list=36%, signal=111%
REACTOME_DEFECTIVE GALNT3 CAUSES FAMILIAL HYPERPHOSPHATEMIC TUMORAL CALCINOSIS (HFTC)	REACTOME_DEFECTIVE GALNT3 CAUSES FAMILIAL HYPERPHOSPHATEMIC TUMORAL CALCINOSIS (HFTC)	17	0.34331393	0.34331393	0.0214724	0.44657028	1	17758	tags=71%, list=36%, signal=111%
REACTOME_INTERLEUKIN-10 SIGNALING	REACTOME_INTERLEUKIN-10 SIGNALING	46	0.34318265	0.34318265	0	0.4457489	1	16209	tags=67%, list=33%, signal=101%
REACTOME_DEREGULATED CDK5 TRIGGERS MULTIPLE NEURODEGENERATIVE PATHWAYS IN ALZHEIMER'S	REACTOME_DEREGULATED CDK5 TRIGGERS MULTIPLE NEURODEGENERATIVE PATHWAYS IN ALZHEIMER'S DISEASE MODELS	22	0.3409848	0.3409848	0.0112936	0.45264742	1	5567	tags=45%, list=11%, signal=51%
REACTOME_NEURODEGENERATIVE DISEASES	REACTOME_NEURODEGENERATIVE DISEASES	22	0.3409848	0.3409848	0.0050201	0.45131215	1	5567	tags=45%, list=11%, signal=51%
REACTOME_ZINC	REACTOME_ZINC TRANSPORTERS	17	0.3400884	0.3400884	0.0317164	0.45329422	1	9275	tags=53%, list=19%, signal=65%
REACTOME_INTERLEUKIN-9 SIGNALING	REACTOME_INTERLEUKIN-9 SIGNALING	3	0.33973968	0.33973968	0.7805826	0.45340908	1	16008	tags=67%, list=33%, signal=99%
REACTOME_SIGNALING BY PTK6	REACTOME_SIGNALING BY PTK6	59	0.3393712	0.3393712	0	0.45344183	1	9959	tags=54%, list=20%, signal=68%
REACTOME_PROTON/OLIGOPEPTIDE COTRANSPORTERS	REACTOME_PROTON/OLIGOPEPTIDE COTRANSPORTERS	4	0.33852455	0.33852455	0.626601	0.45519406	1	7907	tags=50%, list=16%, signal=60%
REACTOME_LIGAND-RECEPTOR INTERACTIONS	REACTOME_LIGAND-RECEPTOR INTERACTIONS	8	0.33820495	0.33820495	0.2634731	0.45502344	1	7924	tags=50%, list=16%, signal=60%
REACTOME_HS-GAG	REACTOME_HS-GAG BIOSYNTHESIS	31	0.3377551	0.3377551	0.002006	0.45540106	1	4005	tags=42%, list=8%, signal=46%
REACTOME_RESPONSE TO ELEVATED PLATELET CYTOSOLIC CA2+	REACTOME_RESPONSE TO ELEVATED PLATELET CYTOSOLIC CA2+	133	0.33683047	0.33683047	0	0.45763665	1	7113	tags=48%, list=15%, signal=56%
REACTOME_CELL JUNCTION ORGANIZATION	REACTOME_CELL JUNCTION ORGANIZATION	87	0.33623007	0.33623007	0	0.4585941	1	13394	tags=61%, list=27%, signal=84%
REACTOME_SYNTHESIS OF EPOXY (EET) AND DIHYDROXYEICOSATRIENOIC ACIDS (DHET)	REACTOME_SYNTHESIS OF EPOXY (EET) AND DIHYDROXYEICOSATRIENOIC ACIDS (DHET)	8	0.335907	0.335907	0.2512219	0.45855284	1	32519	tags=100%, list=66%, signal=298%
REACTOME_LEADING STRAND SYNTHESIS	REACTOME_LEADING STRAND SYNTHESIS	14	0.33503914	0.33503914	0.0704663	0.46068445	1	15076	tags=64%, list=31%, signal=93%
REACTOME_POLYMERASE SWITCHING	REACTOME_POLYMERASE SWITCHING	14	0.33503914	0.33503914	0.0549793	0.4593682	1	15076	tags=64%, list=31%, signal=93%

REACTOME_POLYMERASE SWITCHING ON THE C-STRAND OF THE TELOMERE	REACTOME_POLYMERASE SWITCHING ON THE C-STRAND OF THE TELOMERE	14	0.33503914	0.33503914	0.0720632	0.45805946	1	15076	tags=64%, list=31%, signal=93%
REACTOME_REMOVAL OF THE FLAP INTERMEDIATE	REACTOME_REMOVAL OF THE FLAP INTERMEDIATE	14	0.33503914	0.33503914	0.0414079	0.45675814	1	15076	tags=64%, list=31%, signal=93%
REACTOME_ACYL CHAIN REMODELLING OF PI	REACTOME_ACYL CHAIN REMODELLING OF PI	17	0.33278054	0.33278054	0.026	0.4642657	1	12513	tags=59%, list=26%, signal=79%
REACTOME_GAP JUNCTION TRAFFICKING	REACTOME_GAP JUNCTION TRAFFICKING	28	0.33046573	0.33046573	9.75E-04	0.4724442	1	11807	tags=57%, list=24%, signal=75%
REACTOME_VLDL ASSEMBLY	REACTOME_VLDL ASSEMBLY	5	0.33018792	0.33018792	0.547379	0.4721829	1	13212	tags=60%, list=27%, signal=82%
REACTOME_TP53 REGULATES TRANSCRIPTION OF CELL DEATH GENES	REACTOME_TP53 REGULATES TRANSCRIPTION OF CELL DEATH GENES	44	0.3267583	0.3267583	0	0.48499945	1	9609	tags=52%, list=20%, signal=65%
REACTOME_5-PHOSPHORIBOSE 1-DIPHOSPHATE BIOSYNTHESIS	REACTOME_5-PHOSPHORIBOSE 1-DIPHOSPHATE BIOSYNTHESIS	3	0.32643002	0.32643002	0.8092428	0.48495108	1	338	tags=33%, list=1%, signal=34%
REACTOME_METAL ION SLC TRANSPORTERS	REACTOME_METAL ION SLC TRANSPORTERS	26	0.32621774	0.32621774	0.0112016	0.48454	1	10400	tags=54%, list=21%, signal=68%
REACTOME_TP53 REGULATES TRANSCRIPTION OF GENES INVOLVED IN G2 CELL CYCLE ARREST	REACTOME_TP53 REGULATES TRANSCRIPTION OF GENES INVOLVED IN G2 CELL CYCLE ARREST	18	0.3261941	0.3261941	0.0241206	0.48327252	1	5795	tags=44%, list=12%, signal=50%
REACTOME_ZBP1(DAI) MEDIATED INDUCTION OF TYPE I IFNS	REACTOME_ZBP1(DAI) MEDIATED INDUCTION OF TYPE I IFNS	27	0.32601872	0.32601872	0.008	0.4826433	1	3993	tags=41%, list=8%, signal=44%
REACTOME_KERATAN SULFATE BIOSYNTHESIS	REACTOME_KERATAN SULFATE BIOSYNTHESIS	28	0.3259446	0.3259446	0.007992	0.4816153	1	6782	tags=46%, list=14%, signal=54%
REACTOME_ACTIVATION, MYRISTOLYATION OF BID AND TRANSLOCATION TO	REACTOME_ACTIVATION, MYRISTOLYATION OF BID AND TRANSLOCATION TO MITOCHONDRIA	4	0.3257593	0.3257593	0.6922301	0.48105133	1	8532	tags=50%, list=17%, signal=61%
REACTOME_SIGNALING BY MET	REACTOME_SIGNALING BY MET	67	0.3257112	0.3257112	0	0.47992882	1	5997	tags=45%, list=12%, signal=51%
REACTOME_DISEASES ASSOCIATED WITH O-GLYCOSYLATION OF PROTEINS	REACTOME_DISEASES ASSOCIATED WITH O-GLYCOSYLATION OF PROTEINS	59	0.3248688	0.3248688	0	0.48215875	1	13158	tags=59%, list=27%, signal=81%
REACTOME_P130CAS LINKAGE TO MAPK SIGNALING FOR	REACTOME_P130CAS LINKAGE TO MAPK SIGNALING FOR INTEGRINS	15	0.32432416	0.32432416	0.0572597	0.48316708	1	20031	tags=73%, list=41%, signal=124%
REACTOME_SEMA4D INDUCED CELL MIGRATION AND GROWTH-CONE COLLAPSE	REACTOME_SEMA4D INDUCED CELL MIGRATION AND GROWTH-CONE COLLAPSE	24	0.32306078	0.32306078	0.0154589	0.48703265	1	12751	tags=58%, list=26%, signal=79%
REACTOME_ATF6 (ATF6-ALPHA) ACTIVATES CHAPERONE GENES	REACTOME_ATF6 (ATF6-ALPHA) ACTIVATES CHAPERONE GENES	9	0.32244602	0.32244602	0.229572	0.48819318	1	535	tags=33%, list=1%, signal=34%
REACTOME_CONSTITUTIVE SIGNALING BY EGFRVIII	REACTOME_CONSTITUTIVE SIGNALING BY EGFRVIII	15	0.32221317	0.32221317	0.0761079	0.4878116	1	7077	tags=47%, list=14%, signal=55%
REACTOME_SIGNALING BY EGFRVIII IN CANCER	REACTOME_SIGNALING BY EGFRVIII IN CANCER	15	0.32221317	0.32221317	0.0884692	0.4864896	1	7077	tags=47%, list=14%, signal=55%
REACTOME_ACTIVATION OF CASPASES THROUGH APOPTOSOME-MEDIATED	REACTOME_ACTIVATION OF CASPASES THROUGH APOPTOSOME-MEDIATED CLEAVAGE	5	0.3221201	0.3221201	0.5777549	0.48554355	1	3814	tags=40%, list=8%, signal=43%
REACTOME_CYTOCHROME C-MEDIATED APOPTOTIC	REACTOME_CYTOCHROME C-MEDIATED APOPTOTIC RESPONSE	5	0.3221201	0.3221201	0.5888224	0.4842348	1	3814	tags=40%, list=8%, signal=43%
REACTOME_SIGNALING BY [illegible]	REACTOME_SIGNALING BY HIPPO	20	0.32204005	0.32204005	0.0256158	0.48329425	1	16064	tags=65%, list=33%, signal=97%
REACTOME_MET RECEPTOR ACTIVATION	REACTOME_MET RECEPTOR ACTIVATION	6	0.3215446	0.3215446	0.4699507	0.48419136	1	8739	tags=50%, list=18%, signal=61%
REACTOME_CHEMOKINE RECEPTORS BIND CHEMOKINES	REACTOME_CHEMOKINE RECEPTORS BIND CHEMOKINES	48	0.32017118	0.32017118	0	0.488584	1	11880	tags=56%, list=24%, signal=74%

REACTOME_P75NTR RECRUITS SIGNALLING COMPLEXES	REACTOME_P75NTR RECRUITS SIGNALLING COMPLEXES	13	0.31900167	0.31900167	0.1118744	0.4921977	1	10749	tags=54%, list=22%, signal=69%	
REACTOME_RORA ACTIVATES GENE EXPRESSION	REACTOME_RORA ACTIVATES GENE EXPRESSION	27	0.31864506	0.31864506	0.0049702	0.49239174	1	18862	tags=70%, list=39%, signal=114%	
REACTOME_FMO OXIDISES NUCLEOPHILES	REACTOME_FMO OXIDISES NUCLEOPHILES	3	0.3183898	0.3183898	0.8087649	0.492166	1	33375	tags=100%, list=68%, signal=314%	
REACTOME_TGF-BETA RECEPTOR SIGNALING IN EMT (EPITHELIAL TO MESENCHYMAL	REACTOME_TGF-BETA RECEPTOR SIGNALING IN EMT (EPITHELIAL TO MESENCHYMAL TRANSITION)	16	0.3183556	0.3183556	0.0634441	0.49099642	1	5838	tags=44%, list=12%, signal=50%	
REACTOME_PRE-NOTCH EXPRESSION AND PROCESSING	REACTOME_PRE-NOTCH EXPRESSION AND PROCESSING	48	0.31807962	0.31807962	0	0.4908356	1	8922	tags=50%, list=18%, signal=61%	
REACTOME_IL-6-TYPE CYTOKINE RECEPTOR LIGAND	REACTOME_IL-6-TYPE CYTOKINE RECEPTOR LIGAND INTERACTIONS	17	0.3177586	0.3177586	0.0471888	0.49087837	1	10368	tags=53%, list=21%, signal=67%	
REACTOME_DNA STRAND ELONGATION	REACTOME_DNA STRAND ELONGATION	32	0.31729355	0.31729355	0.004012	0.49152935	1	15076	tags=63%, list=31%, signal=90%	
REACTOME_INTERLEUKIN-4 AND 13 SIGNALING	REACTOME_INTERLEUKIN-4 AND 13 SIGNALING	111	0.31725067	0.31725067	0	0.49039626	1	19349	tags=71%, list=40%, signal=117%	
REACTOME_DEFECTS IN COBALAMIN (B12) METABOLISM	REACTOME_DEFECTS IN COBALAMIN (B12) METABOLISM	11	0.31695434	0.31695434	0.1525424	0.49033982	1	11191	tags=55%, list=23%, signal=71%	
REACTOME_DNA DAMAGE REVERSAL	REACTOME_DNA DAMAGE REVERSAL	8	0.31692863	0.31692863	0.3190377	0.48916465	1	2845	tags=38%, list=6%, signal=40%	
REACTOME_P75NTR NEGATIVELY REGULATES CELL [illegible]	REACTOME_P75NTR NEGATIVELY REGULATES CELL CYCLE VIA SC1	6	0.3162306	0.3162306	0.4829015	0.4908527	1	17160	tags=67%, list=35%, signal=103%	
REACTOME_GLYCOGEN	REACTOME_GLYCOGEN SYNTHESIS	16	0.3148966	0.3148966	0.0800801	0.49507472	1	2947	tags=38%, list=6%, signal=40%	
REACTOME_POST-TRANSCRIPTIONAL SILENCING BY SMALL RNAS	REACTOME_POST-TRANSCRIPTIONAL SILENCING BY SMALL RNAS	3	0.3145773	0.3145773	0.8481519	0.49507976	1	17240	tags=67%, list=35%, signal=103%	
REACTOME_PLASMA LIPOPROTEIN ASSEMBLY, REMODELING, AND CLEARANCE	REACTOME_PLASMA LIPOPROTEIN ASSEMBLY, REMODELING, AND CLEARANCE	71	0.3134579	0.3134579	0	0.49850306	1	13638	tags=59%, list=28%, signal=82%	
REACTOME_RESOLUTION OF D-LOOP STRUCTURES	REACTOME_RESOLUTION OF D-LOOP STRUCTURES	32	0.31262007	0.31262007	0.003012	0.50095123	1	18365	tags=69%, list=38%, signal=110%	
REACTOME_JOSEPHIN DOMAIN DUBS	REACTOME_JOSEPHIN DOMAIN DUBS	12	0.31214637	0.31214637	0.1538462	0.50158936	1	9201	tags=50%, list=19%, signal=62%	
REACTOME_INTERFERON ALPHA/BETA SIGNALING	REACTOME_INTERFERON ALPHA/BETA SIGNALING	67	0.31174183	0.31174183	0	0.50209254	1	11065	tags=54%, list=23%, signal=69%	
REACTOME_PURINE	REACTOME_PURINE CATABOLISM	18	0.3101972	0.3101972	0.0506823	0.5071841	1	6578	tags=44%, list=13%, signal=51%	
REACTOME_GAMMA-CARBOXYLATION, TRANSPORT, AND AMINO-TERMINAL CLEAVAGE OF PROTEINS	REACTOME_GAMMA-CARBOXYLATION, TRANSPORT, AND AMINO-TERMINAL CLEAVAGE OF PROTEINS	11	0.30968776	0.30968776	0.1880597	0.5080683	1	2644	tags=36%, list=5%, signal=38%	
REACTOME_LOSS OF FUNCTION OF SMAD2/3 IN CANCER	REACTOME_LOSS OF FUNCTION OF SMAD2/3 IN CANCER	7	0.30936724	0.30936724	0.4157303	0.5081307	1	5838	tags=43%, list=12%, signal=49%	
REACTOME_SMAD2/3 MH2 DOMAIN MUTANTS IN CANCER	REACTOME_SMAD2/3 MH2 DOMAIN MUTANTS IN CANCER	7	0.30936724	0.30936724	0.4249513	0.50684434	1	5838	tags=43%, list=12%, signal=49%	
REACTOME_GROWTH HORMONE RECEPTOR SIGNALING	REACTOME_GROWTH HORMONE RECEPTOR SIGNALING	22	0.30915186	0.30915186	0.0258373	0.5064254	1	7125	tags=45%, list=15%, signal=53%	
REACTOME_PTK6 REGULATES CELL CYCLE	REACTOME_PTK6 REGULATES CELL CYCLE	6	0.30687925	0.30687925	0.5341797	0.51457417	1	9457	tags=50%, list=19%, signal=62%	
REACTOME_O-LINKED GLYCOSYLATION OF MUCINS	REACTOME_O-LINKED GLYCOSYLATION OF MUCINS	65	0.30573383	0.30573383	0	0.51821655	1	12921	tags=57%, list=26%, signal=77%	

REACTOME_CYTOSOLIC TRNA AMINOACYLATION	REACTOME_CYTOSOLIC TRNA AMINOACYLATION	24	0.30567676	0.30567676	0.019861	0.51715195	1	15642	tags=63%, list=32%, signal=92%
REACTOME_GAP JUNCTION TRAFFICKING AND REGULATION	REACTOME_GAP JUNCTION TRAFFICKING AND REGULATION	30	0.305429	0.305429	0.0070922	0.5168886	1	16064	tags=63%, list=33%, signal=94%
REACTOME_HIGHLY CALCIUM PERMEABLE POSTSYNAPTIC NICOTINIC ACETYLCHOLINE RECEPTORS	REACTOME_HIGHLY CALCIUM PERMEABLE POSTSYNAPTIC NICOTINIC ACETYLCHOLINE RECEPTORS	11	0.30426154	0.30426154	0.1935484	0.5204392	1	7361	tags=45%, list=15%, signal=53%
REACTOME_DOWNREGULATION OF ERBB2 SIGNALING	REACTOME_DOWNREGULATION OF ERBB2 SIGNALING	29	0.3039036	0.3039036	0.0069583	0.52065074	1	7077	tags=45%, list=14%, signal=52%
REACTOME_P38MAPK EVENTS	REACTOME_P38MAPK EVENTS	13	0.30358154	0.30358154	0.1329423	0.5206759	1	26570	tags=85%, list=54%, signal=185%
REACTOME_MRNA EDITING	REACTOME_MRNA EDITING	9	0.3032973	0.3032973	0.2979146	0.5206047	1	6913	tags=44%, list=14%, signal=52%
REACTOME_RESOLUTION OF D-LOOP STRUCTURES THROUGH HOLLIDAY JUNCTION	REACTOME_RESOLUTION OF D-LOOP STRUCTURES THROUGH HOLLIDAY JUNCTION INTERMEDIATES	31	0.30252662	0.30252662	0.0076923	0.52266186	1	18365	tags=68%, list=38%, signal=108%
REACTOME_RHO GTPASES ACTIVATE ROCKS	REACTOME_RHO GTPASES ACTIVATE ROCKS	18	0.3017595	0.3017595	0.0574018	0.52467215	1	6991	tags=44%, list=14%, signal=52%
REACTOME_OTHER INTERLEUKIN SIGNALING	REACTOME_OTHER INTERLEUKIN SIGNALING	34	0.3012928	0.3012928	0.0020222	0.5254317	1	29901	tags=91%, list=61%, signal=234%
REACTOME_CARGO CONCENTRATION IN THE ER	REACTOME_CARGO CONCENTRATION IN THE ER	33	0.3010963	0.3010963	0.0038835	0.52499	1	4555	tags=39%, list=9%, signal=43%
REACTOME_WAX BIOSYNTHESIS	REACTOME_WAX BIOSYNTHESIS	4	0.3010917	0.3010917	0.7664974	0.5237187	1	21981	tags=75%, list=45%, signal=136%
REACTOME_REGULATION OF TP53 ACTIVITY THROUGH ASSOCIATION WITH CO-	REACTOME_REGULATION OF TP53 ACTIVITY THROUGH ASSOCIATION WITH CO-FACTORS	14	0.30081105	0.30081105	0.13381	0.5236963	1	20249	tags=71%, list=41%, signal=122%
REACTOME_ESTABLISHMENT OF SISTER CHROMATID COHESION	REACTOME_ESTABLISHMENT OF SISTER CHROMATID COHESION	11	0.3005326	0.3005326	0.208498	0.5236051	1	25349	tags=82%, list=52%, signal=170%
REACTOME_NUCLEOTIDE-LIKE (PURINERGIC) RECEPTORS	REACTOME_NUCLEOTIDE-LIKE (PURINERGIC) RECEPTORS	15	0.30039498	0.30039498	0.1009901	0.5229677	1	8145	tags=47%, list=17%, signal=56%
REACTOME_GAP JUNCTION ASSEMBLY	REACTOME_GAP JUNCTION ASSEMBLY	19	0.30032992	0.30032992	0.0571705	0.52198696	1	8493	tags=47%, list=17%, signal=57%
REACTOME_REGULATION OF RUNX1 EXPRESSION AND	REACTOME_REGULATION OF RUNX1 EXPRESSION AND ACTIVITY	17	0.2999582	0.2999582	0.075	0.52229303	1	8359	tags=47%, list=17%, signal=57%
REACTOME_DDX58/IFIH1-MEDIATED INDUCTION OF INTERFERON-ALPHA/BETA	REACTOME_DDX58/IFIH1-MEDIATED INDUCTION OF INTERFERON-ALPHA/BETA	80	0.29988238	0.29988238	0	0.52132076	1	12270	tags=55%, list=25%, signal=73%
REACTOME_TRNA	REACTOME_TRNA AMINOACYLATION	42	0.29983172	0.29983172	0	0.5203068	1	15642	tags=62%, list=32%, signal=91%
REACTOME_EPH-EPHRIN	REACTOME_EPH-EPHRIN SIGNALING	95	0.29930213	0.29930213	0	0.52134675	1	12174	tags=55%, list=25%, signal=73%
REACTOME_GAMMA CARBOXYLATION, HYPUSINE FORMATION AND ARYLSULFATASE ACTIVATION	REACTOME_GAMMA CARBOXYLATION, HYPUSINE FORMATION AND ARYLSULFATASE ACTIVATION	39	0.29914984	0.29914984	0	0.5206644	1	5451	tags=41%, list=11%, signal=46%
REACTOME_METABOLISM OF CARBOHYDRATES	REACTOME_METABOLISM OF CARBOHYDRATES	271	0.29678404	0.29678404	0	0.5294922	1	7410	tags=45%, list=15%, signal=52%
REACTOME_DISEASES OF GLYCOSYLATION	REACTOME_DISEASES OF GLYCOSYLATION	85	0.2964776	0.2964776	0	0.5295876	1	13158	tags=56%, list=27%, signal=77%
REACTOME_UNFOLDED PROTEIN RESPONSE (UPR)	REACTOME_UNFOLDED PROTEIN RESPONSE (UPR)	93	0.2958283	0.2958283	0	0.5311535	1	7128	tags=44%, list=15%, signal=51%
REACTOME_ARACHIDONATE PRODUCTION FROM DAG	REACTOME_ARACHIDONATE PRODUCTION FROM DAG	5	0.2956495	0.2956495	0.6901408	0.5307092	1	24696	tags=80%, list=50%, signal=161%
REACTOME_CYTOSOLIC IRON-SULFUR CLUSTER ASSEMBLY	REACTOME_CYTOSOLIC IRON-SULFUR CLUSTER ASSEMBLY	13	0.2951714	0.2951714	0.1914894	0.5316405	1	8149	tags=46%, list=17%, signal=55%
REACTOME_NECTIN/NECL TRANS HETERODIMERIZATION	REACTOME_NECTIN/NECL TRANS HETERODIMERIZATION	7	0.29433396	0.29433396	0.4744233	0.5341229	1	20564	tags=71%, list=42%, signal=123%
REACTOME_GLYCOLYSIS	REACTOME_GLYCOLYSIS	70	0.29320994	0.29320994	0	0.53766733	1	7347	tags=44%, list=15%, signal=52%

REACTOME_CLASSICAL ANTIBODY-MEDIATED COMPLEMENT ACTIVATION	REACTOME_CLASSICAL ANTIBODY-MEDIATED COMPLEMENT ACTIVATION	83	0.29315418	0.29315418	0	0.5366456	1	25195	tags=81%, list=51%, signal=166%
REACTOME_RHO GTPASES ACTIVATE PAKS	REACTOME_RHO GTPASES ACTIVATE PAKS	21	0.29294705	0.29294705	0.0360531	0.5362289	1	20636	tags=71%, list=42%, signal=123%
REACTOME_LAGGING STRAND SYNTHESIS	REACTOME_LAGGING STRAND SYNTHESIS	20	0.29220554	0.29220554	0.0540541	0.53817743	1	15076	tags=60%, list=31%, signal=87%
REACTOME_WNT MEDIATED ACTIVATION OF DVL	REACTOME_WNT MEDIATED ACTIVATION OF DVL	8	0.29210836	0.29210836	0.4041096	0.53731424	1	22422	tags=75%, list=46%, signal=138%
REACTOME_REGULATION OF CHOLESTEROL BIOSYNTHESIS BY SREBP (SREBF)	REACTOME_REGULATION OF CHOLESTEROL BIOSYNTHESIS BY SREBP (SREBF)	55	0.29066187	0.29066187	0	0.5421619	1	6259	tags=42%, list=13%, signal=48%
REACTOME_INTERLEUKIN-35 SIGNALLING	REACTOME_INTERLEUKIN-35 SIGNALLING	7	0.29002416	0.29002416	0.5093966	0.54367155	1	34765	tags=100%, list=71%, signal=345%
REACTOME_ACYL CHAIN REMODELLING OF PS	REACTOME_ACYL CHAIN REMODELLING OF PS	22	0.29001456	0.29001456	0.0392562	0.54246366	1	12513	tags=55%, list=26%, signal=73%
REACTOME_MET ACTIVATES	REACTOME_MET ACTIVATES PTPN11	5	0.28968546	0.28968546	0.6900585	0.54264116	1	34781	tags=100%, list=71%, signal=345%
REACTOME_ORGANIC CATION TRANSPORT	REACTOME_ORGANIC CATION TRANSPORT	9	0.28930515	0.28930515	0.3564357	0.54304224	1	7598	tags=44%, list=16%, signal=53%
REACTOME_SIGNALING BY	REACTOME_SIGNALING BY ACTIVIN	13	0.28858253	0.28858253	0.1863231	0.5449764	1	4705	tags=38%, list=10%, signal=43%
REACTOME_RECYCLING PATHWAY OF L1	REACTOME_RECYCLING PATHWAY OF L1	28	0.28842187	0.28842187	0.0110664	0.54445636	1	10367	tags=50%, list=21%, signal=63%
REACTOME_O-LINKED GLYCOSYLATION	REACTOME_O-LINKED GLYCOSYLATION	114	0.28835642	0.28835642	0	0.5435201	1	10395	tags=50%, list=21%, signal=63%
REACTOME_REGULATION OF KIT SIGNALING	REACTOME_REGULATION OF KIT SIGNALING	16	0.28815323	0.28815323	0.118593	0.5431579	1	13437	tags=56%, list=27%, signal=77%
REACTOME_REACTIONS SPECIFIC TO THE COMPLEX N-GLYCAN SYNTHESIS PATHWAY	REACTOME_REACTIONS SPECIFIC TO THE COMPLEX N-GLYCAN SYNTHESIS PATHWAY	10	0.2870289	0.2870289	0.3346939	0.5467845	1	20223	tags=70%, list=41%, signal=119%
REACTOME_PRE-NOTCH TRANSCRIPTION AND	REACTOME_PRE-NOTCH TRANSCRIPTION AND TRANSLATION	32	0.28670517	0.28670517	0.009901	0.54692364	1	8922	tags=47%, list=18%, signal=57%
REACTOME_SIGNALING BY	REACTOME_SIGNALING BY NOTCH2	32	0.28670517	0.28670517	0.0133333	0.54568344	1	8922	tags=47%, list=18%, signal=57%
REACTOME_VITAMINS B6 ACTIVATION TO PYRIDOXAL PHOSPHATE	REACTOME_VITAMINS B6 ACTIVATION TO PYRIDOXAL PHOSPHATE	3	0.28639898	0.28639898	0.9170041	0.5457979	1	2298	tags=33%, list=5%, signal=35%
REACTOME_TAK1 ACTIVATES NFKB BY PHOSPHORYLATION AND ACTIVATION OF IKKS	REACTOME_TAK1 ACTIVATES NFKB BY PHOSPHORYLATION AND ACTIVATION OF IKKS COMPLEX	30	0.28527302	0.28527302	0.0092402	0.54937786	1	3993	tags=37%, list=8%, signal=40%
REACTOME_MET ACTIVATES RAP1 AND RAC1	REACTOME_MET ACTIVATES RAP1 AND RAC1	11	0.28508767	0.28508767	0.2602339	0.5489587	1	12751	tags=55%, list=26%, signal=74%
REACTOME_INTEGRIN ALPHAIIB BETA3 SIGNALING	REACTOME_INTEGRIN ALPHAIIB BETA3 SIGNALING	27	0.28506893	0.28506893	0.0219342	0.5477947	1	5997	tags=41%, list=12%, signal=46%
REACTOME_GRB7 EVENTS IN ERBB2 SIGNALING	REACTOME_GRB7 EVENTS IN ERBB2 SIGNALING	5	0.2850286	0.2850286	0.7175421	0.5467253	1	5630	tags=40%, list=11%, signal=45%
REACTOME_ER QUALITY CONTROL COMPARTMENT	REACTOME_ER QUALITY CONTROL COMPARTMENT (ERQC)	21	0.28476763	0.28476763	0.0561685	0.5466526	1	9378	tags=48%, list=19%, signal=59%
REACTOME_HSF1-DEPENDENT TRANSACTIVATION	REACTOME_HSF1-DEPENDENT TRANSACTIVATION	34	0.28333655	0.28333655	0.0112821	0.5517544	1	14938	tags=59%, list=31%, signal=85%
REACTOME_INTERFERON GAMMA SIGNALING	REACTOME_INTERFERON GAMMA SIGNALING	91	0.2830245	0.2830245	0	0.5519354	1	10918	tags=51%, list=22%, signal=65%
REACTOME_REGULATION OF INNATE IMMUNE RESPONSES TO CYTOSOLIC DNA	REACTOME_REGULATION OF INNATE IMMUNE RESPONSES TO CYTOSOLIC DNA	14	0.28219038	0.28219038	0.1831276	0.55434406	1	3673	tags=36%, list=8%, signal=39%
REACTOME_MTF1 ACTIVATES GENE EXPRESSION	REACTOME_MTF1 ACTIVATES GENE EXPRESSION	3	0.2820691	0.2820691	0.9322866	0.5536595	1	2510	tags=33%, list=5%, signal=35%

REACTOME_INITIAL TRIGGERING OF COMPLEMENT	REACTOME_INITIAL TRIGGERING OF COMPLEMENT	98	0.28191096	0.28191096	0	0.5530564	1	25195	tags=80%, list=51%, signal=164%
REACTOME_EGFR TRANSACTIVATION BY GASTRIN	REACTOME_EGFR TRANSACTIVATION BY GASTRIN	9	0.28156805	0.28156805	0.3985944	0.55336887	1	29739	tags=89%, list=61%, signal=226%
REACTOME_EPH-EPHRIN MEDIATED REPULSION OF CELLS	REACTOME_EPH-EPHRIN MEDIATED REPULSION OF CELLS	48	0.28151172	0.28151172	0.0019666	0.55239636	1	12751	tags=54%, list=26%, signal=73%
REACTOME_ADVANCED GLYCOSYLATION ENDPRODUCT RECEPTOR SIGNALING	REACTOME_ADVANCED GLYCOSYLATION ENDPRODUCT RECEPTOR SIGNALING	13	0.28119698	0.28119698	0.2077922	0.5525913	1	1300	tags=31%, list=3%, signal=32%
REACTOME_CHOLINE	REACTOME_CHOLINE CATABOLISM	6	0.28053066	0.28053066	0.6632653	0.5541742	1	10747	tags=50%, list=22%, signal=64%
REACTOME_MAP3K8 (TPL2)-DEPENDENT MAPK1/3	REACTOME_MAP3K8 (TPL2)-DEPENDENT MAPK1/3 ACTIVATION	16	0.28041813	0.28041813	0.1189516	0.55345047	1	7695	tags=44%, list=16%, signal=52%
REACTOME_METABOLISM OF NITRIC OXIDE	REACTOME_METABOLISM OF NITRIC OXIDE	20	0.2802993	0.2802993	0.0651068	0.5527724	1	8314	tags=45%, list=17%, signal=54%
REACTOME_ENOS ACTIVATION AND REGULATION	REACTOME_ENOS ACTIVATION AND REGULATION	20	0.2802993	0.2802993	0.0618661	0.5515681	1	8314	tags=45%, list=17%, signal=54%
REACTOME_PECAM1	REACTOME_PECAM1 INTERACTIONS	12	0.2802263	0.2802263	0.2589852	0.55068994	1	14844	tags=58%, list=30%, signal=84%
REACTOME_RUNX1 REGULATES TRANSCRIPTION OF GENES INVOLVED IN BCR SIGNALING	REACTOME_RUNX1 REGULATES TRANSCRIPTION OF GENES INVOLVED IN BCR SIGNALING	6	0.28016642	0.28016642	0.6669951	0.54973775	1	2604	tags=33%, list=5%, signal=35%
REACTOME_PKMTS METHYLATE HISTONE LYSINES	REACTOME_PKMTS METHYLATE HISTONE LYSINES	45	0.2800945	0.2800945	9.90E-04	0.54886705	1	8059	tags=44%, list=16%, signal=53%
REACTOME_RESOLUTION OF D-LOOP STRUCTURES THROUGH SYNTHESIS-DEPENDENT STRAND ANNEALING (SDSA)	REACTOME_RESOLUTION OF D-LOOP STRUCTURES THROUGH SYNTHESIS-DEPENDENT STRAND ANNEALING (SDSA)	26	0.27891004	0.27891004	0.025641	0.55281675	1	18365	tags=65%, list=38%, signal=105%
REACTOME_ACTIVATED NOTCH1 TRANSMITS SIGNAL TO THE NUCLEUS	REACTOME_ACTIVATED NOTCH1 TRANSMITS SIGNAL TO THE NUCLEUS	30	0.27886313	0.27886313	0.0091556	0.5518093	1	7571	tags=43%, list=15%, signal=51%
REACTOME_TIGHT JUNCTION INTERACTIONS	REACTOME_TIGHT JUNCTION INTERACTIONS	29	0.27865317	0.27865317	0.0173077	0.55161065	1	13378	tags=55%, list=27%, signal=76%
REACTOME_CRMPS IN SEMA3A SIGNALING	REACTOME_CRMPS IN SEMA3A SIGNALING	16	0.27849138	0.27849138	0.1325301	0.5511438	1	4729	tags=38%, list=10%, signal=41%
REACTOME_REGULATION OF THYROID HORMONE ACTIVITY	REACTOME_REGULATION OF THYROID HORMONE ACTIVITY	3	0.27817494	0.27817494	0.9381339	0.5514128	1	35344	tags=100%, list=72%, signal=359%
REACTOME_ASSEMBLY OF ACTIVE LPL AND LIPC LIPASE	REACTOME_ASSEMBLY OF ACTIVE LPL AND LIPC LIPASE COMPLEXES	19	0.277797	0.277797	0.0861723	0.55191493	1	12173	tags=53%, list=25%, signal=70%
REACTOME_TP53 REGULATES TRANSCRIPTION OF CELL CYCLE GENES	REACTOME_TP53 REGULATES TRANSCRIPTION OF CELL CYCLE GENES	48	0.27777836	0.27777836	9.71E-04	0.55081356	1	6813	tags=42%, list=14%, signal=48%
REACTOME_AUTOINTEGRATION RESULTS IN VIRAL DNA CIRCLES	REACTOME_AUTOINTEGRATION RESULTS IN VIRAL DNA CIRCLES	3	0.27757582	0.27757582	0.9324727	0.5505357	1	2730	tags=33%, list=6%, signal=35%
REACTOME_INTEGRATION OF VIRAL DNA INTO HOST GENOMIC	REACTOME_INTEGRATION OF VIRAL DNA INTO HOST GENOMIC DNA	3	0.27757582	0.27757582	0.9424032	0.5493668	1	2730	tags=33%, list=6%, signal=35%
REACTOME_METABOLISM OF FOLATE AND PTERINES	REACTOME_METABOLISM OF FOLATE AND PTERINES	17	0.27753222	0.27753222	0.1166181	0.54839236	1	12337	tags=53%, list=25%, signal=71%
REACTOME_SMAD2/SMAD3:SMAD4 HETEROTRIMER REGULATES TRANSCRIPTION	REACTOME_SMAD2/SMAD3:SMAD4 HETEROTRIMER REGULATES TRANSCRIPTION	32	0.2773595	0.2773595	0.0117073	0.54801863	1	6319	tags=41%, list=13%, signal=47%
REACTOME_BASIGIN	REACTOME_BASIGIN INTERACTIONS	25	0.27693093	0.27693093	0.0404858	0.5486556	1	25618	tags=80%, list=52%, signal=168%
REACTOME_O-GLYCOSYLATION OF TSR DOMAIN-CONTAINING	REACTOME_O-GLYCOSYLATION OF TSR DOMAIN-CONTAINING PROTEINS	39	0.2767691	0.2767691	0.0071795	0.5482207	1	6546	tags=41%, list=13%, signal=47%
REACTOME_TNFR1-MEDIATED CERAMIDE PRODUCTION	REACTOME_TNFR1-MEDIATED CERAMIDE PRODUCTION	6	0.27662945	0.27662945	0.6653307	0.54765534	1	10938	tags=50%, list=22%, signal=64%

REACTOME_CONSTITUTIVE SIGNALING BY LIGAND-RESPONSIVE EGFR CANCER	REACTOME_CONSTITUTIVE SIGNALING BY LIGAND-RESPONSIVE EGFR CANCER VARIANTS	19	0.27660775	0.27660775	0.0961338	0.5466043	1	7077	tags=42%, list=14%, signal=49%
REACTOME_SIGNALING BY EGFR IN CANCER	REACTOME_SIGNALING BY EGFR IN CANCER	19	0.27660775	0.27660775	0.1126761	0.54546076	1	7077	tags=42%, list=14%, signal=49%
REACTOME_SIGNALING BY LIGAND-RESPONSIVE EGFR VARIANTS IN CANCER	REACTOME_SIGNALING BY LIGAND-RESPONSIVE EGFR VARIANTS IN CANCER	19	0.27660775	0.27660775	0.0869141	0.544322	1	7077	tags=42%, list=14%, signal=49%
REACTOME_ARYL HYDROCARBON RECEPTOR	REACTOME_ARYL HYDROCARBON RECEPTOR SIGNALLING	7	0.2765636	0.2765636	0.5693359	0.54337424	1	7444	tags=43%, list=15%, signal=51%
REACTOME_GENERATION OF SECOND MESSENGER	REACTOME_GENERATION OF SECOND MESSENGER MOLECULES	34	0.27598825	0.27598825	0.0088583	0.54474473	1	19618	tags=68%, list=40%, signal=113%
REACTOME_DEFECTIVE B3GALTL CAUSES PETERS-PLUS SYNDROME (PPS)	REACTOME_DEFECTIVE B3GALTL CAUSES PETERS-PLUS SYNDROME (PPS)	37	0.2757842	0.2757842	0.0088933	0.54449314	1	12973	tags=54%, list=26%, signal=73%
REACTOME_TELOMERE C-STRAND (LAGGING STRAND)	REACTOME_TELOMERE C-STRAND (LAGGING STRAND) SYNTHESIS	24	0.2755546	0.2755546	0.0433498	0.5443826	1	15076	tags=58%, list=31%, signal=84%
REACTOME_REGULATION OF PYRUVATE DEHYDROGENASE (PDH) COMPLEX	REACTOME_REGULATION OF PYRUVATE DEHYDROGENASE (PDH) COMPLEX	15	0.27529454	0.27529454	0.1756098	0.5443478	1	12638	tags=53%, list=26%, signal=72%
REACTOME_CATION-COUPLED CHLORIDE COTRANSPORTERS	REACTOME_CATION-COUPLED CHLORIDE COTRANSPORTERS	7	0.27460274	0.27460274	0.5625	0.5460637	1	545	tags=29%, list=1%, signal=29%
REACTOME_FICOLINS BIND TO REPETITIVE CARBOHYDRATE STRUCTURES ON THE TARGET CELL SURFACE	REACTOME_FICOLINS BIND TO REPETITIVE CARBOHYDRATE STRUCTURES ON THE TARGET CELL SURFACE	5	0.27422386	0.27422386	0.7517803	0.54651946	1	6159	tags=40%, list=13%, signal=46%
REACTOME_ION INFLUX/EFFLUX AT HOST-PATHOGEN INTERFACE	REACTOME_ION INFLUX/EFFLUX AT HOST-PATHOGEN INTERFACE	4	0.27405488	0.27405488	0.8305253	0.546125	1	35546	tags=100%, list=73%, signal=365%
REACTOME_TFAP2 (AP-2) FAMILY REGULATES TRANSCRIPTION OF GROWTH FACTORS AND THEIR RECEPTORS	REACTOME_TFAP2 (AP-2) FAMILY REGULATES TRANSCRIPTION OF GROWTH FACTORS AND THEIR RECEPTORS	13	0.27396855	0.27396855	0.2317881	0.5453385	1	16720	tags=62%, list=34%, signal=93%
REACTOME_APOPTOTIC CLEAVAGE OF CELLULAR	REACTOME_APOPTOTIC CLEAVAGE OF CELLULAR PROTEINS	37	0.27364534	0.27364534	0.0069238	0.5456442	1	3814	tags=35%, list=8%, signal=38%
REACTOME_GLYCOGEN METABOLISM	REACTOME_GLYCOGEN METABOLISM	27	0.27327776	0.27327776	0.0240848	0.5460979	1	2947	tags=33%, list=6%, signal=35%
REACTOME_FCGR ACTIVATION	REACTOME_FCGR ACTIVATION	89	0.2724404	0.2724404	0	0.5486019	1	25195	tags=79%, list=51%, signal=162%
REACTOME_ATTENUATION	REACTOME_ATTENUATION PHASE	26	0.27197203	0.27197203	0.0284035	0.54950166	1	14938	tags=58%, list=31%, signal=83%
REACTOME_DOWNREGULATION OF ERBB2:ERBB3 SIGNALING	REACTOME_DOWNREGULATION OF ERBB2:ERBB3 SIGNALING	13	0.27185187	0.27185187	0.2345906	0.54892075	1	5524	tags=38%, list=11%, signal=43%
REACTOME_PHASE 3 - RAPID REPOLARISATION	REACTOME_PHASE 3 - RAPID REPOLARISATION	8	0.27174357	0.27174357	0.5055388	0.5482534	1	23419	tags=75%, list=48%, signal=144%
REACTOME_DOWNREGULATION OF ERBB4 SIGNALING	REACTOME_DOWNREGULATION OF ERBB4 SIGNALING	8	0.27110526	0.27110526	0.5315228	0.5499185	1	11209	tags=50%, list=23%, signal=65%
REACTOME_INTRA-GOLGI	REACTOME_INTRA-GOLGI TRAFFIC	44	0.27082488	0.27082488	0.0020161	0.5499557	1	14571	tags=57%, list=30%, signal=81%
REACTOME_SYNTHESIS OF SUBSTRATES IN N-GLYCAN BIOSYTHESIS	REACTOME_SYNTHESIS OF SUBSTRATES IN N-GLYCAN BIOSYTHESIS	63	0.2708206	0.2708206	0	0.54886997	1	10072	tags=48%, list=21%, signal=60%
REACTOME_REGULATION OF COMPLEMENT CASCADE	REACTOME_REGULATION OF COMPLEMENT CASCADE	121	0.27009073	0.27009073	0	0.5507581	1	32939	tags=94%, list=67%, signal=287%
REACTOME_NOTCH2 INTRACELLULAR DOMAIN REGULATES TRANSCRIPTION	REACTOME_NOTCH2 INTRACELLULAR DOMAIN REGULATES TRANSCRIPTION	12	0.2697877	0.2697877	0.2865672	0.55092525	1	15355	tags=58%, list=31%, signal=85%
REACTOME_RHO GTPASES ACTIVATE NADPH OXIDASES	REACTOME_RHO GTPASES ACTIVATE NADPH OXIDASES	13	0.26926064	0.26926064	0.2414487	0.5520265	1	20717	tags=69%, list=42%, signal=120%

REACTOME_BICARBONATE TRANSPORTERS	REACTOME_BICARBONATE TRANSPORTERS	10	0.26833826	0.26833826	0.3830898	0.5547749	1	1552	tags=30%, list=3%, signal=31%
REACTOME_METABOLIC DISORDERS OF BIOLOGICAL OXIDATION ENZYMES	REACTOME_METABOLIC DISORDERS OF BIOLOGICAL OXIDATION ENZYMES	6	0.26832658	0.26832658	0.7105263	0.55372846	1	35827	tags=100%, list=73%, signal=373%
REACTOME_DEFECTIVE CYP11A1 CAUSES ADRENAL INSUFFICIENCY, CONGENITAL, WITH 46,XY SEX REVERSAL	REACTOME_DEFECTIVE CYP11A1 CAUSES ADRENAL INSUFFICIENCY, CONGENITAL, WITH 46,XY SEX REVERSAL (AICSR)	4	0.2683156	0.2683156	0.860396	0.5526799	1	35827	tags=100%, list=73%, signal=373%
REACTOME_ELECTRON TRANSPORT FROM NADPH TO	REACTOME_ELECTRON TRANSPORT FROM NADPH TO FERREDOXIN	3	0.26831016	0.26831016	0.9412361	0.5516113	1	35827	tags=100%, list=73%, signal=373%
REACTOME_BIOSYNTHESIS OF THE N-GLYCAN PRECURSOR (DOLICHOL LIPID-LINKED OLIGOSACCHARIDE, LLO) AND TRANSFER TO A NASCENT	REACTOME_BIOSYNTHESIS OF THE N-GLYCAN PRECURSOR (DOLICHOL LIPID-LINKED OLIGOSACCHARIDE, LLO) AND TRANSFER TO A NASCENT PROTEIN	78	0.26760536	0.26760536	9.94E-04	0.5535588	1	3866	tags=35%, list=8%, signal=38%
REACTOME_NEPHRIN FAMILY INTERACTIONS	REACTOME_NEPHRIN FAMILY INTERACTIONS	22	0.26699984	0.26699984	0.0653218	0.55512196	1	2511	tags=32%, list=5%, signal=34%
REACTOME_RECYCLING OF EIF2:GDP	REACTOME_RECYCLING OF EIF2:GDP	8	0.26681578	0.26681578	0.5262626	0.5548495	1	11419	tags=50%, list=23%, signal=65%
REACTOME_CELL-CELL COMMUNICATION	REACTOME_CELL-CELL COMMUNICATION	121	0.266668	0.266668	0	0.55438644	1	6398	tags=40%, list=13%, signal=46%
REACTOME_CREATION OF C4 AND C2 ACTIVATORS	REACTOME_CREATION OF C4 AND C2 ACTIVATORS	91	0.2661428	0.2661428	0	0.55565304	1	25195	tags=78%, list=51%, signal=160%
REACTOME_REMOVAL OF AMINOTERMINAL PROPEPTIDES FROM GAMMA-CARBOXYLATED PROTEINS	REACTOME_REMOVAL OF AMINOTERMINAL PROPEPTIDES FROM GAMMA-CARBOXYLATED PROTEINS	10	0.26607087	0.26607087	0.4031311	0.5548687	1	1663	tags=30%, list=3%, signal=31%
REACTOME_LYSOSOME VESICLE BIOGENESIS	REACTOME_LYSOSOME VESICLE BIOGENESIS	35	0.26591048	0.26591048	0.0133745	0.55446416	1	6574	tags=40%, list=13%, signal=46%
REACTOME_PHENYLALANINE AND TYROSINE CATABOLISM	REACTOME_PHENYLALANINE AND TYROSINE CATABOLISM	11	0.26563343	0.26563343	0.3648781	0.55455357	1	9252	tags=45%, list=19%, signal=56%
REACTOME_NICOTINATE METABOLISM	REACTOME_NICOTINATE METABOLISM	31	0.26537192	0.26537192	0.0225641	0.5545783	1	7547	tags=42%, list=15%, signal=50%
REACTOME_TP53 REGULATES TRANSCRIPTION OF GENES INVOLVED IN CYTOCHROME C RELEASE	REACTOME_TP53 REGULATES TRANSCRIPTION OF GENES INVOLVED IN CYTOCHROME C RELEASE	20	0.2647921	0.2647921	0.0923226	0.555979	1	18866	tags=65%, list=39%, signal=106%
REACTOME_CIRCADIAN CLOCK	REACTOME_CIRCADIAN CLOCK	69	0.26473698	0.26473698	0	0.5551289	1	5505	tags=38%, list=11%, signal=42%
REACTOME_ASTROCYTIC GLUTAMATE-GLUTAMINE UPTAKE AND METABOLISM	REACTOME_ASTROCYTIC GLUTAMATE-GLUTAMINE UPTAKE AND METABOLISM	4	0.2639754	0.2639754	0.8773006	0.5573486	1	11557	tags=50%, list=24%, signal=65%
REACTOME_NEUROTRANSMITTER UPTAKE AND METABOLISM IN GLIAL CELLS	REACTOME_NEUROTRANSMITTER UPTAKE AND METABOLISM IN GLIAL CELLS	4	0.2639754	0.2639754	0.8384845	0.5562706	1	11557	tags=50%, list=24%, signal=65%
REACTOME_PCNA-DEPENDENT LONG PATCH BASE EXCISION REPAIR	REACTOME_PCNA-DEPENDENT LONG PATCH BASE EXCISION REPAIR	21	0.26362783	0.26362783	0.0901961	0.5566774	1	15076	tags=57%, list=31%, signal=83%
REACTOME_ELEVATION OF CYTOSOLIC CA2+ LEVELS	REACTOME_ELEVATION OF CYTOSOLIC CA2+ LEVELS	16	0.2635562	0.2635562	0.1674926	0.5558915	1	14641	tags=56%, list=30%, signal=80%
REACTOME_ACYL CHAIN REMODELLING OF PC	REACTOME_ACYL CHAIN REMODELLING OF PC	27	0.2630933	0.2630933	0.0468594	0.5569238	1	12513	tags=52%, list=26%, signal=70%
REACTOME_ADHERENS JUNCTIONS INTERACTIONS	REACTOME_ADHERENS JUNCTIONS INTERACTIONS	31	0.26282734	0.26282734	0.0281974	0.5570977	1	6092	tags=39%, list=12%, signal=44%

REACTOME_ZINC EFFLUX AND COMPARTMENTALIZATION BY THE SLC30 FAMILY	REACTOME_ZINC EFFLUX AND COMPARTMENTALIZATION BY THE SLC30 FAMILY	7	0.262715	0.262715	0.6169154	0.55651456	1	8122	tags=43%, list=17%, signal=51%
REACTOME_METABOLISM OF FAT-SOLUBLE VITAMINS	REACTOME_METABOLISM OF FAT-SOLUBLE VITAMINS	48	0.26236236	0.26236236	0.0020367	0.55695564	1	6547	tags=40%, list=13%, signal=46%
REACTOME_IRF3-MEDIATED INDUCTION OF TYPE I IFN	REACTOME_IRF3-MEDIATED INDUCTION OF TYPE I IFN	12	0.26225325	0.26225325	0.3382789	0.55635124	1	7563	tags=42%, list=15%, signal=49%
REACTOME_CREB PHOSPHORYLATION THROUGH THE ACTIVATION OF ADENYLATE CYCLASE	REACTOME_CREB PHOSPHORYLATION THROUGH THE ACTIVATION OF ADENYLATE CYCLASE	7	0.26189798	0.26189798	0.6359127	0.55680704	1	15157	tags=57%, list=31%, signal=83%
REACTOME_REGULATION OF GLUCOKINASE BY GLUCOKINASE REGULATORY PROTEIN	REACTOME_REGULATION OF GLUCOKINASE BY GLUCOKINASE REGULATORY PROTEIN	31	0.26136586	0.26136586	0.0282132	0.55795354	1	4584	tags=35%, list=9%, signal=39%
REACTOME_REGULATION OF IFNG SIGNALING	REACTOME_REGULATION OF IFNG SIGNALING	14	0.26081184	0.26081184	0.2557443	0.55930835	1	1222	tags=29%, list=2%, signal=29%
REACTOME_WNT5A-DEPENDENT INTERNALIZATION OF FZD4	REACTOME_WNT5A-DEPENDENT INTERNALIZATION OF FZD4	15	0.26060608	0.26060608	0.2400398	0.5591063	1	13357	tags=53%, list=27%, signal=73%
REACTOME_CD22 MEDIATED BCR REGULATION	REACTOME_CD22 MEDIATED BCR REGULATION	65	0.25988203	0.25988203	0	0.56118786	1	29476	tags=86%, list=60%, signal=216%
REACTOME_COMPLEMENT CASCADE	REACTOME_COMPLEMENT CASCADE	132	0.25979316	0.25979316	0	0.5605626	1	32939	tags=93%, list=67%, signal=284%
REACTOME_EXTENSION OF TELOMERES	REACTOME_EXTENSION OF TELOMERES	30	0.25891152	0.25891152	0.0326087	0.56334364	1	15076	tags=57%, list=31%, signal=82%
REACTOME_LOSS OF FUNCTION OF SMAD4 IN CANCER	REACTOME_LOSS OF FUNCTION OF SMAD4 IN CANCER	3	0.258847	0.258847	0.9607251	0.56253666	1	3647	tags=33%, list=7%, signal=36%
REACTOME_SMAD4 MH2 DOMAIN MUTANTS IN CANCER	REACTOME_SMAD4 MH2 DOMAIN MUTANTS IN CANCER	3	0.258847	0.258847	0.9739218	0.56148124	1	3647	tags=33%, list=7%, signal=36%
REACTOME_EGFR DOWNREGULATION	REACTOME_EGFR DOWNREGULATION	25	0.25875765	0.25875765	0.0498961	0.5608509	1	8880	tags=44%, list=18%, signal=54%
REACTOME_ACYL CHAIN REMODELLING OF PE	REACTOME_ACYL CHAIN REMODELLING OF PE	29	0.25865874	0.25865874	0.0512295	0.56023574	1	12668	tags=52%, list=26%, signal=70%
REACTOME_COMMON PATHWAY OF FIBRIN CLOT FORMATION	REACTOME_COMMON PATHWAY OF FIBRIN CLOT FORMATION	22	0.25834692	0.25834692	0.1035176	0.5605394	1	27417	tags=82%, list=56%, signal=186%
REACTOME_CLEC7A (DECTIN-1) INDUCES NFAT ACTIVATION	REACTOME_CLEC7A (DECTIN-1) INDUCES NFAT ACTIVATION	11	0.25779676	0.25779676	0.3817635	0.5618556	1	14087	tags=55%, list=29%, signal=77%
REACTOME_HDR THROUGH MMEJ (ALT-NHEJ)	REACTOME_HDR THROUGH MMEJ (ALT-NHEJ)	10	0.2576039	0.2576039	0.4438928	0.5616447	1	16767	tags=60%, list=34%, signal=91%
REACTOME_RUNX3 REGULATES WNT SIGNALING	REACTOME_RUNX3 REGULATES WNT SIGNALING	9	0.2574693	0.2574693	0.5010482	0.5611517	1	3716	tags=33%, list=8%, signal=36%
REACTOME_BMAL1:CLOCK,NPAS2 ACTIVATES CIRCADIAN GENE EXPRESSION	REACTOME_BMAL1:CLOCK,NPAS2 ACTIVATES CIRCADIAN GENE EXPRESSION	46	0.25735933	0.25735933	0.0049652	0.56057584	1	5505	tags=37%, list=11%, signal=42%
REACTOME_TRAFFICKING AND PROCESSING OF ENDOSOMAL	REACTOME_TRAFFICKING AND PROCESSING OF ENDOSOMAL TLR	13	0.25719544	0.25719544	0.3088843	0.5602639	1	10008	tags=46%, list=20%, signal=58%
REACTOME_CLEAVAGE OF THE DAMAGED PURINE	REACTOME_CLEAVAGE OF THE DAMAGED PURINE	3	0.2561306	0.2561306	0.9642857	0.5638821	1	3780	tags=33%, list=8%, signal=36%
REACTOME_DEPURINATION	REACTOME_DEPURINATION	3	0.2561306	0.2561306	0.9625247	0.5628437	1	3780	tags=33%, list=8%, signal=36%
REACTOME_RECOGNITION AND ASSOCIATION OF DNA GLYCOSYLASE WITH SITE CONTAINING AN AFFECTED	REACTOME_RECOGNITION AND ASSOCIATION OF DNA GLYCOSYLASE WITH SITE CONTAINING AN AFFECTED PURINE	3	0.2561306	0.2561306	0.9719439	0.56180906	1	3780	tags=33%, list=8%, signal=36%
REACTOME_SCAVENGING OF HEME FROM PLASMA	REACTOME_SCAVENGING OF HEME FROM PLASMA	87	0.25599816	0.25599816	0	0.5613695	1	25195	tags=77%, list=51%, signal=158%

REACTOME_NEGATIVE REGULATION OF TCF-DEPENDENT SIGNALING BY WNT	REACTOME_NEGATIVE REGULATION OF TCF-DEPENDENT SIGNALING BY WNT LIGAND ANTAGONISTS	14	0.2559192	0.2559192	0.2631058	0.56065726	1	4959	tags=36%, list=10%, signal=40%
REACTOME_NADE MODULATES DEATH SIGNALLING	REACTOME_NADE MODULATES DEATH SIGNALLING	6	0.25545186	0.25545186	0.7376238	0.5616175	1	3814	tags=33%, list=8%, signal=36%
REACTOME_ACTIVATED TAK1 MEDIATES P38 MAPK	REACTOME_ACTIVATED TAK1 MEDIATES P38 MAPK ACTIVATION	19	0.2548039	0.2548039	0.1298315	0.56340915	1	2990	tags=32%, list=6%, signal=34%
REACTOME_REGULATION OF TLR BY ENDOGENOUS LIGAND	REACTOME_REGULATION OF TLR BY ENDOGENOUS LIGAND	16	0.2532585	0.2532585	0.2520492	0.5691655	1	24326	tags=75%, list=50%, signal=149%
REACTOME_RETINOID METABOLISM AND TRANSPORT	REACTOME_RETINOID METABOLISM AND TRANSPORT	44	0.2528627	0.2528627	0.0048309	0.5698803	1	6547	tags=39%, list=13%, signal=45%
REACTOME_GLYCOGEN BREAKDOWN	REACTOME_GLYCOGEN BREAKDOWN (GLYCOGENOLYSIS)	16	0.2523762	0.2523762	0.2346939	0.5709547	1	2947	tags=31%, list=6%, signal=33%
REACTOME_TETRAHYDROBIOPTERIN (BH4) SYNTHESIS, RECYCLING, SALVAGE AND	REACTOME_TETRAHYDROBIOPTERIN (BH4) SYNTHESIS, RECYCLING, SALVAGE AND REGULATION	10	0.25159842	0.25159842	0.473	0.57329327	1	7268	tags=40%, list=15%, signal=47%
REACTOME_REGULATION OF PTEN LOCALIZATION	REACTOME_REGULATION OF PTEN LOCALIZATION	9	0.25145483	0.25145483	0.5403226	0.5728931	1	9451	tags=44%, list=19%, signal=55%
REACTOME_ROLE OF PHOSPHOLIPIDS IN	REACTOME_ROLE OF PHOSPHOLIPIDS IN PHAGOCYTOSIS	102	0.25065643	0.25065643	0	0.5754253	1	25195	tags=76%, list=51%, signal=157%
REACTOME_RELEASE OF HH-NP FROM THE SECRETING CELL	REACTOME_RELEASE OF HH-NP FROM THE SECRETING CELL	8	0.25054383	0.25054383	0.6097319	0.574904	1	6095	tags=38%, list=12%, signal=43%
REACTOME_FORMATION OF FIBRIN CLOT (CLOTTING	REACTOME_FORMATION OF FIBRIN CLOT (CLOTTING CASCADE)	38	0.25020844	0.25020844	0.0153061	0.57543015	1	10951	tags=47%, list=22%, signal=61%
REACTOME_BILE SALT AND ORGANIC ANION SLC TRANSPORTERS	REACTOME_BILE SALT AND ORGANIC ANION SLC TRANSPORTERS	14	0.25015834	0.25015834	0.2945892	0.5746008	1	12236	tags=50%, list=25%, signal=67%
REACTOME_LECTIN PATHWAY OF COMPLEMENT ACTIVATION	REACTOME_LECTIN PATHWAY OF COMPLEMENT ACTIVATION	8	0.24923657	0.24923657	0.6438632	0.57768977	1	6159	tags=38%, list=13%, signal=43%
REACTOME_UREA CYCLE	REACTOME_UREA CYCLE	10	0.24921867	0.24921867	0.495229	0.5767321	1	2488	tags=30%, list=5%, signal=32%
REACTOME_CLATHRIN DERIVED VESICLE BUDDING	REACTOME_CLATHRIN DERIVED VESICLE BUDDING	72	0.24906144	0.24906144	0	0.5764233	1	10944	tags=47%, list=22%, signal=61%
REACTOME_TRANS-GOLGI NETWORK VESICLE BUDDING	REACTOME_TRANS-GOLGI NETWORK VESICLE BUDDING	72	0.24906144	0.24906144	0	0.57539576	1	10944	tags=47%, list=22%, signal=61%
REACTOME_SHC-RELATED EVENTS TRIGGERED BY IGF1R	REACTOME_SHC-RELATED EVENTS TRIGGERED BY IGF1R	9	0.24856333	0.24856333	0.5447236	0.576553	1	4152	tags=33%, list=8%, signal=36%
REACTOME_NEGATIVE REGULATORS OF DDX58/IFIH1 SIGNALING	REACTOME_NEGATIVE REGULATORS OF DDX58/IFIH1 SIGNALING	34	0.24838278	0.24838278	0.0220662	0.57636786	1	3687	tags=32%, list=8%, signal=35%
REACTOME_RAF-INDEPENDENT MAPK1/3 ACTIVATION	REACTOME_RAF-INDEPENDENT MAPK1/3 ACTIVATION	23	0.2481358	0.2481358	0.1149425	0.5764811	1	30433	tags=87%, list=62%, signal=230%
REACTOME_OPSINS	REACTOME_OPSINS	7	0.24786556	0.24786556	0.7130178	0.5766536	1	15844	tags=57%, list=32%, signal=84%
REACTOME_MYD88-INDEPENDENT TLR3/TLR4	REACTOME_MYD88-INDEPENDENT TLR3/TLR4 CASCADE	100	0.24781437	0.24781437	0	0.57586014	1	4048	tags=33%, list=8%, signal=36%
REACTOME_TRIF-MEDIATED TLR3/TLR4 SIGNALING	REACTOME_TRIF-MEDIATED TLR3/TLR4 SIGNALING	100	0.24781437	0.24781437	0	0.57484454	1	4048	tags=33%, list=8%, signal=36%
REACTOME_TOLL LIKE RECEPTOR 3 (TLR3) CASCADE	REACTOME_TOLL LIKE RECEPTOR 3 (TLR3) CASCADE	100	0.24781437	0.24781437	0	0.5738325	1	4048	tags=33%, list=8%, signal=36%
REACTOME_SIGNALING BY TGF-BETA RECEPTOR COMPLEX	REACTOME_SIGNALING BY TGF-BETA RECEPTOR COMPLEX	73	0.24764983	0.24764983	9.84E-04	0.5735693	1	6672	tags=38%, list=14%, signal=44%
REACTOME_NUCLEOTIDE-BINDING DOMAIN, LEUCINE RICH REPEAT CONTAINING RECEPTOR (NLR) SIGNALING PATHWAYS	REACTOME_NUCLEOTIDE-BINDING DOMAIN, LEUCINE RICH REPEAT CONTAINING RECEPTOR (NLR) SIGNALING PATHWAYS	47	0.24731922	0.24731922	0.0048309	0.5740517	1	7695	tags=40%, list=16%, signal=48%
REACTOME_NEGATIVE REGULATION OF MET ACTIVITY	REACTOME_NEGATIVE REGULATION OF MET ACTIVITY	21	0.24730305	0.24730305	0.1200397	0.57312846	1	8880	tags=43%, list=18%, signal=52%

REACTOME_GOLGI CISTERNAE PERICENTRIOLAR STACK REORGANIZATION	REACTOME_GOLGI CISTERNAE PERICENTRIOLAR STACK REORGANIZATION	14	0.24728814	0.24728814	0.2921811	0.5721988	1	22869	tags=71%, list=47%, signal=134%
REACTOME_SYNTHESIS OF PROSTAGLANDINS (PG) AND THROMBOXANES (TX)	REACTOME_SYNTHESIS OF PROSTAGLANDINS (PG) AND THROMBOXANES (TX)	15	0.24721146	0.24721146	0.2893924	0.5715363	1	7484	tags=40%, list=15%, signal=47%
REACTOME_NUCLEOTIDE	REACTOME_NUCLEOTIDE SALVAGE	23	0.24719146	0.24719146	0.1082164	0.5706227	1	11319	tags=48%, list=23%, signal=62%
REACTOME_HSF1 ACTIVATION	REACTOME_HSF1 ACTIVATION	29	0.24677481	0.24677481	0.0477137	0.57147634	1	14938	tags=55%, list=31%, signal=79%
REACTOME_GAMMA-CARBOXYLATION OF PROTEIN PRECURSORS	REACTOME_GAMMA-CARBOXYLATION OF PROTEIN PRECURSORS	10	0.24603207	0.24603207	0.4940594	0.5737237	1	2644	tags=30%, list=5%, signal=32%
REACTOME_ERBB2 REGULATES CELL MOTILITY	REACTOME_ERBB2 REGULATES CELL MOTILITY	15	0.24599253	0.24599253	0.2650841	0.57288164	1	1015	tags=27%, list=2%, signal=27%
REACTOME_SEMET INCORPORATION INTO PROTEINS	REACTOME_SEMET INCORPORATION INTO PROTEINS	11	0.24588019	0.24588019	0.4444445	0.5723862	1	10219	tags=45%, list=21%, signal=57%
REACTOME_ACYL CHAIN REMODELLING OF PG	REACTOME_ACYL CHAIN REMODELLING OF PG	18	0.24541852	0.24541852	0.172	0.57347876	1	12469	tags=50%, list=25%, signal=67%
REACTOME_EPHB-MEDIATED FORWARD SIGNALING	REACTOME_EPHB-MEDIATED FORWARD SIGNALING	42	0.2453822	0.2453822	0.0136187	0.5726487	1	10145	tags=45%, list=21%, signal=57%
REACTOME_REGULATION OF LIPID METABOLISM BY PEROXISOME PROLIFERATOR-ACTIVATED RECEPTOR ALPHA	REACTOME_REGULATION OF LIPID METABOLISM BY PEROXISOME PROLIFERATOR-ACTIVATED RECEPTOR ALPHA (PPARALPHA)	121	0.2452324	0.2452324	0	0.57227373	1	7445	tags=40%, list=15%, signal=47%
REACTOME_LIPID PARTICLE ORGANIZATION	REACTOME_LIPID PARTICLE ORGANIZATION	6	0.24493967	0.24493967	0.7736585	0.57255524	1	36972	tags=100%, list=76%, signal=408%
REACTOME_THE NLRP3 INFLAMMASOME	REACTOME_THE NLRP3 INFLAMMASOME	12	0.24489479	0.24489479	0.3767396	0.5717445	1	20654	tags=67%, list=42%, signal=115%
REACTOME_SEMAPHORIN INTERACTIONS	REACTOME_SEMAPHORIN INTERACTIONS	67	0.24488044	0.24488044	0	0.5708258	1	7757	tags=40%, list=16%, signal=48%
REACTOME_BINDING AND UPTAKE OF LIGANDS BY SCAVENGER RECEPTORS	REACTOME_BINDING AND UPTAKE OF LIGANDS BY SCAVENGER RECEPTORS	116	0.24462858	0.24462858	0	0.57101923	1	25195	tags=76%, list=51%, signal=156%
REACTOME_SYNTHESIS OF PIPS AT THE ER MEMBRANE	REACTOME_SYNTHESIS OF PIPS AT THE ER MEMBRANE	5	0.24389297	0.24389297	0.8801611	0.5733009	1	17437	tags=60%, list=36%, signal=93%
REACTOME_TRAF6 MEDIATED INDUCTION OF PROINFLAMMATORY CYTOKINES	REACTOME_TRAF6 MEDIATED INDUCTION OF PROINFLAMMATORY CYTOKINES	83	0.24302141	0.24302141	0	0.5761061	1	4048	tags=33%, list=8%, signal=35%
REACTOME_DEFECTIVE B4GALT1 CAUSES B4GALT1-CDG	REACTOME_DEFECTIVE B4GALT1 CAUSES B4GALT1-CDG (CDG-2D)	7	0.24255492	0.24255492	0.71139	0.5771926	1	37089	tags=100%, list=76%, signal=412%
REACTOME_DEFECTIVE CHST6 CAUSES MCDC1	REACTOME_DEFECTIVE CHST6 CAUSES MCDC1	7	0.24255492	0.24255492	0.7241379	0.5762126	1	37089	tags=100%, list=76%, signal=412%
REACTOME_DEFECTIVE ST3GAL3 CAUSES MCT12 AND	REACTOME_DEFECTIVE ST3GAL3 CAUSES MCT12 AND EIEE15	7	0.24255492	0.24255492	0.7376238	0.575236	1	37089	tags=100%, list=76%, signal=412%
REACTOME_AXONAL GROWTH STIMULATION	REACTOME_AXONAL GROWTH STIMULATION	4	0.24158514	0.24158514	0.9268051	0.57849807	1	412	tags=25%, list=1%, signal=25%
REACTOME_FORMATION OF THE EDITOSOME	REACTOME_FORMATION OF THE EDITOSOME	7	0.24132936	0.24132936	0.7359438	0.5786689	1	30154	tags=86%, list=62%, signal=223%
REACTOME_MRNA EDITING: C TO U CONVERSION	REACTOME_MRNA EDITING: C TO U CONVERSION	7	0.24132936	0.24132936	0.7051282	0.57769305	1	30154	tags=86%, list=62%, signal=223%
REACTOME_SHC1 EVENTS IN ERBB2 SIGNALING	REACTOME_SHC1 EVENTS IN ERBB2 SIGNALING	22	0.2409965	0.2409965	0.1502392	0.5782344	1	1558	tags=27%, list=3%, signal=28%
REACTOME_GLUCONEOGENESIS	REACTOME_GLUCONEOGENESIS	33	0.24024807	0.24024807	0.0352823	0.58055145	1	10500	tags=45%, list=21%, signal=58%
REACTOME_TGF-BETA RECEPTOR SIGNALING	REACTOME_TGF-BETA RECEPTOR SIGNALING ACTIVATES SMADS	32	0.2388751	0.2388751	0.037037	0.585508	1	6672	tags=38%, list=14%, signal=43%
REACTOME_CELL-CELL JUNCTION ORGANIZATION	REACTOME_CELL-CELL JUNCTION ORGANIZATION	62	0.23866071	0.23866071	0.0010163	0.5854925	1	10441	tags=45%, list=21%, signal=57%

REACTOME_TRANSPORT OF RIBONUCLEOPROTEINS INTO THE HOST NUCLEUS	REACTOME_TRANSPORT OF RIBONUCLEOPROTEINS INTO THE HOST NUCLEUS	31	0.23828341	0.23828341	0.0573123	0.58613485	1	4134	tags=32%, list=8%, signal=35%
REACTOME_PROCESSING OF INTRONLESS PRE-MRNAS	REACTOME_PROCESSING OF INTRONLESS PRE-MRNAS	19	0.23820668	0.23820668	0.2056452	0.58549464	1	19265	tags=63%, list=39%, signal=104%
REACTOME_VLDL CLEARANCE	REACTOME_VLDL CLEARANCE	6	0.23819251	0.23819251	0.814096	0.5845938	1	4659	tags=33%, list=10%, signal=37%
REACTOME_GLUCOSE	REACTOME_GLUCOSE METABOLISM	90	0.23797329	0.23797329	0	0.58451897	1	7410	tags=39%, list=15%, signal=46%
REACTOME_EPHA-MEDIATED GROWTH CONE COLLAPSE	REACTOME_EPHA-MEDIATED GROWTH CONE COLLAPSE	34	0.23796594	0.23796594	0.0254065	0.58357584	1	7077	tags=38%, list=14%, signal=45%
REACTOME_CHYLOMICRON CLEARANCE	REACTOME_CHYLOMICRON CLEARANCE	5	0.23764297	0.23764297	0.8960396	0.58401763	1	7950	tags=40%, list=16%, signal=48%
REACTOME_PEPTIDE HORMONE BIOSYNTHESIS	REACTOME_PEPTIDE HORMONE BIOSYNTHESIS	12	0.23728204	0.23728204	0.4368635	0.5846737	1	4705	tags=33%, list=10%, signal=37%
REACTOME_FERTILIZATION	REACTOME_FERTILIZATION	26	0.23717126	0.23717126	0.0938144	0.58424026	1	29824	tags=85%, list=61%, signal=216%
REACTOME_REPRODUCTION	REACTOME_REPRODUCTION	26	0.23717126	0.23717126	0.0885312	0.5832762	1	29824	tags=85%, list=61%, signal=216%
REACTOME_FCERI MEDIATED CA+2 MOBILIZATION	REACTOME_FCERI MEDIATED CA+2 MOBILIZATION	102	0.23625684	0.23625684	0	0.5863765	1	29739	tags=84%, list=61%, signal=214%
REACTOME_TRAF6 MEDIATED IRF7 ACTIVATION IN TLR7/8 OR 9 SIGNALING	REACTOME_TRAF6 MEDIATED IRF7 ACTIVATION IN TLR7/8 OR 9 SIGNALING	11	0.2350537	0.2350537	0.4736318	0.590628	1	10749	tags=45%, list=22%, signal=58%
REACTOME_TRANSCRIPTIONAL ACTIVITY OF SMAD2/SMAD3:SMAD4	REACTOME_TRANSCRIPTIONAL ACTIVITY OF SMAD2/SMAD3:SMAD4 HETEROTRIMER	44	0.23477556	0.23477556	0.0119403	0.5908679	1	6319	tags=36%, list=13%, signal=42%
REACTOME_FGFRL1 MODULATION OF FGFR1	REACTOME_FGFRL1 MODULATION OF FGFR1 SIGNALING	13	0.23428603	0.23428603	0.3889458	0.59214324	1	14896	tags=54%, list=30%, signal=77%
REACTOME_SIGNALING BY	REACTOME_SIGNALING BY NOTCH	113	0.23423755	0.23423755	0	0.59138227	1	8922	tags=42%, list=18%, signal=51%
REACTOME_DEPOLYMERISATION OF THE NUCLEAR LAMINA	REACTOME_DEPOLYMERISATION OF THE NUCLEAR LAMINA	14	0.23349884	0.23349884	0.3874756	0.5937327	1	16549	tags=57%, list=34%, signal=86%
REACTOME_COPII (COAT PROTEIN 2) MEDIATED VESICLE	REACTOME_COPII (COAT PROTEIN 2) MEDIATED VESICLE TRANSPORT	68	0.23193021	0.23193021	0	0.5997067	1	5220	tags=34%, list=11%, signal=38%
REACTOME_ACTIVATION OF E2F1 TARGET GENES AT G1/S	REACTOME_ACTIVATION OF E2F1 TARGET GENES AT G1/S	28	0.23167033	0.23167033	0.0898204	0.5999436	1	7898	tags=39%, list=16%, signal=47%
REACTOME_G1/S-SPECIFIC TRANSCRIPTION	REACTOME_G1/S-SPECIFIC TRANSCRIPTION	28	0.23167033	0.23167033	0.0850851	0.598968	1	7898	tags=39%, list=16%, signal=47%
REACTOME_ERK/MAPK TARGETS	REACTOME_ERK/MAPK TARGETS	22	0.23150307	0.23150307	0.1888889	0.59875625	1	6474	tags=36%, list=13%, signal=42%
REACTOME_CENTROSOME MATURATION	REACTOME_CENTROSOME MATURATION	80	0.23131585	0.23131585	0	0.5985999	1	16846	tags=58%, list=34%, signal=88%
REACTOME_RECRUITMENT OF MITOTIC CENTROSOME PROTEINS AND COMPLEXES	REACTOME_RECRUITMENT OF MITOTIC CENTROSOME PROTEINS AND COMPLEXES	80	0.23131585	0.23131585	0	0.59763134	1	16846	tags=58%, list=34%, signal=88%
REACTOME_THE CANONICAL RETINOID CYCLE IN RODS (TWILIGHT VISION)	REACTOME_THE CANONICAL RETINOID CYCLE IN RODS (TWILIGHT VISION)	23	0.2309513	0.2309513	0.1465863	0.59833145	1	7856	tags=39%, list=16%, signal=47%
REACTOME_CD28 DEPENDENT PI3K/AKT SIGNALING	REACTOME_CD28 DEPENDENT PI3K/AKT SIGNALING	22	0.23093934	0.23093934	0.161689	0.59742016	1	17630	tags=59%, list=36%, signal=92%
REACTOME_SCAVENGING BY CLASS B RECEPTORS	REACTOME_SCAVENGING BY CLASS B RECEPTORS	6	0.23055346	0.23055346	0.8394161	0.5981262	1	5033	tags=33%, list=10%, signal=37%
REACTOME_IRAK4 DEFICIENCY (TLR2/4)	REACTOME_IRAK4 DEFICIENCY (TLR2/4)	11	0.23050027	0.23050027	0.5257937	0.59738934	1	24326	tags=73%, list=50%, signal=144%
REACTOME_MYD88 DEFICIENCY (TLR2/4)	REACTOME_MYD88 DEFICIENCY (TLR2/4)	11	0.23050027	0.23050027	0.5043902	0.5964305	1	24326	tags=73%, list=50%, signal=144%
REACTOME_NUCLEAR PORE COMPLEX (NPC) DISASSEMBLY	REACTOME_NUCLEAR PORE COMPLEX (NPC) DISASSEMBLY	35	0.23000205	0.23000205	0.0353288	0.5977316	1	4134	tags=31%, list=8%, signal=34%
REACTOME_DOWNREGULATION OF SMAD2/3:SMAD4 TRANSCRIPTIONAL ACTIVITY	REACTOME_DOWNREGULATION OF SMAD2/3:SMAD4 TRANSCRIPTIONAL ACTIVITY	23	0.22995365	0.22995365	0.1415753	0.5969758	1	3647	tags=30%, list=7%, signal=33%

REACTOME_IRAK2 MEDIATED ACTIVATION OF TAK1 COMPLEX UPON TLR7/8 OR 9 STIMULATION	REACTOME_IRAK2 MEDIATED ACTIVATION OF TAK1 COMPLEX UPON TLR7/8 OR 9 STIMULATION	16	0.2298834	0.2298834	0.3441624	0.5963209	1	4048	tags=31%, list=8%, signal=34%
REACTOME_BUTYROPHILIN (BTN) FAMILY INTERACTIONS	REACTOME_BUTYROPHILIN (BTN) FAMILY INTERACTIONS	12	0.22984293	0.22984293	0.4603175	0.59554	1	13230	tags=50%, list=27%, signal=68%
REACTOME_PPARA ACTIVATES GENE EXPRESSION	REACTOME_PPARA ACTIVATES GENE EXPRESSION	118	0.22984202	0.22984202	0	0.5945948	1	7445	tags=38%, list=15%, signal=45%
REACTOME_ACTIVATION OF GENE EXPRESSION BY SREBF	REACTOME_ACTIVATION OF GENE EXPRESSION BY SREBF (SREBP)	42	0.22949329	0.22949329	0.0227273	0.5952159	1	6259	tags=36%, list=13%, signal=41%
REACTOME_ER-PHAGOSOME PATHWAY	REACTOME_ER-PHAGOSOME PATHWAY	83	0.2293901	0.2293901	9.99E-04	0.5947214	1	17693	tags=59%, list=36%, signal=92%
REACTOME_INTERFERON	REACTOME_INTERFERON SIGNALING	194	0.22923732	0.22923732	0	0.594492	1	10525	tags=44%, list=21%, signal=56%
REACTOME_NUCLEAR IMPORT OF REV PROTEIN	REACTOME_NUCLEAR IMPORT OF REV PROTEIN	33	0.22838746	0.22838746	0.0517578	0.5974049	1	6629	tags=36%, list=14%, signal=42%
REACTOME_EXPORT OF VIRAL RIBONUCLEOPROTEINS FROM NUCLEUS	REACTOME_EXPORT OF VIRAL RIBONUCLEOPROTEINS FROM NUCLEUS	32	0.22820106	0.22820106	0.0553907	0.5972385	1	4134	tags=31%, list=8%, signal=34%
REACTOME_NUCLEAR EVENTS (KINASE AND TRANSCRIPTION FACTOR ACTIVATION)	REACTOME_NUCLEAR EVENTS (KINASE AND TRANSCRIPTION FACTOR ACTIVATION)	25	0.22787903	0.22787903	0.1384768	0.5977263	1	6474	tags=36%, list=13%, signal=41%
REACTOME_CONSTITUTIVE SIGNALING BY NOTCH1 HD+PEST DOMAIN MUTANTS	REACTOME_CONSTITUTIVE SIGNALING BY NOTCH1 HD+PEST DOMAIN MUTANTS	57	0.22734344	0.22734344	9.81E-04	0.59921867	1	11215	tags=46%, list=23%, signal=59%
REACTOME_CONSTITUTIVE SIGNALING BY NOTCH1 PEST DOMAIN MUTANTS	REACTOME_CONSTITUTIVE SIGNALING BY NOTCH1 PEST DOMAIN MUTANTS	57	0.22734344	0.22734344	0.0050403	0.59827656	1	11215	tags=46%, list=23%, signal=59%
REACTOME_SIGNALING BY NOTCH1 HD+PEST DOMAIN MUTANTS IN CANCER	REACTOME_SIGNALING BY NOTCH1 HD+PEST DOMAIN MUTANTS IN CANCER	57	0.22734344	0.22734344	0.0058537	0.5973373	1	11215	tags=46%, list=23%, signal=59%
REACTOME_SIGNALING BY NOTCH1 PEST DOMAIN MUTANTS	REACTOME_SIGNALING BY NOTCH1 PEST DOMAIN MUTANTS IN CANCER	57	0.22734344	0.22734344	0.0050201	0.5964011	1	11215	tags=46%, list=23%, signal=59%
REACTOME_SIGNALING BY NOTCH1 IN CANCER	REACTOME_SIGNALING BY NOTCH1 IN CANCER	57	0.22734344	0.22734344	0.0060302	0.5954677	1	11215	tags=46%, list=23%, signal=59%
REACTOME_PLATELET AGGREGATION (PLUG	REACTOME_PLATELET AGGREGATION (PLUG FORMATION)	36	0.22729874	0.22729874	0.0383858	0.59474635	1	13360	tags=50%, list=27%, signal=69%
REACTOME_NUCLEOBASE CATABOLISM	REACTOME_NUCLEOBASE CATABOLISM	36	0.22691667	0.22691667	0.0507614	0.5954889	1	6578	tags=36%, list=13%, signal=42%
REACTOME_VXPX CARGO-TARGETING TO CILIUM	REACTOME_VXPX CARGO-TARGETING TO CILIUM	21	0.2261292	0.2261292	0.2213198	0.5979896	1	12248	tags=48%, list=25%, signal=63%
REACTOME_RECRUITMENT OF NUMA TO MITOTIC	REACTOME_RECRUITMENT OF NUMA TO MITOTIC CENTROSOMES	79	0.22592267	0.22592267	0.00199	0.5979668	1	16846	tags=57%, list=34%, signal=87%
REACTOME_RECOGNITION OF DNA DAMAGE BY PCNA-CONTAINING REPLICATION	REACTOME_RECOGNITION OF DNA DAMAGE BY PCNA-CONTAINING REPLICATION COMPLEX	30	0.22566333	0.22566333	0.1007905	0.5981964	1	16703	tags=57%, list=34%, signal=86%
REACTOME_SIGNALING BY	REACTOME_SIGNALING BY ERBB2	50	0.22522947	0.22522947	0.0148305	0.5991615	1	5630	tags=34%, list=11%, signal=38%
REACTOME_SIGNALING BY BRAF AND RAF FUSIONS	REACTOME_SIGNALING BY BRAF AND RAF FUSIONS	58	0.22487226	0.22487226	0.0059701	0.5997875	1	10951	tags=45%, list=22%, signal=58%
REACTOME_ANTIGEN PROCESSING-CROSS	REACTOME_ANTIGEN PROCESSING-CROSS PRESENTATION	99	0.22483048	0.22483048	0	0.59900767	1	17699	tags=59%, list=36%, signal=92%
REACTOME_NEUTROPHIL DEGRANULATION	REACTOME_NEUTROPHIL DEGRANULATION	474	0.22426558	0.22426558	0	0.6006854	1	11334	tags=45%, list=23%, signal=58%
REACTOME_AURKA ACTIVATION BY TPX2	REACTOME_AURKA ACTIVATION BY TPX2	72	0.22335365	0.22335365	0.0019743	0.6038068	1	12881	tags=49%, list=26%, signal=66%
REACTOME_AKT PHOSPHORYLATES TARGETS IN	REACTOME_AKT PHOSPHORYLATES TARGETS IN THE CYTOSOL	14	0.22285552	0.22285552	0.4436475	0.6050608	1	10075	tags=43%, list=21%, signal=54%

REACTOME_MAPK TARGETS/ NUCLEAR EVENTS MEDIATED BY MAP KINASES	REACTOME_MAPK TARGETS/ NUCLEAR EVENTS MEDIATED BY MAP KINASES	31	0.22274241	0.22274241	0.0798817	0.60463727	1	6474	tags=35%, list=13%, signal=41%
REACTOME_CYTOSOLIC SENSORS OF PATHOGEN-	REACTOME_CYTOSOLIC SENSORS OF PATHOGEN-ASSOCIATED DNA	68	0.22264989	0.22264989	9.95E-04	0.60413104	1	7834	tags=38%, list=16%, signal=45%
REACTOME_SPHINGOLIPID METABOLISM	REACTOME_SPHINGOLIPID METABOLISM	90	0.22239728	0.22239728	0	0.60432595	1	5451	tags=33%, list=11%, signal=37%
REACTOME_TNFR2 NON-CANONICAL NF-KB PATHWAY	REACTOME_TNFR2 NON-CANONICAL NF-KB PATHWAY	98	0.2223674	0.2223674	0	0.6035315	1	8119	tags=39%, list=17%, signal=46%
REACTOME_CDO IN	REACTOME_CDO IN MYOGENESIS	29	0.2223298	0.2223298	0.1117479	0.6027765	1	7692	tags=38%, list=16%, signal=45%
REACTOME_MYOGENESIS	REACTOME_MYOGENESIS	29	0.2223298	0.2223298	0.1154219	0.60185766	1	7692	tags=38%, list=16%, signal=45%
REACTOME_MOLYBDENUM COFACTOR BIOSYNTHESIS	REACTOME_MOLYBDENUM COFACTOR BIOSYNTHESIS	6	0.2223187	0.2223187	0.865458	0.60099554	1	13597	tags=50%, list=28%, signal=69%
REACTOME_CELLULAR HEXOSE TRANSPORT	REACTOME_CELLULAR HEXOSE TRANSPORT	22	0.22227435	0.22227435	0.1901544	0.6002853	1	4700	tags=32%, list=10%, signal=35%
REACTOME_ACTIVATED TLR4 SIGNALLING	REACTOME_ACTIVATED TLR4 SIGNALLING	115	0.22217791	0.22217791	0	0.5997934	1	4048	tags=30%, list=8%, signal=33%
REACTOME_PLATELET ACTIVATION, SIGNALING AND	REACTOME_PLATELET ACTIVATION, SIGNALING AND AGGREGATION	275	0.22214605	0.22214605	0	0.5990131	1	6454	tags=35%, list=13%, signal=40%
REACTOME_PAUSING AND RECOVERY OF TAT-MEDIATED HIV ELONGATION	REACTOME_PAUSING AND RECOVERY OF TAT-MEDIATED HIV ELONGATION	27	0.22199896	0.22199896	0.129285	0.5987477	1	7270	tags=37%, list=15%, signal=43%
REACTOME_TAT-MEDIATED HIV ELONGATION ARREST AND RECOVERY	REACTOME_TAT-MEDIATED HIV ELONGATION ARREST AND RECOVERY	27	0.22199896	0.22199896	0.1230159	0.5978432	1	7270	tags=37%, list=15%, signal=43%
REACTOME_MET RECEPTOR RECYCLING	REACTOME_MET RECEPTOR RECYCLING	10	0.2215504	0.2215504	0.6334341	0.598873	1	8739	tags=40%, list=18%, signal=49%
REACTOME_TFAP2A ACTS AS A TRANSCRIPTIONAL REPRESSOR DURING RETINOIC ACID INDUCED CELL DIFFERENTIATION	REACTOME_TFAP2A ACTS AS A TRANSCRIPTIONAL REPRESSOR DURING RETINOIC ACID INDUCED CELL DIFFERENTIATION	5	0.22017974	0.22017974	0.9195737	0.6040217	1	28391	tags=80%, list=58%, signal=190%
REACTOME_NRAGE SIGNALS DEATH THROUGH JNK	REACTOME_NRAGE SIGNALS DEATH THROUGH JNK	59	0.2198971	0.2198971	0.0061665	0.6043967	1	15802	tags=54%, list=32%, signal=80%
REACTOME_POU5F1 (OCT4), SOX2, NANOG ACTIVATE GENES RELATED TO PROLIFERATION	REACTOME_POU5F1 (OCT4), SOX2, NANOG ACTIVATE GENES RELATED TO PROLIFERATION	13	0.21943161	0.21943161	0.465641	0.60554314	1	557	tags=23%, list=1%, signal=23%
REACTOME_SIGNALING BY CYTOSOLIC FGFR1 FUSION	REACTOME_SIGNALING BY CYTOSOLIC FGFR1 FUSION MUTANTS	18	0.2189443	0.2189443	0.3065476	0.6068245	1	5604	tags=33%, list=11%, signal=38%
REACTOME_DNA REPLICATION INITIATION	REACTOME_DNA REPLICATION INITIATION	8	0.21883489	0.21883489	0.7694672	0.6064164	1	13768	tags=50%, list=28%, signal=70%
REACTOME_TELOMERE C-STRAND SYNTHESIS INITIATION	REACTOME_TELOMERE C-STRAND SYNTHESIS INITIATION	8	0.21883489	0.21883489	0.8011869	0.60551	1	13768	tags=50%, list=28%, signal=70%
REACTOME_VPR-MEDIATED NUCLEAR IMPORT OF PICS	REACTOME_VPR-MEDIATED NUCLEAR IMPORT OF PICS	33	0.21872965	0.21872965	0.063745	0.60507405	1	4134	tags=30%, list=8%, signal=33%
REACTOME_INLB-MEDIATED ENTRY OF LISTERIA MONOCYTOGENES INTO HOST	REACTOME_INLB-MEDIATED ENTRY OF LISTERIA MONOCYTOGENES INTO HOST CELL	15	0.21869256	0.21869256	0.4158607	0.6043324	1	8880	tags=40%, list=18%, signal=49%
REACTOME_REGULATION OF PTEN GENE TRANSCRIPTION	REACTOME_REGULATION OF PTEN GENE TRANSCRIPTION	61	0.21868135	0.21868135	0.0039604	0.6034791	1	4556	tags=31%, list=9%, signal=34%
REACTOME_ASSOCIATION OF TRIC/CCT WITH TARGET PROTEINS DURING	REACTOME_ASSOCIATION OF TRIC/CCT WITH TARGET PROTEINS DURING BIOSYNTHESIS	39	0.21861011	0.21861011	0.041958	0.6028697	1	10647	tags=44%, list=22%, signal=56%
REACTOME_TIE2 SIGNALING	REACTOME_TIE2 SIGNALING	18	0.21841311	0.21841311	0.3029126	0.60280174	1	5630	tags=33%, list=11%, signal=38%
REACTOME_GRB2 EVENTS IN ERBB2 SIGNALING	REACTOME_GRB2 EVENTS IN ERBB2 SIGNALING	16	0.21823224	0.21823224	0.3834661	0.60268843	1	1558	tags=25%, list=3%, signal=26%

REACTOME_GPVI-MEDIATED ACTIVATION CASCADE	REACTOME_GPVI-MEDIATED ACTIVATION CASCADE	53	0.21821323	0.21821323	0.0107422	0.6018977	1	22585	tags=68%, list=46%, signal=126%
REACTOME_REGULATION OF ACTIN DYNAMICS FOR PHAGOCYTIC CUP FORMATION	REACTOME_REGULATION OF ACTIN DYNAMICS FOR PHAGOCYTIC CUP FORMATION	138	0.21792665	0.21792665	0	0.60225576	1	25195	tags=73%, list=51%, signal=150%
REACTOME_TRANSPORT OF MATURE MRNAS DERIVED FROM INTRONLESS TRANSCRIPTS	REACTOME_TRANSPORT OF MATURE MRNAS DERIVED FROM INTRONLESS TRANSCRIPTS	42	0.21779463	0.21779463	0.0370732	0.6019584	1	9163	tags=40%, list=19%, signal=50%
REACTOME_RESOLUTION OF AP SITES VIA THE MULTIPLE-NUCLEOTIDE PATCH REPLACEMENT PATHWAY	REACTOME_RESOLUTION OF AP SITES VIA THE MULTIPLE-NUCLEOTIDE PATCH REPLACEMENT PATHWAY	25	0.21766244	0.21766244	0.1652004	0.6017	1	16767	tags=56%, list=34%, signal=85%
REACTOME_SIGNALING BY HIGH-KINASE ACTIVITY BRAF	REACTOME_SIGNALING BY HIGH-KINASE ACTIVITY BRAF MUTANTS	34	0.21765763	0.21765763	0.0682927	0.60083973	1	10951	tags=44%, list=22%, signal=57%
REACTOME_LOSS OF NLP FROM MITOTIC CENTROSOMES	REACTOME_LOSS OF NLP FROM MITOTIC CENTROSOMES	69	0.217589	0.217589	0	0.60026646	1	16326	tags=55%, list=33%, signal=83%
REACTOME_LOSS OF PROTEINS REQUIRED FOR INTERPHASE MICROTUBULE ORGANIZATION FROM THE CENTROSOME	REACTOME_LOSS OF PROTEINS REQUIRED FOR INTERPHASE MICROTUBULE ORGANIZATION FROM THE CENTROSOME	69	0.217589	0.217589	0.0040568	0.5993863	1	16326	tags=55%, list=33%, signal=83%
REACTOME_SIGNALING BY RETINOIC ACID	REACTOME_SIGNALING BY RETINOIC ACID	43	0.21677315	0.21677315	0.0341709	0.6020451	1	9891	tags=42%, list=20%, signal=52%
REACTOME_SYNTHESIS OF PIPS AT THE GOLGI MEMBRANE	REACTOME_SYNTHESIS OF PIPS AT THE GOLGI MEMBRANE	18	0.21670492	0.21670492	0.2950655	0.60148376	1	19315	tags=61%, list=39%, signal=101%
REACTOME_RHO GTPASES ACTIVATE CIT	REACTOME_RHO GTPASES ACTIVATE CIT	19	0.21635552	0.21635552	0.326572	0.60209703	1	7449	tags=37%, list=15%, signal=43%
REACTOME_HISTIDINE, LYSINE, PHENYLALANINE, TYROSINE, PROLINE AND TRYPTOPHAN CATABOLISM	REACTOME_HISTIDINE, LYSINE, PHENYLALANINE, TYROSINE, PROLINE AND TRYPTOPHAN CATABOLISM	46	0.21548131	0.21548131	0.0200803	0.6050752	1	10747	tags=43%, list=22%, signal=56%
REACTOME_P75 NTR RECEPTOR-MEDIATED SIGNALLING	REACTOME_P75 NTR RECEPTOR-MEDIATED SIGNALLING	96	0.2136846	0.2136846	0	0.61211705	1	14549	tags=51%, list=30%, signal=72%
REACTOME_DEADENYLATION OF MRNA	REACTOME_DEADENYLATION OF MRNA	24	0.21335214	0.21335214	0.2007952	0.6127406	1	5879	tags=33%, list=12%, signal=38%
REACTOME_ONCOGENIC MAPK SIGNALING	REACTOME_ONCOGENIC MAPK SIGNALING	71	0.21325897	0.21325897	9.87E-04	0.6122489	1	10951	tags=44%, list=22%, signal=56%
REACTOME_TRANSCRIPTIONAL REGULATION BY RUNX3	REACTOME_TRANSCRIPTIONAL REGULATION BY RUNX3	108	0.21318413	0.21318413	0	0.6116824	1	17693	tags=57%, list=36%, signal=90%
REACTOME_COENZYME A BIOSYNTHESIS	REACTOME_COENZYME A BIOSYNTHESIS	8	0.21294707	0.21294707	0.8055832	0.6118375	1	1815	tags=25%, list=4%, signal=26%
REACTOME_GAP-FILLING DNA REPAIR SYNTHESIS AND LIGATION IN GG-NER	REACTOME_GAP-FILLING DNA REPAIR SYNTHESIS AND LIGATION IN GG-NER	25	0.21219452	0.21219452	0.1819096	0.614318	1	15076	tags=52%, list=31%, signal=75%
REACTOME_OTHER SEMAPHORIN INTERACTIONS	REACTOME_OTHER SEMAPHORIN INTERACTIONS	19	0.21186078	0.21186078	0.3320197	0.6149147	1	7669	tags=37%, list=16%, signal=44%
REACTOME_PYRUVATE METABOLISM	REACTOME_PYRUVATE METABOLISM	28	0.21154752	0.21154752	0.1275304	0.61536264	1	7134	tags=36%, list=15%, signal=42%
REACTOME_TRAF6 MEDIATED INDUCTION OF TAK1 COMPLEX	REACTOME_TRAF6 MEDIATED INDUCTION OF TAK1 COMPLEX	17	0.21149936	0.21149936	0.3898477	0.6146749	1	4048	tags=29%, list=8%, signal=32%
REACTOME_SYNTHESIS OF GLYCOSYLPHOSPHATIDYLINOSITOL (GPI)	REACTOME_SYNTHESIS OF GLYCOSYLPHOSPHATIDYLINOSITOL (GPI)	16	0.2113386	0.2113386	0.4246988	0.61454934	1	17197	tags=56%, list=35%, signal=87%
REACTOME_TRAF6 MEDIATED INDUCTION OF NFKB AND MAP KINASES UPON TLR7/8 OR 9 ACTIVATION	REACTOME_TRAF6 MEDIATED INDUCTION OF NFKB AND MAP KINASES UPON TLR7/8 OR 9 ACTIVATION	92	0.21118334	0.21118334	0	0.6143658	1	4048	tags=29%, list=8%, signal=32%

REACTOME_POLB-DEPENDENT LONG PATCH BASE EXCISION REPAIR	REACTOME_POLB-DEPENDENT LONG PATCH BASE EXCISION REPAIR	8	0.21091723	0.21091723	0.7985758	0.6146747	1	8035	tags=38%, list=16%, signal=45%
REACTOME_CELL DEATH SIGNALLING VIA NRAGE, NRIF AND NADE	REACTOME_CELL DEATH SIGNALLING VIA NRAGE, NRIF AND NADE	75	0.21091567	0.21091567	0.0039216	0.613798	1	15802	tags=53%, list=32%, signal=79%
REACTOME_TRIGLYCERIDE CATABOLISM	REACTOME_TRIGLYCERIDE CATABOLISM	24	0.21080741	0.21080741	0.2134831	0.6133974	1	20285	tags=63%, list=41%, signal=107%
REACTOME_METABOLISM OF INGESTED SEMET, SEC, MESEC INTO H2SE	REACTOME_METABOLISM OF INGESTED SEMET, SEC, MESEC INTO H2SE	8	0.21077168	0.21077168	0.811608	0.6127001	1	26404	tags=75%, list=54%, signal=163%
REACTOME_INTRINSIC PATHWAY OF FIBRIN CLOT FORMATION	REACTOME_INTRINSIC PATHWAY OF FIBRIN CLOT FORMATION	21	0.21054676	0.21054676	0.2736521	0.6128348	1	10679	tags=43%, list=22%, signal=55%
REACTOME_METHYLATION	REACTOME_METHYLATION	14	0.21041453	0.21041453	0.5010374	0.6125121	1	17679	tags=57%, list=36%, signal=89%
REACTOME_CD209 (DC-SIGN) SIGNALING	REACTOME_CD209 (DC-SIGN) SIGNALING	21	0.21014495	0.21014495	0.264151	0.6128288	1	15362	tags=52%, list=31%, signal=76%
REACTOME_ERBB2 ACTIVATES PTK6 SIGNALING	REACTOME_ERBB2 ACTIVATES PTK6 SIGNALING	13	0.2100755	0.2100755	0.5474227	0.6122736	1	1015	tags=23%, list=2%, signal=24%
REACTOME_ASPARAGINE N-LINKED GLYCOSYLATION	REACTOME_ASPARAGINE N-LINKED GLYCOSYLATION	286	0.20999297	0.20999297	0	0.6117315	1	7411	tags=36%, list=15%, signal=42%
REACTOME_GRB2:SOS PROVIDES LINKAGE TO MAPK SIGNALING FOR INTEGRINS	REACTOME_GRB2:SOS PROVIDES LINKAGE TO MAPK SIGNALING FOR INTEGRINS	15	0.20995575	0.20995575	0.449	0.6110143	1	2779	tags=27%, list=6%, signal=28%
REACTOME_PLASMA LIPOPROTEIN REMODELING	REACTOME_PLASMA LIPOPROTEIN REMODELING	32	0.209829	0.209829	0.0923695	0.610695	1	5033	tags=31%, list=10%, signal=35%
REACTOME_HDR THROUGH HOMOLOGOUS RECOMBINATION (HRR)	REACTOME_HDR THROUGH HOMOLOGOUS RECOMBINATION (HRR)	65	0.20980966	0.20980966	0.0072314	0.60992134	1	18365	tags=58%, list=38%, signal=93%
REACTOME_ERKS ARE	REACTOME_ERKS ARE INACTIVATED	13	0.20934008	0.20934008	0.5503489	0.6110787	1	1051	tags=23%, list=2%, signal=24%
REACTOME_INACTIVATION OF CDC42 AND RAC	REACTOME_INACTIVATION OF CDC42 AND RAC	9	0.20914246	0.20914246	0.7619048	0.61111337	1	16963	tags=56%, list=35%, signal=85%
REACTOME_IKK COMPLEX RECRUITMENT MEDIATED BY	REACTOME_IKK COMPLEX RECRUITMENT MEDIATED BY RIP1	24	0.20907743	0.20907743	0.2058824	0.610547	1	4048	tags=29%, list=8%, signal=32%
REACTOME_APEX1-INDEPENDENT RESOLUTION OF AP SITES VIA THE SINGLE NUCLEOTIDE REPLACEMENT	REACTOME_APEX1-INDEPENDENT RESOLUTION OF AP SITES VIA THE SINGLE NUCLEOTIDE REPLACEMENT PATHWAY	7	0.20881166	0.20881166	0.8657172	0.6108825	1	3766	tags=29%, list=8%, signal=31%
REACTOME_PARADOXICAL ACTIVATION OF RAF SIGNALING BY KINASE INACTIVE BRAF	REACTOME_PARADOXICAL ACTIVATION OF RAF SIGNALING BY KINASE INACTIVE BRAF	38	0.20867985	0.20867985	0.0626896	0.6105861	1	3963	tags=29%, list=8%, signal=31%
REACTOME_SIGNALING BY MODERATE KINASE ACTIVITY BRAF MUTANTS	REACTOME_SIGNALING BY MODERATE KINASE ACTIVITY BRAF MUTANTS	38	0.20867985	0.20867985	0.077381	0.60973215	1	3963	tags=29%, list=8%, signal=31%
REACTOME_COSTIMULATION BY THE CD28 FAMILY	REACTOME_COSTIMULATION BY THE CD28 FAMILY	72	0.20763537	0.20763537	0.003	0.6135485	1	18410	tags=58%, list=38%, signal=93%
REACTOME_MAP KINASE ACTIVATION IN TLR CASCADE	REACTOME_MAP KINASE ACTIVATION IN TLR CASCADE	63	0.20761934	0.20761934	0.006135	0.6127543	1	3836	tags=29%, list=8%, signal=31%
REACTOME_COBALAMIN (CBL, VITAMIN B12) TRANSPORT AND METABOLISM	REACTOME_COBALAMIN (CBL, VITAMIN B12) TRANSPORT AND METABOLISM	21	0.20759101	0.20759101	0.2919075	0.61204106	1	15487	tags=52%, list=32%, signal=77%
REACTOME_L1CAM	REACTOME_L1CAM INTERACTIONS	100	0.20752582	0.20752582	0	0.61146307	1	6996	tags=35%, list=14%, signal=41%
REACTOME_ANTIGEN ACTIVATES B CELL RECEPTOR (BCR) LEADING TO GENERATION OF SECOND MESSENGERS	REACTOME_ANTIGEN ACTIVATES B CELL RECEPTOR (BCR) LEADING TO GENERATION OF SECOND MESSENGERS	97	0.20747136	0.20747136	9.89E-04	0.61085105	1	29739	tags=81%, list=61%, signal=207%

REACTOME_RA BIOSYNTHESIS PATHWAY	REACTOME_RA BIOSYNTHESIS PATHWAY	22	0.20716214	0.20716214	0.2644466	0.61139363	1	9891	tags=41%, list=20%, signal=51%
REACTOME_NRIF SIGNALS CELL DEATH FROM THE NUCLEUS	REACTOME_NRIF SIGNALS CELL DEATH FROM THE NUCLEUS	15	0.20693907	0.20693907	0.4907317	0.6115472	1	6191	tags=33%, list=13%, signal=38%
REACTOME_ACETYLCHOLINE BINDING AND DOWNSTREAM EVENTS	REACTOME_ACETYLCHOLINE BINDING AND DOWNSTREAM EVENTS	14	0.20684972	0.20684972	0.5436508	0.61109215	1	7361	tags=36%, list=15%, signal=42%
REACTOME_ACTIVATION OF NICOTINIC ACETYLCHOLINE RECEPTORS	REACTOME_ACTIVATION OF NICOTINIC ACETYLCHOLINE RECEPTORS	14	0.20684972	0.20684972	0.505988	0.6102481	1	7361	tags=36%, list=15%, signal=42%
REACTOME_POSTSYNAPTIC NICOTINIC ACETYLCHOLINE RECEPTORS	REACTOME_POSTSYNAPTIC NICOTINIC ACETYLCHOLINE RECEPTORS	14	0.20684972	0.20684972	0.5024438	0.6094064	1	7361	tags=36%, list=15%, signal=42%
REACTOME_MAP2K AND MAPK ACTIVATION	REACTOME_MAP2K AND MAPK ACTIVATION	38	0.20667687	0.20667687	0.0646817	0.6093413	1	4061	tags=29%, list=8%, signal=32%
REACTOME_NEP/NS2 INTERACTS WITH THE CELLULAR EXPORT MACHINERY	REACTOME_NEP/NS2 INTERACTS WITH THE CELLULAR EXPORT MACHINERY	31	0.20600492	0.20600492	0.1031434	0.61148894	1	4134	tags=29%, list=8%, signal=32%
REACTOME_GOLGI ASSOCIATED VESICLE BIOGENESIS	REACTOME_GOLGI ASSOCIATED VESICLE BIOGENESIS	56	0.20527922	0.20527922	0.0143296	0.61388785	1	10944	tags=43%, list=22%, signal=55%
REACTOME_HOST INTERACTIONS WITH INFLUENZA	REACTOME_HOST INTERACTIONS WITH INFLUENZA FACTORS	42	0.20515573	0.20515573	0.0439122	0.61356056	1	5118	tags=31%, list=10%, signal=35%
REACTOME_MYD88 DEPENDENT CASCADE INITIATED ON	REACTOME_MYD88 DEPENDENT CASCADE INITIATED ON ENDOSOME	94	0.20493574	0.20493574	9.99E-04	0.6136908	1	4048	tags=29%, list=8%, signal=31%
REACTOME_TOLL LIKE RECEPTOR 7/8 (TLR7/8)	REACTOME_TOLL LIKE RECEPTOR 7/8 (TLR7/8) CASCADE	94	0.20493574	0.20493574	9.70E-04	0.61285126	1	4048	tags=29%, list=8%, signal=31%
REACTOME_IMMUNOREGULATORY INTERACTIONS BETWEEN A LYMPHOID AND A NON-LYMPHOID CELL	REACTOME_IMMUNOREGULATORY INTERACTIONS BETWEEN A LYMPHOID AND A NON-LYMPHOID CELL	199	0.20485389	0.20485389	0	0.61236954	1	25195	tags=72%, list=51%, signal=147%
REACTOME_GENE AND PROTEIN EXPRESSION BY JAK-STAT SIGNALING AFTER INTERLEUKIN-12 STIMULATION	REACTOME_GENE AND PROTEIN EXPRESSION BY JAK-STAT SIGNALING AFTER INTERLEUKIN-12 STIMULATION	37	0.20484598	0.20484598	0.0683168	0.61157167	1	20414	tags=62%, list=42%, signal=107%
REACTOME_TRANSPORT OF MATURE MRNA DERIVED FROM AN INTRONLESS TRANSCRIPT	REACTOME_TRANSPORT OF MATURE MRNA DERIVED FROM AN INTRONLESS TRANSCRIPT	41	0.2046474	0.2046474	0.0491968	0.61159736	1	17455	tags=56%, list=36%, signal=87%
REACTOME_COMPETING ENDOGENOUS RNAS (CERNAS) REGULATE PTEN TRANSLATION	REACTOME_COMPETING ENDOGENOUS RNAS (CERNAS) REGULATE PTEN TRANSLATION	8	0.20429918	0.20429918	0.8214634	0.61238545	1	8359	tags=38%, list=17%, signal=45%
REACTOME_RUNX1 REGULATES GENES INVOLVED IN MEGAKARYOCYTE DIFFERENTIATION AND	REACTOME_RUNX1 REGULATES GENES INVOLVED IN MEGAKARYOCYTE DIFFERENTIATION AND PLATELET FUNCTION	64	0.20419349	0.20419349	0.0070423	0.6119884	1	12201	tags=45%, list=25%, signal=60%
REACTOME_GLYCOPROTEIN HORMONES	REACTOME_GLYCOPROTEIN HORMONES	10	0.20393218	0.20393218	0.7261331	0.6123117	1	4705	tags=30%, list=10%, signal=33%
REACTOME_TRYPTOPHAN CATABOLISM	REACTOME_TRYPTOPHAN CATABOLISM	14	0.20350967	0.20350967	0.5351239	0.6133587	1	25012	tags=71%, list=51%, signal=146%
REACTOME_TOLL LIKE RECEPTOR 9 (TLR9) CASCADE	REACTOME_TOLL LIKE RECEPTOR 9 (TLR9) CASCADE	98	0.20342971	0.20342971	0	0.6128814	1	4048	tags=29%, list=8%, signal=31%
REACTOME_SUMOYLATION OF TRANSCRIPTION FACTORS	REACTOME_SUMOYLATION OF TRANSCRIPTION FACTORS	20	0.20324855	0.20324855	0.3352999	0.6128387	1	9637	tags=40%, list=20%, signal=50%
REACTOME_ALPHA-LINOLENIC (OMEGA3) AND LINOLEIC (OMEGA6) ACID METABOLISM	REACTOME_ALPHA-LINOLENIC (OMEGA3) AND LINOLEIC (OMEGA6) ACID METABOLISM	13	0.20319277	0.20319277	0.554337	0.61228603	1	8885	tags=38%, list=18%, signal=47%

REACTOME_ALPHA-LINOLENIC ACID (ALA) METABOLISM	REACTOME_ALPHA-LINOLENIC ACID (ALA) METABOLISM	13	0.20319277	0.20319277	0.5733064	0.6114608	1	8885	tags=38%, list=18%, signal=47%
REACTOME_THROMBOXANE SIGNALLING THROUGH TP RECEPTOR	REACTOME_THROMBOXANE SIGNALLING THROUGH TP RECEPTOR	23	0.20318373	0.20318373	0.2540816	0.61066455	1	9215	tags=39%, list=19%, signal=48%
REACTOME_ENDOGENOUS STEROLS	REACTOME_ENDOGENOUS STEROLS	27	0.20287271	0.20287271	0.1928721	0.61123693	1	8206	tags=37%, list=17%, signal=44%
REACTOME_INITIATION OF NUCLEAR ENVELOPE	REACTOME_INITIATION OF NUCLEAR ENVELOPE REFORMATION	12	0.20221949	0.20221949	0.6432806	0.613288	1	2341	tags=25%, list=5%, signal=26%
REACTOME_NUCLEAR ENVELOPE REASSEMBLY	REACTOME_NUCLEAR ENVELOPE REASSEMBLY	12	0.20221949	0.20221949	0.6286008	0.6124659	1	2341	tags=25%, list=5%, signal=26%
REACTOME_ORGANIC CATION/ANION/ZWITTERION TRANSPORT	REACTOME_ORGANIC CATION/ANION/ZWITTERION TRANSPORT	14	0.20200814	0.20200814	0.545005	0.612578	1	7598	tags=36%, list=16%, signal=42%
REACTOME_MYD88:MAL CASCADE INITIATED ON PLASMA	REACTOME_MYD88:MAL CASCADE INITIATED ON PLASMA MEMBRANE	95	0.20191056	0.20191056	0	0.6121624	1	4048	tags=28%, list=8%, signal=31%
REACTOME_TOLL LIKE RECEPTOR 2 (TLR2) CASCADE	REACTOME_TOLL LIKE RECEPTOR 2 (TLR2) CASCADE	95	0.20191056	0.20191056	0	0.6113452	1	4048	tags=28%, list=8%, signal=31%
REACTOME_TOLL LIKE RECEPTOR TLR1:TLR2 CASCADE	REACTOME_TOLL LIKE RECEPTOR TLR1:TLR2 CASCADE	95	0.20191056	0.20191056	9.49E-04	0.61052996	1	4048	tags=28%, list=8%, signal=31%
REACTOME_TOLL LIKE RECEPTOR TLR6:TLR2 CASCADE	REACTOME_TOLL LIKE RECEPTOR TLR6:TLR2 CASCADE	95	0.20191056	0.20191056	0.0031881	0.6097171	1	4048	tags=28%, list=8%, signal=31%
REACTOME_SIGNALING BY	REACTOME_SIGNALING BY NOTCH1	72	0.2017907	0.2017907	0.004065	0.609409	1	11215	tags=43%, list=23%, signal=56%
REACTOME_NUCLEAR RECEPTOR TRANSCRIPTION	REACTOME_NUCLEAR RECEPTOR TRANSCRIPTION PATHWAY	51	0.20119947	0.20119947	0.0336301	0.6112269	1	4559	tags=29%, list=9%, signal=32%
REACTOME_MYD88 CASCADE INITIATED ON PLASMA	REACTOME_MYD88 CASCADE INITIATED ON PLASMA MEMBRANE	85	0.20113364	0.20113364	9.63E-04	0.610718	1	3993	tags=28%, list=8%, signal=31%
REACTOME_TOLL LIKE RECEPTOR 10 (TLR10) CASCADE	REACTOME_TOLL LIKE RECEPTOR 10 (TLR10) CASCADE	85	0.20113364	0.20113364	0.0020263	0.6099091	1	3993	tags=28%, list=8%, signal=31%
REACTOME_TOLL LIKE RECEPTOR 5 (TLR5) CASCADE	REACTOME_TOLL LIKE RECEPTOR 5 (TLR5) CASCADE	85	0.20113364	0.20113364	0.002947	0.6091024	1	3993	tags=28%, list=8%, signal=31%
REACTOME_INTERLEUKIN-12 FAMILY SIGNALING	REACTOME_INTERLEUKIN-12 FAMILY SIGNALING	56	0.20092678	0.20092678	0.0162437	0.6091641	1	34765	tags=91%, list=71%, signal=314%
REACTOME_VITAMIN C (ASCORBATE) METABOLISM	REACTOME_VITAMIN C (ASCORBATE) METABOLISM	8	0.20058928	0.20058928	0.8377581	0.6098457	1	2420	tags=25%, list=5%, signal=26%
REACTOME_RHO GTPASE CYCLE	REACTOME_RHO GTPASE CYCLE	139	0.20057012	0.20057012	0	0.6091298	1	12751	tags=46%, list=26%, signal=62%
REACTOME_TOLL LIKE RECEPTOR 4 (TLR4) CASCADE	REACTOME_TOLL LIKE RECEPTOR 4 (TLR4) CASCADE	126	0.20048529	0.20048529	0	0.6087183	1	7695	tags=36%, list=16%, signal=42%
REACTOME_REGULATION OF TP53 ACTIVITY THROUGH	REACTOME_REGULATION OF TP53 ACTIVITY THROUGH ACETYLATION	30	0.19988765	0.19988765	0.1524574	0.61046207	1	24493	tags=70%, list=50%, signal=140%
REACTOME_REGULATION OF HSF1-MEDIATED HEAT SHOCK	REACTOME_REGULATION OF HSF1-MEDIATED HEAT SHOCK RESPONSE	81	0.19983311	0.19983311	0.001998	0.60989404	1	4134	tags=28%, list=8%, signal=31%
REACTOME_TRIGLYCERIDE BIOSYNTHESIS	REACTOME_TRIGLYCERIDE BIOSYNTHESIS	14	0.19967932	0.19967932	0.5184079	0.60975945	1	35692	tags=93%, list=73%, signal=342%
REACTOME_ACTIVATION OF THE PRE-REPLICATIVE COMPLEX	REACTOME_ACTIVATION OF THE PRE-REPLICATIVE COMPLEX	32	0.19948706	0.19948706	0.1375661	0.6098376	1	14720	tags=50%, list=30%, signal=71%
REACTOME_CYTOKINE SIGNALING IN IMMUNE SYSTEM	REACTOME_CYTOKINE SIGNALING IN IMMUNE SYSTEM	900	0.19887434	0.19887434	0	0.61164993	1	10951	tags=42%, list=22%, signal=53%
REACTOME_INTERACTIONS OF REV WITH HOST CELLULAR	REACTOME_INTERACTIONS OF REV WITH HOST CELLULAR PROTEINS	36	0.19807613	0.19807613	0.1056676	0.6143297	1	6629	tags=33%, list=14%, signal=39%
REACTOME_HEDGEHOG 'ON'	REACTOME_HEDGEHOG 'ON' STATE	85	0.19773637	0.19773637	0.0010417	0.61503464	1	13376	tags=47%, list=27%, signal=65%
REACTOME_FREE FATTY ACID RECEPTORS	REACTOME_FREE FATTY ACID RECEPTORS	5	0.19750817	0.19750817	0.9692155	0.6152658	1	29501	tags=80%, list=60%, signal=201%
REACTOME_RHO GTPASES ACTIVATE KTN1	REACTOME_RHO GTPASES ACTIVATE KTN1	11	0.19743954	0.19743954	0.7129001	0.61475843	1	8139	tags=36%, list=17%, signal=44%

REACTOME_CHYLOMICRON REMODELING	REACTOME_CHYLOMICRON REMODELING	10	0.19723216	0.19723216	0.7638749	0.61488307	1	5033	tags=30%, list=10%, signal=33%
REACTOME_FATTY ACYL-COA BIOSYNTHESIS	REACTOME_FATTY ACYL-COA BIOSYNTHESIS	37	0.19705074	0.19705074	0.1010204	0.61484516	1	8885	tags=38%, list=18%, signal=46%
REACTOME_APOPTOTIC EXECUTION PHASE	REACTOME_APOPTOTIC EXECUTION PHASE	51	0.196802	0.196802	0.0269231	0.6151448	1	3814	tags=27%, list=8%, signal=30%
REACTOME_HIV ELONGATION ARREST AND RECOVERY	REACTOME_HIV ELONGATION ARREST AND RECOVERY	29	0.19645011	0.19645011	0.1961962	0.6159152	1	7270	tags=34%, list=15%, signal=40%
REACTOME_PAUSING AND RECOVERY OF HIV ELONGATION	REACTOME_PAUSING AND RECOVERY OF HIV ELONGATION	29	0.19645011	0.19645011	0.182721	0.6151194	1	7270	tags=34%, list=15%, signal=40%
REACTOME_TRANSPORT OF THE SLBP DEPENDANT MATURE	REACTOME_TRANSPORT OF THE SLBP DEPENDANT MATURE MRNA	35	0.1964439	0.1964439	0.1286008	0.61435723	1	7175	tags=34%, list=15%, signal=40%
REACTOME_DOPAMINE	REACTOME_DOPAMINE RECEPTORS	5	0.19609885	0.19609885	0.9611273	0.61503774	1	39363	tags=100%, list=80%, signal=510%
REACTOME_CELLULAR RESPONSE TO HEAT STRESS	REACTOME_CELLULAR RESPONSE TO HEAT STRESS	97	0.19584675	0.19584675	0.0010142	0.6153469	1	3553	tags=27%, list=7%, signal=29%
REACTOME_NS1 MEDIATED EFFECTS ON HOST PATHWAYS	REACTOME_NS1 MEDIATED EFFECTS ON HOST PATHWAYS	40	0.19561575	0.19561575	0.0796813	0.61557454	1	5118	tags=30%, list=10%, signal=33%
REACTOME_N-GLYCAN ANTENNAE ELONGATION IN THE MEDIAL/TRANS-GOLGI	REACTOME_N-GLYCAN ANTENNAE ELONGATION IN THE MEDIAL/TRANS-GOLGI	26	0.19432828	0.19432828	0.2418236	0.6205534	1	13088	tags=46%, list=27%, signal=63%
REACTOME_STRIATED MUSCLE CONTRACTION	REACTOME_STRIATED MUSCLE CONTRACTION	34	0.19389188	0.19389188	0.1469388	0.62166846	1	3473	tags=26%, list=7%, signal=28%
REACTOME_NUCLEAR SIGNALING BY ERBB4	REACTOME_NUCLEAR SIGNALING BY ERBB4	25	0.19364119	0.19364119	0.2540323	0.62198275	1	6191	tags=32%, list=13%, signal=37%
REACTOME_SYNTHESIS OF VERY LONG-CHAIN FATTY ACYL-	REACTOME_SYNTHESIS OF VERY LONG-CHAIN FATTY ACYL-COAS	24	0.19361834	0.19361834	0.2625128	0.621293	1	8885	tags=38%, list=18%, signal=46%
REACTOME_INTERACTIONS OF VPR WITH HOST CELLULAR	REACTOME_INTERACTIONS OF VPR WITH HOST CELLULAR PROTEINS	36	0.19347195	0.19347195	0.1163491	0.62109095	1	4134	tags=28%, list=8%, signal=30%
REACTOME_CELL SURFACE INTERACTIONS AT THE VASCULAR WALL	REACTOME_CELL SURFACE INTERACTIONS AT THE VASCULAR WALL	211	0.19326219	0.19326219	0	0.6211872	1	25618	tags=72%, list=52%, signal=149%
REACTOME_SIGNAL REGULATORY PROTEIN FAMILY	REACTOME_SIGNAL REGULATORY PROTEIN FAMILY INTERACTIONS	13	0.19325997	0.19325997	0.6403326	0.6204017	1	35737	tags=92%, list=73%, signal=342%
REACTOME_SIGNALING BY RAS MUTANTS	REACTOME_SIGNALING BY RAS MUTANTS	48	0.19318609	0.19318609	0.0558276	0.6199697	1	10951	tags=42%, list=22%, signal=54%
REACTOME_EARLY PHASE OF HIV LIFE CYCLE	REACTOME_EARLY PHASE OF HIV LIFE CYCLE	14	0.19308086	0.19308086	0.5890688	0.6196916	1	8035	tags=36%, list=16%, signal=43%
REACTOME_NUCLEAR ENVELOPE BREAKDOWN	REACTOME_NUCLEAR ENVELOPE BREAKDOWN	51	0.19272083	0.19272083	0.0396432	0.62042695	1	17455	tags=55%, list=36%, signal=85%
REACTOME_ANCHORING OF THE BASAL BODY TO THE PLASMA MEMBRANE	REACTOME_ANCHORING OF THE BASAL BODY TO THE PLASMA MEMBRANE	97	0.1927127	0.1927127	0.003009	0.6196901	1	16326	tags=53%, list=33%, signal=79%
REACTOME_TRANSCRIPTIONAL ACTIVATION OF MITOCHONDRIAL BIOGENESIS	REACTOME_TRANSCRIPTIONAL ACTIVATION OF MITOCHONDRIAL BIOGENESIS	53	0.19232269	0.19232269	0.0303327	0.62061876	1	5374	tags=30%, list=11%, signal=34%
REACTOME_DETOXIFICATION OF REACTIVE OXYGEN SPECIES	REACTOME_DETOXIFICATION OF REACTIVE OXYGEN SPECIES	37	0.19225606	0.19225606	0.1061508	0.62011725	1	21030	tags=62%, list=43%, signal=109%
REACTOME_RRNA MODIFICATION IN THE NUCLEUS	REACTOME_RRNA MODIFICATION IN THE NUCLEUS AND CYTOSOL	61	0.19210568	0.19210568	0.0212551	0.62001103	1	13080	tags=46%, list=27%, signal=63%
REACTOME_EPHRIN SIGNALING	REACTOME_EPHRIN SIGNALING	19	0.19165267	0.19165267	0.437811	0.6211928	1	6081	tags=32%, list=12%, signal=36%
REACTOME_FCGAMMA RECEPTOR (FCGR) DEPENDENT	REACTOME_FCGAMMA RECEPTOR (FCGR) DEPENDENT PHAGOCYTOSIS	163	0.19158761	0.19158761	0	0.62067825	1	25195	tags=71%, list=51%, signal=145%
REACTOME_COPI-DEPENDENT GOLGI-TO-ER RETROGRADE	REACTOME_COPI-DEPENDENT GOLGI-TO-ER RETROGRADE TRAFFIC	82	0.1914277	0.1914277	0.0091556	0.620591	1	10944	tags=41%, list=22%, signal=53%

REACTOME_SUMOYLATION OF DNA DAMAGE RESPONSE AND REPAIR PROTEINS	REACTOME_SUMOYLATION OF DNA DAMAGE RESPONSE AND REPAIR PROTEINS	76	0.19061305	0.19061305	0.0118812	0.6233621	1	18384	tags=57%, list=38%, signal=90%
REACTOME_BH3-ONLY PROTEINS ASSOCIATE WITH AND INACTIVATE ANTI-APOPTOTIC	REACTOME_BH3-ONLY PROTEINS ASSOCIATE WITH AND INACTIVATE ANTI-APOPTOTIC BCL-2 MEMBERS	8	0.19005454	0.19005454	0.8687068	0.6250303	1	15177	tags=50%, list=31%, signal=72%
REACTOME_CITRIC ACID CYCLE (TCA CYCLE)	REACTOME_CITRIC ACID CYCLE (TCA CYCLE)	19	0.18891497	0.18891497	0.464497	0.6293805	1	6215	tags=32%, list=13%, signal=36%
REACTOME_DOWNREGULATION OF TGF-BETA RECEPTOR	REACTOME_DOWNREGULATION OF TGF-BETA RECEPTOR SIGNALING	26	0.18854399	0.18854399	0.2689162	0.6302131	1	5838	tags=31%, list=12%, signal=35%
REACTOME_REV-MEDIATED NUCLEAR EXPORT OF HIV RNA	REACTOME_REV-MEDIATED NUCLEAR EXPORT OF HIV RNA	34	0.18825729	0.18825729	0.1561866	0.63074136	1	6629	tags=32%, list=14%, signal=37%
REACTOME_REGULATION OF FZD BY UBIQUITINATION	REACTOME_REGULATION OF FZD BY UBIQUITINATION	21	0.18811296	0.18811296	0.4089069	0.6305979	1	11777	tags=43%, list=24%, signal=56%
REACTOME_SYNTHESIS OF PA	REACTOME_SYNTHESIS OF PA	39	0.18696053	0.18696053	0.1190717	0.63481396	1	13451	tags=46%, list=27%, signal=64%
REACTOME_SIGNAL	REACTOME_SIGNAL AMPLIFICATION	32	0.18690608	0.18690608	0.180198	0.6342525	1	9215	tags=38%, list=19%, signal=46%
REACTOME_THROMBIN SIGNALLING THROUGH PROTEINASE ACTIVATED	REACTOME_THROMBIN SIGNALLING THROUGH PROTEINASE ACTIVATED RECEPTORS (PARS)	32	0.18690608	0.18690608	0.1971545	0.6334636	1	9215	tags=38%, list=19%, signal=46%
REACTOME_POST-CHAPERONIN TUBULIN FOLDING PATHWAY	REACTOME_POST-CHAPERONIN TUBULIN FOLDING PATHWAY	22	0.18554516	0.18554516	0.3804453	0.6387284	1	10949	tags=41%, list=22%, signal=53%
REACTOME_REGULATION OF PLK1 ACTIVITY AT G2/M	REACTOME_REGULATION OF PLK1 ACTIVITY AT G2/M TRANSITION	87	0.18551962	0.18551962	0.0078355	0.63804746	1	12881	tags=45%, list=26%, signal=61%
REACTOME_RHO GTPASES ACTIVATE WASPS AND WAVES	REACTOME_RHO GTPASES ACTIVATE WASPS AND WAVES	36	0.18523553	0.18523553	0.1570248	0.6385075	1	4537	tags=28%, list=9%, signal=31%
REACTOME_TP53 REGULATES TRANSCRIPTION OF DNA REPAIR GENES	REACTOME_TP53 REGULATES TRANSCRIPTION OF DNA REPAIR GENES	58	0.18485296	0.18485296	0.035122	0.6394572	1	12064	tags=43%, list=25%, signal=57%
REACTOME_FANCONI ANEMIA PATHWAY	REACTOME_FANCONI ANEMIA PATHWAY	36	0.184376	0.184376	0.1481113	0.64078283	1	23621	tags=67%, list=48%, signal=129%
REACTOME_DISASSEMBLY OF THE DESTRUCTION COMPLEX AND RECRUITMENT OF AXIN TO THE MEMBRANE	REACTOME_DISASSEMBLY OF THE DESTRUCTION COMPLEX AND RECRUITMENT OF AXIN TO THE MEMBRANE	31	0.18386318	0.18386318	0.238477	0.6423547	1	6797	tags=32%, list=14%, signal=37%
REACTOME_PKA ACTIVATION	REACTOME_PKA ACTIVATION	16	0.1837065	0.1837065	0.6087805	0.64226264	1	3248	tags=25%, list=7%, signal=27%
REACTOME_CD28 CO-	REACTOME_CD28 CO-STIMULATION	35	0.18291438	0.18291438	0.1829026	0.6450965	1	17630	tags=54%, list=36%, signal=85%
REACTOME_PYRUVATE METABOLISM AND CITRIC ACID (TCA) CYCLE	REACTOME_PYRUVATE METABOLISM AND CITRIC ACID (TCA) CYCLE	49	0.1809954	0.1809954	0.0733198	0.65293294	1	7134	tags=33%, list=15%, signal=38%
REACTOME_METABOLISM OF NUCLEOTIDES	REACTOME_METABOLISM OF NUCLEOTIDES	102	0.18093166	0.18093166	0.0050302	0.6524365	1	9400	tags=37%, list=19%, signal=46%
REACTOME_TRANSCRIPTIONAL REGULATION BY THE AP-2 (TFAP2) FAMILY OF TRANSCRIPTION FACTORS	REACTOME_TRANSCRIPTIONAL REGULATION BY THE AP-2 (TFAP2) FAMILY OF TRANSCRIPTION FACTORS	36	0.18071537	0.18071537	0.1549439	0.652562	1	12919	tags=44%, list=26%, signal=60%
REACTOME_TRANSPORT OF THE SLBP INDEPENDENT MATURE	REACTOME_TRANSPORT OF THE SLBP INDEPENDENT MATURE MRNA	34	0.18038306	0.18038306	0.1757451	0.65324825	1	4134	tags=26%, list=8%, signal=29%
REACTOME_SUMOYLATION OF DNA REPLICATION PROTEINS	REACTOME_SUMOYLATION OF DNA REPLICATION PROTEINS	45	0.18024895	0.18024895	0.1170635	0.65306807	1	18384	tags=56%, list=38%, signal=89%
REACTOME_UPTAKE AND ACTIONS OF BACTERIAL TOXINS	REACTOME_UPTAKE AND ACTIONS OF BACTERIAL TOXINS	29	0.18019313	0.18019313	0.2914172	0.65252155	1	9754	tags=38%, list=20%, signal=47%
REACTOME_TP53 REGULATES TRANSCRIPTION OF ADDITIONAL CELL CYCLE GENES WHOSE EXACT ROLE IN THE P53 PATHWAY REMAIN UNCERTAIN	REACTOME_TP53 REGULATES TRANSCRIPTION OF ADDITIONAL CELL CYCLE GENES WHOSE EXACT ROLE IN THE P53 PATHWAY REMAIN UNCERTAIN	20	0.17998774	0.17998774	0.4557823	0.65265083	1	5879	tags=30%, list=12%, signal=34%

REACTOME_TRANSCRIPTIONAL REGULATION OF WHITE ADIPOCYTE DIFFERENTIATION	REACTOME_TRANSCRIPTIONAL REGULATION OF WHITE ADIPOCYTE DIFFERENTIATION	83	0.17942297	0.17942297	0.01002	0.65436465	1	5387	tags=29%, list=11%, signal=32%
REACTOME_SIGNALING BY INTERLEUKINS	REACTOME_SIGNALING BY INTERLEUKINS	687	0.17913449	0.17913449	0	0.65491956	1	10951	tags=40%, list=22%, signal=51%
REACTOME_TOLL-LIKE RECEPTORS CASCADES	REACTOME_TOLL-LIKE RECEPTORS CASCADES	152	0.1789082	0.1789082	0	0.655176	1	7695	tags=34%, list=16%, signal=40%
REACTOME_SYNTHESIS OF PIPS AT THE EARLY ENDOSOME	REACTOME_SYNTHESIS OF PIPS AT THE EARLY ENDOSOME MEMBRANE	16	0.17864384	0.17864384	0.6290323	0.6555626	1	24918	tags=69%, list=51%, signal=140%
REACTOME_PCP/CE PATHWAY	REACTOME_PCP/CE PATHWAY	91	0.17863518	0.17863518	0.007992	0.6548081	1	16558	tags=52%, list=34%, signal=78%
REACTOME_SYNTHESIS OF LEUKOTRIENES (LT) AND EOXINS	REACTOME_SYNTHESIS OF LEUKOTRIENES (LT) AND EOXINS (EX)	21	0.17800617	0.17800617	0.4747475	0.6567849	1	16935	tags=52%, list=35%, signal=80%
REACTOME_TBC/RABGAPS	REACTOME_TBC/RABGAPS	45	0.17711684	0.17711684	0.1155779	0.66005325	1	6568	tags=31%, list=13%, signal=36%
REACTOME_G ALPHA (12/13) SIGNALLING EVENTS	REACTOME_G ALPHA (12/13) SIGNALLING EVENTS	87	0.17666459	0.17666459	0.0111902	0.66126907	1	12751	tags=44%, list=26%, signal=59%
REACTOME_NOTCH1 INTRACELLULAR DOMAIN REGULATES TRANSCRIPTION	REACTOME_NOTCH1 INTRACELLULAR DOMAIN REGULATES TRANSCRIPTION	47	0.17650293	0.17650293	0.0880881	0.6612204	1	12201	tags=43%, list=25%, signal=57%
REACTOME_FORMATION OF TUBULIN FOLDING INTERMEDIATES BY CCT/TRIC	REACTOME_FORMATION OF TUBULIN FOLDING INTERMEDIATES BY CCT/TRIC	25	0.17646097	0.17646097	0.3642931	0.6605905	1	10949	tags=40%, list=22%, signal=51%
REACTOME_POST-ELONGATION PROCESSING OF INTRONLESS PRE-MRNA	REACTOME_POST-ELONGATION PROCESSING OF INTRONLESS PRE-MRNA	28	0.17630324	0.17630324	0.2910373	0.6605212	1	21100	tags=61%, list=43%, signal=107%
REACTOME_PROCESSING OF CAPPED INTRONLESS PRE-MRNA	REACTOME_PROCESSING OF CAPPED INTRONLESS PRE-MRNA	28	0.17630324	0.17630324	0.3106893	0.6597263	1	21100	tags=61%, list=43%, signal=107%
REACTOME_REGULATION OF RUNX3 EXPRESSION AND	REACTOME_REGULATION OF RUNX3 EXPRESSION AND ACTIVITY	54	0.17587084	0.17587084	0.0639594	0.6608591	1	17693	tags=54%, list=36%, signal=84%
REACTOME_SYNTHESIS OF IP3 AND IP4 IN THE CYTOSOL	REACTOME_SYNTHESIS OF IP3 AND IP4 IN THE CYTOSOL	26	0.17504288	0.17504288	0.3550107	0.66374475	1	8382	tags=35%, list=17%, signal=42%
REACTOME_TRP CHANNELS	REACTOME_TRP CHANNELS	25	0.1735758	0.1735758	0.4048096	0.6696438	1	16966	tags=52%, list=35%, signal=80%
REACTOME_MAPK6/MAPK4 SIGNALING	REACTOME_MAPK6/MAPK4 SIGNALING	88	0.17304175	0.17304175	9.96E-04	0.67135364	1	17693	tags=53%, list=36%, signal=83%
REACTOME_TRNA MODIFICATION IN THE NUCLEUS AND CYTOSOL	REACTOME_TRNA MODIFICATION IN THE NUCLEUS AND CYTOSOL	43	0.17226139	0.17226139	0.1372162	0.6741192	1	6375	tags=30%, list=13%, signal=35%
REACTOME_RETROGRADE TRANSPORT AT THE TRANS-GOLGI-NETWORK	REACTOME_RETROGRADE TRANSPORT AT THE TRANS-GOLGI-NETWORK	49	0.1719595	0.1719595	0.0954774	0.67474914	1	14571	tags=47%, list=30%, signal=67%
REACTOME_ADP SIGNALLING THROUGH P2Y PURINOCEPTOR 1	REACTOME_ADP SIGNALLING THROUGH P2Y PURINOCEPTOR 1	25	0.17187168	0.17187168	0.4228395	0.67434597	1	9215	tags=36%, list=19%, signal=44%
REACTOME_HOMOLOGY DIRECTED REPAIR	REACTOME_HOMOLOGY DIRECTED REPAIR	118	0.17176585	0.17176585	9.79E-04	0.6740035	1	18581	tags=55%, list=38%, signal=89%
REACTOME_TRANSCRIPTION OF E2F TARGETS UNDER NEGATIVE CONTROL BY DREAM COMPLEX	REACTOME_TRANSCRIPTION OF E2F TARGETS UNDER NEGATIVE CONTROL BY DREAM COMPLEX	19	0.17154889	0.17154889	0.5656155	0.67419946	1	7065	tags=32%, list=14%, signal=37%
REACTOME_SIGNALING BY HEDGEHOG	REACTOME_SIGNALING BY HEDGEHOG	136	0.1713793	0.1713793	0.001007	0.6742128	1	13953	tags=46%, list=28%, signal=64%
REACTOME_THE ROLE OF GTSE1 IN G2/M PROGRESSION AFTER G2 CHECKPOINT	REACTOME_THE ROLE OF GTSE1 IN G2/M PROGRESSION AFTER G2 CHECKPOINT	59	0.17110445	0.17110445	0.0544355	0.67466	1	20678	tags=59%, list=42%, signal=103%
REACTOME_INTERLEUKIN-1 FAMILY SIGNALING	REACTOME_INTERLEUKIN-1 FAMILY SIGNALING	94	0.17088279	0.17088279	0.0083074	0.67486113	1	17693	tags=53%, list=36%, signal=83%
REACTOME_REPRESSION OF WNT TARGET GENES	REACTOME_REPRESSION OF WNT TARGET GENES	14	0.17083411	0.17083411	0.7619522	0.6742898	1	2129	tags=21%, list=4%, signal=22%
REACTOME_EFFECTS OF PIP2 HYDROLYSIS	REACTOME_EFFECTS OF PIP2 HYDROLYSIS	27	0.17053701	0.17053701	0.3580875	0.674829	1	15229	tags=48%, list=31%, signal=70%

REACTOME_SYNTHESIS OF DNA	REACTOME_SYNTHESIS OF DNA	99	0.17030185	0.17030185	0.0030519	0.67514706	1	19869	tags=58%, list=41%, signal=97%
REACTOME_KINESINS	REACTOME_KINESINS	43	0.17028767	0.17028767	0.1589069	0.67440933	1	8749	tags=35%, list=18%, signal=42%
REACTOME_ACTIVATION OF NF-KAPPAB IN B CELLS	REACTOME_ACTIVATION OF NF-KAPPAB IN B CELLS	66	0.17018694	0.17018694	0.0433031	0.67408895	1	19869	tags=58%, list=41%, signal=97%
REACTOME_MHC CLASS II ANTIGEN PRESENTATION	REACTOME_MHC CLASS II ANTIGEN PRESENTATION	105	0.16957295	0.16957295	0.0081716	0.67610073	1	10367	tags=38%, list=21%, signal=48%
REACTOME_VIRAL MESSENGER RNA SYNTHESIS	REACTOME_VIRAL MESSENGER RNA SYNTHESIS	43	0.1693089	0.1693089	0.1565907	0.67653674	1	4242	tags=26%, list=9%, signal=28%
REACTOME_EGFR INTERACTS WITH PHOSPHOLIPASE C-GAMMA	REACTOME_EGFR INTERACTS WITH PHOSPHOLIPASE C-GAMMA	34	0.169058	0.169058	0.2547835	0.6769248	1	3248	tags=24%, list=7%, signal=25%
REACTOME_PKA ACTIVATION IN GLUCAGON SIGNALLING	REACTOME_PKA ACTIVATION IN GLUCAGON SIGNALLING	17	0.16899928	0.16899928	0.6443089	0.6763964	1	3248	tags=24%, list=7%, signal=25%
REACTOME_PKA-MEDIATED PHOSPHORYLATION OF CREB	REACTOME_PKA-MEDIATED PHOSPHORYLATION OF CREB	17	0.16899928	0.16899928	0.6564103	0.6756034	1	3248	tags=24%, list=7%, signal=25%
REACTOME_DEACTIVATION OF THE BETA-CATENIN TRANSACTIVATING COMPLEX	REACTOME_DEACTIVATION OF THE BETA-CATENIN TRANSACTIVATING COMPLEX	42	0.16813837	0.16813837	0.168932	0.67872983	1	6929	tags=31%, list=14%, signal=36%
REACTOME_DNA DOUBLE STRAND BREAK RESPONSE	REACTOME_DNA DOUBLE STRAND BREAK RESPONSE	59	0.1675265	0.1675265	0.0679012	0.6807684	1	10894	tags=39%, list=22%, signal=50%
REACTOME_ORC1 REMOVAL FROM CHROMATIN	REACTOME_ORC1 REMOVAL FROM CHROMATIN	70	0.16745065	0.16745065	0.0371859	0.6803058	1	17693	tags=53%, list=36%, signal=83%
REACTOME_SWITCHING OF ORIGINS TO A POST-	REACTOME_SWITCHING OF ORIGINS TO A POST-REPLICATIVE STATE	70	0.16745065	0.16745065	0.0333333	0.679512	1	17693	tags=53%, list=36%, signal=83%
REACTOME_SUMO E3 LIGASES SUMOYLATE TARGET PROTEINS	REACTOME_SUMO E3 LIGASES SUMOYLATE TARGET PROTEINS	112	0.16686158	0.16686158	0.0019157	0.6813761	1	16767	tags=51%, list=34%, signal=77%
REACTOME_PI3K EVENTS IN ERBB2 SIGNALING	REACTOME_PI3K EVENTS IN ERBB2 SIGNALING	16	0.16680498	0.16680498	0.672888	0.68081784	1	1015	tags=19%, list=2%, signal=19%
REACTOME_HOST INTERACTIONS OF HIV FACTORS	REACTOME_HOST INTERACTIONS OF HIV FACTORS	127	0.16663107	0.16663107	0.0010183	0.6808053	1	17693	tags=53%, list=36%, signal=82%
REACTOME_ASYMMETRIC LOCALIZATION OF PCP	REACTOME_ASYMMETRIC LOCALIZATION OF PCP PROTEINS	63	0.16584194	0.16584194	0.0493827	0.6836172	1	19869	tags=57%, list=41%, signal=96%
REACTOME_HDR THROUGH HOMOLOGOUS RECOMBINATION (HR) OR SINGLE STRAND ANNEALING (SSA)	REACTOME_HDR THROUGH HOMOLOGOUS RECOMBINATION (HR) OR SINGLE STRAND ANNEALING (SSA)	112	0.16552594	0.16552594	0.002907	0.68428534	1	18581	tags=54%, list=38%, signal=88%
REACTOME_PLATELET CALCIUM HOMEOSTASIS	REACTOME_PLATELET CALCIUM HOMEOSTASIS	28	0.16535038	0.16535038	0.3899497	0.68427914	1	14641	tags=46%, list=30%, signal=66%
REACTOME_FACTORS INVOLVED IN MEGAKARYOCYTE DEVELOPMENT AND PLATELET	REACTOME_FACTORS INVOLVED IN MEGAKARYOCYTE DEVELOPMENT AND PLATELET PRODUCTION	140	0.16462877	0.16462877	0.0019881	0.686792	1	8749	tags=34%, list=18%, signal=42%
REACTOME_METABOLISM OF VITAMINS AND COFACTORS	REACTOME_METABOLISM OF VITAMINS AND COFACTORS	180	0.16311538	0.16311538	0	0.6929649	1	7547	tags=32%, list=15%, signal=37%
REACTOME_IMMUNE SYSTEM	REACTOME_IMMUNE SYSTEM	2213	0.1629798	0.1629798	0	0.6927681	1	10567	tags=37%, list=22%, signal=45%
REACTOME_TCR SIGNALING	REACTOME_TCR SIGNALING	118	0.16238791	0.16238791	0.003	0.6947047	1	19869	tags=57%, list=41%, signal=95%
REACTOME_REGULATION OF MRNA STABILITY BY PROTEINS THAT BIND AU-RICH ELEMENTS	REACTOME_REGULATION OF MRNA STABILITY BY PROTEINS THAT BIND AU-RICH ELEMENTS	88	0.16214658	0.16214658	0.025974	0.69507205	1	14887	tags=47%, list=30%, signal=67%
REACTOME_DNA DOUBLE-STRAND BREAK REPAIR	REACTOME_DNA DOUBLE-STRAND BREAK REPAIR	146	0.1620836	0.1620836	0	0.6945904	1	18581	tags=54%, list=38%, signal=87%
REACTOME_DOWNSTREAM TCR SIGNALING	REACTOME_DOWNSTREAM TCR SIGNALING	97	0.16153023	0.16153023	0.011022	0.69638044	1	19869	tags=57%, list=41%, signal=95%
REACTOME_SIGNALING BY	REACTOME_SIGNALING BY ERBB4	43	0.16152431	0.16152431	0.1979695	0.69560814	1	8039	tags=33%, list=16%, signal=39%
REACTOME_ASSEMBLY OF THE PRE-REPLICATIVE COMPLEX	REACTOME_ASSEMBLY OF THE PRE-REPLICATIVE COMPLEX	67	0.16124853	0.16124853	0.0462366	0.6960464	1	17693	tags=52%, list=36%, signal=82%
REACTOME_POST NMDA RECEPTOR ACTIVATION EVENTS	REACTOME_POST NMDA RECEPTOR ACTIVATION EVENTS	35	0.16106683	0.16106683	0.2834171	0.6961157	1	3310	tags=23%, list=7%, signal=24%

REACTOME_FORMATION OF THE BETA-CATENIN:TCF TRANSACTIVATING COMPLEX	REACTOME_FORMATION OF THE BETA-CATENIN:TCF TRANSACTIVATING COMPLEX	61	0.160838	0.160838	0.0626808	0.6964105	1	12201	tags=41%, list=25%, signal=55%
REACTOME_GLUTATHIONE CONJUGATION	REACTOME_GLUTATHIONE CONJUGATION	35	0.15943184	0.15943184	0.3115079	0.7022076	1	3390	tags=23%, list=7%, signal=25%
REACTOME_CDK-MEDIATED PHOSPHORYLATION AND REMOVAL OF CDC6	REACTOME_CDK-MEDIATED PHOSPHORYLATION AND REMOVAL OF CDC6	50	0.15880197	0.15880197	0.1600408	0.7043601	1	17693	tags=52%, list=36%, signal=81%
REACTOME_SUMOYLATION OF RNA BINDING PROTEINS	REACTOME_SUMOYLATION OF RNA BINDING PROTEINS	46	0.15872289	0.15872289	0.1819106	0.7039214	1	2879	tags=22%, list=6%, signal=23%
REACTOME_POST-TRANSLATIONAL PROTEIN	REACTOME_POST-TRANSLATIONAL PROTEIN MODIFICATION	1327	0.15847869	0.15847869	0	0.70425695	1	7209	tags=30%, list=15%, signal=34%
REACTOME_REGULATION OF APOPTOSIS	REACTOME_REGULATION OF APOPTOSIS	52	0.15803847	0.15803847	0.1325536	0.7054041	1	17693	tags=52%, list=36%, signal=81%
REACTOME_AUF1 (HNRNP D0) BINDS AND DESTABILIZES MRNA	REACTOME_AUF1 (HNRNP D0) BINDS AND DESTABILIZES MRNA	54	0.15733187	0.15733187	0.1138614	0.7079125	1	17693	tags=52%, list=36%, signal=81%
REACTOME_RECRUITMENT AND ATM-MEDIATED PHOSPHORYLATION OF REPAIR AND SIGNALING PROTEINS AT	REACTOME_RECRUITMENT AND ATM-MEDIATED PHOSPHORYLATION OF REPAIR AND SIGNALING PROTEINS AT DNA DOUBLE STRAND BREAKS	58	0.15699044	0.15699044	0.1019368	0.70870197	1	10894	tags=38%, list=22%, signal=49%
REACTOME_DEGRADATION OF	REACTOME_DEGRADATION OF DVL	56	0.1566762	0.1566762	0.1237425	0.7093791	1	17693	tags=52%, list=36%, signal=81%
REACTOME_PACKAGING OF TELOMERE ENDS	REACTOME_PACKAGING OF TELOMERE ENDS	33	0.1563289	0.1563289	0.3460076	0.71022326	1	30928	tags=79%, list=63%, signal=214%
REACTOME_SYNTHESIS OF (16-20)-HYDROXYEICOSATETRAENOIC	REACTOME_SYNTHESIS OF (16-20)-HYDROXYEICOSATETRAENOIC ACIDS (HETE)	9	0.1561039	0.1561039	0.9641077	0.7104686	1	14119	tags=44%, list=29%, signal=62%
REACTOME_TRANSPORT TO THE GOLGI AND SUBSEQUENT MODIFICATION	REACTOME_TRANSPORT TO THE GOLGI AND SUBSEQUENT MODIFICATION	167	0.15566997	0.15566997	0.001003	0.7117581	1	7356	tags=31%, list=15%, signal=36%
REACTOME_PERK REGULATES GENE EXPRESSION	REACTOME_PERK REGULATES GENE EXPRESSION	28	0.15539369	0.15539369	0.4872549	0.71228296	1	9882	tags=36%, list=20%, signal=45%
REACTOME_MET ACTIVATES PI3K/AKT SIGNALING	REACTOME_MET ACTIVATES PI3K/AKT SIGNALING	6	0.15485747	0.15485747	0.9959391	0.7139793	1	8739	tags=33%, list=18%, signal=41%
REACTOME_FCERI MEDIATED NF-KB ACTIVATION	REACTOME_FCERI MEDIATED NF-KB ACTIVATION	151	0.15477985	0.15477985	0.005123	0.7135574	1	25195	tags=67%, list=51%, signal=137%
REACTOME_HEDGEHOG LIGAND BIOGENESIS	REACTOME_HEDGEHOG LIGAND BIOGENESIS	64	0.15446675	0.15446675	0.0909091	0.71423596	1	17693	tags=52%, list=36%, signal=81%
REACTOME_PROGRAMMED CELL DEATH	REACTOME_PROGRAMMED CELL DEATH	169	0.15418413	0.15418413	0	0.714748	1	17972	tags=52%, list=37%, signal=82%
REACTOME_GLYCEROPHOSPHO LIPID BIOSYNTHESIS	REACTOME_GLYCEROPHOSPHOLIPID BIOSYNTHESIS	129	0.15365061	0.15365061	0.0030151	0.7164516	1	11474	tags=39%, list=23%, signal=50%
REACTOME_ACTIVATION OF THE TFAP2 (AP-2) FAMILY OF TRANSCRIPTION FACTORS	REACTOME_ACTIVATION OF THE TFAP2 (AP-2) FAMILY OF TRANSCRIPTION FACTORS	12	0.15284221	0.15284221	0.8959366	0.7194929	1	12919	tags=42%, list=26%, signal=57%
REACTOME_CELLULAR RESPONSE TO HYPOXIA	REACTOME_CELLULAR RESPONSE TO HYPOXIA	72	0.15275334	0.15275334	0.0636183	0.7191186	1	17693	tags=51%, list=36%, signal=80%
REACTOME_REGULATION OF HYPOXIA-INDUCIBLE FACTOR (HIF) BY OXYGEN	REACTOME_REGULATION OF HYPOXIA-INDUCIBLE FACTOR (HIF) BY OXYGEN	72	0.15275334	0.15275334	0.0814664	0.7183142	1	17693	tags=51%, list=36%, signal=80%
REACTOME_REMOVAL OF LICENSING FACTORS FROM	REACTOME_REMOVAL OF LICENSING FACTORS FROM ORIGINS	72	0.15275334	0.15275334	0.0661841	0.71751165	1	17693	tags=51%, list=36%, signal=80%
REACTOME_DAG AND IP3	REACTOME_DAG AND IP3 SIGNALING	32	0.15249614	0.15249614	0.4092742	0.7178915	1	3248	tags=22%, list=7%, signal=23%
REACTOME_CYTOSOLIC SULFONATION OF SMALL MOLECULES	REACTOME_CYTOSOLIC SULFONATION OF SMALL MOLECULES	24	0.15232883	0.15232883	0.5659068	0.7178828	1	23147	tags=63%, list=47%, signal=118%
REACTOME_G0 AND EARLY G1	REACTOME_G0 AND EARLY G1	27	0.152073	0.152073	0.5071428	0.71827114	1	7065	tags=30%, list=14%, signal=35%

REACTOME_ER TO GOLGI ANTEROGRADE TRANSPORT	REACTOME_ER TO GOLGI ANTEROGRADE TRANSPORT	136	0.15194653	0.15194653	0.0069652	0.718073	1	6261	tags=28%, list=13%, signal=32%
REACTOME_APOPTOSIS	REACTOME_APOPTOSIS	166	0.15152787	0.15152787	0.0020346	0.7192581	1	17972	tags=52%, list=37%, signal=82%
REACTOME_INTERCONVERSION OF NUCLEOTIDE DI- AND TRIPHOSPHATES	REACTOME_INTERCONVERSION OF NUCLEOTIDE DI- AND TRIPHOSPHATES	32	0.15127955	0.15127955	0.3897959	0.7195847	1	7898	tags=31%, list=16%, signal=37%
REACTOME_HEDGEHOG 'OFF'	REACTOME_HEDGEHOG 'OFF' STATE	99	0.15123796	0.15123796	0.0152439	0.7189687	1	17833	tags=52%, list=36%, signal=81%
REACTOME_TRIGLYCERIDE METABOLISM	REACTOME_TRIGLYCERIDE METABOLISM	38	0.15092196	0.15092196	0.3280633	0.71973175	1	20963	tags=58%, list=43%, signal=101%
REACTOME_CILIUM ASSEMBLY	REACTOME_CILIUM ASSEMBLY	187	0.1503117	0.1503117	0	0.7218668	1	13091	tags=42%, list=27%, signal=57%
REACTOME_INTRA-GOLGI AND RETROGRADE GOLGI-TO-ER	REACTOME_INTRA-GOLGI AND RETROGRADE GOLGI-TO-ER TRAFFIC	185	0.15001275	0.15001275	9.90E-04	0.7224751	1	10944	tags=37%, list=22%, signal=48%
REACTOME_SUMOYLATION	REACTOME_SUMOYLATION	118	0.14943688	0.14943688	0.008	0.724475	1	16767	tags=49%, list=34%, signal=75%
REACTOME_CROSS-PRESENTATION OF SOLUBLE EXOGENOUS ANTIGENS	REACTOME_CROSS-PRESENTATION OF SOLUBLE EXOGENOUS ANTIGENS (ENDOSOMES)	49	0.14899302	0.14899302	0.1952895	0.72581947	1	17693	tags=51%, list=36%, signal=80%
REACTOME_REGULATION OF ACTIVATED PAK-2P34 BY PROTEASOME MEDIATED DEGRADATION	REACTOME_REGULATION OF ACTIVATED PAK-2P34 BY PROTEASOME MEDIATED DEGRADATION	49	0.14899302	0.14899302	0.203668	0.7250201	1	17693	tags=51%, list=36%, signal=80%
REACTOME_HOMOLOGOUS DNA PAIRING AND STRAND	REACTOME_HOMOLOGOUS DNA PAIRING AND STRAND EXCHANGE	42	0.14885296	0.14885296	0.2958877	0.724875	1	18365	tags=52%, list=38%, signal=84%
REACTOME_ONCOGENE INDUCED SENESCENCE	REACTOME_ONCOGENE INDUCED SENESCENCE	31	0.14852856	0.14852856	0.4576772	0.725574	1	2208	tags=19%, list=5%, signal=20%
REACTOME_BASE EXCISION	REACTOME_BASE EXCISION REPAIR	38	0.14845641	0.14845641	0.3366734	0.7251114	1	5621	tags=26%, list=11%, signal=30%
REACTOME_RESOLUTION OF ABASIC SITES (AP SITES)	REACTOME_RESOLUTION OF ABASIC SITES (AP SITES)	38	0.14845641	0.14845641	0.3253968	0.72431636	1	5621	tags=26%, list=11%, signal=30%
REACTOME_C-TYPE LECTIN RECEPTORS (CLRS)	REACTOME_C-TYPE LECTIN RECEPTORS (CLRS)	141	0.14837793	0.14837793	0.0019512	0.72388697	1	17758	tags=51%, list=36%, signal=80%
REACTOME_INFLAMMASOMES	REACTOME_INFLAMMASOMES	17	0.14830986	0.14830986	0.7782139	0.7234016	1	7141	tags=29%, list=15%, signal=34%
REACTOME_DEPOSITION OF NEW CENPA-CONTAINING NUCLEOSOMES AT THE	REACTOME_DEPOSITION OF NEW CENPA-CONTAINING NUCLEOSOMES AT THE CENTROMERE	52	0.14820234	0.14820234	0.1631274	0.7231317	1	31357	tags=79%, list=64%, signal=219%
REACTOME_NUCLEOSOME ASSEMBLY	REACTOME_NUCLEOSOME ASSEMBLY	52	0.14820234	0.14820234	0.1565041	0.72234225	1	31357	tags=79%, list=64%, signal=219%
REACTOME_DISEASES OF SIGNAL TRANSDUCTION	REACTOME_DISEASES OF SIGNAL TRANSDUCTION	356	0.14819399	0.14819399	0	0.72159255	1	10951	tags=37%, list=22%, signal=47%
REACTOME_DNA REPLICATION	REACTOME_DNA REPLICATION	107	0.14812464	0.14812464	0.0234834	0.7210996	1	16558	tags=49%, list=34%, signal=73%
REACTOME_HH MUTANTS THAT DON'T UNDERGO AUTOCATALYTIC PROCESSING ARE DEGRADED BY ERAD	REACTOME_HH MUTANTS THAT DON'T UNDERGO AUTOCATALYTIC PROCESSING ARE DEGRADED BY ERAD	55	0.14789686	0.14789686	0.1767677	0.72141564	1	17693	tags=51%, list=36%, signal=80%
REACTOME_HH MUTANTS ABROGATE LIGAND SECRETION	REACTOME_HH MUTANTS ABROGATE LIGAND SECRETION	57	0.14758359	0.14758359	0.1620513	0.7221274	1	17693	tags=51%, list=36%, signal=80%
REACTOME_INNATE IMMUNE SYSTEM	REACTOME_INNATE IMMUNE SYSTEM	1394	0.1474198	0.1474198	0	0.72212654	1	11427	tags=38%, list=23%, signal=48%
REACTOME_REGULATION OF PTEN MRNA TRANSLATION	REACTOME_REGULATION OF PTEN MRNA TRANSLATION	13	0.14734057	0.14734057	0.8932927	0.72170454	1	4086	tags=23%, list=8%, signal=25%
REACTOME_DEGRADATION OF GLI1 BY THE PROTEASOME	REACTOME_DEGRADATION OF GLI1 BY THE PROTEASOME	59	0.1472919	0.1472919	0.1349125	0.72115564	1	17693	tags=51%, list=36%, signal=80%
REACTOME_METABOLISM OF WATER-SOLUBLE VITAMINS AND COFACTORS	REACTOME_METABOLISM OF WATER-SOLUBLE VITAMINS AND COFACTORS	123	0.14703141	0.14703141	0.0062893	0.72160506	1	7547	tags=30%, list=15%, signal=35%
REACTOME_HEMOSTASIS	REACTOME_HEMOSTASIS	684	0.14644608	0.14644608	0	0.7235838	1	8749	tags=32%, list=18%, signal=39%

REACTOME_CONSTITUTIVE SIGNALING BY AKT1 E17K IN CANCER	REACTOME_CONSTITUTIVE SIGNALING BY AKT1 E17K IN CANCER	25	0.14574173	0.14574173	0.611002	0.7262173	1	14411	tags=44%, list=29%, signal=62%
REACTOME_REGULATION OF DNA REPLICATION	REACTOME_REGULATION OF DNA REPLICATION	75	0.14552942	0.14552942	0.0625621	0.7264237	1	17693	tags=51%, list=36%, signal=79%
REACTOME_AXON GUIDANCE	REACTOME_AXON GUIDANCE	555	0.14538394	0.14538394	0	0.72634673	1	7077	tags=29%, list=14%, signal=33%
REACTOME_INTRAFLAGELLAR TRANSPORT	REACTOME_INTRAFLAGELLAR TRANSPORT	41	0.14532794	0.14532794	0.3357216	0.7258359	1	10803	tags=37%, list=22%, signal=47%
REACTOME_S PHASE	REACTOME_S PHASE	138	0.14527453	0.14527453	0.0049164	0.7253025	1	17743	tags=51%, list=36%, signal=79%
REACTOME_CHAPERONIN-MEDIATED PROTEIN FOLDING	REACTOME_CHAPERONIN-MEDIATED PROTEIN FOLDING	95	0.1450734	0.1450734	0.0339157	0.7254069	1	10949	tags=37%, list=22%, signal=47%
REACTOME_METABOLISM OF	REACTOME_METABOLISM OF LIPIDS	733	0.14474992	0.14474992	0	0.7261285	1	7580	tags=30%, list=15%, signal=35%
REACTOME_ACTIVATION OF ATR IN RESPONSE TO REPLICATION STRESS	REACTOME_ACTIVATION OF ATR IN RESPONSE TO REPLICATION STRESS	37	0.14401361	0.14401361	0.3914767	0.7288602	1	11480	tags=38%, list=23%, signal=49%
REACTOME_ANTIVIRAL MECHANISM BY IFN-STIMULATED	REACTOME_ANTIVIRAL MECHANISM BY IFN-STIMULATED GENES	73	0.14400372	0.14400372	0.1036168	0.7281259	1	18447	tags=52%, list=38%, signal=83%
REACTOME_ISG15 ANTIVIRAL MECHANISM	REACTOME_ISG15 ANTIVIRAL MECHANISM	73	0.14400372	0.14400372	0.0987167	0.7273471	1	18447	tags=52%, list=38%, signal=83%
REACTOME_CLEC7A (DECTIN-1) SIGNALING	REACTOME_CLEC7A (DECTIN-1) SIGNALING	99	0.14398147	0.14398147	0.0306748	0.72666967	1	17693	tags=51%, list=36%, signal=79%
REACTOME_SPRY REGULATION OF FGF SIGNALING	REACTOME_SPRY REGULATION OF FGF SIGNALING	16	0.1436061	0.1436061	0.8529412	0.7277334	1	35814	tags=88%, list=73%, signal=326%
REACTOME_G2/M TRANSITION	REACTOME_G2/M TRANSITION	181	0.14357756	0.14357756	0	0.72707665	1	17883	tags=51%, list=37%, signal=80%
REACTOME_MITOTIC G2-G2/M PHASES	REACTOME_MITOTIC G2-G2/M PHASES	183	0.1434925	0.1434925	0	0.7266975	1	17883	tags=51%, list=37%, signal=80%
REACTOME_SIGNALING BY ROBO RECEPTOR	REACTOME_SIGNALING BY ROBO RECEPTOR	32	0.14308469	0.14308469	0.4833006	0.72791535	1	8299	tags=31%, list=17%, signal=38%
REACTOME_PTEN REGULATION	REACTOME_PTEN REGULATION	142	0.14211178	0.14211178	0.0059642	0.73173076	1	17888	tags=51%, list=37%, signal=80%
REACTOME_DIGESTION	REACTOME_DIGESTION	22	0.14178368	0.14178368	0.7159329	0.73253024	1	39799	tags=95%, list=81%, signal=510%
REACTOME_MITOTIC PROMETAPHASE	REACTOME_MITOTIC PROMETAPHASE	181	0.140902	0.140902	0	0.7360072	1	17743	tags=50%, list=36%, signal=79%
REACTOME_DNA REPLICATION PRE-INITIATION	REACTOME_DNA REPLICATION PRE-INITIATION	84	0.1405744	0.1405744	0.0472362	0.7368732	1	18193	tags=51%, list=37%, signal=81%
REACTOME_M/G1 TRANSITION	REACTOME_M/G1 TRANSITION	84	0.1405744	0.1405744	0.053554	0.73609346	1	18193	tags=51%, list=37%, signal=81%
REACTOME_SIGNALING BY VEGF	REACTOME_SIGNALING BY VEGF	331	0.14010817	0.14010817	0	0.7374899	1	16558	tags=48%, list=34%, signal=72%
REACTOME_REGULATION OF SIGNALING BY CBL	REACTOME_REGULATION OF SIGNALING BY CBL	18	0.13997793	0.13997793	0.814664	0.7372836	1	17630	tags=50%, list=36%, signal=78%
REACTOME_PLC-GAMMA1 SIGNALLING	REACTOME_PLC-GAMMA1 SIGNALLING	34	0.13962579	0.13962579	0.4683664	0.738176	1	3248	tags=21%, list=7%, signal=22%
REACTOME_NIK-->NONCANONICAL NF-KB	REACTOME_NIK-->NONCANONICAL NF-KB SIGNALING	58	0.13880426	0.13880426	0.1886051	0.74129134	1	17693	tags=50%, list=36%, signal=78%
REACTOME_FBXL7 DOWN-REGULATES AURKA DURING MITOTIC ENTRY AND IN EARLY	REACTOME_FBXL7 DOWN-REGULATES AURKA DURING MITOTIC ENTRY AND IN EARLY MITOSIS	54	0.1387929	0.1387929	0.2332016	0.74057305	1	17693	tags=50%, list=36%, signal=78%
REACTOME_SCF-BETA-TRCP MEDIATED DEGRADATION OF	REACTOME_SCF-BETA-TRCP MEDIATED DEGRADATION OF EMI1	54	0.1387929	0.1387929	0.2243461	0.7397944	1	17693	tags=50%, list=36%, signal=78%
REACTOME_KSRP (KHSRP) BINDS AND DESTABILIZES MRNA	REACTOME_KSRP (KHSRP) BINDS AND DESTABILIZES MRNA	17	0.1378618	0.1378618	0.8620343	0.74334556	1	13413	tags=41%, list=27%, signal=57%
REACTOME_DECTIN-2 FAMILY	REACTOME_DECTIN-2 FAMILY	28	0.13739096	0.13739096	0.6272991	0.7447658	1	17758	tags=50%, list=36%, signal=78%
REACTOME_TRANSPORT OF BILE SALTS AND ORGANIC ACIDS, METAL IONS AND AMINE	REACTOME_TRANSPORT OF BILE SALTS AND ORGANIC ACIDS, METAL IONS AND AMINE COMPOUNDS	85	0.13692236	0.13692236	0.0791583	0.74617565	1	10588	tags=35%, list=22%, signal=45%
REACTOME_SIGNALLING BY NGF	REACTOME_SIGNALLING BY NGF	545	0.13686493	0.13686493	0	0.7456583	1	12958	tags=40%, list=26%, signal=54%
REACTOME_TELOMERE MAINTENANCE	REACTOME_TELOMERE MAINTENANCE	63	0.13670656	0.13670656	0.1624016	0.74561757	1	15076	tags=44%, list=31%, signal=64%

REACTOME_TRNA PROCESSING IN THE NUCLEUS	REACTOME_TRNA PROCESSING IN THE NUCLEUS	58	0.1359297	0.1359297	0.1805274	0.7485804	1	4326	tags=22%, list=9%, signal=25%
REACTOME_CONDENSATION OF PROPHASE CHROMOSOMES	REACTOME_CONDENSATION OF PROPHASE CHROMOSOMES	43	0.13590592	0.13590592	0.3631011	0.7479152	1	30928	tags=77%, list=63%, signal=208%
REACTOME_SIGNALING BY RHO GTPASES	REACTOME_SIGNALING BY RHO GTPASES	391	0.13461123	0.13461123	0	0.7532782	1	11744	tags=37%, list=24%, signal=49%
REACTOME_SUMOYLATION OF CHROMATIN ORGANIZATION PROTEINS	REACTOME_SUMOYLATION OF CHROMATIN ORGANIZATION PROTEINS	57	0.1343213	0.1343213	0.2303207	0.75385755	1	2879	tags=19%, list=6%, signal=20%
REACTOME_IMPORT OF PALMITOYL-COA INTO THE MITOCHONDRIAL MATRIX	REACTOME_IMPORT OF PALMITOYL-COA INTO THE MITOCHONDRIAL MATRIX	12	0.13412698	0.13412698	0.9611273	0.7540432	1	21996	tags=58%, list=45%, signal=106%
REACTOME_FORMATION OF HIV-1 ELONGATION COMPLEX CONTAINING HIV-1 TAT	REACTOME_FORMATION OF HIV-1 ELONGATION COMPLEX CONTAINING HIV-1 TAT	39	0.13366392	0.13366392	0.4717899	0.7554281	1	7270	tags=28%, list=15%, signal=33%
REACTOME_HIV TRANSCRIPTION ELONGATION	REACTOME_HIV TRANSCRIPTION ELONGATION	39	0.13366392	0.13366392	0.4580078	0.7546437	1	7270	tags=28%, list=15%, signal=33%
REACTOME_TAT-MEDIATED ELONGATION OF THE HIV-1 TRANSCRIPT	REACTOME_TAT-MEDIATED ELONGATION OF THE HIV-1 TRANSCRIPT	39	0.13366392	0.13366392	0.4605769	0.75386083	1	7270	tags=28%, list=15%, signal=33%
REACTOME_DNA METHYLATION	REACTOME_DNA METHYLATION	34	0.13314305	0.13314305	0.5423228	0.7555342	1	30928	tags=76%, list=63%, signal=207%
REACTOME_REGULATION OF RAS BY GAPS	REACTOME_REGULATION OF RAS BY GAPS	67	0.13169356	0.13169356	0.1866538	0.76165855	1	19869	tags=54%, list=41%, signal=90%
REACTOME_DECTIN-1 MEDIATED NONCANONICAL NF-KB	REACTOME_DECTIN-1 MEDIATED NONCANONICAL NF-KB SIGNALING	61	0.13060579	0.13060579	0.2313278	0.76606786	1	17693	tags=49%, list=36%, signal=77%
REACTOME_RHO GTPASES ACTIVATE FORMINS	REACTOME_RHO GTPASES ACTIVATE FORMINS	119	0.1305857	0.1305857	0.0359343	0.7653754	1	13371	tags=40%, list=27%, signal=55%
REACTOME_FORMATION OF RNA POL II ELONGATION COMPLEX	REACTOME_FORMATION OF RNA POL II ELONGATION COMPLEX	54	0.1303312	0.1303312	0.2796117	0.7658201	1	6319	tags=26%, list=13%, signal=30%
REACTOME_RNA POLYMERASE II TRANSCRIPTION ELONGATION	REACTOME_RNA POLYMERASE II TRANSCRIPTION ELONGATION	54	0.1303312	0.1303312	0.2857143	0.7650306	1	6319	tags=26%, list=13%, signal=30%
REACTOME_DEGRADATION OF GLI2 BY THE PROTEASOME	REACTOME_DEGRADATION OF GLI2 BY THE PROTEASOME	59	0.13032229	0.13032229	0.2303823	0.7642791	1	17693	tags=49%, list=36%, signal=77%
REACTOME_GLI3 IS PROCESSED TO GLI3R BY THE PROTEASOME	REACTOME_GLI3 IS PROCESSED TO GLI3R BY THE PROTEASOME	59	0.13032229	0.13032229	0.2340842	0.7634929	1	17693	tags=49%, list=36%, signal=77%
REACTOME_VEGFA-VEGFR2 PATHWAY	REACTOME_VEGFA-VEGFR2 PATHWAY	323	0.13017058	0.13017058	0	0.7634159	1	16558	tags=47%, list=34%, signal=70%
REACTOME_SIGNALING BY PDGF	REACTOME_SIGNALING BY PDGF	451	0.12990381	0.12990381	0	0.76384497	1	12588	tags=39%, list=26%, signal=51%
REACTOME_AUTODEGRADATION OF THE E3 UBIQUITIN LIGASE	REACTOME_AUTODEGRADATION OF THE E3 UBIQUITIN LIGASE COP1	51	0.12897028	0.12897028	0.3349609	0.7675277	1	17693	tags=49%, list=36%, signal=77%
REACTOME_VIF-MEDIATED DEGRADATION OF APOBEC3G	REACTOME_VIF-MEDIATED DEGRADATION OF APOBEC3G	51	0.12897028	0.12897028	0.3343284	0.7667413	1	17693	tags=49%, list=36%, signal=77%
REACTOME_VPU MEDIATED DEGRADATION OF CD4	REACTOME_VPU MEDIATED DEGRADATION OF CD4	51	0.12897028	0.12897028	0.3258537	0.76595646	1	17693	tags=49%, list=36%, signal=77%
REACTOME_EPIGENETIC REGULATION OF GENE	REACTOME_EPIGENETIC REGULATION OF GENE EXPRESSION	117	0.1289346	0.1289346	0.0467381	0.76535	1	12952	tags=39%, list=26%, signal=53%
REACTOME_HDMS DEMETHYLATE HISTONES	REACTOME_HDMS DEMETHYLATE HISTONES	27	0.12887053	0.12887053	0.7293087	0.76487356	1	10014	tags=33%, list=20%, signal=42%
REACTOME_TRANSPORT OF MATURE TRANSCRIPT TO CYTOPLASM	REACTOME_TRANSPORT OF MATURE TRANSCRIPT TO CYTOPLASM	82	0.12884821	0.12884821	0.1090555	0.7642023	1	9226	tags=32%, list=19%, signal=39%
REACTOME_MEMBRANE TRAFFICKING	REACTOME_MEMBRANE TRAFFICKING	614	0.12877911	0.12877911	0	0.7637413	1	6851	tags=27%, list=14%, signal=31%
REACTOME_TRANSCRIPTIONAL REGULATION BY RUNX1	REACTOME_TRANSCRIPTIONAL REGULATION BY RUNX1	206	0.12866412	0.12866412	9.79E-04	0.7634985	1	12741	tags=39%, list=26%, signal=52%

REACTOME_DIGESTION AND ABSORPTION	REACTOME_DIGESTION AND ABSORPTION	26	0.12860912	0.12860912	0.753878	0.76300657	1	42670	tags=100%, list=87%, signal=777%
REACTOME_SIGNALING BY	REACTOME_SIGNALING BY FGFR1	50	0.12829193	0.12829193	0.3359684	0.76370627	1	1558	tags=16%, list=3%, signal=17%
REACTOME_SIGNALING BY SCF-	REACTOME_SIGNALING BY SCF-KIT	411	0.1280941	0.1280941	0	0.76390547	1	13437	tags=40%, list=27%, signal=55%
REACTOME_RNA POLYMERASE II PRE-TRANSCRIPTION EVENTS	REACTOME_RNA POLYMERASE II PRE-TRANSCRIPTION EVENTS	76	0.12802067	0.12802067	0.1589267	0.76347804	1	7270	tags=28%, list=15%, signal=32%
REACTOME_COPI-MEDIATED ANTEROGRADE TRANSPORT	REACTOME_COPI-MEDIATED ANTEROGRADE TRANSPORT	83	0.1275523	0.1275523	0.1278119	0.7649213	1	13232	tags=40%, list=27%, signal=54%
REACTOME_PHASE I - FUNCTIONALIZATION OF COMPOUNDS	REACTOME_PHASE I - FUNCTIONALIZATION OF COMPOUNDS	106	0.12753063	0.12753063	0.0542714	0.7642597	1	7626	tags=28%, list=16%, signal=33%
REACTOME_INTERLEUKIN-17 SIGNALING	REACTOME_INTERLEUKIN-17 SIGNALING	300	0.12710881	0.12710881	0	0.76546776	1	10951	tags=35%, list=22%, signal=45%
REACTOME_CLASS I MHC MEDIATED ANTIGEN PROCESSING & PRESENTATION	REACTOME_CLASS I MHC MEDIATED ANTIGEN PROCESSING & PRESENTATION	367	0.12635598	0.12635598	0	0.76830244	1	11870	tags=37%, list=24%, signal=48%
REACTOME_DISEASE	REACTOME_DISEASE	836	0.12542656	0.12542656	0	0.77195793	1	7785	tags=28%, list=16%, signal=33%
REACTOME_ABC-FAMILY PROTEINS MEDIATED	REACTOME_ABC-FAMILY PROTEINS MEDIATED TRANSPORT	103	0.12507984	0.12507984	0.0685773	0.77278715	1	8149	tags=29%, list=17%, signal=35%
REACTOME_INTERLEUKIN-7 SIGNALING	REACTOME_INTERLEUKIN-7 SIGNALING	274	0.12473932	0.12473932	0	0.7736933	1	17693	tags=49%, list=36%, signal=76%
REACTOME_ACTIVATION OF HOX GENES DURING	REACTOME_ACTIVATION OF HOX GENES DURING DIFFERENTIATION	90	0.12375562	0.12375562	0.0959446	0.77763695	1	18977	tags=51%, list=39%, signal=83%
REACTOME_ACTIVATION OF ANTERIOR HOX GENES IN HINDBRAIN DEVELOPMENT DURING EARLY	REACTOME_ACTIVATION OF ANTERIOR HOX GENES IN HINDBRAIN DEVELOPMENT DURING EARLY EMBRYOGENESIS	90	0.12375562	0.12375562	0.1126214	0.77685535	1	18977	tags=51%, list=39%, signal=83%
REACTOME_DOWNSTREAM SIGNALING EVENTS OF B CELL RECEPTOR (BCR)	REACTOME_DOWNSTREAM SIGNALING EVENTS OF B CELL RECEPTOR (BCR)	279	0.12353016	0.12353016	0	0.77716976	1	12588	tags=38%, list=26%, signal=51%
REACTOME_PHOSPHOLIPID METABOLISM	REACTOME_PHOSPHOLIPID METABOLISM	212	0.12223013	0.12223013	0.002045	0.78259164	1	7436	tags=27%, list=15%, signal=32%
REACTOME_DAP12	REACTOME_DAP12 INTERACTIONS	442	0.12220477	0.12220477	0	0.78193074	1	14896	tags=43%, list=30%, signal=61%
REACTOME_DEFECTIVE CFTR CAUSES CYSTIC FIBROSIS	REACTOME_DEFECTIVE CFTR CAUSES CYSTIC FIBROSIS	60	0.12212284	0.12212284	0.2876579	0.7815365	1	17693	tags=48%, list=36%, signal=76%
REACTOME_VISUAL PHOTOTRANSDUCTION	REACTOME_VISUAL PHOTOTRANSDUCTION	98	0.12161727	0.12161727	0.0811359	0.7831764	1	6547	tags=26%, list=13%, signal=29%
REACTOME_CDT1 ASSOCIATION WITH THE CDC6:ORC:ORIGIN COMPLEX	REACTOME_CDT1 ASSOCIATION WITH THE CDC6:ORC:ORIGIN COMPLEX	58	0.12154243	0.12154243	0.3298647	0.78277713	1	17693	tags=48%, list=36%, signal=75%
REACTOME_M PHASE	REACTOME_M PHASE	344	0.12102712	0.12102712	0	0.7844737	1	17743	tags=48%, list=36%, signal=75%
REACTOME_TERMINATION OF TRANSLESION DNA SYNTHESIS	REACTOME_TERMINATION OF TRANSLESION DNA SYNTHESIS	31	0.12044134	0.12044134	0.7175197	0.7865051	1	11480	tags=35%, list=23%, signal=46%
REACTOME_CELL CYCLE,	REACTOME_CELL CYCLE, MITOTIC	485	0.12038592	0.12038592	0	0.78595215	1	17888	tags=48%, list=37%, signal=76%
REACTOME_DEGRADATION OF	REACTOME_DEGRADATION OF AXIN	54	0.12025394	0.12025394	0.3594059	0.78578115	1	17693	tags=48%, list=36%, signal=75%
REACTOME_FORMATION OF HIV ELONGATION COMPLEX IN THE ABSENCE OF HIV TAT	REACTOME_FORMATION OF HIV ELONGATION COMPLEX IN THE ABSENCE OF HIV TAT	41	0.11989925	0.11989925	0.5864136	0.7867292	1	7270	tags=27%, list=15%, signal=31%
REACTOME_SCF(SKP2)-MEDIATED DEGRADATION OF	REACTOME_SCF(SKP2)-MEDIATED DEGRADATION OF P27/P21	59	0.11976798	0.11976798	0.3537207	0.786558	1	19869	tags=53%, list=41%, signal=88%
REACTOME_NGF SIGNALLING VIA TRKA FROM THE PLASMA MEMBRANE	REACTOME_NGF SIGNALLING VIA TRKA FROM THE PLASMA MEMBRANE	462	0.11970925	0.11970925	0	0.7860648	1	13376	tags=39%, list=27%, signal=53%
REACTOME_UBIQUITIN MEDIATED DEGRADATION OF PHOSPHORYLATED CDC25A	REACTOME_UBIQUITIN MEDIATED DEGRADATION OF PHOSPHORYLATED CDC25A	52	0.11953604	0.11953604	0.4455348	0.7861363	1	17693	tags=48%, list=36%, signal=75%

REACTOME_P53-INDEPENDENT DNA DAMAGE RESPONSE	REACTOME_P53-INDEPENDENT DNA DAMAGE RESPONSE	52	0.11953604	0.11953604	0.4121757	0.78535795	1	17693	tags=48%, list=36%, signal=75%
REACTOME_P53-INDEPENDENT G1/S DNA DAMAGE CHECKPOINT	REACTOME_P53-INDEPENDENT G1/S DNA DAMAGE CHECKPOINT	52	0.11953604	0.11953604	0.4074074	0.7845812	1	17693	tags=48%, list=36%, signal=75%
REACTOME_CELLULAR SENESCENCE	REACTOME_CELLULAR SENESCENCE	161	0.11942001	0.11942001	0.01417	0.7843876	1	10898	tags=34%, list=22%, signal=44%
REACTOME_G1/S TRANSITION	REACTOME_G1/S TRANSITION	127	0.11926412	0.11926412	0.045954	0.78435916	1	17693	tags=48%, list=36%, signal=75%
REACTOME_HDACS DEACETYLATE HISTONES	REACTOME_HDACS DEACETYLATE HISTONES	59	0.119262	0.119262	0.3277662	0.78359264	1	17404	tags=47%, list=36%, signal=74%
REACTOME_METABOLISM OF STEROIDS	REACTOME_METABOLISM OF STEROIDS	146	0.11908399	0.11908399	0.0310982	0.7836217	1	6259	tags=25%, list=13%, signal=28%
REACTOME_PEROXISOMAL LIPID METABOLISM	REACTOME_PEROXISOMAL LIPID METABOLISM	29	0.11879062	0.11879062	0.7671756	0.78425616	1	6005	tags=24%, list=12%, signal=27%
REACTOME_REGULATION OF ORNITHINE DECARBOXYLASE	REACTOME_REGULATION OF ORNITHINE DECARBOXYLASE (ODC)	50	0.11876109	0.11876109	0.4542714	0.7836366	1	17693	tags=48%, list=36%, signal=75%
REACTOME_UBIQUITIN-DEPENDENT DEGRADATION OF	REACTOME_UBIQUITIN-DEPENDENT DEGRADATION OF CYCLIN D	50	0.11876109	0.11876109	0.4462312	0.7828669	1	17693	tags=48%, list=36%, signal=75%
REACTOME_UBIQUITIN-DEPENDENT DEGRADATION OF	REACTOME_UBIQUITIN-DEPENDENT DEGRADATION OF CYCLIN D1	50	0.11876109	0.11876109	0.4736842	0.7820986	1	17693	tags=48%, list=36%, signal=75%
REACTOME_TRANSCRIPTIONAL REGULATION BY TP53	REACTOME_TRANSCRIPTIONAL REGULATION BY TP53	355	0.11861234	0.11861234	0	0.78205913	1	8440	tags=29%, list=17%, signal=35%
REACTOME_FORMATION OF THE CORNIFIED ENVELOPE	REACTOME_FORMATION OF THE CORNIFIED ENVELOPE	65	0.11855907	0.11855907	0.3026052	0.7815399	1	7761	tags=28%, list=16%, signal=33%
REACTOME_RNA POLYMERASE I PROMOTER OPENING	REACTOME_RNA POLYMERASE I PROMOTER OPENING	32	0.11842215	0.11842215	0.7121662	0.7814053	1	30928	tags=75%, list=63%, signal=203%
REACTOME_NEGATIVE REGULATION OF MAPK	REACTOME_NEGATIVE REGULATION OF MAPK PATHWAY	40	0.11825754	0.11825754	0.5890547	0.7814301	1	1558	tags=15%, list=3%, signal=15%
REACTOME_G-PROTEIN BETA:GAMMA SIGNALLING	REACTOME_G-PROTEIN BETA:GAMMA SIGNALLING	51	0.11823153	0.11823153	0.4519231	0.78077763	1	5737	tags=24%, list=12%, signal=27%
REACTOME_PI3K/AKT SIGNALING IN CANCER	REACTOME_PI3K/AKT SIGNALING IN CANCER	90	0.11820177	0.11820177	0.1336684	0.7801685	1	14896	tags=42%, list=30%, signal=61%
REACTOME_DNA DAMAGE/TELOMERE STRESS INDUCED SENESCENCE	REACTOME_DNA DAMAGE/TELOMERE STRESS INDUCED SENESCENCE	61	0.11788231	0.11788231	0.2947799	0.7809195	1	13499	tags=39%, list=28%, signal=54%
REACTOME_SIGNALING BY EGFR	REACTOME_SIGNALING BY EGFR	439	0.11772945	0.11772945	0	0.78089464	1	11909	tags=36%, list=24%, signal=47%
REACTOME_RAB GEFS EXCHANGE GTP FOR GDP ON	REACTOME_RAB GEFS EXCHANGE GTP FOR GDP ON RABS	90	0.1176914	0.1176914	0.1497006	0.7803251	1	15465	tags=43%, list=32%, signal=63%
REACTOME_TRANSPORT OF VITAMINS, NUCLEOSIDES, AND RELATED MOLECULES	REACTOME_TRANSPORT OF VITAMINS, NUCLEOSIDES, AND RELATED MOLECULES	39	0.11760828	0.11760828	0.5934718	0.7799607	1	4289	tags=21%, list=9%, signal=22%
REACTOME_BETA-CATENIN INDEPENDENT WNT SIGNALING	REACTOME_BETA-CATENIN INDEPENDENT WNT SIGNALING	140	0.11718893	0.11718893	0.0404449	0.7811953	1	15612	tags=44%, list=32%, signal=64%
REACTOME_DNA DAMAGE	REACTOME_DNA DAMAGE BYPASS	47	0.11694647	0.11694647	0.5040984	0.781549	1	17198	tags=47%, list=35%, signal=72%
REACTOME_INTERLEUKIN-19,20,22,24,26,28 AND 29	REACTOME_INTERLEUKIN-19,20,22,24,26,28 AND 29 SIGNALING	256	0.11678435	0.11678435	0.001	0.7815507	1	10951	tags=34%, list=22%, signal=44%
REACTOME_DOWNSTREAM SIGNAL TRANSDUCTION	REACTOME_DOWNSTREAM SIGNAL TRANSDUCTION	427	0.11658027	0.11658027	0	0.78178805	1	13376	tags=39%, list=27%, signal=53%
REACTOME_MAPK FAMILY SIGNALING CASCADES	REACTOME_MAPK FAMILY SIGNALING CASCADES	283	0.11634982	0.11634982	9.79E-04	0.78212816	1	11810	tags=36%, list=24%, signal=47%
REACTOME_SIGNAL	REACTOME_SIGNAL TRANSDUCTION	2434	0.11630812	0.11630812	0	0.7815568	1	11928	tags=35%, list=24%, signal=44%
REACTOME_GAB1	REACTOME_GAB1 SIGNALOSOME	260	0.11617981	0.11617981	0.0020305	0.7813989	1	17693	tags=48%, list=36%, signal=74%
REACTOME_HIV LIFE CYCLE	REACTOME_HIV LIFE CYCLE	143	0.11594164	0.11594164	0.0276923	0.7817837	1	8035	tags=28%, list=16%, signal=33%
REACTOME_PROCESSING OF DNA DOUBLE-STRAND BREAK	REACTOME_PROCESSING OF DNA DOUBLE-STRAND BREAK ENDS	80	0.11571546	0.11571546	0.2310838	0.78209627	1	11480	tags=35%, list=23%, signal=46%
REACTOME_MRNA SPLICING - MAJOR PATHWAY	REACTOME_MRNA SPLICING - MAJOR PATHWAY	180	0.11566511	0.11566511	0.0149254	0.78158826	1	11222	tags=34%, list=23%, signal=45%

REACTOME_CELLULAR RESPONSES TO STRESS	REACTOME_CELLULAR RESPONSES TO STRESS	382	0.11517048	0.11517048	0	0.7830389	1	8119	tags=28%, list=17%, signal=33%
REACTOME_METABOLISM OF PROTEINS	REACTOME_METABOLISM OF PROTEINS	1976	0.11445591	0.11445591	0	0.78565395	1	7209	tags=26%, list=15%, signal=29%
REACTOME_SENESCENCE-ASSOCIATED SECRETORY PHENOTYPE (SASP)	REACTOME_SENESCENCE-ASSOCIATED SECRETORY PHENOTYPE (SASP)	79	0.11443364	0.11443364	0.234714	0.7850109	1	6801	tags=25%, list=14%, signal=29%
REACTOME_VESICLE-MEDIATED TRANSPORT	REACTOME_VESICLE-MEDIATED TRANSPORT	727	0.11425342	0.11425342	0	0.7850608	1	8901	tags=29%, list=18%, signal=35%
REACTOME_MEIOTIC SYNAPSIS	REACTOME_MEIOTIC SYNAPSIS	59	0.1142451	0.1142451	0.3932039	0.78435206	1	30928	tags=75%, list=63%, signal=202%
REACTOME_DEVELOPMENTAL BIOLOGY	REACTOME_DEVELOPMENTAL BIOLOGY	1000	0.11401054	0.11401054	0	0.78465253	1	7800	tags=27%, list=16%, signal=32%
REACTOME_PIP3 ACTIVATES AKT SIGNALING	REACTOME_PIP3 ACTIVATES AKT SIGNALING	257	0.11394609	0.11394609	0.0010593	0.7842124	1	17693	tags=47%, list=36%, signal=74%
REACTOME_LATE PHASE OF HIV LIFE CYCLE	REACTOME_LATE PHASE OF HIV LIFE CYCLE	130	0.11334557	0.11334557	0.0643863	0.7861972	1	7270	tags=26%, list=15%, signal=31%
REACTOME_CONSTITUTIVE SIGNALING BY ABERRANT PI3K IN CANCER	REACTOME_CONSTITUTIVE SIGNALING BY ABERRANT PI3K IN CANCER	65	0.1129731	0.1129731	0.3644401	0.7871362	1	12554	tags=37%, list=26%, signal=50%
REACTOME_G ALPHA (Q) SIGNALLING EVENTS	REACTOME_G ALPHA (Q) SIGNALLING EVENTS	192	0.11269578	0.11269578	0.0108374	0.78764266	1	9294	tags=30%, list=19%, signal=37%
REACTOME_RHO GTPASE EFFECTORS	REACTOME_RHO GTPASE EFFECTORS	262	0.11231678	0.11231678	0.002064	0.7886644	1	17890	tags=48%, list=37%, signal=75%
REACTOME_PI3K/AKT	REACTOME_PI3K/AKT ACTIVATION	260	0.11231313	0.11231313	0.001002	0.787927	1	17693	tags=47%, list=36%, signal=74%
REACTOME_G BETA:GAMMA SIGNALLING THROUGH	REACTOME_G BETA:GAMMA SIGNALLING THROUGH PI3KGAMMA	48	0.11209081	0.11209081	0.537092	0.78824794	1	5737	tags=23%, list=12%, signal=26%
REACTOME_DISORDERS OF TRANSMEMBRANE	REACTOME_DISORDERS OF TRANSMEMBRANE TRANSPORTERS	74	0.11178175	0.11178175	0.3145246	0.78894824	1	17693	tags=47%, list=36%, signal=74%
REACTOME_POSITIVE EPIGENETIC REGULATION OF	REACTOME_POSITIVE EPIGENETIC REGULATION OF RRNA EXPRESSION	75	0.11169701	0.11169701	0.2855721	0.7886012	1	14777	tags=41%, list=30%, signal=59%
REACTOME_RNA POLYMERASE I TRANSCRIPTION INITIATION	REACTOME_RNA POLYMERASE I TRANSCRIPTION INITIATION	47	0.11169191	0.11169191	0.5572968	0.78787446	1	12246	tags=36%, list=25%, signal=48%
REACTOME_PEPTIDE LIGAND-BINDING RECEPTORS	REACTOME_PEPTIDE LIGAND-BINDING RECEPTORS	188	0.11087988	0.11087988	0.0253807	0.79088056	1	11520	tags=35%, list=24%, signal=45%
REACTOME_CELLULAR RESPONSES TO EXTERNAL	REACTOME_CELLULAR RESPONSES TO EXTERNAL STIMULI	459	0.11061188	0.11061188	0	0.7913759	1	7755	tags=27%, list=16%, signal=32%
REACTOME_ADAPTIVE IMMUNE SYSTEM	REACTOME_ADAPTIVE IMMUNE SYSTEM	961	0.11059002	0.11059002	0	0.79073006	1	14103	tags=40%, list=29%, signal=55%
REACTOME_ORGANELLE BIOGENESIS AND MAINTENANCE	REACTOME_ORGANELLE BIOGENESIS AND MAINTENANCE	278	0.10999464	0.10999464	0.003996	0.79274744	1	12257	tags=36%, list=25%, signal=48%
REACTOME_G2/M CHECKPOINTS	REACTOME_G2/M CHECKPOINTS	149	0.10928422	0.10928422	0.0550363	0.7952446	1	16682	tags=45%, list=34%, signal=68%
REACTOME_GAP-FILLING DNA REPAIR SYNTHESIS AND LIGATION IN TC-NER	REACTOME_GAP-FILLING DNA REPAIR SYNTHESIS AND LIGATION IN TC-NER	64	0.10898359	0.10898359	0.3850051	0.7958672	1	8441	tags=28%, list=17%, signal=34%
REACTOME_DAP12 SIGNALING	REACTOME_DAP12 SIGNALING	430	0.10834163	0.10834163	0	0.7980836	1	14896	tags=41%, list=30%, signal=59%
REACTOME_METABOLISM	REACTOME_METABOLISM	2073	0.10830744	0.10830744	0	0.79748935	1	7628	tags=26%, list=16%, signal=29%
REACTOME_SIGNALLING TO	REACTOME_SIGNALLING TO ERKS	256	0.10824383	0.10824383	0.0049554	0.79702914	1	11367	tags=34%, list=23%, signal=44%
REACTOME_DNA REPAIR	REACTOME_DNA REPAIR	289	0.10786337	0.10786337	0.0031381	0.79806256	1	18977	tags=49%, list=39%, signal=80%
REACTOME_DUAL INCISION IN GG-NER	REACTOME_DUAL INCISION IN GG-NER	41	0.10680975	0.10680975	0.6821121	0.80214125	1	15076	tags=41%, list=31%, signal=60%
REACTOME_PRC2 METHYLATES HISTONES AND DNA	REACTOME_PRC2 METHYLATES HISTONES AND DNA	42	0.10653135	0.10653135	0.7028451	0.8026518	1	30928	tags=74%, list=63%, signal=200%
REACTOME_DEUBIQUITINATION	REACTOME_DEUBIQUITINATION	265	0.10644145	0.10644145	0.0052966	0.80232126	1	8119	tags=27%, list=17%, signal=32%
REACTOME_MRNA SPLICING	REACTOME_MRNA SPLICING	191	0.10628861	0.10628861	0.0237859	0.8022706	1	11222	tags=34%, list=23%, signal=43%
REACTOME_FATTY ACID METABOLISM	REACTOME_FATTY ACID METABOLISM	176	0.10565336	0.10565336	0.0301455	0.8043917	1	9033	tags=29%, list=18%, signal=35%

REACTOME_CHROMOSOME MAINTENANCE	REACTOME_CHROMOSOME MAINTENANCE	88	0.10555206	0.10555206	0.2522523	0.8040948	1	29338	tags=70%, list=60%, signal=175%
REACTOME_SPHINGOLIPID DE NOVO BIOSYNTHESIS	REACTOME_SPHINGOLIPID DE NOVO BIOSYNTHESIS	44	0.10551149	0.10551149	0.6693069	0.8035202	1	3740	tags=18%, list=8%, signal=20%
REACTOME_PI5P, PP2A AND IER3 REGULATE PI3K/AKT SIGNALING	REACTOME_PI5P, PP2A AND IER3 REGULATE PI3K/AKT SIGNALING	94	0.10549702	0.10549702	0.2280355	0.80284196	1	12554	tags=36%, list=26%, signal=49%
REACTOME_METABOLISM OF NON-CODING RNA	REACTOME_METABOLISM OF NON-CODING RNA	52	0.10515061	0.10515061	0.5884146	0.80367845	1	17455	tags=46%, list=36%, signal=72%
REACTOME_SNRNP ASSEMBLY	REACTOME_SNRNP ASSEMBLY	52	0.10515061	0.10515061	0.5991903	0.8029309	1	17455	tags=46%, list=36%, signal=72%
REACTOME_RNA POLYMERASE I TRANSCRIPTION TERMINATION	REACTOME_RNA POLYMERASE I TRANSCRIPTION TERMINATION	31	0.1047876	0.1047876	0.8366081	0.8038922	1	12246	tags=35%, list=25%, signal=47%
REACTOME_RESOLUTION OF SISTER CHROMATID COHESION	REACTOME_RESOLUTION OF SISTER CHROMATID COHESION	105	0.1045095	0.1045095	0.2045455	0.8044288	1	17743	tags=47%, list=36%, signal=73%
REACTOME_CELL CYCLE	REACTOME_CELL CYCLE	589	0.10450535	0.10450535	0	0.8037011	1	17888	tags=47%, list=37%, signal=73%
REACTOME_STABILIZATION OF	REACTOME_STABILIZATION OF P53	56	0.10441589	0.10441589	0.5625	0.80335945	1	20249	tags=52%, list=41%, signal=88%
REACTOME_SIGNALING BY THE B CELL RECEPTOR (BCR)	REACTOME_SIGNALING BY THE B CELL RECEPTOR (BCR)	369	0.10386639	0.10386639	0	0.80512315	1	17908	tags=47%, list=37%, signal=73%
REACTOME_APC/C:CDH1 MEDIATED DEGRADATION OF CDC20 AND OTHER APC/C:CDH1 TARGETED PROTEINS IN LATE MITOSIS/EARLY G1	REACTOME_APC/C:CDH1 MEDIATED DEGRADATION OF CDC20 AND OTHER APC/C:CDH1 TARGETED PROTEINS IN LATE MITOSIS/EARLY G1	71	0.10357875	0.10357875	0.3849903	0.8057116	1	17693	tags=46%, list=36%, signal=73%
REACTOME_TCF DEPENDENT SIGNALING IN RESPONSE TO	REACTOME_TCF DEPENDENT SIGNALING IN RESPONSE TO WNT	201	0.10301334	0.10301334	0.01998	0.8075861	1	13977	tags=39%, list=29%, signal=54%
REACTOME_SLC-MEDIATED TRANSMEMBRANE TRANSPORT	REACTOME_SLC-MEDIATED TRANSMEMBRANE TRANSPORT	245	0.10262432	0.10262432	0.0110887	0.8085989	1	10588	tags=32%, list=22%, signal=40%
REACTOME_POLO-LIKE KINASE MEDIATED EVENTS	REACTOME_POLO-LIKE KINASE MEDIATED EVENTS	16	0.10249443	0.10249443	0.9881423	0.8084496	1	43947	tags=100%, list=90%, signal=975%
REACTOME_HIV INFECTION	REACTOME_HIV INFECTION	224	0.10249329	0.10249329	0.0145349	0.80770874	1	8119	tags=27%, list=17%, signal=32%
REACTOME_MITOTIC PROPHASE	REACTOME_MITOTIC PROPHASE	110	0.1022841	0.1022841	0.1883495	0.8079171	1	18149	tags=47%, list=37%, signal=75%
REACTOME_OXYGEN-DEPENDENT PROLINE HYDROXYLATION OF HYPOXIA-	REACTOME_OXYGEN-DEPENDENT PROLINE HYDROXYLATION OF HYPOXIA-INDUCIBLE FACTOR ALPHA	63	0.10226807	0.10226807	0.4822335	0.8072502	1	19869	tags=51%, list=41%, signal=85%
REACTOME_VEGFR2 MEDIATED CELL PROLIFERATION	REACTOME_VEGFR2 MEDIATED CELL PROLIFERATION	251	0.10182885	0.10182885	0.0110331	0.8085002	1	13376	tags=37%, list=27%, signal=51%
REACTOME_ERCC6 (CSB) AND EHMT2 (G9A) POSITIVELY REGULATE RRNA EXPRESSION	REACTOME_ERCC6 (CSB) AND EHMT2 (G9A) POSITIVELY REGULATE RRNA EXPRESSION	45	0.10177162	0.10177162	0.7026749	0.8080167	1	30928	tags=73%, list=63%, signal=199%
REACTOME_SIGNALLING TO RAS	REACTOME_SIGNALLING TO RAS	249	0.10168458	0.10168458	0.0102987	0.8076756	1	11367	tags=33%, list=23%, signal=43%
REACTOME_CYCLIN A:CDK2-ASSOCIATED EVENTS AT S PHASE ENTRY	REACTOME_CYCLIN A:CDK2-ASSOCIATED EVENTS AT S PHASE ENTRY	80	0.10130536	0.10130536	0.3753878	0.80861074	1	17693	tags=46%, list=36%, signal=72%
REACTOME_MAPK1/MAPK3 SIGNALING	REACTOME_MAPK1/MAPK3 SIGNALING	244	0.10060182	0.10060182	0.0217166	0.8111162	1	10951	tags=32%, list=22%, signal=41%
REACTOME_PROCESSING OF CAPPED INTRON-CONTAINING PRE-MRNA	REACTOME_PROCESSING OF CAPPED INTRON-CONTAINING PRE-MRNA	243	0.10051238	0.10051238	0.0145833	0.81078964	1	11222	tags=33%, list=23%, signal=42%
REACTOME_UB-SPECIFIC PROCESSING PROTEASES	REACTOME_UB-SPECIFIC PROCESSING PROTEASES	188	0.099972	0.099972	0.0484027	0.8125168	1	17782	tags=46%, list=36%, signal=72%
REACTOME_NEGATIVE REGULATION OF THE PI3K/AKT	REACTOME_NEGATIVE REGULATION OF THE PI3K/AKT NETWORK	101	0.09953895	0.09953895	0.277666	0.8137426	1	12588	tags=36%, list=26%, signal=48%
REACTOME_TRANSPORT OF MATURE MRNA DERIVED FROM AN INTRON-CONTAINING	REACTOME_TRANSPORT OF MATURE MRNA DERIVED FROM AN INTRON-CONTAINING TRANSCRIPT	73	0.09937866	0.09937866	0.4120553	0.8137138	1	9226	tags=29%, list=19%, signal=35%
REACTOME_PROLONGED ERK ACTIVATION EVENTS	REACTOME_PROLONGED ERK ACTIVATION EVENTS	245	0.09927574	0.09927574	0.0096432	0.81344503	1	10951	tags=32%, list=22%, signal=41%

REACTOME_NCAM SIGNALING FOR NEURITE OUT-GROWTH	REACTOME_NCAM SIGNALING FOR NEURITE OUT-GROWTH	271	0.09881721	0.09881721	0.0099108	0.81473917	1	11810	tags=34%, list=24%, signal=44%
REACTOME_GASTRIN-CREB SIGNALLING PATHWAY VIA PKC AND MAPK	REACTOME_GASTRIN-CREB SIGNALLING PATHWAY VIA PKC AND MAPK	436	0.09830628	0.09830628	9.88E-04	0.8162959	1	10951	tags=32%, list=22%, signal=41%
REACTOME_RUNX1 REGULATES TRANSCRIPTION OF GENES INVOLVED IN DIFFERENTIATION OF HSCS	REACTOME_RUNX1 REGULATES TRANSCRIPTION OF GENES INVOLVED IN DIFFERENTIATION OF HSCS	98	0.0980197	0.0980197	0.2813765	0.8168317	1	17693	tags=46%, list=36%, signal=72%
REACTOME_FRS2-MEDIATED ACTIVATION	REACTOME_FRS2-MEDIATED ACTIVATION	243	0.09780305	0.09780305	0.022022	0.81701726	1	10951	tags=32%, list=22%, signal=41%
REACTOME_SIGNALING BY	REACTOME_SIGNALING BY LEPTIN	246	0.09759275	0.09759275	0.0130522	0.81719446	1	11367	tags=33%, list=23%, signal=43%
REACTOME_INTERLEUKIN-2 FAMILY SIGNALING	REACTOME_INTERLEUKIN-2 FAMILY SIGNALING	259	0.09730806	0.09730806	0.0159521	0.8176994	1	10951	tags=32%, list=22%, signal=41%
REACTOME_SEPARATION OF SISTER CHROMATIDS	REACTOME_SEPARATION OF SISTER CHROMATIDS	168	0.09628239	0.09628239	0.0809191	0.82153124	1	17743	tags=46%, list=36%, signal=72%
REACTOME_UCH PROTEINASES	REACTOME_UCH PROTEINASES	94	0.09627147	0.09627147	0.3517636	0.82084316	1	17693	tags=46%, list=36%, signal=71%
REACTOME_RET SIGNALING	REACTOME_RET SIGNALING	263	0.09623858	0.09623858	0.014437	0.8202369	1	10951	tags=32%, list=22%, signal=41%
REACTOME_ARACHIDONIC ACID METABOLISM	REACTOME_ARACHIDONIC ACID METABOLISM	59	0.09621813	0.09621813	0.5997994	0.81958866	1	8572	tags=27%, list=18%, signal=33%
REACTOME_CYCLIN E ASSOCIATED EVENTS DURING	REACTOME_CYCLIN E ASSOCIATED EVENTS DURING G1/S TRANSITION	81	0.0955881	0.0955881	0.418	0.8216258	1	17693	tags=46%, list=36%, signal=71%
REACTOME_ROLE OF LAT2/NTAL/LAB ON CALCIUM	REACTOME_ROLE OF LAT2/NTAL/LAB ON CALCIUM MOBILIZATION	337	0.0952089	0.0952089	0.0050813	0.8225873	1	21232	tags=53%, list=43%, signal=93%
REACTOME_INTERLEUKIN-3, 5 AND GM-CSF SIGNALING	REACTOME_INTERLEUKIN-3, 5 AND GM-CSF SIGNALING	264	0.09502418	0.09502418	0.015	0.8227275	1	10951	tags=32%, list=22%, signal=41%
REACTOME_ARMS-MEDIATED ACTIVATION	REACTOME_ARMS-MEDIATED ACTIVATION	242	0.09498125	0.09498125	0.0256663	0.82219046	1	10951	tags=32%, list=22%, signal=41%
REACTOME_SIGNALLING TO P38 VIA RIT AND RIN	REACTOME_SIGNALLING TO P38 VIA RIT AND RIN	242	0.09498125	0.09498125	0.0241935	0.8214511	1	10951	tags=32%, list=22%, signal=41%
REACTOME_REGULATION OF PTEN STABILITY AND ACTIVITY	REACTOME_REGULATION OF PTEN STABILITY AND ACTIVITY	68	0.09465365	0.09465365	0.5361744	0.8221687	1	17693	tags=46%, list=36%, signal=71%
REACTOME_P53-DEPENDENT G1 DNA DAMAGE RESPONSE	REACTOME_P53-DEPENDENT G1 DNA DAMAGE RESPONSE	65	0.09425673	0.09425673	0.5909548	0.82321787	1	20249	tags=51%, list=41%, signal=86%
REACTOME_P53-DEPENDENT G1/S DNA DAMAGE CHECKPOINT	REACTOME_P53-DEPENDENT G1/S DNA DAMAGE CHECKPOINT	65	0.09425673	0.09425673	0.5936571	0.82247955	1	20249	tags=51%, list=41%, signal=86%
REACTOME_G1/S DNA DAMAGE CHECKPOINTS	REACTOME_G1/S DNA DAMAGE CHECKPOINTS	67	0.09403063	0.09403063	0.5477099	0.82271767	1	20249	tags=51%, list=41%, signal=86%
REACTOME_MITOTIC ANAPHASE	REACTOME_MITOTIC ANAPHASE	178	0.09301301	0.09301301	0.0918367	0.8263691	1	17743	tags=46%, list=36%, signal=71%
REACTOME_POST-ELONGATION PROCESSING OF INTRON-CONTAINING PRE-MRNA	REACTOME_POST-ELONGATION PROCESSING OF INTRON-CONTAINING PRE-MRNA	57	0.09236857	0.09236857	0.7118812	0.82845414	1	9226	tags=28%, list=19%, signal=35%
REACTOME_MRNA 3'-END PROCESSING	REACTOME_MRNA 3'-END PROCESSING	57	0.09236857	0.09236857	0.7112887	0.8277138	1	9226	tags=28%, list=19%, signal=35%
REACTOME_GRB2 EVENTS IN EGFR SIGNALING	REACTOME_GRB2 EVENTS IN EGFR SIGNALING	238	0.09190281	0.09190281	0.0417515	0.8289345	1	10951	tags=32%, list=22%, signal=40%
REACTOME_RAF/MAP KINASE CASCADE	REACTOME_RAF/MAP KINASE CASCADE	238	0.09190281	0.09190281	0.024728	0.82819504	1	10951	tags=32%, list=22%, signal=40%
REACTOME_SHC1 EVENTS IN EGFR SIGNALING	REACTOME_SHC1 EVENTS IN EGFR SIGNALING	238	0.09190281	0.09190281	0.0372233	0.8274569	1	10951	tags=32%, list=22%, signal=40%
REACTOME_SOS-MEDIATED SIGNALLING	REACTOME_SOS-MEDIATED SIGNALLING	238	0.09190281	0.09190281	0.0421569	0.82672006	1	10951	tags=32%, list=22%, signal=40%
REACTOME_APC/C-MEDIATED DEGRADATION OF CELL CYCLE PROTEINS	REACTOME_APC/C-MEDIATED DEGRADATION OF CELL CYCLE PROTEINS	85	0.0912511	0.0912511	0.4421769	0.82879275	1	18581	tags=47%, list=38%, signal=76%

REACTOME_REGULATION OF MITOTIC CELL CYCLE	REACTOME_REGULATION OF MITOTIC CELL CYCLE	85	0.0912511	0.0912511	0.4485219	0.828056	1	18581	tags=47%, list=38%, signal=76%
REACTOME_METABOLISM OF POLYAMINES	REACTOME_METABOLISM OF POLYAMINES	84	0.09117723	0.09117723	0.4753623	0.8276129	1	17693	tags=45%, list=36%, signal=71%
REACTOME_B-WICH COMPLEX POSITIVELY REGULATES RRNA EXPRESSION	REACTOME_B-WICH COMPLEX POSITIVELY REGULATES RRNA EXPRESSION	60	0.09107114	0.09107114	0.6513292	0.82735544	1	13499	tags=37%, list=28%, signal=51%
REACTOME_MITOTIC METAPHASE AND ANAPHASE	REACTOME_MITOTIC METAPHASE AND ANAPHASE	179	0.09046336	0.09046336	0.1130604	0.829201	1	17743	tags=45%, list=36%, signal=71%
REACTOME_MITOTIC G1-G1/S PHASES	REACTOME_MITOTIC G1-G1/S PHASES	145	0.08967141	0.08967141	0.1684518	0.83193505	1	16558	tags=43%, list=34%, signal=64%
REACTOME_CLASS A/1 (RHODOPSIN-LIKE RECEPTORS)	REACTOME_CLASS A/1 (RHODOPSIN-LIKE RECEPTORS)	320	0.08907615	0.08907615	0.0107738	0.8337646	1	6530	tags=22%, list=13%, signal=25%
REACTOME_ACTIVATION OF APC/C AND APC/C:CDC20 MEDIATED DEGRADATION OF MITOTIC PROTEINS	REACTOME_ACTIVATION OF APC/C AND APC/C:CDC20 MEDIATED DEGRADATION OF MITOTIC PROTEINS	75	0.08766756	0.08766756	0.5880567	0.8391659	1	19869	tags=49%, list=41%, signal=83%
REACTOME_SIGNALING BY WNT	REACTOME_SIGNALING BY WNT	292	0.08752456	0.08752456	0.0254324	0.8390464	1	11837	tags=33%, list=24%, signal=43%
REACTOME_INTERLEUKIN RECEPTOR SHC SIGNALING	REACTOME_INTERLEUKIN RECEPTOR SHC SIGNALING	248	0.08725584	0.08725584	0.0456853	0.83943546	1	10951	tags=31%, list=22%, signal=40%
REACTOME_OXIDATIVE STRESS INDUCED SENESCENCE	REACTOME_OXIDATIVE STRESS INDUCED SENESCENCE	91	0.08706219	0.08706219	0.4853516	0.8395257	1	17267	tags=44%, list=35%, signal=68%
REACTOME_CHROMATIN MODIFYING ENZYMES	REACTOME_CHROMATIN MODIFYING ENZYMES	238	0.08677158	0.08677158	0.0551724	0.84005195	1	8115	tags=25%, list=17%, signal=30%
REACTOME_CHROMATIN ORGANIZATION	REACTOME_CHROMATIN ORGANIZATION	238	0.08677158	0.08677158	0.0583333	0.8393125	1	8115	tags=25%, list=17%, signal=30%
REACTOME_AMPLIFICATION OF SIGNAL FROM UNATTACHED KINETOCHORES VIA A MAD2 INHIBITORY SIGNAL	REACTOME_AMPLIFICATION OF SIGNAL FROM UNATTACHED KINETOCHORES VIA A MAD2 INHIBITORY SIGNAL	92	0.08653775	0.08653775	0.464497	0.8395838	1	17591	tags=45%, list=36%, signal=69%
REACTOME_AMPLIFICATION OF SIGNAL FROM THE	REACTOME_AMPLIFICATION OF SIGNAL FROM THE KINETOCHORES	92	0.08653775	0.08653775	0.4501953	0.8388461	1	17591	tags=45%, list=36%, signal=69%
REACTOME_ABC TRANSPORTER DISORDERS	REACTOME_ABC TRANSPORTER DISORDERS	67	0.08651941	0.08651941	0.6423712	0.8381914	1	17693	tags=45%, list=36%, signal=70%
REACTOME_AMYLOID FIBER FORMATION	REACTOME_AMYLOID FIBER FORMATION	69	0.08622234	0.08622234	0.6702439	0.8387336	1	5718	tags=20%, list=12%, signal=23%
REACTOME_KERATINIZATION	REACTOME_KERATINIZATION	171	0.08597071	0.08597071	0.1581582	0.8390596	1	33029	tags=76%, list=67%, signal=233%
REACTOME_FC EPSILON RECEPTOR (FCERI) SIGNALING	REACTOME_FC EPSILON RECEPTOR (FCERI) SIGNALING	519	0.0848435	0.0848435	0.0019608	0.84303117	1	20324	tags=50%, list=42%, signal=84%
REACTOME_CLEAVAGE OF GROWING TRANSCRIPT IN THE TERMINATION REGION	REACTOME_CLEAVAGE OF GROWING TRANSCRIPT IN THE TERMINATION REGION	66	0.08365544	0.08365544	0.7018634	0.8472203	1	12230	tags=33%, list=25%, signal=44%
REACTOME_POST-ELONGATION PROCESSING OF THE	REACTOME_POST-ELONGATION PROCESSING OF THE TRANSCRIPT	66	0.08365544	0.08365544	0.7288136	0.8464797	1	12230	tags=33%, list=25%, signal=44%
REACTOME_RNA POLYMERASE II TRANSCRIPTION TERMINATION	REACTOME_RNA POLYMERASE II TRANSCRIPTION TERMINATION	66	0.08365544	0.08365544	0.7212787	0.84574044	1	12230	tags=33%, list=25%, signal=44%
REACTOME_NEGATIVE EPIGENETIC REGULATION OF	REACTOME_NEGATIVE EPIGENETIC REGULATION OF RRNA EXPRESSION	78	0.08334868	0.08334868	0.6390593	0.84630275	1	12246	tags=33%, list=25%, signal=44%
REACTOME_BIOLOGICAL OXIDATIONS	REACTOME_BIOLOGICAL OXIDATIONS	215	0.08180418	0.08180418	0.1056676	0.85195166	1	7626	tags=24%, list=16%, signal=28%
REACTOME_APC/C:CDC20 MEDIATED DEGRADATION OF MITOTIC PROTEINS	REACTOME_APC/C:CDC20 MEDIATED DEGRADATION OF MITOTIC PROTEINS	74	0.08080855	0.08080855	0.7220039	0.855291	1	19869	tags=49%, list=41%, signal=82%
REACTOME_INSULIN RECEPTOR SIGNALLING CASCADE	REACTOME_INSULIN RECEPTOR SIGNALLING CASCADE	291	0.07977558	0.07977558	0.0480962	0.85872036	1	1837	tags=12%, list=4%, signal=12%

REACTOME_RHO GTPASES ACTIVATE PKNS	REACTOME_RHO GTPASES ACTIVATE PKNS	63	0.07924696	0.07924696	0.8168168	0.86009264	1	14777	tags=38%, list=30%, signal=54%
REACTOME_APC/C:CDC20 MEDIATED DEGRADATION OF	REACTOME_APC/C:CDC20 MEDIATED DEGRADATION OF SECURIN	66	0.07915513	0.07915513	0.7778865	0.85971844	1	19869	tags=48%, list=41%, signal=81%
REACTOME_FCERI MEDIATED MAPK ACTIVATION	REACTOME_FCERI MEDIATED MAPK ACTIVATION	332	0.07894721	0.07894721	0.0306122	0.8597906	1	21232	tags=51%, list=43%, signal=90%
REACTOME_TRANSCRIPTIONAL REGULATION BY SMALL RNAS	REACTOME_TRANSCRIPTIONAL REGULATION BY SMALL RNAS	73	0.07828716	0.07828716	0.72167	0.861703	1	18977	tags=47%, list=39%, signal=76%
REACTOME_IRS-MEDIATED SIGNALLING	REACTOME_IRS-MEDIATED SIGNALLING	288	0.07750217	0.07750217	0.0558882	0.8640499	1	1837	tags=11%, list=4%, signal=12%
REACTOME_SIGNALING BY TYPE 1 INSULIN-LIKE GROWTH FACTOR 1 RECEPTOR (IGF1R)	REACTOME_SIGNALING BY TYPE 1 INSULIN-LIKE GROWTH FACTOR 1 RECEPTOR (IGF1R)	293	0.07713287	0.07713287	0.0472441	0.86474866	1	10951	tags=30%, list=22%, signal=38%
REACTOME_REGULATION OF APC/C ACTIVATORS BETWEEN G1/S AND EARLY ANAPHASE	REACTOME_REGULATION OF APC/C ACTIVATORS BETWEEN G1/S AND EARLY ANAPHASE	79	0.07632379	0.07632379	0.7099792	0.86727977	1	18581	tags=46%, list=38%, signal=73%
REACTOME_GPCR LIGAND	REACTOME_GPCR LIGAND BINDING	451	0.07574237	0.07574237	0.0099404	0.8688629	1	6530	tags=21%, list=13%, signal=24%
REACTOME_RNA POLYMERASE I TRANSCRIPTION	REACTOME_RNA POLYMERASE I TRANSCRIPTION	80	0.07500511	0.07500511	0.7277277	0.8709809	1	12246	tags=33%, list=25%, signal=43%
REACTOME_CELL CYCLE CHECKPOINTS	REACTOME_CELL CYCLE CHECKPOINTS	270	0.07237223	0.07237223	0.118593	0.8803273	1	17693	tags=43%, list=36%, signal=67%
REACTOME_RNA POLYMERASE I PROMOTER CLEARANCE	REACTOME_RNA POLYMERASE I PROMOTER CLEARANCE	78	0.07050771	0.07050771	0.7992203	0.88646805	1	12246	tags=32%, list=25%, signal=43%
REACTOME_NORC NEGATIVELY REGULATES RRNA EXPRESSION	REACTOME_NORC NEGATIVELY REGULATES RRNA EXPRESSION	75	0.06998978	0.06998978	0.8228856	0.8875867	1	12246	tags=32%, list=25%, signal=43%
REACTOME_POST-TRANSLATIONAL MODIFICATION: SYNTHESIS OF GPI-ANCHORED	REACTOME_POST-TRANSLATIONAL MODIFICATION: SYNTHESIS OF GPI-ANCHORED PROTEINS	92	0.05890001	0.05890001	0.8786982	0.92497164	1	12555	tags=32%, list=26%, signal=42%
REACTOME_ANTIMICROBIAL PEPTIDES	REACTOME_ANTIMICROBIAL PEPTIDES	75	0.0585914	0.0585914	0.949495	0.92515135	1	23902	tags=55%, list=49%, signal=107%
REACTOME_MEIOSIS	REACTOME_MEIOSIS	87	0.05810317	0.05810317	0.9057171	0.92587537	1	30928	tags=69%, list=63%, signal=187%
