# Supplementary material for: Role of Lamin A/C as Candidate Biomarker of Aggressiveness and Tumorigenicity in Glioblastoma Multiforme
Source: Biomedicines. 2021 Sep 28;9(10):1343. doi: 10.3390/biomedicines9101343 (PMC8533312; doi:10.3390/biomedicines9101343)
Supplement: Supplementary file 1 [file biomedicines-09-01343-s001.zip › biomedicines-1353075-suppl/biomedicines-1353075-suppl(tables).zip!/supplementary tables/Supplementary Table 4.pdf]

Table S4. List of genes correlated with LMNA gene expression in GBM patients from microarray dataset.

var1	var2	corr	corr_CI5%	corr_CI95%	pvalue	FDR_corrected_Pval	Bonferroni_corrected_P
AACS	LMNA	-0.107659701	-0.2421123	0.030849806	1.27E-01	1.87E-01	1.00E+00
FSTL1	LMNA	0.619586736	0.526576204	0.69793976	8.34E-23	7.50E-21	9.90E-19
ELMO2	LMNA	-0.0631694	-0.199480808	0.075540358	3.72E-01	4.60E-01	1.00E+00
CREB3L1	LMNA	0.410451237	0.288762441	0.519088992	1.30E-09	1.05E-08	1.55E-05
RPS11	LMNA	-0.221779939	-0.3491413	-0.086373428	1.51E-03	3.78E-03	1.00E+00
PNMA1	LMNA	-0.224641487	-0.351783	-0.089361772	1.31E-03	3.33E-03	1.00E+00
MMP2	LMNA	0.406849219	0.284793938	0.515922923	1.87E-09	1.47E-08	2.22E-05
SAMD4A	LMNA	0.150245084	0.012452364	0.282437903	3.28E-02	5.81E-02	1.00E+00
SMARCD3	LMNA	-0.418176163	-0.52586894	-0.297287459	5.92E-10	5.10E-09	7.02E-06
A4GNT	LMNA	0.011521386	-0.126731185	0.149334864	8.71E-01	9.04E-01	1.00E+00
C9orf39	LMNA	-0.015341176	-0.153067999	0.122970259	8.28E-01	8.71E-01	1.00E+00
PKNOX2	LMNA	-0.360282627	-0.474722191	-0.233863378	1.39E-07	7.77E-07	1.65E-03
RALYL	LMNA	-0.466529739	-0.567998833	-0.351090699	2.60E-12	3.39E-11	3.08E-08
ZHX3	LMNA	0.084979773	-0.053701226	0.220444618	2.29E-01	3.07E-01	1.00E+00
ERCC5	LMNA	-0.098093992	-0.23298985	0.040505534	1.65E-01	2.33E-01	1.00E+00
RXFP3	LMNA	-0.055862295	-0.19242931	0.082827459	4.30E-01	5.16E-01	1.00E+00
APBB2	LMNA	-0.291739736	-0.413151092	-0.160138299	2.52E-05	9.27E-05	2.99E-01
BBOX1	LMNA	0.017878112	-0.12047022	0.155545213	8.01E-01	8.50E-01	1.00E+00
PRO0478	LMNA	-0.266826366	-0.390493294	-0.133700303	1.24E-04	3.98E-04	1.00E+00
GCSH	LMNA	-0.341372226	-0.457846401	-0.213376996	6.63E-07	3.28E-06	7.87E-03
XDH	LMNA	0.08768326	-0.0509849	0.223034482	2.15E-01	2.90E-01	1.00E+00
EDN1	LMNA	-0.002298278	-0.140304762	0.135795808	9.74E-01	9.80E-01	1.00E+00
MTERF	LMNA	-0.034787827	-0.172012737	0.103761488	6.23E-01	6.98E-01	1.00E+00
PDCL3	LMNA	0.216173873	0.080526012	0.343960066	2.00E-03	4.86E-03	1.00E+00
CLK4	LMNA	-0.117301818	-0.251283623	0.021090718	9.64E-02	1.47E-01	1.00E+00
KCNG1	LMNA	0.084814964	-0.053866751	0.220286674	2.30E-01	3.08E-01	1.00E+00
CXCR4	LMNA	0.499214405	0.387896055	0.59617854	3.96E-14	6.71E-13	4.70E-10
DECR1	LMNA	0.021795943	-0.116605919	0.159367415	7.58E-01	8.14E-01	1.00E+00
SALL1	LMNA	-0.02878089	-0.166171653	0.109705997	6.84E-01	7.52E-01	1.00E+00
PTPRR	LMNA	-0.027480548	-0.164905942	0.110991523	6.98E-01	7.63E-01	1.00E+00
CADM4	LMNA	-0.211075047	-0.339240867	-0.075215768	2.57E-03	6.05E-03	1.00E+00

IRAK1	LMNA	0.596593461	0.499697728	0.678742971	7.33E-21	4.20E-19	8.69E-17
CFHR5	LMNA	0.036733492	-0.101833918	0.173902616	6.04E-01	6.81E-01	1.00E+00
HNRPD	LMNA	-0.218022496	-0.34566946	-0.08245319	1.83E-03	4.48E-03	1.00E+00
TMSB10	LMNA	0.433547757	0.314308665	0.539319564	1.15E-10	1.14E-09	1.37E-06
CXCL3	LMNA	0.29934002	0.168241462	0.420033478	1.51E-05	5.77E-05	1.79E-01
LMAN1	LMNA	0.560944392	0.458390676	0.648756389	3.87E-18	1.43E-16	4.59E-14
SUHW1	LMNA	-0.005567716	-0.143508412	0.132585194	9.37E-01	9.54E-01	1.00E+00
CHD8	LMNA	-0.085992112	-0.221414634	0.05268432	2.24E-01	3.01E-01	1.00E+00
SUMO1	LMNA	-0.248378656	-0.373618675	-0.114244993	3.65E-04	1.06E-03	1.00E+00
GP1BA	LMNA	-0.076036919	-0.211863989	0.062671947	2.82E-01	3.65E-01	1.00E+00
OR7A10	LMNA	-0.120188092	-0.2540243	0.018164292	8.84E-02	1.37E-01	1.00E+00
DDB1	LMNA	-0.203430871	-0.332153721	-0.067269043	3.69E-03	8.33E-03	1.00E+00
CHRNA10	LMNA	-0.012098689	-0.149899322	0.126163034	8.64E-01	9.00E-01	1.00E+00
STYK1	LMNA	-0.242350963	-0.368086955	-0.107910295	5.11E-04	1.44E-03	1.00E+00
MYO9B	LMNA	0.339204765	0.211036068	0.455906766	7.88E-07	3.85E-06	9.35E-03
CCNI	LMNA	-0.287043108	-0.408891148	-0.155139767	3.45E-05	1.23E-04	4.09E-01
MMP7	LMNA	0.334320626	0.205766425	0.451531939	1.16E-06	5.49E-06	1.37E-02
EP300	LMNA	-0.043674522	-0.180636409	0.094948956	5.37E-01	6.19E-01	1.00E+00
CRNKL1	LMNA	0.066191394	-0.0725223	0.20239297	3.49E-01	4.36E-01	1.00E+00
C9orf45	LMNA	-0.453932603	-0.557074083	-0.336999994	1.16E-11	1.35E-10	1.38E-07
XAB2	LMNA	0.07234421	-0.066369637	0.208314732	3.06E-01	3.91E-01	1.00E+00
RTN1	LMNA	-0.410682419	-0.519292093	-0.289017288	1.27E-09	1.03E-08	1.51E-05
HIC2	LMNA	-0.221787217	-0.349148022	-0.086381027	1.51E-03	3.78E-03	1.00E+00
TBX10	LMNA	0.101032147	-0.03754248	0.235794385	1.53E-01	2.18E-01	1.00E+00
CENPQ	LMNA	-0.298240405	-0.419038587	-0.167067992	1.63E-05	6.19E-05	1.93E-01
UTY	LMNA	0.020881081	-0.117508656	0.158475252	7.68E-01	8.22E-01	1.00E+00
OR2W1	LMNA	0.104530227	-0.034011578	0.239130455	1.39E-01	2.01E-01	1.00E+00
ATP5G2	LMNA	-0.154083465	-0.286049793	-0.016380909	2.86E-02	5.12E-02	1.00E+00
ZEB1	LMNA	-0.259981526	-0.384241815	-0.126469609	1.87E-04	5.77E-04	1.00E+00
ZG16	LMNA	-0.047232869	-0.184083545	0.091414202	5.04E-01	5.88E-01	1.00E+00
ERG	LMNA	0.13318184	-0.004960361	0.266336017	5.88E-02	9.67E-02	1.00E+00
PARN	LMNA	0.299301484	0.168200331	0.419998616	1.51E-05	5.79E-05	1.79E-01
SOD2	LMNA	0.450318545	0.332967084	0.55393322	1.76E-11	1.98E-10	2.09E-07
JOSD3	LMNA	-0.061306293	-0.197684214	0.077399772	3.86E-01	4.73E-01	1.00E+00
ADAM5P	LMNA	0.079425749	-0.059275193	0.215118023	2.61E-01	3.43E-01	1.00E+00

CHD9	LMNA	-0.264318197	-0.388203875	-0.131049099	1.44E-04	4.58E-04	1.00E+00
hCG_40738	LMNA	-0.217494219	-0.345181057	-0.081902361	1.88E-03	4.58E-03	1.00E+00
STK16	LMNA	0.095046104	-0.043576676	0.2300782	1.78E-01	2.49E-01	1.00E+00
PDE1C	LMNA	0.201624328	0.06539352	0.330476695	4.01E-03	8.98E-03	1.00E+00
SEMA4D	LMNA	-0.170230678	-0.301203247	-0.032954116	1.54E-02	2.96E-02	1.00E+00
AGPAT1	LMNA	0.074066407	-0.064645591	0.209970472	2.95E-01	3.79E-01	1.00E+00
TOB2	LMNA	-0.146650528	-0.27905205	-0.008777225	3.73E-02	6.48E-02	1.00E+00
BANK1	LMNA	0.131527823	-0.006643813	0.264771227	6.21E-02	1.01E-01	1.00E+00
MAP3K3	LMNA	0.160723361	0.02318683	0.292289046	2.23E-02	4.12E-02	1.00E+00
MAX	LMNA	0.162222394	0.024725111	0.293696081	2.11E-02	3.92E-02	1.00E+00
GRM2	LMNA	-0.185092109	-0.315091835	-0.048274629	8.36E-03	1.73E-02	1.00E+00
OSBPL8	LMNA	-0.148610062	-0.28089822	-0.010780226	3.48E-02	6.09E-02	1.00E+00
PROSC	LMNA	-0.042096582	-0.179106708	0.096515315	5.52E-01	6.33E-01	1.00E+00
NR4A2	LMNA	-0.002708505	-0.140706893	0.135393121	9.69E-01	9.77E-01	1.00E+00
RICS	LMNA	-0.307930122	-0.42779551	-0.177421313	8.27E-06	3.31E-05	9.81E-02
PIR	LMNA	0.154024027	0.016320042	0.285993891	2.86E-02	5.13E-02	1.00E+00
PPCS	LMNA	0.31660212	0.186711776	0.435613651	4.42E-06	1.87E-05	5.25E-02
IPO9	LMNA	-0.165511733	-0.296781554	-0.028102856	1.86E-02	3.49E-02	1.00E+00
LONP1	LMNA	0.313388211	0.183265947	0.43271828	5.59E-06	2.32E-05	6.63E-02
EVC	LMNA	0.643070694	0.554221575	0.717430673	5.80E-25	7.73E-23	6.88E-21
CXCL13	LMNA	0.252298989	0.118370861	0.377211659	2.92E-04	8.67E-04	1.00E+00
SCYL3	LMNA	-0.225053291	-0.352162998	-0.089792023	1.28E-03	3.27E-03	1.00E+00
KIAA1199	LMNA	0.450731632	0.333427829	0.55429237	1.68E-11	1.89E-10	2.00E-07
SORL1	LMNA	-0.094607874	-0.22965936	0.044018034	1.80E-01	2.51E-01	1.00E+00
NAT10	LMNA	0.25541326	0.121651706	0.380063211	2.44E-04	7.37E-04	1.00E+00
CHD1	LMNA	0.203742839	0.06759302	0.33244324	3.63E-03	8.23E-03	1.00E+00
SYN3	LMNA	-0.537448055	-0.628841973	-0.431405272	1.63E-16	4.55E-15	1.93E-12
DMC1	LMNA	-0.042932411	-0.179917068	0.095685705	5.44E-01	6.25E-01	1.00E+00
SLC22A2	LMNA	0.125264176	-0.013011836	0.258839112	7.57E-02	1.20E-01	1.00E+00
SERPINF1	LMNA	0.341687658	0.213717795	0.458128585	6.47E-07	3.20E-06	7.67E-03
C20orf27	LMNA	0.207484471	0.07148093	0.335913746	3.05E-03	7.05E-03	1.00E+00
OR7A17	LMNA	0.088703234	-0.04995955	0.224011091	2.09E-01	2.84E-01	1.00E+00
RPS6KA5	LMNA	-0.534322127	-0.626183512	-0.427829408	2.62E-16	6.98E-15	3.11E-12
TAOK3	LMNA	-0.262886659	-0.386896502	-0.129536776	1.57E-04	4.94E-04	1.00E+00
STK25	LMNA	-0.002610919	-0.140611238	0.135488917	9.71E-01	9.77E-01	1.00E+00

SLC12A4	LMNA	0.419798291	0.299080048	0.527290899	5.00E-10	4.37E-09	5.93E-06
BRCA1	LMNA	-0.098620755	-0.233492822	0.039974486	1.63E-01	2.30E-01	1.00E+00
GBL	LMNA	-0.031390362	-0.168710262	0.107124861	6.57E-01	7.28E-01	1.00E+00
C14orf108	LMNA	-0.250257283	-0.375340906	-0.116221535	3.28E-04	9.66E-04	1.00E+00
CDC25B	LMNA	0.361755528	0.235463755	0.476033097	1.22E-07	6.91E-07	1.45E-03
BMP3	LMNA	0.120964775	-0.0173764	0.254761436	8.64E-02	1.34E-01	1.00E+00
TMEM180	LMNA	-0.140559543	-0.273307185	-0.002558182	4.60E-02	7.80E-02	1.00E+00
CRYGC	LMNA	0.0908446	-0.047805944	0.226060533	1.99E-01	2.72E-01	1.00E+00
SLK	LMNA	0.434208532	0.315042073	0.539896555	1.07E-10	1.07E-09	1.27E-06
POU3F1	LMNA	-0.060224806	-0.196640915	0.078478674	3.95E-01	4.82E-01	1.00E+00
C20orf32	LMNA	0.279691351	0.147328968	0.402212264	5.55E-05	1.91E-04	6.58E-01
USP52	LMNA	-0.227630909	-0.354540587	-0.092486259	1.12E-03	2.91E-03	1.00E+00
HIGD1B	LMNA	-0.219981055	-0.347479585	-0.084496082	1.66E-03	4.10E-03	1.00E+00
BAZ1B	LMNA	0.294478397	0.163056123	0.415632668	2.10E-05	7.84E-05	2.49E-01
USP6NL	LMNA	-0.117780607	-0.251738409	0.020605432	9.50E-02	1.46E-01	1.00E+00
SLCO2B1	LMNA	0.21142425	0.075579204	0.339564276	2.52E-03	5.96E-03	1.00E+00
ABCD4	LMNA	0.03160474	-0.106912728	0.168918737	6.55E-01	7.27E-01	1.00E+00
DIMT1L	LMNA	-0.217660701	-0.345334981	-0.082075942	1.86E-03	4.55E-03	1.00E+00
SLC25A46	LMNA	0.086611193	-0.052062304	0.222007701	2.20E-01	2.97E-01	1.00E+00
LARP7	LMNA	0.109667508	-0.028819816	0.244024056	1.20E-01	1.78E-01	1.00E+00
TEK	LMNA	0.020057472	-0.118321154	0.157671886	7.77E-01	8.30E-01	1.00E+00
CD160	LMNA	-0.22159267	-0.348968348	-0.086177946	1.53E-03	3.81E-03	1.00E+00
TERF2IP	LMNA	-0.328054274	-0.445910769	-0.199016371	1.88E-06	8.55E-06	2.23E-02
PHF20	LMNA	0.043201244	-0.095418832	0.18017767	5.42E-01	6.23E-01	1.00E+00
COL1A1	LMNA	0.664719034	0.579880876	0.735295545	3.99E-27	7.63E-25	4.73E-23
KIAA0090	LMNA	0.382380334	0.257945772	0.494336316	1.96E-08	1.27E-07	2.32E-04
GTPBP1	LMNA	-0.133259687	-0.266409647	0.00488111	5.87E-02	9.64E-02	1.00E+00
GRK5	LMNA	0.246793812	0.112578372	0.372165099	3.99E-04	1.15E-03	1.00E+00
AP1S2	LMNA	0.179523369	0.042526314	0.309894145	1.06E-02	2.13E-02	1.00E+00
RAB33B	LMNA	-0.002735842	-0.140733689	0.135366285	9.69E-01	9.77E-01	1.00E+00
CA11	LMNA	-0.216139312	-0.3439281	-0.080489992	2.01E-03	4.86E-03	1.00E+00
ALDOC	LMNA	-0.530148728	-0.622630891	-0.423060494	4.90E-16	1.27E-14	5.82E-12
NUDT1	LMNA	0.001418164	-0.136659591	0.139441865	9.84E-01	9.87E-01	1.00E+00
ZNF212	LMNA	0.05484189	-0.083843891	0.191443476	4.38E-01	5.24E-01	1.00E+00
ACBD3	LMNA	0.337954996	0.209686945	0.454787857	8.70E-07	4.21E-06	1.03E-02

ZNF83	LMNA	-0.042951545	-0.179935617	0.095666712	5.44E-01	6.25E-01	1.00E+00
PRDM2	LMNA	-0.29873405	-0.419485255	-0.167594747	1.57E-05	6.00E-05	1.86E-01
GDPD5	LMNA	0.042020699	-0.096590624	0.179033129	5.53E-01	6.34E-01	1.00E+00
PDCD4	LMNA	-0.27913212	-0.401703683	-0.146735494	5.75E-05	1.97E-04	6.82E-01
CEP350	LMNA	-0.175386917	-0.306028258	-0.038262337	1.25E-02	2.48E-02	1.00E+00
FOXP3	LMNA	0.140126467	0.002116407	0.272898358	4.67E-02	7.90E-02	1.00E+00
SMYD3	LMNA	-0.206300634	-0.33481607	-0.070250364	3.22E-03	7.41E-03	1.00E+00
CST7	LMNA	0.347857707	0.220390313	0.463643567	3.92E-07	2.03E-06	4.65E-03
SELL	LMNA	-0.000224548	-0.138271263	0.137830725	9.97E-01	9.98E-01	1.00E+00
CIAO1	LMNA	0.082367007	-0.05632445	0.217939847	2.44E-01	3.23E-01	1.00E+00
LGI2	LMNA	0.063798133	-0.074912653	0.200086889	3.67E-01	4.54E-01	1.00E+00
WDR45	LMNA	0.177246904	0.040179049	0.307767117	1.16E-02	2.32E-02	1.00E+00
ANKRD6	LMNA	-0.433313071	-0.539114611	-0.314048216	1.18E-10	1.17E-09	1.41E-06
LTBP4	LMNA	0.14721045	0.009349455	0.279579679	3.66E-02	6.37E-02	1.00E+00
PSCD3	LMNA	0.420309449	0.299645098	0.527738857	4.74E-10	4.16E-09	5.62E-06
PYY	LMNA	0.056181772	-0.082509165	0.192737907	4.27E-01	5.14E-01	1.00E+00
KCNC1	LMNA	-0.460013043	-0.562351749	-0.343794822	5.68E-12	6.99E-11	6.74E-08
SIRT6	LMNA	0.078926738	-0.059775572	0.214639049	2.64E-01	3.46E-01	1.00E+00
CCL19	LMNA	-0.047877092	-0.184707273	0.090773877	4.99E-01	5.83E-01	1.00E+00
ARHGEF9	LMNA	-0.42643719	-0.533104297	-0.306425482	2.48E-10	2.30E-09	2.95E-06
ABR	LMNA	-0.119358611	-0.253236882	0.019005555	9.07E-02	1.40E-01	1.00E+00
C10orf22	LMNA	-0.171436056	-0.302331794	-0.034194329	1.47E-02	2.84E-02	1.00E+00
STK17A	LMNA	0.436520549	0.317609355	0.541914637	8.34E-11	8.50E-10	9.89E-07
FOXE1	LMNA	-0.011334157	-0.149151781	0.126915427	8.73E-01	9.06E-01	1.00E+00
GML	LMNA	-0.098003508	-0.232903445	0.040596746	1.65E-01	2.33E-01	1.00E+00
CNGA3	LMNA	0.02789621	-0.110580648	0.165310583	6.94E-01	7.59E-01	1.00E+00
CD38	LMNA	-0.187945397	-0.317751989	-0.051223442	7.39E-03	1.55E-02	1.00E+00
ZDHHC6	LMNA	0.013802345	-0.124485855	0.15156455	8.45E-01	8.85E-01	1.00E+00
NEFH	LMNA	-0.255141969	-0.379814899	-0.121365787	2.48E-04	7.47E-04	1.00E+00
PGBD5	LMNA	-0.264133141	-0.388034897	-0.130853564	1.46E-04	4.63E-04	1.00E+00
CTDSP2	LMNA	0.006403197	-0.131764279	0.144326618	9.28E-01	9.48E-01	1.00E+00
RMND5B	LMNA	-0.131750552	-0.264981982	0.006417166	6.16E-02	1.01E-01	1.00E+00
ZNF257	LMNA	-0.392960535	-0.503687212	-0.269531225	7.26E-09	5.11E-08	8.61E-05
DUSP4	LMNA	0.48695022	0.374043936	0.585632671	2.01E-13	3.10E-12	2.38E-09
FLJ22167	LMNA	-0.03226821	-0.169563862	0.106256125	6.48E-01	7.21E-01	1.00E+00

EXOSC7	LMNA	-0.038067315	-0.175197611	0.100511899	5.91E-01	6.68E-01	1.00E+00
ROR2	LMNA	0.296894063	0.165631731	0.417820072	1.78E-05	6.74E-05	2.11E-01
FOXM1	LMNA	0.055200225	-0.083486983	0.191789703	4.35E-01	5.21E-01	1.00E+00
ZBTB48	LMNA	0.06742998	-0.071284593	0.203585846	3.40E-01	4.26E-01	1.00E+00
BUD31	LMNA	0.116722326	-0.021677986	0.250733105	9.81E-02	1.50E-01	1.00E+00
MAOA	LMNA	-0.091495405	-0.226683163	0.047151163	1.95E-01	2.69E-01	1.00E+00
CCDC41	LMNA	-0.177644657	-0.308138854	-0.040589064	1.14E-02	2.28E-02	1.00E+00
TNNT3	LMNA	0.132537936	-0.005615817	0.265726933	6.01E-02	9.85E-02	1.00E+00
FBXO11	LMNA	-0.304261576	-0.424482766	-0.173498123	1.07E-05	4.21E-05	1.27E-01
GYPC	LMNA	0.287677203	0.155814228	0.409466596	3.30E-05	1.19E-04	3.92E-01
PLIN	LMNA	-0.215013951	-0.342887072	-0.079317317	2.12E-03	5.10E-03	1.00E+00
RFXAP	LMNA	-0.44785364	-0.551789357	-0.330218969	2.34E-11	2.57E-10	2.78E-07
C6orf15	LMNA	0.161217202	0.023693529	0.292752642	2.19E-02	4.05E-02	1.00E+00
RNF4	LMNA	0.121653958	-0.016677126	0.255415398	8.46E-02	1.32E-01	1.00E+00
PPP1R3D	LMNA	0.219172961	0.083653055	0.346732849	1.73E-03	4.25E-03	1.00E+00
GRB2	LMNA	-0.012094075	-0.149894811	0.126167575	8.64E-01	9.00E-01	1.00E+00
TPM3	LMNA	-0.062971865	-0.199290369	0.075737547	3.73E-01	4.61E-01	1.00E+00
SYT13	LMNA	-0.437327143	-0.542618399	-0.318505413	7.63E-11	7.83E-10	9.05E-07
EPB42	LMNA	-0.052725381	-0.189397795	0.085951241	4.56E-01	5.42E-01	1.00E+00
RP5-1077B	LMNA	-0.221402675	-0.34879287	-0.08597963	1.54E-03	3.84E-03	1.00E+00
EIF1	LMNA	0.090476184	-0.048176557	0.225708018	2.00E-01	2.74E-01	1.00E+00
CETN3	LMNA	-0.166014412	-0.297252837	-0.028619322	1.82E-02	3.44E-02	1.00E+00
FPGT	LMNA	-0.02249181	-0.160045865	0.115919122	7.51E-01	8.08E-01	1.00E+00
PRY	LMNA	-0.247653706	-0.372953847	-0.113482542	3.80E-04	1.10E-03	1.00E+00
NTHL1	LMNA	-0.238052291	-0.364136542	-0.103399339	6.46E-04	1.78E-03	1.00E+00
POLR2B	LMNA	-0.165838739	-0.297088143	-0.028438822	1.83E-02	3.45E-02	1.00E+00
RPS28	LMNA	0.053422814	-0.085256963	0.19007202	4.50E-01	5.36E-01	1.00E+00
GDF10	LMNA	-0.274461161	-0.397452818	-0.141782238	7.72E-05	2.58E-04	9.16E-01
COQ9	LMNA	0.004823146	-0.133316622	0.142779079	9.46E-01	9.59E-01	1.00E+00
P2RX3	LMNA	-0.107436728	-0.24189993	0.031075172	1.28E-01	1.88E-01	1.00E+00
GCC2	LMNA	-0.084687048	-0.220164081	0.053995218	2.31E-01	3.09E-01	1.00E+00
RARRES3	LMNA	0.22826735	0.093151799	0.355127386	1.09E-03	2.83E-03	1.00E+00
PLXNA1	LMNA	0.484115482	0.370849316	0.583190341	2.89E-13	4.34E-12	3.43E-09
WBP4	LMNA	-0.16504252	-0.296341589	-0.02762084	1.89E-02	3.55E-02	1.00E+00
KIAA0100	LMNA	0.033591348	-0.104946322	0.170850062	6.35E-01	7.09E-01	1.00E+00

PMF1	LMNA	-0.209570037	-0.337846676	-0.073649826	2.76E-03	6.45E-03	1.00E+00
HS2ST1	LMNA	0.423653404	0.303343695	0.530667881	3.34E-10	3.00E-09	3.96E-06
CRELD2	LMNA	0.43033607	0.310745967	0.536513691	1.64E-10	1.57E-09	1.94E-06
C8G	LMNA	0.068784876	-0.069930169	0.204890275	3.31E-01	4.16E-01	1.00E+00
CD82	LMNA	0.309808772	0.179431971	0.429490708	7.23E-06	2.94E-05	8.58E-02
PMM1	LMNA	0.067338428	-0.071376095	0.203497686	3.41E-01	4.27E-01	1.00E+00
CBLL1	LMNA	0.109032288	-0.029462178	0.243419337	1.22E-01	1.81E-01	1.00E+00
LIM2	LMNA	0.120251791	-0.01809968	0.254084761	8.83E-02	1.37E-01	1.00E+00
VAX2	LMNA	-0.339614516	-0.456273532	-0.211478499	7.63E-07	3.73E-06	9.05E-03
SETDB1	LMNA	-0.260045172	-0.384299996	-0.126536778	1.86E-04	5.75E-04	1.00E+00
LRAP	LMNA	0.187898682	0.051175144	0.317708452	7.41E-03	1.55E-02	1.00E+00
GCLM	LMNA	0.372071132	0.246691383	0.485199939	4.97E-08	3.01E-07	5.90E-04
CPEB3	LMNA	-0.451079084	-0.554594426	-0.33381541	1.62E-11	1.83E-10	1.92E-07
PPM1A	LMNA	-0.188236609	-0.318023374	-0.051524538	7.30E-03	1.53E-02	1.00E+00
INTS1	LMNA	0.233968087	0.099118569	0.360379015	8.04E-04	2.16E-03	1.00E+00
MMP16	LMNA	-0.348888311	-0.46456388	-0.22150601	3.61E-07	1.88E-06	4.28E-03
CAMTA1	LMNA	-0.610432267	-0.690310278	-0.515852567	5.18E-22	3.63E-20	6.14E-18
SAMSN1	LMNA	0.151488706	0.013724733	0.283608553	3.14E-02	5.58E-02	1.00E+00
DRD3	LMNA	0.040320555	-0.098277487	0.177384183	5.69E-01	6.48E-01	1.00E+00
GMPPA	LMNA	0.637474575	0.547615972	0.71279662	1.97E-24	2.34E-22	2.34E-20
C11orf73	LMNA	-0.178250155	-0.308704671	-0.041213316	1.11E-02	2.23E-02	1.00E+00
AIPL1	LMNA	0.000717941	-0.137346672	0.13875519	9.92E-01	9.93E-01	1.00E+00
PTP4A2	LMNA	0.457174556	0.340621328	0.559889053	7.95E-12	9.50E-11	9.43E-08
IL24	LMNA	0.207077832	0.071058194	0.335536743	3.11E-03	7.17E-03	1.00E+00
BDKRB1	LMNA	0.3841587	0.259890606	0.495909868	1.66E-08	1.09E-07	1.97E-04
HPCA	LMNA	-0.355846816	-0.470771178	-0.22904779	2.02E-07	1.10E-06	2.40E-03
MLF1	LMNA	-0.119621925	-0.253486862	0.018738522	8.99E-02	1.39E-01	1.00E+00
SEC14L1	LMNA	0.172803202	0.035601499	0.303611355	1.39E-02	2.71E-02	1.00E+00
CHFR	LMNA	0.112847042	-0.025602816	0.247049348	1.10E-01	1.65E-01	1.00E+00
EMILIN1	LMNA	0.647171246	0.55906894	0.720822099	2.33E-25	3.33E-23	2.76E-21
THADA	LMNA	0.206399468	0.070353084	0.334907724	3.21E-03	7.38E-03	1.00E+00
TAF12	LMNA	0.259189838	0.125634208	0.383518021	1.95E-04	6.01E-04	1.00E+00
NDUFS4	LMNA	-0.091487439	-0.226675542	0.047159179	1.95E-01	2.69E-01	1.00E+00
ID1	LMNA	0.11763995	-0.020748005	0.25160481	9.54E-02	1.46E-01	1.00E+00
PIK3CB	LMNA	-0.1741518	-0.304873093	-0.036990109	1.32E-02	2.59E-02	1.00E+00

COL18A1	LMNA	0.589468726	0.491406813	0.67277175	2.73E-20	1.43E-18	3.24E-16
PDZD3	LMNA	0.184596875	0.047763058	0.314629915	8.54E-03	1.76E-02	1.00E+00
TBC1D17	LMNA	-0.051062684	-0.187789906	0.087605873	4.70E-01	5.56E-01	1.00E+00
COX8A	LMNA	-0.143245266	-0.27584146	-0.005299055	4.20E-02	7.19E-02	1.00E+00
CDCA4	LMNA	0.243536501	0.109155354	0.369175648	4.78E-04	1.35E-03	1.00E+00
C2orf44	LMNA	-0.278681162	-0.401293513	-0.146256992	5.92E-05	2.02E-04	7.02E-01
C9orf16	LMNA	-0.127865614	-0.261304082	0.010368409	6.98E-02	1.12E-01	1.00E+00
ERBB2IP	LMNA	0.160482286	0.022939504	0.292062713	2.25E-02	4.15E-02	1.00E+00
EMX2	LMNA	0.039757254	-0.098836212	0.176837676	5.74E-01	6.53E-01	1.00E+00
FUS	LMNA	0.017523852	-0.120819433	0.155199397	8.04E-01	8.52E-01	1.00E+00
NIPSNAP3E	LMNA	-0.218243668	-0.345873918	-0.082683829	1.81E-03	4.43E-03	1.00E+00
TF	LMNA	-0.264319849	-0.388205383	-0.131050844	1.44E-04	4.58E-04	1.00E+00
CXorf56	LMNA	0.007388746	-0.130795669	0.145291546	9.17E-01	9.40E-01	1.00E+00
CLCN4	LMNA	-0.244485952	-0.37004729	-0.110152776	4.54E-04	1.29E-03	1.00E+00
PWP2	LMNA	0.119610363	-0.018750248	0.253475886	9.00E-02	1.39E-01	1.00E+00
MMP15	LMNA	-0.244763325	-0.370301891	-0.110444215	4.47E-04	1.27E-03	1.00E+00
MTMR14	LMNA	0.22569151	0.090458933	0.352751843	1.24E-03	3.17E-03	1.00E+00
NPR1	LMNA	0.197909215	0.061539572	0.327025371	4.75E-03	1.04E-02	1.00E+00
DNAL4	LMNA	0.076155329	-0.062553314	0.21197774	2.81E-01	3.64E-01	1.00E+00
ELAC2	LMNA	-0.07537254	-0.211225683	0.063337504	2.86E-01	3.70E-01	1.00E+00
ASS1	LMNA	0.329705186	0.20079353	0.447392606	1.65E-06	7.63E-06	1.96E-02
IPP	LMNA	0.062919905	-0.075789415	0.199240273	3.74E-01	4.61E-01	1.00E+00
TMEM59	LMNA	0.071760582	-0.066953703	0.207753448	3.10E-01	3.95E-01	1.00E+00
POLR2J	LMNA	0.072766865	-0.065946605	0.208721149	3.03E-01	3.88E-01	1.00E+00
SEC23IP	LMNA	0.21323755	0.077466997	0.341243152	2.31E-03	5.53E-03	1.00E+00
RGS4	LMNA	0.12367832	-0.014622341	0.257335594	7.95E-02	1.25E-01	1.00E+00
UQCRC1	LMNA	0.041143394	-0.097461175	0.178182339	5.61E-01	6.41E-01	1.00E+00
SNX6	LMNA	0.128149042	-0.01008029	0.261572536	6.91E-02	1.11E-01	1.00E+00
DDX18	LMNA	-0.049420745	-0.186201371	0.089239097	4.85E-01	5.70E-01	1.00E+00
POLG	LMNA	0.269660004	0.136697831	0.393077957	1.04E-04	3.39E-04	1.00E+00
ADAM23	LMNA	-0.116768987	-0.250777436	0.021630703	9.79E-02	1.49E-01	1.00E+00
ZNHIT2	LMNA	0.142876193	0.00492228	0.275493308	4.25E-02	7.27E-02	1.00E+00
TFR2	LMNA	0.204845342	0.068738196	0.333466217	3.45E-03	7.87E-03	1.00E+00
NCDN	LMNA	-0.118940473	-0.25283988	0.019429558	9.18E-02	1.41E-01	1.00E+00
RAG1	LMNA	0.330835271	0.202010523	0.448406583	1.52E-06	7.03E-06	1.80E-02

POMT1	LMNA	-0.260889741	-0.385071956	-0.127428206	1.77E-04	5.50E-04	1.00E+00
CYP1A1	LMNA	-0.093998605	-0.229076965	0.044631561	1.83E-01	2.54E-01	1.00E+00
SNAPC1	LMNA	0.297500552	0.166278657	0.418369032	1.71E-05	6.48E-05	2.03E-01
GNA11	LMNA	0.172805546	0.035603912	0.303613549	1.39E-02	2.71E-02	1.00E+00
CCDC52	LMNA	0.077124757	-0.061581915	0.212908885	2.75E-01	3.58E-01	1.00E+00
CAPN1	LMNA	0.437762914	0.318989607	0.542998553	7.27E-11	7.48E-10	8.63E-07
DDHD2	LMNA	-0.310406911	-0.430030261	-0.180072369	6.93E-06	2.83E-05	8.22E-02
UPF3A	LMNA	-0.154901907	-0.286819456	-0.017219127	2.77E-02	4.99E-02	1.00E+00
LAGE3	LMNA	-0.055956153	-0.192519975	0.082733951	4.29E-01	5.15E-01	1.00E+00
GNRHR	LMNA	-0.271095332	-0.394386427	-0.138217104	9.52E-05	3.12E-04	1.00E+00
UNC13B	LMNA	0.098202887	-0.040395759	0.233093833	1.64E-01	2.32E-01	1.00E+00
TTLL4	LMNA	0.147441775	0.009585891	0.279797639	3.63E-02	6.32E-02	1.00E+00
PPBP	LMNA	0.170106491	0.032826364	0.301086954	1.55E-02	2.97E-02	1.00E+00
HTR3A	LMNA	0.016684795	-0.1216464	0.154380203	8.14E-01	8.59E-01	1.00E+00
SDC2	LMNA	0.424543724	0.30432905	0.531447297	3.04E-10	2.76E-09	3.60E-06
ANKRD49	LMNA	-0.250684716	-0.375732634	-0.116671395	3.20E-04	9.44E-04	1.00E+00
BTF3	LMNA	-0.083794519	-0.219308576	0.054891461	2.36E-01	3.14E-01	1.00E+00
COX7A2	LMNA	-0.370349056	-0.483671363	-0.244814711	5.79E-08	3.47E-07	6.87E-04
SARS	LMNA	-0.073949575	-0.209858173	0.064762574	2.96E-01	3.80E-01	1.00E+00
C13orf18	LMNA	0.247015614	0.112811573	0.372368566	3.94E-04	1.14E-03	1.00E+00
CACNB1	LMNA	-0.288366936	-0.410092426	-0.156548009	3.16E-05	1.14E-04	3.75E-01
QKI	LMNA	-0.358827408	-0.473426524	-0.232282885	1.57E-07	8.71E-07	1.86E-03
SETMAR	LMNA	-0.366320879	-0.480093109	-0.240428584	8.25E-08	4.80E-07	9.78E-04
LAMB4	LMNA	0.022743359	-0.115670818	0.160291087	7.48E-01	8.06E-01	1.00E+00
MAN2B1	LMNA	0.536731784	0.43058561	0.628233006	1.82E-16	5.04E-15	2.15E-12
EML3	LMNA	0.365314468	0.239333544	0.479198516	9.00E-08	5.20E-07	1.07E-03
ACADL	LMNA	-0.058642178	-0.195113614	0.080056933	4.07E-01	4.95E-01	1.00E+00
OFD1	LMNA	-0.120309631	-0.25413966	0.018041011	8.81E-02	1.37E-01	1.00E+00
CGA	LMNA	-0.04184645	-0.178864162	0.096763548	5.54E-01	6.35E-01	1.00E+00
EIF3EIP	LMNA	-0.206120241	-0.334648776	-0.070062888	3.25E-03	7.46E-03	1.00E+00
PEX16	LMNA	0.152610076	0.014872407	0.284663787	3.01E-02	5.38E-02	1.00E+00
GNG12	LMNA	0.390328111	0.266645344	0.501363069	9.32E-09	6.40E-08	1.11E-04
HABP4	LMNA	-0.144917117	-0.277418104	-0.007006284	3.96E-02	6.84E-02	1.00E+00
CNTN2	LMNA	-0.060387257	-0.19679765	0.078316632	3.93E-01	4.81E-01	1.00E+00
ASNSD1	LMNA	-0.332903623	-0.450261644	-0.204238971	1.29E-06	6.08E-06	1.53E-02

FUT4	LMNA	0.371831082	0.246429727	0.484986903	5.08E-08	3.08E-07	6.02E-04
DBH	LMNA	-0.068157506	-0.204286333	0.070557384	3.35E-01	4.21E-01	1.00E+00
CD53	LMNA	0.151044946	0.013270664	0.283190877	3.19E-02	5.66E-02	1.00E+00
HIST1H2BJ	LMNA	0.064006323	-0.074704779	0.200287556	3.65E-01	4.53E-01	1.00E+00
TFAP2B	LMNA	-0.066308857	-0.202506116	0.072404938	3.48E-01	4.35E-01	1.00E+00
ACF	LMNA	-0.092888522	-0.228015598	0.045749136	1.89E-01	2.60E-01	1.00E+00
TMEM11	LMNA	0.01231146	-0.125953613	0.150107337	8.62E-01	8.98E-01	1.00E+00
CDH8	LMNA	-0.422087355	-0.529296472	-0.301611108	3.93E-10	3.49E-09	4.67E-06
C3orf32	LMNA	-0.104234535	-0.23884858	0.034310178	1.40E-01	2.03E-01	1.00E+00
AGPS	LMNA	0.288826998	0.157037532	0.4105098	3.06E-05	1.11E-04	3.63E-01
C4orf18	LMNA	0.233346863	0.098467884	0.359807121	8.31E-04	2.23E-03	1.00E+00
PCCB	LMNA	-0.281562516	-0.403913407	-0.149315403	4.92E-05	1.71E-04	5.84E-01
IPO13	LMNA	-0.040459823	-0.177519286	0.098139337	5.68E-01	6.47E-01	1.00E+00
PECI	LMNA	0.088104818	-0.050561156	0.223438149	2.12E-01	2.88E-01	1.00E+00
UNG	LMNA	0.012839561	-0.125433773	0.150623579	8.56E-01	8.94E-01	1.00E+00
C6orf105	LMNA	0.233395511	0.098518835	0.359851909	8.29E-04	2.22E-03	1.00E+00
GSTP1	LMNA	0.130692082	-0.007494129	0.263980304	6.38E-02	1.04E-01	1.00E+00
SUV420H1	LMNA	-0.315210661	-0.434360405	-0.185219515	4.90E-06	2.05E-05	5.81E-02
ARHGAP15	LMNA	0.151453399	0.013688604	0.283575324	3.14E-02	5.58E-02	1.00E+00
LTBR	LMNA	0.455492371	0.338741858	0.558428717	9.68E-12	1.14E-10	1.15E-07
TDRKH	LMNA	-0.513913775	-0.608774473	-0.404565181	5.20E-15	1.07E-13	6.16E-11
PSG4	LMNA	0.2533614	0.119489768	0.378184711	2.75E-04	8.21E-04	1.00E+00
SLC9A3R1	LMNA	0.046202299	-0.092438298	0.18308553	5.14E-01	5.98E-01	1.00E+00
NBPF3	LMNA	-0.146176435	-0.278605237	-0.008292781	3.79E-02	6.57E-02	1.00E+00
SLC9A3	LMNA	-0.024943236	-0.162434902	0.11349859	7.25E-01	7.85E-01	1.00E+00
OSBP	LMNA	0.133768966	-0.004362598	0.266891301	5.77E-02	9.50E-02	1.00E+00
PRR5	LMNA	0.271100713	0.138222801	0.394391332	9.52E-05	3.12E-04	1.00E+00
CHMP7	LMNA	0.029043526	-0.109446299	0.166427238	6.82E-01	7.50E-01	1.00E+00
ADRA1A	LMNA	-0.218658232	-0.34625712	-0.083116176	1.77E-03	4.35E-03	1.00E+00
ASAH1	LMNA	-0.05572808	-0.192299659	0.082961167	4.31E-01	5.17E-01	1.00E+00
FKBP4	LMNA	-0.011408678	-0.149224653	0.126842096	8.72E-01	9.05E-01	1.00E+00
ZNF350	LMNA	-0.094497846	-0.229554192	0.044128839	1.81E-01	2.51E-01	1.00E+00
DOM3Z	LMNA	-0.072286149	-0.208258898	0.066427745	3.07E-01	3.91E-01	1.00E+00
GIPR	LMNA	-0.196794394	-0.325989034	-0.060383882	5.00E-03	1.09E-02	1.00E+00
AHI1	LMNA	-0.088889374	-0.224189288	0.049772397	2.08E-01	2.83E-01	1.00E+00

BST1	LMNA	0.376396168	0.25140884	0.489035947	3.38E-08	2.09E-07	4.00E-04
NCR2	LMNA	-0.142329562	-0.2749776	-0.004364315	4.33E-02	7.39E-02	1.00E+00
NADSYN1	LMNA	0.47442146	0.359944814	0.574824688	9.85E-13	1.37E-11	1.17E-08
UBE2W	LMNA	-0.044990101	-0.181911264	0.093642506	5.25E-01	6.08E-01	1.00E+00
RGS14	LMNA	0.210506399	0.074624018	0.338714156	2.64E-03	6.20E-03	1.00E+00
KRT15	LMNA	0.235467606	0.100689678	0.361759071	7.42E-04	2.01E-03	1.00E+00
SCN11A	LMNA	-0.232581867	-0.35910274	-0.097666766	8.66E-04	2.31E-03	1.00E+00
GFI1	LMNA	0.038449927	-0.100132582	0.175568998	5.87E-01	6.65E-01	1.00E+00
FHL1	LMNA	-0.262459636	-0.386506421	-0.129085776	1.61E-04	5.06E-04	1.00E+00
SMC6	LMNA	0.083430894	-0.055256536	0.218959975	2.38E-01	3.17E-01	1.00E+00
GATA1	LMNA	0.128125862	-0.010103854	0.261550581	6.92E-02	1.11E-01	1.00E+00
OSGEP	LMNA	0.046415152	-0.092226806	0.183291682	5.12E-01	5.96E-01	1.00E+00
ARMC6	LMNA	-0.056024246	-0.192585749	0.082666111	4.28E-01	5.15E-01	1.00E+00
HK3	LMNA	0.39848685	0.275596848	0.508561136	4.27E-09	3.14E-08	5.06E-05
PSMD5	LMNA	0.258863905	0.125290332	0.383219994	1.99E-04	6.12E-04	1.00E+00
RSU1	LMNA	0.236688152	0.101968992	0.362881971	6.95E-04	1.90E-03	1.00E+00
RPL4	LMNA	0.034932043	-0.103618649	0.172152853	6.22E-01	6.97E-01	1.00E+00
MAD2L1	LMNA	-0.149121413	-0.281379827	-0.011303102	3.42E-02	6.00E-02	1.00E+00
RBPMS	LMNA	0.500459808	0.389305528	0.597247584	3.35E-14	5.77E-13	3.97E-10
EIF4A3	LMNA	0.090163481	-0.048491096	0.225408783	2.02E-01	2.76E-01	1.00E+00
E4F1	LMNA	0.035902261	-0.102657545	0.173095342	6.12E-01	6.88E-01	1.00E+00
DLEC1	LMNA	-0.110436444	-0.244755929	0.028042083	1.18E-01	1.75E-01	1.00E+00
PRPF3	LMNA	-0.169926172	-0.300918092	-0.032640876	1.56E-02	2.99E-02	1.00E+00
EMR1	LMNA	0.180101951	0.043123131	0.310434538	1.03E-02	2.09E-02	1.00E+00
CHMP2B	LMNA	-0.047563601	-0.184403769	0.091085486	5.01E-01	5.86E-01	1.00E+00
CAMSAP1	LMNA	-0.226435597	-0.353438238	-0.091236622	1.19E-03	3.06E-03	1.00E+00
RPS21	LMNA	0.12627063	-0.011989367	0.259792974	7.33E-02	1.17E-01	1.00E+00
XCR1	LMNA	0.121449275	-0.01688482	0.255221187	8.51E-02	1.33E-01	1.00E+00
ARID5A	LMNA	0.379494926	0.254792412	0.491781656	2.55E-08	1.62E-07	3.02E-04
RBM6	LMNA	-0.185788869	-0.315741622	-0.048994496	8.11E-03	1.69E-02	1.00E+00
UBE2N	LMNA	-0.400758103	-0.51056222	-0.278092591	3.42E-09	2.56E-08	4.06E-05
KLF4	LMNA	0.280300674	0.14797571	0.402766315	5.34E-05	1.84E-04	6.33E-01
MGC4172	LMNA	-0.021993677	-0.159560213	0.116410776	7.56E-01	8.12E-01	1.00E+00
LMO4	LMNA	-0.097031919	-0.231975525	0.041576007	1.70E-01	2.38E-01	1.00E+00
KLKB1	LMNA	-0.124422417	-0.258041138	0.013866768	7.77E-02	1.23E-01	1.00E+00

HDAC3	LMNA	0.25073738	0.116726826	0.375780896	3.19E-04	9.42E-04	1.00E+00
HP	LMNA	0.144285769	0.006361481	0.276822793	4.05E-02	6.97E-02	1.00E+00
SCHIP1	LMNA	-0.546445489	-0.636481978	-0.441716402	4.03E-17	1.27E-15	4.77E-13
BTK	LMNA	0.099369968	-0.039219044	0.234208074	1.59E-01	2.26E-01	1.00E+00
KRCC1	LMNA	-0.197522219	-0.326665655	-0.061138347	4.84E-03	1.06E-02	1.00E+00
PRND	LMNA	0.073075663	-0.0656375	0.209018054	3.01E-01	3.86E-01	1.00E+00
C6orf27	LMNA	-0.144335202	-0.276869408	-0.006411963	4.04E-02	6.96E-02	1.00E+00
PIGL	LMNA	0.203596287	0.067440823	0.332307237	3.66E-03	8.27E-03	1.00E+00
CLCA1	LMNA	-0.019942233	-0.157559466	0.118434824	7.78E-01	8.31E-01	1.00E+00
C1orf112	LMNA	-0.061542767	-0.197912297	0.077163821	3.84E-01	4.72E-01	1.00E+00
OLFML2A	LMNA	0.539660643	0.433938349	0.630722389	1.16E-16	3.34E-15	1.37E-12
HUS1	LMNA	0.426976163	0.307022442	0.533575811	2.35E-10	2.19E-09	2.78E-06
SFRS6	LMNA	-0.136696512	-0.269658757	0.001380541	5.24E-02	8.75E-02	1.00E+00
CXCR7	LMNA	-0.12851687	-0.261920901	0.009706339	6.83E-02	1.10E-01	1.00E+00
UIMC1	LMNA	0.056892993	-0.08180048	0.193424809	4.21E-01	5.09E-01	1.00E+00
FXYD2	LMNA	0.020399732	-0.117983534	0.158005756	7.73E-01	8.27E-01	1.00E+00
GOT2	LMNA	-0.109584878	-0.243945399	0.028903382	1.21E-01	1.78E-01	1.00E+00
ZNF667	LMNA	-0.261890666	-0.385986603	-0.128484944	1.67E-04	5.21E-04	1.00E+00
TFEC	LMNA	0.127731317	-0.010504922	0.261176872	7.01E-02	1.12E-01	1.00E+00
ATP7B	LMNA	-0.393000082	-0.503722115	-0.269574596	7.23E-09	5.10E-08	8.58E-05
RAB38	LMNA	0.426612402	0.306619535	0.533257587	2.44E-10	2.26E-09	2.89E-06
POLD2	LMNA	0.263794944	0.130496246	0.387726064	1.49E-04	4.70E-04	1.00E+00
PPM1H	LMNA	-0.385759925	-0.497326054	-0.261642581	1.43E-08	9.53E-08	1.70E-04
DCX	LMNA	-0.504766597	-0.600941846	-0.39418371	1.86E-14	3.44E-13	2.21E-10
NFYC	LMNA	0.021550072	-0.116848554	0.159127666	7.61E-01	8.16E-01	1.00E+00
ZNF228	LMNA	-0.182903266	-0.313049766	-0.046014121	9.18E-03	1.88E-02	1.00E+00
KIN	LMNA	-0.151053573	-0.283198998	-0.013279491	3.19E-02	5.66E-02	1.00E+00
PLSCR4	LMNA	0.002228906	-0.1358639	0.140236755	9.75E-01	9.81E-01	1.00E+00
HIST1H4I	LMNA	-0.184859423	-0.314874809	-0.048034258	8.44E-03	1.75E-02	1.00E+00
PPARGC1A	LMNA	-0.136356511	-0.269337462	0.001726999	5.30E-02	8.83E-02	1.00E+00
HIG2	LMNA	0.205350512	0.069263039	0.333934846	3.37E-03	7.70E-03	1.00E+00
KCNK10	LMNA	-0.256648725	-0.381193796	-0.122954062	2.27E-04	6.90E-04	1.00E+00
EXOC1	LMNA	-0.181672466	-0.31190097	-0.044743639	9.66E-03	1.97E-02	1.00E+00
OR6A2	LMNA	-0.054696989	-0.191303462	0.083988204	4.39E-01	5.26E-01	1.00E+00
ETFA	LMNA	-0.076382115	-0.212195592	0.062326091	2.80E-01	3.63E-01	1.00E+00

PDAP1	LMNA	0.397135037	0.274112208	0.507369558	4.86E-09	3.55E-08	5.77E-05
C19orf6	LMNA	0.256387882	0.122679058	0.380955127	2.30E-04	6.99E-04	1.00E+00
POLRMT	LMNA	0.23673152	0.102014456	0.362921862	6.94E-04	1.89E-03	1.00E+00
ZNF146	LMNA	-0.004819313	-0.142775324	0.133320387	9.46E-01	9.59E-01	1.00E+00
MIA2	LMNA	-0.071742493	-0.207736051	0.066971804	3.10E-01	3.95E-01	1.00E+00
LY6G6E	LMNA	0.260526986	0.127045298	0.384740409	1.81E-04	5.61E-04	1.00E+00
EIF4E2	LMNA	0.391397994	0.267817971	0.502307852	8.42E-09	5.84E-08	9.99E-05
NENF	LMNA	0.001824369	-0.13626095	0.13984015	9.79E-01	9.84E-01	1.00E+00
HOXB5	LMNA	0.200432401	0.064156607	0.329369775	4.24E-03	9.44E-03	1.00E+00
ZNF324	LMNA	-0.096718628	-0.231676263	0.041891715	1.71E-01	2.40E-01	1.00E+00
CUGBP1	LMNA	-0.085203745	-0.220659249	0.053476269	2.28E-01	3.05E-01	1.00E+00
ENTPD7	LMNA	0.453548226	0.336570865	0.556740173	1.21E-11	1.41E-10	1.44E-07
TTC13	LMNA	-0.334121589	-0.451353538	-0.205551835	1.18E-06	5.57E-06	1.39E-02
GJB5	LMNA	-0.062366136	-0.198706334	0.076342148	3.78E-01	4.65E-01	1.00E+00
PRSS22	LMNA	0.088183334	-0.050482227	0.223513328	2.12E-01	2.87E-01	1.00E+00
CYP3A5	LMNA	-0.176845933	-0.307392331	-0.039765764	1.18E-02	2.35E-02	1.00E+00
MBOAT5	LMNA	0.285133301	0.153109125	0.40715739	3.90E-05	1.38E-04	4.63E-01
CUL4B	LMNA	0.297122895	0.165875807	0.418027208	1.75E-05	6.64E-05	2.08E-01
CENPJ	LMNA	-0.289264783	-0.410906918	-0.157503412	2.98E-05	1.08E-04	3.53E-01
KIAA0664	LMNA	-0.022580297	-0.160132129	0.115831778	7.50E-01	8.07E-01	1.00E+00
PITX1	LMNA	0.37328522	0.248015028	0.486277191	4.46E-08	2.72E-07	5.29E-04
PDGFRB	LMNA	0.462163303	0.346200632	0.564216126	4.40E-12	5.51E-11	5.21E-08
RDX	LMNA	0.005897065	-0.132261609	0.143830973	9.34E-01	9.52E-01	1.00E+00
CELSR3	LMNA	-0.427990513	-0.534463023	-0.308146181	2.10E-10	1.98E-09	2.50E-06
JUND	LMNA	0.118943582	-0.019426406	0.252842832	9.18E-02	1.41E-01	1.00E+00
ITSN1	LMNA	0.001104845	-0.136967047	0.139134624	9.88E-01	9.90E-01	1.00E+00
CHRNB1	LMNA	-0.07945478	-0.215145886	0.05924608	2.61E-01	3.42E-01	1.00E+00
EHBP1L1	LMNA	0.545135914	0.440213897	0.635371075	4.95E-17	1.53E-15	5.87E-13
C19orf2	LMNA	-0.088100933	-0.22343443	0.050565061	2.12E-01	2.88E-01	1.00E+00
FETUB	LMNA	-0.035411077	-0.172618228	0.103144147	6.17E-01	6.92E-01	1.00E+00
CAMK2B	LMNA	-0.014951311	-0.152687158	0.123354299	8.33E-01	8.74E-01	1.00E+00
DCTN1	LMNA	-0.037395941	-0.17454584	0.101177393	5.97E-01	6.74E-01	1.00E+00
TNKS2	LMNA	-0.131311292	-0.264566325	0.00686414	6.25E-02	1.02E-01	1.00E+00
MRTO4	LMNA	0.16324602	0.025775912	0.294656559	2.03E-02	3.78E-02	1.00E+00
C1QBP	LMNA	-0.221454978	-0.348841178	-0.086034223	1.54E-03	3.83E-03	1.00E+00

TTC3	LMNA	-0.366087624	-0.47988579	-0.240174758	8.42E-08	4.89E-07	9.98E-04
NDUFB8	LMNA	-0.189654005	-0.319343966	-0.052990395	6.86E-03	1.45E-02	1.00E+00
CADPS2	LMNA	0.211961771	0.076138706	0.340062034	2.46E-03	5.83E-03	1.00E+00
EDG2	LMNA	0.116807931	-0.021591238	0.250814436	9.78E-02	1.49E-01	1.00E+00
SEMA3G	LMNA	-0.116270048	-0.25030338	0.022136266	9.94E-02	1.51E-01	1.00E+00
IL23A	LMNA	0.125429839	-0.012843557	0.258996136	7.53E-02	1.20E-01	1.00E+00
GJA9	LMNA	0.121960481	-0.016366071	0.255706216	8.38E-02	1.31E-01	1.00E+00
R3HDM2	LMNA	-0.102757576	-0.2374403	0.035801294	1.46E-01	2.10E-01	1.00E+00
C5	LMNA	0.064849393	-0.073862866	0.201100041	3.59E-01	4.46E-01	1.00E+00
SLC2A10	LMNA	0.586018645	0.4873984	0.669876342	5.10E-20	2.61E-18	6.05E-16
TRIM45	LMNA	0.256881742	0.123199747	0.38140699	2.24E-04	6.81E-04	1.00E+00
TSP50	LMNA	0.153925122	0.016218762	0.285900867	2.87E-02	5.14E-02	1.00E+00
PAQR3	LMNA	-0.009550726	-0.147407372	0.128669924	8.93E-01	9.22E-01	1.00E+00
ANKRD26	LMNA	-0.38409161	-0.495850518	-0.259817217	1.67E-08	1.10E-07	1.98E-04
TCP1	LMNA	-0.143388398	-0.275976469	-0.005445184	4.18E-02	7.16E-02	1.00E+00
TMED7	LMNA	0.248406602	0.114274387	0.3736443	3.64E-04	1.06E-03	1.00E+00
CMA1	LMNA	0.156664037	0.019024491	0.288475987	2.60E-02	4.71E-02	1.00E+00
PTCRA	LMNA	-0.039140305	-0.176239023	0.099448049	5.80E-01	6.59E-01	1.00E+00
HCRTR1	LMNA	-0.155270196	-0.287165739	-0.017596377	2.73E-02	4.93E-02	1.00E+00
FST	LMNA	0.409794239	0.288038285	0.518511732	1.39E-09	1.12E-08	1.65E-05
PAWR	LMNA	0.178615017	0.041589531	0.309045578	1.10E-02	2.21E-02	1.00E+00
LDLR	LMNA	0.505000028	0.394448289	0.60114196	1.80E-14	3.34E-13	2.14E-10
ASTN2	LMNA	-0.358176707	-0.472847006	-0.231576384	1.66E-07	9.18E-07	1.97E-03
SCRN1	LMNA	0.115404528	-0.023013111	0.249480872	1.02E-01	1.54E-01	1.00E+00
GPATCH8	LMNA	-0.145476689	-0.277945651	-0.007577876	3.88E-02	6.72E-02	1.00E+00
KIF4A	LMNA	0.063671254	-0.075039334	0.19996459	3.68E-01	4.56E-01	1.00E+00
TANC2	LMNA	-0.190960447	-0.320560735	-0.054342026	6.48E-03	1.38E-02	1.00E+00
ZMYM5	LMNA	0.059590835	-0.079110978	0.196029187	4.00E-01	4.87E-01	1.00E+00
PGM1	LMNA	-0.056302424	-0.192854443	0.082388954	4.26E-01	5.13E-01	1.00E+00
ZNF586	LMNA	-0.064109191	-0.200386702	0.074602063	3.65E-01	4.52E-01	1.00E+00
POLR3G	LMNA	-0.098512665	-0.23338962	0.040083462	1.63E-01	2.31E-01	1.00E+00
CPD	LMNA	0.395530328	0.272350583	0.505954513	5.68E-09	4.09E-08	6.73E-05
SNCAIP	LMNA	-0.356020001	-0.470925522	-0.229235686	1.99E-07	1.09E-06	2.36E-03
DCT	LMNA	-0.367802541	-0.481409718	-0.242041311	7.25E-08	4.28E-07	8.59E-04
HLA-DOA	LMNA	0.255433496	0.121673033	0.380081731	2.44E-04	7.36E-04	1.00E+00

TANK	LMNA	0.336353765	0.207959133	0.45335375	9.87E-07	4.73E-06	1.17E-02
RCAN1	LMNA	0.332137621	0.203413523	0.449574751	1.37E-06	6.40E-06	1.63E-02
UPK1B	LMNA	-0.054890893	-0.191490826	0.083795085	4.38E-01	5.24E-01	1.00E+00
DNAJB4	LMNA	-0.022897384	-0.160441229	0.115518772	7.46E-01	8.04E-01	1.00E+00
LIMD2	LMNA	-0.198990147	-0.328029907	-0.062660479	4.52E-03	1.00E-02	1.00E+00
HIST1H4L	LMNA	-0.224632713	-0.351774903	-0.089352606	1.31E-03	3.33E-03	1.00E+00
PECR	LMNA	0.119453419	-0.01890941	0.253326891	9.04E-02	1.40E-01	1.00E+00
HSPA2	LMNA	0.065835473	-0.072877889	0.202050111	3.52E-01	4.39E-01	1.00E+00
SERP1	LMNA	-0.16721514	-0.298378311	-0.029853279	1.74E-02	3.29E-02	1.00E+00
TACR2	LMNA	0.265914051	0.13273574	0.389660723	1.31E-04	4.19E-04	1.00E+00
NUP85	LMNA	0.08931904	-0.049340356	0.224600585	2.06E-01	2.81E-01	1.00E+00
CD177	LMNA	0.157410791	0.019789838	0.289177752	2.53E-02	4.60E-02	1.00E+00
GPR135	LMNA	0.164192771	0.026748069	0.295544668	1.95E-02	3.66E-02	1.00E+00
PIGG	LMNA	0.206896135	0.07086932	0.335368274	3.13E-03	7.23E-03	1.00E+00
LGR5	LMNA	-0.411215449	-0.519760329	-0.289604946	1.21E-09	9.82E-09	1.43E-05
JAK2	LMNA	0.043334711	-0.095286331	0.180307042	5.40E-01	6.22E-01	1.00E+00
DPYSL4	LMNA	-0.365793636	-0.479624474	-0.239854869	8.63E-08	5.01E-07	1.02E-03
TM9SF4	LMNA	0.477374207	0.363262957	0.577375045	6.81E-13	9.77E-12	8.08E-09
PTHR1	LMNA	-0.116443249	-0.25046795	0.021960774	9.89E-02	1.51E-01	1.00E+00
SIRPG	LMNA	0.334899771	0.206390892	0.452050986	1.11E-06	5.26E-06	1.31E-02
ZNF264	LMNA	0.095247917	-0.043373403	0.230271067	1.78E-01	2.48E-01	1.00E+00
BICD1	LMNA	0.276025249	0.143440115	0.398876824	7.00E-05	2.36E-04	8.30E-01
RAB5A	LMNA	-0.011498947	-0.149312923	0.126753267	8.71E-01	9.05E-01	1.00E+00
FLJ13224	LMNA	0.146770936	0.008900271	0.279165519	3.71E-02	6.45E-02	1.00E+00
METTL5	LMNA	-0.193814191	-0.323217116	-0.057296222	5.71E-03	1.23E-02	1.00E+00
HERC6	LMNA	-0.031887769	-0.169193955	0.106632643	6.52E-01	7.24E-01	1.00E+00
CASP1	LMNA	0.403715558	0.281344844	0.513166099	2.56E-09	1.96E-08	3.03E-05
USP9Y	LMNA	-0.020611442	-0.15821226	0.117774679	7.71E-01	8.25E-01	1.00E+00
LRP1B	LMNA	-0.332385361	-0.449796921	-0.20368047	1.35E-06	6.29E-06	1.60E-02
XAF1	LMNA	0.239336174	0.104746045	0.365316886	6.02E-04	1.67E-03	1.00E+00
PLA2G4C	LMNA	-0.125566498	-0.259125664	0.012704733	7.50E-02	1.19E-01	1.00E+00
APOA2	LMNA	-0.10524396	-0.239810742	0.033290723	1.36E-01	1.98E-01	1.00E+00
SPAG6	LMNA	-0.233250898	-0.359718768	-0.098367379	8.36E-04	2.24E-03	1.00E+00
KALRN	LMNA	-0.210164425	-0.338397364	-0.074268198	2.68E-03	6.29E-03	1.00E+00
SECTM1	LMNA	0.48730809	0.374447429	0.585940874	1.91E-13	2.97E-12	2.27E-09

THOC7	LMNA	0.031317029	-0.107197423	0.168638945	6.58E-01	7.29E-01	1.00E+00
IFNAR1	LMNA	0.389826804	0.266096022	0.500920288	9.78E-09	6.69E-08	1.16E-04
TCTA	LMNA	0.157728277	0.020115277	0.289476067	2.50E-02	4.55E-02	1.00E+00
NY-REN-7	LMNA	-0.346393062	-0.462335234	-0.218805311	4.42E-07	2.26E-06	5.24E-03
TALDO1	LMNA	0.059052774	-0.079647537	0.19550992	4.04E-01	4.91E-01	1.00E+00
B2M	LMNA	0.209254038	0.073321118	0.337553873	2.80E-03	6.53E-03	1.00E+00
LPPR4	LMNA	-0.197405303	-0.326556973	-0.061017142	4.86E-03	1.07E-02	1.00E+00
SQLE	LMNA	-0.111881606	-0.246131022	0.026579932	1.13E-01	1.69E-01	1.00E+00
SEPHS1	LMNA	-0.360009346	-0.474478911	-0.23356652	1.42E-07	7.93E-07	1.69E-03
FAM49A	LMNA	0.082108963	-0.056583423	0.217692372	2.45E-01	3.25E-01	1.00E+00
YTHDC2	LMNA	-0.075540442	-0.211387007	0.063169316	2.85E-01	3.69E-01	1.00E+00
EHD2	LMNA	0.694806597	0.615824733	0.759962909	1.89E-30	6.59E-28	2.24E-26
NCF1	LMNA	0.227796496	0.092659405	0.354693268	1.11E-03	2.89E-03	1.00E+00
SPG7	LMNA	-0.024184818	-0.161695958	0.114247626	7.33E-01	7.92E-01	1.00E+00
ZNF614	LMNA	-0.090964422	-0.226175176	0.047685399	1.98E-01	2.72E-01	1.00E+00
HOXA5	LMNA	-0.072644391	-0.208603385	0.066069193	3.04E-01	3.89E-01	1.00E+00
NUP133	LMNA	-0.310248874	-0.429887712	-0.179903156	7.01E-06	2.85E-05	8.31E-02
FGF12	LMNA	-0.426433336	-0.533100926	-0.306421214	2.49E-10	2.30E-09	2.95E-06
SLMO2	LMNA	0.123462862	-0.014841093	0.257131273	8.00E-02	1.26E-01	1.00E+00
INPP5B	LMNA	0.154263444	0.01656522	0.28621906	2.84E-02	5.09E-02	1.00E+00
PPID	LMNA	-0.005722119	-0.143659637	0.132433496	9.36E-01	9.53E-01	1.00E+00
SNTA1	LMNA	0.078703522	-0.059999378	0.214424776	2.66E-01	3.47E-01	1.00E+00
IL20RA	LMNA	-0.018090738	-0.155752755	0.120260607	7.98E-01	8.48E-01	1.00E+00
UBE2J1	LMNA	0.124659877	-0.013625613	0.258266265	7.71E-02	1.22E-01	1.00E+00
CACNG2	LMNA	-0.40920459	-0.517993563	-0.287388482	1.48E-09	1.19E-08	1.75E-05
GCM1	LMNA	0.082962132	-0.055727112	0.21851053	2.40E-01	3.20E-01	1.00E+00
ELF1	LMNA	0.291796356	0.1601986	0.413202415	2.51E-05	9.25E-05	2.98E-01
TLR5	LMNA	0.115004038	-0.023418769	0.249100218	1.03E-01	1.56E-01	1.00E+00
TCFL5	LMNA	-0.00601811	-0.143949517	0.132142675	9.32E-01	9.51E-01	1.00E+00
C1orf107	LMNA	0.236589194	0.101865253	0.362790943	6.99E-04	1.91E-03	1.00E+00
C19orf22	LMNA	0.322778016	0.193342316	0.441170534	2.80E-06	1.23E-05	3.32E-02
SAFB2	LMNA	-0.057664835	-0.194170108	0.08103123	4.15E-01	5.03E-01	1.00E+00
MAP3K7IP1	LMNA	-0.217372873	-0.34506886	-0.081775847	1.89E-03	4.61E-03	1.00E+00
NCK2	LMNA	0.134596215	-0.003520195	0.267673535	5.62E-02	9.28E-02	1.00E+00
OXA1L	LMNA	0.276241178	0.14366905	0.399073368	6.91E-05	2.33E-04	8.19E-01

KIAA0652	LMNA	-0.038391889	-0.175512666	0.100190123	5.87E-01	6.66E-01	1.00E+00
KLRG1	LMNA	-0.502273582	-0.598803898	-0.391359181	2.62E-14	4.60E-13	3.10E-10
FRAG1	LMNA	0.210897841	0.075031353	0.339076738	2.59E-03	6.10E-03	1.00E+00
ZSCAN12	LMNA	-0.286466509	-0.408367793	-0.154526569	3.58E-05	1.28E-04	4.24E-01
PSMD12	LMNA	0.147967041	0.010122819	0.280292507	3.56E-02	6.22E-02	1.00E+00
E2F4	LMNA	0.291697908	0.160093753	0.413113177	2.53E-05	9.29E-05	3.00E-01
CLEC4M	LMNA	0.057817473	-0.080879085	0.194317478	4.14E-01	5.02E-01	1.00E+00
KIAA0999	LMNA	-0.020458741	-0.158063315	0.117925322	7.73E-01	8.26E-01	1.00E+00
CLDN10	LMNA	-0.048762831	-0.185564656	0.089893309	4.91E-01	5.76E-01	1.00E+00
MGC13053	LMNA	-0.0778713	-0.213625778	0.060833676	2.71E-01	3.53E-01	1.00E+00
TAC1	LMNA	-0.222443331	-0.349753906	-0.087065999	1.46E-03	3.67E-03	1.00E+00
GYPA	LMNA	-0.076464634	-0.212274857	0.062243409	2.79E-01	3.62E-01	1.00E+00
HPCAL4	LMNA	-0.466066387	-0.567597628	-0.350571482	2.75E-12	3.57E-11	3.26E-08
TRAIP	LMNA	-0.208284762	-0.336655595	-0.072313045	2.93E-03	6.81E-03	1.00E+00
MEX3D	LMNA	0.270870906	0.137979511	0.394181869	9.66E-05	3.16E-04	1.00E+00
KIAA0232	LMNA	-0.297669055	-0.418521536	-0.166458415	1.69E-05	6.42E-05	2.00E-01
ERCC8	LMNA	-0.073751417	-0.209667695	0.064960981	2.97E-01	3.81E-01	1.00E+00
NFAT5	LMNA	0.129684046	-0.008519479	0.263026088	6.58E-02	1.07E-01	1.00E+00
GPX4	LMNA	-0.029698987	-0.167065025	0.108798083	6.75E-01	7.44E-01	1.00E+00
CSPG4LYP	LMNA	0.056168802	-0.082522088	0.192725379	4.27E-01	5.14E-01	1.00E+00
KIAA0368	LMNA	0.295392089	0.164030101	0.416460188	1.97E-05	7.40E-05	2.34E-01
FBXO3	LMNA	-0.192190811	-0.321706258	-0.055615405	6.14E-03	1.31E-02	1.00E+00
DVL1	LMNA	0.024042994	-0.114387677	0.161557759	7.34E-01	7.93E-01	1.00E+00
CMKLR1	LMNA	0.29311876	0.161607251	0.414400888	2.30E-05	8.53E-05	2.73E-01
GPR157	LMNA	0.302689428	0.171818118	0.42306211	1.20E-05	4.67E-05	1.42E-01
TYMS	LMNA	0.120474453	-0.017873818	0.254296098	8.77E-02	1.36E-01	1.00E+00
OR52A1	LMNA	0.023003239	-0.115414274	0.160544412	7.45E-01	8.03E-01	1.00E+00
PEF1	LMNA	0.20038489	0.064107312	0.329325646	4.24E-03	9.46E-03	1.00E+00
ZNF750	LMNA	0.025305219	-0.11314103	0.162787536	7.21E-01	7.83E-01	1.00E+00
ALG9	LMNA	0.114610512	-0.023817329	0.248726144	1.04E-01	1.58E-01	1.00E+00
MCM5	LMNA	0.136058714	-0.002030424	0.269056024	5.35E-02	8.91E-02	1.00E+00
SDK2	LMNA	-0.018484388	-0.15613696	0.119872502	7.94E-01	8.44E-01	1.00E+00
BAIAP3	LMNA	-0.276784227	-0.399567614	-0.144244872	6.67E-05	2.26E-04	7.92E-01
RABGGTB	LMNA	-0.069455632	-0.205535868	0.069259457	3.26E-01	4.12E-01	1.00E+00
KCNK7	LMNA	-0.1248124	-0.258410858	0.013470707	7.68E-02	1.22E-01	1.00E+00

PTP4A3	LMNA	0.065490037	-0.07322297	0.201717318	3.54E-01	4.41E-01	1.00E+00
ANKRD40	LMNA	0.186675893	0.049911142	0.316568668	7.81E-03	1.63E-02	1.00E+00
CALCOCO2	LMNA	0.251546147	0.11757819	0.376521971	3.05E-04	9.03E-04	1.00E+00
TMSL8	LMNA	-0.348633863	-0.464336685	-0.221230522	3.68E-07	1.92E-06	4.37E-03
SNCA	LMNA	-0.302908852	-0.423260426	-0.17205255	1.18E-05	4.60E-05	1.40E-01
ZNF551	LMNA	-0.13987504	-0.272660988	-0.001859955	4.71E-02	7.96E-02	1.00E+00
HBQ1	LMNA	-0.164742121	-0.296059886	-0.027312278	1.91E-02	3.59E-02	1.00E+00
ARHGAP26	LMNA	0.168615264	0.031292679	0.299690224	1.64E-02	3.14E-02	1.00E+00
GEMIN6	LMNA	0.142823853	0.004868851	0.275443932	4.26E-02	7.28E-02	1.00E+00
HRAS	LMNA	0.172530208	0.035320469	0.303355888	1.41E-02	2.74E-02	1.00E+00
KLRC4	LMNA	-0.468137769	-0.569390813	-0.352893154	2.14E-12	2.81E-11	2.53E-08
BSDC1	LMNA	-0.165140308	-0.296433287	-0.027721292	1.88E-02	3.54E-02	1.00E+00
DSC1	LMNA	-0.021220448	-0.158806225	0.117173813	7.64E-01	8.19E-01	1.00E+00
RNF43	LMNA	-0.18124706	-0.311503818	-0.04430462	9.84E-03	2.00E-02	1.00E+00
NDUFAF1	LMNA	0.002385036	-0.135710648	0.140389813	9.73E-01	9.79E-01	1.00E+00
MAP2K4	LMNA	-0.395096553	-0.505571906	-0.271874534	5.92E-09	4.24E-08	7.02E-05
FOLR2	LMNA	0.184262029	0.047417207	0.314317559	8.66E-03	1.79E-02	1.00E+00
LYZL6	LMNA	0.074657776	-0.064053396	0.210538841	2.91E-01	3.75E-01	1.00E+00
EPB41L3	LMNA	-0.086500287	-0.221901462	0.052173744	2.21E-01	2.98E-01	1.00E+00
TEKT2	LMNA	-0.052401641	-0.189084784	0.08627347	4.59E-01	5.45E-01	1.00E+00
CDKN2B	LMNA	-0.114656131	-0.24876951	0.023771129	1.04E-01	1.58E-01	1.00E+00
WSB1	LMNA	-0.218488795	-0.346100505	-0.082939464	1.78E-03	4.38E-03	1.00E+00
ZNF480	LMNA	-0.112784209	-0.246989589	0.025666417	1.10E-01	1.65E-01	1.00E+00
MAP3K6	LMNA	0.639506723	0.550013405	0.714480169	1.27E-24	1.57E-22	1.50E-20
PROS1	LMNA	0.539584206	0.433850813	0.630657446	1.17E-16	3.37E-15	1.39E-12
PSMB8	LMNA	0.315870939	0.185927551	0.434955155	4.67E-06	1.97E-05	5.53E-02
HN1	LMNA	-0.261848725	-0.385948282	-0.128440659	1.67E-04	5.22E-04	1.00E+00
MAS1	LMNA	-0.063507016	-0.199806274	0.075203307	3.69E-01	4.57E-01	1.00E+00
APOL1	LMNA	0.486619178	0.373670729	0.585347547	2.09E-13	3.22E-12	2.48E-09
CSHL1	LMNA	-0.035654962	-0.172855135	0.102902546	6.14E-01	6.90E-01	1.00E+00
ZBTB7C	LMNA	0.262131237	0.128738976	0.386206402	1.64E-04	5.14E-04	1.00E+00
AHCTF1	LMNA	0.196765161	0.060353583	0.325961856	5.01E-03	1.09E-02	1.00E+00
SAE2	LMNA	-0.193780495	-0.323185762	-0.057261326	5.72E-03	1.23E-02	1.00E+00
ITGA2	LMNA	0.525201277	0.417414749	0.618414411	1.02E-15	2.44E-14	1.21E-11
AP2S1	LMNA	0.195682183	0.059231263	0.324954815	5.26E-03	1.14E-02	1.00E+00

P15RS	LMNA	-0.529206276	-0.621828098	-0.421984381	5.64E-16	1.43E-14	6.69E-12
MME	LMNA	0.470903615	0.355995419	0.571783692	1.52E-12	2.05E-11	1.81E-08
VAT1	LMNA	0.342539776	0.214638597	0.458890769	6.04E-07	3.00E-06	7.16E-03
MAST4	LMNA	0.228543936	0.093441068	0.355382367	1.07E-03	2.79E-03	1.00E+00
TUFM	LMNA	0.00471618	-0.133421688	0.142674289	9.47E-01	9.60E-01	1.00E+00
KRT33B	LMNA	-0.135577854	-0.268601532	0.002520318	5.44E-02	9.02E-02	1.00E+00
THEG	LMNA	-0.202183785	-0.330996129	-0.065974235	3.91E-03	8.78E-03	1.00E+00
KCTD2	LMNA	-0.135257322	-0.268298543	0.002846835	5.50E-02	9.11E-02	1.00E+00
WDR26	LMNA	0.112298601	-0.026157924	0.246527699	1.12E-01	1.67E-01	1.00E+00
MFI2	LMNA	0.480297673	0.366551051	0.579898191	4.71E-13	6.89E-12	5.58E-09
KRT34	LMNA	0.030656969	-0.107850479	0.167996968	6.65E-01	7.35E-01	1.00E+00
NR4A3	LMNA	0.19115975	0.054548267	0.320746321	6.43E-03	1.37E-02	1.00E+00
SGSM3	LMNA	-0.08249006	-0.218057854	0.056200948	2.43E-01	3.23E-01	1.00E+00
ARSA	LMNA	0.385377399	0.261223967	0.496987787	1.48E-08	9.84E-08	1.76E-04
TOMM22	LMNA	-0.059778718	-0.196210489	0.078923601	3.98E-01	4.85E-01	1.00E+00
SOCS3	LMNA	0.533378371	0.426750466	0.62538047	3.02E-16	7.98E-15	3.58E-12
UNKL	LMNA	-0.211330725	-0.339477662	-0.075481863	2.54E-03	5.99E-03	1.00E+00
POP4	LMNA	0.183407305	0.046534538	0.313520113	8.98E-03	1.84E-02	1.00E+00
BHLHB3	LMNA	0.23304469	0.098151423	0.359528908	8.45E-04	2.26E-03	1.00E+00
MALL	LMNA	0.511435071	0.401749222	0.606653811	7.37E-15	1.47E-13	8.74E-11
SOX15	LMNA	-0.276763491	-0.399548743	-0.144222883	6.68E-05	2.26E-04	7.93E-01
CCNA2	LMNA	-0.017251861	-0.154933865	0.121087526	8.07E-01	8.54E-01	1.00E+00
PARK2	LMNA	-0.285615754	-0.407595453	-0.153621998	3.78E-05	1.34E-04	4.49E-01
GPR124	LMNA	0.442966666	0.324776388	0.547534822	4.07E-11	4.34E-10	4.83E-07
CGRRF1	LMNA	-0.188109517	-0.317904937	-0.05139313	7.34E-03	1.54E-02	1.00E+00
RUVBL2	LMNA	0.023419407	-0.115003408	0.160950044	7.41E-01	7.99E-01	1.00E+00
MCAT	LMNA	0.040186517	-0.098410444	0.177254149	5.70E-01	6.49E-01	1.00E+00
WNT10B	LMNA	-0.364226584	-0.478231236	-0.238150215	9.89E-08	5.67E-07	1.17E-03
ISCA1	LMNA	-0.215530831	-0.343365257	-0.079855882	2.07E-03	4.99E-03	1.00E+00
RPAP1	LMNA	-0.027703778	-0.165123259	0.11077087	6.96E-01	7.61E-01	1.00E+00
C12orf48	LMNA	-0.066484874	-0.202675656	0.072229067	3.47E-01	4.33E-01	1.00E+00
RAI16	LMNA	0.069610213	-0.069104869	0.205684634	3.25E-01	4.11E-01	1.00E+00
EPN1	LMNA	0.167452144	0.030096891	0.298600417	1.72E-02	3.27E-02	1.00E+00
MAGI1	LMNA	-0.590754063	-0.67384979	-0.492901222	2.16E-20	1.16E-18	2.56E-16
GBX2	LMNA	-0.296100001	-0.417101197	-0.164784897	1.88E-05	7.09E-05	2.23E-01

SLC35A1	LMNA	-0.198310925	-0.327398724	-0.061956098	4.67E-03	1.03E-02	1.00E+00
GAL	LMNA	0.233878379	0.0990246	0.360296437	8.08E-04	2.17E-03	1.00E+00
SLC14A2	LMNA	-0.144639021	-0.277155894	-0.006722247	4.00E-02	6.89E-02	1.00E+00
RDH11	LMNA	-0.22135459	-0.348748458	-0.085929441	1.55E-03	3.85E-03	1.00E+00
ZNF518	LMNA	-0.247311009	-0.372639526	-0.113122173	3.88E-04	1.12E-03	1.00E+00
PCYT1B	LMNA	-0.393840056	-0.504463376	-0.270495922	6.68E-09	4.73E-08	7.92E-05
AUH	LMNA	-0.212647552	-0.340696981	-0.076852653	2.38E-03	5.66E-03	1.00E+00
EIF3H	LMNA	-0.13781943	-0.270719688	0.000236062	5.05E-02	8.46E-02	1.00E+00
KIF1B	LMNA	-0.412096568	-0.520534199	-0.290576568	1.10E-09	9.04E-09	1.31E-05
MBD2	LMNA	0.412246564	0.290741996	0.52066592	1.09E-09	8.92E-09	1.29E-05
AMOTL2	LMNA	-0.286248299	-0.408169712	-0.154294534	3.63E-05	1.29E-04	4.31E-01
C6orf120	LMNA	0.042023339	-0.096588004	0.179035689	5.53E-01	6.34E-01	1.00E+00
PIGT	LMNA	0.64694605	0.558802573	0.720635938	2.45E-25	3.46E-23	2.91E-21
PSRC1	LMNA	0.014524188	-0.123774991	0.152269876	8.37E-01	8.78E-01	1.00E+00
PLA2G10	LMNA	-0.051094704	-0.187820877	0.087574016	4.70E-01	5.56E-01	1.00E+00
ALAS1	LMNA	0.052566349	-0.086109535	0.189244036	4.57E-01	5.44E-01	1.00E+00
FOXO1	LMNA	0.231502051	0.096536261	0.358108238	9.16E-04	2.43E-03	1.00E+00
C17orf62	LMNA	0.331147613	0.202346956	0.448686781	1.48E-06	6.87E-06	1.76E-02
KIF5C	LMNA	-0.502086025	-0.598642997	-0.391146767	2.68E-14	4.72E-13	3.18E-10
DUSP10	LMNA	0.165084638	0.027664106	0.296381085	1.89E-02	3.54E-02	1.00E+00
CRLF3	LMNA	0.0583686	-0.080329685	0.194849533	4.09E-01	4.97E-01	1.00E+00
CLCNKB	LMNA	-0.085631076	-0.221068723	0.053047017	2.26E-01	3.03E-01	1.00E+00
PSMA5	LMNA	0.129970753	-0.008227877	0.263297514	6.52E-02	1.06E-01	1.00E+00
FARS2	LMNA	0.175544273	0.038424453	0.3061754	1.25E-02	2.46E-02	1.00E+00
CCDC28A	LMNA	0.079481422	-0.059219362	0.215171456	2.61E-01	3.42E-01	1.00E+00
AMPD3	LMNA	0.34457703	0.216840983	0.460712313	5.12E-07	2.58E-06	6.08E-03
PIAS1	LMNA	-0.23326759	-0.359734136	-0.09838486	8.35E-04	2.23E-03	1.00E+00
ADCYAP1R	LMNA	-0.237417185	-0.363552505	-0.10273334	6.69E-04	1.83E-03	1.00E+00
TMEM134	LMNA	0.239339012	0.104749022	0.365319494	6.02E-04	1.67E-03	1.00E+00
CDH20	LMNA	-0.568544451	-0.65517221	-0.467159963	1.08E-18	4.58E-17	1.29E-14
FBXO7	LMNA	0.00387438	-0.134248425	0.141849511	9.56E-01	9.68E-01	1.00E+00
FLJ14213	LMNA	0.108726731	-0.02977113	0.243128414	1.23E-01	1.82E-01	1.00E+00
ZNF3	LMNA	-0.219733885	-0.3472512	-0.084238207	1.68E-03	4.14E-03	1.00E+00
LRRFIP1	LMNA	0.630849454	0.539810199	0.707301953	8.12E-24	8.75E-22	9.63E-20
TMEM49	LMNA	0.266243086	0.133083589	0.389961021	1.28E-04	4.11E-04	1.00E+00

CNOT2	LMNA	0.127143629	-0.011102242	0.260620145	7.14E-02	1.14E-01	1.00E+00
CBX2	LMNA	-0.06763619	-0.203784406	0.071078487	3.39E-01	4.25E-01	1.00E+00
ZC3H14	LMNA	0.06376412	-0.074946613	0.200054104	3.67E-01	4.55E-01	1.00E+00
ALDH5A1	LMNA	-0.475460954	-0.575722746	-0.361112613	8.65E-13	1.22E-11	1.03E-08
HNT	LMNA	-0.324106058	-0.442364281	-0.194769669	2.53E-06	1.13E-05	3.00E-02
SERPINA4	LMNA	-0.00699831	-0.14490931	0.131179426	9.21E-01	9.44E-01	1.00E+00
FLJ20920	LMNA	0.183547787	0.046679598	0.313651194	8.93E-03	1.83E-02	1.00E+00
CRTAP	LMNA	0.501091002	0.390020074	0.597789264	3.07E-14	5.34E-13	3.65E-10
DDX50	LMNA	-0.072307008	-0.208278956	0.06640687	3.06E-01	3.91E-01	1.00E+00
STYXL1	LMNA	0.243049355	0.108643699	0.368728338	4.91E-04	1.39E-03	1.00E+00
TK2	LMNA	0.256409821	0.122702187	0.380975201	2.30E-04	6.98E-04	1.00E+00
BLVRB	LMNA	0.407689568	0.285719413	0.516661831	1.72E-09	1.37E-08	2.04E-05
STMN1	LMNA	-0.50365279	-0.599986842	-0.392921534	2.17E-14	3.92E-13	2.57E-10
GUCA2A	LMNA	-0.101915158	-0.236636799	0.036651511	1.49E-01	2.14E-01	1.00E+00
DPP6	LMNA	-0.412702945	-0.521066665	-0.291245374	1.04E-09	8.56E-09	1.23E-05
GALNT10	LMNA	0.448053956	0.330442227	0.551963634	2.29E-11	2.51E-10	2.71E-07
STK39	LMNA	-0.068104131	-0.204234946	0.070610741	3.36E-01	4.21E-01	1.00E+00
MMP24	LMNA	0.215444418	0.079765839	0.343285318	2.08E-03	5.01E-03	1.00E+00
CKS2	LMNA	0.045391018	-0.093244277	0.182299678	5.21E-01	6.04E-01	1.00E+00
RHO	LMNA	0.02659784	-0.111863909	0.164046485	7.07E-01	7.70E-01	1.00E+00
BANF1	LMNA	-0.116886402	-0.250888986	0.021511718	9.76E-02	1.49E-01	1.00E+00
CR1	LMNA	0.253296117	0.119421004	0.378124927	2.76E-04	8.24E-04	1.00E+00
RPS6KA2	LMNA	0.271406973	0.138547054	0.394670459	9.34E-05	3.07E-04	1.00E+00
HMBS	LMNA	0.005457927	-0.132693054	0.143400879	9.39E-01	9.55E-01	1.00E+00
C20orf112	LMNA	-0.230093595	-0.35681063	-0.095062212	9.87E-04	2.59E-03	1.00E+00
SLC25A24	LMNA	0.557172346	0.454045725	0.64556746	7.20E-18	2.55E-16	8.54E-14
MRPL22	LMNA	0.103160216	-0.035394856	0.237824273	1.44E-01	2.08E-01	1.00E+00
SLC25A15	LMNA	-0.001754131	-0.139771285	0.136329883	9.80E-01	9.84E-01	1.00E+00
GADD45B	LMNA	0.433973739	0.314781456	0.539691545	1.10E-10	1.10E-09	1.31E-06
TDP1	LMNA	0.210765572	0.074893709	0.338954226	2.61E-03	6.13E-03	1.00E+00
ZNF287	LMNA	-0.216854673	-0.34458969	-0.081235624	1.94E-03	4.72E-03	1.00E+00
DAAM2	LMNA	-0.388918478	-0.500117858	-0.265100899	1.06E-08	7.23E-08	1.26E-04
C11orf57	LMNA	-0.084231145	-0.219727116	0.054453046	2.33E-01	3.11E-01	1.00E+00
RFK	LMNA	0.020120302	-0.118259178	0.157733179	7.76E-01	8.30E-01	1.00E+00
ZFYVE9	LMNA	-0.148601937	-0.280890567	-0.010771919	3.48E-02	6.10E-02	1.00E+00

TCTN3	LMNA	0.333979066	0.205398183	0.451225785	1.19E-06	5.62E-06	1.41E-02
STCH	LMNA	0.097736698	-0.040865689	0.232648652	1.66E-01	2.34E-01	1.00E+00
LOC283871	LMNA	-0.268567238	-0.392081437	-0.135541573	1.11E-04	3.60E-04	1.00E+00
NDUFB3	LMNA	-0.148019105	-0.280341555	-0.010176044	3.55E-02	6.21E-02	1.00E+00
DEFB4	LMNA	0.047170345	-0.091476341	0.184023004	5.05E-01	5.89E-01	1.00E+00
FPR1	LMNA	0.247246466	0.113054305	0.372580323	3.89E-04	1.12E-03	1.00E+00
FMNL1	LMNA	0.457581522	0.341076162	0.560242251	7.57E-12	9.12E-11	8.98E-08
Sep-07	LMNA	-0.150897033	-0.283051647	-0.013119328	3.21E-02	5.68E-02	1.00E+00
PTCD2	LMNA	-0.018080544	-0.155742805	0.120270656	7.98E-01	8.48E-01	1.00E+00
GNLY	LMNA	0.051966265	-0.08670677	0.188663792	4.63E-01	5.49E-01	1.00E+00
GRAMD1C	LMNA	-0.160293459	-0.291885423	-0.022745792	2.27E-02	4.18E-02	1.00E+00
ZNF165	LMNA	-0.044123696	-0.18107173	0.094502953	5.33E-01	6.15E-01	1.00E+00
ARMCX3	LMNA	0.025921822	-0.112531878	0.163388133	7.14E-01	7.76E-01	1.00E+00
NDE1	LMNA	0.112106552	-0.026352288	0.246345013	1.12E-01	1.68E-01	1.00E+00
MAGEF1	LMNA	-0.244840656	-0.370372869	-0.110525471	4.45E-04	1.27E-03	1.00E+00
ITGA10	LMNA	0.24421827	0.109871542	0.369801568	4.61E-04	1.31E-03	1.00E+00
ARHGDIB	LMNA	0.231654121	0.096695449	0.358248311	9.09E-04	2.41E-03	1.00E+00
FSHB	LMNA	0.171400686	0.034157932	0.302298684	1.47E-02	2.84E-02	1.00E+00
ANXA2	LMNA	0.732010217	0.660729017	0.790207048	3.56E-35	3.02E-32	4.22E-31
HLCS	LMNA	0.459108542	0.34278328	0.561567193	6.32E-12	7.71E-11	7.50E-08
MCF2L	LMNA	-0.462554412	-0.564555124	-0.346638386	4.20E-12	5.27E-11	4.98E-08
AK1	LMNA	-0.170967883	-0.301893507	-0.033712577	1.50E-02	2.89E-02	1.00E+00
FH	LMNA	0.02820287	-0.110277489	0.165609082	6.90E-01	7.57E-01	1.00E+00
LGALS2	LMNA	0.065315709	-0.073397106	0.20154936	3.56E-01	4.43E-01	1.00E+00
SYNPO2L	LMNA	-0.150510074	-0.282687377	-0.012723442	3.25E-02	5.76E-02	1.00E+00
KIAA1045	LMNA	-0.343353009	-0.459618011	-0.215517591	5.66E-07	2.83E-06	6.71E-03
C1orf183	LMNA	-0.267584776	-0.391185258	-0.134502338	1.18E-04	3.81E-04	1.00E+00
MAGEA8	LMNA	0.035808039	-0.102750894	0.173003823	6.13E-01	6.89E-01	1.00E+00
DGCR8	LMNA	-0.131830437	-0.265057569	0.006335872	6.15E-02	1.00E-01	1.00E+00
GSR	LMNA	0.276412186	0.143850368	0.399229015	6.83E-05	2.31E-04	8.10E-01
CACNA1C	LMNA	-0.006346024	-0.144270632	0.131820462	9.29E-01	9.48E-01	1.00E+00
FAM20B	LMNA	-0.101989146	-0.236707377	0.036576846	1.49E-01	2.13E-01	1.00E+00
HES2	LMNA	0.130945063	-0.007236758	0.264219737	6.32E-02	1.03E-01	1.00E+00
PDCD6	LMNA	-0.066673456	-0.202857289	0.072040631	3.46E-01	4.32E-01	1.00E+00
INTS7	LMNA	-0.198444122	-0.327522509	-0.062094217	4.64E-03	1.02E-02	1.00E+00

AMPH	LMNA	-0.388395385	-0.499655663	-0.264527942	1.12E-08	7.56E-08	1.33E-04
UCKL1	LMNA	0.218011361	0.082441579	0.345659166	1.83E-03	4.48E-03	1.00E+00
ASB4	LMNA	-0.262578805	-0.386615285	-0.129211631	1.60E-04	5.02E-04	1.00E+00
C10orf97	LMNA	-0.419541693	-0.527066005	-0.298796429	5.13E-10	4.47E-09	6.09E-06
ALDH1L1	LMNA	-0.041224321	-0.178260829	0.09738088	5.60E-01	6.41E-01	1.00E+00
CCL23	LMNA	0.126299562	-0.01195997	0.259820391	7.33E-02	1.17E-01	1.00E+00
OBSL1	LMNA	0.160231483	0.022682215	0.291827231	2.27E-02	4.19E-02	1.00E+00
SLC12A7	LMNA	0.589250231	0.491152835	0.672588458	2.84E-20	1.48E-18	3.37E-16
THAP4	LMNA	0.271009319	0.138126043	0.394308031	9.57E-05	3.14E-04	1.00E+00
RBM4B	LMNA	-0.427160612	-0.533737159	-0.307226758	2.30E-10	2.15E-09	2.73E-06
OGFRL1	LMNA	0.252375542	0.118451473	0.377281782	2.91E-04	8.64E-04	1.00E+00
KIAA0831	LMNA	-0.057934686	-0.194430642	0.080762246	4.13E-01	5.01E-01	1.00E+00
C12orf11	LMNA	0.058147277	-0.080550325	0.194635879	4.11E-01	4.99E-01	1.00E+00
PPP1R15A	LMNA	0.567195671	0.465602222	0.654034507	1.36E-18	5.56E-17	1.62E-14
KIAA0240	LMNA	-0.156950396	-0.28874511	-0.019317961	2.57E-02	4.67E-02	1.00E+00
CD1B	LMNA	-0.102008739	-0.236726066	0.036557074	1.49E-01	2.13E-01	1.00E+00
FCGR2A	LMNA	0.378228865	0.253409615	0.49066011	2.86E-08	1.80E-07	3.39E-04
MDC1	LMNA	-0.107464173	-0.24192607	0.031047433	1.28E-01	1.88E-01	1.00E+00
MAN1A1	LMNA	0.427645035	0.307763409	0.534160874	2.18E-10	2.04E-09	2.59E-06
OCEL1	LMNA	0.2825354	0.150348642	0.404797557	4.62E-05	1.61E-04	5.48E-01
ATP11B	LMNA	0.177061153	0.039987588	0.307593501	1.17E-02	2.33E-02	1.00E+00
NLGN4X	LMNA	-0.099321124	-0.234161448	0.039268299	1.60E-01	2.27E-01	1.00E+00
FBXO34	LMNA	-0.142787712	-0.275409838	-0.004831959	4.26E-02	7.28E-02	1.00E+00
ALOX12	LMNA	0.061392799	-0.077313459	0.197767652	3.85E-01	4.73E-01	1.00E+00
RB1CC1	LMNA	-0.346905914	-0.46279341	-0.21936023	4.24E-07	2.17E-06	5.03E-03
PCDH12	LMNA	0.236450645	0.101720014	0.362663494	7.04E-04	1.92E-03	1.00E+00
RPE	LMNA	0.270621149	0.137715119	0.393954209	9.81E-05	3.21E-04	1.00E+00
EIF4E	LMNA	-0.143658854	-0.276231562	-0.00572132	4.14E-02	7.10E-02	1.00E+00
CSDC2	LMNA	-0.362390898	-0.476598429	-0.236154326	1.16E-07	6.57E-07	1.37E-03
ABHD5	LMNA	0.19004198	0.053391737	0.319705355	6.75E-03	1.43E-02	1.00E+00
TMBIM1	LMNA	0.51082131	0.401052272	0.606128497	8.03E-15	1.59E-13	9.53E-11
PET112L	LMNA	-0.203465645	-0.332185994	-0.067305155	3.68E-03	8.32E-03	1.00E+00
P2RXL1	LMNA	0.182883688	0.045993908	0.313031496	9.18E-03	1.88E-02	1.00E+00
F2RL2	LMNA	0.310398001	0.180062828	0.430022224	6.93E-06	2.83E-05	8.22E-02
TRMT1	LMNA	0.115690148	-0.022723777	0.249752319	1.01E-01	1.53E-01	1.00E+00

IMPG2	LMNA	-0.179039653	-0.309442291	-0.042027427	1.08E-02	2.17E-02	1.00E+00
LRRC32	LMNA	0.405853203	0.283697321	0.515046927	2.07E-09	1.61E-08	2.45E-05
BGLAP	LMNA	-0.19078351	-0.320395969	-0.05415894	6.53E-03	1.39E-02	1.00E+00
HTR4	LMNA	0.086669225	-0.052003991	0.222063289	2.20E-01	2.97E-01	1.00E+00
MRAS	LMNA	-0.172435281	-0.303267051	-0.035222754	1.41E-02	2.75E-02	1.00E+00
TRAF6	LMNA	0.320961691	0.191391053	0.439537202	3.21E-06	1.39E-05	3.80E-02
AXL	LMNA	0.355413668	0.22857789	0.470385122	2.10E-07	1.14E-06	2.49E-03
ATP2C2	LMNA	-0.338990663	-0.455715108	-0.210804911	8.02E-07	3.90E-06	9.51E-03
LMNB1	LMNA	-0.044698762	-0.181628983	0.093931864	5.28E-01	6.10E-01	1.00E+00
TELO2	LMNA	0.135075549	-0.00303199	0.268126706	5.53E-02	9.16E-02	1.00E+00
PNPLA3	LMNA	0.184720728	0.04789099	0.314745443	8.49E-03	1.76E-02	1.00E+00
CSNK1E	LMNA	-0.277941063	-0.400620248	-0.145471822	6.20E-05	2.11E-04	7.36E-01
SRP14	LMNA	0.003717306	-0.134402667	0.141695592	9.58E-01	9.69E-01	1.00E+00
KCNQ4	LMNA	0.062884283	-0.075824972	0.199205929	3.74E-01	4.62E-01	1.00E+00
PIK3C2B	LMNA	-0.295837739	-0.416863735	-0.164505248	1.91E-05	7.20E-05	2.27E-01
C15orf15	LMNA	-0.191284293	-0.320862287	-0.054677151	6.39E-03	1.36E-02	1.00E+00
TUBB2B	LMNA	-0.268888891	-0.392374791	-0.135881878	1.09E-04	3.54E-04	1.00E+00
USP15	LMNA	0.017632486	-0.120712351	0.155305444	8.03E-01	8.52E-01	1.00E+00
C12orf41	LMNA	-0.183598553	-0.313698561	-0.046732021	8.91E-03	1.83E-02	1.00E+00
CEND1	LMNA	-0.236576596	-0.362779355	-0.101852047	7.00E-04	1.91E-03	1.00E+00
RNF31	LMNA	0.203791871	0.067643943	0.332488742	3.63E-03	8.21E-03	1.00E+00
TCEAL2	LMNA	-0.501947724	-0.598524347	-0.390990145	2.74E-14	4.80E-13	3.24E-10
PTGER2	LMNA	0.402291853	0.279778874	0.511912855	2.94E-09	2.23E-08	3.49E-05
UBN1	LMNA	0.306755956	0.176165193	0.426735573	8.98E-06	3.58E-05	1.07E-01
SLC5A7	LMNA	-0.231750118	-0.358336732	-0.096795943	9.05E-04	2.40E-03	1.00E+00
SLC31A1	LMNA	0.227848737	0.092714032	0.354741436	1.11E-03	2.88E-03	1.00E+00
ADAM29	LMNA	-0.093501051	-0.228601286	0.045132517	1.86E-01	2.57E-01	1.00E+00
ST7L	LMNA	-0.051739761	-0.188444752	0.086932173	4.65E-01	5.50E-01	1.00E+00
GFOD1	LMNA	-0.358068273	-0.472750425	-0.231458664	1.68E-07	9.26E-07	1.99E-03
CTNNA1	LMNA	0.454923718	0.338106724	0.557934914	1.03E-11	1.21E-10	1.23E-07
HRBL	LMNA	0.069766693	-0.068948375	0.20583522	3.24E-01	4.09E-01	1.00E+00
CBX4	LMNA	-0.143511582	-0.276092658	-0.005570953	4.16E-02	7.13E-02	1.00E+00
NTRK2	LMNA	-0.366303598	-0.480077749	-0.240409778	8.26E-08	4.81E-07	9.80E-04
FOXN3	LMNA	-0.109013145	-0.243401111	0.029481534	1.23E-01	1.81E-01	1.00E+00
MFGE8	LMNA	0.457676197	0.34118198	0.560324412	7.49E-12	9.03E-11	8.88E-08

PFKFB2	LMNA	-0.295494006	-0.41655248	-0.164138758	1.96E-05	7.36E-05	2.32E-01
TAS2R4	LMNA	-0.186495078	-0.316400095	-0.04972427	7.87E-03	1.64E-02	1.00E+00
SH3TC2	LMNA	0.023990097	-0.114439913	0.161506212	7.35E-01	7.94E-01	1.00E+00
IL10	LMNA	0.149788329	0.011985163	0.282007851	3.34E-02	5.89E-02	1.00E+00
PXMP4	LMNA	0.272377238	0.139574512	0.395554614	8.80E-05	2.90E-04	1.00E+00
RNF167	LMNA	0.029779885	-0.108718071	0.167143734	6.74E-01	7.43E-01	1.00E+00
PRMT5	LMNA	0.020033507	-0.118344793	0.157648508	7.77E-01	8.30E-01	1.00E+00
TSKU	LMNA	0.595212725	0.498089604	0.677586628	9.48E-21	5.33E-19	1.12E-16
ATPIF1	LMNA	-0.101377846	-0.236124214	0.037193692	1.51E-01	2.16E-01	1.00E+00
ETV3	LMNA	-0.12611529	-0.259645769	0.012147198	7.37E-02	1.18E-01	1.00E+00
PAK7	LMNA	-0.614669649	-0.693843991	-0.520812597	2.24E-22	1.74E-20	2.66E-18
PDLIM1	LMNA	0.497813952	0.386311728	0.594975992	4.78E-14	8.00E-13	5.67E-10
CEP63	LMNA	-0.003523553	-0.141505721	0.134592918	9.60E-01	9.70E-01	1.00E+00
WDFY3	LMNA	-0.314596122	-0.433806761	-0.184560647	5.12E-06	2.14E-05	6.07E-02
RNF126	LMNA	0.115489682	-0.022926852	0.249561803	1.02E-01	1.54E-01	1.00E+00
DPM1	LMNA	0.136280227	-0.001804727	0.269265371	5.31E-02	8.85E-02	1.00E+00
CLIC4	LMNA	0.443389747	0.32524726	0.547903364	3.88E-11	4.14E-10	4.61E-07
ACR	LMNA	0.076593133	-0.062114653	0.212398285	2.79E-01	3.61E-01	1.00E+00
KLK7	LMNA	-0.20437798	-0.333032604	-0.068252701	3.53E-03	8.02E-03	1.00E+00
ALOX5AP	LMNA	0.291773883	0.160174666	0.413182045	2.52E-05	9.25E-05	2.99E-01
LRP1	LMNA	0.411752457	0.290197082	0.520231994	1.14E-09	9.33E-09	1.36E-05
TAS2R9	LMNA	0.082119251	-0.056573099	0.217702239	2.45E-01	3.25E-01	1.00E+00
ZNF16	LMNA	0.003709435	-0.134410396	0.141687878	9.58E-01	9.69E-01	1.00E+00
POLR1B	LMNA	-0.105405796	-0.239964977	0.033127251	1.35E-01	1.97E-01	1.00E+00
SLC8A2	LMNA	-0.39463142	-0.50516159	-0.271364137	6.19E-09	4.41E-08	7.34E-05
OLFM4	LMNA	-0.197876907	-0.326995342	-0.061506075	4.76E-03	1.05E-02	1.00E+00
TACC1	LMNA	0.410735748	0.289076079	0.519338942	1.27E-09	1.03E-08	1.50E-05
SAMHD1	LMNA	0.138677147	0.000638368	0.271529839	4.90E-02	8.26E-02	1.00E+00
ATP13A2	LMNA	-0.035597627	-0.172799442	0.102959345	6.15E-01	6.91E-01	1.00E+00
KRT4	LMNA	0.029647582	-0.108848924	0.167015011	6.75E-01	7.44E-01	1.00E+00
PAX6	LMNA	-0.082565742	-0.21813043	0.056124987	2.43E-01	3.22E-01	1.00E+00
SCG2	LMNA	0.365730571	0.239786251	0.479568416	8.68E-08	5.03E-07	1.03E-03
SLC17A6	LMNA	-0.414125117	-0.522315158	-0.292814431	8.98E-10	7.49E-09	1.06E-05
TUBB4	LMNA	-0.417010833	-0.524847039	-0.296000198	6.67E-10	5.70E-09	7.92E-06
PADI4	LMNA	-0.109896612	-0.244242133	0.028588108	1.19E-01	1.77E-01	1.00E+00

FMO3	LMNA	0.079414193	-0.059286781	0.215106931	2.61E-01	3.43E-01	1.00E+00
NLK	LMNA	-0.110173742	-0.244505907	0.028307809	1.19E-01	1.76E-01	1.00E+00
POU4F3	LMNA	-0.089514636	-0.224787802	0.049143661	2.05E-01	2.80E-01	1.00E+00
MDM2	LMNA	0.007613105	-0.13057513	0.145511174	9.14E-01	9.38E-01	1.00E+00
CAPNS1	LMNA	0.440097888	0.321585097	0.545034781	5.61E-11	5.86E-10	6.66E-07
SDF4	LMNA	0.563851465	0.461742631	0.651211959	2.39E-18	9.29E-17	2.83E-14
ITGBL1	LMNA	0.433563308	0.314325924	0.539333145	1.15E-10	1.14E-09	1.37E-06
TAP2	LMNA	0.328793001	0.199811484	0.446573921	1.77E-06	8.13E-06	2.10E-02
OR2C1	LMNA	0.073838492	-0.064873798	0.209751397	2.96E-01	3.80E-01	1.00E+00
KLHDC3	LMNA	-0.509172562	-0.604716932	-0.399180682	1.01E-14	1.96E-13	1.20E-10
EFHD2	LMNA	0.288446856	0.156633043	0.410164935	3.14E-05	1.14E-04	3.73E-01
GALR3	LMNA	0.244558038	0.110228515	0.37011346	4.52E-04	1.29E-03	1.00E+00
NBEA	LMNA	-0.525422394	-0.618602974	-0.417666898	9.88E-16	2.39E-14	1.17E-11
ABCA6	LMNA	0.178032628	0.040989039	0.308501409	1.12E-02	2.25E-02	1.00E+00
ABBA-1	LMNA	-0.279051382	-0.401630251	-0.14664982	5.78E-05	1.98E-04	6.86E-01
GNAI1	LMNA	-0.358266576	-0.47292705	-0.231673951	1.65E-07	9.12E-07	1.96E-03
CLDN3	LMNA	-0.038537307	-0.175653809	0.100045949	5.86E-01	6.64E-01	1.00E+00
AKT2	LMNA	0.032882126	-0.105648457	0.170160697	6.42E-01	7.15E-01	1.00E+00
EGFR	LMNA	0.038807206	-0.099778345	0.17591576	5.83E-01	6.62E-01	1.00E+00
VPS4B	LMNA	0.041349877	-0.0972563	0.1783826	5.59E-01	6.40E-01	1.00E+00
RBM16	LMNA	-0.052695764	-0.18936916	0.085980721	4.56E-01	5.43E-01	1.00E+00
GALNT6	LMNA	0.344491302	0.216748279	0.460635682	5.16E-07	2.60E-06	6.12E-03
ZDHHC3	LMNA	0.204658529	0.068544127	0.3332929	3.48E-03	7.93E-03	1.00E+00
SLC25A4	LMNA	-0.425942418	-0.532671394	-0.305877561	2.62E-10	2.42E-09	3.11E-06
CYB5B	LMNA	0.152384232	0.014641236	0.284451289	3.04E-02	5.42E-02	1.00E+00
NDRG1	LMNA	0.463821467	0.348056904	0.565653123	3.60E-12	4.59E-11	4.27E-08
SESN1	LMNA	-0.226907117	-0.353873129	-0.091729521	1.16E-03	3.00E-03	1.00E+00
GBE1	LMNA	0.470041848	0.355028561	0.571038316	1.69E-12	2.27E-11	2.01E-08
MRPL46	LMNA	-0.183424764	-0.313536404	-0.046552566	8.98E-03	1.84E-02	1.00E+00
CLASP1	LMNA	-0.080770379	-0.216408333	0.057926527	2.53E-01	3.34E-01	1.00E+00
FARP2	LMNA	-0.233539346	-0.359984329	-0.098669483	8.23E-04	2.21E-03	1.00E+00
ACOT11	LMNA	0.185376134	0.048568056	0.315356727	8.26E-03	1.71E-02	1.00E+00
LDHAL6B	LMNA	0.069611028	-0.069104054	0.205685418	3.25E-01	4.11E-01	1.00E+00
MAPKAPK3	LMNA	0.323655284	0.194285124	0.441959137	2.62E-06	1.16E-05	3.11E-02
NCAM2	LMNA	-0.424470987	-0.531383627	-0.304248538	3.06E-10	2.77E-09	3.63E-06

AFAP1	LMNA	0.163591599	0.026130734	0.294980759	2.00E-02	3.74E-02	1.00E+00
PRKD2	LMNA	0.425829622	0.30575266	0.532572695	2.65E-10	2.44E-09	3.14E-06
OR2H2	LMNA	-0.067327026	-0.203486706	0.07138749	3.41E-01	4.27E-01	1.00E+00
ZFP36L1	LMNA	0.327579685	0.198505646	0.445484665	1.95E-06	8.84E-06	2.31E-02
DZIP1	LMNA	-0.176994374	-0.307531083	-0.039918759	1.17E-02	2.34E-02	1.00E+00
GPR161	LMNA	-0.059339594	-0.195786731	0.079361527	4.02E-01	4.89E-01	1.00E+00
RNF146	LMNA	-0.253946635	-0.378720605	-0.12010627	2.65E-04	7.96E-04	1.00E+00
CCNB2	LMNA	0.046840757	-0.091803885	0.183703853	5.08E-01	5.92E-01	1.00E+00
ZNF10	LMNA	-0.449695718	-0.553391643	-0.332272508	1.90E-11	2.11E-10	2.25E-07
WDR74	LMNA	0.058751662	-0.079947773	0.195219292	4.06E-01	4.94E-01	1.00E+00
FAM134A	LMNA	-0.062362073	-0.198702415	0.076346204	3.78E-01	4.65E-01	1.00E+00
PCNP	LMNA	-0.181104309	-0.311370538	-0.044157313	9.90E-03	2.01E-02	1.00E+00
PURA	LMNA	-0.058253096	-0.194738033	0.080444834	4.10E-01	4.98E-01	1.00E+00
DNPEP	LMNA	0.533008547	0.42632775	0.625065734	3.19E-16	8.42E-15	3.79E-12
ERBB2	LMNA	0.435042826	0.315968279	0.540624922	9.81E-11	9.88E-10	1.16E-06
CREB1	LMNA	-0.1421799	-0.27483639	-0.004211564	4.35E-02	7.42E-02	1.00E+00
NEO1	LMNA	0.107394768	-0.031117581	0.241859963	1.28E-01	1.88E-01	1.00E+00
DDX3Y	LMNA	0.103260327	-0.035293793	0.237919736	1.44E-01	2.07E-01	1.00E+00
RPS3A	LMNA	-0.210682716	-0.338877479	-0.074807488	2.62E-03	6.15E-03	1.00E+00
MXRA7	LMNA	0.441319195	0.322943377	0.546099336	4.90E-11	5.17E-10	5.81E-07
LGALS3	LMNA	0.616653729	0.523137225	0.69549728	1.51E-22	1.23E-20	1.79E-18
GLT8D1	LMNA	0.345817477	0.218182612	0.461820939	4.63E-07	2.36E-06	5.50E-03
TMPRSS3	LMNA	-0.015418052	-0.153143091	0.122894527	8.28E-01	8.70E-01	1.00E+00
UPB1	LMNA	-0.07608459	-0.211909785	0.062624187	2.82E-01	3.65E-01	1.00E+00
LAT	LMNA	0.004025167	-0.134100351	0.141997263	9.55E-01	9.66E-01	1.00E+00
CLDN14	LMNA	0.124099096	-0.014195095	0.257734587	7.85E-02	1.24E-01	1.00E+00
DHX38	LMNA	0.075562576	-0.063147143	0.211408274	2.85E-01	3.68E-01	1.00E+00
KCNA3	LMNA	-0.405697823	-0.51491025	-0.283526276	2.10E-09	1.64E-08	2.49E-05
BTBD1	LMNA	0.01802149	-0.120328875	0.155685164	7.99E-01	8.49E-01	1.00E+00
TARS2	LMNA	-0.082502122	-0.218069421	0.056188841	2.43E-01	3.23E-01	1.00E+00
SKIV2L2	LMNA	-0.038336146	-0.17545856	0.100245387	5.88E-01	6.66E-01	1.00E+00
ABCF1	LMNA	0.061073002	-0.077632533	0.197459187	3.88E-01	4.75E-01	1.00E+00
CDT1	LMNA	0.009524089	-0.128696123	0.147381311	8.93E-01	9.22E-01	1.00E+00
ZHX2	LMNA	-0.403261644	-0.512766583	-0.280845501	2.67E-09	2.04E-08	3.17E-05
FCF1	LMNA	0.272464402	0.139666827	0.395634031	8.75E-05	2.89E-04	1.00E+00

LRRC49	LMNA	-0.46476337	-0.566469118	-0.349111744	3.22E-12	4.14E-11	3.82E-08
GUCY1B2	LMNA	-0.12719216	-0.260666123	0.011052919	7.13E-02	1.14E-01	1.00E+00
CD28	LMNA	0.383571393	0.259248211	0.495390282	1.75E-08	1.14E-07	2.08E-04
SMARCA4	LMNA	-0.070472134	-0.206514012	0.068242783	3.19E-01	4.05E-01	1.00E+00
Sep-02	LMNA	-0.068604605	-0.204716747	0.070110407	3.32E-01	4.18E-01	1.00E+00
SOHLH2	LMNA	-0.048119418	-0.184941862	0.090532987	4.96E-01	5.81E-01	1.00E+00
MCOLN3	LMNA	0.300354908	0.169324843	0.420951453	1.41E-05	5.41E-05	1.67E-01
LRP8	LMNA	-0.203741502	-0.332442	-0.067591632	3.63E-03	8.23E-03	1.00E+00
TAGLN3	LMNA	-0.566570373	-0.653506932	-0.464880264	1.51E-18	6.13E-17	1.80E-14
MASP2	LMNA	-0.274328909	-0.397332384	-0.14164209	7.79E-05	2.60E-04	9.24E-01
GPATCH3	LMNA	0.020789476	-0.117599034	0.158385908	7.69E-01	8.23E-01	1.00E+00
TTRAP	LMNA	-0.09839117	-0.233273615	0.040205948	1.64E-01	2.31E-01	1.00E+00
AGL	LMNA	-0.24207619	-0.367834578	-0.107621787	5.19E-04	1.46E-03	1.00E+00
NFE2L3	LMNA	0.418570346	0.297722987	0.526214537	5.68E-10	4.92E-09	6.74E-06
PSD4	LMNA	0.392419499	0.268937916	0.503209667	7.65E-09	5.35E-08	9.07E-05
PALMD	LMNA	0.127194248	-0.011050797	0.260668101	7.12E-02	1.14E-01	1.00E+00
CCNB1IP1	LMNA	-0.183980944	-0.314055331	-0.047126908	8.77E-03	1.80E-02	1.00E+00
TMEM43	LMNA	0.639833136	0.550398629	0.714750507	1.18E-24	1.49E-22	1.40E-20
OBFC1	LMNA	0.397568317	0.274587998	0.507751526	4.66E-09	3.41E-08	5.53E-05
STAT5B	LMNA	-0.0920836	-0.227245799	0.046559273	1.92E-01	2.65E-01	1.00E+00
C14orf79	LMNA	0.257029272	0.123355306	0.381541962	2.22E-04	6.76E-04	1.00E+00
ENPEP	LMNA	0.269201506	0.136212649	0.392659877	1.07E-04	3.48E-04	1.00E+00
SSB	LMNA	0.077740094	-0.060965191	0.213499794	2.71E-01	3.54E-01	1.00E+00
SCT	LMNA	0.140158892	0.002149482	0.27292897	4.66E-02	7.90E-02	1.00E+00
TIMM23	LMNA	-0.215220554	-0.343078216	-0.079532578	2.10E-03	5.06E-03	1.00E+00
SKI	LMNA	0.431062434	0.311551423	0.537148481	1.51E-10	1.46E-09	1.79E-06
SLC22A13	LMNA	-0.118310996	-0.252242138	0.02006777	9.35E-02	1.44E-01	1.00E+00
AKAP3	LMNA	-0.227301712	-0.354237028	-0.092142058	1.14E-03	2.95E-03	1.00E+00
OAS2	LMNA	0.226863437	0.091683858	0.353832844	1.17E-03	3.00E-03	1.00E+00
KIAA0423	LMNA	-0.112536278	-0.246753774	0.025917369	1.11E-01	1.66E-01	1.00E+00
SEC61G	LMNA	0.192381888	0.055813203	0.321884125	6.09E-03	1.30E-02	1.00E+00
CAPN11	LMNA	0.158432185	0.020836923	0.290137381	2.43E-02	4.45E-02	1.00E+00
TMEM50B	LMNA	0.129414687	-0.008793415	0.262771066	6.64E-02	1.07E-01	1.00E+00
DEGS1	LMNA	0.18623223	0.049452634	0.316155028	7.96E-03	1.66E-02	1.00E+00
ARHGEF4	LMNA	-0.312235755	-0.431679447	-0.182031106	6.07E-06	2.50E-05	7.20E-02

DBNDD1	LMNA	0.199364654	0.063048917	0.328377878	4.45E-03	9.87E-03	1.00E+00
TBL1XR1	LMNA	-0.172918564	-0.303719305	-0.035720263	1.39E-02	2.70E-02	1.00E+00
FAIM	LMNA	0.018947046	-0.119416307	0.156588465	7.89E-01	8.40E-01	1.00E+00
SP140	LMNA	0.237506245	0.102826724	0.363634409	6.65E-04	1.82E-03	1.00E+00
G6PD	LMNA	0.553812345	0.450179527	0.64272428	1.24E-17	4.24E-16	1.47E-13
NUDC	LMNA	0.116281259	-0.022124907	0.250314032	9.94E-02	1.51E-01	1.00E+00
GABRP	LMNA	-0.01677447	-0.154467765	0.121558026	8.13E-01	8.59E-01	1.00E+00
SCARA3	LMNA	0.162017848	0.024515171	0.293504122	2.12E-02	3.95E-02	1.00E+00
TACSTD2	LMNA	0.278710319	0.146287927	0.401320034	5.91E-05	2.02E-04	7.01E-01
EIF3J	LMNA	0.014347878	-0.123948633	0.152097613	8.39E-01	8.80E-01	1.00E+00
PPP2R2A	LMNA	-0.208152196	-0.336532721	-0.072175194	2.95E-03	6.85E-03	1.00E+00
CPA3	LMNA	0.3344235	0.205877341	0.451624143	1.15E-06	5.45E-06	1.36E-02
PVALB	LMNA	-0.235106892	-0.361427145	-0.100311682	7.57E-04	2.05E-03	1.00E+00
BCAT2	LMNA	0.394292915	0.270992735	0.504862947	6.39E-09	4.54E-08	7.58E-05
MFN1	LMNA	-0.041610902	-0.178635742	0.096997293	5.57E-01	6.37E-01	1.00E+00
F10	LMNA	0.125171222	-0.013106255	0.258751002	7.59E-02	1.20E-01	1.00E+00
FAM134C	LMNA	0.212020332	0.076199666	0.340116259	2.45E-03	5.81E-03	1.00E+00
SNX11	LMNA	-0.158727906	-0.290415171	-0.02114014	2.41E-02	4.40E-02	1.00E+00
COMP	LMNA	0.259888603	0.126371545	0.384156869	1.88E-04	5.79E-04	1.00E+00
GPR177	LMNA	0.0831361	-0.055552478	0.218677336	2.39E-01	3.19E-01	1.00E+00
TAL1	LMNA	-0.351166774	-0.466597623	-0.223973781	2.99E-07	1.58E-06	3.54E-03
ACSL1	LMNA	0.200977096	0.064721807	0.329875666	4.13E-03	9.24E-03	1.00E+00
ABCC5	LMNA	-0.26923582	-0.392691167	-0.136248958	1.07E-04	3.47E-04	1.00E+00
HCFC2	LMNA	-0.050300417	-0.187052521	0.088364186	4.77E-01	5.62E-01	1.00E+00
TCAP	LMNA	-0.197780288	-0.326905536	-0.061405899	4.78E-03	1.05E-02	1.00E+00
ABL1	LMNA	0.184742384	0.047913359	0.314765642	8.49E-03	1.75E-02	1.00E+00
RBBP7	LMNA	0.100674115	-0.037903677	0.235452755	1.54E-01	2.20E-01	1.00E+00
PTPRG	LMNA	-0.031638002	-0.168951082	0.106879814	6.55E-01	7.26E-01	1.00E+00
NCOR1	LMNA	0.003023933	-0.135083459	0.141016065	9.66E-01	9.75E-01	1.00E+00
MOCOS	LMNA	0.404533179	0.282244462	0.513885616	2.36E-09	1.81E-08	2.80E-05
C14orf93	LMNA	-0.280143625	-0.40262352	-0.147809006	5.39E-05	1.86E-04	6.39E-01
PRDM10	LMNA	-0.168391562	-0.299480649	-0.031062664	1.66E-02	3.17E-02	1.00E+00
TNRC15	LMNA	-0.050891546	-0.187624368	0.087776137	4.72E-01	5.58E-01	1.00E+00
SPINK4	LMNA	-0.003976671	-0.141949744	0.134147974	9.55E-01	9.67E-01	1.00E+00
TXNRD1	LMNA	0.175705698	0.038590768	0.30632634	1.24E-02	2.45E-02	1.00E+00

UBE2H	LMNA	0.35908341	0.232560877	0.473654494	1.54E-07	8.53E-07	1.82E-03
BRDT	LMNA	0.233974211	0.099124985	0.360384653	8.04E-04	2.16E-03	1.00E+00
SLC16A4	LMNA	0.238170999	0.103523836	0.364245694	6.42E-04	1.77E-03	1.00E+00
VIP	LMNA	-0.309895348	-0.429568809	-0.179524657	7.19E-06	2.92E-05	8.52E-02
CALCR	LMNA	0.038349503	-0.100232146	0.175471524	5.88E-01	6.66E-01	1.00E+00
CCNE2	LMNA	-0.229171318	-0.355960674	-0.094097305	1.04E-03	2.71E-03	1.00E+00
SLC6A8	LMNA	0.211979069	0.076156712	0.340078052	2.46E-03	5.82E-03	1.00E+00
LILRA1	LMNA	0.087567078	-0.051101676	0.222923222	2.15E-01	2.91E-01	1.00E+00
MC2R	LMNA	0.105853486	-0.032675001	0.240391601	1.34E-01	1.95E-01	1.00E+00
MBTD1	LMNA	-0.350362189	-0.465879595	-0.22310216	3.19E-07	1.68E-06	3.79E-03
USP33	LMNA	-0.468293763	-0.569525818	-0.353068055	2.10E-12	2.77E-11	2.49E-08
PPP1CB	LMNA	0.231794817	0.096842736	0.358377902	9.02E-04	2.40E-03	1.00E+00
C15orf39	LMNA	0.423729828	0.303428266	0.530734792	3.31E-10	2.98E-09	3.93E-06
ABHD8	LMNA	0.070783164	-0.067931643	0.20681325	3.17E-01	4.02E-01	1.00E+00
MAP3K12	LMNA	0.025586793	-0.112862871	0.163061813	7.18E-01	7.80E-01	1.00E+00
PAAF1	LMNA	-0.472707187	-0.573343134	-0.358019731	1.22E-12	1.66E-11	1.45E-08
ARF5	LMNA	0.198762122	0.062423992	0.327818022	4.57E-03	1.01E-02	1.00E+00
CCT3	LMNA	-0.22466888	-0.351808278	-0.08939039	1.31E-03	3.33E-03	1.00E+00
SLC3A2	LMNA	0.344878834	0.21716736	0.460982078	5.00E-07	2.53E-06	5.93E-03
PIWIL2	LMNA	-0.166423858	-0.297636662	-0.029040052	1.79E-02	3.39E-02	1.00E+00
RAD50	LMNA	0.276166217	0.143589572	0.399005137	6.94E-05	2.34E-04	8.23E-01
IER5	LMNA	0.476799861	0.36261731	0.576879122	7.32E-13	1.05E-11	8.68E-09
SYNE1	LMNA	-0.321651009	-0.440157164	-0.19213146	3.04E-06	1.33E-05	3.61E-02
MBTPS2	LMNA	0.170259531	0.032983798	0.301230265	1.54E-02	2.96E-02	1.00E+00
MVK	LMNA	0.099125111	-0.039465955	0.233974332	1.60E-01	2.27E-01	1.00E+00
TSPYL1	LMNA	-0.272394609	-0.395570441	-0.139592909	8.79E-05	2.90E-04	1.00E+00
NCL	LMNA	0.202925281	0.066744051	0.33168446	3.77E-03	8.51E-03	1.00E+00
PSMD10	LMNA	-0.204876372	-0.333495004	-0.068770432	3.45E-03	7.86E-03	1.00E+00
MOBP	LMNA	-0.303005355	-0.423347642	-0.172155658	1.17E-05	4.57E-05	1.39E-01
GUF1	LMNA	-0.09314221	-0.228258181	0.045493766	1.87E-01	2.59E-01	1.00E+00
WDR44	LMNA	0.203591727	0.067436088	0.332303006	3.66E-03	8.27E-03	1.00E+00
HRH1	LMNA	0.528987231	0.421734313	0.621641484	5.83E-16	1.47E-14	6.92E-12
HIST1H3C	LMNA	-0.021118115	-0.158706427	0.117274785	7.65E-01	8.20E-01	1.00E+00
C5orf30	LMNA	-0.508226864	-0.603907008	-0.39810758	1.15E-14	2.21E-13	1.37E-10
RASGRP3	LMNA	-0.061215653	-0.197596787	0.077490208	3.87E-01	4.74E-01	1.00E+00

RNPEP	LMNA	0.300259828	0.169223332	0.420865462	1.41E-05	5.45E-05	1.68E-01
PRKRIP1	LMNA	0.203379625	0.067215827	0.33210616	3.70E-03	8.35E-03	1.00E+00
MED21	LMNA	-0.038389893	-0.175510729	0.100192102	5.88E-01	6.66E-01	1.00E+00
GRPR	LMNA	0.153772305	0.016062281	0.285757133	2.89E-02	5.17E-02	1.00E+00
SYT11	LMNA	-0.292743839	-0.414061146	-0.161207823	2.36E-05	8.73E-05	2.80E-01
NTSR2	LMNA	-0.348479452	-0.464198806	-0.221063353	3.73E-07	1.94E-06	4.42E-03
Gcom1	LMNA	-0.102522359	-0.237215968	0.036038709	1.47E-01	2.11E-01	1.00E+00
SPTBN2	LMNA	-0.48386985	-0.582978626	-0.370572625	2.98E-13	4.47E-12	3.54E-09
LRMP	LMNA	-0.135227778	-0.268270614	0.00287693	5.50E-02	9.12E-02	1.00E+00
HTRA1	LMNA	0.271479567	0.138623917	0.394736618	9.30E-05	3.06E-04	1.00E+00
RNF111	LMNA	-0.113022616	-0.247216329	0.025425089	1.09E-01	1.64E-01	1.00E+00
SLC26A2	LMNA	0.350216696	0.222944567	0.465749738	3.23E-07	1.70E-06	3.83E-03
WISP1	LMNA	0.389630624	0.265881073	0.500746995	9.96E-09	6.81E-08	1.18E-04
ATP2B4	LMNA	0.090721475	-0.047929808	0.225942726	1.99E-01	2.73E-01	1.00E+00
FLJ10769	LMNA	-0.238781865	-0.364807329	-0.104164552	6.21E-04	1.72E-03	1.00E+00
PTH	LMNA	-0.070440999	-0.206484055	0.068273928	3.19E-01	4.05E-01	1.00E+00
KIAA0895	LMNA	-0.118010686	-0.251956932	0.020372208	9.44E-02	1.45E-01	1.00E+00
CHST12	LMNA	0.37742823	0.252535422	0.489950672	3.07E-08	1.92E-07	3.65E-04
RAB22A	LMNA	0.31936639	0.189678078	0.438101979	3.61E-06	1.55E-05	4.28E-02
TARDBP	LMNA	-0.096465795	-0.231434733	0.042146479	1.72E-01	2.41E-01	1.00E+00
RANBP5	LMNA	-0.238910982	-0.364926029	-0.104299993	6.17E-04	1.71E-03	1.00E+00
P2RY10	LMNA	0.212129488	0.076313298	0.34021733	2.44E-03	5.79E-03	1.00E+00
STAU1	LMNA	0.255594502	0.121842731	0.38022909	2.41E-04	7.30E-04	1.00E+00
NME5	LMNA	-0.06883359	-0.204937166	0.069881462	3.30E-01	4.16E-01	1.00E+00
DDX21	LMNA	0.143693204	0.005756393	0.276263959	4.13E-02	7.10E-02	1.00E+00
CHMP1A	LMNA	0.232617566	0.097704147	0.359135613	8.64E-04	2.30E-03	1.00E+00
BARX1	LMNA	0.156587233	0.018945784	0.288403802	2.61E-02	4.72E-02	1.00E+00
HDAC11	LMNA	-0.152291398	-0.284363937	-0.014546217	3.05E-02	5.44E-02	1.00E+00
NOLA2	LMNA	-0.227865045	-0.354756472	-0.092731086	1.11E-03	2.88E-03	1.00E+00
MTO1	LMNA	-0.046684911	-0.183552932	0.091958754	5.09E-01	5.93E-01	1.00E+00
DHX29	LMNA	0.262119269	0.128726338	0.386195467	1.64E-04	5.15E-04	1.00E+00
HADHB	LMNA	-0.131580534	-0.264821106	0.006590176	6.20E-02	1.01E-01	1.00E+00
ADAR	LMNA	0.051885081	-0.08678756	0.188585286	4.63E-01	5.49E-01	1.00E+00
SF4	LMNA	-0.117879457	-0.251832296	0.020505233	9.48E-02	1.45E-01	1.00E+00
PLXNB2	LMNA	0.500833803	0.389728895	0.597568551	3.18E-14	5.51E-13	3.77E-10

P2RX1	LMNA	-0.11772447	-0.251685089	0.020662335	9.52E-02	1.46E-01	1.00E+00
SLC15A3	LMNA	0.375259581	0.250168553	0.488028295	3.74E-08	2.29E-07	4.43E-04
GAS2	LMNA	-0.003007658	-0.141000114	0.135099437	9.66E-01	9.75E-01	1.00E+00
EN2	LMNA	0.104954208	-0.033583384	0.239534584	1.37E-01	1.99E-01	1.00E+00
NUMB	LMNA	0.424342958	0.30410683	0.531271555	3.10E-10	2.81E-09	3.68E-06
C14orf172	LMNA	0.204265564	0.068135933	0.332928298	3.55E-03	8.05E-03	1.00E+00
TNIP1	LMNA	0.536006504	0.429755815	0.627616264	2.03E-16	5.57E-15	2.41E-12
INHBE	LMNA	0.02981049	-0.108687801	0.16717351	6.74E-01	7.43E-01	1.00E+00
TM9SF2	LMNA	0.174446998	0.037294137	0.305149217	1.30E-02	2.56E-02	1.00E+00
PSKH1	LMNA	0.185677052	0.048878961	0.315637351	8.15E-03	1.69E-02	1.00E+00
NSFL1C	LMNA	0.174825152	0.037683638	0.305502905	1.28E-02	2.53E-02	1.00E+00
RHOG	LMNA	0.434577006	0.315451113	0.540218265	1.03E-10	1.04E-09	1.22E-06
HBB	LMNA	0.004856159	-0.133284195	0.142811419	9.45E-01	9.59E-01	1.00E+00
MED15	LMNA	0.366845471	0.2409995	0.480559321	7.88E-08	4.61E-07	9.34E-04
HEY1	LMNA	-0.241905086	-0.367677412	-0.107442142	5.23E-04	1.47E-03	1.00E+00
KNG1	LMNA	-0.013659064	-0.151424532	0.124626939	8.47E-01	8.86E-01	1.00E+00
CASR	LMNA	-0.048328444	-0.185144201	0.090325188	4.95E-01	5.79E-01	1.00E+00
ITGAX	LMNA	0.164784259	0.02735556	0.296099403	1.91E-02	3.58E-02	1.00E+00
CANT1	LMNA	0.428832573	0.309079303	0.535199361	1.92E-10	1.82E-09	2.28E-06
C6orf66	LMNA	0.021647022	-0.116752883	0.159222204	7.60E-01	8.15E-01	1.00E+00
UNC50	LMNA	-0.073214719	-0.209151747	0.065498297	3.00E-01	3.85E-01	1.00E+00
C21orf33	LMNA	0.005329623	-0.132819101	0.143275207	9.40E-01	9.56E-01	1.00E+00
IRF2	LMNA	0.295227388	0.163854513	0.416311034	2.00E-05	7.48E-05	2.37E-01
HEATR6	LMNA	-0.11124563	-0.245525948	0.027223457	1.15E-01	1.71E-01	1.00E+00
GNG7	LMNA	-0.123430581	-0.25710066	0.014873866	8.01E-02	1.26E-01	1.00E+00
RUNX2	LMNA	0.445875742	0.328015259	0.550068077	2.93E-11	3.17E-10	3.48E-07
PGR	LMNA	0.166873531	0.029502174	0.298058149	1.76E-02	3.33E-02	1.00E+00
SOX1	LMNA	-0.160749849	-0.292313913	-0.023214006	2.23E-02	4.12E-02	1.00E+00
CROCCL1	LMNA	-0.264057054	-0.387965419	-0.130773172	1.46E-04	4.64E-04	1.00E+00
BRE	LMNA	0.088071699	-0.050594448	0.223406438	2.13E-01	2.88E-01	1.00E+00
SRPX	LMNA	0.582765005	0.48362202	0.667143452	9.14E-20	4.51E-18	1.08E-15
MBNL3	LMNA	0.137114275	-0.000954799	0.270053497	5.17E-02	8.64E-02	1.00E+00
ODC1	LMNA	-0.128922441	-0.262304971	0.009293972	6.75E-02	1.09E-01	1.00E+00
SDC3	LMNA	-0.022120439	-0.159683806	0.116285668	7.55E-01	8.11E-01	1.00E+00
NR2F6	LMNA	0.502717629	0.391862123	0.599184802	2.46E-14	4.37E-13	2.92E-10

ADORA2B	LMNA	0.281636974	0.14939447	0.403981083	4.90E-05	1.70E-04	5.81E-01
ZRSR1	LMNA	-0.134602622	-0.267679593	0.00351367	5.61E-02	9.28E-02	1.00E+00
ZFYVE16	LMNA	-0.357657917	-0.472384897	-0.231013201	1.74E-07	9.57E-07	2.06E-03
RPUSD2	LMNA	-0.047773396	-0.184606884	0.090876953	5.00E-01	5.84E-01	1.00E+00
SYNJ2BP	LMNA	0.005033492	-0.133110004	0.142985136	9.43E-01	9.58E-01	1.00E+00
POLE	LMNA	-0.08842155	-0.22374141	0.050242748	2.11E-01	2.86E-01	1.00E+00
E2F2	LMNA	-0.156736609	-0.288544193	-0.019098863	2.59E-02	4.70E-02	1.00E+00
C14orf173	LMNA	0.425834024	0.305757534	0.532576546	2.65E-10	2.44E-09	3.14E-06
THRA	LMNA	-0.57071092	-0.656998814	-0.469663389	7.49E-19	3.22E-17	8.89E-15
MAPK11	LMNA	0.113748992	-0.024689716	0.247907067	1.07E-01	1.61E-01	1.00E+00
TBC1D22A	LMNA	0.234659796	0.099843218	0.361015686	7.75E-04	2.09E-03	1.00E+00
PTGES2	LMNA	-0.034272127	-0.171511654	0.104272217	6.28E-01	7.03E-01	1.00E+00
HIP1R	LMNA	-0.384173317	-0.495922798	-0.259906595	1.66E-08	1.09E-07	1.97E-04
ENO3	LMNA	-0.116708244	-0.250719726	0.021692256	9.81E-02	1.50E-01	1.00E+00
COL4A6	LMNA	0.213732609	0.077982562	0.341701369	2.26E-03	5.40E-03	1.00E+00
TOMM70A	LMNA	-0.168664206	-0.299736073	-0.031343004	1.64E-02	3.13E-02	1.00E+00
IRGC	LMNA	0.052086214	-0.086587398	0.188779783	4.62E-01	5.47E-01	1.00E+00
NAB1	LMNA	-0.177099153	-0.307629019	-0.040026755	1.17E-02	2.33E-02	1.00E+00
DET1	LMNA	-0.21557409	-0.343405275	-0.079900959	2.06E-03	4.98E-03	1.00E+00
TRPM3	LMNA	0.205641149	0.069565027	0.334204431	3.32E-03	7.61E-03	1.00E+00
ZNF532	LMNA	-0.098729266	-0.233596424	0.039865082	1.62E-01	2.29E-01	1.00E+00
RAP2A	LMNA	-0.532640112	-0.62475215	-0.425906668	3.38E-16	8.86E-15	4.00E-12
PLG	LMNA	0.132163132	-0.005997291	0.265372347	6.08E-02	9.95E-02	1.00E+00
FECH	LMNA	0.01679097	-0.121541765	0.154483876	8.13E-01	8.59E-01	1.00E+00
CRIP1	LMNA	0.557406878	0.454315735	0.645765825	6.93E-18	2.46E-16	8.22E-14
AZIN1	LMNA	-0.290364364	-0.41190415	-0.158673818	2.77E-05	1.01E-04	3.28E-01
SLC7A7	LMNA	0.308695631	0.178240481	0.428486368	7.83E-06	3.16E-05	9.29E-02
CA8	LMNA	-0.388393647	-0.499654127	-0.264526039	1.12E-08	7.56E-08	1.33E-04
IL10RA	LMNA	0.324382198	0.195066527	0.442612443	2.48E-06	1.11E-05	2.94E-02
CDC42EP4	LMNA	0.024536634	-0.113900181	0.16203876	7.29E-01	7.89E-01	1.00E+00
C16orf58	LMNA	0.206386774	0.070339891	0.334895952	3.21E-03	7.38E-03	1.00E+00
ARG2	LMNA	-0.100293629	-0.235089664	0.038287486	1.56E-01	2.22E-01	1.00E+00
NOL7	LMNA	0.201557269	0.065323918	0.330414427	4.02E-03	9.01E-03	1.00E+00
JARID1A	LMNA	-0.021365242	-0.158947429	0.11703094	7.63E-01	8.18E-01	1.00E+00
PANK2	LMNA	0.404952057	0.282705431	0.514254173	2.26E-09	1.75E-08	2.68E-05

ASH2L	LMNA	-0.20453764	-0.333180741	-0.068418549	3.50E-03	7.97E-03	1.00E+00
ICAM3	LMNA	0.554794487	0.45130923	0.643555606	1.06E-17	3.66E-16	1.26E-13
TMEM16C	LMNA	-0.45788785	-0.560508083	-0.341418556	7.31E-12	8.84E-11	8.67E-08
MDS1	LMNA	-0.065549763	-0.20177486	0.073163308	3.54E-01	4.41E-01	1.00E+00
CABYR	LMNA	-0.376116193	-0.48878776	-0.251103282	3.46E-08	2.14E-07	4.11E-04
SLC1A3	LMNA	-0.005710405	-0.143648164	0.132445006	9.36E-01	9.53E-01	1.00E+00
RNF139	LMNA	-0.274933069	-0.397882524	-0.142282365	7.50E-05	2.51E-04	8.89E-01
ADAM8	LMNA	0.447498184	0.329822838	0.551480084	2.44E-11	2.67E-10	2.89E-07
SFTPC	LMNA	-0.354855606	-0.469887669	-0.227972565	2.20E-07	1.19E-06	2.61E-03
BCL7B	LMNA	0.321049038	0.191484866	0.439615767	3.18E-06	1.38E-05	3.78E-02
MAN2B2	LMNA	0.408901258	0.287054248	0.51772697	1.52E-09	1.22E-08	1.81E-05
RGS12	LMNA	-0.184399759	-0.314446042	-0.04755946	8.61E-03	1.78E-02	1.00E+00
EIF1AY	LMNA	0.060047373	-0.078655651	0.196469719	3.96E-01	4.83E-01	1.00E+00
NUDT13	LMNA	-0.225730952	-0.35278823	-0.090500152	1.24E-03	3.17E-03	1.00E+00
ARL6IP4	LMNA	0.279446531	0.147069145	0.401989627	5.64E-05	1.93E-04	6.68E-01
RPL35A	LMNA	-0.007856854	-0.145749767	0.130335515	9.12E-01	9.37E-01	1.00E+00
CCDC21	LMNA	-0.116832504	-0.250837781	0.021566337	9.77E-02	1.49E-01	1.00E+00
RAB40C	LMNA	-0.059041101	-0.195498654	0.079659176	4.04E-01	4.91E-01	1.00E+00
EMR3	LMNA	0.254638565	0.1208353	0.37935409	2.55E-04	7.67E-04	1.00E+00
PRRG3	LMNA	-0.087262348	-0.222631385	0.051407946	2.17E-01	2.93E-01	1.00E+00
LRCH1	LMNA	-0.198041675	-0.327148485	-0.061676912	4.72E-03	1.04E-02	1.00E+00
SAA4	LMNA	0.126898516	-0.011351343	0.260387919	7.19E-02	1.15E-01	1.00E+00
RAPGEF5	LMNA	-0.248557557	-0.373782719	-0.114433172	3.61E-04	1.05E-03	1.00E+00
ZCCHC2	LMNA	0.13366936	-0.004464016	0.266797103	5.79E-02	9.53E-02	1.00E+00
CLIP3	LMNA	-0.409447735	-0.518207243	-0.287656419	1.44E-09	1.16E-08	1.71E-05
MAP4K2	LMNA	0.113168763	-0.025277144	0.247355317	1.09E-01	1.63E-01	1.00E+00
C20orf30	LMNA	0.183070448	0.046186728	0.313205781	9.11E-03	1.87E-02	1.00E+00
SULF1	LMNA	0.07042788	-0.068287051	0.206471434	3.19E-01	4.05E-01	1.00E+00
C11orf48	LMNA	-0.246214691	-0.371633791	-0.111969556	4.12E-04	1.18E-03	1.00E+00
PRDM5	LMNA	0.174185285	0.037024594	0.304904416	1.32E-02	2.59E-02	1.00E+00
ELOVL1	LMNA	0.458649336	0.342269835	0.561168812	6.68E-12	8.11E-11	7.92E-08
PPP4R2	LMNA	0.207824325	0.071834276	0.336228802	3.00E-03	6.95E-03	1.00E+00
KCNV1	LMNA	-0.294293443	-0.415465133	-0.162858997	2.13E-05	7.93E-05	2.52E-01
ACP1	LMNA	0.013893954	-0.124395647	0.151654071	8.44E-01	8.85E-01	1.00E+00
SIGLEC5	LMNA	0.244761426	0.11044222	0.370300148	4.47E-04	1.27E-03	1.00E+00

ZMYM2	LMNA	-0.341073314	-0.457578974	-0.213054073	6.79E-07	3.35E-06	8.06E-03
C19orf61	LMNA	0.02618719	-0.112269684	0.163646581	7.11E-01	7.74E-01	1.00E+00
DAGLA	LMNA	-0.18069336	-0.310986826	-0.043733282	1.01E-02	2.04E-02	1.00E+00
GPR32	LMNA	0.145965162	0.008076917	0.278406103	3.82E-02	6.61E-02	1.00E+00
EDG7	LMNA	-0.079955611	-0.215626536	0.058743801	2.58E-01	3.40E-01	1.00E+00
NEUROD6	LMNA	-0.425276515	-0.53208867	-0.305140252	2.81E-10	2.57E-09	3.33E-06
NEURL	LMNA	-0.125503653	-0.259066099	0.012768575	7.51E-02	1.19E-01	1.00E+00
SLC2A4RG	LMNA	0.692771455	0.613383056	0.75830029	3.27E-30	1.05E-27	3.87E-26
LPL	LMNA	0.147441447	0.009585555	0.27979733	3.63E-02	6.32E-02	1.00E+00
CA5B	LMNA	0.022299571	-0.116108868	0.159858451	7.53E-01	8.09E-01	1.00E+00
MPHOSPH9	LMNA	-0.14940634	-0.281648152	-0.011594484	3.38E-02	5.95E-02	1.00E+00
CLEC2D	LMNA	-0.059169513	-0.195622588	0.07953113	4.03E-01	4.90E-01	1.00E+00
HMG2L1	LMNA	-0.149466298	-0.281704614	-0.011655803	3.37E-02	5.94E-02	1.00E+00
GRRP1	LMNA	0.143858713	0.00592539	0.276420056	4.11E-02	7.06E-02	1.00E+00
HCN4	LMNA	0.018091143	-0.120260208	0.15575315	7.98E-01	8.48E-01	1.00E+00
CD8B	LMNA	0.185944965	0.049155788	0.315887177	8.06E-03	1.68E-02	1.00E+00
HIST1H3D	LMNA	0.040751718	-0.097849767	0.177802435	5.65E-01	6.45E-01	1.00E+00
TGM4	LMNA	-0.149348878	-0.28159404	-0.011535718	3.39E-02	5.96E-02	1.00E+00
SLC6A12	LMNA	-0.249945124	-0.375054794	-0.115893033	3.34E-04	9.81E-04	1.00E+00
CD84	LMNA	0.123980918	-0.014315096	0.257622531	7.88E-02	1.24E-01	1.00E+00
TBC1D15	LMNA	-0.05081515	-0.187550468	0.087852141	4.73E-01	5.58E-01	1.00E+00
RASA1	LMNA	0.161731474	0.024221267	0.293235353	2.15E-02	3.98E-02	1.00E+00
PHKG1	LMNA	0.085478862	-0.053199921	0.220922875	2.26E-01	3.04E-01	1.00E+00
MAGEA11	LMNA	0.051499722	-0.087171029	0.188212609	4.67E-01	5.52E-01	1.00E+00
NONO	LMNA	0.040593651	-0.098006577	0.177649107	5.66E-01	6.46E-01	1.00E+00
IMPA1	LMNA	-0.285824767	-0.407785218	-0.153844212	3.73E-05	1.33E-04	4.43E-01
SH3BP1	LMNA	0.053890034	-0.084791781	0.19052362	4.46E-01	5.33E-01	1.00E+00
RARRES1	LMNA	0.330564243	0.201718616	0.448163429	1.55E-06	7.17E-06	1.84E-02
NPM3	LMNA	0.046399775	-0.092242085	0.18327679	5.12E-01	5.96E-01	1.00E+00
CLEC5A	LMNA	0.552394843	0.448549637	0.641524077	1.56E-17	5.23E-16	1.85E-13
B3GNTL1	LMNA	0.318489861	0.188737229	0.437313145	3.85E-06	1.65E-05	4.57E-02
ITCH	LMNA	0.308224542	0.177736349	0.428061235	8.10E-06	3.25E-05	9.60E-02
MGAT3	LMNA	-0.384870682	-0.496539646	-0.260669517	1.55E-08	1.03E-07	1.84E-04
ARPC5L	LMNA	0.170387797	0.033115752	0.301350372	1.53E-02	2.95E-02	1.00E+00
KLHL26	LMNA	0.205495201	0.069413375	0.334069057	3.35E-03	7.65E-03	1.00E+00

MBP	LMNA	-0.26350924	-0.387465144	-0.130194414	1.51E-04	4.78E-04	1.00E+00
SIM2	LMNA	-0.272872453	-0.39600579	-0.140099026	8.53E-05	2.83E-04	1.00E+00
RPP25	LMNA	0.192497748	0.055933143	0.321991971	6.06E-03	1.30E-02	1.00E+00
SLC2A2	LMNA	0.119023063	-0.019345814	0.252918299	9.16E-02	1.41E-01	1.00E+00
EXO1	LMNA	-0.094859331	-0.229899697	0.043764789	1.79E-01	2.49E-01	1.00E+00
SOSTDC1	LMNA	-0.117272141	-0.251255433	0.021120796	9.65E-02	1.47E-01	1.00E+00
HRC	LMNA	-0.078455287	-0.21418647	0.060248253	2.67E-01	3.49E-01	1.00E+00
TRIM48	LMNA	-0.315741994	-0.434839015	-0.185789269	4.71E-06	1.98E-05	5.59E-02
KIRREL	LMNA	0.544494466	0.439478162	0.634826802	5.47E-17	1.67E-15	6.49E-13
PIK3R1	LMNA	-0.464153303	-0.565940624	-0.348428496	3.46E-12	4.43E-11	4.11E-08
HOXC11	LMNA	0.008781162	-0.129426736	0.146654381	9.01E-01	9.28E-01	1.00E+00
MAF	LMNA	0.315806657	0.185858614	0.434897257	4.69E-06	1.98E-05	5.56E-02
DOK5	LMNA	0.016520067	-0.121808732	0.154219353	8.15E-01	8.61E-01	1.00E+00
HELZ	LMNA	-0.076376653	-0.212190346	0.062331563	2.80E-01	3.63E-01	1.00E+00
ZMIZ2	LMNA	0.076461871	-0.062246178	0.212272203	2.79E-01	3.62E-01	1.00E+00
SLC46A3	LMNA	0.079331005	-0.059370202	0.215027088	2.62E-01	3.43E-01	1.00E+00
ADRB3	LMNA	-0.092119573	-0.227280206	0.046523071	1.92E-01	2.65E-01	1.00E+00
PTRH2	LMNA	0.09956077	-0.039026631	0.234390203	1.59E-01	2.25E-01	1.00E+00
DMD	LMNA	0.035273413	-0.103280515	0.172484495	6.18E-01	6.94E-01	1.00E+00
STAMBP	LMNA	-0.212829982	-0.340865869	-0.077042601	2.36E-03	5.62E-03	1.00E+00
KRTAP2-4	LMNA	0.327254206	0.198155426	0.445192407	2.00E-06	9.05E-06	2.37E-02
ADCY2	LMNA	-0.314934534	-0.43411165	-0.184923455	5.00E-06	2.09E-05	5.92E-02
MS4A12	LMNA	0.128247366	-0.009980333	0.261665661	6.89E-02	1.11E-01	1.00E+00
TCF20	LMNA	-0.220448163	-0.347911152	-0.084983472	1.62E-03	4.01E-03	1.00E+00
KLHL20	LMNA	-0.074124255	-0.210026074	0.064587667	2.94E-01	3.78E-01	1.00E+00
SRM	LMNA	0.230160506	0.095132226	0.356872286	9.83E-04	2.58E-03	1.00E+00
OTC	LMNA	-0.125924037	-0.259464522	0.012341507	7.41E-02	1.18E-01	1.00E+00
ABCB11	LMNA	0.060005368	-0.078697547	0.196429189	3.96E-01	4.83E-01	1.00E+00
KCNC2	LMNA	-0.332719744	-0.450096768	-0.204040806	1.31E-06	6.15E-06	1.56E-02
CDH19	LMNA	-0.017103807	-0.15478932	0.12123345	8.09E-01	8.56E-01	1.00E+00
SSH3	LMNA	0.440227273	0.32172897	0.545147576	5.53E-11	5.79E-10	6.56E-07
ZNF135	LMNA	-0.238348197	-0.364408621	-0.103709681	6.36E-04	1.75E-03	1.00E+00
FLJ12529	LMNA	-0.177725317	-0.308214233	-0.040672216	1.14E-02	2.28E-02	1.00E+00
PER1	LMNA	0.123738004	-0.014561742	0.257392191	7.93E-02	1.25E-01	1.00E+00
KLC2	LMNA	-0.0798007	-0.215477874	0.058899167	2.59E-01	3.40E-01	1.00E+00

HDAC1	LMNA	0.419888894	0.299180197	0.527370304	4.95E-10	4.33E-09	5.87E-06
FAM128A	LMNA	-0.023660782	-0.161185288	0.114765087	7.38E-01	7.97E-01	1.00E+00
FNDC3B	LMNA	0.668466418	0.584339723	0.738378075	1.61E-27	3.19E-25	1.91E-23
MTCP1	LMNA	0.277815018	0.145338118	0.400505572	6.25E-05	2.13E-04	7.42E-01
GPR63	LMNA	-0.32119765	-0.439749432	-0.191644483	3.15E-06	1.37E-05	3.74E-02
FLJ21986	LMNA	0.273305827	0.140558103	0.396400577	8.30E-05	2.76E-04	9.85E-01
LMCD1	LMNA	0.328449466	0.199441706	0.446265547	1.82E-06	8.32E-06	2.16E-02
MICAL1	LMNA	-0.107208266	-0.241682318	0.031306071	1.29E-01	1.89E-01	1.00E+00
BLZF1	LMNA	0.066754323	-0.071959824	0.202935174	3.45E-01	4.31E-01	1.00E+00
IQCA	LMNA	-0.188493254	-0.318262529	-0.051789914	7.22E-03	1.52E-02	1.00E+00
PCDHGC3	LMNA	-0.028963873	-0.166349725	0.109525063	6.82E-01	7.50E-01	1.00E+00
ATRNL1	LMNA	-0.532085984	-0.624280462	-0.425273445	3.67E-16	9.60E-15	4.35E-12
CAV2	LMNA	0.404348787	0.282041559	0.513723362	2.40E-09	1.84E-08	2.85E-05
SAC	LMNA	0.089186506	-0.049473628	0.224473722	2.07E-01	2.82E-01	1.00E+00
DUS1L	LMNA	0.377557796	0.252676878	0.490065489	3.04E-08	1.90E-07	3.60E-04
NEK9	LMNA	0.210194048	0.074299019	0.338424808	2.68E-03	6.28E-03	1.00E+00
WARS2	LMNA	0.317326459	0.187488826	0.436265859	4.19E-06	1.78E-05	4.97E-02
DDO	LMNA	0.159118424	0.021540594	0.290781975	2.37E-02	4.35E-02	1.00E+00
MARCO	LMNA	0.351232087	0.224044546	0.466655904	2.97E-07	1.57E-06	3.53E-03
DCHS1	LMNA	0.067172857	-0.071541568	0.203338245	3.42E-01	4.28E-01	1.00E+00
TOMM40	LMNA	0.259628448	0.126097013	0.383919035	1.90E-04	5.87E-04	1.00E+00
RP6-213H1	LMNA	0.306557459	0.175952883	0.426556354	9.11E-06	3.63E-05	1.08E-01
TUBGCP5	LMNA	-0.049885881	-0.186651451	0.088776505	4.81E-01	5.66E-01	1.00E+00
IGSF6	LMNA	-0.007839884	-0.145733157	0.130352197	9.12E-01	9.37E-01	1.00E+00
TPPP	LMNA	-0.161289269	-0.29282029	-0.023767478	2.18E-02	4.04E-02	1.00E+00
Sep-11	LMNA	0.289468181	0.157719884	0.411091405	2.94E-05	1.07E-04	3.48E-01
ADNP	LMNA	-0.24956368	-0.374705146	-0.115491658	3.41E-04	1.00E-03	1.00E+00
UST	LMNA	-0.414960529	-0.523048332	-0.293736431	8.24E-10	6.92E-09	9.78E-06
C13orf34	LMNA	0.033465633	-0.105070791	0.170727877	6.36E-01	7.10E-01	1.00E+00
GSTM5	LMNA	0.015511512	-0.122802455	0.15323438	8.27E-01	8.70E-01	1.00E+00
BTD	LMNA	-0.206450567	-0.33495511	-0.070406193	3.20E-03	7.36E-03	1.00E+00
PDCD1LG2	LMNA	0.430280054	0.310683858	0.536464732	1.65E-10	1.58E-09	1.95E-06
SNRPB2	LMNA	0.186113965	0.049330422	0.316044759	8.00E-03	1.67E-02	1.00E+00
APOA4	LMNA	-0.057860886	-0.194359392	0.080835811	4.13E-01	5.01E-01	1.00E+00
APBA3	LMNA	0.312279544	0.182078018	0.431718924	6.06E-06	2.49E-05	7.18E-02

CUTL1	LMNA	0.13584252	-0.002250688	0.268851692	5.39E-02	8.97E-02	1.00E+00
PMS1	LMNA	-0.335935109	-0.45297869	-0.207507514	1.02E-06	4.88E-06	1.21E-02
EIF3E	LMNA	-0.133083772	-0.266243259	0.005060196	5.90E-02	9.70E-02	1.00E+00
IL9	LMNA	0.020473315	-0.117910945	0.158077531	7.72E-01	8.26E-01	1.00E+00
HOXA11	LMNA	-0.062746449	-0.199073037	0.075962555	3.75E-01	4.63E-01	1.00E+00
NARG1	LMNA	0.071588017	-0.06712638	0.207587473	3.11E-01	3.96E-01	1.00E+00
RPL31	LMNA	-0.163385461	-0.294787377	-0.025919079	2.02E-02	3.76E-02	1.00E+00
RAB28	LMNA	-0.115166504	-0.249254643	0.023254211	1.03E-01	1.55E-01	1.00E+00
LY9	LMNA	0.09271661	-0.045922177	0.227851201	1.89E-01	2.61E-01	1.00E+00
TFCP2	LMNA	-0.079747493	-0.215426812	0.058952529	2.59E-01	3.41E-01	1.00E+00
ATP2B3	LMNA	-0.277933902	-0.400613733	-0.145464227	6.21E-05	2.11E-04	7.36E-01
KDELR2	LMNA	0.631327583	0.540373011	0.707698808	7.34E-24	7.99E-22	8.70E-20
TLE4	LMNA	0.131569029	-0.006601883	0.264810219	6.20E-02	1.01E-01	1.00E+00
FLJ43806	LMNA	-0.097702157	-0.232615665	0.040900505	1.67E-01	2.34E-01	1.00E+00
TLK2	LMNA	-0.112837789	-0.247040548	0.025612182	1.10E-01	1.65E-01	1.00E+00
PKP3	LMNA	0.088419571	-0.050244737	0.223739516	2.11E-01	2.86E-01	1.00E+00
CIR	LMNA	-0.048511677	-0.185321563	0.090143019	4.93E-01	5.78E-01	1.00E+00
RPN2	LMNA	0.545584578	0.440728593	0.635751715	4.61E-17	1.43E-15	5.47E-13
SH3GL2	LMNA	-0.561936905	-0.649594959	-0.459534751	3.29E-18	1.24E-16	3.90E-14
CTSO	LMNA	0.172789154	0.035587036	0.303598209	1.39E-02	2.72E-02	1.00E+00
SFMBT1	LMNA	-0.003614757	-0.141595098	0.134503364	9.59E-01	9.70E-01	1.00E+00
WDR57	LMNA	0.023061779	-0.115356482	0.160601472	7.45E-01	8.03E-01	1.00E+00
SDF2L1	LMNA	0.412688541	0.291229486	0.521054018	1.04E-09	8.56E-09	1.23E-05
IGFBP3	LMNA	0.459900518	0.343668966	0.562254156	5.76E-12	7.08E-11	6.83E-08
FER1L3	LMNA	0.743872114	0.675154249	0.799790769	7.50E-37	8.89E-34	8.89E-33
C20orf43	LMNA	0.059406486	-0.079294822	0.195851285	4.01E-01	4.89E-01	1.00E+00
ARHGAP24	LMNA	0.131536139	-0.006635351	0.264779097	6.20E-02	1.01E-01	1.00E+00
ALB	LMNA	-0.091853771	-0.227025968	0.046790558	1.94E-01	2.66E-01	1.00E+00
SORBS3	LMNA	0.183843578	0.046985049	0.313927173	8.82E-03	1.81E-02	1.00E+00
C4BPA	LMNA	0.099855711	-0.038729178	0.23467172	1.57E-01	2.24E-01	1.00E+00
UPF2	LMNA	-0.156534089	-0.288353852	-0.018891324	2.61E-02	4.73E-02	1.00E+00
AGBL2	LMNA	0.147912439	0.010067001	0.280241068	3.57E-02	6.23E-02	1.00E+00
C1QA	LMNA	0.282631393	0.150450606	0.404884782	4.59E-05	1.60E-04	5.45E-01
DOPEY1	LMNA	-0.344455072	-0.460603295	-0.216709102	5.17E-07	2.60E-06	6.14E-03
TERF1	LMNA	-0.376163054	-0.488829302	-0.251154423	3.45E-08	2.13E-07	4.09E-04

KIF22	LMNA	-0.177165066	-0.307690627	-0.040094694	1.17E-02	2.32E-02	1.00E+00
DNTT	LMNA	0.09540135	-0.043218854	0.230417691	1.77E-01	2.47E-01	1.00E+00
C10orf6	LMNA	-0.019999719	-0.157615546	0.118378121	7.78E-01	8.30E-01	1.00E+00
C11orf41	LMNA	-0.322071028	-0.440534867	-0.192582683	2.95E-06	1.29E-05	3.50E-02
NINJ1	LMNA	0.011243633	-0.127004504	0.149063258	8.74E-01	9.06E-01	1.00E+00
SEC61A2	LMNA	-0.592201033	-0.675062972	-0.494584246	1.66E-20	9.05E-19	1.96E-16
HNRPF	LMNA	0.272527858	0.139734035	0.395691846	8.71E-05	2.88E-04	1.00E+00
COL11A1	LMNA	-0.002671405	-0.140670528	0.13542954	9.70E-01	9.77E-01	1.00E+00
HIST1H1D	LMNA	0.097240907	-0.041365391	0.232175142	1.69E-01	2.37E-01	1.00E+00
UCP3	LMNA	-0.010779837	-0.148609681	0.127460847	8.79E-01	9.11E-01	1.00E+00
MYL6B	LMNA	-0.291820731	-0.41322451	-0.16022456	2.51E-05	9.24E-05	2.98E-01
UBAP2	LMNA	-0.260133081	-0.384380356	-0.126629554	1.85E-04	5.72E-04	1.00E+00
TREM1	LMNA	0.492698128	0.380529802	0.590579412	9.46E-14	1.52E-12	1.12E-09
CKMT2	LMNA	0.031259815	-0.107254035	0.168583303	6.59E-01	7.29E-01	1.00E+00
HLA-C	LMNA	0.407245333	0.28523015	0.516271242	1.80E-09	1.42E-08	2.14E-05
SLC13A3	LMNA	0.00578249	-0.132374182	0.143718763	9.35E-01	9.52E-01	1.00E+00
PLA2G12A	LMNA	0.219715933	0.084219478	0.347234612	1.68E-03	4.15E-03	1.00E+00
SPRED2	LMNA	0.246578426	0.11235193	0.371967504	4.04E-04	1.16E-03	1.00E+00
SCN10A	LMNA	-0.055994623	-0.192557135	0.082695625	4.29E-01	5.15E-01	1.00E+00
TIMP4	LMNA	-0.188847176	-0.318592303	-0.052155909	7.11E-03	1.49E-02	1.00E+00
PITPNC1	LMNA	-0.004837979	-0.14279361	0.133302052	9.46E-01	9.59E-01	1.00E+00
SLIT2	LMNA	0.175074091	0.03794007	0.305735718	1.27E-02	2.51E-02	1.00E+00
RSF1	LMNA	-0.230192704	-0.356901955	-0.095165917	9.82E-04	2.58E-03	1.00E+00
POU3F3	LMNA	-0.04730672	-0.184155052	0.091340804	5.04E-01	5.88E-01	1.00E+00
LIN7C	LMNA	-0.09775157	-0.232662855	0.040850698	1.66E-01	2.34E-01	1.00E+00
NKX2-2	LMNA	-0.398362484	-0.508451529	-0.275460237	4.32E-09	3.18E-08	5.12E-05
DPPA4	LMNA	-0.035152814	-0.172367337	0.103399974	6.19E-01	6.95E-01	1.00E+00
ZNF804A	LMNA	-0.42856703	-0.534967176	-0.30878502	1.98E-10	1.87E-09	2.35E-06
CCIN	LMNA	0.234419061	0.099591003	0.360794119	7.85E-04	2.12E-03	1.00E+00
SLC25A31	LMNA	0.09685761	-0.041751664	0.231809025	1.70E-01	2.39E-01	1.00E+00
KCNMB4	LMNA	-0.08642805	-0.221832264	0.052246326	2.21E-01	2.98E-01	1.00E+00
FUT2	LMNA	0.024649212	-0.113788995	0.162148446	7.28E-01	7.88E-01	1.00E+00
RABL5	LMNA	0.194174577	0.057669464	0.323552433	5.62E-03	1.21E-02	1.00E+00
FZD4	LMNA	0.324180549	0.194849746	0.442431226	2.52E-06	1.12E-05	2.99E-02
GALNS	LMNA	0.611407662	0.516993746	0.691124035	4.28E-22	3.08E-20	5.07E-18

PNMA3	LMNA	-0.490781421	-0.58893068	-0.378365787	1.22E-13	1.93E-12	1.44E-09
STX6	LMNA	-0.108331316	-0.242751902	0.030170898	1.25E-01	1.84E-01	1.00E+00
HIST1H1C	LMNA	-0.085787749	-0.221218837	0.052889628	2.25E-01	3.02E-01	1.00E+00
CIDEB	LMNA	0.261846701	0.128438521	0.385946433	1.67E-04	5.22E-04	1.00E+00
CASP4	LMNA	0.474310868	0.359820592	0.574729128	9.99E-13	1.39E-11	1.18E-08
PDK3	LMNA	0.422280215	0.301824434	0.529465392	3.86E-10	3.43E-09	4.57E-06
WIT1	LMNA	-0.005376955	-0.143321569	0.132772602	9.39E-01	9.55E-01	1.00E+00
TPR	LMNA	0.120633052	-0.017712931	0.254446622	8.72E-02	1.35E-01	1.00E+00
MTX2	LMNA	-0.045307776	-0.182219036	0.093326964	5.22E-01	6.05E-01	1.00E+00
BRD3	LMNA	-0.20844723	-0.336806179	-0.072481997	2.91E-03	6.76E-03	1.00E+00
HIST1H2BC	LMNA	0.149253704	0.011438387	0.281504413	3.40E-02	5.98E-02	1.00E+00
SERPINA3	LMNA	0.221509302	0.086090926	0.348891351	1.53E-03	3.82E-03	1.00E+00
UBXD6	LMNA	-0.00263226	-0.140632157	0.135467968	9.70E-01	9.77E-01	1.00E+00
HOXB7	LMNA	0.209835334	0.073925815	0.338092479	2.72E-03	6.38E-03	1.00E+00
C7orf23	LMNA	0.364269932	0.238197359	0.478269784	9.86E-08	5.65E-07	1.17E-03
KIAA0143	LMNA	0.089627569	-0.049030089	0.224895894	2.05E-01	2.79E-01	1.00E+00
KCNJ16	LMNA	-0.230989987	-0.357636531	-0.096000281	9.41E-04	2.48E-03	1.00E+00
STAB2	LMNA	0.187438301	0.050699195	0.317279367	7.56E-03	1.58E-02	1.00E+00
TNPO2	LMNA	-0.083574562	-0.219097711	0.0551123	2.37E-01	3.16E-01	1.00E+00
CENTG1	LMNA	-0.359713458	-0.474215485	-0.233245131	1.46E-07	8.12E-07	1.73E-03
IRF9	LMNA	0.047954591	-0.090696839	0.1847823	4.98E-01	5.83E-01	1.00E+00
MCPH1	LMNA	-0.286636527	-0.40852212	-0.154707368	3.54E-05	1.26E-04	4.20E-01
FABP2	LMNA	-0.0004295	-0.138472291	0.137629661	9.95E-01	9.96E-01	1.00E+00
C9orf3	LMNA	0.125961236	-0.012303715	0.259499776	7.41E-02	1.18E-01	1.00E+00
FBXL4	LMNA	-0.008835333	-0.146707391	0.129373468	9.01E-01	9.28E-01	1.00E+00
LBH	LMNA	0.21630713	0.080664897	0.344083316	1.99E-03	4.83E-03	1.00E+00
MYO1D	LMNA	0.292674706	0.161134176	0.413998496	2.37E-05	8.76E-05	2.81E-01
PTDSS2	LMNA	-0.016973642	-0.154662233	0.121361737	8.11E-01	8.57E-01	1.00E+00
BMP2K	LMNA	0.184045737	0.047193823	0.314115779	8.74E-03	1.80E-02	1.00E+00
NFU1	LMNA	-0.198046746	-0.327153197	-0.06168217	4.72E-03	1.04E-02	1.00E+00
UGT8	LMNA	-0.437304352	-0.542598516	-0.318480092	7.65E-11	7.84E-10	9.07E-07
SLC25A23	LMNA	-0.334030193	-0.451271614	-0.205453302	1.18E-06	5.60E-06	1.40E-02
MAPK4	LMNA	-0.039122483	-0.176221728	0.099465722	5.80E-01	6.59E-01	1.00E+00
HINT1	LMNA	-0.055695253	-0.192267946	0.08299387	4.31E-01	5.18E-01	1.00E+00
GLP2R	LMNA	-0.018695963	-0.156343442	0.11966389	7.92E-01	8.43E-01	1.00E+00

WNT7B	LMNA	-0.126568309	-0.260075049	0.011686895	7.27E-02	1.16E-01	1.00E+00
SETD4	LMNA	-0.053432471	-0.190081354	0.085247349	4.50E-01	5.36E-01	1.00E+00
CHCHD2	LMNA	-0.006830301	-0.144744817	0.131344548	9.23E-01	9.45E-01	1.00E+00
DYNLT3	LMNA	0.512542672	0.403007269	0.60760159	6.31E-15	1.28E-13	7.48E-11
FKBP11	LMNA	0.582749987	0.483604598	0.667130833	9.17E-20	4.51E-18	1.09E-15
NDP	LMNA	0.0632587	-0.075451211	0.199566897	3.71E-01	4.59E-01	1.00E+00
RHOBTB1	LMNA	0.278952445	0.146544837	0.401540265	5.82E-05	1.99E-04	6.90E-01
FLII	LMNA	0.562588626	0.460286176	0.650145478	2.95E-18	1.12E-16	3.50E-14
SLC4A4	LMNA	-0.000869226	-0.138903559	0.137198238	9.90E-01	9.92E-01	1.00E+00
RPL38	LMNA	-0.184292081	-0.314345594	-0.047448245	8.65E-03	1.78E-02	1.00E+00
HTF9C	LMNA	0.070282351	-0.068432621	0.206331411	3.20E-01	4.06E-01	1.00E+00
RPS16	LMNA	-0.077005268	-0.212794128	0.061701661	2.76E-01	3.58E-01	1.00E+00
AP2A2	LMNA	0.08336049	-0.055327216	0.218892476	2.38E-01	3.17E-01	1.00E+00
CHPF	LMNA	0.645006233	0.556508872	0.71903193	3.78E-25	5.15E-23	4.48E-21
CSNK2A1	LMNA	0.043083128	-0.095536089	0.180063173	5.43E-01	6.24E-01	1.00E+00
MAP7D1	LMNA	0.323841455	0.194485235	0.442126468	2.58E-06	1.14E-05	3.06E-02
FKBP6	LMNA	0.11409258	-0.024341818	0.248233752	1.06E-01	1.60E-01	1.00E+00
ZNF214	LMNA	-0.003695954	-0.141674668	0.134423633	9.58E-01	9.69E-01	1.00E+00
DDX56	LMNA	0.288694527	0.156896572	0.410389626	3.09E-05	1.12E-04	3.67E-01
TWIST1	LMNA	0.447467752	0.329788925	0.551453605	2.45E-11	2.67E-10	2.90E-07
EPO	LMNA	0.070272776	-0.068442198	0.206322198	3.20E-01	4.06E-01	1.00E+00
MRPS18B	LMNA	-0.030511134	-0.167855113	0.107994751	6.66E-01	7.36E-01	1.00E+00
ZNF682	LMNA	-0.35121595	-0.466641505	-0.224027062	2.98E-07	1.57E-06	3.53E-03
AOX1	LMNA	0.223207671	0.087864119	0.350459595	1.41E-03	3.55E-03	1.00E+00
RPL14	LMNA	0.074806641	-0.063904308	0.210681902	2.90E-01	3.74E-01	1.00E+00
CYR61	LMNA	0.543918399	0.43881754	0.634337929	5.98E-17	1.82E-15	7.10E-13
JRKL	LMNA	0.061729532	-0.076977456	0.198092424	3.83E-01	4.70E-01	1.00E+00
DTNA	LMNA	-0.12449521	-0.258110152	0.013792843	7.75E-02	1.23E-01	1.00E+00
MAFF	LMNA	0.56326301	0.461063886	0.650715045	2.64E-18	1.01E-16	3.13E-14
LOC51136	LMNA	-0.049182122	-0.18597045	0.089476391	4.87E-01	5.72E-01	1.00E+00
TMOD3	LMNA	0.571631135	0.47072722	0.657774365	6.40E-19	2.79E-17	7.59E-15
LY96	LMNA	0.46872696	0.353553799	0.569900698	1.99E-12	2.64E-11	2.36E-08
EEA1	LMNA	0.289155192	0.157386782	0.410807511	3.00E-05	1.09E-04	3.56E-01
KLK3	LMNA	-0.01336556	-0.151137692	0.124915927	8.50E-01	8.89E-01	1.00E+00
IL1R1	LMNA	0.514692579	0.405450379	0.609440501	4.65E-15	9.66E-14	5.52E-11

HRSP12	LMNA	-0.337706778	-0.454565586	-0.209419052	8.87E-07	4.28E-06	1.05E-02
KTN1	LMNA	0.215350081	0.07966754	0.343198046	2.08E-03	5.03E-03	1.00E+00
LOH11CR2.	LMNA	0.326987114	0.197868056	0.444952557	2.04E-06	9.22E-06	2.42E-02
ARL5A	LMNA	-0.208809839	-0.337142241	-0.072859105	2.86E-03	6.66E-03	1.00E+00
MAB21L1	LMNA	0.20422712	0.068096002	0.332892626	3.55E-03	8.07E-03	1.00E+00
C20orf59	LMNA	0.520932813	0.412550504	0.614772283	1.90E-15	4.30E-14	2.26E-11
ADAM2	LMNA	-0.100333141	-0.235127371	0.038247631	1.55E-01	2.21E-01	1.00E+00
PHKB	LMNA	-0.137001943	-0.269947361	0.001069281	5.19E-02	8.67E-02	1.00E+00
CCT6A	LMNA	0.140799883	0.002803373	0.273534048	4.56E-02	7.75E-02	1.00E+00
TBC1D8B	LMNA	0.532819161	0.426111295	0.624904546	3.29E-16	8.64E-15	3.90E-12
FAM13A1	LMNA	-0.338615263	-0.455379035	-0.210399641	8.26E-07	4.01E-06	9.80E-03
EHHADH	LMNA	-0.081709203	-0.217308951	0.056984586	2.48E-01	3.28E-01	1.00E+00
LAPTM4B	LMNA	-0.335029519	-0.45216726	-0.206530808	1.10E-06	5.21E-06	1.30E-02
TMEM147	LMNA	0.21117876	0.075323705	0.339336923	2.55E-03	6.03E-03	1.00E+00
ITGB5	LMNA	0.565884043	0.464087995	0.652927766	1.70E-18	6.83E-17	2.02E-14
YIPF3	LMNA	0.199312865	0.062995199	0.32832976	4.46E-03	9.88E-03	1.00E+00
FKBP2	LMNA	0.30754451	0.177008739	0.427447448	8.50E-06	3.40E-05	1.01E-01
XPO7	LMNA	-0.188002119	-0.31780485	-0.051282087	7.38E-03	1.55E-02	1.00E+00
GPR75	LMNA	-0.179860472	-0.310209008	-0.04287403	1.04E-02	2.11E-02	1.00E+00
NR1D1	LMNA	0.235631637	0.100861581	0.361910001	7.36E-04	2.00E-03	1.00E+00
TRIM5	LMNA	0.411022892	0.289392644	0.519591186	1.23E-09	1.00E-08	1.46E-05
TMEM110	LMNA	0.252405123	0.118482623	0.377308878	2.90E-04	8.63E-04	1.00E+00
APOC1	LMNA	-0.035452927	-0.172658881	0.10310269	6.16E-01	6.92E-01	1.00E+00
RNASE4	LMNA	0.503237099	0.392450576	0.599630349	2.29E-14	4.12E-13	2.72E-10
PARD6B	LMNA	-0.137981617	-0.270872895	7.07E-05	5.02E-02	8.42E-02	1.00E+00
ARID1A	LMNA	-0.14966055	-0.281887532	-0.011854472	3.35E-02	5.91E-02	1.00E+00
NEK2	LMNA	-0.080879398	-0.216512927	0.057817159	2.53E-01	3.33E-01	1.00E+00
RRAGB	LMNA	-0.212079895	-0.340171411	-0.076261672	2.45E-03	5.80E-03	1.00E+00
PCDHGA3	LMNA	-0.146199169	-0.278626664	-0.00831601	3.79E-02	6.57E-02	1.00E+00
HIST2H2BE	LMNA	-0.082893591	-0.218444809	0.055795913	2.41E-01	3.20E-01	1.00E+00
RCN2	LMNA	-0.17936148	-0.309742926	-0.042359341	1.06E-02	2.14E-02	1.00E+00
STARD7	LMNA	-0.243484873	-0.369128244	-0.109101124	4.80E-04	1.36E-03	1.00E+00
SHMT2	LMNA	0.176260843	0.03916279	0.306845379	1.21E-02	2.40E-02	1.00E+00
MLYCD	LMNA	-0.068723111	-0.204830821	0.069991924	3.31E-01	4.17E-01	1.00E+00
KIAA1751	LMNA	-0.278927523	-0.401517597	-0.146518392	5.83E-05	1.99E-04	6.91E-01

NDUFA5	LMNA	-0.252919096	-0.377779643	-0.119023902	2.82E-04	8.40E-04	1.00E+00
THAP9	LMNA	-0.365685248	-0.479528128	-0.239736939	8.72E-08	5.05E-07	1.03E-03
FLVCR2	LMNA	0.289263482	0.157502028	0.410905738	2.98E-05	1.08E-04	3.53E-01
RP11-217H	LMNA	0.326946379	0.19782423	0.444915975	2.04E-06	9.25E-06	2.42E-02
AP1S1	LMNA	0.044855634	-0.093776062	0.181780981	5.26E-01	6.09E-01	1.00E+00
NCLN	LMNA	0.499097013	0.387763225	0.596077755	4.03E-14	6.81E-13	4.77E-10
SDHA	LMNA	0.127601994	-0.010636372	0.26105437	7.03E-02	1.13E-01	1.00E+00
SMAD6	LMNA	0.27312814	0.140369872	0.396238717	8.40E-05	2.79E-04	9.96E-01
SAV1	LMNA	0.22790863	0.092776663	0.354796658	1.11E-03	2.87E-03	1.00E+00
SAT1	LMNA	0.40861637	0.286740364	0.51747657	1.57E-09	1.25E-08	1.86E-05
ADAMTS7	LMNA	0.024550389	-0.113886597	0.162052161	7.29E-01	7.89E-01	1.00E+00
RPP38	LMNA	-0.098791082	-0.233655441	0.039802757	1.62E-01	2.29E-01	1.00E+00
RCBTB2	LMNA	-0.153306852	-0.285319308	-0.01558571	2.94E-02	5.25E-02	1.00E+00
VEGFB	LMNA	0.123123685	-0.015185428	0.256809603	8.09E-02	1.27E-01	1.00E+00
COLQ	LMNA	-0.071987825	-0.207972002	0.066726301	3.09E-01	3.93E-01	1.00E+00
RBMS1	LMNA	0.649719945	0.562084859	0.722928265	1.31E-25	2.07E-23	1.56E-21
NRXN2	LMNA	-0.562557098	-0.650118848	-0.460249822	2.96E-18	1.12E-16	3.52E-14
WBSCR16	LMNA	0.520306232	0.411836987	0.614237307	2.08E-15	4.63E-14	2.47E-11
DRG2	LMNA	0.326866245	0.197738016	0.44484401	2.06E-06	9.30E-06	2.44E-02
KLRB1	LMNA	0.171753209	0.034520719	0.302628671	1.45E-02	2.81E-02	1.00E+00
DNASE2B	LMNA	-0.091223654	-0.22642319	0.047424589	1.97E-01	2.70E-01	1.00E+00
SAFB	LMNA	-0.019991795	-0.157607816	0.118385937	7.78E-01	8.30E-01	1.00E+00
MAPK8IP1	LMNA	-0.283083556	-0.405295617	-0.150930931	4.46E-05	1.56E-04	5.29E-01
RFX2	LMNA	0.215600518	0.079928499	0.343429722	2.06E-03	4.98E-03	1.00E+00
MLLT4	LMNA	-0.274512818	-0.397499858	-0.14183698	7.70E-05	2.57E-04	9.13E-01
ANKZF1	LMNA	-0.211097056	-0.339261251	-0.075238672	2.56E-03	6.05E-03	1.00E+00
DUSP8	LMNA	-0.480389039	-0.579977015	-0.366653858	4.65E-13	6.82E-12	5.52E-09
MEF2C	LMNA	-0.327751378	-0.445638824	-0.198690404	1.92E-06	8.74E-06	2.28E-02
SENP5	LMNA	0.054719102	-0.083966181	0.19132483	4.39E-01	5.26E-01	1.00E+00
NFKBIL2	LMNA	-0.082956677	-0.2185053	0.055732587	2.40E-01	3.20E-01	1.00E+00
LBR	LMNA	-0.148372457	-0.280674413	-0.010537291	3.51E-02	6.14E-02	1.00E+00
CBFA2T3	LMNA	-0.378328101	-0.490748032	-0.253517983	2.83E-08	1.78E-07	3.36E-04
AFF3	LMNA	-0.390927075	-0.50189203	-0.267301784	8.81E-09	6.09E-08	1.04E-04
IL2RG	LMNA	0.299361574	0.168264467	0.420052976	1.50E-05	5.77E-05	1.78E-01
CCDC51	LMNA	-0.095524163	-0.23053505	0.043095143	1.76E-01	2.46E-01	1.00E+00

COG7	LMNA	0.184844846	0.0480192	0.314861213	8.45E-03	1.75E-02	1.00E+00
MYB	LMNA	-0.277832461	-0.400521442	-0.14535662	6.25E-05	2.12E-04	7.41E-01
KLF3	LMNA	0.290943838	0.159290765	0.412429569	2.66E-05	9.74E-05	3.16E-01
PLXNA3	LMNA	0.348577074	0.221169041	0.464285977	3.70E-07	1.92E-06	4.39E-03
KCTD14	LMNA	0.234930855	0.100127225	0.361265146	7.64E-04	2.07E-03	1.00E+00
FZR1	LMNA	0.171948377	0.034721585	0.302811349	1.44E-02	2.79E-02	1.00E+00
XRCC2	LMNA	-0.157929402	-0.289665035	-0.020321456	2.48E-02	4.52E-02	1.00E+00
SLC44A4	LMNA	0.123957217	-0.014339161	0.257600058	7.88E-02	1.24E-01	1.00E+00
ESPL1	LMNA	-0.043130264	-0.180108864	0.095489297	5.42E-01	6.24E-01	1.00E+00
MMS19	LMNA	0.098275722	-0.040322334	0.233163381	1.64E-01	2.32E-01	1.00E+00
GMPR2	LMNA	-0.051623813	-0.18833262	0.087047551	4.66E-01	5.51E-01	1.00E+00
ST8SIA5	LMNA	-0.087865401	-0.223208899	0.050801821	2.14E-01	2.89E-01	1.00E+00
TBC1D19	LMNA	0.254406075	0.120590329	0.379141251	2.59E-04	7.77E-04	1.00E+00
CHPT1	LMNA	0.328070968	0.199034337	0.445925757	1.88E-06	8.55E-06	2.22E-02
ERBB4	LMNA	-0.465286475	-0.566922212	-0.349697699	3.02E-12	3.90E-11	3.58E-08
TSPAN32	LMNA	0.171418081	0.034175832	0.302314968	1.47E-02	2.84E-02	1.00E+00
MAP4	LMNA	0.253886385	0.120042797	0.378665439	2.66E-04	7.98E-04	1.00E+00
ACTR3	LMNA	0.368201601	0.24247579	0.481764237	7.00E-08	4.14E-07	8.30E-04
UGCG	LMNA	0.385401551	0.261250396	0.497009146	1.48E-08	9.83E-08	1.75E-04
GPHN	LMNA	-0.332522807	-0.449920174	-0.203828579	1.33E-06	6.23E-06	1.58E-02
ZAP70	LMNA	0.053824378	-0.084857155	0.190460162	4.47E-01	5.33E-01	1.00E+00
SLC6A2	LMNA	0.036623781	-0.101942636	0.173796077	6.05E-01	6.82E-01	1.00E+00
LPP	LMNA	0.551393875	0.447399108	0.640676292	1.83E-17	6.03E-16	2.18E-13
PTPRCAP	LMNA	-0.064483871	-0.200747802	0.074227911	3.62E-01	4.49E-01	1.00E+00
IL12RB1	LMNA	0.044287418	-0.094340372	0.18123039	5.31E-01	6.14E-01	1.00E+00
HIVEP1	LMNA	-0.132733495	-0.265911927	0.005416763	5.97E-02	9.79E-02	1.00E+00
ATRX	LMNA	0.040402962	-0.098195742	0.177464126	5.68E-01	6.48E-01	1.00E+00
EIF4EBP2	LMNA	-0.087692067	-0.223042915	0.050976048	2.15E-01	2.90E-01	1.00E+00
DFFB	LMNA	-0.185688587	-0.315648107	-0.048890879	8.15E-03	1.69E-02	1.00E+00
DMRT1	LMNA	0.078570439	-0.060132806	0.214297018	2.66E-01	3.48E-01	1.00E+00
CHST8	LMNA	-0.06495343	-0.20120029	0.073758959	3.58E-01	4.45E-01	1.00E+00
HSPB6	LMNA	0.337162607	0.208831816	0.454078247	9.26E-07	4.46E-06	1.10E-02
C14orf109	LMNA	-0.01700506	-0.154692909	0.121330772	8.10E-01	8.57E-01	1.00E+00
IER2	LMNA	0.324100049	0.194763209	0.44235888	2.53E-06	1.13E-05	3.01E-02
NMB	LMNA	-0.114286006	-0.248417647	0.024145953	1.05E-01	1.59E-01	1.00E+00

COX5A	LMNA	-0.08664123	-0.222036473	0.052032122	2.20E-01	2.97E-01	1.00E+00
EIF6	LMNA	0.379414087	0.254704103	0.491710055	2.57E-08	1.63E-07	3.04E-04
AIFM1	LMNA	0.229008779	0.093927279	0.355810858	1.04E-03	2.73E-03	1.00E+00
WWC2	LMNA	0.255332731	0.121566832	0.379989505	2.45E-04	7.39E-04	1.00E+00
MRPL4	LMNA	0.153382625	0.015663288	0.285390587	2.93E-02	5.24E-02	1.00E+00
SEMG1	LMNA	-0.011796898	-0.149604257	0.126460053	8.68E-01	9.02E-01	1.00E+00
MPPED2	LMNA	-0.517483456	-0.611826142	-0.408624204	3.13E-15	6.67E-14	3.71E-11
FLJ21062	LMNA	0.118201248	-0.02017903	0.252137913	9.39E-02	1.44E-01	1.00E+00
TRAF3IP2	LMNA	0.077352971	-0.061353199	0.213128051	2.74E-01	3.56E-01	1.00E+00
EPB41L4A	LMNA	-0.135981997	-0.268983518	0.002108587	5.37E-02	8.93E-02	1.00E+00
LILRA4	LMNA	-0.12555576	-0.259115486	0.012715643	7.50E-02	1.19E-01	1.00E+00
LAMA2	LMNA	0.476717201	0.362524398	0.576807742	7.40E-13	1.05E-11	8.77E-09
TAF4B	LMNA	-0.230850786	-0.35750829	-0.095854593	9.48E-04	2.50E-03	1.00E+00
RLBP1	LMNA	-0.250592202	-0.375647851	-0.116574022	3.22E-04	9.48E-04	1.00E+00
SLTM	LMNA	0.040656741	-0.09794399	0.177710306	5.66E-01	6.46E-01	1.00E+00
CD300C	LMNA	0.344451919	0.216705692	0.460600477	5.18E-07	2.60E-06	6.14E-03
FLJ10404	LMNA	-0.059440522	-0.195884132	0.079260879	4.01E-01	4.88E-01	1.00E+00
RTDR1	LMNA	0.067690258	-0.071024445	0.203836466	3.38E-01	4.25E-01	1.00E+00
MALT1	LMNA	0.418821388	0.298000386	0.526434617	5.53E-10	4.80E-09	6.56E-06
ARVCF	LMNA	-0.40151249	-0.511226608	-0.278921908	3.18E-09	2.39E-08	3.77E-05
RENBP	LMNA	0.278732341	0.146311293	0.401340065	5.90E-05	2.02E-04	7.00E-01
ATXN7L1	LMNA	0.202385508	0.066183647	0.331183403	3.87E-03	8.70E-03	1.00E+00
NID1	LMNA	0.454732454	0.337893124	0.557768809	1.06E-11	1.24E-10	1.25E-07
TUBGCP3	LMNA	-0.233772844	-0.360199286	-0.098914054	8.13E-04	2.18E-03	1.00E+00
ZNF783	LMNA	0.147592895	0.009740357	0.279940021	3.61E-02	6.30E-02	1.00E+00
ITIH5	LMNA	-0.021265161	-0.15884983	0.117129694	7.64E-01	8.19E-01	1.00E+00
ZNF85	LMNA	-0.302940611	-0.423289129	-0.172086483	1.17E-05	4.59E-05	1.39E-01
MMP13	LMNA	0.388273182	0.264394102	0.499547677	1.13E-08	7.63E-08	1.34E-04
KIAA0329	LMNA	-0.131006779	-0.264278147	0.007173967	6.31E-02	1.03E-01	1.00E+00
C8A	LMNA	-0.05157608	-0.188286457	0.087095048	4.66E-01	5.52E-01	1.00E+00
MGC87042	LMNA	0.089120588	-0.049539912	0.224410623	2.07E-01	2.82E-01	1.00E+00
HOXC13	LMNA	-0.070250335	-0.206300606	0.068464644	3.20E-01	4.06E-01	1.00E+00
CLGN	LMNA	-0.400124627	-0.510004216	-0.277396336	3.64E-09	2.71E-08	4.31E-05
SLC25A37	LMNA	0.283613567	0.151494033	0.40577712	4.31E-05	1.51E-04	5.11E-01
SMARCC2	LMNA	-0.335188604	-0.452309817	-0.206702367	1.08E-06	5.15E-06	1.28E-02

TFDP2	LMNA	-0.191996984	-0.321525822	-0.055414773	6.19E-03	1.32E-02	1.00E+00
POLI	LMNA	-0.037224367	-0.174379257	0.101347444	5.99E-01	6.76E-01	1.00E+00
HCP5	LMNA	0.219893905	0.084405155	0.34739906	1.66E-03	4.12E-03	1.00E+00
UCN	LMNA	-0.246283589	-0.371697004	-0.112041981	4.11E-04	1.18E-03	1.00E+00
ZNF764	LMNA	-0.039369259	-0.176461199	0.099221004	5.78E-01	6.57E-01	1.00E+00
USF2	LMNA	0.050309391	-0.08835526	0.187061203	4.77E-01	5.62E-01	1.00E+00
C20orf24	LMNA	0.307351651	0.176802413	0.427273356	8.61E-06	3.44E-05	1.02E-01
FHL3	LMNA	0.540602973	0.435017682	0.631522925	1.00E-16	2.93E-15	1.19E-12
SPATA5L1	LMNA	0.086457611	-0.052216624	0.221860582	2.21E-01	2.98E-01	1.00E+00
JMJD3	LMNA	-0.098596656	-0.233469813	0.039998783	1.63E-01	2.30E-01	1.00E+00
MMRN2	LMNA	0.192177203	0.055601319	0.32169359	6.14E-03	1.31E-02	1.00E+00
DSP	LMNA	0.232518393	0.097600303	0.359044288	8.69E-04	2.31E-03	1.00E+00
NDST1	LMNA	0.106861941	-0.031656062	0.241352415	1.30E-01	1.90E-01	1.00E+00
ZNF248	LMNA	-0.570901199	-0.657159196	-0.469883341	7.25E-19	3.13E-17	8.60E-15
PELP1	LMNA	-0.142010583	-0.27467663	-0.004038761	4.38E-02	7.46E-02	1.00E+00
MBL2	LMNA	0.003979887	-0.134144817	0.141952895	9.55E-01	9.67E-01	1.00E+00
ZNF230	LMNA	-0.150106273	-0.282307213	-0.012310372	3.30E-02	5.83E-02	1.00E+00
RNF41	LMNA	-0.403476876	-0.512956028	-0.281082267	2.62E-09	2.00E-08	3.10E-05
THOC5	LMNA	-0.05548465	-0.192064492	0.083203667	4.33E-01	5.19E-01	1.00E+00
MSN	LMNA	0.635221122	0.544959178	0.710928714	3.20E-24	3.62E-22	3.80E-20
SLC9A5	LMNA	-0.207342017	-0.335781679	-0.071332831	3.07E-03	7.09E-03	1.00E+00
SERINC3	LMNA	0.285128906	0.153104453	0.407153399	3.91E-05	1.38E-04	4.63E-01
ZNF434	LMNA	-0.119559126	-0.253427245	0.018802209	9.01E-02	1.39E-01	1.00E+00
MUSK	LMNA	0.004978078	-0.133164437	0.142930852	9.44E-01	9.58E-01	1.00E+00
SMARCD1	LMNA	-0.115089941	-0.24918187	0.023331761	1.03E-01	1.56E-01	1.00E+00
C11orf75	LMNA	0.128247935	-0.009979755	0.261666199	6.89E-02	1.11E-01	1.00E+00
ZFP106	LMNA	0.135120553	-0.00298615	0.268169251	5.52E-02	9.15E-02	1.00E+00
GTF2E1	LMNA	-0.150299049	-0.28248871	-0.012507567	3.28E-02	5.80E-02	1.00E+00
RY1	LMNA	-0.171787298	-0.302660579	-0.034555802	1.45E-02	2.81E-02	1.00E+00
ATAD2B	LMNA	-0.2410368	-0.366879741	-0.106530647	5.49E-04	1.53E-03	1.00E+00
DDOST	LMNA	0.45877591	0.342411352	0.561278624	6.58E-12	7.99E-11	7.80E-08
ARHGAP17	LMNA	0.182142227	0.045228493	0.312339477	9.48E-03	1.93E-02	1.00E+00
GPNMB	LMNA	0.344981893	0.217278817	0.461074191	4.96E-07	2.51E-06	5.88E-03
TTF2	LMNA	0.168681812	0.031361108	0.299752567	1.64E-02	3.13E-02	1.00E+00
SFPQ	LMNA	-0.138220358	-0.271098405	-0.000172652	4.98E-02	8.37E-02	1.00E+00

GFRA4	LMNA	0.256929626	0.123250236	0.381450799	2.23E-04	6.79E-04	1.00E+00
TIGD6	LMNA	0.220685885	0.08523154	0.348130766	1.60E-03	3.97E-03	1.00E+00
SLC39A14	LMNA	0.431077387	0.311568005	0.537161547	1.51E-10	1.46E-09	1.79E-06
AKR1B10	LMNA	0.17050537	0.033236709	0.301460462	1.53E-02	2.93E-02	1.00E+00
NGRN	LMNA	-0.338940867	-0.455670531	-0.21075115	8.05E-07	3.92E-06	9.55E-03
RGS16	LMNA	0.161890692	0.024384668	0.293384785	2.13E-02	3.96E-02	1.00E+00
URB1	LMNA	0.030538936	-0.107967247	0.167882157	6.66E-01	7.36E-01	1.00E+00
GPR137B	LMNA	-0.157068725	-0.288856311	-0.019439235	2.56E-02	4.65E-02	1.00E+00
MECP2	LMNA	-0.01111942	-0.148941788	0.127126726	8.75E-01	9.08E-01	1.00E+00
PSMA1	LMNA	0.177290072	0.040223545	0.307807463	1.16E-02	2.31E-02	1.00E+00
TOP3B	LMNA	-0.146401933	-0.278817767	-0.008523194	3.76E-02	6.52E-02	1.00E+00
NFATC4	LMNA	0.383076339	0.258706808	0.494952249	1.84E-08	1.19E-07	2.18E-04
RAB7L1	LMNA	0.109779297	-0.028706759	0.244130466	1.20E-01	1.78E-01	1.00E+00
CSN3	LMNA	-0.148930654	-0.281200172	-0.011108035	3.44E-02	6.03E-02	1.00E+00
NOS1	LMNA	-0.188056216	-0.317855266	-0.05133802	7.36E-03	1.54E-02	1.00E+00
CA14	LMNA	-0.287087289	-0.408931245	-0.155186756	3.44E-05	1.23E-04	4.07E-01
BMPR1A	LMNA	-0.220724343	-0.348166293	-0.085271674	1.60E-03	3.96E-03	1.00E+00
YBX2	LMNA	-0.211374638	-0.339518331	-0.075527568	2.53E-03	5.98E-03	1.00E+00
PRL	LMNA	-0.053146911	-0.189805313	0.085531634	4.53E-01	5.39E-01	1.00E+00
SNRP70	LMNA	0.012143306	-0.12611912	0.149942943	8.64E-01	9.00E-01	1.00E+00
C6orf130	LMNA	-0.229267964	-0.356049751	-0.094198406	1.03E-03	2.69E-03	1.00E+00
KIAA1166	LMNA	-0.43443783	-0.540096756	-0.31529661	1.05E-10	1.05E-09	1.24E-06
FUBP3	LMNA	0.076674924	-0.062032696	0.212476847	2.78E-01	3.61E-01	1.00E+00
STAG2	LMNA	-0.078870902	-0.214585452	0.059831557	2.65E-01	3.46E-01	1.00E+00
ACACA	LMNA	-0.344209627	-0.460383884	-0.216443703	5.28E-07	2.65E-06	6.26E-03
ABCG1	LMNA	0.008600889	-0.129603999	0.146477967	9.03E-01	9.30E-01	1.00E+00
ODF1	LMNA	-0.164210845	-0.29556162	-0.026766629	1.95E-02	3.66E-02	1.00E+00
TMEM127	LMNA	0.435206184	0.31614966	0.540767521	9.63E-11	9.73E-10	1.14E-06
CD55	LMNA	0.322216172	0.192738622	0.440665379	2.92E-06	1.28E-05	3.46E-02
PLN	LMNA	0.17481846	0.037676745	0.305496647	1.28E-02	2.53E-02	1.00E+00
RPS23	LMNA	-0.163120028	-0.294538354	-0.02564656	2.04E-02	3.80E-02	1.00E+00
LYPLA1	LMNA	0.033126024	-0.105407012	0.170397781	6.40E-01	7.13E-01	1.00E+00
PRDM9	LMNA	-0.043871387	-0.180827209	0.094753488	5.35E-01	6.17E-01	1.00E+00
SSX2	LMNA	0.109699882	-0.028787075	0.244054872	1.20E-01	1.78E-01	1.00E+00
PLUNC	LMNA	-0.00417396	-0.142143055	0.133954228	9.53E-01	9.65E-01	1.00E+00

SYNGR3	LMNA	-0.278864028	-0.401459845	-0.146451019	5.85E-05	2.00E-04	6.94E-01
EML1	LMNA	0.444570426	0.32656162	0.548931631	3.40E-11	3.65E-10	4.03E-07
LNPEP	LMNA	0.518768596	0.41008657	0.612924103	2.60E-15	5.63E-14	3.08E-11
SMAD3	LMNA	0.210797074	0.07492649	0.338983404	2.60E-03	6.12E-03	1.00E+00
NUP93	LMNA	0.021356569	-0.117039499	0.15893897	7.63E-01	8.18E-01	1.00E+00
HISPPD1	LMNA	-0.074047053	-0.209951869	0.06466497	2.95E-01	3.79E-01	1.00E+00
HNRPUL2	LMNA	0.079665251	-0.059035009	0.215347884	2.60E-01	3.41E-01	1.00E+00
YARS2	LMNA	0.212259224	0.076448359	0.340337453	2.42E-03	5.75E-03	1.00E+00
TBC1D12	LMNA	-0.31177611	-0.431265031	-0.181538718	6.28E-06	2.58E-05	7.45E-02
ZCCHC4	LMNA	0.012313217	-0.125951883	0.150109055	8.62E-01	8.98E-01	1.00E+00
FBXO22	LMNA	0.181802987	0.044878347	0.312022813	9.61E-03	1.96E-02	1.00E+00
C16orf24	LMNA	-0.287504491	-0.409309867	-0.155630509	3.34E-05	1.20E-04	3.96E-01
ZNF669	LMNA	-0.252297718	-0.377210495	-0.118369522	2.92E-04	8.67E-04	1.00E+00
PTGDR	LMNA	0.194203422	0.057699339	0.32357927	5.62E-03	1.21E-02	1.00E+00
DDX27	LMNA	0.332446222	0.203746051	0.449851498	1.34E-06	6.27E-06	1.59E-02
KIAA0409	LMNA	0.387064477	0.263070556	0.49847941	1.27E-08	8.51E-08	1.50E-04
ASB8	LMNA	-0.182974078	-0.313115849	-0.046087229	9.15E-03	1.87E-02	1.00E+00
MEPE	LMNA	-0.088449147	-0.223767832	0.050215003	2.11E-01	2.86E-01	1.00E+00
PLP2	LMNA	0.688870942	0.608707624	0.755111363	9.21E-30	2.48E-27	1.09E-25
COQ7	LMNA	-0.239077442	-0.365079053	-0.104474614	6.11E-04	1.69E-03	1.00E+00
CHMP5	LMNA	-0.200171268	-0.329127218	-0.063885674	4.29E-03	9.54E-03	1.00E+00
CACNB3	LMNA	-0.234200191	-0.360592664	-0.09936171	7.94E-04	2.14E-03	1.00E+00
C10orf84	LMNA	-0.216093794	-0.343886	-0.080442553	2.01E-03	4.87E-03	1.00E+00
SPO11	LMNA	-0.103166264	-0.237830041	0.03538875	1.44E-01	2.08E-01	1.00E+00
NEDD4	LMNA	0.303085869	0.172241686	0.423420406	1.16E-05	4.55E-05	1.38E-01
THAP11	LMNA	-0.222784634	-0.350069037	-0.087422365	1.44E-03	3.61E-03	1.00E+00
ZNF43	LMNA	-0.268854828	-0.392343725	-0.135845838	1.09E-04	3.55E-04	1.00E+00
KRT13	LMNA	0.046615384	-0.092027844	0.183485599	5.10E-01	5.94E-01	1.00E+00
NEK4	LMNA	-0.115249056	-0.249333105	0.023170594	1.02E-01	1.55E-01	1.00E+00
MRPL19	LMNA	0.051386438	-0.087283749	0.188103045	4.68E-01	5.53E-01	1.00E+00
RBBP9	LMNA	-0.177322611	-0.307837875	-0.040257086	1.16E-02	2.31E-02	1.00E+00
PRKAR2A	LMNA	0.207013925	0.070991761	0.335477489	3.12E-03	7.19E-03	1.00E+00
IHPK1	LMNA	-0.057943475	-0.194439127	0.080753485	4.13E-01	5.01E-01	1.00E+00
CACNA1E	LMNA	-0.107234912	-0.241707699	0.031279142	1.29E-01	1.89E-01	1.00E+00
CEACAM8	LMNA	0.089608052	-0.049049717	0.224877213	2.05E-01	2.79E-01	1.00E+00

PEX14	LMNA	0.032810026	-0.105719828	0.170090609	6.43E-01	7.16E-01	1.00E+00
BXDC5	LMNA	-0.107475347	-0.241936714	0.031036139	1.28E-01	1.88E-01	1.00E+00
ZBTB38	LMNA	0.447121325	0.329402898	0.551152158	2.55E-11	2.78E-10	3.02E-07
PAFAH1B1	LMNA	-0.151292059	-0.283423471	-0.013523511	3.16E-02	5.61E-02	1.00E+00
PCTK2	LMNA	-0.188415484	-0.31819006	-0.051709496	7.25E-03	1.52E-02	1.00E+00
LRRC16	LMNA	0.236875721	0.102165632	0.363054502	6.88E-04	1.88E-03	1.00E+00
OSTF1	LMNA	0.355439147	0.228605529	0.470407833	2.09E-07	1.14E-06	2.48E-03
KIAA0323	LMNA	0.539275975	0.433497843	0.630395547	1.23E-16	3.51E-15	1.46E-12
FYCO1	LMNA	0.441839701	0.323522406	0.546552933	4.62E-11	4.89E-10	5.48E-07
TXNDC13	LMNA	-0.037811347	-0.174949132	0.100765639	5.93E-01	6.71E-01	1.00E+00
PLTP	LMNA	0.460742431	0.344610724	0.562984279	5.21E-12	6.45E-11	6.18E-08
SLC25A1	LMNA	0.125655618	-0.0126142	0.25921013	7.48E-02	1.19E-01	1.00E+00
EZH2	LMNA	-0.082257985	-0.217835293	0.056433867	2.44E-01	3.24E-01	1.00E+00
PLEKHB1	LMNA	-0.422643035	-0.529783152	-0.302225789	3.71E-10	3.31E-09	4.40E-06
GRB7	LMNA	0.116340165	-0.022065223	0.250370004	9.92E-02	1.51E-01	1.00E+00
ZFP37	LMNA	-0.323822041	-0.442109019	-0.194464367	2.59E-06	1.15E-05	3.07E-02
MRPL33	LMNA	-0.053588164	-0.190231849	0.085092341	4.49E-01	5.35E-01	1.00E+00
PELO	LMNA	0.524149975	0.416216136	0.617517745	1.19E-15	2.81E-14	1.41E-11
ARMC1	LMNA	-0.431238198	-0.537302068	-0.311746351	1.48E-10	1.44E-09	1.76E-06
ZNF154	LMNA	-0.136710089	-0.269671588	0.001366705	5.24E-02	8.75E-02	1.00E+00
C3orf64	LMNA	0.478820628	0.364889426	0.578623641	5.68E-13	8.24E-12	6.73E-09
FLJ10357	LMNA	0.242550721	0.108120052	0.368270419	5.05E-04	1.42E-03	1.00E+00
PON1	LMNA	-0.286501035	-0.408399133	-0.154563283	3.57E-05	1.27E-04	4.23E-01
SYT5	LMNA	-0.344216171	-0.460389734	-0.216450779	5.28E-07	2.65E-06	6.26E-03
TMEM66	LMNA	-0.191638473	-0.321192054	-0.055043702	6.29E-03	1.34E-02	1.00E+00
ATP4A	LMNA	-0.00760217	-0.14550047	0.130585879	9.14E-01	9.38E-01	1.00E+00
HBEGF	LMNA	0.512846913	0.403352907	0.607861883	6.04E-15	1.24E-13	7.17E-11
PI4KA	LMNA	-0.290450153	-0.411981941	-0.158765148	2.75E-05	1.00E-04	3.26E-01
LEPRE1	LMNA	0.713307965	0.63809169	0.775038676	1.05E-32	6.55E-30	1.24E-28
POU2AF1	LMNA	0.135780726	-0.002313643	0.268793287	5.40E-02	8.98E-02	1.00E+00
MRPL12	LMNA	-0.008874015	-0.146745243	0.12933543	9.00E-01	9.28E-01	1.00E+00
GNPDA1	LMNA	0.10825377	-0.030249292	0.242678058	1.25E-01	1.84E-01	1.00E+00
RASAL1	LMNA	-0.200057219	-0.329021278	-0.063767352	4.31E-03	9.59E-03	1.00E+00
ZC3H3	LMNA	-0.006520993	-0.144441963	0.131648522	9.27E-01	9.47E-01	1.00E+00
DDAH1	LMNA	-0.302243272	-0.422658838	-0.171341493	1.23E-05	4.81E-05	1.46E-01

MGAT1	LMNA	0.582020639	0.482758581	0.666517904	1.04E-19	5.07E-18	1.24E-15
TIPARP	LMNA	0.303823202	0.173029598	0.424086693	1.10E-05	4.33E-05	1.31E-01
UGT2B15	LMNA	-0.087686058	-0.223037161	0.050982088	2.15E-01	2.90E-01	1.00E+00
DNASE1L3	LMNA	-0.06173804	-0.19810063	0.076968966	3.83E-01	4.70E-01	1.00E+00
CHEK1	LMNA	0.110269616	-0.028210833	0.244597156	1.18E-01	1.76E-01	1.00E+00
ZNF8	LMNA	-0.142460486	-0.275101124	-0.004497945	4.31E-02	7.36E-02	1.00E+00
TXNDC1	LMNA	0.200738808	0.064474538	0.329654363	4.18E-03	9.32E-03	1.00E+00
CKB	LMNA	-0.403735921	-0.513184021	-0.281367247	2.55E-09	1.95E-08	3.03E-05
RTN3	LMNA	-0.551151169	-0.640470695	-0.447120189	1.91E-17	6.25E-16	2.26E-13
IPO8	LMNA	-0.014070923	-0.151826999	0.124221378	8.42E-01	8.83E-01	1.00E+00
FZD2	LMNA	0.244485756	0.11015257	0.37004711	4.54E-04	1.29E-03	1.00E+00
PART1	LMNA	-0.260622639	-0.384827835	-0.127146261	1.80E-04	5.58E-04	1.00E+00
PSMB6	LMNA	-0.01093675	-0.148763144	0.127306461	8.77E-01	9.09E-01	1.00E+00
MAP3K14	LMNA	0.203764122	0.067615124	0.332462992	3.63E-03	8.22E-03	1.00E+00
PCDHB8	LMNA	0.040372864	-0.098225598	0.177434929	5.68E-01	6.48E-01	1.00E+00
PHC3	LMNA	0.125515141	-0.012756905	0.259076987	7.51E-02	1.19E-01	1.00E+00
PPP1R8	LMNA	-0.111113738	-0.245400452	0.0273569	1.15E-01	1.72E-01	1.00E+00
NOVA2	LMNA	-0.321643901	-0.440150771	-0.192123825	3.05E-06	1.33E-05	3.61E-02
TNFRSF11I	LMNA	0.377716235	0.252849864	0.490205889	3.00E-08	1.87E-07	3.55E-04
GOLPH3	LMNA	0.304842798	0.174119414	0.425007832	1.03E-05	4.06E-05	1.22E-01
LOC51233	LMNA	0.488513003	0.375806268	0.586978355	1.64E-13	2.56E-12	1.94E-09
GGTLA4	LMNA	-0.131895041	-0.265118696	0.006270128	6.13E-02	1.00E-01	1.00E+00
PDE6D	LMNA	-0.428140092	-0.534593834	-0.308311919	2.07E-10	1.95E-09	2.46E-06
TTLL1	LMNA	-0.199055197	-0.32809035	-0.062727945	4.51E-03	9.99E-03	1.00E+00
ZNF117	LMNA	-0.177681203	-0.308173007	-0.040626738	1.14E-02	2.28E-02	1.00E+00
NKRF	LMNA	-0.367401675	-0.481053558	-0.241604916	7.50E-08	4.40E-07	8.90E-04
CLK2	LMNA	0.036405972	-0.102158465	0.173584558	6.07E-01	6.83E-01	1.00E+00
TNFSF15	LMNA	0.287027463	0.155123128	0.408876949	3.45E-05	1.23E-04	4.09E-01
DUSP2	LMNA	0.143021347	0.005070459	0.275630238	4.23E-02	7.24E-02	1.00E+00
HSD17B11	LMNA	-0.054959531	-0.191557146	0.083726723	4.37E-01	5.23E-01	1.00E+00
SECISBP2	LMNA	-0.241676294	-0.367467245	-0.107201942	5.30E-04	1.49E-03	1.00E+00
PPAP2C	LMNA	0.149574226	0.011766184	0.281806246	3.36E-02	5.92E-02	1.00E+00
GABRR2	LMNA	0.068889077	-0.069825983	0.204990575	3.30E-01	4.16E-01	1.00E+00
LOC51145	LMNA	-0.204529832	-0.333173496	-0.068410437	3.50E-03	7.97E-03	1.00E+00
PIK3C3	LMNA	0.002144424	-0.135946823	0.140153933	9.76E-01	9.81E-01	1.00E+00

DHDDS	LMNA	0.305726305	0.175064026	0.425805821	9.66E-06	3.83E-05	1.15E-01
CTSW	LMNA	0.24842873	0.114297663	0.373664592	3.64E-04	1.06E-03	1.00E+00
NEFM	LMNA	-0.220207058	-0.347688399	-0.08473189	1.64E-03	4.06E-03	1.00E+00
ANKRD28	LMNA	-0.208527497	-0.336880572	-0.07256547	2.90E-03	6.74E-03	1.00E+00
MRPL28	LMNA	-0.051084868	-0.187811363	0.087583802	4.70E-01	5.56E-01	1.00E+00
STMN3	LMNA	-0.13034467	-0.26365147	0.007847541	6.45E-02	1.05E-01	1.00E+00
SYN1	LMNA	-0.376510431	-0.489137232	-0.251533551	3.34E-08	2.07E-07	3.96E-04
RAB14	LMNA	-0.129020944	-0.262398246	0.009193812	6.72E-02	1.08E-01	1.00E+00
PIGV	LMNA	0.163879841	0.026426712	0.295251145	1.98E-02	3.70E-02	1.00E+00
CDK2AP2	LMNA	0.331970343	0.203233288	0.44942473	1.39E-06	6.48E-06	1.65E-02
ZIM2	LMNA	-0.382722579	-0.494639201	-0.258319975	1.90E-08	1.23E-07	2.25E-04
APBB1	LMNA	-0.413983287	-0.522190669	-0.292657922	9.11E-10	7.58E-09	1.08E-05
SND1	LMNA	0.459024561	0.342689375	0.56149434	6.39E-12	7.78E-11	7.58E-08
C1orf123	LMNA	-0.124052735	-0.257690628	0.014242171	7.86E-02	1.24E-01	1.00E+00
CHD3	LMNA	-0.146804212	-0.279196878	-0.008934278	3.71E-02	6.45E-02	1.00E+00
RNASE2	LMNA	0.332767176	0.204091922	0.450139299	1.31E-06	6.13E-06	1.55E-02
BCAP31	LMNA	0.499381753	0.38808542	0.596322211	3.87E-14	6.59E-13	4.59E-10
SLC25A44	LMNA	-0.261814191	-0.385916729	-0.128404194	1.67E-04	5.23E-04	1.00E+00
CXXC1	LMNA	-0.085315155	-0.220766009	0.053364362	2.27E-01	3.05E-01	1.00E+00
PIB5PA	LMNA	-0.181282014	-0.311536453	-0.044340691	9.82E-03	2.00E-02	1.00E+00
CD40	LMNA	0.396933355	0.273890759	0.507191746	4.96E-09	3.62E-08	5.88E-05
FAT2	LMNA	-0.079519837	-0.215208326	0.059180838	2.61E-01	3.42E-01	1.00E+00
DYNC1LI2	LMNA	-0.175384075	-0.3060256	-0.038259409	1.25E-02	2.48E-02	1.00E+00
GDI1	LMNA	-0.240602021	-0.366480252	-0.106074315	5.62E-04	1.57E-03	1.00E+00
ZNF81	LMNA	0.006155094	-0.132008076	0.144083664	9.31E-01	9.50E-01	1.00E+00
VSNL1	LMNA	-0.141682198	-0.274366758	-0.003703639	4.43E-02	7.54E-02	1.00E+00
PIH1D1	LMNA	-0.216132838	-0.343922113	-0.080483245	2.01E-03	4.86E-03	1.00E+00
KRTAP5-9	LMNA	0.027794453	-0.110681237	0.165211529	6.95E-01	7.60E-01	1.00E+00
FLJ10081	LMNA	0.05081819	-0.087849116	0.18755341	4.73E-01	5.58E-01	1.00E+00
CS	LMNA	-0.094018068	-0.229095571	0.044611965	1.83E-01	2.54E-01	1.00E+00
ZNF318	LMNA	-0.184909943	-0.31492193	-0.048086445	8.43E-03	1.74E-02	1.00E+00
IGFBP7	LMNA	0.307494511	0.176955248	0.427402316	8.53E-06	3.41E-05	1.01E-01
ZNF609	LMNA	-0.261855289	-0.38595428	-0.128447589	1.67E-04	5.22E-04	1.00E+00
SIRT4	LMNA	-0.497251627	-0.59449301	-0.385675757	5.16E-14	8.57E-13	6.12E-10
SCN4A	LMNA	0.150121744	0.012326197	0.282321779	3.30E-02	5.82E-02	1.00E+00

ARHGAP4	LMNA	0.319868707	0.190217362	0.438553956	3.48E-06	1.50E-05	4.12E-02
EHMT2	LMNA	-0.209285933	-0.337583428	-0.073354294	2.80E-03	6.53E-03	1.00E+00
UFD1L	LMNA	0.018534467	-0.119823125	0.156185834	7.93E-01	8.44E-01	1.00E+00
EXOSC10	LMNA	-0.0068043	-0.14471936	0.131370102	9.23E-01	9.45E-01	1.00E+00
ECE2	LMNA	-0.077097437	-0.212882647	0.061609294	2.75E-01	3.58E-01	1.00E+00
ERMP1	LMNA	-0.134383206	-0.267472134	0.003737125	5.66E-02	9.34E-02	1.00E+00
OVGP1	LMNA	-0.266712284	-0.390389194	-0.133579673	1.25E-04	4.01E-04	1.00E+00
GTPBP3	LMNA	0.172263189	0.035045612	0.303105994	1.42E-02	2.76E-02	1.00E+00
FAM82C	LMNA	0.07938841	-0.059312636	0.215082185	2.61E-01	3.43E-01	1.00E+00
PACS2	LMNA	-0.013572044	-0.15133949	0.124712622	8.48E-01	8.87E-01	1.00E+00
C19orf36	LMNA	-0.102279178	-0.236984025	0.036284145	1.47E-01	2.12E-01	1.00E+00
UNC84A	LMNA	0.075107947	-0.063602531	0.210971443	2.88E-01	3.72E-01	1.00E+00
ARL4C	LMNA	0.476556507	0.362343778	0.576668973	7.55E-13	1.07E-11	8.95E-09
SCD5	LMNA	-0.463255334	-0.565162571	-0.347423031	3.86E-12	4.87E-11	4.58E-08
ATG4B	LMNA	0.013691761	-0.124594744	0.151456484	8.47E-01	8.86E-01	1.00E+00
LASS6	LMNA	-0.133977499	-0.267088503	0.004150264	5.73E-02	9.44E-02	1.00E+00
LSG1	LMNA	0.091870752	-0.046773469	0.227042211	1.93E-01	2.66E-01	1.00E+00
MAL	LMNA	-0.139147374	-0.27197391	-0.001117847	4.83E-02	8.14E-02	1.00E+00
GPR22	LMNA	-0.4158795	-0.523854653	-0.294750909	7.50E-10	6.35E-09	8.90E-06
WDR5B	LMNA	-0.239257121	-0.36524422	-0.10466311	6.05E-04	1.68E-03	1.00E+00
GALR1	LMNA	0.003202371	-0.134908271	0.141190953	9.64E-01	9.73E-01	1.00E+00
AQP4	LMNA	-0.19910981	-0.328141095	-0.062784589	4.50E-03	9.96E-03	1.00E+00
C17orf60	LMNA	0.215112997	0.079420512	0.342978708	2.11E-03	5.08E-03	1.00E+00
HDAC7A	LMNA	0.52537896	0.417617368	0.618565936	9.94E-16	2.40E-14	1.18E-11
GRIN2C	LMNA	-0.231231358	-0.357858888	-0.096252916	9.30E-04	2.46E-03	1.00E+00
ARMC8	LMNA	-0.175519539	-0.306152271	-0.03839897	1.25E-02	2.47E-02	1.00E+00
SLC47A1	LMNA	0.096167557	-0.04244697	0.231149807	1.73E-01	2.43E-01	1.00E+00
DMPK	LMNA	0.0688497	-0.069865354	0.204952673	3.30E-01	4.16E-01	1.00E+00
CCRN4L	LMNA	0.471350647	0.356497064	0.572170283	1.44E-12	1.95E-11	1.71E-08
CBR4	LMNA	-0.496430795	-0.59378787	-0.384747614	5.76E-14	9.47E-13	6.83E-10
KIFC1	LMNA	-0.087120244	-0.222495286	0.05155076	2.18E-01	2.94E-01	1.00E+00
LHX5	LMNA	0.005037244	-0.133106318	0.142988811	9.43E-01	9.58E-01	1.00E+00
SMC1A	LMNA	0.00915139	-0.129062663	0.147016655	8.97E-01	9.26E-01	1.00E+00
LPXN	LMNA	0.285413097	0.153406555	0.40741145	3.83E-05	1.36E-04	4.55E-01
TRPC7	LMNA	0.129440135	-0.008767535	0.26279516	6.64E-02	1.07E-01	1.00E+00

SERPINA1	LMNA	0.43854639	0.319860297	0.543681925	6.67E-11	6.91E-10	7.91E-07
SMYD5	LMNA	-0.050099721	-0.18685835	0.088563815	4.79E-01	5.64E-01	1.00E+00
RPS13	LMNA	-0.102109803	-0.236822469	0.036455082	1.48E-01	2.13E-01	1.00E+00
CRHR2	LMNA	-0.202729032	-0.331502296	-0.066540291	3.81E-03	8.58E-03	1.00E+00
TUSC2	LMNA	-0.125269912	-0.258844549	0.01300601	7.57E-02	1.20E-01	1.00E+00
PRKAA1	LMNA	0.345131141	0.217440233	0.461207584	4.90E-07	2.48E-06	5.81E-03
FADS2	LMNA	-0.350708936	-0.466189058	-0.223477773	3.10E-07	1.63E-06	3.68E-03
ENAH	LMNA	-0.316383344	-0.435416636	-0.186477111	4.49E-06	1.90E-05	5.33E-02
PRO1768	LMNA	-0.119105922	-0.252996971	0.019261794	9.13E-02	1.41E-01	1.00E+00
KIR3DL2	LMNA	0.049821577	-0.088840461	0.186589232	4.81E-01	5.67E-01	1.00E+00
APBA2BP	LMNA	0.106046295	-0.032480209	0.240575322	1.33E-01	1.94E-01	1.00E+00
ATP2C1	LMNA	0.049894295	-0.088768137	0.186659592	4.81E-01	5.66E-01	1.00E+00
ALDH1A2	LMNA	-0.098468496	-0.233347447	0.040127992	1.63E-01	2.31E-01	1.00E+00
RNF103	LMNA	-0.16669971	-0.297895229	-0.029323533	1.77E-02	3.35E-02	1.00E+00
AHCY	LMNA	0.197993111	0.061626558	0.327103348	4.73E-03	1.04E-02	1.00E+00
CROT	LMNA	0.300321013	0.169288655	0.420920799	1.41E-05	5.43E-05	1.67E-01
PABPC3	LMNA	0.067435875	-0.071278701	0.203591522	3.40E-01	4.26E-01	1.00E+00
ALG12	LMNA	0.310870917	0.180569233	0.430448761	6.70E-06	2.74E-05	7.95E-02
EGR1	LMNA	0.34336756	0.21553332	0.459631022	5.65E-07	2.83E-06	6.70E-03
THSD1	LMNA	0.004181926	-0.133946404	0.14215086	9.53E-01	9.65E-01	1.00E+00
CCL17	LMNA	0.138332763	0.000287248	0.271204577	4.96E-02	8.34E-02	1.00E+00
KHK	LMNA	-0.074744414	-0.210622102	0.063966629	2.90E-01	3.74E-01	1.00E+00
ESRRG	LMNA	-0.18236061	-0.312543312	-0.045453914	9.39E-03	1.92E-02	1.00E+00
SLC12A2	LMNA	0.168290828	0.030959091	0.299386272	1.67E-02	3.18E-02	1.00E+00
CNGA1	LMNA	0.011350527	-0.126899318	0.149167789	8.73E-01	9.06E-01	1.00E+00
RDH5	LMNA	0.388871977	0.265049962	0.500076773	1.07E-08	7.25E-08	1.27E-04
CD58	LMNA	0.390927028	0.267301732	0.501891988	8.81E-09	6.09E-08	1.04E-04
PTGFR	LMNA	0.378973916	0.254223301	0.491320162	2.67E-08	1.69E-07	3.17E-04
CCDC48	LMNA	-0.068395791	-0.204515732	0.070319171	3.33E-01	4.19E-01	1.00E+00
CCDC76	LMNA	-0.241446019	-0.367255703	-0.106960202	5.37E-04	1.50E-03	1.00E+00
CDR2	LMNA	0.512205058	0.402623752	0.607312721	6.61E-15	1.33E-13	7.84E-11
ZIC4	LMNA	0.018618128	-0.119740636	0.156267482	7.93E-01	8.43E-01	1.00E+00
SELE	LMNA	0.304184882	0.173416151	0.424413476	1.08E-05	4.23E-05	1.28E-01
OR1G1	LMNA	-0.07637952	-0.212193099	0.062328691	2.80E-01	3.63E-01	1.00E+00
EXOSC9	LMNA	-0.027603493	-0.165025632	0.110869999	6.97E-01	7.62E-01	1.00E+00

PSMC6	LMNA	-0.179600276	-0.309965981	-0.042605639	1.05E-02	2.13E-02	1.00E+00
ABCB1	LMNA	-0.17322569	-0.304006681	-0.036036465	1.37E-02	2.68E-02	1.00E+00
GPR12	LMNA	-0.17204978	-0.302906259	-0.034825953	1.43E-02	2.78E-02	1.00E+00
KIAA0196	LMNA	0.030153513	-0.108348512	0.167507227	6.70E-01	7.39E-01	1.00E+00
SSR3	LMNA	0.432563295	0.313216252	0.538459744	1.29E-10	1.26E-09	1.52E-06
MSI1	LMNA	0.06531551	-0.073397304	0.201549168	3.56E-01	4.43E-01	1.00E+00
TRAT1	LMNA	0.186295744	0.04951827	0.316214248	7.94E-03	1.65E-02	1.00E+00
CC2D1A	LMNA	0.165065147	0.027644083	0.296362807	1.89E-02	3.55E-02	1.00E+00
PLAGL1	LMNA	0.367481004	0.241691272	0.481124043	7.45E-08	4.38E-07	8.84E-04
LLGL1	LMNA	-0.209899091	-0.338151548	-0.073992144	2.72E-03	6.36E-03	1.00E+00
KLF6	LMNA	0.582148299	0.482906649	0.666625196	1.02E-19	4.98E-18	1.21E-15
MTF1	LMNA	0.213957958	0.078217269	0.341909927	2.23E-03	5.35E-03	1.00E+00
RNF2	LMNA	-0.22424075	-0.351413172	-0.088943132	1.34E-03	3.39E-03	1.00E+00
tcag7.1314	LMNA	0.413193537	0.291786562	0.521497397	9.87E-10	8.17E-09	1.17E-05
NR5A1	LMNA	-0.052603734	-0.189280183	0.086072323	4.57E-01	5.43E-01	1.00E+00
THBD	LMNA	0.499236718	0.387921304	0.596197697	3.95E-14	6.70E-13	4.69E-10
ABCD2	LMNA	-0.085564154	-0.2210046	0.053114244	2.26E-01	3.03E-01	1.00E+00
ITGB7	LMNA	0.315280258	0.18529414	0.4344231	4.87E-06	2.05E-05	5.78E-02
DNAJC7	LMNA	-0.061572354	-0.197940833	0.077134298	3.84E-01	4.71E-01	1.00E+00
CCDC81	LMNA	-0.108335006	-0.242755416	0.030167167	1.25E-01	1.84E-01	1.00E+00
TM2D3	LMNA	-0.357513955	-0.472256653	-0.230856935	1.76E-07	9.68E-07	2.08E-03
HUWE1	LMNA	0.061442409	-0.077263959	0.197815502	3.85E-01	4.72E-01	1.00E+00
CDH17	LMNA	0.177194276	0.040124802	0.307717928	1.16E-02	2.32E-02	1.00E+00
CD180	LMNA	0.212648328	0.076853462	0.3406977	2.38E-03	5.66E-03	1.00E+00
FAAH	LMNA	-0.470004954	-0.571006401	-0.354987173	1.70E-12	2.28E-11	2.02E-08
IL17A	LMNA	-0.029106826	-0.166488837	0.109383703	6.81E-01	7.49E-01	1.00E+00
POLA1	LMNA	-0.073009583	-0.208954522	0.065703647	3.02E-01	3.86E-01	1.00E+00
TMPO	LMNA	-0.20092857	-0.329830601	-0.064671452	4.14E-03	9.25E-03	1.00E+00
TM7SF2	LMNA	-0.252152877	-0.377077815	-0.118217005	2.94E-04	8.74E-04	1.00E+00
FLOT2	LMNA	0.213337327	0.077570901	0.341335508	2.30E-03	5.50E-03	1.00E+00
MAP4K1	LMNA	0.144919344	0.007008558	0.277420204	3.96E-02	6.84E-02	1.00E+00
HDGFRP3	LMNA	-0.464683907	-0.566400285	-0.349022742	3.25E-12	4.17E-11	3.85E-08
RRAS2	LMNA	0.176396253	0.03930233	0.306971971	1.20E-02	2.39E-02	1.00E+00
OXCT1	LMNA	-0.301490668	-0.421978466	-0.170537629	1.30E-05	5.05E-05	1.54E-01
LTBP2	LMNA	0.570640152	0.469581589	0.656939164	7.59E-19	3.25E-17	9.00E-15

ARPC5	LMNA	0.21830432	0.082747078	0.345929983	1.80E-03	4.42E-03	1.00E+00
SV2B	LMNA	-0.251885126	-0.376832531	-0.117935081	2.99E-04	8.86E-04	1.00E+00
ZDHHC4	LMNA	0.151299893	0.013531527	0.283430845	3.16E-02	5.61E-02	1.00E+00
CYP2A6	LMNA	-0.085578147	-0.221018008	0.053100187	2.26E-01	3.03E-01	1.00E+00
PDE9A	LMNA	-0.354598158	-0.469658156	-0.227693346	2.25E-07	1.21E-06	2.66E-03
ABCA8	LMNA	0.187716653	0.050986952	0.317538803	7.47E-03	1.56E-02	1.00E+00
NDUFS2	LMNA	-0.068979409	-0.205077522	0.069735662	3.29E-01	4.15E-01	1.00E+00
UBR5	LMNA	-0.150177591	-0.28237436	-0.012383324	3.29E-02	5.82E-02	1.00E+00
FLJ22662	LMNA	0.455686946	0.338959204	0.558597663	9.46E-12	1.12E-10	1.12E-07
GP6	LMNA	-0.006598278	-0.144517638	0.131572572	9.26E-01	9.47E-01	1.00E+00
CRYBA2	LMNA	-0.144699785	-0.277213189	-0.006784307	3.99E-02	6.88E-02	1.00E+00
LEF1	LMNA	0.383408609	0.259070178	0.495246254	1.78E-08	1.16E-07	2.11E-04
ZNF20	LMNA	-0.117936792	-0.251886751	0.020447114	9.46E-02	1.45E-01	1.00E+00
CTPS	LMNA	-0.050670039	-0.187410097	0.087996501	4.74E-01	5.59E-01	1.00E+00
EPS8L1	LMNA	0.076061309	-0.062647512	0.211887419	2.82E-01	3.65E-01	1.00E+00
EYA1	LMNA	-0.405622719	-0.514844184	-0.283443603	2.12E-09	1.65E-08	2.51E-05
SERPINB2	LMNA	-0.157165919	-0.288947648	-0.019538851	2.55E-02	4.63E-02	1.00E+00
MAPK14	LMNA	0.168770714	0.031452524	0.299835849	1.63E-02	3.12E-02	1.00E+00
GTF2F2	LMNA	-0.120995507	-0.2547906	0.017345221	8.63E-02	1.34E-01	1.00E+00
ZNHIT4	LMNA	0.007050104	-0.13112852	0.144960019	9.21E-01	9.43E-01	1.00E+00
PLA1A	LMNA	0.109088315	-0.029405526	0.243472678	1.22E-01	1.81E-01	1.00E+00
RNF113A	LMNA	0.01635448	-0.121971903	0.154057657	8.17E-01	8.62E-01	1.00E+00
HPR	LMNA	-0.072294212	-0.208266652	0.066419676	3.07E-01	3.91E-01	1.00E+00
CLCN2	LMNA	0.017830755	-0.120516904	0.155498987	8.01E-01	8.50E-01	1.00E+00
SATB2	LMNA	0.016033247	-0.122288427	0.15374395	8.21E-01	8.65E-01	1.00E+00
ANXA6	LMNA	-0.015950191	-0.153662835	0.122370261	8.22E-01	8.66E-01	1.00E+00
KCNJ9	LMNA	-0.400696404	-0.510507875	-0.278024771	3.44E-09	2.57E-08	4.08E-05
EMID1	LMNA	0.173395017	0.036210807	0.304165109	1.36E-02	2.66E-02	1.00E+00
DPM3	LMNA	-0.057649008	-0.194154827	0.081047005	4.15E-01	5.03E-01	1.00E+00
SLC25A13	LMNA	0.14599095	0.008103265	0.27843041	3.82E-02	6.61E-02	1.00E+00
KRT24	LMNA	0.044251192	-0.094376347	0.181195284	5.32E-01	6.14E-01	1.00E+00
SMPD1	LMNA	0.474289145	0.359796192	0.574710358	1.00E-12	1.39E-11	1.19E-08
COL6A2	LMNA	0.700667461	0.622864833	0.76474621	3.81E-31	1.41E-28	4.51E-27
TH	LMNA	0.193505707	0.056976765	0.322930065	5.79E-03	1.24E-02	1.00E+00
MOCS3	LMNA	0.137961198	-9.15E-05	0.270853607	5.02E-02	8.42E-02	1.00E+00

ANKS1B	LMNA	-0.454987412	-0.557990228	-0.338177859	1.03E-11	1.20E-10	1.22E-07
GPR126	LMNA	0.172283391	0.035066406	0.303124901	1.42E-02	2.76E-02	1.00E+00
ZC3H12A	LMNA	0.537594739	0.431573152	0.628966669	1.59E-16	4.46E-15	1.89E-12
TMEM47	LMNA	0.107470486	-0.031041052	0.241932083	1.28E-01	1.88E-01	1.00E+00
C17orf71	LMNA	-0.106231735	-0.240752012	0.032292853	1.32E-01	1.94E-01	1.00E+00
HIST1H2BN	LMNA	0.030730493	-0.107777741	0.168068484	6.64E-01	7.34E-01	1.00E+00
RAPGEF1	LMNA	0.130973523	-0.007207802	0.264246673	6.32E-02	1.03E-01	1.00E+00
HSF2BP	LMNA	-0.183557378	-0.313660143	-0.046689503	8.93E-03	1.83E-02	1.00E+00
MAP3K8	LMNA	0.265062015	0.131835136	0.38888298	1.38E-04	4.40E-04	1.00E+00
AKAP10	LMNA	-0.11006636	-0.244403702	0.028416422	1.19E-01	1.76E-01	1.00E+00
DLG4	LMNA	-0.343601236	-0.459839961	-0.215785932	5.54E-07	2.78E-06	6.58E-03
STC1	LMNA	0.474587962	0.360131843	0.574968551	9.65E-13	1.35E-11	1.14E-08
RPAP3	LMNA	0.290553075	0.158874721	0.412075266	2.73E-05	9.98E-05	3.24E-01
STOM	LMNA	0.295179308	0.163803257	0.416267492	2.00E-05	7.50E-05	2.37E-01
MUPCDH	LMNA	-0.006261195	-0.144187565	0.131903819	9.30E-01	9.49E-01	1.00E+00
TMEM14A	LMNA	-0.150095796	-0.282297348	-0.012299655	3.30E-02	5.83E-02	1.00E+00
TCP11L1	LMNA	0.253510533	0.119646859	0.378321279	2.72E-04	8.15E-04	1.00E+00
CWF19L1	LMNA	-0.22642257	-0.353426222	-0.091223006	1.19E-03	3.06E-03	1.00E+00
MYH3	LMNA	-0.227700963	-0.354605181	-0.09255951	1.12E-03	2.90E-03	1.00E+00
GPKOW	LMNA	-0.028053145	-0.165463345	0.110425507	6.92E-01	7.58E-01	1.00E+00
YY1AP1	LMNA	0.057014883	-0.08167901	0.193542518	4.20E-01	5.08E-01	1.00E+00
SPON1	LMNA	-0.27017382	-0.393546418	-0.137241625	1.01E-04	3.29E-04	1.00E+00
SULT1A1	LMNA	-0.021753739	-0.159326263	0.116647569	7.59E-01	8.14E-01	1.00E+00
RAB23	LMNA	0.111786034	-0.026676645	0.246040101	1.13E-01	1.69E-01	1.00E+00
PLA2G4A	LMNA	-0.051889294	-0.188589359	0.086783368	4.63E-01	5.49E-01	1.00E+00
MAPRE3	LMNA	-0.320425476	-0.43905486	-0.190815197	3.34E-06	1.44E-05	3.96E-02
SPEF1	LMNA	-0.026972651	-0.164411449	0.111493507	7.03E-01	7.67E-01	1.00E+00
GGPS1	LMNA	-0.191069631	-0.320662406	-0.05445501	6.45E-03	1.37E-02	1.00E+00
C19orf42	LMNA	0.043240536	-0.095379825	0.180215757	5.41E-01	6.23E-01	1.00E+00
MAP2K2	LMNA	0.27749104	0.144994475	0.400210799	6.38E-05	2.17E-04	7.57E-01
HIST1H2BE	LMNA	-0.080484693	-0.216134228	0.058213113	2.55E-01	3.36E-01	1.00E+00
ELN	LMNA	0.229230346	0.094159053	0.356015079	1.03E-03	2.70E-03	1.00E+00
RNF19B	LMNA	0.28232367	0.150123753	0.404605159	4.69E-05	1.63E-04	5.56E-01
MPI	LMNA	0.05873441	-0.079964974	0.195202641	4.06E-01	4.94E-01	1.00E+00
MEPCE	LMNA	0.300532155	0.169514084	0.421111749	1.39E-05	5.35E-05	1.65E-01

TLR8	LMNA	0.211993354	0.076171583	0.340091279	2.46E-03	5.82E-03	1.00E+00
PCDHA9	LMNA	-0.463791729	-0.565627356	-0.348023605	3.62E-12	4.60E-11	4.29E-08
ABCC3	LMNA	0.560953789	0.458401507	0.648764329	3.87E-18	1.43E-16	4.59E-14
HLA-DMB	LMNA	0.088774404	-0.049887993	0.224079225	2.09E-01	2.84E-01	1.00E+00
RRAGA	LMNA	-0.009096405	-0.146962854	0.129116737	8.98E-01	9.26E-01	1.00E+00
CARS2	LMNA	0.243738987	0.109368047	0.369361558	4.73E-04	1.34E-03	1.00E+00
ANGEL1	LMNA	-0.050626832	-0.187368299	0.088039484	4.74E-01	5.60E-01	1.00E+00
CLUL1	LMNA	-0.027425686	-0.164852532	0.111045749	6.98E-01	7.63E-01	1.00E+00
RHAG	LMNA	-0.038486846	-0.175604832	0.100095979	5.87E-01	6.65E-01	1.00E+00
C9orf95	LMNA	0.309652414	0.179264584	0.42934965	7.31E-06	2.97E-05	8.67E-02
C14orf143	LMNA	0.056301245	-0.082390128	0.192853305	4.26E-01	5.13E-01	1.00E+00
CPN1	LMNA	0.042561533	-0.096053848	0.179557514	5.48E-01	6.28E-01	1.00E+00
HLA-A	LMNA	0.453858946	0.336917757	0.557010099	1.17E-11	1.36E-10	1.39E-07
EDNRB	LMNA	-0.317832918	-0.43672181	-0.188032237	4.04E-06	1.72E-05	4.79E-02
LDHA	LMNA	0.481786588	0.368226762	0.58118249	3.90E-13	5.75E-12	4.62E-09
MRPL20	LMNA	-0.03183203	-0.169139756	0.106687804	6.53E-01	7.24E-01	1.00E+00
SCD	LMNA	0.034480602	-0.10406576	0.171714229	6.26E-01	7.01E-01	1.00E+00
C8orf55	LMNA	-0.110029119	-0.244368256	0.028454088	1.19E-01	1.77E-01	1.00E+00
ATP5S	LMNA	-0.353610362	-0.468777401	-0.226622211	2.44E-07	1.31E-06	2.89E-03
RABL4	LMNA	-0.222850258	-0.350129625	-0.087490888	1.43E-03	3.61E-03	1.00E+00
SILV	LMNA	0.133816692	-0.004314003	0.266936435	5.76E-02	9.49E-02	1.00E+00
RCVRN	LMNA	0.148850318	0.011025887	0.281124509	3.45E-02	6.05E-02	1.00E+00
TEX28	LMNA	0.535569909	0.429256393	0.627244951	2.17E-16	5.91E-15	2.57E-12
SHANK1	LMNA	-0.330794595	-0.448370092	-0.201966713	1.52E-06	7.05E-06	1.80E-02
CD226	LMNA	0.108722203	-0.029775707	0.243124103	1.24E-01	1.82E-01	1.00E+00
TCTN1	LMNA	0.261412927	0.127980529	0.385550075	1.71E-04	5.34E-04	1.00E+00
STAT3	LMNA	0.425277277	0.305141095	0.532089337	2.81E-10	2.57E-09	3.33E-06
PPP2R5C	LMNA	-0.097815577	-0.23272398	0.040786181	1.66E-01	2.34E-01	1.00E+00
SYNJ2	LMNA	0.32673014	0.197591591	0.444721777	2.08E-06	9.38E-06	2.46E-02
CAPG	LMNA	0.518176055	0.409412245	0.61241791	2.83E-15	6.09E-14	3.36E-11
MBD4	LMNA	0.275238643	0.142606248	0.398160742	7.36E-05	2.47E-04	8.73E-01
SLAMF8	LMNA	0.421703906	0.301187003	0.528960595	4.10E-10	3.63E-09	4.86E-06
ROBO1	LMNA	-0.059291264	-0.195740089	0.079409722	4.02E-01	4.89E-01	1.00E+00
ST3GAL6	LMNA	-0.355692289	-0.470633457	-0.228880145	2.05E-07	1.11E-06	2.43E-03
ATN1	LMNA	0.045928674	-0.092710155	0.182820502	5.16E-01	6.00E-01	1.00E+00

MPZL1	LMNA	0.018290391	-0.120063771	0.155947623	7.96E-01	8.46E-01	1.00E+00
ARSB	LMNA	0.388442683	0.264579745	0.499697457	1.11E-08	7.53E-08	1.32E-04
KRT36	LMNA	-0.081188652	-0.216809613	0.057506896	2.51E-01	3.31E-01	1.00E+00
MAD1L1	LMNA	0.230170438	0.095142619	0.356881439	9.83E-04	2.58E-03	1.00E+00
DDX52	LMNA	-0.235453778	-0.361746348	-0.100675187	7.43E-04	2.01E-03	1.00E+00
AMPD1	LMNA	-0.111034957	-0.245325489	0.027436605	1.16E-01	1.72E-01	1.00E+00
INPP1	LMNA	-0.45895149	-0.561430948	-0.34260767	6.44E-12	7.84E-11	7.64E-08
DPEP3	LMNA	0.126482738	-0.011773846	0.259993966	7.29E-02	1.16E-01	1.00E+00
DENND4A	LMNA	-0.159862	-0.29148029	-0.022303211	2.31E-02	4.24E-02	1.00E+00
ANKRD11	LMNA	0.09609926	-0.042515781	0.231084554	1.74E-01	2.43E-01	1.00E+00
EIF5A2	LMNA	0.174268707	0.03711051	0.304982449	1.31E-02	2.58E-02	1.00E+00
CAP1	LMNA	0.387589803	0.263645737	0.498943741	1.21E-08	8.11E-08	1.43E-04
NT5DC3	LMNA	0.064865438	-0.073846842	0.201115502	3.59E-01	4.46E-01	1.00E+00
SEZ6L2	LMNA	0.16146998	0.023952916	0.292989916	2.17E-02	4.02E-02	1.00E+00
Sep-09	LMNA	0.266087465	0.132919065	0.389818994	1.29E-04	4.15E-04	1.00E+00
GUCY2D	LMNA	0.247665363	0.1134948	0.372964537	3.80E-04	1.10E-03	1.00E+00
VPS11	LMNA	-0.057138707	-0.19366209	0.081555609	4.19E-01	5.07E-01	1.00E+00
CPM	LMNA	0.056354652	-0.082336915	0.192904888	4.26E-01	5.12E-01	1.00E+00
NDUFB5	LMNA	-0.233452568	-0.359904438	-0.098578593	8.27E-04	2.22E-03	1.00E+00
CIDEA	LMNA	-0.157690851	-0.289440903	-0.020076913	2.50E-02	4.56E-02	1.00E+00
SLC26A4	LMNA	-0.042572385	-0.179568035	0.096043077	5.47E-01	6.28E-01	1.00E+00
FAM59A	LMNA	-0.119054281	-0.25294794	0.019314159	9.15E-02	1.41E-01	1.00E+00
N6AMT1	LMNA	0.058468326	-0.080230262	0.1949458	4.08E-01	4.96E-01	1.00E+00
FBXO5	LMNA	-0.078918188	-0.214630843	0.059784145	2.64E-01	3.46E-01	1.00E+00
SIPA1L1	LMNA	0.462669569	0.346767288	0.564654932	4.14E-12	5.20E-11	4.91E-08
OXR1	LMNA	-0.118739336	-0.252648895	0.019633498	9.24E-02	1.42E-01	1.00E+00
DGKB	LMNA	-0.601773832	-0.683077815	-0.505737191	2.76E-21	1.69E-19	3.27E-17
DPYS	LMNA	-0.122465724	-0.25618552	0.015853301	8.25E-02	1.29E-01	1.00E+00
GCN5L2	LMNA	-0.237737199	-0.3638468	-0.103068906	6.57E-04	1.80E-03	1.00E+00
MIR16	LMNA	-0.024548464	-0.162050286	0.113888498	7.29E-01	7.89E-01	1.00E+00
TGM3	LMNA	0.102049297	-0.036516143	0.236764754	1.48E-01	2.13E-01	1.00E+00
MTCH1	LMNA	-0.138567812	-0.271426578	-0.00052689	4.92E-02	8.28E-02	1.00E+00
WDR4	LMNA	0.425875977	0.30580399	0.532613257	2.64E-10	2.43E-09	3.13E-06
HK1	LMNA	0.34072674	0.212679697	0.45726888	6.98E-07	3.44E-06	8.28E-03
PDC	LMNA	0.07204539	-0.066668693	0.208027363	3.08E-01	3.93E-01	1.00E+00

VPS33B	LMNA	-0.15633061	-0.288162601	-0.018682814	2.63E-02	4.76E-02	1.00E+00
HEXB	LMNA	0.563962051	0.461870198	0.651305333	2.35E-18	9.18E-17	2.78E-14
GALNT12	LMNA	0.28886364	0.157076524	0.41054304	3.06E-05	1.11E-04	3.62E-01
LOC339229	LMNA	-0.063050884	-0.199366551	0.075658668	3.73E-01	4.60E-01	1.00E+00
MRPL35	LMNA	0.24421643	0.109869609	0.369799878	4.61E-04	1.31E-03	1.00E+00
ORC4L	LMNA	-0.472869535	-0.57348347	-0.358202001	1.19E-12	1.63E-11	1.42E-08
TCEB3	LMNA	0.310604427	0.180283864	0.430208413	6.83E-06	2.79E-05	8.10E-02
TNKS	LMNA	-0.157550209	-0.289308755	-0.019932745	2.51E-02	4.58E-02	1.00E+00
C2orf24	LMNA	0.173821329	0.036649784	0.30456395	1.34E-02	2.62E-02	1.00E+00
CRLF1	LMNA	-0.173850854	-0.304591571	-0.036680189	1.33E-02	2.62E-02	1.00E+00
GGTLA1	LMNA	0.368146498	0.242415794	0.481715286	7.03E-08	4.16E-07	8.34E-04
PYCR1	LMNA	0.060314936	-0.078388771	0.196727875	3.94E-01	4.81E-01	1.00E+00
CDK5R2	LMNA	-0.167981548	-0.299096495	-0.030641115	1.69E-02	3.21E-02	1.00E+00
WAS	LMNA	0.314869931	0.184854193	0.434053449	5.02E-06	2.10E-05	5.95E-02
CCBL2	LMNA	0.015766943	-0.122550807	0.153483864	8.24E-01	8.67E-01	1.00E+00
MADD	LMNA	-0.216144485	-0.343932886	-0.080495383	2.00E-03	4.86E-03	1.00E+00
SLC30A4	LMNA	0.025959751	-0.112494403	0.163425074	7.14E-01	7.76E-01	1.00E+00
WDR42A	LMNA	-0.172062448	-0.302918115	-0.034838991	1.43E-02	2.78E-02	1.00E+00
TUB	LMNA	-0.52867477	-0.621375267	-0.421377628	6.11E-16	1.53E-14	7.24E-12
KLF12	LMNA	-0.450762246	-0.554318986	-0.333461977	1.68E-11	1.89E-10	1.99E-07
ARHGEF18	LMNA	0.214285304	0.078558237	0.342212861	2.20E-03	5.27E-03	1.00E+00
ARRB1	LMNA	-0.149513009	-0.2817486	-0.011703575	3.37E-02	5.93E-02	1.00E+00
KCNK1	LMNA	-0.353948396	-0.46907883	-0.22698873	2.37E-07	1.27E-06	2.81E-03
HSPA1A	LMNA	0.26320026	0.129868021	0.387182945	1.54E-04	4.86E-04	1.00E+00
ITM2C	LMNA	-0.239489126	-0.365457477	-0.104906515	5.97E-04	1.66E-03	1.00E+00
DAPK2	LMNA	-0.128532979	-0.261936157	0.009689961	6.83E-02	1.10E-01	1.00E+00
RUNDC3B	LMNA	-0.51978529	-0.613792459	-0.411243866	2.24E-15	4.94E-14	2.66E-11
SCAMP5	LMNA	-0.561233508	-0.649000684	-0.458723907	3.69E-18	1.37E-16	4.38E-14
EREG	LMNA	0.433575781	0.314339767	0.539344037	1.15E-10	1.14E-09	1.37E-06
IL17RB	LMNA	-0.254543018	-0.379266621	-0.120734622	2.56E-04	7.71E-04	1.00E+00
CENPA	LMNA	0.025077149	-0.113366317	0.162565361	7.23E-01	7.84E-01	1.00E+00
TMED5	LMNA	0.165874526	0.028475592	0.297121694	1.83E-02	3.45E-02	1.00E+00
MCAM	LMNA	0.332726872	0.204048487	0.450103159	1.31E-06	6.15E-06	1.55E-02
FLJ20323	LMNA	0.073641108	-0.065071424	0.209561657	2.98E-01	3.82E-01	1.00E+00
CDH6	LMNA	0.290962373	0.1593105	0.412446373	2.66E-05	9.73E-05	3.15E-01

POLR3E	LMNA	0.022129094	-0.116277126	0.159692244	7.55E-01	8.11E-01	1.00E+00
BRP44	LMNA	-0.135798296	-0.268809893	0.002295743	5.40E-02	8.98E-02	1.00E+00
AQR	LMNA	0.192066884	0.055487126	0.321590894	6.17E-03	1.32E-02	1.00E+00
THG1L	LMNA	0.299625297	0.16854596	0.420291539	1.48E-05	5.67E-05	1.75E-01
CAMP	LMNA	0.213111328	0.077335558	0.341126313	2.33E-03	5.55E-03	1.00E+00
GABRA2	LMNA	-0.290031758	-0.411602533	-0.158319749	2.83E-05	1.03E-04	3.35E-01
C14orf166	LMNA	0.003658722	-0.134460194	0.141638182	9.59E-01	9.69E-01	1.00E+00
ADAMTSL2	LMNA	-0.075051774	-0.210917465	0.063658793	2.88E-01	3.72E-01	1.00E+00
MYL1	LMNA	0.013332866	-0.124948116	0.151105739	8.51E-01	8.89E-01	1.00E+00
TNFSF18	LMNA	-0.061991982	-0.19834553	0.076715555	3.81E-01	4.68E-01	1.00E+00
PPIB	LMNA	0.579529356	0.47987018	0.664423421	1.62E-19	7.57E-18	1.92E-15
KLHL4	LMNA	0.296618853	0.165338209	0.417570938	1.82E-05	6.85E-05	2.15E-01
SFN	LMNA	0.247567835	0.11339224	0.372875089	3.82E-04	1.11E-03	1.00E+00
FRAP1	LMNA	-0.095415438	-0.230431154	0.043204663	1.77E-01	2.47E-01	1.00E+00
CLMN	LMNA	0.323620807	0.194248067	0.441928148	2.63E-06	1.16E-05	3.12E-02
GOLGA5	LMNA	0.245284833	0.110992231	0.370780531	4.34E-04	1.24E-03	1.00E+00
SDCCAG1	LMNA	-0.208755214	-0.337091617	-0.072802293	2.87E-03	6.67E-03	1.00E+00
SSTR3	LMNA	0.001537118	-0.136542857	0.139558504	9.83E-01	9.86E-01	1.00E+00
MAGEA5	LMNA	0.003618467	-0.134499721	0.141598734	9.59E-01	9.70E-01	1.00E+00
OVOL2	LMNA	-0.171385665	-0.302284622	-0.034142474	1.47E-02	2.85E-02	1.00E+00
C10orf95	LMNA	-0.11755158	-0.251520871	0.020837575	9.57E-02	1.46E-01	1.00E+00
RBL2	LMNA	-0.22752175	-0.354439932	-0.092372121	1.13E-03	2.92E-03	1.00E+00
JMJD1B	LMNA	-0.297833334	-0.418670211	-0.166633675	1.67E-05	6.35E-05	1.98E-01
PPP1R10	LMNA	-0.072031419	-0.208013927	0.066682675	3.08E-01	3.93E-01	1.00E+00
C1orf163	LMNA	0.027847175	-0.110629121	0.16526285	6.94E-01	7.60E-01	1.00E+00
CSE1L	LMNA	0.00131054	-0.136765204	0.139336332	9.85E-01	9.88E-01	1.00E+00
ASRGL1	LMNA	-0.556133716	-0.644688849	-0.452850206	8.53E-18	2.98E-16	1.01E-13
RDH16	LMNA	-0.138046765	-0.270934434	4.32E-06	5.01E-02	8.41E-02	1.00E+00
TOM1	LMNA	0.277148454	0.14463113	0.399899067	6.52E-05	2.21E-04	7.74E-01
PTX3	LMNA	0.516575184	0.407591015	0.61104994	3.56E-15	7.56E-14	4.22E-11
CENPN	LMNA	-0.011839298	-0.149645713	0.126418324	8.67E-01	9.02E-01	1.00E+00
TTC15	LMNA	-0.022185471	-0.15974721	0.116221484	7.54E-01	8.10E-01	1.00E+00
TMED2	LMNA	0.223600644	0.088274527	0.350822357	1.38E-03	3.49E-03	1.00E+00
ANG	LMNA	0.523738727	0.415747365	0.617166921	1.26E-15	2.94E-14	1.50E-11
RCAN3	LMNA	0.402484096	0.279990289	0.512082109	2.89E-09	2.19E-08	3.42E-05

CINP	LMNA	0.090543241	-0.048109104	0.225772183	2.00E-01	2.74E-01	1.00E+00
U2AF1	LMNA	0.196271409	0.059841852	0.325502762	5.12E-03	1.12E-02	1.00E+00
NMT2	LMNA	-0.332901561	-0.450259795	-0.204236749	1.29E-06	6.08E-06	1.53E-02
OSGEPL1	LMNA	-0.365680147	-0.479523593	-0.239731388	8.72E-08	5.05E-07	1.03E-03
DFNB31	LMNA	0.380420369	0.255803507	0.492601228	2.34E-08	1.50E-07	2.78E-04
Mar-07	LMNA	-0.061201767	-0.197583393	0.077504062	3.87E-01	4.74E-01	1.00E+00
SLC6A20	LMNA	-0.131766805	-0.26499736	0.006400627	6.16E-02	1.01E-01	1.00E+00
PFKM	LMNA	-0.26296646	-0.386969395	-0.129621065	1.56E-04	4.92E-04	1.00E+00
SGMS1	LMNA	-0.243954724	-0.369559624	-0.109594675	4.67E-04	1.33E-03	1.00E+00
DKC1	LMNA	0.111461098	-0.027005444	0.245730959	1.14E-01	1.70E-01	1.00E+00
MGC5590	LMNA	-0.076731986	-0.212531655	0.061975516	2.78E-01	3.61E-01	1.00E+00
CREBZF	LMNA	-0.128184222	-0.261605855	0.010044527	6.91E-02	1.11E-01	1.00E+00
RIOK3	LMNA	0.217485619	0.081893394	0.345173105	1.88E-03	4.58E-03	1.00E+00
PRPSAP1	LMNA	-0.339830136	-0.456466516	-0.211711337	7.50E-07	3.67E-06	8.89E-03
GCHFR	LMNA	0.312940068	0.182785719	0.432314358	5.77E-06	2.38E-05	6.85E-02
LOC196993	LMNA	-0.092681714	-0.227817831	0.045957301	1.90E-01	2.62E-01	1.00E+00
UBD	LMNA	0.194888247	0.058408704	0.324216363	5.45E-03	1.18E-02	1.00E+00
ATG9A	LMNA	0.069307948	-0.069407142	0.205393735	3.27E-01	4.13E-01	1.00E+00
S100A1	LMNA	-0.290211598	-0.41176562	-0.15851119	2.79E-05	1.02E-04	3.31E-01
RPL6	LMNA	0.078366681	-0.060337082	0.214101405	2.68E-01	3.49E-01	1.00E+00
TMEM40	LMNA	-0.055669263	-0.192242839	0.083019761	4.31E-01	5.18E-01	1.00E+00
DNAJB6	LMNA	-0.035991634	-0.173182147	0.102569	6.11E-01	6.87E-01	1.00E+00
ELP3	LMNA	-0.173366016	-0.304137976	-0.036180947	1.36E-02	2.66E-02	1.00E+00
ZNF787	LMNA	0.164633077	0.027200278	0.295957623	1.92E-02	3.60E-02	1.00E+00
KERA	LMNA	0.244519413	0.110187932	0.370078005	4.53E-04	1.29E-03	1.00E+00
PELI1	LMNA	-0.419792299	-0.527285648	-0.299073425	5.00E-10	4.37E-09	5.93E-06
PPT1	LMNA	-0.051399003	-0.188115198	0.087271246	4.68E-01	5.53E-01	1.00E+00
SLC35C2	LMNA	0.369957115	0.244387716	0.483323366	6.00E-08	3.59E-07	7.11E-04
MT1X	LMNA	0.327622575	0.198551799	0.445523175	1.94E-06	8.82E-06	2.30E-02
UBE2B	LMNA	-0.037601708	-0.174745612	0.100973441	5.95E-01	6.73E-01	1.00E+00
KEAP1	LMNA	0.212683707	0.076890297	0.340730453	2.37E-03	5.65E-03	1.00E+00
MST1	LMNA	-0.07442713	-0.210317176	0.064284376	2.92E-01	3.76E-01	1.00E+00
MUC4	LMNA	-0.170189758	-0.301164929	-0.032912021	1.55E-02	2.97E-02	1.00E+00
RFC4	LMNA	-0.125035503	-0.258622352	0.013244108	7.62E-02	1.21E-01	1.00E+00
BCL2L13	LMNA	0.150723868	0.012942162	0.28288864	3.23E-02	5.71E-02	1.00E+00

GNB2	LMNA	0.491648149	0.379344194	0.589676332	1.09E-13	1.73E-12	1.29E-09
NUP50	LMNA	0.005963832	-0.132196008	0.143896361	9.33E-01	9.51E-01	1.00E+00
SULT4A1	LMNA	-0.259401532	-0.383711575	-0.125857571	1.93E-04	5.95E-04	1.00E+00
S100A8	LMNA	0.267416929	0.134324822	0.391032129	1.19E-04	3.85E-04	1.00E+00
C7	LMNA	0.155266761	0.017592858	0.28716251	2.74E-02	4.93E-02	1.00E+00
CCDC130	LMNA	0.082510938	-0.056179993	0.218077875	2.43E-01	3.23E-01	1.00E+00
RQCD1	LMNA	-0.177726733	-0.308215556	-0.040673675	1.14E-02	2.28E-02	1.00E+00
AYTL2	LMNA	0.345098661	0.217405104	0.461178555	4.91E-07	2.49E-06	5.83E-03
MTUS1	LMNA	-0.115891149	-0.249943334	0.022520149	1.00E-01	1.53E-01	1.00E+00
ARFIP2	LMNA	0.134070312	-0.004055755	0.267176269	5.71E-02	9.42E-02	1.00E+00
UROS	LMNA	-0.244657616	-0.370204863	-0.110333143	4.50E-04	1.28E-03	1.00E+00
LEMD3	LMNA	-0.233506661	-0.359954239	-0.098635249	8.24E-04	2.21E-03	1.00E+00
PLEKHF2	LMNA	0.012846893	-0.125426555	0.150630746	8.56E-01	8.94E-01	1.00E+00
KHDRBS2	LMNA	-0.458111816	-0.560702428	-0.341668912	7.12E-12	8.62E-11	8.44E-08
HOXA7	LMNA	-0.097751534	-0.232662821	0.040850735	1.66E-01	2.34E-01	1.00E+00
POLQ	LMNA	-0.13679098	-0.269748024	0.001284272	5.22E-02	8.72E-02	1.00E+00
GTF3C2	LMNA	-0.026263686	-0.163721078	0.1121941	7.11E-01	7.73E-01	1.00E+00
SOAT1	LMNA	0.453967246	0.337038672	0.557104175	1.16E-11	1.34E-10	1.37E-07
SPAG4	LMNA	0.515491693	0.406358874	0.610123758	4.15E-15	8.70E-14	4.93E-11
MRPS30	LMNA	0.276900597	0.144368277	0.399673516	6.63E-05	2.24E-04	7.86E-01
MR1	LMNA	0.439047233	0.320416999	0.544118704	6.31E-11	6.55E-10	7.48E-07
UBE1L2	LMNA	-0.024650633	-0.16214983	0.113787592	7.28E-01	7.88E-01	1.00E+00
F8	LMNA	0.19822178	0.061863661	0.327315875	4.69E-03	1.03E-02	1.00E+00
KPNA5	LMNA	-0.307268377	-0.427198183	-0.176713327	8.66E-06	3.46E-05	1.03E-01
ACHE	LMNA	0.138032021	-1.93E-05	0.270920507	5.01E-02	8.41E-02	1.00E+00
TNFRSF12/	LMNA	0.719670336	0.645778179	0.780206843	1.60E-33	1.05E-30	1.89E-29
EGR3	LMNA	-0.003020864	-0.141013057	0.135086473	9.66E-01	9.75E-01	1.00E+00
TCEB3B	LMNA	0.272304259	0.139497221	0.39548812	8.84E-05	2.91E-04	1.00E+00
ZNF184	LMNA	-0.391918193	-0.50276713	-0.268388259	8.02E-09	5.58E-08	9.51E-05
OASL	LMNA	0.22693404	0.091757667	0.353897959	1.16E-03	2.99E-03	1.00E+00
SERPIND1	LMNA	0.167333324	0.029974756	0.298489068	1.73E-02	3.28E-02	1.00E+00
IFRD1	LMNA	0.159964924	0.022408784	0.291576939	2.30E-02	4.22E-02	1.00E+00
SPINLW1	LMNA	0.104763459	-0.033776035	0.239352772	1.38E-01	2.00E-01	1.00E+00
KCTD9	LMNA	0.508387782	0.398290155	0.604044837	1.13E-14	2.17E-13	1.34E-10
PRR14	LMNA	-0.024116739	-0.16162962	0.114314855	7.33E-01	7.93E-01	1.00E+00

ZNF267	LMNA	0.2769496	0.144420243	0.399718111	6.60E-05	2.24E-04	7.83E-01
PPP1R3A	LMNA	-0.062742165	-0.199068907	0.075966831	3.75E-01	4.63E-01	1.00E+00
ZBTB6	LMNA	-0.224360711	-0.351523885	-0.089068447	1.33E-03	3.37E-03	1.00E+00
ACTN2	LMNA	-0.26496368	-0.388793208	-0.13173121	1.39E-04	4.42E-04	1.00E+00
TXNIP	LMNA	-0.056818932	-0.193353287	0.081874283	4.22E-01	5.09E-01	1.00E+00
NUDT3	LMNA	-0.314217971	-0.433466036	-0.184155277	5.26E-06	2.19E-05	6.24E-02
DFNA5	LMNA	0.200780714	0.064518022	0.329693283	4.17E-03	9.30E-03	1.00E+00
RPL36AL	LMNA	0.156473242	0.018828972	0.288296664	2.62E-02	4.74E-02	1.00E+00
KLK15	LMNA	0.267939939	0.134877991	0.391509256	1.16E-04	3.74E-04	1.00E+00
RAP2B	LMNA	0.4143895	0.293106192	0.522547203	8.74E-10	7.30E-09	1.04E-05
CHAD	LMNA	-0.111525813	-0.24579253	0.026939962	1.14E-01	1.70E-01	1.00E+00
HEBP2	LMNA	0.375941693	0.250912851	0.488633065	3.52E-08	2.17E-07	4.17E-04
GABPA	LMNA	0.207632982	0.071635332	0.336051424	3.03E-03	7.00E-03	1.00E+00
CAMK2G	LMNA	-0.469370296	-0.57045735	-0.354275285	1.84E-12	2.46E-11	2.18E-08
TLR3	LMNA	0.121578033	-0.016754169	0.255343359	8.48E-02	1.32E-01	1.00E+00
FGF14	LMNA	-0.38707935	-0.498492557	-0.263086839	1.27E-08	8.50E-08	1.50E-04
HMGB2	LMNA	-0.019265296	-0.156899009	0.119102469	7.86E-01	8.37E-01	1.00E+00
POLB	LMNA	-0.423035975	-0.530127257	-0.302660511	3.56E-10	3.18E-09	4.22E-06
KIF3B	LMNA	0.128977866	-0.009237615	0.262357455	6.73E-02	1.09E-01	1.00E+00
C3orf27	LMNA	-0.039230088	-0.17632615	0.099359017	5.79E-01	6.58E-01	1.00E+00
MTMR9	LMNA	-0.480367172	-0.57995815	-0.366629252	4.67E-13	6.83E-12	5.54E-09
SEC31A	LMNA	0.560494684	0.457872409	0.648376363	4.17E-18	1.53E-16	4.95E-14
TRIM58	LMNA	-0.097954688	-0.232856825	0.040645958	1.65E-01	2.33E-01	1.00E+00
TAS2R14	LMNA	-0.30009994	-0.420720855	-0.169052637	1.43E-05	5.50E-05	1.70E-01
NSDHL	LMNA	0.174214772	0.037054962	0.304931998	1.31E-02	2.58E-02	1.00E+00
GLRA3	LMNA	-0.355993266	-0.470901696	-0.229206679	2.00E-07	1.09E-06	2.37E-03
VPS8	LMNA	-0.231226775	-0.357854666	-0.096248119	9.30E-04	2.46E-03	1.00E+00
H1F0	LMNA	-0.099188355	-0.234034707	0.039402181	1.60E-01	2.27E-01	1.00E+00
PRKCB1	LMNA	-0.184239233	-0.314296292	-0.047393662	8.67E-03	1.79E-02	1.00E+00
POLR2D	LMNA	-0.027330002	-0.164759376	0.111140324	6.99E-01	7.64E-01	1.00E+00
UGT2A1	LMNA	-0.00881851	-0.146690928	0.129390011	9.01E-01	9.28E-01	1.00E+00
TOR1B	LMNA	0.408839642	0.286986359	0.517672816	1.53E-09	1.23E-08	1.82E-05
LSS	LMNA	0.233949029	0.099098606	0.360361472	8.05E-04	2.16E-03	1.00E+00
TOPORS	LMNA	-0.211603104	-0.339729907	-0.075765362	2.50E-03	5.92E-03	1.00E+00
HNRNPC	LMNA	-0.164119599	-0.295476036	-0.026672924	1.96E-02	3.66E-02	1.00E+00

TMEM100	LMNA	-0.312838242	-0.432222573	-0.182676612	5.82E-06	2.40E-05	6.90E-02
DHRS9	LMNA	-0.384725736	-0.496411445	-0.260510933	1.58E-08	1.04E-07	1.87E-04
ISG15	LMNA	-0.012756204	-0.150542099	0.125515831	8.57E-01	8.95E-01	1.00E+00
DNAH2	LMNA	-0.216249029	-0.344029578	-0.080604341	1.99E-03	4.84E-03	1.00E+00
ZCCHC14	LMNA	-0.232976997	-0.359466579	-0.098080532	8.48E-04	2.26E-03	1.00E+00
FXR1	LMNA	0.076201338	-0.062507218	0.212021938	2.81E-01	3.64E-01	1.00E+00
ZMYM3	LMNA	-0.103481713	-0.238130835	0.035070293	1.43E-01	2.06E-01	1.00E+00
CREBL2	LMNA	0.157811245	0.020200329	0.289554021	2.49E-02	4.54E-02	1.00E+00
TGDS	LMNA	-0.024071141	-0.161585186	0.114359883	7.34E-01	7.93E-01	1.00E+00
CASP3	LMNA	0.302887152	0.172029366	0.423240815	1.18E-05	4.61E-05	1.40E-01
FAM120C	LMNA	0.007819408	-0.130372326	0.145713115	9.12E-01	9.37E-01	1.00E+00
SCLY	LMNA	-0.099824686	-0.234642108	0.038760468	1.58E-01	2.24E-01	1.00E+00
CACNA2D3	LMNA	-0.218503885	-0.346114454	-0.082955201	1.78E-03	4.38E-03	1.00E+00
CA7	LMNA	-0.178648692	-0.309077039	-0.041624255	1.10E-02	2.20E-02	1.00E+00
ENTPD5	LMNA	0.353572038	0.22658066	0.468743225	2.45E-07	1.31E-06	2.90E-03
SUCLG1	LMNA	-0.329719406	-0.447405367	-0.200808841	1.65E-06	7.63E-06	1.96E-02
PDIA5	LMNA	0.597004429	0.500176505	0.67908707	6.79E-21	3.93E-19	8.05E-17
KCTD20	LMNA	-0.158422464	-0.29012825	-0.020826957	2.43E-02	4.45E-02	1.00E+00
WDR47	LMNA	-0.471671405	-0.572447643	-0.356857047	1.39E-12	1.88E-11	1.64E-08
KLRF1	LMNA	0.04712196	-0.091524429	0.183976153	5.05E-01	5.89E-01	1.00E+00
MAGEH1	LMNA	-0.438128868	-0.543317767	-0.319396272	6.98E-11	7.20E-10	8.28E-07
PRPF40A	LMNA	0.108786344	-0.029710856	0.243185174	1.23E-01	1.82E-01	1.00E+00
SMR3A	LMNA	0.324743135	0.195454581	0.442936785	2.41E-06	1.08E-05	2.86E-02
TAS2R16	LMNA	0.051830236	-0.086842139	0.188532248	4.64E-01	5.50E-01	1.00E+00
SPINK2	LMNA	0.034850695	-0.103699221	0.172073818	6.22E-01	6.97E-01	1.00E+00
NPY5R	LMNA	-0.097423726	-0.232349752	0.041181138	1.68E-01	2.36E-01	1.00E+00
IRF4	LMNA	0.234506552	0.099682664	0.360874646	7.82E-04	2.11E-03	1.00E+00
PRKCE	LMNA	-0.496117368	-0.593518578	-0.38439327	6.00E-14	9.81E-13	7.12E-10
ITGAM	LMNA	0.392303564	0.268810791	0.503107328	7.73E-09	5.40E-08	9.17E-05
RGS2	LMNA	0.294589213	0.163174237	0.415733042	2.08E-05	7.78E-05	2.47E-01
DDX19A	LMNA	0.178159151	0.041119486	0.308619636	1.12E-02	2.24E-02	1.00E+00
PDPN	LMNA	0.50380865	0.393098131	0.600120497	2.12E-14	3.85E-13	2.52E-10
TMEM34	LMNA	-0.020597645	-0.158198803	0.11778829	7.71E-01	8.25E-01	1.00E+00
MST1R	LMNA	-0.136204855	-0.26919414	0.001881524	5.33E-02	8.87E-02	1.00E+00
MGAM	LMNA	0.02421788	-0.114214975	0.161728175	7.32E-01	7.92E-01	1.00E+00

COL3A1	LMNA	0.606826252	0.511636567	0.687300083	1.05E-21	6.76E-20	1.24E-17
PTMA	LMNA	0.01174876	-0.126507427	0.149557189	8.68E-01	9.02E-01	1.00E+00
PRDM11	LMNA	-0.277008633	-0.399771832	-0.144482847	6.58E-05	2.23E-04	7.80E-01
NAPA	LMNA	0.074346255	-0.064365365	0.210239447	2.93E-01	3.77E-01	1.00E+00
DIRAS3	LMNA	0.516384809	0.407374493	0.610887224	3.66E-15	7.76E-14	4.34E-11
LIPF	LMNA	0.089823596	-0.048832944	0.225083508	2.04E-01	2.78E-01	1.00E+00
PSG9	LMNA	0.019012882	-0.119351386	0.156652709	7.88E-01	8.40E-01	1.00E+00
ASGR2	LMNA	0.235412284	0.100631704	0.361708167	7.45E-04	2.02E-03	1.00E+00
ARHGEF11	LMNA	0.005525429	-0.132626738	0.143466994	9.38E-01	9.54E-01	1.00E+00
PIK3R4	LMNA	0.055142921	-0.083544062	0.191734337	4.36E-01	5.22E-01	1.00E+00
IVNS1ABP	LMNA	-0.029016761	-0.166401193	0.109472765	6.82E-01	7.50E-01	1.00E+00
SIGIRR	LMNA	0.094063241	-0.044566478	0.229138755	1.83E-01	2.54E-01	1.00E+00
BTBD3	LMNA	-0.196137081	-0.325377851	-0.059702644	5.15E-03	1.12E-02	1.00E+00
UBE2NL	LMNA	-0.222769859	-0.350055395	-0.087406936	1.44E-03	3.62E-03	1.00E+00
PPP1R2	LMNA	-0.039073167	-0.17617387	0.099514624	5.81E-01	6.59E-01	1.00E+00
FOSL2	LMNA	0.613748916	0.519734302	0.693076481	2.69E-22	2.03E-20	3.19E-18
IL1RAPL2	LMNA	-0.206182731	-0.334706729	-0.07012783	3.24E-03	7.44E-03	1.00E+00
C4orf30	LMNA	-0.211443176	-0.339581803	-0.075598903	2.52E-03	5.96E-03	1.00E+00
TUBA4A	LMNA	0.116122628	-0.02228563	0.250163299	9.98E-02	1.52E-01	1.00E+00
Sep-04	LMNA	-0.196536269	-0.325749037	-0.060116347	5.06E-03	1.10E-02	1.00E+00
SFRS2	LMNA	-0.081356722	-0.216970842	0.057338266	2.50E-01	3.30E-01	1.00E+00
EEF2	LMNA	0.186271152	0.049492856	0.316191318	7.95E-03	1.66E-02	1.00E+00
ZDHHC11	LMNA	-0.26447963	-0.388351274	-0.13121968	1.43E-04	4.54E-04	1.00E+00
HNF1A	LMNA	-0.009699257	-0.147552686	0.128523836	8.91E-01	9.21E-01	1.00E+00
EPHA3	LMNA	0.375050352	0.249940279	0.487842769	3.81E-08	2.34E-07	4.52E-04
PRDX3	LMNA	-0.087819791	-0.223165223	0.050847667	2.14E-01	2.90E-01	1.00E+00
P4HA2	LMNA	0.663933575	0.578946937	0.734649071	4.81E-27	8.92E-25	5.71E-23
RFWD3	LMNA	0.08199263	-0.056700169	0.217580798	2.46E-01	3.26E-01	1.00E+00
RBM12	LMNA	-0.236143347	-0.362380797	-0.1013979	7.16E-04	1.95E-03	1.00E+00
H2AFJ	LMNA	0.318741189	0.189006975	0.437539347	3.78E-06	1.62E-05	4.48E-02
EDIL3	LMNA	-0.0771692	-0.212951567	0.061537376	2.75E-01	3.57E-01	1.00E+00
LOC20038:	LMNA	-0.294463252	-0.415618949	-0.163039981	2.10E-05	7.85E-05	2.49E-01
SERGEF	LMNA	0.052859486	-0.085817753	0.189527447	4.55E-01	5.41E-01	1.00E+00
B3GALT4	LMNA	0.219510456	0.084005116	0.347044739	1.70E-03	4.19E-03	1.00E+00
SMC2	LMNA	-0.05066587	-0.187406064	0.088000648	4.74E-01	5.59E-01	1.00E+00

LOC90925	LMNA	-0.216915673	-0.344646099	-0.081299212	1.93E-03	4.70E-03	1.00E+00
NPFF	LMNA	-0.043075066	-0.180055358	0.095544092	5.43E-01	6.24E-01	1.00E+00
DEDD	LMNA	0.027858997	-0.110617434	0.165274359	6.94E-01	7.60E-01	1.00E+00
SCYL2	LMNA	0.112829272	-0.025620803	0.247032447	1.10E-01	1.65E-01	1.00E+00
PTPN1	LMNA	0.538127094	0.432182492	0.629419183	1.47E-16	4.14E-15	1.74E-12
ZNF174	LMNA	-0.139216306	-0.272039002	-0.00118814	4.82E-02	8.12E-02	1.00E+00
MYH14	LMNA	-0.158072012	-0.289799019	-0.020467657	2.47E-02	4.50E-02	1.00E+00
CCR8	LMNA	-0.059267493	-0.195717148	0.079433427	4.02E-01	4.90E-01	1.00E+00
SKAP1	LMNA	0.114395816	-0.024034752	0.248522043	1.05E-01	1.59E-01	1.00E+00
GADD45G	LMNA	-0.465501391	-0.567108346	-0.349938462	2.94E-12	3.81E-11	3.49E-08
PILRB	LMNA	-0.048167228	-0.184988144	0.090485459	4.96E-01	5.81E-01	1.00E+00
IFITM3	LMNA	0.556620419	0.453410382	0.645100596	7.88E-18	2.77E-16	9.35E-14
DNMT3L	LMNA	0.111834943	-0.026627152	0.24608663	1.13E-01	1.69E-01	1.00E+00
GPATCH1	LMNA	0.023933535	-0.114495766	0.161451093	7.35E-01	7.94E-01	1.00E+00
VPS37C	LMNA	0.415641771	0.294488448	0.523646084	7.69E-10	6.50E-09	9.12E-06
DSC3	LMNA	0.018488624	-0.119868325	0.156141094	7.94E-01	8.44E-01	1.00E+00
TBX4	LMNA	0.250596969	0.11657904	0.375652221	3.22E-04	9.48E-04	1.00E+00
B3GNT3	LMNA	0.252386837	0.118463368	0.377292129	2.90E-04	8.64E-04	1.00E+00
LHCGR	LMNA	-0.05244761	-0.189129231	0.086227718	4.59E-01	5.44E-01	1.00E+00
SAP130	LMNA	-0.215565058	-0.34339692	-0.079891548	2.06E-03	4.99E-03	1.00E+00
UBE2S	LMNA	-0.009960794	-0.147808544	0.128266585	8.88E-01	9.19E-01	1.00E+00
CNR2	LMNA	0.105196046	-0.03333912	0.239765077	1.36E-01	1.98E-01	1.00E+00
PCDH1	LMNA	0.270061216	0.137122445	0.39344376	1.02E-04	3.31E-04	1.00E+00
ATP6V1B2	LMNA	-0.026099177	-0.163560865	0.112356646	7.12E-01	7.75E-01	1.00E+00
PLCG2	LMNA	0.193631674	0.057107209	0.323047282	5.76E-03	1.24E-02	1.00E+00
CAPZA1	LMNA	0.299986193	0.168931208	0.420617976	1.44E-05	5.54E-05	1.71E-01
GLRX3	LMNA	0.011724868	-0.126530939	0.149533829	8.68E-01	9.03E-01	1.00E+00
HRH3	LMNA	-0.338574002	-0.455342095	-0.2103551	8.29E-07	4.02E-06	9.83E-03
KIAA0247	LMNA	0.415792584	0.294654949	0.523778399	7.57E-10	6.40E-09	8.98E-06
MGC15523	LMNA	0.322819685	0.193387093	0.441207995	2.79E-06	1.23E-05	3.31E-02
CRYGD	LMNA	-0.157693714	-0.289443593	-0.020079847	2.50E-02	4.56E-02	1.00E+00
HTR2B	LMNA	0.204232679	0.068101776	0.332897785	3.55E-03	8.07E-03	1.00E+00
CCR1	LMNA	0.259145304	0.12558722	0.383477301	1.96E-04	6.02E-04	1.00E+00
NUS1	LMNA	0.161001489	0.023472191	0.292550148	2.21E-02	4.09E-02	1.00E+00
DNAJA2	LMNA	-0.062059608	-0.198410746	0.076648067	3.80E-01	4.68E-01	1.00E+00

PGRMC1	LMNA	-0.099683887	-0.234507719	0.038902468	1.58E-01	2.25E-01	1.00E+00
UVRAG	LMNA	-0.21217797	-0.340262221	-0.07636377	2.43E-03	5.78E-03	1.00E+00
ITGA2B	LMNA	0.010352095	-0.127881664	0.148191313	8.84E-01	9.15E-01	1.00E+00
CLDN5	LMNA	-0.312820821	-0.43220687	-0.182657945	5.82E-06	2.40E-05	6.91E-02
PTPRN2	LMNA	-0.246934209	-0.372293892	-0.112725983	3.96E-04	1.14E-03	1.00E+00
PSAP	LMNA	0.150617017	0.012832848	0.282788054	3.24E-02	5.74E-02	1.00E+00
MYOM2	LMNA	-0.421533656	-0.528811456	-0.300998717	4.17E-10	3.69E-09	4.95E-06
CHM	LMNA	0.120515653	-0.017832025	0.254335201	8.76E-02	1.36E-01	1.00E+00
CCNL1	LMNA	-0.006013129	-0.143944638	0.13214757	9.32E-01	9.51E-01	1.00E+00
FAM105A	LMNA	-0.138765702	-0.271613473	-0.00072866	4.89E-02	8.23E-02	1.00E+00
LYN	LMNA	0.254592574	0.12078684	0.379311988	2.56E-04	7.69E-04	1.00E+00
DUSP6	LMNA	0.396978093	0.27393988	0.507231189	4.94E-09	3.60E-08	5.86E-05
TGFB3	LMNA	0.45150041	0.334285449	0.554960666	1.54E-11	1.75E-10	1.83E-07
PCDH11Y	LMNA	-0.499837058	-0.596713063	-0.388600672	3.64E-14	6.24E-13	4.32E-10
NAP1L3	LMNA	-0.544713682	-0.635012818	-0.439729585	5.28E-17	1.62E-15	6.27E-13
ELK1	LMNA	0.262062166	0.128666038	0.386143296	1.65E-04	5.16E-04	1.00E+00
HGD	LMNA	0.20143416	0.065196146	0.330300113	4.04E-03	9.05E-03	1.00E+00
C10orf57	LMNA	-0.133396033	-0.266538604	0.004742299	5.84E-02	9.61E-02	1.00E+00
B3GALNT1	LMNA	-0.022283042	-0.159842336	0.116125182	7.53E-01	8.09E-01	1.00E+00
AARSD1	LMNA	-0.247263901	-0.372596315	-0.113072638	3.89E-04	1.12E-03	1.00E+00
MYO3A	LMNA	-0.030959461	-0.168291188	0.107551212	6.62E-01	7.32E-01	1.00E+00
SERPINE2	LMNA	-0.172351836	-0.303188958	-0.035136859	1.42E-02	2.75E-02	1.00E+00
GDAP2	LMNA	-0.007037037	-0.144947226	0.131141364	9.21E-01	9.43E-01	1.00E+00
MAPK6	LMNA	-0.041928628	-0.17894385	0.096681996	5.54E-01	6.34E-01	1.00E+00
COX6B1	LMNA	-0.029587921	-0.166956962	0.10890793	6.76E-01	7.45E-01	1.00E+00
DNASE2	LMNA	0.400817745	0.278158151	0.510614751	3.40E-09	2.54E-08	4.03E-05
MSH5	LMNA	-0.189105128	-0.318832635	-0.052422682	7.03E-03	1.48E-02	1.00E+00
LGMN	LMNA	0.220485979	0.085022933	0.347946089	1.62E-03	4.00E-03	1.00E+00
TCN1	LMNA	0.323135127	0.193726078	0.441491574	2.72E-06	1.20E-05	3.23E-02
SLC24A6	LMNA	0.413711278	0.292357782	0.521951906	9.37E-10	7.77E-09	1.11E-05
TATDN2	LMNA	0.062142806	-0.076565037	0.198490977	3.80E-01	4.67E-01	1.00E+00
SDCBP	LMNA	0.306277544	0.175653511	0.426303609	9.29E-06	3.69E-05	1.10E-01
NUDT11	LMNA	-0.463340611	-0.565236467	-0.347518505	3.82E-12	4.83E-11	4.53E-08
LILRB1	LMNA	0.283314855	0.151176661	0.405505755	4.39E-05	1.54E-04	5.21E-01
PYGL	LMNA	0.589838403	0.491836563	0.673081842	2.55E-20	1.35E-18	3.03E-16

P2RY5	LMNA	0.163005027	0.025528495	0.294430457	2.05E-02	3.81E-02	1.00E+00
SNPH	LMNA	-0.162820523	-0.294257344	-0.025339082	2.06E-02	3.84E-02	1.00E+00
NUCB2	LMNA	0.41456335	0.293298057	0.522699779	8.58E-10	7.19E-09	1.02E-05
B3GNT4	LMNA	-0.20932019	-0.337615172	-0.073389928	2.79E-03	6.52E-03	1.00E+00
SNX27	LMNA	-0.172637629	-0.303456414	-0.035431049	1.40E-02	2.73E-02	1.00E+00
C2orf37	LMNA	0.129630464	-0.008573973	0.262975359	6.60E-02	1.07E-01	1.00E+00
MIZF	LMNA	-0.343669355	-0.459900865	-0.215859574	5.51E-07	2.76E-06	6.54E-03
FOXO4	LMNA	-0.288750975	-0.410440835	-0.156956637	3.08E-05	1.11E-04	3.65E-01
NUBPL	LMNA	-0.183648349	-0.313745023	-0.046783441	8.89E-03	1.83E-02	1.00E+00
NOD1	LMNA	0.505819819	0.395377615	0.601844651	1.61E-14	3.01E-13	1.91E-10
ID4	LMNA	-0.335133766	-0.452260677	-0.206643228	1.09E-06	5.17E-06	1.29E-02
CDH22	LMNA	-0.352218167	-0.46753568	-0.225113081	2.74E-07	1.46E-06	3.25E-03
PXMP3	LMNA	-0.227672315	-0.354578766	-0.092529554	1.12E-03	2.90E-03	1.00E+00
SOX5	LMNA	-0.367290418	-0.480954702	-0.241483808	7.58E-08	4.44E-07	8.99E-04
NUBP1	LMNA	0.116017776	-0.022391861	0.250063664	1.00E-01	1.52E-01	1.00E+00
DSCAM	LMNA	-0.462003229	-0.564077371	-0.346021482	4.48E-12	5.61E-11	5.32E-08
INA	LMNA	-0.519816065	-0.61381874	-0.411278902	2.23E-15	4.93E-14	2.65E-11
DGKI	LMNA	-0.524100947	-0.617475922	-0.416160247	1.20E-15	2.82E-14	1.42E-11
MCOLN1	LMNA	0.3107065	0.180393165	0.430300475	6.78E-06	2.77E-05	8.04E-02
FAM136A	LMNA	0.209181807	0.073245987	0.337486942	2.81E-03	6.55E-03	1.00E+00
RIN3	LMNA	0.428819743	0.309065085	0.535188144	1.93E-10	1.82E-09	2.28E-06
NFIX	LMNA	-0.199511506	-0.328514315	-0.063201242	4.42E-03	9.81E-03	1.00E+00
SLC16A6	LMNA	0.276839521	0.144303508	0.399617934	6.65E-05	2.25E-04	7.89E-01
AKAP1	LMNA	-0.405125989	-0.514407199	-0.282896857	2.22E-09	1.72E-08	2.64E-05
PSG2	LMNA	0.127944844	-0.01028787	0.261379128	6.96E-02	1.12E-01	1.00E+00
HIBCH	LMNA	-0.227681973	-0.354587672	-0.092539653	1.12E-03	2.90E-03	1.00E+00
PLA2G5	LMNA	0.401480307	0.278886524	0.511198267	3.19E-09	2.40E-08	3.78E-05
TIMM10	LMNA	-0.094651993	-0.229701529	0.043973602	1.80E-01	2.51E-01	1.00E+00
MED17	LMNA	-0.066347083	-0.202542936	0.072366745	3.48E-01	4.34E-01	1.00E+00
PSORS1C1	LMNA	0.314229253	0.184167371	0.433476202	5.26E-06	2.19E-05	6.24E-02
KIAA0495	LMNA	0.239606864	0.105030044	0.365565694	5.94E-04	1.65E-03	1.00E+00
LPA	LMNA	-0.196061765	-0.325307814	-0.059624595	5.17E-03	1.12E-02	1.00E+00
PIGA	LMNA	0.127922576	-0.010310506	0.261358036	6.96E-02	1.12E-01	1.00E+00
COL4A4	LMNA	-0.178161292	-0.308621637	-0.041121694	1.12E-02	2.24E-02	1.00E+00
LY75	LMNA	0.4066427	0.284566534	0.515741309	1.91E-09	1.50E-08	2.27E-05

TPP1	LMNA	0.525313175	0.41754235	0.618509837	1.00E-15	2.41E-14	1.19E-11
UTS2	LMNA	0.00755273	-0.130634478	0.145452073	9.15E-01	9.39E-01	1.00E+00
GJA3	LMNA	0.446380634	0.328577672	0.550507547	2.77E-11	3.01E-10	3.28E-07
GALNACT-:	LMNA	0.100363476	-0.038217032	0.23515632	1.55E-01	2.21E-01	1.00E+00
RREB1	LMNA	0.512463203	0.402916992	0.607533597	6.38E-15	1.29E-13	7.56E-11
TMPRSS5	LMNA	-0.404132471	-0.513533007	-0.281803542	2.45E-09	1.88E-08	2.91E-05
MGC3196	LMNA	0.006891643	-0.131284262	0.144804876	9.22E-01	9.44E-01	1.00E+00
FLJ31568	LMNA	-0.080241115	-0.215900506	0.05845744	2.56E-01	3.38E-01	1.00E+00
DNMBP	LMNA	0.395638871	0.272469714	0.506050246	5.62E-09	4.05E-08	6.66E-05
LPHN1	LMNA	-0.348965166	-0.4646325	-0.221589224	3.58E-07	1.87E-06	4.25E-03
NQO1	LMNA	0.384637031	0.260413885	0.496332985	1.59E-08	1.05E-07	1.88E-04
ZSCAN2	LMNA	-0.273719907	-0.396777745	-0.140996796	8.09E-05	2.69E-04	9.60E-01
SP1	LMNA	0.538250083	0.43232328	0.629523718	1.44E-16	4.07E-15	1.71E-12
TOX4	LMNA	-0.031500302	-0.168817176	0.107016074	6.56E-01	7.27E-01	1.00E+00
SEC24C	LMNA	0.015821429	-0.122497125	0.153537079	8.23E-01	8.67E-01	1.00E+00
HSPA9	LMNA	0.322442339	0.192981623	0.440868734	2.87E-06	1.26E-05	3.40E-02
APOBEC1	LMNA	0.172467887	0.035256317	0.303297566	1.41E-02	2.75E-02	1.00E+00
SURF1	LMNA	-0.091886084	-0.227056876	0.046758041	1.93E-01	2.66E-01	1.00E+00
LSM5	LMNA	0.095331536	-0.043289177	0.230350976	1.77E-01	2.47E-01	1.00E+00
ZBTB1	LMNA	0.082510755	-0.056180177	0.2180777	2.43E-01	3.23E-01	1.00E+00
GTF2F1	LMNA	0.287432445	0.155553874	0.409244486	3.36E-05	1.21E-04	3.98E-01
DUSP21	LMNA	0.012415897	-0.125850815	0.150209435	8.61E-01	8.98E-01	1.00E+00
RPS15A	LMNA	-0.010534202	-0.148369436	0.127702511	8.82E-01	9.13E-01	1.00E+00
TAF1	LMNA	0.11738057	-0.021010902	0.251358432	9.62E-02	1.47E-01	1.00E+00
GINS4	LMNA	0.128835897	-0.009381971	0.262223019	6.76E-02	1.09E-01	1.00E+00
MYO15A	LMNA	-0.210926818	-0.339103578	-0.075061508	2.59E-03	6.09E-03	1.00E+00
AP1G2	LMNA	-0.176961806	-0.307500642	-0.039885191	1.18E-02	2.34E-02	1.00E+00
RBM42	LMNA	0.28390414	0.151802784	0.406041071	4.23E-05	1.49E-04	5.02E-01
HCN2	LMNA	0.12359351	-0.014708449	0.25725517	7.97E-02	1.25E-01	1.00E+00
UTP3	LMNA	0.245941828	0.111682737	0.371383427	4.18E-04	1.20E-03	1.00E+00
HNRPA3	LMNA	-0.10759023	-0.242046134	0.030920024	1.27E-01	1.87E-01	1.00E+00
CKLF	LMNA	0.117876436	-0.020508296	0.251829426	9.48E-02	1.45E-01	1.00E+00
U1SNRNPE	LMNA	-0.06979707	-0.205864452	0.068917994	3.24E-01	4.09E-01	1.00E+00
RUSC1	LMNA	0.33170082	0.202942906	0.449182999	1.42E-06	6.59E-06	1.68E-02
MBNL1	LMNA	0.327513272	0.198434182	0.445425033	1.96E-06	8.89E-06	2.32E-02

NUP160	LMNA	-0.121175361	-0.254961273	0.017162744	8.58E-02	1.34E-01	1.00E+00
GRIK5	LMNA	-0.083521307	-0.219046656	0.055165766	2.37E-01	3.16E-01	1.00E+00
USP21	LMNA	-0.235148321	-0.361465269	-0.100355094	7.55E-04	2.04E-03	1.00E+00
FLJ22639	LMNA	-0.143807253	-0.276371523	-0.005872844	4.12E-02	7.07E-02	1.00E+00
HAND1	LMNA	0.112279398	-0.026177359	0.246509432	1.12E-01	1.67E-01	1.00E+00
ORMDL2	LMNA	0.241036548	0.106530382	0.36687951	5.49E-04	1.53E-03	1.00E+00
SERPINB1	LMNA	0.386875307	0.262863455	0.498312189	1.29E-08	8.65E-08	1.53E-04
FGA	LMNA	-0.067602424	-0.203751893	0.071112237	3.39E-01	4.25E-01	1.00E+00
PRSS7	LMNA	0.162787823	0.025305513	0.294226661	2.06E-02	3.84E-02	1.00E+00
IGFBP1	LMNA	0.170055667	0.032774082	0.301039361	1.55E-02	2.98E-02	1.00E+00
SLC1A1	LMNA	-0.322328279	-0.44076618	-0.192859071	2.89E-06	1.27E-05	3.43E-02
DHX57	LMNA	-0.213436323	-0.34142714	-0.077673995	2.29E-03	5.48E-03	1.00E+00
PSAT1	LMNA	-0.487954801	-0.586497759	-0.375176697	1.76E-13	2.73E-12	2.09E-09
FLJ13195	LMNA	-0.047868873	-0.184699317	0.090782046	4.99E-01	5.83E-01	1.00E+00
GDPD3	LMNA	-0.091021412	-0.226229702	0.047628063	1.98E-01	2.71E-01	1.00E+00
PTPN21	LMNA	0.261228123	0.127785423	0.385381197	1.73E-04	5.40E-04	1.00E+00
TBR1	LMNA	-0.259364421	-0.383677645	-0.125818414	1.93E-04	5.96E-04	1.00E+00
TBC1D1	LMNA	0.38625369	0.262182997	0.497762639	1.37E-08	9.13E-08	1.62E-04
IFNG	LMNA	0.163589912	0.026129002	0.294979176	2.00E-02	3.74E-02	1.00E+00
FOS	LMNA	0.292463224	0.160908894	0.413806839	2.40E-05	8.88E-05	2.85E-01
IQGAP1	LMNA	0.744305504	0.675682291	0.800140384	6.48E-37	8.54E-34	7.69E-33
ZNF226	LMNA	-0.106174989	-0.240697945	0.032350186	1.33E-01	1.94E-01	1.00E+00
EMD	LMNA	0.43195295	0.312539139	0.537926563	1.37E-10	1.34E-09	1.63E-06
RETN	LMNA	0.329853759	0.200953506	0.447525931	1.64E-06	7.56E-06	1.94E-02
APH1A	LMNA	0.184672652	0.04784133	0.314700598	8.51E-03	1.76E-02	1.00E+00
C14orf1	LMNA	0.078061596	-0.060642922	0.213808493	2.69E-01	3.51E-01	1.00E+00
PUS7	LMNA	0.22516157	0.089905162	0.352262908	1.27E-03	3.25E-03	1.00E+00
PAFAH2	LMNA	0.177643584	0.040587958	0.308137851	1.14E-02	2.28E-02	1.00E+00
NPEPL1	LMNA	0.407492743	0.28550263	0.516488781	1.76E-09	1.38E-08	2.08E-05
RP11-114G	LMNA	-0.060042817	-0.196465323	0.078660195	3.96E-01	4.83E-01	1.00E+00
TUBB6	LMNA	0.551795738	0.447860974	0.641016682	1.72E-17	5.68E-16	2.04E-13
FOXL2	LMNA	0.034626996	-0.103920776	0.171856473	6.25E-01	6.99E-01	1.00E+00
LATS1	LMNA	-0.227615385	-0.354526272	-0.092470026	1.12E-03	2.91E-03	1.00E+00
KCNQ2	LMNA	-0.236801387	-0.362986129	-0.102087702	6.91E-04	1.89E-03	1.00E+00
SPOCK2	LMNA	0.265994252	0.132820524	0.389733922	1.30E-04	4.17E-04	1.00E+00

Mar-06	LMNA	-0.176212508	-0.306800191	-0.039112982	1.21E-02	2.41E-02	1.00E+00
MGC10334	LMNA	0.201842589	0.065620064	0.330679352	3.97E-03	8.90E-03	1.00E+00
HTATIP2	LMNA	0.247011017	0.11280674	0.372364349	3.94E-04	1.14E-03	1.00E+00
CENTA2	LMNA	0.222244121	0.086858013	0.349569958	1.48E-03	3.70E-03	1.00E+00
FGF2	LMNA	0.002429353	-0.135667147	0.140433256	9.73E-01	9.79E-01	1.00E+00
FXYD7	LMNA	-0.327230511	-0.445171129	-0.198129931	2.00E-06	9.06E-06	2.37E-02
CCDC33	LMNA	0.053571875	-0.085108558	0.190216104	4.49E-01	5.35E-01	1.00E+00
PRODH	LMNA	-0.236668368	-0.362863773	-0.101948252	6.96E-04	1.90E-03	1.00E+00
DDX6	LMNA	-0.010249973	-0.148091422	0.127982124	8.85E-01	9.16E-01	1.00E+00
SLC43A1	LMNA	0.123451449	-0.01485268	0.25712045	8.01E-02	1.26E-01	1.00E+00
NTN1	LMNA	0.105601118	-0.032929947	0.240151114	1.35E-01	1.96E-01	1.00E+00
ING4	LMNA	-0.494401429	-0.592043888	-0.382453907	7.55E-14	1.22E-12	8.95E-10
DPH2	LMNA	-0.001350035	-0.13937506	0.136726448	9.85E-01	9.88E-01	1.00E+00
ZNF313	LMNA	0.232366345	0.0974411	0.358904269	8.76E-04	2.33E-03	1.00E+00
VPS28	LMNA	-0.12823243	-0.261651515	0.009995517	6.90E-02	1.11E-01	1.00E+00
DIAPH1	LMNA	0.406378286	0.284275399	0.515508766	1.96E-09	1.53E-08	2.33E-05
CEP76	LMNA	-0.325204346	-0.44335119	-0.195950501	2.33E-06	1.04E-05	2.77E-02
CORO1A	LMNA	0.148137404	0.010296982	0.280452995	3.54E-02	6.19E-02	1.00E+00
TRAF4	LMNA	-0.231280147	-0.357903831	-0.096303984	9.27E-04	2.45E-03	1.00E+00
RRM2	LMNA	0.237359117	0.102672453	0.363499102	6.71E-04	1.84E-03	1.00E+00
EDG4	LMNA	-0.191566335	-0.321124891	-0.054969041	6.31E-03	1.34E-02	1.00E+00
OS9	LMNA	0.061827548	-0.076879648	0.198186953	3.82E-01	4.70E-01	1.00E+00
SLC4A1AP	LMNA	0.180345758	0.043374652	0.310662228	1.02E-02	2.07E-02	1.00E+00
COG5	LMNA	0.187430843	0.050691485	0.317272415	7.56E-03	1.58E-02	1.00E+00
COPS8	LMNA	0.104247285	-0.034297303	0.238860735	1.40E-01	2.02E-01	1.00E+00
NGLY1	LMNA	0.139695849	0.001677193	0.272491803	4.74E-02	8.00E-02	1.00E+00
AKAP9	LMNA	0.029088635	-0.109401692	0.166471135	6.81E-01	7.49E-01	1.00E+00
NCBP2	LMNA	0.101936308	-0.036630167	0.236656975	1.49E-01	2.13E-01	1.00E+00
C17orf42	LMNA	-0.199432221	-0.328440654	-0.063119001	4.43E-03	9.84E-03	1.00E+00
GPSM3	LMNA	0.227999234	0.09287141	0.354880195	1.10E-03	2.86E-03	1.00E+00
SLC20A1	LMNA	0.533677591	0.427092514	0.625635098	2.89E-16	7.66E-15	3.42E-12
SIL1	LMNA	0.56255395	0.460246192	0.650116189	2.97E-18	1.12E-16	3.52E-14
LDB2	LMNA	-0.08954114	-0.22481317	0.049117008	2.05E-01	2.80E-01	1.00E+00
ASB6	LMNA	0.268742193	0.135726669	0.392241002	1.10E-04	3.57E-04	1.00E+00
KLK5	LMNA	-0.233241227	-0.359709863	-0.098357249	8.36E-04	2.24E-03	1.00E+00

SMAD5OS	LMNA	0.124749976	-0.013534107	0.25835168	7.69E-02	1.22E-01	1.00E+00
UNC93A	LMNA	0.034472858	-0.104073429	0.171706705	6.26E-01	7.01E-01	1.00E+00
SPTB	LMNA	-0.120367809	-0.25419488	0.017981996	8.79E-02	1.36E-01	1.00E+00
EFEMP2	LMNA	0.662446513	0.577179377	0.733424787	6.86E-27	1.23E-24	8.13E-23
EFNB2	LMNA	0.474750434	0.360314354	0.575108926	9.46E-13	1.32E-11	1.12E-08
C21orf62	LMNA	0.296903673	0.165641981	0.417828771	1.78E-05	6.74E-05	2.11E-01
PCM1	LMNA	-0.112101895	-0.246340584	0.026357	1.12E-01	1.68E-01	1.00E+00
FMO5	LMNA	-0.23223787	-0.358785953	-0.097306584	8.82E-04	2.34E-03	1.00E+00
TSG101	LMNA	-0.132828477	-0.266001776	0.005320078	5.95E-02	9.77E-02	1.00E+00
CEBPG	LMNA	0.191762191	0.05517175	0.321307237	6.26E-03	1.33E-02	1.00E+00
GTF3C4	LMNA	0.095870195	-0.042746555	0.230865694	1.75E-01	2.44E-01	1.00E+00
ABHD3	LMNA	0.032287062	-0.106237467	0.169582191	6.48E-01	7.21E-01	1.00E+00
TNFRSF1B	LMNA	0.42685504	0.30688828	0.533469854	2.38E-10	2.21E-09	2.82E-06
CLEC1A	LMNA	0.110467358	-0.028010811	0.24478535	1.18E-01	1.75E-01	1.00E+00
IQSEC1	LMNA	0.189907953	0.053253088	0.319580517	6.79E-03	1.44E-02	1.00E+00
PIGN	LMNA	0.089450966	-0.04920769	0.224726861	2.06E-01	2.80E-01	1.00E+00
PATZ1	LMNA	-0.390475815	-0.501493518	-0.266807211	9.19E-09	6.31E-08	1.09E-04
RBM22	LMNA	-0.10890181	-0.243295111	0.029594108	1.23E-01	1.81E-01	1.00E+00
AEBP1	LMNA	0.433821909	0.314612935	0.539558967	1.12E-10	1.11E-09	1.33E-06
BAG2	LMNA	0.089690886	-0.048966413	0.224956494	2.04E-01	2.79E-01	1.00E+00
PAQR5	LMNA	0.228813315	0.093722823	0.355630687	1.06E-03	2.75E-03	1.00E+00
C9orf127	LMNA	-0.230144979	-0.356857979	-0.095115979	9.84E-04	2.59E-03	1.00E+00
THNSL1	LMNA	-0.432547338	-0.538445805	-0.313198548	1.29E-10	1.26E-09	1.53E-06
ANKRD2	LMNA	0.010228951	-0.128002804	0.148070859	8.85E-01	9.16E-01	1.00E+00
JAM2	LMNA	0.122688144	-0.015627543	0.256396501	8.19E-02	1.28E-01	1.00E+00
SNRPN	LMNA	-0.328587936	-0.446389848	-0.19959075	1.80E-06	8.24E-06	2.14E-02
ALX4	LMNA	-0.007336478	-0.145240378	0.130847046	9.17E-01	9.41E-01	1.00E+00
FAM130A1	LMNA	-0.153750635	-0.285736751	-0.016040092	2.89E-02	5.17E-02	1.00E+00
CACNA1S	LMNA	0.101757436	-0.036810671	0.236486344	1.50E-01	2.14E-01	1.00E+00
TRIM38	LMNA	0.466829473	0.351426609	0.56825834	2.51E-12	3.28E-11	2.97E-08
CORIN	LMNA	0.209218826	0.073284492	0.337521245	2.81E-03	6.54E-03	1.00E+00
TMCC1	LMNA	-0.290817879	-0.412315366	-0.159156651	2.68E-05	9.82E-05	3.18E-01
PCLKC	LMNA	0.147118703	0.009255685	0.279493229	3.67E-02	6.39E-02	1.00E+00
PLEKHG6	LMNA	0.200539786	0.064268028	0.329469516	4.21E-03	9.40E-03	1.00E+00
LRRC40	LMNA	-0.298169628	-0.418974541	-0.166992475	1.63E-05	6.22E-05	1.94E-01

SMG5	LMNA	0.175851298	0.038740786	0.306462478	1.23E-02	2.44E-02	1.00E+00
NCAPD2	LMNA	-0.016901945	-0.154592231	0.121432397	8.11E-01	8.58E-01	1.00E+00
WNT2B	LMNA	0.099566245	-0.039021109	0.234395429	1.59E-01	2.25E-01	1.00E+00
DCP2	LMNA	-0.189236712	-0.318955225	-0.052558773	6.99E-03	1.47E-02	1.00E+00
ASNS	LMNA	-0.16750359	-0.298648628	-0.030149774	1.72E-02	3.26E-02	1.00E+00
MRPL49	LMNA	-0.013824043	-0.151585754	0.124464488	8.45E-01	8.85E-01	1.00E+00
TMEM24	LMNA	-0.294229232	-0.415406966	-0.162790562	2.13E-05	7.96E-05	2.53E-01
RPL18	LMNA	-0.058823209	-0.195288351	0.079876436	4.06E-01	4.93E-01	1.00E+00
SMU1	LMNA	-0.024946907	-0.162438479	0.113494964	7.25E-01	7.85E-01	1.00E+00
ISG20	LMNA	0.557606705	0.454545806	0.645934829	6.71E-18	2.39E-16	7.96E-14
CASZ1	LMNA	0.322538219	0.193084645	0.44095494	2.85E-06	1.26E-05	3.38E-02
POLR1D	LMNA	0.164192768	0.026748065	0.295544664	1.95E-02	3.66E-02	1.00E+00
GIN1	LMNA	-0.133922557	-0.267036548	0.004206208	5.74E-02	9.46E-02	1.00E+00
TMEM177	LMNA	-0.216439108	-0.344205378	-0.080802456	1.98E-03	4.80E-03	1.00E+00
CDKN2AIP	LMNA	-0.227530332	-0.354447845	-0.092381094	1.13E-03	2.92E-03	1.00E+00
KRR1	LMNA	0.009385751	-0.128832179	0.147245963	8.95E-01	9.24E-01	1.00E+00
CXCL1	LMNA	0.318060162	0.188276086	0.436926371	3.97E-06	1.70E-05	4.71E-02
C1D	LMNA	-0.150923399	-0.283076465	-0.013146303	3.20E-02	5.68E-02	1.00E+00
EPM2A	LMNA	-0.319939825	-0.438617943	-0.19029372	3.46E-06	1.49E-05	4.10E-02
LDHC	LMNA	0.119509585	-0.018852451	0.253380213	9.02E-02	1.39E-01	1.00E+00
PC	LMNA	-0.204206491	-0.332873485	-0.068074574	3.56E-03	8.07E-03	1.00E+00
PDZRN4	LMNA	-0.574078873	-0.659836411	-0.473558399	4.20E-19	1.85E-17	4.98E-15
FAH	LMNA	0.503720972	0.392998787	0.600045312	2.15E-14	3.89E-13	2.55E-10
UBE4B	LMNA	-0.195072974	-0.324388194	-0.058600074	5.40E-03	1.17E-02	1.00E+00
NIT1	LMNA	-0.034850611	-0.172073737	0.103699303	6.22E-01	6.97E-01	1.00E+00
CDC2L6	LMNA	-0.211323682	-0.33947114	-0.075474534	2.54E-03	5.99E-03	1.00E+00
BTN3A3	LMNA	0.122688997	-0.015626678	0.256397309	8.19E-02	1.28E-01	1.00E+00
PAX8	LMNA	0.231314396	0.096339833	0.35793538	9.26E-04	2.45E-03	1.00E+00
PRO2012	LMNA	-0.191594731	-0.321151329	-0.054998431	6.30E-03	1.34E-02	1.00E+00
BEX1	LMNA	-0.579534947	-0.664428124	-0.479876661	1.62E-19	7.57E-18	1.92E-15
COMMD3	LMNA	-0.35135493	-0.466765516	-0.224177644	2.94E-07	1.56E-06	3.49E-03
MRPL40	LMNA	0.006895473	-0.131280497	0.144808627	9.22E-01	9.44E-01	1.00E+00
SLC2A4	LMNA	-0.034357836	-0.171594939	0.104187339	6.27E-01	7.02E-01	1.00E+00
SHFM1	LMNA	0.456268976	0.339609426	0.559102981	8.84E-12	1.05E-10	1.05E-07
PKMYT1	LMNA	-0.056432344	-0.192979926	0.082259503	4.25E-01	5.12E-01	1.00E+00

FEZF2	LMNA	-0.402624316	-0.512205554	-0.2801445	2.85E-09	2.16E-08	3.38E-05
DUT	LMNA	-0.188243011	-0.31802934	-0.051531157	7.30E-03	1.53E-02	1.00E+00
LRRC59	LMNA	0.355327339	0.228484243	0.470308174	2.11E-07	1.15E-06	2.51E-03
LY6H	LMNA	-0.352170583	-0.467493231	-0.225061511	2.75E-07	1.46E-06	3.26E-03
MAP2	LMNA	-0.541575295	-0.632348735	-0.436131684	8.62E-17	2.56E-15	1.02E-12
LYL1	LMNA	0.094777634	-0.043847068	0.229821615	1.80E-01	2.50E-01	1.00E+00
EDEM1	LMNA	0.544574852	0.439570356	0.634895015	5.40E-17	1.66E-15	6.41E-13
NOS3	LMNA	0.359821917	0.233362934	0.474312047	1.44E-07	8.06E-07	1.71E-03
ZNF34	LMNA	-0.383250074	-0.495105979	-0.258896799	1.81E-08	1.17E-07	2.14E-04
ADH1A	LMNA	0.069686237	-0.069028838	0.205757795	3.24E-01	4.10E-01	1.00E+00
CYP11B1	LMNA	0.124479606	-0.013808691	0.258095358	7.76E-02	1.23E-01	1.00E+00
CDC73	LMNA	0.143602037	0.005663308	0.276177973	4.15E-02	7.11E-02	1.00E+00
GPR172A	LMNA	0.490560727	0.378116697	0.588740789	1.25E-13	1.99E-12	1.49E-09
GSTM3	LMNA	-0.345610282	-0.461635787	-0.217958483	4.71E-07	2.40E-06	5.59E-03
KCNA5	LMNA	-0.417747622	-0.52549318	-0.296814028	6.19E-10	5.32E-09	7.34E-06
FLRT2	LMNA	0.016272931	-0.12205226	0.153978021	8.18E-01	8.63E-01	1.00E+00
WRN	LMNA	0.128331028	-0.00989528	0.261744897	6.87E-02	1.11E-01	1.00E+00
ANAPC5	LMNA	-0.228346589	-0.355200437	-0.093234669	1.08E-03	2.81E-03	1.00E+00
SDF2	LMNA	-0.03288268	-0.170161236	0.105647908	6.42E-01	7.15E-01	1.00E+00
KRT8P12	LMNA	0.428064668	0.308228346	0.534527874	2.09E-10	1.96E-09	2.48E-06
DZIP3	LMNA	-0.513580692	-0.608489582	-0.404186658	5.45E-15	1.12E-13	6.46E-11
C15orf24	LMNA	0.149211536	0.011395265	0.281464701	3.41E-02	5.98E-02	1.00E+00
NFATC2IP	LMNA	0.093121244	-0.045514872	0.228238133	1.87E-01	2.59E-01	1.00E+00
RIT1	LMNA	0.174816226	0.037674444	0.305494557	1.28E-02	2.53E-02	1.00E+00
RHBDF2	LMNA	0.346769618	0.219212746	0.462671651	4.29E-07	2.20E-06	5.09E-03
SCML1	LMNA	0.234767375	0.099955933	0.361114695	7.71E-04	2.08E-03	1.00E+00
MED9	LMNA	0.057397396	-0.08129779	0.193911883	4.17E-01	5.05E-01	1.00E+00
RAB13	LMNA	0.628667789	0.537243165	0.705490525	1.28E-23	1.30E-21	1.52E-19
FBXW11	LMNA	-0.118067593	-0.252010979	0.020314521	9.42E-02	1.45E-01	1.00E+00
ETAA1	LMNA	0.167676408	0.030327424	0.298810573	1.71E-02	3.24E-02	1.00E+00
C14orf131	LMNA	-0.197359941	-0.326514805	-0.060970116	4.87E-03	1.07E-02	1.00E+00
SLC12A5	LMNA	-0.356017685	-0.470923458	-0.229233174	1.99E-07	1.09E-06	2.36E-03
PHF14	LMNA	-0.094908215	-0.229946418	0.043715554	1.79E-01	2.49E-01	1.00E+00
NRIP3	LMNA	-0.196634714	-0.325840571	-0.060218379	5.03E-03	1.10E-02	1.00E+00
TMEM121	LMNA	-0.34012914	-0.456734113	-0.212034243	7.32E-07	3.60E-06	8.69E-03

NOS1AP	LMNA	-0.372323923	-0.485424267	-0.246966947	4.86E-08	2.95E-07	5.77E-04
FAM3A	LMNA	0.35679651	0.230078272	0.471617467	1.87E-07	1.02E-06	2.21E-03
RPL13	LMNA	0.122788554	-0.015525622	0.256491742	8.17E-02	1.28E-01	1.00E+00
PRDX2	LMNA	-0.355491685	-0.470454661	-0.228662523	2.08E-07	1.13E-06	2.47E-03
SAP30BP	LMNA	-0.161801092	-0.293300693	-0.024292713	2.14E-02	3.97E-02	1.00E+00
WDR76	LMNA	-0.044135546	-0.181083215	0.094491185	5.33E-01	6.15E-01	1.00E+00
PRMT3	LMNA	-0.150861247	-0.283017961	-0.013082714	3.21E-02	5.69E-02	1.00E+00
TRIM10	LMNA	-0.075775965	-0.21161329	0.062933378	2.84E-01	3.67E-01	1.00E+00
FAM82B	LMNA	0.256424456	0.122717616	0.380988592	2.30E-04	6.98E-04	1.00E+00
GCNT4	LMNA	-0.102379419	-0.237079636	0.036182976	1.47E-01	2.11E-01	1.00E+00
MAP9	LMNA	-0.076157405	-0.211979734	0.062551235	2.81E-01	3.64E-01	1.00E+00
GPRASP1	LMNA	-0.224478996	-0.351633046	-0.089192015	1.32E-03	3.36E-03	1.00E+00
CXorf36	LMNA	0.134073019	-0.004052998	0.267178829	5.71E-02	9.42E-02	1.00E+00
CDKN1C	LMNA	-0.308381713	-0.428203079	-0.177904536	8.01E-06	3.22E-05	9.50E-02
DSCR3	LMNA	0.09925339	-0.039336602	0.23409679	1.60E-01	2.27E-01	1.00E+00
ZFAND3	LMNA	0.202631901	0.066439447	0.331412133	3.83E-03	8.62E-03	1.00E+00
C7orf43	LMNA	0.13127698	-0.006899051	0.264533855	6.26E-02	1.02E-01	1.00E+00
HSPH1	LMNA	0.063785469	-0.074925298	0.200074682	3.67E-01	4.55E-01	1.00E+00
SPSB3	LMNA	-0.295450876	-0.416513423	-0.164092775	1.97E-05	7.38E-05	2.33E-01
AQP1	LMNA	-0.11351537	-0.247684923	0.024926247	1.08E-01	1.62E-01	1.00E+00
COL17A1	LMNA	0.165950501	0.028553654	0.297192921	1.83E-02	3.44E-02	1.00E+00
GFAP	LMNA	-0.219214995	-0.346771696	-0.083696901	1.72E-03	4.25E-03	1.00E+00
CDC16	LMNA	0.011855033	-0.126402839	0.149661098	8.67E-01	9.02E-01	1.00E+00
KIAA1614	LMNA	0.234046507	0.099200717	0.360451201	8.01E-04	2.16E-03	1.00E+00
PRSS16	LMNA	-0.065320662	-0.201554132	0.073392159	3.56E-01	4.43E-01	1.00E+00
KIF13B	LMNA	0.367831801	0.242073167	0.481435714	7.23E-08	4.27E-07	8.57E-04
PCDH9	LMNA	-0.060440861	-0.196849366	0.078263161	3.93E-01	4.80E-01	1.00E+00
MKRN3	LMNA	-0.488564768	-0.587022919	-0.375864656	1.63E-13	2.55E-12	1.93E-09
ADAMTS13	LMNA	0.097259614	-0.041346538	0.232193008	1.69E-01	2.37E-01	1.00E+00
ITFG1	LMNA	0.206635854	0.070598774	0.335126928	3.17E-03	7.31E-03	1.00E+00
TUBG2	LMNA	-0.191009497	-0.32060641	-0.054392782	6.47E-03	1.37E-02	1.00E+00
TTYH1	LMNA	-0.415220795	-0.523276713	-0.294023717	8.02E-10	6.76E-09	9.52E-06
SFRS7	LMNA	-0.223588573	-0.350811215	-0.08826192	1.38E-03	3.49E-03	1.00E+00
C3orf18	LMNA	-0.149203091	-0.281456748	-0.011386628	3.41E-02	5.98E-02	1.00E+00
FLJ13236	LMNA	0.469117946	0.353992264	0.570239013	1.90E-12	2.52E-11	2.25E-08

C18orf25	LMNA	0.151695031	0.013935871	0.283802734	3.12E-02	5.54E-02	1.00E+00
EXTL1	LMNA	-0.338497833	-0.4552739	-0.210272876	8.34E-07	4.04E-06	9.89E-03
RANBP10	LMNA	0.065839129	-0.072874237	0.202053632	3.52E-01	4.39E-01	1.00E+00
RARG	LMNA	0.300497912	0.169477524	0.421080782	1.39E-05	5.36E-05	1.65E-01
MYO7A	LMNA	0.162770991	0.025288235	0.294210868	2.06E-02	3.84E-02	1.00E+00
SFRS18	LMNA	-0.269170009	-0.392631154	-0.136179322	1.07E-04	3.48E-04	1.00E+00
CD96	LMNA	0.294287618	0.162852789	0.415459856	2.13E-05	7.93E-05	2.52E-01
ACVR1B	LMNA	-0.277412346	-0.400139195	-0.14491101	6.41E-05	2.17E-04	7.61E-01
DENND1C	LMNA	0.240210277	0.105663202	0.366120265	5.74E-04	1.60E-03	1.00E+00
RBMS3	LMNA	-0.055736445	-0.192307739	0.082952834	4.31E-01	5.17E-01	1.00E+00
SLC41A3	LMNA	-0.102441212	-0.237138572	0.036120611	1.47E-01	2.11E-01	1.00E+00
PSMD1	LMNA	0.136178996	-0.001907872	0.269169702	5.33E-02	8.88E-02	1.00E+00
DGCR6L	LMNA	0.075055701	-0.06365486	0.210921238	2.88E-01	3.72E-01	1.00E+00
ILVBL	LMNA	0.137319396	-0.000745742	0.270247297	5.13E-02	8.59E-02	1.00E+00
CSNK1G3	LMNA	-0.001345287	-0.139370404	0.136731108	9.85E-01	9.88E-01	1.00E+00
RNF186	LMNA	-0.095404007	-0.230420231	0.043216177	1.77E-01	2.47E-01	1.00E+00
IFNGR1	LMNA	-0.050743049	-0.187480723	0.08792387	4.73E-01	5.59E-01	1.00E+00
MAGEL2	LMNA	-0.336491947	-0.453477534	-0.208108208	9.77E-07	4.69E-06	1.16E-02
TSPAN6	LMNA	-0.062741521	-0.199068286	0.075967474	3.75E-01	4.63E-01	1.00E+00
SLC29A2	LMNA	-0.046006773	-0.182896149	0.092632563	5.16E-01	5.99E-01	1.00E+00
MSL2L1	LMNA	-0.166261209	-0.297484196	-0.028872915	1.80E-02	3.41E-02	1.00E+00
MATN2	LMNA	-0.030828331	-0.168163647	0.107680947	6.63E-01	7.33E-01	1.00E+00
ST6GALNA	LMNA	0.31474064	0.184715579	0.433936966	5.07E-06	2.12E-05	6.01E-02
RBMX	LMNA	-0.308889469	-0.42866128	-0.178447934	7.72E-06	3.12E-05	9.16E-02
MPP3	LMNA	0.048748106	-0.08990795	0.185550404	4.91E-01	5.76E-01	1.00E+00
FGL1	LMNA	-0.063150986	-0.199463057	0.07555874	3.72E-01	4.60E-01	1.00E+00
PSORS1C2	LMNA	-0.04389732	-0.180852343	0.094727737	5.35E-01	6.17E-01	1.00E+00
RAD51L3	LMNA	-0.194330266	-0.323697282	-0.057830719	5.58E-03	1.20E-02	1.00E+00
ARHGEF6	LMNA	0.061291498	-0.077414534	0.197669944	3.86E-01	4.74E-01	1.00E+00
TGFBR2	LMNA	0.508215012	0.398094133	0.603896856	1.16E-14	2.21E-13	1.37E-10
ORAI2	LMNA	0.449476857	0.332028465	0.553201312	1.94E-11	2.16E-10	2.31E-07
CDC123	LMNA	-0.221677982	-0.349047139	-0.086266999	1.52E-03	3.80E-03	1.00E+00
IL33	LMNA	-0.076341021	-0.212156118	0.062367265	2.80E-01	3.63E-01	1.00E+00
DEAF1	LMNA	-0.205565605	-0.334134361	-0.06948653	3.34E-03	7.63E-03	1.00E+00
AMN	LMNA	0.123280033	-0.015026706	0.256957885	8.05E-02	1.26E-01	1.00E+00

MSLN	LMNA	0.098546232	-0.04004962	0.23342167	1.63E-01	2.30E-01	1.00E+00
DEFA6	LMNA	-0.116081426	-0.250124148	0.022327374	9.99E-02	1.52E-01	1.00E+00
WWTR1	LMNA	0.675324219	0.592512693	0.744011623	2.98E-28	6.79E-26	3.53E-24
COBL	LMNA	-0.109769517	-0.244121156	0.02871665	1.20E-01	1.78E-01	1.00E+00
PPP1R16B	LMNA	-0.351672301	-0.467048689	-0.224521532	2.87E-07	1.52E-06	3.40E-03
CIB1	LMNA	0.465670245	0.350127634	0.567254579	2.88E-12	3.74E-11	3.42E-08
TSSK1B	LMNA	-0.006998083	-0.144909088	0.131179649	9.21E-01	9.44E-01	1.00E+00
GAS7	LMNA	0.177834668	0.040784948	0.308316422	1.13E-02	2.27E-02	1.00E+00
MDN1	LMNA	-0.287415121	-0.409228765	-0.155535447	3.36E-05	1.21E-04	3.99E-01
HAAO	LMNA	0.029034343	-0.109455379	0.166418303	6.82E-01	7.50E-01	1.00E+00
CTNNAL1	LMNA	0.272312125	0.139505551	0.395495287	8.83E-05	2.91E-04	1.00E+00
TLE6	LMNA	-0.027405187	-0.164832574	0.111066011	6.99E-01	7.63E-01	1.00E+00
CIT	LMNA	-0.316807534	-0.435798622	-0.186932121	4.36E-06	1.85E-05	5.17E-02
TNFAIP2	LMNA	0.42740916	0.307502094	0.533954566	2.24E-10	2.09E-09	2.66E-06
FOXN1	LMNA	0.12296699	-0.015344494	0.256660987	8.13E-02	1.27E-01	1.00E+00
HERC4	LMNA	-0.109510935	-0.24387501	0.02897816	1.21E-01	1.79E-01	1.00E+00
DRAP1	LMNA	0.402737568	0.280269057	0.512305255	2.82E-09	2.14E-08	3.34E-05
PEMT	LMNA	-0.185380244	-0.31536056	-0.048572302	8.26E-03	1.71E-02	1.00E+00
SLC38A1	LMNA	-0.246931367	-0.372291284	-0.112722994	3.96E-04	1.14E-03	1.00E+00
ARHGAP6	LMNA	0.340091909	0.211994035	0.456700795	7.34E-07	3.60E-06	8.71E-03
PHF20L1	LMNA	-0.218255648	-0.345884992	-0.082696321	1.81E-03	4.43E-03	1.00E+00
MCM3AP	LMNA	0.137151012	-0.000917358	0.270088207	5.16E-02	8.63E-02	1.00E+00
MYO1F	LMNA	0.375908852	0.250877012	0.488603949	3.53E-08	2.17E-07	4.18E-04
RAB11A	LMNA	-0.066532198	-0.202721237	0.072181781	3.47E-01	4.33E-01	1.00E+00
PTBP2	LMNA	-0.586720255	-0.67046536	-0.488213216	4.50E-20	2.31E-18	5.33E-16
SNX1	LMNA	-0.000566298	-0.138606463	0.137495452	9.94E-01	9.95E-01	1.00E+00
CTB-1048E	LMNA	0.101195818	-0.037377351	0.235950546	1.52E-01	2.17E-01	1.00E+00
C19orf60	LMNA	0.054127407	-0.084555421	0.190753035	4.44E-01	5.31E-01	1.00E+00
C7orf25	LMNA	0.160865047	0.023332198	0.292422061	2.22E-02	4.10E-02	1.00E+00
ELF5	LMNA	0.063262086	-0.07544783	0.199570162	3.71E-01	4.59E-01	1.00E+00
HOXB9	LMNA	0.156030428	0.018375233	0.287880439	2.66E-02	4.81E-02	1.00E+00
RBM8A	LMNA	-0.146458692	-0.278871259	-0.008581192	3.75E-02	6.51E-02	1.00E+00
PARP3	LMNA	0.365437872	0.239467797	0.479308221	8.91E-08	5.15E-07	1.06E-03
ITGA9	LMNA	0.088438772	-0.050225434	0.223757899	2.11E-01	2.86E-01	1.00E+00
ZFR	LMNA	-0.198965465	-0.328006973	-0.062634881	4.53E-03	1.00E-02	1.00E+00

VANGL1	LMNA	0.165464997	0.028054842	0.296737734	1.86E-02	3.50E-02	1.00E+00
FLJ20699	LMNA	0.376656414	0.251692889	0.489266629	3.30E-08	2.05E-07	3.91E-04
ACSL6	LMNA	-0.445110579	-0.54940195	-0.327163082	3.20E-11	3.44E-10	3.79E-07
CHAF1B	LMNA	0.14897036	0.011148637	0.281237567	3.43E-02	6.03E-02	1.00E+00
NDUFA3	LMNA	-0.236711132	-0.362903109	-0.101993082	6.94E-04	1.90E-03	1.00E+00
FABP4	LMNA	0.066473551	-0.072240381	0.20266475	3.47E-01	4.33E-01	1.00E+00
KCNB1	LMNA	-0.529597638	-0.622161489	-0.422431209	5.32E-16	1.36E-14	6.32E-12
CANX	LMNA	0.21853822	0.08299101	0.34614619	1.78E-03	4.38E-03	1.00E+00
SLC25A28	LMNA	-0.024678615	-0.162177094	0.113759955	7.27E-01	7.88E-01	1.00E+00
SHARPIN	LMNA	0.320696722	0.191106484	0.439298863	3.27E-06	1.42E-05	3.88E-02
ADIPOR2	LMNA	-0.054189889	-0.190813421	0.084493202	4.44E-01	5.30E-01	1.00E+00
TTC23	LMNA	0.289087218	0.157314445	0.410745853	3.01E-05	1.09E-04	3.57E-01
UGP2	LMNA	0.234959076	0.100156795	0.361291117	7.63E-04	2.06E-03	1.00E+00
ECHDC2	LMNA	0.260628477	0.127152423	0.384833171	1.80E-04	5.58E-04	1.00E+00
CIRBP	LMNA	-0.206299429	-0.334814953	-0.070249112	3.22E-03	7.41E-03	1.00E+00
SEC14L4	LMNA	0.055526717	-0.083161761	0.192105132	4.33E-01	5.19E-01	1.00E+00
SMA4	LMNA	-0.255166137	-0.379837021	-0.121391257	2.47E-04	7.46E-04	1.00E+00
FRZB	LMNA	0.081043798	-0.057652226	0.216670649	2.52E-01	3.32E-01	1.00E+00
PABPC1	LMNA	0.128387236	-0.009838135	0.26179813	6.86E-02	1.10E-01	1.00E+00
APOBEC3G	LMNA	0.418466809	0.297608586	0.526123765	5.74E-10	4.96E-09	6.81E-06
CUEDC1	LMNA	-0.050703389	-0.187442358	0.087963324	4.74E-01	5.59E-01	1.00E+00
CLDN8	LMNA	-0.095734666	-0.230736196	0.042883089	1.75E-01	2.45E-01	1.00E+00
VPS52	LMNA	-0.230426794	-0.35711765	-0.095410875	9.70E-04	2.55E-03	1.00E+00
LOC23117	LMNA	-0.053689135	-0.190329444	0.084991811	4.48E-01	5.34E-01	1.00E+00
FAT	LMNA	0.159693424	0.022130304	0.291321987	2.32E-02	4.26E-02	1.00E+00
M6PRBP1	LMNA	0.61939321	0.526349199	0.697778657	8.68E-23	7.72E-21	1.03E-18
PPM1F	LMNA	0.407550795	0.285566568	0.516539821	1.75E-09	1.38E-08	2.07E-05
TSR1	LMNA	0.115507613	-0.022908689	0.249578844	1.02E-01	1.54E-01	1.00E+00
E2F6	LMNA	0.002981669	-0.135124952	0.140974641	9.66E-01	9.75E-01	1.00E+00
TMEM126B	LMNA	-0.156029519	-0.287879584	-0.018374301	2.66E-02	4.81E-02	1.00E+00
ZFHX3	LMNA	0.32809024	0.199055078	0.445943058	1.87E-06	8.54E-06	2.22E-02
PCSK5	LMNA	0.47223431	0.357488874	0.572934335	1.29E-12	1.76E-11	1.53E-08
HEMK1	LMNA	0.035527006	-0.103029306	0.172730841	6.16E-01	6.91E-01	1.00E+00
GIMAP5	LMNA	0.209470938	0.073546738	0.337754855	2.77E-03	6.48E-03	1.00E+00
DPY19L4	LMNA	0.112287165	-0.026169498	0.246516821	1.12E-01	1.67E-01	1.00E+00

FAM77C	LMNA	-0.517300435	-0.611669748	-0.40841599	3.21E-15	6.83E-14	3.81E-11
CTPS2	LMNA	0.220673648	0.08521877	0.348119461	1.60E-03	3.97E-03	1.00E+00
NDUFA9	LMNA	-0.175165882	-0.305821559	-0.038034628	1.27E-02	2.50E-02	1.00E+00
SCRIB	LMNA	-0.078913907	-0.214626734	0.059788437	2.64E-01	3.46E-01	1.00E+00
AURKC	LMNA	0.001032091	-0.137038435	0.139063278	9.88E-01	9.91E-01	1.00E+00
LOC51035	LMNA	-0.243053511	-0.368732154	-0.108648063	4.91E-04	1.39E-03	1.00E+00
RSRC2	LMNA	-0.039318827	-0.176412261	0.099271017	5.79E-01	6.57E-01	1.00E+00
ORM2	LMNA	0.025062722	-0.113380568	0.162551306	7.23E-01	7.84E-01	1.00E+00
FAM115A	LMNA	-0.07764715	-0.213410546	0.061058351	2.72E-01	3.54E-01	1.00E+00
EGLN3	LMNA	0.065097033	-0.073615529	0.201338662	3.57E-01	4.44E-01	1.00E+00
FZD6	LMNA	0.178439614	0.041408665	0.308881695	1.11E-02	2.22E-02	1.00E+00
PITX3	LMNA	0.177389464	0.040325998	0.307900357	1.16E-02	2.31E-02	1.00E+00
GPX3	LMNA	0.145722534	0.007829031	0.2781774	3.85E-02	6.66E-02	1.00E+00
UNC119	LMNA	-0.209928373	-0.338178678	-0.074022608	2.71E-03	6.36E-03	1.00E+00
NOV	LMNA	0.295701609	0.1643601	0.41674047	1.93E-05	7.26E-05	2.29E-01
GRM4	LMNA	0.017966341	-0.120383243	0.155631334	8.00E-01	8.49E-01	1.00E+00
CABC1	LMNA	0.016716071	-0.121615578	0.154410743	8.13E-01	8.59E-01	1.00E+00
KLK1	LMNA	0.100007216	-0.038576374	0.23481632	1.57E-01	2.23E-01	1.00E+00
GPM6B	LMNA	-0.316927649	-0.435906779	-0.187060974	4.32E-06	1.83E-05	5.12E-02
RRAGD	LMNA	-0.070973115	-0.206995988	0.067741611	3.16E-01	4.01E-01	1.00E+00
EIF2S2	LMNA	0.201542789	0.065308889	0.330400982	4.02E-03	9.01E-03	1.00E+00
RNF130	LMNA	0.209439643	0.073514184	0.337725857	2.78E-03	6.49E-03	1.00E+00
CKAP5	LMNA	-0.149970013	-0.282178919	-0.012170994	3.31E-02	5.85E-02	1.00E+00
UCHL5	LMNA	-0.05231439	-0.18900042	0.086360309	4.60E-01	5.45E-01	1.00E+00
C10orf18	LMNA	-0.223109611	-0.350369067	-0.087761715	1.41E-03	3.57E-03	1.00E+00
TMEM93	LMNA	-0.096046903	-0.231034531	0.04256853	1.74E-01	2.43E-01	1.00E+00
KCNMB2	LMNA	-0.34927596	-0.464909979	-0.221925752	3.49E-07	1.82E-06	4.14E-03
AIM2	LMNA	0.136053998	-0.002035229	0.269051567	5.35E-02	8.91E-02	1.00E+00
PNO1	LMNA	0.121569668	-0.016762658	0.255335421	8.48E-02	1.32E-01	1.00E+00
ANK3	LMNA	-0.335848527	-0.452901118	-0.207414122	1.03E-06	4.91E-06	1.22E-02
OR2F2	LMNA	0.065572846	-0.073140249	0.201797099	3.54E-01	4.41E-01	1.00E+00
GNAT2	LMNA	0.146449348	0.008571645	0.278862453	3.75E-02	6.52E-02	1.00E+00
KRT5	LMNA	-0.092587387	-0.227727623	0.046052243	1.90E-01	2.62E-01	1.00E+00
ST13	LMNA	-0.234089985	-0.360491223	-0.099246262	7.99E-04	2.15E-03	1.00E+00
SIX1	LMNA	-0.143613533	-0.276188816	-0.005675046	4.14E-02	7.11E-02	1.00E+00

TIMP2	LMNA	0.425564314	0.305458895	0.532340532	2.73E-10	2.50E-09	3.23E-06
CDH12	LMNA	-0.27746272	-0.40018503	-0.144964438	6.39E-05	2.17E-04	7.58E-01
ZMAT4	LMNA	-0.009281	-0.147143473	0.128935199	8.96E-01	9.24E-01	1.00E+00
QRSL1	LMNA	-0.269295778	-0.392745842	-0.136312402	1.06E-04	3.46E-04	1.00E+00
CCL15	LMNA	0.031634414	-0.106883364	0.168947593	6.55E-01	7.26E-01	1.00E+00
GTF2IRD1	LMNA	0.112147252	-0.026311098	0.24638373	1.12E-01	1.68E-01	1.00E+00
CCDC22	LMNA	0.392590051	0.269124936	0.503360212	7.52E-09	5.27E-08	8.92E-05
SNX24	LMNA	0.129061606	-0.009152465	0.262436749	6.72E-02	1.08E-01	1.00E+00
RARS	LMNA	0.440345029	0.321859915	0.545250229	5.46E-11	5.72E-10	6.48E-07
MORC2	LMNA	-0.045966566	-0.182857204	0.092672509	5.16E-01	6.00E-01	1.00E+00
FAM48A	LMNA	-0.095239758	-0.23026327	0.043381622	1.78E-01	2.48E-01	1.00E+00
FOXD1	LMNA	0.040694669	-0.097906364	0.177747097	5.65E-01	6.45E-01	1.00E+00
COX4I1	LMNA	-0.246141402	-0.371566547	-0.111892517	4.14E-04	1.19E-03	1.00E+00
DSN1	LMNA	0.109252547	-0.029239455	0.243629032	1.22E-01	1.80E-01	1.00E+00
AMT	LMNA	-0.031891036	-0.169197131	0.10662941	6.52E-01	7.24E-01	1.00E+00
GPRC5B	LMNA	-0.258067292	-0.382491479	-0.124450003	2.09E-04	6.40E-04	1.00E+00
CTAGE1	LMNA	-0.04606297	-0.182950581	0.092576729	5.15E-01	5.99E-01	1.00E+00
DTWD1	LMNA	0.219770171	0.084276063	0.347284729	1.67E-03	4.14E-03	1.00E+00
STRN4	LMNA	-0.009440955	-0.147299975	0.128777886	8.94E-01	9.23E-01	1.00E+00
KITLG	LMNA	0.341470296	0.213482949	0.457934137	6.58E-07	3.25E-06	7.80E-03
HSD11B1	LMNA	0.03452665	-0.104020156	0.171758972	6.26E-01	7.00E-01	1.00E+00
HDGF	LMNA	0.104552024	-0.033989565	0.239151233	1.39E-01	2.01E-01	1.00E+00
KRT6B	LMNA	0.057252387	-0.081442313	0.193771863	4.18E-01	5.06E-01	1.00E+00
ARID4B	LMNA	-0.284561662	-0.406638275	-0.152501535	4.05E-05	1.43E-04	4.81E-01
SETX	LMNA	0.166162906	0.028771903	0.297392044	1.81E-02	3.42E-02	1.00E+00
DDR2	LMNA	0.402413006	0.279912108	0.512019521	2.91E-09	2.20E-08	3.45E-05
KCTD12	LMNA	0.317809835	0.188007468	0.43670103	4.05E-06	1.73E-05	4.80E-02
WWP2	LMNA	-0.107512724	-0.241972313	0.030998362	1.28E-01	1.88E-01	1.00E+00
CTSH	LMNA	0.048388434	-0.090265547	0.18520227	4.94E-01	5.79E-01	1.00E+00
FASTKD1	LMNA	-0.329027862	-0.446784727	-0.200064307	1.74E-06	8.00E-06	2.07E-02
PAF1	LMNA	0.033984878	-0.104556665	0.171232516	6.31E-01	7.05E-01	1.00E+00
IFT57	LMNA	0.209491746	0.073568383	0.337774134	2.77E-03	6.48E-03	1.00E+00
TMEM2	LMNA	0.486380635	0.373401824	0.585142076	2.16E-13	3.30E-12	2.56E-09
ZNF592	LMNA	0.079051959	-0.059650015	0.214759248	2.63E-01	3.45E-01	1.00E+00
TNFSF9	LMNA	0.42483167	0.304647786	0.531699335	2.95E-10	2.68E-09	3.49E-06

PPFIA4	LMNA	-0.204688728	-0.333320919	-0.068575499	3.48E-03	7.92E-03	1.00E+00
CFP	LMNA	0.137766189	-0.000290333	0.270669394	5.06E-02	8.47E-02	1.00E+00
DCTD	LMNA	0.372546658	0.247209764	0.485621911	4.77E-08	2.90E-07	5.65E-04
CNIH3	LMNA	0.499759596	0.388513006	0.59664657	3.68E-14	6.30E-13	4.37E-10
MAP4K4	LMNA	0.067104209	-0.071610174	0.203272136	3.43E-01	4.29E-01	1.00E+00
ROD1	LMNA	0.43285629	0.313541343	0.538715666	1.25E-10	1.22E-09	1.48E-06
MFNG	LMNA	-0.184223696	-0.314281798	-0.047377615	8.68E-03	1.79E-02	1.00E+00
JMJD2B	LMNA	-0.090967065	-0.226177705	0.04768274	1.98E-01	2.72E-01	1.00E+00
DOCK3	LMNA	-0.581768775	-0.666306217	-0.482466471	1.09E-19	5.28E-18	1.29E-15
STX16	LMNA	0.157037369	0.019407098	0.288826844	2.56E-02	4.65E-02	1.00E+00
ALDH3B1	LMNA	0.477530184	0.363438317	0.577509712	6.68E-13	9.59E-12	7.92E-09
THSD4	LMNA	0.001505952	-0.136573442	0.139527945	9.83E-01	9.87E-01	1.00E+00
DLAT	LMNA	0.17051091	0.033242409	0.30146565	1.53E-02	2.93E-02	1.00E+00
KIF21B	LMNA	-0.501430232	-0.598080349	-0.390404157	2.93E-14	5.11E-13	3.48E-10
FEZ2	LMNA	0.034003405	-0.10453832	0.17125052	6.31E-01	7.05E-01	1.00E+00
CDC5L	LMNA	-0.238539826	-0.364584807	-0.103910672	6.29E-04	1.74E-03	1.00E+00
KCNJ5	LMNA	0.135814337	-0.002279401	0.268825054	5.39E-02	8.97E-02	1.00E+00
LMNA	LMNA	1	1	1	0.00E+00	0.00E+00	0.00E+00
TBCD	LMNA	-0.131581219	-0.264821754	0.006589479	6.20E-02	1.01E-01	1.00E+00
ZNF250	LMNA	-0.36318782	-0.477307373	-0.237020667	1.08E-07	6.16E-07	1.28E-03
CASQ2	LMNA	-0.266651395	-0.390333632	-0.133515292	1.25E-04	4.02E-04	1.00E+00
PEG10	LMNA	-0.062487613	-0.198823467	0.076220906	3.77E-01	4.64E-01	1.00E+00
TPSD1	LMNA	0.016553405	-0.12177588	0.154251907	8.15E-01	8.61E-01	1.00E+00
PRAME	LMNA	-0.104136016	-0.23875466	0.03440966	1.40E-01	2.03E-01	1.00E+00
NP	LMNA	0.221220727	0.085789722	0.348624815	1.56E-03	3.87E-03	1.00E+00
ZNF12	LMNA	0.286779703	0.15485963	0.408652076	3.51E-05	1.25E-04	4.16E-01
XTP3TPA	LMNA	-0.168762413	-0.299828073	-0.031443989	1.64E-02	3.12E-02	1.00E+00
SIGLEC7	LMNA	0.322530667	0.19307653	0.44094815	2.85E-06	1.26E-05	3.38E-02
FAM70A	LMNA	-0.166599631	-0.297801424	-0.029220684	1.78E-02	3.37E-02	1.00E+00
PANK4	LMNA	-0.097358589	-0.232287541	0.041246787	1.68E-01	2.36E-01	1.00E+00
SNED1	LMNA	0.438500969	0.319809814	0.543642311	6.70E-11	6.94E-10	7.95E-07
HIP1	LMNA	0.091618036	-0.04702777	0.226800473	1.95E-01	2.68E-01	1.00E+00
WNK1	LMNA	-0.001675346	-0.139694038	0.136407203	9.81E-01	9.85E-01	1.00E+00
AHNAK2	LMNA	0.624222811	0.532018263	0.701796768	3.23E-23	3.14E-21	3.84E-19
FLJ10490	LMNA	-0.124382586	-0.258003374	0.013907217	7.78E-02	1.23E-01	1.00E+00

TOE1	LMNA	0.0133294	-0.124951529	0.151102351	8.51E-01	8.89E-01	1.00E+00
RECQL4	LMNA	-0.027543615	-0.16496734	0.110929185	6.97E-01	7.62E-01	1.00E+00
PPA1	LMNA	-0.308046147	-0.427900229	-0.177545458	8.20E-06	3.29E-05	9.72E-02
DPAGT1	LMNA	0.348486088	0.221070537	0.464204731	3.73E-07	1.94E-06	4.42E-03
HAS1	LMNA	0.113747828	-0.024690894	0.247905961	1.07E-01	1.61E-01	1.00E+00
ST7	LMNA	-0.100426844	-0.235216793	0.038153113	1.55E-01	2.21E-01	1.00E+00
MAGED2	LMNA	-0.039095354	-0.176195402	0.099492623	5.81E-01	6.59E-01	1.00E+00
ANKRD55	LMNA	-0.349635588	-0.46523103	-0.222315195	3.39E-07	1.77E-06	4.02E-03
TRPS1	LMNA	0.104646327	-0.033894329	0.239241123	1.38E-01	2.01E-01	1.00E+00
POPDC3	LMNA	0.138311132	0.000265195	0.271184145	4.96E-02	8.35E-02	1.00E+00
ACOX2	LMNA	0.438404761	0.319702888	0.543558403	6.77E-11	7.00E-10	8.03E-07
TFPI2	LMNA	0.502676231	0.391815231	0.599149292	2.48E-14	4.38E-13	2.94E-10
CYP4F11	LMNA	0.157635369	0.020020039	0.289388772	2.51E-02	4.57E-02	1.00E+00
PDHB	LMNA	-0.173390255	-0.304160654	-0.036205904	1.36E-02	2.66E-02	1.00E+00
ERN1	LMNA	0.044187297	-0.094439796	0.181133366	5.32E-01	6.15E-01	1.00E+00
LHX2	LMNA	-0.120798254	-0.254603407	0.017545339	8.68E-02	1.35E-01	1.00E+00
B3GALT1	LMNA	0.125908548	-0.012357244	0.259449843	7.42E-02	1.18E-01	1.00E+00
ADAMTS5	LMNA	0.043733105	-0.094890789	0.180693189	5.37E-01	6.19E-01	1.00E+00
NOTCH3	LMNA	0.551907098	0.447988972	0.641111001	1.69E-17	5.60E-16	2.00E-13
C1orf116	LMNA	-0.001053482	-0.139084255	0.137017446	9.88E-01	9.91E-01	1.00E+00
UGCGL1	LMNA	0.557994527	0.454992363	0.646262803	6.29E-18	2.26E-16	7.47E-14
NF2	LMNA	0.10307453	-0.035481354	0.237742563	1.44E-01	2.08E-01	1.00E+00
POM121	LMNA	0.109912354	-0.028572187	0.244257117	1.19E-01	1.77E-01	1.00E+00
CLPP	LMNA	0.101417842	-0.037153336	0.236162372	1.51E-01	2.16E-01	1.00E+00
MTMR3	LMNA	-0.130864595	-0.26414358	0.007318624	6.34E-02	1.03E-01	1.00E+00
ATP1B3	LMNA	0.226402257	0.091201773	0.353407486	1.20E-03	3.07E-03	1.00E+00
TMEM16A	LMNA	0.529596339	0.422429727	0.622160383	5.33E-16	1.36E-14	6.32E-12
HIST1H3F	LMNA	-0.132997206	-0.266161378	0.00514832	5.92E-02	9.72E-02	1.00E+00
TRIM25	LMNA	0.263255581	0.129926458	0.387233473	1.54E-04	4.85E-04	1.00E+00
CRKL	LMNA	-0.18461635	-0.314648081	-0.047783174	8.53E-03	1.76E-02	1.00E+00
HOXB2	LMNA	0.110207414	-0.028273751	0.244537954	1.18E-01	1.76E-01	1.00E+00
ANP32B	LMNA	0.028978159	-0.109510936	0.166363628	6.82E-01	7.50E-01	1.00E+00
NUP62	LMNA	0.14177339	0.003796698	0.274452812	4.42E-02	7.52E-02	1.00E+00
GATM	LMNA	-0.103852483	-0.238484348	0.034695951	1.41E-01	2.04E-01	1.00E+00
AP4E1	LMNA	0.091698692	-0.046946611	0.226877627	1.94E-01	2.67E-01	1.00E+00

EDG5	LMNA	0.344559351	0.216821865	0.46069651	5.13E-07	2.59E-06	6.09E-03
CDKN3	LMNA	0.135005731	-0.003103104	0.268060703	5.54E-02	9.17E-02	1.00E+00
CDH4	LMNA	0.143314518	0.005369757	0.275906783	4.19E-02	7.17E-02	1.00E+00
PGD	LMNA	0.057188476	-0.081506009	0.193710149	4.19E-01	5.06E-01	1.00E+00
TMEM168	LMNA	0.01302024	-0.125255903	0.150800183	8.54E-01	8.92E-01	1.00E+00
RND1	LMNA	-0.514274347	-0.609082848	-0.404974986	4.94E-15	1.02E-13	5.86E-11
GAD1	LMNA	-0.455839834	-0.558730407	-0.339129994	9.30E-12	1.10E-10	1.10E-07
MPG	LMNA	0.188766763	0.052072749	0.318517379	7.14E-03	1.50E-02	1.00E+00
ZNF133	LMNA	-0.140308384	-0.273070096	-0.002301972	4.64E-02	7.86E-02	1.00E+00
LOC44035C	LMNA	-0.128905829	-0.262289241	0.009310864	6.75E-02	1.09E-01	1.00E+00
FJX1	LMNA	0.228852536	0.093763847	0.35566684	1.05E-03	2.75E-03	1.00E+00
CLCF1	LMNA	0.673330644	0.590135038	0.742374947	4.89E-28	1.08E-25	5.80E-24
AMELY	LMNA	0.105147767	-0.033387884	0.239719064	1.36E-01	1.98E-01	1.00E+00
PHTF2	LMNA	0.106189992	-0.032335028	0.240712239	1.33E-01	1.94E-01	1.00E+00
IGSF2	LMNA	-0.090102482	-0.225350408	0.04855245	2.02E-01	2.76E-01	1.00E+00
TFF3	LMNA	-0.055569502	-0.192146465	0.083119141	4.32E-01	5.18E-01	1.00E+00
NDFIP1	LMNA	-0.044610418	-0.181543381	0.094019604	5.28E-01	6.11E-01	1.00E+00
IQCC	LMNA	-0.047620894	-0.184459239	0.091028539	5.01E-01	5.85E-01	1.00E+00
CUTA	LMNA	-0.156331852	-0.288163768	-0.018684087	2.63E-02	4.76E-02	1.00E+00
HEYL	LMNA	0.204826506	0.068718628	0.333448742	3.45E-03	7.87E-03	1.00E+00
FTSJ2	LMNA	0.320090465	0.190455465	0.438753473	3.42E-06	1.48E-05	4.06E-02
APPL1	LMNA	-0.360886246	-0.475259483	-0.234519155	1.32E-07	7.40E-07	1.56E-03
TNR	LMNA	-0.495749485	-0.593202472	-0.383977403	6.31E-14	1.03E-12	7.48E-10
WAC	LMNA	-0.333066647	-0.450407814	-0.204414671	1.28E-06	6.01E-06	1.51E-02
ADCY9	LMNA	0.201738456	0.065511976	0.330582664	3.99E-03	8.94E-03	1.00E+00
PYCRL	LMNA	0.088905743	-0.049755938	0.224204958	2.08E-01	2.83E-01	1.00E+00
PCGF1	LMNA	0.282913969	0.150750774	0.405141536	4.51E-05	1.58E-04	5.35E-01
PTPN13	LMNA	0.000996319	-0.137073535	0.139028198	9.89E-01	9.91E-01	1.00E+00
AGPAT7	LMNA	-0.089640517	-0.224908285	0.049017068	2.05E-01	2.79E-01	1.00E+00
CDKAL1	LMNA	-0.269395434	-0.392836716	-0.136417855	1.06E-04	3.44E-04	1.00E+00
PDYN	LMNA	-0.251553535	-0.376528741	-0.117585968	3.05E-04	9.02E-04	1.00E+00
SFRP1	LMNA	-0.230269485	-0.356972704	-0.095246261	9.78E-04	2.57E-03	1.00E+00
VAV3	LMNA	0.278945562	0.146537533	0.401534004	5.82E-05	1.99E-04	6.90E-01
MTMR11	LMNA	0.519423975	0.410832543	0.613483887	2.36E-15	5.18E-14	2.81E-11
SUOX	LMNA	-0.239453745	-0.365424956	-0.104869395	5.99E-04	1.66E-03	1.00E+00

IDH3B	LMNA	0.015578337	-0.122736621	0.153299651	8.26E-01	8.69E-01	1.00E+00
STXBP3	LMNA	-0.050484412	-0.187230524	0.08818116	4.76E-01	5.61E-01	1.00E+00
EIF3G	LMNA	0.143152812	0.005204668	0.27575425	4.21E-02	7.21E-02	1.00E+00
GOLT1B	LMNA	0.194574156	0.058083341	0.323924178	5.52E-03	1.19E-02	1.00E+00
ARHGAP22	LMNA	-0.005420065	-0.143363794	0.132730251	9.39E-01	9.55E-01	1.00E+00
NPFFR1	LMNA	0.135729054	-0.002366285	0.268744447	5.41E-02	8.99E-02	1.00E+00
NPC1	LMNA	0.388597103	0.264748879	0.499833905	1.10E-08	7.43E-08	1.30E-04
ALDH9A1	LMNA	-0.057753844	-0.194256046	0.080942509	4.14E-01	5.02E-01	1.00E+00
ICAM5	LMNA	-0.201287367	-0.330163802	-0.065043799	4.07E-03	9.11E-03	1.00E+00
ADD2	LMNA	-0.34061946	-0.457172885	-0.212563817	7.04E-07	3.47E-06	8.35E-03
RASSF1	LMNA	0.337670364	0.209379753	0.454532977	8.90E-07	4.29E-06	1.06E-02
PITPNB	LMNA	-0.118523469	-0.25244391	0.019852362	9.30E-02	1.43E-01	1.00E+00
PAPOLB	LMNA	0.007981361	-0.130213113	0.145871635	9.10E-01	9.36E-01	1.00E+00
URM1	LMNA	0.098864969	-0.039728259	0.233725982	1.62E-01	2.29E-01	1.00E+00
TMEM106B	LMNA	-0.022801017	-0.160347292	0.115613902	7.47E-01	8.05E-01	1.00E+00
LRIG2	LMNA	-0.089040237	-0.224333707	0.049620706	2.08E-01	2.82E-01	1.00E+00
SLC27A5	LMNA	-0.259171824	-0.38350155	-0.125615201	1.96E-04	6.02E-04	1.00E+00
CDKL1	LMNA	0.017751967	-0.120594572	0.155422079	8.02E-01	8.51E-01	1.00E+00
PRODH2	LMNA	0.01837879	-0.119976617	0.1560339	7.95E-01	8.45E-01	1.00E+00
SFRS11	LMNA	-0.197608922	-0.326746249	-0.061228234	4.82E-03	1.06E-02	1.00E+00
IL7	LMNA	0.336153923	0.20774355	0.453174723	1.00E-06	4.81E-06	1.19E-02
SCN9A	LMNA	-0.049916119	-0.186680709	0.08874643	4.81E-01	5.66E-01	1.00E+00
BTG3	LMNA	0.146579889	0.00870504	0.278985479	3.74E-02	6.49E-02	1.00E+00
PAK2	LMNA	0.093561701	-0.045071455	0.228659273	1.85E-01	2.57E-01	1.00E+00
ATP2B1	LMNA	0.169779869	0.032490387	0.300781079	1.57E-02	3.01E-02	1.00E+00
GATA4	LMNA	0.009649239	-0.128573032	0.147503752	8.92E-01	9.22E-01	1.00E+00
PRKAG1	LMNA	0.013353166	-0.124928129	0.151125579	8.50E-01	8.89E-01	1.00E+00
FAM64A	LMNA	-0.105756762	-0.240299433	0.032772714	1.34E-01	1.96E-01	1.00E+00
ATF7IP2	LMNA	-0.219762284	-0.347277441	-0.084267835	1.67E-03	4.14E-03	1.00E+00
EEF1G	LMNA	0.018115418	-0.120236275	0.155776844	7.98E-01	8.48E-01	1.00E+00
INCENP	LMNA	-0.075889884	-0.211722734	0.062819254	2.83E-01	3.66E-01	1.00E+00
SMAD5	LMNA	-0.039197729	-0.176294748	0.099391106	5.80E-01	6.58E-01	1.00E+00
GARS	LMNA	0.347698413	0.220217897	0.463501299	3.98E-07	2.05E-06	4.71E-03
CDK10	LMNA	-0.069535069	-0.205612318	0.069180017	3.25E-01	4.11E-01	1.00E+00
HLX	LMNA	0.408568767	0.286687918	0.517434727	1.58E-09	1.26E-08	1.87E-05

ELAVL3	LMNA	-0.459194043	-0.561641364	-0.342878889	6.26E-12	7.65E-11	7.43E-08
MDM4	LMNA	-0.059590263	-0.196028635	0.079111548	4.00E-01	4.87E-01	1.00E+00
GNL2	LMNA	0.426385631	0.306368381	0.533059188	2.50E-10	2.31E-09	2.96E-06
PFDN2	LMNA	-0.078357977	-0.214093048	0.060345808	2.68E-01	3.49E-01	1.00E+00
ZNF200	LMNA	0.070750391	-0.06796443	0.206781721	3.17E-01	4.02E-01	1.00E+00
NDN	LMNA	-0.342498835	-0.458854153	-0.21459435	6.06E-07	3.01E-06	7.19E-03
PRR3	LMNA	-0.248136394	-0.373396519	-0.113990182	3.70E-04	1.07E-03	1.00E+00
FBLN5	LMNA	0.272165757	0.139350541	0.395361921	8.91E-05	2.94E-04	1.00E+00
PUM1	LMNA	-0.176394382	-0.306970221	-0.039300402	1.20E-02	2.39E-02	1.00E+00
CCDC88A	LMNA	-0.29363802	-0.414871371	-0.162160525	2.22E-05	8.26E-05	2.64E-01
TNNT1	LMNA	-0.011770483	-0.14957843	0.126486048	8.68E-01	9.02E-01	1.00E+00
KLHL24	LMNA	-0.311438577	-0.430960679	-0.181177182	6.43E-06	2.64E-05	7.63E-02
HNRPH2	LMNA	0.043662606	-0.094960787	0.18062486	5.37E-01	6.19E-01	1.00E+00
RAB7A	LMNA	0.17344754	0.036264888	0.30421425	1.36E-02	2.66E-02	1.00E+00
SGPP1	LMNA	0.069008117	-0.069706956	0.205105155	3.29E-01	4.15E-01	1.00E+00
PMS2	LMNA	0.130341772	-0.007850489	0.263648727	6.45E-02	1.05E-01	1.00E+00
ARNT2	LMNA	-0.348409133	-0.464136013	-0.220987227	3.75E-07	1.95E-06	4.45E-03
CBR1	LMNA	0.248851885	0.114742787	0.374052589	3.55E-04	1.04E-03	1.00E+00
ITPR3	LMNA	0.623782486	0.531501059	0.701430632	3.54E-23	3.42E-21	4.20E-19
BIRC3	LMNA	0.455384404	0.33862126	0.558334967	9.80E-12	1.15E-10	1.16E-07
NRSN2	LMNA	0.134826892	-0.003285257	0.26789163	5.57E-02	9.22E-02	1.00E+00
SPOCK1	LMNA	-0.093953355	-0.229033708	0.044677123	1.84E-01	2.55E-01	1.00E+00
H2AFY	LMNA	-0.079105657	-0.214810792	0.059596171	2.63E-01	3.45E-01	1.00E+00
RXRB	LMNA	-0.347989949	-0.463761672	-0.220533455	3.88E-07	2.01E-06	4.60E-03
ZNF638	LMNA	-0.22693416	-0.353898069	-0.091757792	1.16E-03	2.99E-03	1.00E+00
APBA1	LMNA	-0.401095993	-0.510859815	-0.278464019	3.31E-09	2.48E-08	3.92E-05
RAB2A	LMNA	-0.079677551	-0.215359688	0.059022674	2.60E-01	3.41E-01	1.00E+00
ACTN4	LMNA	0.594746575	0.497546837	0.677196143	1.03E-20	5.79E-19	1.23E-16
FXC1	LMNA	0.150182622	0.01238847	0.282379096	3.29E-02	5.82E-02	1.00E+00
C6orf162	LMNA	-0.448564841	-0.552408069	-0.331011681	2.16E-11	2.38E-10	2.56E-07
HPSE2	LMNA	-0.270767119	-0.394087267	-0.137869641	9.72E-05	3.18E-04	1.00E+00
PLCE1	LMNA	-0.035908195	-0.173101105	0.102651667	6.12E-01	6.88E-01	1.00E+00
INSL3	LMNA	0.020778139	-0.117610219	0.158374851	7.69E-01	8.23E-01	1.00E+00
PTPLA	LMNA	0.106905273	-0.031612273	0.241393694	1.30E-01	1.90E-01	1.00E+00
EIF2B5	LMNA	-0.124194671	-0.257825208	0.014098043	7.82E-02	1.23E-01	1.00E+00

DLG1	LMNA	0.035814726	-0.102744268	0.173010319	6.13E-01	6.89E-01	1.00E+00
PINK1	LMNA	-0.179582717	-0.30994958	-0.042587529	1.05E-02	2.13E-02	1.00E+00
TFIP11	LMNA	0.067407297	-0.071307264	0.203564003	3.41E-01	4.27E-01	1.00E+00
VPS33A	LMNA	0.017497761	-0.120845151	0.155173926	8.05E-01	8.52E-01	1.00E+00
SKIP	LMNA	-0.391629545	-0.502512294	-0.268071807	8.24E-09	5.73E-08	9.78E-05
GRAP2	LMNA	0.287293075	0.155405632	0.409118007	3.39E-05	1.22E-04	4.02E-01
GAPDHS	LMNA	0.011955267	-0.126304191	0.149759099	8.66E-01	9.01E-01	1.00E+00
POLR2G	LMNA	-0.147073025	-0.279450187	-0.009209	3.67E-02	6.39E-02	1.00E+00
CNTNAP1	LMNA	0.073675877	-0.065036613	0.20959508	2.97E-01	3.81E-01	1.00E+00
PSTPIP1	LMNA	0.184075478	0.047224538	0.314143525	8.73E-03	1.80E-02	1.00E+00
CYP26A1	LMNA	0.185481985	0.048677417	0.315455442	8.22E-03	1.71E-02	1.00E+00
APOL2	LMNA	0.204801752	0.068692912	0.333425776	3.46E-03	7.88E-03	1.00E+00
TACC2	LMNA	-0.359284557	-0.473833604	-0.232779316	1.51E-07	8.40E-07	1.79E-03
CNBP	LMNA	-0.098988103	-0.233843537	0.039604104	1.61E-01	2.28E-01	1.00E+00
WASF1	LMNA	-0.539097001	-0.630243467	-0.433292907	1.26E-16	3.60E-15	1.50E-12
HSPB1	LMNA	0.567570412	0.466034958	0.654350643	1.28E-18	5.30E-17	1.52E-14
INPP5E	LMNA	0.055639274	-0.083049635	0.192213869	4.32E-01	5.18E-01	1.00E+00
COX7A2L	LMNA	-0.088049113	-0.223384811	0.050617153	2.13E-01	2.88E-01	1.00E+00
HSD17B1	LMNA	-0.231513635	-0.358118909	-0.096548388	9.16E-04	2.43E-03	1.00E+00
ARRB2	LMNA	0.034844459	-0.103705397	0.172067759	6.23E-01	6.97E-01	1.00E+00
CUBN	LMNA	0.157980382	0.020373719	0.289712932	2.47E-02	4.52E-02	1.00E+00
SLC7A6	LMNA	-0.060806185	-0.197201804	0.077898725	3.90E-01	4.77E-01	1.00E+00
IGF1	LMNA	0.102619049	-0.035941118	0.237308184	1.46E-01	2.10E-01	1.00E+00
ITPK1	LMNA	-0.146880599	-0.279268861	-0.009012341	3.70E-02	6.43E-02	1.00E+00
NAALAD2	LMNA	-0.171934396	-0.302798263	-0.034707196	1.44E-02	2.79E-02	1.00E+00
G3BP1	LMNA	0.259161667	0.125604484	0.383492263	1.96E-04	6.02E-04	1.00E+00
HSD17B10	LMNA	0.127602713	-0.010635642	0.261055051	7.03E-02	1.13E-01	1.00E+00
NUP155	LMNA	0.222613503	0.087243677	0.349911032	1.45E-03	3.64E-03	1.00E+00
CYP39A1	LMNA	-0.05250503	-0.189184749	0.086170567	4.58E-01	5.44E-01	1.00E+00
GLULD1	LMNA	-0.009067869	-0.146934932	0.129144799	8.98E-01	9.26E-01	1.00E+00
RBM15	LMNA	-0.035246567	-0.172458415	0.103307108	6.18E-01	6.94E-01	1.00E+00
AMZ2	LMNA	-0.215165947	-0.343027696	-0.079475682	2.10E-03	5.07E-03	1.00E+00
GDF15	LMNA	0.533741797	0.427165915	0.625689733	2.86E-16	7.60E-15	3.39E-12
CYCS	LMNA	-0.090551394	-0.225779984	0.048100902	2.00E-01	2.74E-01	1.00E+00
TECTA	LMNA	-0.401219031	-0.510968174	-0.278599279	3.27E-09	2.46E-08	3.88E-05

CCDC46	LMNA	0.473851631	0.359304802	0.574332287	1.06E-12	1.46E-11	1.25E-08
GNAL	LMNA	-0.381817837	-0.493838448	-0.257330831	2.06E-08	1.33E-07	2.44E-04
LPO	LMNA	0.184238218	0.047392614	0.314295345	8.67E-03	1.79E-02	1.00E+00
GZMM	LMNA	0.097404758	-0.041200256	0.232331635	1.68E-01	2.36E-01	1.00E+00
PAIP1	LMNA	-0.203980116	-0.332663428	-0.067839451	3.59E-03	8.15E-03	1.00E+00
DDX11	LMNA	-0.146145346	-0.278575934	-0.008261015	3.80E-02	6.58E-02	1.00E+00
TAF9B	LMNA	-0.179683354	-0.310043579	-0.042691332	1.05E-02	2.12E-02	1.00E+00
CACNA2D1	LMNA	-0.008809415	-0.146682028	0.129398954	9.01E-01	9.28E-01	1.00E+00
IMP4	LMNA	0.19598836	0.059548528	0.325239553	5.18E-03	1.13E-02	1.00E+00
SH3BP4	LMNA	-0.012963218	-0.150744449	0.125312039	8.55E-01	8.93E-01	1.00E+00
PADI1	LMNA	-0.04426647	-0.181210091	0.094361174	5.32E-01	6.14E-01	1.00E+00
Dec-01	LMNA	0.068978097	-0.069736973	0.20507626	3.29E-01	4.15E-01	1.00E+00
PON3	LMNA	-0.249685533	-0.374816845	-0.115619873	3.39E-04	9.94E-04	1.00E+00
PROP1	LMNA	0.055915647	-0.082774306	0.192480847	4.29E-01	5.16E-01	1.00E+00
UBB	LMNA	-0.01899558	-0.156635826	0.119368447	7.88E-01	8.40E-01	1.00E+00
RPA4	LMNA	-0.024527037	-0.162029409	0.113909659	7.29E-01	7.89E-01	1.00E+00
TCF25	LMNA	-0.115453385	-0.249527306	0.02296362	1.02E-01	1.54E-01	1.00E+00
NDUFS1	LMNA	-0.190049926	-0.319712756	-0.053399957	6.75E-03	1.43E-02	1.00E+00
UPK1A	LMNA	-0.018813794	-0.156458431	0.119547704	7.90E-01	8.42E-01	1.00E+00
AES	LMNA	-0.069316732	-0.205402189	0.069398358	3.27E-01	4.13E-01	1.00E+00
PPP1R13B	LMNA	-0.163583442	-0.294973107	-0.026122359	2.00E-02	3.74E-02	1.00E+00
TCL6	LMNA	-0.178417684	-0.308861205	-0.041386053	1.11E-02	2.22E-02	1.00E+00
ARL2	LMNA	-0.112894531	-0.247094513	0.025554746	1.10E-01	1.65E-01	1.00E+00
CD2BP2	LMNA	0.328720524	0.199733468	0.446508864	1.78E-06	8.16E-06	2.12E-02
TOP3A	LMNA	-0.01964519	-0.157269669	0.118727805	7.81E-01	8.34E-01	1.00E+00
CHRM5	LMNA	-0.167078862	-0.298250592	-0.029713209	1.75E-02	3.31E-02	1.00E+00
FGFR1	LMNA	0.472448384	0.357729187	0.573119407	1.26E-12	1.72E-11	1.49E-08
CEACAM6	LMNA	-0.060894272	-0.197286779	0.077810846	3.89E-01	4.77E-01	1.00E+00
CERK	LMNA	-0.15528248	-0.287177289	-0.017608961	2.73E-02	4.93E-02	1.00E+00
FXYD6	LMNA	-0.545101508	-0.635341883	-0.44017443	4.97E-17	1.53E-15	5.90E-13
AP3S2	LMNA	0.021880558	-0.116522414	0.159449919	7.57E-01	8.13E-01	1.00E+00
ZNF208	LMNA	-0.308017633	-0.427874493	-0.177514948	8.22E-06	3.30E-05	9.74E-02
CARKL	LMNA	0.08548721	-0.053191535	0.220930874	2.26E-01	3.04E-01	1.00E+00
HIST1H4E	LMNA	-0.181824781	-0.312043157	-0.04490084	9.60E-03	1.96E-02	1.00E+00
GOT1	LMNA	-0.157217641	-0.288996252	-0.019591863	2.54E-02	4.63E-02	1.00E+00

BBC3	LMNA	0.028585802	-0.109898893	0.165981789	6.86E-01	7.53E-01	1.00E+00
CASP6	LMNA	0.373143037	0.247859991	0.486151051	4.52E-08	2.75E-07	5.36E-04
HOXA1	LMNA	0.166241626	0.028852791	0.297465838	1.81E-02	3.41E-02	1.00E+00
RCL1	LMNA	-0.192617448	-0.322103387	-0.056057062	6.03E-03	1.29E-02	1.00E+00
RAB40B	LMNA	-0.224135114	-0.351315678	-0.088832784	1.34E-03	3.41E-03	1.00E+00
PI4KB	LMNA	0.121633144	-0.016698246	0.255395649	8.46E-02	1.32E-01	1.00E+00
LRRN2	LMNA	-0.195899534	-0.325156949	-0.059456482	5.20E-03	1.13E-02	1.00E+00
B3GAT1	LMNA	-0.376996694	-0.489568229	-0.252064324	3.20E-08	1.99E-07	3.79E-04
OAZ2	LMNA	0.01863127	-0.119727679	0.156280307	7.92E-01	8.43E-01	1.00E+00
BET1L	LMNA	0.459213611	0.34290077	0.561658338	6.25E-12	7.64E-11	7.41E-08
NOC4L	LMNA	0.079921998	-0.058777513	0.21559428	2.58E-01	3.40E-01	1.00E+00
FRY	LMNA	-0.524819468	-0.61808879	-0.416979397	1.08E-15	2.57E-14	1.28E-11
C10orf12	LMNA	0.231936024	0.096990564	0.358507958	8.96E-04	2.38E-03	1.00E+00
AK3L1	LMNA	0.341245243	0.213239809	0.457732796	6.70E-07	3.31E-06	7.95E-03
FADS1	LMNA	-0.198612087	-0.3276786	-0.062268398	4.60E-03	1.02E-02	1.00E+00
CSF3R	LMNA	0.203102159	0.066927708	0.331848636	3.74E-03	8.45E-03	1.00E+00
DKK2	LMNA	0.362053554	0.235787658	0.476298282	1.19E-07	6.75E-07	1.42E-03
POLR3K	LMNA	-0.233221613	-0.359691805	-0.098336708	8.37E-04	2.24E-03	1.00E+00
ATG2B	LMNA	0.066986323	-0.071727984	0.203158608	3.44E-01	4.30E-01	1.00E+00
RHOT1	LMNA	-0.509800895	-0.605254946	-0.399893832	9.26E-15	1.81E-13	1.10E-10
DARS	LMNA	0.02979652	-0.108701619	0.167159918	6.74E-01	7.43E-01	1.00E+00
EPS8	LMNA	0.284924499	0.15288718	0.406967784	3.96E-05	1.40E-04	4.69E-01
OXT	LMNA	0.137543244	-0.000517584	0.270458778	5.09E-02	8.53E-02	1.00E+00
C10orf56	LMNA	-0.184370917	-0.314419137	-0.04752967	8.62E-03	1.78E-02	1.00E+00
ARL4A	LMNA	0.291292019	0.159661512	0.412745232	2.60E-05	9.54E-05	3.08E-01
CCDC64	LMNA	-0.015213313	-0.152943101	0.123096215	8.30E-01	8.72E-01	1.00E+00
DAD1	LMNA	0.087248375	-0.051421989	0.222618003	2.17E-01	2.93E-01	1.00E+00
HERC2	LMNA	-0.172668778	-0.303485563	-0.035463115	1.40E-02	2.73E-02	1.00E+00
HSD11B2	LMNA	-0.016449021	-0.154149978	0.121878742	8.16E-01	8.61E-01	1.00E+00
USE1	LMNA	0.006808119	-0.131366348	0.144723099	9.23E-01	9.45E-01	1.00E+00
FAM96B	LMNA	-0.092036801	-0.227201037	0.04660637	1.93E-01	2.65E-01	1.00E+00
HNMT	LMNA	0.037552594	-0.101022123	0.17469793	5.96E-01	6.73E-01	1.00E+00
PCGF3	LMNA	-0.141088169	-0.273806148	-0.003097499	4.52E-02	7.68E-02	1.00E+00
BSN	LMNA	-0.479689581	-0.579373525	-0.365866881	5.09E-13	7.42E-12	6.03E-09
CAND1	LMNA	-0.121160582	-0.254947248	0.01717774	8.59E-02	1.34E-01	1.00E+00

ACTR10	LMNA	-0.139412352	-0.272224123	-0.001388066	4.78E-02	8.07E-02	1.00E+00
NASP	LMNA	-0.13006643	-0.263388086	0.008130562	6.50E-02	1.06E-01	1.00E+00
C20orf4	LMNA	0.217826176	0.08224848	0.345487966	1.84E-03	4.52E-03	1.00E+00
ACSBG2	LMNA	-0.021517986	-0.159096378	0.116880216	7.61E-01	8.16E-01	1.00E+00
COL9A2	LMNA	-0.180349649	-0.310665862	-0.043378667	1.02E-02	2.07E-02	1.00E+00
RWDD2A	LMNA	-0.392495469	-0.503276726	-0.26902122	7.59E-09	5.31E-08	9.00E-05
PALLD	LMNA	0.429600399	0.309930365	0.535870645	1.77E-10	1.69E-09	2.10E-06
GPC5	LMNA	-0.141959155	-0.274628103	-0.003986275	4.39E-02	7.47E-02	1.00E+00
CENTD2	LMNA	0.227311128	0.092151902	0.354245711	1.14E-03	2.95E-03	1.00E+00
TBL3	LMNA	0.242310318	0.107867617	0.368049624	5.12E-04	1.44E-03	1.00E+00
TLR1	LMNA	0.232347118	0.097420968	0.358886562	8.76E-04	2.33E-03	1.00E+00
LMO6	LMNA	0.539762737	0.434055272	0.63080913	1.14E-16	3.30E-15	1.35E-12
CCDC106	LMNA	0.008460892	-0.129741651	0.146340962	9.05E-01	9.31E-01	1.00E+00
KPNA2	LMNA	0.006988306	-0.131189258	0.144899516	9.21E-01	9.44E-01	1.00E+00
PARP16	LMNA	0.03265271	-0.10587555	0.169937676	6.45E-01	7.17E-01	1.00E+00
PDIA3	LMNA	0.61688688	0.523410486	0.695691505	1.44E-22	1.18E-20	1.71E-18
MACROD1	LMNA	-0.213153075	-0.341164957	-0.077379029	2.32E-03	5.54E-03	1.00E+00
SFRS1	LMNA	-0.093393077	-0.22849805	0.045241219	1.86E-01	2.58E-01	1.00E+00
DCP1A	LMNA	0.04451516	-0.094114206	0.181451078	5.29E-01	6.12E-01	1.00E+00
TMCO3	LMNA	0.296061783	0.164744143	0.417066594	1.89E-05	7.10E-05	2.24E-01
NTN2L	LMNA	0.164555978	0.02712109	0.295885317	1.93E-02	3.61E-02	1.00E+00
CLN5	LMNA	0.073960627	-0.064751508	0.209868796	2.96E-01	3.80E-01	1.00E+00
BIRC5	LMNA	-0.04009253	-0.177162966	0.098503671	5.71E-01	6.50E-01	1.00E+00
PRR16	LMNA	0.452279746	0.335155046	0.555638003	1.41E-11	1.61E-10	1.67E-07
FAM63B	LMNA	0.215927968	0.080269734	0.343732618	2.03E-03	4.91E-03	1.00E+00
GLE1L	LMNA	0.124105151	-0.014188947	0.257740328	7.85E-02	1.24E-01	1.00E+00
CES2	LMNA	0.241528559	0.10704685	0.36733153	5.34E-04	1.50E-03	1.00E+00
GNAS	LMNA	0.113994365	-0.024441269	0.248140372	1.06E-01	1.60E-01	1.00E+00
KATNB1	LMNA	-0.284048757	-0.406172431	-0.151956458	4.19E-05	1.47E-04	4.97E-01
CPLX3	LMNA	-0.275315289	-0.398230523	-0.142687491	7.32E-05	2.46E-04	8.68E-01
WNT8B	LMNA	-0.200885517	-0.329790617	-0.064626775	4.15E-03	9.27E-03	1.00E+00
TSPAN13	LMNA	-0.210038734	-0.338280922	-0.074137427	2.70E-03	6.33E-03	1.00E+00
MRPL52	LMNA	-0.056684935	-0.193223879	0.08200781	4.23E-01	5.10E-01	1.00E+00
GHR	LMNA	-0.026035648	-0.163498993	0.112419416	7.13E-01	7.75E-01	1.00E+00
BCLAF1	LMNA	-0.197479326	-0.326625783	-0.06109388	4.85E-03	1.06E-02	1.00E+00

GOLGA3	LMNA	0.29088857	0.159231919	0.41237946	2.67E-05	9.78E-05	3.17E-01
CLEC4E	LMNA	0.171172627	0.033923252	0.302085188	1.49E-02	2.87E-02	1.00E+00
SCUBE3	LMNA	-0.052071007	-0.188765078	0.086602532	4.62E-01	5.48E-01	1.00E+00
UCRC	LMNA	-0.138851266	-0.27169428	-0.000815905	4.88E-02	8.21E-02	1.00E+00
CDKL3	LMNA	-0.312390634	-0.431819074	-0.182197033	6.01E-06	2.47E-05	7.12E-02
MGAT4B	LMNA	0.686420552	0.605773257	0.753106403	1.75E-29	4.51E-27	2.08E-25
BBS7	LMNA	-0.16887508	-0.299933616	-0.031559846	1.63E-02	3.11E-02	1.00E+00
ZNF345	LMNA	-0.09574276	-0.23074393	0.042874935	1.75E-01	2.45E-01	1.00E+00
RTF1	LMNA	-0.076436404	-0.212247741	0.062271695	2.80E-01	3.62E-01	1.00E+00
SERPINB9	LMNA	0.174635258	0.03748804	0.305325301	1.29E-02	2.55E-02	1.00E+00
DHRS7	LMNA	-0.025362061	-0.162842906	0.113084879	7.20E-01	7.82E-01	1.00E+00
RIPK4	LMNA	-0.020407471	-0.158013305	0.1179759	7.73E-01	8.27E-01	1.00E+00
PLEC1	LMNA	0.380232684	0.255598429	0.49243503	2.38E-08	1.52E-07	2.83E-04
PIP3-E	LMNA	-0.339208622	-0.455910218	-0.211040232	7.88E-07	3.85E-06	9.35E-03
KNTC1	LMNA	-0.006177626	-0.144105729	0.131985936	9.30E-01	9.49E-01	1.00E+00
EXOSC2	LMNA	-0.163348539	-0.294752738	-0.025881169	2.02E-02	3.77E-02	1.00E+00
MS4A2	LMNA	0.145173918	0.00726859	0.277660218	3.93E-02	6.79E-02	1.00E+00
FGF17	LMNA	-0.27115823	-0.394443755	-0.138283694	9.49E-05	3.11E-04	1.00E+00
WDR59	LMNA	-0.233069025	-0.359551314	-0.098176907	8.44E-04	2.25E-03	1.00E+00
LAIR1	LMNA	0.326003415	0.196809861	0.444069044	2.19E-06	9.87E-06	2.60E-02
EVI2A	LMNA	-0.117053709	-0.25104793	0.021342168	9.71E-02	1.48E-01	1.00E+00
IL17RC	LMNA	0.52924834	0.422032404	0.621863933	5.61E-16	1.42E-14	6.65E-12
GTF3C3	LMNA	-0.006850378	-0.144764474	0.131324817	9.23E-01	9.45E-01	1.00E+00
HS3ST1	LMNA	-0.173975495	-0.304708169	-0.036808543	1.33E-02	2.61E-02	1.00E+00
ITGB1BP2	LMNA	-0.192710949	-0.322190416	-0.056153863	6.00E-03	1.29E-02	1.00E+00
RBPJ	LMNA	-0.350865089	-0.466328411	-0.223646937	3.06E-07	1.62E-06	3.63E-03
LRRC8D	LMNA	-0.449860175	-0.553534655	-0.332455898	1.86E-11	2.08E-10	2.21E-07
ALDH18A1	LMNA	0.095016135	-0.04360686	0.230049558	1.79E-01	2.49E-01	1.00E+00
INE1	LMNA	-0.189441667	-0.319146162	-0.05277076	6.93E-03	1.46E-02	1.00E+00
TPI1	LMNA	0.374738463	0.249600025	0.487566192	3.92E-08	2.40E-07	4.65E-04
FLJ20035	LMNA	0.24801542	0.113862947	0.373285579	3.72E-04	1.08E-03	1.00E+00
GATA6	LMNA	0.324712412	0.195421549	0.442909179	2.42E-06	1.08E-05	2.87E-02
CABP1	LMNA	-0.369073551	-0.482538734	-0.243425307	6.48E-08	3.85E-07	7.69E-04
RASGRF1	LMNA	-0.289290592	-0.410930327	-0.157530879	2.97E-05	1.08E-04	3.52E-01
C4orf15	LMNA	-0.032252195	-0.169548291	0.106271976	6.49E-01	7.21E-01	1.00E+00

ALDH2	LMNA	-0.321100885	-0.439662399	-0.191540551	3.17E-06	1.38E-05	3.76E-02
DAPK3	LMNA	0.437687999	0.318906362	0.542933202	7.33E-11	7.54E-10	8.70E-07
C3	LMNA	0.205732151	0.06965959	0.334288838	3.31E-03	7.58E-03	1.00E+00
EMP2	LMNA	0.350076693	0.222792926	0.465624775	3.27E-07	1.71E-06	3.88E-03
GJB1	LMNA	-0.216086727	-0.343879463	-0.080435187	2.01E-03	4.87E-03	1.00E+00
PIN1	LMNA	-0.185540104	-0.315509641	-0.048737464	8.20E-03	1.70E-02	1.00E+00
SLC6A15	LMNA	-0.133010388	-0.266173846	0.005134901	5.91E-02	9.72E-02	1.00E+00
POLE3	LMNA	-0.043680195	-0.180641907	0.094943323	5.37E-01	6.19E-01	1.00E+00
RIN2	LMNA	0.083638385	-0.055048222	0.219158898	2.37E-01	3.15E-01	1.00E+00
CTSL2	LMNA	-0.040812262	-0.177861162	0.097789703	5.64E-01	6.44E-01	1.00E+00
APOC4	LMNA	0.082427225	-0.056264013	0.217997596	2.44E-01	3.23E-01	1.00E+00
CYorf15B	LMNA	0.000150273	-0.137903588	0.138198407	9.98E-01	9.99E-01	1.00E+00
TRIM2	LMNA	-0.412084833	-0.520523894	-0.290563627	1.11E-09	9.04E-09	1.31E-05
CP110	LMNA	-0.28141549	-0.403779772	-0.149159282	4.97E-05	1.72E-04	5.89E-01
KIAA1622	LMNA	-0.310864122	-0.430442632	-0.180561956	6.70E-06	2.74E-05	7.95E-02
DNM1	LMNA	-0.236849483	-0.363030367	-0.102138124	6.89E-04	1.88E-03	1.00E+00
HYOU1	LMNA	0.285743222	0.153757515	0.407711183	3.75E-05	1.33E-04	4.45E-01
OTUD4	LMNA	0.190387491	0.05374919	0.320027159	6.65E-03	1.41E-02	1.00E+00
USP7	LMNA	-0.127610855	-0.261062763	0.010627366	7.03E-02	1.13E-01	1.00E+00
ZSCAN16	LMNA	-0.508201529	-0.603885307	-0.398078836	1.16E-14	2.21E-13	1.37E-10
ASCC1	LMNA	-0.405205089	-0.514476789	-0.282983917	2.21E-09	1.71E-08	2.62E-05
OTUD3	LMNA	-0.040141496	-0.177210472	0.0984551	5.71E-01	6.50E-01	1.00E+00
YME1L1	LMNA	0.086345606	-0.052329163	0.221753286	2.22E-01	2.98E-01	1.00E+00
HNRNPR	LMNA	-0.155314237	-0.287207147	-0.017641493	2.73E-02	4.92E-02	1.00E+00
SERPING1	LMNA	0.45264155	0.335558821	0.555952408	1.35E-11	1.55E-10	1.60E-07
TPCN1	LMNA	0.298031856	0.166845478	0.418849867	1.65E-05	6.27E-05	1.96E-01
STARD13	LMNA	0.352540439	0.225462368	0.46782316	2.67E-07	1.42E-06	3.16E-03
PCBP4	LMNA	-0.248876924	-0.374075546	-0.114769128	3.55E-04	1.04E-03	1.00E+00
ADI1	LMNA	0.02242588	-0.115984198	0.159981591	7.51E-01	8.08E-01	1.00E+00
CDH10	LMNA	-0.504727029	-0.600907924	-0.394138864	1.87E-14	3.45E-13	2.22E-10
WBSCR22	LMNA	0.330995292	0.202182883	0.448550139	1.50E-06	6.95E-06	1.78E-02
SCP2	LMNA	0.079189956	-0.059511641	0.214891706	2.63E-01	3.44E-01	1.00E+00
KL	LMNA	0.1805604	0.043596099	0.310862669	1.01E-02	2.05E-02	1.00E+00
SLC35D2	LMNA	0.352365488	0.225272747	0.4676671	2.71E-07	1.44E-06	3.21E-03
MAFK	LMNA	0.117649989	-0.020737829	0.251614346	9.54E-02	1.46E-01	1.00E+00

SON	LMNA	0.059156241	-0.079544365	0.195609779	4.03E-01	4.91E-01	1.00E+00
SBNO2	LMNA	0.567436408	0.46588021	0.654237599	1.31E-18	5.40E-17	1.55E-14
RACGAP1	LMNA	-0.149265366	-0.281515395	-0.011450314	3.40E-02	5.97E-02	1.00E+00
SC4MOL	LMNA	-0.057562925	-0.194071711	0.081132807	4.16E-01	5.03E-01	1.00E+00
SLC6A6	LMNA	0.407650202	0.285676055	0.516627221	1.73E-09	1.37E-08	2.05E-05
OBP2A	LMNA	0.036896215	-0.101672661	0.174060628	6.02E-01	6.79E-01	1.00E+00
PSMD3	LMNA	-0.03630206	-0.173483641	0.102261427	6.08E-01	6.84E-01	1.00E+00
ERLIN2	LMNA	0.187599966	0.05086632	0.317430048	7.51E-03	1.57E-02	1.00E+00
PPP1R9A	LMNA	-0.318966035	-0.437741702	-0.189248316	3.72E-06	1.60E-05	4.41E-02
RAB35	LMNA	0.315643078	0.185683193	0.434749919	4.74E-06	2.00E-05	5.63E-02
PDZRN3	LMNA	-0.18050439	-0.310810366	-0.043538312	1.01E-02	2.06E-02	1.00E+00
AVEN	LMNA	0.536150887	0.42992099	0.62773905	1.98E-16	5.48E-15	2.35E-12
FLJ21075	LMNA	-0.176561892	-0.307126816	-0.039473028	1.19E-02	2.38E-02	1.00E+00
BCAM	LMNA	0.382398156	0.257965257	0.494352088	1.95E-08	1.26E-07	2.32E-04
C14orf132	LMNA	-0.300884526	-0.421430399	-0.169890329	1.35E-05	5.24E-05	1.61E-01
PCK2	LMNA	0.38418018	0.259914103	0.49592887	1.66E-08	1.09E-07	1.97E-04
FKRP	LMNA	0.199265529	0.062946101	0.32828578	4.47E-03	9.90E-03	1.00E+00
HLA-DRA	LMNA	0.231063873	0.096077614	0.357704598	9.38E-04	2.48E-03	1.00E+00
GUCY2C	LMNA	0.25538434	0.121621225	0.380036741	2.44E-04	7.37E-04	1.00E+00
RP11-125A	LMNA	-0.085336192	-0.220786166	0.053343232	2.27E-01	3.05E-01	1.00E+00
BARX2	LMNA	0.135665149	-0.002431389	0.268684044	5.42E-02	9.01E-02	1.00E+00
DAO	LMNA	-0.097961596	-0.232863422	0.040638994	1.65E-01	2.33E-01	1.00E+00
EDNRA	LMNA	0.320645717	0.191051708	0.439252981	3.28E-06	1.42E-05	3.89E-02
NIPBL	LMNA	0.024346592	-0.114087867	0.161853591	7.31E-01	7.91E-01	1.00E+00
ZNF331	LMNA	-0.080904342	-0.216536859	0.057792135	2.52E-01	3.33E-01	1.00E+00
PPP2R5A	LMNA	-0.1368682	-0.269820989	0.00120558	5.21E-02	8.70E-02	1.00E+00
HMGN4	LMNA	-0.079816592	-0.215493125	0.058883229	2.59E-01	3.40E-01	1.00E+00
DDX39	LMNA	0.061158483	-0.077547248	0.197541642	3.87E-01	4.75E-01	1.00E+00
EFHC1	LMNA	0.101632835	-0.036936402	0.236367478	1.50E-01	2.15E-01	1.00E+00
EIF3M	LMNA	0.259163688	0.125606617	0.383494111	1.96E-04	6.02E-04	1.00E+00
SLC17A3	LMNA	0.149735038	0.011930657	0.281957671	3.34E-02	5.90E-02	1.00E+00
ZNF24	LMNA	-0.366990649	-0.480688332	-0.241157515	7.78E-08	4.56E-07	9.23E-04
ASCIZ	LMNA	-0.18314716	-0.313277365	-0.04626593	9.08E-03	1.86E-02	1.00E+00
FNDC8	LMNA	-0.029575679	-0.166945051	0.108920037	6.76E-01	7.45E-01	1.00E+00
ESRRA	LMNA	0.227590883	0.092444407	0.35450368	1.12E-03	2.91E-03	1.00E+00

PTMS	LMNA	-0.015114995	-0.152847058	0.123193066	8.31E-01	8.73E-01	1.00E+00
PHF7	LMNA	0.202073106	0.065859344	0.330893375	3.93E-03	8.82E-03	1.00E+00
PIP4K2B	LMNA	-0.354177975	-0.469283534	-0.227237675	2.33E-07	1.25E-06	2.76E-03
IRF3	LMNA	0.32910111	0.200143162	0.446850471	1.73E-06	7.96E-06	2.06E-02
GPR19	LMNA	-0.498794013	-0.595817602	-0.387420399	4.19E-14	7.08E-13	4.97E-10
HHLA2	LMNA	-0.024320303	-0.161827975	0.114113829	7.31E-01	7.91E-01	1.00E+00
GMFG	LMNA	0.184964736	0.048143047	0.314973036	8.41E-03	1.74E-02	1.00E+00
BDH2	LMNA	0.003702028	-0.134417669	0.14168062	9.58E-01	9.69E-01	1.00E+00
APOBEC2	LMNA	-0.049392685	-0.186174217	0.089267002	4.85E-01	5.70E-01	1.00E+00
PRSS21	LMNA	0.128353593	-0.009872339	0.261766268	6.87E-02	1.11E-01	1.00E+00
PENK	LMNA	-0.078938787	-0.214650616	0.059763491	2.64E-01	3.46E-01	1.00E+00
PHF16	LMNA	-0.421993546	-0.529214304	-0.301507348	3.97E-10	3.53E-09	4.71E-06
ZMAT5	LMNA	-0.020909591	-0.158503058	0.117480528	7.68E-01	8.22E-01	1.00E+00
SMAD9	LMNA	-0.375369458	-0.48812572	-0.250288436	3.70E-08	2.28E-07	4.39E-04
SLAMF1	LMNA	0.292466425	0.160912304	0.413809741	2.40E-05	8.88E-05	2.85E-01
RGL1	LMNA	0.036659277	-0.101907462	0.173830547	6.04E-01	6.81E-01	1.00E+00
DLGAP4	LMNA	0.436225359	0.317281476	0.541657043	8.62E-11	8.73E-10	1.02E-06
MBD5	LMNA	0.432356482	0.312986801	0.538279087	1.31E-10	1.28E-09	1.56E-06
PHLDA1	LMNA	0.132071764	-0.006090278	0.265285903	6.10E-02	9.97E-02	1.00E+00
BACE2	LMNA	0.638869113	0.549261024	0.713952029	1.46E-24	1.74E-22	1.73E-20
APPBP2	LMNA	-0.368595382	-0.482114029	-0.242904571	6.76E-08	4.01E-07	8.02E-04
LIF	LMNA	0.531823771	0.424973841	0.624057236	3.82E-16	9.95E-15	4.53E-12
OXTR	LMNA	0.242783158	0.10836414	0.368483884	4.99E-04	1.41E-03	1.00E+00
ACTC1	LMNA	0.056994059	-0.081699762	0.193522409	4.20E-01	5.08E-01	1.00E+00
USP19	LMNA	-0.033196889	-0.170466664	0.105336856	6.39E-01	7.13E-01	1.00E+00
SUV39H2	LMNA	-0.422362352	-0.52953733	-0.30191529	3.82E-10	3.40E-09	4.53E-06
ERO1L	LMNA	0.424329987	0.304092474	0.531260201	3.11E-10	2.81E-09	3.68E-06
ZNF395	LMNA	0.334234351	0.205673406	0.45145461	1.17E-06	5.52E-06	1.38E-02
CNTFR	LMNA	-0.460412135	-0.56269786	-0.34424123	5.42E-12	6.69E-11	6.43E-08
HIST1H2BL	LMNA	0.092066892	-0.046576088	0.227229818	1.93E-01	2.65E-01	1.00E+00
WNT3	LMNA	-0.191122448	-0.320711587	-0.054509665	6.44E-03	1.37E-02	1.00E+00
ZNF549	LMNA	-0.037153428	-0.174310379	0.101417751	6.00E-01	6.77E-01	1.00E+00
ZNF467	LMNA	0.106805059	-0.031713543	0.241298227	1.30E-01	1.91E-01	1.00E+00
SLC25A21	LMNA	-0.210762687	-0.338951553	-0.074890706	2.61E-03	6.13E-03	1.00E+00
IL5	LMNA	-0.223267501	-0.350514828	-0.087926601	1.40E-03	3.54E-03	1.00E+00

CLSTN2	LMNA	-0.416069918	-0.524021705	-0.294961153	7.35E-10	6.24E-09	8.72E-06
LSM12	LMNA	0.085394813	-0.053284348	0.220842339	2.27E-01	3.04E-01	1.00E+00
KRT37	LMNA	0.216834539	0.081214636	0.344571072	1.94E-03	4.72E-03	1.00E+00
NACAD	LMNA	-0.059403162	-0.195848078	0.079298136	4.01E-01	4.89E-01	1.00E+00
NAG18	LMNA	-0.332800147	-0.450168863	-0.204127454	1.30E-06	6.12E-06	1.55E-02
PTGES	LMNA	0.439836282	0.321294214	0.544806707	5.78E-11	6.02E-10	6.85E-07
GDAP1L1	LMNA	-0.580863154	-0.665544944	-0.481416321	1.28E-19	6.10E-18	1.52E-15
MFAP5	LMNA	0.425995068	0.305935864	0.532717463	2.60E-10	2.41E-09	3.09E-06
OPRK1	LMNA	-0.085536778	-0.220978369	0.053141744	2.26E-01	3.03E-01	1.00E+00
C12orf4	LMNA	-0.110848835	-0.245148381	0.027624904	1.16E-01	1.73E-01	1.00E+00
CST3	LMNA	0.137379439	-0.000684544	0.270304024	5.12E-02	8.57E-02	1.00E+00
WDR6	LMNA	-0.209185945	-0.337490776	-0.07325029	2.81E-03	6.55E-03	1.00E+00
NUP88	LMNA	-0.136141471	-0.269134237	0.001946106	5.34E-02	8.89E-02	1.00E+00
CD300A	LMNA	0.17792479	0.04087786	0.308400639	1.13E-02	2.26E-02	1.00E+00
FCGBP	LMNA	0.463614783	0.347825477	0.56547404	3.69E-12	4.69E-11	4.38E-08
CNIH	LMNA	-0.018759151	-0.156405106	0.119601584	7.91E-01	8.42E-01	1.00E+00
VASH1	LMNA	-0.090158327	-0.225403851	0.04849628	2.02E-01	2.76E-01	1.00E+00
DHX16	LMNA	0.180847333	0.04389215	0.311130599	1.00E-02	2.03E-02	1.00E+00
SLC35A5	LMNA	-0.254217897	-0.378968968	-0.12039206	2.61E-04	7.84E-04	1.00E+00
CLEC3B	LMNA	0.011190057	-0.127057222	0.149010866	8.74E-01	9.07E-01	1.00E+00
ARL2BP	LMNA	-0.039226721	-0.176322882	0.099362356	5.79E-01	6.58E-01	1.00E+00
C10orf79	LMNA	-0.152197799	-0.284275863	-0.014450417	3.06E-02	5.45E-02	1.00E+00
ZNF79	LMNA	0.211324304	0.07547518	0.339471715	2.54E-03	5.99E-03	1.00E+00
CRP	LMNA	0.071896259	-0.066817933	0.207883939	3.09E-01	3.94E-01	1.00E+00
OCRL	LMNA	-0.032171898	-0.16947022	0.106351448	6.49E-01	7.21E-01	1.00E+00
GLA	LMNA	0.370498288	0.244977303	0.483803854	5.72E-08	3.43E-07	6.78E-04
NEBL	LMNA	-0.17528944	-0.305937104	-0.038161915	1.26E-02	2.49E-02	1.00E+00
HSPA8	LMNA	-0.025015856	-0.162505649	0.11342686	7.24E-01	7.85E-01	1.00E+00
DIDO1	LMNA	0.176153547	0.039052225	0.306745067	1.22E-02	2.41E-02	1.00E+00
PLA2R1	LMNA	0.371584239	0.246160684	0.484767825	5.19E-08	3.14E-07	6.16E-04
NGDN	LMNA	-0.188843293	-0.318588685	-0.052151893	7.11E-03	1.49E-02	1.00E+00
CBFA2T2	LMNA	-0.167542226	-0.298684834	-0.030189489	1.72E-02	3.26E-02	1.00E+00
SAC3D1	LMNA	-0.007481301	-0.14538215	0.130704692	9.16E-01	9.39E-01	1.00E+00
PNOC	LMNA	-0.187597536	-0.317427783	-0.050863808	7.51E-03	1.57E-02	1.00E+00
PIGP	LMNA	0.129191438	-0.009020443	0.262559684	6.69E-02	1.08E-01	1.00E+00

AGGF1	LMNA	-0.013815691	-0.151577592	0.124472712	8.45E-01	8.85E-01	1.00E+00
PRRG1	LMNA	0.047886349	-0.090764675	0.184716235	4.99E-01	5.83E-01	1.00E+00
DPF2	LMNA	-0.047489244	-0.184331777	0.091159392	5.02E-01	5.86E-01	1.00E+00
MYH13	LMNA	0.207664845	0.07166846	0.336080963	3.02E-03	6.99E-03	1.00E+00
TMED9	LMNA	0.620210455	0.527307914	0.698458932	7.35E-23	6.66E-21	8.72E-19
TRPV5	LMNA	0.041119231	-0.097485149	0.178158903	5.61E-01	6.42E-01	1.00E+00
UGT2B4	LMNA	0.223571292	0.088243872	0.350795264	1.38E-03	3.49E-03	1.00E+00
PJA2	LMNA	0.10999456	-0.028489043	0.244335363	1.19E-01	1.77E-01	1.00E+00
COLEC11	LMNA	-0.119295209	-0.253176687	0.01906985	9.08E-02	1.40E-01	1.00E+00
SCYE1	LMNA	0.332001	0.203266319	0.449452226	1.39E-06	6.47E-06	1.64E-02
MED12	LMNA	0.02613075	-0.11232545	0.163591614	7.12E-01	7.75E-01	1.00E+00
CARS	LMNA	0.357006368	0.230306019	0.471804446	1.83E-07	1.01E-06	2.18E-03
MYH1	LMNA	0.192890094	0.056339335	0.322357152	5.95E-03	1.28E-02	1.00E+00
CYP7A1	LMNA	0.069311105	-0.069403986	0.205396773	3.27E-01	4.13E-01	1.00E+00
PHF1	LMNA	-0.066309279	-0.202506522	0.072404517	3.48E-01	4.35E-01	1.00E+00
LOC644096	LMNA	-0.201360796	-0.330231988	-0.065120005	4.06E-03	9.08E-03	1.00E+00
FRYL	LMNA	-0.024378576	-0.161884756	0.114056279	7.31E-01	7.91E-01	1.00E+00
RHOBTB2	LMNA	-0.199201669	-0.328226446	-0.062879864	4.48E-03	9.93E-03	1.00E+00
MMP27	LMNA	0.395524016	0.272343656	0.505948946	5.68E-09	4.09E-08	6.74E-05
ZNF432	LMNA	-0.110475927	-0.244793505	0.028002143	1.18E-01	1.75E-01	1.00E+00
SRD5A2	LMNA	0.013942306	-0.124348034	0.151701319	8.44E-01	8.84E-01	1.00E+00
UTP14C	LMNA	-0.182685952	-0.312846959	-0.045789769	9.26E-03	1.89E-02	1.00E+00
RABEP2	LMNA	0.340016865	0.21191299	0.456633634	7.39E-07	3.62E-06	8.76E-03
VWA1	LMNA	0.387655518	0.263717695	0.499001821	1.20E-08	8.07E-08	1.42E-04
FUBP1	LMNA	-0.102729106	-0.237413147	0.035830031	1.46E-01	2.10E-01	1.00E+00
IGLL1	LMNA	0.105757277	-0.032772194	0.240299923	1.34E-01	1.96E-01	1.00E+00
KIAA0586	LMNA	0.002458025	-0.135639002	0.140461363	9.72E-01	9.79E-01	1.00E+00
IL27RA	LMNA	0.347637594	0.22015207	0.463446978	3.99E-07	2.06E-06	4.74E-03
STON1	LMNA	0.032503861	-0.106022885	0.169792968	6.46E-01	7.19E-01	1.00E+00
IL5RA	LMNA	-0.070817649	-0.206846427	0.067897144	3.17E-01	4.02E-01	1.00E+00
PERP	LMNA	0.400885186	0.278232284	0.510674151	3.38E-09	2.53E-08	4.01E-05
SMPD2	LMNA	0.096020655	-0.042594974	0.231009452	1.74E-01	2.43E-01	1.00E+00
TNFSF12	LMNA	0.230062997	0.095030196	0.356782435	9.88E-04	2.59E-03	1.00E+00
FN1	LMNA	0.561904454	0.459497338	0.649567544	3.30E-18	1.24E-16	3.92E-14
MTR	LMNA	0.333056107	0.20440331	0.450398363	1.28E-06	6.01E-06	1.52E-02

CSRP3	LMNA	0.082540143	-0.05615068	0.218105882	2.43E-01	3.22E-01	1.00E+00
MMP20	LMNA	0.113965772	-0.02447022	0.248113186	1.06E-01	1.60E-01	1.00E+00
PHLPPL	LMNA	-0.204446149	-0.333095854	-0.068323511	3.52E-03	8.00E-03	1.00E+00
CBX6	LMNA	0.037548166	-0.101026511	0.174693632	5.96E-01	6.73E-01	1.00E+00
DHFR	LMNA	-0.0953929	-0.230409617	0.043227366	1.77E-01	2.47E-01	1.00E+00
PRG3	LMNA	0.191194618	0.05458435	0.320778788	6.42E-03	1.36E-02	1.00E+00
ALPP	LMNA	-0.247087881	-0.372434857	-0.112887557	3.92E-04	1.13E-03	1.00E+00
ASH1L	LMNA	-0.225057301	-0.352166698	-0.089796213	1.28E-03	3.27E-03	1.00E+00
CHRNA2	LMNA	3.14E-06	-0.138047916	0.138054085	1.00E+00	1.00E+00	1.00E+00
PPP1R12A	LMNA	0.073163002	-0.065550069	0.209102025	3.01E-01	3.85E-01	1.00E+00
RBM38	LMNA	0.347917514	0.220455049	0.463696981	3.90E-07	2.02E-06	4.63E-03
ZNF7	LMNA	-0.040694145	-0.177746589	0.097906883	5.65E-01	6.45E-01	1.00E+00
RDH8	LMNA	0.149584706	0.011776902	0.281816114	3.36E-02	5.92E-02	1.00E+00
GPR132	LMNA	0.376630876	0.251665014	0.489243992	3.30E-08	2.05E-07	3.92E-04
DGKD	LMNA	-0.042652021	-0.179645243	0.09596403	5.47E-01	6.28E-01	1.00E+00
TPMT	LMNA	-0.11169	-0.245948738	0.026773823	1.14E-01	1.69E-01	1.00E+00
ATP1A1	LMNA	0.464701714	0.349042687	0.56641571	3.24E-12	4.17E-11	3.84E-08
SERTAD2	LMNA	0.36519845	0.239207329	0.479095373	9.09E-08	5.25E-07	1.08E-03
C5orf4	LMNA	-0.042352762	-0.179355102	0.096261062	5.50E-01	6.31E-01	1.00E+00
ZNF672	LMNA	0.000440697	-0.137618676	0.138483273	9.95E-01	9.96E-01	1.00E+00
PLXNB3	LMNA	-0.139964605	-0.272745547	-0.001951308	4.70E-02	7.94E-02	1.00E+00
FAIM3	LMNA	0.124982164	-0.013298284	0.25857179	7.64E-02	1.21E-01	1.00E+00
UBQLN2	LMNA	-0.171059904	-0.301979658	-0.033807262	1.49E-02	2.88E-02	1.00E+00
NR1I3	LMNA	-0.017473381	-0.155150126	0.120869182	8.05E-01	8.52E-01	1.00E+00
ACVR2A	LMNA	-0.184538286	-0.314575263	-0.04770254	8.56E-03	1.77E-02	1.00E+00
YWHAZ	LMNA	0.089241151	-0.04941868	0.224526029	2.07E-01	2.81E-01	1.00E+00
LILRP2	LMNA	0.259324806	0.125776614	0.383641424	1.94E-04	5.97E-04	1.00E+00
GNAO1	LMNA	-0.608622693	-0.688800044	-0.513736317	7.38E-22	5.03E-20	8.75E-18
OPA1	LMNA	-0.20086127	-0.329768098	-0.064601614	4.15E-03	9.27E-03	1.00E+00
RPS6KA6	LMNA	-0.45542518	-0.558370373	-0.338666805	9.76E-12	1.15E-10	1.16E-07
RPL10	LMNA	0.110116618	-0.028365588	0.244451537	1.19E-01	1.76E-01	1.00E+00
MMP23B	LMNA	0.380213676	0.25557766	0.492418197	2.39E-08	1.52E-07	2.83E-04
PFKL	LMNA	0.484346181	0.371109203	0.583389171	2.81E-13	4.23E-12	3.33E-09
TMEM140	LMNA	0.291256811	0.15962402	0.412713313	2.61E-05	9.56E-05	3.09E-01
AURKB	LMNA	-0.043943434	-0.180897035	0.094681948	5.35E-01	6.17E-01	1.00E+00

IFI16	LMNA	0.346282318	0.218685495	0.462236289	4.46E-07	2.28E-06	5.29E-03
CSTA	LMNA	0.414440671	0.293162664	0.522592113	8.69E-10	7.27E-09	1.03E-05
PRPF39	LMNA	-0.222566699	-0.349867816	-0.087194807	1.45E-03	3.65E-03	1.00E+00
DISC1	LMNA	-0.260017733	-0.384274914	-0.12650782	1.86E-04	5.76E-04	1.00E+00
USP4	LMNA	0.15705386	0.019424	0.288842342	2.56E-02	4.65E-02	1.00E+00
OSBPL10	LMNA	0.524932428	0.417108193	0.618185131	1.06E-15	2.53E-14	1.26E-11
CAPN6	LMNA	0.224204231	0.088904983	0.351379468	1.34E-03	3.39E-03	1.00E+00
CLTCL1	LMNA	-0.180890611	-0.311171009	-0.043936806	9.99E-03	2.03E-02	1.00E+00
NUAK1	LMNA	-0.052131216	-0.1888233	0.086542611	4.61E-01	5.47E-01	1.00E+00
ALG6	LMNA	-0.094661079	-0.229710213	0.043964452	1.80E-01	2.51E-01	1.00E+00
NPPA	LMNA	-0.412138184	-0.520570745	-0.290622465	1.10E-09	9.01E-09	1.30E-05
LAMB3	LMNA	0.339199639	0.211030534	0.455902177	7.88E-07	3.85E-06	9.35E-03
PPL	LMNA	0.414462955	0.293187257	0.52261167	8.67E-10	7.26E-09	1.03E-05
LRTM1	LMNA	0.080933512	-0.057762871	0.216564844	2.52E-01	3.33E-01	1.00E+00
RRAD	LMNA	0.370043021	0.244481301	0.483399643	5.95E-08	3.57E-07	7.06E-04
TIPIN	LMNA	0.195479734	0.059021498	0.324766529	5.30E-03	1.15E-02	1.00E+00
CARD14	LMNA	-0.150807819	-0.282967667	-0.013028051	3.22E-02	5.70E-02	1.00E+00
RBM9	LMNA	-0.141768322	-0.274448029	-0.003791526	4.42E-02	7.52E-02	1.00E+00
LCN1	LMNA	-0.006425139	-0.144348103	0.131742717	9.28E-01	9.47E-01	1.00E+00
RALGPS1	LMNA	-0.664490249	-0.735107256	-0.57960882	4.21E-27	7.93E-25	4.99E-23
NCOA3	LMNA	0.215317797	0.079633902	0.34316818	2.09E-03	5.03E-03	1.00E+00
CCDC6	LMNA	0.123433802	-0.014870596	0.257103714	8.01E-02	1.26E-01	1.00E+00
RASSF4	LMNA	-0.16793607	-0.299053882	-0.030594361	1.69E-02	3.22E-02	1.00E+00
MTHFD1	LMNA	0.283044448	0.150889385	0.405260085	4.47E-05	1.56E-04	5.30E-01
FCMD	LMNA	0.006122687	-0.132039919	0.144051929	9.31E-01	9.50E-01	1.00E+00
IGHD	LMNA	0.118478855	-0.019897593	0.252401544	9.31E-02	1.43E-01	1.00E+00
PLA2G6	LMNA	-0.444924008	-0.549239507	-0.326955324	3.27E-11	3.51E-10	3.87E-07
TPT1	LMNA	0.116489645	-0.021913763	0.250512033	9.87E-02	1.50E-01	1.00E+00
S100A3	LMNA	0.178196776	0.041158279	0.308654794	1.12E-02	2.24E-02	1.00E+00
SEC63	LMNA	0.089886713	-0.048769466	0.225143914	2.03E-01	2.78E-01	1.00E+00
C10orf59	LMNA	0.084354557	-0.054329119	0.219845407	2.33E-01	3.11E-01	1.00E+00
TOR1AIP1	LMNA	0.203730588	0.067580298	0.332431872	3.64E-03	8.23E-03	1.00E+00
PAFAH1B3	LMNA	-0.441854152	-0.546565526	-0.323538484	4.61E-11	4.89E-10	5.47E-07
CEBPD	LMNA	0.517948287	0.409153073	0.612223313	2.92E-15	6.27E-14	3.47E-11
ZNF107	LMNA	-0.184221887	-0.314280111	-0.047375747	8.68E-03	1.79E-02	1.00E+00

ALDH6A1	LMNA	-0.195444923	-0.324734153	-0.05898543	5.31E-03	1.15E-02	1.00E+00
G6PC2	LMNA	0.094067171	-0.044562522	0.229142512	1.83E-01	2.54E-01	1.00E+00
SNTG2	LMNA	-0.086138049	-0.221554447	0.052537701	2.23E-01	3.00E-01	1.00E+00
GRWD1	LMNA	0.335184266	0.206697689	0.45230593	1.08E-06	5.15E-06	1.28E-02
FLJ22222	LMNA	0.347737515	0.220260219	0.463536222	3.96E-07	2.04E-06	4.70E-03
BCKDK	LMNA	0.399879272	0.2771267	0.509788068	3.73E-09	2.77E-08	4.42E-05
CTSB	LMNA	0.515453393	0.406315326	0.610091015	4.18E-15	8.72E-14	4.95E-11
PFKFB1	LMNA	-0.169419723	-0.300443776	-0.032119962	1.59E-02	3.05E-02	1.00E+00
ZFP36	LMNA	0.421451329	0.300907671	0.528739335	4.21E-10	3.71E-09	4.99E-06
SVEP1	LMNA	0.164114858	0.026668055	0.295471589	1.96E-02	3.66E-02	1.00E+00
PXN	LMNA	0.591808998	0.494128183	0.674734323	1.78E-20	9.64E-19	2.11E-16
TNF	LMNA	-0.129753738	-0.263092067	0.008448599	6.57E-02	1.06E-01	1.00E+00
SIRT2	LMNA	-0.431580187	-0.537600886	-0.312125657	1.43E-10	1.39E-09	1.70E-06
C5orf21	LMNA	-0.060059456	-0.196481377	0.0786436	3.96E-01	4.83E-01	1.00E+00
VIL2	LMNA	-0.002992941	-0.14098569	0.135113886	9.66E-01	9.75E-01	1.00E+00
DIXDC1	LMNA	0.009069964	-0.129142739	0.146936982	8.98E-01	9.26E-01	1.00E+00
GANAB	LMNA	0.520560031	0.412125984	0.614454011	2.01E-15	4.48E-14	2.38E-11
PDSS1	LMNA	-0.401826799	-0.511503381	-0.27926749	3.08E-09	2.32E-08	3.65E-05
GRM6	LMNA	0.04747111	-0.091177416	0.18431422	5.02E-01	5.86E-01	1.00E+00
NGFR	LMNA	0.093153898	-0.045482001	0.228269357	1.87E-01	2.59E-01	1.00E+00
ATP8B4	LMNA	0.120656945	-0.017688693	0.254469298	8.72E-02	1.35E-01	1.00E+00
MEIS1	LMNA	0.114004837	-0.024430666	0.248150328	1.06E-01	1.60E-01	1.00E+00
BMP8A	LMNA	0.100435241	-0.038144643	0.235224806	1.55E-01	2.21E-01	1.00E+00
NGFRAP1	LMNA	-0.321917787	-0.440397069	-0.192418051	2.98E-06	1.31E-05	3.54E-02
EDAR	LMNA	-0.192977869	-0.322438845	-0.056430214	5.93E-03	1.27E-02	1.00E+00
NEDD4L	LMNA	-0.159044379	-0.290712429	-0.021464662	2.38E-02	4.36E-02	1.00E+00
CRYBB3	LMNA	-0.082512361	-0.21807924	0.056178565	2.43E-01	3.23E-01	1.00E+00
C6orf60	LMNA	-0.208925491	-0.337249418	-0.072979389	2.85E-03	6.63E-03	1.00E+00
IL1A	LMNA	0.080189399	-0.058509313	0.215850881	2.57E-01	3.38E-01	1.00E+00
GNRH1	LMNA	-0.044910825	-0.181834455	0.093721246	5.26E-01	6.09E-01	1.00E+00
PDGFD	LMNA	0.347645787	0.220160937	0.463454296	3.99E-07	2.06E-06	4.74E-03
CACNA1H	LMNA	0.036238829	-0.102324079	0.173422231	6.09E-01	6.85E-01	1.00E+00
TXNDC3	LMNA	0.190787037	0.05416259	0.320399253	6.53E-03	1.39E-02	1.00E+00
ERCC1	LMNA	0.099452205	-0.039136114	0.234286573	1.59E-01	2.26E-01	1.00E+00
CAV3	LMNA	0.30497717	0.174263064	0.425129209	1.02E-05	4.02E-05	1.21E-01

HARS	LMNA	0.195082756	0.058610208	0.324397293	5.40E-03	1.17E-02	1.00E+00
AQP8	LMNA	-0.067965862	-0.204101825	0.070748958	3.37E-01	4.23E-01	1.00E+00
CREBBP	LMNA	-0.055667013	-0.192240666	0.083022002	4.31E-01	5.18E-01	1.00E+00
ITGB1	LMNA	0.567875757	0.466387592	0.654608212	1.21E-18	5.05E-17	1.44E-14
SPACA1	LMNA	-0.162689255	-0.294134175	-0.025204329	2.07E-02	3.85E-02	1.00E+00
ZNF254	LMNA	-0.24638101	-0.371786387	-0.112144393	4.08E-04	1.17E-03	1.00E+00
PARK7	LMNA	-0.138231498	-0.271108927	-0.000184008	4.98E-02	8.37E-02	1.00E+00
PAX1	LMNA	-0.064229226	-0.20050239	0.074482202	3.64E-01	4.51E-01	1.00E+00
PSMC4	LMNA	0.20927249	0.073340311	0.337570972	2.80E-03	6.53E-03	1.00E+00
KCNH4	LMNA	0.011976201	-0.126283587	0.149779567	8.66E-01	9.01E-01	1.00E+00
TUBA1A	LMNA	-0.311007058	-0.430571541	-0.180715028	6.64E-06	2.71E-05	7.87E-02
PRMT8	LMNA	-0.415528483	-0.523546687	-0.294363379	7.78E-10	6.57E-09	9.22E-06
SELP	LMNA	0.293416786	0.161924789	0.414670927	2.26E-05	8.37E-05	2.67E-01
PSMD8	LMNA	0.158606784	0.021015945	0.290301396	2.42E-02	4.42E-02	1.00E+00
SOX10	LMNA	-0.370018129	-0.483377541	-0.244454183	5.96E-08	3.57E-07	7.07E-04
HTR1E	LMNA	-0.28075366	-0.403178152	-0.148456588	5.18E-05	1.79E-04	6.15E-01
RABGEF1	LMNA	0.199584864	0.063277337	0.328582468	4.40E-03	9.78E-03	1.00E+00
EPS8L3	LMNA	0.006396885	-0.131770482	0.144320436	9.28E-01	9.48E-01	1.00E+00
MAP1LC3B	LMNA	-0.078234833	-0.21397482	0.06046926	2.68E-01	3.50E-01	1.00E+00
AGXT2L1	LMNA	-0.449821675	-0.553501176	-0.332412965	1.87E-11	2.09E-10	2.22E-07
CYB5R4	LMNA	-0.110906069	-0.245202844	0.027567002	1.16E-01	1.73E-01	1.00E+00
MEMO1	LMNA	-0.029201418	-0.166580884	0.109290162	6.80E-01	7.48E-01	1.00E+00
LRBA	LMNA	0.265532296	0.132332197	0.389312277	1.34E-04	4.28E-04	1.00E+00
TRAPPC2	LMNA	-0.407571994	-0.51655846	-0.285589916	1.74E-09	1.38E-08	2.07E-05
FLJ14107	LMNA	0.135864846	-0.002227942	0.268872794	5.39E-02	8.96E-02	1.00E+00
FNTB	LMNA	0.157841107	0.020230941	0.289582078	2.49E-02	4.54E-02	1.00E+00
MYST3	LMNA	-0.21802879	-0.345675278	-0.082459753	1.83E-03	4.48E-03	1.00E+00
KRT8	LMNA	0.21292502	0.077141557	0.340953848	2.35E-03	5.59E-03	1.00E+00
AURKAIP1	LMNA	0.119571098	-0.018790068	0.253438611	9.01E-02	1.39E-01	1.00E+00
LMAN2L	LMNA	0.013300346	-0.124980133	0.151073956	8.51E-01	8.89E-01	1.00E+00
C1GALT1C	LMNA	0.339396084	0.211242639	0.456078019	7.76E-07	3.80E-06	9.21E-03
DSE	LMNA	0.467135194	0.351769259	0.56852301	2.41E-12	3.16E-11	2.86E-08
FHIT	LMNA	-0.405182201	-0.514456653	-0.282958725	2.21E-09	1.71E-08	2.62E-05
OSBPL3	LMNA	0.495802242	0.384037039	0.593247806	6.26E-14	1.02E-12	7.43E-10
PPOX	LMNA	-0.269870802	-0.393270156	-0.136920919	1.03E-04	3.35E-04	1.00E+00

SLC39A9	LMNA	-0.112914407	-0.247113417	0.025534626	1.10E-01	1.64E-01	1.00E+00
EPB49	LMNA	-0.154753032	-0.286679467	-0.01706664	2.79E-02	5.01E-02	1.00E+00
LOC137886	LMNA	-0.228055071	-0.354931676	-0.092929801	1.10E-03	2.85E-03	1.00E+00
C7orf49	LMNA	0.375395429	0.250316773	0.488148748	3.69E-08	2.27E-07	4.38E-04
RHCE	LMNA	0.039852363	-0.098741881	0.176929956	5.73E-01	6.53E-01	1.00E+00
CST8	LMNA	0.115795427	-0.022617123	0.249852369	1.01E-01	1.53E-01	1.00E+00
ATG7	LMNA	0.256698356	0.12300639	0.381239206	2.26E-04	6.88E-04	1.00E+00
SPP1	LMNA	0.255315847	0.121549038	0.379974051	2.45E-04	7.40E-04	1.00E+00
GLI1	LMNA	0.142235599	0.004268412	0.274888945	4.35E-02	7.41E-02	1.00E+00
HYPK	LMNA	-0.026776468	-0.164220425	0.111687387	7.05E-01	7.69E-01	1.00E+00
SFTPD	LMNA	-0.163063054	-0.2944849	-0.025588067	2.04E-02	3.80E-02	1.00E+00
MCFD2	LMNA	0.540281541	0.434649483	0.63124988	1.05E-16	3.07E-15	1.25E-12
ZNF157	LMNA	-0.043153488	-0.180131377	0.095466241	5.42E-01	6.24E-01	1.00E+00
EPS15	LMNA	-0.006011756	-0.143943294	0.132148919	9.32E-01	9.51E-01	1.00E+00
SFRS2B	LMNA	-0.200594242	-0.329520094	-0.064324531	4.20E-03	9.37E-03	1.00E+00
BTNL3	LMNA	-0.090871103	-0.22608589	0.047779282	1.98E-01	2.72E-01	1.00E+00
GPR23	LMNA	-0.372114829	-0.485238716	-0.246739015	4.95E-08	3.00E-07	5.87E-04
TRPA1	LMNA	0.030339652	-0.108164387	0.167688303	6.68E-01	7.38E-01	1.00E+00
ASPSCR1	LMNA	-0.127585836	-0.261039063	0.010652797	7.04E-02	1.13E-01	1.00E+00
GNS	LMNA	0.505887896	0.395454798	0.601902996	1.59E-14	2.99E-13	1.89E-10
FDFT1	LMNA	-0.301317899	-0.421822259	-0.170353118	1.31E-05	5.10E-05	1.56E-01
ENO2	LMNA	0.043220638	-0.095399579	0.180196468	5.41E-01	6.23E-01	1.00E+00
CBX1	LMNA	-0.338862454	-0.455600334	-0.210666495	8.10E-07	3.94E-06	9.61E-03
PTGS2	LMNA	0.271834973	0.139000247	0.395060503	9.10E-05	2.99E-04	1.00E+00
BMP7	LMNA	-0.186966528	-0.316839608	-0.050211534	7.71E-03	1.61E-02	1.00E+00
CCDC90B	LMNA	-0.213931576	-0.341885512	-0.078189791	2.23E-03	5.35E-03	1.00E+00
LRP5	LMNA	-0.126032671	-0.259567474	0.012231138	7.39E-02	1.18E-01	1.00E+00
UBE2D3	LMNA	0.049970021	-0.088692819	0.186732861	4.80E-01	5.65E-01	1.00E+00
ARFGEF2	LMNA	0.274743363	0.142081307	0.397709789	7.59E-05	2.54E-04	9.00E-01
ARR3	LMNA	0.03446306	-0.104083133	0.171697184	6.26E-01	7.01E-01	1.00E+00
MAP1A	LMNA	-0.131442831	-0.264690801	0.006730297	6.22E-02	1.01E-01	1.00E+00
NEFL	LMNA	-0.168051811	-0.299162329	-0.030713351	1.68E-02	3.20E-02	1.00E+00
CD2	LMNA	0.286178539	0.154220358	0.408106385	3.65E-05	1.30E-04	4.33E-01
FLJ23861	LMNA	-0.352571417	-0.467850793	-0.225495944	2.66E-07	1.42E-06	3.15E-03
NAV2	LMNA	-0.02130009	-0.158883893	0.117095229	7.64E-01	8.19E-01	1.00E+00

ENTPD2	LMNA	-0.055828145	-0.192396322	0.08286148	4.30E-01	5.17E-01	1.00E+00
COX7B	LMNA	-0.039431728	-0.176521816	0.099159054	5.77E-01	6.56E-01	1.00E+00
ATP6V1A	LMNA	-0.308821356	-0.428599818	-0.178375035	7.76E-06	3.13E-05	9.20E-02
ANKRD17	LMNA	-0.060284559	-0.196698566	0.078419072	3.94E-01	4.81E-01	1.00E+00
ADH1C	LMNA	0.094175318	-0.044453624	0.229245894	1.82E-01	2.53E-01	1.00E+00
ATXN7	LMNA	0.043244348	-0.095376041	0.180219452	5.41E-01	6.23E-01	1.00E+00
IL21R	LMNA	0.237426583	0.102743194	0.363561148	6.68E-04	1.83E-03	1.00E+00
CHN2	LMNA	-0.139889815	-0.272674937	-0.001875025	4.71E-02	7.96E-02	1.00E+00
HAS2	LMNA	0.155188526	0.017512716	0.287088952	2.74E-02	4.94E-02	1.00E+00
C6orf48	LMNA	-0.162922564	-0.294353086	-0.025443837	2.05E-02	3.82E-02	1.00E+00
TGIF2	LMNA	0.102511079	-0.036050094	0.237205209	1.47E-01	2.11E-01	1.00E+00
KIAA0241	LMNA	0.3813777	0.256849727	0.49344883	2.15E-08	1.39E-07	2.54E-04
IGF2AS	LMNA	-0.055957968	-0.192521728	0.082732143	4.29E-01	5.15E-01	1.00E+00
CYP2C9	LMNA	-0.144646353	-0.277162808	-0.006729736	4.00E-02	6.89E-02	1.00E+00
DNMT3A	LMNA	0.021475975	-0.116921673	0.159055411	7.62E-01	8.17E-01	1.00E+00
CNOT7	LMNA	-0.361459233	-0.47576943	-0.235141762	1.26E-07	7.09E-07	1.49E-03
FCAR	LMNA	0.098384101	-0.040213075	0.233266865	1.64E-01	2.31E-01	1.00E+00
SFRS10	LMNA	-0.079694456	-0.215375912	0.05900572	2.60E-01	3.41E-01	1.00E+00
Mar-03	LMNA	-0.208861164	-0.337189805	-0.072912485	2.85E-03	6.64E-03	1.00E+00
RUNX1T1	LMNA	-0.261697818	-0.385810397	-0.12828132	1.68E-04	5.26E-04	1.00E+00
CTSK	LMNA	0.301655944	0.170714147	0.422127891	1.28E-05	4.99E-05	1.52E-01
LRRC61	LMNA	-0.002200065	-0.140208481	0.135892209	9.75E-01	9.81E-01	1.00E+00
HNF4G	LMNA	0.115723718	-0.022689769	0.249784222	1.01E-01	1.53E-01	1.00E+00
PSIP1	LMNA	-0.321106746	-0.439667671	-0.191546847	3.17E-06	1.38E-05	3.76E-02
AP2B1	LMNA	-0.183318026	-0.313436807	-0.046442353	9.02E-03	1.85E-02	1.00E+00
C7orf28A	LMNA	0.082405945	-0.05628537	0.217977189	2.44E-01	3.23E-01	1.00E+00
SMCHD1	LMNA	0.030843566	-0.107665874	0.168178466	6.63E-01	7.33E-01	1.00E+00
EIF2C3	LMNA	-0.164020047	-0.295382659	-0.02657069	1.97E-02	3.68E-02	1.00E+00
S100B	LMNA	-0.258832469	-0.383191249	-0.125257168	2.00E-04	6.13E-04	1.00E+00
BMP2	LMNA	-0.079596528	-0.215281929	0.059103929	2.60E-01	3.42E-01	1.00E+00
POP7	LMNA	-0.02519318	-0.162678395	0.113251703	7.22E-01	7.83E-01	1.00E+00
UGT2A3	LMNA	0.095226576	-0.043394899	0.230250673	1.78E-01	2.48E-01	1.00E+00
ESR1	LMNA	0.246040739	0.111786704	0.371474184	4.16E-04	1.19E-03	1.00E+00
ZFPL1	LMNA	0.214298652	0.078572141	0.342225213	2.19E-03	5.27E-03	1.00E+00
ARHGAP12	LMNA	-0.258870008	-0.383225576	-0.125296772	1.99E-04	6.12E-04	1.00E+00

PGGT1B	LMNA	0.287686332	0.155823939	0.409474879	3.30E-05	1.19E-04	3.92E-01
LRRC19	LMNA	-0.230848753	-0.357506417	-0.095852465	9.48E-04	2.50E-03	1.00E+00
ZNF767	LMNA	-0.120068402	-0.25391069	0.018285694	8.87E-02	1.37E-01	1.00E+00
DEPDC6	LMNA	-0.142743601	-0.275368224	-0.004786932	4.27E-02	7.29E-02	1.00E+00
SLCO1B1	LMNA	-0.002814259	-0.140810553	0.135289303	9.68E-01	9.76E-01	1.00E+00
SST	LMNA	-0.351564442	-0.466952454	-0.224404657	2.89E-07	1.53E-06	3.43E-03
NACA	LMNA	-0.015292278	-0.153020235	0.123018429	8.29E-01	8.71E-01	1.00E+00
KCNN3	LMNA	-0.315761245	-0.434856354	-0.185809913	4.70E-06	1.98E-05	5.58E-02
GLOD4	LMNA	-0.283650056	-0.405810268	-0.151532803	4.30E-05	1.51E-04	5.10E-01
SCCPDH	LMNA	-0.095800661	-0.230799255	0.042816605	1.75E-01	2.45E-01	1.00E+00
PRF1	LMNA	0.417098604	0.296097137	0.524924018	6.61E-10	5.66E-09	7.85E-06
LST1	LMNA	0.039121239	-0.099466955	0.176220521	5.80E-01	6.59E-01	1.00E+00
CDK4	LMNA	-0.165618105	-0.296881287	-0.028212139	1.85E-02	3.48E-02	1.00E+00
TCF15	LMNA	-0.223597391	-0.350819354	-0.088271129	1.38E-03	3.49E-03	1.00E+00
PARC	LMNA	-0.136378109	-0.269357873	0.001704991	5.30E-02	8.83E-02	1.00E+00
PRMT1	LMNA	-0.002858462	-0.140853879	0.135245909	9.68E-01	9.76E-01	1.00E+00
ITIH3	LMNA	0.096214707	-0.042399466	0.231194853	1.73E-01	2.42E-01	1.00E+00
PPM2C	LMNA	0.061516597	-0.077189934	0.197887056	3.84E-01	4.72E-01	1.00E+00
TEX10	LMNA	-0.214099872	-0.342041261	-0.078365085	2.22E-03	5.31E-03	1.00E+00
EDA2R	LMNA	0.285794593	0.153812131	0.407757823	3.74E-05	1.33E-04	4.44E-01
LOC28334[illegible]	LMNA	0.123975352	-0.014320747	0.257617254	7.88E-02	1.24E-01	1.00E+00
NARFL	LMNA	-0.202769522	-0.331539881	-0.066582329	3.80E-03	8.56E-03	1.00E+00
DENND2A	LMNA	0.198889958	0.06255657	0.327936811	4.55E-03	1.00E-02	1.00E+00
C1orf103	LMNA	-0.054866332	-0.191467094	0.083819547	4.38E-01	5.24E-01	1.00E+00
DMXL1	LMNA	-0.041214895	-0.178251687	0.097390232	5.60E-01	6.41E-01	1.00E+00
KCNAB1	LMNA	-0.410738213	-0.519341108	-0.289078796	1.27E-09	1.03E-08	1.50E-05
FLJ20254	LMNA	0.574866416	0.474469754	0.660499585	3.66E-19	1.64E-17	4.35E-15
RBM3	LMNA	0.219111447	0.083588889	0.346676	1.73E-03	4.27E-03	1.00E+00
GPR1	LMNA	0.088007278	-0.050659206	0.223344753	2.13E-01	2.88E-01	1.00E+00
DMTF1	LMNA	-0.130729314	-0.264015544	0.007456252	6.37E-02	1.03E-01	1.00E+00
HTR5A	LMNA	-0.316874413	-0.435858843	-0.187003865	4.33E-06	1.84E-05	5.14E-02
MXRA5	LMNA	0.474329303	0.359841299	0.574745057	9.97E-13	1.39E-11	1.18E-08
GRM1	LMNA	-0.294726307	-0.415857217	-0.163320365	2.06E-05	7.72E-05	2.45E-01
SCFD1	LMNA	0.077152437	-0.061554175	0.212935468	2.75E-01	3.58E-01	1.00E+00
RAPSN	LMNA	0.317575622	0.187756158	0.43649018	4.12E-06	1.75E-05	4.88E-02

ACOT9	LMNA	0.562789307	0.460517588	0.650314978	2.85E-18	1.09E-16	3.38E-14
PDE4D	LMNA	0.132335473	-0.005821888	0.265535396	6.05E-02	9.90E-02	1.00E+00
TRPC4	LMNA	-0.160854873	-0.29241251	-0.023321759	2.22E-02	4.10E-02	1.00E+00
EPHB3	LMNA	-0.19242916	-0.321928127	-0.055862139	6.08E-03	1.30E-02	1.00E+00
GEMIN4	LMNA	-0.023821923	-0.161342326	0.114605976	7.36E-01	7.95E-01	1.00E+00
RAMP3	LMNA	0.085522302	-0.053156285	0.220964499	2.26E-01	3.03E-01	1.00E+00
CNTN5	LMNA	-0.069417102	-0.205498787	0.069297988	3.26E-01	4.12E-01	1.00E+00
TSPYL5	LMNA	-0.088573821	-0.223887195	0.050089661	2.10E-01	2.85E-01	1.00E+00
GRTP1	LMNA	0.051511071	-0.087159736	0.188223585	4.67E-01	5.52E-01	1.00E+00
ITGB8	LMNA	0.247977122	0.113822669	0.373250457	3.73E-04	1.08E-03	1.00E+00
GAP43	LMNA	0.089792512	-0.048864206	0.225053759	2.04E-01	2.78E-01	1.00E+00
FRS3	LMNA	-0.486349568	-0.585115315	-0.373366804	2.17E-13	3.31E-12	2.57E-09
PDE3A	LMNA	0.155006099	0.01732585	0.286917426	2.76E-02	4.97E-02	1.00E+00
TNFRSF10(	LMNA	0.2616945	0.128277817	0.385807366	1.69E-04	5.26E-04	1.00E+00
JMJD5	LMNA	-0.026321268	-0.163777155	0.112137203	7.10E-01	7.73E-01	1.00E+00
OTOF	LMNA	-0.112228892	-0.24646139	0.026228474	1.12E-01	1.67E-01	1.00E+00
TEX14	LMNA	-0.266327202	-0.390037786	-0.13317252	1.28E-04	4.09E-04	1.00E+00
XYLT2	LMNA	0.464280739	0.348571208	0.566051027	3.41E-12	4.37E-11	4.04E-08
HIPK3	LMNA	0.296833079	0.165566687	0.417764867	1.79E-05	6.76E-05	2.12E-01
XPOT	LMNA	0.017882163	-0.120466226	0.155549168	8.01E-01	8.50E-01	1.00E+00
RRH	LMNA	-0.273054477	-0.396171613	-0.14029184	8.43E-05	2.80E-04	1.00E+00
CDR2L	LMNA	0.319546841	0.1898718	0.438264353	3.56E-06	1.53E-05	4.22E-02
GAL3ST1	LMNA	-0.343201102	-0.459482179	-0.215353384	5.73E-07	2.85E-06	6.79E-03
DHCR7	LMNA	0.161429146	0.023911013	0.292951587	2.17E-02	4.03E-02	1.00E+00
PDZD7	LMNA	0.073699963	-0.065012497	0.209618234	2.97E-01	3.81E-01	1.00E+00
FGFR4	LMNA	0.015443645	-0.122869314	0.15316809	8.27E-01	8.70E-01	1.00E+00
CRAT	LMNA	0.303297549	0.172467869	0.423611703	1.15E-05	4.49E-05	1.36E-01
C1orf217	LMNA	-0.115053468	-0.249147203	0.023368703	1.03E-01	1.56E-01	1.00E+00
SLC19A1	LMNA	0.169858751	0.032571526	0.300854952	1.57E-02	3.00E-02	1.00E+00
PPP1R14D	LMNA	-0.083371516	-0.218903047	0.055316147	2.38E-01	3.17E-01	1.00E+00
LIMS1	LMNA	0.579331872	0.47964131	0.664257335	1.68E-19	7.81E-18	1.99E-15
TMPRSS11	LMNA	-0.05317422	-0.189831712	0.085504448	4.52E-01	5.39E-01	1.00E+00
TRIM14	LMNA	0.353872058	0.226905956	0.469010761	2.39E-07	1.28E-06	2.83E-03
PIK3CD	LMNA	0.314149629	0.184082021	0.433404455	5.29E-06	2.20E-05	6.27E-02
MFAP3L	LMNA	-0.015063235	-0.152796496	0.123244051	8.32E-01	8.73E-01	1.00E+00

DERL2	LMNA	0.439955564	0.321426842	0.544910702	5.70E-11	5.94E-10	6.76E-07
SLC7A11	LMNA	-0.013474135	-0.151243804	0.124809025	8.49E-01	8.88E-01	1.00E+00
FHL5	LMNA	-0.143605816	-0.276181538	-0.005667166	4.15E-02	7.11E-02	1.00E+00
ACAN	LMNA	0.004728279	-0.133409804	0.142686142	9.47E-01	9.60E-01	1.00E+00
BRWD2	LMNA	-0.097736986	-0.232648927	0.040865398	1.66E-01	2.34E-01	1.00E+00
PPM1E	LMNA	-0.610759951	-0.690583682	-0.516235909	4.86E-22	3.43E-20	5.76E-18
DCUN1D2	LMNA	-0.384051438	-0.495814981	-0.259773275	1.68E-08	1.10E-07	1.99E-04
TINAGL1	LMNA	0.303844839	0.173052723	0.424106244	1.10E-05	4.33E-05	1.31E-01
C3orf36	LMNA	-0.089255703	-0.224539958	0.049404047	2.07E-01	2.81E-01	1.00E+00
DOCK4	LMNA	-0.193791055	-0.323195588	-0.057272262	5.72E-03	1.23E-02	1.00E+00
ENOPH1	LMNA	-0.404247907	-0.513634591	-0.281930557	2.43E-09	1.86E-08	2.88E-05
FAM127A	LMNA	-0.032250157	-0.169546309	0.106273993	6.49E-01	7.21E-01	1.00E+00
SLC5A3	LMNA	0.329292829	0.20034956	0.447022541	1.71E-06	7.85E-06	2.03E-02
ARPC1A	LMNA	0.342429769	0.21451971	0.458792382	6.09E-07	3.03E-06	7.23E-03
CHST2	LMNA	0.55569234	0.452342272	0.644315404	9.16E-18	3.20E-16	1.09E-13
SPATA2	LMNA	-0.228503324	-0.355344928	-0.093398592	1.07E-03	2.79E-03	1.00E+00
PGLYRP4	LMNA	-0.027222534	-0.164654746	0.111246542	7.01E-01	7.65E-01	1.00E+00
RUFY1	LMNA	0.414768123	0.293524062	0.522879486	8.41E-10	7.06E-09	9.97E-06
NTS	LMNA	-0.076849809	-0.21264482	0.061857449	2.77E-01	3.60E-01	1.00E+00
FRMD4A	LMNA	-0.187236977	-0.317091711	-0.050491083	7.62E-03	1.59E-02	1.00E+00
RPS4Y1	LMNA	0.093031238	-0.045605476	0.228152069	1.88E-01	2.60E-01	1.00E+00
BCL11B	LMNA	-0.367893242	-0.481490299	-0.242140058	7.19E-08	4.25E-07	8.53E-04
TNFRSF8	LMNA	0.087889855	-0.05077724	0.223232315	2.14E-01	2.89E-01	1.00E+00
FOXE3	LMNA	-0.068587242	-0.204700033	0.070127766	3.32E-01	4.18E-01	1.00E+00
PTGIR	LMNA	0.425815055	0.30573653	0.532559948	2.65E-10	2.44E-09	3.15E-06
ART4	LMNA	-0.146549557	-0.278956893	-0.008674043	3.74E-02	6.50E-02	1.00E+00
EVX1	LMNA	-0.123506121	-0.257172297	0.014797173	7.99E-02	1.26E-01	1.00E+00
PRM1	LMNA	-0.093014851	-0.2281364	0.045621971	1.88E-01	2.60E-01	1.00E+00
GLCE	LMNA	-0.084666331	-0.220144226	0.054016023	2.31E-01	3.09E-01	1.00E+00
GPR18	LMNA	0.266887677	0.133765134	0.390549238	1.23E-04	3.97E-04	1.00E+00
ACAA2	LMNA	0.14346722	0.00552566	0.276050816	4.17E-02	7.14E-02	1.00E+00
PIGK	LMNA	0.09428179	-0.04434641	0.229347671	1.82E-01	2.53E-01	1.00E+00
HIST1H2AC	LMNA	-0.009904823	-0.147753789	0.128321641	8.89E-01	9.19E-01	1.00E+00
C16orf67	LMNA	-0.431298833	-0.53735505	-0.311813599	1.47E-10	1.43E-09	1.75E-06
UQCC	LMNA	0.005589903	-0.132563395	0.143530143	9.37E-01	9.54E-01	1.00E+00

PLS3	LMNA	0.605999764	0.510670917	0.686609765	1.23E-21	7.78E-20	1.46E-17
DAG1	LMNA	0.248927735	0.114822581	0.374122132	3.54E-04	1.03E-03	1.00E+00
WWOX	LMNA	-0.227353181	-0.354284491	-0.092195871	1.14E-03	2.94E-03	1.00E+00
GTSE1	LMNA	-0.008378136	-0.146259971	0.129823019	9.06E-01	9.32E-01	1.00E+00
GP2	LMNA	0.180282292	0.043309176	0.310602959	1.02E-02	2.07E-02	1.00E+00
CHIT1	LMNA	0.202811397	0.066625807	0.331578751	3.79E-03	8.55E-03	1.00E+00
PLOD2	LMNA	0.598956903	0.502451945	0.680721368	4.70E-21	2.76E-19	5.58E-17
RPS24	LMNA	-0.119542607	-0.253411562	0.018818963	9.02E-02	1.39E-01	1.00E+00
KLF9	LMNA	0.255963958	0.122232163	0.380567205	2.36E-04	7.16E-04	1.00E+00
TTC27	LMNA	0.260046793	0.126538489	0.384301479	1.86E-04	5.75E-04	1.00E+00
PYROXD1	LMNA	0.179841726	0.042854693	0.310191499	1.04E-02	2.11E-02	1.00E+00
MIA	LMNA	-0.005838524	-0.143773641	0.132319128	9.34E-01	9.52E-01	1.00E+00
TSPAN2	LMNA	0.221572301	0.086156685	0.348949536	1.53E-03	3.81E-03	1.00E+00
MRPL9	LMNA	-0.062679075	-0.199008077	0.076029804	3.76E-01	4.63E-01	1.00E+00
PCSK2	LMNA	-0.52928158	-0.62189225	-0.422070353	5.58E-16	1.42E-14	6.62E-12
PI3	LMNA	0.317907155	0.188111896	0.436788637	4.02E-06	1.72E-05	4.77E-02
RPL24	LMNA	-0.132166225	-0.265375274	0.005994143	6.08E-02	9.95E-02	1.00E+00
HIST1H2BE	LMNA	0.152194629	0.014447173	0.28427288	3.06E-02	5.45E-02	1.00E+00
CD86	LMNA	0.222721812	0.087356767	0.350011034	1.44E-03	3.62E-03	1.00E+00
CALM2	LMNA	-0.271412207	-0.394675229	-0.138552595	9.34E-05	3.07E-04	1.00E+00
LFNG	LMNA	0.022335451	-0.116073455	0.159893431	7.52E-01	8.09E-01	1.00E+00
GYG2	LMNA	-0.042078311	-0.179088992	0.096533448	5.52E-01	6.33E-01	1.00E+00
INTS12	LMNA	-0.165836954	-0.297086469	-0.028436988	1.83E-02	3.45E-02	1.00E+00
RAB4B	LMNA	-0.173134268	-0.30392114	-0.035942338	1.37E-02	2.68E-02	1.00E+00
CTSF	LMNA	-0.041825542	-0.178843887	0.096784297	5.55E-01	6.35E-01	1.00E+00
HUNK	LMNA	-0.200077601	-0.329040211	-0.063788497	4.30E-03	9.58E-03	1.00E+00
GNA13	LMNA	0.052813616	-0.085863412	0.1894831	4.55E-01	5.42E-01	1.00E+00
B4GALT4	LMNA	0.273768302	0.141048071	0.396821824	8.07E-05	2.69E-04	9.57E-01
TSPAN31	LMNA	-0.190565023	-0.320192498	-0.053932871	6.60E-03	1.40E-02	1.00E+00
CHD1L	LMNA	0.082863333	-0.055826285	0.218415795	2.41E-01	3.20E-01	1.00E+00
CNKSR2	LMNA	-0.395950502	-0.506325083	-0.272811764	5.45E-09	3.94E-08	6.47E-05
C1orf156	LMNA	-0.104295123	-0.238906339	0.034248996	1.40E-01	2.02E-01	1.00E+00
IBSP	LMNA	0.342198028	0.214269275	0.45858511	6.21E-07	3.08E-06	7.36E-03
FUT8	LMNA	0.069172669	-0.069542417	0.205263535	3.28E-01	4.14E-01	1.00E+00
TRMT11	LMNA	-0.133408077	-0.266549994	0.004730038	5.84E-02	9.60E-02	1.00E+00

AGA	LMNA	0.271269671	0.138401681	0.394545324	9.42E-05	3.09E-04	1.00E+00
GFRA2	LMNA	-0.166981213	-0.298159073	-0.029612845	1.75E-02	3.32E-02	1.00E+00
WWP1	LMNA	0.136433347	-0.001648706	0.269410074	5.29E-02	8.81E-02	1.00E+00
B9D2	LMNA	0.146515329	0.008639067	0.278924636	3.75E-02	6.50E-02	1.00E+00
CPZ	LMNA	0.419479085	0.298727231	0.52701113	5.17E-10	4.50E-09	6.13E-06
STAT1	LMNA	0.115163963	-0.023256786	0.249252227	1.03E-01	1.55E-01	1.00E+00
PTTG1	LMNA	0.082900349	-0.055789129	0.218451289	2.41E-01	3.20E-01	1.00E+00
TMEM62	LMNA	0.005184733	-0.132961435	0.143133285	9.42E-01	9.57E-01	1.00E+00
COL8A1	LMNA	0.555505619	0.452127414	0.644157408	9.45E-18	3.28E-16	1.12E-13
RBPJL	LMNA	0.051569848	-0.08710125	0.188280429	4.66E-01	5.52E-01	1.00E+00
CASP8AP2	LMNA	-0.230864709	-0.357521117	-0.095869164	9.48E-04	2.50E-03	1.00E+00
SSBP2	LMNA	0.209052245	0.073111225	0.33736688	2.83E-03	6.59E-03	1.00E+00
MMP12	LMNA	0.105342844	-0.033190841	0.239904982	1.36E-01	1.97E-01	1.00E+00
NME4	LMNA	0.078956065	-0.059746167	0.214667201	2.64E-01	3.46E-01	1.00E+00
LOC55565	LMNA	-0.55233175	-0.641470646	-0.448477107	1.58E-17	5.26E-16	1.87E-13
GABRA1	LMNA	-0.347471699	-0.463298805	-0.21997252	4.05E-07	2.08E-06	4.80E-03
PEX11B	LMNA	-0.132867835	-0.266039005	0.005280015	5.94E-02	9.76E-02	1.00E+00
HABP2	LMNA	0.213065692	0.077288036	0.341084069	2.33E-03	5.56E-03	1.00E+00
REEP1	LMNA	-0.613296643	-0.692699407	-0.519204743	2.94E-22	2.18E-20	3.49E-18
C9orf156	LMNA	-0.133496359	-0.266633489	0.004640157	5.82E-02	9.58E-02	1.00E+00
SMARCA1	LMNA	0.070766089	-0.067948725	0.206796823	3.17E-01	4.02E-01	1.00E+00
WEE1	LMNA	0.409918248	0.28817496	0.518620698	1.38E-09	1.11E-08	1.63E-05
APOF	LMNA	-0.04332551	-0.180298124	0.095295465	5.40E-01	6.22E-01	1.00E+00
GCDH	LMNA	-0.156369614	-0.288199262	-0.018722782	2.63E-02	4.76E-02	1.00E+00
SSR4	LMNA	0.162426131	0.024934233	0.293887271	2.09E-02	3.89E-02	1.00E+00
SPAST	LMNA	-0.529830862	-0.62236015	-0.422697513	5.14E-16	1.32E-14	6.10E-12
RGS1	LMNA	0.302816212	0.171953572	0.423176699	1.18E-05	4.63E-05	1.41E-01
ACCN4	LMNA	-0.239876506	-0.365813522	-0.105312964	5.85E-04	1.63E-03	1.00E+00
PLXND1	LMNA	0.550510942	0.446384535	0.639928297	2.11E-17	6.83E-16	2.51E-13
FLJ20489	LMNA	-0.241400579	-0.367213957	-0.106912501	5.38E-04	1.51E-03	1.00E+00
MLCK	LMNA	-0.062843712	-0.199166813	0.07586547	3.74E-01	4.62E-01	1.00E+00
INTS5	LMNA	0.106198375	-0.032326559	0.240720226	1.33E-01	1.94E-01	1.00E+00
BSG	LMNA	0.321785968	0.19227644	0.440278531	3.01E-06	1.32E-05	3.57E-02
PARP8	LMNA	-0.03418876	-0.171430644	0.104354773	6.29E-01	7.03E-01	1.00E+00
ZNF215	LMNA	0.164146241	0.026700284	0.295501025	1.96E-02	3.66E-02	1.00E+00

TEAD4	LMNA	0.429980493	0.310351734	0.536202898	1.70E-10	1.63E-09	2.02E-06
PDE7B	LMNA	-0.122819054	-0.256520671	0.015494663	8.16E-02	1.28E-01	1.00E+00
MS4A3	LMNA	-0.041875672	-0.178892499	0.096734549	5.54E-01	6.35E-01	1.00E+00
DTX4	LMNA	-0.399021866	-0.509032617	-0.276184595	4.05E-09	2.99E-08	4.80E-05
EFEMP1	LMNA	0.363578649	0.237445613	0.477655001	1.05E-07	5.97E-07	1.24E-03
TNRC6B	LMNA	-0.255595102	-0.380229639	-0.121843364	2.41E-04	7.30E-04	1.00E+00
TULP2	LMNA	0.156395242	0.018749043	0.288223351	2.62E-02	4.75E-02	1.00E+00
RERE	LMNA	-0.099883699	-0.234698432	0.038700951	1.57E-01	2.24E-01	1.00E+00
BNC1	LMNA	0.182741519	0.045847134	0.312898818	9.24E-03	1.89E-02	1.00E+00
FGFBP1	LMNA	-0.016246797	-0.153952499	0.122078011	8.18E-01	8.63E-01	1.00E+00
TIMM8A	LMNA	0.012548646	-0.125720146	0.150339206	8.59E-01	8.96E-01	1.00E+00
PIGB	LMNA	0.434236676	0.315073315	0.539921129	1.07E-10	1.07E-09	1.27E-06
AJAP1	LMNA	-0.45623647	-0.559074761	-0.339573108	8.87E-12	1.05E-10	1.05E-07
COMMD8	LMNA	-0.036470468	-0.173647192	0.102094557	6.06E-01	6.83E-01	1.00E+00
TRIP11	LMNA	0.441011169	0.322600759	0.545830876	5.07E-11	5.33E-10	6.01E-07
SLC25A42	LMNA	-0.027836711	-0.165252664	0.110639465	6.94E-01	7.60E-01	1.00E+00
SYP	LMNA	-0.492374981	-0.590301501	-0.380164873	9.87E-14	1.58E-12	1.17E-09
PCDHB6	LMNA	-0.107417986	-0.241882079	0.031094114	1.28E-01	1.88E-01	1.00E+00
FLJ12716	LMNA	-0.19970656	-0.328695527	-0.063403577	4.38E-03	9.73E-03	1.00E+00
FKBP8	LMNA	0.006677837	-0.131494386	0.144595538	9.25E-01	9.46E-01	1.00E+00
KIAA1109	LMNA	-0.216480225	-0.344243404	-0.080845312	1.97E-03	4.80E-03	1.00E+00
PTPRC	LMNA	0.255819387	0.122079771	0.380434902	2.38E-04	7.21E-04	1.00E+00
POT1	LMNA	0.07370089	-0.065011569	0.209619125	2.97E-01	3.81E-01	1.00E+00
CCT7	LMNA	-0.051262682	-0.18798335	0.087406885	4.69E-01	5.54E-01	1.00E+00
MMP11	LMNA	0.546150533	0.441377941	0.636231801	4.22E-17	1.32E-15	5.00E-13
MYO1E	LMNA	0.611640698	0.517266439	0.691318423	4.08E-22	2.97E-20	4.84E-18
EEF1A2	LMNA	-0.246990342	-0.372345384	-0.112785001	3.95E-04	1.14E-03	1.00E+00
MIPEP	LMNA	0.169770924	0.032481187	0.300772701	1.57E-02	3.01E-02	1.00E+00
ZFX	LMNA	0.275268746	0.142638156	0.398188149	7.34E-05	2.47E-04	8.71E-01
UCHL3	LMNA	-0.057000253	-0.19352839	0.081693589	4.20E-01	5.08E-01	1.00E+00
LRFN4	LMNA	-0.096938851	-0.231886627	0.041669796	1.70E-01	2.38E-01	1.00E+00
XCL1	LMNA	0.282199543	0.149991917	0.404492359	4.72E-05	1.65E-04	5.60E-01
CARHSP1	LMNA	0.319501436	0.189823055	0.438223497	3.57E-06	1.54E-05	4.24E-02
GREM2	LMNA	-0.247941831	-0.373218092	-0.113785552	3.74E-04	1.08E-03	1.00E+00
CCDC102B	LMNA	0.353088796	0.226056765	0.468312261	2.55E-07	1.36E-06	3.02E-03

HDDC2	LMNA	-0.301068412	-0.421596676	-0.17008669	1.34E-05	5.18E-05	1.59E-01
SHC2	LMNA	-0.200661921	-0.329582954	-0.064394757	4.19E-03	9.35E-03	1.00E+00
SDS	LMNA	0.07910848	-0.05959334	0.214813501	2.63E-01	3.45E-01	1.00E+00
CASQ1	LMNA	-0.336665957	-0.453633405	-0.208295942	9.63E-07	4.64E-06	1.14E-02
LYPLA3	LMNA	0.412667601	0.291206388	0.521035632	1.04E-09	8.57E-09	1.24E-05
INPPL1	LMNA	0.294855885	0.163458487	0.415974578	2.05E-05	7.66E-05	2.43E-01
SLC25A40	LMNA	0.238287292	0.103645803	0.364352622	6.38E-04	1.76E-03	1.00E+00
IHH	LMNA	0.052682792	-0.085993633	0.189356619	4.56E-01	5.43E-01	1.00E+00
CHGB	LMNA	-0.395079083	-0.505556495	-0.271855362	5.93E-09	4.24E-08	7.03E-05
COL9A3	LMNA	0.028647204	-0.109838182	0.166041547	6.86E-01	7.53E-01	1.00E+00
C2orf18	LMNA	0.486328135	0.373342645	0.585096853	2.17E-13	3.31E-12	2.58E-09
DDEF2	LMNA	0.242549045	0.108118292	0.36826888	5.05E-04	1.42E-03	1.00E+00
BUB3	LMNA	-0.144046033	-0.276596715	-0.006116667	4.08E-02	7.02E-02	1.00E+00
C6orf211	LMNA	0.151487058	0.013723047	0.283607002	3.14E-02	5.58E-02	1.00E+00
FOXD2	LMNA	-0.045517307	-0.182422021	0.093118825	5.20E-01	6.04E-01	1.00E+00
GGH	LMNA	0.007245522	-0.130936448	0.145151335	9.18E-01	9.42E-01	1.00E+00
GGT1	LMNA	-0.143743929	-0.2763118	-0.005808186	4.13E-02	7.09E-02	1.00E+00
ADARB1	LMNA	0.040479701	-0.098119618	0.17753857	5.67E-01	6.47E-01	1.00E+00
VPS35	LMNA	-0.063390069	-0.199693539	0.075320062	3.70E-01	4.58E-01	1.00E+00
CNN2	LMNA	0.592507727	0.494941067	0.675320056	1.56E-20	8.63E-19	1.86E-16
DBP	LMNA	-0.081866379	-0.217459708	0.056826864	2.47E-01	3.27E-01	1.00E+00
WNT1	LMNA	0.080569733	-0.058127808	0.216215823	2.54E-01	3.35E-01	1.00E+00
COL5A3	LMNA	0.350106886	0.222825629	0.465651726	3.26E-07	1.71E-06	3.87E-03
RHOD	LMNA	0.455643259	0.338910403	0.558559731	9.51E-12	1.12E-10	1.13E-07
ASNA1	LMNA	-0.062589349	-0.198921564	0.076119362	3.76E-01	4.64E-01	1.00E+00
WDTC1	LMNA	-0.089519321	-0.224792286	0.04913895	2.05E-01	2.80E-01	1.00E+00
COL4A2	LMNA	0.480449392	0.36672177	0.580029083	4.62E-13	6.78E-12	5.48E-09
HEBP1	LMNA	0.354245407	0.227310799	0.469343657	2.31E-07	1.25E-06	2.74E-03
SLC1A6	LMNA	-0.389564128	-0.500688256	-0.265808219	1.00E-08	6.85E-08	1.19E-04
LUM	LMNA	0.579752959	0.480129338	0.664611464	1.56E-19	7.34E-18	1.85E-15
C1S	LMNA	0.48101602	0.367359428	0.58051788	4.30E-13	6.32E-12	5.10E-09
TCF7L1	LMNA	-0.243020278	-0.368701637	-0.108613162	4.92E-04	1.39E-03	1.00E+00
ZCCHC6	LMNA	0.523385139	0.415344365	0.616865255	1.33E-15	3.08E-14	1.58E-11
ME2	LMNA	-0.048348845	-0.185163949	0.090304906	4.94E-01	5.79E-01	1.00E+00
PAGE1	LMNA	0.113378334	-0.025064983	0.247554612	1.08E-01	1.63E-01	1.00E+00

DTX2	LMNA	0.552573598	0.448755137	0.641675453	1.52E-17	5.11E-16	1.80E-13
KPNA4	LMNA	0.345281014	0.21760233	0.461341529	4.84E-07	2.46E-06	5.74E-03
H3F3A	LMNA	-0.396230655	-0.50657214	-0.27311929	5.31E-09	3.85E-08	6.30E-05
GLO1	LMNA	-0.124721563	-0.258324745	0.013562963	7.70E-02	1.22E-01	1.00E+00
WDR61	LMNA	-0.037917211	-0.175051901	0.100660699	5.92E-01	6.70E-01	1.00E+00
CD302	LMNA	0.196221173	0.059789789	0.325456048	5.13E-03	1.12E-02	1.00E+00
SIRT7	LMNA	0.0951724	-0.043449468	0.230198899	1.78E-01	2.48E-01	1.00E+00
D4S234E	LMNA	-0.260031677	-0.38428766	-0.126522536	1.86E-04	5.75E-04	1.00E+00
RABIF	LMNA	-0.143518236	-0.276098934	-0.005577747	4.16E-02	7.13E-02	1.00E+00
DYRK3	LMNA	0.40207717	0.279542795	0.511723836	3.00E-09	2.27E-08	3.56E-05
PKIG	LMNA	0.133262732	-0.004878009	0.266412527	5.87E-02	9.64E-02	1.00E+00
PFAS	LMNA	-0.065547946	-0.20177311	0.073165122	3.54E-01	4.41E-01	1.00E+00
ALOXE3	LMNA	-0.104922567	-0.239504426	0.03361534	1.37E-01	1.99E-01	1.00E+00
RPLP0	LMNA	0.16055414	0.023013219	0.292130174	2.25E-02	4.14E-02	1.00E+00
RBM34	LMNA	-0.113530648	-0.247699451	0.024910779	1.08E-01	1.62E-01	1.00E+00
MKNK2	LMNA	0.5741681	0.473661643	0.659911554	4.14E-19	1.83E-17	4.90E-15
ZNF528	LMNA	-0.246727614	-0.37210437	-0.112508775	4.00E-04	1.15E-03	1.00E+00
U2AF2	LMNA	-0.078535582	-0.214263555	0.060167752	2.67E-01	3.48E-01	1.00E+00
SEC16A	LMNA	0.098813601	-0.039780052	0.23367694	1.62E-01	2.29E-01	1.00E+00
EFNA5	LMNA	0.038133438	-0.100446348	0.175261798	5.90E-01	6.68E-01	1.00E+00
ZNF44	LMNA	-0.184736155	-0.314759832	-0.047906925	8.49E-03	1.75E-02	1.00E+00
FCGRT	LMNA	0.361083371	0.234733339	0.475434929	1.30E-07	7.30E-07	1.54E-03
NOL4	LMNA	-0.662928092	-0.733821317	-0.577751706	6.11E-27	1.12E-24	7.25E-23
CCS	LMNA	0.307833916	0.177318376	0.427708676	8.32E-06	3.34E-05	9.87E-02
IGF2BP2	LMNA	0.337761841	0.209478478	0.454614895	8.84E-07	4.27E-06	1.05E-02
MFSD7	LMNA	0.436422482	0.317500425	0.541829062	8.43E-11	8.57E-10	1.00E-06
SIX6	LMNA	-0.209191435	-0.337495863	-0.073256001	2.81E-03	6.55E-03	1.00E+00
CCR6	LMNA	0.28186869	0.14964054	0.404191681	4.82E-05	1.68E-04	5.72E-01
TSSC4	LMNA	0.259534043	0.125997395	0.383832726	1.92E-04	5.91E-04	1.00E+00
COL11A2	LMNA	-0.308082279	-0.427932839	-0.17758412	8.18E-06	3.28E-05	9.70E-02
CHRNA6	LMNA	0.193423529	0.056891667	0.322853592	5.81E-03	1.25E-02	1.00E+00
PLD2	LMNA	0.292343011	0.160780843	0.413697891	2.42E-05	8.94E-05	2.87E-01
PALM	LMNA	-0.355793584	-0.470723736	-0.228990039	2.03E-07	1.11E-06	2.41E-03
ORC1L	LMNA	-0.114703508	-0.248814547	0.023723147	1.04E-01	1.57E-01	1.00E+00
SASH1	LMNA	-0.133964267	-0.267075991	0.004163737	5.73E-02	9.45E-02	1.00E+00

PUM2	LMNA	-0.347436897	-0.46326772	-0.219934854	4.06E-07	2.09E-06	4.82E-03
ZNF365	LMNA	-0.067205093	-0.203369288	0.071509352	3.42E-01	4.28E-01	1.00E+00
CDC14B	LMNA	-0.305767547	-0.425843067	-0.175108126	9.63E-06	3.82E-05	1.14E-01
PHC1	LMNA	-0.321302372	-0.439843619	-0.191756965	3.12E-06	1.36E-05	3.71E-02
KIAA0913	LMNA	-0.122728277	-0.256434568	0.015586807	8.18E-02	1.28E-01	1.00E+00
LDLRAP1	LMNA	0.457714466	0.341224755	0.560357623	7.46E-12	9.01E-11	8.84E-08
NAT8B	LMNA	0.074244852	-0.064466908	0.210141986	2.94E-01	3.78E-01	1.00E+00
PPP3CB	LMNA	-0.4392307	-0.544278689	-0.320620948	6.18E-11	6.43E-10	7.33E-07
ANGPTL4	LMNA	0.466155164	0.350670957	0.567674501	2.72E-12	3.54E-11	3.23E-08
HHEX	LMNA	0.022930743	-0.115485841	0.160473746	7.46E-01	8.04E-01	1.00E+00
LSM14B	LMNA	0.011296472	-0.12695251	0.149114929	8.73E-01	9.06E-01	1.00E+00
PLCH1	LMNA	-0.011639113	-0.149449979	0.126615332	8.69E-01	9.03E-01	1.00E+00
INSM1	LMNA	-0.264817191	-0.388659471	-0.131576397	1.40E-04	4.46E-04	1.00E+00
TLN2	LMNA	-0.050836286	-0.187570914	0.087831113	4.72E-01	5.58E-01	1.00E+00
ZNF493	LMNA	-0.450089971	-0.553734475	-0.332712164	1.81E-11	2.03E-10	2.15E-07
HDAC4	LMNA	-0.273425814	-0.396509872	-0.140685217	8.24E-05	2.74E-04	9.77E-01
GLRA1	LMNA	-0.079835871	-0.215511627	0.058863893	2.59E-01	3.40E-01	1.00E+00
RPS6	LMNA	-0.124104722	-0.257739921	0.014189382	7.85E-02	1.24E-01	1.00E+00
SFXN1	LMNA	-0.053836162	-0.190471552	0.084845422	4.47E-01	5.33E-01	1.00E+00
FAM102A	LMNA	0.318668825	0.188929307	0.437474219	3.80E-06	1.63E-05	4.51E-02
KLHL1	LMNA	-0.219038551	-0.34660863	-0.083512853	1.74E-03	4.28E-03	1.00E+00
SAPS2	LMNA	-0.309390067	-0.429112962	-0.17898375	7.45E-06	3.02E-05	8.84E-02
CTNNBIP1	LMNA	-0.187730108	-0.317551344	-0.051000863	7.46E-03	1.56E-02	1.00E+00
SCGB1A1	LMNA	-0.159892498	-0.291508929	-0.022334494	2.30E-02	4.23E-02	1.00E+00
SCAND2	LMNA	-0.261891856	-0.385987691	-0.128486202	1.67E-04	5.21E-04	1.00E+00
HMGN2	LMNA	-0.238318407	-0.36438123	-0.103678436	6.37E-04	1.75E-03	1.00E+00
NEUROD2	LMNA	-0.415283469	-0.523331707	-0.294092902	7.97E-10	6.72E-09	9.46E-06
YAF2	LMNA	-0.322418011	-0.440846861	-0.192955484	2.87E-06	1.26E-05	3.41E-02
BRPF1	LMNA	0.064693724	-0.074018337	0.200950033	3.60E-01	4.47E-01	1.00E+00
RICH2	LMNA	-0.335980836	-0.453019657	-0.207556839	1.02E-06	4.87E-06	1.21E-02
TBCE	LMNA	-0.072768164	-0.208722398	0.065945305	3.03E-01	3.88E-01	1.00E+00
LIAS	LMNA	-0.273742135	-0.39679799	-0.141020346	8.08E-05	2.69E-04	9.58E-01
MAPK1	LMNA	0.070273728	-0.068441246	0.206323114	3.20E-01	4.06E-01	1.00E+00
MRM1	LMNA	0.19437935	0.057881559	0.323742947	5.57E-03	1.20E-02	1.00E+00
HDHD1A	LMNA	0.112726901	-0.025724425	0.246935082	1.10E-01	1.65E-01	1.00E+00

CTA-246H3	LMNA	0.019184484	-0.119182163	0.156820157	7.86E-01	8.38E-01	1.00E+00
ATP9A	LMNA	-0.415882701	-0.523857461	-0.294754444	7.50E-10	6.35E-09	8.89E-06
HSD17B3	LMNA	-0.062075046	-0.198425634	0.07663266	3.80E-01	4.68E-01	1.00E+00
HN1L	LMNA	0.277480871	0.14498369	0.400201546	6.39E-05	2.17E-04	7.57E-01
SAG	LMNA	0.063514463	-0.075195873	0.199813452	3.69E-01	4.57E-01	1.00E+00
C20orf10	LMNA	-0.213878965	-0.341836821	-0.078134994	2.24E-03	5.36E-03	1.00E+00
HNRNPA2E	LMNA	0.019319884	-0.119048634	0.156952273	7.85E-01	8.37E-01	1.00E+00
RNF216	LMNA	0.264962329	0.131729781	0.388791974	1.39E-04	4.42E-04	1.00E+00
GADD45A	LMNA	0.501425873	0.390399221	0.598076609	2.94E-14	5.11E-13	3.48E-10
MSH4	LMNA	-0.035335031	-0.172544354	0.103219478	6.18E-01	6.93E-01	1.00E+00
HOXD12	LMNA	-0.055427073	-0.192008868	0.083261021	4.33E-01	5.19E-01	1.00E+00
TMEM70	LMNA	0.077092537	-0.061614205	0.212877941	2.75E-01	3.58E-01	1.00E+00
PPP1R14B	LMNA	0.154452711	0.016759053	0.286397053	2.82E-02	5.06E-02	1.00E+00
SBF1	LMNA	-0.125327025	-0.258898684	0.012947996	7.55E-02	1.20E-01	1.00E+00
HIST1H2AM	LMNA	0.006373636	-0.131793327	0.144297671	9.28E-01	9.48E-01	1.00E+00
GNG3	LMNA	-0.41681099	-0.524671762	-0.295779489	6.81E-10	5.81E-09	8.08E-06
C1orf50	LMNA	-0.038088355	-0.175218035	0.100491041	5.90E-01	6.68E-01	1.00E+00
FTO	LMNA	-0.332385189	-0.449796767	-0.203680284	1.35E-06	6.29E-06	1.60E-02
CALCB	LMNA	-0.166312493	-0.29753227	-0.028925613	1.80E-02	3.40E-02	1.00E+00
PPP3R1	LMNA	-0.313780046	-0.433071412	-0.183685887	5.43E-06	2.26E-05	6.44E-02
USP46	LMNA	-0.266358144	-0.390066023	-0.133205234	1.27E-04	4.09E-04	1.00E+00
CCNJ	LMNA	-0.183478531	-0.313586574	-0.046608085	8.96E-03	1.84E-02	1.00E+00
PSD	LMNA	-0.390894714	-0.501863453	-0.267266314	8.84E-09	6.09E-08	1.05E-04
GNAZ	LMNA	-0.396863836	-0.507130452	-0.273814429	4.99E-09	3.64E-08	5.92E-05
FAM57A	LMNA	0.173900332	0.03673114	0.304637857	1.33E-02	2.61E-02	1.00E+00
LILRB3	LMNA	0.454975905	0.338165007	0.557980234	1.03E-11	1.20E-10	1.22E-07
DHX8	LMNA	0.129852239	-0.008348417	0.263185319	6.55E-02	1.06E-01	1.00E+00
SPI1	LMNA	0.309843795	0.179469465	0.429522303	7.21E-06	2.93E-05	8.56E-02
OXSM	LMNA	-0.129647968	-0.262991931	0.008556171	6.59E-02	1.07E-01	1.00E+00
GYS2	LMNA	-0.185626203	-0.315589933	-0.048826422	8.17E-03	1.70E-02	1.00E+00
UBE3B	LMNA	0.123502049	-0.014801308	0.257168435	7.99E-02	1.26E-01	1.00E+00
PLAT	LMNA	0.338993066	0.210807505	0.455717259	8.01E-07	3.90E-06	9.51E-03
NUPL2	LMNA	-0.218424672	-0.346041234	-0.08287259	1.79E-03	4.40E-03	1.00E+00
SF3A1	LMNA	0.008585038	-0.129619585	0.146462455	9.03E-01	9.30E-01	1.00E+00
COPE	LMNA	0.06516708	-0.073545565	0.201406154	3.57E-01	4.44E-01	1.00E+00

EIF3A	LMNA	0.215633837	0.079963219	0.343460544	2.06E-03	4.97E-03	1.00E+00
IQCE	LMNA	0.122492724	-0.015825896	0.256211132	8.24E-02	1.29E-01	1.00E+00
KIAA0182	LMNA	-0.196456698	-0.325675051	-0.060033879	5.08E-03	1.11E-02	1.00E+00
YRDC	LMNA	0.039161906	-0.099426629	0.176259985	5.80E-01	6.59E-01	1.00E+00
GPRC5D	LMNA	-0.147225837	-0.279594177	-0.00936518	3.65E-02	6.36E-02	1.00E+00
LRRC23	LMNA	-0.04453127	-0.181466688	0.094098208	5.29E-01	6.12E-01	1.00E+00
BLVRA	LMNA	0.336398632	0.208007536	0.453393942	9.84E-07	4.72E-06	1.17E-02
SLC22A7	LMNA	-0.178156324	-0.308616995	-0.041116571	1.12E-02	2.24E-02	1.00E+00
RASL12	LMNA	-0.085792466	-0.221223355	0.052884889	2.25E-01	3.02E-01	1.00E+00
DAZAP2	LMNA	-0.049186076	-0.185974277	0.089472459	4.87E-01	5.72E-01	1.00E+00
IKBKB	LMNA	0.164836126	0.027408835	0.296148043	1.91E-02	3.58E-02	1.00E+00
PPFIA2	LMNA	-0.241058981	-0.36690012	-0.106553928	5.48E-04	1.53E-03	1.00E+00
ZNF271	LMNA	-0.267495152	-0.391103493	-0.13440755	1.19E-04	3.83E-04	1.00E+00
CXorf6	LMNA	-0.21617965	-0.34396541	-0.080532032	2.00E-03	4.86E-03	1.00E+00
FRG1	LMNA	-0.221245507	-0.348647704	-0.085815586	1.55E-03	3.87E-03	1.00E+00
BTN2A2	LMNA	0.172339209	0.035123861	0.30317714	1.42E-02	2.76E-02	1.00E+00
THBS4	LMNA	0.072005993	-0.066708119	0.207989475	3.09E-01	3.93E-01	1.00E+00
ARHGAP1	LMNA	0.239081531	0.104478903	0.365082811	6.11E-04	1.69E-03	1.00E+00
ENOX1	LMNA	-0.28442249	-0.406511879	-0.152353626	4.09E-05	1.44E-04	4.85E-01
ZNF706	LMNA	-0.170165071	-0.301141812	-0.032886625	1.55E-02	2.97E-02	1.00E+00
DOK1	LMNA	0.507817206	0.397642826	0.603556104	1.22E-14	2.32E-13	1.45E-10
FCHO1	LMNA	-0.144895076	-0.277397323	-0.006983771	3.96E-02	6.84E-02	1.00E+00
PGAP1	LMNA	-0.305928961	-0.425988835	-0.175280732	9.52E-06	3.78E-05	1.13E-01
HOXD10	LMNA	0.049944416	-0.088718286	0.186708087	4.80E-01	5.66E-01	1.00E+00
CXCR3	LMNA	0.291811526	0.160214757	0.413216166	2.51E-05	9.24E-05	2.98E-01
FSCN1	LMNA	0.125264545	-0.013011461	0.258839462	7.57E-02	1.20E-01	1.00E+00
KIF17	LMNA	-0.223969621	-0.351162934	-0.088659917	1.35E-03	3.43E-03	1.00E+00
TRIM66	LMNA	0.019847232	-0.118528528	0.157466785	7.79E-01	8.32E-01	1.00E+00
CHI3L2	LMNA	0.169143823	0.031836213	0.300185354	1.61E-02	3.08E-02	1.00E+00
SRPX2	LMNA	0.676064011	0.593395381	0.744618765	2.47E-28	5.75E-26	2.93E-24
C13orf24	LMNA	-0.049657343	-0.186430318	0.0890038	4.83E-01	5.68E-01	1.00E+00
CBR3	LMNA	0.165677668	0.028273334	0.296937132	1.85E-02	3.47E-02	1.00E+00
ZNF132	LMNA	-0.082924434	-0.218474383	0.055764953	2.41E-01	3.20E-01	1.00E+00
AQP3	LMNA	0.094724379	-0.043900703	0.229770715	1.80E-01	2.50E-01	1.00E+00
BNIP1	LMNA	-0.117908721	-0.25186009	0.020475569	9.47E-02	1.45E-01	1.00E+00

ST6GALNA	LMNA	0.415326201	0.294140074	0.523369202	7.94E-10	6.69E-09	9.42E-06
KIAA0391	LMNA	-0.089084492	-0.22437607	0.049576207	2.07E-01	2.82E-01	1.00E+00
KRTAP5-8	LMNA	-0.075436471	-0.211287111	0.063273464	2.86E-01	3.69E-01	1.00E+00
SIRPA	LMNA	0.187906583	0.051183313	0.317715815	7.41E-03	1.55E-02	1.00E+00
LYVE1	LMNA	0.296833601	0.165567244	0.41776534	1.79E-05	6.76E-05	2.12E-01
IGFBP6	LMNA	0.504552702	0.39394129	0.600758469	1.92E-14	3.52E-13	2.27E-10
APOH	LMNA	-0.160422777	-0.292006841	-0.022878455	2.26E-02	4.16E-02	1.00E+00
TSC22D2	LMNA	-0.132346206	-0.26554555	0.005810964	6.04E-02	9.90E-02	1.00E+00
PLCD1	LMNA	0.050559112	-0.088106851	0.187302789	4.75E-01	5.60E-01	1.00E+00
NPHS2	LMNA	-0.01813832	-0.155799197	0.120213697	7.98E-01	8.48E-01	1.00E+00
ARHGEF15	LMNA	0.361821569	0.235535528	0.476091862	1.22E-07	6.88E-07	1.44E-03
M-RIP	LMNA	0.052349554	-0.086325312	0.18903442	4.59E-01	5.45E-01	1.00E+00
POLDIP2	LMNA	-0.062641484	-0.198971832	0.076067326	3.76E-01	4.63E-01	1.00E+00
NDUFV1	LMNA	-0.067068676	-0.203237918	0.071645685	3.43E-01	4.29E-01	1.00E+00
SRD5A1	LMNA	0.390993304	0.267374374	0.501950513	8.75E-09	6.05E-08	1.04E-04
RAB3GAP2	LMNA	0.232408807	0.097485559	0.358943372	8.74E-04	2.33E-03	1.00E+00
CLEC7A	LMNA	0.26216311	0.128772633	0.386235521	1.64E-04	5.14E-04	1.00E+00
REXO4	LMNA	0.167775218	0.030428999	0.298903162	1.70E-02	3.23E-02	1.00E+00
HSPA14	LMNA	-0.246929077	-0.372289183	-0.112720586	3.96E-04	1.14E-03	1.00E+00
TAAR5	LMNA	-0.069699498	-0.205770557	0.069015576	3.24E-01	4.10E-01	1.00E+00
ZNF142	LMNA	-0.243422196	-0.369070695	-0.109035291	4.81E-04	1.36E-03	1.00E+00
MUT	LMNA	-0.211929951	-0.34003257	-0.076105582	2.46E-03	5.83E-03	1.00E+00
C2orf43	LMNA	-0.20586621	-0.334413176	-0.069798896	3.29E-03	7.54E-03	1.00E+00
SELPLG	LMNA	0.088602927	-0.050060399	0.223915061	2.10E-01	2.85E-01	1.00E+00
BAZ2B	LMNA	-0.382400516	-0.494354177	-0.257967837	1.95E-08	1.26E-07	2.32E-04
SLC38A3	LMNA	-0.192750681	-0.322227396	-0.056194996	5.99E-03	1.28E-02	1.00E+00
BRD7	LMNA	-0.074738043	-0.210615979	0.06397301	2.90E-01	3.74E-01	1.00E+00
POU6F2	LMNA	-0.169301943	-0.300333459	-0.031998828	1.60E-02	3.06E-02	1.00E+00
NISCH	LMNA	-0.154609399	-0.286544401	-0.016919528	2.80E-02	5.03E-02	1.00E+00
TCEB1	LMNA	-0.041617249	-0.178641898	0.096990994	5.56E-01	6.37E-01	1.00E+00
OPCML	LMNA	-0.519548806	-0.613590499	-0.410974647	2.32E-15	5.10E-14	2.76E-11
DTYMK	LMNA	0.085722498	-0.052955178	0.221156318	2.25E-01	3.02E-01	1.00E+00
TAX1BP3	LMNA	0.361345694	0.235018382	0.475668389	1.27E-07	7.15E-07	1.50E-03
F13B	LMNA	-0.087099394	-0.222475316	0.051571713	2.18E-01	2.94E-01	1.00E+00
RPL34	LMNA	-0.024979725	-0.162470451	0.113462548	7.24E-01	7.85E-01	1.00E+00

AKAP12	LMNA	0.462672843	0.346770952	0.564657769	4.14E-12	5.20E-11	4.91E-08
MARK2	LMNA	-0.013883657	-0.151644009	0.124405786	8.45E-01	8.85E-01	1.00E+00
AMBN	LMNA	0.126116989	-0.012145471	0.25964738	7.37E-02	1.18E-01	1.00E+00
C14orf32	LMNA	0.214825664	0.079121151	0.342712864	2.14E-03	5.15E-03	1.00E+00
FLJ21865	LMNA	-0.02780648	-0.165223237	0.110669348	6.94E-01	7.60E-01	1.00E+00
HSD3B2	LMNA	-0.032366783	-0.169659699	0.106158562	6.47E-01	7.20E-01	1.00E+00
HMG20A	LMNA	-0.261774121	-0.385880117	-0.128361885	1.68E-04	5.24E-04	1.00E+00
WDR77	LMNA	-0.052770434	-0.189441352	0.085906396	4.56E-01	5.42E-01	1.00E+00
ATF2	LMNA	-0.138278078	-0.271152925	-0.000231496	4.97E-02	8.35E-02	1.00E+00
C10orf137	LMNA	-0.355837805	-0.470763147	-0.229038014	2.02E-07	1.10E-06	2.40E-03
ARL6IP5	LMNA	0.107929153	-0.030577443	0.242368923	1.26E-01	1.86E-01	1.00E+00
QTRT1	LMNA	0.201878646	0.06565749	0.33071283	3.96E-03	8.89E-03	1.00E+00
CCNT1	LMNA	-0.140058752	-0.27283443	-0.002047337	4.68E-02	7.92E-02	1.00E+00
AP1B1	LMNA	0.19823714	0.061879588	0.32733015	4.68E-03	1.03E-02	1.00E+00
CD74	LMNA	0.303762217	0.172964424	0.42403159	1.11E-05	4.35E-05	1.32E-01
DYNLL1	LMNA	-0.088377249	-0.223698994	0.050287285	2.11E-01	2.86E-01	1.00E+00
PLSCR1	LMNA	0.362785798	0.2365836	0.476949751	1.12E-07	6.36E-07	1.33E-03
LIPG	LMNA	0.201778643	0.065553689	0.330619978	3.98E-03	8.92E-03	1.00E+00
PHACTR2	LMNA	0.325236411	0.195984981	0.443379999	2.33E-06	1.04E-05	2.76E-02
SLC35E1	LMNA	0.410390555	0.288695551	0.519035679	1.31E-09	1.06E-08	1.56E-05
VENTX	LMNA	0.407553219	0.285569238	0.516541953	1.75E-09	1.38E-08	2.07E-05
FEZ1	LMNA	-0.446802316	-0.550874544	-0.329047458	2.64E-11	2.87E-10	3.13E-07
APOD	LMNA	-0.202490139	-0.331280534	-0.06629227	3.85E-03	8.67E-03	1.00E+00
LAD1	LMNA	0.015347158	-0.122964366	0.153073843	8.28E-01	8.71E-01	1.00E+00
C16orf44	LMNA	0.442649876	0.324423851	0.547258842	4.22E-11	4.48E-10	5.01E-07
C1orf166	LMNA	0.476820784	0.362640829	0.576897189	7.30E-13	1.04E-11	8.66E-09
PAOX	LMNA	0.118504025	-0.019872075	0.252425446	9.30E-02	1.43E-01	1.00E+00
MAPK8	LMNA	-0.475974994	-0.576166754	-0.361690233	8.12E-13	1.15E-11	9.63E-09
NELF	LMNA	-0.2269653	-0.353926788	-0.091790347	1.16E-03	2.99E-03	1.00E+00
RBBP8	LMNA	0.132925778	-0.005221031	0.266093814	5.93E-02	9.74E-02	1.00E+00
DNAJC8	LMNA	-0.243586036	-0.369221128	-0.109207384	4.77E-04	1.35E-03	1.00E+00
KCNJ12	LMNA	-0.135385009	-0.268419244	0.002716768	5.47E-02	9.07E-02	1.00E+00
WNT11	LMNA	0.181869331	0.044946821	0.312084744	9.59E-03	1.95E-02	1.00E+00
SRP54	LMNA	0.109275871	-0.029215869	0.243651236	1.22E-01	1.80E-01	1.00E+00
GPR35	LMNA	-0.038001852	-0.175134065	0.100576794	5.91E-01	6.69E-01	1.00E+00

NRGN	LMNA	-0.231090775	-0.357729381	-0.096105771	9.37E-04	2.47E-03	1.00E+00
IFNW1	LMNA	-0.053298714	-0.189952059	0.085380511	4.51E-01	5.37E-01	1.00E+00
ACVR1	LMNA	0.26724712	0.134145238	0.390877202	1.21E-04	3.89E-04	1.00E+00
SCN1B	LMNA	0.136736236	-0.00134006	0.269696295	5.23E-02	8.74E-02	1.00E+00
RNASEH2B	LMNA	-0.33267009	-0.450052243	-0.203987295	1.32E-06	6.17E-06	1.56E-02
STAR	LMNA	-0.234532849	-0.360898849	-0.099710216	7.80E-04	2.11E-03	1.00E+00
C14orf65	LMNA	-0.218596789	-0.346200328	-0.083052094	1.78E-03	4.37E-03	1.00E+00
TAAR2	LMNA	-0.068531901	-0.204646759	0.070183095	3.32E-01	4.18E-01	1.00E+00
VAMP5	LMNA	0.474086289	0.359568349	0.574535069	1.03E-12	1.42E-11	1.22E-08
TUBA1C	LMNA	0.476641421	0.36243922	0.576742302	7.47E-13	1.06E-11	8.86E-09
PIK3R2	LMNA	-0.142550365	-0.275185921	-0.004589686	4.30E-02	7.34E-02	1.00E+00
ARD1A	LMNA	0.196455129	0.060032252	0.325673592	5.08E-03	1.11E-02	1.00E+00
SYTL2	LMNA	0.218951238	0.083421781	0.346527934	1.74E-03	4.29E-03	1.00E+00
UBXD2	LMNA	0.109756302	-0.028730015	0.244108578	1.20E-01	1.78E-01	1.00E+00
EBF2	LMNA	0.241851013	0.107385371	0.367627741	5.25E-04	1.47E-03	1.00E+00
CAMSAP1L	LMNA	-0.50983387	-0.605283178	-0.399931261	9.22E-15	1.80E-13	1.09E-10
CYP3A43	LMNA	-0.212133194	-0.340220762	-0.076317156	2.44E-03	5.79E-03	1.00E+00
CCDC91	LMNA	-0.031355858	-0.168676706	0.107159003	6.58E-01	7.28E-01	1.00E+00
AKR1B1	LMNA	0.001939752	-0.136147707	0.139953275	9.78E-01	9.83E-01	1.00E+00
KAL1	LMNA	-0.197793648	-0.326917954	-0.061419751	4.78E-03	1.05E-02	1.00E+00
GRID2	LMNA	-0.511981236	-0.607121201	-0.402369521	6.82E-15	1.36E-13	8.09E-11
ZNF423	LMNA	-0.249036635	-0.374221975	-0.114937146	3.52E-04	1.03E-03	1.00E+00
PSMB4	LMNA	0.070174624	-0.068540374	0.206227757	3.21E-01	4.06E-01	1.00E+00
ARPP-21	LMNA	-0.583934839	-0.668126314	-0.484979379	7.42E-20	3.71E-18	8.80E-16
XPNPEP3	LMNA	-0.005053026	-0.143004271	0.133090815	9.43E-01	9.58E-01	1.00E+00
CYBB	LMNA	0.315126198	0.185128953	0.434284317	4.93E-06	2.07E-05	5.84E-02
UXS1	LMNA	0.119796388	-0.018561584	0.253652481	8.95E-02	1.38E-01	1.00E+00
C7orf16	LMNA	-0.249193538	-0.374365824	-0.115102217	3.48E-04	1.02E-03	1.00E+00
SPTA1	LMNA	0.108811236	-0.029685688	0.243208874	1.23E-01	1.82E-01	1.00E+00
SHB	LMNA	0.326746553	0.197609249	0.444736518	2.07E-06	9.37E-06	2.46E-02
CHST7	LMNA	0.346065923	0.218451382	0.462042939	4.54E-07	2.32E-06	5.39E-03
SEMA6D	LMNA	0.017938839	-0.120410355	0.155604489	8.00E-01	8.49E-01	1.00E+00
IKZF4	LMNA	-0.123950033	-0.257593246	0.014346455	7.88E-02	1.24E-01	1.00E+00
NDUFA1	LMNA	0.088181918	-0.05048365	0.223511973	2.12E-01	2.87E-01	1.00E+00
HSPE1	LMNA	-0.077022334	-0.212810518	0.061684559	2.76E-01	3.58E-01	1.00E+00

MAPK13	LMNA	0.416252808	0.295163094	0.524182144	7.22E-10	6.13E-09	8.56E-06
MYCN	LMNA	-0.508959947	-0.60453486	-0.398939398	1.04E-14	2.01E-13	1.24E-10
KCNJ3	LMNA	-0.46081529	-0.563047457	-0.344692235	5.16E-12	6.40E-11	6.13E-08
ZNF573	LMNA	-0.24705398	-0.372403759	-0.112851912	3.93E-04	1.14E-03	1.00E+00
PCDHGA8	LMNA	-0.153970268	-0.285943329	-0.016264992	2.87E-02	5.14E-02	1.00E+00
ERO1LB	LMNA	0.040307641	-0.098290297	0.177371655	5.69E-01	6.48E-01	1.00E+00
NTF3	LMNA	0.054428145	-0.084255943	0.191043671	4.42E-01	5.28E-01	1.00E+00
GTF2B	LMNA	-0.184804356	-0.314823446	-0.047977374	8.46E-03	1.75E-02	1.00E+00
GSPT1	LMNA	0.338755268	0.210550781	0.455504377	8.17E-07	3.97E-06	9.69E-03
GUSB	LMNA	0.630505448	0.539405315	0.707016393	8.73E-24	9.33E-22	1.04E-19
EXTL3	LMNA	0.295848382	0.164516595	0.416873371	1.91E-05	7.20E-05	2.27E-01
PMPCB	LMNA	0.137996908	-5.51E-05	0.270887339	5.02E-02	8.41E-02	1.00E+00
COPS3	LMNA	-0.130558277	-0.263853658	0.007630249	6.40E-02	1.04E-01	1.00E+00
LIG1	LMNA	-0.11188586	-0.24613507	0.026575626	1.13E-01	1.69E-01	1.00E+00
NID2	LMNA	0.344888931	0.21717828	0.460991103	5.00E-07	2.52E-06	5.93E-03
LSM8	LMNA	0.321502216	0.191971627	0.440023351	3.08E-06	1.34E-05	3.65E-02
TMEM97	LMNA	-0.27488226	-0.397836262	-0.142228515	7.52E-05	2.52E-04	8.92E-01
SUZ12	LMNA	-0.257335891	-0.381822467	-0.123678633	2.18E-04	6.66E-04	1.00E+00
EXTL2	LMNA	-0.326121201	-0.444174847	-0.196936552	2.18E-06	9.79E-06	2.58E-02
PDE6B	LMNA	0.015340891	-0.122970539	0.153067721	8.28E-01	8.71E-01	1.00E+00
MRPS16	LMNA	-0.012711099	-0.150498009	0.125560232	8.58E-01	8.95E-01	1.00E+00
KRT18	LMNA	0.486241049	0.373244481	0.585021836	2.20E-13	3.34E-12	2.61E-09
C10orf118	LMNA	0.0057976	-0.132359336	0.143733562	9.35E-01	9.52E-01	1.00E+00
ZDHHC13	LMNA	-0.032838309	-0.170118103	0.105691831	6.43E-01	7.16E-01	1.00E+00
JMJD2C	LMNA	-0.062860796	-0.199183285	0.075848417	3.74E-01	4.62E-01	1.00E+00
OR2F1	LMNA	-0.059720013	-0.196153841	0.078982149	3.99E-01	4.86E-01	1.00E+00
ZNF415	LMNA	-0.249540236	-0.374683655	-0.11546699	3.42E-04	1.00E-03	1.00E+00
CDK5RAP3	LMNA	-0.048712349	-0.185515796	0.089943502	4.91E-01	5.76E-01	1.00E+00
YTHDF2	LMNA	0.060868785	-0.077836273	0.197262193	3.89E-01	4.77E-01	1.00E+00
GGCX	LMNA	0.486391152	0.373413679	0.585151135	2.16E-13	3.30E-12	2.56E-09
FZD8	LMNA	0.412472107	0.290990756	0.520863973	1.06E-09	8.73E-09	1.26E-05
TCEA1	LMNA	-0.026107839	-0.163569301	0.112348088	7.12E-01	7.75E-01	1.00E+00
ARPC4	LMNA	0.239338669	0.104748663	0.36531918	6.02E-04	1.67E-03	1.00E+00
SUSD4	LMNA	-0.418032548	-0.525743018	-0.297128794	6.01E-10	5.18E-09	7.12E-06
C22orf24	LMNA	-0.201094145	-0.329984368	-0.064843274	4.11E-03	9.19E-03	1.00E+00

EGLN2	LMNA	0.25247638	0.118557662	0.37737415	2.89E-04	8.60E-04	1.00E+00
KBTBD4	LMNA	-0.177311384	-0.307827382	-0.040245514	1.16E-02	2.31E-02	1.00E+00
TNFRSF14	LMNA	0.550665107	0.446561666	0.640058913	2.06E-17	6.70E-16	2.44E-13
ROBO3	LMNA	-0.040831519	-0.177879841	0.097770598	5.64E-01	6.44E-01	1.00E+00
TRIM28	LMNA	-0.002415773	-0.140419944	0.135680477	9.73E-01	9.79E-01	1.00E+00
FGF5	LMNA	0.292956856	0.161434759	0.41425418	2.33E-05	8.62E-05	2.76E-01
RTCD1	LMNA	0.440619215	0.322164832	0.545489238	5.30E-11	5.55E-10	6.28E-07
MZF1	LMNA	-0.087097668	-0.222473663	0.051573448	2.18E-01	2.94E-01	1.00E+00
CNIH4	LMNA	0.24456713	0.110238068	0.370121806	4.52E-04	1.29E-03	1.00E+00
ZFP2	LMNA	-0.364042399	-0.478067443	-0.237949908	1.01E-07	5.75E-07	1.19E-03
CSPG5	LMNA	-0.456607503	-0.559396854	-0.339987667	8.49E-12	1.01E-10	1.01E-07
FKBP15	LMNA	0.394059611	0.270736779	0.504657102	6.54E-09	4.64E-08	7.75E-05
BZW2	LMNA	0.008662546	-0.129543372	0.146538305	9.03E-01	9.29E-01	1.00E+00
PTPRN	LMNA	-0.097869223	-0.232775211	0.040732106	1.66E-01	2.34E-01	1.00E+00
HTATSF1	LMNA	-0.107491272	-0.241951881	0.031020044	1.28E-01	1.88E-01	1.00E+00
WFDC2	LMNA	0.02151949	-0.116878733	0.159097845	7.61E-01	8.16E-01	1.00E+00
TST	LMNA	-0.114336901	-0.248466034	0.024094413	1.05E-01	1.59E-01	1.00E+00
NDUFA7	LMNA	-0.078690841	-0.214412603	0.060012092	2.66E-01	3.47E-01	1.00E+00
TTC22	LMNA	-0.000988494	-0.139020523	0.137081213	9.89E-01	9.91E-01	1.00E+00
RNF24	LMNA	0.394716528	0.271457523	0.505236671	6.14E-09	4.38E-08	7.28E-05
SFRS4	LMNA	0.072953955	-0.065759332	0.208901037	3.02E-01	3.86E-01	1.00E+00
CD70	LMNA	0.528584346	0.421274413	0.621298223	6.19E-16	1.55E-14	7.34E-12
DCPS	LMNA	0.416482827	0.29541709	0.524383918	7.05E-10	6.00E-09	8.36E-06
PDXDC1	LMNA	0.311736368	0.181496148	0.431229197	6.30E-06	2.58E-05	7.47E-02
SRC	LMNA	-0.142799065	-0.275420548	-0.004843548	4.26E-02	7.28E-02	1.00E+00
NTNG1	LMNA	0.128294556	-0.009932358	0.261710354	6.88E-02	1.11E-01	1.00E+00
SETD1B	LMNA	0.062817482	-0.075891652	0.199141524	3.74E-01	4.62E-01	1.00E+00
TINP1	LMNA	0.128190431	-0.010038214	0.261611737	6.90E-02	1.11E-01	1.00E+00
ZNF606	LMNA	-0.268128783	-0.391681517	-0.135077746	1.14E-04	3.70E-04	1.00E+00
SSR1	LMNA	0.326215644	0.197038137	0.444259679	2.16E-06	9.73E-06	2.56E-02
NFS1	LMNA	0.074917445	-0.063793334	0.210788381	2.89E-01	3.73E-01	1.00E+00
CRMP1	LMNA	-0.504017417	-0.600299512	-0.393334687	2.06E-14	3.76E-13	2.45E-10
NUP107	LMNA	-0.062003166	-0.198356316	0.076704394	3.81E-01	4.68E-01	1.00E+00
OSBPL9	LMNA	0.194697733	0.05821135	0.32403914	5.49E-03	1.19E-02	1.00E+00
MYOZ3	LMNA	-0.131264078	-0.264521646	0.006912179	6.26E-02	1.02E-01	1.00E+00

PDE4B	LMNA	-0.216213789	-0.343996985	-0.080567613	2.00E-03	4.85E-03	1.00E+00
ADAM18	LMNA	-0.053317468	-0.189970188	0.085361841	4.51E-01	5.37E-01	1.00E+00
FBXL7	LMNA	0.209666793	0.073750479	0.337936325	2.75E-03	6.43E-03	1.00E+00
IDH3G	LMNA	0.108546786	-0.029953061	0.242957076	1.24E-01	1.83E-01	1.00E+00
CCDC87	LMNA	0.069514761	-0.069200327	0.205592773	3.26E-01	4.11E-01	1.00E+00
ARFGAP3	LMNA	0.15714652	0.019518969	0.288929418	2.55E-02	4.64E-02	1.00E+00
MAPRE2	LMNA	-0.154031667	-0.286001076	-0.016327866	2.86E-02	5.13E-02	1.00E+00
CSNK1G1	LMNA	0.165461046	0.028050784	0.29673403	1.86E-02	3.50E-02	1.00E+00
IL1RN	LMNA	0.339112951	0.210936939	0.455824578	7.94E-07	3.87E-06	9.42E-03
MAFB	LMNA	0.338450082	0.210221331	0.455231148	8.37E-07	4.06E-06	9.92E-03
RAC3	LMNA	-0.30768871	-0.42757761	-0.177163015	8.41E-06	3.37E-05	9.97E-02
C1orf54	LMNA	0.293512665	0.162026951	0.414757797	2.24E-05	8.33E-05	2.66E-01
TMEM14B	LMNA	-0.217734222	-0.345402953	-0.0821526	1.85E-03	4.53E-03	1.00E+00
ADIPOR1	LMNA	0.366564672	0.240693894	0.480309779	8.07E-08	4.71E-07	9.58E-04
GRINA	LMNA	0.107697759	-0.030811338	0.242148548	1.27E-01	1.87E-01	1.00E+00
CLIP4	LMNA	-0.125588917	-0.259146913	0.012681959	7.49E-02	1.19E-01	1.00E+00
TFPI	LMNA	0.329725259	0.200815143	0.447410619	1.65E-06	7.63E-06	1.96E-02
DPEP1	LMNA	-0.025925493	-0.163391708	0.112528251	7.14E-01	7.76E-01	1.00E+00
C14orf118	LMNA	0.099109506	-0.03948169	0.233959435	1.61E-01	2.28E-01	1.00E+00
FABP6	LMNA	-0.453041849	-0.556300231	-0.336005607	1.29E-11	1.49E-10	1.53E-07
TMEM87A	LMNA	0.140617327	0.002617132	0.273361731	4.59E-02	7.79E-02	1.00E+00
BBS5	LMNA	-0.032441686	-0.169732521	0.106084425	6.47E-01	7.19E-01	1.00E+00
SMTN	LMNA	0.237063164	0.10236215	0.363226908	6.81E-04	1.86E-03	1.00E+00
CYP17A1	LMNA	-0.022351075	-0.159908664	0.116058032	7.52E-01	8.09E-01	1.00E+00
SCG3	LMNA	-0.657241068	-0.729135567	-0.570998338	2.33E-26	4.01E-24	2.77E-22
GFER	LMNA	0.305388429	0.174702753	0.425500671	9.89E-06	3.92E-05	1.17E-01
CD209	LMNA	0.378977372	0.254227076	0.491323223	2.67E-08	1.69E-07	3.17E-04
NRIP2	LMNA	-0.147227527	-0.27959577	-0.009366908	3.65E-02	6.36E-02	1.00E+00
CYB5R2	LMNA	0.40002564	0.27728755	0.509917014	3.67E-09	2.73E-08	4.36E-05
RIC8B	LMNA	-0.348420487	-0.464146152	-0.220999519	3.75E-07	1.95E-06	4.44E-03
CSGlcA-T	LMNA	0.678344249	0.596117311	0.746489425	1.39E-28	3.31E-26	1.65E-24
DNTTIP2	LMNA	0.281621028	0.149377537	0.403966589	4.90E-05	1.70E-04	5.81E-01
TNNI1	LMNA	-0.062427346	-0.198765356	0.076281056	3.77E-01	4.65E-01	1.00E+00
GABRB3	LMNA	-0.519542885	-0.613585442	-0.410967906	2.32E-15	5.10E-14	2.76E-11
PCBD1	LMNA	0.112864095	-0.025585554	0.247065567	1.10E-01	1.65E-01	1.00E+00

KTELC1	LMNA	0.127783388	-0.010451992	0.261226195	6.99E-02	1.12E-01	1.00E+00
HOXD3	LMNA	-0.35591161	-0.470828924	-0.229118087	2.01E-07	1.10E-06	2.39E-03
GPR85	LMNA	-0.546019127	-0.636120339	-0.441227162	4.31E-17	1.34E-15	5.11E-13
P11	LMNA	-0.280767322	-0.403190572	-0.148471092	5.18E-05	1.79E-04	6.14E-01
SP3	LMNA	0.080061856	-0.05863724	0.215728491	2.57E-01	3.39E-01	1.00E+00
GOSR2	LMNA	0.118760698	-0.01961184	0.252669179	9.23E-02	1.42E-01	1.00E+00
DDX1	LMNA	-0.122010483	-0.255753654	0.016315328	8.37E-02	1.31E-01	1.00E+00
BFSP1	LMNA	0.44125182	0.322868432	0.546040617	4.94E-11	5.20E-10	5.85E-07
FLRT3	LMNA	-0.12821839	-0.261638217	0.010009791	6.90E-02	1.11E-01	1.00E+00
RNPS1	LMNA	-0.097728918	-0.232641222	0.040873531	1.66E-01	2.34E-01	1.00E+00
LCP2	LMNA	0.365182773	0.239190275	0.479081435	9.11E-08	5.26E-07	1.08E-03
TAS2R8	LMNA	-0.019512873	-0.157140573	0.118858305	7.83E-01	8.35E-01	1.00E+00
SEZ6L	LMNA	-0.515458478	-0.610095362	-0.406321108	4.17E-15	8.72E-14	4.95E-11
NR2C1	LMNA	-0.318217429	-0.437067933	-0.188444855	3.93E-06	1.68E-05	4.66E-02
EXDL2	LMNA	-0.007310253	-0.145214705	0.130872823	9.18E-01	9.41E-01	1.00E+00
SLC25A10	LMNA	-0.171964676	-0.302826604	-0.03473836	1.44E-02	2.79E-02	1.00E+00
ADAMTS3	LMNA	0.157420362	0.019799649	0.289186746	2.53E-02	4.60E-02	1.00E+00
PCYOX1L	LMNA	0.036906038	-0.101662927	0.174070166	6.02E-01	6.79E-01	1.00E+00
TNFRSF13I	LMNA	-0.066866111	-0.203042836	0.071848115	3.44E-01	4.30E-01	1.00E+00
VPS54	LMNA	0.178555243	0.041527894	0.30898973	1.10E-02	2.21E-02	1.00E+00
MKI67	LMNA	0.058120465	-0.080577052	0.194609995	4.11E-01	4.99E-01	1.00E+00
C7orf54	LMNA	0.046158749	-0.092481569	0.183043349	5.14E-01	5.98E-01	1.00E+00
GLS	LMNA	-0.053985849	-0.190616225	0.084696377	4.45E-01	5.32E-01	1.00E+00
PCDHB12	LMNA	0.033958561	-0.104582725	0.171206941	6.31E-01	7.05E-01	1.00E+00
LGALS13	LMNA	-0.195095792	-0.324409418	-0.058623713	5.40E-03	1.17E-02	1.00E+00
IL4R	LMNA	0.523550343	0.41553265	0.617006203	1.30E-15	3.01E-14	1.54E-11
SEC11A	LMNA	0.281865486	0.149637138	0.404188769	4.83E-05	1.68E-04	5.72E-01
C4orf6	LMNA	0.288577425	0.156771969	0.410283391	3.11E-05	1.13E-04	3.69E-01
SPP2	LMNA	0.282204015	0.149996666	0.404496423	4.72E-05	1.65E-04	5.60E-01
CCL5	LMNA	0.36417033	0.238089037	0.478181211	9.94E-08	5.69E-07	1.18E-03
PEX5	LMNA	-0.206181273	-0.334705376	-0.070126315	3.24E-03	7.44E-03	1.00E+00
CLSPN	LMNA	-0.001451619	-0.139474669	0.136626761	9.84E-01	9.87E-01	1.00E+00
LOC51336	LMNA	-0.244656085	-0.370203457	-0.110331534	4.50E-04	1.28E-03	1.00E+00
SPAG1	LMNA	0.052169138	-0.086504871	0.188859969	4.61E-01	5.47E-01	1.00E+00
C9orf82	LMNA	-0.040753107	-0.177803782	0.097848389	5.65E-01	6.45E-01	1.00E+00

TM4SF1	LMNA	0.199686035	0.063382285	0.328676459	4.38E-03	9.74E-03	1.00E+00
SMG7	LMNA	0.045792661	-0.092845281	0.182688754	5.18E-01	6.01E-01	1.00E+00
WDR45L	LMNA	-0.088548522	-0.223862975	0.050115096	2.10E-01	2.85E-01	1.00E+00
TAS2R13	LMNA	-0.005044954	-0.142996363	0.133098745	9.43E-01	9.58E-01	1.00E+00
SPAG8	LMNA	-0.193766409	-0.323172655	-0.057246738	5.73E-03	1.23E-02	1.00E+00
VASP	LMNA	0.583310475	0.48425487	0.667601778	8.29E-20	4.13E-18	9.84E-16
ZCCHC11	LMNA	-0.232995765	-0.35948386	-0.098100186	8.47E-04	2.26E-03	1.00E+00
LOX	LMNA	0.590192306	0.492248019	0.673378676	2.39E-20	1.27E-18	2.84E-16
SYPL1	LMNA	0.39792485	0.274979555	0.508065802	4.51E-09	3.30E-08	5.34E-05
BAG5	LMNA	0.108102107	-0.03040261	0.242533632	1.26E-01	1.85E-01	1.00E+00
RPS27L	LMNA	0.283350886	0.151214941	0.405538489	4.38E-05	1.54E-04	5.20E-01
MGC2752	LMNA	0.086553527	-0.052120248	0.221952462	2.21E-01	2.97E-01	1.00E+00
IQSEC3	LMNA	-0.31129652	-0.430832579	-0.181025034	6.50E-06	2.66E-05	7.71E-02
TGFBR3	LMNA	-0.113490761	-0.247661521	0.024951163	1.08E-01	1.62E-01	1.00E+00
PPA2	LMNA	-0.072516314	-0.208480229	0.066197385	3.05E-01	3.90E-01	1.00E+00
MED24	LMNA	-0.277484068	-0.400204455	-0.144987081	6.38E-05	2.17E-04	7.57E-01
CASP9	LMNA	-0.179756959	-0.310112327	-0.042767255	1.05E-02	2.11E-02	1.00E+00
PDCD1	LMNA	0.126040987	-0.012222689	0.259575355	7.39E-02	1.18E-01	1.00E+00
MAP3K7	LMNA	0.050553413	-0.088112521	0.187297275	4.75E-01	5.60E-01	1.00E+00
C17orf81	LMNA	0.029270897	-0.109221454	0.166648492	6.79E-01	7.48E-01	1.00E+00
SRPR	LMNA	0.590024195	0.492052564	0.673237678	2.47E-20	1.31E-18	2.93E-16
BAG4	LMNA	0.110662853	-0.027813051	0.244971397	1.17E-01	1.74E-01	1.00E+00
ZNF32	LMNA	-0.434418643	-0.540080004	-0.31527531	1.05E-10	1.05E-09	1.25E-06
BRD2	LMNA	0.132437574	-0.005717969	0.265631988	6.03E-02	9.87E-02	1.00E+00
IL32	LMNA	0.392370216	0.268883875	0.503166164	7.68E-09	5.37E-08	9.11E-05
LAMP2	LMNA	0.400053867	0.277318571	0.50994188	3.66E-09	2.73E-08	4.34E-05
FAM53B	LMNA	-0.075729471	-0.211568621	0.062979955	2.84E-01	3.67E-01	1.00E+00
CAT	LMNA	0.013094213	-0.125183077	0.150872487	8.53E-01	8.92E-01	1.00E+00
C16orf80	LMNA	-0.360683554	-0.475079072	-0.234298936	1.34E-07	7.52E-07	1.59E-03
SLC7A1	LMNA	-0.070174096	-0.206227249	0.068540902	3.21E-01	4.06E-01	1.00E+00
C1orf76	LMNA	-0.050419776	-0.187167994	0.088245457	4.76E-01	5.61E-01	1.00E+00
PRKCQ	LMNA	-0.138212121	-0.271090626	-0.000164255	4.98E-02	8.37E-02	1.00E+00
ATXN1	LMNA	0.139438266	0.001414494	0.272248593	4.78E-02	8.07E-02	1.00E+00
LAMC2	LMNA	0.327288497	0.198192321	0.445223198	1.99E-06	9.03E-06	2.36E-02
CPOX	LMNA	0.160955096	0.02342459	0.292506597	2.21E-02	4.09E-02	1.00E+00

SPSB1	LMNA	0.38954456	0.26578678	0.50067097	1.00E-08	6.86E-08	1.19E-04
APH1B	LMNA	0.368943257	0.243283407	0.482423013	6.56E-08	3.89E-07	7.78E-04
USP36	LMNA	0.147845626	0.009998701	0.280178125	3.57E-02	6.25E-02	1.00E+00
CTNND1	LMNA	0.300972153	0.1699839	0.421509636	1.35E-05	5.21E-05	1.60E-01
MADCAM1	LMNA	-0.122204411	-0.255937631	0.016118516	8.32E-02	1.30E-01	1.00E+00
GABRG2	LMNA	-0.385664936	-0.497242059	-0.261538626	1.44E-08	9.60E-08	1.71E-04
LYZ	LMNA	0.316967761	0.187104004	0.435942896	4.31E-06	1.83E-05	5.11E-02
F5	LMNA	-0.076188176	-0.212009294	0.062520405	2.81E-01	3.64E-01	1.00E+00
TMEM186	LMNA	-0.155309089	-0.287202307	-0.017636219	2.73E-02	4.92E-02	1.00E+00
TPM2	LMNA	0.53502309	0.428630976	0.626779836	2.35E-16	6.39E-15	2.79E-12
SEMA4F	LMNA	0.135845356	-0.002247799	0.268854373	5.39E-02	8.97E-02	1.00E+00
NUDCD3	LMNA	0.072587386	-0.06612625	0.208548571	3.05E-01	3.89E-01	1.00E+00
OLFML3	LMNA	0.284167438	0.152082576	0.406280228	4.16E-05	1.46E-04	4.93E-01
PPP1R11	LMNA	0.008286656	-0.129912962	0.146170441	9.07E-01	9.33E-01	1.00E+00
ELAVL1	LMNA	-0.03236165	-0.169654708	0.106163643	6.48E-01	7.20E-01	1.00E+00
GCNT3	LMNA	0.173193836	0.036003668	0.303976876	1.37E-02	2.68E-02	1.00E+00
DNAJC17	LMNA	0.203685811	0.067533796	0.332390319	3.64E-03	8.25E-03	1.00E+00
N4BP1	LMNA	0.137164181	-0.000903936	0.270100649	5.16E-02	8.62E-02	1.00E+00
ABCA2	LMNA	-0.10360634	-0.238249665	0.03494447	1.42E-01	2.05E-01	1.00E+00
BNIP3L	LMNA	0.048097623	-0.090554654	0.184920764	4.97E-01	5.81E-01	1.00E+00
SLC35F2	LMNA	0.392601271	0.26913724	0.503370116	7.51E-09	5.27E-08	8.91E-05
ATP10D	LMNA	0.31316472	0.183026448	0.432516848	5.68E-06	2.35E-05	6.74E-02
LCP1	LMNA	0.32747157	0.19838931	0.445387588	1.96E-06	8.91E-06	2.33E-02
IGBP1	LMNA	-0.038048703	-0.175179544	0.10053035	5.91E-01	6.69E-01	1.00E+00
GALNT8	LMNA	-0.382469394	-0.494415136	-0.258043144	1.94E-08	1.26E-07	2.30E-04
PRKCH	LMNA	0.278065597	0.145603929	0.400733545	6.15E-05	2.10E-04	7.30E-01
DCAKD	LMNA	-0.160969266	-0.292519898	-0.023439128	2.21E-02	4.09E-02	1.00E+00
ELA2A	LMNA	-0.020974701	-0.15856656	0.117416287	7.67E-01	8.21E-01	1.00E+00
PITRM1	LMNA	0.039815367	-0.098778575	0.176894061	5.74E-01	6.53E-01	1.00E+00
RASSF8	LMNA	0.547153784	0.44252929	0.637082664	3.60E-17	1.15E-15	4.27E-13
GUK1	LMNA	-0.064117283	-0.2003945	0.074593983	3.65E-01	4.52E-01	1.00E+00
USP12	LMNA	0.107005943	-0.03151054	0.241489593	1.30E-01	1.90E-01	1.00E+00
STXBP1	LMNA	-0.414638751	-0.522765951	-0.293381274	8.52E-10	7.14E-09	1.01E-05
ADM	LMNA	0.53062874	0.423608698	0.623039697	4.56E-16	1.18E-14	5.41E-12
LSM2	LMNA	-0.2392805	-0.36526571	-0.104687637	6.04E-04	1.67E-03	1.00E+00

GHRHR	LMNA	0.129163237	-0.009049119	0.262532982	6.69E-02	1.08E-01	1.00E+00
SERF2	LMNA	0.310184341	0.179834061	0.429829501	7.04E-06	2.87E-05	8.35E-02
VPS72	LMNA	-0.418724431	-0.52634962	-0.297893247	5.59E-10	4.85E-09	6.63E-06
CD22	LMNA	-0.053873627	-0.190507763	0.084808117	4.46E-01	5.33E-01	1.00E+00
CD47	LMNA	0.027797487	-0.110678238	0.165214482	6.95E-01	7.60E-01	1.00E+00
LAP3	LMNA	0.264030481	0.130745096	0.387941153	1.47E-04	4.65E-04	1.00E+00
IMPDH1	LMNA	0.531422178	0.424515027	0.623715324	4.05E-16	1.05E-14	4.81E-12
PPIC	LMNA	0.609381332	0.514623385	0.689433274	6.36E-22	4.36E-20	7.55E-18
ACP6	LMNA	0.466874344	0.351476898	0.568297187	2.49E-12	3.26E-11	2.96E-08
PRKACA	LMNA	0.367742559	0.241976009	0.481356428	7.28E-08	4.29E-07	8.64E-04
PPP1R1A	LMNA	-0.385688086	-0.49726253	-0.261563961	1.44E-08	9.59E-08	1.71E-04
C9orf40	LMNA	-0.040576068	-0.177632051	0.098024021	5.66E-01	6.46E-01	1.00E+00
ASXL1	LMNA	0.013765041	-0.124522587	0.151528096	8.46E-01	8.85E-01	1.00E+00
IDH1	LMNA	0.09905627	-0.039535369	0.233908613	1.61E-01	2.28E-01	1.00E+00
XRCC5	LMNA	-0.168930823	-0.299985834	-0.031617169	1.62E-02	3.10E-02	1.00E+00
TBRG4	LMNA	0.352529851	0.225450891	0.467813715	2.67E-07	1.42E-06	3.17E-03
RAB1A	LMNA	0.116621801	-0.02177985	0.250637597	9.84E-02	1.50E-01	1.00E+00
INHA	LMNA	-0.013352081	-0.151124519	0.124929197	8.50E-01	8.89E-01	1.00E+00
MLL2	LMNA	-0.322957674	-0.441332048	-0.193535376	2.76E-06	1.22E-05	3.27E-02
FXYD1	LMNA	-0.108973154	-0.243363037	0.029521971	1.23E-01	1.81E-01	1.00E+00
DKFZP566E	LMNA	-0.206418055	-0.334924961	-0.070372402	3.21E-03	7.37E-03	1.00E+00
KMO	LMNA	0.21035685	0.07446841	0.338575623	2.66E-03	6.24E-03	1.00E+00
KIAA1128	LMNA	-0.355515028	-0.470475467	-0.228687845	2.08E-07	1.13E-06	2.47E-03
NUDT4	LMNA	-0.152561992	-0.284618546	-0.014823188	3.02E-02	5.39E-02	1.00E+00
USO1	LMNA	0.311901427	0.181672956	0.431378022	6.22E-06	2.56E-05	7.38E-02
RAB9A	LMNA	-0.0802377	-0.21589723	0.058460865	2.56E-01	3.38E-01	1.00E+00
RUFY3	LMNA	-0.524624309	-0.617922339	-0.416756889	1.11E-15	2.63E-14	1.32E-11
CLDN1	LMNA	0.338869231	0.210673811	0.455606401	8.09E-07	3.94E-06	9.60E-03
FBXO38	LMNA	0.02796864	-0.110509047	0.165381088	6.93E-01	7.59E-01	1.00E+00
VRK3	LMNA	0.048393676	-0.090260336	0.185207344	4.94E-01	5.79E-01	1.00E+00
ICOS	LMNA	0.202984326	0.066805358	0.331739266	3.76E-03	8.49E-03	1.00E+00
NFKB1	LMNA	0.505318255	0.394809009	0.601414749	1.72E-14	3.20E-13	2.05E-10
FASTK	LMNA	0.391768502	0.268224145	0.502634975	8.13E-09	5.66E-08	9.65E-05
LDB1	LMNA	-0.241114814	-0.366951418	-0.106612533	5.47E-04	1.53E-03	1.00E+00
ABCC1	LMNA	0.3510484	0.223845532	0.466491994	3.02E-07	1.59E-06	3.58E-03

IFNA6	LMNA	-0.125229706	-0.258806439	0.01304685	7.58E-02	1.20E-01	1.00E+00
PCBP2	LMNA	-0.041538745	-0.178565767	0.097068894	5.57E-01	6.38E-01	1.00E+00
RBP3	LMNA	0.187490059	0.0507527	0.317327609	7.54E-03	1.58E-02	1.00E+00
MUC13	LMNA	-0.056495724	-0.193041141	0.08219635	4.25E-01	5.11E-01	1.00E+00
C8orf30A	LMNA	0.280459221	0.148144012	0.402910464	5.28E-05	1.82E-04	6.27E-01
NUP205	LMNA	0.140399034	0.002394443	0.273155669	4.63E-02	7.84E-02	1.00E+00
MFAP1	LMNA	0.073212336	-0.065500683	0.209149455	3.00E-01	3.85E-01	1.00E+00
ACTA1	LMNA	0.187445436	0.050706571	0.317286017	7.56E-03	1.58E-02	1.00E+00
NHLH1	LMNA	-0.264698215	-0.388550848	-0.131450666	1.41E-04	4.49E-04	1.00E+00
GABBR2	LMNA	-0.318295277	-0.437138004	-0.188528399	3.90E-06	1.67E-05	4.63E-02
CXorf34	LMNA	0.245056508	0.11075229	0.370570983	4.40E-04	1.25E-03	1.00E+00
TDRD7	LMNA	-0.077915177	-0.213667908	0.060789694	2.70E-01	3.53E-01	1.00E+00
KCND2	LMNA	-0.539392715	-0.630494742	-0.433631524	1.21E-16	3.46E-15	1.43E-12
WIPF1	LMNA	0.513648081	0.404263238	0.608547223	5.39E-15	1.11E-13	6.40E-11
TIMM17B	LMNA	-0.072511202	-0.208475313	0.066202502	3.05E-01	3.90E-01	1.00E+00
SNX15	LMNA	0.013579387	-0.124705392	0.151346666	8.48E-01	8.87E-01	1.00E+00
AGXT	LMNA	-0.007160479	-0.145068079	0.131020036	9.19E-01	9.42E-01	1.00E+00
IGF2R	LMNA	0.442927713	0.324733038	0.547500888	4.09E-11	4.35E-10	4.85E-07
PIP5K1B	LMNA	-0.313222172	-0.43256863	-0.183088013	5.66E-06	2.34E-05	6.71E-02
ATP8A2	LMNA	-0.332594144	-0.449984143	-0.203905453	1.32E-06	6.20E-06	1.57E-02
FGF21	LMNA	-0.017560301	-0.155234979	0.120783505	8.04E-01	8.52E-01	1.00E+00
AFTPH	LMNA	0.127285601	-0.010957952	0.260754646	7.10E-02	1.14E-01	1.00E+00
RBM15B	LMNA	0.16267571	0.025190424	0.294121465	2.07E-02	3.85E-02	1.00E+00
FCER1G	LMNA	0.296094087	0.16477859	0.417095842	1.88E-05	7.09E-05	2.23E-01
AGBL5	LMNA	0.027349574	-0.111120979	0.164778431	6.99E-01	7.64E-01	1.00E+00
SNTB1	LMNA	-0.016384517	-0.154086988	0.121942305	8.17E-01	8.62E-01	1.00E+00
APEX2	LMNA	0.389175256	0.265382188	0.500344719	1.04E-08	7.07E-08	1.23E-04
C17orf39	LMNA	0.242779821	0.108360635	0.368480819	4.99E-04	1.41E-03	1.00E+00
SLC24A3	LMNA	-0.337857009	-0.454700115	-0.209581189	8.77E-07	4.24E-06	1.04E-02
TXNL4B	LMNA	-0.069436358	-0.205517319	0.069278732	3.26E-01	4.12E-01	1.00E+00
UBE3A	LMNA	0.007871811	-0.130320811	0.145764407	9.11E-01	9.37E-01	1.00E+00
TGFB1I1	LMNA	0.697120008	0.618602108	0.761851826	1.01E-30	3.62E-28	1.20E-26
RPL10L	LMNA	0.053735632	-0.084945516	0.190374386	4.48E-01	5.34E-01	1.00E+00
RBM13	LMNA	-0.012706726	-0.150493734	0.125564537	8.58E-01	8.95E-01	1.00E+00
LOC389517	LMNA	-0.096882599	-0.231832895	0.041726483	1.70E-01	2.39E-01	1.00E+00

TOP2B	LMNA	-0.418488428	-0.526142719	-0.297632473	5.73E-10	4.96E-09	6.79E-06
NPVF	LMNA	-0.132981345	-0.266146375	0.005164466	5.92E-02	9.72E-02	1.00E+00
SHOX2	LMNA	0.202417571	0.066216933	0.331213168	3.86E-03	8.69E-03	1.00E+00
ITGA7	LMNA	0.520781746	0.412378465	0.61464331	1.94E-15	4.37E-14	2.31E-11
RAD54L2	LMNA	0.071369965	-0.067344561	0.207377736	3.13E-01	3.98E-01	1.00E+00
KCNIP2	LMNA	-0.503105753	-0.599517699	-0.392301779	2.34E-14	4.18E-13	2.77E-10
C2orf47	LMNA	0.021128467	-0.117264571	0.158716523	7.65E-01	8.20E-01	1.00E+00
KLF13	LMNA	-0.026344199	-0.163799487	0.112114545	7.10E-01	7.73E-01	1.00E+00
TSPAN4	LMNA	0.690231264	0.610337571	0.756223873	6.43E-30	1.86E-27	7.62E-26
NLE1	LMNA	-0.075252965	-0.211110789	0.063457277	2.87E-01	3.71E-01	1.00E+00
TPST1	LMNA	0.197670161	0.061291723	0.326803172	4.80E-03	1.05E-02	1.00E+00
CEACAM1	LMNA	0.119691361	-0.018668102	0.253552779	8.98E-02	1.39E-01	1.00E+00
PFKFB4	LMNA	0.309716523	0.179333214	0.429407487	7.28E-06	2.96E-05	8.63E-02
SREBF1	LMNA	0.261693784	0.12827706	0.385806711	1.69E-04	5.26E-04	1.00E+00
RNF11	LMNA	-0.152194445	-0.284272707	-0.014446985	3.06E-02	5.45E-02	1.00E+00
IL21	LMNA	-0.115287389	-0.249369539	0.023131766	1.02E-01	1.55E-01	1.00E+00
LTK	LMNA	0.048415499	-0.090238639	0.185228468	4.94E-01	5.79E-01	1.00E+00
DKKL1	LMNA	0.077882883	-0.060822065	0.2136369	2.71E-01	3.53E-01	1.00E+00
EPAS1	LMNA	0.354315938	0.227387284	0.469406542	2.30E-07	1.24E-06	2.73E-03
POLD3	LMNA	-0.027538936	-0.164962785	0.11093381	6.97E-01	7.62E-01	1.00E+00
UBTF	LMNA	-0.070141876	-0.206196246	0.068573129	3.21E-01	4.07E-01	1.00E+00
PHB	LMNA	-0.067907285	-0.204045428	0.070807512	3.37E-01	4.23E-01	1.00E+00
KIAA0427	LMNA	-0.385214938	-0.496844112	-0.261046193	1.51E-08	9.98E-08	1.79E-04
FPRL2	LMNA	0.376861775	0.251917047	0.489448649	3.24E-08	2.01E-07	3.84E-04
HSDL2	LMNA	-0.121747359	-0.255504016	0.016582346	8.43E-02	1.32E-01	1.00E+00
SEMA6B	LMNA	-0.198777852	-0.32783264	-0.062440306	4.57E-03	1.01E-02	1.00E+00
AKR1A1	LMNA	0.143190315	0.005242955	0.275789626	4.21E-02	7.20E-02	1.00E+00
CLTB	LMNA	0.051161394	-0.087507663	0.187885383	4.70E-01	5.55E-01	1.00E+00
NXT2	LMNA	-0.208366097	-0.336730981	-0.072397624	2.92E-03	6.79E-03	1.00E+00
JDP2	LMNA	0.185329929	0.04852032	0.315313636	8.28E-03	1.72E-02	1.00E+00
MORF4L1	LMNA	-0.167143046	-0.298310745	-0.029779178	1.74E-02	3.30E-02	1.00E+00
HSPB7	LMNA	0.174185528	0.037024844	0.304904643	1.32E-02	2.59E-02	1.00E+00
POU2F1	LMNA	-0.274598126	-0.39757754	-0.141927386	7.66E-05	2.56E-04	9.08E-01
MLLT11	LMNA	-0.403997164	-0.513413934	-0.281654667	2.49E-09	1.91E-08	2.95E-05
CNNM2	LMNA	-0.267447818	-0.391060309	-0.134357489	1.19E-04	3.84E-04	1.00E+00

ZC3H15	LMNA	-0.106377467	-0.240890861	0.032145608	1.32E-01	1.93E-01	1.00E+00
ELK3	LMNA	0.617438029	0.524056525	0.696150589	1.29E-22	1.07E-20	1.53E-18
CBLC	LMNA	0.238310901	0.103670564	0.364374329	6.37E-04	1.75E-03	1.00E+00
SEC61B	LMNA	0.246279173	0.11203734	0.371692953	4.11E-04	1.18E-03	1.00E+00
RRP15	LMNA	0.040608033	-0.09799231	0.177663059	5.66E-01	6.46E-01	1.00E+00
OR3A2	LMNA	-0.120814774	-0.254619085	0.017528579	8.68E-02	1.35E-01	1.00E+00
RSL1D1	LMNA	0.003844271	-0.134277992	0.141820007	9.57E-01	9.68E-01	1.00E+00
P2RX7	LMNA	-0.378086838	-0.490534271	-0.253254524	2.90E-08	1.82E-07	3.44E-04
ANKMY2	LMNA	-0.054147564	-0.190772516	0.084535349	4.44E-01	5.31E-01	1.00E+00
PSME2	LMNA	0.430655897	0.311100597	0.536793211	1.58E-10	1.52E-09	1.87E-06
ADNP2	LMNA	-0.095489828	-0.23050224	0.043129729	1.76E-01	2.46E-01	1.00E+00
RBM25	LMNA	-0.022192018	-0.159753593	0.116215022	7.54E-01	8.10E-01	1.00E+00
PLEKHA5	LMNA	-0.062885427	-0.199207032	0.07582383	3.74E-01	4.62E-01	1.00E+00
DHX58	LMNA	0.170590561	0.033324355	0.301540229	1.52E-02	2.93E-02	1.00E+00
ARCN1	LMNA	0.310066019	0.179707379	0.429722769	7.10E-06	2.89E-05	8.42E-02
POLR2E	LMNA	0.13267039	-0.005480996	0.265852233	5.98E-02	9.81E-02	1.00E+00
SEC24A	LMNA	0.386028742	0.261936786	0.497563747	1.40E-08	9.31E-08	1.66E-04
IFITM1	LMNA	0.263900436	0.130607698	0.3878224	1.48E-04	4.68E-04	1.00E+00
ZNF643	LMNA	-0.321087505	-0.439650365	-0.19152618	3.18E-06	1.38E-05	3.77E-02
DNA2L	LMNA	-0.380429486	-0.492609301	-0.255813469	2.34E-08	1.50E-07	2.78E-04
ZBTB25	LMNA	0.223269646	0.08792884	0.350516808	1.40E-03	3.54E-03	1.00E+00
HIGD2A	LMNA	0.014900396	-0.123404449	0.15263742	8.33E-01	8.75E-01	1.00E+00
TTLL5	LMNA	0.081002701	-0.057693456	0.216631223	2.52E-01	3.33E-01	1.00E+00
TBX6	LMNA	-0.008430503	-0.146311221	0.129771531	9.05E-01	9.32E-01	1.00E+00
SPTBN4	LMNA	-0.186187981	-0.316113771	-0.049406908	7.98E-03	1.66E-02	1.00E+00
SGK3	LMNA	-0.244623803	-0.370173826	-0.110297615	4.50E-04	1.28E-03	1.00E+00
FBXO28	LMNA	-0.079680638	-0.215362651	0.059019577	2.60E-01	3.41E-01	1.00E+00
GCN1L1	LMNA	0.126928578	-0.011320792	0.260416402	7.18E-02	1.15E-01	1.00E+00
CLEC10A	LMNA	0.30046676	0.169444263	0.421052609	1.39E-05	5.37E-05	1.65E-01
GAS6	LMNA	0.357136454	0.230447202	0.471920345	1.81E-07	9.98E-07	2.15E-03
AMOT	LMNA	-0.300608766	-0.421181031	-0.169595882	1.38E-05	5.33E-05	1.64E-01
TSHR	LMNA	-0.271576224	-0.394824706	-0.138726261	9.24E-05	3.04E-04	1.00E+00
ETV1	LMNA	-0.215331569	-0.34318092	-0.079648251	2.09E-03	5.03E-03	1.00E+00
LDOC1	LMNA	-0.194191747	-0.323568408	-0.057687247	5.62E-03	1.21E-02	1.00E+00
ERGIC2	LMNA	-0.105601487	-0.240151465	0.032929574	1.35E-01	1.96E-01	1.00E+00

ADAM11	LMNA	-0.309148726	-0.428895212	-0.178725421	7.58E-06	3.07E-05	8.99E-02
NAT1	LMNA	0.224857465	0.089587419	0.351982302	1.29E-03	3.30E-03	1.00E+00
ASB9	LMNA	0.291195986	0.159559251	0.412658171	2.62E-05	9.59E-05	3.10E-01
ATP6V0E2	LMNA	-0.203905934	-0.332594591	-0.067762406	3.61E-03	8.18E-03	1.00E+00
HGFAC	LMNA	-0.051259602	-0.187980371	0.08740995	4.69E-01	5.54E-01	1.00E+00
TRAFD1	LMNA	-0.011884624	-0.149690031	0.126373716	8.67E-01	9.02E-01	1.00E+00
MTCH2	LMNA	0.045475099	-0.093160754	0.182381132	5.20E-01	6.04E-01	1.00E+00
BACH2	LMNA	-0.321070923	-0.439635451	-0.191508371	3.18E-06	1.38E-05	3.77E-02
AUTS2	LMNA	-0.023138876	-0.16067662	0.11528037	7.44E-01	8.02E-01	1.00E+00
PGS1	LMNA	0.034944204	-0.103606603	0.172164668	6.22E-01	6.97E-01	1.00E+00
LEPREL1	LMNA	0.481118738	0.367475033	0.580606481	4.24E-13	6.25E-12	5.03E-09
TFF1	LMNA	-0.092965644	-0.228089346	0.045671505	1.88E-01	2.60E-01	1.00E+00
RPRM	LMNA	-0.535483342	-0.627171322	-0.429157376	2.20E-16	5.97E-15	2.60E-12
PPP2R3A	LMNA	-0.008795108	-0.146668028	0.129413023	9.01E-01	9.28E-01	1.00E+00
HAP1	LMNA	0.100785323	-0.03779149	0.235558871	1.54E-01	2.19E-01	1.00E+00
BAT2	LMNA	0.003237826	-0.134873461	0.141225701	9.64E-01	9.73E-01	1.00E+00
EPHB2	LMNA	-0.032163519	-0.169462073	0.10635974	6.50E-01	7.21E-01	1.00E+00
LPHN2	LMNA	0.222799236	0.087437611	0.350082518	1.44E-03	3.61E-03	1.00E+00
ACTG1	LMNA	0.410406753	0.288713406	0.51904991	1.31E-09	1.06E-08	1.55E-05
CENPT	LMNA	-0.140724638	-0.273463024	-0.002726608	4.58E-02	7.76E-02	1.00E+00
RNF123	LMNA	0.015862813	-0.122456352	0.153577498	8.23E-01	8.66E-01	1.00E+00
ZP2	LMNA	-0.040793577	-0.177843038	0.09780824	5.64E-01	6.45E-01	1.00E+00
PLAUR	LMNA	0.649182041	0.561448153	0.722483871	1.48E-25	2.28E-23	1.76E-21
BMP5	LMNA	-0.007625067	-0.145522883	0.130563371	9.14E-01	9.38E-01	1.00E+00
MUM1	LMNA	-0.349488516	-0.465099738	-0.222155925	3.43E-07	1.79E-06	4.07E-03
SNX26	LMNA	-0.445564631	-0.549797249	-0.327668746	3.04E-11	3.28E-10	3.60E-07
MID2	LMNA	0.191437801	0.054836016	0.321005218	6.35E-03	1.35E-02	1.00E+00
ISG20L1	LMNA	0.376477994	0.251498148	0.48910848	3.35E-08	2.07E-07	3.97E-04
RBM28	LMNA	0.170059514	0.03277804	0.301042963	1.55E-02	2.98E-02	1.00E+00
SRRM2	LMNA	-0.044105625	-0.181054218	0.094520897	5.33E-01	6.15E-01	1.00E+00
MEP1B	LMNA	-0.047794526	-0.18462734	0.09085595	4.99E-01	5.84E-01	1.00E+00
PCSK7	LMNA	0.363234744	0.237071684	0.477349112	1.08E-07	6.14E-07	1.28E-03
HNRNPA1	LMNA	-0.2078087	-0.336214317	-0.071818029	3.00E-03	6.95E-03	1.00E+00
PBX2	LMNA	0.141333722	0.003348046	0.274037897	4.48E-02	7.62E-02	1.00E+00
CENTB1	LMNA	0.424561143	0.30434833	0.531462544	3.03E-10	2.76E-09	3.60E-06

BCAS3	LMNA	-0.324859095	-0.443040982	-0.195579262	2.39E-06	1.07E-05	2.84E-02
HGS	LMNA	0.191524925	0.054926184	0.321086336	6.32E-03	1.35E-02	1.00E+00
FLJ20184	LMNA	-0.084318705	-0.219811043	0.054365121	2.33E-01	3.11E-01	1.00E+00
TMC7	LMNA	0.323065885	0.193651665	0.441429328	2.74E-06	1.21E-05	3.25E-02
POLA2	LMNA	-0.007660894	-0.145557953	0.130528152	9.14E-01	9.38E-01	1.00E+00
NOL10	LMNA	0.121165778	-0.017172468	0.254952179	8.58E-02	1.34E-01	1.00E+00
KCNJ8	LMNA	0.08686822	-0.051804027	0.222253898	2.19E-01	2.95E-01	1.00E+00
HSD17B6	LMNA	-0.07297078	-0.208917213	0.06574249	3.02E-01	3.86E-01	1.00E+00
EDEM3	LMNA	0.313274369	0.183143948	0.432615676	5.64E-06	2.34E-05	6.68E-02
SLC16A1	LMNA	0.271869717	0.139037039	0.395092164	9.08E-05	2.99E-04	1.00E+00
TCOF1	LMNA	0.215950164	0.080292865	0.343753149	2.02E-03	4.90E-03	1.00E+00
SF3B3	LMNA	-0.037167819	-0.174324352	0.101403488	5.99E-01	6.77E-01	1.00E+00
RANBP9	LMNA	0.090214434	-0.048439846	0.225457543	2.02E-01	2.76E-01	1.00E+00
CPNE7	LMNA	-0.04283469	-0.179822334	0.09578271	5.45E-01	6.26E-01	1.00E+00
EVL	LMNA	-0.461408543	-0.563561832	-0.345355998	4.81E-12	5.99E-11	5.71E-08
NUDT21	LMNA	-0.002078589	-0.140089391	0.13601144	9.77E-01	9.82E-01	1.00E+00
IFNA21	LMNA	-0.049115422	-0.1859059	0.089542717	4.88E-01	5.73E-01	1.00E+00
CFD	LMNA	0.263765354	0.130464984	0.387699042	1.49E-04	4.71E-04	1.00E+00
PYCARD	LMNA	0.257426956	0.123774665	0.381905771	2.17E-04	6.63E-04	1.00E+00
MYBPC2	LMNA	0.006750531	-0.131422945	0.144666715	9.24E-01	9.45E-01	1.00E+00
ZNF235	LMNA	-0.317531454	-0.436450417	-0.187708767	4.13E-06	1.76E-05	4.90E-02
ACSL4	LMNA	0.18333085	0.046455595	0.313448774	9.01E-03	1.85E-02	1.00E+00
KIAA0644	LMNA	-0.135282114	-0.268321979	0.002821582	5.49E-02	9.10E-02	1.00E+00
ERC1	LMNA	-0.120100686	-0.253941334	0.018252949	8.87E-02	1.37E-01	1.00E+00
NKIRAS2	LMNA	-0.033788809	-0.171041971	0.10475081	6.33E-01	7.07E-01	1.00E+00
TRMT5	LMNA	-0.256164034	-0.380750295	-0.122443073	2.33E-04	7.08E-04	1.00E+00
TMEM176B	LMNA	0.230559952	0.095550223	0.357240339	9.63E-04	2.53E-03	1.00E+00
PPP1R7	LMNA	0.080779035	-0.057917844	0.216416638	2.53E-01	3.34E-01	1.00E+00
SOX2	LMNA	-0.310086898	-0.429741604	-0.179729734	7.09E-06	2.89E-05	8.41E-02
C16orf30	LMNA	0.047580665	-0.091068524	0.18442029	5.01E-01	5.86E-01	1.00E+00
COMT	LMNA	0.298909243	0.167781708	0.419643761	1.55E-05	5.93E-05	1.84E-01
AOC2	LMNA	-0.022302455	-0.159861263	0.116106022	7.53E-01	8.09E-01	1.00E+00
PDLIM5	LMNA	0.080491225	-0.058206561	0.216140495	2.55E-01	3.36E-01	1.00E+00
SPHK2	LMNA	-0.150832413	-0.282990819	-0.013053214	3.21E-02	5.70E-02	1.00E+00
THNSL2	LMNA	0.182748075	0.045853902	0.312904936	9.24E-03	1.89E-02	1.00E+00

LARP5	LMNA	-0.072904325	-0.208853319	0.065809011	3.02E-01	3.87E-01	1.00E+00
C1QTNF1	LMNA	0.346029033	0.218411473	0.462009977	4.55E-07	2.32E-06	5.40E-03
TRADD	LMNA	0.641976807	0.55292948	0.71652536	7.38E-25	9.73E-23	8.76E-21
C1orf43	LMNA	-0.067276171	-0.203437735	0.071438316	3.41E-01	4.28E-01	1.00E+00
SCARF1	LMNA	0.257948035	0.124324218	0.382382404	2.10E-04	6.44E-04	1.00E+00
RAD51L1	LMNA	0.010754868	-0.127485413	0.148585261	8.79E-01	9.11E-01	1.00E+00
LETMD1	LMNA	-0.103284576	-0.237942859	0.035269314	1.44E-01	2.07E-01	1.00E+00
KRT75	LMNA	0.190308924	0.053667905	0.319953986	6.67E-03	1.41E-02	1.00E+00
CD3EAP	LMNA	0.2412903	0.106796737	0.367112643	5.41E-04	1.51E-03	1.00E+00
B3GNT2	LMNA	0.322555663	0.193103389	0.440970625	2.85E-06	1.25E-05	3.38E-02
TMEM63A	LMNA	0.034310174	-0.104234539	0.171548626	6.28E-01	7.02E-01	1.00E+00
DUSP13	LMNA	-0.101779391	-0.236507288	0.036788516	1.49E-01	2.14E-01	1.00E+00
FNBP1	LMNA	0.251393595	0.117417588	0.376382199	3.07E-04	9.10E-04	1.00E+00
MAGEB2	LMNA	0.095129895	-0.04349228	0.230158278	1.78E-01	2.48E-01	1.00E+00
EYA2	LMNA	0.171805016	0.034574037	0.302677163	1.45E-02	2.81E-02	1.00E+00
FANCG	LMNA	-0.175880986	-0.306490236	-0.038771376	1.23E-02	2.43E-02	1.00E+00
CD1C	LMNA	0.195813645	0.059367482	0.325077074	5.22E-03	1.14E-02	1.00E+00
LASS2	LMNA	0.569849429	0.468667713	0.656272591	8.68E-19	3.70E-17	1.03E-14
BCL2L14	LMNA	-0.183559155	-0.313661801	-0.046691337	8.92E-03	1.83E-02	1.00E+00
ZNF471	LMNA	-0.115464444	-0.249537817	0.022952417	1.02E-01	1.54E-01	1.00E+00
ZNF224	LMNA	0.01586539	-0.122453813	0.153580015	8.23E-01	8.66E-01	1.00E+00
AVP	LMNA	0.18378555	0.046925123	0.313873034	8.84E-03	1.82E-02	1.00E+00
CKAP4	LMNA	0.655697539	0.569167402	0.727862632	3.34E-26	5.66E-24	3.96E-22
EFS	LMNA	-0.196551764	-0.325763445	-0.060132407	5.05E-03	1.10E-02	1.00E+00
PITPNM1	LMNA	0.339385192	0.211230878	0.456068269	7.77E-07	3.80E-06	9.22E-03
TRIM68	LMNA	0.030221865	-0.1082809	0.167573722	6.69E-01	7.39E-01	1.00E+00
ZNF652	LMNA	-0.294969947	-0.416077883	-0.163580074	2.03E-05	7.60E-05	2.41E-01
UCK2	LMNA	0.213198361	0.077426187	0.341206876	2.32E-03	5.53E-03	1.00E+00
TMEM4	LMNA	-0.047749658	-0.184583902	0.090900549	5.00E-01	5.84E-01	1.00E+00
SCN3B	LMNA	-0.423579554	-0.530603223	-0.303261974	3.36E-10	3.02E-09	3.99E-06
RNASEH1	LMNA	0.201919359	0.06569975	0.33075063	3.95E-03	8.88E-03	1.00E+00
FAM50A	LMNA	0.540079461	0.434418021	0.631078211	1.09E-16	3.16E-15	1.29E-12
DRD1	LMNA	-0.295367178	-0.416437628	-0.164003542	1.98E-05	7.41E-05	2.34E-01
OAT	LMNA	-0.156612428	-0.288427481	-0.018971603	2.60E-02	4.72E-02	1.00E+00
IQGAP2	LMNA	0.243042178	0.108636162	0.368721747	4.92E-04	1.39E-03	1.00E+00

CDYL	LMNA	-0.168349494	-0.299441236	-0.03101941	1.66E-02	3.17E-02	1.00E+00
C20orf149	LMNA	0.384619807	0.26039504	0.49631775	1.59E-08	1.05E-07	1.89E-04
ANKS1A	LMNA	-0.205989761	-0.334527764	-0.069927289	3.27E-03	7.50E-03	1.00E+00
HYAL3	LMNA	0.10569286	-0.032837269	0.240238539	1.34E-01	1.96E-01	1.00E+00
CLPB	LMNA	0.371048224	0.245576532	0.484292052	5.44E-08	3.28E-07	6.46E-04
SMNDC1	LMNA	-0.070154364	-0.206208262	0.068560638	3.21E-01	4.07E-01	1.00E+00
COBLL1	LMNA	0.121266573	-0.017070199	0.255047825	8.56E-02	1.33E-01	1.00E+00
DONSON	LMNA	0.185546885	0.048744471	0.315515965	8.20E-03	1.70E-02	1.00E+00
SLC30A9	LMNA	-0.279628168	-0.402154808	-0.147261912	5.57E-05	1.91E-04	6.61E-01
E2F8	LMNA	0.201079358	0.064827929	0.329970635	4.11E-03	9.20E-03	1.00E+00
CCDC25	LMNA	-0.129228897	-0.262595153	0.00898235	6.68E-02	1.08E-01	1.00E+00
C20orf116	LMNA	0.366025807	0.240107493	0.479830845	8.46E-08	4.91E-07	1.00E-03
C2orf55	LMNA	-0.004700607	-0.142659033	0.133436984	9.47E-01	9.60E-01	1.00E+00
PRCP	LMNA	-0.118016505	-0.251962458	0.02036631	9.44E-02	1.45E-01	1.00E+00
SPINK1	LMNA	0.331958161	0.203220162	0.449413805	1.39E-06	6.48E-06	1.65E-02
FAM12B	LMNA	-0.094887172	-0.229926306	0.043736748	1.79E-01	2.49E-01	1.00E+00
NDUFB1	LMNA	-0.195581401	-0.324861085	-0.059126837	5.28E-03	1.15E-02	1.00E+00
DIO3	LMNA	0.198982089	0.062652122	0.32802242	4.53E-03	1.00E-02	1.00E+00
HPN	LMNA	-0.063822947	-0.200110807	0.074887878	3.67E-01	4.54E-01	1.00E+00
NBN	LMNA	-0.113499733	-0.247670053	0.024942079	1.08E-01	1.62E-01	1.00E+00
C14orf94	LMNA	0.009958025	-0.128269309	0.147805835	8.88E-01	9.19E-01	1.00E+00
PVRL1	LMNA	-0.294034052	-0.415230157	-0.162582553	2.16E-05	8.05E-05	2.57E-01
CNTD2	LMNA	-0.02192619	-0.159494411	0.11647738	7.57E-01	8.13E-01	1.00E+00
OCLM	LMNA	-0.124433732	-0.258051866	0.013855277	7.77E-02	1.23E-01	1.00E+00
ZSCAN18	LMNA	-0.197468705	-0.32661591	-0.061082869	4.85E-03	1.06E-02	1.00E+00
MYL4	LMNA	0.198548785	0.062202753	0.327619774	4.62E-03	1.02E-02	1.00E+00
SLC17A1	LMNA	0.026904526	-0.111560834	0.164345116	7.04E-01	7.68E-01	1.00E+00
TAF5L	LMNA	-0.103218254	-0.237879617	0.035336266	1.44E-01	2.07E-01	1.00E+00
L3MBTL	LMNA	-0.237835474	-0.363937171	-0.103171962	6.54E-04	1.79E-03	1.00E+00
TSTA3	LMNA	0.250723746	0.116712475	0.375768401	3.19E-04	9.42E-04	1.00E+00
CCDC59	LMNA	-0.007974408	-0.14586483	0.130219949	9.10E-01	9.36E-01	1.00E+00
RAC1	LMNA	0.194413099	0.057916516	0.323774345	5.56E-03	1.20E-02	1.00E+00
C19orf15	LMNA	0.13299275	-0.005152856	0.266157163	5.92E-02	9.72E-02	1.00E+00
MED20	LMNA	-0.180930864	-0.311208593	-0.04397834	9.97E-03	2.02E-02	1.00E+00
CHMP4A	LMNA	0.224326166	0.089032359	0.351492003	1.33E-03	3.38E-03	1.00E+00

NFE2	LMNA	0.072028479	-0.066685617	0.208011099	3.08E-01	3.93E-01	1.00E+00
KLK14	LMNA	-0.07097451	-0.20699733	0.067740216	3.16E-01	4.01E-01	1.00E+00
FBXL12	LMNA	0.222892629	0.087535133	0.350168745	1.43E-03	3.60E-03	1.00E+00
ZNF364	LMNA	-0.067271073	-0.203432826	0.071443411	3.41E-01	4.28E-01	1.00E+00
TOMM20	LMNA	-0.20881629	-0.337148219	-0.072865814	2.86E-03	6.65E-03	1.00E+00
ARSF	LMNA	0.160085818	0.022532793	0.291690458	2.29E-02	4.21E-02	1.00E+00
MAST2	LMNA	0.1366392	-0.001438944	0.269604601	5.25E-02	8.76E-02	1.00E+00
GTF2A1	LMNA	-0.091557514	-0.226742577	0.047088669	1.95E-01	2.68E-01	1.00E+00
ATP1A3	LMNA	-0.612649818	-0.692160053	-0.518447511	3.35E-22	2.47E-20	3.97E-18
PNKP	LMNA	0.34661511	0.219045563	0.462533617	4.34E-07	2.22E-06	5.15E-03
MRPS35	LMNA	-0.116570961	-0.250589294	0.021831366	9.85E-02	1.50E-01	1.00E+00
MATR3	LMNA	-0.510838941	-0.606143588	-0.401072291	8.01E-15	1.59E-13	9.50E-11
S100P	LMNA	-0.003685172	-0.141664103	0.134434221	9.58E-01	9.69E-01	1.00E+00
MSC	LMNA	0.360758606	0.234380476	0.475145875	1.33E-07	7.48E-07	1.58E-03
CILP	LMNA	0.206416279	0.070370556	0.334923314	3.21E-03	7.37E-03	1.00E+00
PROZ	LMNA	0.102601656	-0.035958673	0.237291596	1.46E-01	2.10E-01	1.00E+00
SEC23B	LMNA	0.034081228	-0.104461257	0.171326149	6.30E-01	7.04E-01	1.00E+00
FAM5C	LMNA	-0.436457132	-0.541859298	-0.317538913	8.40E-11	8.55E-10	9.96E-07
C19orf40	LMNA	0.246976776	0.112770738	0.372332939	3.95E-04	1.14E-03	1.00E+00
DCK	LMNA	-0.299192761	-0.41990026	-0.168084291	1.52E-05	5.83E-05	1.81E-01
C17orf63	LMNA	-0.109529185	-0.243892383	0.028959705	1.21E-01	1.79E-01	1.00E+00
TIA1	LMNA	-0.286827933	-0.408695851	-0.154910922	3.49E-05	1.25E-04	4.14E-01
SPAR	LMNA	-0.171325101	-0.302227927	-0.034080151	1.48E-02	2.85E-02	1.00E+00
SLC6A4	LMNA	0.150221585	0.012428326	0.28241578	3.28E-02	5.81E-02	1.00E+00
SPTLC1	LMNA	0.118552106	-0.019823328	0.252471105	9.29E-02	1.43E-01	1.00E+00
HMGB3	LMNA	-0.217534061	-0.345217894	-0.0819439	1.87E-03	4.58E-03	1.00E+00
TOPBP1	LMNA	-0.145899748	-0.278344445	-0.008010084	3.83E-02	6.63E-02	1.00E+00
NAT8	LMNA	-0.11334222	-0.24752027	0.025101544	1.08E-01	1.63E-01	1.00E+00
MID1IP1	LMNA	0.028704522	-0.109781509	0.16609733	6.85E-01	7.53E-01	1.00E+00
KLF11	LMNA	0.117382144	-0.021009307	0.251359926	9.62E-02	1.47E-01	1.00E+00
HOMER3	LMNA	0.456006413	0.339316087	0.558875033	9.12E-12	1.08E-10	1.08E-07
KCNAB3	LMNA	-0.259710568	-0.383994111	-0.126183668	1.90E-04	5.85E-04	1.00E+00
KLHDC4	LMNA	0.083220391	-0.055467861	0.218758154	2.39E-01	3.18E-01	1.00E+00
PHLDA3	LMNA	0.456988326	0.34041321	0.559727414	8.12E-12	9.69E-11	9.63E-08
DOCK2	LMNA	0.26368543	0.130380547	0.387626052	1.50E-04	4.73E-04	1.00E+00

TUG1	LMNA	-0.089972936	-0.225226432	0.048682746	2.03E-01	2.77E-01	1.00E+00
NUP214	LMNA	0.162849068	0.025368386	0.294284127	2.06E-02	3.83E-02	1.00E+00
GABARAP	LMNA	-0.170908351	-0.30183777	-0.033651322	1.50E-02	2.89E-02	1.00E+00
GOLM1	LMNA	-0.216626892	-0.344379045	-0.080998188	1.96E-03	4.76E-03	1.00E+00
DPYSL2	LMNA	-0.205310565	-0.33389779	-0.069221533	3.38E-03	7.71E-03	1.00E+00
SDCCAG8	LMNA	-0.23765126	-0.363767769	-0.102978786	6.60E-04	1.81E-03	1.00E+00
SOX13	LMNA	-0.103746263	-0.238383075	0.034803199	1.42E-01	2.05E-01	1.00E+00
KEL	LMNA	0.043901019	-0.094724064	0.180855928	5.35E-01	6.17E-01	1.00E+00
EXOC5	LMNA	-0.050458269	-0.187205233	0.088207166	4.76E-01	5.61E-01	1.00E+00
GK	LMNA	0.161727802	0.024217498	0.293231906	2.15E-02	3.98E-02	1.00E+00
SLCO1B3	LMNA	0.126036973	-0.012226768	0.259571551	7.39E-02	1.18E-01	1.00E+00
RPL36A	LMNA	0.085252664	-0.053427132	0.220706127	2.28E-01	3.05E-01	1.00E+00
DNAJB1	LMNA	0.449105841	0.331614795	0.552878636	2.03E-11	2.24E-10	2.41E-07
ALPK3	LMNA	0.249379454	0.115297822	0.374536264	3.45E-04	1.01E-03	1.00E+00
IKZF5	LMNA	-0.321990754	-0.440462684	-0.192496441	2.97E-06	1.30E-05	3.52E-02
CYLC2	LMNA	0.120036644	-0.018317906	0.253880545	8.88E-02	1.37E-01	1.00E+00
C8orf51	LMNA	0.140349573	0.002343988	0.273108978	4.63E-02	7.85E-02	1.00E+00
NRP1	LMNA	0.630463224	0.539355622	0.706981341	8.81E-24	9.33E-22	1.04E-19
NR2F1	LMNA	-0.119027552	-0.252922562	0.019341261	9.16E-02	1.41E-01	1.00E+00
ACAD8	LMNA	-0.297509038	-0.418376713	-0.16628771	1.71E-05	6.48E-05	2.03E-01
PLEK	LMNA	0.191457935	0.054856854	0.321023965	6.34E-03	1.35E-02	1.00E+00
NLRP3	LMNA	0.222499105	0.087124232	0.349805404	1.46E-03	3.66E-03	1.00E+00
RBM35A	LMNA	-0.035938532	-0.173130572	0.10262161	6.12E-01	6.88E-01	1.00E+00
GPR3	LMNA	0.337057941	0.208718877	0.453984504	9.34E-07	4.50E-06	1.11E-02
RAB8B	LMNA	0.059986058	-0.078716808	0.196410556	3.96E-01	4.83E-01	1.00E+00
UBE2E3	LMNA	-0.291892265	-0.41328935	-0.160300747	2.50E-05	9.20E-05	2.96E-01
C20orf111	LMNA	0.036682543	-0.101884406	0.173853141	6.04E-01	6.81E-01	1.00E+00
GNAI2	LMNA	0.461253515	0.345182533	0.563427424	4.90E-12	6.09E-11	5.81E-08
METTL8	LMNA	0.113374963	-0.025068397	0.247551406	1.08E-01	1.63E-01	1.00E+00
SLC39A7	LMNA	0.436755251	0.317870067	0.542119431	8.13E-11	8.30E-10	9.64E-07
CAMK1	LMNA	0.19423545	0.057732512	0.323609068	5.61E-03	1.21E-02	1.00E+00
PRDX6	LMNA	0.457467167	0.34094835	0.560143008	7.68E-12	9.24E-11	9.11E-08
RFC3	LMNA	-0.194349726	-0.323715386	-0.057850874	5.58E-03	1.20E-02	1.00E+00
FAM129A	LMNA	0.578246325	0.478383473	0.663344225	2.03E-19	9.34E-18	2.41E-15
BCAN	LMNA	-0.518834198	-0.612980141	-0.410161234	2.57E-15	5.58E-14	3.05E-11

TETRAN	LMNA	0.495165033	0.383316814	0.592700215	6.82E-14	1.11E-12	8.09E-10
ILF2	LMNA	-0.157529107	-0.289288926	-0.019911114	2.52E-02	4.58E-02	1.00E+00
SMPD4	LMNA	-0.038145777	-0.175273774	0.100434116	5.90E-01	6.68E-01	1.00E+00
AKAP7	LMNA	-0.368299546	-0.481851244	-0.242582436	6.94E-08	4.11E-07	8.23E-04
ZNF500	LMNA	-0.196057042	-0.325303423	-0.059619701	5.17E-03	1.12E-02	1.00E+00
FLJ11151	LMNA	0.314062465	0.183988591	0.433325911	5.32E-06	2.22E-05	6.31E-02
PSMA3	LMNA	0.161284549	0.023762636	0.29281586	2.18E-02	4.04E-02	1.00E+00
ASCC3	LMNA	0.125588251	-0.012682635	0.259146282	7.49E-02	1.19E-01	1.00E+00
ACYP2	LMNA	-0.381131397	-0.493230778	-0.256580527	2.19E-08	1.41E-07	2.60E-04
SOX21	LMNA	-0.060514192	-0.196920113	0.078190012	3.92E-01	4.80E-01	1.00E+00
CLDN4	LMNA	0.204429416	0.06830613	0.333080329	3.52E-03	8.00E-03	1.00E+00
LYRM1	LMNA	-0.227183283	-0.354127816	-0.09201824	1.15E-03	2.96E-03	1.00E+00
DEFB1	LMNA	0.013407219	-0.12487491	0.151178406	8.50E-01	8.89E-01	1.00E+00
GRM8	LMNA	-0.263916787	-0.387837331	-0.130624974	1.48E-04	4.68E-04	1.00E+00
SLC22A18	LMNA	0.576176378	0.475986136	0.661602384	2.92E-19	1.32E-17	3.46E-15
RNF141	LMNA	-0.075143478	-0.211005584	0.063566943	2.88E-01	3.71E-01	1.00E+00
OR7C2	LMNA	-0.205927187	-0.33446973	-0.069862262	3.28E-03	7.52E-03	1.00E+00
GRK6	LMNA	0.16786812	0.030524505	0.298990213	1.69E-02	3.22E-02	1.00E+00
PIGZ	LMNA	-0.189101725	-0.318829465	-0.052419162	7.03E-03	1.48E-02	1.00E+00
VPS26A	LMNA	-0.203444221	-0.332166112	-0.067282907	3.68E-03	8.33E-03	1.00E+00
EPHA2	LMNA	0.541939469	0.436549008	0.632657982	8.15E-17	2.43E-15	9.66E-13
KIAA0562	LMNA	0.132125605	-0.006035483	0.265336843	6.09E-02	9.95E-02	1.00E+00
TCHH	LMNA	0.108519376	-0.029980773	0.242930977	1.24E-01	1.83E-01	1.00E+00
MGC16824	LMNA	-0.106095979	-0.240622662	0.032430013	1.33E-01	1.94E-01	1.00E+00
SARS2	LMNA	-0.182369253	-0.312551379	-0.045462836	9.39E-03	1.92E-02	1.00E+00
ZCWPW1	LMNA	-0.072072211	-0.208053158	0.066641852	3.08E-01	3.93E-01	1.00E+00
WDR8	LMNA	-0.06022127	-0.196637504	0.078482201	3.95E-01	4.82E-01	1.00E+00
MTHFR	LMNA	0.226434306	0.091235273	0.353437048	1.19E-03	3.06E-03	1.00E+00
RPGRIP1	LMNA	-0.033198622	-0.170468349	0.10533514	6.39E-01	7.13E-01	1.00E+00
ACAT1	LMNA	-0.182227184	-0.312418776	-0.045316187	9.44E-03	1.93E-02	1.00E+00
MEOX1	LMNA	0.251256255	0.117273007	0.376256361	3.10E-04	9.16E-04	1.00E+00
DACH1	LMNA	-0.197085372	-0.326259557	-0.060685492	4.93E-03	1.08E-02	1.00E+00
ADAMDEC1	LMNA	0.089443237	-0.049215462	0.224719463	2.06E-01	2.80E-01	1.00E+00
PLK3	LMNA	0.582447295	0.483253465	0.666876471	9.67E-20	4.74E-18	1.15E-15
PHKA2	LMNA	0.197773839	0.061399213	0.326899542	4.78E-03	1.05E-02	1.00E+00

CALML4	LMNA	-0.074155652	-0.210056252	0.064556227	2.94E-01	3.78E-01	1.00E+00
TSSC1	LMNA	-0.292294391	-0.413653826	-0.160729054	2.43E-05	8.97E-05	2.88E-01
TMEM45A	LMNA	0.406060217	0.283925217	0.515229015	2.03E-09	1.58E-08	2.40E-05
ATP5I	LMNA	-0.172428879	-0.30326106	-0.035216163	1.41E-02	2.75E-02	1.00E+00
MRPS34	LMNA	0.057654894	-0.081041139	0.19416051	4.15E-01	5.03E-01	1.00E+00
POU1F1	LMNA	0.058296316	-0.080401747	0.194779755	4.10E-01	4.98E-01	1.00E+00
TMEM28	LMNA	-0.376567158	-0.489187515	-0.251595467	3.32E-08	2.06E-07	3.94E-04
FBN2	LMNA	0.165938878	0.028541712	0.297182025	1.83E-02	3.44E-02	1.00E+00
DAP3	LMNA	0.275207242	0.142572964	0.398132153	7.37E-05	2.47E-04	8.74E-01
ZC3H7A	LMNA	-0.080085377	-0.215751062	0.058613648	2.57E-01	3.39E-01	1.00E+00
LAIR2	LMNA	0.11950447	-0.018857638	0.253375357	9.03E-02	1.39E-01	1.00E+00
ST3GAL1	LMNA	0.546312821	0.441564161	0.636369453	4.11E-17	1.29E-15	4.88E-13
PHF3	LMNA	-0.026440158	-0.163892934	0.112019725	7.09E-01	7.72E-01	1.00E+00
DVL2	LMNA	-0.004345627	-0.142311252	0.133785634	9.51E-01	9.64E-01	1.00E+00
LCT	LMNA	0.093454257	-0.045179626	0.228556546	1.86E-01	2.57E-01	1.00E+00
GEMIN8	LMNA	-0.124538357	-0.258151058	0.013749026	7.74E-02	1.22E-01	1.00E+00
DICER1	LMNA	0.042628117	-0.095987758	0.179622068	5.47E-01	6.28E-01	1.00E+00
ARMCX5	LMNA	-0.049451771	-0.186231395	0.089208242	4.85E-01	5.70E-01	1.00E+00
HIF3A	LMNA	-0.195367038	-0.324661713	-0.058904735	5.33E-03	1.16E-02	1.00E+00
CAPZA2	LMNA	0.012030139	-0.126230501	0.149832303	8.65E-01	9.01E-01	1.00E+00
IFNA2	LMNA	0.110863597	-0.02760997	0.245162428	1.16E-01	1.73E-01	1.00E+00
PLA2G2A	LMNA	0.295391885	0.164029883	0.416460003	1.97E-05	7.40E-05	2.34E-01
FOLH1	LMNA	-0.151824484	-0.283924561	-0.014068349	3.10E-02	5.52E-02	1.00E+00
MAPKAP1	LMNA	0.228559522	0.09345737	0.355396735	1.07E-03	2.79E-03	1.00E+00
CYFIP1	LMNA	0.178101291	0.041059831	0.308565571	1.12E-02	2.25E-02	1.00E+00
NPAS1	LMNA	0.035296003	-0.103258139	0.17250644	6.18E-01	6.93E-01	1.00E+00
TRPV6	LMNA	0.033847879	-0.104692321	0.171099378	6.32E-01	7.06E-01	1.00E+00
RSHL1	LMNA	0.00475737	-0.13338123	0.142714641	9.46E-01	9.60E-01	1.00E+00
PIAS3	LMNA	0.355159262	0.228301926	0.470158356	2.14E-07	1.16E-06	2.54E-03
SOCS6	LMNA	-0.014552858	-0.152297887	0.123746754	8.37E-01	8.78E-01	1.00E+00
TAF7L	LMNA	-0.120204715	-0.254040078	0.018147431	8.84E-02	1.37E-01	1.00E+00
MRPL24	LMNA	0.062315075	-0.07639311	0.198657096	3.78E-01	4.66E-01	1.00E+00
YWHAE	LMNA	-0.407750226	-0.516715162	-0.285786226	1.71E-09	1.36E-08	2.03E-05
GREB1	LMNA	-0.227306969	-0.354241875	-0.092147554	1.14E-03	2.95E-03	1.00E+00
NUP62CL	LMNA	0.036779608	-0.101788219	0.173947397	6.03E-01	6.80E-01	1.00E+00

MOSPD2	LMNA	0.184619335	0.047786257	0.314650866	8.53E-03	1.76E-02	1.00E+00
NEUROG3	LMNA	0.037801672	-0.10077523	0.17493974	5.93E-01	6.71E-01	1.00E+00
MARK1	LMNA	-0.428489495	-0.534899377	-0.308699097	2.00E-10	1.88E-09	2.37E-06
MGST3	LMNA	-0.099268839	-0.234111537	0.039321024	1.60E-01	2.27E-01	1.00E+00
BDNF	LMNA	0.2124224	0.076618238	0.340488532	2.40E-03	5.72E-03	1.00E+00
LMBRD1	LMNA	-0.067116261	-0.203283743	0.07159813	3.43E-01	4.29E-01	1.00E+00
PNMT	LMNA	-0.1866042	-0.316501829	-0.049837046	7.84E-03	1.63E-02	1.00E+00
PRPF19	LMNA	0.01185765	-0.126400263	0.149663657	8.67E-01	9.02E-01	1.00E+00
NDUFS8	LMNA	0.055383153	-0.08330477	0.191966436	4.34E-01	5.20E-01	1.00E+00
TFCP2L1	LMNA	0.273130102	0.140371949	0.396240504	8.39E-05	2.79E-04	9.96E-01
SLC25A16	LMNA	-0.216458046	-0.344222892	-0.080822194	1.97E-03	4.80E-03	1.00E+00
EIF2B3	LMNA	0.082130119	-0.056562192	0.217712662	2.45E-01	3.25E-01	1.00E+00
HSPB3	LMNA	-0.275463899	-0.398365818	-0.142845018	7.25E-05	2.44E-04	8.60E-01
RPA2	LMNA	-0.107524701	-0.241983721	0.030986256	1.28E-01	1.88E-01	1.00E+00
PAK6	LMNA	-0.069779694	-0.205847731	0.068935372	3.24E-01	4.09E-01	1.00E+00
RBM4	LMNA	-0.265736493	-0.389498662	-0.132548043	1.32E-04	4.23E-04	1.00E+00
CSF1	LMNA	0.43714924	0.318307759	0.542463189	7.78E-11	7.95E-10	9.23E-07
SEMA3E	LMNA	0.074788513	-0.063922464	0.210664481	2.90E-01	3.74E-01	1.00E+00
MXD4	LMNA	-0.239933622	-0.365866014	-0.105372896	5.83E-04	1.62E-03	1.00E+00
TNFSF10	LMNA	0.299646308	0.168568387	0.420310544	1.48E-05	5.67E-05	1.75E-01
SMARCB1	LMNA	-0.141117581	-0.273833907	-0.003127509	4.52E-02	7.67E-02	1.00E+00
TADA2L	LMNA	-0.270448752	-0.393797056	-0.137532631	9.91E-05	3.24E-04	1.00E+00
SCIN	LMNA	0.067107102	-0.071607283	0.203274922	3.43E-01	4.29E-01	1.00E+00
PPAP2A	LMNA	0.082757964	-0.05593205	0.218314758	2.42E-01	3.21E-01	1.00E+00
ULK1	LMNA	-0.141278737	-0.273986005	-0.003291942	4.49E-02	7.64E-02	1.00E+00
NPAL2	LMNA	0.12467473	-0.013610528	0.258280346	7.71E-02	1.22E-01	1.00E+00
MRPS11	LMNA	-0.0267972	-0.164240612	0.111666899	7.05E-01	7.69E-01	1.00E+00
SLC2A3	LMNA	0.473341137	0.358731526	0.573891099	1.13E-12	1.55E-11	1.34E-08
ARID3A	LMNA	0.258397047	0.124797831	0.382793065	2.05E-04	6.28E-04	1.00E+00
FEM1B	LMNA	0.31987325	0.190222239	0.438558044	3.47E-06	1.50E-05	4.12E-02
GNG5	LMNA	0.421463045	0.300920628	0.528749599	4.20E-10	3.71E-09	4.98E-06
ACOX1	LMNA	-0.047861341	-0.184692025	0.090789534	4.99E-01	5.83E-01	1.00E+00
MTHFSD	LMNA	0.160673254	0.023135422	0.292242004	2.24E-02	4.13E-02	1.00E+00
TLX2	LMNA	0.037409426	-0.101164027	0.174558933	5.97E-01	6.74E-01	1.00E+00
KIF5B	LMNA	-0.057014895	-0.19354253	0.081678998	4.20E-01	5.08E-01	1.00E+00

POLM	LMNA	0.324008233	0.19466451	0.442276362	2.55E-06	1.13E-05	3.03E-02
C1orf181	LMNA	0.032100815	-0.106421799	0.169401106	6.50E-01	7.22E-01	1.00E+00
C10orf92	LMNA	-0.057341728	-0.193858132	0.081353271	4.18E-01	5.05E-01	1.00E+00
MUC7	LMNA	-0.07520363	-0.211063384	0.063506693	2.87E-01	3.71E-01	1.00E+00
NUP153	LMNA	0.017580291	-0.1207638	0.155254493	8.04E-01	8.52E-01	1.00E+00
CSDE1	LMNA	-0.034765172	-0.171990727	0.103783925	6.23E-01	6.98E-01	1.00E+00
CLPTM1	LMNA	0.158273387	0.020674111	0.289988203	2.45E-02	4.47E-02	1.00E+00
LRRC17	LMNA	0.194297078	0.057796343	0.323666405	5.59E-03	1.21E-02	1.00E+00
ATP5B	LMNA	-0.088707711	-0.224015377	0.049955048	2.09E-01	2.84E-01	1.00E+00
S100A5	LMNA	0.231717038	0.096761313	0.358306263	9.06E-04	2.40E-03	1.00E+00
ZNHIT1	LMNA	0.147534814	0.00968099	0.279885299	3.61E-02	6.31E-02	1.00E+00
EFHD1	LMNA	-0.303154054	-0.423482027	-0.172314541	1.16E-05	4.53E-05	1.37E-01
HIST1H4G	LMNA	0.002258371	-0.135834979	0.140265641	9.75E-01	9.80E-01	1.00E+00
GOLGA2L1	LMNA	-0.056696635	-0.193235178	0.081996152	4.23E-01	5.10E-01	1.00E+00
COPZ2	LMNA	0.555513198	0.452136135	0.644163821	9.44E-18	3.28E-16	1.12E-13
ELF3	LMNA	-0.027852897	-0.165268421	0.110623464	6.94E-01	7.60E-01	1.00E+00
CPSF3L	LMNA	0.239321972	0.104731146	0.365303832	6.03E-04	1.67E-03	1.00E+00
POLR2I	LMNA	-0.148927978	-0.281197652	-0.011105299	3.44E-02	6.03E-02	1.00E+00
ZNF665	LMNA	-0.050367878	-0.187117785	0.088297082	4.77E-01	5.62E-01	1.00E+00
TLR6	LMNA	0.159563481	0.021997029	0.291199959	2.33E-02	4.28E-02	1.00E+00
GPI	LMNA	0.429964769	0.310334302	0.536189154	1.70E-10	1.63E-09	2.02E-06
ANGPTL7	LMNA	0.077211498	-0.061494986	0.212992188	2.75E-01	3.57E-01	1.00E+00
RAD9A	LMNA	0.087587169	-0.051081483	0.222942462	2.15E-01	2.91E-01	1.00E+00
NDST4	LMNA	-0.341659935	-0.458103785	-0.213687841	6.48E-07	3.21E-06	7.69E-03
AGPAT3	LMNA	0.236608591	0.101885587	0.362808787	6.98E-04	1.90E-03	1.00E+00
ADORA2A	LMNA	-0.139135018	-0.271962242	-0.001105247	4.83E-02	8.14E-02	1.00E+00
TNRC5	LMNA	0.054542913	-0.084141649	0.191154576	4.41E-01	5.27E-01	1.00E+00
KCNH2	LMNA	-0.159131521	-0.290794276	-0.021554025	2.37E-02	4.35E-02	1.00E+00
CCHCR1	LMNA	0.030409513	-0.108095278	0.167756262	6.67E-01	7.37E-01	1.00E+00
RPS27A	LMNA	-0.004081741	-0.142052696	0.134044793	9.54E-01	9.66E-01	1.00E+00
GCM2	LMNA	0.047635343	-0.091014176	0.184473228	5.01E-01	5.85E-01	1.00E+00
TUBA3C	LMNA	0.084591899	-0.054090773	0.220072889	2.31E-01	3.09E-01	1.00E+00
HOM-TES-1	LMNA	0.236226134	0.101484676	0.362456959	7.13E-04	1.94E-03	1.00E+00
IGFALS	LMNA	-0.21228849	-0.34036455	-0.076478827	2.42E-03	5.75E-03	1.00E+00
E2F1	LMNA	-0.016755122	-0.154448873	0.121577094	8.13E-01	8.59E-01	1.00E+00

PLCB3	LMNA	0.433571285	0.314334777	0.539340111	1.15E-10	1.14E-09	1.37E-06
NPAT	LMNA	-0.287171049	-0.409007263	-0.155275843	3.42E-05	1.22E-04	4.05E-01
NR0B1	LMNA	-0.335616526	-0.452693254	-0.207163884	1.05E-06	4.99E-06	1.24E-02
COIL	LMNA	-0.400012752	-0.509905661	-0.277273387	3.68E-09	2.73E-08	4.36E-05
RASGRP2	LMNA	-0.174533226	-0.30522987	-0.037382948	1.30E-02	2.56E-02	1.00E+00
APOB	LMNA	0.244384954	0.110046662	0.369954579	4.56E-04	1.30E-03	1.00E+00
RAB11FIP2	LMNA	-0.166933614	-0.298114461	-0.029563924	1.76E-02	3.33E-02	1.00E+00
PIGR	LMNA	0.05343931	-0.08524054	0.190087965	4.50E-01	5.36E-01	1.00E+00
CDC25C	LMNA	-0.125330636	-0.258902107	0.012944328	7.55E-02	1.20E-01	1.00E+00
RCOR3	LMNA	-0.032154803	-0.169453598	0.106368367	6.50E-01	7.22E-01	1.00E+00
TSC2	LMNA	-0.107054986	-0.241536309	0.031460978	1.29E-01	1.90E-01	1.00E+00
NRP2	LMNA	0.593605813	0.496218898	0.676240353	1.28E-20	7.08E-19	1.52E-16
FUT6	LMNA	-0.182710068	-0.312869466	-0.045814665	9.25E-03	1.89E-02	1.00E+00
CDH2	LMNA	0.209251135	0.073318099	0.337551184	2.80E-03	6.53E-03	1.00E+00
PTPRB	LMNA	-0.003813244	-0.141789604	0.134308459	9.57E-01	9.68E-01	1.00E+00
ACP2	LMNA	0.478060494	0.364034585	0.577967528	6.25E-13	9.02E-12	7.41E-09
GTF3A	LMNA	-0.107479165	-0.24194035	0.03103228	1.28E-01	1.88E-01	1.00E+00
LAG3	LMNA	0.072169081	-0.066544907	0.208146317	3.07E-01	3.92E-01	1.00E+00
AIM1L	LMNA	0.007119786	-0.131060032	0.14502824	9.20E-01	9.43E-01	1.00E+00
ARID5B	LMNA	0.351900463	0.224768777	0.467252251	2.81E-07	1.49E-06	3.34E-03
KIAA0256	LMNA	-0.211637182	-0.339761465	-0.075800833	2.50E-03	5.91E-03	1.00E+00
FLNC	LMNA	0.396696167	0.273630342	0.506982618	5.07E-09	3.69E-08	6.02E-05
PRAF2	LMNA	0.031220988	-0.107292453	0.168545542	6.59E-01	7.30E-01	1.00E+00
ITGB1BP3	LMNA	0.169000402	0.031688721	0.30005101	1.62E-02	3.10E-02	1.00E+00
C14orf147	LMNA	0.070882169	-0.067832597	0.206908497	3.16E-01	4.02E-01	1.00E+00
ZRSR2	LMNA	0.162212666	0.024715126	0.293686952	2.11E-02	3.92E-02	1.00E+00
KRAS	LMNA	-0.261173895	-0.385331641	-0.127728174	1.74E-04	5.42E-04	1.00E+00
SPTAN1	LMNA	-0.240364726	-0.366262198	-0.105825283	5.70E-04	1.59E-03	1.00E+00
FLJ14803	LMNA	0.306652609	0.176054654	0.426642265	9.05E-06	3.60E-05	1.07E-01
RCAN2	LMNA	0.166713378	0.029337581	0.297908041	1.77E-02	3.35E-02	1.00E+00
NECAP2	LMNA	0.245267081	0.110973575	0.370764239	4.35E-04	1.24E-03	1.00E+00
CDX2	LMNA	0.163315736	0.02584749	0.294721964	2.02E-02	3.77E-02	1.00E+00
ECOP	LMNA	0.243683296	0.109309547	0.369310427	4.75E-04	1.34E-03	1.00E+00
ACTR1A	LMNA	-0.233349022	-0.359809108	-0.098470145	8.31E-04	2.23E-03	1.00E+00
PPARG	LMNA	0.112018864	-0.026441029	0.246261597	1.12E-01	1.68E-01	1.00E+00

BBS10	LMNA	-0.463135699	-0.565058898	-0.347289095	3.91E-12	4.94E-11	4.64E-08
ISOC2	LMNA	0.14930283	0.011488627	0.281550676	3.39E-02	5.97E-02	1.00E+00
CITED1	LMNA	0.076596786	-0.062110992	0.212401794	2.79E-01	3.61E-01	1.00E+00
PRDM4	LMNA	0.152002599	0.014250635	0.284092178	3.08E-02	5.49E-02	1.00E+00
HSPA4	LMNA	0.423881804	0.30359645	0.530867848	3.26E-10	2.94E-09	3.86E-06
SERPINB7	LMNA	0.23503635	0.100237765	0.361362229	7.60E-04	2.06E-03	1.00E+00
DHX40	LMNA	-0.238626273	-0.364664286	-0.104001347	6.26E-04	1.73E-03	1.00E+00
RAB26	LMNA	-0.483673134	-0.582809064	-0.37035105	3.06E-13	4.57E-12	3.63E-09
RRP9	LMNA	0.220017269	0.084533867	0.347513046	1.65E-03	4.09E-03	1.00E+00
EVI5	LMNA	-0.14746967	-0.279823922	-0.009614403	3.62E-02	6.32E-02	1.00E+00
CAPN9	LMNA	0.066276784	-0.072436984	0.202475222	3.49E-01	4.35E-01	1.00E+00
HIP2	LMNA	0.002814288	-0.135289274	0.140810582	9.68E-01	9.76E-01	1.00E+00
GNB1L	LMNA	-0.045177239	-0.182092571	0.093456629	5.23E-01	6.06E-01	1.00E+00
MYBL2	LMNA	-0.012104207	-0.149904717	0.126157603	8.64E-01	9.00E-01	1.00E+00
ZNF407	LMNA	-0.122209212	-0.255942185	0.016113643	8.32E-02	1.30E-01	1.00E+00
PPIG	LMNA	0.104145842	-0.034399739	0.238764027	1.40E-01	2.03E-01	1.00E+00
C12orf10	LMNA	-0.0467036	-0.18357103	0.091940183	5.09E-01	5.93E-01	1.00E+00
MYL6	LMNA	0.340500078	0.212434871	0.457066059	7.11E-07	3.50E-06	8.43E-03
NAGA	LMNA	0.475931166	0.361640981	0.5761289	8.16E-13	1.15E-11	9.68E-09
MAPT	LMNA	-0.593869688	-0.676461466	-0.496526029	1.22E-20	6.77E-19	1.44E-16
MRE11A	LMNA	0.042826282	-0.095791056	0.179814183	5.45E-01	6.26E-01	1.00E+00
HSPA4L	LMNA	-0.033386727	-0.170651184	0.105148912	6.37E-01	7.11E-01	1.00E+00
PLXNC1	LMNA	-0.05048238	-0.187228558	0.088183181	4.76E-01	5.61E-01	1.00E+00
STBD1	LMNA	0.584570545	0.485717188	0.668660293	6.62E-20	3.34E-18	7.85E-16
CTAG2	LMNA	-0.118446528	-0.252370846	0.019930367	9.32E-02	1.43E-01	1.00E+00
C14orf169	LMNA	0.284300908	0.152224415	0.406401454	4.12E-05	1.45E-04	4.89E-01
MTTP	LMNA	0.180090128	0.043110936	0.310423497	1.03E-02	2.09E-02	1.00E+00
KCTD5	LMNA	0.082657001	-0.05603339	0.218217943	2.42E-01	3.22E-01	1.00E+00
ECM1	LMNA	0.580298901	0.480762163	0.66507054	1.42E-19	6.69E-18	1.68E-15
CHRNB4	LMNA	0.021094936	-0.117297656	0.158683822	7.66E-01	8.20E-01	1.00E+00
NYX	LMNA	0.039096297	-0.099491688	0.176196316	5.81E-01	6.59E-01	1.00E+00
RLN1	LMNA	-0.067357665	-0.203516211	0.071356868	3.41E-01	4.27E-01	1.00E+00
GZMK	LMNA	0.319751968	0.190092025	0.438448922	3.51E-06	1.51E-05	4.16E-02
C1orf21	LMNA	-0.287208732	-0.409041463	-0.155315923	3.41E-05	1.22E-04	4.04E-01
EPB41L1	LMNA	-0.22429493	-0.351463176	-0.088999729	1.33E-03	3.38E-03	1.00E+00

HOXA3	LMNA	0.085733614	-0.052944011	0.221166969	2.25E-01	3.02E-01	1.00E+00
TOM1L2	LMNA	-0.122636081	-0.256347116	0.015680389	8.21E-02	1.28E-01	1.00E+00
TMEM165	LMNA	0.137448554	-0.000614099	0.270369321	5.11E-02	8.55E-02	1.00E+00
MNDA	LMNA	0.223848189	0.088533079	0.351050852	1.36E-03	3.45E-03	1.00E+00
RAB21	LMNA	0.245539667	0.111260048	0.371014394	4.28E-04	1.22E-03	1.00E+00
RPS8	LMNA	-0.014108408	-0.151863627	0.124184464	8.42E-01	8.83E-01	1.00E+00
FOXJ2	LMNA	0.225485318	0.090243459	0.352561614	1.25E-03	3.20E-03	1.00E+00
MSX2	LMNA	-0.093204714	-0.228317947	0.045430845	1.87E-01	2.59E-01	1.00E+00
C10orf76	LMNA	-0.184011879	-0.314084191	-0.047158856	8.75E-03	1.80E-02	1.00E+00
PBRM1	LMNA	-0.191383797	-0.320954936	-0.054780127	6.36E-03	1.35E-02	1.00E+00
ZNF343	LMNA	0.321720082	0.192205661	0.44021928	3.03E-06	1.32E-05	3.59E-02
CPB2	LMNA	-0.154554731	-0.286492993	-0.016863539	2.81E-02	5.04E-02	1.00E+00
LY6G6C	LMNA	-0.181833519	-0.312051314	-0.044909859	9.60E-03	1.96E-02	1.00E+00
PIM1	LMNA	0.329617839	0.200699482	0.447314221	1.67E-06	7.68E-06	1.98E-02
CTF1	LMNA	0.312951014	0.182797448	0.432324225	5.77E-06	2.38E-05	6.84E-02
GRLF1	LMNA	-0.195536259	-0.324819101	-0.059080065	5.29E-03	1.15E-02	1.00E+00
EGFL7	LMNA	-0.065288934	-0.201523562	0.07342385	3.56E-01	4.43E-01	1.00E+00
USP9X	LMNA	0.185189729	0.048375478	0.315182881	8.33E-03	1.73E-02	1.00E+00
IFIT2	LMNA	-0.04947644	-0.186255266	0.08918371	4.84E-01	5.70E-01	1.00E+00
S100G	LMNA	0.054258112	-0.084425266	0.190879353	4.43E-01	5.30E-01	1.00E+00
GUCY1B3	LMNA	-0.131165535	-0.264428389	0.007012444	6.28E-02	1.02E-01	1.00E+00
NR3C1	LMNA	0.171305793	0.034060282	0.302209852	1.48E-02	2.85E-02	1.00E+00
FCN2	LMNA	0.086096768	-0.052579176	0.221514899	2.23E-01	3.00E-01	1.00E+00
CORO1B	LMNA	0.477618349	0.363537441	0.577585829	6.60E-13	9.49E-12	7.83E-09
PARP11	LMNA	-0.174688662	-0.30537525	-0.037543047	1.29E-02	2.54E-02	1.00E+00
F11	LMNA	-0.140477347	-0.273229596	-0.002474332	4.61E-02	7.82E-02	1.00E+00
DNALI1	LMNA	0.233850727	0.098995635	0.360270982	8.09E-04	2.17E-03	1.00E+00
MAP2K6	LMNA	-0.484138968	-0.583210583	-0.370875772	2.88E-13	4.33E-12	3.42E-09
LEFTY1	LMNA	-0.369315945	-0.482754005	-0.243689306	6.34E-08	3.78E-07	7.52E-04
ATHL1	LMNA	0.23108952	0.096104458	0.357728225	9.37E-04	2.47E-03	1.00E+00
FSTL4	LMNA	-0.242302614	-0.368042548	-0.107859528	5.12E-04	1.44E-03	1.00E+00
ANKRD47	LMNA	-0.165196915	-0.296486366	-0.027779441	1.88E-02	3.53E-02	1.00E+00
ATP2A1	LMNA	0.015527602	-0.122786604	0.153250095	8.26E-01	8.70E-01	1.00E+00
SERINC2	LMNA	0.127170898	-0.011074528	0.26064598	7.13E-02	1.14E-01	1.00E+00
PAXIP1	LMNA	0.157697606	0.020083836	0.289447249	2.50E-02	4.56E-02	1.00E+00

ZC3HAV1	LMNA	0.496197412	0.38448376	0.593587353	5.94E-14	9.72E-13	7.05E-10
YY1	LMNA	0.109321275	-0.029169956	0.24369446	1.21E-01	1.80E-01	1.00E+00
PNLIP	LMNA	0.146883095	0.009014892	0.279271213	3.70E-02	6.43E-02	1.00E+00
C14orf105	LMNA	0.075483727	-0.063226128	0.211332515	2.86E-01	3.69E-01	1.00E+00
ZNF80	LMNA	0.097503322	-0.041100915	0.232425771	1.67E-01	2.35E-01	1.00E+00
SLBP	LMNA	0.026110683	-0.112345278	0.163572071	7.12E-01	7.75E-01	1.00E+00
AASDHPPT	LMNA	-0.367683648	-0.481304088	-0.241911875	7.32E-08	4.31E-07	8.68E-04
UBL4A	LMNA	0.359720525	0.233252807	0.474221777	1.46E-07	8.12E-07	1.73E-03
CKS1B	LMNA	0.035123518	-0.103428993	0.172338875	6.20E-01	6.95E-01	1.00E+00
MCM3	LMNA	0.089384556	-0.049274473	0.224663296	2.06E-01	2.80E-01	1.00E+00
KAZALD1	LMNA	0.16579973	0.028398743	0.297051571	1.84E-02	3.46E-02	1.00E+00
SLC19A3	LMNA	0.025383094	-0.113064102	0.162863395	7.20E-01	7.82E-01	1.00E+00
CDC37L1	LMNA	-0.048758666	-0.185560624	0.08989745	4.91E-01	5.76E-01	1.00E+00
NDUFA4L2	LMNA	0.289162849	0.157394931	0.410814456	3.00E-05	1.09E-04	3.55E-01
THTPA	LMNA	-0.384005268	-0.495774136	-0.259722772	1.68E-08	1.10E-07	2.00E-04
BNIP3	LMNA	0.059074585	-0.079625788	0.195530971	4.04E-01	4.91E-01	1.00E+00
HIST3H2A	LMNA	-0.325788135	-0.44387566	-0.196578318	2.23E-06	1.00E-05	2.65E-02
STEAP4	LMNA	0.322148867	0.19266631	0.440604859	2.93E-06	1.29E-05	3.48E-02
HTR3B	LMNA	-0.154439201	-0.286384348	-0.016745217	2.82E-02	5.06E-02	1.00E+00
FES	LMNA	0.505640458	0.39517427	0.601690922	1.65E-14	3.08E-13	1.96E-10
C11orf71	LMNA	-0.365146325	-0.479049032	-0.239150624	9.13E-08	5.27E-07	1.08E-03
NPTX2	LMNA	0.084519828	-0.054163151	0.220003813	2.32E-01	3.10E-01	1.00E+00
CBLB	LMNA	0.052889114	-0.08578826	0.189556091	4.55E-01	5.41E-01	1.00E+00
NME6	LMNA	0.038529781	-0.100053411	0.175646505	5.86E-01	6.64E-01	1.00E+00
CETN1	LMNA	-0.191307814	-0.320884187	-0.054701492	6.38E-03	1.36E-02	1.00E+00
RORB	LMNA	0.089293416	-0.049366123	0.224576057	2.06E-01	2.81E-01	1.00E+00
AP2M1	LMNA	0.201903766	0.065683564	0.330736152	3.96E-03	8.88E-03	1.00E+00
SNAPC4	LMNA	-0.113978625	-0.248125406	0.024457206	1.06E-01	1.60E-01	1.00E+00
FAF1	LMNA	0.009258472	-0.128957354	0.147121431	8.96E-01	9.25E-01	1.00E+00
SLC9A7	LMNA	0.233804239	0.098946939	0.360228187	8.11E-04	2.18E-03	1.00E+00
CDK6	LMNA	0.158851649	0.021267027	0.290531404	2.39E-02	4.38E-02	1.00E+00
P2RY1	LMNA	0.164464718	0.027027361	0.295799728	1.93E-02	3.62E-02	1.00E+00
VAPB	LMNA	0.272776294	0.139997173	0.395918188	8.58E-05	2.84E-04	1.00E+00
CSK	LMNA	0.227901398	0.0927691	0.35478999	1.11E-03	2.87E-03	1.00E+00
IGHM	LMNA	0.208050584	0.072069535	0.336438535	2.97E-03	6.88E-03	1.00E+00

RAB27B	LMNA	-0.116590716	-0.250608063	0.021811349	9.84E-02	1.50E-01	1.00E+00
C2orf33	LMNA	-0.520067484	-0.61403344	-0.411565147	2.16E-15	4.77E-14	2.56E-11
CTSS	LMNA	0.2546989	0.120898877	0.379409323	2.54E-04	7.64E-04	1.00E+00
SNAP25	LMNA	-0.422223317	-0.529415558	-0.301761497	3.88E-10	3.45E-09	4.60E-06
TUFT1	LMNA	0.164253845	0.02681079	0.295601951	1.95E-02	3.65E-02	1.00E+00
LILRA2	LMNA	0.086454995	-0.052219253	0.221858075	2.21E-01	2.98E-01	1.00E+00
TLL2	LMNA	-0.008916669	-0.146786981	0.129293486	9.00E-01	9.27E-01	1.00E+00
LUC7L	LMNA	-0.301468014	-0.421957984	-0.170513435	1.30E-05	5.05E-05	1.54E-01
LCK	LMNA	0.181027509	0.044078064	0.311298832	9.93E-03	2.02E-02	1.00E+00
PRPF6	LMNA	0.116005811	-0.022403983	0.250052295	1.00E-01	1.52E-01	1.00E+00
AKTIP	LMNA	-0.308188991	-0.428029149	-0.177698306	8.12E-06	3.26E-05	9.63E-02
FURIN	LMNA	0.708049115	0.63174968	0.770760659	4.78E-32	2.84E-29	5.67E-28
SOX12	LMNA	-0.130918037	-0.26419416	0.007264253	6.33E-02	1.03E-01	1.00E+00
UQCRFS1	LMNA	-0.178873818	-0.309287366	-0.041856407	1.09E-02	2.18E-02	1.00E+00
ADH7	LMNA	0.062160407	-0.076547471	0.19850795	3.79E-01	4.67E-01	1.00E+00
RAMP1	LMNA	0.076541931	-0.062165958	0.212349104	2.79E-01	3.62E-01	1.00E+00
KIR3DX1	LMNA	0.084529042	-0.054153898	0.220012645	2.32E-01	3.10E-01	1.00E+00
INSL5	LMNA	-0.026775738	-0.164219714	0.111688108	7.05E-01	7.69E-01	1.00E+00
PDE8A	LMNA	-0.012472465	-0.150264734	0.125795134	8.60E-01	8.97E-01	1.00E+00
NAT6	LMNA	-0.104789495	-0.239377589	0.03374974	1.38E-01	2.00E-01	1.00E+00
EDN3	LMNA	-0.025197377	-0.162682482	0.113247558	7.22E-01	7.83E-01	1.00E+00
BLM	LMNA	0.076812518	-0.061894818	0.212609003	2.77E-01	3.60E-01	1.00E+00
GMIP	LMNA	0.211071284	0.075211852	0.339237382	2.57E-03	6.05E-03	1.00E+00
CHST4	LMNA	0.203890577	0.067746456	0.332580339	3.61E-03	8.18E-03	1.00E+00
SF3A2	LMNA	0.13435045	-0.003770483	0.267441162	5.66E-02	9.34E-02	1.00E+00
FN3KRP	LMNA	-0.113822849	-0.247977293	0.024614935	1.07E-01	1.61E-01	1.00E+00
PRUNE	LMNA	-0.157374841	-0.289143971	-0.019752989	2.53E-02	4.60E-02	1.00E+00
SMAD7	LMNA	-0.039036263	-0.176138057	0.099551218	5.81E-01	6.59E-01	1.00E+00
SDC4	LMNA	0.538459548	0.432563072	0.629701746	1.39E-16	3.96E-15	1.65E-12
IQWD1	LMNA	0.221805802	0.086400427	0.349165186	1.51E-03	3.78E-03	1.00E+00
FHL2	LMNA	0.502716972	0.391861379	0.599184239	2.46E-14	4.37E-13	2.92E-10
KIAA1107	LMNA	-0.556376352	-0.644894124	-0.453129461	8.20E-18	2.88E-16	9.73E-14
PSMB2	LMNA	0.288550482	0.156743301	0.410258948	3.12E-05	1.13E-04	3.70E-01
GOLGA8A	LMNA	-0.230945764	-0.357595791	-0.095953997	9.44E-04	2.49E-03	1.00E+00
TMEM57	LMNA	-0.061279921	-0.197658777	0.077426085	3.86E-01	4.74E-01	1.00E+00

WARS	LMNA	0.333025654	0.204370489	0.450371059	1.28E-06	6.02E-06	1.52E-02
PHOX2A	LMNA	0.218717522	0.083178012	0.34631192	1.76E-03	4.34E-03	1.00E+00
S100A14	LMNA	-0.044482463	-0.181419394	0.094146678	5.30E-01	6.12E-01	1.00E+00
FGFR2	LMNA	-0.299036086	-0.419758518	-0.167917077	1.54E-05	5.89E-05	1.83E-01
ASPA	LMNA	-0.170787407	-0.301724536	-0.033526882	1.51E-02	2.90E-02	1.00E+00
CLDND1	LMNA	-0.238475618	-0.364525775	-0.103843326	6.31E-04	1.74E-03	1.00E+00
XRCC3	LMNA	-0.022504246	-0.160057989	0.115906847	7.51E-01	8.08E-01	1.00E+00
ITPKA	LMNA	0.009893808	-0.128332476	0.147743013	8.89E-01	9.19E-01	1.00E+00
BTBD2	LMNA	0.047518866	-0.091129949	0.184360457	5.02E-01	5.86E-01	1.00E+00
GH2	LMNA	0.055201899	-0.083485316	0.19179132	4.35E-01	5.21E-01	1.00E+00
RDBP	LMNA	-0.183574897	-0.313676489	-0.046707593	8.92E-03	1.83E-02	1.00E+00
CSF3	LMNA	0.359588578	0.233109497	0.4741043	1.47E-07	8.20E-07	1.75E-03
LMO2	LMNA	0.226515275	0.091319908	0.35351173	1.19E-03	3.05E-03	1.00E+00
GPATCH4	LMNA	-0.03298586	-0.170261536	0.105545767	6.41E-01	7.14E-01	1.00E+00
PDPR	LMNA	0.048282955	-0.090370411	0.185100168	4.95E-01	5.80E-01	1.00E+00
PTK7	LMNA	0.508370055	0.398270042	0.604029653	1.13E-14	2.17E-13	1.34E-10
PPP2CB	LMNA	-0.154344909	-0.286295673	-0.016648649	2.83E-02	5.07E-02	1.00E+00
TWF2	LMNA	0.587015097	0.488555682	0.670712855	4.26E-20	2.20E-18	5.06E-16
B4GALT6	LMNA	-0.412493505	-0.520882763	-0.291014358	1.06E-09	8.72E-09	1.26E-05
DOLPP1	LMNA	-0.165135811	-0.29642907	-0.027716672	1.88E-02	3.54E-02	1.00E+00
C4orf8	LMNA	-0.209750779	-0.338014139	-0.073837849	2.74E-03	6.41E-03	1.00E+00
GLT25D1	LMNA	0.703287808	0.626016488	0.766882504	1.84E-31	8.07E-29	2.18E-27
TNNI2	LMNA	0.0680441	-0.070670751	0.204177151	3.36E-01	4.22E-01	1.00E+00
JHDM1D	LMNA	-0.175908242	-0.30651572	-0.03879946	1.23E-02	2.43E-02	1.00E+00
CD7	LMNA	0.157975033	0.020368235	0.289707906	2.47E-02	4.52E-02	1.00E+00
RPL22	LMNA	-0.04571015	-0.182608828	0.092927252	5.18E-01	6.02E-01	1.00E+00
CYC1	LMNA	-0.178388425	-0.308833866	-0.041355883	1.11E-02	2.22E-02	1.00E+00
EPRS	LMNA	0.047822277	-0.090828365	0.184654206	4.99E-01	5.84E-01	1.00E+00
B4GALT2	LMNA	-0.013842146	-0.151603444	0.124446663	8.45E-01	8.85E-01	1.00E+00
CHUK	LMNA	-0.05867617	-0.195146426	0.080023041	4.07E-01	4.94E-01	1.00E+00
CHAT	LMNA	0.262577145	0.129209878	0.386613769	1.60E-04	5.02E-04	1.00E+00
RAB6B	LMNA	-0.553210215	-0.642214508	-0.449487094	1.37E-17	4.63E-16	1.62E-13
HR	LMNA	-0.373671384	-0.48661976	-0.248436138	4.31E-08	2.63E-07	5.11E-04
CCDC134	LMNA	0.149467592	0.011657126	0.281705832	3.37E-02	5.94E-02	1.00E+00
PDPK1	LMNA	-0.088425257	-0.223744959	0.050239021	2.11E-01	2.86E-01	1.00E+00

C14orf130	LMNA	0.123920148	-0.014376801	0.257564909	7.89E-02	1.24E-01	1.00E+00
RAB33A	LMNA	-0.577183861	-0.662450287	-0.477152781	2.45E-19	1.12E-17	2.90E-15
DENND4B	LMNA	-0.095214808	-0.230239427	0.043406752	1.78E-01	2.48E-01	1.00E+00
CPT1B	LMNA	-0.2408843	-0.366739624	-0.10637058	5.54E-04	1.54E-03	1.00E+00
KYNU	LMNA	0.473819589	0.359268817	0.574304597	1.06E-12	1.46E-11	1.26E-08
MS4A5	LMNA	0.179349255	0.042346732	0.309731507	1.07E-02	2.14E-02	1.00E+00
TNMD	LMNA	0.055630352	-0.083058524	0.19220525	4.32E-01	5.18E-01	1.00E+00
PEX7	LMNA	-0.132038921	-0.265254828	0.006123703	6.10E-02	9.98E-02	1.00E+00
IL22	LMNA	0.285929682	0.153955759	0.407880467	3.71E-05	1.32E-04	4.40E-01
SRD5A2L	LMNA	0.227317447	0.09215851	0.354251538	1.14E-03	2.95E-03	1.00E+00
FAM62A	LMNA	0.563864357	0.461757501	0.651222843	2.38E-18	9.29E-17	2.83E-14
HOXC5	LMNA	0.013555796	-0.12472862	0.151323611	8.48E-01	8.87E-01	1.00E+00
SPATA6	LMNA	0.141559652	0.003578585	0.274251113	4.45E-02	7.57E-02	1.00E+00
C18orf22	LMNA	0.01785405	-0.12049394	0.155521726	8.01E-01	8.50E-01	1.00E+00
STX11	LMNA	0.452329892	0.335211007	0.555681582	1.40E-11	1.60E-10	1.66E-07
KCNC3	LMNA	0.049555179	-0.089105404	0.18633146	4.84E-01	5.69E-01	1.00E+00
CYP7B1	LMNA	-0.113333604	-0.247512076	0.025110267	1.08E-01	1.63E-01	1.00E+00
THOC1	LMNA	-0.089489351	-0.2247636	0.04916909	2.05E-01	2.80E-01	1.00E+00
C14orf159	LMNA	0.11363995	-0.024800117	0.247803384	1.07E-01	1.61E-01	1.00E+00
TBXAS1	LMNA	0.246927257	0.112718673	0.372287514	3.96E-04	1.14E-03	1.00E+00
HSF4	LMNA	-0.010922492	-0.1487492	0.12732049	8.77E-01	9.09E-01	1.00E+00
ALDH1B1	LMNA	0.225027802	0.089765391	0.352139479	1.28E-03	3.27E-03	1.00E+00
USP25	LMNA	0.214110022	0.078375657	0.342050654	2.22E-03	5.31E-03	1.00E+00
ZNF124	LMNA	-0.417040092	-0.524872701	-0.296032513	6.65E-10	5.69E-09	7.89E-06
PCYOX1	LMNA	0.301474692	0.170520567	0.421964021	1.30E-05	5.05E-05	1.54E-01
FBXO17	LMNA	0.310281701	0.179938304	0.429917322	6.99E-06	2.85E-05	8.29E-02
C19orf29	LMNA	0.229598289	0.09454398	0.35635419	1.01E-03	2.65E-03	1.00E+00
RAD51C	LMNA	-0.239130432	-0.365127764	-0.104530203	6.09E-04	1.69E-03	1.00E+00
SLPI	LMNA	0.274909441	0.142257322	0.39786101	7.51E-05	2.51E-04	8.91E-01
GPR45	LMNA	-0.107964511	-0.242402595	0.030541702	1.26E-01	1.86E-01	1.00E+00
PARP6	LMNA	-0.112156886	-0.246392894	0.026301348	1.12E-01	1.68E-01	1.00E+00
C1orf159	LMNA	-0.082820347	-0.218374577	0.055869433	2.41E-01	3.21E-01	1.00E+00
REV1	LMNA	-0.138867791	-0.271709886	-0.000832755	4.87E-02	8.21E-02	1.00E+00
SULT2A1	LMNA	-0.010575657	-0.148409982	0.127661727	8.81E-01	9.13E-01	1.00E+00
SPEN	LMNA	-0.176071827	-0.306668666	-0.038968018	1.22E-02	2.42E-02	1.00E+00

PRPS1	LMNA	-0.092352501	-0.227502985	0.04628865	1.91E-01	2.64E-01	1.00E+00
GNA15	LMNA	0.412088665	0.290567853	0.520527259	1.10E-09	9.04E-09	1.31E-05
NIP30	LMNA	-0.424333226	-0.531263036	-0.304096059	3.11E-10	2.81E-09	3.68E-06
GAMT	LMNA	0.103679407	-0.0348707	0.238319332	1.42E-01	2.05E-01	1.00E+00
SMCP	LMNA	-0.265773306	-0.389532263	-0.132586957	1.32E-04	4.22E-04	1.00E+00
ZNF217	LMNA	0.609387329	0.514630399	0.68943828	6.35E-22	4.36E-20	7.54E-18
GJA7	LMNA	0.257845838	0.12421643	0.382288929	2.12E-04	6.47E-04	1.00E+00
NOX4	LMNA	0.304460086	0.173710307	0.424662105	1.06E-05	4.16E-05	1.25E-01
FRAT2	LMNA	-0.249399117	-0.374554289	-0.115318509	3.44E-04	1.01E-03	1.00E+00
RNASEN	LMNA	0.032024861	-0.106496969	0.169327255	6.51E-01	7.23E-01	1.00E+00
MCHR1	LMNA	0.259366767	0.125820889	0.38367979	1.93E-04	5.96E-04	1.00E+00
ACCN2	LMNA	-0.561257723	-0.649021144	-0.458751818	3.68E-18	1.37E-16	4.36E-14
TBX1	LMNA	0.195657695	0.059205889	0.324932041	5.26E-03	1.14E-02	1.00E+00
OPRS1	LMNA	0.007495542	-0.130690693	0.145396092	9.16E-01	9.39E-01	1.00E+00
KCNG2	LMNA	0.14531033	0.007407934	0.277788821	3.91E-02	6.76E-02	1.00E+00
HIRIP3	LMNA	-0.279224483	-0.401787686	-0.146833506	5.72E-05	1.96E-04	6.78E-01
SALL2	LMNA	-0.368589478	-0.482108785	-0.242898142	6.76E-08	4.01E-07	8.02E-04
MPHOSPH8	LMNA	-0.057619292	-0.194126135	0.081076625	4.15E-01	5.03E-01	1.00E+00
CYP2E1	LMNA	-0.389384037	-0.500529164	-0.265610913	1.02E-08	6.95E-08	1.21E-04
ACO1	LMNA	0.049390838	-0.089268839	0.18617243	4.85E-01	5.70E-01	1.00E+00
THEM2	LMNA	0.111123881	-0.027346638	0.245410103	1.15E-01	1.72E-01	1.00E+00
OPRL1	LMNA	-0.029018573	-0.166402956	0.109470973	6.82E-01	7.50E-01	1.00E+00
CTH	LMNA	-0.055765025	-0.192335348	0.082924362	4.31E-01	5.17E-01	1.00E+00
ATF5	LMNA	0.177667532	0.040612645	0.308160231	1.14E-02	2.28E-02	1.00E+00
IQCG	LMNA	0.248640904	0.114520845	0.373859142	3.60E-04	1.05E-03	1.00E+00
TULP4	LMNA	-0.207277534	-0.335721896	-0.071265795	3.08E-03	7.11E-03	1.00E+00
MEGF9	LMNA	-0.229948758	-0.356677165	-0.094910664	9.94E-04	2.61E-03	1.00E+00
TM7SF4	LMNA	0.248475009	0.114346343	0.373707028	3.63E-04	1.06E-03	1.00E+00
PLEKHA1	LMNA	-0.281272436	-0.403649741	-0.149007384	5.01E-05	1.74E-04	5.95E-01
PAPPA2	LMNA	0.104164058	-0.034381345	0.238781393	1.40E-01	2.03E-01	1.00E+00
SLC4A2	LMNA	0.542831263	0.437571144	0.633415147	7.09E-17	2.14E-15	8.41E-13
SNUPN	LMNA	0.229606353	0.094552417	0.356361622	1.01E-03	2.65E-03	1.00E+00
PFKFB3	LMNA	0.186315009	0.049538178	0.316232209	7.93E-03	1.65E-02	1.00E+00
PKNOX1	LMNA	-0.061310908	-0.197688666	0.077395167	3.86E-01	4.73E-01	1.00E+00
KIAA0406	LMNA	0.00263338	-0.135466868	0.140633255	9.70E-01	9.77E-01	1.00E+00

C20orf29	LMNA	0.273981124	0.141273565	0.397015655	7.96E-05	2.66E-04	9.44E-01
FLJ20581	LMNA	-0.229586852	-0.356343649	-0.094532015	1.01E-03	2.65E-03	1.00E+00
SFRP4	LMNA	0.376312482	0.251317505	0.488961765	3.40E-08	2.10E-07	4.03E-04
OSTM1	LMNA	0.169255071	0.031950623	0.300289556	1.60E-02	3.07E-02	1.00E+00
CLCN7	LMNA	0.281358176	0.149098423	0.403727676	4.99E-05	1.73E-04	5.91E-01
AGTR1	LMNA	0.224365406	0.089073352	0.351528218	1.33E-03	3.37E-03	1.00E+00
FLJ23049	LMNA	-0.142773257	-0.275396201	-0.004817204	4.27E-02	7.29E-02	1.00E+00
HAPLN2	LMNA	-0.222987625	-0.350256449	-0.08763433	1.42E-03	3.59E-03	1.00E+00
PCDHGA9	LMNA	0.125255601	-0.013020547	0.258830984	7.57E-02	1.20E-01	1.00E+00
MAP3K10	LMNA	-0.003221168	-0.141209375	0.134889816	9.64E-01	9.73E-01	1.00E+00
AMDHD2	LMNA	0.445812901	0.327945265	0.550013374	2.95E-11	3.19E-10	3.50E-07
USP2	LMNA	-0.407359822	-0.516371909	-0.285356237	1.78E-09	1.40E-08	2.11E-05
MAN2A1	LMNA	0.45398081	0.337053816	0.557115958	1.15E-11	1.34E-10	1.37E-07
ABCG4	LMNA	-0.35289506	-0.468139468	-0.225846752	2.59E-07	1.38E-06	3.07E-03
CLCA2	LMNA	0.218256779	0.082697501	0.345886037	1.81E-03	4.43E-03	1.00E+00
CBX8	LMNA	-0.046136152	-0.183021462	0.09250402	5.14E-01	5.98E-01	1.00E+00
STRAP	LMNA	-0.221529084	-0.348909622	-0.086111575	1.53E-03	3.82E-03	1.00E+00
RND3	LMNA	0.180942307	0.043990148	0.311219279	9.97E-03	2.02E-02	1.00E+00
COQ3	LMNA	-0.41431716	-0.522483713	-0.293026358	8.80E-10	7.35E-09	1.04E-05
RRBP1	LMNA	0.66180673	0.57641916	0.732897919	7.98E-27	1.41E-24	9.46E-23
NAT13	LMNA	0.069754018	-0.06896105	0.205823023	3.24E-01	4.10E-01	1.00E+00
IL1RAP	LMNA	0.402676501	0.280201894	0.512251495	2.83E-09	2.16E-08	3.36E-05
MAT2B	LMNA	-0.04265658	-0.179649663	0.095959505	5.47E-01	6.28E-01	1.00E+00
NDOR1	LMNA	0.315430167	0.185454883	0.434558138	4.82E-06	2.03E-05	5.71E-02
CSNK1D	LMNA	0.619202512	0.526125524	0.697619899	9.02E-23	7.86E-21	1.07E-18
KIR3DL1	LMNA	0.045029387	-0.093603486	0.181949327	5.25E-01	6.08E-01	1.00E+00
TJP1	LMNA	-0.086948317	-0.222330616	0.051723537	2.19E-01	2.95E-01	1.00E+00
RALGDS	LMNA	0.006588456	-0.131582224	0.14450802	9.26E-01	9.47E-01	1.00E+00
PTPRT	LMNA	-0.389232432	-0.500395232	-0.265444824	1.03E-08	7.04E-08	1.23E-04
ASPHD1	LMNA	0.121806685	-0.016522145	0.255560302	8.42E-02	1.31E-01	1.00E+00
GPR44	LMNA	-0.120501323	-0.2543216	0.017846561	8.76E-02	1.36E-01	1.00E+00
PER2	LMNA	0.104826682	-0.033712183	0.239413034	1.38E-01	2.00E-01	1.00E+00
ZKSCAN4	LMNA	-0.074215489	-0.210113764	0.06449631	2.94E-01	3.78E-01	1.00E+00
KCNE1L	LMNA	-0.046844618	-0.183707592	0.091800048	5.08E-01	5.92E-01	1.00E+00
CCKBR	LMNA	-0.294188454	-0.415370027	-0.162747103	2.14E-05	7.97E-05	2.54E-01

RBM12B	LMNA	-0.263864856	-0.387789909	-0.130570108	1.48E-04	4.69E-04	1.00E+00
FAM118A	LMNA	0.108973815	-0.029521302	0.243363666	1.23E-01	1.81E-01	1.00E+00
TAF4	LMNA	-0.043282257	-0.180256198	0.095338406	5.41E-01	6.22E-01	1.00E+00
NDUFB6	LMNA	-0.227578565	-0.354492322	-0.092431528	1.12E-03	2.91E-03	1.00E+00
ADRB2	LMNA	-0.188496017	-0.318265103	-0.051792771	7.22E-03	1.52E-02	1.00E+00
PMFBP1	LMNA	-0.056029619	-0.19259094	0.082660758	4.28E-01	5.15E-01	1.00E+00
TRIM9	LMNA	-0.169908578	-0.300901616	-0.032622779	1.56E-02	2.99E-02	1.00E+00
PRSS3	LMNA	-0.153513258	-0.285513469	-0.015797038	2.92E-02	5.22E-02	1.00E+00
DKFZp762E	LMNA	0.107428487	-0.031083501	0.241892081	1.28E-01	1.88E-01	1.00E+00
CD3D	LMNA	0.282040593	0.149823103	0.404347909	4.77E-05	1.66E-04	5.66E-01
TBP	LMNA	-0.285886166	-0.40784096	-0.153909491	3.72E-05	1.32E-04	4.41E-01
CTSD	LMNA	0.42950984	0.309829979	0.535791479	1.79E-10	1.70E-09	2.12E-06
HPGD	LMNA	-0.137169439	-0.270105618	0.000898577	5.16E-02	8.62E-02	1.00E+00
DNAJC12	LMNA	-0.467604111	-0.56892892	-0.352294878	2.28E-12	3.00E-11	2.70E-08
SEMA3B	LMNA	0.147448372	0.009592633	0.279803854	3.63E-02	6.32E-02	1.00E+00
MRPL17	LMNA	0.399064695	0.276231649	0.509070357	4.03E-09	2.98E-08	4.78E-05
FKBP1B	LMNA	-0.088357266	-0.223679862	0.050307374	2.11E-01	2.86E-01	1.00E+00
IL3	LMNA	0.076523992	-0.062183933	0.212331873	2.79E-01	3.62E-01	1.00E+00
DRAM	LMNA	0.542089427	0.436720864	0.632785314	7.96E-17	2.38E-15	9.44E-13
ARHGAP19	LMNA	0.016683421	-0.121647754	0.154378862	8.14E-01	8.59E-01	1.00E+00
GLI3	LMNA	0.412168793	0.290656223	0.520597626	1.10E-09	8.98E-09	1.30E-05
VAV2	LMNA	0.380726237	0.256137743	0.49287206	2.28E-08	1.46E-07	2.70E-04
PTCH1	LMNA	-0.375395513	-0.488148823	-0.250316865	3.69E-08	2.27E-07	4.38E-04
TP53BP1	LMNA	-0.011607934	-0.149419492	0.126646015	8.70E-01	9.04E-01	1.00E+00
ERCC3	LMNA	-0.108961294	-0.243351746	0.029533962	1.23E-01	1.81E-01	1.00E+00
SLC17A7	LMNA	-0.312877702	-0.432258143	-0.182718894	5.80E-06	2.39E-05	6.88E-02
AMACR	LMNA	0.098570881	-0.040024769	0.233445204	1.63E-01	2.30E-01	1.00E+00
TFRC	LMNA	0.116495245	-0.021908088	0.250517354	9.87E-02	1.50E-01	1.00E+00
RHCG	LMNA	-0.103732972	-0.238370403	0.034816618	1.42E-01	2.05E-01	1.00E+00
TMEM80	LMNA	-0.083669479	-0.219188707	0.055017004	2.36E-01	3.15E-01	1.00E+00
DDX46	LMNA	-0.220917979	-0.348345167	-0.085473752	1.58E-03	3.93E-03	1.00E+00
TCEAL4	LMNA	-0.01440081	-0.15214933	0.123896503	8.39E-01	8.80E-01	1.00E+00
AK2	LMNA	0.313415563	0.18329526	0.432742932	5.58E-06	2.31E-05	6.62E-02
VPS13A	LMNA	-0.155561847	-0.28743994	-0.017895153	2.71E-02	4.88E-02	1.00E+00
LHPP	LMNA	-0.226756672	-0.353734377	-0.091572248	1.17E-03	3.02E-03	1.00E+00

FAM55D	LMNA	-0.214026054	-0.341972947	-0.078288197	2.22E-03	5.33E-03	1.00E+00
PRPF38B	LMNA	-0.221391528	-0.348782574	-0.085967995	1.54E-03	3.84E-03	1.00E+00
BCOR	LMNA	-0.256847396	-0.381375566	-0.123163533	2.24E-04	6.82E-04	1.00E+00
AVPR2	LMNA	0.080991906	-0.057704287	0.216620866	2.52E-01	3.33E-01	1.00E+00
PFDN5	LMNA	-0.123563204	-0.25722643	0.014739218	7.98E-02	1.25E-01	1.00E+00
NSUN3	LMNA	-0.084018258	-0.219523053	0.054666813	2.35E-01	3.13E-01	1.00E+00
CMTM6	LMNA	0.302080009	0.171167095	0.422511256	1.25E-05	4.86E-05	1.48E-01
KCNK12	LMNA	-0.191474318	-0.321039218	-0.054873809	6.34E-03	1.35E-02	1.00E+00
GRB14	LMNA	-0.176620392	-0.307181502	-0.039533317	1.19E-02	2.37E-02	1.00E+00
RP2	LMNA	0.158870004	0.021285848	0.290548644	2.39E-02	4.38E-02	1.00E+00
SERPINF2	LMNA	0.246197352	0.11195133	0.371617882	4.13E-04	1.18E-03	1.00E+00
PICK1	LMNA	0.164146312	0.026700357	0.295501092	1.96E-02	3.66E-02	1.00E+00
DYNC1I2	LMNA	-0.185818369	-0.31576913	-0.049024977	8.10E-03	1.69E-02	1.00E+00
TYRO3	LMNA	0.008903678	-0.12930626	0.14677427	9.00E-01	9.28E-01	1.00E+00
OR12D2	LMNA	-0.05446161	-0.19107601	0.084222617	4.41E-01	5.28E-01	1.00E+00
FANCF	LMNA	0.024824018	-0.113616343	0.162318756	7.26E-01	7.87E-01	1.00E+00
CSNK1A1	LMNA	0.350662279	0.22342723	0.46614742	3.12E-07	1.64E-06	3.70E-03
TBL1Y	LMNA	0.222503148	0.087128453	0.349809137	1.46E-03	3.66E-03	1.00E+00
CCNC	LMNA	-0.217722099	-0.345391745	-0.082139959	1.85E-03	4.54E-03	1.00E+00
C3orf60	LMNA	0.079768517	-0.058931444	0.215446989	2.59E-01	3.41E-01	1.00E+00
SLC10A2	LMNA	0.016362338	-0.12196416	0.15406533	8.17E-01	8.62E-01	1.00E+00
ZBP1	LMNA	0.185099188	0.048281942	0.315098438	8.36E-03	1.73E-02	1.00E+00
CHKA	LMNA	-0.137497269	-0.270415344	0.000564446	5.10E-02	8.54E-02	1.00E+00
UBAP1	LMNA	-0.277741357	-0.400438553	-0.145259983	6.28E-05	2.13E-04	7.45E-01
DHRS3	LMNA	0.186611602	0.049844697	0.316508731	7.83E-03	1.63E-02	1.00E+00
MAP3K1	LMNA	-0.18539941	-0.315378434	-0.048592104	8.25E-03	1.71E-02	1.00E+00
PBK	LMNA	-0.02344684	-0.160976781	0.114976323	7.40E-01	7.99E-01	1.00E+00
ALDOA	LMNA	0.430005189	0.310379114	0.536224485	1.70E-10	1.63E-09	2.01E-06
EXOSC5	LMNA	-0.199577498	-0.328575625	-0.063269696	4.40E-03	9.78E-03	1.00E+00
AZU1	LMNA	0.24267927	0.108255043	0.368388477	5.02E-04	1.41E-03	1.00E+00
GAB2	LMNA	-0.097435825	-0.232361307	0.041168944	1.68E-01	2.36E-01	1.00E+00
DIS3	LMNA	-0.224916472	-0.352036751	-0.08964907	1.29E-03	3.29E-03	1.00E+00
THAP3	LMNA	-0.202566936	-0.331351826	-0.066371999	3.84E-03	8.64E-03	1.00E+00
VPS13D	LMNA	-0.190958903	-0.320559297	-0.054340428	6.48E-03	1.38E-02	1.00E+00
IQCB1	LMNA	-0.040383429	-0.177445177	0.098215118	5.68E-01	6.48E-01	1.00E+00

SPATS2	LMNA	-0.235506513	-0.361794871	-0.100730452	7.41E-04	2.01E-03	1.00E+00
SKIV2L	LMNA	0.146419587	0.008541234	0.278834405	3.76E-02	6.52E-02	1.00E+00
ATF4	LMNA	0.138580541	0.000539868	0.2714386	4.92E-02	8.28E-02	1.00E+00
C19orf62	LMNA	-0.005588597	-0.143528863	0.132564679	9.37E-01	9.54E-01	1.00E+00
SPIN1	LMNA	-0.19080794	-0.320418719	-0.054184218	6.53E-03	1.38E-02	1.00E+00
IL12A	LMNA	-0.006213798	-0.144141152	0.131950392	9.30E-01	9.49E-01	1.00E+00
PRB3	LMNA	-0.073488541	-0.209414992	0.065224169	2.99E-01	3.83E-01	1.00E+00
RAPGEF4	LMNA	-0.599849874	-0.681468549	-0.503493072	3.97E-21	2.41E-19	4.71E-17
FUZ	LMNA	-0.106074192	-0.240601903	0.032452024	1.33E-01	1.94E-01	1.00E+00
CST5	LMNA	0.058861715	-0.079838043	0.195325516	4.05E-01	4.93E-01	1.00E+00
C3orf37	LMNA	0.280765839	0.148469518	0.403189224	5.18E-05	1.79E-04	6.14E-01
CROP	LMNA	-0.303448121	-0.42374777	-0.172628767	1.13E-05	4.44E-05	1.34E-01
ZNF696	LMNA	-0.049977902	-0.186740487	0.088684981	4.80E-01	5.65E-01	1.00E+00
HEG1	LMNA	0.460117319	0.343911456	0.562442185	5.61E-12	6.91E-11	6.66E-08
LIN28	LMNA	-0.01754235	-0.155217455	0.120801199	8.04E-01	8.52E-01	1.00E+00
SURF2	LMNA	-0.118480045	-0.252402675	0.019896386	9.31E-02	1.43E-01	1.00E+00
KIAA1539	LMNA	0.306984934	0.17641012	0.426942301	8.84E-06	3.53E-05	1.05E-01
WHSC2	LMNA	-0.11195346	-0.246199379	0.026507217	1.13E-01	1.68E-01	1.00E+00
PSMC1	LMNA	0.215951708	0.080294475	0.343754577	2.02E-03	4.90E-03	1.00E+00
RFXANK	LMNA	0.240148824	0.105598715	0.366063791	5.76E-04	1.60E-03	1.00E+00
SLC7A8	LMNA	0.263970989	0.13068224	0.387886827	1.47E-04	4.66E-04	1.00E+00
SPATA7	LMNA	-0.13335691	-0.266501602	0.00478213	5.85E-02	9.62E-02	1.00E+00
ADA	LMNA	0.243654243	0.10927903	0.369283752	4.75E-04	1.34E-03	1.00E+00
MAZ	LMNA	-0.138225935	-0.271103674	-0.000178338	4.98E-02	8.37E-02	1.00E+00
PIN4	LMNA	-0.211139344	-0.339300417	-0.075282683	2.56E-03	6.03E-03	1.00E+00
PDCD10	LMNA	0.005871931	-0.132286304	0.143806359	9.34E-01	9.52E-01	1.00E+00
PDE1A	LMNA	-0.259783597	-0.384060874	-0.126260734	1.89E-04	5.83E-04	1.00E+00
TAF6L	LMNA	-0.15142515	-0.283548736	-0.013659697	3.15E-02	5.59E-02	1.00E+00
KIAA0284	LMNA	0.080657378	-0.058039887	0.216299915	2.54E-01	3.35E-01	1.00E+00
DCTN6	LMNA	-0.394315562	-0.504882928	-0.271017582	6.38E-09	4.53E-08	7.57E-05
ACADS	LMNA	0.268486931	0.135456614	0.392008191	1.12E-04	3.62E-04	1.00E+00
MKRN2	LMNA	-0.10057496	-0.235358137	0.038003702	1.54E-01	2.20E-01	1.00E+00
GALNT4	LMNA	0.56090187	0.458341669	0.648720458	3.90E-18	1.44E-16	4.63E-14
C22orf31	LMNA	-0.136569922	-0.269539136	0.001509539	5.26E-02	8.78E-02	1.00E+00
PSME4	LMNA	0.366148515	0.240241018	0.479939912	8.37E-08	4.87E-07	9.93E-04

TFG	LMNA	0.139406356	0.001381952	0.272218462	4.78E-02	8.07E-02	1.00E+00
SSNA1	LMNA	0.119124647	-0.019242807	0.25301475	9.13E-02	1.41E-01	1.00E+00
EPHX2	LMNA	0.078086751	-0.060617706	0.213832645	2.69E-01	3.51E-01	1.00E+00
ANXA5	LMNA	0.499265368	0.387953722	0.596222293	3.93E-14	6.69E-13	4.67E-10
ELOVL4	LMNA	-0.307273041	-0.427202393	-0.176718316	8.66E-06	3.46E-05	1.03E-01
CCL24	LMNA	0.144766974	0.00685293	0.27727654	3.98E-02	6.87E-02	1.00E+00
KRTAP1-1	LMNA	0.106149066	-0.032376378	0.240673244	1.33E-01	1.94E-01	1.00E+00
ZMAT3	LMNA	0.077257715	-0.061448666	0.213036573	2.74E-01	3.57E-01	1.00E+00
BATF	LMNA	0.353433527	0.226430489	0.468619704	2.48E-07	1.33E-06	2.94E-03
ATF7IP	LMNA	-0.134077709	-0.267183265	0.004048222	5.71E-02	9.42E-02	1.00E+00
MSTP9	LMNA	-0.103831508	-0.23846435	0.03471713	1.41E-01	2.04E-01	1.00E+00
FAM131A	LMNA	-0.18911821	-0.318844824	-0.052436212	7.03E-03	1.48E-02	1.00E+00
VIPR2	LMNA	-0.414658831	-0.522783574	-0.293403436	8.50E-10	7.13E-09	1.01E-05
CCDC72	LMNA	-0.063635949	-0.199930558	0.075074583	3.68E-01	4.56E-01	1.00E+00
TEF	LMNA	-0.186627036	-0.31652312	-0.049860648	7.83E-03	1.63E-02	1.00E+00
LYK5	LMNA	-0.395089852	-0.505565995	-0.271867181	5.92E-09	4.24E-08	7.03E-05
MRPL44	LMNA	-0.139003128	-0.271837694	-0.000970756	4.85E-02	8.18E-02	1.00E+00
LIMK2	LMNA	0.206802455	0.070771943	0.335281411	3.15E-03	7.26E-03	1.00E+00
ETF1	LMNA	0.51211556	0.402522093	0.60723614	6.70E-15	1.34E-13	7.94E-11
HHAT	LMNA	0.257890375	0.124263403	0.382329665	2.11E-04	6.46E-04	1.00E+00
PROL1	LMNA	-0.071617569	-0.207615897	0.067096809	3.11E-01	3.96E-01	1.00E+00
KCNJ14	LMNA	0.017386796	-0.120954527	0.155065598	8.06E-01	8.53E-01	1.00E+00
UBE4A	LMNA	-0.105111086	-0.239684105	0.033424933	1.37E-01	1.98E-01	1.00E+00
MYST1	LMNA	-0.32250772	-0.440927519	-0.193051874	2.86E-06	1.26E-05	3.39E-02
EMX1	LMNA	-0.026853877	-0.164295799	0.111610888	7.04E-01	7.68E-01	1.00E+00
MX2	LMNA	0.216957754	0.081343079	0.344685012	1.93E-03	4.70E-03	1.00E+00
C11orf30	LMNA	-0.33278561	-0.450155828	-0.204111788	1.30E-06	6.12E-06	1.55E-02
HSP90AA1	LMNA	0.079590769	-0.059109705	0.215276402	2.60E-01	3.42E-01	1.00E+00
ICK	LMNA	-0.297208807	-0.418104972	-0.165967446	1.74E-05	6.61E-05	2.07E-01
CCDC68	LMNA	0.101155054	-0.037418479	0.235911653	1.52E-01	2.17E-01	1.00E+00
EIF2C1	LMNA	-0.130751335	-0.264036385	0.00743385	6.36E-02	1.03E-01	1.00E+00
NDUFC2	LMNA	-0.329642929	-0.447336737	-0.200726497	1.66E-06	7.67E-06	1.97E-02
SEL1L	LMNA	0.282376426	0.150179786	0.404653098	4.67E-05	1.63E-04	5.54E-01
DUOX1	LMNA	0.086219311	-0.052456057	0.221632297	2.22E-01	2.99E-01	1.00E+00
UBE2G2	LMNA	-0.00307144	-0.141062628	0.135036818	9.65E-01	9.74E-01	1.00E+00

PARP1	LMNA	-0.254241355	-0.378990445	-0.120416775	2.61E-04	7.84E-04	1.00E+00
FAM60A	LMNA	-0.166521324	-0.297728023	-0.029140211	1.79E-02	3.38E-02	1.00E+00
SIAH1	LMNA	-0.360083879	-0.474545263	-0.233647481	1.41E-07	7.89E-07	1.68E-03
SH2D4A	LMNA	0.426202109	0.306165138	0.532898619	2.55E-10	2.35E-09	3.02E-06
LHX1	LMNA	-0.30203696	-0.42247234	-0.171121111	1.25E-05	4.87E-05	1.48E-01
EIF4B	LMNA	-0.027618622	-0.16504036	0.110855044	6.96E-01	7.62E-01	1.00E+00
KRT2	LMNA	-0.075338805	-0.211193269	0.063371295	2.87E-01	3.70E-01	1.00E+00
RPS15	LMNA	-0.014879599	-0.152617102	0.123424934	8.34E-01	8.75E-01	1.00E+00
GOLGA7	LMNA	-0.299092148	-0.419809237	-0.167976909	1.53E-05	5.87E-05	1.82E-01
MAGEC2	LMNA	-0.06263292	-0.198963574	0.076075874	3.76E-01	4.63E-01	1.00E+00
JAG2	LMNA	-0.160696815	-0.292264125	-0.023159596	2.23E-02	4.12E-02	1.00E+00
PPBPL2	LMNA	0.056647114	-0.082045498	0.193187353	4.23E-01	5.10E-01	1.00E+00
BLOC1S1	LMNA	-0.133741042	-0.266864894	0.00439103	5.78E-02	9.51E-02	1.00E+00
CD34	LMNA	0.329467358	0.200537463	0.447179175	1.69E-06	7.76E-06	2.00E-02
STX3	LMNA	0.355846036	0.229046944	0.470770483	2.02E-07	1.10E-06	2.40E-03
SLCO4A1	LMNA	0.168359441	0.031029637	0.299450555	1.66E-02	3.17E-02	1.00E+00
NXPH4	LMNA	0.136074378	-0.002014465	0.269070828	5.35E-02	8.91E-02	1.00E+00
MCTS1	LMNA	0.221357091	0.085932051	0.348750768	1.55E-03	3.85E-03	1.00E+00
SLC37A4	LMNA	0.009356099	-0.128861341	0.147216952	8.95E-01	9.24E-01	1.00E+00
TGM1	LMNA	-0.160120407	-0.291722936	-0.022568274	2.28E-02	4.20E-02	1.00E+00
RP13-122B	LMNA	0.172394036	0.035180297	0.303228451	1.42E-02	2.75E-02	1.00E+00
ZC3H13	LMNA	-0.068880738	-0.204982548	0.069834321	3.30E-01	4.16E-01	1.00E+00
PHACTR4	LMNA	0.001572867	-0.136507774	0.139593557	9.82E-01	9.86E-01	1.00E+00
ARPC2	LMNA	0.28783235	0.155979271	0.409607379	3.27E-05	1.18E-04	3.88E-01
ACYP1	LMNA	-0.2114381	-0.339577103	-0.07559362	2.52E-03	5.96E-03	1.00E+00
P2RY13	LMNA	-0.090641944	-0.225866628	0.048009813	2.00E-01	2.74E-01	1.00E+00
PRKCSH	LMNA	0.523125818	0.415048832	0.616643996	1.38E-15	3.19E-14	1.64E-11
ENG	LMNA	0.518001061	0.409213122	0.612268403	2.90E-15	6.24E-14	3.44E-11
GAPVD1	LMNA	-0.006020525	-0.143951882	0.132140303	9.32E-01	9.51E-01	1.00E+00
DPH5	LMNA	-0.289937759	-0.411517287	-0.158219691	2.85E-05	1.04E-04	3.38E-01
CCNO	LMNA	0.252071857	0.118131694	0.377003595	2.96E-04	8.78E-04	1.00E+00
HLA-F	LMNA	0.385760212	0.261642894	0.497326307	1.43E-08	9.53E-08	1.70E-04
RXRG	LMNA	-0.366201257	-0.479986789	-0.24029841	8.33E-08	4.85E-07	9.88E-04
ZNF140	LMNA	-0.096606709	-0.231569349	0.042004491	1.71E-01	2.40E-01	1.00E+00
SULT1E1	LMNA	0.150391732	0.012602378	0.282575967	3.26E-02	5.78E-02	1.00E+00

BRD9	LMNA	-0.005924881	-0.143858215	0.132234279	9.33E-01	9.51E-01	1.00E+00
TBC1D4	LMNA	-0.176321371	-0.306901966	-0.039225163	1.21E-02	2.40E-02	1.00E+00
RIG	LMNA	-0.077035853	-0.212823502	0.061671011	2.76E-01	3.58E-01	1.00E+00
GLUD1	LMNA	-0.316772315	-0.435766908	-0.186894341	4.37E-06	1.85E-05	5.18E-02
OGT	LMNA	-0.082795214	-0.218350477	0.05589466	2.41E-01	3.21E-01	1.00E+00
HBXIP	LMNA	-0.073691081	-0.209609695	0.065021391	2.97E-01	3.81E-01	1.00E+00
SYT1	LMNA	-0.344340837	-0.460501179	-0.216585578	5.22E-07	2.62E-06	6.19E-03
PLA2G3	LMNA	-0.098166336	-0.233058931	0.040432605	1.65E-01	2.32E-01	1.00E+00
APIP	LMNA	0.01745113	-0.120891115	0.155128404	8.05E-01	8.53E-01	1.00E+00
ACRV1	LMNA	-0.436712479	-0.542082111	-0.317822554	8.17E-11	8.33E-10	9.69E-07
RNF207	LMNA	0.061718807	-0.076988159	0.19808208	3.83E-01	4.70E-01	1.00E+00
CMPK	LMNA	0.241868776	0.10740402	0.367644058	5.25E-04	1.47E-03	1.00E+00
Mar-02	LMNA	0.042957103	-0.095661195	0.179941005	5.44E-01	6.25E-01	1.00E+00
MYLIP	LMNA	-0.045160914	-0.182076755	0.093472844	5.23E-01	6.06E-01	1.00E+00
RPIA	LMNA	0.199068379	0.062741618	0.328102599	4.51E-03	9.98E-03	1.00E+00
PHIP	LMNA	-0.112169521	-0.246404913	0.026288561	1.12E-01	1.67E-01	1.00E+00
ZNF701	LMNA	0.123897629	-0.014399667	0.257543555	7.90E-02	1.24E-01	1.00E+00
HIST1H1B	LMNA	-0.11295427	-0.247151329	0.025494274	1.09E-01	1.64E-01	1.00E+00
SLC11A2	LMNA	-0.197107732	-0.326280345	-0.06070867	4.93E-03	1.08E-02	1.00E+00
DHX32	LMNA	0.181168091	0.04422313	0.311430089	9.87E-03	2.01E-02	1.00E+00
NBEAL2	LMNA	0.326851125	0.19772175	0.444830431	2.06E-06	9.31E-06	2.44E-02
SCAPER	LMNA	-0.43445926	-0.540115466	-0.3153204	1.05E-10	1.05E-09	1.24E-06
RP4-691N2	LMNA	-0.174134728	-0.304857124	-0.036972528	1.32E-02	2.59E-02	1.00E+00
PLA2G2F	LMNA	0.167646404	0.03029658	0.298782457	1.71E-02	3.25E-02	1.00E+00
MEN1	LMNA	0.247263502	0.113072219	0.37259595	3.89E-04	1.12E-03	1.00E+00
TYROBP	LMNA	0.213347477	0.07758147	0.341344903	2.30E-03	5.50E-03	1.00E+00
NIP7	LMNA	0.216526947	0.080894011	0.344286614	1.97E-03	4.79E-03	1.00E+00
TCP11	LMNA	0.056520091	-0.08217207	0.193064675	4.24E-01	5.11E-01	1.00E+00
FASTKD3	LMNA	-0.152472886	-0.284534706	-0.014731979	3.03E-02	5.41E-02	1.00E+00
TMEM158	LMNA	0.512223029	0.402644166	0.607328098	6.60E-15	1.33E-13	7.82E-11
RARA	LMNA	0.108514199	-0.029986006	0.242926048	1.24E-01	1.83E-01	1.00E+00
BDH1	LMNA	0.065542392	-0.073170671	0.201767759	3.54E-01	4.41E-01	1.00E+00
CARM1	LMNA	0.299499535	0.16841172	0.420177776	1.49E-05	5.72E-05	1.77E-01
SS18	LMNA	0.234984769	0.100183716	0.361314761	7.62E-04	2.06E-03	1.00E+00
NPHP4	LMNA	-0.134758548	-0.267827015	0.003354865	5.59E-02	9.24E-02	1.00E+00

SFRP5	LMNA	0.001861777	-0.136224236	0.139876827	9.79E-01	9.84E-01	1.00E+00
IKZF2	LMNA	-0.134729674	-0.267799716	0.003384273	5.59E-02	9.25E-02	1.00E+00
MYD88	LMNA	0.557699222	0.454652329	0.646013072	6.61E-18	2.36E-16	7.84E-14
PML	LMNA	0.59902026	0.502525805	0.680774387	4.65E-21	2.74E-19	5.51E-17
TAF1A	LMNA	-0.064850612	-0.201101215	0.073861649	3.59E-01	4.46E-01	1.00E+00
CBFB	LMNA	0.312331957	0.18213417	0.431766176	6.03E-06	2.48E-05	7.16E-02
EBAG9	LMNA	-0.204997081	-0.333606987	-0.068895836	3.43E-03	7.82E-03	1.00E+00
HIST1H3H	LMNA	-0.046363821	-0.183241968	0.09227781	5.12E-01	5.96E-01	1.00E+00
UROD	LMNA	0.252846816	0.118947778	0.377713444	2.83E-04	8.43E-04	1.00E+00
DPP3	LMNA	0.368522198	0.242824878	0.482049023	6.80E-08	4.03E-07	8.07E-04
COMMD4	LMNA	0.251828268	0.117875217	0.376780442	3.00E-04	8.89E-04	1.00E+00
PDE2A	LMNA	-0.419556003	-0.527078548	-0.298812246	5.13E-10	4.47E-09	6.08E-06
DAB2	LMNA	0.395328729	0.27212933	0.5057767	5.79E-09	4.16E-08	6.87E-05
TUBB2A	LMNA	-0.143175674	-0.275775815	-0.005228007	4.21E-02	7.20E-02	1.00E+00
RPL36	LMNA	0.173168043	0.035977112	0.303952742	1.37E-02	2.68E-02	1.00E+00
ASPM	LMNA	0.023929741	-0.114499511	0.161447396	7.35E-01	7.94E-01	1.00E+00
LRP6	LMNA	-0.369693601	-0.483089376	-0.244100662	6.14E-08	3.66E-07	7.28E-04
SERPINH1	LMNA	0.777547321	0.71639501	0.826843915	3.67E-42	1.45E-38	4.35E-38
RBCK1	LMNA	0.340225899	0.212138743	0.456820705	7.27E-07	3.57E-06	8.62E-03
AFF2	LMNA	-0.304759916	-0.424932962	-0.174030811	1.03E-05	4.08E-05	1.23E-01
EXOD1	LMNA	-0.13175094	-0.264982349	0.006416771	6.16E-02	1.01E-01	1.00E+00
TLE1	LMNA	0.176595707	0.039507877	0.307158426	1.19E-02	2.37E-02	1.00E+00
CD244	LMNA	0.102522484	-0.036038583	0.237216087	1.47E-01	2.11E-01	1.00E+00
PLXNA2	LMNA	0.24702219	0.112818487	0.372374598	3.94E-04	1.14E-03	1.00E+00
MGLL	LMNA	0.017015021	-0.121320955	0.154702634	8.10E-01	8.57E-01	1.00E+00
ACTL6B	LMNA	-0.50937197	-0.604887686	-0.399406994	9.84E-15	1.91E-13	1.17E-10
PNRC2	LMNA	-0.252858722	-0.377724348	-0.118960317	2.83E-04	8.43E-04	1.00E+00
IL2RA	LMNA	0.450821097	0.333527623	0.554370149	1.67E-11	1.88E-10	1.98E-07
STXBP5L	LMNA	-0.475360413	-0.575635895	-0.360999646	8.76E-13	1.23E-11	1.04E-08
FAP	LMNA	0.525319622	0.417549701	0.618515334	1.00E-15	2.41E-14	1.19E-11
TBCC	LMNA	-0.213048931	-0.341068553	-0.077270583	2.33E-03	5.56E-03	1.00E+00
NSF	LMNA	-0.379110404	-0.491441065	-0.254372382	2.64E-08	1.67E-07	3.13E-04
GPR37	LMNA	0.162458028	0.024966974	0.293917203	2.09E-02	3.89E-02	1.00E+00
KCNJ1	LMNA	-0.165908696	-0.297153728	-0.0285107	1.83E-02	3.45E-02	1.00E+00
NFASC	LMNA	-0.061643575	-0.198009523	0.077063231	3.83E-01	4.71E-01	1.00E+00

SFRS15	LMNA	0.127031584	-0.011216112	0.260513993	7.16E-02	1.15E-01	1.00E+00
CLCA4	LMNA	-0.230753142	-0.357418331	-0.095752401	9.53E-04	2.51E-03	1.00E+00
LONRF3	LMNA	0.053937414	-0.084744605	0.190569413	4.46E-01	5.32E-01	1.00E+00
SCGB1D1	LMNA	0.033248892	-0.105285373	0.170517212	6.39E-01	7.12E-01	1.00E+00
OR2J3	LMNA	-0.15750366	-0.289265015	-0.01988503	2.52E-02	4.58E-02	1.00E+00
LSM6	LMNA	-0.101665327	-0.236398475	0.036903616	1.50E-01	2.15E-01	1.00E+00
SMURF1	LMNA	0.382474222	0.258048423	0.494419409	1.94E-08	1.26E-07	2.30E-04
MLLT1	LMNA	0.088584861	-0.050078561	0.223897765	2.10E-01	2.85E-01	1.00E+00
A2BP1	LMNA	-0.386078363	-0.497607621	-0.261991096	1.39E-08	9.27E-08	1.65E-04
HNRPDL	LMNA	-0.345691783	-0.461708619	-0.218046644	4.68E-07	2.38E-06	5.55E-03
BTNL8	LMNA	0.158212704	0.020611897	0.289931195	2.45E-02	4.48E-02	1.00E+00
TPM4	LMNA	0.671953953	0.588493956	0.741244236	6.87E-28	1.45E-25	8.15E-24
TNFSF4	LMNA	0.544483665	0.439465776	0.634817637	5.48E-17	1.67E-15	6.50E-13
COPS5	LMNA	-0.232056653	-0.358619058	-0.097116854	8.90E-04	2.37E-03	1.00E+00
CSTF2	LMNA	0.180881594	0.043927502	0.311162589	9.99E-03	2.03E-02	1.00E+00
ACADSB	LMNA	-0.144849627	-0.277354471	-0.00693735	3.97E-02	6.85E-02	1.00E+00
HERPUD1	LMNA	0.263579407	0.13026854	0.387529226	1.51E-04	4.76E-04	1.00E+00
BCL2L11	LMNA	-0.015830749	-0.153546182	0.122487943	8.23E-01	8.67E-01	1.00E+00
HTR1F	LMNA	0.070065119	-0.068649902	0.206122388	3.22E-01	4.07E-01	1.00E+00
SCPEP1	LMNA	0.311717173	0.181475587	0.431211889	6.31E-06	2.59E-05	7.48E-02
PRSS12	LMNA	-0.166367202	-0.297583554	-0.028981832	1.80E-02	3.39E-02	1.00E+00
SLC28A2	LMNA	0.141610497	0.00363047	0.274299095	4.44E-02	7.56E-02	1.00E+00
INHBA	LMNA	0.424452719	0.304228319	0.531367637	3.07E-10	2.78E-09	3.64E-06
CDCA3	LMNA	0.043117351	-0.095502116	0.180096347	5.42E-01	6.24E-01	1.00E+00
UGDH	LMNA	0.133671319	-0.004462021	0.266798956	5.79E-02	9.53E-02	1.00E+00
RP11-298P.	LMNA	-0.1970586	-0.326234668	-0.060657741	4.94E-03	1.08E-02	1.00E+00
MYO1A	LMNA	0.060855922	-0.077849107	0.197249783	3.90E-01	4.77E-01	1.00E+00
SLC36A1	LMNA	0.379518598	0.254818271	0.491802622	2.54E-08	1.62E-07	3.02E-04
PLCB1	LMNA	-0.545276885	-0.635490677	-0.440375607	4.84E-17	1.50E-15	5.74E-13
PPEF1	LMNA	0.173167377	0.035976426	0.303952119	1.37E-02	2.68E-02	1.00E+00
SEPP1	LMNA	-0.025863975	-0.163331792	0.11258903	7.15E-01	7.77E-01	1.00E+00
LOC440348	LMNA	-0.080865405	-0.216499502	0.057831197	2.53E-01	3.33E-01	1.00E+00
LOXL2	LMNA	0.629680069	0.538434043	0.706331142	1.04E-23	1.08E-21	1.23E-19
BPTF	LMNA	-0.257345824	-0.381831553	-0.123689107	2.18E-04	6.66E-04	1.00E+00
SERPINA7	LMNA	-0.070224227	-0.206275484	0.068490759	3.21E-01	4.06E-01	1.00E+00

LOC201229	LMNA	-0.337199515	-0.454111303	-0.208871642	9.24E-07	4.45E-06	1.10E-02
DENR	LMNA	0.114278233	-0.024153824	0.248410258	1.05E-01	1.59E-01	1.00E+00
CHRNA1	LMNA	0.139543348	0.001521661	0.272347813	4.76E-02	8.04E-02	1.00E+00
RARRES2	LMNA	0.381085095	0.256529923	0.493189786	2.20E-08	1.42E-07	2.61E-04
RPL21	LMNA	-0.150580878	-0.282754032	-0.012795876	3.24E-02	5.74E-02	1.00E+00
SENP2	LMNA	-0.120687978	-0.254498751	0.017657211	8.71E-02	1.35E-01	1.00E+00
XPNPEP1	LMNA	0.045187513	-0.093446424	0.182102525	5.23E-01	6.06E-01	1.00E+00
ADPRH	LMNA	0.451976624	0.334816794	0.55537457	1.46E-11	1.67E-10	1.73E-07
C17orf68	LMNA	-0.032864427	-0.170143492	0.105665977	6.42E-01	7.15E-01	1.00E+00
KCNS1	LMNA	-0.101668749	-0.236401739	0.036900163	1.50E-01	2.15E-01	1.00E+00
ADAMTS1	LMNA	0.54085662	0.435308258	0.631738372	9.63E-17	2.82E-15	1.14E-12
CDK5RAP1	LMNA	-0.048216896	-0.185036223	0.090436083	4.96E-01	5.81E-01	1.00E+00
ZNF571	LMNA	-0.292097634	-0.413475496	-0.160519481	2.46E-05	9.08E-05	2.92E-01
PRKG1	LMNA	0.084416351	-0.054267065	0.219904636	2.32E-01	3.10E-01	1.00E+00
P2RY6	LMNA	0.337916205	0.209645078	0.454753122	8.73E-07	4.22E-06	1.04E-02
RASGRP1	LMNA	-0.067541476	-0.203693207	0.071173155	3.40E-01	4.26E-01	1.00E+00
TRIM21	LMNA	0.417725844	0.29678997	0.525474083	6.20E-10	5.33E-09	7.35E-06
CADM3	LMNA	-0.250585879	-0.375642056	-0.116567366	3.22E-04	9.49E-04	1.00E+00
CFI	LMNA	0.517551292	0.408701381	0.611884107	3.10E-15	6.61E-14	3.67E-11
ADRA2B	LMNA	0.174244836	0.037085925	0.30496012	1.31E-02	2.58E-02	1.00E+00
PCF11	LMNA	-0.154413985	-0.286360634	-0.016719392	2.82E-02	5.06E-02	1.00E+00
FOXRED2	LMNA	-0.24833848	-0.373581835	-0.114202735	3.66E-04	1.06E-03	1.00E+00
LOC400451	LMNA	-0.008380303	-0.146262092	0.129820888	9.06E-01	9.32E-01	1.00E+00
CYP20A1	LMNA	0.246186492	0.111939914	0.371607918	4.13E-04	1.18E-03	1.00E+00
FABP1	LMNA	-0.123522982	-0.257188287	0.014780055	7.99E-02	1.26E-01	1.00E+00
GLTSCR1	LMNA	0.086738144	-0.051934738	0.222129304	2.20E-01	2.96E-01	1.00E+00
C17orf88	LMNA	-0.011625537	-0.149436704	0.126628692	8.70E-01	9.03E-01	1.00E+00
CDH16	LMNA	-0.141022008	-0.273743703	-0.003029996	4.53E-02	7.69E-02	1.00E+00
CYTL1	LMNA	-0.111997818	-0.246241577	0.026462327	1.13E-01	1.68E-01	1.00E+00
SORBS1	LMNA	-0.212959523	-0.340985789	-0.077177485	2.34E-03	5.58E-03	1.00E+00
PEA15	LMNA	-0.404496754	-0.513853564	-0.282204379	2.37E-09	1.82E-08	2.81E-05
FGF7	LMNA	0.223557953	0.08822994	0.35078295	1.38E-03	3.49E-03	1.00E+00
GUCY1A2	LMNA	-0.033005386	-0.170280516	0.105526438	6.41E-01	7.14E-01	1.00E+00
NPC1L1	LMNA	0.077615392	-0.061090182	0.213380051	2.72E-01	3.55E-01	1.00E+00
PH-4	LMNA	0.087885761	-0.050781355	0.223228395	2.14E-01	2.89E-01	1.00E+00

TAT	LMNA	0.225776905	0.090548176	0.352830624	1.23E-03	3.16E-03	1.00E+00
TBCA	LMNA	-0.017087645	-0.15477354	0.121249379	8.09E-01	8.56E-01	1.00E+00
FNBP4	LMNA	0.008707039	-0.129499622	0.146581846	9.02E-01	9.29E-01	1.00E+00
C12orf43	LMNA	-0.118470556	-0.252393664	0.019906007	9.31E-02	1.43E-01	1.00E+00
STXBP6	LMNA	-0.213070518	-0.341088536	-0.077293061	2.33E-03	5.56E-03	1.00E+00
SNAP23	LMNA	0.229822067	0.094778107	0.356560416	1.00E-03	2.63E-03	1.00E+00
CBL	LMNA	0.060019297	-0.078683655	0.196442628	3.96E-01	4.83E-01	1.00E+00
WDR25	LMNA	-0.060372538	-0.196783449	0.078331314	3.93E-01	4.81E-01	1.00E+00
ZBTB33	LMNA	-0.21077952	-0.338967145	-0.074908224	2.60E-03	6.13E-03	1.00E+00
MGA	LMNA	-0.109318071	-0.243691411	0.029173196	1.21E-01	1.80E-01	1.00E+00
FAM128B	LMNA	-0.1714838	-0.302376488	-0.034243463	1.47E-02	2.84E-02	1.00E+00
GPR4	LMNA	0.28801928	0.156178132	0.409776994	3.23E-05	1.17E-04	3.83E-01
GSTK1	LMNA	0.488865742	0.37620416	0.587282016	1.56E-13	2.46E-12	1.85E-09
CLCN5	LMNA	-0.009809801	-0.147660832	0.128415106	8.90E-01	9.20E-01	1.00E+00
SGCA	LMNA	0.067529819	-0.071184806	0.203681982	3.40E-01	4.26E-01	1.00E+00
FUSIP1	LMNA	-0.042666086	-0.179658879	0.09595007	5.47E-01	6.28E-01	1.00E+00
C22orf29	LMNA	0.126694409	-0.011558758	0.260194532	7.24E-02	1.16E-01	1.00E+00
YIPF1	LMNA	0.25411207	0.120280563	0.378872077	2.63E-04	7.89E-04	1.00E+00
MAG	LMNA	-0.149244405	-0.281495655	-0.011428877	3.40E-02	5.98E-02	1.00E+00
FLT3	LMNA	0.057101207	-0.081592981	0.193625879	4.20E-01	5.07E-01	1.00E+00
GALK2	LMNA	0.101284729	-0.037287644	0.236035375	1.51E-01	2.17E-01	1.00E+00
DLK2	LMNA	-0.348361484	-0.464093464	-0.220935643	3.77E-07	1.95E-06	4.47E-03
ZNF451	LMNA	-0.30628219	-0.426307804	-0.17565848	9.29E-06	3.69E-05	1.10E-01
RAB3B	LMNA	-0.358210166	-0.472876807	-0.231612708	1.66E-07	9.16E-07	1.96E-03
UCP1	LMNA	0.138189749	0.000141446	0.271069493	4.98E-02	8.37E-02	1.00E+00
REEP5	LMNA	0.126381387	-0.011876829	0.259897929	7.31E-02	1.17E-01	1.00E+00
FGF9	LMNA	-0.623117899	-0.700877942	-0.530720569	4.06E-23	3.82E-21	4.82E-19
FADD	LMNA	0.446130446	0.32829897	0.550289784	2.85E-11	3.09E-10	3.38E-07
VNN1	LMNA	0.350335942	0.22307373	0.465856169	3.20E-07	1.68E-06	3.80E-03
SRPK2	LMNA	-0.377994302	-0.49045228	-0.25315348	2.92E-08	1.83E-07	3.46E-04
ABI1	LMNA	-0.288718536	-0.410411407	-0.156922119	3.09E-05	1.12E-04	3.66E-01
CACNA1A	LMNA	-0.172303799	-0.303144	-0.035087412	1.42E-02	2.76E-02	1.00E+00
ALDH3A1	LMNA	0.170406292	0.033134779	0.30136769	1.53E-02	2.95E-02	1.00E+00
GRIN2D	LMNA	0.132453599	-0.005701659	0.265647148	6.02E-02	9.87E-02	1.00E+00
SLC1A4	LMNA	-0.284561756	-0.406638361	-0.152501635	4.05E-05	1.43E-04	4.81E-01

CDX4	LMNA	0.092927024	-0.04571038	0.228052416	1.88E-01	2.60E-01	1.00E+00
SPG21	LMNA	0.182770669	0.045877227	0.312926022	9.23E-03	1.89E-02	1.00E+00
ZNF286A	LMNA	-0.353321339	-0.468519654	-0.226308862	2.50E-07	1.34E-06	2.96E-03
IL1F6	LMNA	-0.227159855	-0.35410621	-0.091993745	1.15E-03	2.96E-03	1.00E+00
DOK3	LMNA	0.385670013	0.261544182	0.497246548	1.44E-08	9.60E-08	1.71E-04
ZNF302	LMNA	-0.295978882	-0.416991533	-0.164655745	1.90E-05	7.14E-05	2.25E-01
ENY2	LMNA	0.046220388	-0.092420325	0.18310305	5.14E-01	5.98E-01	1.00E+00
GRIN1	LMNA	-0.290990978	-0.412472307	-0.159340957	2.65E-05	9.72E-05	3.15E-01
OR1A1	LMNA	0.142390767	0.004426784	0.275035346	4.32E-02	7.38E-02	1.00E+00
CALU	LMNA	0.649074574	0.56132096	0.722395079	1.52E-25	2.31E-23	1.80E-21
ANKFY1	LMNA	-0.145117391	-0.277606926	-0.00721085	3.93E-02	6.80E-02	1.00E+00
LIMCH1	LMNA	-0.315670314	-0.434774451	-0.1857124	4.73E-06	1.99E-05	5.62E-02
DENND3	LMNA	0.156105337	0.018451986	0.287950854	2.65E-02	4.80E-02	1.00E+00
JARID1D	LMNA	0.031682951	-0.106835333	0.168994791	6.54E-01	7.26E-01	1.00E+00
RWDD1	LMNA	-0.054662096	-0.191269746	0.084022955	4.40E-01	5.26E-01	1.00E+00
HIST1H2AK	LMNA	-0.161553762	-0.293068554	-0.024038892	2.16E-02	4.01E-02	1.00E+00
SLC18A2	LMNA	0.111401719	-0.027065525	0.245674463	1.14E-01	1.71E-01	1.00E+00
UPK3A	LMNA	-0.146294539	-0.27871655	-0.008413457	3.78E-02	6.55E-02	1.00E+00
LOC93349	LMNA	0.476072676	0.361800008	0.576251122	8.02E-13	1.13E-11	9.51E-09
FIP1L1	LMNA	-0.215716113	-0.343536653	-0.080048957	2.05E-03	4.96E-03	1.00E+00
SSH1	LMNA	0.333772268	0.205175249	0.451040411	1.21E-06	5.70E-06	1.43E-02
LENEP	LMNA	0.079685572	-0.059014629	0.215367387	2.60E-01	3.41E-01	1.00E+00
RHOB	LMNA	0.015265546	-0.123044762	0.152994123	8.29E-01	8.71E-01	1.00E+00
RIBC2	LMNA	0.085288434	-0.053391203	0.220740403	2.27E-01	3.05E-01	1.00E+00
ENSA	LMNA	-0.126170138	-0.259697746	0.012091471	7.36E-02	1.17E-01	1.00E+00
CKAP2	LMNA	-0.248146078	-0.373405399	-0.114000367	3.70E-04	1.07E-03	1.00E+00
HOOK2	LMNA	0.075184127	-0.063526228	0.211044643	2.88E-01	3.71E-01	1.00E+00
INTS9	LMNA	-0.152023918	-0.28411224	-0.014272454	3.08E-02	5.48E-02	1.00E+00
DKFZP564J	LMNA	-0.291524925	-0.412956369	-0.159909533	2.56E-05	9.40E-05	3.04E-01
CCNG1	LMNA	0.18699491	0.05024087	0.316866066	7.70E-03	1.61E-02	1.00E+00
HNRPAB	LMNA	0.217350901	0.081752939	0.345048544	1.89E-03	4.61E-03	1.00E+00
AMH	LMNA	-0.222579543	-0.349879676	-0.087208218	1.45E-03	3.65E-03	1.00E+00
HADH	LMNA	-0.222937092	-0.350209795	-0.087581562	1.43E-03	3.59E-03	1.00E+00
TRPM1	LMNA	0.130047166	-0.008150156	0.26336985	6.51E-02	1.06E-01	1.00E+00
CACNG3	LMNA	-0.328174731	-0.446018912	-0.199146011	1.86E-06	8.48E-06	2.21E-02

PPP2R1A	LMNA	-0.028690389	-0.166083577	0.109795482	6.85E-01	7.53E-01	1.00E+00
CCKAR	LMNA	0.318762091	0.18902941	0.437558159	3.77E-06	1.62E-05	4.47E-02
COL2A1	LMNA	0.040353315	-0.09824499	0.177415964	5.69E-01	6.48E-01	1.00E+00
PTH2R	LMNA	-0.285404558	-0.407403696	-0.153397477	3.84E-05	1.36E-04	4.55E-01
IFI30	LMNA	0.407879126	0.285928206	0.516828486	1.69E-09	1.34E-08	2.00E-05
DDN	LMNA	-0.288368185	-0.410093559	-0.156549338	3.16E-05	1.14E-04	3.75E-01
GLUL	LMNA	0.237677907	0.10300673	0.363792275	6.59E-04	1.81E-03	1.00E+00
HK2	LMNA	0.257374636	0.123719491	0.38185791	2.18E-04	6.65E-04	1.00E+00
ELOVL6	LMNA	0.344958074	0.217253057	0.461052903	4.97E-07	2.51E-06	5.89E-03
MDK	LMNA	0.512223792	0.402645032	0.60732875	6.60E-15	1.33E-13	7.82E-11
EPHX1	LMNA	0.124365824	-0.01392424	0.257987482	7.78E-02	1.23E-01	1.00E+00
RASSF2	LMNA	-0.124877054	-0.258472149	0.013405042	7.66E-02	1.21E-01	1.00E+00
BFAR	LMNA	0.30867617	0.178219654	0.428468807	7.84E-06	3.16E-05	9.30E-02
ZNF14	LMNA	-0.22306502	-0.350327901	-0.087715151	1.42E-03	3.57E-03	1.00E+00
PPIL2	LMNA	-0.027508714	-0.164933363	0.110963683	6.98E-01	7.62E-01	1.00E+00
PRTN3	LMNA	0.070744256	-0.067970566	0.206775819	3.17E-01	4.02E-01	1.00E+00
CTA-126B4	LMNA	0.012161654	-0.126101061	0.149960881	8.64E-01	9.00E-01	1.00E+00
AVPR1A	LMNA	0.24423691	0.109891125	0.369818679	4.60E-04	1.31E-03	1.00E+00
KLHL22	LMNA	-0.154427359	-0.286373212	-0.016733089	2.82E-02	5.06E-02	1.00E+00
HSPBP1	LMNA	-0.056043253	-0.192604109	0.082647174	4.28E-01	5.15E-01	1.00E+00
PRKD1	LMNA	-0.062731191	-0.199058326	0.075977785	3.75E-01	4.63E-01	1.00E+00
TNFAIP8	LMNA	0.450099288	0.332722554	0.553742576	1.81E-11	2.03E-10	2.15E-07
KIAA0195	LMNA	-0.177461519	-0.307967699	-0.040400275	1.15E-02	2.30E-02	1.00E+00
GNB2L1	LMNA	0.291198485	0.159561912	0.412660436	2.62E-05	9.59E-05	3.10E-01
SCGB2A2	LMNA	0.027907154	-0.11056983	0.165321236	6.93E-01	7.59E-01	1.00E+00
CALCRL	LMNA	-0.143310469	-0.275902963	-0.005365623	4.19E-02	7.17E-02	1.00E+00
ICAM1	LMNA	0.550739063	0.446646642	0.64012157	2.04E-17	6.64E-16	2.42E-13
UBXD7	LMNA	-0.078212375	-0.213953259	0.060491773	2.69E-01	3.50E-01	1.00E+00
TMEM35	LMNA	-0.599388427	-0.681082461	-0.50295503	4.34E-21	2.58E-19	5.14E-17
ZNF674	LMNA	-0.034732685	-0.171959162	0.103816102	6.24E-01	6.98E-01	1.00E+00
BCL2L1	LMNA	0.396416644	0.273323465	0.506736147	5.21E-09	3.78E-08	6.18E-05
HECTD3	LMNA	0.399318633	0.276510652	0.509294115	3.94E-09	2.92E-08	4.67E-05
BRSK2	LMNA	-0.534806168	-0.626595307	-0.428382902	2.43E-16	6.57E-15	2.89E-12
CD19	LMNA	-0.100039556	-0.234847186	0.038543755	1.57E-01	2.23E-01	1.00E+00
RAPGEF3	LMNA	-0.270982005	-0.394283135	-0.138097127	9.59E-05	3.14E-04	1.00E+00

LGALS9	LMNA	0.191680227	0.055086917	0.321230928	6.28E-03	1.34E-02	1.00E+00
SCARB2	LMNA	0.125528884	-0.012742944	0.259090013	7.51E-02	1.19E-01	1.00E+00
KIAA0974	LMNA	-0.28769845	-0.409485876	-0.15583683	3.30E-05	1.19E-04	3.91E-01
MAPK3	LMNA	-0.089733932	-0.224997693	0.048923121	2.04E-01	2.79E-01	1.00E+00
USP34	LMNA	-0.202068068	-0.330888697	-0.065854114	3.93E-03	8.82E-03	1.00E+00
MAP2K1IP1	LMNA	0.014558356	-0.12374134	0.152303258	8.37E-01	8.78E-01	1.00E+00
CHIC2	LMNA	-0.132677128	-0.265858606	0.005474139	5.98E-02	9.81E-02	1.00E+00
SLC16A3	LMNA	0.605027879	0.509535697	0.68579782	1.48E-21	9.29E-20	1.76E-17
STK4	LMNA	0.108397303	-0.030104188	0.242814737	1.25E-01	1.84E-01	1.00E+00
APLP2	LMNA	0.528125355	0.420750532	0.620907114	6.63E-16	1.64E-14	7.86E-12
DLX5	LMNA	-0.147247826	-0.279614896	-0.009387655	3.65E-02	6.36E-02	1.00E+00
FBXO41	LMNA	-0.324640632	-0.442844678	-0.195344373	2.43E-06	1.09E-05	2.89E-02
MICAL3	LMNA	-0.280897471	-0.403308888	-0.148609267	5.14E-05	1.78E-04	6.09E-01
ITIH2	LMNA	-0.021003103	-0.15859426	0.117388265	7.67E-01	8.21E-01	1.00E+00
ADK	LMNA	-0.344468485	-0.460615286	-0.216723606	5.17E-07	2.60E-06	6.13E-03
TNNI3K	LMNA	-0.368459525	-0.481993352	-0.242756633	6.84E-08	4.05E-07	8.11E-04
HDAC2	LMNA	-0.257238552	-0.381733421	-0.123575987	2.19E-04	6.69E-04	1.00E+00
PRR7	LMNA	0.257747475	0.124112689	0.382198959	2.13E-04	6.51E-04	1.00E+00
THBS2	LMNA	0.297484441	0.16626147	0.418354451	1.71E-05	6.49E-05	2.03E-01
CA2	LMNA	-0.030391526	-0.167738765	0.108113072	6.68E-01	7.37E-01	1.00E+00
RANBP17	LMNA	-0.391390703	-0.502301415	-0.267809979	8.43E-09	5.84E-08	1.00E-04
CRYZ	LMNA	0.334218762	0.205656599	0.451440637	1.17E-06	5.53E-06	1.38E-02
GBAS	LMNA	-0.083596067	-0.219118328	0.055090709	2.37E-01	3.16E-01	1.00E+00
TGOLN2	LMNA	0.541309188	0.43582677	0.632122746	8.98E-17	2.64E-15	1.07E-12
CTBS	LMNA	0.389914797	0.266192437	0.500998013	9.69E-09	6.64E-08	1.15E-04
ETS1	LMNA	0.314582407	0.184545945	0.433794404	5.12E-06	2.14E-05	6.08E-02
FGD1	LMNA	-0.116236761	-0.250271751	0.022169992	9.95E-02	1.51E-01	1.00E+00
EDC4	LMNA	-0.110261967	-0.244589876	0.02821857	1.18E-01	1.76E-01	1.00E+00
PCDH8	LMNA	-0.059262822	-0.19571264	0.079438085	4.02E-01	4.90E-01	1.00E+00
KCNK15	LMNA	0.20097122	0.06471571	0.329870209	4.13E-03	9.24E-03	1.00E+00
HSP90B1	LMNA	0.520547919	0.412112191	0.61444367	2.01E-15	4.48E-14	2.38E-11
GSTA3	LMNA	-0.062481011	-0.198817101	0.076227495	3.77E-01	4.64E-01	1.00E+00
TNIP2	LMNA	0.319799871	0.190143455	0.438492022	3.49E-06	1.51E-05	4.14E-02
BCAS1	LMNA	-0.313116984	-0.432473823	-0.182975294	5.70E-06	2.36E-05	6.76E-02
HOXB6	LMNA	0.359424795	0.232931617	0.473958472	1.49E-07	8.30E-07	1.77E-03

NOL6	LMNA	0.089430526	-0.049228245	0.224707296	2.06E-01	2.80E-01	1.00E+00
PDHA2	LMNA	0.210578341	0.074698878	0.338780797	2.63E-03	6.18E-03	1.00E+00
SORD	LMNA	-0.118830124	-0.252735102	0.019541447	9.21E-02	1.42E-01	1.00E+00
SPC25	LMNA	-0.02040848	-0.158014289	0.117974905	7.73E-01	8.27E-01	1.00E+00
VAMP3	LMNA	0.282930528	0.150768366	0.405156582	4.51E-05	1.57E-04	5.34E-01
WDHD1	LMNA	0.098546091	-0.040049762	0.233421535	1.63E-01	2.30E-01	1.00E+00
TCIRG1	LMNA	0.631573603	0.540662636	0.70790299	6.97E-24	7.65E-22	8.26E-20
STAP2	LMNA	0.21863733	0.083094375	0.346237799	1.77E-03	4.36E-03	1.00E+00
CHCHD8	LMNA	-0.149619177	-0.281848573	-0.011812157	3.36E-02	5.92E-02	1.00E+00
TRIM44	LMNA	0.204968813	0.068866468	0.333580763	3.43E-03	7.83E-03	1.00E+00
KIAA1305	LMNA	0.009640752	-0.12858138	0.147495449	8.92E-01	9.22E-01	1.00E+00
PARL	LMNA	-0.149525939	-0.281760776	-0.011716799	3.37E-02	5.93E-02	1.00E+00
CRNN	LMNA	0.156872279	0.019237901	0.288671697	2.58E-02	4.68E-02	1.00E+00
SIDT2	LMNA	0.253170779	0.119288986	0.378010144	2.78E-04	8.29E-04	1.00E+00
RYR2	LMNA	-0.301121923	-0.421645061	-0.170143833	1.33E-05	5.16E-05	1.58E-01
TRRAP	LMNA	0.161798648	0.024290204	0.293298399	2.14E-02	3.97E-02	1.00E+00
TRAF1	LMNA	0.493844001	0.38182411	0.591564689	8.13E-14	1.31E-12	9.64E-10
GRN	LMNA	0.550598579	0.446485226	0.640002548	2.08E-17	6.75E-16	2.47E-13
SCAMP1	LMNA	-0.345447761	-0.46149055	-0.217782688	4.78E-07	2.43E-06	5.66E-03
TRIM32	LMNA	0.055529806	-0.083158685	0.192108116	4.32E-01	5.19E-01	1.00E+00
PTS	LMNA	-0.005497938	-0.143440068	0.132653746	9.38E-01	9.54E-01	1.00E+00
BANP	LMNA	-0.015498402	-0.153221574	0.122815371	8.27E-01	8.70E-01	1.00E+00
ATP6V1G1	LMNA	0.003360557	-0.13475296	0.141345983	9.62E-01	9.72E-01	1.00E+00
PRKACG	LMNA	-0.204420363	-0.333071929	-0.068296726	3.52E-03	8.00E-03	1.00E+00
ADCY6	LMNA	0.097309519	-0.041296241	0.232240674	1.68E-01	2.36E-01	1.00E+00
PRKY	LMNA	-0.110661952	-0.24497054	0.027813962	1.17E-01	1.74E-01	1.00E+00
CYP51A1	LMNA	0.221797302	0.086391554	0.349157336	1.51E-03	3.78E-03	1.00E+00
DDC	LMNA	-0.083434115	-0.218963064	0.055253302	2.38E-01	3.17E-01	1.00E+00
ANPEP	LMNA	0.592400985	0.494816874	0.675230583	1.60E-20	8.76E-19	1.89E-16
NPR2	LMNA	0.228025946	0.092899343	0.354904823	1.10E-03	2.86E-03	1.00E+00
PROM1	LMNA	-0.283188544	-0.405391001	-0.151042467	4.43E-05	1.55E-04	5.26E-01
COPG	LMNA	0.433814897	0.314605153	0.539552844	1.12E-10	1.11E-09	1.33E-06
OR5I1	LMNA	-0.142385422	-0.275030303	-0.004421329	4.32E-02	7.38E-02	1.00E+00
TRIP4	LMNA	0.451826366	0.334649133	0.555243978	1.48E-11	1.69E-10	1.76E-07
ZFYVE26	LMNA	0.1191847	-0.019181912	0.253071767	9.11E-02	1.41E-01	1.00E+00

SNX3	LMNA	-0.090531091	-0.225760557	0.048121325	2.00E-01	2.74E-01	1.00E+00
C1orf175	LMNA	-0.03667799	-0.17384872	0.101888918	6.04E-01	6.81E-01	1.00E+00
CSH2	LMNA	0.124663126	-0.013622313	0.258269345	7.71E-02	1.22E-01	1.00E+00
PPY2	LMNA	-0.058437034	-0.194915594	0.080261459	4.09E-01	4.96E-01	1.00E+00
STOML2	LMNA	-0.077057511	-0.212844303	0.061649306	2.76E-01	3.58E-01	1.00E+00
ALPI	LMNA	0.081888151	-0.056805016	0.21748059	2.47E-01	3.27E-01	1.00E+00
FTSJ1	LMNA	0.334052887	0.205477768	0.451291957	1.18E-06	5.59E-06	1.40E-02
ZNF273	LMNA	-0.373290449	-0.48628183	-0.248020731	4.46E-08	2.72E-07	5.29E-04
DST	LMNA	-0.019523874	-0.157151307	0.118847455	7.83E-01	8.35E-01	1.00E+00
GLRX5	LMNA	-0.212699127	-0.340744728	-0.076906352	2.37E-03	5.65E-03	1.00E+00
C20orf12	LMNA	-0.151605387	-0.283718368	-0.013844134	3.13E-02	5.56E-02	1.00E+00
CAB39	LMNA	-0.037970878	-0.175103998	0.100607499	5.92E-01	6.69E-01	1.00E+00
RAB5C	LMNA	0.073596323	-0.065116262	0.209518605	2.98E-01	3.82E-01	1.00E+00
FLAD1	LMNA	-0.108171004	-0.242599243	0.030332962	1.25E-01	1.85E-01	1.00E+00
TTLL3	LMNA	0.023445467	-0.114977679	0.160975443	7.40E-01	7.99E-01	1.00E+00
MSH2	LMNA	-0.158917096	-0.290592877	-0.021334138	2.39E-02	4.37E-02	1.00E+00
NPPC	LMNA	0.036029266	-0.102531715	0.173218698	6.11E-01	6.87E-01	1.00E+00
PIP4K2C	LMNA	-0.175225167	-0.305877	-0.038095702	1.26E-02	2.49E-02	1.00E+00
CYLD	LMNA	0.20586806	0.069800819	0.334414892	3.29E-03	7.54E-03	1.00E+00
ZEB2	LMNA	-0.263085228	-0.387077878	-0.129746514	1.55E-04	4.89E-04	1.00E+00
MRP63	LMNA	-0.125599364	-0.259156814	0.012671347	7.49E-02	1.19E-01	1.00E+00
WTAP	LMNA	0.129252632	-0.008958214	0.262617627	6.68E-02	1.08E-01	1.00E+00
NEUROD4	LMNA	-0.202301455	-0.331105372	-0.066096389	3.89E-03	8.73E-03	1.00E+00
MGAT4A	LMNA	0.159014717	0.021434243	0.290684569	2.38E-02	4.36E-02	1.00E+00
MTMR2	LMNA	-0.082332491	-0.217906746	0.056359092	2.44E-01	3.24E-01	1.00E+00
CHRM2	LMNA	-0.212690921	-0.340737132	-0.076897809	2.37E-03	5.65E-03	1.00E+00
TSC22D4	LMNA	-0.199832108	-0.32881216	-0.063533816	4.35E-03	9.68E-03	1.00E+00
PPYR1	LMNA	0.083248404	-0.055439739	0.218785013	2.39E-01	3.18E-01	1.00E+00
CCNH	LMNA	-0.135586166	-0.268609389	0.002511851	5.44E-02	9.02E-02	1.00E+00
USP48	LMNA	0.04792409	-0.090727159	0.184752772	4.98E-01	5.83E-01	1.00E+00
NR1H4	LMNA	-0.103274689	-0.237933432	0.035279294	1.44E-01	2.07E-01	1.00E+00
RASL10A	LMNA	-0.498432872	-0.595507503	-0.387011828	4.40E-14	7.40E-13	5.22E-10
RRM1	LMNA	0.115321872	-0.023096838	0.249402313	1.02E-01	1.55E-01	1.00E+00
GABRA4	LMNA	-0.304860706	-0.425024008	-0.174138557	1.03E-05	4.05E-05	1.22E-01
C1orf35	LMNA	-0.04241521	-0.179415649	0.096199081	5.49E-01	6.30E-01	1.00E+00

SSTR1	LMNA	-0.225288353	-0.352379888	-0.090037639	1.27E-03	3.23E-03	1.00E+00
APOBEC3C	LMNA	0.529335287	0.422131671	0.621938003	5.54E-16	1.41E-14	6.57E-12
CTDSPL	LMNA	0.14660327	0.008728932	0.279007514	3.73E-02	6.49E-02	1.00E+00
RUSC2	LMNA	-0.273888391	-0.396931198	-0.141175309	8.01E-05	2.67E-04	9.50E-01
PDE11A	LMNA	-0.336501842	-0.453486396	-0.208118882	9.76E-07	4.69E-06	1.16E-02
ZCCHC8	LMNA	0.02815426	-0.110325546	0.165561768	6.91E-01	7.57E-01	1.00E+00
RAD17	LMNA	-0.117588236	-0.251555689	0.020800421	9.56E-02	1.46E-01	1.00E+00
TEX12	LMNA	-0.062685887	-0.199014645	0.076023005	3.75E-01	4.63E-01	1.00E+00
LILRB5	LMNA	0.26180361	0.128393022	0.385907061	1.67E-04	5.23E-04	1.00E+00
CD5	LMNA	0.308349144	0.177869683	0.428173686	8.02E-06	3.23E-05	9.52E-02
ARHGEF1	LMNA	0.451720477	0.334530984	0.555151945	1.50E-11	1.71E-10	1.78E-07
ABCA4	LMNA	0.272062012	0.139240673	0.395267389	8.97E-05	2.96E-04	1.00E+00
TSPAN9	LMNA	0.619173306	0.526091269	0.697595584	9.07E-23	7.86E-21	1.08E-18
WDR67	LMNA	-0.223902824	-0.351101281	-0.088590145	1.36E-03	3.44E-03	1.00E+00
THUMPD1	LMNA	-0.163527621	-0.294920741	-0.026065043	2.00E-02	3.75E-02	1.00E+00
ARID4A	LMNA	-0.33649715	-0.453482194	-0.20811382	9.76E-07	4.69E-06	1.16E-02
OPRM1	LMNA	-0.063850889	-0.20013774	0.074859978	3.67E-01	4.54E-01	1.00E+00
SYCP2	LMNA	-0.147324233	-0.27968689	-0.00946575	3.64E-02	6.35E-02	1.00E+00
CYP26B1	LMNA	-0.121748228	-0.25550484	0.016581465	8.43E-02	1.32E-01	1.00E+00
FLJ13231	LMNA	-0.146425734	-0.278840198	-0.008547515	3.76E-02	6.52E-02	1.00E+00
LECT2	LMNA	-0.167329304	-0.298485301	-0.029970624	1.73E-02	3.28E-02	1.00E+00
PCCA	LMNA	-0.401589725	-0.511294621	-0.279006824	3.15E-09	2.38E-08	3.74E-05
AQP5	LMNA	0.069728557	-0.068986515	0.205798521	3.24E-01	4.10E-01	1.00E+00
RAB30	LMNA	-0.229253705	-0.356036609	-0.09418349	1.03E-03	2.70E-03	1.00E+00
OSBPL11	LMNA	-0.282968914	-0.405191458	-0.150809143	4.49E-05	1.57E-04	5.33E-01
YLPM1	LMNA	-0.10345914	-0.238109312	0.035093082	1.43E-01	2.06E-01	1.00E+00
GNB5	LMNA	-0.026715298	-0.164160861	0.111747837	7.06E-01	7.69E-01	1.00E+00
CCL21	LMNA	-0.063257841	-0.199566069	0.075452068	3.71E-01	4.59E-01	1.00E+00
PRKAR1B	LMNA	-0.26376802	-0.387701476	-0.1304678	1.49E-04	4.71E-04	1.00E+00
C1orf121	LMNA	0.253742856	0.119891593	0.378534016	2.69E-04	8.05E-04	1.00E+00
FMO2	LMNA	0.074517436	-0.064193941	0.210403967	2.92E-01	3.76E-01	1.00E+00
IL16	LMNA	0.187026817	0.050273849	0.316895809	7.69E-03	1.61E-02	1.00E+00
MSTN	LMNA	-0.483609806	-0.582754476	-0.370279724	3.09E-13	4.60E-12	3.66E-09
VCL	LMNA	0.57507106	0.474706607	0.660671891	3.54E-19	1.59E-17	4.19E-15
TCF3	LMNA	0.126441957	-0.011815285	0.259955323	7.30E-02	1.16E-01	1.00E+00

CCDC88C	LMNA	-0.073143188	-0.209082976	0.065569904	3.01E-01	3.85E-01	1.00E+00
HFE	LMNA	0.515555574	0.406431508	0.610178372	4.12E-15	8.64E-14	4.88E-11
SERPINE1	LMNA	0.692103826	0.612582393	0.757754681	3.90E-30	1.22E-27	4.63E-26
FAM125B	LMNA	-0.40198932	-0.511646485	-0.279446194	3.03E-09	2.29E-08	3.59E-05
BAI2	LMNA	-0.211840418	-0.339949665	-0.076012383	2.47E-03	5.86E-03	1.00E+00
SMC5	LMNA	0.300794678	0.169794391	0.421349152	1.36E-05	5.26E-05	1.62E-01
AKAP5	LMNA	-0.325843394	-0.4439253	-0.19663775	2.22E-06	9.98E-06	2.63E-02
POU2F3	LMNA	0.203109607	0.066935442	0.331855549	3.74E-03	8.45E-03	1.00E+00
BACH1	LMNA	0.400073109	0.277339717	0.509958832	3.66E-09	2.72E-08	4.34E-05
PPP2R2D	LMNA	-0.161416776	-0.292939976	-0.023898319	2.17E-02	4.03E-02	1.00E+00
C11orf63	LMNA	0.185336031	0.048526624	0.315319326	8.27E-03	1.72E-02	1.00E+00
SSSCA1	LMNA	0.231514277	0.096549059	0.3581195	9.16E-04	2.43E-03	1.00E+00
LRRC51	LMNA	-0.235994712	-0.362244053	-0.101242109	7.22E-04	1.96E-03	1.00E+00
LRRC3	LMNA	-0.030071965	-0.167427895	0.108429175	6.71E-01	7.40E-01	1.00E+00
PORCN	LMNA	0.142773094	0.004817037	0.275396047	4.27E-02	7.29E-02	1.00E+00
DTL	LMNA	-0.03425846	-0.171498373	0.104285752	6.28E-01	7.03E-01	1.00E+00
ACTG2	LMNA	0.436361976	0.317433219	0.541776262	8.49E-11	8.61E-10	1.01E-06
FAM124B	LMNA	-0.07210255	-0.208082335	0.06661149	3.08E-01	3.93E-01	1.00E+00
RSAD2	LMNA	0.089488924	-0.049169519	0.224763192	2.05E-01	2.80E-01	1.00E+00
CTBP2	LMNA	-0.243870572	-0.369482366	-0.109506273	4.70E-04	1.33E-03	1.00E+00
ZMYND11	LMNA	-0.358725507	-0.473335776	-0.232172236	1.59E-07	8.78E-07	1.88E-03
CRTC3	LMNA	0.2732009	0.140446948	0.396304997	8.36E-05	2.77E-04	9.91E-01
MYH8	LMNA	0.066557446	-0.072156552	0.202745555	3.47E-01	4.33E-01	1.00E+00
LRRFIP2	LMNA	0.23588137	0.101123315	0.362139775	7.26E-04	1.97E-03	1.00E+00
TTN	LMNA	-0.205051898	-0.33365784	-0.068952787	3.42E-03	7.80E-03	1.00E+00
RGS6	LMNA	0.174681238	0.037535401	0.305368307	1.29E-02	2.54E-02	1.00E+00
PLLP	LMNA	-0.353089045	-0.468312483	-0.226057035	2.55E-07	1.36E-06	3.02E-03
SRGAP3	LMNA	-0.58174951	-0.666290025	-0.48244413	1.10E-19	5.28E-18	1.30E-15
PDK1	LMNA	0.153402213	0.015683343	0.285409013	2.93E-02	5.24E-02	1.00E+00
NBR2	LMNA	-0.273892447	-0.396934893	-0.141179607	8.00E-05	2.67E-04	9.49E-01
ZFYVE21	LMNA	0.051698759	-0.086972973	0.1884051	4.65E-01	5.51E-01	1.00E+00
C13orf1	LMNA	-0.104718555	-0.239309971	0.033821385	1.38E-01	2.00E-01	1.00E+00
ZNF137	LMNA	-0.160661155	-0.292230645	-0.023123009	2.24E-02	4.13E-02	1.00E+00
CEP27	LMNA	0.260993856	0.127538113	0.385167108	1.76E-04	5.47E-04	1.00E+00
WDR41	LMNA	0.126881855	-0.011368274	0.260372134	7.20E-02	1.15E-01	1.00E+00

BEST2	LMNA	0.136461158	-0.001620368	0.269436355	5.28E-02	8.81E-02	1.00E+00
RNF121	LMNA	0.101956648	-0.036609641	0.236676377	1.49E-01	2.13E-01	1.00E+00
MEG3	LMNA	-0.170510625	-0.301465383	-0.033242116	1.53E-02	2.93E-02	1.00E+00
BCCIP	LMNA	-0.225639514	-0.352703873	-0.090404595	1.24E-03	3.18E-03	1.00E+00
OXSR1	LMNA	0.189891791	0.053236369	0.319565463	6.79E-03	1.44E-02	1.00E+00
RAD51	LMNA	-0.046466636	-0.183341544	0.09217565	5.11E-01	5.95E-01	1.00E+00
RPL13A	LMNA	-0.008569355	-0.146447108	0.129635005	9.04E-01	9.30E-01	1.00E+00
MEST	LMNA	0.04596169	-0.092677354	0.182852481	5.16E-01	6.00E-01	1.00E+00
DYRK1A	LMNA	-0.177948887	-0.308423157	-0.040902703	1.13E-02	2.26E-02	1.00E+00
HTRA2	LMNA	-0.040516032	-0.177573812	0.098083578	5.67E-01	6.47E-01	1.00E+00
SARDH	LMNA	0.154436231	0.016742175	0.286381555	2.82E-02	5.06E-02	1.00E+00
ILKAP	LMNA	-0.23680183	-0.362986536	-0.102088166	6.91E-04	1.89E-03	1.00E+00
ANGPTL2	LMNA	0.199930609	0.063636001	0.328903664	4.33E-03	9.64E-03	1.00E+00
RBBP5	LMNA	-0.018450407	-0.156103796	0.119906006	7.94E-01	8.45E-01	1.00E+00
ORC2L	LMNA	0.011266952	-0.126981557	0.149086063	8.74E-01	9.06E-01	1.00E+00
HBS1L	LMNA	-0.098614332	-0.23348669	0.039980962	1.63E-01	2.30E-01	1.00E+00
TMEM30A	LMNA	0.127827128	-0.010407531	0.261267626	6.98E-02	1.12E-01	1.00E+00
PDS5A	LMNA	0.036756182	-0.101811433	0.17392465	6.04E-01	6.81E-01	1.00E+00
WISP3	LMNA	-0.068213176	-0.204339929	0.070501732	3.35E-01	4.21E-01	1.00E+00
CRK	LMNA	0.132342536	-0.0058147	0.265542077	6.04E-02	9.90E-02	1.00E+00
NCAPH2	LMNA	-0.147670995	-0.280013602	-0.00982019	3.60E-02	6.28E-02	1.00E+00
RNASET2	LMNA	0.184260182	0.047415298	0.314315835	8.66E-03	1.79E-02	1.00E+00
NEDD9	LMNA	0.521680772	0.413402415	0.615410779	1.71E-15	3.89E-14	2.02E-11
WDR79	LMNA	-0.132244185	-0.26544903	0.005914799	6.06E-02	9.93E-02	1.00E+00
PSG6	LMNA	0.011826548	-0.126430872	0.149633247	8.67E-01	9.02E-01	1.00E+00
SMPDL3B	LMNA	0.011905652	-0.126353021	0.14971059	8.66E-01	9.02E-01	1.00E+00
MORC4	LMNA	0.437895051	0.319136439	0.543113817	7.17E-11	7.38E-10	8.50E-07
PSMD13	LMNA	0.129852137	-0.008348521	0.263185222	6.55E-02	1.06E-01	1.00E+00
DDX23	LMNA	-0.033613541	-0.170871631	0.104924349	6.35E-01	7.09E-01	1.00E+00
ETV5	LMNA	0.228154119	0.093033382	0.355022994	1.09E-03	2.84E-03	1.00E+00
MYRIP	LMNA	-0.25233114	-0.377241111	-0.118404717	2.91E-04	8.66E-04	1.00E+00
LY6E	LMNA	0.260573758	0.127094666	0.384783159	1.80E-04	5.60E-04	1.00E+00
ATP12A	LMNA	0.196593241	0.060175394	0.32580201	5.04E-03	1.10E-02	1.00E+00
BTBD7	LMNA	0.142943515	0.004991005	0.275556817	4.24E-02	7.26E-02	1.00E+00
AUP1	LMNA	0.230124346	0.09509439	0.356838967	9.85E-04	2.59E-03	1.00E+00

PIP	LMNA	0.092677879	-0.045961161	0.227814163	1.90E-01	2.62E-01	1.00E+00
GSTO1	LMNA	0.19904645	0.062718874	0.328082223	4.51E-03	9.99E-03	1.00E+00
CORO7	LMNA	-0.113882853	-0.248034346	0.02455418	1.07E-01	1.61E-01	1.00E+00
COX6A2	LMNA	-0.091135934	-0.226339269	0.047512845	1.97E-01	2.71E-01	1.00E+00
MAD2L1BP	LMNA	-0.149223432	-0.281475904	-0.01140743	3.40E-02	5.98E-02	1.00E+00
PITPNM3	LMNA	-0.108297338	-0.242719547	0.030205247	1.25E-01	1.84E-01	1.00E+00
ENPP1	LMNA	0.279475407	0.14709979	0.402015887	5.63E-05	1.93E-04	6.67E-01
SCNN1A	LMNA	0.246058756	0.111805643	0.371490716	4.16E-04	1.19E-03	1.00E+00
LSM1	LMNA	-0.141015036	-0.273737123	-0.003022883	4.53E-02	7.69E-02	1.00E+00
KCNE2	LMNA	-0.088897293	-0.224196869	0.049764435	2.08E-01	2.83E-01	1.00E+00
IDUA	LMNA	0.415467987	0.294296595	0.523493608	7.82E-10	6.60E-09	9.28E-06
P2RX4	LMNA	0.306334817	0.175714764	0.426355324	9.26E-06	3.68E-05	1.10E-01
PPP2R3C	LMNA	-0.227976836	-0.354859544	-0.092847987	1.10E-03	2.86E-03	1.00E+00
CCND2	LMNA	-0.159696009	-0.291324415	-0.022132956	2.32E-02	4.26E-02	1.00E+00
COX11	LMNA	-0.322184551	-0.440636946	-0.192704648	2.93E-06	1.28E-05	3.47E-02
CUL4A	LMNA	0.174467894	0.037315658	0.305168762	1.30E-02	2.56E-02	1.00E+00
CFB	LMNA	0.426384409	0.306367027	0.533058119	2.50E-10	2.31E-09	2.96E-06
PDZK1	LMNA	0.159205584	0.021629978	0.290863837	2.36E-02	4.33E-02	1.00E+00
PCP4	LMNA	-0.281972648	-0.404286161	-0.149750944	4.79E-05	1.67E-04	5.68E-01
UBIAD1	LMNA	0.362225454	0.235974496	0.476451231	1.18E-07	6.66E-07	1.39E-03
CDC42BPB	LMNA	0.014570514	-0.123729365	0.152315137	8.37E-01	8.78E-01	1.00E+00
ZNF323	LMNA	-0.356308744	-0.471182837	-0.229548978	1.95E-07	1.07E-06	2.31E-03
RIMS2	LMNA	-0.489783157	-0.58807166	-0.377239206	1.39E-13	2.19E-12	1.65E-09
CXCL6	LMNA	0.287130219	0.155232416	0.408970207	3.43E-05	1.23E-04	4.06E-01
SLC34A2	LMNA	0.123972579	-0.014323563	0.257614624	7.88E-02	1.24E-01	1.00E+00
RNMTL1	LMNA	-0.03449897	-0.171732077	0.104047569	6.26E-01	7.01E-01	1.00E+00
NPTN	LMNA	0.145865141	0.007974726	0.278311824	3.83E-02	6.63E-02	1.00E+00
SART3	LMNA	-0.08837714	-0.223698891	0.050287395	2.11E-01	2.86E-01	1.00E+00
UPP1	LMNA	0.584567533	0.485713692	0.668657763	6.62E-20	3.34E-18	7.86E-16
CAPN10	LMNA	-0.03667879	-0.173849496	0.101888126	6.04E-01	6.81E-01	1.00E+00
SLC6A9	LMNA	0.002811576	-0.135291937	0.140807923	9.68E-01	9.76E-01	1.00E+00
CCR5	LMNA	0.348648176	0.221246019	0.464349466	3.68E-07	1.91E-06	4.36E-03
NDUFS5	LMNA	0.002744771	-0.135357519	0.140742441	9.69E-01	9.76E-01	1.00E+00
CISD1	LMNA	-0.356902462	-0.471711869	-0.230193254	1.85E-07	1.02E-06	2.19E-03
PDLIM3	LMNA	0.162022466	0.024519912	0.293508457	2.12E-02	3.95E-02	1.00E+00

ZNHIT3	LMNA	-0.393991196	-0.504596737	-0.270661725	6.58E-09	4.67E-08	7.81E-05
SNRPD3	LMNA	0.114346631	-0.02408456	0.248475284	1.05E-01	1.59E-01	1.00E+00
VPS24	LMNA	0.255791393	0.122050262	0.380409283	2.39E-04	7.22E-04	1.00E+00
SCN8A	LMNA	-0.373489678	-0.486458571	-0.248237983	4.38E-08	2.67E-07	5.20E-04
KIAA0701	LMNA	-0.219984061	-0.347482363	-0.084499219	1.66E-03	4.10E-03	1.00E+00
UNC93B1	LMNA	0.425769345	0.305685916	0.53251995	2.67E-10	2.45E-09	3.16E-06
GMNN	LMNA	-0.249870557	-0.374986445	-0.115814566	3.35E-04	9.85E-04	1.00E+00
FDXR	LMNA	0.178116154	0.041075155	0.308579459	1.12E-02	2.25E-02	1.00E+00
SPCS2	LMNA	-0.105955907	-0.240489196	0.032571527	1.33E-01	1.95E-01	1.00E+00
CDCP1	LMNA	0.560527737	0.457910499	0.648404296	4.15E-18	1.52E-16	4.92E-14
PAX3	LMNA	0.031874812	-0.106645466	0.169181356	6.52E-01	7.24E-01	1.00E+00
PNLIPRP1	LMNA	0.158418556	0.02082295	0.290124578	2.43E-02	4.45E-02	1.00E+00
LASS4	LMNA	-0.234343213	-0.360724308	-0.099511542	7.88E-04	2.12E-03	1.00E+00
HSD17B8	LMNA	0.088557629	-0.05010594	0.223871693	2.10E-01	2.85E-01	1.00E+00
EYA4	LMNA	0.28772118	0.15586101	0.409506502	3.30E-05	1.19E-04	3.91E-01
PRKAB1	LMNA	0.033230831	-0.105303253	0.170499656	6.39E-01	7.12E-01	1.00E+00
KIF20A	LMNA	0.200974382	0.064718991	0.329873146	4.13E-03	9.24E-03	1.00E+00
YAP1	LMNA	0.611367201	0.516946401	0.691090283	4.31E-22	3.08E-20	5.11E-18
SC5DL	LMNA	-0.402897093	-0.512445687	-0.280444513	2.77E-09	2.11E-08	3.29E-05
NNT	LMNA	-0.173535765	-0.304296793	-0.036355731	1.35E-02	2.65E-02	1.00E+00
DKFZp566H	LMNA	-0.135077912	-0.268128941	0.003029583	5.53E-02	9.16E-02	1.00E+00
ITPKC	LMNA	0.393875889	0.270535231	0.504494995	6.65E-09	4.71E-08	7.89E-05
ST8SIA2	LMNA	-0.067789654	-0.20393217	0.070925094	3.38E-01	4.24E-01	1.00E+00
LMX1B	LMNA	0.028421978	-0.110060866	0.165822345	6.88E-01	7.55E-01	1.00E+00
RAD21	LMNA	-0.50724155	-0.603062945	-0.396989844	1.32E-14	2.50E-13	1.57E-10
SNRPB	LMNA	0.100894539	-0.037681309	0.235663085	1.53E-01	2.19E-01	1.00E+00
MIF	LMNA	0.167513235	0.030159688	0.298657666	1.72E-02	3.26E-02	1.00E+00
DLGAP2	LMNA	-0.317233577	-0.436182233	-0.187389176	4.22E-06	1.79E-05	5.01E-02
PFN1	LMNA	0.528162889	0.42079337	0.620939099	6.59E-16	1.64E-14	7.82E-12
IRF8	LMNA	-0.034557355	-0.171788806	0.103989748	6.25E-01	7.00E-01	1.00E+00
PRO0132	LMNA	0.088980747	-0.049680523	0.224276759	2.08E-01	2.83E-01	1.00E+00
MICALL2	LMNA	0.508952696	0.39893117	0.60452865	1.04E-14	2.01E-13	1.24E-10
ZNF654	LMNA	0.115407334	-0.023010268	0.249483539	1.02E-01	1.54E-01	1.00E+00
SS18L1	LMNA	-0.243722671	-0.369346578	-0.109350908	4.74E-04	1.34E-03	1.00E+00
ACD	LMNA	0.020621913	-0.117764348	0.158222474	7.71E-01	8.25E-01	1.00E+00

BCL3	LMNA	0.619371511	0.526323747	0.697760592	8.72E-23	7.72E-21	1.03E-18
SLC16A8	LMNA	-0.081410111	-0.217022056	0.057284697	2.49E-01	3.30E-01	1.00E+00
ITGB3	LMNA	0.543283247	0.438089292	0.633798831	6.61E-17	2.00E-15	7.84E-13
MRLC2	LMNA	0.390905483	0.267278117	0.501872963	8.83E-09	6.09E-08	1.05E-04
CEP152	LMNA	-0.166005979	-0.29724493	-0.028610657	1.82E-02	3.44E-02	1.00E+00
F2RL3	LMNA	0.072981207	-0.065732053	0.208927239	3.02E-01	3.86E-01	1.00E+00
CLIP1	LMNA	0.279049556	0.146647882	0.40162859	5.78E-05	1.98E-04	6.86E-01
ZNF75	LMNA	0.01474646	-0.123556071	0.152487033	8.35E-01	8.76E-01	1.00E+00
SF3B4	LMNA	0.226913232	0.091735913	0.353878768	1.16E-03	3.00E-03	1.00E+00
ATP5C1	LMNA	-0.280401472	-0.40285796	-0.148082709	5.30E-05	1.83E-04	6.29E-01
MAP7D3	LMNA	0.542646894	0.437359805	0.633258625	7.30E-17	2.20E-15	8.66E-13
SEMA5A	LMNA	0.052787389	-0.085889519	0.189457744	4.56E-01	5.42E-01	1.00E+00
PSCDBP	LMNA	0.373090581	0.247802794	0.486104512	4.54E-08	2.76E-07	5.38E-04
UBP1	LMNA	-0.04211877	-0.179128223	0.096493295	5.52E-01	6.33E-01	1.00E+00
XYLB	LMNA	0.073220083	-0.065492927	0.209156903	3.00E-01	3.85E-01	1.00E+00
C13orf15	LMNA	-0.370861693	-0.48412647	-0.24537327	5.53E-08	3.33E-07	6.56E-04
ZNF282	LMNA	0.300537507	0.169519799	0.421116589	1.39E-05	5.35E-05	1.65E-01
ZNF222	LMNA	-0.265956022	-0.38969903	-0.132780108	1.30E-04	4.18E-04	1.00E+00
COL10A1	LMNA	0.534642491	0.428195732	0.626456066	2.49E-16	6.69E-15	2.96E-12
MFSD5	LMNA	0.383610789	0.259291299	0.495425138	1.75E-08	1.14E-07	2.07E-04
C20orf67	LMNA	-0.102582206	-0.237273046	0.035978304	1.46E-01	2.10E-01	1.00E+00
NLGN4Y	LMNA	0.00964166	-0.128580486	0.147496337	8.92E-01	9.22E-01	1.00E+00
INHBC	LMNA	-0.102325247	-0.237027966	0.03623765	1.47E-01	2.12E-01	1.00E+00
GNG13	LMNA	-0.311597525	-0.431104005	-0.181347429	6.36E-06	2.61E-05	7.54E-02
NUMA1	LMNA	-0.039531113	-0.176618253	0.099060491	5.76E-01	6.56E-01	1.00E+00
F12	LMNA	-0.057269501	-0.193788388	0.081425257	4.18E-01	5.06E-01	1.00E+00
C1orf41	LMNA	0.071203376	-0.067511239	0.207217492	3.14E-01	3.99E-01	1.00E+00
SUPT6H	LMNA	0.143838822	0.005905079	0.276401296	4.11E-02	7.07E-02	1.00E+00
GSK3A	LMNA	-0.065291457	-0.201525993	0.07342133	3.56E-01	4.43E-01	1.00E+00
GIPC1	LMNA	0.136651407	-0.001426505	0.269616136	5.25E-02	8.76E-02	1.00E+00
ELL2	LMNA	0.273338027	0.140592216	0.396429908	8.29E-05	2.75E-04	9.83E-01
C9orf167	LMNA	0.503519822	0.392770881	0.599872814	2.21E-14	3.99E-13	2.62E-10
PVRL3	LMNA	0.300018505	0.168965701	0.420647201	1.44E-05	5.53E-05	1.71E-01
ADAM20	LMNA	0.006443974	-0.131724208	0.144366547	9.27E-01	9.47E-01	1.00E+00
GPR87	LMNA	0.129584176	-0.008621048	0.262931535	6.61E-02	1.07E-01	1.00E+00

ZNF770	LMNA	-0.1357759	-0.268788725	0.002318559	5.40E-02	8.98E-02	1.00E+00
SMR3B	LMNA	-0.097541397	-0.232462135	0.041062539	1.67E-01	2.35E-01	1.00E+00
FZD1	LMNA	0.6729265	0.589653208	0.742043054	5.40E-28	1.17E-25	6.41E-24
TRPC4AP	LMNA	0.144376427	0.006454064	0.276908283	4.04E-02	6.95E-02	1.00E+00
MKL1	LMNA	0.134938523	-0.003171559	0.267997165	5.55E-02	9.19E-02	1.00E+00
C2orf28	LMNA	0.21096813	0.0751045	0.339141841	2.58E-03	6.08E-03	1.00E+00
LAMA4	LMNA	0.478352079	0.364362477	0.578219227	6.02E-13	8.72E-12	7.14E-09
EIF4G2	LMNA	0.157501045	0.01988235	0.289262558	2.52E-02	4.58E-02	1.00E+00
ZZZ3	LMNA	-0.037921678	-0.175056237	0.100656271	5.92E-01	6.70E-01	1.00E+00
C16orf5	LMNA	-0.281435123	-0.403797617	-0.149180129	4.96E-05	1.72E-04	5.88E-01
GALNAC4S	LMNA	0.095604694	-0.04301402	0.230612002	1.76E-01	2.46E-01	1.00E+00
IGFBP4	LMNA	0.482801611	0.369369551	0.582057738	3.42E-13	5.09E-12	4.06E-09
NDUFA10	LMNA	0.047257199	-0.091390021	0.184107103	5.04E-01	5.88E-01	1.00E+00
CTNNA2	LMNA	-0.307941779	-0.427806031	-0.177433785	8.26E-06	3.31E-05	9.80E-02
NUDT15	LMNA	0.141318034	0.003332039	0.274023092	4.48E-02	7.63E-02	1.00E+00
CEPT1	LMNA	-0.113801222	-0.24795673	0.024636832	1.07E-01	1.61E-01	1.00E+00
CLIC2	LMNA	0.259946237	0.126432368	0.384209556	1.87E-04	5.78E-04	1.00E+00
RNF13	LMNA	-0.252497155	-0.377393178	-0.118579539	2.89E-04	8.59E-04	1.00E+00
TDRD1	LMNA	-0.065197691	-0.201435648	0.07351499	3.57E-01	4.43E-01	1.00E+00
KCNK5	LMNA	0.098238331	-0.040360029	0.233127677	1.64E-01	2.32E-01	1.00E+00
CCDC92	LMNA	-0.06119506	-0.197576923	0.077510754	3.87E-01	4.74E-01	1.00E+00
ETNK1	LMNA	-0.186941429	-0.316816211	-0.05018559	7.72E-03	1.61E-02	1.00E+00
CD69	LMNA	0.076122282	-0.062586424	0.211945994	2.82E-01	3.64E-01	1.00E+00
LTA	LMNA	0.154362911	0.016667085	0.286312603	2.83E-02	5.07E-02	1.00E+00
TTPA	LMNA	-0.207689421	-0.336103745	-0.071694012	3.02E-03	6.99E-03	1.00E+00
MYOZ1	LMNA	-0.23848115	-0.364530861	-0.103849129	6.31E-04	1.74E-03	1.00E+00
IFNB1	LMNA	-0.143725955	-0.276294848	-0.005789833	4.13E-02	7.09E-02	1.00E+00
CLNS1A	LMNA	-0.377990719	-0.490449106	-0.253149569	2.92E-08	1.83E-07	3.47E-04
SC65	LMNA	0.483542674	0.370204112	0.582696608	3.11E-13	4.64E-12	3.69E-09
CXorf45	LMNA	-0.314676936	-0.433879572	-0.184647284	5.09E-06	2.13E-05	6.04E-02
ZXDB	LMNA	0.046503479	-0.092139041	0.183377225	5.11E-01	5.95E-01	1.00E+00
PEX5L	LMNA	-0.357976911	-0.472669047	-0.231359481	1.69E-07	9.32E-07	2.00E-03
EPS15L1	LMNA	-0.011767367	-0.149575383	0.126489114	8.68E-01	9.02E-01	1.00E+00
GPA33	LMNA	0.130752009	-0.007433163	0.264037024	6.36E-02	1.03E-01	1.00E+00
MGEA5	LMNA	-0.277188815	-0.399935795	-0.144673935	6.51E-05	2.20E-04	7.72E-01

HIST1H3A	LMNA	-0.297556753	-0.418419898	-0.166338611	1.70E-05	6.47E-05	2.02E-01
BCAT1	LMNA	0.473778705	0.359222903	0.574269266	1.07E-12	1.47E-11	1.27E-08
ING1	LMNA	-0.116523923	-0.250544602	0.021879029	9.86E-02	1.50E-01	1.00E+00
SLC2A9	LMNA	0.191245772	0.054637287	0.320826419	6.40E-03	1.36E-02	1.00E+00
ORC6L	LMNA	-0.191975769	-0.321506072	-0.055392814	6.20E-03	1.32E-02	1.00E+00
KLK11	LMNA	0.174510629	0.037359674	0.305208734	1.30E-02	2.56E-02	1.00E+00
C19orf28	LMNA	0.557731238	0.454689194	0.646040148	6.57E-18	2.36E-16	7.80E-14
MAGEB1	LMNA	-0.124001369	-0.257641923	0.014294329	7.87E-02	1.24E-01	1.00E+00
MED22	LMNA	-0.131469592	-0.264716125	0.006703066	6.22E-02	1.01E-01	1.00E+00
ETV6	LMNA	0.386227406	0.262154228	0.4977394	1.37E-08	9.15E-08	1.62E-04
CEBPB	LMNA	0.621191193	0.528458735	0.699275112	6.02E-23	5.58E-21	7.14E-19
KRT85	LMNA	-0.134345304	-0.267436296	0.003775723	5.66E-02	9.34E-02	1.00E+00
CRYGA	LMNA	-0.273970659	-0.397006124	-0.141262477	7.97E-05	2.66E-04	9.45E-01
HMGA1	LMNA	0.225509365	0.090268588	0.3525838	1.25E-03	3.20E-03	1.00E+00
CAPN7	LMNA	-0.017448015	-0.155125363	0.120894185	8.05E-01	8.53E-01	1.00E+00
MGC5566	LMNA	-0.318306328	-0.437147952	-0.188540259	3.90E-06	1.67E-05	4.63E-02
GEM	LMNA	0.454079956	0.337164515	0.55720208	1.14E-11	1.33E-10	1.35E-07
NANOS1	LMNA	-0.306298262	-0.426322317	-0.175675669	9.28E-06	3.69E-05	1.10E-01
RANBP2	LMNA	0.134446396	-0.003672772	0.267531882	5.64E-02	9.32E-02	1.00E+00
APITD1	LMNA	0.076206055	-0.062502492	0.212026469	2.81E-01	3.64E-01	1.00E+00
LIG3	LMNA	-0.161281156	-0.292812675	-0.023759153	2.18E-02	4.04E-02	1.00E+00
IRAK4	LMNA	0.287058766	0.15515642	0.408905358	3.44E-05	1.23E-04	4.08E-01
PARD3	LMNA	-0.147172745	-0.279544151	-0.009310918	3.66E-02	6.37E-02	1.00E+00
SERPINI2	LMNA	-0.095821814	-0.230819467	0.042795295	1.75E-01	2.45E-01	1.00E+00
TTC9	LMNA	-0.237336907	-0.363478675	-0.102649165	6.71E-04	1.84E-03	1.00E+00
MLPH	LMNA	0.448352937	0.330775473	0.552223734	2.21E-11	2.44E-10	2.62E-07
RETSAT	LMNA	0.264420624	0.131157329	0.388297398	1.43E-04	4.56E-04	1.00E+00
NRG1	LMNA	-0.039085008	-0.176185361	0.099502883	5.81E-01	6.59E-01	1.00E+00
CAST	LMNA	0.734870912	0.664203092	0.79252093	1.43E-35	1.30E-32	1.70E-31
TBC1D9	LMNA	-0.053504457	-0.190150938	0.08517568	4.49E-01	5.36E-01	1.00E+00
TTK	LMNA	-0.077297546	-0.213074825	0.061408747	2.74E-01	3.57E-01	1.00E+00
ZNF557	LMNA	0.251105286	0.117114085	0.376118029	3.13E-04	9.23E-04	1.00E+00
DDX41	LMNA	0.352394797	0.225304512	0.467693244	2.70E-07	1.44E-06	3.20E-03
TGFBI	LMNA	0.637295946	0.547405304	0.712648592	2.05E-24	2.41E-22	2.43E-20
PGM3	LMNA	0.139090601	0.001059953	0.271920297	4.84E-02	8.15E-02	1.00E+00

PSMB10	LMNA	0.354937457	0.228061341	0.469960635	2.18E-07	1.18E-06	2.59E-03
UBE2D2	LMNA	-0.140232059	-0.272998042	-0.002224115	4.65E-02	7.88E-02	1.00E+00
GABARAPL	LMNA	-0.387977242	-0.499286154	-0.264070002	1.16E-08	7.83E-08	1.38E-04
DGCR2	LMNA	-0.16103435	-0.292580996	-0.023505908	2.21E-02	4.08E-02	1.00E+00
UNC45A	LMNA	0.574556555	0.474111154	0.660238674	3.87E-19	1.72E-17	4.59E-15
CISH	LMNA	0.459737477	0.343486617	0.562112745	5.87E-12	7.20E-11	6.96E-08
OGDHL	LMNA	-0.065249762	-0.20148582	0.073462978	3.56E-01	4.43E-01	1.00E+00
SPINT2	LMNA	0.031520061	-0.106996522	0.168836391	6.56E-01	7.27E-01	1.00E+00
CASK	LMNA	-0.210283942	-0.338508084	-0.07439255	2.67E-03	6.26E-03	1.00E+00
GIT2	LMNA	0.319628448	0.189959411	0.438337782	3.54E-06	1.53E-05	4.20E-02
CLDN18	LMNA	0.113276407	-0.025168172	0.247457684	1.08E-01	1.63E-01	1.00E+00
RNF128	LMNA	0.321069389	0.191506723	0.439634071	3.18E-06	1.38E-05	3.77E-02
ABHD6	LMNA	-0.420209026	-0.527650855	-0.29953408	4.79E-10	4.20E-09	5.68E-06
C14orf161	LMNA	0.159005672	0.021424968	0.290676073	2.38E-02	4.36E-02	1.00E+00
UTP11L	LMNA	0.017648235	-0.120696827	0.155320818	8.03E-01	8.51E-01	1.00E+00
GCNT1	LMNA	0.546436652	0.441706261	0.636474483	4.03E-17	1.27E-15	4.78E-13
DUSP9	LMNA	-0.104100479	-0.238720781	0.034445545	1.40E-01	2.03E-01	1.00E+00
TM4SF5	LMNA	0.235714752	0.100948688	0.361986474	7.33E-04	1.99E-03	1.00E+00
SUCLA2	LMNA	-0.267797529	-0.391379346	-0.134727361	1.17E-04	3.77E-04	1.00E+00
NANS	LMNA	0.393877614	0.270537123	0.504496516	6.65E-09	4.71E-08	7.89E-05
OLFML1	LMNA	0.174645174	0.037498254	0.305334576	1.29E-02	2.55E-02	1.00E+00
ATP10B	LMNA	-0.294649627	-0.415787763	-0.163238631	2.08E-05	7.76E-05	2.46E-01
SCNM1	LMNA	-0.196048997	-0.325295941	-0.059611364	5.17E-03	1.12E-02	1.00E+00
NPAS3	LMNA	-0.15177447	-0.283877494	-0.014017166	3.11E-02	5.53E-02	1.00E+00
AMD1	LMNA	0.050687778	-0.087978855	0.187427257	4.74E-01	5.59E-01	1.00E+00
GGA2	LMNA	0.043598343	-0.095024591	0.180562575	5.38E-01	6.20E-01	1.00E+00
PRKCA	LMNA	-0.119325108	-0.253205074	0.019039529	9.08E-02	1.40E-01	1.00E+00
TRIB2	LMNA	0.067789165	-0.070925582	0.203931699	3.38E-01	4.24E-01	1.00E+00
SDCCAG3	LMNA	0.178583652	0.041557188	0.309016273	1.10E-02	2.21E-02	1.00E+00
TTC1	LMNA	0.024649656	-0.113788557	0.162148878	7.28E-01	7.88E-01	1.00E+00
GHSR	LMNA	-0.070255236	-0.206305321	0.068459742	3.20E-01	4.06E-01	1.00E+00
ATP8B1	LMNA	0.450405313	0.333063858	0.554008662	1.75E-11	1.96E-10	2.07E-07
HIPK1	LMNA	-0.01604122	-0.153751736	0.122280572	8.21E-01	8.65E-01	1.00E+00
C1orf78	LMNA	0.616517092	0.52297709	0.69538345	1.55E-22	1.25E-20	1.84E-18
CLN3	LMNA	0.191794588	0.055205281	0.321337398	6.25E-03	1.33E-02	1.00E+00

STX4	LMNA	0.486598396	0.3736473	0.585329646	2.10E-13	3.22E-12	2.49E-09
WAPAL	LMNA	0.045698262	-0.092939062	0.182597313	5.18E-01	6.02E-01	1.00E+00
TUBB1	LMNA	0.062151846	-0.076556015	0.198499694	3.80E-01	4.67E-01	1.00E+00
SCN5A	LMNA	-0.180018515	-0.310356615	-0.04303706	1.04E-02	2.09E-02	1.00E+00
ZNF192	LMNA	-0.21752268	-0.345207372	-0.081932035	1.87E-03	4.58E-03	1.00E+00
SEC22A	LMNA	0.071122832	-0.067591824	0.207140012	3.14E-01	4.00E-01	1.00E+00
DPY19L1	LMNA	0.414149302	0.292841119	0.522336385	8.96E-10	7.48E-09	1.06E-05
C11orf9	LMNA	-0.194148102	-0.323527801	-0.057642043	5.63E-03	1.21E-02	1.00E+00
GRIA2	LMNA	-0.599700753	-0.681343786	-0.50331919	4.09E-21	2.46E-19	4.85E-17
VDAC2	LMNA	-0.094265679	-0.229332271	0.044362633	1.82E-01	2.53E-01	1.00E+00
C8orf44	LMNA	-0.459610215	-0.562002362	-0.343344289	5.96E-12	7.29E-11	7.07E-08
ZPBP	LMNA	-0.118154589	-0.2520936	0.020226331	9.40E-02	1.44E-01	1.00E+00
NUSAP1	LMNA	0.008888108	-0.129321572	0.146759034	9.00E-01	9.28E-01	1.00E+00
LANCL1	LMNA	-0.47594132	-0.57613767	-0.361652391	8.15E-13	1.15E-11	9.67E-09
FGF23	LMNA	-0.012351461	-0.150146442	0.12591424	8.62E-01	8.98E-01	1.00E+00
PCNT	LMNA	-0.104781254	-0.239369734	0.033758063	1.38E-01	2.00E-01	1.00E+00
BCKDHB	LMNA	-0.061907806	-0.198264354	0.076799557	3.81E-01	4.69E-01	1.00E+00
IFNA8	LMNA	-0.004406839	-0.142371226	0.133725516	9.50E-01	9.63E-01	1.00E+00
BET1	LMNA	0.356853063	0.230139644	0.471667855	1.86E-07	1.02E-06	2.20E-03
SPRR1B	LMNA	-0.143870338	-0.276431019	-0.00593726	4.11E-02	7.06E-02	1.00E+00
FLRT1	LMNA	-0.497513148	-0.59471764	-0.385971516	4.98E-14	8.31E-13	5.91E-10
HDAC6	LMNA	0.026373874	-0.112085222	0.163828385	7.09E-01	7.73E-01	1.00E+00
SNX17	LMNA	0.084242778	-0.054441364	0.219738267	2.33E-01	3.11E-01	1.00E+00
N4BP3	LMNA	-0.100665503	-0.235444537	0.037912364	1.54E-01	2.20E-01	1.00E+00
PLSCR3	LMNA	0.49949346	0.388211828	0.596418109	3.82E-14	6.50E-13	4.53E-10
TTC30A	LMNA	-0.008824816	-0.1466971	0.129383809	9.01E-01	9.28E-01	1.00E+00
CRISP1	LMNA	-0.174406615	-0.305111445	-0.037252545	1.30E-02	2.57E-02	1.00E+00
KRT32	LMNA	-0.004890701	-0.142845257	0.133250266	9.45E-01	9.59E-01	1.00E+00
GHRH	LMNA	-0.155734553	-0.287602303	-0.01807209	2.69E-02	4.86E-02	1.00E+00
IL11RA	LMNA	-0.180082259	-0.310416148	-0.043102818	1.03E-02	2.09E-02	1.00E+00
GDF3	LMNA	0.022069061	-0.116336376	0.159633713	7.55E-01	8.11E-01	1.00E+00
RPS6KB1	LMNA	-0.058120914	-0.194610429	0.080576605	4.11E-01	4.99E-01	1.00E+00
POLR3B	LMNA	-0.419634544	-0.527147386	-0.298899055	5.08E-10	4.44E-09	6.03E-06
TOP1	LMNA	0.278552537	0.146120521	0.401176513	5.97E-05	2.04E-04	7.08E-01
DNAJC10	LMNA	0.418232888	0.297350132	0.525918676	5.88E-10	5.08E-09	6.98E-06

CRCT1	LMNA	0.010003427	-0.128224649	0.147850249	8.88E-01	9.18E-01	1.00E+00
ADIPOQ	LMNA	-0.17427077	-0.304984379	-0.037112635	1.31E-02	2.58E-02	1.00E+00
MPST	LMNA	-0.170869891	-0.301801763	-0.03361175	1.50E-02	2.90E-02	1.00E+00
DPM2	LMNA	0.185395175	0.048587729	0.315374485	8.25E-03	1.71E-02	1.00E+00
FAM38B	LMNA	0.006023695	-0.132137188	0.143954986	9.32E-01	9.51E-01	1.00E+00
RIT2	LMNA	-0.33326723	-0.45058765	-0.20463086	1.26E-06	5.92E-06	1.49E-02
SLC18A1	LMNA	-0.097037565	-0.231980918	0.041570318	1.69E-01	2.38E-01	1.00E+00
RPS17	LMNA	0.149708804	0.011903825	0.281932969	3.35E-02	5.90E-02	1.00E+00
ABCA3	LMNA	-0.205459158	-0.334035625	-0.069375925	3.35E-03	7.67E-03	1.00E+00
CD44	LMNA	0.587114054	0.48867063	0.670795917	4.19E-20	2.17E-18	4.97E-16
FARP1	LMNA	-0.040059012	-0.177130448	0.098536916	5.71E-01	6.51E-01	1.00E+00
KCNMA1	LMNA	-0.117475576	-0.251448678	0.02091461	9.59E-02	1.47E-01	1.00E+00
PAX7	LMNA	-0.090143483	-0.225389645	0.048511211	2.02E-01	2.76E-01	1.00E+00
TUBD1	LMNA	-0.374038929	-0.486945779	-0.248836987	4.17E-08	2.55E-07	4.95E-04
NARG2	LMNA	-0.142096758	-0.274757942	-0.004126709	4.37E-02	7.44E-02	1.00E+00
GNL3	LMNA	0.205141376	0.06904575	0.333740846	3.40E-03	7.77E-03	1.00E+00
BTG2	LMNA	-0.068535082	-0.204649821	0.070179915	3.32E-01	4.18E-01	1.00E+00
ITGA5	LMNA	0.769314996	0.706273571	0.820251435	8.83E-41	2.62E-37	1.05E-36
NDUFS6	LMNA	0.322128446	0.19264437	0.440586497	2.94E-06	1.29E-05	3.48E-02
MEFV	LMNA	0.08445395	-0.054229307	0.219940673	2.32E-01	3.10E-01	1.00E+00
TUT1	LMNA	-0.004506843	-0.142469203	0.133627297	9.49E-01	9.62E-01	1.00E+00
ERAL1	LMNA	-0.180900042	-0.311179815	-0.043946537	9.98E-03	2.03E-02	1.00E+00
ECHS1	LMNA	-0.039253883	-0.17634924	0.09933542	5.79E-01	6.58E-01	1.00E+00
NMBR	LMNA	-0.050637849	-0.187378957	0.088028524	4.74E-01	5.60E-01	1.00E+00
VPS4A	LMNA	-0.088851089	-0.224152637	0.049810891	2.09E-01	2.83E-01	1.00E+00
ABCB7	LMNA	0.292819834	0.161288782	0.414130013	2.35E-05	8.69E-05	2.78E-01
CYP11A1	LMNA	0.048810665	-0.089845749	0.185610952	4.90E-01	5.75E-01	1.00E+00
PFKP	LMNA	0.119183894	-0.019182729	0.253071002	9.11E-02	1.41E-01	1.00E+00
C9orf91	LMNA	-0.221031159	-0.348449714	-0.085591873	1.57E-03	3.90E-03	1.00E+00
ABCC6	LMNA	0.013022003	-0.125254167	0.150801907	8.54E-01	8.92E-01	1.00E+00
LRRC41	LMNA	0.366136698	0.240228158	0.479929408	8.38E-08	4.87E-07	9.94E-04
ATP5F1	LMNA	-0.215411241	-0.343254625	-0.079731267	2.08E-03	5.01E-03	1.00E+00
GFOD2	LMNA	-0.155864197	-0.287724177	-0.018204915	2.68E-02	4.83E-02	1.00E+00
MOSC1	LMNA	-0.341796129	-0.458225618	-0.213834996	6.41E-07	3.18E-06	7.60E-03
NCF4	LMNA	0.301242909	0.170273034	0.421754456	1.32E-05	5.12E-05	1.57E-01

HYMAI	LMNA	0.142929221	0.004976413	0.275543333	4.24E-02	7.26E-02	1.00E+00
SLC25A3	LMNA	0.011046647	-0.127198331	0.14887062	8.76E-01	9.08E-01	1.00E+00
NAGPA	LMNA	0.4295832	0.3099113	0.53585561	1.77E-10	1.69E-09	2.10E-06
MTHFD2L	LMNA	-0.502962326	-0.599394685	-0.392139303	2.38E-14	4.25E-13	2.83E-10
ZNF646	LMNA	-0.13172404	-0.264956896	0.006444145	6.17E-02	1.01E-01	1.00E+00
RAB1B	LMNA	0.264388466	0.131123348	0.388268035	1.43E-04	4.56E-04	1.00E+00
IFT122	LMNA	0.214731793	0.079023356	0.342626009	2.15E-03	5.17E-03	1.00E+00
GALNT3	LMNA	-0.093152881	-0.228268385	0.045483025	1.87E-01	2.59E-01	1.00E+00
LDB3	LMNA	-0.26486616	-0.388704177	-0.131628147	1.39E-04	4.45E-04	1.00E+00
GARNL1	LMNA	-0.423806689	-0.530802085	-0.303513323	3.28E-10	2.96E-09	3.89E-06
ALDOAP2	LMNA	0.468507864	0.353308118	0.569711102	2.04E-12	2.70E-11	2.42E-08
LRRC6	LMNA	0.033046222	-0.105486013	0.170320211	6.41E-01	7.14E-01	1.00E+00
NTRK1	LMNA	0.364256182	0.238182405	0.478257556	9.87E-08	5.66E-07	1.17E-03
ARTS-1	LMNA	0.415005733	0.293786326	0.523087999	8.20E-10	6.90E-09	9.73E-06
UBAC1	LMNA	-0.03302178	-0.170296452	0.105510209	6.41E-01	7.14E-01	1.00E+00
SLC6A11	LMNA	-0.107728741	-0.242178055	0.030780022	1.27E-01	1.87E-01	1.00E+00
NAP1L2	LMNA	-0.400216343	-0.510085011	-0.277497133	3.61E-09	2.69E-08	4.28E-05
EPB41L4B	LMNA	-0.240643784	-0.366518628	-0.106118147	5.61E-04	1.56E-03	1.00E+00
RPL19	LMNA	-0.118716694	-0.252627395	0.019656456	9.24E-02	1.42E-01	1.00E+00
CNGB1	LMNA	-0.047540091	-0.184381007	0.091108853	5.02E-01	5.86E-01	1.00E+00
LOC643641	LMNA	-0.026380387	-0.163834728	0.112078787	7.09E-01	7.73E-01	1.00E+00
FAM134B	LMNA	0.026473051	-0.111987222	0.163924966	7.08E-01	7.72E-01	1.00E+00
WISP2	LMNA	0.083942354	-0.054743026	0.219450293	2.35E-01	3.13E-01	1.00E+00
NTSR1	LMNA	0.100857594	-0.037718581	0.235627833	1.53E-01	2.19E-01	1.00E+00
HS3ST3A1	LMNA	0.452540119	0.335445619	0.555864269	1.37E-11	1.57E-10	1.62E-07
GPSM2	LMNA	-0.359478471	-0.474006264	-0.232989912	1.49E-07	8.27E-07	1.76E-03
PRKCG	LMNA	-0.176673369	-0.307231024	-0.039587915	1.19E-02	2.37E-02	1.00E+00
CHORDC1	LMNA	0.066552305	-0.07216169	0.202740603	3.47E-01	4.33E-01	1.00E+00
MON1B	LMNA	0.193323157	0.056787733	0.322760186	5.84E-03	1.25E-02	1.00E+00
TRIB3	LMNA	0.255964323	0.122232548	0.380567539	2.36E-04	7.16E-04	1.00E+00
SLC2A5	LMNA	0.155621627	0.017956396	0.28749614	2.70E-02	4.87E-02	1.00E+00
C2orf49	LMNA	0.008637305	-0.129568191	0.146513605	9.03E-01	9.30E-01	1.00E+00
DDX5	LMNA	-0.060576331	-0.196980062	0.078128025	3.92E-01	4.79E-01	1.00E+00
ZNF446	LMNA	0.007512817	-0.130673712	0.145413003	9.15E-01	9.39E-01	1.00E+00
MLF1IP	LMNA	-0.053954556	-0.19058598	0.084727537	4.46E-01	5.32E-01	1.00E+00

SLC26A1	LMNA	0.068539227	-0.07017577	0.204653812	3.32E-01	4.18E-01	1.00E+00
RGN	LMNA	-0.023320213	-0.160853365	0.115101343	7.42E-01	8.00E-01	1.00E+00
COL4A5	LMNA	0.165633689	0.028228151	0.296895899	1.85E-02	3.48E-02	1.00E+00
CCNB1	LMNA	0.093857398	-0.044773741	0.228941972	1.84E-01	2.55E-01	1.00E+00
CCDC28B	LMNA	-0.401197814	-0.510949489	-0.278575954	3.28E-09	2.46E-08	3.88E-05
RP11-35N6	LMNA	-0.613646534	-0.692991126	-0.519614419	2.75E-22	2.06E-20	3.26E-18
KCNK3	LMNA	-0.37167008	-0.484844012	-0.246254243	5.15E-08	3.12E-07	6.11E-04
ZFP161	LMNA	-0.298254155	-0.419051029	-0.167082664	1.62E-05	6.19E-05	1.93E-01
FGR	LMNA	0.338259004	0.210015076	0.455060067	8.49E-07	4.12E-06	1.01E-02
COX15	LMNA	-0.218270323	-0.345898557	-0.082711625	1.80E-03	4.43E-03	1.00E+00
EPN2	LMNA	-0.228432766	-0.355279884	-0.093324798	1.08E-03	2.80E-03	1.00E+00
AQP9	LMNA	0.303312859	0.172484229	0.423625539	1.14E-05	4.49E-05	1.36E-01
XK	LMNA	-0.125187514	-0.258766445	0.013089707	7.59E-02	1.20E-01	1.00E+00
SLC15A2	LMNA	-0.287022646	-0.408872577	-0.155118005	3.45E-05	1.23E-04	4.09E-01
MREG	LMNA	0.267877382	0.134811822	0.39145219	1.16E-04	3.75E-04	1.00E+00
HEPH	LMNA	0.292757271	0.161222133	0.414073319	2.36E-05	8.72E-05	2.80E-01
THRAP3	LMNA	0.129205711	-0.009005928	0.262573199	6.69E-02	1.08E-01	1.00E+00
MET	LMNA	0.293828118	0.162363095	0.415043595	2.19E-05	8.16E-05	2.60E-01
PDLIM2	LMNA	0.332831348	0.20416108	0.450196839	1.30E-06	6.11E-06	1.54E-02
ING3	LMNA	-0.388826741	-0.500036806	-0.265000412	1.07E-08	7.28E-08	1.27E-04
PHYHIP	LMNA	-0.276478597	-0.399289459	-0.143920786	6.80E-05	2.30E-04	8.07E-01
ADAM7	LMNA	-0.081702509	-0.217302529	0.056991303	2.48E-01	3.28E-01	1.00E+00
COPS7A	LMNA	0.052230903	-0.0864434	0.188919693	4.60E-01	5.46E-01	1.00E+00
WSCD1	LMNA	-0.19130957	-0.320885823	-0.05470331	6.38E-03	1.36E-02	1.00E+00
SLC12A8	LMNA	0.242662115	0.108237028	0.368372723	5.02E-04	1.41E-03	1.00E+00
RNF185	LMNA	-0.061352599	-0.197728878	0.07735357	3.86E-01	4.73E-01	1.00E+00
TNS3	LMNA	-0.038021516	-0.175153153	0.100557301	5.91E-01	6.69E-01	1.00E+00
IGSF3	LMNA	-0.063130806	-0.199443601	0.075578885	3.72E-01	4.60E-01	1.00E+00
SRPK1	LMNA	-0.257663176	-0.382121851	-0.124023783	2.14E-04	6.54E-04	1.00E+00
MED13L	LMNA	-0.182995059	-0.313135428	-0.046108891	9.14E-03	1.87E-02	1.00E+00
C7orf44	LMNA	-0.157632498	-0.289386074	-0.020017096	2.51E-02	4.57E-02	1.00E+00
PTCD3	LMNA	-0.386989521	-0.498413153	-0.262988494	1.28E-08	8.56E-08	1.51E-04
RPL7A	LMNA	-0.079201591	-0.214902874	0.059499974	2.63E-01	3.44E-01	1.00E+00
TEAD1	LMNA	0.235348913	0.100565294	0.361649855	7.47E-04	2.02E-03	1.00E+00
ARL6IP1	LMNA	-0.319355956	-0.43809259	-0.189666877	3.61E-06	1.55E-05	4.28E-02

CTAGE5	LMNA	-0.105458849	-0.240015535	0.033073661	1.35E-01	1.97E-01	1.00E+00
SLC25A14	LMNA	-0.177838169	-0.308319694	-0.040788558	1.13E-02	2.27E-02	1.00E+00
LEP	LMNA	0.129145668	-0.009066985	0.262516346	6.70E-02	1.08E-01	1.00E+00
PCDH21	LMNA	-0.488730254	-0.587165382	-0.376051324	1.59E-13	2.50E-12	1.89E-09
CACNG5	LMNA	-0.141333361	-0.274037556	-0.003347677	4.48E-02	7.62E-02	1.00E+00
ECH1	LMNA	-0.023538941	-0.161066544	0.114885388	7.39E-01	7.98E-01	1.00E+00
MAPKAPK2	LMNA	0.547975655	0.443472741	0.637779535	3.16E-17	1.01E-15	3.75E-13
ATXN10	LMNA	-0.445836418	-0.550033846	-0.327971459	2.95E-11	3.18E-10	3.49E-07
LHFPL2	LMNA	0.412865778	0.291424992	0.521209636	1.02E-09	8.43E-09	1.21E-05
CCL22	LMNA	0.172662723	0.035456882	0.303479897	1.40E-02	2.73E-02	1.00E+00
ACTR8	LMNA	-0.144036865	-0.276588068	-0.006107305	4.08E-02	7.03E-02	1.00E+00
PTPN22	LMNA	0.400425091	0.27772656	0.510268894	3.53E-09	2.64E-08	4.19E-05
CYP2F1	LMNA	-0.050518227	-0.187263237	0.088147523	4.75E-01	5.61E-01	1.00E+00
ITGA3	LMNA	0.597286896	0.500505613	0.679323557	6.44E-21	3.74E-19	7.64E-17
RABGGTA	LMNA	0.285276708	0.153261567	0.407287609	3.87E-05	1.37E-04	4.59E-01
KIAA1033	LMNA	0.210349533	0.074460797	0.338568846	2.66E-03	6.24E-03	1.00E+00
ZNF510	LMNA	-0.410536162	-0.519163603	-0.288856057	1.29E-09	1.05E-08	1.53E-05
SLC26A10	LMNA	-0.26255322	-0.386591913	-0.129184611	1.60E-04	5.03E-04	1.00E+00
STX8	LMNA	-0.232919034	-0.359413208	-0.098019832	8.50E-04	2.27E-03	1.00E+00
RUNX3	LMNA	0.365340308	0.239361655	0.479221488	8.98E-08	5.19E-07	1.07E-03
EFNB1	LMNA	0.457055682	0.340488481	0.559785876	8.06E-12	9.63E-11	9.56E-08
EIF4G3	LMNA	-0.080406382	-0.216059087	0.058291667	2.55E-01	3.36E-01	1.00E+00
ACSM3	LMNA	0.322378548	0.192913083	0.440811379	2.88E-06	1.27E-05	3.42E-02
C5AR1	LMNA	0.506503681	0.396153023	0.602430714	1.46E-14	2.75E-13	1.74E-10
SIGLEC8	LMNA	-0.063964907	-0.200247637	0.074746134	3.66E-01	4.53E-01	1.00E+00
ASCC3L1	LMNA	-0.023775793	-0.161297372	0.114651525	7.37E-01	7.96E-01	1.00E+00
ZNF623	LMNA	-0.169311575	-0.300342481	-0.032008735	1.60E-02	3.06E-02	1.00E+00
A2M	LMNA	0.264577572	0.131323177	0.388440699	1.42E-04	4.52E-04	1.00E+00
NOL8	LMNA	0.180476554	0.043509594	0.310784372	1.02E-02	2.06E-02	1.00E+00
GRPEL1	LMNA	0.309122127	0.178696951	0.428871212	7.59E-06	3.07E-05	9.01E-02
LMNB2	LMNA	0.168073071	0.030735208	0.299182249	1.68E-02	3.20E-02	1.00E+00
ROCK2	LMNA	0.152253178	0.014507098	0.284327974	3.05E-02	5.44E-02	1.00E+00
SNX16	LMNA	-0.140715555	-0.27345445	-0.002717341	4.58E-02	7.76E-02	1.00E+00
Magmas	LMNA	-0.180000856	-0.310340122	-0.043018844	1.04E-02	2.09E-02	1.00E+00
ANXA3	LMNA	-0.081323174	-0.21693866	0.057371926	2.50E-01	3.30E-01	1.00E+00

C11orf2	LMNA	-0.179234773	-0.309624566	-0.042228659	1.07E-02	2.15E-02	1.00E+00
VKORC1	LMNA	0.490224977	0.377737777	0.58845188	1.31E-13	2.07E-12	1.55E-09
TRPM6	LMNA	-0.048436932	-0.185249215	0.09021733	4.94E-01	5.79E-01	1.00E+00
KIAA1609	LMNA	0.434946104	0.315860889	0.540540488	9.91E-11	9.97E-10	1.18E-06
FOXK2	LMNA	0.079106642	-0.059595182	0.214811737	2.63E-01	3.45E-01	1.00E+00
FEV	LMNA	-0.241532112	-0.367334793	-0.107050579	5.34E-04	1.50E-03	1.00E+00
EED	LMNA	-0.211432475	-0.339571893	-0.075587765	2.52E-03	5.96E-03	1.00E+00
RNF32	LMNA	-0.066690788	-0.202873983	0.072023312	3.46E-01	4.32E-01	1.00E+00
PDCD5	LMNA	0.229846417	0.094803584	0.356582856	1.00E-03	2.62E-03	1.00E+00
SLC8A1	LMNA	0.006836352	-0.131338601	0.144750742	9.23E-01	9.45E-01	1.00E+00
HES1	LMNA	0.180453346	0.04348565	0.3107627	1.02E-02	2.06E-02	1.00E+00
DGUOK	LMNA	-0.151775616	-0.283878573	-0.014018339	3.11E-02	5.53E-02	1.00E+00
CLDN16	LMNA	-0.053433816	-0.190082654	0.08524601	4.50E-01	5.36E-01	1.00E+00
CLC	LMNA	-0.098426808	-0.233307643	0.040170019	1.63E-01	2.31E-01	1.00E+00
ISL1	LMNA	-0.258989494	-0.383334833	-0.125422831	1.98E-04	6.08E-04	1.00E+00
C1QTNF3	LMNA	-0.055375417	-0.191958962	0.083312477	4.34E-01	5.20E-01	1.00E+00
GAGE1	LMNA	0.071607866	-0.067106519	0.207606564	3.11E-01	3.96E-01	1.00E+00
KIAA0528	LMNA	-0.356930283	-0.471736657	-0.230223447	1.85E-07	1.01E-06	2.19E-03
VGLL3	LMNA	0.452070383	0.334921415	0.555456055	1.44E-11	1.65E-10	1.71E-07
MANEA	LMNA	0.098720251	-0.039874172	0.233587817	1.62E-01	2.30E-01	1.00E+00
C1orf61	LMNA	-0.549407704	-0.638993428	-0.445117188	2.52E-17	8.12E-16	2.99E-13
SUPT4H1	LMNA	-0.278395943	-0.401034066	-0.145954384	6.03E-05	2.05E-04	7.15E-01
CD1A	LMNA	0.115291126	-0.023127981	0.249373091	1.02E-01	1.55E-01	1.00E+00
ASAHL	LMNA	0.343095457	0.21523919	0.45938771	5.77E-07	2.88E-06	6.85E-03
TRAF5	LMNA	0.173952704	0.036785073	0.30468685	1.33E-02	2.61E-02	1.00E+00
RAPGEF6	LMNA	-0.332214141	-0.449643375	-0.203495974	1.36E-06	6.37E-06	1.62E-02
WHSC1	LMNA	-0.233721396	-0.360151924	-0.098860165	8.15E-04	2.19E-03	1.00E+00
NEIL1	LMNA	-0.107342428	-0.24181011	0.03117048	1.28E-01	1.88E-01	1.00E+00
KIAA0020	LMNA	0.302088938	0.171176633	0.422519328	1.25E-05	4.86E-05	1.48E-01
C16orf45	LMNA	-0.274550545	-0.397534213	-0.141876962	7.68E-05	2.57E-04	9.11E-01
GFPT2	LMNA	0.521392777	0.413074372	0.615164947	1.78E-15	4.04E-14	2.11E-11
RBM10	LMNA	0.031424671	-0.107090912	0.168743627	6.57E-01	7.28E-01	1.00E+00
MRS2L	LMNA	-0.004922542	-0.142876449	0.13321899	9.45E-01	9.59E-01	1.00E+00
DNAH17	LMNA	-0.188208623	-0.317997294	-0.051495601	7.31E-03	1.53E-02	1.00E+00
STARD5	LMNA	0.297015547	0.165761305	0.417930039	1.77E-05	6.69E-05	2.10E-01

C19orf10	LMNA	0.514198385	0.404888649	0.609017885	4.99E-15	1.03E-13	5.92E-11
SIP1	LMNA	-0.271164586	-0.394449548	-0.138290424	9.48E-05	3.11E-04	1.00E+00
DNAJC15	LMNA	0.113274021	-0.025170587	0.247455414	1.08E-01	1.63E-01	1.00E+00
C1orf160	LMNA	0.112649956	-0.025802308	0.246861899	1.10E-01	1.65E-01	1.00E+00
SLFN12	LMNA	0.285135026	0.153110959	0.407158957	3.90E-05	1.38E-04	4.63E-01
STAU2	LMNA	-0.537780415	-0.629124505	-0.431785668	1.55E-16	4.36E-15	1.83E-12
FAM98A	LMNA	0.196074251	0.059637534	0.325319425	5.16E-03	1.12E-02	1.00E+00
EXOC3	LMNA	0.165712719	0.028309346	0.296969994	1.84E-02	3.47E-02	1.00E+00
RAD23B	LMNA	0.078505369	-0.060198042	0.214234551	2.67E-01	3.48E-01	1.00E+00
HIST3H3	LMNA	-0.132580865	-0.265767544	0.005572121	6.00E-02	9.84E-02	1.00E+00
NCOR2	LMNA	0.227421461	0.092267261	0.354347454	1.13E-03	2.93E-03	1.00E+00
TNFRSF9	LMNA	0.420591178	0.299956565	0.527985726	4.60E-10	4.06E-09	5.46E-06
KLK8	LMNA	-0.108267789	-0.242691408	0.03023512	1.25E-01	1.84E-01	1.00E+00
NOL1	LMNA	0.195116729	0.058645404	0.324428893	5.39E-03	1.17E-02	1.00E+00
ALX1	LMNA	-0.144870935	-0.277374561	-0.006959114	3.97E-02	6.85E-02	1.00E+00
CCNE1	LMNA	0.135663	-0.002433578	0.268682013	5.42E-02	9.01E-02	1.00E+00
PKDREJ	LMNA	0.072756353	-0.065957126	0.208711042	3.03E-01	3.88E-01	1.00E+00
TIAL1	LMNA	-0.199541754	-0.328542417	-0.063232618	4.41E-03	9.79E-03	1.00E+00
WHSC1L1	LMNA	-0.270491905	-0.393836393	-0.137578309	9.89E-05	3.23E-04	1.00E+00
HIST1H1E	LMNA	-0.241005555	-0.366851033	-0.10649785	5.50E-04	1.54E-03	1.00E+00
SMUG1	LMNA	-0.036326777	-0.173507646	0.102236936	6.08E-01	6.84E-01	1.00E+00
TM4SF4	LMNA	0.160068996	0.022515537	0.291674662	2.29E-02	4.21E-02	1.00E+00
NPY6R	LMNA	-0.099034932	-0.233888243	0.039556885	1.61E-01	2.28E-01	1.00E+00
UFM1	LMNA	0.180364005	0.043393477	0.310679269	1.02E-02	2.07E-02	1.00E+00
AP3M2	LMNA	-0.375013759	-0.487810319	-0.249900356	3.82E-08	2.34E-07	4.53E-04
USP14	LMNA	-0.019874887	-0.157493764	0.118501252	7.79E-01	8.31E-01	1.00E+00
CORO2A	LMNA	0.123347431	-0.014958283	0.257021803	8.03E-02	1.26E-01	1.00E+00
ETNK2	LMNA	0.009201411	-0.129013471	0.1470656	8.97E-01	9.25E-01	1.00E+00
APOE	LMNA	-0.143941355	-0.276497996	-0.006009776	4.10E-02	7.05E-02	1.00E+00
ANGPT4	LMNA	0.117723972	-0.02066284	0.251684616	9.52E-02	1.46E-01	1.00E+00
DSTN	LMNA	0.182954718	0.046067242	0.313097782	9.16E-03	1.87E-02	1.00E+00
SFRS14	LMNA	-0.24086446	-0.366721395	-0.106349757	5.54E-04	1.55E-03	1.00E+00
FRS2	LMNA	0.085106306	-0.053574138	0.220565876	2.28E-01	3.06E-01	1.00E+00
NR2E3	LMNA	-0.008401375	-0.146282715	0.12980017	9.06E-01	9.32E-01	1.00E+00
ST8SIA4	LMNA	0.380199451	0.255562118	0.492405601	2.39E-08	1.52E-07	2.83E-04

GMPR	LMNA	0.018118919	-0.120232824	0.155780261	7.98E-01	8.48E-01	1.00E+00
TUBB2C	LMNA	0.222438348	0.087060796	0.349749305	1.46E-03	3.67E-03	1.00E+00
LOC730092	LMNA	-0.193230935	-0.322674364	-0.056692241	5.86E-03	1.26E-02	1.00E+00
ORM1	LMNA	0.281216337	0.148947817	0.403598747	5.03E-05	1.74E-04	5.97E-01
HSPD1	LMNA	0.093948897	-0.044681612	0.229029446	1.84E-01	2.55E-01	1.00E+00
C5orf13	LMNA	-0.251503958	-0.376483317	-0.117533774	3.06E-04	9.05E-04	1.00E+00
F2RL1	LMNA	0.322448873	0.192988644	0.440874609	2.87E-06	1.26E-05	3.40E-02
PLEKHA9	LMNA	0.213117859	0.077342358	0.341132359	2.32E-03	5.55E-03	1.00E+00
ZNF232	LMNA	-0.383133825	-0.495003117	-0.258769672	1.83E-08	1.19E-07	2.16E-04
SLC6A7	LMNA	-0.037900091	-0.175035282	0.10067767	5.92E-01	6.70E-01	1.00E+00
ADH5	LMNA	-0.219602619	-0.347129904	-0.084101263	1.69E-03	4.17E-03	1.00E+00
SHBG	LMNA	0.039133685	-0.099454614	0.176232599	5.80E-01	6.59E-01	1.00E+00
DSCR6	LMNA	-0.089704107	-0.224969148	0.048953116	2.04E-01	2.79E-01	1.00E+00
CROCCL2	LMNA	-0.283634016	-0.405795696	-0.15151576	4.30E-05	1.51E-04	5.11E-01
CDIPT	LMNA	0.236402831	0.101669894	0.36261951	7.06E-04	1.92E-03	1.00E+00
SLC16A5	LMNA	0.300978453	0.169990627	0.421515333	1.35E-05	5.21E-05	1.60E-01
TUBB	LMNA	0.085188742	-0.053491337	0.220644873	2.28E-01	3.05E-01	1.00E+00
GAS2L1	LMNA	0.155720139	0.018057322	0.287588752	2.69E-02	4.86E-02	1.00E+00
TOR3A	LMNA	0.177102466	0.04003017	0.307632116	1.17E-02	2.33E-02	1.00E+00
PREP	LMNA	-0.01428308	-0.152034301	0.124012447	8.40E-01	8.81E-01	1.00E+00
RFX3	LMNA	-0.243340667	-0.368995835	-0.108949658	4.84E-04	1.37E-03	1.00E+00
CHMP1B	LMNA	0.045923461	-0.092715334	0.182815452	5.16E-01	6.00E-01	1.00E+00
COPS4	LMNA	-0.240091489	-0.366011099	-0.10553855	5.78E-04	1.61E-03	1.00E+00
MYH6	LMNA	-0.031511464	-0.168828031	0.107005028	6.56E-01	7.27E-01	1.00E+00
BCHE	LMNA	-0.378088199	-0.490535477	-0.25325601	2.90E-08	1.82E-07	3.44E-04
MAP3K13	LMNA	-0.45301653	-0.556278232	-0.335977347	1.29E-11	1.49E-10	1.53E-07
LCMT1	LMNA	-0.179548219	-0.309917357	-0.042551946	1.06E-02	2.13E-02	1.00E+00
EIF1AX	LMNA	-0.126128454	-0.259658244	0.012133823	7.37E-02	1.17E-01	1.00E+00
SLC24A5	LMNA	-0.305226112	-0.425354066	-0.174529209	1.00E-05	3.96E-05	1.19E-01
BCL2	LMNA	-0.19475978	-0.324096859	-0.058275623	5.48E-03	1.18E-02	1.00E+00
HBZ	LMNA	-0.205082427	-0.33368616	-0.068984504	3.41E-03	7.79E-03	1.00E+00
TJAP1	LMNA	-0.101128871	-0.235886672	0.037444896	1.52E-01	2.18E-01	1.00E+00
MAPBPIP	LMNA	0.226155523	0.090943881	0.353179896	1.21E-03	3.10E-03	1.00E+00
TMEM156	LMNA	-0.206452283	-0.334956701	-0.070407976	3.20E-03	7.36E-03	1.00E+00
MYO15B	LMNA	0.009745191	-0.128478656	0.147597624	8.91E-01	9.21E-01	1.00E+00

ZNF239	LMNA	-0.396082659	-0.50644163	-0.27295683	5.38E-09	3.89E-08	6.39E-05
KIAA1324	LMNA	-0.222849866	-0.350129264	-0.087490479	1.43E-03	3.61E-03	1.00E+00
PLCL2	LMNA	-0.227694348	-0.354599083	-0.092552594	1.12E-03	2.90E-03	1.00E+00
C4orf29	LMNA	-0.32596857	-0.444037745	-0.196772383	2.20E-06	9.89E-06	2.61E-02
SNX19	LMNA	0.142835306	0.004880543	0.275454737	4.26E-02	7.28E-02	1.00E+00
NAALADL1	LMNA	0.239165965	0.104567479	0.365160427	6.08E-04	1.68E-03	1.00E+00
DUSP5	LMNA	0.521424445	0.413110442	0.615191979	1.77E-15	4.03E-14	2.10E-11
GKN1	LMNA	0.053856598	-0.084825073	0.190491304	4.47E-01	5.33E-01	1.00E+00
PXDN	LMNA	0.362952625	0.236764964	0.477098159	1.10E-07	6.28E-07	1.31E-03
FCN1	LMNA	0.225745574	0.090515433	0.35280172	1.24E-03	3.16E-03	1.00E+00
SLMO1	LMNA	-0.138175974	-0.271056482	-0.000127403	4.99E-02	8.37E-02	1.00E+00
TNXB	LMNA	0.213202849	0.077430861	0.341211031	2.32E-03	5.53E-03	1.00E+00
C1QL1	LMNA	-0.316983627	-0.435957182	-0.187121025	4.30E-06	1.82E-05	5.10E-02
BIRC7	LMNA	0.041421582	-0.097185151	0.178452142	5.58E-01	6.39E-01	1.00E+00
A4GALT	LMNA	0.327772303	0.198712922	0.445657611	1.92E-06	8.73E-06	2.28E-02
TIMM22	LMNA	0.025696015	-0.112754968	0.163168199	7.17E-01	7.79E-01	1.00E+00
ABHD9	LMNA	0.097844552	-0.040756975	0.23275165	1.66E-01	2.34E-01	1.00E+00
TOMM34	LMNA	0.357695904	0.231054435	0.472418736	1.73E-07	9.54E-07	2.05E-03
ZNF35	LMNA	-0.19793785	-0.327051985	-0.061569261	4.75E-03	1.04E-02	1.00E+00
LIMD1	LMNA	0.021171714	-0.1172219	0.158758699	7.65E-01	8.20E-01	1.00E+00
CARD8	LMNA	0.095684077	-0.042934052	0.230687856	1.76E-01	2.45E-01	1.00E+00
KIAA0286	LMNA	0.051474299	-0.087196326	0.188188021	4.67E-01	5.53E-01	1.00E+00
CD6	LMNA	0.221438841	0.086017379	0.348826274	1.54E-03	3.84E-03	1.00E+00
RSRC1	LMNA	-0.097254838	-0.232188447	0.041351351	1.69E-01	2.37E-01	1.00E+00
TOX3	LMNA	-0.499920686	-0.596784848	-0.388695318	3.60E-14	6.19E-13	4.27E-10
C16orf35	LMNA	-0.020051666	-0.157666223	0.118326881	7.77E-01	8.30E-01	1.00E+00
CRHBP	LMNA	-0.184753386	-0.314775905	-0.047924725	8.48E-03	1.75E-02	1.00E+00
PTRF	LMNA	0.80366019	0.748671352	0.847665964	5.77E-47	3.42E-43	6.84E-43
FBXO24	LMNA	0.229142326	0.094066977	0.355933952	1.04E-03	2.71E-03	1.00E+00
CHST11	LMNA	0.197084771	0.060684869	0.326258998	4.93E-03	1.08E-02	1.00E+00
THRB	LMNA	0.077685949	-0.061019462	0.213447802	2.72E-01	3.54E-01	1.00E+00
RNF39	LMNA	0.146597303	0.008722834	0.27900189	3.74E-02	6.49E-02	1.00E+00
MYBPC1	LMNA	-0.215325373	-0.343175188	-0.079641795	2.09E-03	5.03E-03	1.00E+00
PSMD11	LMNA	0.090766363	-0.047884651	0.225985675	1.99E-01	2.73E-01	1.00E+00
ALAD	LMNA	0.122711256	-0.015604084	0.256418423	8.19E-02	1.28E-01	1.00E+00

AQP2	LMNA	0.060612911	-0.078091534	0.197015351	3.91E-01	4.79E-01	1.00E+00
EN1	LMNA	0.047227025	-0.09142001	0.184077886	5.04E-01	5.88E-01	1.00E+00
GSTM4	LMNA	0.063482569	-0.075227714	0.199782708	3.69E-01	4.57E-01	1.00E+00
ZNF187	LMNA	-0.463437636	-0.565320542	-0.347627134	3.77E-12	4.78E-11	4.48E-08
CDC42BPA	LMNA	0.108803143	-0.029693871	0.243201169	1.23E-01	1.82E-01	1.00E+00
F7	LMNA	-0.253395862	-0.37821627	-0.119526068	2.74E-04	8.20E-04	1.00E+00
CNOT1	LMNA	0.038137468	-0.100442353	0.175265709	5.90E-01	6.68E-01	1.00E+00
SLC13A4	LMNA	-0.074076035	-0.209979726	0.064635951	2.95E-01	3.79E-01	1.00E+00
ZBTB11	LMNA	-0.182491262	-0.312665255	-0.045588784	9.34E-03	1.91E-02	1.00E+00
B3GALT5	LMNA	0.056554192	-0.082138091	0.19309761	4.24E-01	5.11E-01	1.00E+00
LUC7L2	LMNA	-0.045102147	-0.18201982	0.093531216	5.24E-01	6.07E-01	1.00E+00
SPTBN1	LMNA	-0.113162408	-0.247349273	0.025283577	1.09E-01	1.63E-01	1.00E+00
EXOC2	LMNA	-0.113708544	-0.247868608	0.024730668	1.07E-01	1.61E-01	1.00E+00
LSM4	LMNA	-0.132007393	-0.265224999	0.006155789	6.11E-02	9.99E-02	1.00E+00
IRS1	LMNA	0.251563588	0.117596551	0.37653795	3.04E-04	9.02E-04	1.00E+00
TMEM1	LMNA	0.05664858	-0.082044037	0.193188769	4.23E-01	5.10E-01	1.00E+00
MRPL34	LMNA	0.013012744	-0.125263283	0.150792857	8.54E-01	8.92E-01	1.00E+00
SAMM50	LMNA	-0.118233213	-0.25216827	0.020146625	9.38E-02	1.44E-01	1.00E+00
CDC42EP3	LMNA	0.34320387	0.215356377	0.459484654	5.72E-07	2.85E-06	6.79E-03
HSF2	LMNA	-0.485665524	-0.584526034	-0.37259581	2.37E-13	3.59E-12	2.81E-09
SRP72	LMNA	-0.042227027	-0.179233191	0.096385853	5.51E-01	6.32E-01	1.00E+00
MFN2	LMNA	-0.008094339	-0.145982215	0.130102041	9.09E-01	9.35E-01	1.00E+00
TSPAN7	LMNA	-0.496738106	-0.594051884	-0.385095074	5.53E-14	9.12E-13	6.55E-10
SGK269	LMNA	-0.102441156	-0.237138519	0.036120667	1.47E-01	2.11E-01	1.00E+00
NUCB1	LMNA	0.499861733	0.388628597	0.596734244	3.63E-14	6.23E-13	4.31E-10
RHOH	LMNA	0.309417245	0.179012842	0.429137483	7.44E-06	3.02E-05	8.82E-02
MTX1	LMNA	0.211963674	0.076140686	0.340063796	2.46E-03	5.83E-03	1.00E+00
CENTA1	LMNA	-0.227030978	-0.353987359	-0.091859009	1.16E-03	2.98E-03	1.00E+00
ATR	LMNA	0.110006731	-0.028476732	0.244346947	1.19E-01	1.77E-01	1.00E+00
DDX49	LMNA	-0.002866407	-0.140861667	0.135238109	9.68E-01	9.76E-01	1.00E+00
TACR1	LMNA	0.033385808	-0.105149822	0.170650291	6.37E-01	7.11E-01	1.00E+00
SFRS5	LMNA	0.023017809	-0.11539989	0.160558614	7.45E-01	8.03E-01	1.00E+00
THAP1	LMNA	-0.128718725	-0.26211206	0.009501108	6.79E-02	1.09E-01	1.00E+00
RHBDF1	LMNA	0.415941412	0.294819266	0.523908968	7.45E-10	6.32E-09	8.84E-06
RASIP1	LMNA	0.032008576	-0.106513086	0.169311421	6.51E-01	7.23E-01	1.00E+00

DPYD	LMNA	0.524042644	0.416093786	0.617426187	1.21E-15	2.83E-14	1.43E-11
MYO5C	LMNA	0.079984919	-0.058714406	0.215654661	2.58E-01	3.39E-01	1.00E+00
DOHH	LMNA	0.146059103	0.008172898	0.278494648	3.81E-02	6.59E-02	1.00E+00
POF1B	LMNA	0.246814628	0.112600256	0.372184194	3.99E-04	1.15E-03	1.00E+00
MAPK10	LMNA	-0.516369988	-0.610874556	-0.407357637	3.66E-15	7.76E-14	4.35E-11
ZNF552	LMNA	0.001596143	-0.136484932	0.13961638	9.82E-01	9.86E-01	1.00E+00
USP32	LMNA	0.097676513	-0.040926352	0.232591175	1.67E-01	2.34E-01	1.00E+00
MED27	LMNA	-0.134968221	-0.268025242	0.00314131	5.55E-02	9.18E-02	1.00E+00
NCAM1	LMNA	-0.519234534	-0.613322088	-0.4106169	2.43E-15	5.30E-14	2.88E-11
PRDX4	LMNA	0.393128228	0.269715139	0.503835213	7.15E-09	5.04E-08	8.48E-05
AGT	LMNA	-0.208214365	-0.336590345	-0.072239841	2.94E-03	6.83E-03	1.00E+00
SLC22A14	LMNA	-0.331610448	-0.449101941	-0.202845544	1.43E-06	6.64E-06	1.69E-02
DAPP1	LMNA	0.027242491	-0.111226818	0.164674176	7.00E-01	7.65E-01	1.00E+00
PILRA	LMNA	0.189271707	0.052594968	0.318987827	6.98E-03	1.47E-02	1.00E+00
ATF7	LMNA	0.280272553	0.14794586	0.402740746	5.35E-05	1.84E-04	6.34E-01
ABCF2	LMNA	0.376529852	0.251554749	0.489154447	3.33E-08	2.07E-07	3.96E-04
KIAA0748	LMNA	-0.166041572	-0.297278298	-0.028647229	1.82E-02	3.43E-02	1.00E+00
C17orf85	LMNA	-0.096483306	-0.231451462	0.042128835	1.72E-01	2.41E-01	1.00E+00
TKTL1	LMNA	-0.233422298	-0.359876571	-0.098546891	8.28E-04	2.22E-03	1.00E+00
FGF1	LMNA	0.080242625	-0.058455926	0.215901955	2.56E-01	3.38E-01	1.00E+00
IL6R	LMNA	0.344340362	0.216585064	0.460500754	5.22E-07	2.62E-06	6.20E-03
CHRNB2	LMNA	-0.404947137	-0.514249844	-0.282700016	2.26E-09	1.75E-08	2.68E-05
COL7A1	LMNA	0.260238388	0.126740695	0.384476616	1.84E-04	5.69E-04	1.00E+00
NFIL3	LMNA	0.172918542	0.03572024	0.303719285	1.39E-02	2.70E-02	1.00E+00
LRRC48	LMNA	-0.01970456	-0.157327593	0.118669248	7.81E-01	8.33E-01	1.00E+00
TM6SF1	LMNA	-0.144169829	-0.27671346	-0.006243083	4.07E-02	7.00E-02	1.00E+00
SPG20	LMNA	-0.120544132	-0.25436223	0.017803135	8.75E-02	1.36E-01	1.00E+00
COX10	LMNA	-0.034093676	-0.171338245	0.104448931	6.30E-01	7.04E-01	1.00E+00
DNAJA3	LMNA	-0.035878241	-0.173072011	0.102681343	6.12E-01	6.88E-01	1.00E+00
ECEL1	LMNA	-0.179247519	-0.309636472	-0.042241804	1.07E-02	2.15E-02	1.00E+00
GCA	LMNA	0.005237294	-0.132909802	0.14318477	9.41E-01	9.56E-01	1.00E+00
GLG1	LMNA	0.49855298	0.387147706	0.595610639	4.33E-14	7.29E-13	5.14E-10
CIITA	LMNA	0.321490551	0.191959097	0.44001286	3.08E-06	1.34E-05	3.65E-02
CLDN6	LMNA	0.247284859	0.113094676	0.372615539	3.88E-04	1.12E-03	1.00E+00
EPHA4	LMNA	-0.044463304	-0.18140083	0.094165705	5.30E-01	6.12E-01	1.00E+00

FANCC	LMNA	-0.113823895	-0.247978288	0.024613876	1.07E-01	1.61E-01	1.00E+00
MUTYH	LMNA	-0.053691431	-0.190331663	0.084989526	4.48E-01	5.34E-01	1.00E+00
L2HGDH	LMNA	-0.380615573	-0.492774075	-0.256016813	2.30E-08	1.48E-07	2.73E-04
GPATCH2	LMNA	0.18560204	0.048801457	0.3155674	8.18E-03	1.70E-02	1.00E+00
PSG3	LMNA	-0.175954224	-0.306558712	-0.038846839	1.23E-02	2.43E-02	1.00E+00
ZNF227	LMNA	-0.125448104	-0.259013449	0.012825003	7.53E-02	1.20E-01	1.00E+00
MRPL15	LMNA	0.096527276	-0.04208453	0.231493467	1.72E-01	2.41E-01	1.00E+00
NQO2	LMNA	0.176617804	0.03953065	0.307179083	1.19E-02	2.37E-02	1.00E+00
C21orf59	LMNA	0.335052393	0.206555475	0.452187757	1.09E-06	5.20E-06	1.30E-02
ZBTB20	LMNA	-0.125476986	-0.259040823	0.012795664	7.52E-02	1.19E-01	1.00E+00
NEU1	LMNA	0.349562127	0.222235641	0.465165452	3.41E-07	1.78E-06	4.05E-03
QRICH1	LMNA	-0.128944729	-0.262326076	0.00927131	6.74E-02	1.09E-01	1.00E+00
C2orf34	LMNA	-0.117910238	-0.251861531	0.020474031	9.47E-02	1.45E-01	1.00E+00
UBE2L6	LMNA	0.116197697	-0.022209572	0.250234632	9.96E-02	1.51E-01	1.00E+00
HP1BP3	LMNA	-0.185890931	-0.315836792	-0.049099954	8.08E-03	1.68E-02	1.00E+00
MED14	LMNA	0.192320824	0.055749991	0.321827284	6.11E-03	1.31E-02	1.00E+00
TSN	LMNA	-0.104166326	-0.238783556	0.034379055	1.40E-01	2.03E-01	1.00E+00
TPBG	LMNA	0.418817423	0.297996004	0.526431141	5.54E-10	4.80E-09	6.57E-06
SPRY2	LMNA	0.317991461	0.188202363	0.436864528	3.99E-06	1.71E-05	4.74E-02
LZTFL1	LMNA	-0.129287008	-0.262650176	0.008923256	6.67E-02	1.08E-01	1.00E+00
OSR2	LMNA	0.198481047	0.062132509	0.327556825	4.63E-03	1.02E-02	1.00E+00
KLHL7	LMNA	-0.23582434	-0.362087304	-0.101063542	7.28E-04	1.98E-03	1.00E+00
XPC	LMNA	0.224323062	0.089029117	0.351489139	1.33E-03	3.38E-03	1.00E+00
GMFB	LMNA	-0.191139525	-0.320727488	-0.054527337	6.43E-03	1.37E-02	1.00E+00
PBEF1	LMNA	0.480777178	0.367090633	0.580311854	4.43E-13	6.51E-12	5.25E-09
CCR3	LMNA	0.132925147	-0.005221673	0.266093218	5.93E-02	9.74E-02	1.00E+00
AGTPBP1	LMNA	-0.237424156	-0.363558916	-0.102740649	6.68E-04	1.83E-03	1.00E+00
TMEM8	LMNA	0.43294536	0.313640175	0.538793462	1.23E-10	1.21E-09	1.46E-06
PCSK6	LMNA	-0.239504366	-0.365471484	-0.104922504	5.97E-04	1.66E-03	1.00E+00
STAT5A	LMNA	0.504575726	0.393967384	0.600778209	1.91E-14	3.52E-13	2.27E-10
FAM18B	LMNA	0.252926794	0.11903201	0.377786694	2.82E-04	8.40E-04	1.00E+00
PTPN2	LMNA	0.341317208	0.213317557	0.45779718	6.66E-07	3.29E-06	7.90E-03
SF3A3	LMNA	0.08195108	-0.056741865	0.217540947	2.46E-01	3.26E-01	1.00E+00
EFCBP2	LMNA	-0.453508401	-0.556705575	-0.336526406	1.22E-11	1.41E-10	1.45E-07
HCFC1	LMNA	0.017218101	-0.121120801	0.154900906	8.08E-01	8.55E-01	1.00E+00

NFYA	LMNA	0.263871099	0.130576703	0.38779561	1.48E-04	4.69E-04	1.00E+00
PBLD	LMNA	-0.229502186	-0.35626562	-0.094443437	1.02E-03	2.66E-03	1.00E+00
PIGF	LMNA	-0.23429925	-0.360683843	-0.099465485	7.90E-04	2.13E-03	1.00E+00
AHNAK	LMNA	0.56863869	0.467268825	0.655251686	1.07E-18	4.52E-17	1.27E-14
ACTR5	LMNA	-0.056135967	-0.192693664	0.082554803	4.27E-01	5.14E-01	1.00E+00
KIF14	LMNA	-0.036787331	-0.173954897	0.101780565	6.03E-01	6.80E-01	1.00E+00
PTGER1	LMNA	0.260627729	0.127151634	0.384832488	1.80E-04	5.58E-04	1.00E+00
NOS2A	LMNA	0.173327476	0.036141265	0.304101916	1.36E-02	2.67E-02	1.00E+00
TENC1	LMNA	0.085420331	-0.053258715	0.220866791	2.27E-01	3.04E-01	1.00E+00
YIPF2	LMNA	0.441419487	0.323054939	0.546186741	4.84E-11	5.12E-10	5.75E-07
ETV2	LMNA	-0.017426429	-0.15510429	0.120915462	8.06E-01	8.53E-01	1.00E+00
KIAA1012	LMNA	-0.347844369	-0.463631656	-0.220375876	3.93E-07	2.03E-06	4.66E-03
C17orf53	LMNA	0.027108144	-0.111359599	0.164543373	7.02E-01	7.66E-01	1.00E+00
AHR	LMNA	0.260608653	0.127131499	0.384815053	1.80E-04	5.59E-04	1.00E+00
PTPRH	LMNA	0.424526215	0.304309668	0.531431971	3.04E-10	2.76E-09	3.61E-06
ATP10A	LMNA	0.099228783	-0.039361415	0.2340733	1.60E-01	2.27E-01	1.00E+00
ATP6V1C1	LMNA	-0.09083599	-0.226052295	0.047814606	1.99E-01	2.72E-01	1.00E+00
DPH4	LMNA	-0.235486644	-0.361776589	-0.10070963	7.42E-04	2.01E-03	1.00E+00
TAS2R3	LMNA	-0.212502	-0.340562228	-0.076701112	2.40E-03	5.70E-03	1.00E+00
C5orf5	LMNA	-0.214771511	-0.342662759	-0.079064734	2.14E-03	5.16E-03	1.00E+00
KCNA4	LMNA	-0.286211809	-0.408136587	-0.154255734	3.64E-05	1.30E-04	4.32E-01
COQ10B	LMNA	0.219755908	0.084261183	0.34727155	1.68E-03	4.14E-03	1.00E+00
NMNAT2	LMNA	-0.389452502	-0.500589647	-0.265685921	1.01E-08	6.91E-08	1.20E-04
PSMF1	LMNA	0.271164407	0.138290235	0.394449385	9.48E-05	3.11E-04	1.00E+00
SORBS2	LMNA	-0.253688161	-0.378483934	-0.119833975	2.69E-04	8.07E-04	1.00E+00
NFE2L2	LMNA	0.28664246	0.154713677	0.408527505	3.54E-05	1.26E-04	4.20E-01
SH3PXD2A	LMNA	0.259465129	0.125924678	0.383769721	1.92E-04	5.93E-04	1.00E+00
NRF1	LMNA	-0.040981918	-0.178025721	0.097621385	5.63E-01	6.43E-01	1.00E+00
SPINK5	LMNA	-0.12008053	-0.253922202	0.018273393	8.87E-02	1.37E-01	1.00E+00
SH2D2A	LMNA	0.3829998	0.25862311	0.494884521	1.85E-08	1.20E-07	2.19E-04
PRDM12	LMNA	0.162179722	0.024681313	0.293656036	2.11E-02	3.92E-02	1.00E+00
RBBP6	LMNA	-0.025272679	-0.162755838	0.113173174	7.21E-01	7.83E-01	1.00E+00
OPA3	LMNA	0.385549225	0.261411998	0.497139737	1.46E-08	9.70E-08	1.73E-04
PAQR4	LMNA	-0.214415186	-0.342333049	-0.078693532	2.18E-03	5.24E-03	1.00E+00
IFI27	LMNA	0.008727475	-0.129479527	0.146601845	9.02E-01	9.29E-01	1.00E+00

SKAP2	LMNA	0.089302356	-0.049357133	0.224584615	2.06E-01	2.81E-01	1.00E+00
TJP3	LMNA	0.105091471	-0.033444746	0.23966541	1.37E-01	1.98E-01	1.00E+00
C9orf61	LMNA	-0.287269411	-0.409096531	-0.155380462	3.39E-05	1.22E-04	4.03E-01
MT1G	LMNA	0.394776811	0.27152367	0.505289851	6.10E-09	4.36E-08	7.24E-05
HS1BP3	LMNA	0.291950539	0.160362811	0.41334217	2.49E-05	9.17E-05	2.95E-01
IDS	LMNA	0.320547118	0.190945824	0.439164287	3.31E-06	1.43E-05	3.92E-02
PARG	LMNA	-0.360294525	-0.474732783	-0.233876303	1.39E-07	7.76E-07	1.65E-03
OR2B2	LMNA	0.074468569	-0.064242878	0.210357003	2.92E-01	3.76E-01	1.00E+00
DYRK4	LMNA	-0.079326036	-0.215022318	0.059375185	2.62E-01	3.43E-01	1.00E+00
MICALL1	LMNA	0.399236548	0.276420463	0.509221788	3.97E-09	2.94E-08	4.70E-05
GALR2	LMNA	-0.067821033	-0.203962382	0.070893729	3.38E-01	4.24E-01	1.00E+00
CHRM4	LMNA	-0.045391912	-0.182300545	0.093243389	5.21E-01	6.04E-01	1.00E+00
RIC3	LMNA	-0.5188535	-0.612996628	-0.410183202	2.57E-15	5.58E-14	3.05E-11
GPBP1L1	LMNA	0.11412852	-0.024305427	0.248267921	1.06E-01	1.60E-01	1.00E+00
ART1	LMNA	-0.274990424	-0.397934746	-0.142343154	7.47E-05	2.50E-04	8.86E-01
TBX21	LMNA	-0.060079291	-0.196500515	0.078623816	3.96E-01	4.83E-01	1.00E+00
DUS4L	LMNA	0.073550434	-0.065162205	0.209474491	2.98E-01	3.82E-01	1.00E+00
KCNJ6	LMNA	0.036608099	-0.101958176	0.173780849	6.05E-01	6.82E-01	1.00E+00
TNFAIP6	LMNA	0.42535752	0.305229936	0.532159563	2.79E-10	2.56E-09	3.30E-06
CCT4	LMNA	-0.0666982	-0.202881121	0.072015906	3.46E-01	4.32E-01	1.00E+00
RTEL1	LMNA	0.226354083	0.09115142	0.353363051	1.20E-03	3.07E-03	1.00E+00
CASP5	LMNA	0.411446767	0.289859999	0.519963508	1.18E-09	9.61E-09	1.40E-05
CHMP6	LMNA	0.015753525	-0.122564027	0.153470759	8.24E-01	8.67E-01	1.00E+00
BRD4	LMNA	0.331464363	0.202688167	0.448970909	1.45E-06	6.71E-06	1.71E-02
NDUFA13	LMNA	-0.169991632	-0.300979394	-0.032708212	1.56E-02	2.99E-02	1.00E+00
CYP19A1	LMNA	0.220908159	0.085463504	0.348336096	1.58E-03	3.93E-03	1.00E+00
CD151	LMNA	0.705002251	0.628079924	0.768279478	1.13E-31	5.61E-29	1.35E-27
DOK4	LMNA	-0.112100327	-0.246339092	0.026358587	1.12E-01	1.68E-01	1.00E+00
FAM26B	LMNA	0.536105005	0.4298685	0.627700031	2.00E-16	5.51E-15	2.37E-12
ARFRP1	LMNA	0.420387559	0.29973145	0.527807303	4.70E-10	4.14E-09	5.58E-06
MRPL41	LMNA	0.012671853	-0.125598866	0.150459645	8.58E-01	8.95E-01	1.00E+00
CRYBA1	LMNA	0.067079922	-0.071634446	0.203248747	3.43E-01	4.29E-01	1.00E+00
HRH2	LMNA	0.118547094	-0.019828409	0.252466345	9.29E-02	1.43E-01	1.00E+00
MTMR6	LMNA	-0.172446117	-0.303277192	-0.035233907	1.41E-02	2.75E-02	1.00E+00
SCAMP3	LMNA	0.275223813	0.142590528	0.39814724	7.36E-05	2.47E-04	8.73E-01

MTG1	LMNA	-0.030340236	-0.167688872	0.108163809	6.68E-01	7.38E-01	1.00E+00
UBTD1	LMNA	0.449562641	0.332124118	0.553275915	1.93E-11	2.14E-10	2.28E-07
SOX9	LMNA	0.001983518	-0.136104752	0.139996183	9.78E-01	9.83E-01	1.00E+00
CRABP1	LMNA	-0.221228133	-0.348631657	-0.085797453	1.56E-03	3.87E-03	1.00E+00
FLJ33790	LMNA	-0.365330798	-0.479213033	-0.239351309	8.99E-08	5.20E-07	1.07E-03
FAU	LMNA	-0.144139323	-0.276684691	-0.006211931	4.07E-02	7.00E-02	1.00E+00
DTNB	LMNA	-0.391506895	-0.502404005	-0.267937351	8.34E-09	5.79E-08	9.89E-05
CARD9	LMNA	0.320794173	0.191211141	0.439386521	3.25E-06	1.41E-05	3.85E-02
PACSIN3	LMNA	0.360275271	0.233855386	0.474715643	1.39E-07	7.77E-07	1.65E-03
OMD	LMNA	0.160627938	0.023088931	0.29219946	2.24E-02	4.13E-02	1.00E+00
HOXB8	LMNA	0.132316499	-0.005841199	0.265517445	6.05E-02	9.91E-02	1.00E+00
NSBP1	LMNA	-0.350007042	-0.465562606	-0.222717488	3.29E-07	1.72E-06	3.90E-03
SLC4A5	LMNA	-0.207753792	-0.336163418	-0.07176094	3.01E-03	6.97E-03	1.00E+00
FBXO46	LMNA	-0.030653361	-0.16799346	0.107854048	6.65E-01	7.35E-01	1.00E+00
UGCGL2	LMNA	0.080490446	-0.058207342	0.216139748	2.55E-01	3.36E-01	1.00E+00
SVIL	LMNA	0.568226842	0.466793091	0.65490434	1.14E-18	4.81E-17	1.36E-14
OAS1	LMNA	0.166130807	0.02873892	0.297361953	1.81E-02	3.42E-02	1.00E+00
PHB2	LMNA	-0.104008116	-0.238632727	0.034538807	1.41E-01	2.04E-01	1.00E+00
NDRG2	LMNA	-0.497390288	-0.594612113	-0.385832568	5.06E-14	8.42E-13	6.01E-10
ERMAP	LMNA	0.271249455	0.138380278	0.394526899	9.43E-05	3.10E-04	1.00E+00
APBA2	LMNA	-0.523807538	-0.617225623	-0.415825796	1.25E-15	2.92E-14	1.48E-11
TCF21	LMNA	0.197424242	0.061036775	0.326574578	4.86E-03	1.06E-02	1.00E+00
JPH2	LMNA	0.40755565	0.285571915	0.51654409	1.75E-09	1.38E-08	2.07E-05
DLST	LMNA	0.244639319	0.110313918	0.370188068	4.50E-04	1.28E-03	1.00E+00
SLA	LMNA	0.306106784	0.175470894	0.426149415	9.41E-06	3.74E-05	1.12E-01
AMELX	LMNA	0.189516827	0.052848501	0.319216178	6.91E-03	1.46E-02	1.00E+00
WNT6	LMNA	-0.094928823	-0.229966114	0.043694799	1.79E-01	2.49E-01	1.00E+00
ATP2B2	LMNA	-0.554310928	-0.643146327	-0.450752978	1.15E-17	3.92E-16	1.36E-13
CPVL	LMNA	0.343306487	0.215467302	0.459576413	5.68E-07	2.84E-06	6.73E-03
RGS20	LMNA	0.044798734	-0.093832576	0.181725849	5.27E-01	6.10E-01	1.00E+00
TRAM2	LMNA	0.684173064	0.603083795	0.751266372	3.14E-29	7.76E-27	3.72E-25
ZNRF4	LMNA	0.051393612	-0.087276611	0.188109983	4.68E-01	5.53E-01	1.00E+00
ZNF419	LMNA	-0.12917738	-0.262546373	0.009034738	6.69E-02	1.08E-01	1.00E+00
LXN	LMNA	0.106906075	-0.031611464	0.241394457	1.30E-01	1.90E-01	1.00E+00
TLK1	LMNA	0.213562958	0.077805876	0.34154435	2.28E-03	5.44E-03	1.00E+00

UPK3B	LMNA	-0.16149565	-0.29301401	-0.023979258	2.17E-02	4.02E-02	1.00E+00
MTMR12	LMNA	0.127866601	-0.010367406	0.261305016	6.98E-02	1.12E-01	1.00E+00
KLHL21	LMNA	0.442619513	0.324390064	0.547232389	4.23E-11	4.49E-10	5.02E-07
ZNF384	LMNA	-0.032634303	-0.169919781	0.10589377	6.45E-01	7.17E-01	1.00E+00
PI15	LMNA	0.223154927	0.087809038	0.350410902	1.41E-03	3.56E-03	1.00E+00
RER1	LMNA	0.484721479	0.371532021	0.5837126	2.68E-13	4.03E-12	3.17E-09
ELAVL2	LMNA	-0.451909643	-0.555316356	-0.334742053	1.47E-11	1.68E-10	1.74E-07
KLF2	LMNA	0.133646695	-0.004487093	0.266775669	5.79E-02	9.53E-02	1.00E+00
RPN1	LMNA	0.373859388	0.248641172	0.486786527	4.24E-08	2.59E-07	5.03E-04
PMVK	LMNA	-0.043565883	-0.180531113	0.095056819	5.38E-01	6.20E-01	1.00E+00
SIX2	LMNA	0.043922864	-0.094702373	0.1808771	5.35E-01	6.17E-01	1.00E+00
HPS1	LMNA	0.380552186	0.255947546	0.492717949	2.31E-08	1.48E-07	2.74E-04
RNF7	LMNA	0.320370464	0.190756123	0.439005371	3.35E-06	1.45E-05	3.97E-02
TFE3	LMNA	0.466764448	0.351353733	0.568202043	2.53E-12	3.30E-11	3.00E-08
C11orf17	LMNA	0.231184298	0.096203659	0.357815536	9.32E-04	2.46E-03	1.00E+00
SNRPC	LMNA	-0.033870395	-0.171121259	0.104670026	6.32E-01	7.06E-01	1.00E+00
KCTD13	LMNA	-0.10480284	-0.239390309	0.033736261	1.38E-01	2.00E-01	1.00E+00
DLGAP1	LMNA	-0.488492584	-0.586960775	-0.375783236	1.64E-13	2.57E-12	1.95E-09
PGLYRP1	LMNA	-0.09015822	-0.225403748	0.048496387	2.02E-01	2.76E-01	1.00E+00
IL8	LMNA	0.32794295	0.198896564	0.445810823	1.89E-06	8.62E-06	2.25E-02
IRF7	LMNA	0.29872403	0.167584054	0.419476189	1.57E-05	6.00E-05	1.86E-01
SERPINB13	LMNA	-0.064566269	-0.200827209	0.074145624	3.61E-01	4.48E-01	1.00E+00
SET	LMNA	-0.032029544	-0.169331809	0.106492334	6.51E-01	7.23E-01	1.00E+00
NAB2	LMNA	0.449907998	0.332509228	0.55357624	1.85E-11	2.07E-10	2.19E-07
BLR1	LMNA	-0.02003917	-0.157654032	0.118339208	7.77E-01	8.30E-01	1.00E+00
LRP5L	LMNA	0.025083434	-0.113360109	0.162571483	7.23E-01	7.84E-01	1.00E+00
FAM120A	LMNA	0.463791074	0.348022872	0.565626789	3.62E-12	4.60E-11	4.29E-08
ASCL2	LMNA	0.278408869	0.145968097	0.401045824	6.02E-05	2.05E-04	7.14E-01
SHH	LMNA	0.084330994	-0.054352781	0.219822822	2.33E-01	3.11E-01	1.00E+00
SH2B1	LMNA	-0.111716112	-0.24597358	0.026747401	1.13E-01	1.69E-01	1.00E+00
ATP5H	LMNA	0.027320956	-0.111149265	0.164750569	7.00E-01	7.64E-01	1.00E+00
THPO	LMNA	-0.137409099	-0.270332045	0.000654314	5.12E-02	8.56E-02	1.00E+00
HIST1H3E	LMNA	-0.028169639	-0.165576737	0.110310342	6.91E-01	7.57E-01	1.00E+00
TYRP1	LMNA	-0.172081971	-0.302936388	-0.034859085	1.43E-02	2.78E-02	1.00E+00
EIF2S1	LMNA	0.085387652	-0.053291542	0.220835477	2.27E-01	3.04E-01	1.00E+00

RFNG	LMNA	0.36992155	0.244348972	0.483291786	6.01E-08	3.60E-07	7.13E-04
RAB20	LMNA	0.465424424	0.349852237	0.567041688	2.97E-12	3.85E-11	3.52E-08
TNFRSF17	LMNA	0.005233428	-0.1329136	0.143180983	9.41E-01	9.56E-01	1.00E+00
RBM7	LMNA	0.0908498	-0.047800713	0.226065508	1.98E-01	2.72E-01	1.00E+00
TMPRSS4	LMNA	-0.097067247	-0.23200927	0.041540405	1.69E-01	2.38E-01	1.00E+00
NKX2-8	LMNA	-0.025683241	-0.163155757	0.112767588	7.17E-01	7.79E-01	1.00E+00
C1orf115	LMNA	-0.283183099	-0.405386054	-0.151036682	4.43E-05	1.55E-04	5.26E-01
TAF9	LMNA	0.028657592	-0.10982791	0.166051658	6.86E-01	7.53E-01	1.00E+00
TERF2	LMNA	-0.329990909	-0.447649002	-0.201101188	1.62E-06	7.48E-06	1.92E-02
TNFRSF1A	LMNA	0.620831451	0.528036566	0.698975754	6.48E-23	5.96E-21	7.69E-19
ACADVL	LMNA	0.205385387	0.069299275	0.333967196	3.36E-03	7.69E-03	1.00E+00
ISCU	LMNA	-0.143394942	-0.275982642	-0.005451866	4.18E-02	7.16E-02	1.00E+00
KIAA0841	LMNA	-0.023166513	-0.160703558	0.115253086	7.43E-01	8.02E-01	1.00E+00
GTF2H5	LMNA	-0.053141332	-0.18979992	0.085537188	4.53E-01	5.39E-01	1.00E+00
EDG8	LMNA	0.004810904	-0.133328647	0.142767086	9.46E-01	9.59E-01	1.00E+00
RAB15	LMNA	-0.043137527	-0.180115905	0.095482086	5.42E-01	6.24E-01	1.00E+00
HBP1	LMNA	0.101179751	-0.037393562	0.235935217	1.52E-01	2.17E-01	1.00E+00
TNNT2	LMNA	-0.279568122	-0.402100203	-0.147198185	5.59E-05	1.92E-04	6.63E-01
CECR5	LMNA	-0.277467124	-0.400189038	-0.14496911	6.39E-05	2.17E-04	7.58E-01
JRK	LMNA	-0.282483324	-0.404750237	-0.150293328	4.64E-05	1.62E-04	5.50E-01
PHGDH	LMNA	-0.415402323	-0.523435993	-0.294224106	7.88E-10	6.64E-09	9.34E-06
XPO4	LMNA	0.021007307	-0.117384117	0.15859836	7.67E-01	8.21E-01	1.00E+00
ARHGAP25	LMNA	0.165993508	0.028597843	0.297233239	1.82E-02	3.44E-02	1.00E+00
CA9	LMNA	0.479649013	0.365821241	0.579338519	5.11E-13	7.45E-12	6.06E-09
TLX1	LMNA	-0.271051832	-0.394346779	-0.13817105	9.55E-05	3.13E-04	1.00E+00
GPS1	LMNA	-0.248293931	-0.373540983	-0.114155877	3.67E-04	1.07E-03	1.00E+00
RPS29	LMNA	-0.127669715	-0.261118519	0.010567538	7.02E-02	1.12E-01	1.00E+00
MKLN1	LMNA	0.017202371	-0.121136305	0.154885548	8.08E-01	8.55E-01	1.00E+00
ATP6V0A2	LMNA	0.134769597	-0.003343612	0.267837461	5.58E-02	9.24E-02	1.00E+00
KIAA0319L	LMNA	0.160617779	0.023078508	0.292189922	2.24E-02	4.13E-02	1.00E+00
DOPEY2	LMNA	0.019830689	-0.118544845	0.157450646	7.79E-01	8.32E-01	1.00E+00
SLC29A3	LMNA	-0.109899224	-0.244244619	0.028585467	1.19E-01	1.77E-01	1.00E+00
LGALS4	LMNA	-0.165619548	-0.29688264	-0.028213622	1.85E-02	3.48E-02	1.00E+00
SDHD	LMNA	0.002020145	-0.136068802	0.140032094	9.77E-01	9.82E-01	1.00E+00
USH2A	LMNA	0.12400286	-0.014292815	0.257643337	7.87E-02	1.24E-01	1.00E+00

NF1	LMNA	-0.464006375	-0.565813329	-0.34826396	3.52E-12	4.50E-11	4.18E-08
IMPAD1	LMNA	0.35374664	0.226769968	0.468898925	2.41E-07	1.29E-06	2.86E-03
APOBEC3A	LMNA	0.352575854	0.225500752	0.46785475	2.66E-07	1.42E-06	3.15E-03
OLR1	LMNA	0.022639313	-0.115773524	0.16018966	7.49E-01	8.07E-01	1.00E+00
HCFC1R1	LMNA	0.001942272	-0.136145233	0.139955746	9.78E-01	9.83E-01	1.00E+00
TAOK2	LMNA	0.078548899	-0.060154401	0.21427634	2.66E-01	3.48E-01	1.00E+00
NRAP	LMNA	0.03619687	-0.102365653	0.173381481	6.09E-01	6.85E-01	1.00E+00
PPFIBP1	LMNA	0.363205322	0.237039695	0.47732294	1.08E-07	6.15E-07	1.28E-03
LYPD3	LMNA	0.09647565	-0.042136549	0.231444148	1.72E-01	2.41E-01	1.00E+00
BCL7A	LMNA	-0.600189951	-0.681753059	-0.503889646	3.73E-21	2.27E-19	4.42E-17
AGER	LMNA	-0.16500436	-0.296305806	-0.027581642	1.89E-02	3.55E-02	1.00E+00
MCM10	LMNA	-0.171478038	-0.302371094	-0.034237533	1.47E-02	2.84E-02	1.00E+00
MAP4K3	LMNA	-0.259665224	-0.383952657	-0.126135819	1.90E-04	5.86E-04	1.00E+00
PFTK1	LMNA	0.022950668	-0.115466171	0.160493169	7.46E-01	8.04E-01	1.00E+00
CBS	LMNA	-0.335552192	-0.45263561	-0.207094495	1.05E-06	5.02E-06	1.25E-02
CLK3	LMNA	0.268785141	0.135772108	0.392280171	1.10E-04	3.56E-04	1.00E+00
GABRE	LMNA	0.099983909	-0.038599881	0.234794075	1.57E-01	2.23E-01	1.00E+00
FIS1	LMNA	-0.078524533	-0.214252948	0.06017883	2.67E-01	3.48E-01	1.00E+00
ELF4	LMNA	0.615803614	0.522141026	0.694789	1.79E-22	1.42E-20	2.12E-18
C11orf49	LMNA	-0.243036934	-0.368716931	-0.108630654	4.92E-04	1.39E-03	1.00E+00
CLIP2	LMNA	0.010904783	-0.127337914	0.148731881	8.78E-01	9.10E-01	1.00E+00
CLPS	LMNA	0.03134426	-0.107170479	0.168665427	6.58E-01	7.28E-01	1.00E+00
PPCDC	LMNA	0.262764951	0.129408229	0.386785327	1.58E-04	4.97E-04	1.00E+00
KDELR1	LMNA	0.495144528	0.38329364	0.592682593	6.84E-14	1.11E-12	8.11E-10
NT5E	LMNA	0.420408668	0.299754787	0.527825801	4.69E-10	4.13E-09	5.56E-06
FOXN2	LMNA	-0.324004277	-0.442272807	-0.194660258	2.55E-06	1.13E-05	3.03E-02
NCSTN	LMNA	0.474837785	0.360412482	0.575184395	9.35E-13	1.31E-11	1.11E-08
PARP4	LMNA	0.414103804	0.292790911	0.522296451	9.00E-10	7.50E-09	1.07E-05
CD83	LMNA	0.119193446	-0.019173043	0.253080071	9.11E-02	1.41E-01	1.00E+00
NPY	LMNA	-0.283952835	-0.406085303	-0.151854528	4.22E-05	1.48E-04	5.00E-01
IL18	LMNA	-0.007965048	-0.145855669	0.13022915	9.10E-01	9.36E-01	1.00E+00
BEGAIN	LMNA	-0.21485109	-0.34273639	-0.079147641	2.14E-03	5.14E-03	1.00E+00
VPS16	LMNA	0.200225748	0.063942197	0.329177824	4.28E-03	9.52E-03	1.00E+00
SLC16A2	LMNA	0.081252728	-0.057442607	0.216871081	2.50E-01	3.31E-01	1.00E+00
SLC35B1	LMNA	0.040040589	-0.098555189	0.177112575	5.72E-01	6.51E-01	1.00E+00

C4orf20	LMNA	-0.026691043	-0.164137243	0.111771806	7.06E-01	7.70E-01	1.00E+00
IGFBP2	LMNA	0.503372299	0.392603744	0.5997463	2.25E-14	4.05E-13	2.67E-10
NOTCH2	LMNA	0.261707081	0.128291099	0.385818861	1.68E-04	5.26E-04	1.00E+00
SIGLEC1	LMNA	0.280788051	0.148493099	0.403209416	5.17E-05	1.79E-04	6.13E-01
SLC9A2	LMNA	-0.045155212	-0.182071231	0.093478507	5.23E-01	6.06E-01	1.00E+00
CD93	LMNA	0.506218378	0.395829508	0.602186224	1.52E-14	2.86E-13	1.81E-10
CEP164	LMNA	0.113758693	-0.024679893	0.247916292	1.07E-01	1.61E-01	1.00E+00
P53AIP1	LMNA	-0.274451335	-0.397443871	-0.141771826	7.73E-05	2.58E-04	9.17E-01
ADD1	LMNA	-0.220681742	-0.348126939	-0.085227218	1.60E-03	3.97E-03	1.00E+00
ZNF136	LMNA	-0.077859248	-0.213614207	0.060845756	2.71E-01	3.53E-01	1.00E+00
MGP	LMNA	0.323847869	0.194492129	0.442132233	2.58E-06	1.14E-05	3.06E-02
DNAJC1	LMNA	0.325365633	0.196123941	0.443496096	2.30E-06	1.03E-05	2.73E-02
CCDC144A	LMNA	-0.324144319	-0.442398666	-0.194810799	2.53E-06	1.12E-05	3.00E-02
ACLY	LMNA	0.081744874	-0.056948792	0.217343165	2.47E-01	3.27E-01	1.00E+00
TRPC1	LMNA	-0.134199642	-0.267298563	0.003924058	5.69E-02	9.38E-02	1.00E+00
CYP4F12	LMNA	-0.099906361	-0.234720062	0.038678095	1.57E-01	2.24E-01	1.00E+00
FKBP5	LMNA	0.367895118	0.2421421	0.481491965	7.19E-08	4.25E-07	8.52E-04
SMS	LMNA	0.524204754	0.416278581	0.617564472	1.18E-15	2.79E-14	1.40E-11
MAPK7	LMNA	0.245484299	0.111201858	0.370963584	4.29E-04	1.23E-03	1.00E+00
RRAGC	LMNA	0.308536347	0.178070016	0.428342628	7.92E-06	3.19E-05	9.39E-02
PARD6A	LMNA	-0.430714845	-0.536844727	-0.311165964	1.57E-10	1.52E-09	1.86E-06
CCDC85B	LMNA	0.093255372	-0.045379848	0.228366385	1.87E-01	2.59E-01	1.00E+00
SYNGR4	LMNA	0.119912009	-0.018444318	0.253762237	8.92E-02	1.38E-01	1.00E+00
WIF1	LMNA	-0.233305946	-0.359769449	-0.098425031	8.33E-04	2.23E-03	1.00E+00
GCH1	LMNA	0.226025165	0.090807635	0.353059645	1.22E-03	3.12E-03	1.00E+00
STRN3	LMNA	0.094933037	-0.043690554	0.229970141	1.79E-01	2.49E-01	1.00E+00
TMOD2	LMNA	-0.396474334	-0.506787017	-0.273386798	5.18E-09	3.76E-08	6.15E-05
FLI1	LMNA	0.20688759	0.070860437	0.335360351	3.13E-03	7.23E-03	1.00E+00
DGKQ	LMNA	0.028099252	-0.110379927	0.165508224	6.91E-01	7.58E-01	1.00E+00
MAB21L2	LMNA	0.11168828	-0.026775565	0.245947101	1.14E-01	1.69E-01	1.00E+00
SCNN1B	LMNA	0.313392387	0.183270423	0.432722044	5.59E-06	2.32E-05	6.63E-02
ECHDC3	LMNA	0.050376103	-0.0882889	0.187125743	4.76E-01	5.62E-01	1.00E+00
TMEM106C	LMNA	0.073349031	-0.065363838	0.209280873	3.00E-01	3.84E-01	1.00E+00
CSNK2A2	LMNA	0.144617812	0.006700586	0.277135896	4.00E-02	6.90E-02	1.00E+00
GPRIN2	LMNA	-0.186247191	-0.316168978	-0.049468095	7.96E-03	1.66E-02	1.00E+00

CPA4	LMNA	0.421813983	0.301308745	0.529057018	4.05E-10	3.59E-09	4.80E-06
RPL39	LMNA	0.042273942	-0.096339291	0.179278679	5.50E-01	6.31E-01	1.00E+00
HERC3	LMNA	-0.013533722	-0.151302038	0.124750355	8.48E-01	8.87E-01	1.00E+00
MELK	LMNA	0.138572036	0.000531198	0.271430568	4.92E-02	8.28E-02	1.00E+00
IL15RA	LMNA	0.447199797	0.329490337	0.551220443	2.52E-11	2.76E-10	2.99E-07
HMBOX1	LMNA	-0.15073318	-0.282897406	-0.012951689	3.23E-02	5.71E-02	1.00E+00
CUL3	LMNA	-0.411966693	-0.520420144	-0.290433338	1.12E-09	9.14E-09	1.33E-05
PODXL	LMNA	0.25706441	0.123392357	0.381574109	2.21E-04	6.75E-04	1.00E+00
CCT6B	LMNA	-0.118374483	-0.252302429	0.020003408	9.34E-02	1.43E-01	1.00E+00
GPX2	LMNA	-0.01392731	-0.151686666	0.1243628	8.44E-01	8.84E-01	1.00E+00
ITK	LMNA	0.250293584	0.116259738	0.375374175	3.27E-04	9.64E-04	1.00E+00
CLIC5	LMNA	-0.155102064	-0.287007658	-0.017424149	2.75E-02	4.96E-02	1.00E+00
RABAC1	LMNA	0.02817322	-0.110306802	0.165580222	6.91E-01	7.57E-01	1.00E+00
YPEL1	LMNA	-0.527374883	-0.620267535	-0.419894118	7.41E-16	1.83E-14	8.78E-12
DNAJC4	LMNA	0.338625484	0.210410675	0.455388186	8.25E-07	4.01E-06	9.79E-03
NR1D2	LMNA	-0.268447289	-0.391972035	-0.135414677	1.12E-04	3.63E-04	1.00E+00
KIAA0776	LMNA	-0.081749737	-0.217347829	0.056943912	2.47E-01	3.27E-01	1.00E+00
FBXL5	LMNA	-0.285801223	-0.407763842	-0.153819181	3.74E-05	1.33E-04	4.43E-01
C13orf27	LMNA	-0.114695543	-0.248806975	0.023731214	1.04E-01	1.57E-01	1.00E+00
DEFA5	LMNA	0.258561999	0.124971836	0.382943915	2.03E-04	6.22E-04	1.00E+00
TRHDE	LMNA	-0.287375057	-0.409192407	-0.155492832	3.37E-05	1.21E-04	4.00E-01
ZNF536	LMNA	-0.351941581	-0.467288934	-0.224813336	2.80E-07	1.49E-06	3.32E-03
MEF2B	LMNA	-0.410447164	-0.519085413	-0.288757951	1.30E-09	1.05E-08	1.55E-05
UQCRQ	LMNA	-0.063571026	-0.199867977	0.075139401	3.69E-01	4.56E-01	1.00E+00
ITGB2	LMNA	0.367495995	0.241707592	0.481137362	7.44E-08	4.37E-07	8.83E-04
PTPN4	LMNA	-0.553345111	-0.642328719	-0.44964221	1.34E-17	4.54E-16	1.59E-13
STX5	LMNA	0.068112123	-0.070602752	0.204242641	3.35E-01	4.21E-01	1.00E+00
CD72	LMNA	0.384978328	0.260787296	0.496634853	1.54E-08	1.02E-07	1.83E-04
ERH	LMNA	-0.158784172	-0.290468023	-0.021197835	2.40E-02	4.39E-02	1.00E+00
XRCC1	LMNA	0.107272534	-0.031241119	0.241743535	1.29E-01	1.89E-01	1.00E+00
VEGFA	LMNA	0.361309301	0.234978835	0.475636001	1.27E-07	7.17E-07	1.51E-03
MPHOSPH1	LMNA	-0.001119005	-0.13914851	0.136953152	9.87E-01	9.90E-01	1.00E+00
MORC1	LMNA	-0.200933476	-0.329835157	-0.064676542	4.14E-03	9.25E-03	1.00E+00
PARVB	LMNA	0.528788244	0.421507159	0.62147195	6.01E-16	1.52E-14	7.12E-12
ELL	LMNA	0.504263594	0.393613651	0.600510594	1.99E-14	3.65E-13	2.36E-10

SETBP1	LMNA	-0.093759587	-0.228848464	0.044872222	1.84E-01	2.56E-01	1.00E+00
CDH11	LMNA	0.317900039	0.188104261	0.436782232	4.02E-06	1.72E-05	4.77E-02
NDC80	LMNA	0.030872124	-0.10763762	0.168206242	6.63E-01	7.33E-01	1.00E+00
GIMAP6	LMNA	0.22361158	0.088285949	0.350832452	1.38E-03	3.49E-03	1.00E+00
AREG	LMNA	0.365065085	0.239062248	0.478976804	9.20E-08	5.30E-07	1.09E-03
BAT2D1	LMNA	0.112982397	-0.025465803	0.247178078	1.09E-01	1.64E-01	1.00E+00
CDS2	LMNA	0.133318533	-0.004821201	0.266465304	5.86E-02	9.63E-02	1.00E+00
LIPT1	LMNA	-0.214085932	-0.342028361	-0.078350565	2.22E-03	5.32E-03	1.00E+00
SENP3	LMNA	-0.272299909	-0.395484156	-0.139492614	8.84E-05	2.91E-04	1.00E+00
IL1F9	LMNA	-0.058989168	-0.195448531	0.07971096	4.04E-01	4.92E-01	1.00E+00
GRIN2A	LMNA	-0.228370072	-0.355222086	-0.093259228	1.08E-03	2.81E-03	1.00E+00
MAN2C1	LMNA	0.067239647	-0.071474818	0.203402564	3.42E-01	4.28E-01	1.00E+00
NSUN5	LMNA	0.366575165	0.240705314	0.480319104	8.07E-08	4.71E-07	9.57E-04
SF3B5	LMNA	-0.049922449	-0.186686833	0.088740135	4.80E-01	5.66E-01	1.00E+00
CEP72	LMNA	-0.09778916	-0.232698753	0.040812809	1.66E-01	2.34E-01	1.00E+00
MYC	LMNA	0.222659278	0.087291472	0.349953297	1.45E-03	3.63E-03	1.00E+00
NRXN1	LMNA	-0.706206992	-0.769260773	-0.62953055	8.07E-32	4.16E-29	9.58E-28
DECR2	LMNA	0.002229422	-0.135863394	0.140237261	9.75E-01	9.81E-01	1.00E+00
SLC37A1	LMNA	-0.107334044	-0.241802124	0.031178953	1.28E-01	1.88E-01	1.00E+00
SUPT16H	LMNA	0.038213276	-0.100367199	0.175339295	5.89E-01	6.67E-01	1.00E+00
MUS81	LMNA	0.016329317	-0.121996698	0.154033084	8.18E-01	8.62E-01	1.00E+00
PHYH	LMNA	0.025217636	-0.113227545	0.162702219	7.22E-01	7.83E-01	1.00E+00
ULBP1	LMNA	-0.150882394	-0.283037867	-0.01310435	3.21E-02	5.69E-02	1.00E+00
OIP5	LMNA	-0.055543933	-0.192121764	0.083144612	4.32E-01	5.18E-01	1.00E+00
IL10RB	LMNA	0.496233329	0.384524365	0.593618213	5.91E-14	9.69E-13	7.01E-10
OTUB2	LMNA	-0.167020396	-0.298195797	-0.029653117	1.75E-02	3.32E-02	1.00E+00
NELL2	LMNA	-0.160710259	-0.292276746	-0.023173388	2.23E-02	4.12E-02	1.00E+00
MAPK8IP2	LMNA	-0.448952118	-0.552744934	-0.331443414	2.06E-11	2.28E-10	2.45E-07
POU3F2	LMNA	0.040707032	-0.097894099	0.17775909	5.65E-01	6.45E-01	1.00E+00
RPLP2	LMNA	0.080579562	-0.058117948	0.216225254	2.54E-01	3.35E-01	1.00E+00
FGF22	LMNA	0.254235794	0.120410916	0.378985354	2.61E-04	7.84E-04	1.00E+00
PSCD4	LMNA	0.317218001	0.187372464	0.436168209	4.23E-06	1.80E-05	5.01E-02
TRAPPC6A	LMNA	-0.091929266	-0.22709818	0.046714586	1.93E-01	2.66E-01	1.00E+00
C21orf2	LMNA	-0.007244652	-0.145150483	0.130937303	9.18E-01	9.42E-01	1.00E+00
CEMP1	LMNA	0.338547472	0.210326461	0.455318343	8.30E-07	4.03E-06	9.85E-03

IL1RAPL1	LMNA	-0.518495901	-0.612691156	-0.409776222	2.70E-15	5.84E-14	3.21E-11
LIN7B	LMNA	-0.312936569	-0.432311204	-0.18278197	5.77E-06	2.38E-05	6.85E-02
VCP	LMNA	0.173740248	0.03656629	0.304488097	1.34E-02	2.63E-02	1.00E+00
NKX2-1	LMNA	-0.260888114	-0.385070468	-0.127426488	1.77E-04	5.50E-04	1.00E+00
BGN	LMNA	0.498148538	0.386690184	0.595263336	4.57E-14	7.67E-13	5.42E-10
LAMA3	LMNA	0.206282779	0.070231808	0.334799512	3.23E-03	7.41E-03	1.00E+00
APOBEC3F	LMNA	0.553433487	0.449743836	0.642403543	1.32E-17	4.49E-16	1.57E-13
COCH	LMNA	-0.230706013	-0.35737491	-0.095703078	9.56E-04	2.52E-03	1.00E+00
SCGB1D2	LMNA	-0.138798328	-0.271644285	-0.000761927	4.88E-02	8.23E-02	1.00E+00
SP4	LMNA	-0.297741226	-0.418586853	-0.166535409	1.68E-05	6.39E-05	1.99E-01
SLC11A1	LMNA	0.397090919	0.274063765	0.507330663	4.88E-09	3.57E-08	5.79E-05
ICAM2	LMNA	-0.080837376	-0.216472611	0.057859317	2.53E-01	3.34E-01	1.00E+00
C21orf25	LMNA	0.356021093	0.229236871	0.470926495	1.99E-07	1.09E-06	2.36E-03
SH3GL1	LMNA	0.374228975	0.24904427	0.487114341	4.10E-08	2.51E-07	4.86E-04
RALB	LMNA	0.326244434	0.197069105	0.444285538	2.15E-06	9.71E-06	2.56E-02
GSK3B	LMNA	-0.193371784	-0.322805439	-0.056838085	5.83E-03	1.25E-02	1.00E+00
GNGT1	LMNA	-0.080307018	-0.215963744	0.058391336	2.56E-01	3.37E-01	1.00E+00
GPD1L	LMNA	-0.157221549	-0.288999924	-0.019595868	2.54E-02	4.63E-02	1.00E+00
VPS37B	LMNA	-0.070912881	-0.206938042	0.067801873	3.16E-01	4.01E-01	1.00E+00
TNFAIP3	LMNA	0.50183506	0.390862561	0.598427689	2.78E-14	4.85E-13	3.29E-10
C6orf32	LMNA	0.040280391	-0.098317328	0.17734522	5.69E-01	6.48E-01	1.00E+00
ATG3	LMNA	0.038868719	-0.099717352	0.175975459	5.83E-01	6.61E-01	1.00E+00
KLHDC2	LMNA	-0.398203287	-0.508311219	-0.275285373	4.39E-09	3.22E-08	5.20E-05
NDUFV2	LMNA	-0.032991709	-0.170267221	0.105539977	6.41E-01	7.14E-01	1.00E+00
PANK3	LMNA	-0.099142465	-0.233990898	0.039448456	1.60E-01	2.27E-01	1.00E+00
BLK	LMNA	-0.075688871	-0.211529614	0.063020628	2.84E-01	3.67E-01	1.00E+00
MATN4	LMNA	-0.122095547	-0.255834354	0.016229	8.34E-02	1.30E-01	1.00E+00
GPM6A	LMNA	-0.604036724	-0.684969569	-0.50837831	1.79E-21	1.11E-19	2.12E-17
WDR82	LMNA	-0.075134009	-0.210996485	0.063576427	2.88E-01	3.71E-01	1.00E+00
APOM	LMNA	-0.188099078	-0.317895209	-0.051382336	7.35E-03	1.54E-02	1.00E+00
PKP2	LMNA	-0.076457682	-0.21226818	0.062250375	2.79E-01	3.62E-01	1.00E+00
C1orf135	LMNA	-0.13173783	-0.264969944	0.006430112	6.16E-02	1.01E-01	1.00E+00
TRIP10	LMNA	0.635245726	0.544988177	0.710949114	3.19E-24	3.62E-22	3.78E-20
HRASLS	LMNA	-0.403222818	-0.512732409	-0.280802793	2.68E-09	2.05E-08	3.18E-05
TESK1	LMNA	0.105045259	-0.033491421	0.239621365	1.37E-01	1.99E-01	1.00E+00

FSD1	LMNA	-0.471916827	-0.572659846	-0.357132505	1.34E-12	1.83E-11	1.59E-08
SFXN3	LMNA	0.365157899	0.239163215	0.479059321	9.13E-08	5.26E-07	1.08E-03
MMP1	LMNA	0.470379211	0.355407036	0.571330135	1.62E-12	2.18E-11	1.93E-08
CA12	LMNA	0.652697288	0.565610953	0.725386927	6.66E-26	1.08E-23	7.90E-22
ZNF611	LMNA	-0.070727719	-0.206759909	0.06798711	3.17E-01	4.03E-01	1.00E+00
C19orf58	LMNA	0.005618038	-0.132535754	0.143557698	9.37E-01	9.53E-01	1.00E+00
NCOA6	LMNA	0.038660916	-0.099923393	0.175773781	5.85E-01	6.63E-01	1.00E+00
PDE6G	LMNA	0.113720098	-0.02471897	0.247879594	1.07E-01	1.61E-01	1.00E+00
PPP4R1	LMNA	-0.023443698	-0.160973719	0.114979425	7.41E-01	7.99E-01	1.00E+00
GCLC	LMNA	-0.286890577	-0.408752709	-0.154977544	3.48E-05	1.24E-04	4.13E-01
MAN1A2	LMNA	0.097822695	-0.040779007	0.232730778	1.66E-01	2.34E-01	1.00E+00
SEC61A1	LMNA	0.457455527	0.340935341	0.560132906	7.69E-12	9.24E-11	9.12E-08
IKBKAP	LMNA	-0.172652757	-0.303470571	-0.035446623	1.40E-02	2.73E-02	1.00E+00
TWSG1	LMNA	0.461843061	0.345842234	0.563938528	4.57E-12	5.71E-11	5.42E-08
UPF1	LMNA	0.266804558	0.133677242	0.390473394	1.24E-04	3.99E-04	1.00E+00
KIAA1219	LMNA	0.177700635	0.04064677	0.308191166	1.14E-02	2.28E-02	1.00E+00
CTDP1	LMNA	0.242615005	0.108187556	0.368329457	5.03E-04	1.42E-03	1.00E+00
ZMYND10	LMNA	0.15887852	0.02129458	0.290556643	2.39E-02	4.38E-02	1.00E+00
WNT16	LMNA	0.005886518	-0.132271972	0.143820645	9.34E-01	9.52E-01	1.00E+00
ADAMTS6	LMNA	-0.176580461	-0.307144175	-0.039492165	1.19E-02	2.37E-02	1.00E+00
SNW1	LMNA	0.209183191	0.073247425	0.337488224	2.81E-03	6.55E-03	1.00E+00
IL18RAP	LMNA	0.135648019	-0.002448839	0.268667853	5.42E-02	9.01E-02	1.00E+00
RPP30	LMNA	-0.007086335	-0.14499549	0.131092911	9.20E-01	9.43E-01	1.00E+00
KRT86	LMNA	0.023575327	-0.114849462	0.161102006	7.39E-01	7.98E-01	1.00E+00
CDC40	LMNA	-0.184127592	-0.314192143	-0.04727836	8.71E-03	1.79E-02	1.00E+00
SLIT1	LMNA	-0.40774623	-0.516711649	-0.285781824	1.71E-09	1.36E-08	2.03E-05
KIAA0574	LMNA	-0.077559298	-0.213326185	0.061146406	2.73E-01	3.55E-01	1.00E+00
GTPBP2	LMNA	0.085964353	-0.052712208	0.221388038	2.24E-01	3.01E-01	1.00E+00
SETD3	LMNA	0.03645116	-0.102113689	0.173628441	6.07E-01	6.83E-01	1.00E+00
C15orf44	LMNA	0.180867397	0.043912853	0.311149333	1.00E-02	2.03E-02	1.00E+00
PRRX2	LMNA	0.45505245	0.338250497	0.558046708	1.02E-11	1.20E-10	1.21E-07
GPR110	LMNA	0.181232302	0.044289391	0.31149004	9.85E-03	2.00E-02	1.00E+00
C11orf10	LMNA	-0.030742579	-0.16808024	0.107765784	6.64E-01	7.34E-01	1.00E+00
MKKS	LMNA	-0.204139809	-0.332811611	-0.068005315	3.57E-03	8.09E-03	1.00E+00
ELK4	LMNA	0.264193195	0.130917017	0.388089734	1.45E-04	4.61E-04	1.00E+00

ACOX3	LMNA	0.142886861	0.00493317	0.275503372	4.25E-02	7.27E-02	1.00E+00
ADCY1	LMNA	-0.234332106	-0.360714085	-0.099499905	7.89E-04	2.13E-03	1.00E+00
KIAA0372	LMNA	0.087554286	-0.051114533	0.222910972	2.15E-01	2.91E-01	1.00E+00
TP53AP1	LMNA	0.224265944	0.08896945	0.351436424	1.33E-03	3.39E-03	1.00E+00
RHBG	LMNA	0.114198617	-0.024234446	0.248334565	1.06E-01	1.59E-01	1.00E+00
SMURF2	LMNA	0.022028593	-0.116376316	0.159594257	7.56E-01	8.12E-01	1.00E+00
TP53I3	LMNA	0.296955799	0.165697578	0.417875956	1.77E-05	6.72E-05	2.10E-01
EBP	LMNA	0.129867476	-0.00833292	0.263199743	6.55E-02	1.06E-01	1.00E+00
SLC22A3	LMNA	-0.178834802	-0.309250916	-0.041816172	1.09E-02	2.19E-02	1.00E+00
TOR1A	LMNA	0.311824819	0.181590893	0.431308949	6.26E-06	2.57E-05	7.42E-02
P2RY4	LMNA	-0.001105516	-0.139135282	0.136966388	9.88E-01	9.90E-01	1.00E+00
TRPV1	LMNA	-0.324338282	-0.442572978	-0.195019315	2.49E-06	1.11E-05	2.95E-02
ADAMTS12	LMNA	0.542108074	0.436742234	0.632801147	7.94E-17	2.38E-15	9.41E-13
PES1	LMNA	0.111348204	-0.027119673	0.245623546	1.15E-01	1.71E-01	1.00E+00
UCP2	LMNA	0.00282223	-0.135281477	0.140818367	9.68E-01	9.76E-01	1.00E+00
ATG4A	LMNA	0.089345085	-0.049314165	0.224625515	2.06E-01	2.81E-01	1.00E+00
FOXG1	LMNA	0.138383229	0.000338699	0.271252242	4.95E-02	8.33E-02	1.00E+00
MAGEA10	LMNA	-0.087246519	-0.222616225	0.051423855	2.17E-01	2.93E-01	1.00E+00
WFS1	LMNA	-0.071980834	-0.207965279	0.066733297	3.09E-01	3.93E-01	1.00E+00
TRIM24	LMNA	0.011442589	-0.126808726	0.149257813	8.72E-01	9.05E-01	1.00E+00
PABPN1	LMNA	-0.149283358	-0.281532339	-0.011468713	3.40E-02	5.97E-02	1.00E+00
PROC	LMNA	-0.135993784	-0.268994658	0.002096578	5.36E-02	8.93E-02	1.00E+00
SLCO1C1	LMNA	-0.239896313	-0.365831726	-0.105333748	5.84E-04	1.62E-03	1.00E+00
SLC25A30	LMNA	0.189529039	0.052861133	0.319227555	6.90E-03	1.46E-02	1.00E+00
SLC22A5	LMNA	-0.015448361	-0.153172696	0.122864669	8.27E-01	8.70E-01	1.00E+00
DEPDC1	LMNA	0.099566601	-0.03902075	0.234395769	1.59E-01	2.25E-01	1.00E+00
KIF23	LMNA	0.11619259	-0.022214746	0.250229779	9.96E-02	1.51E-01	1.00E+00
SYN2	LMNA	-0.363224013	-0.477339567	-0.237060017	1.08E-07	6.15E-07	1.28E-03
ASPN	LMNA	0.494872487	0.382986201	0.592448784	7.09E-14	1.15E-12	8.41E-10
CENTG2	LMNA	-0.129179422	-0.262548307	0.009032661	6.69E-02	1.08E-01	1.00E+00
ZDHHC17	LMNA	-0.40104481	-0.510814738	-0.278407753	3.32E-09	2.49E-08	3.94E-05
VNN3	LMNA	0.154852551	0.017168573	0.286773047	2.78E-02	4.99E-02	1.00E+00
KCNH1	LMNA	-0.355851376	-0.470775242	-0.229052737	2.02E-07	1.10E-06	2.40E-03
PPARD	LMNA	0.296158034	0.16484678	0.41715374	1.87E-05	7.06E-05	2.22E-01
PSMAL	LMNA	-0.155726408	-0.287594645	-0.018063745	2.69E-02	4.86E-02	1.00E+00

FLJ10815	LMNA	0.275699528	0.143094801	0.398580324	7.15E-05	2.41E-04	8.48E-01
YBX1	LMNA	0.036847098	-0.101721336	0.174012934	6.03E-01	6.80E-01	1.00E+00
STK24	LMNA	0.218528984	0.082981377	0.346137654	1.78E-03	4.38E-03	1.00E+00
SPEG	LMNA	0.250847907	0.116843162	0.375882181	3.17E-04	9.36E-04	1.00E+00
STK10	LMNA	0.377694154	0.252825755	0.490186322	3.00E-08	1.88E-07	3.56E-04
ZNF695	LMNA	-0.205665249	-0.334226785	-0.06959007	3.32E-03	7.60E-03	1.00E+00
SCTR	LMNA	0.009562849	-0.128658001	0.147419233	8.93E-01	9.22E-01	1.00E+00
AAAS	LMNA	-0.096143559	-0.231126879	0.042471149	1.73E-01	2.43E-01	1.00E+00
SSX3	LMNA	-0.104506318	-0.239107665	0.034035722	1.39E-01	2.01E-01	1.00E+00
ABCD3	LMNA	-0.309387709	-0.429110835	-0.178981226	7.45E-06	3.02E-05	8.84E-02
MTF2	LMNA	-0.206488856	-0.334990616	-0.070445988	3.19E-03	7.36E-03	1.00E+00
FIG4	LMNA	-0.303552141	-0.423841766	-0.172739924	1.13E-05	4.41E-05	1.33E-01
TMCO6	LMNA	0.073108992	-0.065604136	0.209050098	3.01E-01	3.85E-01	1.00E+00
C9orf46	LMNA	0.032394846	-0.106130787	0.169686982	6.47E-01	7.20E-01	1.00E+00
ATP6V1F	LMNA	-0.036704411	-0.173874376	0.101862737	6.04E-01	6.81E-01	1.00E+00
IGHMBP2	LMNA	0.012206594	-0.126056829	0.150004817	8.63E-01	9.00E-01	1.00E+00
CCDC94	LMNA	-0.027948627	-0.165361607	0.110528831	6.93E-01	7.59E-01	1.00E+00
DUS2L	LMNA	-0.05663569	-0.19317632	0.082056882	4.23E-01	5.10E-01	1.00E+00
FAM3C	LMNA	0.224292829	0.088997535	0.351461237	1.33E-03	3.38E-03	1.00E+00
DCTN4	LMNA	-0.073798207	-0.209712673	0.064914133	2.97E-01	3.81E-01	1.00E+00
EIF2AK2	LMNA	0.024420006	-0.114015364	0.161925124	7.30E-01	7.90E-01	1.00E+00
CST4	LMNA	0.034623187	-0.103924549	0.171852772	6.25E-01	6.99E-01	1.00E+00
CCL11	LMNA	0.337995385	0.209730538	0.454824024	8.67E-07	4.20E-06	1.03E-02
LMO7	LMNA	-0.121086866	-0.254877297	0.01725253	8.61E-02	1.34E-01	1.00E+00
PAM	LMNA	0.455963653	0.339268317	0.558837909	9.16E-12	1.08E-10	1.09E-07
NUTF2	LMNA	0.067466454	-0.071248138	0.203620968	3.40E-01	4.26E-01	1.00E+00
ADRBK1	LMNA	0.242133874	0.107682352	0.367887562	5.17E-04	1.45E-03	1.00E+00
ZNF84	LMNA	-0.02139958	-0.158980914	0.116997058	7.62E-01	8.18E-01	1.00E+00
SLC39A4	LMNA	0.25725757	0.123596042	0.381750819	2.19E-04	6.68E-04	1.00E+00
CITED2	LMNA	0.350738201	0.223509476	0.466215175	3.10E-07	1.63E-06	3.67E-03
C12orf49	LMNA	0.253702894	0.119849495	0.378497424	2.69E-04	8.06E-04	1.00E+00
GRM3	LMNA	-0.376762182	-0.489360377	-0.251808337	3.27E-08	2.03E-07	3.87E-04
CCDC49	LMNA	-0.089319355	-0.224600886	0.04934004	2.06E-01	2.81E-01	1.00E+00
STARD8	LMNA	0.091132349	-0.047516452	0.226335838	1.97E-01	2.71E-01	1.00E+00
FNDC4	LMNA	0.220669223	0.085214153	0.348115374	1.60E-03	3.97E-03	1.00E+00

SIAH2	LMNA	0.265248227	0.132031943	0.38905297	1.36E-04	4.35E-04	1.00E+00
CCDC103	LMNA	-0.117990463	-0.251937725	0.020392709	9.44E-02	1.45E-01	1.00E+00
ATP5A1	LMNA	-0.292424878	-0.413772087	-0.160868047	2.41E-05	8.90E-05	2.86E-01
HTR7P	LMNA	0.052951397	-0.085726263	0.189616303	4.54E-01	5.41E-01	1.00E+00
LALBA	LMNA	-0.219240395	-0.34679517	-0.083723397	1.72E-03	4.24E-03	1.00E+00
RMND5A	LMNA	-0.242499327	-0.368223217	-0.108066084	5.07E-04	1.42E-03	1.00E+00
ATP5J2	LMNA	0.029089015	-0.109401316	0.166471504	6.81E-01	7.49E-01	1.00E+00
PSCD2	LMNA	0.148445615	0.010612089	0.280743324	3.50E-02	6.13E-02	1.00E+00
MMP3	LMNA	0.152620576	0.014883155	0.284673667	3.01E-02	5.38E-02	1.00E+00
ZNF409	LMNA	-0.247960569	-0.373235277	-0.11380526	3.74E-04	1.08E-03	1.00E+00
CRHR1	LMNA	-0.126069377	-0.259602259	0.012193846	7.38E-02	1.18E-01	1.00E+00
WDR70	LMNA	-0.121214935	-0.254998826	0.017122592	8.57E-02	1.33E-01	1.00E+00
ZFAND1	LMNA	-0.08393566	-0.219443877	0.054749748	2.35E-01	3.13E-01	1.00E+00
CCL18	LMNA	0.28583631	0.153856485	0.407795697	3.73E-05	1.33E-04	4.42E-01
KRTAP1-3	LMNA	-0.225516746	-0.352590609	-0.0902763	1.25E-03	3.20E-03	1.00E+00
RINT1	LMNA	0.141362366	0.003377274	0.27406493	4.48E-02	7.62E-02	1.00E+00
NRN1	LMNA	0.26231576	0.128933834	0.386374981	1.62E-04	5.09E-04	1.00E+00
SPAG5	LMNA	-0.052450071	-0.18913161	0.086225269	4.58E-01	5.44E-01	1.00E+00
KIAA0408	LMNA	-0.354358264	-0.469444279	-0.227433184	2.29E-07	1.24E-06	2.72E-03
F13A1	LMNA	0.473021585	0.358372719	0.5736149	1.17E-12	1.61E-11	1.39E-08
DNAH7	LMNA	-0.245516237	-0.370992893	-0.111235424	4.29E-04	1.23E-03	1.00E+00
SLC10A1	LMNA	0.305600268	0.174929257	0.425691995	9.75E-06	3.86E-05	1.16E-01
BRCA2	LMNA	0.029251258	-0.109240875	0.166629382	6.79E-01	7.48E-01	1.00E+00
ACADM	LMNA	-0.218716684	-0.346311147	-0.083177139	1.76E-03	4.34E-03	1.00E+00
GIPC2	LMNA	0.194060846	0.057551671	0.323446616	5.65E-03	1.22E-02	1.00E+00
OGN	LMNA	0.167378623	0.030021318	0.298531519	1.73E-02	3.28E-02	1.00E+00
RAGE	LMNA	-0.135971383	-0.268973487	0.0021194	5.37E-02	8.93E-02	1.00E+00
XPO6	LMNA	0.091897552	-0.0467465	0.227067845	1.93E-01	2.66E-01	1.00E+00
FMR1	LMNA	-0.205887425	-0.334432852	-0.069820942	3.29E-03	7.53E-03	1.00E+00
DUSP3	LMNA	0.474069123	0.359549069	0.574520235	1.03E-12	1.42E-11	1.22E-08
ANKMY1	LMNA	0.121064441	-0.017275282	0.254856016	8.61E-02	1.34E-01	1.00E+00
CHMP2A	LMNA	0.193261962	0.056724368	0.322703238	5.86E-03	1.26E-02	1.00E+00
FAM8A1	LMNA	-0.328265545	-0.44610044	-0.199243751	1.85E-06	8.43E-06	2.19E-02
GPR21	LMNA	-0.068159505	-0.204288258	0.070555385	3.35E-01	4.21E-01	1.00E+00
BBS9	LMNA	0.00084261	-0.137224353	0.138877456	9.91E-01	9.92E-01	1.00E+00

UNC119B	LMNA	0.18276111	0.045867359	0.312917102	9.23E-03	1.89E-02	1.00E+00
SLC12A3	LMNA	0.047789257	-0.090861187	0.184622239	4.99E-01	5.84E-01	1.00E+00
ZDHHC7	LMNA	0.408508496	0.286621518	0.517381749	1.59E-09	1.27E-08	1.88E-05
FVT1	LMNA	0.275969948	0.143381484	0.398826486	7.03E-05	2.37E-04	8.33E-01
ENTPD6	LMNA	0.065763329	-0.072949962	0.201980609	3.52E-01	4.39E-01	1.00E+00
PPP1R2P9	LMNA	0.164720542	0.027290114	0.29603965	1.91E-02	3.59E-02	1.00E+00
ERCC4	LMNA	-0.062145232	-0.198493317	0.076562615	3.80E-01	4.67E-01	1.00E+00
MMP8	LMNA	0.089853321	-0.048803049	0.225111956	2.03E-01	2.78E-01	1.00E+00
RAD23A	LMNA	0.23288296	0.097982056	0.359379993	8.52E-04	2.27E-03	1.00E+00
ACY1	LMNA	0.196478006	0.060055963	0.325694864	5.07E-03	1.11E-02	1.00E+00
MT1E	LMNA	0.40786309	0.285910542	0.516814387	1.69E-09	1.35E-08	2.01E-05
PPP5C	LMNA	-0.10772286	-0.242172454	0.030785966	1.27E-01	1.87E-01	1.00E+00
GPR20	LMNA	0.039330375	-0.099259565	0.176423467	5.78E-01	6.57E-01	1.00E+00
GHITM	LMNA	-0.161392237	-0.292916942	-0.023873138	2.18E-02	4.03E-02	1.00E+00
SCAMP4	LMNA	0.329318149	0.20037682	0.447045265	1.70E-06	7.84E-06	2.02E-02
NARG1L	LMNA	-0.33608747	-0.45311519	-0.207671865	1.01E-06	4.83E-06	1.20E-02
MYO9A	LMNA	-0.164542589	-0.295872761	-0.027107339	1.93E-02	3.61E-02	1.00E+00
BLMH	LMNA	-0.070537656	-0.206577052	0.06817724	3.18E-01	4.04E-01	1.00E+00
NDUFB7	LMNA	-0.118710801	-0.252621799	0.01966243	9.24E-02	1.42E-01	1.00E+00
ADCYAP1	LMNA	-0.329580063	-0.44728032	-0.200658809	1.67E-06	7.70E-06	1.98E-02
SP110	LMNA	0.311164899	0.180884068	0.430713885	6.56E-06	2.68E-05	7.78E-02
MIER2	LMNA	0.114760534	-0.023665393	0.248868755	1.04E-01	1.57E-01	1.00E+00
KIAA0513	LMNA	-0.214040055	-0.341985904	-0.07830278	2.22E-03	5.33E-03	1.00E+00
SH3BP2	LMNA	0.280041573	0.147700684	0.402530728	5.43E-05	1.87E-04	6.44E-01
PNMA2	LMNA	-0.324034835	-0.44230027	-0.194693106	2.55E-06	1.13E-05	3.02E-02
MAP3K7IP2	LMNA	-0.090468385	-0.225700554	0.048184403	2.00E-01	2.74E-01	1.00E+00
AGRN	LMNA	0.14549089	0.007592384	0.277959038	3.88E-02	6.71E-02	1.00E+00
CEP290	LMNA	-0.04232144	-0.179324733	0.096292149	5.50E-01	6.31E-01	1.00E+00
ANXA10	LMNA	-0.003453849	-0.141437411	0.134661359	9.61E-01	9.71E-01	1.00E+00
RTN2	LMNA	-0.303999695	-0.424246161	-0.173218223	1.09E-05	4.28E-05	1.29E-01
C14orf133	LMNA	-0.106192418	-0.24071455	0.032332577	1.33E-01	1.94E-01	1.00E+00
PRPS1L1	LMNA	0.056275733	-0.082415548	0.192828663	4.26E-01	5.13E-01	1.00E+00
PRPH2	LMNA	-0.129887706	-0.263218895	0.008312344	6.54E-02	1.06E-01	1.00E+00
KLRA1	LMNA	-0.060791673	-0.197187804	0.077913203	3.90E-01	4.77E-01	1.00E+00
IRX4	LMNA	0.049327979	-0.089331348	0.186111601	4.86E-01	5.71E-01	1.00E+00

GOLGB1	LMNA	0.094537188	-0.044089219	0.229591797	1.81E-01	2.51E-01	1.00E+00
GPR97	LMNA	0.196392463	0.059967307	0.325615324	5.09E-03	1.11E-02	1.00E+00
NFKBIL1	LMNA	0.04905063	-0.089607145	0.185843195	4.88E-01	5.73E-01	1.00E+00
CHD7	LMNA	-0.468236641	-0.569476382	-0.353004009	2.11E-12	2.78E-11	2.50E-08
APBB3	LMNA	-0.211174891	-0.339333339	-0.075319677	2.55E-03	6.03E-03	1.00E+00
RPS10	LMNA	-0.116151322	-0.250190565	0.022256559	9.97E-02	1.52E-01	1.00E+00
HIC1	LMNA	0.413482222	0.292105055	0.521750832	9.59E-10	7.94E-09	1.14E-05
TLE3	LMNA	-0.165951914	-0.297194246	-0.028555106	1.83E-02	3.44E-02	1.00E+00
PSMB7	LMNA	-0.034867942	-0.172090575	0.103682138	6.22E-01	6.97E-01	1.00E+00
IAPP	LMNA	-0.240544863	-0.36642773	-0.106014329	5.64E-04	1.57E-03	1.00E+00
SHQ1	LMNA	0.304057683	0.173280199	0.424298553	1.09E-05	4.27E-05	1.29E-01
API5	LMNA	0.052550657	-0.086125153	0.189228864	4.58E-01	5.44E-01	1.00E+00
GP1BB	LMNA	-0.001839148	-0.13985464	0.136246445	9.79E-01	9.84E-01	1.00E+00
SLC6A1	LMNA	-0.487241497	-0.585883525	-0.374372344	1.93E-13	2.99E-12	2.29E-09
OCLN	LMNA	-0.252988661	-0.377843355	-0.119097169	2.81E-04	8.37E-04	1.00E+00
NAGLU	LMNA	0.384061183	0.259783934	0.495823601	1.68E-08	1.10E-07	1.99E-04
FAM117A	LMNA	0.055796835	-0.082892672	0.192366077	4.30E-01	5.17E-01	1.00E+00
SPRY1	LMNA	0.396852978	0.273802508	0.507120879	5.00E-09	3.64E-08	5.93E-05
FLJ10781	LMNA	-0.434675753	-0.540304475	-0.315560739	1.02E-10	1.03E-09	1.21E-06
CDON	LMNA	-0.015667446	-0.153386685	0.122648833	8.25E-01	8.68E-01	1.00E+00
SH3YL1	LMNA	-0.370890377	-0.484151933	-0.245404527	5.52E-08	3.32E-07	6.55E-04
IGKC	LMNA	0.175911033	0.038802335	0.306518329	1.23E-02	2.43E-02	1.00E+00
TREM2	LMNA	0.029305242	-0.10918749	0.166681911	6.79E-01	7.47E-01	1.00E+00
MMP14	LMNA	0.756580551	0.690667492	0.810026912	9.39E-39	1.59E-35	1.11E-34
SERPINI1	LMNA	-0.184409298	-0.31445494	-0.047569311	8.61E-03	1.78E-02	1.00E+00
HDHD3	LMNA	0.262655387	0.129292512	0.386685243	1.59E-04	5.00E-04	1.00E+00
DYNC1LI1	LMNA	-0.218519885	-0.346129243	-0.082971888	1.78E-03	4.38E-03	1.00E+00
FASTKD5	LMNA	0.133260042	-0.004880748	0.266409983	5.87E-02	9.64E-02	1.00E+00
TDRD3	LMNA	-0.149737	-0.281959519	-0.011932663	3.34E-02	5.90E-02	1.00E+00
THSD7A	LMNA	-0.360159515	-0.474612597	-0.233729642	1.40E-07	7.84E-07	1.66E-03
NDST3	LMNA	-0.272341637	-0.395522176	-0.139536806	8.82E-05	2.91E-04	1.00E+00
CXCL2	LMNA	0.270748057	0.137849461	0.394069891	9.73E-05	3.18E-04	1.00E+00
DHRS12	LMNA	0.078750942	-0.059951834	0.214470298	2.65E-01	3.47E-01	1.00E+00
MAP3K15	LMNA	-0.359503205	-0.474028288	-0.233016775	1.48E-07	8.26E-07	1.76E-03
FBXO9	LMNA	-0.348130597	-0.463887278	-0.220685702	3.84E-07	1.99E-06	4.55E-03

APPL2	LMNA	-0.241947433	-0.367716309	-0.107486601	5.22E-04	1.47E-03	1.00E+00
TNPO1	LMNA	0.20023961	0.063956579	0.329190701	4.27E-03	9.52E-03	1.00E+00
CARD10	LMNA	0.156265997	0.018616606	0.288101869	2.64E-02	4.77E-02	1.00E+00
MRPL13	LMNA	-0.017322708	-0.155003031	0.121017697	8.07E-01	8.54E-01	1.00E+00
SNX5	LMNA	0.137456383	-0.00060612	0.270376717	5.11E-02	8.55E-02	1.00E+00
EEF1D	LMNA	0.079192914	-0.059508675	0.214894545	2.63E-01	3.44E-01	1.00E+00
RAB6A	LMNA	-0.174703944	-0.305389543	-0.037558788	1.29E-02	2.54E-02	1.00E+00
SOD1	LMNA	0.079588286	-0.059112195	0.215274019	2.60E-01	3.42E-01	1.00E+00
C12orf5	LMNA	0.119638758	-0.018721451	0.253502842	8.99E-02	1.39E-01	1.00E+00
PACS1	LMNA	0.233297539	0.098416226	0.359761709	8.33E-04	2.23E-03	1.00E+00
PAPOLG	LMNA	-0.181350164	-0.311600079	-0.044411019	9.80E-03	1.99E-02	1.00E+00
CHML	LMNA	-0.157592734	-0.289348712	-0.019976335	2.51E-02	4.57E-02	1.00E+00
SIRT5	LMNA	-0.167290211	-0.298448664	-0.029930441	1.73E-02	3.28E-02	1.00E+00
MSRB2	LMNA	-0.250062946	-0.375162787	-0.11601702	3.32E-04	9.75E-04	1.00E+00
BCR	LMNA	-0.197535177	-0.3266777	-0.061151781	4.83E-03	1.06E-02	1.00E+00
PUS3	LMNA	-0.118582045	-0.252499535	0.019792974	9.28E-02	1.43E-01	1.00E+00
CAPN2	LMNA	0.534726593	0.428291904	0.626527613	2.46E-16	6.64E-15	2.92E-12
FXYD5	LMNA	0.52412139	0.416183551	0.617493361	1.20E-15	2.81E-14	1.42E-11
TWISTNB	LMNA	0.441054112	0.322648522	0.545868304	5.05E-11	5.31E-10	5.98E-07
UBE1L	LMNA	0.262361951	0.128982614	0.38641718	1.62E-04	5.08E-04	1.00E+00
UBE1C	LMNA	-0.191016336	-0.320612779	-0.05439986	6.47E-03	1.37E-02	1.00E+00
CHD2	LMNA	0.142516634	0.004555256	0.275154098	4.30E-02	7.35E-02	1.00E+00
C15orf2	LMNA	-0.088662616	-0.223972205	0.050000387	2.10E-01	2.85E-01	1.00E+00
FZD5	LMNA	0.398731186	0.275865255	0.508776465	4.17E-09	3.07E-08	4.94E-05
NR4A1	LMNA	-0.011803131	-0.149610351	0.126453918	8.68E-01	9.02E-01	1.00E+00
LOC339047	LMNA	-0.093772727	-0.228861026	0.044858992	1.84E-01	2.56E-01	1.00E+00
TRIM17	LMNA	-0.149589179	-0.281820326	-0.011781477	3.36E-02	5.92E-02	1.00E+00
RPL15	LMNA	-0.293508569	-0.414754086	-0.162022586	2.24E-05	8.33E-05	2.66E-01
ATP5G3	LMNA	-0.06027079	-0.196685282	0.078432806	3.94E-01	4.81E-01	1.00E+00
PRC1	LMNA	0.10439373	-0.034149421	0.239000339	1.39E-01	2.02E-01	1.00E+00
ADAMTS8	LMNA	-0.121046138	-0.254838647	0.017293852	8.62E-02	1.34E-01	1.00E+00
ABCB9	LMNA	0.170821473	0.033561932	0.301756431	1.51E-02	2.90E-02	1.00E+00
HOXC4	LMNA	-0.043930174	-0.180884183	0.094695116	5.35E-01	6.17E-01	1.00E+00
TRAK2	LMNA	0.10183984	-0.036727516	0.236564953	1.49E-01	2.14E-01	1.00E+00
STAB1	LMNA	0.434463135	0.315324702	0.540118849	1.05E-10	1.05E-09	1.24E-06

CEACAM5	LMNA	-0.026724591	-0.16416991	0.111738654	7.06E-01	7.69E-01	1.00E+00
MYT1L	LMNA	-0.46478747	-0.566489994	-0.349138738	3.21E-12	4.14E-11	3.81E-08
RASA2	LMNA	0.162175496	0.024676976	0.293652071	2.11E-02	3.92E-02	1.00E+00
LRRTM2	LMNA	-0.370678205	-0.483963581	-0.245173336	5.63E-08	3.38E-07	6.67E-04
STAG1	LMNA	0.11362012	-0.024820194	0.247784529	1.07E-01	1.62E-01	1.00E+00
OSBPL7	LMNA	-0.02589486	-0.163361872	0.112558517	7.15E-01	7.77E-01	1.00E+00
GIMAP4	LMNA	0.176974978	0.039898767	0.307512954	1.17E-02	2.34E-02	1.00E+00
FUT3	LMNA	-0.149338197	-0.281583981	-0.011524794	3.39E-02	5.96E-02	1.00E+00
DBI	LMNA	-0.171795323	-0.302668091	-0.034564062	1.45E-02	2.81E-02	1.00E+00
LPIN2	LMNA	0.065491622	-0.073221386	0.201718846	3.54E-01	4.41E-01	1.00E+00
PAPD1	LMNA	-0.34294106	-0.459249641	-0.215072304	5.85E-07	2.91E-06	6.94E-03
APOOL	LMNA	-0.086859789	-0.222245822	0.0518125	2.19E-01	2.95E-01	1.00E+00
DIABLO	LMNA	-0.208212807	-0.336588901	-0.07223822	2.94E-03	6.83E-03	1.00E+00
ARMC9	LMNA	0.236333472	0.101597188	0.362555704	7.09E-04	1.93E-03	1.00E+00
RPS6KA4	LMNA	0.296604578	0.165322984	0.417558015	1.82E-05	6.86E-05	2.16E-01
TRHR	LMNA	-0.092545901	-0.227687948	0.046093999	1.90E-01	2.62E-01	1.00E+00
SLITRK3	LMNA	-0.279068181	-0.40164553	-0.146667646	5.77E-05	1.98E-04	6.85E-01
TGFBRAP1	LMNA	-0.001718326	-0.139736179	0.136365023	9.81E-01	9.85E-01	1.00E+00
FAHD2A	LMNA	0.116643093	-0.021758274	0.250657827	9.83E-02	1.50E-01	1.00E+00
CNTN1	LMNA	-0.537001107	-0.628461995	-0.430893788	1.74E-16	4.85E-15	2.07E-12
ZNF211	LMNA	-0.000616237	-0.138655442	0.137446457	9.93E-01	9.94E-01	1.00E+00
BBS4	LMNA	-0.123351232	-0.257025409	0.014954424	8.03E-02	1.26E-01	1.00E+00
TMEM181	LMNA	-0.100448415	-0.235237378	0.038131353	1.55E-01	2.21E-01	1.00E+00
PFDN1	LMNA	0.017598551	-0.120745801	0.155272318	8.04E-01	8.52E-01	1.00E+00
MINPP1	LMNA	0.099393765	-0.039195047	0.234230789	1.59E-01	2.26E-01	1.00E+00
MPHOSPH(	LMNA	-0.155006135	-0.28691746	-0.017325886	2.76E-02	4.97E-02	1.00E+00
RGS11	LMNA	-0.147187538	-0.279558089	-0.009326036	3.66E-02	6.37E-02	1.00E+00
HOXC10	LMNA	-0.015496189	-0.153219413	0.122817551	8.27E-01	8.70E-01	1.00E+00
ITPKB	LMNA	0.149154222	0.011336653	0.281410725	3.41E-02	5.99E-02	1.00E+00
HS6ST1	LMNA	0.359665977	0.233193561	0.474173212	1.46E-07	8.15E-07	1.74E-03
AKR1D1	LMNA	0.135519655	-0.002579606	0.26854652	5.45E-02	9.04E-02	1.00E+00
TNP2	LMNA	-0.098165645	-0.233058271	0.040433302	1.65E-01	2.32E-01	1.00E+00
MEOX2	LMNA	0.251966227	0.118020473	0.376906829	2.98E-04	8.83E-04	1.00E+00
EML4	LMNA	0.264307857	0.131038173	0.388194433	1.44E-04	4.58E-04	1.00E+00
SGTA	LMNA	-0.154434554	-0.286379978	-0.016740458	2.82E-02	5.06E-02	1.00E+00

ATP6V0C	LMNA	-0.102827592	-0.237507072	0.035730621	1.45E-01	2.09E-01	1.00E+00
PRPF8	LMNA	-0.021894298	-0.159463316	0.116508854	7.57E-01	8.13E-01	1.00E+00
PSMD6	LMNA	-0.011246685	-0.149066243	0.1270015	8.74E-01	9.06E-01	1.00E+00
HIST1H2BI	LMNA	0.174900303	0.037761049	0.305573189	1.28E-02	2.52E-02	1.00E+00
TMC5	LMNA	0.11408787	-0.024346588	0.248229273	1.06E-01	1.60E-01	1.00E+00
FKBP3	LMNA	-0.190687424	-0.320306488	-0.054059518	6.56E-03	1.39E-02	1.00E+00
FLJ20273	LMNA	0.497197672	0.385614741	0.594446664	5.20E-14	8.62E-13	6.16E-10
PLEKHB2	LMNA	-0.067835042	-0.203975871	0.070879726	3.37E-01	4.24E-01	1.00E+00
RPL28	LMNA	0.056869693	-0.081823699	0.193402308	4.21E-01	5.09E-01	1.00E+00
NOX3	LMNA	-0.263006691	-0.387006142	-0.129663558	1.56E-04	4.91E-04	1.00E+00
ZNF544	LMNA	-0.095552098	-0.230561744	0.043067002	1.76E-01	2.46E-01	1.00E+00
EPYC	LMNA	0.346086767	0.218473932	0.462061564	4.53E-07	2.31E-06	5.38E-03
ELAC1	LMNA	-0.182691504	-0.312852141	-0.0457955	9.26E-03	1.89E-02	1.00E+00
METT11D1	LMNA	-0.111012712	-0.245304322	0.027459111	1.16E-01	1.72E-01	1.00E+00
BIN2	LMNA	0.164299435	0.026857611	0.295644711	1.95E-02	3.64E-02	1.00E+00
RPL18A	LMNA	-0.012914613	-0.15069694	0.125359889	8.55E-01	8.93E-01	1.00E+00
UBOX5	LMNA	-0.010269487	-0.148110509	0.127962928	8.85E-01	9.16E-01	1.00E+00
TCERG1	LMNA	-0.218852905	-0.346437051	-0.083319217	1.75E-03	4.32E-03	1.00E+00
MPP6	LMNA	0.087160838	-0.051509963	0.222534165	2.17E-01	2.93E-01	1.00E+00
KRT16	LMNA	0.115238826	-0.023180957	0.249323382	1.02E-01	1.55E-01	1.00E+00
UBE2O	LMNA	-0.287723882	-0.409508954	-0.155863884	3.29E-05	1.19E-04	3.91E-01
KLF5	LMNA	0.457290493	0.340750896	0.559989676	7.84E-12	9.38E-11	9.30E-08
C9orf31	LMNA	0.248069733	0.11392007	0.373335387	3.71E-04	1.08E-03	1.00E+00
APOLD1	LMNA	-0.133535761	-0.266670754	0.004600041	5.81E-02	9.57E-02	1.00E+00
UBL5	LMNA	-0.100654896	-0.235434416	0.037923065	1.54E-01	2.20E-01	1.00E+00
ARHGAP29	LMNA	0.53433052	0.427839005	0.626190653	2.62E-16	6.98E-15	3.10E-12
TNFSF8	LMNA	0.187169483	0.050421316	0.317028797	7.65E-03	1.60E-02	1.00E+00
PDE5A	LMNA	0.099423921	-0.039164636	0.234259575	1.59E-01	2.26E-01	1.00E+00
CDR1	LMNA	-0.44392128	-0.548366321	-0.325838919	3.66E-11	3.90E-10	4.34E-07
FBLN2	LMNA	0.252867413	0.11896947	0.377732307	2.83E-04	8.42E-04	1.00E+00
C14orf104	LMNA	-0.334394204	-0.451597886	-0.205845754	1.15E-06	5.46E-06	1.37E-02
HBE1	LMNA	-0.106810826	-0.241303721	0.031707716	1.30E-01	1.91E-01	1.00E+00
ROBO4	LMNA	0.091828751	-0.046815735	0.227002035	1.94E-01	2.67E-01	1.00E+00
C1orf108	LMNA	0.087197558	-0.051473061	0.222569333	2.17E-01	2.93E-01	1.00E+00
FOXI1	LMNA	0.103985443	-0.034561701	0.238611111	1.41E-01	2.04E-01	1.00E+00

BCKDHA	LMNA	-0.073009145	-0.2089541	0.065704086	3.02E-01	3.86E-01	1.00E+00
RAB4A	LMNA	-0.272816761	-0.395955054	-0.140040036	8.56E-05	2.84E-04	1.00E+00
C11orf80	LMNA	0.049775894	-0.088885896	0.18654503	4.82E-01	5.67E-01	1.00E+00
MYOC	LMNA	-0.113891721	-0.248042778	0.024545201	1.07E-01	1.61E-01	1.00E+00
GIF	LMNA	-0.171896664	-0.302762946	-0.034668361	1.44E-02	2.80E-02	1.00E+00
TMEM39B	LMNA	0.113002198	-0.025445758	0.24719691	1.09E-01	1.64E-01	1.00E+00
RPL3	LMNA	-0.04713903	-0.183992681	0.091507464	5.05E-01	5.89E-01	1.00E+00
THBS1	LMNA	0.60756756	0.512502899	0.687919131	9.06E-22	6.04E-20	1.07E-17
APOO	LMNA	-0.063901731	-0.200186745	0.074809214	3.66E-01	4.54E-01	1.00E+00
ATPBD1C	LMNA	-0.148786531	-0.281064432	-0.010960664	3.46E-02	6.06E-02	1.00E+00
FARSA	LMNA	0.019096099	-0.119269324	0.156733912	7.87E-01	8.39E-01	1.00E+00
ARMCX1	LMNA	-0.313612984	-0.432920855	-0.183506836	5.50E-06	2.28E-05	6.52E-02
EIF4ENIF1	LMNA	-0.233834363	-0.360255918	-0.098978494	8.10E-04	2.18E-03	1.00E+00
CMAS	LMNA	0.062993456	-0.075715994	0.199311185	3.73E-01	4.61E-01	1.00E+00
OR7E24	LMNA	0.103982889	-0.03456428	0.238608676	1.41E-01	2.04E-01	1.00E+00
TNFRSF21	LMNA	-0.022395489	-0.159951963	0.116014195	7.52E-01	8.09E-01	1.00E+00
PLAC1	LMNA	-0.019913103	-0.157531047	0.118463557	7.78E-01	8.31E-01	1.00E+00
KLHL18	LMNA	0.090310524	-0.048343193	0.225549496	2.01E-01	2.76E-01	1.00E+00
LBA1	LMNA	0.308213961	0.177725026	0.428051685	8.10E-06	3.26E-05	9.61E-02
MKL2	LMNA	0.022604844	-0.115807548	0.160156058	7.49E-01	8.07E-01	1.00E+00
TBX3	LMNA	-0.117865756	-0.251819283	0.020519121	9.48E-02	1.45E-01	1.00E+00
TAZ	LMNA	0.053307352	-0.085371912	0.189960409	4.51E-01	5.37E-01	1.00E+00
CRIP2	LMNA	0.16775018	0.03040326	0.2988797	1.70E-02	3.24E-02	1.00E+00
DAXX	LMNA	0.123581693	-0.014720447	0.257243963	7.97E-02	1.25E-01	1.00E+00
ELMO1	LMNA	-0.434431166	-0.540090938	-0.315289213	1.05E-10	1.05E-09	1.24E-06
RGS13	LMNA	0.159114872	0.021536952	0.290778639	2.37E-02	4.35E-02	1.00E+00
DIO2	LMNA	0.052817403	-0.085859643	0.189486761	4.55E-01	5.42E-01	1.00E+00
UNC13A	LMNA	-0.428506709	-0.534914429	-0.308718173	1.99E-10	1.88E-09	2.36E-06
TAF11	LMNA	-0.256955353	-0.381474336	-0.123277363	2.23E-04	6.79E-04	1.00E+00
ORC3L	LMNA	-0.226602864	-0.353592518	-0.091411466	1.18E-03	3.04E-03	1.00E+00
PRX	LMNA	0.242970138	0.108560503	0.368655593	4.94E-04	1.39E-03	1.00E+00
TARBP2	LMNA	-0.031367886	-0.168688404	0.107147101	6.58E-01	7.28E-01	1.00E+00
CABIN1	LMNA	-0.023870668	-0.16138983	0.114557843	7.36E-01	7.95E-01	1.00E+00
TRIOBP	LMNA	0.501351468	0.390314976	0.598012766	2.97E-14	5.16E-13	3.52E-10
HIST1H2AC	LMNA	-0.020793873	-0.158390196	0.117594697	7.69E-01	8.23E-01	1.00E+00

RBM5	LMNA	-0.214027432	-0.341974223	-0.078289632	2.22E-03	5.33E-03	1.00E+00
TUBGCP4	LMNA	-0.151612098	-0.283724684	-0.013851002	3.12E-02	5.56E-02	1.00E+00
NCOA1	LMNA	-0.263945875	-0.387863894	-0.130655706	1.47E-04	4.67E-04	1.00E+00
CDK7	LMNA	0.276436354	0.143875994	0.399251011	6.82E-05	2.30E-04	8.09E-01
SSR2	LMNA	0.122551156	-0.015766588	0.25626656	8.23E-02	1.29E-01	1.00E+00
IL25	LMNA	-7.29E-05	-0.138122511	0.137979489	9.99E-01	9.99E-01	1.00E+00
CRELD1	LMNA	0.173470305	0.036288328	0.304235549	1.36E-02	2.65E-02	1.00E+00
GPR6	LMNA	-0.149604599	-0.281834847	-0.011797248	3.36E-02	5.92E-02	1.00E+00
SNCG	LMNA	-0.262296268	-0.386357174	-0.128913249	1.63E-04	5.10E-04	1.00E+00
CHEK2	LMNA	0.003910777	-0.134212683	0.141885175	9.56E-01	9.67E-01	1.00E+00
GAL3ST4	LMNA	0.378450439	0.253651583	0.490856419	2.80E-08	1.77E-07	3.32E-04
KBTBD10	LMNA	-0.183604807	-0.313704396	-0.046738478	8.91E-03	1.83E-02	1.00E+00
DRD4	LMNA	0.005678701	-0.132476154	0.143617113	9.36E-01	9.53E-01	1.00E+00
GDF11	LMNA	-0.036818159	-0.173984832	0.101750015	6.03E-01	6.80E-01	1.00E+00
SEMG2	LMNA	-0.00685291	-0.144766954	0.131322328	9.23E-01	9.45E-01	1.00E+00
MCM2	LMNA	0.057397826	-0.081297361	0.193912299	4.17E-01	5.05E-01	1.00E+00
CD247	LMNA	0.103735822	-0.03481374	0.238373121	1.42E-01	2.05E-01	1.00E+00
SOX14	LMNA	0.065556776	-0.073156302	0.201781617	3.54E-01	4.41E-01	1.00E+00
RBMX2	LMNA	0.015999348	-0.122321828	0.153710844	8.21E-01	8.65E-01	1.00E+00
KIAA0922	LMNA	-0.014967652	-0.152703123	0.123338202	8.33E-01	8.74E-01	1.00E+00
TGS1	LMNA	-0.10245924	-0.237155767	0.036102414	1.47E-01	2.11E-01	1.00E+00
C16orf57	LMNA	0.370934375	0.245452471	0.484190991	5.50E-08	3.31E-07	6.52E-04
SYF2	LMNA	0.161809056	0.024300886	0.293308167	2.14E-02	3.97E-02	1.00E+00
MCM4	LMNA	0.043340927	-0.09528016	0.180313068	5.40E-01	6.22E-01	1.00E+00
PDZD2	LMNA	0.1003046	-0.03827642	0.235100134	1.56E-01	2.22E-01	1.00E+00
CEP192	LMNA	-0.266310731	-0.390022754	-0.133155106	1.28E-04	4.10E-04	1.00E+00
IFT88	LMNA	-0.207051147	-0.335512001	-0.071030454	3.11E-03	7.18E-03	1.00E+00
MCC	LMNA	0.377947865	0.253102775	0.490411134	2.93E-08	1.84E-07	3.48E-04
RPL9	LMNA	-0.126625987	-0.260129701	0.011628286	7.25E-02	1.16E-01	1.00E+00
RAB32	LMNA	0.444011157	0.325938972	0.548444596	3.62E-11	3.87E-10	4.29E-07
P2RX2	LMNA	0.06405586	-0.074655316	0.200335301	3.65E-01	4.52E-01	1.00E+00
DDX43	LMNA	0.006741897	-0.13143143	0.144658261	9.24E-01	9.45E-01	1.00E+00
HHLA3	LMNA	0.125971125	-0.012293668	0.259509148	7.40E-02	1.18E-01	1.00E+00
ID2	LMNA	-0.304663091	-0.424845496	-0.173927307	1.04E-05	4.10E-05	1.23E-01
UBE1	LMNA	0.242673867	0.108249369	0.368383516	5.02E-04	1.41E-03	1.00E+00

SLC24A1	LMNA	0.383018474	0.25864353	0.494901045	1.84E-08	1.20E-07	2.19E-04
ARHGAP5	LMNA	-0.159310745	-0.290962603	-0.021737825	2.35E-02	4.32E-02	1.00E+00
C20orf23	LMNA	0.380806137	0.256225059	0.492942805	2.26E-08	1.45E-07	2.68E-04
CETP	LMNA	0.084037471	-0.05464752	0.219541471	2.34E-01	3.13E-01	1.00E+00
ZNF688	LMNA	-0.156381135	-0.288210091	-0.018734588	2.63E-02	4.75E-02	1.00E+00
APOC2	LMNA	-0.039816024	-0.176894697	0.098777924	5.74E-01	6.53E-01	1.00E+00
PWP1	LMNA	0.058640128	-0.080058977	0.195111636	4.07E-01	4.95E-01	1.00E+00
FAM50B	LMNA	0.224796347	0.089523563	0.351925904	1.30E-03	3.31E-03	1.00E+00
PTPN6	LMNA	0.330488309	0.201636836	0.448095301	1.56E-06	7.21E-06	1.85E-02
BAHD1	LMNA	0.046653035	-0.09199043	0.183522062	5.10E-01	5.94E-01	1.00E+00
GPR56	LMNA	-0.225185395	-0.352284891	-0.089930056	1.27E-03	3.25E-03	1.00E+00
GRIK3	LMNA	-0.122852296	-0.256552201	0.01546092	8.15E-02	1.28E-01	1.00E+00
DGCR14	LMNA	-0.106714309	-0.241211774	0.031805247	1.31E-01	1.91E-01	1.00E+00
CACNB2	LMNA	-0.287959877	-0.409723094	-0.156114936	3.24E-05	1.17E-04	3.85E-01
PDE10A	LMNA	-0.277925312	-0.400605918	-0.145455114	6.21E-05	2.11E-04	7.36E-01
METAP2	LMNA	-0.156262242	-0.28809834	-0.018612759	2.64E-02	4.77E-02	1.00E+00
RHOQ	LMNA	0.209606441	0.073687695	0.337880406	2.75E-03	6.44E-03	1.00E+00
MAP3K4	LMNA	-0.102507633	-0.237201923	0.036053571	1.47E-01	2.11E-01	1.00E+00
PDHX	LMNA	-0.212497948	-0.340558477	-0.076696893	2.40E-03	5.70E-03	1.00E+00
RPL23AP13	LMNA	-0.204048663	-0.332727035	-0.067910645	3.58E-03	8.13E-03	1.00E+00
MTA1	LMNA	-0.155973723	-0.287827135	-0.018317133	2.66E-02	4.82E-02	1.00E+00
GNG11	LMNA	0.450752422	0.333451019	0.554310445	1.68E-11	1.89E-10	1.99E-07
ZBED4	LMNA	-0.199280533	-0.32829972	-0.062961663	4.46E-03	9.90E-03	1.00E+00
CLCN3	LMNA	-0.148669096	-0.280953824	-0.010840587	3.47E-02	6.08E-02	1.00E+00
CDK2	LMNA	0.194950924	0.058473635	0.324274666	5.43E-03	1.18E-02	1.00E+00
SLAMF7	LMNA	0.207668718	0.071672487	0.336084553	3.02E-03	6.99E-03	1.00E+00
CDC37	LMNA	0.349646016	0.222326489	0.465240339	3.39E-07	1.77E-06	4.02E-03
ZER1	LMNA	-0.159358742	-0.29100768	-0.021787048	2.35E-02	4.31E-02	1.00E+00
GRK4	LMNA	-0.433379197	-0.539172361	-0.314121599	1.18E-10	1.16E-09	1.40E-06
PRPH	LMNA	0.061067967	-0.077637557	0.19745433	3.88E-01	4.75E-01	1.00E+00
PPP2CA	LMNA	-0.140231476	-0.272997492	-0.002223521	4.65E-02	7.88E-02	1.00E+00
LRP12	LMNA	0.072255259	-0.066458661	0.208229192	3.07E-01	3.91E-01	1.00E+00
POLR2A	LMNA	-0.020553566	-0.158155809	0.117831775	7.72E-01	8.25E-01	1.00E+00
SEC14L2	LMNA	0.36624656	0.240347709	0.480027055	8.30E-08	4.83E-07	9.85E-04
OGFOD1	LMNA	0.177365325	0.040301116	0.307877796	1.16E-02	2.31E-02	1.00E+00

DKFZP586I	LMNA	0.257708898	0.124072004	0.382163673	2.13E-04	6.52E-04	1.00E+00
MC3R	LMNA	0.121749979	-0.016579688	0.255506502	8.43E-02	1.32E-01	1.00E+00
NOL5A	LMNA	-0.022529372	-0.160082483	0.115882045	7.50E-01	8.07E-01	1.00E+00
PHEX	LMNA	0.148783524	0.010957589	0.281061599	3.46E-02	6.06E-02	1.00E+00
HIST1H2AE	LMNA	-0.213721865	-0.341691425	-0.077971372	2.26E-03	5.40E-03	1.00E+00
C20orf117	LMNA	-0.292822423	-0.414132359	-0.16129154	2.35E-05	8.69E-05	2.78E-01
POLH	LMNA	-0.039799456	-0.176878623	0.098794355	5.74E-01	6.53E-01	1.00E+00
DDX17	LMNA	-0.131608313	-0.264847391	0.006561909	6.19E-02	1.01E-01	1.00E+00
CAMTA2	LMNA	-0.064374483	-0.200642383	0.074337149	3.63E-01	4.50E-01	1.00E+00
PLEKHA2	LMNA	0.18519179	0.048377607	0.315184803	8.32E-03	1.73E-02	1.00E+00
SNAPC2	LMNA	0.503114011	0.392311134	0.599524783	2.33E-14	4.18E-13	2.77E-10
FILIP1L	LMNA	0.428523932	0.308737259	0.53492949	1.99E-10	1.88E-09	2.36E-06
PDE4DIP	LMNA	-0.17265202	-0.303469881	-0.035445863	1.40E-02	2.73E-02	1.00E+00
LRRC1	LMNA	-0.328956404	-0.446720589	-0.199987382	1.75E-06	8.04E-06	2.08E-02
SCN7A	LMNA	0.129919258	-0.008280253	0.263248765	6.53E-02	1.06E-01	1.00E+00
GAS1	LMNA	0.082295553	-0.056396163	0.217871321	2.44E-01	3.24E-01	1.00E+00
DGKA	LMNA	0.088687069	-0.049975803	0.223995615	2.09E-01	2.84E-01	1.00E+00
PEG3	LMNA	-0.438388202	-0.54354396	-0.319684484	6.79E-11	7.01E-10	8.05E-07
NADK	LMNA	0.471352831	0.356499514	0.572172171	1.44E-12	1.95E-11	1.71E-08
SGSH	LMNA	0.521713007	0.413439134	0.615438294	1.70E-15	3.88E-14	2.01E-11
CXCL10	LMNA	0.214373986	0.078650615	0.342294925	2.19E-03	5.25E-03	1.00E+00
GALT	LMNA	-0.232492014	-0.359019997	-0.097572683	8.70E-04	2.32E-03	1.00E+00
MCF2	LMNA	-0.395460283	-0.505892734	-0.272273708	5.72E-09	4.11E-08	6.78E-05
ZMYM4	LMNA	-0.208004592	-0.336395903	-0.072021712	2.97E-03	6.89E-03	1.00E+00
ZNF263	LMNA	0.1507566	0.01297565	0.282919453	3.22E-02	5.71E-02	1.00E+00
STK32B	LMNA	0.028637229	-0.109848045	0.166031839	6.86E-01	7.53E-01	1.00E+00
KIAA0888	LMNA	-0.541401869	-0.632201456	-0.435932965	8.85E-17	2.61E-15	1.05E-12
TACSTD1	LMNA	-0.223373148	-0.350612356	-0.088036933	1.40E-03	3.52E-03	1.00E+00
ATP6AP2	LMNA	0.245047958	0.110743305	0.370563135	4.40E-04	1.26E-03	1.00E+00
TYR	LMNA	-0.120065989	-0.253908399	0.018288142	8.87E-02	1.37E-01	1.00E+00
GDPD2	LMNA	0.183251511	0.046373674	0.31337474	9.04E-03	1.85E-02	1.00E+00
ABHD10	LMNA	-0.509581747	-0.605067309	-0.399645087	9.55E-15	1.86E-13	1.13E-10
TACR3	LMNA	-0.07796658	-0.213717263	0.060738168	2.70E-01	3.52E-01	1.00E+00
SLC35C1	LMNA	0.574807571	0.474401651	0.660450038	3.70E-19	1.65E-17	4.39E-15
KIF1A	LMNA	-0.534529928	-0.626360304	-0.428067017	2.54E-16	6.79E-15	3.01E-12

VCAN	LMNA	0.119223119	-0.019142953	0.253108244	9.10E-02	1.40E-01	1.00E+00
HERC5	LMNA	0.130038972	-0.00815849	0.263362094	6.51E-02	1.06E-01	1.00E+00
UBE2A	LMNA	0.206287623	0.070236842	0.334804004	3.22E-03	7.41E-03	1.00E+00
SYDE1	LMNA	0.627216394	0.535536334	0.70428488	1.74E-23	1.72E-21	2.06E-19
ELA3A	LMNA	0.090117565	-0.04853728	0.225364842	2.02E-01	2.76E-01	1.00E+00
TM9SF3	LMNA	0.224570997	0.089288129	0.351717949	1.31E-03	3.34E-03	1.00E+00
HAO2	LMNA	-0.026855381	-0.164297264	0.111609402	7.04E-01	7.68E-01	1.00E+00
RNH1	LMNA	0.434418829	0.315275517	0.540080167	1.05E-10	1.05E-09	1.25E-06
MAFG	LMNA	0.226369364	0.091167392	0.353377146	1.20E-03	3.07E-03	1.00E+00
BICD2	LMNA	0.289107336	0.157335854	0.410764101	3.01E-05	1.09E-04	3.57E-01
C14orf43	LMNA	0.389492473	0.265729712	0.500624957	1.01E-08	6.88E-08	1.20E-04
CDH7	LMNA	-0.280910706	-0.403320919	-0.148623318	5.13E-05	1.78E-04	6.09E-01
JUP	LMNA	0.270423204	0.137505588	0.393773766	9.93E-05	3.24E-04	1.00E+00
SHANK2	LMNA	-0.575535501	-0.661062904	-0.475244197	3.26E-19	1.47E-17	3.87E-15
OSBP2	LMNA	0.18334606	0.0464713	0.313462966	9.01E-03	1.85E-02	1.00E+00
DAK	LMNA	0.104852791	-0.033685813	0.23943792	1.38E-01	2.00E-01	1.00E+00
CBX3	LMNA	0.002503218	-0.135594641	0.140505663	9.72E-01	9.78E-01	1.00E+00
C3orf58	LMNA	0.291102063	0.15945924	0.41257302	2.63E-05	9.65E-05	3.12E-01
RBMXL2	LMNA	-0.080787614	-0.216424869	0.057909238	2.53E-01	3.34E-01	1.00E+00
TCL1B	LMNA	0.014433817	-0.123863995	0.15218158	8.38E-01	8.79E-01	1.00E+00
FGF13	LMNA	-0.431347923	-0.537397944	-0.311868045	1.47E-10	1.42E-09	1.74E-06
KBTBD2	LMNA	0.047791111	-0.090859344	0.184624035	4.99E-01	5.84E-01	1.00E+00
KIF3A	LMNA	-0.510623056	-0.605958795	-0.400827174	8.26E-15	1.63E-13	9.79E-11
DCXR	LMNA	-0.354578563	-0.469640687	-0.227672095	2.25E-07	1.22E-06	2.67E-03
MYL9	LMNA	0.629211241	0.537882454	0.705941845	1.15E-23	1.17E-21	1.36E-19
PDIA6	LMNA	0.437217545	0.318383646	0.542522782	7.72E-11	7.90E-10	9.16E-07
CDC23	LMNA	-0.046032335	-0.182920908	0.092607167	5.15E-01	5.99E-01	1.00E+00
CASKIN2	LMNA	-0.179405521	-0.309784065	-0.042404764	1.06E-02	2.14E-02	1.00E+00
PBXIP1	LMNA	-0.002335493	-0.140341245	0.135759279	9.74E-01	9.80E-01	1.00E+00
EHD1	LMNA	0.175154179	0.038022572	0.305810615	1.27E-02	2.50E-02	1.00E+00
NBL1	LMNA	0.222075198	0.086681658	0.349413969	1.49E-03	3.73E-03	1.00E+00
MARCKSL1	LMNA	-0.502432503	-0.598940225	-0.391539172	2.56E-14	4.51E-13	3.04E-10
RAB11FIP5	LMNA	0.380952673	0.2563852	0.493072545	2.23E-08	1.44E-07	2.65E-04
CDKN1A	LMNA	0.460957258	0.344851066	0.563170556	5.08E-12	6.30E-11	6.02E-08
NCK1	LMNA	-0.06140065	-0.197775224	0.077305626	3.85E-01	4.73E-01	1.00E+00

SAPS3	LMNA	-0.146733093	-0.279129857	-0.008861598	3.72E-02	6.46E-02	1.00E+00
ZNF550	LMNA	-0.08464975	-0.220128335	0.054032675	2.31E-01	3.09E-01	1.00E+00
TRAF3IP3	LMNA	0.15828654	0.020687597	0.29000056	2.45E-02	4.47E-02	1.00E+00
LSR	LMNA	0.011723251	-0.126532531	0.149532248	8.68E-01	9.03E-01	1.00E+00
MIS12	LMNA	-0.315843849	-0.434930756	-0.185898499	4.67E-06	1.97E-05	5.54E-02
KIAA0774	LMNA	-0.241716594	-0.367504265	-0.10724425	5.29E-04	1.48E-03	1.00E+00
CXorf1	LMNA	-0.510354928	-0.605729268	-0.400522761	8.57E-15	1.69E-13	1.02E-10
CUL7	LMNA	0.59118993	0.493408118	0.674215281	1.99E-20	1.07E-18	2.36E-16
DIO1	LMNA	0.060240666	-0.078462854	0.196656218	3.94E-01	4.82E-01	1.00E+00
LIPC	LMNA	0.036828918	-0.101739353	0.17399528	6.03E-01	6.80E-01	1.00E+00
EIF2B1	LMNA	-0.093063304	-0.228182732	0.045573197	1.88E-01	2.60E-01	1.00E+00
HS3ST2	LMNA	-0.07276588	-0.208720202	0.065947591	3.03E-01	3.88E-01	1.00E+00
C20orf11	LMNA	0.297687517	0.166478111	0.418538245	1.69E-05	6.41E-05	2.00E-01
CTRL	LMNA	-0.113440513	-0.24761374	0.025002034	1.08E-01	1.62E-01	1.00E+00
GSTZ1	LMNA	0.011319084	-0.126930259	0.149137042	8.73E-01	9.06E-01	1.00E+00
PAK4	LMNA	0.314773111	0.184750392	0.433966221	5.05E-06	2.11E-05	5.99E-02
CCRL1	LMNA	0.005179769	-0.132966313	0.143128422	9.42E-01	9.57E-01	1.00E+00
RNF10	LMNA	0.251299958	0.117319013	0.376296404	3.09E-04	9.14E-04	1.00E+00
MAGOH	LMNA	0.176077508	0.038973872	0.306673977	1.22E-02	2.42E-02	1.00E+00
CENPB	LMNA	0.39463866	0.271372082	0.505167977	6.19E-09	4.41E-08	7.34E-05
C19orf7	LMNA	-0.074806367	-0.210681638	0.063904583	2.90E-01	3.74E-01	1.00E+00
JMJD1A	LMNA	-0.075034688	-0.210901046	0.063675907	2.89E-01	3.72E-01	1.00E+00
GATA2	LMNA	-0.010740209	-0.148570924	0.127499835	8.79E-01	9.11E-01	1.00E+00
HIST1H4H	LMNA	-0.0364453	-0.173622751	0.102119496	6.07E-01	6.83E-01	1.00E+00
CELSR2	LMNA	-0.175736158	-0.306354822	-0.038622153	1.24E-02	2.45E-02	1.00E+00
GABRA5	LMNA	-0.264105662	-0.388009805	-0.13082453	1.46E-04	4.63E-04	1.00E+00
STAM2	LMNA	0.221009035	0.085568782	0.348429277	1.57E-03	3.91E-03	1.00E+00
GPC3	LMNA	-0.079604391	-0.215289475	0.059096044	2.60E-01	3.42E-01	1.00E+00
GRP	LMNA	0.002433872	-0.135662711	0.140437686	9.73E-01	9.79E-01	1.00E+00
SV2A	LMNA	-0.281411834	-0.403776449	-0.149155399	4.97E-05	1.72E-04	5.89E-01
EBNA1BP2	LMNA	0.087498375	-0.051170729	0.222857428	2.16E-01	2.91E-01	1.00E+00
TAF1C	LMNA	-0.20389727	-0.332586551	-0.067753407	3.61E-03	8.18E-03	1.00E+00
MAGEA12	LMNA	-0.081950677	-0.21754056	0.05674227	2.46E-01	3.26E-01	1.00E+00
GSG1	LMNA	0.127114907	-0.011131432	0.260592934	7.14E-02	1.14E-01	1.00E+00
PDGFRA	LMNA	-0.272661588	-0.395813685	-0.139875677	8.64E-05	2.86E-04	1.00E+00

CACNG1	LMNA	-0.219329254	-0.346877288	-0.083816089	1.71E-03	4.22E-03	1.00E+00
CIAPIN1	LMNA	-0.240520296	-0.366405155	-0.105988546	5.65E-04	1.57E-03	1.00E+00
CSTB	LMNA	0.354181574	0.227241578	0.469286743	2.33E-07	1.25E-06	2.76E-03
FRMPD1	LMNA	-0.236041185	-0.362286808	-0.101290819	7.20E-04	1.96E-03	1.00E+00
PTPRJ	LMNA	0.148952939	0.011130823	0.28122116	3.44E-02	6.03E-02	1.00E+00
ZNF668	LMNA	0.204735818	0.068624417	0.333364606	3.47E-03	7.90E-03	1.00E+00
PLEKHJ1	LMNA	-0.038953294	-0.176057538	0.09963349	5.82E-01	6.60E-01	1.00E+00
ADAT1	LMNA	-0.044743168	-0.181672009	0.093887763	5.27E-01	6.10E-01	1.00E+00
TMEM50A	LMNA	0.194475313	0.057980956	0.323832224	5.55E-03	1.20E-02	1.00E+00
CENPI	LMNA	0.04519878	-0.093435232	0.182113441	5.23E-01	6.06E-01	1.00E+00
HOOK1	LMNA	-0.230360216	-0.357056306	-0.095341205	9.73E-04	2.56E-03	1.00E+00
RPP21	LMNA	-0.146074389	-0.278509055	-0.008188516	3.80E-02	6.59E-02	1.00E+00
GTF2E2	LMNA	0.388888802	0.265068393	0.500091639	1.07E-08	7.24E-08	1.27E-04
IL17B	LMNA	-0.009149964	-0.14701526	0.129064066	8.97E-01	9.26E-01	1.00E+00
CCNF	LMNA	-0.056191753	-0.192747548	0.082499221	4.27E-01	5.14E-01	1.00E+00
CRYL1	LMNA	-0.320381859	-0.439015622	-0.190768359	3.35E-06	1.45E-05	3.97E-02
FOXH1	LMNA	0.11831831	-0.020060356	0.252249084	9.35E-02	1.44E-01	1.00E+00
NFYB	LMNA	-0.189415598	-0.319121876	-0.052743795	6.94E-03	1.46E-02	1.00E+00
KCNQ3	LMNA	-0.1191038	-0.252994957	0.019263946	9.14E-02	1.41E-01	1.00E+00
PPM1G	LMNA	0.26345283	0.130134824	0.387413626	1.52E-04	4.79E-04	1.00E+00
NOVA1	LMNA	-0.436446277	-0.541849826	-0.317526855	8.41E-11	8.55E-10	9.97E-07
FZD3	LMNA	-0.091353413	-0.226547328	0.047294033	1.96E-01	2.69E-01	1.00E+00
NMT1	LMNA	0.047726143	-0.090923922	0.184561137	5.00E-01	5.84E-01	1.00E+00
AKAP8	LMNA	0.292344995	0.160782956	0.413699689	2.42E-05	8.94E-05	2.87E-01
SOCS5	LMNA	0.204916592	0.068812216	0.333532316	3.44E-03	7.84E-03	1.00E+00
HADHA	LMNA	0.117931032	-0.020452953	0.25188128	9.46E-02	1.45E-01	1.00E+00
PLEKHF1	LMNA	0.407460876	0.285467533	0.516460762	1.76E-09	1.39E-08	2.09E-05
DLG5	LMNA	0.075854524	-0.062854678	0.211688763	2.83E-01	3.66E-01	1.00E+00
CFDP1	LMNA	-0.074724114	-0.210602593	0.06398696	2.91E-01	3.74E-01	1.00E+00
PGM5	LMNA	-0.143837086	-0.276399659	-0.005903307	4.11E-02	7.07E-02	1.00E+00
C1orf144	LMNA	0.255459398	0.121700333	0.380105438	2.43E-04	7.35E-04	1.00E+00
SUHW4	LMNA	-0.371683525	-0.484855945	-0.246268897	5.15E-08	3.12E-07	6.10E-04
TFEB	LMNA	0.049081936	-0.089576014	0.185873493	4.88E-01	5.73E-01	1.00E+00
RND2	LMNA	-0.340657748	-0.457207146	-0.212605174	7.02E-07	3.46E-06	8.33E-03
ATG12	LMNA	0.158642487	0.021052553	0.290334933	2.41E-02	4.41E-02	1.00E+00

BMI1	LMNA	-0.401738142	-0.511425314	-0.279170008	3.11E-09	2.34E-08	3.68E-05
RNMT	LMNA	-0.079234085	-0.214934063	0.05946739	2.62E-01	3.44E-01	1.00E+00
MASP1	LMNA	-0.324328506	-0.442564193	-0.195008805	2.49E-06	1.11E-05	2.95E-02
MYH4	LMNA	0.143198507	0.005251318	0.275797353	4.20E-02	7.20E-02	1.00E+00
SLURP1	LMNA	0.202378428	0.066176297	0.33117683	3.87E-03	8.71E-03	1.00E+00
KIAA0984	LMNA	-0.343220465	-0.459499494	-0.215374315	5.72E-07	2.85E-06	6.78E-03
ASTN1	LMNA	-0.325036496	-0.44320038	-0.195770011	2.36E-06	1.06E-05	2.80E-02
MYCL1	LMNA	-0.449368426	-0.553107012	-0.331907564	1.97E-11	2.18E-10	2.33E-07
SH3BGR	LMNA	0.065798427	-0.072914899	0.202014421	3.52E-01	4.39E-01	1.00E+00
RPAP2	LMNA	-0.291495653	-0.412929834	-0.15987836	2.57E-05	9.41E-05	3.04E-01
FOSB	LMNA	0.24285693	0.108441612	0.368551632	4.97E-04	1.40E-03	1.00E+00
KRT35	LMNA	0.135725039	-0.002370376	0.268740652	5.41E-02	8.99E-02	1.00E+00
MIA3	LMNA	-0.204823746	-0.333446181	-0.06871576	3.45E-03	7.87E-03	1.00E+00
KIR3DL3	LMNA	-0.268644256	-0.39215168	-0.135623054	1.11E-04	3.59E-04	1.00E+00
SEMA3F	LMNA	0.457357729	0.340826038	0.560048029	7.78E-12	9.33E-11	9.22E-08
TMEM135	LMNA	-0.061514737	-0.197885262	0.07719179	3.84E-01	4.72E-01	1.00E+00
SLC27A2	LMNA	-0.206450807	-0.334955333	-0.070406442	3.20E-03	7.36E-03	1.00E+00
NDUFB2	LMNA	-0.022323466	-0.159881746	0.116085284	7.52E-01	8.09E-01	1.00E+00
FAM46C	LMNA	0.071476163	-0.067238302	0.207479885	3.12E-01	3.97E-01	1.00E+00
G6PC	LMNA	-0.176242388	-0.306828126	-0.039143773	1.21E-02	2.40E-02	1.00E+00
KRT33A	LMNA	-0.047260165	-0.184109974	0.091387074	5.04E-01	5.88E-01	1.00E+00
OVOL1	LMNA	0.175475208	0.038353298	0.306110819	1.25E-02	2.47E-02	1.00E+00
PAMCI	LMNA	0.254085205	0.120252258	0.37884748	2.63E-04	7.90E-04	1.00E+00
S100A7	LMNA	-0.034854027	-0.172077056	0.103695921	6.22E-01	6.97E-01	1.00E+00
MAPKBP1	LMNA	-0.004600399	-0.142560861	0.133535408	9.48E-01	9.61E-01	1.00E+00
CPE	LMNA	-0.222695836	-0.349987051	-0.087329645	1.44E-03	3.63E-03	1.00E+00
HARS2	LMNA	0.364097802	0.23801016	0.478116713	1.00E-07	5.73E-07	1.19E-03
GNB1	LMNA	0.041655852	-0.096952688	0.178679333	5.56E-01	6.37E-01	1.00E+00
TRIM46	LMNA	-0.023885529	-0.161404312	0.114543169	7.36E-01	7.95E-01	1.00E+00
UPF3B	LMNA	-0.078173793	-0.213916216	0.060530451	2.69E-01	3.51E-01	1.00E+00
CXCR6	LMNA	0.273332705	0.140586578	0.39642506	8.29E-05	2.75E-04	9.83E-01
C16orf3	LMNA	-0.016175096	-0.153882479	0.122148661	8.19E-01	8.64E-01	1.00E+00
HLA-DOB	LMNA	-0.003109996	-0.141100417	0.134998965	9.65E-01	9.74E-01	1.00E+00
HPSE	LMNA	0.2142422	0.078513337	0.342172973	2.20E-03	5.28E-03	1.00E+00
GSCL	LMNA	-0.027865828	-0.165281009	0.110610681	6.94E-01	7.60E-01	1.00E+00

POLD1	LMNA	0.090209264	-0.048445046	0.225452596	2.02E-01	2.76E-01	1.00E+00
DMWD	LMNA	-0.062953979	-0.199273125	0.075755401	3.73E-01	4.61E-01	1.00E+00
CALD1	LMNA	0.573198155	0.472539477	0.659094617	4.89E-19	2.15E-17	5.80E-15
LCAT	LMNA	-0.025087778	-0.162575715	0.113355818	7.23E-01	7.84E-01	1.00E+00
SCRT1	LMNA	-0.188853998	-0.318598659	-0.052162963	7.11E-03	1.49E-02	1.00E+00
ST6GAL1	LMNA	-0.221427334	-0.348815646	-0.086005368	1.54E-03	3.84E-03	1.00E+00
POMC	LMNA	-0.070382561	-0.20642783	0.068332383	3.20E-01	4.05E-01	1.00E+00
UNC84B	LMNA	0.202062216	0.065848039	0.330883264	3.93E-03	8.82E-03	1.00E+00
NSMAF	LMNA	0.2319102	0.096963529	0.358484174	8.97E-04	2.38E-03	1.00E+00
ADSS	LMNA	0.192358942	0.05578945	0.321862766	6.09E-03	1.30E-02	1.00E+00
SKIL	LMNA	0.064437848	-0.074273871	0.200703449	3.62E-01	4.49E-01	1.00E+00
HMGCS1	LMNA	-0.217642479	-0.345318133	-0.082056942	1.86E-03	4.55E-03	1.00E+00
PYGO1	LMNA	-0.221664263	-0.349034469	-0.086252678	1.52E-03	3.80E-03	1.00E+00
POLR3F	LMNA	-0.13805157	-0.270938973	-5.80E-07	5.01E-02	8.41E-02	1.00E+00
ZNF277P	LMNA	0.068596717	-0.070118293	0.204709153	3.32E-01	4.18E-01	1.00E+00
CNNM3	LMNA	-0.145718596	-0.278173689	-0.007825008	3.85E-02	6.66E-02	1.00E+00
WRNIP1	LMNA	-0.016371161	-0.154073946	0.121955466	8.17E-01	8.62E-01	1.00E+00
ALAS2	LMNA	0.044554897	-0.094074743	0.181489583	5.29E-01	6.12E-01	1.00E+00
MRPL23	LMNA	0.157343739	0.019721109	0.289114745	2.53E-02	4.61E-02	1.00E+00
ST3GAL5	LMNA	-0.193772059	-0.323177913	-0.05725259	5.72E-03	1.23E-02	1.00E+00
ZBTB7B	LMNA	0.420868075	0.300262716	0.528228344	4.47E-10	3.94E-09	5.30E-06
DHRS1	LMNA	0.125658673	-0.012611096	0.259213026	7.48E-02	1.19E-01	1.00E+00
NFKBIA	LMNA	0.35049628	0.223247409	0.465999271	3.16E-07	1.66E-06	3.75E-03
CNOT3	LMNA	0.097408706	-0.041196276	0.232335407	1.68E-01	2.36E-01	1.00E+00
GAK	LMNA	0.100714616	-0.03786282	0.235491403	1.54E-01	2.20E-01	1.00E+00
SFT2D2	LMNA	0.507737713	0.397552648	0.603488007	1.23E-14	2.34E-13	1.46E-10
HOXA6	LMNA	-0.152391979	-0.284458578	-0.014649165	3.04E-02	5.42E-02	1.00E+00
PSMA6	LMNA	-0.005216455	-0.143164358	0.132930274	9.41E-01	9.56E-01	1.00E+00
CRTC1	LMNA	-0.10666623	-0.24116597	0.031853831	1.31E-01	1.91E-01	1.00E+00
LY6D	LMNA	0.029839007	-0.108659596	0.167201255	6.73E-01	7.43E-01	1.00E+00
CPT1A	LMNA	0.23816269	0.103515121	0.364238054	6.42E-04	1.77E-03	1.00E+00
ALMS1	LMNA	-0.077255003	-0.213033968	0.061451385	2.74E-01	3.57E-01	1.00E+00
LMO1	LMNA	-0.203893984	-0.332583501	-0.067749994	3.61E-03	8.18E-03	1.00E+00
EIF3I	LMNA	0.229023506	0.093942684	0.355824432	1.04E-03	2.73E-03	1.00E+00
DCN	LMNA	0.36898361	0.243327353	0.482458853	6.53E-08	3.88E-07	7.75E-04

PRB4	LMNA	0.028643865	-0.109841483	0.166038298	6.86E-01	7.53E-01	1.00E+00
MCM3APAS	LMNA	-0.411628002	-0.520122689	-0.290059842	1.16E-09	9.44E-09	1.37E-05
SUCLG2	LMNA	0.369172854	0.243533459	0.482626927	6.42E-08	3.82E-07	7.62E-04
FOXF2	LMNA	0.108873109	-0.029623129	0.243267785	1.23E-01	1.81E-01	1.00E+00
MLH1	LMNA	-0.108200583	-0.24262741	0.030303061	1.25E-01	1.84E-01	1.00E+00
S100A12	LMNA	0.092574624	-0.046065089	0.227715417	1.90E-01	2.62E-01	1.00E+00
ABHD14A	LMNA	-0.370502833	-0.483807889	-0.244982255	5.71E-08	3.43E-07	6.78E-04
DEXI	LMNA	0.016338192	-0.121987953	0.154041751	8.17E-01	8.62E-01	1.00E+00
ACTN1	LMNA	0.578390076	0.478550016	0.663465156	1.98E-19	9.15E-18	2.35E-15
AMPD2	LMNA	0.251298053	0.117317008	0.376294659	3.09E-04	9.14E-04	1.00E+00
IFNAR2	LMNA	0.056328055	-0.082363415	0.192879199	4.26E-01	5.13E-01	1.00E+00
CYB5A	LMNA	-0.247885321	-0.373166267	-0.113726121	3.75E-04	1.09E-03	1.00E+00
TLOC1	LMNA	-0.156109074	-0.287954366	-0.018455815	2.65E-02	4.80E-02	1.00E+00
NRBF2	LMNA	0.334026316	0.205449122	0.451268139	1.18E-06	5.60E-06	1.40E-02
MKS1	LMNA	-0.206111346	-0.334640526	-0.070053643	3.25E-03	7.46E-03	1.00E+00
CX3CR1	LMNA	-0.133662346	-0.26679047	0.004471157	5.79E-02	9.53E-02	1.00E+00
PDE1B	LMNA	-0.207325412	-0.335766284	-0.071315568	3.07E-03	7.10E-03	1.00E+00
KCTD3	LMNA	-0.092971043	-0.228094508	0.04566607	1.88E-01	2.60E-01	1.00E+00
ITGAE	LMNA	-0.248370463	-0.373611162	-0.114236375	3.65E-04	1.06E-03	1.00E+00
SLC30A3	LMNA	-0.429320421	-0.535625886	-0.309620015	1.82E-10	1.74E-09	2.16E-06
KISS1	LMNA	-0.036299663	-0.173481314	0.102263802	6.08E-01	6.84E-01	1.00E+00
PLP1	LMNA	-0.464387189	-0.566143246	-0.348690423	3.37E-12	4.32E-11	3.99E-08
C14orf2	LMNA	-0.251178798	-0.376185388	-0.117191469	3.11E-04	9.20E-04	1.00E+00
IFRD2	LMNA	0.448815247	0.331290825	0.552625882	2.10E-11	2.32E-10	2.49E-07
ANKRD7	LMNA	-0.08555652	-0.220997286	0.053121912	2.26E-01	3.03E-01	1.00E+00
XAB1	LMNA	0.070528213	-0.068186686	0.206567967	3.19E-01	4.04E-01	1.00E+00
NARS2	LMNA	-0.345968507	-0.461955895	-0.218345995	4.58E-07	2.33E-06	5.43E-03
TMLHE	LMNA	-0.245554987	-0.371028453	-0.111276149	4.28E-04	1.22E-03	1.00E+00
SERPINB3	LMNA	0.038037414	-0.100541541	0.175168586	5.91E-01	6.69E-01	1.00E+00
FAM127B	LMNA	0.119589252	-0.018771658	0.253455845	9.00E-02	1.39E-01	1.00E+00
ZNF391	LMNA	-0.278937824	-0.401526967	-0.146529323	5.82E-05	1.99E-04	6.90E-01
ABCA5	LMNA	0.03889124	-0.099695021	0.175997315	5.83E-01	6.61E-01	1.00E+00
DNAJB14	LMNA	-0.113213019	-0.247397403	0.025232342	1.09E-01	1.63E-01	1.00E+00
TSFM	LMNA	-0.202803891	-0.331571783	-0.066618013	3.80E-03	8.55E-03	1.00E+00
WRB	LMNA	-0.294271306	-0.415445079	-0.162835404	2.13E-05	7.94E-05	2.53E-01

ABCC8	LMNA	-0.434085513	-0.539789142	-0.314905521	1.09E-10	1.08E-09	1.29E-06
MAP1S	LMNA	0.175445265	0.038322449	0.306082819	1.25E-02	2.47E-02	1.00E+00
BPI	LMNA	0.192042578	0.055461966	0.321568267	6.18E-03	1.32E-02	1.00E+00
TTC4	LMNA	0.174538585	0.037388468	0.305234882	1.30E-02	2.56E-02	1.00E+00
BNC2	LMNA	0.499572174	0.388300904	0.596485682	3.78E-14	6.44E-13	4.48E-10
HIST1H4A	LMNA	-0.207451635	-0.335883306	-0.071446793	3.05E-03	7.06E-03	1.00E+00
NDUFS3	LMNA	-0.22650944	-0.353506349	-0.091313809	1.19E-03	3.05E-03	1.00E+00
WDR3	LMNA	-0.047405718	-0.184250905	0.09124241	5.03E-01	5.87E-01	1.00E+00
MAGED1	LMNA	-0.074168652	-0.210068747	0.06454321	2.94E-01	3.78E-01	1.00E+00
TTC33	LMNA	-0.421840509	-0.529080254	-0.301338084	4.04E-10	3.58E-09	4.79E-06
CPN2	LMNA	-0.106999845	-0.241483784	0.031516702	1.30E-01	1.90E-01	1.00E+00
STMN2	LMNA	-0.471371188	-0.572188045	-0.356520115	1.44E-12	1.95E-11	1.70E-08
PCOLCE2	LMNA	0.215587063	0.079914477	0.343417275	2.06E-03	4.98E-03	1.00E+00
ROR1	LMNA	0.497875303	0.386381121	0.595028683	4.74E-14	7.95E-13	5.63E-10
HSD17B14	LMNA	0.10569515	-0.032834956	0.240240721	1.34E-01	1.96E-01	1.00E+00
SAMD4B	LMNA	0.278075883	0.14561484	0.400742903	6.15E-05	2.10E-04	7.29E-01
HEXA	LMNA	0.552339886	0.448486459	0.641477535	1.58E-17	5.26E-16	1.87E-13
USP39	LMNA	0.002640773	-0.135459611	0.140640502	9.70E-01	9.77E-01	1.00E+00
HNRNPU	LMNA	0.010516063	-0.127720356	0.148351694	8.82E-01	9.13E-01	1.00E+00
NRD1	LMNA	0.274881007	0.142227186	0.39783512	7.52E-05	2.52E-04	8.92E-01
ATXN2L	LMNA	-0.007649645	-0.145546942	0.13053921	9.14E-01	9.38E-01	1.00E+00
MAP2K3	LMNA	0.706745992	0.630179732	0.769699708	6.93E-32	3.74E-29	8.22E-28
FLT4	LMNA	0.090221419	-0.04843282	0.225464227	2.02E-01	2.76E-01	1.00E+00
OMG	LMNA	-0.618552026	-0.697078307	-0.525362649	1.03E-22	8.72E-21	1.22E-18
SCAP	LMNA	0.057962305	-0.080734715	0.194457306	4.13E-01	5.00E-01	1.00E+00
ELA2	LMNA	0.18136271	0.044423965	0.311611791	9.79E-03	1.99E-02	1.00E+00
NARS	LMNA	-0.098587643	-0.233461208	0.04000787	1.63E-01	2.30E-01	1.00E+00
PRCC	LMNA	0.036308681	-0.102254867	0.173490071	6.08E-01	6.84E-01	1.00E+00
ZNF675	LMNA	-0.429477665	-0.535763352	-0.309794313	1.79E-10	1.71E-09	2.13E-06
VENTXP1	LMNA	0.151960848	0.014207905	0.284052888	3.09E-02	5.49E-02	1.00E+00
CALCOCO1	LMNA	-0.313267392	-0.432609388	-0.183136472	5.64E-06	2.34E-05	6.69E-02
ZBTB32	LMNA	0.071256683	-0.067457905	0.207268769	3.14E-01	3.99E-01	1.00E+00
RFC5	LMNA	-0.171470012	-0.302363581	-0.034229273	1.47E-02	2.84E-02	1.00E+00
BRAF	LMNA	0.068378856	-0.070336101	0.204499429	3.34E-01	4.19E-01	1.00E+00
PAX5	LMNA	0.285358329	0.153348333	0.407361721	3.85E-05	1.36E-04	4.56E-01

MGC14376	LMNA	0.46754791	0.352231877	0.568880273	2.30E-12	3.01E-11	2.72E-08
TNFSF5IP1	LMNA	-0.100974735	-0.235739605	0.037600403	1.53E-01	2.18E-01	1.00E+00
PEBP1	LMNA	-0.188044471	-0.31784432	-0.051325876	7.36E-03	1.54E-02	1.00E+00
AAK1	LMNA	-0.207408584	-0.335843394	-0.071402035	3.06E-03	7.07E-03	1.00E+00
FBXO2	LMNA	-0.309196095	-0.428937952	-0.178776123	7.55E-06	3.06E-05	8.96E-02
RMND1	LMNA	-0.153784226	-0.285768346	-0.016074488	2.89E-02	5.17E-02	1.00E+00
IGKV1-5	LMNA	0.134973853	-0.003135573	0.268030567	5.55E-02	9.18E-02	1.00E+00
COL1A2	LMNA	0.701804176	0.624231722	0.765673114	2.78E-31	1.06E-28	3.29E-27
SERPINA5	LMNA	0.198585044	0.062240354	0.327653469	4.61E-03	1.02E-02	1.00E+00
GMEB1	LMNA	-0.020046012	-0.157660706	0.118332459	7.77E-01	8.30E-01	1.00E+00
AKT3	LMNA	-0.313624815	-0.432931518	-0.183519516	5.49E-06	2.28E-05	6.52E-02
AANAT	LMNA	0.004527543	-0.133606966	0.142489483	9.49E-01	9.62E-01	1.00E+00
CRB1	LMNA	-0.63011947	-0.706695962	-0.538951082	9.47E-24	9.94E-22	1.12E-19
C19orf21	LMNA	-0.112589838	-0.246804718	0.025863157	1.11E-01	1.66E-01	1.00E+00
UBR4	LMNA	0.002824322	-0.135279424	0.140820417	9.68E-01	9.76E-01	1.00E+00
LTBP3	LMNA	0.512772048	0.403267852	0.607797833	6.11E-15	1.25E-13	7.24E-11
AMHR2	LMNA	0.263036634	0.129695186	0.387033492	1.56E-04	4.90E-04	1.00E+00
CTTN	LMNA	0.375164856	0.250065203	0.487944302	3.77E-08	2.31E-07	4.47E-04
UTP15	LMNA	0.20093863	0.064681891	0.329839944	4.14E-03	9.25E-03	1.00E+00
C20orf39	LMNA	-0.331250745	-0.448779294	-0.202458049	1.47E-06	6.82E-06	1.74E-02
MYBBP1A	LMNA	0.057778567	-0.080917866	0.194279915	4.14E-01	5.02E-01	1.00E+00
HSBP1	LMNA	-0.39529181	-0.505744135	-0.272088812	5.81E-09	4.17E-08	6.89E-05
PROCR	LMNA	0.488460278	0.375746798	0.586932963	1.65E-13	2.57E-12	1.96E-09
PHF11	LMNA	0.2431983	0.108800129	0.368865109	4.87E-04	1.38E-03	1.00E+00
PASK	LMNA	-0.195034928	-0.324352805	-0.058560659	5.41E-03	1.17E-02	1.00E+00
RFXDC2	LMNA	-0.193040678	-0.3224973	-0.056495245	5.91E-03	1.27E-02	1.00E+00
KIAA0467	LMNA	0.111845977	-0.026615987	0.246097127	1.13E-01	1.69E-01	1.00E+00
NDEL1	LMNA	0.250326743	0.116294636	0.375404566	3.27E-04	9.62E-04	1.00E+00
USP8	LMNA	0.004573824	-0.13356151	0.142534825	9.48E-01	9.61E-01	1.00E+00
BAIAP2	LMNA	0.202271265	0.066065048	0.331077344	3.89E-03	8.75E-03	1.00E+00
SI	LMNA	0.205308995	0.069219902	0.333896335	3.38E-03	7.71E-03	1.00E+00
ARSJ	LMNA	0.631863432	0.541003863	0.708143514	6.55E-24	7.26E-22	7.77E-20
GULP1	LMNA	-0.006470773	-0.144392788	0.131697873	9.27E-01	9.47E-01	1.00E+00
KCNE4	LMNA	0.447559276	0.329890918	0.551533241	2.42E-11	2.65E-10	2.87E-07
KCNS3	LMNA	-0.189455581	-0.319159124	-0.052785151	6.92E-03	1.46E-02	1.00E+00

BAAT	LMNA	-0.060883569	-0.197276453	0.077821525	3.89E-01	4.77E-01	1.00E+00
DKFZp434k	LMNA	0.127095631	-0.011151022	0.260574672	7.15E-02	1.14E-01	1.00E+00
MBD1	LMNA	0.07174715	-0.066967144	0.207740529	3.10E-01	3.95E-01	1.00E+00
ITGAL	LMNA	0.175947132	0.038839531	0.306552081	1.23E-02	2.43E-02	1.00E+00
WDR73	LMNA	0.13942291	0.001398833	0.272234092	4.78E-02	8.07E-02	1.00E+00
ARFGAP1	LMNA	0.340125994	0.212030845	0.456731298	7.32E-07	3.60E-06	8.69E-03
ZBTB40	LMNA	-0.08830492	-0.223629744	0.050359999	2.11E-01	2.87E-01	1.00E+00
CH25H	LMNA	0.22964377	0.094591563	0.356396104	1.01E-03	2.65E-03	1.00E+00
LOC81691	LMNA	-0.407938004	-0.516880248	-0.28599306	1.68E-09	1.34E-08	1.99E-05
CYP4B1	LMNA	-0.115942544	-0.249992174	0.022468081	1.00E-01	1.52E-01	1.00E+00
ALPL	LMNA	0.090664788	-0.047986833	0.225888486	1.99E-01	2.73E-01	1.00E+00
FOLR3	LMNA	0.285938409	0.153965037	0.40788839	3.70E-05	1.32E-04	4.39E-01
CHST3	LMNA	0.253742403	0.119891115	0.378533601	2.69E-04	8.05E-04	1.00E+00
TRIM62	LMNA	-0.108888798	-0.243282723	0.029607265	1.23E-01	1.81E-01	1.00E+00
MAP3K9	LMNA	-0.256744091	-0.38128105	-0.12305461	2.26E-04	6.86E-04	1.00E+00
ABLIM1	LMNA	-0.188658141	-0.31841617	-0.051960421	7.17E-03	1.51E-02	1.00E+00
BTAF1	LMNA	-0.219058287	-0.34662687	-0.083533439	1.73E-03	4.28E-03	1.00E+00
MAST3	LMNA	-0.255636094	-0.380267155	-0.12188657	2.41E-04	7.28E-04	1.00E+00
RBM35B	LMNA	-0.143933538	-0.276490623	-0.006001794	4.10E-02	7.05E-02	1.00E+00
ANKRD25	LMNA	0.640337853	0.550994358	0.715168475	1.06E-24	1.35E-22	1.25E-20
RYBP	LMNA	0.102151944	-0.036412553	0.236862665	1.48E-01	2.12E-01	1.00E+00
UQCRC2	LMNA	-0.284422975	-0.40651232	-0.152354141	4.09E-05	1.44E-04	4.85E-01
TTC26	LMNA	0.34527212	0.217592711	0.461333581	4.84E-07	2.46E-06	5.74E-03
ZNF22	LMNA	-0.433145511	-0.538968273	-0.313862273	1.21E-10	1.18E-09	1.43E-06
MAEA	LMNA	0.006867351	-0.131308136	0.144781092	9.23E-01	9.45E-01	1.00E+00
RNPEPL1	LMNA	0.538384493	0.432477148	0.629637956	1.41E-16	4.00E-15	1.67E-12
HYAL1	LMNA	-0.009366208	-0.147226842	0.128851399	8.95E-01	9.24E-01	1.00E+00
PSD3	LMNA	-0.214263614	-0.342192789	-0.078535643	2.20E-03	5.28E-03	1.00E+00
AGR2	LMNA	-0.013975144	-0.151733408	0.124315697	8.44E-01	8.84E-01	1.00E+00
HDLBP	LMNA	0.584839114	0.486028935	0.668885859	6.31E-20	3.21E-18	7.48E-16
GNB3	LMNA	-0.226034892	-0.353068618	-0.090817801	1.22E-03	3.12E-03	1.00E+00
HECW1	LMNA	-0.394672625	-0.50519794	-0.271409349	6.17E-09	4.40E-08	7.31E-05
ACTR2	LMNA	0.27783731	0.145361764	0.400525854	6.24E-05	2.12E-04	7.41E-01
HMGB1	LMNA	-0.249692009	-0.374822781	-0.115626687	3.39E-04	9.94E-04	1.00E+00
EDG1	LMNA	-0.295656872	-0.416699961	-0.164312401	1.94E-05	7.28E-05	2.30E-01

HOPX	LMNA	0.013011804	-0.125264208	0.150791938	8.54E-01	8.92E-01	1.00E+00
ZNF304	LMNA	-0.134444282	-0.267529883	0.003674926	5.64E-02	9.32E-02	1.00E+00
OR12D3	LMNA	-0.014336643	-0.152086636	0.123959697	8.40E-01	8.80E-01	1.00E+00
METTL1	LMNA	-0.056469366	-0.193015683	0.082222614	4.25E-01	5.12E-01	1.00E+00
PSPH	LMNA	-0.023613953	-0.161139649	0.114811325	7.39E-01	7.97E-01	1.00E+00
SOAT2	LMNA	0.205649692	0.069573905	0.334212355	3.32E-03	7.61E-03	1.00E+00
RAP1GDS1	LMNA	-0.284394461	-0.406486423	-0.152323838	4.10E-05	1.44E-04	4.86E-01
CLCA3	LMNA	0.380708121	0.256117946	0.49285602	2.28E-08	1.46E-07	2.71E-04
DARS2	LMNA	-0.007881281	-0.145773677	0.130311501	9.11E-01	9.37E-01	1.00E+00
CDC25A	LMNA	-0.223197486	-0.350450192	-0.087853483	1.41E-03	3.55E-03	1.00E+00
RCN3	LMNA	0.61601696	0.522391008	0.694966765	1.71E-22	1.37E-20	2.03E-18
CREBL1	LMNA	0.02395961	-0.114470018	0.161476503	7.35E-01	7.94E-01	1.00E+00
PTGER3	LMNA	-0.146878459	-0.279266845	-0.009010155	3.70E-02	6.43E-02	1.00E+00
USP29	LMNA	-0.125118651	-0.25870117	0.013159653	7.60E-02	1.21E-01	1.00E+00
ARHGEF10	LMNA	-0.149353507	-0.281598399	-0.011540452	3.39E-02	5.96E-02	1.00E+00
ADAM30	LMNA	-0.056877135	-0.193409495	0.081816283	4.21E-01	5.09E-01	1.00E+00
ATOX1	LMNA	0.116712894	-0.021687543	0.250724144	9.81E-02	1.50E-01	1.00E+00
KIF26B	LMNA	0.214556298	0.078840531	0.342463625	2.17E-03	5.21E-03	1.00E+00
C17orf70	LMNA	0.117675656	-0.020711813	0.251638725	9.53E-02	1.46E-01	1.00E+00
STT3A	LMNA	0.481629003	0.368049372	0.581046585	3.97E-13	5.86E-12	4.71E-09
ZNF225	LMNA	-0.114172316	-0.24830956	0.024261078	1.06E-01	1.59E-01	1.00E+00
DNASE1	LMNA	-0.130828631	-0.264109543	0.007355212	6.35E-02	1.03E-01	1.00E+00
C1orf106	LMNA	-0.405568425	-0.514796423	-0.283383838	2.13E-09	1.65E-08	2.52E-05
PTN	LMNA	0.027588091	-0.110885223	0.165010638	6.97E-01	7.62E-01	1.00E+00
RAB6IP1	LMNA	-0.174367719	-0.305075063	-0.037212484	1.31E-02	2.57E-02	1.00E+00
HECA	LMNA	-0.003484409	-0.14146736	0.134631353	9.61E-01	9.71E-01	1.00E+00
RAE1	LMNA	0.075156486	-0.063553913	0.211018084	2.88E-01	3.71E-01	1.00E+00
TNIK	LMNA	-0.322663691	-0.44106775	-0.193219467	2.82E-06	1.25E-05	3.35E-02
RNF122	LMNA	-0.131003546	-0.264275087	0.007177256	6.31E-02	1.03E-01	1.00E+00
ACTN3	LMNA	0.449375204	0.331915122	0.553112907	1.97E-11	2.18E-10	2.33E-07
SLC22A18A	LMNA	0.179084652	0.042073835	0.309484329	1.08E-02	2.17E-02	1.00E+00
CCNA1	LMNA	-0.051809058	-0.188511767	0.086863214	4.64E-01	5.50E-01	1.00E+00
ELP4	LMNA	-0.098494683	-0.233372451	0.04010159	1.63E-01	2.31E-01	1.00E+00
FAM65A	LMNA	0.250358572	0.116328134	0.375433738	3.26E-04	9.61E-04	1.00E+00
IL26	LMNA	-0.041479577	-0.178508386	0.097127605	5.58E-01	6.38E-01	1.00E+00

RYK	LMNA	0.105736481	-0.032793203	0.240280106	1.34E-01	1.96E-01	1.00E+00
QARS	LMNA	0.297899207	0.166703954	0.418729825	1.66E-05	6.33E-05	1.97E-01
RPL10A	LMNA	-0.069871998	-0.205936555	0.068843056	3.23E-01	4.09E-01	1.00E+00
LRP3	LMNA	-0.103825306	-0.238458437	0.034723391	1.41E-01	2.04E-01	1.00E+00
OR2S2	LMNA	-0.22151224	-0.348894064	-0.086093992	1.53E-03	3.82E-03	1.00E+00
BID	LMNA	-0.197147949	-0.326317733	-0.060750359	4.92E-03	1.08E-02	1.00E+00
IRS4	LMNA	-0.026173125	-0.163632883	0.112283581	7.12E-01	7.74E-01	1.00E+00
MACF1	LMNA	0.280397104	0.148078072	0.402853989	5.30E-05	1.83E-04	6.29E-01
CXCL14	LMNA	0.392715593	0.269262605	0.503471022	7.43E-09	5.22E-08	8.82E-05
SEC24D	LMNA	0.612161021	0.51787538	0.691752412	3.69E-22	2.70E-20	4.37E-18
LOC374395	LMNA	0.260326413	0.126833599	0.384557078	1.83E-04	5.67E-04	1.00E+00
TGFB2	LMNA	0.345673127	0.218026463	0.461691947	4.69E-07	2.38E-06	5.56E-03
CHRNE	LMNA	0.024213972	-0.114218835	0.161724366	7.32E-01	7.92E-01	1.00E+00
MDFIC	LMNA	0.636279595	0.54620688	0.711806224	2.55E-24	2.97E-22	3.03E-20
HSPB8	LMNA	-0.170813223	-0.301748706	-0.033553444	1.51E-02	2.90E-02	1.00E+00
PRDM14	LMNA	0.057607201	-0.081088676	0.194114461	4.15E-01	5.03E-01	1.00E+00
MNAT1	LMNA	-0.165946426	-0.297189101	-0.028549468	1.83E-02	3.44E-02	1.00E+00
ADD3	LMNA	-0.438489901	-0.543632659	-0.319797514	6.71E-11	6.95E-10	7.96E-07
MBNL2	LMNA	-0.070967135	-0.206990235	0.067747595	3.16E-01	4.01E-01	1.00E+00
KLK6	LMNA	-0.130789241	-0.264072262	0.007395286	6.36E-02	1.03E-01	1.00E+00
ATP6V0D1	LMNA	0.031844604	-0.106675361	0.169151982	6.53E-01	7.24E-01	1.00E+00
MTA2	LMNA	-0.077262373	-0.213041047	0.061443998	2.74E-01	3.57E-01	1.00E+00
TMEM111	LMNA	0.128837825	-0.00938001	0.262224844	6.76E-02	1.09E-01	1.00E+00
KIAA1279	LMNA	-0.257132434	-0.381636341	-0.123464087	2.21E-04	6.72E-04	1.00E+00
LZTR1	LMNA	0.106558664	-0.031962522	0.241063493	1.31E-01	1.92E-01	1.00E+00
RAP1A	LMNA	0.103025967	-0.035530377	0.237696252	1.45E-01	2.08E-01	1.00E+00
AXIN1	LMNA	0.250675453	0.116661645	0.375724145	3.20E-04	9.44E-04	1.00E+00
POLR1C	LMNA	-0.07143699	-0.207442206	0.067277498	3.12E-01	3.98E-01	1.00E+00
TRIO	LMNA	0.206176144	0.070120985	0.33470062	3.24E-03	7.44E-03	1.00E+00
NUBP2	LMNA	-0.189623837	-0.319315863	-0.052959189	6.87E-03	1.45E-02	1.00E+00
ID3	LMNA	-0.061447443	-0.197820357	0.077258936	3.85E-01	4.72E-01	1.00E+00
TM9SF1	LMNA	0.50257803	0.391704001	0.599065058	2.51E-14	4.44E-13	2.98E-10
POU4F2	LMNA	-0.290286145	-0.411833221	-0.158590549	2.78E-05	1.01E-04	3.30E-01
ZNF473	LMNA	-0.021698865	-0.159272757	0.116701721	7.59E-01	8.15E-01	1.00E+00
IQCK	LMNA	-0.092736871	-0.227870577	0.045901783	1.89E-01	2.61E-01	1.00E+00

MTM1	LMNA	0.053511957	-0.085168214	0.190158187	4.49E-01	5.36E-01	1.00E+00
GPR107	LMNA	0.392945477	0.269514711	0.503673922	7.27E-09	5.12E-08	8.63E-05
CAPN3	LMNA	-0.249465386	-0.374615039	-0.115388234	3.43E-04	1.01E-03	1.00E+00
FLJ14154	LMNA	-0.013563738	-0.151331373	0.124720801	8.48E-01	8.87E-01	1.00E+00
RECQL	LMNA	0.445925963	0.328071198	0.550111793	2.92E-11	3.16E-10	3.46E-07
AP1G1	LMNA	-0.009342245	-0.147203397	0.128874966	8.95E-01	9.24E-01	1.00E+00
CTNNBL1	LMNA	0.21926677	0.083750909	0.346819544	1.72E-03	4.24E-03	1.00E+00
ENPP4	LMNA	-0.13448041	-0.267564043	0.003638133	5.64E-02	9.31E-02	1.00E+00
PADI3	LMNA	-0.09983291	-0.234649957	0.038752174	1.57E-01	2.24E-01	1.00E+00
ECHDC1	LMNA	0.159666278	0.022102462	0.291296495	2.32E-02	4.27E-02	1.00E+00
RNF170	LMNA	-0.137279176	-0.270209298	0.000786734	5.14E-02	8.60E-02	1.00E+00
SMARCC1	LMNA	-0.075058891	-0.210924304	0.063651665	2.88E-01	3.72E-01	1.00E+00
C16orf7	LMNA	0.181766432	0.044840618	0.311988688	9.63E-03	1.96E-02	1.00E+00
CG018	LMNA	-0.293484318	-0.414732114	-0.161996746	2.24E-05	8.34E-05	2.66E-01
GNAI3	LMNA	0.20288665	0.06670394	0.331648603	3.78E-03	8.53E-03	1.00E+00
KCNE1	LMNA	0.328619806	0.199625054	0.446418456	1.80E-06	8.22E-06	2.13E-02
POLG2	LMNA	-0.341700467	-0.458140044	-0.213731635	6.46E-07	3.20E-06	7.66E-03
CD4	LMNA	0.288656998	0.156856638	0.41035558	3.10E-05	1.12E-04	3.67E-01
EBI2	LMNA	0.377690443	0.252821704	0.490183034	3.00E-08	1.88E-07	3.56E-04
IRF1	LMNA	0.520909849	0.412524351	0.614752677	1.91E-15	4.30E-14	2.26E-11
TPD52L2	LMNA	0.599144422	0.502670553	0.680878287	4.54E-21	2.69E-19	5.39E-17
PTPRE	LMNA	0.277016847	0.144491558	0.399779306	6.58E-05	2.23E-04	7.80E-01
PTK2B	LMNA	0.235476159	0.100698641	0.361766941	7.42E-04	2.01E-03	1.00E+00
SDHB	LMNA	0.025059731	-0.113383522	0.162548392	7.23E-01	7.84E-01	1.00E+00
SOX4	LMNA	-0.362318153	-0.476533708	-0.236075255	1.17E-07	6.61E-07	1.38E-03
TSPAN3	LMNA	-0.372986687	-0.486012337	-0.247689513	4.58E-08	2.79E-07	5.43E-04
RHOF	LMNA	0.091733344	-0.046911741	0.226910774	1.94E-01	2.67E-01	1.00E+00
SH2D1A	LMNA	0.104049061	-0.034497464	0.238671762	1.41E-01	2.03E-01	1.00E+00
PTPLAD1	LMNA	-0.054426361	-0.191041946	0.08425772	4.42E-01	5.28E-01	1.00E+00
DUSP22	LMNA	0.125824491	-0.01244264	0.259370181	7.44E-02	1.18E-01	1.00E+00
USP20	LMNA	-0.17329679	-0.304073206	-0.03610967	1.36E-02	2.67E-02	1.00E+00
CALB1	LMNA	0.157523476	0.019905343	0.289283636	2.52E-02	4.58E-02	1.00E+00
DERA	LMNA	-0.102096997	-0.236810254	0.036468005	1.48E-01	2.13E-01	1.00E+00
TMEM118	LMNA	-0.397981027	-0.508115319	-0.275041255	4.48E-09	3.29E-08	5.32E-05
MCRS1	LMNA	-0.105475007	-0.240030934	0.03305734	1.35E-01	1.97E-01	1.00E+00

C18orf8	LMNA	0.18728956	0.050545437	0.317140725	7.61E-03	1.59E-02	1.00E+00
FLJ10241	LMNA	-0.029977696	-0.167336184	0.108522419	6.72E-01	7.41E-01	1.00E+00
GINS3	LMNA	-0.196765582	-0.325962247	-0.060354019	5.01E-03	1.09E-02	1.00E+00
C19orf53	LMNA	0.111513196	-0.026952728	0.245780527	1.14E-01	1.70E-01	1.00E+00
TPP2	LMNA	-0.159225617	-0.290882651	-0.021650522	2.36E-02	4.33E-02	1.00E+00
GJA12	LMNA	-0.134667311	-0.267740755	0.003447787	5.60E-02	9.26E-02	1.00E+00
PKD1	LMNA	0.100246003	-0.038335526	0.235044212	1.56E-01	2.22E-01	1.00E+00
ST6GALNA	LMNA	0.147377748	0.009520447	0.279737313	3.63E-02	6.34E-02	1.00E+00
JAM3	LMNA	0.246769479	0.112552788	0.372142776	4.00E-04	1.15E-03	1.00E+00
CHSY1	LMNA	0.571292038	0.470335167	0.657488598	6.78E-19	2.94E-17	8.05E-15
LAPTM4A	LMNA	0.353793182	0.226820432	0.468940428	2.40E-07	1.29E-06	2.85E-03
TRPM8	LMNA	0.245911971	0.111651355	0.371356031	4.19E-04	1.20E-03	1.00E+00
MYOM1	LMNA	-0.160876085	-0.292432423	-0.023343523	2.22E-02	4.10E-02	1.00E+00
PHKG2	LMNA	-0.025966904	-0.16343204	0.112487337	7.14E-01	7.76E-01	1.00E+00
EXT2	LMNA	0.464074626	0.348340389	0.56587246	3.50E-12	4.46E-11	4.15E-08
MOBKL1B	LMNA	0.334087273	0.205514839	0.451322779	1.18E-06	5.58E-06	1.40E-02
DIAPH2	LMNA	0.104054123	-0.034492352	0.238676588	1.41E-01	2.03E-01	1.00E+00
DOLK	LMNA	0.309090932	0.178663561	0.428843065	7.61E-06	3.07E-05	9.03E-02
TUBAL3	LMNA	-0.01181797	-0.14962486	0.126439314	8.67E-01	9.02E-01	1.00E+00
CRYZL1	LMNA	-0.272645879	-0.395799372	-0.139859037	8.65E-05	2.86E-04	1.00E+00
CLN8	LMNA	-0.167609498	-0.298747874	-0.030258642	1.71E-02	3.25E-02	1.00E+00
PTPN12	LMNA	0.512670441	0.403152418	0.607710904	6.19E-15	1.26E-13	7.35E-11
ABL2	LMNA	0.496409065	0.384723046	0.5937692	5.77E-14	9.47E-13	6.85E-10
ACVRL1	LMNA	0.280133174	0.147797912	0.402614017	5.39E-05	1.86E-04	6.40E-01
C14orf156	LMNA	0.006541058	-0.131628804	0.14446161	9.26E-01	9.47E-01	1.00E+00
CRY2	LMNA	-0.286819903	-0.408688563	-0.154902382	3.50E-05	1.25E-04	4.15E-01
PTPRZ1	LMNA	-0.29055529	-0.412077274	-0.158877079	2.73E-05	9.98E-05	3.24E-01
LAMP1	LMNA	0.33194192	0.203202664	0.449399239	1.39E-06	6.49E-06	1.65E-02
PNPLA4	LMNA	0.232173434	0.09723912	0.35872661	8.85E-04	2.35E-03	1.00E+00
FCGR2B	LMNA	0.505421411	0.394925947	0.601503171	1.70E-14	3.17E-13	2.02E-10
DIP2C	LMNA	-0.302463364	-0.422857781	-0.171576608	1.21E-05	4.74E-05	1.44E-01
RXRA	LMNA	0.208835602	0.072885899	0.337166115	2.86E-03	6.65E-03	1.00E+00
MAP3K5	LMNA	0.044304214	-0.094323693	0.181246667	5.31E-01	6.14E-01	1.00E+00
PDLIM7	LMNA	0.628240727	0.536740865	0.705135819	1.40E-23	1.41E-21	1.67E-19
ALKBH1	LMNA	-0.049093827	-0.185885001	0.089564191	4.88E-01	5.73E-01	1.00E+00

ARL14	LMNA	0.172742514	0.035539022	0.303554565	1.40E-02	2.72E-02	1.00E+00
SNIP1	LMNA	0.160804929	0.023270517	0.292365622	2.22E-02	4.11E-02	1.00E+00
CLDN11	LMNA	-0.153046043	-0.285073955	-0.015318698	2.97E-02	5.30E-02	1.00E+00
TIMP3	LMNA	0.142138219	0.004169024	0.274797063	4.36E-02	7.43E-02	1.00E+00
CIB2	LMNA	-0.089303668	-0.22458587	0.049355814	2.06E-01	2.81E-01	1.00E+00
HRK	LMNA	-0.22053016	-0.347986905	-0.085069036	1.61E-03	3.99E-03	1.00E+00
MXRA8	LMNA	0.579016021	0.479275287	0.663991682	1.78E-19	8.22E-18	2.11E-15
RGS3	LMNA	0.483769543	0.370459641	0.582892166	3.02E-13	4.52E-12	3.59E-09
SPAG16	LMNA	-0.164699474	-0.296019892	-0.027268474	1.92E-02	3.59E-02	1.00E+00
C4orf31	LMNA	0.029870994	-0.108627958	0.167232375	6.73E-01	7.42E-01	1.00E+00
FAM5B	LMNA	-0.30854246	-0.428348145	-0.178076559	7.91E-06	3.19E-05	9.39E-02
ABHD4	LMNA	0.149609594	0.011802356	0.28183955	3.36E-02	5.92E-02	1.00E+00
ARHGEF12	LMNA	-0.096454064	-0.231423527	0.042158298	1.72E-01	2.41E-01	1.00E+00
NFATC1	LMNA	0.158353371	0.020756116	0.290063342	2.44E-02	4.46E-02	1.00E+00
RAC2	LMNA	0.485992806	0.372964674	0.58480799	2.27E-13	3.45E-12	2.69E-09
GLUD2	LMNA	0.001888305	-0.1361982	0.139902836	9.79E-01	9.84E-01	1.00E+00
Sep-10	LMNA	0.154614749	0.016925008	0.286549432	2.80E-02	5.03E-02	1.00E+00
UAP1L1	LMNA	0.366787187	0.240936066	0.480507527	7.92E-08	4.63E-07	9.39E-04
RPP40	LMNA	0.120102822	-0.018250782	0.253943362	8.87E-02	1.37E-01	1.00E+00
SLC18A3	LMNA	-0.044602603	-0.181535809	0.094027365	5.28E-01	6.11E-01	1.00E+00
YOD1	LMNA	-0.154942291	-0.286857429	-0.017260491	2.77E-02	4.98E-02	1.00E+00
RALY	LMNA	0.325137197	0.195878295	0.443290859	2.34E-06	1.05E-05	2.78E-02
TBX5	LMNA	-0.19850301	-0.327577235	-0.062155285	4.63E-03	1.02E-02	1.00E+00
NAPG	LMNA	0.094867489	-0.043756572	0.229907494	1.79E-01	2.49E-01	1.00E+00
C14orf45	LMNA	0.287680497	0.155817733	0.409469585	3.30E-05	1.19E-04	3.92E-01
RHD	LMNA	-0.132654985	-0.26583766	0.005496677	5.98E-02	9.81E-02	1.00E+00
HMOX2	LMNA	-0.025226293	-0.162710651	0.113218995	7.22E-01	7.83E-01	1.00E+00
DBNDD2	LMNA	-0.150187169	-0.282383377	-0.012393121	3.29E-02	5.82E-02	1.00E+00
YIPF4	LMNA	0.092915601	-0.045721878	0.228041493	1.88E-01	2.60E-01	1.00E+00
ZBTB22	LMNA	-0.078596156	-0.214321706	0.060107023	2.66E-01	3.48E-01	1.00E+00
THAP10	LMNA	-0.305031038	-0.425177867	-0.174320652	1.01E-05	4.01E-05	1.20E-01
ANKRD10	LMNA	-0.152148993	-0.284229937	-0.014400464	3.06E-02	5.46E-02	1.00E+00
PLCG1	LMNA	0.198177762	0.061818018	0.327274965	4.69E-03	1.03E-02	1.00E+00
TAGLN2	LMNA	0.704388424	0.627341023	0.767779385	1.35E-31	6.15E-29	1.60E-27
SLC16A7	LMNA	-0.170384334	-0.301347129	-0.033112189	1.53E-02	2.95E-02	1.00E+00

HTR2C	LMNA	-0.284435479	-0.406523676	-0.152367429	4.09E-05	1.44E-04	4.85E-01
TSGA14	LMNA	0.017556003	-0.120787742	0.155230783	8.04E-01	8.52E-01	1.00E+00
MDH1	LMNA	-0.249711441	-0.374840594	-0.115647134	3.38E-04	9.93E-04	1.00E+00
ITGB4	LMNA	0.285357331	0.153347272	0.407360814	3.85E-05	1.36E-04	4.56E-01
DCBLD2	LMNA	0.641080312	0.551870865	0.715783224	8.99E-25	1.16E-22	1.07E-20
C5orf28	LMNA	0.09564606	-0.042972349	0.23065153	1.76E-01	2.46E-01	1.00E+00
YTHDF3	LMNA	-0.070259501	-0.206309426	0.068455476	3.20E-01	4.06E-01	1.00E+00
FMO4	LMNA	0.221378502	0.085954398	0.348770543	1.54E-03	3.84E-03	1.00E+00
THYN1	LMNA	-0.37154009	-0.484728641	-0.246112568	5.21E-08	3.15E-07	6.18E-04
OTOR	LMNA	0.013762616	-0.124524975	0.151525726	8.46E-01	8.85E-01	1.00E+00
PGRMC2	LMNA	0.280203194	0.147872236	0.402677683	5.37E-05	1.85E-04	6.37E-01
DRD5	LMNA	-0.302190822	-0.422611427	-0.171285465	1.24E-05	4.83E-05	1.47E-01
TMEM30B	LMNA	0.019095112	-0.119270297	0.156732949	7.87E-01	8.39E-01	1.00E+00
PAQR6	LMNA	-0.29860086	-0.419364745	-0.167452616	1.59E-05	6.05E-05	1.88E-01
UBE2I	LMNA	0.058058182	-0.08063914	0.194549867	4.12E-01	5.00E-01	1.00E+00
TBC1D5	LMNA	-0.106463401	-0.240972734	0.03205878	1.32E-01	1.92E-01	1.00E+00
C8orf70	LMNA	-0.302446337	-0.422842391	-0.171558418	1.22E-05	4.74E-05	1.44E-01
HRH4	LMNA	0.013978478	-0.124312413	0.151736666	8.43E-01	8.84E-01	1.00E+00
ANGPTL3	LMNA	0.043867987	-0.094756863	0.180823915	5.35E-01	6.17E-01	1.00E+00
MYO6	LMNA	-0.207458597	-0.335889759	-0.07145403	3.05E-03	7.06E-03	1.00E+00
GLRX2	LMNA	0.256934163	0.12325502	0.381454949	2.23E-04	6.79E-04	1.00E+00
ATP11A	LMNA	0.167608621	0.03025774	0.298747051	1.71E-02	3.25E-02	1.00E+00
MUC16	LMNA	0.086322841	-0.052352036	0.221731478	2.22E-01	2.99E-01	1.00E+00
SLC25A5	LMNA	-0.017124904	-0.154809917	0.121212657	8.09E-01	8.56E-01	1.00E+00
MYO1C	LMNA	0.590656643	0.492787936	0.673768095	2.20E-20	1.17E-18	2.61E-16
RBM23	LMNA	-0.175427338	-0.306066055	-0.03830398	1.25E-02	2.47E-02	1.00E+00
FAM89B	LMNA	-0.005280345	-0.143226939	0.13286751	9.41E-01	9.56E-01	1.00E+00
FAS	LMNA	0.447882701	0.330251358	0.551814641	2.33E-11	2.56E-10	2.77E-07
KIFAP3	LMNA	-0.22629159	-0.353305408	-0.091086099	1.20E-03	3.08E-03	1.00E+00
NGFB	LMNA	0.000357094	-0.137700695	0.138401272	9.96E-01	9.97E-01	1.00E+00
C1orf63	LMNA	-0.045349173	-0.18225914	0.093285844	5.22E-01	6.05E-01	1.00E+00
PERLD1	LMNA	0.169914268	0.032628631	0.300906944	1.56E-02	2.99E-02	1.00E+00
GLRA2	LMNA	-0.254815022	-0.379515624	-0.121021243	2.52E-04	7.60E-04	1.00E+00
BTN3A2	LMNA	0.202243905	0.066036646	0.331051944	3.90E-03	8.75E-03	1.00E+00
C17orf59	LMNA	0.107585375	-0.030924931	0.24204151	1.28E-01	1.87E-01	1.00E+00

HSPBAP1	LMNA	-0.189152113	-0.318876409	-0.052471276	7.02E-03	1.48E-02	1.00E+00
CSTF3	LMNA	-0.278513016	-0.401140563	-0.146078591	5.98E-05	2.04E-04	7.09E-01
PRKCI	LMNA	0.079930474	-0.058769012	0.215602414	2.58E-01	3.40E-01	1.00E+00
OSMR	LMNA	0.615796332	0.522132493	0.694782932	1.79E-22	1.42E-20	2.12E-18
SOD3	LMNA	0.391295103	0.267705183	0.502217004	8.51E-09	5.89E-08	1.01E-04
ZNF574	LMNA	-0.087552993	-0.222909733	0.051115833	2.15E-01	2.91E-01	1.00E+00
CYSLTR2	LMNA	-0.090512235	-0.225742514	0.048140294	2.00E-01	2.74E-01	1.00E+00
RPL12	LMNA	0.238253507	0.103610369	0.364321558	6.39E-04	1.76E-03	1.00E+00
WIZ	LMNA	0.124269396	-0.014022162	0.257896057	7.81E-02	1.23E-01	1.00E+00
ALDH4A1	LMNA	-0.097711651	-0.232624732	0.040890935	1.67E-01	2.34E-01	1.00E+00
CRYAB	LMNA	-0.210216402	-0.338445516	-0.074322277	2.67E-03	6.28E-03	1.00E+00
RNF17	LMNA	0.01174596	-0.126510183	0.149554451	8.68E-01	9.02E-01	1.00E+00
NEIL3	LMNA	-0.10588844	-0.240424908	0.032639688	1.34E-01	1.95E-01	1.00E+00
COPA	LMNA	0.167827265	0.030482505	0.298951931	1.70E-02	3.23E-02	1.00E+00
KIF11	LMNA	-0.0643535	-0.20062216	0.074358103	3.63E-01	4.50E-01	1.00E+00
SLC26A3	LMNA	-0.047484558	-0.18432724	0.09116405	5.02E-01	5.86E-01	1.00E+00
SKP2	LMNA	-0.070679714	-0.206713725	0.068035133	3.18E-01	4.03E-01	1.00E+00
ZNF175	LMNA	-0.207370294	-0.335807895	-0.071362228	3.06E-03	7.08E-03	1.00E+00
PARVA	LMNA	0.563357369	0.461172715	0.65079473	2.59E-18	9.99E-17	3.08E-14
JAKMIP2	LMNA	-0.347428299	-0.463260039	-0.219925549	4.06E-07	2.09E-06	4.82E-03
C8orf4	LMNA	0.405555299	0.28336939	0.514784877	2.13E-09	1.65E-08	2.53E-05
PTHLH	LMNA	0.461217338	0.345142055	0.563396058	4.92E-12	6.11E-11	5.84E-08
RNF34	LMNA	-0.095303282	-0.230323977	0.043317636	1.77E-01	2.47E-01	1.00E+00
PKLR	LMNA	0.038085583	-0.100493789	0.175215345	5.90E-01	6.68E-01	1.00E+00
PCDHB17	LMNA	0.050299783	-0.088364817	0.187051907	4.77E-01	5.62E-01	1.00E+00
CD36	LMNA	0.146758687	0.008887754	0.279153977	3.71E-02	6.46E-02	1.00E+00
CCT2	LMNA	-0.056504217	-0.193049343	0.082187888	4.24E-01	5.11E-01	1.00E+00
PRKG2	LMNA	-0.344661733	-0.460788026	-0.216932579	5.09E-07	2.57E-06	6.04E-03
ARF4	LMNA	0.232949789	0.098052039	0.359441527	8.49E-04	2.27E-03	1.00E+00
SIKE	LMNA	-0.147753041	-0.2800909	-0.009904058	3.59E-02	6.26E-02	1.00E+00
ANAPC13	LMNA	-0.315443158	-0.43456984	-0.185468813	4.81E-06	2.02E-05	5.71E-02
MBTPS1	LMNA	0.174385005	0.037230287	0.305091231	1.31E-02	2.57E-02	1.00E+00
SLCO3A1	LMNA	0.081633983	-0.057060065	0.2172368	2.48E-01	3.28E-01	1.00E+00
ZNF692	LMNA	-0.158258277	-0.289974009	-0.02065862	2.45E-02	4.47E-02	1.00E+00
GPSN2	LMNA	0.2170906	0.081481568	0.344807856	1.91E-03	4.67E-03	1.00E+00

NCF2	LMNA	0.369889872	0.244314464	0.483263658	6.03E-08	3.60E-07	7.15E-04
SH3GLB1	LMNA	0.464587922	0.348915238	0.566317138	3.29E-12	4.22E-11	3.90E-08
SLC12A6	LMNA	-0.069959736	-0.206020984	0.068755304	3.22E-01	4.08E-01	1.00E+00
MRPL48	LMNA	-0.31749575	-0.436418273	-0.18767046	4.14E-06	1.76E-05	4.91E-02
HMGN3	LMNA	-0.395455041	-0.50588811	-0.272267955	5.72E-09	4.11E-08	6.78E-05
SUPT5H	LMNA	0.044116272	-0.094510324	0.181064536	5.33E-01	6.15E-01	1.00E+00
XRCC6	LMNA	-0.072812227	-0.208764766	0.0659012	3.03E-01	3.88E-01	1.00E+00
SOS2	LMNA	-0.207777629	-0.336185514	-0.071785723	3.01E-03	6.96E-03	1.00E+00
PAX9	LMNA	0.07540309	-0.063306902	0.211255037	2.86E-01	3.70E-01	1.00E+00
CCDC99	LMNA	0.031365676	-0.107149287	0.168686255	6.58E-01	7.28E-01	1.00E+00
NNAT	LMNA	-0.150960629	-0.283111511	-0.013184395	3.20E-02	5.67E-02	1.00E+00
USP16	LMNA	0.032894124	-0.105636579	0.170172361	6.42E-01	7.15E-01	1.00E+00
C20orf42	LMNA	-0.386339834	-0.497838801	-0.262277287	1.36E-08	9.07E-08	1.61E-04
LARS	LMNA	-0.146099564	-0.278532783	-0.008214238	3.80E-02	6.59E-02	1.00E+00
SCML2	LMNA	-0.396723792	-0.507006975	-0.27366067	5.06E-09	3.68E-08	6.00E-05
BCL9	LMNA	-0.028008708	-0.16542009	0.110469437	6.92E-01	7.58E-01	1.00E+00
ABO	LMNA	0.106510656	-0.032011032	0.241017755	1.31E-01	1.92E-01	1.00E+00
TRAF3	LMNA	0.355766552	0.228960711	0.470699644	2.04E-07	1.11E-06	2.41E-03
LETM1	LMNA	0.151460328	0.013695694	0.283581845	3.14E-02	5.58E-02	1.00E+00
PLXNB1	LMNA	-0.420285393	-0.527717776	-0.299618503	4.75E-10	4.17E-09	5.64E-06
NIPSNAP1	LMNA	-0.207158977	-0.335611977	-0.071142546	3.09E-03	7.15E-03	1.00E+00
USP10	LMNA	-0.116094923	-0.250136974	0.022313699	9.99E-02	1.52E-01	1.00E+00
F9	LMNA	-0.046498331	-0.183372239	0.092144156	5.11E-01	5.95E-01	1.00E+00
CNGB3	LMNA	-0.22611289	-0.353140569	-0.090899322	1.21E-03	3.11E-03	1.00E+00
LIPE	LMNA	-0.444208972	-0.548616869	-0.326159192	3.54E-11	3.79E-10	4.20E-07
C12orf52	LMNA	0.161720593	0.0242101	0.29322514	2.15E-02	3.98E-02	1.00E+00
PI4K2A	LMNA	0.270587989	0.137680018	0.393923982	9.83E-05	3.21E-04	1.00E+00
KIAA0515	LMNA	-0.070117861	-0.206173138	0.068597149	3.21E-01	4.07E-01	1.00E+00
MED8	LMNA	0.367720488	0.241951982	0.481336819	7.30E-08	4.30E-07	8.66E-04
TEX261	LMNA	0.391988372	0.268465201	0.502829085	7.97E-09	5.55E-08	9.45E-05
STAT4	LMNA	0.032802402	-0.105727375	0.170083197	6.43E-01	7.16E-01	1.00E+00
LY86	LMNA	0.008297562	-0.12990224	0.146181114	9.07E-01	9.33E-01	1.00E+00
WDR32	LMNA	-0.039565152	-0.176651281	0.099026734	5.76E-01	6.55E-01	1.00E+00
FLJ13611	LMNA	-0.360939929	-0.475307263	-0.234577483	1.31E-07	7.38E-07	1.56E-03
SNRPA	LMNA	-0.058250227	-0.194735264	0.080447694	4.10E-01	4.98E-01	1.00E+00

ZMYND8	LMNA	-0.031554699	-0.168870075	0.106962246	6.56E-01	7.27E-01	1.00E+00
GATAD2A	LMNA	0.373736603	0.248507263	0.486677612	4.29E-08	2.62E-07	5.08E-04
PDXK	LMNA	0.2724612	0.139663436	0.395631114	8.75E-05	2.89E-04	1.00E+00
PTGES3	LMNA	-0.08330123	-0.21883566	0.055386708	2.39E-01	3.18E-01	1.00E+00
C7orf42	LMNA	0.629538971	0.538268029	0.706213984	1.07E-23	1.10E-21	1.27E-19
TAP1	LMNA	0.389051884	0.265247037	0.500235724	1.05E-08	7.14E-08	1.25E-04
CYP11B2	LMNA	0.111450877	-0.027015786	0.245721234	1.14E-01	1.70E-01	1.00E+00
ETS2	LMNA	-0.007923328	-0.145814833	0.130270165	9.11E-01	9.36E-01	1.00E+00
C6orf166	LMNA	-0.09151974	-0.226706442	0.047126678	1.95E-01	2.68E-01	1.00E+00
PRMT2	LMNA	0.20022288	0.063939221	0.32917516	4.28E-03	9.52E-03	1.00E+00
PRDX1	LMNA	0.089110155	-0.049550402	0.224400636	2.07E-01	2.82E-01	1.00E+00
FGB	LMNA	0.027141329	-0.1113268	0.164575683	7.01E-01	7.66E-01	1.00E+00
INTS8	LMNA	-0.089936019	-0.225191102	0.048719875	2.03E-01	2.77E-01	1.00E+00
COX17	LMNA	0.116321062	-0.022084578	0.250351853	9.92E-02	1.51E-01	1.00E+00
C16orf33	LMNA	-0.369915038	-0.483286004	-0.244341879	6.02E-08	3.60E-07	7.14E-04
EPHA5	LMNA	-0.078456834	-0.214187955	0.060246702	2.67E-01	3.49E-01	1.00E+00
PIWIL1	LMNA	-0.226614086	-0.353602868	-0.091423196	1.18E-03	3.04E-03	1.00E+00
FOLR1	LMNA	0.134145797	-0.003978889	0.267247649	5.70E-02	9.40E-02	1.00E+00
FREQ	LMNA	-0.025720255	-0.163191809	0.11273102	7.16E-01	7.78E-01	1.00E+00
KIAA0082	LMNA	-0.074377952	-0.210269912	0.064333623	2.93E-01	3.77E-01	1.00E+00
TSGA10	LMNA	-0.147825358	-0.28015903	-0.009977982	3.58E-02	6.25E-02	1.00E+00
TMCC2	LMNA	-0.440192605	-0.545117353	-0.321690419	5.55E-11	5.80E-10	6.59E-07
TCF12	LMNA	-0.322615712	-0.441024613	-0.193167912	2.83E-06	1.25E-05	3.36E-02
FAM130A2	LMNA	-0.566344218	-0.6533161	-0.464619183	1.57E-18	6.34E-17	1.87E-14
MYOT	LMNA	-0.437250689	-0.542551698	-0.31842047	7.70E-11	7.88E-10	9.13E-07
HAL	LMNA	0.06967184	-0.069043237	0.20574394	3.24E-01	4.10E-01	1.00E+00
POU4F1	LMNA	-0.300005451	-0.420635394	-0.168951765	1.44E-05	5.54E-05	1.71E-01
SNRPF	LMNA	0.059056594	-0.079643728	0.195513607	4.04E-01	4.91E-01	1.00E+00
BCL2L2	LMNA	0.120143693	-0.018209327	0.253982157	8.85E-02	1.37E-01	1.00E+00
CUGBP2	LMNA	-0.341903442	-0.458321611	-0.21395095	6.35E-07	3.15E-06	7.54E-03
EMG1	LMNA	0.06491854	-0.073793806	0.201166671	3.59E-01	4.45E-01	1.00E+00
PRRG4	LMNA	0.130390085	-0.007801343	0.263694458	6.44E-02	1.04E-01	1.00E+00
HIRA	LMNA	-0.103402596	-0.238055395	0.035150168	1.43E-01	2.06E-01	1.00E+00
MERTK	LMNA	0.076889385	-0.06181779	0.21268283	2.77E-01	3.59E-01	1.00E+00
BAG1	LMNA	-0.194090229	-0.323473955	-0.057582104	5.64E-03	1.22E-02	1.00E+00

MYNN	LMNA	-0.198895502	-0.327941963	-0.06256232	4.54E-03	1.00E-02	1.00E+00
CORO2B	LMNA	0.167407815	0.030051325	0.298558876	1.72E-02	3.27E-02	1.00E+00
ALOX15	LMNA	0.072801221	-0.065912216	0.208754183	3.03E-01	3.88E-01	1.00E+00
TBXA2R	LMNA	0.369384551	0.24376403	0.482814932	6.31E-08	3.76E-07	7.48E-04
RNF144A	LMNA	-0.201078611	-0.329969942	-0.064827154	4.11E-03	9.20E-03	1.00E+00
Mar-05	LMNA	-0.208608666	-0.336955799	-0.072649882	2.89E-03	6.71E-03	1.00E+00
CST1	LMNA	0.061729312	-0.076977676	0.198092212	3.83E-01	4.70E-01	1.00E+00
NUPR1	LMNA	0.28718531	0.155291011	0.409020206	3.41E-05	1.22E-04	4.05E-01
DULLARD	LMNA	0.215505154	0.079829126	0.343341504	2.07E-03	5.00E-03	1.00E+00
DCLRE1B	LMNA	0.060635893	-0.078068608	0.197037523	3.91E-01	4.79E-01	1.00E+00
ITGA8	LMNA	0.136549087	-0.001530769	0.269519447	5.27E-02	8.78E-02	1.00E+00
CCL7	LMNA	0.26901397	0.136014217	0.392488857	1.08E-04	3.52E-04	1.00E+00
TP73	LMNA	0.063038824	-0.075670707	0.199354924	3.73E-01	4.60E-01	1.00E+00
PRKCD	LMNA	0.307279045	0.176724739	0.427207813	8.66E-06	3.46E-05	1.03E-01
NDUFB4	LMNA	-0.00634807	-0.144272636	0.131818451	9.29E-01	9.48E-01	1.00E+00
DSC2	LMNA	0.262405718	0.129028835	0.386457164	1.62E-04	5.07E-04	1.00E+00
RBMS2	LMNA	0.202807048	0.066621292	0.331574714	3.80E-03	8.55E-03	1.00E+00
GRIK4	LMNA	-0.42344463	-0.530485087	-0.303112673	3.41E-10	3.05E-09	4.05E-06
TMPRSS6	LMNA	-0.005804196	-0.143740022	0.132352855	9.35E-01	9.52E-01	1.00E+00
GLB1L	LMNA	0.363637821	0.237509954	0.477707629	1.04E-07	5.95E-07	1.23E-03
COL4A3	LMNA	-0.194052056	-0.323438439	-0.057542568	5.65E-03	1.22E-02	1.00E+00
PUS1	LMNA	0.321684988	0.192167962	0.440187721	3.04E-06	1.33E-05	3.60E-02
MYO10	LMNA	-0.310509296	-0.43012261	-0.180181999	6.88E-06	2.81E-05	8.16E-02
PEX1	LMNA	-0.139942334	-0.272724521	-0.001928592	4.70E-02	7.94E-02	1.00E+00
OLFM1	LMNA	-0.48246744	-0.58176961	-0.368993278	3.57E-13	5.29E-12	4.24E-09
RBM19	LMNA	0.094895679	-0.04372818	0.229934437	1.79E-01	2.49E-01	1.00E+00
RET	LMNA	-0.003174476	-0.141163613	0.134935659	9.64E-01	9.73E-01	1.00E+00
TAPBP	LMNA	0.563450605	0.461280252	0.650873465	2.55E-18	9.87E-17	3.03E-14
RUNX1	LMNA	0.660313625	0.574645565	0.731667998	1.14E-26	1.98E-24	1.35E-22
IL1RL1	LMNA	0.290490799	0.15880842	0.412018797	2.74E-05	1.00E-04	3.25E-01
ALX3	LMNA	-0.015887156	-0.153601272	0.122432368	8.22E-01	8.66E-01	1.00E+00
MID1	LMNA	0.245800912	0.111534623	0.371254124	4.22E-04	1.21E-03	1.00E+00
C14orf138	LMNA	-0.154076936	-0.286043653	-0.016374223	2.86E-02	5.12E-02	1.00E+00
GPR64	LMNA	-0.022619711	-0.160170551	0.115792873	7.49E-01	8.07E-01	1.00E+00
SNX10	LMNA	0.095123172	-0.043499052	0.230151853	1.78E-01	2.48E-01	1.00E+00

MYO16	LMNA	-0.380205993	-0.492411394	-0.255569265	2.39E-08	1.52E-07	2.83E-04
DBF4	LMNA	0.011360651	-0.126889356	0.149177689	8.73E-01	9.06E-01	1.00E+00
POGK	LMNA	-0.265966777	-0.389708845	-0.132791478	1.30E-04	4.18E-04	1.00E+00
MAPK9	LMNA	0.112470928	-0.025983511	0.246691616	1.11E-01	1.66E-01	1.00E+00
RIPK2	LMNA	0.058467754	-0.080230833	0.194945248	4.09E-01	4.96E-01	1.00E+00
LRRC31	LMNA	-0.024812415	-0.162307451	0.113627804	7.26E-01	7.87E-01	1.00E+00
C8orf79	LMNA	-0.019511861	-0.157139585	0.118859303	7.83E-01	8.35E-01	1.00E+00
CLDN7	LMNA	0.340827756	0.212788813	0.457359266	6.93E-07	3.41E-06	8.21E-03
EFNB3	LMNA	-0.39135137	-0.502266685	-0.267766861	8.46E-09	5.86E-08	1.00E-04
GLP1R	LMNA	-0.114321549	-0.248451438	0.02410996	1.05E-01	1.59E-01	1.00E+00
CSTF2T	LMNA	-0.309260591	-0.428996144	-0.178845158	7.52E-06	3.05E-05	8.92E-02
TRIM37	LMNA	-0.551098898	-0.640426414	-0.447060121	1.92E-17	6.28E-16	2.28E-13
GRHL2	LMNA	-0.107656234	-0.242108998	0.03085331	1.27E-01	1.87E-01	1.00E+00
IREB2	LMNA	-0.02404697	-0.161561633	0.114383751	7.34E-01	7.93E-01	1.00E+00
GRSF1	LMNA	-0.16615211	-0.297381924	-0.02876081	1.81E-02	3.42E-02	1.00E+00
CNR1	LMNA	0.157617422	0.020001642	0.289371909	2.51E-02	4.57E-02	1.00E+00
ABCC4	LMNA	-0.055559159	-0.192136473	0.083129444	4.32E-01	5.18E-01	1.00E+00
DLG3	LMNA	-0.204915666	-0.333531458	-0.068811255	3.44E-03	7.84E-03	1.00E+00
ZFP36L2	LMNA	0.417709363	0.296771764	0.525459631	6.21E-10	5.34E-09	7.37E-06
VGLL1	LMNA	0.0154339	-0.122878914	0.153158571	8.27E-01	8.70E-01	1.00E+00
IL1F7	LMNA	0.034235015	-0.104308968	0.171475591	6.29E-01	7.03E-01	1.00E+00
NR2E1	LMNA	0.22731206	0.092152877	0.354246571	1.14E-03	2.95E-03	1.00E+00
MS4A6A	LMNA	0.189107043	0.052424663	0.31883442	7.03E-03	1.48E-02	1.00E+00
FTL	LMNA	0.295453328	0.164095389	0.416515644	1.97E-05	7.38E-05	2.33E-01
DYRK2	LMNA	0.004871001	-0.133269616	0.142825959	9.45E-01	9.59E-01	1.00E+00
PCLO	LMNA	-0.283329448	-0.405519013	-0.151192165	4.39E-05	1.54E-04	5.21E-01
ARIH2	LMNA	-0.038536659	-0.175653181	0.100046592	5.86E-01	6.64E-01	1.00E+00
PCNX	LMNA	0.217783269	0.08220374	0.345448298	1.85E-03	4.52E-03	1.00E+00
ANXA9	LMNA	0.122711701	-0.015603633	0.256418845	8.19E-02	1.28E-01	1.00E+00
SRR	LMNA	-0.346574867	-0.462497663	-0.219002019	4.36E-07	2.23E-06	5.17E-03
SCNN1D	LMNA	-0.207157008	-0.335610152	-0.0711405	3.10E-03	7.15E-03	1.00E+00
NOL3	LMNA	0.349430618	0.222093227	0.465048051	3.45E-07	1.80E-06	4.09E-03
IFITM2	LMNA	0.561768354	0.459340437	0.649452566	3.38E-18	1.27E-16	4.01E-14
ARNTL2	LMNA	0.42352275	0.303199116	0.530553488	3.38E-10	3.03E-09	4.01E-06
MRPS18A	LMNA	0.05104666	-0.087621816	0.187774406	4.71E-01	5.56E-01	1.00E+00

ASPH	LMNA	0.097769018	-0.040833112	0.232679517	1.66E-01	2.34E-01	1.00E+00
PVRIG	LMNA	0.131909782	-0.006255127	0.265132643	6.13E-02	1.00E-01	1.00E+00
CPA2	LMNA	0.04037402	-0.098224452	0.177436049	5.68E-01	6.48E-01	1.00E+00
LEPR	LMNA	0.103957689	-0.034589725	0.23858465	1.41E-01	2.04E-01	1.00E+00
SH3BGRL	LMNA	-0.024872922	-0.1623664	0.113568041	7.25E-01	7.86E-01	1.00E+00
C16orf42	LMNA	0.189685722	0.053023203	0.319373511	6.86E-03	1.45E-02	1.00E+00
C9orf6	LMNA	0.18484586	0.048020248	0.314862159	8.45E-03	1.75E-02	1.00E+00
ERN2	LMNA	-0.115248895	-0.249332952	0.023170758	1.02E-01	1.55E-01	1.00E+00
SYNGR2	LMNA	0.247824856	0.113662531	0.373110814	3.77E-04	1.09E-03	1.00E+00
CDKN2D	LMNA	-0.198094602	-0.327197676	-0.06173179	4.71E-03	1.04E-02	1.00E+00
PITPNA	LMNA	0.009523898	-0.12869631	0.147381124	8.93E-01	9.22E-01	1.00E+00
PRPF4B	LMNA	-0.089187043	-0.224474236	0.049473088	2.07E-01	2.82E-01	1.00E+00
NRBP1	LMNA	0.500767312	0.389653623	0.59751149	3.21E-14	5.54E-13	3.81E-10
SLC43A3	LMNA	0.567299696	0.465722342	0.654122267	1.34E-18	5.51E-17	1.59E-14
SLC25A22	LMNA	-0.122754447	-0.256459391	0.015560244	8.18E-02	1.28E-01	1.00E+00
ILK	LMNA	0.485160279	0.372026442	0.584090716	2.53E-13	3.82E-12	3.00E-09
SLC22A8	LMNA	0.129050569	-0.009163687	0.262426299	6.72E-02	1.08E-01	1.00E+00
SEC14L3	LMNA	0.267953825	0.134892679	0.391521923	1.16E-04	3.74E-04	1.00E+00
MRPS7	LMNA	-0.170538263	-0.301491261	-0.03327055	1.52E-02	2.93E-02	1.00E+00
SLC22A1	LMNA	0.026478403	-0.111981933	0.163930178	7.08E-01	7.72E-01	1.00E+00
BTN2A3	LMNA	0.032732485	-0.105796584	0.170015229	6.44E-01	7.16E-01	1.00E+00
RASA4	LMNA	-0.250040061	-0.375141811	-0.115992937	3.32E-04	9.76E-04	1.00E+00
PITX2	LMNA	0.089590154	-0.049067717	0.224860083	2.05E-01	2.79E-01	1.00E+00
CCNL2	LMNA	-0.024189696	-0.161700711	0.114242809	7.33E-01	7.92E-01	1.00E+00
GJA4	LMNA	0.190714635	0.054087673	0.320331828	6.55E-03	1.39E-02	1.00E+00
FABP3	LMNA	-0.032931732	-0.170208919	0.10559935	6.42E-01	7.15E-01	1.00E+00
MYBPC3	LMNA	-0.051846156	-0.188547643	0.086826297	4.64E-01	5.50E-01	1.00E+00
LOC72821	LMNA	-0.273277594	-0.396374859	-0.140528195	8.32E-05	2.76E-04	9.87E-01
TRPV2	LMNA	0.426726705	0.306746134	0.533357584	2.41E-10	2.24E-09	2.86E-06
NIBP	LMNA	-0.322254522	-0.440699861	-0.192779825	2.91E-06	1.28E-05	3.45E-02
CDADC1	LMNA	-0.191585339	-0.321142584	-0.05498871	6.31E-03	1.34E-02	1.00E+00
ZFHX4	LMNA	0.156123953	0.018471061	0.287968353	2.65E-02	4.79E-02	1.00E+00
TFPT	LMNA	0.064793403	-0.073918786	0.201046088	3.60E-01	4.46E-01	1.00E+00
TSPYL2	LMNA	-0.241478574	-0.36728561	-0.106994376	5.36E-04	1.50E-03	1.00E+00
EIF2S3	LMNA	0.181191383	0.044247166	0.311451836	9.86E-03	2.01E-02	1.00E+00

ABTB2	LMNA	0.213190773	0.077418285	0.341199853	2.32E-03	5.54E-03	1.00E+00
SOX30	LMNA	0.095247337	-0.043373988	0.230270513	1.78E-01	2.48E-01	1.00E+00
VBP1	LMNA	-0.299850422	-0.420495172	-0.16878627	1.45E-05	5.59E-05	1.73E-01
DKFZP564(	LMNA	-0.212283814	-0.34036022	-0.076473958	2.42E-03	5.75E-03	1.00E+00
MORC3	LMNA	-0.147381601	-0.279740943	-0.009524385	3.63E-02	6.34E-02	1.00E+00
BHMT2	LMNA	0.179627343	0.042633559	0.309991263	1.05E-02	2.12E-02	1.00E+00
FZD7	LMNA	0.558278788	0.455319708	0.646503178	6.01E-18	2.17E-16	7.13E-14
CDH18	LMNA	-0.446503997	-0.550614916	-0.328715101	2.73E-11	2.97E-10	3.24E-07
CHL1	LMNA	0.198391557	0.06203971	0.327473659	4.65E-03	1.03E-02	1.00E+00
PMS2CL	LMNA	-0.073168587	-0.209107395	0.065544478	3.01E-01	3.85E-01	1.00E+00
TBC1D2B	LMNA	0.413598746	0.292233619	0.521853123	9.47E-10	7.86E-09	1.12E-05
FA2H	LMNA	-0.395719019	-0.506120933	-0.272557683	5.58E-09	4.02E-08	6.61E-05
TTLL7	LMNA	-0.066403609	-0.202597382	0.072310266	3.48E-01	4.34E-01	1.00E+00
SPOCK3	LMNA	-0.292631687	-0.413959511	-0.161088349	2.38E-05	8.79E-05	2.82E-01
SLC13A2	LMNA	-0.071405249	-0.207411675	0.067309257	3.13E-01	3.98E-01	1.00E+00
AIM1	LMNA	0.546608278	0.441903214	0.636620045	3.92E-17	1.24E-15	4.65E-13
GSN	LMNA	0.434378822	0.315231105	0.540045237	1.05E-10	1.05E-09	1.25E-06
EGR2	LMNA	0.367466547	0.241675534	0.481111197	7.46E-08	4.38E-07	8.85E-04
SMARCA5	LMNA	0.058332387	-0.080365787	0.194814576	4.10E-01	4.97E-01	1.00E+00
GARNL4	LMNA	0.017545614	-0.120797982	0.155220641	8.04E-01	8.52E-01	1.00E+00
WWC1	LMNA	-0.148826465	-0.281102043	-0.011001497	3.45E-02	6.05E-02	1.00E+00
PLEKHG3	LMNA	-0.263940098	-0.387858619	-0.130649603	1.47E-04	4.67E-04	1.00E+00
PLS1	LMNA	-0.260402097	-0.384626257	-0.12691348	1.82E-04	5.65E-04	1.00E+00
DGKZ	LMNA	0.094566902	-0.044059295	0.229620198	1.81E-01	2.51E-01	1.00E+00
EFNA1	LMNA	0.089662476	-0.048994985	0.224929303	2.04E-01	2.79E-01	1.00E+00
ANK2	LMNA	-0.103141053	-0.237806	0.0354142	1.44E-01	2.08E-01	1.00E+00
PAGE4	LMNA	-0.222836893	-0.350117286	-0.087476933	1.43E-03	3.61E-03	1.00E+00
SH3GL3	LMNA	-0.487876592	-0.586430418	-0.375088496	1.78E-13	2.76E-12	2.11E-09
SENP6	LMNA	-0.177403224	-0.307913217	-0.040340183	1.15E-02	2.30E-02	1.00E+00
AKR7A2	LMNA	0.147927567	0.010082466	0.28025532	3.56E-02	6.23E-02	1.00E+00
FKBP10	LMNA	0.653218114	0.5662281	0.72581683	5.91E-26	9.74E-24	7.01E-22
RIF1	LMNA	0.002874389	-0.135230273	0.140869491	9.68E-01	9.76E-01	1.00E+00
PRLH	LMNA	-0.175330761	-0.305975745	-0.038204484	1.26E-02	2.48E-02	1.00E+00
VLDLR	LMNA	0.180938498	0.043986218	0.311215722	9.97E-03	2.02E-02	1.00E+00
VEGFC	LMNA	0.515429474	0.406288131	0.610070565	4.19E-15	8.73E-14	4.97E-11

LARP1	LMNA	0.294124355	0.162678791	0.415311962	2.15E-05	8.00E-05	2.55E-01
SRBD1	LMNA	0.005541341	-0.132611105	0.14348258	9.38E-01	9.54E-01	1.00E+00
DBT	LMNA	-0.346194761	-0.462158057	-0.218590767	4.49E-07	2.29E-06	5.33E-03
ITGB6	LMNA	-0.116304599	-0.25033621	0.022101259	9.93E-02	1.51E-01	1.00E+00
CGGBP1	LMNA	-0.006425122	-0.144348087	0.131742734	9.28E-01	9.47E-01	1.00E+00
SLC1A2	LMNA	-0.172028892	-0.302886709	-0.034804454	1.44E-02	2.79E-02	1.00E+00
INVS	LMNA	0.334246055	0.205686025	0.4514651	1.16E-06	5.52E-06	1.38E-02
MPO	LMNA	-0.189467464	-0.319170194	-0.052797442	6.92E-03	1.46E-02	1.00E+00
ZBTB16	LMNA	-0.092226055	-0.22738205	0.046415909	1.92E-01	2.64E-01	1.00E+00
TADA3L	LMNA	0.278550894	0.146118778	0.401175018	5.97E-05	2.04E-04	7.08E-01
MOBKL3	LMNA	-0.232978901	-0.359468333	-0.098082526	8.48E-04	2.26E-03	1.00E+00
PDGFB	LMNA	0.172457768	0.0352459	0.303288095	1.41E-02	2.75E-02	1.00E+00
CUTL2	LMNA	-0.451238503	-0.554733007	-0.333993254	1.59E-11	1.80E-10	1.88E-07
RFX1	LMNA	0.383551997	0.259226998	0.495373121	1.76E-08	1.15E-07	2.08E-04
KLK2	LMNA	-0.046067204	-0.182954682	0.092572523	5.15E-01	5.99E-01	1.00E+00
VIM	LMNA	0.644441225	0.555841043	0.718564584	4.28E-25	5.77E-23	5.08E-21
REG1B	LMNA	0.144053734	0.006124531	0.276603978	4.08E-02	7.02E-02	1.00E+00
SUMO3	LMNA	0.053078459	-0.085599777	0.189739139	4.53E-01	5.39E-01	1.00E+00
UQCRB	LMNA	-0.423396188	-0.530442673	-0.303059071	3.43E-10	3.07E-09	4.07E-06
MLL4	LMNA	0.082855018	-0.055834632	0.218407822	2.41E-01	3.20E-01	1.00E+00
LGALS3BP	LMNA	0.609441692	0.514693972	0.689483651	6.29E-22	4.36E-20	7.46E-18
DKFZP564(	LMNA	0.005711303	-0.132444123	0.143649043	9.36E-01	9.53E-01	1.00E+00
MRFAP1L1	LMNA	-0.322374134	-0.44080741	-0.19290834	2.88E-06	1.27E-05	3.42E-02
SPR	LMNA	0.216195684	0.080548743	0.34398024	2.00E-03	4.85E-03	1.00E+00
HOXA10	LMNA	-0.080080768	-0.21574664	0.058618271	2.57E-01	3.39E-01	1.00E+00
NGB	LMNA	-0.019528477	-0.157155798	0.118842915	7.83E-01	8.35E-01	1.00E+00
LZTS1	LMNA	0.457339718	0.34080591	0.560032398	7.79E-12	9.34E-11	9.24E-08
ABCE1	LMNA	0.007777941	-0.130413091	0.145672525	9.13E-01	9.37E-01	1.00E+00
ARHGEF3	LMNA	0.106985389	-0.031531311	0.241470013	1.30E-01	1.90E-01	1.00E+00
ChGn	LMNA	-0.286069327	-0.408007242	-0.154104235	3.67E-05	1.31E-04	4.36E-01
CSNK1G2	LMNA	0.413085709	0.291667607	0.521402731	9.98E-10	8.26E-09	1.18E-05
SUPT3H	LMNA	-0.148966659	-0.281234082	-0.011144852	3.44E-02	6.03E-02	1.00E+00
GABRB2	LMNA	-0.300928861	-0.421470489	-0.169937671	1.35E-05	5.22E-05	1.60E-01
MGC72080	LMNA	0.121194881	-0.017142939	0.254979796	8.58E-02	1.33E-01	1.00E+00
SLIT3	LMNA	0.275627083	0.143018003	0.398514375	7.18E-05	2.42E-04	8.52E-01

CD27	LMNA	0.234506379	0.099682483	0.360874486	7.82E-04	2.11E-03	1.00E+00
EGLN1	LMNA	0.213736725	0.077986848	0.341705178	2.26E-03	5.40E-03	1.00E+00
PEX13	LMNA	0.330601227	0.201758448	0.448196611	1.54E-06	7.15E-06	1.83E-02
MAN1C1	LMNA	0.389359392	0.265583913	0.500507393	1.02E-08	6.96E-08	1.21E-04
RWDD3	LMNA	-0.105621577	-0.24017061	0.03290928	1.35E-01	1.96E-01	1.00E+00
GRIN2B	LMNA	-0.340019525	-0.456636015	-0.211915863	7.39E-07	3.62E-06	8.76E-03
HSPA6	LMNA	0.518426068	0.409696751	0.612631499	2.73E-15	5.89E-14	3.24E-11
ATP6AP1	LMNA	0.383378492	0.25903724	0.495219606	1.78E-08	1.16E-07	2.12E-04
NR1H2	LMNA	0.264396605	0.131131948	0.388275467	1.43E-04	4.56E-04	1.00E+00
PDK2	LMNA	-0.122760476	-0.256465109	0.015554124	8.18E-02	1.28E-01	1.00E+00
PHC2	LMNA	0.417370612	0.296397574	0.52516257	6.43E-10	5.52E-09	7.63E-06
LIN7A	LMNA	0.183684499	0.046820771	0.313778752	8.88E-03	1.82E-02	1.00E+00
SLC38A2	LMNA	0.194597563	0.058107587	0.323945954	5.52E-03	1.19E-02	1.00E+00
FAM83E	LMNA	0.158298019	0.020699366	0.290011344	2.44E-02	4.47E-02	1.00E+00
SPHK1	LMNA	0.488366235	0.375640725	0.586851999	1.67E-13	2.60E-12	1.98E-09
TRIM26	LMNA	0.183558997	0.046691174	0.313661653	8.92E-03	1.83E-02	1.00E+00
C18orf24	LMNA	0.035812562	-0.102746413	0.173008217	6.13E-01	6.89E-01	1.00E+00
C15orf29	LMNA	-0.346314999	-0.462265488	-0.218720852	4.45E-07	2.27E-06	5.28E-03
ADAM9	LMNA	0.556990056	0.453835873	0.645413272	7.42E-18	2.62E-16	8.80E-14
RUVBL1	LMNA	0.016216491	-0.122107873	0.153922904	8.19E-01	8.63E-01	1.00E+00
TMUB2	LMNA	-0.070928415	-0.206952986	0.067786332	3.16E-01	4.01E-01	1.00E+00
APAF1	LMNA	-0.044890707	-0.181814962	0.093741228	5.26E-01	6.09E-01	1.00E+00
GPR176	LMNA	0.066310734	-0.072403063	0.202507924	3.48E-01	4.35E-01	1.00E+00
MYH11	LMNA	0.15661613	0.018975396	0.288430961	2.60E-02	4.72E-02	1.00E+00
NEK1	LMNA	-0.219868436	-0.347375527	-0.084378583	1.67E-03	4.12E-03	1.00E+00
MPP2	LMNA	-0.340894147	-0.457418669	-0.212860529	6.89E-07	3.40E-06	8.17E-03
TNK2	LMNA	-0.375345028	-0.488104059	-0.250261781	3.71E-08	2.28E-07	4.40E-04
C12orf24	LMNA	-0.378554952	-0.490949011	-0.25376572	2.78E-08	1.75E-07	3.29E-04
MATN3	LMNA	0.227284857	0.092124436	0.354221485	1.14E-03	2.95E-03	1.00E+00
ZNF289	LMNA	0.006455495	-0.131712886	0.144377828	9.27E-01	9.47E-01	1.00E+00
AGK	LMNA	0.262911366	0.129562873	0.386919071	1.57E-04	4.93E-04	1.00E+00
IFNGR2	LMNA	0.515994581	0.406930707	0.610553666	3.87E-15	8.16E-14	4.59E-11
NDUFA4	LMNA	-0.191275398	-0.320854005	-0.054667946	6.39E-03	1.36E-02	1.00E+00
ITPR1	LMNA	0.206158221	0.070102358	0.334683998	3.24E-03	7.45E-03	1.00E+00
DUSP1	LMNA	0.438186884	0.319460747	0.54336837	6.94E-11	7.16E-10	8.23E-07

DDAH2	LMNA	0.090259952	-0.048394061	0.225501102	2.01E-01	2.76E-01	1.00E+00
ATXN3	LMNA	0.024176012	-0.114256322	0.161687377	7.33E-01	7.92E-01	1.00E+00
TRIM27	LMNA	0.004548461	-0.133586421	0.142509977	9.49E-01	9.62E-01	1.00E+00
LUZP4	LMNA	0.118908101	-0.019462382	0.252809143	9.19E-02	1.42E-01	1.00E+00
SETD6	LMNA	-0.316513678	-0.435534007	-0.186616909	4.45E-06	1.88E-05	5.28E-02
CDC42EP2	LMNA	0.157816355	0.020205567	0.289558822	2.49E-02	4.54E-02	1.00E+00
P2RY2	LMNA	0.282525892	0.150338543	0.404788918	4.62E-05	1.61E-04	5.49E-01
SLC45A2	LMNA	-0.172091079	-0.302944912	-0.03486846	1.43E-02	2.78E-02	1.00E+00
RABGAP1	LMNA	-0.360910641	-0.475281196	-0.234545661	1.32E-07	7.39E-07	1.56E-03
CHP	LMNA	0.209332292	0.073402516	0.337626385	2.79E-03	6.52E-03	1.00E+00
GPRC5A	LMNA	0.485444826	0.372347092	0.584335889	2.44E-13	3.69E-12	2.89E-09
SOX17	LMNA	0.029312803	-0.109180013	0.166689268	6.79E-01	7.47E-01	1.00E+00
PAK3	LMNA	-0.504424643	-0.600648676	-0.39379616	1.95E-14	3.58E-13	2.31E-10
ZNF259	LMNA	0.410513103	0.288830638	0.519143344	1.30E-09	1.05E-08	1.54E-05
LOC63920	LMNA	-0.336414285	-0.453407964	-0.208024423	9.83E-07	4.72E-06	1.17E-02
TGFBR1	LMNA	0.190076716	0.053427672	0.319737709	6.74E-03	1.43E-02	1.00E+00
GBP2	LMNA	0.434302047	0.315145879	0.539978205	1.06E-10	1.06E-09	1.26E-06
MRC2	LMNA	0.690465692	0.610618533	0.756415556	6.04E-30	1.79E-27	7.16E-26
CDC42	LMNA	-0.267191895	-0.390826815	-0.134086836	1.21E-04	3.90E-04	1.00E+00
AOF2	LMNA	-0.316461677	-0.435487178	-0.186561131	4.47E-06	1.89E-05	5.30E-02
LRPPRC	LMNA	-0.165821904	-0.29707236	-0.028421526	1.83E-02	3.46E-02	1.00E+00
CD97	LMNA	0.648986598	0.561216837	0.722322389	1.55E-25	2.32E-23	1.84E-21
SETD5	LMNA	-0.244567694	-0.370122323	-0.110238661	4.52E-04	1.29E-03	1.00E+00
NINJ2	LMNA	-0.167986837	-0.299101451	-0.030646553	1.69E-02	3.21E-02	1.00E+00
PTER	LMNA	0.14080967	0.002813358	0.273543285	4.56E-02	7.74E-02	1.00E+00
POMGNT1	LMNA	0.240482164	0.105948527	0.366370115	5.66E-04	1.58E-03	1.00E+00
RRS1	LMNA	0.245887117	0.111625231	0.371333225	4.20E-04	1.20E-03	1.00E+00
HRB	LMNA	0.075666528	-0.06304301	0.211508148	2.84E-01	3.68E-01	1.00E+00
SNX2	LMNA	0.171828169	0.034597867	0.302698836	1.45E-02	2.80E-02	1.00E+00
ECGF1	LMNA	0.513347421	0.403921586	0.608290047	5.63E-15	1.15E-13	6.68E-11
ATP1B2	LMNA	-0.085330086	-0.220780316	0.053349365	2.27E-01	3.05E-01	1.00E+00
RBX1	LMNA	-0.184360741	-0.314409643	-0.047519159	8.63E-03	1.78E-02	1.00E+00
LOC40050€	LMNA	-0.271939015	-0.395155311	-0.139110422	9.04E-05	2.98E-04	1.00E+00
COL4A3BP	LMNA	0.229819967	0.09477591	0.35655848	1.00E-03	2.63E-03	1.00E+00
C6orf97	LMNA	0.280903717	0.148615898	0.403314566	5.13E-05	1.78E-04	6.09E-01

GRHPR	LMNA	-0.227686922	-0.354592235	-0.092544828	1.12E-03	2.90E-03	1.00E+00
TSNAX	LMNA	-0.193845109	-0.323245884	-0.057328241	5.71E-03	1.23E-02	1.00E+00
TAS2R1	LMNA	-0.076052552	-0.211879007	0.062656285	2.82E-01	3.65E-01	1.00E+00
SEMA7A	LMNA	0.298514391	0.167360346	0.419286507	1.59E-05	6.08E-05	1.89E-01
ZBTB7A	LMNA	0.429954085	0.310322458	0.536179816	1.70E-10	1.63E-09	2.02E-06
ATM	LMNA	0.0413947	-0.097211826	0.17842607	5.59E-01	6.39E-01	1.00E+00
TIE1	LMNA	0.035172792	-0.103380186	0.172386745	6.19E-01	6.95E-01	1.00E+00
PCID2	LMNA	-0.069457638	-0.205537799	0.069257451	3.26E-01	4.12E-01	1.00E+00
HIST1H3G	LMNA	0.142803696	0.004848276	0.275424917	4.26E-02	7.28E-02	1.00E+00
EDF1	LMNA	0.193736586	0.057215854	0.323144905	5.73E-03	1.23E-02	1.00E+00
GTF2H4	LMNA	0.032995136	-0.105536585	0.170270552	6.41E-01	7.14E-01	1.00E+00
NEUROD1	LMNA	-0.313030681	-0.432396034	-0.182882815	5.74E-06	2.37E-05	6.80E-02
LRRC15	LMNA	0.47764608	0.363568619	0.57760977	6.58E-13	9.48E-12	7.81E-09
TFF2	LMNA	0.212139505	0.076323727	0.340226605	2.44E-03	5.79E-03	1.00E+00
PARP2	LMNA	-0.31181123	-0.431296697	-0.181576338	6.26E-06	2.57E-05	7.43E-02
WDR60	LMNA	0.023989504	-0.114440497	0.161505635	7.35E-01	7.94E-01	1.00E+00
IFNA5	LMNA	0.008983481	-0.129227785	0.146852359	8.99E-01	9.27E-01	1.00E+00
ZNF134	LMNA	0.012011712	-0.126248637	0.149814287	8.65E-01	9.01E-01	1.00E+00
GSDML	LMNA	-0.246283468	-0.371696893	-0.112041855	4.11E-04	1.18E-03	1.00E+00
SMEK1	LMNA	0.066893095	-0.071821149	0.203068825	3.44E-01	4.30E-01	1.00E+00
PCGF2	LMNA	-0.248360671	-0.373602183	-0.114226076	3.65E-04	1.06E-03	1.00E+00
CYP2A13	LMNA	0.015057516	-0.123249685	0.152790909	8.32E-01	8.73E-01	1.00E+00
TNS1	LMNA	0.299038375	0.16791952	0.419760589	1.54E-05	5.89E-05	1.82E-01
MDM1	LMNA	-0.125924374	-0.259464842	0.012341165	7.41E-02	1.18E-01	1.00E+00
KCNH6	LMNA	-0.329022267	-0.446779705	-0.200058284	1.74E-06	8.00E-06	2.07E-02
SMPX	LMNA	-0.227735087	-0.354636646	-0.092595192	1.12E-03	2.89E-03	1.00E+00
CD9	LMNA	0.252752869	0.118848836	0.377627398	2.84E-04	8.47E-04	1.00E+00
SRGN	LMNA	0.238854972	0.10424124	0.364874539	6.18E-04	1.71E-03	1.00E+00
ALDH7A1	LMNA	0.121120064	-0.017218849	0.254908799	8.60E-02	1.34E-01	1.00E+00
EIF3F	LMNA	0.08770874	-0.05095929	0.223058881	2.15E-01	2.90E-01	1.00E+00
VDAC3	LMNA	-0.377508345	-0.490021668	-0.252622889	3.05E-08	1.91E-07	3.62E-04
CASP7	LMNA	0.577015258	0.476957517	0.662308405	2.52E-19	1.15E-17	2.99E-15
KCNJ10	LMNA	-0.475118776	-0.575427152	-0.36072816	9.03E-13	1.27E-11	1.07E-08
EDEM2	LMNA	0.527237313	0.419737148	0.620150279	7.56E-16	1.86E-14	8.97E-12
CCNJL	LMNA	-0.041921721	-0.178937152	0.096688851	5.54E-01	6.34E-01	1.00E+00

CAMK1G	LMNA	-0.201669556	-0.33051869	-0.065440462	4.00E-03	8.97E-03	1.00E+00
AATF	LMNA	0.086540304	-0.052133535	0.221939795	2.21E-01	2.97E-01	1.00E+00
CDC20	LMNA	0.180437986	0.043469803	0.310748356	1.02E-02	2.06E-02	1.00E+00
E2F5	LMNA	-0.079029878	-0.214738054	0.059672155	2.64E-01	3.45E-01	1.00E+00
ACSL5	LMNA	0.245238709	0.11094376	0.370738202	4.35E-04	1.24E-03	1.00E+00
FTSJ3	LMNA	0.242839935	0.108423765	0.368536025	4.97E-04	1.40E-03	1.00E+00
PRUNE2	LMNA	0.172581417	0.035373183	0.30340381	1.40E-02	2.73E-02	1.00E+00
LYPLA2	LMNA	0.289566429	0.157824451	0.411180516	2.92E-05	1.06E-04	3.46E-01
C19orf56	LMNA	-0.00182156	-0.139837395	0.136263707	9.79E-01	9.84E-01	1.00E+00
FAM30A	LMNA	-0.058974444	-0.19543432	0.079725641	4.04E-01	4.92E-01	1.00E+00
SMAD4	LMNA	-0.136192386	-0.269182355	0.001894229	5.33E-02	8.88E-02	1.00E+00
AFM	LMNA	-0.020256109	-0.157865658	0.118125214	7.75E-01	8.28E-01	1.00E+00
ACPP	LMNA	0.418273972	0.297395523	0.525954697	5.86E-10	5.06E-09	6.95E-06
G0S2	LMNA	0.313607724	0.183501199	0.432916115	5.50E-06	2.28E-05	6.52E-02
FCHSD2	LMNA	-0.567261452	-0.654090003	-0.465678181	1.35E-18	5.53E-17	1.60E-14
SLC4A7	LMNA	0.260249391	0.126752308	0.384486674	1.84E-04	5.69E-04	1.00E+00
RRP1B	LMNA	0.191839147	0.055251401	0.321378882	6.24E-03	1.33E-02	1.00E+00
STAT6	LMNA	0.40408044	0.281746293	0.513487219	2.47E-09	1.89E-08	2.92E-05
CCDC40	LMNA	0.012101449	-0.126160317	0.149902021	8.64E-01	9.00E-01	1.00E+00
GNL1	LMNA	0.198919679	0.062587395	0.327964429	4.54E-03	1.00E-02	1.00E+00
ZNF195	LMNA	-0.3895033	-0.500634522	-0.265741575	1.01E-08	6.88E-08	1.20E-04
GART	LMNA	0.035871419	-0.102688101	0.173065385	6.12E-01	6.88E-01	1.00E+00
THOP1	LMNA	-0.051514309	-0.188226716	0.087156514	4.67E-01	5.52E-01	1.00E+00
PER3	LMNA	-0.13113072	-0.264395442	0.007047866	6.29E-02	1.02E-01	1.00E+00
TRIAP1	LMNA	0.138001929	-5.00E-05	0.270892082	5.02E-02	8.41E-02	1.00E+00
SCARB1	LMNA	0.131823279	-0.006343157	0.265050796	6.15E-02	1.00E-01	1.00E+00
ADCY8	LMNA	-0.152253721	-0.284328484	-0.014507653	3.05E-02	5.44E-02	1.00E+00
CACNA1F	LMNA	0.082208272	-0.056483759	0.217787615	2.45E-01	3.24E-01	1.00E+00
C10orf10	LMNA	0.379124212	0.254387464	0.491453296	2.64E-08	1.67E-07	3.13E-04
SFRS8	LMNA	-0.102518941	-0.237212707	0.036042159	1.47E-01	2.11E-01	1.00E+00
PBOV1	LMNA	-0.180712675	-0.311004862	-0.043753211	1.01E-02	2.04E-02	1.00E+00
GOLSYN	LMNA	-0.216197334	-0.343981766	-0.080550463	2.00E-03	4.85E-03	1.00E+00
PSG1	LMNA	0.091041517	-0.047607836	0.226248937	1.98E-01	2.71E-01	1.00E+00
DHX34	LMNA	-0.014822744	-0.152561559	0.123480935	8.34E-01	8.76E-01	1.00E+00
CAMK2N1	LMNA	0.111483429	-0.026982848	0.245752205	1.14E-01	1.70E-01	1.00E+00

FLJ20433	LMNA	0.135621733	-0.002475618	0.268643007	5.43E-02	9.02E-02	1.00E+00
GREM1	LMNA	0.007366078	-0.130817951	0.145269355	9.17E-01	9.41E-01	1.00E+00
NFIC	LMNA	0.52191379	0.413667857	0.61560967	1.65E-15	3.78E-14	1.96E-11
ITPR2	LMNA	0.101419672	-0.037151489	0.236164119	1.51E-01	2.16E-01	1.00E+00
QPCT	LMNA	0.085369284	-0.053309991	0.220817877	2.27E-01	3.04E-01	1.00E+00
PRKAG2	LMNA	-0.204763933	-0.33339069	-0.068653624	3.46E-03	7.89E-03	1.00E+00
H2AFZ	LMNA	-0.105629377	-0.240178044	0.0329014	1.35E-01	1.96E-01	1.00E+00
MLLT3	LMNA	-0.19079816	-0.320409611	-0.054174099	6.53E-03	1.38E-02	1.00E+00
CCNT2	LMNA	-0.3472454	-0.463096669	-0.219727608	4.13E-07	2.12E-06	4.89E-03
PLK4	LMNA	-0.106392269	-0.240904963	0.032130653	1.32E-01	1.93E-01	1.00E+00
H2AFX	LMNA	-0.227049799	-0.354004716	-0.091878686	1.16E-03	2.98E-03	1.00E+00
MED16	LMNA	0.179010626	0.041997493	0.309415175	1.08E-02	2.17E-02	1.00E+00
PLEKHQ1	LMNA	0.433341964	0.31408028	0.539139845	1.18E-10	1.16E-09	1.40E-06
GOSR1	LMNA	-0.083574608	-0.219097756	0.055112253	2.37E-01	3.16E-01	1.00E+00
BTG4	LMNA	0.060560436	-0.078143881	0.196964727	3.92E-01	4.79E-01	1.00E+00
RPL30	LMNA	-0.142270817	-0.274922173	-0.004304356	4.34E-02	7.40E-02	1.00E+00
PLOD3	LMNA	0.731347166	0.659924233	0.789670499	4.39E-35	3.47E-32	5.21E-31
ZBTB39	LMNA	-0.116416383	-0.250442424	0.021987996	9.90E-02	1.51E-01	1.00E+00
SHC3	LMNA	-0.28115832	-0.40354601	-0.148886217	5.05E-05	1.75E-04	5.99E-01
HAVCR1	LMNA	-0.100590826	-0.235373277	0.037987697	1.54E-01	2.20E-01	1.00E+00
DYNC2H1	LMNA	-0.066717114	-0.202899338	0.071997005	3.45E-01	4.32E-01	1.00E+00
WASF3	LMNA	-0.401915642	-0.51158161	-0.279365178	3.05E-09	2.31E-08	3.62E-05
DRG1	LMNA	-0.14511339	-0.277603153	-0.007206763	3.93E-02	6.80E-02	1.00E+00
SPCS1	LMNA	-0.052351629	-0.189036427	0.086323246	4.59E-01	5.45E-01	1.00E+00
PRR4	LMNA	-0.2702454	-0.393611675	-0.137317388	1.00E-04	3.28E-04	1.00E+00
KDELR3	LMNA	0.738489966	0.668602508	0.795445837	4.43E-36	4.38E-33	5.26E-32
SRP19	LMNA	0.173804867	0.036632832	0.304548549	1.34E-02	2.62E-02	1.00E+00
GABRA6	LMNA	-0.062998621	-0.199316165	0.075710838	3.73E-01	4.61E-01	1.00E+00
RNF5	LMNA	-0.405376989	-0.514628016	-0.28317312	2.17E-09	1.68E-08	2.57E-05
MFSD1	LMNA	0.41122133	0.28961143	0.519765495	1.21E-09	9.82E-09	1.43E-05
KRI1	LMNA	0.070467117	-0.068247803	0.206509184	3.19E-01	4.05E-01	1.00E+00
LRP10	LMNA	0.595746539	0.49871125	0.678033736	8.59E-21	4.85E-19	1.02E-16
KLHL25	LMNA	-0.260099495	-0.384349655	-0.126594109	1.85E-04	5.73E-04	1.00E+00
PUS7L	LMNA	-0.209229395	-0.337531039	-0.073295486	2.80E-03	6.54E-03	1.00E+00
MGMT	LMNA	0.058991287	-0.079708847	0.195450576	4.04E-01	4.92E-01	1.00E+00

BUB1	LMNA	0.04260137	-0.096014307	0.179596136	5.47E-01	6.28E-01	1.00E+00
RNF138	LMNA	-0.141566787	-0.274257847	-0.003585866	4.45E-02	7.57E-02	1.00E+00
MYLPF	LMNA	0.06200809	-0.076699479	0.198361065	3.81E-01	4.68E-01	1.00E+00
HOXD1	LMNA	-0.337657421	-0.454521387	-0.209365785	8.91E-07	4.30E-06	1.06E-02
DYNLRB1	LMNA	-0.014614453	-0.152358065	0.123686089	8.36E-01	8.78E-01	1.00E+00
AIF1	LMNA	0.125178934	-0.013098422	0.258758312	7.59E-02	1.20E-01	1.00E+00
HCN3	LMNA	-0.474099018	-0.574546068	-0.359582645	1.03E-12	1.42E-11	1.22E-08
MAP4K5	LMNA	-0.169329963	-0.300359704	-0.032027647	1.60E-02	3.06E-02	1.00E+00
CHODL	LMNA	0.122648615	-0.015667667	0.256359005	8.20E-02	1.28E-01	1.00E+00
EXOSC8	LMNA	-0.198270722	-0.327361361	-0.06191441	4.68E-03	1.03E-02	1.00E+00
LASP1	LMNA	0.302910508	0.17205432	0.423261923	1.18E-05	4.60E-05	1.40E-01
SLC28A1	LMNA	-0.05385627	-0.190490987	0.0848254	4.47E-01	5.33E-01	1.00E+00
PLAA	LMNA	-0.078424044	-0.214156476	0.060279575	2.67E-01	3.49E-01	1.00E+00
KRT6A	LMNA	0.027562515	-0.110910503	0.16498574	6.97E-01	7.62E-01	1.00E+00
MYO7B	LMNA	0.034373324	-0.104172001	0.171609989	6.27E-01	7.02E-01	1.00E+00
SEH1L	LMNA	-0.000585093	-0.138624897	0.137477012	9.93E-01	9.95E-01	1.00E+00
MTNR1A	LMNA	0.017864808	-0.120483335	0.155532227	8.01E-01	8.50E-01	1.00E+00
TSPAN5	LMNA	-0.040273217	-0.177338259	0.098324444	5.69E-01	6.48E-01	1.00E+00
MCL1	LMNA	0.567187867	0.465593212	0.654027924	1.36E-18	5.56E-17	1.62E-14
EHBP1	LMNA	0.216483592	0.080848821	0.344246518	1.97E-03	4.80E-03	1.00E+00
CDC45L	LMNA	-0.070932635	-0.206957046	0.06778211	3.16E-01	4.01E-01	1.00E+00
ATAD3A	LMNA	0.309002358	0.178568759	0.428763144	7.66E-06	3.09E-05	9.08E-02
PRNP	LMNA	0.15894747	0.021365285	0.290621407	2.39E-02	4.37E-02	1.00E+00
OSGIN2	LMNA	-0.089633432	-0.224901505	0.049024193	2.05E-01	2.79E-01	1.00E+00
DARC	LMNA	-0.059030631	-0.195488549	0.079669616	4.04E-01	4.91E-01	1.00E+00
SHMT1	LMNA	0.165818658	0.028418191	0.297069317	1.84E-02	3.46E-02	1.00E+00
POPDC2	LMNA	0.337193213	0.208864841	0.454105658	9.24E-07	4.45E-06	1.10E-02
CRISP3	LMNA	0.028378624	-0.110103729	0.165780149	6.88E-01	7.55E-01	1.00E+00
FBXL8	LMNA	0.208663474	0.072706883	0.337006595	2.88E-03	6.70E-03	1.00E+00
TRIP13	LMNA	0.102635211	-0.035924805	0.237323599	1.46E-01	2.10E-01	1.00E+00
C1orf113	LMNA	0.472755233	0.358073671	0.573384666	1.21E-12	1.66E-11	1.44E-08
NEB	LMNA	-0.204606815	-0.333244921	-0.068490406	3.49E-03	7.94E-03	1.00E+00
ASGR1	LMNA	-0.168505386	-0.299587287	-0.031179698	1.65E-02	3.15E-02	1.00E+00
IL6	LMNA	0.385231192	0.261063979	0.496858488	1.50E-08	9.97E-08	1.78E-04
CTGF	LMNA	0.512347773	0.402785866	0.607434833	6.48E-15	1.31E-13	7.69E-11

RAB17	LMNA	0.200692943	0.064426947	0.329611766	4.18E-03	9.34E-03	1.00E+00
JARID1B	LMNA	-0.191799201	-0.321341693	-0.055210056	6.25E-03	1.33E-02	1.00E+00
FBXL2	LMNA	-0.243828098	-0.369443371	-0.109461655	4.71E-04	1.33E-03	1.00E+00
PTBP1	LMNA	0.390663256	0.267012634	0.501659054	9.03E-09	6.22E-08	1.07E-04
PTPN7	LMNA	0.351685746	0.2245361	0.467060684	2.86E-07	1.52E-06	3.40E-03
BRD1	LMNA	-0.108826205	-0.243223126	0.029670553	1.23E-01	1.82E-01	1.00E+00
STATH	LMNA	0.097806626	-0.040795204	0.232715432	1.66E-01	2.34E-01	1.00E+00
CDC7	LMNA	-0.351704778	-0.467077665	-0.224556724	2.86E-07	1.51E-06	3.39E-03
ZNF335	LMNA	0.311824818	0.181590893	0.431308949	6.26E-06	2.57E-05	7.42E-02
SNX7	LMNA	0.280393349	0.148074087	0.402850575	5.30E-05	1.83E-04	6.29E-01
MCCC2	LMNA	0.122483032	-0.015835733	0.256201939	8.25E-02	1.29E-01	1.00E+00
CPT2	LMNA	0.097864595	-0.040736771	0.232770791	1.66E-01	2.34E-01	1.00E+00
CDC2	LMNA	0.014910851	-0.123394151	0.152647633	8.33E-01	8.75E-01	1.00E+00
C5orf23	LMNA	-0.122722557	-0.256429142	0.015592614	8.19E-02	1.28E-01	1.00E+00
IVD	LMNA	-0.017102204	-0.154787755	0.121235029	8.09E-01	8.56E-01	1.00E+00
HEATR1	LMNA	0.326059319	0.19686999	0.444119261	2.19E-06	9.83E-06	2.59E-02
HSPC152	LMNA	0.132152056	-0.006008563	0.265361868	6.08E-02	9.95E-02	1.00E+00
PSME1	LMNA	0.231900847	0.096953737	0.358475559	8.97E-04	2.38E-03	1.00E+00
STAG3	LMNA	0.044119463	-0.094507155	0.181067629	5.33E-01	6.15E-01	1.00E+00
MSL3L1	LMNA	0.135141526	-0.002964787	0.268189078	5.52E-02	9.14E-02	1.00E+00
MVP	LMNA	0.669658128	0.585758752	0.739357743	1.21E-27	2.47E-25	1.43E-23
EPOR	LMNA	0.272743495	0.139962432	0.395888306	8.60E-05	2.85E-04	1.00E+00
ZMYM1	LMNA	-0.08621865	-0.221631664	0.052456721	2.22E-01	2.99E-01	1.00E+00
BCL7C	LMNA	0.327944412	0.198898138	0.445812136	1.89E-06	8.62E-06	2.25E-02
PSTPIP2	LMNA	0.35146778	0.224299919	0.466866209	2.91E-07	1.54E-06	3.46E-03
SF1	LMNA	-0.114051644	-0.248194831	0.02438327	1.06E-01	1.60E-01	1.00E+00
PNLIPRP2	LMNA	-0.023323351	-0.160856424	0.115098245	7.42E-01	8.00E-01	1.00E+00
BCAS4	LMNA	0.036258104	-0.102304981	0.173440951	6.08E-01	6.85E-01	1.00E+00
TRSPAP1	LMNA	0.001537163	-0.136542813	0.139558548	9.83E-01	9.86E-01	1.00E+00
NUP210	LMNA	-0.005829438	-0.143764742	0.132328055	9.34E-01	9.52E-01	1.00E+00
RAB11B	LMNA	-0.104604369	-0.239201129	0.033936703	1.38E-01	2.01E-01	1.00E+00
ANP32C	LMNA	-0.143720983	-0.276290159	-0.005784757	4.13E-02	7.09E-02	1.00E+00
ITGB3BP	LMNA	0.034725779	-0.103822941	0.171952452	6.24E-01	6.98E-01	1.00E+00
EDG6	LMNA	0.248167507	0.114022905	0.373425051	3.69E-04	1.07E-03	1.00E+00
UBASH3A	LMNA	0.189331558	0.052656872	0.319043585	6.96E-03	1.47E-02	1.00E+00

YWHAB	LMNA	-0.077301158	-0.213078293	0.061405128	2.74E-01	3.57E-01	1.00E+00
CUL1	LMNA	0.239197305	0.104600358	0.365189236	6.07E-04	1.68E-03	1.00E+00
CCRK	LMNA	0.044332948	-0.094295159	0.181274511	5.31E-01	6.14E-01	1.00E+00
LY6G5C	LMNA	-0.254462681	-0.379193073	-0.120649972	2.58E-04	7.74E-04	1.00E+00
DHX9	LMNA	-0.247682989	-0.372980704	-0.113513337	3.80E-04	1.10E-03	1.00E+00
SLC7A2	LMNA	-0.056338751	-0.19288953	0.082352758	4.26E-01	5.13E-01	1.00E+00
CLK1	LMNA	-0.213053553	-0.341072832	-0.077275396	2.33E-03	5.56E-03	1.00E+00
NCKAP1	LMNA	-0.09620377	-0.231184404	0.042410486	1.73E-01	2.42E-01	1.00E+00
PKN2	LMNA	-0.060477653	-0.196884861	0.078226461	3.93E-01	4.80E-01	1.00E+00
VDR	LMNA	0.61913631	0.526047877	0.697564783	9.14E-23	7.86E-21	1.08E-18
PODNL1	LMNA	0.559326556	0.456526521	0.647389031	5.06E-18	1.84E-16	6.00E-14
ACE	LMNA	0.369057181	0.243407478	0.482524195	6.49E-08	3.86E-07	7.70E-04
DALRD3	LMNA	0.221841358	0.086437543	0.349198022	1.51E-03	3.77E-03	1.00E+00
PSMA2	LMNA	-0.094926107	-0.229963518	0.043697534	1.79E-01	2.49E-01	1.00E+00
OPN1SW	LMNA	-0.060853741	-0.19724768	0.077851282	3.90E-01	4.77E-01	1.00E+00
CCDC131	LMNA	-0.08107253	-0.216698213	0.0576234	2.51E-01	3.32E-01	1.00E+00
EML2	LMNA	0.248150455	0.114004971	0.373409413	3.70E-04	1.07E-03	1.00E+00
DUSP11	LMNA	-0.010040277	-0.147886297	0.128188402	8.87E-01	9.18E-01	1.00E+00
DSPP	LMNA	-0.132273962	-0.265477202	0.005884493	6.06E-02	9.92E-02	1.00E+00
L1CAM	LMNA	-0.232413999	-0.358948153	-0.097490995	8.73E-04	2.33E-03	1.00E+00
NEK11	LMNA	0.036691711	-0.101875322	0.173862043	6.04E-01	6.81E-01	1.00E+00
DLL3	LMNA	-0.541099117	-0.631944335	-0.435586081	9.28E-17	2.72E-15	1.10E-12
CREB3L2	LMNA	0.537665531	0.431654175	0.629026847	1.57E-16	4.42E-15	1.87E-12
GFRA3	LMNA	0.003231515	-0.134879658	0.141219515	9.64E-01	9.73E-01	1.00E+00
CPA1	LMNA	0.237935249	0.103276596	0.364028919	6.50E-04	1.79E-03	1.00E+00
PPT2	LMNA	-0.139756734	-0.272549289	-0.00173929	4.73E-02	7.99E-02	1.00E+00
FASLG	LMNA	0.310532042	0.180206355	0.430143126	6.87E-06	2.80E-05	8.14E-02
RTN4	LMNA	-0.115847345	-0.249901707	0.022564527	1.01E-01	1.53E-01	1.00E+00
GNL3L	LMNA	0.148242902	0.010404837	0.280552376	3.52E-02	6.17E-02	1.00E+00
NUDT2	LMNA	-0.234318658	-0.360701706	-0.099485817	7.89E-04	2.13E-03	1.00E+00
GTPBP8	LMNA	-0.266434116	-0.390135355	-0.133285558	1.27E-04	4.07E-04	1.00E+00
CACNA1D	LMNA	-0.407657323	-0.516633482	-0.285683898	1.73E-09	1.37E-08	2.05E-05
PRKAA2	LMNA	-0.236875601	-0.363054391	-0.102165505	6.88E-04	1.88E-03	1.00E+00
CD163	LMNA	0.473061583	0.358417628	0.573649473	1.17E-12	1.60E-11	1.38E-08
CD37	LMNA	0.218392455	0.082838992	0.346011454	1.79E-03	4.40E-03	1.00E+00

NBLA00301	LMNA	-0.172228062	-0.303073119	-0.035009456	1.42E-02	2.77E-02	1.00E+00
DPT	LMNA	0.482600523	0.369143124	0.58188436	3.51E-13	5.21E-12	4.16E-09
RGS5	LMNA	-0.260200646	-0.384442117	-0.126700862	1.84E-04	5.70E-04	1.00E+00
ACTL8	LMNA	-0.025843732	-0.163312076	0.112609029	7.15E-01	7.77E-01	1.00E+00
PRDM8	LMNA	0.003106699	-0.135002202	0.141097185	9.65E-01	9.74E-01	1.00E+00
PRKAR2B	LMNA	-0.208466198	-0.336823759	-0.072501722	2.91E-03	6.76E-03	1.00E+00
OPLAH	LMNA	0.321517652	0.191988208	0.440037233	3.07E-06	1.34E-05	3.65E-02
KRT14	LMNA	-0.018546261	-0.156197345	0.119811496	7.93E-01	8.44E-01	1.00E+00
MRPL18	LMNA	-0.043530082	-0.180496413	0.095092364	5.38E-01	6.20E-01	1.00E+00
PACRG	LMNA	-0.263227137	-0.387207494	-0.129896413	1.54E-04	4.85E-04	1.00E+00
ABCG2	LMNA	-0.398076919	-0.508199839	-0.275146575	4.44E-09	3.26E-08	5.27E-05
KREMEN2	LMNA	0.025121744	-0.113322267	0.162608804	7.23E-01	7.84E-01	1.00E+00
HNRPUL1	LMNA	-0.061595114	-0.197962784	0.077111587	3.84E-01	4.71E-01	1.00E+00
FBXO21	LMNA	-0.433086936	-0.538917114	-0.313797273	1.21E-10	1.19E-09	1.44E-06
GRB10	LMNA	0.568176314	0.466734729	0.654861723	1.15E-18	4.82E-17	1.37E-14
CLSTN1	LMNA	0.0055698	-0.132583146	0.143510453	9.37E-01	9.54E-01	1.00E+00
TBL1X	LMNA	0.269794743	0.136840423	0.393200808	1.03E-04	3.36E-04	1.00E+00
CHCHD3	LMNA	0.336216611	0.207811175	0.453230883	9.98E-07	4.78E-06	1.18E-02
LMAN2	LMNA	0.587761124	0.489422347	0.671338999	3.72E-20	1.94E-18	4.42E-16
CRKRS	LMNA	0.112932622	-0.025516188	0.24713074	1.10E-01	1.64E-01	1.00E+00
INSIG2	LMNA	0.238750342	0.104131486	0.364778349	6.22E-04	1.72E-03	1.00E+00
GPR65	LMNA	0.152040315	0.014289235	0.284127669	3.08E-02	5.48E-02	1.00E+00
STXBP2	LMNA	0.315964852	0.186028268	0.43503974	4.63E-06	1.96E-05	5.50E-02
CMAH	LMNA	0.286411862	0.154468458	0.408318188	3.59E-05	1.28E-04	4.26E-01
ZNF155	LMNA	-0.100463083	-0.235251375	0.038116558	1.55E-01	2.21E-01	1.00E+00
DFFA	LMNA	0.112223559	-0.026233872	0.246456317	1.12E-01	1.67E-01	1.00E+00
FUT1	LMNA	-0.218415055	-0.346032345	-0.082862561	1.79E-03	4.40E-03	1.00E+00
COQ6	LMNA	-0.127946817	-0.261380997	0.010285864	6.96E-02	1.12E-01	1.00E+00
TSPAN15	LMNA	-0.187394995	-0.317239002	-0.050654428	7.57E-03	1.58E-02	1.00E+00
PRPF4	LMNA	0.192378366	0.055809557	0.321880846	6.09E-03	1.30E-02	1.00E+00
PGK2	LMNA	0.161264743	0.023742312	0.292797268	2.19E-02	4.05E-02	1.00E+00
MS4A1	LMNA	-0.002106659	-0.14011691	0.135983889	9.76E-01	9.82E-01	1.00E+00
TMEM51	LMNA	0.365056886	0.239053329	0.478969514	9.21E-08	5.30E-07	1.09E-03
ZNF580	LMNA	-0.18516084	-0.315155938	-0.048345633	8.34E-03	1.73E-02	1.00E+00
DDX28	LMNA	-0.109886626	-0.244232628	0.028598209	1.20E-01	1.77E-01	1.00E+00

BTN1A1	LMNA	0.02032495	-0.118057305	0.15793281	7.74E-01	8.27E-01	1.00E+00
EZH1	LMNA	-0.227197754	-0.354141161	-0.092033369	1.15E-03	2.96E-03	1.00E+00
TTC31	LMNA	0.097897938	-0.040703162	0.232802632	1.66E-01	2.34E-01	1.00E+00
LSP1	LMNA	0.405558574	0.283372994	0.514787757	2.13E-09	1.65E-08	2.53E-05
PCAF	LMNA	-0.314634094	-0.433840973	-0.184601355	5.11E-06	2.13E-05	6.06E-02
CHP2	LMNA	-0.33193132	-0.449389733	-0.203191244	1.39E-06	6.49E-06	1.65E-02
WDR46	LMNA	0.221567894	0.086152085	0.348945466	1.53E-03	3.81E-03	1.00E+00
ALOX15B	LMNA	0.272437764	0.139638615	0.395609761	8.76E-05	2.89E-04	1.00E+00
CDK9	LMNA	0.179795456	0.042806965	0.310148284	1.05E-02	2.11E-02	1.00E+00
CD59	LMNA	0.363514833	0.237376222	0.477598241	1.05E-07	6.00E-07	1.25E-03
ERP29	LMNA	0.331914019	0.203172603	0.449374216	1.40E-06	6.50E-06	1.66E-02
LRRTM4	LMNA	-0.477705839	-0.577661361	-0.363635808	6.53E-13	9.43E-12	7.75E-09
BCMO1	LMNA	-0.028261929	-0.165666567	0.110219101	6.90E-01	7.56E-01	1.00E+00
TTR	LMNA	0.009866296	-0.128359537	0.1477161	8.89E-01	9.20E-01	1.00E+00
DDIT4	LMNA	0.181274179	0.044332606	0.311529138	9.83E-03	2.00E-02	1.00E+00
MAOB	LMNA	0.171042318	0.033789166	0.301963193	1.49E-02	2.88E-02	1.00E+00
CACNB4	LMNA	-0.578193307	-0.663299623	-0.478322052	2.05E-19	9.39E-18	2.43E-15
PTGDS	LMNA	-0.154462457	-0.286406218	-0.016769034	2.82E-02	5.06E-02	1.00E+00
C3orf63	LMNA	-0.033327267	-0.170593391	0.10520778	6.38E-01	7.12E-01	1.00E+00
BST2	LMNA	0.345220594	0.217536982	0.461287532	4.86E-07	2.47E-06	5.77E-03
ATP5L	LMNA	-0.214606864	-0.342510414	-0.078893208	2.16E-03	5.20E-03	1.00E+00
CYP1A2	LMNA	-0.231259856	-0.357885139	-0.096282745	9.28E-04	2.45E-03	1.00E+00
ONECUT1	LMNA	-0.142046863	-0.274710863	-0.004075787	4.37E-02	7.45E-02	1.00E+00
NUDT9	LMNA	0.193836711	0.057319544	0.32323807	5.71E-03	1.23E-02	1.00E+00
TMEM149	LMNA	0.144457492	0.006536853	0.276984725	4.03E-02	6.93E-02	1.00E+00
STX1A	LMNA	-0.101992244	-0.236710332	0.036573719	1.49E-01	2.13E-01	1.00E+00
STX17	LMNA	0.128347023	-0.009879018	0.261760045	6.87E-02	1.11E-01	1.00E+00
USP6	LMNA	-0.409020755	-0.517831997	-0.287185915	1.51E-09	1.21E-08	1.79E-05
MRPL3	LMNA	0.046086266	-0.092553584	0.182973145	5.15E-01	5.99E-01	1.00E+00
POMP	LMNA	0.065041204	-0.073671291	0.201284867	3.58E-01	4.45E-01	1.00E+00
INPP4B	LMNA	0.147255411	0.009395408	0.279622044	3.65E-02	6.36E-02	1.00E+00
GMPPB	LMNA	0.515981896	0.406916281	0.610542822	3.87E-15	8.16E-14	4.59E-11
ALLC	LMNA	0.077251244	-0.061455152	0.213030359	2.74E-01	3.57E-01	1.00E+00
BMPR2	LMNA	-0.059401565	-0.195846537	0.079299729	4.01E-01	4.89E-01	1.00E+00
KLF7	LMNA	0.301988975	0.171069857	0.422428963	1.25E-05	4.89E-05	1.49E-01

EAPP	LMNA	-0.215420253	-0.343262963	-0.079740659	2.08E-03	5.01E-03	1.00E+00
AHSA1	LMNA	0.011983501	-0.126276403	0.149786705	8.66E-01	9.01E-01	1.00E+00
GCC1	LMNA	0.356318649	0.229559725	0.471191663	1.94E-07	1.06E-06	2.31E-03
TIMM9	LMNA	-0.070115602	-0.206170965	0.068599408	3.21E-01	4.07E-01	1.00E+00
CDO1	LMNA	-0.214119231	-0.342059177	-0.07838525	2.21E-03	5.31E-03	1.00E+00
IFI6	LMNA	-0.006507797	-0.144429042	0.131661489	9.27E-01	9.47E-01	1.00E+00
MGAT2	LMNA	0.342271496	0.214348667	0.458650822	6.17E-07	3.06E-06	7.32E-03
WBP5	LMNA	0.448497627	0.330936756	0.5523496	2.18E-11	2.40E-10	2.58E-07
SLC5A6	LMNA	0.256010831	0.122281573	0.3806101	2.36E-04	7.14E-04	1.00E+00
HIVEP2	LMNA	-0.024343896	-0.161850964	0.114090528	7.31E-01	7.91E-01	1.00E+00
KIAA0907	LMNA	-0.295739677	-0.416774941	-0.16440069	1.93E-05	7.25E-05	2.29E-01
SUMO2	LMNA	-0.399137675	-0.509134666	-0.276311831	4.01E-09	2.96E-08	4.75E-05
KIAA1822L	LMNA	0.18939262	0.052720029	0.319100471	6.94E-03	1.46E-02	1.00E+00
C11orf67	LMNA	0.189407167	0.052735075	0.319114022	6.94E-03	1.46E-02	1.00E+00
CTSG	LMNA	0.123117102	-0.01519211	0.25680336	8.09E-02	1.27E-01	1.00E+00
TXK	LMNA	0.345073451	0.217377838	0.461156023	4.92E-07	2.49E-06	5.84E-03
GRIK1	LMNA	-0.026881994	-0.164323177	0.111583101	7.04E-01	7.68E-01	1.00E+00
CUL5	LMNA	-0.121254911	-0.255036759	0.017082031	8.56E-02	1.33E-01	1.00E+00
FRMD1	LMNA	0.094114132	-0.044515235	0.229187404	1.83E-01	2.54E-01	1.00E+00
ICAM4	LMNA	0.057784213	-0.080912238	0.194285367	4.14E-01	5.02E-01	1.00E+00
FPGS	LMNA	0.312434276	0.18224379	0.431858418	5.99E-06	2.47E-05	7.10E-02
ANAPC2	LMNA	0.082659262	-0.056031121	0.218220111	2.42E-01	3.22E-01	1.00E+00
SNRPA1	LMNA	0.030573616	-0.107932939	0.167915891	6.66E-01	7.36E-01	1.00E+00
TAF13	LMNA	0.135395323	-0.002706262	0.268428993	5.47E-02	9.07E-02	1.00E+00
KCNJ4	LMNA	-0.301415461	-0.421910469	-0.170457309	1.31E-05	5.07E-05	1.55E-01
HIST1H2BC	LMNA	-0.074381381	-0.210273207	0.06433019	2.93E-01	3.77E-01	1.00E+00
SLC5A5	LMNA	0.133883496	-0.004245982	0.26699961	5.75E-02	9.47E-02	1.00E+00
IL12RB2	LMNA	-0.120042073	-0.253885698	0.0183124	8.88E-02	1.37E-01	1.00E+00
EIF5	LMNA	-0.056903439	-0.193434897	0.081790069	4.21E-01	5.09E-01	1.00E+00
SYNC1	LMNA	0.290074584	0.158365337	0.411641371	2.82E-05	1.03E-04	3.34E-01
PALB2	LMNA	-0.117724749	-0.251685355	0.020662051	9.52E-02	1.46E-01	1.00E+00
UBL3	LMNA	-0.29249241	-0.41383329	-0.160939984	2.40E-05	8.87E-05	2.85E-01
RNASE3	LMNA	0.188920088	0.052231312	0.318660236	7.09E-03	1.49E-02	1.00E+00
PIK3CG	LMNA	0.267906274	0.134842383	0.391478547	1.16E-04	3.74E-04	1.00E+00
BCORL1	LMNA	0.030165621	-0.108336536	0.167519007	6.70E-01	7.39E-01	1.00E+00

CD5L	LMNA	-0.093112176	-0.228229463	0.045524	1.87E-01	2.59E-01	1.00E+00
ZNF238	LMNA	-0.523717172	-0.617148531	-0.415722796	1.27E-15	2.94E-14	1.50E-11
TRIM49	LMNA	-0.011635468	-0.149446415	0.126618919	8.69E-01	9.03E-01	1.00E+00
POLR2K	LMNA	-0.227533471	-0.35445074	-0.092384377	1.13E-03	2.92E-03	1.00E+00
C8B	LMNA	0.081348984	-0.05734603	0.216963419	2.50E-01	3.30E-01	1.00E+00
PREB	LMNA	0.240107087	0.105554918	0.366025434	5.78E-04	1.61E-03	1.00E+00
TUBA8	LMNA	-0.365118502	-0.479024295	-0.239120356	9.16E-08	5.28E-07	1.09E-03
IGLV2-14	LMNA	0.253953046	0.120113024	0.378726475	2.65E-04	7.96E-04	1.00E+00
STIL	LMNA	-0.018722505	-0.156369344	0.119637719	7.91E-01	8.42E-01	1.00E+00
NME7	LMNA	-0.041027804	-0.178070227	0.09757586	5.62E-01	6.42E-01	1.00E+00
UBE2C	LMNA	0.124355318	-0.013934909	0.257977521	7.78E-02	1.23E-01	1.00E+00
FHOD1	LMNA	0.627909877	0.536351772	0.704860998	1.51E-23	1.50E-21	1.79E-19
CDK2AP1	LMNA	-0.160561443	-0.292137032	-0.023020712	2.24E-02	4.14E-02	1.00E+00
CYP3A7	LMNA	-0.024248752	-0.161758256	0.114184488	7.32E-01	7.92E-01	1.00E+00
DHPS	LMNA	-0.235323181	-0.361626177	-0.100538329	7.48E-04	2.03E-03	1.00E+00
RPL5	LMNA	-0.249360938	-0.374519289	-0.115278339	3.45E-04	1.01E-03	1.00E+00
TFDP1	LMNA	0.041266759	-0.097338773	0.178301988	5.60E-01	6.40E-01	1.00E+00
TRGV5	LMNA	-0.058269089	-0.194753472	0.08042889	4.10E-01	4.98E-01	1.00E+00
HCCS	LMNA	0.27284578	0.140070774	0.395981491	8.54E-05	2.83E-04	1.00E+00
LHX3	LMNA	-0.324167364	-0.442419376	-0.194835572	2.52E-06	1.12E-05	2.99E-02
GTPBP4	LMNA	-0.064930972	-0.20117865	0.073781389	3.59E-01	4.45E-01	1.00E+00
TUBGCP2	LMNA	0.171507283	0.034267628	0.30239847	1.47E-02	2.84E-02	1.00E+00
CXorf57	LMNA	-0.25659444	-0.381144127	-0.122896828	2.28E-04	6.91E-04	1.00E+00
SLITRK5	LMNA	-0.328896094	-0.446666456	-0.19992246	1.76E-06	8.07E-06	2.09E-02
IRF2BP1	LMNA	0.020603124	-0.117782885	0.158204147	7.71E-01	8.25E-01	1.00E+00
NDST2	LMNA	0.017347048	-0.120993706	0.155026794	8.06E-01	8.53E-01	1.00E+00
CD79A	LMNA	-0.028904584	-0.166292028	0.10958369	6.83E-01	7.51E-01	1.00E+00
CIC	LMNA	0.085886197	-0.052790727	0.221313159	2.24E-01	3.01E-01	1.00E+00
IL1R2	LMNA	0.29476087	0.163357206	0.415888521	2.06E-05	7.70E-05	2.44E-01
PPME1	LMNA	-0.02987619	-0.16723743	0.108622819	6.73E-01	7.42E-01	1.00E+00
PIK3CA	LMNA	0.077033982	-0.061672886	0.212821705	2.76E-01	3.58E-01	1.00E+00
TNPO3	LMNA	0.104276671	-0.034267629	0.238888749	1.40E-01	2.02E-01	1.00E+00
COLEC10	LMNA	0.039157344	-0.099431153	0.176255558	5.80E-01	6.59E-01	1.00E+00
SLC9A6	LMNA	-0.364531384	-0.478502273	-0.238481719	9.64E-08	5.53E-07	1.14E-03
CCDC53	LMNA	0.032296366	-0.106228258	0.169591237	6.48E-01	7.20E-01	1.00E+00

DCUN1D1	LMNA	-0.309128823	-0.428877254	-0.178704118	7.59E-06	3.07E-05	9.00E-02
GK3P	LMNA	0.328211616	0.199185708	0.446052025	1.86E-06	8.46E-06	2.20E-02
CYB5R1	LMNA	0.264137253	0.130857909	0.388038653	1.46E-04	4.63E-04	1.00E+00
TSR2	LMNA	0.062919252	-0.075790066	0.199239644	3.74E-01	4.61E-01	1.00E+00
SLC6A5	LMNA	-0.035733898	-0.172931809	0.102824345	6.14E-01	6.89E-01	1.00E+00
RAB3D	LMNA	0.333914848	0.205328953	0.451168221	1.19E-06	5.64E-06	1.42E-02
ERBB3	LMNA	-0.416306783	-0.524229493	-0.295222694	7.18E-10	6.10E-09	8.51E-06
DAZL	LMNA	0.24047842	0.105944598	0.366366674	5.66E-04	1.58E-03	1.00E+00
DCUN1D4	LMNA	-0.198560197	-0.327630379	-0.062214588	4.61E-03	1.02E-02	1.00E+00
CYP2C18	LMNA	0.004524101	-0.133610347	0.142486111	9.49E-01	9.62E-01	1.00E+00
SDC1	LMNA	0.606909887	0.511734297	0.68736993	1.03E-21	6.71E-20	1.22E-17
ATP6V1H	LMNA	-0.205749909	-0.334305308	-0.069678042	3.31E-03	7.58E-03	1.00E+00
SYK	LMNA	0.247880653	0.113721211	0.373161986	3.75E-04	1.09E-03	1.00E+00
IFI44L	LMNA	-0.079505875	-0.215194925	0.05919484	2.61E-01	3.42E-01	1.00E+00
RPL3L	LMNA	0.077556048	-0.061149663	0.213323065	2.73E-01	3.55E-01	1.00E+00
ST20	LMNA	0.105843875	-0.032684709	0.240382443	1.34E-01	1.95E-01	1.00E+00
FUT9	LMNA	-0.611246918	-0.690989942	-0.516805657	4.41E-22	3.13E-20	5.23E-18
ACSM1	LMNA	0.051192876	-0.08747634	0.187915833	4.69E-01	5.55E-01	1.00E+00
ADMR	LMNA	-0.103672455	-0.238312703	0.034877719	1.42E-01	2.05E-01	1.00E+00
TDG	LMNA	-0.134877165	-0.267939158	0.003234054	5.56E-02	9.21E-02	1.00E+00
PMP2	LMNA	-0.352745001	-0.468005625	-0.225684093	2.62E-07	1.40E-06	3.11E-03
PLAG1	LMNA	0.326770637	0.197635159	0.444758147	2.07E-06	9.36E-06	2.46E-02
MYCBP2	LMNA	-0.380314954	-0.492507882	-0.255688321	2.36E-08	1.51E-07	2.80E-04
AGPAT2	LMNA	0.682622785	0.601229724	0.749996542	4.68E-29	1.13E-26	5.55E-25
SLC12A1	LMNA	-0.165307787	-0.296590328	-0.027893337	1.87E-02	3.52E-02	1.00E+00
ENTPD4	LMNA	0.01100537	-0.127238945	0.148830253	8.76E-01	9.09E-01	1.00E+00
BRP44L	LMNA	-0.176900791	-0.30744361	-0.039822304	1.18E-02	2.35E-02	1.00E+00
CYP27A1	LMNA	0.378409392	0.253606757	0.490820054	2.81E-08	1.77E-07	3.34E-04
THAP7	LMNA	-0.063156228	-0.19946811	0.075553507	3.72E-01	4.60E-01	1.00E+00
XPO1	LMNA	-0.168059418	-0.299169457	-0.030721172	1.68E-02	3.20E-02	1.00E+00
KCNN1	LMNA	-0.354203612	-0.469306392	-0.227265476	2.32E-07	1.25E-06	2.75E-03
C1orf2	LMNA	0.289777071	0.158048651	0.411371557	2.88E-05	1.05E-04	3.41E-01
SLC7A9	LMNA	-0.098773826	-0.233638966	0.039820155	1.62E-01	2.29E-01	1.00E+00
CAMK2A	LMNA	-0.198001913	-0.327111528	-0.061635684	4.73E-03	1.04E-02	1.00E+00
DBC1	LMNA	-0.312014419	-0.431479896	-0.181793995	6.17E-06	2.54E-05	7.32E-02

IHPK2	LMNA	-0.202841264	-0.331606475	-0.066656817	3.79E-03	8.54E-03	1.00E+00
FADS3	LMNA	0.195455532	0.058996422	0.32474402	5.31E-03	1.15E-02	1.00E+00
GRM5	LMNA	-0.413516815	-0.5217812	-0.292143222	9.55E-10	7.92E-09	1.13E-05
PEX3	LMNA	-0.121927726	-0.25567514	0.016399312	8.39E-02	1.31E-01	1.00E+00
AZGP1	LMNA	0.049885037	-0.088777345	0.186650635	4.81E-01	5.66E-01	1.00E+00
MED1	LMNA	-0.009457698	-0.147316355	0.12876142	8.94E-01	9.23E-01	1.00E+00
CLU	LMNA	0.13769598	-0.0003619	0.270603069	5.07E-02	8.49E-02	1.00E+00
PABPC4	LMNA	0.306869282	0.176286411	0.426837889	8.91E-06	3.55E-05	1.06E-01
CXCL12	LMNA	0.15578079	0.018119461	0.287645769	2.68E-02	4.85E-02	1.00E+00
HNRPH3	LMNA	-0.205717824	-0.334275549	-0.069644702	3.31E-03	7.59E-03	1.00E+00
TFAP2C	LMNA	0.416472282	0.295405444	0.524374667	7.06E-10	6.00E-09	8.37E-06
ENDOD1	LMNA	0.362962058	0.236775218	0.477106549	1.10E-07	6.27E-07	1.31E-03
IDI1	LMNA	-0.39449532	-0.50504152	-0.271214806	6.27E-09	4.46E-08	7.44E-05
ULBP2	LMNA	0.275938316	0.14334795	0.398797693	7.04E-05	2.37E-04	8.35E-01
CA10	LMNA	-0.496411738	-0.593771496	-0.384726067	5.77E-14	9.47E-13	6.85E-10
OPRD1	LMNA	-0.068182717	-0.204310605	0.070532181	3.35E-01	4.21E-01	1.00E+00
CCL16	LMNA	0.276131408	0.143552666	0.398973454	6.95E-05	2.35E-04	8.25E-01
COMMD10	LMNA	-0.057942636	-0.194438317	0.080754322	4.13E-01	5.01E-01	1.00E+00
SACM1L	LMNA	-0.217321917	-0.345021745	-0.081722721	1.89E-03	4.62E-03	1.00E+00
CST6	LMNA	0.417805801	0.296878298	0.525544196	6.15E-10	5.30E-09	7.29E-06
KLHL12	LMNA	0.011988407	-0.126271574	0.149791501	8.66E-01	9.01E-01	1.00E+00
CD63	LMNA	0.563624733	0.461481096	0.651020506	2.48E-18	9.62E-17	2.94E-14
LGI1	LMNA	-0.096886189	-0.231836324	0.041722865	1.70E-01	2.39E-01	1.00E+00
CRYBB1	LMNA	0.048621892	-0.09003344	0.185428243	4.92E-01	5.77E-01	1.00E+00
TOP2A	LMNA	-0.039439113	-0.176528982	0.09915173	5.77E-01	6.56E-01	1.00E+00
CX3CL1	LMNA	0.032747805	-0.10578142	0.170030122	6.44E-01	7.16E-01	1.00E+00
GPR50	LMNA	0.00763269	-0.130555877	0.145530346	9.14E-01	9.38E-01	1.00E+00
GYPB	LMNA	-0.230104581	-0.356820753	-0.095073707	9.86E-04	2.59E-03	1.00E+00
NR5A2	LMNA	-0.105552688	-0.240104962	0.03297887	1.35E-01	1.96E-01	1.00E+00
GADD45GII	LMNA	0.128763473	-0.00945561	0.262154436	6.78E-02	1.09E-01	1.00E+00
FEN1	LMNA	0.084697117	-0.053985105	0.220173732	2.31E-01	3.09E-01	1.00E+00
EHD3	LMNA	-0.274532902	-0.397518147	-0.141858264	7.69E-05	2.57E-04	9.12E-01
IGF1R	LMNA	0.114380213	-0.024050554	0.248507209	1.05E-01	1.59E-01	1.00E+00
CAPRIN2	LMNA	0.24559598	0.111319232	0.37106607	4.27E-04	1.22E-03	1.00E+00
KLRC3	LMNA	-0.525884608	-0.618997102	-0.418194032	9.23E-16	2.24E-14	1.09E-11

PURG	LMNA	-0.618416945	-0.696965828	-0.525204248	1.06E-22	8.89E-21	1.25E-18
SF3B1	LMNA	-0.111625878	-0.245887733	0.026838709	1.14E-01	1.70E-01	1.00E+00
DEFB126	LMNA	-0.128360782	-0.261773077	0.00986503	6.87E-02	1.11E-01	1.00E+00
CAV1	LMNA	0.569775524	0.468582308	0.656210282	8.79E-19	3.74E-17	1.04E-14
ALDH1A1	LMNA	0.090837649	-0.047812937	0.226053882	1.99E-01	2.72E-01	1.00E+00
ANKRD5	LMNA	0.209375633	0.0734476	0.337666546	2.78E-03	6.50E-03	1.00E+00
NLGN1	LMNA	-0.258207178	-0.382619419	-0.124597552	2.07E-04	6.35E-04	1.00E+00
DDEFL1	LMNA	0.139795906	0.001779243	0.272586273	4.72E-02	7.98E-02	1.00E+00
HPRT1	LMNA	0.170642236	0.033377521	0.301588614	1.52E-02	2.92E-02	1.00E+00
RNASEL	LMNA	0.166445601	0.029062395	0.297657043	1.79E-02	3.39E-02	1.00E+00
DNAH9	LMNA	0.24428799	0.10994479	0.36986557	4.59E-04	1.30E-03	1.00E+00
TNS4	LMNA	0.056684039	-0.082008704	0.193223014	4.23E-01	5.10E-01	1.00E+00
FANCI	LMNA	-0.016511718	-0.154211201	0.121816959	8.16E-01	8.61E-01	1.00E+00
PSMA7	LMNA	0.305350078	0.174661749	0.425466033	9.92E-06	3.93E-05	1.18E-01
TTF1	LMNA	-0.192648227	-0.322132036	-0.056088927	6.02E-03	1.29E-02	1.00E+00
DBF4B	LMNA	-0.429226862	-0.535544091	-0.309516311	1.84E-10	1.75E-09	2.19E-06
TUBG1	LMNA	0.04675357	-0.091890527	0.183619421	5.09E-01	5.93E-01	1.00E+00
RAD54L	LMNA	-0.062289691	-0.198632619	0.076418443	3.78E-01	4.66E-01	1.00E+00
PLAGL2	LMNA	0.295316525	0.163949541	0.416391757	1.98E-05	7.43E-05	2.35E-01
HMGCL	LMNA	0.368998881	0.243343984	0.482472416	6.52E-08	3.88E-07	7.74E-04
C11orf60	LMNA	-0.218020059	-0.345667207	-0.082450649	1.83E-03	4.48E-03	1.00E+00
ABCD1	LMNA	0.525584076	0.417851281	0.618740845	9.65E-16	2.34E-14	1.14E-11
ACAA1	LMNA	-0.004142934	-0.142112655	0.133984697	9.53E-01	9.65E-01	1.00E+00
SPARCL1	LMNA	-0.252768103	-0.37764135	-0.118864879	2.84E-04	8.47E-04	1.00E+00
TXNDC14	LMNA	-0.1003509	-0.235144319	0.038229717	1.55E-01	2.21E-01	1.00E+00
FAM32A	LMNA	-0.031579998	-0.168894676	0.106937212	6.55E-01	7.27E-01	1.00E+00
IL6ST	LMNA	0.324156581	0.194823981	0.442409686	2.52E-06	1.12E-05	2.99E-02
TMEM109	LMNA	0.534700825	0.428262438	0.626505692	2.47E-16	6.65E-15	2.93E-12
CCBL1	LMNA	-0.340860742	-0.45738878	-0.212824444	6.91E-07	3.41E-06	8.19E-03
FAM90A1	LMNA	0.129926953	-0.008272426	0.26325605	6.53E-02	1.06E-01	1.00E+00
PRSS23	LMNA	0.596018859	0.499028416	0.678261801	8.16E-21	4.65E-19	9.68E-17
ANK1	LMNA	0.115893516	-0.022517751	0.249945583	1.00E-01	1.53E-01	1.00E+00
IL22RA1	LMNA	0.077573358	-0.061132314	0.213339687	2.72E-01	3.55E-01	1.00E+00
SPATA20	LMNA	0.335946687	0.207520003	0.452989063	1.02E-06	4.87E-06	1.21E-02
ATP4B	LMNA	0.014604207	-0.12369618	0.152348055	8.37E-01	8.78E-01	1.00E+00

TEC	LMNA	0.314535624	0.184495792	0.433752253	5.14E-06	2.14E-05	6.10E-02
C14orf122	LMNA	-0.123893374	-0.25753952	0.014403987	7.90E-02	1.24E-01	1.00E+00
APCS	LMNA	0.10197326	-0.036592877	0.236692224	1.49E-01	2.13E-01	1.00E+00
PSMB5	LMNA	0.02812654	-0.11035295	0.165534786	6.91E-01	7.57E-01	1.00E+00
ABCB4	LMNA	0.067541694	-0.071172937	0.203693417	3.40E-01	4.26E-01	1.00E+00
C9orf38	LMNA	-0.000505544	-0.138546875	0.137555057	9.94E-01	9.95E-01	1.00E+00
C6orf10	LMNA	-0.124484517	-0.258100014	0.013803703	7.75E-02	1.23E-01	1.00E+00
MXD3	LMNA	-0.171357877	-0.30225861	-0.034113879	1.48E-02	2.85E-02	1.00E+00
SFTPB	LMNA	0.008360109	-0.129840743	0.146242329	9.06E-01	9.32E-01	1.00E+00
CARTPT	LMNA	-0.238444821	-0.36449746	-0.103811025	6.32E-04	1.74E-03	1.00E+00
MON2	LMNA	-0.128495611	-0.261900768	0.009727953	6.84E-02	1.10E-01	1.00E+00
HNF4A	LMNA	0.122763057	-0.015551504	0.256467558	8.18E-02	1.28E-01	1.00E+00
CNTNAP2	LMNA	-0.402408879	-0.512015888	-0.279907569	2.91E-09	2.20E-08	3.45E-05
RABEP1	LMNA	-0.187749202	-0.317569139	-0.051020603	7.46E-03	1.56E-02	1.00E+00
TNFRSF10I	LMNA	0.516210513	0.407176269	0.610738244	3.75E-15	7.93E-14	4.45E-11
FRK	LMNA	0.121405574	-0.016929163	0.255179721	8.52E-02	1.33E-01	1.00E+00
TBX19	LMNA	0.379834134	0.255162983	0.492082081	2.47E-08	1.57E-07	2.93E-04
PPAP2B	LMNA	-0.084227681	-0.219723796	0.054456525	2.33E-01	3.11E-01	1.00E+00
TMEM16K	LMNA	0.167709644	0.03036159	0.298841717	1.70E-02	3.24E-02	1.00E+00
CTDSP1	LMNA	0.514312271	0.405018091	0.609115281	4.91E-15	1.02E-13	5.82E-11
CDK5R1	LMNA	-0.63613694	-0.711687972	-0.546038698	2.63E-24	3.03E-22	3.12E-20
CHD4	LMNA	0.036164706	-0.102397522	0.173350243	6.09E-01	6.85E-01	1.00E+00
GABRR1	LMNA	0.136947122	-0.001125151	0.269895561	5.20E-02	8.68E-02	1.00E+00
MOSC2	LMNA	0.085037901	-0.053642844	0.220500323	2.29E-01	3.06E-01	1.00E+00
C6orf26	LMNA	-0.15900732	-0.290677622	-0.021426659	2.38E-02	4.36E-02	1.00E+00
IKBKE	LMNA	0.412424316	0.290938045	0.520822009	1.07E-09	8.77E-09	1.27E-05
HIF1A	LMNA	0.315265329	0.185278132	0.434409651	4.88E-06	2.05E-05	5.78E-02
RELA	LMNA	0.379866698	0.255198559	0.492110921	2.46E-08	1.57E-07	2.92E-04
DBN1	LMNA	-0.188602217	-0.318364061	-0.05190259	7.19E-03	1.51E-02	1.00E+00
TMEM16B	LMNA	-0.027859687	-0.16527503	0.110616752	6.94E-01	7.60E-01	1.00E+00
ACAD10	LMNA	-0.042635904	-0.179629618	0.095980028	5.47E-01	6.28E-01	1.00E+00
QTRTD1	LMNA	0.340247595	0.212162174	0.45684012	7.25E-07	3.57E-06	8.60E-03
TSEN34	LMNA	0.160603675	0.023064038	0.292176681	2.24E-02	4.13E-02	1.00E+00
RPS19	LMNA	-0.013733292	-0.15149707	0.12455385	8.46E-01	8.86E-01	1.00E+00
KIF18A	LMNA	-0.040674756	-0.177727781	0.097926118	5.65E-01	6.45E-01	1.00E+00

C1QB	LMNA	0.207269373	0.071257311	0.33571433	3.08E-03	7.11E-03	1.00E+00
TXNDC9	LMNA	0.071018055	-0.067696651	0.20703922	3.15E-01	4.01E-01	1.00E+00
TMEM22	LMNA	0.223404145	0.088069305	0.35064097	1.39E-03	3.52E-03	1.00E+00
KHSRP	LMNA	0.045662152	-0.092974936	0.182562333	5.19E-01	6.02E-01	1.00E+00
SPATA2L	LMNA	0.180480822	0.043513997	0.310788357	1.02E-02	2.06E-02	1.00E+00
TNFRSF11/	LMNA	0.410685805	0.28902102	0.519295067	1.27E-09	1.03E-08	1.51E-05
SEMA4G	LMNA	-0.386640165	-0.498104319	-0.262606041	1.32E-08	8.83E-08	1.56E-04
IBTK	LMNA	0.034452507	-0.104093584	0.171686929	6.26E-01	7.01E-01	1.00E+00
FBL	LMNA	0.115981315	-0.0224288	0.250029017	1.00E-01	1.52E-01	1.00E+00
C21orf91	LMNA	-0.006384938	-0.144308738	0.131782221	9.28E-01	9.48E-01	1.00E+00
MATN1	LMNA	0.153633718	0.015920378	0.285626778	2.90E-02	5.19E-02	1.00E+00
GPR172B	LMNA	0.145252532	0.007348894	0.277734333	3.92E-02	6.77E-02	1.00E+00
CFTR	LMNA	0.05745003	-0.08124533	0.193962706	4.17E-01	5.04E-01	1.00E+00
VSX1	LMNA	-0.392006702	-0.502845267	-0.268485299	7.95E-09	5.54E-08	9.43E-05
CAMK1D	LMNA	-0.3287473	-0.446532899	-0.19976229	1.78E-06	8.15E-06	2.11E-02
RTP4	LMNA	0.152616746	0.014879235	0.284670063	3.01E-02	5.38E-02	1.00E+00
ARMCX6	LMNA	0.069508769	-0.069206319	0.205587007	3.26E-01	4.11E-01	1.00E+00
DYNC1I1	LMNA	-0.377369339	-0.489898483	-0.252471129	3.09E-08	1.93E-07	3.67E-04
PPP4C	LMNA	0.102588228	-0.035972226	0.23727879	1.46E-01	2.10E-01	1.00E+00
KIAA0828	LMNA	-0.16730287	-0.298460528	-0.029943453	1.73E-02	3.28E-02	1.00E+00
TREH	LMNA	0.003605203	-0.134512745	0.141585736	9.59E-01	9.70E-01	1.00E+00
PAR5	LMNA	-0.425215297	-0.532035095	-0.305072477	2.83E-10	2.58E-09	3.36E-06
CD48	LMNA	0.216729571	0.081105217	0.344474001	1.95E-03	4.74E-03	1.00E+00
ST14	LMNA	0.325782393	0.196572143	0.443870502	2.23E-06	1.00E-05	2.65E-02
GDI2	LMNA	-0.099949003	-0.234760761	0.038635086	1.57E-01	2.23E-01	1.00E+00
PKN1	LMNA	0.329619195	0.200700942	0.447315437	1.67E-06	7.68E-06	1.98E-02
SPON2	LMNA	0.472869393	0.358201842	0.573483347	1.19E-12	1.63E-11	1.42E-08
XBP1	LMNA	0.402496987	0.280004466	0.512093458	2.88E-09	2.19E-08	3.42E-05
MLF2	LMNA	-0.25976805	-0.384046661	-0.126244327	1.89E-04	5.83E-04	1.00E+00
CEBPA	LMNA	-0.005409135	-0.143353089	0.132740988	9.39E-01	9.55E-01	1.00E+00
SFRS12	LMNA	0.137994336	-5.78E-05	0.270884909	5.02E-02	8.41E-02	1.00E+00
EFCAB6	LMNA	-0.071655493	-0.207652373	0.067058861	3.11E-01	3.96E-01	1.00E+00
SELT	LMNA	-0.044841718	-0.181767497	0.093789884	5.26E-01	6.09E-01	1.00E+00
SLC39A2	LMNA	0.093062415	-0.045574092	0.228181881	1.88E-01	2.60E-01	1.00E+00
ALOX12B	LMNA	-0.022942707	-0.160485408	0.11547403	7.46E-01	8.04E-01	1.00E+00

ERF	LMNA	0.116627929	-0.021773641	0.250643419	9.83E-02	1.50E-01	1.00E+00
ARL3	LMNA	-0.525327503	-0.618522055	-0.417558688	1.00E-15	2.41E-14	1.19E-11
MLLT10	LMNA	-0.229499689	-0.356263319	-0.094440825	1.02E-03	2.66E-03	1.00E+00
BPHL	LMNA	-0.227834617	-0.354728417	-0.092699267	1.11E-03	2.88E-03	1.00E+00
COX5B	LMNA	-0.174011226	-0.304741595	-0.03684534	1.33E-02	2.60E-02	1.00E+00
S100A10	LMNA	0.565111383	0.463196265	0.652275628	1.93E-18	7.70E-17	2.29E-14
TDGF1	LMNA	-0.260392712	-0.384617679	-0.126903575	1.82E-04	5.65E-04	1.00E+00
ERCC6	LMNA	-0.320664209	-0.439269616	-0.191071567	3.28E-06	1.42E-05	3.89E-02
THOC6	LMNA	0.200476643	0.064202511	0.329410868	4.23E-03	9.42E-03	1.00E+00
BAZ1A	LMNA	0.113996546	-0.02443906	0.248142446	1.06E-01	1.60E-01	1.00E+00
RRP12	LMNA	0.188512207	0.051809513	0.318280189	7.21E-03	1.52E-02	1.00E+00
LRRN3	LMNA	-0.060673548	-0.197073849	0.078031044	3.91E-01	4.78E-01	1.00E+00
EFTUD1	LMNA	0.288849245	0.157061205	0.410529982	3.06E-05	1.11E-04	3.63E-01
PTPRO	LMNA	-0.369697041	-0.483092431	-0.244104408	6.13E-08	3.66E-07	7.28E-04
ARID3B	LMNA	0.08173773	-0.05695596	0.217336313	2.48E-01	3.27E-01	1.00E+00
ACBD4	LMNA	0.095538667	-0.043080532	0.23054891	1.76E-01	2.46E-01	1.00E+00
KIAA0133	LMNA	-0.049039183	-0.185832117	0.089618527	4.88E-01	5.73E-01	1.00E+00
CD3G	LMNA	0.254925439	0.121137599	0.379616698	2.51E-04	7.55E-04	1.00E+00
NAT11	LMNA	-0.210031783	-0.338274482	-0.074130195	2.70E-03	6.33E-03	1.00E+00
PPAT	LMNA	-0.064637039	-0.200895408	0.074074948	3.61E-01	4.48E-01	1.00E+00
SIRT3	LMNA	0.056434152	-0.082257702	0.192981672	4.25E-01	5.12E-01	1.00E+00
DPP8	LMNA	-0.083184122	-0.21872338	0.05550427	2.39E-01	3.18E-01	1.00E+00
ZDHHC14	LMNA	-0.043631953	-0.18059515	0.094991221	5.38E-01	6.20E-01	1.00E+00
OTUD7B	LMNA	0.239104771	0.104503283	0.365104174	6.10E-04	1.69E-03	1.00E+00
ZFP64	LMNA	0.120162001	-0.018190756	0.253999535	8.85E-02	1.37E-01	1.00E+00
ACTB	LMNA	0.525987281	0.418311136	0.619084644	9.09E-16	2.22E-14	1.08E-11
IL7R	LMNA	0.461430944	0.345381063	0.563581253	4.80E-12	5.98E-11	5.69E-08
MSRA	LMNA	-0.009143132	-0.147008576	0.129070784	8.97E-01	9.26E-01	1.00E+00
TRMT12	LMNA	-0.222522393	-0.349826907	-0.087148547	1.46E-03	3.65E-03	1.00E+00
TLR4	LMNA	0.049110346	-0.089547764	0.185900988	4.88E-01	5.73E-01	1.00E+00
IFI35	LMNA	0.349083259	0.221717092	0.464737938	3.55E-07	1.85E-06	4.21E-03
BSCL2	LMNA	-0.016198938	-0.153905762	0.122125169	8.19E-01	8.63E-01	1.00E+00
ANKRD12	LMNA	-0.149393983	-0.281636515	-0.011581846	3.38E-02	5.95E-02	1.00E+00
CFHR2	LMNA	0.251334759	0.117355649	0.376328291	3.08E-04	9.13E-04	1.00E+00
DDX51	LMNA	-0.005681521	-0.143619875	0.132473383	9.36E-01	9.53E-01	1.00E+00

KIAA1086	LMNA	-0.222542833	-0.349845781	-0.087169889	1.46E-03	3.65E-03	1.00E+00
SLC30A5	LMNA	0.371543505	0.246116289	0.484731672	5.21E-08	3.15E-07	6.18E-04
IMPG1	LMNA	-0.04308915	-0.18006901	0.095530112	5.43E-01	6.24E-01	1.00E+00
ACVR2B	LMNA	-0.384688124	-0.496378177	-0.260469783	1.58E-08	1.05E-07	1.88E-04
ZNF185	LMNA	0.295972928	0.164649396	0.416986142	1.90E-05	7.14E-05	2.25E-01
DNASE1L2	LMNA	-0.463000305	-0.564941566	-0.347137521	3.98E-12	5.01E-11	4.72E-08
LMO3	LMNA	-0.166600797	-0.297802517	-0.029221883	1.78E-02	3.37E-02	1.00E+00
NEUROG1	LMNA	0.03105925	-0.107452481	0.168388242	6.61E-01	7.31E-01	1.00E+00
LIN37	LMNA	-0.092765164	-0.227897634	0.045873305	1.89E-01	2.61E-01	1.00E+00
SOCS2	LMNA	0.192310722	0.055739533	0.32181788	6.11E-03	1.31E-02	1.00E+00
DSCR4	LMNA	-0.12775788	-0.261202033	0.010477921	7.00E-02	1.12E-01	1.00E+00
ZNF587	LMNA	-0.123600745	-0.25726203	0.014701104	7.97E-02	1.25E-01	1.00E+00
PPP2R5D	LMNA	-0.223939628	-0.35113525	-0.088628588	1.36E-03	3.43E-03	1.00E+00
CYP2C8	LMNA	-0.143504127	-0.276085626	-0.005563341	4.16E-02	7.13E-02	1.00E+00
C1orf80	LMNA	0.149752498	0.011948515	0.281974112	3.34E-02	5.90E-02	1.00E+00
DOCK5	LMNA	0.181977148	0.045058103	0.312185388	9.54E-03	1.95E-02	1.00E+00
C11orf24	LMNA	0.464275715	0.348565582	0.566046675	3.41E-12	4.37E-11	4.05E-08
CCDC15	LMNA	-0.074698054	-0.210577549	0.064013059	2.91E-01	3.74E-01	1.00E+00
CAP2	LMNA	-0.137812449	-0.270713093	0.000243178	5.05E-02	8.46E-02	1.00E+00
TIMM44	LMNA	0.07825707	-0.060446968	0.21399617	2.68E-01	3.50E-01	1.00E+00
ROM1	LMNA	-0.002539585	-0.140541313	0.135558941	9.71E-01	9.78E-01	1.00E+00
MYLC2PL	LMNA	0.092969203	-0.045667922	0.228092749	1.88E-01	2.60E-01	1.00E+00
MOS	LMNA	-0.075859156	-0.211693214	0.062850037	2.83E-01	3.66E-01	1.00E+00
MLH3	LMNA	0.108918408	-0.029577326	0.243310914	1.23E-01	1.81E-01	1.00E+00
CD1E	LMNA	0.13208209	-0.00607977	0.265295672	6.10E-02	9.97E-02	1.00E+00
NOX1	LMNA	0.134727877	-0.003386103	0.267798017	5.59E-02	9.25E-02	1.00E+00
DPEP2	LMNA	0.171177574	0.033928342	0.302089819	1.49E-02	2.87E-02	1.00E+00
DNAJB5	LMNA	-0.30442218	-0.424627861	-0.173669789	1.06E-05	4.17E-05	1.26E-01
MBIP	LMNA	-0.328743001	-0.44652904	-0.199757662	1.78E-06	8.15E-06	2.11E-02
COPB1	LMNA	0.35196537	0.224839116	0.467310158	2.80E-07	1.49E-06	3.32E-03
CCDC90A	LMNA	0.174669654	0.037523469	0.305357472	1.29E-02	2.54E-02	1.00E+00
NOLA1	LMNA	0.251009652	0.117013416	0.376030397	3.14E-04	9.28E-04	1.00E+00
CD320	LMNA	0.276811248	0.144273526	0.399592204	6.66E-05	2.25E-04	7.90E-01
RABL3	LMNA	0.171568021	0.034330134	0.302455326	1.46E-02	2.83E-02	1.00E+00
ANGEL2	LMNA	-0.296600514	-0.417554336	-0.165318651	1.82E-05	6.86E-05	2.16E-01

C19orf24	LMNA	0.399216125	0.276398024	0.509203792	3.97E-09	2.94E-08	4.71E-05
SMG6	LMNA	0.097651364	-0.040951701	0.232567158	1.67E-01	2.35E-01	1.00E+00
INSR	LMNA	0.08385026	-0.054835495	0.219362011	2.35E-01	3.14E-01	1.00E+00
GLRB	LMNA	-0.396123741	-0.506477858	-0.273001926	5.36E-09	3.88E-08	6.36E-05
HLA-DMA	LMNA	0.257196776	0.123531934	0.381695203	2.20E-04	6.70E-04	1.00E+00
HCRP1	LMNA	-0.301363867	-0.421863821	-0.170402209	1.31E-05	5.08E-05	1.55E-01
GPR137	LMNA	0.180298553	0.043325952	0.310618145	1.02E-02	2.07E-02	1.00E+00
URG4	LMNA	0.243398727	0.109010641	0.369049146	4.82E-04	1.36E-03	1.00E+00
CIZ1	LMNA	0.235535888	0.100761236	0.361821901	7.40E-04	2.01E-03	1.00E+00
MARK4	LMNA	-0.053523806	-0.19016964	0.085156417	4.49E-01	5.36E-01	1.00E+00
LRDD	LMNA	-0.056517896	-0.193062555	0.082174257	4.24E-01	5.11E-01	1.00E+00
METTL4	LMNA	-0.154954688	-0.286869086	-0.017273189	2.77E-02	4.98E-02	1.00E+00
CBY1	LMNA	-0.013792459	-0.151554889	0.124495589	8.46E-01	8.85E-01	1.00E+00
NFX1	LMNA	-0.120524922	-0.254343998	0.017822622	8.75E-02	1.36E-01	1.00E+00
MBD3	LMNA	0.126750995	-0.011501256	0.260248147	7.22E-02	1.15E-01	1.00E+00
MTERFD2	LMNA	-0.180501675	-0.31080783	-0.043535511	1.02E-02	2.06E-02	1.00E+00
PGC	LMNA	-0.117490722	-0.251463064	0.020899259	9.59E-02	1.47E-01	1.00E+00
FGF3	LMNA	-0.121039379	-0.254832233	0.01730071	8.62E-02	1.34E-01	1.00E+00
SLC35A3	LMNA	0.019772084	-0.118602649	0.157393471	7.80E-01	8.32E-01	1.00E+00
CLEC16A	LMNA	-0.238213531	-0.364284801	-0.103568442	6.40E-04	1.76E-03	1.00E+00
IER3IP1	LMNA	0.14883419	0.011009396	0.281109319	3.45E-02	6.05E-02	1.00E+00
RASAL2	LMNA	0.159500589	0.021932525	0.291140895	2.34E-02	4.29E-02	1.00E+00
C1orf89	LMNA	0.239460316	0.104876288	0.365430996	5.98E-04	1.66E-03	1.00E+00
PAICS	LMNA	0.108727029	-0.029770828	0.243128699	1.23E-01	1.82E-01	1.00E+00
SYNJ1	LMNA	-0.326273217	-0.444311391	-0.197100065	2.15E-06	9.70E-06	2.55E-02
MMD	LMNA	-0.247727464	-0.373021494	-0.113560109	3.79E-04	1.10E-03	1.00E+00
NFKBIE	LMNA	0.279711863	0.147350738	0.402230917	5.54E-05	1.90E-04	6.57E-01
KLK10	LMNA	0.073873333	-0.064838913	0.209784887	2.96E-01	3.80E-01	1.00E+00
TCEB2	LMNA	-0.346058317	-0.462036143	-0.218443153	4.54E-07	2.32E-06	5.39E-03
NIT2	LMNA	0.112557182	-0.02589621	0.246773658	1.11E-01	1.66E-01	1.00E+00
SERPINB1(	LMNA	0.069267641	-0.069447448	0.205354942	3.27E-01	4.13E-01	1.00E+00
KLF15	LMNA	-0.397978399	-0.508113002	-0.275038368	4.48E-09	3.29E-08	5.32E-05
HLA-B	LMNA	0.456082212	0.339400768	0.558940841	9.03E-12	1.07E-10	1.07E-07
PPP2R2B	LMNA	-0.386161317	-0.497680967	-0.26208189	1.38E-08	9.20E-08	1.64E-04
ARHGEF17	LMNA	0.017703857	-0.120641997	0.155375115	8.03E-01	8.51E-01	1.00E+00

TCF7L2	LMNA	-0.093413905	-0.228517965	0.04522025	1.86E-01	2.58E-01	1.00E+00
WSB2	LMNA	-0.352830905	-0.468082247	-0.22577721	2.60E-07	1.39E-06	3.09E-03
CHD5	LMNA	-0.338139531	-0.454953093	-0.209886119	8.58E-07	4.15E-06	1.02E-02
ME3	LMNA	-0.110434828	-0.244754392	0.028043717	1.18E-01	1.75E-01	1.00E+00
CACYBP	LMNA	-0.321685234	-0.440187942	-0.192168226	3.04E-06	1.33E-05	3.60E-02
TCTN2	LMNA	0.347261092	0.21974459	0.463110686	4.12E-07	2.12E-06	4.89E-03
C2orf25	LMNA	-0.031595869	-0.168910111	0.106921506	6.55E-01	7.27E-01	1.00E+00
JAK1	LMNA	0.079121015	-0.059580771	0.214825533	2.63E-01	3.45E-01	1.00E+00
GPD2	LMNA	0.060405876	-0.07829806	0.196815613	3.93E-01	4.80E-01	1.00E+00
FBXL11	LMNA	0.295640937	0.164295412	0.416685531	1.94E-05	7.29E-05	2.30E-01
CDV3	LMNA	0.407464658	0.285471699	0.516464087	1.76E-09	1.39E-08	2.09E-05
SCUBE2	LMNA	-0.095235477	-0.230259179	0.043385934	1.78E-01	2.48E-01	1.00E+00
GALNT11	LMNA	0.339354196	0.211197411	0.456040525	7.79E-07	3.81E-06	9.24E-03
MYCBP	LMNA	0.323773767	0.194412478	0.442065631	2.60E-06	1.15E-05	3.08E-02
GPX5	LMNA	-0.20926394	-0.337563049	-0.073331418	2.80E-03	6.53E-03	1.00E+00
QSER1	LMNA	0.135392913	-0.002708717	0.268426716	5.47E-02	9.07E-02	1.00E+00
FLOT1	LMNA	0.343261759	0.215418952	0.459536418	5.70E-07	2.84E-06	6.76E-03
C1orf164	LMNA	-0.166895616	-0.298078848	-0.029524872	1.76E-02	3.33E-02	1.00E+00
MMP25	LMNA	-0.016540588	-0.154239392	0.12178851	8.15E-01	8.61E-01	1.00E+00
ULK2	LMNA	-0.283637875	-0.405799202	-0.151519861	4.30E-05	1.51E-04	5.10E-01
PIGO	LMNA	0.204576623	0.068459043	0.333216909	3.49E-03	7.95E-03	1.00E+00
CHST5	LMNA	-0.112883042	-0.247083586	0.025566376	1.10E-01	1.65E-01	1.00E+00
NRCAM	LMNA	0.322493278	0.193036356	0.440914534	2.86E-06	1.26E-05	3.39E-02
SLC35E3	LMNA	-0.060810124	-0.197205604	0.077894795	3.90E-01	4.77E-01	1.00E+00
LYRM4	LMNA	-0.241998115	-0.367762864	-0.107539813	5.21E-04	1.46E-03	1.00E+00
CSRP2	LMNA	0.283799169	0.151691243	0.40594572	4.26E-05	1.50E-04	5.05E-01
HYPE	LMNA	0.182040153	0.045123134	0.312244199	9.52E-03	1.94E-02	1.00E+00
HIPK2	LMNA	-0.229698815	-0.356446832	-0.094649153	1.01E-03	2.64E-03	1.00E+00
GPER	LMNA	-0.068275923	-0.204400336	0.070439004	3.34E-01	4.20E-01	1.00E+00
DAP	LMNA	0.5524122	0.44856959	0.641538775	1.56E-17	5.23E-16	1.85E-13
ZMIZ1	LMNA	-0.206032928	-0.3345678	-0.069972149	3.26E-03	7.49E-03	1.00E+00
DDX58	LMNA	0.168620543	0.031298107	0.299695169	1.64E-02	3.14E-02	1.00E+00
TIMM13	LMNA	-0.062854529	-0.199177243	0.075854673	3.74E-01	4.62E-01	1.00E+00
DCC1	LMNA	-0.121549703	-0.255316479	0.016782916	8.48E-02	1.32E-01	1.00E+00
AKT1	LMNA	0.358793958	0.232246563	0.473396736	1.58E-07	8.73E-07	1.87E-03

GBA3	LMNA	-0.079153555	-0.214856767	0.059548141	2.63E-01	3.44E-01	1.00E+00
CDC34	LMNA	0.066970593	-0.071743704	0.20314346	3.44E-01	4.30E-01	1.00E+00
TEX13A	LMNA	-0.105628218	-0.240176939	0.032902571	1.35E-01	1.96E-01	1.00E+00
MTRF1	LMNA	-0.211884963	-0.339990913	-0.076058752	2.47E-03	5.84E-03	1.00E+00
MCM6	LMNA	0.097886253	-0.040714941	0.232791474	1.66E-01	2.34E-01	1.00E+00
RNF125	LMNA	-0.152260846	-0.284335189	-0.014514946	3.05E-02	5.44E-02	1.00E+00
ABCA7	LMNA	0.189474441	0.052804659	0.319176693	6.92E-03	1.46E-02	1.00E+00
CASC1	LMNA	-0.169578122	-0.300592132	-0.032282878	1.58E-02	3.03E-02	1.00E+00
EIF4A2	LMNA	-0.454167409	-0.557278043	-0.337262161	1.13E-11	1.32E-10	1.34E-07
SHROOM2	LMNA	0.100817541	-0.037758988	0.235589614	1.53E-01	2.19E-01	1.00E+00
RPGR	LMNA	0.288575946	0.156770395	0.410282049	3.11E-05	1.13E-04	3.69E-01
COL6A3	LMNA	0.619237224	0.526166237	0.697648797	8.96E-23	7.86E-21	1.06E-18
TMEFF1	LMNA	-0.52794938	-0.620757153	-0.420549698	6.80E-16	1.68E-14	8.07E-12
GPR125	LMNA	-0.069428202	-0.20550947	0.069286888	3.26E-01	4.12E-01	1.00E+00
PLEKHA4	LMNA	0.509215388	0.399229286	0.604753605	1.01E-14	1.95E-13	1.19E-10
USP22	LMNA	-0.173327248	-0.304101703	-0.03614103	1.36E-02	2.67E-02	1.00E+00
PTCD1	LMNA	0.205701912	0.069628168	0.334260791	3.32E-03	7.59E-03	1.00E+00
GNPAT	LMNA	-0.171170464	-0.302083163	-0.033921026	1.49E-02	2.87E-02	1.00E+00
CRTAC1	LMNA	-0.396016469	-0.506383259	-0.272884174	5.42E-09	3.91E-08	6.43E-05
BXDC2	LMNA	0.064427568	-0.074284137	0.200693542	3.62E-01	4.49E-01	1.00E+00
C18orf1	LMNA	-0.362943719	-0.477090235	-0.236755281	1.11E-07	6.28E-07	1.31E-03
WDR18	LMNA	-0.049117601	-0.185908009	0.08954055	4.88E-01	5.73E-01	1.00E+00
HSD17B12	LMNA	-0.012531486	-0.150322431	0.125737038	8.60E-01	8.96E-01	1.00E+00
HIST1H2BN	LMNA	0.04334412	-0.09527699	0.180316163	5.40E-01	6.22E-01	1.00E+00
FAM107A	LMNA	-0.341910549	-0.458327969	-0.21395863	6.35E-07	3.15E-06	7.53E-03
EFNA3	LMNA	-0.323045943	-0.441411401	-0.193630234	2.74E-06	1.21E-05	3.25E-02
P18SRP	LMNA	-0.155379491	-0.287268498	-0.017708339	2.72E-02	4.91E-02	1.00E+00
CYP2B6	LMNA	0.148592516	0.010762286	0.280881694	3.48E-02	6.10E-02	1.00E+00
CAMKK2	LMNA	-0.218231272	-0.345862458	-0.082670901	1.81E-03	4.43E-03	1.00E+00
NES	LMNA	0.315778853	0.185828796	0.434872214	4.70E-06	1.98E-05	5.57E-02
KIAA0649	LMNA	-0.114406044	-0.248531767	0.024024394	1.05E-01	1.59E-01	1.00E+00
TBC1D2	LMNA	0.286459937	0.15451958	0.408361828	3.58E-05	1.28E-04	4.25E-01
SLC25A12	LMNA	-0.211519388	-0.339652382	-0.075678227	2.51E-03	5.94E-03	1.00E+00
ERCC6L	LMNA	0.155403724	0.017733165	0.287291281	2.72E-02	4.91E-02	1.00E+00
POLR2C	LMNA	0.009521248	-0.128698917	0.147378531	8.93E-01	9.22E-01	1.00E+00

PON2	LMNA	-0.037527827	-0.174673885	0.101046671	5.96E-01	6.73E-01	1.00E+00
ANKRD27	LMNA	0.117049003	-0.021346937	0.25104346	9.71E-02	1.48E-01	1.00E+00
HPX	LMNA	0.060904138	-0.077801003	0.197296296	3.89E-01	4.77E-01	1.00E+00
EIF3K	LMNA	-0.004109754	-0.142080145	0.134017282	9.54E-01	9.66E-01	1.00E+00
CLEC4A	LMNA	0.115508055	-0.02290824	0.249579264	1.02E-01	1.54E-01	1.00E+00
CPSF4	LMNA	0.079886995	-0.05881262	0.215560689	2.58E-01	3.40E-01	1.00E+00
MLXIPL	LMNA	-0.179749952	-0.310105783	-0.042760027	1.05E-02	2.11E-02	1.00E+00
ENC1	LMNA	-0.197049888	-0.326226569	-0.06064871	4.94E-03	1.08E-02	1.00E+00
MAP2K1	LMNA	0.109647493	-0.028840058	0.244005003	1.20E-01	1.78E-01	1.00E+00
GH1	LMNA	0.092888129	-0.045749532	0.228015222	1.89E-01	2.60E-01	1.00E+00
FKSG2	LMNA	0.139174806	0.00114582	0.271999814	4.82E-02	8.13E-02	1.00E+00
HPS5	LMNA	0.138894542	0.000860032	0.271735149	4.87E-02	8.20E-02	1.00E+00
KIAA0430	LMNA	-0.304813048	-0.424980958	-0.17408761	1.03E-05	4.06E-05	1.22E-01
POP5	LMNA	-0.118513481	-0.252434426	0.019862488	9.30E-02	1.43E-01	1.00E+00
PTP4A1	LMNA	-0.031262996	-0.168586397	0.107250887	6.59E-01	7.29E-01	1.00E+00
MARS	LMNA	-0.089668523	-0.224935091	0.048988902	2.04E-01	2.79E-01	1.00E+00
ARSE	LMNA	0.258585434	0.124996557	0.382965346	2.03E-04	6.21E-04	1.00E+00
PPIE	LMNA	0.031927154	-0.106593666	0.169232251	6.52E-01	7.24E-01	1.00E+00
UBR2	LMNA	-0.003433677	-0.141417643	0.134681165	9.61E-01	9.71E-01	1.00E+00
GP5	LMNA	0.228638074	0.093539529	0.355469148	1.06E-03	2.78E-03	1.00E+00
IL8RB	LMNA	0.107577492	-0.030932899	0.242034002	1.28E-01	1.87E-01	1.00E+00
MAK	LMNA	0.037632728	-0.100942694	0.174775727	5.95E-01	6.72E-01	1.00E+00
OR11A1	LMNA	-0.039494322	-0.176582554	0.099096978	5.77E-01	6.56E-01	1.00E+00
STC2	LMNA	0.146868131	0.0089996	0.279257112	3.70E-02	6.44E-02	1.00E+00
PGLS	LMNA	0.423663991	0.303355411	0.530677151	3.33E-10	3.00E-09	3.95E-06
SAE1	LMNA	0.046286421	-0.092354716	0.183167005	5.13E-01	5.97E-01	1.00E+00
COL6A1	LMNA	0.702810933	0.625442734	0.766493826	2.10E-31	8.58E-29	2.49E-27
STMN4	LMNA	-0.598307494	-0.680177878	-0.501694967	5.32E-21	3.11E-19	6.31E-17
OAZ1	LMNA	0.140370403	0.002365236	0.273128642	4.63E-02	7.85E-02	1.00E+00
SGCE	LMNA	0.106740601	-0.031778679	0.241236821	1.31E-01	1.91E-01	1.00E+00
YIPF5	LMNA	0.260212908	0.126713803	0.384453326	1.84E-04	5.70E-04	1.00E+00
PRSS8	LMNA	-0.027009573	-0.164447399	0.111457017	7.03E-01	7.67E-01	1.00E+00
IL11	LMNA	0.34700914	0.219471933	0.462885624	4.21E-07	2.16E-06	4.99E-03
WNT4	LMNA	0.045445098	-0.093190556	0.182352069	5.21E-01	6.04E-01	1.00E+00
CSN2	LMNA	0.390479006	0.266810708	0.501496336	9.19E-09	6.31E-08	1.09E-04

TCF7	LMNA	0.093515539	-0.04511793	0.228615138	1.86E-01	2.57E-01	1.00E+00
SAMD9	LMNA	0.34524178	0.217559895	0.461306465	4.86E-07	2.46E-06	5.76E-03
TDO2	LMNA	0.40057873	0.277895428	0.510404226	3.48E-09	2.60E-08	4.13E-05
MMRN1	LMNA	-0.012572047	-0.150362081	0.125697112	8.59E-01	8.96E-01	1.00E+00
SV2C	LMNA	-0.249614184	-0.374751442	-0.115544798	3.40E-04	9.98E-04	1.00E+00
LCN2	LMNA	0.049933015	-0.088729626	0.186697056	4.80E-01	5.66E-01	1.00E+00
AKR1C3	LMNA	-0.206496983	-0.334998153	-0.070454435	3.19E-03	7.35E-03	1.00E+00
RNF19A	LMNA	-0.028818624	-0.166208375	0.109668686	6.84E-01	7.51E-01	1.00E+00
YKT6	LMNA	0.511986596	0.402375609	0.607125787	6.82E-15	1.36E-13	8.09E-11
GMDS	LMNA	0.331837956	0.203090652	0.449305996	1.40E-06	6.53E-06	1.67E-02
SPARC	LMNA	0.370391248	0.24486068	0.483708822	5.77E-08	3.46E-07	6.84E-04
CBX5	LMNA	-0.192048366	-0.321573655	-0.055467958	6.18E-03	1.32E-02	1.00E+00
MAGEB4	LMNA	-0.152086688	-0.284171308	-0.014336697	3.07E-02	5.47E-02	1.00E+00
UBE2V2	LMNA	-0.189165213	-0.318888614	-0.052484825	7.01E-03	1.48E-02	1.00E+00
ASB7	LMNA	0.484066088	0.370793674	0.583147768	2.91E-13	4.36E-12	3.45E-09
BOLA1	LMNA	-0.291811079	-0.41321576	-0.16021428	2.51E-05	9.24E-05	2.98E-01
PPP2R5E	LMNA	-0.079672814	-0.215355143	0.059027424	2.60E-01	3.41E-01	1.00E+00
COL5A1	LMNA	0.702893891	0.625542539	0.766561445	2.05E-31	8.58E-29	2.43E-27
DERL1	LMNA	0.329163384	0.200210202	0.446906363	1.73E-06	7.93E-06	2.05E-02
FBXL18	LMNA	0.194998625	0.058523051	0.324319037	5.42E-03	1.17E-02	1.00E+00
KIAA0460	LMNA	-0.24637253	-0.371778606	-0.112135478	4.09E-04	1.17E-03	1.00E+00
ADAM22	LMNA	-0.30847878	-0.428290677	-0.17800841	7.95E-06	3.20E-05	9.43E-02
SERPINC1	LMNA	0.051832422	-0.086839964	0.188534361	4.64E-01	5.50E-01	1.00E+00
MOAP1	LMNA	-0.433213366	-0.539027534	-0.313937571	1.20E-10	1.18E-09	1.42E-06
F3	LMNA	0.25702704	0.123352952	0.381539921	2.22E-04	6.76E-04	1.00E+00
MYOHD1	LMNA	0.185584669	0.048783509	0.315551201	8.19E-03	1.70E-02	1.00E+00
ZNF37A	LMNA	-0.177783562	-0.308268664	-0.040732262	1.14E-02	2.27E-02	1.00E+00
GTF3C1	LMNA	0.062569934	-0.076138741	0.198902844	3.76E-01	4.64E-01	1.00E+00
CTSZ	LMNA	0.520268591	0.411794127	0.614205166	2.09E-15	4.65E-14	2.48E-11
PLEKHM2	LMNA	0.266638904	0.133502084	0.390322234	1.25E-04	4.02E-04	1.00E+00
PRNPIP	LMNA	-0.216376077	-0.344147083	-0.080736759	1.98E-03	4.82E-03	1.00E+00
DRD1IP	LMNA	-0.321076561	-0.439640522	-0.191514426	3.18E-06	1.38E-05	3.77E-02
NR1I2	LMNA	0.100294468	-0.03828664	0.235090464	1.56E-01	2.22E-01	1.00E+00
ZNF266	LMNA	-0.199276668	-0.32829613	-0.062957654	4.47E-03	9.90E-03	1.00E+00
EFCAB1	LMNA	-0.048608376	-0.18541516	0.090046878	4.92E-01	5.77E-01	1.00E+00

COX4NB	LMNA	0.006474592	-0.13169412	0.144396527	9.27E-01	9.47E-01	1.00E+00
TMEM48	LMNA	0.290725259	0.159058039	0.412231389	2.70E-05	9.88E-05	3.20E-01
SAPS1	LMNA	0.338241796	0.209996502	0.45504466	8.51E-07	4.12E-06	1.01E-02
SEPHS2	LMNA	0.181821517	0.044897472	0.312040111	9.60E-03	1.96E-02	1.00E+00
APOA1	LMNA	-0.068019561	-0.204153526	0.07069528	3.36E-01	4.22E-01	1.00E+00
PYGM	LMNA	-0.095482643	-0.230495375	0.043136967	1.76E-01	2.46E-01	1.00E+00
KPNB1	LMNA	0.145714386	0.007820707	0.27816972	3.85E-02	6.66E-02	1.00E+00
WNT5B	LMNA	0.146845728	0.008976705	0.279236001	3.70E-02	6.44E-02	1.00E+00
FOXO3	LMNA	-0.014570459	-0.152315083	0.123729419	8.37E-01	8.78E-01	1.00E+00
KHDRBS1	LMNA	-0.067656625	-0.203804083	0.071058062	3.39E-01	4.25E-01	1.00E+00
CRYBB2	LMNA	0.048699063	-0.089956712	0.185502936	4.91E-01	5.76E-01	1.00E+00
LARS2	LMNA	0.006422331	-0.131745476	0.144345354	9.28E-01	9.47E-01	1.00E+00
C3orf28	LMNA	0.128229296	-0.009998703	0.261648547	6.90E-02	1.11E-01	1.00E+00
ZBTB5	LMNA	-0.500770221	-0.597513987	-0.389656917	3.21E-14	5.54E-13	3.81E-10
SLC25A38	LMNA	0.054788705	-0.083896861	0.191392085	4.39E-01	5.25E-01	1.00E+00
C10orf68	LMNA	-0.328677056	-0.446469846	-0.199686678	1.79E-06	8.19E-06	2.12E-02
FTCD	LMNA	-0.147575374	-0.279923513	-0.009722448	3.61E-02	6.30E-02	1.00E+00
DCTN2	LMNA	-0.22853502	-0.355374148	-0.093431743	1.07E-03	2.79E-03	1.00E+00
PSEN1	LMNA	0.041386069	-0.09722039	0.1784177	5.59E-01	6.39E-01	1.00E+00
PLA2G4B	LMNA	-0.287461427	-0.409270787	-0.155584702	3.35E-05	1.21E-04	3.98E-01
MCF2L2	LMNA	-0.532076153	-0.624272092	-0.425262212	3.67E-16	9.60E-15	4.36E-12
CDKN2A	LMNA	-0.119037707	-0.252932203	0.019330965	9.15E-02	1.41E-01	1.00E+00
OLA1	LMNA	-0.240914284	-0.366767174	-0.106402052	5.53E-04	1.54E-03	1.00E+00
TSHB	LMNA	-0.039432824	-0.17652288	0.099157966	5.77E-01	6.56E-01	1.00E+00
RELN	LMNA	-0.378883765	-0.491240303	-0.254124836	2.69E-08	1.70E-07	3.20E-04
SCN2B	LMNA	-0.237812204	-0.363915772	-0.10314756	6.54E-04	1.80E-03	1.00E+00
MFHAS1	LMNA	0.216657238	0.081029819	0.344407109	1.95E-03	4.76E-03	1.00E+00
NKX3-2	LMNA	-0.060497109	-0.196903633	0.078207052	3.92E-01	4.80E-01	1.00E+00
MEX3C	LMNA	0.227246893	0.092084744	0.354186476	1.14E-03	2.95E-03	1.00E+00
SSBP1	LMNA	0.223922792	0.088611002	0.351119711	1.36E-03	3.44E-03	1.00E+00
KPNA6	LMNA	0.241969727	0.107510008	0.367736788	5.22E-04	1.46E-03	1.00E+00
ATP5E	LMNA	0.287986121	0.156142856	0.409746907	3.24E-05	1.17E-04	3.84E-01
SUPT7L	LMNA	-0.180650668	-0.31094696	-0.043689233	1.01E-02	2.05E-02	1.00E+00
CASP2	LMNA	0.224012249	0.088704443	0.351202278	1.35E-03	3.43E-03	1.00E+00
PDIA4	LMNA	0.63949757	0.550002603	0.714472588	1.27E-24	1.57E-22	1.51E-20

SERBP1	LMNA	0.263339724	0.130015341	0.387310324	1.53E-04	4.82E-04	1.00E+00
TESC	LMNA	-0.160170077	-0.291769574	-0.022619224	2.28E-02	4.20E-02	1.00E+00
YTHDC1	LMNA	-0.370560327	-0.483858932	-0.245044898	5.68E-08	3.42E-07	6.74E-04
KIAA1641	LMNA	-0.282458211	-0.404727417	-0.150266653	4.64E-05	1.62E-04	5.51E-01
CSF1R	LMNA	0.236971155	0.102265684	0.363142281	6.85E-04	1.87E-03	1.00E+00
JUN	LMNA	0.510221051	0.400370777	0.605614658	8.74E-15	1.71E-13	1.04E-10
NAGK	LMNA	0.10577585	-0.032753431	0.240317622	1.34E-01	1.95E-01	1.00E+00
PCNXL2	LMNA	-0.096293831	-0.231270447	0.042319746	1.73E-01	2.42E-01	1.00E+00
ATAD5	LMNA	-0.255016045	-0.379699636	-0.121233082	2.50E-04	7.51E-04	1.00E+00
MYL2	LMNA	0.150991158	0.013215631	0.283140248	3.20E-02	5.67E-02	1.00E+00
AZI1	LMNA	-0.148536383	-0.280828822	-0.010704893	3.49E-02	6.11E-02	1.00E+00
STK38	LMNA	0.238000288	0.103344803	0.364088725	6.48E-04	1.78E-03	1.00E+00
RBP1	LMNA	0.285800634	0.153818554	0.407763307	3.74E-05	1.33E-04	4.43E-01
KIAA1026	LMNA	0.073084992	-0.065628161	0.209027024	3.01E-01	3.86E-01	1.00E+00
HIST1H4C	LMNA	-0.449367083	-0.553105844	-0.331906066	1.97E-11	2.18E-10	2.34E-07
ADAMTSL3	LMNA	-0.17636995	-0.306947381	-0.039275224	1.20E-02	2.39E-02	1.00E+00
TOMM7	LMNA	0.149733444	0.011929027	0.281956171	3.34E-02	5.90E-02	1.00E+00
HSFX1	LMNA	-0.174473781	-0.305174269	-0.037321722	1.30E-02	2.56E-02	1.00E+00
TREX1	LMNA	0.026607361	-0.1118545	0.164055757	7.07E-01	7.70E-01	1.00E+00
TNFSF14	LMNA	0.124485992	-0.013802205	0.258101413	7.75E-02	1.23E-01	1.00E+00
C18orf10	LMNA	-0.151957349	-0.284049596	-0.014204325	3.09E-02	5.49E-02	1.00E+00
CRISPLD2	LMNA	0.463640053	0.347853771	0.565495936	3.68E-12	4.68E-11	4.37E-08
AOAH	LMNA	0.077414723	-0.061291309	0.213187352	2.73E-01	3.56E-01	1.00E+00
CA6	LMNA	-0.092572598	-0.227713479	0.046067129	1.90E-01	2.62E-01	1.00E+00
TRIM15	LMNA	-0.006680544	-0.144598188	0.131491726	9.25E-01	9.46E-01	1.00E+00
PNN	LMNA	-0.100746119	-0.235521463	0.037831039	1.54E-01	2.19E-01	1.00E+00
CEP57	LMNA	-0.321505321	-0.440026144	-0.191974962	3.08E-06	1.34E-05	3.65E-02
AR	LMNA	0.178059823	0.041017077	0.308526822	1.12E-02	2.25E-02	1.00E+00
DDX3X	LMNA	0.123814777	-0.014483791	0.257464992	7.92E-02	1.25E-01	1.00E+00
PLXDC1	LMNA	0.135742987	-0.002352091	0.268757616	5.41E-02	8.99E-02	1.00E+00
HNRNPL	LMNA	-0.163353348	-0.29475725	-0.025886107	2.02E-02	3.77E-02	1.00E+00
KIF3C	LMNA	-0.208288661	-0.336659208	-0.072317099	2.93E-03	6.81E-03	1.00E+00
EPB41L5	LMNA	-0.541522549	-0.632303942	-0.436071244	8.69E-17	2.57E-15	1.03E-12
RUNDC3A	LMNA	-0.632238281	-0.708454568	-0.541445231	6.05E-24	6.77E-22	7.18E-20
ARHGEF10	LMNA	0.041105167	-0.097499103	0.178145263	5.61E-01	6.42E-01	1.00E+00

POLR3D	LMNA	0.103950134	-0.034597354	0.238577448	1.41E-01	2.04E-01	1.00E+00
INDO	LMNA	0.335976658	0.207552332	0.453015913	1.02E-06	4.87E-06	1.21E-02
GABRA3	LMNA	-0.524062123	-0.617442803	-0.416115991	1.21E-15	2.83E-14	1.43E-11
E2F3	LMNA	-0.165291282	-0.296574851	-0.027876382	1.87E-02	3.52E-02	1.00E+00
SCG5	LMNA	-0.009322041	-0.147183629	0.128894836	8.95E-01	9.24E-01	1.00E+00
HDC	LMNA	0.044831562	-0.093799971	0.181757657	5.26E-01	6.09E-01	1.00E+00
KLHL3	LMNA	-0.313701269	-0.433000418	-0.183601455	5.46E-06	2.27E-05	6.48E-02
FGD6	LMNA	-0.054236053	-0.190858034	0.084447233	4.43E-01	5.30E-01	1.00E+00
ATP6V1B1	LMNA	-0.100522978	-0.235308532	0.038056139	1.55E-01	2.20E-01	1.00E+00
GOLGA4	LMNA	0.206771526	0.070739794	0.335252732	3.15E-03	7.27E-03	1.00E+00
NOTCH1	LMNA	-0.093433575	-0.228536772	0.045200448	1.86E-01	2.58E-01	1.00E+00
ATPAF2	LMNA	0.012634924	-0.125635217	0.150423546	8.58E-01	8.96E-01	1.00E+00
ECD	LMNA	0.11401898	-0.024416345	0.248163775	1.06E-01	1.60E-01	1.00E+00
SSX5	LMNA	0.011818079	-0.126439207	0.149624967	8.67E-01	9.02E-01	1.00E+00
SNAP91	LMNA	-0.581191603	-0.665821061	-0.481797155	1.21E-19	5.78E-18	1.43E-15
OCA2	LMNA	-0.19764521	-0.32677998	-0.061265856	4.81E-03	1.06E-02	1.00E+00
PNPO	LMNA	0.023067603	-0.115350733	0.160607149	7.45E-01	8.03E-01	1.00E+00
TMF1	LMNA	0.424782456	0.304593308	0.53165626	2.96E-10	2.69E-09	3.51E-06
DAPK1	LMNA	-0.199120651	-0.328151168	-0.062795832	4.50E-03	9.96E-03	1.00E+00
RPS6KC1	LMNA	-0.249284659	-0.374449361	-0.115198085	3.47E-04	1.01E-03	1.00E+00
CDH5	LMNA	0.329118496	0.200161878	0.446866075	1.73E-06	7.95E-06	2.05E-02
DAAM1	LMNA	-0.076597934	-0.212402897	0.062109842	2.79E-01	3.61E-01	1.00E+00
SELENBP1	LMNA	0.006220072	-0.131944227	0.144147295	9.30E-01	9.49E-01	1.00E+00
NEK3	LMNA	-0.270073449	-0.393454912	-0.137135392	1.01E-04	3.31E-04	1.00E+00
SSTR4	LMNA	0.309179057	0.178757886	0.428922579	7.56E-06	3.06E-05	8.97E-02
TNFRSF10I	LMNA	0.439723946	0.321169313	0.544708765	5.85E-11	6.09E-10	6.94E-07
FOSL1	LMNA	0.669233514	0.585253084	0.739008714	1.34E-27	2.69E-25	1.59E-23
GSTT1	LMNA	0.048947189	-0.089710001	0.185743085	4.89E-01	5.74E-01	1.00E+00
INPP5A	LMNA	-0.222877414	-0.350154697	-0.087519244	1.43E-03	3.60E-03	1.00E+00
CD40LG	LMNA	-0.012619471	-0.15040844	0.125650429	8.59E-01	8.96E-01	1.00E+00
CES1	LMNA	0.17185757	0.034628126	0.302726355	1.45E-02	2.80E-02	1.00E+00
DCI	LMNA	-0.11021969	-0.244549639	0.028261333	1.18E-01	1.76E-01	1.00E+00
TRAF3IP1	LMNA	0.371420097	0.245981792	0.484622138	5.27E-08	3.18E-07	6.25E-04
SMARCE1	LMNA	-0.135393331	-0.268427111	0.002708291	5.47E-02	9.07E-02	1.00E+00
B3GAT3	LMNA	0.402571188	0.28008607	0.512158782	2.86E-09	2.17E-08	3.39E-05

VRK1	LMNA	-0.159946028	-0.291559195	-0.022389401	2.30E-02	4.23E-02	1.00E+00
STK17B	LMNA	0.129500839	-0.008705802	0.262852634	6.62E-02	1.07E-01	1.00E+00
AARS	LMNA	-0.022808471	-0.160354558	0.115606544	7.47E-01	8.05E-01	1.00E+00
CNTN6	LMNA	-0.326234463	-0.444276582	-0.19705838	2.16E-06	9.72E-06	2.56E-02
CYP3A4	LMNA	-0.14311799	-0.275721402	-0.005169118	4.22E-02	7.22E-02	1.00E+00
PISD	LMNA	-0.074082483	-0.209985924	0.064629493	2.95E-01	3.79E-01	1.00E+00
ZAK	LMNA	0.53641328	0.430221186	0.62796218	1.91E-16	5.28E-15	2.26E-12
USP1	LMNA	-0.068658475	-0.204768603	0.070056548	3.32E-01	4.17E-01	1.00E+00
TRAP1	LMNA	-0.152367185	-0.284435249	-0.014623787	3.04E-02	5.43E-02	1.00E+00
RNF44	LMNA	-0.160128084	-0.291730144	-0.022576148	2.28E-02	4.20E-02	1.00E+00
CENPM	LMNA	-0.039622923	-0.176707337	0.098969439	5.76E-01	6.55E-01	1.00E+00
NPTXR	LMNA	-0.176755175	-0.307307495	-0.039672225	1.19E-02	2.36E-02	1.00E+00
SAR1B	LMNA	0.021178403	-0.1172153	0.158765222	7.65E-01	8.20E-01	1.00E+00
DPYSL3	LMNA	0.189447285	0.05277657	0.319151396	6.93E-03	1.46E-02	1.00E+00
GPC1	LMNA	0.43545642	0.316427521	0.540985945	9.37E-11	9.47E-10	1.11E-06
APP	LMNA	0.280624456	0.148319422	0.403060689	5.23E-05	1.81E-04	6.20E-01
GLS2	LMNA	-0.34491033	-0.461010229	-0.217201422	4.99E-07	2.52E-06	5.92E-03
TOB1	LMNA	0.074485439	-0.064225983	0.210373217	2.92E-01	3.76E-01	1.00E+00
MNX1	LMNA	-0.387853042	-0.499176391	-0.263933991	1.18E-08	7.92E-08	1.40E-04
TCEAL1	LMNA	-0.306719849	-0.426702973	-0.176126572	9.01E-06	3.59E-05	1.07E-01
ORC5L	LMNA	-0.016717032	-0.154411681	0.121614631	8.13E-01	8.59E-01	1.00E+00
CENPF	LMNA	0.012639038	-0.125631168	0.150427567	8.58E-01	8.96E-01	1.00E+00
C6	LMNA	0.048123693	-0.090528737	0.184946	4.96E-01	5.81E-01	1.00E+00
LOC441601	LMNA	-0.033013404	-0.17028831	0.105518501	6.41E-01	7.14E-01	1.00E+00
PRSS1	LMNA	0.068379764	-0.070335194	0.204500303	3.34E-01	4.19E-01	1.00E+00
PTGIS	LMNA	0.347640366	0.22015507	0.463449454	3.99E-07	2.06E-06	4.74E-03
C6orf124	LMNA	-0.411745005	-0.52022545	-0.290188865	1.14E-09	9.33E-09	1.36E-05
CEACAM3	LMNA	0.193453165	0.056922355	0.322881171	5.81E-03	1.25E-02	1.00E+00
KRT23	LMNA	0.102780574	-0.03577808	0.237462233	1.46E-01	2.10E-01	1.00E+00
ODZ4	LMNA	0.112471668	-0.025982763	0.246692319	1.11E-01	1.66E-01	1.00E+00
UBC	LMNA	0.384558744	0.260328237	0.496263739	1.60E-08	1.06E-07	1.90E-04
ATRN	LMNA	0.201816941	0.065593441	0.330655537	3.97E-03	8.91E-03	1.00E+00
HAPLN1	LMNA	-0.075947758	-0.211778334	0.062761273	2.83E-01	3.66E-01	1.00E+00
RANGAP1	LMNA	-0.15553519	-0.287414879	-0.017867844	2.71E-02	4.89E-02	1.00E+00
SNCB	LMNA	-0.319560661	-0.438276788	-0.189886637	3.56E-06	1.53E-05	4.22E-02

S100A9	LMNA	0.307523367	0.176986119	0.427428363	8.51E-06	3.41E-05	1.01E-01
C10orf26	LMNA	0.08616602	-0.052509598	0.221581244	2.23E-01	3.00E-01	1.00E+00
SRY	LMNA	0.147848261	0.010001395	0.280180608	3.57E-02	6.25E-02	1.00E+00
RNGTT	LMNA	-0.260317077	-0.384548544	-0.126823746	1.83E-04	5.67E-04	1.00E+00
CDCA8	LMNA	0.00981285	-0.128412107	0.147663814	8.90E-01	9.20E-01	1.00E+00
CEP250	LMNA	0.173098955	0.035905981	0.303888099	1.38E-02	2.69E-02	1.00E+00
MLC1	LMNA	0.070051524	-0.068663499	0.206109307	3.22E-01	4.07E-01	1.00E+00
ATPBD1B	LMNA	0.362760557	0.236556161	0.476927297	1.12E-07	6.37E-07	1.33E-03
TNIP3	LMNA	0.319064421	0.189353923	0.437830241	3.69E-06	1.59E-05	4.38E-02
KCNJ2	LMNA	0.075937427	-0.062771623	0.211768409	2.83E-01	3.66E-01	1.00E+00
IFT52	LMNA	-0.065668881	-0.20188962	0.073044314	3.53E-01	4.40E-01	1.00E+00
UTP20	LMNA	0.21954032	0.08403627	0.347072335	1.69E-03	4.18E-03	1.00E+00
ZNF492	LMNA	-0.450970482	-0.554500016	-0.33369426	1.64E-11	1.85E-10	1.94E-07
PDHA1	LMNA	0.119652635	-0.018707377	0.253516016	8.99E-02	1.39E-01	1.00E+00
BYSL	LMNA	-0.010097534	-0.147942307	0.128132079	8.87E-01	9.18E-01	1.00E+00
TKT	LMNA	0.304401903	0.173648115	0.424609542	1.06E-05	4.17E-05	1.26E-01
PMPCA	LMNA	0.04719948	-0.091447386	0.184051215	5.05E-01	5.89E-01	1.00E+00
RNF38	LMNA	-0.301925944	-0.422371982	-0.17100253	1.26E-05	4.91E-05	1.49E-01
KLHL2	LMNA	-0.111399781	-0.245672619	0.027067486	1.14E-01	1.71E-01	1.00E+00
AHDC1	LMNA	0.151301328	0.013532995	0.283432196	3.16E-02	5.61E-02	1.00E+00
APOB48R	LMNA	0.430682708	0.311130328	0.536816642	1.58E-10	1.52E-09	1.87E-06
GLTP	LMNA	-0.079882271	-0.215556155	0.058817357	2.58E-01	3.40E-01	1.00E+00
MPL	LMNA	-0.046636806	-0.183506345	0.092006557	5.10E-01	5.94E-01	1.00E+00
DMP1	LMNA	0.262342659	0.128962241	0.386399556	1.62E-04	5.09E-04	1.00E+00
C9orf78	LMNA	0.018596639	-0.119761824	0.156246511	7.93E-01	8.44E-01	1.00E+00
ADAM12	LMNA	0.687295648	0.606820942	0.753822567	1.39E-29	3.67E-27	1.65E-25
CRTAM	LMNA	0.275679814	0.143073902	0.398562378	7.16E-05	2.41E-04	8.49E-01
CSPG4	LMNA	0.144819678	0.006906761	0.277326233	3.97E-02	6.86E-02	1.00E+00
HSD17B2	LMNA	0.08800334	-0.050663165	0.223340982	2.13E-01	2.88E-01	1.00E+00
UBE2G1	LMNA	-0.160691303	-0.292258949	-0.02315394	2.23E-02	4.12E-02	1.00E+00
ADCY7	LMNA	0.184518811	0.047682426	0.314557097	8.57E-03	1.77E-02	1.00E+00
PAK1	LMNA	0.053394424	-0.085285227	0.190044577	4.50E-01	5.37E-01	1.00E+00
FRAS1	LMNA	-0.124722483	-0.258325616	0.01356203	7.70E-02	1.22E-01	1.00E+00
PELI2	LMNA	-0.318566398	-0.437382033	-0.188819374	3.83E-06	1.64E-05	4.54E-02
ATP13A1	LMNA	0.317176579	0.187328025	0.436130914	4.24E-06	1.80E-05	5.03E-02

OR2B6	LMNA	-0.156049265	-0.287898146	-0.018394534	2.66E-02	4.80E-02	1.00E+00
SIDT1	LMNA	-0.271759024	-0.394991292	-0.138919824	9.14E-05	3.01E-04	1.00E+00
C1RL	LMNA	0.524557698	0.416680946	0.617865524	1.12E-15	2.65E-14	1.33E-11
ATP9B	LMNA	-0.160069432	-0.291675071	-0.022515984	2.29E-02	4.21E-02	1.00E+00
TPX2	LMNA	0.140498128	0.00249553	0.273249212	4.61E-02	7.82E-02	1.00E+00
DAZAP1	LMNA	0.202244456	0.066037218	0.331052456	3.90E-03	8.75E-03	1.00E+00
HMGCS2	LMNA	0.110203498	-0.028277711	0.244534228	1.18E-01	1.76E-01	1.00E+00
PRKRA	LMNA	-0.190916924	-0.320520207	-0.05429699	6.50E-03	1.38E-02	1.00E+00
TLN1	LMNA	0.504082283	0.39340819	0.600355133	2.04E-14	3.73E-13	2.42E-10
B9D1	LMNA	0.057789164	-0.080907303	0.194290147	4.14E-01	5.02E-01	1.00E+00
NKX2-5	LMNA	0.199357978	0.063041991	0.328371675	4.45E-03	9.87E-03	1.00E+00
MITF	LMNA	0.261923943	0.128520082	0.386017008	1.66E-04	5.20E-04	1.00E+00
LILRB2	LMNA	0.488696853	0.376013647	0.587136628	1.60E-13	2.51E-12	1.90E-09
CSTF1	LMNA	0.176493655	0.039402707	0.307063027	1.20E-02	2.38E-02	1.00E+00
GYS1	LMNA	0.432533983	0.313183731	0.53843414	1.29E-10	1.26E-09	1.53E-06
BTN2A1	LMNA	0.20877726	0.072825221	0.337112047	2.87E-03	6.66E-03	1.00E+00
NSMCE4A	LMNA	-0.28953553	-0.41115249	-0.157791564	2.92E-05	1.06E-04	3.47E-01
DNAI1	LMNA	-0.231973652	-0.358542614	-0.097029958	8.94E-04	2.38E-03	1.00E+00
IGF2BP3	LMNA	0.096968671	-0.041639745	0.231915112	1.70E-01	2.38E-01	1.00E+00
HOXD11	LMNA	0.053048093	-0.085630006	0.189709784	4.53E-01	5.40E-01	1.00E+00
FNBP1L	LMNA	-0.169585536	-0.300599075	-0.032290503	1.58E-02	3.03E-02	1.00E+00
DHX35	LMNA	-0.017880768	-0.155547805	0.120467602	8.01E-01	8.50E-01	1.00E+00
SLC33A1	LMNA	0.364032436	0.237939073	0.478058582	1.01E-07	5.75E-07	1.19E-03
HRG	LMNA	-0.211774664	-0.339888777	-0.075943938	2.48E-03	5.87E-03	1.00E+00
ZNF131	LMNA	-0.166196112	-0.297423172	-0.028806023	1.81E-02	3.42E-02	1.00E+00
USP47	LMNA	-0.101494583	-0.236235585	0.037075904	1.51E-01	2.16E-01	1.00E+00
TRIM33	LMNA	-0.321201516	-0.439752909	-0.191648636	3.15E-06	1.37E-05	3.73E-02
FBXO42	LMNA	-0.140141978	-0.272913002	-0.002132229	4.67E-02	7.90E-02	1.00E+00
HTN1	LMNA	-0.026214904	-0.163673571	0.112242301	7.11E-01	7.74E-01	1.00E+00
C13orf23	LMNA	-0.105066446	-0.239641559	0.033470022	1.37E-01	1.99E-01	1.00E+00
PAPOLA	LMNA	0.226029889	0.090812572	0.353064003	1.22E-03	3.12E-03	1.00E+00
C1orf27	LMNA	0.118375664	-0.02000221	0.252303551	9.34E-02	1.43E-01	1.00E+00
AATK	LMNA	-0.352176749	-0.467498731	-0.225068193	2.75E-07	1.46E-06	3.26E-03
MSH3	LMNA	0.291785534	0.160187075	0.413192606	2.52E-05	9.25E-05	2.98E-01
RNF14	LMNA	0.109946769	-0.02853738	0.244289874	1.19E-01	1.77E-01	1.00E+00

NDUFAB1	LMNA	-0.244466283	-0.370029236	-0.110132111	4.54E-04	1.29E-03	1.00E+00
SLC4A8	LMNA	-0.173552781	-0.304312712	-0.036373252	1.35E-02	2.65E-02	1.00E+00
BAK1	LMNA	0.392887709	0.269451357	0.503622936	7.31E-09	5.14E-08	8.67E-05
COX6C	LMNA	-0.211667326	-0.33978938	-0.07583221	2.49E-03	5.90E-03	1.00E+00
MTNR1B	LMNA	0.143586698	0.005647647	0.276163507	4.15E-02	7.12E-02	1.00E+00
SPECC1L	LMNA	0.006993401	-0.131184251	0.144904504	9.21E-01	9.44E-01	1.00E+00
PGCP	LMNA	0.476764777	0.362577874	0.576848825	7.35E-13	1.05E-11	8.72E-09
SPN	LMNA	-0.067210253	-0.203374257	0.071504195	3.42E-01	4.28E-01	1.00E+00
GPR143	LMNA	-0.064882103	-0.201131561	0.073830197	3.59E-01	4.46E-01	1.00E+00
ZNF576	LMNA	0.110211014	-0.028270109	0.244541381	1.18E-01	1.76E-01	1.00E+00
TMEM39A	LMNA	0.305780635	0.175122121	0.425854886	9.62E-06	3.82E-05	1.14E-01
PCSK1	LMNA	0.315073282	0.185072216	0.434236647	4.95E-06	2.07E-05	5.87E-02
ATP5D	LMNA	-0.049262734	-0.186048462	0.08939623	4.86E-01	5.72E-01	1.00E+00
MAGEB3	LMNA	-0.136634766	-0.269600411	0.001443462	5.25E-02	8.76E-02	1.00E+00
SLC13A1	LMNA	-0.180092984	-0.310426164	-0.043113882	1.03E-02	2.09E-02	1.00E+00
RPS5	LMNA	-0.057234936	-0.193755012	0.081459706	4.18E-01	5.06E-01	1.00E+00
ARF6	LMNA	0.536030578	0.429783356	0.627636738	2.02E-16	5.56E-15	2.40E-12
KIAA1009	LMNA	-0.113408125	-0.247582941	0.025034824	1.08E-01	1.62E-01	1.00E+00
ANP32E	LMNA	0.022091498	-0.116314232	0.159655588	7.55E-01	8.11E-01	1.00E+00
YEATS4	LMNA	-0.169670951	-0.300679072	-0.032378357	1.58E-02	3.02E-02	1.00E+00
MTMR1	LMNA	-0.263221371	-0.387202228	-0.129890322	1.54E-04	4.85E-04	1.00E+00
PCOLCE	LMNA	0.693377847	0.614110421	0.758795774	2.78E-30	9.41E-28	3.29E-26
MNS1	LMNA	0.022212833	-0.116194478	0.159773886	7.54E-01	8.10E-01	1.00E+00
HMGN1	LMNA	-0.047879243	-0.184709356	0.090771739	4.99E-01	5.83E-01	1.00E+00
CXADR	LMNA	-0.103863546	-0.238494896	0.034684781	1.41E-01	2.04E-01	1.00E+00
PCYT2	LMNA	0.103127983	-0.035427395	0.237793536	1.44E-01	2.08E-01	1.00E+00
TSC22D1	LMNA	-0.416287899	-0.524212927	-0.295201841	7.19E-10	6.11E-09	8.53E-06
UTF1	LMNA	-0.016841228	-0.154532947	0.121492236	8.12E-01	8.58E-01	1.00E+00
BZRAP1	LMNA	-0.514909816	-0.609626256	-0.405697329	4.51E-15	9.39E-14	5.35E-11
LAX1	LMNA	0.137678682	-0.000379532	0.270586728	5.07E-02	8.49E-02	1.00E+00
PUF60	LMNA	-0.259277822	-0.383598467	-0.12572704	1.94E-04	5.98E-04	1.00E+00
ELOVL5	LMNA	0.105522752	-0.03300911	0.240076434	1.35E-01	1.96E-01	1.00E+00
SPPL2B	LMNA	0.176979044	0.039902957	0.307516754	1.17E-02	2.34E-02	1.00E+00
SHC1	LMNA	0.76173454	0.696976326	0.814168983	1.47E-39	3.17E-36	1.75E-35
B4GALNT1	LMNA	-0.328898793	-0.446668879	-0.199925365	1.76E-06	8.07E-06	2.09E-02

ZNF673	LMNA	-0.187785257	-0.317602742	-0.051057877	7.45E-03	1.56E-02	1.00E+00
IRX5	LMNA	0.102432422	-0.036129482	0.237130189	1.47E-01	2.11E-01	1.00E+00
LIMS2	LMNA	-0.192019747	-0.321547013	-0.055438335	6.19E-03	1.32E-02	1.00E+00
ITM2B	LMNA	0.109126104	-0.029367314	0.243508655	1.22E-01	1.80E-01	1.00E+00
GINS1	LMNA	-0.032835889	-0.17011575	0.105694226	6.43E-01	7.16E-01	1.00E+00
BAHCC1	LMNA	-0.25592855	-0.380534803	-0.122194839	2.37E-04	7.17E-04	1.00E+00
PAPSS2	LMNA	0.304697112	0.173963674	0.424876229	1.04E-05	4.10E-05	1.23E-01
ENTPD3	LMNA	-0.12287939	-0.2565779	0.015433418	8.15E-02	1.28E-01	1.00E+00
CLCC1	LMNA	0.255120928	0.121343613	0.379795641	2.48E-04	7.47E-04	1.00E+00
SMO	LMNA	0.215499568	0.079823305	0.343336336	2.07E-03	5.00E-03	1.00E+00
ACSL3	LMNA	0.1494838	0.011673702	0.281721095	3.37E-02	5.94E-02	1.00E+00
PIK3R5	LMNA	0.378711309	0.253936482	0.491087529	2.74E-08	1.73E-07	3.25E-04
GLT8D2	LMNA	0.448309481	0.330727035	0.55218593	2.22E-11	2.45E-10	2.64E-07
CDC14A	LMNA	-0.366751893	-0.480476161	-0.240897652	7.94E-08	4.64E-07	9.42E-04
UTRN	LMNA	0.36935688	0.243733891	0.482790358	6.32E-08	3.77E-07	7.50E-04
CNN1	LMNA	0.462815126	0.346930223	0.564781082	4.07E-12	5.12E-11	4.82E-08
KRT1	LMNA	0.027779632	-0.110695888	0.1651971	6.95E-01	7.60E-01	1.00E+00
HISPPD2A	LMNA	-0.10733187	-0.241800054	0.03118115	1.28E-01	1.88E-01	1.00E+00
SDAD1	LMNA	0.145578412	0.007681794	0.278041543	3.87E-02	6.70E-02	1.00E+00
SIGLEC9	LMNA	0.366937694	0.241099877	0.480641275	7.82E-08	4.57E-07	9.27E-04
C22orf26	LMNA	0.064873897	-0.073838394	0.201123653	3.59E-01	4.46E-01	1.00E+00
RPL35	LMNA	0.056678906	-0.082013818	0.193218057	4.23E-01	5.10E-01	1.00E+00
IMPDH2	LMNA	0.13247733	-0.005677504	0.265669598	6.02E-02	9.86E-02	1.00E+00
FLJ22655	LMNA	-0.526582679	-0.619592256	-0.418990289	8.33E-16	2.04E-14	9.88E-12
ENOX2	LMNA	0.272521704	0.139727517	0.395686238	8.72E-05	2.88E-04	1.00E+00
INTS6	LMNA	0.11429879	-0.024133006	0.248429802	1.05E-01	1.59E-01	1.00E+00
FPRL1	LMNA	0.317832167	0.188031431	0.436721133	4.04E-06	1.72E-05	4.79E-02
CLTA	LMNA	-0.03195053	-0.16925498	0.106570532	6.52E-01	7.23E-01	1.00E+00
SEC14L5	LMNA	-0.244207805	-0.36979196	-0.109860547	4.61E-04	1.31E-03	1.00E+00
SMC3	LMNA	-0.116191897	-0.250229121	0.022215448	9.96E-02	1.51E-01	1.00E+00
C6orf123	LMNA	-0.054275819	-0.190896465	0.084407634	4.43E-01	5.30E-01	1.00E+00
OGDH	LMNA	0.386306	0.262240254	0.497808888	1.36E-08	9.09E-08	1.61E-04
GPR37L1	LMNA	-0.008105101	-0.145992748	0.130091461	9.09E-01	9.35E-01	1.00E+00
FLJ20160	LMNA	-0.175862785	-0.306473219	-0.038752622	1.23E-02	2.44E-02	1.00E+00
ASB13	LMNA	-0.435456854	-0.540986324	-0.316428003	9.37E-11	9.47E-10	1.11E-06

GSS	LMNA	0.406351598	0.284246016	0.515485294	1.97E-09	1.54E-08	2.33E-05
NT5M	LMNA	-0.25007873	-0.375177254	-0.116033631	3.31E-04	9.74E-04	1.00E+00
SIX5	LMNA	0.423552889	0.303232467	0.530579876	3.37E-10	3.02E-09	4.00E-06
KPNA3	LMNA	-0.194946651	-0.32427069	-0.058469207	5.43E-03	1.18E-02	1.00E+00
TAF5	LMNA	-0.257213923	-0.38171089	-0.123550016	2.20E-04	6.70E-04	1.00E+00
KCNA1	LMNA	-0.092897455	-0.228024141	0.045740144	1.89E-01	2.60E-01	1.00E+00
HSPA1L	LMNA	-0.215125134	-0.342989937	-0.079433159	2.11E-03	5.08E-03	1.00E+00
RHOC	LMNA	0.393279262	0.26988079	0.503968504	7.04E-09	4.97E-08	8.36E-05
TH1L	LMNA	0.110794132	-0.027680245	0.245096325	1.16E-01	1.73E-01	1.00E+00
SSTR2	LMNA	-0.463552929	-0.565420444	-0.347756221	3.72E-12	4.72E-11	4.41E-08
PPP3CA	LMNA	-0.254836612	-0.379535387	-0.121043994	2.52E-04	7.59E-04	1.00E+00
CHRNA5	LMNA	0.06631123	-0.072402568	0.202508401	3.48E-01	4.35E-01	1.00E+00
DLX2	LMNA	-0.165616673	-0.296879945	-0.028210668	1.85E-02	3.48E-02	1.00E+00
SLC1A7	LMNA	0.072648158	-0.066065423	0.208607008	3.04E-01	3.89E-01	1.00E+00
C6orf108	LMNA	0.050331993	-0.088332778	0.187083069	4.77E-01	5.62E-01	1.00E+00
ALDH3A2	LMNA	-0.077723601	-0.213483957	0.060981723	2.72E-01	3.54E-01	1.00E+00
DDB2	LMNA	0.425333372	0.3052032	0.532138429	2.79E-10	2.56E-09	3.31E-06
FLJ11286	LMNA	0.441274141	0.322893261	0.54606007	4.92E-11	5.19E-10	5.84E-07
SPATA1	LMNA	0.091049426	-0.047599879	0.226256504	1.98E-01	2.71E-01	1.00E+00
CLEC1B	LMNA	0.063192869	-0.075516929	0.199503434	3.72E-01	4.59E-01	1.00E+00
MKNK1	LMNA	0.161973859	0.024470025	0.293462839	2.13E-02	3.95E-02	1.00E+00
DYSF	LMNA	0.262552633	0.12918399	0.386591376	1.60E-04	5.03E-04	1.00E+00
FOXJ1	LMNA	0.056636708	-0.082055868	0.193177302	4.23E-01	5.10E-01	1.00E+00
LEPREL2	LMNA	0.602697469	0.50681499	0.683850098	2.31E-21	1.42E-19	2.74E-17
SLC27A3	LMNA	0.447083663	0.329360933	0.551119384	2.56E-11	2.79E-10	3.03E-07
C1orf174	LMNA	0.000341413	-0.137716079	0.138385891	9.96E-01	9.97E-01	1.00E+00
MTRR	LMNA	0.174410399	0.037256442	0.305114985	1.30E-02	2.57E-02	1.00E+00
PLEKHO1	LMNA	0.006628524	-0.131542848	0.144547253	9.25E-01	9.46E-01	1.00E+00
HTR7	LMNA	0.294221816	0.162782659	0.415400249	2.14E-05	7.96E-05	2.53E-01
RING1	LMNA	-0.231298066	-0.357920338	-0.09632274	9.26E-04	2.45E-03	1.00E+00
NPC2	LMNA	0.396629271	0.273556897	0.506923634	5.11E-09	3.71E-08	6.06E-05
BHMT	LMNA	-0.221065814	-0.348481725	-0.08562804	1.57E-03	3.90E-03	1.00E+00
YTHDF1	LMNA	0.206065524	0.070006024	0.33459803	3.26E-03	7.48E-03	1.00E+00
MSMB	LMNA	-0.197110171	-0.326282612	-0.060711199	4.93E-03	1.08E-02	1.00E+00
LMAN1L	LMNA	0.045314803	-0.093319985	0.182225843	5.22E-01	6.05E-01	1.00E+00

CEP170	LMNA	-0.327814421	-0.445695426	-0.198758246	1.91E-06	8.70E-06	2.27E-02
PF4	LMNA	0.069255959	-0.069459131	0.205343698	3.27E-01	4.13E-01	1.00E+00
MSH6	LMNA	0.011848516	-0.126409252	0.149654726	8.67E-01	9.02E-01	1.00E+00
IL1F5	LMNA	0.065343406	-0.07336944	0.201576045	3.56E-01	4.42E-01	1.00E+00
ZNF556	LMNA	-0.278784434	-0.401387449	-0.146366566	5.88E-05	2.01E-04	6.97E-01
PLEKHC1	LMNA	0.131009246	-0.007171458	0.264280481	6.31E-02	1.03E-01	1.00E+00
BCL2L10	LMNA	-0.24112547	-0.366961208	-0.106623718	5.46E-04	1.53E-03	1.00E+00
AIRE	LMNA	0.106179907	-0.032345217	0.24070263	1.33E-01	1.94E-01	1.00E+00
IPO4	LMNA	0.507982126	0.39782992	0.603697375	1.19E-14	2.27E-13	1.42E-10
FIGF	LMNA	-0.09825125	-0.233140013	0.040347005	1.64E-01	2.32E-01	1.00E+00
QDPR	LMNA	-0.212763588	-0.340804405	-0.07697347	2.37E-03	5.63E-03	1.00E+00
SLCO2A1	LMNA	0.238781532	0.104164203	0.364807023	6.21E-04	1.72E-03	1.00E+00
FOXA2	LMNA	-0.012376886	-0.150171298	0.125889214	8.61E-01	8.98E-01	1.00E+00
MAPK12	LMNA	-0.077717261	-0.213477869	0.060988078	2.72E-01	3.54E-01	1.00E+00
BOP1	LMNA	-0.097704689	-0.232618083	0.040897953	1.67E-01	2.34E-01	1.00E+00
PRDM16	LMNA	0.099214976	-0.039375338	0.234060119	1.60E-01	2.27E-01	1.00E+00
POLR1E	LMNA	0.109282061	-0.02920961	0.243657129	1.22E-01	1.80E-01	1.00E+00
INSRR	LMNA	0.240419182	0.105882431	0.36631224	5.68E-04	1.58E-03	1.00E+00
PDE6C	LMNA	-0.052084241	-0.188777876	0.086589361	4.62E-01	5.47E-01	1.00E+00
C1orf216	LMNA	-0.073929904	-0.209839265	0.064782271	2.96E-01	3.80E-01	1.00E+00
SIPA1	LMNA	0.385389775	0.261237509	0.496998731	1.48E-08	9.83E-08	1.76E-04
EP400	LMNA	-0.00524775	-0.143195012	0.13289953	9.41E-01	9.56E-01	1.00E+00
ULK4	LMNA	-0.019247005	-0.156881162	0.119120507	7.86E-01	8.37E-01	1.00E+00
PTK2	LMNA	-0.409127031	-0.5179254	-0.287303018	1.49E-09	1.19E-08	1.77E-05
BTN3A1	LMNA	0.028093541	-0.110385573	0.165502665	6.91E-01	7.58E-01	1.00E+00
NDUFA8	LMNA	-0.088742679	-0.224048854	0.04991989	2.09E-01	2.84E-01	1.00E+00
LAMP3	LMNA	0.380019727	0.255365751	0.492246444	2.43E-08	1.55E-07	2.88E-04
FABP5	LMNA	0.270088349	0.137151162	0.393468496	1.01E-04	3.31E-04	1.00E+00
GLTSCR2	LMNA	0.052556734	-0.086119105	0.18923474	4.58E-01	5.44E-01	1.00E+00
SORT1	LMNA	0.233446105	0.098571825	0.359898489	8.27E-04	2.22E-03	1.00E+00
LLGL2	LMNA	-0.032709545	-0.169992928	0.105819291	6.44E-01	7.17E-01	1.00E+00
YWHAQ	LMNA	-0.24960288	-0.374741079	-0.115532903	3.40E-04	9.98E-04	1.00E+00
LRIT1	LMNA	0.118621184	-0.019753292	0.252536701	9.27E-02	1.43E-01	1.00E+00
KIAA1704	LMNA	-0.183003611	-0.313143409	-0.046117721	9.14E-03	1.87E-02	1.00E+00
ENOSF1	LMNA	0.111472082	-0.02699433	0.245741409	1.14E-01	1.70E-01	1.00E+00

MEIS2	LMNA	-0.084039416	-0.219543335	0.054645567	2.34E-01	3.13E-01	1.00E+00
KIR2DL4	LMNA	0.083856603	-0.054829126	0.219368092	2.35E-01	3.14E-01	1.00E+00
PCDH7	LMNA	-0.287421325	-0.409234395	-0.155542046	3.36E-05	1.21E-04	3.99E-01
NFKB2	LMNA	0.565022332	0.463093504	0.652200459	1.96E-18	7.79E-17	2.33E-14
RPLP1	LMNA	0.12556212	-0.012709181	0.259121514	7.50E-02	1.19E-01	1.00E+00
FZD9	LMNA	-0.236693808	-0.362887174	-0.101974922	6.95E-04	1.90E-03	1.00E+00
HESX1	LMNA	-0.138209365	-0.271088022	-0.000161445	4.98E-02	8.37E-02	1.00E+00
GNAT1	LMNA	0.183586204	0.046719269	0.313687039	8.91E-03	1.83E-02	1.00E+00
NKX6-1	LMNA	-0.037427259	-0.174576247	0.101146351	5.97E-01	6.74E-01	1.00E+00
CTA-216E1	LMNA	0.109220419	-0.029271943	0.243598445	1.22E-01	1.80E-01	1.00E+00
IFT140	LMNA	-0.042466589	-0.179465464	0.096148085	5.48E-01	6.29E-01	1.00E+00
ORAI3	LMNA	0.309004893	0.178571472	0.428765431	7.66E-06	3.09E-05	9.08E-02
DENND1A	LMNA	0.195585868	0.059131465	0.324865239	5.28E-03	1.15E-02	1.00E+00
ABHD2	LMNA	0.23041398	0.095397466	0.357105843	9.70E-04	2.55E-03	1.00E+00
ALCAM	LMNA	-0.399214058	-0.50920197	-0.276395752	3.98E-09	2.94E-08	4.72E-05
QPRT	LMNA	0.150124321	0.012328833	0.282324205	3.30E-02	5.82E-02	1.00E+00
TRAM1	LMNA	0.445299169	0.327373101	0.549566143	3.13E-11	3.37E-10	3.71E-07
TRIM29	LMNA	0.088167761	-0.050497883	0.223498417	2.12E-01	2.87E-01	1.00E+00
ATP1B4	LMNA	0.129063937	-0.009150095	0.262438956	6.72E-02	1.08E-01	1.00E+00
NUP37	LMNA	0.259323491	0.125775227	0.383640222	1.94E-04	5.97E-04	1.00E+00
CDS1	LMNA	-0.143345348	-0.275935862	-0.005401232	4.18E-02	7.17E-02	1.00E+00
RHEB	LMNA	-0.084731244	-0.220206438	0.053950832	2.31E-01	3.08E-01	1.00E+00
C4orf27	LMNA	-0.250127001	-0.375221497	-0.116084429	3.30E-04	9.72E-04	1.00E+00
RAB3A	LMNA	-0.505369887	-0.601459007	-0.394867539	1.71E-14	3.18E-13	2.03E-10
SAA3P	LMNA	0.036957141	-0.101612283	0.174119787	6.02E-01	6.79E-01	1.00E+00
SLC22A6	LMNA	-0.148250741	-0.28055976	-0.010412851	3.52E-02	6.17E-02	1.00E+00
GPD1	LMNA	-0.305625471	-0.425714756	-0.174956205	9.73E-06	3.86E-05	1.15E-01
KIAA0265	LMNA	0.242366928	0.107927058	0.368101617	5.10E-04	1.43E-03	1.00E+00
CDH15	LMNA	0.157122974	0.019494835	0.288907291	2.55E-02	4.64E-02	1.00E+00
NPM1	LMNA	0.252092065	0.118152972	0.377022107	2.95E-04	8.77E-04	1.00E+00
ERAF	LMNA	-0.021044552	-0.158634684	0.117347369	7.66E-01	8.21E-01	1.00E+00
ADAM19	LMNA	0.438741108	0.320076722	0.543851743	6.53E-11	6.77E-10	7.74E-07
PRPS2	LMNA	0.433177785	0.313898087	0.53899646	1.20E-10	1.18E-09	1.43E-06
GCK	LMNA	0.082633018	-0.056057462	0.218194945	2.42E-01	3.22E-01	1.00E+00
DNMT3B	LMNA	-0.132226587	-0.265432381	0.005932709	6.07E-02	9.93E-02	1.00E+00

SNF1LK2	LMNA	0.353153884	0.226127325	0.468370311	2.53E-07	1.36E-06	3.01E-03
ADRA2A	LMNA	0.026232254	-0.112225157	0.163690468	7.11E-01	7.74E-01	1.00E+00
TSPYL4	LMNA	-0.496428225	-0.593785662	-0.384744708	5.76E-14	9.47E-13	6.83E-10
MGC24039	LMNA	-0.367909414	-0.481504666	-0.242157664	7.18E-08	4.24E-07	8.51E-04
TASP1	LMNA	0.281907525	0.149681782	0.404226975	4.81E-05	1.67E-04	5.71E-01
WDR19	LMNA	0.094676635	-0.043948785	0.229725082	1.80E-01	2.50E-01	1.00E+00
C10orf38	LMNA	-0.392338512	-0.503138178	-0.268849112	7.70E-09	5.39E-08	9.14E-05
CCT8	LMNA	0.108089743	-0.030415108	0.242521858	1.26E-01	1.85E-01	1.00E+00
PDE4C	LMNA	-0.156796437	-0.28860042	-0.019160176	2.58E-02	4.69E-02	1.00E+00
N-PAC	LMNA	-0.114460691	-0.248583718	0.023969055	1.05E-01	1.58E-01	1.00E+00
POGZ	LMNA	-0.266679638	-0.390359404	-0.133545154	1.25E-04	4.01E-04	1.00E+00
FYB	LMNA	0.152130297	0.014381329	0.284212344	3.07E-02	5.46E-02	1.00E+00
GUCA1B	LMNA	-0.275194261	-0.398120334	-0.142559205	7.38E-05	2.48E-04	8.75E-01
ZZEF1	LMNA	-0.068730482	-0.204837916	0.069984554	3.31E-01	4.17E-01	1.00E+00
PPFIA3	LMNA	-0.355420098	-0.470390854	-0.228584866	2.10E-07	1.14E-06	2.49E-03
ZNF334	LMNA	0.010054558	-0.128174354	0.147900267	8.87E-01	9.18E-01	1.00E+00
C12orf44	LMNA	0.140943743	0.002950145	0.273669833	4.54E-02	7.71E-02	1.00E+00
RANBP6	LMNA	-0.275915932	-0.398777317	-0.143324219	7.05E-05	2.37E-04	8.36E-01
LDHB	LMNA	-0.362570764	-0.476758452	-0.236349843	1.14E-07	6.47E-07	1.35E-03
CRYBA4	LMNA	0.058580116	-0.080118809	0.195053709	4.08E-01	4.95E-01	1.00E+00
BAMBI	LMNA	-0.18262855	-0.312793387	-0.045730509	9.28E-03	1.90E-02	1.00E+00
RAB5B	LMNA	-0.138483199	-0.271346663	-0.000440622	4.94E-02	8.30E-02	1.00E+00
FOXB1	LMNA	-0.099308443	-0.234149343	0.039281087	1.60E-01	2.27E-01	1.00E+00
MRPS12	LMNA	0.193781444	0.057262309	0.323186645	5.72E-03	1.23E-02	1.00E+00
CRIPT	LMNA	-0.384381959	-0.496107363	-0.260134835	1.63E-08	1.07E-07	1.93E-04
DEPDC5	LMNA	-0.219045069	-0.346614653	-0.083519651	1.74E-03	4.28E-03	1.00E+00
RYR1	LMNA	-0.320084765	-0.438748344	-0.190449344	3.42E-06	1.48E-05	4.06E-02
LTBP1	LMNA	0.535790622	0.429508859	0.627432667	2.10E-16	5.74E-15	2.49E-12
MAPRE1	LMNA	0.021785922	-0.116615809	0.159357644	7.58E-01	8.14E-01	1.00E+00
TRIP12	LMNA	0.25749694	0.123848469	0.381969789	2.16E-04	6.60E-04	1.00E+00
FGF8	LMNA	0.049900301	-0.088762163	0.186665404	4.81E-01	5.66E-01	1.00E+00
NDUFA2	LMNA	-0.127204408	-0.260677726	0.011040471	7.12E-02	1.14E-01	1.00E+00
C3orf52	LMNA	0.344786925	0.217067965	0.460899928	5.04E-07	2.54E-06	5.98E-03
SENP7	LMNA	-0.406962667	-0.516022685	-0.284918865	1.85E-09	1.45E-08	2.20E-05
MOBKL2B	LMNA	-0.454821494	-0.557846138	-0.337992561	1.05E-11	1.22E-10	1.24E-07

KIAA0355	LMNA	0.071082739	-0.067631937	0.207101444	3.15E-01	4.00E-01	1.00E+00
CPEB1	LMNA	-0.027799118	-0.165216069	0.110676626	6.95E-01	7.60E-01	1.00E+00
ACOT7	LMNA	-0.003584401	-0.14156535	0.134533171	9.60E-01	9.70E-01	1.00E+00
FGF6	LMNA	0.142367479	0.004403015	0.275013374	4.33E-02	7.38E-02	1.00E+00
PPEF2	LMNA	0.197577694	0.061195859	0.326717222	4.82E-03	1.06E-02	1.00E+00
ABI2	LMNA	0.134828062	-0.003284066	0.267892735	5.57E-02	9.22E-02	1.00E+00
KIAA0317	LMNA	0.073217904	-0.065495108	0.209154809	3.00E-01	3.85E-01	1.00E+00
IKZF3	LMNA	-0.063520813	-0.199819574	0.075189533	3.69E-01	4.57E-01	1.00E+00
H3F3B	LMNA	-0.173491638	-0.304255508	-0.036310295	1.35E-02	2.65E-02	1.00E+00
SLC38A4	LMNA	0.121996209	-0.016329814	0.255740111	8.37E-02	1.31E-01	1.00E+00
ATF1	LMNA	0.218985709	0.083457736	0.346559793	1.74E-03	4.29E-03	1.00E+00
FGFR3	LMNA	-0.09237362	-0.227523183	0.046267395	1.91E-01	2.63E-01	1.00E+00
DYNC1H1	LMNA	-0.103934082	-0.238562144	0.034613561	1.41E-01	2.04E-01	1.00E+00
DIP	LMNA	0.074286135	-0.064425568	0.210181665	2.93E-01	3.77E-01	1.00E+00
TMEM33	LMNA	0.018481038	-0.119875805	0.15613369	7.94E-01	8.44E-01	1.00E+00
ARIH1	LMNA	0.300147717	0.169103643	0.420764067	1.43E-05	5.49E-05	1.69E-01
POLDIP3	LMNA	-0.177517741	-0.308020243	-0.04045823	1.15E-02	2.29E-02	1.00E+00
C1orf129	LMNA	0.160375313	0.022829763	0.291962277	2.26E-02	4.17E-02	1.00E+00
POU6F1	LMNA	-0.221631449	-0.349004164	-0.086218426	1.52E-03	3.80E-03	1.00E+00
BBS1	LMNA	0.003604026	-0.134513901	0.141584582	9.59E-01	9.70E-01	1.00E+00
RGPD5	LMNA	-0.143024241	-0.275632968	-0.005073414	4.23E-02	7.24E-02	1.00E+00
C7orf24	LMNA	0.214647613	0.078935658	0.342548118	2.16E-03	5.19E-03	1.00E+00
GPR171	LMNA	0.228505183	0.093400536	0.355346642	1.07E-03	2.79E-03	1.00E+00
CDC6	LMNA	-0.013872403	-0.151633011	0.124416868	8.45E-01	8.85E-01	1.00E+00
PLD1	LMNA	0.261466647	0.128037244	0.385599163	1.71E-04	5.33E-04	1.00E+00
KDELC1	LMNA	0.215442151	0.079763476	0.34328322	2.08E-03	5.01E-03	1.00E+00
SULT1C2	LMNA	0.084296876	-0.054387041	0.21979012	2.33E-01	3.11E-01	1.00E+00
CHI3L1	LMNA	0.555104586	0.451665989	0.643818043	1.01E-17	3.49E-16	1.20E-13
PIP5K3	LMNA	-0.253147451	-0.37798878	-0.119264414	2.78E-04	8.30E-04	1.00E+00
CSDA	LMNA	0.607136644	0.511999283	0.687559297	9.85E-22	6.49E-20	1.17E-17
ITFG2	LMNA	-0.033687068	-0.170943092	0.104851548	6.34E-01	7.08E-01	1.00E+00
VTCN1	LMNA	-0.099616614	-0.234443507	0.038970313	1.58E-01	2.25E-01	1.00E+00
WDR62	LMNA	-0.108483058	-0.242896395	0.030017491	1.24E-01	1.83E-01	1.00E+00
NDUFC1	LMNA	0.053196542	-0.085482226	0.189853291	4.52E-01	5.38E-01	1.00E+00
NBR1	LMNA	-0.065352326	-0.20158464	0.07336053	3.55E-01	4.42E-01	1.00E+00

HPS4	LMNA	-0.24757092	-0.372877918	-0.113395484	3.82E-04	1.11E-03	1.00E+00
KIF2A	LMNA	-0.383812512	-0.495603608	-0.259511933	1.71E-08	1.12E-07	2.03E-04
RARB	LMNA	-0.118329278	-0.252259499	0.020049237	9.35E-02	1.44E-01	1.00E+00
CEL	LMNA	0.144624017	0.006706924	0.277141747	4.00E-02	6.90E-02	1.00E+00
IMMT	LMNA	0.045965897	-0.092673174	0.182856556	5.16E-01	6.00E-01	1.00E+00
CNOT6	LMNA	-0.193637404	-0.323052614	-0.057113142	5.76E-03	1.24E-02	1.00E+00
PICALM	LMNA	0.171956283	0.034729722	0.302818749	1.44E-02	2.79E-02	1.00E+00
MARK3	LMNA	-0.000938207	-0.138971208	0.137130554	9.89E-01	9.91E-01	1.00E+00
DHX15	LMNA	-0.111867265	-0.24611738	0.026594444	1.13E-01	1.69E-01	1.00E+00
ZNF702	LMNA	-0.132388602	-0.265585658	0.005767814	6.04E-02	9.89E-02	1.00E+00
HIST1H1A	LMNA	0.084847212	-0.053834364	0.220317579	2.30E-01	3.08E-01	1.00E+00
HLF	LMNA	-0.430114292	-0.536319849	-0.310500074	1.68E-10	1.61E-09	1.99E-06
ADAMTS2	LMNA	0.433450978	0.31420126	0.539235048	1.17E-10	1.15E-09	1.38E-06
ARPC3	LMNA	0.157314567	0.019691209	0.289087332	2.54E-02	4.61E-02	1.00E+00
BRD8	LMNA	-0.469305612	-0.570401386	-0.354202738	1.85E-12	2.47E-11	2.20E-08
NPHS1	LMNA	-0.002203429	-0.14021178	0.135888907	9.75E-01	9.81E-01	1.00E+00
WDR12	LMNA	-0.009136287	-0.147001878	0.129077516	8.97E-01	9.26E-01	1.00E+00
HOXD13	LMNA	-0.151196991	-0.28333399	-0.013426235	3.17E-02	5.63E-02	1.00E+00
KIAA0494	LMNA	0.225824388	0.0905978	0.352874429	1.23E-03	3.15E-03	1.00E+00
GABRQ	LMNA	-0.088361362	-0.223683784	0.050303257	2.11E-01	2.86E-01	1.00E+00
TCF4	LMNA	-0.432711902	-0.53858955	-0.313381135	1.26E-10	1.24E-09	1.50E-06
APOBEC3B	LMNA	0.510213808	0.400362555	0.605608458	8.74E-15	1.71E-13	1.04E-10
NR2C2	LMNA	-0.180037112	-0.310373983	-0.043056244	1.03E-02	2.09E-02	1.00E+00
NKTR	LMNA	-0.209623261	-0.337895991	-0.073705193	2.75E-03	6.44E-03	1.00E+00
MYH2	LMNA	0.019742221	-0.118632103	0.157364336	7.80E-01	8.33E-01	1.00E+00
TLE2	LMNA	-0.143448138	-0.276032817	-0.005506177	4.17E-02	7.15E-02	1.00E+00
FXN	LMNA	0.086986093	-0.051685575	0.222366798	2.18E-01	2.95E-01	1.00E+00
AURKA	LMNA	0.162038869	0.024536746	0.29352385	2.12E-02	3.94E-02	1.00E+00
KIAA0892	LMNA	0.086440819	-0.052233496	0.221844496	2.21E-01	2.98E-01	1.00E+00
GPRC5C	LMNA	-0.043430084	-0.180399488	0.095191644	5.39E-01	6.21E-01	1.00E+00
TBC1D9B	LMNA	0.377132547	0.252212625	0.489688631	3.16E-08	1.97E-07	3.75E-04
PAEP	LMNA	0.309197018	0.17877711	0.428938784	7.55E-06	3.06E-05	8.96E-02
PNPLA6	LMNA	0.335392921	0.206922717	0.4524929	1.06E-06	5.07E-06	1.26E-02
SPG11	LMNA	0.030398148	-0.108106521	0.167745207	6.68E-01	7.37E-01	1.00E+00
KCNJ13	LMNA	-0.251149505	-0.376158547	-0.117160632	3.12E-04	9.21E-04	1.00E+00

NOC3L	LMNA	-0.051879625	-0.188580009	0.08679299	4.63E-01	5.49E-01	1.00E+00
AP3B1	LMNA	0.371674375	0.246258924	0.484847824	5.15E-08	3.12E-07	6.11E-04
C11orf68	LMNA	0.09174328	-0.046901743	0.226920278	1.94E-01	2.67E-01	1.00E+00
NAG	LMNA	0.000946638	-0.137122283	0.138979476	9.89E-01	9.91E-01	1.00E+00
AKR7A3	LMNA	0.067753401	-0.070961331	0.203897264	3.38E-01	4.24E-01	1.00E+00
AHCYL1	LMNA	-0.246080623	-0.37151078	-0.111828628	4.15E-04	1.19E-03	1.00E+00
FLJ11184	LMNA	-0.022556471	-0.160108901	0.115855297	7.50E-01	8.07E-01	1.00E+00
MLN	LMNA	0.036554309	-0.102011478	0.173728613	6.06E-01	6.82E-01	1.00E+00
RPP14	LMNA	-0.040361722	-0.17742412	0.098236651	5.68E-01	6.48E-01	1.00E+00
TIAM1	LMNA	-0.033526552	-0.170787086	0.105010476	6.36E-01	7.10E-01	1.00E+00
PBX1	LMNA	-0.391618499	-0.502502541	-0.268059697	8.25E-09	5.73E-08	9.79E-05
PXMP2	LMNA	-0.283166161	-0.405370666	-0.151018688	4.44E-05	1.55E-04	5.26E-01
KRT17	LMNA	0.016489296	-0.121839055	0.154189305	8.16E-01	8.61E-01	1.00E+00
LACTB2	LMNA	0.024475758	-0.113960304	0.161979445	7.30E-01	7.90E-01	1.00E+00
ZNF711	LMNA	-0.521844893	-0.615550865	-0.413589371	1.67E-15	3.81E-14	1.98E-11
GGA1	LMNA	-0.130488464	-0.263787579	0.007701268	6.42E-02	1.04E-01	1.00E+00
VAMP4	LMNA	0.149739041	0.011934751	0.28196144	3.34E-02	5.90E-02	1.00E+00
C20orf19	LMNA	-0.355015321	-0.470030046	-0.228145796	2.17E-07	1.17E-06	2.57E-03
BCAP29	LMNA	0.383727076	0.259418487	0.495528022	1.73E-08	1.13E-07	2.05E-04
DDX24	LMNA	-0.041570413	-0.178596477	0.09703747	5.57E-01	6.38E-01	1.00E+00
PHACTR1	LMNA	-0.410919989	-0.519500793	-0.289279194	1.24E-09	1.01E-08	1.48E-05
SLC35E2	LMNA	-0.2552098	-0.379876987	-0.121437274	2.47E-04	7.44E-04	1.00E+00
LOXL1	LMNA	0.72708463	0.654754451	0.786219079	1.67E-34	1.23E-31	1.98E-30
IQSEC2	LMNA	0.011621487	-0.126632677	0.149432744	8.70E-01	9.03E-01	1.00E+00
RGSL1	LMNA	0.213160558	0.077386822	0.341171884	2.32E-03	5.54E-03	1.00E+00
SETD8	LMNA	0.332234977	0.203518424	0.44966206	1.36E-06	6.36E-06	1.61E-02
HEXIM1	LMNA	0.121639578	-0.016691718	0.255401753	8.46E-02	1.32E-01	1.00E+00
PRRX1	LMNA	0.50282358	0.391982136	0.59927568	2.43E-14	4.32E-13	2.88E-10
SULT1A2	LMNA	-0.118734633	-0.252644429	0.019638267	9.24E-02	1.42E-01	1.00E+00
ASTE1	LMNA	0.056369602	-0.082322019	0.192919327	4.26E-01	5.12E-01	1.00E+00
KLHL9	LMNA	-0.248317333	-0.373562443	-0.114180492	3.66E-04	1.07E-03	1.00E+00
GAA	LMNA	0.478101044	0.364080183	0.578002532	6.21E-13	8.99E-12	7.37E-09
MYOD1	LMNA	0.224370642	0.089078821	0.35153305	1.33E-03	3.37E-03	1.00E+00
ZNF747	LMNA	0.198076989	0.061713528	0.327181306	4.72E-03	1.04E-02	1.00E+00
ARHGEF16	LMNA	-0.155947529	-0.287802513	-0.018290295	2.67E-02	4.82E-02	1.00E+00

SCN1A	LMNA	-0.445711912	-0.549925463	-0.327832782	2.99E-11	3.22E-10	3.54E-07
GDF9	LMNA	-0.40791456	-0.516859637	-0.285967237	1.68E-09	1.34E-08	2.00E-05
IL1RL2	LMNA	0.003995955	-0.134129038	0.141968639	9.55E-01	9.67E-01	1.00E+00
HNRPH1	LMNA	-0.154723199	-0.286651414	-0.017036084	2.79E-02	5.02E-02	1.00E+00
MED18	LMNA	0.041034035	-0.097569678	0.178076271	5.62E-01	6.42E-01	1.00E+00
TRAF2	LMNA	0.222256319	0.086870748	0.349581222	1.48E-03	3.70E-03	1.00E+00
ECE1	LMNA	0.645670213	0.557293832	0.719581055	3.26E-25	4.49E-23	3.86E-21
TEX13B	LMNA	0.040655512	-0.097945209	0.177709114	5.66E-01	6.46E-01	1.00E+00
SNN	LMNA	-0.379101499	-0.491433176	-0.254362654	2.64E-08	1.67E-07	3.13E-04
HCK	LMNA	0.287183021	0.155288576	0.409018128	3.41E-05	1.22E-04	4.05E-01
C6orf62	LMNA	0.066817925	-0.071896267	0.20299643	3.45E-01	4.31E-01	1.00E+00
GABBR1	LMNA	-0.496770644	-0.594079837	-0.385131865	5.50E-14	9.09E-13	6.53E-10
YIPF6	LMNA	-0.124452944	-0.25807008	0.013835767	7.76E-02	1.23E-01	1.00E+00
BMP6	LMNA	-0.17231046	-0.303150235	-0.035094269	1.42E-02	2.76E-02	1.00E+00
PROX1	LMNA	-0.105581455	-0.240132376	0.03294981	1.35E-01	1.96E-01	1.00E+00
LANCL2	LMNA	0.028253004	-0.110227925	0.16565788	6.90E-01	7.57E-01	1.00E+00
SCN3A	LMNA	-0.599509853	-0.681184061	-0.503096604	4.24E-21	2.54E-19	5.03E-17
IL8RA	LMNA	0.104480677	-0.034061617	0.239083222	1.39E-01	2.01E-01	1.00E+00
LSM3	LMNA	-0.240658167	-0.366531843	-0.106133242	5.61E-04	1.56E-03	1.00E+00
CDSN	LMNA	0.015073308	-0.123234129	0.152806336	8.31E-01	8.73E-01	1.00E+00
EFHA1	LMNA	-0.197373823	-0.32652771	-0.060984507	4.87E-03	1.07E-02	1.00E+00
SSRP1	LMNA	0.036769334	-0.1017984	0.173937421	6.03E-01	6.81E-01	1.00E+00
ASXL2	LMNA	-0.019534683	-0.157161853	0.118836794	7.83E-01	8.35E-01	1.00E+00
RPE65	LMNA	-0.134052208	-0.26715915	0.00407419	5.72E-02	9.42E-02	1.00E+00
SNAI1	LMNA	0.48807259	0.375309537	0.586599178	1.73E-13	2.70E-12	2.06E-09
SPCS3	LMNA	0.12238602	-0.015934197	0.256109912	8.27E-02	1.29E-01	1.00E+00
EFNA2	LMNA	-0.223002223	-0.350269925	-0.087649574	1.42E-03	3.58E-03	1.00E+00
CLDN9	LMNA	-0.044835757	-0.181761722	0.093795804	5.26E-01	6.09E-01	1.00E+00
DEF8	LMNA	-0.202889914	-0.331651633	-0.066707329	3.78E-03	8.53E-03	1.00E+00
CHAF1A	LMNA	-0.017881461	-0.155548482	0.120466919	8.01E-01	8.50E-01	1.00E+00
C1orf165	LMNA	-0.413719704	-0.521959302	-0.292367079	9.36E-10	7.77E-09	1.11E-05
ZFPM2	LMNA	-0.422475216	-0.529636179	-0.30204014	3.78E-10	3.36E-09	4.48E-06
C9orf7	LMNA	-0.090811531	-0.226028893	0.047839212	1.99E-01	2.73E-01	1.00E+00
GC	LMNA	-0.000755767	-0.138792287	0.137309559	9.91E-01	9.93E-01	1.00E+00
FTH1	LMNA	0.246056193	0.111802949	0.371488364	4.16E-04	1.19E-03	1.00E+00

IER3	LMNA	0.406611353	0.284532018	0.515713741	1.92E-09	1.50E-08	2.27E-05
YWHAH	LMNA	-0.083010144	-0.218556566	0.055678917	2.40E-01	3.20E-01	1.00E+00
ADRB1	LMNA	0.02953467	-0.108960595	0.166905149	6.76E-01	7.45E-01	1.00E+00
FOXL1	LMNA	0.098477275	-0.040119141	0.233355829	1.63E-01	2.31E-01	1.00E+00
MGC31957	LMNA	0.173154131	0.035962789	0.303939726	1.37E-02	2.68E-02	1.00E+00
MUC5B	LMNA	0.11095316	-0.02751936	0.245247654	1.16E-01	1.73E-01	1.00E+00
ZNF193	LMNA	-0.163269684	-0.29467876	-0.025800209	2.02E-02	3.78E-02	1.00E+00
CSRP1	LMNA	0.279262065	0.146873387	0.401821865	5.70E-05	1.96E-04	6.76E-01
MOSPD1	LMNA	-0.01927386	-0.156907365	0.119094023	7.85E-01	8.37E-01	1.00E+00
UMPS	LMNA	0.227032413	0.091860509	0.353988682	1.16E-03	2.98E-03	1.00E+00
RAD1	LMNA	-0.000418153	-0.138461161	0.137640793	9.95E-01	9.96E-01	1.00E+00
RCP9	LMNA	0.164907503	0.027482151	0.296214978	1.90E-02	3.57E-02	1.00E+00
COG4	LMNA	-0.06186126	-0.198219465	0.076846006	3.82E-01	4.69E-01	1.00E+00
NFRKB	LMNA	-0.084493207	-0.219978299	0.054189885	2.32E-01	3.10E-01	1.00E+00
FANCA	LMNA	-0.086602282	-0.221999165	0.052071258	2.20E-01	2.97E-01	1.00E+00
CDC2L5	LMNA	0.090554592	-0.048097685	0.225783044	2.00E-01	2.74E-01	1.00E+00
GDF2	LMNA	-0.076207977	-0.212028315	0.062500566	2.81E-01	3.64E-01	1.00E+00
PCBP3	LMNA	-0.278306635	-0.400952824	-0.145859636	6.06E-05	2.07E-04	7.19E-01
TIMM17A	LMNA	0.049066205	-0.089591657	0.185858269	4.88E-01	5.73E-01	1.00E+00
BFSP2	LMNA	-0.033180381	-0.170450618	0.105353199	6.39E-01	7.13E-01	1.00E+00
OR2H1	LMNA	-0.022995198	-0.160536573	0.115422212	7.45E-01	8.03E-01	1.00E+00
HNRNPA0	LMNA	-0.083114575	-0.218656697	0.055574086	2.40E-01	3.19E-01	1.00E+00
IKBKG	LMNA	0.508064944	0.397923876	0.603768315	1.18E-14	2.25E-13	1.40E-10
TM4SF20	LMNA	0.02157846	-0.116820541	0.159155348	7.61E-01	8.16E-01	1.00E+00
PCBP1	LMNA	0.248910022	0.114803947	0.374105892	3.54E-04	1.03E-03	1.00E+00
LRRC2	LMNA	0.1540687	0.016365789	0.286035906	2.86E-02	5.12E-02	1.00E+00
NSD1	LMNA	0.090525299	-0.048127152	0.225755015	2.00E-01	2.74E-01	1.00E+00
AMMECR1	LMNA	0.265082974	0.131857287	0.388902114	1.38E-04	4.40E-04	1.00E+00
MAGEC1	LMNA	-0.078778772	-0.214497013	0.059923931	2.65E-01	3.47E-01	1.00E+00
SEC13	LMNA	0.205894365	0.069828154	0.334439289	3.29E-03	7.53E-03	1.00E+00
WDR91	LMNA	0.217909294	0.082335148	0.345564808	1.84E-03	4.50E-03	1.00E+00
HAGH	LMNA	-0.304396869	-0.424604995	-0.173642735	1.06E-05	4.17E-05	1.26E-01
EGF	LMNA	0.260497759	0.127014449	0.384713695	1.81E-04	5.62E-04	1.00E+00
NHLH2	LMNA	-0.16082401	-0.292383536	-0.023290094	2.22E-02	4.11E-02	1.00E+00
NCAPD3	LMNA	-0.060321582	-0.196734286	0.078382143	3.94E-01	4.81E-01	1.00E+00

BRCC3	LMNA	0.241516069	0.107033738	0.367320056	5.35E-04	1.50E-03	1.00E+00
CNDP2	LMNA	0.099391181	-0.039197653	0.234228322	1.59E-01	2.26E-01	1.00E+00
FYN	LMNA	-0.214482659	-0.342395485	-0.078763819	2.18E-03	5.23E-03	1.00E+00
YPEL5	LMNA	-0.246371374	-0.371777546	-0.112134263	4.09E-04	1.17E-03	1.00E+00
KCND3	LMNA	-0.187855969	-0.317668645	-0.051130984	7.42E-03	1.55E-02	1.00E+00
LRRC42	LMNA	0.100919611	-0.037656015	0.235687008	1.53E-01	2.19E-01	1.00E+00
GTF3C5	LMNA	-0.122241213	-0.255972544	0.016081165	8.31E-02	1.30E-01	1.00E+00
AKR1C4	LMNA	0.009093314	-0.129119776	0.14695983	8.98E-01	9.26E-01	1.00E+00
RBM26	LMNA	-0.268033349	-0.391594465	-0.134976797	1.15E-04	3.72E-04	1.00E+00
DUSP14	LMNA	-0.015379792	-0.153105719	0.122932218	8.28E-01	8.71E-01	1.00E+00
AP4M1	LMNA	0.405468971	0.283274365	0.514708934	2.15E-09	1.67E-08	2.55E-05
RIMBP2	LMNA	-0.325999771	-0.444065772	-0.196805942	2.20E-06	9.87E-06	2.60E-02
ABCC2	LMNA	0.024385134	-0.114049803	0.161891146	7.30E-01	7.91E-01	1.00E+00
COL5A2	LMNA	0.704534284	0.62751659	0.767898226	1.29E-31	6.14E-29	1.54E-27
DNAJC16	LMNA	0.074096485	-0.064615474	0.209999382	2.95E-01	3.79E-01	1.00E+00
TTC12	LMNA	0.335782028	0.207342394	0.452841539	1.03E-06	4.93E-06	1.22E-02
SNX13	LMNA	0.173458788	0.036276469	0.304224773	1.36E-02	2.66E-02	1.00E+00
ELA2B	LMNA	0.062532593	-0.076176011	0.198866839	3.77E-01	4.64E-01	1.00E+00
CSPP1	LMNA	-0.221839405	-0.349196219	-0.086435505	1.51E-03	3.77E-03	1.00E+00
NAIP	LMNA	0.001855694	-0.136230206	0.139870863	9.79E-01	9.84E-01	1.00E+00
MYOZ2	LMNA	0.135620198	-0.002477182	0.268641556	5.43E-02	9.02E-02	1.00E+00
XRCC4	LMNA	0.00462726	-0.133509026	0.142587176	9.48E-01	9.61E-01	1.00E+00
CYB561	LMNA	0.480061062	0.366284823	0.579694051	4.85E-13	7.09E-12	5.75E-09
CHST10	LMNA	-0.051400599	-0.188116742	0.087269658	4.68E-01	5.53E-01	1.00E+00
BAI1	LMNA	-0.32543651	-0.443559774	-0.196200162	2.29E-06	1.03E-05	2.72E-02
THY1	LMNA	0.165834301	0.028434263	0.297083982	1.83E-02	3.45E-02	1.00E+00
KIT	LMNA	-0.471400461	-0.572213358	-0.356552966	1.43E-12	1.94E-11	1.70E-08
TBC1D8	LMNA	0.253290767	0.119415368	0.378120028	2.76E-04	8.24E-04	1.00E+00
PDE6H	LMNA	-0.26973467	-0.393146036	-0.136776848	1.04E-04	3.37E-04	1.00E+00
EPHA7	LMNA	-0.306017098	-0.426068427	-0.175374984	9.46E-06	3.76E-05	1.12E-01
SOLH	LMNA	0.105365844	-0.033167608	0.239926901	1.36E-01	1.97E-01	1.00E+00
FLJ20309	LMNA	-0.090009576	-0.225261497	0.048645895	2.03E-01	2.77E-01	1.00E+00
MAP7	LMNA	-0.14934907	-0.28159422	-0.011535914	3.39E-02	5.96E-02	1.00E+00
SPAG9	LMNA	-0.30727765	-0.427206554	-0.176723247	8.66E-06	3.46E-05	1.03E-01
ZNF294	LMNA	-0.153850458	-0.285830642	-0.016142308	2.88E-02	5.16E-02	1.00E+00

CENTB2	LMNA	-0.072391646	-0.208360348	0.066322161	3.06E-01	3.91E-01	1.00E+00
FLJ21963	LMNA	0.493395032	0.38131693	0.591178679	8.62E-14	1.38E-12	1.02E-09
ANTXR1	LMNA	-0.068504173	-0.204620068	0.070210816	3.33E-01	4.18E-01	1.00E+00
CREB3	LMNA	0.045869984	-0.092768464	0.182763652	5.17E-01	6.00E-01	1.00E+00
ETV7	LMNA	0.383655517	0.259340219	0.495464711	1.74E-08	1.14E-07	2.06E-04
DGAT1	LMNA	0.035928534	-0.102631516	0.17312086	6.12E-01	6.88E-01	1.00E+00
TAC3	LMNA	-0.184685165	-0.314712271	-0.047854255	8.51E-03	1.76E-02	1.00E+00
CORO1C	LMNA	-0.095784275	-0.230783598	0.042833113	1.75E-01	2.45E-01	1.00E+00
RAD54B	LMNA	-0.180939023	-0.311216212	-0.043986759	9.97E-03	2.02E-02	1.00E+00
HRASLS3	LMNA	0.325428904	0.196191983	0.443552941	2.29E-06	1.03E-05	2.72E-02
MED25	LMNA	-0.02800913	-0.165420501	0.11046902	6.92E-01	7.58E-01	1.00E+00
BARD1	LMNA	0.108273988	-0.030228854	0.242697311	1.25E-01	1.84E-01	1.00E+00
ZNF177	LMNA	-0.393280387	-0.503969497	-0.269882024	7.04E-09	4.97E-08	8.35E-05
MIP	LMNA	-0.106732231	-0.241228848	0.031787137	1.31E-01	1.91E-01	1.00E+00
ZNF442	LMNA	-0.037537929	-0.174683693	0.101036659	5.96E-01	6.73E-01	1.00E+00
F2	LMNA	0.217103718	0.081495242	0.344819986	1.91E-03	4.67E-03	1.00E+00
FDX1	LMNA	0.174312599	0.037155714	0.305023505	1.31E-02	2.58E-02	1.00E+00
GRIA1	LMNA	-0.0255195	-0.162996266	0.11292935	7.18E-01	7.80E-01	1.00E+00
IL13RA1	LMNA	0.441182607	0.322791445	0.545980295	4.97E-11	5.23E-10	5.90E-07
EIF2B2	LMNA	0.126936607	-0.011312634	0.260424008	7.18E-02	1.15E-01	1.00E+00
NHP2L1	LMNA	-0.220616142	-0.348066337	-0.085158761	1.60E-03	3.98E-03	1.00E+00
WNT5A	LMNA	0.065096858	-0.073615704	0.201338493	3.57E-01	4.44E-01	1.00E+00
ZNF593	LMNA	0.283599134	0.151478697	0.405764009	4.31E-05	1.51E-04	5.12E-01
TRA@	LMNA	0.342364981	0.214449694	0.458734436	6.12E-07	3.04E-06	7.26E-03
WIPI2	LMNA	0.035105616	-0.103446726	0.172321483	6.20E-01	6.95E-01	1.00E+00
TAS2R7	LMNA	0.052629487	-0.086046691	0.189305081	4.57E-01	5.43E-01	1.00E+00
RANBP1	LMNA	-0.120539288	-0.254357633	0.017808048	8.75E-02	1.36E-01	1.00E+00
CSNK2B	LMNA	0.022321396	-0.116087327	0.159879729	7.53E-01	8.09E-01	1.00E+00
DKFZP434(	LMNA	0.052933189	-0.085744387	0.189598701	4.54E-01	5.41E-01	1.00E+00
SLC7A4	LMNA	-0.283002691	-0.405222146	-0.150845025	4.48E-05	1.57E-04	5.32E-01
WBP11	LMNA	-0.001778671	-0.139795345	0.136305799	9.80E-01	9.84E-01	1.00E+00
TEX2	LMNA	0.100177764	-0.038404355	0.234979089	1.56E-01	2.22E-01	1.00E+00
C17orf80	LMNA	-0.332903869	-0.450261865	-0.204239237	1.29E-06	6.08E-06	1.53E-02
TMEM176A	LMNA	0.187704131	0.050974007	0.317527133	7.47E-03	1.56E-02	1.00E+00
GALNT2	LMNA	0.62238953	0.529865354	0.700272103	4.71E-23	4.40E-21	5.59E-19

CTNNB1	LMNA	-0.010503579	-0.148339483	0.127732637	8.82E-01	9.13E-01	1.00E+00
BHLHB2	LMNA	0.580782588	0.481322911	0.665477211	1.30E-19	6.17E-18	1.54E-15
TMEM185B	LMNA	0.424285435	0.304043163	0.5312212	3.12E-10	2.82E-09	3.70E-06
ARD1B	LMNA	0.040689039	-0.097911949	0.177741636	5.65E-01	6.45E-01	1.00E+00
STK3	LMNA	0.290428452	0.158742045	0.411962263	2.75E-05	1.01E-04	3.27E-01
REPS2	LMNA	-0.356394638	-0.471259377	-0.229642179	1.93E-07	1.06E-06	2.29E-03
LOC541469	LMNA	0.001050373	-0.137020497	0.139081206	9.88E-01	9.91E-01	1.00E+00
H2AFY2	LMNA	-0.336686205	-0.453651542	-0.208317787	9.62E-07	4.63E-06	1.14E-02
CCNK	LMNA	0.122462628	-0.015856443	0.256182584	8.25E-02	1.29E-01	1.00E+00
FSHR	LMNA	0.110175413	-0.028306119	0.244507497	1.19E-01	1.76E-01	1.00E+00
GAS8	LMNA	0.317454497	0.187626197	0.436381133	4.15E-06	1.77E-05	4.93E-02
UPK2	LMNA	-0.216006218	-0.343804997	-0.080351283	2.02E-03	4.89E-03	1.00E+00
C1orf66	LMNA	-0.082713982	-0.218272583	0.055976196	2.42E-01	3.21E-01	1.00E+00
LCE2B	LMNA	0.079612372	-0.05908804	0.215297135	2.60E-01	3.42E-01	1.00E+00
CD200	LMNA	-0.5239899	-0.617381194	-0.416033664	1.22E-15	2.85E-14	1.45E-11
IMPACT	LMNA	0.029795878	-0.108702253	0.167159294	6.74E-01	7.43E-01	1.00E+00
KRT83	LMNA	-0.01901433	-0.156654121	0.119349958	7.88E-01	8.40E-01	1.00E+00
COL19A1	LMNA	0.209423206	0.073497085	0.337710626	2.78E-03	6.49E-03	1.00E+00
BMP15	LMNA	-0.085408959	-0.220855894	0.053270138	2.27E-01	3.04E-01	1.00E+00
POL3S	LMNA	0.195259396	0.058793211	0.324561594	5.36E-03	1.16E-02	1.00E+00
ITGA6	LMNA	0.105794728	-0.03273436	0.24033561	1.34E-01	1.95E-01	1.00E+00
GAD2	LMNA	-0.339760562	-0.456404247	-0.211636206	7.54E-07	3.69E-06	8.94E-03
C1GALT1	LMNA	0.158782625	0.021196248	0.290466569	2.40E-02	4.39E-02	1.00E+00
BAG3	LMNA	0.346575983	0.219003227	0.46249866	4.36E-07	2.23E-06	5.17E-03
ZNF468	LMNA	0.046604994	-0.092038168	0.183475537	5.10E-01	5.94E-01	1.00E+00
RIC8A	LMNA	0.393581431	0.270212225	0.504235162	6.84E-09	4.84E-08	8.12E-05
CA5A	LMNA	-0.221983367	-0.349329166	-0.08658579	1.50E-03	3.75E-03	1.00E+00
CCND1	LMNA	-0.051178653	-0.187902076	0.087490491	4.69E-01	5.55E-01	1.00E+00
DKK4	LMNA	-0.158176732	-0.289897401	-0.020575017	2.46E-02	4.48E-02	1.00E+00
SGK2	LMNA	-0.097560396	-0.23248028	0.041043389	1.67E-01	2.35E-01	1.00E+00
PIK3C2G	LMNA	-0.176331282	-0.306911231	-0.039235376	1.21E-02	2.40E-02	1.00E+00
USP11	LMNA	-0.32709919	-0.445053204	-0.197988638	2.02E-06	9.15E-06	2.40E-02
IMPA2	LMNA	0.429556131	0.309881294	0.535831947	1.78E-10	1.70E-09	2.11E-06
PRKDC	LMNA	-0.044146159	-0.1810935	0.094480646	5.33E-01	6.15E-01	1.00E+00
DSCR2	LMNA	0.144643171	0.006726485	0.277159807	4.00E-02	6.89E-02	1.00E+00

MSR1	LMNA	0.320314007	0.190695498	0.438954581	3.36E-06	1.45E-05	3.99E-02
NOL11	LMNA	-0.094881452	-0.22992084	0.043742509	1.79E-01	2.49E-01	1.00E+00
ZCCHC10	LMNA	-0.153504572	-0.285505298	-0.015788145	2.92E-02	5.22E-02	1.00E+00
PDCD6IP	LMNA	0.363149833	0.236979366	0.477273583	1.09E-07	6.18E-07	1.29E-03
TRPM2	LMNA	0.274268189	0.141577746	0.397277088	7.82E-05	2.61E-04	9.27E-01
PSMD2	LMNA	0.161662596	0.02415058	0.293170705	2.15E-02	3.99E-02	1.00E+00
USP18	LMNA	0.037511981	-0.101062378	0.174658501	5.96E-01	6.73E-01	1.00E+00
ATXN2	LMNA	-0.174894621	-0.305567875	-0.037755196	1.28E-02	2.52E-02	1.00E+00
SLC17A4	LMNA	0.02325371	-0.115166999	0.160788548	7.43E-01	8.01E-01	1.00E+00
RS1	LMNA	-0.136970084	-0.269917258	0.001101749	5.19E-02	8.68E-02	1.00E+00
RRAS	LMNA	0.596646585	0.499759613	0.678787453	7.26E-21	4.18E-19	8.61E-17
LAMC3	LMNA	0.172625584	0.03541865	0.303445142	1.40E-02	2.73E-02	1.00E+00
NET1	LMNA	-0.379588521	-0.491864553	-0.254894657	2.53E-08	1.61E-07	3.00E-04
NPY1R	LMNA	-0.142978866	-0.275590164	-0.005027092	4.24E-02	7.25E-02	1.00E+00
TOX	LMNA	-0.294871605	-0.415988816	-0.163475244	2.04E-05	7.65E-05	2.42E-01
MVD	LMNA	0.043984029	-0.09464164	0.180936377	5.34E-01	6.17E-01	1.00E+00
C11orf61	LMNA	-0.155030551	-0.286940418	-0.017350897	2.76E-02	4.97E-02	1.00E+00
IK	LMNA	0.016121694	-0.12220128	0.153830328	8.20E-01	8.64E-01	1.00E+00
GCNT2	LMNA	-0.289234806	-0.410879727	-0.15747151	2.98E-05	1.08E-04	3.54E-01
GABRG3	LMNA	-0.156432737	-0.288258593	-0.018787465	2.62E-02	4.75E-02	1.00E+00
BCS1L	LMNA	-0.26490277	-0.3887376	-0.131666837	1.39E-04	4.44E-04	1.00E+00
BCAS2	LMNA	-0.199660134	-0.328652397	-0.063355417	4.39E-03	9.75E-03	1.00E+00
ACE2	LMNA	0.006495136	-0.131673932	0.144416644	9.27E-01	9.47E-01	1.00E+00
KCTD17	LMNA	0.05125464	-0.087414887	0.187975571	4.69E-01	5.54E-01	1.00E+00
TPPP3	LMNA	0.112141543	-0.026316876	0.246378299	1.12E-01	1.68E-01	1.00E+00
CALB2	LMNA	-0.194516343	-0.323870395	-0.058023456	5.54E-03	1.20E-02	1.00E+00
ICT1	LMNA	-0.101246967	-0.235999347	0.037325744	1.52E-01	2.17E-01	1.00E+00
CD79B	LMNA	0.103958147	-0.034589263	0.238585087	1.41E-01	2.04E-01	1.00E+00
CEBPZ	LMNA	0.01065585	-0.127582831	0.148488417	8.80E-01	9.12E-01	1.00E+00
FMOD	LMNA	0.366986452	0.241152946	0.480684602	7.78E-08	4.56E-07	9.23E-04
IRS2	LMNA	0.258735361	0.125154722	0.38310245	2.01E-04	6.16E-04	1.00E+00
C21orf66	LMNA	-0.14958891	-0.281820073	-0.011781202	3.36E-02	5.92E-02	1.00E+00
LUZP2	LMNA	-0.441547964	-0.546298706	-0.323197857	4.77E-11	5.05E-10	5.66E-07
CLN6	LMNA	0.475569272	0.361234321	0.575816311	8.54E-13	1.20E-11	1.01E-08
ANAPC1	LMNA	0.179067422	0.042056065	0.309468232	1.08E-02	2.17E-02	1.00E+00

PTPN14	LMNA	0.654644399	0.567918658	0.726993831	4.26E-26	7.11E-24	5.05E-22
APTX	LMNA	-0.168256884	-0.29935447	-0.030924191	1.67E-02	3.18E-02	1.00E+00
SNRPG	LMNA	-0.128446126	-0.261853903	0.009778264	6.85E-02	1.10E-01	1.00E+00
BMS1	LMNA	0.124003791	-0.01429187	0.257644219	7.87E-02	1.24E-01	1.00E+00
NFATC3	LMNA	0.029321283	-0.109171626	0.16669752	6.79E-01	7.47E-01	1.00E+00
ADCK2	LMNA	0.260963534	0.127506103	0.385139396	1.76E-04	5.48E-04	1.00E+00
ZKSCAN1	LMNA	-0.210302914	-0.338525659	-0.07441229	2.66E-03	6.25E-03	1.00E+00
FASTKD2	LMNA	0.042590255	-0.096025339	0.179585361	5.47E-01	6.28E-01	1.00E+00
ARS2	LMNA	0.021248473	-0.117146161	0.158833556	7.64E-01	8.19E-01	1.00E+00
LRIG1	LMNA	-0.160772701	-0.292335367	-0.023237452	2.23E-02	4.11E-02	1.00E+00
KCNMB3	LMNA	-0.073230674	-0.209167086	0.065482325	3.00E-01	3.85E-01	1.00E+00
ERC2	LMNA	-0.502702153	-0.599171527	-0.391844593	2.47E-14	4.38E-13	2.93E-10
POFUT2	LMNA	0.614637866	0.520775371	0.6938175	2.26E-22	1.74E-20	2.68E-18
PRKACB	LMNA	-0.422822127	-0.529939991	-0.302423918	3.64E-10	3.25E-09	4.32E-06
PRDM13	LMNA	-0.209577519	-0.337853608	-0.073657609	2.76E-03	6.45E-03	1.00E+00
KLK12	LMNA	0.036604062	-0.101962177	0.173776928	6.05E-01	6.82E-01	1.00E+00
ZNF354A	LMNA	-0.031509308	-0.168825934	0.107007162	6.56E-01	7.27E-01	1.00E+00
PCDH11X	LMNA	-0.46154054	-0.563676269	-0.3455037	4.74E-12	5.91E-11	5.62E-08
TMC6	LMNA	0.105617091	-0.032913811	0.240166336	1.35E-01	1.96E-01	1.00E+00
CAND2	LMNA	-0.238859434	-0.36487864	-0.10424592	6.18E-04	1.71E-03	1.00E+00
RIMS1	LMNA	-0.470802763	-0.57169647	-0.355882255	1.54E-12	2.08E-11	1.83E-08
SF3B2	LMNA	0.056754679	-0.081938312	0.193291235	4.22E-01	5.10E-01	1.00E+00
RCN1	LMNA	0.534899649	0.428489807	0.62667483	2.40E-16	6.50E-15	2.85E-12
CPB1	LMNA	-0.006526151	-0.144447014	0.131643452	9.27E-01	9.47E-01	1.00E+00
BCAR3	LMNA	0.241730075	0.107258403	0.367516648	5.29E-04	1.48E-03	1.00E+00
BCL6	LMNA	0.026946797	-0.111519058	0.164386275	7.03E-01	7.68E-01	1.00E+00
MDH2	LMNA	0.235780689	0.101017793	0.362047142	7.30E-04	1.98E-03	1.00E+00
DRP2	LMNA	-0.520741811	-0.614609214	-0.412332987	1.96E-15	4.39E-14	2.32E-11
TPD52L1	LMNA	-0.009664525	-0.147518707	0.128557997	8.91E-01	9.21E-01	1.00E+00
TXNL4A	LMNA	-0.028897181	-0.166284824	0.109591009	6.83E-01	7.51E-01	1.00E+00
OR3A1	LMNA	0.156882617	0.019248496	0.288681412	2.58E-02	4.68E-02	1.00E+00
RAB25	LMNA	-0.118820306	-0.252725779	0.019551401	9.21E-02	1.42E-01	1.00E+00
C22orf9	LMNA	0.031378003	-0.10713709	0.168698242	6.58E-01	7.28E-01	1.00E+00
PCTK3	LMNA	0.033946012	-0.104595151	0.171194746	6.32E-01	7.06E-01	1.00E+00
POR	LMNA	0.359628362	0.233152706	0.474139721	1.47E-07	8.18E-07	1.74E-03

ARPP-19	LMNA	-0.25318843	-0.378026309	-0.119307577	2.77E-04	8.29E-04	1.00E+00
SREBF2	LMNA	-0.181208091	-0.311467435	-0.044264406	9.86E-03	2.00E-02	1.00E+00
ZWINT	LMNA	-0.029274949	-0.166652435	0.109217447	6.79E-01	7.48E-01	1.00E+00
PAK1IP1	LMNA	-0.103453486	-0.23810392	0.035098791	1.43E-01	2.06E-01	1.00E+00
OR10H1	LMNA	-0.232234586	-0.358782928	-0.097303145	8.82E-04	2.34E-03	1.00E+00
ENPP2	LMNA	-0.052257742	-0.188945644	0.086416689	4.60E-01	5.46E-01	1.00E+00
UXT	LMNA	0.046262776	-0.092378209	0.183144105	5.13E-01	5.97E-01	1.00E+00
ZXDC	LMNA	0.04921195	-0.089446731	0.185999316	4.87E-01	5.72E-01	1.00E+00
TGFB1	LMNA	0.615467267	0.521746951	0.694508728	1.91E-22	1.50E-20	2.27E-18
SLC6A16	LMNA	-0.048352667	-0.185167648	0.090301106	4.94E-01	5.79E-01	1.00E+00
SLC31A2	LMNA	0.130292217	-0.007900897	0.263601819	6.46E-02	1.05E-01	1.00E+00
LRRC8E	LMNA	0.477640552	0.363562404	0.577604997	6.59E-13	9.48E-12	7.81E-09
ZNF780B	LMNA	0.198580831	0.062235986	0.327649555	4.61E-03	1.02E-02	1.00E+00
APEX1	LMNA	-0.139825307	-0.272614032	-0.00180923	4.72E-02	7.97E-02	1.00E+00
PPIAL4	LMNA	0.114563143	-0.023865301	0.248681113	1.04E-01	1.58E-01	1.00E+00
ZIC3	LMNA	-0.293312732	-0.414576647	-0.16181392	2.27E-05	8.43E-05	2.69E-01
CEP68	LMNA	-0.459116814	-0.561574369	-0.342792531	6.32E-12	7.71E-11	7.49E-08
PAX2	LMNA	0.283844837	0.151739769	0.405987203	4.25E-05	1.49E-04	5.04E-01
MRPL11	LMNA	0.060749879	-0.077954897	0.197147486	3.90E-01	4.78E-01	1.00E+00
LPAL2	LMNA	-0.105229933	-0.239797374	0.033304891	1.36E-01	1.98E-01	1.00E+00
VPS53	LMNA	-0.367794339	-0.481402431	-0.242032382	7.25E-08	4.28E-07	8.60E-04
MPDU1	LMNA	0.061789355	-0.07691776	0.198150119	3.82E-01	4.70E-01	1.00E+00
PSEN2	LMNA	0.333434295	0.204810934	0.450737429	1.24E-06	5.85E-06	1.47E-02
GAST	LMNA	-0.124066103	-0.257703304	0.014228597	7.85E-02	1.24E-01	1.00E+00
DHRS7B	LMNA	-0.045859233	-0.182753239	0.092779144	5.17E-01	6.00E-01	1.00E+00
C10orf28	LMNA	-0.156161366	-0.28800352	-0.018509395	2.65E-02	4.79E-02	1.00E+00
UBE2L3	LMNA	0.074585039	-0.06412624	0.210468938	2.91E-01	3.75E-01	1.00E+00
ADRA1D	LMNA	0.361463458	0.235146352	0.47577319	1.26E-07	7.09E-07	1.49E-03
FZD10	LMNA	0.078598867	-0.060104305	0.214324309	2.66E-01	3.48E-01	1.00E+00
ATP6V1E1	LMNA	-0.04140324	-0.178434353	0.097203351	5.59E-01	6.39E-01	1.00E+00
SAR1A	LMNA	0.048854608	-0.089802056	0.185653482	4.90E-01	5.75E-01	1.00E+00
ASB1	LMNA	0.039093356	-0.099494605	0.176193462	5.81E-01	6.59E-01	1.00E+00
MCTP2	LMNA	0.438294588	0.319580443	0.543462311	6.86E-11	7.08E-10	8.13E-07
TMEM5	LMNA	-0.00255085	-0.140552355	0.135547883	9.71E-01	9.78E-01	1.00E+00
LCMT2	LMNA	-0.184076113	-0.314144117	-0.047225194	8.73E-03	1.80E-02	1.00E+00

ZNF223	LMNA	-0.316708232	-0.435709204	-0.1868256	4.39E-06	1.86E-05	5.20E-02
BIRC2	LMNA	0.085728207	-0.052949443	0.221161787	2.25E-01	3.02E-01	1.00E+00
NLGN3	LMNA	-0.352630712	-0.467903683	-0.225560214	2.65E-07	1.41E-06	3.14E-03
MEP1A	LMNA	-0.048550678	-0.185359314	0.090104243	4.93E-01	5.78E-01	1.00E+00
LOH3CR2A	LMNA	0.127933674	-0.010299225	0.261368547	6.96E-02	1.12E-01	1.00E+00
TMEM53	LMNA	0.224712575	0.089436041	0.3518486	1.30E-03	3.32E-03	1.00E+00
SLC9A3R2	LMNA	0.221763146	0.086355899	0.349125792	1.51E-03	3.78E-03	1.00E+00
TIMP1	LMNA	0.671890188	0.588417962	0.741191855	6.98E-28	1.45E-25	8.28E-24
PTPN9	LMNA	0.521066956	0.412703276	0.614886803	1.87E-15	4.23E-14	2.21E-11
ABCA12	LMNA	0.178627957	0.041602874	0.309057667	1.10E-02	2.20E-02	1.00E+00
TPST2	LMNA	0.46601101	0.350509433	0.567549675	2.77E-12	3.59E-11	3.28E-08
AQP6	LMNA	-0.178401464	-0.308846049	-0.041369328	1.11E-02	2.22E-02	1.00E+00
KCNIP1	LMNA	-0.098445445	-0.233325438	0.040151231	1.63E-01	2.31E-01	1.00E+00
YES1	LMNA	0.05928124	-0.079419718	0.195730416	4.02E-01	4.90E-01	1.00E+00
ABT1	LMNA	0.122326268	-0.015994842	0.25605323	8.29E-02	1.30E-01	1.00E+00
RIPK5	LMNA	-0.067246035	-0.203408714	0.071468435	3.42E-01	4.28E-01	1.00E+00
SMG1	LMNA	-0.189765592	-0.31944791	-0.053105822	6.83E-03	1.44E-02	1.00E+00
IL13	LMNA	0.055832255	-0.082857386	0.192400292	4.30E-01	5.17E-01	1.00E+00
MDFI	LMNA	-0.100647281	-0.235427149	0.037930747	1.54E-01	2.20E-01	1.00E+00
PIP5K1C	LMNA	0.208569016	0.072608648	0.336919052	2.89E-03	6.73E-03	1.00E+00
POU2F2	LMNA	0.19245433	0.055888196	0.321951557	6.07E-03	1.30E-02	1.00E+00
TSPAN14	LMNA	0.150430783	0.012642327	0.282612731	3.26E-02	5.77E-02	1.00E+00
GPT	LMNA	-0.12954238	-0.262891964	0.008663555	6.61E-02	1.07E-01	1.00E+00
PDK4	LMNA	-0.194950413	-0.32427419	-0.058473105	5.43E-03	1.18E-02	1.00E+00
CLIC1	LMNA	0.690043383	0.61011241	0.756070241	6.75E-30	1.91E-27	8.01E-26
LILRA5	LMNA	0.341622665	0.213647573	0.458070445	6.50E-07	3.22E-06	7.71E-03
TREML2	LMNA	0.22907514	0.093996695	0.355872025	1.04E-03	2.72E-03	1.00E+00
ELL3	LMNA	0.116240298	-0.022166409	0.250275112	9.95E-02	1.51E-01	1.00E+00
NNMT	LMNA	0.525350688	0.417585127	0.618541826	9.98E-16	2.41E-14	1.18E-11
NUFIP1	LMNA	-0.13187116	-0.265096101	0.00629443	6.14E-02	1.00E-01	1.00E+00
ZNF74	LMNA	-0.388774352	-0.499990517	-0.264943026	1.08E-08	7.31E-08	1.28E-04
RHBDL1	LMNA	0.167587757	0.030236292	0.2987275	1.71E-02	3.25E-02	1.00E+00
HYAL2	LMNA	0.379206369	0.254477204	0.491526069	2.62E-08	1.66E-07	3.10E-04
IGFBP5	LMNA	0.347424457	0.219921391	0.463256608	4.07E-07	2.09E-06	4.82E-03
FAM29A	LMNA	-0.170835156	-0.301769242	-0.033576011	1.51E-02	2.90E-02	1.00E+00

NRTN	LMNA	-0.116501896	-0.250523674	0.021901348	9.87E-02	1.50E-01	1.00E+00
KIAA0556	LMNA	-0.190824527	-0.320434164	-0.054201381	6.52E-03	1.38E-02	1.00E+00
JMJD2A	LMNA	-0.076402072	-0.212214762	0.062306095	2.80E-01	3.63E-01	1.00E+00
ERGIC3	LMNA	0.248789526	0.114677187	0.373995414	3.57E-04	1.04E-03	1.00E+00
POLD4	LMNA	0.501892493	0.390927599	0.598476963	2.76E-14	4.82E-13	3.27E-10
EPHB1	LMNA	-0.511025904	-0.606303617	-0.401284583	7.80E-15	1.55E-13	9.26E-11
LRRC36	LMNA	-0.253192975	-0.378030471	-0.119312364	2.77E-04	8.29E-04	1.00E+00
TARBP1	LMNA	-0.28187997	-0.404201932	-0.149652519	4.82E-05	1.68E-04	5.72E-01
ANAPC10	LMNA	0.065020088	-0.073692382	0.201264521	3.58E-01	4.45E-01	1.00E+00
C1orf9	LMNA	0.102248558	-0.036315048	0.236954819	1.48E-01	2.12E-01	1.00E+00
COLEC12	LMNA	0.297210331	0.165969071	0.418106351	1.74E-05	6.61E-05	2.07E-01
TNFRSF25	LMNA	-0.074150861	-0.210051647	0.064561025	2.94E-01	3.78E-01	1.00E+00
MEGF6	LMNA	-0.153914619	-0.285890989	-0.016208007	2.87E-02	5.15E-02	1.00E+00
LPHN3	LMNA	-0.445398268	-0.549652418	-0.327483464	3.10E-11	3.33E-10	3.67E-07
BMP10	LMNA	-0.174221903	-0.304938669	-0.037062307	1.31E-02	2.58E-02	1.00E+00
C21orf55	LMNA	-0.275057854	-0.39799614	-0.142414623	7.44E-05	2.50E-04	8.83E-01
SLC2A1	LMNA	0.380078873	0.255430372	0.492298822	2.42E-08	1.54E-07	2.87E-04
CER1	LMNA	0.009503346	-0.128716523	0.147361017	8.93E-01	9.23E-01	1.00E+00
LSAMP	LMNA	-0.462380804	-0.564404652	-0.346444067	4.28E-12	5.37E-11	5.08E-08
RPH3A	LMNA	-0.145135875	-0.277624352	-0.007229731	3.93E-02	6.79E-02	1.00E+00
CREM	LMNA	0.124569416	-0.013717484	0.258180504	7.73E-02	1.22E-01	1.00E+00
SGK	LMNA	0.002856981	-0.135247363	0.140852428	9.68E-01	9.76E-01	1.00E+00
ATP2A3	LMNA	0.311748716	0.181509375	0.431240331	6.29E-06	2.58E-05	7.46E-02
PTGER4	LMNA	0.419547397	0.298802733	0.527071005	5.13E-10	4.47E-09	6.09E-06
CCR7	LMNA	0.348394406	0.220971283	0.464122862	3.75E-07	1.95E-06	4.45E-03
METAP1	LMNA	-0.068884308	-0.204985984	0.069830752	3.30E-01	4.16E-01	1.00E+00
KCNQ1	LMNA	0.313568062	0.183458692	0.432880371	5.52E-06	2.29E-05	6.54E-02
RECQL5	LMNA	-0.080558091	-0.216204653	0.058139486	2.54E-01	3.35E-01	1.00E+00
SSFA2	LMNA	0.224127452	0.088824781	0.351308607	1.34E-03	3.41E-03	1.00E+00
NR2F2	LMNA	0.128030689	-0.010200605	0.261460438	6.94E-02	1.11E-01	1.00E+00
MTERFD1	LMNA	-0.032453233	-0.169743747	0.106072996	6.47E-01	7.19E-01	1.00E+00
BCL2A1	LMNA	0.275146216	0.142508279	0.398076591	7.40E-05	2.48E-04	8.78E-01
ZBTB24	LMNA	-0.006621637	-0.14454051	0.131549616	9.25E-01	9.46E-01	1.00E+00
NLRX1	LMNA	0.339342495	0.211184777	0.456030052	7.80E-07	3.81E-06	9.25E-03
FHOD3	LMNA	-0.264404135	-0.388282343	-0.131139905	1.43E-04	4.56E-04	1.00E+00

PRDM1	LMNA	0.52773988	0.420310617	0.620578614	7.02E-16	1.73E-14	8.32E-12
PSG7	LMNA	-0.0060606	-0.143991128	0.132100925	9.32E-01	9.50E-01	1.00E+00
ARHGEF5	LMNA	0.314920026	0.184907901	0.43409858	5.00E-06	2.09E-05	5.93E-02
CCDC47	LMNA	0.167366307	0.030008659	0.298519978	1.73E-02	3.28E-02	1.00E+00
FGD2	LMNA	0.135549723	-0.002548976	0.268574941	5.44E-02	9.03E-02	1.00E+00
SLC26A6	LMNA	0.195738892	0.059290024	0.325007555	5.24E-03	1.14E-02	1.00E+00
BIN1	LMNA	-0.187384325	-0.317229056	-0.050643398	7.58E-03	1.58E-02	1.00E+00
SRRM1	LMNA	0.028596726	-0.109888092	0.16599242	6.86E-01	7.53E-01	1.00E+00
P2RY14	LMNA	-0.35726769	-0.472037264	-0.230589638	1.79E-07	9.88E-07	2.13E-03
PCSK1N	LMNA	-0.404676979	-0.514012145	-0.282402705	2.32E-09	1.79E-08	2.76E-05
PPP1CA	LMNA	0.250739283	0.116728829	0.37578264	3.19E-04	9.42E-04	1.00E+00
ZNF33B	LMNA	-0.280975775	-0.403380071	-0.148692402	5.11E-05	1.77E-04	6.06E-01
MOCS1	LMNA	0.143706847	0.005770323	0.276276827	4.13E-02	7.09E-02	1.00E+00
NAP1L1	LMNA	-0.166049605	-0.297285829	-0.028655483	1.82E-02	3.43E-02	1.00E+00
ECT2	LMNA	-0.048029358	-0.184854679	0.090622516	4.97E-01	5.82E-01	1.00E+00
CACNA2D2	LMNA	-0.476618114	-0.576722176	-0.362413024	7.49E-13	1.06E-11	8.88E-09
PTDSS1	LMNA	0.015463646	-0.12284961	0.153187626	8.27E-01	8.70E-01	1.00E+00
DOCK6	LMNA	0.360887999	0.23452106	0.475261043	1.32E-07	7.40E-07	1.56E-03
SLC38A6	LMNA	0.370986323	0.245509078	0.484237104	5.47E-08	3.30E-07	6.49E-04
C10orf119	LMNA	0.030219606	-0.108283135	0.167571524	6.69E-01	7.39E-01	1.00E+00
CCR4	LMNA	0.290669955	0.158999158	0.412181244	2.71E-05	9.91E-05	3.21E-01
COX6A1	LMNA	-0.410258595	-0.518919741	-0.288550094	1.33E-09	1.07E-08	1.58E-05
B3GALT2	LMNA	-0.546326862	-0.636381363	-0.441580274	4.10E-17	1.29E-15	4.86E-13
CD14	LMNA	0.348683328	0.221284075	0.464380853	3.67E-07	1.91E-06	4.35E-03
ABCC9	LMNA	-0.030302809	-0.167652464	0.108200832	6.69E-01	7.38E-01	1.00E+00
SNAP29	LMNA	0.099181828	-0.039408763	0.234028476	1.60E-01	2.27E-01	1.00E+00
TRIP6	LMNA	0.565110446	0.463195183	0.652274837	1.93E-18	7.70E-17	2.30E-14
HMGCR	LMNA	-0.285749264	-0.407716669	-0.153763939	3.75E-05	1.33E-04	4.45E-01
CDH3	LMNA	-0.001041815	-0.139072814	0.137028893	9.88E-01	9.91E-01	1.00E+00
KLHDC8A	LMNA	0.034547349	-0.103999657	0.171779084	6.25E-01	7.00E-01	1.00E+00
IFT74	LMNA	-0.096689107	-0.231648062	0.041921463	1.71E-01	2.40E-01	1.00E+00
C9orf116	LMNA	0.189222291	0.052543858	0.31894179	7.00E-03	1.47E-02	1.00E+00
EI24	LMNA	0.088420072	-0.050244234	0.223739995	2.11E-01	2.86E-01	1.00E+00
CENTD1	LMNA	0.011241168	-0.127006929	0.149060848	8.74E-01	9.06E-01	1.00E+00
RWDD2B	LMNA	-0.041563992	-0.17859025	0.097043842	5.57E-01	6.38E-01	1.00E+00

INSL6	LMNA	0.004715722	-0.133422137	0.142673841	9.47E-01	9.60E-01	1.00E+00
HERC1	LMNA	-0.327499281	-0.44541247	-0.198419128	1.96E-06	8.89E-06	2.32E-02
NPAS2	LMNA	0.405777657	0.283614158	0.514980476	2.08E-09	1.62E-08	2.47E-05
NR3C2	LMNA	-0.109527731	-0.243890999	0.028961175	1.21E-01	1.79E-01	1.00E+00
DOCK1	LMNA	0.160859283	0.023326284	0.29241665	2.22E-02	4.10E-02	1.00E+00
FAM63A	LMNA	-0.201436031	-0.330301851	-0.065198088	4.04E-03	9.05E-03	1.00E+00
FAM111A	LMNA	0.184497211	0.047660115	0.314536948	8.58E-03	1.77E-02	1.00E+00
INPP5F	LMNA	-0.132123251	-0.265334615	0.006037879	6.09E-02	9.95E-02	1.00E+00
MYBL1	LMNA	-0.173305757	-0.304081595	-0.036118902	1.36E-02	2.67E-02	1.00E+00
HOXB1	LMNA	0.313741359	0.183644422	0.433036547	5.45E-06	2.27E-05	6.46E-02
CRADD	LMNA	-0.128016265	-0.261446776	0.010215268	6.94E-02	1.11E-01	1.00E+00
EMCN	LMNA	-0.097837347	-0.23274477	0.040764238	1.66E-01	2.34E-01	1.00E+00
DUSP12	LMNA	-0.071103575	-0.207121488	0.067611091	3.15E-01	4.00E-01	1.00E+00
BLNK	LMNA	-0.036421109	-0.173599258	0.102143466	6.07E-01	6.83E-01	1.00E+00
VASH2	LMNA	-0.141148469	-0.27386306	-0.003159024	4.51E-02	7.67E-02	1.00E+00
PDZK1IP1	LMNA	0.164615654	0.027182383	0.295941284	1.92E-02	3.60E-02	1.00E+00
SKP1A	LMNA	-0.215710223	-0.343531203	-0.080042818	2.05E-03	4.96E-03	1.00E+00
IL19	LMNA	-0.036189952	-0.173374762	0.102372508	6.09E-01	6.85E-01	1.00E+00
DOC2A	LMNA	-0.332293388	-0.449714443	-0.203581364	1.36E-06	6.33E-06	1.61E-02
COPB2	LMNA	0.497085477	0.385487867	0.59435029	5.27E-14	8.74E-13	6.26E-10
CTR9	LMNA	0.188790157	0.052096941	0.318539176	7.13E-03	1.50E-02	1.00E+00
VIL1	LMNA	0.123091839	-0.015217756	0.2567794	8.09E-02	1.27E-01	1.00E+00
CDC27	LMNA	-0.014778919	-0.152518744	0.123524101	8.35E-01	8.76E-01	1.00E+00
PTTG1IP	LMNA	0.309106049	0.178679742	0.428856705	7.60E-06	3.07E-05	9.02E-02
LECT1	LMNA	0.007551397	-0.130635788	0.145450769	9.15E-01	9.39E-01	1.00E+00
COPS6	LMNA	0.231603438	0.096642393	0.358201627	9.12E-04	2.42E-03	1.00E+00
COL9A1	LMNA	-0.273758827	-0.396813193	-0.141038031	8.07E-05	2.69E-04	9.57E-01
MCCC1	LMNA	-0.233475309	-0.359925375	-0.098602412	8.26E-04	2.21E-03	1.00E+00
GPC4	LMNA	0.406626504	0.2845487	0.515727065	1.91E-09	1.50E-08	2.27E-05
VAC14	LMNA	0.227768267	0.092629887	0.35466724	1.11E-03	2.89E-03	1.00E+00
PSMD9	LMNA	0.223946275	0.088635531	0.351141386	1.36E-03	3.43E-03	1.00E+00
PPY	LMNA	0.098542445	-0.040053438	0.233418053	1.63E-01	2.30E-01	1.00E+00
SRCAP	LMNA	0.098764377	-0.039829682	0.233629945	1.62E-01	2.29E-01	1.00E+00
PPP1R13L	LMNA	0.33002344	0.201136218	0.447678192	1.61E-06	7.47E-06	1.92E-02
BPGM	LMNA	-0.138045343	-0.270933091	5.77E-06	5.01E-02	8.41E-02	1.00E+00

TTC19	LMNA	-0.258704586	-0.383074307	-0.125122255	2.01E-04	6.17E-04	1.00E+00
GFPT1	LMNA	0.260736227	0.127266158	0.384931652	1.78E-04	5.55E-04	1.00E+00
C1orf114	LMNA	-0.243693527	-0.36931982	-0.109320294	4.74E-04	1.34E-03	1.00E+00
HMOX1	LMNA	0.35816662	0.231565433	0.472838022	1.66E-07	9.18E-07	1.97E-03
SLC27A6	LMNA	0.20318018	0.067008722	0.331921051	3.73E-03	8.42E-03	1.00E+00
CALML5	LMNA	-0.061846417	-0.19820515	0.076860818	3.82E-01	4.69E-01	1.00E+00
MRPS10	LMNA	-0.120928193	-0.25472672	0.017413514	8.65E-02	1.34E-01	1.00E+00
PRR13	LMNA	0.308218886	0.177730296	0.42805613	8.10E-06	3.26E-05	9.61E-02
INS	LMNA	0.317571102	0.187751308	0.436486112	4.12E-06	1.75E-05	4.89E-02
CECR1	LMNA	0.268674611	0.135655169	0.392179366	1.11E-04	3.58E-04	1.00E+00
SERPINB8	LMNA	0.395256914	0.272050516	0.505713356	5.83E-09	4.18E-08	6.91E-05
FLT1	LMNA	0.287445555	0.155567819	0.409256384	3.36E-05	1.21E-04	3.98E-01
FEM1C	LMNA	0.430157643	0.310548138	0.53635774	1.67E-10	1.60E-09	1.98E-06
PDIA2	LMNA	-0.280949354	-0.403356053	-0.148664351	5.12E-05	1.77E-04	6.07E-01
TMED3	LMNA	0.390086452	0.266380529	0.501149631	9.54E-09	6.54E-08	1.13E-04
NUCKS1	LMNA	-0.029851945	-0.167213842	0.108646799	6.73E-01	7.42E-01	1.00E+00
EXT1	LMNA	0.366695443	0.240836215	0.480425996	7.98E-08	4.66E-07	9.47E-04
RCOR1	LMNA	0.17847298	0.041443069	0.30891287	1.10E-02	2.22E-02	1.00E+00
EBI3	LMNA	0.08896239	-0.049698981	0.224259186	2.08E-01	2.83E-01	1.00E+00
SMAD2	LMNA	0.043948662	-0.094676757	0.180902101	5.35E-01	6.17E-01	1.00E+00
ODZ3	LMNA	0.173478865	0.036297142	0.304243557	1.35E-02	2.65E-02	1.00E+00
POLS	LMNA	0.009037099	-0.129175058	0.146904824	8.98E-01	9.26E-01	1.00E+00
NXN	LMNA	-0.247084094	-0.372431384	-0.112883576	3.93E-04	1.13E-03	1.00E+00
PPIH	LMNA	-0.130598609	-0.263891833	0.007589219	6.39E-02	1.04E-01	1.00E+00
FOXJ3	LMNA	0.132508064	-0.005646222	0.265698673	6.01E-02	9.86E-02	1.00E+00
EIF5B	LMNA	0.057478485	-0.081216969	0.193990181	4.16E-01	5.04E-01	1.00E+00
EIF2B4	LMNA	0.045508271	-0.093127802	0.182413267	5.20E-01	6.04E-01	1.00E+00
C20orf121	LMNA	0.20942424	0.073498161	0.337711585	2.78E-03	6.49E-03	1.00E+00
CENPE	LMNA	0.01853802	-0.119819622	0.156189302	7.93E-01	8.44E-01	1.00E+00
KIAA0415	LMNA	0.360945762	0.234583821	0.475312455	1.31E-07	7.38E-07	1.56E-03
CRABP2	LMNA	0.451642903	0.33444443	0.55508452	1.51E-11	1.72E-10	1.80E-07
TSPAN12	LMNA	-0.296022447	-0.417030978	-0.164702198	1.89E-05	7.12E-05	2.24E-01
CXorf15	LMNA	0.028617833	-0.109867223	0.166012963	6.86E-01	7.53E-01	1.00E+00
LOC57228	LMNA	0.499981379	0.388764009	0.596836945	3.57E-14	6.15E-13	4.24E-10
ACTR3B	LMNA	-0.237778184	-0.363884488	-0.103111884	6.56E-04	1.80E-03	1.00E+00

ASL	LMNA	0.571621581	0.470716174	0.657766314	6.41E-19	2.79E-17	7.61E-15
GATA3	LMNA	0.347823868	0.220353686	0.463613346	3.93E-07	2.03E-06	4.67E-03
TFAM	LMNA	-0.304970595	-0.42512327	-0.174256034	1.02E-05	4.02E-05	1.21E-01
PCDHA5	LMNA	-0.19596302	-0.325215988	-0.059522269	5.19E-03	1.13E-02	1.00E+00
PARP12	LMNA	0.317519065	0.187695475	0.436439264	4.13E-06	1.76E-05	4.90E-02
PIGH	LMNA	-0.171633178	-0.302516317	-0.034397188	1.46E-02	2.82E-02	1.00E+00
ENDOGL1	LMNA	-0.183921401	-0.31399978	-0.047065417	8.79E-03	1.81E-02	1.00E+00
GTF2H1	LMNA	0.076435787	-0.062272314	0.212247148	2.80E-01	3.62E-01	1.00E+00
MPZ	LMNA	-0.053380643	-0.190031255	0.085298947	4.51E-01	5.37E-01	1.00E+00
BIRC4	LMNA	0.191533307	0.054934859	0.321094141	6.32E-03	1.35E-02	1.00E+00
SSBP3	LMNA	-0.395228912	-0.505688656	-0.272019786	5.84E-09	4.19E-08	6.93E-05
BPESC1	LMNA	0.134426547	-0.003692987	0.267513115	5.65E-02	9.33E-02	1.00E+00
FOXC2	LMNA	0.299584777	0.168502708	0.420254885	1.48E-05	5.69E-05	1.76E-01
HS3ST3B1	LMNA	0.40753671	0.285551055	0.516527438	1.75E-09	1.38E-08	2.07E-05
GDNF	LMNA	-0.056626916	-0.193167845	0.082065625	4.23E-01	5.10E-01	1.00E+00
CTNS	LMNA	0.210632448	0.07475518	0.338830917	2.62E-03	6.16E-03	1.00E+00
KIAA0859	LMNA	0.094858504	-0.043765621	0.229898907	1.79E-01	2.49E-01	1.00E+00
TRPC5	LMNA	-0.236057959	-0.362302241	-0.101308401	7.19E-04	1.96E-03	1.00E+00
PMS2L3	LMNA	0.018249914	-0.120103678	0.155908117	7.97E-01	8.47E-01	1.00E+00
TEP1	LMNA	0.418965306	0.298159422	0.526560779	5.45E-10	4.74E-09	6.47E-06
KIDINS220	LMNA	-0.204139747	-0.332811553	-0.068005249	3.57E-03	8.09E-03	1.00E+00
CRH	LMNA	-0.099318975	-0.234159397	0.039270466	1.60E-01	2.27E-01	1.00E+00
CES3	LMNA	0.123450122	-0.014854028	0.257119191	8.01E-02	1.26E-01	1.00E+00
ACP5	LMNA	0.355981579	0.229193999	0.47089128	2.00E-07	1.09E-06	2.37E-03
AMFR	LMNA	0.125087243	-0.013191555	0.258671397	7.61E-02	1.21E-01	1.00E+00
CA4	LMNA	-0.248337505	-0.37358094	-0.114201709	3.66E-04	1.06E-03	1.00E+00
PLCB4	LMNA	-0.511625605	-0.606816871	-0.401965606	7.17E-15	1.43E-13	8.51E-11
MPHOSPH:	LMNA	0.207708973	0.071714341	0.33612187	3.02E-03	6.98E-03	1.00E+00
PGF	LMNA	-0.025983458	-0.163448163	0.112470981	7.14E-01	7.76E-01	1.00E+00
G3BP2	LMNA	-0.200378389	-0.329319606	-0.064100566	4.25E-03	9.46E-03	1.00E+00
SR140	LMNA	-0.149229201	-0.281481337	-0.011413329	3.40E-02	5.98E-02	1.00E+00
ISLR	LMNA	0.348245759	0.220810366	0.463990122	3.80E-07	1.97E-06	4.51E-03
HOXA2	LMNA	-0.171149119	-0.30206318	-0.033899062	1.49E-02	2.87E-02	1.00E+00
PYGB	LMNA	0.326354066	0.197187032	0.444384009	2.14E-06	9.65E-06	2.53E-02
BAT1	LMNA	-0.123207258	-0.256888865	0.015100587	8.07E-02	1.27E-01	1.00E+00

TSC1	LMNA	-0.209738325	-0.3380026	-0.073824893	2.74E-03	6.41E-03	1.00E+00
NARF	LMNA	-0.167887954	-0.299008798	-0.030544895	1.69E-02	3.22E-02	1.00E+00
DKK3	LMNA	0.147091006	0.009227377	0.279467131	3.67E-02	6.39E-02	1.00E+00
UTP18	LMNA	-0.191305982	-0.320882482	-0.054699597	6.38E-03	1.36E-02	1.00E+00
TSKS	LMNA	0.192978838	0.056431218	0.322439747	5.93E-03	1.27E-02	1.00E+00
DDX31	LMNA	0.160371879	0.02282624	0.291959052	2.26E-02	4.17E-02	1.00E+00
TULP1	LMNA	0.176793153	0.039711367	0.307342996	1.18E-02	2.36E-02	1.00E+00
TNRC4	LMNA	-0.560206202	-0.648132558	-0.457539983	4.38E-18	1.60E-16	5.19E-14
ZNF430	LMNA	-0.06545387	-0.201682473	0.073259098	3.55E-01	4.41E-01	1.00E+00
POSTN	LMNA	0.582916563	0.483797847	0.667270804	8.90E-20	4.42E-18	1.06E-15
AASS	LMNA	0.059446582	-0.079254836	0.19588998	4.01E-01	4.88E-01	1.00E+00
APOL5	LMNA	-0.044778568	-0.18170631	0.093852604	5.27E-01	6.10E-01	1.00E+00
PLA2G1B	LMNA	-0.086846065	-0.222232677	0.05182629	2.19E-01	2.95E-01	1.00E+00
FLJ11506	LMNA	0.312084215	0.181868764	0.431542824	6.14E-06	2.53E-05	7.28E-02
GTF2A2	LMNA	-0.094278454	-0.229344483	0.044349769	1.82E-01	2.53E-01	1.00E+00
RCHY1	LMNA	-0.300821498	-0.421373406	-0.169823029	1.36E-05	5.26E-05	1.61E-01
CYP27B1	LMNA	-0.154507011	-0.286448117	-0.016814664	2.81E-02	5.05E-02	1.00E+00
VPREB1	LMNA	-0.140661968	-0.273403869	-0.002662672	4.59E-02	7.78E-02	1.00E+00
MGC4294	LMNA	0.440659604	0.32220975	0.545524444	5.27E-11	5.53E-10	6.25E-07
TACC3	LMNA	0.171397146	0.034154289	0.30229537	1.47E-02	2.84E-02	1.00E+00
PDCL	LMNA	-0.112361794	-0.246587809	0.026093967	1.11E-01	1.67E-01	1.00E+00
DMXL2	LMNA	-0.412550188	-0.520932535	-0.291076879	1.05E-09	8.67E-09	1.25E-05
EID1	LMNA	-0.09764536	-0.232561424	0.040957753	1.67E-01	2.35E-01	1.00E+00
MS4A4A	LMNA	0.264332485	0.131064196	0.388216921	1.44E-04	4.58E-04	1.00E+00
RBM14	LMNA	-0.108323811	-0.242744755	0.030178485	1.25E-01	1.84E-01	1.00E+00
MGC29506	LMNA	0.034203797	-0.104339883	0.171445256	6.29E-01	7.03E-01	1.00E+00
PPP2R5B	LMNA	-0.087777424	-0.223124653	0.050890252	2.14E-01	2.90E-01	1.00E+00
TNFSF11	LMNA	0.246904859	0.112695123	0.372266967	3.96E-04	1.14E-03	1.00E+00
ATG2A	LMNA	0.086130558	-0.052545227	0.221547271	2.23E-01	3.00E-01	1.00E+00
RPGRIP1L	LMNA	-0.061498087	-0.197869203	0.077208403	3.85E-01	4.72E-01	1.00E+00
SPOP	LMNA	-0.397749491	-0.507911231	-0.274786965	4.58E-09	3.35E-08	5.44E-05
OSGIN1	LMNA	0.273561772	0.140829256	0.396633712	8.17E-05	2.72E-04	9.69E-01
PTPRF	LMNA	0.093890332	-0.04474058	0.228973458	1.84E-01	2.55E-01	1.00E+00
ICMT	LMNA	0.468164607	0.352923245	0.569414041	2.13E-12	2.80E-11	2.53E-08
SEC24B	LMNA	0.055683776	-0.083005303	0.192256859	4.31E-01	5.18E-01	1.00E+00

MAML1	LMNA	0.214406355	0.078684333	0.342324878	2.18E-03	5.25E-03	1.00E+00
POLL	LMNA	-0.09805304	-0.232950744	0.040546816	1.65E-01	2.33E-01	1.00E+00
FXR2	LMNA	-0.230754311	-0.357419407	-0.095753624	9.53E-04	2.51E-03	1.00E+00
TYK2	LMNA	0.251405952	0.117430597	0.376393522	3.07E-04	9.09E-04	1.00E+00
MUC6	LMNA	-0.029478268	-0.166850271	0.109016375	6.77E-01	7.46E-01	1.00E+00
ADAM28	LMNA	0.056717724	-0.081975137	0.193255546	4.23E-01	5.10E-01	1.00E+00
MYL3	LMNA	-0.236219389	-0.362450753	-0.101477606	7.13E-04	1.94E-03	1.00E+00
NRL	LMNA	-0.2143045	-0.342230625	-0.078578233	2.19E-03	5.27E-03	1.00E+00
PIP4K2A	LMNA	-0.097833536	-0.232741131	0.040768079	1.66E-01	2.34E-01	1.00E+00
MED7	LMNA	0.057366601	-0.081328482	0.193882149	4.17E-01	5.05E-01	1.00E+00
MYLK	LMNA	0.332579289	0.203889444	0.449970822	1.33E-06	6.21E-06	1.57E-02
RRP1	LMNA	0.301892652	0.17096697	0.422341886	1.26E-05	4.91E-05	1.50E-01
TFAP4	LMNA	-0.165102381	-0.296397722	-0.027682331	1.89E-02	3.54E-02	1.00E+00
CYP4F2	LMNA	0.116866756	-0.021531627	0.250870322	9.76E-02	1.49E-01	1.00E+00
UNC5C	LMNA	0.281339	0.149078062	0.403710246	4.99E-05	1.73E-04	5.92E-01
ARL4D	LMNA	0.016764341	-0.121568009	0.154457874	8.13E-01	8.59E-01	1.00E+00
SH3BP5	LMNA	0.432604142	0.313261572	0.538495424	1.28E-10	1.25E-09	1.52E-06
AKAP6	LMNA	-0.514360973	-0.609156929	-0.405073446	4.88E-15	1.01E-13	5.78E-11
CTLA4	LMNA	0.077533438	-0.061172325	0.213301353	2.73E-01	3.55E-01	1.00E+00
ACSBG1	LMNA	-0.047843364	-0.184674621	0.090807403	4.99E-01	5.84E-01	1.00E+00
MTMR4	LMNA	-0.276915853	-0.3996874	-0.144384456	6.62E-05	2.24E-04	7.85E-01
NECAP1	LMNA	-0.419126952	-0.526702476	-0.298338058	5.36E-10	4.66E-09	6.36E-06
SLC25A17	LMNA	-0.156828056	-0.288630136	-0.01919258	2.58E-02	4.68E-02	1.00E+00
POLR2F	LMNA	-0.402048536	-0.511698624	-0.279511308	3.01E-09	2.28E-08	3.57E-05
PLA2G2D	LMNA	-0.112196843	-0.246430904	0.026260909	1.12E-01	1.67E-01	1.00E+00
WNT2	LMNA	0.323761259	0.194399033	0.442054389	2.60E-06	1.15E-05	3.08E-02
GLMN	LMNA	-0.097546135	-0.23246666	0.041057764	1.67E-01	2.35E-01	1.00E+00
MCM7	LMNA	-0.02917561	-0.16655577	0.109315684	6.80E-01	7.48E-01	1.00E+00
TRIM52	LMNA	-0.066799282	-0.202978474	0.071914897	3.45E-01	4.31E-01	1.00E+00
DCLRE1A	LMNA	-0.392601086	-0.503369952	-0.269137036	7.51E-09	5.27E-08	8.91E-05
PDX1	LMNA	0.127711356	-0.010525211	0.261157964	7.01E-02	1.12E-01	1.00E+00
UBQLN3	LMNA	0.004108879	-0.134018141	0.142079287	9.54E-01	9.66E-01	1.00E+00
PRPF31	LMNA	0.107937003	-0.030569508	0.242376399	1.26E-01	1.86E-01	1.00E+00
TLR7	LMNA	0.098491944	-0.040104352	0.233369836	1.63E-01	2.31E-01	1.00E+00
SMAD1	LMNA	0.244873505	0.110559988	0.370403019	4.44E-04	1.27E-03	1.00E+00

CLCN1	LMNA	0.086601889	-0.052071654	0.221998788	2.20E-01	2.97E-01	1.00E+00
RIOK2	LMNA	0.247510668	0.113332123	0.372822656	3.83E-04	1.11E-03	1.00E+00
CEACAM21	LMNA	0.129973436	-0.008225148	0.263300054	6.52E-02	1.06E-01	1.00E+00
PDLIM4	LMNA	0.475917941	0.361626119	0.576117477	8.17E-13	1.15E-11	9.70E-09
STX18	LMNA	-0.038542724	-0.175659067	0.100040578	5.86E-01	6.64E-01	1.00E+00
CCPG1	LMNA	0.230928239	0.095935655	0.357579645	9.45E-04	2.49E-03	1.00E+00
SLC2A6	LMNA	0.027506716	-0.110965658	0.164931418	6.98E-01	7.62E-01	1.00E+00
SORCS3	LMNA	-0.457880981	-0.560502122	-0.341410879	7.31E-12	8.84E-11	8.67E-08
NOLA3	LMNA	0.12600596	-0.012258276	0.25954216	7.40E-02	1.18E-01	1.00E+00
PDGFA	LMNA	0.289128773	0.157358668	0.410783547	3.00E-05	1.09E-04	3.56E-01
TRDMT1	LMNA	-0.192018452	-0.321545807	-0.055436994	6.19E-03	1.32E-02	1.00E+00
CFL1	LMNA	0.082405379	-0.056285938	0.217976646	2.44E-01	3.23E-01	1.00E+00
IL4	LMNA	0.316897707	0.187028853	0.435879817	4.33E-06	1.83E-05	5.13E-02
NAT2	LMNA	-0.197971092	-0.327082883	-0.061603728	4.74E-03	1.04E-02	1.00E+00
CPSF6	LMNA	-0.056232588	-0.19278699	0.082458535	4.27E-01	5.13E-01	1.00E+00
PRG2	LMNA	-0.168062413	-0.299172263	-0.030724252	1.68E-02	3.20E-02	1.00E+00
PIGQ	LMNA	0.159103677	0.021525471	0.290768124	2.37E-02	4.35E-02	1.00E+00
CLSTN3	LMNA	-0.18965687	-0.319346634	-0.052993358	6.86E-03	1.45E-02	1.00E+00
KIAA0146	LMNA	-0.045607441	-0.182509335	0.093029286	5.19E-01	6.03E-01	1.00E+00
GBP1	LMNA	0.378287704	0.253473868	0.490712241	2.84E-08	1.79E-07	3.37E-04
CEP55	LMNA	0.360850796	0.23448064	0.475227931	1.32E-07	7.42E-07	1.57E-03
ZNF408	LMNA	0.199760609	0.063459644	0.328745738	4.37E-03	9.71E-03	1.00E+00
KRT20	LMNA	-0.035748563	-0.172946053	0.102809817	6.13E-01	6.89E-01	1.00E+00
EYA3	LMNA	0.170277497	0.03300228	0.301247089	1.54E-02	2.96E-02	1.00E+00
KRT38	LMNA	0.054234188	-0.08444909	0.190856232	4.43E-01	5.30E-01	1.00E+00
WDR7	LMNA	-0.293131073	-0.414412046	-0.16162037	2.30E-05	8.53E-05	2.73E-01
BLCAP	LMNA	-0.256607033	-0.381155649	-0.122910105	2.27E-04	6.91E-04	1.00E+00
HLA-DPB1	LMNA	0.250876738	0.116873509	0.375908601	3.17E-04	9.35E-04	1.00E+00
SFI1	LMNA	-0.17583326	-0.306445612	-0.0387222	1.23E-02	2.44E-02	1.00E+00
GNE	LMNA	-0.169872195	-0.300867543	-0.032585354	1.57E-02	3.00E-02	1.00E+00
MGAT4C	LMNA	-0.360085069	-0.474546323	-0.233648774	1.41E-07	7.89E-07	1.68E-03
SLC35A2	LMNA	0.595954761	0.49895376	0.678208121	8.26E-21	4.69E-19	9.80E-17
TUSC3	LMNA	-0.179032546	-0.309435652	-0.042020098	1.08E-02	2.17E-02	1.00E+00
GABRD	LMNA	-0.278423582	-0.401059209	-0.145983707	6.02E-05	2.05E-04	7.13E-01
IARS	LMNA	0.029659108	-0.108837525	0.167026224	6.75E-01	7.44E-01	1.00E+00

ARFIP1	LMNA	0.173027018	0.035831918	0.303820787	1.38E-02	2.69E-02	1.00E+00
SFRS9	LMNA	0.120065246	-0.018288895	0.253907694	8.88E-02	1.37E-01	1.00E+00
CEP110	LMNA	-0.08365163	-0.219171595	0.055034924	2.37E-01	3.15E-01	1.00E+00
MYF6	LMNA	0.127850118	-0.010384162	0.261289403	6.98E-02	1.12E-01	1.00E+00
MGST2	LMNA	0.234779908	0.099969064	0.361126229	7.70E-04	2.08E-03	1.00E+00
EVI2B	LMNA	0.173866306	0.036696101	0.304606026	1.33E-02	2.62E-02	1.00E+00
TRPV4	LMNA	0.189503059	0.05283426	0.319203353	6.91E-03	1.46E-02	1.00E+00
SLC25A11	LMNA	-0.130862031	-0.264141154	0.007321232	6.34E-02	1.03E-01	1.00E+00
EHD4	LMNA	0.581548657	0.482211199	0.666121201	1.13E-19	5.45E-18	1.35E-15
NOX5	LMNA	-0.003728709	-0.141706766	0.13439147	9.58E-01	9.69E-01	1.00E+00
NCKAP1L	LMNA	0.264511669	0.131253536	0.388380528	1.42E-04	4.54E-04	1.00E+00
EMP3	LMNA	0.673299388	0.590097771	0.74234928	4.93E-28	1.08E-25	5.84E-24
SYNCRIP	LMNA	0.167850527	0.030506419	0.298973729	1.70E-02	3.23E-02	1.00E+00
C1orf38	LMNA	0.366615337	0.240749033	0.480354805	8.04E-08	4.69E-07	9.53E-04
ZNF426	LMNA	0.059934534	-0.078768196	0.196360841	3.97E-01	4.84E-01	1.00E+00
ELOVL2	LMNA	0.179454325	0.0424551	0.309829652	1.06E-02	2.14E-02	1.00E+00
ATP5J	LMNA	-0.117786408	-0.251743919	0.020599552	9.50E-02	1.46E-01	1.00E+00
CBX7	LMNA	-0.109891584	-0.244237347	0.028593194	1.19E-01	1.77E-01	1.00E+00
OSBPL1A	LMNA	-0.10614489	-0.240669265	0.032380597	1.33E-01	1.94E-01	1.00E+00
MED13	LMNA	-0.238444104	-0.364496801	-0.103810273	6.32E-04	1.74E-03	1.00E+00
ZNF589	LMNA	-0.155056953	-0.286965242	-0.01737794	2.76E-02	4.96E-02	1.00E+00
CHRNB3	LMNA	0.014293936	-0.124001757	0.152044907	8.40E-01	8.81E-01	1.00E+00
GOLGA2	LMNA	0.322032729	0.192541536	0.440500428	2.96E-06	1.30E-05	3.51E-02
NIF3L1	LMNA	-0.328296827	-0.446128522	-0.199277418	1.84E-06	8.42E-06	2.19E-02
TRA2A	LMNA	-0.136621193	-0.269587585	0.001457293	5.25E-02	8.76E-02	1.00E+00
F2R	LMNA	0.336446041	0.208058682	0.453436411	9.80E-07	4.71E-06	1.16E-02
PHF2	LMNA	-0.135344555	-0.268381004	0.002757977	5.48E-02	9.09E-02	1.00E+00
PID1	LMNA	-0.489194691	-0.587565173	-0.376575254	1.50E-13	2.36E-12	1.78E-09
MTAP	LMNA	-0.026688411	-0.16413468	0.111774407	7.06E-01	7.70E-01	1.00E+00
RFC1	LMNA	0.054603575	-0.084081236	0.191213196	4.40E-01	5.27E-01	1.00E+00
C5orf3	LMNA	0.134556374	-0.003560771	0.267635866	5.62E-02	9.29E-02	1.00E+00
ADORA3	LMNA	0.095976011	-0.042639951	0.230966798	1.74E-01	2.44E-01	1.00E+00
ACTL7A	LMNA	0.045286357	-0.09334824	0.182198286	5.22E-01	6.05E-01	1.00E+00
RAI2	LMNA	-0.468846959	-0.570004535	-0.353688364	1.96E-12	2.60E-11	2.32E-08
MAP2K7	LMNA	0.28733516	0.155450396	0.4091562	3.38E-05	1.21E-04	4.01E-01

HYAL4	LMNA	-0.283765415	-0.405915059	-0.151655377	4.27E-05	1.50E-04	5.06E-01
GZMB	LMNA	0.325523305	0.196293503	0.44363775	2.28E-06	1.02E-05	2.70E-02
BMP1	LMNA	0.606104443	0.510793209	0.686697206	1.20E-21	7.71E-20	1.43E-17
CPNE6	LMNA	-0.275777405	-0.398651217	-0.143177359	7.11E-05	2.39E-04	8.44E-01
SP2	LMNA	-0.160093771	-0.291697925	-0.02254095	2.28E-02	4.21E-02	1.00E+00
DPF1	LMNA	-0.491543505	-0.589586314	-0.379226053	1.10E-13	1.76E-12	1.31E-09
SMPD3	LMNA	-0.522987325	-0.616525825	-0.414891008	1.41E-15	3.25E-14	1.67E-11
TMEM38B	LMNA	0.006448044	-0.131720209	0.144370532	9.27E-01	9.47E-01	1.00E+00
CCDC56	LMNA	-0.416742557	-0.524611738	-0.295703913	6.86E-10	5.85E-09	8.14E-06
NFE2L1	LMNA	0.363611031	0.237480823	0.477683801	1.04E-07	5.96E-07	1.24E-03
MRPS31	LMNA	-0.238329711	-0.364391624	-0.103690293	6.36E-04	1.75E-03	1.00E+00
STIP1	LMNA	0.141255482	0.003268213	0.273964058	4.49E-02	7.64E-02	1.00E+00
MMP26	LMNA	0.067172184	-0.071542241	0.203337597	3.42E-01	4.28E-01	1.00E+00
RASL11B	LMNA	-0.314577183	-0.433789697	-0.184540345	5.13E-06	2.14E-05	6.08E-02
NT5DC2	LMNA	0.117236895	-0.021156516	0.251221951	9.66E-02	1.48E-01	1.00E+00
HLA-G	LMNA	0.445901476	0.328043923	0.550090478	2.92E-11	3.17E-10	3.47E-07
LRP2	LMNA	-0.13024778	-0.263559756	0.007946098	6.47E-02	1.05E-01	1.00E+00
MTDH	LMNA	0.35292535	0.225879586	0.468166485	2.58E-07	1.38E-06	3.06E-03
HSP90AB1	LMNA	0.100799074	-0.037777618	0.235571993	1.53E-01	2.19E-01	1.00E+00
PMAIP1	LMNA	0.367703819	0.241933834	0.481322009	7.31E-08	4.30E-07	8.67E-04
LMBR1L	LMNA	-0.226699965	-0.353682076	-0.091512968	1.18E-03	3.03E-03	1.00E+00
SLC25A20	LMNA	0.151671547	0.013911838	0.283780632	3.12E-02	5.54E-02	1.00E+00
RSBN1	LMNA	-0.450642086	-0.554214519	-0.333327947	1.70E-11	1.91E-10	2.02E-07
S100A2	LMNA	0.354217548	0.227280588	0.469318818	2.32E-07	1.25E-06	2.75E-03
C2	LMNA	0.334504551	0.205964731	0.451696787	1.14E-06	5.42E-06	1.35E-02
RHOT2	LMNA	0.056468302	-0.082223673	0.193014656	4.25E-01	5.12E-01	1.00E+00
RALGPS2	LMNA	-0.375931719	-0.488624222	-0.250901966	3.52E-08	2.17E-07	4.17E-04
EIF4EBP1	LMNA	0.268965019	0.135962425	0.392444217	1.09E-04	3.53E-04	1.00E+00
C2orf27	LMNA	-0.453035125	-0.556294388	-0.335998101	1.29E-11	1.49E-10	1.53E-07
GCKR	LMNA	0.311311856	0.181041458	0.430846408	6.49E-06	2.66E-05	7.70E-02
FER	LMNA	0.081041842	-0.057654188	0.216668773	2.52E-01	3.32E-01	1.00E+00
TRPC6	LMNA	0.223339219	0.088001498	0.350581034	1.40E-03	3.53E-03	1.00E+00
RGL2	LMNA	-0.010662118	-0.148494548	0.127576664	8.80E-01	9.12E-01	1.00E+00
ARPC1B	LMNA	0.615345571	0.521604378	0.694407315	1.96E-22	1.53E-20	2.32E-18
SNRK	LMNA	-0.279431833	-0.40197626	-0.147053547	5.64E-05	1.94E-04	6.69E-01

UTP6	LMNA	-0.047145836	-0.183999271	0.0915007	5.05E-01	5.89E-01	1.00E+00
DNM2	LMNA	0.358686334	0.232129701	0.473300891	1.59E-07	8.80E-07	1.89E-03
GCS1	LMNA	0.405119002	0.282889168	0.514401052	2.22E-09	1.72E-08	2.64E-05
EHMT1	LMNA	0.029279344	-0.109213101	0.166656711	6.79E-01	7.48E-01	1.00E+00
GLDC	LMNA	-0.079719949	-0.215400378	0.058980153	2.59E-01	3.41E-01	1.00E+00
FBP1	LMNA	0.336465916	0.208080124	0.453454215	9.79E-07	4.70E-06	1.16E-02
VARS	LMNA	0.093899894	-0.044730953	0.228982599	1.84E-01	2.55E-01	1.00E+00
PLA2G7	LMNA	-0.05247422	-0.18915496	0.086201232	4.58E-01	5.44E-01	1.00E+00
RAX	LMNA	0.024182327	-0.114250085	0.161693531	7.33E-01	7.92E-01	1.00E+00
TERT	LMNA	0.051653853	-0.087017659	0.188361672	4.65E-01	5.51E-01	1.00E+00
CCL1	LMNA	-0.045092565	-0.182010537	0.093540733	5.24E-01	6.07E-01	1.00E+00
FUCA1	LMNA	0.343014059	0.215151206	0.45931492	5.81E-07	2.90E-06	6.89E-03
ALS2CR8	LMNA	-0.257257869	-0.381751091	-0.123596356	2.19E-04	6.68E-04	1.00E+00
CXorf21	LMNA	0.094870541	-0.043753498	0.229910411	1.79E-01	2.49E-01	1.00E+00
KCMF1	LMNA	0.233930798	0.099079509	0.36034469	8.06E-04	2.17E-03	1.00E+00
MFAP2	LMNA	0.391244892	0.267650143	0.502172668	8.55E-09	5.92E-08	1.01E-04
OXCT2	LMNA	0.125078062	-0.01320088	0.258662695	7.61E-02	1.21E-01	1.00E+00
WWC3	LMNA	0.347298894	0.219785501	0.463144453	4.11E-07	2.11E-06	4.87E-03
SOCS1	LMNA	0.395342797	0.272144769	0.505789109	5.78E-09	4.15E-08	6.86E-05
IL17RA	LMNA	0.357928668	0.231307109	0.472626075	1.70E-07	9.36E-07	2.01E-03
C14orf115	LMNA	0.039709048	-0.098884022	0.176790903	5.75E-01	6.54E-01	1.00E+00
ST5	LMNA	0.28490133	0.152862553	0.406946743	3.96E-05	1.40E-04	4.70E-01
STRA6	LMNA	0.466698984	0.351280366	0.568145366	2.55E-12	3.32E-11	3.02E-08
MPP5	LMNA	0.056265725	-0.08242552	0.192818996	4.26E-01	5.13E-01	1.00E+00
SPA17	LMNA	-0.028568801	-0.165965243	0.109915701	6.87E-01	7.53E-01	1.00E+00
C21orf7	LMNA	0.369996438	0.244430553	0.483358281	5.97E-08	3.58E-07	7.09E-04
FLJ10986	LMNA	0.19184886	0.055261455	0.321387925	6.23E-03	1.33E-02	1.00E+00
LHFP	LMNA	0.147573617	0.009720652	0.279921858	3.61E-02	6.30E-02	1.00E+00
CAMK4	LMNA	-0.233556774	-0.360000374	-0.098687738	8.22E-04	2.21E-03	1.00E+00
GALNT14	LMNA	-0.383313062	-0.495161713	-0.258965685	1.80E-08	1.17E-07	2.13E-04
CLPX	LMNA	0.015529576	-0.12278466	0.153252023	8.26E-01	8.70E-01	1.00E+00
NPLOC4	LMNA	0.252524281	0.118608104	0.377418025	2.88E-04	8.58E-04	1.00E+00
PJA1	LMNA	-0.294179534	-0.415361947	-0.162737597	2.14E-05	7.97E-05	2.54E-01
CPLX2	LMNA	-0.406936367	-0.515999559	-0.284889904	1.86E-09	1.46E-08	2.20E-05
ARSD	LMNA	0.502462547	0.3915732	0.598965997	2.55E-14	4.50E-13	3.02E-10

WHDC1L1	LMNA	0.221250298	0.085820586	0.348652129	1.55E-03	3.87E-03	1.00E+00
RB1	LMNA	-0.026515593	-0.163966394	0.111945184	7.08E-01	7.71E-01	1.00E+00
PHLDA2	LMNA	0.512526612	0.402989024	0.607587849	6.32E-15	1.28E-13	7.50E-11
C2orf56	LMNA	-0.006450548	-0.144372984	0.131717748	9.27E-01	9.47E-01	1.00E+00
GUCY2F	LMNA	0.007669543	-0.13051965	0.14556642	9.14E-01	9.38E-01	1.00E+00
MPV17	LMNA	0.134211605	-0.003911875	0.267309876	5.69E-02	9.38E-02	1.00E+00
PSMD4	LMNA	-0.002681346	-0.140680272	0.135419782	9.70E-01	9.77E-01	1.00E+00
SLC35D1	LMNA	0.565610654	0.463772451	0.652697035	1.78E-18	7.13E-17	2.11E-14
C20orf103	LMNA	-0.148186197	-0.280498959	-0.010346864	3.53E-02	6.18E-02	1.00E+00
COL16A1	LMNA	0.242769417	0.108349709	0.368471265	4.99E-04	1.41E-03	1.00E+00
ERLIN1	LMNA	0.298519106	0.167365376	0.419290772	1.59E-05	6.08E-05	1.89E-01
JMJD4	LMNA	0.194849945	0.058369026	0.324180734	5.46E-03	1.18E-02	1.00E+00
SLC29A1	LMNA	0.232760935	0.097854274	0.359267632	8.58E-04	2.29E-03	1.00E+00
GLRX	LMNA	0.21077171	0.074900096	0.338959911	2.60E-03	6.13E-03	1.00E+00
TP53I11	LMNA	0.062309327	-0.076398847	0.198651553	3.78E-01	4.66E-01	1.00E+00
ST3GAL4	LMNA	0.404735867	0.28246751	0.514063959	2.31E-09	1.78E-08	2.74E-05
ALG8	LMNA	0.142323532	0.00435816	0.27497191	4.33E-02	7.39E-02	1.00E+00
PF4V1	LMNA	0.061342983	-0.077363164	0.197719603	3.86E-01	4.73E-01	1.00E+00
SHOC2	LMNA	-0.105271518	-0.239837006	0.033262887	1.36E-01	1.98E-01	1.00E+00
REG1A	LMNA	0.145160415	0.007254797	0.277647487	3.93E-02	6.79E-02	1.00E+00
FBXW7	LMNA	-0.409739056	-0.518463242	-0.287977468	1.40E-09	1.13E-08	1.66E-05
CYB5R3	LMNA	0.231027722	0.096039776	0.357671294	9.40E-04	2.48E-03	1.00E+00
MINA	LMNA	0.146258619	0.008376754	0.278682696	3.78E-02	6.55E-02	1.00E+00
HHLA1	LMNA	0.179809707	0.042821664	0.310161594	1.04E-02	2.11E-02	1.00E+00
MYST4	LMNA	-0.367592461	-0.481223072	-0.241812605	7.38E-08	4.34E-07	8.75E-04
NR0B2	LMNA	-0.121728257	-0.255485892	0.016601731	8.44E-02	1.32E-01	1.00E+00
TFAP2A	LMNA	-0.234622924	-0.360981752	-0.099804587	7.77E-04	2.10E-03	1.00E+00
UCHL5IP	LMNA	0.011307894	-0.12694127	0.149126099	8.73E-01	9.06E-01	1.00E+00
TIMELESS	LMNA	-0.049538549	-0.186315367	0.089121943	4.84E-01	5.69E-01	1.00E+00
DDX10	LMNA	0.200072678	0.063783389	0.329035637	4.31E-03	9.58E-03	1.00E+00
SLC25A36	LMNA	-0.222724708	-0.350013708	-0.087359791	1.44E-03	3.62E-03	1.00E+00
TMED10	LMNA	0.272858558	0.140084309	0.395993132	8.54E-05	2.83E-04	1.00E+00
ZNF507	LMNA	-0.169304133	-0.30033551	-0.032001081	1.60E-02	3.06E-02	1.00E+00
LOC90379	LMNA	0.121355238	-0.016980236	0.255131959	8.54E-02	1.33E-01	1.00E+00
RAB11FIP1	LMNA	0.400744049	0.278077143	0.510549841	3.42E-09	2.56E-08	4.06E-05

ATAD4	LMNA	-0.076390242	-0.212203399	0.062317948	2.80E-01	3.63E-01	1.00E+00
PHF15	LMNA	0.383251843	0.258898734	0.495107545	1.81E-08	1.17E-07	2.14E-04
ZNF26	LMNA	-0.170637323	-0.301584013	-0.033372466	1.52E-02	2.92E-02	1.00E+00
KRT7	LMNA	0.399116421	0.276288479	0.509115937	4.01E-09	2.96E-08	4.76E-05
RPA3	LMNA	0.146551378	0.008675904	0.278958609	3.74E-02	6.50E-02	1.00E+00
YIF1A	LMNA	0.160364794	0.022818972	0.291952401	2.26E-02	4.17E-02	1.00E+00
PPRC1	LMNA	0.211545085	0.075704973	0.339676179	2.51E-03	5.93E-03	1.00E+00
PCDH17	LMNA	-0.279893829	-0.402396385	-0.147543867	5.48E-05	1.88E-04	6.50E-01
PHF8	LMNA	0.003081024	-0.135027409	0.141072022	9.65E-01	9.74E-01	1.00E+00
RDH14	LMNA	0.153005604	0.015277299	0.285035912	2.97E-02	5.31E-02	1.00E+00
DNAJB2	LMNA	-0.02483832	-0.162332689	0.113602218	7.26E-01	7.86E-01	1.00E+00
HNRPK	LMNA	-0.098414598	-0.233295985	0.040182329	1.63E-01	2.31E-01	1.00E+00
PTPLB	LMNA	0.198428498	0.062078016	0.327507989	4.64E-03	1.02E-02	1.00E+00
RABEPK	LMNA	0.026325567	-0.112132955	0.163781342	7.10E-01	7.73E-01	1.00E+00
SNF8	LMNA	0.066255223	-0.072458527	0.202454454	3.49E-01	4.35E-01	1.00E+00
CBARA1	LMNA	-0.29463135	-0.415771209	-0.16321915	2.08E-05	7.76E-05	2.46E-01
RAD51AP1	LMNA	-0.142702333	-0.275329291	-0.004744806	4.28E-02	7.30E-02	1.00E+00
HAT1	LMNA	0.163464253	0.025999978	0.294861294	2.01E-02	3.75E-02	1.00E+00
H2AFV	LMNA	-0.172505892	-0.303333132	-0.035295439	1.41E-02	2.74E-02	1.00E+00
OAZ3	LMNA	-0.068813879	-0.204918193	0.06990117	3.30E-01	4.16E-01	1.00E+00
CXorf48	LMNA	-0.016813583	-0.154505955	0.12151948	8.12E-01	8.58E-01	1.00E+00
RC3H2	LMNA	0.077383389	-0.061322714	0.213157261	2.74E-01	3.56E-01	1.00E+00
SGCD	LMNA	0.023574092	-0.114850682	0.161100801	7.39E-01	7.98E-01	1.00E+00
PHTF1	LMNA	0.189030719	0.052345726	0.318763311	7.05E-03	1.48E-02	1.00E+00
CA3	LMNA	0.086723223	-0.051949732	0.222115012	2.20E-01	2.96E-01	1.00E+00
PKIA	LMNA	-0.461767895	-0.563873367	-0.345758117	4.61E-12	5.76E-11	5.47E-08
STX10	LMNA	0.212324064	0.076515862	0.340397487	2.42E-03	5.74E-03	1.00E+00
PEO1	LMNA	-0.2749714	-0.397917424	-0.142322991	7.48E-05	2.51E-04	8.87E-01
JMJD2D	LMNA	-0.252618563	-0.377504383	-0.118707393	2.87E-04	8.54E-04	1.00E+00
KRT19	LMNA	0.184061255	0.047209849	0.314130256	8.74E-03	1.80E-02	1.00E+00
ZNF143	LMNA	-0.168952329	-0.300005979	-0.031639284	1.62E-02	3.10E-02	1.00E+00
ENO1	LMNA	0.313105836	0.182963348	0.432463774	5.70E-06	2.36E-05	6.77E-02
TIPRL	LMNA	-0.124944538	-0.258536122	0.0133365	7.64E-02	1.21E-01	1.00E+00
MAN1B1	LMNA	0.564238485	0.462189098	0.651538732	2.24E-18	8.79E-17	2.66E-14
P4HA1	LMNA	0.47650039	0.362280705	0.576620512	7.60E-13	1.08E-11	9.01E-09

TPTE	LMNA	-0.227173911	-0.354119172	-0.092008441	1.15E-03	2.96E-03	1.00E+00
AKAP8L	LMNA	-0.055411279	-0.191993609	0.083276754	4.33E-01	5.19E-01	1.00E+00
GPR17	LMNA	-0.427762326	-0.534263457	-0.307893357	2.16E-10	2.02E-09	2.56E-06
LRRC20	LMNA	-0.428692667	-0.535077032	-0.308924252	1.95E-10	1.85E-09	2.32E-06
UBE2Z	LMNA	0.451683242	0.334489439	0.555119582	1.51E-11	1.71E-10	1.79E-07
COL4A1	LMNA	0.425200907	0.305056545	0.5320225	2.83E-10	2.58E-09	3.36E-06
ISOC1	LMNA	-0.016701654	-0.154396665	0.121629786	8.13E-01	8.59E-01	1.00E+00
RNASE1	LMNA	0.353063591	0.226029442	0.468289781	2.55E-07	1.37E-06	3.03E-03
C20orf20	LMNA	0.518843354	0.410171655	0.612987962	2.57E-15	5.58E-14	3.05E-11
NDUFB11	LMNA	-0.219932416	-0.347434644	-0.084445336	1.66E-03	4.11E-03	1.00E+00
STK19	LMNA	0.050591482	-0.08807465	0.187334103	4.75E-01	5.60E-01	1.00E+00
OSBPL2	LMNA	-0.055493246	-0.192072797	0.083195104	4.33E-01	5.19E-01	1.00E+00
GRM7	LMNA	-0.280356188	-0.402816788	-0.148034638	5.32E-05	1.83E-04	6.31E-01
SLC39A8	LMNA	0.396838121	0.273786195	0.50710778	5.01E-09	3.64E-08	5.94E-05
APPBP1	LMNA	-0.238520768	-0.364567286	-0.103890683	6.30E-04	1.74E-03	1.00E+00
STS	LMNA	0.228363498	0.093252353	0.355216025	1.08E-03	2.81E-03	1.00E+00
TMEM123	LMNA	0.24442505	0.110088789	0.369991386	4.55E-04	1.29E-03	1.00E+00
GLI2	LMNA	0.216813338	0.081192535	0.344551466	1.94E-03	4.72E-03	1.00E+00
BTNL2	LMNA	-0.156096501	-0.287942548	-0.018442933	2.65E-02	4.80E-02	1.00E+00
ALDH1A3	LMNA	0.255383283	0.121620111	0.380035774	2.44E-04	7.37E-04	1.00E+00
TGIF1	LMNA	0.437483844	0.318679519	0.542755105	7.50E-11	7.70E-10	8.90E-07
SCO2	LMNA	0.171295423	0.034049611	0.302200145	1.48E-02	2.85E-02	1.00E+00
TP53	LMNA	0.200759918	0.064496444	0.329673969	4.17E-03	9.31E-03	1.00E+00
CCDC69	LMNA	0.139417283	0.001393095	0.272228779	4.78E-02	8.07E-02	1.00E+00
ZFAND5	LMNA	0.18567019	0.048871871	0.315630952	8.16E-03	1.69E-02	1.00E+00
ICA1	LMNA	-0.458178322	-0.560760136	-0.341743258	7.06E-12	8.56E-11	8.37E-08
RAPGEF2	LMNA	-0.446930361	-0.550985977	-0.329190122	2.60E-11	2.83E-10	3.09E-07
NAV3	LMNA	-0.263834012	-0.387761742	-0.130537521	1.48E-04	4.69E-04	1.00E+00
EPS8L2	LMNA	0.348288436	0.220856565	0.464028232	3.79E-07	1.96E-06	4.49E-03
ATP6V1G2	LMNA	-0.623128451	-0.700886719	-0.53073296	4.05E-23	3.82E-21	4.81E-19
MNT	LMNA	0.087397219	-0.051272397	0.222760552	2.16E-01	2.92E-01	1.00E+00
C6orf145	LMNA	0.237779739	0.103113515	0.363885919	6.56E-04	1.80E-03	1.00E+00
PPP6C	LMNA	0.204275408	0.068146158	0.332937431	3.54E-03	8.05E-03	1.00E+00
OR51E2	LMNA	0.314588008	0.184551949	0.43379945	5.12E-06	2.14E-05	6.08E-02
OTUB1	LMNA	-0.110682544	-0.244990136	0.027793131	1.17E-01	1.74E-01	1.00E+00

ENTPD1	LMNA	-0.005885933	-0.143820072	0.132272546	9.34E-01	9.52E-01	1.00E+00
TMEM115	LMNA	0.408302635	0.286394727	0.517200792	1.62E-09	1.29E-08	1.92E-05
STK11	LMNA	0.307951607	0.177444301	0.427814901	8.25E-06	3.31E-05	9.79E-02
PRPSAP2	LMNA	-0.520573533	-0.614465539	-0.412141358	2.00E-15	4.48E-14	2.38E-11
SH3BGRL3	LMNA	0.608133355	0.513164247	0.688391534	8.12E-22	5.50E-20	9.63E-18
MX1	LMNA	0.12362766	-0.014673777	0.257287554	7.96E-02	1.25E-01	1.00E+00
PSMC5	LMNA	0.132662908	-0.005488613	0.265845155	5.98E-02	9.81E-02	1.00E+00
TTTY9A	LMNA	0.211013632	0.075151853	0.339183985	2.57E-03	6.07E-03	1.00E+00
EXOSC4	LMNA	-0.004962257	-0.142915355	0.133179977	9.44E-01	9.58E-01	1.00E+00
SETD1A	LMNA	0.167817789	0.030472764	0.298943053	1.70E-02	3.23E-02	1.00E+00
YARS	LMNA	0.084886132	-0.053795275	0.220354879	2.30E-01	3.07E-01	1.00E+00
SLN	LMNA	0.182231821	0.045320973	0.312423104	9.44E-03	1.93E-02	1.00E+00
RELB	LMNA	0.616617502	0.523094767	0.6954671	1.52E-22	1.23E-20	1.80E-18
NLRP1	LMNA	-0.230722239	-0.35738986	-0.09572006	9.55E-04	2.51E-03	1.00E+00
LAMB2	LMNA	0.528430935	0.421099305	0.621167506	6.33E-16	1.58E-14	7.51E-12
FNTA	LMNA	-0.265232184	-0.389038325	-0.132014987	1.36E-04	4.36E-04	1.00E+00
HNF1B	LMNA	-0.17089923	-0.301829231	-0.033641938	1.50E-02	2.89E-02	1.00E+00
C14orf139	LMNA	0.266062501	0.132892674	0.389796211	1.30E-04	4.15E-04	1.00E+00
UMOD	LMNA	-0.013408808	-0.151179959	0.124873346	8.50E-01	8.89E-01	1.00E+00
ZNF512B	LMNA	0.061086549	-0.077619017	0.197472255	3.88E-01	4.75E-01	1.00E+00
GPR25	LMNA	0.028699993	-0.109785987	0.166092923	6.85E-01	7.53E-01	1.00E+00
C6orf49	LMNA	-0.224859121	-0.35198383	-0.089589149	1.29E-03	3.30E-03	1.00E+00
ATP6V0A1	LMNA	-0.378163583	-0.49060227	-0.253338328	2.88E-08	1.81E-07	3.41E-04
SNFT	LMNA	0.299534417	0.168448954	0.420209331	1.49E-05	5.70E-05	1.76E-01
ZNF768	LMNA	0.181325817	0.044385893	0.311577348	9.81E-03	2.00E-02	1.00E+00
GTF2I	LMNA	-0.269525214	-0.392955054	-0.13655519	1.05E-04	3.42E-04	1.00E+00
KCNN2	LMNA	-0.324003419	-0.442272036	-0.194659335	2.55E-06	1.13E-05	3.03E-02
DYNC2LI1	LMNA	-0.046113916	-0.182999926	0.092526113	5.15E-01	5.98E-01	1.00E+00
DGKE	LMNA	-0.212619263	-0.340670792	-0.076823201	2.38E-03	5.67E-03	1.00E+00
DNAH3	LMNA	0.024545637	-0.11389129	0.162047532	7.29E-01	7.89E-01	1.00E+00
RPS6KA1	LMNA	0.168448072	0.031120767	0.299533591	1.66E-02	3.16E-02	1.00E+00
C9orf53	LMNA	-0.019974368	-0.157590815	0.118403127	7.78E-01	8.31E-01	1.00E+00
PTPRM	LMNA	-0.171463373	-0.302357366	-0.034222441	1.47E-02	2.84E-02	1.00E+00
MRPS15	LMNA	0.186556406	0.049787652	0.316457272	7.85E-03	1.64E-02	1.00E+00
TMEM59L	LMNA	-0.405097927	-0.514382511	-0.282865972	2.23E-09	1.72E-08	2.64E-05

SSPN	LMNA	0.138628798	0.000589071	0.271484177	4.91E-02	8.27E-02	1.00E+00
CNOT8	LMNA	-0.263454061	-0.387414749	-0.130136124	1.52E-04	4.79E-04	1.00E+00
GORASP2	LMNA	0.094724785	-0.043900293	0.229771103	1.80E-01	2.50E-01	1.00E+00
ADAM17	LMNA	0.180590521	0.043627176	0.310890796	1.01E-02	2.05E-02	1.00E+00
CHRNA3	LMNA	-0.158591297	-0.290286848	-0.021000065	2.42E-02	4.42E-02	1.00E+00
MYOG	LMNA	-0.032408571	-0.169700327	0.106117202	6.47E-01	7.19E-01	1.00E+00
UBE2D4	LMNA	0.01179104	-0.126465817	0.149598529	8.68E-01	9.02E-01	1.00E+00
CPNE1	LMNA	0.202436403	0.066236484	0.331230651	3.86E-03	8.69E-03	1.00E+00
AK5	LMNA	-0.25887006	-0.383225623	-0.125296826	1.99E-04	6.12E-04	1.00E+00
RPL37A	LMNA	-0.235329985	-0.361632437	-0.100545459	7.48E-04	2.03E-03	1.00E+00
CPS1	LMNA	-0.195499389	-0.324784809	-0.059041862	5.30E-03	1.15E-02	1.00E+00
NDRG4	LMNA	-0.367408599	-0.48105971	-0.241612454	7.50E-08	4.40E-07	8.90E-04
ALG5	LMNA	-0.007330296	-0.145234326	0.130853122	9.18E-01	9.41E-01	1.00E+00
NCOA2	LMNA	-0.315131387	-0.434288991	-0.185134516	4.92E-06	2.07E-05	5.84E-02
LOC130074	LMNA	-0.06170294	-0.198066778	0.077003992	3.83E-01	4.70E-01	1.00E+00
PIAS4	LMNA	0.167379301	0.030022015	0.298532155	1.73E-02	3.28E-02	1.00E+00
S100PBP	LMNA	-0.110348857	-0.244672572	0.02813068	1.18E-01	1.75E-01	1.00E+00
TEGT	LMNA	0.356110472	0.229333846	0.471006148	1.98E-07	1.08E-06	2.35E-03
USP5	LMNA	-0.06671353	-0.202895886	0.072000587	3.46E-01	4.32E-01	1.00E+00
CFLAR	LMNA	0.405487562	0.283294829	0.514725289	2.14E-09	1.66E-08	2.54E-05
KIAA0692	LMNA	0.276575136	0.144023152	0.399377322	6.76E-05	2.28E-04	8.02E-01
WDR48	LMNA	-0.430727869	-0.53685611	-0.311180406	1.57E-10	1.52E-09	1.86E-06
PCDHGB6	LMNA	-0.090068488	-0.225317876	0.048586641	2.02E-01	2.77E-01	1.00E+00
NRXN3	LMNA	-0.178507021	-0.308944675	-0.04147817	1.10E-02	2.21E-02	1.00E+00
ACACB	LMNA	0.099263077	-0.039326834	0.234106037	1.60E-01	2.27E-01	1.00E+00
CDKN2C	LMNA	-0.032834983	-0.170114869	0.105695123	6.43E-01	7.16E-01	1.00E+00
KIAA0226	LMNA	-0.12427674	-0.257903021	0.014014704	7.80E-02	1.23E-01	1.00E+00
TRAK1	LMNA	-0.294349016	-0.415515472	-0.162918226	2.12E-05	7.90E-05	2.51E-01
CUTC	LMNA	-0.257064813	-0.381574478	-0.123392782	2.21E-04	6.75E-04	1.00E+00
AGPAT5	LMNA	-0.072289196	-0.208261828	0.066424696	3.07E-01	3.91E-01	1.00E+00
WDR68	LMNA	-0.224238337	-0.351410946	-0.088940611	1.34E-03	3.39E-03	1.00E+00
PREPL	LMNA	-0.274845623	-0.397802902	-0.142189685	7.54E-05	2.52E-04	8.94E-01
ABCB6	LMNA	0.011028994	-0.127215701	0.148853356	8.76E-01	9.08E-01	1.00E+00
RABGAP1L	LMNA	0.270983764	0.138098988	0.394284738	9.59E-05	3.14E-04	1.00E+00
FCGR1A	LMNA	0.105256546	-0.03327801	0.239822737	1.36E-01	1.98E-01	1.00E+00

EIF4H	LMNA	0.350814104	0.223591703	0.466282912	3.08E-07	1.62E-06	3.65E-03
DLC1	LMNA	0.401413293	0.278812848	0.511139253	3.21E-09	2.41E-08	3.80E-05
MAPK8IP3	LMNA	-0.376519386	-0.48914517	-0.251543325	3.34E-08	2.07E-07	3.96E-04
SPRY4	LMNA	0.348752512	0.22135898	0.464442628	3.65E-07	1.90E-06	4.32E-03
RAP2C	LMNA	0.398755067	0.275891489	0.50879751	4.16E-09	3.06E-08	4.93E-05
ETFB	LMNA	0.181161059	0.044215874	0.311423524	9.87E-03	2.01E-02	1.00E+00
RORC	LMNA	0.067158819	-0.071555598	0.203324726	3.42E-01	4.28E-01	1.00E+00
GRAP	LMNA	0.044601327	-0.094028632	0.181534572	5.29E-01	6.11E-01	1.00E+00
SEPW1	LMNA	-0.325349111	-0.443481253	-0.196106174	2.31E-06	1.03E-05	2.74E-02
NMU	LMNA	-0.246033086	-0.371467162	-0.11177866	4.16E-04	1.19E-03	1.00E+00
MXD1	LMNA	0.017671465	-0.120673927	0.155343496	8.03E-01	8.51E-01	1.00E+00
IFIH1	LMNA	0.209902141	0.073995317	0.338154374	2.72E-03	6.36E-03	1.00E+00
AZI2	LMNA	-0.047250553	-0.184100668	0.091396626	5.04E-01	5.88E-01	1.00E+00
WDR37	LMNA	-0.252259964	-0.377175911	-0.118329767	2.93E-04	8.69E-04	1.00E+00
SEMA4A	LMNA	-0.12570896	-0.259260687	0.01256001	7.46E-02	1.19E-01	1.00E+00
RPL8	LMNA	0.104808555	-0.033730489	0.239395756	1.38E-01	2.00E-01	1.00E+00
NUAK2	LMNA	0.449297208	0.331828158	0.553045074	1.98E-11	2.20E-10	2.35E-07
AHSG	LMNA	-0.274989678	-0.397934067	-0.142342364	7.47E-05	2.50E-04	8.86E-01
RBM39	LMNA	0.009296564	-0.128919892	0.147158701	8.96E-01	9.24E-01	1.00E+00
MANSC1	LMNA	-0.163115663	-0.294534259	-0.025642078	2.04E-02	3.80E-02	1.00E+00
IMP3	LMNA	-0.078595361	-0.214320943	0.060107819	2.66E-01	3.48E-01	1.00E+00
ARHGDIG	LMNA	-0.392076787	-0.502907138	-0.26856214	7.90E-09	5.51E-08	9.37E-05
ELTD1	LMNA	0.213091666	0.077315083	0.341108113	2.33E-03	5.56E-03	1.00E+00
PRAMEF10	LMNA	0.057371098	-0.081323999	0.193886491	4.17E-01	5.05E-01	1.00E+00
RGS17	LMNA	0.259319826	0.12577136	0.383636872	1.94E-04	5.97E-04	1.00E+00
KPTN	LMNA	-0.065857365	-0.2020712	0.072856019	3.52E-01	4.38E-01	1.00E+00
C2orf3	LMNA	-0.107640608	-0.242094116	0.030869104	1.27E-01	1.87E-01	1.00E+00
PCTP	LMNA	0.064566923	-0.074144971	0.200827839	3.61E-01	4.48E-01	1.00E+00
MRPL42	LMNA	0.011948643	-0.12631071	0.149752623	8.66E-01	9.01E-01	1.00E+00
SIRT1	LMNA	-0.384582656	-0.496284889	-0.260354397	1.60E-08	1.05E-07	1.89E-04
MANBA	LMNA	0.377650829	0.252778452	0.49014793	3.01E-08	1.88E-07	3.57E-04
CD164	LMNA	0.420350624	0.299690617	0.527774938	4.72E-10	4.15E-09	5.60E-06
ZNF671	LMNA	-0.272823567	-0.395961255	-0.140047245	8.56E-05	2.83E-04	1.00E+00
GFRA1	LMNA	-0.396202588	-0.50654739	-0.27308848	5.32E-09	3.85E-08	6.31E-05
PRM2	LMNA	0.326852282	0.197722995	0.444831471	2.06E-06	9.31E-06	2.44E-02

IL1B	LMNA	0.164120069	0.026673406	0.295476477	1.96E-02	3.66E-02	1.00E+00
HAX1	LMNA	-0.154571878	-0.286509118	-0.0168811	2.81E-02	5.04E-02	1.00E+00
REN	LMNA	0.093356372	-0.045278171	0.228462955	1.86E-01	2.58E-01	1.00E+00
ZKSCAN3	LMNA	-0.179275804	-0.309662895	-0.042270977	1.07E-02	2.15E-02	1.00E+00
CTSA	LMNA	0.666736884	0.582281196	0.736955755	2.45E-27	4.77E-25	2.91E-23
TPRKB	LMNA	-0.07310074	-0.209042165	0.065612396	3.01E-01	3.85E-01	1.00E+00
UBFD1	LMNA	0.141149249	0.00315982	0.273863796	4.51E-02	7.67E-02	1.00E+00
CDKL5	LMNA	-0.215792603	-0.343607406	-0.080128666	2.04E-03	4.94E-03	1.00E+00
HIST1H2BD	LMNA	0.132781046	-0.005368359	0.265956909	5.96E-02	9.78E-02	1.00E+00
COQ4	LMNA	0.285716232	0.153728821	0.407686679	3.76E-05	1.33E-04	4.46E-01
TXNDC4	LMNA	0.362621913	0.236405444	0.476803956	1.14E-07	6.45E-07	1.35E-03
C5orf22	LMNA	0.140547514	0.002545911	0.273295831	4.60E-02	7.80E-02	1.00E+00
VGF	LMNA	-0.157504562	-0.289265863	-0.019885955	2.52E-02	4.58E-02	1.00E+00
INPP4A	LMNA	-0.069452004	-0.205532377	0.069263086	3.26E-01	4.12E-01	1.00E+00
RNF8	LMNA	-0.179712606	-0.310070901	-0.042721505	1.05E-02	2.12E-02	1.00E+00
BMX	LMNA	0.294312769	0.162879595	0.415482639	2.12E-05	7.92E-05	2.52E-01
SLCO5A1	LMNA	-0.436410476	-0.541818585	-0.317487089	8.44E-11	8.57E-10	1.00E-06
PTPRU	LMNA	0.344355501	0.216601434	0.460514288	5.22E-07	2.62E-06	6.19E-03
ATP8B3	LMNA	0.019791696	-0.118583305	0.157412604	7.80E-01	8.32E-01	1.00E+00
HOXC8	LMNA	0.089566208	-0.049091798	0.224837163	2.05E-01	2.80E-01	1.00E+00
ADPGK	LMNA	0.388309396	0.264433764	0.499579678	1.13E-08	7.61E-08	1.34E-04
IL12B	LMNA	0.057722476	-0.080973776	0.19422576	4.15E-01	5.02E-01	1.00E+00
LARP4	LMNA	0.101338551	-0.03723334	0.236086724	1.51E-01	2.17E-01	1.00E+00
ZMPSTE24	LMNA	0.203285312	0.067117891	0.332018628	3.71E-03	8.38E-03	1.00E+00
PFDN4	LMNA	-0.035568412	-0.172771063	0.102988287	6.15E-01	6.91E-01	1.00E+00
KLHL11	LMNA	-0.255807136	-0.38042369	-0.122066857	2.38E-04	7.22E-04	1.00E+00
PSMB3	LMNA	-0.02765201	-0.165072864	0.110822041	6.96E-01	7.61E-01	1.00E+00
KIAA0157	LMNA	-0.272376865	-0.395554274	-0.139574117	8.80E-05	2.90E-04	1.00E+00
DDX25	LMNA	-0.520083031	-0.614046716	-0.411582848	2.15E-15	4.77E-14	2.55E-11
NCAN	LMNA	-0.455624113	-0.558543107	-0.338889016	9.53E-12	1.12E-10	1.13E-07
RPS25	LMNA	-0.072126816	-0.208105671	0.066587206	3.08E-01	3.92E-01	1.00E+00
SOBP	LMNA	-0.392306718	-0.503110113	-0.268814249	7.73E-09	5.40E-08	9.17E-05
TESK2	LMNA	-0.14834201	-0.280645733	-0.010506162	3.51E-02	6.15E-02	1.00E+00
DNM1L	LMNA	-0.070028719	-0.206087362	0.068686309	3.22E-01	4.07E-01	1.00E+00
LOC55908	LMNA	-0.025583478	-0.163058585	0.112866146	7.18E-01	7.80E-01	1.00E+00

MTIF2	LMNA	0.155676138	0.018012242	0.287547386	2.69E-02	4.86E-02	1.00E+00
ZNF207	LMNA	-0.090146759	-0.22539278	0.048507916	2.02E-01	2.76E-01	1.00E+00
EXOC7	LMNA	-0.020946466	-0.158539022	0.117444146	7.67E-01	8.22E-01	1.00E+00
CLEC11A	LMNA	0.054665508	-0.084019557	0.191273042	4.40E-01	5.26E-01	1.00E+00
TXN2	LMNA	-0.156894006	-0.288692116	-0.019260168	2.58E-02	4.67E-02	1.00E+00
TOLLIP	LMNA	0.072637878	-0.066075712	0.208597123	3.04E-01	3.89E-01	1.00E+00
TRAPPC3	LMNA	0.26951998	0.136549651	0.392950282	1.05E-04	3.42E-04	1.00E+00
TAF15	LMNA	-0.120466129	-0.254288197	0.017882262	8.77E-02	1.36E-01	1.00E+00
HAMP	LMNA	0.114940125	-0.023483502	0.249039467	1.03E-01	1.56E-01	1.00E+00
GRIA4	LMNA	-0.364631559	-0.478591347	-0.238590676	9.55E-08	5.48E-07	1.13E-03
IDE	LMNA	0.373490304	0.248238665	0.486459126	4.38E-08	2.67E-07	5.20E-04
DLK1	LMNA	-0.000320561	-0.138365438	0.137736535	9.96E-01	9.97E-01	1.00E+00
PLVAP	LMNA	0.358925773	0.232389696	0.47351412	1.56E-07	8.64E-07	1.85E-03
NOD2	LMNA	0.396101653	0.272977681	0.50645838	5.37E-09	3.89E-08	6.37E-05
SCMH1	LMNA	0.262054731	0.128658187	0.386136503	1.65E-04	5.16E-04	1.00E+00
ELMO3	LMNA	0.041180113	-0.097424743	0.178217952	5.61E-01	6.41E-01	1.00E+00
GPR68	LMNA	0.140448479	0.002444883	0.273202345	4.62E-02	7.83E-02	1.00E+00
HTR2A	LMNA	-0.199363892	-0.32837717	-0.063048127	4.45E-03	9.87E-03	1.00E+00
FUT7	LMNA	-0.091498839	-0.226686448	0.047147708	1.95E-01	2.69E-01	1.00E+00
PRELP	LMNA	-0.072633353	-0.208592772	0.066080241	3.04E-01	3.89E-01	1.00E+00
COL8A2	LMNA	0.479318892	0.365449875	0.579053651	5.33E-13	7.76E-12	6.32E-09
GYG1	LMNA	-0.138196491	-0.271075862	-0.00014832	4.98E-02	8.37E-02	1.00E+00
C16orf68	LMNA	-0.067862141	-0.204001962	0.070852638	3.37E-01	4.23E-01	1.00E+00
R3HDM1	LMNA	-0.429025343	-0.535367906	-0.309292953	1.88E-10	1.79E-09	2.23E-06
ZNF91	LMNA	-0.202604063	-0.331386291	-0.066410545	3.83E-03	8.62E-03	1.00E+00
LPIN1	LMNA	-0.191877046	-0.321414165	-0.055290629	6.23E-03	1.33E-02	1.00E+00
KRT12	LMNA	0.042184772	-0.09642779	0.17919222	5.51E-01	6.32E-01	1.00E+00
BAALC	LMNA	-0.346512599	-0.462442032	-0.218934646	4.38E-07	2.24E-06	5.19E-03
MKRN1	LMNA	-0.155403423	-0.287290998	-0.017732856	2.72E-02	4.91E-02	1.00E+00
KIAA1598	LMNA	-0.277692218	-0.400393845	-0.145207861	6.30E-05	2.14E-04	7.47E-01
ANXA7	LMNA	-0.002799814	-0.140796395	0.135303484	9.68E-01	9.76E-01	1.00E+00
TNP1	LMNA	-0.070852267	-0.20687973	0.067862512	3.16E-01	4.02E-01	1.00E+00
WDR13	LMNA	0.292227308	0.1606576	0.413593026	2.44E-05	9.00E-05	2.90E-01
GAPDH	LMNA	0.364949571	0.238936591	0.478874102	9.29E-08	5.34E-07	1.10E-03
BSPRY	LMNA	-0.209753782	-0.338016921	-0.073840974	2.73E-03	6.41E-03	1.00E+00

COX7C	LMNA	-0.204390003	-0.333043759	-0.06826519	3.53E-03	8.01E-03	1.00E+00
FAM108B1	LMNA	-0.301570963	-0.422051061	-0.170623385	1.29E-05	5.02E-05	1.53E-01
PGAM2	LMNA	-0.042086506	-0.179096938	0.096525315	5.52E-01	6.33E-01	1.00E+00
PEX12	LMNA	-0.198155661	-0.327254425	-0.061795102	4.70E-03	1.03E-02	1.00E+00
APC	LMNA	-0.416900772	-0.524750508	-0.295878643	6.75E-10	5.76E-09	8.01E-06
PMP22	LMNA	0.506917058	0.396621813	0.602784924	1.38E-14	2.61E-13	1.64E-10
TLR2	LMNA	0.315245513	0.185256884	0.4343918	4.88E-06	2.05E-05	5.79E-02
NPBWR2	LMNA	0.056938888	-0.081754743	0.193469131	4.21E-01	5.08E-01	1.00E+00
WFDC1	LMNA	-0.131807672	-0.265036029	0.006359039	6.15E-02	1.00E-01	1.00E+00
MTL5	LMNA	0.26910186	0.136107212	0.392569008	1.08E-04	3.50E-04	1.00E+00
COMMD9	LMNA	-0.002826996	-0.140823038	0.135276799	9.68E-01	9.76E-01	1.00E+00
INADL	LMNA	0.037018684	-0.101551291	0.174179546	6.01E-01	6.78E-01	1.00E+00
GPX1	LMNA	0.159311996	0.021739107	0.290963777	2.35E-02	4.32E-02	1.00E+00
PSG11	LMNA	0.030159899	-0.108342196	0.16751344	6.70E-01	7.39E-01	1.00E+00
SLC39A1	LMNA	0.608001787	0.51301045	0.688281689	8.33E-22	5.61E-20	9.88E-18
SNAPC3	LMNA	-0.126869251	-0.260360192	0.011381083	7.20E-02	1.15E-01	1.00E+00
C4orf16	LMNA	-0.031223337	-0.168547827	0.107290128	6.59E-01	7.30E-01	1.00E+00
GNA12	LMNA	0.255767486	0.122025064	0.380387404	2.39E-04	7.23E-04	1.00E+00
LIMK1	LMNA	0.512691885	0.403176781	0.607729251	6.17E-15	1.26E-13	7.32E-11
MECR	LMNA	0.017602789	-0.120741624	0.155276455	8.04E-01	8.52E-01	1.00E+00
CDC42EP1	LMNA	0.280339845	0.148017291	0.40280193	5.32E-05	1.83E-04	6.31E-01
KIAA0101	LMNA	0.061743277	-0.076963741	0.19810568	3.83E-01	4.70E-01	1.00E+00
B4GALT5	LMNA	0.375906776	0.250874746	0.488602109	3.53E-08	2.17E-07	4.18E-04
PIGC	LMNA	0.007695501	-0.130494133	0.145591829	9.13E-01	9.38E-01	1.00E+00
LRAT	LMNA	0.144547456	0.006628731	0.277069556	4.01E-02	6.91E-02	1.00E+00
MMP19	LMNA	0.464935393	0.349304425	0.566618124	3.15E-12	4.07E-11	3.74E-08
IL18R1	LMNA	0.361147019	0.234802498	0.475491575	1.29E-07	7.26E-07	1.53E-03
VNN2	LMNA	0.129350371	-0.008858821	0.26271017	6.65E-02	1.08E-01	1.00E+00
AKAP11	LMNA	-0.412970194	-0.521301313	-0.291540176	1.01E-09	8.34E-09	1.20E-05
BCL10	LMNA	0.410162525	0.288444201	0.518835333	1.34E-09	1.08E-08	1.59E-05
GLB1	LMNA	0.519274305	0.410662172	0.613356057	2.42E-15	5.28E-14	2.87E-11
DTX3	LMNA	-0.324948419	-0.443121243	-0.195675306	2.38E-06	1.06E-05	2.82E-02
ACCN3	LMNA	-0.36641907	-0.480180377	-0.240535438	8.18E-08	4.77E-07	9.70E-04
MOCS2	LMNA	-0.17831552	-0.308765746	-0.041280711	1.11E-02	2.23E-02	1.00E+00
HCRT	LMNA	-0.047323222	-0.18417103	0.091324402	5.04E-01	5.88E-01	1.00E+00

CYBRD1	LMNA	0.435002605	0.315923621	0.540589811	9.85E-11	9.92E-10	1.17E-06
REG3A	LMNA	0.018500627	-0.119856491	0.156152809	7.94E-01	8.44E-01	1.00E+00
SULT2B1	LMNA	0.081192042	-0.057503495	0.216812865	2.51E-01	3.31E-01	1.00E+00
Sep-06	LMNA	0.325416366	0.196178499	0.443541676	2.29E-06	1.03E-05	2.72E-02
MMP10	LMNA	0.30215977	0.171252295	0.422583357	1.24E-05	4.83E-05	1.47E-01
CDA	LMNA	0.09744604	-0.041158648	0.232371064	1.68E-01	2.36E-01	1.00E+00
ME1	LMNA	0.060829388	-0.077875577	0.197224187	3.90E-01	4.77E-01	1.00E+00
TCN2	LMNA	0.364684264	0.238648004	0.478638211	9.51E-08	5.46E-07	1.13E-03
MGRN1	LMNA	0.062040532	-0.076667104	0.19839235	3.80E-01	4.68E-01	1.00E+00
ZFY	LMNA	0.029373657	-0.109119832	0.166748482	6.78E-01	7.47E-01	1.00E+00
CHRNG	LMNA	0.034635818	-0.103912039	0.171865044	6.25E-01	6.99E-01	1.00E+00
IVL	LMNA	0.04760565	-0.091043691	0.18444448	5.01E-01	5.85E-01	1.00E+00
PLEKHA6	LMNA	-0.173181634	-0.303965459	-0.035991105	1.37E-02	2.68E-02	1.00E+00
CALM1	LMNA	-0.167854859	-0.298977788	-0.030510873	1.69E-02	3.23E-02	1.00E+00
PGDS	LMNA	-0.016893559	-0.154584043	0.121440662	8.11E-01	8.58E-01	1.00E+00
C8orf33	LMNA	-0.105324994	-0.239887971	0.033208871	1.36E-01	1.97E-01	1.00E+00
CYFIP2	LMNA	-0.479263936	-0.579006225	-0.365388056	5.37E-13	7.80E-12	6.37E-09
TEX11	LMNA	-0.074494645	-0.210382064	0.064216765	2.92E-01	3.76E-01	1.00E+00
CKM	LMNA	0.051082808	-0.087585851	0.187809371	4.70E-01	5.56E-01	1.00E+00
MAP3K11	LMNA	0.485851189	0.37280506	0.584685989	2.31E-13	3.51E-12	2.74E-09
CEBPE	LMNA	0.306763749	0.176173528	0.426742609	8.98E-06	3.58E-05	1.06E-01
ACOT8	LMNA	-0.025050355	-0.162539258	0.113392783	7.23E-01	7.84E-01	1.00E+00
ESR2	LMNA	0.382573285	0.258156735	0.494507079	1.92E-08	1.25E-07	2.28E-04
OLIG2	LMNA	-0.484869809	-0.583840422	-0.371699146	2.62E-13	3.96E-12	3.11E-09
AGTR2	LMNA	-0.06867378	-0.204783335	0.070041246	3.31E-01	4.17E-01	1.00E+00
DNAI2	LMNA	-0.195387687	-0.324680918	-0.058926129	5.33E-03	1.16E-02	1.00E+00
EPM2AIP1	LMNA	-0.345302108	-0.461360382	-0.217625146	4.83E-07	2.45E-06	5.73E-03
C14orf106	LMNA	-0.078187212	-0.2139291	0.060516998	2.69E-01	3.51E-01	1.00E+00
PZP	LMNA	0.298806371	0.167671925	0.419550689	1.56E-05	5.97E-05	1.85E-01
RPS9	LMNA	-0.055549602	-0.192127241	0.083138964	4.32E-01	5.18E-01	1.00E+00
SIVA1	LMNA	0.167062193	0.029696076	0.29823497	1.75E-02	3.31E-02	1.00E+00
HEATR2	LMNA	0.419572831	0.298830845	0.527093297	5.12E-10	4.47E-09	6.07E-06
CD3E	LMNA	0.340262083	0.212177822	0.456853086	7.25E-07	3.56E-06	8.59E-03
APRT	LMNA	0.409723201	0.287959994	0.51844931	1.40E-09	1.13E-08	1.66E-05
AMIGO2	LMNA	0.235461436	0.100683213	0.361753394	7.43E-04	2.01E-03	1.00E+00

GPS2	LMNA	-0.002245826	-0.140253343	0.135847292	9.75E-01	9.80E-01	1.00E+00
NOL14	LMNA	0.25405868	0.120224314	0.378823194	2.64E-04	7.91E-04	1.00E+00
TRIM36	LMNA	-0.109460964	-0.243827441	0.029028695	1.21E-01	1.79E-01	1.00E+00
RALBP1	LMNA	0.298393055	0.167230873	0.419176716	1.61E-05	6.13E-05	1.91E-01
EVPL	LMNA	-0.058801614	-0.195267507	0.079897968	4.06E-01	4.93E-01	1.00E+00
ITIH4	LMNA	-0.247989587	-0.373261888	-0.113835778	3.73E-04	1.08E-03	1.00E+00
ADARB2	LMNA	-0.427693614	-0.534203362	-0.30781723	2.17E-10	2.03E-09	2.58E-06
PIM2	LMNA	0.091935141	-0.046708674	0.2271038	1.93E-01	2.66E-01	1.00E+00
REGL	LMNA	0.007878008	-0.130314718	0.145770474	9.11E-01	9.37E-01	1.00E+00
GJA5	LMNA	0.222796188	0.087434428	0.350079704	1.44E-03	3.61E-03	1.00E+00
SLC17A5	LMNA	0.217013652	0.08140135	0.344736702	1.92E-03	4.68E-03	1.00E+00
PIPOX	LMNA	0.319265894	0.189570195	0.438011546	3.63E-06	1.56E-05	4.31E-02
HGF	LMNA	0.337816995	0.209538003	0.454664284	8.80E-07	4.25E-06	1.04E-02
EPHB4	LMNA	0.689735405	0.609743351	0.755818389	7.33E-30	2.02E-27	8.69E-26
SOX18	LMNA	0.160051363	0.022497449	0.291658104	2.29E-02	4.21E-02	1.00E+00
SERPINA1(	LMNA	0.172992121	0.035795991	0.303788134	1.38E-02	2.70E-02	1.00E+00
INSIG1	LMNA	0.245842377	0.111578206	0.371292172	4.21E-04	1.21E-03	1.00E+00
WDR23	LMNA	-0.030234102	-0.167585626	0.108268796	6.69E-01	7.39E-01	1.00E+00
SYNGR1	LMNA	-0.296865951	-0.417794624	-0.165601747	1.79E-05	6.75E-05	2.12E-01
TEX15	LMNA	-0.167925578	-0.299044051	-0.030583575	1.69E-02	3.22E-02	1.00E+00
REEP2	LMNA	-0.138477964	-0.271341719	-0.000435285	4.94E-02	8.30E-02	1.00E+00
CDK3	LMNA	-0.027101293	-0.164536702	0.11136637	7.02E-01	7.66E-01	1.00E+00
HSPA12A	LMNA	-0.35642235	-0.471284072	-0.229672249	1.93E-07	1.06E-06	2.29E-03
REPIN1	LMNA	-0.175759833	-0.306376958	-0.038646545	1.23E-02	2.44E-02	1.00E+00
ARL8B	LMNA	0.021817704	-0.116584444	0.159388633	7.58E-01	8.14E-01	1.00E+00
PDE4A	LMNA	0.03269793	-0.105830789	0.169981636	6.44E-01	7.17E-01	1.00E+00
CAPZB	LMNA	0.317142023	0.187290952	0.436099801	4.25E-06	1.80E-05	5.04E-02
SATB1	LMNA	-0.5249303	-0.618183315	-0.417105766	1.06E-15	2.53E-14	1.26E-11
PPM1D	LMNA	-0.402638526	-0.512218064	-0.280160128	2.84E-09	2.16E-08	3.37E-05
VPS45	LMNA	-0.504876544	-0.601036103	-0.394308326	1.83E-14	3.39E-13	2.17E-10
TP53BP2	LMNA	-0.208823801	-0.33715518	-0.072873626	2.86E-03	6.65E-03	1.00E+00
GINS2	LMNA	-0.033032327	-0.170306704	0.105499768	6.41E-01	7.14E-01	1.00E+00
CYP1B1	LMNA	0.420307614	0.299643069	0.527737248	4.74E-10	4.16E-09	5.62E-06
CACNA1G	LMNA	-0.17321611	-0.303997717	-0.036026601	1.37E-02	2.68E-02	1.00E+00
VGLL4	LMNA	0.261156226	0.127709521	0.385315494	1.74E-04	5.42E-04	1.00E+00

XYLT1	LMNA	-0.171454835	-0.302349374	-0.034213655	1.47E-02	2.84E-02	1.00E+00
BRWD1	LMNA	-0.43360937	-0.539373369	-0.314377045	1.15E-10	1.14E-09	1.36E-06
ROS1	LMNA	0.05692044	-0.081773127	0.193451315	4.21E-01	5.08E-01	1.00E+00
BMP8B	LMNA	0.009483532	-0.128736011	0.147341631	8.93E-01	9.23E-01	1.00E+00
SLC5A4	LMNA	-0.090119662	-0.225366849	0.04853517	2.02E-01	2.76E-01	1.00E+00
SLC6A3	LMNA	-0.000712433	-0.138749787	0.137352077	9.92E-01	9.93E-01	1.00E+00
C16orf53	LMNA	0.136356973	-0.001726528	0.269337899	5.30E-02	8.83E-02	1.00E+00
APC2	LMNA	-0.260302832	-0.384535523	-0.12680871	1.83E-04	5.67E-04	1.00E+00
GOLPH3L	LMNA	0.019396751	-0.118972828	0.157027274	7.84E-01	8.36E-01	1.00E+00
ZBTB17	LMNA	-0.085640585	-0.221077834	0.053037465	2.26E-01	3.03E-01	1.00E+00
C6orf54	LMNA	-0.191822736	-0.321363604	-0.055234416	6.24E-03	1.33E-02	1.00E+00
SLC19A2	LMNA	0.005289797	-0.132858225	0.143236197	9.40E-01	9.56E-01	1.00E+00
C6orf134	LMNA	-0.545689928	-0.635841087	-0.440849458	4.53E-17	1.41E-15	5.38E-13
C9	LMNA	-0.073557812	-0.209481584	0.065154818	2.98E-01	3.82E-01	1.00E+00
ZBED5	LMNA	-0.349427808	-0.465045543	-0.222090185	3.45E-07	1.80E-06	4.09E-03
PVR	LMNA	0.505383858	0.394883376	0.601470982	1.71E-14	3.18E-13	2.03E-10
ARTN	LMNA	-0.036397201	-0.173576039	0.102167156	6.07E-01	6.83E-01	1.00E+00
LTA4H	LMNA	0.232463734	0.097543071	0.358993954	8.71E-04	2.32E-03	1.00E+00
MAGEA4	LMNA	0.079219295	-0.059482222	0.214919866	2.62E-01	3.44E-01	1.00E+00
PDE3B	LMNA	-0.37694874	-0.489525727	-0.252011976	3.21E-08	2.00E-07	3.81E-04
GNRH2	LMNA	-0.011341626	-0.149159085	0.126908077	8.73E-01	9.06E-01	1.00E+00
STEAP1	LMNA	0.443394606	0.325252669	0.547907597	3.88E-11	4.14E-10	4.60E-07
FLJ10292	LMNA	0.079547846	-0.05915275	0.215235207	2.60E-01	3.42E-01	1.00E+00
RPL39L	LMNA	0.173877315	0.036707438	0.304616325	1.33E-02	2.62E-02	1.00E+00
PGK1	LMNA	0.366308563	0.240415181	0.480082162	8.26E-08	4.81E-07	9.79E-04
CCL13	LMNA	0.347242976	0.219724984	0.463094504	4.13E-07	2.12E-06	4.89E-03
RLF	LMNA	-0.264656455	-0.38851272	-0.131406535	1.41E-04	4.50E-04	1.00E+00
MLXIP	LMNA	0.236335776	0.101599603	0.362557823	7.09E-04	1.93E-03	1.00E+00
PLOD1	LMNA	0.739162816	0.669420982	0.79598934	3.56E-36	3.84E-33	4.22E-32
MTFR1	LMNA	0.074771005	-0.063939998	0.210647656	2.90E-01	3.74E-01	1.00E+00
NPDC1	LMNA	-0.170098816	-0.301079768	-0.032818469	1.55E-02	2.98E-02	1.00E+00
C11orf16	LMNA	0.017786501	-0.120560529	0.155455789	8.02E-01	8.50E-01	1.00E+00
RRN3	LMNA	-0.115720088	-0.249780772	0.022693447	1.01E-01	1.53E-01	1.00E+00
GPAA1	LMNA	0.297269288	0.166031961	0.418159716	1.74E-05	6.58E-05	2.06E-01
C3orf14	LMNA	-0.087756166	-0.223104297	0.05091162	2.14E-01	2.90E-01	1.00E+00

TEX264	LMNA	0.343905776	0.216115175	0.460112241	5.41E-07	2.71E-06	6.42E-03
C22orf28	LMNA	-0.110992277	-0.245284877	0.027479785	1.16E-01	1.72E-01	1.00E+00
RYR3	LMNA	0.084026197	-0.054658841	0.219530664	2.34E-01	3.13E-01	1.00E+00
VAMP2	LMNA	-0.357226241	-0.472000337	-0.230544651	1.80E-07	9.91E-07	2.14E-03
XPNPEP2	LMNA	0.315243125	0.185254323	0.434389649	4.88E-06	2.05E-05	5.79E-02
PDE6A	LMNA	-0.148862884	-0.281136344	-0.011038736	3.45E-02	6.05E-02	1.00E+00
SUPV3L1	LMNA	-0.212868695	-0.340901707	-0.07708291	2.35E-03	5.61E-03	1.00E+00
FIBP	LMNA	0.109852336	-0.028632888	0.244199989	1.20E-01	1.77E-01	1.00E+00
SPIB	LMNA	0.032243727	-0.106280357	0.169540058	6.49E-01	7.21E-01	1.00E+00
TBCB	LMNA	-0.126394158	-0.25991003	0.011863853	7.31E-02	1.17E-01	1.00E+00
DHCR24	LMNA	0.155212089	0.017536853	0.287111107	2.74E-02	4.94E-02	1.00E+00
ADRA2C	LMNA	-0.311300525	-0.43083619	-0.181029323	6.50E-06	2.66E-05	7.71E-02
MEF2D	LMNA	0.162971699	0.025494279	0.294399187	2.05E-02	3.82E-02	1.00E+00
MYO1B	LMNA	0.380069716	0.255420368	0.492290713	2.42E-08	1.54E-07	2.87E-04
VAMP8	LMNA	0.211287427	0.0754368	0.339437563	2.54E-03	6.00E-03	1.00E+00
ANKRA2	LMNA	-0.305696764	-0.425779143	-0.175032438	9.68E-06	3.84E-05	1.15E-01
PANX1	LMNA	0.342395704	0.214482895	0.458761914	6.11E-07	3.04E-06	7.25E-03
RCBTB1	LMNA	-0.361258624	-0.475590901	-0.234923768	1.28E-07	7.20E-07	1.52E-03
FGL2	LMNA	0.234413314	0.099584982	0.36078883	7.85E-04	2.12E-03	1.00E+00
CEP70	LMNA	-0.392158969	-0.502979686	-0.268652247	7.84E-09	5.47E-08	9.30E-05
WASL	LMNA	0.219659957	0.08416108	0.347182887	1.68E-03	4.16E-03	1.00E+00
DCHS2	LMNA	-0.094904864	-0.229943215	0.04371893	1.79E-01	2.49E-01	1.00E+00
RNF25	LMNA	0.118436139	-0.0199409	0.25236098	9.32E-02	1.43E-01	1.00E+00
CYBA	LMNA	0.24385418	0.109489053	0.369467317	4.70E-04	1.33E-03	1.00E+00
ARHGAP11	LMNA	0.066279086	-0.072434685	0.202477439	3.49E-01	4.35E-01	1.00E+00
PLAU	LMNA	0.617500571	0.524129842	0.69620268	1.27E-22	1.06E-20	1.51E-18
MPZL2	LMNA	0.404949078	0.282702153	0.514251553	2.26E-09	1.75E-08	2.68E-05
MATK	LMNA	-0.17276337	-0.303574081	-0.035560492	1.39E-02	2.72E-02	1.00E+00
EHF	LMNA	0.160300584	0.022753101	0.291892112	2.27E-02	4.18E-02	1.00E+00
CTNND2	LMNA	-0.442277778	-0.54693465	-0.324009808	4.40E-11	4.66E-10	5.22E-07
PTEN	LMNA	0.05180184	-0.086870396	0.188504787	4.64E-01	5.50E-01	1.00E+00
ANXA1	LMNA	0.647390831	0.559328687	0.721003611	2.22E-25	3.25E-23	2.63E-21
AFF1	LMNA	0.325316264	0.196070852	0.443451743	2.31E-06	1.04E-05	2.74E-02
ZNF189	LMNA	-0.280367474	-0.402827049	-0.148046619	5.31E-05	1.83E-04	6.30E-01
SUSD5	LMNA	-0.282806004	-0.40504344	-0.150636085	4.54E-05	1.59E-04	5.39E-01

SLC28A3	LMNA	0.271469189	0.138612929	0.39472716	9.31E-05	3.06E-04	1.00E+00
GUCY1A3	LMNA	0.095155513	-0.043466477	0.230182761	1.78E-01	2.48E-01	1.00E+00
RASSF7	LMNA	0.148937946	0.011115491	0.28120704	3.44E-02	6.03E-02	1.00E+00
SETD2	LMNA	-0.111009359	-0.245301131	0.027462503	1.16E-01	1.72E-01	1.00E+00
ROGDI	LMNA	-0.350920945	-0.466378256	-0.223707448	3.05E-07	1.61E-06	3.62E-03
C20orf195	LMNA	0.176427958	0.039335003	0.30700161	1.20E-02	2.39E-02	1.00E+00
TICAM1	LMNA	0.427192807	0.307262422	0.533765321	2.29E-10	2.14E-09	2.72E-06
PACSIN2	LMNA	0.241201901	0.106703946	0.367031429	5.44E-04	1.52E-03	1.00E+00
SERPINB5	LMNA	0.090813722	-0.047837008	0.226030989	1.99E-01	2.73E-01	1.00E+00
PRKCDBP	LMNA	0.470380895	0.355408925	0.571331592	1.62E-12	2.18E-11	1.93E-08
TFDP3	LMNA	-0.04672552	-0.183592257	0.091918401	5.09E-01	5.93E-01	1.00E+00
PLCB2	LMNA	0.178183594	0.041144688	0.308642476	1.12E-02	2.24E-02	1.00E+00
RFX5	LMNA	-0.039462772	-0.17655194	0.099128267	5.77E-01	6.56E-01	1.00E+00
TXNDC15	LMNA	0.520673055	0.412254689	0.614550511	1.97E-15	4.43E-14	2.34E-11
PTPN18	LMNA	0.389265619	0.265481181	0.500424551	1.03E-08	7.02E-08	1.22E-04
HLTF	LMNA	-0.342644231	-0.458984188	-0.214751487	5.99E-07	2.98E-06	7.10E-03
PKP1	LMNA	0.163299399	0.025830716	0.294706637	2.02E-02	3.77E-02	1.00E+00
NDUFA6	LMNA	-0.102607902	-0.237297553	0.035952369	1.46E-01	2.10E-01	1.00E+00
KCNF1	LMNA	0.244666034	0.110341988	0.37021259	4.49E-04	1.28E-03	1.00E+00
HMG20B	LMNA	0.338320862	0.210081847	0.455115453	8.45E-07	4.10E-06	1.00E-02
SYNE2	LMNA	-0.041700726	-0.17872285	0.096908158	5.56E-01	6.37E-01	1.00E+00
SLC22A4	LMNA	0.292936309	0.161412869	0.414235561	2.33E-05	8.63E-05	2.76E-01
VCPIP1	LMNA	0.376765069	0.251811487	0.489362935	3.26E-08	2.03E-07	3.87E-04
NETO2	LMNA	0.011572261	-0.126681121	0.14938461	8.70E-01	9.04E-01	1.00E+00
SQRDL	LMNA	0.469629697	0.354566236	0.570681772	1.78E-12	2.38E-11	2.11E-08
BAI3	LMNA	-0.604308975	-0.685197094	-0.508696187	1.70E-21	1.06E-19	2.02E-17
GALK1	LMNA	0.307386629	0.176839833	0.427304932	8.59E-06	3.44E-05	1.02E-01
SERPINA6	LMNA	-0.095683948	-0.230687734	0.042934181	1.76E-01	2.45E-01	1.00E+00
ASCL3	LMNA	-0.136486348	-0.269460159	0.001594701	5.28E-02	8.80E-02	1.00E+00
NOSIP	LMNA	-0.089099926	-0.224390844	0.049560687	2.07E-01	2.82E-01	1.00E+00
HD	LMNA	-0.005093695	-0.143044109	0.133050866	9.43E-01	9.58E-01	1.00E+00
TRIM23	LMNA	-0.426737006	-0.533366596	-0.306757543	2.41E-10	2.24E-09	2.85E-06
FBXL6	LMNA	0.20079302	0.064530792	0.329704712	4.17E-03	9.30E-03	1.00E+00
FABP7	LMNA	0.143981013	0.006050272	0.276535396	4.09E-02	7.04E-02	1.00E+00
ARL1	LMNA	0.267984992	0.134925646	0.391550354	1.15E-04	3.73E-04	1.00E+00

MAGEC3	LMNA	-0.027151945	-0.164586019	0.111316308	7.01E-01	7.66E-01	1.00E+00
CDK5RAP2	LMNA	0.073414603	-0.065298192	0.209343911	2.99E-01	3.83E-01	1.00E+00
KCTD15	LMNA	-0.163242918	-0.294653649	-0.025772728	2.03E-02	3.78E-02	1.00E+00
CD1D	LMNA	0.227706488	0.092565287	0.354610276	1.12E-03	2.90E-03	1.00E+00
EIF3B	LMNA	0.315081528	0.185081058	0.434244075	4.94E-06	2.07E-05	5.86E-02
KIAA0141	LMNA	-0.007772474	-0.145667174	0.130418465	9.13E-01	9.37E-01	1.00E+00
BIK	LMNA	-0.029802955	-0.167166179	0.108695254	6.74E-01	7.43E-01	1.00E+00
PAX4	LMNA	-0.367551358	-0.481186552	-0.24176786	7.41E-08	4.36E-07	8.79E-04
NANOG	LMNA	-0.198833289	-0.327884153	-0.062497799	4.56E-03	1.01E-02	1.00E+00
CEP135	LMNA	-0.089310795	-0.224592693	0.049348647	2.06E-01	2.81E-01	1.00E+00
ALOX5	LMNA	0.372146191	0.246773201	0.485266548	4.94E-08	3.00E-07	5.86E-04
TRIM22	LMNA	0.300866439	0.169871016	0.421414044	1.36E-05	5.24E-05	1.61E-01
LYRM2	LMNA	-0.214586951	-0.342491989	-0.078872464	2.16E-03	5.20E-03	1.00E+00
GPR162	LMNA	-0.452156516	-0.555530911	-0.33501753	1.43E-11	1.63E-10	1.69E-07
RNASE6	LMNA	0.271182831	0.138309741	0.394466177	9.47E-05	3.11E-04	1.00E+00
GJA1	LMNA	0.127303109	-0.010940158	0.260771232	7.10E-02	1.14E-01	1.00E+00
TAS2R10	LMNA	-0.289617455	-0.411226795	-0.15787876	2.91E-05	1.06E-04	3.45E-01
FASN	LMNA	-0.161575584	-0.293089037	-0.024061287	2.16E-02	4.01E-02	1.00E+00
MRPS14	LMNA	0.027818886	-0.110657085	0.165235313	6.94E-01	7.60E-01	1.00E+00
HMHB1	LMNA	0.043588169	-0.095034692	0.180552714	5.38E-01	6.20E-01	1.00E+00
GPR116	LMNA	0.127269912	-0.010973897	0.260739783	7.11E-02	1.14E-01	1.00E+00
TAF7	LMNA	-0.105309506	-0.23987321	0.033224515	1.36E-01	1.97E-01	1.00E+00
GK2	LMNA	0.012573183	-0.125695993	0.150363192	8.59E-01	8.96E-01	1.00E+00
ZNF219	LMNA	-0.085189664	-0.220645756	0.053490412	2.28E-01	3.05E-01	1.00E+00
AMBP	LMNA	0.069155506	-0.069559579	0.205247016	3.28E-01	4.14E-01	1.00E+00
CD33	LMNA	0.223845246	0.088530005	0.351048136	1.36E-03	3.45E-03	1.00E+00
RAB3GAP1	LMNA	0.017724837	-0.120621316	0.155395595	8.02E-01	8.51E-01	1.00E+00
NXPH3	LMNA	-0.283908378	-0.406044921	-0.151807288	4.23E-05	1.49E-04	5.02E-01
KIAA0953	LMNA	-0.062621442	-0.198952507	0.07608733	3.76E-01	4.63E-01	1.00E+00
CROCC	LMNA	-0.102006635	-0.236724059	0.036559197	1.49E-01	2.13E-01	1.00E+00
CCBP2	LMNA	-0.263459678	-0.38741988	-0.130142058	1.52E-04	4.79E-04	1.00E+00
GPX7	LMNA	0.275420925	0.142799464	0.398326694	7.27E-05	2.45E-04	8.63E-01
TGM2	LMNA	0.521009703	0.412638071	0.614837925	1.88E-15	4.26E-14	2.23E-11
STAM	LMNA	-0.241847132	-0.367624176	-0.107381296	5.25E-04	1.47E-03	1.00E+00
BASP1	LMNA	-0.417269895	-0.525074242	-0.296286328	6.50E-10	5.57E-09	7.71E-06

ZNF202	LMNA	-0.200247373	-0.329197911	-0.063964633	4.27E-03	9.51E-03	1.00E+00
ACTL6A	LMNA	-0.031053452	-0.168382603	0.107458217	6.61E-01	7.31E-01	1.00E+00
ARHGEF7	LMNA	-0.469738883	-0.570776231	-0.354688708	1.76E-12	2.35E-11	2.08E-08
SLC23A2	LMNA	-0.129500255	-0.26285208	0.008706396	6.62E-02	1.07E-01	1.00E+00
TBK1	LMNA	0.043771211	-0.094852953	0.180730121	5.36E-01	6.18E-01	1.00E+00
CRYM	LMNA	-0.313204619	-0.43255281	-0.183069204	5.66E-06	2.34E-05	6.72E-02
PKD2	LMNA	0.437213424	0.318379067	0.542519186	7.73E-11	7.90E-10	9.17E-07
STX2	LMNA	0.135294279	-0.002809191	0.268333478	5.49E-02	9.10E-02	1.00E+00
ACTL7B	LMNA	0.204992137	0.0688907	0.3336024	3.43E-03	7.82E-03	1.00E+00
RPL29	LMNA	0.012763531	-0.125508618	0.150549261	8.57E-01	8.95E-01	1.00E+00
NR1H3	LMNA	0.182518364	0.045616761	0.31269055	9.33E-03	1.90E-02	1.00E+00
MPPE1	LMNA	-0.022071452	-0.159636043	0.116334017	7.55E-01	8.11E-01	1.00E+00
CLCN6	LMNA	-0.319609719	-0.43832093	-0.189939304	3.54E-06	1.53E-05	4.20E-02
C16orf59	LMNA	-0.184666558	-0.314694914	-0.047835035	8.51E-03	1.76E-02	1.00E+00
AADAC	LMNA	0.00570479	-0.132450523	0.143642664	9.36E-01	9.53E-01	1.00E+00
SQSTM1	LMNA	0.498618618	0.387221963	0.595667	4.29E-14	7.23E-13	5.09E-10
TCP10	LMNA	0.123109042	-0.015200293	0.256795715	8.09E-02	1.27E-01	1.00E+00
BIN3	LMNA	0.373045264	0.247753382	0.486064307	4.56E-08	2.77E-07	5.41E-04
DEFA4	LMNA	0.043073468	-0.095545679	0.180053808	5.43E-01	6.24E-01	1.00E+00
HPS6	LMNA	0.156186368	0.018535014	0.288027021	2.64E-02	4.78E-02	1.00E+00
ZNF197	LMNA	-0.154699343	-0.286628981	-0.01701165	2.79E-02	5.02E-02	1.00E+00
MAN2A2	LMNA	-0.125218114	-0.258795451	0.013058624	7.58E-02	1.20E-01	1.00E+00
PTOV1	LMNA	-0.235442789	-0.361736236	-0.100663671	7.43E-04	2.01E-03	1.00E+00
GABPB2	LMNA	0.225333856	0.090085186	0.352421871	1.26E-03	3.23E-03	1.00E+00
KCND1	LMNA	0.087072659	-0.051598581	0.22244971	2.18E-01	2.94E-01	1.00E+00
RNF208	LMNA	-0.26717721	-0.390813417	-0.134071307	1.21E-04	3.90E-04	1.00E+00
PTPN11	LMNA	-0.099021323	-0.233875251	0.039570607	1.61E-01	2.28E-01	1.00E+00
ZNF274	LMNA	-0.04100618	-0.178049253	0.097597314	5.62E-01	6.43E-01	1.00E+00
C7orf26	LMNA	0.103581979	-0.034969065	0.238226438	1.42E-01	2.06E-01	1.00E+00
ATF3	LMNA	0.388323135	0.264448811	0.499591819	1.13E-08	7.60E-08	1.34E-04
UCHL1	LMNA	-0.309811807	-0.429493446	-0.17943522	7.23E-06	2.94E-05	8.57E-02
APOL6	LMNA	0.335940353	0.207513171	0.452983388	1.02E-06	4.88E-06	1.21E-02
PLK1	LMNA	0.042896743	-0.095721113	0.17988249	5.44E-01	6.26E-01	1.00E+00
NPHP1	LMNA	0.078270664	-0.06043334	0.214009222	2.68E-01	3.50E-01	1.00E+00
DAB1	LMNA	-0.298954165	-0.419684404	-0.167829649	1.55E-05	5.92E-05	1.84E-01

MLL	LMNA	-0.266122532	-0.389850998	-0.132956138	1.29E-04	4.14E-04	1.00E+00
ANKRD15	LMNA	-0.201872974	-0.330707563	-0.065651603	3.96E-03	8.89E-03	1.00E+00
C1orf218	LMNA	0.008370615	-0.129830414	0.14625261	9.06E-01	9.32E-01	1.00E+00
DDX47	LMNA	-0.072338382	-0.208309127	0.06637547	3.06E-01	3.91E-01	1.00E+00
ATP8B2	LMNA	0.267822994	0.134754295	0.391402576	1.16E-04	3.76E-04	1.00E+00
ANXA13	LMNA	0.263222662	0.129891685	0.387203406	1.54E-04	4.85E-04	1.00E+00
RFTN1	LMNA	0.346380664	0.218791897	0.462324157	4.43E-07	2.26E-06	5.25E-03
TAPBPL	LMNA	0.423847638	0.30355864	0.530837936	3.27E-10	2.95E-09	3.88E-06
BTG1	LMNA	0.123576633	-0.014725584	0.257239165	7.97E-02	1.25E-01	1.00E+00
DPP4	LMNA	0.457366145	0.340835444	0.560055333	7.77E-12	9.33E-11	9.22E-08
KLHL23	LMNA	-0.639050622	-0.714102384	-0.54947519	1.40E-24	1.69E-22	1.66E-20
APOC3	LMNA	-0.031341591	-0.168662831	0.10717312	6.58E-01	7.28E-01	1.00E+00
NPPB	LMNA	0.067919231	-0.070795571	0.204056929	3.37E-01	4.23E-01	1.00E+00
ZNF148	LMNA	-0.081070077	-0.21669586	0.057625861	2.51E-01	3.32E-01	1.00E+00
CNOT4	LMNA	-0.035129162	-0.172344359	0.103423402	6.20E-01	6.95E-01	1.00E+00
HIST1H3I	LMNA	-0.037538758	-0.174684498	0.101035837	5.96E-01	6.73E-01	1.00E+00
IKZF1	LMNA	0.16243909	0.024947535	0.293899431	2.09E-02	3.89E-02	1.00E+00
ZNF141	LMNA	-0.259923957	-0.384189189	-0.126408856	1.87E-04	5.78E-04	1.00E+00
PSMC2	LMNA	0.321944796	0.192447067	0.440421357	2.98E-06	1.30E-05	3.53E-02
APEH	LMNA	0.267672706	0.134595338	0.391265475	1.18E-04	3.79E-04	1.00E+00
GGA3	LMNA	-0.245166967	-0.370672359	-0.110868366	4.37E-04	1.25E-03	1.00E+00
KCNA2	LMNA	-0.459854156	-0.562213945	-0.343617112	5.79E-12	7.11E-11	6.87E-08
LPGAT1	LMNA	0.097115319	-0.041491959	0.232055187	1.69E-01	2.37E-01	1.00E+00
TMEM159	LMNA	0.26604846	0.13287783	0.389783396	1.30E-04	4.16E-04	1.00E+00
PCYT1A	LMNA	0.413054785	0.291633492	0.521375581	1.00E-09	8.28E-09	1.19E-05
BRMS1	LMNA	0.429311903	0.309610573	0.535618439	1.83E-10	1.74E-09	2.17E-06
CHST1	LMNA	-0.007638341	-0.145535877	0.130550323	9.14E-01	9.38E-01	1.00E+00
LGALS1	LMNA	0.707718154	0.631350893	0.770491237	5.26E-32	2.97E-29	6.23E-28
THOC2	LMNA	0.126474261	-0.01178246	0.259985934	7.29E-02	1.16E-01	1.00E+00
TAF1B	LMNA	-0.185477887	-0.31545162	-0.048673183	8.22E-03	1.71E-02	1.00E+00
GPR173	LMNA	0.00136345	-0.136713284	0.139388214	9.85E-01	9.88E-01	1.00E+00
CASP10	LMNA	0.140814051	0.002817828	0.273547421	4.56E-02	7.74E-02	1.00E+00
COL15A1	LMNA	0.531701912	0.424834613	0.62395349	3.89E-16	1.01E-14	4.61E-12
ALK	LMNA	0.21547448	0.079797163	0.343313128	2.07E-03	5.00E-03	1.00E+00
GAN	LMNA	-0.144661176	-0.277176785	-0.006744875	4.00E-02	6.89E-02	1.00E+00

EXOC6B	LMNA	0.086296479	-0.052378523	0.221706224	2.22E-01	2.99E-01	1.00E+00
PCMT1	LMNA	-0.171383462	-0.302282561	-0.034140207	1.47E-02	2.85E-02	1.00E+00
HIST1H2AE	LMNA	-0.039156541	-0.176254779	0.099431949	5.80E-01	6.59E-01	1.00E+00
VAMP1	LMNA	-0.296419068	-0.417390071	-0.165125145	1.84E-05	6.94E-05	2.18E-01
HDAC5	LMNA	-0.284103849	-0.406222471	-0.152015002	4.18E-05	1.47E-04	4.95E-01
SRI	LMNA	-0.086294484	-0.221704312	0.052380528	2.22E-01	2.99E-01	1.00E+00
HLA-E	LMNA	0.359241172	0.2327322	0.473794973	1.52E-07	8.43E-07	1.80E-03
SLC25A32	LMNA	0.190165239	0.053519253	0.319820161	6.71E-03	1.42E-02	1.00E+00
FLT3LG	LMNA	0.353522054	0.226526467	0.468698651	2.46E-07	1.32E-06	2.91E-03
WDR1	LMNA	0.618765955	0.525613524	0.697256433	9.85E-23	8.41E-21	1.17E-18
ATP1B1	LMNA	-0.345564453	-0.461594833	-0.21790891	4.73E-07	2.40E-06	5.61E-03
SGPL1	LMNA	0.043349277	-0.095271869	0.180321162	5.40E-01	6.22E-01	1.00E+00
AFG3L2	LMNA	0.225144688	0.089887521	0.352247331	1.28E-03	3.25E-03	1.00E+00
C5orf15	LMNA	0.255978621	0.12224762	0.380580624	2.36E-04	7.15E-04	1.00E+00
SWAP70	LMNA	0.572654638	0.471910803	0.658636751	5.37E-19	2.35E-17	6.37E-15
UBXD1	LMNA	0.118151658	-0.020229302	0.252090816	9.40E-02	1.44E-01	1.00E+00
TRIM31	LMNA	0.11494723	-0.023476306	0.249046221	1.03E-01	1.56E-01	1.00E+00
LILRB4	LMNA	0.149772788	0.011969267	0.281993217	3.34E-02	5.89E-02	1.00E+00
GSTA4	LMNA	-0.639447282	-0.714430936	-0.549943258	1.28E-24	1.57E-22	1.52E-20
ARNT	LMNA	0.277821942	0.145345463	0.400511872	6.25E-05	2.13E-04	7.41E-01
CDKN1B	LMNA	-0.486642685	-0.585367794	-0.373697228	2.09E-13	3.21E-12	2.48E-09
SEMA3A	LMNA	0.375920602	0.250889835	0.488614367	3.52E-08	2.17E-07	4.18E-04
FOXC1	LMNA	0.36184272	0.235558514	0.476110683	1.21E-07	6.87E-07	1.44E-03
BTRC	LMNA	-0.495606081	-0.593079243	-0.383815307	6.43E-14	1.05E-12	7.62E-10
LSM14A	LMNA	0.120806697	-0.017536773	0.25461142	8.68E-02	1.35E-01	1.00E+00
IQCH	LMNA	0.06023047	-0.078473024	0.196646381	3.94E-01	4.82E-01	1.00E+00
STEAP3	LMNA	0.607151853	0.512017058	0.687571998	9.82E-22	6.49E-20	1.16E-17
YEATS2	LMNA	-0.144458945	-0.276986094	-0.006538336	4.02E-02	6.93E-02	1.00E+00
CABP5	LMNA	-0.001038636	-0.139069697	0.137032012	9.88E-01	9.91E-01	1.00E+00
TRIM3	LMNA	-0.150930685	-0.283083325	-0.013153758	3.20E-02	5.68E-02	1.00E+00
FGG	LMNA	-0.020512133	-0.158115395	0.11787265	7.72E-01	8.26E-01	1.00E+00
ABCA1	LMNA	0.393358375	0.269967563	0.504038322	6.99E-09	4.94E-08	8.29E-05
HNRPM	LMNA	0.077351389	-0.061354785	0.213126531	2.74E-01	3.56E-01	1.00E+00
PLSCR2	LMNA	0.223556098	0.088228003	0.350781238	1.38E-03	3.49E-03	1.00E+00
JTB	LMNA	-0.131351659	-0.264604526	0.006823066	6.24E-02	1.02E-01	1.00E+00

PQBP1	LMNA	-0.205550437	-0.334120292	-0.06947077	3.34E-03	7.64E-03	1.00E+00
CLEC2B	LMNA	0.455539051	0.338793999	0.558469248	9.63E-12	1.13E-10	1.14E-07
EDC3	LMNA	-0.029090597	-0.166473044	0.109399752	6.81E-01	7.49E-01	1.00E+00
REST	LMNA	0.388946013	0.265131062	0.500142186	1.06E-08	7.21E-08	1.26E-04
THBS3	LMNA	0.537373017	0.431319395	0.628778182	1.65E-16	4.59E-15	1.95E-12
EVI1	LMNA	0.25833225	0.12472948	0.382733805	2.06E-04	6.30E-04	1.00E+00
MGAT5	LMNA	-0.078572073	-0.214298586	0.060131168	2.66E-01	3.48E-01	1.00E+00
TSPAN8	LMNA	-0.083619259	-0.219140562	0.055067425	2.37E-01	3.16E-01	1.00E+00
DYNLT1	LMNA	-0.048097584	-0.184920726	0.090554692	4.97E-01	5.81E-01	1.00E+00
MUC1	LMNA	0.503094949	0.39228954	0.599508434	2.34E-14	4.18E-13	2.77E-10
IGSF1	LMNA	-0.077274619	-0.213052807	0.061431725	2.74E-01	3.57E-01	1.00E+00
TEAD3	LMNA	0.649600247	0.561943166	0.722829381	1.35E-25	2.10E-23	1.60E-21
ATP13A3	LMNA	0.407845527	0.285891196	0.516798947	1.70E-09	1.35E-08	2.01E-05
C3AR1	LMNA	0.216626071	0.080997332	0.344378286	1.96E-03	4.76E-03	1.00E+00
STOML1	LMNA	0.110172671	-0.028308892	0.244504887	1.19E-01	1.76E-01	1.00E+00
EFNA4	LMNA	0.314147846	0.18408011	0.433402848	5.29E-06	2.20E-05	6.27E-02
HAO1	LMNA	0.141094601	0.003104062	0.273812219	4.52E-02	7.68E-02	1.00E+00
USP24	LMNA	-0.218589864	-0.346193927	-0.083044871	1.78E-03	4.37E-03	1.00E+00
TWF1	LMNA	0.082136825	-0.056555462	0.217719093	2.45E-01	3.25E-01	1.00E+00
MRPS17	LMNA	0.172810687	0.035609204	0.303618359	1.39E-02	2.71E-02	1.00E+00
MYH9	LMNA	0.69112792	0.611412319	0.756956974	5.06E-30	1.54E-27	6.01E-26
C9orf9	LMNA	0.031515209	-0.107001323	0.168831672	6.56E-01	7.27E-01	1.00E+00
LBP	LMNA	0.255176931	0.121402633	0.379846901	2.47E-04	7.46E-04	1.00E+00
FSCN3	LMNA	0.128470827	-0.009753151	0.261877296	6.84E-02	1.10E-01	1.00E+00
BDKRB2	LMNA	0.510052923	0.400179917	0.605470722	8.94E-15	1.75E-13	1.06E-10
HSD17B4	LMNA	-0.250099302	-0.37519611	-0.11605528	3.31E-04	9.73E-04	1.00E+00
SEC31B	LMNA	-0.396234693	-0.506575701	-0.273123723	5.31E-09	3.85E-08	6.29E-05
IDH2	LMNA	-0.081881836	-0.217474533	0.056811352	2.47E-01	3.27E-01	1.00E+00
SFRS16	LMNA	-0.01456491	-0.152309662	0.123734884	8.37E-01	8.78E-01	1.00E+00
AICDA	LMNA	0.263197669	0.129865285	0.387180579	1.54E-04	4.86E-04	1.00E+00
RAP1GAP	LMNA	-0.360366715	-0.474797045	-0.233954725	1.38E-07	7.72E-07	1.64E-03
C1orf56	LMNA	-0.05388584	-0.190519567	0.084795957	4.46E-01	5.33E-01	1.00E+00
DCLK1	LMNA	-0.255079558	-0.379757773	-0.121300015	2.49E-04	7.49E-04	1.00E+00
MEF2A	LMNA	0.279295357	0.146908716	0.401852143	5.69E-05	1.95E-04	6.75E-01
ASF1B	LMNA	0.180701361	0.043741537	0.310994296	1.01E-02	2.04E-02	1.00E+00

HTN3	LMNA	0.112735467	-0.025715754	0.24694323	1.10E-01	1.65E-01	1.00E+00
ADAMTS9	LMNA	0.290574601	0.158897638	0.412094784	2.73E-05	9.97E-05	3.24E-01
COPZ1	LMNA	0.073517193	-0.065195485	0.209442536	2.98E-01	3.83E-01	1.00E+00
SLC4A3	LMNA	0.309329734	0.178919169	0.429058529	7.48E-06	3.03E-05	8.88E-02
TAF2	LMNA	-0.196005167	-0.325255182	-0.059565944	5.18E-03	1.13E-02	1.00E+00
KATNA1	LMNA	0.030458335	-0.108046982	0.167803754	6.67E-01	7.37E-01	1.00E+00
STIM1	LMNA	0.403557132	0.281170555	0.513026665	2.60E-09	1.98E-08	3.08E-05
TBX2	LMNA	0.289832796	0.158107965	0.411422096	2.87E-05	1.04E-04	3.40E-01
FOXD3	LMNA	-0.04702322	-0.183880542	0.091622557	5.06E-01	5.90E-01	1.00E+00
RPS4X	LMNA	0.027057542	-0.111409609	0.164494105	7.02E-01	7.66E-01	1.00E+00
PODXL2	LMNA	-0.490278656	-0.588498072	-0.377798355	1.30E-13	2.06E-12	1.54E-09
C1orf176	LMNA	-0.24368941	-0.36931604	-0.10931597	4.74E-04	1.34E-03	1.00E+00
RPS3	LMNA	-0.119415112	-0.253290523	0.018948258	9.05E-02	1.40E-01	1.00E+00
COL21A1	LMNA	0.280603053	0.1482967	0.403041231	5.23E-05	1.81E-04	6.21E-01
RAI14	LMNA	0.498788783	0.387414482	0.595813112	4.20E-14	7.08E-13	4.98E-10
LRFN3	LMNA	0.124536879	-0.013750526	0.258149657	7.74E-02	1.22E-01	1.00E+00
SRPK3	LMNA	-0.275233729	-0.398156268	-0.142601039	7.36E-05	2.47E-04	8.73E-01
FKBP14	LMNA	0.309374489	0.178967075	0.429098908	7.46E-06	3.02E-05	8.85E-02
TNNI3	LMNA	-0.119704058	-0.253564833	0.018655225	8.97E-02	1.39E-01	1.00E+00
CXorf9	LMNA	0.248855035	0.114746101	0.374055477	3.55E-04	1.04E-03	1.00E+00
REC8	LMNA	-0.305188935	-0.425320486	-0.174489461	1.00E-05	3.97E-05	1.19E-01
HOXB3	LMNA	0.324469584	0.195160475	0.442690972	2.46E-06	1.10E-05	2.92E-02
SGCB	LMNA	0.081821945	-0.056871453	0.217417089	2.47E-01	3.27E-01	1.00E+00
FRAT1	LMNA	-0.102356899	-0.237058156	0.036205705	1.47E-01	2.11E-01	1.00E+00
CLP1	LMNA	0.233383827	0.098506598	0.359841152	8.30E-04	2.22E-03	1.00E+00
MORN1	LMNA	0.128134955	-0.010094611	0.261559193	6.92E-02	1.11E-01	1.00E+00
DUOX2	LMNA	0.114373541	-0.02405731	0.248500866	1.05E-01	1.59E-01	1.00E+00
TSC22D3	LMNA	0.234640549	0.099823053	0.360997972	7.76E-04	2.10E-03	1.00E+00
ARHGEF2	LMNA	0.050939999	-0.087727933	0.187671236	4.72E-01	5.57E-01	1.00E+00
CASP8	LMNA	0.539652997	0.433929593	0.630715893	1.16E-16	3.34E-15	1.38E-12
PRKD3	LMNA	-0.044512307	-0.181448313	0.09411704	5.29E-01	6.12E-01	1.00E+00
CFH	LMNA	0.379061331	0.25431878	0.491397596	2.65E-08	1.68E-07	3.14E-04
NRIP1	LMNA	0.301175822	0.170201391	0.421693797	1.33E-05	5.14E-05	1.57E-01
TRO	LMNA	-0.424958241	-0.531810117	-0.3047879	2.91E-10	2.65E-09	3.45E-06
BNIP2	LMNA	0.343271352	0.215429322	0.459544996	5.69E-07	2.84E-06	6.75E-03

DHX30	LMNA	-0.064954391	-0.201201217	0.073757999	3.58E-01	4.45E-01	1.00E+00
TBC1D22B	LMNA	-0.019319913	-0.156952301	0.119048606	7.85E-01	8.37E-01	1.00E+00
GPR52	LMNA	0.070304865	-0.0684101	0.206353074	3.20E-01	4.06E-01	1.00E+00
FGF20	LMNA	-0.31087584	-0.430453201	-0.180574505	6.70E-06	2.74E-05	7.95E-02
CASC3	LMNA	-0.431908584	-0.537887802	-0.312489923	1.38E-10	1.34E-09	1.64E-06
METRN	LMNA	-0.180602456	-0.310901941	-0.043639491	1.01E-02	2.05E-02	1.00E+00
KRT3	LMNA	-0.029935306	-0.167294944	0.108564348	6.72E-01	7.42E-01	1.00E+00
ARF1	LMNA	0.22936658	0.094301572	0.356140642	1.03E-03	2.68E-03	1.00E+00
MOG	LMNA	-0.253151858	-0.377992816	-0.119269057	2.78E-04	8.30E-04	1.00E+00
ATP7A	LMNA	0.161983935	0.024480365	0.293472295	2.13E-02	3.95E-02	1.00E+00
CCNG2	LMNA	-0.355907313	-0.470825095	-0.229113425	2.01E-07	1.10E-06	2.39E-03
PLCH2	LMNA	-0.343488768	-0.459739401	-0.215664349	5.59E-07	2.80E-06	6.64E-03
ITSN2	LMNA	0.146036243	0.00814954	0.2784731	3.81E-02	6.60E-02	1.00E+00
GIP	LMNA	0.081345108	-0.057349919	0.2169597	2.50E-01	3.30E-01	1.00E+00
LOC89944	LMNA	-0.182347856	-0.312531409	-0.045440749	9.39E-03	1.92E-02	1.00E+00
PSPN	LMNA	0.210991839	0.075129173	0.3391638	2.58E-03	6.07E-03	1.00E+00
HOXB13	LMNA	-0.056959041	-0.193488592	0.08173466	4.21E-01	5.08E-01	1.00E+00
MTMR8	LMNA	-0.109570488	-0.243931701	0.028917934	1.21E-01	1.78E-01	1.00E+00
UBXD8	LMNA	0.300544287	0.169527037	0.421122721	1.39E-05	5.35E-05	1.64E-01
GYPE	LMNA	-0.253248043	-0.378080902	-0.119370367	2.76E-04	8.26E-04	1.00E+00
SPAM1	LMNA	-0.053903089	-0.190536238	0.084778782	4.46E-01	5.33E-01	1.00E+00
PPP2R1B	LMNA	0.260657776	0.12718335	0.384859951	1.79E-04	5.58E-04	1.00E+00
CNN3	LMNA	0.223937437	0.0886263	0.351133229	1.36E-03	3.43E-03	1.00E+00
JAG1	LMNA	0.333736575	0.205136773	0.451008415	1.21E-06	5.71E-06	1.44E-02
HIST1H2AL	LMNA	0.04199309	-0.096618024	0.179006357	5.53E-01	6.34E-01	1.00E+00
CHGA	LMNA	-0.417608743	-0.525371397	-0.296660614	6.27E-10	5.39E-09	7.44E-06
CACNA1B	LMNA	-0.221977212	-0.349323482	-0.086579365	1.50E-03	3.75E-03	1.00E+00
PAPPA	LMNA	0.448154217	0.330553976	0.552050859	2.26E-11	2.49E-10	2.68E-07
RAPGEFL1	LMNA	-0.504262916	-0.600510013	-0.393612883	1.99E-14	3.65E-13	2.36E-10
RHOA	LMNA	0.112958016	-0.025490482	0.247154892	1.09E-01	1.64E-01	1.00E+00
CYP4F8	LMNA	0.356593684	0.229858168	0.471436743	1.90E-07	1.04E-06	2.25E-03
TRH	LMNA	0.126370682	-0.011887707	0.259887784	7.31E-02	1.17E-01	1.00E+00
DCTN3	LMNA	-0.369398459	-0.482827283	-0.24377918	6.30E-08	3.76E-07	7.47E-04
NT5C	LMNA	0.076111195	-0.062597532	0.211935343	2.82E-01	3.64E-01	1.00E+00
SLC1A5	LMNA	0.494304092	0.382343925	0.591960216	7.64E-14	1.24E-12	9.07E-10

CALCA	LMNA	0.027332933	-0.111137426	0.16476223	6.99E-01	7.64E-01	1.00E+00
VPS41	LMNA	-0.198581272	-0.327649964	-0.062236442	4.61E-03	1.02E-02	1.00E+00
SYCP1	LMNA	-0.074871591	-0.210744318	0.063839258	2.90E-01	3.73E-01	1.00E+00
KIAA0174	LMNA	-0.082447909	-0.218017431	0.056243254	2.43E-01	3.23E-01	1.00E+00
CXCL11	LMNA	0.203602744	0.067447529	0.33231323	3.66E-03	8.27E-03	1.00E+00
ZNF639	LMNA	-0.235181424	-0.361495732	-0.100389782	7.54E-04	2.04E-03	1.00E+00
CACNG4	LMNA	-0.316084301	-0.43514732	-0.186156374	4.59E-06	1.94E-05	5.45E-02
TNNC1	LMNA	-0.065555894	-0.201780768	0.073157183	3.54E-01	4.41E-01	1.00E+00
GFI1B	LMNA	-0.041360729	-0.178393124	0.097245533	5.59E-01	6.39E-01	1.00E+00
C11orf58	LMNA	-0.060217066	-0.196633448	0.078486394	3.95E-01	4.82E-01	1.00E+00
PSCD1	LMNA	-0.236034968	-0.362281089	-0.101284302	7.20E-04	1.96E-03	1.00E+00
NUDT18	LMNA	0.149215957	0.011399785	0.281468865	3.41E-02	5.98E-02	1.00E+00
CASD1	LMNA	-0.31866327	-0.43746922	-0.188923345	3.80E-06	1.63E-05	4.51E-02
LPPR2	LMNA	0.001659514	-0.136422741	0.139678515	9.81E-01	9.85E-01	1.00E+00
TTC35	LMNA	0.030450706	-0.108054529	0.167796332	6.67E-01	7.37E-01	1.00E+00
SMC4	LMNA	0.183673945	0.046809873	0.313768905	8.88E-03	1.83E-02	1.00E+00
ZNF771	LMNA	0.007808774	-0.13038278	0.145702706	9.12E-01	9.37E-01	1.00E+00
TTBK2	LMNA	-0.460344383	-0.562639105	-0.344165442	5.46E-12	6.74E-11	6.48E-08
GJB3	LMNA	0.060195226	-0.078508178	0.196612376	3.95E-01	4.82E-01	1.00E+00
RGS19	LMNA	0.268882392	0.135875001	0.392368863	1.09E-04	3.54E-04	1.00E+00
SFRS3	LMNA	-0.237155583	-0.36331191	-0.102459047	6.78E-04	1.85E-03	1.00E+00
SCRG1	LMNA	-0.376587917	-0.489205915	-0.251618125	3.32E-08	2.06E-07	3.93E-04
NRAS	LMNA	0.074936726	-0.063774023	0.21080691	2.89E-01	3.73E-01	1.00E+00
FBXW2	LMNA	0.308733299	0.178280794	0.428520359	7.81E-06	3.15E-05	9.26E-02
SIX3	LMNA	-0.101462177	-0.236204669	0.037108603	1.51E-01	2.16E-01	1.00E+00
DUSP26	LMNA	-0.647737665	-0.721290288	-0.559738991	2.05E-25	3.04E-23	2.43E-21
HDAC9	LMNA	-0.123875246	-0.257522331	0.014422394	7.90E-02	1.24E-01	1.00E+00
ZDHHC24	LMNA	0.51165639	0.40200057	0.606843217	7.14E-15	1.43E-13	8.47E-11
OGG1	LMNA	-0.100577809	-0.235360856	0.038000828	1.54E-01	2.20E-01	1.00E+00
DNAJC3	LMNA	0.384406155	0.260161304	0.496128766	1.62E-08	1.07E-07	1.92E-04
LITAF	LMNA	0.364858945	0.23883801	0.478793526	9.37E-08	5.38E-07	1.11E-03
ZNF410	LMNA	0.026895358	-0.111569894	0.16433619	7.04E-01	7.68E-01	1.00E+00
APLP1	LMNA	-0.124638948	-0.258246423	0.013646868	7.72E-02	1.22E-01	1.00E+00
AFP	LMNA	0.227939899	0.092809361	0.354825489	1.10E-03	2.87E-03	1.00E+00
OR7A5	LMNA	-0.129018396	-0.262395834	0.009196402	6.73E-02	1.08E-01	1.00E+00

ZW10	LMNA	0.195073465	0.058600584	0.324388651	5.40E-03	1.17E-02	1.00E+00
DLX4	LMNA	-0.143664558	-0.276236942	-0.005727144	4.14E-02	7.10E-02	1.00E+00
TUBA1B	LMNA	-0.087284853	-0.222652939	0.051385329	2.17E-01	2.93E-01	1.00E+00
MGC70863	LMNA	0.055603592	-0.083085182	0.192179398	4.32E-01	5.18E-01	1.00E+00
C12orf29	LMNA	-0.328742293	-0.446528404	-0.1997569	1.78E-06	8.15E-06	2.11E-02
CRY1	LMNA	-0.067512702	-0.203665501	0.071201914	3.40E-01	4.26E-01	1.00E+00
OR1D2	LMNA	-0.065173511	-0.201412351	0.073539141	3.57E-01	4.44E-01	1.00E+00
C1orf25	LMNA	-0.225496438	-0.352571873	-0.090255079	1.25E-03	3.20E-03	1.00E+00
PHOX2B	LMNA	0.039605898	-0.098986323	0.176690818	5.76E-01	6.55E-01	1.00E+00
CUZD1	LMNA	-0.179395572	-0.309774772	-0.042394503	1.06E-02	2.14E-02	1.00E+00
SCAND1	LMNA	0.134437229	-0.003682108	0.267523215	5.65E-02	9.32E-02	1.00E+00
MYT1	LMNA	-0.508457851	-0.604104851	-0.398369658	1.12E-14	2.15E-13	1.32E-10
VILL	LMNA	-0.114605164	-0.24872106	0.023822744	1.04E-01	1.58E-01	1.00E+00
PPP3CC	LMNA	0.071265445	-0.067449138	0.207277198	3.14E-01	3.99E-01	1.00E+00
GOLGA1	LMNA	0.020462501	-0.117921613	0.158066983	7.73E-01	8.26E-01	1.00E+00
ZBTB43	LMNA	0.057069294	-0.081624786	0.193595061	4.20E-01	5.07E-01	1.00E+00
VAPA	LMNA	-0.127625424	-0.261076565	0.010612557	7.03E-02	1.13E-01	1.00E+00
MEA1	LMNA	0.021068378	-0.117323861	0.15865792	7.66E-01	8.21E-01	1.00E+00
STAP1	LMNA	0.025171082	-0.113273532	0.162656867	7.22E-01	7.84E-01	1.00E+00
PIK3R3	LMNA	-0.334131532	-0.45136245	-0.205562555	1.17E-06	5.57E-06	1.39E-02
TGM5	LMNA	0.177481187	0.040420549	0.30798608	1.15E-02	2.30E-02	1.00E+00
SLC34A1	LMNA	-0.15099551	-0.283144344	-0.013220083	3.19E-02	5.67E-02	1.00E+00
USPL1	LMNA	-0.19434504	-0.323711026	-0.05784602	5.58E-03	1.20E-02	1.00E+00
DLX6	LMNA	-0.187015675	-0.316885423	-0.050262333	7.70E-03	1.61E-02	1.00E+00
FBXO40	LMNA	-0.198196575	-0.32729245	-0.061837525	4.69E-03	1.03E-02	1.00E+00
NKG7	LMNA	0.245507622	0.111226369	0.370984987	4.29E-04	1.23E-03	1.00E+00
BRF1	LMNA	-0.108503065	-0.242915445	0.029997264	1.24E-01	1.83E-01	1.00E+00
CCL27	LMNA	-0.04681553	-0.183679423	0.091828955	5.08E-01	5.92E-01	1.00E+00
EMP1	LMNA	0.607088542	0.51194307	0.687519127	9.94E-22	6.51E-20	1.18E-17
ACTR6	LMNA	-0.421667577	-0.528928771	-0.301146824	4.11E-10	3.64E-09	4.88E-06
PFN2	LMNA	-0.490911897	-0.58904294	-0.378513059	1.20E-13	1.90E-12	1.42E-09
MYBPH	LMNA	0.356223455	0.229456434	0.471106833	1.96E-07	1.07E-06	2.32E-03
CHCHD7	LMNA	0.250194894	0.116155877	0.375283724	3.29E-04	9.69E-04	1.00E+00
TCEA2	LMNA	-0.203842544	-0.332535766	-0.06769657	3.62E-03	8.19E-03	1.00E+00
PPP1CC	LMNA	-0.255584072	-0.380219544	-0.121831738	2.41E-04	7.30E-04	1.00E+00

COG2	LMNA	-0.428474899	-0.534886614	-0.308682922	2.00E-10	1.88E-09	2.37E-06
FLJ20294	LMNA	0.030519955	-0.107986025	0.167863693	6.66E-01	7.36E-01	1.00E+00
TARS	LMNA	0.29686373	0.165599379	0.417792614	1.79E-05	6.75E-05	2.12E-01
ARHGAP28	LMNA	-0.07965969	-0.215342547	0.059040586	2.60E-01	3.41E-01	1.00E+00
TRAPPC2L	LMNA	-0.028754683	-0.166146148	0.109731911	6.85E-01	7.52E-01	1.00E+00
CCDC109B	LMNA	0.544360557	0.439324588	0.634713168	5.59E-17	1.70E-15	6.62E-13
LGTN	LMNA	0.316562133	0.186668883	0.435577641	4.44E-06	1.88E-05	5.26E-02
KCNB2	LMNA	-0.386336995	-0.497836291	-0.26227418	1.36E-08	9.07E-08	1.61E-04
USP13	LMNA	-0.068977498	-0.205075683	0.069737573	3.29E-01	4.15E-01	1.00E+00
RPS2	LMNA	0.197270707	0.060877612	0.326431852	4.89E-03	1.07E-02	1.00E+00
C17orf75	LMNA	-0.326244068	-0.444285209	-0.197068711	2.16E-06	9.71E-06	2.56E-02
FBXW4	LMNA	0.004860412	-0.133280017	0.142815586	9.45E-01	9.59E-01	1.00E+00
SLC2A8	LMNA	0.084303832	-0.054380057	0.219796787	2.33E-01	3.11E-01	1.00E+00
WT1	LMNA	0.192831534	0.056278706	0.322302649	5.97E-03	1.28E-02	1.00E+00
SNRPE	LMNA	-0.075477012	-0.211326064	0.063232854	2.86E-01	3.69E-01	1.00E+00
LEPROT	LMNA	0.318368032	0.18860648	0.437203491	3.88E-06	1.66E-05	4.61E-02
STK38L	LMNA	0.019070762	-0.119294309	0.156709189	7.88E-01	8.39E-01	1.00E+00
CUEDC2	LMNA	-0.377739755	-0.49022673	-0.252875545	2.99E-08	1.87E-07	3.55E-04
IL13RA2	LMNA	0.134357041	-0.00376377	0.267447395	5.66E-02	9.34E-02	1.00E+00
DDX42	LMNA	0.022030366	-0.116374566	0.159595985	7.56E-01	8.12E-01	1.00E+00
TXNRD2	LMNA	0.083654874	-0.055031667	0.219174705	2.37E-01	3.15E-01	1.00E+00
C4orf19	LMNA	-0.039801882	-0.176880976	0.09879195	5.74E-01	6.53E-01	1.00E+00
TNFRSF4	LMNA	0.127613765	-0.010624408	0.26106552	7.03E-02	1.13E-01	1.00E+00
AOC3	LMNA	0.217479757	0.081887283	0.345167685	1.88E-03	4.58E-03	1.00E+00
MTHFD2	LMNA	-0.057409914	-0.193923971	0.081285313	4.17E-01	5.05E-01	1.00E+00
KSR1	LMNA	0.100905559	-0.037670192	0.2356736	1.53E-01	2.19E-01	1.00E+00
SS18L2	LMNA	-0.299409068	-0.42009594	-0.168315159	1.50E-05	5.75E-05	1.78E-01
OAS3	LMNA	0.277745665	0.145264553	0.400442473	6.28E-05	2.13E-04	7.45E-01
SLC22A11	LMNA	-0.025437589	-0.162916478	0.113010268	7.19E-01	7.81E-01	1.00E+00
LARGE	LMNA	-0.365089696	-0.478998685	-0.239089021	9.18E-08	5.29E-07	1.09E-03
LIMA1	LMNA	0.028582503	-0.109902154	0.165978578	6.86E-01	7.53E-01	1.00E+00
STARD3	LMNA	0.139381839	0.001356948	0.272195311	4.79E-02	8.08E-02	1.00E+00
VPS39	LMNA	-0.017608058	-0.155281598	0.12073643	8.04E-01	8.52E-01	1.00E+00
ZNF236	LMNA	-0.243763958	-0.369384484	-0.109394278	4.72E-04	1.34E-03	1.00E+00
C8orf32	LMNA	-0.245416072	-0.370900972	-0.111130154	4.31E-04	1.23E-03	1.00E+00

GABRB1	LMNA	-0.29688481	-0.417811696	-0.165621862	1.78E-05	6.74E-05	2.11E-01
ODAM	LMNA	0.07114407	-0.067570576	0.207160442	3.14E-01	4.00E-01	1.00E+00
MORF4L2	LMNA	-0.049411046	-0.186191986	0.089248742	4.85E-01	5.70E-01	1.00E+00
FBLN1	LMNA	0.425289166	0.305154258	0.532099742	2.81E-10	2.57E-09	3.33E-06
PRKAB2	LMNA	-0.084548211	-0.220031017	0.054134648	2.32E-01	3.10E-01	1.00E+00
AFF4	LMNA	0.268133921	0.13508318	0.391686204	1.14E-04	3.70E-04	1.00E+00
HSPB2	LMNA	0.107365649	-0.031147011	0.241832228	1.28E-01	1.88E-01	1.00E+00
ZNF76	LMNA	-0.176207523	-0.30679553	-0.039107845	1.21E-02	2.41E-02	1.00E+00
RAF1	LMNA	-0.196076142	-0.325321184	-0.059639494	5.16E-03	1.12E-02	1.00E+00
SUB1	LMNA	-0.09912522	-0.233974436	0.039465844	1.60E-01	2.27E-01	1.00E+00
MRPS33	LMNA	-0.039449702	-0.176539257	0.099141229	5.77E-01	6.56E-01	1.00E+00
ZIC1	LMNA	0.016143044	-0.122180243	0.153851177	8.20E-01	8.64E-01	1.00E+00
KLRK1	LMNA	-0.336144463	-0.453166248	-0.207733344	1.00E-06	4.81E-06	1.19E-02
LYST	LMNA	0.055101288	-0.08358553	0.191694112	4.36E-01	5.22E-01	1.00E+00
UBE2M	LMNA	0.09106418	-0.047585036	0.226270619	1.97E-01	2.71E-01	1.00E+00
RAG1AP1	LMNA	0.425368878	0.305242511	0.532169502	2.78E-10	2.55E-09	3.30E-06
ZNF281	LMNA	-0.099713373	-0.234535863	0.038872732	1.58E-01	2.25E-01	1.00E+00
P2RX5	LMNA	-0.292821128	-0.414131186	-0.161290161	2.35E-05	8.69E-05	2.78E-01
NCR3	LMNA	0.00522694	-0.132919974	0.143174628	9.41E-01	9.56E-01	1.00E+00
ST8SIA1	LMNA	-0.159368515	-0.291016859	-0.021797071	2.35E-02	4.31E-02	1.00E+00
HLA-DPA1	LMNA	0.209096019	0.073156755	0.337407445	2.82E-03	6.57E-03	1.00E+00
FKBPL	LMNA	-0.035079523	-0.172296135	0.103472571	6.20E-01	6.95E-01	1.00E+00
ANKRD46	LMNA	-0.701969557	-0.765807948	-0.62443063	2.65E-31	1.05E-28	3.15E-27
CD248	LMNA	0.533596861	0.427000225	0.6255664	2.92E-16	7.74E-15	3.47E-12
SNX4	LMNA	-0.271043881	-0.394339533	-0.138162633	9.55E-05	3.13E-04	1.00E+00
CCR2	LMNA	0.418494342	0.297639007	0.526147903	5.72E-10	4.96E-09	6.79E-06
ZYX	LMNA	0.641163295	0.551968842	0.715851926	8.83E-25	1.15E-22	1.05E-20
SMOX	LMNA	0.194358281	0.057859735	0.323723345	5.58E-03	1.20E-02	1.00E+00
ZSCAN5	LMNA	-0.000860165	-0.138894672	0.137207129	9.90E-01	9.92E-01	1.00E+00
RIMS3	LMNA	-0.525962815	-0.619063784	-0.418283232	9.12E-16	2.22E-14	1.08E-11
NACAP1	LMNA	-0.029347034	-0.166722577	0.10914616	6.78E-01	7.47E-01	1.00E+00
DRD2	LMNA	-0.026644232	-0.16409166	0.111818065	7.07E-01	7.70E-01	1.00E+00
COPS2	LMNA	0.228824632	0.093734659	0.355641118	1.05E-03	2.75E-03	1.00E+00
CEACAM4	LMNA	0.316060073	0.18613039	0.4351255	4.60E-06	1.94E-05	5.46E-02
KRT76	LMNA	-0.02509986	-0.162587485	0.113343884	7.23E-01	7.84E-01	1.00E+00

SOX3	LMNA	-0.093562385	-0.228659927	0.045070767	1.85E-01	2.57E-01	1.00E+00
GATAD1	LMNA	0.248432183	0.114301295	0.373667758	3.64E-04	1.06E-03	1.00E+00
AVIL	LMNA	-0.122151817	-0.255887737	0.016171893	8.33E-02	1.30E-01	1.00E+00
LMOD1	LMNA	0.122221509	-0.016101163	0.255953851	8.31E-02	1.30E-01	1.00E+00
FCER1A	LMNA	0.146336877	0.008456719	0.278756453	3.77E-02	6.54E-02	1.00E+00
TMEM112B	LMNA	0.529546838	0.422373207	0.622118215	5.36E-16	1.37E-14	6.36E-12
HIGD1A	LMNA	-0.199337523	-0.328352671	-0.063020775	4.45E-03	9.87E-03	1.00E+00
CALR	LMNA	0.55459741	0.451082518	0.643388809	1.09E-17	3.76E-16	1.30E-13
ADRA1B	LMNA	0.124910965	-0.013370599	0.258504296	7.65E-02	1.21E-01	1.00E+00
SNRPD1	LMNA	-0.232414991	-0.358949067	-0.097492034	8.73E-04	2.33E-03	1.00E+00
LTB	LMNA	0.301392631	0.170432928	0.421889828	1.31E-05	5.08E-05	1.55E-01
NCAPG2	LMNA	0.153328142	0.015607507	0.285339336	2.94E-02	5.25E-02	1.00E+00
NEU3	LMNA	-0.221411354	-0.348800886	-0.085988689	1.54E-03	3.84E-03	1.00E+00
KCNMB1	LMNA	0.379413957	0.254703962	0.49170994	2.57E-08	1.63E-07	3.04E-04
DES	LMNA	0.416595752	0.295541792	0.524482971	6.97E-10	5.94E-09	8.26E-06
BZW1	LMNA	0.150194431	0.012400549	0.282390214	3.29E-02	5.81E-02	1.00E+00
ITGAV	LMNA	0.198356933	0.062003806	0.327441482	4.66E-03	1.03E-02	1.00E+00
ZNF221	LMNA	-0.202843033	-0.331608116	-0.066658654	3.79E-03	8.54E-03	1.00E+00
LENG4	LMNA	0.371169004	0.245708151	0.484399264	5.39E-08	3.25E-07	6.39E-04
C20orf3	LMNA	0.071926029	-0.066788142	0.20791257	3.09E-01	3.94E-01	1.00E+00
GDAP1	LMNA	-0.481796828	-0.581191321	-0.368238289	3.89E-13	5.75E-12	4.61E-09
PIP5K1A	LMNA	0.115762602	-0.022650377	0.249821175	1.01E-01	1.53E-01	1.00E+00
PCNA	LMNA	-0.000515822	-0.138556956	0.137544973	9.94E-01	9.95E-01	1.00E+00
C1orf34	LMNA	0.297815066	0.166614185	0.418653678	1.67E-05	6.36E-05	1.98E-01
BEST1	LMNA	-0.199621258	-0.328616279	-0.063315089	4.40E-03	9.76E-03	1.00E+00
RBBP4	LMNA	-0.223950584	-0.351145363	-0.088640032	1.36E-03	3.43E-03	1.00E+00
MMACHC	LMNA	0.125389528	-0.012884506	0.258957928	7.54E-02	1.20E-01	1.00E+00
REV3L	LMNA	-0.394323961	-0.504890338	-0.271026797	6.37E-09	4.53E-08	7.56E-05
PHKA1	LMNA	0.176194612	0.03909454	0.30678346	1.21E-02	2.41E-02	1.00E+00
PRKAR1A	LMNA	0.092497975	-0.046142236	0.227642113	1.90E-01	2.63E-01	1.00E+00
AVPI1	LMNA	0.035234727	-0.103318836	0.172446913	6.19E-01	6.94E-01	1.00E+00
HSD3B1	LMNA	-0.05816708	-0.194654996	0.080530583	4.11E-01	4.99E-01	1.00E+00
ATG5	LMNA	0.00905013	-0.129162243	0.146917575	8.98E-01	9.26E-01	1.00E+00
SARM1	LMNA	-0.230975459	-0.357623147	-0.095985076	9.42E-04	2.49E-03	1.00E+00
RAD52	LMNA	-0.189093977	-0.318822246	-0.052411149	7.03E-03	1.48E-02	1.00E+00

RGS7	LMNA	-0.503809563	-0.60012128	-0.393099165	2.12E-14	3.85E-13	2.52E-10
CD207	LMNA	0.105513998	-0.033017953	0.240068091	1.35E-01	1.97E-01	1.00E+00
HMP19	LMNA	-0.56821121	-0.654891156	-0.466775036	1.15E-18	4.81E-17	1.36E-14
TMEPAI	LMNA	0.609489468	0.514749844	0.689523524	6.23E-22	4.35E-20	7.39E-18
MYCT1	LMNA	0.035356939	-0.103197776	0.172565636	6.17E-01	6.93E-01	1.00E+00
GM2A	LMNA	0.333872631	0.205283442	0.451130378	1.20E-06	5.66E-06	1.42E-02
SCGN	LMNA	-0.106141179	-0.24066573	0.032384346	1.33E-01	1.94E-01	1.00E+00
ETV4	LMNA	0.312994652	0.182844208	0.432363559	5.75E-06	2.38E-05	6.82E-02
MPP1	LMNA	0.224300446	0.089005491	0.351468266	1.33E-03	3.38E-03	1.00E+00
CD2AP	LMNA	0.493418164	0.38134306	0.591198568	8.60E-14	1.38E-12	1.02E-09
CCL20	LMNA	0.347747201	0.220270702	0.463544873	3.96E-07	2.04E-06	4.70E-03
CCDC86	LMNA	0.359157331	0.23264115	0.473720317	1.53E-07	8.48E-07	1.81E-03
ZFP30	LMNA	-0.360733456	-0.47512349	-0.234353152	1.34E-07	7.49E-07	1.58E-03
MYH10	LMNA	-0.284456488	-0.406542756	-0.152389757	4.08E-05	1.44E-04	4.84E-01
CTBP1	LMNA	-0.063093272	-0.199407416	0.075616354	3.72E-01	4.60E-01	1.00E+00
MAK10	LMNA	-0.208305425	-0.336674747	-0.072334533	2.93E-03	6.80E-03	1.00E+00
OR10J1	LMNA	-0.128839339	-0.262226278	0.009378471	6.76E-02	1.09E-01	1.00E+00
TMEM9B	LMNA	-0.109131726	-0.243514007	0.029361629	1.22E-01	1.80E-01	1.00E+00
DNAJA1	LMNA	0.067156505	-0.071557911	0.203322498	3.42E-01	4.28E-01	1.00E+00
LOR	LMNA	-0.266740939	-0.390415342	-0.133609972	1.24E-04	4.00E-04	1.00E+00
MAP6D1	LMNA	-0.333662902	-0.45094237	-0.205057355	1.22E-06	5.74E-06	1.45E-02
LRRC50	LMNA	-0.06595398	-0.202164272	0.072759497	3.51E-01	4.38E-01	1.00E+00
PRKX	LMNA	-0.376851455	-0.489439502	-0.251905782	3.24E-08	2.01E-07	3.84E-04
ARMC7	LMNA	-0.032698539	-0.169982228	0.105830186	6.44E-01	7.17E-01	1.00E+00
KIF5A	LMNA	-0.454606514	-0.557659431	-0.337752482	1.07E-11	1.25E-10	1.27E-07
ARG1	LMNA	-0.057253019	-0.193772473	0.081441684	4.18E-01	5.06E-01	1.00E+00
PCTK1	LMNA	0.293051508	0.161535601	0.41433995	2.31E-05	8.57E-05	2.74E-01
NSL1	LMNA	-0.398845992	-0.508877636	-0.275991377	4.12E-09	3.04E-08	4.89E-05
ASCC2	LMNA	0.32638708	0.197222545	0.444413662	2.13E-06	9.63E-06	2.53E-02
KIF2C	LMNA	-0.003386512	-0.14137142	0.134727475	9.62E-01	9.72E-01	1.00E+00
PSENEN	LMNA	0.141604345	0.003624193	0.27429329	4.44E-02	7.56E-02	1.00E+00
FCRL2	LMNA	0.045597761	-0.093038902	0.182499958	5.19E-01	6.03E-01	1.00E+00
RAB11FIP3	LMNA	-0.046447023	-0.183322549	0.092195138	5.12E-01	5.96E-01	1.00E+00
NPR3	LMNA	-0.085124445	-0.220583258	0.053555919	2.28E-01	3.06E-01	1.00E+00
CENTD3	LMNA	0.390289309	0.266602823	0.5013288	9.36E-09	6.42E-08	1.11E-04

KBTBD11	LMNA	-0.355118051	-0.470121621	-0.228257225	2.15E-07	1.16E-06	2.55E-03
HBD	LMNA	-0.04063178	-0.177686094	0.097968752	5.66E-01	6.46E-01	1.00E+00
PCK1	LMNA	0.0163041	-0.122021546	0.154008459	8.18E-01	8.63E-01	1.00E+00
IRAK3	LMNA	0.226312614	0.091108074	0.3533248	1.20E-03	3.08E-03	1.00E+00
OLAH	LMNA	-0.23435056	-0.36073107	-0.099519238	7.88E-04	2.12E-03	1.00E+00
CNNM4	LMNA	0.185823742	0.049030529	0.31577414	8.10E-03	1.69E-02	1.00E+00
MYO5A	LMNA	-0.018072029	-0.155734493	0.120279052	7.99E-01	8.48E-01	1.00E+00
CYB561D2	LMNA	0.32065782	0.191064706	0.439263868	3.28E-06	1.42E-05	3.89E-02
MEGF8	LMNA	0.08361247	-0.05507424	0.219134054	2.37E-01	3.16E-01	1.00E+00
SIPA1L3	LMNA	0.068757175	-0.069957865	0.204863611	3.31E-01	4.17E-01	1.00E+00
ADAM10	LMNA	0.476709505	0.362515747	0.576801096	7.40E-13	1.05E-11	8.78E-09
ALPPL2	LMNA	-0.196510977	-0.325725521	-0.060090134	5.06E-03	1.10E-02	1.00E+00
OBFC2B	LMNA	-0.210309202	-0.338531484	-0.074418833	2.66E-03	6.25E-03	1.00E+00
GALC	LMNA	0.203622526	0.067468073	0.332331588	3.65E-03	8.27E-03	1.00E+00
LIPA	LMNA	-0.095456465	-0.230470359	0.043163336	1.77E-01	2.47E-01	1.00E+00
NAP1L4	LMNA	0.069518247	-0.06919684	0.205596129	3.26E-01	4.11E-01	1.00E+00
MRPS22	LMNA	-0.081934844	-0.217525374	0.056758159	2.46E-01	3.26E-01	1.00E+00
GNG4	LMNA	-0.528663218	-0.621365425	-0.421364442	6.12E-16	1.53E-14	7.26E-12
TBKBP1	LMNA	-0.238009515	-0.364097209	-0.103354479	6.47E-04	1.78E-03	1.00E+00
PSG5	LMNA	0.09216786	-0.046474475	0.227326391	1.92E-01	2.65E-01	1.00E+00
CAMLG	LMNA	-0.292783749	-0.414097313	-0.16125034	2.35E-05	8.71E-05	2.79E-01
RSAD1	LMNA	-0.192521036	-0.322013648	-0.055957252	6.05E-03	1.30E-02	1.00E+00
SLC6A13	LMNA	-0.30939012	-0.429113011	-0.178983807	7.45E-06	3.02E-05	8.84E-02
AGPAT4	LMNA	-0.27351374	-0.396589962	-0.140778368	8.20E-05	2.73E-04	9.72E-01
ZNF167	LMNA	-0.344712391	-0.460833307	-0.216987362	5.07E-07	2.56E-06	6.01E-03
FAM53C	LMNA	-0.04184206	-0.178859906	0.096767904	5.54E-01	6.35E-01	1.00E+00
VWF	LMNA	0.206569204	0.070529499	0.335065124	3.18E-03	7.33E-03	1.00E+00
VTN	LMNA	-0.137339313	-0.270266115	0.000725441	5.13E-02	8.58E-02	1.00E+00
BAD	LMNA	-0.008156154	-0.146042715	0.130041269	9.08E-01	9.34E-01	1.00E+00
TPM1	LMNA	0.347967968	0.220509662	0.463742041	3.89E-07	2.01E-06	4.61E-03
PYHIN1	LMNA	0.229805685	0.094760967	0.356545319	1.00E-03	2.63E-03	1.00E+00
PDS5B	LMNA	-0.215475174	-0.34331377	-0.079797886	2.07E-03	5.00E-03	1.00E+00
CIDEC	LMNA	-0.10040929	-0.235200041	0.038170819	1.55E-01	2.21E-01	1.00E+00
CRIM1	LMNA	0.390598301	0.266941447	0.501601691	9.09E-09	6.25E-08	1.08E-04
DHTKD1	LMNA	-0.344062327	-0.4602522	-0.216284437	5.34E-07	2.68E-06	6.34E-03

SH3GLB2	LMNA	-0.249759178	-0.374884351	-0.115697365	3.37E-04	9.91E-04	1.00E+00
SMPDL3A	LMNA	0.312726511	0.182556895	0.432121857	5.86E-06	2.42E-05	6.95E-02
SFRS2IP	LMNA	0.255113288	0.121335562	0.379788647	2.48E-04	7.48E-04	1.00E+00
FLNB	LMNA	0.394536838	0.27126036	0.505078149	6.25E-09	4.45E-08	7.41E-05
NOC2L	LMNA	0.147834409	0.009987235	0.280167557	3.58E-02	6.25E-02	1.00E+00
NRG2	LMNA	-0.273854968	-0.396900757	-0.141139895	8.02E-05	2.67E-04	9.52E-01
C14orf162	LMNA	-0.314245659	-0.433490985	-0.184184957	5.25E-06	2.19E-05	6.23E-02
HMG4L	LMNA	-0.057327753	-0.193844637	0.0813672	4.18E-01	5.05E-01	1.00E+00
IL15	LMNA	0.428147316	0.308319924	0.534600152	2.07E-10	1.95E-09	2.45E-06
GABARAPL	LMNA	-0.138428193	-0.271294711	-0.000384541	4.94E-02	8.32E-02	1.00E+00
SPTBN5	LMNA	0.19305614	0.056511254	0.32251169	5.91E-03	1.27E-02	1.00E+00
C1orf77	LMNA	-0.129123574	-0.262495426	0.009089452	6.70E-02	1.08E-01	1.00E+00
LAT2	LMNA	0.093308692	-0.045326172	0.228417366	1.87E-01	2.58E-01	1.00E+00
WDR78	LMNA	0.115720239	-0.022693294	0.249780916	1.01E-01	1.53E-01	1.00E+00
SLCO1A2	LMNA	-0.542303764	-0.632967302	-0.436966513	7.70E-17	2.31E-15	9.13E-13
LIG4	LMNA	-0.213116418	-0.341131025	-0.077340858	2.33E-03	5.55E-03	1.00E+00
GSDMDC1	LMNA	0.486249876	0.37325443	0.58502944	2.20E-13	3.34E-12	2.61E-09
METT10D	LMNA	-0.212361131	-0.340431806	-0.076554452	2.41E-03	5.73E-03	1.00E+00
ECSIT	LMNA	-0.304627385	-0.424813241	-0.173889138	1.04E-05	4.11E-05	1.24E-01
BMP4	LMNA	0.027520836	-0.110951701	0.164945164	6.97E-01	7.62E-01	1.00E+00
VSIG4	LMNA	0.231199421	0.096219487	0.357829467	9.31E-04	2.46E-03	1.00E+00
DIRAS2	LMNA	-0.361920485	-0.476179879	-0.235643031	1.21E-07	6.83E-07	1.43E-03
SLC12A9	LMNA	0.380462658	0.255849716	0.492638674	2.33E-08	1.49E-07	2.77E-04
MC1R	LMNA	0.390902244	0.267274568	0.501870103	8.83E-09	6.09E-08	1.05E-04
TXNL1	LMNA	-0.045500253	-0.1824055	0.093135767	5.20E-01	6.04E-01	1.00E+00
GALNT7	LMNA	0.351607387	0.224451192	0.466990772	2.88E-07	1.52E-06	3.42E-03
ISG20L2	LMNA	0.105883691	-0.032644486	0.240420383	1.34E-01	1.95E-01	1.00E+00
OBSCN	LMNA	0.017480728	-0.12086194	0.155157298	8.05E-01	8.52E-01	1.00E+00
GBA	LMNA	0.406770475	0.284707229	0.515853675	1.89E-09	1.48E-08	2.24E-05
C6orf64	LMNA	-0.135597182	-0.2686198	0.002500629	5.43E-02	9.02E-02	1.00E+00
ESD	LMNA	0.017771464	-0.120575352	0.15544111	8.02E-01	8.50E-01	1.00E+00
PNRC1	LMNA	0.00701877	-0.131159318	0.144929341	9.21E-01	9.44E-01	1.00E+00
PPIA	LMNA	0.164824677	0.027397075	0.296137307	1.91E-02	3.58E-02	1.00E+00
VDAC1	LMNA	0.303829097	0.173035898	0.424092019	1.10E-05	4.33E-05	1.31E-01
CLDN17	LMNA	-0.087768437	-0.223116048	0.050899285	2.14E-01	2.90E-01	1.00E+00

TRIB1	LMNA	0.401896501	0.279344131	0.511564756	3.06E-09	2.31E-08	3.63E-05
MED6	LMNA	-0.069315288	-0.2054008	0.069399802	3.27E-01	4.13E-01	1.00E+00
TXNDC5	LMNA	0.68628488	0.605610853	0.752995358	1.81E-29	4.58E-27	2.15E-25
CD46	LMNA	0.384159403	0.259891375	0.49591049	1.66E-08	1.09E-07	1.97E-04
ICOSLG	LMNA	0.30902066	0.178588348	0.428779659	7.65E-06	3.09E-05	9.07E-02
RGR	LMNA	-0.338708702	-0.455462689	-0.210500511	8.20E-07	3.98E-06	9.72E-03
DSG1	LMNA	0.159620328	0.022055333	0.291253344	2.33E-02	4.27E-02	1.00E+00
CCK	LMNA	-0.380311985	-0.492505253	-0.255685077	2.37E-08	1.51E-07	2.81E-04
C17orf48	LMNA	-0.18771384	-0.317536181	-0.050984044	7.47E-03	1.56E-02	1.00E+00
C1orf69	LMNA	-0.150945539	-0.283097307	-0.013168956	3.20E-02	5.68E-02	1.00E+00
DEF6	LMNA	0.260718568	0.127247519	0.384915513	1.79E-04	5.56E-04	1.00E+00
SIT1	LMNA	0.231917278	0.096970939	0.358490693	8.97E-04	2.38E-03	1.00E+00
UTP14A	LMNA	0.379033704	0.254288604	0.491373123	2.66E-08	1.68E-07	3.15E-04
RPH3AL	LMNA	-0.12189182	-0.255641075	0.01643575	8.40E-02	1.31E-01	1.00E+00
NXF1	LMNA	0.053658197	-0.085022614	0.190299541	4.48E-01	5.35E-01	1.00E+00
C20orf46	LMNA	-0.245406921	-0.370892574	-0.111120537	4.31E-04	1.23E-03	1.00E+00
NHEJ1	LMNA	0.071757172	-0.066957116	0.207750169	3.10E-01	3.95E-01	1.00E+00
SLC24A2	LMNA	-0.117675217	-0.251638307	0.020712258	9.53E-02	1.46E-01	1.00E+00
TUBB3	LMNA	-0.090251321	-0.225492842	0.048402744	2.01E-01	2.76E-01	1.00E+00
SEC22B	LMNA	0.172397129	0.035183481	0.303231346	1.42E-02	2.75E-02	1.00E+00
S100A6	LMNA	0.515555139	0.406431013	0.610178	4.12E-15	8.64E-14	4.88E-11
CDKL2	LMNA	-0.248152184	-0.373410998	-0.114006789	3.70E-04	1.07E-03	1.00E+00
TINF2	LMNA	0.067784291	-0.070930455	0.203927006	3.38E-01	4.24E-01	1.00E+00
SLC7A10	LMNA	-0.100888262	-0.235657095	0.037687642	1.53E-01	2.19E-01	1.00E+00
SPRR1A	LMNA	-0.104960589	-0.239540666	0.033576939	1.37E-01	1.99E-01	1.00E+00
CYP4A11	LMNA	-0.137292106	-0.270221514	0.000773556	5.14E-02	8.59E-02	1.00E+00
SCEL	LMNA	-0.065142563	-0.201382531	0.073570053	3.57E-01	4.44E-01	1.00E+00
TES	LMNA	0.492307178	0.380088309	0.590243187	9.96E-14	1.59E-12	1.18E-09
CCDC70	LMNA	0.241669698	0.107195018	0.367461186	5.30E-04	1.49E-03	1.00E+00
SH3TC1	LMNA	0.33914117	0.210967405	0.455849838	7.92E-07	3.86E-06	9.40E-03
RAB36	LMNA	0.383435452	0.259099534	0.495270004	1.78E-08	1.16E-07	2.11E-04
GRIA3	LMNA	-0.364632183	-0.478591902	-0.238591356	9.55E-08	5.48E-07	1.13E-03
CRYGB	LMNA	-0.237961963	-0.364053484	-0.10330461	6.49E-04	1.78E-03	1.00E+00
BHLHB9	LMNA	-0.262723553	-0.386747511	-0.129364506	1.58E-04	4.99E-04	1.00E+00
CRISP2	LMNA	0.047832883	-0.090817822	0.184664474	4.99E-01	5.84E-01	1.00E+00

ILF3	LMNA	-0.103781771	-0.23841693	0.034767347	1.42E-01	2.05E-01	1.00E+00
NTRK3	LMNA	-0.430568711	-0.536717015	-0.31100392	1.60E-10	1.54E-09	1.89E-06
B3GNT1	LMNA	-0.306812119	-0.426786279	-0.176225266	8.95E-06	3.57E-05	1.06E-01
ZNF444	LMNA	-0.138592565	-0.271449956	-0.000552128	4.92E-02	8.28E-02	1.00E+00
LARP6	LMNA	0.106224762	-0.032299898	0.240745368	1.32E-01	1.94E-01	1.00E+00
FMO1	LMNA	0.329871243	0.200972332	0.447541621	1.63E-06	7.55E-06	1.94E-02
POLR3C	LMNA	-0.00569503	-0.143633106	0.132460111	9.36E-01	9.53E-01	1.00E+00
FBN1	LMNA	0.646467783	0.55823693	0.720240539	2.73E-25	3.80E-23	3.23E-21
SGCG	LMNA	-0.222244578	-0.349570379	-0.08685849	1.48E-03	3.70E-03	1.00E+00
JOSD1	LMNA	0.193773129	0.057253697	0.323178908	5.72E-03	1.23E-02	1.00E+00
BEX4	LMNA	-0.528447828	-0.6211819	-0.421118587	6.32E-16	1.58E-14	7.49E-12
INHBB	LMNA	0.129372347	-0.008836473	0.262730977	6.65E-02	1.08E-01	1.00E+00
TBL2	LMNA	0.474210047	0.35970735	0.57464201	1.01E-12	1.40E-11	1.20E-08
HYDIN	LMNA	-0.125087169	-0.258671327	0.01319163	7.61E-02	1.21E-01	1.00E+00
RPS6KB2	LMNA	0.248984431	0.114882225	0.374174113	3.53E-04	1.03E-03	1.00E+00
ADRM1	LMNA	0.561497231	0.459027894	0.649223506	3.53E-18	1.32E-16	4.19E-14
DDEF1	LMNA	0.093951943	-0.044678545	0.229032357	1.84E-01	2.55E-01	1.00E+00
PEX6	LMNA	0.028195891	-0.110284389	0.165602289	6.90E-01	7.57E-01	1.00E+00
BAT3	LMNA	0.061725548	-0.076981433	0.198088581	3.83E-01	4.70E-01	1.00E+00
TXLNA	LMNA	0.471574882	0.356748717	0.572364182	1.40E-12	1.90E-11	1.66E-08
RAB31	LMNA	0.22671058	0.091524065	0.353691866	1.18E-03	3.03E-03	1.00E+00
SCGB2A1	LMNA	-0.076251209	-0.212069844	0.062457252	2.81E-01	3.64E-01	1.00E+00
TMEM187	LMNA	0.063456924	-0.075253318	0.199757986	3.70E-01	4.57E-01	1.00E+00
AIP	LMNA	-0.052971451	-0.189635691	0.085706299	4.54E-01	5.40E-01	1.00E+00
LGALS14	LMNA	-0.090492843	-0.225723958	0.0481598	2.00E-01	2.74E-01	1.00E+00
SLC6A14	LMNA	-0.157731049	-0.289478672	-0.020118119	2.50E-02	4.55E-02	1.00E+00
DDX4	LMNA	-0.206931552	-0.335401113	-0.070906135	3.13E-03	7.22E-03	1.00E+00
CTNNA3	LMNA	-0.383824026	-0.495613794	-0.259524527	1.71E-08	1.12E-07	2.03E-04
PRRC1	LMNA	0.468619628	0.353433441	0.569807818	2.01E-12	2.67E-11	2.39E-08
AP3B2	LMNA	-0.440261697	-0.545177585	-0.321767249	5.51E-11	5.77E-10	6.54E-07
TRGV7	LMNA	0.141212092	0.00322394	0.273923106	4.50E-02	7.65E-02	1.00E+00
LAMA5	LMNA	0.546641895	0.441941793	0.636648556	3.90E-17	1.24E-15	4.63E-13
TMEM184B	LMNA	0.157183313	0.019556679	0.288963994	2.55E-02	4.63E-02	1.00E+00
AKAP4	LMNA	0.019246453	-0.119121051	0.156880623	7.86E-01	8.37E-01	1.00E+00
ZNF292	LMNA	-0.343441488	-0.459697125	-0.215613237	5.62E-07	2.81E-06	6.66E-03

C21orf45	LMNA	0.005241493	-0.132905678	0.143188883	9.41E-01	9.56E-01	1.00E+00
ARNTL	LMNA	0.098094929	-0.040504589	0.232990745	1.65E-01	2.33E-01	1.00E+00
CTCF	LMNA	-0.171613606	-0.302497997	-0.034377047	1.46E-02	2.83E-02	1.00E+00
CCL2	LMNA	0.457681382	0.341187776	0.560328912	7.49E-12	9.03E-11	8.88E-08
SNTB2	LMNA	0.499735223	0.388485423	0.596625648	3.69E-14	6.31E-13	4.38E-10
KPNA1	LMNA	-0.033890285	-0.171140589	0.104650331	6.32E-01	7.06E-01	1.00E+00
KIAA0746	LMNA	0.324336162	0.195017036	0.442571073	2.49E-06	1.11E-05	2.95E-02
KRT81	LMNA	-0.124420218	-0.258039054	0.013869001	7.77E-02	1.23E-01	1.00E+00
ALDH3B2	LMNA	0.093542524	-0.045090762	0.228640938	1.85E-01	2.57E-01	1.00E+00
TMEM41B	LMNA	-0.131551634	-0.264793759	0.006619584	6.20E-02	1.01E-01	1.00E+00
M6PR	LMNA	0.309646085	0.179257809	0.429343941	7.32E-06	2.97E-05	8.68E-02
S100A11	LMNA	0.614253551	0.520325257	0.69349716	2.43E-22	1.86E-20	2.89E-18
LAMC1	LMNA	0.722356493	0.649027874	0.782386335	7.10E-34	4.95E-31	8.42E-30
CCND3	LMNA	0.154735456	0.017048638	0.286662939	2.79E-02	5.01E-02	1.00E+00
COASY	LMNA	0.111544526	-0.026921028	0.245810334	1.14E-01	1.70E-01	1.00E+00
EFHC2	LMNA	0.129236268	-0.008974854	0.262602133	6.68E-02	1.08E-01	1.00E+00
DOT1L	LMNA	0.003911194	-0.134212274	0.141885584	9.56E-01	9.67E-01	1.00E+00
CLTC	LMNA	0.020068469	-0.118310307	0.157682614	7.77E-01	8.30E-01	1.00E+00
CLASP2	LMNA	-0.747408156	-0.80264218	-0.679464616	2.28E-37	3.37E-34	2.70E-33
SRP9	LMNA	-0.329366832	-0.447088957	-0.200429233	1.70E-06	7.81E-06	2.01E-02
EIF2AK3	LMNA	0.205800978	0.06973111	0.334352674	3.30E-03	7.56E-03	1.00E+00
GPR88	LMNA	-0.173142823	-0.303929145	-0.035951146	1.37E-02	2.68E-02	1.00E+00
COL13A1	LMNA	0.503250154	0.392465366	0.599641546	2.29E-14	4.12E-13	2.72E-10
SMYD2	LMNA	0.224246561	0.088949202	0.351418536	1.34E-03	3.39E-03	1.00E+00
TMPRSS2	LMNA	0.140343885	0.002338186	0.273103609	4.64E-02	7.85E-02	1.00E+00
PBX3	LMNA	0.121499265	-0.016834095	0.255268621	8.50E-02	1.32E-01	1.00E+00
SIGLEC6	LMNA	-0.306972448	-0.426931028	-0.176396763	8.85E-06	3.53E-05	1.05E-01
PVRL2	LMNA	0.592045086	0.494402823	0.674932243	1.70E-20	9.27E-19	2.02E-16
ALKBH4	LMNA	0.082945618	-0.055743688	0.218494696	2.41E-01	3.20E-01	1.00E+00
ZNF629	LMNA	0.060980282	-0.077725038	0.197369747	3.89E-01	4.76E-01	1.00E+00
NXT1	LMNA	0.191367626	0.054763391	0.320939879	6.37E-03	1.35E-02	1.00E+00
CCDC93	LMNA	-0.016526642	-0.154225773	0.121802253	8.15E-01	8.61E-01	1.00E+00
TROAP	LMNA	-0.121293185	-0.255073077	0.017043198	8.55E-02	1.33E-01	1.00E+00
KCNA10	LMNA	-0.052460699	-0.189141886	0.08621469	4.58E-01	5.44E-01	1.00E+00
FLJ10154	LMNA	-0.259751513	-0.384031544	-0.126226877	1.89E-04	5.84E-04	1.00E+00

ANGPT1	LMNA	0.301902476	0.170977463	0.422350767	1.26E-05	4.91E-05	1.50E-01
MED23	LMNA	-0.120664166	-0.254476152	0.017681367	8.72E-02	1.35E-01	1.00E+00
RAN	LMNA	-0.077239155	-0.213018749	0.061467268	2.75E-01	3.57E-01	1.00E+00
LMTK2	LMNA	-0.099151539	-0.233999561	0.039439306	1.60E-01	2.27E-01	1.00E+00
SEMA6A	LMNA	-0.171986575	-0.302847102	-0.0347609	1.44E-02	2.79E-02	1.00E+00
UFC1	LMNA	-0.21755385	-0.34523619	-0.081964533	1.87E-03	4.57E-03	1.00E+00
UBE1DC1	LMNA	-0.016978667	-0.15466714	0.121356784	8.10E-01	8.57E-01	1.00E+00
GMEB2	LMNA	0.293870337	0.162408085	0.415081843	2.19E-05	8.13E-05	2.59E-01
EEF1A1	LMNA	-0.092514579	-0.227657993	0.046125524	1.90E-01	2.63E-01	1.00E+00
PSMD14	LMNA	-0.028830897	-0.166220319	0.109656551	6.84E-01	7.51E-01	1.00E+00
FLJ10213	LMNA	-0.090022417	-0.225273786	0.048632979	2.03E-01	2.77E-01	1.00E+00
CHAC1	LMNA	0.0994978	-0.039090133	0.234330096	1.59E-01	2.26E-01	1.00E+00
PDCD2	LMNA	0.050180521	-0.088483446	0.186936525	4.78E-01	5.63E-01	1.00E+00
MAST1	LMNA	-0.611375255	-0.691097001	-0.516955825	4.30E-22	3.08E-20	5.10E-18
EPHA1	LMNA	0.183382576	0.046509004	0.313497039	8.99E-03	1.84E-02	1.00E+00
HMGA2	LMNA	0.410286437	0.288580783	0.518944203	1.33E-09	1.07E-08	1.57E-05
EIF4G1	LMNA	0.344875856	0.21716414	0.460979416	5.00E-07	2.53E-06	5.93E-03
ING2	LMNA	0.022575221	-0.115836789	0.16012718	7.50E-01	8.07E-01	1.00E+00
C1orf109	LMNA	-0.092236584	-0.22739212	0.046405312	1.92E-01	2.64E-01	1.00E+00
INTS3	LMNA	0.013327011	-0.124953881	0.151100017	8.51E-01	8.89E-01	1.00E+00
TRPM4	LMNA	0.503447208	0.392688612	0.599810542	2.23E-14	4.02E-13	2.64E-10
LTB4R	LMNA	0.059057025	-0.079643298	0.195514023	4.04E-01	4.91E-01	1.00E+00
ISYNA1	LMNA	0.145799531	0.007907695	0.27824998	3.84E-02	6.65E-02	1.00E+00
UBE2D1	LMNA	-0.138866452	-0.271708621	-0.00083139	4.87E-02	8.21E-02	1.00E+00
LSM7	LMNA	-0.141020547	-0.273742324	-0.003028505	4.53E-02	7.69E-02	1.00E+00
IDH3A	LMNA	0.08645997	-0.052214254	0.221862841	2.21E-01	2.98E-01	1.00E+00
FLJ21511	LMNA	0.095306321	-0.043314576	0.23032688	1.77E-01	2.47E-01	1.00E+00
CREB5	LMNA	-0.070404077	-0.206448532	0.068310861	3.19E-01	4.05E-01	1.00E+00
LRRC47	LMNA	-0.193139638	-0.322589399	-0.056597709	5.89E-03	1.26E-02	1.00E+00
ANGPT2	LMNA	0.285511256	0.153510905	0.407500575	3.81E-05	1.35E-04	4.52E-01
RANBP3	LMNA	0.023778418	-0.114648933	0.16129993	7.37E-01	7.96E-01	1.00E+00
DYRK1B	LMNA	-0.055048323	-0.191642938	0.083638285	4.36E-01	5.23E-01	1.00E+00
HLA-DRB6	LMNA	0.234132213	0.099290499	0.360530093	7.97E-04	2.15E-03	1.00E+00
ATP6V0A4	LMNA	-0.312065756	-0.431526182	-0.18184899	6.15E-06	2.53E-05	7.29E-02
COPS7B	LMNA	-0.073424611	-0.209353533	0.065288173	2.99E-01	3.83E-01	1.00E+00

TMSB4Y	LMNA	-0.00415887	-0.14212827	0.133969047	9.53E-01	9.65E-01	1.00E+00
RBKS	LMNA	0.198039975	0.061675149	0.327146905	4.72E-03	1.04E-02	1.00E+00
ITGA4	LMNA	0.564345291	0.462312318	0.651628905	2.20E-18	8.67E-17	2.61E-14
RIN1	LMNA	0.620337624	0.527457117	0.698564774	7.16E-23	6.54E-21	8.50E-19
DNAJC6	LMNA	-0.453152873	-0.556396694	-0.336129533	1.27E-11	1.47E-10	1.51E-07
SEC23A	LMNA	0.326246529	0.197071358	0.44428742	2.15E-06	9.71E-06	2.56E-02
PHLDB1	LMNA	0.067475072	-0.071239525	0.203629265	3.40E-01	4.26E-01	1.00E+00
CLOCK	LMNA	-0.0708253	-0.206853787	0.067889491	3.17E-01	4.02E-01	1.00E+00
LOC26010	LMNA	0.605906656	0.510562147	0.686531988	1.25E-21	7.88E-20	1.48E-17
MLX	LMNA	0.097020384	-0.041587632	0.231964507	1.70E-01	2.38E-01	1.00E+00
TPD52	LMNA	-0.006191256	-0.144119076	0.131972543	9.30E-01	9.49E-01	1.00E+00
PSMA4	LMNA	0.0995926	-0.038994531	0.234420585	1.58E-01	2.25E-01	1.00E+00
C1orf149	LMNA	-0.098731794	-0.233598837	0.039862534	1.62E-01	2.29E-01	1.00E+00
C14orf135	LMNA	-0.074699638	-0.210579071	0.064011472	2.91E-01	3.74E-01	1.00E+00
KIFC3	LMNA	0.379409951	0.254699586	0.491706392	2.57E-08	1.63E-07	3.05E-04
ROCK1	LMNA	0.216925895	0.081309868	0.344655552	1.93E-03	4.70E-03	1.00E+00
NCAPH	LMNA	-0.085795671	-0.221226426	0.05288167	2.25E-01	3.02E-01	1.00E+00
TAGLN	LMNA	0.508254249	0.398138649	0.603930464	1.15E-14	2.20E-13	1.36E-10
PTPRK	LMNA	-0.215336511	-0.343185492	-0.0796534	2.09E-03	5.03E-03	1.00E+00
CCDC19	LMNA	0.248686366	0.114568668	0.373900827	3.59E-04	1.05E-03	1.00E+00
ZNF45	LMNA	0.057751746	-0.080944601	0.19425402	4.14E-01	5.02E-01	1.00E+00
ZNF329	LMNA	-0.194472573	-0.323829675	-0.057978118	5.55E-03	1.20E-02	1.00E+00
TK1	LMNA	0.233627255	0.098761559	0.360065259	8.19E-04	2.20E-03	1.00E+00
TAX1BP1	LMNA	0.085926744	-0.052749991	0.221352007	2.24E-01	3.01E-01	1.00E+00
ZDHHC18	LMNA	0.541894265	0.436497204	0.632619598	8.20E-17	2.44E-15	9.73E-13
C10orf88	LMNA	-0.350329048	-0.465850016	-0.223066262	3.20E-07	1.68E-06	3.80E-03
TMBIM4	LMNA	0.153837379	0.016128914	0.28581834	2.88E-02	5.16E-02	1.00E+00
KLF1	LMNA	-0.137996154	-0.270886627	5.59E-05	5.02E-02	8.41E-02	1.00E+00
NMUR1	LMNA	0.256229697	0.122512296	0.380810382	2.33E-04	7.05E-04	1.00E+00
KIR2DS4	LMNA	-0.079254863	-0.214954006	0.059446555	2.62E-01	3.44E-01	1.00E+00
SAP30L	LMNA	0.488259503	0.375520343	0.586760107	1.69E-13	2.63E-12	2.01E-09
KDR	LMNA	0.252067913	0.118127542	0.376999983	2.96E-04	8.78E-04	1.00E+00
KCNK2	LMNA	-0.370946454	-0.484201713	-0.245465633	5.49E-08	3.31E-07	6.52E-04
VEZF1	LMNA	-0.392842483	-0.503583019	-0.269401758	7.34E-09	5.16E-08	8.71E-05
GIT1	LMNA	-0.172692418	-0.303507686	-0.035487451	1.40E-02	2.72E-02	1.00E+00

RLN2	LMNA	-0.17358521	-0.304343052	-0.036406644	1.35E-02	2.64E-02	1.00E+00
ST3GAL2	LMNA	0.390632989	0.266979463	0.501632325	9.06E-09	6.23E-08	1.07E-04
DNM3	LMNA	-0.506904326	-0.602774015	-0.396607374	1.39E-14	2.61E-13	1.64E-10
C7orf10	LMNA	0.277709569	0.145226265	0.400409632	6.29E-05	2.14E-04	7.47E-01
MMP28	LMNA	-0.162872969	-0.294306553	-0.025392923	2.06E-02	3.83E-02	1.00E+00
ZNF394	LMNA	0.412691775	0.291233053	0.521056857	1.04E-09	8.56E-09	1.23E-05
HPD	LMNA	0.380755209	0.256169404	0.492897713	2.27E-08	1.46E-07	2.69E-04
MOXD1	LMNA	0.483084654	0.369688283	0.582301763	3.30E-13	4.91E-12	3.91E-09
PDGFRL	LMNA	0.284718881	0.152668632	0.406781057	4.01E-05	1.42E-04	4.76E-01
ODF2	LMNA	0.2163461	0.080705514	0.344119358	1.98E-03	4.82E-03	1.00E+00
TREX2	LMNA	0.170179631	0.032901603	0.301155446	1.55E-02	2.97E-02	1.00E+00
MFAP4	LMNA	0.347833429	0.220364035	0.463621885	3.93E-07	2.03E-06	4.66E-03
SMCR7L	LMNA	-0.099015159	-0.233869366	0.039576823	1.61E-01	2.28E-01	1.00E+00
EPB41	LMNA	-0.398216162	-0.508322567	-0.275299516	4.38E-09	3.22E-08	5.20E-05
GZMH	LMNA	0.267301593	0.134202845	0.390926901	1.20E-04	3.87E-04	1.00E+00
CNNM1	LMNA	-0.375342946	-0.488102213	-0.250259509	3.71E-08	2.28E-07	4.40E-04
PHF17	LMNA	-0.224001801	-0.351192635	-0.08869353	1.35E-03	3.43E-03	1.00E+00
CBLN1	LMNA	-0.473113679	-0.573694502	-0.358476122	1.16E-12	1.59E-11	1.37E-08
NUP98	LMNA	0.401070611	0.278436117	0.510837461	3.32E-09	2.49E-08	3.93E-05
PRKRIR	LMNA	-0.417056384	-0.52488699	-0.296050507	6.64E-10	5.69E-09	7.88E-06
RMI1	LMNA	-0.317351629	-0.43628852	-0.18751583	4.19E-06	1.78E-05	4.96E-02
MED4	LMNA	0.055602316	-0.083086452	0.192178166	4.32E-01	5.18E-01	1.00E+00
TRABD	LMNA	0.335290081	0.206811805	0.452400749	1.07E-06	5.11E-06	1.27E-02
C11orf21	LMNA	0.055041695	-0.083644886	0.191636534	4.37E-01	5.23E-01	1.00E+00
SHCBP1	LMNA	0.273777875	0.141058213	0.396830543	8.06E-05	2.69E-04	9.56E-01
ECM2	LMNA	0.165294815	0.027880012	0.296578165	1.87E-02	3.52E-02	1.00E+00
PTPRS	LMNA	-0.318373274	-0.437208209	-0.188612105	3.88E-06	1.66E-05	4.61E-02
COTL1	LMNA	0.197969599	0.06160218	0.327081495	4.74E-03	1.04E-02	1.00E+00
ANKRD57	LMNA	0.110046199	-0.028436813	0.244384513	1.19E-01	1.76E-01	1.00E+00
CLDN15	LMNA	0.138948661	0.000915216	0.271786258	4.86E-02	8.19E-02	1.00E+00
ZNF659	LMNA	0.394405515	0.271116274	0.50496229	6.32E-09	4.50E-08	7.50E-05
TNC	LMNA	0.547788723	0.443258136	0.637621047	3.26E-17	1.04E-15	3.86E-13
GUCA2B	LMNA	-0.486639097	-0.585364703	-0.373693184	2.09E-13	3.21E-12	2.48E-09
DOCK9	LMNA	-0.29243653	-0.413782647	-0.160880459	2.41E-05	8.89E-05	2.86E-01
ITGB1BP1	LMNA	0.167064223	0.029698163	0.298236873	1.75E-02	3.31E-02	1.00E+00

DLG2	LMNA	-0.460133755	-0.56245644	-0.34392984	5.60E-12	6.90E-11	6.64E-08
BRAP	LMNA	0.178301153	0.041265899	0.308752323	1.11E-02	2.23E-02	1.00E+00
C13orf7	LMNA	-0.369886209	-0.483260406	-0.244310474	6.03E-08	3.60E-07	7.16E-04
ZC3H7B	LMNA	-0.080739425	-0.216378635	0.05795758	2.53E-01	3.34E-01	1.00E+00
PPP1R12B	LMNA	-0.426375975	-0.53305074	-0.306357687	2.50E-10	2.31E-09	2.97E-06
SOCS7	LMNA	-0.204990644	-0.333601015	-0.068889149	3.43E-03	7.82E-03	1.00E+00
MARCKS	LMNA	-0.420089478	-0.527546091	-0.299401925	4.85E-10	4.25E-09	5.75E-06
SACS	LMNA	-0.147682997	-0.28002491	-0.009832459	3.60E-02	6.28E-02	1.00E+00
TTLL12	LMNA	0.070686651	-0.068028193	0.206720399	3.17E-01	4.03E-01	1.00E+00
SH2D3A	LMNA	0.000410787	-0.137648019	0.138453936	9.95E-01	9.96E-01	1.00E+00
RFC2	LMNA	0.197043965	0.060642571	0.326221063	4.94E-03	1.08E-02	1.00E+00
PPARA	LMNA	0.015055311	-0.123251857	0.152788755	8.32E-01	8.73E-01	1.00E+00
DVL3	LMNA	0.06558344	-0.073129667	0.201807305	3.54E-01	4.41E-01	1.00E+00
ADFP	LMNA	0.396558776	0.273479503	0.506861475	5.14E-09	3.73E-08	6.10E-05
KRIT1	LMNA	-0.048630011	-0.185436101	0.090025367	4.92E-01	5.77E-01	1.00E+00
SERTAD3	LMNA	0.507769179	0.397588343	0.603514962	1.23E-14	2.33E-13	1.46E-10
LEFTY2	LMNA	0.137184105	-0.000883631	0.270119474	5.16E-02	8.62E-02	1.00E+00
MN1	LMNA	-0.331753608	-0.449230345	-0.202999778	1.41E-06	6.57E-06	1.68E-02
PTPRD	LMNA	-0.519847592	-0.613845664	-0.411314796	2.22E-15	4.91E-14	2.64E-11
RORA	LMNA	0.059655122	-0.079046865	0.196091223	3.99E-01	4.86E-01	1.00E+00
PIAS2	LMNA	-0.060029971	-0.196452927	0.078673008	3.96E-01	4.83E-01	1.00E+00
MOSPD3	LMNA	0.117198738	-0.021195188	0.251185704	9.67E-02	1.48E-01	1.00E+00
FBXL15	LMNA	-0.199657955	-0.328650372	-0.063353156	4.39E-03	9.75E-03	1.00E+00
MYH15	LMNA	-0.201932839	-0.330763145	-0.065713742	3.95E-03	8.87E-03	1.00E+00
CRX	LMNA	0.221685234	0.086274569	0.349053837	1.52E-03	3.80E-03	1.00E+00
SLC22A17	LMNA	-0.476906207	-0.576970952	-0.36273685	7.22E-13	1.03E-11	8.57E-09
TBC1D13	LMNA	0.107391109	-0.031121279	0.241856478	1.28E-01	1.88E-01	1.00E+00
PLK2	LMNA	0.15192522	0.014171443	0.284019361	3.09E-02	5.50E-02	1.00E+00
EIF1B	LMNA	-0.381273275	-0.493356384	-0.256735592	2.17E-08	1.40E-07	2.57E-04
PRIM1	LMNA	-0.239846796	-0.365786217	-0.105281791	5.86E-04	1.63E-03	1.00E+00
ATP1A2	LMNA	-0.354252675	-0.469350137	-0.22731868	2.31E-07	1.25E-06	2.74E-03
CRYAA	LMNA	0.227477388	0.092325737	0.354399026	1.13E-03	2.92E-03	1.00E+00
PLEK2	LMNA	0.504216065	0.39355979	0.600469842	2.01E-14	3.67E-13	2.38E-10
BACE1	LMNA	0.16986145	0.032574301	0.30085748	1.57E-02	3.00E-02	1.00E+00
FAM12A	LMNA	0.017868622	-0.120479575	0.15553595	8.01E-01	8.50E-01	1.00E+00

TG	LMNA	-0.05627791	-0.192830765	0.082413379	4.26E-01	5.13E-01	1.00E+00
AGTRL1	LMNA	0.040812602	-0.097789366	0.177861492	5.64E-01	6.44E-01	1.00E+00
OPTN	LMNA	0.07035189	-0.068363062	0.20639832	3.20E-01	4.05E-01	1.00E+00
MAPKAPK5	LMNA	0.033003235	-0.105528568	0.170278425	6.41E-01	7.14E-01	1.00E+00
DGKG	LMNA	-0.125991589	-0.259528541	0.012272877	7.40E-02	1.18E-01	1.00E+00
RBP4	LMNA	-0.159851114	-0.291470068	-0.022292045	2.31E-02	4.24E-02	1.00E+00
ZNF428	LMNA	-0.235531964	-0.36181829	-0.100757124	7.40E-04	2.01E-03	1.00E+00
TFB2M	LMNA	0.040457403	-0.098141737	0.177516939	5.68E-01	6.47E-01	1.00E+00
METTL9	LMNA	-0.390728499	-0.501716671	-0.26708414	8.98E-09	6.18E-08	1.06E-04
ATP5O	LMNA	-0.186522614	-0.316425767	-0.049752728	7.86E-03	1.64E-02	1.00E+00
SP100	LMNA	0.510529803	0.400721299	0.605878969	8.37E-15	1.65E-13	9.92E-11
CPSF1	LMNA	0.056670805	-0.082021891	0.193210233	4.23E-01	5.10E-01	1.00E+00
LIME1	LMNA	-0.017961395	-0.155626506	0.120388119	8.00E-01	8.49E-01	1.00E+00
S100A4	LMNA	0.623685252	0.531386859	0.701349776	3.61E-23	3.46E-21	4.29E-19
BTC	LMNA	0.238610678	0.103984989	0.364649948	6.27E-04	1.73E-03	1.00E+00
MAP2K5	LMNA	0.102614688	-0.035945519	0.237304025	1.46E-01	2.10E-01	1.00E+00
NUP188	LMNA	0.109345524	-0.029145434	0.243717545	1.21E-01	1.79E-01	1.00E+00
SDPR	LMNA	-0.066802752	-0.202981816	0.07191143	3.45E-01	4.31E-01	1.00E+00
RPS20	LMNA	0.075485319	-0.063224533	0.211334045	2.86E-01	3.69E-01	1.00E+00
LAMB1	LMNA	0.650644932	0.563179988	0.723692309	1.06E-25	1.70E-23	1.26E-21
IGF2	LMNA	-0.013866328	-0.151627075	0.12442285	8.45E-01	8.85E-01	1.00E+00
ATAD2	LMNA	-0.134288517	-0.267382601	0.003833553	5.67E-02	9.36E-02	1.00E+00
ADM2	LMNA	0.064241829	-0.074469616	0.200514537	3.64E-01	4.51E-01	1.00E+00
NPEPPS	LMNA	-0.284221673	-0.406329488	-0.152140211	4.14E-05	1.46E-04	4.91E-01
DMN	LMNA	0.206673885	0.070638303	0.335162193	3.17E-03	7.30E-03	1.00E+00
EPN3	LMNA	-0.170345475	-0.301310743	-0.033072212	1.54E-02	2.95E-02	1.00E+00
CD80	LMNA	0.304219	0.173452617	0.424444301	1.07E-05	4.22E-05	1.27E-01
GPR77	LMNA	0.052564871	-0.086111006	0.189242607	4.58E-01	5.44E-01	1.00E+00
CLIC3	LMNA	0.381542122	0.257029446	0.493594385	2.11E-08	1.37E-07	2.51E-04
HOMER2	LMNA	0.425287591	0.305152515	0.532098364	2.81E-10	2.57E-09	3.33E-06
KLF8	LMNA	0.162594871	0.025107442	0.294045611	2.08E-02	3.87E-02	1.00E+00
DNMT1	LMNA	0.006920072	-0.131256321	0.144832711	9.22E-01	9.44E-01	1.00E+00
HTR1B	LMNA	-0.108346783	-0.24276663	0.030155262	1.25E-01	1.84E-01	1.00E+00
EPB41L2	LMNA	-0.127833684	-0.261273837	0.010400867	6.98E-02	1.12E-01	1.00E+00
JMJD6	LMNA	0.469091166	0.353962231	0.570215842	1.90E-12	2.53E-11	2.26E-08

CTSL1	LMNA	0.465322229	0.349737752	0.566953178	3.01E-12	3.89E-11	3.57E-08
BRIP1	LMNA	-0.196887454	-0.326075555	-0.06048034	4.98E-03	1.09E-02	1.00E+00
SMARCD2	LMNA	0.451508782	0.33429479	0.554967943	1.54E-11	1.75E-10	1.82E-07
WIPF2	LMNA	-0.097466383	-0.232390493	0.041138145	1.68E-01	2.36E-01	1.00E+00
GPR27	LMNA	-0.116414759	-0.250440881	0.021989641	9.90E-02	1.51E-01	1.00E+00
ELAVL4	LMNA	-0.604608377	-0.685447291	-0.509045795	1.60E-21	1.00E-19	1.90E-17
CDH9	LMNA	-0.324855868	-0.443038082	-0.195575792	2.39E-06	1.07E-05	2.84E-02
PPM1B	LMNA	-0.237204355	-0.363356766	-0.102510183	6.76E-04	1.85E-03	1.00E+00
SUV39H1	LMNA	0.217369866	0.081772712	0.34506608	1.89E-03	4.61E-03	1.00E+00
SLC7A5	LMNA	-0.079551472	-0.215238686	0.059149114	2.60E-01	3.42E-01	1.00E+00
DLG7	LMNA	0.116023987	-0.022385568	0.250069567	1.00E-01	1.52E-01	1.00E+00
AAMP	LMNA	0.054188176	-0.084494908	0.190811765	4.44E-01	5.30E-01	1.00E+00
T	LMNA	-0.2229152	-0.350189584	-0.087558702	1.43E-03	3.60E-03	1.00E+00
NFIB	LMNA	-0.395063375	-0.505542639	-0.271838125	5.94E-09	4.25E-08	7.04E-05
MBD6	LMNA	-0.057468788	-0.193980817	0.081226634	4.17E-01	5.04E-01	1.00E+00
TUSC4	LMNA	-0.203173327	-0.331914691	-0.067001607	3.73E-03	8.43E-03	1.00E+00
CAPRIN1	LMNA	0.040545591	-0.098054255	0.177602487	5.67E-01	6.46E-01	1.00E+00
ETFDH	LMNA	0.035418655	-0.10313664	0.172625589	6.17E-01	6.92E-01	1.00E+00
SLC15A1	LMNA	0.267314198	0.134216176	0.390938402	1.20E-04	3.87E-04	1.00E+00
KLK13	LMNA	0.111436483	-0.027030351	0.245707539	1.14E-01	1.70E-01	1.00E+00
GSPT2	LMNA	-0.060910471	-0.197302405	0.077794685	3.89E-01	4.77E-01	1.00E+00
TRIM13	LMNA	-0.339780554	-0.456422141	-0.211657795	7.53E-07	3.69E-06	8.93E-03
NAT9	LMNA	-0.063347819	-0.19965281	0.075362242	3.70E-01	4.58E-01	1.00E+00
MB	LMNA	-0.111969402	-0.246214544	0.026491084	1.13E-01	1.68E-01	1.00E+00
C15orf5	LMNA	-0.189401401	-0.31910865	-0.052729111	6.94E-03	1.46E-02	1.00E+00
LIFR	LMNA	-0.21856713	-0.346172913	-0.083021161	1.78E-03	4.37E-03	1.00E+00
DMBT1	LMNA	0.189192137	0.052512671	0.318913698	7.00E-03	1.48E-02	1.00E+00
KCNAB2	LMNA	-0.026368787	-0.163823431	0.112090249	7.10E-01	7.73E-01	1.00E+00
EIF4A1	LMNA	0.237321894	0.102633424	0.363464868	6.72E-04	1.84E-03	1.00E+00
MXI1	LMNA	-0.426695688	-0.53333045	-0.30671178	2.42E-10	2.25E-09	2.87E-06
SPTLC2	LMNA	0.195229459	0.058762194	0.324533748	5.36E-03	1.16E-02	1.00E+00
TTC28	LMNA	-0.095134438	-0.23016262	0.043487704	1.78E-01	2.48E-01	1.00E+00
TSEN2	LMNA	-0.224669686	-0.351809022	-0.089391233	1.31E-03	3.33E-03	1.00E+00
MAGI2	LMNA	-0.254723938	-0.379432244	-0.120925262	2.54E-04	7.64E-04	1.00E+00
NLRP2	LMNA	0.047849838	-0.090800968	0.184680889	4.99E-01	5.84E-01	1.00E+00

EXPH5	LMNA	-0.146767946	-0.279162702	-0.008897216	3.71E-02	6.45E-02	1.00E+00
NELL1	LMNA	-0.271596073	-0.394842795	-0.138747278	9.23E-05	3.04E-04	1.00E+00
MAP3K2	LMNA	0.135091095	-0.003016155	0.268141403	5.53E-02	9.15E-02	1.00E+00
SEPX1	LMNA	0.174687648	0.037542003	0.305374302	1.29E-02	2.54E-02	1.00E+00
TSPO	LMNA	0.427761353	0.307892279	0.534262607	2.16E-10	2.02E-09	2.56E-06
ADORA1	LMNA	0.209677998	0.073762135	0.337946707	2.74E-03	6.43E-03	1.00E+00
CLINT1	LMNA	0.269439644	0.136464639	0.392877029	1.05E-04	3.43E-04	1.00E+00
SYMPK	LMNA	0.132237293	-0.005921813	0.26544251	6.07E-02	9.93E-02	1.00E+00
LEPROTL1	LMNA	-0.394535265	-0.505076761	-0.271258634	6.25E-09	4.45E-08	7.41E-05
C2orf54	LMNA	0.052037411	-0.086635966	0.188732592	4.62E-01	5.48E-01	1.00E+00
SEMA6C	LMNA	-0.37529482	-0.488059541	-0.250207	3.73E-08	2.29E-07	4.42E-04
POLE2	LMNA	0.028265522	-0.110215548	0.165670065	6.90E-01	7.56E-01	1.00E+00
IL2	LMNA	0.028145823	-0.110333887	0.165553555	6.91E-01	7.57E-01	1.00E+00
TBPL1	LMNA	-0.384321617	-0.496053985	-0.260068823	1.64E-08	1.08E-07	1.94E-04
STX12	LMNA	-0.249525736	-0.374670362	-0.115451733	3.42E-04	1.00E-03	1.00E+00
MRPL39	LMNA	0.08699511	-0.051676513	0.222375435	2.18E-01	2.95E-01	1.00E+00
PLCL1	LMNA	-0.42375287	-0.530754966	-0.303453765	3.30E-10	2.97E-09	3.92E-06
CASC5	LMNA	-0.04841457	-0.185227569	0.090239562	4.94E-01	5.79E-01	1.00E+00
IFIT5	LMNA	-0.348665609	-0.464365032	-0.221264892	3.67E-07	1.91E-06	4.36E-03
DDIT3	LMNA	0.043603955	-0.095019019	0.180568014	5.38E-01	6.20E-01	1.00E+00
FAM46A	LMNA	0.478629378	0.364674331	0.578458576	5.81E-13	8.43E-12	6.90E-09
CYLC1	LMNA	-0.170276707	-0.301246349	-0.033001467	1.54E-02	2.96E-02	1.00E+00
HPCAL1	LMNA	0.189584579	0.052918582	0.319279294	6.89E-03	1.45E-02	1.00E+00
HOMER1	LMNA	-0.063817281	-0.200105345	0.074893535	3.67E-01	4.54E-01	1.00E+00
CDH1	LMNA	-0.166400265	-0.297614547	-0.029015808	1.79E-02	3.39E-02	1.00E+00
CCDC101	LMNA	-0.222454056	-0.349763809	-0.087077197	1.46E-03	3.67E-03	1.00E+00
ITPA	LMNA	0.282076264	0.149860986	0.404380326	4.76E-05	1.66E-04	5.65E-01
D15Wsu75e	LMNA	0.226624125	0.091433691	0.353612128	1.18E-03	3.04E-03	1.00E+00
EDA	LMNA	-0.033297034	-0.170564005	0.105237712	6.38E-01	7.12E-01	1.00E+00
CREG1	LMNA	0.158363084	0.020766075	0.290072468	2.44E-02	4.46E-02	1.00E+00
SAP18	LMNA	-0.198296335	-0.327385165	-0.061940969	4.67E-03	1.03E-02	1.00E+00
IFIT1	LMNA	-0.231496847	-0.358103444	-0.096530814	9.17E-04	2.43E-03	1.00E+00
CHRNA4	LMNA	-0.147430671	-0.279787177	-0.009574541	3.63E-02	6.33E-02	1.00E+00
CALML3	LMNA	-0.043976241	-0.180928829	0.094649372	5.34E-01	6.17E-01	1.00E+00
HSPA5	LMNA	0.614169084	0.520226335	0.693426749	2.48E-22	1.88E-20	2.94E-18

JUNB	LMNA	0.486526802	0.373566593	0.585267979	2.12E-13	3.25E-12	2.51E-09
SERPINB6	LMNA	0.48694611	0.374039302	0.58562913	2.01E-13	3.10E-12	2.38E-09
RAB40A	LMNA	-0.120101196	-0.253941818	0.018252432	8.87E-02	1.37E-01	1.00E+00
SCN2A	LMNA	-0.551421838	-0.640699978	-0.447431244	1.83E-17	6.01E-16	2.17E-13
ALDH8A1	LMNA	-0.257270307	-0.38176247	-0.123609473	2.19E-04	6.68E-04	1.00E+00
ZKSCAN5	LMNA	0.056704261	-0.081988553	0.193242543	4.23E-01	5.10E-01	1.00E+00
WNT7A	LMNA	-0.112406737	-0.246630558	0.026048481	1.11E-01	1.66E-01	1.00E+00
PRRG2	LMNA	0.035615169	-0.102941968	0.172816481	6.15E-01	6.91E-01	1.00E+00
RALA	LMNA	0.215659437	0.079989895	0.343484225	2.05E-03	4.97E-03	1.00E+00
H6PD	LMNA	0.482733632	0.369293005	0.581999128	3.45E-13	5.12E-12	4.09E-09
CAD	LMNA	0.324383951	0.195068412	0.442614019	2.48E-06	1.11E-05	2.94E-02
SAP30	LMNA	0.084439633	-0.054243685	0.219926951	2.32E-01	3.10E-01	1.00E+00
DOCK10	LMNA	-0.203626264	-0.332335057	-0.067471955	3.65E-03	8.27E-03	1.00E+00
XPA	LMNA	-0.244644882	-0.370193174	-0.110319762	4.50E-04	1.28E-03	1.00E+00
FAIM2	LMNA	-0.415034884	-0.523113579	-0.293818503	8.18E-10	6.88E-09	9.70E-06
PRB1	LMNA	-0.076479565	-0.212289199	0.062228449	2.79E-01	3.62E-01	1.00E+00
SPDEF	LMNA	-0.09177328	-0.226948975	0.046871554	1.94E-01	2.67E-01	1.00E+00
ETHE1	LMNA	0.350737852	0.223509097	0.466214863	3.10E-07	1.63E-06	3.67E-03
GNPTAB	LMNA	-0.226988053	-0.353947772	-0.091814133	1.16E-03	2.99E-03	1.00E+00
IRF5	LMNA	0.112435578	-0.02601929	0.246657992	1.11E-01	1.66E-01	1.00E+00
ABCC10	LMNA	0.180863002	0.043908318	0.31114523	1.00E-02	2.03E-02	1.00E+00
INSL4	LMNA	-0.03088198	-0.168215828	0.107627869	6.63E-01	7.33E-01	1.00E+00
GNMT	LMNA	-0.386726545	-0.498180682	-0.262700601	1.31E-08	8.76E-08	1.55E-04
PFDN6	LMNA	0.013986554	-0.124304461	0.151744558	8.43E-01	8.84E-01	1.00E+00
RPA1	LMNA	0.017676923	-0.120668547	0.155348823	8.03E-01	8.51E-01	1.00E+00
ACTR1B	LMNA	0.105899191	-0.032628826	0.240435153	1.34E-01	1.95E-01	1.00E+00
TROVE2	LMNA	-0.152001354	-0.284091006	-0.014249361	3.08E-02	5.49E-02	1.00E+00
C12orf35	LMNA	0.234766355	0.099954864	0.361113756	7.71E-04	2.08E-03	1.00E+00
EEF1E1	LMNA	-0.08720814	-0.222579468	0.051462426	2.17E-01	2.93E-01	1.00E+00
PLEKHM1	LMNA	0.216478044	0.080843038	0.344241387	1.97E-03	4.80E-03	1.00E+00
MSX1	LMNA	0.075361381	-0.063348682	0.211214961	2.86E-01	3.70E-01	1.00E+00
FNDC3A	LMNA	0.106024841	-0.032501885	0.240554879	1.33E-01	1.94E-01	1.00E+00
ESF1	LMNA	0.196732403	0.060319629	0.325931398	5.01E-03	1.09E-02	1.00E+00
HSPC171	LMNA	0.282221156	0.150014871	0.404511999	4.72E-05	1.64E-04	5.59E-01
MRPL2	LMNA	0.070175013	-0.068539984	0.206228131	3.21E-01	4.06E-01	1.00E+00

MICB	LMNA	0.400875326	0.278221445	0.510665466	3.38E-09	2.53E-08	4.01E-05
CELP	LMNA	0.008824479	-0.129384141	0.146696769	9.01E-01	9.28E-01	1.00E+00
ST8SIA3	LMNA	-0.440170196	-0.545097818	-0.321665501	5.57E-11	5.81E-10	6.60E-07
C10orf116	LMNA	-0.094056701	-0.229132503	0.044573064	1.83E-01	2.54E-01	1.00E+00
MYL7	LMNA	0.05256305	-0.086112818	0.189240847	4.58E-01	5.44E-01	1.00E+00
NCR1	LMNA	0.178688217	0.041665013	0.309113968	1.09E-02	2.20E-02	1.00E+00
CD52	LMNA	0.2624513	0.129076973	0.386498805	1.61E-04	5.06E-04	1.00E+00
KHDRBS3	LMNA	-0.206950315	-0.33541851	-0.070925639	3.13E-03	7.21E-03	1.00E+00
SLC30A10	LMNA	-0.429971637	-0.536195157	-0.310341916	1.70E-10	1.63E-09	2.02E-06
PMS2L5	LMNA	-0.024606973	-0.162107293	0.113830712	7.28E-01	7.89E-01	1.00E+00
UBE2E1	LMNA	-0.221763861	-0.349126452	-0.086356645	1.51E-03	3.78E-03	1.00E+00
AP4S1	LMNA	-0.136135454	-0.26912855	0.001952237	5.34E-02	8.89E-02	1.00E+00
TPK1	LMNA	0.11681604	-0.02158302	0.25082214	9.78E-02	1.49E-01	1.00E+00
MICAL2	LMNA	0.3308771	0.202055576	0.448444108	1.51E-06	7.01E-06	1.79E-02
UBA52	LMNA	0.15489501	0.017212062	0.286812971	2.77E-02	4.99E-02	1.00E+00
GEMIN7	LMNA	0.041113315	-0.097491019	0.178153165	5.61E-01	6.42E-01	1.00E+00
PPIF	LMNA	-0.129176229	-0.262545284	0.009035908	6.69E-02	1.08E-01	1.00E+00
RIPK1	LMNA	0.521955306	0.413715152	0.615645105	1.64E-15	3.77E-14	1.94E-11
COL14A1	LMNA	0.403570151	0.281184877	0.513038124	2.59E-09	1.98E-08	3.08E-05
HSPC159	LMNA	-0.099925961	-0.234738769	0.038658326	1.57E-01	2.24E-01	1.00E+00
CPNE3	LMNA	0.004980942	-0.133161624	0.142933658	9.44E-01	9.58E-01	1.00E+00
ATP8A1	LMNA	-0.493564782	-0.59132463	-0.381508681	8.43E-14	1.36E-12	1.00E-09
ALOX12P2	LMNA	-0.063860087	-0.200146605	0.074850795	3.67E-01	4.54E-01	1.00E+00
CSAD	LMNA	-0.102216409	-0.236924155	0.036347493	1.48E-01	2.12E-01	1.00E+00
MTHFS	LMNA	0.354091296	0.227143682	0.469206248	2.34E-07	1.26E-06	2.78E-03
RECK	LMNA	0.101222353	-0.037350579	0.235975863	1.52E-01	2.17E-01	1.00E+00
CXCL9	LMNA	0.303138815	0.172298258	0.423468255	1.16E-05	4.54E-05	1.37E-01
ABAT	LMNA	-0.552298269	-0.641442291	-0.448438618	1.59E-17	5.27E-16	1.88E-13
VPREB3	LMNA	0.093942269	-0.044688286	0.229023109	1.84E-01	2.55E-01	1.00E+00
EGFL6	LMNA	0.387278004	0.263304337	0.498668153	1.24E-08	8.35E-08	1.47E-04
C1orf14	LMNA	0.163290639	0.025821723	0.294698419	2.02E-02	3.78E-02	1.00E+00
RAB3IL1	LMNA	0.238590592	0.103963921	0.364631482	6.27E-04	1.73E-03	1.00E+00
FGF18	LMNA	0.121304265	-0.017031956	0.255083591	8.55E-02	1.33E-01	1.00E+00
LHX6	LMNA	-0.079709331	-0.215390188	0.058990802	2.59E-01	3.41E-01	1.00E+00
SLC20A2	LMNA	-0.03016154	-0.167515036	0.108340572	6.70E-01	7.39E-01	1.00E+00

JARID2	LMNA	-0.196143151	-0.325383496	-0.059708934	5.15E-03	1.12E-02	1.00E+00
CRBN	LMNA	-0.348809102	-0.464493157	-0.221420249	3.63E-07	1.89E-06	4.30E-03
SPINT1	LMNA	0.489077663	0.376443228	0.587464439	1.52E-13	2.40E-12	1.80E-09
PIN1L	LMNA	-0.062930646	-0.199250629	0.075778692	3.74E-01	4.61E-01	1.00E+00
ABCF3	LMNA	0.150130024	0.012334667	0.282329575	3.30E-02	5.82E-02	1.00E+00
NCBP1	LMNA	-0.028523144	-0.165920807	0.109960844	6.87E-01	7.54E-01	1.00E+00
SSX1	LMNA	0.043361178	-0.095260055	0.180332697	5.40E-01	6.22E-01	1.00E+00
CELSR1	LMNA	0.343949066	0.216161979	0.460150944	5.39E-07	2.70E-06	6.39E-03
SLC9A1	LMNA	0.509664141	0.399738607	0.605137857	9.44E-15	1.84E-13	1.12E-10
CHRND	LMNA	0.022526542	-0.115884838	0.160079725	7.50E-01	8.07E-01	1.00E+00
ELSPBP1	LMNA	0.036349952	-0.102213973	0.173530153	6.08E-01	6.84E-01	1.00E+00
SRPRB	LMNA	0.081399382	-0.057295463	0.217011763	2.49E-01	3.30E-01	1.00E+00
FOXF1	LMNA	0.116829799	-0.021569077	0.250835212	9.78E-02	1.49E-01	1.00E+00
LTF	LMNA	0.315381724	0.185402938	0.434514501	4.84E-06	2.03E-05	5.73E-02
TPO	LMNA	-0.202926335	-0.331685438	-0.066745145	3.77E-03	8.51E-03	1.00E+00
KIAA1467	LMNA	-0.351817396	-0.467178141	-0.22467876	2.83E-07	1.50E-06	3.36E-03
DNAJB9	LMNA	0.010651797	-0.127586819	0.148484453	8.80E-01	9.12E-01	1.00E+00
PAFAH1B2	LMNA	0.372987217	0.247690091	0.486012806	4.58E-08	2.79E-07	5.43E-04
MRPS27	LMNA	-0.194518031	-0.323871965	-0.058025205	5.54E-03	1.20E-02	1.00E+00
TNN	LMNA	0.132236308	-0.005922816	0.265441579	6.07E-02	9.93E-02	1.00E+00
UBAP2L	LMNA	0.036093995	-0.102467582	0.173281566	6.10E-01	6.86E-01	1.00E+00
WBP2	LMNA	-0.101501607	-0.236242287	0.037068816	1.51E-01	2.16E-01	1.00E+00
AGRP	LMNA	0.154400851	0.016705941	0.286348283	2.82E-02	5.06E-02	1.00E+00
PRPF18	LMNA	-0.110845313	-0.245145029	0.027628467	1.16E-01	1.73E-01	1.00E+00
GTDC1	LMNA	-0.449540525	-0.553256682	-0.332099457	1.93E-11	2.15E-10	2.29E-07
TMEM131	LMNA	0.210862419	0.07499449	0.339043929	2.59E-03	6.10E-03	1.00E+00
RCE1	LMNA	0.404403461	0.28210172	0.513771472	2.39E-09	1.84E-08	2.83E-05
CD81	LMNA	0.228553364	0.093450929	0.355391059	1.07E-03	2.79E-03	1.00E+00
TIMM8B	LMNA	-0.170857751	-0.301790396	-0.033599258	1.50E-02	2.90E-02	1.00E+00
MYH7	LMNA	-0.209998394	-0.338243549	-0.074095457	2.70E-03	6.34E-03	1.00E+00
SCRN3	LMNA	-0.316650632	-0.435657336	-0.186763813	4.41E-06	1.86E-05	5.23E-02
SH2B3	LMNA	0.61339163	0.519315956	0.692778604	2.89E-22	2.16E-20	3.43E-18
KIF1C	LMNA	0.130771167	-0.007413673	0.264055156	6.36E-02	1.03E-01	1.00E+00
TMCO1	LMNA	0.08429315	-0.054390782	0.219786549	2.33E-01	3.11E-01	1.00E+00
NEUROG2	LMNA	0.124047421	-0.014247567	0.257685589	7.86E-02	1.24E-01	1.00E+00

SUHW2	LMNA	-0.297512424	-0.418379777	-0.166291321	1.71E-05	6.48E-05	2.03E-01
PAPSS1	LMNA	-0.249075298	-0.374257422	-0.114977821	3.51E-04	1.03E-03	1.00E+00
CABP2	LMNA	-0.043008012	-0.179990356	0.095610657	5.43E-01	6.25E-01	1.00E+00
H1FX	LMNA	-0.232874634	-0.359372326	-0.097973337	8.52E-04	2.27E-03	1.00E+00
ELF2	LMNA	-0.15027948	-0.282470286	-0.012487549	3.28E-02	5.80E-02	1.00E+00
HOXA4	LMNA	-0.009762551	-0.147614607	0.128461581	8.90E-01	9.21E-01	1.00E+00
KCNK13	LMNA	0.078441743	-0.060261831	0.214173467	2.67E-01	3.49E-01	1.00E+00
SEMA3D	LMNA	-0.370521814	-0.48382474	-0.245002936	5.70E-08	3.43E-07	6.76E-04
AP3D1	LMNA	0.231691165	0.096734227	0.358282431	9.07E-04	2.41E-03	1.00E+00
GLYAT	LMNA	-0.078478254	-0.214208519	0.060225227	2.67E-01	3.49E-01	1.00E+00
OGFR	LMNA	0.287455524	0.155578423	0.409265431	3.35E-05	1.21E-04	3.98E-01
MC5R	LMNA	-0.018675587	-0.156323557	0.11968398	7.92E-01	8.43E-01	1.00E+00
FLJ30092	LMNA	-0.442689095	-0.54729301	-0.324467494	4.20E-11	4.47E-10	4.98E-07
TGFA	LMNA	0.05462507	-0.08405983	0.191233967	4.40E-01	5.26E-01	1.00E+00
C8orf17	LMNA	0.01205529	-0.126205747	0.149856893	8.65E-01	9.01E-01	1.00E+00
DDX54	LMNA	0.365493835	0.239528683	0.479357971	8.86E-08	5.13E-07	1.05E-03
NPAL3	LMNA	0.086585732	-0.052087888	0.221983311	2.20E-01	2.97E-01	1.00E+00
NXF3	LMNA	0.321960691	0.192464144	0.44043565	2.97E-06	1.30E-05	3.53E-02
MMP17	LMNA	-0.216613278	-0.344366455	-0.080983997	1.96E-03	4.77E-03	1.00E+00
KIF15	LMNA	-0.155378037	-0.287267131	-0.01770685	2.72E-02	4.91E-02	1.00E+00
MYL5	LMNA	0.121695498	-0.016634973	0.255454811	8.45E-02	1.32E-01	1.00E+00
PRLR	LMNA	0.1311317	-0.007046869	0.264396369	6.29E-02	1.02E-01	1.00E+00
AP3S1	LMNA	0.270708183	0.137807251	0.394033545	9.75E-05	3.19E-04	1.00E+00
CHIA	LMNA	0.082003318	-0.056689443	0.217591049	2.46E-01	3.26E-01	1.00E+00
FGFR1OP	LMNA	0.231292542	0.096316958	0.357915249	9.27E-04	2.45E-03	1.00E+00
MED28	LMNA	0.029012412	-0.109477065	0.166396961	6.82E-01	7.50E-01	1.00E+00
PTPRA	LMNA	0.069067288	-0.069647791	0.205162107	3.29E-01	4.14E-01	1.00E+00
CEACAM7	LMNA	0.00053202	-0.137529082	0.138572843	9.94E-01	9.95E-01	1.00E+00
GOLIM4	LMNA	0.116235365	-0.022171408	0.250270424	9.95E-02	1.51E-01	1.00E+00
PADI2	LMNA	-0.072343586	-0.208314132	0.066370261	3.06E-01	3.91E-01	1.00E+00
ADSL	LMNA	-0.101520765	-0.236260563	0.037049486	1.51E-01	2.16E-01	1.00E+00
HSF1	LMNA	0.184927996	0.048105094	0.314938769	8.42E-03	1.74E-02	1.00E+00
LAS1L	LMNA	0.232971076	0.098074331	0.359461127	8.48E-04	2.26E-03	1.00E+00
DR1	LMNA	0.003523271	-0.134593195	0.141505444	9.60E-01	9.70E-01	1.00E+00
BAP1	LMNA	0.062434801	-0.076273616	0.198772544	3.77E-01	4.65E-01	1.00E+00

C14orf140	LMNA	0.093996208	-0.044633975	0.229074674	1.83E-01	2.54E-01	1.00E+00
SLC17A2	LMNA	-0.052484261	-0.189164668	0.086191239	4.58E-01	5.44E-01	1.00E+00
HOXA9	LMNA	-0.127821524	-0.261262319	0.010413227	6.99E-02	1.12E-01	1.00E+00
TMEM161A	LMNA	0.287833853	0.155980869	0.409608742	3.27E-05	1.18E-04	3.88E-01
TMEM144	LMNA	-0.19072156	-0.320338277	-0.054094839	6.55E-03	1.39E-02	1.00E+00
HIF1AN	LMNA	-0.073235369	-0.2091716	0.065477624	3.00E-01	3.85E-01	1.00E+00
METTL7A	LMNA	-0.075174059	-0.21103497	0.063536312	2.88E-01	3.71E-01	1.00E+00
NCKIPSD	LMNA	0.024052568	-0.114378223	0.161567089	7.34E-01	7.93E-01	1.00E+00
ITM2A	LMNA	-0.460609414	-0.562868935	-0.344461916	5.29E-12	6.55E-11	6.28E-08
POLR2H	LMNA	-0.010375581	-0.148214286	0.127858559	8.83E-01	9.15E-01	1.00E+00
ABCG5	LMNA	-0.037666069	-0.174808095	0.100909646	5.95E-01	6.72E-01	1.00E+00
PCDHA3	LMNA	-0.160888046	-0.292443652	-0.023355795	2.22E-02	4.10E-02	1.00E+00
DSG3	LMNA	0.341532976	0.21355067	0.457990211	6.55E-07	3.24E-06	7.76E-03
ZNF180	LMNA	-0.10076109	-0.235535748	0.037815937	1.54E-01	2.19E-01	1.00E+00
BUB1B	LMNA	-0.020062058	-0.15767636	0.118316631	7.77E-01	8.30E-01	1.00E+00
JMJD1C	LMNA	-0.317685405	-0.436589014	-0.187873952	4.08E-06	1.74E-05	4.84E-02
NFKBIB	LMNA	0.264117082	0.130836597	0.388020233	1.46E-04	4.63E-04	1.00E+00
DKK1	LMNA	0.456468886	0.339832784	0.559276525	8.63E-12	1.03E-10	1.02E-07
CAPN5	LMNA	0.085869495	-0.052807505	0.221297158	2.24E-01	3.01E-01	1.00E+00
ZNF205	LMNA	0.26271394	0.129354353	0.38673873	1.59E-04	4.99E-04	1.00E+00
COX7A1	LMNA	-8.00E-05	-0.138129495	0.137972505	9.99E-01	9.99E-01	1.00E+00
OLFML2B	LMNA	0.444078915	0.326014402	0.548503606	3.59E-11	3.84E-10	4.26E-07
MAGEA1	LMNA	-0.003932407	-0.141906371	0.134191442	9.56E-01	9.67E-01	1.00E+00
PA2G4	LMNA	-0.004208605	-0.142177001	0.133920203	9.53E-01	9.65E-01	1.00E+00
NEDD8	LMNA	-0.111411731	-0.245683989	0.027055395	1.14E-01	1.71E-01	1.00E+00
MRPS2	LMNA	0.142847079	0.004892561	0.275465843	4.26E-02	7.28E-02	1.00E+00
ABHD11	LMNA	0.3899408	0.266220929	0.501020981	9.67E-09	6.63E-08	1.15E-04
NOTCH4	LMNA	0.046399872	-0.092241989	0.183276884	5.12E-01	5.96E-01	1.00E+00
CADM1	LMNA	0.13422922	-0.003893938	0.267326532	5.68E-02	9.38E-02	1.00E+00
PAIP2B	LMNA	-0.497556896	-0.594755215	-0.386020993	4.95E-14	8.27E-13	5.87E-10
MAT1A	LMNA	0.1171744	-0.021219854	0.251162584	9.68E-02	1.48E-01	1.00E+00
THUMPD2	LMNA	-0.13244096	-0.265635191	0.005714522	6.03E-02	9.87E-02	1.00E+00
CALM3	LMNA	-0.298111123	-0.418921598	-0.166930051	1.64E-05	6.24E-05	1.94E-01
KCNC4	LMNA	0.056651929	-0.0820407	0.193192003	4.23E-01	5.10E-01	1.00E+00
TSPAN1	LMNA	0.223438946	0.08810565	0.350673095	1.39E-03	3.51E-03	1.00E+00

NMI	LMNA	0.424466639	0.304243726	0.531379821	3.06E-10	2.77E-09	3.63E-06
ACIN1	LMNA	0.040546646	-0.098053208	0.17760351	5.67E-01	6.46E-01	1.00E+00
RGS9	LMNA	-0.397870541	-0.508017932	-0.274919908	4.53E-09	3.32E-08	5.37E-05
XKR8	LMNA	0.432532719	0.313182328	0.538433035	1.29E-10	1.26E-09	1.53E-06
RPL23	LMNA	-0.130945669	-0.264220312	0.00723614	6.32E-02	1.03E-01	1.00E+00
B4GALT7	LMNA	0.471018225	0.356124024	0.57188281	1.50E-12	2.03E-11	1.78E-08
CNKSR1	LMNA	-0.196713425	-0.325913754	-0.060299959	5.02E-03	1.10E-02	1.00E+00
MPDZ	LMNA	-0.240219613	-0.366128844	-0.105672999	5.74E-04	1.60E-03	1.00E+00
SDHC	LMNA	-0.039791571	-0.176870972	0.098802176	5.74E-01	6.53E-01	1.00E+00
ATF6	LMNA	0.413981324	0.292655757	0.522188946	9.11E-10	7.58E-09	1.08E-05
GBF1	LMNA	0.022416242	-0.115993711	0.159972195	7.52E-01	8.08E-01	1.00E+00
ITIH1	LMNA	0.044308288	-0.094319647	0.181250615	5.31E-01	6.14E-01	1.00E+00
ZC3H11A	LMNA	0.001111345	-0.136960668	0.139140999	9.87E-01	9.90E-01	1.00E+00
RNASEH2A	LMNA	-0.005065144	-0.143016141	0.133078912	9.43E-01	9.58E-01	1.00E+00
CCR10	LMNA	0.101120367	-0.037453476	0.235878558	1.52E-01	2.18E-01	1.00E+00
EIF2AK1	LMNA	0.043029859	-0.09558897	0.180011534	5.43E-01	6.24E-01	1.00E+00
B4GALT3	LMNA	0.077173579	-0.061532987	0.212955772	2.75E-01	3.57E-01	1.00E+00
TMEM112	LMNA	0.090262787	-0.04839121	0.225503815	2.01E-01	2.76E-01	1.00E+00
MAP1B	LMNA	0.220160795	0.084683619	0.347645656	1.64E-03	4.07E-03	1.00E+00
NVL	LMNA	-0.180011821	-0.310350363	-0.043030154	1.04E-02	2.09E-02	1.00E+00
PKM2	LMNA	0.606813478	0.51162164	0.687289414	1.05E-21	6.76E-20	1.24E-17
GGNBP2	LMNA	-0.071047887	-0.207067917	0.067666806	3.15E-01	4.00E-01	1.00E+00
ARC	LMNA	-0.088693498	-0.22400177	0.049969339	2.09E-01	2.84E-01	1.00E+00
NUP54	LMNA	0.031486525	-0.107029707	0.168803778	6.56E-01	7.27E-01	1.00E+00
PPFIBP2	LMNA	0.050379023	-0.088285996	0.187128568	4.76E-01	5.62E-01	1.00E+00
GPR175	LMNA	0.417359533	0.296385337	0.525152853	6.44E-10	5.52E-09	7.64E-06
VCAM1	LMNA	0.172452138	0.035240105	0.303282827	1.41E-02	2.75E-02	1.00E+00
STAT2	LMNA	0.335329856	0.206854702	0.45243639	1.07E-06	5.10E-06	1.27E-02
Sep-08	LMNA	-0.18285322	-0.313003063	-0.045962453	9.20E-03	1.88E-02	1.00E+00
PTAFR	LMNA	0.162869004	0.025388852	0.294302832	2.06E-02	3.83E-02	1.00E+00
ALG3	LMNA	0.444173637	0.326119854	0.548586098	3.55E-11	3.81E-10	4.22E-07
REL	LMNA	0.13197882	-0.006184867	0.265197965	6.12E-02	9.99E-02	1.00E+00
GNA14	LMNA	0.23142165	0.0964521	0.358034178	9.20E-04	2.44E-03	1.00E+00
CR2	LMNA	0.116102101	-0.022306428	0.250143794	9.99E-02	1.52E-01	1.00E+00
RNF40	LMNA	0.196091658	0.059655572	0.325335612	5.16E-03	1.12E-02	1.00E+00

EWSR1	LMNA	-0.022760071	-0.160307378	0.115654321	7.48E-01	8.05E-01	1.00E+00
CSN1S1	LMNA	-0.12436424	-0.257985981	0.013925848	7.78E-02	1.23E-01	1.00E+00
PLEKHH3	LMNA	0.196418338	0.059994123	0.325639383	5.08E-03	1.11E-02	1.00E+00
IFT81	LMNA	-0.124665326	-0.258271431	0.013620078	7.71E-02	1.22E-01	1.00E+00
PDCD11	LMNA	0.048131808	-0.09052067	0.184953857	4.96E-01	5.81E-01	1.00E+00
PCDHB1	LMNA	-0.053759554	-0.190397508	0.084921698	4.47E-01	5.34E-01	1.00E+00
TM7SF3	LMNA	-0.017500625	-0.155176722	0.120842328	8.05E-01	8.52E-01	1.00E+00
OR10H3	LMNA	0.017792634	-0.120554484	0.155461775	8.02E-01	8.50E-01	1.00E+00
ABP1	LMNA	-0.049160013	-0.185949054	0.089498377	4.87E-01	5.72E-01	1.00E+00
GLT25D2	LMNA	0.05703583	-0.081658135	0.193562746	4.20E-01	5.08E-01	1.00E+00
CHRD	LMNA	-0.088437613	-0.223756789	0.050226599	2.11E-01	2.86E-01	1.00E+00
PEX10	LMNA	0.146245051	0.008362891	0.278669908	3.78E-02	6.56E-02	1.00E+00
C19orf57	LMNA	-0.280031041	-0.402521151	-0.147689505	5.43E-05	1.87E-04	6.44E-01
KLC1	LMNA	-0.116625291	-0.250640913	0.021776313	9.83E-02	1.50E-01	1.00E+00
GALE	LMNA	0.218997862	0.083470411	0.346571024	1.74E-03	4.29E-03	1.00E+00
NT5C2	LMNA	-0.307396173	-0.427313547	-0.176850043	8.59E-06	3.43E-05	1.02E-01
TBC1D10B	LMNA	0.170186111	0.032908269	0.301161514	1.55E-02	2.97E-02	1.00E+00
EFCAB2	LMNA	-0.229737888	-0.356482841	-0.094690033	1.01E-03	2.64E-03	1.00E+00
NCALD	LMNA	-0.526517707	-0.619536867	-0.418916171	8.41E-16	2.06E-14	9.97E-12
AKAP13	LMNA	0.435156639	0.316094648	0.540724273	9.69E-11	9.77E-10	1.15E-06
FLG	LMNA	0.097184632	-0.041422106	0.232121391	1.69E-01	2.37E-01	1.00E+00
ACCN1	LMNA	-0.15228192	-0.284355018	-0.014536515	3.05E-02	5.44E-02	1.00E+00
ZNF337	LMNA	-0.12349395	-0.257160755	0.01480953	7.99E-02	1.26E-01	1.00E+00
ARMET	LMNA	0.496900954	0.385279211	0.594191781	5.41E-14	8.94E-13	6.41E-10
ALS2CL	LMNA	0.192341857	0.055771764	0.321846863	6.10E-03	1.30E-02	1.00E+00
REEP4	LMNA	0.549341299	0.445040918	0.638937149	2.54E-17	8.18E-16	3.02E-13
MTSS1	LMNA	-0.528664417	-0.621366446	-0.42136581	6.12E-16	1.53E-14	7.26E-12
ACN9	LMNA	-0.123442855	-0.2571123	0.014861405	8.01E-02	1.26E-01	1.00E+00
ADH1B	LMNA	-0.086864428	-0.222250265	0.051807838	2.19E-01	2.95E-01	1.00E+00
DLD	LMNA	0.123357606	-0.014947952	0.257031454	8.03E-02	1.26E-01	1.00E+00
CDK5	LMNA	-0.13809556	-0.270980525	-4.54E-05	5.00E-02	8.39E-02	1.00E+00
PPFIA1	LMNA	0.049206578	-0.089452072	0.185994117	4.87E-01	5.72E-01	1.00E+00
DNAJB12	LMNA	0.139431769	0.001407868	0.272242457	4.78E-02	8.07E-02	1.00E+00
MLANA	LMNA	-0.023176512	-0.160713303	0.115243214	7.43E-01	8.02E-01	1.00E+00
HMMR	LMNA	0.139975314	0.001962231	0.272755657	4.69E-02	7.94E-02	1.00E+00

CUL2	LMNA	-0.238049998	-0.364134434	-0.103396935	6.46E-04	1.78E-03	1.00E+00
DENND4C	LMNA	0.078471396	-0.060232102	0.214201936	2.67E-01	3.49E-01	1.00E+00
KIAA0319	LMNA	-0.318905877	-0.437687563	-0.189183743	3.73E-06	1.60E-05	4.43E-02
RPS7	LMNA	-0.103311686	-0.23796871	0.035241945	1.43E-01	2.07E-01	1.00E+00
JAK3	LMNA	0.331829092	0.203081103	0.449298047	1.40E-06	6.53E-06	1.67E-02
C6orf106	LMNA	0.274189186	0.141494031	0.397205141	7.86E-05	2.62E-04	9.32E-01
HEY2	LMNA	-0.260541169	-0.384753372	-0.127060268	1.80E-04	5.61E-04	1.00E+00
GCG	LMNA	-0.183954172	-0.314030353	-0.047099259	8.78E-03	1.81E-02	1.00E+00
FCER2	LMNA	-0.125818474	-0.259364478	0.012448753	7.44E-02	1.18E-01	1.00E+00
CAMKV	LMNA	-0.564527322	-0.651782585	-0.462522335	2.13E-18	8.44E-17	2.53E-14
ARHGDIA	LMNA	0.407169845	0.285147016	0.516204865	1.81E-09	1.43E-08	2.15E-05
ARFGEF1	LMNA	0.019439196	-0.118930968	0.157068687	7.84E-01	8.36E-01	1.00E+00
CXCL5	LMNA	0.270098188	0.137161576	0.393477466	1.01E-04	3.30E-04	1.00E+00
AP1M2	LMNA	-0.028864249	-0.166252776	0.109623572	6.83E-01	7.51E-01	1.00E+00
GCAT	LMNA	-0.117244942	-0.251229596	0.021148361	9.66E-02	1.48E-01	1.00E+00
TRAPPC4	LMNA	-0.0961791	-0.231160835	0.042435341	1.73E-01	2.43E-01	1.00E+00
LL22NC03-'	LMNA	-0.350596993	-0.466089155	-0.223356507	3.13E-07	1.65E-06	3.72E-03
SPRR3	LMNA	-0.065250032	-0.20148608	0.073462708	3.56E-01	4.43E-01	1.00E+00
LAPTM5	LMNA	0.280781588	0.148486237	0.403203541	5.17E-05	1.79E-04	6.14E-01
CETN2	LMNA	0.122738492	-0.015576439	0.256444257	8.18E-02	1.28E-01	1.00E+00
NOLC1	LMNA	-0.082190097	-0.217770184	0.056502	2.45E-01	3.25E-01	1.00E+00
IARS2	LMNA	0.26406252	0.130778947	0.38797041	1.46E-04	4.64E-04	1.00E+00
HSPC111	LMNA	0.388088672	0.264192032	0.499384627	1.15E-08	7.76E-08	1.37E-04
C16orf61	LMNA	-0.230735888	-0.357402434	-0.095734344	9.54E-04	2.51E-03	1.00E+00
RHOBTB3	LMNA	-0.198145149	-0.327244655	-0.061784202	4.70E-03	1.03E-02	1.00E+00
RGS10	LMNA	0.034773214	-0.103775961	0.171998539	6.23E-01	6.98E-01	1.00E+00
PHLPP	LMNA	-0.459715865	-0.562094	-0.343462446	5.89E-12	7.21E-11	6.98E-08
CAB39L	LMNA	-0.39281113	-0.503555346	-0.269367375	7.37E-09	5.17E-08	8.74E-05
CHERP	LMNA	0.034361695	-0.104183517	0.171598689	6.27E-01	7.02E-01	1.00E+00
FSTL3	LMNA	0.450939296	0.333659472	0.554472905	1.64E-11	1.86E-10	1.95E-07
QSOX1	LMNA	0.558205676	0.455235512	0.646441355	6.08E-18	2.19E-16	7.21E-14
PEX11A	LMNA	-0.038564069	-0.175679784	0.100019416	5.86E-01	6.64E-01	1.00E+00
FCN3	LMNA	0.27244307	0.139644234	0.395614595	8.76E-05	2.89E-04	1.00E+00
NPTX1	LMNA	-0.140046257	-0.272822635	-0.002034593	4.68E-02	7.92E-02	1.00E+00
PTPN3	LMNA	-0.146667618	-0.279068155	-0.008794689	3.73E-02	6.47E-02	1.00E+00

C11orf51	LMNA	-0.093976477	-0.229055811	0.044653842	1.83E-01	2.54E-01	1.00E+00
ZBED2	LMNA	0.045569573	-0.093066904	0.182472652	5.20E-01	6.03E-01	1.00E+00
NPY2R	LMNA	0.150119161	0.012323555	0.282319347	3.30E-02	5.82E-02	1.00E+00
SCAMP2	LMNA	0.299911737	0.168851724	0.420550632	1.45E-05	5.57E-05	1.72E-01
SYT17	LMNA	-0.23142389	-0.358036241	-0.096454444	9.20E-04	2.44E-03	1.00E+00
PLD3	LMNA	0.393364795	0.269974604	0.504043987	6.99E-09	4.94E-08	8.29E-05
ART3	LMNA	-0.119350629	-0.253229303	0.01901365	9.07E-02	1.40E-01	1.00E+00
SGSM2	LMNA	-0.249986041	-0.375092298	-0.11593609	3.33E-04	9.79E-04	1.00E+00
SLC5A1	LMNA	0.106501367	-0.032020418	0.241008905	1.31E-01	1.92E-01	1.00E+00
MLNR	LMNA	0.172883821	0.035684494	0.303686794	1.39E-02	2.71E-02	1.00E+00
EPHB6	LMNA	-0.248186679	-0.373442631	-0.114043069	3.69E-04	1.07E-03	1.00E+00
POFUT1	LMNA	0.607609452	0.512551862	0.68795411	8.99E-22	6.02E-20	1.07E-17
C6orf25	LMNA	-0.111577112	-0.245841337	0.026888054	1.14E-01	1.70E-01	1.00E+00
STAC	LMNA	0.435754984	0.316759072	0.541246535	9.07E-11	9.18E-10	1.08E-06
CXXC4	LMNA	-0.624351302	-0.701903602	-0.532169201	3.15E-23	3.09E-21	3.74E-19
TJP2	LMNA	-0.237636283	-0.363753996	-0.102963081	6.61E-04	1.81E-03	1.00E+00
JARID1C	LMNA	0.145033246	0.0071249	0.277527593	3.95E-02	6.81E-02	1.00E+00
LILRA3	LMNA	0.251566332	0.117599441	0.376540465	3.04E-04	9.02E-04	1.00E+00
SERINC1	LMNA	-0.089564816	-0.224835831	0.049093198	2.05E-01	2.80E-01	1.00E+00
CCT5	LMNA	0.079944934	-0.058754509	0.21561629	2.58E-01	3.40E-01	1.00E+00
ARF3	LMNA	-0.101183862	-0.235939139	0.037389414	1.52E-01	2.17E-01	1.00E+00
RAB27A	LMNA	0.584093056	0.485162995	0.668259221	7.21E-20	3.62E-18	8.55E-16
SLC9A8	LMNA	0.057595427	-0.081100412	0.194103093	4.16E-01	5.03E-01	1.00E+00
PEX19	LMNA	-0.204032165	-0.332711726	-0.06789351	3.58E-03	8.13E-03	1.00E+00
CNP	LMNA	-0.320446492	-0.439073766	-0.190837765	3.33E-06	1.44E-05	3.95E-02
EDN2	LMNA	0.210314057	0.074423885	0.338535982	2.66E-03	6.25E-03	1.00E+00
PSMD7	LMNA	0.011870077	-0.126388033	0.149675807	8.67E-01	9.02E-01	1.00E+00
UQCR	LMNA	-0.124145309	-0.257778404	0.014148169	7.84E-02	1.24E-01	1.00E+00
CCDC121	LMNA	-0.380712176	-0.492859611	-0.256122378	2.28E-08	1.46E-07	2.70E-04
SSX2IP	LMNA	0.095200668	-0.043420995	0.230225914	1.78E-01	2.48E-01	1.00E+00
PPP1R3C	LMNA	0.080337357	-0.058360904	0.215992856	2.56E-01	3.37E-01	1.00E+00
TM2D1	LMNA	-0.055791981	-0.192361387	0.082897508	4.30E-01	5.17E-01	1.00E+00
LRP4	LMNA	-0.339959559	-0.456582348	-0.211851103	7.42E-07	3.64E-06	8.80E-03
TTC17	LMNA	0.060437616	-0.078266398	0.196846235	3.93E-01	4.80E-01	1.00E+00
C4BPB	LMNA	0.154440752	0.016746805	0.286385807	2.82E-02	5.06E-02	1.00E+00

POMT2	LMNA	0.043616814	-0.095006252	0.180580478	5.38E-01	6.20E-01	1.00E+00
ARL15	LMNA	-0.344054927	-0.460245585	-0.216276436	5.34E-07	2.68E-06	6.34E-03
ZNF253	LMNA	-0.430129722	-0.536333336	-0.310517182	1.67E-10	1.61E-09	1.98E-06
CHRNA9	LMNA	0.497448625	0.385898544	0.594662221	5.02E-14	8.37E-13	5.96E-10
SOX11	LMNA	-0.381297077	-0.493377456	-0.256761607	2.16E-08	1.39E-07	2.56E-04
HIVEP3	LMNA	0.517719754	0.408893047	0.612028051	3.02E-15	6.47E-14	3.58E-11
Sep-15	LMNA	0.097395867	-0.041209216	0.232323144	1.68E-01	2.36E-01	1.00E+00
MRPL16	LMNA	-0.016219711	-0.153926049	0.1221047	8.19E-01	8.63E-01	1.00E+00
PKD2L1	LMNA	-0.037812744	-0.174950488	0.100764255	5.93E-01	6.71E-01	1.00E+00
RHBDD3	LMNA	0.211890689	0.076064712	0.339996215	2.47E-03	5.84E-03	1.00E+00
BMPR1B	LMNA	-0.129233673	-0.262599675	0.008977494	6.68E-02	1.08E-01	1.00E+00
PDE8B	LMNA	-0.200693266	-0.329612065	-0.064427281	4.18E-03	9.34E-03	1.00E+00
ABLIM3	LMNA	0.139692209	0.001673481	0.272488367	4.74E-02	8.00E-02	1.00E+00
CENPC1	LMNA	-0.171516778	-0.302407359	-0.0342774	1.47E-02	2.84E-02	1.00E+00
C2orf42	LMNA	-0.129594621	-0.262941424	0.008610426	6.60E-02	1.07E-01	1.00E+00
LTC4S	LMNA	0.090567064	-0.048085139	0.225794979	2.00E-01	2.74E-01	1.00E+00
PSMC3	LMNA	0.144733147	0.006818382	0.277244646	3.99E-02	6.88E-02	1.00E+00
SMARCA2	LMNA	-0.039486307	-0.176574777	0.099104926	5.77E-01	6.56E-01	1.00E+00
FUT5	LMNA	0.051791438	-0.086880747	0.188494728	4.64E-01	5.50E-01	1.00E+00
P4HB	LMNA	0.647247336	0.559158944	0.720884997	2.29E-25	3.31E-23	2.72E-21
ADH6	LMNA	0.178660703	0.041636641	0.309088262	1.10E-02	2.20E-02	1.00E+00
CST2	LMNA	0.072540277	-0.066173401	0.208503272	3.05E-01	3.89E-01	1.00E+00
PLAC4	LMNA	-0.228052608	-0.354929405	-0.092927225	1.10E-03	2.85E-03	1.00E+00
BRF2	LMNA	-0.032046616	-0.169348408	0.106475439	6.51E-01	7.23E-01	1.00E+00
RAMP2	LMNA	-0.267286793	-0.390913399	-0.134187194	1.20E-04	3.88E-04	1.00E+00
BCL11A	LMNA	-0.360934715	-0.475302622	-0.234571817	1.31E-07	7.38E-07	1.56E-03
F11R	LMNA	0.454311515	0.337423068	0.557403212	1.11E-11	1.29E-10	1.32E-07
CLUAP1	LMNA	-0.069303057	-0.205389028	0.069412033	3.27E-01	4.13E-01	1.00E+00
ZNF330	LMNA	0.186722489	0.0499593	0.316612107	7.80E-03	1.63E-02	1.00E+00
PSMB1	LMNA	0.12424776	-0.014044133	0.257875543	7.81E-02	1.23E-01	1.00E+00
VIPR1	LMNA	-0.341411693	-0.457881709	-0.213419634	6.61E-07	3.27E-06	7.84E-03
TXN	LMNA	0.393687593	0.270328677	0.504328843	6.77E-09	4.79E-08	8.04E-05
ACTA2	LMNA	0.456168178	0.33949681	0.559015473	8.94E-12	1.06E-10	1.06E-07
KIAA0947	LMNA	0.183187371	0.046307449	0.313314889	9.07E-03	1.86E-02	1.00E+00
REM1	LMNA	-0.115814696	-0.24987068	0.022597603	1.01E-01	1.53E-01	1.00E+00

FANCE	LMNA	-0.068852829	-0.204955685	0.069862226	3.30E-01	4.16E-01	1.00E+00
PLAC8	LMNA	-0.033850988	-0.171102399	0.104689243	6.32E-01	7.06E-01	1.00E+00
BECN1	LMNA	0.144264085	0.006339337	0.276802345	4.05E-02	6.98E-02	1.00E+00
GMPS	LMNA	0.39562685	0.27245652	0.506039644	5.63E-09	4.05E-08	6.67E-05
LGALS8	LMNA	0.428653248	0.308880566	0.535042564	1.96E-10	1.85E-09	2.33E-06
ANKRD1	LMNA	0.275738179	0.143135775	0.398615509	7.13E-05	2.40E-04	8.46E-01
DDR1	LMNA	-0.044081944	-0.181031268	0.094544412	5.33E-01	6.16E-01	1.00E+00
ATP6V1D	LMNA	0.087645424	-0.051022931	0.222998249	2.15E-01	2.90E-01	1.00E+00
PTGS1	LMNA	0.35286888	0.225818374	0.468116118	2.59E-07	1.39E-06	3.08E-03
ALDOB	LMNA	-0.104719761	-0.23931112	0.033820166	1.38E-01	2.00E-01	1.00E+00
DCC	LMNA	-0.380664941	-0.492817787	-0.25607076	2.29E-08	1.47E-07	2.72E-04
SPAG7	LMNA	0.018291243	-0.120062932	0.155948454	7.96E-01	8.46E-01	1.00E+00
HOXD9	LMNA	-0.16741513	-0.298565731	-0.030058844	1.72E-02	3.27E-02	1.00E+00
LOC440295	LMNA	-0.185403145	-0.315381917	-0.048595963	8.25E-03	1.71E-02	1.00E+00
SYNPO	LMNA	0.567132961	0.465529813	0.653981601	1.38E-18	5.59E-17	1.63E-14
C6orf47	LMNA	0.288293842	0.15647024	0.41002611	3.17E-05	1.15E-04	3.76E-01
UBE3C	LMNA	0.43621915	0.317274581	0.541651625	8.62E-11	8.73E-10	1.02E-06
TRIT1	LMNA	-0.226428082	-0.353431306	-0.091228767	1.19E-03	3.06E-03	1.00E+00
HOXC6	LMNA	0.091378243	-0.04726905	0.226571081	1.96E-01	2.69E-01	1.00E+00
LRP2BP	LMNA	-0.36337378	-0.477472782	-0.237222854	1.06E-07	6.07E-07	1.26E-03
PDSS2	LMNA	-0.015556975	-0.153278785	0.122757667	8.26E-01	8.69E-01	1.00E+00
MYST2	LMNA	-0.301318647	-0.421822935	-0.170353916	1.31E-05	5.10E-05	1.56E-01
GABARAPL	LMNA	-0.238077284	-0.364159524	-0.103425552	6.45E-04	1.77E-03	1.00E+00
ARAF	LMNA	0.529899687	0.422776104	0.622418774	5.09E-16	1.31E-14	6.04E-12
PLA2G2E	LMNA	0.253003391	0.119112684	0.377856846	2.80E-04	8.37E-04	1.00E+00
KLF10	LMNA	0.390810155	0.267173635	0.501788781	8.91E-09	6.14E-08	1.06E-04
DCTN5	LMNA	0.056304615	-0.082386771	0.192856559	4.26E-01	5.13E-01	1.00E+00
ASCL1	LMNA	-0.554388987	-0.6432124	-0.450842767	1.13E-17	3.88E-16	1.34E-13
TSNAXIP1	LMNA	-0.073864114	-0.209776026	0.064848144	2.96E-01	3.80E-01	1.00E+00
HKDC1	LMNA	0.329501088	0.200573778	0.447209445	1.68E-06	7.74E-06	1.99E-02
PHF10	LMNA	0.433667526	0.314441589	0.539424154	1.14E-10	1.13E-09	1.35E-06
FAM131B	LMNA	-0.17561545	-0.306241954	-0.038497785	1.24E-02	2.46E-02	1.00E+00
PSME3	LMNA	-0.128928877	-0.262311066	0.009287428	6.74E-02	1.09E-01	1.00E+00
IFNA10	LMNA	0.031037353	-0.107474146	0.168366945	6.61E-01	7.31E-01	1.00E+00
NUP43	LMNA	-0.16549181	-0.296762874	-0.028082389	1.86E-02	3.49E-02	1.00E+00

DBR1	LMNA	-0.109355917	-0.243727439	0.029134925	1.21E-01	1.79E-01	1.00E+00
NME3	LMNA	0.164781454	0.027352679	0.296096773	1.91E-02	3.58E-02	1.00E+00
CYP46A1	LMNA	-0.211267833	-0.339419417	-0.075416408	2.54E-03	6.00E-03	1.00E+00
L1TD1	LMNA	-0.249115161	-0.374293968	-0.115019758	3.50E-04	1.02E-03	1.00E+00
NMD3	LMNA	0.134594092	-0.003522357	0.267671529	5.62E-02	9.28E-02	1.00E+00
CHN1	LMNA	-0.17648957	-0.307059208	-0.039398497	1.20E-02	2.38E-02	1.00E+00
HGSNAT	LMNA	0.423288863	0.302940317	0.530348699	3.47E-10	3.10E-09	4.11E-06
RAG2	LMNA	0.016129165	-0.122193918	0.153837623	8.20E-01	8.64E-01	1.00E+00
KIAA0754	LMNA	0.090839082	-0.047811495	0.226055253	1.99E-01	2.72E-01	1.00E+00
TMED1	LMNA	0.3355377	0.207078865	0.452622625	1.05E-06	5.02E-06	1.25E-02
PMM2	LMNA	0.501921162	0.390960065	0.598501559	2.75E-14	4.81E-13	3.26E-10
VPS13C	LMNA	0.140726026	0.002728024	0.273464334	4.58E-02	7.76E-02	1.00E+00
METTL3	LMNA	-0.079916654	-0.215589152	0.058782873	2.58E-01	3.40E-01	1.00E+00
REXO2	LMNA	0.558518904	0.455596239	0.646706209	5.78E-18	2.10E-16	6.85E-14
SLC14A1	LMNA	-0.23520069	-0.361513461	-0.10040997	7.53E-04	2.04E-03	1.00E+00
ANXA4	LMNA	0.489211286	0.376593977	0.587579457	1.49E-13	2.36E-12	1.77E-09
CA1	LMNA	-0.08838344	-0.223704923	0.050281061	2.11E-01	2.86E-01	1.00E+00
UAP1	LMNA	0.422734331	0.302326788	0.529863105	3.68E-10	3.28E-09	4.36E-06
KCNJ15	LMNA	0.424831553	0.304647657	0.531699233	2.95E-10	2.68E-09	3.49E-06
DHODH	LMNA	0.305438751	0.174756557	0.425546121	9.86E-06	3.90E-05	1.17E-01
TULP3	LMNA	0.332328383	0.203619073	0.449745825	1.35E-06	6.32E-06	1.60E-02
RPS14	LMNA	-0.044587138	-0.181520824	0.094042723	5.29E-01	6.11E-01	1.00E+00
ATP2A2	LMNA	-0.131316919	-0.26457165	0.006858414	6.25E-02	1.02E-01	1.00E+00
APBB1IP	LMNA	0.011084888	-0.127160704	0.148908018	8.76E-01	9.08E-01	1.00E+00
ATIC	LMNA	0.076324828	-0.06238349	0.212140563	2.80E-01	3.63E-01	1.00E+00
ONECUT2	LMNA	-0.414010649	-0.522214686	-0.292688116	9.08E-10	7.57E-09	1.08E-05
ADAM15	LMNA	0.493564951	0.381508873	0.591324776	8.43E-14	1.36E-12	1.00E-09
FAM110B	LMNA	-0.452353026	-0.555701686	-0.335236824	1.40E-11	1.60E-10	1.66E-07
NPL	LMNA	0.103445881	-0.035106468	0.238096669	1.43E-01	2.06E-01	1.00E+00
LGR4	LMNA	-0.007192149	-0.145099084	0.130988908	9.19E-01	9.42E-01	1.00E+00
STRN	LMNA	-0.083002261	-0.218549008	0.05568683	2.40E-01	3.20E-01	1.00E+00
UEVLD	LMNA	0.233149968	0.098261675	0.35962584	8.40E-04	2.25E-03	1.00E+00
GAB1	LMNA	-0.301261648	-0.421771399	-0.170293046	1.32E-05	5.12E-05	1.57E-01
SULT1B1	LMNA	0.242908592	0.108495867	0.368599074	4.95E-04	1.40E-03	1.00E+00
SNAI2	LMNA	0.526827674	0.419269782	0.619801105	8.03E-16	1.97E-14	9.52E-12

ZGPAT	LMNA	0.469171152	0.354051935	0.570285049	1.88E-12	2.51E-11	2.23E-08
KCNN4	LMNA	0.535715717	0.429423176	0.627368962	2.12E-16	5.79E-15	2.51E-12
SNF1LK	LMNA	0.501061789	0.389987001	0.597764196	3.09E-14	5.35E-13	3.66E-10
DLEU1	LMNA	-0.195531987	-0.324815127	-0.059075638	5.29E-03	1.15E-02	1.00E+00
UBE2Q1	LMNA	0.20434372	0.068217114	0.333000816	3.53E-03	8.03E-03	1.00E+00
ZMYM6	LMNA	0.229639445	0.094587039	0.356392119	1.01E-03	2.65E-03	1.00E+00
JPH3	LMNA	-0.474285684	-0.574707367	-0.359792305	1.00E-12	1.39E-11	1.19E-08
HEATR3	LMNA	0.140058921	0.00204751	0.27283459	4.68E-02	7.92E-02	1.00E+00
CYP2J2	LMNA	-0.121149638	-0.254936863	0.017188843	8.59E-02	1.34E-01	1.00E+00
FAM119B	LMNA	-0.030445638	-0.167791403	0.108059542	6.67E-01	7.37E-01	1.00E+00
FAM38A	LMNA	0.606013875	0.510687402	0.686621552	1.22E-21	7.78E-20	1.45E-17
APOL3	LMNA	0.399201562	0.276382023	0.509190959	3.98E-09	2.94E-08	4.72E-05
FLNA	LMNA	0.693237557	0.613942132	0.75868115	2.88E-30	9.50E-28	3.42E-26
IL2RB	LMNA	0.444570691	0.326561915	0.548931861	3.40E-11	3.65E-10	4.03E-07
SLCO4C1	LMNA	0.012363597	-0.125902294	0.150158307	8.61E-01	8.98E-01	1.00E+00
DENND2D	LMNA	0.251966426	0.118020683	0.376907012	2.98E-04	8.83E-04	1.00E+00
KIAA0152	LMNA	0.451952716	0.334790116	0.555353792	1.46E-11	1.67E-10	1.73E-07
BAX	LMNA	0.313648023	0.183544389	0.432952433	5.48E-06	2.28E-05	6.51E-02
CP	LMNA	0.363624349	0.237495305	0.477695647	1.04E-07	5.95E-07	1.24E-03
LRPAP1	LMNA	0.298611259	0.167463713	0.419374154	1.58E-05	6.05E-05	1.88E-01
G6PC3	LMNA	0.212767643	0.076977691	0.340808158	2.36E-03	5.63E-03	1.00E+00
RPL37	LMNA	0.115424718	-0.022992659	0.249500061	1.02E-01	1.54E-01	1.00E+00
NCOA4	LMNA	-0.220363648	-0.347833072	-0.084895283	1.63E-03	4.03E-03	1.00E+00
LRRC14	LMNA	-0.123994943	-0.25763583	0.014300854	7.87E-02	1.24E-01	1.00E+00
GORASP1	LMNA	-0.05896164	-0.195421961	0.079738409	4.05E-01	4.92E-01	1.00E+00
FKBP1A	LMNA	0.379530391	0.254831154	0.491813067	2.54E-08	1.61E-07	3.01E-04
FXYD3	LMNA	-0.283952605	-0.406085094	-0.151854284	4.22E-05	1.48E-04	5.00E-01
CYP24A1	LMNA	0.159491079	0.021922772	0.291131964	2.34E-02	4.29E-02	1.00E+00
SMARCAL1	LMNA	0.383903593	0.259611557	0.495684187	1.70E-08	1.11E-07	2.02E-04
NDUFS7	LMNA	-0.045849976	-0.182744272	0.092788341	5.17E-01	6.00E-01	1.00E+00
ABCB8	LMNA	0.348007824	0.220552804	0.463777636	3.88E-07	2.01E-06	4.60E-03
CCDC44	LMNA	-0.007607952	-0.145506129	0.130580195	9.14E-01	9.38E-01	1.00E+00
PRMT7	LMNA	-0.119236207	-0.25312067	0.019129681	9.10E-02	1.40E-01	1.00E+00
ZNF562	LMNA	-0.119847206	-0.253700721	0.018510044	8.93E-02	1.38E-01	1.00E+00
COQ2	LMNA	0.22283597	0.087475969	0.350116434	1.43E-03	3.61E-03	1.00E+00

USH1C	LMNA	-0.511228765	-0.606477244	-0.401514939	7.59E-15	1.51E-13	9.00E-11
SRF	LMNA	0.427822154	0.307959643	0.534315783	2.14E-10	2.01E-09	2.54E-06
MAT2A	LMNA	-0.122226817	-0.255958887	0.016095776	8.31E-02	1.30E-01	1.00E+00
PGPEP1	LMNA	0.35447111	0.227555562	0.469544889	2.27E-07	1.23E-06	2.69E-03
TRPC3	LMNA	-0.206706875	-0.335192784	-0.070672594	3.16E-03	7.29E-03	1.00E+00
SEMA4C	LMNA	-0.005976539	-0.143908805	0.132183522	9.33E-01	9.51E-01	1.00E+00
SIN3B	LMNA	0.120986424	-0.017354435	0.254781981	8.63E-02	1.34E-01	1.00E+00
KLRD1	LMNA	0.070898188	-0.067816572	0.206923907	3.16E-01	4.01E-01	1.00E+00
UTX	LMNA	-0.016464633	-0.154165223	0.121863358	8.16E-01	8.61E-01	1.00E+00
TRIM8	LMNA	-0.015413076	-0.153138231	0.122899429	8.28E-01	8.70E-01	1.00E+00
NDRG3	LMNA	-0.469022291	-0.570156247	-0.353884989	1.92E-12	2.55E-11	2.27E-08
SLC10A3	LMNA	0.761497504	0.696685957	0.813978603	1.60E-39	3.17E-36	1.90E-35
SEMA3C	LMNA	0.226924145	0.091747322	0.353888833	1.16E-03	3.00E-03	1.00E+00
RNF6	LMNA	0.147802273	0.009954384	0.280137282	3.58E-02	6.25E-02	1.00E+00
GRAMD3	LMNA	-0.230025511	-0.356747892	-0.094990973	9.90E-04	2.60E-03	1.00E+00
VAV1	LMNA	0.240318894	0.105777186	0.366220081	5.71E-04	1.59E-03	1.00E+00
PDGFC	LMNA	0.389063876	0.265260174	0.500246318	1.05E-08	7.14E-08	1.25E-04
CACNA1I	LMNA	-0.212969947	-0.340995438	-0.077188338	2.34E-03	5.58E-03	1.00E+00
PEPD	LMNA	0.256412409	0.122704916	0.38097757	2.30E-04	6.98E-04	1.00E+00
S100A13	LMNA	0.399330114	0.276523266	0.509304231	3.93E-09	2.92E-08	4.66E-05
ARMCX2	LMNA	0.305050904	0.174341891	0.425195811	1.01E-05	4.01E-05	1.20E-01
TP63	LMNA	0.137793803	-0.000262185	0.270695479	5.05E-02	8.46E-02	1.00E+00
ANXA11	LMNA	0.39187592	0.268341911	0.502729809	8.05E-09	5.60E-08	9.55E-05
CSF2RB	LMNA	0.468283695	0.353056767	0.569517105	2.10E-12	2.77E-11	2.49E-08
ZNF358	LMNA	0.023910379	-0.114518631	0.161428528	7.36E-01	7.94E-01	1.00E+00
IFI44	LMNA	0.118948142	-0.019421782	0.252847162	9.18E-02	1.41E-01	1.00E+00
EIF2C4	LMNA	-0.299781341	-0.420432687	-0.168712528	1.46E-05	5.62E-05	1.73E-01
RPS6KA3	LMNA	0.503429401	0.392668438	0.599795271	2.23E-14	4.03E-13	2.65E-10
PHF21A	LMNA	-0.149463997	-0.281702447	-0.01165345	3.38E-02	5.94E-02	1.00E+00
FAM49B	LMNA	-0.298398636	-0.419181766	-0.167236828	1.61E-05	6.13E-05	1.91E-01
DACT1	LMNA	0.175520968	0.038400442	0.306153608	1.25E-02	2.47E-02	1.00E+00
ATP5G1	LMNA	-0.285857563	-0.407814992	-0.153879081	3.72E-05	1.32E-04	4.42E-01
PPWD1	LMNA	0.018566916	-0.119791131	0.156217503	7.93E-01	8.44E-01	1.00E+00
PNPLA2	LMNA	0.539828155	0.434130193	0.630864709	1.13E-16	3.27E-15	1.34E-12
DNAJC13	LMNA	0.220811779	0.08536292	0.348247064	1.59E-03	3.94E-03	1.00E+00

PAH	LMNA	-0.212341966	-0.340414062	-0.0765345	2.41E-03	5.74E-03	1.00E+00
PTCH2	LMNA	-0.052754	-0.189425464	0.085922754	4.56E-01	5.42E-01	1.00E+00
EAF2	LMNA	0.092937039	-0.045700299	0.228061993	1.88E-01	2.60E-01	1.00E+00
ERCC2	LMNA	0.11793575	-0.02044817	0.251885761	9.46E-02	1.45E-01	1.00E+00
TRMU	LMNA	-0.116096287	-0.250138269	0.022312318	9.99E-02	1.52E-01	1.00E+00
USP3	LMNA	-0.227211885	-0.354154192	-0.092048143	1.15E-03	2.96E-03	1.00E+00
C14orf101	LMNA	0.10349938	-0.035052457	0.23814768	1.43E-01	2.06E-01	1.00E+00
CCDC9	LMNA	0.31848292	0.18872978	0.437306898	3.85E-06	1.65E-05	4.57E-02
VPS13B	LMNA	-0.162528674	-0.293983495	-0.025039491	2.08E-02	3.88E-02	1.00E+00
DCLRE1C	LMNA	-0.286126675	-0.408059303	-0.154165211	3.66E-05	1.30E-04	4.34E-01
ST18	LMNA	-0.333803008	-0.451067967	-0.205208388	1.21E-06	5.69E-06	1.43E-02
FRMD4B	LMNA	0.038277297	-0.10030373	0.175401438	5.89E-01	6.67E-01	1.00E+00
PSMB9	LMNA	0.330653181	0.201814403	0.448243222	1.54E-06	7.13E-06	1.82E-02
RFPL1	LMNA	-0.399935773	-0.509837844	-0.277188791	3.71E-09	2.75E-08	4.39E-05
LOC552889	LMNA	-0.367495565	-0.48113698	-0.241707123	7.44E-08	4.37E-07	8.83E-04
CDC2L2	LMNA	0.085070748	-0.053609853	0.2205318	2.29E-01	3.06E-01	1.00E+00
ProSAPiP1	LMNA	-0.409615308	-0.518354501	-0.287841089	1.42E-09	1.14E-08	1.68E-05
ADRBK2	LMNA	-0.291701788	-0.413116693	-0.160097884	2.53E-05	9.29E-05	3.00E-01
HCLS1	LMNA	0.284690999	0.152638998	0.406755736	4.02E-05	1.42E-04	4.77E-01
GPR15	LMNA	-0.020392173	-0.157998383	0.117990991	7.73E-01	8.27E-01	1.00E+00
CSF2	LMNA	0.223532171	0.088203013	0.350759151	1.38E-03	3.50E-03	1.00E+00
SLC2A11	LMNA	-0.314800533	-0.433990927	-0.184779791	5.04E-06	2.11E-05	5.98E-02
GRIP2	LMNA	-0.154430466	-0.286376133	-0.01673627	2.82E-02	5.06E-02	1.00E+00
MMP9	LMNA	0.3063413	0.175721697	0.426361178	9.25E-06	3.68E-05	1.10E-01
GPLD1	LMNA	-0.45036402	-0.55397276	-0.333017803	1.76E-11	1.97E-10	2.08E-07
KIAA0802	LMNA	0.190091528	0.053442996	0.319751505	6.73E-03	1.43E-02	1.00E+00
DHRS2	LMNA	-0.231641239	-0.358236446	-0.096681964	9.10E-04	2.41E-03	1.00E+00
RAB8A	LMNA	0.413762375	0.292414162	0.521996759	9.32E-10	7.74E-09	1.11E-05
SGEF	LMNA	-0.003297393	-0.14128408	0.134814977	9.63E-01	9.72E-01	1.00E+00
PIK3IP1	LMNA	-0.115866078	-0.249919509	0.022545548	1.01E-01	1.53E-01	1.00E+00
RPS27	LMNA	0.101878279	-0.036688727	0.23660162	1.49E-01	2.14E-01	1.00E+00
SNRPD2	LMNA	0.011081877	-0.127163667	0.148905073	8.76E-01	9.08E-01	1.00E+00
SLC39A6	LMNA	-0.157224199	-0.289002414	-0.019598585	2.54E-02	4.63E-02	1.00E+00
CTSC	LMNA	0.407108404	0.285079354	0.516150839	1.82E-09	1.43E-08	2.16E-05
AQP7	LMNA	-0.045447583	-0.182354476	0.093188088	5.21E-01	6.04E-01	1.00E+00
