# Supplementary material for: Role of Lamin A/C as Candidate Biomarker of Aggressiveness and Tumorigenicity in Glioblastoma Multiforme
Source: Biomedicines. 2021 Sep 28;9(10):1343. doi: 10.3390/biomedicines9101343 (PMC8533312; doi:10.3390/biomedicines9101343)
Supplement: Supplementary file 1 [file biomedicines-09-01343-s001.zip › biomedicines-1353075-suppl/biomedicines-1353075-suppl(tables).zip!/supplementary tables/Supplementary Table 5.pdf]

Table S5. Venn diagram breakdown of the core molecular KEGG pathways in relation to the GBM subtypes from microarray dataset.

Names	total	elements
Classical Mesenchymal Neural Proneural	43	KEGG_GLYCOSPHINGOLIPID_BIOSYNTHESIS_GANGLIO_SERIES
		KEGG_LYSOSOME
		KEGG_GLYCOSAMINOGLYCAN_DEGRADATION
		KEGG_DNA_REPLICATION
		KEGG_GLUTATHIONE_METABOLISM
		KEGG_FOCAL_ADHESION
		KEGG_NOD_LIKE_RECEPTOR_SIGNALING_PATHWAY
		KEGG_FRUCTOSE_AND_MANNOSE_METABOLISM
		KEGG_SNARE_INTERACTIONS_IN_VESICULAR_TRANSPORT
		KEGG_GLYCOLYSIS_GLUCONEOGENESIS
		KEGG_PATHOGENIC_ESCHERICHIA_COLI_INFECTION
		KEGG_PROTEIN_EXPORT
		KEGG_GALACTOSE_METABOLISM
		KEGG_N_GLYCAN_BIOSYNTHESIS
		KEGG_OTHER_GLYCAN_DEGRADATION
		KEGG_NICOTINATE_AND_NICOTINAMIDE_METABOLISM
		KEGG_BASE_EXCISION_REPAIR
		KEGG_PROTEASOME
		KEGG_APOPTOSIS
		KEGG_P53_SIGNALING_PATHWAY
		KEGG_REGULATION_OF_ACTIN_CYTOSKELETON
		KEGG_ECM_RECEPTOR_INTERACTION
		KEGG_SMALL_CELL_LUNG_CANCER
		KEGG_VALINE_LEUCINE_AND_ISOLEUCINE_BIOSYNTHESIS
		KEGG_JAK_STAT_SIGNALING_PATHWAY
		KEGG_PYRIMIDINE_METABOLISM
		KEGG_DRUG_METABOLISM_OTHER_ENZYMES
		KEGG_ONE_CARBON_POOL_BY_FOLATE
		KEGG_PORPHYRIN_AND_CHLOROPHYLL_METABOLISM
		KEGG_PANCREATIC_CANCER
		KEGG_VIRAL_MYOCARDITIS
		KEGG_BLADDER_CANCER
		KEGG_PENTOSE_PHOSPHATE_PATHWAY

		KEGG_GLYCOSPHINGOLIPID_BIOSYNTHESIS_GLOBO_SERIES
		KEGG_STARCH_AND_SUCROSE_METABOLISM
		KEGG_CELL_CYCLE
		KEGG_GLYCOSAMINOGLYCAN_BIOSYNTHESIS_KERATAN_SULFATE
		KEGG_AMINO_SUGAR_AND_NUCLEOTIDE_SUGAR_METABOLISM
		KEGG_O_GLYCAN_BIOSYNTHESIS
		KEGG_GLYCOSAMINOGLYCAN_BIOSYNTHESIS_HEPARAN_SULFATE
		KEGG_PATHWAYS_IN_CANCER
		KEGG_PANTOTHENATE_AND_COA_BIOSYNTHESIS
		KEGG_GLYCOSAMINOGLYCAN_BIOSYNTHESIS_CHONDROITIN_SULFATE
Classical Mesenchymal Proneural	17	KEGG_HYPERTROPHIC_CARDIOMYOPATHY_HCM
		KEGG_COMPLEMENT_AND_COAGULATION_CASCADES
		KEGG_PHENYLALANINE_METABOLISM
		KEGG_INTESTINAL_IMMUNE_NETWORK_FOR_IGA_PRODUCTION
		KEGG_STEROID_BIOSYNTHESIS
		KEGG_FOLATE_BIOSYNTHESIS
		KEGG_RIBOFLAVIN_METABOLISM
		KEGG_RIBOSOME
		KEGG_THYROID_CANCER
		KEGG_CYTOKINE_CYTOKINE_RECEPTOR_INTERACTION
		KEGG_TYROSINE_METABOLISM
		KEGG_ALLOGRAFT_REJECTION
		KEGG_OOCYTE_MEIOSIS
		KEGG_AXON_GUIDANCE
		KEGG_HEMATOPOIETIC_CELL_LINEAGE
		KEGG_RENIN_ANGIOTENSIN_SYSTEM
		KEGG_PROGESTERONE_MEDIATED_OOCYTE_MATURATION
Mesenchymal Neural Proneural	19	KEGG_CYSTEINE_AND_METHIONINE_METABOLISM
		KEGG_CITRATE_CYCLE_TCA_CYCLE
		KEGG_RNA_DEGRADATION
		KEGG_ASCORBATE_AND_ALDARATE_METABOLISM
		KEGG_GLYCOSYLPHOSPHATIDYLINOSITOL_GPI_ANCHOR_BIOSYNTHESIS
		KEGG_RNA_POLYMERASE
		KEGG_SPLICEOSOME
		KEGG_UBIQUITIN_MEDIATED_PROTEOLYSIS
		KEGG_GLYOXYLATE_AND_DICARBOXYLATE_METABOLISM

		KEGG_MISMATCH_REPAIR
		KEGG_BASAL_TRANSCRIPTION_FACTORS
		KEGG_NUCLEOTIDE_EXCISION_REPAIR
		KEGG_HOMOLOGOUS_RECOMBINATION
		KEGG_NON_HOMOLOGOUS_END_JOINING
		KEGG_TERPENOID_BACKBONE_BIOSYNTHESIS
		KEGG_TGF_BETA_SIGNALING_PATHWAY
		KEGG_AMINOACYL_TRNA_BIOSYNTHESIS
		KEGG_RIG_I_LIKE_RECEPTOR_SIGNALING_PATHWAY
		KEGG_SELENOAMINO_ACID_METABOLISM
Classical Neural	29	KEGG_GRAFT_VERSUS_HOST_DISEASE
		KEGG_TIGHT_JUNCTION
		KEGG_ENDOCYTOSIS
		KEGG_SPHINGOLIPID_METABOLISM
		KEGG_NEUROTROPHIN_SIGNALING_PATHWAY
		KEGG_ACUTE_MYELOID_LEUKEMIA
		KEGG_ENDOMETRIAL_CANCER
		KEGG_COLORECTAL_CANCER
		KEGG_VEGF_SIGNALING_PATHWAY
		KEGG_CELL_ADHESION_MOLECULES_CAMS
		KEGG_ADHERENS_JUNCTION
		KEGG_TOLL_LIKE_RECEPTOR_SIGNALING_PATHWAY
		KEGG_CHRONIC_MYELOID_LEUKEMIA
		KEGG_ERBB_SIGNALING_PATHWAY
		KEGG_LEUKOCYTE_TRANSENDOTHELIAL_MIGRATION
		KEGG_INSULIN_SIGNALING_PATHWAY
		KEGG_PRION_DISEASES
		KEGG_BIOSYNTHESIS_OF_UNSATURATED_FATTY_ACIDS
		KEGG_ARRHYTHMOGENIC_RIGHT_VENTRICULAR_CARDIOMYOPATHY_ARVC
		KEGG_ADIPOCYTOKINE_SIGNALING_PATHWAY
		KEGG_B_CELL_RECEPTOR_SIGNALING_PATHWAY
		KEGG_PROSTATE_CANCER
		KEGG_EPITHELIAL_CELL_SIGNALING_IN_HELICOBACTER_PYLORI_INFECTION
		KEGG_LEISHMANIA_INFECTION
		KEGG_FC_GAMMA_R_MEDIATED_PHAGOCYTOSIS
		KEGG_VIBRIO_CHOLERAE_INFECTION

		KEGG_CYTOSOLIC_DNA_SENSING_PATHWAY
		KEGG_RENAL_CELL_CARCINOMA
		KEGG_ANTIGEN_PROCESSING_AND_PRESENTATION
Mesenchymal Proneural	9	KEGG_GLYCOSPHINGOLIPID_BIOSYNTHESIS_LACTO_AND_NEOLACTO_SERIES
		KEGG_SULFUR_METABOLISM
		KEGG_PENTOSE_AND_GLUCURONATE_INTERCONVERSIONS
		KEGG_ALANINE_ASPARTATE_AND_GLUTAMATE_METABOLISM
		KEGG_HEDGEHOG_SIGNALING_PATHWAY
		KEGG_PURINE_METABOLISM
		KEGG_AUTOIMMUNE_THYROID_DISEASE
		KEGG_PARKINSONS_DISEASE
		KEGG_HUNTINGTONS_DISEASE
Classical	21	KEGG_TYPE_II_DIABETES_MELLITUS
		KEGG_MELANOMA
		KEGG_PRIMARY_BILE_ACID_BIOSYNTHESIS
		KEGG_GNRH_SIGNALING_PATHWAY
		KEGG_FC_EPSILON_RI_SIGNALING_PATHWAY
		KEGG_VASCULAR_SMOOTH_MUSCLE_CONTRACTION
		KEGG_T_CELL_RECEPTOR_SIGNALING_PATHWAY
		KEGG_ALZHEIMERS_DISEASE
		KEGG_BASAL_CELL_CARCINOMA
		KEGG_SYSTEMIC_LUPUS_ERYTHEMATOSUS
		KEGG_ARACHIDONIC_ACID_METABOLISM
		KEGG_STEROID_HORMONE_BIOSYNTHESIS
		KEGG_PRIMARY_IMMUNODEFICIENCY
		KEGG_MAPK_SIGNALING_PATHWAY
		KEGG_GLIOMA
		KEGG_NATURAL_KILLER_CELL_MEDIATED_CYTOTOXICITY
		KEGG_MELANOGENESIS
		KEGG_AMYOTROPHIC_LATERAL_SCLEROSIS_ALS
		KEGG_DILATED_CARDIOMYOPATHY
		KEGG_GAP_JUNCTION
		KEGG_CHEMOKINE_SIGNALING_PATHWAY
Neural	18	KEGG_FATTY_ACID_METABOLISM
		KEGG_ARGININE_AND_PROLINE_METABOLISM
		KEGG_NON_SMALL_CELL_LUNG_CANCER

KEGG_ETHER_LIPID_METABOLISM
KEGG_GLYCINE_SERINE_AND_THREONINE_METABOLISM
KEGG_PEROXISOME
KEGG_MTOR_SIGNALING_PATHWAY
KEGG_DORSO_VENTRAL_AXIS_FORMATION
KEGG_LIMONENE_AND_PINENE_DEGRADATION
KEGG_BUTANOATE_METABOLISM
KEGG_VALINE_LEUCINE_AND_ISOLEUCINE_DEGRADATION
KEGG_NOTCH_SIGNALING_PATHWAY
KEGG_TRYPTOPHAN_METABOLISM
KEGG_PROPANOATE_METABOLISM
KEGG_CIRCADIAN_RHYTHM_MAMMAL
KEGG_ABC_TRANSPORTERS
KEGG_LYSINE_DEGRADATION
KEGG_PYRUVATE_METABOLISM
