# Supplementary material for: Role of Lamin A/C as Candidate Biomarker of Aggressiveness and Tumorigenicity in Glioblastoma Multiforme
Source: Biomedicines. 2021 Sep 28;9(10):1343. doi: 10.3390/biomedicines9101343 (PMC8533312; doi:10.3390/biomedicines9101343)
Supplement: Supplementary file 1 [file biomedicines-09-01343-s001.zip › biomedicines-1353075-suppl/biomedicines-1353075-suppl(tables).zip!/supplementary tables/Supplementary Table 6.pdf]

Table S6. Gene set enrichment analysis of the core molecular KEGG pathways in the GBM subtype patients from microarray dataset.

All subtype

NAME	GS<br>follow link to MSigDB	SIZE	ES	NES	NOM p-val	FDR q-val	FWER p-val	RANK AT MAX	LEADING EDGE
KEGG_OTHER_GLYCAN_DEGRADATION	KEGG_OTHER_GLYCAN_DEGRADATION	12	0.6504333	0.6504333	0	6.69E-04	5.00E-04	2176	tags=83%, list=18%, signal=102%
KEGG_GLYCOSAMINOGLYCAN_DEGRADATION	KEGG_GLYCOSAMINOGLYCAN_DEGRADATION	21	0.57187504	0.571875	0	0.01139325	0.0175	1700	tags=71%, list=14%, signal=83%
KEGG_GLYCOSAMINOGLYCAN_BIOSYNTHESIS_CHONDROITIN_SULFATE	KEGG_GLYCOSAMINOGLYCAN_BIOSYNTHESIS_CHONDROITIN_SULFATE	13	0.5108532	0.5108532	0	0.02235264	0.051	3070	tags=77%, list=26%, signal=104%
KEGG_O_GLYCAN_BIOSYNTHESI	KEGG_O_GLYCAN_BIOSYNTHESI	18	0.49904296	0.499043	0	0.02259652	0.0675	1996	tags=67%, list=17%, signal=80%
KEGG_AMINO_SUGAR_AND_NUCLEOTIDE_SUGAR_METABOLISM	KEGG_AMINO_SUGAR_AND_NUCLEOTIDE_SUGAR_METABOLISM	39	0.461686	0.461686	0	0.04082201	0.149	5185	tags=90%, list=44%, signal=159%
KEGG_GRAFT_VERSUS_HOST_DISEASE	KEGG_GRAFT_VERSUS_HOST_DISEASE	29	0.45085528	0.4508553	0	0.04291353	0.1825	5707	tags=93%, list=48%, signal=179%
KEGG_GLYCOSPHINGOLIPID_BIOSYNTHESIS_GANGLIO_SERIES	KEGG_GLYCOSPHINGOLIPID_BIOSYNTHESIS_GANGLIO_SERIES	13	0.4267877	0.4267877	0.0090634	0.05598099	0.267	1329	tags=54%, list=11%, signal=61%
KEGG_ALLOGRAFT_REJECTION	KEGG_ALLOGRAFT_REJECTION	30	0.4224289	0.4224289	0	0.05337475	0.288	3699	tags=73%, list=31%, signal=106%
KEGG_LEISHMANIA_INFECTION	KEGG_LEISHMANIA_INFECTION	58	0.4215913	0.4215913	0	0.04787578	0.2905	5452	tags=88%, list=46%, signal=162%
KEGG_GLYCOSPHINGOLIPID_BIOSYNTHESIS_GLOBO_SERIES	KEGG_GLYCOSPHINGOLIPID_BIOSYNTHESIS_GLOBO_SERIES	13	0.4018869	0.4018869	0.019833	0.06172565	0.3945	1624	tags=54%, list=14%, signal=62%
KEGG_ECM_RECEPTOR_INTERACTION	KEGG_ECM_RECEPTOR_INTERACTION	78	0.38625634	0.3862563	0	0.07510629	0.492	3963	tags=72%, list=33%, signal=107%
KEGG_PANTOTHENATE_AND_COA_BIOSYNTHESIS	KEGG_PANTOTHENATE_AND_COA_BIOSYNTHESIS	14	0.38140666	0.3814067	0.0181452	0.07536735	0.5265	2258	tags=57%, list=19%, signal=70%
KEGG_NOD_LIKE_RECEPTOR_SIGNALING_PATHWAY	KEGG_NOD_LIKE_RECEPTOR_SIGNALING_PATHWAY	52	0.37892175	0.3789218	0	0.07283738	0.5445	5332	tags=83%, list=45%, signal=150%
KEGG_INTESTINAL_IMMUNE_NETWORK_FOR_IGA_PRODUCTION	KEGG_INTESTINAL_IMMUNE_NETWORK_FOR_IGA_PRODUCTION	35	0.37732407	0.3773241	0	0.06971845	0.5535	2992	tags=63%, list=25%, signal=84%
KEGG_GLYCOSAMINOGLYCAN_BIOSYNTHESIS_KERATAN_SULFAT[illegible]	KEGG_GLYCOSAMINOGLYCAN_BIOSYNTHESIS_KERATAN_SULFAT[illegible]	11	0.36237812	0.3623781	0.0839921	0.08559142	0.65	6487	tags=91%, list=55%, signal=200%
KEGG_PROTEASOME	KEGG_PROTEASOME	42	0.34953946	0.3495395	0	0.10107853	0.728	6881	tags=93%, list=58%, signal=220%
KEGG_LYSOSOME	KEGG_LYSOSOME	106	0.34621534	0.3462153	0	0.10116799	0.746	3426	tags=63%, list=29%, signal=88%
KEGG_HEMATOPOIETIC_CELL_LINEAGE	KEGG_HEMATOPOIETIC_CELL_LINEAGE	77	0.34588242	0.3458824	0	0.09617624	0.749	5011	tags=77%, list=42%, signal=132%
KEGG_ASTHMA	KEGG_ASTHMA	22	0.31869635	0.3186964	0.0166503	0.1455133	0.896	6469	tags=86%, list=55%, signal=190%
KEGG_GLYCOSAMINOGLYCAN_BI	KEGG_GLYCOSAMINOGLYCAN_BI	21	0.3143726	0.3143726	0.0192503	0.14897138	0.913	1360	tags=43%, list=11%, signal=48%
KEGG_TOLL_LIKE_RECEPTOR_SI	KEGG_TOLL_LIKE_RECEPTOR_SI	88	0.30999985	0.3099999	0	0.15189648	0.928	5649	tags=78%, list=48%, signal=149%
KEGG_RENIN_ANGIOTENSIN_SYS	KEGG_RENIN_ANGIOTENSIN_SYS	16	0.30747637	0.3074764	0.0665343	0.15156728	0.9385	7476	tags=94%, list=63%, signal=253%
KEGG_LEUKOCYTE_TRANSENDO	KEGG_LEUKOCYTE_TRANSENDO	99	0.3067483	0.3067483	0	0.14683111	0.9405	4298	tags=67%, list=36%, signal=104%
KEGG_TYPE_I_DIABETES_MELLIT	KEGG_TYPE_I_DIABETES_MELLIT	36	0.30393928	0.3039393	0.0020471	0.14782451	0.9535	3653	tags=61%, list=31%, signal=88%
KEGG_APOPTOSIS	KEGG_APOPTOSIS	79	0.3038426	0.3038426	0	0.14211078	0.9535	3025	tags=56%, list=26%, signal=74%
KEGG_NICOTINATE_AND_NICOTI	KEGG_NICOTINATE_AND_NICOTI	15	0.3023498	0.3023498	0.1214575	0.14022781	0.9555	2743	tags=53%, list=23%, signal=69%
KEGG_PRIMARY_IMMUNODEFICIE	KEGG_PRIMARY_IMMUNODEFICIE	33	0.30087808	0.3008781	0.002994	0.13841012	0.9605	3269	tags=58%, list=28%, signal=79%
KEGG_ANTIGEN_PROCESSING_A	KEGG_ANTIGEN_PROCESSING_A	63	0.2950441	0.2950441	0	0.14708565	0.969	2919	tags=54%, list=25%, signal=71%
KEGG_VIRAL_MYOCARDITIS	KEGG_VIRAL_MYOCARDITIS	62	0.2942899	0.2942899	0	0.14373218	0.9715	3796	tags=61%, list=32%, signal=90%

KEGG_N_GLYCAN_BIOSYNTHESI	KEGG_N_GLYCAN_BIOSYNTHESI	34	0.2909989	0.2909989	0.002004	0.14672317	0.975	1790	tags=44%, list=15%, signal=52%
KEGG_GLYCOSYLPHOSPHATIDYL	KEGG_GLYCOSYLPHOSPHATIDYL	19	0.29007155	0.2900716	0.0613254	0.14435332	0.9755	4679	tags=68%, list=39%, signal=113%
KEGG_VALINE_LEUCINE_AND_IS	KEGG_VALINE_LEUCINE_AND_IS	10	0.28481013	0.2848101	0.3277487	0.15344657	0.9815	6112	tags=80%, list=52%, signal=165%
KEGG_AUTOIMMUNE_THYROID_D	KEGG_AUTOIMMUNE_THYROID_D	38	0.2846878	0.2846878	0.0040775	0.1490374	0.9815	7245	tags=89%, list=61%, signal=229%
KEGG_COMPLEMENT_AND_COA	KEGG_COMPLEMENT_AND_COA	65	0.28208172	0.2820817	0	0.15131813	0.984	3058	tags=54%, list=26%, signal=72%
KEGG_FOCAL_ADHESION	KEGG_FOCAL_ADHESION	182	0.27775812	0.2777581	0	0.15809517	0.9865	2946	tags=52%, list=25%, signal=68%
KEGG_CYTOKINE_CYTOKINE_RE	KEGG_CYTOKINE_CYTOKINE_RE	214	0.27579722	0.2757972	0	0.15841691	0.987	4158	tags=62%, list=35%, signal=94%
KEGG_PENTOSE_PHOSPHATE_P	KEGG_PENTOSE_PHOSPHATE_P	23	0.2640725	0.2640725	0.0737463	0.18685716	0.9965	3061	tags=52%, list=26%, signal=70%
KEGG_STEROID_BIOSYNTHESIS	KEGG_STEROID_BIOSYNTHESIS	15	0.26365557	0.2636556	0.2232232	0.18304078	0.9965	3992	tags=60%, list=34%, signal=90%
KEGG_PROTEIN_EXPORT	KEGG_PROTEIN_EXPORT	18	0.26006305	0.2600631	0.1354705	0.18862303	0.9975	4826	tags=67%, list=41%, signal=112%
KEGG_PORPHYRIN_AND_CHLOR	KEGG_PORPHYRIN_AND_CHLOR	24	0.25619578	0.2561958	0.0591233	0.19525264	0.999	6850	tags=83%, list=58%, signal=197%
KEGG_PRION_DISEASES	KEGG_PRION_DISEASES	34	0.25399667	0.2539967	0.0246792	0.19718362	0.999	5367	tags=71%, list=45%, signal=129%
KEGG_STARCH_AND_SUCROSE_	KEGG_STARCH_AND_SUCROSE_	29	0.25064194	0.2506419	0.0444874	0.20332973	0.9995	4804	tags=66%, list=41%, signal=110%
KEGG_GALACTOSE_METABOLIS	KEGG_GALACTOSE_METABOLIS	23	0.24751057	0.2475106	0.1015936	0.20916049	1	4804	tags=65%, list=41%, signal=109%
KEGG_PATHOGENIC_ESCHERICH	KEGG_PATHOGENIC_ESCHERICH	49	0.24353465	0.2435347	0.0078201	0.2177758	1	5352	tags=69%, list=45%, signal=126%
KEGG_CELL_ADHESION_MOLECU	KEGG_CELL_ADHESION_MOLECU	113	0.24311009	0.2431101	0	0.21452758	1	4700	tags=64%, list=40%, signal=105%
KEGG_ASCORBATE_AND_ALDAR	KEGG_ASCORBATE_AND_ALDAR	10	0.2421941	0.2421941	0.5059171	0.21285544	1	7803	tags=90%, list=66%, signal=263%
KEGG_RIBOFLAVIN_METABOLISM	KEGG_RIBOFLAVIN_METABOLISM	12	0.2401812	0.2401812	0.4093162	0.21559373	1	2095	tags=42%, list=18%, signal=51%
KEGG_PHENYLALANINE_METABO	KEGG_PHENYLALANINE_METABO	17	0.23734051	0.2373405	0.2411067	0.2211757	1	3467	tags=53%, list=29%, signal=75%
KEGG_GLYCOLYSIS_GLUCONEO	KEGG_GLYCOLYSIS_GLUCONEO	53	0.23279601	0.232796	0.0061038	0.23312409	1	5530	tags=70%, list=47%, signal=130%
KEGG_FRUCTOSE_AND_MANNO	KEGG_FRUCTOSE_AND_MANNO	30	0.2307129	0.2307129	0.0667996	0.23618205	1	2409	tags=43%, list=20%, signal=54%
KEGG_SMALL_CELL_LUNG_CANC	KEGG_SMALL_CELL_LUNG_CANC	81	0.22948039	0.2294804	0	0.23626943	1	3592	tags=53%, list=30%, signal=76%
KEGG_RIG_I_LIKE_RECEPTOR_SI	KEGG_RIG_I_LIKE_RECEPTOR_SI	58	0.22876602	0.228766	0	0.23463325	1	4456	tags=60%, list=38%, signal=96%
KEGG_DRUG_METABOLISM_OTH	KEGG_DRUG_METABOLISM_OTH	32	0.22814506	0.2281451	0.0612648	0.2325377	1	4713	tags=63%, list=40%, signal=103%
KEGG_AMINOACYL_TRNA_BIOSY	KEGG_AMINOACYL_TRNA_BIOSY	29	0.22196801	0.221968	0.0856327	0.25222948	1	8006	tags=90%, list=68%, signal=275%
KEGG_T_CELL_RECEPTOR_SIGN	KEGG_T_CELL_RECEPTOR_SIGN	103	0.21965514	0.2196551	0	0.25711808	1	5707	tags=70%, list=48%, signal=134%
KEGG_SNARE_INTERACTIONS_IN	KEGG_SNARE_INTERACTIONS_IN	30	0.21918854	0.2191885	0.0910931	0.25443926	1	4522	tags=60%, list=38%, signal=97%
KEGG_CHEMOKINE_SIGNALING_	KEGG_CHEMOKINE_SIGNALING_	154	0.21597427	0.2159743	0	0.2634302	1	4556	tags=60%, list=38%, signal=96%
KEGG_REGULATION_OF_ACTIN_	KEGG_REGULATION_OF_ACTIN_	181	0.21577051	0.2157705	0	0.2597474	1	5342	tags=66%, list=45%, signal=119%
KEGG_CYTOSOLIC_DNA_SENSIN	KEGG_CYTOSOLIC_DNA_SENSIN	40	0.21319799	0.213198	0.045	0.26651338	1	8746	tags=95%, list=74%, signal=361%
KEGG_JAK_STAT_SIGNALING_PA	KEGG_JAK_STAT_SIGNALING_PA	126	0.21153969	0.2115397	0	0.26945445	1	7306	tags=83%, list=62%, signal=213%
KEGG_B_CELL_RECEPTOR_SIGN	KEGG_B_CELL_RECEPTOR_SIGN	68	0.21105036	0.2110504	0.0030864	0.26694298	1	5010	tags=63%, list=42%, signal=109%
KEGG_PENTOSE_AND_GLUCURO	KEGG_PENTOSE_AND_GLUCURO	12	0.20982446	0.2098245	0.6070409	0.26784703	1	6408	tags=75%, list=54%, signal=163%
KEGG_MISMATCH_REPAIR	KEGG_MISMATCH_REPAIR	23	0.20157135	0.2015714	0.2623457	0.29972202	1	9473	tags=100%, list=80%, signal=496%
KEGG_ONE_CARBON_POOL_BY_	KEGG_ONE_CARBON_POOL_BY_	15	0.19743918	0.1974392	0.570702	0.31417155	1	7939	tags=87%, list=67%, signal=262%
KEGG_FC_GAMMA_R_MEDIATED	KEGG_FC_GAMMA_R_MEDIATED	83	0.19638951	0.1963895	0.0040201	0.31410462	1	3116	tags=46%, list=26%, signal=62%
KEGG_GLYCEROLIPID_METABOLI	KEGG_GLYCEROLIPID_METABOLI	38	0.18849336	0.1884934	0.1281534	0.34969535	1	7758	tags=84%, list=65%, signal=243%
KEGG_NATURAL_KILLER_CELL_M	KEGG_NATURAL_KILLER_CELL_M	106	0.18600492	0.1860049	0	0.358113	1	5421	tags=64%, list=46%, signal=117%
KEGG_GLUTATHIONE_METABOLI	KEGG_GLUTATHIONE_METABOLI	35	0.17924497	0.179245	0.2017375	0.3914354	1	6012	tags=69%, list=51%, signal=139%
KEGG_ARRHYTHMOGENIC_RIGH	KEGG_ARRHYTHMOGENIC_RIGH	66	0.17550014	0.1755001	0.0270793	0.4083895	1	984	tags=26%, list=8%, signal=28%
KEGG_HYPERTROPHIC_CARDIO	KEGG_HYPERTROPHIC_CARDIO	75	0.17029558	0.1702956	0.0376238	0.43534496	1	1471	tags=29%, list=12%, signal=33%
KEGG_HISTIDINE_METABOLISM	KEGG_HISTIDINE_METABOLISM	23	0.17000487	0.1700049	0.4564565	0.43134567	1	9331	tags=96%, list=79%, signal=448%
KEGG_HOMOLOGOUS_RECOMBI	KEGG_HOMOLOGOUS_RECOMBI	25	0.16794251	0.1679425	0.438634	0.43904647	1	9397	tags=96%, list=79%, signal=461%
KEGG_ETHER_LIPID_METABOLIS	KEGG_ETHER_LIPID_METABOLIS	25	0.16762145	0.1676215	0.4253807	0.4349315	1	3708	tags=48%, list=31%, signal=70%
KEGG_CITRATE_CYCLE_TCA_CY	KEGG_CITRATE_CYCLE_TCA_CY	29	0.16687603	0.166876	0.365	0.43393576	1	6204	tags=69%, list=52%, signal=144%
KEGG_STEROID_HORMONE_BIO	KEGG_STEROID_HORMONE_BIO	36	0.16613106	0.1661311	0.2408325	0.43301067	1	7918	tags=83%, list=67%, signal=250%

KEGG_SPHINGOLIPID_METABOLI	KEGG_SPHINGOLIPID_METABOLI	28	0.16553174	0.1655317	0.3682132	0.43096438		1	1429	tags=29%, list=12%, signal=32%
KEGG_EPITHELIAL_CELL_SIGNAL	KEGG_EPITHELIAL_CELL_SIGNAL	59	0.16341476	0.1634148	0.0743243	0.43887734		1	3498	tags=46%, list=29%, signal=65%
KEGG_CIRCADIAN_RHYTHM_MA	KEGG_CIRCADIAN_RHYTHM_MA	11	0.16272183	0.1627218	0.8921283	0.43767095		1	4540	tags=55%, list=38%, signal=88%
KEGG_THYROID_CANCER	KEGG_THYROID_CANCER	29	0.16139074	0.1613907	0.3855422	0.44053638		1	5042	tags=59%, list=43%, signal=102%
KEGG_ACUTE_MYELOID_LEUKEM	KEGG_ACUTE_MYELOID_LEUKEM	55	0.16050208	0.1605021	0.1032505	0.44066873		1	2417	tags=36%, list=20%, signal=45%
KEGG_TRYPTOPHAN_METABOLI	KEGG_TRYPTOPHAN_METABOLI	29	0.16027153	0.1602715	0.4153355	0.43670845		1	7918	tags=83%, list=67%, signal=248%
KEGG_ABC_TRANSPORTERS	KEGG_ABC_TRANSPORTERS	36	0.15558752	0.1555875	0.3168016	0.4615865		1	6066	tags=67%, list=51%, signal=136%
KEGG_ADIPOCYTOKINE_SIGNALI	KEGG_ADIPOCYTOKINE_SIGNALI	63	0.15229595	0.152296	0.0948104	0.47848165		1	4603	tags=54%, list=39%, signal=88%
KEGG_P53_SIGNALING_PATHWA	KEGG_P53_SIGNALING_PATHWA	59	0.1491916	0.1491916	0.1298969	0.4947111		1	5475	tags=61%, list=46%, signal=113%
KEGG_BLADDER_CANCER	KEGG_BLADDER_CANCER	41	0.14855368	0.1485537	0.3053892	0.49308875		1	2293	tags=34%, list=19%, signal=42%
KEGG_SULFUR_METABOLISM	KEGG_SULFUR_METABOLISM	9	0.14833255	0.1483326	0.9631841	0.48894066		1	4830	tags=56%, list=41%, signal=94%
KEGG_PATHWAYS_IN_CANCER	KEGG_PATHWAYS_IN_CANCER	300	0.14669551	0.1466955	0	0.49434823		1	3403	tags=43%, list=29%, signal=59%
KEGG_ADHERENS_JUNCTION	KEGG_ADHERENS_JUNCTION	71	0.14623204	0.146232	0.1087613	0.49201593		1	1950	tags=31%, list=16%, signal=37%
KEGG_TYROSINE_METABOLISM	KEGG_TYROSINE_METABOLISM	38	0.14412914	0.1441291	0.3524004	0.5010654		1	9843	tags=97%, list=83%, signal=571%
KEGG_NUCLEOTIDE_EXCISION_R	KEGG_NUCLEOTIDE_EXCISION_R	41	0.14226778	0.1422678	0.3514852	0.5085982		1	9599	tags=95%, list=81%, signal=497%
KEGG_NOTCH_SIGNALING_PATH	KEGG_NOTCH_SIGNALING_PATH	36	0.14036424	0.1403642	0.4370664	0.5169664		1	3281	tags=42%, list=28%, signal=57%
KEGG_ARACHIDONIC_ACID_MET	KEGG_ARACHIDONIC_ACID_MET	45	0.13848686	0.1384869	0.3066802	0.52478033		1	8905	tags=89%, list=75%, signal=355%
KEGG_VIBRIO_CHOLERAE_INFEC	KEGG_VIBRIO_CHOLERAE_INFEC	45	0.13669065	0.1366907	0.321393	0.53255266		1	3128	tags=40%, list=26%, signal=54%
KEGG_PANCREATIC_CANCER	KEGG_PANCREATIC_CANCER	70	0.13591422	0.1359142	0.1348089	0.53266853		1	5682	tags=61%, list=48%, signal=117%
KEGG_VEGF_SIGNALING_PATHW	KEGG_VEGF_SIGNALING_PATHW	69	0.13200933	0.1320093	0.172549	0.5559288		1	3599	tags=43%, list=30%, signal=62%
KEGG_RENAL_CELL_CARCINOMA	KEGG_RENAL_CELL_CARCINOMA	66	0.13087037	0.1308704	0.1997961	0.559038		1	5284	tags=58%, list=45%, signal=103%
KEGG_PRIMARY_BILE_ACID_BIOS	KEGG_PRIMARY_BILE_ACID_BIOS	14	0.13050826	0.1305083	0.942058	0.55608493		1	10313	tags=100%, list=87%, signal=766%
KEGG_DILATED_CARDIOMYOPAT	KEGG_DILATED_CARDIOMYOPAT	80	0.13032682	0.1303268	0.1119247	0.5517663		1	984	tags=21%, list=8%, signal=23%
KEGG_MTOR_SIGNALING_PATH	KEGG_MTOR_SIGNALING_PATH	45	0.12994781	0.1299478	0.4040404	0.5487855		1	2417	tags=33%, list=20%, signal=42%
KEGG_ENDOCYTOSIS	KEGG_ENDOCYTOSIS	145	0.12978058	0.1297806	0.0128968	0.544604		1	3468	tags=42%, list=29%, signal=59%
KEGG_DORSO_VENTRAL_AXIS_F	KEGG_DORSO_VENTRAL_AXIS_F	18	0.12844117	0.1284412	0.8943248	0.549278		1	10338	tags=100%, list=87%, signal=778%
KEGG_PEROXISOME	KEGG_PEROXISOME	65	0.12540518	0.1254052	0.2565657	0.5668539		1	9650	tags=94%, list=81%, signal=501%
KEGG_VASCULAR_SMOOTH_MUS	KEGG_VASCULAR_SMOOTH_MUS	99	0.12125593	0.1212559	0.0805825	0.593619		1	5042	tags=55%, list=43%, signal=94%
KEGG_FC_EPSILON_RI_SIGNALIN	KEGG_FC_EPSILON_RI_SIGNALIN	72	0.11988179	0.1198818	0.2367632	0.5986766		1	5010	tags=54%, list=42%, signal=93%
KEGG_CHRONIC_MYELOID_LEUK	KEGG_CHRONIC_MYELOID_LEUK	70	0.11987156	0.1198716	0.232333	0.59303725		1	1466	tags=24%, list=12%, signal=28%
KEGG_NEUROTROPHIN_SIGNALI	KEGG_NEUROTROPHIN_SIGNALI	116	0.11713979	0.1171398	0.0721747	0.6086843		1	5269	tags=56%, list=44%, signal=100%
KEGG_PROSTATE_CANCER	KEGG_PROSTATE_CANCER	84	0.11622509	0.1162251	0.2070352	0.6103652		1	6537	tags=67%, list=55%, signal=147%
KEGG_BASAL_CELL_CARCINOMA	KEGG_BASAL_CELL_CARCINOMA	46	0.10985128	0.1098513	0.6061204	0.65435857		1	6436	tags=65%, list=54%, signal=142%
KEGG_TYPE_II_DIABETES_MELLI	KEGG_TYPE_II_DIABETES_MELLI	42	0.10788225	0.1078823	0.6572864	0.6639901		1	2395	tags=31%, list=20%, signal=39%
KEGG_PYRIMIDINE_METABOLISM	KEGG_PYRIMIDINE_METABOLISM	71	0.10217448	0.1021745	0.4192932	0.70429695		1	8817	tags=85%, list=74%, signal=327%
KEGG_MELANOMA	KEGG_MELANOMA	66	0.10137927	0.1013793	0.4782609	0.70469165		1	6710	tags=67%, list=57%, signal=153%
KEGG_MAPK_SIGNALING_PATHW	KEGG_MAPK_SIGNALING_PATHW	236	0.10004287	0.1000429	0.0197239	0.7092463		1	3560	tags=40%, list=30%, signal=56%
KEGG_COLORECTAL_CANCER	KEGG_COLORECTAL_CANCER	61	0.09495524	0.0949552	0.6028513	0.74403375		1	3156	tags=36%, list=27%, signal=49%
KEGG_TGF_BETA_SIGNALING_PA	KEGG_TGF_BETA_SIGNALING_PA	77	0.09443057	0.0944306	0.4727273	0.74166983		1	2891	tags=34%, list=24%, signal=44%
KEGG_ARGININE_AND_PROLINE_	KEGG_ARGININE_AND_PROLINE_	45	0.08972587	0.0897259	0.8266932	0.7717876		1	8954	tags=84%, list=75%, signal=343%
KEGG_AXON_GUIDANCE	KEGG_AXON_GUIDANCE	110	0.08897485	0.0889749	0.3346856	0.7710512		1	4021	tags=43%, list=34%, signal=64%
KEGG_INSULIN_SIGNALING_PAT	KEGG_INSULIN_SIGNALING_PAT	123	0.08789587	0.0878959	0.2753479	0.7721664		1	5042	tags=51%, list=43%, signal=88%
KEGG_PPAR_SIGNALING_PATHW	KEGG_PPAR_SIGNALING_PATHW	62	0.08301024	0.0830102	0.744489	0.80154556		1	7819	tags=74%, list=66%, signal=217%
KEGG_SYSTEMIC_LUPUS_ERYTH	KEGG_SYSTEMIC_LUPUS_ERYTH	86	0.08069813	0.0806981	0.5957011	0.8113548		1	7737	tags=73%, list=65%, signal=209%

Classical

NAME	GS<br>follow link to MSigDB	SIZE	ES	NES	NOM p-val	FDR q-val	FWER p-val	RANK AT MAX	LEADING EDGE
KEGG_GLYCOSPHINGOLIPID_BIOSYNTHESIS_GANGLIO_SERIES	KEGG_GLYCOSPHINGOLIPID_BIOSYNTHESIS_GANGLIO_SERIES	13	0.5320075	0.532008	0.002024292	0.04138072	0.035	4644	tags=92%, list=39%, signal=152%
KEGG_RIBOFLAVIN_METABOLISM	KEGG_RIBOFLAVIN_METABOLISM	12	0.4307056	0.430706	0.024048096	0.17041734	0.2495	3791	tags=75%, list=32%, signal=110%
KEGG_GLYCOSAMINOGLYCAN_BIOSYNTHESIS_CHONDROITIN_SULFATE	KEGG_GLYCOSAMINOGLYCAN_BIOSYNTHESIS_CHONDROITIN_SULFATE	13	0.416769	0.416769	0.017408123	0.1495961	0.313	2360	tags=62%, list=20%, signal=77%
KEGG_O_GLYCAN_BIOSYNTHESI	KEGG_O_GLYCAN_BIOSYNTHESI	18	0.4035824	0.403582	0.003937008	0.14098983	0.3745	5762	tags=89%, list=49%, signal=173%
KEGG_PROTEASOME	KEGG_PROTEASOME	42	0.401345	0.401345	0	0.11796489	0.3895	5704	tags=88%, list=48%, signal=169%
KEGG_AMINO_SUGAR_AND_NUCLEOTIDE_SUGAR_METABOLISM	KEGG_AMINO_SUGAR_AND_NUCLEOTIDE_SUGAR_METABOLISM	39	0.3932897	0.39329	0	0.11445503	0.435	4473	tags=77%, list=38%, signal=123%
KEGG_OTHER_GLYCAN_DEGRADATION	KEGG_OTHER_GLYCAN_DEGRADATION	12	0.3771101	0.37711	0.05320197	0.13142918	0.5325	1461	tags=50%, list=12%, signal=57%
KEGG_PANTOTHENATE_AND_COA_BIOSYNTHESIS	KEGG_PANTOTHENATE_AND_COA_BIOSYNTHESIS	14	0.3756302	0.37563	0.027272727	0.11875249	0.546	5715	tags=86%, list=48%, signal=165%
KEGG_GLYCOSAMINOGLYCAN_DEGRADATION	KEGG_GLYCOSAMINOGLYCAN_DEGRADATION	21	0.3542288	0.354229	0.01004016	0.15362799	0.6945	5971	tags=86%, list=50%, signal=172%
KEGG_GLYCOSAMINOGLYCAN_BIOSYNTHESIS_KERATAN_SULFAT	KEGG_GLYCOSAMINOGLYCAN_BIOSYNTHESIS_KERATAN_SULFAT	11	0.3527417	0.352742	0.0805169	0.14217733	0.704	5523	tags=82%, list=47%, signal=153%
KEGG_GLYCOSPHINGOLIPID_BIOSYNTHESIS_GLOBO_SERIES	KEGG_GLYCOSPHINGOLIPID_BIOSYNTHESIS_GLOBO_SERIES	13	0.3492998	0.3493	0.054080628	0.13683459	0.722	2247	tags=54%, list=19%, signal=66%
KEGG_PHENYLALANINE_METABOLISM	KEGG_PHENYLALANINE_METABOLISM	17	0.3278531	0.327853	0.041194644	0.1864644	0.859	4488	tags=71%, list=38%, signal=113%
KEGG_PRION_DISEASES	KEGG_PRION_DISEASES	34	0.3012555	0.301255	0.00404449	0.27044004	0.957	3064	tags=56%, list=26%, signal=75%
KEGG_FRUCTOSE_AND_MANNOSE_METABOLISM	KEGG_FRUCTOSE_AND_MANNOSE_METABOLISM	30	0.2977177	0.297718	0.003067485	0.2666962	0.967	3593	tags=60%, list=30%, signal=86%
KEGG_RENIN_ANGIOTENSIN_SYSTEM	KEGG_RENIN_ANGIOTENSIN_SYSTEM	16	0.2892815	0.289282	0.10965795	0.28593805	0.9795	6209	tags=81%, list=52%, signal=170%
KEGG_N_GLYCAN_BIOSYNTHESI	KEGG_N_GLYCAN_BIOSYNTHESIS	34	0.2692721	0.269272	0.012121212	0.3717479	0.9955	5884	tags=76%, list=50%, signal=151%
KEGG_GLYCOSAMINOGLYCAN_BIOSYNTHESIS_HEPARAN_SULFAT	KEGG_GLYCOSAMINOGLYCAN_BIOSYNTHESIS_HEPARAN_SULFAT	21	0.2658727	0.265873	0.08250497	0.3703569	0.998	4758	tags=67%, list=40%, signal=111%
KEGG_COMPLEMENT_AND_COAGULATION_CASCADES	KEGG_COMPLEMENT_AND_COAGULATION_CASCADES	65	0.2580037	0.258004	0	0.39880326	0.9995	5714	tags=74%, list=48%, signal=142%
KEGG_FOLATE_BIOSYNTHESIS	KEGG_FOLATE_BIOSYNTHESIS	11	0.25047	0.25047	0.42942345	0.42751563	0.9995	7813	tags=91%, list=66%, signal=266%
KEGG_ECM_RECEPTOR_INTERA	KEGG_ECM_RECEPTOR_INTERAC	78	0.248617	0.248617	0	0.41915464	0.9995	3608	tags=55%, list=30%, signal=79%
KEGG_NOD_LIKE_RECEPTOR_SI	KEGG_NOD_LIKE_RECEPTOR_SIG	52	0.2485603	0.24856	0.00203252	0.39947456	0.9995	2994	tags=50%, list=25%, signal=67%
KEGG_PATHOGENIC_ESCHERICH	KEGG_PATHOGENIC_ESCHERICHI	49	0.2470388	0.247039	0.003976143	0.390754	0.9995	1680	tags=39%, list=14%, signal=45%
KEGG_SYSTEMIC_LUPUS_ERYTH	KEGG_SYSTEMIC_LUPUS_ERYTH	86	0.2415768	0.241577	0	0.4079572	0.9995	5567	tags=71%, list=47%, signal=133%
KEGG_PENTOSE_PHOSPHATE_P	KEGG_PENTOSE_PHOSPHATE_P	23	0.2378798	0.23788	0.122266404	0.4152108	1	4918	tags=65%, list=41%, signal=111%
KEGG_LEISHMANIA_INFECTION	KEGG_LEISHMANIA_INFECTION	58	0.2373677	0.237368	0	0.40194649	1	4150	tags=59%, list=35%, signal=90%
KEGG_PORPHYRIN_AND_CHLOR	KEGG_PORPHYRIN_AND_CHLOR	24	0.2370311	0.237031	0.106167845	0.38825697	1	1641	tags=38%, list=14%, signal=43%
KEGG_RIBOSOME	KEGG_RIBOSOME	71	0.2347593	0.234759	0.001034126	0.3884177	1	7421	tags=86%, list=63%, signal=228%
KEGG_HEMATOPOIETIC_CELL_LI	KEGG_HEMATOPOIETIC_CELL_LI	77	0.2320347	0.232035	0	0.3912809	1	3580	tags=53%, list=30%, signal=76%
KEGG_GRAFT_VERSUS_HOST_DI	KEGG_GRAFT_VERSUS_HOST_DI	29	0.2276282	0.227628	0.09207921	0.405082	1	8348	tags=93%, list=70%, signal=314%
KEGG_GALACTOSE_METABOLIS	KEGG_GALACTOSE_METABOLISM	23	0.2231287	0.223129	0.19009902	0.4212613	1	3030	tags=48%, list=26%, signal=64%

KEGG_PROTEIN_EXPORT	KEGG_PROTEIN_EXPORT	18	0.2132617	0.213262	0.31451613	0.4743865	1	768	tags=28%, list=6%, signal=30%
KEGG_VIBRIO_CHOLERAE_INFEC	KEGG_VIBRIO_CHOLERAE_INFEC	45	0.2088776	0.208878	0.032520324	0.49258736	1	1748	tags=36%, list=15%, signal=42%
KEGG_THYROID_CANCER	KEGG_THYROID_CANCER	29	0.2025421	0.202542	0.1640706	0.5256945	1	6191	tags=72%, list=52%, signal=151%
KEGG_STARCH_AND_SUCROSE_	KEGG_STARCH_AND_SUCROSE_	29	0.2021341	0.202134	0.1437008	0.5133235	1	4151	tags=55%, list=35%, signal=85%
KEGG_ALLOGRAFT_REJECTION	KEGG_ALLOGRAFT_REJECTION	30	0.1965343	0.196534	0.16950853	0.5439476	1	8348	tags=90%, list=70%, signal=303%
KEGG_INTESTINAL_IMMUNE_NET	KEGG_INTESTINAL_IMMUNE_NET	35	0.1903111	0.190311	0.15724564	0.5805959	1	3848	tags=51%, list=32%, signal=76%
KEGG_ACUTE_MYELOID_LEUKEM	KEGG_ACUTE_MYELOID_LEUKEMI	55	0.1873782	0.187378	0.036511157	0.59065455	1	2531	tags=40%, list=21%, signal=51%
KEGG_PRIMARY_BILE_ACID_BIOS	KEGG_PRIMARY_BILE_ACID_BIOS	14	0.1870312	0.187031	0.64070106	0.57803196	1	7102	tags=79%, list=60%, signal=196%
KEGG_LYSOSOME	KEGG_LYSOSOME	106	0.1855298	0.18553	9.50E-04	0.57661325	1	6993	tags=77%, list=59%, signal=187%
KEGG_HYPERTROPHIC_CARDIO	KEGG_HYPERTROPHIC_CARDIOM	75	0.1846019	0.184602	0.014127145	0.56982416	1	7944	tags=85%, list=67%, signal=257%
KEGG_FOCAL_ADHESION	KEGG_FOCAL_ADHESION	182	0.1772178	0.177218	0	0.6216877	1	2100	tags=35%, list=18%, signal=42%
KEGG_APOPTOSIS	KEGG_APOPTOSIS	79	0.1752995	0.175299	0.010731707	0.62583697	1	5290	tags=62%, list=45%, signal=111%
KEGG_GLYCOLYSIS_GLUCONEO	KEGG_GLYCOLYSIS_GLUCONEO	53	0.1744983	0.174498	0.08059701	0.6185865	1	5547	tags=64%, list=47%, signal=120%
KEGG_BASE_EXCISION_REPAIR	KEGG_BASE_EXCISION_REPAIR	31	0.1723675	0.172368	0.29196337	0.62427557	1	7142	tags=77%, list=60%, signal=194%
KEGG_STEROID_BIOSYNTHESIS	KEGG_STEROID_BIOSYNTHESIS	15	0.1692416	0.169242	0.72167	0.6404888	1	5901	tags=67%, list=50%, signal=133%
KEGG_VIRAL_MYOCARDITIS	KEGG_VIRAL_MYOCARDITIS	62	0.165665	0.165665	0.053946055	0.66020435	1	7609	tags=81%, list=64%, signal=224%
KEGG_DILATED_CARDIOMYOPAT	KEGG_DILATED_CARDIOMYOPAT	80	0.1644949	0.164495	0.024340771	0.6577474	1	5029	tags=59%, list=42%, signal=101%
KEGG_TYROSINE_METABOLISM	KEGG_TYROSINE_METABOLISM	38	0.1643457	0.164346	0.24475524	0.64528507	1	9604	tags=97%, list=81%, signal=510%
KEGG_ADHERENS_JUNCTION	KEGG_ADHERENS_JUNCTION	71	0.1637944	0.163794	0.046248715	0.63743657	1	1910	tags=32%, list=16%, signal=38%
KEGG_SNARE_INTERACTIONS_IN	KEGG_SNARE_INTERACTIONS_IN	30	0.1635391	0.163539	0.36091724	0.62722397	1	3599	tags=47%, list=30%, signal=67%
KEGG_CHEMOKINE_SIGNALING_	KEGG_CHEMOKINE_SIGNALING_P	154	0.1634893	0.163489	0	0.6153745	1	2860	tags=40%, list=24%, signal=52%
KEGG_JAK_STAT_SIGNALING_PA	KEGG_JAK_STAT_SIGNALING_PA	126	0.1628087	0.162809	9.72E-04	0.609315	1	5054	tags=59%, list=43%, signal=101%
KEGG_GLUTATHIONE_METABOLI	KEGG_GLUTATHIONE_METABOLI	35	0.1621263	0.162126	0.28429425	0.6035569	1	2487	tags=37%, list=21%, signal=47%
KEGG_ANTIGEN_PROCESSING_A	KEGG_ANTIGEN_PROCESSING_A	63	0.1605036	0.160504	0.06981318	0.60574627	1	8083	tags=84%, list=68%, signal=263%
KEGG_SPHINGOLIPID_METABOLI	KEGG_SPHINGOLIPID_METABOLIS	28	0.1597846	0.159785	0.42021805	0.60130715	1	4462	tags=54%, list=38%, signal=86%
KEGG_ARACHIDONIC_ACID_MET	KEGG_ARACHIDONIC_ACID_META	45	0.1573706	0.157371	0.1955684	0.6117735	1	6837	tags=73%, list=58%, signal=172%
KEGG_DNA_REPLICATION	KEGG_DNA_REPLICATION	32	0.1550875	0.155087	0.37333333	0.6212616	1	9654	tags=97%, list=81%, signal=519%
KEGG_CYTOKINE_CYTOKINE_RE	KEGG_CYTOKINE_CYTOKINE_RE	214	0.1538966	0.153897	0.001023541	0.6214397	1	8127	tags=84%, list=69%, signal=261%
KEGG_ARRHYTHMOGENIC_RIGH	KEGG_ARRHYTHMOGENIC_RIGHT	66	0.1530851	0.153085	0.09174312	0.61783403	1	5022	tags=58%, list=42%, signal=99%
KEGG_BIOSYNTHESIS_OF_UNSA	KEGG_BIOSYNTHESIS_OF_UNSAT	18	0.1526675	0.152668	0.7238193	0.61115026	1	4780	tags=56%, list=40%, signal=93%
KEGG_DRUG_METABOLISM_OTH	KEGG_DRUG_METABOLISM_OTH	32	0.1511984	0.151198	0.4016227	0.6140086	1	9700	tags=97%, list=82%, signal=530%
KEGG_TYPE_II_DIABETES_MELLI	KEGG_TYPE_II_DIABETES_MELLIT	42	0.148458	0.148458	0.3026973	0.6282049	1	3045	tags=40%, list=26%, signal=54%
KEGG_NICOTINATE_AND_NICOTI	KEGG_NICOTINATE_AND_NICOTI	15	0.1471226	0.147123	0.859127	0.6303139	1	3791	tags=47%, list=32%, signal=69%
KEGG_B_CELL_RECEPTOR_SIGN	KEGG_B_CELL_RECEPTOR_SIGN	68	0.1458217	0.145822	0.08840864	0.6321447	1	3163	tags=41%, list=27%, signal=56%
KEGG_STEROID_HORMONE_BIO	KEGG_STEROID_HORMONE_BIOS	36	0.1450816	0.145082	0.39041096	0.6286671	1	7508	tags=78%, list=63%, signal=211%
KEGG_ERBB_SIGNALING_PATHW	KEGG_ERBB_SIGNALING_PATHW	80	0.1432301	0.14323	0.039235413	0.6351722	1	3204	tags=41%, list=27%, signal=56%
KEGG_CYTOSOLIC_DNA_SENSIN	KEGG_CYTOSOLIC_DNA_SENSIN	40	0.1412437	0.141244	0.3550356	0.64429075	1	3963	tags=48%, list=33%, signal=71%
KEGG_PYRIMIDINE_METABOLISM	KEGG_PYRIMIDINE_METABOLISM	71	0.1395225	0.139523	0.13654225	0.65094054	1	9546	tags=94%, list=80%, signal=481%
KEGG_SMALL_CELL_LUNG_CANC	KEGG_SMALL_CELL_LUNG_CANC	81	0.1377478	0.137748	0.07354346	0.657306	1	3794	tags=46%, list=32%, signal=67%
KEGG_TOLL_LIKE_RECEPTOR_SI	KEGG_TOLL_LIKE_RECEPTOR_SI	88	0.1371938	0.137194	0.065656565	0.653265	1	3101	tags=40%, list=26%, signal=53%
KEGG_VALINE_LEUCINE_AND_IS	KEGG_VALINE_LEUCINE_AND_IS	10	0.1350211	0.135021	0.9816248	0.6644247	1	1957	tags=30%, list=17%, signal=36%
KEGG_T_CELL_RECEPTOR_SIGN	KEGG_T_CELL_RECEPTOR_SIGN	103	0.1328347	0.132835	0.05029586	0.6750597	1	5346	tags=58%, list=45%, signal=105%
KEGG_BLADDER_CANCER	KEGG_BLADDER_CANCER	41	0.1312995	0.1313	0.42345923	0.67982244	1	3654	tags=44%, list=31%, signal=63%
KEGG_ADIPOCYTOKINE_SIGNALI	KEGG_ADIPOCYTOKINE_SIGNALI	63	0.130761	0.130761	0.21768707	0.67549855	1	2598	tags=35%, list=22%, signal=44%
KEGG_PROSTATE_CANCER	KEGG_PROSTATE_CANCER	84	0.1296665	0.129666	0.10376399	0.6768511	1	8073	tags=81%, list=68%, signal=252%
KEGG_ONE_CARBON_POOL_BY_	KEGG_ONE_CARBON_POOL_BY_	15	0.129478	0.129478	0.9312064	0.66982085	1	8744	tags=87%, list=74%, signal=329%

KEGG_CHRONIC_MYELOID_LEUK	KEGG_CHRONIC_MYELOID_LEUK	70	0.1244154	0.124415	0.19483101	0.70687306	1	8359	tags=83%, list=70%, signal=279%
KEGG_NEUROTROPHIN_SIGNALI	KEGG_NEUROTROPHIN_SIGNALIN	116	0.1240927	0.124093	0.049079753	0.7008359	1	3347	tags=41%, list=28%, signal=56%
KEGG_GLIOMA	KEGG_GLIOMA	59	0.1239582	0.123958	0.30041152	0.6933494	1	8386	tags=83%, list=71%, signal=282%
KEGG_RENAL_CELL_CARCINOMA	KEGG_RENAL_CELL_CARCINOMA	66	0.1236967	0.123697	0.23138034	0.6867854	1	5728	tags=61%, list=48%, signal=117%
KEGG_VASCULAR_SMOOTH_MUS	KEGG_VASCULAR_SMOOTH_MUS	99	0.1206393	0.120639	0.092762485	0.70576346	1	8164	tags=81%, list=69%, signal=257%
KEGG_EPITHELIAL_CELL_SIGNAL	KEGG_EPITHELIAL_CELL_SIGNALI	59	0.1179791	0.117979	0.3573544	0.72179383	1	1823	tags=27%, list=15%, signal=32%
KEGG_LEUKOCYTE_TRANSENDO	KEGG_LEUKOCYTE_TRANSENDO	99	0.1156399	0.11564	0.12578617	0.7342439	1	8103	tags=80%, list=68%, signal=250%
KEGG_REGULATION_OF_ACTIN_	KEGG_REGULATION_OF_ACTIN_C	181	0.1149322	0.114932	0.01489573	0.731911	1	2064	tags=29%, list=17%, signal=34%
KEGG_NATURAL_KILLER_CELL_M	KEGG_NATURAL_KILLER_CELL_M	106	0.1126842	0.112684	0.12706612	0.7438827	1	8073	tags=79%, list=68%, signal=246%
KEGG_CELL_ADHESION_MOLECU	KEGG_CELL_ADHESION_MOLECU	113	0.1083259	0.108326	0.14035088	0.7755521	1	7018	tags=70%, list=59%, signal=170%
KEGG_CELL_CYCLE	KEGG_CELL_CYCLE	104	0.1045588	0.104559	0.1875	0.80155617	1	8235	tags=80%, list=69%, signal=259%
KEGG_MELANOMA	KEGG_MELANOMA	66	0.1031573	0.103157	0.48129424	0.80532694	1	8486	tags=82%, list=72%, signal=286%
KEGG_PRIMARY_IMMUNODEFICIE	KEGG_PRIMARY_IMMUNODEFICIE	33	0.1030027	0.103003	0.86336637	0.79763037	1	9922	tags=94%, list=84%, signal=573%
KEGG_P53_SIGNALING_PATHWA	KEGG_P53_SIGNALING_PATHWAY	59	0.1013014	0.101301	0.56643355	0.8041973	1	4231	tags=46%, list=36%, signal=71%
KEGG_OOCYTE_MEIOSIS	KEGG_OOCYTE_MEIOSIS	91	0.0985416	0.098542	0.31445906	0.8210289	1	8223	tags=79%, list=69%, signal=256%
KEGG_PROGESTERONE_MEDIAT	KEGG_PROGESTERONE_MEDIAT	73	0.0981206	0.098121	0.50775194	0.8158023	1	8103	tags=78%, list=68%, signal=245%
KEGG_INSULIN_SIGNALING_PAT	KEGG_INSULIN_SIGNALING_PATH	123	0.0965573	0.096557	0.18018018	0.82136625	1	3494	tags=39%, list=29%, signal=55%
KEGG_MELANOGENESIS	KEGG_MELANOGENESIS	86	0.0911133	0.091113	0.43213573	0.86055034	1	8166	tags=78%, list=69%, signal=248%
KEGG_ENDOMETRIAL_CANCER	KEGG_ENDOMETRIAL_CANCER	50	0.0908383	0.090838	0.7917526	0.8541496	1	1061	tags=18%, list=9%, signal=20%
KEGG_GAP_JUNCTION	KEGG_GAP_JUNCTION	75	0.0905275	0.090528	0.57566464	0.8479305	1	1146	tags=19%, list=10%, signal=21%
KEGG_AMYOTROPHIC_LATERAL_	KEGG_AMYOTROPHIC_LATERAL_	50	0.090381	0.090381	0.77744806	0.8404271	1	4150	tags=44%, list=35%, signal=67%
KEGG_PATHWAYS_IN_CANCER	KEGG_PATHWAYS_IN_CANCER	300	0.088714	0.088714	0.021934198	0.8464406	1	2571	tags=30%, list=22%, signal=38%
KEGG_GNRH_SIGNALING_PATH	KEGG_GNRH_SIGNALING_PATHW	88	0.0871212	0.087121	0.48873654	0.8514461	1	995	tags=17%, list=8%, signal=18%
KEGG_AXON_GUIDANCE	KEGG_AXON_GUIDANCE	110	0.0866615	0.086661	0.39070228	0.84671044	1	6528	tags=64%, list=55%, signal=140%
KEGG_COLORECTAL_CANCER	KEGG_COLORECTAL_CANCER	61	0.0857047	0.085705	0.7184369	0.8463291	1	3654	tags=39%, list=31%, signal=57%
KEGG_BASAL_CELL_CARCINOMA	KEGG_BASAL_CELL_CARCINOMA	46	0.0854145	0.085415	0.85796547	0.8402329	1	7756	tags=74%, list=65%, signal=213%
KEGG_MAPK_SIGNALING_PATHW	KEGG_MAPK_SIGNALING_PATHW	236	0.0820001	0.082	0.08020833	0.858624	1	1056	tags=17%, list=9%, signal=18%
KEGG_PANCREATIC_CANCER	KEGG_PANCREATIC_CANCER	70	0.0813038	0.081304	0.72410357	0.855534	1	8359	tags=79%, list=70%, signal=265%
KEGG_TIGHT_JUNCTION	KEGG_TIGHT_JUNCTION	113	0.0775389	0.077539	0.48466864	0.8755345	1	1817	tags=23%, list=15%, signal=27%
KEGG_VEGF_SIGNALING_PATHW	KEGG_VEGF_SIGNALING_PATHW	69	0.07534	0.07534	0.7942387	0.8827539	1	3064	tags=33%, list=26%, signal=45%
KEGG_FC_EPSILON_RI_SIGNALIN	KEGG_FC_EPSILON_RI_SIGNALIN	72	0.067088	0.067088	0.89823467	0.9279877	1	7609	tags=71%, list=64%, signal=196%
KEGG_ENDOCYTOSIS	KEGG_ENDOCYTOSIS	145	0.0659161	0.065916	0.54104847	0.92606807	1	8633	tags=79%, list=73%, signal=288%
KEGG_FC_GAMMA_R_MEDIATED	KEGG_FC_GAMMA_R_MEDIATED_	83	0.0636589	0.063659	0.8470705	0.9294233	1	7537	tags=70%, list=64%, signal=190%
KEGG_ALZHEIMERS_DISEASE	KEGG_ALZHEIMERS_DISEASE	142	0.0549666	0.054967	0.7823937	0.95924735	1	9628	tags=87%, list=81%, signal=455%

Neural

NAME	GS<br>follow link to MSigDB	SIZE	ES	NES	NOM p-val	FDR q-val	FWER p-val	RANK AT MAX	LEADING EDGE
KEGG_OTHER_GLYCAN_DEGRADATION	KEGG_OTHER_GLYCAN_DEGRADATION	12	0.5606291	0.56062907	0	0.02560054	0.021	3240	tags=83%, list=27%, signal=115%
KEGG_MISMATCH_REPAIR	KEGG_MISMATCH_REPAIR	23	0.4728688	0.4728688	0	0.07291422	0.1195	5746	tags=96%, list=48%, signal=185%
KEGG_GLYCOSAMINOGLYCAN_BIOSYNTHESIS_CHONDROITIN_SULFATE	KEGG_GLYCOSAMINOGLYCAN_BIOSYNTHESIS_CHONDROITIN_SULFATE	13	0.4655252	0.4655252	0.0068695	0.05515797	0.134	3607	tags=77%, list=30%, signal=110%
KEGG_VALINE_LEUCINE_AND_ISOLEUCINE_BIOSYNTHESIS	KEGG_VALINE_LEUCINE_AND_ISOLEUCINE_BIOSYNTHESIS	10	0.3971308	0.39713082	0.0676768	0.15089335	0.402	3595	tags=70%, list=30%, signal=100%
KEGG_AMINO_SUGAR_AND_NUCLEOTIDE_SUGAR_METABOLISM	KEGG_AMINO_SUGAR_AND_NUCLEOTIDE_SUGAR_METABOLISM	39	0.3781146	0.3781146	0	0.17715015	0.5405	6477	tags=92%, list=55%, signal=203%
KEGG_ONE_CARBON_POOL_BY_FOLATE	KEGG_ONE_CARBON_POOL_BY_FOLATE	15	0.3749825	0.37498245	0.0228175	0.15621778	0.563	3464	tags=67%, list=29%, signal=94%
KEGG_HOMOLOGOUS_RECOMBINATION	KEGG_HOMOLOGOUS_RECOMBINATION	25	0.3718124	0.3718124	9.80E-04	0.14089197	0.581	5561	tags=84%, list=47%, signal=158%
KEGG_DNA_REPLICATION	KEGG_DNA_REPLICATION	32	0.3695997	0.36959967	0.001	0.12771326	0.5935	4893	tags=78%, list=41%, signal=133%
KEGG_AMINOACYL_TRNA_BIOSYNTHESIS	KEGG_AMINOACYL_TRNA_BIOSYNTHESIS	29	0.3575528	0.35755277	0.0010246	0.14198957	0.682	5175	tags=79%, list=44%, signal=140%
KEGG_GLYCOSAMINOGLYCAN_DEGRADATION	KEGG_GLYCOSAMINOGLYCAN_DEGRADATION	21	0.3473186	0.3473186	0.0057034	0.15332825	0.7475	3229	tags=62%, list=27%, signal=85%
KEGG_N_GLYCAN_BIOSYNTHESI	KEGG_N_GLYCAN_BIOSYNTHESI	34	0.3361188	0.33611882	9.80E-04	0.16877104	0.8175	4047	tags=68%, list=34%, signal=102%
KEGG_LYSINE_DEGRADATION	KEGG_LYSINE_DEGRADATION	38	0.3303965	0.3303965	0	0.17024064	0.843	4832	tags=74%, list=41%, signal=124%
KEGG_PENTOSE_PHOSPHATE_PATHWAY	KEGG_PENTOSE_PHOSPHATE_PATHWAY	23	0.3207812	0.3207812	0.0127077	0.18863559	0.8905	4968	tags=74%, list=42%, signal=127%
KEGG_O_GLYCAN_BIOSYNTHESI	KEGG_O_GLYCAN_BIOSYNTHESI	18	0.317148	0.31714803	0.0438247	0.18728852	0.9045	4150	tags=67%, list=35%, signal=102%
KEGG_SPLICEOSOME	KEGG_SPLICEOSOME	86	0.3146429	0.31464285	0	0.18245992	0.9125	4293	tags=67%, list=36%, signal=105%
KEGG_BIOSYNTHESIS_OF_UNSATURATED_FATTY_ACIDS	KEGG_BIOSYNTHESIS_OF_UNSATURATED_FATTY_ACIDS	18	0.2883241	0.28832406	0.0736739	0.26669562	0.982	6468	tags=83%, list=55%, signal=183%
KEGG_CIRCADIAN_RHYTHM_MAMMAL	KEGG_CIRCADIAN_RHYTHM_MAMMAL	11	0.2867446	0.28674456	0.2621951	0.25726885	0.9855	6305	tags=82%, list=53%, signal=175%
KEGG_NOTCH_SIGNALING_PATHWAY	KEGG_NOTCH_SIGNALING_PATHWAY	36	0.2866299	0.28662986	0.0019685	0.24340713	0.9855	4846	tags=69%, list=41%, signal=117%
KEGG_ECM_RECEPTOR_INTERACTION	KEGG_ECM_RECEPTOR_INTERACTION	78	0.28406	0.28406	0	0.2412446	0.988	1974	tags=45%, list=17%, signal=53%
KEGG_NUCLEOTIDE_EXCISION_	KEGG_NUCLEOTIDE_EXCISION_	41	0.2827134	0.2827134	9.92E-04	0.23461892	0.9895	6782	tags=85%, list=57%, signal=199%
KEGG_CITRATE_CYCLE_TCA_CY	KEGG_CITRATE_CYCLE_TCA_CY	29	0.2763488	0.2763488	0.0223577	0.2483506	0.9935	2864	tags=52%, list=24%, signal=68%
KEGG_VALINE_LEUCINE_AND_IS	KEGG_VALINE_LEUCINE_AND_IS	40	0.2712352	0.2712352	0.005923	0.2574707	0.995	4502	tags=65%, list=38%, signal=104%
KEGG_GLYCOSPHINGOLIPID_BI	KEGG_GLYCOSPHINGOLIPID_BI	13	0.2684354	0.2684354	0.2497532	0.257821	0.9965	3205	tags=54%, list=27%, signal=74%
KEGG_GLYCOSPHINGOLIPID_BI	KEGG_GLYCOSPHINGOLIPID_BI	13	0.264637	0.26463696	0.2589372	0.26363966	0.997	3250	tags=54%, list=27%, signal=74%
KEGG_PORPHYRIN_AND_CHLOR	KEGG_PORPHYRIN_AND_CHLOR	24	0.2568717	0.2568717	0.0712831	0.28714463	0.998	6842	tags=83%, list=58%, signal=197%
KEGG_LIMONENE_AND_PINENE_	KEGG_LIMONENE_AND_PINENE_	10	0.255865	0.25586498	0.4298781	0.28018638	0.998	525	tags=30%, list=4%, signal=31%
KEGG_PROTEIN_EXPORT	KEGG_PROTEIN_EXPORT	18	0.2529884	0.25298843	0.1535394	0.28266996	0.9985	3592	tags=56%, list=30%, signal=80%
KEGG_SELENOAMINO_ACID_ME	KEGG_SELENOAMINO_ACID_ME	19	0.2392668	0.2392668	0.1898734	0.34237796	1	6529	tags=79%, list=55%, signal=175%
KEGG_ANTIGEN_PROCESSING_	KEGG_ANTIGEN_PROCESSING_	63	0.2392484	0.23924838	0	0.33065182	1	4895	tags=65%, list=41%, signal=110%
KEGG_GALACTOSE_METABOLIS	KEGG_GALACTOSE_METABOLIS	23	0.2368623	0.2368623	0.1347305	0.3320987	1	6477	tags=78%, list=55%, signal=172%
KEGG_LYSOSOME	KEGG_LYSOSOME	106	0.2358972	0.23589721	0	0.32605606	1	5394	tags=69%, list=45%, signal=125%
KEGG_NON_HOMOLOGOUS_EN	KEGG_NON_HOMOLOGOUS_EN	13	0.2348729	0.23487285	0.3751234	0.32082915	1	1778	tags=38%, list=15%, signal=45%
KEGG_BASE_EXCISION_REPAIR	KEGG_BASE_EXCISION_REPAIR	31	0.2348329	0.23483291	0.0492462	0.31136522	1	8316	tags=94%, list=70%, signal=312%

KEGG_FRUCTOSE_AND_MANNO	KEGG_FRUCTOSE_AND_MANNO	30	0.2314173	0.23141734	0.0693756	0.31916654	1	6354	tags=77%, list=54%, signal=165%
KEGG_GLYCOSAMINOGLYCAN_B	KEGG_GLYCOSAMINOGLYCAN_B	11	0.2256807	0.22568074	0.5729167	0.33966482	1	3794	tags=55%, list=32%, signal=80%
KEGG_ASCORBATE_AND_ALDAR	KEGG_ASCORBATE_AND_ALDAR	10	0.2232068	0.22320676	0.6083418	0.3435712	1	6842	tags=80%, list=58%, signal=189%
KEGG_PROTEASOME	KEGG_PROTEASOME	42	0.2214943	0.22149426	0.0273438	0.34341377	1	8959	tags=98%, list=76%, signal=398%
KEGG_CHRONIC_MYELOID_LEU	KEGG_CHRONIC_MYELOID_LEU	70	0.2211075	0.22110748	0.0010163	0.3364376	1	6033	tags=73%, list=51%, signal=147%
KEGG_PANTOTHENATE_AND_C	KEGG_PANTOTHENATE_AND_C	14	0.2188443	0.21884426	0.4452333	0.3395866	1	5878	tags=71%, list=50%, signal=141%
KEGG_SMALL_CELL_LUNG_CAN	KEGG_SMALL_CELL_LUNG_CAN	81	0.2100642	0.21006417	9.95E-04	0.3789426	1	6017	tags=72%, list=51%, signal=144%
KEGG_PROPANOATE_METABOLI	KEGG_PROPANOATE_METABOLI	26	0.2081551	0.20815514	0.187	0.38108468	1	4378	tags=58%, list=37%, signal=91%
KEGG_CELL_CYCLE	KEGG_CELL_CYCLE	104	0.2072232	0.2072232	0	0.37794977	1	6800	tags=78%, list=57%, signal=181%
KEGG_GLYCOSYLPHOSPHATIDY	KEGG_GLYCOSYLPHOSPHATIDY	19	0.2060192	0.20601924	0.302	0.37597337	1	1929	tags=37%, list=16%, signal=44%
KEGG_GLUTATHIONE_METABOLI	KEGG_GLUTATHIONE_METABOLI	35	0.1990335	0.19903354	0.1174129	0.41011155	1	3406	tags=49%, list=29%, signal=68%
KEGG_SPHINGOLIPID_METABOLI	KEGG_SPHINGOLIPID_METABOLI	28	0.1969116	0.19691156	0.2058824	0.4141635	1	5717	tags=68%, list=48%, signal=131%
KEGG_BLADDER_CANCER	KEGG_BLADDER_CANCER	41	0.1953221	0.1953221	0.0860534	0.4159254	1	5790	tags=68%, list=49%, signal=133%
KEGG_APOPTOSIS	KEGG_APOPTOSIS	79	0.1908652	0.19086517	0.0059761	0.43655795	1	5557	tags=66%, list=47%, signal=123%
KEGG_ENDOMETRIAL_CANCER	KEGG_ENDOMETRIAL_CANCER	50	0.1899068	0.1899068	0.0527404	0.43381184	1	9379	tags=98%, list=79%, signal=466%
KEGG_PEROXISOME	KEGG_PEROXISOME	65	0.1888023	0.18880232	0.0129611	0.4324817	1	7625	tags=83%, list=64%, signal=231%
KEGG_SNARE_INTERACTIONS_I	KEGG_SNARE_INTERACTIONS_I	30	0.1862779	0.18627787	0.2027559	0.44048807	1	6888	tags=77%, list=58%, signal=182%
KEGG_PANCREATIC_CANCER	KEGG_PANCREATIC_CANCER	70	0.184236	0.18423603	0.0172414	0.44516417	1	5790	tags=67%, list=49%, signal=130%
KEGG_P53_SIGNALING_PATHWA	KEGG_P53_SIGNALING_PATHWA	59	0.1808034	0.1808034	0.0258193	0.45956057	1	5705	tags=66%, list=48%, signal=127%
KEGG_TERPENOID_BACKBONE_	KEGG_TERPENOID_BACKBONE_	12	0.1794958	0.17949584	0.7642586	0.4598801	1	5779	tags=67%, list=49%, signal=130%
KEGG_GLYOXYLATE_AND_DICA	KEGG_GLYOXYLATE_AND_DICA	10	0.1757806	0.1757806	0.8786653	0.47782075	1	6218	tags=70%, list=52%, signal=147%
KEGG_STARCH_AND_SUCROSE	KEGG_STARCH_AND_SUCROSE	29	0.1737137	0.17371371	0.3037249	0.48421177	1	7350	tags=79%, list=62%, signal=208%
KEGG_NICOTINATE_AND_NICOTI	KEGG_NICOTINATE_AND_NICOTI	15	0.1729844	0.1729844	0.7150206	0.48114347	1	7438	tags=80%, list=63%, signal=214%
KEGG_ADHERENS_JUNCTION	KEGG_ADHERENS_JUNCTION	71	0.1729351	0.17293513	0.0237643	0.47300085	1	4809	tags=58%, list=41%, signal=97%
KEGG_PYRIMIDINE_METABOLIS	KEGG_PYRIMIDINE_METABOLIS	71	0.1717715	0.17177148	0.0237358	0.4731246	1	1983	tags=34%, list=17%, signal=40%
KEGG_COLORECTAL_CANCER	KEGG_COLORECTAL_CANCER	61	0.1712148	0.1712148	0.0444225	0.46921167	1	8089	tags=85%, list=68%, signal=267%
KEGG_RNA_DEGRADATION	KEGG_RNA_DEGRADATION	43	0.1707178	0.17071778	0.1455446	0.46475595	1	9290	tags=95%, list=78%, signal=438%
KEGG_FOCAL_ADHESION	KEGG_FOCAL_ADHESION	182	0.1692842	0.16928422	0	0.46675816	1	1541	tags=30%, list=13%, signal=34%
KEGG_RENAL_CELL_CARCINOM	KEGG_RENAL_CELL_CARCINOM	66	0.1689586	0.16895856	0.0467662	0.46145502	1	4116	tags=52%, list=35%, signal=78%
KEGG_LEISHMANIA_INFECTION	KEGG_LEISHMANIA_INFECTION	58	0.1658047	0.16580474	0.075122	0.47597185	1	6017	tags=67%, list=51%, signal=136%
KEGG_CYTOSOLIC_DNA_SENSIN	KEGG_CYTOSOLIC_DNA_SENSIN	40	0.1639594	0.16395941	0.1881288	0.4809873	1	8735	tags=90%, list=74%, signal=340%
KEGG_GLYCOSAMINOGLYCAN_B	KEGG_GLYCOSAMINOGLYCAN_B	21	0.1639175	0.1639175	0.5490798	0.4738781	1	3706	tags=48%, list=31%, signal=69%
KEGG_RNA_POLYMERASE	KEGG_RNA_POLYMERASE	21	0.161476	0.16147602	0.5834166	0.483486	1	8253	tags=86%, list=70%, signal=281%
KEGG_ACUTE_MYELOID_LEUKE	KEGG_ACUTE_MYELOID_LEUKE	55	0.1589773	0.1589773	0.1106758	0.49497572	1	4807	tags=56%, list=41%, signal=94%
KEGG_VIRAL_MYOCARDITIS	KEGG_VIRAL_MYOCARDITIS	62	0.1583511	0.15835105	0.0985916	0.49228764	1	1574	tags=29%, list=13%, signal=33%
KEGG_PROSTATE_CANCER	KEGG_PROSTATE_CANCER	84	0.1574227	0.15742269	0.0187192	0.4918108	1	6899	tags=74%, list=58%, signal=175%
KEGG_MTOR_SIGNALING_PATH	KEGG_MTOR_SIGNALING_PATH	45	0.154728	0.154728	0.202544	0.5042386	1	8186	tags=84%, list=69%, signal=272%
KEGG_ADIPOCYTOKINE_SIGNALI	KEGG_ADIPOCYTOKINE_SIGNALI	63	0.1546667	0.15466674	0.098117	0.49754077	1	3069	tags=41%, list=26%, signal=55%
KEGG_FATTY_ACID_METABOLIS	KEGG_FATTY_ACID_METABOLIS	36	0.1485491	0.14854911	0.3660263	0.53557	1	4502	tags=53%, list=38%, signal=85%
KEGG_FC_GAMMA_R_MEDIATED	KEGG_FC_GAMMA_R_MEDIATED	83	0.1421946	0.14219461	0.0625	0.5787713	1	8041	tags=82%, list=68%, signal=253%
KEGG_PATHOGENIC_ESCHERIC	KEGG_PATHOGENIC_ESCHERIC	49	0.1414506	0.14145064	0.2416244	0.577006	1	8252	tags=84%, list=70%, signal=274%
KEGG_EPITHELIAL_CELL_SIGNA	KEGG_EPITHELIAL_CELL_SIGNA	59	0.1405282	0.14052817	0.1865121	0.5772047	1	2763	tags=37%, list=23%, signal=48%
KEGG_PYRUVATE_METABOLISM	KEGG_PYRUVATE_METABOLISM	33	0.1401134	0.14011341	0.5174604	0.57295156	1	3014	tags=39%, list=25%, signal=53%
KEGG_DRUG_METABOLISM_OTH	KEGG_DRUG_METABOLISM_OTH	32	0.1399539	0.1399539	0.5031579	0.5668265	1	6868	tags=72%, list=58%, signal=170%
KEGG_DORSO_VENTRAL_AXIS_	KEGG_DORSO_VENTRAL_AXIS_	18	0.1387716	0.13877161	0.8338369	0.56890357	1	2309	tags=33%, list=19%, signal=41%
KEGG_TRYPTOPHAN_METABOLI	KEGG_TRYPTOPHAN_METABOLI	29	0.138409	0.13840903	0.5836694	0.5645827	1	4087	tags=48%, list=34%, signal=73%

KEGG_GLYCINE_SERINE_AND_T	KEGG_GLYCINE_SERINE_AND_T	27	0.1377003	0.13770027	0.6633761	0.56292385	1	5837	tags=63%, list=49%, signal=124%
KEGG_ABC_TRANSPORTERS	KEGG_ABC_TRANSPORTERS	36	0.1373102	0.1373102	0.4584616	0.55910796	1	6941	tags=72%, list=59%, signal=174%
KEGG_NON_SMALL_CELL_LUNG	KEGG_NON_SMALL_CELL_LUNG	51	0.1338013	0.13380128	0.2795157	0.58014727	1	8186	tags=82%, list=69%, signal=265%
KEGG_TOLL_LIKE_RECEPTOR_S	KEGG_TOLL_LIKE_RECEPTOR_S	88	0.1290234	0.1290234	0.0982143	0.61078095	1	8049	tags=81%, list=68%, signal=249%
KEGG_NOD_LIKE_RECEPTOR_SI	KEGG_NOD_LIKE_RECEPTOR_SI	52	0.127912	0.127912	0.3457031	0.6127686	1	8524	tags=85%, list=72%, signal=300%
KEGG_B_CELL_RECEPTOR_SIG	KEGG_B_CELL_RECEPTOR_SIG	68	0.1262471	0.12624711	0.2167832	0.61892134	1	3045	tags=38%, list=26%, signal=51%
KEGG_VIBRIO_CHOLERAE_INFE	KEGG_VIBRIO_CHOLERAE_INFE	45	0.1256689	0.12566888	0.4639279	0.61642927	1	4576	tags=51%, list=39%, signal=83%
KEGG_GLYCOLYSIS_GLUCONEO	KEGG_GLYCOLYSIS_GLUCONEO	53	0.1248476	0.12484758	0.3596914	0.6159114	1	6357	tags=66%, list=54%, signal=142%
KEGG_UBIQUITIN_MEDIATED_PR	KEGG_UBIQUITIN_MEDIATED_PR	105	0.1232951	0.12329507	0.0705765	0.62192	1	4875	tags=53%, list=41%, signal=90%
KEGG_BUTANOATE_METABOLIS	KEGG_BUTANOATE_METABOLIS	30	0.1216963	0.12169627	0.735529	0.6273449	1	1722	tags=27%, list=15%, signal=31%
KEGG_ARRHYTHMOGENIC_RIGH	KEGG_ARRHYTHMOGENIC_RIGH	66	0.1214742	0.1214742	0.2691924	0.62232363	1	543	tags=17%, list=5%, signal=17%
KEGG_CYSTEINE_AND_METHIO	KEGG_CYSTEINE_AND_METHIO	31	0.1163461	0.11634609	0.7572314	0.6571583	1	9335	tags=90%, list=79%, signal=423%
KEGG_ETHER_LIPID_METABOLIS	KEGG_ETHER_LIPID_METABOLIS	25	0.1142036	0.11420362	0.8578337	0.6677701	1	2917	tags=36%, list=25%, signal=48%
KEGG_TGF_BETA_SIGNALING_P	KEGG_TGF_BETA_SIGNALING_P	77	0.112071	0.112071	0.2913223	0.67864245	1	7766	tags=77%, list=65%, signal=221%
KEGG_PATHWAYS_IN_CANCER	KEGG_PATHWAYS_IN_CANCER	300	0.1096482	0.10964822	0.0039526	0.69121283	1	6480	tags=65%, list=55%, signal=140%
KEGG_GRAFT_VERSUS_HOST_D	KEGG_GRAFT_VERSUS_HOST_D	29	0.1070741	0.10707405	0.8554217	0.7058597	1	1186	tags=21%, list=10%, signal=23%
KEGG_LEUKOCYTE_TRANSEND	KEGG_LEUKOCYTE_TRANSEND	99	0.1042557	0.10425572	0.2102564	0.7224068	1	1648	tags=24%, list=14%, signal=28%
KEGG_RIG_I_LIKE_RECEPTOR_S	KEGG_RIG_I_LIKE_RECEPTOR_S	58	0.0985631	0.09856308	0.5997994	0.7631471	1	1903	tags=26%, list=16%, signal=31%
KEGG_INSULIN_SIGNALING_PAT	KEGG_INSULIN_SIGNALING_PAT	123	0.0959179	0.0959179	0.2235529	0.7774404	1	4755	tags=50%, list=40%, signal=82%
KEGG_PRION_DISEASES	KEGG_PRION_DISEASES	34	0.093672	0.09367197	0.8885512	0.7890143	1	1333	tags=21%, list=11%, signal=23%
KEGG_REGULATION_OF_ACTIN_	KEGG_REGULATION_OF_ACTIN_	181	0.0923337	0.09233367	0.1013514	0.7928215	1	3835	tags=41%, list=32%, signal=60%
KEGG_ENDOCYTOSIS	KEGG_ENDOCYTOSIS	145	0.0914478	0.09144776	0.1961962	0.79201716	1	7107	tags=69%, list=60%, signal=170%
KEGG_BASAL_TRANSCRIPTION_	KEGG_BASAL_TRANSCRIPTION_	28	0.090795	0.09079498	0.9534413	0.789443	1	6549	tags=64%, list=55%, signal=143%
KEGG_NEUROTROPHIN_SIGNALI	KEGG_NEUROTROPHIN_SIGNALI	116	0.0894896	0.08948958	0.2957746	0.79236174	1	8252	tags=78%, list=70%, signal=255%
KEGG_CELL_ADHESION_MOLEC	KEGG_CELL_ADHESION_MOLEC	113	0.0836681	0.08366814	0.400767	0.83021337	1	1745	tags=23%, list=15%, signal=27%
KEGG_ERBB_SIGNALING_PATH	KEGG_ERBB_SIGNALING_PATH	80	0.0822793	0.08227929	0.6313132	0.83235365	1	1550	tags=21%, list=13%, signal=24%
KEGG_JAK_STAT_SIGNALING_P	KEGG_JAK_STAT_SIGNALING_P	126	0.0815565	0.08155654	0.3548387	0.83010936	1	10149	tags=94%, list=86%, signal=642%
KEGG_ARGININE_AND_PROLINE	KEGG_ARGININE_AND_PROLINE	45	0.0801241	0.08012414	0.9067882	0.83284765	1	4587	tags=47%, list=39%, signal=76%
KEGG_TIGHT_JUNCTION	KEGG_TIGHT_JUNCTION	113	0.0801229	0.08012289	0.4726912	0.82514125	1	947	tags=16%, list=8%, signal=17%
KEGG_VEGF_SIGNALING_PATH	KEGG_VEGF_SIGNALING_PATH	69	0.0638758	0.06387582	0.9175662	0.91990954	1	9387	tags=86%, list=79%, signal=408%

Proneural

NAME	GS<br>follow link to MSigDB	SIZE	ES	NES	NOM p-val	FDR q-val	FWER p-val	RANK AT MAX	LEADING EDGE
KEGG_PROTEASOME	KEGG_PROTEASOME	42	0.5947465	0.59474653	0	0.00720335	0.008	3136	tags=86%, list=26%, signal=116%
KEGG_DNA_REPLICATION	KEGG_DNA_REPLICATION	32	0.5138865	0.5138865	0	0.02091735	0.0435	3557	tags=81%, list=30%, signal=116%
KEGG_RIBOSOME	KEGG_RIBOSOME	71	0.4610086	0.46100864	0	0.04774645	0.1395	5756	tags=94%, list=49%, signal=182%
KEGG_GLYCOSAMINOGLYCAN_BI OSYNTHESIS_KERATAN_SULFATE	KEGG_GLYCOSAMINOGLYCAN_BI OSYNTHESIS_KERATAN_SULFATE	11	0.4236875	0.4236875	0.02313883	0.07297315	0.268	2526	tags=64%, list=21%, signal=81%
KEGG_GLYCOSAMINOGLYCAN_BI OSYNTHESIS_CHONDROITIN_SUL [illegible]	KEGG_GLYCOSAMINOGLYCAN_BI OSYNTHESIS_CHONDROITIN_SUL [illegible]	13	0.4226192	0.4226192	0.01408451	0.0597183	0.274	3203	tags=69%, list=27%, signal=95%
KEGG_GLYCOSAMINOGLYCAN_D EGRADATION	KEGG_GLYCOSAMINOGLYCAN_D EGRADATION	21	0.4175144	0.41751438	0	0.05477012	0.297	4657	tags=81%, list=39%, signal=133%
KEGG_NON_HOMOLOGOUS_END _JOINING	KEGG_NON_HOMOLOGOUS_END _JOINING	13	0.4029258	0.4029258	0.02439024	0.06288511	0.3815	2524	tags=62%, list=21%, signal=78%
KEGG_VALINE_LEUCINE_AND_IS OLEUCINE BIOSYNTHESIS	KEGG_VALINE_LEUCINE_AND_IS OLEUCINE BIOSYNTHESIS	10	0.3900422	0.39004222	0.07715134	0.07101039	0.4585	3679	tags=70%, list=31%, signal=101%
KEGG_RIBOFLAVIN_METABOLISM	KEGG_RIBOFLAVIN_METABOLISM	12	0.3828495	0.38284945	0.04295704	0.07237185	0.506	4358	tags=75%, list=37%, signal=118%
KEGG_PENTOSE_AND_GLUCURO NATE_INTERCONVERSIONS	KEGG_PENTOSE_AND_GLUCURO NATE_INTERCONVERSIONS	12	0.358007	0.35800698	0.06489676	0.10238796	0.6795	6629	tags=92%, list=56%, signal=208%
KEGG_STEROID_BIOSYNTHESIS	KEGG_STEROID_BIOSYNTHESIS	15	0.352188	0.35218802	0.0278607	0.10511861	0.725	3734	tags=67%, list=31%, signal=97%
KEGG_HOMOLOGOUS_RECOMBIN ATION	KEGG_HOMOLOGOUS_RECOMBIN ATION	25	0.3484748	0.34847483	0.003	0.10321117	0.758	6786	tags=92%, list=57%, signal=215%
KEGG_GLYCOSPHINGOLIPID_BIO SYNTHESIS_GLOBO_SERIES	KEGG_GLYCOSPHINGOLIPID_BIO SYNTHESIS_GLOBO_SERIES	13	0.3432742	0.3432742	0.06461233	0.10369723	0.786	4143	tags=69%, list=35%, signal=106%
KEGG_GLYCOSPHINGOLIPID_BIO SYNTHESIS_GANGLIO_SERIES	KEGG_GLYCOSPHINGOLIPID_BIO SYNTHESIS_GANGLIO_SERIES	13	0.3432742	0.3432742	0.07046332	0.09629028	0.786	4143	tags=69%, list=35%, signal=106%
KEGG_PENTOSE_PHOSPHATE_P ATHWAY	KEGG_PENTOSE_PHOSPHATE_P ATHWAY	23	0.3333101	0.33331007	0.01029866	0.10570349	0.8325	5851	tags=83%, list=49%, signal=163%
KEGG_RENIN_ANGIOTENSIN_SYS	KEGG_RENIN_ANGIOTENSIN_SYS	16	0.3240248	0.32402483	0.05533199	0.11601582	0.876	4315	tags=69%, list=36%, signal=108%
KEGG_MISMATCH_REPAIR	KEGG_MISMATCH_REPAIR	23	0.3238996	0.32389963	0.01	0.10936172	0.8775	8025	tags=100%, list=68%, signal=309%
KEGG_FOLATE_BIOSYNTHESIS	KEGG_FOLATE_BIOSYNTHESIS	11	0.3178711	0.3178711	0.18606965	0.1153074	0.9015	4858	tags=73%, list=41%, signal=123%
KEGG CELL CYCLE	KEGG CELL CYCLE	104	0.3119619	0.31196186	0	0.12093648	0.9285	3516	tags=61%, list=30%, signal=85%
KEGG_NUCLEOTIDE_EXCISION_R	KEGG_NUCLEOTIDE_EXCISION_R	41	0.3088062	0.30880618	0	0.12066864	0.935	4738	tags=71%, list=40%, signal=117%
KEGG_RNA_POLYMERASE	KEGG_RNA_POLYMERASE	21	0.3004879	0.30048794	0.03405573	0.13213226	0.955	7172	tags=90%, list=60%, signal=228%
KEGG_N_GLYCAN_BIOSYNTHESIS	KEGG_N_GLYCAN_BIOSYNTHESIS	34	0.2978979	0.29789793	0.00292969	0.13139574	0.9585	4499	tags=68%, list=38%, signal=109%
KEGG_BASE_EXCISION_REPAIR	KEGG_BASE_EXCISION_REPAIR	31	0.2939468	0.2939468	0.00487329	0.13429524	0.966	6469	tags=84%, list=55%, signal=184%
KEGG_PYRIMIDINE_METABOLISM	KEGG_PYRIMIDINE_METABOLISM	71	0.2791394	0.2791394	0	0.165831	0.99	3724	tags=59%, list=31%, signal=86%
KEGG_PROTEIN_EXPORT	KEGG_PROTEIN_EXPORT	18	0.2786035	0.2786035	0.10019841	0.16069345	0.9905	7242	tags=89%, list=61%, signal=228%
KEGG_AMINOACYL_TRNA_BIOSY	KEGG_AMINOACYL_TRNA_BIOSY	29	0.2721984	0.2721984	0.02628918	0.17158982	0.995	4140	tags=62%, list=35%, signal=95%
KEGG_PANTOTHENATE_AND_CO	KEGG_PANTOTHENATE_AND_CO	14	0.2714117	0.27141172	0.21026157	0.16733769	0.9955	3561	tags=57%, list=30%, signal=82%
KEGG_OTHER_GLYCAN_DEGRAD	KEGG_OTHER_GLYCAN_DEGRAD	12	0.2704535	0.27045354	0.30653265	0.16458249	0.996	6678	tags=83%, list=56%, signal=191%
KEGG_SULFUR_METABOLISM	KEGG_SULFUR_METABOLISM	9	0.2696538	0.26965377	0.444	0.16092871	0.9965	4710	tags=67%, list=40%, signal=110%
KEGG_NICOTINATE_AND_NICOTIN	KEGG_NICOTINATE_AND_NICOTIN	15	0.2641621	0.26416212	0.20259482	0.17026478	0.9985	1614	tags=40%, list=14%, signal=46%
KEGG_O_GLYCAN_BIOSYNTHESIS	KEGG_O_GLYCAN_BIOSYNTHESIS	18	0.2600912	0.26009122	0.15331355	0.1754667	0.9985	2849	tags=50%, list=24%, signal=66%
KEGG_PHENYLALANINE_METABO	KEGG_PHENYLALANINE_METABO	17	0.2583606	0.25836062	0.18625498	0.17499591	0.9985	5311	tags=71%, list=45%, signal=128%
KEGG_TYROSINE_METABOLISM	KEGG_TYROSINE_METABOLISM	38	0.2572056	0.25720558	0.00811359	0.17303294	0.999	7882	tags=92%, list=66%, signal=274%
KEGG_AMINO_SUGAR_AND_NUCL	KEGG_AMINO_SUGAR_AND_NUCL	39	0.2419965	0.24199654	0.01980198	0.21457748	0.9995	2004	tags=41%, list=17%, signal=49%
KEGG_ASCORBATE_AND_ALDAR	KEGG_ASCORBATE_AND_ALDAR	10	0.2412658	0.24126583	0.53892213	0.21120821	0.9995	698	tags=30%, list=6%, signal=32%
KEGG_TERPENOID_BACKBONE_B	KEGG_TERPENOID_BACKBONE_B	12	0.238465	0.23846501	0.45210728	0.2152002	0.9995	7057	tags=83%, list=60%, signal=206%
KEGG_DRUG_METABOLISM_OTHE	KEGG_DRUG_METABOLISM_OTHE	32	0.2378889	0.2378889	0.03503504	0.21152076	0.9995	2374	tags=44%, list=20%, signal=55%

KEGG_ECM_RECEPTOR_INTERAC	KEGG_ECM_RECEPTOR_INTERAC	78	0.2376702	0.23767024	9.89E-04	0.20672812	0.9995	848	tags=31%, list=7%, signal=33%
KEGG_RNA_DEGRADATION	KEGG_RNA_DEGRADATION	43	0.2359884	0.23598835	0.0110664	0.20733972	0.9995	6588	tags=79%, list=56%, signal=177%
KEGG_GLYOXYLATE_AND_DICAR	KEGG_GLYOXYLATE_AND_DICAR	10	0.2181435	0.21814346	0.6382766	0.2689407	1	9274	tags=100%, list=78%, signal=458%
KEGG_BASAL_TRANSCRIPTION_F	KEGG_BASAL_TRANSCRIPTION_F	28	0.2098426	0.20984262	0.14638783	0.2997913	1	8529	tags=93%, list=72%, signal=330%
KEGG_SPLICEOSOME	KEGG_SPLICEOSOME	86	0.2096855	0.20968552	0	0.2931935	1	8149	tags=90%, list=69%, signal=284%
KEGG_PORPHYRIN_AND_CHLOR	KEGG_PORPHYRIN_AND_CHLOR	24	0.2063901	0.20639011	0.24537487	0.30200398	1	8922	tags=96%, list=75%, signal=386%
KEGG_GALACTOSE_METABOLISM	KEGG_GALACTOSE_METABOLISM	23	0.2014942	0.2014942	0.27110228	0.3193017	1	6380	tags=74%, list=54%, signal=160%
KEGG_GLYCOSYLPHOSPHATIDYLI	KEGG_GLYCOSYLPHOSPHATIDYLI	19	0.2008365	0.20083654	0.3816568	0.3151969	1	3863	tags=53%, list=33%, signal=78%
KEGG_ALANINE_ASPARTATE_AN	KEGG_ALANINE_ASPARTATE_AN	27	0.1943623	0.19436227	0.22662602	0.34060314	1	4288	tags=56%, list=36%, signal=87%
KEGG_SNARE_INTERACTIONS_IN	KEGG_SNARE_INTERACTIONS_IN	30	0.1858834	0.18588339	0.2199211	0.38034326	1	6102	tags=70%, list=51%, signal=144%
KEGG_CYSTEINE_AND_METHIONI	KEGG_CYSTEINE_AND_METHIONI	31	0.1857736	0.18577361	0.20788178	0.3731017	1	7366	tags=81%, list=62%, signal=212%
KEGG_ONE_CARBON_POOL_BY_	KEGG_ONE_CARBON_POOL_BY_	15	0.1769805	0.17698047	0.6944162	0.41826034	1	4228	tags=53%, list=36%, signal=83%
KEGG_GLYCOLYSIS_GLUCONEOG	KEGG_GLYCOLYSIS_GLUCONEOG	53	0.1762849	0.17628492	0.07645875	0.41444194	1	6421	tags=72%, list=54%, signal=156%
KEGG_SELENOAMINO_ACID_MET	KEGG_SELENOAMINO_ACID_MET	19	0.1649977	0.16499765	0.59237534	0.48012003	1	6160	tags=68%, list=52%, signal=142%
KEGG_PATHOGENIC_ESCHERICHI	KEGG_PATHOGENIC_ESCHERICHI	49	0.1616446	0.16164455	0.1546287	0.4946477	1	5109	tags=59%, list=43%, signal=104%
KEGG_FOCAL_ADHESION	KEGG_FOCAL_ADHESION	182	0.1593501	0.15935008	0	0.5020122	1	2048	tags=33%, list=17%, signal=39%
KEGG_GLYCOSPHINGOLIPID_BIO	KEGG_GLYCOSPHINGOLIPID_BIO	23	0.1588424	0.1588424	0.54332316	0.49660644	1	2244	tags=35%, list=19%, signal=43%
KEGG_STARCH_AND_SUCROSE_	KEGG_STARCH_AND_SUCROSE_	29	0.1557043	0.15570433	0.443669	0.51011556	1	8381	tags=86%, list=71%, signal=293%
KEGG_CITRATE_CYCLE_TCA_CYC	KEGG_CITRATE_CYCLE_TCA_CYC	29	0.1529967	0.15299666	0.45277208	0.52119964	1	8822	tags=90%, list=74%, signal=349%
KEGG_P53_SIGNALING_PATHWAY	KEGG_P53_SIGNALING_PATHWAY	59	0.1520095	0.15200952	0.10616785	0.5195573	1	8457	tags=86%, list=71%, signal=300%
KEGG_GLYCOSAMINOGLYCAN_BI	KEGG_GLYCOSAMINOGLYCAN_BI	21	0.151336	0.151336	0.6772592	0.51561224	1	6114	tags=67%, list=52%, signal=137%
KEGG_PROGESTERONE_MEDIAT	KEGG_PROGESTERONE_MEDIAT	73	0.1512207	0.1512207	0.07249256	0.5077625	1	3253	tags=42%, list=27%, signal=58%
KEGG_AUTOIMMUNE_THYROID_DI	KEGG_AUTOIMMUNE_THYROID_DI	38	0.1489507	0.14895067	0.32681018	0.5153803	1	9786	tags=97%, list=83%, signal=555%
KEGG_GLUTATHIONE_METABOLIS	KEGG_GLUTATHIONE_METABOLIS	35	0.1473754	0.14737542	0.40634596	0.5187149	1	1306	tags=26%, list=11%, signal=29%
KEGG_ALLOGRAFT_REJECTION	KEGG_ALLOGRAFT_REJECTION	30	0.1449705	0.14497048	0.52935225	0.52758235	1	10144	tags=100%, list=86%, signal=689%
KEGG_COMPLEMENT_AND_COAG	KEGG_COMPLEMENT_AND_COAG	65	0.1362181	0.1362181	0.14859438	0.586848	1	6968	tags=72%, list=59%, signal=174%
KEGG_OOCYTE_MEIOSIS	KEGG_OOCYTE_MEIOSIS	91	0.1337627	0.13376266	0.05694981	0.597654	1	1292	tags=24%, list=11%, signal=27%
KEGG_UBIQUITIN_MEDIATED_PR	KEGG_UBIQUITIN_MEDIATED_PR	105	0.1329607	0.13296066	0.03937008	0.5948053	1	5665	tags=61%, list=48%, signal=116%
KEGG_PARKINSONS_DISEASE	KEGG_PARKINSONS_DISEASE	96	0.1318854	0.13188544	0.06804734	0.59467596	1	8825	tags=88%, list=74%, signal=339%
KEGG_PURINE_METABOLISM	KEGG_PURINE_METABOLISM	127	0.1276446	0.12764455	0.02628918	0.6198387	1	7373	tags=75%, list=62%, signal=196%
KEGG_THYROID_CANCER	KEGG_THYROID_CANCER	29	0.1167331	0.11673307	0.7912088	0.7038497	1	9660	tags=93%, list=81%, signal=501%
KEGG_RIG_I_LIKE_RECEPTOR_SI	KEGG_RIG_I_LIKE_RECEPTOR_SI	58	0.1144809	0.1144809	0.41902834	0.71396565	1	9281	tags=90%, list=78%, signal=410%
KEGG_HEMATOPOIETIC_CELL_LI	KEGG_HEMATOPOIETIC_CELL_LI	77	0.1123598	0.11235977	0.25478348	0.7224358	1	4220	tags=47%, list=36%, signal=72%
KEGG_INTESTINAL_IMMUNE_NET	KEGG_INTESTINAL_IMMUNE_NET	35	0.1115434	0.11154337	0.7356784	0.7192447	1	8168	tags=80%, list=69%, signal=256%
KEGG_FRUCTOSE_AND_MANNOS	KEGG_FRUCTOSE_AND_MANNOS	30	0.1109327	0.11093267	0.8235887	0.71451056	1	1454	tags=23%, list=12%, signal=27%
KEGG_CYTOKINE_CYTOKINE_RE	KEGG_CYTOKINE_CYTOKINE_RE	214	0.1093753	0.10937531	0.01390268	0.71852875	1	5154	tags=54%, list=43%, signal=94%
KEGG_TGF_BETA_SIGNALING_PA	KEGG_TGF_BETA_SIGNALING_PA	77	0.0999735	0.09997345	0.4003984	0.7928197	1	4674	tags=49%, list=39%, signal=81%
KEGG_HYPERTROPHIC_CARDIOM	KEGG_HYPERTROPHIC_CARDIOM	75	0.0940348	0.09403479	0.4883041	0.83507	1	314	tags=12%, list=3%, signal=12%
KEGG_HEDGEHOG_SIGNALING_P	KEGG_HEDGEHOG_SIGNALING_P	45	0.0935628	0.0935628	0.82095623	0.8281675	1	3374	tags=38%, list=28%, signal=53%
KEGG_HUNTINGTONS_DISEASE	KEGG_HUNTINGTONS_DISEASE	148	0.0910201	0.09102011	0.16083916	0.8398837	1	9030	tags=85%, list=76%, signal=352%
KEGG_BLADDER_CANCER	KEGG_BLADDER_CANCER	41	0.0892403	0.08924031	0.87643677	0.84498394	1	10515	tags=98%, list=89%, signal=857%
KEGG_REGULATION_OF_ACTIN_C	KEGG_REGULATION_OF_ACTIN_C	181	0.0886329	0.08863291	0.11660079	0.839218	1	1978	tags=25%, list=17%, signal=30%
KEGG_LYSOSOME	KEGG_LYSOSOME	106	0.0868705	0.08687046	0.37000975	0.8424339	1	6810	tags=66%, list=57%, signal=154%
KEGG_AXON_GUIDANCE	KEGG_AXON_GUIDANCE	110	0.0850367	0.08503674	0.35384616	0.84718907	1	4822	tags=49%, list=41%, signal=82%
KEGG_VIRAL_MYOCARDITIS	KEGG_VIRAL_MYOCARDITIS	62	0.0811619	0.08116192	0.7744511	0.86751324	1	9945	tags=92%, list=84%, signal=566%
KEGG_APOPTOSIS	KEGG_APOPTOSIS	79	0.0786377	0.0786377	0.69815195	0.87618613	1	4327	tags=44%, list=36%, signal=69%
KEGG_SMALL_CELL_LUNG_CANC	KEGG_SMALL_CELL_LUNG_CANC	81	0.0784699	0.07846985	0.67034066	0.8671144	1	1271	tags=19%, list=11%, signal=21%

KEGG_JAK_STAT_SIGNALING_PA	KEGG_JAK_STAT_SIGNALING_PA	126	0.0738121	0.07381213	0.5014837	0.88901925	1	10334	tags=94%, list=87%, signal=726%
KEGG_PANCREATIC_CANCER	KEGG_PANCREATIC_CANCER	70	0.0708833	0.07088332	0.84691846	0.8983455	1	10515	tags=96%, list=89%, signal=839%
KEGG_NOD_LIKE_RECEPTOR_SIG	KEGG_NOD_LIKE_RECEPTOR_SIG	52	0.0581093	0.05810925	0.98913044	0.957805	1	8664	tags=79%, list=73%, signal=291%
KEGG_PATHWAYS_IN_CANCER	KEGG_PATHWAYS_IN_CANCER	300	0.0552826	0.05528259	0.33164302	0.9581545	1	3590	tags=36%, list=30%, signal=50%

Mesenchymal

NAME	GS<br>follow link to MSigDB	SIZE	ES	NES	NOM p-val	FDR q-val	FWER p-val	RANK AT MAX	LEADING EDGE
KEGG_PROTEASOME	KEGG_PROTEASOME	42	0.59474653	0.59474653	0	0.00962011	0.01	3136	tags=86%, list=26%, signal=116%
KEGG_DNA_REPLICATION	KEGG_DNA_REPLICATION	32	0.5138865	0.5138865	0	0.026044514	0.055	3557	tags=81%, list=30%, signal=116%
KEGG_RIBOSOME	KEGG_RIBOSOME	71	0.46100864	0.46100864	0	0.047453124	0.1445	5756	tags=94%, list=49%, signal=182%
KEGG_GLYCOSAMINOGLYCAN_BIOSYNTHESIS_KERATAN_SULFATE	KEGG_GLYCOSAMINOGLYCAN_BIOSYNTHESIS_KERATAN_SULFATE	11	0.4236875	0.4236875	0.022772277	0.07159363	0.2745	2526	tags=64%, list=21%, signal=81%
KEGG_GLYCOSAMINOGLYCAN_BIOSYNTHESIS_CHONDROITIN_SULFATE	KEGG_GLYCOSAMINOGLYCAN_BIOSYNTHESIS_CHONDROITIN_SULFATE	13	0.4226192	0.4226192	0.014227643	0.058455955	0.2795	3203	tags=69%, list=27%, signal=95%
KEGG_GLYCOSAMINOGLYCAN_DEGRADATION	KEGG_GLYCOSAMINOGLYCAN_DEGRADATION	21	0.41751438	0.41751438	9.95E-04	0.055178963	0.307	4657	tags=81%, list=39%, signal=133%
KEGG_NON_HOMOLOGOUS_END_JOI[illegible]	KEGG_NON_HOMOLOGOUS_END_JO[illegible]	13	0.4029258	0.4029258	0.02112676	0.06459014	0.391	2524	tags=62%, list=21%, signal=78%
KEGG_VALINE_LEUCINE_AND_ISOLEUCINE_BIOSYNTHESIS	KEGG_VALINE_LEUCINE_AND_ISOLEUCINE_BIOSYNTHESIS	10	0.39004222	0.39004222	0.07492507	0.07231504	0.474	3679	tags=70%, list=31%, signal=101%
KEGG_RIBOFLAVIN_METABOLISM	KEGG_RIBOFLAVIN_METABOLISM	12	0.38284945	0.38284945	0.043613706	0.07340552	0.5215	4358	tags=75%, list=37%, signal=118%
KEGG_PENTOSE_AND_GLUCURONATE_INTERCONVERSIONS	KEGG_PENTOSE_AND_GLUCURONATE_INTERCONVERSIONS	12	0.35800698	0.35800698	0.07008885	0.10271946	0.68	6629	tags=92%, list=56%, signal=208%
KEGG_STEROID_BIOSYNTHESIS	KEGG_STEROID_BIOSYNTHESIS	15	0.35218802	0.35218802	0.042639595	0.103682645	0.721	3734	tags=67%, list=31%, signal=97%
KEGG_HOMOLOGOUS_RECOMBINATI[illegible]	KEGG_HOMOLOGOUS_RECOMBINAT[illegible]	25	0.34847483	0.34847483	0.003061225	0.10218766	0.7455	6786	tags=92%, list=57%, signal=215%
KEGG_GLYCOSPHINGOLIPID_BIOSYNTHESIS_GLOBO_SERIES	KEGG_GLYCOSPHINGOLIPID_BIOSYNTHESIS_GLOBO_SERIES	13	0.3432742	0.3432742	0.07556428	0.1036095	0.7765	4143	tags=69%, list=35%, signal=106%
KEGG_GLYCOSPHINGOLIPID_BIOSYNTHESIS_GANGLIO_SERIES	KEGG_GLYCOSPHINGOLIPID_BIOSYNTHESIS_GANGLIO_SERIES	13	0.3432742	0.3432742	0.07258064	0.09620882	0.7765	4143	tags=69%, list=35%, signal=106%
KEGG_PENTOSE_PHOSPHATE_PATH[illegible]	KEGG_PENTOSE_PHOSPHATE_PAT[illegible]	23	0.33331007	0.33331007	0.008179959	0.1047559	0.8305	5851	tags=83%, list=49%, signal=163%
KEGG_RENIN_ANGIOTENSIN_SYSTEM	KEGG_RENIN_ANGIOTENSIN_SYSTE[illegible]	16	0.32402483	0.32402483	0.053359684	0.11630112	0.8785	4315	tags=69%, list=36%, signal=108%
KEGG_MISMATCH_REPAIR	KEGG_MISMATCH_REPAIR	23	0.32389963	0.32389963	0.009287925	0.10962738	0.8795	8025	tags=100%, list=68%, signal=309%
KEGG_FOLATE_BIOSYNTHESIS	KEGG_FOLATE_BIOSYNTHESIS	11	0.3178711	0.3178711	0.18118812	0.11574933	0.907	4858	tags=73%, list=41%, signal=123%
KEGG_CELL_CYCLE	KEGG_CELL_CYCLE	104	0.31196186	0.31196186	0	0.1223285	0.928	3516	tags=61%, list=30%, signal=85%
KEGG_NUCLEOTIDE_EXCISION_REPA	KEGG_NUCLEOTIDE_EXCISION_REP	41	0.30880618	0.30880618	0	0.12273602	0.9355	4738	tags=71%, list=40%, signal=117%
KEGG_RNA_POLYMERASE	KEGG_RNA_POLYMERASE	21	0.30048794	0.30048794	0.03160041	0.13441338	0.9585	7172	tags=90%, list=60%, signal=228%
KEGG_N_GLYCAN_BIOSYNTHESIS	KEGG_N_GLYCAN_BIOSYNTHESIS	34	0.29789793	0.29789793	0.002051282	0.13353059	0.9635	4499	tags=68%, list=38%, signal=109%
KEGG_BASE_EXCISION_REPAIR	KEGG_BASE_EXCISION_REPAIR	31	0.2939468	0.2939468	0.006910168	0.13537721	0.9715	6469	tags=84%, list=55%, signal=184%
KEGG_PYRIMIDINE_METABOLISM	KEGG_PYRIMIDINE_METABOLISM	71	0.2791394	0.2791394	0	0.16508707	0.99	3724	tags=59%, list=31%, signal=86%
KEGG_PROTEIN_EXPORT	KEGG_PROTEIN_EXPORT	18	0.2786035	0.2786035	0.09724951	0.16033931	0.9905	7242	tags=89%, list=61%, signal=228%
KEGG_AMINOACYL_TRNA_BIOSYNTH	KEGG_AMINOACYL_TRNA_BIOSYNT	29	0.2721984	0.2721984	0.012820513	0.17220083	0.9945	4140	tags=62%, list=35%, signal=95%
KEGG_PANTOTHENATE_AND_COA_BI	KEGG_PANTOTHENATE_AND_COA_	14	0.27141172	0.27141172	0.20543806	0.16769114	0.9945	3561	tags=57%, list=30%, signal=82%
KEGG_OTHER_GLYCAN_DEGRADATI	KEGG_OTHER_GLYCAN_DEGRADATI	12	0.27045354	0.27045354	0.27318045	0.16410978	0.9945	6678	tags=83%, list=56%, signal=191%
KEGG_SULFUR_METABOLISM	KEGG_SULFUR_METABOLISM	9	0.26965377	0.26965377	0.48243803	0.16070656	0.995	4710	tags=67%, list=40%, signal=110%
KEGG_NICOTINATE_AND_NICOTINAMI	KEGG_NICOTINATE_AND_NICOTINA	15	0.26416212	0.26416212	0.21919192	0.17049184	0.9965	1614	tags=40%, list=14%, signal=46%
KEGG_O_GLYCAN_BIOSYNTHESIS	KEGG_O_GLYCAN_BIOSYNTHESIS	18	0.26009122	0.26009122	0.15004936	0.17592153	0.998	2849	tags=50%, list=24%, signal=66%
KEGG_PHENYLALANINE_METABOLIS	KEGG_PHENYLALANINE_METABOLIS	17	0.25836062	0.25836062	0.15384616	0.17513557	0.998	5311	tags=71%, list=45%, signal=128%
KEGG_TYROSINE_METABOLISM	KEGG_TYROSINE_METABOLISM	38	0.25720558	0.25720558	0.010901883	0.17306502	0.998	7882	tags=92%, list=66%, signal=274%
KEGG_AMINO_SUGAR_AND_NUCLEO	KEGG_AMINO_SUGAR_AND_NUCLE	39	0.24199654	0.24199654	0.018556701	0.2162996	1	2004	tags=41%, list=17%, signal=49%
KEGG_ASCORBATE_AND_ALDARATE	KEGG_ASCORBATE_AND_ALDARAT	10	0.24126583	0.24126583	0.49259624	0.21262644	1	698	tags=30%, list=6%, signal=32%
KEGG_TERPENOID_BACKBONE_BIOS	KEGG_TERPENOID_BACKBONE_BIO	12	0.23846501	0.23846501	0.4486653	0.21707888	1	7057	tags=83%, list=60%, signal=206%

KEGG_DRUG_METABOLISM_OTHER_	KEGG_DRUG_METABOLISM_OTHER_	32	0.2378889	0.2378889	0.04682927	0.21287635	1	2374	tags=44%, list=20%, signal=55%
KEGG_ECM_RECEPTOR_INTERACTIO	KEGG_ECM_RECEPTOR_INTERACTI	78	0.23767024	0.23767024	0	0.208026	1	848	tags=31%, list=7%, signal=33%
KEGG_RNA_DEGRADATION	KEGG_RNA_DEGRADATION	43	0.23598835	0.23598835	0.014271152	0.20826896	1	6588	tags=79%, list=56%, signal=177%
KEGG_GLYOXYLATE_AND_DICARBOX	KEGG_GLYOXYLATE_AND_DICARBO	10	0.21814346	0.21814346	0.6531219	0.27014282	1	9274	tags=100%, list=78%, signal=458%
KEGG_BASAL_TRANSCRIPTION_FACT	KEGG_BASAL_TRANSCRIPTION_FAC	28	0.20984262	0.20984262	0.14772727	0.30084065	1	8529	tags=93%, list=72%, signal=330%
KEGG_SPLICEOSOME	KEGG_SPLICEOSOME	86	0.20968552	0.20968552	9.91E-04	0.29445854	1	8149	tags=90%, list=69%, signal=284%
KEGG_PORPHYRIN_AND_CHLOROPH	KEGG_PORPHYRIN_AND_CHLOROP	24	0.20639011	0.20639011	0.17824773	0.30300948	1	8922	tags=96%, list=75%, signal=386%
KEGG_GALACTOSE_METABOLISM	KEGG_GALACTOSE_METABOLISM	23	0.2014942	0.2014942	0.2646213	0.31947938	1	6380	tags=74%, list=54%, signal=160%
KEGG_GLYCOSYLPHOSPHATIDYLINO	KEGG_GLYCOSYLPHOSPHATIDYLIN	19	0.20083654	0.20083654	0.39735773	0.3154583	1	3863	tags=53%, list=33%, signal=78%
KEGG_ALANINE_ASPARTATE_AND_G	KEGG_ALANINE_ASPARTATE_AND_	27	0.19436227	0.19436227	0.22178988	0.3420203	1	4288	tags=56%, list=36%, signal=87%
KEGG_SNARE_INTERACTIONS_IN_VE	KEGG_SNARE_INTERACTIONS_IN_V	30	0.18588339	0.18588339	0.22902271	0.37953335	1	6102	tags=70%, list=51%, signal=144%
KEGG_CYSTEINE_AND_METHIONINE_	KEGG_CYSTEINE_AND_METHIONINE	31	0.18577361	0.18577361	0.22109534	0.3722906	1	7366	tags=81%, list=62%, signal=212%
KEGG_ONE_CARBON_POOL_BY_FOL	KEGG_ONE_CARBON_POOL_BY_FOL	15	0.17698047	0.17698047	0.6587902	0.4174282	1	4228	tags=53%, list=36%, signal=83%
KEGG_GLYCOLYSIS_GLUCONEOGEN	KEGG_GLYCOLYSIS_GLUCONEOGE	53	0.17628492	0.17628492	0.071428575	0.41298383	1	6421	tags=72%, list=54%, signal=156%
KEGG_SELENOAMINO_ACID_METABO	KEGG_SELENOAMINO_ACID_METAB	19	0.16499765	0.16499765	0.59336495	0.47868395	1	6160	tags=68%, list=52%, signal=142%
KEGG_PATHOGENIC_ESCHERICHIA_	KEGG_PATHOGENIC_ESCHERICHIA_	49	0.16164455	0.16164455	0.16876818	0.49395663	1	5109	tags=59%, list=43%, signal=104%
KEGG_FOCAL_ADHESION	KEGG_FOCAL_ADHESION	182	0.15935008	0.15935008	0	0.50115776	1	2048	tags=33%, list=17%, signal=39%
KEGG_GLYCOSPHINGOLIPID_BIOSYN	KEGG_GLYCOSPHINGOLIPID_BIOSY	23	0.1588424	0.1588424	0.57185626	0.4955047	1	2244	tags=35%, list=19%, signal=43%
KEGG_STARCH_AND_SUCROSE_MET	KEGG_STARCH_AND_SUCROSE_ME	29	0.15570433	0.15570433	0.435468	0.51001805	1	8381	tags=86%, list=71%, signal=293%
KEGG_CITRATE_CYCLE_TCA_CYCLE	KEGG_CITRATE_CYCLE_TCA_CYCLE	29	0.15299666	0.15299666	0.4512195	0.520981	1	8822	tags=90%, list=74%, signal=349%
KEGG_P53_SIGNALING_PATHWAY	KEGG_P53_SIGNALING_PATHWAY	59	0.15200952	0.15200952	0.102564104	0.51825887	1	8457	tags=86%, list=71%, signal=300%
KEGG_GLYCOSAMINOGLYCAN_BIOSY	KEGG_GLYCOSAMINOGLYCAN_BIOS	21	0.151336	0.151336	0.659	0.51449263	1	6114	tags=67%, list=52%, signal=137%
KEGG_PROGESTERONE_MEDIATED_	KEGG_PROGESTERONE_MEDIATED	73	0.1512207	0.1512207	0.06647399	0.506577	1	3253	tags=42%, list=27%, signal=58%
KEGG_AUTOIMMUNE_THYROID_DISE	KEGG_AUTOIMMUNE_THYROID_DISE	38	0.14895067	0.14895067	0.34611502	0.5155661	1	9786	tags=97%, list=83%, signal=555%
KEGG_GLUTATHIONE_METABOLISM	KEGG_GLUTATHIONE_METABOLISM	35	0.14737542	0.14737542	0.39476237	0.51886934	1	1306	tags=26%, list=11%, signal=29%
KEGG_ALLOGRAFT_REJECTION	KEGG_ALLOGRAFT_REJECTION	30	0.14497048	0.14497048	0.5264735	0.5289548	1	10144	tags=100%, list=86%, signal=689%
KEGG_COMPLEMENT_AND_COAGULA	KEGG_COMPLEMENT_AND_COAGUL	65	0.1362181	0.1362181	0.15763052	0.58969384	1	6968	tags=72%, list=59%, signal=174%
KEGG_OOCYTE_MEIOSIS	KEGG_OOCYTE_MEIOSIS	91	0.13376266	0.13376266	0.08414873	0.60049295	1	1292	tags=24%, list=11%, signal=27%
KEGG_UBIQUITIN_MEDIATED_PROTE	KEGG_UBIQUITIN_MEDIATED_PROT	105	0.13296066	0.13296066	0.055045873	0.5975005	1	5665	tags=61%, list=48%, signal=116%
KEGG_PARKINSONS_DISEASE	KEGG_PARKINSONS_DISEASE	96	0.13188544	0.13188544	0.07003891	0.5973296	1	8825	tags=88%, list=74%, signal=339%
KEGG_PURINE_METABOLISM	KEGG_PURINE_METABOLISM	127	0.12764455	0.12764455	0.024415055	0.6231358	1	7373	tags=75%, list=62%, signal=196%
KEGG_THYROID_CANCER	KEGG_THYROID_CANCER	29	0.11673307	0.11673307	0.775428	0.7091308	1	9660	tags=93%, list=81%, signal=501%
KEGG_RIG_I_LIKE_RECEPTOR_SIGNA	KEGG_RIG_I_LIKE_RECEPTOR_SIGN	58	0.1144809	0.1144809	0.4135864	0.718769	1	9281	tags=90%, list=78%, signal=410%
KEGG_HEMATOPOIETIC_CELL_LINEA	KEGG_HEMATOPOIETIC_CELL_LINE	77	0.11235977	0.11235977	0.28965518	0.7269024	1	4220	tags=47%, list=36%, signal=72%
KEGG_INTESTINAL_IMMUNE_NETWO	KEGG_INTESTINAL_IMMUNE_NETWO	35	0.11154337	0.11154337	0.715534	0.723869	1	8168	tags=80%, list=69%, signal=256%
KEGG_FRUCTOSE_AND_MANNOSE_M	KEGG_FRUCTOSE_AND_MANNOSE_	30	0.11093267	0.11093267	0.8124373	0.7192628	1	1454	tags=23%, list=12%, signal=27%
KEGG_CYTOKINE_CYTOKINE_RECEP	KEGG_CYTOKINE_CYTOKINE_RECE	214	0.10937531	0.10937531	0.005830904	0.7227593	1	5154	tags=54%, list=43%, signal=94%
KEGG_TGF_BETA_SIGNALING_PATH	KEGG_TGF_BETA_SIGNALING_PATH	77	0.09997345	0.09997345	0.4028777	0.7971936	1	4674	tags=49%, list=39%, signal=81%
KEGG_HYPERTROPHIC_CARDIOMYO	KEGG_HYPERTROPHIC_CARDIOMYO	75	0.09403479	0.09403479	0.528866	0.8384965	1	314	tags=12%, list=3%, signal=12%
KEGG_HEDGEHOG_SIGNALING_PATH	KEGG_HEDGEHOG_SIGNALING_PAT	45	0.0935628	0.0935628	0.7915811	0.8318491	1	3374	tags=38%, list=28%, signal=53%
KEGG_HUNTINGTONS_DISEASE	KEGG_HUNTINGTONS_DISEASE	148	0.09102011	0.09102011	0.16787565	0.8431987	1	9030	tags=85%, list=76%, signal=352%
KEGG_BLADDER_CANCER	KEGG_BLADDER_CANCER	41	0.08924031	0.08924031	0.87189674	0.8472744	1	10515	tags=98%, list=89%, signal=857%
KEGG_REGULATION_OF_ACTIN_CYT	KEGG_REGULATION_OF_ACTIN_CYT	181	0.08863291	0.08863291	0.1194907	0.841756	1	1978	tags=25%, list=17%, signal=30%
KEGG_LYSOSOME	KEGG_LYSOSOME	106	0.08687046	0.08687046	0.3934263	0.8459618	1	6810	tags=66%, list=57%, signal=154%
KEGG_AXON_GUIDANCE	KEGG_AXON_GUIDANCE	110	0.08503674	0.08503674	0.39898476	0.84971166	1	4822	tags=49%, list=41%, signal=82%
KEGG_VIRAL_MYOCARDITIS	KEGG_VIRAL_MYOCARDITIS	62	0.08116192	0.08116192	0.776329	0.8692608	1	9945	tags=92%, list=84%, signal=566%

KEGG_APOPTOSIS	KEGG_APOPTOSIS	79	0.0786377	0.0786377	0.693837	0.87765706	1	4327	tags=44%, list=36%, signal=69%
KEGG_SMALL_CELL_LUNG_CANCER	KEGG_SMALL_CELL_LUNG_CANCER	81	0.07846985	0.07846985	0.66863906	0.8685714	1	1271	tags=19%, list=11%, signal=21%
KEGG_JAK_STAT_SIGNALING_PATHW	KEGG_JAK_STAT_SIGNALING_PATH	126	0.07381213	0.07381213	0.472167	0.8910851	1	10334	tags=94%, list=87%, signal=726%
KEGG_PANCREATIC_CANCER	KEGG_PANCREATIC_CANCER	70	0.07088332	0.07088332	0.84740883	0.89990073	1	10515	tags=96%, list=89%, signal=839%
KEGG_NOD_LIKE_RECEPTOR_SIGNA	KEGG_NOD_LIKE_RECEPTOR_SIGN	52	0.05810925	0.05810925	0.9900398	0.9578714	1	8664	tags=79%, list=73%, signal=291%
KEGG_PATHWAYS_IN_CANCER	KEGG_PATHWAYS_IN_CANCER	300	0.05528259	0.05528259	0.29493546	0.95814866	1	3590	tags=36%, list=30%, signal=50%
